# The transcriptome of syncytia induced by the cyst nematode *Heterodera schachtii* in Arabidopsis roots

**DOI:** 10.1111/j.1365-313X.2008.03727.x

**Published:** 2008-12-09

**Authors:** Dagmar Szakasits, Petra Heinen, Krzysztof Wieczorek, Julia Hofmann, Florian Wagner, David P Kreil, Peter Sykacek, Florian M W Grundler, Holger Bohlmann

**Affiliations:** 1 Institute of Plant Protection, Department of Applied Plant Sciences and Plant Biotechnology, University of Natural Resources and Applied Life SciencesVienna, Austria; 2 RZPD German Resource Center for Genome ResearchBerlin, Germany; 3 WWTF Chair of Bioinformatics, Department of Biotechnology, University of Natural Resources and Applied Life SciencesVienna, Austria

**Keywords:** Arabidopsis, plant pathogen, *Heterodera schachtii*, syncytium, transcriptome, Affymetrix GeneChip

## Abstract

*Arabidopsis thaliana* is a host for the sugar beet cyst nematode *Heterodera schachtii*. Juvenile nematodes invade the roots and induce the development of a syncytium, which functions as a feeding site for the nematode. Here, we report on the transcriptome of syncytia induced in the roots of Arabidopsis. Microaspiration was employed to harvest pure syncytium material, which was then used to prepare RNA for hybridization to Affymetrix GeneChips. Initial data analysis showed that the gene expression in syncytia at 5 and 15 days post-infection did not differ greatly, and so both time points were compared together with control roots. Out of a total of 21 138 genes, 18.4% (3893) had a higher expression level and 15.8% (3338) had a lower expression level in syncytia, as compared with control roots, using a multiple-testing corrected false discovery rate of below 5%. A gene ontology (GO) analysis of up- and downregulated genes showed that categories related to high metabolic activity were preferentially upregulated. A principal component analysis was applied to compare the transcriptome of syncytia with the transcriptome of different Arabidopsis organs (obtained by the AtGenExpress project), and with specific root tissues. This analysis revealed that syncytia are transcriptionally clearly different from roots (and all other organs), as well as from other root tissues.

## Introduction

Biotrophic plant parasites derive all of their nutrients from living plant tissues. Such a lifestyle has been developed by bacteria, fungi and oomycetes, and animals. All of them face similar problems: to be successful, they have to make intimate contact with their host while avoiding a resistance response, and they have to produce specific structures for the uptake of nutrients, such as the haustoria produced by powdery mildews. Plant parasitic nematodes of the family *Heteroderidae* induce the development of specialized feeding structures in the roots of their host plants, which consist of a syncytial fusion of hypertrophied cells. The syncytia are the only nutrient source for these nematodes, and are thus a severe nutrient sink for the host. The nematodes feed from the syncytium through a feeding tube that is produced at the tip of the stylet during each feeding cycle (Davis *et al.*, 2004; Williamson and Kumar, 2006). The nematodes invade the roots with the help of their stylet, assisted by secretions produced from two subventral pharyngeal gland cells that have been shown to contain cell wall degrading enzymes, such as cellulases and pectinases, as well as a putative expansin (Kudla *et al.*, 2005; Smant *et al.*, 1998; Vanholme *et al.*, 2004). Having reached the central cylinder, the nematode selects a single cell that is carefully pierced by the stylet. In Arabidopsis, the initial syncytial cells are preferably procambium or pericycle cells within the central cylinder (Golinowski *et al.*, 1996; Sobczak *et al.*, 1997). From this cell the development of the syncytium is initiated through secretions of the nematode, and by a coordinated expression of plant genes. Such plant genes include, for instance, expansins and cellulases, which are important for the degradation of cell walls to incorporate new cells into the growing syncytium (Goellner *et al.*, 2001; Wieczorek *et al.*, 2006, 2008). Cells incorporated into the syncytium undergo drastic changes in structure and activity. The central vacuole is fragmented into many smaller ones and the cells become metabolically active, as indicated by the dense granular cytoplasm, large numbers of mitochondria, ribosomes, and a proliferation of the endoplasmic reticulum (Golinowski *et al.*, 1996; Sobczak *et al.*, 1997). To cope with this high metabolic acivity, the nuclei and nucleoli are enlarged, and contain endoreduplicated DNA (Niebel *et al.*, 1996). Although the syncytium is a plant-derived structure, it is also dependent on the continuous activity of the nematode, because death or artificial removal of the nematode leads to a degradation of the syncytium. How exactly the nematode induces the development of the syncytium is currently unknown, although a few proteins that are produced by the dorsal pharyngeal gland cell that might be involved in this activity have been identified (Jaubert *et al.*, 2002; Vanholme *et al.*, 2004).

The structurally visible drastic changes in cell morphology imply an underlying global change in gene expression. Indeed, a number of genes have been identified that are particularly induced in syncytia or in giant cells, using a variety of methods such as differential display and promoter tagging, as reviewed by Gheysen and Fenoll (2002). During recent years the methodology has shifted towards microarrays and GeneChips, which allow a global view on the changes in gene expression in feeding sites. As a first approach, whole roots or dissected root segments of Arabidopsis roots infected with *Heterodera schachtii* (Puthoff *et al.*, 2003) and soybean roots infected with *Heterodera glycines* (Alkharouf *et al.*, 2006; Ithal *et al.*, 2007a; Klink *et al.*, 2007a) were used. Recently, laser capture microdissection and Affymetrix GeneChips have been used to study the transcriptome of syncytia induced in soybean roots by *H. glycines* (Ithal *et al.*, 2007b; Klink *et al.*, 2007b).

The rather wide host range of the sugarbeet cyst nematode *H. schachtii* has been exploited to use the interaction with *Arabidopsis thaliana* roots as a model system. The translucent roots growing on artificial media have made it possible to study the behavior of this and other nematode species inside the root (Wyss and Grundler, 1992). *H. schachtii* can complete its whole life cycle on Arabidopsis plants *in vitro* within 6 weeks (Sijmons *et al.*, 1991). Now, the availability of microarrays makes it possible to study the transcriptome of feeding sites induced in Arabidopsis roots. Puthoff *et al.* (2003) used the first-generation Affymetrix Arabidopsis GeneChip, which covers ∼30% of the genome. They compared whole roots infected with *H. schachtii* or *H. glycines* at 3 days post-infection (dpi) with control roots (Puthoff *et al.*, 2003), and identified 128 and 12 genes, respectively, with altered steady-state mRNA levels after nematode infection.

The second-generation ATH1 Arabidopsis GeneChip, which contains probes covering ∼75% of the genome, was used by Hammes *et al.* to study *Meloidogyne incognita* galls on Arabidopsis roots, but only the expression of 1400 genes coding for transport proteins was reported (Hammes *et al.*, 2005). Similarly, Jammes *et al.* studied *M. incognita* galls using the CATMA microarrays, which contain probes for 22 089 genes (Jammes *et al.*, 2005).

Previous reports concerning studies of gene expression in Arabidopsis feeding sites were hampered by the fact that the material used contained not only material from the feeding cells, but also included surrounding tissue. It is thus difficult to differentiate between gene expression in feeding cells and in the surrounding tissue. In studies that used the whole-root system, these expression patterns were even overlain with systemic expression from elsewhere in the root system. To avoid these problems, our approach has been to isolate pure syncytium material by microaspiration, thereby enabling a transcriptome analysis of syncytia alone. In this way, we were able to monitor and analyze the expression of 21 138 genes at different time points during the interaction of *H. schachtii* with Arabidopsis roots. Our results reveal the transcriptome of syncytia, and show that they are clearly different from roots and all other organs.

## Results

Syncytia that develop inside the roots can be microaspirated to obtain pure syncytium material, without contaminating root tissues (Juergensen *et al.*, 2003). We have used this technique to obtain material for a transcriptome analysis of syncytia at 5 and 15 dpi.

The development of the syncytium starts from an initial syncytial cell in the central cylinder of the root, selected by the nematode (Golinowski *et al.*, 1996; Wyss and Grundler, 1992). Therefore, the preferred control would have been material from such cells before induction. This was, however, technically impossible. We have therefore used root segments from 12-day-old plants (0 dpi), corresponding to the elongation zone, and have excluded root tips and secondary root primordia.

Total RNA was isolated, amplified, and hybridized to Affymetrix ATH1 GeneChips (as described in detail in Experimental procedures).

Our initial strategy was to analyze the development of the syncytium over time. However, in line with analysis results showing that only a few transcripts were significantly different between syncytia at 5 and 15 dpi, instead of treating the data as a time series, we used a linear model of effects, with one contrast giving the differences between both (combined) syncytia tissues and the controls, and another contrast examining the remaining differences between syncytia at 5 and 15 dpi.

Broad trends in gene expression, in comparison with previously published data sets, were visualized using principal component analysis (PCA).

In an analysis of differential expression, the transcriptome of syncytia was observed to be very different from the control root samples. Table S1 presents the complete results from comparing the syncytium samples (at both 5 and 15 dpi) with controls. A total of 7231 genes (34.2%) were differentially expressed for a false discovery rate cut-off of *q* < 5%, after correction for the multiple testing of 21 138 genes. Compared with the control, in syncytia 18.4% (3893) of all genes had a higher expression level, and 15.8% (3338) had a lower expression level. The average expression levels, and differences between syncytia and controls, for the 100 most significantly differentially expressed genes, are shown in Figure S1.

### Upregulated genes

Table S2 takes an alternative view, showing the list of 100 genes that have the highest increase in expression compared with the controls. Among these upregulated genes, several genes encode proteins that are probably involved in the degradation of cell walls, a process that is important for the expansion of the syncytium: pectate lyase family proteins *At3g27400* and *At4g24780*, as well as expansins ATEXPA6 (*At2g28950*) and ATEXPA1 (*At1g69530*) (Wieczorek *et al.*, 2006). Several other genes code for chloroplast proteins such as glyceraldehyde 3-phosphate dehydrogenase A (*At3g26650*), cytochrome B6-F complex iron-sulfur subunit (*At4g03280*) and several chlorophyll *a*–*b* binding proteins (*At3g54890*, *At5g54270*, *At2g40100*, *At4g10340*, *At1g15820* and *At5g01530*).

### Downregulated genes

On the other hand, if we look at the list of genes that showed a strong decrease in expression level, we find two prominent groups of genes (Table S3). One strongly over-represented group comprises genes coding for peroxidases. Among the 3338 downregulated genes were 35 peroxidases, representing an odds ratio of 4.6 (95% confidence interval, CI, 2.8–7.3), *P* < 10^−9^, Fisher's exact test, compared with the number of peroxidase genes assessed on the chip. The effect was even more pronounced when we focussed on the 100 differentially expressed genes with the strongest decrease in expression. These include 14 peroxidases, corresponding to an odds ratio of 47 (CI 24–89), *P* < 10^−15^. In contrast, only one gene coding for a (chloroplast) peroxidase was found among the 100 genes with the strongest significant increase in expression (Table S2), a number compatible with the representation of peroxidases on the chip. The second prominent group of genes over-represented are those that code for major intrinsic proteins (MIPs), which include aquaporins (Wallace and Roberts, 2004). Arabidopsis has 35 MIP genes, and nine of them were among the list of 100 genes with a strong decrease in expression level, corresponding to an odds ratio of 73 (CI 29–164), *P* < 10^−12^. Contingency tables for all tests are provided in the Table S4(a,b).

### Highly expressed genes

Genes can also be viewed according to their expression level in the syncytium (Table S5). The most strongly expressed genes typically had only slightly higher expression levels in the syncytia, compared with the control roots. As we go down the list, more and more genes show no significant differences. The genes most strongly expressed included those coding for proteins involved in primary metabolism, such as ribosomal proteins.

### Differences between 5- and 15-day-old syncytia

In a comparison of 5- and 15-day-old syncytia, only 22 genes were differentially expressed with a false discovery rate cut-off of *q* < 5%, after correction for multiple testing. Of these, 19 genes were more highly expressed in 15-dpi syncytia, as compared with 5-dpi syncytia, whereas only three genes were more highly expressed in 5-dpi syncytia than in 15-dpi syncytia (Table S6). Results for all genes are shown in Table S7, and the high degree of similarity of 5- and 15-dpi samples is also reflected in MA plots (Figure S2). Whereas many of the differentially expressed genes have no known function, two of the genes that were more highly expressed in 15-dpi syncytia code for phytosulfokines (Yang *et al.*, 2001).

### Validation of GeneChip data

**Figure 1:**
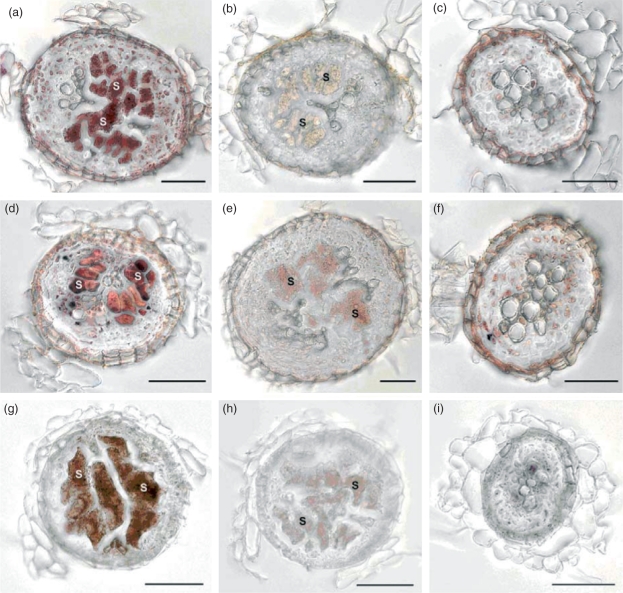
*In situ* RT-PCR analysis of three induced genes The expression of *At3g63140*, *At5g64080* and *At1g10010* was analyzed on cross sections of infected (15-dpi syncytia) and uninfected control roots of Arabidopsis (scale bar = 50 μm). (a) Purple stained transcripts of *At3g63140* are visible within the syncytium (S), and only visible to a small extent in the surrounding tissue outside of the vascular cylinder. (b) A control reaction for (a) on a syncytium performed without Taq polymerase shows neither specific staining in the infected part of the root (S) nor staining in any other root cells. (c) Uninfected root sections show no staining of transcripts. (d) Transcripts of *At5g64080* are mainly stained within the syncytium (S), with some staining in small cells adjacent to the syncytium. (e) A control reaction for (d) without Taq polymerase on another syncytium shows no specific (purple) staining. (f) In an uninfected root there is some typical transcript staining visible in the phloem. (g) For *At1g10010*-specific transcripts, an intensive staining is restricted to cells within the syncytium (S) only. (h) In a control reaction for (g) excluding Taq polymerase the whole cross-section through a syncytium does not show any specific staining. (i) In an uninfected root staining of *At1g10010* is restricted to phloem cells.

We have already published a detailed expression analysis of expansins (Wieczorek *et al.*, 2006) and endo-1,4-β-glucanases (Wieczorek *et al.*, 2008), in relation to the formation of syncytia induced in Arabidopsis roots by *H. schachtii*. In these studies, the differential expression of 29 and 25 genes, respectively, was validated using *in situ* RT-PCR, RT-PCR, and promoter:*gus* lines. Furthermore, several genes involved in starch metabolism in syncytia have also been validated recently (Hofmann *et al.*, 2008). Therefore, we have only selected three additional genes for further analysis using qPCR and *in situ* RT-PCR in the present study: *At3g63140* codes for a putative mRNA binding protein (84-fold upregulated, significance rank 40), *At5g64080* codes for a lipid transfer protein (30-fold upregulated, significance rank 1159), and *At1g10010* codes for an amino acid permease (AAP8, 23-fold upregulated, significance rank 259). All three genes showed a strong upregulation in syncytia as compared with roots. Of these, only expression of *At1g10010* was detectable in uninfected control root segments by real-time PCR, but transcripts for all three genes were detected in syncytia (Table 1). As no expression was detected for *At3g63140* and *At5g64080* in control root segments, it was not possible to formally calculate a fold change value. The *in situ* RT-PCR revealed that all genes showed a strong expression in syncytia. *At3g63140* and *At5g64080*, but not *At1g10010*, were found to be slightly expressed in the surrounding tissue. For the last two genes, specific staining was also detected in the phloem of uninfected roots, whereas in control reactions without polymerase only non-specific background staining was found (Figure 1a–i).

**Table 1:** qPCR validation

		Chip data-fold change (log_2_)		qPCR-fold change (log_2_)	
			
Accession number	Function	5-dpi versus ct	15-dpi versus ct	5-dpi versus ct	15-dpi versus ct
*At3g63140*	Putative mRNA-binding protein	6.3	6.5	–	∞
*At5g64080*	Lipid transfer protein	5.3	4.5	–	∞
*At1g10010*	AAP8; amino acid permease	2.9	6.1	–	6.2
*At1g22710*^a^	AtSUC2; phloemspec. sucrose transporter	−0.6	0.0	−1.5	−1.0
*At1g09960*^a^	AtSUC4; phloemspec. sucrose transporter	0.8	0.9	−0.9	0.3
*At4g05320*^b^	UBQ10; polyubiquitin	−0.5	−0.4	−1.1	−0.3
*At3g18780*^b^	ACT2; actin 2	−1.0	−1.6	−2.3	−1.1
*At5g10790*^b^	UBP22; ubiquitin-specific protease	−0.4	−0.4	−0.7	−0.6
*At1g32900*^c^	GBSS1; starch synthase	4.4	6.1	3.6	3.5
*At5g24300*^c^	SS1; starch synthase	3.7	4.4	2.6	2.6
*At3g29320*^c^	PHS1; starch phosphorylase	5.2	4.7	2.3	2.1
*At5g03650*^c^	SBE2; branching enzyme	3.3	4.1	1.2	2.0
*At4g39210*^c^	APL3; ADP-glc pyrophosphorylase	3.2	3.1	3.3	2.8
*At3g46970*^c^	PHS2; starch phosphorylase	3.2	3.1	1.7	1.9

The fold change of 5- and 15-dpi syncytia, as compared to control roots, is shown on a log_2_ scale. For the first three genes, only one time point was measured via qPCR, whereas for the remaining genes investigated in previous studies (^a^Hofmann *et al.*, 2007; ^b^Hofmann and Grundler, 2007; ^c^Hofmann *et al.*, 2008), both time points were measured. For both *At3g63140* and *At5g64080* no RNA was detected in the control, making it impossible to calculate a fold change value (indicated by ∞).

### Genes involved in syncytium formation and maintenance

**Figure 2:**
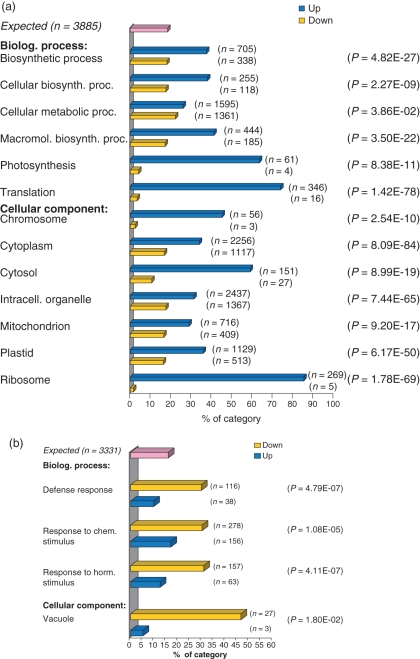
Preferential regulation in gene ontology (GO) categories with relevance to syncytium function Preferential regulation of differentially expressed genes for selected GO categories of the two domains ‘biological process’ and ‘cellular component’. The percentage of genes found in the examined subset is shown on the *x*-axis. The first (pink) bar at the top of each panel thus plots the size of the examined gene set (3331 and 3385, respectively) relative to the chip size. This represents the ratio expected on average if the distribution of examined genes across GO categories matched that of all the genes tested on the chip. For each GO category, a pair of bars compares the numbers of repressed genes (yellow) and induced genes (blue), and the *P* value for this comparison is displayed on the right. (a) Over-representation of upregulated genes compared with downregulated genes for a representative random selection of categories. (b) Over-representation of downregulated genes compared with upregulated genes in four different subcategories of particular interest.

**Figure 3:**
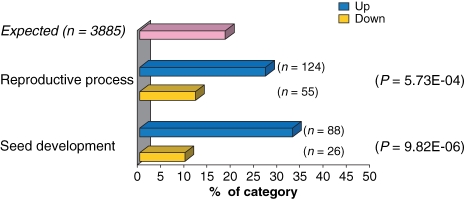
Preferential regulation in gene ontology (GO) categories related to reproduction GO categories showing over-representation of upregulated genes compared with downregulated genes within the domain ‘biological process’. For details, see Figure 2.

In analogy with the procedure described by Jammes *et al.* (2005), we explored the regulation of ‘biological processes’ and ‘molecular functions’, and their distribution across ‘cellular components’, according to the gene ontology classification (GO; http://www.geneontology.org), by comparing their representations in significantly up- and downregulated genes. To this end, for each of the 4278 GO categories used, we compared the prevalence in the 3885 GO annotated significantly upregulated genes, with the prevalence in the same number of downregulated genes (Fisher's exact test with Bonferroni correction). Conversely, its prevalence in the 3331 GO annotated significantly downregulated genes was compared with its prevalence in the same number of upregulated genes (full results in Table S8a,b). Categories of special interest are shown in Figures 2 and 3, and are outlined below. In our comparison of significantly up- and downregulated genes, for the domain ‘cellular component’, significantly more genes were upregulated for the categories ‘chromosome’, ‘cytoplasm’, ‘intracellular organelle’, ‘mitochondrion’, ‘plastid’, and ‘ribosome’. Similarly, we found more upregulated genes than downregulated genes belonging to the ‘biological process’ categories ‘biosynthetic process’, ‘cellular biosynthetic process’, ‘cellular metabolic process’, ‘macromolecular biosynthetic process’, ‘photosynthesis’, and ‘translation’ (Figure 2a). On the other hand, this comparison identified categories of the ‘biological process’ domain, such as ‘defense response’, ‘response to chemical stimulus’, and ‘response to hormone stimulus’, with a significant over-representation of downregulated genes. Within the ‘cellular component’ domain, the category ‘vacuole’ included significantly more genes that were downregulated rather than upregulated (Figure 2b).

### The transcriptome of syncytia is distinct from that of roots or other organs

**Figure 4:**
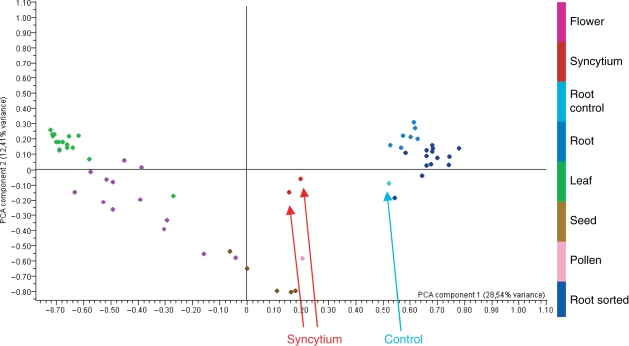
Principal component analysis PCA was applied to a total of 185 samples from three different studies. Each dot represents a condition (i.e. a specific tissue type), and colours code for specific plant organs. The control sample for the current study is indicated in turquoise, and the two different infection stages of the syncytium samples at 5 and 15 dpi are shown in red.

We analyzed the tissue-specific expression of the one hundred most strongly induced genes in Genevestigator (Zimmermann *et al.*, 2004) (Table S9), and noted that some strongly induced genes are not root-specific, but are instead expressed in seeds (such as *Pdf2.1*) or pollen (*MIOX4* and *MIOX5*). This is also reflected in a comparison of up- and downregulated genes in the categories ‘reproductive process’ and ‘seed development’ within the domain ‘biological process’ (Figure 3), where upregulated genes were significantly over-represented. To examine data with a global view, we compared the transcriptomes of syncytia with the transcriptomes of different Arabidopsis organs (flower, leaf, pollen, root and seed) obtained by the AtGenExpress project (Schmid *et al.*, 2005) by PCA. We also included transcriptome data from a project that used fluorescence-activated cell sorting to isolate specific root tissues (Birnbaum *et al.*, 2003). Whereas the transcriptome of our root samples clustered together with the transcriptomes of whole roots of different stages and different root tissues, the syncytial transcriptomes were clearly separated (Figure 4). The separation on PCA component 1 clearly differentiates syncytia from root tissue. On the other hand, the samples did not cluster with any other organ tissues. Thus, although derived from root cells and inside the root, the transcriptome of the syncytium is clearly different from roots (and all other organs), as well as from other root tissues.

## Discussion

A problem in analyzing gene expression in nematode feeding sites is that a plant root can only support a limited number of these feeding sites. Thus, if sampling total roots, the feeding sites will comprise only a small quantity of the root material, and it will therefore not be possible to differentiate gene expression in the syncytia from systemic expression in the root induced through nematode infection. For a thorough analysis it is therefore necessary to isolate pure material from feeding sites. We have used microaspiration of syncytia induced by the cyst nematode *H. schachtii* in Arabidopsis roots to isolate such pure material for a transcriptome analysis with Affymetrix GeneChips. Using the latest generation of Affymetrix Arabidopsis chips (ATH1), together with the current annotation by TAIR (http://www.arabidopsis.org), allowed a clear assessment of expression levels for 21 138 genes (Dai *et al.*, 2005).

### Validation

For validation, we have compared the GeneChip results of three genes (*At3g63140, At5g64080* and *At1g10010*) with *in situ* RT-PCR and qPCR (Figure 1a–i and Table 1). It should be noted that for qPCR we are routinely using syncytia that are cut out from the roots, and thus also contain the surrounding root tissues, in comparison with the pure syncytium material that was obtained through microaspiration for GeneChip analysis. Expression levels obtained by these methods may therefore show quantitative differences, but the time needed to obtain material through microaspiration precludes their use as a routine method. In addition to the three genes validated in this paper, we have recently published a comprehensive analysis of expansin gene expression in syncytia, in which we have also validated this GeneChip dataset with *in situ* RT-PCR, promoter:*gus* lines, and semi-quantitative RT-PCR (Wieczorek *et al.*, 2006). Furthermore, the expression of endo-1,4-β-glucanases observed in this data set has been validated using *in situ* RT-PCR and qPCR (Wieczorek *et al.*, 2008). In addition, the expression of genes involved in sugar transport and starch metabolism has also been validated (Hofmann *et al.*, 2007, 2008). In all cases examined, the results of the GeneChip analysis were found to be reliable. For reference, we have also included the published qPCR values for these other genes in Table 1.

### Cell wall degradation and synthesis in syncytia

Starting from a single initial cell, the syncytium expands by incorporating surrounding cells. The cell walls between these cells are partly degraded. It is known that nematodes produce a range of cell wall degrading enzymes in their gland cells (Vanholme *et al.*, 2004, 2006), which can be secreted through the stylet, but to what extent these enzymes might be involved in the cell wall degradation within the syncytium is not yet known. On the other hand, it is known that plant-derived genes for cell wall degrading enzymes (Goellner *et al.*, 2001), as well as expansin genes (Wieczorek *et al.*, 2006), are upregulated in syncytia. Analysis of the syncytial transcriptome showed that several expansin genes were specifically upregulated (Wieczorek *et al.*, 2006). Genes for putative cell wall degrading enzymes were also upregulated, and some of them were among the most strongly induced (Table S2), such as those coding for pectate lyases and for a β-glucanase (Wieczorek *et al.*, 2008). Thus, it is possible that the degradation of cell walls within the syncytium is solely achieved through endogenous plant proteins, and that the nematode-specific enzymes are only responsible for cell wall degradation and loosening during the passage of the nematodes through the plant root towards the central cylinder.

The formation of syncytia is, on the one hand, accompanied by a degradation of cell walls; on the other hand, cell wall synthesis is also needed, for instance, for producing cell wall ingrowths (Jones and Northcote, 1972; Sobczak *et al.*, 1997) that are commonly found close to the xylem, and for the thickening of the outer cell wall of the syncytium (Golinowski *et al.*, 1996). Cell wall ingrowths are also characteristic for transfer cells, which are thought to facilitate solute transport at the interface between the apoplast and the symplast (Offler *et al.*, 2002). In this regard it is also noteworthy that genes coding for myo-inositol oxygenase (MIOX) (Kanter *et al.*, 2005) were strongly expressed in syncytia. Three of the four Arabidopsis *MIOX* genes are represented on the GeneChip. Of these, *MIOX2* was approximately eightfold upregulated, and *MIOX4* and *MIOX5* were among the most strongly upregulated genes in syncytia (Table S2). The function of these genes in general is not totally clear, but MIOX4 and MIOX5 are otherwise highly expressed in pollen (Table S9). *MIOX* genes are probably involved in the production of UDP-glucuronic acid, an important precursor for several nucleotide sugars used in cell wall biosynthesis, but there are also indications from overexpression studies that they might be involved in the synthesis of ascorbate (Lorence *et al.*, 2004). UDP-glucuronic acid can also be produced through a second pathway, which uses UDP-glucose dehydrogenase, but the corresponding genes are expressed at the same level in syncytia and in control roots. Thus, the *MIOX* genes seem to play an important role for the function of syncytia. This issue is currently under further investigation in our laboratory.

### Metabolic activity

For the host plant, the syncytia are sinks of nutrients. Other particularly important sinks are seeds and pollen. It is therefore interesting that genes of the GO categories ‘seed development’ and ‘reproductive process’ were preferentially upregulated in syncytia (Figure 3; Table S8a). Seeds and pollen are also characterized by high metabolic activity, with an overexpression of components of protein synthesis and metabolism observed in seeds and pollen (Schmid *et al.*, 2005). The same holds true for syncytia, which are also characterized by high metabolic activity. An examination of gene regulation for the category ‘ribosome’ of the GO domain ‘cellular component’ found a strong over-representation of upregulated genes (Figure 2a; Table S8a). Highlighting the biological significance of this observation, many ribosomal genes belong to the most strongly expressed genes (Table S5). Moreover, genes of the GO category ‘translation’ were preferentially upregulated, indicating a strong increase of protein biosynthesis. The high metabolic activity of syncytia was also reflected in the preferential upregulation of genes within the categories ‘biosynthetic process’, ‘cellular biosynthetic process’, ‘cellular metabolic process’, and ‘macromolecule biosynthetic process’ (Figure 2a). Although these are clear trends, many other GO categories and gene families have both up- and downregulated members. The expansin gene family is a nice example of this (Wieczorek *et al.*, 2006). High metabolic activity with similar numbers of upregulated and downregulated genes has also been observed for galls induced by the root-knot nematode *M. incognita* in Arabidopsis roots (Jammes *et al.*, 2005).

### Transport of nutrients into syncytia

Syncytia are the only source of nutrients for the cyst nematodes throughout their life, and are therefore a severe nutrient sink for the plant. Up to recently, it was thought that syncytia are symplastically isolated, and that sucrose is taken up by the sucrose transporter *Suc2* (Juergensen *et al.*, 2003). Analysis of the transcriptome data presented here gave no indication that the *Suc2* gene was induced in syncytia. *Suc2* was expressed at low levels in both syncytia and control roots. This is in agreement with recent data implying the existence of plasmodesmata between syncytia and the phloem (Hofmann and Grundler, 2006; Hofmann *et al.*, 2007; Hoth *et al.*, 2005). Similarly, many other sugar transporters and sugar-metabolizing enzymes were not strongly induced in our experiment (J. Hofmann, P. Hess, D. Szakasits, A. Blöchl, A. van Bell, H. Bohlmann and F. Grundler, unpublished data).

Contrary to the moderate regulation of sugar transporters, we found a strong upregulation of an amino acid transporter (AAP6; Table S2). A second amino acid transporter of the same group (AAP8) was also upregulated in syncytia, and was among the few genes that were significantly more strongly expressed in 15-dpi syncytia than in 5-dpi syncytia (Table S6). These transporters are proton symporters, and seem to be especially needed for the transport of acidic amino acids (Okumoto *et al.*, 2002). It is not yet known if syncytia have a specific demand for acidic amino acids, or if these transporters might have a different function. The gene coding for AAP6 has been found to be only slightly upregulated in gall segments of Arabidopsis roots infected with *M. incognita* (Hammes *et al.*, 2005). Whether this is caused by a dilution effect of the giant cells with the surrounding material, or if there are fundamental differences between the amino acid transport into syncytia and giant cells, remains an open question. However, it once again highlights the importance of using pure material for a transcriptome analysis of nematode feeding sites.

### Suppression of defense responses

Pathogens in general face the problem of coping with defense reactions of their hosts. Bacteria, for instance, produce a variety of effectors for this purpose (Debroy *et al.*, 2004; Jamir *et al.*, 2004). It has recently been shown that these can be delivered through a kind of molecular injection needle, the type-III secretion system, into the host cells (Galan and Wolf-Watz, 2006). By analogy, it can be expected that nematodes use their stylet to deliver effectors into syncytia. A range of different proteins that might act as such has been purified from nematodes *in vitro* (Vanholme *et al.*, 2004), but in most cases there is no clue yet as to their possible functions. Microneedles have been used to demonstrate that a mechanical stimulus comparable with the piercing of the cell wall by a nematode stylet induces defense reactions in plant cells (Gus-Mayer *et al.*, 1998). However, the transcriptome analysis of the syncytia presented in this paper shows that defense gene expression is repressed (Figure 2b), similar to the results obtained for galls induced by the root-knot nematode *M. incognita* in Arabidopsis roots (Jammes *et al.*, 2005). Whether the peroxidase genes, which were among the most strongly downregulated genes (Table S3), are also involved in defense responses, remains an open question.

An exception is the expression of a group of plant defensin genes (for a review see Thomma *et al.*, 2002). *Pdf2.2* and *Pdf2.3* were strongly expressed both in control root segments and in syncytia, and *Pdf2.1* was among the most strongly upregulated genes in syncytia (Table S2). It is not known whether these peptides are taken up by the nematode, although their small size indicates that this might be the case (Böckenhoff and Grundler, 1994). This would imply that at least these defensins have no effect on *H. schachtii* and, probably, other cyst nematodes.

### Plastids in syncytia

Arabidopsis plants inoculated with nematodes are routinely grown in Petri dishes in a dark/night cycle to assist the observation and manipulation (such as microaspiration) of the infected plants (Sijmons *et al.*, 1991; Wyss and Grundler, 1992). It has been known for a long time that syncytia formed under these conditions contain chloroplasts (Golinowski *et al.*, 1996; Sijmons *et al.*, 1991). In accordance with these observations, we found that genes coding for chloroplast proteins were among the most strongly upregulated genes (Table S2), and that genes in the GO category ‘plastid’ were preferentially upregulated (Figure 2a). Furthermore, genes assigned to the GO category ‘photosynthesis’ were also preferentially upregulated (Figure 2a). Thus, at least under these growth conditions, syncytia contain plastids that seem to perform active photosynthesis. This would, of course, not be possible under natural conditions. We have therefore looked at syncytia from roots kept in the dark after infection. These syncytia have a comparable number of plastids that show a similar fluorescence in confocal microscopy as those from plants kept under a light/dark cycle (D. Szakasits, M. Sobczak and H. Bohlmann, unpublished data). Plastids within the syncytia are known to be different from those found in cells surrounding the syncytia (Golinowski *et al.*, 1996). Hence, our results corroborate that the differentiation of plastids is influenced by the syncytium.

### The syncytial transcriptome

We noted that among the most strongly upregulated genes in syncytia were several that are otherwise specifically expressed in pollen or seeds (Table S9). This motivated us to perform a PCA (Figure 4) to compare the transcriptome data from syncytia with those for different Arabidopsis organs and tissues. A similar approach has been reported for Arabidopsis organs (Schmid *et al.*, 2005), and for Arabidopsis root tissues obtained through cell sorting (Birnbaum *et al.*, 2003). Root tissues and root organs also clustered together in our analysis. In addition, the root control segments used in our work fell into the same cluster. Syncytia, however, were clearly located outside the root cluster, which also agreed with the global differences between syncytium and control root sections (18.4% of all genes analyzed were upregulated in syncytia, and 15.8% were downregulated for *q* < 5%). Moreover, syncytia did not cluster with any other tissue types, including samples from flowers, leaves, pollen and seeds. This analysis therefore indicated that syncytia, although formed within the root, have a characteristic, unique transcriptional profile that is different from that of any other organ, and also from that of any other root tissue.

In a previous study (Puthoff *et al.*, 2003), a comparison of total roots infected with *H. schachtii* at 3 dpi with control roots identified 116 differentially expressed genes (71 upregulated and 45 downregulated), using the first generation Arabidopsis GeneChip, which covered approximately one third of all genes. There are several possible explanations of why that study has identified a much lower number of genes than our analysis. First, the current GeneChip probes more than twice the number of genes. Second, we have isolated pure syncytium material, and have specifically analyzed the changes within syncytia. Third, the differences in sampling time points might also affect results: syncytium material for this study was obtained at both 5 and 15 dpi. The expression differences between 3- and 5-dpi time points, however, seem to be only marginal (initial, sample count limited comparisons in this laboratory have identified only four genes showing significantly different expression levels; D. Szakasits, D. Kreil and H. Bohlmann, unpublished data).

If we compare the analyses of whole infected roots with those of the aspirated syncytia performed for this study, we find that 56 of the genes (34 up- and 22 downregulated) identified by Puthoff *et al.* (2003) are also differentially regulated in syncytia, for an agreement of almost 50%. The genes only identified by Puthoff *et al.* (2003) are probably genes that are systemically induced or repressed through nematode infection. The fact that no genes coding for ribosomal proteins were found in the Puthoff *et al.* (2003) study provides evidence corroborating this interpretation. Such genes have, however, been shown to be strongly expressed in nematode feeding sites in our analysis, and in other transcriptome studies (Ithal *et al.*, 2007b; Jammes *et al.*, 2005), and are indicative of the high metabolic activity in these feeding sites.

Other laboratories have also reported differences comparing excised infection sites versus syncytium material for transcriptome analysis (Ithal *et al.*, 2007b; Klink *et al.*, 2007b). The second study for instance found only two genes in common between the 77 genes induced in syncytia and the 502 genes induced in infected root samples at 3 dpi. These data also suggest that the majority of induced genes in infected whole root samples (Puthoff *et al.*, 2003) probably represent systemically induced genes.

Recently, laser capture microdissection has been applied to study syncytia induced by *H. glycines* in soybean roots, using Affymetrix GeneChips containing 37 744 probe sets (Ithal *et al.*, 2007b; Klink *et al.*, 2007b). In the first study, 1116 genes were induced at 2 dpi, and 649 genes were suppressed. In the second study, 77 genes were induced and 210 were suppressed at 3 dpi in a compatible interaction, whereas 206 were induced and 63 were suppressed at 8 dpi. Both studies used a –fold change cut-off of 1.5, and a 0.5 and 5% false discovery rate threshold, respectively. At present, it is not clear why more induced and repressed genes were identified in the first study. Whereas some small differences could probably be explained by the difference between the 2- and 3-dpi time points, the fact that the first study identified so many more genes is unexpected, particularly as it used a more stringent statistical cut-off. In addition, the second study identified three times more suppressed than induced genes at 3 dpi, whereas the relationship was completely different and reversed at 8 dpi. We currently have no explanation for this.

Ithal *et al.* identified 1116 upregulated and 649 downregulated genes in syncytia at 2 dpi. As expected, this study also revealed a high metabolic activity in syncytia, as shown by the upregulation of 35 genes coding for ribosomal proteins. These authors also found both up- and downregulated genes within gene families, matching our observations in the present study. Of the 1765 differentially regulated genes, 833 upregulated and 449 downregulated genes had homologs in Arabidopsis (collapsing many-to-one mappings). Expecting few gene expression differences between 3- and 5-dpi samples of syncytia induced by *H. schachtii* in Arabidopsis roots (D. Szakasits, D. Kreil and H. Bohlmann, unpublished data), we compared the gene lists for upregulated or downregulated genes from the Ithal *et al.* (2007b) study with our data. We found that of the 833 Arabidopsis homologs of upregulated soybean genes, 312 were also upregulated in syncytia induced by *H. schachtii* in the Arabidopsis roots. Of the 449 Arabidopsis homologs of downregulated soybean genes, 146 were also downregulated in syncytia induced by *H. schachtii* in Arabidopsis roots.

From a biological point of view, syncytia induced by related nematodes in different hosts should be quite similar in relation to their basic metabolism. This is reflected in the comparison here. The list of homologous genes that were upregulated in both systems includes, for instance, almost all of the genes coding for ribosomal proteins (all except two). In soybean, eight genes homologous to six Arabidopsis expansin genes were upregulated. Of these, four genes were also upregulated in Arabidopsis syncytia (a 66–75% agreement). A detailed gene-level comparison of the differences between data sets is also likely to highlight the subtle effects of choice of sampling time point and differences in statistical analysis, and would require a comprehensive analysis of Arabidopsis and soybean homologs. Of course, paralogs may have diverged in both function and transcriptional regulation. Considering the many non-unique mappings, a reliable identification of orthologs would thus be part of the challenge. (For instance, the present data bases map seven soybean genes to the same putative peroxidase *At5g05340.*)

## Conclusion

Our analysis has identified syncytia as having a characteristic, unique transcriptional profile. The expression of a large range of genes is changed in syncytia, compared with control roots, and the fundamental question that remains to be answered is how the formation of this organ is induced by the nematode. It is generally agreed that proteins secreted by the nematode are involved. Future work will be focused on linking the genes that are up- and downregulated in the syncytium to developmental pathways, and on linking these to the activity of nematode-derived effectors.

## Experimental procedures

### Plant cultivation

Seeds of Arabidopsis (cv. Columbia) were surface-sterilized for 10 min in 5% (w/v) calcium hypochlorite, submerged for 5 min in 70% (v/v) ethanol and were then washed three times in sterile water (Sijmons *et al.*, 1991). The sterilized seeds were then placed into sterile Petri dishes (Ø 9 cm) on a modified 0.2 concentrated Knop medium supplemented with 2% sucrose (Sijmons *et al.*, 1991). Seeds were kept at 4°C for 3 days prior to incubation in a growth chamber at 25°C, with a 16-h light and 8-h dark cycle.

### Nematode infection

*Heterodera schachtii* was multiplied *in vitro* on mustard (*Sinapsis alba* cv. Albatros) roots growing on 0.2 concentrated Knop medium supplemented with 2% sucrose (Sijmons *et al.*, 1991). Hatching of L2 larvae was stimulated by soaking the cysts in sterile 3 mm ZnCl_2_. The juveniles were washed four times in sterile water and resuspended in 0.5% (w/v) Gelrite for inoculation. Twelve-day-old roots of *A. thaliana* plants were inoculated with about 30 juveniles under axenic conditions.

### RNA isolation

RNA was isolated from aspirated syncytia and root segments using the RNeasy Mini Kit (Qiagen, http://www.qiagen.com). The quality of all RNA samples was controlled by an Agilent 2100 Bioanalyzer (Agilent Technologies, http://www.home.agilent.com).

### qPCR

RNA was transcribed into cDNA using random primers [oligo(dN)_6_] and SuperScript III reverse transcriptase (Invitrogen, http://www.invitrogen.com), following the manufacturer's instructions. Gene-specific primers were selected using Primer Express v2.0 (Applied BioSystems, http://www.appliedbiosystems.com), and were checked for gene specificity within the Arabidopsis genome by a Blast search of the Arabidopsis gene data base. Primer sequences can be found in Appendix S1. *18S* RNA and *UBP22* were used as internal references, as described previously (Hofmann and Grundler, 2007).

Quantitative real-time PCR was performed in an ABI PRISM 7300 Sequence Detector (Applied BioSystems) using SYBR Green to monitor double-stranded DNA synthesis. The final PCR reaction volume was 25 μl, containing 12.5 μl 2× Platinum SYBR Green qPCR SuperMix (Invitrogen), with UDG and ROX as reference dyes, primers and MgCl_2_, dependent on primer pairs, water and 2 μl of cDNA template. Primer efficiencies and PCR conditions can be found in Appendix S1. cDNA was diluted 1:100 for *18S* RNA, and 1:2 for all other primers. As a control, water was added instead of cDNA, resulting in no detectable fluorescent signal. PCR was carried out at 50°C for 2 min and 95°C for 5 min, followed by 43 cycles at 95°C for 15 sec, at 60°C for 30 sec and at 72°C for 60 sec. Data analysis was carried out using the Sequence Detection Software (sds) v2.0 (Applied BioSystems). Changes in transcript levels were related to the expression of *18S* RNA and *UBP22* using the formula (1+*E*)^−ΔΔ*C*t^ (Livak and Schmittgen, 2001).

### *In situ* RT-PCR

*In situ* RT-PCR was performed exactly as described previously (Wieczorek *et al.*, 2006), using the same primers as for the real-time RT-PCR analysis (see Appendix S1).

### Affymetrix GeneChip analysis

**Figure 5:**
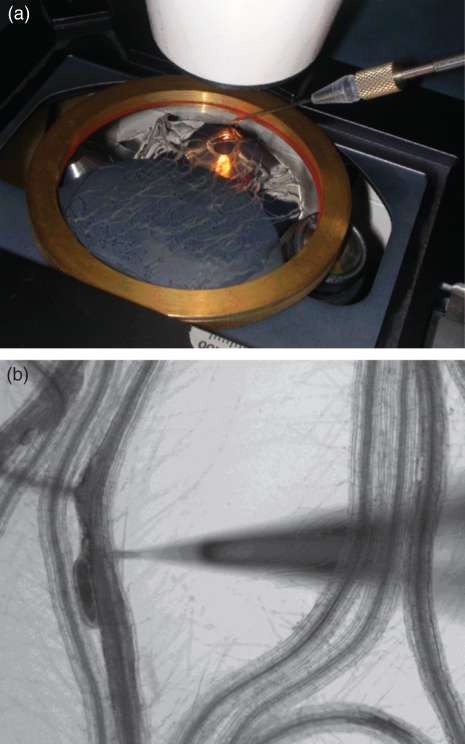
Experimental set-up for the microaspiration of infected Arabidopsis roots (a) A metal ring fixed under an inverse microscope (Zeiss, http://www.zeiss.com) holds a thin glass plate covered with medium enclosing the roots. (b) A microcapillary is navigated towards the roots by a micromanipulator (Eppendorf, http://www.eppendorf.com) for piercing a single syncytium.

Arabidopsis plants were grown in Knop medium on thin glass plates, which were kept in Petri dishes under the conditions described above. They were inoculated with *H. schachtii* larvae after 12 days. The glass plates supporting the roots could then be removed from the Petri dishes for microscopy (Figure 5a). Cytoplasm from syncytia was obtained through microaspiration using an inverse microscope equipped with a microinjector (Figure 5b). The number of syncytia microaspirated was 346 for 5-dpi syncytia, and 191 for 15-dpi syncytia. These syncytia were collected from seven and five independent inoculations, respectively, but were pooled to obtain enough material for RNA amplification. Control root segments were cut from the elongation zone of uninfected roots, which were grown under the same conditions as described above. Special care was taken to avoid any root tips or lateral root primordia. Similarly as for infected material, uninfected root segments were collected as a pool from approximately 1000 plants that were grown in four independent batches.

RNA was isolated as described above. As the most variable part of the whole procedure is the amplification of the RNA using T7 RNA polymerase, we have performed at least three independent amplification experiments for each RNA pool, and have used each amplified copy RNA for the labeling and hybridization of one GeneChip. We hybridized four chips for 5-dpi samples, three chips for 15-dpi samples and four chips for control samples, with individual microarrays representing independent technical replicates. Biotin-labeled probes were prepared according to the Affymetrix protocol, with some modifications. For further details see Appendix S1.

### Statistical analysis of microarray data

Affymetrix CEL files were read into the R statistical analysis environment (http://www.r-project.org) using the affy package of the Bioconductor suite (http://www.bioconductor.org). As 10–40% of probe sets are affected by updated gene annotation, chips were processed with the current TAIR v8 probe-set annotation (Dai *et al.*, 2005). Probe sequence-specific ‘background correction’ (Wu *et al.*, 2004) was performed using routines available in the Bioconductor *gcrma* package. Using the ‘affinity’ model, although ‘MM’ probes were employed for the determination of affinity parameters, only ‘PM’ probes were used for the probe-specific background correction. An inspection of exploratory pairwise scatter and ‘MA’ plots confirmed the need for inter-chip normalization. Thus, the explicit normalization steps required made a subtraction of the heuristic estimate for optical instrument background, as offered in gcrma, unnecessary. Defaults were used for all other gcrma parameters. As an examination of pairwise quantile-quantile plots showed only random fluctuations, interchip normalization could be achieved using quantile-quantile normalization (Bolstad *et al.*, 2003). See the ‘Low-level microarray analysis and diagnostic plots’ section (Appendix S3) for diagnostic plots and figures.

After normalization, robust summaries of probe-set signals were obtained for each gene using an iterative weighted least-squares fit of a linear probe level model (Bolstad, 2004), through the *fitPLM* function of the Bioconductor package *affyPLM*. This process automatically identifies unreliable chip areas, and correspondingly downweights outlier probes. See Appendix S2 and S3.

The normalized data on a log2 scale were then fitted gene by gene with a linear model including hybridization batch effects, using the *lmFit* function (Smyth, 2004) of the Bioconductor package *limma*. The result tables also include *q* values as indicators of significance of contrasts, after correction for multiple testing controlling the false discovery rate (Benjamini and Hochberg, 1995). For the statistical tests, individual gene variances have been moderated using an Empirical Bayes approach that draws strength from transferring variance characteristics from the set of all genes to the test for each individual gene (Smyth, 2004).

Full GO annotation was downloaded from TAIR on 6 Jan 2007 (http://www.arabidopsis.org). Annotation (including ‘unknown’ assignments) was available for almost all genes on the chip (99.6%, 21 053). To permit analyses of arbitrary GO categories, GO-IDs were processed resolving obsolete IDs (http://www.geneontology.org; rev. 1.287, 6 Jan 2007), secondary IDs/aliases (rev. 1.48, 5 Jan 2007), and annotation was revised for consistency by the fully recursive propagation of category membership to parent nodes. For each category, we then tested for relative enrichment of genes in the test set by comparison with the distribution of genes on the chip by Fisher's exact test and Bonferroni correction for multiple testing of the *N* = 4279 examined categories. This corresponds and is equivalent to the commonly employed tests using the hypergeometric distribution. Results are provided in the ‘Analysis’ section of Appendix S1.

To further characterize the nature of regulatory changes, in this paper we tested whether significantly regulated genes were preferentially up- or downregulated. In an assessment of the over-representation of upregulated genes in comparison with downregulated genes, we compared the distribution across GO categories of the 3885 annotated genes that were upregulated significantly for *q* < 5%, with that of an equal number of the most significantly downregulated genes. Similarly, examining the over-representation of downregulated genes in comparison with upregulated genes, we compared the distribution across GO categories of the 3331 annotated genes that were downregulated significantly for *q* < 5% with that of an equal number of most significantly upregulated genes. *P* values for a significance assessment of the observed differences from the binomial distribution were Bonferroni corrected for testing of all GO categories (*N* = 4279), and are also provided in the ‘Analysis’ section of Appendix S1. Results for selected categories are presented in Figures 2(a,b) and 3.

The additional online material providing large, comprehensive tables and plots, and detailed technical analysis is archived at http://bioinf.boku.ac.at/pub/Szakasits2008/.

### Principal component analysis

In total, 185 Affymetrix.CEL files from three different studies (Birnbaum *et al.*, 2003; Schmid *et al.*, 2005, this work) were directly loaded from the Affymetrix GeneChip Operating Software (GCOS) into GeneSpring v7.2 (Silicon Genetics; Agilent Technologies, http://www.home.agilent.com) applying the GeneSpring GCRMA probe summarization (robust multi-chip average, with GC-content background correction algorithm). After preprocessing the files, the following GeneSpring standard normalization steps for one-colour data were performed: (i) data transformation (set measurements from less than 0.01 to 0.01), (ii) per chip (normalized to the 50th percentile) and (iii) per gene (normalized to median).

In order to compare expression patterns of different tissue types, we performed a Principal Components Analysis of log-ratios for all of the different tissue samples. The data were then visualized by plotting samples in principal component space, utilizing the first two components, thereby explaining ∼41% of the total expression variance. The relationship between the samples was then investigated by visually examining clusters in this reduced two-dimensional space.
