# Supplementary material for: The transcriptome of syncytia induced by the cyst nematode Heterodera schachtii in Arabidopsis roots
Source: Plant J. 2008 Dec 9;57(5):771–84. doi: 10.1111/j.1365-313X.2008.03727.x (PMC2667683; doi:10.1111/j.1365-313X.2008.03727.x)
Supplement: Supporting Information [file Szakasits2008.html]

Supplement for Szakasits et al. (2008)


### Online Supplement for Szakasits *et al.* (2008)

These pages contain supplementary material for the manuscript.

# The Transcriptome of Syncytia Induced by the Cyst Nematode *Heterodera schachtii* in *Arabidopsis* Roots

Dagmar Szakasits,1
Petra Heinen,1
Krzysztof Wieczorek,1
Julia Hofmann,1
Florian Wagner,2
David P. Kreil,3
Peter Sykacek,3
Florian M. W. Grundler1
Holger Bohlmann,1

1. Institute of Plant Protection, Department of Applied Plant
   Sciences and Plant Biotechnology, University of Natural Resources and
   Applied Life Sciences, Vienna, Austria
2. RZPD, Germany; *present address:* ATLAS Biolabs GmbH,
   Berlin, Germany
3. WWTF Chair of Bioinformatics, Department of Biotechnology, University
   of Natural Resources and Applied Life Sciences, Vienna, Austria

**Add link to original journal article once published.**

These pages provide a comprehensive archive of supplementary data and
results for the above journal manuscript. The tables comprise
thousands of pages and are provided so that readers can easily search
for particular genes in the list of results.

This page lists content sorted by type and in the context of a short
description. To complement this, for easier reference by figure and
table number (following the order of appearance in the manuscript
text), please use the below links:

- Table 1: Syncytium *vs* Root, all
- Table 2: Syncytium *vs* Root, strongly up
- Table 3: Syncytium *vs* Root, strongly down
- Table 4: Contingency tables (on this page)
- Table 5: Highly expressed genes in Syncytia
- Table 6: 15 dpi *vs* 5 dpi Syncytium, up/down
- Table 7: 15 dpi *vs* 5 dpi Syncytium, all
- Table 8: Gene Ontology tables (on this page)
- Table 9: Genevestigator
- Supplementary Figure 1: Syncytium *vs* Root, *M(A)*)
- Supplementary Figure 2: 15 dpi *vs* 5 dpi Syncytium, *M(A)*


## Appendix S1: Methods

We provide supplementary documents providing 
further details on the microarray experiments and data analysis methods
employed as well as particulars about the performed
quantitative real-time and *in situ*
PCR.

## Appendix S2: Archive of data and sample description tables

- An archive containing the raw CEL data for the 11 hybridizations
  analysed in the paper will be made available here from
  the publication date. 
  [ 37.3MB zip ]
- We provide an extensive description of the performed sample
  measurements through a table of target meta
  data (one row per sample).
- After normalization and weighted summarization using
  reannotated probesets as detailed in the Methods section we obtained
  the normalized transcript signals on log2-equivalent scale
  underlying subsequent statistical analysis. 
  [ 3.3MB zip ]

## Appendix S3: Low-level microarray analysis and diagnostic plots

Diagnostic plots are provided of the raw, unnormalized data. These
have been used to determine an appropriate normalization
method. Subsequent diagnostics verify successful normalization and
examine data set characteristics for systematic trends and artefacts.

- Traditional *M(A)* plots for all pairs of chips showing
  the need for normalization after probe sequence specific background
  subtraction. Some summary statistics are included in panels below the
  diagonal. Coloured curves track a loess fit of *M(A)*. 
  [ 8.1MB pdf ]
- Exploratory scatter plots (panels above diagonal) and
  quantile-quantile plots (panels below diagonal), both after probe
  sequence specific background subtraction. The lack of systematic
  trends in the quantile-quantile plots mapping to biological classes
  indicates the suitability of quantile-quantile normalization for this
  data set. 
  [ 11.2MB pdf ]
- Residuals in a robust fit of a linear probe level model after
  probe sequence specific background subtraction and quantile-quantile
  normalization. These show some random spatial artefacts, which have
  been dealt with by appropriate downweighting of the affected probes in
  a robust iterative weighted least squares fit in the probe signal
  summarization process. 
  [ 23.9MB pdf ]

## Analysis results

- Main contrasts in a batch detrending linear model are listed in the comprehensive tables below.

  [**Supplementary Table 1**] *Contrast:* Syncytium *vs* Root. Listing differential expression for all genes.
   
  [ 5.0MB pdf ]  
  Subsets of this table showing differential expression for a
  multiple-testing corrected FDR<5% are available as
  **Supplementary Table 2** (upregulation) and
  **Supplementary Table 3** (downregulation).

  [**Supplementary Table 7**] *Contrast:* 15 dpi Syncytium *vs*
  5 dpi Syncytium. Listing differential expression for all genes.
   
  [ 4.3MB pdf ]  
  A subset of this table showing differential expression for a
  multiple-testing corrected FDR<5% is available as
  **Supplementary Table 6**, showing (a) upregulated and
  (b) downregulated genes.
- [**Supplementary Table 5**] The 10% most highly expressed genes in Syncytia.
   
  [ 490kB pdf ]
- [**Supplementary Table 4**] Contingency Tables for Fisher's
  Exact test.

  (a) Contingency tables and Fisher's Exact test for the
  overrepresentation of peroxidase
  genes in downregulated transcripts.

  (b) Contingency tables and Fisher's Exact test for the
  overrepresentation of MIPS genes in
  downregulated transcripts.
- [**Supplementary Table 8**] Gene Ontology Analysis
  Results.

  (a) GO categories
  preferentially
  upregulated.

  (b) GO categories
  preferentially
  downregulated.

  Tables are tab-delimited and are easily viewed in all popular
  spreadsheet programs.
- [**Supplementary Table 9**] Genevestigator
  table. Expression of the 100 strongest upregulated genes according
  to Genevestigator.
- *M(A)* plots for main contrasts including standard
  errors (grey bars).

  [**Supplementary Figure 1**] *Contrast:* Syncytium *vs* Root.
  See Table S1 for a full list of gene names
  (5.0 MB pdf).
   
  [ 824kB pdf ]

  [**Supplementary Figure 2**] *Contrast:* 15 dpi Syncytium *vs* 5 dpi Syncytium.
   
  [ 814kB pdf ]

---

*Last updated 4th March, 2008 –
D. Kreil*
