# Supplementary material for: The transcriptome of syncytia induced by the cyst nematode Heterodera schachtii in Arabidopsis roots
Source: Plant J. 2008 Dec 9;57(5):771–84. doi: 10.1111/j.1365-313X.2008.03727.x (PMC2667683; doi:10.1111/j.1365-313X.2008.03727.x)
Supplement: Supporting Information [file TableS2.pdf]

List of affected genes, confidential preview.

Statistics are testing for differential expression (two-sided non-null).

Ranks refer to this test.

Results are grouped by

Differentially expressed with *q*<5%	*M*>0	Up-regulation
Differentially expressed with *q*<5%	*M*<0	Down-regulation
Non-differentially expressed, *q* of 5% or higher		Insignificant

Additional summary views:

5 dpi and 15 dpi Syncytium vs Root Up regulation Strongest 100
meeting a 5% adj.q cutoff for the original test of
(5 dpi and 15 dpi Syncytium) vs Root
on the entire set of all genes. **... Table 1**

Contact: D. Kreil
http://bioinf.boku.ac.at/

Rank	Description	Sync	Root	M	t
36	AT5G56640.1, myo-inositol oxygenase (MIOX5)	10.9	2.1	8.8	27.6
222	AT5G49630.1, amino acid permease 6 (AAP6)	11.5	3.8	7.8	15.9
18	AT2G02120.1, plant defensin (PDF2.1)	11.0	3.3	7.7	33.0
47	AT1G64110.2, ATPase family protein	11.0	3.3	7.7	26.6
23	AT5G64870.1, expressed protein	10.4	2.9	7.5	30.0
104	AT3G26650.1, lyceraldehyde 3-phosphate dehydrogenase A, chloroplast (GAPA)	10.4	2.9	7.4	20.4
26	AT5G56600.1, profilin 5 (PRO5)	11.5	4.1	7.4	29.8
255	AT3G27400.1, similar to pectate lyase	9.5	2.2	7.3	15.2
38	AT2G21330.3, similar to fructose-bisphosphate aldolase	9.9	2.7	7.3	27.3
93	AT3G09390.1, metallothionein protein (MT2A)	12.7	5.5	7.2	21.1
388	AT3G26080.1, plastid-lipid associated protein PAP	8.3	1.2	7.2	13.0
387	AT3G26070.1, plastid-lipid associated protein PAP MPE11.24	8.3	1.2	7.2	13.0
154	AT4G03280.2, cytochrome B6-F complex iron-sulfur subunit, chloroplast	11.1	4.1	7.0	17.9
66	AT3G54890.3, chlorophyll A-B binding protein LHCA1	11.5	4.7	6.9	24.0
356	AT4G24780.1, similar to pectate lyase	11.3	4.5	6.8	13.4
149	AT5G54270.1, chlorophyll A-B binding protein LHCB3	11.3	4.5	6.8	18.0
137	AT2G26500.2, cytochrome b6f complex subunit (petM)	10.5	3.8	6.7	18.6
289	AT2G41300.1, strictosidine synthase family protein	10.3	3.6	6.7	14.5
169	AT4G17600.1, chlorophyll A-B binding protein LIL3:1	9.8	3.2	6.7	17.2
99	AT5G01300.1, phosphatidylethanolamine-binding family protein	9.1	2.5	6.6	20.9
281	AT2G40100.1, chlorophyll A-B binding protein (LHCB4.3)	9.5	3.1	6.4	14.7
146	AT2G43360.1, biotin synthase (BIO2)	11.9	5.5	6.4	18.3
68	AT2G30570.2, photosystem II reaction center W (PsbW) protein-related	12.0	5.6	6.4	23.5
40	AT3G63140.1, mRNA-binding protein, putative	8.9	2.5	6.4	27.1
956	AT1G09200.1, histone H3	10.3	3.9	6.3	9.7
1018	AT4G10340.1, chlorophyll A-B binding protein (LHCB5)	12.7	6.5	6.3	9.5
853	AT2G44460.1, glycosyl hydrolase family 1 protein	9.5	3.3	6.2	10.1
445	AT1G30760.1, FAD-binding domain-containing protein	8.9	2.7	6.2	12.4
78	AT1G68470.1, exostosin family protein	9.6	3.5	6.2	22.2
51	AT4G38970.2, fructose-bisphosphate aldolase, putative	9.9	3.8	6.2	25.6
77	AT5G66110.1, heavy-metal-associated domain-containing protein	8.5	2.3	6.1	22.3
355	AT3G50610.1, expressed protein	8.2	2.1	6.1	13.4
37	AT5G16970.1, NADP-dependent oxidoreductase, putative (P1)	9.2	3.2	6.1	27.4
85	AT5G16990.1, NADP-dependent oxidoreductase, putative	9.7	3.7	6.1	21.5
96	AT4G24040.1, Trehalase1 ATTRE1	9.9	3.8	6.0	21.1
223	AT4G12800.1, photosystem I reaction center subunit XI (PSI-L)	8.4	2.4	6.0	15.8
531	AT2G21130.1, cyclophilin (CYP2)	11.0	5.0	6.0	11.7
462	AT2G28950.1, expansin (EXP6)	8.8	2.8	5.9	12.2
90	AT4G03210.2, xyloglucan endotransglycosylase XTH9	10.0	4.1	5.9	21.2
489	AT1G80820.1, cinnamoyl-CoA reductase (CCR2)	9.1	3.4	5.7	12.1
2701	AT1G69530.3, expansin (EXP1)	7.9	2.2	5.7	6.5
209	AT4G30950.1, omega-6 fatty acid desaturase (FAD6)	10.2	4.5	5.7	16.4
331	AT1G15820.1, chlorophyll A-B binding protein (LHCB6)	9.7	4.0	5.7	13.8
343	AT2G16890.2, UDP-glucoronosyl/UDP-glucosyl transferase	9.8	4.1	5.7	13.7
201	AT2G24270.2, NADP-dependent glyceraldehyde-3-phosphate dehydrogenase, putative	8.2	2.6	5.6	16.6
162	AT1G11330.1, S-locus lectin protein kinase family protein	8.9	3.3	5.6	17.4
551	AT3G51030.1, thioredoxin (ATTRX1)	10.8	5.2	5.6	11.6
725	AT3G13470.1, RuBisCO subunit binding-protein beta subunit	11.9	6.3	5.6	10.6
410	AT5G01530.1, chlorophyll A-B binding protein (LHCB4)	10.2	4.7	5.5	12.7
632	AT4G05180.1, oxygen-evolving enhancer protein 3 (PSBQ2)	9.1	3.5	5.5	11.1
190	AT4G28750.1, photosystem I reaction center subunit IV (PSAE1)	9.2	3.7	5.5	16.7
310	AT1G79260.1, expressed protein	7.8	2.3	5.5	14.2
989	AT5G54770.1, thiazole biosynthetic enzyme (THI1)	12.1	6.6	5.5	9.6
213	AT1G03630.2, NADPH-protochlorophyllide oxidoreductase A (PORA)	7.6	2.2	5.4	16.2
896	AT3G21055.1, photosystem II 5 kD protein	7.5	2.1	5.4	9.9
1036	AT3G02480.1, ABA-responsive protein	8.6	3.2	5.4	9.4
756	AT5G02120.1, thylakoid membrane one helix protein (OHP)	7.6	2.2	5.4	10.5
106	AT2G28900.1, mitochondrial import inner membrane translocase subunit Tim17	10.9	5.5	5.4	20.4
208	AT1G62290.2, similar to aspartyl protease family protein	8.5	3.1	5.4	16.4
373	AT5G53880.1, expressed protein	9.5	4.1	5.4	13.2

168	AT3G23325.1, similar to Splicing factor 3B subunit 10	9.8	4.4	5.3	17.2
339	AT3G48610.1, phosphoesterase family protein	8.1	2.8	5.3	13.7
3435	AT5G09810.1, actin 7	9.7	4.3	5.3	5.7
758	AT1G42970.1, glyceraldehyde-3-phosphate dehydrogenase B (GAPB)	8.8	3.5	5.3	10.5
923	AT4G26260.1, myo-inositol oxygenase (MIOX4)	7.7	2.4	5.3	9.8
282	AT1G76450.1, oxygen-evolving complex-related	7.0	1.8	5.3	14.7
229	AT1G55490.2, beta subunit of chloroplast chaperonin 60 (CPN60B)	11.1	5.9	5.3	15.7
194	AT5G07800.1, flavin-containing monooxygenase family protein	9.8	4.5	5.3	16.7
124	AT1G68010.1, NADH-dependent hydroxypyruvate reductase (HPR)	7.8	2.6	5.3	19.4
1069	AT1G55670.1, photosystem I reaction center subunit V (PSAG)	8.2	3.0	5.3	9.3
866	AT2G35370.1, glycine cleavage system H protein 1(GCDH)	7.7	2.5	5.2	10.0
1962	AT5G43330.1, malate dehydrogenase	11.1	5.8	5.2	7.5
200	AT1G32900.1, starch synthase, putative	8.3	3.1	5.2	16.6
514	AT5G50800.1, nodulin MtN3 family protein	9.8	4.6	5.2	11.9
176	AT3G15850.1, fatty acid desaturase family protein (FAD5)	8.8	3.6	5.2	17.0
166	AT4G38370.1, phosphoglycerate/bisphosphoglycerate mutase family protein	8.6	3.5	5.2	17.3
202	AT4G09010.1, L-ascorbate peroxidase, chloroplast	9.0	3.9	5.2	16.6
1275	AT2G17280.1, phosphoglycerate/bisphosphoglycerate mutase family protein	9.3	4.1	5.1	8.8
203	AT2G38140.1, chloroplast 30S ribosomal protein S31 (PSRP4)	9.2	4.1	5.1	16.6
135	AT2G36230.1, N'-5'-phosphoribosyl-formimino-5-aminoimidazole-4-carboxamide ribonucleotide isomerase (BBM)	8.6	3.5	5.1	18.6
506	AT1G24735.1, caffeoyl-CoA 3-O-methyltransferase, putative	10.0	4.9	5.1	11.9
594	AT5G17870.1, plastid-specific ribosomal protein-related	8.0	3.0	5.1	11.4
485	AT3G61100.1, expressed protein	7.6	2.5	5.1	12.1
734	AT4G25810.1, endo-xyloglucan transferase, putative (XTR6)	9.6	4.6	5.1	10.6
662	AT1G03600.1, photosystem II family protein	8.4	3.4	5.0	10.9
284	AT2G46110.1, ketopantoate hydroxymethyltransferase family protein	8.6	3.6	5.0	14.7
1337	AT5G59890.2, actin-depolymerizing factor 4 (ADF4)	12.2	7.3	5.0	8.6
493	AT1G80560.1, 3-isopropylmalate dehydrogenase, chloroplast	10.4	5.4	5.0	12.0
375	AT3G29320.1, glucan phosphorylase, putative	9.4	4.4	4.9	13.2
827	AT2G35040.1, AICARFT/IMPCHase bienzyme family protein	10.0	5.1	4.9	10.2
140	AT3G52180.2, similar to protein phosphatase-related	9.1	4.2	4.9	18.5
1168	AT4G24830.1, arginosuccinate synthase family	12.1	7.2	4.9	9.1
1159	AT5G64080.2, lipid transfer protein (LTP)	7.5	2.6	4.9	9.1
893	AT5G17310.2, UTP--glucose-1-phosphate uridylyltransferase	9.1	4.3	4.9	9.9
511	AT5G52970.1, thylakoid lumen 15.0 kDa protein	7.9	3.1	4.8	11.9
240	AT5G17170.1, rubredoxin family protein	8.4	3.6	4.8	15.5
661	AT1G01940.1, peptidyl-prolyl cis-trans isomerase cyclophilin-type family protein,	9.3	4.5	4.8	10.9
297	AT5G04620.2, aminotransferase class I and II family protein	8.4	3.6	4.8	14.4
894	AT3G08030.2, expressed protein	8.2	3.4	4.8	9.9
480	AT3G47070.1, expressed protein	7.9	3.1	4.8	12.1
