# Supplementary material for: The transcriptome of syncytia induced by the cyst nematode Heterodera schachtii in Arabidopsis roots
Source: Plant J. 2008 Dec 9;57(5):771–84. doi: 10.1111/j.1365-313X.2008.03727.x (PMC2667683; doi:10.1111/j.1365-313X.2008.03727.x)
Supplement: Supporting Information [file TableS3.pdf]

List of affected genes, confidential preview.

Statistics are testing for differential expression (two-sided non-null).

Ranks refer to this test.

Results are grouped by

Differentially expressed with q<5%	M>0	Up-regulation
Differentially expressed with q<5%	M<0	Down-regulation
Non-differentially expressed, q of 5% or higher		Insignificant

Additional summary views:

5 dpi and 15 dpi Syncytium vs Root Down regulation Strongest 100
meeting a 5% adj.q cutoff for the original test of
(5 dpi and 15 dpi Syncytium) vs Root
on the entire set of all genes. **... Table 2**

Contact: D. Kreil
http://bioinf.boku.ac.at/

Rank	Description	Sync	Root	M	t
11	AT3G61430.1, plasma membrane intrinsic protein 1A (PIP1A)	4.5	15.3	-10.8	-41.0
7	AT1G09090.2, respiratory burst oxidase protein B (RbohB)	1.9	12.0	-10.0	-42.9
28	AT4G15390.1, similar to alcohol acyltransferase	3.7	13.5	-9.8	-29.6
16	AT5G47450.1, major intrinsic family protein (AtTIP2;3)	2.8	12.3	-9.4	-34.0
2	AT5G59090.3, similar to subtilase family protein	2.8	12.1	-9.3	-56.8
9	AT2G32270.1, zinc transporter (ZIP3)	3.4	12.7	-9.3	-42.2
17	AT3G53420.2, plasma membrane intrinsic protein (PIP2A)	4.3	13.6	-9.2	-33.4
5	AT1G43160.1, ethylene response factor RAP2.6	2.2	11.3	-9.1	-43.7
46	AT4G23400.1, major intrinsic family protein (PIP1D)	3.4	12.4	-9.0	-26.6
1	AT1G61590.1, protein kinase, putative	2.6	11.6	-9.0	-57.3
3	AT1G05260.1, peroxidase 3 (PER3)	2.9	11.9	-9.0	-44.1
62	AT5G54040.1, DC1 domain-containing protein	2.6	11.5	-8.9	-24.3
13	AT3G01190.1, peroxidase 27 (PER27)	3.5	12.4	-8.9	-36.6
42	AT4G02270.1, pollen Ole e 1 allergen and extensin family protein	2.5	11.2	-8.7	-27.0
25	AT1G01620.1, plasma membrane intrinsic protein 1C (PIP1C)	3.6	12.2	-8.6	-29.8
27	AT5G64100.1, peroxidase ATP3a	3.2	11.8	-8.6	-29.7
35	AT5G63600.2, similar to flavonol synthase 1	4.4	13.0	-8.6	-28.0
50	AT3G26520.1, tonoplast intrinsic protein (TIP2)	3.9	12.4	-8.5	-25.6
48	AT5G19890.1, peroxidase, putative	3.4	11.9	-8.5	-26.5
20	AT4G20110.1, vacuolar sorting receptor, putative,	3.5	12.0	-8.5	-32.0
21	AT3G22600.1, lipid transfer protein	4.2	12.7	-8.5	-31.7
57	AT4G17340.1, major intrinsic family protein (TIP2;2)	5.3	13.7	-8.4	-24.8
8	AT2G16750.1, protein kinase family protein	2.5	10.9	-8.4	-42.8
214	AT3G54590.1, proline-rich extensin-like protein (ATHRGP1)	3.0	11.2	-8.2	-16.1
10	AT1G74760.1, zinc finger (C3HC4-type RING finger) protein	2.7	10.9	-8.2	-41.8
43	AT5G67400.1, peroxidase 73 (PER73)	2.9	11.0	-8.1	-26.9
122	AT2G37130.1, peroxidase 21 (PER21)	4.0	12.1	-8.1	-19.4
24	AT2G28160.1, basic helix-loop-helix (bHLH) protein	2.8	10.8	-8.1	-29.8
70	AT5G66390.1, peroxidase 72 (PER72)	2.9	11.0	-8.0	-23.4
91	AT4G13580.1, disease resistance-responsive protein	3.7	11.7	-8.0	-21.2
31	AT1G72200.1, zinc finger (C3HC4-type RING finger) family protein	3.0	10.9	-7.9	-28.7
4	AT3G25930.1, universal stress protein (USP)	3.1	10.9	-7.8	-44.0
80	AT1G13080.2, cytochrome P450 protein (CYP71B2)	2.9	10.7	-7.7	-22.1
33	AT5G44480.1, NAD-dependent epimerase/dehydratase (DUR)	3.6	11.3	-7.7	-28.3
22	AT3G53480.1, PDR5-like ABC transporter	3.4	11.1	-7.7	-31.1
92	AT2G22170.1, lipid-associated family protein	3.8	11.5	-7.7	-21.1
19	AT4G05200.1, protein kinase family protein	2.9	10.6	-7.7	-32.2
111	AT1G62660.1, beta-fructosidase (BFRUCT3) / invertase, vacuolar	3.0	10.7	-7.7	-20.1
103	AT4G33020.1, metal transporter, putative (ZIP9)	2.9	10.5	-7.7	-20.5
6	AT5G44020.1, acid phosphatase class B family protein	3.3	11.0	-7.7	-42.9
65	AT5G56870.1, beta-galactosidase, putative / lactase	4.3	12.0	-7.7	-24.0
73	AT5G52790.1, CBS domain-containing protein-related	3.0	10.7	-7.6	-23.0
41	AT4G16260.1, glycosyl hydrolase family 17 protein	3.8	11.4	-7.6	-27.0
72	AT4G30170.1, peroxidase ATP8a	2.4	10.0	-7.6	-23.2
30	AT3G11340.1, UDP-glucosyl transferase family protein	3.0	10.5	-7.4	-28.8
108	AT1G30870.1, cationic peroxidase	3.1	10.6	-7.4	-20.3
249	AT5G42180.1, peroxidase 64 (PER64)	3.1	10.6	-7.4	-15.3
15	AT1G18100.1, mother of FT and TF1 protein	2.3	9.8	-7.4	-35.4
105	AT2G45960.1, plasma membrane intrinsic protein 1B (PIP1B)	6.0	13.4	-7.4	-20.4
12	AT1G08320.3, similar to bZIP family transcription factor	3.2	10.5	-7.3	-38.9
58	AT5G44380.1, similar to Berberine-bridge-forming enzyme (BBE)	3.7	11.0	-7.3	-24.8
14	AT3G48920.1, myb family transcription factor (MYB45)	2.4	9.7	-7.3	-35.8
102	AT4G28890.1, zinc finger (C3HC4-type RING finger) family protein	3.3	10.5	-7.3	-20.6
69	AT4G32650.3, ATKC1, similar to inward rectifying potassium channel (KAT2)	2.8	10.0	-7.3	-23.4
60	AT4G25820.1, endo-xyloglucan transferase (XTR9)	4.1	11.4	-7.2	-24.6
34	AT5G09530.1, hydroxyproline-rich glycoprotein f	3.2	10.4	-7.2	-28.2
283	AT4G26010.1, peroxidase ATP13a	3.3	10.5	-7.2	-14.7
152	AT5G03570.1, iron-responsive transporter-related	3.6	10.8	-7.2	-18.0
52	AT3G29035.1, no apical meristem (NAM) family protein	3.4	10.6	-7.2	-25.5
130	AT4G34580.1, phosphoglyceride transfer protein, putative	3.2	10.4	-7.2	-19.2

45	AT2G26820.1, avirulence-responsive family protein (ATPP2 A3)	3.1	10.2	-7.2	-26.7
114	AT5G48290.2, similar to heavy-metal-associated domain-containing protein	2.3	9.5	-7.1	-20.0
88	AT2G01880.1, purple acid phosphatase (PAP7)	2.8	9.9	-7.1	-21.4
98	AT4G24130.1, expressed protein	3.5	10.5	-7.0	-20.9
81	AT3G21260.2, glycolipid transfer protein-related	3.3	10.3	-7.0	-21.8
126	AT2G37750.1, expressed protein	3.8	10.8	-7.0	-19.3
49	AT4G11290.1, peroxidase ATP19a	2.0	9.0	-7.0	-25.9
107	AT3G13610.1, 2OG-Fe(II) oxygenase family protein	3.0	10.0	-6.9	-20.3
314	AT5G17820.1, peroxidase 57 (PER57) (ATP13a)	2.9	9.8	-6.9	-14.0
211	AT5G04960.1, pectinesterase family protein	2.9	9.8	-6.9	-16.3
147	AT1G08090.1, high-affinity nitrate transporter (ATNRT2:1)	6.4	13.3	-6.9	-18.2
67	AT1G13110.1, cytochrome P450 71B7 (CYP71B7)	2.9	9.9	-6.9	-24.0
53	AT5G26280.1, meprin and TRAF homology domain-containing protein	4.5	11.4	-6.9	-25.2
55	AT5G19970.1, expressed protein	3.3	10.2	-6.9	-25.1
899	AT3G05920.1, heavy-metal-associated domain-containing protein	2.9	9.8	-6.9	-9.9
112	AT3G29410.1, terpene synthase/cyclase family protein	2.8	9.7	-6.8	-20.0
64	AT1G44050.1, DC1 domain-containing protein	2.4	9.2	-6.8	-24.1
75	AT1G30750.1, expressed protein	2.6	9.4	-6.8	-22.5
116	AT2G28110.1, glycosyl transferase family 47 protein	3.1	9.9	-6.8	-19.9
170	AT5G04950.1, nicotianamine synthase, putative	3.1	9.9	-6.7	-17.2
59	AT2G36100.1, integral membrane family protein	4.4	11.1	-6.7	-24.7
177	AT1G49570.1, peroxidase ATP5a	3.1	9.8	-6.7	-17.0
1328	AT1G21310.1, extensin ATEXT3	4.3	11.0	-6.7	-8.6
74	AT2G35585.1, expressed protein	2.8	9.4	-6.7	-22.6
128	AT2G21045.1, similar to senescence-associated family protein	4.5	11.2	-6.7	-19.2
299	AT5G60660.1, major intrinsic family protein (PIP2F)	3.7	10.3	-6.7	-14.3
63	AT3G61390.2, U-box domain-containing protein	2.6	9.2	-6.6	-24.2
150	AT5G41040.2, similar to hypersensitivity-related gene product HSR201	2.8	9.4	-6.6	-18.0
129	AT3G14680.1, cytochrome P450 (CYP72A14)	3.4	10.1	-6.6	-19.2
131	AT4G18510.1, CLE2(CLAVATA3/ESR RELATED)	2.9	9.5	-6.6	-19.1
119	AT4G35060.1, heavy-metal-associated domain-containing protein	3.3	9.8	-6.6	-19.8
1866	AT5G49080.1, proline-rich extensin-like family protein	4.1	10.6	-6.6	-7.6
257	AT3G61940.1, zinc transporter, putative (MTPA1)	3.9	10.5	-6.6	-15.2
163	AT2G44380.1, DC1 domain-containing protein	3.2	9.8	-6.5	-17.4
54	AT1G10970.1, metal transporter, putative (ZIP4)	4.8	11.4	-6.5	-25.2
285	AT1G52200.1, expressed protein	3.1	9.6	-6.5	-14.7
56	AT1G49500.1, expressed protein	4.4	10.9	-6.5	-25.0
294	AT4G13770.1, cytochrome P450 (CYP83A1)	3.2	9.7	-6.5	-14.4
265	AT5G42250.1, alcohol dehydrogenase, putative	4.1	10.5	-6.5	-15.0
44	AT5G63590.1, flavonol synthase, putative	4.4	10.8	-6.5	-26.8
