# Supplementary material for: The transcriptome of syncytia induced by the cyst nematode Heterodera schachtii in Arabidopsis roots
Source: Plant J. 2008 Dec 9;57(5):771–84. doi: 10.1111/j.1365-313X.2008.03727.x (PMC2667683; doi:10.1111/j.1365-313X.2008.03727.x)
Supplement: Supporting Information [file TableS5.pdf]

List of affected genes, confidential preview.

Statistics are testing for differential expression (two-sided non-null).

Ranks refer to this test.

Results are grouped by

Differentially expressed with q<5%	M>0	Up-regulation
Differentially expressed with q<5%	M<0	Down-regulation
Non-differentially expressed, q of 5% or higher		Insignificant

Additional summary views:

5 dpi and 15 dpi Syncytium   Strongest expressed

Colour coding is according to the original test of
(5 dpi and 15 dpi Syncytium) vs Root
on the entire set of all genes. **... Table S2**

Contact: D. Kreil
http://bioinf.boku.ac.at/

Rank	Description	Sync	Root	M	t
892	AT5G02380.1 | Symbol: MT2B | metallothionein protein 2B (MT-2B), identical to SWISS-PROT:Q38805 metallothionein-like protein 2B (MT-2B) (Arabidopsis thaliana) | chr5:506545-507242 REVERSE | Aliases: METALLOTHIONEIN 2B, T1E22.140, T1E22_140	14.0	12.2	1.8	9.9
1114	AT4G29350.1 | Symbol: PRF2 | profilin 2 (PRO2) (PFN2) (PRF2), identical to profilin 2 SP:Q42418 GI:1353772 from (Arabidopsis thaliana); identical to cDNA profilin (PRF2) GI:9965570 | chr4:14450035-14451383 FORWARD | Aliases: F17A13.170, F17A13_170, PFN2, PROFILIN 2	13.9	12.3	1.6	9.2
1390	AT3G60245.1 | 60S ribosomal protein L37a (RPL37aC) | chr3:22279721-22280981 FORWARD | Aliases: None	13.7	12.6	1.2	8.5
7528	AT4G27960.2 | Symbol: UBC9 | ubiquitin-conjugating enzyme E2-17 kDa 9 (UBC9), E2; identical to gi:297883, SP:P35132; identical to cDNA UBC9 for ubiquitin conjugating enzyme homolog GI:297883 | chr4:13915839-13917426 REVERSE | Aliases: T13J8.70, T13J8_70, UBIQUITIN CONJUGATING ENZYME 9, UBIQUITIN PROTEIN LIGASE	13.5	12.3	1.2	3.3
7529	AT5G53300.2 | Symbol: UBC10 | ubiquitin-conjugating enzyme 10 (UBC10), E2; identical to gi:297877, SP:P35133 | chr5:21649740-21651389 REVERSE | Aliases: None	13.5	12.3	1.2	3.3
3923	AT4G05050.2 | Symbol: UBQ11 | similar to polyubiquitin (UBQ14) [Arabidopsis thaliana] (TAIR:At4g02890.1); similar to polyubiquitin (UBQ3) [Arabidopsis thaliana] (TAIR:At5g03240.2); similar to polyubiquitin (UBQ4) [Arabidopsis thaliana] (TAIR:At5g20620.1); similar to polyubiquitin (UBQ14) [Arabidopsis thaliana] (TAIR:At4g02890.2); similar to polyubiquitin (UBQ14) [Arabidopsis thaliana] (TAIR:At4g02890.3); similar to ubiquitin-like protein [Phaseolus vulgaris] (GB:AAB36545.1); similar to polyubiquitin [Fragaria x ananassa] (GB:AAB68045.1); similar to pentameric polyubiquitin (GB:AAA34124.1); contains InterPro domain Ubiquitin domain (InterPro:IPR000626) | chr4:2588002-2589137 REVERSE | Aliases: C17L7.6, UBIQUITIN 11	13.5	12.4	1.1	5.3
12680	AT1G54410.1 | dehydrin family protein, contains Pfam domain, PF00257: Dehydrin	13.4	13.7	-0.3	-1.7
306	AT1G66410.1 | Symbol: CAM4 | calmodulin-1/4 (CAM4), identical to calmodulin (Arabidopsis thaliana) GI:16223; nearly identical to SP:P25854 Calmodulin-1/4 {Arabidopsis thaliana} | chr1:24777880-24779516 REVERSE | Aliases: ACAM 4, CALMODULIN 4, T27F4.1, T27F4_1	13.4	10.9	2.5	14.2
3060	ATCG00470.1 | Symbol: ATPE | ATPase epsilon subunit | chrC:52265-52663 REVERSE | Aliases: ATPE	13.4	12.4	1.0	6.1
2755	AT5G39570.1 | expressed protein | chr5:15861187-15863406 FORWARD | Aliases: MIJ24.6, MIJ24_6	13.4	12.6	0.8	6.4
667	AT3G11940.2 | Symbol: ATRPS5A | 40S ribosomal protein S5 (RPS5B), similar to 40S ribosomal protein S5 GB:AAC98068 GI:4056502 from (Arabidopsis thaliana) | chr3:3777901-3779509 REVERSE | Aliases: AML1, ARABIDOPSIS MINUTE LIKE 1, MEC18.11, RIBOSOMAL PROTEIN 5A	13.3	11.9	1.4	10.9
666	AT2G37270.2 | Symbol: ATRPS5B | similar to 40S ribosomal protein S5 (RPS5B) [Arabidopsis thaliana] (TAIR:At3g11940.1); similar to 40S ribosomal protein S5 (RPS5B) [Arabidopsis thaliana] (TAIR:At3g11940.2); similar to putative 40S ribosomal protein S5 [Oryza sativa (japonica cultivar-group)] (GB:NP_908322.1); contains InterPro domain Ribosomal protein S7, eukaryotic and archaeal form (InterPro:IPR005716); contains InterPro domain Ribosomal protein S7 (InterPro:IPR000235) | chr2:15654756-15656282 REVERSE | Aliases: F3G5.6, F3G5_6, RIBOSOMAL PROTEIN 5B	13.3	11.9	1.4	10.9
2776	AT3G10860.1 | ubiquinol-cytochrome C reductase complex ubiquinone-binding protein, putative / ubiquinol-cytochrome C reductase complex 8.2 kDa protein, putative, similar to ubiquinol--cytochrome c reductase GI:633687 from (Solanum tuberosum) | chr3:3399741-3400732 FORWARD | Aliases: T7M13.6	13.3	12.7	0.6	6.4
1691	AT5G42980.1 | Symbol: ATTRX3 |	13.3	12.2	1.1	7.9
3490	AT5G42300.1 | ubiquitin family protein, contains INTERPRO:IPR000626 ubiquitin domain | chr5:16929561-16931158 REVERSE | Aliases: K5J14.10, K5J14_10	13.3	12.7	0.6	5.7
3109	AT5G64350.1 | Symbol: FKBP12 |	13.2	12.2	1.0	6.0
4159	AT3G52300.2 | similar to putative mitochondrial F0 ATP synthase D chain [Oryza sativa (japonica cultivar-group)] (GB:XP_482965.1) | chr3:19407543-19409355 FORWARD | Aliases: T25B15.70	13.2	11.6	1.6	5.1
1117	AT2G02100.1 | Symbol: LCR69/PDF2.2 | plant defensin-fusion protein, putative (PDF2.2), plant defensin protein family member, personal communication, Bart Thomma (Bart.Thomma@agr.kuleuven.ac.be); similar to SWISS-PROT:O65740 | chr2:528349-529107 FORWARD | Aliases: F5O4.13, F5O4_13, LCR69, Low molecular weight cysteine rich 69, PDF2.2	13.2	11.1	2.0	9.2
4802	AT4G05320.5 | Symbol: UBQ10 | polyubiquitin (UBQ10) (SEN3), senescence-associated protein; identical to GI:870791 | chr4:2718168-2720130 FORWARD | Aliases: C17L7.240, C17L7_240, POLYUBIQUITIN 10	13.1	13.6	-0.5	-4.7
2608	AT1G14320.1 | 60S ribosomal protein L10 (RPL10A) / Wilm's tumor suppressor protein-related, similar to tumor suppressor GI:575354 from (Oryza sativa) | chr1:4888209-4889656 FORWARD | Aliases: F14L17.9, F14L17_9	13.1	12.0	1.1	6.6

330	AT5G40370.1 | glutaredoxin, putative, similar to glutaredoxin (Ricinus communis) SWISS-PROT:P55143 | chr5:16164833-16166382 REVERSE | Aliases: MPO12.80, MPO12_80	13.1	10.5	2.5	13.8
15619	AT1G76200.1 | expressed protein | chr1:28598144-28599636 FORWARD | Aliases: T23E18.30, T23E18_30	13.1	13.2	-0.1	-1.0
2309	AT1G52300.1 | 60S ribosomal protein L37 (RPL37B), similar to SP:Q43292 from (Arabidopsis thaliana) | chr1:19478716-19479887 REVERSE | Aliases: F19K6.12, F19K6_12	13.0	11.8	1.2	7.0
2719	AT3G43980.1 | 40S ribosomal protein S29 (RPS29A), ribosomal protein S29, rat, PIR:S30298 | chr3:15789389-15790298 REVERSE | Aliases: T15B3.120, T15B3_120	13.0	11.8	1.2	6.4
2720	AT3G44010.1 | 40S ribosomal protein S29 (RPS29B), ribosomal protein S29, rat, PIR:S30298 | chr3:15810918-15812187 REVERSE | Aliases: T15B3.150	13.0	11.8	1.2	6.4
84	AT1G28400.1 | expressed protein, similar to E6 (GI:1000090) (Gossypium barbadense) | chr1:9972370-9973779 REVERSE | Aliases: F3M18.16, F3M18_16	12.9	10.0	3.0	21.5
983	AT1G77120.1 | Symbol: ADH1 | alcohol dehydrogenase (ADH), identical to alcohol dehydrogenase GI:469467 from (Arabidopsis thaliana) | chr1:28980345-28982311 FORWARD | Aliases: ADH, ALCOHOL DEHYDROGENASE, ALCOHOL DEHYDROGENASE 1, F22K20.19, F22K20_19	12.9	9.5	3.4	9.6
835	AT5G59880.2 | Symbol: ADF3 | actin-depolymerizing factor 3 (ADF3), identical to SP:Q9ZSK4 Actin-depolymerizing factor 3 (ADF 3) (AtADF3) {Arabidopsis thaliana} | chr5:24137457-24139105 FORWARD | Aliases: ACTIN DEPOLYMERIZING FACTOR 3, MMN10.4, MMN10_4	12.9	10.4	2.5	10.1
1238	AT3G49010.3 | Symbol: ATBBC1 |	12.9	10.8	2.1	8.9
1859	AT4G20150.1 | expressed protein | chr4:10888309-10889606 REVERSE | Aliases: F1C12.70, F1C12_70	12.9	11.6	1.2	7.6
1342	AT4G27090.1 | 60S ribosomal protein L14 (RPL14B), ribosomal protein L14 - Human,PIR3:JC5954 | chr4:13593911-13595257 REVERSE | Aliases: T24A18.40, T24A18_40	12.8	11.4	1.4	8.6
12758	AT3G55440.1 | Symbol: ATCTIMC | triosephosphate isomerase, cytosolic, putative, strong similarity to triosephosphate isomerase, cytosolic from Petunia hybrida (SP:P48495), from Coptis japonica (SP:P21820) | chr3:20564671-20567537 FORWARD | Aliases: CYTOSOLIC TRIOSE PHOSPHATE ISOMERASE, T22E16.100	12.8	12.2	0.7	1.6
7983	NA	12.8	13.2	-0.4	-3.1
2617	AT1G31812.1 | acyl-CoA binding protein / ACBP, identical to acyl-CoA-binding protein (ACBP) (Arabidopsis thaliana) SWISS-PROT:P57752 | chr1:11410857-11412170 REVERSE | Aliases: F5M6.27, F5M6_27	12.8	11.6	1.1	6.6
2479	AT3G12490.2 | similar to cysteine protease inhibitor, putative / cystatin, putative [Arabidopsis thaliana] (TAIR:At5g05110.1); similar to cysteine protease inhibitor CPI-1 [Brassica oleracea] (GB:AAL59842.1); contains InterPro domain Cystatin C/M (InterPro:IPR003243); contains InterPro domain Cysteine protease inhibitor (InterPro:IPR000010)	12.7	10.6	2.1	6.7
1018	AT4G10340.1 | Symbol: LHCB5 | chlorophyll A-B binding protein CP26, chloroplast / light-harvesting complex II protein 5 / LHCIIc (LHCB5), identical to SP:Q9XF89 Chlorophyll A/B-binding protein CP26, chloroplast precursor (Light-harvesting complex II protein 5) (LHCB5) (LHCIIc) {Arabidopsis thaliana}; contains Pfam profile: PF00504 chlorophyll A-B binding protein; chlorophyll a/b-binding protein CP26 in PS II, Brassica juncea, gb:X95727 | chr4:6408012-6409673 FORWARD | Aliases: CP 26, CP26, F24G24.140, F24G24_140, LIGHT HARVESTING COMPLEX OF PHOTOSYSTEM II 5	12.7	6.5	6.3	9.5
9708	AT3G14990.3 | similar to DJ-1 family protein [Arabidopsis thaliana] (TAIR:At1g53280.1); similar to putative 4-methyl-5(B-hydroxyethyl)-thiazol monophosphate biosynthesis enzyme [Oryza sativa (japonica cultivar-group)] (GB:BAD54224.1); contains InterPro domain Protein of unknown function ThiJ/PfpI (InterPro:IPR002818); contains InterPro domain DJ-1 protein (InterPro:IPR006287) | chr3:5047407-5049884 FORWARD | Aliases: K15M2.13, K15M2_13	12.7	11.8	0.9	2.5
93	AT3G09390.1 | Symbol: MT2A | metallothionein protein, putative (MT2A), identical to Swiss-Prot:P25860 metallothionein-like protein 2A (MT-2A) (MT-K) (MT-1G) (Arabidopsis thaliana) | chr3:2889492-2890235 REVERSE | Aliases: ATMT 1, ATMT K, F3L24.28, METALLOTHIONEIN 2A, MT2	12.7	5.5	7.2	21.1
8748	AT5G37600.1 | Symbol: ATGSR1 |	12.7	13.4	-0.7	-2.8
20417	AT4G21960.1 | Symbol: PRXR1 | peroxidase 42 (PER42) (P42) (PRXR1), identical to SP:Q9SB81 Peroxidase 42 precursor (EC 1.11.1.7) (Atperox P42) (PRXR1) (ATP1a/ATP1b) {Arabidopsis thaliana} | chr4:11646186-11648373 REVERSE | Aliases: F1N20.3, PEROXIDASE PRXR1	12.7	12.6	0.0	0.1
15411	AT5G54160.1 | Symbol: ATOMT1 |	12.6	12.3	0.3	1.1
1976	AT2G19760.1 | Symbol: PRF1 | profilin 1 (PRO1) (PFN1) (PRF1) / allergen Ara t 8, identical to profilin 1 (Allergen Ara t 8) SP:Q42449 GI:1353770 from (Arabidopsis thaliana) | chr2:8523869-8525249 REVERSE | Aliases: F6F22.21, F6F22_21, PFN1, PROFILIN 1	12.6	11.0	1.6	7.4

5598	AT4G04830.1 | methionine sulfoxide reductase domain-containing protein / SelR domain-containing protein, low similarity to pilin-like transcription factor (Homo sapiens) GI:5059062, SP:P14930 Peptide methionine sulfoxide reductase msrA/msrB (EC 1.8.4.6) {Neisseria gonorrhoeae}; contains Pfam profile PF01641: SelR domain | chr4:2445857-2447082 FORWARD | Aliases: T4B21.5, T4B21_5	12.6	12.1	0.5	4.2
8263	AT5G53560.1 | Symbol: ATB5 A |	12.6	12.1	0.5	3.0
1193	AT1G07600.1 | Symbol: MT1A | metallothionein-like protein 1A (MT-1A) (MT-Q) (MT-2), identical to Metallothionein-like protein 1A (MT-1A) (MT-Q) (MT-2) SP:P43392 from (Arabidopsis thaliana) | chr1:2338901-2339318 REVERSE | Aliases: ATMT 2, ATMT Q, F22G5.2, LIGHT STRESS REGULATED 4, LSR4, METALLOTHIONEIN 1A, MT Q, MT1	12.5	14.3	-1.8	-9.0
6902	NA	12.5	13.1	-0.5	-3.6
684	AT3G16080.1 | 60S ribosomal protein L37 (RPL37C), similar to ribosomal protein L37 GB:BAA04888 from (Homo sapiens) | chr3:5454824-5455933 FORWARD | Aliases: MSL1.12	12.5	10.4	2.1	10.8
1456	AT3G58610.1 | ketol-acid reductoisomerase, identical to ketol-acid reductoisomerase, chloroplast precursor (EC 1.1.1.86) (Acetohydroxy-acid reductoisomerase) (Alpha-keto-beta-hydroxylacil reductoisomerase) (Swiss-Prot:Q05758) (Arabidopsis thaliana) | chr3:21682429-21685818 FORWARD | Aliases: F14P22.200, F14P22_200	12.5	10.3	2.2	8.3
6425	AT1G47420.1 | expressed protein, identical to hypothetical protein GB:AAD46040 GI:5668814 from (Arabidopsis thaliana) | chr1:17397958-17399693 REVERSE | Aliases: T3F24.12	12.5	10.4	2.1	3.8
10913	AT5G65020.1 | Symbol: ANNAT2 |	12.5	11.7	0.8	2.1
9552	AT5G41700.4 | Symbol: UBC8 | ubiquitin-conjugating enzyme 8 (UBC8), E2; identical to gi:297882, SP:P35131 | chr5:16693070-16694737 FORWARD | Aliases: ATUBC8, MBK23.24, MBK23_24, UBC8, UBIQUITIN CONJUGATING ENZYME, UBIQUITIN CONJUGATING ENZYME 8	12.5	12.0	0.5	2.5
12625	AT3G57870.1 | Symbol: AHUS5 | ubiquitin-conjugating enzyme, putative, strong similarity to SP:P50550 Ubiquitin-like protein SUMO-1 conjugating enzyme (EC 6.3.2.19) (SUMO- 1-protein ligase) (Ubiquitin carrier protein) (Ubiquitin-conjugating enzyme UbcE2A) {Xenopus laevis}; contains Pfam profile PF00179: Ubiquitin-conjugating enzyme | chr3:21439624-21441155 REVERSE | Aliases: EMB1637, EMBRYO DEFECTIVE 1637, T10K17.80	12.5	12.0	0.5	1.7
7396	AT3G08610.1 | expressed protein | chr3:2615432-2616809 REVERSE | Aliases: F17O14.8	12.5	11.9	0.6	3.4
1820	AT5G18800.2 | NADH-ubiquinone oxidoreductase 19 kDa subunit (NDUFA8) family protein, contains Pfam profile: PF05850 NADH-ubiquinone oxidoreductase 19 kDa subunit | chr5:6267067-6268616 FORWARD | Aliases: F17K4.50, F17K4_50	12.4	11.0	1.4	7.7
5101	AT1G65980.2 | Symbol: TPX1 | similar to peroxiredoxin type 2, putative [Arabidopsis thaliana] (TAIR:At1g65970.1); similar to thioredoxin peroxidase 1 [Lycopersicon esculentum] (GB:AAP34571.1); similar to thioredoxin-dependent peroxidase [Plantago major] (GB:CAH58634.1); contains InterPro domain Alkyl hydroperoxide reductase/ Thiol specific antioxidant/ Mal allergen (InterPro:IPR000866) | chr1:24562969-24564599 REVERSE | Aliases: F12P19.14, F12P19_14, PEROXIREDOXIN TPX1	12.4	11.9	0.6	4.5
12456	AT2G02130.1 | Symbol: LCR68/PDF2.3 | plant defensin-fusion protein, putative (PDF2.3), plant defensin protein family member, personal communication, Bart Thomma (Bart.Thomma@agr.kuleuven.ac.be) | chr2:540017-540615 FORWARD | Aliases: F5O4.10, F5O4_10, LCR68, Low molecular weight cysteine rich 68, PDF2.3	12.4	12.2	0.2	1.7
12100	AT1G08830.1 | Symbol: CSD1 | superoxide dismutase (Cu-Zn) (SODCC) / copper/zinc superoxide dismutase (CSD1), identical to SWISS-PROT: P24704 | chr1:2827089-2829260 FORWARD | Aliases: F22O13.32, F22O13_32	12.4	11.8	0.6	1.8
1472	AT1G18210.2 | calcium-binding protein, putative, similar to SP:Q9M7R0 Calcium-binding allergen Ole e 8 (PCA18/PCA23) {Olea europaea}; contains INTERPRO:IPR002048 calcium-binding EF-hand domain | chr1:6266602-6268821 REVERSE | Aliases: T10F20.22	12.4	8.8	3.6	8.3
5849	AT4G33865.1 | 40S ribosomal protein S29 (RPS29C) | chr4:16233214-16234201 REVERSE | Aliases: None	12.4	11.8	0.6	4.1
18782	NA	12.4	12.5	-0.1	-0.4
2064	AT2G24940.1 | cytochrome b5 domain-containing protein, similar to SP:P70580 Membrane associated progesterone receptor component 1 {Rattus norvegicus}; contains Pfam profile PF00173: Heme/Steroid binding domain | chr2:10616473-10619311 FORWARD | Aliases: F27C12.14, F27C12_14	12.4	10.1	2.3	7.3
2065	AT2G24945.1 | expressed protein | chr2:10616492-10619311 FORWARD | Aliases: None	12.4	10.1	2.3	7.3
19546	AT1G13440.1 | glyceraldehyde 3-phosphate dehydrogenase, cytosolic, putative / NAD-dependent glyceraldehyde-3-phosphate dehydrogenase, putative, very strong similarity to SP:P25858 Glyceraldehyde 3-phosphate dehydrogenase, cytosolic (EC 1.2.1.12) {Arabidopsis thaliana}; contains Pfam profiles PF02800: Glyceraldehyde 3-phosphate dehydrogenase C-terminal domain, PF00044: Glyceraldehyde 3-phosphate dehydrogenase NAD binding domain	12.4	12.3	0.1	0.3

922	AT3G46010.1 | Symbol: ADF1 | actin-depolymerizing factor 1 (ADF1), identical to SP:Q39250 Actin-depolymerizing factor 1 (ADF-1) (AtADF1) {Arabidopsis thaliana} | chr3:16920391-16921805 REVERSE | Aliases: ACTIN DEPOLYMERIZING FACTOR 1, ATADF, ATADF1, F16L2.220	12.4	10.0	2.4	9.8
1576	AT5G56795.1 | Symbol: MT1B | one of the five metallothioneins (MTs) genes identified in Arabidopsis. MTs are cysteine-rich proteins required for heavy metal tolerance. The MT1b gene, however, is indicated to be inactive. | chr5:22988283-22990379 REVERSE | Aliases: None	12.4	14.3	-1.9	-8.1
505	AT3G05560.2 | 60S ribosomal protein L22-2 (RPL22B), identical to 60S ribosomal protein L22-2 SP:Q9M9W1 from (Arabidopsis thaliana) | chr3:1614221-1615449 FORWARD | Aliases: F18C1.17, F18C1_17	12.4	10.0	2.4	11.9
18455	AT3G02360.2 | 6-phosphogluconate dehydrogenase family protein, contains Pfam profiles: PF00393 6-phosphogluconate dehydrogenase C-terminal domain, PF03446 NAD binding domain of 6-phosphogluconate ;similar to 6-phosphogluconate dehydrogenase GB:BAA22812 GI:2529229 (Glycine max) | chr3:482035-484154 FORWARD | Aliases: F11A12.5, F11A12_5	12.4	12.5	-0.1	-0.5
12330	AT1G04410.1 | malate dehydrogenase, cytosolic, putative, strong similarity to malate dehydrogenase from Mesembryanthemum crystallinum (SP:O24047), Medicago sativa (SP:O48905), Prunus persica (GI:15982948); contains InterPro entry IPR001236: Lactate/malate dehydrogenase | chr1:1189077-1191411 REVERSE | Aliases: F19P19.13, F19P19_13	12.4	11.6	0.8	1.7
4590	AT3G09630.2 | similar to 60S ribosomal protein L4/L1 (RPL4D) [Arabidopsis thaliana] (TAIR:At5g02870.1); similar to PREDICTED OJ1014_E09.28 gene product [Oryza sativa (japonica cultivar-group)] (GB:XP_507356.1); contains InterPro domain Ribosomal protein L4/L1e (InterPro:IPR002136) | chr3:2953742-2955753 FORWARD | Aliases: F11F8.22	12.3	11.2	1.2	4.8
1106	AT2G46540.1 | expressed protein | chr2:19117544-19118841 REVERSE | Aliases: F13A10.7, F13A10_7	12.3	11.5	0.8	9.2
15380	NA	12.3	12.2	0.1	1.1
19776	AT3G08580.2 | Symbol: AAC1 | ADP, ATP carrier protein 1, mitochondrial / ADP/ATP translocase 1 / adenine nucleotide translocator 1 (ANT1), identical to SWISS-PROT:P31167 ADP,ATP carrier protein 1 (Adenine nucleotide translocator 1) (Arabidopsis thaliana) | chr3:2605448-2607793 REVERSE | Aliases: ADP/ATP CARRIER 1, F17O14.5	12.3	12.4	-0.1	-0.2
10998	NA	12.3	12.7	-0.3	-2.1
5373	AT4G35460.1 | Symbol: NTR1 | thioredoxin reductase 1 / NADPH-dependent thioredoxin reductase 1 (NTR1), identical to SP:Q39243	12.3	12.9	-0.5	-4.4
5372	AT2G17420.1 | Symbol: NTRA | similar to thioredoxin reductase 1 / NADPH-dependent thioredoxin reductase 1 (NTR1) [Arabidopsis thaliana] (TAIR:At4g35460.1); similar to NADPH-thioredoxin reductase [Triticum aestivum] (GB:CAD19162.1); contains InterPro domain Thioredoxin reductase (InterPro:IPR005982); contains InterPro domain Pyridine nucleotide-disulphide oxidoreductase, class I (InterPro:IPR001100); contains InterPro domain FAD-dependent pyridine nucleotide-disulphide oxidoreductase (InterPro:IPR001327); contains InterPro domain Pyridine nucleotide-disulphide oxidoreductase, class-II, active site (InterPro:IPR008255); contains InterPro domain Adrenodoxin reductase (InterPro:IPR000759); contains InterPro domain Pyridine nucleotide-disulphide oxidoreductase, class-II (InterPro:IPR000103) | chr2:7571427-7573621 FORWARD | Aliases: ATNTRA, F5J6.18, F5J6_18	12.3	12.9	-0.5	-4.4
206	AT5G02240.1 | expressed protein | chr5:451421-453155 FORWARD | Aliases: T1E22.5	12.3	8.6	3.7	16.5
429	AT3G52590.1 | Symbol: UBQ1 | ubiquitin extension protein 1 (UBQ1) / 60S ribosomal protein L40 (RPL40B), identical to GI:166929, GI:166930 | chr3:19516614-19517910 FORWARD | Aliases: EMB2167, EMBRYO DEFECTIVE 2167, F3C22.8, UBIQUITIN EXTENSION PROTEIN 1	12.3	10.0	2.3	12.6
4674	AT2G42680.1 | ethylene-responsive transcriptional coactivator, putative, similar to ethylene-responsive transcriptional coactivator (Lycopersicon esculentum) gi:5669634:gb:AAD46402 | chr2:17781980-17783387 FORWARD | Aliases: F14N22.5, F14N22_5	12.3	11.2	1.1	4.8
651	AT1G15930.2 | 40S ribosomal protein S12 (RPS12A), similar to 40S ribosomal protein S12 GI:4263712 from (Arabidopsis thaliana) | chr1:5471431-5472978 FORWARD | Aliases: T24D18.3, T24D18_3	12.3	10.3	2.0	11.0
5859	AT1G01470.1 | Symbol: LEA14 | late embryogenesis abundant protein, putative / LEA protein, putative, similar to SP:P46518 Late embryogenesis abundant protein Lea14-A {Gossypium hirsutum}; contains Pfam profile PF03168: Late embryogenesis abundant protein | chr1:172146-172923 REVERSE | Aliases: F22L4.3, F22L4_3, LATE EMBRYOGENESIS ABUNDANT 14, LIGHT STRESS REGULATED 3, LSR3	12.3	10.4	2.0	4.1
3854	AT1G54630.2 | Symbol: ACP3 | similar to acyl carrier protein, chloroplast, putative / ACP, putative [Arabidopsis thaliana] (TAIR:At1g54580.1); similar to acyl carrier protein [Brassica napus] (GB:CAA34248.1); contains InterPro domain Phosphopantetheine-binding domain (InterPro:IPR006163); contains InterPro domain Acyl carrier protein (ACP) (InterPro:IPR003231) | chr1:20405044-20406671 REVERSE | Aliases: ACYL CARRIER PROTEIN 3, T22H22.7, T22H22_7	12.3	11.2	1.1	5.4
3853	AT1G54580.1 | Symbol: ACP2 | acyl carrier protein, chloroplast, putative / ACP, putative, strong similarity to SP:P25701 Acyl carrier protein 2, chloroplast precursor (ACP) {Arabidopsis thaliana}; contains InterPro accession IPR003881: Isochorismatase | chr1:20393100-20394682 FORWARD | Aliases: ACYL CARRIER PROTEIN 2, T22H22.3, T22H22_3	12.3	11.2	1.1	5.4

8793	AT2G18960.1 | Symbol: AHA1 | ATPase 1, plasma membrane-type, putative / proton pump 1, putative / proton-exporting ATPase, putative, strong similarity to SP:P20649 ATPase 1, plasma membrane-type (EC 3.6.3.6) (Proton pump 1) {Arabidopsis thaliana}; contains InterPro accession IPR001757: ATPase, E1-E2 type; contains Pfam profile PF00690: Cation transporter/ATPase, N-terminus | chr2:8228713-8234701 FORWARD | Aliases: F19F24.16, F19F24_16, PLASMA MEMBRANE PROTON ATPASE, PMA	12.3	11.4	0.9	2.8
3445	AT3G01130.1 | expressed protein | chr3:44491-45597 REVERSE | Aliases: T4P13.18, T4P13_18	12.3	11.2	1.1	5.7
7375	AT5G64400.2 | expressed protein, similar to expressed protein [Arabidopsis thaliana] (TAIR:At5g09570.1); similar to unknown protein [Oryza sativa] (GB:XP_469336.1); contains InterPro domain Protein of unknown function DUF657 (InterPro:IPR007017) | chr5:25766066-25767582 FORWARD | Aliases: MSJ1.24, MSJ1_24	12.3	11.4	0.9	3.4
10839	AT1G65930.1 | isocitrate dehydrogenase, putative / NADP+ isocitrate dehydrogenase, putative, strong similarity to isocitrate dehydrogenase SP:Q40345 from (Medicago sativa) | chr1:24542587-24545790 FORWARD | Aliases: F12P19.10, F12P19_10	12.3	12.7	-0.4	-2.1
7635	NA	12.2	12.9	-0.6	-3.3
904	AT1G69620.1 | Symbol: RPL34 | 60S ribosomal protein L34 (RPL34B), similar to SP:Q42351 from (Arabidopsis thaliana) | chr1:26193501-26194986 FORWARD | Aliases: F24J1.23, RIBOSOMAL PROTEIN L34, RPL34	12.2	10.7	1.6	9.9
630	AT4G09320.1 | Symbol: NDPK1 | nucleoside diphosphate kinase 1 (NDK1), identical to identical to Nucleoside diphosphate kinase I (NDK I) (NDP kinase I) (NDPK I) (SP:P39207) (Arabidopsis thaliana); contains Pfam PF00334 : Nucleoside diphosphate kinase domain; | chr4:5923397-5924529 FORWARD | Aliases: T30A10.80, T30A10_80	12.2	10.7	1.5	11.1
438	AT1G18540.1 | 60S ribosomal protein L6 (RPL6A), similar to 60S ribosomal protein L6 GI:7208784 from (Cicer arietinum) | chr1:6377314-6378571 REVERSE | Aliases: F25I16.12, F25I16_12	12.2	10.3	2.0	12.5
17718	AT3G48140.1 | senescence-associated protein, putative, similar to B12D protein (Ipomoea batatas) GB:AAD22104 | chr3:17789385-17790534 FORWARD | Aliases: T24C20.20	12.2	12.1	0.1	0.6
1337	AT5G59890.2 | Symbol: ADF4 | actin-depolymerizing factor 4 (ADF4), identical to SP:Q9ZSK3 Actin-depolymerizing factor 4 (ADF-4) (AtADF4) {Arabidopsis thaliana} | chr5:24139827-24141138 FORWARD | Aliases: ACTIN DEPOLYMERIZING FACTOR 4, MMN10.8, MMN10_8	12.2	7.3	5.0	8.6
4151	AT5G47570.1 | expressed protein | chr5:19309839-19311962 REVERSE | Aliases: MNJ7.16, MNJ7_16	12.2	11.0	1.2	5.1
2805	AT3G53730.1 | histone H4, identical to histone H4 from Lycopersicon esculentum GI:297150, Lolium temulentum SP:P02308, Acropora formosa GI:455652, Citrus jambhiri GI:16797797	12.2	10.3	1.9	6.4
4079	AT1G13060.1 | Symbol: PBE1 | 20S proteasome beta subunit E1 (PBE1) (PRCE), identical to GB:O23717; identical to cDNA proteasome subunit prce GI:2511595 | chr1:4452269-4454872 FORWARD | Aliases: 20S PROTEASOME BETA SUBUNIT PBE1, F3F19.8, F3F19_8	12.2	10.5	1.7	5.2
4080	AT3G26340.1 | 20S proteasome beta subunit E, putative, very strong similarity to SP:O23717 Proteasome subunit beta type 5 precursor (EC 3.4.25.1) (20S proteasome subunit E) (Proteasome epsilon chain) {Arabidopsis thaliana}	12.2	10.5	1.7	5.2
15871	AT2G36530.1 | Symbol: LOS2 | enolase, identical to SWISS-PROT:P25696 enolase (EC 4.2.1.11) (2-phosphoglycerate dehydratase)(2-phospho-D- glycerate hydro-lyase) (Arabidopsis thaliana) | chr2:15327835-15330945 REVERSE | Aliases: F1O11.16, F1O11_16	12.2	11.8	0.4	1.0
11681	AT2G47380.1 | cytochrome c oxidase subunit Vc family protein / COX5C family protein, contains Pfam profile: PF05799 cytochrome c oxidase subunit Vc (COX5C) | chr2:19448025-19449123 FORWARD | Aliases: T8I13.22	12.2	11.8	0.4	1.9
3104	AT1G23820.2 | Symbol: SPDS1 | spermidine synthase 1 (SPDSYN1) / putrescine aminopropyltransferase 1, identical to SP:Q9ZUB3 Spermidine synthase 1 (EC 2.5.1.16) (Putrescine aminopropyltransferase 1) (SPDSY 1) {Arabidopsis thaliana} | chr1:8420276-8422928 FORWARD | Aliases: F5O8.38, F5O8_38, SPERMIDINE SYNTHASE 1	12.2	10.5	1.7	6.0
212	AT2G36170.1 | ubiquitin extension protein 2 (UBQ2) / 60S ribosomal protein L40 (RPL40A), identical to GI:166930, GI:166931 | chr2:15179196-15180358 FORWARD | Aliases: F9C22.10, F9C22_10	12.2	8.5	3.6	16.2
2534	AT1G48830.2 | 40S ribosomal protein S7 (RPS7A), similar to 40S ribosomal protein S7 homolog GI:5532505 from (Brassica oleracea) | chr1:18063304-18064925 REVERSE | Aliases: F11I4.1, F11I4_1	12.2	10.5	1.6	6.6
5663	AT5G08300.1 | succinyl-CoA ligase (GDP-forming) alpha-chain, mitochondrial, putative / succinyl-CoA synthetase, alpha chain, putative / SCS-alpha, putative, identical to SP:P53586 Succinyl-CoA ligase (GDP-forming) alpha-chain, mitochondrial precursor (EC 6.2.1.4) (Succinyl-CoA synthetase, alpha chain) (SCS-alpha) {Arabidopsis thaliana}; strong similarity to SP:P13086 Succinyl-CoA ligase (GDP-forming) alpha-chain, mitochondrial precursor {Rattus norvegicus}; contains Pfam profiles PF00549: CoA-ligase, PF02629: CoA binding domain | chr5:2667471-2669918 FORWARD | Aliases: F8L15.30, F8L15_30	12.1	10.3	1.9	4.2

3288	AT1G51650.1 | ATP synthase epsilon chain, mitochondrial, identical to ATP synthase epsilon chain, mitochondrial SP:Q96253 from (Arabidopsis thaliana) | chr1:19156283-19157597 FORWARD | Aliases: F19C24.25, F19C24_25	12.1	11.5	0.6	5.8
16369	AT3G62290.1 | Gene encoding ADP-ribosylation factor and similar to other ARFs and ARF-like proteins. Members of this family are known to be essential for vesicle coating and uncoating and functions in GTP-binding. The gene is shown to play a role in cell division, cell expansion and cellulose production using antisense construct. | chr3:23062627-23064719 FORWARD | Aliases: T17J13.250	12.1	12.4	-0.2	-0.9
2644	AT4G29480.1 | mitochondrial ATP synthase g subunit family protein, contains Pfam profile: PF04718 mitochondrial ATP synthase g subunit	12.1	10.4	1.7	6.5
4422	AT2G47170.1 | Symbol: ARF1 | Gene encoding ADP-ribosylation factor and similar to other ARFs and ARF-like proteins. Members of this family are known to be essential for vesicle coating and uncoating and functions in GTP-binding. The gene is shown to play a role in cell division, cell expansion and cellulose production using antisense construct. | chr2:19373694-19375870 FORWARD | Aliases: ADP RIBOSYLATION FACTOR, ADP RIBOSYLATION FACTOR 1, T8I13.1	12.1	10.6	1.6	4.9
9374	AT1G31910.1 | GHMP kinase family protein, contains TIGRFAM profile TIGR01219: phosphomevalonate kinase; contains Pfam PF00288: GHMP kinases putative ATP-binding protein domain; similar to Phosphomevalonate kinase (EC 2.7.4.2) (Swiss-Prot:P24521) (Saccharomyces cerevisiae) | chr1:11459030-11461805 FORWARD | Aliases: F5M6.9, F5M6_9	12.1	11.8	0.3	2.6
914	AT1G22840.1 | cytochrome c, putative, similar to cytochrome c (Pumpkin, Winter squash) SWISS-PROT:P00051 | chr1:8079279-8080563 FORWARD | Aliases: F19G10.20, F19G10_20	12.1	10.3	1.9	9.8
17203	AT1G45145.1 | Symbol: ATTRX5 |	12.1	11.9	0.2	0.7
1463	AT1G67430.1 | 60S ribosomal protein L17 (RPL17B), similar to ribosomal protein GI:19101 from (Hordeum vulgare)	12.1	10.0	2.1	8.3
6481	AT5G51970.2 | sorbitol dehydrogenase, putative / L-iditol 2-dehydrogenase, putative, similar to NAD-dependent sorbitol dehydrogenase from Malus x domestica (gi:4519539) | chr5:21128820-21130629 FORWARD | Aliases: MSG15.7, MSG15_7	12.1	10.9	1.2	3.8
996	AT3G12780.1 | Symbol: PGK1 | phosphoglycerate kinase, putative, similar to SP:P41758 Phosphoglycerate kinase, chloroplast precursor (EC 2.7.2.3) {Chlamydomonas reinhardtii}; contains Pfam profile PF00162: phosphoglycerate kinase | chr3:4060978-4063230 REVERSE | Aliases: MBK21.15, PHOSPHOGLYCERATE KINASE 1	12.1	7.9	4.2	9.6
1445	AT2G45070.3 | Symbol: SEC61 BETA | similar to sec61beta family protein [Arabidopsis thaliana] (TAIR:At3g60540.1); similar to sec61beta family protein [Arabidopsis thaliana] (TAIR:At3g60540.2); similar to GA10096-PA [Drosophila pseudoobscura] (GB:EAL25183.1); contains InterPro domain Sec61beta (InterPro:IPR005609) | chr2:18594092-18595334 REVERSE | Aliases: SEC 61 BETA SUBUNIT, T14P1.12	12.1	10.6	1.5	8.3
1869	AT5G47930.1 | 40S ribosomal protein S27 (RPS27D) | chr5:19423403-19424624 REVERSE | Aliases: K16F13.2, K16F13_2	12.1	10.2	1.9	7.6
989	AT5G54770.1 | Symbol: THI1 | thiazole biosynthetic enzyme, chloroplast (ARA6) (THI1) (THI4), identical to SP:Q38814 Thiazole biosynthetic enzyme, chloroplast precursor (ARA6) {Arabidopsis thaliana}	12.1	6.6	5.5	9.6
12173	AT1G48750.1 | protease inhibitor/seed storage/lipid transfer protein (LTP) family protein, similar to TED4 (Zinnia elegans) GI:493721; contains Pfam protease inhibitor/seed storage/LTP family domain PF00234	12.1	11.6	0.5	1.8
3232	AT5G03850.1 | 40S ribosomal protein S28 (RPS28B), ribosomal protein S28, Arabidopsis thaliana, EMBL:ATRP28A | chr5:1028339-1029120 REVERSE | Aliases: MED24.14	12.1	11.0	1.1	5.9
775	AT1G77940.1 | 60S ribosomal protein L30 (RPL30B), similar to ribosomal protein L30 GI:388034 from (Homo sapiens) | chr1:29308791-29310259 REVERSE | Aliases: F28K19.15, F28K19_15	12.1	10.3	1.8	10.4
10994	AT5G08290.1 | Symbol: YLS8 | yellow-leaf-specific protein 8 (YLS8) / mitosis protein DIM1, putative, contains Pfam domain PF02966: Mitosis protein DIM1; identical to cDNA YLS8 mRNA for Dim1 homolog GI:13122293 | chr5:2665977-2667156 FORWARD | Aliases: F8L15.20, F8L15_20	12.1	11.6	0.5	2.1
3676	AT4G32470.2 | ubiquinol-cytochrome C reductase complex 14 kDa protein, putative, similar to SP:P48502 Ubiquinol-cytochrome C reductase complex 14 kDa protein (EC 1.10.2.2) (CR14) {Solanum tuberosum}; contains Pfam profile PF02271: Ubiquinol-cytochrome C reductase complex 14kD subunit | chr4:15669225-15671200 REVERSE | Aliases: F8B4.170, F8B4_170	12.1	10.7	1.4	5.5
1074	AT3G15353.1 | Symbol: MT3 | metallothionein protein, putative	12.1	8.0	4.1	9.3
1168	AT4G24830.1 | arginosuccinate synthase family, contains Pfam profile: PF00764 arginosuccinate synthase	12.1	7.2	4.9	9.1
8232	AT2G33040.1 | ATP synthase gamma chain, mitochondrial (ATPC), identical to SP:Q96250 ATP synthase gamma chain, mitochondrial precursor (EC 3.6.3.14) {Arabidopsis thaliana}; contains Pfam profile: PF00231 ATP synthase | chr2:14025857-14028257 REVERSE | Aliases: F25I18.22, F25I18_22, T21L14.5	12.0	11.0	1.0	3.0

7475	NA	12.0	12.6	-0.5	-3.3
3711	NA	12.0	10.7	1.3	5.5
68	AT2G30570.2 | photosystem II reaction center W (PsbW) protein-related, similar to photosystem II reaction center W protein SP:Q41387 from (Spinacia oleracea) | chr2:13025520-13027271 REVERSE | Aliases: T6B20.8, T6B20_8	12.0	5.6	6.4	23.5
1134	AT1G74050.1 | 60S ribosomal protein L6 (RPL6C), similar to 60S ribosomal protein L6 (YL 16 like) GB:CAB57309 from (Cyanophora paradoxa) | chr1:27850710-27852467 REVERSE | Aliases: F2P9.8, F2P9_8	12.0	11.1	1.0	9.1
1135	AT1G74060.1 | 60S ribosomal protein L6 (RPL6B), similar to 60S ribosomal protein L6 (YL 16 like) GB:CAB57309 from (Cyanophora paradoxa) | chr1:27853497-27855028 REVERSE | Aliases: F2P9.7, F2P9_7	12.0	11.1	1.0	9.1
19676	AT3G13520.1 | Symbol: AGP12 | arabinogalactan-protein (AGP12), identical to gi:10880501:gb:AAG24280 | chr3:4408932-4409447 FORWARD | Aliases: ARABINOGALACTAN PROTEIN 12, MRP15.18	12.0	12.1	-0.0	-0.3
2354	AT5G56710.1 | 60S ribosomal protein L31 (RPL31C) | chr5:22961062-22962196 REVERSE | Aliases: MIK19.16, MIK19_16	12.0	10.2	1.8	6.9
5093	AT2G23090.1 | expressed protein | chr2:9836530-9837490 REVERSE | Aliases: F21P24.15	12.0	10.9	1.1	4.5
11643	AT3G01120.1 | Symbol: MTO1 | cystathionine gamma-synthase, chloroplast / O-succinylhomoserine (Thiol)-lyase (CGS), identical to SP:P55217 Cystathionine gamma-synthase, chloroplast precursor (EC 4.2.99.9) (CGS) (O-succinylhomoserine (Thiol)-lyase) {Arabidopsis thaliana}	12.0	11.4	0.6	1.9
5309	AT5G12140.1 | Symbol: ATCYS1 |	12.0	10.8	1.1	4.4
1198	AT2G31490.1 | expressed protein | chr2:13419060-13420323 FORWARD | Aliases: T28P16.2, T28P16_2	12.0	10.4	1.5	9.0
8294	AT2G28910.1 | Symbol: CXIP4 | CAX-interacting protein 4 (CAXIP4), contains Pfam domain PF00098: Zinc knuckle; identical to cDNA CAX-interacting protein 4 GI:27651998	12.0	12.7	-0.7	-3.0
579	AT3G53020.1 | 60S ribosomal protein L24 (RPL24B), 60S ribosomal protein L24, Arabidopsis thaliana, EMBL:AC006282 | chr3:19671545-19673003 REVERSE | Aliases: F8J2.190	12.0	10.3	1.6	11.4
13048	AT3G12760.1 | expressed protein, similar to RP42 protein (Homo sapiens) GI:9896486; contains Pfam profile PF00627: UBA/TS-N domain, PF03556: Domain of unknown function (DUF298) | chr3:4054746-4056987 FORWARD | Aliases: MBK21.14	12.0	11.7	0.3	1.6
527	AT4G29390.1 | 40S ribosomal protein S30 (RPS30B), RIBOSOMAL PROTEIN S30 - Arabidopsis thaliana,PID:e1358183 | chr4:14464993-14465989 REVERSE | Aliases: F17A13.210, F17A13_210	12.0	10.1	1.9	11.8
7524	AT1G23190.1 | phosphoglucomutase, cytoplasmic, putative / glucose phosphomutase, putative, strong similarity to SP:P93805 Phosphoglucomutase, cytoplasmic 2 (EC 5.4.2.2) (Glucose phosphomutase 2) (PGM 2) {Zea mays}; contains InterPro accession IPR006352: Phosphoglucosamine mutase | chr1:8219879-8224458 FORWARD | Aliases: T26J12.5, T26J12_5	11.9	9.6	2.3	3.3
1482	AT4G10100.2 | Symbol: CNX7/SIR5 | molybdenum cofactor synthesis family protein, similar to Molybdenum cofactor synthesis protein 2 small subunit (Molybdopterin-synthase small subunit) (MOCS2A) (MOCO1-A) (Swiss-Prot:O96033) (Homo sapiens); contains TIGRFAM TIGR01682: molybdopterin converting factor, subunit 1; contains Pfam PF02597: ThiS family | chr4:6308712-6309504 FORWARD | Aliases: CNX7, F28M11.20, F28M11_20, MOLYBDOPTERIN SYNTHASE, SIR5	11.9	10.8	1.1	8.3
14026	AT4G26970.1 | aconitate hydratase, cytoplasmic, putative / citrate hydro-lyase/aconitase, putative, strong similarity to SP:P49608 Aconitate hydratase, cytoplasmic (EC 4.2.1.3) (Citrate hydro-lyase) (Aconitase) {Cucurbita maxima}; contains Pfam profiles PF00330: Aconitase family (aconitate hydratase), PF00694: Aconitase C-terminal domain | chr4:13542924-13549101 FORWARD | Aliases: F10M23.310, F10M23_310	11.9	12.1	-0.2	-1.4
3652	AT1G66580.1 | 60S ribosomal protein L10 (RPL10C), contains Pfam profile: PF00826: Ribosomal L10 | chr1:24842828-24844275 FORWARD | Aliases: T12I7.3, T12I7_3	11.9	10.7	1.2	5.5
3200	AT5G24660.1 | expressed protein | chr5:8443282-8443837 REVERSE | Aliases: MXC17.2, MXC17_2	11.9	8.8	3.1	5.9
393	AT3G62870.1 | 60S ribosomal protein L7A (RPL7aB), 60S RIBOSOMAL PROTEIN L7A - Oryza sativa, SWISSPROT:RL7A_ORYSA | chr3:23253640-23255328 REVERSE | Aliases: F26K9.300	11.9	9.9	2.0	12.9
6161	AT5G20290.1 | 40S ribosomal protein S8 (RPS8A), ribosomal protein S8 - Zea mays, PIR:T04088 | chr5:6851483-6853065 REVERSE | Aliases: F5O24.180, F5O24_180	11.9	10.9	1.0	3.9

725	AT3G13470.1 | chaperonin, putative, similar SWISS-PROT:P21240- RuBisCO subunit binding-protein beta subunit, chloroplast precursor (60 kDa chaperonin beta subunit, CPN-60 beta) (Arabidopsis thaliana); contains Pfam:PF00118 domain, TCP-1/cpn60 chaperonin family | chr3:4389692-4392762 FORWARD | Aliases: MRP15.11	11.9	6.3	5.6	10.6
2506	AT5G37780.1 | Symbol: CAM1 | calmodulin-1/4 (CAM1), identical to calmodulin 4 (Arabidopsis thaliana) GI:16223, SP:P25854 Calmodulin-1/4 {Arabidopsis thaliana} | chr5:15021763-15023435 REVERSE | Aliases: ACAM 1, CALMODULIN 1, CAM1, K22F20.20, K22F20_20, TCH1	11.9	9.5	2.4	6.7
2913	AT1G27530.1 | expressed protein, Similar to gb:AF151884 CGI-126 protein from Homo sapiens. EST gb:Z18048 comes from this gene | chr1:9562586-9563926 REVERSE | Aliases: T17H3.3, T17H3_3	11.9	10.3	1.5	6.2
8204	AT1G43170.3 | Symbol: ARP1 | similar to 60S ribosomal protein L3 (RPL3B) [Arabidopsis thaliana] (TAIR:At1g61580.1); similar to ribosomal protein L3 [Triticum aestivum] (GB:AAQ62076.1); similar to ribosomal protein L3 [Triticum aestivum] (GB:AAQ62074.1); similar to ribosomal protein L3 [Lycopersicon esculentum] (GB:AAR17783.1); similar to ribosomal protein L3A [Nicotiana tabacum] (GB:AAQ96335.1); similar to ribosomal protein L3 [Triticum aestivum] (GB:AAQ21399.1); contains InterPro domain Ribosomal protein L3 (InterPro:IPR000597) | chr1:16269213-16271310 FORWARD | Aliases: ARABIDOPSIS RIBOSOMAL PROTEIN 1, EMB2207, EMBRYO DEFECTIVE 2207, F1I21.1, F1I21_1, RPL3A	11.9	11.2	0.7	3.0
8173	AT4G13430.1 | aconitase family protein / aconitate hydratase family protein, contains Pfam profile PF00330: Aconitase family (aconitate hydratase | chr4:7803929-7807865 REVERSE | Aliases: T9E8.170, T9E8_170	11.9	10.7	1.2	3.0
555	AT5G64130.2 | expressed protein | chr5:25681625-25683425 REVERSE | Aliases: MHJ24.11, MHJ24_11	11.9	10.4	1.5	11.6
146	AT2G43360.1 | Symbol: BIO2 | biotin synthase (BioB) (BIO2), identical to SP:P54967 Pfam profile PF04055: radical SAM domain protein | chr2:18017828-18020315 REVERSE | Aliases: BIOB, BIOTIN AUXOTROPH 2, BIOTIN AUXOTROPH B, BIOTIN SYNTHASE, T1O24.10	11.9	5.5	6.4	18.3
11889	AT3G03100.2 | similar to NADH:ubiquinone oxidoreductase B17.2-like subunit [Chlamydomonas reinhardtii] (GB:AAQ64638.1); contains InterPro domain NADH:ubiquinone oxidoreductase 17.2 kD subunit (InterPro:IPR007763) | chr3:705388-707657 REVERSE | Aliases: T17B22.21, T17B22_21	11.9	11.4	0.5	1.8
3179	AT3G52730.1 | ubiquinol-cytochrome C reductase UQCRX/QCR9-like family protein, contains Pfam profile: PF05365 ubiquinol-cytochrome C reductase, UQCRX/QCR9 like | chr3:19553909-19555242 REVERSE | Aliases: F3C22.130	11.9	10.8	1.0	5.9
9290	AT1G79550.2 | Symbol: PGK | phosphoglycerate kinase, putative, similar to SP:P41758 Phosphoglycerate kinase, chloroplast precursor (EC 2.7.2.3) {Chlamydomonas reinhardtii}; contains Pfam profile PF00162: phosphoglycerate kinase | chr1:29928916-29931431 REVERSE | Aliases: PHOSPHOGLYCERATE KINASE, T8K14.3, T8K14_3	11.9	10.7	1.2	2.6
1425	AT4G16720.1 | 60S ribosomal protein L15 (RPL15A) | chr4:9399987-9401404 REVERSE | Aliases: DL4385C, FCAALL.416	11.9	10.6	1.3	8.4
1068	AT3G43810.1 | Symbol: CAM7 | calmodulin-7 (CAM7), almost identical to calmodulin GI:16227 from (Arabidopsis thaliana), SP:P59220 Calmodulin-7 {Arabidopsis thaliana} | chr3:15675358-15677445 REVERSE | Aliases: CALMODULIN 7, T28A8.100	11.9	8.0	3.9	9.3
4821	AT1G67350.1 | expressed protein, contains similarity to MHC class II antigen GI:9502037 from (Aotus nancymaae)	11.8	11.2	0.6	4.7
8746	AT2G23120.1 | expressed protein | chr2:9849123-9849714 FORWARD | Aliases: F21P24.18, F21P24_18	11.8	12.4	-0.6	-2.8
4888	AT1G11840.5 | Symbol: ATGLX1 |	11.8	10.3	1.6	4.6
1164	AT1G01100.2 | 60S acidic ribosomal protein P1 (RPP1A), similar to 60S ACIDIC RIBOSOMAL PROTEIN P1 GB:O23095 from (Arabidopsis thaliana) | chr1:50091-51182 REVERSE | Aliases: T25K16.9, T25K16_9	11.8	9.6	2.2	9.1
3328	AT5G10980.1 | histone H3, identical to HISTONE H3.2, MINOR, Medicago sativa, SWISSPROT:P11105, histone H3 variant H3.3 Lycopersicon esculentum GI:1435157; contains Pfam profile PF00125 Core histone H2A/H2B/H3/H4 | chr5:3472429-3473442 REVERSE | Aliases: T30N20.250, T30N20_250	11.8	10.8	1.0	5.8
10219	AT4G24620.1 | Symbol: PGI1 | glucose-6-phosphate isomerase, putative, similar to glucose-6-phosphate isomerase (Spinacia oleracea) GI:3413511; contains Pfam profile PF00342: glucose-6-phosphate isomerase | chr4:12708767-12712835 REVERSE | Aliases: CHLOROPLASTIC PHOSPHOGLUCOSE ISOMERASE, F22K18.180, F22K18_180, PGI, PHOSPHO GLC ISOMERASE, PHOSPHOGLUCOSE ISOMERASE PRECURSOR	11.8	10.9	0.9	2.3
7219	AT3G02470.2 | Symbol: SAMDC | similar to adenosylmethionine decarboxylase family protein [Arabidopsis thaliana] (TAIR:At5g15950.1); similar to S-adenosyl-L-methionine decarboxylase [Brassica juncea] (GB:AAF20160.1); contains InterPro domain S-adenosylmethionine decarboxylase (InterPro:IPR001985) | chr3:509428-511583 FORWARD | Aliases: ADOMETDC, F16B3.10, F16B3_10, S ADENOSYLMETHIONINE DECARBOXYLASE	11.8	12.4	-0.6	-3.4
871	AT4G26230.1 | 60S ribosomal protein L31 (RPL31B), ribosomal protein L31, Nicotiana glutinosa, U23784 | chr4:13285837-13286800 FORWARD | Aliases: T25K17.40, T25K17_40	11.8	10.1	1.7	10.0
6385	AT4G00860.1 | Symbol: ATOZI1 |	11.8	10.8	1.0	3.8

6072	AT1G70310.1 | Symbol: SPDS2 | spermidine synthase 2 (SPDSYN2) / putrescine aminopropyltransferase 2, identical to SP:O48661 Spermidine synthase 2 (EC 2.5.1.16) (Putrescine aminopropyltransferase 2) (SPDSY 2) {Arabidopsis thaliana} | chr1:26488969-26491076 REVERSE | Aliases: F17O7.16, F17O7_16, SPERMIDINE SYNTHASE 2	11.8	10.6	1.1	4.0
3062	AT5G35530.1 | 40S ribosomal protein S3 (RPS3C)	11.8	10.6	1.1	6.1
12467	AT4G14880.2 | Symbol: OASA1 | cysteine synthase / O-acetylserine (thiol)-lyase / O-acetylserine sulfhydrylase (OAS1), nearly identical to SP:P47998 Cysteine synthase (EC 4.2.99.8) (O-acetylserine sulfhydrylase) (O-acetylserine (Thiol)-lyase) {Arabidopsis thaliana}; identical to cDNA O-acetylserine lyase (At.OAS.5-8) GI:6983573 | chr4:8517955-8520406 REVERSE | Aliases: DL3480C, FCAALL.34	11.8	11.1	0.7	1.7
1130	AT3G60770.1 | 40S ribosomal protein S13 (RPS13A), AtRPS13A mRNA for cytoplasmic ribosomal protein S13, Arabidopsis thaliana,AB031739 | chr3:22471265-22472718 REVERSE | Aliases: T4C21.180	11.8	9.2	2.5	9.2
5846	AT2G03680.1 | Symbol: SPR1 | expressed protein, Alternative splicing exists based on EST evidence | chr2:1120797-1122004 FORWARD | Aliases: F19B11.13, F19B11_13, SKU6, SPIRAL1	11.8	10.7	1.1	4.1
4044	AT5G47210.1 | nuclear RNA-binding protein, putative, similar to nuclear RNA binding protein GI:6492264 from (Arabidopsis thaliana) | chr5:19186249-19188446 REVERSE | Aliases: MQL5.6, MQL5_6	11.7	10.6	1.2	5.2
376	AT3G17820.1 | Symbol: ATGSKB6 | glutamine synthetase (GS1), identical to glutamine synthetase, cytosolic isozyme (glutamate-- ammonia ligase, GS1) (Arabidopsis thaliana) SWISS-PROT:Q9LVI8 | chr3:6097420-6099601 FORWARD | Aliases: GLN1;3, MEB5.4	11.7	7.6	4.1	13.1
3932	AT1G75280.1 | isoflavone reductase, putative, identical to SP:P52577 Isoflavone reductase homolog P3 (EC 1.3.1.-) {Arabidopsis thaliana}; contains Pfam profile PF02716: isoflavone reductase. Involved in response to oxidative stress. | chr1:28255622-28257280 FORWARD | Aliases: F22H5.17, F22H5_17, P3	11.7	8.2	3.6	5.3
5699	AT4G34700.1 | complex 1 family protein / LVR family protein, contains Pfam PF05347: Complex 1 protein (LYR family) | chr4:16556850-16558851 FORWARD | Aliases: T4L20.280, T4L20_280	11.7	10.4	1.3	4.2
3724	ATCG00380.1 | Symbol: RPS4 | Chloroplast encoded ribosomal protein S4	11.7	9.2	2.6	5.5
5221	AT3G12120.1 | Symbol: FAD2 | omega-6 fatty acid desaturase, endoplasmic reticulum (FAD2) / delta-12 desaturase, identical to omega-6 fatty acid desaturase, endoplasmic reticulum (FAD2) SP:P46313 (Arabidopsis thaliana (Mouse-ear cress)) (Plant Cell 6:147-158(1994)) | chr3:3860291-3863036 REVERSE | Aliases: DELTA 12 DESATURASE, FATTY ACID DESATURASE 2, T21B14.6	11.7	9.6	2.2	4.4
7497	AT5G20090.1 | expressed protein, contains Pfam domain, PF03650: Uncharacterized protein family (UPF0041) | chr5:6787004-6788656 REVERSE | Aliases: F28I16.240, F28I16_240	11.7	11.0	0.7	3.3
9123	AT1G64230.2 | similar to ubiquitin-conjugating enzyme E2-17 kDa 9 (UBC9) [Arabidopsis thaliana] (TAIR:At4g27960.1); similar to ubiquitin-conjugating enzyme E2-17 kDa 9 (UBC9) [Arabidopsis thaliana] (TAIR:At4g27960.2); similar to ubiquitin-conjugating enzyme OsUBC5b [Oryza sativa (japonica cultivar-group)] (GB:XP_464900.1); similar to ubiquitin-conjugating enzyme E2 [Gossypium raimondii] (GB:AAL99225.1); similar to ubiquitin-conjugating enzyme 8 [Capsicum annuum] (GB:AAR83891.1); similar to ubiquitin carrier protein (GB:AAA34125.1); contains InterPro domain Ubiquitin-conjugating enzymes (InterPro:IPR000608) | chr1:23837231-23839199 FORWARD | Aliases: F22C12.2, F22C12_2	11.7	11.1	0.6	2.7
2987	AT2G27720.1 | 60S acidic ribosomal protein P2 (RPP2A) | chr2:11825568-11826655 FORWARD | Aliases: F15K20.18, F15K20_18	11.7	10.4	1.3	6.1
2773	AT4G10480.1 | nascent polypeptide associated complex alpha chain protein, putative / alpha-NAC, putative, similar to alpha-NAC, non-muscle form (Mus musculus) GI:1666690; contains Pfam profiles PF01849: NAC domain, PF00627: UBA/TS-N domain | chr4:6477872-6479160 REVERSE | Aliases: F7L13.60, F7L13_60	11.7	9.9	1.8	6.4
3570	AT3G25530.2 | similar to 6-phosphogluconate dehydrogenase NAD-binding domain-containing protein [Arabidopsis thaliana] (TAIR:At1g17650.1); similar to oxidoreductase-like [Oryza sativa (japonica cultivar-group)] (GB:BAD45192.1); contains InterPro domain 3-hydroxyisobutyrate dehydrogenase (InterPro:IPR002204); contains InterPro domain 6-phosphogluconate dehydrogenase, NAD binding domain (InterPro:IPR006115) | chr3:9273037-9274830 REVERSE | Aliases: MWL2.23	11.7	10.2	1.5	5.6
3636	AT4G37870.2 | similar to phosphoenolpyruvate carboxykinase (ATP), putative / PEP carboxykinase, putative / PEPCK, putative [Arabidopsis thaliana] (TAIR:At5g65690.1); similar to phosphoenolpyruvate carboxykinase [Flaveria pringlei] (GB:BAB43909.1); similar to phosphoenolpyruvate carboxykinase [Zoysia japonica] (GB:BAD88616.1); similar to phosphoenolpyruvate carboxykinase [Flaveria trinervia] (GB:BAB43907.1); similar to PEPCK [Cucumis sativus] (GB:AAM00814.1); similar to phosphoenolpyruvate carboxykinase [Lycopersicon esculentum] (GB:AAG01894.2); contains InterPro domain Phosphoenolpyruvate carboxykinase (ATP) (InterPro:IPR001272); contains InterPro domain Phosphoenolpyruvate carboxykinase, N-terminal (InterPro:IPR008210) | chr4:17802688-17806533 REVERSE | Aliases: T28I19.150, T28I19_150	11.7	8.6	3.1	5.5
2450	AT2G01250.1 | 60S ribosomal protein L7 (RPL7B)	11.7	10.2	1.5	6.8

3657	AT4G33680.1 | Symbol: AGD2 | aminotransferase class I and II family protein, low similarity to Aromatic Aminotransferase from Pyrococcus horikoshii GP:14278621; contains Pfam profile PF00155 aminotransferase, classes I and II | chr4:16171697-16174681 REVERSE | Aliases: ABERRANT GROWTH AND DEATH 2, T16L1.170, T16L1_170	11.7	8.5	3.2	5.5
19762	AT1G56700.1 | pyrrolidone-carboxylate peptidase family protein, similar to Pyrrolidone-carboxylate peptidase (5-oxoprolyl- peptidase) (Pyroglutamyl-peptidase I) (PGP-I) (Pyrase). (Swiss-Prot:O73944) (Pyrococcus furiosus); similar to Pyrrolidone-carboxylate peptidase (5-oxoprolyl- peptidase) (Pyroglutamyl-peptidase I) (PGP-I). (Swiss-Prot:O07883) (Thermococcus litoralis); contains Pfam PF01470: pyrrolidone-carboxylate peptidase	11.7	11.6	0.1	0.3
3479	AT1G02780.1 | Symbol: EMB2386 | 60S ribosomal protein L19 (RPL19A), similar to ribosomal protein L19 GI:36127 from (Homo sapiens) | chr1:607821-609435 REVERSE | Aliases: EMB2386, EMBRYO DEFECTIVE 2386, T14P4.34	11.7	9.1	2.5	5.7
13022	AT3G47810.3 | calcineurin-like phosphoesterase family protein, contains Pfam profile: PF00149 calcineurin-like phosphoesterase	11.7	11.1	0.5	1.6
11258	AT1G07890.5 | Symbol: APX1 | similar to L-ascorbate peroxidase 1b (APX1b) [Arabidopsis thaliana] (TAIR:At3g09640.1); similar to ascorbate peroxidase [Brassica juncea] (GB:AAN60794.1); contains InterPro domain Haem peroxidase (InterPro:IPR002016); contains InterPro domain Plant ascorbate peroxidase (InterPro:IPR002207) | chr1:2437327-2439649 FORWARD | Aliases: ASCORBATE PEROXIDASE, CS1, F24B9.2, F24B9_2	11.7	10.6	1.0	2.0
5119	AT5G11520.1 | Symbol: ASP3 | aspartate aminotransferase, chloroplast / transaminase A (ASP3) (YLS4), identical to SP:P46644 Aspartate aminotransferase, chloroplast precursor (EC 2.6.1.1) (Transaminase A) {Arabidopsis thaliana}; identical to cDNA YLS4 mRNA for aspartate aminotransferase (ASP3), partial cds GI:13122285 | chr5:3685090-3687765 REVERSE | Aliases: ASPARTATE AMINOTRANSFERASE 3, ASPARTATE AMINOTRANSFERASE, CHLOROPLAST PRECURSOR, F15N18.110, F15N18_110, YELLOW LEAF SPECIFIC GENE 4, YLS4	11.7	9.7	1.9	4.5
359	AT3G09500.1 | 60S ribosomal protein L35 (RPL35A), similar to 60S ribosomal protein L35 GB:AAC27830 | chr3:2916986-2918036 FORWARD | Aliases: F11F8.7	11.6	9.3	2.3	13.4
3227	AT3G20390.1 | endoribonuclease L-PSP family protein, contains Pfam domain PF01042: Endoribonuclease L-PSP | chr3:7109955-7111830 REVERSE | Aliases: MQC12.17	11.6	9.8	1.8	5.9
14748	AT5G43940.1 | alcohol dehydrogenase class III / glutathione-dependent formaldehyde dehydrogenase / GSH-FDH (ADHIII), identical to gi:1143388 | chr5:17701421-17704165 FORWARD | Aliases: MRH10.4, MRH10_4	11.6	11.1	0.5	1.2
11032	AT2G27510.1 | ferredoxin, putative, similar to non-photosynthetic ferredoxin from Citrus sinensis (GI:1360725), Ferredoxin, root R-B2 from Raphanus sativus (SP:P14937); contains Pfam profile PF00111 2Fe-2S iron-sulfur cluster binding domain | chr2:11765157-11766554 REVERSE | Aliases: F10A12.19, F10A12_19	11.6	11.2	0.4	2.1
10289	AT5G20010.1 | Symbol: RAN 1 | Ras-related GTP-binding nuclear protein (RAN-1), identical to GTP-binding nuclear protein RAN-1 SP:P41916 from (Arabidopsis thaliana) | chr5:6760286-6762096 FORWARD | Aliases: ATRAN1, F28I16.160, F28I16_160, RAN1	11.6	10.5	1.1	2.3
10290	AT5G20020.1 | Symbol: RAN2 | Ras-related GTP-binding nuclear protein (RAN-2), identical to GTP-binding nuclear protein RAN-2 SP:P41917 from (Arabidopsis thaliana) | chr5:6762754-6764673 FORWARD | Aliases: F28I16.170, F28I16_170	11.6	10.5	1.1	2.3
10000	AT3G02090.2 | Symbol: MPPBETA | mitochondrial processing peptidase beta subunit, putative, similar to mitochondrial processing peptidase beta subunit, mitochondrial precursor, Beta-MPP (Human) SWISS-PROT:O75439 | chr3:365556-368918 FORWARD | Aliases: F1C9.12, F1C9_12, MPPBETA	11.6	10.4	1.2	2.4
11952	AT4G38800.1 | phosphorylase family protein, contains weak similarity to Swiss-Prot:O51931 nucleosidase (Includes: 5'-methylthioadenosine nucleosidase (EC 3.2.2.16); S-adenosylhomocysteine nucleosidase (Buchnera aphidicola)	11.6	11.1	0.5	1.8
2704	AT2G46390.1 | expressed protein | chr2:19049374-19050133 REVERSE | Aliases: F11C10.8	11.6	10.4	1.2	6.5
6396	AT1G01800.1 | short-chain dehydrogenase/reductase (SDR) family protein, similar to carbonyl reductase GI:1049108 from (Mus musculus) | chr1:293342-295040 FORWARD | Aliases: T1N6.22, T1N6_22	11.6	10.0	1.5	3.8
4540	AT4G24190.2 | Symbol: SHD | shepherd protein (SHD) / clavata formation protein, putative, nearly identical to SHEPHERD (Arabidopsis thaliana) GI:19570872; contains Pfam profiles PF02518: ATPase, histidine kinase-, DNA gyrase B-, and HSP90-like domain protein, PF00183: Hsp90 protein | chr4:12551731-12555915 REVERSE | Aliases: SHEPHERD, T22A6.20, T22A6_20	11.6	10.0	1.6	4.9
296	AT3G48930.1 | Symbol: EMB1080 | 40S ribosomal protein S11 (RPS11A) | chr3:18151756-18153251 REVERSE | Aliases: EMB1080, EMBRYO DEFECTIVE 1080, T2I13.230	11.6	9.1	2.5	14.4
2676	AT5G15200.1 | 40S ribosomal protein S9 (RPS9B), 40S ribosomal protein S9, Chlamydomonas sp., EMBL:AU066528 | chr5:4934886-4936384 REVERSE | Aliases: F8M21.90, F8M21_90	11.6	10.1	1.5	6.5
7479	AT5G60640.2 | Symbol: ATPDIL1 4 | thioredoxin family protein, similar to protein disulfide isomerase GI:5902592 from (Volvox carteri f. nagariensis), GI:2708314 from Chlamydomonas reinhardtii; contains Pfam profile: PF00085 Thioredoxin | chr5:24388186-24391264 REVERSE | Aliases: ATPDIL1 4, MUP24.6, MUP24_6, PDI LIKE 1 4	11.6	11.1	0.4	3.3

14807	AT3G15580.1 | Symbol: APG8H | autophagy 8i (APG8i), identical to autophagy 8i (Arabidopsis thaliana) GI:19912167; contains Pfam profile PF02991: Microtubule associated protein 1A/1B, light chain 3; supporting cDNA gi:21636957:gb:AF492760.1: | chr3:5273902-5275102 REVERSE | Aliases: MQD17.3	11.6	11.8	-0.2	-1.2
10067	AT3G48170.1 | Symbol: ALDH10A9 | betaine-aldehyde dehydrogenase, putative, similar to betaine-aldehyde dehydrogenase, chloroplast precursor (BADH) (Arabidopsis thaliana) SWISS-PROT:Q9S795 | chr3:17797129-17800967 REVERSE | Aliases: T24C20.50	11.6	11.2	0.4	2.3
2307	AT4G33010.1 | glycine dehydrogenase (decarboxylating), putative / glycine decarboxylase, putative / glycine cleavage system P-protein, putative, strong similarity to SP:P49361 Glycine dehydrogenase (decarboxylating) A, mitochondrial precursor (EC 1.4.4.2) {Flaveria pringlei}; contains Pfam profile PF02347: Glycine cleavage system P-protein | chr4:15926673-15931335 REVERSE | Aliases: F4I10.7	11.6	7.3	4.3	7.0
9025	AT5G50850.1 | pyruvate dehydrogenase E1 component beta subunit, mitochondrial / PDHE1-B (PDH2), identical to SP:Q38799 Pyruvate dehydrogenase E1 component beta subunit, mitochondrial precursor (EC 1.2.4.1) (PDHE1-B) {Arabidopsis thaliana}	11.6	10.9	0.7	2.7
6574	AT1G29850.2 | double-stranded DNA-binding family protein, contains Pfam profile: PF01984 double-stranded DNA-binding domain | chr1:10447747-10449930 FORWARD | Aliases: F1N18.11, F1N18_11	11.6	10.3	1.2	3.7
7349	NA	11.6	12.2	-0.6	-3.4
1249	AT5G02870.1 | 60S ribosomal protein L4/L1 (RPL4D), 60S roibosomal protein L4, Arabidopsis thaliana, EMBL:CAA79104 | chr5:657784-659716 FORWARD | Aliases: F9G14.180, F9G14_180	11.6	9.9	1.6	8.8
4088	AT1G48420.1 | desulfhydrase family, similar to similar to D-cysteine desulfhydrase (EC 4.4.1.15). (Swiss-Prot:P59329) (Escherichia coli O6); contains TIGRFAM TIGR01275: pyridoxal phosphate-dependent enzymes, D-cysteine desulfhydrase family profile | chr1:17900267-17902472 REVERSE | Aliases: T1N15.3, T1N15_3	11.6	10.1	1.4	5.2
18385	AT1G07140.1 | Symbol: SIRANBP | Ran-binding protein 1a (RanBP1a), identical to Ran-binding protein (atranbp1a) GI:2058282 from (Arabidopsis thaliana)	11.5	11.4	0.2	0.5
4233	AT1G48600.2 | phosphoethanolamine N-methyltransferase 2, putative (NMT2), very similar to :PEM2_ARATH Putative phosphoethanolamine N-methyltransferase 2 (EC 2.1.1.103) (SP:Q944H0){Arabidopsis thaliana}; very similar to Halotolerance protein Hal3b (SP:P94063)(Arabidopsis thaliana); to similar to GB:AAF61950 from (Spinacia oleracea) | chr1:17969539-17973005 FORWARD | Aliases: T1N15.23, T1N15_23	11.5	12.3	-0.7	-5.1
1373	AT3G56340.1 | 40S ribosomal protein S26 (RPS26C), several 40S ribosomal protein S26 | chr3:20903116-20904395 REVERSE | Aliases: T5P19.4	11.5	8.6	2.9	8.5
205	AT5G16060.1 | expressed protein | chr5:5246125-5247434 FORWARD | Aliases: F1N13.200, F1N13_200	11.5	7.9	3.6	16.5
4620	AT2G20820.2 | expressed protein, . | chr2:8971430-8972505 FORWARD | Aliases: F5H14.21, F5H14_21	11.5	10.2	1.3	4.8
606	AT3G53740.3 | similar to 60S ribosomal protein L36 (RPL36A) [Arabidopsis thaliana] (TAIR:At2g37600.1); similar to putative 60S ribosomal protein L36 [Oryza sativa (japonica cultivar-group)] (GB:XP_475364.1); contains InterPro domain Ribosomal protein L36E (InterPro:IPR000509) | chr3:19924705-19925969 REVERSE | Aliases: F5K20.40	11.5	9.1	2.5	11.3
889	AT5G19510.1 | elongation factor 1B alpha-subunit 2 (eEF1Balpha2), identical to elongation factor 1B alpha-subunit (Arabidopsis thaliana) GI:6686821 | chr5:6581660-6583213 REVERSE | Aliases: T20D1.30, T20D1_30	11.5	10.0	1.5	9.9
4759	AT4G16520.2 | autophagy 8f (APG8f), identical to autophagy 8f (Arabidopsis thaliana) GI:19912161; contains Pfam profile PF02991: Microtubule associated protein 1A/1B, light chain 3 | chr4:9306708-9308465 REVERSE | Aliases: DL4285C, FCAALL.383	11.5	12.2	-0.7	-4.7
978	AT2G03870.2 | small nuclear ribonucleoprotein, putative / snRNP, putative / Sm protein, putative, similar to U6 snRNA-associated Sm-like protein LSm7 (Homo sapiens) SWISS-PROT:Q9UK45	11.5	10.0	1.5	9.6
66	AT3G54890.3 | Symbol: LHCA1 | chlorophyll A-B binding protein / LHCI type I (CAB), identical to chlorophyll A/B-binding protein (Arabidopsis thaliana) GI:16207; contains Pfam profile: PF00504 chlorophyll A-B binding protein | chr3:20350482-20351968 REVERSE | Aliases: F28P10.130, F28P10_130, LHCA1	11.5	4.7	6.9	24.0
1384	AT4G37930.1 | Symbol: SHM1 | glycine hydroxymethyltransferase / serine hydroxymethyltransferase / serine/threonine aldolase (SHM1), identical to serine hydroxymethyl transferase (Arabidopsis thaliana) GI:6899945 | chr4:17831740-17834859 REVERSE | Aliases: F20D10.50, F20D10_50, SERINE HYDROXYMETHYLTRANSFERASE 1, SERINE TRANSHYDROXYMETHYLASE, SERINE TRANSHYDROXYMETHYLTRANSFERASE, STM	11.5	8.3	3.2	8.5
2419	AT1G41880.1 | 60S ribosomal protein L35a (RPL35aB), identical to GB:CAB81600 from (Arabidopsis thaliana) | chr1:15653564-15654932 REVERSE | Aliases: F5A13.4, F5A13_4	11.5	9.5	2.0	6.8
2420	AT3G55750.1 | 60S ribosomal protein L35a (RPL35aD), ribosomal protein L35a.e.c15, Saccharomyces cerevisiae, PIR:S44069	11.5	9.5	2.0	6.8

222	AT5G49630.1 | Symbol: AAP6 | amino acid permease 6 (AAP6), identical to amino acid permease 6 (AAP6) (Arabidopsis thaliana) GI:1769887 | chr5:20159696-20163712 REVERSE | Aliases: AMINO ACID PERMEASE 6, MNI5.1, MNI5_1	11.5	3.8	7.8	15.9
9806	AT1G54100.2 | Symbol: ALDH7B4 | aldehyde dehydrogenase, putative / antiquitin, putative, strong similarity to SP:Q41247 Aldehyde dehydrogenase family 7 member A1 (EC 1.2.1.3) (Antiquitin 1) (Brassica turgor-responsive/drought-induced gene 26 protein) (Btg-26) {Brassica napus}; similar to turgor-responsive protein 26G (aldehyde dehydrogenase family 7 member A1) (Pisum sativum) SWISS-PROT:P25795 | chr1:20198932-20202640 REVERSE | Aliases: F15I1.19, F15I1_19	11.5	11.0	0.5	2.4
13144	NA	11.5	10.8	0.7	1.6
2501	AT1G78040.1 | pollen Ole e 1 allergen and extensin family protein, contains Pfam domain, PF01190: Pollen proteins Ole e I family | chr1:29350732-29352001 FORWARD | Aliases: F28K19.26, F28K19_26	11.5	10.0	1.5	6.7
8770	ATCG00020.1 | Symbol: PSBA | Encodes chlorophyll binding protein D1, a part of the photosystem II reaction center core | chrC:383-1444 REVERSE | Aliases: PSBA	11.5	9.4	2.1	2.8
1376	AT5G56670.1 | 40S ribosomal protein S30 (RPS30C) | chr5:22952639-22953207 REVERSE | Aliases: MIK19.12	11.5	9.4	2.1	8.5
11550	AT5G48810.1 | Symbol: ATB5 B |	11.5	11.1	0.4	1.9
3628	AT4G19410.1 | pectinacetylesterase, putative, similar to pectinacetylesterase precursor GI:1431629 from (Vigna radiata) | chr4:10582032-10585769 REVERSE | Aliases: T5K18.190, T5K18_190	11.5	9.7	1.8	5.5
452	AT5G23740.1 | Symbol: RPS11 BETA | 40S ribosomal protein S11 (RPS11C)	11.5	8.8	2.7	12.4
9638	AT3G48000.1 | Symbol: ALDH2B4 | aldehyde dehydrogenase (ALDH2), identical to aldehyde dehydrogenase (Arabidopsis thaliana) GI:8574427; similar to mitochondrial aldehyde dehydrogenase (Arabidopsis thaliana) gi:19850249:gb:AAL99612; identical to cDNA aldehyde dehydrogenase AtALDH2a GI:20530140 | chr3:17727852-17730999 REVERSE | Aliases: ALDEHYDE DEHYDROGENASE 2, ALDH2, T17F15.130	11.5	10.7	0.8	2.5
1347	AT3G07110.2 | similar to 60S ribosomal protein L13A (RPL13aD) [Arabidopsis thaliana] (TAIR:At5g48760.1); similar to ribosomal protein L13a [Lupinus luteus] (GB:CAA11283.1); contains InterPro domain Ribosomal protein L13, bacterial and organelle form (InterPro:IPR005823); contains InterPro domain Ribosomal protein L13, archea and eukaryotic form (InterPro:IPR005755); contains InterPro domain Ribosomal protein L13 (InterPro:IPR005822) | chr3:2252025-2253534 FORWARD | Aliases: T1B9.24, T1B9_24	11.5	9.2	2.3	8.6
788	AT1G77710.1 | expressed protein, similar to hypothetical protein GB:P34661 (Caenorhabditis elegans) | chr1:29211577-29212943 FORWARD | Aliases: T32E8.4, T32E8_4	11.5	8.8	2.7	10.3
26	AT5G56600.1 | Symbol: PRF3 | profilin 5 (PRO5) (PRF3), identical to SP:Q9FE63 Profilin 5 {Arabidopsis thaliana} | chr5:22926914-22928047 REVERSE | Aliases: MIK19.4, MIK19_4, PFN3, PROFILIN, PROFILIN 3	11.5	4.1	7.4	29.8
15784	AT3G22200.1 | Symbol: POP2 | 4-aminobutyrate aminotransferase / gamma-amino-N-butyrate transaminase / GABA transaminase / beta-alanine--oxoglutarate aminotransferase, identical to gamma-aminobutyrate transaminase subunit precursor (Arabidopsis thaliana) (EC 2.6.1.19) GI:14030435; contains Pfam profile PF00202: aminotransferase, class III; identical to cDNA gamma-aminobutyrate transaminase subunit precursor, nuclear gene for mitochondrial product GI:14030434	11.5	11.0	0.4	1.0
1125	AT3G47836.1 | expressed protein | chr3:17661246-17662750 REVERSE | Aliases: None	11.5	9.5	1.9	9.2
76	AT3G02790.1 | zinc finger (C2H2 type) family protein, contains Pfam profile: PF00096 zinc finger, C2H2 type | chr3:604852-605428 FORWARD | Aliases: F13E7.27, F13E7_27	11.5	7.0	4.4	22.4
2862	AT4G00100.1 | Symbol: ATRPS13A | 40S ribosomal protein S13 (RPS13A), similar to ribosomal protein S13; PF00312 (View Sanger Pfam): ribosomal protein S15; identical to cDNA AtRPS13A mRNA for cytoplasmic ribosomal protein S13 GI:6521011 | chr4:37096-38312 FORWARD | Aliases: F6N15.7, F6N15_7, PFL2, POINTED FIRST LEAF 2, RIBOSOMAL PROTEIN S13, RIBOSOMAL PROTEIN S13A, RPS13	11.5	9.0	2.4	6.3
1294	AT2G19730.2 | similar to 60S ribosomal protein L28 (RPL28C) [Arabidopsis thaliana] (TAIR:At4g29410.1); similar to putative 60S ribosomal L28 protein [Oryza sativa (japonica cultivar-group)] (GB:AAV67824.1); contains InterPro domain Ribosomal L28e protein (InterPro:IPR002672) | chr2:8518589-8520303 FORWARD | Aliases: F6F22.24, F6F22_24	11.5	9.5	1.9	8.7
4117	AT3G01910.2 | Symbol: SOX | similar to nitrate reductase 1 (NR1) [Arabidopsis thaliana] (TAIR:At1g77760.1); similar to B Chain B, Sulfite Oxidase From Chicken Liver (GB:1SOX); contains InterPro domain Eukaryotic molybdopterin oxidoreductase (InterPro:IPR008335); contains InterPro domain Mo-co oxidoreductase dimerisation domain (InterPro:IPR005066); contains InterPro domain Oxidoreductase, molybdopterin binding (InterPro:IPR000572) | chr3:314703-316719 REVERSE | Aliases: F28J7.38, F28J7_38, SULFITE OXIDASE	11.4	10.5	1.0	5.2
151	AT4G15910.1 | Symbol: ATDI21 |	11.4	7.6	3.8	18.0

12883	AT4G14710.2 | similar to iron-deficiency-responsive protein, putative [Arabidopsis thaliana] (TAIR:At4g14716.1); similar to submergence induced protein 2A [Oryza sativa] (GB:AAC19375.1); contains InterPro domain Acireductone dioxygenase, ARD (InterPro:IPR004313); contains InterPro domain Cupin domain (InterPro:IPR007113) | chr4:8424675-8426550 REVERSE | Aliases: DL3395C, FCAALL.141	11.4	10.5	1.0	1.6
12884	AT4G14716.1 | iron-deficiency-responsive protein, putative, strong similarity to iron-deficiency induced gene (Hordeum vulgare) GI:14522834; contains Pfam profile PF03079: ARD/ARD' family | chr4:8430202-8431940 REVERSE | Aliases: None	11.4	10.5	1.0	1.6
3165	AT1G26550.1 | peptidyl-prolyl cis-trans isomerase PPIC-type family protein, similar to SP:Q9Y237 Peptidyl-prolyl cis-trans isomerase NIMA-interacting 4 (EC 5.2.1.8) (Rotamase Pin4) (PPIase Pin4) (Parvulin 14) {Homo sapiens}; contains Pfam profile PF00639: PPIC-type PPIASE domain | chr1:9171119-9172926 FORWARD | Aliases: T1K7.8, T1K7_8	11.4	10.6	0.8	5.9
5596	AT3G13930.1 | dihydrolipoamide S-acetyltransferase, putative, similar to dihydrolipoamide S-acetyltransferase (Zea mays) GI:5669871; contains Pfam profiles PF00198: 2-oxo acid dehydrogenases acyltransferase (catalytic domain), PF00364: Biotin-requiring enzyme, PF02817: e3 binding domain | chr3:4595883-4600476 FORWARD | Aliases: MDC16.18	11.4	10.8	0.7	4.2
2741	AT4G31700.1 | Symbol: RPS6 | 40S ribosomal protein S6 (RPS6A), ribosomal protein S6, Arabidopsis thaliana, PID:g2662469 | chr4:15346083-15347731 REVERSE | Aliases: F28M20.110, F28M20_110, RIBOSOMAL PROTEIN S6	11.4	9.5	1.9	6.4
9400	AT2G31390.1 | pfkB-type carbohydrate kinase family protein, contains Pfam profile: PF00294 pfkB family carbohydrate kinase | chr2:13390140-13393286 REVERSE | Aliases: T28P16.12, T28P16_12	11.4	10.9	0.5	2.6
17773	AT3G17390.1 | Symbol: MTO3 | S-adenosylmethionine synthetase, putative, similar to S-adenosylmethionine synthetase 2 (Methionine adenosyltransferase 2, AdoMet synthetase 2) (Catharanthus roseus) SWISS-PROT:Q96552 | chr3:5952193-5954088 REVERSE | Aliases: MGD8.26, S ADENOSYLMETHIONINE SYNTHETASE, SAMS3	11.4	11.3	0.1	0.6
1601	AT4G33070.1 | pyruvate decarboxylase, putative, strong similarity to SP:P51846 Pyruvate decarboxylase isozyme 2 (EC 4.1.1.1) (PDC) {Nicotiana tabacum}; contains InterPro entry IPR000399: Pyruvate decarboxylase | chr4:15952292-15954774 REVERSE | Aliases: F4I10.4	11.4	7.1	4.4	8.0
11558	AT1G60680.1 | aldo/keto reductase family protein, contains Pfam profile PF00248: oxidoreductase, aldo/keto reductase family | chr1:22350775-22352913 REVERSE | Aliases: F8A5.20, F8A5_20	11.4	11.1	0.3	1.9
9983	ATCG00820.1 | Symbol: RPS19 | Encodes a 6.8-kDa protein of the small ribosomal subunit. | chrC:84005-84283 REVERSE | Aliases: RPS19	11.4	9.7	1.7	2.4
3344	AT1G08360.1 | 60S ribosomal protein L10A (RPL10aA), similar to 60S ribosomal protein L10A GB:AAC73045 GI:3860277 from (Arabidopsis thaliana) | chr1:2636027-2637912 FORWARD | Aliases: T27G7.6, T27G7_6	11.4	9.6	1.8	5.8
3731	AT3G22110.1 | Symbol: PAC1 | 20S proteasome alpha subunit C (PAC1) (PRC9), identical to GB:AAC32057 from (Arabidopsis thaliana) (Genetics (1998) 149 (2), 677-692); identical to cDNA proteasome subunit prc9 GI:2511583 | chr3:7792645-7794161 REVERSE | Aliases: 20S PROTEASOME SUBUNIT PAC1, MKA23.2	11.4	9.5	1.9	5.5
1748	AT3G57610.1 | adenylosuccinate synthetase (ADSS), identical to adenylosuccinate synthetase, chloroplast precursor (EC 6.3.4.4) (IMP-- aspartate ligase) (AdSS) (AMPSase) (Swiss-Prot:Q96529) (Arabidopsis thaliana) | chr3:21345261-21347635 REVERSE | Aliases: F15B8.200, F15B8_200	11.4	8.9	2.5	7.8
7750	AT3G12050.2 | Aha1 domain-containing protein, contains Pfam PF05146: Aha1 domain; similar to Protein C14orf3 (HSPC322) (Swiss-Prot:O95433) (Homo sapiens) | chr3:3839108-3841472 FORWARD | Aliases: MEC18.18	11.4	10.8	0.6	3.2
20084	AT3G61440.1 | Symbol: ATCYSC1 | encodes a cysteine synthase isomer. The isomer is however less effective in cysteine biosynthesis. It is involved in beta-cyanoalanine biosynthesis, a intermediate of cyanide detoxification pathway. | chr3:22746722-22748953 FORWARD | Aliases: ARATH;BSAS3;1, CYSTEINE SYNTHASE, F2A19.40	11.4	11.3	0.1	0.2
16533	AT2G43970.2 | La domain-containing protein, contains Pfam profile PF05383: La domain | chr2:18212311-18215294 REVERSE | Aliases: F6E13.10	11.4	11.2	0.2	0.9
3276	AT4G34670.1 | 40S ribosomal protein S3A (RPS3aB) | chr4:16548651-16550453 FORWARD | Aliases: T4L20.250, T4L20_250	11.4	9.9	1.5	5.8
7291	AT1G26630.1 | eukaryotic translation initiation factor 5A, putative / eIF-5A, putative, strong similariy to SP:Q9AXQ6 Eukaryotic translation initiation factor 5A-1 (eIF-5A 1) {Lycopersicon esculentum} | chr1:9205823-9207402 FORWARD | Aliases: T24P13.1, T24P13_1	11.4	9.7	1.7	3.4
19671	AT3G02780.1 | Symbol: IPP2 | isopentenyl-diphosphate delta-isomerase II / isopentenyl diphosphate:dimethylallyl diphosphate isomerase II (IPP2), identical to isopentenyl diphosphate:dimethylallyl diphosphate isomerase (IPP2) GB:U49259 (Arabidopsis thaliana) | chr3:602440-604682 REVERSE | Aliases: F13E7.28, F13E7_28, IDI2, IPIAT1, ISOPENTENYL DIPHOSPHATE ISOMERASE 2, ISOPENTENYL PYROPHOSPHATE:DIMETHYLLALLYL PYROPHOSPHATE ISOMERASE, ISOPENTENYL PYROPHOSPHATE:DIMETHYLLALLYL PYROPHOSPHATE ISOMERASE 2	11.4	11.4	-0.1	-0.3
7309	AT4G37830.1 | cytochrome c oxidase-related, contains weak similarity to cytochrome c oxidase polypeptide VIa-liver, mitochondrial precursor (EC 1.9.3.1) (Swiss-Prot:P10818) (Rattus norvegicus) | chr4:17787473-17788821 REVERSE | Aliases: None	11.4	10.7	0.7	3.4

11262	AT2G02760.1 | Symbol: ATUBC2 |	11.4	11.0	0.4	2.0
3580	AT1G75330.1 | Symbol: OTC | ornithine carbamoyltransferase, chloroplast / ornithine transcarbamylase / OTCase (OTC), identical to SP:O50039 Ornithine carbamoyltransferase, chloroplast precursor (EC 2.1.3.3) (OTCase) (Ornithine transcarbamylase) {Arabidopsis thaliana} | chr1:28269920-28272068 REVERSE | Aliases: F1B16.13, F1B16_13, ORNITHINE CARBAMOYLTRANSFERASE	11.3	9.1	2.2	5.6
3976	AT1G07770.2 | Symbol: RPS15A |	11.3	10.5	0.9	5.3
10529	ATCG00340.1 | Symbol: PSAB | Encodes the D1 subunit of photosystem I and II reaction centers. | chrC:37375-39579 REVERSE | Aliases: PSAB	11.3	10.5	0.8	2.2
2411	AT2G21170.1 | Symbol: TIM | triosephosphate isomerase, chloroplast, putative, similar to Triosephosphate isomerase, chloroplast precursor: SP:P48496 from Spinacia oleracea, SP:P46225 from Secale cereale | chr2:9077835-9080304 REVERSE | Aliases: F26H11.7, F26H11_7, TRIOSEPHOSPHATE ISOMERASE	11.3	9.3	2.0	6.8
1108	AT2G36060.2 | ubiquitin-conjugating enzyme family protein, similar to DNA-binding protein CROC-1B (Homo sapiens) GI:1066082; contains Pfam profile PF00179: Ubiquitin-conjugating enzyme | chr2:15149876-15151261 REVERSE | Aliases: F11F19.3, F11F19_3	11.3	10.2	1.1	9.2
5177	AT1G35160.1 | Symbol: GRF4 | 14-3-3 protein GF14 phi (GRF4), identical to GF14 protein phi chain GI:1493805, SP:P46077 from (Arabidopsis thaliana) | chr1:12867159-12868771 FORWARD | Aliases: GF14 PHI, GF14 PROTEIN PHI CHAIN, GRF4, T32G9.30, T32G9_30	11.3	6.7	4.6	4.5
5178	AT4G09000.1 | Symbol: GRF1 | 14-3-3-like protein GF14 chi / general regulatory factor 1 (GRF1), identical to 14-3-3 protein GF14 chi chain GI:1702986, SP:P42643 from (Arabidopsis thaliana)	11.3	6.7	4.6	4.5
1895	AT5G20720.2 | Symbol: CPN20 | 20 kDa chaperonin, chloroplast (CPN21) (CHCPN10) (CPN20), identical to chloroplast 20 kDa chaperonin, chloroplast precursor (Protein Cpn21), chloroplast protein Cpn10, chloroplast chaperonin 10 (Ch-CPN10), SP:O65282 from (Arabidopsis thaliana); identical to cDNA chaperonin 20 GI:14587372 | chr5:7014688-7016476 FORWARD | Aliases: ATCPN21, CHAPERONIN 10, CHAPERONIN 20, CHCPN10, CHLOROPLAST CHAPERONIN 10, CPN10, CPN21, T1M15.120, T1M15_120	11.3	9.3	2.1	7.6
3789	AT1G79040.1 | photosystem II 10 kDa polypeptide, identical to photosystem II 10 kDa polypeptide, chloroplast (precursor) SP:P27202 from (Arabidopsis thaliana); contains Pfam profile: PF04725 photosystem II 10 kDa polypeptide PsbR | chr1:29740911-29741831 FORWARD | Aliases: YUP8H12R.34, YUP8H12R_34	11.3	9.9	1.5	5.4
5588	AT2G01140.1 | fructose-bisphosphate aldolase, putative, similar to plastidic aldolase NPALDP1 from Nicotiana paniculata (GI:4827251); contains Pfam profile PF00274 Fructose-bisphosphate aldolase class-I | chr2:94810-96634 REVERSE | Aliases: F10A8.2, F10A8_2	11.3	9.8	1.5	4.2
1095	AT1G04270.2 | Symbol: RPS15 | similar to 40S ribosomal protein S15 (RPS15D) [Arabidopsis thaliana] (TAIR:At5g09510.1); similar to ribosomal S15 protein [Retama raetam] (GB:AAL32040.1); contains InterPro domain Ribosomal protein S19/S15 (InterPro:IPR002222); contains InterPro domain Ribosomal protein S15, eukaryotic and archaeal form (InterPro:IPR005713) | chr1:1141603-1143050 REVERSE | Aliases: F19P19.29, F19P19_29, RIBOSOMAL PROTEIN S15	11.3	9.6	1.8	9.3
16437	AT2G41430.4 | Symbol: ERD15 | dehydration-induced protein (ERD15), identical to dehydration-induced protein ERD15 GI:710626 from (Arabidopsis thaliana) | chr2:17276388-17277620 FORWARD | Aliases: CID1, DEHYDRATION INDUCED PROTEIN, EARLY RESPONSIVE TO DEHYDRATION 15, LIGHT STRESS REGULATED 1, LSR1, T26J13.2	11.3	11.5	-0.2	-0.9
2730	AT4G39200.1 | 40S ribosomal protein S25 (RPS25E), ribosomal protein S25, Lycopersicon esculentum, PIR2:S40089 | chr4:18257330-18258670 FORWARD | Aliases: T22F8.100, T22F8_100	11.3	9.6	1.7	6.4
596	AT1G07610.1 | Symbol: MT1C | metallothionein-like protein 1C (MT-1C), identical to Metallothionein-like protein 1C (MT-1C). (SP:Q38804) (Arabidopsis thaliana) | chr1:2341539-2342120 FORWARD | Aliases: F22G5.40	11.3	14.1	-2.7	-11.3
6193	AT3G09820.2 | Symbol: ADK1 | adenosine kinase 1 (ADK1) / adenosine 5'-phosphotransferase 1, identical to adenosine kinase 1 /adenosine 5'-phosphotransferase 1 SP:Q9SF85 from (Arabidopsis thaliana) | chr3:3012099-3014949 FORWARD | Aliases: ADENOSINE KINASE 1, F8A24.13	11.3	9.3	2.0	3.9
11183	AT5G19760.1 | dicarboxylate/tricarboxylate carrier (DTC), identical to dicarboxylate/tricarboxylate carrier (Arabidopsis thaliana) GI:19913113	11.3	10.5	0.8	2.0
1183	AT4G00585.1 | expressed protein | chr4:250955-252532 REVERSE | Aliases: None	11.3	9.2	2.1	9.0
4649	AT3G61110.1 | Symbol: ARS27A |	11.3	9.3	2.0	4.8
149	AT5G54270.1 | Symbol: LHCB3 | chlorophyll A-B binding protein / LHCII type III (LHCB3), identical to Lhcb3 protein (Arabidopsis thaliana) GI:4741952; contains Pfam profile PF00504: Chlorophyll A-B binding protein | chr5:22055555-22056794 FORWARD | Aliases: LHCB3*1, LIGHT HARVESTING CHLOROPHYLL BINDING PROTEIN 3, MDK4.9, MDK4_9	11.3	4.5	6.8	18.0
3330	AT1G64190.1 | 6-phosphogluconate dehydrogenase family protein, contains Pfam profiles: PF00393 6-phosphogluconate dehydrogenase C-terminal domain, PF03446 NAD binding domain of 6-phosphogluconate | chr1:23828991-23830799 REVERSE | Aliases: F22C12.5, F22C12_5	11.3	12.2	-0.9	-5.8

1981	ATCG00800.1 | Symbol: RPS3 | encodes a chloroplast ribosomal protein S3, a constituent of the small subunit of the ribosomal complex | chrC:82826-83482 REVERSE | Aliases: RPS3	11.3	9.8	1.5	7.4
12905	AT1G56070.1 | Symbol: LOS1 | elongation factor 2, putative / EF-2, putative, similar to ELONGATION FACTOR 2 GB:O14460 from (Schizosaccharomyces pombe) | chr1:20971595-20975407 REVERSE | Aliases: AT1G56075, AT1G56075.1, T6H22.13, T6H22.24, T6H22_13	11.3	10.8	0.5	1.6
5297	AT5G35590.1 | Symbol: PAA1 | 20S proteasome alpha subunit A1 (PAA1) (PRC1), identical to proteasome subunit alpha type 6-1 SP:O81146 GI:12643647 from (Arabidopsis thaliana); identical to cDNA proteasome subunit prc1 GI:2511587 | chr5:13782400-13785047 REVERSE | Aliases: 20S PROTEASOME SUBUNIT PAA1, K2K18.4, K2K18_4	11.3	10.4	0.9	4.4
5296	AT2G05840.2 | Symbol: PAA2 | similar to 20S proteasome alpha subunit A1 (PAA1) (PRC1) [Arabidopsis thaliana] (TAIR:At5g35590.1); similar to proteasome IOTA subunit [Glycine max] (GB:AAC28135.1); contains InterPro domain Proteasome subunit, A-type (InterPro:IPR000426); contains InterPro domain Multispecific proteasome protease (InterPro:IPR001353) | chr2:2234089-2236287 FORWARD | Aliases: 20S PROTEASOME SUBUNIT PAA2, T6P5.4, T6P5_4	11.3	10.4	0.9	4.4
2683	AT2G32520.1 | dienelactone hydrolase family protein, low similarity to dienelactone hydrolase (Pseudomonas resinovorans) GI:13094163; contains Pfam profile PF01738: Dienelactone hydrolase family | chr2:13812596-13814613 REVERSE | Aliases: T26B15.8, T26B15_8	11.3	9.4	1.9	6.5
13662	AT2G21660.2 | Symbol: ATGRP7 |	11.3	10.4	0.9	1.4
3550	AT5G58590.1 | Symbol: RANBP1 |	11.3	8.9	2.4	5.6
8940	AT4G35630.1 | Symbol: PSAT | phosphoserine aminotransferase, chloroplast (PSAT), identical to Phosphoserine aminotransferase, chloroplast precursor (PSAT) (SP:Q96255)(Arabidopsis thaliana); contains TIGRFAM TIGR01364: phosphoserine aminotransferase; contains Pfam PF00266: aminotransferase, class V | chr4:16904064-16905727 FORWARD | Aliases: PHOSPHOSERINE AMINOTRANSFERASE	11.3	10.1	1.2	2.7
4356	AT1G53240.1 | malate dehydrogenase (NAD), mitochondrial, identical to mitochondrial NAD-dependent malate dehydrogenase GI:3929649 SP:Q9ZP06 from (Arabidopsis thaliana); contains InterPro entry IPR001236: Lactate/malate dehydrogenase | chr1:19858283-19860605 REVERSE | Aliases: F12M16.14, F12M16_14	11.3	9.0	2.3	5.0
6410	NA	11.3	12.0	-0.7	-3.8
2153	AT5G56500.2 | similar to chaperonin, putative [Arabidopsis thaliana] (TAIR:At1g26230.1); similar to RuBisCO subunit binding-protein beta subunit, chloroplast / 60 kDa chaperonin beta subunit / CPN-60 beta [Arabidopsis thaliana] (TAIR:At1g55490.2); similar to chaperonin, putative [Arabidopsis thaliana] (TAIR:At3g13470.1); similar to RuBisCO subunit binding-protein beta subunit, chloroplast / 60 kDa chaperonin beta subunit / CPN-60 beta [Arabidopsis thaliana] (TAIR:At1g55490.1); similar to putative RuBisCO subunit binding-protein beta subunit, chloroplast precursor [Oryza sativa (japonica cultivar-group)] (GB:XP_463795.1); similar to chaperonin [Secale cereale] (GB:CAA93139.1); similar to chaperonin 62.5K beta chain - rape (GB:PW0007); similar to putative chaperonin 60 beta precursor [Oryza sativa (japonica cultivar-group)] (GB:NP_910308.1); similar to chaperonin precursor [Pisum sativum] (GB:AAA66365.1); contains InterPro domain Chaperonin Cpn60 (InterPro:IPR001844); contains InterPro domain Chaperonin Cpn60/TCP-1 (InterPro:IPR002423) | chr5:22891017-22894622 FORWARD | Aliases: MCD7.27, MCD7_27	11.3	8.2	3.1	7.2
7086	AT5G56150.2 | ubiquitin-conjugating enzyme, putative, strong similarity to ubiquitin-conjugating enzyme UBC2 (Mesembryanthemum crystallinum) GI:5762457, UBC4 (Pisum sativum) GI:456568; contains Pfam profile PF00179: Ubiquitin-conjugating enzyme | chr5:22746408-22748409 FORWARD | Aliases: MDA7.21, MDA7_21	11.3	10.6	0.7	3.5
10479	AT1G10670.2 | Symbol: ACLA 1 |	11.3	10.6	0.7	2.2
801	AT1G10960.1 | ferredoxin, chloroplast, putative, strong similarity to FERREDOXIN PRECURSOR GB:P16972 (SP:P16972) from (Arabidopsis thaliana) | chr1:3664386-3665039 FORWARD | Aliases: T19D16.12, T19D16_12	11.3	7.2	4.1	10.3
356	AT4G24780.1 | pectate lyase family protein, similar to pectate lyase GP:14289169 from (Salix gilgiana)	11.3	4.5	6.8	13.4
426	AT1G61570.1 | Symbol: TIM13 | mitochondrial import inner membrane translocase (TIM13), identical to mitochondrial import inner membrane translocase subunit Tim13 (Arabidopsis thaliana) Swiss-Prot:Q9XH48; contains Pfam domain, PF02953: Tim10/DDP family zinc finger | chr1:22722370-22723181 REVERSE | Aliases: T25B24.8, T25B24_8, TIM13, translocase of the inner membrane 13	11.3	8.8	2.4	12.6
16213	AT3G62400.2 | expressed protein, cytochrome c oxidase subunit 5c - Oryza sativa, EMBL:AB027123	11.3	11.1	0.2	0.9
10970	AT1G06530.1 | myosin heavy chain-related, similar to myosin heavy chain (GI:1408194) {Placopecten magellanicus}; similar to Myosin heavy chain, clone 203 (Fragment) (SP:P39922){Hydra attenuata}; contains one transmembrane domain | chr1:2001593-2002595 FORWARD | Aliases: F12K11.14, F12K11_14	11.3	10.7	0.6	2.1
13561	AT1G09780.1 | 2,3-biphosphoglycerate-independent phosphoglycerate mutase, putative / phosphoglyceromutase, putative, strong similarity to SP:Q42908 2,3-bisphosphoglycerate-independent phosphoglycerate mutase (EC 5.4.2.1) (Phosphoglyceromutase) {Mesembryanthemum crystallinum}; contains Pfam profile PF01676: Metalloenzyme superfamily | chr1:3165372-3167871 REVERSE | Aliases: F21M12.16, F21M12_16	11.3	11.8	-0.6	-1.5

804	AT5G16130.1 | 40S ribosomal protein S7 (RPS7C), 40S ribosomal protein S7 homolog - Brassica oleracea, EMBL:AF144752 | chr5:5268424-5270091 FORWARD | Aliases: T21H19.50, T21H19_50	11.3	8.8	2.4	10.2
3261	AT1G25275.3 | expressed protein | chr1:8860659-8861349 FORWARD | Aliases: None	11.3	10.4	0.9	5.8
13056	AT1G48850.3 | Symbol: EMB1144 | similar to 3-phosphoshikimate 1-carboxyvinyltransferase, putative / 5-enolpyruvylshikimate-3-phosphate, putative / EPSP synthase, putative [Arabidopsis thaliana] (TAIR:At1g48860.1); similar to chorismate synthase 1 [Lycopersicon esculentum] (GB:CAA79859.1); contains InterPro domain Chorismate synthase (InterPro:IPR000453) | chr1:18068245-18071758 REVERSE | Aliases: EMB1144, EMBRYO DEFECTIVE 1144, T24P22.3, T24P22_3	11.2	10.8	0.4	1.6
16713	AT5G04750.1 | F1F0-ATPase inhibitor protein, putative, similar to F1F0-ATPase inhibitor protein (Oryza sativa (japonica cultivar-group)) gi:5106371:dbj:BAA81661 | chr5:1372148-1374006 FORWARD | Aliases: MUK11.3	11.2	11.3	-0.1	-0.8
9728	AT5G35630.2 | Symbol: GS2 | similar to glutamine synthetase, putative [Arabidopsis thaliana] (TAIR:At5g16570.1); similar to glutamine synthetase, putative [Arabidopsis thaliana] (TAIR:At5g37600.1); similar to glutamine synthetase, putative [Arabidopsis thaliana] (TAIR:At1g66200.1); similar to glutamine synthetase GS58 [Nicotiana attenuata] (GB:AAR86719.1); similar to glutamine synthetase precursor [Juglans nigra] (GB:AAD49734.1); similar to GLNA2_DAUCA Glutamine synthetase, chloroplast precursor (Glutamate--ammonia ligase) (GS2) (GB:O22506); similar to plastidic glutamine synthetase precursor [Brassica napus] (GB:CAA73062.1); similar to glutamine synthetase [Brassica napus] (GB:CAB72423.1); contains InterPro domain Glutamine synthetase, beta-Grasp domain (InterPro:IPR008147); contains InterPro domain Glutamine synthetase, catalytic domain (InterPro:IPR008146) | chr5:13848250-13850772 FORWARD | Aliases: ATGSL1, GLN2, GLUTAMINE SYNTHETASE 2, MJE4.9, MJE4_9	11.2	11.8	-0.6	-2.4
4429	AT4G32240.1 | expressed protein | chr4:15568306-15569194 FORWARD | Aliases: F10M6.120, F10M6_120	11.2	10.4	0.8	4.9
3373	AT5G52470.1 | Symbol: FIB1 | fibrillarin 1 (FBR1) (FIB1) (SKIP7), identical to fibrillarin 1 GI:9965653 from (Arabidopsis thaliana); C-terminus identical to SKP1 interacting partner 7 GI:10716959 from (Arabidopsis thaliana); contains Pfam domain PF01269: Fibrillarin | chr5:21311454-21313902 FORWARD | Aliases: ATFBR1, ATFIB1, FBR1, FIBRILLARIN 1, K24M7.22, K24M7_22, SKIP7, SKP1 INTERACTING PARTNER 7	11.2	10.4	0.8	5.7
4192	AT1G69510.3 | expressed protein | chr1:26130049-26131596 FORWARD | Aliases: F10D13.16, F10D13_16	11.2	9.9	1.3	5.1
8289	AT3G08590.2 | 2,3-biphosphoglycerate-independent phosphoglycerate mutase, putative / phosphoglyceromutase, putative, strong similarity to SP:Q42908 2,3-bisphosphoglycerate-independent phosphoglycerate mutase (EC 5.4.2.1) (Phosphoglyceromutase) {Mesembryanthemum crystallinum}; contains Pfam profile PF01676: Metalloenzyme superfamily | chr3:2608483-2611333 REVERSE | Aliases: F17O14.6	11.2	10.1	1.2	3.0
16097	AT2G04350.2 | long-chain-fatty-acid--CoA ligase family protein / long-chain acyl-CoA synthetase family protein (LACS8), similar to LACS 4 (SP:O35547) from Rattus norvegicus, LACS 4 (SP:O60488) from Homo sapiens; contains Pfam HMM hit: AMP-binding enzymes PF00501	11.2	11.0	0.3	1.0
3836	AT3G25140.1 | Symbol: QUA1 | glycosyl transferase family 8 protein, contains Pfam profile: PF01501 glycosyl transferase family 8 | chr3:9154718-9156851 FORWARD | Aliases: MJL12.8, QUASIMODO1	11.2	10.3	0.9	5.4
7255	AT3G01280.1 | porin, putative, similar to SP:P42055 34 kDa outer mitochondrial membrane protein porin (Voltage-dependent anion-selective channel protein) (VDAC) {Solanum tuberosum}; contains Pfam profile PF01459: Eukaryotic porin | chr3:85619-87865 FORWARD | Aliases: T22N4.9, T22N4_9	11.2	9.1	2.1	3.4
720	AT5G43150.1 | expressed protein | chr5:17342246-17343126 FORWARD | Aliases: MMG4.18, MMG4_18	11.2	10.1	1.1	10.7
718	AT3G05590.1 | Symbol: RPL18 | 60S ribosomal protein L18 (RPL18B), similar to GB:P42791 | chr3:1621486-1623020 FORWARD | Aliases: CYTOPLASMIC RIBOSOMAL PROTEIN L18, F18C1.14, F18C1_14, RIBOSOMAL PROTEIN L18	11.2	9.1	2.1	10.7
13808	AT2G44350.2 | Symbol: ATCS | citrate synthase, mitochondrial, putative, strong similarity to SP:P20115 Citrate synthase, mitochondrial precursor {Arabidopsis thaliana}; contains Pfam profile PF00285: Citrate synthase | chr2:18323354-18328158 FORWARD | Aliases: F4I1.16	11.2	10.7	0.5	1.4
2735	AT5G59870.1 | histone H2A, putative, similar to histone H2A Petroselinum crispum SP:P19177, Lycopersicon esculentum SP:P25469, Zea mays SP:P40280; contains Pfam profile PF00125 Core histone H2A/H2B/H3/H4 | chr5:24132608-24133444 REVERSE | Aliases: MMN10.22, MMN10_22	11.2	8.3	2.9	6.4
862	AT5G52650.1 | 40S ribosomal protein S10 (RPS10C), contains similarity to 40S ribosomal protein S10 | chr5:21372841-21374301 REVERSE | Aliases: F6N7.14, F6N7_14	11.2	9.4	1.7	10.0
5497	AT2G42210.4 | expressed protein, similar to hypothetical protein [Oryza sativa (japonica cultivar-group)] (GB:XP_450480.1); contains InterPro domain Mitochondrial import inner membrane translocase, subunit Tim17/22 (InterPro:IPR003397) | chr2:17597291-17598930 FORWARD | Aliases: T24P15.12, T24P15_12	11.2	10.0	1.2	4.3
472	AT3G56800.1 | Symbol: CAM3 | calmodulin-2/3/5 (CAM3), identical to calmodulin GI:474183 from (Arabidopsis thaliana); almost identical to calmodulin-2/3/5 SP:P25069 (Arabidopsis thaliana) | chr3:21045656-21047053 REVERSE | Aliases: ACAM 3, CALMODULIN 3, T8M16.130	11.2	8.6	2.6	12.2

13917	AT1G22410.1 | 2-dehydro-3-deoxyphosphoheptonate aldolase, putative / 3-deoxy-D-arabino-heptulosonate 7-phosphate synthase, putative / DAHP synthetase, putative, similar to 3-deoxy-D-arabino-heptulosonate 7-phosphate GI:170224 from (Nicotiana tabacum), SP:P21357 from Solanum tuberosum; contains Pfam Class-II DAHP synthetase family domain PF01474 | chr1:7912043-7914992 FORWARD | Aliases: F12K8.24, F12K8_24	11.2	10.7	0.5	1.4
449	AT4G29410.1 | 60S ribosomal protein L28 (RPL28C), unknown protein chromosome II BAC F6F22 - Arabidopsis thaliana,PID:g3687251 | chr4:14468250-14470173 REVERSE | Aliases: F17A13.230, F17A13_230	11.2	8.2	3.0	12.4
19962	AT3G21240.1 | Symbol: 4CL2 | 4-coumarate--CoA ligase 2 / 4-coumaroyl-CoA synthase 2 (4CL2), identical to SP:Q9S725 4-coumarate--CoA ligase 2 (EC 6.2.1.12) (4CL 2) (4-coumaroyl-CoA synthase 2) {Arabidopsis thaliana} | chr3:7454282-7457385 REVERSE | Aliases: COUMARATE:COA LIGASE, AT4CL2, MXL8.10	11.2	11.1	0.1	0.2
2542	AT3G48990.1 | AMP-dependent synthetase and ligase family protein, similar to peroxisomal-coenzyme A synthetase (FAT2) (gi:586339) from Saccharomyces cerevisiae; contains Pfam AMP-binding enzyme domain PF00501; identical to cDNA; identical to cDNA adenosine monophosphate binding protein 3 AMPBP3 (AMPBP3)GI:20799714 | chr3:18169732-18172334 REVERSE | Aliases: T2J13.170	11.2	12.3	-1.2	-6.6
5323	AT1G74020.1 | Symbol: SS2 | strictosidine synthase family protein, similar to strictosidine synthase (Rauvolfia serpentina)(SP:P15324); contains strictosidine synthase domain PF03088 | chr1:27838792-27840970 REVERSE | Aliases: ATSS 2 STRICTOSIDINE SYNTHASE, F2P9.11, F2P9_11, STRICTOSIDINE SYNTHASE 2	11.2	10.4	0.8	4.4
2449	AT4G16500.1 | cysteine protease inhibitor family protein / cystatin family protein, similar to SP:Q06445 Cysteine proteinase inhibitor (Cystatin) {Vigna unguiculata}; contains Pfam profile PF00031: Cystatin domain | chr4:9301413-9302045 REVERSE | Aliases: DL4275C, FCAALL.171	11.2	10.0	1.2	6.8
7536	AT3G09200.1 | 60S acidic ribosomal protein P0 (RPP0B), similar to putative 60S acidic ribosomal protein P0 GB:P50346 (Glycine max) | chr3:2823096-2825096 REVERSE | Aliases: F3L24.7	11.2	9.9	1.3	3.3
4542	AT2G24765.1 | ADP-ribosylation factor 3 (ARF3), identical to GP:453191 ADP-ribosylation factor 3 {Arabidopsis thaliana}; contains domain PF00025: ADP-ribosylation factor family | chr2:10569805-10572274 FORWARD | Aliases: F27A10.8	11.2	10.2	0.9	4.9
1678	AT3G18740.1 | 60S ribosomal protein L30 (RPL30C), similar to 60S RIBOSOMAL PROTEIN L30 GB:O49884 from (Lupinus luteus)	11.2	9.9	1.2	7.9
2682	AT5G27700.1 | 40S ribosomal protein S21 (RPS21C), ribosomal protein S21, Zea mays, PIR:T03945 | chr5:9807323-9808480 REVERSE | Aliases: T1G16.30, T1G16_30	11.2	8.9	2.3	6.5
3589	AT2G34480.1 | 60S ribosomal protein L18A (RPL18aB) | chr2:14539787-14541280 REVERSE | Aliases: F13P17.34	11.2	9.4	1.7	5.6
2967	AT5G02450.1 | 60S ribosomal protein L36 (RPL36C), 60S ribosomal protein L36, Arabidopsis thaliana, EMBL:AC004684 | chr5:533119-534635 FORWARD | Aliases: T22P11.40, T22P11_40	11.2	9.9	1.3	6.2
690	AT3G53890.1 | 40S ribosomal protein S21 (RPS21B), ribosomal protein S21, cytosolic - Oryza sativa, PIR:S38357 | chr3:19966322-19967327 REVERSE | Aliases: F5K20.190	11.2	8.1	3.0	10.8
1394	AT4G09800.1 | Symbol: RPS18C |	11.2	10.0	1.1	8.5
229	AT1G55490.2 | Symbol: CPN60B |	11.1	5.9	5.3	15.7
350	AT4G02380.1 | Symbol: SAG21 | late embryogenesis abundant 3 family protein / LEA3 family protein, similar to several small proteins (~100 aa) that are induced by heat, auxin, ethylene and wounding such as Phaseolus aureus indole-3-acetic acid induced protein ARG (SW:32292); contains Pfam profile PF03242: Late embryogenesis abundant protein	11.1	12.7	-1.5	-13.5
154	AT4G03280.2 | Symbol: PETC | cytochrome B6-F complex iron-sulfur subunit, chloroplast / Rieske iron-sulfur protein / plastoquinol-plastocyanin reductase (petC), identical to gi:9843639; identical to cDNA rieske iron-sulfur protein precursor (petC) GI:5725449 | chr4:1440177-1441861 FORWARD | Aliases: F4C21.21, F4C21_21, PGR1, PHOTOSYNTHETIC ELECTRON TRANSFER C, PROTON GRADIENT REGULATION 1, RIESKE IRON SULFUR PROTEIN PRECURSOR	11.1	4.1	7.0	17.9
3314	AT3G06700.3 | similar to 60S ribosomal protein L29 (RPL29B) [Arabidopsis thaliana] (TAIR:At3g06680.1); similar to P0475H04.11 [Oryza sativa (japonica cultivar-group)] (GB:NP_914175.1); contains InterPro domain Ribosomal L29e protein (InterPro:IPR002673) | chr3:2117361-2117960 REVERSE | Aliases: T8E24.8	11.1	9.0	2.2	5.8
5636	AT4G23100.2 | Symbol: RML1 | similar to gamma-glutamylcysteine synthetase [Brassica juncea] (GB:CAA06613.1); similar to glutamate-cysteine ligase [Brassica juncea] (GB:CAD91714.1); similar to gamma-glutamylcysteine synthetase precursor [Pisum sativum] (GB:AAF22137.1); similar to gamma-glutamylcysteine synthetase [Lotus corniculatus var. japonicus] (GB:AAO27827.1); similar to glutamate-cysteine ligase [Brassica juncea] (GB:CAD91713.1); contains InterPro domain Glutamate--cysteine ligase, plant (InterPro:IPR006336) | chr4:12103172-12107310 REVERSE | Aliases: CAD2, CADMIUM SENSITIVE 2, F7H19.290, F7H19_290, GAMMA GLUTAMYL CYSTEINE SYNTHETASE, GAMMA GLUTAMYL CYSTEINE SYNTHETASE 1, GAMMA GLUTAMYLCYSTEINE SYNTHETASE, GSH1, ROOT MERISTEMLESS 1	11.1	8.4	2.7	4.2
13107	AT1G35720.1 | Symbol: ANNAT1 |	11.1	10.0	1.1	1.6

15917	AT5G03290.1 | isocitrate dehydrogenase, putative / NAD+ isocitrate dehydrogenase, putative, strong similarity to isocitrate dehydrogenase (NAD+) (Nicotiana tabacum) GI:3021506 | chr5:793984-795995 FORWARD | Aliases: F12E4.20, F12E4_20	11.1	10.7	0.4	1.0
7302	AT2G28190.1 | Symbol: CSD2 | superoxide dismutase (Cu-Zn), chloroplast (SODCP) / copper/zinc superoxide dismutase (CSD2), identical to GP:3273753:AF061519 | chr2:12021579-12023620 FORWARD | Aliases: COPPER/ZINC SUPEROXIDE DISMUTASE, COPPER/ZINC SUPEROXIDE DISMUTASE 2, CZSOD2, F24D13.2, F24D13_2	11.1	9.4	1.7	3.4
1640	AT5G01600.1 | Symbol: ATFER1 |	11.1	8.5	2.6	8.0
7735	AT3G07230.1 | wound-responsive protein-related, similar to wound-induced basic protein SP:Q09020 (Phaseolus vulgaris) (Plant Physiol. 101 (4), 1409 (1993)) | chr3:2299780-2300273 FORWARD | Aliases: T1B9.10	11.1	9.6	1.5	3.2
736	AT1G22780.1 | Symbol: PFL | 40S ribosomal protein S18 (RPS18A), Match to ribosomal S18 gene mRNA gb:Z28701, DNA gb:Z23165 from A. thaliana. ESTs gb:T21121, gb:Z17755, gb:R64776 and gb:R30430 come from this gene | chr1:8067853-8069319 FORWARD | Aliases: 40S RIBOSOMAL PROTEIN S18, PFL1, POINTED FIRST LEAVES, RPS18A, T22J18.5, T22J18_5	11.1	9.0	2.1	10.6
187	AT3G23940.1 | dehydratase family, contains Pfam profile: PF00920 dehydratase family | chr3:8648712-8652564 FORWARD | Aliases: F14O13.18	11.1	7.2	4.0	16.8
980	AT2G32060.3 | 40S ribosomal protein S12 (RPS12C) | chr2:13646122-13647257 REVERSE | Aliases: F22D22.19, F22D22_19	11.1	8.5	2.6	9.6
685	AT1G04480.1 | 60S ribosomal protein L23 (RPL23A), identical to GB:AAB80655 | chr1:1216094-1217457 FORWARD | Aliases: F19P19.5, F19P19_5	11.1	9.5	1.6	10.8
380	AT4G18280.1 | glycine-rich cell wall protein-related, glycine-rich protein 1.0 precursor, Phaseolus vulgaris, PIR1:S01821 | chr4:10104007-10104735 FORWARD | Aliases: T9A21.130, T9A21_130	11.1	7.0	4.1	13.1
7188	AT5G09810.1 | Symbol: ACT7 | actin 7 (ACT7) / actin 2, identical to SP:P53492 Actin 7 (Actin-2) {Arabidopsis thaliana} | chr5:3052167-3054615 FORWARD | Aliases: ACTIN_2, MYH9.2, MYH9_2	11.1	9.4	1.7	3.5
12996	AT3G04120.1 | Symbol: GAPC | glyceraldehyde-3-phosphate dehydrogenase, cytosolic (GAPC) / NAD-dependent glyceraldehyde-3-phosphate dehydrogenase, identical to SP:P25858 Glyceraldehyde 3-phosphate dehydrogenase, cytosolic (EC 1.2.1.12) {Arabidopsis thaliana} | chr3:1080960-1083537 FORWARD | Aliases: GLYCERALDEHYDE 3 PHOSPHATE DEHYDROGENASE C SUBUNIT, T6K12.26, T6K12_26	11.1	10.1	1.0	1.6
7013	AT2G17390.1 | ankyrin repeat family protein, contains ankyrin repeats, Pfam:PF00023 | chr2:7562732-7565120 FORWARD | Aliases: F5J6.15, F5J6_15	11.1	10.4	0.7	3.5
6486	AT4G11600.1 | Symbol: ATGPX6 |	11.1	9.8	1.3	3.8
422	AT3G23390.1 | 60S ribosomal protein L36a/L44 (RPL36aA), similar to ribosomal protein L41 GB:AAA34366 from (Candida maltosa) | chr3:8375382-8376397 FORWARD | Aliases: MLM24.22	11.1	9.1	2.0	12.6
2201	AT5G01210.1 | transferase family protein, contains Pfam profile PF02458 transferase family | chr5:84473-86274 FORWARD | Aliases: F7J8.190, F7J8_190	11.1	9.0	2.1	7.1
3022	AT4G27130.1 | eukaryotic translation initiation factor SUI1, putative, similar to SP:P32911 Protein translation factor SUI1 {Saccharomyces cerevisiae}; contains Pfam profile PF01253: Translation initiation factor SUI1 | chr4:13604601-13606454 REVERSE | Aliases: T24A18.80, T24A18_80	11.1	9.9	1.2	6.1
1962	AT5G43330.1 | malate dehydrogenase, cytosolic, putative, strong similarity to cytosolic malate dehydrogenase (EC 1.1.1.37) SP:O24047 {Mesembryanthemum crystallinum}, SP:O48905 {Medicago sativa}, (Prunus persica) GI:15982948; contains InterPro entry IPR001236: Lactate/malate dehydrogenase | chr5:17407661-17409867 FORWARD | Aliases: MWF20.2, MWF20_2	11.1	5.8	5.2	7.5
6601	AT5G37510.2 | Symbol: EMB1467 | NADH-ubiquinone dehydrogenase, mitochondrial, putative, similar to NADH-ubiquinone oxidoreductase 75 kDa subunit, mitochondrial from Solanum tuberosum (SP:Q43644) | chr5:14914640-14917877 FORWARD | Aliases: EMB1467, EMBRYO DEFECTIVE 1467, MPA22.5, MPA22_5	11.1	8.7	2.4	3.7
1443	AT1G14810.1 | semialdehyde dehydrogenase family protein, similar to SP:O31219 Aspartate-semialdehyde dehydrogenase (EC 1.2.1.11) (ASA dehydrogenase) (ASADH) {Legionella pneumophila}; contains Pfam profiles PF02774: Semialdehyde dehydrogenase dimerisation domain, PF01118: Semialdehyde dehydrogenase NAD binding domain | chr1:5102419-5104684 REVERSE | Aliases: F10B6.22, F10B6_22	11.1	7.5	3.5	8.4
4568	AT5G02500.1 | Symbol: HSC70 1 | heat shock cognate 70 kDa protein 1 (HSC70-1) (HSP70-1), identical to SP:P22953 Heat shock cognate 70 kDa protein 1 (Hsc70.1) {Arabidopsis thaliana} | chr5:553743-556437 REVERSE | Aliases: AT HSC70 1, HSC70, HSP70 1, T22P11.90, T22P11_90	11.1	10.2	0.9	4.8
1087	AT5G64140.1 | Symbol: RPS28 | 40S ribosomal protein S28 (RPS28C) | chr5:25684550-25685518 REVERSE | Aliases: MHJ24.12, MHJ24_12, RIBOSOMAL PROTEIN S28	11.1	9.0	2.1	9.3
11162	AT3G60300.1 | RWD domain-containing protein, contains weak similarity to RING finger protein 25 (RING finger protein AO7) (Swiss-Prot:Q9QZR0) (Mus musculus) | chr3:22296306-22298454 FORWARD | Aliases: F27H5.90	11.1	10.8	0.3	2.0

1623	AT5G55190.1 | Symbol: RAN3 | Ras-related GTP-binding protein (RAN3), identical to atran3 (Arabidopsis thaliana) GI:2058280 | chr5:22409402-22411392 FORWARD | Aliases: MCO15.14, MCO15_14	11.1	9.8	1.3	8.0
10684	AT3G05880.1 | Symbol: RCI2A | hydrophobic protein (RCI2A) / low temperature and salt responsive protein (LTI6A), identical to SP:Q9ZNQ7 Hydrophobic protein RCI2A (Low temperature and salt responsive protein LTI6A) {Arabidopsis thaliana} | chr3:1755503-1756546 REVERSE | Aliases: F10A16.18, F10A16_18, RARE COLD INDUCIBLE 2A	11.1	11.6	-0.5	-2.2
15389	AT3G23600.2 | similar to dienelactone hydrolase family protein [Arabidopsis thaliana] (TAIR:At3g23570.1); similar to 'putative endo-1,3;1,4-beta-D-glucanase' [Oryza sativa (japonica cultivar-group)] (GB:AAU10803.1); contains InterPro domain Dienelactone hydrolase (InterPro:IPR002925) | chr3:8473802-8475895 FORWARD | Aliases: MDB19.8	11.1	10.7	0.4	1.1
3660	AT5G01350.1 | expressed protein | chr5:145635-147199 REVERSE | Aliases: T10O8.60, T10O8_60	11.1	9.9	1.1	5.5
338	AT3G62840.1 | small nuclear ribonucleoprotein D2, putative / snRNP core protein D2, putative / Sm protein D2, putative, similar to small nuclear ribonucleoprotein Sm D2 (snRNP core protein D2) (Sm-D2) (Mus musculus) SWISS-PROT:P43330 | chr3:23246086-23247791 REVERSE | Aliases: F26K9.270	11.1	8.2	2.8	13.7
1655	AT3G51800.2 | Symbol: ATG2 | metallopeptidase M24 family protein, similar to SP:P50580 Proliferation-associated protein 2G4 {Mus musculus}; contains Pfam profile PF00557: metallopeptidase family M24 | chr3:19222004-19224646 REVERSE | Aliases: ATEM1.5	11.1	9.2	1.8	7.9
705	AT2G36160.1 | 40S ribosomal protein S14 (RPS14A) | chr2:15176897-15178428 FORWARD | Aliases: F9C22.9, F9C22_9	11.0	9.4	1.6	10.7
20602	AT3G23820.1 | Symbol: GAE6 | NAD-dependent epimerase/dehydratase family protein, similar to nucleotide sugar epimerase from Vibrio vulnificus GI:3093975 (PID:g3093975), WbnF (Escherichia coli) GI:5739472, CAPI protein {Staphylococcus aureus} SP:P39858; contains Pfam profile: PF01370 NAD dependent epimerase/dehydratase family | chr3:8603451-8605469 FORWARD | Aliases: F14O13.9, UDP D GLUCURONATE 4 EPIMERASE 6	11.0	11.1	-0.0	-0.1
13251	AT5G09220.1 | Symbol: AAP2 | amino acid permease 2 (AAP2), identical to amine acid permease AAP2 (Arabidopsis thaliana) GI:510236 | chr5:2866253-2869055 FORWARD | Aliases: AMINO ACID PERMEASE 2, T2K12.6	11.0	9.9	1.2	1.5
2648	AT4G35450.4 | Symbol: AKR2 | ankyrin repeat family protein / AFT protein (AFT), contains ankyrin repeats, Pfam:PF00023; identical to cDNA AFT protein (AFT) GI:3478699 | chr4:16839933-16842082 FORWARD | Aliases: AFT, ANKYRIN REPEAT CONTAINING PROTEIN 2, F15J1.20, F15J1_20	11.0	8.6	2.4	6.5
18	AT2G02120.1 | Symbol: LCR70/PDF2.1 | plant defensin-fusion protein, putative (PDF2.1), plant defensin protein family member, personal communication, Bart Thomma (Bart.Thomma@agr.kuleuven.ac.be); contains a gamma-thionin family signature (PDOC00725) | chr2:538254-539054 FORWARD | Aliases: F5O4.11, F5O4_11, LCR70, Low molecular weight cysteine rich 70, PDF2.1	11.0	3.3	7.7	33.0
7767	AT2G33470.2 | glycolipid transfer protein-related, similar to phosphoinositol 4-phosphate adaptor protein-2 (GI:14165198) (Homo sapiens); similar to Glycolipid transfer protein (GLTP) (Swiss-Prot:Q9JL62) (Mus musculus); similar to Glycolipid transfer protein (GLTP) (Swiss-Prot:Q9NZD2) (Homo sapiens) | chr2:14183492-14185543 REVERSE | Aliases: F4P9.24, F4P9_24	11.0	9.8	1.3	3.2
8586	AT1G78380.1 | Symbol: ATGSTU19 | glutathione S-transferase, putative, similar to glutathione transferase GI:2853219 from (Carica papaya) | chr1:29491306-29492799 REVERSE | Aliases: F3F9.11, F3F9_11, GLUTATHIONE TRANSFERASE 8, GST8	11.0	10.2	0.8	2.9
12366	AT2G44100.1 | Symbol: ATGDI1 |	11.0	10.2	0.8	1.7
16889	AT5G45130.1 | Symbol: RHA1 | Ras-related protein (RHA1) / small GTP-binding protein, identical to Ras-related protein RHA1 SP:P31582 from (Arabidopsis thaliana) | chr5:18261493-18263670 FORWARD | Aliases: K17O22.15, K17O22_15	11.0	11.2	-0.2	-0.8
19785	AT1G60710.1 | Symbol: ATB2 | aldo/keto reductase family protein, contains Pfam profile PF00248: oxidoreductase, aldo/keto reductase family | chr1:22358418-22360407 REVERSE | Aliases: F8A5.23, F8A5_23	11.0	11.1	-0.1	-0.2
2614	AT5G09590.1 | Symbol: mtHSC70 2 | heat shock protein 70 / HSP70 (HSC70-5), identical to heat shock protein 70 (Arabidopsis thaliana) GI:6746590 | chr5:2975576-2978751 FORWARD | Aliases: F17I14.220, F17I14_220, HEAT SHOCK PROTEIN 70, HSC70 5	11.0	8.4	2.7	6.6
8776	AT3G48680.1 | bacterial transferase hexapeptide repeat-containing protein, contains Pfam profile PF00132: Bacterial transferase hexapeptide (four repeats); ferripyochelin binding protein - Methanobacterium thermoautotrophicum, EMBL:AE000918.1 | chr3:18046092-18047789 FORWARD | Aliases: T8P19.190	11.0	9.9	1.2	2.8
1143	AT2G47710.1 | universal stress protein (USP) family protein, similar to ER6 protein (Lycopersicon esculentum) GI:5669654; contains Pfam profile PF00582: universal stress protein family | chr2:19561904-19563135 REVERSE | Aliases: F17A22.10	11.0	7.1	3.9	9.1
9446	AT5G54760.1 | eukaryotic translation initiation factor SUI1, putative, similar to SP:P32911 Protein translation factor SUI1 {Saccharomyces cerevisiae}; contains Pfam profile PF01253: Translation initiation factor SUI1 | chr5:22261112-22262961 FORWARD | Aliases: MBG8.2, MBG8_2	11.0	10.4	0.7	2.6

7339	AT4G39090.1 | Symbol: RD19 | cysteine proteinase RD19a (RD19A) / thiol protease, identical to cysteine proteinase RD19a, thiol protease SP:P43296, GI:435618 from (Arabidopsis thaliana) | chr4:18214569-18217476 REVERSE | Aliases: EMB3005, EMBRYO DEFECTIVE 3005, F19H22.190, F19H22_190, RD19A, RESPONSIVE TO DEHYDRATION 19	11.0	12.7	-1.7	-3.4
5984	AT4G31300.2 | Symbol: PBA1 | similar to 20S proteasome beta subunit E1 (PBE1) (PRCE) [Arabidopsis thaliana] (TAIR:At1g13060.1); similar to PREDICTED OJ1079_F11.26 gene product [Oryza sativa (japonica cultivar-group)] (GB:XP_507536.1); contains InterPro domain Proteasome B-type subunit (InterPro:IPR000243); contains InterPro domain Multispecific proteasome protease (InterPro:IPR001353) | chr4:15188769-15191159 FORWARD | Aliases: 20S PROTEASOME SUBUNIT PBA1, F8F16.120, F8F16_120	11.0	9.2	1.8	4.0
16165	AT2G05710.1 | aconitate hydratase, cytoplasmic, putative / citrate hydro-lyase/aconitase, putative, nearly identical to SP:P49608 Aconitate hydratase, cytoplasmic (EC 4.2.1.3) (Citrate hydro-lyase) (Aconitase) {Cucurbita maxima}; contains Pfam profiles PF00330: Aconitase family (aconitate hydratase), PF00694: Aconitase C-terminal domain | chr2:2141396-2146675 FORWARD | Aliases: T3P4.5, T3P4_5	11.0	10.7	0.3	0.9
4276	AT5G47435.2 | formyltetrahydrofolate deformylase, putative, similar to formyltetrahydrofolate deformylase (strain PCC 6803- Synechocystis sp.) SWISS-PROT:Q55135 | chr5:19258930-19261181 FORWARD | Aliases: None	11.0	9.9	1.1	5.0
3849	AT1G24360.1 | 3-oxoacyl-(acyl-carrier protein) reductase, chloroplast / 3-ketoacyl-acyl carrier protein reductase, identical to 3-oxoacyl-(acyl-carrier protein) reductase SP:P33207 from (Arabidopsis thaliana) | chr1:8640725-8643467 FORWARD | Aliases: F21J9.2, F21J9.34, F21J9_34	11.0	9.8	1.2	5.4
2699	AT1G16740.1 | ribosomal protein L20 family protein, similar to ribosomal protein L20 GI:3603025 from (Guillardia theta) | chr1:5727683-5729072 FORWARD | Aliases: F19K19.15	11.0	9.0	2.0	6.5
1282	AT4G34620.1 | Symbol: SSR16 | ribosomal protein S16 family protein, ribosomal protein S16, Neurospora crassa, PIR2:A29927 | chr4:16534867-16536176 REVERSE | Aliases: RIBOSOMAL PROTEIN S16, T4L20.200, T4L20_200	11.0	9.0	2.0	8.8
6854	AT3G46440.2 | Symbol: UXS5 | similar to NAD-dependent epimerase/dehydratase family protein [Arabidopsis thaliana] (TAIR:At2g28760.2); similar to NAD-dependent epimerase/dehydratase family protein [Arabidopsis thaliana] (TAIR:At2g28760.1); similar to UDP-glucuronic acid decarboxylase (UXS3) [Arabidopsis thaliana] (TAIR:At5g59290.1); similar to UDP-D-glucuronate carboxy-lyase [Pisum sativum] (GB:BAB40967.1); contains InterPro domain NAD-dependent epimerase/dehydratase (InterPro:IPR001509) | chr3:17100030-17102811 REVERSE | Aliases: F18L15.160	11.0	8.5	2.5	3.6
1112	AT1G34030.1 | 40S ribosomal protein S18 (RPS18B), similar to ribosomal protein S18 GI:38422 from (Homo sapiens) | chr1:12370055-12371530 REVERSE | Aliases: F12G12.15, F12G12_15	11.0	7.7	3.3	9.2
8932	AT4G15470.1 | expressed protein, low similarity to N-methyl-D-aspartate receptor-associated protein (Drosophila melanogaster) GI:567104; contains Pfam profile PF01027: Uncharacterized protein family UPF0005	11.0	9.8	1.2	2.7
7228	AT5G13450.2 | similar to F1-ATP synthase delta subunit [Ipomoea batatas] (GB:BAA77508.1); similar to oligomycin sensitivity conferring protein [Silene latifolia] (GB:AAN38066.1); contains InterPro domain H+-transporting two-sector ATPase, delta (OSCP) subunit (InterPro:IPR000711) | chr5:4310317-4312015 REVERSE | Aliases: T22N19.100, T22N19_100	11.0	9.5	1.5	3.4
2014	NA	11.0	9.8	1.2	7.4
3856	AT4G31990.3 | Symbol: ASP5 | similar to aspartate aminotransferase, cytoplasmic isozyme 1 / transaminase A (ASP2) [Arabidopsis thaliana] (TAIR:At5g19550.1); similar to Asp aminotransferase (GB:1908424A); similar to aspartate transaminase (EC 2.6.1.1) AAT5 precursor - soybean (GB:S33528); similar to aspartate aminotransferase [Lotus corniculatus] (GB:AAC12674.1); similar to aspartate aminotransferase [Phaseolus vulgaris] (GB:AAN76499.1); similar to aspartate aminotransferase isozyme 5 [Glycine max] (GB:AAB26677.2); contains InterPro domain Aminotransferase, class I and II (InterPro:IPR004839); contains InterPro domain Aminotransferases class-I pyridoxal-phosphate-binding site (InterPro:IPR004838); contains InterPro domain Aspartate/other aminotransferase (InterPro:IPR000796) | chr4:15470767-15473859 REVERSE | Aliases: AAT3, ASPARTATE AMINOTRANSFERASE, ASPARTATE AMINOTRANSFERASE 5, ASPARTATE AMINOTRANSFERASE DEFICIENT 3, F11C18.15	11.0	9.3	1.7	5.4
7306	AT2G27730.1 | expressed protein, contains 1 transmembrane domain; similar to Unknown mitochondrial protein At2g27730 (Swiss-Prot:Q9ZUX4) (Arabidopsis thaliana) similar to F1F0-ATPase inhibitor protein (GI:5821432) (Oryza sativa) | chr2:11826906-11828955 REVERSE | Aliases: F15K20.17, F15K20_17	11.0	10.2	0.8	3.4
10142	AT5G39950.1 | Symbol: ATTRX2 |	11.0	10.1	0.9	2.3
250	AT1G27435.1 | expressed protein | chr1:9527561-9528865 REVERSE | Aliases: None	11.0	8.3	2.7	15.3
816	AT3G03640.1 | Symbol: GLUC | glycosyl hydrolase family 1 protein, contains Pfam PF00232 : Glycosyl hydrolase family 1 domain; TIGRFAM TIGR01233: 6-phospho-beta-galactosidase; identical to beta-glucosidase GB:AAC31962 (Arabidopsis thaliana); similar to thioglucoside glucohydrolase (GI:984052) (Arabidopsis thaliana) | chr3:881031-884163 FORWARD | Aliases: BETA GLUCOSIDASE, T12J13.8, T12J13_8	11.0	6.7	4.3	10.2

531	AT2G21130.1 | peptidyl-prolyl cis-trans isomerase / cyclophilin (CYP2) / rotamase, identical to cyclophilin (Arabidopsis thaliana) gi:2443757:gb:AAB71402; similar to peptidyl-prolyl cis-trans isomerase, PPIase (cyclophilin, cyclosporin A-binding protein) (Arabidopsis thaliana) SWISS-PROT:P34790 | chr2:9062479-9063313 REVERSE | Aliases: F26H11.11, F26H11_11	11.0	5.0	6.0	11.7
47	AT1G64110.2 | AAA-type ATPase family protein, contains Pfam domain, PF00004: ATPase, AAA family | chr1:23800382-23805388 REVERSE | Aliases: F22C12.12, F22C12_12	11.0	3.3	7.7	26.6
4706	AT3G16770.1 | Symbol: ATEBP/RAP2.3 | Encodes a member of the ERF (ethylene response factor) subfamily B-2 of ERF/AP2 transcription factor family (RAP2.3). The protein contains one AP2 domain. There are 5 members in this subfamily including RAP2.2 AND RAP2.12. | chr3:5705721-5707029 FORWARD | Aliases: ATEBP, MGL6.1, RAP2.3, RELATED TO AP2 3	11.0	9.2	1.8	4.8
7269	AT5G05370.1 | ubiquinol-cytochrome C reductase complex ubiquinone-binding protein, putative / ubiquinol-cytochrome C reductase complex 8.2 kDa protein, putative, strong similarity to SP:P46269 Ubiquinol-cytochrome C reductase complex ubiquinone-binding protein QP-C (EC 1.10.2.2) (Ubiquinol-cytochrome C reductase complex 8.2 kDa protein) {Solanum tuberosum}	11.0	10.5	0.4	3.4
8917	AT5G11500.1 | expressed protein, contains Pfam profile PF05670: Domain of unknown function (DUF814) | chr5:3676641-3679347 FORWARD | Aliases: F15N18.90, F15N18_90	11.0	10.3	0.7	2.7
3793	AT5G42190.1 | Symbol: ASK2 | E3 ubiquitin ligase SCF complex subunit SKP1/ASK1 (At2) / UFO-binding protein (UIP2), E3 ubiquitin ligase; skp1b; identical to UIP2 GI:3719211 from (Arabidopsis thaliana); contains Pfam profiles PF01466: Skp1 family, dimerisation domain and PF03931:Skp1 family, tetramerisation domain; identical to cDNA UFO binding protein UIP2 mRNA, partial cds GI:3719210 | chr5:16870883-16872793 REVERSE | Aliases: ARABIDOPSIS SKP1 LIKE 2, MJC20.30, MJC20_30, SKP1B	11.0	8.6	2.4	5.4
3707	AT1G48440.1 | expressed protein | chr1:17910669-17912191 FORWARD | Aliases: T1N15.5, T1N15_5	11.0	9.8	1.2	5.5
6613	AT5G03300.1 | Symbol: ADK2 | adenosine kinase 2 (ADK2), contains Pfam profile: PF00294 pfkB family carbohydrate kinase; identical to cDNA adenosine kinase 2 (ADK2) GI:12017763 | chr5:796441-799153 FORWARD | Aliases: ADENOSINE KINASE 2, F12E4.30, F12E4_30	11.0	9.8	1.1	3.7
4467	AT4G30190.1 | Symbol: AHA2 | ATPase 2, plasma membrane-type, putative / proton pump 2, putative / proton-exporting ATPase, putative, strong similarity to SP:P19456 ATPase 2, plasma membrane-type (EC 3.6.3.6) (Proton pump 2) {Arabidopsis thaliana}; contains InterPro accession IPR001757: ATPase, E1-E2 type; contains Pfam profile PF00690: Cation transporter/ATPase, N-terminus | chr4:14770505-14776059 REVERSE | Aliases: F9N11.40, F9N11_40, P TYPE H(+) ATPASE ISOFORM 2, PMA2	10.9	12.2	-1.2	-4.9
4830	AT4G17390.1 | 60S ribosomal protein L15 (RPL15B) | chr4:9714225-9715624 REVERSE | Aliases: DL4730C, FCAALL.32	10.9	9.9	1.0	4.7
6610	AT1G15950.1 | Symbol: CCR1 | cinnamoyl-CoA reductase, putative, nearly identical to CCR1 (GI:12034897), similar to cinnamoyl CoA reductase GI:2058310 from (Eucalyptus gunnii) | chr1:5478748-5482159 FORWARD | Aliases: CINNAMOYL COA REDUCTASE, CINNAMOYL COA REDUCTASE 1, IRREGULAR XYLEM 4, IRX4, T24D18.5, T24D18_5	10.9	9.3	1.7	3.7
18957	AT1G13930.1 | expressed protein, weakly similar to drought-induced protein SDi-6 (PIR:S71562) common sunflower (fragment) | chr1:4761039-4761722 FORWARD | Aliases: F16A14.14	10.9	10.8	0.1	0.4
1102	AT1G10760.1 | Symbol: SEX1 | starch excess protein (SEX1), identical to SEX1 (Arabidopsis thaliana) GI:12044358; supporting cDNA gi:12044357:gb:AF312027.1:AF312027 | chr1:3581035-3590044 REVERSE | Aliases: GWD1, SEX1, SOP, SOP1, STARCH EXCESS 1 PROTEIN, STARCH EXCESS 1, T16B5.10, T16B5_10	10.9	8.8	2.2	9.2
3931	AT4G14800.1 | Symbol: PBD2 | 20S proteasome beta subunit D2 (PBD2) (PRCGA), identical to SP:O24633 Proteasome subunit beta type 2-2 (EC 3.4.25.1) (20S proteasome alpha subunit D2) {Arabidopsis thaliana}, cDNA proteasome subunit prcga GI:2511571 | chr4:8500283-8502224 FORWARD | Aliases: 20S PROTEASOME BETA SUBUNIT 2, 20S PROTEASOME BETA SUBUNIT PBD2, DL3440W, FCAALL.135	10.9	8.6	2.4	5.3
9420	AT1G53320.1 | F-box family protein / tubby family protein (TULP7), similar to Tubby related protein 2 (Tubby-like protein 2) (P4-6 protein) (Fragment) (SP:P46686) (Mus musculus); similar to phosphodiesterase (GI:467578) (Mus musculus); similar to Tubby protein homolog 1. (Swiss-Prot:Q09306) (Caenorhabditis elegans) contains Pfam profile: PF01167: Tub family; contains Pfam PF00646: F-box domain | chr1:19894682-19897159 REVERSE | Aliases: F12M16.22, F12M16_22	10.9	10.5	0.5	2.6
1931	AT5G27850.1 | 60S ribosomal protein L18 (RPL18C), 60S ribosomal protein L18, Arabidopsis thaliana, SWISSPROT:RL18_ARATH | chr5:9873160-9874602 FORWARD | Aliases: F14I23.10, F14I23_10	10.9	9.1	1.8	7.5
6256	AT1G74920.1 | Symbol: ALDH10A8 | betaine-aldehyde dehydrogenase, putative, identical to betaine-aldehyde dehydrogenase, chloroplast precursor (BADH) (Arabidopsis thaliana) SWISS-PROT:Q9S795; strong similarity to betaine aldehyde dehydrogenase (Amaranthus hypochondriacus) GI:2388710 | chr1:28142686-28146405 REVERSE | Aliases: F25A4.11, F25A4_11	10.9	9.5	1.4	3.9
4868	AT4G13520.1 | expressed protein | chr4:7864390-7864920 FORWARD | Aliases: T6G15.70, T6G15_70	10.9	9.8	1.1	4.6
610	AT5G04850.1 | SNF7 family protein, contains Pfam domain, PF03357: SNF7 family | chr5:1408063-1409893 REVERSE | Aliases: MUK11.17, MUK11_17	10.9	7.8	3.1	11.2

36	AT5G56640.1 | Symbol: MIOX5 | expressed protein, similar to myo-inositol oxygenase (Sus scrofa) gi:17432544:gb:AAL39076 | chr5:22944555-22946869 REVERSE | Aliases: MIK19.9, MIK19_9	10.9	2.1	8.8	27.6
2518	AT1G67250.1 | proteasome maturation factor UMP1 family protein, contains Pfam profile PF05348: Proteasome maturation factor UMP1 | chr1:25167305-25168715 REVERSE | Aliases: F1N21.7	10.9	8.8	2.1	6.7
8366	ATCG00550.1 | Symbol: PSBJ | PSII component | chrC:63538-63660 REVERSE | Aliases: PSBJ	10.9	10.3	0.6	3.0
12958	AT5G60360.2 | Symbol: AALP | similar to cysteine proteinase, putative [Arabidopsis thaliana] (TAIR:At3g45310.1); similar to cysteine protease [Nicotiana tabacum] (GB:BAA96501.1); contains InterPro domain Papain cysteine protease (C1) (InterPro:IPR000668); contains InterPro domain Eukaryotic thiol (cysteine) protease (InterPro:IPR000169) | chr5:24297123-24299623 FORWARD | Aliases: MUF9.4, MUF9_4	10.9	11.8	-0.8	-1.6
8524	AT1G27350.1 | expressed protein, contains 1 transmembrane domain; similar to ribosome associated membrane protein RAMP4 GI:4585827 (Rattus norvegicus); similar to ESTs gb:T20610 and gb:AA586199 | chr1:9498138-9499412 REVERSE | Aliases: F17L21.31	10.9	10.4	0.6	2.9
8523	AT1G27330.1 | expressed protein, similar to EST gb:AA650671 and gb:T20610 | chr1:9492965-9494051 FORWARD | Aliases: F17L21.12, F17L21_12	10.9	10.4	0.6	2.9
106	AT2G28900.1 | mitochondrial import inner membrane translocase subunit Tim17/Tim22/Tim23 family protein, contains Pfam PF02466: Mitochondrial import inner membrane translocase subunit Tim17 | chr2:12421191-12422655 REVERSE | Aliases: F8N16.19, F8N16_19	10.9	5.5	5.4	20.4
4297	AT3G56310.2 | alpha-galactosidase, putative / melibiase, putative / alpha-D-galactoside galactohydrolase, putative, similar to alpha-galactosidase SP:Q42656 from (Coffea arabica) | chr3:20893806-20897302 FORWARD | Aliases: F18O21.270	10.9	11.9	-1.0	-5.0
7380	AT3G15660.2 | similar to thioredoxin family protein [Arabidopsis thaliana] (TAIR:At4g04950.1); similar to glutaredoxin-related protein -like [Oryza sativa (japonica cultivar-group)] (GB:XP_550301.1); contains InterPro domain Glutaredoxin (InterPro:IPR002109); contains InterPro domain Glutaredoxin-related protein (InterPro:IPR004480)	10.9	9.0	1.9	3.4
4010	AT3G54110.1 | Symbol: ATPUMP1 | plant uncoupling mitochondrial protein (PUMP), identical to plant uncoupling mitochondrial protein (Arabidopsis thaliana) GI:3115108 | chr3:20049670-20052179 FORWARD | Aliases: F24B22.70, PUMP, UCP, UCP2, UNCOUPLING PROTEIN, UNCOUPLING PROTEIN PUMP2	10.9	8.6	2.3	5.3
856	AT4G33640.1 | expressed protein | chr4:16159551-16160306 REVERSE | Aliases: T16L1.130, T16L1_130	10.9	9.8	1.1	10.1
20294	AT4G19640.1 | Symbol: ARA7 | Ras-related GTP-binding protein, putative, similar to GTP-binding protein RAB5A GI:1370178 from (Lotus japonicus) | chr4:10687258-10689621 REVERSE | Aliases: F24J7.190, F24J7_190	10.9	10.8	0.1	0.2
9895	ATCG01020.1 | Symbol: RPL32 | encodes a chloroplast ribosomal protein L32, a constituent of the large subunit of the ribosomal complex | chrC:113449-113607 FORWARD | Aliases: RPL32	10.9	9.3	1.6	2.4
777	AT1G59900.1 | Symbol: AT E1 ALPHA | pyruvate dehydrogenase E1 component alpha subunit, mitochondrial (PDHE1-A), identical to SP:P52901 Pyruvate dehydrogenase E1 component alpha subunit, mitochondrial precursor (EC 1.2.4.1) (PDHE1-A) {Arabidopsis thaliana} | chr1:22054960-22057550 FORWARD | Aliases: PYRUVATE DEHYDROGENASE E1 ALPHA SUBUNIT	10.9	8.9	2.0	10.4
446	AT2G30000.1 | expressed protein, contains Pfam domain PF03660: Uncharacterised protein family (UPF0123) | chr2:12810932-12812054 REVERSE | Aliases: F23F1.8, F23F1_8	10.9	8.8	2.1	12.4
2474	AT1G80600.1 | acetylornithine aminotransferase, mitochondrial, putative / acetylornithine transaminase, putative / AOTA, putative / ACOAT, putative, similar to SP:O04866 Acetylornithine aminotransferase, mitochondrial precursor (EC 2.6.1.11) (ACOAT) (Acetylornithine transaminase) (AOTA) {Alnus glutinosa}; contains Pfam profile PF00202: aminotransferase, class III | chr1:30303410-30305445 REVERSE | Aliases: T21F11.7, T21F11_7	10.9	7.6	3.2	6.7
4818	AT1G26270.1 | phosphatidylinositol 3- and 4-kinase family protein, similar to phosphatidylinositol 4-kinase type-II beta (Homo sapiens) GI:20159767; contains Pfam profile PF00454: Phosphatidylinositol 3- and 4-kinase | chr1:9089511-9092444 REVERSE | Aliases: F28B23.7, F28B23_7	10.9	8.2	2.6	4.7
945	AT5G16450.2 | dimethylmenaquinone methyltransferase family protein, similar to bacterial S-adenosylmethionine:2-demethylmenaquinone methyltransferases; contains Pfam profile PF03737: Dimethylmenaquinone methyltransferase | chr5:5374062-5375462 FORWARD | Aliases: MQK4.18, MQK4_18	10.9	8.2	2.7	9.7
15023	AT3G57450.1 | expressed protein | chr3:21272911-21273599 FORWARD | Aliases: T8H10.50	10.9	10.5	0.3	1.2
6523	AT3G04400.1 | Symbol: EMB2171 | 60S ribosomal protein L23 (RPL23C), similar to ribosomal protein L17 GB:AAA34113.1 from (Nicotiana tabacum) | chr3:1167282-1168663 FORWARD | Aliases: EMB2171, EMBRYO DEFECTIVE 2171, T27C4.4, T27C4_4	10.9	10.1	0.8	3.8
243	AT2G43780.1 | expressed protein | chr2:18143521-18143724 REVERSE | Aliases: F18O19.11	10.9	8.6	2.2	15.5

1802	AT3G53580.1 | diaminopimelate epimerase family protein, contains Pfam profile PF01678: Diaminopimelate epimerase | chr3:19875742-19878112 FORWARD | Aliases: F4P12.280	10.9	9.5	1.3	7.7
17180	AT5G13420.1 | transaldolase, putative, similar to transaldolase (Solanum tuberosum) gi:2078350:gb:AAB54016 | chr5:4301795-4304315 REVERSE | Aliases: T22N19.70, T22N19_70	10.9	11.1	-0.3	-0.7
655	AT2G39460.1 | Symbol: ATRPL23A | 60S ribosomal protein L23A (RPL23aA), identical to GB:AF034694 | chr2:16482001-16483163 FORWARD | Aliases: F12L6.12, F12L6_12, RIBOSOMAL PROTEIN L23A, RPL23A	10.9	6.8	4.0	11.0
9013	ATCG00440.1 | Symbol: NDHC | Encodes NADH dehydrogenase D3 subunit of the chloroplast NAD(P)H dehydrogenase complex | chrC:50001-50363 REVERSE | Aliases: NDHC	10.9	9.9	1.0	2.7
12980	NA	10.9	11.2	-0.4	-1.6
195	AT5G51940.1 | DNA-directed RNA polymerase II, putative, similar to SP:O88828 DNA-directed RNA polymerase II 14.4 kDa polypeptide (EC 2.7.7.6) (RPB6) (RPB14.4) {Rattus norvegicus}; contains Pfam profile PF01192: RNA polymerases K / 14 to 18 kDa subunit | chr5:21121813-21123157 FORWARD | Aliases: MSG15.2, MSG15_2	10.8	9.2	1.7	16.7
950	AT3G48560.1 | Symbol: CSR1 | acetolactate synthase, chloroplast / acetohydroxy-acid synthase (ALS), nearly identical to SP:P17597 Acetolactate synthase, chloroplast precursor (EC 2.2.1.6, formerly EC 4.1.3.18) (Acetohydroxy-acid synthase) (ALS) {Arabidopsis thaliana} | chr3:18012297-18014566 REVERSE | Aliases: ACETOHYDROXY ACID SYNTHASE, ACETOLACTATE SYNTHASE, ACETOLACTATE SYNTHASE DEFICIENT, AHAS, ALS, CHLORSULFURON/IMIDAZOLINONE RESISTANT 1, IMIDAZOLE RESISTANT 1, IMR1, T8P19.70, TZP5	10.8	8.5	2.3	9.7
19624	AT4G39260.4 | Symbol: ATGRP8 |	10.8	11.0	-0.2	-0.3
10640	AT3G24170.2 | similar to gluthatione reductase, chloroplast [Arabidopsis thaliana] (TAIR:At3g54660.1); similar to glutathione reductase [Mesembryanthemum crystallinum] (GB:CAC13956.1); similar to glutathione-disulfide reductase (EC 1.8.1.7) - turnip (GB:T14394); similar to glutathione reductase [Pisum sativum] (GB:CAA66924.1); similar to Glutathione Reductase precursor [Spinacia oleracea] (GB:BAA07108.1); similar to glutathione reductase [Brassica oleracea] (GB:BAD14936.1); contains InterPro domain Pyridine nucleotide-disulphide oxidoreductase dimerisation domain (InterPro:IPR004099); contains InterPro domain Glutathione reductase, plant (InterPro:IPR006324); contains InterPro domain Mercuric reductase (InterPro:IPR000815); contains InterPro domain Pyridine nucleotide-disulphide oxidoreductase, class I (InterPro:IPR001100); contains InterPro domain FAD-dependent pyridine nucleotide-disulphide oxidoreductase (InterPro:IPR001327); contains InterPro domain Trypanothione reductase (InterPro:IPR001864); contains InterPro domain Pyridine nucleotide-disulphide oxidoreductase, class-II (InterPro:IPR000103) | chr3:8729517-8734548 REVERSE | Aliases: MUJ8.3, MUJ8_3	10.8	9.8	1.0	2.2
948	AT3G12390.1 | nascent polypeptide associated complex alpha chain protein, putative / alpha-NAC, putative, similar to alpha-NAC, non-muscle form (Mus musculus) GI:1666690; contains Pfam profiles PF01849: NAC domain, PF00627: UBA/TS-N domain | chr3:3942266-3943789 FORWARD | Aliases: T2E22.29	10.8	9.2	1.7	9.7
2725	AT1G56450.1 | Symbol: PBG1 | 20S proteasome beta subunit G1 (PBG1) (PRCH), identical to 20S proteasome beta subunit (PBG1) GI:3421123 (Arabidopsis thaliana); identical to cDNA proteasome subunit prch GI:2511597 | chr1:21145558-21148065 FORWARD | Aliases: F13N6.3, F13N6_3, PROTEASOME BETA SUBUNIT PBG1	10.8	8.0	2.8	6.4
7014	AT2G21290.1 | expressed protein | chr2:9119937-9120411 FORWARD | Aliases: F3K23.5, F3K23_5	10.8	10.1	0.7	3.5
11370	AT3G45980.1 | histone H2B, identical to histone H2B Arabidopsis thaliana GI:2407802; contains Pfam profile PF00125 Core histone H2A/H2B/H3/H4 | chr3:16907801-16908998 REVERSE | Aliases: F16L2.190	10.8	10.3	0.6	2.0
2655	AT2G21580.2 | similar to 40S ribosomal protein S25 (RPS25E) [Arabidopsis thaliana] (TAIR:At4g39200.1); similar to PREDICTED P0562A06.14 gene product [Oryza sativa (japonica cultivar-group)] (GB:XP_507607.1); contains InterPro domain S25 ribosomal protein (InterPro:IPR004977) | chr2:9243633-9244891 FORWARD | Aliases: F2G1.15, F2G1_15	10.8	8.3	2.6	6.5
18881	AT1G32460.1 | expressed protein | chr1:11738190-11739306 FORWARD | Aliases: F5D14.24, F5D14_24	10.8	10.9	-0.1	-0.4
6216	AT3G62790.1 | NADH-ubiquinone oxidoreductase-related, contains weak similarity to NADH-ubiquinone oxidoreductase 15 kDa subunit (EC 1.6.5.3) (EC 1.6.99.3) (Complex I-15 kDa) (CI-15 kDa) (Swiss-Prot:O43920) (Homo sapiens) | chr3:23234059-23235283 REVERSE | Aliases: F26K9.220	10.8	10.3	0.5	3.9
10206	AT1G76180.1 | Symbol: ERD14 | dehydrin (ERD14), identical to SP:P42763 Dehydrin ERD14 {Arabidopsis thaliana} | chr1:28591667-28592771 REVERSE | Aliases: EARLY RESPONSE TO DEHYDRATION 14, ERD14, T23E18.12, T23E18_12	10.8	11.7	-0.9	-2.3
6765	AT1G01090.1 | Symbol: PDH E1 ALPHA | pyruvate dehydrogenase E1 component alpha subunit, chloroplast, identical to pyruvate dehydrogenase E1 alpha subunit GB:AAB86803 GI:2454182 from (Arabidopsis thaliana); identical to cDNA pyruvate dehydrogenase E1 alpha subunit mRNA, nuclear gene encoding plastid protein GI:2454181 | chr1:47485-49279 REVERSE | Aliases: PYRUVATE DEHYDROGENASE E1 ALPHA, T25K16.8, T25K16_8	10.8	9.5	1.4	3.6

7982	AT2G14170.1 | Symbol: ALDH6B2 | methylmalonate-semialdehyde dehydrogenase, putative, similar to methylmalonate-semialdehyde dehydrogenase (acylating), mitochondrial precursor (MMSDH) (Rattus norvegicus) SWISS-PROT:Q02253 | chr2:5984438-5988981 REVERSE | Aliases: T22C12.10, T22C12_10	10.8	10.4	0.4	3.1
8521	AT5G66120.2 | 3-dehydroquinate synthase, putative, similar to aroB (Neisseria gonorrhoeae)(GI:2661441); contains 3-dehydroquinate synthase domain PF01761 | chr5:26448602-26451004 REVERSE | Aliases: K2A18.20, K2A18_20	10.8	9.3	1.5	2.9
1109	AT2G39390.1 | 60S ribosomal protein L35 (RPL35B) | chr2:16457738-16458956 REVERSE | Aliases: F12L6.5, F12L6_5	10.8	8.9	1.9	9.2
1694	AT4G19120.2 | early-responsive to dehydration stress protein (ERD3), identical to ERD3 protein (Arabidopsis thaliana) GI:15320410; contains Pfam profile PF03141: Putative methyltransferase; identical to cDNA ERD3 GI:15320409 | chr4:10460306-10463113 REVERSE | Aliases: T18B16.90, T18B16_90	10.8	9.0	1.8	7.9
4252	AT4G31985.1 | 60S ribosomal protein L39 (RPL39C) | chr4:15469901-15470565 FORWARD | Aliases: None	10.8	8.9	1.9	5.1
8308	AT5G52840.1 | NADH-ubiquinone oxidoreductase-related, contains weak similarity to NADH-ubiquinone oxidoreductase 13 kDa-B subunit (EC 1.6.5.3) (EC 1.6.99.3) (Complex I-13Kd-B) (CI-13Kd-B) (Complex I subunit B13) (Swiss-Prot:Q63362) (Rattus norvegicus) | chr5:21430880-21432232 FORWARD | Aliases: MXC20.6, MXC20_6	10.8	9.9	0.9	3.0
1187	AT2G40765.1 | expressed protein | chr2:17019276-17020403 FORWARD | Aliases: None	10.8	9.1	1.7	9.0
13642	AT2G24200.1 | cytosol aminopeptidase, identical to cytosol aminopeptidase SP:P30184 from (Arabidopsis thaliana); contains Pfam profiles: PF00883 cytosol aminopeptidase family catalytic domain, PF02789: cytosol aminopeptidase family N-terminal domain | chr2:10293889-10296635 REVERSE | Aliases: F27D4.11, F27D4_11	10.8	10.2	0.6	1.4
3912	AT1G48630.1 | guanine nucleotide-binding family protein / activated protein kinase C receptor, putative / RACK, putative, contains 7 WD-40 repeats (PF00400); very similar to guanine nucleotide-binding protein; activated protein kinase C receptor; RACK1 (GI:9294068) {Arabidopsis thaliana}; similar to WD-40 repeat auxin-dependent protein ARCA (SP:O24456) (Arabidopsis thaliana); | chr1:17985448-17986995 REVERSE | Aliases: F11I4.18, F11I4_18	10.8	8.9	1.9	5.3
4555	AT1G30630.1 | coatomer protein epsilon subunit family protein / COPE family protein, similar to SP:O14579 Coatomer epsilon subunit (Epsilon-coat protein) (Epsilon-COP) from Homo sapiens, SP:Q60445 from Cricetulus griseus; ESTs gb:Z17908, gb:AA728673, gb:N96555, gb:H76335, gb:AA712463, gb:W43247, gb:T45611, gb:T21160, gb:T14119 and AI100483 come from this gene | chr1:10858296-10860243 REVERSE | Aliases: T5I8.8, T5I8_8	10.8	8.8	2.0	4.8
1968	AT2G31200.1 | Symbol: ADF6 | actin-depolymerizing factor 6 (ADF6), identical to SP:Q9ZSK2 Actin-depolymerizing factor 6 (ADF-6) (AtADF6) {Arabidopsis thaliana} | chr2:13301130-13302487 FORWARD | Aliases: ACTIN DEPOLYMERIZING FACTOR 6, F16D14.4, F16D14_4	10.8	7.9	2.9	7.5
9483	AT2G01720.1 | ribophorin I family protein, similar to SP:P04843 Dolichyl-diphosphooligosaccharide--protein glycosyltransferase 67 kDa subunit precursor (EC 2.4.1.119) (Ribophorin I) {Homo sapiens}; contains Pfam profile PF04597: Ribophorin I | chr2:317032-320249 REVERSE | Aliases: T8O11.11, T8O11_11	10.8	10.2	0.6	2.5
3216	AT1G15120.1 | ubiquinol-cytochrome C reductase complex 7.8 kDa protein, putative / mitochondrial hinge protein, putative, similar to SP:P48504 Ubiquinol-cytochrome C reductase complex 7.8 kDa protein (EC 1.10.2.2) (Mitochondrial hinge protein) (CR7) {Solanum tuberosum}; contains Pfam profile PF02320: Ubiquinol-cytochrome C reductase hinge protein | chr1:5202849-5204188 FORWARD | Aliases: F9L1.5, F9L1_5	10.8	9.6	1.2	5.9
2798	AT5G38650.1 | proteasome maturation factor UMP1 family protein, contains Pfam profile PF05348: Proteasome maturation factor UMP1 | chr5:15488192-15490025 REVERSE | Aliases: MBB18.20, MBB18_20	10.8	8.7	2.1	6.4
1550	AT5G05730.1 | Symbol: ASA1 | anthranilate synthase, alpha subunit, component I-1 (ASA1), identical to SP:P32068 | chr5:1719574-1722904 REVERSE | Aliases: A METHYL TRYPTOPHAN RESISTANT 1, AMT1, ANTHRANILATE SYNTHASE ALPHA SUBUNIT, ANTHRANILATE SYNTHASE ALPHA SUBUNIT 1, MJJ3.14, MJJ3_14, TRP5, TRYPTOPHAN BIOSYNTHESIS 5, WEI2	10.8	9.5	1.2	8.1
12879	AT1G48900.1 | signal recognition particle 54 kDa protein 3 / SRP54 (SRP-54C), identical to SP:P49967 Signal recognition particle 54 kDa protein 3 (SRP54) {Arabidopsis thaliana} | chr1:18088450-18091547 REVERSE | Aliases: F27K7.8, F27K7_8	10.8	10.1	0.7	1.6
935	AT5G61170.1 | 40S ribosomal protein S19 (RPS19C), 40S ribsomal protein S19, Oryza sativa, SWISSPROT:RS19_ORYSA	10.8	8.1	2.7	9.7
4454	AT5G35360.2 | Symbol: CAC2 | similar to methylcrotonyl-CoA carboxylase alpha chain, mitochondrial / 3-methylcrotonyl-CoA carboxylase 1 (MCCA) [Arabidopsis thaliana] (TAIR:At1g03090.2); similar to biotin carboxylase precursor [Glycine max] (GB:AAC23573.1); similar to biotin carboxylase precursor [Glycine max] (GB:AAC02267.1); similar to acetyl-CoA carboxylase [Glycine max] (GB:AAF80469.1); similar to biotin carboxylase subunit (GB:AAC41659.1); contains InterPro domain Carbamoyl-phosphate synthetase large chain, N-terminal (InterPro:IPR005481); contains InterPro domain Acetyl-CoA carboxylase, biotin carboxylase (InterPro:IPR004549); contains InterPro domain Biotin carboxylase, C-terminal (InterPro:IPR005482); contains InterPro domain Carbamoyl-phosphate synthase L chain, ATP-binding (InterPro:IPR005479) | chr5:13601388-13606337 FORWARD | Aliases: ACETYL COA CARBOXYLASE, T26D22.8, T26D22_8	10.8	9.7	1.0	4.9
551	AT3G51030.1 | Symbol: ATTRX1 |	10.8	5.2	5.6	11.6

10467	AT1G61780.1 | postsynaptic protein-related, similar to postsynaptic protein CRIPT GI:3098551 from (Rattus norvegicus) | chr1:22816280-22817806 REVERSE | Aliases: T13M11.14, T13M11_14	10.8	10.4	0.4	2.2
7976	AT2G22670.2 | Symbol: IAA8 | auxin-responsive protein / indoleacetic acid-induced protein 8 (IAA8), identical to SP:Q38826 Auxin-responsive protein IAA8 (Indoleacetic acid-induced protein 8) {Arabidopsis thaliana}	10.8	9.9	0.8	3.1
5395	AT3G10090.1 | 40S ribosomal protein S28 (RPS28A), similar to ribosomal protein S28 GB:P34789 (Arabidopsis thaliana) | chr3:3108815-3110022 REVERSE | Aliases: T22K18.8	10.8	8.7	2.1	4.3
616	AT5G23250.1 | succinyl-CoA ligase (GDP-forming) alpha-chain, mitochondrial, putative / succinyl-CoA synthetase, alpha chain, putative / SCS-alpha, putative, similar to SP:P36967 Succinyl-CoA ligase (GDP-forming) alpha-chain, mitochondrial precursor (EC 6.2.1.4) (Succinyl-CoA synthetase, alpha chain) (SCS-alpha) {Dictyostelium discoideum}; contains Pfam profiles PF00549: CoA-ligase, PF02629: CoA binding domain | chr5:7830392-7832846 FORWARD | Aliases: MKD15.11, MKD15_11	10.8	7.0	3.8	11.2
1638	AT5G27760.1 | hypoxia-responsive family protein, contains Pfam profile: PF04588 hypoxia induced protein conserved region | chr5:9830368-9831787 FORWARD | Aliases: T1G16.90, T1G16_90	10.8	8.0	2.7	8.0
3415	AT2G13560.1 | malate oxidoreductase, putative, similar to NAD-dependent malic enzyme 62 kDa isoform, mitochondrial precursor (EC 1.1.1.39) (NAD-ME) (SP:P37221) {Solanum tuberosum} | chr2:5657046-5662301 FORWARD | Aliases: T10F5.10, T10F5_10	10.7	9.6	1.2	5.7
1948	AT5G10360.1 | Symbol: EMB3010 | 40S ribosomal protein S6 (RPS6B) | chr5:3258475-3260228 REVERSE | Aliases: EMB3010, EMBRYO DEFECTIVE 3010, F12B17.290, F12B17_290	10.7	9.2	1.5	7.5
6643	AT1G63770.2 | peptidase M1 family protein, similar to SP:P04825 Aminopeptidase N (EC 3.4.11.2) (Alpha-aminoacylpeptide hydrolase) {Escherichia coli}; contains Pfam profile PF01433: Peptidase family M1 | chr1:23661169-23667906 REVERSE | Aliases: F24D7.4, F24D7_4	10.7	9.5	1.3	3.7
6269	AT3G57150.1 | Symbol: NAP57 | dyskerin, putative / nucleolar protein NAP57, putative, similar to SP:P40615 Dyskerin (Nucleolar protein NAP57) {Rattus norvegicus}; contains Pfam profiles PF01509: TruB family pseudouridylate synthase (N terminal domain), PF01472: PUA domain; supporting cDNA gi:8901185:gb:AF234984.2:AF234984 | chr3:21164952-21166988 REVERSE | Aliases: ATNAP57, F24I3.230	10.7	9.4	1.4	3.9
2503	AT5G26710.1 | glutamate-tRNA ligase, putative / glutamyl-tRNA synthetase, putatuve / GluRS, putative, identical to gi:3435196 | chr5:9305600-9308774 FORWARD | Aliases: None	10.7	8.8	1.9	6.7
1403	AT5G64460.5 | similar to phosphoglycerate/bisphosphoglycerate mutase family protein [Arabidopsis thaliana] (TAIR:At2g17280.1); similar to phosphoglycerate mutase-like protein [Glycine max] (GB:AAG38145.1) | chr5:25790075-25792761 REVERSE | Aliases: T12B11.5, T12B11_5	10.7	7.4	3.3	8.5
4692	AT5G19440.1 | similar to Eucalyptus gunnii alcohol dehydrogenase of unknown physiological function (GI:1143445), apple tree, PIR:T16995; NOT a cinnamyl-alcohol dehydrogenase | chr5:6556422-6558344 FORWARD | Aliases: F7K24.190, F7K24_190	10.7	8.4	2.3	4.8
12068	AT1G74030.1 | enolase, putative, similar to Swiss-Prot:P15007 enolase (EC 4.2.1.11) (2-phosphoglycerate dehydratase)(2-phospho-D- glycerate hydro-lyase) (Drosophila melanogaster) | chr1:27842845-27845592 REVERSE | Aliases: F2P9.10, F2P9_10	10.7	9.9	0.8	1.8
10502	AT5G53120.3 | Symbol: SPDS3 | spermidine synthase, putative / putrescine aminopropyltransferase, putative, similar to SP:O82147 Spermidine synthase (EC 2.5.1.16) (Putrescine aminopropyltransferase) (SPDSY) {Coffea arabica}; contains Pfam profile PF01564: Spermine/spermidine synthase | chr5:21552058-21555474 FORWARD | Aliases: MFH8.5, MFH8_5, SPERMIDINE SYNTHASE 3, SPMS	10.7	10.1	0.6	2.2
4354	AT3G02080.1 | 40S ribosomal protein S19 (RPS19A), similar to 40S ribosomal protein S19 GB:P40978 (Oryza sativa) | chr3:363918-365255 REVERSE | Aliases: F1C9.13, F1C9_13	10.7	9.2	1.5	5.0
9709	AT5G58070.1 | lipocalin, putative, similar to temperature stress-induced lipocalin (Triticum aestivum) GI:18650668 | chr5:23517287-23518435 REVERSE | Aliases: K21L19.9, K21L19_9	10.7	9.1	1.6	2.5
433	AT3G01740.1 | expressed protein | chr3:267836-268845 FORWARD | Aliases: F4P13.29	10.7	7.7	3.0	12.5
2311	AT2G27530.2 | 60S ribosomal protein L10A (RPL10aB) | chr2:11770332-11771821 REVERSE | Aliases: F10A12.22	10.7	8.3	2.4	7.0
6473	AT1G06640.2 | 2-oxoglutarate-dependent dioxygenase, putative, similar to 2A6 (GI:599622) and tomato ethylene synthesis regulatory protein E8 (SP:P10967); contains Pfam profile: PF00671 Iron/Ascorbate oxidoreductase family | chr1:2032344-2034013 FORWARD | Aliases: F12K11.27, F12K11_27	10.7	11.6	-0.9	-3.8
1674	AT1G03330.1 | small nuclear ribonucleoprotein D, putative / snRNP core SM-like protein, putative / U6 snRNA-associated Sm-like protein, putative, similar to SWISS-PROT:Q9Y333 U6 snRNA-associated Sm-like protein LSm2 (Small nuclear ribonuclear protein D homolog, G7b, SnRNP core SM-like protein SM-x5) (Homo sapiens) | chr1:817983-819563 REVERSE | Aliases: F15K9.7, F15K9_7	10.7	8.8	1.9	7.9

12408	AT5G11770.1 | NADH-ubiquinone oxidoreductase 20 kDa subunit, mitochondrial, identical to NADH-ubiquinone oxidoreductase 20 kDa subunit mitochondrial (precursor) SP:Q42577 from (Arabidopsis thaliana); contains Pfam profile: PF01058 NADH ubiquinone oxidoreductase, 20 Kd subunit | chr5:3790807-3793001 REVERSE | Aliases: T22P22.160, T22P22_160	10.7	9.5	1.2	1.7
4326	AT1G70730.1 | phosphoglucomutase, cytoplasmic, putative / glucose phosphomutase, putative, strong similarity to SP:P93804 Phosphoglucomutase, cytoplasmic 1 (EC 5.4.2.2) (Glucose phosphomutase 1) (PGM 1) {Zea mays}; contains InterPro accession IPR006352: Phosphoglucosamine mutase | chr1:26672527-26676418 REVERSE | Aliases: F5A18.9, F5A18_9	10.7	8.5	2.2	5.0
4113	AT4G29735.1 | expressed protein, contains Pfam domain PF05251: Uncharacterised protein family (UPF0197) | chr4:14562645-14564384 REVERSE | Aliases: None	10.7	8.9	1.8	5.2
11395	AT1G75950.1 | Symbol: SKP1 | E3 ubiquitin ligase SCF complex subunit SKP1/ASK1 (At1), E3 ubiquitin ligase; skp1a; identical to Skp1a GI:3068807, Skp1p GI:1432083 and UIP1 GI:3719209 from (Arabidopsis thaliana); contains Pfam profiles PF01466: Skp1 family, dimerisation domain and PF03931: Skp1 family, tetramerisation domain; | chr1:28520258-28521360 FORWARD | Aliases: ARABIDOPSIS SKP1 HOMOLOGUE, ASK1, ATSKP1, SKP1A, T4O12.17, T4O12_17, UFO INTERACTING PROTEIN 1, UIP1	10.7	9.9	0.8	2.0
5166	AT2G27020.1 | Symbol: PAG1 | 20S proteasome alpha subunit G (PAG1) (PRC8), identical to proteasome subunit alpha type 3 SP:O23715, GI:12644056 from (Arabidopsis thaliana); identical to cDNA proteasome subunit prc8 GI:2511591 | chr2:11535437-11538054 REVERSE | Aliases: 20S PROTEASOME SUBUNIT PAG1, T20P8.7, T20P8_7	10.7	7.8	2.9	4.5
9501	AT3G16640.1 | Symbol: TCTP | translationally controlled tumor family protein, similar to translationally controlled tumor protein GB:AAD10032 from (Hevea brasiliensis) | chr3:5669379-5670823 REVERSE | Aliases: MGL6.19, TRANSLATIONALLY CONTROLLED TUMOR PROTEIN	10.7	11.3	-0.6	-2.5
17748	AT1G18880.1 | proton-dependent oligopeptide transport (POT) family protein, contains Pfam profile: PF00854 POT family | chr1:6520744-6523359 FORWARD | Aliases: F6A14.2, F6A14_2	10.7	10.4	0.3	0.6
13667	AT3G06400.1 | DNA-dependent ATPase, putative, similar to DNA-dependent ATPase SNF2H (Mus musculus) GI:14028669; contains Pfam profiles PF00271: Helicase conserved C-terminal domain, PF00176: SNF2 family N-terminal domain, PF00249: Myb-like DNA-binding domain | chr3:1941007-1946930 FORWARD | Aliases: F24P17.13, F24P17_13	10.7	10.9	-0.3	-1.4
1082	AT5G61220.1 | complex 1 family protein / LVR family protein, contains Pfam PF05347: Complex 1 protein (LYR family) | chr5:24642802-24644149 REVERSE | Aliases: MAF19.16, MAF19_16	10.7	6.7	4.0	9.3
7955	AT5G11670.1 | malate oxidoreductase, putative, similar to NADP-dependent malic enzyme (EC 1.1.1.40) (NADP-ME) (SP:P12628) {Phaseolus vulgaris}	10.7	11.4	-0.7	-3.1
19130	AT4G38920.1 | vacuolar ATP synthase 16 kDa proteolipid subunit 3 / V-ATPase 16 kDa proteolipid subunit 3 (AVAP3) (AVA-P3), identical to SP:P59227 Vacuolar ATP synthase 16 kDa proteolipid subunit 1/3/5 (EC 3.6.3.14) (V-ATPase 16 kDa proteolipid subunit 1/3/5) {Arabidopsis thaliana}; contains Pfam profile PF00137: ATP synthase subunit C | chr4:18147205-18149261 FORWARD | Aliases: F19H22.20	10.7	10.8	-0.1	-0.4
7516	AT5G47890.1 | NADH-ubiquinone oxidoreductase B8 subunit, putative, similar to SP:O43678 NADH-ubiquinone oxidoreductase B8 subunit (EC 1.6.5.3) (EC 1.6.99.3) (Complex I-B8) (CI-B8) {Homo sapiens}; contains Pfam profile PF05047: Mitochondrial ribosomal protein L51 / S25 / CI-B8 domain | chr5:19405963-19407852 FORWARD | Aliases: MCA23.23, MCA23_23	10.7	10.0	0.6	3.3
9714	AT3G59920.1 | Symbol: ATGDI2 |	10.7	9.6	1.0	2.5
4311	AT5G58710.1 | Symbol: ROC7 | peptidyl-prolyl cis-trans isomerase, putative / cyclophilin, putative / rotamase, putative (ROC7), similar to cyclophilin (Arabidopsis thaliana) gi:2443755:gb:AAB71401 | chr5:23735018-23736975 FORWARD | Aliases: CYCLOPHILIN, MZN1.23, MZN1_23	10.7	9.1	1.5	5.0
920	AT2G25210.1 | 60S ribosomal protein L39 (RPL39A) | chr2:10746934-10747631 FORWARD | Aliases: T22F11.20, T22F11_20	10.7	7.4	3.3	9.8
6925	AT1G72370.2 | Symbol: P40 | similar to 40S ribosomal protein SA (RPSaB) [Arabidopsis thaliana] (TAIR:At3g04770.2); similar to RSSA_DAUCA 40S ribosomal protein SA (p40) (GB:O80377); contains InterPro domain Ribosomal protein S2, eukaryotic and archaeal form (InterPro:IPR005707); contains InterPro domain Ribosomal protein S2 (InterPro:IPR001865) | chr1:27246607-27248589 REVERSE | Aliases: AP40, P40, RP40, RPSAA, T10D10.16, T10D10_16	10.7	8.8	1.9	3.6
11045	AT3G52880.1 | monodehydroascorbate reductase, putative, monodehydroascorbate reductase (NADH), Lycoperison esculentum, PIR:T06407 | chr3:19612190-19615431 REVERSE | Aliases: F8J2.50	10.7	9.6	1.0	2.1
3883	AT2G30970.1 | Symbol: ASP1 | aspartate aminotransferase, mitochondrial / transaminase A (ASP1), identical to SP:P46643 Aspartate aminotransferase, mitochondrial precursor (EC 2.6.1.1) (Transaminase A) {Arabidopsis thaliana} | chr2:13185926-13188984 FORWARD | Aliases: ASPARTATE AMINOTRANSFERASE 1, F7F1.18, F7F1_18	10.7	6.3	4.3	5.4
10505	AT4G30010.1 | expressed protein | chr4:14672877-14673225 FORWARD | Aliases: F6G3.40, F6G3_40	10.7	9.9	0.8	2.2
5939	AT2G22425.1 | expressed protein, weak similarity to Swiss-Prot:Q9Y6A9 microsomal signal peptidase 12 kDa subunit (SPase 12 kDa subunit, SPC12, HSPC033) (Homo sapiens)	10.7	10.2	0.5	4.1

535	AT1G13690.1 | Symbol: ATE1 | RNA recognition motif (RRM)-containing protein, contains Pfam profile: PF00076 RNA recognition motif | chr1:4693286-4694404 FORWARD | Aliases: F21F23.13, F21F23_13, GRPE HOMOLOG	10.7	7.5	3.2	11.7
2532	AT4G11010.1 | Symbol: NDPK3 | nucleoside diphosphate kinase 3, mitochondrial (NDK3), identical to Nucleoside diphosphate kinase III, mitochondrial precursor (NDK III) (NDP kinase III) (NDPK III) (SP:O49203) (Arabidopsis thaliana); contains Pfam PF00334 : Nucleoside diphosphate kinase domain;	10.7	8.5	2.2	6.6
4200	AT5G46020.1 | expressed protein | chr5:18680104-18682054 REVERSE | Aliases: MCL19.6, MCL19_6	10.6	9.4	1.2	5.1
1724	AT5G14800.1 | Symbol: P5CR | pyrroline-5-carboxylate reductase, identical to pyrroline-5-carboxylate reductase SP:P54904 from (Arabidopsis thaliana) | chr5:4785965-4787840 REVERSE | Aliases: AT P5C1, AT P5R, PYRROLINE 5 CARBOXYLATE (P5C) REDUCTASE, T9L3.100, T9L3_100	10.6	8.2	2.4	7.8
3188	AT5G52310.1 | Symbol: COR78 | low-temperature-responsive protein 78 (LTI78) / desiccation-responsive protein 29A (RD29A) | chr5:21258075-21260839 FORWARD | Aliases: COLD REGULATED 78, K24M7.4, K24M7_4, LTI140, LTI78, RD29A, RESPONSIVE TO DESSICATION 29A	10.6	12.7	-2.0	-5.9
8707	AT4G16240.1 | expressed protein | chr4:9192351-9192583 REVERSE | Aliases: DL4160C, FCAALL.320	10.6	9.8	0.9	2.8
12353	AT1G29150.1 | Symbol: ATS9 | 26S proteasome regulatory subunit, putative (RPN6), similar to 19S proteosome subunit 9 GB:AAC34120 GI:3450889 from (Arabidopsis thaliana) | chr1:10180505-10182689 FORWARD | Aliases: 19S PROTEOSOME SUBUNIT 9, F28N24.15, F28N24_15, RPN6	10.6	9.8	0.8	1.7
4531	AT1G65290.1 | acyl carrier family protein / ACP family protein, similar to SP:P53665 Acyl carrier protein, mitochondrial precursor (ACP) (NADH-ubiquinone oxidoreductase 9.6 kDa subunit) (MtACP-1) {Arabidopsis thaliana}; contains InterPro accession IPR003881: Isochorismatase | chr1:24252515-24254065 REVERSE | Aliases: T8F5.6, T8F5_6	10.6	9.1	1.5	4.9
6645	AT5G58290.1 | Symbol: RPT3 | 26S proteasome AAA-ATPase subunit (RPT3), identical to 26S proteasome AAA-ATPase subunit RPT3 GI:6652882 from (Arabidopsis thaliana) | chr5:23586304-23588556 FORWARD | Aliases: 26S PROTEASOME AAA ATPASE SUBUNIT RPT3, MCK7.16, MCK7_16	10.6	8.7	2.0	3.7
9126	AT5G58740.1 | nuclear movement family protein, contains Pfam profile: PF03593 nuclear movement protein	10.6	9.9	0.7	2.7
368	AT2G36620.1 | 60S ribosomal protein L24 (RPL24A) | chr2:15357486-15358944 REVERSE | Aliases: F13K3.2, F13K3_2	10.6	8.0	2.6	13.3
2117	AT1G33040.1 | nascent polypeptide-associated complex (NAC) domain-containing protein, similar to alpha-NAC, non-muscle form (Mus musculus) GI:1666690; contains Pfam profile PF01849: NAC domain | chr1:11966479-11967823 FORWARD | Aliases: F9L11.19, F9L11_19	10.6	7.1	3.5	7.2
20639	AT1G11650.2 | Symbol: ATRBP45B | RNA-binding protein 45 (RBP45), putative, similar to gb:U90212 DNA binding protein ACBF from Nicotiana tabacum and contains 3 PF:00076 RNA recognition motif domains. ESTs gb:T44278, gb:R65195, gb:N65904, gb:H37499, gb:R90487, gb:N95952, gb:T44278, gb:Z20166, gb:N96891, gb:W43137, gb:F15504, gb:F1 | chr1:3914774-3918163 FORWARD | Aliases: ATRBP45B, F25C20.21, F25C20_21	10.6	10.7	-0.0	-0.1
4968	AT5G54940.2 | eukaryotic translation initiation factor SUI1, putative, similar to SP:P32911 Protein translation factor SUI1 {Saccharomyces cerevisiae}; contains Pfam profile PF01253: Translation initiation factor SUI1 | chr5:22325497-22326633 REVERSE | Aliases: MBG8.21, MBG8_21	10.6	11.3	-0.7	-4.6
5347	AT2G27450.2 | Symbol: NLP1 | carbon-nitrogen hydrolase family protein, low similarity to beta-alanine synthase (Drosophila melanogaster) GI:14334063; contains Pfam profile PF00795: hydrolase, carbon-nitrogen family | chr2:11744510-11746676 REVERSE | Aliases: ATNLP1, CPA, F10A12.13, F10A12_13, N CARBAMOYLPUTRESCINE AMIDOHYDROLASE, NITRILASE LIKE PROTEIN 1	10.6	8.7	1.9	4.4
14866	AT1G48030.2 | dihydrolipoamide dehydrogenase 1, mitochondrial / lipoamide dehydrogenase 1 (MTLPD1), identical to GB:AAF34795 (gi:12704696) from (Arabidopsis thaliana)	10.6	10.1	0.5	1.2
508	AT2G30410.1 | Symbol: KIS | tubulin folding cofactor A (KIESEL), identical to cDNA tubulin folding cofactor A, GI:20514256, SP:O04350 Tubulin-specific chaperone A (Tubulin-folding cofactor A) (CFA) (TCP1-chaperonin cofactor A homolog) {Arabidopsis thaliana} | chr2:12966403-12967845 FORWARD | Aliases: KIESEL, T9D9.22, T9D9_22	10.6	8.4	2.2	11.9
5532	AT1G12230.1 | transaldolase, putative, similar to Swiss-Prot:P30148 transaldolase B (EC 2.2.1.2) (Escherichia coli O157:H7) | chr1:4148032-4151023 FORWARD | Aliases: T28K15.4, T28K15_4	10.6	8.3	2.3	4.3
9755	AT4G40030.1 | histone H3.2, identical to Histone H3.2, minor Lolium temulentum SP:P11105, nearly identical to histone H3.2 Mus pahari GI:515005; contains Pfam profile PF00125 Core histone H2A/H2B/H3/H4 | chr4:18555571-18556964 REVERSE | Aliases: T5J17.200, T5J17_200	10.6	9.8	0.8	2.4
12556	AT4G29040.1 | Symbol: RPT2A | 26S proteasome AAA-ATPase subunit (RPT2a), almost identical to 26S proteasome AAA-ATPase subunit RPT2a (GI:6652880) {Arabidopsis thaliana}; Drosophila melanogaster 26S proteasome subunit 4 ATPase, PID:g1066065 | chr4:14312309-14314568 FORWARD | Aliases: 26S PROTEASOME AAA ATPASE SUBUNIT RPT2A, F19B15.70, F19B15_70	10.6	10.1	0.5	1.7

2763 AT5G59910.1 | histone H2B, nearly identical to histone H2B Arabidopsis thaliana GI:2407802; contains Pfam profile PF00125 Core histone H2A/H2B/H3/H4 | chr5:24144354-24145161 FORWARD | Aliases: MMN10.15, MMN10_15	10.6	9.3	1.3	6.4
1041 AT4G14615.1 | expressed protein | chr4:8383765-8385074 FORWARD | Aliases: None	10.6	8.7	1.9	9.4
4236 AT4G34870.1 | Symbol: ROC5 | peptidyl-prolyl cis-trans isomerase / cyclophilin (CYP1) / rotamase, identical to cyclophilin (CYP1) gi:992643:gb:AAA75512; similar to peptidyl-prolyl cis-trans isomerase, PPIase (cyclophilin, cyclosporin A-binding protein) (Catharanthus roseus) SWISS-PROT:Q39613 | chr4:16614332-16615318 FORWARD | Aliases: ATCYP1, CYCLOPHILIN, F11I11.110, F11I11_110, ROTAMASE CYP	10.6	7.9	2.7	5.1
14615 AT1G11580.1 | pectin methylesterase, putative, similar to pectin methylesterase GI:1617583 from (Lycopersicon esculentum) | chr1:3888690-3890811 FORWARD | Aliases: ATPMEPCRA, T23J18.24, T23J18_24	10.6	9.7	0.9	1.2
13821 AT2G31570.1 | Symbol: ATGPX2 |	10.6	11.1	-0.5	-1.4
14271 AT4G16330.1 | oxidoreductase, 2OG-Fe(II) oxygenase family protein, similar to flavonone-3-hydroxylase (naringenin,2-oxoglutarate 3-dioxygenase) from Malus domestica (SP:Q06942), Pyrus communis (GI:20269881); contains Pfam domain PF03171, 2OG-Fe(II) oxygenase superfamily	10.6	10.3	0.3	1.3
8768 AT2G30490.1 | Symbol: ATC4H | trans-cinnamate 4-monooxygenase / cinnamic acid 4-hydroxylase (C4H) (CA4H) / cytochrome P450 73 (CYP73) (CYP73A5), identical to SP:P92994: Trans-cinnamate 4-monooxygenase (EC 1.14.13.11) (Cinnamic acid 4-hydroxylase) (CA4H) (C4H) (P450C4H) (Cytochrome P450 73). {Arabidopsis thaliana}; molecular marker C4H (GB:U71080) | chr2:13000740-13002847 REVERSE | Aliases: C4H, CINNAMATE 4 HYDROXYLASE, CINNAMATE 4 HYDROXYLASE, CYP73A5, T6B20.16, T6B20_16	10.6	11.4	-0.9	-2.8
3376 AT4G02230.1 | 60S ribosomal protein L19 (RPL19C), similar to L19 from several species | chr4:979229-980667 REVERSE | Aliases: T2H3.3, T2H3_3	10.6	8.3	2.3	5.7
7373 AT5G12470.1 | expressed protein | chr5:4044625-4047439 REVERSE | Aliases: None	10.6	9.6	1.0	3.4
15598 AT3G52220.1 | expressed protein | chr3:19380340-19382033 REVERSE | Aliases: T25B15.5	10.6	10.3	0.3	1.0
4055 AT2G20860.1 | Symbol: LIP1 | lipoic acid synthase (LIP1), identical to gi:3928758 contains Pfam profile PF04055: radical SAM domain protein | chr2:8986660-8988286 FORWARD | Aliases: F5H14.17, F5H14_17, LIPOIC ACID SYNTHASE	10.6	8.3	2.3	5.2
497 AT3G09970.1 | calcineurin-like phosphoesterase family protein, contains Pfam profile: PF00149 Calcineurin-like phosphoesterase	10.6	8.4	2.2	12.0
571 AT3G52580.1 | 40S ribosomal protein S14 (RPS14C), ribosomal protein S14 -Zea mays,PIR2:A30097 | chr3:19514203-19515902 FORWARD | Aliases: F3C22.6	10.6	6.5	4.1	11.5
1503 AT2G43460.1 | 60S ribosomal protein L38 (RPL38A) | chr2:18053081-18054478 REVERSE | Aliases: T1O24.20	10.6	8.5	2.1	8.2
227 AT5G41010.1 | DNA-directed RNA polymerases I, II, and III 7 kDa subunit, putative, similar to SP:P53803 DNA-directed RNA polymerases I, II, and III 7.0 kDa polypeptide (EC 2.7.7.6) (ABC10-alpha) (RPB7.0) (RPB10alpha) {Homo sapiens}; contains Pfam profile PF03604: DNA directed RNA polymerase, 7 kDa subunit | chr5:16441278-16442180 FORWARD | Aliases: MEE6.8, MEE6_8	10.6	8.6	2.0	15.8
3019 AT3G09300.1 | oxysterol-binding family protein, similar to SP:P22059 Oxysterol-binding protein 1 {Homo sapiens}; contains Pfam profile PF01237: Oxysterol-binding protein | chr3:2857883-2860674 FORWARD | Aliases: F3L24.17	10.6	9.2	1.4	6.1
4315 AT5G66680.1 | Symbol: DGL1 | dolichyl-diphosphooligosaccharide-protein glycosyltransferase 48kDa subunit family protein, similar to SP:Q05052 Dolichyl-diphosphooligosaccharide--protein glycosyltransferase 48 kDa subunit precursor (EC 2.4.1.119) (Oligosaccharyl transferase 48 kDa subunit) {Canis familiaris}; contains Pfam profile PF03345: Dolichyl-diphosphooligosaccharide-protein glycosyltransferase 48kD subunit | chr5:26634770-26637893 REVERSE | Aliases: MSN2.7, MSN2_7	10.6	9.5	1.1	5.0
383 AT5G19370.1 | rhodanese-like domain-containing protein / PPIC-type PPIASE domain-containing protein, low similarity to MPT-synthase sulfurylase (Synechococcus sp. PCC 7942) GI:2950364; contains Pfam profiles PF00581: Rhodanese-like domain, PF00639: PPIC-type PPIASE domain; identical to cDNA peptidyl-prolyl cis-trans isomerase GI:2246379 | chr5:6524148-6526668 REVERSE | Aliases: F7K24.120, F7K24_120	10.6	7.8	2.7	13.0
11043 AT3G12290.1 | tetrahydrofolate dehydrogenase/cyclohydrolase, putative, similar to SP:P07245 C-1-tetrahydrofolate synthase, cytoplasmic (C1-THF synthase) (Includes: Methylenetetrahydrofolate dehydrogenase (EC 1.5.1.5); Methenyltetrahydrofolate cyclohydrolase (EC 3.5.4.9); Formyltetrahydrofolate synthetase (EC 6.3.4.3)) {Saccharomyces cerevisiae}; contains Pfam profiles PF02882: Tetrahydrofolate dehydrogenase/cyclohydrolase, NAD(P)-binding domain, PF00763: Tetrahydrofolate dehydrogenase/cyclohydrolase, catalytic domain | chr3:3919509-3921526 FORWARD | Aliases: F28J15.8	10.6	9.9	0.7	2.1
16318 AT4G20850.1 | Symbol: TPP2 | subtilase family protein, contains similarity to Tripeptidyl-peptidase II (EC 3.4.14.10) (TPP-II) (Tripeptidyl aminopeptidase) (Swiss-Prot:P29144) (Homo sapiens) | chr4:11160732-11169900 REVERSE | Aliases: T13K14.10, T13K14_10, TRIPEPTIDYL PEPTIDASE II	10.6	10.7	-0.2	-0.9

20806	AT4G39980.1 | Symbol: DHS1 | 2-dehydro-3-deoxyphosphoheptonate aldolase 1 / 3-deoxy-D-arabino-heptulosonate 7-phosphate synthase 1 / DAHP synthetase 1 (DHS1), nearly identical to SP:P29965 | chr4:18539575-18542087 FORWARD | Aliases: 3 DEOXY D ARABINO HEPTULOSONATE 7 PHOSPHATE SYNTHASE 1, 3 DEOXY D ARABINO HEPTULOSONATE Y PHOSPHATE SYNTHASE, T5J17.150, T5J17_150	10.6	10.6	-0.0	-0.1
6213	AT4G16450.1 | expressed protein | chr4:9280104-9280740 FORWARD | Aliases: DL4250W, FCAALL.388	10.6	9.8	0.7	3.9
3033	AT5G45010.1 | DSS1/SEM1 family protein, contains Pfam profile PF05160: DSS1/SEM1 family | chr5:18184471-18185601 REVERSE | Aliases: K21C13.20, K21C13_20	10.6	8.9	1.7	6.1
2187	AT1G01050.1 | inorganic pyrophosphatase, putative (soluble) / pyrophosphate phospho-hydrolase, putative / PPase, putative, strong similarity to SP:Q43187 Soluble inorganic pyrophosphatase (EC 3.6.1.1) (Pyrophosphate phospho- hydrolase) (PPase) {Solanum tuberosum}; contains Pfam profile PF00719: inorganic pyrophosphatase | chr1:31181-33148 REVERSE | Aliases: T25K16.5, T25K16_5	10.6	8.4	2.2	7.1
20647	AT4G34110.1 | Symbol: PAB2 | polyadenylate-binding protein 2 (PABP2), non-consensus TA donor splice site at exon 2, polyadenylate-binding protein - Triticum aestivum (common wheat),PIR:T06979 | chr4:16336392-16340102 FORWARD | Aliases: F28A23.130, F28A23_130, POLY(A) BINDING PROTEIN 2	10.5	10.5	0.0	0.1
5066	AT5G54900.1 | Symbol: ATRBP45A | RNA-binding protein 45 (RBP45), putative, contains similarity to polyadenylate-binding protein 5 | chr5:22312609-22315572 FORWARD | Aliases: ATRBP45A, MBG8.17, MBG8_17	10.5	9.2	1.4	4.5
2191	AT1G74470.1 | geranylgeranyl reductase, identical to geranylgeranyl reductase GB:Y14044 (Arabidopsis thaliana) (involvement: chlorophyll, the tocopherol and the phylloquinone pathways Eur J Biochem 1998 Jan 15;251(1-2):413-7) | chr1:27994826-27996667 FORWARD | Aliases: F1M20.15, F1M20_15	10.5	6.3	4.3	7.1
7734	AT4G10320.1 | isoleucyl-tRNA synthetase, putative / isoleucine--tRNA ligase, putative, similar to SP:P41252 Isoleucyl-tRNA synthetase, cytoplasmic (EC 6.1.1.5) (Isoleucine--tRNA ligase) (IleRS) (IRS) {Homo sapiens}; contains Pfam profile PF00133: tRNA synthetases class I (I, L, M and V) | chr4:6397326-6404505 REVERSE | Aliases: F24G24.120, F24G24_120	10.5	9.7	0.8	3.2
14977	AT4G22310.1 | expressed protein, contains Pfam domain, PF03650: Uncharacterized protein family (UPF0041) | chr4:11791357-11792848 FORWARD | Aliases: T10I14.140, T10I14_140	10.5	9.9	0.6	1.2
4161	AT3G57050.3 | Symbol: CBL | cystathionine beta-lyase, chloroplast / beta-cystathionase / cysteine lyase (CBL), identical to SP:P53780 Cystathionine beta-lyase, chloroplast precursor (EC 4.4.1.8) (CBL) (Beta-cystathionase) (Cysteine lyase) {Arabidopsis thaliana}	10.5	9.2	1.3	5.1
1485	AT2G35635.1 | Symbol: UBQ7 | ubiquitin family protein, contains INTERPRO:IPR000626 ubiquitin domain | chr2:14988073-14989195 FORWARD | Aliases: None	10.5	6.6	3.9	8.3
7692	AT5G43060.1 | cysteine proteinase, putative / thiol protease, putative, similar to cysteine proteinase RD21A precursor (thiol protease) GI:435619, SP:P43297 from (Arabidopsis thaliana) | chr5:17286772-17289388 REVERSE | Aliases: MMG4.7, MMG4_7	10.5	11.8	-1.3	-3.2
5308	AT3G58680.1 | ethylene-responsive transcriptional coactivator, putative, similar to ethylene-responsive transcriptional coactivator (Lycopersicon esculentum) gi:5669634:gb:AAD46402 | chr3:21718253-21719881 FORWARD | Aliases: T20N10.30, T20N10_30	10.5	8.9	1.6	4.4
7231	AT5G66760.1 | Symbol: SDH1 1 |	10.5	9.1	1.4	3.4
2992	AT3G55520.1 | immunophilin, putative / FKBP-type peptidyl-prolyl cis-trans isomerase, putative, POSSIBLE PEPTIDYL-PROLYL CIS-TRANS ISOMERASE) (EC 5.2.1.8) (PPIASE) (ROTAMASE) SP:P30416(Mouse);P59 PROTEIN (HSP BINDING IMMUNOPHILIN), rabbit, SWISSPROT:P27124:FKB4_RABBIT | chr3:20604691-20606324 FORWARD | Aliases: T22E16.180	10.5	8.6	2.0	6.1
1867	AT2G19680.1 | mitochondrial ATP synthase g subunit family protein, contains Pfam profile: PF04718 mitochondrial ATP synthase g subunit	10.5	7.0	3.5	7.6
7522	AT1G65820.1 | microsomal glutathione s-transferase, putative, similar to MGST3_HUMAN SP:O14880	10.5	11.4	-0.9	-3.3
6586	AT5G67590.1 | Symbol: FRO1 | NADH-ubiquinone oxidoreductase-related, contains weak similarity to NADH-ubiquinone oxidoreductase 21 kDa subunit, mitochondrial precursor (EC 1.6.5.3) (EC 1.6.99.3) (Complex I-21KD) (CI-21KD). (Swiss-Prot:P25711) (Neurospora crassa); contains Pfam PF04800: ETC complex I subunit conserved region	10.5	8.8	1.7	3.7
20584	AT3G52930.1 | fructose-bisphosphate aldolase, putative, similar to SP:O65735:ALF_CICAR Fructose-bisphosphate aldolase, cytoplasmic isozyme {Cicer arietinum}, cytosolic aldolase (Fragaria x ananassa) GI:10645188; contains Pfam profile PF00274 Fructose-bisphosphate aldolase class-I | chr3:19637726-19639920 REVERSE | Aliases: F8J2.100	10.5	10.5	0.1	0.1
2001	AT1G06680.1 | Symbol: PSBP | photosystem II oxygen-evolving complex 23 (OEC23), JBC 14:211-238 (2002); identical to 23 kDa polypeptide of oxygen-evolving comlex (OEC) GB:CAA66785 GI:1769905 (Arabidopsis thaliana) | chr1:2047878-2049417 FORWARD | Aliases: F12K11.3, F12K11_3, OE23, OEE2, OXYGEN EVOLVING ENHANCER PROTEIN 2	10.5	6.7	3.8	7.4

3476	AT3G07568.1 | expressed protein | chr3:2416249-2417796 REVERSE | Aliases: None	10.5	8.4	2.1	5.7
2494	AT3G09860.1 | expressed protein | chr3:3026125-3027213 FORWARD | Aliases: F8A24.9	10.5	7.5	3.0	6.7
10048	AT1G79660.1 | expressed protein | chr1:29980756-29981666 FORWARD | Aliases: F20B17.9, F20B17_9	10.5	9.7	0.8	2.3
17849	AT1G59960.1 | aldo/keto reductase, putative, similar to NADPH-dependent codeinone reductase GI:6478210 (Papaver somniferum), NAD(P)H dependent 6'-deoxychalcone synthase (Glycine max)(GI:18728) | chr1:22074961-22076812 REVERSE | Aliases: F23H11.27, F23H11_27	10.5	10.7	-0.2	-0.6
10155	AT4G11150.1 | Symbol: TUF | vacuolar ATP synthase subunit E / V-ATPase E subunit / vacuolar proton pump E subunit (VATE), identical to SP:Q39258 Vacuolar ATP synthase subunit E (EC 3.6.3.14) (V-ATPase E subunit) (Vacuolar proton pump E subunit) {Arabidopsis thaliana} | chr4:6799958-6801927 FORWARD | Aliases: EMB2448, EMBRYO DEFECTIVE 2448, T22B4.130, T22B4_130, TUFF, VACUOLAR H+ ATPASE SUBUNIT E ISOFORM 1, VHA E1	10.5	11.1	-0.6	-2.3
4517	AT3G59280.1 | Symbol: TXR1 | signaling molecule-related, contains similarity to mitochondria-associated granulocyte macrophage CSF signaling molecule, mitochondrial precursor (CGI-136) (Mus musculus) SWISS-PROT:Q9CQV1 | chr3:21919890-21921699 REVERSE | Aliases: F25L23.140, THAXTOMIN A RESISTANT 1	10.5	9.1	1.4	4.9
1063	AT3G47370.3 | similar to 40S ribosomal protein S20 (RPS20A) [Arabidopsis thaliana] (TAIR:At3g45030.1); similar to 40S ribosomal protein S20 (RPS20C) [Arabidopsis thaliana] (TAIR:At5g62300.1); similar to 40S subunit ribosomal protein [Oryza sativa (japonica cultivar-group)] (GB:XP_550614.1); contains InterPro domain Ribosomal protein S10, eukaryotic and archaeal form (InterPro:IPR005729); contains InterPro domain Ribosomal protein S10 (InterPro:IPR001848) | chr3:17464463-17465496 REVERSE | Aliases: T21L8.120	10.5	8.2	2.3	9.3
2319	AT1G62750.1 | elongation factor Tu family protein, similar to elongation factor G SP:P34811 (Glycine max (Soybean)) | chr1:23237099-23240112 REVERSE | Aliases: F23N19.11, F23N19_11	10.5	6.9	3.6	6.9
3454	AT3G62810.1 | complex 1 family protein / LVR family protein, contains Pfam PF05347: Complex 1 protein (LYR family) | chr3:23238683-23239341 FORWARD | Aliases: F26K9.240	10.5	7.4	3.1	5.7
12204	AT3G24830.1 | 60S ribosomal protein L13A (RPL13aB), similar to 60S RIBOSOMAL PROTEIN L13A GB:P35427 from (Rattus norvegicus) | chr3:9064570-9066089 FORWARD | Aliases: K7P8.13	10.5	9.9	0.6	1.8
137	AT2G26500.2 | cytochrome b6f complex subunit (petM), putative, nearly identical to cytochrome b6f complex subunit (GI:3090403) (Arabidopsis thaliana); alternative splice forms exist | chr2:11277353-11278031 FORWARD | Aliases: T9J22.17, T9J22_17	10.5	3.8	6.7	18.6
20117	NA	10.5	10.4	0.1	0.2
495	AT1G61520.2 | Symbol: LHCA3*1 | similar to chlorophyll A-B binding protein, putative (LHCA5) [Arabidopsis thaliana] (TAIR:At1g45474.1); similar to chlorophyll A-B binding protein, putative (LHCA5) [Arabidopsis thaliana] (TAIR:At1g45474.2); similar to probable chlorophyll a/b-binding protein type III precursor - garden pea chloroplast (GB:T06411); contains InterPro domain Chlorophyll A-B binding protein (InterPro:IPR001344) | chr1:22703738-22705048 FORWARD | Aliases: LHCA3*1, T25B24.12, T25B24_12	10.5	6.3	4.2	12.0
5583	AT4G32940.1 | Symbol: GAMMA VPE | vacuolar processing enzyme gamma / gamma-VPE, nearly identical to SP:Q39119 Vacuolar processing enzyme, gamma-isozyme precursor (EC 3.4.22.-) (Gamma-VPE) {Arabidopsis thaliana} | chr4:15900273-15903271 REVERSE | Aliases: F26P21.60, F26P21_60, GAMMAVPE	10.5	11.8	-1.3	-4.2
5748	AT3G12260.1 | complex 1 family protein / LVR family protein, contains Pfam PF05347: Complex 1 protein (LYR family) | chr3:3908947-3910403 REVERSE | Aliases: F28J15.12	10.5	8.2	2.3	4.2
9136	AT5G23540.1 | 26S proteasome regulatory subunit, putative, similar to 26S proteasome-associated pad1 homolog (Homo sapiens) GI:1923256, 26S proteasome, non-ATPase subunit (Mus musculus) GI:2505940; contains Pfam profile PF01398: Mov34/MPN/PAD-1 family | chr5:7937672-7939578 FORWARD | Aliases: MQM1.19, MQM1_19	10.5	8.7	1.7	2.7
4393	AT1G02160.1 | expressed protein | chr1:410785-411590 FORWARD | Aliases: T6A9.18	10.5	9.4	1.0	5.0
8790	AT4G02890.3 | Symbol: UBQ14 | polyubiquitin (UBQ14), identical to GI:166795; similar to N. sylvestris hexameric polyubiquitin, GenBank accession number M74101 | chr4:1278530-1280028 REVERSE | Aliases: T5J8.21, T5J8_21	10.5	9.7	0.7	2.8
3470	AT3G11630.1 | 2-cys peroxiredoxin, chloroplast (BAS1), identical to SP:Q96291 2-cys peroxiredoxin BAS1, chloroplast precursor {Arabidopsis thaliana}; contains Pfam profile: PF00578 AhpC/TSA (alkyl hydroperoxide reductase and thiol-specific antioxidant) family | chr3:3672126-3674081 FORWARD | Aliases: F24K9.28, F24K9_28, T19F11.3	10.5	7.1	3.3	5.7
8077	AT2G21870.2 | expressed protein | chr2:9327278-9329814 REVERSE | Aliases: F7D8.19, F7D8_19	10.5	8.9	1.6	3.1

2944	AT3G59760.3 | Symbol: OASC | cysteine synthase, mitochondrial, putative / O-acetylserine (thiol)-lyase, putative / O-acetylserine sulfhydrylase, putative, similar to SP:Q43725 Cysteine synthase, mitochondrial precursor (EC 4.2.99.8) (O- acetylserine sulfhydrylase) (O-acetylserine (Thiol)-lyase) {Arabidopsis thaliana} | chr3:22083451-22086393 REVERSE | Aliases: ATCS C, CSASE C, F24G16.30	10.5	7.6	2.9	6.2
11436	AT1G14400.2 | Symbol: UBC1 | ubiquitin-conjugating enzyme 1 (UBC1), E2; identical to gi:431259, SP:P25865 | chr1:4927021-4928528 REVERSE | Aliases: ATUBC1, F14L17.17, F14L17_17, UBIQUITIN CARRIER PROTEIN 1	10.5	10.0	0.5	2.0
8749	AT1G16810.2 | similar to CGI-144-like protein [Lycopersicon esculentum] (GB:CAC81814.1) | chr1:5755610-5756888 FORWARD | Aliases: F17F16.25	10.5	10.0	0.5	2.8
1879	AT4G21110.1 | G10 family protein, contains Pfam profile: PF01125 G10 protein | chr4:11267196-11268968 FORWARD | Aliases: F7J7.50, F7J7_50	10.5	8.9	1.5	7.6
9092	AT5G61210.1 | Symbol: SNAP33 |	10.5	9.3	1.2	2.7
7005	AT2G41840.1 | 40S ribosomal protein S2 (RPS2C)	10.5	9.2	1.2	3.5
18846	NA	10.5	10.2	0.2	0.4
15367	ATCG00220.1 | Symbol: PSBM | PSII low MW protein | chrC:28707-28811 REVERSE | Aliases: PSBM	10.5	9.8	0.6	1.1
8930	AT1G72020.1 | expressed protein | chr1:27112954-27114093 REVERSE | Aliases: F28P5.9, F28P5_9	10.5	9.2	1.2	2.7
9479	AT2G35810.1 | expressed protein | chr2:15056229-15057324 FORWARD | Aliases: F11F19.28, F11F19_28	10.5	9.1	1.4	2.5
10043	AT1G51200.1 | zinc finger (AN1-like) family protein, contains Pfam domains, PF01428: AN1-like Zinc finger and PF01754: A20-like zinc finger | chr1:18988102-18990236 FORWARD | Aliases: F11M15.7, F11M15_7	10.4	9.6	0.8	2.3
1258	AT1G60950.1 | Symbol: FED A | ferredoxin, chloroplast (PETF), identical to FERREDOXIN PRECURSOR GB:P16972 (SP:P16972) from (Arabidopsis thaliana) | chr1:22448185-22448826 FORWARD | Aliases: FERRODOXIN A	10.4	6.0	4.4	8.8
5837	AT2G41530.1 | esterase, putative, similar to SP:P10768 Esterase D (EC 3.1.1.1) {Homo sapiens}; contains Pfam profile: PF00756 putative esterase | chr2:17330573-17332689 REVERSE | Aliases: T32G6.5, T32G6_5	10.4	8.9	1.5	4.1
6500	AT2G26080.1 | glycine dehydrogenase (decarboxylating), putative / glycine decarboxylase, putative / glycine cleavage system P-protein, putative, strong similarity to SP:P26969 Glycine dehydrogenase (decarboxylating), mitochondrial precursor (EC 1.4.4.2) {Pisum sativum}; contains Pfam profile PF02347: Glycine cleavage system P-protein | chr2:11116098-11120906 REVERSE | Aliases: T19L18.11, T19L18_11	10.4	8.8	1.7	3.8
7978	AT5G43460.1 | lesion inducing protein-related, similar to ORF, able to induce HR-like lesions (Nicotiana tabacum) | chr5:17477112-17478979 FORWARD | Aliases: MWF20.18, MWF20_18	10.4	9.9	0.5	3.1
13258	AT1G68300.1 | universal stress protein (USP) family protein, similar to ER6 protein (Lycopersicon esculentum) GI:5669654; contains Pfam profile PF00582: universal stress protein family | chr1:25602007-25603007 REVERSE | Aliases: T22E19.7, T22E19_7	10.4	9.9	0.6	1.5
8238	ATCG00300.1 | Symbol: YCF9 | encodes PsbZ, which is a subunit of photosystem II. In Chlamydomonas, this protein has been shown to be essential in the interaction between PS II and the light harvesting complex II. | chrC:35751-35939 FORWARD | Aliases: YCF9	10.4	9.5	1.0	3.0
8919	AT4G05160.1 | Encodes a peroxisomal protein involved in the activation of fatty acids through esterification with CoA. At4g05160 preferentially activates fatty acids with medium chain length (C6:0 and C7:0) as well as even-numbered long-chain fatty acids (C14:0,	10.4	10.8	-0.4	-2.7
10237	ATCG00540.1 | Symbol: PETA | Encodes cytochrome f apoprotein; involved in photosynthetic electron transport chain; encoded by the chloroplast genome and is transcriptionally repressed by a nuclear gene HCF2. | chrC:61657-62619 FORWARD | Aliases: PETA	10.4	9.1	1.4	2.3
9084	AT5G41670.2 | 6-phosphogluconate dehydrogenase family protein, contains Pfam profiles: PF00393 6-phosphogluconate dehydrogenase C-terminal domain, PF03446 NAD binding domain of 6-phosphogluconate | chr5:16682558-16684399 REVERSE | Aliases: MBK23.20, MBK23_20	10.4	10.9	-0.5	-2.7
20751	AT1G24180.1 | Symbol: IAR4 | pyruvate dehydrogenase E1 component alpha subunit, mitochondrial, putative, similar to SP:P52901 Pyruvate dehydrogenase E1 component alpha subunit, mitochondrial precursor (EC 1.2.4.1) (PDHE1-A) {Arabidopsis thaliana}; contains Pfam profile PF00676: Dehydrogenase E1 component | chr1:8560519-8563484 REVERSE | Aliases: F3I6.11, F3I6_11	10.4	10.4	0.0	0.1
1746	AT1G73230.1 | nascent polypeptide-associated complex (NAC) domain-containing protein, similar to SP:P20290 Transcription factor BTF3 (RNA polymerase B transcription factor 3) {Homo sapiens}; contains Pfam profile PF01849: NAC domain | chr1:27543969-27545603 REVERSE | Aliases: T18K17.10, T18K17_10	10.4	7.5	2.9	7.8
18106	AT3G10410.1 | Symbol: SCPL49 |	10.4	10.1	0.3	0.6

2797	AT3G02560.2 | 40S ribosomal protein S7 (RPS7B), similar to ribosomal protein S7 GB:AAD26256 from (Secale cereale) | chr3:541807-543341 FORWARD | Aliases: F16B3.19, F16B3_19	10.4	8.4	2.1	6.4
10725	AT3G50000.1 | Symbol: CKA2 | casein kinase II alpha chain 2, identical to casein kinase II, alpha chain 2 (CK II) (Arabidopsis thaliana) SWISS-PROT:Q08466 | chr3:18545470-18547878 FORWARD | Aliases: ATCKA2, ATPK15D, CASEIN KINASE II, ALPHA CHAIN 2, F3A4.80	10.4	9.5	0.9	2.2
16185	AT1G56580.1 | expressed protein, contains Pfam profile PF04398: Protein of unknown function, DUF538 | chr1:21201924-21202621 REVERSE | Aliases: F25P12.97, F25P12_97	10.4	10.2	0.3	0.9
1648	AT1G17510.1 | expressed protein | chr1:6023497-6024302 REVERSE | Aliases: F1L3.22, F1L3_22	10.4	7.6	2.8	8.0
19917	AT2G23070.1 | casein kinase II alpha chain, putative, similar to casein kinase II, alpha chain (CK II) (Zea mays) SWISS-PROT:P28523; contains protein kinase domain, Pfam:PF00069 | chr2:9831052-9833978 REVERSE | Aliases: F21P24.13, F21P24_13	10.4	10.5	-0.1	-0.2
17214	AT1G37130.1 | Symbol: NIA2 | nitrate reductase 2 (NR2), identical to SP:P11035 Nitrate reductase 2 (formerly EC 1.6.6.1) (NR2) {Arabidopsis thaliana}	10.4	10.7	-0.3	-0.7
23	AT5G64870.1 | expressed protein | chr5:25946764-25948610 REVERSE | Aliases: MXK3.10, MXK3_10	10.4	2.9	7.5	30.0
4799	AT5G63000.1 | expressed protein | chr5:25297238-25298759 REVERSE | Aliases: MJH22.6, MJH22_6	10.4	9.0	1.4	4.7
2063	AT1G26880.1 | 60S ribosomal protein L34 (RPL34A), identical to GB:Q42351, location of EST 105E2T7, gb:T22624 | chr1:9315370-9316716 REVERSE | Aliases: T2P11.7, T2P11_7	10.4	7.9	2.5	7.3
15500	AT3G14595.1 | expressed protein | chr3:4908957-4910483 FORWARD | Aliases: None	10.4	10.2	0.2	1.1
1943	AT5G02270.1 | Symbol: ATNAP9 |	10.4	12.0	-1.6	-7.5
2919	AT3G46430.1 | expressed protein | chr3:17098672-17099482 FORWARD | Aliases: F18L15.150, F18L15_150	10.4	8.6	1.8	6.2
1886	AT5G58420.1 | 40S ribosomal protein S4 (RPS4D), ribosomal protein S4, Arabidopsis thaliana, PIR:T48480	10.4	8.5	1.9	7.6
4344	AT3G15590.1 | DNA-binding protein, putative, similar to DNA-binding protein (Triticum aestivum) GI:6958202; contains Pfam profile: PF01535 PPR repeat | chr3:5275408-5277994 REVERSE | Aliases: MQD17.5	10.4	9.6	0.8	5.0
2062	AT3G23990.1 | Symbol: HSP60 | chaperonin (CPN60) (HSP60), identical to SWISS-PROT:P29197- chaperonin CPN60, mitochondrial precursor (HSP60) (Arabidopsis thaliana) | chr3:8668935-8672585 FORWARD | Aliases: F14O13.1	10.4	6.9	3.5	7.3
9408	AT1G51980.1 | mitochondrial processing peptidase alpha subunit, putative, similar to mitochondrial processing peptidase alpha subunit, mitochondrial precursor, Alpha-MPP (Ubiquinol-cytochrome C reductase subunit II) (Potato) SWISS-PROT:P29677 | chr1:19327071-19330551 REVERSE | Aliases: F5F19.4, F5F19_4	10.4	8.5	1.9	2.6
5436	AT3G17090.2 | similar to protein phosphatase 2C family protein / PP2C family protein [Arabidopsis thaliana] (TAIR:At4g38520.2); similar to protein phosphatase 2C family protein / PP2C family protein [Arabidopsis thaliana] (TAIR:At4g38520.1); similar to protein phosphatase 2c-like protein [Thellungiella halophila] (GB:AAM19705.1); contains InterPro domain Protein phosphatase 2C-like (InterPro:IPR001932) | chr3:5826809-5829627 FORWARD | Aliases: K14A17.4	10.4	9.3	1.1	4.3
4864	AT1G49670.1 | ARP protein (REF), identical to ARP protein GB:CAA89858 GI:886434 from (Arabidopsis thaliana); contains Pfam profile PF00107: oxidoreductase, zinc-binding dehydrogenase family | chr1:18384930-18389775 REVERSE | Aliases: F14J22.10, F14J22_10	10.4	9.0	1.4	4.7
4570	AT1G73880.1 | UDP-glucoronosyl/UDP-glucosyl transferase family protein, contains Pfam profile: PF00201 UDP-glucoronosyl and UDP-glucosyl transferase | chr1:27788642-27790465 FORWARD | Aliases: F2P9.25, F2P9_25	10.4	7.4	3.0	4.8
1107	AT3G63410.1 | Symbol: APG1 | chloroplast inner envelope membrane protein, putative (APG1), similar to SP:P23525 37 kDa inner envelope membrane protein, chloroplast precursor (E37) {Spinacia oleracea}; contains Pfam profile PF01209: methlytransferase, UbiE/COQ5 family | chr3:23426190-23428093 REVERSE | Aliases: ALBINO OR PALE GREEN MUTANT 1, MAA21.40, VITAMIN E DEFECTIVE, VTE3	10.4	5.7	4.7	9.2
8416	AT2G02050.1 | NADH-ubiquinone oxidoreductase B18 subunit, putative, contains Pfam PF05676: NADH-ubiquinone oxidoreductase B18 subunit (NDUFB7); similar to NADH-ubiquinone oxidoreductase B18 subunit (EC 1.6.5.3) (EC 1.6.99.3) (Complex I-B18) (CI-B18) (Cell adhesion protein SQM1) (Swiss-Prot:P17568) (Homo sapiens); similar to NADH:ubiquinoe oxidoreductase NDUFB7 subunit (GI:9651635) (Homo sapiens)	10.4	9.1	1.2	2.9
798	AT3G08690.1 | ubiquitin-conjugating enzyme 11 (UBC11), E2; identical to gi:12643427, SP:P35134	10.4	6.9	3.4	10.3
2573	AT3G04840.1 | 40S ribosomal protein S3A (RPS3aA), similar to 40S ribosomal protein S3A (S phase specific protein GBIS289) GB:P49396 (Brassica rapa) | chr3:1329699-1331581 FORWARD | Aliases: T9J14.21, T9J14_21	10.4	8.2	2.2	6.6

164	AT1G48160.1 | signal recognition particle 19 kDa protein, putative / SRP19, putative, similar to signal recognition particle 19 kDa protein subunit SRP19 GI:624221 (Oryza sativa (japonica cultivar-group)); contains Pfam profile: PF01922 SRP19 protein | chr1:17790214-17792693 REVERSE | Aliases: F21D18.11, F21D18_11	10.4	6.7	3.7	17.4
18999	AT5G03345.1 | expressed protein | chr5:814122-815618 FORWARD | Aliases: None	10.4	10.4	-0.1	-0.4
4129	AT2G27860.1 | Symbol: AXS1 | expressed protein | chr2:11871470-11873975 REVERSE | Aliases: F15K20.4, F15K20_4, UDP D APIOSE/UDP D XYLOSE SYNTHASE 1	10.4	8.3	2.1	5.1
4128	AT1G08200.1 | Symbol: AXS2 | expressed protein | chr1:2573857-2576709 REVERSE | Aliases: T23G18.6, T23G18_6, UDP D APIOSE/UDP D XYLOSE SYNTHASE 2	10.4	8.3	2.1	5.1
10430	ATCG01040.1 | Symbol: YCF5 | hypothetical protein | chrC:114461-115447 FORWARD | Aliases: YCF5	10.4	9.0	1.4	2.2
7383	AT3G56460.1 | oxidoreductase, zinc-binding dehydrogenase family protein, low similarity to probable NADP-dependent oxidoreductase (zeta-crystallin homolog) P1 (SP:Q39172)(gi:886428) and P2 (SP:Q39173)(gi:886430), zeta-crystallin / quinone reductase (NADPH) - Mus musculus, PIR:A54932; contains Pfam profile PF00107: oxidoreductase, zinc-binding dehydrogenase family | chr3:20943674-20945466 REVERSE | Aliases: T5P19.110	10.4	9.4	1.0	3.4
11676	AT5G20000.1 | Symbol: RPT6A | 26S proteasome AAA-ATPase subunit, putative, almost identical to 26S proteasome AAA-ATPase subunit RPT6a GI:6652888 from (Arabidopsis thaliana); almost identical to a member of conserved Sug1 CAD family AtSUG1 GI:13537115 from (Arabidopsis thaliana) | chr5:6756635-6759751 FORWARD | Aliases: 26S PROTEASOME AAA ATPASE SUBUNIT, F28I16.150, F28I16_150	10.4	8.7	1.6	1.9
11675	AT5G19990.1 | Symbol: ATSUG1 |	10.4	8.7	1.6	1.9
3728	AT4G15000.1 | 60S ribosomal protein L27 (RPL27C) | chr4:8571829-8572485 FORWARD | Aliases: DL3545W, FCAALL.99	10.4	8.0	2.4	5.5
4937	AT3G46040.1 | 40S ribosomal protein S15A (RPS15aD), cytoplasmic ribosomal protein S15a, Arabidopsis thaliana, EMBL:ATAF1412 | chr3:16925554-16926744 FORWARD | Aliases: F12M12.10	10.4	9.2	1.1	4.6
493	AT1G80560.1 | 3-isopropylmalate dehydrogenase, chloroplast, putative, strong similarity to 3-ISOPROPYLMALATE DEHYDROGENASE PRECURSOR GB:P29102 SP:P29102 from (Brassica napus)	10.4	5.4	5.0	12.0
5628	AT5G16470.1 | zinc finger (C2H2 type) family protein, contains Pfam profile: PF00096 zinc finger, C2H2 type | chr5:5379419-5380043 FORWARD | Aliases: MQK4.20, MQK4_20	10.4	9.2	1.2	4.2
18477	AT4G02620.1 | vacuolar ATPase subunit F family protein, contains weak similarity to vacuolar ATP synthase subunit F (EC 3.6.3.14) (V-ATPase F subunit) (Vacuolar proton pump F subunit) (V-ATPase 14 kDa subunit) (Swiss-Prot:P50408) (Rattus norvegicus); contains Pfam PF01990: ATP synthase (F/14-kDa) subunit | chr4:1149248-1151346 REVERSE | Aliases: T10P11.25, T10P11_25	10.4	10.5	-0.1	-0.5
8983	AT2G01470.1 | Symbol: STL2P | St12p protein (ST12p) / SEC12p protein, putative, 99.8% identical to St12p protein (GI:166878) {Arabidopsis thaliana} | chr2:211969-214464 REVERSE | Aliases: ATSEC12, F2I9.9, F2I9_9, SEC12P LIKE PROTEIN	10.4	9.6	0.7	2.7
104	AT3G26650.1 | Symbol: GAPA | glyceraldehyde 3-phosphate dehydrogenase A, chloroplast (GAPA) / NADP-dependent glyceraldehydephosphate dehydrogenase subunit A, identical to SP:P25856 Glyceraldehyde 3-phosphate dehydrogenase A, chloroplast precursor (EC 1.2.1.13) (NADP-dependent glyceraldehydephosphate dehydrogenase subunit A) {Arabidopsis thaliana} | chr3:9796330-9798282 FORWARD | Aliases: GAPA 1, GLYCERALDEHYDE 3 PHOSPHATE DEHYDROGENASE A SUBUNIT, GLYCERALDEHYDE 3 PHOSPHATE DEHYDROGENASE A SUBUNIT, MLJ15.3	10.4	2.9	7.4	20.4
5259	AT1G45000.1 | 26S proteasome regulatory complex subunit p42D, putative, similar to 26S proteasome regulatory complex subunit p42D (Drosophila melanogaster) gi:6434958:gb:AAF08391 | chr1:17011584-17014326 FORWARD | Aliases: F27F5.8, F27F5_8	10.3	9.4	1.0	4.4
4181	AT4G34030.1 | Symbol: MCCB | methylcrotonyl-CoA carboxylase beta chain, mitochondrial / 3-methylcrotonyl-CoA carboxylase 2 (MCCB), identical to SP:Q9LDD8 Methylcrotonyl-CoA carboxylase beta chain, mitochondrial precursor (EC 6.4.1.4) (3-Methylcrotonyl-CoA carboxylase 2) (MCCase beta subunit) (3-methylcrotonyl-CoA:carbon dioxide ligase beta subunit) {Arabidopsis thaliana}; contains Pfam profile: PF01039 carboxyl transferase domain | chr4:16301198-16304031 FORWARD | Aliases: 3 METHYLCROTONYL COA CARBOXYLASE, F28A23.210, F28A23_210	10.3	9.5	0.9	5.1
5827	AT4G18040.1 | Symbol: EIF4E | eukaryotic translation initiation factor 4E 1 / eIF-4E1 / mRNA cap-binding protein 1 (EIF4E1), identical to SP:O23252 Eukaryotic translation initiation factor 4E (eIF-4E) (eIF4E) (mRNA cap-binding protein) (eIF-4F 25 kDa subunit) (eIF-4F P26 subunit) {Arabidopsis thaliana} | chr4:10016583-10018198 REVERSE | Aliases: AT.EIF4E1, CUCUMOVIRUS MULTIPLICATION 1, CUM1, EUKARYOTIC TRANSLATION INITATION FACTOR 4E, F15J5.10, F15J5_10, LSP1	10.3	8.1	2.2	4.1
1081	AT5G27770.1 | 60S ribosomal protein L22 (RPL22C), ribosomal protein L22 (cytosolic), Rattus norvegicus, PIR:S52084 | chr5:9835890-9837332 FORWARD | Aliases: T1G16.100, T1G16_100	10.3	8.0	2.3	9.3
14225	AT3G46000.1 | Symbol: ADF2 | actin-depolymerizing factor, putative (ADF2), strong similarity to SP:Q9ZSK3 Actin-depolymerizing factor 4 (ADF-4) (AtADF4) {Arabidopsis thaliana}; contains Pfam profile PF00241: Cofilin/tropomyosin-type actin-binding protein | chr3:16918466-16919980 REVERSE | Aliases: ACTIN DEPOLYMERIZING FACTOR 2, F16L2.210	10.3	9.9	0.4	1.3

4001	AT2G16060.1 | Symbol: AHB1 | non-symbiotic hemoglobin 1 (HB1) (GLB1), identical to SP:O24520 Non-symbiotic hemoglobin 1 (Hb1) (ARAth GLB1) {Arabidopsis thaliana} | chr2:6989703-6990713 REVERSE | Aliases: ARATH GLB1, F7H1.8, F7H1_8, GLB1, HEMOGLOBIN, NSHB1	10.3	9.1	1.3	5.3
10963	AT2G44060.2 | late embryogenesis abundant family protein / LEA family protein, similar to ethylene-responsive late embryogenesis-like protein (Lycopersicon esculentum) GI:1684830; contains Pfam profile PF03168: Late embryogenesis abundant protein	10.3	9.2	1.1	2.1
10937	ATCG00040.1 | Symbol: MATK | Encodes a maturase located in the trnK intron in the chloroplast genome.	10.3	8.9	1.5	2.1
5470	AT5G65760.1 | serine carboxypeptidase S28 family protein, similar to SP:P42785 Lysosomal Pro-X carboxypeptidase precursor (EC 3.4.16.2) (Prolylcarboxypeptidase) (PRCP) (Proline carboxypeptidase) {Homo sapiens}; contains Pfam profile PF05577: Serine carboxypeptidase S28	10.3	8.8	1.5	4.3
1035	AT4G00810.2 | 60S acidic ribosomal protein P1 (RPP1B), similar to acidic ribosomal protein p1 | chr4:345952-347192 REVERSE | Aliases: A_TM018A10.9, A_TM018A10_9, T18A10.8, T18A10_8	10.3	6.5	3.9	9.4
3115	AT5G24800.1 | Symbol: BZO2H2 |	10.3	8.7	1.7	6.0
1696	AT3G06610.1 | DNA-binding enhancer protein-related, similar to huntingtin interacting protein HYPK (GI:3329429) (Homo sapiens); identical to Egd2p (GI:172043) (Saccharomyces cerevisiae) similar to EGD2 protein (GAL4 DNA-binding enhancer protein 2) (Swiss-Prot:P38879) (Saccharomyces cerevisiae)	10.3	8.4	1.9	7.9
1842	AT3G14600.1 | 60S ribosomal protein L18A (RPL18aC), similar to GB:CAA08791 from (Podocoryne carnea) | chr3:4910704-4912180 FORWARD | Aliases: MIE1.10	10.3	7.7	2.6	7.6
13371	AT1G56190.1 | phosphoglycerate kinase, putative, similar to SP:P41758 Phosphoglycerate kinase, chloroplast precursor (EC 2.7.2.3) {Chlamydomonas reinhardtii}; contains Pfam profile PF00162: phosphoglycerate kinase | chr1:21032030-21034314 FORWARD | Aliases: F14G9.19, F14G9_19	10.3	9.8	0.5	1.5
3892	AT1G23310.2 | Symbol: GGT1 | similar to glutamate:glyoxylate aminotransferase 2 (GGT2) [Arabidopsis thaliana] (TAIR:At1g70580.1); similar to glutamate:glyoxylate aminotransferase 2 (GGT2) [Arabidopsis thaliana] (TAIR:At1g70580.2); similar to alanine aminotransferase [Oryza sativa (indica cultivar-group)] (GB:AAO84040.1); contains InterPro domain 1-aminocyclopropane-1-carboxylate synthase (InterPro:IPR001176) | chr1:8268393-8271922 REVERSE | Aliases: ALANINE 2 OXOGLUTARATE AMINOTRANSFERASE 1, AOAT1, F26F24.16, F26F24_16, GLUTAMATE:GLYOXYLATE AMINOTRANSFERASE	10.3	9.0	1.3	5.3
1622	AT3G22630.1 | Symbol: PBD1 | 20S proteasome beta subunit D (PBD1) (PRGB), identical to GB:CAA74026 from (Arabidopsis thaliana) (FEBS Lett. (1997) 416 (3), 281-285); identical to cDNA proteasome subunit prgb GI:2511589 | chr3:8009547-8010851 REVERSE | Aliases: F16J14.20, MULTICATALYTIC ENDOPEPTIDASE COMPLEX, PROTEASOME COMPONENT, BETA SUBUNIT, PRCGB, PROTEASOME SUBUNIT PRGB	10.3	7.2	3.1	8.0
2228	AT5G60540.1 | Symbol: EMB2407 | SNO glutamine amidotransferase family protein, similar to pyridoxine synthesis protein PDX2 (Cercospora nicotianae) GI:9954418; contains Pfam profile PF01174: SNO glutamine amidotransferase family | chr5:24353849-24356028 REVERSE | Aliases: EMB2407, EMBRYO DEFECTIVE 2407, MUF9.1, MUF9_1	10.3	6.7	3.6	7.1
2028	AT1G18500.1 | 2-isopropylmalate synthase, putative, strong similarity to 2-isopropylmalate synthase (IMS1) (Arabidopsis thaliana) GI:12330687; contains Pfam profile PF00682: HMGL-like | chr1:6369340-6373018 FORWARD | Aliases: F15H18.3, F15H18_3	10.3	7.5	2.8	7.3
1480	AT1G07700.3 | thioredoxin family protein, low similarity to thioredoxin (Gallus gallus) GI:212766; contains Pfam profile: PF00085 Thioredoxin | chr1:2379644-2381357 FORWARD | Aliases: F24B9.21, F24B9_21	10.3	7.2	3.1	8.3
2417	AT3G23570.1 | dienelactone hydrolase family protein, similar to SP:Q9ZT66 Endo-1,3;1,4-beta-D-glucanase precursor (EC 3.2.1.-) {Zea mays}; contains Pfam profile: PF01738 dienelactone hydrolase family | chr3:8457785-8459698 REVERSE | Aliases: MDB19.5	10.3	6.6	3.7	6.8
1170	AT4G10040.1 | cytochrome c, putative, similar to cytochrome c (Pumpkin, Winter squash) SWISS-PROT:P00051 | chr4:6276995-6278501 FORWARD | Aliases: F28M11.5	10.3	7.5	2.8	9.0
2629	AT4G22740.2 | glycine-rich protein | chr4:11942812-11945967 REVERSE | Aliases: T12H17.1	10.3	8.0	2.3	6.5
13842	AT2G46800.2 | Symbol: ZAT1 | zinc transporter (ZAT), identical to zinc transporter ZAT (Arabidopsis thaliana) gi:4206640:gb:AAD11757; member of the cation diffusion facilitator (CDF) family, or cation efflux (CE) family, PMID:11500563 | chr2:19244503-19246474 FORWARD | Aliases: ATMTP1, F19D11.8, MTP1, ZINC TRANSPORTER OF ARABIDOPSIS THALIANA, ZINC TRANSPORTER ZAT	10.3	10.6	-0.3	-1.4
2480	AT5G14590.1 | isocitrate dehydrogenase, putative / NADP+ isocitrate dehydrogenase, putative, strong similarity to isocitrate dehydrogenase (NADP+) (Nicotiana tabacum) GI:3021512; contains Pfam domain PF00180: dehydrogenase, isocitrate/isopropylmalate family | chr5:4703301-4706738 REVERSE | Aliases: T15N1.80, T15N1_80	10.3	8.1	2.2	6.7
16513	AT2G40890.1 | Symbol: CYP98A3 | cytochrome P450 98A3, putative (CYP98A3), identical to Cytochrome P450 98A3 (SP:O22203) (Arabidopsis thaliana); similar to gi:17978651 from Pinus taeda | chr2:17065131-17067730 REVERSE | Aliases: REF8, T20B5.9, T20B5_9	10.3	10.7	-0.4	-0.9

16121	AT1G53280.1 | DJ-1 family protein, similar to DJ-1 protein (Homo sapiens) GI:1780755; similar to DJ-1 beta (GI:18642508) (Drosophila melanogaster); contains Pfam profile: PF01965 ThiJ/PfpI family; TIGRFAM TIGR01383: DJ-1 family protein | chr1:19867968-19871057 REVERSE | Aliases: F12M16.18, F12M16_18	10.3	10.4	-0.2	-0.9
7509	AT5G19550.1 | Symbol: ASP2 | aspartate aminotransferase, cytoplasmic isozyme 1 / transaminase A (ASP2), identical to SP:P46645 Aspartate aminotransferase, cytoplasmic isozyme 1 (EC 2.6.1.1) (Transaminase A) {Arabidopsis thaliana}	10.3	8.4	1.9	3.3
3519	AT3G59540.1 | 60S ribosomal protein L38 (RPL38B), 60S RIBOSOMAL PROTEIN L38 - Lycopersicon esculentum, EMBL:X69979 | chr3:22006655-22007783 REVERSE | Aliases: T16L24.90	10.3	7.7	2.6	5.6
8613	AT5G53350.1 | Symbol: CLPX | ATP-dependent Clp protease ATP-binding subunit ClpX1 (CLPX), identical to CLP protease regulatory subunit CLPX GI:2674203 from (Arabidopsis thaliana) | chr5:21661124-21664933 FORWARD | Aliases: K19E1.15, K19E1_15	10.3	10.8	-0.5	-2.9
5187	AT3G60820.2 | Symbol: PBF1 | similar to 20S proteasome beta subunit C1 (PBC1) (PRCT) [Arabidopsis thaliana] (TAIR:At1g21720.1); similar to beta 6 subunit of 20S proteasome [Oryza sativa (japonica cultivar-group)] (GB:BAA28276.1); contains InterPro domain Proteasome B-type subunit (InterPro:IPR000243); contains InterPro domain Multispecific proteasome protease (InterPro:IPR001353) | chr3:22482730-22484904 REVERSE | Aliases: 20S PROTEASOME BETA SUBUNIT PBF1, T4C21.230	10.3	8.2	2.1	4.5
8953	ATCG00790.1 | Symbol: RPL16 | chloroplast gene encoding a ribosomal protein L16, which is a constituent of 50S large ribosomal subunit | chrC:81189-82652 REVERSE | Aliases: RIBOSOMAL PROTEIN L16, RPL16	10.3	8.1	2.1	2.7
3248	AT5G20180.2 | ribosomal protein L36 family protein, contains Pfam profile: PF00444 ribosomal protein L36 | chr5:6810966-6812148 FORWARD | Aliases: F5O24.70, F5O24_70	10.3	8.8	1.5	5.9
9531	AT4G34230.2 | Symbol: CAD5 | similar to cinnamyl-alcohol dehydrogenase (CAD) [Arabidopsis thaliana] (TAIR:At3g19450.1); similar to cinnamyl alcohol dehydrogenase [Aralia cordata] (GB:BAA03099.1); contains InterPro domain Zinc-containing alcohol dehydrogenase superfamily (InterPro:IPR002085); contains InterPro domain Zinc-containing alcohol dehydrogenase (InterPro:IPR002328) | chr4:16386723-16388723 REVERSE | Aliases: ATCAD5, CAD 5, CINNAMYL ALCOHOL DEHYDROGENASE 5, F10M10.11	10.3	9.1	1.2	2.5
4136	AT5G15320.1 | expressed protein, predicted protein, Arabidopsis thaliana | chr5:4977541-4978897 FORWARD | Aliases: F8M21.210, F8M21_210	10.3	9.3	1.0	5.1
289	AT2G41300.1 | strictosidine synthase family protein, similar to strictosidine synthase (Rauvolfia serpentina)(SP:P15324); contains strictosidine synthase domain PF03088; protein alignments support a CG non-consensus donor splice site. | chr2:17221315-17223370 REVERSE | Aliases: F13H10.15, F13H10_15	10.3	3.6	6.7	14.5
1141	AT5G67490.1 | expressed protein | chr5:26951379-26952178 REVERSE | Aliases: K9I9.5, K9I9_5	10.3	7.7	2.6	9.1
4114	AT2G45790.1 | eukaryotic phosphomannomutase family protein, contains Pfam profile: PF03332 eukaryotic phosphomannomutase | chr2:18862853-18865442 FORWARD | Aliases: F4I18.23	10.3	8.7	1.6	5.2
642	AT5G30510.1 | 30S ribosomal protein S1, putative, similar to Swiss-Prot:P29344 30S ribosomal protein S1, chloroplast precursor (CS1) (Spinacia oleracea) | chr5:11636256-11638528 REVERSE | Aliases: None	10.3	7.9	2.4	11.0
956	AT1G09200.1 | histone H3, identical to histone H3 from Zea mays SP:P05203, Medicago sativa GI:166384, Encephalartos altensteinii SP:P08903, Pisum sativum SP:P02300; contains Pfam profile PF00125 Core histone H2A/H2B/H3/H4 | chr1:2971595-2972201 REVERSE | Aliases: T12M4.9	10.3	3.9	6.3	9.7
1423	AT4G15930.1 | dynein light chain, putative, similar to dynein light chain 2 (Mus musculus) GI:15545995; contains Pfam profile PF01221: Dynein light chain type 1 | chr4:9036236-9038077 FORWARD | Aliases: DL4005W, FCAALL.238	10.3	8.8	1.4	8.4
17168	AT5G46730.1 | glycine-rich protein | chr5:18981239-18982295 FORWARD | Aliases: MZA15.15, MZA15_15	10.3	9.9	0.3	0.7
5790	AT1G56340.2 | Symbol: CRT1 | similar to calreticulin 2 (CRT2) [Arabidopsis thaliana] (TAIR:At1g09210.1); similar to calreticulin [Beta vulgaris subsp. vulgaris] (GB:CAA05161.1); similar to calreticulin [Nicotiana plumbaginifolia] (GB:CAA95999.1); similar to calreticulin (GB:AAA80652.1); similar to calcium-binding protein calreticulin [Prunus armeniaca] (GB:AAD32207.1); similar to CRTC_RICCO Calreticulin precursor (GB:P93508); contains InterPro domain Calreticulin (InterPro:IPR001580) | chr1:21093545-21096322 REVERSE | Aliases: CALRETICULIN 1, F14G9.5, F14G9_5	10.3	9.0	1.3	4.1
658	AT1G05205.1 | expressed protein | chr1:1509212-1510280 REVERSE | Aliases: None	10.3	8.1	2.1	10.9
3032	AT1G47260.1 | Symbol: APFI | bacterial transferase hexapeptide repeat-containing protein, contains Pfam profile PF00132: Bacterial transferase hexapeptide (four repeats) | chr1:17323048-17325924 REVERSE | Aliases: F8G22.2, F8G22_2	10.3	8.7	1.6	6.1
13733	AT5G10450.2 | Symbol: GRF6 | similar to 14-3-3 protein GF14 kappa (GRF8) [Arabidopsis thaliana] (TAIR:At5g65430.2); similar to 14-3-3 g-1 protein [Nicotiana tabacum] (GB:BAD12179.1); similar to 14-3-3 protein [Solanum tuberosum] (GB:CAA72384.1); similar to GF14 lambda [Brassica napus] (GB:AAK26636.1); contains InterPro domain 14-3-3 protein (InterPro:IPR000308) | chr5:3283868-3286348 REVERSE | Aliases: 14 3 3 LIKE PROTEIN 1, AFT1, F12B17.200, F12B17_200, GF14 LAMBDA	10.3	10.7	-0.5	-1.4

8251	AT2G34470.2 | Symbol: UREG | similar to urease accessory protein G [Solanum tuberosum] (GB:CAC33002.1); similar to urease accessory protein G [Solanum tuberosum] (GB:CAC33000.1); contains InterPro domain Urease accessory protein UreG (InterPro:IPR004400); contains InterPro domain HypB/UreG, nucleotide-binding (InterPro:IPR002894) | chr2:14537814-14539584 REVERSE | Aliases: PSKF109, T31E10.19, T31E10_19, UREASE ACCESSORY PROTEIN UREG	10.3	11.1	-0.8	-3.0
13428	AT4G26710.2 | ATP synthase subunit H family protein, contains similarity to Swiss-Prot:O15342 Vacuolar ATP synthase subunit H (V-ATPase H subunit) (Vacuolar proton pump H subunit) (V-ATPase M9.2 subunit) (V-ATPase 9.2 kDa membrane accessory protein) (Homo sapiens) | chr4:13468603-13469941 FORWARD | Aliases: F10M23.50, F10M23_50	10.3	9.8	0.5	1.5
228	AT5G13280.1 | Symbol: AK LYS1 | aspartate kinase, identical to aspartate kinase (Arabidopsis thaliana) GI:4376158 | chr5:4249441-4252839 FORWARD | Aliases: AK, ASPARTATE KINASE, T31B5.100, T31B5_100	10.3	5.5	4.7	15.7
2860	AT3G13580.3 | 60S ribosomal protein L7 (RPL7D), similar to 60S ribosomal protein L7 GB:AAD14525 GI:4262232 from (Arabidopsis thaliana)	10.3	8.7	1.5	6.3
1389	AT5G47700.1 | 60S acidic ribosomal protein P1 (RPP1C) | chr5:19345029-19346209 REVERSE | Aliases: MCA23.2, MCA23_2	10.3	8.2	2.0	8.5
846	AT1G20450.2 | Symbol: ERD10 | dehydrin (ERD10), identical to dehydrin ERD10 (Low-temperature-induced protein LTI45) (Arabidopsis thaliana) SWISS-PROT:P42759 | chr1:7087993-7089596 REVERSE | Aliases: ER10, ERD10, F5M15.21, F5M15_21, LTI45	10.3	12.8	-2.5	-10.1
1055	AT4G13170.1 | 60S ribosomal protein L13A (RPL13aC), ribosomal protein L13a -Lupinus luteus,PID:e1237871 | chr4:7654940-7656561 REVERSE | Aliases: F17N18.60, F17N18_60	10.3	6.9	3.3	9.4
4962	AT3G53430.1 | 60S ribosomal protein L12 (RPL12B), 60S RIBOSOMAL PROTEIN L12, Prunus armeniaca, SWISSPROT:RL12_PRUAR | chr3:19820665-19821447 REVERSE | Aliases: F4P12.130	10.2	8.2	2.0	4.6
11065	AT5G27380.1 | Symbol: GSH2 | glutathione synthetase (GSH2), non-consensus AT donor splice site at exon 6, AC acceptor splice site at exon 7; identical to Swiss-Prot:P46416 glutathione synthetase, chloroplast precursor (Glutathione synthase) (GSH synthetase) (GSH-S) (Arabidopsis thaliana) | chr5:9668039-9670858 REVERSE | Aliases: F21A20.90, F21A20_90, GLUTATHIONE SYNTHETASE, GSHB	10.2	10.5	-0.3	-2.1
599	AT4G12600.1 | ribosomal protein L7Ae/L30e/S12e/Gadd45 family protein, Similar to NHP2/L7Ae family proteins, see SWISSPROT:P32495 and PMID:2063628. | chr4:7453199-7454478 FORWARD | Aliases: T1P17.190, T1P17_190	10.2	6.8	3.5	11.3
360	AT1G70780.1 | expressed protein | chr1:26698845-26700041 REVERSE | Aliases: F5A18.4, F5A18_4	10.2	11.8	-1.5	-13.4
6264	AT3G61220.1 | short-chain dehydrogenase/reductase (SDR) family protein, similar to carbonyl reductase GI:1049108 from (Mus musculus) | chr3:22673962-22675468 FORWARD | Aliases: T20K12.120	10.2	9.2	1.1	3.9
15659	AT3G55770.5 | similar to LIM domain-containing protein [Arabidopsis thaliana] (TAIR:At2g39900.1); similar to LIM domain protein [Gossypium hirsutum] (GB:AAL38006.1); contains InterPro domain Zn-binding protein, LIM (InterPro:IPR001781) | chr3:20714379-20716299 FORWARD | Aliases: F1I16.180	10.2	10.5	-0.3	-1.0
973	AT2G32090.2 | lactoylglutathione lyase family protein / glyoxalase I family protein, contains glyoxalase family protein domain, Pfam:PF00903 | chr2:13651704-13652906 FORWARD | Aliases: F22D22.16, F22D22_16	10.2	7.0	3.2	9.6
5531	AT1G42960.1 | expressed protein localized to the inner membrane of the chloroplast. | chr1:16128235-16129727 FORWARD | Aliases: F13A11.2, F13A11_2	10.2	9.1	1.1	4.3
5917	AT5G20570.1 | Symbol: RBX1 | ring-box protein-related, similar to ring-box protein 1 GI:4769004 from (Homo sapiens) | chr5:6956662-6958313 REVERSE | Aliases: F7C8.160, F7C8_160, HRT1, ROC1	10.2	9.2	1.1	4.1
735	AT4G21620.1 | glycine-rich protein | chr4:11491495-11492179 FORWARD | Aliases: F17L22.80, F17L22_80	10.2	6.4	3.8	10.6
11062	AT3G01770.1 | DNA-binding bromodomain-containing protein, contains bromodomain, INTERPRO:IPR001487 | chr3:275418-278792 REVERSE | Aliases: F28J7.10	10.2	9.7	0.5	2.1
664	AT5G01650.1 | macrophage migration inhibitory factor family protein / MIF family protein, contains pfam profile: PF001187 Macrophage migration inhibitory factor | chr5:242547-244096 REVERSE | Aliases: F7A7.170, F7A7_170	10.2	7.0	3.3	10.9
953	AT5G02610.1 | 60S ribosomal protein L35 (RPL35D), ribosomal protein L35- cytosolic, Arabidopsis thaliana, PIR:T00549 | chr5:587528-588731 FORWARD | Aliases: T22P11.200, T22P11_200	10.2	6.9	3.3	9.7
3733	AT5G08040.1 | expressed protein | chr5:2577203-2578120 REVERSE | Aliases: T22D6.2	10.2	8.3	1.9	5.5

976	AT1G49970.1 | Symbol: CLPR1 | ATP-dependent Clp protease proteolytic subunit (ClpR1) (nClpP5), identical to nClpP5 GB:BAA82069 GI:5360595 from (Arabidopsis thaliana); identical to cDNA nClpP5 (nuclear encoded ClpP5) GI:5360594 | chr1:18505201-18508277 REVERSE | Aliases: F2J10.14, F2J10_14, NCLPP5	10.2	7.7	2.5	9.6
11140	AT3G07680.1 | emp24/gp25L/p24 family protein, similar to SP:Q15363 Cop-coated vesicle membrane protein p24 precursor (p24A) {Homo sapiens}; contains Pfam profile PF01105: emp24/gp25L/p24 family | chr3:2455308-2456885 FORWARD | Aliases: MLP3.13	10.2	9.6	0.6	2.0
410	AT5G01530.1 | chlorophyll A-B binding protein CP29 (LHCB4), identical to CP29 (Arabidopsis thaliana) GI:298036; contains Pfam profile: PF00504 chlorophyll A-B binding protein	10.2	4.7	5.5	12.7
3608	AT5G48570.1 | peptidyl-prolyl cis-trans isomerase, putative / FK506-binding protein, putative, similar to rof1 (Arabidopsis thaliana) GI:1373396 | chr5:19707768-19711071 REVERSE | Aliases: K15N18.12, K15N18_12	10.2	8.2	2.0	5.6
19356	AT2G25670.2 | expressed protein | chr2:10935742-10938135 REVERSE | Aliases: F3N11.12, F3N11_12	10.2	10.1	0.1	0.3
3438	AT5G03455.1 | Symbol: CDC25 | rhodanese-like domain-containing protein, contains Rhodanese-like domain PF:00581 | chr5:862558-864107 FORWARD | Aliases: ARATH;CDC25	10.2	9.0	1.2	5.7
1876	AT2G37870.1 | protease inhibitor/seed storage/lipid transfer protein (LTP) family protein, contains Pfam protease inhibitor/seed storage/LTP family domain PF00234 | chr2:15866317-15866985 FORWARD | Aliases: T8P21.22, T8P21_22	10.2	6.6	3.7	7.6
14043	AT2G21560.1 | expressed protein, contains weak similarity to reticulocyte-binding protein 2 homolog A (Plasmodium falciparum) gi:9754767:gb:AAF98066 | chr2:9237348-9238588 REVERSE | Aliases: F2G1.17, F2G1_17	10.2	9.6	0.6	1.4
941	AT5G67220.1 | nitrogen regulation family protein, similar to unknown protein (gb:AAF51525.1) ; contains Pfam domain PF01207: Dihydrouridine synthase (Dus); similar to (SP:P45672) NIFR3-like protein (SP:P45672) (Azospirillum brasilense) | chr5:26836684-26839190 REVERSE | Aliases: K21H1.18, K21H1_18	10.2	8.3	1.9	9.7
3254	AT5G46250.2 | RNA recognition motif (RRM)-containing protein, contains similarity to RNA-binding protein; contains InterPro entry IPR000504: RNA-binding region RNP-1 (RNA recognition motif) (RRM) | chr5:18772535-18775505 FORWARD | Aliases: MPL12.3, MPL12_3	10.2	11.1	-0.9	-5.9
244	AT5G05710.1 | pleckstrin homology (PH) domain-containing protein, similar to AtPH1 (Arabidopsis thaliana) GI:5926716; contains Pfam profile PF00169: PH domain	10.2	5.9	4.3	15.4
2005	AT3G14290.1 | Symbol: PAE2 | 20S proteasome alpha subunit E2 (PAE2), identical to 20S proteasome subunit PAE2 GB:AAC32061 from (Arabidopsis thaliana)	10.2	8.9	1.4	7.4
688	AT3G54260.1 | expressed protein, various predicted proteins, Arabidopsis thaliana | chr3:20095988-20097741 REVERSE | Aliases: F24B22.220	10.2	7.7	2.5	10.8
209	AT4G30950.1 | Symbol: FAD6 | omega-6 fatty acid desaturase, chloroplast (FAD6) (FADC), identical to GI:493068	10.2	4.5	5.7	16.4
5702	AT4G33250.1 | Symbol: EIF3K | eukaryotic translation initiation factor 3 subunit 11 / eIF-3 p25 / eIF3k (TIF3K1), identical to Swiss-Prot:Q9SZA3 eukaryotic translation initiation factor 3 subunit 11 (eIF-3 p25) (eIF3k) (Arabidopsis thaliana); identical to cDNA initiation factor 3k GI:12407752	10.2	8.3	1.9	4.2
2595	AT5G61030.1 | RNA-binding protein, putative, similar to RNA-binding protein from (Solanum tuberosum) GI:15822705, (Nicotiana tabacum) GI:15822703, (Nicotiana sylvestris) GI:624925; contains InterPro entry IPR000504: RNA-binding region RNP-1 (RNA recognition motif) (RRM)	10.2	8.3	1.9	6.6
523	AT1G54320.1 | LEM3 (ligand-effect modulator 3) family protein / CDC50 family protein, Similar to GI:11994416; GI:4966357; GI:4835763; GI:9757735 from (Arabidopsis thaliana) | chr1:20279202-20281624 REVERSE | Aliases: F20D21.14, F20D21_14	10.2	11.9	-1.7	-11.8
5150	AT2G47640.3 | small nuclear ribonucleoprotein D2, putative / snRNP core protein D2, putative / Sm protein D2, putative, similar to small nuclear ribonucleoprotein Sm D2 (snRNP core protein D2) (Sm-D2) (Mus musculus) SWISS-PROT:P43330 | chr2:19544275-19545669 FORWARD | Aliases: F17A22.3	10.2	9.2	1.0	4.5
13568	AT3G12580.1 | Symbol: HSP70 | heat shock protein 70, putative / HSP70, putative, strong similarity to heat shock protein GI:425194 (Spinacia oleracea) | chr3:3991268-3993798 REVERSE | Aliases: HSP70, T2E22.11	10.2	9.8	0.4	1.5
609	AT4G20260.4 | similar to DREPP plasma membrane polypeptide-related [Arabidopsis thaliana] (TAIR:At5g44610.1); similar to DREPP2 protein [Nicotiana tabacum] (GB:CAB91552.1); contains InterPro domain DREPP plasma membrane polypeptide (InterPro:IPR008469) | chr4:10940749-10943512 FORWARD | Aliases: F1C12.180, F1C12_180	10.2	12.2	-2.0	-11.2
1762	AT1G27400.1 | 60S ribosomal protein L17 (RPL17A), similar to GB:P51413 from (Arabidopsis thaliana); similar to ESTs gb:L33542 and gb:AA660016 | chr1:9515122-9516912 FORWARD | Aliases: F17L21.19, F17L21_19	10.2	6.6	3.5	7.8
1989	AT4G37910.1 | Symbol: MTHSC70 1 | heat shock protein 70, mitochondrial, putative / HSP70, mitochondrial, putative, strong similarity to SP:Q01899 Heat shock 70 kDa protein, mitochondrial precursor {Phaseolus vulgaris} | chr4:17825074-17828171 REVERSE | Aliases: F20D10.30, F20D10_30, MTHSC70 1	10.2	7.7	2.5	7.4

301	AT3G51820.1 | chlorophyll synthetase, putative, identical to gi:972938 putative chlorophyll synthetase from Arabidopsis thaliana | chr3:19226980-19230027 REVERSE | Aliases: ATEM1.7, ATG4	10.2	5.6	4.6	14.3
12198	AT2G37410.2 | Symbol: ATTIM17 2 | mitochondrial import inner membrane translocase (TIM17), nearly identical to SP:Q9SP35 Mitochondrial import inner membrane translocase subunit TIM17 {Arabidopsis thaliana}	10.2	9.3	0.8	1.8
1218	AT4G32520.1 | glycine hydroxymethyltransferase, putative / serine hydroxymethyltransferase, putative / serine/threonine aldolase, putative, similar to serine hydroxymethyltransferase (Chlamydomonas reinhardtii) GI:17066746; contains Pfam profile PF00464: serine hydroxymethyltransferase	10.2	6.7	3.5	8.9
512	AT3G44590.2 | 60S acidic ribosomal protein P2 (RPP2D), acidic ribosomal protein P2, maize, PIR:S54179	10.2	7.7	2.5	11.9
11240	AT1G26850.3 | dehydration-responsive family protein, similar to early-responsive to dehydration stress ERD3 protein (Arabidopsis thaliana) GI:15320410; contains Pfam profile PF03141: Putative methyltransferase | chr1:9300860-9304120 REVERSE | Aliases: T2P11.4, T2P11_4	10.2	9.7	0.5	2.0
4513	AT4G17520.1 | nuclear RNA-binding protein, putative, similar to nuclear RNA binding protein GI:6492264 from (Arabidopsis thaliana) | chr4:9771343-9773605 FORWARD | Aliases: DL4795W, FCAALL.142	10.2	8.8	1.4	4.9
8684	AT3G57650.1 | Symbol: LPAT2 | acyl-CoA:1-acylglycerol-3-phosphate acyltransferase, putative, similar to acyl-CoA:1-acylglycerol-3-phosphate acyltransferase GI:4583544 from (Brassica napus) | chr3:21360491-21364151 FORWARD | Aliases: F15B8.160	10.2	9.7	0.5	2.8
13765	AT4G32870.1 | expressed protein, hypothetical protein F17H15.20 Arabidopsis thaliana chromosome II BAC F17H15, PID:g3643606 | chr4:15862147-15862745 FORWARD | Aliases: T16I18.80, T16I18_80	10.2	9.9	0.3	1.4
1447	AT5G50870.1 | ubiquitin-conjugating enzyme, putative, strong similarity to ubiquitin conjugating enzyme (Lycopersicon esculentum) GI:886679; contains Pfam profile PF00179: Ubiquitin-conjugating enzyme | chr5:20716709-20718302 REVERSE | Aliases: K3K7.1, K3K7_1	10.2	6.0	4.2	8.3
1211	AT5G61820.1 | expressed protein, MtN19, Medicago truncatula, EMBL:MTY15367 | chr5:24851544-24853917 REVERSE | Aliases: MAC9.6, MAC9_6	10.2	11.8	-1.6	-9.0
4284	AT5G40930.1 | Symbol: TOM20 4 | mitochondrial import receptor subunit TOM20-4 / translocase of outer membrane 20 kDa subunit 4, identical to mitochondrial import receptor subunit TOM20-4 SP:P82805 from (Arabidopsis thaliana) | chr5:16421688-16423224 FORWARD | Aliases: MMG1.2, MMG1_2, TOM20, TRANSLOCASE OF OUTER MEMBRANE 20 4	10.2	8.5	1.7	5.0
14065	AT1G22300.3 | Symbol: GRF10 | 14-3-3 protein GF14 epsilon (GRF10), identical to 14-3-3 protein GF14 epsilon GI:5802798, SP:P48347 from (Arabidopsis thaliana)	10.2	9.4	0.7	1.4
436	AT2G30200.2 | similar to malonyl-CoA:ACP transacylase [Perilla frutescens] (GB:AAG43518.1); contains InterPro domain Acyl transferase domain (InterPro:IPR001227) | chr2:12890074-12892845 REVERSE | Aliases: T27E13.6, T27E13_6	10.2	5.5	4.6	12.5
3678	AT5G14030.1 | translocon-associated protein beta (TRAPB) family protein, low similarity to SP:P23438 Translocon-associated protein, beta subunit precursor (TRAP-beta) (Signal sequence receptor beta subunit) {Canis familiaris}; contains Pfam profile PF05753: Translocon-associated protein beta (TRAPB) | chr5:4526813-4528411 FORWARD | Aliases: MUA22.2, MUA22_2	10.2	8.9	1.3	5.5
3363	AT4G33510.1 | Symbol: DHS2 | 2-dehydro-3-deoxyphosphoheptonate aldolase 2 / 3-deoxy-D-arabino-heptulosonate 7-phosphate synthase 2 / DAHP synthetase 2 (DHS2), nearly identical to SP:Q00218 | chr4:16116450-16118800 FORWARD | Aliases: 3 DEOXY D ARABINO HEPTULOSONATE 7 PHOSPHATE SYNTHASE, F17M5.270, F17M5_270	10.2	8.8	1.4	5.8
10960	AT5G52920.1 | pyruvate kinase, putative, similar to pyruvate kinase isozyme G, chloroplast precursor (Nicotiana tabacum) SWISS-PROT:Q40546 | chr5:21480769-21484043 FORWARD | Aliases: MXC20.15, MXC20_15	10.2	9.5	0.6	2.1
10742	AT4G20360.1 | elongation factor Tu / EF-Tu (TUFA), identical to SWISS-PROT:P17745 elongation factor Tu, chloroplast precursor (EF-Tu) (Arabidopsis thaliana) | chr4:10989963-10991720 FORWARD | Aliases: F9F13.10, F9F13_10	10.2	9.3	0.9	2.1
7092	AT2G06050.2 | Symbol: OPR3 | 12-oxophytodienoate reductase (OPR3) / delayed dehiscence1 (DDE1), nearly identical to DELAYED DEHISCENCE1 (GI:7688991) and to OPR3 (GI:10242314); contains Pfam profile PF00724:oxidoreductase, FAD/FMN-binding; identical to cDNA OPDA-reductase homolog GI:5059114 | chr2:2359112-2362124 REVERSE | Aliases: 12 OXO PHYTODIENOATE REDUCTASE, 12 OXOPHYTODIENOATE 10,11 REDUCTASE, F5K7.19, F5K7_19	10.2	8.7	1.5	3.5
8218	AT4G05390.1 | ferredoxin--NADP(+) reductase, putative / adrenodoxin reductase, putative, strong similarity to SP:P41345 Ferredoxin--NADP reductase, root isozyme, chloroplast precursor (EC 1.18.1.2) (FNR) {Oryza sativa}, ferredoxin-NADP reductase precursor (Zea mays) GI:500751 | chr4:2738712-2740562 REVERSE | Aliases: C6L9.70, C6L9_70	10.2	9.2	0.9	3.0
4119	AT3G47520.1 | Symbol: MDH | malate dehydrogenase (NAD), chloroplast (MDH), identical to chloroplast NAD-malate dehydrogenase (Arabidopsis thaliana) GI:3256066; contains InterPro entry IPR001236: Lactate/malate dehydrogenase; contains Pfam profiles PF00056: lactate/malate dehydrogenase, NAD binding domain and PF02866: lactate/malate dehydrogenase, alpha/beta C-terminal domain | chr3:17524259-17526026 FORWARD | Aliases: F1P2.70, MALATE DEHYDROGENASE	10.2	8.5	1.7	5.2

12175	AT4G03520.1 | Symbol: ATHM2 | thioredoxin M-type 2, chloroplast (TRX-M2), nearly identical to SP:Q9SEU8 Thioredoxin M-type 2, chloroplast precursor (TRX-M2) {Arabidopsis thaliana} | chr4:1562357-1564164 REVERSE | Aliases: F9H3.15, F9H3_15, T5L23.1	10.2	9.5	0.6	1.8
15646	AT1G15690.1 | Symbol: AVP1 | pyrophosphate-energized vacuolar membrane proton pump / pyrophosphate-energized inorganic pyrophosphatase (AVP-3), identical to pyrophosphate-energized vacuolar membrane proton pump (pyrophosphate-energized inorganic pyrophosphatase) SP:P31414 from (Arabidopsis thaliana) | chr1:5398985-5402949 FORWARD | Aliases: ATAVP3, AVP 3, F7H2.3, F7H2_3, VACUOLAR H+ PYROPHOSPHATASE AVP 3	10.2	9.8	0.4	1.0
3721	AT1G10590.3 | DNA-binding protein-related, contains weak similarity to G-quartet DNA binding protein 3 (Tetrahymena thermophila) gi:4583503:gb:AAD25098 | chr1:3502051-3503410 REVERSE | Aliases: F20B24.1	10.2	9.2	1.0	5.5
905	AT2G41650.1 | expressed protein | chr2:17370985-17371512 FORWARD | Aliases: T32G6.17, T32G6_17	10.2	8.1	2.1	9.9
2081	AT5G58110.1 | expressed protein, predicted proteins, Homo sapiens and Drosophila melanogaster | chr5:23532412-23533860 FORWARD | Aliases: K21L19.11, K21L19_11	10.2	8.7	1.5	7.3
11839	AT2G30620.1 | histone H1.2, nearly identical to SP:P26569 Histone H1.2 {Arabidopsis thaliana} | chr2:13052008-13053588 FORWARD | Aliases: T6B20.3, T6B20_3	10.2	9.7	0.5	1.9
1619	AT3G46560.1 | Symbol: TIM9 | mitochondrial import inner membrane translocase (TIM9), identical to mitochondrial import inner membrane translocase subunit Tim9 (Arabidopsis thaliana) Swiss-Prot:Q9XGX9; contains Pfam domain, PF02953: Tim10/DDP family zinc finger | chr3:17149572-17150473 FORWARD | Aliases: EMB2474, EMBRYO DEFECTIVE 2474, F12A12.80	10.2	7.6	2.6	8.0
5300	AT5G42790.1 | Symbol: PAF1 | 20S proteasome alpha subunit F1 (PAF1), (gb:AAC32062.1) | chr5:17176278-17178298 REVERSE | Aliases: 20S PROTEASOME SUBUNIT PAF1, MJB21.17, MJB21_17, PAF1	10.1	8.1	2.1	4.4
295	AT1G73940.1 | expressed protein | chr1:27801974-27803248 REVERSE | Aliases: F2P9.19, F2P9_19	10.1	5.5	4.7	14.4
20948	AT2G29700.1 | Symbol: ATPH1 | pleckstrin homology (PH) domain-containing protein (PH1), identical to AtPH1 (Arabidopsis thaliana) GI:5926716; contains Pfam profile PF00169: PH domain | chr2:12704497-12705244 FORWARD | Aliases: ARABIDOPSIS THALIANA PLECKSTRIN HOMOLOGUE 1, T27A16.20, T27A16_20	10.1	10.2	-0.0	-0.0
7265	AT4G13010.1 | oxidoreductase, zinc-binding dehydrogenase family protein, low similarity to probable NADP-dependent oxidoreductase (zeta-crystallin homolog) P1 (SP:Q39172)(gi:886428) and P2 (SP:Q39173)(gi:886430); contains Pfam profile PF00107: oxidoreductase, zinc-binding dehydrogenase family | chr4:7600548-7602726 FORWARD | Aliases: F25G13.100, F25G13_100	10.1	8.8	1.3	3.4
14428	AT4G24330.1 | expressed protein, hypothetical protein - Caenorhabditis elegans,PID:e1350884 | chr4:12603531-12606338 REVERSE | Aliases: T22A6.160, T22A6_160	10.1	9.8	0.3	1.3
12704	AT1G16890.2 | ubiquitin-conjugating enzyme, putative, nearly identical to ubiquitin-conjugating enzyme E2 (Catharanthus roseus) GI:5381319; contains Pfam profile PF00179: Ubiquitin-conjugating enzyme | chr1:5776339-5778448 REVERSE | Aliases: F17F16.19	10.1	9.5	0.6	1.7
12705	AT1G78870.3 | similar to ubiquitin-conjugating enzyme, putative [Arabidopsis thaliana] (TAIR:At1g16890.2); similar to ubiquitin-conjugating enzyme E2 [Catharanthus roseus] (GB:AAD42941.1); contains InterPro domain Ubiquitin-conjugating enzymes (InterPro:IPR000608) | chr1:29655348-29657410 FORWARD | Aliases: F9K20.8, F9K20_8	10.1	9.5	0.6	1.7
15686	AT1G02305.1 | cathepsin B-like cysteine protease, putative, similar to cathepsin B-like cysteine proteinase (Nicotiana rustica) GI:609175; contains Pfam profile PF00112: Papain family cysteine protease | chr1:455778-458124 FORWARD | Aliases: None	10.1	10.6	-0.5	-1.0
18116	AT1G21440.1 | mutase family protein, similar to carboxyvinyl-carboxyphosphonate phosphorylmutase GB:O49290 from (Arabidopsis thaliana); similar to carboxyphosphonoenolpyruvate mutase (GI:47149) (Streptomyces hygroscopicus); contains Prosite PS00161: Isocitrate lyase signature | chr1:7502159-7504140 REVERSE | Aliases: F24J8.7, F24J8_7	10.1	9.9	0.3	0.6
6310	AT5G26880.1 | tRNA/rRNA methyltransferase (SpoU) family protein, low similarity to tRNA (Gm18) methyltransferase (Thermus thermophilus) GI:11079208; contains Pfam profile PF00588: SpoU rRNA Methylase (RNA methyltransferase, TrmH) family | chr5:9457700-9459233 REVERSE | Aliases: F2P16.25, F2P16_25	10.1	9.5	0.6	3.9
5432	AT2G33220.1 | expressed protein | chr2:14085948-14087213 FORWARD | Aliases: F25I18.4, F25I18_4	10.1	9.0	1.2	4.3
435	AT1G31230.1 | bifunctional aspartate kinase/homoserine dehydrogenase / AK-HSDH, nearly identical to gb:X71364 (PIR:S46497) aspartate kinase / homoserine dehydrogenase from Arabidopsis thaliana; contains ACT domain	10.1	6.5	3.7	12.5
20458	AT1G64950.1 | Symbol: CYP89A5 | cytochrome P450, putative, similar to cytochrome P450 89A2 (CYPLXXXIX) (SP:Q42602) (Arabidopsis thaliana);similar to cytochrome P450 (GI:438242) (Solanum melongena) | chr1:24131224-24133121 FORWARD | Aliases: F13O11.25, F13O11_25	10.1	10.1	0.0	0.1

1047	AT4G22570.1 | adenine phosphoribosyltransferase, putative, strong similarity to Adenine phosphoribosyltransferase (Hordeum vulgare subsp. vulgare) GI:9711921; contains Pfam profile PF00156: Phosphoribosyl transferase domain | chr4:11882161-11885414 REVERSE | Aliases: F7K2.150, F7K2_150	10.1	6.2	3.9	9.4
2821	AT4G01850.1 | S-adenosylmethionine synthetase 2 (SAM2), identical to S-adenosylmethionine synthetase 2 (Methionine adenosyltransferase 2, AdoMet synthetase 2) (Arabidopsis thaliana) SWISS-PROT:P17562 | chr4:796097-798285 REVERSE | Aliases: T7B11.11, T7B11_11	10.1	11.5	-1.3	-6.3
5593	AT5G51040.2 | expressed protein, contains InterPro domain Protein of unknown function DUF339 (InterPro:IPR005631) | chr5:20767886-20769250 FORWARD | Aliases: K3K7.22, K3K7_22	10.1	9.1	1.0	4.2
3747	AT3G20500.1 | calcineurin-like phosphoesterase family protein, contains Pfam profile: PF00149 calcineurin-like phosphoesterase	10.1	8.3	1.8	5.4
2643	AT5G02790.1 | In2-1 protein, putative, similar to In2-1, Zea mays, EMBL:X58573	10.1	6.3	3.8	6.5
4847	AT3G25230.1 | Symbol: ROF1 | peptidyl-prolyl cis-trans isomerase / FK506-binding protein (ROF1), identical to rotamase FKBP (ROF1) GB:U49453 (Arabidopsis thaliana) (Mol. Gen. Genet. 252 (5), 510-517 (1996)) | chr3:9189444-9192642 FORWARD | Aliases: MJL12.21, ROTAMASE FKBP 1	10.1	7.9	2.2	4.7
843	AT2G28000.1 | Symbol: CPN60A |	10.1	7.3	2.9	10.1
9632	AT4G13180.1 | short-chain dehydrogenase/reductase (SDR) family protein, similar to short-chain type dehydrogenase/reductase SP:Q08632 (Picea abies) | chr4:7657192-7658193 REVERSE | Aliases: F17N18.70, F17N18_70	10.1	8.0	2.1	2.5
2700	AT2G18230.1 | inorganic pyrophosphatase (soluble) (PPA) / pyrophosphate phospho-hydrolase / PPase, nearly identical to SP:P21216 Soluble inorganic pyrophosphatase (EC 3.6.1.1) (Pyrophosphate phospho- hydrolase) (PPase) {Arabidopsis thaliana} | chr2:7939009-7941411 REVERSE | Aliases: T30D6.26, T30D6_26	10.1	5.5	4.6	6.5
1471	AT4G01150.1 | expressed protein | chr4:493548-494795 FORWARD | Aliases: F2N1.18, F2N1_18	10.1	6.7	3.4	8.3
11164	AT1G79230.1 | Symbol: ST1 | mercaptopyruvate sulfurtransferase (MST1) (RDH1), identical to mercaptopyruvate sulfurtransferase GI:6009981 and thiosulfate sulfurtransferase GI:5834508 from (Arabidopsis thaliana) | chr1:29805586-29808832 FORWARD | Aliases: ARABIDOPSIS THALIANA RHODANESE HOMOLOGUE 1, ATMST1, ATRDH1, MERCAPTOPYRUVATE SULFURTRANSFERASE, MERCAPTOPYRUVATE SULFURTRANSFERASE 1, MST1, YUP8H12R.17, YUP8H12R_17	10.1	8.5	1.6	2.0
696	AT2G44860.1 | 60S ribosomal protein L24, putative | chr2:18507444-18509617 REVERSE | Aliases: T13E15.13	10.1	6.7	3.4	10.7
3429	AT4G18730.1 | Symbol: RPL16B |	10.1	8.0	2.1	5.7
3430	AT5G45775.2 | 60S ribosomal protein L11 (RPL11D) | chr5:18582292-18583785 REVERSE | Aliases: None	10.1	8.0	2.1	5.7
15457	AT3G10770.2 | expressed protein | chr3:3372275-3374276 REVERSE | Aliases: T7M13.15	10.1	9.8	0.3	1.1
8064	AT3G06050.1 | Symbol: ATPRXIIF/PRXIIF | Encodes a mitochondrial matrix localized peroxiredoxin involved in redox homeostasis. Knockout mutants have reduced root growth under certain oxidative stress conditions. | chr3:1826160-1827867 REVERSE | Aliases: ATPRXIIF, F24F17.3, F24F17_3, PRXIIF	10.1	7.7	2.4	3.1
18418	AT1G29470.2 | similar to dehydration-responsive protein-related [Arabidopsis thaliana] (TAIR:At5g64030.1); similar to dehydration-responsive protein-related [Arabidopsis thaliana] (TAIR:At3g51070.1); similar to dehydration-responsive protein-related [Arabidopsis thaliana] (TAIR:At2g34300.1); similar to OSJNBb0020J19.14 [Oryza sativa (japonica cultivar-group)] (GB:XP_474482.1); similar to ankyrin-like protein [Oryza sativa (japonica cultivar-group)] (GB:BAD82580.1); contains InterPro domain Putative methyltransferase DUF248 (InterPro:IPR004159) | chr1:10310231-10313741 REVERSE | Aliases: F15D2.5, F15D2_5	10.1	10.0	0.1	0.5
354	AT1G53460.1 | expressed protein | chr1:19958158-19960300 REVERSE | Aliases: T3F20.22, T3F20_22	10.1	7.1	3.0	13.5
479	AT3G58700.1 | 60S ribosomal protein L11 (RPL11B), ribosomal protein L11, cytosolic, Arabidopsis thaliana, PIR:S49033 | chr3:21722540-21723930 FORWARD | Aliases: T20N10.50	10.1	7.7	2.4	12.1
2212	AT3G07910.1 | expressed protein | chr3:2523163-2524171 REVERSE | Aliases: F17A17.25	10.1	8.5	1.6	7.1
8517	AT1G80460.2 | Symbol: NHO1 | similar to glycerol kinase [Pandanus amaryllifolius] (GB:AAR88660.1); contains InterPro domain Carbohydrate kinase, FGGY (InterPro:IPR000577); contains InterPro domain Glycerol kinase (InterPro:IPR005999) | chr1:30251637-30254197 REVERSE | Aliases: GLI1, T21F11.21, T21F11_21	10.1	10.8	-0.7	-2.9
537	AT5G48870.1 | Symbol: SAD1 | small nuclear ribonucleoprotein, putative / snRNP, putative / Sm protein, putative, similar to U6 snRNA-associated Sm-like protein LSm5 (Homo sapiens) SWISS-PROT:Q9Y4Y9	10.1	8.2	1.9	11.7
7461	AT1G16240.2 | Symbol: SYP51 | similar to syntaxin 52 (SYP52) [Arabidopsis thaliana] (TAIR:At1g79590.1); similar to putative syntaxin of plants 52 [Oryza sativa (japonica cultivar-group)] (GB:XP_463898.1); contains InterPro domain Target SNARE coiled-coil domain (InterPro:IPR000727) | chr1:5555001-5556797 REVERSE | Aliases: ATSYP51, F3O9.4, F3O9_4, SYNTAXIN OF PLANTS 51	10.1	10.7	-0.6	-3.3

5912	AT3G11710.1 | lysyl-tRNA synthetase, putative / lysine--tRNA ligase, putative, similar to SP:Q43776 Lysyl-tRNA synthetase (EC 6.1.1.6) (Lysine--tRNA ligase) {Lycopersicon esculentum}; contains Pfam profile PF00152: tRNA synthetases class II (D, K and N) | chr3:3702179-3705707 REVERSE | Aliases: T19F11.11, T19F11_11	10.1	9.1	0.9	4.1
7815	AT5G59150.1 | Ras-related GTP-binding protein, putative, similar to Ras-related protein Rab11C SP:Q40193 from (Lotus japonicus) | chr5:23893835-23895655 FORWARD | Aliases: MNC17.6, MNC17_6	10.1	10.5	-0.4	-3.2
2922	AT2G27710.3 | 60S acidic ribosomal protein P2 (RPP2B) | chr2:11823868-11824944 FORWARD | Aliases: F15K20.19, F15K20_19	10.1	7.6	2.5	6.2
9894	AT4G34050.2 | caffeoyl-CoA 3-O-methyltransferase, putative, nearly identical to GI:2960356 (Populus balsamifera subsp. trichocarpa), GI:684942 (Medicago sativa subsp. sativa) isoform contains a GT-TG intron which removes an internal segment of the protein. | chr4:16310820-16311978 FORWARD | Aliases: F28A23.190, F28A23_190	10.1	11.1	-1.0	-2.4
10610	AT1G14610.1 | Symbol: TWN2 | valyl-tRNA synthetase / valine--tRNA ligase (VALRS), nearly identical to SP:P93736 Valyl-tRNA synthetase (EC 6.1.1.9) (Valine--tRNA ligase) (ValRS) {Arabidopsis thaliana} | chr1:5008239-5014512 REVERSE | Aliases: T5E21.11, T5E21_11, TWIN 2, VALRS, VALYL TRNA SYNTHETASE	10.1	9.5	0.6	2.2
9829	AT5G65620.1 | peptidase M3 family protein / thimet oligopeptidase family protein, similar to SP:P27237 Oligopeptidase A (EC 3.4.24.70) {Salmonella typhimurium}; contains Pfam profile PF01432: Peptidase family M3 | chr5:26239174-26243549 FORWARD | Aliases: K21L13.14, K21L13_14	10.1	9.3	0.7	2.4
6592	AT5G55290.1 | ATP synthase subunit H family protein, contains weak similarity to Vacuolar ATP synthase subunit H (EC 3.6.3.14) (V-ATPase H subunit) (Vacuolar proton pump H subunit) (V-ATPase M9.2 subunit) (V-ATPase 9.2 kDa membrane accessory protein) (Swiss-Prot:P81103) (Bos taurus); contains Pfam profile PF05493: ATP synthase subunit H | chr5:22440263-22441312 REVERSE | Aliases: MCO15.24, MCO15_24	10.1	9.5	0.6	3.7
1028	AT3G02180.2 | Symbol: SP1L | expressed protein | chr3:404891-405656 FORWARD | Aliases: F1C9.3, SPIRAL 1 LIKE	10.1	8.1	2.0	9.5
8820	AT3G13070.1 | CBS domain-containing protein / transporter associated domain-containing protein, similar to SP:Q54318 Hemolysin C (Serpulina hyodysenteriae) {Treponema hyodysenteriae}; contains Pfam profiles PF00571: CBS domain, PF03471: Transporter associated domain, PF01595: Domain of unknown function | chr3:4191518-4195119 REVERSE | Aliases: MJG19.2	10.1	9.3	0.8	2.8
3659	AT1G74270.1 | 60S ribosomal protein L35a (RPL35aC), similar to ribosomal protein L33B GB:NP_014877 from (Saccharomyces cerevisiae) | chr1:27931917-27933158 REVERSE | Aliases: F1O17.6, F1O17_6	10.1	8.1	2.0	5.5
1566	AT3G52090.1 | Symbol: ATRPB13.6 | DNA-directed RNA polymerase II 13.6 kDa subunit (RPB13.6), identical to SP:Q38859 DNA-directed RNA polymerase II 13.6 kDa polypeptide (EC 2.7.7.6) {Arabidopsis thaliana} | chr3:19328832-19330030 REVERSE | Aliases: F4F15.200, RNA POLYMERASE II 13.6 KDA SUBUNIT	10.1	8.0	2.1	8.1
3743	AT1G16310.1 | cation efflux family protein, contains cation efflux family protein domain, Pfam:PF01545	10.1	7.0	3.1	5.5
859	AT1G14980.1 | Symbol: CPN10 | 10 kDa chaperonin (CPN10), identical to SP:P34893 from (Arabidopsis thaliana) | chr1:5165623-5166740 REVERSE | Aliases: CHAPERONIN 10, MITOCHONDRIAL CHAPERONIN 10, T15D22.2, T15D22_2	10.1	6.9	3.2	10.1
3986	AT3G55620.1 | Symbol: EMB1624 | eukaryotic translation initiation factor 6, putative / eIF-6, putative, similar to SP:O55135 Eukaryotic translation initiation factor 6 (eIF-6) (B4 integrin interactor) {Mus musculus}; contains Pfam profile PF01912: eIF-6 family | chr3:20645182-20647485 FORWARD | Aliases: EMB1624, EMBRYO DEFECTIVE 1624, F1I16.30	10.1	8.2	1.9	5.3
622	AT3G13860.1 | chaperonin, putative, similar to SWISS-PROT:P29197- chaperonin CPN60, mitochondrial precursor (HSP60) (Arabidopsis thaliana) ; contains Pfam:PF00118 domain, TCP-1/cpn60 chaperonin family | chr3:4561524-4565256 REVERSE | Aliases: MCP4.9	10.1	7.1	3.0	11.1
3020	AT3G47470.1 | Symbol: CAB4 | chlorophyll A-B binding protein 4, chloroplast / LHCI type III CAB-4 (CAB4), identical to SP:P27521 Chlorophyll A-B binding protein 4, chloroplast precursor (LHCI type III CAB-4) (LHCP) {Arabidopsis thaliana}	10.1	6.5	3.6	6.1
11235	AT1G04750.2 | Symbol: VAMP7B |	10.1	9.0	1.1	2.0
5910	AT1G74210.1 | glycerophosphoryl diester phosphodiesterase family protein, low similarity to glycerophosphodiester phosphodiesterase (Borrelia hermsii) GI:1399038; contains Pfam profile PF03009: Glycerophosphoryl diester phosphodiesterase family | chr1:27913979-27916602 FORWARD | Aliases: F1O17.12, F1O17_12	10.1	8.7	1.3	4.1
13687	AT2G34690.1 | Symbol: ACD11 | expressed protein | chr2:14637430-14639029 FORWARD | Aliases: ACCELERATED CELL DEATH 11, T29F13.10, T29F13_10	10.1	9.8	0.2	1.4
5265	AT3G25520.1 | Symbol: ATL5 | 60S ribosomal protein L5, similar to 60S ribosomal protein L5 GB:P49625 from (Oryza sativa) | chr3:9270524-9272626 REVERSE | Aliases: A. THALIANA RIBOSOMAL PROTEIN L5, MWL2.17, RIBOSOMAL PROTEIN L5	10.1	7.7	2.4	4.4
7016	AT5G61510.1 | NADP-dependent oxidoreductase, putative, similar to zeta-crystallin homolog TED2 from Zinnia elegans (gi:531096); contains Pfam zinc-binding dehydrogenase domain PF00107 | chr5:24754171-24756224 REVERSE | Aliases: K11J9.5, K11J9_5	10.1	8.8	1.3	3.5

5681	ATCG00080.1 | Symbol: PSBI | PSII I protein | chrC:7583-7693 FORWARD | Aliases: PSBI	10.1	7.3	2.8	4.2
5755	AT5G01410.1 | stress-responsive protein, putative, similar to ethylene-inducible protein HEVER (Hevea brasiliensis) SWISS-PROT:Q39963 | chr5:172317-173613 REVERSE | Aliases: T10O8.120, T10O8_120	10.1	7.9	2.2	4.2
831	AT3G02870.2 | similar to inositol monophosphatase family protein [Arabidopsis thaliana] (TAIR:At1g31190.1); similar to L-galactose-1-phosphate phosphatase [Malus x domestica] (GB:AAV49507.1); contains InterPro domain Inositol monophosphatase (InterPro:IPR000760)	10.0	7.7	2.3	10.1
3606	AT4G22212.1 | Encodes a defensin-like (DEFL) family protein. | chr4:11753998-11754700 FORWARD | Aliases: None	10.0	11.4	-1.4	-5.6
18487	AT5G48930.1 | transferase family protein, similar to anthranilate N-hydroxycinnamoyl/benzoyltransferase from Dianthus caryophyllus (GI:3288180, GI:2239091); contains Pfam profile PF02458 transferase family | chr5:19853525-19855371 REVERSE | Aliases: K19E20.4, K19E20_4	10.0	10.3	-0.3	-0.5
14177	AT3G17810.1 | dihydroorotate dehydrogenase family protein / dihydroorotate oxidase family protein, low similarity to SP:Q12882 Dihydropyrimidine dehydrogenase (NADP+) precursor (EC 1.3.1.2) (DPD) (DHPDHase) (Dihydrouracil dehydrogenase) (Dihydrothymine dehydrogenase) {Homo sapiens}; contains Pfam profile PF01180: Dihydroorotate dehydrogenase | chr3:6094157-6096572 FORWARD | Aliases: MEB5.3	10.0	9.0	1.0	1.3
7527	AT2G18390.1 | Symbol: TTN5 | ADP-ribosylation factor-like protein 2 (ARL2), identical to ARL2 G-protein (Halimasch; HAL; TITAN5) GI:20514265 from (Arabidopsis thaliana); identical to cDNA ARL2 G-protein mRNA GI:20514264; contains Pfam profile PF00025: ADP-ribosylation factor family; contains TIGRfam profile TIGR00231: small GTP-binding protein domain | chr2:7995247-7996943 FORWARD | Aliases: ARG1 LIKE 2, ARL2, ATARLC1, HAL, HALLIMASCH, T30D6.10, T30D6_10, TITAN 5	10.0	9.5	0.5	3.3
6043	AT3G15640.1 | cytochrome c oxidase family protein, contains Pfam domain, PF01215: Cytochrome c oxidase subunit Vb | chr3:5299117-5301752 FORWARD | Aliases: MSJ11.5	10.0	9.1	1.0	4.0
9606	AT4G30600.1 | signal recognition particle receptor alpha subunit family protein, similar to Signal recognition particle receptor alpha subunit (SR-alpha) (Docking protein alpha) (DP-alpha) (SP:P08240) (Homo sapiens}; similar to Signal recognition particle receptor alpha subunit	10.0	10.6	-0.5	-2.5
815	AT1G23220.1 | dynein light chain type 1 family protein, similar to SP:O02414 Dynein light chain LC6, flagellar outer arm {Anthocidaris crassispina}; contains Pfam profile PF01221: Dynein light chain type 1 | chr1:8242499-8244307 FORWARD | Aliases: F26F24.7, F26F24_7	10.0	6.6	3.4	10.2
827	AT2G35040.1 | AICARFT/IMPCHase bienzyme family protein, similar to SP:P12048 Bifunctional purine biosynthesis protein purH (Includes: Phosphoribosylaminoimidazolecarboxamide formyltransferase (EC 2.1.2.3) (AICAR transformylase); IMP cyclohydrolase (EC 3.5.4.10) (Inosinicase) (IMP synthetase) (ATIC)) {Bacillus subtilis}; contains Pfam profiles PF01808: AICARFT/IMPCHase bienzyme, PF02142: MGS-like domain | chr2:14772239-14775367 REVERSE | Aliases: F19I3.27, F19I3_27	10.0	5.1	4.9	10.2
14485	AT3G08710.1 | thioredoxin family protein, similar to thioredoxin H-type GB:P29448 SP:P29448 (Arabidopsis thaliana), Thioredoxin H-type 2 (TRX-H2) SP:Q07090 {Nicotiana tabacum}; contains Pfam profile: PF00085 Thioredoxin | chr3:2645223-2646496 FORWARD | Aliases: F17O14.18	10.0	9.6	0.4	1.3
7881	AT5G54855.1 | pollen Ole e 1 allergen and extensin family protein, contains Pfam domain, PF01190: Pollen proteins Ole e I family | chr5:22300026-22301613 FORWARD | Aliases: None	10.0	10.5	-0.4	-3.2
1729	AT3G22320.1 | Symbol: ATRPABC24.3 | DNA-directed RNA polymerase, putative, similar to SP:P19388 DNA-directed RNA polymerase II 23 kDa polypeptide (EC 2.7.7.6) {Homo sapiens}; contains Pfam profiles PF03871: RNA polymerase Rpb5 N-terminal domain, PF01191: RNA polymerase Rpb5 C-terminal domain | chr3:7890893-7892330 REVERSE | Aliases: MCB17.20	10.0	8.2	1.8	7.8
17445	AT3G15610.1 | transducin family protein / WD-40 repeat family protein, contains 7 WD-40 repeats (PF00400); similar to serine/threonine kinase receptor associated protein GB:NP_035629 (SP:Q9Z1Z2) (Mus musculus); UNR-interacting protein GB:NP_009109 (Homo sapiens)	10.0	10.1	-0.1	-0.7
10341	NA	10.0	10.5	-0.4	-2.3
2290	AT1G54690.1 | histone H2A, putative, strong similarity to histone H2A GI:3204129 SP:O65759 from Cicer arietinum, Picea abies SP:P35063; contains Pfam profile PF00125 Core histone H2A/H2B/H3/H4 | chr1:20417983-20418954 REVERSE | Aliases: T22H22.12, T22H22_12	10.0	6.4	3.6	7.0
5248	AT5G50460.1 | protein transport protein SEC61 gamma subunit, putative, similar to Swiss-Prot:Q19967 protein transport protein SEC61 gamma subunit (Caenorhabditis elegans) | chr5:20568663-20569792 REVERSE | Aliases: MBA10.8	10.0	7.9	2.1	4.4
17758	AT2G30950.1 | Symbol: VAR2 | FtsH protease (VAR2), identical to zinc dependent protease VAR2 GI:7650138 from (Arabidopsis thaliana) | chr2:13181402-13184300 FORWARD | Aliases: F7F1.16, F7F1_16, FTSH2, VAR2, VARIEGATED 2, YELLOW VARIEGATED	10.0	10.2	-0.2	-0.6
6466	AT1G08110.4 | similar to lactoylglutathione lyase, putative / glyoxalase I, putative [Arabidopsis thaliana] (TAIR:At1g67280.1); similar to Glyoxalase I [Cicer arietinum] (GB:CAA12028.1); contains InterPro domain Glyoxalase I (InterPro:IPR004361); contains InterPro domain Glyoxalase/Bleomycin resistance protein/dioxygenase domain (InterPro:IPR004360) | chr1:2535368-2537928 FORWARD | Aliases: T6D22.20, T6D22_20	10.0	7.8	2.2	3.8

10327	AT2G47320.1 | peptidyl-prolyl cis-trans isomerase cyclophilin-type family protein, contains Pfam domain, PF00160: peptidyl-prolyl cis-trans isomerase, cyclophilin-type | chr2:19434442-19435987 FORWARD | Aliases: T8I13.16	10.0	8.9	1.1	2.3
20619	AT4G10840.2 | kinesin light chain-related, low similarity to kinesin light chain (Plectonema boryanum) GI:2645229; contains Pfam profile PF00515 TPR Domain | chr4:6656512-6659252 FORWARD | Aliases: F25I24.50, F25I24_50	10.0	10.0	0.0	0.1
4215	AT2G22860.1 | Symbol: ATPSK2 |	10.0	8.9	1.2	5.1
11985	AT1G63460.1 | glutathione peroxidase, putative, contains Pfam profile: PF00255 glutathione peroxidases	10.0	9.7	0.3	1.8
396	AT3G30775.1 | Symbol: ERD5 | proline oxidase, mitochondrial / osmotic stress-responsive proline dehydrogenase (POX) (PRO1) (ERD5), nearly identical to SP:P92983 Proline oxidase, mitochondrial precursor (EC 1.5.3.-) (Osmotic stress- induced proline dehydrogenase) (Arabidopsis thaliana); identical to cDNA proline oxidase precursor GI:1817543	10.0	6.5	3.5	12.9
5787	AT3G45970.1 | Symbol: ATEXLA1 | expansin family protein (EXPL1), similar to cim1 induced allergen, Glycine max, EMBL:U03860; expansin-like gene, PMID:11641069, www.bio.psu.edu/expansins | chr3:16907151-16908293 FORWARD | Aliases: ARABIDOPSIS THALIANA EXPANSIN LIKE A1, ATEXPL1, ATHEXP BETA 2.1, EXPL1, F16L2.180, F16L2_180	10.0	10.7	-0.8	-4.1
767	AT5G16710.1 | dehydroascorbate reductase, putative, Strong similarity to dehydroascorbate reductase (Spinacia oleracea) gi:10952512 gb:AAG24945 | chr5:5483291-5485069 FORWARD | Aliases: F5E19.50, F5E19_50	10.0	7.2	2.8	10.4
14779	AT1G16210.1 | expressed protein, ESTs gb:T04357 and gb:AA595092 come from this gene | chr1:5546127-5547546 REVERSE | Aliases: F3O9.2, F3O9_2	10.0	9.5	0.5	1.2
1772	AT5G53400.1 | nuclear movement family protein, contains Pfam profile: PF03593 nuclear movement protein	10.0	6.7	3.3	7.8
14896	AT1G02840.3 | Symbol: SR1 | pre-mRNA splicing factor SF2 (SF2) / SR1 protein, identical to SP:O22315 Pre-mRNA splicing factor SF2 (SR1 protein) {Arabidopsis thaliana} | chr1:626741-629819 FORWARD | Aliases: ATSRP34, F22D16.16, F22D16_16, SRP34	10.0	9.8	0.2	1.2
786	AT3G06310.1 | NADH-ubiquinone oxidoreductase 19 kDa subunit (NDUFA8) family protein, contains Pfam profile PF05850: NADH-ubiquinone oxidoreductase 19 kDa subunit (NDUFA8); similar to NADH-ubiquinone oxidoreductase 19 kDa subunit (EC 1.6.5.3) (EC 1.6.99.3) (Complex I-19KD) (CI-19KD) (Complex I-PGIV) (CI-PGIV) (Swiss-Prot:P51970) (Homo sapiens) | chr3:1912978-1914387 REVERSE | Aliases: F24P17.23, F24P17_23	10.0	8.0	2.0	10.3
90	AT4G03210.2 | Symbol: XTH9 | similar to xyloglucan:xyloglucosyl transferase / xyloglucan endotransglycosylase / endo-xyloglucan transferase (EXGT-A4) [Arabidopsis thaliana] (TAIR:At5g13870.1); similar to sadtomato protein [Capsicum annuum] (GB:AAS77347.1); contains InterPro domain Glycoside hydrolase, family 16 (InterPro:IPR000757); contains InterPro domain Beta-glucanase (InterPro:IPR008264) | chr4:1415953-1417352 FORWARD | Aliases: F4C21.14, F4C21_14, XYLOGLUCAN ENDOTRANSGLUCOSYLASE/HYDROLASE 9	10.0	4.1	5.9	21.2
1552	AT1G28350.1 | similar to tRNA synthetase class I (W and Y) family protein [Arabidopsis thaliana] (TAIR:At2g33840.1); similar to putative tyrosyl-tRNA synthetase [Oryza sativa (japonica cultivar-group)] (GB:XP_480120.1); contains InterPro domain Aminoacyl-tRNA synthetase, class Ib (InterPro:IPR002305) | chr1:9944470-9949786 FORWARD | Aliases: F3M18.22, F3M18_22	10.0	7.5	2.5	8.1
1553	AT2G33840.1 | tRNA synthetase class I (W and Y) family protein, similar to SP:P54577 Tyrosyl-tRNA synthetase (EC 6.1.1.1) (Tyrosyl--tRNA ligase) (TyrRS) {Homo sapiens}; contains Pfam profile PF00579: tRNA synthetases class I (W and Y) | chr2:14322044-14324575 FORWARD | Aliases: T1B8.14, T1B8_14	10.0	7.5	2.5	8.1
8794	AT4G23650.1 | Symbol: CDPK6 | calcium-dependent protein kinase, putative / CDPK, putative, similar to calcium-dependent protein kinase (Marchantia polymorpha) gi:5162877:dbj:BAA81748; contains protein kinase domain, Pfam:PF00069; contains EF hand domain (calcium-binding EF-hand), Pfam:PF00036, INTERPRO:IPR002048 | chr4:12324779-12327469 REVERSE | Aliases: CALCIUM DEPENDENT PROTEIN KINASE 6, CPK3, F9D16.120, F9D16_120	10.0	9.3	0.7	2.8
1079	AT4G02510.1 | Symbol: TOC159 |	10.0	8.2	1.8	9.3
11707	AT5G41520.1 | 40S ribosomal protein S10 (RPS10B), contains similarity to 40S ribosomal protein S10 | chr5:16626419-16627889 REVERSE | Aliases: MBK23.4, MBK23_4	10.0	9.4	0.6	1.9
16690	AT5G56630.1 | phosphofructokinase family protein, similar to phosphofructokinase (Amycolatopsis methanolica) GI:17432243; contains Pfam profile PF00365: Phosphofructokinase | chr5:22941148-22944189 FORWARD | Aliases: MIK19.8, MIK19_8	10.0	9.7	0.3	0.8
1367	AT3G18130.1 | guanine nucleotide-binding family protein / activated protein kinase C receptor (RACK1), identical to guanine nucleotide-binding protein; activated protein kinase C receptor; RACK1 (GI:9294068) {Arabidopsis thaliana}; contains Pfam profile: PF00400 WD domain, G-beta repeat (7 copies) | chr3:6210912-6212439 REVERSE | Aliases: MRC8.11	10.0	6.2	3.7	8.5
16119	AT5G20660.1 | 24 kDa vacuolar protein, putative, similar to 24 kDa vacuolar protein VP24 (Ipomoea batatas) gi:5821406:dbj:BAA83809 | chr5:6986237-6991045 FORWARD | Aliases: T1M15.60, T1M15_60	10.0	10.1	-0.2	-0.9

5723	AT1G21720.1 | Symbol: PBC1 | 20S proteasome beta subunit C1 (PBC1) (PRCT), almost identical to GB:AAC32069 from (Arabidopsis thaliana), EST gb:T76747 comes from this gene; identical to cDNA proteasome subunit prct GI:2511567 | chr1:7626272-7628251 FORWARD | Aliases: 20S PROTEASOME BETA SUBUNIT PBC1, F8K7.15, F8K7_15	10.0	6.1	3.9	4.2
7845	AT1G36980.1 | expressed protein | chr1:14027484-14029382 FORWARD | Aliases: T32E20.29, T32E20_29	10.0	10.5	-0.5	-3.2
1583	AT3G05060.1 | SAR DNA-binding protein, putative, strong similarity to SAR DNA-binding protein-1 (Pisum sativum) GI:3132696; contains Pfam profile PF01798: Putative snoRNA binding domain; encodes NOP58-like protein | chr3:1412873-1415958 REVERSE | Aliases: T12H1.2, T12H1_2	10.0	7.9	2.1	8.1
2697	AT4G02930.1 | elongation factor Tu, putative / EF-Tu, putative, similar to mitochondrial elongation factor Tu (Arabidopsis thaliana) gi:1149571:emb:CAA61511 | chr4:1295409-1298397 REVERSE | Aliases: T4I9.19	10.0	7.1	2.9	6.5
16958	AT1G06760.1 | histone H1, putative, similar to histone H1-1 GB:CAA44312 GI:16314 from (Arabidopsis thaliana); identical to cDNA H1-1C mRNA for histone H1-1 (partial) GI:732560 | chr1:2076503-2077697 REVERSE | Aliases: F4H5.15, F4H5_15	10.0	10.2	-0.2	-0.8
6098	AT1G16700.1 | NADH-ubiquinone oxidoreductase 23 kDa subunit, mitochondrial, putative, very strong similarity to SP:Q42599 NADH-ubiquinone oxidoreductase 23 kDa subunit, mitochondrial precursor (EC 1.6.5.3) (EC 1.6.99.3) (Complex I-23KD) (CI-23KD) (Complex I- 28.5KD) (CI-28.5KD) {Arabidopsis thaliana}; contains Pfam profile PF00037: iron-sulfur cluster-binding protein | chr1:5709718-5711889 FORWARD | Aliases: F19K19.1, F19K19_1	10.0	6.9	3.1	4.0
6099	AT1G79010.1 | NADH-ubiquinone oxidoreductase 23 kDa subunit, mitochondrial (TYKY), identical to SP:Q42599 NADH-ubiquinone oxidoreductase 23 kDa subunit, mitochondrial precursor (EC 1.6.5.3) (EC 1.6.99.3) (Complex I-23KD) (CI-23KD) (Complex I- 28.5KD) (CI-28.5KD) {Arabidopsis thaliana} | chr1:29729814-29731910 REVERSE | Aliases: YUP8H12R.37, YUP8H12R_37	10.0	6.9	3.1	4.0
8406	AT4G24470.2 | Symbol: ZIM | zinc finger (GATA type) protein ZIM (ZIM), identical to zinc-finger protein expressed in Inflorescence Meristem, ZIM gi:8918533 from (Arabidopsis thaliana) | chr4:12645600-12647950 FORWARD | Aliases: T22A6.300, T22A6_300	10.0	8.9	1.0	2.9
506	AT1G24735.1 | caffeoyl-CoA 3-O-methyltransferase, putative, similar to SP:Q43237 (Vitis vinifera), GI:684942 (Medicago sativa subsp. sativa) | chr1:8758685-8759434 FORWARD | Aliases: F5A9.20, F5A9_20	10.0	4.9	5.1	11.9
3683	AT4G27500.1 | Symbol: PPI1 | expressed protein, non-consensus GA donor splice site at exon 6 | chr4:13743178-13745906 FORWARD | Aliases: F27G19.100, F27G19_100, PROTON PUMP INTERACTOR 1	10.0	11.3	-1.3	-5.5
901	AT3G25220.1 | Symbol: FKBP15 1 | FK506-binding protein 2-1 (FKBP15-1) / immunophilin / peptidyl-prolyl cis-trans isomerase / rotamase, identical to SP:Q38935 FK506-binding protein 2-1 precursor (EC 5.2.1.8) (Peptidyl-prolyl cis- trans isomerase) (PPiase) (Rotamase) (15 kDa FKBP) (FKBP-15-1) {Arabidopsis thaliana}, immunophilin (FKBP15-1) GB:U52046 (Arabidopsis thaliana) (Proc. Natl. Acad. Sci. U.S.A. 93 (14), 6964-6969 (1996)) | chr3:9183855-9185849 FORWARD | Aliases: MJL12.19	9.9	8.3	1.7	9.9
38	AT2G21330.3 | similar to fructose-bisphosphate aldolase, putative [Arabidopsis thaliana] (TAIR:At4g38970.1); similar to plastidic aldolase NPALDP1 [Nicotiana paniculata] (GB:BAA77604.1); similar to latex plastidic aldolase-like protein [Hevea brasiliensis] (GB:AAM46780.1); contains InterPro domain Fructose-bisphosphate aldolase, class-I (InterPro:IPR000741) | chr2:9135228-9137293 REVERSE | Aliases: F3K23.9, F3K23_9	9.9	2.7	7.3	27.3
12138	AT1G80780.2 | CCR4-NOT transcription complex protein, putative, similar to SWISS-PROT:Q60809 CCR4-NOT transcription complex, subunit 7 (CCR4-associated factor 1, (CAF1) (Mus musculus) | chr1:30363007-30364265 FORWARD | Aliases: F23A5.13, F23A5_13	9.9	10.4	-0.4	-1.8
6329	AT4G38740.1 | Symbol: ROC1 | peptidyl-prolyl cis-trans isomerase / cyclophilin / rotamase / cyclosporin A-binding protein (ROC1), identical to SP:P34790 Peptidyl-prolyl cis-trans isomerase (EC 5.2.1.8) (PPIase) (Rotamase) (Cyclophilin) (Cyclosporin A-binding protein) {Arabidopsis thaliana} | chr4:18083389-18084245 REVERSE | Aliases: CYCLOPHILIN, T9A14.20, T9A14_20	9.9	6.7	3.2	3.8
1361	AT3G01690.1 | expressed protein | chr3:256562-258554 FORWARD | Aliases: F4P13.23, F4P13_23	9.9	11.6	-1.6	-8.6
17678	AT5G67600.1 | expressed protein | chr5:26976797-26977535 REVERSE | Aliases: K9I9.17, K9I9_17	9.9	10.2	-0.2	-0.6
12942	AT3G57280.1 | expressed protein, contains Pfam profile PF03647: Uncharacterised protein family (UPF0136) | chr3:21204820-21206695 FORWARD | Aliases: F28O9.130	9.9	9.6	0.3	1.6
4103	AT5G47620.3 | heterogeneous nuclear ribonucleoprotein, putative / hnRNP, putative | chr5:19319496-19321316 REVERSE | Aliases: MNJ7.21, MNJ7_21	9.9	10.8	-0.9	-5.2
1127	AT4G25740.2 | similar to 40S ribosomal protein S10 (RPS10C) [Arabidopsis thaliana] (TAIR:At5g52650.1); similar to RS10_ORYSA 40S ribosomal protein S10 (GB:Q9AYP4); contains InterPro domain Plectin/S10, N-terminal (InterPro:IPR005326) | chr4:13107273-13108822 REVERSE | Aliases: F14M19.20, F14M19_20	9.9	7.2	2.7	9.2
19896	AT1G51680.2 | Symbol: 4CL1 | 4-coumarate--CoA ligase 1 / 4-coumaroyl-CoA synthase 1 (4CL1), identical to SP:Q42524 4-coumarate--CoA ligase 1 (EC 6.2.1.12) (4CL 1) (4-coumaroyl-CoA synthase 1) {Arabidopsis thaliana} | chr1:19163097-19165220 REVERSE | Aliases: 4 COUMARATE:COA LIGASE, 4 COUMARATE:COA LIGASE 1, 4CL.1, AT4CL1, F19C24.11, F19C24_11	9.9	9.8	0.2	0.2

3048	AT1G50200.1 | Symbol: ALATS | aminoacyl-tRNA synthetase family protein, contains Pfam profiles: PF01411 tRNA synthetases class II (A), PF02272 DHHA1 domain | chr1:18594882-18602149 REVERSE | Aliases: ACD, ALANYL TRNA SYNTHETASE, ALARS, F14I3.17, F14I3_17	9.9	10.9	-1.0	-6.1
4648	AT3G43520.1 | expressed protein, contains Pfam profile PF03647: Uncharacterised protein family (UPF0136) | chr3:15417012-15418896 FORWARD | Aliases: T18D12.90	9.9	8.3	1.6	4.8
18066	AT1G01550.2 | Symbol: BPS1 | similar to expressed protein [Arabidopsis thaliana] (TAIR:At2g46080.1); similar to OSJNBa0018M05.6 [Oryza sativa (japonica cultivar-group)] (GB:XP_474319.1) | chr1:199792-201775 FORWARD | Aliases: BYPASS 1, F22L4.9, F22L4_9	9.9	10.1	-0.2	-0.6
51	AT4G38970.2 | fructose-bisphosphate aldolase, putative, strong similarity to plastidic fructose-bisphosphate aldolase (EC 4.1.2.13) from Nicotiana paniculata (NPALDP1) (GI:4827251), Oryza sativa, PIR2:T02057 (SP:Q40677) | chr4:18163490-18165734 REVERSE | Aliases: F19H22.70, F19H22_70	9.9	3.8	6.2	25.6
14118	AT2G16710.1 | hesB-like domain-containing protein, similar to IscA (putative iron-sulfur cluster assembly protein) (Azotobacter vinelandii) GI:2271523; contains Pfam profile PF01521: HesB-like domain | chr2:7255255-7257367 FORWARD | Aliases: T24I21.12, T24I21_12	9.9	9.5	0.4	1.4
3428	AT4G25130.1 | peptide methionine sulfoxide reductase, putative, strong similarity to SP:P54151 Peptide methionine sulfoxide reductase (EC 1.8.4.6) {Brassica napus}; contains Pfam profile PF01625: Peptide methionine sulfoxide reductase | chr4:12898603-12900074 REVERSE | Aliases: F24A6.2	9.9	6.8	3.2	5.7
10159	AT1G64850.1 | calcium-binding EF hand family protein, contains INTERPRO:IPR002048 calcium-binding EF-hand domain | chr1:24100098-24101271 REVERSE | Aliases: F13O11.15, F13O11_15	9.9	9.6	0.3	2.3
4329	AT3G13845.1 | expressed protein | chr3:4557333-4558586 REVERSE | Aliases: None	9.9	8.7	1.2	5.0
16676	AT3G19640.1 | magnesium transporter CorA-like family protein (MRS2-3), low similarity to SP:Q01926 RNA splicing protein MRS2, mitochondrial precursor {Saccharomyces cerevisiae}; contains Pfam profile PF01544: CorA-like Mg2+ transporter protein | chr3:6820936-6823312 FORWARD | Aliases: MMB12.11	9.9	9.7	0.2	0.8
2106	AT2G44310.1 | calcium-binding EF hand family protein, contains INTERPRO:IPR002048 calcium-binding EF-hand domain | chr2:18316288-18316902 FORWARD | Aliases: F4I1.12	9.9	7.5	2.3	7.2
16741	AT4G27750.1 | expressed protein | chr4:13841694-13843639 FORWARD | Aliases: T29A15.240, T29A15_240	9.9	9.6	0.3	0.8
9213	AT2G47470.3 | Symbol: ATPDIL2 1 | similar to thioredoxin family protein [Arabidopsis thaliana] (TAIR:At2g32920.1); similar to thioredoxin family protein [Arabidopsis thaliana] (TAIR:At1g04980.1); similar to protein disulfide-isomerase precursor [Nicotiana tabacum] (GB:CAA72092.1); contains InterPro domain Disulphide isomerase (InterPro:IPR005788); contains InterPro domain Thioredoxin type domain (InterPro:IPR006662); contains InterPro domain Thioredoxin domain 2 (InterPro:IPR006663) | chr2:19488492-19491085 FORWARD | Aliases: ATPDIL2 1, PDI LIKE 2 1, T30B22.23	9.9	8.8	1.1	2.6
406	AT1G48350.1 | ribosomal protein L18 family protein, similar to ribosomal protein L18 GI:3980238 from (Thermotoga maritima) | chr1:17870876-17872010 FORWARD | Aliases: F11A17.10, F11A17_10	9.9	5.7	4.2	12.8
13837	AT3G11930.4 | similar to universal stress protein (USP) family protein [Arabidopsis thaliana] (TAIR:At3g58450.1); similar to putative ethylene-responsive protein [Oryza sativa (japonica cultivar-group)] (GB:AAP53941.1); contains InterPro domain Usp domain (InterPro:IPR006016); contains InterPro domain Universal stress protein (Usp) (InterPro:IPR006015) | chr3:3776265-3777700 FORWARD | Aliases: MEC18.3	9.9	10.2	-0.3	-1.4
96	AT4G24040.1 | Symbol: ATTRE1 |	9.9	3.8	6.0	21.1
18295	AT5G49540.1 | expressed protein, contains Pfam profile PF05646: Protein of unknown function (DUF786) | chr5:20121810-20123334 REVERSE | Aliases: K6M13.9, K6M13_9	9.9	9.8	0.1	0.5
716	AT2G33800.1 | ribosomal protein S5 family protein, contains Pfam profiles PF03719: Ribosomal protein S5, C-terminal domain, PF00333: Ribosomal protein S5, N-terminal domain | chr2:14307848-14309506 REVERSE | Aliases: T1B8.10, T1B8_10	9.9	7.4	2.5	10.7
6376	AT4G02580.1 | NADH-ubiquinone oxidoreductase 24 kDa subunit, putative, similar to NADH-ubiquinone oxidoreductase 24 kDa subunit, mitochondrial precursor (EC 1.6.5.3) (EC 1.6.99.3) (Polypeptide II) (Swiss-Prot:P04394) (Bos taurus) | chr4:1134494-1137156 FORWARD | Aliases: T10P11.14, T10P11_14	9.9	7.2	2.7	3.8
8779	AT2G01350.2 | quinolinate phosphoribosyl transferase family protein, contains Pfam profile: PF01729 quinolinate phosphoribosyl transferase, C-terminal domain | chr2:165145-167246 REVERSE | Aliases: F10A8.23, F10A8_23	9.9	9.3	0.6	2.8
7565	AT1G30230.1 | elongation factor 1-beta / EF-1-beta, identical to SP:P48006 Elongation factor 1-beta (EF-1-beta) {Arabidopsis thaliana}	9.9	8.2	1.7	3.3
7566	AT2G18110.1 | elongation factor 1-beta, putative / EF-1-beta, putative, nearly identical to eEF-1beta (Arabidopsis thaliana) GI:398606	9.9	8.2	1.7	3.3

20049	AT1G60940.2 | serine/threonine protein kinase, putative, similar to serine/threonine-protein kinase ASK1 from (Arabidopsis thaliana), SWISS-PROT:P43291 | chr1:22442804-22445882 REVERSE | Aliases: T7P1.8, T7P1_8	9.9	9.9	-0.1	-0.2
14253	NA	9.9	9.0	0.8	1.3
1003	AT2G37760.3 | aldo/keto reductase family protein, similar to chalcone reductase (Sesbania rostrata)(GI:2792155), and aldose reductase ALDRXV4 (Xerophyta viscosa)(GI:4539944), (Hordeum vulgare)(GI:728592) | chr2:15838948-15840909 FORWARD | Aliases: T8P21.6	9.9	6.7	3.2	9.5
19811	AT4G31180.2 | aspartyl-tRNA synthetase, putative / aspartate--tRNA ligase, putative, similar to Aspartyl-tRNA synthetase (Aspartate--tRNA ligase) (AspRS) (Homo sapiens) GI:20178330 | chr4:15155916-15159568 FORWARD | Aliases: F6E21.100, F6E21_100	9.9	9.9	-0.0	-0.2
11100	AT2G21160.1 | translocon-associated protein alpha (TRAP alpha) family protein, contains Pfam profile: PF03896 translocon-associated protein (TRAP), alpha subunit | chr2:9075412-9077508 FORWARD | Aliases: F26H11.8, F26H11_8	9.9	9.1	0.7	2.0
3268	AT5G23900.1 | 60S ribosomal protein L13 (RPL13D) | chr5:8064016-8065520 REVERSE | Aliases: MRO11.6, MRO11_6	9.9	8.0	1.9	5.8
2538	AT1G75270.1 | dehydroascorbate reductase, putative, similar to GI:6939839 from (Oryza sativa) | chr1:28253736-28255010 REVERSE | Aliases: F22H5.1, F22H5_1	9.9	7.7	2.2	6.6
17402	AT5G65430.2 | Symbol: GRF8 | 14-3-3 protein GF14 kappa (GRF8), identical to 14-3-3 protein GF14 kappa GI:5802794, SP:P48348 from (Arabidopsis thaliana) | chr5:26165427-26167568 REVERSE | Aliases: GF14 KAPPA, GF14 KAPPA ISOFORM, MNA5.16, MNA5_16	9.9	10.2	-0.4	-0.7
5812	AT5G42890.1 | sterol carrier protein 2 (SCP-2) family protein, similar to sterol carrier protein-2 (Homo sapiens) GI:432973; contains Pfam profile PF02036: SCP-2 sterol transfer family | chr5:17211500-17213260 REVERSE | Aliases: MBD2.8, MBD2_8	9.9	8.7	1.1	4.1
930	AT4G14320.1 | 60S ribosomal protein L36a/L44 (RPL36aB)	9.9	7.8	2.1	9.8
16704	AT1G62790.2 | protease inhibitor/seed storage/lipid transfer protein (LTP) family protein, contains Pfam protease inhibitor/seed storage/LTP family domain PF00234 | chr1:23256003-23257547 FORWARD | Aliases: F23N19.16, F23N19_16	9.9	9.6	0.2	0.8
7907	AT5G63850.1 | Symbol: AAP4 | amino acid transporter 4, putative (AAP4), identical to amino acid transporter GI:608671 from (Arabidopsis thaliana); | chr5:25568281-25570746 FORWARD | Aliases: MGI19.6, MGI19_6	9.8	6.6	3.2	3.2
5735	AT5G43850.1 | acireductone dioxygenase (ARD/ARD') family protein, similar to iron-deficiency induced gene (Hordeum vulgare) GI:14522834, SIPL (Homo sapiens) GI:16551383; contains Pfam profile PF03079: ARD/ARD' family | chr5:17644412-17646385 REVERSE | Aliases: MQD19.21, MQD19_21	9.8	9.0	0.9	4.2
805	AT2G30140.1 | UDP-glucoronosyl/UDP-glucosyl transferase family protein, contains Pfam profile: PF00201 UDP-glucoronosyl and UDP-glucosyl transferase | chr2:12879211-12880897 FORWARD | Aliases: T27E13.12, T27E13_12	9.8	7.1	2.8	10.2
2999	AT3G03160.1 | expressed protein | chr3:729775-730474 FORWARD | Aliases: T17B22.15, T17B22_15	9.8	8.8	1.1	6.1
8696	AT4G02450.1 | glycine-rich protein, similar to several proteins containing a tandem repeat region such as Plasmodium falciparum GGM tandem repeat protein (GB:U27807) | chr4:1073774-1075878 REVERSE | Aliases: T14P8.5, T14P8_5	9.8	9.3	0.6	2.8
19425	ATCG00490.1 | Symbol: RBCL | large subunit of RUBISCO. | chrC:54958-56397 FORWARD | Aliases: RBCL	9.8	9.6	0.2	0.3
7886	AT3G62600.1 | DNAJ heat shock family protein, similar to DnaJ homolog subfamily B member 11 precursor (SP:Q99KV1){Mus musculus}; contains Pfam PF00226: DnaJ domain; contains PfaPF01556: DnaJ C terminal regionm | chr3:23161766-23164486 REVERSE | Aliases: F26K9.30	9.8	8.7	1.1	3.2
17288	AT3G29075.1 | glycine-rich protein | chr3:11052726-11053931 REVERSE | Aliases: MRI12.9	9.8	9.5	0.3	0.7
6480	AT5G09650.1 | inorganic pyrophosphatase family protein, similar to SP:Q15181 Inorganic pyrophosphatase (EC 3.6.1.1) (Pyrophosphate {Homo sapiens}; contains Pfam profile PF00719: inorganic pyrophosphatase | chr5:2991156-2993214 REVERSE | Aliases: F17I14.160, F17I14_160	9.8	8.5	1.4	3.8
510	AT3G49470.1 | nascent polypeptide-associated complex (NAC) domain-containing protein, similar to alpha-NAC, non-muscle form (Mus musculus) GI:1666690; contains Pfam profile PF01849: NAC domain | chr3:18351974-18353459 FORWARD | Aliases: T9C5.70	9.8	5.8	4.0	11.9
1759	AT3G63250.2 | Symbol: ATHMT 2/HMT 2 | homocysteine S-methyltransferase 2 (HMT-2), 99.7% identical to homocysteine S-methyltransferase AtHMT-2 (GI:6685163) (Arabidopsis thaliana) | chr3:23381313-23383591 REVERSE | Aliases: ATHMT 2, F16M2.100, HMT 2	9.8	7.6	2.3	7.8
169	AT4G17600.1 | Symbol: LIL3:1 |	9.8	3.2	6.7	17.2
15234	AT1G21770.1 | expressed protein | chr1:7651635-7652275 REVERSE | Aliases: F8K7.21, F8K7_21	9.8	9.5	0.4	1.1

4285	AT1G62180.1 | Symbol: APR2 | 5'-adenylylsulfate reductase 2, chloroplast (APR2) (APSR) / adenosine 5'-phosphosulfate 5'-adenylylsulfate (APS) sulfotransferase 2 / 3'-phosphoadenosine-5'-phosphosulfate (PAPS) reductase homolog 43 (PRH-43), identical to SP:P92981 5'-adenylylsulfate reductase 2, chloroplast precursor (EC 1.8.4.9) (Adenosine 5'-phosphosulfate 5'-adenylylsulfate sulfotransferase 2) (APS sulfotransferase 2) (Thioredoxin independent APS reductase 2) (3'-phosphoadenosine-5'-phosphosulfate reductase homolog 43) (PAPS reductase homolog 43) (Prh-43) {Arabidopsis thaliana}; identical to cDNA PAPS reductase homolog (PRH43) GI:1710115	9.8	8.4	1.5	5.0
7999	AT3G61860.1 | Symbol: ATRSP31 | arginine/serine-rich splicing factor RSP31 (RSP31), identical to SP:P92964 Arginine/serine-rich splicing factor RSP31 {Arabidopsis thaliana} | chr3:22911047-22913309 REVERSE | Aliases: ARGININE/SERINE RICH SPLICING FACTOR, F21F14.30	9.8	10.6	-0.7	-3.1
425	AT5G23020.1 | Symbol: MAM L | 2-isopropylmalate synthase 2 (IMS2), identical to 2-isopropylmalate synthase (IMS2) (Arabidopsis thaliana) GI:12330689 | chr5:7718206-7721842 FORWARD | Aliases: 2 ISOPROPYLMALATE SYNTHASE 2, IMS2, METHYLTHIOALKYMALATE SYNTHASE LIKE, MYJ24.1, MYJ24_1	9.8	11.1	-1.3	-12.6
10719	AT2G16860.1 | GCIP-interacting family protein, similar to GCIP-interacting protein mp29 (GI:27372623) (Mus musculus); similar to GCIP-interacting protein P29 (GI:11967379) (Homo sapiens) | chr2:7311002-7313735 REVERSE | Aliases: F12A24.4, F12A24_4	9.8	9.3	0.5	2.2
5923	AT3G24200.2 | similar to putative oxygenase [Oryza sativa (japonica cultivar-group)] (GB:XP_470364.1); contains InterPro domain Flavoprotein monooxygenase (InterPro:IPR000733); contains InterPro domain NAD-binding site (InterPro:IPR000205); contains InterPro domain Aromatic-ring hydroxylase (InterPro:IPR003042); contains InterPro domain UbiH/COQ6 monooxygenase (InterPro:IPR000689) | chr3:8747838-8751376 REVERSE | Aliases: MUJ8.10	9.8	8.4	1.4	4.1
1398	AT1G17880.1 | nascent polypeptide-associated complex (NAC) domain-containing protein / BTF3b-like transcription factor, putative, similar to SP:P20290 Transcription factor BTF3 (RNA polymerase B transcription factor 3) {Homo sapiens}; contains Pfam profile PF01849: NAC domain; identical to cDNA BTF3b-like factor GI:5912423 | chr1:6152420-6153946 REVERSE | Aliases: F2H15.11, F2H15_11	9.8	6.4	3.4	8.5
3674	AT5G58930.1 | expressed protein, contains Pfam PF05340: Protein of unknown function (DUF740) | chr5:23811541-23813687 REVERSE | Aliases: K19M22.12, K19M22_12	9.8	9.2	0.7	5.5
11743	AT1G64720.1 | Symbol: CP5 | expressed protein, weak similarity to SP:P53809 Phosphatidylcholine transfer protein (PC-TP) {Rattus norvegicus} | chr1:24050216-24052261 REVERSE | Aliases: CP5, F13O11.4, F13O11_4	9.8	9.2	0.6	1.9
19695	AT5G47200.1 | Ras-related GTP-binding protein, putative, similar to GTP-binding protein GI:303750 from (Pisum sativum)	9.8	9.7	0.1	0.3
2888	AT1G12200.1 | flavin-containing monooxygenase family protein / FMO family protein, low similarity to FMO2 from Homo sapiens (SP:Q99518); contains Pfam profile: PF00743 Flavin-binding monooxygenase-like | chr1:4137531-4140026 FORWARD | Aliases: T28K15.7, T28K15_7	9.8	7.5	2.3	6.3
703	AT2G25720.1 | expressed protein | chr2:10962597-10963130 REVERSE | Aliases: F3N11.17, F3N11_17	9.8	6.9	2.9	10.7
4399	AT5G20500.1 | glutaredoxin, putative, similar to glutaredoxin (Populus tremula x Populus tremuloides) gi:19548658:gb:AAL90750 | chr5:6938600-6940269 FORWARD | Aliases: F7C8.90, F7C8_90	9.8	8.0	1.9	5.0
17176	AT2G37340.3 | Symbol: RSZ33 | splicing factor RSZ33 (RSZ33), nearly identical to splicing factor RSZ33 (Arabidopsis thaliana) GI:9843663; contains Pfam profiles PF00076: RNA recognition motif. (a.k.a. RRM, RBD, or RNP domain), PF00098: Zinc knuckle | chr2:15677226-15679750 REVERSE | Aliases: F3G5.13, F3G5_13	9.8	9.7	0.1	0.7
3269	AT2G26990.1 | Symbol: FUS12 | COP9 signalosome complex subunit 2 / CSN complex subunit 2 (CSN2), proteasome, COP9-complex and eIF3-domain protein; identical to CSN complex subunit 2 (Arabidopsis thaliana) GI:18056655; identical to cDNA CSN complex subunit 2 (CSN2) GI:18056654 | chr2:11526585-11529665 REVERSE | Aliases: ATCSN2, CONSTITUTIVE PHOTOMORPHOGENIC 12, COP12, CSN2, FUSCA 12, T20P8.4, T20P8_4	9.8	7.8	2.0	5.8
2127	AT1G47960.1 | Symbol: C/VIF1 |	9.8	7.4	2.4	7.2
4135	AT5G16150.3 | hexose transporter, putative, strong similarity to hexose transporter (Arabidopsis thaliana) GI:8347250; contains Pfam profile PF00083: major facilitator superfamily protein | chr5:5272690-5275818 FORWARD | Aliases: T21H19.70, T21H19_70	9.8	8.6	1.2	5.1
739	AT1G16000.1 | expressed protein | chr1:5494530-5495317 FORWARD | Aliases: T24D18.10, T24D18_10	9.8	6.4	3.5	10.6
3163	AT2G18040.1 | Symbol: PIN1AT |	9.8	7.6	2.3	5.9
293	AT2G41290.1 | strictosidine synthase family protein, similar to strictosidine synthase (Rauvolfia serpentina)(SP:P15324); contains strictosidine synthase domain PF03088 | chr2:17217533-17220037 REVERSE | Aliases: F13H10.16, F13H10_16	9.8	6.1	3.7	14.4
13749	ATCG00740.1 | Symbol: RPOA | RNA polymerase alpha subunit | chrC:77901-78890 REVERSE | Aliases: RPOA	9.8	9.0	0.8	1.4
20039	AT1G63940.4 | monodehydroascorbate reductase, putative, similar to monodehydroascorbate reductase GB:AAD28178 (Brassica juncea) | chr1:23733769-23737555 FORWARD | Aliases: T12P18.4, T12P18_4	9.8	9.9	-0.1	-0.2

343	AT2G16890.2 | UDP-glucoronosyl/UDP-glucosyl transferase family protein, contains Pfam profile: PF00201 UDP-glucoronosyl and UDP-glucosyl transferase | chr2:7323971-7326263 FORWARD | Aliases: F12A24.7, F12A24_7	9.8	4.1	5.7	13.7
8432	AT1G32210.1 | Symbol: ATDAD1 |	9.8	8.9	0.9	2.9
514	AT5G50800.1 | nodulin MtN3 family protein, similar to MtN3 GI:1619602 (root nodule development) from (Medicago truncatula) | chr5:20682303-20684530 REVERSE | Aliases: K7B16.1, K7B16_1	9.8	4.6	5.2	11.9
11654	AT1G26670.1 | Symbol: VTI12 | vesical transport v-SNARE 12 (VTI12) / vesicle soluble NSF attachment protein receptor VTI1b (VTI1B) receptor VTI1b, identical to SP:Q9SEL5 Vesicle transport v-SNARE 12 (AtVTI12) (Vesicle transport v-SNARE protein VTI1b) (Vesicle soluble NSF attachment protein receptor VTI1b) (AtVTI1b) {Arabidopsis thaliana} | chr1:9215951-9217990 FORWARD | Aliases: ATVTI12, T24P13.5, T24P13_5, VTI1B	9.8	10.1	-0.3	-1.9
1927	AT1G77380.1 | Symbol: AAP3 | amino acid carrier, putative / amino acid permease, putative, strong similarity to amino acid carrier GI:3293031 from (Ricinus communis); contains Pfam profile PF01490: Transmembrane amino acid transporter protein; identical to cDNA AAP3 (Amino Acid Permease) GI:3970651 | chr1:29079782-29082284 REVERSE | Aliases: F2P24.9, F2P24_9	9.8	7.1	2.7	7.5
120	AT2G07340.2 | prefoldin-related KE2 family protein, contains similarity to Swiss-Prot:O60925 prefoldin subunit 1 (Homo sapiens); contains Pfam domain, PF01920: KE2 family protein | chr2:3045556-3046716 FORWARD | Aliases: T13E11.11, T13E11_11	9.8	5.9	3.9	19.7
18934	AT1G66240.1 | copper homeostasis factor, putative / copper chaperone, putative (CCH), similar to gi:3168840 contains Pfam profile PF00403: Heavy-metal-associated domain | chr1:24689940-24690995 REVERSE | Aliases: T6J19.6, T6J19_6	9.8	9.7	0.1	0.4
18680	AT3G06350.1 | Symbol: EMB3004 | dehydroquinate dehydratase, putative / shikimate dehydrogenase, putative, similar to dehydroquinate dehydratase/shikimate dehydrogenase (Nicotiana tabacum)(GI:535771), dehydroquinate dehydratase/shikimate:NADP oxidoreductase (Lycopersicon esculentum)(GI:3169883) | chr3:1924368-1927759 REVERSE | Aliases: EMB3004, EMBRYO DEFECTIVE 3004, F24P17.18, F24P17_18	9.8	9.7	0.1	0.5
5477	AT3G45770.2 | oxidoreductase, zinc-binding dehydrogenase family protein, similar to nuclear receptor binding factor-1 NRBF-1 - Rattus norvegicus, EMBL:AB015724; contains Pfam zinc-binding dehydrogenase domain PF00107 | chr3:16816547-16818780 REVERSE | Aliases: F16L2.2	9.8	7.9	1.9	4.3
351	AT1G11430.1 | plastid developmental protein DAG, putative, similar to DAG protein, chloroplast precursor (Garden snapdragon) SWISS-PROT:Q38732	9.8	6.5	3.3	13.5
2560	AT5G55160.1 | Symbol: SUM2 | small ubiquitin-like modifier 2 (SUMO), similar to ubiquitin-like protein SMT3 SP:P55852 from (Arabidopsis thaliana); identical to cDNA small ubiquitin-like modifier 2 (SUMO) GI:22652843; contains Pfam profile PF00240: Ubiquitin family | chr5:22400795-22402151 REVERSE | Aliases: MCO15.11, MCO15_11, SMALL UBIQUITIN LIKE MODIFIER 2, SUMO 2, SUMO2	9.8	8.3	1.4	6.6
3599	AT4G12340.1 | expressed protein | chr4:7321276-7322448 REVERSE | Aliases: T4C9.180, T4C9_180	9.8	8.1	1.7	5.6
2458	AT1G04430.1 | dehydration-responsive protein-related, similar to early-responsive to dehydration stress ERD3 protein (Arabidopsis thaliana) GI:15320410; contains Pfam profile PF03141: Putative methyltransferase | chr1:1198136-1201526 FORWARD | Aliases: F19P19.11, F19P19_11	9.8	7.6	2.2	6.7
4199	AT5G64310.1 | Symbol: AGP1 | arabinogalactan-protein (AGP1), identical to gi:3883120 gb:AAC77823 | chr5:25739159-25739872 FORWARD | Aliases: ARABINOGALACTAN PROTEIN 1, MSJ1.15, MSJ1_15	9.8	7.9	1.9	5.1
8909	AT1G43310.1 | triose phosphate/phosphate translocator-related, similar to SP:P52178 Triose phosphate/phosphate translocator, non-green plastid, chloroplast precursor (CTPT) {Brassica oleracea} | chr1:16348414-16348915 REVERSE | Aliases: F1I21.17, F1I21_17	9.8	7.4	2.4	2.7
8910	AT5G33320.1 | Symbol: CUE1 | triose phosphate/phosphate translocator, putative, similar to SWISS-PROT:P52178 triose phosphate/phosphate translocator (Cauliflower) {Brassica oleracea} | chr5:12606068-12608978 FORWARD | Aliases: ARAPPT, CAB UNDEREXPRESSED 1, F19N2.40, F19N2_40, PHOSPHATE/PHOSPHOENOLPYRUVATE TRANSLOCATOR, PHOSPHOENOLPYRUVATE/PHOSPHATE TRANSLOCATOR, PPT	9.8	7.4	2.4	2.7
4771	AT5G25940.1 | early nodulin-related, contains weak similarity to Swiss-Prot:Q02921 early nodulin 93 (N-93) (Glycine max) | chr5:9053704-9055217 REVERSE | Aliases: T1N24.3, T1N24_3	9.8	8.2	1.6	4.7
168	AT3G23325.1 | splicing factor, putative, similar to Splicing factor 3B subunit 10 (SF3b10) (Pre-mRNA splicing factor SF3b 10 kDa subunit) (Swiss-Prot:Q9BWJ5) (Homo sapiens) | chr3:8345778-8347056 FORWARD | Aliases: None	9.8	4.4	5.3	17.2
809	AT2G35795.1 | DNAJ heat shock N-terminal domain-containing protein, contains Pfam profile PF00226 DnaJ domain | chr2:15049289-15050646 FORWARD | Aliases: T20F21.1, T20F21_1	9.8	7.4	2.3	10.2
2122	AT5G47810.1 | phosphofructokinase family protein, similar to phosphofructokinase (Amycolatopsis methanolica) GI:17432243; contains Pfam profile PF00365: Phosphofructokinase | chr5:19373582-19375351 REVERSE | Aliases: MCA23.13, MCA23_13	9.8	8.0	1.8	7.2

5978	AT1G66260.1 | RNA and export factor-binding protein, putative, similar to GI:7159943 from (Mus musculus) (RNA 6 (4), 638-650 (2000)) | chr1:24699303-24702093 REVERSE | Aliases: T6J19.1, T6J19_1	9.8	10.6	-0.8	-4.0
18919	AT3G60450.1 | expressed protein | chr3:22351940-22353285 FORWARD | Aliases: T8B10.110	9.8	9.6	0.2	0.4
2348	AT1G25260.1 | acidic ribosomal protein P0-related, contains similarity to 60S acidic ribosomal protein GI:5815233 from (Homo sapiens) | chr1:8853946-8855847 REVERSE | Aliases: F4F7.35, F4F7_35	9.8	7.0	2.8	6.9
7771	AT5G63840.1 | Symbol: RSW3 | alpha-glucosidase, putative, similar to alpha-glucosidase GI:2648032 from (Solanum tuberosum) | chr5:25562282-25566425 FORWARD | Aliases: MGI19.5, MGI19_5, RADIAL SWELLING 3	9.8	8.7	1.0	3.2
7614	AT5G60160.1 | aspartyl aminopeptidase, putative, similar to SP:Q9ULA0 Aspartyl aminopeptidase (EC 3.4.11.21) {Homo sapiens}; contains Pfam profile PF02127: Aminopeptidase I zinc metalloprotease (M18) | chr5:24240942-24244094 REVERSE | Aliases: F15L12.1, F15L12_1	9.8	8.5	1.2	3.3
3223	AT3G12920.1 | expressed protein | chr3:4121890-4123503 REVERSE | Aliases: MGH6.3	9.8	10.5	-0.8	-5.9
4095	AT3G02630.1 | acyl-(acyl-carrier-protein) desaturase, putative / stearoyl-ACP desaturase, putative, similar to Acyl-(acyl-carrier protein) desaturase from Sesamum indicum GI:575942, Cucumis sativus SP:P32061, Ricinus communis SP:P22337; contains Pfam profile PF03405 Fatty acid desaturase | chr3:562038-564877 FORWARD | Aliases: F16B3.26, F16B3_26	9.8	6.2	3.5	5.2
194	AT5G07800.1 | flavin-containing monooxygenase family protein / FMO family protein, similar to flavin-containing monooxygenase 2 (FMO2) from Homo sapiens (GI:1834493); contains Pfam profile: PF00743 Flavin-binding monooxygenase-like | chr5:2486607-2489297 REVERSE | Aliases: MXM12.4, MXM12_4	9.8	4.5	5.3	16.7
3291	AT5G63400.2 | Symbol: ADK1 | similar to adenylate kinase, putative [Arabidopsis thaliana] (TAIR:At5g50370.1); similar to adenylate kinase-a [Oryza sativa] (GB:BAA01180.1); similar to adenylate kinase [Oryza sativa] (GB:BAA94761.1); contains InterPro domain Adenylate kinase, active site lid (InterPro:IPR007862); contains InterPro domain Adenylate kinase (InterPro:IPR000850) | chr5:25410227-25412090 REVERSE | Aliases: ADENYLATE KINASE 1, MLE2.3, MLE2_3	9.8	7.7	2.1	5.8
4437	AT1G72680.1 | cinnamyl-alcohol dehydrogenase, putative, similar to cinnamyl-alcohol dehydrogenase GB:AAC35846 (Medicago sativa), SP:Q08350 (Picea abies) | chr1:27362894-27364678 REVERSE | Aliases: F28P22.13, F28P22_13	9.7	6.4	3.3	4.9
883	AT2G04630.1 | DNA-directed RNA polymerase II, putative, similar to SP:Q24320 DNA-directed RNA polymerase II 14.4 kDa polypeptide (EC 2.7.7.6) (RPB6) {Drosophila melanogaster}; contains Pfam profile PF01192: RNA polymerases K / 14 to 18 kDa subunit | chr2:1618933-1620308 REVERSE | Aliases: F28I8.33, F28I8_33	9.7	8.1	1.7	9.9
16054	AT2G38710.2 | similar to DUF51 family protein [Dictyostelium discoideum] (GB:EAL65703.1); contains InterPro domain Protein of unknown function DUF51 (InterPro:IPR002733) | chr2:16191447-16194213 REVERSE | Aliases: T6A23.9, T6A23_9	9.7	10.0	-0.3	-1.0
331	AT1G15820.1 | Symbol: LHCB6 | chlorophyll A-B binding protein, chloroplast (LHCB6), nearly identical to Lhcb6 protein (Arabidopsis thaliana) GI:4741960; contains Pfam profile PF00504: Chlorophyll A-B binding protein | chr1:5446123-5447776 REVERSE | Aliases: CP24, F7H2.16, F7H2_16, LHCB6	9.7	4.0	5.7	13.8
761	AT4G09550.1 | expressed protein | chr4:6039132-6040171 FORWARD | Aliases: T15G18.30, T15G18_30	9.7	6.2	3.6	10.5
17035	AT2G17870.1 | cold-shock DNA-binding family protein, contains Pfam domains, PF00313: 'Cold-shock' DNA-binding domain and PF00098: Zinc knuckle	9.7	9.9	-0.2	-0.8
9590	AT1G64520.1 | 26S proteasome regulatory subunit, putative (RPN12), similar to 26S proteasome regulatory complex subunit p30 GB:AAF08395 GI:6434966 from (Drosophila melanogaster) | chr1:23960061-23961981 FORWARD | Aliases: F1N19.9, F1N19_9	9.7	8.3	1.5	2.5
3202	AT5G57120.1 | expressed protein, weak similarity to SP:Q14978 Nucleolar phosphoprotein p130 {Homo sapiens} | chr5:23139766-23141739 REVERSE | Aliases: MUL3.6, MUL3_6	9.7	8.2	1.5	5.9
19131	AT1G15340.1 | Symbol: MBD10 | methyl-CpG-binding domain-containing protein, contains Pfam profile PF01429: Methyl-CpG binding domain | chr1:5275672-5277657 REVERSE | Aliases: F9L1.28, F9L1_28	9.7	9.6	0.1	0.4
8811	AT2G43750.1 | Symbol: OASB | cysteine synthase, chloroplast / O-acetylserine (thiol)-lyase / O-acetylserine sulfhydrylase / cpACS1 (OASB), identical to SP:P47999 Cysteine synthase, chloroplast precursor (EC 4.2.99.8) (O-acetylserine sulfhydrylase) (O-acetylserine (Thiol)-lyase) (cpACS1) {Arabidopsis thaliana}; identical to cDNA O-acetylserine lyase (At.OAS.7-4) GI:6983575 | chr2:18136488-18139629 REVERSE | Aliases: ACS1, ATCS B, CPACS1, CSASE B, CYSTEINE SYNTHASE, CYSTEINE SYNTHASE 1, F18O19.14	9.7	8.9	0.8	2.8
13534	AT4G22670.1 | tetratricopeptide repeat (TPR)-containing protein, similar to Hsc70-interacting protein (Hip) from {Homo sapiens} SP:P50502, {Rattus norvegicus} SP:P50503; contains Pfam profile PF00515: tetratricopeptide repeat (TPR) domain	9.7	9.4	0.3	1.5
1753	AT3G09735.1 | DNA-binding S1FA family protein, contains Pfam profile: PF04689 DNA binding protein S1FA	9.7	8.2	1.5	7.8

3762	AT4G34350.1 | Symbol: CLB6 | Arabidopsis ISPH is involved in the plastid nonmevalonate pathway of isoprenoid biosynthesis. It was shown to complement the lethal phenotype of E.coli ispH mutant and is therefore most likely encodes a protein with 4-hydroxy-3-methylbut-2-en-1-yl diphosphate reductase activity involved in the last step of mevalonate-independent isopentenyl biosynthesis. | chr4:16428484-16431117 REVERSE | Aliases: CHLOROPLAST BIOGENESIS, F10M10.120, F10M10_120, ISPH	9.7	8.1	1.6	5.4
10383	AT5G63030.1 | glutaredoxin, putative, similar to glutaredoxin (Ricinus communis) gi:1732424:emb:CAA89699 | chr5:25303511-25304906 FORWARD | Aliases: MJH22.9, MJH22_9	9.7	9.3	0.4	2.2
3117	AT3G50685.1 | expressed protein | chr3:18845200-18845815 REVERSE | Aliases: None	9.7	8.5	1.2	6.0
2349	AT1G18640.2 | Symbol: PSP | 3-phosphoserine phosphatase (PSP), nearly identical to 3-phosphoserine phosphatase GI:3759177 from (Arabidopsis thaliana)	9.7	7.7	2.0	6.9
3058	AT5G01340.1 | mitochondrial substrate carrier family protein, contains Pfam profile: PF00153 mitochondrial carrier protein | chr5:143054-144719 REVERSE | Aliases: T10O8.50, T10O8_50	9.7	6.9	2.8	6.1
8443	AT3G46030.1 | histone H2B, putative, strong similarity to histone H2B Arabidopsis thaliana GI:2407802, Gossypium hirsutum SP:O22582, Lycopersicon esculentum GI:3021489; contains Pfam profile PF00125 Core histone H2A/H2B/H3/H4 | chr3:16924433-16925116 REVERSE | Aliases: F16L2.240	9.7	8.9	0.8	2.9
85	AT5G16990.1 | NADP-dependent oxidoreductase, putative, strong similarity to probable NADP-dependent oxidoreductase (zeta-crystallin homolog) P1 (SP:Q39172)(gi:886428) and P2 (SP:Q39173)(gi:886430), Arabidopsis thaliana | chr5:5581661-5584032 REVERSE | Aliases: F2K13.140, F2K13_140	9.7	3.7	6.1	21.5
5766	AT5G20890.1 | chaperonin, putative, similar to SWISS-PROT:P78371- T-complex protein 1, beta subunit (TCP-1-beta) (Homo sapiens); contains Pfam:PF00118 domain, TCP-1/cpn60 chaperonin family | chr5:7086655-7090083 REVERSE | Aliases: F22D1.60, F22D1_60	9.7	8.6	1.1	4.2
15378	AT5G56030.1 | Symbol: HSP81 2 | heat shock protein 81-2 (HSP81-2), nearly identical to SP:P55737 Heat shock protein 81-2 (HSP81-2) {Arabidopsis thaliana} | chr5:22704058-22706876 FORWARD | Aliases: HEAT SHOCK PROTEIN 81 2, MDA7.7, MDA7_7	9.7	9.5	0.2	1.1
7342	AT3G26770.1 | short-chain dehydrogenase/reductase (SDR) family protein, similar to sex determination protein tasselseed 2 SP:P50160 from (Zea mays) | chr3:9846721-9848385 FORWARD | Aliases: MDJ14.1	9.7	8.8	0.9	3.4
9282	AT4G35830.1 | aconitate hydratase, cytoplasmic / citrate hydro-lyase / aconitase (ACO), identical to SP:Q42560 Aconitate hydratase, cytoplasmic (EC 4.2.1.3) (Citrate hydro-lyase) (Aconitase) {Arabidopsis thaliana}; contains Pfam profiles PF00330: Aconitase family (aconitate hydratase), PF00694: Aconitase C-terminal domain | chr4:16972602-16978061 REVERSE | Aliases: F4B14.100, F4B14_100	9.7	10.2	-0.5	-2.6
241	AT1G28110.2 | Symbol: SCPL45 |	9.7	5.2	4.5	15.5
16059	AT3G06580.1 | Symbol: GAL1 | galactokinase (GAL1), identical to galactokinase (Galactose kinase) (Arabidopsis thaliana) SWISS-PROT:Q9SEE5 | chr3:2048911-2052031 REVERSE | Aliases: F5E6.9, F5E6_9, GALACTOSE KINASE 1	9.7	9.2	0.5	1.0
15217	AT1G01630.1 | SEC14 cytosolic factor, putative / phosphoglyceride transfer protein, putative, contains Pfam PF00650 : CRAL/TRIO domain and PF03765 : CRAL/TRIO, N-terminus; similar to polyphosphoinositide binding protein Ssh2p GB:AAB94599 GI:2739046 from (Glycine max) | chr1:229057-230917 FORWARD | Aliases: T1N6.1, T1N6_1	9.7	9.3	0.4	1.1
1996	AT5G11340.1 | GCN5-related N-acetyltransferase (GNAT) family protein, low similarity to SP:Q03503 L-A virus GAG protein N-acetyltransferase (EC 2.3.1.-) {Saccharomyces cerevisiae}; contains Pfam profile PF00583: acetyltransferase, GNAT family | chr5:3619156-3621232 FORWARD | Aliases: F2I11.230, F2I11_230	9.7	7.6	2.1	7.4
18648	AT5G43830.1 | expressed protein, similar to auxin down-regulated protein ARG10 (Vigna radiata) GI:2970051, wali7 (aluminum-induced protein) (Triticum aestivum) GI:451193 | chr5:17639563-17641545 REVERSE | Aliases: MQD19.19, MQD19_19	9.7	9.5	0.2	0.5
12847	AT5G20070.1 | MutT/nudix family protein, low similarity to SP:Q19427 NADH pyrophosphatase (EC 3.6.1.-) {Caenorhabditis elegans}; contains Pfam profile PF00293: NUDIX domain | chr5:6779862-6782484 FORWARD | Aliases: F28I16.220, F28I16_220	9.7	9.3	0.4	1.6
1353	AT2G37990.1 | ribosome biogenesis regulatory protein (RRS1) family protein, contains Pfam profile PF04939: Ribosome biogenesis regulatory protein (RRS1); similar to Ribosome biogenesis regulatory protein homolog (Swiss-Prot:Q15050) (Homo sapiens)	9.7	6.6	3.1	8.6
20006	AT5G46210.1 | Symbol: CUL4 | cullin, putative, similar to SP:Q13619 Cullin homolog 4A (CUL-4A) {Homo sapiens}; contains Pfam profile PF00888: Cullin family | chr5:18748645-18754037 REVERSE | Aliases: CUL4, MDE13.3, MDE13_3	9.7	9.6	0.0	0.2
10933	AT5G14440.2 | similar to surfeit locus protein 2 family protein / SURF2 family protein [Arabidopsis thaliana] (TAIR:At5g40570.1); similar to hypothetical protein DDB0206414 [Dictyostelium discoideum] (GB:EAL67308.1); contains InterPro domain Surfeit locus 2 (InterPro:IPR008833)	9.7	9.0	0.7	2.1
2241	AT2G27960.1 | Symbol: CKS1 | cyclin-dependent kinase / CDK (CKS1), identical to Cks1 protein (Arabidopsis thaliana) gi:2274859:emb:CAA03859 | chr2:11917803-11918703 REVERSE | Aliases: CATALYTIC SUBUNIT OF CYCLIN DEPENDENT KINASE, CDK subunit 1, CKS1, CKS1AT, P10CKS1AT, SUC1, T1E2.12, T1E2_12	9.7	7.2	2.5	7.0

14491	AT5G35680.2 | eukaryotic translation initiation factor 1A, putative / eIF-1A, putative / eIF-4C, putative, strong similarity to translation initiation factor (eIF-1A) (Beta vulgaris) GI:17977975; contains Pfam profile PF01176: Eukaryotic initiation factor 1A | chr5:13875151-13876207 REVERSE | Aliases: MXH1.2, MXH1_2	9.7	9.2	0.5	1.3
16173	AT3G27770.1 | expressed protein | chr3:10286072-10288979 REVERSE | Aliases: MGF10.17	9.7	10.1	-0.4	-0.9
260	AT5G25770.2 | expressed protein | chr5:8969219-8972049 REVERSE | Aliases: F18A17.20, F18A17_20	9.7	11.7	-2.1	-15.1
18998	AT1G04120.1 | Symbol: ATMRP5 |	9.7	9.6	0.1	0.4
7134	AT1G54270.1 | Symbol: EIF4A 2 | eukaryotic translation initiation factor 4A-2 / eIF-4A-2, similar to eukaryotic translation initiation factor 4A GI:19696 from (Nicotiana plumbaginifolia) | chr1:20263359-20265933 FORWARD | Aliases: F20D21.9, F20D21_9	9.7	8.6	1.1	3.5
7678	AT2G02510.1 | expressed protein | chr2:673304-674738 FORWARD | Aliases: T8K22.19, T8K22_19	9.7	9.1	0.6	3.3
4084	AT5G22740.1 | Symbol: ATCSLA02 | glycosyl transferase family 2 protein, similar to beta-(1-3)-glucosyl transferase GB:AAC62210 GI:3687658 from (Bradyrhizobium japonicum), cellulose synthase from Agrobacterium tumeficiens (gi:710492) and Agrobacterium radiobacter (gi:710493); contains Pfam glycosyl transferase, group 2 family protein domain PF00535	9.7	7.1	2.6	5.2
6956	AT3G15020.1 | malate dehydrogenase (NAD), mitochondrial, putative, similar to mitochondrial NAD-dependent malate dehydrogenase GB:CAA10320 SP:Q9ZP06 (Arabidopsis thaliana); contains InterPro entry IPR001236: Lactate/malate dehydrogenase | chr3:5056083-5058255 FORWARD | Aliases: K15M2.16	9.7	7.9	1.7	3.6
1269	AT3G49720.1 | expressed protein | chr3:18450988-18453182 REVERSE | Aliases: T16K5.70	9.7	7.0	2.7	8.8
16169	AT2G04900.1 | expressed protein | chr2:1722478-1723828 FORWARD | Aliases: F1O13.3	9.7	9.5	0.2	0.9
550	AT5G18040.1 | expressed protein | chr5:5972421-5974158 FORWARD | Aliases: MCM23.14, MCM23_14	9.7	7.4	2.3	11.6
6528	AT3G03250.1 | Symbol: UGP | Is thought to encodes a cytosolic UDP-glucose pyrophosphorylase with strong similarity to UTP--glucose-1-phosphate uridylyltransferase (SwissProt P19595, EC 2.7.7.9, UDP-glucose pyrophosphorylase <i>Solanum tuberosum</i>). | chr3:749486-754166 REVERSE | Aliases: T17B22.6, T17B22_6, UGP, UGPASE	9.7	7.6	2.1	3.8
14029	AT5G17380.1 | pyruvate decarboxylase family protein, similar to 2-hydroxyphytanoyl-CoA lyase (Homo sapiens) GI:6273457; contains InterPro entry IPR000399: Pyruvate decarboxylase | chr5:5724770-5726748 REVERSE | Aliases: T10B6.40, T10B6_40	9.7	10.2	-0.5	-1.4
17707	AT1G49820.1 | 5-methylthioribose kinase family, contains TIGRfam TIGR01767: 5-methylthioribose kinase profile | chr1:18446600-18448766 FORWARD | Aliases: F10F5.1, F10F5_1	9.7	9.3	0.3	0.6
10189	AT1G24050.1 | expressed protein, similar to hypothetical protein [Arabidopsis thaliana] (TAIR:At1g70220.1); similar to hypothetical protein DDB0191031 [Dictyostelium discoideum] (GB:EAL72551.1) | chr1:8507009-8508853 FORWARD | Aliases: T23E23.20, T23E23_20	9.7	8.8	0.8	2.3
3988	AT1G02140.1 | mago nashi family protein, similar to Mago Nashi, Genbank Accession Number U03559; contains Pfam PF02792: Mago nashi protein domain | chr1:403195-404439 REVERSE | Aliases: T7I23.7, T7I23_7	9.7	6.9	2.7	5.3
253	AT5G06210.1 | RNA-binding protein, putative, contains similarity to RNA-binding protein from (Nicotiana tabacum) GI:15822703, (Nicotiana sylvestris) GI:624925, (Solanum tuberosum) GI:15822705; contains InterPro entry IPR000504: RNA-binding region RNP-1 (RNA recognition motif) (RRM) | chr5:1878472-1879741 FORWARD | Aliases: MBL20.9, MBL20_9	9.7	6.1	3.5	15.2
20557	AT1G79940.2 | similar to DNAJ heat shock N-terminal domain-containing protein / sec63 domain-containing protein [Arabidopsis thaliana] (TAIR:At4g21180.1); similar to DNAJ-like Sec63 homologue [Plasmodium falciparum 3D7] (GB:NP_705096.1); contains InterPro domain Heat shock protein DnaJ (InterPro:IPR003095); contains InterPro domain Heat shock protein DnaJ, N-terminal (InterPro:IPR001623); contains InterPro domain Sec63 domain (InterPro:IPR004179) | chr1:30074497-30078232 FORWARD | Aliases: F19K16.10, F19K16_10	9.7	9.6	0.0	0.1
12792	AT4G33360.1 | terpene cyclase/mutase-related, low similarity to squalene-hopene cyclase from Zymomonas mobilis (SP:P33990) | chr4:16067675-16069377 REVERSE | Aliases: F17M5.120, F17M5_120	9.7	8.9	0.8	1.6
11307	AT5G67380.2 | Symbol: CKA1 | similar to casein kinase II alpha chain 2 [Arabidopsis thaliana] (TAIR:At3g50000.1); similar to casein kinase 2 catalytic subunit [Nicotiana tabacum] (GB:BAC02726.1); similar to casein kinase 2 catalytic subunit [Nicotiana tabacum] (GB:BAC02727.1); similar to protein kinase CK2 alpha chain [Nicotiana tabacum] (GB:CAD27342.1); similar to protein kinase CK2 alpha chain [Nicotiana tabacum] (GB:CAD27341.1); contains InterPro domain Serine/threonine protein kinase, active site (InterPro:IPR008271); contains InterPro domain Serine/threonine protein kinase (InterPro:IPR002290); contains InterPro domain Protein kinase (InterPro:IPR000719) | chr5:26898192-26900634 REVERSE | Aliases: ATCKA1, ATPK12D, K8K14.10, K8K14_10	9.7	9.1	0.6	2.0

575	AT1G57540.2 | expressed protein, contains similarity to T-box domain GI:5931592 from (Cynops pyrrhogaster) | chr1:21313995-21315077 REVERSE | Aliases: F25P12.4	9.7	6.3	3.3	11.4
441	AT2G18400.1 | ribosomal protein L6 family protein | chr2:7996561-7997668 REVERSE | Aliases: T30D6.9, T30D6_9	9.7	6.5	3.1	12.5
873	AT5G63310.1 | Symbol: NDPK2 | nucleotide diphosphate kinase II, chloroplast (NDPK2), identical to SP:O64903 Nucleoside diphosphate kinase II, chloroplast precursor (NDK II) (NDP kinase II) (NDPK II) (NDPK Ia) (Arabidopsis thaliana); contains Pfam PF00334 : Nucleoside diphosphate kinase domain; contains Pfam profile PF00334: Nucleoside diphosphate kinase | chr5:25389130-25391081 REVERSE | Aliases: MDC12.28, MDC12_28, NDPK IA, NDPK IA IA, NDPK1A, NUCLEOSIDE DIPHOSPHATE KINASE 2, NUCLEOSIDE DIPHOSPHATE KINASE IA	9.7	6.1	3.5	10.0
8551	AT1G49300.2 | similar to Ras-related GTP-binding protein, putative [Arabidopsis thaliana] (TAIR:At3g18820.1); similar to putative GTP-binding protein [Cucumis sativus] (GB:AAQ72787.1); contains InterPro domain Small GTP-binding protein domain (InterPro:IPR005225); contains InterPro domain Ras small GTPase, Rab type (InterPro:IPR003579); contains InterPro domain Ras small GTPase, Rho type (InterPro:IPR003578); contains InterPro domain Ras small GTPase, Ras type (InterPro:IPR003577); contains InterPro domain Ras GTPase superfamily (InterPro:IPR001806); contains InterPro domain GTP-binding nuclear protein Ran (InterPro:IPR002041) | chr1:18238417-18241195 FORWARD | Aliases: F13F21.26, F13F21_26	9.7	10.2	-0.5	-2.9
3435	NA	9.7	4.3	5.3	5.7
621	AT1G29250.1 | expressed protein, contains TIGRFAM TIGR00285: conserved hypothetical protein TIGR00285 | chr1:10223252-10224713 REVERSE | Aliases: F28N24.7, F28N24_7	9.7	6.6	3.1	11.1
3916	AT3G17940.1 | aldose 1-epimerase family protein, similar to ALDOSE 1-EPIMERASE PRECURSOR GB:P05149 (SP:P05149) from (Acinetobacter calcoaceticus); contains Pfam profile PF01263 Aldose 1-epimerase | chr3:6143525-6145324 REVERSE | Aliases: MEB5.16	9.7	8.6	1.0	5.3
12592	AT2G25070.1 | protein phosphatase 2C, putative / PP2C, putative | chr2:10670319-10673043 REVERSE | Aliases: F13D4.1	9.7	10.0	-0.3	-1.7
3980	AT3G19820.2 | Symbol: DWF1 | cell elongation protein / DWARF1 / DIMINUTO (DIM), identical to GB:S71189 (SP:Q39085) from (Arabidopsis thaliana); contains Pfam FAD binding domain PF01565 | chr3:6879624-6882265 REVERSE | Aliases: CABBAGE 1, CBB1, DIM, DIM1, DIMINUTIA, DIMINUTO 1, DW1, DWARF 1, EVE1, MPN9.6	9.7	7.0	2.6	5.3
8019	AT4G34720.1 | vacuolar ATP synthase 16 kDa proteolipid subunit 1 / V-ATPase 16 kDa proteolipid subunit 1 (AVAP1) (AVA-P1), identical to SP:P59227 Vacuolar ATP synthase 16 kDa proteolipid subunit 1/3/5 (EC 3.6.3.14) (V-ATPase 16 kDa proteolipid subunit 1/3/5) {Arabidopsis thaliana}; contains Pfam profile PF00137: ATP synthase subunit C | chr4:16567829-16569300 REVERSE | Aliases: T4L20.300	9.6	10.6	-1.0	-3.1
16205	AT1G05180.3 | Symbol: AXR1 | similar to auxin-resistance protein, putative [Arabidopsis thaliana] (TAIR:At2g32410.1); similar to putative ubiquitin-activating enzyme (alternative splicing product) [Oryza sativa (japonica cultivar-group)] (GB:XP_470133.1) | chr1:1498113-1501823 REVERSE | Aliases: AUXIN RESISTANT 1, AUXIN RESISTANT PROTEIN 1, YUP8H12.21, YUP8H12_21	9.6	9.8	-0.2	-0.9
3615	AT2G36950.1 | heavy-metal-associated domain-containing protein, nearly identical to farnesylated protein ATFP2 (GI:4097545) Pfam profile PF00403: Heavy-metal-associated domain | chr2:15522212-15524010 FORWARD | Aliases: T1J8.13, T1J8_13	9.6	11.6	-2.0	-5.5
8426	AT2G20420.1 | succinyl-CoA ligase (GDP-forming) beta-chain, mitochondrial, putative / succinyl-CoA synthetase, beta chain, putative / SCS-beta, putative, identical to SP:O82662 Succinyl-CoA ligase (GDP-forming) beta-chain, mitochondrial precursor (EC 6.2.1.4) (Succinyl-CoA synthetase, beta chain) (SCS- beta) {Arabidopsis thaliana}; similar to SP:O97580 Succinyl-CoA ligase (ADP-forming) beta-chain, mitochondrial precursor (EC 6.2.1.5) {Sus scrofa}; contains Pfam profiles PF00549: CoA-ligase, PF02222: ATP-grasp domain | chr2:8812537-8815187 FORWARD | Aliases: F11A3.3, F11A3_3	9.6	7.8	1.8	2.9
1344	AT1G72170.1 | expressed protein, contains Pfam PF04418: Domain of unknown function (DUF543) | chr1:27159997-27161338 FORWARD | Aliases: T9N14.2	9.6	7.1	2.5	8.6
1488	AT3G04550.1 | expressed protein | chr3:1225928-1227442 FORWARD | Aliases: F7O18.2, F7O18_2	9.6	7.5	2.1	8.3
734	AT4G25810.1 | Symbol: XTR6 | xyloglucan:xyloglucosyl transferase, putative / xyloglucan endotransglycosylase, putative / endo-xyloglucan transferase, putative (XTR6), identical to xyloglucan endotransglycosylase-related protein GI:1244758 from (Arabidopsis thaliana) | chr4:13128655-13129893 FORWARD | Aliases: F14M19.90, F14M19_90, XYLOGLUCAN ENDOTRANSGLYCOSYLASE 6	9.6	4.6	5.1	10.6
1208	AT4G29840.2 | Symbol: MTO2 | threonine synthase, chloroplast, identical to SP:Q9S7B5 Threonine synthase, chloroplast precursor (EC 4.2.3.1) (TS) {Arabidopsis thaliana} | chr4:14597716-14601061 REVERSE | Aliases: F27B13.80, F27B13_80, METHIONINE OVER ACCUMULATOR, THREONINE SYNTHASE, TS	9.6	6.5	3.1	9.0
7507	AT5G12040.2 | carbon-nitrogen hydrolase family protein, similar to Nit protein 2 (Homo sapiens) GI:9367116; contains Pfam profile PF00795: hydrolase, carbon-nitrogen family | chr5:3885155-3888055 FORWARD | Aliases: F14F18.210, F14F18_210	9.6	8.8	0.9	3.3
3027	AT3G47833.1 | expressed protein | chr3:17659618-17661125 FORWARD | Aliases: None	9.6	8.5	1.2	6.1

4935	AT5G38660.1 | Symbol: APE1 | expressed protein, similar to unknown protein (pir::S75762) | chr5:15490363-15492765 REVERSE | Aliases: ACCLIMATION OF PHOTOSYNTHESIS TO ENVIRONMENT, MBB18.21, MBB18_21	9.6	7.4	2.2	4.6
20220	AT3G13410.1 | expressed protein | chr3:4361889-4364242 REVERSE | Aliases: MRP15.4	9.6	9.6	0.1	0.2
3857	AT1G17470.1 | Symbol: ATDRG1 |	9.6	8.0	1.6	5.4
13736	AT5G47120.1 | Symbol: ATBI 1 |	9.6	10.4	-0.7	-1.4
78	AT1G68470.1 | exostosin family protein, contains Pfam profile: PF03016	9.6	3.5	6.2	22.2
1890	AT5G08100.2 | L-asparaginase / L-asparagine amidohydrolase, identical to Swiss-Prot:P50287 L-asparaginase (EC 3.5.1.1) (L-asparagine amidohydrolase) (Arabidopsis thaliana) | chr5:2593051-2594628 REVERSE | Aliases: T22D6.40, T22D6_40	9.6	7.8	1.8	7.6
17705	AT3G44110.2 | Symbol: ATJ3 | DNAJ heat shock protein, putative (J3), identical to AtJ3 (Arabidopsis thaliana) GI:2641638, strong similarity to several plant DnaJ proteins from PGR; contains Pfam profiles PF00226 DnaJ domain, PF00684 DnaJ central domain (4 repeats), PF01556 DnaJ C terminal region | chr3:15879781-15882208 REVERSE | Aliases: ATJ, F26G5.60	9.6	9.2	0.4	0.6
10737	AT3G21865.1 | expressed protein | chr3:7701139-7703328 REVERSE | Aliases: MSD21.24, MSD21_24	9.6	9.2	0.4	2.1
1902	AT3G50910.1 | expressed protein | chr3:18930494-18933219 FORWARD | Aliases: F18B3.190	9.6	7.9	1.7	7.6
5789	AT3G19240.1 | expressed protein | chr3:6664256-6666564 FORWARD | Aliases: MVI11.21	9.6	8.3	1.3	4.1
3464	AT5G46030.1 | expressed protein, contains Pfam profile PF05129: Putative zinc binding domain (DUF701) | chr5:18687356-18688566 FORWARD | Aliases: MCL19.8, MCL19_8	9.6	8.4	1.2	5.7
1789	AT5G13630.1 | Symbol: GUN5 | magnesium-chelatase subunit chlH, chloroplast, putative / Mg-protoporphyrin IX chelatase, putative (CHLH), nearly identical to magnesium chelatase subunit GI:1154627 from (Arabidopsis thaliana); contains Pfam profile: PF02514 CobN/magnesium chelatase family protein | chr5:4387337-4392230 REVERSE | Aliases: CCH, CCH1, CHLH, CONDITIONAL CHLORINA, GENOMES UNCOUPLED 5, T6I14.12	9.6	6.2	3.4	7.7
3987	AT2G31090.1 | expressed protein | chr2:13263215-13263788 FORWARD | Aliases: T16B12.10, T16B12_10	9.6	10.8	-1.2	-5.3
13987	AT1G79690.1 | MutT/nudix family protein, contains Pfam NUDIX domain (PF00293); very low similarity to Chain A and Chain B of Escherichia coli isopentenyl diphosphate:dimethylallyl diphosphate isomerase (gi:15826361) (gi:15826360) | chr1:29990223-29996035 FORWARD | Aliases: F20B17.11, F20B17_11	9.6	9.9	-0.3	-1.4
19350	AT1G74090.1 | sulfotransferase family protein, similar to SP:P52837 Flavonol 4'-sulfotransferase (EC 2.8.2.-) (F4-ST) {Flaveria chloraefolia}; contains Pfam profile PF00685: Sulfotransferase domain | chr1:27866570-27867854 FORWARD | Aliases: F2P9.4, F2P9_4	9.6	9.5	0.1	0.3
885	AT3G04920.1 | 40S ribosomal protein S24 (RPS24A), similar to ribosomal protein S19 GB:445612 (Solanum tuberosum) and similar to ribosomal protein S24 GB:4506703 (Homo sapiens) | chr3:1360888-1362301 FORWARD | Aliases: T9J14.13, T9J14_13	9.6	6.4	3.2	9.9
760	AT5G16570.1 | Symbol: GLN1;4 |	9.6	7.3	2.3	10.5
13580	AT5G53800.1 | expressed protein | chr5:21865361-21867337 FORWARD | Aliases: MGN6.19, MGN6_19	9.6	8.8	0.8	1.5
8626	AT5G63510.1 | bacterial transferase hexapeptide repeat-containing protein, contains similarity to acetyltransferase; contains Pfam profile PF00132: Bacterial transferase hexapeptide (four repeats) | chr5:25441226-25443067 FORWARD | Aliases: MLE2.14, MLE2_14	9.6	8.4	1.3	2.9
6984	AT4G35985.1 | senescence/dehydration-associated protein-related, similar to senescence-associated protein 12 (Hemerocallis hybrid cultivar) gi:3551958:gb:AAC34857; similar to early-responsive to dehydration stress ERD7 protein (Arabidopsis thaliana) gi:15320412:dbj:BAB63916 | chr4:17032271-17033865 REVERSE | Aliases: None	9.6	10.5	-0.9	-3.5
10332	AT1G70490.3 | ADP-ribosylation factor, putative, nearly identical to ADP-ribosylation factor 1 GB:P36397 (Arabidopsis thaliana), ADP-ribosylation factor GI:166586 (Arabidopsis thaliana) | chr1:26567590-26569453 REVERSE | Aliases: F24J13.6, F24J13_6	9.6	8.6	1.0	2.3
7690	AT4G13780.1 | methionine--tRNA ligase, putative / methionyl-tRNA synthetase, putative / MetRS, putative, similar to methionyl-tRNA synthetase (Oryza sativa) GI:4091008; contains Pfam profiles PF00133: tRNA synthetases class I (I, L, M and V), PF01588: Putative tRNA binding domain | chr4:7993198-7998511 REVERSE | Aliases: F18A5.170, F18A5_170	9.6	8.8	0.8	3.2
377	AT2G24190.1 | short-chain dehydrogenase/reductase (SDR) family protein, contains INTERPRO family IPR002198 short-chain dehydrogenase/reductase (SDR) superfamily | chr2:10290820-10292014 REVERSE | Aliases: F27D4.10, F27D4_10	9.6	6.1	3.6	13.1

7973	AT3G23640.1 | glycosyl hydrolase family 31 protein, similar to alpha-glucosidase II from SP:Q9F234 (Bacillus thermoamyloliquefaciens); contains Pfam profile: PF01055 Glycosyl hydrolases family 31 | chr3:8501935-8509591 FORWARD | Aliases: MDB19.14	9.6	9.2	0.4	3.1
8254	AT4G35000.1 | Symbol: APX3 | L-ascorbate peroxidase 3 (APX3), identical to ascorbate peroxidase 3 (Arabidopsis thaliana) GI:2444019, L-ascorbate peroxidase (Arabidopsis thaliana) gi:1523791:emb:CAA66926; similar to ascorbate peroxidase (Gossypium hirsutum) gi:1019946:gb:AAB52954 | chr4:16664827-16667710 REVERSE | Aliases: ASCORBATE PEROXIDASE 3, M4E13.60, M4E13_60	9.6	7.7	1.9	3.0
6531	AT2G47610.1 | 60S ribosomal protein L7A (RPL7aA) | chr2:19536860-19538725 FORWARD | Aliases: T30B22.8	9.6	7.8	1.8	3.7
8084	AT5G16050.1 | Symbol: GRF5 | 14-3-3 protein GF14 upsilon (GRF5), identical to 14-3-3 protein GF14 upsilon GI:2232148 from (Arabidopsis thaliana) | chr5:5243748-5245814 REVERSE | Aliases: F1N13.190, F1N13_190, GF14 UPSILON, GF14 UPSILON CHAIN	9.6	7.7	1.9	3.1
14198	AT3G09810.1 | Symbol: AT3G09805 | isocitrate dehydrogenase, putative / NAD+ isocitrate dehydrogenase, putative, strong similarity to isocitrate dehydrogenase (NAD+) GB:CAA65502 GI:3021506 (Nicotiana tabacum) | chr3:3008699-3011442 FORWARD | Aliases: AT3G09805, F8A24.14, F8A24_14	9.6	8.7	0.9	1.3
5473	AT5G55070.1 | 2-oxoacid dehydrogenase family protein, similar to SP:Q01205 Dihydrolipoamide succinyltransferase component of 2-oxoglutarate dehydrogenase complex, mitochondrial precursor (EC 2.3.1.61) {Rattus norvegicus}; contains Pfam profiles PF00198: 2-oxo acid dehydrogenases acyltransferase (catalytic domain), PF00364: Biotin-requiring enzyme | chr5:22364490-22368043 FORWARD | Aliases: MCO15.2, MCO15_2	9.6	7.3	2.3	4.3
1804	AT3G60210.1 | chloroplast chaperonin 10, putative, similar to chloroplast chaperonin 10 GI:14041813 from (Arabidopsis thaliana) | chr3:22262219-22263800 REVERSE | Aliases: F27H5.5	9.6	6.3	3.3	7.7
9131	AT1G09330.1 | expressed protein, contains 3 transmembrane domains; contains Pfam profile PF05832: Eukaryotic protein of unknown function | chr1:3012764-3015093 REVERSE | Aliases: T31J12.5, T31J12_5	9.6	8.9	0.7	2.7
528	AT2G23930.1 | small nuclear ribonucleoprotein G, putative / snRNP-G, putative / Sm protein G, putative, similar to small nuclear ribonucleoprotein G (snRNP-G, Sm protein G, Sm-G, SmG) (Homo sapiens) SWISS-PROT:Q15357 | chr2:10189345-10190320 FORWARD | Aliases: T29E15.13, T29E15_13	9.6	7.2	2.4	11.8
4407	AT5G65660.1 | hydroxyproline-rich glycoprotein family protein | chr5:26262475-26263605 REVERSE | Aliases: K21L13.18, K21L13_18	9.6	10.8	-1.2	-4.9
3934	AT5G17990.1 | Symbol: TRP1 | anthranilate phosphoribosyltransferase, identical to anthranilate phosphoribosyltransferase, chloroplast precursor (EC 2.4.2.18) SP:Q02166 from (Arabidopsis thaliana) | chr5:5957215-5959906 FORWARD | Aliases: MCM23.6, MCM23_6, PAT1, PHOSPHORIBOSYLANTHRANILATE TRANSFERASE, PHOSPHORIBOSYLANTHRANILATE TRANSFERASE 1, TRYPTOPHAN BIOSYNTHESIS 1	9.6	7.2	2.4	5.3
2809	AT2G40060.1 | expressed protein | chr2:16733562-16735263 FORWARD | Aliases: T28M21.22, T28M21_22	9.6	8.6	1.0	6.4
21047	AT4G34450.1 | coatomer gamma-2 subunit, putative / gamma-2 coat protein, putative / gamma-2 COP, putative, similar to SP:Q9UBF2 Coatomer gamma-2 subunit (Gamma-2 coat protein) (Gamma-2 COP) {Homo sapiens}; contains Pfam profile: PF01602 Adaptin N terminal region	9.6	9.6	0.0	0.0
7878	AT5G52540.1 | expressed protein, contains PF05684: Protein of unknown function (DUF819) | chr5:21338654-21341006 REVERSE | Aliases: F6N7.1, F6N7_1	9.6	7.4	2.2	3.2
6599	AT3G02770.1 | dimethylmenaquinone methyltransferase family protein, similar to bacterial S-adenosylmethionine:2-demethylmenaquinone methyltransferases; contains Pfam profile PF03737: Dimethylmenaquinone methyltransferase | chr3:600900-602218 REVERSE | Aliases: F13E7.29, F13E7_29	9.6	8.4	1.1	3.7
1015	AT2G20060.1 | ribosomal protein L4 family protein, contains Pfam profile PF00573: ribosomal protein L4/L1 family | chr2:8666124-8668562 FORWARD | Aliases: T2G17.14, T2G17_14	9.6	6.4	3.2	9.5
8356	AT3G18780.2 | Symbol: ACT2 | actin 2 (ACT2), identical to SP:Q96292 Actin 2 {Arabidopsis thaliana}; nearly identical to SP:Q96293 Actin 8 (Arabidopsis thaliana) GI:1669387 and to At1g49240 | chr3:6474877-6477210 FORWARD | Aliases: ACTIN 2, ACTIN2, DEFORMED ROOT HAIRS 1, DER1, LIGHT STRESS REGULATED 2, LSR2, MVE11.16	9.6	10.9	-1.3	-3.0
1905	AT3G03150.1 | expressed protein | chr3:727561-729467 REVERSE | Aliases: T17B22.16, T17B22_16	9.6	7.0	2.5	7.5
8408	AT1G77370.1 | glutaredoxin, putative, similar to glutaredoxin (Ricinus communis) gi:1732424:emb:CAA89699 | chr1:29078741-29079683 FORWARD | Aliases: F2P24.8, F2P24_8	9.6	10.3	-0.7	-2.9
17065	AT4G21580.2 | similar to NADP-dependent oxidoreductase, putative [Arabidopsis thaliana] (TAIR:At5g61510.1); similar to Putative quinone oxidoreductase [Oryza sativa] (GB:AAK98702.1); contains InterPro domain Zinc-containing alcohol dehydrogenase superfamily (InterPro:IPR002085)	9.6	9.3	0.3	0.8
2712	AT1G22450.1 | Symbol: COX6B | cytochrome c oxidase subunit 6b, putative (COX6b), nearly identical to subunit 6b of cytochrome c oxidase (Arabidopsis thaliana) GI:6518353 | chr1:7925303-7927267 FORWARD | Aliases: ATCOX6B2, CYTOCHROME C OXIDASE 6B, F12K8.20, F12K8_20, SUBUNIT 6B OF CYTOCHROME C OXIDASE	9.6	7.8	1.8	6.5

2638	AT2G40430.1 | expressed protein, identical to Protein At2g40430 (Swiss-Prot:O22892) (Arabidopsis thaliana); similar to Glioma tumor suppressor candidate region gene 2 protein (p60) (Swiss-Prot:Q9NZM5) (Homo sapiens) | chr2:16886297-16889505 REVERSE | Aliases: T2P4.22	9.6	8.3	1.3	6.5
14831	AT3G52850.1 | Symbol: ATELP1 |	9.6	9.1	0.5	1.2
13464	AT5G23760.1 | heavy-metal-associated domain-containing protein, Pfam profile PF00403: Heavy-metal-associated domain | chr5:8013090-8014231 REVERSE | Aliases: MRO11.20, MRO11_20	9.6	9.2	0.4	1.5
13067	AT2G39780.1 | Symbol: RNS2 | ribonuclease 2 (RNS2), identical to ribonuclease 2 precursor SP:P42814, GI:289210; contains a ribonuclease T2 family histidine active site signature (PDOC00459)	9.6	10.1	-0.6	-1.6
15799	AT2G35120.1 | glycine cleavage system H protein, mitochondrial, putative, similar to SP:Q39732 Glycine cleavage system H protein, mitochondrial precursor {Flaveria anomala}; contains Pfam profile PF01597: Glycine cleavage H-protein | chr2:14812718-14814415 REVERSE | Aliases: T4C15.21, T4C15_21	9.6	8.9	0.7	1.0
14242	AT1G11860.2 | aminomethyltransferase, putative, similar to aminomethyltransferase, mitochondrial precursor SP:O49849 from (Flaveria anomala) | chr1:4001294-4003441 FORWARD | Aliases: F12F1.30, F12F1_30	9.6	9.9	-0.3	-1.3
468	AT5G06290.1 | 2-cys peroxiredoxin, chloroplast, putative, very strong similarity to SP:Q96291 2-cys peroxiredoxin BAS1, chloroplast precursor {Arabidopsis thaliana}; contains Pfam profile: PF00578 AhpC/TSA (alkyl hydroperoxide reductase and thiol-specific antioxidant) family | chr5:1919249-1921404 FORWARD | Aliases: MHF15.19, MHF15_19	9.6	4.9	4.7	12.2
8735	AT3G05970.1 | Symbol: LACS6 | long-chain-fatty-acid--CoA ligase / long-chain acyl-CoA synthetase (LACS6), strong similarity to AMP-binding protein (MF39P) gi:1617274 from Brassica napus, similar to putative long-chain-fatty-acid--CoA ligase (brain isozyme) GB:P33124 (Rattus norvegicus); contains Pfam AMP-binding enzyme domain PF00501; identical to cDNA AtLACS6 for long-chain acyl-CoA synthetase GI:22531705 | chr3:1786324-1791808 REVERSE | Aliases: F2O10.7, F2O10_7, LONG CHAIN ACYL COA SYNTHETASE 6	9.6	9.9	-0.4	-2.8
21058	AT5G28050.2 | similar to cytidine/deoxycytidylate deaminase family protein [Arabidopsis thaliana] (TAIR:At3g05300.1); similar to putative cytidine deaminase; putative deoxycytidylate deaminase [Cicer arietinum] (GB:CAA07230.1); contains InterPro domain Cytidine/deoxycytidylate deaminase, zinc-binding region (InterPro:IPR002125) | chr5:10043918-10045886 REVERSE | Aliases: F15F15.120, F15F15_120	9.6	9.6	-0.0	-0.0
19951	AT2G31400.1 | pentatricopeptide (PPR) repeat-containing protein, low similarity to fertility restorer (Petunia x hybrida) GI:22128587, post-transcriptional control of chloroplast gene expression CRP1 (Zea mays) GI:3289002; contains Pfam profile PF01535: PPR repeat | chr2:13394081-13397783 REVERSE | Aliases: T28P16.11, T28P16_11	9.6	9.6	-0.0	-0.2
2993	AT3G11510.1 | 40S ribosomal protein S14 (RPS14B), similar to 40S ribosomal protein S14 GB:P19950 (Zea mays) | chr3:3623463-3624945 REVERSE | Aliases: F24K9.19	9.6	6.3	3.2	6.1
6872	AT4G35230.1 | protein kinase family protein, contains protein kinase domain, Pfam:PF00069 | chr4:16755123-16758096 REVERSE | Aliases: F23E12.210, F23E12_210	9.6	8.8	0.8	3.6
1925	AT5G58490.1 | cinnamoyl-CoA reductase family, similar to cinnamoyl-CoA reductase from Pinus taeda (GI:17978649), Eucalyptus gunnii (GI:2058311) | chr5:23660248-23661824 FORWARD | Aliases: MQJ2.6, MQJ2_6	9.6	6.3	3.2	7.5
5519	AT2G23340.1 | encodes a member of the DREB subfamily A-5 of ERF/AP2 transcription factor family. The protein contains one AP2 domain. There are 16 members in this subfamily including RAP2.1, RAP2.9 and RAP2.10. | chr2:9945079-9945953 FORWARD | Aliases: T20D16.3, T20D16_3	9.6	11.4	-1.9	-4.3
1703	AT3G19740.1 | similar to AAA-type ATPase family protein [Arabidopsis thaliana] (TAIR:At1g50140.1); similar to spastin-like [Oryza sativa (japonica cultivar-group)] (GB:BAD37292.1); contains InterPro domain AAA ATPase (InterPro:IPR003593); contains InterPro domain AAA ATPase, central region (InterPro:IPR003959) | chr3:6855843-6859040 REVERSE | Aliases: MMB12.22	9.6	8.3	1.3	7.9
18642	AT3G01520.1 | universal stress protein (USP) family protein, similar to ER6 protein (GI:5669654) (Lycopersicon esculentum); contains Pfam profile PF00582: universal stress protein family | chr3:208446-210110 FORWARD | Aliases: F4P13.7, F4P13_7	9.6	9.8	-0.2	-0.5
1229	AT2G01090.1 | ubiquinol-cytochrome C reductase complex 7.8 kDa protein, putative / mitochondrial hinge protein, putative, similar to SP:P48504 Ubiquinol-cytochrome C reductase complex 7.8 kDa protein (EC 1.10.2.2) (Mitochondrial hinge protein) (CR7) {Solanum tuberosum}; contains Pfam profile PF02320: Ubiquinol-cytochrome C reductase hinge protein | chr2:80014-81200 FORWARD | Aliases: F23H14.6, F23H14_6	9.6	6.8	2.8	8.9
15623	AT5G58020.1 | expressed protein, contains PF04641: Protein of unknown function, DUF602	9.6	9.2	0.4	1.0
867	AT3G17300.1 | expressed protein | chr3:5907240-5908898 FORWARD | Aliases: MGD8.14	9.6	6.9	2.7	10.0

14044	AT1G53580.2 | similar to hydroxyacylglutathione hydrolase, cytoplasmic / glyoxalase II (GLX2-2) [Arabidopsis thaliana] (TAIR:At3g10850.1); similar to putative glyoxalase II [Oryza sativa (japonica cultivar-group)] (GB:NP_916812.1); contains InterPro domain Beta-lactamase-like (InterPro:IPR001279) | chr1:19994943-19996898 REVERSE | Aliases: F22G10.9, T3F20.11, T3F20_11	9.6	10.0	-0.4	-1.4
1454	AT5G56940.1 | ribosomal protein S16 family protein, contains Pfam profile PF00886: ribosomal protein S16 | chr5:23048027-23049637 FORWARD | Aliases: MHM17.5, MHM17_5	9.6	7.4	2.2	8.3
14352	AT1G55840.1 | SEC14 cytosolic factor (SEC14) / phosphoglyceride transfer protein, similar to polyphosphoinositide binding protein SEC14 homolog Ssh1p (GB:AAB94598) (Glycine max); identified in Eur J Biochem 1998 Dec 1;258(2):402-10 as AtSEC14, characterized by functional complementation in S. cerevisiae. | chr1:20877268-20879938 FORWARD | Aliases: F14J16.8, F14J16_8	9.6	8.9	0.6	1.3
20810	AT1G08480.1 | expressed protein | chr1:2684237-2685620 FORWARD | Aliases: T27G7.26	9.6	9.6	-0.0	-0.1
10862	AT1G23490.1 | Symbol: ATARF/ATARF1/ATARFA1A | Gene encoding ADP-ribosylation factor and similar to other ARFs and ARF-like proteins. Members of this family are known to be essential for vesicle coating and uncoating and functions in GTP-binding. The gene is shown to play a role in cell division, cell expansion and cellulose production using antisense construct. | chr1:8336693-8338661 FORWARD | Aliases: ATARF, ATARF1, ATARFA1A, F28C11.12, F5O8.5, F5O8_5	9.5	8.9	0.7	2.1
586	AT5G40730.1 | Symbol: AGP24 | arabinogalactan-protein (AGP24)	9.5	12.7	-3.2	-11.4
6843	AT1G68790.1 | expressed protein | chr1:25838372-25842982 REVERSE | Aliases: F14K14.10, F14K14_10	9.5	7.5	2.0	3.6
2161	AT1G34430.1 | Symbol: EMB3003 | dihydrolipoamide S-acetyltransferase, putative, similar to dihydrolipoamide S-acetyltransferase (LTA2) (Arabidopsis thaliana) GI:5881963; contains Pfam profiles PF00198: 2-oxo acid dehydrogenases acyltransferase (catalytic domain), PF00364: Biotin-requiring enzyme, PF02817: e3 binding domain | chr1:12587730-12590120 REVERSE | Aliases: EMB3003, EMBRYO DEFECTIVE 3003, F12K21.24, F12K21_24	9.5	6.7	2.8	7.2
15781	AT2G45950.1 | Symbol: ASK20 | SKP1 family protein, similar to glycoprotein FP21 SP:P52285 from (Dictyostelium discoideum); contains Pfam profile PF01466: Skp1 family, dimerisation domain | chr2:18911397-18914871 REVERSE | Aliases: ARABIDOPSIS SKP1 LIKE 20, ASK20, F4I18.7	9.5	10.2	-0.7	-1.0
15782	AT3G61415.1 | Symbol: ASK21 | SKP1 family protein, low similarity to SP:P52285 Glycoprotein FP21 precursor {Dictyostelium discoideum}; contains Pfam profile PF01466: Skp1 family, dimerisation domain | chr3:22733685-22737383 REVERSE | Aliases: ARABIDOPSIS SKP1 LIKE 21, ASK21	9.5	10.2	-0.7	-1.0
255	AT3G27400.1 | pectate lyase family protein, similar to pectate lyase GP:7547009 from (Vitis vinifera); contains Pfam profile: PF00544 pectate lyase | chr3:10141560-10144462 FORWARD | Aliases: K1G2.22	9.5	2.2	7.3	15.2
853	AT2G44460.1 | glycosyl hydrolase family 1 protein, contains Pfam PF00232 : Glycosyl hydrolase family 1 domain; TIGRFAM TIGR01233: 6-phospho-beta-galactosidase; similar to beta-glucosidase 1 (GI:12043529) (Arabidopsis thaliana) | chr2:18353576-18357042 FORWARD | Aliases: F4I1.27	9.5	3.3	6.2	10.1
4495	AT5G55920.1 | nucleolar protein, putative, similar to SP:P46087 Proliferating-cell nucleolar antigen p120 (Proliferation-associated nucleolar protein p120) {Homo sapiens}, SP:P40991 Nucleolar protein NOP2 {Saccharomyces cerevisiae}; contains Pfam profile PF01189: NOL1/NOP2/sun family | chr5:22662709-22666673 REVERSE | Aliases: None	9.5	8.2	1.3	4.9
8475	AT1G27450.2 | Symbol: APT1 | adenine phosphoribosyltransferase 1 (APT1), nearly identical to SP:P31166 Adenine phosphoribosyltransferase 1 (EC 2.4.2.7) (APRT) {Arabidopsis thaliana}	9.5	7.4	2.1	2.9
439	AT2G37660.1 | expressed protein | chr2:15802392-15804137 REVERSE | Aliases: F13M22.16, F13M22_16	9.5	5.7	3.8	12.5
7985	AT5G65780.1 | branched-chain amino acid aminotransferase 5 / branched-chain amino acid transaminase 5 (BCAT5), nearly identical to SP:Q9FYA6 Branched-chain amino acid aminotransferase 5, chloroplast precursor (EC 2.6.1.42) (Atbcat-5) {Arabidopsis thaliana}; contains Pfam profile: PF01063 aminotransferase class IV | chr5:26332616-26335980 FORWARD | Aliases: MPA24.13, MPA24_13	9.5	7.8	1.7	3.1
19800	AT1G11360.2 | universal stress protein (USP) family protein, contains Pfam PF00582: universal stress protein family domain; similar to ethylene-responsive ER6 protein (GI:5669654) (Lycopersicon esculentum) | chr1:3821529-3823053 REVERSE | Aliases: T23J18.35, T23J18_35	9.5	9.5	0.1	0.2
6373	AT5G55000.2 | Symbol: FIP2 | potassium channel tetramerisation domain-containing protein / pentapeptide repeat-containing protein, contains Pfam profiles PF02214: K+ channel tetramerisation domain, PF00805: Pentapeptide repeats (8 copies) | chr5:22335536-22338973 FORWARD | Aliases: MBG8.27, MBG8_27	9.5	7.4	2.1	3.8
8447	AT5G08530.1 | NADH-ubiquinone oxidoreductase 51 kDa subunit, mitochondrial, putative, similar to NADH-ubiquinone oxidoreductase 51 kDa subunit, mitochondrial precursor (EC 1.6.5.3) (EC 1.6.99.3) from {Homo sapiens} SP:P49821, {Bos taurus} SP:P25708, {Aspergillus niger} SP:Q92406; contains Pfam profile PF01512: Respiratory-chain NADH dehydrogenase 51 Kd subunit | chr5:2759320-2761860 REVERSE | Aliases: MAH20.9, MAH20_9	9.5	8.6	1.0	2.9

277	AT5G53580.1 | aldo/keto reductase family protein, contains Pfam profile PF00248: oxidoreductase, aldo/keto reductase family | chr5:21782305-21784210 REVERSE | Aliases: MNC6.12, MNC6_12	9.5	5.0	4.5	14.8
281	AT2G40100.1 | Symbol: LHCB4.3 | chlorophyll A-B binding protein (LHCB4.3), identical to Lhcb4:3 protein (Arabidopsis thaliana) GI:4741956; contains Pfam profile: PF00504 chlorophyll A-B binding protein | chr2:16752881-16754478 FORWARD | Aliases: F27I1.2, F27I1_2	9.5	3.1	6.4	14.7
4703	AT4G31720.2 | Symbol: TAFII15 | transcription initiation factor IID (TFIID) 23-30kDa subunit (TAF2H) family protein, contains Pfam profile: PF03540 transcription initiation factor TFIID 23-30kDa | chr4:15353958-15356352 REVERSE | Aliases: F28M20.90, F28M20_90	9.5	7.7	1.8	4.8
3787	AT1G06570.1 | Symbol: PDS1 | 4-hydroxyphenylpyruvate dioxygenase (HPD), identical to 4-hydroxyphenylpyruvate dioxygenase (HPD) SP:P93836 (Arabidopsis thaliana (Mouse-ear cress)) | chr1:2011885-2013544 REVERSE | Aliases: 4 HYDROXYPHENYLPYRUVATE DIOXYGENASE, F12K11.9, F12K11_9, HPD, P HYDROXYPHENYLPYRUVATE DIOXYGENASE, PHYTOENE DESATURATION 1	9.5	6.9	2.7	5.4
4240	AT3G05220.2 | heavy-metal-associated domain-containing protein, similar to farnesylated protein 1 (GI:23304411) {Hordeum vulgare subsp. spontaneum}; contains Pfam profile PF00403: Heavy-metal-associated domain | chr3:1489425-1491320 FORWARD | Aliases: T12H1.19, T12H1_19	9.5	10.5	-1.0	-5.1
1559	AT3G22840.1 | Symbol: ELIP1 | chlorophyll A-B binding family protein / early light-induced protein (ELIP), identical to early light-induced protein; ELIP (Arabidopsis thaliana) GI:1872544; contains Pfam profile: PF00504 chlorophyll A-B binding protein; identical to cDNA early light-induced protein GI:1872543	9.5	5.6	4.0	8.1
2180	AT1G47250.1 | Symbol: PAF2 | 20S proteasome alpha subunit F2 (PAF2) (PRC2B) (PRS1), identical to GB:AAC32063 from (Arabidopsis thaliana) (Genetics 149 (2), 677-692 (1998)); identical to cDNA proteasome subunit prc2b GI:2511585 | chr1:17321617-17324100 FORWARD | Aliases: 20S PROTEASOME SUBUNIT PAF2, F8G22.3, F8G22_3	9.5	6.2	3.3	7.1
11653	AT5G05100.1 | expressed protein | chr5:1505123-1507203 REVERSE | Aliases: MUG13.4, MUG13_4	9.5	9.8	-0.3	-1.9
12768	AT5G67330.1 | Symbol: ATNRAMP4 | NRAMP metal ion transporter 4 (NRAMP4), identical to metal transporter Nramp4 (Arabidopsis thaliana) gi:6468014:gb:AAF13279; member of the natural resistance-associated macrophage protein (NRAMP) metal transporter family, PMID:11500563 | chr5:26878955-26881123 FORWARD | Aliases: K8K14.5, K8K14_5, METAL TRANSPORTER NRAMP4, NRAMP4	9.5	10.1	-0.5	-1.6
11591	AT3G52560.2 | ubiquitin-conjugating enzyme family protein, similar to DNA-binding protein CROC-1B (Homo sapiens) GI:1066082; contains Pfam profile PF00179: Ubiquitin-conjugating enzyme | chr3:19505362-19507058 REVERSE | Aliases: F3C22.2	9.5	8.5	1.0	1.9
3263	AT2G27190.1 | Symbol: PAP1 | iron(III)-zinc(II) purple acid phosphatase (PAP12), identical to iron(III)-zinc(II) purple acid phosphatase (precursor) SP:Q38924 from (Arabidopsis thaliana) | chr2:11628304-11630534 REVERSE | Aliases: PURPLE ACID PHOSPHATASE 1, SECRETED PURPLE ACID PHOSPHATASE PRECURSOR, T22O13.4, T22O13_4	9.5	8.7	0.8	5.8
1418	AT4G22890.3 | expressed protein | chr4:12006997-12009529 FORWARD | Aliases: F7H19.70, F7H19_70	9.5	6.6	2.9	8.4
19712	AT3G05760.1 | expressed protein | chr3:1707909-1710529 FORWARD | Aliases: F10A16.5, F10A16_5	9.5	9.6	-0.1	-0.3
13493	AT1G56280.2 | Symbol: ATDI19 |	9.5	10.0	-0.5	-1.5
4487	AT4G35490.1 | ribosomal protein L11 family protein, several ribosomal proteins L11 | chr4:16855907-16856679 FORWARD | Aliases: F15J1.60, F15J1_60	9.5	7.1	2.4	4.9
8021	AT5G04740.1 | ACT domain-containing protein, contains Pfam profile PF01842: ACT domain	9.5	10.7	-1.2	-3.1
1875	AT5G22650.2 | Symbol: HD2B | expressed protein, non-consensus AT donor splice site at exon 3, AC acceptor splice site at exon 4; | chr5:7534067-7536276 FORWARD | Aliases: ATHD2B, HD2, HDA4, HDT02, HDT2, HISTONE DEACETYLASE, HISTONE DEACETYLASE 2, HISTONE DEACETYLASE 2B, MDJ22.7, MDJ22_7	9.5	7.7	1.8	7.6
1295	AT3G56910.1 | expressed protein | chr3:21080395-21081312 REVERSE | Aliases: T8M16.240	9.5	7.6	1.9	8.7
7293	AT2G26170.2 | Symbol: CYP711A1 | thromboxane-A synthase, putative / cytochrome P450 family protein, simiar to Thromboxane-A synthase (TXA synthase) (TXS) (SP:P47787) (Sus scrofa); contains Pfam profile: PF00067: Cytochrome P450; supported by cDNA: gi_15810029_gb_AY054283.1_ | chr2:11147899-11150761 FORWARD | Aliases: MAX1, MORE AXILLARY BRANCHES, MORE AXILLARY BRANCHES 1, T1D16.19, T1D16_19	9.5	8.6	0.9	3.4
2350	AT1G30510.3 | ferredoxin--NADP(+) reductase, putative / adrenodoxin reductase, putative, strong similarity to SP:P41345 Ferredoxin--NADP reductase, root isozyme, chloroplast precursor (EC 1.18.1.2) (FNR) {Oryza sativa}, ferredoxin-NADP reductase precursor (Zea mays) GI:500751 | chr1:10806968-10809144 REVERSE | Aliases: F26G16.13, F26G16_13	9.5	11.5	-2.0	-6.9
5133	AT4G15940.1 | fumarylacetoacetate hydrolase family protein, contains Pfam domain, PF01557: fumarylacetoacetate hydrolase family protein | chr4:9038304-9040758 FORWARD | Aliases: DL4011W, FCAALL.203	9.5	7.9	1.6	4.5

1771	AT2G37120.1 | DNA-binding S1FA family protein, contains Pfam profile: PF04689 DNA binding protein S1FA	9.5	7.7	1.8	7.8
1967	AT1G31970.1 | DEAD/DEAH box helicase, putative, similar to p68 RNA helicase (Schizosaccharomyces pombe) GI:173419 | chr1:11479846-11482870 FORWARD | Aliases: F5M6.3	9.5	7.7	1.8	7.5
9146	AT1G50670.1 | OTU-like cysteine protease family protein, contains Pfam profile PF02338: OTU-like cysteine protease | chr1:18778532-18780621 REVERSE | Aliases: F17J6.19, F17J6_19	9.5	8.9	0.6	2.6
6198	AT5G40650.1 | Symbol: SDH2 2 |	9.5	7.9	1.6	3.9
549	AT3G12740.1 | LEM3 (ligand-effect modulator 3) family protein / CDC50 family protein, Similar to GI:4585976; GI:4966357; GI:4835763; GI:9757735 from (Arabidopsis thaliana) | chr3:4049522-4052050 FORWARD | Aliases: MBK21.12	9.5	11.3	-1.8	-11.6
9986	AT1G27130.1 | Symbol: ATGSTU13 | glutathione S-transferase, putative, similar to glutathione S-transferase GB: AAF22517 GI:6652870 from (Papaver somniferum) | chr1:9425447-9426873 FORWARD | Aliases: GLUTATHIONE S TRANSFERASE 12, GST12, T7N9.190, T7N9_190	9.5	9.1	0.4	2.4
17013	AT1G24510.2 | T-complex protein 1 epsilon subunit, putative / TCP-1-epsilon, putative / chaperonin, putative, identical to SWISS-PROT:O04450- T-complex protein 1, epsilon subunit (TCP-1-epsilon) (Arabidopsis thaliana); strong similarity to SP:P54411 T-complex protein 1, epsilon subunit (TCP-1-epsilon) (CCT-epsilon) (TCP-K36) {Avena sativa}; contains Pfam:PF00118 domain, TCP-1/cpn60 chaperonin family | chr1:8685298-8688231 REVERSE | Aliases: F21J9.17	9.5	9.3	0.2	0.8
1073	AT2G06520.1 | membrane protein, putative, contains 2 transmembrane domains; | chr2:2587781-2588355 REVERSE | Aliases: T12H3.7, T12H3_7	9.5	4.7	4.8	9.3
7104	AT2G45820.1 | DNA-binding protein, putative, identical to DNA-binding protein gi:601843:gb:AAA57124 (Arabidopsis thaliana); contains Pfam domain, PF03766: Remorin, N-terminal region; contains Pfam domain, PF03763: Remorin, C-terminal region | chr2:18870032-18871735 REVERSE | Aliases: F4I18.20	9.5	10.3	-0.8	-3.5
17898	AT4G39730.1 | lipid-associated family protein, contains PLAT/LH2 (Polycystin-1, Lipoxygenase, Alpha-Toxin/Lipoxygenase homology) domain Pfam:PF01477 | chr4:18432893-18433689 FORWARD | Aliases: T19P19.120, T19P19_120	9.5	9.3	0.2	0.6
11701	AT2G20360.1 | expressed protein | chr2:8793096-8796554 FORWARD | Aliases: F11A3.9, F11A3_9	9.5	7.9	1.5	1.9
9605	AT5G09830.1 | BolA-like family protein, contains Pfam profile: PF01722 BolA-like protein | chr5:3057658-3058822 REVERSE | Aliases: MYH9.4, MYH9_4	9.5	9.0	0.5	2.5
10823	AT5G17190.1 | expressed protein, similar to unknown protein (gb:AAF26109.1) | chr5:5652225-5652985 FORWARD | Aliases: MKP11.4, MKP11_4	9.5	8.5	1.0	2.1
3801	AT3G02520.1 | Symbol: GRF7 | 14-3-3 protein GF14 nu (GRF7), identical to 14-3-3 protein GF14 nu GI:1531631 from (Arabidopsis thaliana) | chr3:526444-528320 REVERSE | Aliases: F16B3.15, F16B3_15, GF14 NU	9.5	6.4	3.1	5.4
3779	AT1G31340.1 | Symbol: RUB1 | ubiquitin family protein, contains INTERPRO:IPR000626 ubiquitin domain | chr1:11217865-11219437 REVERSE | Aliases: ATRUB1, NEDD8, RELATED TO UBIQUITIN 1, T19E23.13, T19E23_13	9.5	6.6	2.9	5.4
7156	ATCG00670.1 | Symbol: CLPP1 | Encodes the only ClpP (caseinolytic protease) encoded within the plastid genome. Contains a highly conserved catalytic triad of Ser-type proteases (Ser-His-Asp). Part of the 350 kDa chloroplast Clp complex. The name reflects nomenclature described in Adam et. al (2001).	9.5	7.9	1.6	3.5
6712	AT1G53210.1 | sodium/calcium exchanger family protein / calcium-binding EF hand family protein, contains Pfam profiles: PF01699 sodium/calcium exchanger protein, PF00036 EF hand | chr1:19848300-19851504 FORWARD | Aliases: F12M16.12, F12M16_12	9.5	10.7	-1.2	-3.7
373	AT5G53880.1 | expressed protein | chr5:21890020-21890529 REVERSE | Aliases: K19P17.4, K19P17_4	9.5	4.1	5.4	13.2
1250	AT5G42150.1 | expressed protein | chr5:16863378-16865507 FORWARD | Aliases: MJC20.26, MJC20_26	9.5	6.7	2.8	8.8
21043	AT1G52740.1 | histone H2A, putative, similar to histone H2A.F/Z Arabidopsis thaliana GI:2407800; contains Pfam profile PF00125 Core histone H2A/H2B/H3/H4 | chr1:19648963-19650099 FORWARD | Aliases: F14G24.1, F14G24_1	9.5	9.5	-0.0	-0.0
2416	AT3G57010.1 | strictosidine synthase family protein, similar to strictosidine synthase (Rauvolfia serpentina)(SP:P15324) | chr3:21106633-21108430 REVERSE | Aliases: F24I3.90	9.5	6.6	2.9	6.8
9193	AT1G56330.1 | Symbol: SAR1 | GTP-binding protein (SAR1B), identical to GTP-binding protein (SAR1B) (Arabidopsis thaliana) SP:Q01474 | chr1:21090220-21092214 REVERSE | Aliases: ATSAR1, ATSARA1B, F14G9.6, F14G9_6, SAR LIKE, SARL, SECRETION ASSOCIATED RAS	9.5	8.3	1.2	2.6
3405	AT5G16840.1 | RNA recognition motif (RRM)-containing protein, predicted proteins - Arabidopsis thaliana | chr5:5535923-5538253 FORWARD | Aliases: F5E19.180, F5E19_180	9.5	7.2	2.2	5.7
3829	AT5G05200.1 | ABC1 family protein, contains Pfam domain, PF03109: ABC1 family | chr5:1543606-1547138 REVERSE | Aliases: K2A11.7, K2A11_7	9.5	8.5	1.0	5.4

11246	AT1G10950.1 | endomembrane protein 70, putative | chr1:3659216-3663984 FORWARD | Aliases: T19D16.13, T19D16_13	9.5	8.9	0.6	2.0
814	AT5G14780.1 | Symbol: FDH | formate dehydrogenase (FDH), identical to GI:7677266 | chr5:4776987-4779497 FORWARD | Aliases: FORMATE DEHYDROGENASE, T9L3.80, T9L3_80	9.5	4.9	4.5	10.2
2916	AT4G18100.1 | 60S ribosomal protein L32 (RPL32A), ribosomal protein L32, human, PIR1:R5HU32 | chr4:10035497-10036552 REVERSE | Aliases: F15J5.70, F15J5_70	9.5	6.2	3.2	6.2
2917	AT5G46430.2 | 60S ribosomal protein L32 (RPL32B) | chr5:18850494-18851791 FORWARD | Aliases: K11I1.2, K11I1_2	9.5	6.2	3.2	6.2
10221	AT5G28390.1 | RNA recognition motif (RRM)-containing protein, contains InterPro entry IPR000504: RNA-binding region RNP-1 (RNA recognition motif) (RRM) | chr5:10342028-10343001 REVERSE | Aliases: F21B23.40, F21B23_40	9.5	10.3	-0.9	-2.3
10220	AT3G52660.2 | similar to RNA recognition motif (RRM)-containing protein [Arabidopsis thaliana] (TAIR:At4g00830.1); similar to putative RNA-binding protein [Oryza sativa (japonica cultivar-group)] (GB:AAP52145.1); contains InterPro domain RNA-binding region RNP-1	9.5	10.3	-0.9	-2.3
9713	AT1G60810.1 | Symbol: ACLA 2 |	9.5	8.5	1.0	2.5
9960	NA	9.4	7.3	2.2	2.4
459	AT3G47860.1 | apolipoprotein D-related, contains weak similarity to Apolipoprotein D precursor (ApoD) (Swiss-Prot:P51910) (Mus musculus) | chr3:17667682-17669273 REVERSE | Aliases: T23J7.190	9.4	6.4	3.0	12.3
10170	AT5G40670.1 | PQ-loop repeat family protein / transmembrane family protein, similar to SP:O60931 Cystinosin {Homo sapiens}; contains Pfam profile PF04193: PQ loop repeat | chr5:16302939-16305013 FORWARD | Aliases: MNF13.23, MNF13_23	9.4	10.2	-0.8	-2.3
1195	AT2G20450.1 | 60S ribosomal protein L14 (RPL14A) | chr2:8820947-8822332 FORWARD | Aliases: T13C7.4, T13C7_4	9.4	6.9	2.6	9.0
7863	AT1G75750.1 | Symbol: GASA1 | gibberellin-regulated protein 1 (GASA1) / gibberellin-responsive protein 1, identical to SP:P46689 Gibberellin-regulated protein 1 precursor {Arabidopsis thaliana}; supporting cDNA gi:887938:gb:U11766.1:ATU11766 | chr1:28445202-28446023 REVERSE | Aliases: F10A5.6, F10A5_6, GAST1 PROTEIN HOMOLOG	9.4	10.6	-1.1	-3.2
6204	AT3G51920.1 | Symbol: CAM9 | calmodulin-9 (CAM9), identical to calmodulin 9 GI:5825602 from (Arabidopsis thaliana); contains Pfam profile PF00036: EF hand | chr3:19279026-19280366 REVERSE | Aliases: CALMODULIN 9, F4F15.30	9.4	10.2	-0.8	-3.9
7362	AT1G01490.2 | similar to heavy-metal-associated domain-containing protein [Arabidopsis thaliana] (TAIR:At5g23760.1); similar to unknown [Populus tremuloides] (GB:AAO63778.1); contains InterPro domain Heavy metal transport/detoxification protein (InterPro:IPR006121) | chr1:180059-182293 REVERSE | Aliases: F22L4.5, F22L4_5	9.4	10.8	-1.3	-3.4
4283	AT2G38550.1 | expressed protein, contains Pfam profile PF03647: Uncharacterised protein family (UPF0136) | chr2:16139092-16141493 FORWARD | Aliases: T6A23.25, T6A23_25	9.4	6.9	2.5	5.0
10401	AT5G10780.1 | expressed protein, HSPC184, Homo sapiens, EMBL:AF151018 | chr5:3408347-3410044 FORWARD | Aliases: T30N20.50, T30N20_50	9.4	8.2	1.2	2.2
365	AT5G47680.1 | expressed protein, contains Pfam profile PF04243: Protein of unknown function (DUF425) | chr5:19332309-19334748 REVERSE | Aliases: MNJ7.27, MNJ7_27	9.4	5.6	3.8	13.3
11530	AT1G69410.1 | eukaryotic translation initiation factor 5A, putative / eIF-5A, putative, strong similarity to eukaryotic initiation factor 5A (2) (Nicotiana plumbaginifolia) GI:19702, SP:Q9AXQ6: Eukaryotic translation initiation factor 5A-1 (eIF-5A 1) {Lycopersicon esculentum} | chr1:26092868-26094047 FORWARD | Aliases: F10D13.8, F10D13_8	9.4	8.6	0.8	1.9
17346	AT1G51060.1 | histone H2A, putative, similar to histone H2A GI:7595337 from Arabidopsis thaliana, Triticum aestivum GI:536892, Picea abies SP:P35063; contains Pfam profile PF00125 Core histone H2A/H2B/H3/H4	9.4	9.2	0.2	0.7
11118	AT2G46140.1 | late embryogenesis abundant protein, putative / LEA protein, putative, similar to SP:P46518 Late embryogenesis abundant protein Lea14-A {Gossypium hirsutum}; contains Pfam profile PF03168: Late embryogenesis abundant protein	9.4	8.4	1.0	2.0
14398	AT1G77840.1 | eukaryotic translation initiation factor 5, putative / eIF-5, putative, similar to SP:P55876 Eukaryotic translation initiation factor 5 (eIF-5) {Zea mays}; contains Pfam profiles PF02020: eIF4-gamma/eIF5/eIF2-epsilon, PF01873: Domain found in IF2B/IF5 | chr1:29273381-29275480 FORWARD | Aliases: T32E8.15	9.4	9.0	0.4	1.3
2487	AT5G65480.1 | expressed protein | chr5:26193247-26195140 FORWARD | Aliases: MNA5.18	9.4	11.1	-1.7	-6.7

848	AT3G62030.1 | Symbol: ROC4 | peptidyl-prolyl cis-trans isomerase, chloroplast / cyclophilin / rotamase / cyclosporin A-binding protein (ROC4), identical to peptidyl-prolyl cis-trans isomerase, chloroplast precursor, PPIase (cyclophilin, cyclosporin A-binding protein) (Arabidopsis thaliana) SWISS-PROT:P34791; identical to cDNA nuclear-encoded chloroplast stromal cyclophilin (ROC4) GI:405130 | chr3:22984585-22986345 FORWARD | Aliases: PEPTIDYLPROLYL ISOMERASE ROC4, ROTAMASE CYCLOPHILIN 4, ROTAMASE CYP 4, T17J13.1	9.4	5.6	3.8	10.1
17530	AT4G24550.2 | clathrin adaptor complexes medium subunit family protein, contains Pfam profile: PF00928 adaptor complexes medium subunit family	9.4	9.6	-0.2	-0.7
13755	AT4G21105.1 | expressed protein | chr4:11265662-11266982 FORWARD | Aliases: None	9.4	9.0	0.4	1.4
3266	AT1G07040.1 | expressed protein | chr1:2160944-2163302 REVERSE | Aliases: F10K1.25, F10K1_25	9.4	6.9	2.5	5.8
5241	AT1G02560.1 | Symbol: CLPP5 | ATP-dependent Clp protease proteolytic subunit (ClpP1), identical to nClpP1 GB:BAA82065 GI:5360579 from (Arabidopsis thaliana); contains Pfam profile PF00574: Clp protease; contains TIGRfam profile TIGR00493: ATP-dependent Clp protease, proteolytic subunit ClpP | chr1:537888-540109 FORWARD | Aliases: CLP PROTEASE 1 PROTEOLYTIC SUBUNIT, NCLPP1, NCLPP5, NUCLEAR ENCODED CLP PROTEASE 1, T14P4.12, T14P4_12	9.4	7.2	2.2	4.4
6475	AT5G08570.1 | pyruvate kinase, putative, similar to pyruvate kinase, cytosolic isozyme (Glycine max) SWISS-PROT:Q42806	9.4	7.9	1.6	3.8
9312	AT5G38530.1 | tryptophan synthase-related, low similarity to tryptophan synthases, beta subunit, from Lactococcus lactis (SP:Q01998), Pyrococcus kodakaraensis (SP:Q9YGB0), Thermus thermophilus (SP:P16609); contains Pfam pyridoxal-phosphate dependent enzyme domain PF00291 | chr5:15441269-15443700 FORWARD | Aliases: MBB18.6, MBB18_6	9.4	8.2	1.2	2.6
4413	AT3G18490.1 | aspartyl protease family protein, contains Pfam domain, PF00026: eukaryotic aspartyl protease | chr3:6348761-6350674 REVERSE | Aliases: MYF24.39	9.4	7.9	1.5	4.9
2624	AT5G57290.1 | 60S acidic ribosomal protein P3 (RPP3B) | chr5:23224055-23225121 REVERSE | Aliases: MJB24.10, MJB24_10	9.4	6.9	2.5	6.6
1160	AT3G55280.1 | 60S ribosomal protein L23A (RPL23aB), various ribosomal L23a proteins | chr3:20511312-20512686 FORWARD | Aliases: T26I12.160	9.4	5.9	3.5	9.1
14259	AT1G05850.1 | Symbol: ELP | chitinase-like protein 1 (CTL1), similar to class I chitinase GI:7798656 from (Halimolobos perplexa var. perplexa); contains Pfam profile PF00182: Chitinase class I; identical to cDNA chitinase-like protein 1 (CTL1) CTL1-ELP1 allele GI:17226328 | chr1:1766502-1768662 REVERSE | Aliases: CHITINASE LIKE PROTEIN 1, CTL1, ECTOPIC DEPOSITION OF LIGNIN IN PITH, ELP1, T20M3.12, T20M3_12	9.4	10.1	-0.7	-1.3
10975	AT1G43900.1 | protein phosphatase 2C, putative / PP2C, putative, similar to protein phosphatase type 2C GI:4336436 from (Lotus japonicus) | chr1:16656485-16658885 FORWARD | Aliases: F9C16.6, F9C16_6	9.4	9.0	0.4	2.1
10841	AT4G04770.1 | Symbol: ATABC1 |	9.4	8.8	0.6	2.1
15696	AT2G28840.1 | ankyrin repeat family protein, contains ankyrin repeats, Pfam:PF00023 | chr2:12385436-12387819 FORWARD | Aliases: F8N16.13, F8N16_13	9.4	9.7	-0.3	-1.0
3710	AT4G34710.2 | Symbol: ADC2 | arginine decarboxylase 2 (SPE2), identical to SP:O23141 Arginine decarboxylase 2 (EC 4.1.1.19) (ARGDC 2) (ADC 2) (ADC-N) {Arabidopsis thaliana} | chr4:16560067-16562936 REVERSE | Aliases: ARGININE DECARBOXYLASE, ARGININE DECARBOXYLASE 2, SPE2, T4L20.290, T4L20_290	9.4	7.7	1.7	5.5
4384	AT5G05160.1 | leucine-rich repeat transmembrane protein kinase, putative | chr5:1528001-1530063 FORWARD | Aliases: K2A11.3, K2A11_3	9.4	8.3	1.1	5.0
6146	AT1G16180.1 | TMS membrane family protein / tumour differentially expressed (TDE) family protein, contains Pfam domain, PF03348: TMS membrane protein/tumour differentially expressed protein (TDE) | chr1:5540899-5542896 FORWARD | Aliases: T24D18.26, T24D18_26	9.4	11.2	-1.8	-3.9
9917	AT5G18310.2 | expressed protein, predicted proteins, Drosophila melanogaster | chr5:6061734-6063024 FORWARD | Aliases: F20L16.30, F20L16_30	9.4	10.0	-0.6	-2.4
3467	AT4G23885.1 | expressed protein | chr4:12419404-12420477 FORWARD | Aliases: None	9.4	8.2	1.2	5.7
3633	AT4G16370.1 | Symbol: ATOPT3 |	9.4	6.4	2.9	5.5
9324	AT1G11910.1 | aspartyl protease family protein, contains Pfam profiles: PF00026 eukaryotic aspartyl protease, PF03489 surfactant protein B, PF05184 saposin-like type B, region 1 | chr1:4016789-4020916 REVERSE | Aliases: F12F1.24, F12F1_24	9.4	11.1	-1.7	-2.6
7138	ATCG00810.1 | Symbol: RPL22 | encodes a chloroplast ribosomal protein L22, a constituent of the large subunit of the ribosomal complex | chrC:83467-83949 REVERSE | Aliases: RPL22	9.4	6.8	2.5	3.5
9997	AT4G10050.1 | hydrolase, alpha/beta fold family protein, similar to protein phosphatase methylesterase-1 (Homo sapiens) GI:5533003; contains Pfam profile PF00561: hydrolase, alpha/beta fold family | chr4:6284447-6287287 REVERSE | Aliases: F28M11.4	9.4	8.6	0.8	2.4

7203	AT1G76790.1 | O-methyltransferase family 2 protein, similar to caffeic acid O-methyltransferase (Catharanthus roseus)(GI:18025321), catechol O-methyltransferase GB:CAA55358 (Vanilla planifolia) | chr1:28827080-28828567 REVERSE | Aliases: F28O16.16, F28O16_16	9.4	8.2	1.2	3.4
9934	AT4G17530.1 | Symbol: RAB1C | Ras-related GTP-binding protein, putative, very strong similarity to RAB1C (Lotus corniculatus var. japonicus) GI:1370166; contains Pfam profile PF00071: Ras family | chr4:9773094-9775598 REVERSE | Aliases: DL4800C, FCAALL.87	9.4	9.9	-0.5	-2.4
19443	AT4G25030.2 | expressed protein | chr4:12864838-12866837 FORWARD | Aliases: F13M23.170, F13M23_170	9.4	9.3	0.1	0.3
1077	AT5G60670.1 | 60S ribosomal protein L12 (RPL12C), 60S RIBOSOMAL PROTEIN L12 (like), Arabidopsis thaliana, PIR:T45883 | chr5:24398136-24398819 REVERSE | Aliases: MUP24.13, MUP24_13	9.4	7.3	2.1	9.3
3159	AT2G02230.1 | Symbol: ATPP2 B1 | similar to F-box family protein / SKP1 interacting partner 3-related [Arabidopsis thaliana] (TAIR:At2g02240.1); similar to F-box family protein-like [Oryza sativa (japonica cultivar-group)] (GB:XP_468355.1); contains InterPro domain Cyclin-like F-box (InterPro:IPR001810) | chr2:590222-591649 REVERSE | Aliases: ATPP2 B1, T16F16.2, T16F16_2	9.4	5.5	3.9	5.9
17016	AT3G14310.1 | Symbol: ATPME3 |	9.4	8.9	0.5	0.8
6090	AT1G16460.3 | Symbol: ATRDH2 |	9.4	7.6	1.7	4.0
2541	AT5G46160.2 | ribosomal protein L14 family protein / huellenlos paralog (HLP), contains Pfam profile PF00238: Ribosomal protein L14p/L23e; identical to cDNA HUELLENLOS PARALOG (HLP) nuclear gene for mitochondrial product GU:18140859 | chr5:18728582-18729734 REVERSE | Aliases: MCL19.22, MCL19_22	9.4	7.7	1.6	6.6
7459	AT4G15093.1 | catalytic LigB subunit of aromatic ring-opening dioxygenase family, contains Pfam PF02900: Catalytic LigB subunit of aromatic ring-opening dioxygenase | chr4:8618370-8619643 FORWARD | Aliases: None	9.4	10.4	-1.1	-3.3
12715	AT1G48920.1 | nucleolin, putative, similar to nuM1 protein GI:1279562 from (Medicago sativa) | chr1:18101776-18105291 FORWARD | Aliases: F27K7.6, F27K7_6	9.4	8.9	0.5	1.7
18597	AT5G59420.1 | oxysterol-binding family protein, similar to SP:P16258 Oxysterol-binding protein 1 {Oryctolagus cuniculus}; contains Pfam profile PF01237: Oxysterol-binding protein | chr5:23978806-23982050 FORWARD | Aliases: F2O15.17, F2O15_17	9.4	9.5	-0.2	-0.5
13648	AT2G14750.1 | Symbol: APK | adenylylsulfate kinase 1 (AKN1), identical to adenylylsulfate kinase 1, chloroplast precursor (APS kinase, Adenosine-5'phosphosulfate kinase, ATP adenosine-5'- phosphosulfate 3'-phosphotransferase) (Arabidopsis thaliana) SWISS-PROT:Q43295 | chr2:6321178-6322848 FORWARD | Aliases: ADENOSINE PHOSPHOSULFATE KINASE, APS KINASE, F26C24.11, F26C24_11	9.4	9.7	-0.3	-1.4
2630	AT1G43710.1 | Symbol: EMB1075 | serine decarboxylase, identical to serine decarboxylase (Arabidopsis thaliana) GI:15011302; contains Pfam profile PF00282: Pyridoxal-dependent decarboxylase conserved domain	9.4	10.7	-1.4	-6.5
21115	AT4G18930.1 | cyclic phosphodiesterase, identical to cyclic phosphodiesterase (Arabidopsis thaliana) gi:2065013:emb:CAA72363 | chr4:10370715-10372056 FORWARD | Aliases: F13C5.100, F13C5_100	9.4	9.4	0.0	0.0
375	AT3G29320.1 | glucan phosphorylase, putative, similar to alpha-glucan phosphorylase, L isozyme 1 precursor GB:P04045 from (Solanum tuberosum) (J. Biochem. 106 (4), 691-695 (1989)) | chr3:11254059-11259051 FORWARD | Aliases: MUO10.17	9.4	4.4	4.9	13.2
7268	ATCG00480.1 | Symbol: ATPB | chloroplast-encoded gene for beta subunit of ATP synthase | chrC:52660-54156 REVERSE | Aliases: ATPB	9.4	7.6	1.7	3.4
17088	AT1G48410.2 | Symbol: AGO1 | argonaute protein (AGO1), identical to SP:O04379 Argonaute protein (AGO1) {Arabidopsis thaliana}; contains Pfam profiles PF02171: Piwi domain, PF02170: PAZ domain | chr1:17889766-17896236 REVERSE | Aliases: ARGONAUTE 1, ARGONAUTE1, T1N15.2, T1N15_2	9.4	9.7	-0.3	-0.8
5695	AT2G25950.1 | expressed protein | chr2:11075729-11077738 FORWARD | Aliases: F17H15.2	9.4	7.3	2.0	4.2
4047	AT4G38220.2 | aminoacylase, putative / N-acyl-L-amino-acid amidohydrolase, putative, similar to aminoacylase-1 (N-acyl-L-amino-acid amidohydrolase, ACY-1)(Homo sapiens) SWISS-PROT:Q03154 | chr4:17925174-17927091 FORWARD | Aliases: F20D10.340, F20D10_340	9.4	10.7	-1.3	-5.2
4304	AT5G23590.2 | similar to DNAJ heat shock protein, mitochondrially targeted (GFA2) [Arabidopsis thaliana] (TAIR:At5g48030.1); similar to PREDICTED: similar to hypothetical protein FLJ10634 [Canis familiaris] (GB:XP_535435.1); contains InterPro domain Heat shock protein DnaJ (InterPro:IPR003095); contains InterPro domain Heat shock protein DnaJ, N-terminal (InterPro:IPR001623) | chr5:7953532-7955625 REVERSE | Aliases: MQM1.14, MQM1_14	9.4	7.7	1.6	5.0
4981	AT1G08970.4 | Symbol: HAP5C | CCAAT-box binding transcription factor Hap5a, putative | chr1:2882546-2884285 FORWARD | Aliases: F7G19.16, F7G19_16, HAP5C, HEME ACTIVATED PROTEIN 5C	9.4	7.1	2.3	4.6

669	AT1G65700.2 | similar to small nuclear ribonucleoprotein, putative / snRNP, putative / Sm protein, putative [Arabidopsis thaliana] (TAIR:At1g19120.1); similar to PREDICTED: similar to U6 snRNA-associated Sm-like protein LSm8 [Gallus gallus] (GB:XP_416009.1); contains InterPro domain Small nuclear ribonucleoprotein (Sm protein) (InterPro:IPR001163) | chr1:24437943-24439918 REVERSE | Aliases: F1E22.8, F1E22_8	9.4	6.2	3.2	10.9
10423	AT1G52420.1 | glycosyl transferase family 1 protein, contains Pfam profile: PF00534 Glycosyl transferases group 1 | chr1:19531518-19534956 FORWARD | Aliases: F19K6.13	9.4	9.0	0.4	2.2
7488	AT3G17020.1 | universal stress protein (USP) family protein, similar to early nodulin ENOD18 (Vicia faba) GI:11602747; contains Pfam profile PF00582: universal stress protein family | chr3:5802555-5804130 REVERSE | Aliases: K14A17.9	9.4	11.5	-2.1	-3.3
4622	AT2G29960.1 | Symbol: CYP5 | peptidyl-prolyl cis-trans isomerase / cyclophilin (CYP5) / rotamase, identical to cyclophilin (Arabidopsis thaliana) gi:2443755:gb:AAB71401 | chr2:12776134-12777656 REVERSE | Aliases: ATCYP5, F23F1.12, F23F1_12	9.4	8.1	1.3	4.8
11166	AT3G15730.1 | Symbol: PLDALPHA1 | phospholipase D alpha 1 / PLD alpha 1 (PLDALPHA1) (PLD1) / choline phosphatase 1, identical to SP:Q38882 Phospholipase D alpha 1 (EC 3.1.4.4) (AtPLDalpha1) (PLD alpha 1) (Choline phosphatase 1) (Phosphatidylcholine-hydrolyzing phospholipase D 1) (PLDalpha) (Arabidopsis thaliana) | chr3:5330344-5333752 FORWARD | Aliases: MSJ11.13, PHOSPHOLIPASE D, PHOSPHOLIPASE D ALPHA 1, PLD	9.4	8.2	1.1	2.0
11448	AT1G19660.2 | similar to wound-responsive protein-related [Arabidopsis thaliana] (TAIR:At1g75380.2); similar to wound-responsive protein-related [Arabidopsis thaliana] (TAIR:At1g75380.1); similar to wound-responsive protein-related [Arabidopsis thaliana] (TAIR:At1g75380.3); similar to putative wound inductive gene [Oryza sativa (japonica cultivar-group)] (GB:XP_481914.1) | chr1:6799972-6802643 REVERSE | Aliases: F14P1.1, F14P1_1	9.4	10.7	-1.3	-2.0
11449	AT1G75380.3 | wound-responsive protein-related, similar to wound inducive gene GI:8096273 from (Nicotiana tabacum) | chr1:28285050-28287694 REVERSE | Aliases: F1B16.9, F1B16_9	9.4	10.7	-1.3	-2.0
6808	AT4G39660.1 | Symbol: AGT2 | alanine--glyoxylate aminotransferase, putative / beta-alanine-pyruvate aminotransferase, putative / AGT, putative, similar to SP:Q64565 Alanine--glyoxylate aminotransferase 2, mitochondrial precursor (EC 2.6.1.44) (AGT 2) (Beta-alanine-pyruvate aminotransferase) {Rattus norvegicus}; contains Pfam profile PF00202: aminotransferase, class III | chr4:18406767-18409466 FORWARD | Aliases: ALANINE:GLYOXYLATE AMINOTRANSFERASE 2, T19P19.50, T19P19_50	9.4	8.1	1.3	3.6
1539	AT5G14320.1 | 30S ribosomal protein S13, chloroplast (CS13), ribosomal protein S13 precursor, chloroplast Arabidopsis thaliana, PIR:S59594; identical to cDNA ribosomal protein S13 GI:1515106	9.4	6.7	2.7	8.1
854	AT3G56680.1 | expressed protein | chr3:21002259-21004646 FORWARD | Aliases: T8M16.10	9.4	6.7	2.6	10.1
3517	AT5G40780.2 | Symbol: LHT1 | lysine and histidine specific transporter, putative, strong similarity to lysine and histidine specific transporter GI:2576361 from (Arabidopsis thaliana); contains Pfam profile PF01490: Transmembrane amino acid transporter protein | chr5:16340910-16344502 FORWARD | Aliases: K1B16.3, K1B16_3, LYSINE AND HISTIDINE SPECIFIC TRANSPORTER	9.4	11.2	-1.8	-5.6
8092	AT5G51570.1 | band 7 family protein, similar to hypersensitive-induced response protein (Zea mays) GI:7716468; contains Pfam profile PF01145: SPFH domain / Band 7 family | chr5:20966652-20968723 FORWARD | Aliases: K17N15.12, K17N15_12	9.3	7.4	2.0	3.1
9448	AT5G13000.1 | Symbol: ATGSL12 | glycosyl transferase family 48 protein, contains Pfam profile: PF02364 1,3-beta-glucan synthase | chr5:4110298-4121430 REVERSE | Aliases: GLUCAN SYNTHASE LIKE 12, GSL12, T24H18.170, T24H18_170	9.3	9.7	-0.3	-2.5
14206	AT5G21090.1 | leucine-rich repeat protein, putative, similar to leucine rich repeat protein (LRP) GI:1619300 from (Lycopersicon esculentum); contains leucine rich-repeat (LRR) domains Pfam:PF00560, INTERPRO:IPR001611 | chr5:7164614-7167257 FORWARD | Aliases: T10F18.120, T10F18_120	9.3	8.4	0.9	1.3
16409	AT4G01370.1 | Symbol: ATMPK4 |	9.3	9.0	0.3	0.9
18011	AT4G02860.1 | similar to phenazine biosynthesis PhzC/PhzF family protein [Arabidopsis thaliana] (TAIR:At1g03210.1); similar to phenazine biosynthesis family protein [Oryza sativa (japonica cultivar-group)] (GB:AAU89223.1); contains InterPro domain Phenazine biosynthesis PhzC/PhzF protein (InterPro:IPR003719) | chr4:1268713-1270525 REVERSE | Aliases: T5J8.18, T5J8_18	9.3	9.1	0.3	0.6
661	AT1G01940.1 | peptidyl-prolyl cis-trans isomerase cyclophilin-type family protein, contains Pfam domain, PF00160: peptidyl-prolyl cis-trans isomerase, cyclophilin-type | chr1:323027-324917 FORWARD | Aliases: F22M8.7, F22M8_7	9.3	4.5	4.8	10.9
8100	AT3G04790.1 | ribose 5-phosphate isomerase-related, similar to ribose-5-phosphate isomerase GI:18654317 from (Spinacia oleracea) | chr3:1313324-1314369 FORWARD | Aliases: F7O18.28, F7O18_28	9.3	8.3	1.1	3.1
18866	AT5G59160.3 | Symbol: TOPP2 | similar to serine/threonine protein phosphatase PP1 isozyme 5 (TOPP5) / phosphoprotein phosphatase 1 [Arabidopsis thaliana] (TAIR:At3g46820.1); similar to protein phosphatase type 1 [Nicotiana tabacum] (GB:CAB07804.1); contains InterPro domain Metallo-phosphoesterase (InterPro:IPR004843); contains InterPro domain Serine/threonine-specific protein phosphatase and bis(5-nucleosyl)-tetraphosphatase (InterPro:IPR006186) | chr5:23896563-23898855 FORWARD | Aliases: MNC17.9, MNC17_9, PPO	9.3	9.2	0.2	0.4

2495	AT5G32450.1 | RNA recognition motif (RRM)-containing protein, various predicted proteins, Arabidopsis thaliana and others | chr5:12096848-12098803 FORWARD | Aliases: F18O9.60, F18O9_60	9.3	8.5	0.9	6.7
6635	AT1G45332.1 | mitochondrial elongation factor, putative, similar to mitochondrial elongation factor GI:3917 from (Saccharomyces cerevisiae) | chr1:17174795-17179261 REVERSE | Aliases: AT1G45261, F2G19.2, F2G19_2	9.3	6.8	2.6	3.7
6636	AT2G45030.1 | mitochondrial elongation factor, putative, similar to SP:P25039 Elongation factor G 1, mitochondrial precursor (mEF-G-1) {Saccharomyces cerevisiae}; contains Pfam profiles PF00009: Elongation factor Tu GTP binding domain, PF03764: Elongation factor G domain IV, PF00679: Elongation factor G C-terminus | chr2:18579486-18584045 FORWARD | Aliases: T14P1.16	9.3	6.8	2.6	3.7
1173	AT5G13520.1 | peptidase M1 family protein, similar to SP:P09960 Leukotriene A-4 hydrolase (EC 3.3.2.6) (LTA-4 hydrolase) {Homo sapiens}; contains Pfam profile PF01433: Peptidase family M1 | chr5:4341543-4344674 REVERSE | Aliases: T6I14.50, T6I14_50	9.3	6.0	3.4	9.0
1548	AT3G45160.1 | expressed protein | chr3:16544436-16545063 REVERSE | Aliases: T14D3.100	9.3	5.5	3.8	8.1
4281	AT5G63890.2 | Symbol: ATHDH | histidinol dehydrogenase, putative / HDH, putative, strong similarity to SP:P24226 Histidinol dehydrogenase, chloroplast precursor (EC 1.1.1.23) (HDH) {Brassica oleracea var.capitata}; contains Pfam profile PF00815: histidinol dehydrogenase | chr5:25582657-25585421 REVERSE | Aliases: HISTIDINOL DEHYDROGENASE, MGI19.9, MGI19_9	9.3	7.3	2.0	5.0
13325	AT5G54680.1 | basic helix-loop-helix (bHLH) family protein, similar to unknown protein (pir :B71406) | chr5:22234286-22236830 FORWARD | Aliases: K5F14.2, K5F14_2	9.3	9.6	-0.3	-1.5
2942	AT2G19800.1 | Symbol: MIOX2 | expressed protein, similar to myo-inositol oxygenase (Sus scrofa) gi:17432544:gb:AAL39076 | chr2:8537986-8540533 REVERSE | Aliases: F6F22.17, F6F22_17, MYO INOSITOL OXYGENASE 2	9.3	6.4	2.9	6.2
10049	AT1G12840.1 | Symbol: DET3 | vacuolar ATP synthase subunit C (VATC) / V-ATPase C subunit / vacuolar proton pump C subunit (DET3), identical to vacuolar ATP synthase subunit C SP:Q9SDS7 from (Arabidopsis thaliana) | chr1:4375517-4378509 FORWARD | Aliases: DE ETIOLATED 3, F13K23.9, F13K23_9, VACUOLAR ATP SYNTHASE SUBUNIT C	9.3	8.6	0.8	2.3
1861	AT5G03740.1 | Symbol: HD2C | zinc finger (C2H2 type) family protein, contains Pfam domain, PF00096: Zinc finger, C2H2 type | chr5:981896-984201 FORWARD | Aliases: F17C15.160, F17C15_160, HISTONE DEACETYLASE 2C	9.3	7.8	1.5	7.6
17729	AT2G29020.1 | Rab5-interacting family protein, similar to Protein C20orf24 (Rab5-interacting protein) (RIP5) (PNAS-11) (Swiss-Prot:Q9BUV8) (Homo sapiens); contains transmembrane domains | chr2:12476806-12478742 FORWARD | Aliases: T9I4.10, T9I4_10	9.3	9.4	-0.1	-0.6
5512	AT1G48450.2 | similar to expressed protein [Arabidopsis thaliana] (TAIR:At3g17800.1); similar to unknown [Brassica napus] (GB:AAK30572.1); contains InterPro domain Protein of unknown function DUF760 (InterPro:IPR008479) | chr1:17912171-17914113 REVERSE | Aliases: T1N15.6, T1N15_6	9.3	8.3	1.0	4.3
2261	AT2G20890.1 | Symbol: THF1 | Chloroplast-localized Thylakoid formation1 gene product involved in vesicle-mediated formation of thylakoid membranes. Thf1 antisense lines contain abnormal chloroplasts early in leaf development (chloroplasts have loosely stacked thylakoid membranes). Expression was induced in the light and decreased under dark conditions. | chr2:8994749-8996494 FORWARD | Aliases: F5H14.14, F5H14_14, THF1, THYLAKOID FORMATION1	9.3	7.2	2.1	7.0
11535	AT2G05830.3 | similar to eukaryotic translation initiation factor 2B family protein / eIF-2B family protein [Arabidopsis thaliana] (TAIR:At3g07300.1); similar to IDI2 [Hordeum vulgare subsp. vulgare] (GB:BAB21393.1); contains InterPro domain Initiation factor 2B (InterPro:IPR000649) | chr2:2229704-2232103 FORWARD | Aliases: T6P5.3, T6P5_3	9.3	8.4	0.9	1.9
818	AT3G27080.1 | Symbol: TOM20 3 | mitochondrial import receptor subunit TOM20-3 / translocase of outer membrane 20 kDa subunit 3 (TOM20-3), identical to mitochondrial import receptor subunit TOM20-3 SP:P82874 from (Arabidopsis thaliana) | chr3:9986319-9987866 REVERSE | Aliases: MOJ10.22, TRANSLOCASE OUTER MEMBRANE 20 3	9.3	6.6	2.7	10.2
2863	AT2G29530.1 | Symbol: TIM10 | mitochondrial import inner membrane translocase (TIM10), identical to mitochondrial import inner membrane translocase subunit Tim10 (Arabidopsis thaliana) Swiss-Prot:Q9ZW33; contains Pfam domain, PF02953: Tim10/DDP family zinc finger | chr2:12647901-12649367 REVERSE | Aliases: F16P2.9, F16P2_9, TIM10	9.3	6.0	3.3	6.3
20040	AT4G36515.1 | expressed protein | chr4:17229557-17230299 REVERSE | Aliases: None	9.3	9.2	0.1	0.2
4624	AT4G13940.3 | Symbol: HOG1 | similar to adenosylhomocysteinase, putative / S-adenosyl-L-homocysteine hydrolase, putative / AdoHcyase, putative [Arabidopsis thaliana] (TAIR:At3g23810.1); similar to adenosylhomocysteinase (EC 3.3.1.1) - wheat (GB:T06764); similar to S-adenosyl-L-homocystein hydrolase [Nicotiana sylvestris] (GB:BAA03709.1); similar to adenosylhomocysteinase [Medicago truncatula] (GB:AAO89238.1); similar to S-adenosyl-L-homocysteinase [Lupinus luteus] (GB:AAD56048.1); similar to wheat adenosylhomocysteinase-like protein [Oryza sativa (japonica cultivar-group)] (GB:AAO72664.1); contains InterPro domain S-adenosyl-L-homocysteine hydrolase (InterPro:IPR000043) | chr4:8054856-8057176 FORWARD | Aliases: DL3010W, EMB1395, EMBRYO DEFECTIVE 1395, FCAALL.35, HOMOLOGY DEPENDENT GENE SILENCING 1, S ADENOSYL L HOMOCYSTEINE HYDROLASE, SAHH, SAHH1	9.3	11.8	-2.4	-4.8

3878	AT3G10670.1 | Symbol: ATNAP7 |	9.3	6.6	2.8	5.4
336	AT3G55170.2 | 60S ribosomal protein L35 (RPL35C), various ribosomal L35 proteins | chr3:20463946-20465316 REVERSE | Aliases: T26I12.50	9.3	6.1	3.2	13.8
4644	AT1G45130.1 | beta-galactosidase, putative / lactase, putative, similar to beta-galactosidase (Lycopersicon esculentum) GI:7939619, beta-galactosidase BG1 GI:15081596 from (Vitis vinifera); contains Pfam profile PF01301: Glycosyl hydrolases family 35 | chr1:17067692-17071702 FORWARD | Aliases: F27F5.20, F27F5_20	9.3	10.5	-1.2	-4.8
1412	AT3G07770.1 | heat shock protein-related, strong similarity to heat-shock protein (Secale cereale) GI:556673; contains Pfam profiles PF02518: ATPase, histidine kinase-, DNA gyrase B-, and HSP90-like domain protein, PF00183: Hsp90 protein | chr3:2479561-2484171 FORWARD | Aliases: MLP3.22	9.3	5.2	4.1	8.4
3210	AT3G24315.1 | sec20 family protein, contains Pfam PF03908: Sec20 | chr3:8820464-8822810 REVERSE | Aliases: None	9.3	7.9	1.4	5.9
19848	AT2G06530.1 | SNF7 family protein, contains Pfam domain, PF03357: SNF7 family | chr2:2588542-2590442 REVERSE | Aliases: T12H3.8, T12H3_8	9.3	9.3	0.1	0.2
1773	AT2G14890.2 | Symbol: AGP9 | arabinogalactan-protein (AGP9), identical to gi:10880495:gb:AAG24277 | chr2:6406703-6408064 FORWARD | Aliases: ARABINOGALACTAN PROTEIN 9, T26I20.5, T26I20_5	9.3	7.0	2.3	7.7
7842	AT3G63520.1 | Symbol: CCD1 | 9-cis-epoxycarotenoid dioxygenase / neoxanthin cleavage enzyme / NCED1 / carotenoid cleavage dioxygenase 1 (CCD1), identical to putative 9-cis-epoxy-carotenoid dioxygenase (GI:3096910); contains Pfam profile PF03055: Retinal pigment epithelial membrane protein | chr3:23463915-23466871 FORWARD | Aliases: ATCCD1, ATNCED1, CAROTENOID CLEAVAGE DIOXYGENASE 1, MAA21.150	9.3	10.1	-0.7	-3.2
10650	AT5G14040.1 | mitochondrial phosphate transporter, identical to mitochondrial phosphate transporter GI:3318617 from (Arabidopsis thaliana) | chr5:4530645-4533072 REVERSE | Aliases: MUA22.4, MUA22_4	9.3	7.9	1.5	2.2
1767	AT3G22240.1 | expressed protein | chr3:7863684-7864586 REVERSE | Aliases: MMP21.2	9.3	11.8	-2.5	-7.8
17614	AT4G29010.1 | Symbol: AIM1 | abnormal inflorescence meristem 1 / fatty acid multifunctional protein (AIM1), identical to gi:4337025; contains Pfam profiles PF02737 (3-hydroxyacyl-CoA dehydrogenase, NAD binding domain), PF00378 (enoyl-CoA hydratase/isomerase family protein), PF00725 (3-hydroxyacyl-CoA dehydrogenase) | chr4:14296679-14302066 REVERSE | Aliases: ABNORMAL INFLORESCENCE MERISTEM, F19B15.40, F19B15_40	9.3	9.1	0.3	0.7
1209	AT2G44670.1 | senescence-associated protein-related, similar to senescence-associated protein SAG102 (GI:22331931) (Arabidopsis thaliana); | chr2:18432255-18433135 FORWARD | Aliases: F16B22.16	9.3	5.7	3.7	9.0
18310	AT5G40760.1 | Symbol: G6PD6 | Encodes a cytosolic glucose-6-phosphate dehydrogenase that is insensitive to reduction by DTT and whose mRNA is expressed ubiquitously.	9.3	9.1	0.3	0.5
11955	AT5G20900.1 | expressed protein | chr5:7090751-7092541 FORWARD | Aliases: F22D1.70, F22D1_70	9.3	8.5	0.9	1.8
7007	AT3G20050.1 | Symbol: ATTCP 1 | T-complex protein 1 alpha subunit / TCP-1-alpha / chaperonin (CCT1), identical to SWISS-PROT:P28769- T-complex protein 1, alpha subunit (TCP-1-alpha) (Arabidopsis thaliana) | chr3:6998260-7002407 REVERSE | Aliases: MAL21.5, MAL21_5	9.3	7.5	1.9	3.5
15793	AT3G60600.3 | Symbol: VAP27 1 | similar to vesicle-associated membrane protein, putative / VAMP, putative [Arabidopsis thaliana] (TAIR:At2g45140.1); similar to putative vesicle-associated membrane protein-associated protein [Oryza sativa (japonica cultivar-group)] (GB:XP_480176.1); contains InterPro domain Major sperm protein (MSP) domain (InterPro:IPR000535) | chr3:22411370-22413678 FORWARD | Aliases: T4C21.10, VAMP/SYNAPTOBREVIN ASSOCIATED PROTEIN 27 1	9.3	9.6	-0.3	-1.0
5518	AT4G26410.1 | expressed protein | chr4:13346757-13349025 FORWARD | Aliases: M3E9.160, M3E9_160	9.3	8.1	1.2	4.3
19496	AT2G30870.1 | Symbol: ATGSTF10 | glutathione S-transferase, putative, supported by cDNA GI:443698 GB:D17673 | chr2:13148527-13150296 FORWARD | Aliases: ATGSTF4, EARLY DEHYDRATION INDUCED 13, ERD13, F7F1.8, F7F1_8, GLUTATHIONE S TRANSFERASE	9.3	9.6	-0.3	-0.3
19836	AT3G47000.1 | glycosyl hydrolase family 3 protein, beta-D-glucan exohydrolase, Nicotiana tabacum, TREMBL:AB017502_1 | chr3:17324631-17327547 REVERSE | Aliases: F13I12.50	9.3	9.2	0.1	0.2
19182	AT5G39510.1 | Symbol: VTI11 | vesicle transport v-SNARE 11 (VTI11) / vesicle soluble NSF attachment protein receptor VTI1a (VTI1A), identical to SP:Q9SEL6 Vesicle transport v-SNARE 11 (AtVTI11) (Vesicle transport v-SNARE protein VTI1a) (Vesicle soluble NSF attachment protein receptor VTI1a) (AtVTI1a) {Arabidopsis thaliana} | chr5:15838671-15840992 FORWARD | Aliases: ATVTI11, ATVTI1A, MUL8.190, MUL8_190, SGR4, VTI1A, ZIG	9.3	9.2	0.1	0.4
2665	AT5G13710.1 | Symbol: SMT1 | sterol 24-C-methyltransferase, putative, similar to SP:P25087 Sterol 24-C-methyltransferase, Delta(24)-sterol C- methyltransferase, Saccharomyces cerevisiae | chr5:4423698-4427050 REVERSE | Aliases: CEPHALOPOD, CPH, MSH12.18, MSH12_18, SAM:CYCLOARTENOL C24 METHYLTRANSFERASE, STEROL METHYLTRANSFERASE 1	9.3	7.2	2.1	6.5

304	AT2G43710.2 | Symbol: SSI2 | acyl-(acyl-carrier-protein) desaturase / stearoyl-ACP desaturase (SSI2), identical to gi:15149310; contains Pfam profile PF03405: Fatty acid desaturase; identical to cDNA stearoyl ACP desaturase (SSI2), SSI2-FAB2 allele, GI:15149309 | chr2:18127039-18129966 FORWARD | Aliases: F18O19.18, FAB2	9.3	5.2	4.1	14.2
11230	AT5G17920.1 | Symbol: ATCIMS |	9.3	6.1	3.2	2.0
11229	AT3G03780.2 | 5-methyltetrahydropteroyltriglutamate--homocysteine methyltransferase, putative / vitamin-B12-independent methionine synthase, putative / cobalamin-independent methionine synthase, putative, very strong similarity to SP:O50008 5-methyltetrahydropteroyltriglutamate--homocysteine methyltransferase (EC 2.1.1.14) (Vitamin-B12-independent methionine synthase isozyme) (Cobalamin-independent methionine synthase isozyme) {Arabidopsis thaliana}; contains Pfam profile PF01717: Methionine synthase, vitamin-B12 independent | chr3:957095-960985 FORWARD | Aliases: F20H23.19, F20H23_19	9.3	6.1	3.2	2.0
4927	AT2G16800.1 | high-affinity nickel-transport family protein, contains Pfam domain, PF03824: High-affinity nickel-transport protein | chr2:7292820-7294447 FORWARD | Aliases: T24I21.21, T24I21_21	9.3	8.4	0.9	4.6
1096	AT2G19940.2 | semialdehyde dehydrogenase family protein, similar to N-acetyl-glutamyl-phosphate reductase (Campylobacter jejuni) GI:6650362; contains Pfam profiles PF02774: Semialdehyde dehydrogenase dimerisation domain, PF01118: Semialdehyde dehydrogenase NAD binding domain | chr2:8620116-8623099 FORWARD | Aliases: F6F22.3, F6F22_3	9.3	4.7	4.6	9.3
9848	AT5G50920.1 | Symbol: CLPC | Similar to ATP-dependent Clp protease ATP-binding subunit / ClpC, almost identical to ClpC GI:2921158 from (Arabidopsis thaliana); contains Pfam profile PF02861: Clp amino terminal domain; contains Pfam profile PF00004: ATPase, AAA family; contains Pfam profile PF02151: UvrB/uvrC motif. Involved in protein import into the chloroplast. May provide ATP source that drives the TIC translocation machinery, | chr5:20732698-20737597 REVERSE | Aliases: ATHSP93 V, CLPC, HSP93 V, K3K7.7, K3K7_7	9.3	8.8	0.5	2.4
3991	AT5G13120.1 | peptidyl-prolyl cis-trans isomerase cyclophilin-type family protein, contains Pfam domain, PF00160: peptidyl-prolyl cis-trans isomerase, cyclophilin-type | chr5:4162506-4164787 REVERSE | Aliases: T19L5.80, T19L5_80	9.3	8.1	1.2	5.3
5705	AT1G19920.1 | Symbol: APS2 | sulfate adenylyltransferase 2 / ATP-sulfurylase 2 (ASA1) (MET3-1) (APS2), identical to ATP sulfurylase (APS2) (Arabidopsis thaliana) GI:1575324 | chr1:6914604-6916745 REVERSE | Aliases: ASA1, ATP SULFURYLASE PRECURSOR, F6F9.2, F6F9_2	9.3	6.8	2.5	4.2
7605	AT1G53400.1 | expressed protein | chr1:19928439-19930268 FORWARD | Aliases: F12M16.29, F12M16_29	9.3	10.0	-0.7	-3.3
8083	AT3G09840.1 | Symbol: CDC48 | cell division cycle protein 48 (CDC48A) (CDC48), identical to SP:P54609 Cell division cycle protein 48 homolog {Arabidopsis thaliana} | chr3:3019345-3023050 FORWARD | Aliases: ATCDC48, CDC48A, CELL DIVISION CYCLE 48, F8A24.11	9.3	10.6	-1.3	-3.1
7022	AT3G18280.1 | protease inhibitor/seed storage/lipid transfer protein (LTP) family protein, similar to TED4 (Zinnia elegans) GI:493721; contains Pfam protease inhibitor/seed storage/LTP family domain PF00234	9.3	10.6	-1.3	-3.5
14773	AT3G63310.1 | expressed protein, low similarity to N-methyl-D-aspartate receptor-associated protein (Drosophila melanogaster) GI:567104; contains Pfam profile PF01027: Uncharacterized protein family UPF0005	9.3	8.7	0.5	1.2
2018	AT3G03980.1 | short-chain dehydrogenase/reductase (SDR) family protein, similar to short-chain type dehydrogenase/reductase SP:Q08632 (Picea abies) | chr3:1031749-1033214 FORWARD | Aliases: T11I18.9, T11I18_9	9.3	5.6	3.6	7.4
8730	AT1G63800.1 | ubiquitin-conjugating enzyme 5 (UBC5), E2; identical to gi:431269, SP:P42749 | chr1:23671279-23672743 REVERSE | Aliases: T12P18.18, T12P18_18	9.3	10.3	-1.0	-2.8
1570	AT1G09830.1 | phosphoribosylamine--glycine ligase (PUR2), Identical to phosphoribosylamine--glycine ligase, chloroplast (precursor) SP:P52420 from (Arabidopsis thaliana) | chr1:3192683-3195008 REVERSE | Aliases: F21M12.22, F21M12_22	9.3	6.3	2.9	8.1
10489	AT4G36020.1 | cold-shock DNA-binding family protein, contains Pfam domains, PF00313: 'Cold-shock' DNA-binding domain and PF00098: Zinc knuckle	9.3	8.7	0.6	2.2
11351	AT4G10610.2 | Symbol: RBP37 | similar to RNA-binding protein, putative [Arabidopsis thaliana] (TAIR:At1g32790.1); similar to putative RNA-binding protein RBP37 [Oryza sativa (japonica cultivar-group)] (GB:XP_479783.1); contains InterPro domain RNA-binding region RNP-1 (RNA recognition motif) (InterPro:IPR000504) | chr4:6557173-6559591 FORWARD | Aliases: ATRBP37, CID12, RNA BINDING PROTEIN 37, T4F9.70, T4F9_70	9.3	8.5	0.8	2.0
3119	AT2G45200.1 | Symbol: GOS12 | Golgi SNARE 12 protein / Golgi SNAP receptor complex member 1, identical to Probable 28 kDa Golgi SNARE protein (Golgi SNAP receptor complex member 1) (SP:O22151) {Arabidopsis thaliana} | chr2:18644582-18646858 REVERSE | Aliases: ATGOS12, F4L23.29, GOLGI SNARE 12	9.3	7.5	1.8	6.0
417	AT3G50550.2 | expressed protein, isoform contains a non-consensus AT donor site at intron 1 | chr3:18770444-18771420 FORWARD | Aliases: T20E23.150	9.3	5.6	3.7	12.7
7031	AT5G65270.1 | Ras-related GTP-binding family protein, similar to GTP-binding protein RAB11A GI:1370142 from (Lotus japonicus); contains Pfam profile: PF00071 Ras family | chr5:26100602-26101940 FORWARD | Aliases: MQN23.22, MQN23_22	9.3	7.3	2.0	3.5

1604	AT5G20160.2 | ribosomal protein L7Ae/L30e/S12e/Gadd45 family protein, Similar to NHP2/L7Ae family proteins, see SWISSPROT:P32495 and PMID:2063628. | chr5:6803838-6805197 REVERSE | Aliases: F5O24.50, F5O24_50	9.3	5.8	3.4	8.0
4824	AT4G26840.1 | Symbol: SUM1 | ubiquitin-like protein (SMT3), identical to Ubiquitin-like protein SMT3 SP:P55852 from(Arabidopsis thaliana); identical to cDNA SMT3 protein GI:1707371 | chr4:13497334-13498733 FORWARD | Aliases: F10M23.180, F10M23_180, SMALL UBIQUITIN LIKE MODIFIER 1, SUMO 1, SUMO1	9.3	7.9	1.3	4.7
6963	AT3G27430.2 | Symbol: PBB1 | 20S proteasome beta subunit B (PBB1), identical to 20S proteasome beta subunit PBB1 (PBB1) GB:AAC32066 (Arabidopsis thaliana) (Genetics 149 (2), 677-692 (1998)); contains Pfam profile: PF00227 proteasome A-type and B-type; | chr3:10153754-10156579 FORWARD | Aliases: 20S PROTEASOME BETA SUBUNIT PBB1, K1G2.26	9.3	6.4	2.8	3.5
13814	AT1G60420.1 | DC1 domain-containing protein, contains Pfam domain PF03107: DC1 domain | chr1:22265573-22268324 FORWARD | Aliases: T13D8.29, T13D8_29	9.3	9.8	-0.5	-1.4
1275	AT2G17280.1 | phosphoglycerate/bisphosphoglycerate mutase family protein, contains Pfam profile PF00300: phosphoglycerate mutase family | chr2:7520293-7522122 FORWARD | Aliases: F5J6.4, F5J6_4	9.3	4.1	5.1	8.8
7218	AT2G17360.1 | 40S ribosomal protein S4 (RPS4A), contains ribosomal protein S4 signature from residues 8 to 22 | chr2:7553567-7555395 FORWARD | Aliases: F5J6.12, F5J6_12	9.3	7.6	1.7	3.4
10217	AT1G76140.1 | similar to prolyl oligopeptidase, putative / prolyl endopeptidase, putative / post-proline cleaving enzyme, putative [Arabidopsis thaliana] (TAIR:At1g20380.1); similar to putative prolyl endopeptidase [Oryza sativa (japonica cultivar-group)] (GB:NP_908392.1); similar to OTTHUMP00000040498 [Homo sapiens] (GB:CAH72545.1); similar to OSJNBa0084K11.14 [Oryza sativa (japonica cultivar-group)] (GB:XP_473492.1); similar to similar to Prolyl endopeptidase (Post-proline cleaving enzyme) (PE) [Gallus gallus] (GB:NP_001006410.1); similar to prolyl endopeptidase [Mus musculus] (GB:NP_035286.1); contains InterPro domain Prolyl oligopeptidase serine protease (S9A) (InterPro:IPR002470); contains InterPro domain Prolyl oligopeptidase (InterPro:IPR001375); contains InterPro domain Prolyl oligopeptidase, N-terminal beta-propeller domain (InterPro:IPR004106) | chr1:28576024-28579930 FORWARD | Aliases: T23E18.8, T23E18_8	9.3	10.0	-0.7	-2.3
4369	AT3G56510.2 | similar to unnamed protein product [Yarrowia lipolytica CLIB99] (GB:CAG82708.1) | chr3:20954907-20957640 REVERSE | Aliases: T5P19.160	9.3	8.2	1.1	5.0
2787	AT3G16780.1 | 60S ribosomal protein L19 (RPL19B), similar to ribosomal protein L19 GB:CAA45090 from (Homo sapiens) | chr3:5708931-5710415 FORWARD | Aliases: MGL6.7	9.3	7.3	1.9	6.4
6816	AT4G27450.1 | expressed protein, similar to auxin down-regulated protein ARG10 (Vigna radiata) GI:2970051, wali7 (aluminum-induced protein) (Triticum aestivum) GI:451193 | chr4:13727493-13728892 REVERSE | Aliases: F27G19.50, F27G19_50	9.3	8.2	1.1	3.6
20332	AT4G13670.1 | peptidoglycan-binding domain-containing protein, similar to spore cortex-lytic enzyme prepeptide (GI:1644192) (Bacillus cereus); contains Pfam PF01471: Putative peptidoglycan binding domain; contains Pfam PF00684 : DnaJ central domain (4 repeats) | chr4:7948575-7950881 FORWARD | Aliases: F18A5.60, F18A5_60	9.3	9.3	-0.1	-0.1
15353	AT3G24080.1 | KRR1 family protein, contains Pfam PF05178: Krr1 family | chr3:8694969-8697131 REVERSE | Aliases: F14O13.28	9.3	8.8	0.5	1.1
4319	AT5G10695.1 | expressed protein | chr5:3377664-3378222 FORWARD | Aliases: None	9.3	8.0	1.3	5.0
3509	AT1G01070.2 | nodulin MtN21 family protein, similar to MtN21 GI:2598575 (root nodule development) from (Medicago truncatula) | chr1:38753-40927 REVERSE | Aliases: T25K16.7, T25K16_7	9.3	7.0	2.2	5.6
1310	AT3G07410.1 | Ras-related GTP-binding family protein, contains Pfam profile: PF00071 Ras family | chr3:2372323-2373562 REVERSE | Aliases: F21O3.12	9.3	5.2	4.1	8.7
7432	AT2G32240.1 | expressed protein, contains Pfam profile: PF04508 viral A-type inclusion protein repeat | chr2:13691376-13695401 REVERSE | Aliases: F22D22.1, F22D22_1	9.3	10.3	-1.1	-3.4
1850	AT1G66530.1 | arginyl-tRNA synthetase, putative / arginine--tRNA ligase, putative, similar to SP:P37880 Arginyl-tRNA synthetase (EC 6.1.1.19) (Arginine--tRNA ligase) (ArgRS) {Cricetulus longicaudatus}; contains Pfam profiles PF00750: arginyl-tRNA synthetase, PF03485: arginyl-tRNA synthetase N-terminal domain | chr1:24822574-24826005 REVERSE | Aliases: F28G11.14, F28G11_14	9.3	5.9	3.3	7.6
16907	AT5G11680.1 | expressed protein, predicted proteins, Arabidopsis thaliana | chr5:3758859-3760848 FORWARD | Aliases: T22P22.70, T22P22_70	9.3	9.4	-0.1	-0.8
16421	AT5G54640.1 | Symbol: RAT5 | histone H2A, identical to histone H2A Arabidopsis thaliana GI:7595337 | chr5:22213703-22214713 FORWARD | Aliases: MRB17.14, MRB17_14, RESISTANT TO AGROBACTERIUM TRANSFORMATION 5	9.3	8.9	0.3	0.9
8049	AT2G19790.1 | clathrin adaptor complex small chain family protein, contains Pfam profile: PF01217 clathrin adaptor complex small chain	9.2	7.2	2.0	3.1

323	AT5G49910.1 | Symbol: cpHSC70 2 | heat shock protein 70 / HSP70 (HSC70-7), identical to heat shock protein 70 (Arabidopsis thaliana) GI:6746592 | chr5:20320640-20324039 FORWARD | Aliases: HEAT SHOCK PROTEIN 70, HEAT SHOCK PROTEIN 70 7, HSC70 7, K9P8.5, K9P8_5	9.2	5.6	3.7	14.0
1897	AT5G27670.1 | histone H2A, putative, similar to histone H2A Lycopersicon esculentum SP:P25469, Pisum sativum SP:P25470, Petroselinum crispum SP:P19177; contains Pfam profile PF00125 Core histone H2A/H2B/H3/H4 | chr5:9792579-9793458 REVERSE | Aliases: T1G16.3	9.2	7.6	1.7	7.6
20441	AT1G12520.3 | Symbol: CCS1 | similar to superoxide dismutase (Cu-Zn), chloroplast (SODCP) / copper/zinc superoxide dismutase (CSD2) [Arabidopsis thaliana] (TAIR:At2g28190.1); similar to putative copper/zinc superoxide dismutase copper chaperone precursor [Lycopersicon esculentum] (GB:AAD12307.2); contains InterPro domain Copper/Zinc superoxide dismutase (InterPro:IPR001424) | chr1:4267104-4268884 REVERSE | Aliases: ATCCS, COPPER/ZINC SUPEROXIDE DISMUTASE COPPER CHAPERONE, F5O11.26, F5O11_26	9.2	9.3	-0.0	-0.1
7963	AT3G22960.1 | pyruvate kinase, putative, similar to pyruvate kinase isozyme A, chloroplast precursor (Ricinus communis) SWISS-PROT:Q43117 | chr3:8139242-8141992 FORWARD | Aliases: F5N5.15	9.2	8.2	1.0	3.1
731	AT2G21820.1 | expressed protein | chr2:9309965-9310327 REVERSE | Aliases: F7D8.14, F7D8_14	9.2	6.2	3.0	10.6
9962	AT3G24160.1 | Symbol: PMP | expressed protein, identical to cDNA putative type 1 membrane protein (PMP)GI:4206764 | chr3:8726138-8729396 FORWARD | Aliases: MUJ8.6, PUTATIVE TYPE 1 MEMBRANE PROTEIN	9.2	10.0	-0.7	-2.4
1734	AT5G44200.1 | Symbol: CBP20 | nuclear cap-binding protein, putative, similar to SP:P52298 20 kDa nuclear cap binding protein (CBP20) (NCBP interacting protein 1) {Homo sapiens}; non-consensus AT donor splice site at exon 4, AC acceptor splice site at exon 5; contains InterPro entry IPR000504: RNA-binding region RNP-1 (RNA recognition motif) (RRM) | chr5:17819166-17821154 REVERSE | Aliases: ATCBP20, CAP BINDING PROTEIN 20, MLN1.12, MLN1_12	9.2	7.2	2.0	7.8
879	AT1G04590.2 | Symbol: EMB2748 | expressed protein, isoform contains GG acceptor splice site at intron 7 | chr1:1258577-1261424 REVERSE | Aliases: EMB2748, EMBRYO DEFECTIVE 2748, T1G11.16, T1G11_16	9.2	5.2	4.0	10.0
203	AT2G38140.1 | Symbol: PSRP4 | chloroplast 30S ribosomal protein S31 (PSRP4) | chr2:15987961-15988676 FORWARD | Aliases: 30S RIBOSOMAL PROTEIN S31, F16M14.7, F16M14_7, PLASTID SPECIFIC RIBOSOMAL PROTEIN 4	9.2	4.1	5.1	16.6
2915	AT3G01540.4 | Symbol: DRH1 | similar to DEAD box RNA helicase, putative [Arabidopsis thaliana] (TAIR:At5g14610.1); similar to DEAD box RNA helicase, putative [Arabidopsis thaliana] (TAIR:At3g06480.1); similar to putative p68 RNA helicase [Oryza sativa (japonica cultivar-group)] (GB:XP_550286.1); similar to putative RNA helicase, DRH1 [Oryza sativa (japonica cultivar-group)] (GB:XP_462826.1); similar to putative RNA helicase, DRH1 [Oryza sativa (japonica cultivar-group)] (GB:NP_918275.1); similar to P72 DEAD box protein [Pisum sativum] (GB:AAF04377.1); similar to putative ATP-dependent RNA helicase DB10 [Oryza sativa (japonica cultivar-group)] (GB:BAD88050.1); contains InterPro domain WW/Rsp5/WWP domain (InterPro:IPR001202); contains InterPro domain ATP-dependent helicase, DEAD-box (InterPro:IPR000629); contains InterPro domain Helicase, C-terminal (InterPro:IPR001650); contains InterPro domain DEAD/DEAH box helicase (InterPro:IPR001410) | chr3:212525-216678 REVERSE | Aliases: ATDRH1, F4P13.9, F4P13_9, RNA HELICASE DRH1	9.2	10.7	-1.4	-6.2
6542	AT5G59140.1 | SKP1 family protein, similar to elongin C, Drosophila melanogaster, GI:2780365 PIR:JC5794; contains Pfam profile PF01466: Skp1 family, dimerisation domain | chr5:23891676-23892910 REVERSE | Aliases: MNC17.5, MNC17_5	9.2	8.1	1.2	3.7
7803	AT4G17040.1 | ATP-dependent Clp protease proteolytic subunit, putative, similar to ATP-dependent Clp protease proteolytic subunit GI:7264063 from (Synechococcus sp.PCC 7942) | chr4:9585724-9589381 REVERSE | Aliases: DL4550C, FCAALL.413	9.2	7.3	2.0	3.2
7981	AT3G54640.1 | Symbol: TSA1 | tryptophan synthase, alpha subunit (TSA1), identical to gi:619753 | chr3:20234137-20236501 REVERSE | Aliases: TRP3, TRYPTOPHAN SYNTHASE ALPHA CHAIN	9.2	7.5	1.7	3.1
9809	AT1G56200.1 | Symbol: EMB1303 | expressed protein | chr1:21034480-21035721 FORWARD | Aliases: EMB1303, EMBRYO DEFECTIVE 1303, F14G9.26, F14G9_26	9.2	8.6	0.6	2.4
37	AT5G16970.1 | NADP-dependent oxidoreductase, putative (P1), identical to probable NADP-dependent oxidoreductase P1, zeta-crystallin homolog (SP:Q39172)(gi:886428), Arabidopsis thaliana; similar to allyl alcohol dehydrogenase (Nicotiana tabacum) GI:6692816; contains Pfam profile PF00107: oxidoreductase, zinc-binding dehydrogenase family | chr5:5576171-5578049 REVERSE | Aliases: F2K13.120, F2K13_120	9.2	3.2	6.1	27.4
20355	AT5G22630.1 | prephenate dehydratase family protein, contains Pfam profile PF00800: prephenate dehydratase | chr5:7524345-7526163 FORWARD | Aliases: MDJ22.5, MDJ22_5	9.2	9.1	0.1	0.1
8942	AT1G54360.4 | similar to TATA box-binding protein-associated factor (TAF) family protein [Arabidopsis thaliana] (TAIR:At1g04950.1); similar to TATA box-binding protein-associated factor (TAF) family protein [Arabidopsis thaliana] (TAIR:At1g04950.2); similar to SPCC16C4.18c [Schizosaccharomyces pombe] (GB:CAA20756.1); contains InterPro domain Histone-fold/TFIID-TAF/NF-Y (InterPro:IPR007124); contains InterPro domain TATA box binding protein associated factor (TAF) (InterPro:IPR004823) | chr1:20294267-20296991 FORWARD | Aliases: F20D21.18, F20D21_18	9.2	8.8	0.5	2.7
3622	AT1G20580.1 | small nuclear ribonucleoprotein, putative / snRNP, putative / Sm protein, putative, similar to small nuclear ribonucleoprotein Sm D3 (snRNP core protein D3, Sm-D3) (Mus musculus) SWISS-PROT:P43331 | chr1:7128878-7130631 FORWARD | Aliases: F5M15.9, F5M15_9	9.2	7.7	1.6	5.5

199	AT5G62610.1 | basic helix-loop-helix (bHLH) family protein, contains Pfam profile: PF00010 helix-loop-helix DNA-binding domain	9.2	5.0	4.2	16.6
4543	AT2G17130.2 | Symbol: IDH2 | isocitrate dehydrogenase subunit 2 / NAD+ isocitrate dehydrogenase subunit 2, nearly identical to NAD+ dependent isocitrate dehydrogenase subunit 2 (Arabidopsis thaliana) GI:1766048 | chr2:7467896-7469636 REVERSE | Aliases: F6P23.14, F6P23_14, ISOCITRATE DEHYDROGENASE SUBUNIT 2, NAD+ DEPENDENT ISOCITRATE DEHYDROGENASE SUBUNIT 2	9.2	10.7	-1.5	-4.9
545	AT1G33810.1 | expressed protein | chr1:12265050-12266827 FORWARD | Aliases: F14M2.27	9.2	4.4	4.8	11.7
10148	AT3G02740.1 | aspartyl protease family protein, contains Pfam profile: PF00026 eukaryotic aspartyl protease | chr3:590517-593174 FORWARD | Aliases: F13E7.32, F13E7_32	9.2	9.7	-0.4	-2.3
2823	AT5G53850.3 | haloacid dehalogenase-like hydrolase family protein, low similarity to enolase-phosphatase E-1 enzyme (Klebsiella oxytoca) GI:401712; contains InterPro accession IPR005834: Haloacid dehalogenase-like hydrolase | chr5:21878223-21882133 REVERSE | Aliases: K19P17.1, K19P17_1	9.2	10.5	-1.2	-6.3
10838	AT1G04850.1 | ubiquitin-associated (UBA)/TS-N domain-containing protein, weak similarity to SP:P45974 Ubiquitin carboxyl-terminal hydrolase 5 (EC 3.1.2.15) {Homo sapiens}; contains Pfam profile PF00627: UBA/TS-N domain | chr1:1365118-1368792 REVERSE | Aliases: F13M7.16, F13M7_16	9.2	8.6	0.6	2.1
5576	AT2G23610.1 | esterase, putative, similar to ethylene-induced esterase (Citrus sinensis) GI:14279437, polyneuridine aldehyde esterase (Rauvolfia serpentina) GI:6651393; contains Pfam profile PF00561: hydrolase, alpha/beta fold family | chr2:10051259-10053612 REVERSE | Aliases: F26B6.26, F26B6_26	9.2	6.4	2.8	4.2
8542	AT3G13200.1 | Cwf15 / Cwc15 cell cycle control family protein, contains Pfam profile PF04889: Cwf15/Cwc15 cell cycle control protein; similar to Cell cycle control protein cwf15 (Swiss-Prot:P78794) (Schizosaccharomyces pombe) | chr3:4242132-4244140 FORWARD | Aliases: MJG19.16	9.2	8.2	1.0	2.9
7658	AT3G10920.2 | Symbol: MSD1 | similar to superoxide dismutase (Mn), putative / manganese superoxide dismutase, putative [Arabidopsis thaliana] (TAIR:At3g56350.1); similar to superoxide dismutase [Raphanus sativus] (GB:AAL07333.1); contains InterPro domain Manganese and iron superoxide dismutase (InterPro:IPR001189) | chr3:3417982-3419858 FORWARD | Aliases: F9F8.26, MANGANESE SUPEROXIDE DISMUTASE 1	9.2	7.1	2.1	3.3
9884	AT3G63000.1 | NPL4 family protein, contains Pfam domain, PF05021: NPL4 family | chr3:23294584-23296515 FORWARD | Aliases: T20O10.100	9.2	9.8	-0.6	-2.4
732	AT3G03490.1 | peroxisomal protein PEX19 family protein, contains Pfam profile: PF04614 Pex19 protein family | chr3:830172-831853 REVERSE | Aliases: T21P5.9, T21P5_9	9.2	7.0	2.2	10.6
5466	AT1G70480.2 | expressed protein, similar to expressed protein [Arabidopsis thaliana] (TAIR:At1g23560.1); contains InterPro domain Protein of unknown function DUF220 (InterPro:IPR003863) | chr1:26565722-26567472 FORWARD | Aliases: F24J13.5, F24J13_5	9.2	7.2	2.0	4.3
3181	AT5G41210.1 | Symbol: ATGSTT1 | glutathione S-transferase (GST10), identical to glutathione transferase AtGST 10 (Arabidopsis thaliana) GI:4049401 | chr5:16509603-16511126 REVERSE | Aliases: GST10, MEE6.28, MEE6_28	9.2	7.3	1.9	5.9
20859	AT5G46620.1 | expressed protein, similar to unknown protein (pir::T05082) | chr5:18934886-18936647 REVERSE | Aliases: F10E10.1, F10E10_1	9.2	9.2	-0.0	-0.1
3383	AT5G28060.1 | 40S ribosomal protein S24 (RPS24B), 40S ribosomal protein S19, Cyanophora paradoxa, EMBL:CPA245654 | chr5:10069553-10070897 REVERSE | Aliases: F15F15.130, F15F15_130	9.2	6.9	2.3	5.7
6081	AT2G26240.1 | expressed protein, contains Pfam profile PF03647: Uncharacterised protein family (UPF0136) | chr2:11174335-11175535 REVERSE | Aliases: T1D16.12, T1D16_12	9.2	9.9	-0.7	-4.0
13848	AT5G51410.2 | LUC7 N_terminus domain-containing protein, similar to cisplatin resistance-associated overexpressed protein (Homo sapiens) GI:6899846; contains Pfam profile PF03194: LUC7 N_terminus | chr5:20897967-20900930 REVERSE | Aliases: MFG13.12, MFG13_12	9.2	10.0	-0.8	-1.4
4695	AT4G39280.1 | phenylalanyl-tRNA synthetase, putative / phenylalanine--tRNA ligase, putative, similar to SP:Q9Y285 Phenylalanyl-tRNA synthetase alpha chain (EC 6.1.1.20) (Phenylalanine- -tRNA ligase alpha chain) (PheRS) {Homo sapiens}; contains Pfam profile PF01409: tRNA synthetases class II core domain (F) | chr4:18281456-18284843 REVERSE | Aliases: T22F8.180, T22F8_180	9.2	6.9	2.3	4.8
7570	AT5G47720.5 | similar to acetyl-CoA C-acyltransferase, putative / 3-ketoacyl-CoA thiolase, putative [Arabidopsis thaliana] (TAIR:At5g48230.2); similar to acetyl-CoA C-acyltransferase, putative / 3-ketoacyl-CoA thiolase, putative [Arabidopsis thaliana] (TAIR:At5g48230.1); similar to cytosolic acetoacetyl-coenzyme A thiolase [Nicotiana tabacum] (GB:AAU95618.1); similar to peroxisomal acetoacetyl-coenzyme A thiolase [Nicotiana tabacum] (GB:AAU95619.1); contains InterPro domain Thiolase (InterPro:IPR002155) | chr5:19348883-19352038 FORWARD | Aliases: MCA23.4, MCA23_4	9.2	10.1	-0.9	-3.3
2165	AT1G14450.1 | expressed protein, contains similarity to cytochrome c oxidase subunit I GI:5678701 from (Loligo pealei)	9.2	7.6	1.7	7.2
2653	AT5G64250.2 | 2-nitropropane dioxygenase family / NPD family, contains Pfam profile PF03060: oxidoreductase, 2-nitropropane dioxygenase (NPD) family | chr5:25714450-25716438 REVERSE | Aliases: MSJ1.9, MSJ1_9	9.2	10.6	-1.4	-6.5

7050	AT3G25470.1 | bacterial hemolysin-related, similar to hemolysine GB:AAD36643 from (Thermotoga maritima), contains Pfam profile: PF01479 S4 domain | chr3:9234178-9236450 FORWARD | Aliases: MWL2.14	9.2	8.8	0.4	3.5
3782	AT3G49910.1 | 60S ribosomal protein L26 (RPL26A), 60S RIBOSOMAL PROTEIN L26, Brassica rapa, EMBL:BRD495 | chr3:18515241-18515952 FORWARD | Aliases: F3A4.4	9.2	7.5	1.8	5.4
6314	AT1G80830.1 | Symbol: NRAMP1 |	9.2	10.5	-1.3	-3.9
5970	AT2G33210.1 | chaperonin, putative, similar to SWISS-PROT:Q05046- chaperonin CPN60-2, mitochondrial precursor (HSP60-2) (Cucurbita maxima); contains Pfam:PF00118 domain, TCP-1/cpn60 chaperonin family | chr2:14081986-14085797 REVERSE | Aliases: F25I18.5, F25I18_5	9.2	7.8	1.4	4.0
6536	AT4G22000.1 | expressed protein | chr4:11659650-11660916 REVERSE | Aliases: F1N20.100, F1N20_100	9.2	7.8	1.4	3.7
9220	AT3G17780.1 | expressed protein | chr3:6086954-6088342 FORWARD | Aliases: MIG5.8	9.2	8.2	1.0	2.6
7253	AT1G16470.2 | Symbol: PAB1 | similar to 20S proteasome alpha subunit D2 (PAD2) (PRS1) (PRC6) [Arabidopsis thaliana] (TAIR:At5g66140.1); similar to proteasome subunit alpha type 2 [Oryza sativa (japonica cultivar-group)] (GB:AAT78811.1); contains InterPro domain Proteasome subunit, A-type (InterPro:IPR000426); contains InterPro domain Multispecific proteasome protease (InterPro:IPR001353) | chr1:5622832-5625637 FORWARD | Aliases: F3O9.27, F3O9_27, PROTEASOME SUBUNIT PAB1	9.2	7.6	1.6	3.4
671	AT5G54600.2 | 50S ribosomal protein L24, chloroplast (CL24), identical to SP:P92959 50S ribosomal protein L24, chloroplast precursor {Arabidopsis thaliana} | chr5:22200204-22201793 FORWARD | Aliases: MRB17.10, MRB17_10	9.2	6.5	2.7	10.9
4239	AT2G47400.1 | Symbol: CP12 1 |	9.2	7.7	1.5	5.1
3011	AT2G40360.1 | transducin family protein / WD-40 repeat family protein, contains 4 WD-40 repeats (PF00400); similar to block of proliferation protein Bop1 (GI:1679772) (Mus musculus) | chr2:16859958-16863768 REVERSE | Aliases: T3G21.13, T3G21_13	9.2	7.9	1.3	6.1
14269	AT1G68220.1 | expressed protein | chr1:25573894-25575375 FORWARD | Aliases: T22E19.15, T22E19_15	9.2	9.5	-0.3	-1.3
4658	AT3G17210.1 | stable protein 1-related, similar to stable protein 1 (GI:13445204) (Populus tremula) PMID:12376651; similar to pop3 peptide GB:AAC26526 from (Populus balsamifera subsp. trichocarpa X Populus deltoides) | chr3:5882290-5883206 FORWARD | Aliases: MGD8.2	9.2	7.9	1.3	4.8
17348	AT4G02080.1 | Symbol: ASAR1 | GTP-binding protein (SAR1A), identical to SP:O04834 GTP-binding protein SAR1A. (Arabidopsis thaliana) | chr4:921462-922776 FORWARD | Aliases: ATSAR2, ATSARA1C, T10M13.9, T10M13_9	9.2	9.5	-0.3	-0.7
8462	AT3G45010.1 | Symbol: SCPL48 |	9.2	10.2	-1.0	-2.9
7106	AT4G09570.1 | Symbol: CPK4 | calcium-dependent protein kinase, putative / CDPK, putative, similar to calcium-dependent protein kinase (Arabidopsis thaliana) gi:604881:dbj:BAA04830; contains protein kinase domain, Pfam:PF00069; contains EF hand domain (calcium-binding EF-hand), Pfam:PF00036, INTERPRO:IPR002048 | chr4:6049518-6052332 FORWARD | Aliases: T25P22.10, T25P22_10	9.2	8.3	0.9	3.5
10848	AT1G77350.2 | similar to Krtcap2 protein [Mus musculus] (GB:AAH43030.1); similar to keratinocytes associated protein 2 [Mus musculus] (GB:NP_079603.1); similar to ENSANGP00000016671 [Anopheles gambiae str. PEST] (GB:XP_312812.2) | chr1:29075214-29076793 FORWARD | Aliases: F2P24.6, F2P24_6	9.2	8.6	0.6	2.1
10339	AT1G19600.1 | pfkB-type carbohydrate kinase family protein, contains Pfam profile: PF00294 pfkB family carbohydrate kinase | chr1:6779015-6781164 FORWARD | Aliases: F14P1.7, F14P1_7	9.2	8.4	0.8	2.3
4948	AT5G59850.1 | 40S ribosomal protein S15A (RPS15aF), cytoplasmic ribosomal protein S15a, Arabidopsis thaliana, EMBL:ATAF1412 | chr5:24129426-24130836 REVERSE | Aliases: MMN10.16	9.2	7.0	2.2	4.6
18582	AT4G37120.1 | Symbol: SMP2 | expressed protein | chr4:17486641-17489282 FORWARD | Aliases: AP22.39, AP22_39	9.2	9.1	0.1	0.5
17806	AT2G16510.1 | Symbol: AVA P1 |	9.2	8.9	0.3	0.6
4539	AT5G06360.1 | ribosomal protein S8e family protein, contains Pfam profile PF01201: Ribosomal protein S8e | chr5:1944772-1946725 FORWARD | Aliases: MHF15.12, MHF15_12	9.2	6.9	2.3	4.9
6337	AT5G66140.1 | Symbol: PAD2 | 20S proteasome alpha subunit D2 (PAD2) (PRS1) (PRC6), identical to SP:O24616 Proteasome subunit alpha type 7-2 (EC 3.4.25.1) (20S proteasome alpha subunit D2) {Arabidopsis thaliana} | chr5:26454396-26455947 REVERSE | Aliases: 20S PROTEASOME SUBUNIT PAD2, K2A18.22, K2A18_22	9.2	7.1	2.1	3.8
2050	AT3G22230.1 | 60S ribosomal protein L27 (RPL27B), similar to 60S RIBOSOMAL PROTEIN L27 GB:P41101 from (Solanum tuberosum) | chr3:7843974-7844604 REVERSE | Aliases: MKA23.22	9.2	6.8	2.4	7.3

3418	AT1G63980.2 | similar to aldose 1-epimerase family protein [Arabidopsis thaliana] (TAIR:At5g15140.1); similar to CG11180-PA [Drosophila melanogaster] (GB:NP_611495.1); contains InterPro domain D111/G-patch domain (InterPro:IPR000467) | chr1:23744225-23746965 FORWARD | Aliases: F22C12.25, F22C12_25	9.2	7.4	1.8	5.7
13032	AT5G54870.1 | expressed protein, strong similarity to unknown protein (pir::T04825) | chr5:22306361-22309025 FORWARD | Aliases: MBG8.14, MBG8_14	9.2	9.7	-0.6	-1.6
7039	AT5G62200.1 | embryo-specific protein-related, contains weak similarity to embryo-specific protein 3 (GI:3335171) (Arabidopsis thaliana) | chr5:25001494-25002900 REVERSE | Aliases: MMI9.3, MMI9_3	9.2	7.3	1.9	3.5
2666	AT5G65110.2 | Symbol: ACX2 | similar to acyl-CoA oxidase, putative [Arabidopsis thaliana] (TAIR:At1g06310.1); similar to acyl-CoA oxidase homolog [Phalaenopsis sp. 'True Lady'] (GB:AAB67883.1); similar to acyl-CoA oxidase [Phalaenopsis cv. 'True Lady'] (GB:AAR00586.1); similar to acyl CoA oxidase homolog [Cucurbita sp. cv. Kurokawa Amakuri] (GB:AAC15870.1); contains InterPro domain Acyl-CoA dehydrogenase, C-terminal (InterPro:IPR006090) | chr5:26026861-26029745 REVERSE | Aliases: ACYL COA OXIDASE 2, ATACX2, MQN23.4, MQN23_4	9.2	10.8	-1.7	-6.5
6728	AT1G72500.1 | inter-alpha-trypsin inhibitor heavy chain-related, low similarity to Inter-alpha-inhibitor H4 heavy chain (Rattus norvegicus) GI:2292988; contains Pfam profile PF00092: von Willebrand factor type A domain | chr1:27298998-27302218 REVERSE | Aliases: T10D10.3, T10D10_3	9.2	10.2	-1.0	-3.7
13764	AT5G15460.2 | expressed protein | chr5:5018774-5020656 REVERSE | Aliases: T20K14.70, T20K14_70	9.2	8.5	0.7	1.4
5451	AT2G46100.1 | expressed protein | chr2:18960353-18962069 FORWARD | Aliases: T3F17.25	9.2	7.8	1.3	4.3
5590	AT1G77180.2 | similar to RNA recognition motif (RRM)-containing protein [Arabidopsis thaliana] (TAIR:At2g16940.1); similar to WD-40 repeat family protein [Arabidopsis thaliana] (TAIR:At5g67320.1); similar to GAMYB-binding protein [Hordeum vulgare subsp. vulgare] (GB:AAO25542.1); contains InterPro domain SKIP/SNW domain (InterPro:IPR004015) | chr1:29004502-29007019 REVERSE | Aliases: T14N5.5, T14N5_5	9.2	10.1	-0.9	-4.2
19878	AT3G53110.1 | Symbol: LOS4 | DEAD/DEAH box helicase, putative, RNA helicase, Mus musculus, PIR:I49731	9.2	9.1	0.0	0.2
15091	AT1G53310.3 | Symbol: ATPPC1 |	9.2	10.1	-1.0	-1.1
466	AT1G09815.1 | DNA polymerase delta subunit 4 family, contains similarity to Swiss-Prot:Q9HCU8 DNA polymerase delta subunit 4 (DNA polymerase delta subunit p12) (Homo sapiens)	9.2	7.3	1.9	12.2
190	AT4G28750.1 | Symbol: PSAE1 | photosystem I reaction center subunit IV, chloroplast, putative / PSI-E, putative (PSAE1), identical to SP:Q9S831; similar to SP:P12354 Photosystem I reaction center subunit IV, chloroplast precursor (PSI-E) {Spinacia oleracea}; contains Pfam profile PF02427: Photosystem I reaction centre subunit IV / PsaE | chr4:14202779-14203961 REVERSE | Aliases: F16A16.140, F16A16_140, PSA E1 KNOCKOUT	9.2	3.7	5.5	16.7
955	AT1G60000.2 | 29 kDa ribonucleoprotein, chloroplast, putative / RNA-binding protein cp29, putative, similar to 29 kDa ribonucleoprotein chloroplast precursor {Nicotiana sylvestris} SP:Q08935, SP:Q08937; contains InterPro entry IPR000504: RNA-binding region RNP-1 (RNA recognition motif) (RRM) contains an AG-donor site at intron. | chr1:22097238-22098227 REVERSE | Aliases: T2K10.5, T2K10_5	9.2	5.8	3.4	9.7
15349	AT5G60620.1 | phospholipid/glycerol acyltransferase family protein, contains Pfam PF01553: Acyltransferase | chr5:24384442-24387015 FORWARD | Aliases: MUP24.4, MUP24_4	9.2	9.6	-0.5	-1.1
2433	AT1G47128.1 | cysteine proteinase (RD21A) / thiol protease, identical to SP:P43297 Cysteine proteinase RD21A precursor (EC 3.4.22.-) {Arabidopsis thaliana}, thiol protease RD21A SP:P43297 from (Arabidopsis thaliana) | chr1:17285265-17288110 REVERSE | Aliases: F2G19.31, F2G19_31	9.2	11.7	-2.6	-6.8
706	AT4G32060.1 | calcium-binding EF hand family protein, contains INTERPRO:IPR002048 calcium-binding EF-hand domain | chr4:15501084-15503599 FORWARD | Aliases: F10N7.140, F10N7_140	9.2	11.5	-2.3	-10.7
21060	AT1G23800.1 | Symbol: ALDH2B7 | aldehyde dehydrogenase, mitochondrial (ALDH3), nearly identical to mitochondrial aldehyde dehydrogenase ALDH3 (Arabidopsis thaliana) gi:19850249:gb:AAL99612; contains Pfam profile PF00171: aldehyde dehydrogenase (NAD) family protein | chr1:8412041-8414868 REVERSE | Aliases: ALDH2B, F5O8.35, F5O8_35	9.2	9.2	0.0	0.0
1826	AT1G20340.1 | Symbol: DRT112 |	9.2	4.9	4.3	7.7
20480	AT4G25650.2 | Rieske (2Fe-2S) domain-containing protein, similar to cell death suppressor protein lls1 from Zea mays (gi:1935909), Rieske iron-sulfur protein Tic55 from Pisum sativum (gi:2764524); contains Pfam PF00355 Rieske (2Fe-2S) domain | chr4:13080914-13083206 REVERSE | Aliases: L73G19.30, L73G19_30	9.2	9.1	0.0	0.1
6836	AT5G16070.1 | chaperonin, putative, similar to SWISS-PROT:P80317 T-complex protein 1, zeta subunit (TCP-1-zeta) (Mus musculus); contains Pfam:PF00118 domain, TCP-1/cpn60 chaperonin family | chr5:5247357-5251182 REVERSE | Aliases: F1N13.210, F1N13_210	9.2	7.3	1.8	3.6

3597	AT1G52760.1 | esterase/lipase/thioesterase family protein, low similarity to monoglyceride lipase from (Homo sapiens) GI:14594904, (Mus musculus) GI:2632162; contains Interpro entry IPR000379 | chr1:19655013-19656411 FORWARD | Aliases: F14G24.3, F14G24_3	9.2	11.0	-1.9	-5.6
1177	AT1G16340.2 | similar to 2-dehydro-3-deoxyphosphooctonate aldolase / phospho-2-dehydro-3-deoxyoctonate aldolase / 3-deoxy-D-manno-octulosonic acid 8-phosphate synthetase (KDSA) [Arabidopsis thaliana] (TAIR:At1g79500.1); similar to 2-dehydro-3-deoxyphosphooctonate aldolase / phospho-2-dehydro-3-deoxyoctonate aldolase / 3-deoxy-D-manno-octulosonic acid 8-phosphate synthetase (KDSA) [Arabidopsis thaliana] (TAIR:At1g79500.3); similar to 2-dehydro-3-deoxyphosphooctonate aldolase / phospho-2-dehydro-3-deoxyoctonate aldolase / 3-deoxy-D-manno-octulosonic acid 8-phosphate synthetase (KDSA) [Arabidopsis thaliana] (TAIR:At1g79500.2); similar to 3-desoxy-D-manno octulosonic acid-8-phosphate synthase [Lycopersicon esculentum] (GB:CAC35366.1); similar to putative 2-dehydro-3-deoxyphosphooctonate aldolase [Oryza sativa (japonica cultivar-group)] (GB:AAO72599.1); contains InterPro domain DAHP synthetase I/KDSA superfamily (InterPro:IPR006218); contains InterPro domain 2-dehydro-3-deoxyphosphooctonate aldolase (InterPro:IPR006269)	9.2	5.5	3.7	9.0
5184	AT5G58375.1 | expressed protein | chr5:23613500-23614186 FORWARD | Aliases: None	9.2	10.8	-1.6	-4.5
16377	AT5G10860.1 | CBS domain-containing protein, contains Pfam profile PF00571: CBS domain	9.2	9.8	-0.7	-0.9
5307	AT1G55330.1 | Symbol: AGP21 | arabinogalactan-protein (AGP21)	9.2	10.7	-1.6	-4.4
1271	AT3G16100.1 | Ras-related GTP-binding family protein, contains Pfam profile: PF00071 Ras family | chr3:5459178-5460783 FORWARD | Aliases: MSL1.14	9.2	6.4	2.8	8.8
10378	AT2G01060.2 | myb family transcription factor, contains Pfam profile: PF00249 myb-like DNA-binding domain | chr2:73258-75341 REVERSE | Aliases: F23H14.3, F23H14_3	9.2	9.6	-0.5	-2.3
18188	AT1G21680.1 | expressed protein, similar to TolB protein precursor (SP:Q9ZDM5) {Rickettsia prowazekii}; ESTs gb:N96028, gb:F14286, gb:T20680, gb:F14443, gb:AA657300 and gb:N65244 come from this gene	9.2	9.4	-0.3	-0.5
4954	AT1G06780.1 | glycosyl transferase family 8 protein, contains Pfam profile: PF01501 glycosyl transferase family 8 | chr1:2083423-2086961 FORWARD | Aliases: F4H5.13, F4H5_13	9.2	10.9	-1.7	-4.6
19606	AT5G54540.1 | expressed protein | chr5:22173738-22175280 FORWARD | Aliases: MRB17.4, MRB17_4	9.2	9.3	-0.1	-0.3
18729	AT3G20510.1 | expressed protein, contains Pfam profile PF03647: Uncharacterised protein family (UPF0136) | chr3:7160683-7161997 FORWARD | Aliases: K10D20.5	9.2	9.2	-0.1	-0.4
5784	AT5G38480.2 | Symbol: GRF3 | similar to 14-3-3 protein GF14 nu (GRF7) [Arabidopsis thaliana] (TAIR:At3g02520.1); similar to 14-3-3 e-1 protein [Nicotiana tabacum] (GB:BAD12176.1); similar to 14-3-3 e-2 protein [Nicotiana tabacum] (GB:BAD12177.1); contains InterPro domain 14-3-3 protein (InterPro:IPR000308) | chr5:15426927-15428746 FORWARD | Aliases: GENERAL REGULATORY FACTOR 3, GF14PSI ISOFORM, MXI10.21, MXI10_21, RCI1	9.2	7.0	2.2	4.1
1854	AT3G14430.1 | expressed protein | chr3:4824204-4824844 REVERSE | Aliases: MOA2.3	9.2	10.0	-0.8	-7.6
624	AT2G45710.1 | 40S ribosomal protein S27 (RPS27A) | chr2:18838235-18839281 FORWARD | Aliases: F4I18.31	9.2	5.7	3.5	11.1
17497	AT2G38870.1 | protease inhibitor, putative, similar to SP:P24076 Glu S.griseus protease inhibitor (BGIA) {Momordica charantia}; contains Pfam profile PF00280: Potato inhibitor I family | chr2:16243459-16244358 REVERSE | Aliases: T7F6.4, T7F6_4	9.2	9.4	-0.3	-0.7
3523	AT2G33250.1 | expressed protein | chr2:14104509-14105788 FORWARD | Aliases: F25I18.1, F25I18_1	9.2	7.8	1.3	5.6
6973	AT2G17265.1 | Symbol: HSK | homoserine kinase (HSK), identical to homoserine kinase (Arabidopsis thaliana) gi:4927412:gb:AAD33097 | chr2:7515670-7516837 FORWARD | Aliases: F5J6.24, F5J6_24, HOMOSERINE KINASE	9.2	7.0	2.2	3.5
17749	AT5G12390.1 | expressed protein | chr5:4010444-4012040 REVERSE | Aliases: None	9.2	9.3	-0.2	-0.6
1565	AT3G20790.1 | oxidoreductase family protein, weak similarity to SP:Q07982 Glucose--fructose oxidoreductase precursor (EC 1.1.99.28) {Zymomonas mobilis}; contains Pfam profiles PF01408: Oxidoreductase family NAD-binding Rossmann fold, PF02894: Oxidoreductase family C-terminal alpha/beta domain | chr3:7268768-7271517 FORWARD | Aliases: MOE17.10	9.2	6.9	2.3	8.1
5258	AT3G51260.2 | Symbol: PAD1 | similar to 20S proteasome alpha subunit D2 (PAD2) (PRS1) (PRC6) [Arabidopsis thaliana] (TAIR:At5g66140.1); similar to proteasome alpha subunit [Oryza sativa (japonica cultivar-group)] (GB:XP_483663.1); similar to proteasome alpha subunit [Lycopersicon esculentum] (GB:CAA74725.1); contains InterPro domain Proteasome subunit, A-type (InterPro:IPR000426); contains InterPro domain Multispecific proteasome protease (InterPro:IPR001353) | chr3:19041974-19044043 FORWARD | Aliases: 20S PROTEASOME ALPHA SUBUNIT PAD1, F24M12.300	9.2	6.2	3.0	4.4
20285	AT1G12910.1 | Symbol: AN11 | flower pigmentation protein (AN11), contains 3 WD-40 repeats (PF00400); identical to GB:AAC18912 from (Arabidopsis thaliana) (Genes Dev. 11 (11), 1422-1434 (1997)) | chr1:4394899-4396289 REVERSE | Aliases: ATAN11, F13K23.16, F13K23_16	9.2	9.1	0.1	0.2

5609	AT5G40770.1 | Symbol: ATPHB3 |	9.2	7.4	1.8	4.2
11320	AT5G66530.1 | aldose 1-epimerase family protein, similar to apospory-associated protein C; APOC (Chlamydomonas reinhardtii) GI:6970044; contains Pfam profile PF01263: Aldose 1-epimerase | chr5:26570973-26572902 REVERSE | Aliases: K1F13.19, K1F13_19	9.2	9.8	-0.6	-2.0
16828	AT2G41630.1 | Symbol: TFIIB | transcription initiation factor IIB-1 / general transcription factor TFIIB-1 (TFIIB1), identical to transcription initiation factor IIB-1 (TFIIB1) SP:P48512 from (Arabidopsis thaliana) | chr2:17362410-17364676 REVERSE | Aliases: T32G6.15, T32G6_15, TRANSCRIPTION FACTOR II B, TRANSCRIPTION FACTOR TFIIB	9.2	9.5	-0.3	-0.8
5941	AT2G47450.1 | Symbol: CAO | chloroplast signal recognition particle component (CAO), nearly identical to CAO (Arabidopsis thaliana) GI:4102582 | chr2:19479751-19481384 FORWARD | Aliases: CHAOS, CHLOROPHYLL A/B BINDING PROTEIN HARVESTING ORGANELLE SPECIFIC, CPSRP43, T30B22.25	9.2	7.7	1.4	4.1
1932	AT4G19880.3 | similar to expressed protein [Arabidopsis thaliana] (TAIR:At5g45020.1); similar to putative transferase [Escherichia coli O157:H7 EDL933] (GB:AAG58235.1) | chr4:10784401-10786433 REVERSE | Aliases: T16H5.240, T16H5_240	9.1	10.4	-1.2	-7.5
5645	AT4G30480.3 | expressed protein | chr4:14897352-14899127 FORWARD | Aliases: F17I23.180, F17I23_180	9.1	7.3	1.9	4.2
1556	AT4G04640.1 | Symbol: ATPC1 | ATP synthase gamma chain 1, chloroplast (ATPC1), identical to SP:Q01908 ATP synthase gamma chain 1, chloroplast precursor (EC 3.6.3.14) {Arabidopsis thaliana} | chr4:2350496-2352016 REVERSE | Aliases: T19J18.4, T19J18_4	9.1	6.2	2.9	8.1
7400	AT1G16170.1 | expressed protein | chr1:5538731-5539790 REVERSE | Aliases: T24D18.25, T24D18_25	9.1	9.6	-0.5	-3.4
893	AT5G17310.2 | UTP--glucose-1-phosphate uridylyltransferase, putative / UDP-glucose pyrophosphorylase, putative / UGPase, putative, strong similarity to SP:P19595 UTP--glucose-1-phosphate uridylyltransferase (EC 2.7.7.9) (UDP-glucose pyrophosphorylase) (UDPGP) (UGPase) {Solanum tuberosum}; contains Pfam profile PF01704: UTP--glucose-1-phosphate uridylyltransferase | chr5:5696649-5700924 REVERSE | Aliases: MKP11.26, MKP11_26	9.1	4.3	4.9	9.9
2119	AT3G06680.1 | 60S ribosomal protein L29 (RPL29B), similar to 60S ribosomal protein L29 GB:P25886 from (Rattus norvegicus) | chr3:2114408-2114885 REVERSE | Aliases: T8E24.9	9.1	5.4	3.7	7.2
15338	AT5G23610.2 | similar to meiosis protein-related (DYAD) (SWI1) [Arabidopsis thaliana] (TAIR:At5g51330.1); similar to expressed protein (with alternative splicing) [Oryza sativa (japonica cultivar-group)] (GB:XP_469018.1) | chr5:7957343-7959354 REVERSE | Aliases: MQM1.12, MQM1_12	9.1	9.4	-0.2	-1.1
2167	AT3G03920.1 | Gar1 RNA-binding region family protein, contains Pfam profile PF04410: Gar1 protein RNA binding region | chr3:1009005-1010477 REVERSE | Aliases: F20H23.3, F20H23_3	9.1	6.5	2.6	7.2
6496	AT1G27970.1 | nuclear transport factor 2 (NTF2), putative, similar to Swiss-Prot:P33331 nuclear transport factor 2 (NTF-2) (Nuclear transport factor P10) (Saccharomyces cerevisiae) | chr1:9746798-9748006 FORWARD | Aliases: F13K9.26, F13K9_26	9.1	6.2	2.9	3.8
10576	AT3G49860.1 | Symbol: ATARLA1B | ADP-ribosylation factor, putative, similar to GTP-binding ADP-ribosylation factor homolog 1 protein (SP:P25160) (Drosophila melanogaster) and various ADP-RIBOSYLATION FACTOR (ARF) - like proteins; contains PF00025: ADP-ribosylation factor family domain	9.1	8.8	0.4	2.2
1008	AT5G28750.1 | thylakoid assembly protein, putative, similar to thylakoid assembly 4 (tha4) protein (Pisum sativum) GI:4929305; contains Pfam profile PF02416: mttA/Hcf106 family | chr5:10784003-10785723 REVERSE | Aliases: T32B20.2, T32B20_2	9.1	6.6	2.5	9.5
8830	AT3G05530.1 | Symbol: RPT5A | 26S proteasome AAA-ATPase subunit (RPT5a), identical to GB:AAF22525 GI:6652886 from (Arabidopsis thaliana) | chr3:1603438-1606237 FORWARD | Aliases: 19S PROTEASOME REGULATORY COMPLEX SUBUNIT S6A, 26S PROTEASOME AAA ATPASE SUBUNIT, ATS6A.2, F22F7.1, F22F7_1	9.1	7.9	1.2	2.8
1103	AT5G65360.1 | histone H3, identical to histone H3 from Zea mays SP:P05203, Medicago sativa GI:166384, Encephalartos altensteinii SP:P08903, Pisum sativum SP:P02300; contains Pfam profile PF00125 Core histone H2A/H2B/H3/H4 | chr5:26137085-26137807 REVERSE | Aliases: MNA5.9	9.1	6.3	2.9	9.2
554	AT1G64880.1 | ribosomal protein S5 family protein, contains similarity to 30S ribosomal protein S5 GI:6969105 from (Campylobacter jejuni) | chr1:24109305-24112809 FORWARD | Aliases: F13O11.18, F13O11_18	9.1	5.0	4.1	11.6
1469	AT3G56360.1 | expressed protein, unknown protein 110K5.12 - Sorghum bicolor, TREMBL:AF124045_5	9.1	11.4	-2.2	-8.3
140	AT3G52180.2 | similar to protein phosphatase-related [Arabidopsis thaliana] (TAIR:At3g10940.1); similar to unknown protein [Oryza sativa (japonica cultivar-group)] (GB:XP_482104.1); contains InterPro domain Tyrosine specific protein phosphatase and dual specificity protein phosphatase (InterPro:IPR000387); contains InterPro domain Dual specificity protein phosphatase (InterPro:IPR000340)	9.1	4.2	4.9	18.5
9351	AT2G46170.1 | reticulon family protein (RTNLB5), weak similarity to Nogo-C protein (Rattus norvegicus) GI:6822251; contains Pfam profile PF02453: Reticulon | chr2:18972386-18974259 FORWARD | Aliases: T3F17.18	9.1	7.2	2.0	2.6

11706	ATCG00780.1 | Symbol: RPL14 | encodes a chloroplast ribosomal protein L14, a constituent of the large subunit of the ribosomal complex | chrC:80696-81064 REVERSE | Aliases: RPL14	9.1	7.8	1.4	1.9
143	AT5G50400.1 | calcineurin-like phosphoesterase family protein, contains Pfam profile: PF00149 calcineurin-like phosphoesterase	9.1	12.0	-2.9	-18.5
20114	AT2G44080.1 | expressed protein | chr2:18244261-18245249 FORWARD | Aliases: F6E13.21	9.1	9.2	-0.0	-0.2
15770	AT3G62190.1 | DNAJ heat shock N-terminal domain-containing protein, similar to SP:P39101 CAJ1 protein Saccharomyces cerevisiae; contains Pfam profile PF00226 DnaJ domain | chr3:23032096-23034307 FORWARD | Aliases: T17J13.150	9.1	9.3	-0.2	-1.0
5359	AT4G16830.1 | nuclear RNA-binding protein (RGGA), identical to nuclear RNA binding protein GI:6492264 from (Arabidopsis thaliana) | chr4:9470458-9472613 FORWARD | Aliases: DL4440W, FCAALL.13	9.1	10.4	-1.3	-4.4
9681	AT1G11240.1 | expressed protein | chr1:3766754-3768294 REVERSE | Aliases: T28P6.24, T28P6_24	9.1	7.8	1.3	2.5
326	AT3G25920.1 | Symbol: RPL15 | 50S ribosomal protein L15, chloroplast (CL15), identical to GB:P25873 from (Arabidopsis thaliana) | chr3:9491321-9493855 REVERSE | Aliases: MPE11.32, RIBOSOMAL PROTEIN CL15	9.1	5.2	3.9	13.9
5604	AT3G62450.1 | expressed protein | chr3:23109120-23109606 FORWARD | Aliases: T12C14.150	9.1	8.3	0.8	4.2
15125	AT5G17050.1 | UDP-glucoronosyl/UDP-glucosyl transferase family protein, similar to UDP glucose:flavonoid 3-o-glucosyltransferase, Vitis vinifera, EMBL:AF000372 | chr5:5607791-5609495 REVERSE | Aliases: F2K13.200, F2K13_200	9.1	8.5	0.6	1.1
444	AT1G08780.1 | prefoldin, putative, similar to Swiss-Prot:Q9NQP4 prefoldin subunit 4 (Protein C-1) (Homo sapiens) | chr1:2809783-2811194 REVERSE | Aliases: F22O13.27, F22O13_27	9.1	5.8	3.3	12.5
12898	AT2G22480.1 | phosphofructokinase family protein, similar to phosphofructokinase (Amycolatopsis methanolica) GI:17432243; contains Pfam profile PF00365: Phosphofructokinase | chr2:9552656-9555643 FORWARD | Aliases: F14M13.12, F14M13_12	9.1	9.7	-0.6	-1.6
18838	AT2G27600.1 | AAA-type ATPase family protein / vacuolar sorting protein-related, similar to SP:P46467 SKD1 protein (Vacuolar sorting protein 4b) {Mus musculus}; contains Pfam profiles PF00004: ATPase AAA family, PF04212: MIT domain | chr2:11788115-11791008 FORWARD | Aliases: F10A12.27, F10A12_27	9.1	8.8	0.3	0.4
6375	AT2G31350.2 | hydroxyacylglutathione hydrolase, putative / glyoxalase II, putative, similar to glyoxalase II isozyme (Arabidopsis thaliana) gi:2570338:gb:AAC49865 | chr2:13375370-13378070 FORWARD | Aliases: T28P16.16, T28P16_16	9.1	10.0	-0.9	-3.8
99	AT5G01300.1 | phosphatidylethanolamine-binding family protein, similar to cold-regulated protein (Hordeum vulgare subsp. vulgare) GI:10799810; contains Pfam profile PF01161: Phosphatidylethanolamine-binding protein	9.1	2.5	6.6	20.9
9372	AT1G04690.1 | Symbol: KAB1 | potassium channel protein, putative, nearly identical to K+ channel protein (Arabidopsis thaliana) GI:1063415; contains Pfam profile PF00248: oxidoreductase, aldo/keto reductase family	9.1	8.0	1.1	2.6
3717	AT1G48300.1 | expressed protein | chr1:17851024-17852466 FORWARD | Aliases: F11A17.15, F11A17_15	9.1	11.7	-2.6	-5.5
18892	AT3G23280.2 | zinc finger (C3HC4-type RING finger) family protein / ankyrin repeat family protein, contains Pfam profile: PF00097 zinc finger, C3HC4 type (RING finger) and Pfam profile: PF00023 ankyrin repeat | chr3:8321371-8324435 FORWARD | Aliases: K14B15.19	9.1	9.3	-0.2	-0.4
134	AT2G42570.1 | expressed protein | chr2:17724453-17727158 REVERSE | Aliases: F14N22.16, F14N22_16	9.1	5.2	3.9	18.6
4841	AT5G07090.2 | similar to 40S ribosomal protein S4 (RPS4D) [Arabidopsis thaliana] (TAIR:At5g58420.1); similar to ribosomal protein S4 [Solanum tuberosum] (GB:CAA54095.1); contains InterPro domain RNA-binding S4 (InterPro:IPR002942); contains InterPro domain KOW (Kyrpides, Ouzounis, Woese) motif (InterPro:IPR006646); contains InterPro domain Ribosomal protein S4E (InterPro:IPR000876); contains InterPro domain KOW (InterPro:IPR005824) | chr5:2202398-2204079 FORWARD | Aliases: T28J14.30	9.1	7.0	2.1	4.7
16078	AT3G60340.2 | palmitoyl protein thioesterase family protein, palmitoyl-protein thioesterase precursor, Mus musculus, EMBL:AF071025 | chr3:22315148-22317899 FORWARD | Aliases: T8B10.2	9.1	8.8	0.4	1.0
3097	AT5G03660.1 | expressed protein, low similarity to outer surface protein F (Borrelia burgdorferi) GI:466482; contains Pfam profile PF04949: Family of unknown function (DUF662) | chr5:937976-940057 FORWARD | Aliases: F17C15.80, F17C15_80	9.1	6.5	2.6	6.0
315	AT1G13640.1 | phosphatidylinositol 3- and 4-kinase family protein, low similarity to phosphatidylinositol 4-kinase type-II beta (Homo sapiens) GI:20159767; contains Pfam profile PF00454: Phosphatidylinositol 3- and 4-kinase | chr1:4677024-4679801 REVERSE | Aliases: F21F23.8, F21F23_8	9.1	5.4	3.6	14.0

2298	AT2G37250.1 | Symbol: ADK/ATPADK1 | adenylate kinase family protein, contains Pfam profile: PF00406 adenylate kinase | chr2:15649016-15650797 FORWARD | Aliases: ADK, ATPADK1, F3G5.4, F3G5_4	9.1	7.4	1.7	7.0
13124	AT1G32790.2 | Symbol: CID11 | similar to RNA-binding protein, putative [Arabidopsis thaliana] (TAIR:At4g10610.1); similar to putative RNA-binding protein RBP37 [Oryza sativa (japonica cultivar-group)] (GB:XP_479783.1); contains InterPro domain RNA-binding region RNP-1 (RNA recognition motif) (InterPro:IPR000504) | chr1:11874684-11877367 REVERSE | Aliases: CID11, F6N18.17, F6N18_17	9.1	9.9	-0.8	-1.6
6895	AT1G33700.2 | similar to expressed protein [Arabidopsis thaliana] (TAIR:At5g49900.1); similar to expressed protein [Arabidopsis thaliana] (TAIR:At4g10060.1); similar to expressed protein [Arabidopsis thaliana] (TAIR:At3g24180.1); similar to unknown protein [Oryza sativa (japonica cultivar-group)] (GB:AAP54244.1); similar to At5g49900 [Oryza sativa (japonica cultivar-group)] (GB:AAX95400.1); contains InterPro domain Protein of unknown function DUF608 (InterPro:IPR006775) | chr1:12207208-12214162 REVERSE | Aliases: F14M2.16, F14M2_16	9.1	9.6	-0.5	-3.6
15410	AT1G17080.1 | expressed protein | chr1:5839679-5841559 REVERSE | Aliases: F20D23.22, F20D23_22	9.1	9.5	-0.4	-1.1
3173	AT3G51100.2 | expressed protein | chr3:18991762-18993241 REVERSE | Aliases: F24M12.140	9.1	7.5	1.6	5.9
6997	AT4G24990.1 | Symbol: ATGP4 | ubiquitin family protein, contains INTERPRO:IPR000626 ubiquitin domain | chr4:12849719-12851643 REVERSE | Aliases: F13M23.130, F13M23_130	9.1	10.1	-1.0	-3.5
4123	AT4G08685.1 | Symbol: SAH7 | pollen Ole e 1 allergen and extensin family protein, contains Pfam domain, PF01190: Pollen proteins Ole e I family | chr4:5550426-5551816 FORWARD | Aliases: None	9.1	6.1	2.9	5.2
10477	AT2G45990.4 | expressed protein, similar to unknown protein [Oryza sativa (japonica cultivar-group)] (GB:XP_477679.1) | chr2:18926436-18928302 FORWARD | Aliases: F4I18.49	9.1	8.3	0.8	2.2
14293	AT3G12630.1 | zinc finger (AN1-like) family protein, contains Pfam domain, PF01428: AN1-like Zinc finger | chr3:4012615-4013590 FORWARD | Aliases: T2E22.6	9.1	9.5	-0.4	-1.3
3977	AT1G07170.1 | expressed protein, contains Pfam domain PF03660: Uncharacterised protein family (UPF0123) | chr1:2200133-2201139 FORWARD | Aliases: F10K1.35	9.1	8.1	1.0	5.3
17494	AT5G54960.1 | Symbol: PDC2 | pyruvate decarboxylase, putative, strong similarity to pyruvate decarboxylase 1 (Vitis vinifera) GI:10732644; contains InterPro entry IPR000399: Pyruvate decarboxylase | chr5:22327913-22329987 REVERSE | Aliases: MBG8.23, MBG8_23, PYRUVATE DECARBOXYLASE, PYRUVATE DECARBOXYLASE 2	9.1	9.4	-0.3	-0.7
7770	ATCG00570.1 | Symbol: PSBF | PSII cytochrome b559 | chrC:63942-64061 REVERSE | Aliases: PSBF	9.1	7.3	1.8	3.2
2509	AT2G32480.2 | membrane-associated zinc metalloprotease, putative, similar to Hypothetical zinc metalloprotease All3971 (SP:Q8YQ64) (strain PCC 7120) {Anabaena sp.} Pfam PF00595: PDZ domain (Also known as DHR or GLGF); contains TIGRFAM TIGR00054: membrane-associated zinc metalloprotease, putative | chr2:13795594-13797150 REVERSE | Aliases: T26B15.4, T26B15_4	9.1	7.6	1.5	6.7
12155	AT4G35220.1 | cyclase family protein, contains Pfam profile: PF04199 putative cyclase | chr4:16752618-16754310 FORWARD | Aliases: F23E12.220, F23E12_220	9.1	8.3	0.8	1.8
8672	AT1G32400.3 | Symbol: TOM2A | similar to expressed protein [Arabidopsis thaliana] (TAIR:At2g20230.1); similar to senescence-associated protein-like [Oryza sativa (japonica cultivar-group)] (GB:XP_480197.1); contains InterPro domain CD9/CD37/CD63 antigen (InterPro:IPR000301) | chr1:11689088-11691445 REVERSE | Aliases: F5D14.17, F5D14_17, TOBAMOVIRUS MULTIPLICATION 2A	9.1	10.2	-1.1	-2.8
20773	AT1G72820.1 | mitochondrial substrate carrier family protein, contains Pfam profile: PF00153 mitochondrial carrier protein | chr1:27406540-27408335 FORWARD | Aliases: F3N23.2, F3N23_2	9.1	9.1	-0.0	-0.1
14648	AT3G27240.1 | cytochrome c1, putative, cytochrome c1, heme protein, mitochondrial precursor (Clone PC13III) (Solanum tuberosum) SWISS-PROT:P25076 | chr3:10057026-10059953 REVERSE | Aliases: K17E12.6	9.1	8.0	1.1	1.2
2160	AT5G57300.1 | UbiE/COQ5 methyltransferase family protein, similar to ubiquinone biosynthesis methyltransferase COQ5 (Saccharomyces cerevisiae)(SP:P49017), ubiquinone/menaquinone biosynthesis methyltransferase ubiE (Escherichia coli)(SP:P27851); contains Pfam profile PF01209: methlytransferase, UbiE/COQ5 family | chr5:23225809-23228316 REVERSE | Aliases: MJB24.11, MJB24_11	9.1	6.6	2.5	7.2
2995	AT4G30810.1 | Symbol: SCPL29 |	9.1	7.1	1.9	6.1
3225	AT2G37190.1 | 60S ribosomal protein L12 (RPL12A) | chr2:15626486-15627198 REVERSE | Aliases: T2N18.5, T2N18_5	9.1	6.9	2.2	5.9

1080	AT2G36830.1 | Symbol: GAMMA TIP | major intrinsic family protein / MIP family protein, contains Pfam profile: MIP PF00230 | chr2:15452505-15453653 FORWARD | Aliases: GAMMA TIP1, GAMMA TONOPLAST INTRINSIC PROTEIN, T1J8.1, T1J8_1, TIP1;1	9.1	12.6	-3.6	-9.3
5943	AT1G17160.1 | pfkB-type carbohydrate kinase family protein, contains Pfam profile: PF00294 pfkB family carbohydrate kinase | chr1:5867639-5869323 FORWARD | Aliases: F20D23.14, F20D23_14	9.1	8.0	1.1	4.1
6200	AT2G27970.1 | Symbol: CKS2 | cyclin-dependent kinase, putative / CDK, putative, similar to Cks1 protein (Arabidopsis thaliana) gi:2274859:emb:CAA03859 | chr2:11919295-11920348 REVERSE | Aliases: CDK subunit 2, CKS2, T1E2.11, T1E2_11	9.1	7.4	1.6	3.9
678	AT5G45750.1 | Ras-related GTP-binding protein, putative, similar to GTP-binding protein GI:303744 from (Pisum sativum)	9.1	5.3	3.8	10.8
1618	AT4G30660.1 | hydrophobic protein, putative / low temperature and salt responsive protein, putative, similar to SP:Q9ZNQ7 Hydrophobic protein RCI2A (Low temperature and salt responsive protein LTI6A) {Arabidopsis thaliana}; contains Pfam profile PF01679: Uncharacterized protein family | chr4:14955453-14956069 FORWARD | Aliases: T10C21.10, T10C21_10	9.1	7.1	1.9	8.0
489	AT1G80820.1 | Symbol: CCR2 | cinnamoyl-CoA reductase, putative, identical to CCR2 (GI:12407990), similar to cinnamoyl CoA reductase from Eucalyptus gunnii (GI:2058311) | chr1:30375465-30377562 FORWARD | Aliases: CINNAMOYL COA REDUCTASE, F23A5.17, F23A5_17	9.1	3.4	5.7	12.1
11170	AT5G42270.1 | Symbol: VAR1 | FtsH protease, putative, similar to FtsH protease GI:13183728 from (Medicago sativa) | chr5:16919714-16923100 FORWARD | Aliases: FTSH5, K5J14.13, K5J14_13, VARIEGATED 1	9.1	8.3	0.8	2.0
19302	AT3G22850.1 | expressed protein, similar to auxin down-regulated protein ARG10 (Vigna radiata) GI:2970051, wali7 (aluminum-induced protein) (Triticum aestivum) GI:451193 | chr3:8089010-8090462 FORWARD | Aliases: F5N5.2	9.1	8.9	0.2	0.3
1657	AT5G63670.1 | transcription initiation protein-related, contains weak similarity to Transcription initiation protein SPT4 homolog 1 (Swiss-Prot:Q16550) (Mus musculus)	9.1	6.6	2.5	7.9
10262	AT2G16600.1 | Symbol: ROC3 | peptidyl-prolyl cis-trans isomerase, cytosolic / cyclophilin / rotamase (ROC3), identical to cytosolic cyclophilin (Arabidopsis thaliana) GI:1305455 | chr2:7207889-7208650 FORWARD | Aliases: CYCLOPHILIN, T24I21.1, T24I21_1	9.1	7.4	1.7	2.3
4367	AT1G50920.1 | GTP-binding protein-related, similar to GTP-binding protein SP:Q99ME9 from (Mus musculus) | chr1:18873807-18876598 FORWARD | Aliases: F8A12.14, F8A12_14	9.1	6.3	2.7	5.0
2963	AT3G62420.1 | bZIP transcription factor family protein, similar to common plant regulatory factor 6 GI:9650826 from (Petroselinum crispum) | chr3:23102820-23103260 REVERSE | Aliases: T12C14.120	9.1	10.2	-1.2	-6.2
17560	AT3G11230.1 | yippee family protein, similar to qdgl-1 (Coturnix coturnix) GI:10441650, Yippee protein (Homo sapiens) GI:5713281; contains Pfam profile PF03226: Yippee putative zinc-binding protein | chr3:3516583-3518377 FORWARD | Aliases: F11B9.15	9.1	9.2	-0.1	-0.7
18847	AT5G22060.1 | Symbol: ATJ2 | DNAJ heat shock protein, putative, strong similarity to SP:O60884 DnaJ homolog subfamily A member 2 (Dnj3) Homo sapiens, several plant DnaJ proteins from PGR; contains Pfam profiles PF00226 DnaJ domain, PF00684 DnaJ central domain (4 repeats), PF01556 DnaJ C terminal region | chr5:7303625-7305800 REVERSE | Aliases: None	9.1	9.3	-0.2	-0.4
6540	AT5G66440.1 | expressed protein | chr5:26547461-26548323 REVERSE | Aliases: K1F13.9, K1F13_9	9.1	7.5	1.6	3.7
8003	AT5G20280.1 | sucrose-phosphate synthase, putative, similar to sucrose-phosphate synthase (EC 2.4.1.14) isoform 1 - Citrus unshiu, EMBL:AB005023 | chr5:6844716-6850065 REVERSE | Aliases: F5O24.170, F5O24_170	9.1	6.7	2.4	3.1
9952	AT3G62120.2 | tRNA synthetase class II (G, H, P and S) family protein, similar to SP:P07814 Bifunctional aminoacyl-tRNA synthetase (Includes: Glutamyl-tRNA synthetase (EC 6.1.1.17) (Glutamate--tRNA ligase); Prolyl-tRNA synthetase (EC 6.1.1.15) (Proline--tRNA ligase)) {Homo sapiens}; contains Pfam profiles PF00587: tRNA synthetase class II core domain (G, H, P, S and T), PF03129: Anticodon binding domain	9.1	7.8	1.3	2.4
10350	AT5G50950.2 | fumarate hydratase, putative / fumarase, putative, similar to SP:P55250 Fumarate hydratase, mitochondrial precursor (EC 4.2.1.2) (Fumarase) {Rhizopus oryzae}; contains Pfam profile PF00206: Lyase | chr5:20746870-20751152 FORWARD | Aliases: K3K7.11, K3K7_11	9.1	8.1	1.0	2.3
10349	AT2G47510.1 | Symbol: FUM1 | fumarate hydratase, putative / fumarase, putative, similar to SP:P55250 Fumarate hydratase, mitochondrial precursor (EC 4.2.1.2) (Fumarase) {Rhizopus oryzae}; contains Pfam profile PF00206: Lyase | chr2:19505458-19509316 FORWARD | Aliases: FUMARASE 1, T30B22.19	9.1	8.1	1.0	2.3
7463	AT1G18080.1 | Symbol: ATARCA |	9.1	5.8	3.3	3.3
11434	AT3G19490.1 | Symbol: ATNHD1 |	9.1	8.6	0.5	2.0

632	AT4G05180.1 | oxygen-evolving enhancer protein 3, chloroplast, putative (PSBQ2), identical to SP:Q41932 Oxygen-evolving enhancer protein 3-2, chloroplast precursor (OEE3) (16 kDa subunit of oxygen evolving system of photosystem II) (OEC 16 kDa subunit) {Arabidopsis thaliana}; similar to SP:P12301 Oxygen-evolving enhancer protein 3, chloroplast precursor (OEE3) (16 kDa subunit of oxygen evolving system of photosystem II) (OEC 16 kDa subunit) {Spinacia oleracea}; contains Pfam profile PF05757: Oxygen evolving enhancer protein 3 (PsbQ) | chr4:2671820-2673241 REVERSE | Aliases: C17L7.100, C17L7_100	9.1	3.5	5.5	11.1
4632	AT3G54010.2 | Symbol: PAS1 | peptidyl-prolyl cis-trans isomerase, putative / FK506-binding protein, putative / pasticcino 1-D (PAS1-D), nearly identical to pasticcino 1-D (Arabidopsis thaliana) GI:3080740 | chr3:20011867-20016161 FORWARD | Aliases: F5K20.310, PASTICCINO 1	9.1	10.2	-1.1	-4.8
16268	AT1G50250.1 | Symbol: FTSH1 | encodes an FTSH protease that is localized to the chloroplast | chr1:18617877-18620731 REVERSE | Aliases: F14I3.14, F14I3_14, FTSH1	9.1	8.8	0.2	0.9
1612	AT4G08390.2 | L-ascorbate peroxidase, stromal (sAPX), identical to stromal ascorbate peroxidase (Arabidopsis thaliana) gi:1419388:emb:CAA67425	9.1	5.8	3.2	8.0
3562	AT3G56240.1 | Symbol: CCH | copper homeostasis factor / copper chaperone (CCH) (ATX1), identical to gi:3168840 Pfam profile PF00403: Heavy-metal-associated domain | chr3:20874236-20875470 REVERSE | Aliases: ATX1, COPPER CHAPERONE, COPPER HOMEOSTASIS FACTOR, F18O21.200	9.1	10.7	-1.6	-5.6
1247	AT1G19130.1 | expressed protein | chr1:6609360-6610593 REVERSE | Aliases: F14D16.29, F14D16_29	9.1	6.8	2.3	8.8
6350	AT1G30380.1 | photosystem I reaction center subunit psaK, chloroplast, putative / photosystem I subunit X, putative / PSI-K, putative (PSAK), identical to SP:Q9SUI5; strong similarity to SP:P36886 Photosystem I reaction center subunit psaK, chloroplast precursor (Photosystem I subunit X) (PSI-K) (Light-harvesting complex I 7 kDa protein){Hordeum vulgare}; contains Pfam profile PF01241: Photosystem I psaG / psaK	9.1	8.0	1.1	3.8
938	AT4G36910.1 | CBS domain-containing protein, contains Pfam profile PF00571: CBS domain	9.1	4.6	4.5	9.7
2890	AT3G62970.1 | similar to zinc finger (C3HC4-type RING finger) family protein [Arabidopsis thaliana] (TAIR:At5g18650.1); similar to putative PGPD14 protein (pollen germination related protein) [Oryza sativa (japonica cultivar-group)] (GB:NP_916676.1); similar to zinc finger protein ZFP-like [Oryza sativa (japonica cultivar-group)] (GB:BAD87761.1); contains InterPro domain CHY zinc finger (InterPro:IPR008913); contains InterPro domain Zn-finger, RING (InterPro:IPR001841) | chr3:23281609-23283895 FORWARD | Aliases: T20O10.70	9.1	6.3	2.7	6.3
5754	AT1G28680.1 | transferase family protein, similar to elicitor inducible gene product EIG-I24 (Nicotiana tabacum) (gi:10798748); contains Pfam transferase family domain PF00248 | chr1:10078175-10080015 FORWARD | Aliases: F1K23.12, F1K23_12	9.1	10.3	-1.3	-4.2
1644	AT1G22140.2 | expressed protein | chr1:7814830-7815620 REVERSE | Aliases: F2E2.21, F2E2_21	9.1	6.8	2.2	8.0
10631	AT3G04560.1 | expressed protein | chr3:1226624-1229414 REVERSE | Aliases: F7O18.3, F7O18_3	9.1	8.7	0.4	2.2
1133	AT5G55640.1 | expressed protein | chr5:22550385-22551304 REVERSE | Aliases: MDF20.8, MDF20_8	9.1	7.1	2.0	9.1
413	AT1G15250.1 | 60S ribosomal protein L37 (RPL37A), almost identical to GB:Q43292 | chr1:5248660-5249457 REVERSE | Aliases: F9L1.46, F9L1_46	9.1	4.9	4.1	12.7
7759	AT3G49680.1 | branched-chain amino acid aminotransferase 3 / branched-chain amino acid transaminase 3 (BCAT3), identical to SP:Q9M401 Branched-chain amino acid aminotransferase 3, chloroplast precursor (EC 2.6.1.42) (Atbcat-3){Arabidopsis thaliana} | chr3:18433644-18436674 FORWARD | Aliases: T16K5.30	9.1	7.5	1.5	3.2
16358	AT4G17940.1 | expressed protein | chr4:9965754-9966978 FORWARD | Aliases: T6K21.120, T6K21_120	9.1	8.7	0.3	0.9
19004	AT5G13190.1 | expressed protein | chr5:4204820-4206408 REVERSE | Aliases: T19L5.5	9.1	9.1	-0.1	-0.4
12632	AT2G34585.1 | expressed protein | chr2:14575126-14575716 REVERSE | Aliases: None	9.1	9.4	-0.4	-1.7
560	AT1G15390.1 | Symbol: PDF1A | peptide deformylase, mitochondrial / polypeptide deformylase 1A (PDF1A), nearly identical to SP:Q9FV53 Peptide deformylase, mitochondrial precursor (EC 3.5.1.88) (PDF) (Polypeptide deformylase) {Arabidopsis thaliana}; contains Pfam profile PF01327: polypeptide deformylase; supporting cDNA gi:11320951:gb:AF250959.1:AF250959 | chr1:5294624-5295792 FORWARD | Aliases: PEPTIDE DEFORMYLASE 1A	9.1	5.2	3.8	11.5
3816	AT5G13110.1 | Symbol: G6PD2 | Encodes a plastidic glucose-6-phosphate dehydrogenase that is sensitive to reduction by DTT and whose mRNA is most highly expressed in root. | chr5:4158814-4161823 FORWARD | Aliases: G6PD2, GLUCOSE 6 PHOSPHATE DEHYDROGENASE 2, T19L5.70, T19L5_70	9.1	10.6	-1.5	-5.4
807	AT5G35700.1 | fimbrin-like protein, putative, similar to fimbrin-like protein (ATFIM1) (Arabidopsis thaliana) GI:2905893, fimbrin (Schizosaccharomyces pombe) GI:3057144; contains Pfam profile PF00307: Calponin homology (CH) domain	9.1	5.4	3.7	10.2
534	AT5G06590.1 | expressed protein | chr5:2016646-2018836 FORWARD | Aliases: F15M7.12, F15M7_12	9.1	4.7	4.4	11.7
8397	AT3G13772.1 | endomembrane protein 70, putative, TM4 family; | chr3:4521540-4524401 REVERSE | Aliases: MMM17.22	9.0	9.9	-0.8	-2.9

9185	AT1G06400.1 | Symbol: ARA2 | Ras-related GTP-binding protein (ARA-2), identical to Ras-related protein ARA-2 SP:P28185 from (Arabidopsis thaliana) | chr1:1950843-1952726 REVERSE | Aliases: ARA 2, SMALL GTP BINDING PROTEIN, T2D23.10, T2D23_10	9.0	8.1	0.9	2.6
15804	AT2G27285.1 | expressed protein, weak similarity to maebl (GI:20087019) (Plasmodium falciparum), chimeric erythrocyte-binding protein MAEBL (GI:22086284) (Plasmodium falciparum) | chr2:11683038-11685178 REVERSE | Aliases: None	9.0	8.6	0.4	1.0
14964	AT2G03150.1 | Symbol: EMB1579 | ATP/GTP-binding protein family, contains ATP/GTP-binding site motif A (P-loop), PROSITE:PS00017 | chr2:951930-959162 FORWARD | Aliases: EMB1579, EMBRYO DEFECTIVE 1579, T18E12.18, T18E12_18	9.0	8.4	0.6	1.2
3105	AT5G08560.1 | transducin family protein / WD-40 repeat family protein, contains 7 WD-40 repeats (PF00400); similar to will die slowly protein (WDS) (SP:Q9V3J8) (Drosophila melanogaster) | chr5:2770875-2774673 REVERSE | Aliases: MAH20.12, MAH20_12	9.0	10.1	-1.1	-6.0
202	AT4G09010.1 | L-ascorbate peroxidase, chloroplast, putative, identical to SP:P82281:TL29_ARATH (EC 1.11.1.11) {Arabidopsis thaliana}; ascorbate peroxidase - Spinacia oleracea, (gi:1669585); contains Pfam domain PF00141: Peroxidase; contains TIGRfam domain TIGR01409: Tat (twin-arginine translocation) pathway signal sequence; identical to ascorbate peroxidase APX4 (AT4g09010) mRNA, partial cds GI:31980499 | chr4:5777080-5779418 REVERSE | Aliases: F23J3.40, F23J3_40	9.0	3.9	5.2	16.6
14407	AT5G36880.2 | similar to acyl-activating enzyme 17 (AAE17) [Arabidopsis thaliana] (TAIR:At5g23050.1); similar to Acetyl-CoA synthetase 2, isoform a [Homo sapiens] (GB:AAH12172.1); similar to OTTHUMP00000030713 [Homo sapiens] (GB:CAI19312.1); similar to OSJNBb0118P14.12 [Oryza sativa (japonica cultivar-group)] (GB:XP_472384.1); similar to OJ000315_02.12 [Oryza sativa (japonica cultivar-group)] (GB:CAE05367.3); similar to acetyl-CoA synthetase [Solanum tuberosum] (GB:CAA67130.1); contains InterPro domain AMP-dependent synthetase and ligase (InterPro:IPR000873) | chr5:14551843-14557516 REVERSE | Aliases: F5H8.15, F5H8_15	9.0	9.3	-0.3	-1.3
8413	AT3G58170.1 | Symbol: ATBS14A | Bet1-like SNARE 1-1 / Bet1 / Sft1-like SNARE 14a / BS14a (BET11), identical to SP:Q9M2J9 Bet1-like SNARE 1-1 (AtBET11) (Bet1/Sft1-like SNARE 14a) (AtBS14a) (Mouse-ear cress) {Arabidopsis thaliana}; supporting cDNA gi:14030602:gb:AF368175.1:AF368175 | chr3:21553344-21554918 REVERSE | Aliases: ATBET11, BET11, F9D24.80	9.0	7.9	1.1	2.9
1050	AT1G75350.1 | Symbol: EMB2184 | ribosomal protein L31 family protein, similar to SP:O46917 from (Guillardia theta) | chr1:28275742-28276481 FORWARD | Aliases: EMB2184, EMBRYO DEFECTIVE 2184, F1B16.11, F1B16_11	9.0	7.2	1.8	9.4
6550	AT3G08730.1 | Symbol: ATPK1 | serine/threonine protein kinase (PK1) (PK6), identical to serine/threonine-protein kinase AtPK1/AtPK6 (ribosomal-protein S6 kinase ATPK6) (Arabidopsis thaliana) SWISS-PROT:P42818 | chr3:2651453-2654189 REVERSE | Aliases: ATPK6, F17O14.20, PROTEIN SERINE KINASE	9.0	8.1	0.9	3.7
10280	AT1G75680.1 | glycosyl hydrolase family 9 protein, similar to endo-beta-1,4-glucanase GB:AAC12685 GI:3025470 from (Pinus radiata) | chr1:28420713-28423190 REVERSE | Aliases: F10A5.13, F10A5_13	9.0	9.5	-0.4	-2.3
2304	AT1G09630.1 | Symbol: ATRAB11C | Ras-related GTP-binding protein, putative, similar to GTP-binding protein GI:1370146 from (Lotus japonicus) | chr1:3118205-3119710 REVERSE | Aliases: F21M12.2, F21M12_2, RAS RELATED SMALL GTPASE	9.0	5.4	3.7	7.0
737	AT5G54290.1 | cytochrome c biogenesis protein family, low similarity to cytochrome c biogenesis protein CcdA (Paracoccus pantotrophus) GI:11095328; contains Pfam profile PF02683: Cytochrome C biogenesis protein transmembrane region | chr5:22068263-22071604 FORWARD | Aliases: MDK4.11, MDK4_11	9.0	5.7	3.4	10.6
4669	AT5G45350.1 | proline-rich family protein, contains proline rich extensin domains, INTERPRO:IPR002965 | chr5:18399064-18400926 REVERSE | Aliases: MFC19.1, MFC19_1	9.0	6.9	2.2	4.8
15828	AT2G26800.3 | hydroxymethylglutaryl-CoA lyase, putative / 3-hydroxy-3-methylglutarate-CoA lyase, putative / HMG-CoA lyase, putative, similar to SP:P35915 Hydroxymethylglutaryl-CoA lyase (EC 4.1.3.4) (HMG-CoA lyase) {Gallus gallus}; contains Pfam profile PF00682: HMGL-like | chr2:11436888-11439810 REVERSE | Aliases: F12C20.16, F12C20_16	9.0	8.6	0.4	1.0
9653	AT3G13920.2 | Symbol: EIF4A1 |	9.0	7.6	1.5	2.5
3380	AT5G57330.1 | aldose 1-epimerase family protein, contains Pfam profile PF01263 Aldose 1-epimerase | chr5:23235440-23238315 FORWARD | Aliases: MJB24.14, MJB24_14	9.0	6.7	2.4	5.7
1589	AT1G76860.1 | small nuclear ribonucleoprotein, putative / snRNP, putative / Sm protein, putative, similar to SWISS-PROT:Q9Y4Z1 U6 snRNA-associated Sm-like protein LSm3 (MDS017) (Mouse) | chr1:28859355-28860644 REVERSE | Aliases: F7O12.3, F7O12_3	9.0	7.3	1.7	8.1
778	AT5G14105.1 | expressed protein | chr5:4551991-4553053 REVERSE | Aliases: None	9.0	6.2	2.8	10.4
8030	AT5G12200.1 | dihydropyrimidinase / DHPase / dihydropyrimidine amidohydrolase / hydantoinase (PYD2), identical to dihydropyrimidine amidohydrolase (PYD2) (Arabidopsis thaliana) GI:28194047; similar to SP:Q9EQF5 Dihydropyrimidinase (EC 3.5.2.2) (DHPase) (Hydantoinase) (DHP) {Mus musculus}; contains Pfam profile PF01979: Amidohydrolase family	9.0	10.0	-1.0	-3.1

5024	AT1G29900.1 | Symbol: CARB | carbamoyl-phosphate synthase family protein, similar to carbamoylphosphate synthetase GI:6552726 from (Medicago sativa); contains Pfam profiles PF02786: Carbamoyl-phosphate synthase L chain ATP binding domain, PF00289: Carbamoyl-phosphate synthase L chain N-terminal domain, PF02787: Carbamoyl-phosphate synthetase large chain oligomerisation domain | chr1:10468038-10472195 FORWARD | Aliases: CARBAMOYL PHOSPHATE SYNTHETASE B, CARBAMOYL PHOSPHATE SYNTHETASE LARGE SUBUNIT, F1N18.6, F1N18_6	9.0	6.8	2.3	4.6
5463	AT4G05530.1 | short-chain dehydrogenase/reductase (SDR) family protein, similar to peroxisomal short-chain alcohol dehydrogenase GI:4105190 from (Homo sapiens) | chr4:2816392-2818362 FORWARD | Aliases: T1J24.9, T1J24_9	9.0	6.8	2.2	4.3
5063	AT1G05720.1 | selenoprotein family protein, contains Prosite PS00190: Cytochrome c family heme-binding site signature; similar to 15 kDa selenoprotein (GI:12314088) {Homo sapiens} | chr1:1717461-1718852 REVERSE | Aliases: F3F20.17, F3F20_17	9.0	7.8	1.3	4.5
16158	AT3G18820.1 | Ras-related GTP-binding protein, putative, similar to Ras-related protein RAB7 GI:1370186 from (Pisum sativum), Plant Mol. Biol. 21 (6), 1195-1199 (1993); contains Pfam profile: PF00071 Ras family | chr3:6484107-6486252 FORWARD | Aliases: MVE11.21	9.0	8.6	0.4	0.9
1132	AT5G66410.1 | expressed protein | chr5:26537032-26538922 FORWARD | Aliases: K1F13.6, K1F13_6	9.0	7.0	2.1	9.2
21090	AT2G29420.1 | Symbol: ATGSTU7 | glutathione S-transferase, putative | chr2:12625013-12625976 REVERSE | Aliases: F16P2.20, F16P2_20, GLUTATHIONE S TRANSFERASE 25, GST25	9.0	9.0	0.0	0.0
17339	AT2G14910.2 | expressed protein | chr2:6413558-6416234 REVERSE | Aliases: T26I20.7, T26I20_7	9.0	8.6	0.4	0.7
6430	AT2G41110.1 | Symbol: ATCAL4 |	9.0	6.9	2.2	3.8
6010	AT5G15450.1 | heat shock protein 100, putative / HSP100, putative / heat shock protein clpB, putative / HSP100/ClpB, putative, similar to HSP100/ClpB GI:9651530 (Phaseolus lunatus) | chr5:5014402-5018350 REVERSE | Aliases: T20K14.60, T20K14_60	9.0	6.1	2.9	4.0
20714	AT2G20920.1 | expressed protein | chr2:9005767-9007013 FORWARD | Aliases: F5H14.11, F5H14_11	9.0	9.0	-0.0	-0.1
12151	AT3G56950.1 | small basic membrane integral family protein, contains similarity to small basic membrane integral protein ZmSIP2-1 (GI:13447817) (Zea mays) | chr3:21088985-21090810 REVERSE | Aliases: T8M16.9	9.0	8.4	0.6	1.8
14612	ATMG00660.1 | Symbol: ORF149 |	9.0	10.2	-1.2	-1.2
17118	ATCG00140.1 | Symbol: ATPH | ATPase III subunit | chrC:13262-13507 REVERSE | Aliases: ATPH	9.0	8.7	0.3	0.8
12766	AT3G19130.1 | Symbol: ATRBP47B | RNA-binding protein, putative, similar to RNA Binding Protein 47 (Nicotiana plumbaginifolia) GI:9663769, DNA binding protein ACBF GB:AAC49850 from (Nicotiana tabacum); contains InterPro entry IPR000504: RNA-binding region RNP-1 (RNA recognition motif) (RRM) | chr3:6611219-6614050 REVERSE | Aliases: ATRBP47B, MVI11.3	9.0	9.4	-0.3	-1.6
10559	AT2G42590.3 | Symbol: GRF9 | similar to 14-3-3 protein GF14 iota (GRF12) [Arabidopsis thaliana] (TAIR:At1g26480.1); similar to 14-3-3 protein homolog [Maackia amurensis] (GB:AAC15418.1); similar to SGF14D [Glycine max] (GB:AAB09583.1); contains InterPro domain 14-3-3 protein (InterPro:IPR000308) | chr2:17738933-17741045 REVERSE | Aliases: F14N22.14, F14N22_14, GF14 MU	9.0	8.2	0.8	2.2
9457	AT3G19910.1 | zinc finger (C3HC4-type RING finger) family protein, contains Pfam profile: PF00097 zinc finger, C3HC4 type (RING finger) | chr3:6926314-6929572 FORWARD | Aliases: MPN9.16	9.0	8.2	0.8	2.5
2909	AT1G76260.1 | transducin family protein / WD-40 repeat family protein, contains 6 WD-40 repeats (PF00400); similar to retinoblastoma A associated protein; RbAp48 (GI:3309245) (Xenopus laevis) | chr1:28615206-28618838 FORWARD | Aliases: T23E18.19, T23E18_19	9.0	6.0	3.0	6.2
5843	AT5G08380.1 | alpha-galactosidase, putative / melibiase, putative / alpha-D-galactoside galactohydrolase, putative, similar to alpha-galactosidase SP:Q42656 from (Coffea arabica); contains Pfam profile PF02065: Melibiase | chr5:2694678-2697688 REVERSE | Aliases: F8L15.110, F8L15_110	9.0	7.6	1.5	4.1
3626	AT1G27150.1 | expressed protein | chr1:9429044-9432235 FORWARD | Aliases: T7N9.21, T7N9_21	9.0	9.9	-0.8	-5.5
18124	AT5G07470.1 | Symbol: PMSR3 | peptide methionine sulfoxide reductase (MSR), nearly identical to peptide methionine sulfoxide reductase (msr) (Arabidopsis thaliana) GI:4884033 | chr5:2362571-2364375 REVERSE | Aliases: PEPTIDEMETHIONINE SULFOXIDE REDUCTASE 3, T2I1.180, T2I1_180	9.0	8.8	0.2	0.6
19183	AT5G09760.1 | pectinesterase family protein, contains Pfam profile: PF01095 pectinesterase | chr5:3032376-3034544 FORWARD | Aliases: F17I14.50, F17I14_50	9.0	9.1	-0.1	-0.4

5707	AT2G34750.1 | RNA polymerase I specific transcription initiation factor RRN3 family protein, contains Pfam PF05327: RNA polymerase I specific transcription initiation factor RRN3; similar to RRN3 (GI:7670100) (Homo sapiens) similar to RNA polymerase I specific transcription initiation factor RRN3 (Swiss-Prot:P36070) (Saccharomyces cerevisiae) | chr2:14667387-14671345 REVERSE | Aliases: T29F13.4, T29F13_4	9.0	7.9	1.1	4.2
9866	AT5G11110.1 | similar to sucrose-phosphate synthase, putative [Arabidopsis thaliana] (TAIR:At1g04920.1); similar to sucrose-phosphate synthase, putative [Arabidopsis thaliana] (TAIR:At5g20280.1); similar to sucrose-phosphate synthase, putative [Arabidopsis thaliana] (TAIR:At4g10120.1); similar to sucrose phosphate synthase [Lycopersicon esculentum] (GB:AAU29197.1); similar to sucrose-6-phosphate synthase [Nicotiana tabacum] (GB:AAF06792.1); similar to sucrose-phosphate synthase (EC 2.4.1.14) - spinach (GB:JQ2277); similar to sucrose-phosphate synthase [Solanum tuberosum] (GB:CAA51872.1); similar to sucrose phosphate synthase [Actinidia chinensis] (GB:AAL86360.1); contains InterPro domain Glycosyl transferase, group 1 (InterPro:IPR001296) | chr5:3536227-3541134 FORWARD | Aliases: T5K6.100, T5K6_100	9.0	8.0	1.0	2.4
12118	AT1G28440.1 | leucine-rich repeat transmembrane protein kinase, putative, similar to receptor kinase GI:4105699 from (Arabidopsis thaliana) | chr1:9996810-10000433 FORWARD | Aliases: F3M18.12, F3M18_12	9.0	8.2	0.8	1.8
17805	AT1G50430.1 | Symbol: DWF5 | 7-dehydrocholesterol reductase / 7-DHC reductase / sterol delta-7-reductase (ST7R) / dwarf5 protein (DWF5), identical to SP:Q9LDU6 7-dehydrocholesterol reductase (EC 1.3.1.21) (7-DHC reductase) (Sterol delta-7-reductase) (Dwarf5 protein) {Arabidopsis thaliana} | chr1:18685614-18689307 REVERSE | Aliases: 7RED, DWARF 5, F11F12.21, F11F12_21, LE, LEPIDA, PA, PARVA, ST7R, STEROL DELTA 7 REDUCTASE, STEROL DELTA7 REDUCTASE	9.0	9.1	-0.1	-0.6
9784	AT1G67280.1 | lactoylglutathione lyase, putative / glyoxalase I, putative, similar to putative lactoylglutathione lyase SP:Q39366, GI:2494843 from (Brassica oleracea)	9.0	8.0	1.0	2.4
4378	AT3G48570.1 | protein transport protein SEC61 gamma subunit, putative, similar to Swiss-Prot:Q19967 protein transport protein SEC61 gamma subunit (Caenorhabditis elegans) | chr3:18014898-18015959 REVERSE | Aliases: T8P19.80	9.0	7.1	1.9	5.0
12026	AT3G44100.1 | MD-2-related lipid recognition domain-containing protein / ML domain-containing protein, contains Pfam profile PF02221: ML domain | chr3:15877028-15878291 REVERSE | Aliases: F26G5.50	9.0	8.7	0.3	1.8
2759	AT4G14680.1 | Symbol: APS3 | sulfate adenylyltransferase 3 / ATP-sulfurylase 3 (APS3), identical to ATP sulfurylase (APS3) (Arabidopsis thaliana) GI:1575327 | chr4:8413286-8415349 REVERSE | Aliases: DL3380C, FCAALL.128	9.0	5.6	3.4	6.4
604	AT3G21110.2 | Symbol: PUR7 | similar to SAICAR synthetase [Nicotiana tabacum] (GB:AAR06292.1); contains InterPro domain SAICAR synthetase (InterPro:IPR001636) | chr3:7402141-7405614 REVERSE | Aliases: 5' PHOSPHORIBOSYL 4 (N SUCCINOCARBOXAMIDE) 5 AMINOIMIDAZOLE SYNTHETASE, MSA6.26, PURC, SAIRCAR SYNTHETASE	9.0	5.9	3.1	11.3
2260	AT5G40450.1 | expressed protein | chr5:16202027-16212756 REVERSE | Aliases: MPO12.160, MPO12_160	9.0	11.2	-2.2	-7.0
10481	AT1G07750.1 | cupin family protein, similar to legumin (11S-globulin) from Ginkgo biloba (GI:949869), 11S globulin from Avena sativa (GI:472867) | chr1:2404034-2405939 REVERSE | Aliases: F24B9.13, F24B9_13	9.0	8.2	0.8	2.2
9375	AT3G24010.1 | PHD finger family protein, contains Pfam profile: PF00628 PHD-finger | chr3:8675965-8678227 REVERSE | Aliases: F14O13.20	9.0	8.5	0.5	2.6
1887	AT4G37470.1 | hydrolase, alpha/beta fold family protein, low similarity to SP:Q59093 3-oxoadipate enol-lactonase I (EC 3.1.1.24) (Enol-lactone hydrolase I) (Beta-ketoadipate enol-lactone hydrolase I) {Acinetobacter calcoaceticus}; contains Pfam profile PF00561: hydrolase, alpha/beta fold family | chr4:17616653-17618452 REVERSE | Aliases: F6G17.120, F6G17_120	9.0	10.4	-1.4	-7.6
21005	AT4G30760.2 | expressed protein, contains Pfam profile PF04396: Protein of unknown function, DUF537 | chr4:14982528-14983425 FORWARD | Aliases: T10C21.110, T10C21_110	9.0	9.0	-0.0	-0.0
1235	AT5G39340.1 | Symbol: AHP3 | two-component phosphorelay mediator 2 (HP2), nearly identical to ATHP2 (Arabidopsis thaliana) GI:4156243	9.0	7.6	1.4	8.9
711	AT4G02840.1 | small nuclear ribonucleoprotein D1, putative / snRNP core protein D1, putative / Sm protein D1, putative, similar to small nuclear ribonucleoprotein Sm D1 (snRNP core protein D1, Sm-D1, Sm-D autoantigen) (Mouse) SWISS-PROT:P13641 | chr4:1264627-1266486 FORWARD | Aliases: T5J8.16, T5J8_16	9.0	6.9	2.1	10.7
13978	AT1G51710.1 | Symbol: UBP6 | ubiquitin-specific protease 6, putative (UBP6), similar to GI:11993465 | chr1:19179313-19183631 REVERSE | Aliases: F19C24.8, F19C24_8, UBIQUITIN SPECIFIC PROTEASE 6	9.0	8.4	0.6	1.4
12773	AT2G26280.1 | Symbol: CID7 | smr (Small MutS Related) domain-containing protein, weak similarity to PRLI-interacting factor N (Arabidopsis thaliana) GI:11139276; contains Pfam profile PF01713: Smr domain | chr2:11194811-11198415 REVERSE | Aliases: T1D16.8, T1D16_8	9.0	9.5	-0.5	-1.6
9842	AT5G19860.1 | expressed protein, contains Pfam profile PF04398: Protein of unknown function, DUF538 | chr5:6714266-6715877 REVERSE | Aliases: T29J13.3	9.0	10.2	-1.2	-2.4

5043	AT4G38890.1 | dihydrouridine synthase family protein, contains Pfam domain, PF01207: Dihydrouridine synthase (Dus) | chr4:18135903-18139094 REVERSE | Aliases: F19H22.4	9.0	7.9	1.1	4.5
2440	AT1G66200.2 | Symbol: ATGSR2 |	9.0	12.5	-3.5	-6.8
5419	AT1G12410.1 | Symbol: CLPR2 | ATP-dependent Clp protease proteolytic subunit (ClpP2), identical to nClpP2 GI:5360589 from (Arabidopsis thaliana) | chr1:4223035-4225112 FORWARD | Aliases: CLPP2, F5O11.13, F5O11_13, NCLPP2	9.0	7.4	1.6	4.3
799	AT4G26900.1 | Symbol: AT HF | imidazole glycerol phosphate synthase hisHF, chloroplast / IGP synthase / ImGPP synthase / IGPS, identical to SP:Q9SZ30 Imidazole glycerol phosphate synthase hisHF, chloroplast precursor (IGP synthase) {Arabidopsis thaliana} | chr4:13515488-13520317 FORWARD | Aliases: HISHF	9.0	5.7	3.3	10.3
2536	AT4G15770.1 | 60S ribosome subunit biogenesis protein, putative, contains similarity to 60S ribosome subunit biogenesis protein NIP7 (Swiss-Prot:Q08962) (Saccharomyces cerevisiae) | chr4:8978009-8978877 FORWARD | Aliases: DL3925W, FCAALL.377	9.0	8.2	0.8	6.6
11119	AT5G17770.1 | Symbol: ATCBR | NADH-cytochrome b5 reductase, identical to NADH-cytochrome b5 reductase (Arabidopsis thaliana) GI:4240116 | chr5:5864253-5866650 REVERSE | Aliases: MVA3.120, MVA3_120	9.0	9.7	-0.7	-2.0
6933	AT1G62040.1 | autophagy 8c (APG8c), identical to autophagy 8c (Arabidopsis thaliana) GI:19912155; contains Pfam profile PF02991: Microtubule associated protein 1A/1B, light chain 3 | chr1:22936566-22938088 FORWARD | Aliases: F8K4.23, F8K4_23	9.0	8.2	0.8	3.6
6019	AT5G20060.3 | similar to phospholipase/carboxylesterase family protein [Arabidopsis thaliana] (TAIR:At1g52700.1); similar to biostress-resistance-related protein [Triticum aestivum] (GB:AAM29178.1); contains InterPro domain Phospholipase/Carboxylesterase (InterPro:IPR003140) | chr5:6776277-6779616 FORWARD | Aliases: F28I16.210, F28I16_210	9.0	7.5	1.4	4.0
5833	AT1G53850.1 | Symbol: PAE1 | 20S proteasome alpha subunit E1 (PAE1), identical to 20S proteasome subunit PAE1 GI:3421087 from (Arabidopsis thaliana) | chr1:20107622-20109663 REVERSE | Aliases: 20S PROTEASOME SUBUNIT PAE1, T18A20.8, T18A20_8	9.0	7.7	1.3	4.1
5165	AT3G57090.1 | expressed protein | chr3:21139509-21141167 FORWARD | Aliases: F24I3.170	9.0	7.4	1.6	4.5
158	AT5G05990.1 | mitochondrial glycoprotein family protein / MAM33 family protein, low similarity to SUAPRGA1 (Emericella nidulans) GI:6562379; contains Pfam profile PF02330: Mitochondrial glycoprotein	9.0	4.2	4.7	17.7
280	AT3G16140.1 | photosystem I reaction center subunit VI, chloroplast, putative / PSI-H, putative (PSAH1), identical to SP:Q9SUI7; similar to PSI-H precursor (Nicotiana sylvestris) GI:407353; contains Pfam profile PF03244: Photosystem I reaction centre subunit VI | chr3:5468515-5469482 REVERSE | Aliases: MSL1.18	9.0	4.5	4.5	14.8
447	AT5G23460.1 | expressed protein | chr5:7912772-7913498 FORWARD | Aliases: K19M13.9, K19M13_9	9.0	6.0	2.9	12.4
4572	AT5G05000.3 | Symbol: OEP34 | translocate of chloroplast 34 (TOC34) / GTP-binding protein (OEP34), contains Pfam PF04548: AIG1 family;contains TIGRFAM TIGR00991: GTP-binding protein and TIGR00231: small GTP-binding protein domain; 99.7% identical to atToc34 protein (GI:11557975) (Arabidopsis thaliana); similar to Chain A,	9.0	7.7	1.3	4.8
1151	AT5G44110.2 | Symbol: POP1 | similar to ABC transporter family protein [Arabidopsis thaliana] (TAIR:At1g03905.1); similar to hypothetical protein UM03153.1 [Ustilago maydis 521] (GB:EAK84383.1); contains InterPro domain ABC transporter (InterPro:IPR003439); contains InterPro domain ATP/GTP-binding site motif A (P-loop) (InterPro:IPR001687)	9.0	11.5	-2.5	-9.1
9697	AT1G73990.1 | Symbol: SPPA | peptidase U7 family protein, similar to protease IV GB:AAA57008 from (Escherichia coli); contains Pfam profile PF01343: Peptidase family U7 | chr1:27828094-27832587 FORWARD | Aliases: F2P9.14, F2P9_14, SPPA	9.0	8.5	0.5	2.5
1960	AT5G56360.1 | calmodulin-binding protein, similar to alpha glucosidase II beta subunit from GI:2104691 (Mus musculus) | chr5:22840528-22845223 REVERSE | Aliases: MCD7.9, MCD7_9	9.0	7.0	1.9	7.5
7837	AT1G09770.1 | Symbol: ATCDC5 |	9.0	7.7	1.3	3.2
7350	AT2G39990.1 | Symbol: EIF2 | eukaryotic translation initiation factor 3 subunit 5 / eIF-3 epsilon / eIF3f (TIF3F1), identical to SP:O04202 Eukaryotic translation initiation factor 3 subunit 5 (eIF-3 epsilon) (eIF3 p32 subunit) (eIF3f) {Arabidopsis thaliana}; contains Pfam profile PF01398: Mov34/MPN/PAD-1 family | chr2:16705174-16707090 REVERSE | Aliases: T28M21.15, T28M21_15, TRANSLATION INITIATION FACTOR EIF2 P47 SUBUNIT HOMOLOG	9.0	7.3	1.7	3.4
13204	AT3G42050.1 | vacuolar ATP synthase subunit H family protein, identical to probable vacuolar ATP synthase subunit H (EC 3.6.3.14)(V-ATPase H subunit) (Vacuolar proton pump H subunit) (Vacuolar proton pump subunit SFD) SP:Q9LX65 from (Arabidopsis thaliana); contains Pfam PF03224: V-ATPase subunit H | chr3:14239482-14244216 REVERSE | Aliases: F4M19.10	9.0	9.7	-0.7	-1.5
5195	AT1G19835.1 | expressed protein, contains Pfam PF05911: Plant protein of unknown function (DUF869) | chr1:6855943-6860323 REVERSE | Aliases: F14P1.24, F14P1_24	9.0	9.9	-1.0	-4.5

18693	AT3G03330.1 | short-chain dehydrogenase/reductase (SDR) family protein, contains INTERPRO family IPR002198 Short-chain dehydrogenase/reductase (SDR) superfamily | chr3:783339-786177 REVERSE | Aliases: T21P5.25, T21P5_25	9.0	9.1	-0.1	-0.5
3512	AT2G36930.1 | zinc finger (C2H2 type) family protein, contains Prosite PS00028: Zinc finger, C2H2 type, domain; weak similarity to Zinc finger protein T86 (Swiss-Prot:O00488) (Homo sapiens) | chr2:15516981-15518584 REVERSE | Aliases: T1J8.11, T1J8_11	9.0	7.0	2.0	5.6
817	AT4G19390.1 | expressed protein | chr4:10574779-10576439 REVERSE | Aliases: T5K18.170, T5K18_170	9.0	6.4	2.6	10.2
4153	AT3G49870.1 | Symbol: ATARLA1C | ADP-ribosylation factor, putative, similar to ADP-ribosylation factor-like protein 1 (SP:P40616) (Homo sapiens); ARF3 ADP-RIBOSYLATION FACTOR,GP:453191 Arabidopsis thaliana; contains domain PF00025: ADP-ribosylation factor family | chr3:18503435-18505124 REVERSE | Aliases: ATARLA1C, T16K5.220	9.0	10.1	-1.2	-5.1
7100	AT2G46330.2 | Symbol: AGP16 | arabinogalactan-protein (AGP16), identical to gi:10880509:gb:AAG24284 | chr2:19025581-19026328 REVERSE | Aliases: ARABINOGALACTAN PROTEIN 16, F11C10.2	9.0	10.2	-1.2	-3.5
2243	AT3G16520.3 | UDP-glucoronosyl/UDP-glucosyl transferase family protein, contains Pfam profile: PF00201 UDP-glucoronosyl and UDP-glucosyl transferase | chr3:5619134-5620879 REVERSE | Aliases: MDC8.15	9.0	5.4	3.6	7.0
8535	AT2G31810.3 | acetolactate synthase small subunit, putative, similar to gi:5931761 from Nicotiana plumbaginifolia | chr2:13531299-13535578 FORWARD | Aliases: F20M17.15, F20M17_15	9.0	7.6	1.3	2.9
4274	AT5G54750.1 | transport protein particle (TRAPP) component Bet3, putative, similar to SP:P36149 Transport protein particle 22 kDa subunit (TRAPP 22 kDa subunit) {Saccharomyces cerevisiae}; contains Pfam profile PF04051: Transport protein particle (TRAPP) component, Bet3	9.0	7.3	1.6	5.0
19451	AT5G42520.1 | expressed protein | chr5:17017511-17019835 FORWARD | Aliases: MDH9.22, MDH9_22	9.0	9.0	-0.1	-0.3
11918	AT5G44790.1 | Symbol: RAN1 | copper-exporting ATPase / responsive-to-antagonist 1 / copper-transporting ATPase (RAN1), identical to SP:Q9S7J8	9.0	9.7	-0.7	-1.8
2247	AT4G24280.1 | Symbol: CPHSC70 1 | heat shock protein 70, putative / HSP70, putative, strong similarity to heat shock protein 70 (Arabidopsis thaliana) GI:6746592; similar to heat shock 70 protein - Spinacia oleracea,PID:g2654208 | chr4:12589998-12593640 FORWARD | Aliases: CPHSC70 1, T22A6.110, T22A6_110	9.0	7.3	1.7	7.0
5740	AT4G12720.3 | MutT/nudix family protein, similar to SP:P53370 Nucleoside diphosphate-linked moiety X motif 6 {Homo sapiens}; contains Pfam profile PF00293: NUDIX domain | chr4:7487540-7490073 FORWARD | Aliases: T20K18.70, T20K18_70	9.0	9.5	-0.5	-4.2
6976	AT2G44620.1 | Symbol: mtACP 1 | acyl carrier protein, mitochondrial / ACP / NADH-ubiquinone oxidoreductase 9.6 kDa subunit, identical to SP:P53665 Acyl carrier protein, mitochondrial precursor (ACP) (NADH-ubiquinone oxidoreductase 9.6 kDa subunit) (MtACP-1) {Arabidopsis thaliana}; identical to cDNA acyl carrier protein precursor GI:468265 | chr2:18421359-18422310 FORWARD | Aliases: F16B22.11, F16B22.28, F16B22_28, MITOCHONDRIAL ACYL CARRIER PROTEIN 1	9.0	7.3	1.6	3.5
5201	AT5G04590.1 | Symbol: SIR | sulfite reductase / ferredoxin (SIR), identical to sulfite reductase (Arabidopsis thaliana) GI:804953, GI:2584721	9.0	6.6	2.4	4.5
3895	AT5G54500.1 | Symbol: FQR1 | quinone reductase, putative, similar to 1,4-benzoquinone reductase (Phanerochaete chrysosporium)(GI:4454993); contains flavodoxin domain PF00258	9.0	10.3	-1.3	-5.3
1999	AT1G72340.1 | eukaryotic translation initiation factor 2B family protein / eIF-2B family protein, similar to SP:Q64270 Translation initiation factor eIF-2B alpha subunit {Rattus norvegicus}; contains Pfam profile PF01008: Initiation factor 2 subunit family | chr1:27240543-27242274 FORWARD | Aliases: T10D10.19, T10D10_19	9.0	7.4	1.5	7.4
11894	AT3G61200.1 | thioesterase family protein, contains Pfam profile PF03061: thioesterase family protein | chr3:22668430-22669365 REVERSE | Aliases: T20K12.100	9.0	8.5	0.4	1.8
3235	AT2G27030.3 | Symbol: CAM2 | calmodulin-2/3/5 (CAM5) (TCH1), identical to calmodulin GI:474183 from (Arabidopsis thaliana), SP:P25069 Calmodulin-2/3/5 {Arabidopsis thaliana}	9.0	6.5	2.4	5.9
2677	AT4G33090.1 | Symbol: APM1 | aminopeptidase M, similar to SP:Q11011 Puromycin-sensitive aminopeptidase (EC 3.4.11.-) (PSA) {Mus musculus}; contains Pfam profile PF01433: Peptidase family M1	8.9	10.9	-2.0	-6.5
4691	AT4G27230.1 | histone H2A, putative, strong similarity to histone H2A Arabidopsis thaliana GI:7595337, Triticum aestivum GI:536892, Picea abies SP:P35063; contains Pfam profile PF00125 Core histone H2A/H2B/H3/H4 | chr4:13637345-13638425 REVERSE | Aliases: M4I22.40, M4I22_40	8.9	6.4	2.5	4.8
4849	AT5G62540.1 | ubiquitin-conjugating enzyme 3 (UBC3), E2; identical to gi:431261, SP:P42746 | chr5:25121160-25122845 FORWARD | Aliases: K19B1.15, K19B1_15	8.9	7.3	1.6	4.7

8306	AT3G22890.1 | Symbol: APS1 | sulfate adenylyltransferase 1 / ATP-sulfurylase 1 (APS1), nearly identical to ATP sulfurylase (APS1) (Arabidopsis thaliana) GI:6606509 | chr3:8112730-8114996 FORWARD | Aliases: F5N5.7	8.9	10.4	-1.4	-3.0
6366	AT5G50370.1 | adenylate kinase, putative, similar to adenylate kinase (ATP-AMP transphosphorylase) (Arabidopsis thaliana) SWISS-PROT:O82514 | chr5:20526385-20527926 REVERSE | Aliases: MXI22.8, MXI22_8	8.9	6.4	2.6	3.8
14683	AT4G29160.2 | SNF7 family protein, contains Pfam domain, PF03357: SNF7 family | chr4:14380810-14382672 FORWARD | Aliases: F19B15.190, F19B15_190	8.9	8.7	0.2	1.2
5742	AT3G03070.1 | NADH-ubiquinone oxidoreductase-related, contains weak similarity to NADH-ubiquinone oxidoreductase 13 kDa-A subunit, mitochondrial precursor (EC 1.6.5.3) (EC 1.6.99.3) (Complex I-13KD-A) (CI-13KD-A) (Swiss-Prot:P23934) (Bos taurus) | chr3:696306-698163 REVERSE | Aliases: T17B22.24, T17B22_24	8.9	7.7	1.3	4.2
16106	AT2G29210.1 | splicing factor PWI domain-containing protein, contains Pfam profile PF01480: PWI domain	8.9	8.1	0.8	0.9
1072	AT3G60480.1 | expressed protein | chr3:22358426-22359213 REVERSE | Aliases: T8B10.140	8.9	7.2	1.8	9.3
12814	AT5G07440.1 | Symbol: GDH2 | glutamate dehydrogenase 2 (GDH2), identical to glutamate dehydrogenase 2 (GDH 2) (Arabidopsis thaliana) SWISS-PROT:Q38946 | chr5:2355938-2358195 FORWARD | Aliases: GLUTAMATE DEHYDROGENASE 2, T2I1.150, T2I1_150	8.9	9.4	-0.5	-1.6
1470	AT2G37470.1 | histone H2B, putative, strong similarity to histone H2B from Lycopersicon esculentum GI:3021483, GI:3021485, Capsicum annuum SP:O49118; contains Pfam profile PF00125 Core histone H2A/H2B/H3/H4	8.9	5.4	3.5	8.3
11303	AT3G19170.1 | Zinc metalloprotease pitrilysin subfamily A. Signal peptide degrading enzyme targeted to mitochondria and chloroplasts. Expressed only in siliques and flowers | chr3:6625427-6631946 REVERSE | Aliases: MVI11.6	8.9	7.9	1.0	2.0
3730	AT1G79440.1 | Symbol: ALDH5F1 | succinate-semialdehyde dehydrogenase (SSADH1), similar to succinate-semialdehyde dehydrogenase (NADP+) (SSDH) (Escherichia coli) SWISS-PROT:P25526; identical to succinic semialdehyde dehydrogenase mRNA, nuclear gene encoding mitochondrial protein GI:6684441; contains TIGRfam profile TIGR01780:succinic semialdehyde dehydrogenase; contains Pfam profile PF00171: aldehyde dehydrogenase (NAD) family protein	8.9	6.5	2.5	5.5
4628	AT5G06340.1 | diadenosine 5',5'''-P1,P4-tetraphosphate hydrolase, putative, similar to diadenosine 5',5'''-P1,P4-tetraphosphate hydrolase from (Lupinus angustifolius) GI:1888557, (Hordeum vulgare subsp. vulgare) GI:2564253; contains Pfam profile PF00293: NUDIX domain | chr5:1936407-1938112 FORWARD | Aliases: MHF15.14, MHF15_14	8.9	6.2	2.7	4.8
6906	AT5G15910.1 | dehydrogenase-related, low similarity to SP:Q9R1J0 NAD(P)-dependent steroid dehydrogenase (EC 1.1.1.-) {Mus musculus} | chr5:5193140-5195399 FORWARD | Aliases: F1N13.50, F1N13_50	8.9	8.3	0.6	3.6
13339	ATCG00760.1 | Symbol: RPL36 | encodes a chloroplast ribosomal protein L36, a constituent of the large subunit of the ribosomal complex | chrC:79489-79602 REVERSE | Aliases: RPL36	8.9	7.6	1.3	1.5
15576	AT5G63620.2 | oxidoreductase, zinc-binding dehydrogenase family protein, contains PFAM zinc-binding dehydrogenase domain PF00107 | chr5:25483354-25485619 REVERSE | Aliases: MBK5.9, MBK5_9	8.9	9.2	-0.3	-1.1
162	AT1G11330.1 | S-locus lectin protein kinase family protein, contains Pfam domains, PF00954: S-locus glycoprotein family, PF00069: Protein kinase domain, and PF01453: Lectin (probable mannose binding) | chr1:3810221-3813607 FORWARD | Aliases: T28P6.2, T28P6_2	8.9	3.3	5.6	17.4
9517	AT2G17640.1 | Symbol: AtSerat3;1 | serine O-acetyltransferase, putative (SAT-106), similar to Arabidopsis thaliana serine acetyltransferase GI:905391	8.9	8.3	0.6	2.5
13449	AT2G42790.1 | citrate synthase, glyoxysomal, putative, strong similarity to SP:P49299 Citrate synthase, glyoxysomal precursor {Cucurbita maxima}; contains Pfam profile PF00285: Citrate synthase | chr2:17809933-17813126 REVERSE | Aliases: F7D19.21, F7D19_21	8.9	9.5	-0.6	-1.5
8458	AT3G56880.1 | VQ motif-containing protein, contains PF05678: VQ motif | chr3:21070894-21071944 FORWARD | Aliases: T8M16.210	8.9	8.1	0.8	2.9
8769	AT3G23400.1 | plastid-lipid associated protein PAP / fibrillin family protein, contains Pfam profile PF04755: PAP_fibrillin | chr3:8376454-8378307 REVERSE | Aliases: MLM24.13	8.9	7.6	1.3	2.8
14854	AT3G18430.1 | calcium-binding EF hand family protein, similar to Calcineurin B subunit (Protein phosphatase 2B regulatory subunit) (Calcineurin regulatory subunit) SP:P42322 from (Naegleria gruberi); contains Pfam profile PF00036: EF hand	8.9	8.7	0.2	1.2
1042	AT5G57040.1 | lactoylglutathione lyase family protein / glyoxalase I family protein, contains Pfam PF00903: glyoxalase family protein | chr5:23101131-23102472 REVERSE | Aliases: MHM17.18, MHM17_18	8.9	6.9	2.0	9.4
1965	AT1G77540.1 | expressed protein, similar to GB:AAD41433 | chr1:29142570-29143103 REVERSE | Aliases: T5M16.13, T5M16_13	8.9	6.1	2.8	7.5

4933	AT4G12060.1 | Clp amino terminal domain-containing protein, contains Pfam profile: PF02861 Clp amino terminal domain | chr4:7228001-7229935 REVERSE | Aliases: F16J13.130, F16J13_130	8.9	7.4	1.5	4.6
14941	NA	8.9	9.4	-0.5	-1.2
6260	AT3G11400.1 | Symbol: EIF3G1 |	8.9	7.3	1.6	3.9
7871	AT4G09720.2 | Ras-related GTP-binding protein, putative, similar to GTP-binding protein RAB7A from (Lotus japonicus) | chr4:6133293-6135180 FORWARD | Aliases: F17A8.70, F17A8_70	8.9	8.4	0.5	3.2
11462	AT4G09830.1 | expressed protein | chr4:6188764-6190850 FORWARD | Aliases: F17A8.180, F17A8_180	8.9	8.4	0.5	2.0
6402	AT5G55200.1 | co-chaperone grpE protein, putative, similar to chaperone GrpE type 2 (Nicotiana tabacum) GI:3851640; contains Pfam profile PF01025: co-chaperone GrpE | chr5:22411860-22413777 FORWARD | Aliases: MCO15.15, MCO15_15	8.9	7.8	1.1	3.8
18583	AT2G02160.1 | zinc finger (CCCH-type) family protein, contains Pfam domain, PF00642: Zinc finger C-x8-C-x5-C-x3-H type (and similar) | chr2:553109-556082 REVERSE | Aliases: F5O4.7, F5O4_7	8.9	8.5	0.4	0.5
12936	AT2G04520.1 | eukaryotic translation initiation factor 1A, putative / eIF-1A, putative / eIF-4C, putative, strong similarity to translation initiation factor (eIF-1A) (Beta vulgaris) GI:17977975; contains Pfam profile PF01176: Eukaryotic initiation factor 1A | chr2:1574589-1575856 REVERSE | Aliases: T1O3.7, T1O3_7	8.9	8.4	0.5	1.6
1769	AT3G63390.1 | expressed protein | chr3:23422141-23422943 REVERSE | Aliases: MAA21.20	8.9	7.1	1.8	7.8
3243	AT1G52380.1 | Ran-binding protein 1 domain-containing protein / RanBP1 domain-containing protein, weak similarity to SP:Q09717 Ran-specific GTPase-activating protein 1 (Ran binding protein 1) (RANBP1) (Spi1-binding protein) {Schizosaccharomyces pombe}; contains Pfam profile PF00638: RanBP1 domain | chr1:19512654-19515207 FORWARD | Aliases: F19K6.4, F19K6_4	8.9	7.6	1.3	5.9
2625	AT2G19740.1 | 60S ribosomal protein L31 (RPL31A) | chr2:8520599-8521601 FORWARD | Aliases: F6F22.23, F6F22_23	8.9	6.1	2.8	6.6
4196	AT3G12650.1 | expressed protein | chr3:4017837-4018638 REVERSE | Aliases: T2E22.4	8.9	8.0	0.9	5.1
3431	AT5G62630.1 | expressed protein | chr5:25160821-25163685 REVERSE | Aliases: MRG21.5, MRG21_5	8.9	7.1	1.8	5.7
4382	AT1G14720.1 | Symbol: XTR2 | xyloglucan:xyloglucosyl transferase / xyloglucan endotransglycosylase / endo-xyloglucan transferase (XTR2), identical to endoxyloglucan transferase (Arabidopsis thaliana) GI:5533311 | chr1:5066631-5068629 REVERSE | Aliases: ENDOXYLOGLUCAN TRANSFERASE A2, EXGT A2, F10B6.12, F10B6_12, XYLOGLUCAN ENDOTRANSGLYCOSYLASE 2, XYLOGLUCAN ENDOTRANSGLYCOSYLASE RELATED 2, XYLOGLUCAN ENDOTRANSGLYCOSYLASE RELATED PROTEIN	8.9	7.7	1.2	5.0
11424	AT5G52820.1 | WD-40 repeat family protein / notchless protein, putative, similar to notchless (Xenopus laevis) GI:3687833; contains Pfam PF00400: WD domain, G-beta repeat (8 copies) | chr5:21418582-21421579 FORWARD | Aliases: MXC20.4, MXC20_4	8.9	8.3	0.6	2.0
5769	AT5G04910.1 | expressed protein, ; expression supported by MPSS | chr5:1437696-1438771 FORWARD | Aliases: MUG13.24, MUG13_24	8.9	7.9	1.0	4.2
8548	AT1G63290.1 | ribulose-phosphate 3-epimerase, cytosolic, putative / pentose-5-phosphate 3-epimerase, putative, strong similarity to D-ribulose-5-phosphate 3-epimerase (Oryza sativa) GI:6007803; contains Pfam profile PF00834: Ribulose-phosphate 3 epimerase family	8.9	8.0	0.9	2.9
4690	AT3G07880.1 | Rho GDP-dissociation inhibitor family protein, similar to SP:P52565 Rho GDP-dissociation inhibitor 1 (Rho GDI 1) (Rho-GDI alpha) {Homo sapiens}; contains Pfam profile PF02115: RHO protein GDP dissociation inhibitor | chr3:2513977-2515828 FORWARD | Aliases: F17A17.22	8.9	7.4	1.5	4.8
1202	AT5G09770.1 | ribosomal protein L17 family protein, contains Pfam profile: PF01196 ribosomal protein L17 | chr5:3035047-3036543 REVERSE | Aliases: F17I14.40, F17I14_40	8.9	6.1	2.8	9.0
9721	AT3G28730.1 | Symbol: ATHMG | structure-specific recognition protein 1 / high mobility group protein / HMG protein, nearly identical to SP:Q05153 Structure-specific recognition protein 1 homolog (HMG protein) {Arabidopsis thaliana}; contains Pfam profile PF00505: HMG (high mobility group) box; contains Pfam profile PF03531: Structure-specific recognition protein | chr3:10786126-10790386 FORWARD | Aliases: HIGH MOBILITY GROUP, T19N8.3	8.9	9.2	-0.3	-2.5
14105	AT4G12590.1 | expressed protein, contains Pfam PF05863: Eukaryotic protein of unknown function (DUF850) | chr4:7451001-7453085 REVERSE | Aliases: T1P17.180, T1P17_180	8.9	7.5	1.4	1.4
20265	AT1G74230.1 | glycine-rich RNA-binding protein, similar to RNA-binding protein GB:S46286 from (Nicotiana sylvestris) | chr1:27918367-27920744 FORWARD | Aliases: F1O17.10, F1O17_10	8.9	8.9	-0.0	-0.2

2935	AT4G26300.1 | Symbol: EMB1027 | arginyl-tRNA synthetase, putative / arginine--tRNA ligase, putative, similar to SP:P37880 Arginyl-tRNA synthetase (EC 6.1.1.19) (Arginine--tRNA ligase) (ArgRS) {Cricetulus longicaudatus}; contains Pfam profiles PF00750: arginyl-tRNA synthetase, PF03485: arginyl-tRNA synthetase N-terminal domain | chr4:13307986-13313118 REVERSE | Aliases: EMB1027, EMBRYO DEFECTIVE 1027, T25K17.110, T25K17_110	8.9	6.8	2.0	6.2
14535	AT5G45410.2 | expressed protein, similar to expressed protein [Arabidopsis thaliana] (TAIR:At4g25030.1); similar to expressed protein [Arabidopsis thaliana] (TAIR:At4g25030.2); similar to unknown protein [Oryza sativa (japonica cultivar-group)] (GB:XP_476693.1) | chr5:18419135-18421010 REVERSE | Aliases: MFC19.8, MFC19_8	8.9	7.8	1.1	1.3
11279	AT5G19690.1 | Symbol: STT3A | oligosaccharyl transferase STT3 subunit family protein, similar to SP:P39007 Oligosaccharyl transferase STT3 subunit {Saccharomyces cerevisiae}; contains Pfam profile PF02516: Oligosaccharyl transferase STT3 subunit	8.9	8.3	0.5	2.0
806	AT5G64816.2 | expressed protein | chr5:25930226-25931229 FORWARD | Aliases: None	8.9	6.3	2.6	10.2
4614	AT1G28580.2 | GDSL-motif lipase, putative, similar to lipase (Arabidopsis thaliana) GI:1145627; contains InterPro Entry IPR001087 Lipolytic enzyme, G-D-S-L family | chr1:10044323-10046413 REVERSE | Aliases: F1K23.18, F1K23_18	8.9	10.1	-1.2	-4.8
14318	AT4G01610.2 | cathepsin B-like cysteine protease, putative, similar to cathepsin B-like cysteine proteinase GI:609175 from (Nicotiana rustica); contains an unusually short, 5nt exon | chr4:694695-697126 FORWARD | Aliases: T15B16.17, T15B16_17	8.9	9.8	-0.9	-1.3
15955	AT4G08850.2 | leucine-rich repeat family protein / protein kinase family protein, contains Pfam domains PF00560: Leucine Rich Repeat and PF00069: Protein kinase domain | chr4:5637335-5640633 REVERSE | Aliases: T32A17.160, T32A17_160	8.9	9.1	-0.2	-1.0
3974	AT5G19350.1 | RNA-binding protein 45 (RBP45), putative	8.9	7.9	1.0	5.3
13545	AT1G65720.1 | expressed protein | chr1:24443922-24444736 REVERSE | Aliases: F1E22.9, F1E22_9	8.9	8.2	0.7	1.5
20945	AT1G15920.2 | CCR4-NOT transcription complex protein, putative, similar to SWISS-PROT:Q60809 CCR4-NOT transcription complex, subunit 7 (CCR4-associated factor 1, (CAF1) (Mus musculus) | chr1:5469397-5471143 FORWARD | Aliases: T24D18.2, T24D18_2	8.9	8.9	-0.0	-0.0
6568	AT1G29990.1 | prefoldin, putative, similar to Swiss-Prot:O15212 prefoldin subunit 6 (Protein Ke2) (Homo sapiens) | chr1:10507577-10509029 FORWARD | Aliases: T1P2.3, T1P2_3	8.9	7.2	1.6	3.7
7607	AT3G06035.1 | expressed protein | chr3:1823105-1824188 REVERSE | Aliases: None	8.9	8.1	0.8	3.3
1920	AT5G61310.3 | similar to cytochrome c oxidase subunit Vc family protein / COX5C family protein [Arabidopsis thaliana] (TAIR:At2g47380.1); similar to cytochrome c oxidase subunit 5c [Helianthus annuus] (GB:AAL67939.1); contains InterPro domain Cytochrome c oxidase subunit Vc (InterPro:IPR008432) | chr5:24670635-24672079 REVERSE | Aliases: MFB13.16, MFB13_16	8.9	6.8	2.1	7.5
9356	AT5G24760.2 | alcohol dehydrogenase, putative, similar to alcohol dehydrogenase from Solanum tuberosum (SP:p14673); contains Pfam zinc-binding dehydrogenase domain PF00107 | chr5:8494795-8497303 REVERSE | Aliases: T4C12.30	8.9	8.1	0.8	2.6
9890	AT5G62350.1 | invertase/pectin methylesterase inhibitor family protein / DC 1.2 homolog (FL5-2I22), similar to SP:Q42534 Pectinesterase 2 precursor (EC 3.1.1.11) (Pectin methylesterase 2) (PE 2) {Arabidopsis thaliana}; contains Pfam profile PF04043: Plant invertase/pectin methylesterase inhibitor; FL5-2I22 mRNA for DC 1.2 homolog, partial cds GI:11127598 | chr5:25054652-25055588 FORWARD | Aliases: MMI9.21, MMI9_21	8.9	8.3	0.6	2.4
440	AT1G67820.1 | protein phosphatase 2C, putative / PP2C, putative, similar to protein phosphatase 2C emb:CAA72341.1 | chr1:25433516-25435725 FORWARD | Aliases: F12A21.5, F12A21_5	8.9	4.9	3.9	12.5
5921	AT1G21570.1 | zinc finger (CCCH-type) family protein, contains Pfam domain, PF00642: Zinc finger C-x8-C-x5-C-x3-H type (and similar) | chr1:7557251-7560272 REVERSE | Aliases: F24J8.21, F24J8_21	8.9	10.4	-1.5	-4.1
9095	AT5G56170.1 | expressed protein, contains similarity to GPI-anchored protein | chr5:22753195-22754512 FORWARD | Aliases: MDA7.23, MDA7_23	8.9	9.5	-0.6	-2.7
6386	AT3G51310.1 | vacuolar protein sorting-associated protein 35 family protein / VPS35 family protein, similar to vacuolar protein sorting 35 (Mus musculus) GI:11875394; contains Pfam profile PF03635: Vacuolar protein sorting-associated protein 35 | chr3:19055502-19060300 REVERSE | Aliases: F24M12.350	8.9	8.1	0.8	3.8
2743	AT1G09130.2 | similar to ATP-dependent Clp protease proteolytic subunit (ClpR1) (nClpP5) [Arabidopsis thaliana] (TAIR:At1g49970.1); similar to COG0740: Protease subunit of ATP-dependent Clp proteases [Nostoc punctiforme PCC 73102] (GB:ZP_00108611.1); contains InterPro domain Clp protease (InterPro:IPR001907) | chr1:2939928-2942269 REVERSE | Aliases: F7G19.1, F7G19_1	8.9	5.6	3.3	6.4
40	AT3G63140.1 | mRNA-binding protein, putative, similar to mRNA binding protein precursor (GI:26453355) (Lycopersicon esculentum) | chr3:23337347-23339684 REVERSE | Aliases: T20O10.240	8.9	2.5	6.4	27.1

1815	AT5G59480.2 | haloacid dehalogenase-like hydrolase family protein, low similarity to SP:P53078 SSM1 protein {Saccharomyces cerevisiae}; contains InterPro accession IPR005834: Haloacid dehalogenase-like hydrolase | chr5:23996649-23998793 REVERSE | Aliases: F2O15.10, F2O15_10	8.9	4.3	4.6	7.7
17129	ATCG00660.1 | Symbol: RPL20 | encodes a chloroplast ribosomal protein L20, a constituent of the large subunit of the ribosomal complex | chrC:68512-68865 REVERSE | Aliases: RPL20	8.9	7.9	1.0	0.8
6641	AT5G56290.1 | Symbol: PEX5 | peroxisomal targeting signal type 1 receptor (PEX5), identical to GI:3603353; contains Pfam profile PF00515 TPR Domain | chr5:22803755-22809345 FORWARD | Aliases: MXK23.3, MXK23_3	8.9	7.5	1.3	3.7
536	AT4G19710.2 | bifunctional aspartate kinase/homoserine dehydrogenase, putative / AK-HSDH, putative, similar to gb:X71364 (PIR:S46497) aspartate kinase / homoserine dehydrogenase from Arabidopsis thaliana | chr4:10724865-10729681 FORWARD | Aliases: T16H5.70, T16H5_70	8.9	4.3	4.5	11.7
1230	AT5G17710.2 | Symbol: EMB1241 | co-chaperone grpE family protein, similar to co-chaperone CGE1 precursor isoform a (Chlamydomonas reinhardtii) GI:15384277; contains Pfam profile PF01025: co-chaperone GrpE | chr5:5839345-5841707 REVERSE | Aliases: EMB1241, EMBRYO DEFECTIVE 1241, MVA3.60, MVA3_60	8.9	5.4	3.5	8.9
15095	AT5G44340.1 | Symbol: TUB4 | tubulin beta-4 chain (TUB4), nearly identical to SP:P24636 Tubulin beta-4 chain {Arabidopsis thaliana} | chr5:17876422-17878328 REVERSE | Aliases: K9L2.12, K9L2_12	8.9	9.4	-0.6	-1.1
14372	AT5G48340.1 | expressed protein | chr5:19607765-19610115 FORWARD | Aliases: K23F3.6, K23F3_6	8.9	8.2	0.6	1.3
5535	AT1G74840.1 | myb family transcription factor, similar to myb-related transcription activator GI:9279717 from (Arabidopsis thaliana) | chr1:28119558-28121066 REVERSE | Aliases: F25A4.19, F25A4_19	8.9	10.2	-1.3	-4.3
3981	AT1G72550.2 | tRNA synthetase beta subunit family protein, contains Pfam profiles: PF03484 phenylalanine-tRNA synthetase, B5 domain, PF03483 B3/4 domain; an isoform contains a non-consensus TG acceptor splice site at a terminal exon. | chr1:27323413-27327677 REVERSE | Aliases: F28P22.26, F28P22_26	8.9	7.0	1.9	5.3
763	AT1G78630.1 | Symbol: EMB1473 | ribosomal protein L13 family protein, similar to ribosomal protein L13 GI:170132 from (Spinacia oleracea) | chr1:29580849-29582551 FORWARD | Aliases: EMB1473, EMBRYO DEFECTIVE 1473, T30F21.4, T30F21_4	8.9	4.1	4.7	10.5
7148	AT1G54680.1 | expressed protein | chr1:20416721-20418149 FORWARD | Aliases: T22H22.11, T22H22_11	8.9	7.7	1.2	3.5
445	AT1G30760.1 | FAD-binding domain-containing protein, similar to SP:P30986 reticuline oxidase precursor (Berberine-bridge-forming enzyme) (BBE) (Tetrahydroprotoberberine synthase) (Eschscholzia californica); contains PF01565 FAD binding domain | chr1:10918260-10920659 FORWARD | Aliases: T17H7.1	8.9	2.7	6.2	12.4
2266	AT4G19860.1 | lecithin:cholesterol acyltransferase family protein / LACT family protein, similar to lysosomal phospholipase A2 (Mus musculus) GI:18699602; contains Pfam profile PF02450: Lecithin:cholesterol acyltransferase (phosphatidylcholine-sterol acyltransferase) | chr4:10777522-10780373 REVERSE | Aliases: T16H5.220, T16H5_220	8.9	11.4	-2.5	-7.0
17656	AT5G40490.1 | RNA recognition motif (RRM)-containing protein, ribonucleoprotein, Xenopus laevis, PIR:S40778; contains InterPro entry IPR000504: RNA-binding region RNP-1 (RNA recognition motif) (RRM)	8.9	8.6	0.2	0.6
6082	AT3G22845.1 | emp24/gp25L/p24 protein-related, contains weak similarity to transmembrane protein (GI:1212965) (Homo sapiens) | chr3:8087314-8088754 FORWARD | Aliases: F5N5.1	8.9	7.7	1.2	4.0
7739	AT2G46505.1 | Symbol: SDH4 | expressed protein | chr2:19096801-19097621 REVERSE | Aliases: None	8.9	7.2	1.6	3.2
12961	AT3G62560.1 | GTP-binding protein, putative, similar to GTP-binding protein SAR1A (SP:O04834) (Arabidopsis thaliana); small GTP-binding protein Bsar1a - Brassica campestris, EMBL:U55035 | chr3:23148459-23150021 FORWARD | Aliases: T12C14.260	8.9	8.3	0.6	1.6
2939	AT3G48730.1 | glutamate-1-semialdehyde 2,1-aminomutase 2 (GSA 2) / glutamate-1-semialdehyde aminotransferase 2 (GSA-AT 2), identical to GSA2 (SP:Q42522) | chr3:18060608-18062687 FORWARD | Aliases: T8P19.240	8.9	5.2	3.6	6.2
12836	AT2G24500.1 | Symbol: FZF | zinc finger (C2H2 type) family protein, contains Pfam profile: PF00096 zinc finger, C2H2 type | chr2:10408115-10409948 REVERSE | Aliases: C2H2 ZINC FINGER PROTEIN FZF, T28I24.23, T28I24_23	8.9	8.6	0.3	1.6
14921	AT5G07350.1 | tudor domain-containing protein / nuclease family protein, contains Pfam domains PF00567: Tudor domain and PF00565: Staphylococcal nuclease homologue | chr5:2320082-2325420 REVERSE | Aliases: T2I1.60, T2I1_60	8.8	9.1	-0.3	-1.2
2738	AT3G02190.1 | 60S ribosomal protein L39 (RPL39B), similar to ribosomal protein L39 GB:P51424 (Arabidopsis thaliana) | chr3:405823-406408 REVERSE | Aliases: F1C9.36	8.8	7.3	1.6	6.4

7925	AT3G60240.1 | Symbol: EIF4G | MIF4G domain-containing protein / MA3 domain-containing protein, similar to eukaryotic protein synthesis initiation factor (Homo sapiens) GI:3941724; contains Pfam profiles PF02854: MIF4G domain, PF02847: MA3 domain	8.8	8.1	0.8	3.2
8989	AT5G08535.2 | D111/G-patch domain-containing protein, contains Pfam profile PF01585: G-patch domain; contains a 2nt micro-exon, as identified using GeneSeqer.	8.8	9.4	-0.5	-2.7
2813	AT3G20670.1 | histone H2A, putative, strong similarity to histone H2A GB:AAF64418 GI:7595337 from Arabidopsis thaliana, Triticum aestivum GI:536892; contains Pfam profile PF00125 Core histone H2A/H2B/H3/H4 | chr3:7229417-7230508 FORWARD | Aliases: F3H11.6	8.8	5.9	3.0	6.3
11886	AT1G50380.1 | prolyl oligopeptidase family protein, similar to oligopeptidase B (Leishmania major) GI:4581757; contains Pfam profiles PF00326: prolyl oligopeptidase family, PF02897: Prolyl oligopeptidase, N-terminal beta-propeller domain | chr1:18665977-18670146 FORWARD | Aliases: F14I3.4, F14I3_4	8.8	8.2	0.7	1.8
811	AT1G08580.1 | expressed protein | chr1:2715086-2716387 REVERSE | Aliases: F22O13.6, F22O13_6	8.8	5.9	3.0	10.2
1816	AT4G28830.1 | expressed protein | chr4:14232452-14234005 FORWARD | Aliases: F16A16.60, F16A16_60	8.8	6.5	2.4	7.7
14680	AT2G30930.1 | expressed protein | chr2:13169517-13170473 FORWARD | Aliases: F7F1.14, F7F1_14	8.8	8.4	0.4	1.2
5311	AT1G20540.1 | transducin family protein / WD-40 repeat family protein, contains 6 WD-40 repeats (PF00400); similar to Rbap46 polypeptide (GI:9454362) (Gallus gallus) | chr1:7112611-7115810 FORWARD | Aliases: F5M15.14, F5M15_14	8.8	7.5	1.3	4.4
4040	AT1G21065.1 | expressed protein | chr1:7374199-7375778 FORWARD | Aliases: None	8.8	7.5	1.4	5.2
10103	AT4G24380.2 | expressed protein, similar to expressed protein [Arabidopsis thaliana] (TAIR:At5g65400.1); similar to unknown protein [Oryza sativa (japonica cultivar-group)] (GB:XP_479601.1) | chr4:12612545-12614027 FORWARD | Aliases: T22A6.210, T22A6_210	8.8	8.0	0.8	2.3
20671	AT4G23630.1 | reticulon family protein (RTNLB1), weak similarity to Nogo-C protein (Rattus norvegicus) GI:6822251; contains Pfam profile PF02453: Reticulon | chr4:12317834-12319947 FORWARD | Aliases: F9D16.100, F9D16_100	8.8	8.9	-0.1	-0.1
2858	AT2G05630.1 | Symbol: atg8d | autophagy 8d (APG8d), identical to autophagy 8d (Arabidopsis thaliana) GI:19912157; contains Pfam profile PF02991: Microtubule associated protein 1A/1B, light chain 3 | chr2:2082983-2084879 REVERSE | Aliases: T20G20.2, T20G20_2	8.8	7.2	1.6	6.3
3951	AT1G53290.1 | galactosyltransferase family protein, contains Pfam profile: PF01762 galactosyltransferase ;contains similarity to Avr9 elicitor response protein GI:4138265 from (Nicotiana tabacum) | chr1:19874967-19877158 FORWARD | Aliases: F12M16.19, F12M16_19	8.8	6.8	2.0	5.3
18563	AT2G37110.1 | expressed protein, contains Pfam profile PF04749: Protein of unknown function, DUF614 | chr2:15599636-15601072 FORWARD | Aliases: T2N18.13, T2N18_13	8.8	9.0	-0.1	-0.5
3975	AT1G66670.1 | Symbol: CLPP3 | ATP-dependent Clp protease proteolytic subunit (ClpP3), identical to ATP-dependent Clp protease (nClpP3) GI:5360591 (Arabidopsis thaliana) | chr1:24867448-24869363 REVERSE | Aliases: F4N21.19, F4N21_19, NCLPP3, NCLPP4	8.8	7.2	1.7	5.3
1248	AT3G06790.2 | plastid developmental protein DAG, putative, similar to DAG protein, chloroplast precursor (Garden snapdragon) SWISS-PROT:Q38732	8.8	5.5	3.3	8.8
17351	AT4G24820.2 | 26S proteasome regulatory subunit, putative (RPN7), contains similarity to ubiquitin activating enzyme GI:3647283 from (Lycopersicon esculentum)	8.8	8.1	0.7	0.7
4760	AT5G22290.1 | Symbol: ANAC089 | no apical meristem (NAM) family protein, contains Pfam PF02365: No apical meristem (NAM) domain | chr5:7375926-7377626 REVERSE | Aliases: ANAC089, T6G21.9	8.8	6.9	1.9	4.7
19575	AT2G33150.1 | Symbol: PED1 | acetyl-CoA C-acyltransferase, putative / 3-ketoacyl-CoA thiolase, putative, similar to 3-ketoacyl-CoA thiolase (E.C. 2.3.1.16) from (Arabidopsis thaliana) GI:2981616, (Cucumis sativus) GI:393707, (Cucurbita cv. Kurokawa Amakuri) GI:1694621; contains InterPro accession IPR002155: Thiolase	8.8	8.6	0.2	0.3
342	AT1G07070.1 | 60S ribosomal protein L35a (RPL35aA), similar to ribosomal protein L35a GI:57118 from (Rattus norvegicus) | chr1:2168563-2169850 FORWARD | Aliases: F10K1.22, F10K1_22	8.8	4.6	4.2	13.7
11825	AT5G18420.3 | expressed protein, non-consensus GC donor splice site at exon 1, unknown (C40) protein, Homo sapiens, EMBL:AF103798 | chr5:6105524-6109380 REVERSE | Aliases: F20L16.140, F20L16_140	8.8	8.3	0.6	1.9
839	AT3G52420.1 | outer envelope membrane protein, putative, similar to outer envelope membrane protein gi:703236:gb:AAA63414 | chr3:19440753-19440947 FORWARD | Aliases: F22O6.200	8.8	6.6	2.2	10.1
19413	AT5G03630.1 | monodehydroascorbate reductase, putative, monodehydroascorbate reductase (NADH), cucumber, PIR:JU0182 | chr5:922165-924676 REVERSE | Aliases: F17C15.50, F17C15_50	8.8	9.1	-0.3	-0.3

9994	AT2G21620.2 | Symbol: RD2 | universal stress protein (USP) family protein / responsive to dessication protein (RD2), strong similarity to RD2 protein (Arabidopsis thaliana) GI:15320408; contains Pfam profile PF00582: universal stress protein family; identical to cDNA RD2 GI:15320407 | chr2:9255678-9257319 FORWARD | Aliases: F2G1.11, F2G1_11	8.8	9.9	-1.1	-2.4
8175	AT3G25860.1 | Symbol: LTA2 | dihydrolipoamide S-acetyltransferase (LTA2), identical to dihydrolipoamide S-acetyltransferase (LTA2) (Arabidopsis thaliana) GI:5881963 | chr3:9461797-9464024 FORWARD | Aliases: DIHYDROLIPOAMIDE S ACETYLTRANSFERASE, MPE11.6, PLASTID E2 SUBUNIT OF PYRUVATE DECARBOXYLASE, PLE2	8.8	6.9	2.0	3.0
4586	AT1G41830.1 | Symbol: SKS6 | multi-copper oxidase type I family protein, similar to pollen-specific BP10 protein (SP:Q00624)(Brassica napus); contains Pfam profile: PF00394 Multicopper oxidase | chr1:15606058-15610302 REVERSE | Aliases: F5A13.5, F5A13_5, SKS6	8.8	6.7	2.1	4.8
1629	AT1G04870.2 | protein arginine N-methyltransferase family protein, similar to SP:Q96LA8 Protein arginine N-methyltransferase 6 (EC 2.1.1.-) {Homo sapiens} | chr1:1373233-1375741 REVERSE | Aliases: F13M7.14, F13M7_14	8.8	5.9	2.9	8.0
12759	AT2G24830.1 | zinc finger (CCCH-type) family protein / D111/G-patch domain-containing protein, contains Pfam profiles PF01585: G-patch domain, PF00642: Zinc finger C-x8-C-x5-C-x3-H type (and similar) | chr2:10583408-10586309 REVERSE | Aliases: F27C12.25, F27C12_25	8.8	8.3	0.5	1.6
10212	AT5G11560.1 | PQQ enzyme repeat-containing protein, contains Pfam profile PF01011: PQQ enzyme repeat | chr5:3709482-3714042 REVERSE | Aliases: F15N18.150, F15N18_150	8.8	9.6	-0.8	-2.3
176	AT3G15850.1 | Symbol: FAD5 | fatty acid desaturase family protein, similar to delta 9 acyl-lipid desaturase (ADS1) GI:2970034 from (Arabidopsis thaliana) | chr3:5359016-5361192 FORWARD | Aliases: ADS3, FADB, FATTY ACID DESATURASE 5, FATTY ACID DESATURASE B, JB67, MSJ11.25	8.8	3.6	5.2	17.0
832	AT5G24300.1 | starch synthase, putative, similar to starch synthase I-1 GI:9369334 from (Triticum aestivum) | chr5:8266802-8271199 FORWARD | Aliases: MOP9.12, MOP9_12	8.8	4.8	4.0	10.1
13726	AT5G05210.1 | nucleolar matrix protein-related, contains Pfam domain, PF04935: Surfeit locus protein 6	8.8	8.2	0.6	1.4
758	AT1G42970.1 | Symbol: GAPB | glyceraldehyde-3-phosphate dehydrogenase B, chloroplast (GAPB) / NADP-dependent glyceraldehydephosphate dehydrogenase subunit B, identical to SP:P25857 Glyceraldehyde 3-phosphate dehydrogenase B, chloroplast precursor (EC 1.2.1.13) (NADP-dependent glyceraldehydephosphate dehydrogenase subunit B) {Arabidopsis thaliana} | chr1:16129874-16132283 FORWARD | Aliases: F13A11.3, F13A11_3, GLYCERALDEHYDE 3 PHOSPHATE DEHYDROGENASE B SUBUNIT	8.8	3.5	5.3	10.5
7467	AT3G50210.3 | similar to 2-oxoacid-dependent oxidase, putative (DIN11) [Arabidopsis thaliana] (TAIR:At3g49620.1); similar to putative 2-oxoacid-dependent oxidase [Oryza sativa (japonica cultivar-group)] (GB:XP_450237.1); contains InterPro domain Isopenicillin N synthase (InterPro:IPR002283); contains InterPro domain 2OG-Fe(II) oxygenase superfamily (InterPro:IPR005123) | chr3:18625154-18627417 REVERSE | Aliases: F11C1.50	8.8	9.5	-0.7	-3.3
19678	AT1G50300.1 | zinc finger (Ran-binding) family protein / RNA recognition motif (RRM)-containing protein, similar to SP:Q27294 RNA-binding protein cabeza {Drosophila melanogaster}; contains Pfam profiles: PF00076 RNA recognition motif (aka RRM, RBD, or RNP domain), PF00641: Zn-finger in Ran binding protein and others | chr1:18632356-18635610 REVERSE | Aliases: F14I3.10, F14I3_10	8.8	9.0	-0.2	-0.3
19801	AT1G59610.1 | Symbol: ADL3 | dynamin-like protein, putative (ADL3), strong similarity to dynamin-like protein 6 (ADL6) (Arabidopsis thaliana) GI:6651399; contains Pfam profiles PF01031: Dynamin central region, PF00350: Dynamin family, PF02212: Dynamin GTPase effector domain, PF00169: PH domain; identical to cDNA dynamin-like protein ADL3, GI:4803835 | chr1:21896886-21904750 FORWARD | Aliases: CF1, DYNAMIN LIKE 3, T30E16.17, T30E16_17	8.8	8.9	-0.1	-0.2
12062	AT1G30120.1 | Symbol: PDH E1 BETA | pyruvate dehydrogenase E1 component beta subunit, chloroplast, identical to pyruvate dehydrogenase E1 beta subunit (Arabidopsis thaliana) GI:2454184; identical to cDNA pyruvate dehydrogenase E1 beta subunit mRNA, nuclear gene encoding plastid protein GI:2454183 | chr1:10584144-10586615 REVERSE | Aliases: PYRUVATE DEHYDROGENASE E1 BETA	8.8	8.3	0.5	1.8
1100	AT1G20110.1 | zinc finger (FYVE type) family protein, contains Pfam profile: PF01363 FYVE zinc finger | chr1:6971476-6974812 FORWARD | Aliases: T20H2.10, T20H2_10	8.8	10.7	-1.9	-9.3
335	AT1G67730.1 | b-keto acyl reductase, putative (GLOSSY8), similar to b-keto acyl reductase GI:2586127 from (Hordeum vulgare) | chr1:25395123-25397273 FORWARD | Aliases: F12A21.14, F12A21_14	8.8	11.5	-2.7	-13.8
309	AT5G55280.1 | Symbol: FTSZ1 1 | cell division protein FtsZ, chloroplast, putative (FTSZ), identical to SP:Q42545 Cell division protein ftsZ homolog, chloroplast precursor {Arabidopsis thaliana}; similar to FtsZ1 (Tagetes erecta) GI:8896066; contains Pfam profiles PF00091: Tubulin/FtsZ family, GTPase domain, PF03953: Tubulin/FtsZ family, C-terminal domain | chr5:22437882-22439838 REVERSE | Aliases: ATFTSZ1 1, CPFTSZ, MCO15.23, MCO15_23	8.8	4.5	4.3	14.2
15823	AT5G52240.1 | Symbol: MSBP1 | cytochrome b5 domain-containing protein, similar to SP:P70580 Membrane associated progesterone receptor component 1 {Rattus norvegicus}; contains Pfam profile PF00173: Heme/Steroid binding domain | chr5:21230286-21231965 FORWARD | Aliases: ATMP1, F17P19.14, F17P19_14	8.8	8.4	0.4	1.0
12511	AT5G55400.1 | fimbrin-like protein, putative, similar to fimbrin-like protein (ATFIM1) (Arabidopsis thaliana) GI:2905893; contains Pfam profile PF00307: Calponin homology (CH) domain	8.8	7.7	1.1	1.7

16606	AT4G11220.1 | reticulon family protein (RTNLB2), similar to SP:Q64548 Reticulon 1 (Neuroendocrine-specific protein) {Rattus norvegicus}; contains Pfam profile PF02453: Reticulon | chr4:6837946-6839791 REVERSE | Aliases: F8L21.10, F8L21_10	8.8	9.1	-0.3	-0.8
1438	AT5G44710.1 | expressed protein, similar to unknown protein (ref:NP_011731.1) | chr5:18058153-18059787 FORWARD | Aliases: K23L20.5, K23L20_5	8.8	5.5	3.4	8.4
1429	AT3G50360.1 | Symbol: ATCEN2 |	8.8	6.3	2.5	8.4
4470	AT2G15290.1 | expressed protein | chr2:6649408-6651117 REVERSE | Aliases: F27O10.6, F27O10_6	8.8	6.7	2.1	4.9
19658	AT2G01100.3 | expressed protein | chr2:81455-83217 FORWARD | Aliases: F23H14.7, F23H14_7	8.8	8.9	-0.1	-0.3
13284	AT5G51620.2 | expressed protein | chr5:20984324-20985253 FORWARD | Aliases: K17N15.17, K17N15_17	8.8	8.2	0.6	1.5
7784	AT2G26300.1 | Symbol: GPA1 | guanine nucleotide binding protein (G-protein) alpha-1 subunit / GP-alpha-1 (GPA1), identical to SP:P18064 Guanine nucleotide-binding protein alpha-1 subunit (GP-alpha-1) {Arabidopsis thaliana} | chr2:11204373-11208070 FORWARD | Aliases: G PROTEIN ALPHA SUBUNIT, G PROTEIN ALPHA SUBUNIT 1, GP ALPHA 1, T1D16.6, T1D16_6	8.8	7.9	0.9	3.2
7142	AT3G51880.2 | Symbol: HMGB1 | high mobility group protein alpha (HMGalpha) / HMG protein alpha, nearly identical to HMG protein (HMGalpha) (Arabidopsis thaliana) GI:2832357; contains Pfam profile PF00505: HMG (high mobility group) box | chr3:19257980-19260072 REVERSE | Aliases: ATEM1.13, HIGH MOBILITY GROUP B 1, HMG ALPHA, NFD1	8.8	7.8	1.0	3.5
15122	AT3G26580.1 | expressed protein | chr3:9760124-9761781 FORWARD | Aliases: MFE16.11	8.8	8.4	0.4	1.1
910	AT4G21280.2 | similar to oxygen-evolving enhancer protein 3, chloroplast, putative (PSBQ2) [Arabidopsis thaliana] (TAIR:At4g05180.1); similar to chloroplast oxygen-evolving enhancer protein [Manihot esculenta] (GB:AAV74404.1); contains InterPro domain Twin-arginine translocation pathway signal (InterPro:IPR006311); contains InterPro domain Oxygen evolving enhancer 3 (InterPro:IPR008797) | chr4:11334412-11335783 FORWARD | Aliases: T6K22.20	8.8	5.1	3.7	9.8
8767	AT4G28470.1 | similar to 26S proteasome regulatory subunit S2 (RPN1) [Arabidopsis thaliana] (TAIR:At2g20580.1); similar to Unknown (protein for MGC:83116) [Xenopus laevis] (GB:AAH68911.1); similar to MGC97650 protein [Xenopus tropicalis] (GB:NP_001015819.1); similar to putative 26S proteasome regulatory subunit S2 [Oryza sativa (japonica cultivar-group)] (GB:BAD54002.1); similar to putative proteasome 26S non-ATPase subunit 2 [Oryza sativa (japonica cultivar-group)] (GB:XP_450542.1); similar to MGC83233 protein [Xenopus laevis] (GB:AAH68957.1); contains InterPro domain Proteasome/cyclosome, regulatory subunit (InterPro:IPR002015) | chr4:14066882-14072549 REVERSE | Aliases: F20O9.150, F20O9_150	8.8	8.1	0.7	2.8
20583	AT1G72440.1 | CCAAT-box-binding transcription factor-related, similar to CCAAT-box-binding transcription factor (CCAAT-binding factor) (CBF) (Swiss-Prot:Q03701) (Homo sapiens), GB:P53569 (Mus musculus) | chr1:27271827-27277258 REVERSE | Aliases: T10D10.9, T10D10_9	8.8	8.9	-0.1	-0.1
2138	AT5G49510.2 | similar to unnamed protein product [Mus musculus] (GB:BAC37066.1); similar to PREDICTED: similar to von Hippel-Lindau binding protein 1 [Canis familiaris] (GB:XP_538210.1); similar to PFD3_MOUSE Prefoldin subunit 3 (Von Hippel-Lindau binding protein 1) (VHL binding protein-1) (VBP-1) (GB:P61759); similar to von Hippel-Lindau binding protein 1 [Homo sapiens] (GB:AAV38411.1); similar to von Hippel-Lindau binding protein 1 [synthetic construct] (GB:AAV38448.1); contains InterPro domain Putative prefoldin chaperone (InterPro:IPR004127) | chr5:20097841-20100147 FORWARD | Aliases: K6M13.5, K6M13_5	8.8	7.0	1.8	7.2
13853	AT2G40600.1 | appr-1-p processing enzyme family protein, contains Pfam domain PF01661: Appr-1-p processing enzyme family | chr2:16953948-16955615 REVERSE | Aliases: T2P4.5, T2P4_5	8.8	8.4	0.4	1.4
18000	AT3G55050.2 | serine/threonine protein phosphatase 2C (PP2C6), identical to Ser/Thr protein phosphatase 2C (PP2C6) (GI:15020818) (Arabidopsis thaliana); similar to protein phosphatase 2C (GI:3608412) (Mesembryanthemum crystallinum); contains Pfam PF00481 : Protein phosphatase 2C domain; contains TIGRFAM TIGR01573 : CRISPR-associated protein Cas2 | chr3:20411465-20413572 REVERSE | Aliases: T15C9.50	8.8	8.9	-0.1	-0.6
5151	ATCG01090.1 | Symbol: NDHI | Encodes subunit of the chloroplast NAD(P)H dehydrogenase complex	8.8	7.1	1.7	4.5
16316	AT3G02910.1 | expressed protein, contains Pfam domain PF03674: Uncharacterised protein family (UPF0131) | chr3:649821-650928 FORWARD | Aliases: F13E7.14, F13E7_14	8.8	9.4	-0.6	-0.9
19021	AT4G21470.1 | riboflavin kinase/FAD synthetase family protein, contains Pfam profiles PF01687: Riboflavin kinase / FAD synthetase, PF00702: haloacid dehalogenase-like hydrolase | chr4:11431135-11433332 FORWARD | Aliases: F18E5.90, F18E5_90	8.8	8.7	0.1	0.4
7756	AT5G41600.1 | reticulon family protein (RTNLB4), weak similarity to Nogo-C protein (Rattus norvegicus) GI:6822251, SP:O95197 Reticulon protein 3 (Neuroendocrine-specific protein-like) {Homo sapiens}; contains Pfam profile PF02453: Reticulon | chr5:16653358-16654921 FORWARD | Aliases: MBK23.13, MBK23_13	8.8	6.1	2.6	3.2
12431	AT5G47030.1 | ATP synthase delta' chain, mitochondrial, identical to SP:Q96252 ATP synthase delta' chain, mitochondrial precursor (EC 3.6.3.14) {Arabidopsis thaliana}; contains Pfam profile PF02823: ATP synthase, Delta/Epsilon chain, beta-sandwich domain	8.8	7.1	1.7	1.7

2207	AT5G44000.1 | glutathione S-transferase C-terminal domain-containing protein, contains Pfam domain PF00043: Glutathione S-transferase, C-terminal domain | chr5:17719357-17721178 REVERSE | Aliases: MRH10.11, MRH10_11	8.8	6.6	2.2	7.1
7404	AT1G21670.1 | expressed protein, similar to TolB protein precursor (SP:P50601) {Pseudomonas aeruginosa} | chr1:7610387-7612671 FORWARD | Aliases: F8K7.9, F8K7_9	8.8	6.7	2.1	3.4
4725	AT1G15370.1 | expressed protein | chr1:5287057-5288506 FORWARD | Aliases: F9L1.32, F9L1_32	8.8	7.5	1.3	4.7
10642	AT4G24920.1 | protein transport protein SEC61 gamma subunit, putative, similar to Swiss-Prot:Q19967 protein transport protein SEC61 gamma subunit (Caenorhabditis elegans) | chr4:12819521-12820746 REVERSE | Aliases: F13M23.60, F13M23_60	8.8	8.0	0.8	2.2
3937	AT2G39730.3 | Symbol: RCA | ribulose bisphosphate carboxylase/oxygenase activase / RuBisCO activase, identical to SWISS-PROT:P10896 ribulose bisphosphate carboxylase/oxygenase activase, chloroplast precursor (RuBisCO activase, RA)(Arabidopsis thaliana) | chr2:16577824-16580597 REVERSE | Aliases: RUBISCO ACTIVASE, T5I7.18	8.8	5.1	3.7	5.3
17128	AT3G20870.1 | metal transporter family protein, contains ZIP Zinc transporter domain, Pfam:PF02535 | chr3:7309359-7312708 REVERSE | Aliases: MOE17.18	8.8	9.1	-0.3	-0.8
5895	AT1G31870.2 | similar to splicing factor PWI domain-containing protein [Arabidopsis thaliana] (TAIR:At2g29210.1); similar to unknown protein [Oryza sativa (japonica cultivar-group)] (GB:XP_480359.1); contains InterPro domain Bipartite nuclear localization signal (InterPro:IPR001472) | chr1:11436703-11440089 FORWARD | Aliases: F5M6.12, F5M6_12	8.8	7.4	1.4	4.1
18009	AT3G17240.3 | Symbol: LPD2 | dihydrolipoamide dehydrogenase 2, mitochondrial / lipoamide dehydrogenase 2 (MTLPD2), nearly identical to GB:AAF34796 (gi:6984216) from (Arabidopsis thaliana); alternative splice form exists | chr3:5889883-5892255 REVERSE | Aliases: LIPOAMIDE DEHYDROGENASE 2, MGD8.7	8.8	9.0	-0.2	-0.6
1704	AT1G62480.1 | vacuolar calcium-binding protein-related, contains weak similarity to vacuolar calcium binding protein (Raphanus sativus) gi:9049359:dbj:BAA99394 | chr1:23132370-23133424 FORWARD | Aliases: T3P18.4, T3P18_4	8.8	6.5	2.3	7.9
12090	AT1G25530.1 | lysine and histidine specific transporter, putative, similar to lysine and histidine specific transporter GI:2576361 from (Arabidopsis thaliana); contains Pfam profile PF01490: Transmembrane amino acid transporter protein | chr1:8964531-8967378 REVERSE | Aliases: F2J7.5, F2J7_5	8.8	7.7	1.1	1.8
10218	AT5G48020.1 | expressed protein | chr5:19479884-19482183 REVERSE | Aliases: MDN11.10, MDN11_10	8.8	8.0	0.8	2.3
11796	AT4G17790.1 | expressed protein | chr4:9891064-9892807 FORWARD | Aliases: DL4930W, FCAALL.62	8.8	9.2	-0.4	-1.9
629	AT2G05620.1 | Symbol: PGR5 | expressed protein | chr2:2081050-2081837 REVERSE | Aliases: T20G20.3, T20G20_3	8.8	4.5	4.3	11.1
4428	AT2G47790.1 | transducin family protein / WD-40 repeat family protein, similar to WD-repeat protein 5 (SP:Q9UGP9) (Homo sapiens); The first 3 exons are identical to that of GB:AJ224957. This gene appears to be a truncated version of that in GB:AJ224957; contains 4 WD-40 repeats (PF00400) | chr2:19577641-19580086 REVERSE | Aliases: F17A22.18	8.8	6.8	2.0	4.9
17405	AT4G39240.1 | kelch repeat-containing F-box family protein, similar to SKP1 interacting partner 6 (Arabidopsis thaliana) GI:10716957; contains Pfam profiles PF01344: Kelch motif, PF00646: F-box domain | chr4:18269151-18270781 REVERSE | Aliases: T22F8.140, T22F8_140	8.8	8.6	0.1	0.7
4815	AT5G01710.1 | expressed protein | chr5:263516-265613 REVERSE | Aliases: F7A7.230, F7A7_230	8.8	9.6	-0.9	-4.7
20403	AT5G40250.1 | zinc finger (C3HC4-type RING finger) family protein, similar to RING-H2 finger protein RHX1a (Arabidopsis thaliana) GI:3790591; contains Pfam profile PF00097: Zinc finger, C3HC4 type (RING finger) | chr5:16103284-16104414 FORWARD | Aliases: MSN9.150, MSN9_150	8.8	8.8	-0.0	-0.1
1664	AT1G55805.1 | BolA-like family protein, contains Pfam profile: PF01722 BolA-like protein | chr1:20862397-20863105 REVERSE | Aliases: None	8.8	5.6	3.2	7.9
11878	AT5G35330.2 | Symbol: MBD02 | methyl-CpG-binding domain-containing protein, similar to methyl-CpG binding protein MBD4 (Mus musculus) GI:3800807; contains Pfam profile PF01429: Methyl-CpG binding domain | chr5:13540710-13542990 REVERSE | Aliases: MBD2, T26D22.18, T26D22_18	8.8	8.2	0.5	1.8
8723	AT3G54300.1 | Symbol: ATVAMP727 | synaptobrevin family protein, similar to vesicle-associated membrane protein 7B (At VAMP7B), Arabidopsis thaliana, EMBL:AF025333 | chr3:20119187-20121624 REVERSE | Aliases: F24B22.260, VAMP727	8.8	7.1	1.6	2.8
17787	AT4G22150.1 | Symbol: pux3 | UBX domain-containing protein, similar to XY40 protein (Rattus norvegicus) GI:2547025; contains Pfam profile PF00789: UBX domain	8.8	8.9	-0.2	-0.6
2659	AT1G64750.2 | DSS1/SEM1 family protein, contains Pfam profile PF05160: DSS1/SEM1 family | chr1:24056244-24057568 REVERSE | Aliases: F13O11.6, F13O11_6	8.8	6.6	2.1	6.5

462	AT2G28950.1 | Symbol: ATEXPA6 | expansin, putative (EXP6), similar to expansin GI:2828241 from (Brassica napus); contains Pfam profile PF01357: Pollen allergen | chr2:12438418-12440672 REVERSE | Aliases: ARABIDOPSIS THALIANA EXPANSIN A6, ATEXP6, ATHEXP ALPHA 1.8, EXPANSIN 6, T9I4.3, T9I4_3	8.8	2.8	5.9	12.2
2088	AT3G21420.1 | oxidoreductase, 2OG-Fe(II) oxygenase family protein, similar to SP:Q9ZWQ9 Flavonol synthase (EC 1.14.11.-) {Citrus unshiu}; contains Pfam profile PF03171: oxidoreductase, 2OG-Fe(II) oxygenase family | chr3:7541509-7543524 FORWARD | Aliases: MHC9.10	8.8	5.7	3.0	7.2
1582	AT1G07430.1 | protein phosphatase 2C, putative / PP2C, putative, similar to GB:CAB90633 from (Fagus sylvatica)	8.8	4.2	4.6	8.1
5855	AT1G68660.1 | expressed protein | chr1:25781514-25782824 REVERSE | Aliases: F24J5.10, F24J5_10	8.8	7.6	1.2	4.1
14971	AT4G01320.1 | Symbol: ATSTE24 | CAAX protease, putative (STE24), contains Pfam domain, PF01435: Peptidase family M48 | chr4:545796-549261 FORWARD | Aliases: ATSTE24, F2N1.21, F2N1_21, STE24	8.8	8.4	0.3	1.2
4507	AT5G39740.1 | 60S ribosomal protein L5 (RPL5B), ribosomal protein L5, rice | chr5:15920551-15922688 FORWARD | Aliases: MKM21.30, MKM21_30	8.8	5.6	3.2	4.9
12631	AT5G24980.1 | expressed protein | chr5:8609084-8609531 FORWARD | Aliases: F6A4.190, F6A4_190	8.8	7.8	0.9	1.7
17486	AT3G44140.1 | expressed protein | chr3:15896064-15896367 REVERSE | Aliases: F26G5.90	8.8	8.9	-0.2	-0.7
1530	AT3G26890.3 | expressed protein, similar to expressed protein [Arabidopsis thaliana] (TAIR:At5g41110.1); similar to unknown protein [Oryza sativa (japonica cultivar-group)] (GB:XP_483533.1) | chr3:9908499-9912853 REVERSE | Aliases: MDJ14.23	8.7	9.5	-0.8	-8.2
12643	AT5G64370.1 | Symbol: PYD3 | PYD3 encodes a beta-ureidopropionase which, when expressed in E. coli, has been shown to convert beta-ureidopropionate into beta-alanine. | chr5:25756441-25758438 FORWARD | Aliases: MSJ1.21, MSJ1_21, PYD3	8.7	9.6	-0.8	-1.7
17517	AT5G17330.1 | Symbol: GAD | glutamate decarboxylase 1 (GAD 1), sp:Q42521 | chr5:5711070-5715077 FORWARD | Aliases: GAD1, GLUTAMATE DECARBOXYLASE, MKP11.30, MKP11_30	8.7	9.2	-0.5	-0.7
11679	AT5G04870.1 | Symbol: CPK1 | calcium-dependent protein kinase isoform AK1 (AK1), identical to calcium-dependent protein kinase, isoform AK1 (CDPK) (Arabidopsis thaliana) SWISS-PROT:Q06850; contains protein kinase domain, Pfam:PF00069; contains EF hand domain (calcium-binding EF-hand), Pfam:PF00036, INTERPRO:IPR002048 | chr5:1416784-1420339 REVERSE | Aliases: AK1, ATCPK1, CALCIUM DEPENDENT PROTEIN KINASE, MUK11.19, MUK11_19	8.7	8.3	0.4	1.9
8727	AT2G31040.1 | ATP synthase protein I -related, contains weaks similarity to Swiss-Prot:P08443 ATP synthase protein I (Synechococcus sp.) | chr2:13215983-13218162 REVERSE | Aliases: T16B12.15, T16B12_15	8.7	8.0	0.8	2.8
692	AT4G10270.1 | wound-responsive family protein, similar to wound induced protein (GI:19320) (Lycopersicon esculentum) | chr4:6374730-6375206 FORWARD | Aliases: T9A4.6	8.7	4.2	4.6	10.8
18771	AT5G55850.1 | Symbol: NOI | similar to nitrate-responsive NOI protein, putative [Arabidopsis thaliana] (TAIR:At5g63270.1); similar to nitrate-induced NOI protein [Zea mays] (GB:AAC03022.1); contains InterPro domain Nitrate-induced NOI (InterPro:IPR008700) | chr5:22620603-22622458 FORWARD | Aliases: MWJ3.3, MWJ3_3, NOI PROTEIN	8.7	8.6	0.1	0.4
8190	AT3G29180.1 | expressed protein | chr3:11149452-11152797 FORWARD | Aliases: MUO22.2	8.7	8.1	0.7	3.0
2435	AT2G24360.1 | serine/threonine/tyrosine kinase, putative, similar to serine/threonine/tyrosine kinase (Arachis hypogaea) gi:13124865:gb:AAK11734 | chr2:10371531-10373971 REVERSE | Aliases: T28I24.9, T28I24_9	8.7	10.0	-1.3	-6.8
10180	AT4G27040.1 | expressed protein | chr4:13572444-13575544 REVERSE | Aliases: F10M23.380, F10M23_380	8.7	9.2	-0.4	-2.3
543	AT1G50450.1 | expressed protein | chr1:18691453-18694039 REVERSE | Aliases: F11F12.19, F11F12_19	8.7	4.2	4.5	11.7
6272	AT4G34200.1 | D-3-phosphoglycerate dehydrogenase, putative / 3-PGDH, putative, similar to phosphoglycerate dehydrogenase, Arabidopsis thaliana, SP:O04130 | chr4:16373850-16376626 REVERSE | Aliases: F10M10.7	8.7	6.0	2.7	3.9
11429	AT4G27800.3 | protein phosphatase 2C PPH1 / PP2C PPH1 (PPH1), identical to SP:P49599:P2C3_ARATH Protein phosphatase 2C PPH1 (EC 3.1.3.16) (PP2C) {Arabidopsis thaliana}; similar to protein phosphatase-2C; PP2C (GI:3643090) (Mesembryanthemum crystallinum) | chr4:13851876-13854197 REVERSE | Aliases: T27E11.40, T27E11_40	8.7	8.4	0.3	2.0
12478	AT1G07510.1 | Symbol: FTSH10 |	8.7	9.2	-0.5	-1.7
6243	AT1G10840.2 | Symbol: TIF3H1 |	8.7	7.5	1.2	3.9

15202	AT5G10540.1 | peptidase M3 family protein / thimet oligopeptidase family protein, similar to SP:P27237 Oligopeptidase A (EC 3.4.24.70) {Salmonella typhimurium}; contains Pfam profile PF01432: Peptidase family M3 | chr5:3328093-3334972 FORWARD | Aliases: F12B17.110, F12B17_110	8.7	8.3	0.5	1.1
1736	AT1G62380.1 | Symbol: ACO2 | 1-aminocyclopropane-1-carboxylate oxidase, putative / ACC oxidase, putative, nearly identical to ACC oxidase (ACC ox1) GI:587086 from (Brassica oleracea) | chr1:23085927-23087918 FORWARD | Aliases: ACC OXIDASE, ACC OXIDASE 2, ATACO2, F24O1.40, F24O1_40	8.7	12.1	-3.3	-7.8
16895	AT3G42790.1 | PHD finger family protein, contains PHD-finger domain, INTERPRO:IPR001965 | chr3:14888946-14890755 REVERSE | Aliases: T21C14.10	8.7	8.5	0.2	0.8
7120	AT5G53540.1 | MSP1 protein, putative / intramitochondrial sorting protein, putative, similar to Swiss-Prot:P28737 MSP1 protein (TAT-binding homolog 4) (Saccharomyces cerevisiae); contains Pfam domain, PF00004: ATPase, AAA family | chr5:21766512-21768463 REVERSE | Aliases: MNC6.8, MNC6_8	8.7	8.1	0.6	3.5
1013	AT4G38490.1 | expressed protein | chr4:18006282-18007366 FORWARD | Aliases: F20M13.50, F20M13_50	8.7	6.9	1.9	9.5
8013	AT2G30500.1 | kinase interacting family protein, similar to kinase interacting protein 1 (GI:13936326) (Petunia integrifolia) | chr2:13005236-13007149 REVERSE | Aliases: T6B20.15, T6B20_15	8.7	9.9	-1.1	-3.1
6595	AT5G12080.2 | mechanosensitive ion channel domain-containing protein / MS ion channel domain-containing protein, contains Pfam profile PF00924: Mechanosensitive ion channel | chr5:3898048-3901521 REVERSE | Aliases: F14F18.230, F14F18_230	8.7	7.1	1.6	3.7
3273	AT1G09430.1 | Symbol: ACLA 3 |	8.7	10.3	-1.6	-5.8
9503	AT1G76930.2 | Symbol: ATEXT4 |	8.7	6.8	1.9	2.5
10296	AT2G31510.1 | IBR domain-containing protein / ARIADNE-like protein ARI7 (ARI7), identical to ARIADNE-like protein ARI7 (Arabidopsis thaliana) GI:29125028; contains similarity to Swiss-Prot:Q94981 ariadne-1 protein (Ari-1) (Drosophila melanogaster); contains Pfam profile PF01485: IBR domain | chr2:13423764-13428247 REVERSE | Aliases: T9H9.3, T9H9_3	8.7	9.5	-0.8	-2.3
4599	AT1G08450.2 | Symbol: CRT3 | calreticulin 3 (CRT3), identical to similar to SP:O04153 Calreticulin 3 precursor {Arabidopsis thaliana}	8.7	7.7	1.0	4.8
4852	AT2G33590.1 | cinnamoyl-CoA reductase family, similar to cinnamoyl-CoA reductase from Pinus taeda (GI:17978649), Eucalyptus gunnii (GI:2058311) | chr2:14231344-14233678 FORWARD | Aliases: F4P9.36, F4P9_36	8.7	6.5	2.2	4.7
13813	AT4G04610.1 | Symbol: APR1 | 5'-adenylylsulfate reductase (APR1) / PAPS reductase homolog (PRH19), identical to 5'-adenylylsulfate reductase (Arabidopsis thaliana) GI:2738756; identical to cDNA PAPS reductase homolog (PRH19) GI:1710111 | chr4:2325001-2327011 FORWARD | Aliases: 3' PHOSPHOADENOSINE 5' PHOSPHOSULFATE REDUCTASE, APR, APS REDUCTASE, ATAPR1, F4H6.13, F4H6_13, PAPS REDUCTASE HOMOLOG 19, PRH19	8.7	8.1	0.6	1.4
5313	AT1G79050.1 | DNA repair protein recA, identical to DNA repair protein recA, chloroplast (Precursor) SP:Q39199 from (Arabidopsis thaliana) ;contains Pfam profile: PF00154 recA bacterial DNA recombination protein | chr1:29741854-29745049 REVERSE | Aliases: YUP8H12R.33, YUP8H12R_33	8.7	7.0	1.7	4.4
1239	AT5G47110.1 | lil3 protein, putative, similar to Lil3 protein (Arabidopsis thaliana) gi:4741966:gb:AAD28780 | chr5:19151215-19152544 REVERSE | Aliases: K14A3.6, K14A3_6	8.7	5.9	2.8	8.9
3300	AT5G48240.1 | expressed protein, similar to hypothetical protein [Oryza sativa (japonica cultivar-group)] (GB:AAX96070.1) | chr5:19576469-19578464 FORWARD | Aliases: MIF21.13, MIF21_13	8.7	6.6	2.1	5.8
1563	AT1G51400.1 | photosystem II 5 kD protein, 100% identical to GI:4836947 (F5D21.10) | chr1:19055730-19056250 REVERSE | Aliases: F5D21.10, F5D21_10	8.7	5.3	3.4	8.1
8712	AT5G26610.2 | D111/G-patch domain-containing protein, contains Pfam PF01585: G-patch domain | chr5:9375256-9377128 FORWARD | Aliases: None	8.7	9.4	-0.7	-2.8
11013	AT3G07800.1 | thymidine kinase, putative, similar to thymidine kinase (Oryza sativa) SWISS-PROT:O81263	8.7	7.9	0.8	2.1
2899	AT4G13660.1 | pinoresinol-lariciresinol reductase, putative, similar to pinoresinol-lariciresinol reductase TH1 (Tsuga heterophylla)(GI:7578915); contains isoflavone reductase domain PF02716	8.7	11.0	-2.3	-6.2
12245	AT3G54840.1 | Symbol: ARA6 | Rab GTPase (ARA6), identical to small GTPase Ara6 (Arabidopsis thaliana) GI:13160603 | chr3:20329480-20331970 FORWARD | Aliases: F28P10.180	8.7	7.5	1.2	1.8
7623	AT4G12040.2 | zinc finger (AN1-like) family protein, contains Pfam domains, PF01428: AN1-like Zinc finger and PF01754: A20-like zinc finger | chr4:7214704-7215979 FORWARD | Aliases: F16J13.110, F16J13_110	8.7	9.8	-1.1	-3.3
2996	AT3G23690.1 | basic helix-loop-helix (bHLH) family protein, contains Pfam profile: PF00010 helix-loop-helix DNA-binding domain	8.7	7.7	1.1	6.1

1698	AT5G62670.1 | ATPase, plasma membrane-type, putative / proton pump, putative, strong similarity to P-type H(+)-transporting ATPase from Nicotiana plumbaginifolia (SP:Q08435, SP:Q08436), Lycopersicon esculentum (GI:5901757, SP:P22180), Solanum tuberosum (GI:435003); contains InterPro accession IPR001757: ATPase, E1-E2 type | chr5:25176456-25183574 FORWARD | Aliases: MRG21.9, MRG21_9	8.7	5.3	3.4	7.9
5716	AT1G30880.1 | expressed protein, similar to ESTs gb:R30049 and gb:T46176 | chr1:10993215-10994100 REVERSE | Aliases: F17F8.25	8.7	6.9	1.8	4.2
772	AT4G10300.1 | expressed protein | chr4:6384458-6386185 FORWARD | Aliases: T9A4.16	8.7	5.5	3.2	10.4
1788	AT2G41220.1 | Symbol: GLU2 | glutamate synthase, chloroplast (GLU2) / ferredoxin-dependent glutamate synthase (Fd-GOGAT 2), identical to SP:Q9T0P4 Ferredoxin-dependent glutamate synthase 2, chloroplast precursor (EC 1.4.7.1) (Fd-GOGAT 2) {Arabidopsis thaliana} | chr2:17185012-17195829 FORWARD | Aliases: F13H10.23, F13H10_23, GLUTAMATE SYNTHASE PRECURSOR	8.7	10.8	-2.1	-7.7
9683	AT2G46490.1 | expressed protein (APS2), identical to cDNA Aps2, partial cds GI:4519894	8.7	7.4	1.3	2.5
7942	AT2G04400.1 | indole-3-glycerol phosphate synthase (IGPS), nearly identical to SP:P49572 | chr2:1531159-1534041 FORWARD | Aliases: T1O3.19, T1O3_19	8.7	7.4	1.3	3.1
985	AT3G11500.1 | small nuclear ribonucleoprotein G, putative / snRNP-G, putative / Sm protein G, putative, similar to SWISS-PROT:Q15357 small nuclear ribonucleoprotein G (snRNP-G, Sm protein G, Sm-G, SmG) (Homo sapiens) | chr3:3621415-3622598 REVERSE | Aliases: F24K9.17	8.7	6.9	1.8	9.6
607	AT2G29670.1 | expressed protein | chr2:12689221-12692164 REVERSE | Aliases: T27A16.23, T27A16_23	8.7	4.9	3.8	11.3
4229	AT5G43260.1 | chaperone protein dnaJ-related, similar to Chaperone protein dnaJ (SP:Q9WZV3) (Thermotoga maritima) | chr5:17374615-17375310 REVERSE | Aliases: MNL12.8, MNL12_8	8.7	7.4	1.3	5.1
3480	AT5G16040.1 | regulator of chromosome condensation (RCC1) family protein, similar to UVB-resistance protein UVR8 (Arabidopsis thaliana) GI:5478530; contains Pfam profile PF00415: Regulator of chromosome condensation (RCC1)	8.7	6.6	2.1	5.7
8354	AT3G10850.1 | Symbol: GLX2 2 |	8.7	7.0	1.7	3.0
10764	AT4G32910.1 | expressed protein | chr4:15881259-15885390 FORWARD | Aliases: F26P21.30, F26P21_30	8.7	8.1	0.6	2.1
7597	AT4G17620.2 | similar to glycine-rich protein [Arabidopsis thaliana] (TAIR:At4g30460.1); similar to glycine-rich protein [Haloxylon ammodendron] (GB:AAS99237.1); contains domain GLY_RICH (PS50315); contains domain SER_RICH (PS50324) | chr4:9821927-9825019 FORWARD | Aliases: DL4845W, FCAALL.74	8.7	9.4	-0.7	-3.3
366	AT1G17100.1 | SOUL heme-binding family protein, similar to SOUL protein (Mus musculus) GI:4886906; contains Pfam profile PF04832: SOUL heme-binding protein | chr1:5844729-5846215 FORWARD | Aliases: F20D23.21, F20D23_21	8.7	5.7	3.0	13.3
4828	AT4G10750.1 | HpcH/HpaI aldolase family protein, similar to 2,4-dihydroxydec-2-ene-1,10-dioic acid aldolase (Sphingopyxis macrogoltabida) GI:23330203; contains Pfam profile PF03328: HpcH/HpaI aldolase family | chr4:6618672-6619935 FORWARD | Aliases: T12H20.10, T12H20_10	8.7	7.3	1.4	4.7
2926	AT3G54210.1 | ribosomal protein L17 family protein, contains Pfam profile: PF01196 ribosomal protein L17 | chr3:20078525-20079413 REVERSE | Aliases: F24B22.170	8.7	6.2	2.4	6.2
1725	AT1G17490.1 | expressed protein | chr1:6008582-6009409 FORWARD | Aliases: F28G4.23	8.7	7.5	1.2	7.8
18974	AT2G34590.1 | transketolase family protein, similar to SP:O66113 Pyruvate dehydrogenase E1 component, beta subunit (EC 1.2.4.1). {Zymomonas mobilis}; contains Pfam profiles PF02779: Transketolase, pyridine binding domain, PF02780: Transketolase, C-terminal domain | chr2:14575890-14578048 REVERSE | Aliases: T31E10.7, T31E10_7	8.7	8.8	-0.1	-0.4
1020	AT4G35850.1 | pentatricopeptide (PPR) repeat-containing protein, low similarity to CRP1 (Zea mays) GI:3289002; contains Pfam profile PF01535: PPR repeat | chr4:16983528-16987203 FORWARD | Aliases: F4B14.120, F4B14_120	8.7	4.8	3.9	9.5
4364	AT5G24490.1 | 30S ribosomal protein, putative, similar to SP:P19954 Plastid-specific 30S ribosomal protein 1, chloroplast precursor (CS-S5) (CS5) (S22) (Ribosomal protein 1) (PSRP-1) {Spinacia oleracea}; contains Pfam profile PF02482: Sigma 54 modulation protein / S30EA ribosomal protein | chr5:8365624-8367429 FORWARD | Aliases: T31K7.7, T31K7_7	8.7	7.4	1.3	5.0
6953	AT5G40960.1 | expressed protein | chr5:16429973-16430179 REVERSE | Aliases: MEE6.3, MEE6_3	8.7	7.4	1.3	3.6
8202	AT1G02330.1 | expressed protein, contains similarity to hepatocellular carcinoma-associated antigen 59 GI:7158847 from (Homo sapiens) | chr1:462081-463612 REVERSE | Aliases: T6A9.2, T6A9_2	8.7	7.8	0.9	3.0

16526	AT1G03920.1 | protein kinase, putative, contains protein kinase domain, Pfam:PF00069 | chr1:1001320-1004382 FORWARD | Aliases: F21M11.15, F21M11_15	8.7	8.3	0.4	0.9
1138	AT4G02610.1 | tryptophan synthase, alpha subunit, putative, similar to A. thaliana tryptophan synthase alpha chain (EC 4.2.1.20), GenBank accession number U18993 (gi:619753) | chr4:1147634-1149577 FORWARD | Aliases: T10P11.11, T10P11_11	8.7	5.4	3.3	9.1
18865	AT5G38830.1 | tRNA synthetase class I (C) family protein, similar to SP:Q06752 Cysteinyl-tRNA synthetase (EC 6.1.1.16) (Cysteine--tRNA ligase) (CysRS) {Bacillus subtilis}; contains Pfam profile PF01406: tRNA synthetases class I (C) | chr5:15562994-15565382 REVERSE | Aliases: K15E6.3, K15E6_3	8.7	8.5	0.1	0.4
8716	AT3G07480.1 | expressed protein | chr3:2388958-2389817 FORWARD | Aliases: F21O3.19	8.7	6.9	1.8	2.8
19730	AT5G46630.2 | clathrin adaptor complexes medium subunit family protein, contains Pfam profile: PF00928 adaptor complexes medium subunit family	8.7	8.6	0.1	0.3
7159	AT1G80070.1 | Symbol: SUS2 | splicing factor, putative, strong similarity to splicing factor Prp8 (Homo sapiens) GI:3661610; contains Pfam profile PF01398: Mov34/MPN/PAD-1 family | chr1:30122945-30132704 FORWARD | Aliases: ABNORMAL SUSPENSOR 2, EMB14, EMB158, EMB177, EMB33, EMBRYO DEFECTIVE 14, EMBRYO DEFECTIVE 177, EMBRYO DEFECTIVE 33, F18B13.15, F18B13_15	8.7	7.5	1.2	3.5
10124	AT2G42910.1 | ribose-phosphate pyrophosphokinase 4 / phosphoribosyl diphosphate synthetase 4 (PRS4), identical to phosphoribosyl diphosphate synthase (prs4) (Arabidopsis thaliana) GI:4902472	8.7	6.8	1.9	2.3
8936	AT1G70370.1 | BURP domain-containing protein / polygalacturonase, putative, similar to polygalacturonase isoenzyme 1 beta subunit (Lycopersicon esculentum) GI:170480; contains Pfam profile PF03181: BURP domain | chr1:26516385-26518851 REVERSE | Aliases: F17O7.9, F17O7_9	8.7	9.2	-0.6	-2.7
8832	AT1G79750.1 | malate oxidoreductase, putative, similar to malate oxidoreductase (NADP-dependent malic enzyme) GB:P34105 (Populus balsamifera subsp. trichocarpa) | chr1:30012219-30016279 REVERSE | Aliases: F19K16.27, F19K16_27	8.7	7.7	1.0	2.8
11791	AT1G52600.1 | signal peptidase, putative, similar to SP:P13679 Microsomal signal peptidase 21 kDa subunit (EC 3.4.-.-) {Canis familiaris}; contains Pfam profile PF00461: Signal peptidase I | chr1:19594194-19596343 FORWARD | Aliases: F6D8.18, F6D8_18	8.7	8.2	0.5	1.9
20589	AT1G08700.1 | presenilin family protein, similar to SP:P52166 Presenilin sel-12 {Caenorhabditis elegans}; contains Pfam profile PF01080: Presenilin | chr1:2769818-2771480 REVERSE | Aliases: F22O13.18, F22O13_18	8.7	8.7	-0.0	-0.1
389	AT4G27380.1 | expressed protein | chr4:13701262-13703156 FORWARD | Aliases: F27G19.4	8.7	4.1	4.5	13.0
3809	AT2G30150.1 | UDP-glucoronosyl/UDP-glucosyl transferase family protein, contains Pfam profile: PF00201 UDP-glucoronosyl and UDP-glucosyl transferase | chr2:12881783-12883199 FORWARD | Aliases: T27E13.11, T27E13_11	8.7	7.1	1.6	5.4
3392	AT5G51280.1 | DEAD-box protein abstrakt, putative | chr5:20858474-20861032 FORWARD | Aliases: MWD22.23, MWD22_23	8.7	7.1	1.6	5.7
6910	AT1G48140.1 | dolichol-phosphate mannosyltransferase-related, contains weak similarity to Swiss-Prot:Q9P2X0 dolichol-phosphate mannosyltransferase subunit 3 (Dolichol-phosphate mannose synthase subunit 3, Dolichyl-phosphate beta-D- mannosyltransferase subunit 3, Mannose-P-dolichol synthase subunit 3, MPD synthase subunit 3, DPM synthase complex subunit 3, Prostin 1 (Homo sapiens)	8.7	7.8	0.8	3.6
4761	AT1G60440.1 | eukaryotic pantothenate kinase family protein, similar to pantothenate kinase GI:4191500 from (Aspergillus nidulans); contains Pfam profile PF03630: Fumble | chr1:22270175-22272906 REVERSE | Aliases: T13D8.31, T13D8_31	8.7	9.2	-0.6	-4.7
2887	AT4G25100.4 | Symbol: FSD1 | similar to superoxide dismutase (Fe), putative / iron superoxide dismutase, putative [Arabidopsis thaliana] (TAIR:At5g51100.1); similar to Fe-superoxide dismutase (GB:AAA32791.1); contains InterPro domain Manganese and iron superoxide dismutase (InterPro:IPR001189) | chr4:12884310-12886537 REVERSE | Aliases: F24A6.1, FE SUPEROXIDE DISMUTASE 1	8.7	4.4	4.3	6.3
3487	AT2G36290.1 | hydrolase, alpha/beta fold family protein, low similarity to 2-hydroxy-6-oxo-6-phenylhexa-2,4-dienoate hydrolase (Rhodococcus sp. RHA1) GI:8978311; contains Pfam profile PF00561: hydrolase, alpha/beta fold family | chr2:15215706-15217918 REVERSE | Aliases: F2H17.10, F2H17_10	8.7	10.4	-1.8	-5.7
19114	AT2G44820.2 | expressed protein | chr2:18491426-18492885 FORWARD | Aliases: F16B22.34, F16B22_34	8.7	8.4	0.2	0.4
3640	AT1G24280.1 | Symbol: G6PD3 | Encodes a plastidic glucose-6-phosphate dehydrogenase that is sensitive to reduction by DTT and whose mRNA is most highly expressed in root. | chr1:8609434-8612569 FORWARD | Aliases: F3I6.22, F3I6_22, G6PD3, GLUCOSE 6 PHOSPHATE DEHYDROGENASE 3	8.7	9.8	-1.1	-5.5
966	AT1G34370.3 | similar to zinc finger (C2H2 type) family protein [Arabidopsis thaliana] (TAIR:At5g22890.1); similar to putative zinc finger protein [Oryza sativa (japonica cultivar-group)] (GB:XP_470361.1); contains InterPro domain Zn-finger, C2H2 type (InterPro:IPR007087) | chr1:12550455-12552677 FORWARD | Aliases: F7P12.7, F7P12_7	8.7	11.1	-2.4	-9.6

5016	AT3G04830.2 | expressed protein | chr3:1326255-1329370 FORWARD | Aliases: T9J14.22, T9J14_22	8.7	6.5	2.2	4.6
2314	AT3G16310.1 | mitotic phosphoprotein N' end (MPPN) family protein, contains Pfam profile PF05172: MPPN (Mitotic PhosphoProtein N' end) (rrm-like) domain | chr3:5526424-5528606 REVERSE | Aliases: MYA6.11	8.7	6.2	2.4	6.9
9818	AT1G72360.1 | encodes a member of the ERF (ethylene response factor) subfamily B-2 of ERF/AP2 transcription factor family. The protein contains one AP2 domain. There are 5 members in this subfamily including RAP2.2 AND RAP2.12. | chr1:27245474-27246489 FORWARD | Aliases: T10D10.17, T10D10_17	8.7	9.7	-1.0	-2.4
19582	AT5G05790.1 | myb family transcription factor, contains Pfam profile: PF00249 myb-like DNA-binding domain | chr5:1740573-1742029 REVERSE | Aliases: MJJ3.20, MJJ3_20	8.7	8.7	-0.1	-0.3
1825	AT5G53420.2 | similar to expressed protein [Arabidopsis thaliana] (TAIR:At4g27900.1); similar to expressed protein [Arabidopsis thaliana] (TAIR:At4g27900.2); similar to zinc finger CONSTANS-like protein [Oryza sativa (japonica cultivar-group)] (GB:BAD73395.1) | chr5:21691615-21692959 FORWARD | Aliases: MYN8.3, MYN8_3	8.7	6.6	2.0	7.7
6547	AT4G00570.1 | malate oxidoreductase, putative, similar to NAD-dependent malic enzyme 59 kDa isoform, mitochondrial precursor (EC 1.1.1.39) (NAD-ME) (SP:P37225) {Solanum tuberosum} | chr4:242516-246736 REVERSE | Aliases: F6N23.16, F6N23_16	8.7	7.4	1.3	3.7
18853	AT3G54540.1 | Symbol: ATGCN4 |	8.7	8.8	-0.1	-0.4
9004	AT1G52730.2 | transducin family protein / WD-40 repeat family protein, contains 7 WD-40 repeats (PF00400); similar to UNR-interacting protein (WD-40 repeat protein PT-WD) (SP:Q9Y3F4) (Homo sapiens) | chr1:19646158-19648802 FORWARD | Aliases: F6D8.2, F6D8_2	8.7	9.1	-0.5	-2.7
17539	AT1G68500.1 | expressed protein | chr1:25705666-25706174 FORWARD | Aliases: T26J14.7, T26J14_7	8.6	8.4	0.3	0.7
12084	AT2G35880.1 | expressed protein | chr2:15070067-15072930 REVERSE | Aliases: F11F19.21, F11F19_21	8.6	9.3	-0.7	-1.8
5873	AT5G16010.1 | 3-oxo-5-alpha-steroid 4-dehydrogenase family protein / steroid 5-alpha-reductase family protein, similar to steroid 5alpha-reductase - Rattus norvegicus, PIR:A34239 (SP:24008); contains Pfam 3-oxo-5-alpha-steroid 4-dehydrogenase domain PF02544 | chr5:5227927-5229119 FORWARD | Aliases: F1N13.150, F1N13_150	8.6	6.6	2.0	4.1
166	AT4G38370.1 | phosphoglycerate/bisphosphoglycerate mutase family protein, contains Pfam profile PF00300: phosphoglycerate mutase family | chr4:17969521-17971289 REVERSE | Aliases: F22I13.140, F22I13_140	8.6	3.5	5.2	17.3
8858	AT4G04040.1 | pyrophosphate--fructose-6-phosphate 1-phosphotransferase beta subunit, putative / pyrophosphate-dependent 6-phosphofructose-1-kinase, putative, strong similarity to SP:Q41141 Pyrophosphate--fructose 6-phosphate 1-phosphotransferase beta subunit (EC 2.7.1.90) (PFP) (6-phosphofructokinase, pyrophosphate-dependent) (Pyrophosphate-dependent 6-phosphofructose-1-kinase) (PPI-PFK) {Ricinus communis} | chr4:1939248-1942952 FORWARD | Aliases: T24H24.15, T24H24_15	8.6	9.2	-0.5	-2.8
4365	AT1G65580.1 | endonuclease/exonuclease/phosphatase family protein, similar to inositol polyphosphate 5-phosphatase II isoform (GI:15418718) (Mus musculus); contains 6 (5 weak) Pfam: Pf00400 WD domain, G-beta repeats and Pfam PF03372: Endonuclease/Exonuclease/phosphatase family | chr1:24380977-24387742 REVERSE | Aliases: F5I14.11, F5I14_11	8.6	9.9	-1.3	-5.0
455	AT4G16155.1 | dihydrolipoamide dehydrogenase 2, plastidic / lipoamide dehydrogenase 2 (PTLPD2), identical to plastidic lipoamide dehydrogenase from Arabidopsis thaliana (gi:7159284) | chr4:9153386-9157278 REVERSE | Aliases: None	8.6	5.4	3.2	12.3
15661	AT5G43130.1 | similar to transcription initiation factor IID (TFIID) component TAF4 family protein [Arabidopsis thaliana] (TAIR:At1g27720.1); similar to putative protein [Oryza sativa] (GB:CAC39055.1); contains InterPro domain Transcription initiation factor TFIID component TAF4 (InterPro:IPR007900) | chr5:17332677-17338391 REVERSE | Aliases: MMG4.16, MMG4_16	8.6	9.0	-0.3	-1.0
15225	AT1G20050.1 | Symbol: HYD1 | C-8,7 sterol isomerase, identical to C-8,7 sterol isomerase GI:11279073 from (Arabidopsis thaliana) (Plant Mol. Biol. 38 (5), 807-815 (1998)) | chr1:6949060-6950310 FORWARD | Aliases: HYDRA1	8.6	9.2	-0.6	-1.1
4740	AT1G19110.1 | inter-alpha-trypsin inhibitor heavy chain-related, similar to SP:Q61704 Inter-alpha-trypsin inhibitor heavy chain H3 precursor {Mus musculus}; contains Pfam profile PF00092: von Willebrand factor type A domain | chr1:6602117-6605899 FORWARD | Aliases: F14D16.26, F14D16_26	8.6	9.4	-0.8	-4.7
17734	AT3G58600.1 | expressed protein, hypothetical protein F21M11.17 - Arabidopsis thaliana, EMBL:AC003027 | chr3:21679173-21682140 REVERSE | Aliases: F14P22.190	8.6	8.9	-0.3	-0.6
4009	AT2G42490.1 | copper amine oxidase, putative, similar to copper methylamine oxidase precursor (MAOXII) (Arthrobacter sp.) SWISS-PROT:Q07123 | chr2:17698497-17702926 REVERSE | Aliases: MHK10.21, MHK10_21	8.6	10.1	-1.4	-5.3

5107	AT4G14010.1 | Symbol: RALFL32 | rapid alkalinization factor (RALF) family protein, similar to RALF precursor (Nicotiana tabacum) GI:16566316 | chr4:8092832-8093377 REVERSE | Aliases: DL3045C, FCAALL.140, RALF LIKE 32	8.6	9.8	-1.1	-4.5
5569	AT2G32580.1 | expressed protein | chr2:13834740-13836386 FORWARD | Aliases: T26B15.14, T26B15_14	8.6	6.1	2.6	4.2
10670	AT5G66510.1 | bacterial transferase hexapeptide repeat-containing protein, contains Pfam profile PF00132: Bacterial transferase hexapeptide (four repeats) | chr5:26566930-26568856 REVERSE | Aliases: K1F13.17, K1F13_17	8.6	7.0	1.7	2.2
3416	AT4G35840.1 | zinc finger (C3HC4-type RING finger) family protein, contains a TG non-consensus donor splice site at exon 2; contains Pfam profile PF00097: Zinc finger, C3HC4 type (RING finger) | chr4:16980965-16982368 FORWARD | Aliases: F4B14.110, F4B14_110	8.6	6.3	2.4	5.7
2814	AT4G18593.1 | dual specificity protein phosphatase-related, contains weak similarity to Dual specificity protein phosphatase 12 (EC 3.1.3.48) (EC 3.1.3.16) (Dual-specificity tyrosine phosphatase YVH1) (Swiss-Prot:Q9UNI6) (Homo sapiens) | chr4:10237583-10238847 FORWARD | Aliases: None	8.6	7.1	1.5	6.3
135	AT2G36230.1 | Symbol: BBM II II | N'-5'-phosphoribosyl-formimino-5-aminoimidazole-4-carboxamide ribonucleotide isomerase, identical to N'-5'-phosphoribosyl-formimino-5-aminoimidazole- 4-carboxamide ribonucleotide isomerase GI:3449284 from (Arabidopsis thaliana); contains Pfam profile: PF00977 histidine biosynthesis protein | chr2:15200779-15202617 REVERSE | Aliases: F2H17.16, F2H17_16, PHOSPHORIBOSYLFORMIMINO 5 AMINOIMIDAZOLE CARBOXAMIDE RIBOTIDE ISOMERASE	8.6	3.5	5.1	18.6
1757	AT3G62940.2 | OTU-like cysteine protease family protein, contains Pfam profile PF02338: OTU-like cysteine protease | chr3:23273925-23275646 REVERSE | Aliases: T20O10.40	8.6	6.9	1.7	7.8
17740	AT4G34180.1 | cyclase family protein, contains Pfam profile: PF04199 putative cyclase | chr4:16369361-16371434 REVERSE | Aliases: F10M10.6	8.6	8.3	0.3	0.6
13399	AT3G61990.1 | O-methyltransferase family 3 protein, several O-methyltransferases - different species; contains Pfam 01596 O-methyltransferase domain | chr3:22968048-22969970 REVERSE | Aliases: F21F14.160	8.6	7.7	1.0	1.5
15537	AT2G43630.1 | expressed protein | chr2:18102854-18104565 FORWARD | Aliases: F18O19.26	8.6	8.3	0.3	1.1
15715	AT1G61730.1 | DNA-binding storekeeper protein-related, contains Pfam profile: PF04504 protein of unknown function, DUF573; similar to storekeeper protein GI:14268476 (Solanum tuberosum) | chr1:22796884-22798368 REVERSE | Aliases: T13M11.9, T13M11_9	8.6	8.8	-0.2	-1.0
1419	AT2G46690.1 | auxin-responsive family protein, similar to indole-3-acetic acid induced protein ARG7 (SP:P32295) (Phaseolus aureus) | chr2:19187800-19188537 FORWARD | Aliases: T3A4.7	8.6	10.5	-1.9	-8.4
2870	AT2G36910.1 | Symbol: ATPGP1 |	8.6	10.1	-1.5	-6.3
10785	AT1G32750.1 | Symbol: HAF01 | HAC13 protein (HAC13), identical to HAC13 (Arabidopsis thaliana) gi:21105767:gb:AAM34782; contains Pfam domains, PF00439: Bromodomain and PF00240: Ubiquitin family | chr1:11846227-11856241 REVERSE | Aliases: F6N18.13, F6N18_13, GTD01, GTD1, HAC13, HAF1	8.6	9.2	-0.6	-2.1
19813	AT1G32290.1 | expressed protein | chr1:11650202-11650547 REVERSE | Aliases: F27G20.4	8.6	8.7	-0.1	-0.2
795	AT4G18480.1 | Symbol: CHLI1 | magnesium-chelatase subunit chlI, chloroplast / Mg-protoporphyrin IX chelatase (CHLI) (CS) (CH42), identical to SP:P161127 Magnesium-chelatase subunit chlI, chloroplast precursor (Mg-protoporphyrin IX chelatase) (Protein CS/CH-42) {Arabidopsis thaliana} | chr4:10201683-10203454 REVERSE | Aliases: CH 42, CH42, CHLORATA, CHLORINA 42, F28J12.140, F28J12_140, PROTOPORPHYRIN IX MG CHELATASE	8.6	4.7	3.9	10.3
9640	AT1G77450.1 | Symbol: ANAC032 | no apical meristem (NAM) family protein, contains Pfam PF02365: No apical meristem (NAM) domain; similar to GRAB1 protein GB:CAA09371, a novel member of the NAC domain family | chr1:29104848-29106155 FORWARD | Aliases: ANAC032, T5M16.4, T5M16_4	8.6	8.0	0.6	2.5
19929	AT1G36050.1 | expressed protein | chr1:13451416-13454454 FORWARD | Aliases: F5J5.4, F5J5_4	8.6	8.5	0.1	0.2
1252	AT3G17930.1 | expressed protein | chr3:6141834-6143316 REVERSE | Aliases: MEB5.15	8.6	5.8	2.8	8.8
3566	AT5G10730.1 | expressed protein | chr5:3390615-3393107 REVERSE | Aliases: MAJ23.90, MAJ23_90	8.6	6.6	2.1	5.6
4174	AT3G55430.1 | glycosyl hydrolase family 17 protein / beta-1,3-glucanase, putative, similar to beta-1,3 glucanase GI:7414433 from (Pisum sativum); contains Pfam profile PF00332: Glycosyl hydrolases family 17	8.6	7.0	1.7	5.1
14415	AT3G45310.2 | similar to cysteine proteinase, putative / AALP protein (AALP) [Arabidopsis thaliana] (TAIR:At5g60360.1); similar to cysteine protease [Prunus armeniaca] (GB:AAB97142.1); contains InterPro domain Papain cysteine protease (C1) (InterPro:IPR000668); contains InterPro domain Eukaryotic thiol (cysteine) protease (InterPro:IPR000169) | chr3:16639369-16641506 REVERSE | Aliases: F18N11.70	8.6	9.3	-0.7	-1.3
12011	AT5G53440.1 | expressed protein | chr5:21700905-21705196 REVERSE | Aliases: MYN8.5, MYN8_5	8.6	8.3	0.3	1.8

9271	AT3G49580.1 | expressed protein | chr3:18386254-18386762 REVERSE | Aliases: T9C5.170	8.6	6.3	2.3	2.6
5513	AT4G00630.1 | Symbol: KEA2 | K+ efflux antiporter, putative (KEA2), Monovalent cation:proton antiporter family 2 (CPA2 family) member, PMID:11500563; similar to SWISS-PROT:SPP03819 Glutathione-regulated potassium-efflux system protein kefC (K(+)/H(+) antiporter) (Escherichia coli) | chr4:261655-265958 REVERSE | Aliases: ATKEA2, F6N23.15, F6N23_15	8.6	9.6	-1.0	-4.3
17387	AT2G17120.1 | peptidoglycan-binding LysM domain-containing protein, contains Pfam profile PF01476: LysM domain; supporting cDNA gi:16226688:gb:AF428464.1:AF428464 | chr2:7466129-7468388 FORWARD | Aliases: F6P23.25, F6P23_25	8.6	8.4	0.2	0.7
5102	AT1G08880.1 | histone H2A, putative, Strong similarity to histone H2A Cicer arietinum SP:O65759, Picea abies SP:P35063; contains Pfam profile PF00125 Core histone H2A/H2B/H3/H4; ESTs gb:ATTS3874,gb:T46627,gb:T14194 come from this gene | chr1:2846956-2847784 REVERSE | Aliases: F7G19.24, F7G19_24	8.6	5.8	2.8	4.5
19820	AT3G57230.1 | Symbol: AGL16 | MADS-box protein (AGL16), MADS-box transcription factor DEFH125 - Antirrhinum majus, PIR:T17029; contains Pfam domain PF00319: SRF-type transcription factor (DNA-binding and dimerisation domain); contains Pfam domain PF01486: K-box region | chr3:21188689-21191911 FORWARD | Aliases: F28O9.80	8.6	8.5	0.1	0.2
5215	AT1G14830.1 | Symbol: ADL1C | dynamin-like protein C (DL1C), nearly identical to dynamin-like protein C (Arabidopsis thaliana) GI:19569772 | chr1:5107416-5111665 REVERSE | Aliases: ADL1C, ADL5, DYNAMIN LIKE PROTEIN 5, F10B6.23, F10B6_23	8.6	6.4	2.2	4.4
305	AT4G21610.1 | Symbol: LOL2 | zinc finger protein, putative, similar to zinc-finger protein Lsd1 (Arabidopsis thaliana) gi:1872521:gb:AAC49660	8.6	4.3	4.3	14.2
20255	AT3G20120.2 | Symbol: CYP705A21 | similar to cytochrome P450 family protein [Arabidopsis thaliana] (TAIR:At3g20110.1); similar to C93A2_SOYBN Cytochrome P450 93A2 (GB:Q42799); contains InterPro domain E-class P450, group I (InterPro:IPR002401); contains InterPro domain Cytochrome P450 (InterPro:IPR001128) | chr3:7023317-7025929 FORWARD | Aliases: MAL21.16	8.6	8.6	0.0	0.2
9037	AT5G49530.1 | SIN-like family protein, low similarity to Sex-lethal interactor (Drosophila melanogaster) GI:6049274; contains Pfam profile PF04801: Sin-like protein conserved region | chr5:20117805-20121673 FORWARD | Aliases: K6M13.8, K6M13_8	8.6	7.9	0.7	2.7
1562	AT5G42480.1 | Symbol: ARC6 | DNAJ plastid division protein (ARC6), almost identical to dnaJ plastid division protein ARC6 (GI:33436339) (Arabidopsis thaliana);low similarity to cell division protein Ftn2 (Synechococcus sp. PCC 7942) GI:16226084; contains Pfam profile PF00226: DnaJ domain | chr5:17002410-17005668 FORWARD | Aliases: ACCUMULATION AND REPLICATION OF CHLOROPLASTS 6, MDH9.18, MDH9_18	8.6	7.2	1.4	8.1
300	AT5G65750.1 | 2-oxoglutarate dehydrogenase E1 component, putative / oxoglutarate decarboxylase, putative / alpha-ketoglutaric dehydrogenase, putative, similar to SP:P20967 2-oxoglutarate dehydrogenase E1 component, mitochondrial precursor (EC 1.2.4.2) (Alpha-ketoglutarate dehydrogenase) {Saccharomyces cerevisiae}; contains Pfam profiles PF02779: Transketolase, pyridine binding domain, PF00676: Dehydrogenase E1 component | chr5:26320932-26325415 FORWARD | Aliases: MPA24.10, MPA24_10	8.6	4.7	3.9	14.3
1228	AT3G01470.1 | Symbol: ATHB 1 |	8.6	11.5	-2.9	-8.9
9283	AT5G47840.1 | adenylate kinase, chloroplast, putative / ATP-AMP transphosphorylase, putative, similar to SP:P43188 Adenylate kinase, chloroplast (EC 2.7.4.3) (ATP-AMP transphosphorylase) {Zea mays}; contains Pfam profile PF00406: Adenylate kinase	8.6	7.3	1.3	2.6
15385	AT1G73030.1 | SNF7 family protein, contains Pfam domain, PF03357: SNF7 family | chr1:27477487-27478783 FORWARD | Aliases: F3N23.23, F3N23_23	8.6	8.2	0.4	1.1
13188	AT1G01230.1 | ORMDL family protein, contains Pfam domain PF04061: ORMDL family	8.6	8.1	0.5	1.5
1407	AT5G47320.1 | Symbol: RPS19 | 30S ribosomal protein S19, mitochondrial (RPS19) | chr5:19220379-19222499 FORWARD | Aliases: MQL5.18, MQL5_18	8.6	5.7	2.9	8.4
5188	AT3G50970.1 | Symbol: XERO2 | dehydrin xero2 (XERO2) / low-temperature-induced protein LTI30 (LTI30), identical to dehydrin Xero 2 (Low-temperature-induced protein LTI30) (Arabidopsis thaliana) SWISS-PROT:P42758 | chr3:18951758-18952535 FORWARD | Aliases: F24M12.10	8.6	10.8	-2.2	-4.5
13488	AT3G11730.1 | Symbol: ATFP8 | Ras-related GTP-binding protein, putative, similar to Rab1-like small GTP-binding protein GI:4096662 from (Petunia x hybrida) | chr3:3709332-3711489 REVERSE | Aliases: ATFP8, F26K24.2	8.6	8.3	0.3	1.5
17043	AT2G22250.3 | similar to aminotransferase class I and II family protein [Arabidopsis thaliana] (TAIR:At1g77670.1); similar to aspartate aminotransferase [Pinus pinaster] (GB:CAF31327.1); contains InterPro domain Aminotransferase, class I and II (InterPro:IPR004839); contains InterPro domain Aminotransferases class-I pyridoxal-phosphate-binding site (InterPro:IPR004838) | chr2:9464890-9467999 REVERSE | Aliases: T26C19.9, T26C19_9	8.6	8.9	-0.3	-0.8
1742	AT5G03650.1 | Symbol: SBE2.2 |	8.6	4.9	3.7	7.8
3737	AT5G10810.1 | Symbol: ATER | enhancer of rudimentary protein, putative, identical to enhancer of rudimentary homolog SP:Q96319 from (Arabidopsis thaliana); contains Pfam profile: PF01133 enhancer of rudimentary | chr5:3418667-3420636 REVERSE | Aliases: ENHANCER OF RUDIMENTARY HOMOLOG ATER, T30N20.80, T30N20_80	8.6	6.3	2.4	5.5

572	AT1G22270.1 | expressed protein, contains Pfam domain PF03966: Protein of unknown function (DUF343) | chr1:7865482-7866128 FORWARD | Aliases: T16E15.11, T16E15_11	8.6	4.4	4.2	11.5
16084	AT3G02760.1 | histidyl-tRNA synthetase, putative / histidine--tRNA ligase, putative, similar to SP:P12081 Histidyl-tRNA synthetase (EC 6.1.1.21) (Histidine--tRNA ligase) (HisRS) {Homo sapiens}; contains Pfam profiles PF00587: tRNA synthetase class II core domain (G, H, P, S and T), PF03129: Anticodon binding domain | chr3:597366-599445 REVERSE | Aliases: F13E7.30, F13E7_30	8.6	8.2	0.4	1.0
2239	AT2G28740.1 | Symbol: HIS4 | histone H4, identical to histone H4 from Lycopersicon esculentum GI:297150, Lolium temulentum SP:P02308, Acropora formosa GI:455652, Citrus jambhiri GI:16797797	8.6	5.9	2.7	7.0
7504	AT5G51140.1 | pseudouridine synthase family protein, low similarity to SP:P23851 Ribosomal large subunit pseudouridine synthase C (EC 4.2.1.70) (Pseudouridylate synthase) (Uracil hydrolyase) {Escherichia coli}; contains Pfam profile PF00849: RNA pseudouridylate synthase	8.6	7.9	0.7	3.3
8295	AT5G15090.1 | porin, putative / voltage-dependent anion-selective channel protein, putative, similar to SP:P42055 34 kDa outer mitochondrial membrane protein porin (Voltage-dependent anion-selective channel protein) (VDAC) {Solanum tuberosum}; contains Pfam profile PF01459: Eukaryotic porin; identical to cDNA voltage-dependent anion-selective channel protein GI:4006940 | chr5:4889298-4891475 REVERSE | Aliases: F2G14.210, F2G14_210	8.6	7.4	1.2	3.0
1065	AT5G23120.1 | Symbol: HCF136 |	8.6	5.8	2.8	9.3
8338	AT1G10630.1 | Gene encoding ADP-ribosylation factor and similar to other ARFs and ARF-like proteins. Members of this family are known to be essential for vesicle coating and uncoating and functions in GTP-binding. The gene is shown to play a role in cell division, cell expansion and cellulose production using antisense construct. | chr1:3512796-3514724 REVERSE | Aliases: F20B24.7, F20B24_7	8.6	7.6	0.9	3.0
12014	AT4G15560.1 | Symbol: CLA1 | 1-deoxy-D-xylulose 5-phosphate synthase, putative / 1-deoxyxylulose-5-phosphate synthase, putative / DXP-synthase, putative (DEF) (CLA1), identical to SP:Q38854 Probable 1-deoxy-D-xylulose 5-phosphate synthase, chloroplast precursor (EC 4.1.3.37) (1-deoxyxylulose-5-phosphate synthase) (DXP synthase) (DXPS). (Mouse-ear cress) {Arabidopsis thaliana}, DEF (deficient in photosynthesis) protein (Arabidopsis thaliana) GI:1399261 | chr4:8883905-8887563 FORWARD | Aliases: 1 DEOXY D XYLULOSE 5 PHOSPHATE SYNTHASE, CHLOROPLASTOS ALTERADOS, CLA, CLOROPLASTOS ALTERADOS 1, DEF, DL3821W, DXS, FCAALL.24	8.6	8.0	0.6	1.8
11088	AT3G57290.1 | Symbol: EIF3E | eukaryotic translation initiation factor 3E / eIF3e (TIF3E1), identical to eukaryotic initiation factor 3E subunit (Arabidopsis thaliana) gi:12407658:gb:AAG53613 | chr3:21207533-21210127 REVERSE | Aliases: ATEIF3E 1, EIF3E, F28O9.140, INT 6, TIF3E1	8.6	7.4	1.2	2.0
919	AT2G37790.1 | aldo/keto reductase family protein, similar to chalcone reductase (Sesbania rostrata)(GI:2792155), and aldose reductase ALDRXV4 (Xerophyta viscosa)(GI:4539944),	8.6	5.5	3.1	9.8
9538	AT4G39990.1 | Symbol: ATGB3 | Ras-related GTP-binding protein, putative, similar to GTP-binding protein GI:303738 from (Pisum sativum) | chr4:18542616-18543972 FORWARD | Aliases: GTP BINDING PROTEIN 3, T5J17.160, T5J17_160	8.6	7.3	1.3	2.5
8598	ATCG00640.1 | Symbol: RPL33 | encodes a chloroplast ribosomal protein L33, a constituent of the large subunit of the ribosomal complex | chrC:67488-67688 FORWARD | Aliases: RPL33	8.6	6.2	2.4	2.9
10247	AT5G11740.1 | Symbol: AGP15 | arabinogalactan-protein (AGP15), identical to gi:10880507:gb:AAG24283 | chr5:3784252-3784793 FORWARD | Aliases: ARABINOGALACTAN PROTEIN 15, T22P22.130, T22P22_130	8.6	7.2	1.4	2.3
6791	AT2G43090.1 | aconitase C-terminal domain-containing protein, contains Pfam profile PF00694: Aconitase C-terminal domain | chr2:17926004-17927506 FORWARD | Aliases: MFL8.15	8.6	5.2	3.4	3.6
3141	AT1G62810.1 | copper amine oxidase, putative, similar to copper amine oxidase (Cicer arietinum) gi:3819099:emb:CAA08855 | chr1:23261391-23265631 REVERSE | Aliases: F23N19.18, F23N19_18	8.6	6.7	1.9	6.0
9127	AT5G36230.1 | eIF4-gamma/eIF5/eIF2-epsilon domain-containing protein, low similarity to SP:Q13144 Translation initiation factor eIF-2B epsilon subunit (eIF-2B GDP-GTP exchange factor) {Homo sapiens}; contains Pfam profile PF02020: eIF4-gamma/eIF5/eIF2-epsilon | chr5:14290614-14294232 FORWARD | Aliases: T30G6.9, T30G6_9	8.6	7.9	0.7	2.7
15653	AT2G33340.3 | similar to transducin family protein / WD-40 repeat family protein [Arabidopsis thaliana] (TAIR:At1g04510.1); similar to ENSANGP00000016070 [Anopheles gambiae str. PEST] (GB:XP_308568.2); contains InterPro domain Zn-finger, modified RING (InterPro:IPR003613); contains InterPro domain G-protein beta WD-40 repeat (InterPro:IPR001680) | chr2:14133294-14138219 REVERSE | Aliases: F4P9.11, F4P9_11	8.6	8.4	0.2	1.0
1261	AT3G26980.1 | ubiquitin family protein, contains INTERPRO:IPR000626 ubiquitin domain | chr3:9947403-9949277 REVERSE | Aliases: MOJ10.7	8.6	5.4	3.2	8.8
8405	AT2G02800.2 | Symbol: APK2B | protein kinase (APK2b), identical to protein kinase APK2b (Arabidopsis thaliana) gi:2852449:dbj:BAA24695 | chr2:795514-799441 REVERSE | Aliases: PROTEIN KINASE 2B, T20F6.6, T20F6_6	8.6	9.7	-1.2	-2.9

1359	AT1G24610.1 | SET domain-containing protein, low similarity to SP:Q43088 Ribulose-1,5 bisphosphate carboxylase/oxygenase large subunit N- methyltransferase, chloroplast precursor (EC 2.1.1.127) {Pisum sativum}; contains Pfam profile PF00856: SET domain | chr1:8720177-8722732 REVERSE | Aliases: F21J9.27	8.6	5.7	2.8	8.6
11116	AT4G21160.4 | Symbol: ZAC | zinc finger and C2 domain protein (ZAC), identical to zinc finger and C2 domain protein GI:9957238 from (Arabidopsis thaliana) | chr4:11284387-11286755 FORWARD | Aliases: None	8.6	7.4	1.2	2.0
6629	AT2G17990.1 | expressed protein | chr2:7834004-7835836 FORWARD | Aliases: T27K22.14, T27K22_14	8.6	9.5	-0.9	-3.7
15882	AT5G16830.1 | Symbol: SYP21 | syntaxin 21 (SYP21) / PEP12 homolog, identical to Syntaxin homolog (PEP12 homolog) (SP:Q39233) and syntaxin of plants 21 (GP:899122) {Arabidopsis thaliana}; contains Pfam profiles PF05739:SNARE domain and PF00804: Syntaxin	8.6	8.1	0.4	1.0
19073	AT3G01780.1 | expressed protein, est hit, | chr3:279067-283554 FORWARD | Aliases: F28J7.11, F28J7_11	8.6	8.7	-0.1	-0.4
14603	AT5G20920.2 | Symbol: EIF2 BETA | eukaryotic translation initiation factor 2 subunit 2, putative / eIF-2-beta, putative, similar to SP:P41035 Eukaryotic translation initiation factor 2 subunit (eIF-2-beta) {Oryctolagus cuniculus}; contains Pfam profile PF01873: Domain found in IF2B/IF5 | chr5:7094721-7097077 REVERSE | Aliases: EMB1401, EMBRYO DEFECTIVE 1401, F22D1.90, F22D1_90	8.6	8.3	0.3	1.2
20097	AT5G01750.2 | expressed protein, contains Pfam profile PF04525: Protein of unknown function (DUF567) | chr5:289762-291323 FORWARD | Aliases: T20L15.20, T20L15_20	8.6	8.7	-0.1	-0.2
3197	AT1G26665.2 | expressed protein | chr1:9214164-9215538 FORWARD | Aliases: None	8.6	6.9	1.6	5.9
2771	AT4G21860.1 | methionine sulfoxide reductase domain-containing protein / SelR domain-containing protein, low similarity to pilin-like transcription factor (Homo sapiens) GI:5059062, SP:P14930 Peptide methionine sulfoxide reductase msrA/msrB (EC 1.8.4.6) {Neisseria gonorrhoeae}; contains Pfam profile PF01641: SelR domain | chr4:11600085-11601572 REVERSE | Aliases: T8O5.70, T8O5_70	8.6	6.3	2.3	6.4
1066	AT5G57560.1 | Symbol: TCH4 | xyloglucan:xyloglucosyl transferase / xyloglucan endotransglycosylase / endo-xyloglucan transferase (TCH4), identical to xyloglucan endotransglycosylase TCH4 protein GI:886116 | chr5:23324356-23325507 REVERSE | Aliases: MUA2.13, MUA2_13, TOUCH 4, XTH22, XYLOGLUCAN ENDOTRANSGLYCOSYLASE	8.6	4.2	4.4	9.3
13069	AT4G15545.1 | expressed protein | chr4:8875918-8877755 FORWARD | Aliases: None	8.6	7.6	1.0	1.6
4248	AT5G17000.1 | NADP-dependent oxidoreductase, putative, strong similarity to probable NADP-dependent oxidoreductase (zeta-crystallin homolog) P1 (SP:Q39172)(gi:886428) and P2 (SP:Q39173)(gi:886430), Arabidopsis thaliana | chr5:5584804-5587066 REVERSE | Aliases: F2K13.150, F2K13_150	8.6	6.9	1.7	5.1
8228	AT2G21970.1 | Symbol: SEP2 | stress enhanced protein 2 (SEP2), nearly identical to stress enhanced protein 2; SEP2 (GI:7384980) (Arabidopsis thaliana) | chr2:9364063-9364996 REVERSE | Aliases: F7D8.29, F7D8_29, SEP2, STRESS ENHANCED PROTEIN 2	8.6	7.8	0.7	3.0
4101	AT2G45520.1 | expressed protein | chr2:18761830-18763362 REVERSE | Aliases: F17K2.5	8.6	7.4	1.2	5.2
284	AT2G46110.1 | ketopantoate hydroxymethyltransferase family protein, similar to SP:Q9Y7B6 3-methyl-2-oxobutanoate hydroxymethyltransferase (EC 2.1.2.11) (Ketopantoate hydroxymethyltransferase) {Emericella nidulans}; contains Pfam profile PF02548: Ketopantoate hydroxymethyltransferase | chr2:18961532-18963603 REVERSE | Aliases: T3F17.24	8.6	3.6	5.0	14.7
9338	AT5G09410.1 | Symbol: EICBP.B | calmodulin-binding protein, similar to another ethylene-upregulated calmodulin-binding protein ER1 GI:11612392 from (Nicotiana tabacum) | chr5:2921458-2927417 FORWARD | Aliases: EICBP.B, ETHYLENE INDUCED CALMODULIN BINDING PROTEIN, T5E8.210, T5E8_210	8.6	9.4	-0.8	-2.6
13217	AT5G58380.1 | Symbol: CIPK10 |	8.6	9.3	-0.7	-1.5
2149	AT1G76520.2 | auxin efflux carrier family protein, contains auxin efflux carrier domain, Pfam:PF03547 | chr1:28720049-28722308 FORWARD | Aliases: F14G6.12, F14G6_12	8.6	4.5	4.1	7.2
1036	AT3G02480.1 | ABA-responsive protein-related, similar to ABA-inducible protein (Fagus sylvatica) GI:3901016, cold-induced protein kin1 (Brassica napus) GI:167146 | chr3:512321-513034 FORWARD | Aliases: F16B3.11, F16B3_11	8.6	3.2	5.4	9.4
8018	AT2G20230.1 | expressed protein | chr2:8732622-8735101 FORWARD | Aliases: F11A3.22, F11A3_22	8.6	10.1	-1.5	-3.1
14330	AT3G27380.1 | Symbol: SDH2 1 |	8.6	8.1	0.5	1.3
16935	AT2G44970.1 | lipase-related, contains weak similarity to lipase (Pseudomonas aeruginosa) gi:3550950:gb:AAC34733. | chr2:18555898-18559130 REVERSE | Aliases: T14P1.23, T14P1_23	8.6	8.4	0.2	0.8

13049	AT4G02720.1 | expressed protein, temporary automated functional assignment | chr4:1204303-1205866 REVERSE | Aliases: T5J8.1	8.6	8.0	0.6	1.6
9196	AT1G70160.1 | expressed protein, similar to hypothetical protein GI:4455225 from (Arabidopsis thaliana) | chr1:26423612-26426188 FORWARD | Aliases: F20P5.12, F20P5_12	8.6	9.7	-1.1	-2.6
4700	AT5G05980.2 | Symbol: ATDFB | similar to dihydrofolate synthetase/folylpolyglutamate synthetase (DHFS/FPGS4) [Arabidopsis thaliana] (TAIR:At3g55630.3); similar to folylpoly-gammaglutamate synthetase precursor [Cricetulus griseus] (GB:AAK69546.1); contains InterPro domain Folylpolyglutamate synthetase (InterPro:IPR001645) | chr5:1799491-1804296 REVERSE | Aliases: A. THALIANA DHFS FPGS HOMOLOG B, K18J17.17, K18J17_17	8.6	9.5	-0.9	-4.8
5814	AT3G20000.1 | Symbol: TOM40 | porin family protein, low similarity to haymaker protein (Mus musculus) GI:17834089, mitochondrial outer membrane protein MOM35 (Mus musculus) GI:6650562; contains Pfam profile PF01459: Eukaryotic porin | chr3:6967596-6970571 FORWARD | Aliases: MZE19.5	8.6	7.0	1.5	4.1
1610	AT5G03380.2 | similar to heavy-metal-associated domain-containing protein [Arabidopsis thaliana] (TAIR:At2g36950.1); similar to GMFP5 [Glycine max] (GB:AAD09514.1); contains InterPro domain Heavy metal binding (InterPro:IPR006191); contains InterPro domain Heavy metal transport/detoxification protein (InterPro:IPR006121) | chr5:832103-834284 REVERSE | Aliases: F12E4.120, F12E4_120	8.6	12.3	-3.7	-8.0
1755	AT5G63970.2 | similar to copine-related [Arabidopsis thaliana] (TAIR:At3g01650.1); similar to P0401G10.23 [Oryza sativa (japonica cultivar-group)] (GB:NP_914244.1); contains InterPro domain von Willebrand factor, type A (InterPro:IPR002035) | chr5:25623997-25626213 REVERSE | Aliases: MBM17.7, MBM17_7	8.6	9.7	-1.2	-7.8
2164	AT3G54920.1 | Symbol: PMR6 | pectate lyase, putative / powdery mildew susceptibility protein (PMR6), identical to powdery mildew susceptibility protein (Arabidopsis thaliana) GI:22506901; similar to pectate lyase 2 GP:6606534 from (Musa acuminata) | chr3:20356077-20359507 FORWARD | Aliases: F28P10.100, POWDERY MILDEW RESISTANT 6	8.6	5.9	2.7	7.2
16953	AT2G03510.1 | band 7 family protein, contains Pfam profile PF01145: SPFH domain / Band 7 family | chr2:1066639-1069096 FORWARD | Aliases: T4M8.5, T4M8_5	8.6	8.2	0.4	0.8
4085	AT4G36990.1 | Symbol: HSF4 | heat shock factor protein 4 (HSF4) / heat shock transcription factor 4 (HSTF4), identical to heat shock transcription factor 4 (HSF4) SP:Q96320 from (Arabidopsis thaliana) | chr4:17440287-17442145 FORWARD | Aliases: AP22.9, AP22_9, AT HSFB1, ATHSF4, HEAT SHOCK FACTOR 4, HEAT SHOCK TRANSCRIPTION FACTOR 4, HSFB1	8.6	11.3	-2.7	-5.2
8345	AT3G61070.1 | peroxisomal biogenesis factor 11 family protein / PEX11 family protein, contains Pfam PF05648: Peroxisomal biogenesis factor 11 (PEX11) | chr3:22615577-22617741 REVERSE | Aliases: T27I15.160	8.5	7.5	1.1	3.0
14190	AT1G18980.1 | germin-like protein, putative, similar to germin-like protein subfamily T member 1 (SP:P92995); contains PS00725 germin family signature	8.5	7.8	0.7	1.3
1901	AT1G77670.1 | aminotransferase class I and II family protein, similar to kynurenine aminotransferase /glutamine transaminase K GI:1030066 (Rattus norvegicus) | chr1:29193795-29195869 REVERSE | Aliases: T5M16.26, T5M16_26	8.5	4.7	3.8	7.6
7351	AT4G34490.1 | Symbol: ATCAP1 |	8.5	7.7	0.8	3.4
20668	AT1G58200.2 | mechanosensitive ion channel domain-containing protein / MS ion channel domain-containing protein, contains Pfam profile PF00924: Mechanosensitive ion channel | chr1:21551852-21556763 REVERSE | Aliases: F16M22.2, F16M22_2	8.5	8.6	-0.0	-0.1
1865	AT5G22880.1 | histone H2B, putative, strong similarity to histone H2B-3 Lycopersicon esculentum GI:3021485, H2B Gossypium hirsutum SP:O22582; contains Pfam profile PF00125 Core histone H2A/H2B/H3/H4	8.5	4.5	4.0	7.6
7205	AT5G15350.1 | plastocyanin-like domain-containing protein, contains plastocyanin-like domain Pfam:PF02298 | chr5:4984962-4986221 REVERSE | Aliases: F8M21.240, F8M21_240	8.5	6.8	1.8	3.4
2484	AT2G44120.2 | 60S ribosomal protein L7 (RPL7C)	8.5	6.5	2.0	6.7
13500	AT5G38630.1 | Symbol: ACYB 1 |	8.5	8.9	-0.4	-1.5
2410	AT3G08720.2 | Symbol: ATPK19 |	8.5	9.8	-1.3	-6.8
4053	AT4G29140.1 | MATE efflux protein-related, several hypothetical proteins - Arabidopsis thaliana; contains Pfam profile PF01554: Uncharacterized membrane protein family | chr4:14368925-14370898 FORWARD | Aliases: F19B15.170, F19B15_170	8.5	6.7	1.8	5.2
958	AT2G43640.1 | signal recognition particle 14 kDa family protein / SRP14 family protein, similar to SP:P16254 Signal recognition particle 14 kDa protein (SRP14) {Mus musculus}; contains Pfam profile: PF02290 signal recognition particle 14kD protein | chr2:18104578-18106139 REVERSE | Aliases: F18O19.25	8.5	6.8	1.8	9.7

2463	AT4G04870.1 | CDP-alcohol phosphatidyltransferase family protein, similar to SP:Q07560 Cardiolipin synthetase (EC 2.7.8.-) {Saccharomyces cerevisiae; contains Pfam profile PF01066: CDP-alcohol phosphatidyltransferase | chr4:2461211-2464141 REVERSE | Aliases: T4B21.19, T4B21_19	8.5	6.7	1.8	6.7
7821	AT3G12800.1 | short-chain dehydrogenase/reductase (SDR) family protein, contains Pfam profile PF00106:oxidoreductase, short chain dehydrogenase/reductase family | chr3:4063331-4064795 REVERSE | Aliases: AT3G12790, MBK21.23	8.5	7.1	1.4	3.2
6356	ATCG00720.1 | Symbol: PETB | Encodes the cytochrome b(6) subunit of the cytochrome b6f complex. | chrC:74841-76292 FORWARD | Aliases: PETB	8.5	6.7	1.8	3.8
4449	AT5G60960.1 | pentatricopeptide (PPR) repeat-containing protein, contains Pfam profile PF01535: PPR repeat | chr5:24545476-24547237 REVERSE | Aliases: MSL3.8, MSL3_8	8.5	5.8	2.8	4.9
10961	AT2G18280.2 | similar to F-box family protein / tubby family protein [Arabidopsis thaliana] (TAIR:At1g47270.1); similar to F-box family protein / tubby family protein [Arabidopsis thaliana] (TAIR:At2g47900.1); similar to putative tubby-like protein [Oryza sativa (japonica cultivar-group)] (GB:XP_467371.1); contains InterPro domain Cyclin-like F-box (InterPro:IPR001810); contains InterPro domain Tubby (InterPro:IPR000007) | chr2:7953080-7955550 FORWARD | Aliases: T30D6.21, T30D6_21	8.5	9.3	-0.8	-2.1
15093	AT1G51070.1 | basic helix-loop-helix (bHLH) family protein, similar to bHLH transcription factor GI:3757520 from (Arabidopsis thaliana) | chr1:18931559-18933430 FORWARD | Aliases: F23H24.8, F23H24_8	8.5	9.1	-0.6	-1.1
12541	AT5G44320.1 | eukaryotic translation initiation factor 3 subunit 7, putative / eIF-3 zeta, putative / eIF3d, putative, similar to initiation factor 3d (Arabidopsis thaliana) GI:12407755, SP:O15371 Eukaryotic translation initiation factor 3 subunit 7 (eIF-3 zeta) (eIF3 p66) (eIF3d) {Homo sapiens}; contains Pfam profile PF05091: Eukaryotic translation initiation factor 3 subunit 7 (eIF-3) | chr5:17871816-17873907 REVERSE | Aliases: K9L2.10, K9L2_10	8.5	8.2	0.3	1.7
5010	AT3G13040.2 | myb family transcription factor, contains Pfam profile: PF00249 myb-like DNA-binding domain | chr3:4172149-4175255 REVERSE | Aliases: MGH6.22	8.5	9.7	-1.2	-4.6
2412	AT3G24590.1 | signal peptidase I family protein, contains Pfam profile: PF00461 signal peptidase I | chr3:8970679-8972175 FORWARD | Aliases: MOB24.17	8.5	7.4	1.1	6.8
15032	AT1G76550.1 | pyrophosphate--fructose-6-phosphate 1-phosphotransferase alpha subunit, putative / pyrophosphate-dependent 6-phosphofructose-1-kinase, putative, strong similarity to SP:Q41140 Pyrophosphate--fructose 6-phosphate 1-phosphotransferase alpha subunit (EC 2.7.1.90) (PFP) (PPI-PFK) {Ricinus communis}; contains Pfam profile PF00365: Phosphofructokinase | chr1:28727596-28731900 REVERSE | Aliases: F14G6.15, F14G6_15	8.5	7.8	0.8	1.2
11284	AT1G72770.1 | Symbol: HAB1 | protein phosphatase 2C P2C-HA / PP2C P2C-HA (P2C-HA), identical to protein phosphatase 2C (AtP2C-HA) GB:AJ003119 (Arabidopsis thaliana) (Plant Mol. Biol. 38 (5), 879-883 (1998)) | chr1:27393720-27396943 FORWARD | Aliases: F28P22.4, F28P22_4, HOMOLOGY TO ABI1	8.5	8.9	-0.4	-2.0
14859	ATCG00170.1 | Symbol: RPOC2 | RNA polymerase beta' subunit-2 | chrC:15938-20068 REVERSE | Aliases: RPOC2	8.5	7.5	1.0	1.2
5631	AT2G33740.2 | Symbol: CUTA | copper-binding protein (CUTA), identical to copper-binding protein CUTA GI:12963361 from (Arabidopsis thaliana); contains Pfam profile: PF03091 CutA1 divalent ion tolerance protein | chr2:14276657-14278752 FORWARD | Aliases: T1B8.5, T1B8_5	8.5	7.3	1.2	4.2
16789	AT3G07090.1 | expressed protein | chr3:2243060-2244826 REVERSE | Aliases: T1B9.26	8.5	8.0	0.6	0.8
17239	AT5G13010.1 | Symbol: EMB3011 | RNA helicase, putative, similar to DEAH-box RNA helicase (Chlamydomonas reinhardtii) GI:12044832; contains Pfam profiles PF04408: Helicase associated domain (HA2), PF00271: Helicase conserved C-terminal domain | chr5:4122581-4128864 FORWARD | Aliases: EMB3011, EMBRYO DEFECTIVE 3011, T24H18.180, T24H18_180	8.5	8.8	-0.3	-0.7
9752	AT3G53500.2 | Symbol: RSZ32 | zinc knuckle (CCHC-type) family protein, contains Pfam domain PF00098: Zinc knuckle | chr3:19845324-19847976 REVERSE | Aliases: F4P12.200, RSZ32	8.5	7.8	0.7	2.4
8025	AT5G47090.1 | expressed protein | chr5:19144486-19145996 FORWARD | Aliases: K14A3.4, K14A3_4	8.5	6.7	1.8	3.1
7654	AT3G28720.1 | expressed protein | chr3:10783513-10785684 FORWARD | Aliases: T19N8.1	8.5	7.1	1.4	3.3
3709	AT5G46840.1 | RNA recognition motif (RRM)-containing protein, contains InterPro entry IPR000504: RNA-binding region RNP-1 (RNA recognition motif) (RRM) | chr5:19022828-19024993 FORWARD | Aliases: MZA15.26	8.5	6.0	2.6	5.5
11460	AT5G65720.1 | cysteine desulfurase, mitochondrial (NIFS), identical to Cysteine desulfurase, mitochondrial precursor (SP:O49543) {Arabidopsis thaliana}; identical to cDNA GI:12656131; contains Pfam profile PF00266: aminotransferase, class V | chr5:26313479-26315298 FORWARD | Aliases: MPA24.7, MPA24_7	8.5	8.0	0.5	2.0
3963	AT4G33780.1 | expressed protein | chr4:16201833-16203643 REVERSE | Aliases: T16L1.270, T16L1_270	8.5	6.9	1.6	5.3

11947	AT4G26570.2 | Symbol: ATCBL3 |	8.5	8.0	0.6	1.8
17317	AT1G80040.2 | expressed protein | chr1:30114040-30116163 REVERSE | Aliases: F18B13.13, F18B13_13	8.5	8.4	0.2	0.7
10637	AT5G41790.1 | Symbol: CIP1 | COP1-interactive protein 1 / CIP1, almost identical to CIP1 (GI:836950) (Arabidopsis thaliana) | chr5:16745606-16750004 FORWARD | Aliases: COP1 INTERACTIVE PROTEIN 1, K16L22.7, K16L22_7	8.5	9.8	-1.3	-2.2
1325	AT1G54780.1 | thylakoid lumen 18.3 kDa protein, SP:Q9ZVL6 | chr1:20443101-20444826 FORWARD | Aliases: T22H22.19, T22H22_19	8.5	6.4	2.2	8.6
1420	AT5G56910.1 | expressed protein | chr5:23040148-23041428 FORWARD | Aliases: MHM17.2, MHM17_2	8.5	4.9	3.6	8.4
631	AT5G15750.1 | RNA-binding S4 domain-containing protein, 40S RIBOSOMAL PROTEINs - different species | chr5:5141194-5142861 FORWARD | Aliases: F14F8.130, F14F8_130	8.5	6.4	2.1	11.1
11820	AT1G14730.1 | similar to cytochrome B561 family protein [Arabidopsis thaliana] (TAIR:At4g25570.1); similar to putative cytochrome protein [Oryza sativa (japonica cultivar-group)] (GB:XP_469562.1); contains InterPro domain Cytochrome b561 / ferric reductase transmembrane (InterPro:IPR006593); contains InterPro domain Cytochrome b561 (InterPro:IPR004877) | chr1:5073100-5074774 FORWARD | Aliases: F10B6.13, F10B6_13	8.5	8.8	-0.3	-1.9
9555	AT3G49490.1 | expressed protein | chr3:18355729-18359665 REVERSE | Aliases: T9C5.90	8.5	8.9	-0.4	-2.5
8154	AT5G46180.1 | Symbol: delta OAT | ornithine aminotransferase, putative / ornithine--oxo-acid aminotransferase, putative, similar to SP:Q92413 Ornithine aminotransferase (EC 2.6.1.13) (Ornithine--oxo-acid aminotransferase) (Aspergillus nidulans) {Emericella nidulans}; contains Pfam profile PF00202: aminotransferase, class III | chr5:18735702-18738498 REVERSE | Aliases: MCL19.24, MCL19_24	8.5	7.1	1.5	3.0
2576	AT3G25400.1 | expressed protein | chr3:9214469-9215516 FORWARD | Aliases: MWL2.8	8.5	6.8	1.7	6.6
4940	AT2G36240.1 | similar to pentatricopeptide (PPR) repeat-containing protein [Arabidopsis thaliana] (TAIR:At1g09900.1); similar to putative pentatricopeptide repeat protein [Oryza sativa (japonica cultivar-group)] (GB:NP_909693.1); contains InterPro domain PPR repeat (InterPro:IPR002885); contains InterPro domain Proline-rich region (InterPro:IPR000694) | chr2:15202732-15204310 FORWARD | Aliases: F2H17.15, F2H17_15	8.5	6.8	1.7	4.6
12650	AT3G27570.1 | expressed protein | chr3:10215233-10217973 REVERSE | Aliases: MMJ24.12	8.5	7.8	0.7	1.7
2937	AT5G64500.1 | membrane protein-related, contains weak similarity to spinster type IV (GI:12003976) (Drosophila melanogaster) | chr5:25797608-25800880 FORWARD | Aliases: T12B11.9, T12B11_9	8.5	9.9	-1.4	-6.2
3407	AT1G09210.1 | calreticulin 2 (CRT2), identical to SP:Q38858 Calreticulin 2 precursor {Arabidopsis thaliana} | chr1:2972844-2976731 REVERSE | Aliases: T12M4.8, T12M4_8	8.5	6.6	1.9	5.7
12476	ATCG00130.1 | Symbol: ATPF | ATPase F subunit. | chrC:11529-12798 REVERSE | Aliases: ATPF	8.5	7.0	1.5	1.7
10704	AT4G02150.1 | Symbol: ATIMPALPHA3/MOS6 | Encodes IMPORTIN ALPHA 3. Mutant plants act as suppressors of snc1 response and salicylic acid accumulation. Located in the nucleus. Involved in protein import. | chr4:950649-953689 REVERSE | Aliases: ATIMPALPHA3, MOS6, T10M13.16, T10M13_16	8.5	7.5	1.0	2.2
20340	AT1G72150.1 | Symbol: PATL1 | SEC14 cytosolic factor family protein / phosphoglyceride transfer family protein, similar to SEC14-like protein 2 (Alpha-tocopherol associated protein) (TAP) (bTAP) (Fragment) (SP:P58875) {Bos taurus}; similar to GI:807956 from (Saccharomyces cerevisiae); contains Pfam PF00650 : CRAL/TRIO domain; contains Pfam PF03765 : CRAL/TRIO, N-terminus	8.5	8.6	-0.1	-0.1
13835	AT3G51370.2 | protein phosphatase 2C, putative / PP2C, putative, similar to Ser/Thr protein phosphatase 2C (PP2C6) (GI:15020818) (Arabidopsis thaliana); similar to protein phosphatase 2C (GI:3608412) (Mesembryanthemum crystallinum); contains Pfam PF00481 : Protein phosphatase 2C domain	8.5	9.0	-0.5	-1.4
13591	AT4G29120.1 | 6-phosphogluconate dehydrogenase NAD-binding domain-containing protein, similar to SP:P23523 2-hydroxy-3-oxopropionate reductase (EC 1.1.1.60) (Tartronate semialdehyde reductase) {Escherichia coli}; contains Pfam profile PF03446: NAD binding domain of 6-phosphogluconate dehydrogenase | chr4:14350864-14352018 FORWARD | Aliases: F19B15.150, F19B15_150	8.5	7.9	0.6	1.5
538	AT2G42740.1 | Symbol: RPL16A |	8.5	5.6	2.9	11.7
4156	AT5G28840.1 | NAD-dependent epimerase/dehydratase family protein, similar to sugar epimerase BlmG from Streptomyces verticillus GI:9937230; contains Pfam profile PF01370 NAD dependent epimerase/dehydratase family | chr5:10862177-10864906 REVERSE | Aliases: F7P1.20, F7P1_20	8.5	6.1	2.4	5.1
2553	AT2G28430.1 | expressed protein | chr2:12166639-12167528 REVERSE | Aliases: T1B3.5, T1B3_5	8.5	6.0	2.5	6.6
2170	AT3G12360.1 | ankyrin repeat family protein, contains ankyrin repeat domains, Pfam:PF00023 | chr3:3934085-3936701 FORWARD | Aliases: T2E22.31	8.5	9.8	-1.3	-7.2

9636	AT3G24800.1 | Symbol: PRT1 | PRT1 protein (PRT1), E3, N-end rule ubiquitin ligase, contains two RING finger domain; identical to PRT1 (Arabidopsis thaliana) GI:3319884 | chr3:9055516-9058005 FORWARD | Aliases: K7P8.19, PROTEOLYSIS 1, PRT1	8.5	9.1	-0.6	-2.5
4536	AT4G33670.1 | L-galactose dehydrogenase (L-GalDH), identical to L-galactose dehydrogenase (Arabidopsis thaliana) GI:16555790; similar to L-fucose dehydrogenase (Pseudomonas sp.) GI:829054; contains Pfam profile PF00248: oxidoreductase, aldo/keto reductase family	8.5	6.9	1.6	4.9
5075	AT1G78670.1 | gamma-glutamyl hydrolase, putative / gamma-Glu-X carboxypeptidase, putative / conjugase, putative, similar to gamma glutamyl hydrolase GI:1679658 SP:P93164 from (Glycine max) | chr1:29595848-29598392 FORWARD | Aliases: F9K20.29, F9K20_29	8.5	10.0	-1.5	-4.5
15711	AT3G18035.1 | Symbol: HON4 | histone H1/H5 family protein, contains Pfam domain, PF00538: linker histone H1 and H5 family;similar to HMG I/Y like protein (GI:15706274) (Glycine max);similar to HMR1 protein (GI:4218141) (Antirrhinum majus); similar to high mobility group protein (GI:1483173) (Canavalia gladiata) | chr3:6169096-6171730 REVERSE | Aliases: HISTONE H1, MRC8.1	8.5	8.2	0.3	1.0
5393	AT1G08510.1 | Symbol: FATB | acyl-(acyl carrier protein) thioesterase / acyl-ACP thioesterase / oleoyl-(acyl-carrier protein) hydrolase / S-acyl fatty acid synthase thioesterase, identical to acyl-(acyl carrier protein) thioesterase (Arabidopsis thaliana) GI:804948 | chr1:2691083-2694380 REVERSE | Aliases: T27G7.19, T27G7_19	8.5	10.5	-2.1	-4.3
4597	AT3G62880.2 | similar to mitochondrial import inner membrane translocase subunit Tim17/Tim22/Tim23 family protein [Arabidopsis thaliana] (TAIR:At2g28900.1); similar to B1146F03.20 [Oryza sativa (japonica cultivar-group)] (GB:NP_914110.1); contains InterPro domain Mitochondrial import inner membrane translocase, subunit Tim17/22 (InterPro:IPR003397)	8.5	6.8	1.7	4.8
11441	AT1G04770.1 | male sterility MS5 family protein, similar to male sterility MS5 (Arabidopsis thaliana) GI:3859112; contains Pfam profile PF00515 TPR Domain | chr1:1336370-1337838 REVERSE | Aliases: F13M7.24, F13M7_24	8.5	9.3	-0.8	-2.0
1255	AT4G24440.2 | transcription initiation factor IIA gamma chain / TFIIA-gamma (TFIIA-S), identical to transcription initiation factor IIA gamma chain SP:Q39236 from (Arabidopsis thaliana); | chr4:12633190-12634767 FORWARD | Aliases: T22A6.270, T22A6_270	8.5	5.3	3.1	8.8
17335	AT1G55460.1 | Kin17 DNA-binding protein-related, contains similarity to zinc finger protein rts2 GB:U16133 GI:563244 from (Saccharomyces cerevisiae) | chr1:20711144-20712690 FORWARD | Aliases: T5A14.13, T5A14_13	8.5	8.9	-0.4	-0.7
1476	AT5G57280.1 | expressed protein | chr5:23221696-23223877 FORWARD | Aliases: MJB24.9, MJB24_9	8.5	6.0	2.5	8.3
15697	AT5G08080.2 | Symbol: SYP132 |	8.5	8.7	-0.2	-1.0
14397	AT4G30530.1 | defense-related protein, putative, strong similarity to defense-related protein (Brassica carinata) GI:14009290; contains Pfam profile PF00117: glutamine amidotransferase class-I | chr4:14920540-14922464 FORWARD | Aliases: F17I23.130, F17I23_130	8.5	7.9	0.6	1.3
17408	AT3G22950.1 | Symbol: ATARFC1 | Gene encoding ADP-ribosylation factor and similar to ADP-ribosylation factor GB:P91924 (Dugesia japonica), other ARFs and ARF-like proteins. Members of this family are known to be essential for vesicle coating and uncoating and functions in GTP-binding.	8.5	8.2	0.2	0.7
1153	AT4G18700.1 | Symbol: CIPK12 |	8.5	10.2	-1.8	-9.1
2904	AT1G03090.2 | Symbol: MCCA | methylcrotonyl-CoA carboxylase alpha chain, mitochondrial / 3-methylcrotonyl-CoA carboxylase 1 (MCCA), nearly identical to SP:Q42523 Methylcrotonyl-CoA carboxylase alpha chain, mitochondrial precursor (EC 6.4.1.4) (3-Methylcrotonyl-CoA carboxylase 1) (MCCase alpha subunit) (3-methylcrotonyl-CoA:carbon dioxide ligase alpha subunit) {Arabidopsis thaliana} | chr1:739687-744184 FORWARD | Aliases: None	8.5	9.4	-1.0	-6.2
9074	AT1G71950.1 | expressed protein, similar to Pi starvation-induced protein GB:BAA06151 from (Nicotiana tabacum)	8.5	7.8	0.7	2.7
19798	AT1G16350.1 | inosine-5'-monophosphate dehydrogenase, putative, strong similarity to SP:P47996 gb:L34684 inosine monophosphate dehydrogenase (IMPDH) from Arabidopsis thaliana; member of the PF:00478 IMP dehydrogenase family | chr1:5590945-5592866 FORWARD | Aliases: F3O9.15, F3O9_15	8.5	8.6	-0.1	-0.2
4177	AT1G42990.1 | Symbol: ATBZIP60 | AtbZIP60 consists of a bZIP DNA binding domain followed by a putative transmembrane domain. GFP fusions containing the first 260 amino acids (AtbZIP60deltaC) are nuclear-localized. AtbZIP60 is upregulated by the addition of tunicamycin (ER stress response inductor), DTT (inhibitor of disulfide bond formation) and azetin-2-carboxylate (proline analog perturbing protein structure). It is hypothesized that upon ER stress the protein is proteolyzed and the soluble part translocalized into the nucleus. AtbZIP60deltaC can activate the promoters of the ER chaperones BiP1, BiP2 and BiP3 and CNX1 and CNX2 via binding to the ER stress response element (ERSE) and the plant unfolded protein response element(P-UPRE). It can also activate its own transcription. | chr1:16138292-16139903 REVERSE | Aliases: ATBZIP60, F13A11.5, F13A11_5	8.5	9.7	-1.2	-5.1
900	AT1G09560.1 | Symbol: GLP5 | germin-like protein (GLP4) (GLP5), identical to Arabidopsis germin-like protein subfamily 2 member 1 (SP:P94014); Location of EST 180L10T7, gi:906417 | chr1:3093841-3094864 FORWARD | Aliases: F14J9.22, F14J9_22, GERMIN LIKE PROTEIN 5, GLP5	8.5	12.2	-3.7	-9.9
2216	AT2G02710.3 | PAC motif-containing protein, similar to nonphototropic hypocotyl 1 (Zea mays) GI:2687358; contains Pfam profile PF00785: PAC motif | chr2:758696-760815 REVERSE | Aliases: T20F6.15, T20F6_15	8.5	10.6	-2.2	-7.1

20675	AT5G66030.2	Golgi-localized GRIP domain-containing protein, contains Pfam profile PF01465: GRIP domain; supporting cDNA gi:20303028:gb:AF499634.1: | chr5:26422147-26427114 REVERSE | Aliases: K2A18.10, K2A18_10	8.5	8.5	-0.0	-0.1
20203	AT5G61670.2	expressed protein | chr5:24800807-24803171 FORWARD | Aliases: K11J9.20, K11J9_20	8.5	8.5	-0.0	-0.2
1324	AT3G05070.1	expressed protein | chr3:1416165-1417743 FORWARD | Aliases: T12H1.3, T12H1_3	8.5	4.8	3.7	8.6
