# Supplementary material for: The transcriptome of syncytia induced by the cyst nematode Heterodera schachtii in Arabidopsis roots
Source: Plant J. 2008 Dec 9;57(5):771–84. doi: 10.1111/j.1365-313X.2008.03727.x (PMC2667683; doi:10.1111/j.1365-313X.2008.03727.x)
Supplement: Supporting Information [file TableS6.pdf]

List of affected genes, confidential preview.

Statistics are testing for differential expression (two-sided non-null).

Ranks refer to this test.

Results are grouped by

Differentially expressed with $q<5\%$	$M>0$	Up-regulation
Differentially expressed with $q<5\%$	$M<0$	Down-regulation
Non-differentially expressed, q of 5% or higher		Insignificant

Orthogonal contrasts examined:

15 dpi Syncytium vs 5 dpi Syncytium ... **Table 3**

Contact: D. Kreil
http://bioinf.boku.ac.at/

Rank	Description	15.dpi	5.dpi	M	t	adj.q	B
2	AT3G15840.3, expressed protein	6.4	2.3	4.0	14.9	0.65%	7.4
3	AT4G39675.1, expressed protein	8.8	2.4	6.5	12.4	1.93%	6.1
4	AT5G54940.2, eukaryotic translation initiation factor SUI1	11.7	9.6	2.1	11.1	3.43%	6.3
5	AT5G50790.1, nodulin MtN3 family protein	8.4	3.7	4.7	10.6	3.46%	3.3
6	AT5G10040.1, expressed protein	8.9	3.8	5.1	10.5	3.46%	4.4
7	AT1G08430.1, expressed protein	9.1	5.4	3.7	10.1	3.67%	3.8
9	AT1G27730.1, zinc finger (C2H2 type) family protein (ZAT10)	9.6	5.8	3.7	9.8	3.67%	4.4
10	AT4G33560.1, expressed protein	8.6	4.9	3.7	9.7	3.67%	4.0
11	AT5G65870.1, phytosulfokines 5 precursor (ATPSK5)	7.1	3.6	3.5	9.6	3.67%	4.4
12	AT1G62480.1, vacuolar calcium-binding protein-related	10.6	7.0	3.6	9.5	3.67%	3.6
13	AT3G19680.1, expressed protein	7.5	5.3	2.2	8.7	4.98%	4.4
14	AT5G10695.1, expressed protein	10.7	7.8	2.9	8.7	4.98%	4.2
16	AT3G03910.1, glutamate dehydrogenase, putative	9.8	5.5	4.3	8.6	4.98%	2.0
17	AT1G10010.1, amino acid permease (AAP8)	8.6	5.4	3.2	8.5	4.98%	3.3
18	AT2G18050.2, histone HIS1-3	6.3	3.5	2.9	8.5	4.98%	3.8
19	AT3G48740.1, nodulin MtN3 family protein	9.8	4.4	5.4	8.4	4.98%	1.9
20	AT2G22860.1, phytosulfokines 2 precursor (ATPSK2)	11.2	8.8	2.5	8.4	4.98%	4.1
21	AT2G17710.1, expressed protein	8.3	3.9	4.4	8.4	4.98%	3.1
22	AT1G74880.1, subunit NDH-O of NAD(P)H:plastoquinone dehydrogenase complex	5.4	2.8	2.6	8.4	4.98%	3.6
1	AT3G60140.1, glycosyl hydrolase family 1 protein (DIN2)	3.0	8.6	-5.6	-20.2	0.12%	7.3
8	AT5G05340.1, peroxidase, putative	2.8	8.8	-6.0	-9.8	3.67%	4.9
15	AT3G50300.1, transferase family protein	4.1	7.7	-3.5	-8.6	4.98%	3.0
