# Supplementary figures and images for: The transcriptome of syncytia induced by the cyst nematode Heterodera schachtii in Arabidopsis roots
Source: Plant J. 2008 Dec 9;57(5):771–84. doi: 10.1111/j.1365-313X.2008.03727.x (PMC2667683; doi:10.1111/j.1365-313X.2008.03727.x)

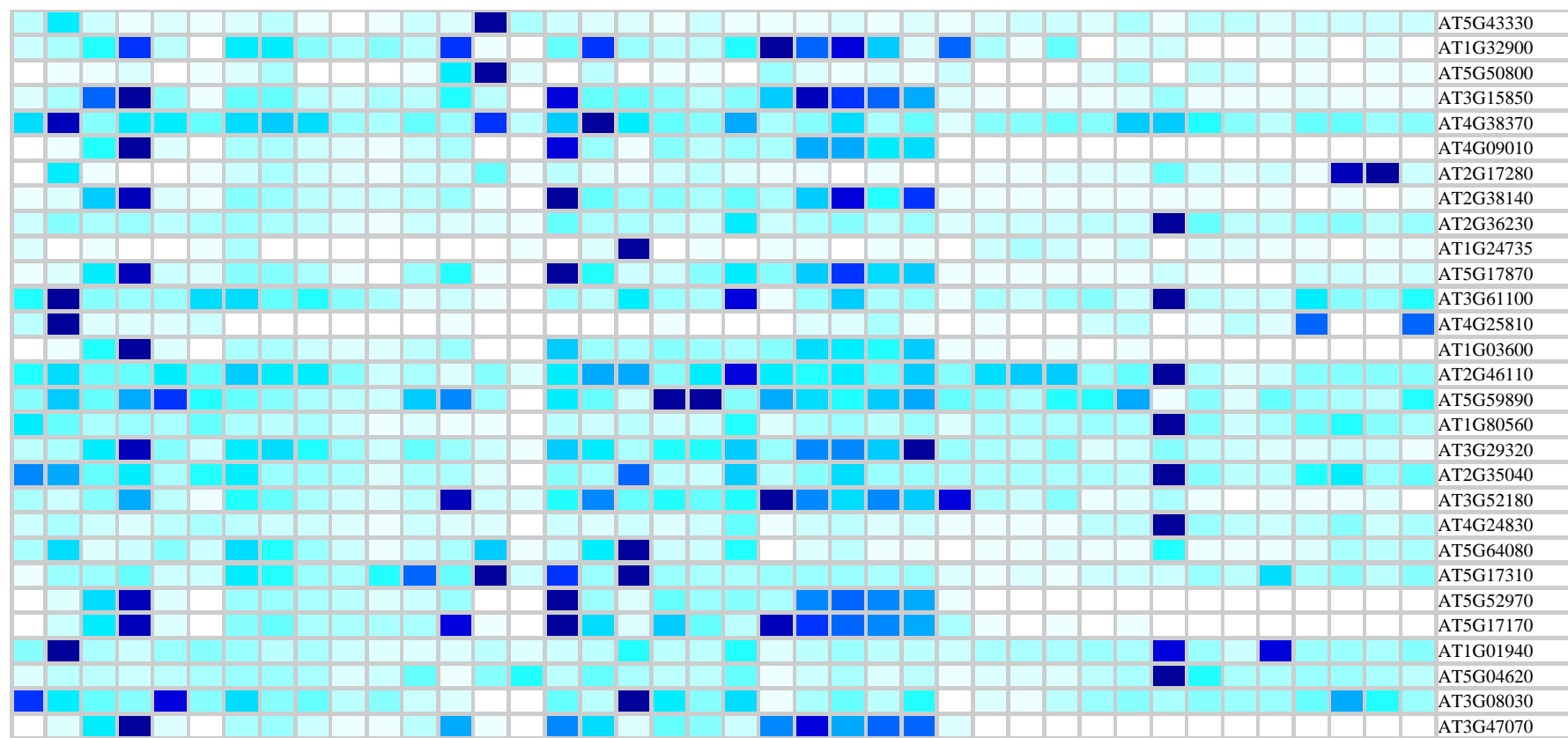

Supplement: Supporting Information [file TableS9.pdf]

Contrast: Syn vs control

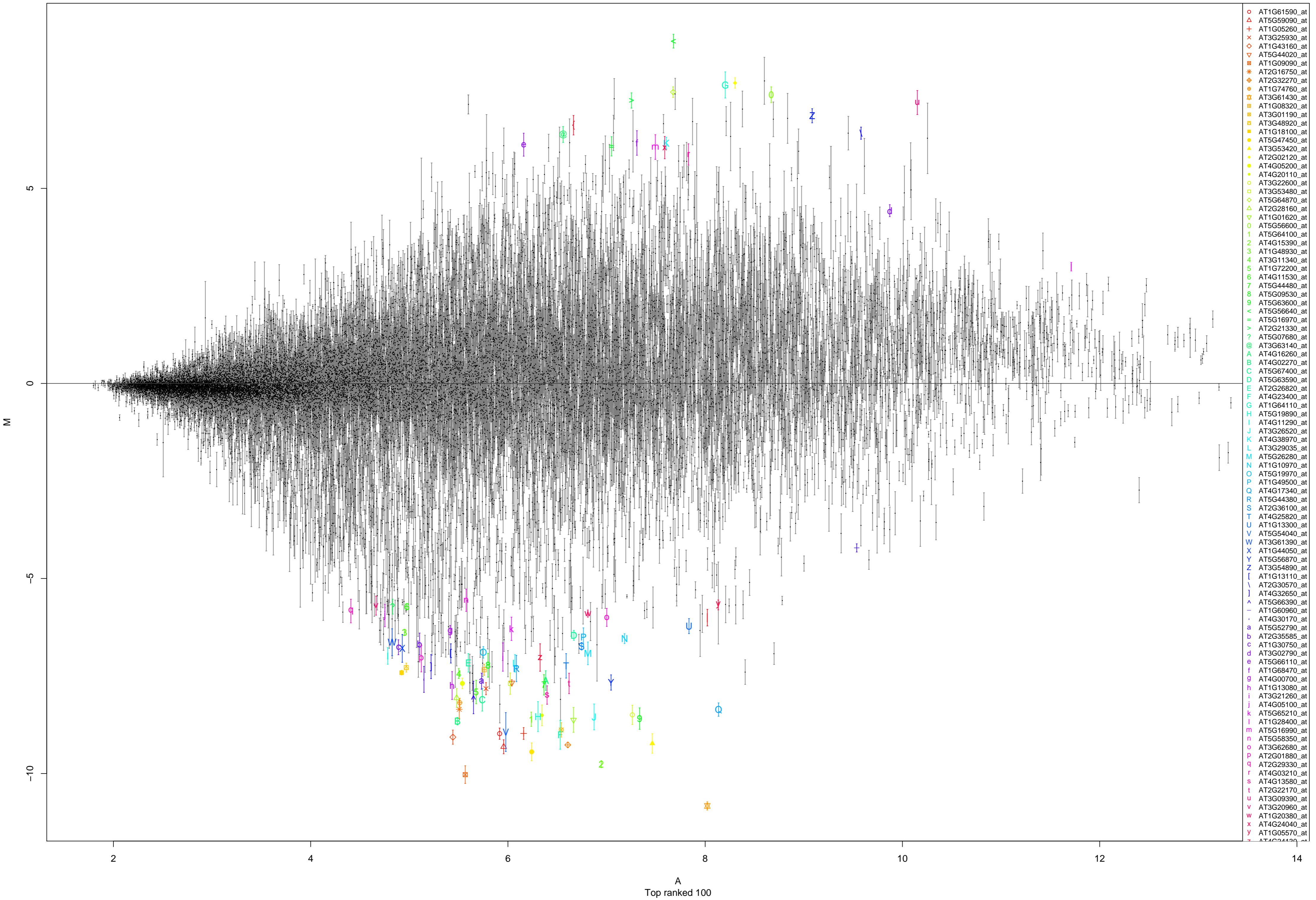

Supplement: Supporting Information [file FigS1.pdf]

Contrast: Syn15 vsSyn5

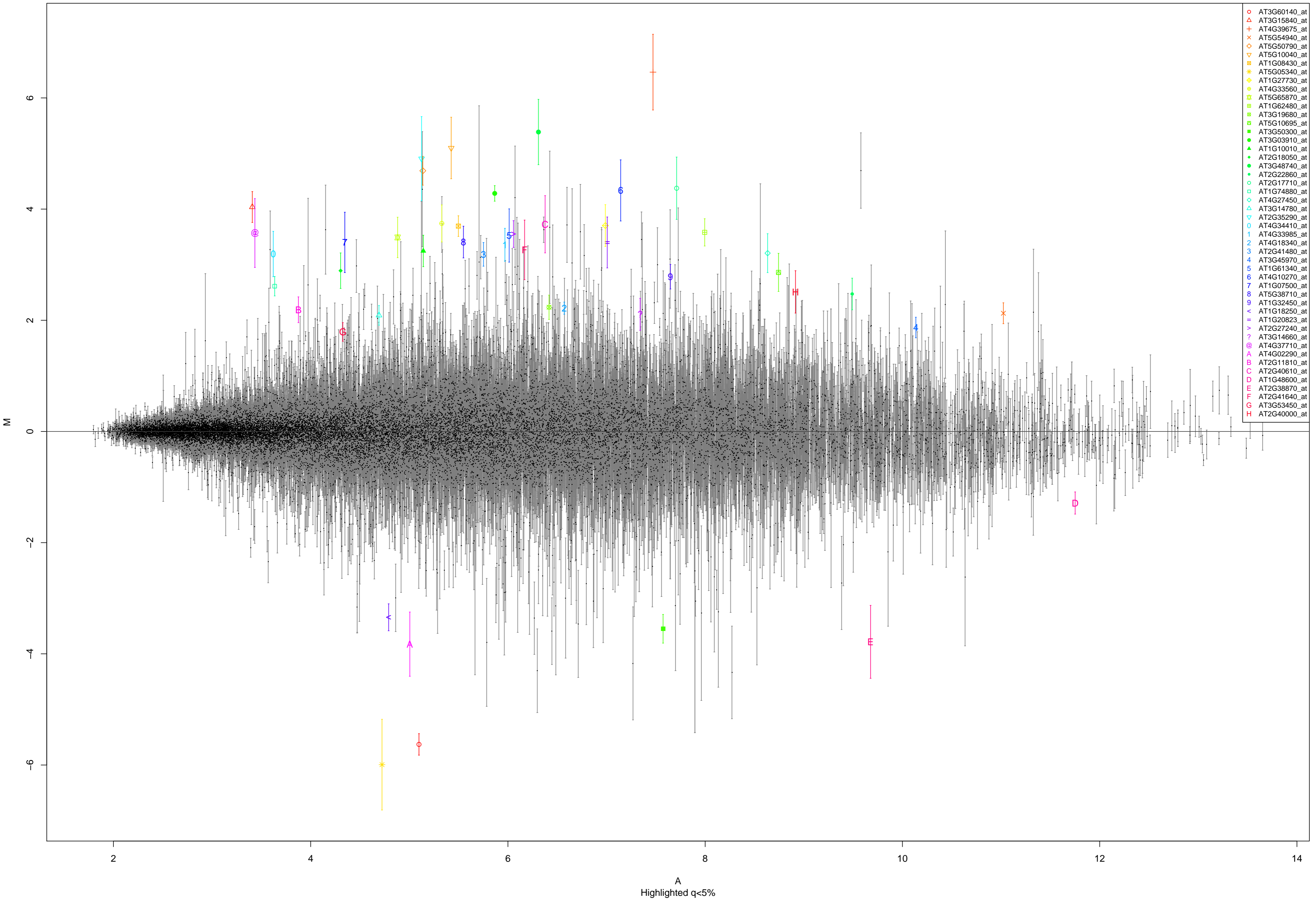

Supplement: Supporting Information [file FigS2.pdf]

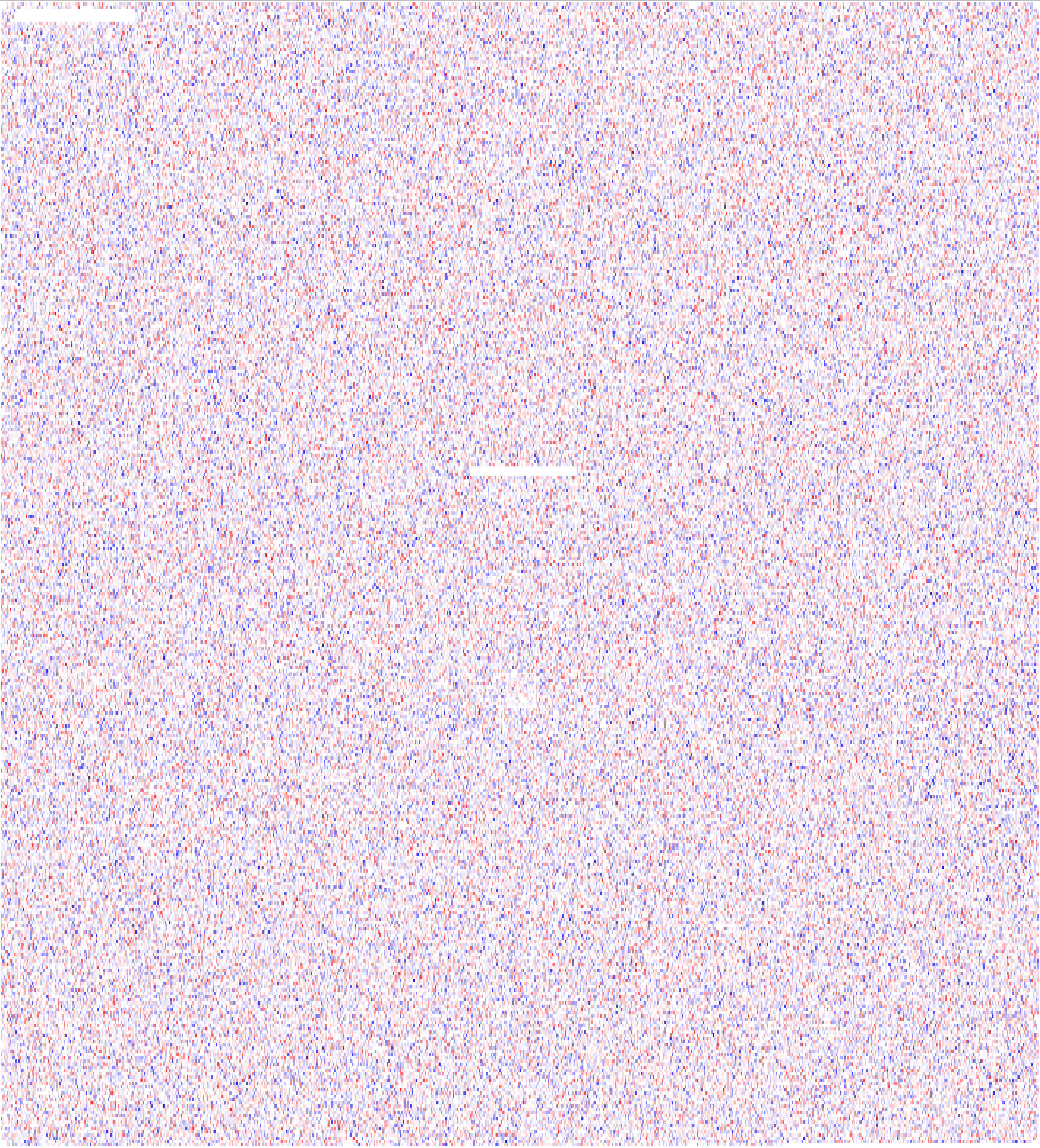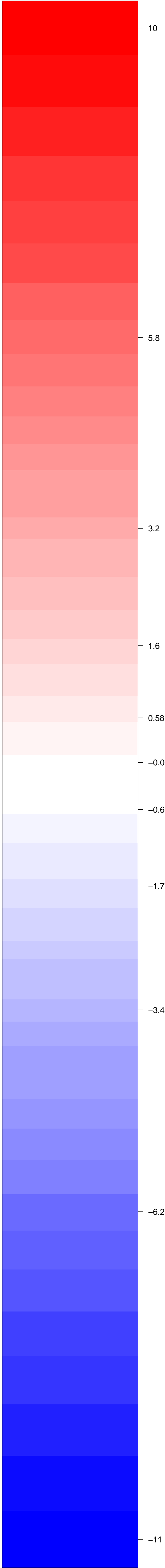

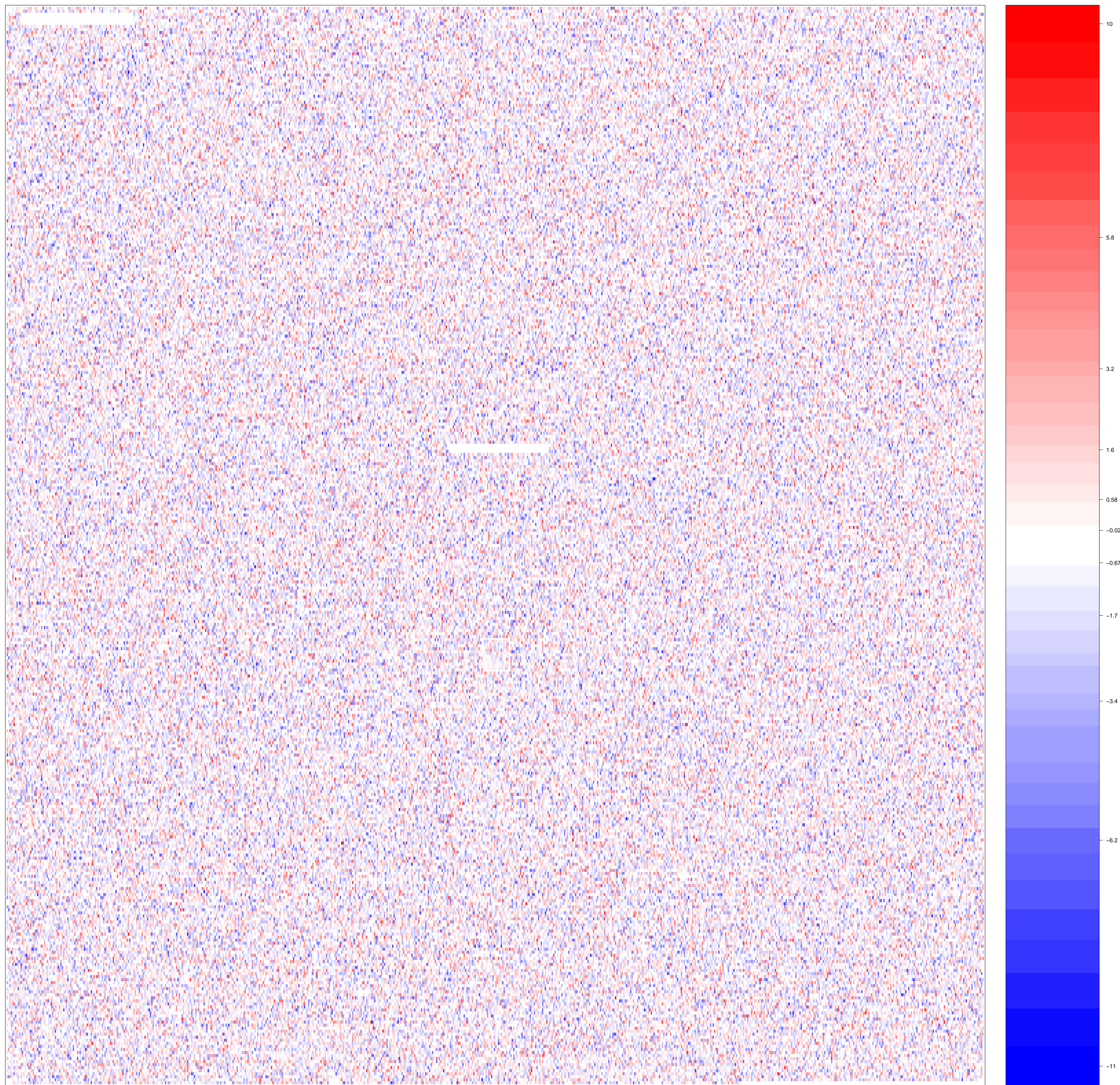

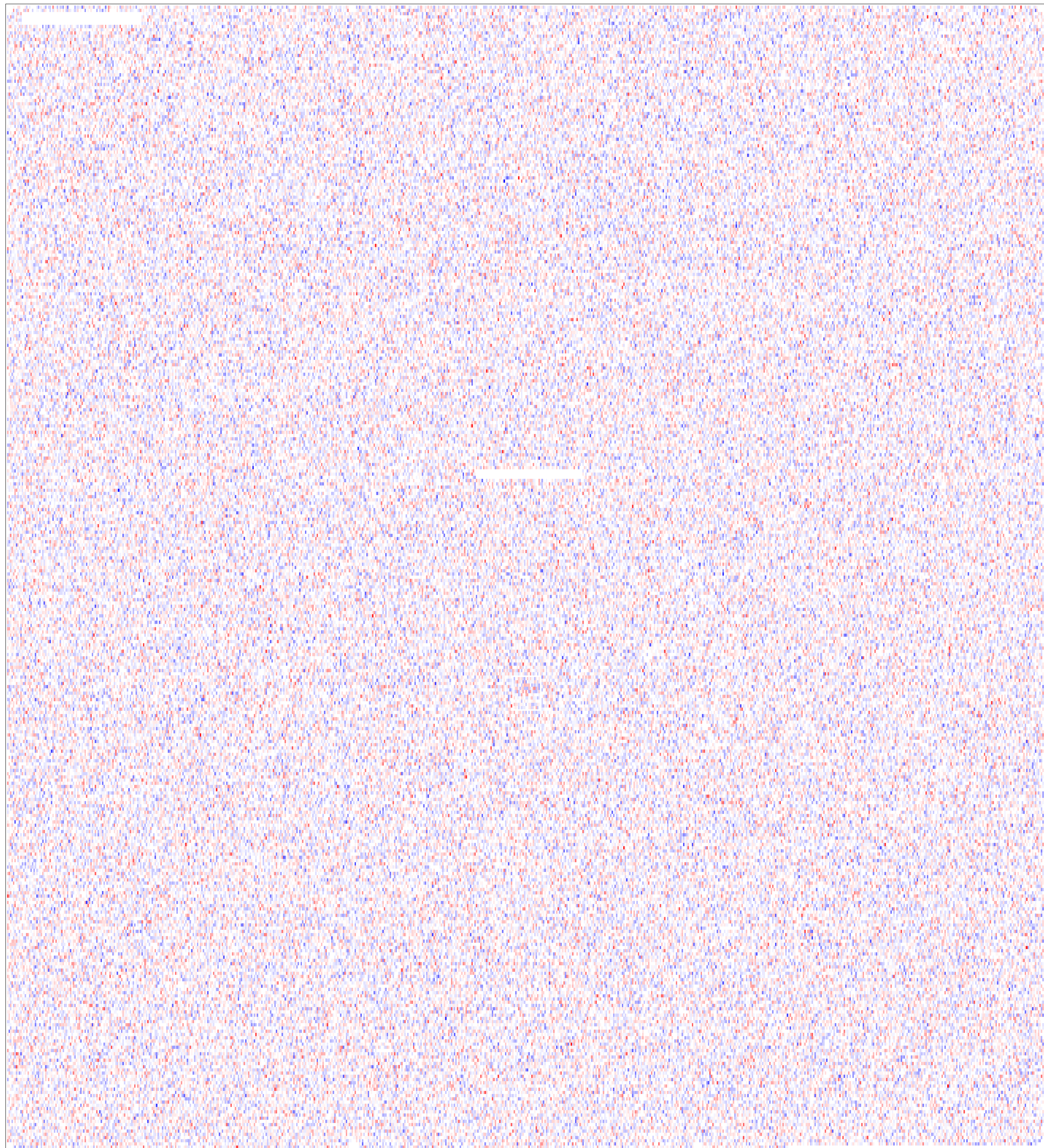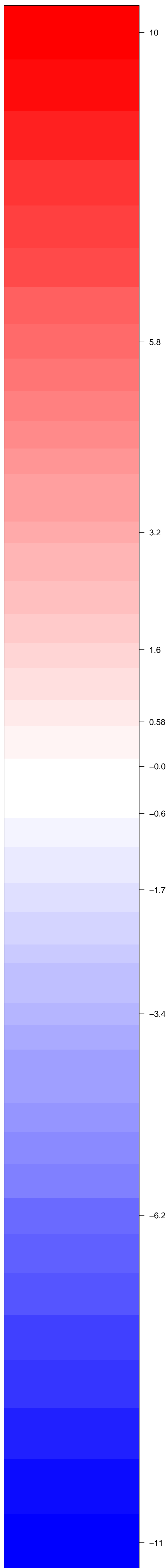

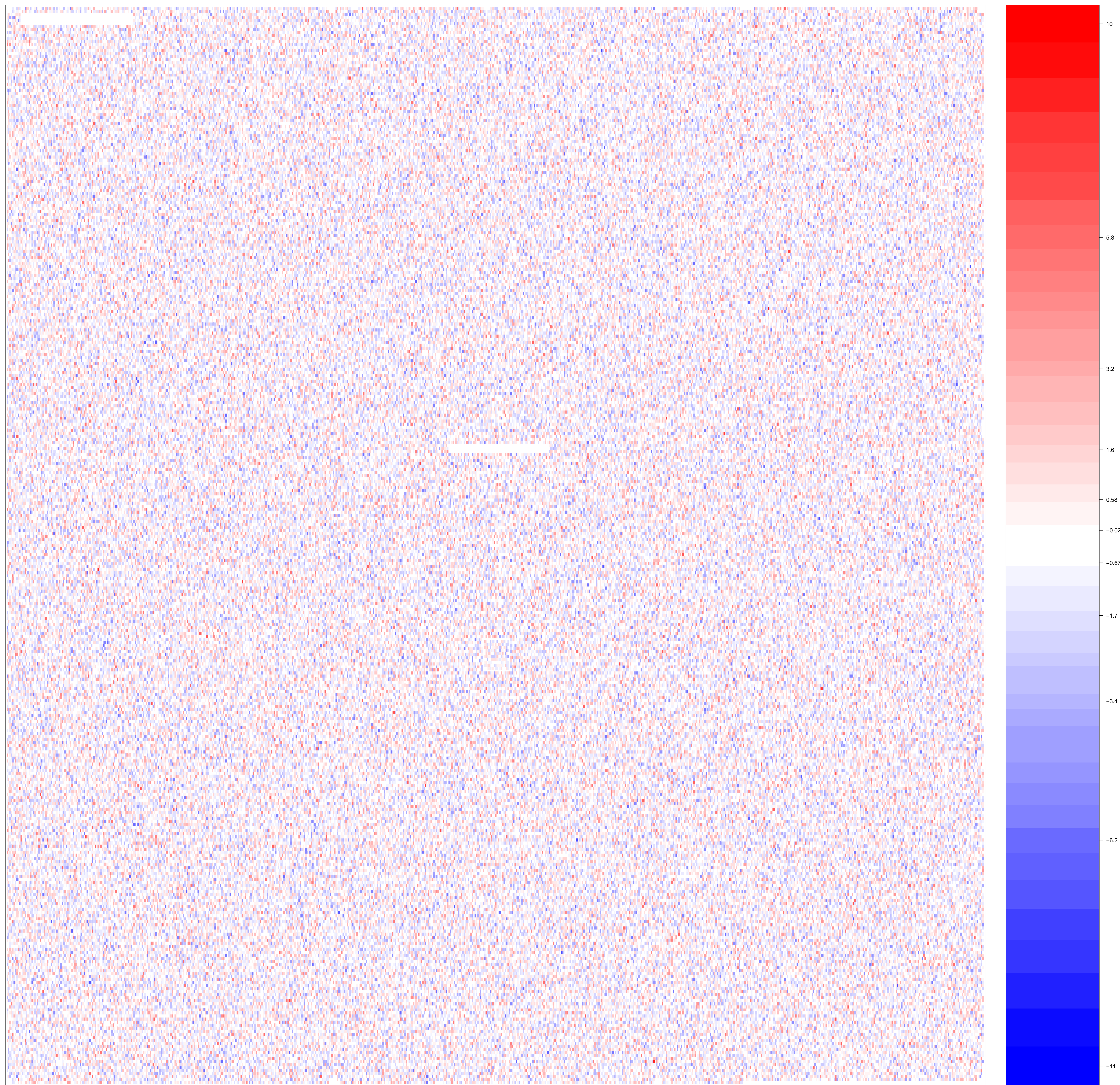

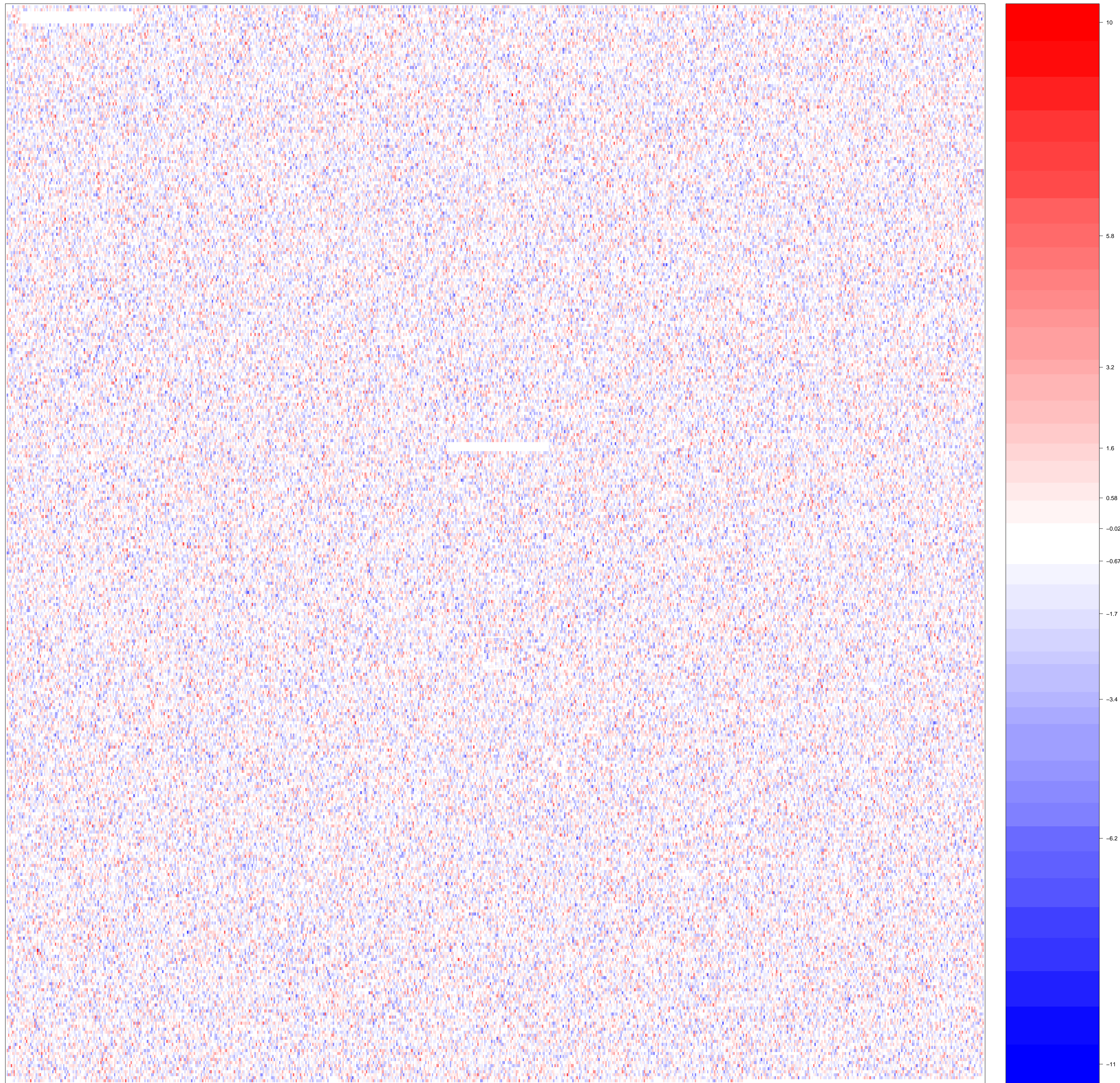

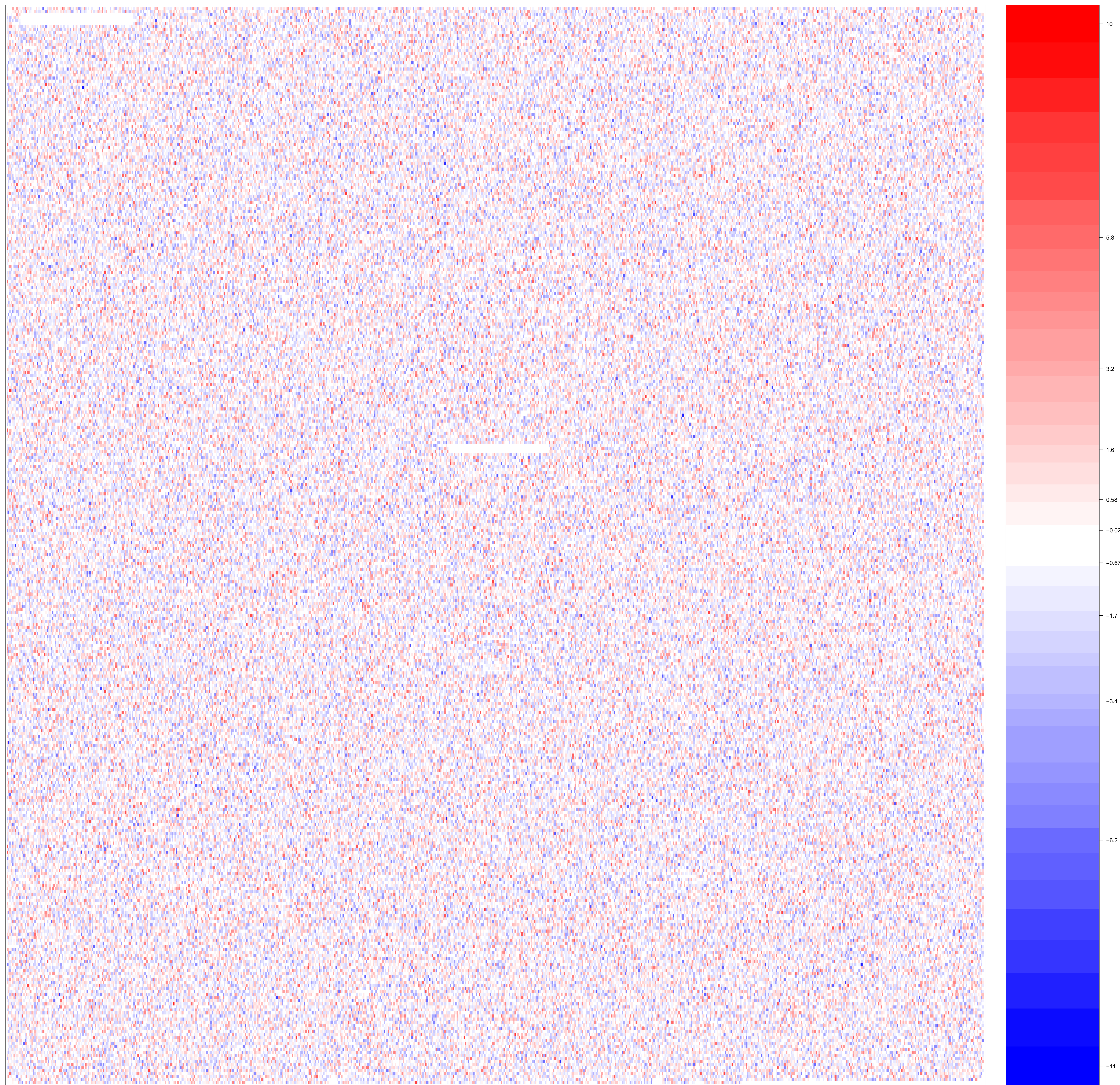

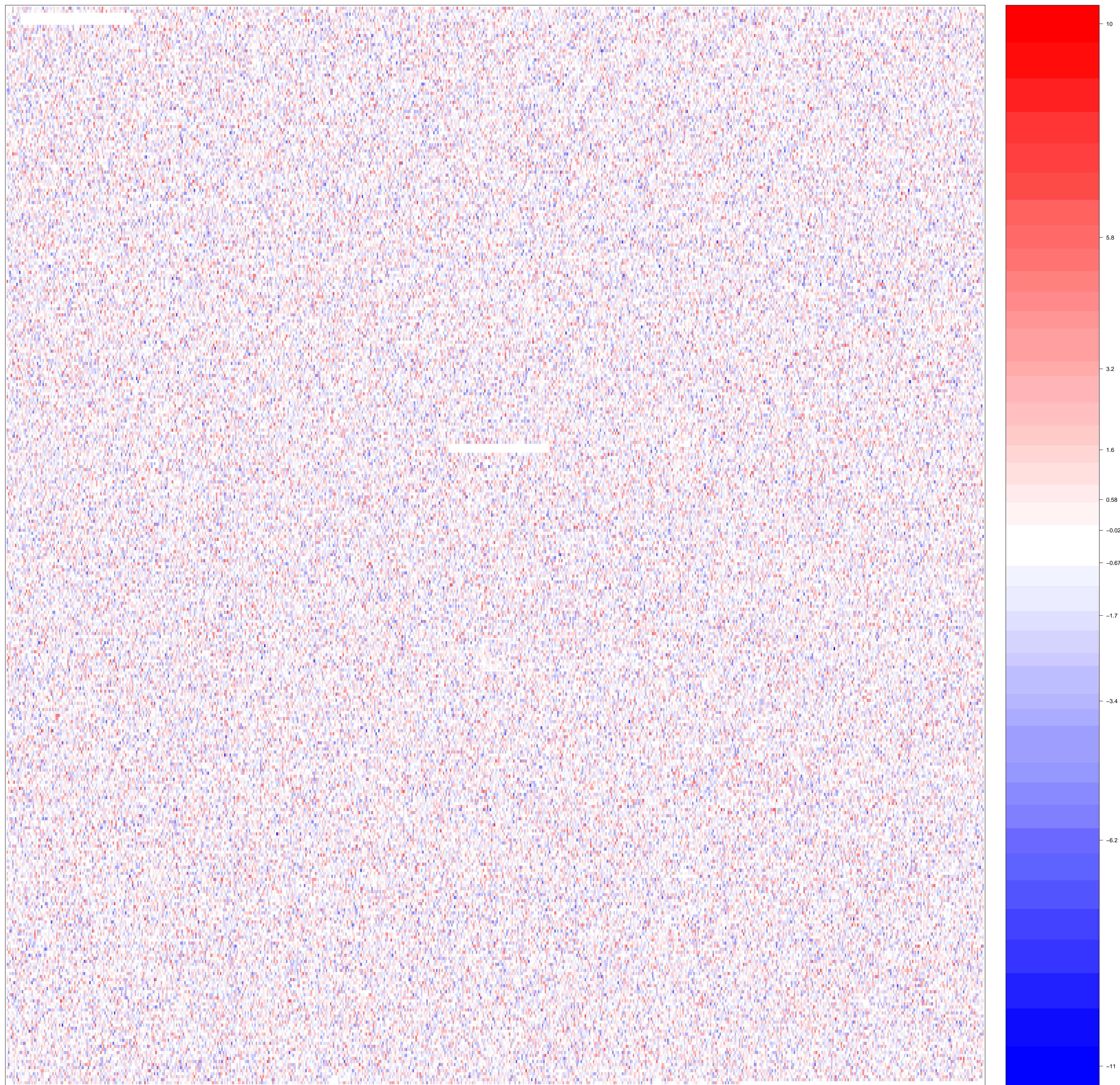

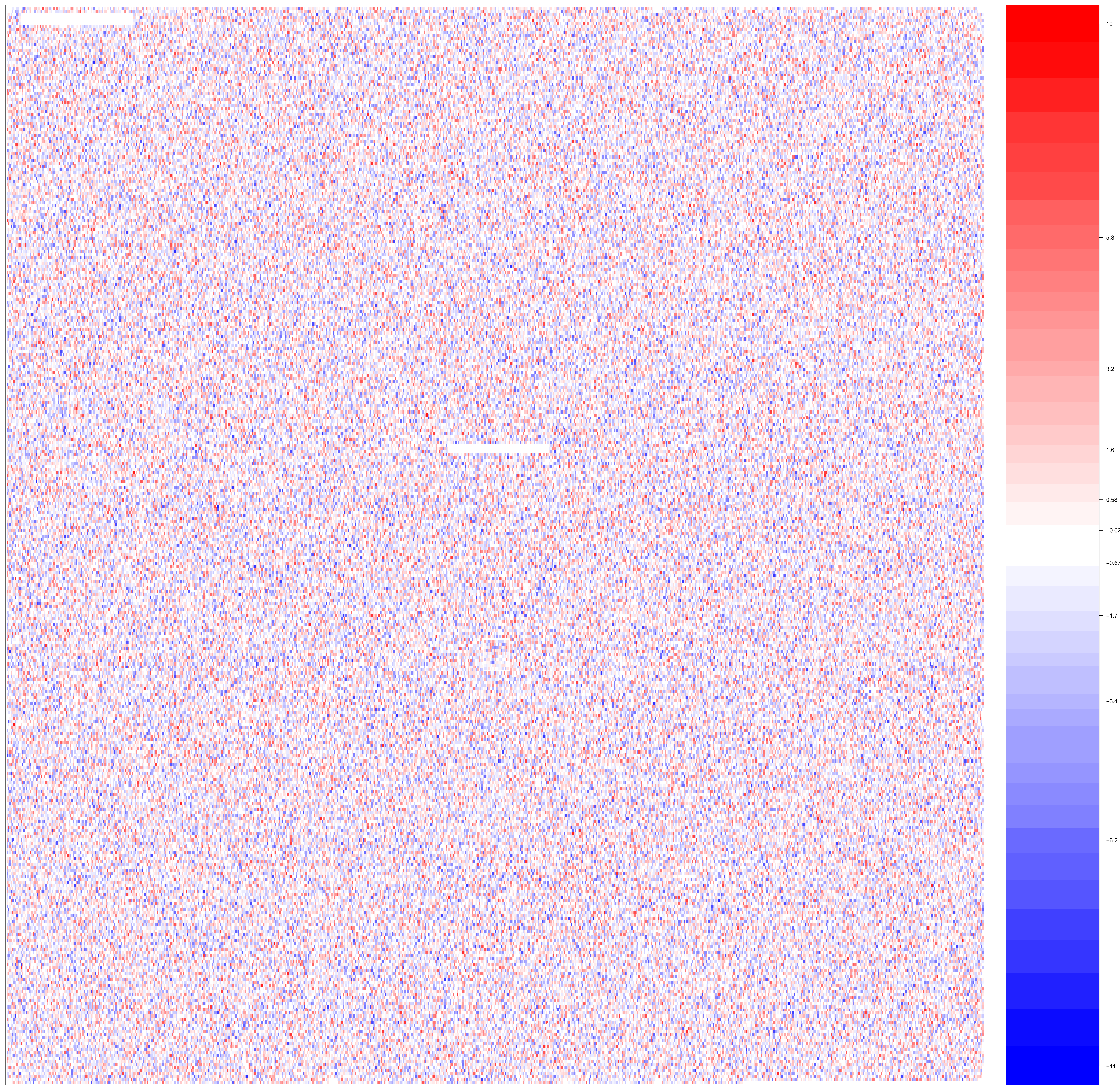

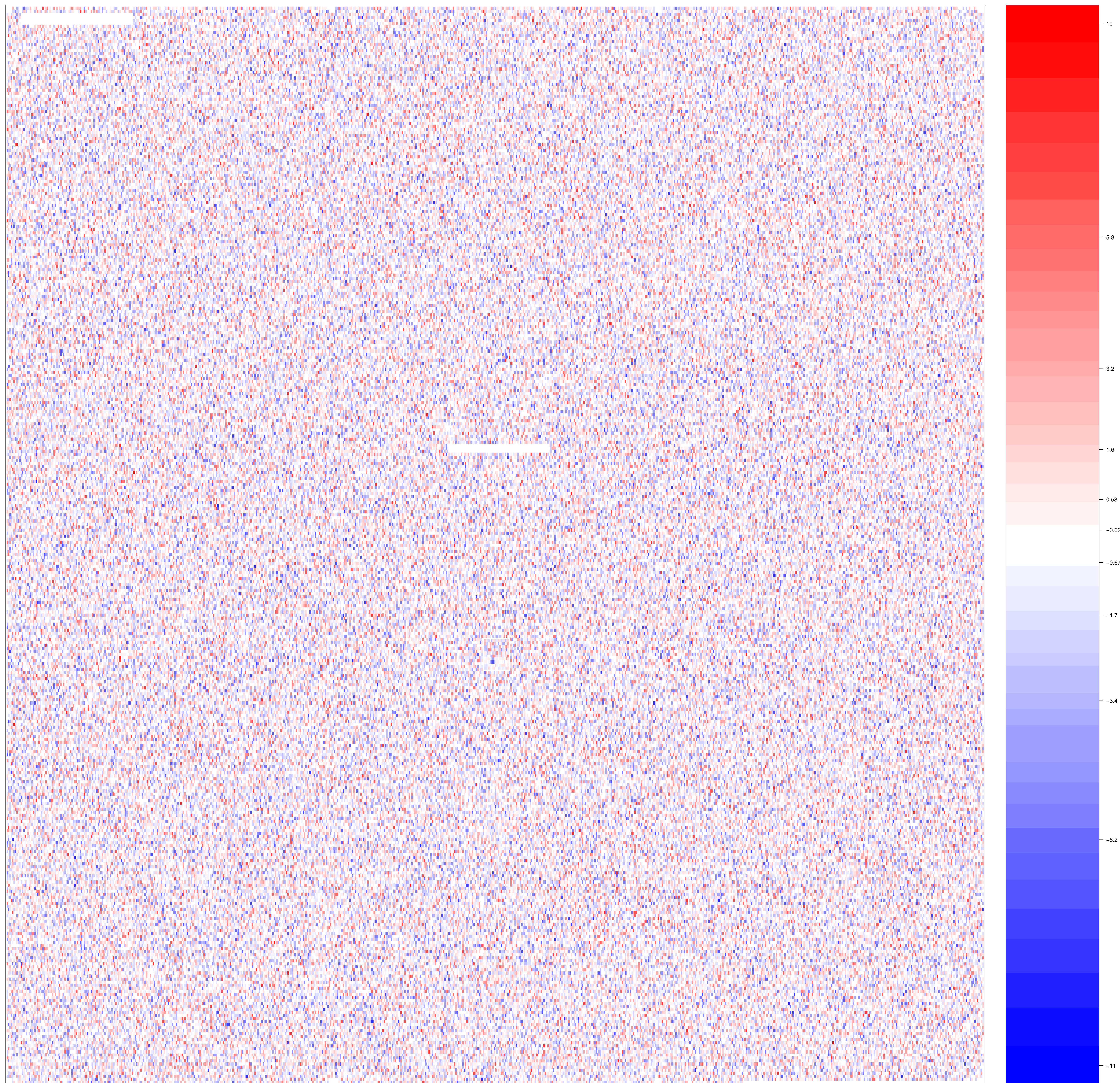

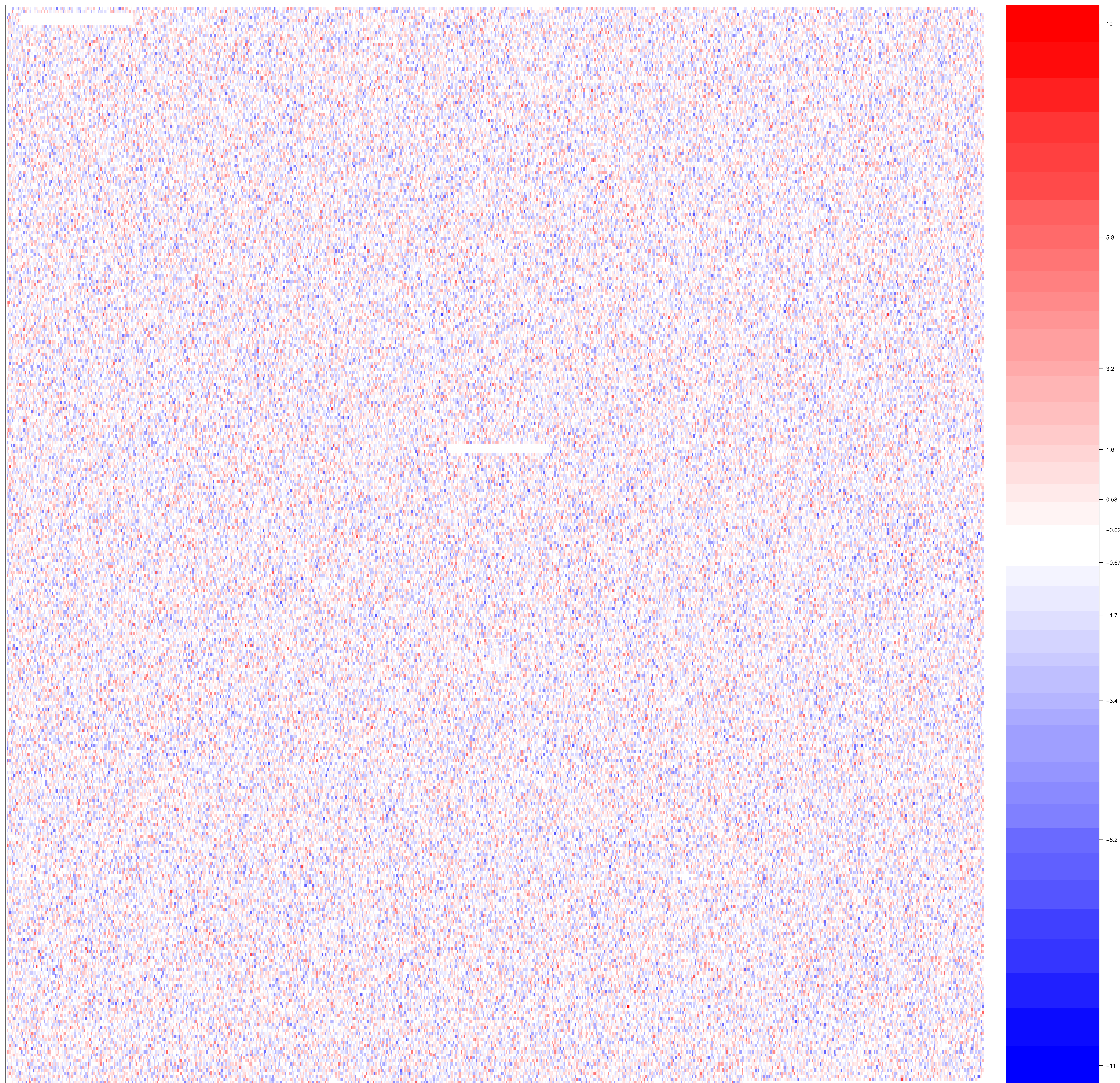

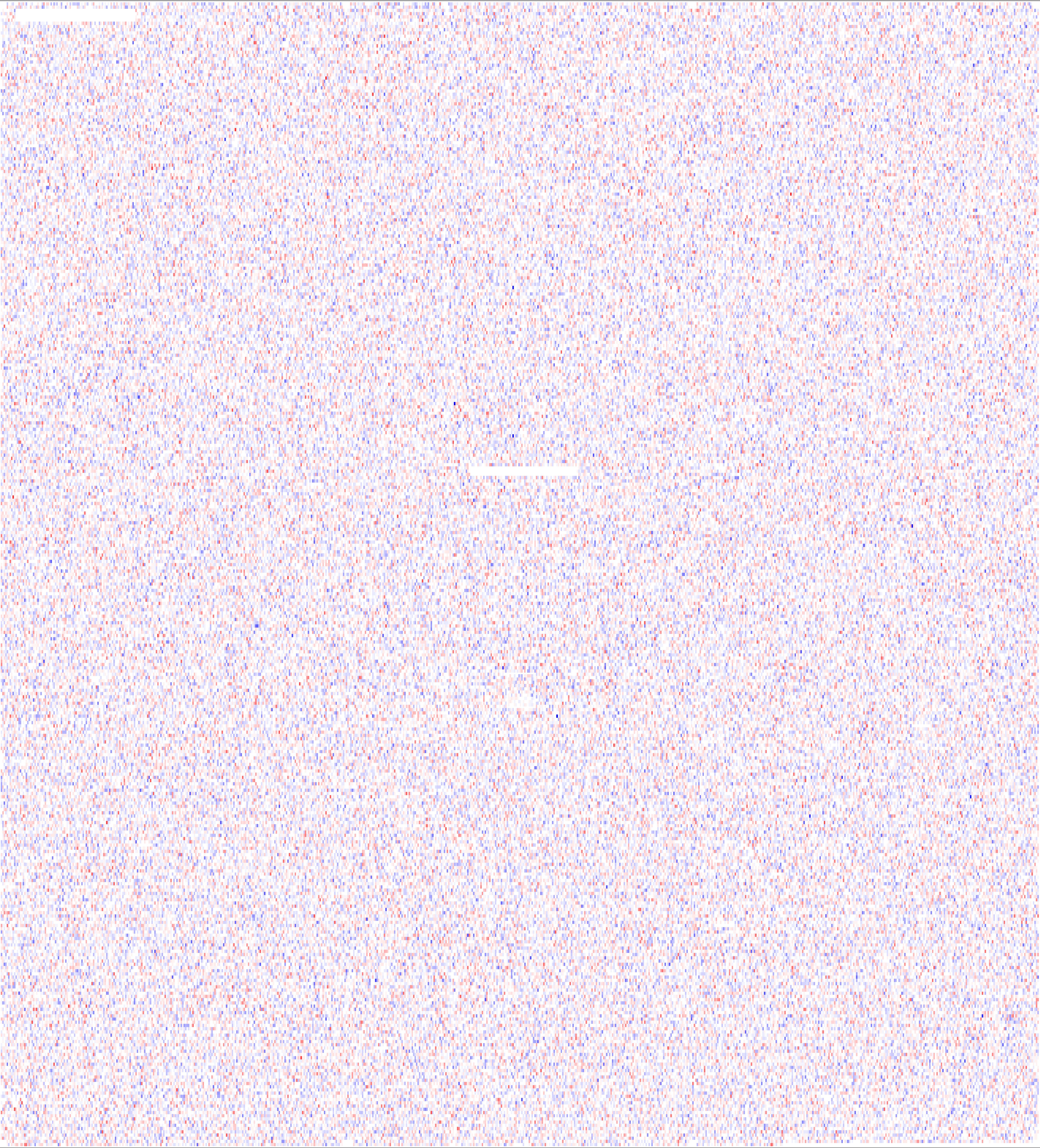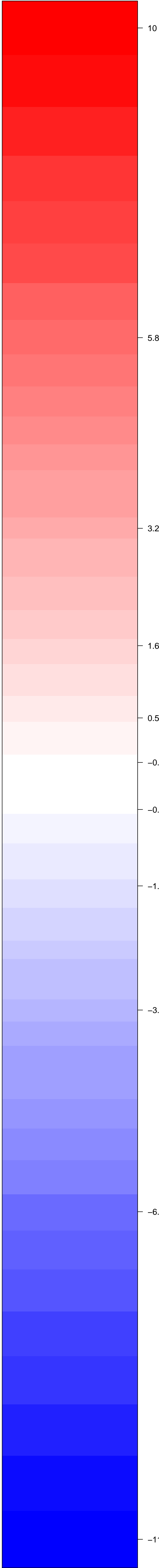

Supplement: Supporting Information [file gcrma.qq.PLM.resids.pdf]

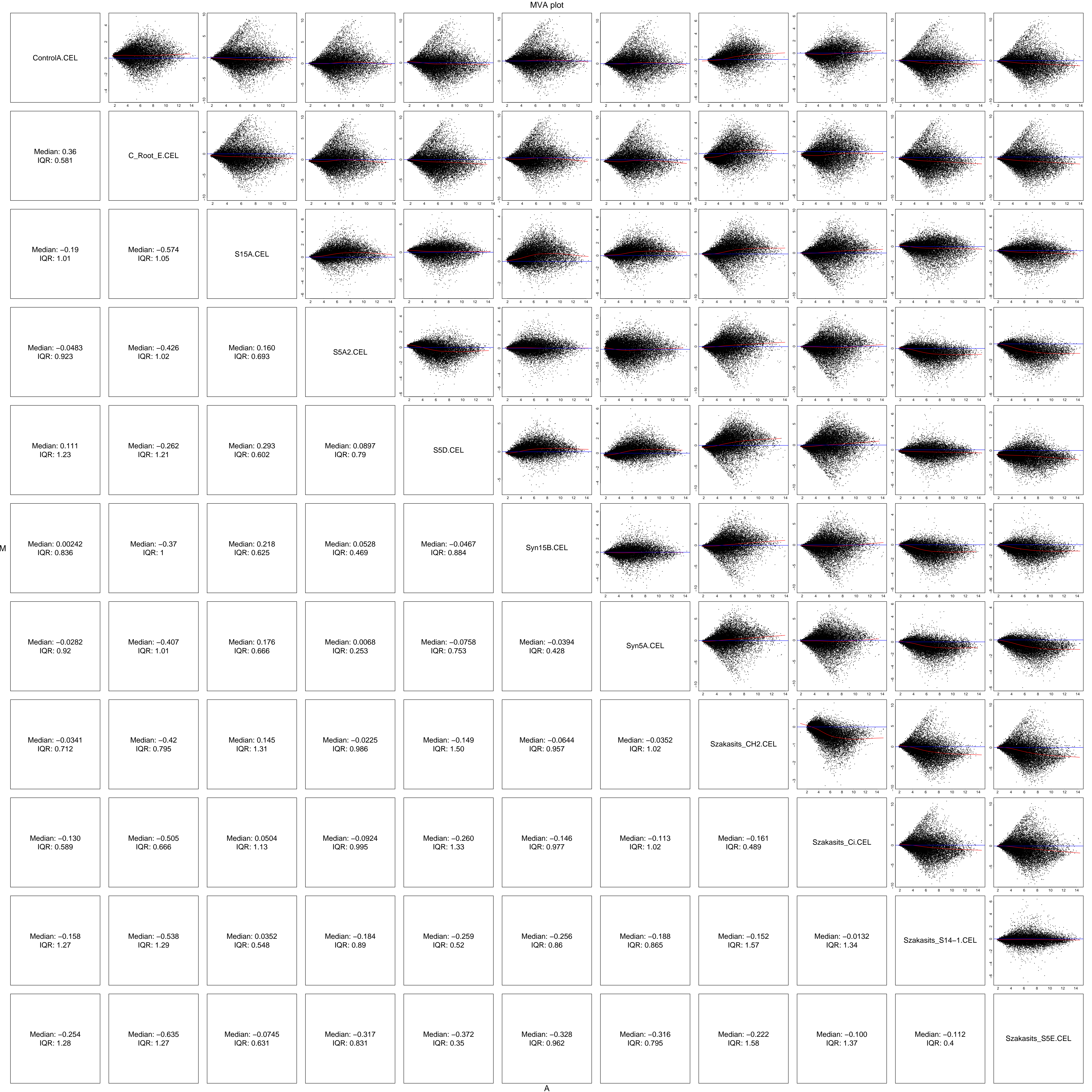

Supplement: Supporting Information [file gcrma.raw.PLM.MAs.pdf]

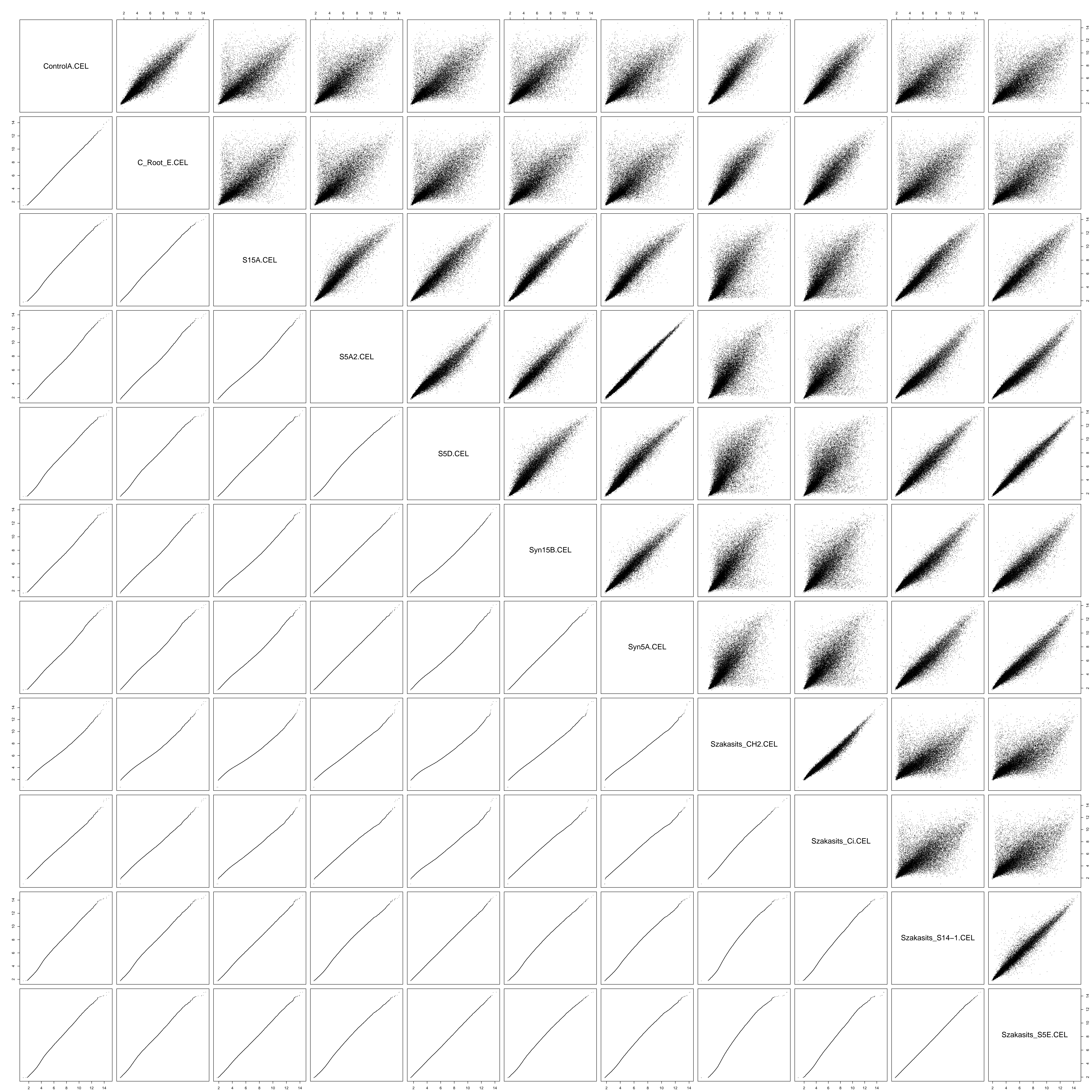

Supplement: Supporting Information [file gcrma.raw.PLM.QQnScatters.pdf]
