# Supplementary material for: The transcriptome of syncytia induced by the cyst nematode Heterodera schachtii in Arabidopsis roots
Source: Plant J. 2008 Dec 9;57(5):771–84. doi: 10.1111/j.1365-313X.2008.03727.x (PMC2667683; doi:10.1111/j.1365-313X.2008.03727.x)
Supplement: Supporting Information [file TableS1.pdf]

**List of affected genes, confidential preview.**

*Statistics are testing for differential expression (two-sided non-null).*

Ranks refer to this test.

Results are grouped by

| | | |
|---|---|---|
| Differentially expressed with *q*<5% | *M*>0 | Up-regulation |
| Differentially expressed with *q*<5% | *M*<0 | Down-regulation |
| Non-differentially expressed, *q* of 5% or higher | | Insignificant |

Orthogonal contrasts examined:



| | | *up* | *down* | *non-diff* |
|---|---|---|---|---|
| (5 dpi and 15 dpi Syncytium) vs Root | ... **Table S1** | page 2 | page 189 | page 355 |



Contact: D. Kreil
http://bioinf.boku.ac.at/

| Rank | Description | Sync | Root | M | t | adj.q | B |
|---|---|---|---|---|---|---|---|
| 18 | AT2G02120.1 \| Symbol: LCR70/PDF2.1 \| plant defensin-fusion protein, putative (PDF2.1), plant defensin protein family member, personal communication, Bart Thomma (Bart.Thomma@agr.kuleuven.ac.be); contains a gamma-thionin family signature (PDOC00725) \| chr2:538254-539054 FORWARD \| Aliases: F5O4.11, F5O4_11, LCR70, Low molecular weight cysteine rich 70, PDF2.1 | 11.0 | 3.3 | 7.7 | 33.0 | 1.2E-6 | 13.0 |
| 23 | AT5G64870.1 \| expressed protein \| chr5:25946764-25948610 REVERSE \| Aliases: MXK3.10, MXK3_10 | 10.4 | 2.9 | 7.5 | 30.0 | 1.9E-6 | 12.4 |
| 26 | AT5G56600.1 \| Symbol: PRF3 \| profilin 5 (PRO5) (PRF3), identical to SP:Q9FE63 Profilin 5 {Arabidopsis thaliana} \| chr5:22926914-22928047 REVERSE \| Aliases: MIK19.4, MIK19_4, PFN3, PROFILIN, PROFILIN 3 | 11.5 | 4.1 | 7.4 | 29.8 | 1.9E-6 | 13.1 |
| 36 | AT5G56640.1 \| Symbol: MIOX5 \| expressed protein, similar to myo-inositol oxygenase (Sus scrofa) gi:17432544:gb:AAL39076 \| chr5:22944555-22946869 REVERSE \| Aliases: MIK19.9, MIK19_9 | 10.9 | 2.1 | 8.8 | 27.6 | 2.6E-6 | 10.8 |
| 37 | AT5G16970.1 \| NADP-dependent oxidoreductase, putative (P1), identical to probable NADP-dependent oxidoreductase P1, zeta-crystallin homolog (SP:Q39172)(gi:886428), Arabidopsis thaliana; similar to allyl alcohol dehydrogenase (Nicotiana tabacum) GI:6692816; contains Pfam profile PF00107: oxidoreductase, zinc-binding dehydrogenase family \| chr5:5576171-5578049 REVERSE \| Aliases: F2K13.120, F2K13_120 | 9.2 | 3.2 | 6.1 | 27.4 | 2.6E-6 | 14.9 |
| 38 | AT2G21330.3 \| similar to fructose-bisphosphate aldolase, putative [Arabidopsis thaliana] (TAIR:At4g38970.1); similar to plastidic aldolase NPALDP1 [Nicotiana paniculata] (GB:BAA77604.1); similar to latex plastidic aldolase-like protein [Hevea brasiliensis] (GB:AAM46780.1); contains InterPro domain Fructose-bisphosphate aldolase, class-I (InterPro:IPR000741) \| chr2:9135228-9137293 REVERSE \| Aliases: F3K23.9, F3K23_9 | 9.9 | 2.7 | 7.3 | 27.3 | 2.6E-6 | 12.3 |
| 40 | AT3G63140.1 \| mRNA-binding protein, putative, similar to mRNA binding protein precursor (GI:26453355) (Lycopersicon esculentum) \| chr3:23337347-23339684 REVERSE \| Aliases: T20O10.240 | 8.9 | 2.5 | 6.4 | 27.1 | 2.6E-6 | 13.4 |
| 47 | AT1G64110.2 \| AAA-type ATPase family protein, contains Pfam domain, PF00004: ATPase, AAA family \| chr1:23800382-23805388 REVERSE \| Aliases: F22C12.12, F22C12_12 | 11.0 | 3.3 | 7.7 | 26.6 | 2.7E-6 | 13.9 |
| 51 | AT4G38970.2 \| fructose-bisphosphate aldolase, putative, strong similarity to plastidic fructose-bisphosphate aldolase (EC 4.1.2.13) from Nicotiana paniculata (NPALDP1) (GI:4827251), Oryza sativa, PIR2:T02057 (SP:Q40677) \| chr4:18163490-18165734 REVERSE \| Aliases: F19H22.70, F19H22_70 | 9.9 | 3.8 | 6.2 | 25.6 | 3.3E-6 | 12.5 |
| 66 | AT3G54890.3 \| Symbol: LHCA1 \| chlorophyll A-B binding protein / LHCI type I (CAB), identical to chlorophyll A/B-binding protein (Arabidopsis thaliana) GI:16207; contains Pfam profile: PF00504 chlorophyll A-B binding protein \| chr3:20350482-20351968 REVERSE \| Aliases: F28P10.130, F28P10_130, LHCA1 | 11.5 | 4.7 | 6.9 | 24.0 | 4.4E-6 | 11.4 |
| 68 | AT2G30570.2 \| photosystem II reaction center W (PsbW) protein-related, similar to photosystem II reaction center W protein SP:Q41387 from (Spinacia oleracea) \| chr2:13025520-13027271 REVERSE \| Aliases: T6B20.8, T6B20_8 | 12.0 | 5.6 | 6.4 | 23.5 | 5.0E-6 | 11.5 |
| 76 | AT3G02790.1 \| zinc finger (C2H2 type) family protein, contains Pfam profile: PF00096 zinc finger, C2H2 type \| chr3:604852-605428 FORWARD \| Aliases: F13E7.27, F13E7_27 | 11.5 | 7.0 | 4.4 | 22.4 | 6.6E-6 | 13.4 |
| 77 | AT5G66110.1 \| similar to heavy-metal-associated domain-containing protein / copper chaperone (CCH)-related [Arabidopsis thaliana] (TAIR:At4g38580.1); similar to Putative atfp6-like protein [Oryza sativa (japonica cultivar-group)] (GB:XP_470243.1); contains InterPro domain Heavy metal binding (InterPro:IPR006191); contains InterPro domain Heavy metal transport/detoxification protein (InterPro:IPR006121) \| chr5:26447428-26448138 FORWARD \| Aliases: K2A18.19, K2A18_19 | 8.5 | 2.3 | 6.1 | 22.3 | 6.6E-6 | 13.1 |
| 78 | AT1G68470.1 \| exostosin family protein, contains Pfam profile: PF03016 | 9.6 | 3.5 | 6.2 | 22.2 | 6.8E-6 | 13.3 |
| 84 | AT1G28400.1 \| expressed protein, similar to E6 (GI:1000090) (Gossypium barbadense) \| chr1:9972370-9973779 REVERSE \| Aliases: F3M18.16, F3M18_16 | 12.9 | 10.0 | 3.0 | 21.5 | 8.1E-6 | 14.5 |
| 85 | AT5G16990.1 \| NADP-dependent oxidoreductase, putative, strong similarity to probable NADP-dependent oxidoreductase (zeta-crystallin homolog) P1 (SP:Q39172)(gi:886428) and P2 (SP:Q39173)(gi:886430), Arabidopsis thaliana \| chr5:5581661-5584032 REVERSE \| Aliases: F2K13.140, F2K13_140 | 9.7 | 3.7 | 6.1 | 21.5 | 8.1E-6 | 13.1 |
| 90 | AT4G03210.2 \| Symbol: XTH9 \| similar to xyloglucan:xyloglucosyl transferase / xyloglucan endotransglycosylase / endo-xyloglucan transferase (EXGT-A4) [Arabidopsis thaliana] (TAIR:At5g13870.1); similar to sadtomato protein [Capsicum annuum] (GB:AAS77347.1); contains InterPro domain Glycoside hydrolase, family 16 (InterPro:IPR000757); contains InterPro domain Beta-glucanase (InterPro:IPR008264) \| chr4:1415953-1417352 FORWARD \| Aliases: F4C21.14, F4C21_14, XYLOGLUCAN ENDOTRANSGLUCOSYLASE/HYDROLASE 9 | 10.0 | 4.1 | 5.9 | 21.2 | 8.5E-6 | 12.4 |
| 93 | AT3G09390.1 \| Symbol: MT2A \| metallothionein protein, putative (MT2A), identical to Swiss-Prot:P25860 metallothionein-like protein 2A (MT-2A) (MT-K) (MT-1G) (Arabidopsis thaliana) \| chr3:2889492-2890235 REVERSE \| Aliases: ATMT 1, ATMT K, F3L24.28, METALLOTHIONEIN 2A, MT2 | 12.7 | 5.5 | 7.2 | 21.1 | 8.5E-6 | 11.0 |
| 96 | AT4G24040.1 \| Symbol: ATTRE1 \| | 9.9 | 3.8 | 6.0 | 21.1 | 8.5E-6 | 12.3 |

| Rank | Description | Sync | Root | M | t | adj.q | B |
|---|---|---|---|---|---|---|---|
| 99 | AT5G01300.1 \| phosphatidylethanolamine-binding family protein, similar to cold-regulated protein (Hordeum vulgare subsp. vulgare) GI:10799810; contains Pfam profile PF01161: Phosphatidylethanolamine-binding protein | 9.1 | 2.5 | 6.6 | 20.9 | 9.0E-6 | 10.9 |
| 104 | AT3G26650.1 \| Symbol: GAPA \| glyceraldehyde 3-phosphate dehydrogenase A, chloroplast (GAPA) / NADP-dependent glyceraldehydephosphate dehydrogenase subunit A, identical to SP:P25856 Glyceraldehyde 3-phosphate dehydrogenase A, chloroplast precursor (EC 1.2.1.13) (NADP-dependent glyceraldehydephosphate dehydrogenase subunit A) {Arabidopsis thaliana} \| chr3:9796330-9798282 FORWARD \| Aliases: GAPA 1, GLYCERALDEHYDE 3 PHOSPHATE DEHYDROGENASE A SUBUNIT, GLYCERALDEHYDE 3 PHOSPHATE DEHYDROGENASE A SUBUNIT, MLJ15.3 | 10.4 | 2.9 | 7.4 | 20.4 | 1.0E-5 | 11.5 |
| 106 | AT2G28900.1 \| mitochondrial import inner membrane translocase subunit Tim17/Tim22/Tim23 family protein, contains Pfam PF02466: Mitochondrial import inner membrane translocase subunit Tim17 \| chr2:12421191-12422655 REVERSE \| Aliases: F8N16.19, F8N16_19 | 10.9 | 5.5 | 5.4 | 20.4 | 1.0E-5 | 11.5 |
| 120 | AT2G07340.2 \| prefoldin-related KE2 family protein, contains similarity to Swiss-Prot:O60925 prefoldin subunit 1 (Homo sapiens); contains Pfam domain, PF01920: KE2 family protein \| chr2:3045556-3046716 FORWARD \| Aliases: T13E11.11, T13E11_11 | 9.8 | 5.9 | 3.9 | 19.7 | 1.2E-5 | 13.0 |
| 124 | AT1G68010.1 \| Symbol: HPR \| glycerate dehydrogenase / NADH-dependent hydroxypyruvate reductase, identical to hydroxypyruvate reductase (HPR) GB:D85339 (Arabidopsis thaliana) (Plant Cell Physiol 1997 Apr;38(4):449-55) \| chr1:25497016-25499651 FORWARD \| Aliases: None | 7.8 | 2.6 | 5.3 | 19.4 | 1.3E-5 | 11.2 |
| 134 | AT2G42570.1 \| expressed protein \| chr2:17724453-17727158 REVERSE \| Aliases: F14N22.16, F14N22_16 | 9.1 | 5.2 | 3.9 | 18.6 | 1.6E-5 | 12.5 |
| 135 | AT2G36230.1 \| Symbol: BBM II II \| N'-5'-phosphoribosyl-formimino-5-aminoimidazole-4-carboxamide ribonucleotide isomerase, identical to N'-5'-phosphoribosyl-formimino-5-aminoimidazole- 4-carboxamide ribonucleotide isomerase GI:3449284 from (Arabidopsis thaliana); contains Pfam profile: PF00977 histidine biosynthesis protein \| chr2:15200779-15202617 REVERSE \| Aliases: F2H17.16, F2H17_16, PHOSPHORIBOSYLFORMIMINO 5 AMINOIMIDAZOLE CARBOXAMIDE RIBOTIDE ISOMERASE | 8.6 | 3.5 | 5.1 | 18.6 | 1.6E-5 | 11.6 |
| 137 | AT2G26500.2 \| cytochrome b6f complex subunit (petM), putative, nearly identical to cytochrome b6f complex subunit (GI:3090403) (Arabidopsis thaliana); alternative splice forms exist \| chr2:11277353-11278031 FORWARD \| Aliases: T9J22.17, T9J22_17 | 10.5 | 3.8 | 6.7 | 18.6 | 1.6E-5 | 10.9 |
| 140 | AT3G52180.2 \| similar to protein phosphatase-related [Arabidopsis thaliana] (TAIR:At3g10940.1); similar to unknown protein [Oryza sativa (japonica cultivar-group)] (GB:XP_482104.1); contains InterPro domain Tyrosine specific protein phosphatase and dual specificity protein phosphatase (InterPro:IPR000387); contains InterPro domain Dual specificity protein phosphatase (InterPro:IPR000340) | 9.1 | 4.2 | 4.9 | 18.5 | 1.6E-5 | 11.7 |
| 146 | AT2G43360.1 \| Symbol: BIO2 \| biotin synthase (BioB) (BIO2), identical to SP:P54967 Pfam profile PF04055: radical SAM domain protein \| chr2:18017828-18020315 REVERSE \| Aliases: BIOB, BIOTIN AUXOTROPH 2, BIOTIN AUXOTROPH B, BIOTIN SYNTHASE, T1O24.10 | 11.9 | 5.5 | 6.4 | 18.3 | 1.7E-5 | 12.4 |
| 149 | AT5G54270.1 \| Symbol: LHCB3 \| chlorophyll A-B binding protein / LHCII type III (LHCB3), identical to Lhcb3 protein (Arabidopsis thaliana) GI:4741952; contains Pfam profile PF00504: Chlorophyll A-B binding protein \| chr5:22055555-22056794 FORWARD \| Aliases: LHCB3*1, LIGHT HARVESTING CHLOROPHYLL BINDING PROTEIN 3, MDK4.9, MDK4_9 | 11.3 | 4.5 | 6.8 | 18.0 | 2.0E-5 | 11.6 |
| 151 | AT4G15910.1 \| Symbol: ATDI21 \| | 11.4 | 7.6 | 3.8 | 18.0 | 2.0E-5 | 12.2 |
| 154 | AT4G03280.2 \| Symbol: PETC \| cytochrome B6-F complex iron-sulfur subunit, chloroplast / Rieske iron-sulfur protein / plastoquinol-plastocyanin reductase (petC), identical to gi:9843639; identical to cDNA rieske iron-sulfur protein precursor (petC) GI:5725449 \| chr4:1440177-1441861 FORWARD \| Aliases: F4C21.21, F4C21_21, PGR1, PHOTOSYNTHETIC ELECTRON TRANSFER C, PROTON GRADIENT REGULATION 1, RIESKE IRON SULFUR PROTEIN PRECURSOR | 11.1 | 4.1 | 7.0 | 17.9 | 2.0E-5 | 11.2 |
| 155 | AT4G29430.1 \| Symbol: RPS15AE \| 40S ribosomal protein S15A (RPS15aE), ribosomal protein S15a - Brassica napus,PIR2:S20945 \| chr4:14472408-14473627 FORWARD \| Aliases: F17A13.250, F17A13_250, RPS15AE | 7.4 | 3.6 | 3.8 | 17.8 | 2.0E-5 | 11.7 |
| 158 | AT5G05990.1 \| mitochondrial glycoprotein family protein / MAM33 family protein, low similarity to SUAPRGA1 (Emericella nidulans) GI:6562379; contains Pfam profile PF02330: Mitochondrial glycoprotein | 9.0 | 4.2 | 4.7 | 17.7 | 2.1E-5 | 11.4 |
| 162 | AT1G11330.1 \| S-locus lectin protein kinase family protein, contains Pfam domains, PF00954: S-locus glycoprotein family, PF00069: Protein kinase domain, and PF01453: Lectin (probable mannose binding) \| chr1:3810221-3813607 FORWARD \| Aliases: T28P6.2, T28P6_2 | 8.9 | 3.3 | 5.6 | 17.4 | 2.4E-5 | 10.2 |
| 164 | AT1G48160.1 \| signal recognition particle 19 kDa protein, putative / SRP19, putative, similar to signal recognition particle 19 kDa protein subunit SRP19 GI:624221 (Oryza sativa (japonica cultivar-group)); contains Pfam profile: PF01922 SRP19 protein \| chr1:17790214-17792693 REVERSE \| Aliases: F21D18.11, F21D18_11 | 10.4 | 6.7 | 3.7 | 17.4 | 2.4E-5 | 12.3 |
| 166 | AT4G38370.1 \| phosphoglycerate/bisphosphoglycerate mutase family protein, contains Pfam profile PF00300: phosphoglycerate mutase family \| chr4:17969521-17971289 REVERSE \| Aliases: F22I13.140, F22I13_140 | 8.6 | 3.5 | 5.2 | 17.3 | 2.4E-5 | 10.7 |
| 168 | AT3G23325.1 \| splicing factor, putative, similar to Splicing factor 3B subunit 10 (SF3b10) (Pre-mRNA splicing factor SF3b 10 kDa subunit) (Swiss-Prot:Q9BWJ5) (Homo sapiens) \| chr3:8345778-8347056 FORWARD \| Aliases: None | 9.8 | 4.4 | 5.3 | 17.2 | 2.5E-5 | 10.3 |

| Rank | Description | Sync | Root | M | t | adj.q | B |
|---|---|---|---|---|---|---|---|
| 169 | AT4G17600.1 \| Symbol: LIL3:1 \| | 9.8 | 3.2 | 6.7 | 17.2 | 2.5E-5 | 9.0 |
| 175 | AT1G68585.1 \| expressed protein \| chr1:25760521-25761532 FORWARD \| Aliases: None | 7.4 | 3.4 | 4.1 | 17.1 | 2.5E-5 | 11.4 |
| 176 | AT3G15850.1 \| Symbol: FAD5 \| fatty acid desaturase family protein, similar to delta 9 acyl-lipid desaturase (ADS1) GI:2970034 from (Arabidopsis thaliana) \| chr3:5359016-5361192 FORWARD \| Aliases: ADS3, FADB, FATTY ACID DESATURASE 5, FATTY ACID DESATURASE B, JB67, MSJ11.25 | 8.8 | 3.6 | 5.2 | 17.0 | 2.6E-5 | 10.4 |
| 187 | AT3G23940.1 \| dehydratase family, contains Pfam profile: PF00920 dehydratase family \| chr3:8648712-8652564 FORWARD \| Aliases: F14O13.18 | 11.1 | 7.2 | 4.0 | 16.8 | 2.8E-5 | 11.9 |
| 189 | AT1G10270.1 \| pentatricopeptide (PPR) repeat-containing protein, contains Pfam profile: PF01535 PPR repeat; similar to ESTs gb:R30192 and gb:AA651017 \| chr1:3363506-3366490 FORWARD \| Aliases: F14N23.15, F14N23_15 | 7.6 | 4.1 | 3.5 | 16.7 | 2.8E-5 | 11.8 |
| 190 | AT4G28750.1 \| Symbol: PSAE1 \| photosystem I reaction center subunit IV, chloroplast, putative / PSI-E, putative (PSAE1), identical to SP:Q9S831; similar to SP:P12354 Photosystem I reaction center subunit IV, chloroplast precursor (PSI-E) {Spinacia oleracea}; contains Pfam profile PF02427: Photosystem I reaction centre subunit IV / PsaE \| chr4:14202779-14203961 REVERSE \| Aliases: F16A16.140, F16A16_140, PSA E1 KNOCKOUT | 9.2 | 3.7 | 5.5 | 16.7 | 2.8E-5 | 10.0 |
| 193 | AT1G73177.1 \| expressed protein \| chr1:27520248-27521515 REVERSE \| Aliases: None | 8.0 | 4.7 | 3.4 | 16.7 | 2.8E-5 | 12.2 |
| 194 | AT5G07800.1 \| flavin-containing monooxygenase family protein / FMO family protein, similar to flavin-containing monooxygenase 2 (FMO2) from Homo sapiens (GI:1834493); contains Pfam profile: PF00743 Flavin-binding monooxygenase-like \| chr5:2486607-2489297 REVERSE \| Aliases: MXM12.4, MXM12_4 | 9.8 | 4.5 | 5.3 | 16.7 | 2.8E-5 | 10.6 |
| 195 | AT5G51940.1 \| DNA-directed RNA polymerase II, putative, similar to SP:O88828 DNA-directed RNA polymerase II 14.4 kDa polypeptide (EC 2.7.7.6) (RPB6) (RPB14.4) {Rattus norvegicus}; contains Pfam profile PF01192: RNA polymerases K / 14 to 18 kDa subunit \| chr5:21121813-21123157 FORWARD \| Aliases: MSG15.2, MSG15_2 | 10.8 | 9.2 | 1.7 | 16.7 | 2.8E-5 | 13.3 |
| 199 | AT5G62610.1 \| basic helix-loop-helix (bHLH) family protein, contains Pfam profile: PF00010 helix-loop-helix DNA-binding domain | 9.2 | 5.0 | 4.2 | 16.6 | 2.8E-5 | 11.7 |
| 200 | AT1G32900.1 \| starch synthase, putative, similar to starch synthase SP:Q42857 from (Ipomoea batatas) \| chr1:11920371-11923746 REVERSE \| Aliases: F9L11.8, F9L11_8 | 8.3 | 3.1 | 5.2 | 16.6 | 2.8E-5 | 11.1 |
| 201 | AT2G24270.2 \| Symbol: ALDH11A3 \| NADP-dependent glyceraldehyde-3-phosphate dehydrogenase, putative, similar to NADP-dependent glyceraldehyde-3-phosphate dehydrogenase (NON-phosphorylating glyceraldehyde 3-phosphate; glyceraldehyde-3-phosphate dehydrogenase (NADP+)) (Nicotiana plumbaginifolia) SWISS-PROT:P93338 \| chr2:10334132-10336826 REVERSE \| Aliases: F27D4.18, F27D4_18 | 8.2 | 2.6 | 5.6 | 16.6 | 2.8E-5 | 10.1 |
| 202 | AT4G09010.1 \| L-ascorbate peroxidase, chloroplast, putative, identical to SP:P82281:TL29_ARATH (EC 1.11.1.11) {Arabidopsis thaliana}; ascorbate peroxidase - Spinacia oleracea, (gi:1669585); contains Pfam domain PF00141: Peroxidase; contains TIGRfam domain TIGR01409: Tat (twin-arginine translocation) pathway signal sequence; identical to ascorbate peroxidase APX4 (AT4g09010) mRNA, partial cds GI:31980499 \| chr4:5777080-5779418 REVERSE \| Aliases: F23J3.40, F23J3_40 | 9.0 | 3.9 | 5.2 | 16.6 | 2.8E-5 | 9.8 |
| 203 | AT2G38140.1 \| Symbol: PSRP4 \| chloroplast 30S ribosomal protein S31 (PSRP4) \| chr2:15987961-15988676 FORWARD \| Aliases: 30S RIBOSOMAL PROTEIN S31, F16M14.7, F16M14_7, PLASTID SPECIFIC RIBOSOMAL PROTEIN 4 | 9.2 | 4.1 | 5.1 | 16.6 | 2.8E-5 | 11.0 |
| 205 | AT5G16060.1 \| expressed protein \| chr5:5246125-5247434 FORWARD \| Aliases: F1N13.200, F1N13_200 | 11.5 | 7.9 | 3.6 | 16.5 | 2.8E-5 | 12.2 |
| 206 | AT5G02240.1 \| expressed protein \| chr5:451421-453155 FORWARD \| Aliases: T1E22.5 | 12.3 | 8.6 | 3.7 | 16.5 | 2.9E-5 | 12.1 |
| 208 | AT1G62290.2 \| similar to aspartyl protease family protein [Arabidopsis thaliana] (TAIR:At1g11910.1); similar to aspartyl protease family protein [Arabidopsis thaliana] (TAIR:At4g04460.1); similar to aspartic proteinase (EC 3.4.23.-) - cowpea (GB:T11686); similar to ASPR_CUCPE Aspartic proteinase precursor (GB:O04057); similar to aspartic proteinase [Vigna unguiculata] (GB:AAB03843.2); similar to aspartic proteinase [Theobroma cacao] (GB:CAC86004.1); similar to aspartic proteinase 1 [Glycine max] (GB:BAB62890.1); contains InterPro domain Eukaryotic/viral aspartic protease, active site (InterPro:IPR001969); contains InterPro domain Saposin B subdomain (InterPro:IPR008140); contains InterPro domain Saposin-like type B, 2 (InterPro:IPR008138); contains InterPro domain Saposin B (InterPro:IPR008139); contains InterPro domain Aspartic protease A1, pepsin (InterPro:IPR001461); contains InterPro domain Saposin-like type B, 1 (InterPro:IPR007856) \| chr1:23013576-23017193 REVERSE \| Aliases: F19K23.21, F19K23_21 | 8.5 | 3.1 | 5.4 | 16.4 | 2.9E-5 | 10.0 |
| 209 | AT4G30950.1 \| Symbol: FAD6 \| omega-6 fatty acid desaturase, chloroplast (FAD6) (FADC), identical to GI:493068 | 10.2 | 4.5 | 5.7 | 16.4 | 3.0E-5 | 11.6 |
| 212 | AT2G36170.1 \| ubiquitin extension protein 2 (UBQ2) / 60S ribosomal protein L40 (RPL40A), identical to GI:166930, GI:166931 \| chr2:15179196-15180358 FORWARD \| Aliases: F9C22.10, F9C22_10 | 12.2 | 8.5 | 3.6 | 16.2 | 3.2E-5 | 11.8 |

| Rank | Description | Sync | Root | M | t | adj.q | B |
|---|---|---|---|---|---|---|---|
| 213 | AT1G03630.2 \| Symbol: POR \| similar to protochlorophyllide reductase A, chloroplast / PCR A / NADPH-protochlorophyllide oxidoreductase A (PORA) [Arabidopsis thaliana] (TAIR:At5g54190.1); similar to protochlorophyllide reductase B, chloroplast / PCR B / NADPH-protochlorophyllide oxidoreductase B (PORB) [Arabidopsis thaliana] (TAIR:At4g27440.1); similar to NADPH-protochlorophyllide oxidoreductase [Cucumis sativus] (GB:BAA21089.1); contains InterPro domain Glucose/ribitol dehydrogenase (InterPro:IPR002347); contains InterPro domain Light-dependent protochlorophyllide reductase (InterPro:IPR005979); contains InterPro domain Short-chain dehydrogenase/reductase SDR (InterPro:IPR002198) \| chr1:907650-909375 FORWARD \| Aliases: F21B7.24, NADPH:PROTOCHLOROPHYLLIDE OXIDOREDUCTASE, PROTOCHLOROPHYLLIDE OXIDOREDUCTASE | 7.6 | 2.2 | 5.4 | 16.2 | 3.2E-5 | 9.4 |
| 222 | AT5G49630.1 \| Symbol: AAP6 \| amino acid permease 6 (AAP6), identical to amino acid permease 6 (AAP6) (Arabidopsis thaliana) GI:1769887 \| chr5:20159696-20163712 REVERSE \| Aliases: AMINO ACID PERMEASE 6, MNI5.1, MNI5_1 | 11.5 | 3.8 | 7.8 | 15.9 | 3.6E-5 | 10.5 |
| 223 | AT4G12800.1 \| photosystem I reaction center subunit XI, chloroplast (PSI-L) / PSI subunit V, identical to Photosystem I reaction center subunit XI, chloroplast precursor (PSI-L) (PSI subunit V) (Swiss-Prot:Q9SUI4) (Arabidopsis thaliana); contains Pfam profile PF02605: photosystem I reaction center subunit XI; contains 2 transmembrane domains \| chr4:7521319-7522667 FORWARD \| Aliases: T20K18.150, T20K18_150 | 8.4 | 2.4 | 6.0 | 15.8 | 3.7E-5 | 9.7 |
| 227 | AT5G41010.1 \| DNA-directed RNA polymerases I, II, and III 7 kDa subunit, putative, similar to SP:P53803 DNA-directed RNA polymerases I, II, and III 7.0 kDa polypeptide (EC 2.7.7.6) (ABC10-alpha) (RPB7.0) (RPB10alpha) {Homo sapiens}; contains Pfam profile PF03604: DNA directed RNA polymerase, 7 kDa subunit \| chr5:16441278-16442180 FORWARD \| Aliases: MEE6.8, MEE6_8 | 10.6 | 8.6 | 2.0 | 15.8 | 3.7E-5 | 12.7 |
| 228 | AT5G13280.1 \| Symbol: AK LYS1 \| aspartate kinase, identical to aspartate kinase (Arabidopsis thaliana) GI:4376158 \| chr5:4249441-4252839 FORWARD \| Aliases: AK, ASPARTATE KINASE, T31B5.100, T31B5_100 | 10.3 | 5.5 | 4.7 | 15.7 | 3.8E-5 | 11.3 |
| 229 | AT1G55490.2 \| Symbol: CPN60B \| | 11.1 | 5.9 | 5.3 | 15.7 | 3.9E-5 | 10.1 |
| 233 | AT5G48220.1 \| indole-3-glycerol phosphate synthase, putative, similar to SP:P49572 \| chr5:19567071-19569460 FORWARD \| Aliases: MIF21.11, MIF21_11 | 8.1 | 4.4 | 3.7 | 15.6 | 3.9E-5 | 11.1 |
| 240 | AT5G17170.1 \| rubredoxin family protein, contains Pfam profile PF00301: Rubredoxin \| chr5:5649205-5651160 FORWARD \| Aliases: MKP11.2, MKP11_2 | 8.4 | 3.6 | 4.8 | 15.5 | 4.1E-5 | 9.6 |
| 241 | AT1G28110.2 \| Symbol: SCPL45 \| | 9.7 | 5.2 | 4.5 | 15.5 | 4.1E-5 | 11.2 |
| 242 | AT2G18600.1 \| RUB1-conjugating enzyme, putative, strong similarity to gi:6635457 RUB1 conjugating enzyme (Arabidopsis thaliana); contains Pfam profile PF00179: Ubiquitin-conjugating enzyme \| chr2:8080282-8082030 REVERSE \| Aliases: F24H14.5, F24H14_5 | 7.6 | 3.3 | 4.2 | 15.5 | 4.1E-5 | 10.6 |
| 243 | AT2G43780.1 \| expressed protein \| chr2:18143521-18143724 REVERSE \| Aliases: F18O19.11 | 10.9 | 8.6 | 2.2 | 15.5 | 4.1E-5 | 12.5 |
| 244 | AT5G05710.1 \| pleckstrin homology (PH) domain-containing protein, similar to AtPH1 (Arabidopsis thaliana) GI:5926716; contains Pfam profile PF00169: PH domain | 10.2 | 5.9 | 4.3 | 15.4 | 4.1E-5 | 10.9 |
| 250 | AT1G27435.1 \| expressed protein \| chr1:9527561-9528865 REVERSE \| Aliases: None | 11.0 | 8.3 | 2.7 | 15.3 | 4.3E-5 | 11.6 |
| 252 | AT3G55400.1 \| methionyl-tRNA synthetase / methionine--tRNA ligase / MetRS (cpMetRS), identical to methionyl-tRNA synthetase MEtRS (Arabidopsis thaliana) GI:2266985 \| chr3:20546608-20550044 REVERSE \| Aliases: T22E16.60 | 8.2 | 3.5 | 4.8 | 15.3 | 4.3E-5 | 9.5 |
| 253 | AT5G06210.1 \| RNA-binding protein, putative, contains similarity to RNA-binding protein from (Nicotiana tabacum) GI:15822703, (Nicotiana sylvestris) GI:624925, (Solanum tuberosum) GI:15822705; contains InterPro entry IPR000504: RNA-binding region RNP-1 (RNA recognition motif) (RRM) \| chr5:1878472-1879741 FORWARD \| Aliases: MBL20.9, MBL20_9 | 9.7 | 6.1 | 3.5 | 15.2 | 4.3E-5 | 11.3 |
| 255 | AT3G27400.1 \| pectate lyase family protein, similar to pectate lyase GP:7547009 from (Vitis vinifera); contains Pfam profile: PF00544 pectate lyase \| chr3:10141560-10144462 FORWARD \| Aliases: K1G2.22 | 9.5 | 2.2 | 7.3 | 15.2 | 4.3E-5 | 9.3 |
| 259 | AT1G10010.1 \| Symbol: AAP8 \| amino acid permease, putative, similar to amino acid permease I GI:22641 from (Arabidopsis thaliana); GC splice site at position 1256 is predicted from alignment and not confirmed experimentally \| chr1:3265978-3268728 FORWARD \| Aliases: T27I1.3, T27I1_3 | 7.0 | 2.5 | 4.5 | 15.1 | 4.5E-5 | 10.3 |
| 267 | AT4G39235.1 \| expressed protein \| chr4:18268043-18269320 FORWARD \| Aliases: None | 7.1 | 3.0 | 4.1 | 15.0 | 4.7E-5 | 10.8 |
| 269 | AT4G30800.1 \| 40S ribosomal protein S11 (RPS11B), ribosomal protein S11, Arabidopsis thaliana,PIR2:C35542 \| chr4:15001173-15002677 FORWARD \| Aliases: T10C21.1 | 8.1 | 3.9 | 4.2 | 15.0 | 4.7E-5 | 10.2 |
| 277 | AT5G53580.1 \| aldo/keto reductase family protein, contains Pfam profile PF00248: oxidoreductase, aldo/keto reductase family \| chr5:21782305-21784210 REVERSE \| Aliases: MNC6.12, MNC6_12 | 9.5 | 5.0 | 4.5 | 14.8 | 5.0E-5 | 10.5 |

| Rank | Description | Sync | Root | M | t | adj.q | B |
|---|---|---|---|---|---|---|---|
| 280 | AT3G16140.1 \| photosystem I reaction center subunit VI, chloroplast, putative / PSI-H, putative (PSAH1), identical to SP:Q9SUI7; similar to PSI-H precursor (Nicotiana sylvestris) GI:407353; contains Pfam profile PF03244: Photosystem I reaction centre subunit VI \| chr3:5468515-5469482 REVERSE \| Aliases: MSL1.18 | 9.0 | 4.5 | 4.5 | 14.8 | 5.1E-5 | 10.0 |
| 281 | AT2G40100.1 \| Symbol: LHCB4.3 \| chlorophyll A-B binding protein (LHCB4.3), identical to Lhcb4:3 protein (Arabidopsis thaliana) GI:4741956; contains Pfam profile: PF00504 chlorophyll A-B binding protein \| chr2:16752881-16754478 FORWARD \| Aliases: F27I1.2, F27I1_2 | 9.5 | 3.1 | 6.4 | 14.7 | 5.2E-5 | 9.4 |
| 282 | AT1G76450.1 \| oxygen-evolving complex-related, SP:Q9S720; contains a PsbP domain \| chr1:28689465-28691614 FORWARD \| Aliases: F15M4.5 | 7.0 | 1.8 | 5.3 | 14.7 | 5.2E-5 | 10.2 |
| 284 | AT2G46110.1 \| ketopantoate hydroxymethyltransferase family protein, similar to SP:Q9Y7B6 3-methyl-2-oxobutanoate hydroxymethyltransferase (EC 2.1.2.11) (Ketopantoate hydroxymethyltransferase) {Emericella nidulans}; contains Pfam profile PF02548: Ketopantoate hydroxymethyltransferase \| chr2:18961532-18963603 REVERSE \| Aliases: T3F17.24 | 8.6 | 3.6 | 5.0 | 14.7 | 5.2E-5 | 9.1 |
| 289 | AT2G41300.1 \| strictosidine synthase family protein, similar to strictosidine synthase (Rauvolfia serpentina)(SP:P15324); contains strictosidine synthase domain PF03088; protein alignments support a CG non-consensus donor splice site. \| chr2:17221315-17223370 REVERSE \| Aliases: F13H10.15, F13H10_15 | 10.3 | 3.6 | 6.7 | 14.5 | 5.6E-5 | 10.9 |
| 293 | AT2G41290.1 \| strictosidine synthase family protein, similar to strictosidine synthase (Rauvolfia serpentina)(SP:P15324); contains strictosidine synthase domain PF03088 \| chr2:17217533-17220037 REVERSE \| Aliases: F13H10.16, F13H10_16 | 9.8 | 6.1 | 3.7 | 14.4 | 5.9E-5 | 10.4 |
| 295 | AT1G73940.1 \| expressed protein \| chr1:27801974-27803248 REVERSE \| Aliases: F2P9.19, F2P9_19 | 10.1 | 5.5 | 4.7 | 14.4 | 5.9E-5 | 11.4 |
| 296 | AT3G48930.1 \| Symbol: EMB1080 \| 40S ribosomal protein S11 (RPS11A) \| chr3:18151756-18153251 REVERSE \| Aliases: EMB1080, EMBRYO DEFECTIVE 1080, T2I13.230 | 11.6 | 9.1 | 2.5 | 14.4 | 5.9E-5 | 12.0 |
| 297 | AT5G04620.2 \| aminotransferase class I and II family protein, similar to 8-amino-7-oxononanoate synthase, Bacillus sphaericus, PIR:JQ0512 (SP:P22806), Bacillus subtilis (SP:P53556); contains Pfam protile PF00155 aminotransferase, classes I and II | 8.4 | 3.6 | 4.8 | 14.4 | 5.9E-5 | 9.4 |
| 298 | AT1G08430.1 \| expressed protein, contains Pfam profile PF01027: Uncharacterized protein family UPF0005 | 7.2 | 3.2 | 4.1 | 14.3 | 6.0E-5 | 9.9 |
| 300 | AT5G65750.1 \| 2-oxoglutarate dehydrogenase E1 component, putative / oxoglutarate decarboxylase, putative / alpha-ketoglutaric dehydrogenase, putative, similar to SP:P20967 2-oxoglutarate dehydrogenase E1 component, mitochondrial precursor (EC 1.2.4.2) (Alpha-ketoglutarate dehydrogenase) {Saccharomyces cerevisiae}; contains Pfam profiles PF02779: Transketolase, pyridine binding domain, PF00676: Dehydrogenase E1 component \| chr5:26320932-26325415 FORWARD \| Aliases: MPA24.10, MPA24_10 | 8.6 | 4.7 | 3.9 | 14.3 | 6.2E-5 | 10.0 |
| 301 | AT3G51820.1 \| chlorophyll synthetase, putative, identical to gi:972938 putative chlorophyll synthetase from Arabidopsis thaliana \| chr3:19226980-19230027 REVERSE \| Aliases: ATEM1.7, ATG4 | 10.2 | 5.6 | 4.6 | 14.3 | 6.2E-5 | 9.8 |
| 304 | AT2G43710.2 \| Symbol: SSI2 \| acyl-(acyl-carrier-protein) desaturase / stearoyl-ACP desaturase (SSI2), identical to gi:15149310; contains Pfam profile PF03405: Fatty acid desaturase; identical to cDNA stearoyl ACP desaturase (SSI2), SSI2-FAB2 allele, GI:15149309 \| chr2:18127039-18129966 FORWARD \| Aliases: F18O19.18, FAB2 | 9.3 | 5.2 | 4.1 | 14.2 | 6.3E-5 | 10.9 |
| 305 | AT4G21610.1 \| Symbol: LOL2 \| zinc finger protein, putative, similar to zinc-finger protein Lsd1 (Arabidopsis thaliana) gi:1872521:gb:AAC49660 | 8.6 | 4.3 | 4.3 | 14.2 | 6.3E-5 | 10.1 |
| 306 | AT1G66410.1 \| Symbol: CAM4 \| calmodulin-1/4 (CAM4), identical to calmodulin (Arabidopsis thaliana) GI:16223; nearly identical to SP:P25854 Calmodulin-1/4 {Arabidopsis thaliana} \| chr1:24777880-24779516 REVERSE \| Aliases: ACAM 4, CALMODULIN 4, T27F4.1, T27F4_1 | 13.4 | 10.9 | 2.5 | 14.2 | 6.3E-5 | 11.8 |
| 309 | AT5G55280.1 \| Symbol: FTSZ1 1 \| cell division protein FtsZ, chloroplast, putative (FTSZ), identical to SP:Q42545 Cell division protein ftsZ homolog, chloroplast precursor {Arabidopsis thaliana}; similar to FtsZ1 (Tagetes erecta) GI:8896066; contains Pfam profiles PF00091: Tubulin/FtsZ family, GTPase domain, PF03953: Tubulin/FtsZ family, C-terminal domain \| chr5:22437882-22439838 REVERSE \| Aliases: ATFTSZ1 1, CPFTSZ, MCO15.23, MCO15_23 | 8.8 | 4.5 | 4.3 | 14.2 | 6.4E-5 | 9.8 |
| 310 | AT1G79260.1 \| expressed protein \| chr1:29818154-29819202 FORWARD \| Aliases: YUP8H12R.14, YUP8H12R_14 | 7.8 | 2.3 | 5.5 | 14.2 | 6.4E-5 | 10.2 |
| 313 | AT4G37550.2 \| similar to formamidase, putative / formamide amidohydrolase, putative [Arabidopsis thaliana] (TAIR:At4g37560.1); similar to formamidase [Methylophilus methylotrophus] (GB:CAA67953.1); contains InterPro domain Acetamidase/Formamidase (InterPro:IPR004304) \| chr4:17643575-17645821 FORWARD \| Aliases: F19F18.40, F19F18_40 | 6.8 | 3.6 | 3.3 | 14.1 | 6.7E-5 | 10.5 |
| 315 | AT1G13640.1 \| phosphatidylinositol 3- and 4-kinase family protein, low similarity to phosphatidylinositol 4-kinase type-II beta (Homo sapiens) GI:20159767; contains Pfam profile PF00454: Phosphatidylinositol 3- and 4-kinase \| chr1:4677024-4679801 REVERSE \| Aliases: F21F23.8, F21F23_8 | 9.1 | 5.4 | 3.6 | 14.0 | 6.7E-5 | 10.6 |
| 323 | AT5G49910.1 \| Symbol: cpHSC70 2 \| heat shock protein 70 / HSP70 (HSC70-7), identical to heat shock protein 70 (Arabidopsis thaliana) GI:6746592 \| chr5:20320640-20324039 FORWARD \| Aliases: HEAT SHOCK PROTEIN 70, HEAT SHOCK PROTEIN 70 7, HSC70 7, K9P8.5, K9P8_5 | 9.2 | 5.6 | 3.7 | 14.0 | 6.7E-5 | 10.6 |

| Rank | Description | Sync | Root | M | t | adj.q | B |
|---|---|---|---|---|---|---|---|
| 326 | AT3G25920.1 \| Symbol: RPL15 \| 50S ribosomal protein L15, chloroplast (CL15), identical to GB:P25873 from (Arabidopsis thaliana) \| chr3:9491321-9493855 REVERSE \| Aliases: MPE11.32, RIBOSOMAL PROTEIN CL15 | 9.1 | 5.2 | 3.9 | 13.9 | 6.9E-5 | 9.9 |
| 330 | AT5G40370.1 \| glutaredoxin, putative, similar to glutaredoxin (Ricinus communis) SWISS-PROT:P55143 \| chr5:16164833-16166382 REVERSE \| Aliases: MPO12.80, MPO12_80 | 13.1 | 10.5 | 2.5 | 13.8 | 7.2E-5 | 11.5 |
| 331 | AT1G15820.1 \| Symbol: LHCB6 \| chlorophyll A-B binding protein, chloroplast (LHCB6), nearly identical to Lhcb6 protein (Arabidopsis thaliana) GI:4741960; contains Pfam profile PF00504: Chlorophyll A-B binding protein \| chr1:5446123-5447776 REVERSE \| Aliases: CP24, F7H2.16, F7H2_16, LHCB6 | 9.7 | 4.0 | 5.7 | 13.8 | 7.2E-5 | 9.2 |
| 336 | AT3G55170.2 \| 60S ribosomal protein L35 (RPL35C), various ribosomal L35 proteins \| chr3:20463946-20465316 REVERSE \| Aliases: T26I12.50 | 9.3 | 6.1 | 3.2 | 13.8 | 7.3E-5 | 10.3 |
| 338 | AT3G62840.1 \| small nuclear ribonucleoprotein D2, putative / snRNP core protein D2, putative / Sm protein D2, putative, similar to small nuclear ribonucleoprotein Sm D2 (snRNP core protein D2) (Sm-D2) (Mus musculus) SWISS-PROT:P43330 \| chr3:23246086-23247791 REVERSE \| Aliases: F26K9.270 | 11.1 | 8.2 | 2.8 | 13.7 | 7.5E-5 | 11.4 |
| 339 | AT3G48610.1 \| phosphoesterase family protein, low similarity to SP:P95246 Phospholipase C 2 precursor (EC 3.1.4.3) {Mycobacterium tuberculosis}; contains Pfam profile PF04185: Phosphoesterase family | 8.1 | 2.8 | 5.3 | 13.7 | 7.5E-5 | 9.9 |
| 341 | AT1G28395.3 \| expressed protein \| chr1:9969428-9971504 REVERSE \| Aliases: None | 8.1 | 3.6 | 4.5 | 13.7 | 7.6E-5 | 10.5 |
| 342 | AT1G07070.1 \| 60S ribosomal protein L35a (RPL35aA), similar to ribosomal protein L35a GI:57118 from (Rattus norvegicus) \| chr1:2168563-2169850 FORWARD \| Aliases: F10K1.22, F10K1_22 | 8.8 | 4.6 | 4.2 | 13.7 | 7.6E-5 | 10.7 |
| 343 | AT2G16890.2 \| UDP-glucoronosyl/UDP-glucosyl transferase family protein, contains Pfam profile: PF00201 UDP-glucoronosyl and UDP-glucosyl transferase \| chr2:7323971-7326263 FORWARD \| Aliases: F12A24.7, F12A24_7 | 9.8 | 4.1 | 5.7 | 13.7 | 7.6E-5 | 10.1 |
| 351 | AT1G11430.1 \| plastid developmental protein DAG, putative, similar to DAG protein, chloroplast precursor (Garden snapdragon) SWISS-PROT:Q38732 | 9.8 | 6.5 | 3.3 | 13.5 | 8.3E-5 | 10.3 |
| 354 | AT1G53460.1 \| expressed protein \| chr1:19958158-19960300 REVERSE \| Aliases: T3F20.22, T3F20_22 | 10.1 | 7.1 | 3.0 | 13.5 | 8.3E-5 | 10.8 |
| 355 | AT3G50610.1 \| expressed protein \| chr3:18790704-18791393 REVERSE \| Aliases: T20E23.210 | 8.2 | 2.1 | 6.1 | 13.4 | 8.5E-5 | 8.8 |
| 356 | AT4G24780.1 \| pectate lyase family protein, similar to pectate lyase GP:14289169 from (Salix gilgiana) | 11.3 | 4.5 | 6.8 | 13.4 | 8.5E-5 | 10.0 |
| 359 | AT3G09500.1 \| 60S ribosomal protein L35 (RPL35A), similar to 60S ribosomal protein L35 GB:AAC27830 \| chr3:2916986-2918036 FORWARD \| Aliases: F11F8.7 | 11.6 | 9.3 | 2.3 | 13.4 | 8.6E-5 | 11.4 |
| 365 | AT5G47680.1 \| expressed protein, contains Pfam profile PF04243: Protein of unknown function (DUF425) \| chr5:19332309-19334748 REVERSE \| Aliases: MNJ7.27, MNJ7_27 | 9.4 | 5.6 | 3.8 | 13.3 | 8.9E-5 | 9.9 |
| 366 | AT1G17100.1 \| SOUL heme-binding family protein, similar to SOUL protein (Mus musculus) GI:4886906; contains Pfam profile PF04832: SOUL heme-binding protein \| chr1:5844729-5846215 FORWARD \| Aliases: F20D23.21, F20D23_21 | 8.7 | 5.7 | 3.0 | 13.3 | 8.9E-5 | 10.3 |
| 368 | AT2G36620.1 \| 60S ribosomal protein L24 (RPL24A) \| chr2:15357486-15358944 REVERSE \| Aliases: F13K3.2, F13K3_2 | 10.6 | 8.0 | 2.6 | 13.3 | 9.0E-5 | 11.0 |
| 369 | AT5G14920.2 \| similar to proline-rich family protein [Arabidopsis thaliana] (TAIR:At2g27380.1); similar to cell wall-plasma membrane linker protein [Brassica napus] (GB:CAA64425.1); contains InterPro domain Proline-rich region (InterPro:IPR000694) \| chr5:4826546-4827983 FORWARD \| Aliases: F2G14.40, F2G14_40 | 7.8 | 3.1 | 4.8 | 13.2 | 9.2E-5 | 9.0 |
| 373 | AT5G53880.1 \| expressed protein \| chr5:21890020-21890529 REVERSE \| Aliases: K19P17.4, K19P17_4 | 9.5 | 4.1 | 5.4 | 13.2 | 9.4E-5 | 10.9 |
| 375 | AT3G29320.1 \| glucan phosphorylase, putative, similar to alpha-glucan phosphorylase, L isozyme 1 precursor GB:P04045 from (Solanum tuberosum) (J. Biochem. 106 (4), 691-695 (1989)) \| chr3:11254059-11259051 FORWARD \| Aliases: MUO10.17 | 9.4 | 4.4 | 4.9 | 13.2 | 9.4E-5 | 9.8 |
| 376 | AT3G17820.1 \| Symbol: ATGSKB6 \| glutamine synthetase (GS1), identical to glutamine synthetase, cytosolic isozyme (glutamate-- ammonia ligase, GS1) (Arabidopsis thaliana) SWISS-PROT:Q9LVI8 \| chr3:6097420-6099601 FORWARD \| Aliases: GLN1;3, MEB5.4 | 11.7 | 7.6 | 4.1 | 13.1 | 9.5E-5 | 9.6 |
| 377 | AT2G24190.1 \| short-chain dehydrogenase/reductase (SDR) family protein, contains INTERPRO family IPR002198 short-chain dehydrogenase/reductase (SDR) superfamily \| chr2:10290820-10292014 REVERSE \| Aliases: F27D4.10, F27D4_10 | 9.6 | 6.1 | 3.6 | 13.1 | 9.6E-5 | 10.1 |
| 379 | AT4G35980.1 \| expressed protein \| chr4:17030575-17031695 REVERSE \| Aliases: T19K4.110 | 8.3 | 5.0 | 3.3 | 13.1 | 9.6E-5 | 10.6 |

| Rank | Description | Sync | Root | M | t | adj.q | B |
|---|---|---|---|---|---|---|---|
| 380 | AT4G18280.1 \| glycine-rich cell wall protein-related, glycine-rich protein 1.0 precursor, Phaseolus vulgaris, PIR1:S01821 \| chr4:10104007-10104735 FORWARD \| Aliases: T9A21.130, T9A21_130 | 11.1 | 7.0 | 4.1 | 13.1 | 9.6E-5 | 9.6 |
| 381 | AT4G14890.1 \| ferredoxin family protein, similar to SP:P00252 Ferredoxin I from Nostoc muscorum, SP:P00248 Ferredoxin from Mastigocladus laminosus, SP:P00244 Ferredoxin I from Aphanizomenon flos-aquae; contains Pfam profile PF00111 2Fe-2S iron-sulfur cluster binding domain \| chr4:8520827-8521448 FORWARD \| Aliases: DL3485W, FCAALL.7 | 8.5 | 3.7 | 4.7 | 13.0 | 9.9E-5 | 8.6 |
| 383 | AT5G19370.1 \| rhodanese-like domain-containing protein / PPIC-type PPIASE domain-containing protein, low similarity to MPT-synthase sulfurylase (Synechococcus sp. PCC 7942) GI:2950364; contains Pfam profiles PF00581: Rhodanese-like domain, PF00639: PPIC-type PPIASE domain; identical to cDNA peptidyl-prolyl cis-trans isomerase GI:2246379 \| chr5:6524148-6526668 REVERSE \| Aliases: F7K24.120, F7K24_120 | 10.6 | 7.8 | 2.7 | 13.0 | 1.0E-4 | 11.0 |
| 386 | AT2G32220.1 \| 60S ribosomal protein L27 (RPL27A) \| chr2:13685996-13686600 FORWARD \| Aliases: F22D22.3, F22D22_3 | 7.2 | 2.9 | 4.3 | 13.0 | 1.0E-4 | 9.8 |
| 387 | AT3G26070.1 \| plastid-lipid associated protein PAP / fibrillin family protein, contains Pfam profile PF04755: PAP_fibrillin \| chr3:9527985-9529820 FORWARD \| Aliases: MPE11.24 | 8.3 | 1.2 | 7.2 | 13.0 | 1.0E-4 | 6.4 |
| 388 | AT3G26080.1 \| plastid-lipid associated protein PAP / fibrillin family protein, low similarity to Plastid-lipid-Associated Protein (Nicotiana tabacum) GI:2632088; contains Pfam profile PF04755: PAP_fibrillin | 8.3 | 1.2 | 7.2 | 13.0 | 1.0E-4 | 6.4 |
| 389 | AT4G27380.1 \| expressed protein \| chr4:13701262-13703156 FORWARD \| Aliases: F27G19.4 | 8.7 | 4.1 | 4.5 | 13.0 | 1.0E-4 | 9.7 |
| 390 | AT1G09415.1 \| NPR1/NIM1-interacting protein 3 (NIMIN-3), identical to NIMIN-3 protein (Arabidopsis thaliana) GI:12057158, cDNA NIMIN-3 protein (nimin-3 gene)GI:12057157 \| chr1:3037756-3038390 REVERSE \| Aliases: None | 7.3 | 3.3 | 4.1 | 13.0 | 1.0E-4 | 9.9 |
| 391 | AT3G24310.1 \| Symbol: MYB305 \| | 8.2 | 3.8 | 4.3 | 13.0 | 1.0E-4 | 9.3 |
| 393 | AT3G62870.1 \| 60S ribosomal protein L7A (RPL7aB), 60S RIBOSOMAL PROTEIN L7A - Oryza sativa, SWISSPROT:RL7A_ORYSA \| chr3:23253640-23255328 REVERSE \| Aliases: F26K9.300 | 11.9 | 9.9 | 2.0 | 12.9 | 1.0E-4 | 11.1 |
| 396 | AT3G30775.1 \| Symbol: ERD5 \| proline oxidase, mitochondrial / osmotic stress-responsive proline dehydrogenase (POX) (PRO1) (ERD5), nearly identical to SP:P92983 Proline oxidase, mitochondrial precursor (EC 1.5.3.-) (Osmotic stress- induced proline dehydrogenase) (Arabidopsis thaliana); identical to cDNA proline oxidase precursor GI:1817543 | 10.0 | 6.5 | 3.5 | 12.9 | 1.0E-4 | 9.7 |
| 398 | AT1G67110.1 \| Symbol: CYP735A2 \| cytochrome P450, putative, similar to Cytochrome P450 72A1 (SP:Q05047) (Catharanthus roseus); \| chr1:25065394-25069080 REVERSE \| Aliases: F5A8.3, F5A8_3 | 6.9 | 2.8 | 4.1 | 12.9 | 1.0E-4 | 10.0 |
| 405 | AT1G74730.1 \| expressed protein \| chr1:28082592-28084009 FORWARD \| Aliases: F25A4.30, F25A4_30 | 8.0 | 4.1 | 3.9 | 12.8 | 1.1E-4 | 9.5 |
| 406 | AT1G48350.1 \| ribosomal protein L18 family protein, similar to ribosomal protein L18 GI:3980238 from (Thermotoga maritima) \| chr1:17870876-17872010 FORWARD \| Aliases: F11A17.10, F11A17_10 | 9.9 | 5.7 | 4.2 | 12.8 | 1.1E-4 | 10.0 |
| 407 | AT5G04310.1 \| pectate lyase family protein, similar to pectate lyase GP:14531296 from (Fragaria x ananassa) \| chr5:1203204-1207353 REVERSE \| Aliases: T19N18.40, T19N18_40 | 8.0 | 5.2 | 2.8 | 12.8 | 1.1E-4 | 10.6 |
| 408 | AT5G48790.1 \| expressed protein \| chr5:19797086-19799266 REVERSE \| Aliases: K24G6.12, K24G6_12 | 7.5 | 3.8 | 3.7 | 12.8 | 1.1E-4 | 9.5 |
| 410 | AT5G01530.1 \| chlorophyll A-B binding protein CP29 (LHCB4), identical to CP29 (Arabidopsis thaliana) GI:298036; contains Pfam profile: PF00504 chlorophyll A-B binding protein | 10.2 | 4.7 | 5.5 | 12.7 | 1.1E-4 | 9.5 |
| 411 | AT2G38465.1 \| expressed protein \| chr2:16113341-16113978 REVERSE \| Aliases: None | 6.6 | 3.7 | 2.8 | 12.7 | 1.1E-4 | 10.2 |
| 413 | AT1G15250.1 \| 60S ribosomal protein L37 (RPL37A), almost identical to GB:Q43292 \| chr1:5248660-5249457 REVERSE \| Aliases: F9L1.46, F9L1_46 | 9.1 | 4.9 | 4.1 | 12.7 | 1.1E-4 | 9.6 |
| 415 | AT3G09480.1 \| histone H2B, putative, similar to histone from Arabidopsis thaliana GI:2407802, Gossypium hirsutum SP:O22582, H2B-3 GB:CAA12231 from (Lycopersicon esculentum); contains Pfam profile PF00125 Core histone H2A/H2B/H3/H4 \| chr3:2914896-2915276 REVERSE \| Aliases: F11F8.5 | 7.7 | 3.3 | 4.4 | 12.7 | 1.1E-4 | 9.7 |
| 417 | AT3G50550.2 \| expressed protein, isoform contains a non-consensus AT donor site at intron 1 \| chr3:18770444-18771420 FORWARD \| Aliases: T20E23.150 | 9.3 | 5.6 | 3.7 | 12.7 | 1.1E-4 | 10.3 |
| 422 | AT3G23390.1 \| 60S ribosomal protein L36a/L44 (RPL36aA), similar to ribosomal protein L41 GB:AAA34366 from (Candida maltosa) \| chr3:8375382-8376397 FORWARD \| Aliases: MLM24.22 | 11.1 | 9.1 | 2.0 | 12.6 | 1.1E-4 | 11.2 |

| Rank | Description | Sync | Root | M | t | adj.q | B |
|---|---|---|---|---|---|---|---|
| 423 | AT3G55800.1 \| Symbol: SBPASE \| | 8.0 | 3.5 | 4.6 | 12.6 | 1.1E-4 | 8.9 |
| 426 | AT1G61570.1 \| Symbol: TIM13 \| mitochondrial import inner membrane translocase (TIM13), identical to mitochondrial import inner membrane translocase subunit Tim13 (Arabidopsis thaliana) Swiss-Prot:Q9XH48; contains Pfam domain, PF02953: Tim10/DDP family zinc finger \| chr1:22722370-22723181 REVERSE \| Aliases: T25B24.8, T25B24_8, TIM13, translocase of the inner membrane 13 | 11.3 | 8.8 | 2.4 | 12.6 | 1.2E-4 | 10.8 |
| 428 | AT2G01520.1 \| major latex protein-related / MLP-related, low similarity to major latex protein {Papaver somniferum}(GI:169000) contains Pfam profile PF00407: Pathogenesis-related protein Bet v I family \| chr2:235928-237130 FORWARD \| Aliases: F2I9.14, F2I9_14 | 8.3 | 4.8 | 3.6 | 12.6 | 1.2E-4 | 9.9 |
| 429 | AT3G52590.1 \| Symbol: UBQ1 \| ubiquitin extension protein 1 (UBQ1) / 60S ribosomal protein L40 (RPL40B), identical to GI:166929, GI:166930 \| chr3:19516614-19517910 FORWARD \| Aliases: EMB2167, EMBRYO DEFECTIVE 2167, F3C22.8, UBIQUITIN EXTENSION PROTEIN 1 | 12.3 | 10.0 | 2.3 | 12.6 | 1.2E-4 | 11.0 |
| 433 | AT3G01740.1 \| expressed protein \| chr3:267836-268845 FORWARD \| Aliases: F4P13.29 | 10.7 | 7.7 | 3.0 | 12.5 | 1.2E-4 | 10.9 |
| 434 | AT5G06720.1 \| peroxidase, putative, identical to peroxidase (Arabidopsis thaliana) gi:1491617:emb:CAA68212 \| chr5:2077430-2079006 REVERSE \| Aliases: MPH15.8, MPH15_8 | 7.4 | 2.8 | 4.6 | 12.5 | 1.2E-4 | 9.3 |
| 435 | AT1G31230.1 \| bifunctional aspartate kinase/homoserine dehydrogenase / AK-HSDH, nearly identical to gb:X71364 (PIR:S46497) aspartate kinase / homoserine dehydrogenase from Arabidopsis thaliana; contains ACT domain | 10.1 | 6.5 | 3.7 | 12.5 | 1.2E-4 | 10.4 |
| 436 | AT2G30200.2 \| similar to malonyl-CoA:ACP transacylase [Perilla frutescens] (GB:AAG43518.1); contains InterPro domain Acyl transferase domain (InterPro:IPR001227) \| chr2:12890074-12892845 REVERSE \| Aliases: T27E13.6, T27E13_6 | 10.2 | 5.5 | 4.6 | 12.5 | 1.2E-4 | 10.1 |
| 438 | AT1G18540.1 \| 60S ribosomal protein L6 (RPL6A), similar to 60S ribosomal protein L6 GI:7208784 from (Cicer arietinum) \| chr1:6377314-6378571 REVERSE \| Aliases: F25I16.12, F25I16_12 | 12.2 | 10.3 | 2.0 | 12.5 | 1.2E-4 | 11.0 |
| 439 | AT2G37660.1 \| expressed protein \| chr2:15802392-15804137 REVERSE \| Aliases: F13M22.16, F13M22_16 | 9.5 | 5.7 | 3.8 | 12.5 | 1.2E-4 | 9.6 |
| 440 | AT1G67820.1 \| protein phosphatase 2C, putative / PP2C, putative, similar to protein phosphatase 2C emb:CAA72341.1 \| chr1:25433516-25435725 FORWARD \| Aliases: F12A21.5, F12A21_5 | 8.9 | 4.9 | 3.9 | 12.5 | 1.2E-4 | 9.9 |
| 441 | AT2G18400.1 \| ribosomal protein L6 family protein \| chr2:7996561-7997668 REVERSE \| Aliases: T30D6.9, T30D6_9 | 9.7 | 6.5 | 3.1 | 12.5 | 1.2E-4 | 10.0 |
| 444 | AT1G08780.1 \| prefoldin, putative, similar to Swiss-Prot:Q9NQP4 prefoldin subunit 4 (Protein C-1) (Homo sapiens) \| chr1:2809783-2811194 REVERSE \| Aliases: F22O13.27, F22O13_27 | 9.1 | 5.8 | 3.3 | 12.5 | 1.2E-4 | 10.2 |
| 445 | AT1G30760.1 \| FAD-binding domain-containing protein, similar to SP:P30986 reticuline oxidase precursor (Berberine-bridge-forming enzyme) (BBE) (Tetrahydroprotoberberine synthase) (Eschscholzia californica); contains PF01565 FAD binding domain \| chr1:10918260-10920659 FORWARD \| Aliases: T17H7.1 | 8.9 | 2.7 | 6.2 | 12.4 | 1.2E-4 | 9.5 |
| 446 | AT2G30000.1 \| expressed protein, contains Pfam domain PF03660: Uncharacterised protein family (UPF0123) \| chr2:12810932-12812054 REVERSE \| Aliases: F23F1.8, F23F1_8 | 10.9 | 8.8 | 2.1 | 12.4 | 1.2E-4 | 11.0 |
| 447 | AT5G23460.1 \| expressed protein \| chr5:7912772-7913498 FORWARD \| Aliases: K19M13.9, K19M13_9 | 9.0 | 6.0 | 2.9 | 12.4 | 1.2E-4 | 9.9 |
| 449 | AT4G29410.1 \| 60S ribosomal protein L28 (RPL28C), unknown protein chromosome II BAC F6F22 - Arabidopsis thaliana,PID:g3687251 \| chr4:14468250-14470173 REVERSE \| Aliases: F17A13.230, F17A13_230 | 11.2 | 8.2 | 3.0 | 12.4 | 1.2E-4 | 10.8 |
| 452 | AT5G23740.1 \| Symbol: RPS11 BETA \| 40S ribosomal protein S11 (RPS11C) | 11.5 | 8.8 | 2.7 | 12.4 | 1.2E-4 | 10.6 |
| 454 | AT4G01897.1 \| expressed protein \| chr4:819957-821484 REVERSE \| Aliases: None | 8.0 | 4.9 | 3.2 | 12.4 | 1.3E-4 | 10.2 |
| 455 | AT4G16155.1 \| dihydrolipoamide dehydrogenase 2, plastidic / lipoamide dehydrogenase 2 (PTLPD2), identical to plastidic lipoamide dehydrogenase from Arabidopsis thaliana (gi:7159284) \| chr4:9153386-9157278 REVERSE \| Aliases: None | 8.6 | 5.4 | 3.2 | 12.3 | 1.3E-4 | 9.7 |
| 457 | AT3G60140.1 \| Symbol: DIN2 \| glycosyl hydrolase family 1 protein, contains Pfam PF00232 : Glycosyl hydrolase family 1 domain; TIGRFAM TIGR01233: 6-phospho-beta-galactosidase; similar to Cyanogenic Beta-Glucosidase (GI:1311386)(pdb:1CBG) (Trifolium Repens); identical beta-glucosidase GI:10834547 \| chr3:22227648-22231792 FORWARD \| Aliases: DARK INDUCIBLE 2, SENESCENCE RELATED GENE 2, SRG2, T2O9.120, T2O9_120 | 5.8 | 3.2 | 2.7 | 12.3 | 1.3E-4 | 10.3 |
| 459 | AT3G47860.1 \| apolipoprotein D-related, contains weak similarity to Apolipoprotein D precursor (ApoD) (Swiss-Prot:P51910) (Mus musculus) \| chr3:17667682-17669273 REVERSE \| Aliases: T23J7.190 | 9.4 | 6.4 | 3.0 | 12.3 | 1.3E-4 | 10.7 |

| Rank | Description | Sync | Root | M | t | adj.q | B |
|---|---|---|---|---|---|---|---|
| 462 | AT2G28950.1 \| Symbol: ATEXPA6 \| expansin, putative (EXP6), similar to expansin GI:2828241 from (Brassica napus); contains Pfam profile PF01357: Pollen allergen \| chr2:12438418-12440672 REVERSE \| Aliases: ARABIDOPSIS THALIANA EXPANSIN A6, ATEXP6, ATHEXP ALPHA 1.8, EXPANSIN 6, T9I4.3, T9I4_3 | 8.8 | 2.8 | 5.9 | 12.2 | 1.3E-4 | 10.3 |
| 463 | AT5G64650.1 \| ribosomal protein L17 family protein, contains Pfam profile: PF01196 ribosomal protein L17 \| chr5:25856586-25858035 REVERSE \| Aliases: MUB3.17, MUB3_17 | 7.5 | 3.6 | 4.0 | 12.2 | 1.3E-4 | 9.6 |
| 466 | AT1G09815.1 \| DNA polymerase delta subunit 4 family, contains similarity to Swiss-Prot:Q9HCU8 DNA polymerase delta subunit 4 (DNA polymerase delta subunit p12) (Homo sapiens) | 9.2 | 7.3 | 1.9 | 12.2 | 1.4E-4 | 10.8 |
| 468 | AT5G06290.1 \| 2-cys peroxiredoxin, chloroplast, putative, very strong similarity to SP:Q96291 2-cys peroxiredoxin BAS1, chloroplast precursor {Arabidopsis thaliana}; contains Pfam profile: PF00578 AhpC/TSA (alkyl hydroperoxide reductase and thiol-specific antioxidant) family \| chr5:1919249-1921404 FORWARD \| Aliases: MHF15.19, MHF15_19 | 9.6 | 4.9 | 4.7 | 12.2 | 1.4E-4 | 8.3 |
| 469 | AT3G15900.1 \| expressed protein \| chr3:5376257-5377089 REVERSE \| Aliases: MVC8.2 | 7.1 | 3.9 | 3.2 | 12.2 | 1.4E-4 | 9.7 |
| 472 | AT3G56800.1 \| Symbol: CAM3 \| calmodulin-2/3/5 (CAM3), identical to calmodulin GI:474183 from (Arabidopsis thaliana); almost identical to calmodulin-2/3/5 SP:P25069 (Arabidopsis thaliana) \| chr3:21045656-21047053 REVERSE \| Aliases: ACAM 3, CALMODULIN 3, T8M16.130 | 11.2 | 8.6 | 2.6 | 12.2 | 1.4E-4 | 10.6 |
| 473 | AT1G14620.1 \| Symbol: DECOY \| expressed protein \| chr1:5014749-5016599 REVERSE \| Aliases: DECOY, EXGT A2, T5E21.12, T5E21_12, XTR2 | 8.2 | 3.6 | 4.6 | 12.2 | 1.4E-4 | 10.0 |
| 475 | AT5G38710.1 \| proline oxidase, putative / osmotic stress-responsive proline dehydrogenase, putative, similar to proline oxidase, mitochondrial precursor (Osmotic stress- induced proline dehydrogenase) (Arabidopsis thaliana) SWISS-PROT:P92983 \| chr5:15518570-15521129 FORWARD \| Aliases: MKD10.10, MKD10_10 | 7.3 | 3.2 | 4.1 | 12.1 | 1.4E-4 | 8.8 |
| 479 | AT3G58700.1 \| 60S ribosomal protein L11 (RPL11B), ribosomal protein L11, cytosolic, Arabidopsis thaliana, PIR:S49033 \| chr3:21722540-21723930 FORWARD \| Aliases: T20N10.50 | 10.1 | 7.7 | 2.4 | 12.1 | 1.4E-4 | 10.2 |
| 480 | AT3G47070.1 \| expressed protein \| chr3:17348050-17348718 REVERSE \| Aliases: F13I12.120 | 7.9 | 3.1 | 4.8 | 12.1 | 1.4E-4 | 8.6 |
| 484 | AT4G23620.1 \| 50S ribosomal protein-related, contains weak similarity to 50S ribosomal protein L25 (TL5). (Swiss-Prot:P56930) (Thermus thermophilus) \| chr4:12314035-12316725 REVERSE \| Aliases: F9D16.90, F9D16_90 | 7.6 | 4.3 | 3.4 | 12.1 | 1.4E-4 | 9.9 |
| 485 | AT3G61100.1 \| expressed protein, contains Pfam profile PF04396: Protein of unknown function, DUF537 \| chr3:22621427-22622296 REVERSE \| Aliases: T27I15.190 | 7.6 | 2.5 | 5.1 | 12.1 | 1.4E-4 | 8.0 |
| 489 | AT1G80820.1 \| Symbol: CCR2 \| cinnamoyl-CoA reductase, putative, identical to CCR2 (GI:12407990), similar to cinnamoyl CoA reductase from Eucalyptus gunnii (GI:2058311) \| chr1:30375465-30377562 FORWARD \| Aliases: CINNAMOYL COA REDUCTASE, F23A5.17, F23A5_17 | 9.1 | 3.4 | 5.7 | 12.1 | 1.4E-4 | 10.1 |
| 493 | AT1G80560.1 \| 3-isopropylmalate dehydrogenase, chloroplast, putative, strong similarity to 3-ISOPROPYLMALATE DEHYDROGENASE PRECURSOR GB:P29102 SP:P29102 from (Brassica napus) | 10.4 | 5.4 | 5.0 | 12.0 | 1.4E-4 | 9.4 |
| 495 | AT1G61520.2 \| Symbol: LHCA3*1 \| similar to chlorophyll A-B binding protein, putative (LHCA5) [Arabidopsis thaliana] (TAIR:At1g45474.1); similar to chlorophyll A-B binding protein, putative (LHCA5) [Arabidopsis thaliana] (TAIR:At1g45474.2); similar to probable chlorophyll a/b-binding protein type III precursor - garden pea chloroplast (GB:T06411); contains InterPro domain Chlorophyll A-B binding protein (InterPro:IPR001344) \| chr1:22703738-22705048 FORWARD \| Aliases: LHCA3*1, T25B24.12, T25B24_12 | 10.5 | 6.3 | 4.2 | 12.0 | 1.5E-4 | 9.2 |
| 496 | AT1G80270.2 \| DNA-binding protein, putative, similar to DNA-binding protein (Triticum aestivum) GI:6958202; contains Pfam profile: PF01535 PPR repeat \| chr1:30185926-30188399 FORWARD \| Aliases: F5I6.2, F5I6_2 | 7.8 | 5.1 | 2.7 | 12.0 | 1.5E-4 | 10.1 |
| 497 | AT3G09970.1 \| calcineurin-like phosphoesterase family protein, contains Pfam profile: PF00149 Calcineurin-like phosphoesterase | 10.6 | 8.4 | 2.2 | 12.0 | 1.5E-4 | 10.4 |
| 504 | AT4G33560.1 \| expressed protein \| chr4:16135403-16135917 FORWARD \| Aliases: T16L1.50, T16L1_50 | 6.7 | 3.2 | 3.6 | 11.9 | 1.5E-4 | 9.5 |
| 505 | AT3G05560.2 \| 60S ribosomal protein L22-2 (RPL22B), identical to 60S ribosomal protein L22-2 SP:Q9M9W1 from (Arabidopsis thaliana) \| chr3:1614221-1615449 FORWARD \| Aliases: F18C1.17, F18C1_17 | 12.4 | 10.0 | 2.4 | 11.9 | 1.5E-4 | 10.8 |
| 506 | AT1G24735.1 \| caffeoyl-CoA 3-O-methyltransferase, putative, similar to SP:Q43237 (Vitis vinifera), GI:684942 (Medicago sativa subsp. sativa) \| chr1:8758685-8759434 FORWARD \| Aliases: F5A9.20, F5A9_20 | 10.0 | 4.9 | 5.1 | 11.9 | 1.5E-4 | 9.0 |

| Rank | Description | Sync | Root | M | t | adj.q | B |
|---|---|---|---|---|---|---|---|
| 508 | AT2G30410.1 \| Symbol: KIS \| tubulin folding cofactor A (KIESEL), identical to cDNA tubulin folding cofactor A, GI:20514256, SP:O04350 Tubulin-specific chaperone A (Tubulin-folding cofactor A) (CFA) (TCP1-chaperonin cofactor A homolog) {Arabidopsis thaliana} \| chr2:12966403-12967845 FORWARD \| Aliases: KIESEL, T9D9.22, T9D9_22 | 10.6 | 8.4 | 2.2 | 11.9 | 0.02% | 10.4 |
| 510 | AT3G49470.1 \| nascent polypeptide-associated complex (NAC) domain-containing protein, similar to alpha-NAC, non-muscle form (Mus musculus) GI:1666690; contains Pfam profile PF01849: NAC domain \| chr3:18351974-18353459 FORWARD \| Aliases: T9C5.70 | 9.8 | 5.8 | 4.0 | 11.9 | 0.02% | 9.3 |
| 511 | AT5G52970.1 \| thylakoid lumen 15.0 kDa protein, SP:Q9LVV5; similar to unknown protein (pir :S77462) \| chr5:21496835-21498452 FORWARD \| Aliases: MNB8.3, MNB8_3 | 7.9 | 3.1 | 4.8 | 11.9 | 0.02% | 8.2 |
| 512 | AT3G44590.2 \| 60S acidic ribosomal protein P2 (RPP2D), acidic ribosomal protein P2, maize, PIR:S54179 | 10.2 | 7.7 | 2.5 | 11.9 | 0.02% | 10.3 |
| 514 | AT5G50800.1 \| nodulin MtN3 family protein, similar to MtN3 GI:1619602 (root nodule development) from (Medicago truncatula) \| chr5:20682303-20684530 REVERSE \| Aliases: K7B16.1, K7B16_1 | 9.8 | 4.6 | 5.2 | 11.9 | 0.02% | 9.4 |
| 524 | AT3G05810.1 \| expressed protein \| chr3:1730982-1732390 REVERSE \| Aliases: F10A16.10, F10A16_10 | 7.7 | 3.4 | 4.3 | 11.8 | 0.02% | 8.7 |
| 527 | AT4G29390.1 \| 40S ribosomal protein S30 (RPS30B), RIBOSOMAL PROTEIN S30 - Arabidopsis thaliana,PID:e1358183 \| chr4:14464993-14465989 REVERSE \| Aliases: F17A13.210, F17A13_210 | 12.0 | 10.1 | 1.9 | 11.8 | 0.02% | 10.6 |
| 528 | AT2G23930.1 \| small nuclear ribonucleoprotein G, putative / snRNP-G, putative / Sm protein G, putative, similar to small nuclear ribonucleoprotein G (snRNP-G, Sm protein G, Sm-G, SmG) (Homo sapiens) SWISS-PROT:Q15357 \| chr2:10189345-10190320 FORWARD \| Aliases: T29E15.13, T29E15_13 | 9.6 | 7.2 | 2.4 | 11.8 | 0.02% | 10.3 |
| 530 | AT3G02720.1 \| DJ-1 family protein / protease-related, similar to Intracellular Protease (Pyrococcus horikoshii) GI:11513902; contains Pfam profile: PF01965 ThiJ/PfpI family \| chr3:586270-588270 FORWARD \| Aliases: F16B3.36 | 7.9 | 5.7 | 2.2 | 11.8 | 0.02% | 10.4 |
| 531 | AT2G21130.1 \| peptidyl-prolyl cis-trans isomerase / cyclophilin (CYP2) / rotamase, identical to cyclophilin (Arabidopsis thaliana) gi:2443757:gb:AAB71402; similar to peptidyl-prolyl cis-trans isomerase, PPIase (cyclophilin, cyclosporin A-binding protein) (Arabidopsis thaliana) SWISS-PROT:P34790 \| chr2:9062479-9063313 REVERSE \| Aliases: F26H11.11, F26H11_11 | 11.0 | 5.0 | 6.0 | 11.7 | 0.02% | 8.5 |
| 534 | AT5G06590.1 \| expressed protein \| chr5:2016646-2018836 FORWARD \| Aliases: F15M7.12, F15M7_12 | 9.1 | 4.7 | 4.4 | 11.7 | 0.02% | 9.2 |
| 535 | AT1G13690.1 \| Symbol: ATE1 \| RNA recognition motif (RRM)-containing protein, contains Pfam profile: PF00076 RNA recognition motif \| chr1:4693286-4694404 FORWARD \| Aliases: F21F23.13, F21F23_13, GRPE HOMOLOG | 10.7 | 7.5 | 3.2 | 11.7 | 0.02% | 9.1 |
| 536 | AT4G19710.2 \| bifunctional aspartate kinase/homoserine dehydrogenase, putative / AK-HSDH, putative, similar to gb:X71364 (PIR:S46497) aspartate kinase / homoserine dehydrogenase from Arabidopsis thaliana \| chr4:10724865-10729681 FORWARD \| Aliases: T16H5.70, T16H5_70 | 8.9 | 4.3 | 4.5 | 11.7 | 0.02% | 9.1 |
| 537 | AT5G48870.1 \| Symbol: SAD1 \| small nuclear ribonucleoprotein, putative / snRNP, putative / Sm protein, putative, similar to U6 snRNA-associated Sm-like protein LSm5 (Homo sapiens) SWISS-PROT:Q9Y4Y9 | 10.1 | 8.2 | 1.9 | 11.7 | 0.02% | 10.4 |
| 538 | AT2G42740.1 \| Symbol: RPL16A \| | 8.5 | 5.6 | 2.9 | 11.7 | 0.02% | 9.8 |
| 542 | AT1G76080.1 \| thioredoxin family protein, low similarity to thioredoxin (TRX) (Fasciola hepatica) GI:6687568; contains Pfam profile PF00085: Thioredoxin \| chr1:28552680-28554313 REVERSE \| Aliases: T23E18.2, T23E18_2 | 8.0 | 4.9 | 3.1 | 11.7 | 0.02% | 9.2 |
| 543 | AT1G50450.1 \| expressed protein \| chr1:18691453-18694039 REVERSE \| Aliases: F11F12.19, F11F12_19 | 8.7 | 4.2 | 4.5 | 11.7 | 0.02% | 8.6 |
| 544 | AT5G23330.1 \| riboflavin biosynthesis protein-related, contains weak similarity to Riboflavin biosynthesis protein ribF. (Swiss-Prot:P08391) (Escherichia coli O157:H7) \| chr5:7854132-7855816 FORWARD \| Aliases: MKD15.19, MKD15_19 | 8.1 | 5.5 | 2.6 | 11.7 | 0.02% | 9.9 |
| 545 | AT1G33810.1 \| expressed protein \| chr1:12265050-12266827 FORWARD \| Aliases: F14M2.27 | 9.2 | 4.4 | 4.8 | 11.7 | 0.02% | 9.4 |
| 550 | AT5G18040.1 \| expressed protein \| chr5:5972421-5974158 FORWARD \| Aliases: MCM23.14, MCM23_14 | 9.7 | 7.4 | 2.3 | 11.6 | 0.02% | 9.9 |
| 551 | AT3G51030.1 \| Symbol: ATTRX1 \| | 10.8 | 5.2 | 5.6 | 11.6 | 0.02% | 8.8 |
| 553 | AT4G14385.3 \| expressed protein \| chr4:8287892-8288669 REVERSE \| Aliases: None | 8.3 | 5.8 | 2.5 | 11.6 | 0.02% | 9.7 |
| 554 | AT1G64880.1 \| ribosomal protein S5 family protein, contains similarity to 30S ribosomal protein S5 GI:6969105 from (Campylobacter jejuni) \| chr1:24109305-24112809 FORWARD \| Aliases: F13O11.18, F13O11_18 | 9.1 | 5.0 | 4.1 | 11.6 | 0.02% | 9.6 |

| Rank | Description | Sync | Root | M | t | adj.q | B |
|---|---|---|---|---|---|---|---|
| 555 | AT5G64130.2 \| expressed protein \| chr5:25681625-25683425 REVERSE \| Aliases: MHJ24.11, MHJ24_11 | 11.9 | 10.4 | 1.5 | 11.6 | 0.02% | 10.5 |
| 560 | AT1G15390.1 \| Symbol: PDF1A \| peptide deformylase, mitochondrial / polypeptide deformylase 1A (PDF1A), nearly identical to SP:Q9FV53 Peptide deformylase, mitochondrial precursor (EC 3.5.1.88) (PDF) (Polypeptide deformylase) {Arabidopsis thaliana}; contains Pfam profile PF01327: polypeptide deformylase; supporting cDNA gi:11320951:gb:AF250959.1:AF250959 \| chr1:5294624-5295792 FORWARD \| Aliases: PEPTIDE DEFORMYLASE 1A | 9.1 | 5.2 | 3.8 | 11.5 | 0.02% | 9.3 |
| 561 | AT5G42290.1 \| transcription activator-related, contains weak similarity to beta-cell E-box transcription activator 1 gi:529197:gb:AAA65702 \| chr5:16928928-16929332 FORWARD \| Aliases: K5J14.9, K5J14_9 | 5.7 | 2.2 | 3.5 | 11.5 | 0.02% | 8.9 |
| 562 | AT2G27240.1 \| expressed protein, contains Pfam profile PF01027: Uncharacterized protein family UPF0005 | 7.8 | 3.5 | 4.3 | 11.5 | 0.02% | 8.1 |
| 563 | AT3G62050.1 \| expressed protein, contains Pfam profile PF04396: Protein of unknown function, DUF537 \| chr3:22989221-22989994 FORWARD \| Aliases: T17J13.10 | 5.3 | 2.5 | 2.8 | 11.5 | 0.02% | 9.6 |
| 567 | AT1G54140.1 \| Symbol: TAFII21 \| transcription initiation factor IID (TFIID) 31 kDa subunit (TAFII-31) family protein, contains Pfam profile: PF02291 transcription initiation factor IID, 31kD subunit \| chr1:20217637-20218887 REVERSE \| Aliases: F15I1.24, F15I1_24, TATA BINDING PROTEIN ASSOCIATED FACTOR 21KDA SUBUNI, TATA BINDING PROTEIN ASSOCIATED FACTOR 21KDA SUBUNIT | 7.5 | 4.2 | 3.3 | 11.5 | 0.02% | 9.3 |
| 571 | AT3G52580.1 \| 40S ribosomal protein S14 (RPS14C), ribosomal protein S14 -Zea mays,PIR2:A30097 \| chr3:19514203-19515902 FORWARD \| Aliases: F3C22.6 | 10.6 | 6.5 | 4.1 | 11.5 | 0.02% | 9.8 |
| 572 | AT1G22270.1 \| expressed protein, contains Pfam domain PF03966: Protein of unknown function (DUF343) \| chr1:7865482-7866128 FORWARD \| Aliases: T16E15.11, T16E15_11 | 8.6 | 4.4 | 4.2 | 11.5 | 0.02% | 9.4 |
| 575 | AT1G57540.2 \| expressed protein, contains similarity to T-box domain GI:5931592 from (Cynops pyrrhogaster) \| chr1:21313995-21315077 REVERSE \| Aliases: F25P12.4 | 9.7 | 6.3 | 3.3 | 11.4 | 0.02% | 9.8 |
| 579 | AT3G53020.1 \| 60S ribosomal protein L24 (RPL24B), 60S ribosomal protein L24, Arabidopsis thaliana, EMBL:AC006282 \| chr3:19671545-19673003 REVERSE \| Aliases: F8J2.190 | 12.0 | 10.3 | 1.6 | 11.4 | 0.02% | 10.4 |
| 584 | AT5G08120.1 \| myosin heavy chain-related, identical to myosin heavy chain-like protein GI:1732515 from (Arabidopsis thaliana) \| chr5:2600492-2602804 REVERSE \| Aliases: T22D6.60, T22D6_60 | 7.7 | 4.2 | 3.5 | 11.4 | 0.02% | 9.9 |
| 589 | AT2G21860.1 \| violaxanthin de-epoxidase-related, contains weak similarity to violaxanthin de-epoxidase precursor gi:1438875:gb:AAC49373 \| chr2:9325338-9327082 REVERSE \| Aliases: F7D8.18, F7D8_18 | 6.7 | 3.1 | 3.6 | 11.4 | 0.02% | 8.7 |
| 594 | AT5G17870.1 \| plastid-specific ribosomal protein-related, contains similarity to plastid-specific ribosomal protein 6 precursor GI:7578927 from (Spinacia oleracea) \| chr5:5907777-5908337 FORWARD \| Aliases: MPI7.3, MPI7_3 | 8.0 | 3.0 | 5.1 | 11.4 | 0.02% | 8.7 |
| 599 | AT4G12600.1 \| ribosomal protein L7Ae/L30e/S12e/Gadd45 family protein, Similar to NHP2/L7Ae family proteins, see SWISSPROT:P32495 and PMID:2063628. \| chr4:7453199-7454478 FORWARD \| Aliases: T1P17.190, T1P17_190 | 10.2 | 6.8 | 3.5 | 11.3 | 0.02% | 9.8 |
| 602 | AT1G17380.1 \| expressed protein \| chr1:5955481-5957205 REVERSE \| Aliases: F28G4.16 | 6.8 | 2.7 | 4.1 | 11.3 | 0.02% | 8.8 |
| 603 | AT2G40880.1 \| Symbol: FL3 27 \| | 8.1 | 5.1 | 3.0 | 11.3 | 0.02% | 9.5 |
| 604 | AT3G21110.2 \| Symbol: PUR7 \| similar to SAICAR synthetase [Nicotiana tabacum] (GB:AAR06292.1); contains InterPro domain SAICAR synthetase (InterPro:IPR001636) \| chr3:7402141-7405614 REVERSE \| Aliases: 5' PHOSPHORIBOSYL 4 (N SUCCINOCARBOXAMIDE) 5 AMINOIMIDAZOLE SYNTHETASE, MSA6.26, PURC, SAIRCAR SYNTHETASE | 9.0 | 5.9 | 3.1 | 11.3 | 0.02% | 9.4 |
| 606 | AT3G53740.3 \| similar to 60S ribosomal protein L36 (RPL36A) [Arabidopsis thaliana] (TAIR:At2g37600.1); similar to putative 60S ribosomal protein L36 [Oryza sativa (japonica cultivar-group)] (GB:XP_475364.1); contains InterPro domain Ribosomal protein L36E (InterPro:IPR000509) \| chr3:19924705-19925969 REVERSE \| Aliases: F5K20.40 | 11.5 | 9.1 | 2.5 | 11.3 | 0.02% | 9.9 |
| 607 | AT2G29670.1 \| expressed protein \| chr2:12689221-12692164 REVERSE \| Aliases: T27A16.23, T27A16_23 | 8.7 | 4.9 | 3.8 | 11.3 | 0.02% | 8.2 |
| 608 | AT3G18940.1 \| clast3-related, contains Prosite PS00626: Regulator of chromosome condensation (RCC1) signature 2; similar to Clast3 protein (GI:9857631) (Mus musculus) | 6.3 | 3.7 | 2.7 | 11.2 | 0.02% | 9.4 |
| 610 | AT5G04850.1 \| SNF7 family protein, contains Pfam domain, PF03357: SNF7 family \| chr5:1408063-1409893 REVERSE \| Aliases: MUK11.17, MUK11_17 | 10.9 | 7.8 | 3.1 | 11.2 | 0.02% | 9.9 |

| Rank | Description | Sync | Root | M | t | adj.q | B |
|---|---|---|---|---|---|---|---|
| 611 | AT5G40610.1 \| glycerol-3-phosphate dehydrogenase (NAD+) / GPDH, strong similarity to SP:P52425 Glycerol-3-phosphate dehydrogenase (NAD+) (EC 1.1.1.8) {Cuphea lanceolata}; contains Pfam profile PF01210: NAD-dependent glycerol-3-phosphate dehydrogenase \| chr5:16282090-16284520 REVERSE \| Aliases: MNF13.130, MNF13_130 | 7.5 | 4.0 | 3.5 | 11.2 | 0.02% | 9.1 |
| 612 | AT2G37240.1 \| expressed protein, weak similarity to fmHP (GI:22902096) (Takifugu rubripes) \| chr2:15647018-15648842 REVERSE \| Aliases: F3G5.3, F3G5_3 | 7.8 | 4.2 | 3.6 | 11.2 | 0.02% | 8.6 |
| 614 | AT4G37090.2 \| expressed protein, similar to hypothetical protein DDB0205993 [Dictyostelium discoideum] (GB:EAL67624.1) | 7.3 | 4.1 | 3.3 | 11.2 | 0.02% | 9.3 |
| 616 | AT5G23250.1 \| succinyl-CoA ligase (GDP-forming) alpha-chain, mitochondrial, putative / succinyl-CoA synthetase, alpha chain, putative / SCS-alpha, putative, similar to SP:P36967 Succinyl-CoA ligase (GDP-forming) alpha-chain, mitochondrial precursor (EC 6.2.1.4) (Succinyl-CoA synthetase, alpha chain) (SCS-alpha) {Dictyostelium discoideum}; contains Pfam profiles PF00549: CoA-ligase, PF02629: CoA binding domain \| chr5:7830392-7832846 FORWARD \| Aliases: MKD15.11, MKD15_11 | 10.8 | 7.0 | 3.8 | 11.2 | 0.02% | 9.7 |
| 618 | AT1G69880.1 \| thioredoxin, putative, similar to SP:Q38879 Thioredoxin H-type 2 (TRX-H-2) {Arabidopsis thaliana}; contains Pfam profile: PF00085 Thioredoxin \| chr1:26325142-26326656 FORWARD \| Aliases: T17F3.9, T17F3_9 | 7.1 | 2.4 | 4.8 | 11.2 | 0.02% | 8.1 |
| 621 | AT1G29250.1 \| expressed protein, contains TIGRFAM TIGR00285: conserved hypothetical protein TIGR00285 \| chr1:10223252-10224713 REVERSE \| Aliases: F28N24.7, F28N24_7 | 9.7 | 6.6 | 3.1 | 11.1 | 0.02% | 9.5 |
| 622 | AT3G13860.1 \| chaperonin, putative, similar to SWISS-PROT:P29197- chaperonin CPN60, mitochondrial precursor (HSP60) (Arabidopsis thaliana) ; contains Pfam:PF00118 domain, TCP-1/cpn60 chaperonin family \| chr3:4561524-4565256 REVERSE \| Aliases: MCP4.9 | 10.1 | 7.1 | 3.0 | 11.1 | 0.02% | 9.4 |
| 623 | AT1G04420.1 \| aldo/keto reductase family protein, Similar to SP:Q46933 Tas protein {Escherichia coli}, Babesia aldo-keto reductase SP:P40690; contains Pfam profile PF00248: oxidoreductase, aldo/keto reductase family \| chr1:1191607-1193883 FORWARD \| Aliases: F19P19.12, F19P19_12 | 7.7 | 3.8 | 3.8 | 11.1 | 0.02% | 8.5 |
| 624 | AT2G45710.1 \| 40S ribosomal protein S27 (RPS27A) \| chr2:18838235-18839281 FORWARD \| Aliases: F4I18.31 | 9.2 | 5.7 | 3.5 | 11.1 | 0.02% | 9.4 |
| 625 | AT3G46970.1 \| Encodes a cytosolic alpha-glucan phosphorylase. \| chr3:17312379-17317431 REVERSE \| Aliases: F13I12.20 | 8.2 | 5.1 | 3.1 | 11.1 | 0.02% | 9.4 |
| 629 | AT2G05620.1 \| Symbol: PGR5 \| expressed protein \| chr2:2081050-2081837 REVERSE \| Aliases: T20G20.3, T20G20_3 | 8.8 | 4.5 | 4.3 | 11.1 | 0.02% | 9.5 |
| 630 | AT4G09320.1 \| Symbol: NDPK1 \| nucleoside diphosphate kinase 1 (NDK1), identical to identical to Nucleoside diphosphate kinase I (NDK I) (NDP kinase I) (NDPK I) (SP:P39207) (Arabidopsis thaliana); contains Pfam PF00334 : Nucleoside diphosphate kinase domain; \| chr4:5923397-5924529 FORWARD \| Aliases: T30A10.80, T30A10_80 | 12.2 | 10.7 | 1.5 | 11.1 | 0.02% | 10.1 |
| 631 | AT5G15750.1 \| RNA-binding S4 domain-containing protein, 40S RIBOSOMAL PROTEINs - different species \| chr5:5141194-5142861 FORWARD \| Aliases: F14F8.130, F14F8_130 | 8.5 | 6.4 | 2.1 | 11.1 | 0.02% | 9.7 |
| 632 | AT4G05180.1 \| oxygen-evolving enhancer protein 3, chloroplast, putative (PSBQ2), identical to SP:Q41932 Oxygen-evolving enhancer protein 3-2, chloroplast precursor (OEE3) (16 kDa subunit of oxygen evolving system of photosystem II) (OEC 16 kDa subunit) {Arabidopsis thaliana}; similar to SP:P12301 Oxygen-evolving enhancer protein 3, chloroplast precursor (OEE3) (16 kDa subunit of oxygen evolving system of photosystem II) (OEC 16 kDa subunit) {Spinacia oleracea}; contains Pfam profile PF05757: Oxygen evolving enhancer protein 3 (PsbQ) \| chr4:2671820-2673241 REVERSE \| Aliases: C17L7.100, C17L7_100 | 9.1 | 3.5 | 5.5 | 11.1 | 0.02% | 8.9 |
| 633 | AT1G74040.1 \| Symbol: IMS1 \| 2-isopropylmalate synthase 1 (IMS1), identical to 2-isopropylmalate synthase (IMS1) (Arabidopsis thaliana) GI:12330687; identical to cDNA 2-isopropylmalate synthase (IMS1) GI:12330686 | 8.0 | 4.7 | 3.3 | 11.1 | 0.02% | 9.6 |
| 640 | AT5G44560.1 \| SNF7 family protein, contains Pfam domain, PF03357: SNF7 family \| chr5:17963206-17965627 FORWARD \| Aliases: MFC16.25, MFC16_25 | 8.2 | 4.2 | 4.0 | 11.0 | 0.02% | 8.5 |
| 641 | AT1G52590.1 \| expressed protein \| chr1:19592678-19593942 REVERSE \| Aliases: F6D8.19, F6D8_19 | 7.0 | 4.3 | 2.7 | 11.0 | 0.02% | 9.3 |
| 642 | AT5G30510.1 \| 30S ribosomal protein S1, putative, similar to Swiss-Prot:P29344 30S ribosomal protein S1, chloroplast precursor (CS1) (Spinacia oleracea) \| chr5:11636256-11638528 REVERSE \| Aliases: None | 10.3 | 7.9 | 2.4 | 11.0 | 0.02% | 10.0 |
| 643 | AT1G08380.1 \| expressed protein \| chr1:2640858-2641825 REVERSE \| Aliases: T27G7.25, T27G7_25 | 7.6 | 3.5 | 4.1 | 11.0 | 0.02% | 8.4 |
| 644 | AT1G72030.1 \| GCN5-related N-acetyltransferase (GNAT) family protein, contains Pfam profile PF00583: acetyltransferase, GNAT family \| chr1:27114527-27115868 FORWARD \| Aliases: F28P5.8, F28P5_8 | 7.9 | 3.2 | 4.7 | 11.0 | 0.02% | 7.1 |

| Rank | Description | Sync | Root | M | t | adj.q | B |
|---|---|---|---|---|---|---|---|
| 647 | AT3G03910.1 \| glutamate dehydrogenase, putative, similar to glutamate dehydrogenase 1 (GDH 1) (Arabidopsis thaliana) SWISS-PROT:Q43314 | 7.7 | 3.5 | 4.2 | 11.0 | 0.02% | 7.5 |
| 651 | AT1G15930.2 \| 40S ribosomal protein S12 (RPS12A), similar to 40S ribosomal protein S12 GI:4263712 from (Arabidopsis thaliana) \| chr1:5471431-5472978 FORWARD \| Aliases: T24D18.3, T24D18_3 | 12.3 | 10.3 | 2.0 | 11.0 | 0.02% | 10.1 |
| 655 | AT2G39460.1 \| Symbol: ATRPL23A \| 60S ribosomal protein L23A (RPL23aA), identical to GB:AF034694 \| chr2:16482001-16483163 FORWARD \| Aliases: F12L6.12, F12L6_12, RIBOSOMAL PROTEIN L23A, RPL23A | 10.9 | 6.8 | 4.0 | 11.0 | 0.02% | 9.0 |
| 658 | AT1G05205.1 \| expressed protein \| chr1:1509212-1510280 REVERSE \| Aliases: None | 10.3 | 8.1 | 2.1 | 10.9 | 0.02% | 10.0 |
| 661 | AT1G01940.1 \| peptidyl-prolyl cis-trans isomerase cyclophilin-type family protein, contains Pfam domain, PF00160: peptidyl-prolyl cis-trans isomerase, cyclophilin-type \| chr1:323027-324917 FORWARD \| Aliases: F22M8.7, F22M8_7 | 9.3 | 4.5 | 4.8 | 10.9 | 0.02% | 8.6 |
| 662 | AT1G03600.1 \| photosystem II family protein, similar to SP:P74367 {Synechocystis sp.}; similar to ESTs emb:Z27038, gb:AA451546, emb:Z29876, gb:T45359 and gb:R90316 \| chr1:898875-899654 FORWARD \| Aliases: F21B7.21, F21B7.37, F21B7_37 | 8.4 | 3.4 | 5.0 | 10.9 | 0.02% | 7.6 |
| 663 | AT3G44890.1 \| Symbol: RPL9 \| 50S ribosomal protein L9, chloroplast (CL9), contains Pfam profile PF03948: Ribosomal protein L9, C-terminal domain; contains Pfam profile PF01281: ribosomal protein L9, N-terminal domain; contains TIGRfam profile TIGR00158: ribosomal protein L9 \| chr3:16397420-16399451 FORWARD \| Aliases: F28D10.80, RP19 | 7.3 | 3.0 | 4.2 | 10.9 | 0.02% | 8.7 |
| 664 | AT5G01650.1 \| macrophage migration inhibitory factor family protein / MIF family protein, contains pfam profile: PF001187 Macrophage migration inhibitory factor \| chr5:242547-244096 REVERSE \| Aliases: F7A7.170, F7A7_170 | 10.2 | 7.0 | 3.3 | 10.9 | 0.02% | 9.3 |
| 666 | AT2G37270.2 \| Symbol: ATRPS5B \| similar to 40S ribosomal protein S5 (RPS5B) [Arabidopsis thaliana] (TAIR:At3g11940.1); similar to 40S ribosomal protein S5 (RPS5B) [Arabidopsis thaliana] (TAIR:At3g11940.2); similar to putative 40S ribosomal protein S5 [Oryza sativa (japonica cultivar-group)] (GB:NP_908322.1); contains InterPro domain Ribosomal protein S7, eukaryotic and archaeal form (InterPro:IPR005716); contains InterPro domain Ribosomal protein S7 (InterPro:IPR000235) \| chr2:15654756-15656282 REVERSE \| Aliases: F3G5.6, F3G5_6, RIBOSOMAL PROTEIN 5B | 13.3 | 11.9 | 1.4 | 10.9 | 0.02% | 10.0 |
| 667 | AT3G11940.2 \| Symbol: ATRPS5A \| 40S ribosomal protein S5 (RPS5B), similar to 40S ribosomal protein S5 GB:AAC98068 GI:4056502 from (Arabidopsis thaliana) \| chr3:3777901-3779509 REVERSE \| Aliases: AML1, ARABIDOPSIS MINUTE LIKE 1, MEC18.11, RIBOSOMAL PROTEIN 5A | 13.3 | 11.9 | 1.4 | 10.9 | 0.02% | 10.0 |
| 669 | AT1G65700.2 \| similar to small nuclear ribonucleoprotein, putative / snRNP, putative / Sm protein, putative [Arabidopsis thaliana] (TAIR:At1g19120.1); similar to PREDICTED: similar to U6 snRNA-associated Sm-like protein LSm8 [Gallus gallus] (GB:XP_416009.1); contains InterPro domain Small nuclear ribonucleoprotein (Sm protein) (InterPro:IPR001163) \| chr1:24437943-24439918 REVERSE \| Aliases: F1E22.8, F1E22_8 | 9.4 | 6.2 | 3.2 | 10.9 | 0.02% | 9.4 |
| 671 | AT5G54600.2 \| 50S ribosomal protein L24, chloroplast (CL24), identical to SP:P92959 50S ribosomal protein L24, chloroplast precursor {Arabidopsis thaliana} \| chr5:22200204-22201793 FORWARD \| Aliases: MRB17.10, MRB17_10 | 9.2 | 6.5 | 2.7 | 10.9 | 0.02% | 9.4 |
| 674 | AT2G22540.1 \| Symbol: SVP \| short vegetative phase protein (SVP), identical to cDNA short vegetative phase protein (SVP) GI:10944319; | 7.9 | 5.9 | 2.0 | 10.9 | 0.02% | 9.7 |
| 676 | AT1G64700.1 \| expressed protein \| chr1:24043473-24044258 REVERSE \| Aliases: F13O11.2, F13O11_2 | 7.0 | 4.6 | 2.4 | 10.9 | 0.02% | 9.7 |
| 678 | AT5G45750.1 \| Ras-related GTP-binding protein, putative, similar to GTP-binding protein GI:303744 from (Pisum sativum) | 9.1 | 5.3 | 3.8 | 10.8 | 0.02% | 9.7 |
| 679 | AT5G28220.1 \| expressed protein, predicted proteins, Arabidopsis thaliana, D.melanogaster, C.elegans and S.pombe \| chr5:10190365-10193123 REVERSE \| Aliases: F26C17.8, F26C17_8 | 6.4 | 3.8 | 2.6 | 10.8 | 0.02% | 9.4 |
| 682 | AT4G33985.1 \| expressed protein \| chr4:16288117-16288918 REVERSE \| Aliases: None | 7.3 | 3.8 | 3.5 | 10.8 | 0.02% | 8.6 |
| 683 | AT3G50790.1 \| late embryogenesis abundant protein, putative / LEA protein, putative, similar to Picea glauca late embryogenesis abundant protein (EMB8), PID:g1350545 SP:Q40863; contains Pfam profile PF00561: hydrolase, alpha/beta fold family \| chr3:18890904-18892963 REVERSE \| Aliases: F18B3.70 | 6.1 | 3.3 | 2.8 | 10.8 | 0.02% | 9.4 |
| 684 | AT3G16080.1 \| 60S ribosomal protein L37 (RPL37C), similar to ribosomal protein L37 GB:BAA04888 from (Homo sapiens) \| chr3:5454824-5455933 FORWARD \| Aliases: MSL1.12 | 12.5 | 10.4 | 2.1 | 10.8 | 0.02% | 10.0 |
| 685 | AT1G04480.1 \| 60S ribosomal protein L23 (RPL23A), identical to GB:AAB80655 \| chr1:1216094-1217457 FORWARD \| Aliases: F19P19.5, F19P19_5 | 11.1 | 9.5 | 1.6 | 10.8 | 0.02% | 10.0 |
| 686 | AT2G47440.1 \| DNAJ heat shock N-terminal domain-containing protein, contains Pfam profiles PF00226: DnaJ domain, PF00515: TPR Domain; similar to GP:2104534:AF001308 (T10M13.11) \| chr2:19476789-19478956 FORWARD \| Aliases: T30B22.26 | 7.6 | 4.9 | 2.7 | 10.8 | 0.02% | 9.5 |
| 688 | AT3G54260.1 \| expressed protein, various predicted proteins, Arabidopsis thaliana \| chr3:20095988-20097741 REVERSE \| Aliases: F24B22.220 | 10.2 | 7.7 | 2.5 | 10.8 | 0.02% | 9.5 |

| Rank | Description | Sync | Root | M | t | adj.q | B |
|---|---|---|---|---|---|---|---|
| 690 | AT3G53890.1 \| 40S ribosomal protein S21 (RPS21B), ribosomal protein S21, cytosolic - Oryza sativa, PIR:S38357 \| chr3:19966322-19967327 REVERSE \| Aliases: F5K20.190 | 11.2 | 8.1 | 3.0 | 10.8 | 0.02% | 9.7 |
| 692 | AT4G10270.1 \| wound-responsive family protein, similar to wound induced protein (GI:19320) (Lycopersicon esculentum) \| chr4:6374730-6375206 FORWARD \| Aliases: T9A4.6 | 8.7 | 4.2 | 4.6 | 10.8 | 0.02% | 8.3 |
| 695 | AT3G12320.1 \| expressed protein \| chr3:3923742-3925442 FORWARD \| Aliases: T2E22.34 | 7.0 | 3.3 | 3.7 | 10.8 | 0.02% | 8.2 |
| 696 | AT2G44860.1 \| 60S ribosomal protein L24, putative \| chr2:18507444-18509617 REVERSE \| Aliases: T13E15.13 | 10.1 | 6.7 | 3.4 | 10.7 | 0.02% | 9.7 |
| 702 | AT4G02790.1 \| GTP-binding family protein, contains Pfam domain, PF01926: GTPase of unknown function \| chr4:1247399-1249588 FORWARD \| Aliases: T5J8.11, T5J8_11 | 6.7 | 3.7 | 3.1 | 10.7 | 0.02% | 8.7 |
| 703 | AT2G25720.1 \| expressed protein \| chr2:10962597-10963130 REVERSE \| Aliases: F3N11.17, F3N11_17 | 9.8 | 6.9 | 2.9 | 10.7 | 0.02% | 9.1 |
| 704 | AT2G42220.1 \| rhodanese-like domain-containing protein, contains rhodanese-like domain PF:00581 \| chr2:17599167-17600783 FORWARD \| Aliases: T24P15.13, T24P15_13 | 8.3 | 3.5 | 4.7 | 10.7 | 0.02% | 7.8 |
| 705 | AT2G36160.1 \| 40S ribosomal protein S14 (RPS14A) \| chr2:15176897-15178428 FORWARD \| Aliases: F9C22.9, F9C22_9 | 11.0 | 9.4 | 1.6 | 10.7 | 0.02% | 9.9 |
| 708 | AT2G40610.1 \| Symbol: ATEXPA8 \| expansin, putative (EXP8), similar to expansin 2 GI:7025493 from (Zinnia elegans); alpha-expansin gene family, PMID:11641069 \| chr2:16955941-16957635 REVERSE \| Aliases: ARABIDOPSIS THALIANA EXPANSIN A8, ATEXP8, ATHEXP ALPHA 1.11, EXP8, T2P4.4, T2P4_4 | 8.1 | 3.8 | 4.3 | 10.7 | 0.03% | 8.3 |
| 709 | AT1G14820.3 \| SEC14 cytosolic factor family protein / phosphoglyceride transfer family protein, contains Pfam PF00650 : CRAL/TRIO domain; similar to phosphatidylinositol/phosphatidylcholine transfer protein SP:P24280 (Saccharomyces cerevisiae (Baker's yeast)) \| chr1:5105109-5106790 REVERSE \| Aliases: F10B6.40, F10B6_40 | 7.4 | 4.6 | 2.8 | 10.7 | 0.03% | 8.8 |
| 711 | AT4G02840.1 \| small nuclear ribonucleoprotein D1, putative / snRNP core protein D1, putative / Sm protein D1, putative, similar to small nuclear ribonucleoprotein Sm D1 (snRNP core protein D1, Sm-D1, Sm-D autoantigen) (Mouse) SWISS-PROT:P13641 \| chr4:1264627-1266486 FORWARD \| Aliases: T5J8.16, T5J8_16 | 9.0 | 6.9 | 2.1 | 10.7 | 0.03% | 9.6 |
| 716 | AT2G33800.1 \| ribosomal protein S5 family protein, contains Pfam profiles PF03719: Ribosomal protein S5, C-terminal domain, PF00333: Ribosomal protein S5, N-terminal domain \| chr2:14307848-14309506 REVERSE \| Aliases: T1B8.10, T1B8_10 | 9.9 | 7.4 | 2.5 | 10.7 | 0.03% | 9.3 |
| 718 | AT3G05590.1 \| Symbol: RPL18 \| 60S ribosomal protein L18 (RPL18B), similar to GB:P42791 \| chr3:1621486-1623020 FORWARD \| Aliases: CYTOPLASMIC RIBOSOMAL PROTEIN L18, F18C1.14, F18C1_14, RIBOSOMAL PROTEIN L18 | 11.2 | 9.1 | 2.1 | 10.7 | 0.03% | 9.7 |
| 720 | AT5G43150.1 \| expressed protein \| chr5:17342246-17343126 FORWARD \| Aliases: MMG4.18, MMG4_18 | 11.2 | 10.1 | 1.1 | 10.7 | 0.03% | 9.9 |
| 724 | AT2G30390.1 \| ferrochelatase II, identical to Swiss-Prot:O04921 ferrochelatase II, chloroplast precursor (EC 4.99.1.1) (Protoheme ferro-lyase) (Heme synthetase) (Arabidopsis thaliana) \| chr2:12958104-12961167 REVERSE \| Aliases: T9D9.1 | 8.1 | 5.0 | 3.0 | 10.6 | 0.03% | 8.9 |
| 725 | AT3G13470.1 \| chaperonin, putative, similar SWISS-PROT:P21240- RuBisCO subunit binding-protein beta subunit, chloroplast precursor (60 kDa chaperonin beta subunit, CPN-60 beta) (Arabidopsis thaliana); contains Pfam:PF00118 domain, TCP-1/cpn60 chaperonin family \| chr3:4389692-4392762 FORWARD \| Aliases: MRP15.11 | 11.9 | 6.3 | 5.6 | 10.6 | 0.03% | 9.5 |
| 728 | AT3G59650.1 \| mitochondrial ribosomal protein L51/S25/CI-B8 family protein, low similarity to mitochondrial ribosomal protein bMRP36a (Mus musculus) GI:13559406; contains Pfam profile PF05047: Mitochondrial ribosomal protein L51 / S25 / CI-B8 domain \| chr3:22044152-22045134 FORWARD \| Aliases: T16L24.200 | 7.4 | 3.9 | 3.5 | 10.6 | 0.03% | 8.5 |
| 729 | AT5G08565.1 \| expressed protein \| chr5:2775899-2777400 FORWARD \| Aliases: None | 6.2 | 2.9 | 3.3 | 10.6 | 0.03% | 8.8 |
| 731 | AT2G21820.1 \| expressed protein \| chr2:9309965-9310327 REVERSE \| Aliases: F7D8.14, F7D8_14 | 9.2 | 6.2 | 3.0 | 10.6 | 0.03% | 9.2 |
| 732 | AT3G03490.1 \| peroxisomal protein PEX19 family protein, contains Pfam profile: PF04614 Pex19 protein family \| chr3:830172-831853 REVERSE \| Aliases: T21P5.9, T21P5_9 | 9.2 | 7.0 | 2.2 | 10.6 | 0.03% | 9.7 |
| 734 | AT4G25810.1 \| Symbol: XTR6 \| xyloglucan:xyloglucosyl transferase, putative / xyloglucan endotransglycosylase, putative / endo-xyloglucan transferase, putative (XTR6), identical to xyloglucan endotransglycosylase-related protein GI:1244758 from (Arabidopsis thaliana) \| chr4:13128655-13129893 FORWARD \| Aliases: F14M19.90, F14M19_90, XYLOGLUCAN ENDOTRANSGLYCOSYLASE 6 | 9.6 | 4.6 | 5.1 | 10.6 | 0.03% | 8.0 |
| 735 | AT4G21620.1 \| glycine-rich protein \| chr4:11491495-11492179 FORWARD \| Aliases: F17L22.80, F17L22_80 | 10.2 | 6.4 | 3.8 | 10.6 | 0.03% | 7.9 |

| Rank | Description | Sync | Root | M | t | adj.q | B |
|---|---|---|---|---|---|---|---|
| 736 | AT1G22780.1 \| Symbol: PFL \| 40S ribosomal protein S18 (RPS18A), Match to ribosomal S18 gene mRNA gb:Z28701, DNA gb:Z23165 from A. thaliana. ESTs gb:T21121, gb:Z17755, gb:R64776 and gb:R30430 come from this gene \| chr1:8067853-8069319 FORWARD \| Aliases: 40S RIBOSOMAL PROTEIN S18, PFL1, POINTED FIRST LEAVES, RPS18A, T22J18.5, T22J18_5 | 11.1 | 9.0 | 2.1 | 10.6 | 0.03% | 9.7 |
| 737 | AT5G54290.1 \| cytochrome c biogenesis protein family, low similarity to cytochrome c biogenesis protein CcdA (Paracoccus pantotrophus) GI:11095328; contains Pfam profile PF02683: Cytochrome C biogenesis protein transmembrane region \| chr5:22068263-22071604 FORWARD \| Aliases: MDK4.11, MDK4_11 | 9.0 | 5.7 | 3.4 | 10.6 | 0.03% | 9.2 |
| 739 | AT1G16000.1 \| expressed protein \| chr1:5494530-5495317 FORWARD \| Aliases: T24D18.10, T24D18_10 | 9.8 | 6.4 | 3.5 | 10.6 | 0.03% | 8.8 |
| 742 | AT5G45170.1 \| CbbY protein-related, low similarity to SP:P40119 CbbY protein, chromosomal {Alcaligenes eutrophus} \| chr5:18287571-18290396 REVERSE \| Aliases: K18C1.5, K18C1_5 | 8.4 | 6.3 | 2.1 | 10.5 | 0.03% | 9.6 |
| 744 | AT1G50575.1 \| lysine decarboxylase family protein, contains Pfam profile PF03641: lysine decarboxylase family \| chr1:18732387-18734055 REVERSE \| Aliases: None | 7.2 | 4.1 | 3.1 | 10.5 | 0.03% | 8.8 |
| 745 | AT1G53120.1 \| RNA-binding S4 domain-containing protein | 6.6 | 4.2 | 2.5 | 10.5 | 0.03% | 9.1 |
| 746 | AT2G29460.1 \| Symbol: ATGSTU4 \| glutathione S-transferase, putative \| chr2:12633624-12634755 REVERSE \| Aliases: F16P2.16, F16P2_16, GLUTATHIONE S TRANSFERASE 22, GST22 | 7.7 | 2.9 | 4.8 | 10.5 | 0.03% | 8.6 |
| 748 | AT4G30270.1 \| Symbol: MERI5B \| | 7.4 | 3.5 | 3.9 | 10.5 | 0.03% | 8.2 |
| 749 | AT1G79500.4 \| Symbol: KDSA \| similar to 2-dehydro-3-deoxyphosphooctonate aldolase, putative / phospho-2-dehydro-3-deoxyoctonate aldolase, putative / 3-deoxy-D-manno-octulosonic acid 8-phosphate synthetase, putative [Arabidopsis thaliana] (TAIR:At1g16340.1); similar to putative 2-dehydro-3-deoxyphosphooctonate aldolase [Oryza sativa (japonica cultivar-group)] (GB:AAO72599.1); contains InterPro domain DAHP synthetase I/KDSA superfamily (InterPro:IPR006218); contains InterPro domain 2-dehydro-3-deoxyphosphooctonate aldolase (InterPro:IPR006269) \| chr1:29908408-29911452 FORWARD \| Aliases: T8K14.8, T8K14_8 | 7.2 | 4.1 | 3.1 | 10.5 | 0.03% | 8.6 |
| 750 | AT1G13270.2 \| Symbol: MAP1C \| metallopeptidase M24 family protein, similar to SP:Q01662 Methionine aminopeptidase 1 precursor (EC 3.4.11.18) {Saccharomyces cerevisiae}; contains Pfam profile PF00557: metallopeptidase family M24 \| chr1:4544953-4547295 FORWARD \| Aliases: MAP1B, METHIONINE AMINOPEPTIDASE 1B | 8.4 | 4.7 | 3.7 | 10.5 | 0.03% | 8.1 |
| 752 | AT5G41970.1 \| similar to expressed protein [Arabidopsis thaliana] (TAIR:At3g49320.1); similar to LOC496075 protein [Xenopus laevis] (GB:AAH87491.1); contains InterPro domain Metal-dependent protein hydrolase (InterPro:IPR003226) \| chr5:16808411-16810485 FORWARD \| Aliases: MJC20.7, MJC20_7 | 8.1 | 5.4 | 2.7 | 10.5 | 0.03% | 9.5 |
| 754 | AT1G63420.1 \| expressed protein \| chr1:23519539-23522575 FORWARD \| Aliases: F2K11.20, F2K11_20 | 8.0 | 5.9 | 2.1 | 10.5 | 0.03% | 9.3 |
| 756 | AT5G02120.1 \| Symbol: OHP \| thylakoid membrane one helix protein (OHP), identical to one helix protein GI:3283057 from (Arabidopsis thaliana) | 7.6 | 2.2 | 5.4 | 10.5 | 0.03% | 7.7 |
| 758 | AT1G42970.1 \| Symbol: GAPB \| glyceraldehyde-3-phosphate dehydrogenase B, chloroplast (GAPB) / NADP-dependent glyceraldehydephosphate dehydrogenase subunit B, identical to SP:P25857 Glyceraldehyde 3-phosphate dehydrogenase B, chloroplast precursor (EC 1.2.1.13) (NADP-dependent glyceraldehydephosphate dehydrogenase subunit B) {Arabidopsis thaliana} \| chr1:16129874-16132283 FORWARD \| Aliases: F13A11.3, F13A11_3, GLYCERALDEHYDE 3 PHOSPHATE DEHYDROGENASE B SUBUNIT | 8.8 | 3.5 | 5.3 | 10.5 | 0.03% | 7.6 |
| 760 | AT5G16570.1 \| Symbol: GLN1;4 \| | 9.6 | 7.3 | 2.3 | 10.5 | 0.03% | 9.4 |
| 761 | AT4G09550.1 \| expressed protein \| chr4:6039132-6040171 FORWARD \| Aliases: T15G18.30, T15G18_30 | 9.7 | 6.2 | 3.6 | 10.5 | 0.03% | 9.6 |
| 762 | AT5G14760.1 \| L-aspartate oxidase family protein, similar to L-aspartate oxidase, Escherichia coli (SP:P10902); contains Pfam profiles PF00890 FAD binding domain, PF02910 Fumarate reductase/succinate dehydrogenase flavoprotein C-terminal domain \| chr5:4768472-4772156 FORWARD \| Aliases: T9L3.60, T9L3_60 | 7.7 | 3.6 | 4.2 | 10.5 | 0.03% | 8.8 |
| 763 | AT1G78630.1 \| Symbol: EMB1473 \| ribosomal protein L13 family protein, similar to ribosomal protein L13 GI:170132 from (Spinacia oleracea) \| chr1:29580849-29582551 FORWARD \| Aliases: EMB1473, EMBRYO DEFECTIVE 1473, T30F21.4, T30F21_4 | 8.9 | 4.1 | 4.7 | 10.5 | 0.03% | 8.4 |
| 764 | AT5G27560.1 \| expressed protein, hypothetical protein slr1702 - Synechocystis sp., PIR:S75312 \| chr5:9731731-9733921 FORWARD \| Aliases: F15A18.20, F15A18_20 | 8.2 | 5.4 | 2.7 | 10.5 | 0.03% | 9.1 |
| 767 | AT5G16710.1 \| dehydroascorbate reductase, putative, Strong similarity to dehydroascorbate reductase (Spinacia oleracea) gi:10952512 gb:AAG24945 \| chr5:5483291-5485069 FORWARD \| Aliases: F5E19.50, F5E19_50 | 10.0 | 7.2 | 2.8 | 10.4 | 0.03% | 9.3 |
| 772 | AT4G10300.1 \| expressed protein \| chr4:6384458-6386185 FORWARD \| Aliases: T9A4.16 | 8.7 | 5.5 | 3.2 | 10.4 | 0.03% | 8.6 |
| 774 | AT2G23530.1 \| expressed protein, ; expression supported by MPSS \| chr2:10027732-10029875 REVERSE \| Aliases: F26B6.18, F26B6_18 | 7.1 | 3.7 | 3.3 | 10.4 | 0.03% | 8.5 |

| Rank | Description | Sync | Root | M | t | adj.q | B |
|---|---|---|---|---|---|---|---|
| 775 | AT1G77940.1 \| 60S ribosomal protein L30 (RPL30B), similar to ribosomal protein L30 GI:388034 from (Homo sapiens) \| chr1:29308791-29310259 REVERSE \| Aliases: F28K19.15, F28K19_15 | 12.1 | 10.3 | 1.8 | 10.4 | 0.03% | 9.7 |
| 777 | AT1G59900.1 \| Symbol: AT E1 ALPHA \| pyruvate dehydrogenase E1 component alpha subunit, mitochondrial (PDHE1-A), identical to SP:P52901 Pyruvate dehydrogenase E1 component alpha subunit, mitochondrial precursor (EC 1.2.4.1) (PDHE1-A) {Arabidopsis thaliana} \| chr1:22054960-22057550 FORWARD \| Aliases: PYRUVATE DEHYDROGENASE E1 ALPHA SUBUNIT | 10.9 | 8.9 | 2.0 | 10.4 | 0.03% | 9.7 |
| 778 | AT5G14105.1 \| expressed protein \| chr5:4551991-4553053 REVERSE \| Aliases: None | 9.0 | 6.2 | 2.8 | 10.4 | 0.03% | 9.2 |
| 781 | AT5G61880.2 \| signaling molecule-related, contains similarity to mitochondria-associated granulocyte macrophage CSF signaling molecule, mitochondrial precursor (CGI-136) (Mus musculus) SWISS-PROT:Q9CQV1 \| chr5:24867542-24868997 FORWARD \| Aliases: MAC9.20, MAC9_20 | 7.3 | 3.1 | 4.2 | 10.4 | 0.03% | 8.5 |
| 785 | AT3G18680.2 \| aspartate/glutamate/uridylate kinase family protein, similar to UMP-kinase GB:CAB38122 gi:4468612 from (Lactococcus lactis) ; contains Pfam profile PF00696: Amino acid kinase family \| chr3:6427413-6429658 FORWARD \| Aliases: MVE11.4 | 7.0 | 3.3 | 3.8 | 10.3 | 0.03% | 8.3 |
| 786 | AT3G06310.1 \| NADH-ubiquinone oxidoreductase 19 kDa subunit (NDUFA8) family protein, contains Pfam profile PF05850: NADH-ubiquinone oxidoreductase 19 kDa subunit (NDUFA8); similar to NADH-ubiquinone oxidoreductase 19 kDa subunit (EC 1.6.5.3) (EC 1.6.99.3) (Complex I-19KD) (CI-19KD) (Complex I-PGIV) (CI-PGIV) (Swiss-Prot:P51970) (Homo sapiens) \| chr3:1912978-1914387 REVERSE \| Aliases: F24P17.23, F24P17_23 | 10.0 | 8.0 | 2.0 | 10.3 | 0.03% | 9.5 |
| 787 | AT1G35420.1 \| dienelactone hydrolase family protein, low similarity to dienelactone hydrolase (Rhodococcus opacus) GI:23094407; contains Pfam profile PF01738: Dienelactone hydrolase family \| chr1:13026292-13027645 FORWARD \| Aliases: F12A4.4, F12A4_4 | 7.3 | 3.6 | 3.7 | 10.3 | 0.03% | 7.6 |
| 788 | AT1G77710.1 \| expressed protein, similar to hypothetical protein GB:P34661 (Caenorhabditis elegans) \| chr1:29211577-29212943 FORWARD \| Aliases: T32E8.4, T32E8_4 | 11.5 | 8.8 | 2.7 | 10.3 | 0.03% | 9.5 |
| 789 | AT3G12110.1 \| Symbol: ACT11 \| actin 11 (ACT11), identical to SP:P53496 Actin 11 {Arabidopsis thaliana} \| chr3:3857860-3859804 FORWARD \| Aliases: ACTIN 11, T21B14.7 | 7.8 | 4.0 | 3.8 | 10.3 | 0.03% | 8.7 |
| 790 | AT5G11690.1 \| Symbol: ATTIM17 3 \| mitochondrial import inner membrane translocase subunit Tim17, putative, similar to SP:Q9SP35 Mitochondrial import inner membrane translocase subunit TIM17 {Arabidopsis thaliana}; contains Pfam profile PF02466: Mitochondrial import inner membrane translocase subunit Tim17 \| chr5:3761245-3762347 FORWARD \| Aliases: T22P22.80, T22P22_80 | 8.2 | 5.3 | 3.0 | 10.3 | 0.03% | 8.7 |
| 791 | AT1G76730.1 \| 5-formyltetrahydrofolate cyclo-ligase family protein, contains Pfam profile PF01812 5-formyltetrahydrofolate cyclo-ligase \| chr1:28807830-28809678 REVERSE \| Aliases: F28O16.10, F28O16_10 | 6.8 | 3.3 | 3.6 | 10.3 | 0.03% | 8.3 |
| 794 | AT5G51820.1 \| Symbol: PGM \| phosphoglucomutase, chloroplast (PGM) (PGMP) / glucose phosphomutase, identical to SP:Q9SCY0 Phosphoglucomutase, chloroplast precursor (EC 5.4.2.2) (Glucose phosphomutase) (PGM) {Arabidopsis thaliana} \| chr5:21080594-21085283 REVERSE \| Aliases: ATPGMP, MIO24.4, MIO24_4, PGM1, PHOSPHOGLUCOMUTASE, STARCH FREE 1, STF1 | 8.4 | 4.8 | 3.6 | 10.3 | 0.03% | 8.7 |
| 795 | AT4G18480.1 \| Symbol: CHLI1 \| magnesium-chelatase subunit chlI, chloroplast / Mg-protoporphyrin IX chelatase (CHLI) (CS) (CH42), identical to SP:P161127 Magnesium-chelatase subunit chlI, chloroplast precursor (Mg-protoporphyrin IX chelatase) (Protein CS/CH-42) {Arabidopsis thaliana} \| chr4:10201683-10203454 REVERSE \| Aliases: CH 42, CH42, CHLORATA, CHLORINA 42, F28J12.140, F28J12_140, PROTOPORPHYRIN IX MG CHELATASE | 8.6 | 4.7 | 3.9 | 10.3 | 0.03% | 8.6 |
| 797 | AT3G23700.1 \| S1 RNA-binding domain-containing protein, contains Pfam domain, PF00575: S1 RNA binding domain \| chr3:8531436-8533774 REVERSE \| Aliases: MYM9.4 | 7.5 | 3.5 | 4.0 | 10.3 | 0.03% | 8.8 |
| 798 | AT3G08690.1 \| ubiquitin-conjugating enzyme 11 (UBC11), E2; identical to gi:12643427, SP:P35134 | 10.4 | 6.9 | 3.4 | 10.3 | 0.03% | 8.8 |
| 799 | AT4G26900.1 \| Symbol: AT HF \| imidazole glycerol phosphate synthase hisHF, chloroplast / IGP synthase / ImGPP synthase / IGPS, identical to SP:Q9SZ30 Imidazole glycerol phosphate synthase hisHF, chloroplast precursor (IGP synthase) {Arabidopsis thaliana} \| chr4:13515488-13520317 FORWARD \| Aliases: HISHF | 9.0 | 5.7 | 3.3 | 10.3 | 0.03% | 8.8 |
| 801 | AT1G10960.1 \| ferredoxin, chloroplast, putative, strong similarity to FERREDOXIN PRECURSOR GB:P16972 (SP:P16972) from (Arabidopsis thaliana) \| chr1:3664386-3665039 FORWARD \| Aliases: T19D16.12, T19D16_12 | 11.3 | 7.2 | 4.1 | 10.3 | 0.03% | 8.4 |
| 804 | AT5G16130.1 \| 40S ribosomal protein S7 (RPS7C), 40S ribosomal protein S7 homolog - Brassica oleracea, EMBL:AF144752 \| chr5:5268424-5270091 FORWARD \| Aliases: T21H19.50, T21H19_50 | 11.3 | 8.8 | 2.4 | 10.2 | 0.03% | 9.3 |
| 805 | AT2G30140.1 \| UDP-glucoronosyl/UDP-glucosyl transferase family protein, contains Pfam profile: PF00201 UDP-glucoronosyl and UDP-glucosyl transferase \| chr2:12879211-12880897 FORWARD \| Aliases: T27E13.12, T27E13_12 | 9.8 | 7.1 | 2.8 | 10.2 | 0.03% | 9.4 |

| Rank | Description | Sync | Root | M | t | adj.q | B |
|---|---|---|---|---|---|---|---|
| 806 | AT5G64816.2 \| expressed protein \| chr5:25930226-25931229 FORWARD \| Aliases: None | 8.9 | 6.3 | 2.6 | 10.2 | 0.03% | 9.1 |
| 807 | AT5G35700.1 \| fimbrin-like protein, putative, similar to fimbrin-like protein (ATFIM1) (Arabidopsis thaliana) GI:2905893, fimbrin (Schizosaccharomyces pombe) GI:3057144; contains Pfam profile PF00307: Calponin homology (CH) domain | 9.1 | 5.4 | 3.7 | 10.2 | 0.03% | 9.3 |
| 809 | AT2G35795.1 \| DNAJ heat shock N-terminal domain-containing protein, contains Pfam profile PF00226 DnaJ domain \| chr2:15049289-15050646 FORWARD \| Aliases: T20F21.1, T20F21_1 | 9.8 | 7.4 | 2.3 | 10.2 | 0.03% | 9.4 |
| 811 | AT1G08580.1 \| expressed protein \| chr1:2715086-2716387 REVERSE \| Aliases: F22O13.6, F22O13_6 | 8.8 | 5.9 | 3.0 | 10.2 | 0.03% | 8.8 |
| 812 | AT5G62980.1 \| dihydroneopterin aldolase, putative, similar to SP:O33725 Dihydroneopterin aldolase (EC 4.1.2.25) (DHNA) {Streptococcus pyogenes}; contains Pfam profile PF02152: dihydroneopterin aldolase \| chr5:25293260-25294418 FORWARD \| Aliases: MJH22.3, MJH22_3 | 7.7 | 4.5 | 3.2 | 10.2 | 0.03% | 8.6 |
| 814 | AT5G14780.1 \| Symbol: FDH \| formate dehydrogenase (FDH), identical to GI:7677266 \| chr5:4776987-4779497 FORWARD \| Aliases: FORMATE DEHYDROGENASE, T9L3.80, T9L3_80 | 9.5 | 4.9 | 4.5 | 10.2 | 0.03% | 8.8 |
| 815 | AT1G23220.1 \| dynein light chain type 1 family protein, similar to SP:O02414 Dynein light chain LC6, flagellar outer arm {Anthocidaris crassispina}; contains Pfam profile PF01221: Dynein light chain type 1 \| chr1:8242499-8244307 FORWARD \| Aliases: F26F24.7, F26F24_7 | 10.0 | 6.6 | 3.4 | 10.2 | 0.03% | 9.4 |
| 816 | AT3G03640.1 \| Symbol: GLUC \| glycosyl hydrolase family 1 protein, contains Pfam PF00232 : Glycosyl hydrolase family 1 domain; TIGRFAM TIGR01233: 6-phospho-beta-galactosidase; identical to beta-glucosidase GB:AAC31962 (Arabidopsis thaliana); similar to thioglucoside glucohydrolase (GI:984052) (Arabidopsis thaliana) \| chr3:881031-884163 FORWARD \| Aliases: BETA GLUCOSIDASE, T12J13.8, T12J13_8 | 11.0 | 6.7 | 4.3 | 10.2 | 0.03% | 8.6 |
| 817 | AT4G19390.1 \| expressed protein \| chr4:10574779-10576439 REVERSE \| Aliases: T5K18.170, T5K18_170 | 9.0 | 6.4 | 2.6 | 10.2 | 0.03% | 8.9 |
| 818 | AT3G27080.1 \| Symbol: TOM20 3 \| mitochondrial import receptor subunit TOM20-3 / translocase of outer membrane 20 kDa subunit 3 (TOM20-3), identical to mitochondrial import receptor subunit TOM20-3 SP:P82874 from (Arabidopsis thaliana) \| chr3:9986319-9987866 REVERSE \| Aliases: MOJ10.22, TRANSLOCASE OUTER MEMBRANE 20 3 | 9.3 | 6.6 | 2.7 | 10.2 | 0.03% | 8.8 |
| 820 | AT4G14690.1 \| Symbol: ELIP2 \| chlorophyll A-B binding family protein / early light-induced protein, putative, strong similarity to early light-induced protein; ELIP (Arabidopsis thaliana) GI:1872544; contains Pfam profile: PF00504 chlorophyll A-B binding protein \| chr4:8418278-8419258 FORWARD \| Aliases: DL3385W, EARLY LIGHT INDUCIBLE PROTEIN 2, FCAALL.232 | 8.5 | 5.0 | 3.4 | 10.2 | 0.03% | 7.9 |
| 821 | AT1G07830.1 \| ribosomal protein L29 family protein, similar to GB:CAA83057 from (Saccharomyces cerevisiae) \| chr1:2422295-2423650 FORWARD \| Aliases: F24B9.7, F24B9_7 | 7.8 | 5.0 | 2.8 | 10.2 | 0.03% | 9.0 |
| 823 | AT3G44450.1 \| expressed protein \| chr3:16085084-16085607 REVERSE \| Aliases: F14L2.1 | 5.5 | 2.2 | 3.3 | 10.2 | 0.03% | 8.2 |
| 824 | AT3G44750.1 \| Symbol: HD2A \| histone deacetylase, putative (HD2A), contains Pfam domain, PF00096: Zinc finger, C2H2 type; identical to cDNA putative histone deacetylase (HD2A) GI:11066134 \| chr3:16308970-16310733 FORWARD \| Aliases: ATHD2A, HDA3, HDT1, HISTONE DEACETYLASE 2A, T32N15.8 | 8.1 | 4.5 | 3.6 | 10.2 | 0.03% | 8.5 |
| 825 | AT2G43810.1 \| small nuclear ribonucleoprotein F, putative / U6 snRNA-associated Sm-like protein, putative / Sm protein F, putative, similar to SWISS-PROT:Q9Y4Y8 U6 snRNA-associated Sm-like protein LSm6 (Mus musculus) \| chr2:18156388-18158371 FORWARD \| Aliases: F18O19.8 | 7.9 | 4.4 | 3.5 | 10.2 | 0.03% | 8.8 |
| 827 | AT2G35040.1 \| AICARFT/IMPCHase bienzyme family protein, similar to SP:P12048 Bifunctional purine biosynthesis protein purH (Includes: Phosphoribosylaminoimidazolecarboxamide formyltransferase (EC 2.1.2.3) (AICAR transformylase); IMP cyclohydrolase (EC 3.5.4.10) (Inosinicase) (IMP synthetase) (ATIC)) {Bacillus subtilis}; contains Pfam profiles PF01808: AICARFT/IMPCHase bienzyme, PF02142: MGS-like domain \| chr2:14772239-14775367 REVERSE \| Aliases: F19I3.27, F19I3_27 | 10.0 | 5.1 | 4.9 | 10.2 | 0.03% | 7.7 |
| 830 | AT4G17540.1 \| expressed protein \| chr4:9775868-9777827 FORWARD \| Aliases: DL4805W, FCAALL.37 | 6.7 | 3.9 | 2.8 | 10.1 | 0.03% | 9.1 |
| 831 | AT3G02870.2 \| similar to inositol monophosphatase family protein [Arabidopsis thaliana] (TAIR:At1g31190.1); similar to L-galactose-1-phosphate phosphatase [Malus x domestica] (GB:AAV49507.1); contains InterPro domain Inositol monophosphatase (InterPro:IPR000760) | 10.0 | 7.7 | 2.3 | 10.1 | 0.03% | 9.4 |
| 832 | AT5G24300.1 \| starch synthase, putative, similar to starch synthase I-1 GI:9369334 from (Triticum aestivum) \| chr5:8266802-8271199 FORWARD \| Aliases: MOP9.12, MOP9_12 | 8.8 | 4.8 | 4.0 | 10.1 | 0.03% | 8.6 |
| 834 | AT5G09300.2 \| 2-oxoisovalerate dehydrogenase, putative / 3-methyl-2-oxobutanoate dehydrogenase, putative / branched-chain alpha-keto acid dehydrogenase E1 alpha subunit, putative, similar to branched-chain alpha-keto acid dehydrogenase E1-alpha subunit (Gallus gallus) GI:12964598; contains Pfam profile PF00676: Dehydrogenase E1 component \| chr5:2884181-2886474 REVERSE \| Aliases: T5E8.100, T5E8_100 | 8.3 | 6.0 | 2.3 | 10.1 | 0.03% | 8.9 |

| Rank | Description | Sync | Root | M | t | adj.q | B |
|---|---|---|---|---|---|---|---|
| 835 | AT5G59880.2 \| Symbol: ADF3 \| actin-depolymerizing factor 3 (ADF3), identical to SP:Q9ZSK4 Actin-depolymerizing factor 3 (ADF 3) (AtADF3) {Arabidopsis thaliana} \| chr5:24137457-24139105 FORWARD \| Aliases: ACTIN DEPOLYMERIZING FACTOR 3, MMN10.4, MMN10_4 | 12.9 | 10.4 | 2.5 | 10.1 | 0.03% | 9.4 |
| 836 | AT4G13250.1 \| short-chain dehydrogenase/reductase (SDR) family protein, contains INTERPRO family IPR002198 Short-chain dehydrogenase/reductase (SDR) superfamily \| chr4:7684238-7686803 REVERSE \| Aliases: F17N18.140, F17N18_140 | 8.2 | 4.6 | 3.6 | 10.1 | 0.03% | 8.0 |
| 839 | AT3G52420.1 \| outer envelope membrane protein, putative, similar to outer envelope membrane protein gi:703236:gb:AAA63414 \| chr3:19440753-19440947 FORWARD \| Aliases: F22O6.200 | 8.8 | 6.6 | 2.2 | 10.1 | 0.03% | 9.2 |
| 843 | AT2G28000.1 \| Symbol: CPN60A \| | 10.1 | 7.3 | 2.9 | 10.1 | 0.03% | 9.1 |
| 844 | AT5G11480.1 \| expressed protein \| chr5:3669198-3671575 REVERSE \| Aliases: F15N18.70, F15N18_70 | 8.4 | 5.5 | 2.9 | 10.1 | 0.03% | 8.8 |
| 848 | AT3G62030.1 \| Symbol: ROC4 \| peptidyl-prolyl cis-trans isomerase, chloroplast / cyclophilin / rotamase / cyclosporin A-binding protein (ROC4), identical to peptidyl-prolyl cis-trans isomerase, chloroplast precursor, PPIase (cyclophilin, cyclosporin A-binding protein) (Arabidopsis thaliana) SWISS-PROT:P34791; identical to cDNA nuclear-encoded chloroplast stromal cyclophilin (ROC4) GI:405130 \| chr3:22984585-22986345 FORWARD \| Aliases: PEPTIDYLPROLYL ISOMERASE ROC4, ROTAMASE CYCLOPHILIN 4, ROTAMASE CYP 4, T17J13.1 | 9.4 | 5.6 | 3.8 | 10.1 | 0.03% | 8.4 |
| 853 | AT2G44460.1 \| glycosyl hydrolase family 1 protein, contains Pfam PF00232 : Glycosyl hydrolase family 1 domain; TIGRFAM TIGR01233: 6-phospho-beta-galactosidase; similar to beta-glucosidase 1 (GI:12043529) (Arabidopsis thaliana) \| chr2:18353576-18357042 FORWARD \| Aliases: F4I1.27 | 9.5 | 3.3 | 6.2 | 10.1 | 0.03% | 8.3 |
| 854 | AT3G56680.1 \| expressed protein \| chr3:21002259-21004646 FORWARD \| Aliases: T8M16.10 | 9.4 | 6.7 | 2.6 | 10.1 | 0.03% | 9.0 |
| 855 | AT2G19750.1 \| 40S ribosomal protein S30 (RPS30A) \| chr2:8522119-8522888 FORWARD \| Aliases: F6F22.22, F6F22_22 | 7.9 | 4.5 | 3.4 | 10.1 | 0.03% | 8.8 |
| 856 | AT4G33640.1 \| expressed protein \| chr4:16159551-16160306 REVERSE \| Aliases: T16L1.130, T16L1_130 | 10.9 | 9.8 | 1.1 | 10.1 | 0.03% | 9.4 |
| 857 | AT1G16520.1 \| expressed protein \| chr1:5648763-5651160 FORWARD \| Aliases: F3O9.32 | 6.9 | 3.5 | 3.4 | 10.1 | 0.03% | 8.8 |
| 858 | AT4G16060.1 \| expressed protein \| chr4:9094437-9096293 FORWARD \| Aliases: DL4070W, FCAALL.269 | 8.1 | 5.7 | 2.4 | 10.1 | 0.03% | 9.0 |
| 859 | AT1G14980.1 \| Symbol: CPN10 \| 10 kDa chaperonin (CPN10), identical to SP:P34893 from (Arabidopsis thaliana) \| chr1:5165623-5166740 REVERSE \| Aliases: CHAPERONIN 10, MITOCHONDRIAL CHAPERONIN 10, T15D22.2, T15D22_2 | 10.1 | 6.9 | 3.2 | 10.1 | 0.03% | 8.9 |
| 860 | AT4G08950.1 \| phosphate-responsive protein, putative (EXO), similar to phi-1 (phosphate-induced gene) (Nicotiana tabacum) GI:3759184; contains Pfam profile PF04674: Phosphate-induced protein 1 conserved region \| chr4:5740294-5741521 FORWARD \| Aliases: T3H13.3, T3H13_3 | 7.5 | 3.7 | 3.8 | 10.1 | 0.03% | 7.7 |
| 862 | AT5G52650.1 \| 40S ribosomal protein S10 (RPS10C), contains similarity to 40S ribosomal protein S10 \| chr5:21372841-21374301 REVERSE \| Aliases: F6N7.14, F6N7_14 | 11.2 | 9.4 | 1.7 | 10.0 | 0.03% | 9.3 |
| 864 | AT3G57320.1 \| expressed protein \| chr3:21221008-21221827 FORWARD \| Aliases: F28O9.170 | 7.8 | 4.0 | 3.8 | 10.0 | 0.03% | 8.5 |
| 866 | AT2G35370.1 \| Symbol: GDCH \| glycine cleavage system H protein 1, mitochondrial (GDCSH) (GCDH), identical to SP:P25855 Glycine cleavage system H protein 1, mitochondrial precursor {Arabidopsis thaliana} \| chr2:14898181-14899297 FORWARD \| Aliases: GLYCINE DECARBOXYLASE COMPLEX H PROTEIN, T32F12.25, T32F12_25 | 7.7 | 2.5 | 5.2 | 10.0 | 0.03% | 6.9 |
| 867 | AT3G17300.1 \| expressed protein \| chr3:5907240-5908898 FORWARD \| Aliases: MGD8.14 | 9.6 | 6.9 | 2.7 | 10.0 | 0.03% | 9.0 |
| 870 | AT3G10610.1 \| 40S ribosomal protein S17 (RPS17C), similar to 40S ribosomal protein S17 GB:AAD50774 (Lycopersicon esculentum) \| chr3:3319041-3320154 FORWARD \| Aliases: F13M14.10 | 8.5 | 5.8 | 2.6 | 10.0 | 0.03% | 9.0 |
| 871 | AT4G26230.1 \| 60S ribosomal protein L31 (RPL31B), ribosomal protein L31, Nicotiana glutinosa, U23784 \| chr4:13285837-13286800 FORWARD \| Aliases: T25K17.40, T25K17_40 | 11.8 | 10.1 | 1.7 | 10.0 | 0.03% | 9.4 |
| 873 | AT5G63310.1 \| Symbol: NDPK2 \| nucleotide diphosphate kinase II, chloroplast (NDPK2), identical to SP:O64903 Nucleoside diphosphate kinase II, chloroplast precursor (NDK II) (NDP kinase II) (NDPK II) (NDPK Ia) (Arabidopsis thaliana); contains Pfam PF00334 : Nucleoside diphosphate kinase domain; contains Pfam profile PF00334: Nucleoside diphosphate kinase \| chr5:25389130-25391081 REVERSE \| Aliases: MDC12.28, MDC12_28, NDPK IA, NDPK IA IA, NDPK1A, NUCLEOSIDE DIPHOSPHATE KINASE 2, NUCLEOSIDE DIPHOSPHATE KINASE IA | 9.7 | 6.1 | 3.5 | 10.0 | 0.03% | 8.3 |
| 876 | AT1G73470.2 \| expressed protein \| chr1:27628983-27631359 FORWARD \| Aliases: T9L24.34, T9L24_34 | 8.0 | 5.8 | 2.2 | 10.0 | 0.03% | 8.8 |

| Rank | Description | Sync | Root | M | t | adj.q | B |
|---|---|---|---|---|---|---|---|
| 878 | AT3G10200.1 \| dehydration-responsive protein-related, similar to early-responsive to dehydration stress ERD3 protein (Arabidopsis thaliana) GI:15320410; contains Pfam profile PF03141: Putative methyltransferase \| chr3:3157613-3160186 FORWARD \| Aliases: F14P13.20 | 6.2 | 3.2 | 3.0 | 10.0 | 0.03% | 8.3 |
| 879 | AT1G04590.2 \| Symbol: EMB2748 \| expressed protein, isoform contains GG acceptor splice site at intron 7 \| chr1:1258577-1261424 REVERSE \| Aliases: EMB2748, EMBRYO DEFECTIVE 2748, T1G11.16, T1G11_16 | 9.2 | 5.2 | 4.0 | 10.0 | 0.03% | 9.0 |
| 880 | AT3G55010.2 \| phosphoribosylformylglycinamidine cyclo-ligase, chloroplast / phosphoribosyl-aminoimidazole synthetase / AIR synthase (PUR5), identical to phosphoribosylformylglycinamidine cyclo-ligase, chloroplast precursor SP:Q05728 from (Arabidopsis thaliana); contains Pfam profiles: PF02769 AIR synthase related protein, C-terminal domain, PF00586 AIR synthase related protein, N-terminal domain | 8.2 | 4.4 | 3.8 | 10.0 | 0.03% | 8.8 |
| 883 | AT2G04630.1 \| DNA-directed RNA polymerase II, putative, similar to SP:Q24320 DNA-directed RNA polymerase II 14.4 kDa polypeptide (EC 2.7.7.6) (RPB6) {Drosophila melanogaster}; contains Pfam profile PF01192: RNA polymerases K / 14 to 18 kDa subunit \| chr2:1618933-1620308 REVERSE \| Aliases: F28I8.33, F28I8_33 | 9.7 | 8.1 | 1.7 | 9.9 | 0.04% | 9.2 |
| 885 | AT3G04920.1 \| 40S ribosomal protein S24 (RPS24A), similar to ribosomal protein S19 GB:445612 (Solanum tuberosum) and similar to ribosomal protein S24 GB:4506703 (Homo sapiens) \| chr3:1360888-1362301 FORWARD \| Aliases: T9J14.13, T9J14_13 | 9.6 | 6.4 | 3.2 | 9.9 | 0.04% | 8.5 |
| 889 | AT5G19510.1 \| elongation factor 1B alpha-subunit 2 (eEF1Balpha2), identical to elongation factor 1B alpha-subunit (Arabidopsis thaliana) GI:6686821 \| chr5:6581660-6583213 REVERSE \| Aliases: T20D1.30, T20D1_30 | 11.5 | 10.0 | 1.5 | 9.9 | 0.04% | 9.3 |
| 891 | AT3G09700.1 \| DNAJ heat shock N-terminal domain-containing protein, contains Pfam profile PF00226 DnaJ domain; similar to a region of DNAJ domain-containing protein MCJ GB:AAD38506 \| chr3:2974886-2976282 REVERSE \| Aliases: F11F8.29 | 6.6 | 4.1 | 2.5 | 9.9 | 0.04% | 8.8 |
| 892 | AT5G02380.1 \| Symbol: MT2B \| metallothionein protein 2B (MT-2B), identical to SWISS-PROT:Q38805 metallothionein-like protein 2B (MT-2B) (Arabidopsis thaliana) \| chr5:506545-507242 REVERSE \| Aliases: METALLOTHIONEIN 2B, T1E22.140, T1E22_140 | 14.0 | 12.2 | 1.8 | 9.9 | 0.04% | 9.2 |
| 893 | AT5G17310.2 \| UTP--glucose-1-phosphate uridylyltransferase, putative / UDP-glucose pyrophosphorylase, putative / UGPase, putative, strong similarity to SP:P19595 UTP--glucose-1-phosphate uridylyltransferase (EC 2.7.7.9) (UDP-glucose pyrophosphorylase) (UDPGP) (UGPase) {Solanum tuberosum}; contains Pfam profile PF01704: UTP--glucose-1-phosphate uridylyltransferase \| chr5:5696649-5700924 REVERSE \| Aliases: MKP11.26, MKP11_26 | 9.1 | 4.3 | 4.9 | 9.9 | 0.04% | 8.4 |
| 894 | AT3G08030.2 \| expressed protein, contains Pfam profile PF04862: Protein of unknown function, DUF642 \| chr3:2564159-2566008 FORWARD \| Aliases: F17A17.37 | 8.2 | 3.4 | 4.8 | 9.9 | 0.04% | 8.2 |
| 895 | AT4G31810.1 \| enoyl-CoA hydratase/isomerase family protein, similar to CHY1 (gi:8572760); contains Pfam profile PF00388 enoyl-CoA hydratase/isomerase family protein \| chr4:15387131-15390340 REVERSE \| Aliases: F11C18.10, F11C18_10 | 8.0 | 3.9 | 4.1 | 9.9 | 0.04% | 8.9 |
| 896 | AT3G21055.1 \| photosystem II 5 kD protein, putative, identical to Swiss-Prot:Q39195 photosystem II 5 kDa protein, chloroplast precursor (PSII-T) (Arabidopsis thaliana) \| chr3:7376643-7377192 REVERSE \| Aliases: None | 7.5 | 2.1 | 5.4 | 9.9 | 0.04% | 7.7 |
| 897 | AT1G03360.1 \| exonuclease family protein, similar to Exosome complex exonuclease RRP4 (Ribosomal RNA processing protein 4)(SP:P38792) {Saccharomyces cerevisiae} \| chr1:824561-826179 FORWARD \| Aliases: F15K9.4, F15K9_4 | 7.7 | 3.7 | 4.0 | 9.9 | 0.04% | 7.8 |
| 901 | AT3G25220.1 \| Symbol: FKBP15 1 \| FK506-binding protein 2-1 (FKBP15-1) / immunophilin / peptidyl-prolyl cis-trans isomerase / rotamase, identical to SP:Q38935 FK506-binding protein 2-1 precursor (EC 5.2.1.8) (Peptidyl-prolyl cis- trans isomerase) (PPiase) (Rotamase) (15 kDa FKBP) (FKBP-15-1) {Arabidopsis thaliana}, immunophilin (FKBP15-1) GB:U52046 (Arabidopsis thaliana) (Proc. Natl. Acad. Sci. U.S.A. 93 (14), 6964-6969 (1996)) \| chr3:9183855-9185849 FORWARD \| Aliases: MJL12.19 | 9.9 | 8.3 | 1.7 | 9.9 | 0.04% | 9.3 |
| 902 | AT5G37290.1 \| armadillo/beta-catenin repeat family protein, contains Pfam profile: PF00514 armadillo/beta-catenin-like repeat \| chr5:14779618-14781574 FORWARD \| Aliases: MNJ8.8, MNJ8_8 | 5.7 | 3.4 | 2.3 | 9.9 | 0.04% | 8.7 |
| 904 | AT1G69620.1 \| Symbol: RPL34 \| 60S ribosomal protein L34 (RPL34B), similar to SP:Q42351 from (Arabidopsis thaliana) \| chr1:26193501-26194986 FORWARD \| Aliases: F24J1.23, RIBOSOMAL PROTEIN L34, RPL34 | 12.2 | 10.7 | 1.6 | 9.9 | 0.04% | 9.3 |
| 905 | AT2G41650.1 \| expressed protein \| chr2:17370985-17371512 FORWARD \| Aliases: T32G6.17, T32G6_17 | 10.2 | 8.1 | 2.1 | 9.9 | 0.04% | 9.1 |
| 907 | AT1G71260.1 \| expressed protein \| chr1:26865327-26867080 REVERSE \| Aliases: F3I17.9, F3I17_9 | 7.8 | 3.5 | 4.3 | 9.9 | 0.04% | 8.6 |
| 908 | AT3G10350.1 \| anion-transporting ATPase family protein, similar to SP:O43681 Arsenical pump-driving ATPase (EC 3.6.3.16) (Arsenite-translocating ATPase) (Arsenical resistance ATPase) (Arsenite-transporting ATPase) (ARSA) (ASNA-I) {Homo sapiens}; contains Pfam profile PF02374: Anion-transporting ATPase; contains non-consensus GA donor splice site at intron 5 \| chr3:3208244-3211299 FORWARD \| Aliases: F14P13.5 | 7.6 | 5.2 | 2.4 | 9.8 | 0.04% | 8.9 |
| 909 | AT3G15780.1 \| expressed protein \| chr3:5341433-5342564 FORWARD \| Aliases: MSJ11.18 | 7.7 | 4.6 | 3.2 | 9.8 | 0.04% | 8.7 |

| Rank | Description | Sync | Root | M | t | adj.q | B |
|---|---|---|---|---|---|---|---|
| 910 | AT4G21280.2 \| similar to oxygen-evolving enhancer protein 3, chloroplast, putative (PSBQ2) [Arabidopsis thaliana] (TAIR:At4g05180.1); similar to chloroplast oxygen-evolving enhancer protein [Manihot esculenta] (GB:AAV74404.1); contains InterPro domain Twin-arginine translocation pathway signal (InterPro:IPR006311); contains InterPro domain Oxygen evolving enhancer 3 (InterPro:IPR008797) \| chr4:11334412-11335783 FORWARD \| Aliases: T6K22.20 | 8.8 | 5.1 | 3.7 | 9.8 | 0.04% | 7.9 |
| 911 | AT2G39220.1 \| patatin family protein, similar to patatin-like latex allergen (Hevea brasiliensis)(PMID:10589016); contains patatin domain PF01734 \| chr2:16381974-16384098 REVERSE \| Aliases: T16B24.14, T16B24_14 | 7.5 | 4.0 | 3.5 | 9.8 | 0.04% | 7.9 |
| 914 | AT1G22840.1 \| cytochrome c, putative, similar to cytochrome c (Pumpkin, Winter squash) SWISS-PROT:P00051 \| chr1:8079279-8080563 FORWARD \| Aliases: F19G10.20, F19G10_20 | 12.1 | 10.3 | 1.9 | 9.8 | 0.04% | 9.3 |
| 919 | AT2G37790.1 \| aldo/keto reductase family protein, similar to chalcone reductase (Sesbania rostrata)(GI:2792155), and aldose reductase ALDRXV4 (Xerophyta viscosa)(GI:4539944), | 8.6 | 5.5 | 3.1 | 9.8 | 0.04% | 8.7 |
| 920 | AT2G25210.1 \| 60S ribosomal protein L39 (RPL39A) \| chr2:10746934-10747631 FORWARD \| Aliases: T22F11.20, T22F11_20 | 10.7 | 7.4 | 3.3 | 9.8 | 0.04% | 8.5 |
| 922 | AT3G46010.1 \| Symbol: ADF1 \| actin-depolymerizing factor 1 (ADF1), identical to SP:Q39250 Actin-depolymerizing factor 1 (ADF-1) (AtADF1) {Arabidopsis thaliana} \| chr3:16920391-16921805 REVERSE \| Aliases: ACTIN DEPOLYMERIZING FACTOR 1, ATADF, ATADF1, F16L2.220 | 12.4 | 10.0 | 2.4 | 9.8 | 0.04% | 9.1 |
| 923 | AT4G26260.1 \| Symbol: MIOX4 \| expressed protein, similar to myo-inositol oxygenase (Sus scrofa) gi:17432544:gb:AAL39076 \| chr4:13297948-13300186 FORWARD \| Aliases: MYO INOSITOL OXYGENASE 4, T25K17.70, T25K17_70 | 7.7 | 2.4 | 5.3 | 9.8 | 0.04% | 6.3 |
| 927 | AT1G19050.1 \| Symbol: ARR7 \| two-component responsive regulator / response regulator 7 (ARR7), identical to response regulator 7 GI:3953603 from (Arabidopsis thaliana) \| chr1:6577824-6579102 REVERSE \| Aliases: ARR7, F14D16.20, F14D16_20, RESPONSE REGULATOR 7 | 7.9 | 4.6 | 3.2 | 9.8 | 0.04% | 8.2 |
| 928 | AT1G55900.1 \| Symbol: TIM50 \| NLI interacting factor (NIF) family protein, contains Pfam profile PF03031: NLI interacting factor \| chr1:20906733-20909393 FORWARD \| Aliases: EMB1860, EMBRYO DEFECTIVE 1860, F14J16.15, F14J16_15 | 6.9 | 3.3 | 3.6 | 9.8 | 0.04% | 8.5 |
| 929 | AT1G31760.1 \| SWIB complex BAF60b domain-containing protein, contains Pfam profile PF02201: BAF60b domain of the SWIB complex \| chr1:11372407-11373463 REVERSE \| Aliases: F5M6.23 | 5.8 | 3.4 | 2.4 | 9.8 | 0.04% | 8.9 |
| 930 | AT4G14320.1 \| 60S ribosomal protein L36a/L44 (RPL36aB) | 9.9 | 7.8 | 2.1 | 9.8 | 0.04% | 9.1 |
| 931 | AT2G38810.3 \| histone H2A, putative, strong similarity to histone H2A.F/Z Arabidopsis thaliana GI:2407800; contains Pfam profile PF00125 Core histone H2A/H2B/H3/H4 \| chr2:16226363-16227907 REVERSE \| Aliases: F13I13.4, F13I13_4 | 8.0 | 3.8 | 4.2 | 9.8 | 0.04% | 8.1 |
| 935 | AT5G61170.1 \| 40S ribosomal protein S19 (RPS19C), 40S ribsomal protein S19, Oryza sativa, SWISSPROT:RS19_ORYSA | 10.8 | 8.1 | 2.7 | 9.7 | 0.04% | 9.0 |
| 938 | AT4G36910.1 \| CBS domain-containing protein, contains Pfam profile PF00571: CBS domain | 9.1 | 4.6 | 4.5 | 9.7 | 0.04% | 7.2 |
| 940 | AT5G10690.1 \| pentatricopeptide (PPR) repeat-containing protein / CBS domain-containing protein, contains CBS and PPR domain repeats \| chr5:3374394-3377431 REVERSE \| Aliases: MAJ23.50, MAJ23_50 | 6.3 | 3.8 | 2.4 | 9.7 | 0.04% | 8.5 |
| 941 | AT5G67220.1 \| nitrogen regulation family protein, similar to unknown protein (gb:AAF51525.1) ; contains Pfam domain PF01207: Dihydrouridine synthase (Dus); similar to (SP:P45672) NIFR3-like protein (SP:P45672) (Azospirillum brasilense) \| chr5:26836684-26839190 REVERSE \| Aliases: K21H1.18, K21H1_18 | 10.2 | 8.3 | 1.9 | 9.7 | 0.04% | 9.1 |
| 942 | AT3G15840.3 \| expressed protein \| chr3:5356657-5358506 REVERSE \| Aliases: MSJ11.24, MSJ11_24 | 4.3 | 2.3 | 2.0 | 9.7 | 0.04% | 9.1 |
| 944 | AT5G10850.1 \| expressed protein \| chr5:3428160-3428825 FORWARD \| Aliases: T30N20.120, T30N20_120 | 8.0 | 4.5 | 3.5 | 9.7 | 0.04% | 7.7 |
| 945 | AT5G16450.2 \| dimethylmenaquinone methyltransferase family protein, similar to bacterial S-adenosylmethionine:2-demethylmenaquinone methyltransferases; contains Pfam profile PF03737: Dimethylmenaquinone methyltransferase \| chr5:5374062-5375462 FORWARD \| Aliases: MQK4.18, MQK4_18 | 10.9 | 8.2 | 2.7 | 9.7 | 0.04% | 8.4 |
| 946 | AT1G30070.1 \| SGS domain-containing protein, similar to calcyclin binding protein (Mus musculus) GI:3142331; contains Pfam profile PF05002: SGS domain | 8.3 | 3.6 | 4.6 | 9.7 | 0.04% | 8.2 |
| 948 | AT3G12390.1 \| nascent polypeptide associated complex alpha chain protein, putative / alpha-NAC, putative, similar to alpha-NAC, non-muscle form (Mus musculus) GI:1666690; contains Pfam profiles PF01849: NAC domain, PF00627: UBA/TS-N domain \| chr3:3942266-3943789 FORWARD \| Aliases: T2E22.29 | 10.8 | 9.2 | 1.7 | 9.7 | 0.04% | 9.1 |

| Rank | Description | Sync | Root | M | t | adj.q | B |
|---|---|---|---|---|---|---|---|
| 950 | AT3G48560.1 \| Symbol: CSR1 \| acetolactate synthase, chloroplast / acetohydroxy-acid synthase (ALS), nearly identical to SP:P17597 Acetolactate synthase, chloroplast precursor (EC 2.2.1.6, formerly EC 4.1.3.18) (Acetohydroxy-acid synthase) (ALS) {Arabidopsis thaliana} \| chr3:18012297-18014566 REVERSE \| Aliases: ACETOHYDROXY ACID SYNTHASE, ACETOLACTATE SYNTHASE, ACETOLACTATE SYNTHASE DEFICIENT, AHAS, ALS, CHLORSULFURON/IMIDAZOLINONE RESISTANT 1, IMIDAZOLE RESISTANT 1, IMR1, T8P19.70, TZP5 | 10.8 | 8.5 | 2.3 | 9.7 | 0.04% | 9.1 |
| 951 | AT4G39860.2 \| expressed protein \| chr4:18499878-18501636 FORWARD \| Aliases: T5J17.30, T5J17_30 | 7.7 | 4.1 | 3.5 | 9.7 | 0.04% | 8.6 |
| 953 | AT5G02610.1 \| 60S ribosomal protein L35 (RPL35D), ribosomal protein L35- cytosolic, Arabidopsis thaliana, PIR:T00549 \| chr5:587528-588731 FORWARD \| Aliases: T22P11.200, T22P11_200 | 10.2 | 6.9 | 3.3 | 9.7 | 0.04% | 9.1 |
| 955 | AT1G60000.2 \| 29 kDa ribonucleoprotein, chloroplast, putative / RNA-binding protein cp29, putative, similar to 29 kDa ribonucleoprotein chloroplast precursor {Nicotiana sylvestris} SP:Q08935, SP:Q08937; contains InterPro entry IPR000504: RNA-binding region RNP-1 (RNA recognition motif) (RRM) contains an AG-donor site at intron. \| chr1:22097238-22098227 REVERSE \| Aliases: T2K10.5, T2K10_5 | 9.2 | 5.8 | 3.4 | 9.7 | 0.04% | 8.4 |
| 956 | AT1G09200.1 \| histone H3, identical to histone H3 from Zea mays SP:P05203, Medicago sativa GI:166384, Encephalartos altensteinii SP:P08903, Pisum sativum SP:P02300; contains Pfam profile PF00125 Core histone H2A/H2B/H3/H4 \| chr1:2971595-2972201 REVERSE \| Aliases: T12M4.9 | 10.3 | 3.9 | 6.3 | 9.7 | 0.04% | 8.5 |
| 957 | AT1G71500.1 \| Rieske (2Fe-2S) domain-containing protein, contains Pfam profile PF00355: iron-sulfur cluster-binding protein, rieske family \| chr1:26939700-26941269 FORWARD \| Aliases: F26A9.12 | 7.2 | 3.8 | 3.4 | 9.7 | 0.04% | 8.0 |
| 958 | AT2G43640.1 \| signal recognition particle 14 kDa family protein / SRP14 family protein, similar to SP:P16254 Signal recognition particle 14 kDa protein (SRP14) {Mus musculus}; contains Pfam profile: PF02290 signal recognition particle 14kD protein \| chr2:18104578-18106139 REVERSE \| Aliases: F18O19.25 | 8.5 | 6.8 | 1.8 | 9.7 | 0.04% | 9.1 |
| 960 | AT2G20585.2 \| expressed protein \| chr2:8872180-8873848 FORWARD \| Aliases: None | 6.6 | 3.0 | 3.6 | 9.7 | 0.04% | 8.4 |
| 962 | AT4G13720.1 \| inosine triphosphate pyrophosphatase, putative / HAM1 family protein, contains Pfam profile PF01725: Ham1 family; similar to inosine triphosphate pyrophosphatase (GI:13398328) (Homo sapiens) \| chr4:7966976-7968900 REVERSE \| Aliases: F18A5.110, F18A5_110 | 8.2 | 5.7 | 2.5 | 9.6 | 0.04% | 8.5 |
| 963 | AT4G34240.2 \| Symbol: ALDH3I1 \| aldehyde dehydrogenase (ALDH3), similar to aldehyde dehydrogenase (Arabidopsis thaliana) gi:17065876:emb:CAC84903; contains Pfam profile PF00171: aldehyde dehydrogenase (NAD) family protein; identical to cDNA aldehyde dehydrogenase (ALDH3 gene) GI:17065875, aldehyde dehydrogenase (Arabidopsis thaliana) GI:17065876 \| chr4:16389782-16392086 FORWARD \| Aliases: ALDH3, F10M10.10, F10M10_10 | 7.1 | 4.2 | 2.9 | 9.6 | 0.04% | 8.2 |
| 964 | AT5G25450.1 \| ubiquinol-cytochrome C reductase complex 14 kDa protein, putative, similar to SP:P48502 Ubiquinol-cytochrome C reductase complex 14 kDa protein (EC 1.10.2.2) (CR14) {Solanum tuberosum}; contains Pfam profile PF02271: Ubiquinol-cytochrome C reductase complex 14kD subunit \| chr5:8857039-8858479 FORWARD \| Aliases: F18G18.190, F18G18_190 | 7.6 | 4.5 | 3.1 | 9.6 | 0.04% | 8.6 |
| 967 | AT3G06020.1 \| expressed protein, ; expression supported by MPSS \| chr3:1813273-1814175 FORWARD \| Aliases: F2O10.2, F2O10_2 | 6.1 | 3.9 | 2.2 | 9.6 | 0.04% | 8.8 |
| 970 | AT1G65370.1 \| meprin and TRAF homology domain-containing protein / MATH domain-containing protein, contains Pfam profile PF00917: MATH domain \| chr1:24288136-24289806 REVERSE \| Aliases: T8F5.15, T8F5_15 | 7.1 | 3.7 | 3.4 | 9.6 | 0.04% | 7.8 |
| 971 | AT1G79850.1 \| Symbol: RPS17 \| 30S ribosomal protein S17, chloroplast / CS17 (RPS17), identical to 30S ribosomal protein S17, chloroplast precursor GB:P16180 (Arabidopsis thaliana) \| chr1:30046136-30046845 REVERSE \| Aliases: CS17, F19K16.19, F19K16_19, ORE4, PRPS17 | 8.4 | 4.5 | 3.9 | 9.6 | 0.04% | 8.0 |
| 973 | AT2G32090.2 \| lactoylglutathione lyase family protein / glyoxalase I family protein, contains glyoxalase family protein domain, Pfam:PF00903 \| chr2:13651704-13652906 FORWARD \| Aliases: F22D22.16, F22D22_16 | 10.2 | 7.0 | 3.2 | 9.6 | 0.04% | 9.1 |
| 975 | AT3G18760.1 \| ribosomal protein S6 family protein, contains TIGRFAM profile TIGR00166: ribosomal protein S6 \| chr3:6457696-6459633 REVERSE \| Aliases: MVE11.12 | 7.7 | 4.8 | 2.8 | 9.6 | 0.04% | 8.6 |
| 976 | AT1G49970.1 \| Symbol: CLPR1 \| ATP-dependent Clp protease proteolytic subunit (ClpR1) (nClpP5), identical to nClpP5 GB:BAA82069 GI:5360595 from (Arabidopsis thaliana); identical to cDNA nClpP5 (nuclear encoded ClpP5) GI:5360594 \| chr1:18505201-18508277 REVERSE \| Aliases: F2J10.14, F2J10_14, NCLPP5 | 10.2 | 7.7 | 2.5 | 9.6 | 0.04% | 9.1 |
| 978 | AT2G03870.2 \| small nuclear ribonucleoprotein, putative / snRNP, putative / Sm protein, putative, similar to U6 snRNA-associated Sm-like protein LSm7 (Homo sapiens) SWISS-PROT:Q9UK45 | 11.5 | 10.0 | 1.5 | 9.6 | 0.04% | 8.9 |
| 980 | AT2G32060.3 \| 40S ribosomal protein S12 (RPS12C) \| chr2:13646122-13647257 REVERSE \| Aliases: F22D22.19, F22D22_19 | 11.1 | 8.5 | 2.6 | 9.6 | 0.04% | 8.9 |
| 983 | AT1G77120.1 \| Symbol: ADH1 \| alcohol dehydrogenase (ADH), identical to alcohol dehydrogenase GI:469467 from (Arabidopsis thaliana) \| chr1:28980345-28982311 FORWARD \| Aliases: ADH, ALCOHOL DEHYDROGENASE, ALCOHOL DEHYDROGENASE 1, F22K20.19, F22K20_19 | 12.9 | 9.5 | 3.4 | 9.6 | 0.04% | 9.0 |

| Rank | Description | Sync | Root | M | t | adj.q | B |
|---|---|---|---|---|---|---|---|
| 985 | AT3G11500.1 \| small nuclear ribonucleoprotein G, putative / snRNP-G, putative / Sm protein G, putative, similar to SWISS-PROT:Q15357 small nuclear ribonucleoprotein G (snRNP-G, Sm protein G, Sm-G, SmG) (Homo sapiens) \| chr3:3621415-3622598 REVERSE \| Aliases: F24K9.17 | 8.7 | 6.9 | 1.8 | 9.6 | 0.04% | 9.0 |
| 987 | AT1G23740.1 \| oxidoreductase, zinc-binding dehydrogenase family protein, contains Pfam profile PF00107: oxidoreductase, zinc-binding dehydrogenase family \| chr1:8398115-8399717 REVERSE \| Aliases: F5O8.29, F5O8_29 | 8.2 | 5.0 | 3.2 | 9.6 | 0.04% | 8.3 |
| 989 | AT5G54770.1 \| Symbol: THI1 \| thiazole biosynthetic enzyme, chloroplast (ARA6) (THI1) (THI4), identical to SP:Q38814 Thiazole biosynthetic enzyme, chloroplast precursor (ARA6) {Arabidopsis thaliana} | 12.1 | 6.6 | 5.5 | 9.6 | 0.04% | 7.9 |
| 990 | AT4G13950.1 \| Symbol: RALFL31 \| rapid alkalinization factor (RALF) family protein, similar to RALF precursor (Nicotiana tabacum) GI:16566316 \| chr4:8058067-8058670 REVERSE \| Aliases: DL3015C, FCAALL.121, RALF LIKE 31 | 6.6 | 3.8 | 2.8 | 9.6 | 0.04% | 8.2 |
| 994 | AT2G26340.1 \| expressed protein \| chr2:11222353-11223701 FORWARD \| Aliases: T1D16.2, T1D16_2 | 7.2 | 4.9 | 2.3 | 9.6 | 0.04% | 8.5 |
| 995 | AT2G32990.1 \| glycosyl hydrolase family 9 protein, similar to endo-beta-1,4-glucanase GI:4972236 from (Fragaria x ananassa) \| chr2:14010327-14013094 FORWARD \| Aliases: T21L14.7, T21L14_7 | 7.6 | 5.0 | 2.6 | 9.6 | 0.04% | 8.5 |
| 996 | AT3G12780.1 \| Symbol: PGK1 \| phosphoglycerate kinase, putative, similar to SP:P41758 Phosphoglycerate kinase, chloroplast precursor (EC 2.7.2.3) {Chlamydomonas reinhardtii}; contains Pfam profile PF00162: phosphoglycerate kinase \| chr3:4060978-4063230 REVERSE \| Aliases: MBK21.15, PHOSPHOGLYCERATE KINASE 1 | 12.1 | 7.9 | 4.2 | 9.6 | 0.04% | 8.6 |
| 999 | AT2G44190.1 \| expressed protein, contains Pfam profile: PF04484 family of unknown function (DUF566) \| chr2:18279267-18281573 FORWARD \| Aliases: F6E13.32 | 7.2 | 3.8 | 3.4 | 9.5 | 0.04% | 8.4 |
| 1002 | AT5G11450.1 \| oxygen-evolving complex-related, 23 kDa polypeptide of water-oxidizing complex of photosystem II, Nicotiana tabacum, EMBL:NT23WOP2B \| chr5:3654431-3656556 FORWARD \| Aliases: F15N18.40, F15N18_40 | 7.3 | 4.0 | 3.3 | 9.5 | 0.04% | 8.1 |
| 1003 | AT2G37760.3 \| aldo/keto reductase family protein, similar to chalcone reductase (Sesbania rostrata)(GI:2792155), and aldose reductase ALDRXV4 (Xerophyta viscosa)(GI:4539944), (Hordeum vulgare)(GI:728592) \| chr2:15838948-15840909 FORWARD \| Aliases: T8P21.6 | 9.9 | 6.7 | 3.2 | 9.5 | 0.04% | 8.8 |
| 1004 | AT2G24060.1 \| translation initiation factor 3 (IF-3) family protein, similar to SP:P33319 Translation initiation factor IF-3 {Proteus vulgaris}; contains Pfam profiles PF00707: Translation initiation factor IF-3, C-terminal domain, PF05198: Translation initiation factor IF-3, N-terminal domain | 8.3 | 4.3 | 4.0 | 9.5 | 0.04% | 8.4 |
| 1005 | AT2G43800.1 \| formin homology 2 domain-containing protein / FH2 domain-containing protein, contains formin homology 2 domain, Pfam:PF02181 \| chr2:18152798-18155974 FORWARD \| Aliases: F18O19.9 | 6.3 | 3.3 | 2.9 | 9.5 | 0.04% | 8.1 |
| 1006 | AT1G02850.3 \| glycosyl hydrolase family 1 protein, contains Pfam PF00232 : Glycosyl hydrolase family 1 domain; TIGRFAM TIGR01233: 6-phospho-beta-galactosidase; similar to hydroxyisourate hydrolase (GI:19569603) (Glycine max) \| chr1:630512-633259 FORWARD \| Aliases: F22D16.15, F22D16_15 | 8.5 | 4.4 | 4.1 | 9.5 | 0.04% | 8.3 |
| 1008 | AT5G28750.1 \| thylakoid assembly protein, putative, similar to thylakoid assembly 4 (tha4) protein (Pisum sativum) GI:4929305; contains Pfam profile PF02416: mttA/Hcf106 family \| chr5:10784003-10785723 REVERSE \| Aliases: T32B20.2, T32B20_2 | 9.1 | 6.6 | 2.5 | 9.5 | 0.04% | 8.7 |
| 1012 | AT4G18810.1 \| expressed protein, similar to UV-B and ozone similarly regulated protein 1 UOS1 (Pisum sativum) GI:20339364 \| chr4:10322449-10325781 REVERSE \| Aliases: F28A21.220, F28A21_220 | 6.1 | 2.7 | 3.5 | 9.5 | 0.04% | 7.4 |
| 1013 | AT4G38490.1 \| expressed protein \| chr4:18006282-18007366 FORWARD \| Aliases: F20M13.50, F20M13_50 | 8.7 | 6.9 | 1.9 | 9.5 | 0.04% | 8.9 |
| 1015 | AT2G20060.1 \| ribosomal protein L4 family protein, contains Pfam profile PF00573: ribosomal protein L4/L1 family \| chr2:8666124-8668562 FORWARD \| Aliases: T2G17.14, T2G17_14 | 9.6 | 6.4 | 3.2 | 9.5 | 0.04% | 8.4 |
| 1018 | AT4G10340.1 \| Symbol: LHCB5 \| chlorophyll A-B binding protein CP26, chloroplast / light-harvesting complex II protein 5 / LHCIIc (LHCB5), identical to SP:Q9XF89 Chlorophyll A/B-binding protein CP26, chloroplast precursor (Light-harvesting complex II protein 5) (LHCB5) (LHCIIc) {Arabidopsis thaliana}; contains Pfam profile: PF00504 chlorophyll A-B binding protein; chlorophyll a/b-binding protein CP26 in PS II, Brassica juncea, gb:X95727 \| chr4:6408012-6409673 FORWARD \| Aliases: CP 26, CP26, F24G24.140, F24G24_140, LIGHT HARVESTING COMPLEX OF PHOTOSYSTEM II 5 | 12.7 | 6.5 | 6.3 | 9.5 | 0.04% | 8.7 |
| 1020 | AT4G35850.1 \| pentatricopeptide (PPR) repeat-containing protein, low similarity to CRP1 (Zea mays) GI:3289002; contains Pfam profile PF01535: PPR repeat \| chr4:16983528-16987203 FORWARD \| Aliases: F4B14.120, F4B14_120 | 8.7 | 4.8 | 3.9 | 9.5 | 0.04% | 8.4 |
| 1021 | AT5G53940.1 \| yippee family protein, similar to mdgl-1 (Mus musculus) GI:10441648, Yippee protein (Drosophila melanogaster) GI:5713279; contains Pfam profile PF03226: Yippee putative zinc-binding protein \| chr5:21914227-21915836 REVERSE \| Aliases: K19P17.11, K19P17_11 | 7.2 | 4.8 | 2.4 | 9.5 | 0.04% | 8.6 |

| Rank | Description | Sync | Root | M | t | adj.q | B |
|---|---|---|---|---|---|---|---|
| 1024 | AT5G02220.1 \| expressed protein \| chr5:441705-442144 REVERSE \| Aliases: T7H20.270, T7H20_270 | 6.0 | 2.7 | 3.3 | 9.5 | 0.04% | 8.3 |
| 1025 | AT2G33530.1 \| Symbol: SCPL46 \| | 7.1 | 4.7 | 2.4 | 9.5 | 0.04% | 8.3 |
| 1028 | AT3G02180.2 \| Symbol: SP1L \| expressed protein \| chr3:404891-405656 FORWARD \| Aliases: F1C9.3, SPIRAL 1 LIKE | 10.1 | 8.1 | 2.0 | 9.5 | 0.04% | 8.9 |
| 1030 | AT5G52520.1 \| tRNA synthetase class II (G, H, P and S) family protein, similar to SP:P07814 Bifunctional aminoacyl-tRNA synthetase (Includes: Glutamyl-tRNA synthetase (EC 6.1.1.17) (Glutamate--tRNA ligase); Prolyl-tRNA synthetase (EC 6.1.1.15) (Proline--tRNA ligase)) {Homo sapiens}; contains Pfam profiles PF00587: tRNA synthetase class II core domain (G, H, P, S and T), PF03129: Anticodon binding domain | 8.0 | 4.3 | 3.7 | 9.5 | 0.04% | 8.2 |
| 1033 | AT1G56500.1 \| haloacid dehalogenase-like hydrolase family protein, low similarity to SP:P95649 CbbY protein {Rhodobacter sphaeroides}; contains InterPro accession IPR005834: Haloacid dehalogenase-like hydrolase \| chr1:21163409-21170986 FORWARD \| Aliases: AT1G56505, F13N6.21, F13N6_21 | 6.7 | 4.6 | 2.2 | 9.4 | 0.04% | 8.8 |
| 1034 | AT2G36250.2 \| Symbol: FTSZ2 1 \| chloroplast division protein FtsZ (FtsZ2-1), identical to chloroplast division protein AtFtsZ2-1 (Arabidopsis thaliana) GI:15636809, plastid division protein FtsZ (Arabidopsis thaliana) GI:14195704 | 7.8 | 4.9 | 2.9 | 9.4 | 0.04% | 8.1 |
| 1035 | AT4G00810.2 \| 60S acidic ribosomal protein P1 (RPP1B), similar to acidic ribosomal protein p1 \| chr4:345952-347192 REVERSE \| Aliases: A_TM018A10.9, A_TM018A10_9, T18A10.8, T18A10_8 | 10.3 | 6.5 | 3.9 | 9.4 | 0.04% | 8.0 |
| 1036 | AT3G02480.1 \| ABA-responsive protein-related, similar to ABA-inducible protein (Fagus sylvatica) GI:3901016, cold-induced protein kin1 (Brassica napus) GI:167146 \| chr3:512321-513034 FORWARD \| Aliases: F16B3.11, F16B3_11 | 8.6 | 3.2 | 5.4 | 9.4 | 0.04% | 8.4 |
| 1037 | AT3G52040.1 \| expressed protein \| chr3:19315151-19315839 REVERSE \| Aliases: F4F15.150 | 8.1 | 4.9 | 3.2 | 9.4 | 0.04% | 8.2 |
| 1039 | AT1G29070.1 \| ribosomal protein L34 family protein, similar to plastid ribosomal protein L34 precursor GB:AAF64157 GI:7578860 from (Spinacia oleracea) | 7.0 | 3.6 | 3.4 | 9.4 | 0.04% | 8.0 |
| 1041 | AT4G14615.1 \| expressed protein \| chr4:8383765-8385074 FORWARD \| Aliases: None | 10.6 | 8.7 | 1.9 | 9.4 | 0.04% | 9.0 |
| 1042 | AT5G57040.1 \| lactoylglutathione lyase family protein / glyoxalase I family protein, contains Pfam PF00903: glyoxalase family protein \| chr5:23101131-23102472 REVERSE \| Aliases: MHM17.18, MHM17_18 | 8.9 | 6.9 | 2.0 | 9.4 | 0.04% | 8.6 |
| 1044 | AT1G16705.2 \| similar to TAZ zinc finger family protein / zinc finger (ZZ type) family protein [Arabidopsis thaliana] (TAIR:At1g16710.1) \| chr1:5712185-5713745 FORWARD \| Aliases: None | 3.5 | 2.0 | 1.5 | 9.4 | 0.05% | 8.9 |
| 1047 | AT4G22570.1 \| adenine phosphoribosyltransferase, putative, strong similarity to Adenine phosphoribosyltransferase (Hordeum vulgare subsp. vulgare) GI:9711921; contains Pfam profile PF00156: Phosphoribosyl transferase domain \| chr4:11882161-11885414 REVERSE \| Aliases: F7K2.150, F7K2_150 | 10.1 | 6.2 | 3.9 | 9.4 | 0.05% | 8.3 |
| 1048 | AT1G60970.1 \| clathrin adaptor complex small chain family protein, contains Pfam profile: PF01217 clathrin adaptor complex small chain | 7.0 | 3.0 | 4.0 | 9.4 | 0.05% | 7.3 |
| 1049 | AT3G59840.1 \| expressed protein \| chr3:22116499-22117175 REVERSE \| Aliases: F24G16.110 | 8.1 | 3.6 | 4.5 | 9.4 | 0.05% | 8.1 |
| 1050 | AT1G75350.1 \| Symbol: EMB2184 \| ribosomal protein L31 family protein, similar to SP:O46917 from (Guillardia theta) \| chr1:28275742-28276481 FORWARD \| Aliases: EMB2184, EMBRYO DEFECTIVE 2184, F1B16.11, F1B16_11 | 9.0 | 7.2 | 1.8 | 9.4 | 0.05% | 8.7 |
| 1052 | AT3G24430.1 \| Symbol: HCF101 \| | 7.2 | 3.7 | 3.5 | 9.4 | 0.05% | 8.0 |
| 1053 | AT4G25890.1 \| 60S acidic ribosomal protein P3 (RPP3A), acidic ribosomal protein P3a - maize, PIR2:T02037 \| chr4:13159500-13160457 REVERSE \| Aliases: F14M19.170, F14M19_170 | 8.2 | 6.1 | 2.2 | 9.4 | 0.05% | 8.6 |
| 1054 | AT3G07750.2 \| 3' exoribonuclease family domain 1-containing protein, similar to SP:Q15024 Exosome complex exonuclease RRP42 (EC 3.1.13.-) (Ribosomal RNA processing protein 42) {Homo sapiens}; contains Pfam profile PF01138: 3' exoribonuclease family, domain 1 \| chr3:2473247-2475398 FORWARD \| Aliases: F17A17.9, F17A17_9, MLP3.20 | 7.3 | 4.0 | 3.3 | 9.4 | 0.05% | 8.2 |
| 1055 | AT4G13170.1 \| 60S ribosomal protein L13A (RPL13aC), ribosomal protein L13a -Lupinus luteus,PID:e1237871 \| chr4:7654940-7656561 REVERSE \| Aliases: F17N18.60, F17N18_60 | 10.3 | 6.9 | 3.3 | 9.4 | 0.05% | 8.6 |

| Rank | Description | Sync | Root | M | t | adj.q | B |
|---|---|---|---|---|---|---|---|
| 1060 | AT5G10240.2 \| Symbol: ASN3 \| similar to asparagine synthetase 1 (glutamine-hydrolyzing) / glutamine-dependent asparagine synthetase 1 (ASN1) [Arabidopsis thaliana] (TAIR:At3g47340.3); similar to asparagine synthetase 1 (glutamine-hydrolyzing) / glutamine-dependent asparagine synthetase 1 (ASN1) [Arabidopsis thaliana] (TAIR:At3g47340.2); similar to asparagine synthetase 2 (ASN2) [Arabidopsis thaliana] (TAIR:At5g65010.1); similar to asparagine synthetase 1 (glutamine-hydrolyzing) / glutamine-dependent asparagine synthetase 1 (ASN1) [Arabidopsis thaliana] (TAIR:At3g47340.1); similar to asparagine synthetase 2 (ASN2) [Arabidopsis thaliana] (TAIR:At5g65010.2); similar to asparagine synthetase [Helianthus annuus] (GB:AAF02776.1); similar to glutamine-dependent asparagine synthetase 1 [Hordeum vulgare subsp. vulgare] (GB:AAK49456.1); similar to glutamine-dependent asparagine synthetase [Triticum aestivum] (GB:AAU89392.1); similar to putative asparagine synthetase [Pinus sylvestris] (GB:CAD43058.1); similar to asparagine synthetase [Striga hermonthica] (GB:AAM94340.1); contains InterPro domain Glutamine amidotransferase, class-II (InterPro:IPR000583); contains InterPro domain Asparagine synthase, glutamine-hydrolyzing (InterPro:IPR006426); contains InterPro domain Asparagine synthase (InterPro:IPR001962) | 8.5 | 5.6 | 2.9 | 9.4 | 0.05% | 8.1 |
| 1063 | AT3G47370.3 \| similar to 40S ribosomal protein S20 (RPS20A) [Arabidopsis thaliana] (TAIR:At3g45030.1); similar to 40S ribosomal protein S20 (RPS20C) [Arabidopsis thaliana] (TAIR:At5g62300.1); similar to 40S subunit ribosomal protein [Oryza sativa (japonica cultivar-group)] (GB:XP_550614.1); contains InterPro domain Ribosomal protein S10, eukaryotic and archaeal form (InterPro:IPR005729); contains InterPro domain Ribosomal protein S10 (InterPro:IPR001848) \| chr3:17464463-17465496 REVERSE \| Aliases: T21L8.120 | 10.5 | 8.2 | 2.3 | 9.3 | 0.05% | 8.8 |
| 1064 | AT4G13480.1 \| myb family transcription factor (MYB79), contains PFASM profile: myb DNA binding domain PF00249; identical to cDNA putative transcription factor (MYB79) mRNA, partial cds GI:3941511 \| chr4:7836671-7837680 FORWARD \| Aliases: T6G15.30, T6G15_30 | 7.9 | 4.5 | 3.4 | 9.3 | 0.05% | 7.4 |
| 1065 | AT5G23120.1 \| Symbol: HCF136 \| | 8.6 | 5.8 | 2.8 | 9.3 | 0.05% | 8.4 |
| 1066 | AT5G57560.1 \| Symbol: TCH4 \| xyloglucan:xyloglucosyl transferase / xyloglucan endotransglycosylase / endo-xyloglucan transferase (TCH4), identical to xyloglucan endotransglycosylase TCH4 protein GI:886116 \| chr5:23324356-23325507 REVERSE \| Aliases: MUA2.13, MUA2_13, TOUCH 4, XTH22, XYLOGLUCAN ENDOTRANSGLYCOSYLASE | 8.6 | 4.2 | 4.4 | 9.3 | 0.05% | 7.9 |
| 1068 | AT3G43810.1 \| Symbol: CAM7 \| calmodulin-7 (CAM7), almost identical to calmodulin GI:16227 from (Arabidopsis thaliana), SP:P59220 Calmodulin-7 {Arabidopsis thaliana} \| chr3:15675358-15677445 REVERSE \| Aliases: CALMODULIN 7, T28A8.100 | 11.9 | 8.0 | 3.9 | 9.3 | 0.05% | 8.3 |
| 1069 | AT1G55670.1 \| photosystem I reaction center subunit V, chloroplast, putative / PSI-G, putative (PSAG), identical to SP:Q9S7N7; similar to SP:Q00327 Photosystem I reaction center subunit V, chloroplast precursor (PSI-G) (Photosystem I 9 kDa protein) {Hordeum vulgare}; contains Pfam profile PF01241: Photosystem I psaG / psaK \| chr1:20806336-20807115 REVERSE \| Aliases: F20N2.33, F20N2_33 | 8.2 | 3.0 | 5.3 | 9.3 | 0.05% | 6.5 |
| 1072 | AT3G60480.1 \| expressed protein \| chr3:22358426-22359213 REVERSE \| Aliases: T8B10.140 | 8.9 | 7.2 | 1.8 | 9.3 | 0.05% | 8.8 |
| 1073 | AT2G06520.1 \| membrane protein, putative, contains 2 transmembrane domains; \| chr2:2587781-2588355 REVERSE \| Aliases: T12H3.7, T12H3_7 | 9.5 | 4.7 | 4.8 | 9.3 | 0.05% | 8.1 |
| 1074 | AT3G15353.1 \| Symbol: MT3 \| metallothionein protein, putative | 12.1 | 8.0 | 4.1 | 9.3 | 0.05% | 8.7 |
| 1076 | AT4G25010.1 \| nodulin MtN3 family protein, similar to MtN3 GI:1619602 (root nodule development) from (Medicago truncatula) \| chr4:12854640-12856361 REVERSE \| Aliases: F13M23.150, F13M23_150 | 8.0 | 4.0 | 4.0 | 9.3 | 0.05% | 6.7 |
| 1077 | AT5G60670.1 \| 60S ribosomal protein L12 (RPL12C), 60S RIBOSOMAL PROTEIN L12 (like), Arabidopsis thaliana, PIR:T45883 \| chr5:24398136-24398819 REVERSE \| Aliases: MUP24.13, MUP24_13 | 9.4 | 7.3 | 2.1 | 9.3 | 0.05% | 8.7 |
| 1079 | AT4G02510.1 \| Symbol: TOC159 \| | 10.0 | 8.2 | 1.8 | 9.3 | 0.05% | 8.8 |
| 1081 | AT5G27770.1 \| 60S ribosomal protein L22 (RPL22C), ribosomal protein L22 (cytosolic), Rattus norvegicus, PIR:S52084 \| chr5:9835890-9837332 FORWARD \| Aliases: T1G16.100, T1G16_100 | 10.3 | 8.0 | 2.3 | 9.3 | 0.05% | 8.5 |
| 1082 | AT5G61220.1 \| complex 1 family protein / LVR family protein, contains Pfam PF05347: Complex 1 protein (LYR family) \| chr5:24642802-24644149 REVERSE \| Aliases: MAF19.16, MAF19_16 | 10.7 | 6.7 | 4.0 | 9.3 | 0.05% | 8.7 |
| 1087 | AT5G64140.1 \| Symbol: RPS28 \| 40S ribosomal protein S28 (RPS28C) \| chr5:25684550-25685518 REVERSE \| Aliases: MHJ24.12, MHJ24_12, RIBOSOMAL PROTEIN S28 | 11.1 | 9.0 | 2.1 | 9.3 | 0.05% | 8.8 |
| 1088 | AT1G32220.1 \| expressed protein \| chr1:11607881-11609713 FORWARD \| Aliases: F3C3.2, F3C3_2 | 8.3 | 3.7 | 4.6 | 9.3 | 0.05% | 8.0 |
| 1089 | AT3G12510.1 \| expressed protein \| chr3:3967134-3967804 FORWARD \| Aliases: T2E22.17 | 6.5 | 3.9 | 2.6 | 9.3 | 0.05% | 8.0 |
| 1091 | AT2G37640.1 \| Symbol: ATEXPA3 \| expansin, putative (EXP3), identical to Alpha-expansin 3 precursor (At-EXP3)(Arabidopsis thaliana) SWISS-PROT:O80932; alpha-expansin gene family, PMID:11641069 \| chr2:15794783-15796931 REVERSE \| Aliases: ARABIDOPSIS THALIANA EXPANSIN A3, ATEXP3, ATHEXP ALPHA 1.9, F13M22.14, F13M22_14 | 7.1 | 3.3 | 3.8 | 9.3 | 0.05% | 7.6 |

| Rank | Description | Sync | Root | M | t | adj.q | B |
|---|---|---|---|---|---|---|---|
| 1095 | AT1G04270.2 \| Symbol: RPS15 \| similar to 40S ribosomal protein S15 (RPS15D) [Arabidopsis thaliana] (TAIR:At5g09510.1); similar to ribosomal S15 protein [Retama raetam] (GB:AAL32040.1); contains InterPro domain Ribosomal protein S19/S15 (InterPro:IPR002222); contains InterPro domain Ribosomal protein S15, eukaryotic and archaeal form (InterPro:IPR005713) \| chr1:1141603-1143050 REVERSE \| Aliases: F19P19.29, F19P19_29, RIBOSOMAL PROTEIN S15 | 11.3 | 9.6 | 1.8 | 9.3 | 0.05% | 8.8 |
| 1096 | AT2G19940.2 \| semialdehyde dehydrogenase family protein, similar to N-acetyl-glutamyl-phosphate reductase (Campylobacter jejuni) GI:6650362; contains Pfam profiles PF02774: Semialdehyde dehydrogenase dimerisation domain, PF01118: Semialdehyde dehydrogenase NAD binding domain \| chr2:8620116-8623099 FORWARD \| Aliases: F6F22.3, F6F22_3 | 9.3 | 4.7 | 4.6 | 9.3 | 0.05% | 7.9 |
| 1102 | AT1G10760.1 \| Symbol: SEX1 \| starch excess protein (SEX1), identical to SEX1 (Arabidopsis thaliana) GI:12044358; supporting cDNA gi:12044357:gb:AF312027.1:AF312027 \| chr1:3581035-3590044 REVERSE \| Aliases: GWD1, SEX1, SOP, SOP1, STARCH EXCESS 1 PROTEIN, STARCH EXCESS 1, T16B5.10, T16B5_10 | 10.9 | 8.8 | 2.2 | 9.2 | 0.05% | 8.8 |
| 1103 | AT5G65360.1 \| histone H3, identical to histone H3 from Zea mays SP:P05203, Medicago sativa GI:166384, Encephalartos altensteinii SP:P08903, Pisum sativum SP:P02300; contains Pfam profile PF00125 Core histone H2A/H2B/H3/H4 \| chr5:26137085-26137807 REVERSE \| Aliases: MNA5.9 | 9.1 | 6.3 | 2.9 | 9.2 | 0.05% | 8.5 |
| 1106 | AT2G46540.1 \| expressed protein \| chr2:19117544-19118841 REVERSE \| Aliases: F13A10.7, F13A10_7 | 12.3 | 11.5 | 0.8 | 9.2 | 0.05% | 8.5 |
| 1107 | AT3G63410.1 \| Symbol: APG1 \| chloroplast inner envelope membrane protein, putative (APG1), similar to SP:P23525 37 kDa inner envelope membrane protein, chloroplast precursor (E37) {Spinacia oleracea}; contains Pfam profile PF01209: methlytransferase, UbiE/COQ5 family \| chr3:23426190-23428093 REVERSE \| Aliases: ALBINO OR PALE GREEN MUTANT 1, MAA21.40, VITAMIN E DEFECTIVE, VTE3 | 10.4 | 5.7 | 4.7 | 9.2 | 0.05% | 7.9 |
| 1108 | AT2G36060.2 \| ubiquitin-conjugating enzyme family protein, similar to DNA-binding protein CROC-1B (Homo sapiens) GI:1066082; contains Pfam profile PF00179: Ubiquitin-conjugating enzyme \| chr2:15149876-15151261 REVERSE \| Aliases: F11F19.3, F11F19_3 | 11.3 | 10.2 | 1.1 | 9.2 | 0.05% | 8.8 |
| 1109 | AT2G39390.1 \| 60S ribosomal protein L35 (RPL35B) \| chr2:16457738-16458956 REVERSE \| Aliases: F12L6.5, F12L6_5 | 10.8 | 8.9 | 1.9 | 9.2 | 0.05% | 8.8 |
| 1111 | AT1G55480.1 \| expressed protein \| chr1:20717350-20719200 FORWARD \| Aliases: T5A14.12, T5A14_12 | 8.4 | 5.3 | 3.1 | 9.2 | 0.05% | 7.6 |
| 1112 | AT1G34030.1 \| 40S ribosomal protein S18 (RPS18B), similar to ribosomal protein S18 GI:38422 from (Homo sapiens) \| chr1:12370055-12371530 REVERSE \| Aliases: F12G12.15, F12G12_15 | 11.0 | 7.7 | 3.3 | 9.2 | 0.05% | 8.5 |
| 1113 | AT1G26470.1 \| expressed protein \| chr1:9155080-9156233 FORWARD \| Aliases: T1K7.16, T1K7_16 | 7.2 | 3.9 | 3.3 | 9.2 | 0.05% | 8.0 |
| 1114 | AT4G29350.1 \| Symbol: PRF2 \| profilin 2 (PRO2) (PFN2) (PRF2), identical to profilin 2 SP:Q42418 GI:1353772 from (Arabidopsis thaliana); identical to cDNA profilin (PRF2) GI:9965570 \| chr4:14450035-14451383 FORWARD \| Aliases: F17A13.170, F17A13_170, PFN2, PROFILIN 2 | 13.9 | 12.3 | 1.6 | 9.2 | 0.05% | 8.8 |
| 1117 | AT2G02100.1 \| Symbol: LCR69/PDF2.2 \| plant defensin-fusion protein, putative (PDF2.2), plant defensin protein family member, personal communication, Bart Thomma (Bart.Thomma@agr.kuleuven.ac.be); similar to SWISS-PROT:O65740 \| chr2:528349-529107 FORWARD \| Aliases: F5O4.13, F5O4_13, LCR69, Low molecular weight cysteine rich 69, PDF2.2 | 13.2 | 11.1 | 2.0 | 9.2 | 0.05% | 8.7 |
| 1118 | AT5G26220.1 \| ChaC-like family protein, contains Pfam profile: PF04752 ChaC-like protein \| chr5:9162972-9164680 REVERSE \| Aliases: T19G15.70, T19G15_70 | 7.8 | 3.6 | 4.2 | 9.2 | 0.05% | 7.5 |
| 1120 | AT4G02530.1 \| chloroplast thylakoid lumen protein, SP:022773 ;TL16_ARATH \| chr4:1112146-1114026 REVERSE \| Aliases: T10P11.17, T10P11_17 | 6.9 | 4.0 | 3.0 | 9.2 | 0.05% | 7.8 |
| 1121 | AT4G34090.2 \| expressed protein \| chr4:16326861-16330183 REVERSE \| Aliases: F28A23.150, F28A23_150 | 7.5 | 4.3 | 3.2 | 9.2 | 0.05% | 8.1 |
| 1122 | AT3G19790.1 \| expressed protein, ; expression supported by MPSS \| chr3:6874943-6875446 REVERSE \| Aliases: MMB12.25 | 8.3 | 5.7 | 2.6 | 9.2 | 0.05% | 8.4 |
| 1124 | AT3G22420.2 \| Symbol: WNK2 \| protein kinase family protein, contains protein kinase domain, Pfam:PF00069 \| chr3:7946533-7949081 FORWARD \| Aliases: MCB17.15, PROTEIN KINASE, WITH NO K 2, ZIK3 | 6.7 | 3.4 | 3.3 | 9.2 | 0.05% | 7.5 |
| 1125 | AT3G47836.1 \| expressed protein \| chr3:17661246-17662750 REVERSE \| Aliases: None | 11.5 | 9.5 | 1.9 | 9.2 | 0.05% | 8.7 |
| 1127 | AT4G25740.2 \| similar to 40S ribosomal protein S10 (RPS10C) [Arabidopsis thaliana] (TAIR:At5g52650.1); similar to RS10_ORYSA 40S ribosomal protein S10 (GB:Q9AYP4); contains InterPro domain Plectin/S10, N-terminal (InterPro:IPR005326) \| chr4:13107273-13108822 REVERSE \| Aliases: F14M19.20, F14M19_20 | 9.9 | 7.2 | 2.7 | 9.2 | 0.05% | 8.1 |
| 1128 | AT5G47630.1 \| acyl carrier family protein / ACP family protein, similar to acyl carrier protein, mitochondrial precursor (ACP) (NADH-ubiquinone oxidoreductase 9.6 kDa subunit) from {Arabidopsis thaliana} SP:P53665, {Neurospora crassa} SP:P11943; contains Pfam profile PF00550: Phosphopantetheine attachment site \| chr5:19323373-19324629 FORWARD \| Aliases: MNJ7.22, MNJ7_22 | 6.7 | 3.0 | 3.7 | 9.2 | 0.05% | 7.0 |

| Rank | Description | Sync | Root | M | t | adj.q | B |
|---|---|---|---|---|---|---|---|
| 1129 | AT5G12980.1 \| rcd1-like cell differentiation protein, putative, similar to protein involved in sexual development (Schizosaccharomyces pombe) GI:1620896; contains Pfam profile PF04078: Cell differentiation family, Rcd1-like \| chr5:4105386-4108179 REVERSE \| Aliases: T24H18.150, T24H18_150 | 6.7 | 3.1 | 3.5 | 9.2 | 0.05% | 7.9 |
| 1130 | AT3G60770.1 \| 40S ribosomal protein S13 (RPS13A), AtRPS13A mRNA for cytoplasmic ribosomal protein S13, Arabidopsis thaliana,AB031739 \| chr3:22471265-22472718 REVERSE \| Aliases: T4C21.180 | 11.8 | 9.2 | 2.5 | 9.2 | 0.05% | 8.7 |
| 1131 | AT3G05020.1 \| Symbol: ACP1 \| acyl carrier protein 1, chloroplast (ACP-1), identical to SP:P11829 Acyl carrier protein 1, chloroplast precursor (ACP) {Arabidopsis thaliana} \| chr3:1391658-1392960 REVERSE \| Aliases: ACP, ACYL CARRIER PROTEIN, T9J14.3, T9J14_3 | 7.7 | 4.0 | 3.7 | 9.2 | 0.05% | 8.2 |
| 1132 | AT5G66410.1 \| expressed protein \| chr5:26537032-26538922 FORWARD \| Aliases: K1F13.6, K1F13_6 | 9.0 | 7.0 | 2.1 | 9.2 | 0.05% | 8.5 |
| 1133 | AT5G55640.1 \| expressed protein \| chr5:22550385-22551304 REVERSE \| Aliases: MDF20.8, MDF20_8 | 9.1 | 7.1 | 2.0 | 9.1 | 0.05% | 8.6 |
| 1134 | AT1G74050.1 \| 60S ribosomal protein L6 (RPL6C), similar to 60S ribosomal protein L6 (YL 16 like) GB:CAB57309 from (Cyanophora paradoxa) \| chr1:27850710-27852467 REVERSE \| Aliases: F2P9.8, F2P9_8 | 12.0 | 11.1 | 1.0 | 9.1 | 0.05% | 8.6 |
| 1135 | AT1G74060.1 \| 60S ribosomal protein L6 (RPL6B), similar to 60S ribosomal protein L6 (YL 16 like) GB:CAB57309 from (Cyanophora paradoxa) \| chr1:27853497-27855028 REVERSE \| Aliases: F2P9.7, F2P9_7 | 12.0 | 11.1 | 1.0 | 9.1 | 0.05% | 8.6 |
| 1137 | AT2G34570.1 \| expressed protein, contains Pfam profile: PF04900 protein of unknown function, DUF652 \| chr2:14569980-14571861 REVERSE \| Aliases: T31E10.9, T31E10_9 | 6.3 | 4.3 | 1.9 | 9.1 | 0.05% | 8.5 |
| 1138 | AT4G02610.1 \| tryptophan synthase, alpha subunit, putative, similar to A. thaliana tryptophan synthase alpha chain (EC 4.2.1.20), GenBank accession number U18993 (gi:619753) \| chr4:1147634-1149577 FORWARD \| Aliases: T10P11.11, T10P11_11 | 8.7 | 5.4 | 3.3 | 9.1 | 0.05% | 8.1 |
| 1141 | AT5G67490.1 \| expressed protein \| chr5:26951379-26952178 REVERSE \| Aliases: K9I9.5, K9I9_5 | 10.3 | 7.7 | 2.6 | 9.1 | 0.05% | 8.3 |
| 1143 | AT2G47710.1 \| universal stress protein (USP) family protein, similar to ER6 protein (Lycopersicon esculentum) GI:5669654; contains Pfam profile PF00582: universal stress protein family \| chr2:19561904-19563135 REVERSE \| Aliases: F17A22.10 | 11.0 | 7.1 | 3.9 | 9.1 | 0.05% | 8.2 |
| 1145 | AT3G44735.1 \| phytosulfokines-related, contains similarities to phytosulfokines from (Arabidopsis thaliana) \| chr3:16302187-16303137 REVERSE \| Aliases: None | 5.9 | 3.3 | 2.6 | 9.1 | 0.05% | 8.1 |
| 1152 | AT5G06860.1 \| Symbol: PGIP1 \| polygalacturonase inhibiting protein 1 (PGIP1), identical to polygalacturonase inhibiting protein 1 (PGIP1) (Arabidopsis thaliana) gi:7800199:gb:AAF69827; contains leucine rich-repeat (LRR) domains Pfam:PF00560, INTERPRO:IPR001611 \| chr5:2132351-2133588 FORWARD \| Aliases: MOJ9.3, MOJ9_3, POLYGALACTURONASE INHIBITING PROTEIN 1 | 7.5 | 4.1 | 3.4 | 9.1 | 0.05% | 7.6 |
| 1154 | AT1G35680.1 \| 50S ribosomal protein L21, chloroplast / CL21 (RPL21), identical to 50S ribosomal protein L21, chloroplast precursor (CL21) (Arabidopsis thaliana) SWISS-PROT:P51412 \| chr1:13209965-13211630 FORWARD \| Aliases: F15O4.7 | 8.3 | 4.6 | 3.7 | 9.1 | 0.05% | 7.6 |
| 1156 | AT3G18610.1 \| nucleolin, putative, contains Pfam profile: PF00076 RNA recognition motif \| chr3:6404276-6407828 REVERSE \| Aliases: K24M9.10 | 6.8 | 4.0 | 2.8 | 9.1 | 0.05% | 8.1 |
| 1158 | AT1G10120.1 \| similar to basic helix-loop-helix (bHLH) family protein [Arabidopsis thaliana] (TAIR:At1g68920.1); similar to TA1 protein-like [Oryza sativa (japonica cultivar-group)] (GB:BAD34345.1); contains InterPro domain Basic helix-loop-helix dimerization domain bHLH (InterPro:IPR001092) \| chr1:3304044-3306190 REVERSE \| Aliases: T27I1.15, T27I1_15 | 6.6 | 4.1 | 2.5 | 9.1 | 0.05% | 8.3 |
| 1159 | AT5G64080.2 \| protease inhibitor/seed storage/lipid transfer protein (LTP) family protein, contains Pfam protease inhibitor/seed storage/LTP family domain PF00234 \| chr5:25662489-25663954 REVERSE \| Aliases: MHJ24.6, MHJ24_6 | 7.5 | 2.6 | 4.9 | 9.1 | 0.05% | 6.6 |
| 1160 | AT3G55280.1 \| 60S ribosomal protein L23A (RPL23aB), various ribosomal L23a proteins \| chr3:20511312-20512686 FORWARD \| Aliases: T26I12.160 | 9.4 | 5.9 | 3.5 | 9.1 | 0.05% | 8.1 |
| 1164 | AT1G01100.2 \| 60S acidic ribosomal protein P1 (RPP1A), similar to 60S ACIDIC RIBOSOMAL PROTEIN P1 GB:O23095 from (Arabidopsis thaliana) \| chr1:50091-51182 REVERSE \| Aliases: T25K16.9, T25K16_9 | 11.8 | 9.6 | 2.2 | 9.1 | 0.05% | 8.7 |
| 1168 | AT4G24830.1 \| arginosuccinate synthase family, contains Pfam profile: PF00764 arginosuccinate synthase | 12.1 | 7.2 | 4.9 | 9.1 | 0.05% | 8.1 |
| 1169 | AT4G30330.1 \| small nuclear ribonucleoprotein E, putative / snRNP-E, putative / Sm protein E, putative, similar to SWISS-PROT:P08578 small nuclear ribonucleoprotein E (snRNP-E) (Sm protein E, Sm-E, SmE) (Chicken) \| chr4:14836558-14838024 REVERSE \| Aliases: F17I23.330, F17I23_330 | 7.6 | 4.5 | 3.1 | 9.1 | 0.05% | 8.1 |
| 1170 | AT4G10040.1 \| cytochrome c, putative, similar to cytochrome c (Pumpkin, Winter squash) SWISS-PROT:P00051 \| chr4:6276995-6278501 FORWARD \| Aliases: F28M11.5 | 10.3 | 7.5 | 2.8 | 9.0 | 0.05% | 8.5 |

| Rank | Description | Sync | Root | M | t | adj.q | B |
|---|---|---|---|---|---|---|---|
| 1173 | AT5G13520.1 \| peptidase M1 family protein, similar to SP:P09960 Leukotriene A-4 hydrolase (EC 3.3.2.6) (LTA-4 hydrolase) {Homo sapiens}; contains Pfam profile PF01433: Peptidase family M1 \| chr5:4341543-4344674 REVERSE \| Aliases: T6I14.50, T6I14_50 | 9.3 | 6.0 | 3.4 | 9.0 | 0.05% | 8.1 |
| 1174 | AT1G26730.1 \| EXS family protein / ERD1/XPR1/SYG1 family protein, similar to PHO1 protein (Arabidopsis thaliana) GI:20069032; contains Pfam profiles PF03105: SPX domain, PF03124: EXS family | 7.2 | 4.6 | 2.6 | 9.0 | 0.05% | 8.0 |
| 1176 | AT2G21385.1 \| expressed protein \| chr2:9156723-9158934 FORWARD \| Aliases: None | 8.0 | 5.0 | 3.0 | 9.0 | 0.05% | 8.2 |
| 1177 | AT1G16340.2 \| similar to 2-dehydro-3-deoxyphosphooctonate aldolase / phospho-2-dehydro-3-deoxyoctonate aldolase / 3-deoxy-D-manno-octulosonic acid 8-phosphate synthetase (KDSA) [Arabidopsis thaliana] (TAIR:At1g79500.1); similar to 2-dehydro-3-deoxyphosphooctonate aldolase / phospho-2-dehydro-3-deoxyoctonate aldolase / 3-deoxy-D-manno-octulosonic acid 8-phosphate synthetase (KDSA) [Arabidopsis thaliana] (TAIR:At1g79500.3); similar to 2-dehydro-3-deoxyphosphooctonate aldolase / phospho-2-dehydro-3-deoxyoctonate aldolase / 3-deoxy-D-manno-octulosonic acid 8-phosphate synthetase (KDSA) [Arabidopsis thaliana] (TAIR:At1g79500.2); similar to 3-desoxy-D-manno octulosonic acid-8-phosphate synthase [Lycopersicon esculentum] (GB:CAC35366.1); similar to putative 2-dehydro-3-deoxyphosphooctonate aldolase [Oryza sativa (japonica cultivar-group)] (GB:AAO72599.1); contains InterPro domain DAHP synthetase I/KDSA superfamily (InterPro:IPR006218); contains InterPro domain 2-dehydro-3-deoxyphosphooctonate aldolase (InterPro:IPR006269) | 9.2 | 5.5 | 3.7 | 9.0 | 0.05% | 7.9 |
| 1178 | AT3G27690.1 \| Symbol: LHCB2:4 \| chlorophyll A-B binding protein (LHCB2:4), nearly identical to Lhcb2 protein (Arabidopsis thaliana) GI:4741950; similar to chlorophyll A-B binding protein 151 precursor (LHCP) GB:P27518 from (Gossypium hirsutum); contains Pfam PF00504: Chlorophyll A-B binding protein \| chr3:10257184-10258248 FORWARD \| Aliases: LHCB2.3, MGF10.10 | 7.0 | 3.3 | 3.7 | 9.0 | 0.05% | 7.4 |
| 1179 | AT1G73180.1 \| eukaryotic translation initiation factor-related, similar to eukaryotic translation initiation factor 2A (GI:21956484) (Homo sapiens); similar to Eukaryotic translation initiation factor 3 subunit 9 (eIF-3 eta) (eIF3 p116) (eIF3 p110) (eIF3b) (Swiss-Prot:P55884) (Homo sapiens) \| chr1:27521577-27525290 FORWARD \| Aliases: T18K17.15, T18K17_15 | 7.9 | 6.0 | 1.9 | 9.0 | 0.06% | 8.4 |
| 1181 | AT2G28600.1 \| expressed protein \| chr2:12258862-12261900 FORWARD \| Aliases: T8O18.11, T8O18_11 | 6.1 | 3.3 | 2.8 | 9.0 | 0.06% | 8.0 |
| 1182 | AT2G34200.1 \| zinc finger (C3HC4-type RING finger) family protein \| chr2:14447727-14449084 FORWARD \| Aliases: F13P17.4, F13P17_4 | 5.9 | 3.4 | 2.5 | 9.0 | 0.06% | 8.1 |
| 1183 | AT4G00585.1 \| expressed protein \| chr4:250955-252532 REVERSE \| Aliases: None | 11.3 | 9.2 | 2.1 | 9.0 | 0.06% | 8.6 |
| 1184 | AT2G29540.2 \| Symbol: ATRPC14 \| similar to DNA-directed RNA polymerase II 13.6 kDa subunit (RPB13.6) [Arabidopsis thaliana] (TAIR:At3g52090.1); similar to Polymerase (RNA) I polypeptide C [Homo sapiens] (GB:CAI16933.1); contains InterPro domain DNA-directed RNA polymerase, 13 to 16 kDa subunit (InterPro:IPR008193) \| chr2:12649598-12651021 FORWARD \| Aliases: ATRPAC14, F16P2.8, F16P2_8, RNA POLYMERASE 14 KDA SUBUNIT | 8.4 | 5.0 | 3.4 | 9.0 | 0.06% | 8.3 |
| 1186 | AT5G58250.1 \| expressed protein \| chr5:23576703-23577739 FORWARD \| Aliases: MCK7.12, MCK7_12 | 6.6 | 3.4 | 3.3 | 9.0 | 0.06% | 7.8 |
| 1187 | AT2G40765.1 \| expressed protein \| chr2:17019276-17020403 FORWARD \| Aliases: None | 10.8 | 9.1 | 1.7 | 9.0 | 0.06% | 8.5 |
| 1191 | AT1G30500.2 \| CCAAT-binding transcription factor (CBF-B/NF-YA) family protein, contains Pfam profile: PF02045 CCAAT-binding transcription factor (CBF-B/NF-YA) | 7.7 | 5.3 | 2.4 | 9.0 | 0.06% | 7.9 |
| 1195 | AT2G20450.1 \| 60S ribosomal protein L14 (RPL14A) \| chr2:8820947-8822332 FORWARD \| Aliases: T13C7.4, T13C7_4 | 9.4 | 6.9 | 2.6 | 9.0 | 0.06% | 8.4 |
| 1197 | AT5G39720.1 \| avirulence-responsive protein-related / avirulence induced gene (AIG) protein-related, similar to SP:P54121 AIG2 protein {Arabidopsis thaliana} \| chr5:15916874-15917881 FORWARD \| Aliases: MKM21.1 | 6.6 | 3.0 | 3.6 | 9.0 | 0.06% | 7.1 |
| 1198 | AT2G31490.1 \| expressed protein \| chr2:13419060-13420323 FORWARD \| Aliases: T28P16.2, T28P16_2 | 12.0 | 10.4 | 1.5 | 9.0 | 0.06% | 8.6 |
| 1202 | AT5G09770.1 \| ribosomal protein L17 family protein, contains Pfam profile: PF01196 ribosomal protein L17 \| chr5:3035047-3036543 REVERSE \| Aliases: F17I14.40, F17I14_40 | 8.9 | 6.1 | 2.8 | 9.0 | 0.06% | 8.3 |
| 1204 | AT1G09340.1 \| expressed protein \| chr1:3015388-3018236 FORWARD \| Aliases: None | 7.6 | 4.2 | 3.4 | 9.0 | 0.06% | 7.2 |
| 1206 | AT1G12400.1 \| expressed protein \| chr1:4221861-4222835 FORWARD \| Aliases: F5O11.12, F5O11_12 | 8.1 | 5.0 | 3.1 | 9.0 | 0.06% | 8.1 |
| 1208 | AT4G29840.2 \| Symbol: MTO2 \| threonine synthase, chloroplast, identical to SP:Q9S7B5 Threonine synthase, chloroplast precursor (EC 4.2.3.1) (TS) {Arabidopsis thaliana} \| chr4:14597716-14601061 REVERSE \| Aliases: F27B13.80, F27B13_80, METHIONINE OVER ACCUMULATOR, THREONINE SYNTHASE, TS | 9.6 | 6.5 | 3.1 | 9.0 | 0.06% | 8.2 |
| 1209 | AT2G44670.1 \| senescence-associated protein-related, similar to senescence-associated protein SAG102 (GI:22331931) (Arabidopsis thaliana); \| chr2:18432255-18433135 FORWARD \| Aliases: F16B22.16 | 9.3 | 5.7 | 3.7 | 9.0 | 0.06% | 8.2 |

| Rank | Description | Sync | Root | M | t | adj.q | B |
|---|---|---|---|---|---|---|---|
| 1212 | AT4G35570.1 \| Symbol: HMGB5 \| high mobility group protein delta (HMGdelta) / HMG protein delta, identical to HMG protein (HMGdelta) (Arabidopsis thaliana) GI:2832363 | 7.8 | 5.7 | 2.1 | 9.0 | 0.06% | 8.4 |
| 1214 | AT5G01670.2 \| aldose reductase, putative, similar to aldose reductase (Hordeum vulgare)(GI:728592), aldose reductase ALDRXV4 (Xerophyta viscosa)(GI:4539944) \| chr5:251975-253957 FORWARD \| Aliases: F7A7.190, F7A7_190 | 6.9 | 4.6 | 2.3 | 8.9 | 0.06% | 8.0 |
| 1216 | AT5G09660.2 \| Symbol: PMDH2 \| similar to malate dehydrogenase, glyoxysomal, putative [Arabidopsis thaliana] (TAIR:At2g22780.1); similar to malate dehydrogenase 1 [Brassica napus] (GB:CAB43994.1); contains InterPro domain Malate dehydrogenase, active site (InterPro:IPR001252); contains InterPro domain Lactate/malate dehydrogenase (InterPro:IPR001236) \| chr5:2993445-2995308 REVERSE \| Aliases: F17I14.150, F17I14_150, PEROXISOMAL NAD MALATE DEHYDROGENASE 2, PMDH2 | 6.8 | 3.1 | 3.8 | 8.9 | 0.06% | 6.8 |
| 1217 | AT4G18370.1 \| Symbol: HHOA \| protease HhoA, chloroplast (SPPA) (HHOA), identical to SP:Q9SEL7 Protease HhoA, chloroplast precursor (EC 3.4.21.-) {Arabidopsis thaliana} \| chr4:10149203-10151722 FORWARD \| Aliases: F28J12.30, F28J12_30 | 8.1 | 5.3 | 2.8 | 8.9 | 0.06% | 8.1 |
| 1218 | AT4G32520.1 \| glycine hydroxymethyltransferase, putative / serine hydroxymethyltransferase, putative / serine/threonine aldolase, putative, similar to serine hydroxymethyltransferase (Chlamydomonas reinhardtii) GI:17066746; contains Pfam profile PF00464: serine hydroxymethyltransferase | 10.2 | 6.7 | 3.5 | 8.9 | 0.06% | 8.0 |
| 1219 | AT2G36990.1 \| Symbol: SIGF \| RNA polymerase sigma subunit SigF (sigF) / sigma-like factor (SIG6), identical to RNA polymerase sigma subunit SigF (Arabidopsis thaliana) GI:7209640; contains Pfam profiles PF04545: Sigma-70, region 4, PF04539: Sigma-70 region 3, PF04542: Sigma-70 region 2; identical to cDNA partial mRNA for putative sigma-like transcription factor (sig6 gene) GI:6273429 \| chr2:15544337-15547218 REVERSE \| Aliases: RNA POLYMERASE SIGMA 70 FACTOR, SIG6, T1J8.17, T1J8_17 | 6.0 | 3.8 | 2.2 | 8.9 | 0.06% | 8.2 |
| 1220 | AT3G09210.1 \| KOW domain-containing transcription factor family protein, ; est match \| chr3:2825290-2826886 REVERSE \| Aliases: F3L24.8 | 8.4 | 5.7 | 2.7 | 8.9 | 0.06% | 7.8 |
| 1221 | AT1G09795.1 \| Symbol: ATATP PRT2 \| ATP phosphoribosyl transferase 2 (ATP-PRT2), identical to ATP phosphoribosyl transferase (AtATP-PRT2) (Arabidopsis thaliana) GI:6683619; supporting cDNA gi:6683618:dbj:AB025250.1: \| chr1:3173536-3176912 FORWARD \| Aliases: ATP PHOSPHORIBOSYL TRANSFERASE 2 | 7.2 | 3.3 | 3.9 | 8.9 | 0.06% | 6.9 |
| 1222 | AT3G60370.1 \| immunophilin / FKBP-type peptidyl-prolyl cis-trans isomerase family protein, SP:Q9M222; similar to FKBP-type peptidyl-prolyl cis-trans isomerase fkpA precursor (PPiase) (Rotamase)(SP:Q8X880) (Escherichia coli O157:H7) ; contains Pfam PF00254: peptidyl-prolyl cis-trans isomerase, FKBP-type \| chr3:22325747-22327514 REVERSE \| Aliases: T8B10.30 | 6.0 | 3.9 | 2.2 | 8.9 | 0.06% | 8.1 |
| 1229 | AT2G01090.1 \| ubiquinol-cytochrome C reductase complex 7.8 kDa protein, putative / mitochondrial hinge protein, putative, similar to SP:P48504 Ubiquinol-cytochrome C reductase complex 7.8 kDa protein (EC 1.10.2.2) (Mitochondrial hinge protein) (CR7) {Solanum tuberosum}; contains Pfam profile PF02320: Ubiquinol-cytochrome C reductase hinge protein \| chr2:80014-81200 FORWARD \| Aliases: F23H14.6, F23H14_6 | 9.6 | 6.8 | 2.8 | 8.9 | 0.06% | 8.3 |
| 1230 | AT5G17710.2 \| Symbol: EMB1241 \| co-chaperone grpE family protein, similar to co-chaperone CGE1 precursor isoform a (Chlamydomonas reinhardtii) GI:15384277; contains Pfam profile PF01025: co-chaperone GrpE \| chr5:5839345-5841707 REVERSE \| Aliases: EMB1241, EMBRYO DEFECTIVE 1241, MVA3.60, MVA3_60 | 8.9 | 5.4 | 3.5 | 8.9 | 0.06% | 7.8 |
| 1231 | AT1G55120.1 \| Symbol: ATFRUCT5 \| beta-fructosidase, putative / beta-fructofuranosidase, putative, similar to beta-fructofuranosidase GI:402740 \| chr1:20570265-20572943 FORWARD \| Aliases: BETA FRUCTOFURANOSIDASE, BETA FRUCTOFURANOSIDASE 5, T7N22.6, T7N22_6 | 6.2 | 3.3 | 2.9 | 8.9 | 0.06% | 7.6 |
| 1233 | AT2G29690.1 \| Symbol: ASA2 \| anthranilate synthase, alpha subunit, component I-2 (ASA2), identical to SP:P32069 \| chr2:12700948-12704042 FORWARD \| Aliases: ATHANSYNAB, T27A16.21, T27A16_21 | 8.0 | 5.2 | 2.7 | 8.9 | 0.06% | 7.9 |
| 1235 | AT5G39340.1 \| Symbol: AHP3 \| two-component phosphorelay mediator 2 (HP2), nearly identical to ATHP2 (Arabidopsis thaliana) GI:4156243 | 9.0 | 7.6 | 1.4 | 8.9 | 0.06% | 8.5 |
| 1236 | AT1G69700.1 \| Symbol: ATHVA22C \| ABA-responsive protein (HVA22c), identical to AtHVA22c (Arabidopsis thaliana) GI:4884936 \| chr1:26223901-26225487 FORWARD \| Aliases: ATHVA22C, T6C23.10, T6C23_10 | 6.7 | 2.8 | 4.0 | 8.9 | 0.06% | 7.4 |
| 1238 | AT3G49010.3 \| Symbol: ATBBC1 \| | 12.9 | 10.8 | 2.1 | 8.9 | 0.06% | 8.5 |
| 1239 | AT5G47110.1 \| lil3 protein, putative, similar to Lil3 protein (Arabidopsis thaliana) gi:4741966:gb:AAD28780 \| chr5:19151215-19152544 REVERSE \| Aliases: K14A3.6, K14A3_6 | 8.7 | 5.9 | 2.8 | 8.9 | 0.06% | 8.1 |
| 1243 | AT3G57160.1 \| expressed protein \| chr3:21168589-21169795 REVERSE \| Aliases: None | 7.2 | 4.8 | 2.4 | 8.9 | 0.06% | 8.0 |
| 1246 | AT1G20430.1 \| expressed protein \| chr1:7083186-7083825 REVERSE \| Aliases: F5M15.30, F5M15_30 | 6.3 | 3.5 | 2.8 | 8.8 | 0.06% | 7.8 |
| 1247 | AT1G19130.1 \| expressed protein \| chr1:6609360-6610593 REVERSE \| Aliases: F14D16.29, F14D16_29 | 9.1 | 6.8 | 2.3 | 8.8 | 0.06% | 8.2 |
| 1248 | AT3G06790.2 \| plastid developmental protein DAG, putative, similar to DAG protein, chloroplast precursor (Garden snapdragon) SWISS-PROT:Q38732 | 8.8 | 5.5 | 3.3 | 8.8 | 0.06% | 8.1 |

| Rank | Description | Sync | Root | M | t | adj.q | B |
|---|---|---|---|---|---|---|---|
| 1249 | AT5G02870.1 \| 60S ribosomal protein L4/L1 (RPL4D), 60S roibosomal protein L4, Arabidopsis thaliana, EMBL:CAA79104 \| chr5:657784-659716 FORWARD \| Aliases: F9G14.180, F9G14_180 | 11.6 | 9.9 | 1.6 | 8.8 | 0.06% | 8.5 |
| 1250 | AT5G42150.1 \| expressed protein \| chr5:16863378-16865507 FORWARD \| Aliases: MJC20.26, MJC20_26 | 9.5 | 6.7 | 2.8 | 8.8 | 0.06% | 8.2 |
| 1252 | AT3G17930.1 \| expressed protein \| chr3:6141834-6143316 REVERSE \| Aliases: MEB5.15 | 8.6 | 5.8 | 2.8 | 8.8 | 0.06% | 7.9 |
| 1253 | AT2G20270.1 \| glutaredoxin family protein, contains glutaredoxin domain, Pfam:PF00462 \| chr2:8744786-8746741 REVERSE \| Aliases: F11A3.18, F11A3_18 | 7.7 | 3.0 | 4.6 | 8.8 | 0.06% | 7.9 |
| 1254 | AT1G15980.1 \| expressed protein \| chr1:5489292-5491303 FORWARD \| Aliases: T24D18.8, T24D18_8 | 7.1 | 3.7 | 3.4 | 8.8 | 0.06% | 7.2 |
| 1255 | AT4G24440.2 \| transcription initiation factor IIA gamma chain / TFIIA-gamma (TFIIA-S), identical to transcription initiation factor IIA gamma chain SP:Q39236 from (Arabidopsis thaliana); \| chr4:12633190-12634767 FORWARD \| Aliases: T22A6.270, T22A6_270 | 8.5 | 5.3 | 3.1 | 8.8 | 0.06% | 8.3 |
| 1256 | AT4G26860.1 \| alanine racemase family protein, contains Pfam domain, PF01168: Alanine racemase, N-terminal domain \| chr4:13503092-13504756 REVERSE \| Aliases: F10M23.200, F10M23_200 | 6.4 | 4.7 | 1.7 | 8.8 | 0.06% | 8.4 |
| 1258 | AT1G60950.1 \| Symbol: FED A \| ferredoxin, chloroplast (PETF), identical to FERREDOXIN PRECURSOR GB:P16972 (SP:P16972) from (Arabidopsis thaliana) \| chr1:22448185-22448826 FORWARD \| Aliases: FERRODOXIN A | 10.4 | 6.0 | 4.4 | 8.8 | 0.06% | 7.7 |
| 1260 | AT1G02280.1 \| Symbol: TOC33 \| GTP-binding protein (TOC33), identical to atToc33 protein (GI:11557973) (Arabidopsis thaliana); Carboxyl-terminal end highly similar to GTP-binding protein SP:U43377, location of EST gb:AA394770 and gb:R30089; identical to cDNA for chloroplast atToc33 protein GI:11557972 | 7.5 | 4.1 | 3.4 | 8.8 | 0.06% | 7.7 |
| 1261 | AT3G26980.1 \| ubiquitin family protein, contains INTERPRO:IPR000626 ubiquitin domain \| chr3:9947403-9949277 REVERSE \| Aliases: MOJ10.7 | 8.6 | 5.4 | 3.2 | 8.8 | 0.06% | 8.4 |
| 1262 | AT4G15530.3 \| similar to pyruvate,orthophosphate dikinase [Flaveria brownii] (GB:CAA55784.1); similar to pyruvate,orthophosphate dikinase [Mesembryanthemum crystallinum] (GB:CAA57872.1); similar to pyruvate,orthophosphate dikinase [Mesembryanthemum crystallinum] (GB:CAA55143.1); similar to cold stable pyruvate, orthophosphate dikinase (GB:AAA86940.1); similar to pyruvate, phosphate dikinase (EC 2.7.9.1) precursor, chloroplast - Flaveria brownii (GB:S56649); contains InterPro domain Pyruvate phosphate dikinase, PEP/pyruvate-binding (InterPro:IPR002192); contains InterPro domain PEP-utilizing enzyme (InterPro:IPR000121); contains InterPro domain PEP-utilising enzyme, mobile region (InterPro:IPR008279) \| chr4:8864826-8870905 REVERSE \| Aliases: DL3805C, FCAALL.325 | 5.3 | 2.7 | 2.6 | 8.8 | 0.06% | 7.7 |
| 1263 | AT1G10230.1 \| Symbol: ASK18 \| E3 ubiquitin ligase SCF complex subunit SKP1/ASK1 (At18), putative, E3 ubiquitin ligase; similar to Skp1 homolog Skp1a GI:3068807 (Arabidopsis thaliana) \| chr1:3355573-3356361 FORWARD \| Aliases: ARABIDOPSIS SKP1 LIKE 18, ASK18, F14N23.11, F14N23_11 | 5.5 | 2.7 | 2.8 | 8.8 | 0.06% | 7.7 |
| 1264 | AT1G17350.1 \| auxin-induced-related / indole-3-acetic acid induced-related, similar to auxin-induced protein TGSAUR22 (GI:10185820) (Tulipa gesnerian) | 7.9 | 4.5 | 3.3 | 8.8 | 0.06% | 8.2 |
| 1265 | AT4G38225.3 \| expressed protein \| chr4:17927216-17928888 FORWARD \| Aliases: None | 6.6 | 3.8 | 2.8 | 8.8 | 0.06% | 7.7 |
| 1266 | AT3G08950.1 \| electron transport SCO1/SenC family protein, similar to SP:P23833 SCO1 protein, mitochondrial precursor {Saccharomyces cerevisiae}; contains Pfam profile PF02630: SCO1/SenC \| chr3:2727220-2729606 FORWARD \| Aliases: T16O11.9 | 7.1 | 4.2 | 2.9 | 8.8 | 0.06% | 7.9 |
| 1267 | AT3G01790.2 \| ribosomal protein L13 family protein, similar to putative ribosomal protein L13 GB:AAC07691 (Aquifex aeolicus) \| chr3:283697-285744 REVERSE \| Aliases: F28J7.12, F28J7_12 | 8.0 | 4.2 | 3.8 | 8.8 | 0.06% | 7.9 |
| 1269 | AT3G49720.1 \| expressed protein \| chr3:18450988-18453182 REVERSE \| Aliases: T16K5.70 | 9.7 | 7.0 | 2.7 | 8.8 | 0.06% | 8.4 |
| 1271 | AT3G16100.1 \| Ras-related GTP-binding family protein, contains Pfam profile: PF00071 Ras family \| chr3:5459178-5460783 FORWARD \| Aliases: MSL1.14 | 9.2 | 6.4 | 2.8 | 8.8 | 0.06% | 7.8 |
| 1273 | AT5G64040.2 \| Symbol: PSI N \| similar to photosystem I subunit N [Hordeum vulgare subsp. vulgare] (GB:CAA47056.1); contains InterPro domain Photosystem I reaction centre subunit N (InterPro:IPR008796) \| chr5:25645814-25646841 REVERSE \| Aliases: MHJ24.2, MHJ24_2 | 8.0 | 3.8 | 4.2 | 8.8 | 0.06% | 7.5 |
| 1274 | AT3G55500.1 \| Symbol: ATEXPA16 \| expansin, putative (EXP16), similar to expansin GI:2828241 from (Brassica napus); alpha-expansin gene family, PMID:11641069 \| chr3:20586052-20587125 REVERSE \| Aliases: ARABIDOPSIS THALIANA EXPANSIN A16, ATEXP16, ATHEXP ALPHA 1.7, EXP16, T22E16.160 | 7.3 | 3.4 | 3.8 | 8.8 | 0.06% | 6.6 |
| 1275 | AT2G17280.1 \| phosphoglycerate/bisphosphoglycerate mutase family protein, contains Pfam profile PF00300: phosphoglycerate mutase family \| chr2:7520293-7522122 FORWARD \| Aliases: F5J6.4, F5J6_4 | 9.3 | 4.1 | 5.1 | 8.8 | 0.06% | 7.0 |
| 1276 | AT2G34900.2 \| Symbol: IMB1 \| DNA-binding bromodomain-containing protein, contains bromodomain, INTERPRO:IPR001487 \| chr2:14730221-14732708 REVERSE \| Aliases: F19I3.13, F19I3_13, IMBIBITION INDUCIBLE 1 | 8.3 | 5.1 | 3.2 | 8.8 | 0.06% | 8.1 |

| Rank | Description | Sync | Root | M | t | adj.q | B |
|---|---|---|---|---|---|---|---|
| 1278 | AT2G30350.2 \| endo/excinuclease amino terminal domain-containing protein, contains Pfam domain PF01541: Endo/excinuclease amino terminal domain \| chr2:12941314-12942824 REVERSE \| Aliases: T9D9.16, T9D9_16 | 6.6 | 2.6 | 3.9 | 8.8 | 0.06% | 7.7 |
| 1281 | AT1G23410.1 \| ubiquitin extension protein, putative / 40S ribosomal protein S27A (RPS27aA), strong similarity to ubiquitin extension protein (UBQ5) GB:AAA32906 GI:166934 from (Arabidopsis thaliana) \| chr1:8314913-8315520 FORWARD \| Aliases: F26F24.28, F26F24_28 | 8.0 | 4.6 | 3.3 | 8.8 | 0.06% | 7.2 |
| 1282 | AT4G34620.1 \| Symbol: SSR16 \| ribosomal protein S16 family protein, ribosomal protein S16, Neurospora crassa, PIR2:A29927 \| chr4:16534867-16536176 REVERSE \| Aliases: RIBOSOMAL PROTEIN S16, T4L20.200, T4L20_200 | 11.0 | 9.0 | 2.0 | 8.8 | 0.06% | 8.3 |
| 1285 | AT1G70560.1 \| alliinase C-terminal domain-containing protein, contains Pfam profiles: PF04864 allinase C-terminal domain, PF04863 alliinase EGF-like domain \| chr1:26608518-26611166 FORWARD \| Aliases: F24J13.13, F24J13_13 | 6.5 | 3.0 | 3.5 | 8.8 | 0.06% | 6.7 |
| 1290 | AT3G29185.2 \| expressed protein \| chr3:11156166-11158444 REVERSE \| Aliases: MXO21.1 | 6.7 | 4.7 | 1.9 | 8.7 | 0.06% | 8.2 |
| 1291 | AT4G36710.1 \| scarecrow transcription factor family protein \| chr4:17305762-17307647 FORWARD \| Aliases: AP22.56, AP22_56 | 7.4 | 4.3 | 3.1 | 8.7 | 0.06% | 7.6 |
| 1292 | AT1G32200.2 \| Symbol: ATS1 \| glycerol-3-phosphate acyltransferase, chloroplast (ATS1), identical to SP:Q43307:PLSB_ARATH Glycerol-3-phosphate acyltransferase, chloroplast precursor (EC 2.3.1.15) (GPAT) (ATS1) {Arabidopsis thaliana}; contains Pfam profile PF01553: Acyltransferase \| chr1:11602064-11605040 REVERSE \| Aliases: ACT1, ACYLTRANSFERASE 1, F3C3.13, F3C3_13, GLYCEROL 3 PHOSPHATE ACYLTRANSFERASE | 6.7 | 4.3 | 2.4 | 8.7 | 0.06% | 8.1 |
| 1294 | AT2G19730.2 \| similar to 60S ribosomal protein L28 (RPL28C) [Arabidopsis thaliana] (TAIR:At4g29410.1); similar to putative 60S ribosomal L28 protein [Oryza sativa (japonica cultivar-group)] (GB:AAV67824.1); contains InterPro domain Ribosomal L28e protein (InterPro:IPR002672) \| chr2:8518589-8520303 FORWARD \| Aliases: F6F22.24, F6F22_24 | 11.5 | 9.5 | 1.9 | 8.7 | 0.06% | 8.4 |
| 1295 | AT3G56910.1 \| expressed protein \| chr3:21080395-21081312 REVERSE \| Aliases: T8M16.240 | 9.5 | 7.6 | 1.9 | 8.7 | 0.06% | 8.3 |
| 1306 | AT1G08390.1 \| expressed protein \| chr1:2642211-2643282 FORWARD \| Aliases: T27G7.27 | 6.2 | 3.7 | 2.5 | 8.7 | 0.06% | 7.7 |
| 1310 | AT3G07410.1 \| Ras-related GTP-binding family protein, contains Pfam profile: PF00071 Ras family \| chr3:2372323-2373562 REVERSE \| Aliases: F21O3.12 | 9.3 | 5.2 | 4.1 | 8.7 | 0.07% | 7.8 |
| 1311 | AT5G64860.1 \| Symbol: DPE1 \| 4-alpha-glucanotransferase, putative / disproportionating enzyme, putative, similar to 4-alpha-glucanotransferase SP:Q06801 from (Solanum tuberosum) \| chr5:25942400-25946034 REVERSE \| Aliases: DISPROPORTIONATING ENZYME, MXK3.9, MXK3_9 | 7.3 | 4.3 | 3.0 | 8.7 | 0.07% | 7.8 |
| 1312 | AT3G15790.1 \| Symbol: MBD11 \| methyl-CpG-binding domain-containing protein, contains Pfam profile PF01429: Methyl-CpG binding domain \| chr3:5343216-5344707 FORWARD \| Aliases: MSJ11.19 | 6.9 | 3.7 | 3.2 | 8.7 | 0.07% | 7.7 |
| 1316 | AT4G30620.1 \| expressed protein, contains Pfam domain PF02575: Uncharacterized BCR, YbaB family COG0718; contains similarity to complex interacting protein 9 (CIP9) GI: 32892212 \| chr4:14948592-14950078 REVERSE \| Aliases: F17I23.40, F17I23_40 | 6.6 | 3.0 | 3.5 | 8.7 | 0.07% | 6.8 |
| 1324 | AT3G05070.1 \| expressed protein \| chr3:1416165-1417743 FORWARD \| Aliases: T12H1.3, T12H1_3 | 8.5 | 4.8 | 3.7 | 8.6 | 0.07% | 7.8 |
| 1325 | AT1G54780.1 \| thylakoid lumen 18.3 kDa protein, SP:Q9ZVL6 \| chr1:20443101-20444826 FORWARD \| Aliases: T22H22.19, T22H22_19 | 8.5 | 6.4 | 2.2 | 8.6 | 0.07% | 8.0 |
| 1326 | AT3G02730.1 \| thioredoxin, putative, similar to SP:P29450 Thioredoxin F-type, chloroplast precursor (TRX-F) {Pisum sativum}; contains Pfam profile: PF00085 Thioredoxin \| chr3:588415-589692 REVERSE \| Aliases: F13E7.33, F13E7_33 | 8.2 | 4.3 | 3.9 | 8.6 | 0.07% | 7.0 |
| 1327 | AT5G03970.2 \| similar to F-box family protein [Arabidopsis thaliana] (TAIR:At5g07610.1); similar to unknown protein [Oryza sativa (japonica cultivar-group)] (GB:AAP52196.1); similar to putative F-Box protein [Solanum demissum] (GB:AAT38719.1); contains InterPro domain Cyclin-like F-box (InterPro:IPR001810); contains InterPro domain F-box protein interaction domain (InterPro:IPR006527) \| chr5:1071568-1073395 REVERSE \| Aliases: F8F6.180, F8F6_180 | 7.1 | 4.5 | 2.6 | 8.6 | 0.07% | 7.7 |
| 1331 | AT3G57490.1 \| 40S ribosomal protein S2 (RPS2D), 40S ribosomal protein S2 - Arabidopsis thaliana, SWISSPROT:RS2_ARATH \| chr3:21290643-21291925 REVERSE \| Aliases: T8H10.90 | 8.0 | 5.0 | 3.0 | 8.6 | 0.07% | 7.7 |
| 1335 | AT1G56150.1 \| auxin-responsive family protein, similar to SP:P33082 Auxin-induced protein X15. (Soybean) {Glycine max} | 5.0 | 1.9 | 3.1 | 8.6 | 0.07% | 7.1 |
| 1336 | AT2G24040.1 \| hydrophobic protein, putative / low temperature and salt responsive protein, putative, similar to SP:Q9ZNQ7 Hydrophobic protein RCI2A (Low temperature and salt responsive protein LTI6A) {Arabidopsis thaliana}; contains Pfam profile PF01679: Uncharacterized protein family \| chr2:10231057-10231686 FORWARD \| Aliases: T29E15.24, T29E15_24 | 7.4 | 5.8 | 1.6 | 8.6 | 0.07% | 8.3 |
| 1337 | AT5G59890.2 \| Symbol: ADF4 \| actin-depolymerizing factor 4 (ADF4), identical to SP:Q9ZSK3 Actin-depolymerizing factor 4 (ADF-4) (AtADF4) {Arabidopsis thaliana} \| chr5:24139827-24141138 FORWARD \| Aliases: ACTIN DEPOLYMERIZING FACTOR 4, MMN10.8, MMN10_8 | 12.2 | 7.3 | 5.0 | 8.6 | 0.07% | 8.0 |

| Rank | Description | Sync | Root | M | t | adj.q | B |
|---|---|---|---|---|---|---|---|
| 1338 | AT2G35410.1 \| 33 kDa ribonucleoprotein, chloroplast, putative / RNA-binding protein cp33, putative, similar to SP:P19684 33 kDa ribonucleoprotein, chloroplast precursor {Nicotiana sylvestris}; contains InterPro entry IPR000504: RNA-binding region RNP-1 (RNA recognition motif) (RRM) \| chr2:14905407-14906829 FORWARD \| Aliases: T32F12.21, T32F12_21 | 7.5 | 4.6 | 2.9 | 8.6 | 0.07% | 7.7 |
| 1339 | AT4G30220.1 \| small nuclear ribonucleoprotein F, putative / snRNP-F, putative / Sm protein F, putative, similar to SWISS-PROT:Q15356 small nuclear ribonucleoprotein F (snRNP-F, Sm protein F, Sm-F, SmF) (Mouse) \| chr4:14802961-14804329 REVERSE \| Aliases: F9N11.70, F9N11_70 | 8.3 | 5.3 | 3.0 | 8.6 | 0.07% | 7.5 |
| 1340 | AT5G64670.1 \| ribosomal protein L15 family protein \| chr5:25869493-25871215 REVERSE \| Aliases: MUB3.19, MUB3_19 | 8.2 | 6.5 | 1.7 | 8.6 | 0.07% | 8.1 |
| 1342 | AT4G27090.1 \| 60S ribosomal protein L14 (RPL14B), ribosomal protein L14 - Human,PIR3:JC5954 \| chr4:13593911-13595257 REVERSE \| Aliases: T24A18.40, T24A18_40 | 12.8 | 11.4 | 1.4 | 8.6 | 0.07% | 8.3 |
| 1344 | AT1G72170.1 \| expressed protein, contains Pfam PF04418: Domain of unknown function (DUF543) \| chr1:27159997-27161338 FORWARD \| Aliases: T9N14.2 | 9.6 | 7.1 | 2.5 | 8.6 | 0.07% | 8.1 |
| 1347 | AT3G07110.2 \| similar to 60S ribosomal protein L13A (RPL13aD) [Arabidopsis thaliana] (TAIR:At5g48760.1); similar to ribosomal protein L13a [Lupinus luteus] (GB:CAA11283.1); contains InterPro domain Ribosomal protein L13, bacterial and organelle form (InterPro:IPR005823); contains InterPro domain Ribosomal protein L13, archea and eukaryotic form (InterPro:IPR005755); contains InterPro domain Ribosomal protein L13 (InterPro:IPR005822) \| chr3:2252025-2253534 FORWARD \| Aliases: T1B9.24, T1B9_24 | 11.5 | 9.2 | 2.3 | 8.6 | 0.07% | 8.3 |
| 1348 | AT4G38400.1 \| Symbol: ATEXLA2 \| expansin family protein (EXPL2), contains Pfam profile: PF01357 pollen allergen; expansin-like gene, PMID:11641069, www.bio.psu.edu/expansins | 6.4 | 3.2 | 3.2 | 8.6 | 0.07% | 6.8 |
| 1349 | AT5G10300.1 \| hydrolase, alpha/beta fold family protein, similar to ethylene-induced esterase (Citrus sinensis) GI:14279437, alpha-hydroxynitrile lyase (Manihot esculenta) GI:2780225; contains Pfam profile PF00561: hydrolase, alpha/beta fold family \| chr5:3239624-3240871 FORWARD \| Aliases: F18D22.70, F18D22_70 | 7.9 | 6.0 | 2.0 | 8.6 | 0.07% | 8.0 |
| 1351 | AT2G47260.1 \| Symbol: WRKY23 \| | 6.3 | 4.4 | 1.9 | 8.6 | 0.07% | 8.1 |
| 1353 | AT2G37990.1 \| ribosome biogenesis regulatory protein (RRS1) family protein, contains Pfam profile PF04939: Ribosome biogenesis regulatory protein (RRS1); similar to Ribosome biogenesis regulatory protein homolog (Swiss-Prot:Q15050) (Homo sapiens) | 9.7 | 6.6 | 3.1 | 8.6 | 0.07% | 7.6 |
| 1354 | AT2G15000.2 \| expressed protein \| chr2:6488440-6489469 REVERSE \| Aliases: T15J14.4, T15J14_4 | 6.6 | 3.9 | 2.8 | 8.6 | 0.07% | 7.9 |
| 1355 | AT3G15190.1 \| chloroplast 30S ribosomal protein S20, putative, contains Pfam profile: PF01649 ribosomal protein S20 \| chr3:5116154-5117622 FORWARD \| Aliases: F4B12.10 | 7.2 | 5.4 | 1.8 | 8.6 | 0.07% | 8.0 |
| 1359 | AT1G24610.1 \| SET domain-containing protein, low similarity to SP:Q43088 Ribulose-1,5 bisphosphate carboxylase/oxygenase large subunit N- methyltransferase, chloroplast precursor (EC 2.1.1.127) {Pisum sativum}; contains Pfam profile PF00856: SET domain \| chr1:8720177-8722732 REVERSE \| Aliases: F21J9.27 | 8.6 | 5.7 | 2.8 | 8.6 | 0.07% | 7.9 |
| 1360 | AT2G16070.2 \| expressed protein \| chr2:6990974-6992544 REVERSE \| Aliases: F7H1.9, F7H1_9 | 8.2 | 5.8 | 2.4 | 8.6 | 0.07% | 8.0 |
| 1367 | AT3G18130.1 \| guanine nucleotide-binding family protein / activated protein kinase C receptor (RACK1), identical to guanine nucleotide-binding protein; activated protein kinase C receptor; RACK1 (GI:9294068) {Arabidopsis thaliana}; contains Pfam profile: PF00400 WD domain, G-beta repeat (7 copies) \| chr3:6210912-6212439 REVERSE \| Aliases: MRC8.11 | 10.0 | 6.2 | 3.7 | 8.5 | 0.07% | 7.7 |
| 1368 | AT3G14780.1 \| similar to glycosyl transferase family 48 protein [Arabidopsis thaliana] (TAIR:At3g14570.1); similar to putative callose synthase 1 catalytic subunit [Oryza sativa (japonica cultivar-group)] (GB:XP_468556.1) \| chr3:4961813-4963513 REVERSE \| Aliases: T21E2.4 | 5.6 | 4.0 | 1.7 | 8.5 | 0.07% | 8.2 |
| 1370 | AT4G32300.1 \| lectin protein kinase family protein, contains Pfam domains, PF01453: Lectin (probable mannose binding) and PF00069: Protein kinase domain \| chr4:15599481-15602601 FORWARD \| Aliases: F10M6.60, F10M6_60 | 7.8 | 5.0 | 2.8 | 8.5 | 0.07% | 7.5 |
| 1373 | AT3G56340.1 \| 40S ribosomal protein S26 (RPS26C), several 40S ribosomal protein S26 \| chr3:20903116-20904395 REVERSE \| Aliases: T5P19.4 | 11.5 | 8.6 | 2.9 | 8.5 | 0.07% | 8.2 |
| 1376 | AT5G56670.1 \| 40S ribosomal protein S30 (RPS30C) \| chr5:22952639-22953207 REVERSE \| Aliases: MIK19.12 | 11.5 | 9.4 | 2.1 | 8.5 | 0.07% | 8.2 |
| 1377 | AT5G57930.2 \| Symbol: APO2 \| expressed protein \| chr5:23471843-23473719 FORWARD \| Aliases: ACCUMULATION OF PHOTOSYSTEM ONE 2, EMB1629, EMBRYO DEFECTIVE 1629, MTI20.19, MTI20_19 | 7.0 | 5.2 | 1.7 | 8.5 | 0.07% | 8.1 |
| 1379 | AT3G54960.1 \| Symbol: ATPDIL1 3 \| Encodes a protein disulfide isomerase-like (PDIL) protein, a member of a multigene family within the thioredoxin (TRX) superfamily. | 8.4 | 5.3 | 3.0 | 8.5 | 0.07% | 7.6 |

| Rank | Description | Sync | Root | M | t | adj.q | B |
|---|---|---|---|---|---|---|---|
| 1384 | AT4G37930.1 \| Symbol: SHM1 \| glycine hydroxymethyltransferase / serine hydroxymethyltransferase / serine/threonine aldolase (SHM1), identical to serine hydroxymethyl transferase (Arabidopsis thaliana) GI:6899945 \| chr4:17831740-17834859 REVERSE \| Aliases: F20D10.50, F20D10_50, SERINE HYDROXYMETHYLTRANSFERASE 1, SERINE TRANSHYDROXYMETHYLASE, SERINE TRANSHYDROXYMETHYLTRANSFERASE, STM | 11.5 | 8.3 | 3.2 | 8.5 | 0.07% | 8.1 |
| 1385 | AT5G07020.1 \| proline-rich family protein \| chr5:2180503-2182387 REVERSE \| Aliases: MOJ9.19, MOJ9_19 | 6.9 | 3.9 | 2.9 | 8.5 | 0.07% | 7.4 |
| 1386 | AT5G42310.1 \| pentatricopeptide (PPR) repeat-containing protein, contains Pfam profile PF01535: PPR repeat \| chr5:16933011-16936156 FORWARD \| Aliases: K5J14.11, K5J14_11 | 5.8 | 2.9 | 2.9 | 8.5 | 0.07% | 7.6 |
| 1389 | AT5G47700.1 \| 60S acidic ribosomal protein P1 (RPP1C) \| chr5:19345029-19346209 REVERSE \| Aliases: MCA23.2, MCA23_2 | 10.3 | 8.2 | 2.0 | 8.5 | 0.07% | 7.9 |
| 1390 | AT3G60245.1 \| 60S ribosomal protein L37a (RPL37aC) \| chr3:22279721-22280981 FORWARD \| Aliases: None | 13.7 | 12.6 | 1.2 | 8.5 | 0.07% | 8.1 |
| 1393 | AT2G47780.1 \| rubber elongation factor (REF) protein-related, similar to Small rubber particle protein (SRPP) (22 kDa rubber particle protein) (22 kDa RPP) (Latex allergen Hev b 3) (27 kDa natural rubber allergen) (Swiss-Prot:O82803) (Hevea brasiliensis); similar to Stress-related protein (Swiss-Prot:Q9SW70) (Vitis riparia) \| chr2:19577132-19578494 FORWARD \| Aliases: F17A22.17 | 6.7 | 3.0 | 3.7 | 8.5 | 0.07% | 6.6 |
| 1394 | AT4G09800.1 \| Symbol: RPS18C \| | 11.2 | 10.0 | 1.1 | 8.5 | 0.07% | 8.0 |
| 1398 | AT1G17880.1 \| nascent polypeptide-associated complex (NAC) domain-containing protein / BTF3b-like transcription factor, putative, similar to SP:P20290 Transcription factor BTF3 (RNA polymerase B transcription factor 3) {Homo sapiens}; contains Pfam profile PF01849: NAC domain; identical to cDNA BTF3b-like factor GI:5912423 \| chr1:6152420-6153946 REVERSE \| Aliases: F2H15.11, F2H15_11 | 9.8 | 6.4 | 3.4 | 8.5 | 0.07% | 7.2 |
| 1402 | AT1G27120.1 \| galactosyltransferase family protein, contains Pfam profile:PF01762 galactosyltransferase \| chr1:9421164-9424054 FORWARD \| Aliases: T7N9.18, T7N9_18 | 6.1 | 2.9 | 3.2 | 8.5 | 0.08% | 7.3 |
| 1403 | AT5G64460.5 \| similar to phosphoglycerate/bisphosphoglycerate mutase family protein [Arabidopsis thaliana] (TAIR:At2g17280.1); similar to phosphoglycerate mutase-like protein [Glycine max] (GB:AAG38145.1) \| chr5:25790075-25792761 REVERSE \| Aliases: T12B11.5, T12B11_5 | 10.7 | 7.4 | 3.3 | 8.5 | 0.08% | 7.8 |
| 1405 | AT5G02590.1 \| chloroplast lumen common family protein, various predicted proteins, Arabidopsis thaliana \| chr5:583084-584177 FORWARD \| Aliases: T22P11.180, T22P11_180 | 5.0 | 2.5 | 2.5 | 8.5 | 0.08% | 7.7 |
| 1406 | AT1G59990.1 \| DEAD/DEAH box helicase, putative (RH22), similar to RNA helicase GI:3776015 from (Arabidopsis thaliana); contains Pfam profiles PF00271: Helicase conserved C-terminal domain, PF00270: DEAD/DEAH box helicase; matches EST OAO811-2 \| chr1:22093904-22096667 REVERSE \| Aliases: T2K10.4, T2K10_4 | 7.9 | 4.6 | 3.2 | 8.4 | 0.08% | 7.7 |
| 1407 | AT5G47320.1 \| Symbol: RPS19 \| 30S ribosomal protein S19, mitochondrial (RPS19) \| chr5:19220379-19222499 FORWARD \| Aliases: MQL5.18, MQL5_18 | 8.6 | 5.7 | 2.9 | 8.4 | 0.08% | 7.9 |
| 1411 | AT5G23850.1 \| expressed protein \| chr5:8038129-8040934 FORWARD \| Aliases: MRO11.11, MRO11_11 | 6.9 | 4.6 | 2.3 | 8.4 | 0.08% | 7.6 |
| 1412 | AT3G07770.1 \| heat shock protein-related, strong similarity to heat-shock protein (Secale cereale) GI:556673; contains Pfam profiles PF02518: ATPase, histidine kinase-, DNA gyrase B-, and HSP90-like domain protein, PF00183: Hsp90 protein \| chr3:2479561-2484171 FORWARD \| Aliases: MLP3.22 | 9.3 | 5.2 | 4.1 | 8.4 | 0.08% | 7.8 |
| 1413 | AT5G08590.1 \| Symbol: ASK2 \| serine/threonine protein kinase (ASK2), identical to serine/threonine-protein kinase ASK2, SWISS-PROT: P43292; contains protein kinase domain, Pfam:PF00069 \| chr5:2783410-2786095 FORWARD \| Aliases: ARABIDOPSIS SERINE/THREONINE KINASE 2, MAH20.15, MAH20_15 | 8.1 | 5.4 | 2.8 | 8.4 | 0.08% | 7.9 |
| 1416 | AT4G24260.1 \| endo-1,4-beta-glucanase, putative / cellulase, putative, similar to endo-1,4-beta-D-glucanase; cellulase GI:5689613 from (Brassica napus) \| chr4:12577881-12580143 REVERSE \| Aliases: T22A6.90, T22A6_90 | 7.2 | 3.7 | 3.5 | 8.4 | 0.08% | 7.0 |
| 1418 | AT4G22890.3 \| expressed protein \| chr4:12006997-12009529 FORWARD \| Aliases: F7H19.70, F7H19_70 | 9.5 | 6.6 | 2.9 | 8.4 | 0.08% | 8.0 |
| 1420 | AT5G56910.1 \| expressed protein \| chr5:23040148-23041428 FORWARD \| Aliases: MHM17.2, MHM17_2 | 8.5 | 4.9 | 3.6 | 8.4 | 0.08% | 7.6 |
| 1421 | AT2G34260.2 \| transducin family protein / WD-40 repeat family protein, similar to Vegetatible incompatibility protein HET-E-1 (SP:Q00808) {Podospora anserina} | 7.3 | 5.0 | 2.3 | 8.4 | 0.08% | 7.9 |
| 1423 | AT4G15930.1 \| dynein light chain, putative, similar to dynein light chain 2 (Mus musculus) GI:15545995; contains Pfam profile PF01221: Dynein light chain type 1 \| chr4:9036236-9038077 FORWARD \| Aliases: DL4005W, FCAALL.238 | 10.3 | 8.8 | 1.4 | 8.4 | 0.08% | 8.1 |
| 1424 | AT1G10360.1 \| Symbol: ATGSTU18 \| glutathione S-transferase, putative, similar to glutathione S-transferase (sp:Q03666:GTX4_TOBAC); similar to EST gb:H36275 gb:AB039930. \| chr1:3395560-3396851 REVERSE \| Aliases: F14N23.24, F14N23_24, GLUTATHIONE S TRANSFERASE 29, GST29 | 5.9 | 2.7 | 3.3 | 8.4 | 0.08% | 7.0 |

| Rank | Description | Sync | Root | M | t | adj.q | B |
|---|---|---|---|---|---|---|---|
| 1425 | AT4G16720.1 \| 60S ribosomal protein L15 (RPL15A) \| chr4:9399987-9401404 REVERSE \| Aliases: DL4385C, FCAALL.416 | 11.9 | 10.6 | 1.3 | 8.4 | 0.08% | 8.1 |
| 1426 | AT4G26780.1 \| Symbol: AR192 \| co-chaperone grpE family protein, similar to chaperone GrpE type 2 (Nicotiana tabacum) GI:3851640; contains Pfam profile PF01025: co-chaperone GrpE \| chr4:13484845-13486777 REVERSE \| Aliases: F10M23.120, F10M23_120 | 7.9 | 5.3 | 2.6 | 8.4 | 0.08% | 7.9 |
| 1427 | AT5G20040.2 \| Symbol: ATIPT9 \| | 8.4 | 5.1 | 3.3 | 8.4 | 0.08% | 7.4 |
| 1429 | AT3G50360.1 \| Symbol: ATCEN2 \| | 8.8 | 6.3 | 2.5 | 8.4 | 0.08% | 8.0 |
| 1430 | AT4G18400.1 \| expressed protein \| chr4:10169877-10171268 REVERSE \| Aliases: F28J12.60, F28J12_60 | 7.8 | 6.0 | 1.8 | 8.4 | 0.08% | 8.1 |
| 1431 | AT1G12900.1 \| glyceraldehyde 3-phosphate dehydrogenase, chloroplast, putative / NADP-dependent glyceraldehydephosphate dehydrogenase, putative, similar to SP:P25856 Glyceraldehyde 3-phosphate dehydrogenase A, chloroplast precursor (EC 1.2.1.13) (NADP-dependent glyceraldehydephosphate dehydrogenase subunit A) {Arabidopsis thaliana}; contains Pfam profiles PF02800: Glyceraldehyde 3-phosphate dehydrogenase C-terminal domain, PF00044: Glyceraldehyde 3-phosphate dehydrogenase NAD binding domain | 7.5 | 2.9 | 4.6 | 8.4 | 0.08% | 6.9 |
| 1438 | AT5G44710.1 \| expressed protein, similar to unknown protein (ref:NP_011731.1) \| chr5:18058153-18059787 FORWARD \| Aliases: K23L20.5, K23L20_5 | 8.8 | 5.5 | 3.4 | 8.4 | 0.08% | 7.7 |
| 1439 | AT1G10310.1 \| short-chain dehydrogenase/reductase (SDR) family protein, contains INTERPRO family IPR002198 short-chain dehydrogenase/reductase (SDR) superfamily \| chr1:3381605-3383916 REVERSE \| Aliases: F14N23.19, F14N23_19 | 8.3 | 6.5 | 1.8 | 8.4 | 0.08% | 7.9 |
| 1442 | AT3G29280.1 \| expressed protein, ; expression supported by MPSS \| chr3:11237942-11239184 REVERSE \| Aliases: MMF24.3 | 6.3 | 4.0 | 2.3 | 8.4 | 0.08% | 7.7 |
| 1443 | AT1G14810.1 \| semialdehyde dehydrogenase family protein, similar to SP:O31219 Aspartate-semialdehyde dehydrogenase (EC 1.2.1.11) (ASA dehydrogenase) (ASADH) {Legionella pneumophila}; contains Pfam profiles PF02774: Semialdehyde dehydrogenase dimerisation domain, PF01118: Semialdehyde dehydrogenase NAD binding domain \| chr1:5102419-5104684 REVERSE \| Aliases: F10B6.22, F10B6_22 | 11.1 | 7.5 | 3.5 | 8.4 | 0.08% | 7.7 |
| 1445 | AT2G45070.3 \| Symbol: SEC61 BETA \| similar to sec61beta family protein [Arabidopsis thaliana] (TAIR:At3g60540.1); similar to sec61beta family protein [Arabidopsis thaliana] (TAIR:At3g60540.2); similar to GA10096-PA [Drosophila pseudoobscura] (GB:EAL25183.1); contains InterPro domain Sec61beta (InterPro:IPR005609) \| chr2:18594092-18595334 REVERSE \| Aliases: SEC 61 BETA SUBUNIT, T14P1.12 | 12.1 | 10.6 | 1.5 | 8.3 | 0.08% | 8.1 |
| 1447 | AT5G50870.1 \| ubiquitin-conjugating enzyme, putative, strong similarity to ubiquitin conjugating enzyme (Lycopersicon esculentum) GI:886679; contains Pfam profile PF00179: Ubiquitin-conjugating enzyme \| chr5:20716709-20718302 REVERSE \| Aliases: K3K7.1, K3K7_1 | 10.2 | 6.0 | 4.2 | 8.3 | 0.08% | 8.0 |
| 1448 | AT5G50720.1 \| Symbol: ATHVA22E \| ABA-responsive protein (HVA22e), identical to AtHVA22e (Arabidopsis thaliana) GI:11225589 \| chr5:20650422-20651778 REVERSE \| Aliases: ATHVA22E, MFB16.12, MFB16_12 | 7.6 | 4.4 | 3.2 | 8.3 | 0.08% | 7.6 |
| 1449 | AT1G15700.1 \| Symbol: ATPC2 \| ATP synthase gamma chain 2, chloroplast (ATPC2), identical to SP:Q01909 ATP synthase gamma chain 2, chloroplast precursor (EC 3.6.3.14) {Arabidopsis thaliana}; contains Pfam profile: PF00231 ATP synthase; similar to ATP synthase gamma-subunit GI:21241 from (Spinacia oleracea) \| chr1:5402564-5403813 REVERSE \| Aliases: F7H2.4, F7H2_4 | 6.7 | 4.3 | 2.5 | 8.3 | 0.08% | 7.8 |
| 1453 | AT2G40480.1 \| expressed protein, similar to expressed protein [Arabidopsis thaliana] (TAIR:At3g56270.1); similar to unknown protein [Oryza sativa (japonica cultivar-group)] (GB:AAU44189.1); contains InterPro domain Plant protein of unknown function DUF827 (InterPro:IPR008545) \| chr2:16917442-16919670 FORWARD \| Aliases: T2P4.17, T2P4_17 | 7.0 | 5.0 | 1.9 | 8.3 | 0.08% | 8.0 |
| 1454 | AT5G56940.1 \| ribosomal protein S16 family protein, contains Pfam profile PF00886: ribosomal protein S16 \| chr5:23048027-23049637 FORWARD \| Aliases: MHM17.5, MHM17_5 | 9.6 | 7.4 | 2.2 | 8.3 | 0.08% | 7.9 |
| 1455 | AT5G38520.1 \| hydrolase, alpha/beta fold family protein, low similarity to hydrolase (Terrabacter sp. DBF63) GI:14196240; contains Pfam profile PF00561: hydrolase, alpha/beta fold family \| chr5:15438804-15440661 FORWARD \| Aliases: MBB18.5, MBB18_5 | 5.0 | 2.2 | 2.8 | 8.3 | 0.08% | 7.3 |
| 1456 | AT3G58610.1 \| ketol-acid reductoisomerase, identical to ketol-acid reductoisomerase, chloroplast precursor (EC 1.1.1.86) (Acetohydroxy-acid reductoisomerase) (Alpha-keto-beta-hydroxylacil reductoisomerase) (Swiss-Prot:Q05758) (Arabidopsis thaliana) \| chr3:21682429-21685818 FORWARD \| Aliases: F14P22.200, F14P22_200 | 12.5 | 10.3 | 2.2 | 8.3 | 0.08% | 8.0 |
| 1458 | AT2G33450.1 \| 50S ribosomal protein L28, chloroplast (CL28) \| chr2:14180655-14181609 FORWARD \| Aliases: F4P9.22, F4P9_22 | 6.1 | 2.8 | 3.3 | 8.3 | 0.08% | 7.1 |
| 1460 | AT2G04530.1 \| Symbol: CPZ \| RNase Z, 97% identical to RNase Z (GI:20975607) (Arabidopsis thaliana); similar to RNase Z (GI:20975609) (Arabidopsis thaliana); identical to cDNA RNase Z (At2g04530) GI:20975606 \| chr2:1576768-1578738 FORWARD \| Aliases: T1O3.6, T1O3_6 | 5.6 | 2.6 | 3.0 | 8.3 | 0.08% | 7.0 |

| Rank | Description | Sync | Root | M | t | adj.q | B |
|---|---|---|---|---|---|---|---|
| 1461 | AT1G06010.1 \| expressed protein \| chr1:1822617-1823996 FORWARD \| Aliases: T21E18.6, T21E18_6 | 6.4 | 3.5 | 2.9 | 8.3 | 0.08% | 7.7 |
| 1462 | AT4G22160.2 \| expressed protein \| chr4:11733135-11734039 FORWARD \| Aliases: T10I14.8 | 6.0 | 3.6 | 2.3 | 8.3 | 0.08% | 7.7 |
| 1463 | AT1G67430.1 \| 60S ribosomal protein L17 (RPL17B), similar to ribosomal protein GI:19101 from (Hordeum vulgare) | 12.1 | 10.0 | 2.1 | 8.3 | 0.08% | 8.0 |
| 1466 | AT1G68540.1 \| oxidoreductase family protein, similar to cinnamoyl CoA reductase (Eucalyptus gunnii, gi:2058311), cinnamyl-alcohol dehydrogenase, E. gunnii (gi:1143445), CPRD14 protein, Vigna unguiculata (gi:1854445) \| chr1:25723725-25725028 FORWARD \| Aliases: T26J14.11, T26J14_11 | 7.1 | 4.0 | 3.0 | 8.3 | 0.08% | 6.8 |
| 1467 | AT1G71720.1 \| S1 RNA-binding domain-containing protein, contains Pfam domain, PF00575: S1 RNA binding domain \| chr1:26987406-26989652 FORWARD \| Aliases: F14O23.10, F14O23_10 | 8.4 | 5.7 | 2.7 | 8.3 | 0.08% | 7.6 |
| 1470 | AT2G37470.1 \| histone H2B, putative, strong similarity to histone H2B from Lycopersicon esculentum GI:3021483, GI:3021485, Capsicum annuum SP:O49118; contains Pfam profile PF00125 Core histone H2A/H2B/H3/H4 | 8.9 | 5.4 | 3.5 | 8.3 | 0.08% | 7.2 |
| 1471 | AT4G01150.1 \| expressed protein \| chr4:493548-494795 FORWARD \| Aliases: F2N1.18, F2N1_18 | 10.1 | 6.7 | 3.4 | 8.3 | 0.08% | 7.7 |
| 1472 | AT1G18210.2 \| calcium-binding protein, putative, similar to SP:Q9M7R0 Calcium-binding allergen Ole e 8 (PCA18/PCA23) {Olea europaea}; contains INTERPRO:IPR002048 calcium-binding EF-hand domain \| chr1:6266602-6268821 REVERSE \| Aliases: T10F20.22 | 12.4 | 8.8 | 3.6 | 8.3 | 0.08% | 8.0 |
| 1473 | AT3G50440.1 \| similar to esterase, putative [Arabidopsis thaliana] (TAIR:At2g23620.1); similar to methyl jasmonate esterase [Solanum tuberosum] (GB:AAV87151.1); contains InterPro domain Esterase/lipase/thioesterase (InterPro:IPR000379); contains InterPro domain Alpha/beta hydrolase fold (InterPro:IPR000073) \| chr3:18728292-18729475 REVERSE \| Aliases: T20E23.40 | 7.5 | 3.7 | 3.8 | 8.3 | 0.08% | 6.3 |
| 1474 | AT1G52220.2 \| expressed protein, similar to expressed protein [Arabidopsis thaliana] (TAIR:At2g46820.1); similar to unknown protein [Oryza sativa (japonica cultivar-group)] (GB:XP_478022.1) \| chr1:19457238-19458366 REVERSE \| Aliases: F9I5.10, F9I5_10 | 6.2 | 3.1 | 3.1 | 8.3 | 0.08% | 6.8 |
| 1476 | AT5G57280.1 \| expressed protein \| chr5:23221696-23223877 FORWARD \| Aliases: MJB24.9, MJB24_9 | 8.5 | 6.0 | 2.5 | 8.3 | 0.08% | 7.7 |
| 1478 | AT2G21280.1 \| Symbol: GC1 \| expressed protein, similar to YfhF (GI:2804536) (Bacillus subtilis) \| chr2:9117394-9119776 REVERSE \| Aliases: GIANT CHLOROPLAST 1 | 7.0 | 3.4 | 3.5 | 8.3 | 0.08% | 7.2 |
| 1479 | AT5G24010.1 \| protein kinase family protein, contains protein kinase domain, Pfam:PF00069 \| chr5:8113798-8116618 FORWARD \| Aliases: MZF18.11, MZF18_11 | 6.3 | 3.9 | 2.4 | 8.3 | 0.08% | 7.9 |
| 1480 | AT1G07700.3 \| thioredoxin family protein, low similarity to thioredoxin (Gallus gallus) GI:212766; contains Pfam profile: PF00085 Thioredoxin \| chr1:2379644-2381357 FORWARD \| Aliases: F24B9.21, F24B9_21 | 10.3 | 7.2 | 3.1 | 8.3 | 0.08% | 7.7 |
| 1481 | AT2G03430.1 \| ankyrin repeat family protein, contains ankyrin repeats, Pfam:PF00023 \| chr2:1036029-1037613 REVERSE \| Aliases: T4M8.14, T4M8_14 | 7.6 | 5.9 | 1.7 | 8.3 | 0.08% | 8.0 |
| 1482 | AT4G10100.2 \| Symbol: CNX7/SIR5 \| molybdenum cofactor synthesis family protein, similar to Molybdenum cofactor synthesis protein 2 small subunit (Molybdopterin-synthase small subunit) (MOCS2A) (MOCO1-A) (Swiss-Prot:O96033) (Homo sapiens); contains TIGRFAM TIGR01682: molybdopterin converting factor, subunit 1; contains Pfam PF02597: ThiS family \| chr4:6308712-6309504 FORWARD \| Aliases: CNX7, F28M11.20, F28M11_20, MOLYBDOPTERIN SYNTHASE, SIR5 | 11.9 | 10.8 | 1.1 | 8.3 | 0.08% | 7.8 |
| 1485 | AT2G35635.1 \| Symbol: UBQ7 \| ubiquitin family protein, contains INTERPRO:IPR000626 ubiquitin domain \| chr2:14988073-14989195 FORWARD \| Aliases: None | 10.5 | 6.6 | 3.9 | 8.3 | 0.08% | 7.7 |
| 1486 | AT4G29660.1 \| Symbol: EMB2752 \| expressed protein, predicted proteins, Arabidopsis thaliana \| chr4:14534269-14535298 REVERSE \| Aliases: EMB2752, EMBRYO DEFECTIVE 2752, T16L4.170, T16L4_170 | 8.3 | 6.3 | 2.0 | 8.3 | 0.08% | 7.9 |
| 1487 | AT5G23290.1 \| c-myc binding protein, putative / prefoldin, putative, similar to Swiss-Prot:Q99471 prefoldin subunit 5 (C-myc binding protein Mm-1) (Myc modulator 1) (Homo sapiens) \| chr5:7846084-7847626 FORWARD \| Aliases: MKD15.15, MKD15_15 | 6.7 | 4.1 | 2.7 | 8.3 | 0.08% | 7.4 |
| 1488 | AT3G04550.1 \| expressed protein \| chr3:1225928-1227442 FORWARD \| Aliases: F7O18.2, F7O18_2 | 9.6 | 7.5 | 2.1 | 8.3 | 0.08% | 8.0 |
| 1489 | AT4G27660.1 \| expressed protein, ; expression supported by MPSS \| chr4:13816890-13818033 REVERSE \| Aliases: T29A15.150, T29A15_150 | 6.4 | 3.3 | 3.1 | 8.3 | 0.08% | 7.0 |
| 1490 | AT5G03050.1 \| expressed protein \| chr5:715028-716320 REVERSE \| Aliases: F15A17.80, F15A17_80 | 6.4 | 2.8 | 3.6 | 8.3 | 0.08% | 7.6 |
| 1491 | AT1G75690.1 \| chaperone protein dnaJ-related, contains Pfam PF00684 : DnaJ central domain (4 repeats); similar to bundle sheath defective protein 2 (GI:4732091) (Zea mays) \| chr1:28425771-28426880 REVERSE \| Aliases: F10A5.12, F10A5_12 | 6.2 | 2.7 | 3.4 | 8.3 | 0.08% | 7.2 |

| Rank | Description | Sync | Root | M | t | adj.q | B |
|---|---|---|---|---|---|---|---|
| 1495 | AT5G54580.1 \| RNA recognition motif (RRM)-containing protein, low similarity to RNA-binding protein RGP-3 (Nicotiana sylvestris) GI:1009363; contains InterPro entry IPR000504: RNA-binding region RNP-1 (RNA recognition motif) (RRM) | 8.4 | 6.4 | 2.0 | 8.3 | 0.08% | 7.9 |
| 1497 | AT2G35520.2 \| defender against cell death 2 (DAD2), identical to defender against cell death 2 (DAD-2, AtDAD2) (Arabidopsis thaliana) SWISS-PROT:O22622 \| chr2:14928572-14929920 FORWARD \| Aliases: T32F12.10, T32F12_10 | 8.3 | 4.7 | 3.6 | 8.2 | 0.09% | 7.7 |
| 1500 | AT1G04290.1 \| thioesterase family protein, contains Pfam profile PF03061: thioesterase family protein; EST gb:T45093 comes from this gene \| chr1:1147566-1148532 REVERSE \| Aliases: F19P19.27, F19P19_27 | 7.6 | 4.7 | 2.9 | 8.2 | 0.09% | 7.7 |
| 1503 | AT2G43460.1 \| 60S ribosomal protein L38 (RPL38A) \| chr2:18053081-18054478 REVERSE \| Aliases: T1O24.20 | 10.6 | 8.5 | 2.1 | 8.2 | 0.09% | 8.0 |
| 1505 | AT4G02770.1 \| photosystem I reaction center subunit II, chloroplast, putative / photosystem I 20 kDa subunit, putative / PSI-D, putative (PSAD1), similar to SP:P12353 Photosystem I reaction center subunit II, chloroplast precursor (Photosystem I 20 kDa subunit) (PSI-D) {Spinacia oleracea}; contains Pfam profile PF02531: PsaD | 8.0 | 4.3 | 3.7 | 8.2 | 0.09% | 7.3 |
| 1508 | AT4G25600.1 \| ShTK domain-containing protein, similar to PBCV-1 prolyl 4-hydroxylase (Paramecium bursaria Chlorella virus 1) GI:1131429; contains Pfam profile PF01549: ShTK domain \| chr4:13060668-13062604 FORWARD \| Aliases: M7J2.30, M7J2_30 | 7.1 | 4.9 | 2.1 | 8.2 | 0.09% | 7.7 |
| 1511 | AT2G34770.1 \| Symbol: FAH1 \| fatty acid hydroxylase (FAH1), identical to fatty acid hydroxylase Fah1p GB:AF021804 GI:2736147 from (Arabidopsis thaliana) \| chr2:14672746-14675329 FORWARD \| Aliases: FAH1P, FATTY ACID HYDROXYLASE 1, T29F13.2, T29F13_2 | 7.7 | 4.2 | 3.5 | 8.2 | 0.09% | 7.8 |
| 1512 | AT2G19385.1 \| expressed protein, weak similarity to Cell growth regulating nucleolar protein (Swiss-Prot:Q08288) (Mus musculus) \| chr2:8394117-8395794 FORWARD \| Aliases: None | 5.7 | 3.4 | 2.3 | 8.2 | 0.09% | 7.6 |
| 1514 | AT1G63970.2 \| Symbol: ISPF \| 2C-methyl-D-erythritol 2,4-cyclodiphosphate synthase, putative, similar to 2C-methyl-D-erythritol 2,4-cyclodiphosphate synthase GI:7621712 from (Catharanthus roseus) \| chr1:23742354-23743999 REVERSE \| Aliases: 2C METHYL D ERYTHRITOL 2,4 CYCLODIPHOSPHATE SYNTHASE, T12P18.1, T12P18_1 | 7.3 | 4.2 | 3.2 | 8.2 | 0.09% | 7.5 |
| 1518 | AT5G08610.1 \| DEAD box RNA helicase (RH26), strong similarity to RNA helicase RH26 (Arabidopsis thaliana) GI:3776025; contains Pfam profiles PF00270: DEAD/DEAH box helicase, PF00271: Helicase conserved C-terminal domain; identical to cDNA DEAD box RNA helicase, RH26 GI:3776024 \| chr5:2790296-2794216 FORWARD \| Aliases: MAH20.17, MAH20_17 | 6.5 | 3.8 | 2.8 | 8.2 | 0.09% | 7.3 |
| 1519 | AT1G73020.1 \| similar to PREDICTED: similar to expressed sequence AI604832 [Rattus norvegicus] (GB:XP_236774.3); contains InterPro domain Protein of unknown function DUF590 (InterPro:IPR007632) \| chr1:27473211-27477145 REVERSE \| Aliases: F3N23.22, F3N23_22 | 7.4 | 4.2 | 3.3 | 8.2 | 0.09% | 6.8 |
| 1521 | AT3G60810.2 \| expressed protein \| chr3:22481494-22483079 FORWARD \| Aliases: T4C21.220 | 6.9 | 5.3 | 1.7 | 8.2 | 0.09% | 7.8 |
| 1522 | AT2G44650.1 \| Symbol: CHL CPN10 \| chloroplast chaperonin 10 (cpn10), identical to chloroplast chaperonin 10 GI:14041813 from (Arabidopsis thaliana) \| chr2:18426478-18428186 REVERSE \| Aliases: CHLOROPLAST CHAPERONIN 10, CPN10, F16B22.14 | 8.4 | 6.5 | 1.8 | 8.2 | 0.09% | 7.9 |
| 1523 | AT3G55250.1 \| expressed protein, predicted pectate-lyase, Arabidopsis thaliana, PIR:T06728 \| chr3:20490266-20491611 FORWARD \| Aliases: T26I12.130 | 8.4 | 4.9 | 3.5 | 8.2 | 0.09% | 7.5 |
| 1531 | AT1G05190.1 \| Symbol: EMB2394 \| ribosomal protein L6 family protein, Similar to Mycobacterium RlpF (gb:Z84395). ESTs gb:T75785,gb:R30580,gb:T04698 come from this gene \| chr1:1502343-1503826 REVERSE \| Aliases: EMB2394, EMBRYO DEFECTIVE 2394, YUP8H12.20, YUP8H12_20 | 7.0 | 3.4 | 3.6 | 8.2 | 0.09% | 7.3 |
| 1532 | AT1G31800.1 \| cytochrome P450 family protein, similar to Cytochrome P450 97B2 (SP:048921) (Glycine max); contains Pfam profile: PF00067: Cytochrome P450 \| chr1:11396383-11400071 FORWARD \| Aliases: F5M6.19, F5M6_19 | 7.0 | 4.6 | 2.4 | 8.2 | 0.09% | 7.7 |
| 1533 | AT3G63420.2 \| Symbol: AGG1 \| heterotrimeric G protein gamma-subunit (AGG1) \| chr3:23428292-23429673 FORWARD \| Aliases: HETEROTRIMERIC G PROTEIN GAMMA SUBUNIT, MAA21.50 | 8.3 | 6.3 | 2.0 | 8.2 | 0.09% | 7.9 |
| 1536 | AT5G50110.1 \| methyltransferase-related, contains weak similarity to Methyltransferase gidB (EC 2.1.-.-) (Glucose inhibited division protein B). (Swiss-Prot:Q93D95) (Streptococcus mutans) \| chr5:20390794-20393433 FORWARD \| Aliases: MPF21.12, MPF21_12 | 7.3 | 4.7 | 2.6 | 8.2 | 0.09% | 7.5 |
| 1539 | AT5G14320.1 \| 30S ribosomal protein S13, chloroplast (CS13), ribosomal protein S13 precursor, chloroplast Arabidopsis thaliana, PIR:S59594; identical to cDNA ribosomal protein S13 GI:1515106 | 9.4 | 6.7 | 2.7 | 8.1 | 0.09% | 7.8 |
| 1543 | AT3G07590.1 \| small nuclear ribonucleoprotein D1, putative / snRNP core protein D1, putative / Sm protein D1, putative, similar to SWISS-PROT:SP:P13641 small nuclear ribonucleoprotein Sm D1 (snRNP core protein D1, Sm-D1, Sm-D autoantigen)(Mouse) \| chr3:2423091-2424170 FORWARD \| Aliases: MLP3.4 | 6.3 | 3.2 | 3.2 | 8.1 | 0.09% | 7.6 |

| Rank | Description | Sync | Root | M | t | adj.q | B |
|---|---|---|---|---|---|---|---|
| 1545 | AT2G20390.1 \| expressed protein \| chr2:8803364-8805299 REVERSE \| Aliases: F11A3.6, F11A3_6 | 7.9 | 5.8 | 2.1 | 8.1 | 0.09% | 7.6 |
| 1546 | AT2G18050.2 \| Symbol: HIS1 3 \| | 4.9 | 2.8 | 2.1 | 8.1 | 0.09% | 7.7 |
| 1548 | AT3G45160.1 \| expressed protein \| chr3:16544436-16545063 REVERSE \| Aliases: T14D3.100 | 9.3 | 5.5 | 3.8 | 8.1 | 0.09% | 7.8 |
| 1550 | AT5G05730.1 \| Symbol: ASA1 \| anthranilate synthase, alpha subunit, component I-1 (ASA1), identical to SP:P32068 \| chr5:1719574-1722904 REVERSE \| Aliases: A METHYL TRYPTOPHAN RESISTANT 1, AMT1, ANTHRANILATE SYNTHASE ALPHA SUBUNIT, ANTHRANILATE SYNTHASE ALPHA SUBUNIT 1, MJJ3.14, MJJ3_14, TRP5, TRYPTOPHAN BIOSYNTHESIS 5, WEI2 | 10.8 | 9.5 | 1.2 | 8.1 | 0.09% | 7.8 |
| 1551 | AT2G43660.2 \| glycosyl hydrolase family protein 17, similar to glucan endo-1,3-beta-glucosidase precursor SP:P52409 from (Triticum aestivum); C terminal homology only | 6.9 | 3.5 | 3.4 | 8.1 | 0.09% | 7.0 |
| 1552 | AT1G28350.1 \| similar to tRNA synthetase class I (W and Y) family protein [Arabidopsis thaliana] (TAIR:At2g33840.1); similar to putative tyrosyl-tRNA synthetase [Oryza sativa (japonica cultivar-group)] (GB:XP_480120.1); contains InterPro domain Aminoacyl-tRNA synthetase, class Ib (InterPro:IPR002305) \| chr1:9944470-9949786 FORWARD \| Aliases: F3M18.22, F3M18_22 | 10.0 | 7.5 | 2.5 | 8.1 | 0.09% | 7.6 |
| 1553 | AT2G33840.1 \| tRNA synthetase class I (W and Y) family protein, similar to SP:P54577 Tyrosyl-tRNA synthetase (EC 6.1.1.1) (Tyrosyl--tRNA ligase) (TyrRS) {Homo sapiens}; contains Pfam profile PF00579: tRNA synthetases class I (W and Y) \| chr2:14322044-14324575 FORWARD \| Aliases: T1B8.14, T1B8_14 | 10.0 | 7.5 | 2.5 | 8.1 | 0.09% | 7.6 |
| 1556 | AT4G04640.1 \| Symbol: ATPC1 \| ATP synthase gamma chain 1, chloroplast (ATPC1), identical to SP:Q01908 ATP synthase gamma chain 1, chloroplast precursor (EC 3.6.3.14) {Arabidopsis thaliana} \| chr4:2350496-2352016 REVERSE \| Aliases: T19J18.4, T19J18_4 | 9.1 | 6.2 | 2.9 | 8.1 | 0.09% | 7.6 |
| 1557 | AT1G77090.1 \| thylakoid lumenal 29.8 kDa protein, identical to SP:O49292:TL30_ARATH (Arabidopsis thaliana);contains a PsbP domain AF370571; SIMILAR TO GI:13926195--F22K20.16 \| chr1:28965291-28966816 REVERSE \| Aliases: F22K20.16, F22K20_16 | 5.8 | 2.9 | 2.9 | 8.1 | 0.09% | 7.0 |
| 1558 | AT1G35910.1 \| trehalose-6-phosphate phosphatase, putative, similar to trehalose-6-phosphate phosphatase (AtTPPB) (Arabidopsis thaliana) GI:2944180; contains Pfam profile PF02358: Trehalose-phosphatase \| chr1:13364219-13366270 REVERSE \| Aliases: F10O5.8, F10O5_8 | 5.6 | 2.8 | 2.8 | 8.1 | 0.09% | 7.2 |
| 1559 | AT3G22840.1 \| Symbol: ELIP1 \| chlorophyll A-B binding family protein / early light-induced protein (ELIP), identical to early light-induced protein; ELIP (Arabidopsis thaliana) GI:1872544; contains Pfam profile: PF00504 chlorophyll A-B binding protein; identical to cDNA early light-induced protein GI:1872543 | 9.5 | 5.6 | 4.0 | 8.1 | 0.09% | 6.5 |
| 1560 | AT4G26310.1 \| elongation factor P (EF-P) family protein, similar to SP:Q45288 Elongation factor P (EF-P) {Corynebacterium glutamicum}; contains Pfam profile PF01132: Elongation factor P (EF-P) \| chr4:13313780-13316114 REVERSE \| Aliases: T25K17.120, T25K17_120 | 6.8 | 4.1 | 2.8 | 8.1 | 0.09% | 7.6 |
| 1562 | AT5G42480.1 \| Symbol: ARC6 \| DNAJ plastid division protein (ARC6), almost identical to dnaJ plastid division protein ARC6 (GI:33436339) (Arabidopsis thaliana);low similarity to cell division protein Ftn2 (Synechococcus sp. PCC 7942) GI:16226084; contains Pfam profile PF00226: DnaJ domain \| chr5:17002410-17005668 FORWARD \| Aliases: ACCUMULATION AND REPLICATION OF CHLOROPLASTS 6, MDH9.18, MDH9_18 | 8.6 | 7.2 | 1.4 | 8.1 | 0.09% | 7.8 |
| 1563 | AT1G51400.1 \| photosystem II 5 kD protein, 100% identical to GI:4836947 (F5D21.10) \| chr1:19055730-19056250 REVERSE \| Aliases: F5D21.10, F5D21_10 | 8.7 | 5.3 | 3.4 | 8.1 | 0.09% | 7.5 |
| 1564 | AT5G50180.1 \| protein kinase, putative, similar to protein kinase ATN1 (Arabidopsis thaliana) gi:1054633:emb:CAA63387 \| chr5:20448164-20450326 FORWARD \| Aliases: K6A12.4, K6A12_4 | 7.1 | 4.2 | 2.9 | 8.1 | 0.09% | 7.4 |
| 1565 | AT3G20790.1 \| oxidoreductase family protein, weak similarity to SP:Q07982 Glucose--fructose oxidoreductase precursor (EC 1.1.99.28) {Zymomonas mobilis}; contains Pfam profiles PF01408: Oxidoreductase family NAD-binding Rossmann fold, PF02894: Oxidoreductase family C-terminal alpha/beta domain \| chr3:7268768-7271517 FORWARD \| Aliases: MOE17.10 | 9.2 | 6.9 | 2.3 | 8.1 | 0.09% | 7.7 |
| 1566 | AT3G52090.1 \| Symbol: ATRPB13.6 \| DNA-directed RNA polymerase II 13.6 kDa subunit (RPB13.6), identical to SP:Q38859 DNA-directed RNA polymerase II 13.6 kDa polypeptide (EC 2.7.7.6) {Arabidopsis thaliana} \| chr3:19328832-19330030 REVERSE \| Aliases: F4F15.200, RNA POLYMERASE II 13.6 KDA SUBUNIT | 10.1 | 8.0 | 2.1 | 8.1 | 0.09% | 7.8 |
| 1567 | AT2G17972.1 \| expressed protein \| chr2:7828667-7829403 FORWARD \| Aliases: None | 6.1 | 3.2 | 2.9 | 8.1 | 0.09% | 6.8 |
| 1568 | AT3G04620.1 \| expressed protein \| chr3:1255532-1256857 REVERSE \| Aliases: F7O18.10, F7O18_10 | 5.8 | 3.0 | 2.8 | 8.1 | 0.09% | 6.9 |
| 1570 | AT1G09830.1 \| phosphoribosylamine--glycine ligase (PUR2), Identical to phosphoribosylamine--glycine ligase, chloroplast (precursor) SP:P52420 from (Arabidopsis thaliana) \| chr1:3192683-3195008 REVERSE \| Aliases: F21M12.22, F21M12_22 | 9.3 | 6.3 | 2.9 | 8.1 | 0.09% | 7.5 |

| Rank | Description | Sync | Root | M | t | adj.q | B |
|---|---|---|---|---|---|---|---|
| 1571 | AT3G08740.1 \| elongation factor P (EF-P) family protein, similar to SP:P33398 Elongation factor P (EF-P) {Escherichia coli O157:H7}; contains Pfam profile PF01132: Elongation factor P (EF-P) | 8.3 | 5.2 | 3.2 | 8.1 | 0.09% | 7.0 |
| 1572 | AT5G50410.1 \| expressed protein \| chr5:20545425-20547053 REVERSE \| Aliases: MXI22.13, MXI22_13 | 6.7 | 4.0 | 2.6 | 8.1 | 0.09% | 7.1 |
| 1573 | AT3G02900.1 \| expressed protein \| chr3:645828-647622 FORWARD \| Aliases: F13E7.15, F13E7_15 | 7.1 | 4.8 | 2.2 | 8.1 | 0.09% | 7.4 |
| 1574 | AT3G19800.1 \| expressed protein \| chr3:6876120-6878156 FORWARD \| Aliases: MPN9.4 | 5.8 | 3.1 | 2.6 | 8.1 | 0.09% | 7.2 |
| 1575 | AT2G29630.2 \| thiamine biosynthesis family protein / thiC family protein, contains Pfam profile: PF01964 ThiC family \| chr2:12674111-12677388 FORWARD \| Aliases: T27A16.27, T27A16_27 | 7.0 | 3.8 | 3.2 | 8.1 | 0.09% | 6.7 |
| 1577 | AT3G59980.1 \| tRNA-binding region domain-containing protein, similar to SP:O54873 Multisynthetase complex auxiliary component p43 (Contains: Endothelial-monocyte activating polypeptide II (EMAP-II) (Small inducible cytokine subfamily E member 1)) {Cricetulus griseus}; contains Pfam profile PF01588: Putative tRNA binding domain \| chr3:22165221-22166618 REVERSE \| Aliases: F24G16.250 | 7.9 | 5.9 | 2.0 | 8.1 | 0.09% | 7.6 |
| 1580 | AT1G52230.1 \| photosystem I reaction center subunit VI, chloroplast, putative / PSI-H, putative (PSAH2), identical to SP:Q9SUI6; similar to PSI-H precursor (Nicotiana sylvestris) GI:407355; contains Pfam profile PF03244: Photosystem I reaction centre subunit VI \| chr1:19458505-19459337 FORWARD \| Aliases: F9I5.11, F9I5_11 | 6.7 | 3.4 | 3.3 | 8.1 | 0.09% | 6.7 |
| 1581 | AT1G67740.1 \| Symbol: PSBY \| photosystem II core complex proteins psbY, chloroplast (PSBY) / L-arginine metabolising enzyme, identical to SP:O49347 Photosystem II core complex proteins psbY, chloroplast precursor (L-arginine metabolising enzyme) (L-AME) (Contains: Photosystem II protein psbY-1 (psbY-A1); Photosystem II protein psbY-2 (psbY-A2)) (Arabidopsis thaliana) \| chr1:25397731-25398817 REVERSE \| Aliases: F12A21.13, F12A21_13, YCF32 | 6.7 | 3.5 | 3.2 | 8.1 | 0.09% | 6.8 |
| 1582 | AT1G07430.1 \| protein phosphatase 2C, putative / PP2C, putative, similar to GB:CAB90633 from (Fagus sylvatica) | 8.8 | 4.2 | 4.6 | 8.1 | 0.09% | 7.5 |
| 1583 | AT3G05060.1 \| SAR DNA-binding protein, putative, strong similarity to SAR DNA-binding protein-1 (Pisum sativum) GI:3132696; contains Pfam profile PF01798: Putative snoRNA binding domain; encodes NOP58-like protein \| chr3:1412873-1415958 REVERSE \| Aliases: T12H1.2, T12H1_2 | 10.0 | 7.9 | 2.1 | 8.1 | 0.09% | 7.7 |
| 1584 | AT3G61870.2 \| expressed protein, hypothetical protein - Synechocystis sp. (strain PCC 6803), PIR:S75899 \| chr3:22913627-22915034 FORWARD \| Aliases: F21F14.40 | 6.5 | 3.8 | 2.7 | 8.1 | 0.09% | 7.1 |
| 1589 | AT1G76860.1 \| small nuclear ribonucleoprotein, putative / snRNP, putative / Sm protein, putative, similar to SWISS-PROT:Q9Y4Z1 U6 snRNA-associated Sm-like protein LSm3 (MDS017) (Mouse) \| chr1:28859355-28860644 REVERSE \| Aliases: F7O12.3, F7O12_3 | 9.0 | 7.3 | 1.7 | 8.1 | 0.10% | 7.8 |
| 1595 | AT2G38450.1 \| expressed protein \| chr2:16109115-16109993 FORWARD \| Aliases: T19C21.6, T19C21_6 | 7.7 | 5.8 | 1.9 | 8.0 | 0.10% | 7.7 |
| 1596 | AT3G16150.1 \| L-asparaginase, putative / L-asparagine amidohydrolase, putative, similar to Swiss-Prot:P30364 L-asparaginase (EC 3.5.1.1) (L-asparagine amidohydrolase) (Lupinus angustifolius) | 8.5 | 5.2 | 3.2 | 8.0 | 0.10% | 7.0 |
| 1601 | AT4G33070.1 \| pyruvate decarboxylase, putative, strong similarity to SP:P51846 Pyruvate decarboxylase isozyme 2 (EC 4.1.1.1) (PDC) {Nicotiana tabacum}; contains InterPro entry IPR000399: Pyruvate decarboxylase \| chr4:15952292-15954774 REVERSE \| Aliases: F4I10.4 | 11.4 | 7.1 | 4.4 | 8.0 | 0.10% | 7.7 |
| 1602 | AT5G17930.1 \| similar to MIF4G domain-containing protein / MA3 domain-containing protein [Arabidopsis thaliana] (TAIR:At1g80930.1); similar to nucleolar protein with MIF4G domain 1 [Homo sapiens] (GB:NP_612409.1); contains InterPro domain Initiation factor eIF-4 gamma, MA3 (InterPro:IPR003891); contains InterPro domain Initiation factor eIF-4 gamma, middle (InterPro:IPR003890) \| chr5:5940000-5944428 REVERSE \| Aliases: MCM23.1, MCM23_1 | 6.5 | 4.1 | 2.4 | 8.0 | 0.10% | 7.7 |
| 1603 | AT3G14190.1 \| expressed protein \| chr3:4710490-4711976 FORWARD \| Aliases: MAG2.18 | 8.3 | 6.1 | 2.3 | 8.0 | 0.10% | 7.5 |
| 1604 | AT5G20160.2 \| ribosomal protein L7Ae/L30e/S12e/Gadd45 family protein, Similar to NHP2/L7Ae family proteins, see SWISSPROT:P32495 and PMID:2063628. \| chr5:6803838-6805197 REVERSE \| Aliases: F5O24.50, F5O24_50 | 9.3 | 5.8 | 3.4 | 8.0 | 0.10% | 7.6 |
| 1612 | AT4G08390.2 \| L-ascorbate peroxidase, stromal (sAPX), identical to stromal ascorbate peroxidase (Arabidopsis thaliana) gi:1419388:emb:CAA67425 | 9.1 | 5.8 | 3.2 | 8.0 | 0.10% | 7.5 |
| 1613 | AT5G50810.1 \| Symbol: TIM8 \| mitochondrial import inner membrane translocase (TIM8), identical to mitochondrial import inner membrane translocase subunit Tim8 (Arabidopsis thaliana) Swiss-Prot:Q9XGY4; contains Pfam domain, PF02953: Tim10/DDP family zinc finger \| chr5:20692860-20693816 REVERSE \| Aliases: K7B16.3, K7B16_3 | 8.1 | 5.4 | 2.7 | 8.0 | 0.10% | 7.2 |
| 1614 | AT1G74390.1 \| exonuclease family protein, contains exonuclease domain, Pfam:PF00929 \| chr1:27965198-27967508 REVERSE \| Aliases: F1M20.7, F1M20_7 | 5.1 | 3.3 | 1.9 | 8.0 | 0.10% | 7.6 |

| Rank | Description | Sync | Root | M | t | adj.q | B |
|---|---|---|---|---|---|---|---|
| 1616 | AT1G33265.1 \| expressed protein, contains Pfam profile PF03647: Uncharacterised protein family (UPF0136); supporting cDNA gi:23198247:gb:BT000332.1: \| chr1:12066575-12068391 FORWARD \| Aliases: None | 7.9 | 3.8 | 4.0 | 8.0 | 0.10% | 7.3 |
| 1617 | AT3G59810.1 \| small nuclear ribonucleoprotein F, putative / U6 snRNA-associated Sm-like protein, putative / Sm protein F, putative, similar to SWISS-PROT:Q9Y4Y8 U6 snRNA-associated Sm-like protein LSm6 (Mus musculus) \| chr3:22107492-22108885 FORWARD \| Aliases: F24G16.80 | 8.0 | 5.8 | 2.2 | 8.0 | 0.10% | 7.7 |
| 1618 | AT4G30660.1 \| hydrophobic protein, putative / low temperature and salt responsive protein, putative, similar to SP:Q9ZNQ7 Hydrophobic protein RCI2A (Low temperature and salt responsive protein LTI6A) {Arabidopsis thaliana}; contains Pfam profile PF01679: Uncharacterized protein family \| chr4:14955453-14956069 FORWARD \| Aliases: T10C21.10, T10C21_10 | 9.1 | 7.1 | 1.9 | 8.0 | 0.10% | 7.7 |
| 1619 | AT3G46560.1 \| Symbol: TIM9 \| mitochondrial import inner membrane translocase (TIM9), identical to mitochondrial import inner membrane translocase subunit Tim9 (Arabidopsis thaliana) Swiss-Prot:Q9XGX9; contains Pfam domain, PF02953: Tim10/DDP family zinc finger \| chr3:17149572-17150473 FORWARD \| Aliases: EMB2474, EMBRYO DEFECTIVE 2474, F12A12.80 | 10.2 | 7.6 | 2.6 | 8.0 | 0.10% | 7.4 |
| 1620 | AT1G45050.1 \| Symbol: ATUBC2 1 \| ubiquitin-conjugating enzyme 15 (UBC15), E2; identical to ubiquitin-conjugating enzyme 15 GI:2801442 from (Arabidopsis thaliana) \| chr1:17033721-17035638 FORWARD \| Aliases: F27F5.13, F27F5_13 | 6.4 | 3.5 | 3.0 | 8.0 | 0.10% | 7.5 |
| 1621 | AT5G06110.1 \| DNAJ heat shock N-terminal domain-containing protein / cell division protein-related, similar to GlsA (Volvox carteri f. nagariensis) GI:4633129; contains Pfam profiles PF00226 DnaJ domain, PF00249 Myb-like DNA-binding domain \| chr5:1840831-1843001 REVERSE \| Aliases: K16F4.7, K16F4_7 | 8.2 | 6.7 | 1.5 | 8.0 | 0.10% | 7.7 |
| 1622 | AT3G22630.1 \| Symbol: PBD1 \| 20S proteasome beta subunit D (PBD1) (PRGB), identical to GB:CAA74026 from (Arabidopsis thaliana) ( FEBS Lett. (1997) 416 (3), 281-285); identical to cDNA proteasome subunit prgb GI:2511589 \| chr3:8009547-8010851 REVERSE \| Aliases: F16J14.20, MULTICATALYTIC ENDOPEPTIDASE COMPLEX, PROTEASOME COMPONENT, BETA SUBUNIT, PRCGB, PROTEASOME SUBUNIT PRGB | 10.3 | 7.2 | 3.1 | 8.0 | 0.10% | 7.7 |
| 1623 | AT5G55190.1 \| Symbol: RAN3 \| Ras-related GTP-binding protein (RAN3), identical to atran3 (Arabidopsis thaliana) GI:2058280 \| chr5:22409402-22411392 FORWARD \| Aliases: MCO15.14, MCO15_14 | 11.1 | 9.8 | 1.3 | 8.0 | 0.10% | 7.8 |
| 1624 | AT2G21640.1 \| expressed protein \| chr2:9261539-9262705 FORWARD \| Aliases: F2G1.9, F2G1_9 | 4.1 | 2.4 | 1.7 | 8.0 | 0.10% | 7.6 |
| 1625 | AT2G39795.1 \| mitochondrial glycoprotein family protein / MAM33 family protein, low similarity to SUAPRGA1 (Emericella nidulans) GI:6562379; contains Pfam profile PF02330: Mitochondrial glycoprotein | 6.6 | 4.8 | 1.9 | 8.0 | 0.10% | 7.6 |
| 1627 | AT3G08940.2 \| Symbol: LHCB4.2 \| chlorophyll A-B binding protein (LHCB4.2), contains Pfam profile: PF00504 chlorophyll A-B binding protein; identical to Lhcb4.2 protein GB:AAD28774 (Arabidopsis thaliana) \| chr3:2717681-2718874 FORWARD \| Aliases: T16O11.12 | 7.7 | 4.2 | 3.5 | 8.0 | 0.10% | 7.4 |
| 1629 | AT1G04870.2 \| protein arginine N-methyltransferase family protein, similar to SP:Q96LA8 Protein arginine N-methyltransferase 6 (EC 2.1.1.-) {Homo sapiens} \| chr1:1373233-1375741 REVERSE \| Aliases: F13M7.14, F13M7_14 | 8.8 | 5.9 | 2.9 | 8.0 | 0.10% | 7.4 |
| 1630 | AT3G12370.1 \| ribosomal protein L10 family protein, similar to 50S ribosomal protein L10 (Oryza sativa) GB:AAC64971 GI:3777602 (Oryza sativa) \| chr3:3937197-3938101 REVERSE \| Aliases: T2E22.37 | 7.9 | 5.6 | 2.4 | 8.0 | 0.10% | 7.3 |
| 1632 | AT2G21320.1 \| zinc finger (B-box type) family protein \| chr2:9133344-9134924 FORWARD \| Aliases: F3K23.8, F3K23_8 | 6.8 | 4.0 | 2.7 | 8.0 | 0.10% | 7.1 |
| 1633 | AT1G32310.1 \| expressed protein \| chr1:11653544-11654771 FORWARD \| Aliases: F27G20.14 | 7.9 | 4.5 | 3.3 | 8.0 | 0.10% | 7.3 |
| 1638 | AT5G27760.1 \| hypoxia-responsive family protein, contains Pfam profile: PF04588 hypoxia induced protein conserved region \| chr5:9830368-9831787 FORWARD \| Aliases: T1G16.90, T1G16_90 | 10.8 | 8.0 | 2.7 | 8.0 | 0.10% | 7.7 |
| 1640 | AT5G01600.1 \| Symbol: ATFER1 \| | 11.1 | 8.5 | 2.6 | 8.0 | 0.10% | 7.6 |
| 1643 | AT4G14540.1 \| CCAAT-box binding transcription factor subunit B (NF-YB) (HAP3 ) (AHAP3) family, contains Pfam PF00808 : Histone-like transcription factor (CBF/NF-Y) and archaeal histone; similar to LEC1-like protein (GI:22536010) (Phaseolus coccineus) \| chr4:8344612-8345214 FORWARD \| Aliases: DL3310W, FCAALL.252 | 6.7 | 3.9 | 2.8 | 8.0 | 0.10% | 7.2 |
| 1644 | AT1G22140.2 \| expressed protein \| chr1:7814830-7815620 REVERSE \| Aliases: F2E2.21, F2E2_21 | 9.1 | 6.8 | 2.2 | 8.0 | 0.10% | 7.6 |
| 1647 | AT4G25080.3 \| magnesium-protoporphyrin O-methyltransferase, putative / magnesium-protoporphyrin IX methyltransferase, putative, similar to SP:Q55467 Magnesium-protoporphyrin O-methyltransferase (EC 2.1.1.1) (Magnesium-protoporphyrin IX methyltransferase) {Synechocystis sp.} \| chr4:12876958-12878639 FORWARD \| Aliases: F24A6.6, MGPIXMT | 8.4 | 6.3 | 2.1 | 8.0 | 0.10% | 7.7 |
| 1648 | AT1G17510.1 \| expressed protein \| chr1:6023497-6024302 REVERSE \| Aliases: F1L3.22, F1L3_22 | 10.4 | 7.6 | 2.8 | 8.0 | 0.10% | 7.7 |

| Rank | Description | Sync | Root | M | t | adj.q | B |
|---|---|---|---|---|---|---|---|
| 1649 | AT4G37200.1 \| Symbol: HCF164 \| | 7.2 | 5.6 | 1.6 | 8.0 | 0.10% | 7.7 |
| 1653 | AT2G39140.1 \| pseudouridine synthase family protein \| chr2:16337261-16340438 FORWARD \| Aliases: AT2G39150, T16B24.22, T16B24_22 | 5.9 | 4.0 | 1.8 | 7.9 | 0.10% | 7.5 |
| 1654 | AT1G56300.1 \| DNAJ heat shock N-terminal domain-containing protein, similar to SP:Q9QYI7 DnaJ homolog subfamily B member 8 Mus musculus; contains Pfam profile: PF00226: DnaJ domain \| chr1:21082485-21083991 REVERSE \| Aliases: F14G9.9, F14G9_9 | 7.4 | 5.4 | 2.0 | 7.9 | 0.10% | 7.6 |
| 1655 | AT3G51800.2 \| Symbol: ATG2 \| metallopeptidase M24 family protein, similar to SP:P50580 Proliferation-associated protein 2G4 {Mus musculus}; contains Pfam profile PF00557: metallopeptidase family M24 \| chr3:19222004-19224646 REVERSE \| Aliases: ATEM1.5 | 11.1 | 9.2 | 1.8 | 7.9 | 0.10% | 7.7 |
| 1657 | AT5G63670.1 \| transcription initiation protein-related, contains weak similarity to Transcription initiation protein SPT4 homolog 1 (Swiss-Prot:Q16550) (Mus musculus) | 9.1 | 6.6 | 2.5 | 7.9 | 0.10% | 7.6 |
| 1662 | AT1G13030.1 \| sphere organelles protein-related, contains weak similarity to Swiss-Prot:Q09003 sphere organelles protein SPH-1 (Sphere protein 1) (Xenopus laevis) \| chr1:4444041-4447404 REVERSE \| Aliases: F3F19.5, F3F19_5 | 5.6 | 4.3 | 1.3 | 7.9 | 0.10% | 7.7 |
| 1664 | AT1G55805.1 \| BolA-like family protein, contains Pfam profile: PF01722 BolA-like protein \| chr1:20862397-20863105 REVERSE \| Aliases: None | 8.8 | 5.6 | 3.2 | 7.9 | 0.10% | 7.3 |
| 1665 | AT3G14110.2 \| Symbol: FLU \| tetratricopeptide repeat (TPR)-containing protein, contains Pfam profile PF00515: TPR Domain \| chr3:4675712-4677665 REVERSE \| Aliases: FLUORESCENT IN BLUE LIGHT, MAG2.7 | 7.5 | 5.1 | 2.3 | 7.9 | 0.10% | 7.3 |
| 1666 | AT1G05350.1 \| thiF family protein, low similarity to SP:P30138 Adenylyltransferase thiF (EC 2.7.7.-) {Escherichia coli}; contains Pfam profile PF00899: ThiF family \| chr1:1560890-1564004 REVERSE \| Aliases: YUP8H12.3, YUP8H12_3 | 8.1 | 5.5 | 2.5 | 7.9 | 0.10% | 7.3 |
| 1669 | AT3G57360.1 \| expressed protein \| chr3:21236549-21237662 FORWARD \| Aliases: F28O9.210 | 7.4 | 4.9 | 2.5 | 7.9 | 0.10% | 7.3 |
| 1670 | AT4G24770.1 \| Symbol: RBP31 \| 31 kDa ribonucleoprotein, chloroplast, putative / RNA-binding protein RNP-T, putative / RNA-binding protein 1/2/3, putative / RNA-binding protein cp31, putative, similar to SP:Q04836 31 kDa ribonucleoprotein, chloroplast precursor (RNA-binding protein RNP-T) (RNA-binding protein 1/2/3) (AtRBP33) (RNA-binding protein cp31) {Arabidopsis thaliana}; contains InterPro entry IPR000504: RNA-binding region RNP-1 (RNA recognition motif) (RRM) \| chr4:12766040-12768033 REVERSE \| Aliases: 31 KDA RNA BINDING PROTEIN, ATRBP31, ATRBP33, CP31, F6I7.11, STEP1 | 7.9 | 4.2 | 3.7 | 7.9 | 0.10% | 6.7 |
| 1671 | AT2G35900.1 \| expressed protein \| chr2:15077076-15078732 REVERSE \| Aliases: F11F19.19, F11F19_19 | 7.3 | 4.1 | 3.3 | 7.9 | 0.10% | 7.2 |
| 1673 | AT3G63440.1 \| similar to FAD-binding domain-containing protein / cytokinin oxidase family protein [Arabidopsis thaliana] (TAIR:At2g41510.1); similar to putative cytokinin oxidase [Oryza sativa (japonica cultivar-group)] (GB:XP_463696.1); similar to putative cytokinin dehydrogenase [Oryza sativa (japonica cultivar-group)] (GB:BAD87612.1); similar to cytokinin oxidase 3 [Zea mays] (GB:CAE55201.1); contains InterPro domain FAD linked oxidase, N-terminal (InterPro:IPR006094) \| chr3:23435132-23437240 FORWARD \| Aliases: MAA21.70 | 6.3 | 3.1 | 3.2 | 7.9 | 0.10% | 6.9 |
| 1674 | AT1G03330.1 \| small nuclear ribonucleoprotein D, putative / snRNP core SM-like protein, putative / U6 snRNA-associated Sm-like protein, putative, similar to SWISS-PROT:Q9Y333 U6 snRNA-associated Sm-like protein LSm2 (Small nuclear ribonuclear protein D homolog, G7b, SnRNP core SM-like protein SM-x5) (Homo sapiens) \| chr1:817983-819563 REVERSE \| Aliases: F15K9.7, F15K9_7 | 10.7 | 8.8 | 1.9 | 7.9 | 0.10% | 7.6 |
| 1675 | AT1G47270.1 \| F-box family protein / tubby family protein, contains Pfam profiles: PF00646 F-box domain, PF01167 Tub family; similar to Chain A, C-Terminal Domain Of Mouse Brain Tubby Protein Length(GI:6730158) (Mus musculus); similar to phosphodiesterase (GI:467578) (Mus musculus); similar to Tubby protein homolog (Swiss-Prot:O88808) (Rattus norvegicus) \| chr1:17328996-17331168 FORWARD \| Aliases: F8G22.1, F8G22_1 | 7.5 | 5.4 | 2.1 | 7.9 | 0.10% | 7.6 |
| 1676 | AT3G25940.1 \| transcription factor S-II (TFIIS) domain-containing protein, similar to SP:O94703 DNA-directed RNA polymerase I 13.1 kDa polypeptide (EC 2.7.7.6) {Schizosaccharomyces pombe}; contains Pfam profile PF01096: Transcription factor S-II (TFIIS) \| chr3:9495934-9497329 FORWARD \| Aliases: MPE11.11 | 7.5 | 5.5 | 2.1 | 7.9 | 0.10% | 7.6 |
| 1678 | AT3G18740.1 \| 60S ribosomal protein L30 (RPL30C), similar to 60S RIBOSOMAL PROTEIN L30 GB:O49884 from (Lupinus luteus) | 11.2 | 9.9 | 1.2 | 7.9 | 0.10% | 7.7 |
| 1679 | AT2G37510.1 \| RNA-binding protein, putative, similar to SP:P10979 Glycine-rich RNA-binding, abscisic acid-inducible protein {Zea mays}; contains InterPro entry IPR000504: RNA-binding region RNP-1 (RNA recognition motif) (RRM) \| chr2:15750070-15751619 REVERSE \| Aliases: F3G5.30, F3G5_30 | 5.3 | 3.1 | 2.2 | 7.9 | 0.10% | 7.4 |
| 1680 | AT2G44610.1 \| Symbol: RAB6 \| Ras-related GTP-binding protein, putative, similar to GTP-binding protein GI:623586 from (Nicotiana tabacum) ; contains an ADP-ribosylation factors family signature for proteins involved in protein trafficking \| chr2:18418507-18421149 REVERSE \| Aliases: F16B22.10 | 7.3 | 4.7 | 2.6 | 7.9 | 0.10% | 7.4 |
| 1681 | AT5G18570.1 \| GTP1/OBG family protein, similar to SP:P20964 Spo0B-associated GTP-binding protein {Bacillus subtilis}; contains Pfam profile PF01018: GTP1/OBG family \| chr5:6171663-6174835 REVERSE \| Aliases: T28N17.50, T28N17_50 | 5.7 | 3.4 | 2.2 | 7.9 | 0.10% | 7.3 |

| Rank | Description | Sync | Root | M | t | adj.q | B |
|---|---|---|---|---|---|---|---|
| 1685 | AT2G46090.1 \| Encodes a putative sphingosine kinase (SphK) containing the five conserved domains (C1-C5) previously identified in SphKs. \| chr2:18957980-18960347 FORWARD \| Aliases: T3F17.26 | 7.5 | 5.2 | 2.4 | 7.9 | 0.10% | 7.6 |
| 1686 | AT1G02475.1 \| expressed protein \| chr1:513916-515343 REVERSE \| Aliases: None | 6.3 | 3.8 | 2.5 | 7.9 | 0.10% | 7.2 |
| 1687 | AT3G26060.1 \| peroxiredoxin Q, putative, similar to peroxiredoxin Q (Sedum lineare) GI:6899842; contains Pfam profile: PF00578 AhpC/TSA (alkyl hydroperoxide reductase and thiol-specific antioxidant) family \| chr3:9526013-9527611 FORWARD \| Aliases: MPE11.21 | 7.6 | 3.4 | 4.2 | 7.9 | 0.10% | 6.3 |
| 1690 | AT5G39960.1 \| GTP-binding family protein, contains Pfam domain, PF01926: GTPase of unknown function \| chr5:16011423-16014048 FORWARD \| Aliases: MYH19.120, MYH19_120 | 5.2 | 3.7 | 1.5 | 7.9 | 0.11% | 7.6 |
| 1691 | AT5G42980.1 \| Symbol: ATTRX3 \| | 13.3 | 12.2 | 1.1 | 7.9 | 0.11% | 7.5 |
| 1693 | AT5G10920.1 \| argininosuccinate lyase, putative / arginosuccinase, putative, similar to argininosuccinate lyase (Nostoc punctiforme) GI:7672743; contains Pfam profile PF00206: Lyase \| chr5:3441753-3444143 FORWARD \| Aliases: T30N20.190, T30N20_190 | 7.9 | 4.2 | 3.7 | 7.9 | 0.11% | 7.0 |
| 1694 | AT4G19120.2 \| early-responsive to dehydration stress protein (ERD3), identical to ERD3 protein (Arabidopsis thaliana) GI:15320410; contains Pfam profile PF03141: Putative methyltransferase; identical to cDNA ERD3 GI:15320409 \| chr4:10460306-10463113 REVERSE \| Aliases: T18B16.90, T18B16_90 | 10.8 | 9.0 | 1.8 | 7.9 | 0.11% | 7.6 |
| 1695 | AT3G22300.1 \| Symbol: RPS10 \| 40S ribosomal protein S10, mitochondrial (RPS10), identical to SP:P42797 40S ribosomal protein S10, mitochondrial precursor {Arabidopsis thaliana} \| chr3:7885675-7886827 FORWARD \| Aliases: MCB17.5, RIBOSOMAL PROTEIN S10 | 6.8 | 4.2 | 2.5 | 7.9 | 0.11% | 7.1 |
| 1696 | AT3G06610.1 \| DNA-binding enhancer protein-related, similar to huntingtin interacting protein HYPK (GI:3329429) (Homo sapiens); identical to Egd2p (GI:172043) (Saccharomyces cerevisiae) similar to EGD2 protein (GAL4 DNA-binding enhancer protein 2) (Swiss-Prot:P38879) (Saccharomyces cerevisiae) | 10.3 | 8.4 | 1.9 | 7.9 | 0.11% | 7.6 |
| 1698 | AT5G62670.1 \| ATPase, plasma membrane-type, putative / proton pump, putative, strong similarity to P-type H(+)-transporting ATPase from Nicotiana plumbaginifolia (SP:Q08435, SP:Q08436), Lycopersicon esculentum (GI:5901757, SP:P22180), Solanum tuberosum (GI:435003); contains InterPro accession IPR001757: ATPase, E1-E2 type \| chr5:25176456-25183574 FORWARD \| Aliases: MRG21.9, MRG21_9 | 8.7 | 5.3 | 3.4 | 7.9 | 0.11% | 6.8 |
| 1699 | AT1G06960.2 \| small nuclear ribonucleoprotein U2B, putative / spliceosomal protein, putative, non-consensus splice donor GC at exon 4; similar to spliceosomal protein (U2B) GI:169588 from (Solanum tuberosum) \| chr1:2135850-2137731 FORWARD \| Aliases: F10K1.32, F10K1_32 | 5.3 | 3.6 | 1.7 | 7.9 | 0.11% | 7.5 |
| 1701 | AT1G20020.1 \| ferredoxin--NADP(+) reductase, putative / adrenodoxin reductase, putative, strong similarity to Ferredoxin--NADP reductase, chloroplast precursor (EC 1.18.1.2) (FNR) from {Pisum sativum} SP:P10933, {Mesembryanthemum crystallinum} SP:P41343, {Spinacia oleracea} SP:P00455, (Capsicum annuum) GI:6899972 \| chr1:6942796-6945006 FORWARD \| Aliases: T20H2.20, T20H2_20 | 5.9 | 3.0 | 2.9 | 7.9 | 0.11% | 7.2 |
| 1702 | AT4G32350.1 \| expressed protein, contains Pfam profile: PF03398 eukaryotic protein of unknown function, DUF292 | 5.1 | 3.4 | 1.7 | 7.9 | 0.11% | 7.5 |
| 1703 | AT3G19740.1 \| similar to AAA-type ATPase family protein [Arabidopsis thaliana] (TAIR:At1g50140.1); similar to spastin-like [Oryza sativa (japonica cultivar-group)] (GB:BAD37292.1); contains InterPro domain AAA ATPase (InterPro:IPR003593); contains InterPro domain AAA ATPase, central region (InterPro:IPR003959) \| chr3:6855843-6859040 REVERSE \| Aliases: MMB12.22 | 9.6 | 8.3 | 1.3 | 7.9 | 0.11% | 7.6 |
| 1704 | AT1G62480.1 \| vacuolar calcium-binding protein-related, contains weak similarity to vacuolar calcium binding protein (Raphanus sativus) gi:9049359:dbj:BAA99394 \| chr1:23132370-23133424 FORWARD \| Aliases: T3P18.4, T3P18_4 | 8.8 | 6.5 | 2.3 | 7.9 | 0.11% | 7.2 |
| 1706 | AT1G66820.1 \| glycine-rich protein \| chr1:24933703-24934214 FORWARD \| Aliases: F4N21.5, F4N21_5 | 7.0 | 5.1 | 2.0 | 7.9 | 0.11% | 7.3 |
| 1708 | AT5G09510.1 \| 40S ribosomal protein S15 (RPS15D), ribosomal protein S15 - Arabidopsis thaliana, EMBL:Z23161 \| chr5:2955114-2956674 REVERSE \| Aliases: T5E8.310, T5E8_310 | 8.2 | 5.5 | 2.7 | 7.9 | 0.11% | 7.6 |
| 1709 | AT2G41950.1 \| expressed protein \| chr2:17520157-17521921 FORWARD \| Aliases: T6D20.26, T6D20_26 | 6.8 | 4.4 | 2.3 | 7.9 | 0.11% | 7.4 |
| 1710 | AT5G02050.1 \| mitochondrial glycoprotein family protein / MAM33 family protein, low similarity to SUAPRGA1 (Emericella nidulans) GI:6562379; contains Pfam profile PF02330: Mitochondrial glycoprotein | 7.9 | 4.6 | 3.3 | 7.9 | 0.11% | 6.8 |
| 1712 | AT2G29650.3 \| inorganic phosphate transporter, putative, similar to brain specific Na+-dependent inorganic phosphate cotransporter (Rattus norvegicus) GI:507415; contains Pfam profile PF00083: major facilitator superfamily protein | 6.3 | 3.6 | 2.8 | 7.8 | 0.11% | 6.8 |
| 1715 | AT5G57230.1 \| expressed protein \| chr5:23207798-23208711 FORWARD \| Aliases: MJB24.4, MJB24_4 | 6.6 | 4.6 | 2.0 | 7.8 | 0.11% | 7.5 |
| 1716 | AT1G28150.1 \| expressed protein \| chr1:9834930-9835886 REVERSE \| Aliases: F13K9.24 | 7.5 | 5.7 | 1.8 | 7.8 | 0.11% | 7.6 |

| Rank | Description | Sync | Root | M | t | adj.q | B |
|---|---|---|---|---|---|---|---|
| 1718 | AT1G72320.3 \| pumilio/Puf RNA-binding domain-containing protein, contains Pfam profile: PF00806 Pumilio-family RNA binding domains (aka PUM-HD, Pumilio homology domain) (5 copies) \| chr1:27231822-27236658 REVERSE \| Aliases: T9N14.7 | 7.9 | 4.0 | 3.9 | 7.8 | 0.11% | 7.5 |
| 1721 | AT1G13380.1 \| expressed protein \| chr1:4589068-4590408 REVERSE \| Aliases: T6J4.13, T6J4_13 | 8.2 | 6.0 | 2.2 | 7.8 | 0.11% | 7.6 |
| 1724 | AT5G14800.1 \| Symbol: P5CR \| pyrroline-5-carboxylate reductase, identical to pyrroline-5-carboxylate reductase SP:P54904 from (Arabidopsis thaliana) \| chr5:4785965-4787840 REVERSE \| Aliases: AT P5C1, AT P5R, PYRROLINE 5 CARBOXYLATE (P5C) REDUCTASE, T9L3.100, T9L3_100 | 10.6 | 8.2 | 2.4 | 7.8 | 0.11% | 7.3 |
| 1725 | AT1G17490.1 \| expressed protein \| chr1:6008582-6009409 FORWARD \| Aliases: F28G4.23 | 8.7 | 7.5 | 1.2 | 7.8 | 0.11% | 7.6 |
| 1728 | AT2G45860.1 \| expressed protein \| chr2:18881834-18882649 REVERSE \| Aliases: F4I18.16 | 6.9 | 5.0 | 1.9 | 7.8 | 0.11% | 7.4 |
| 1729 | AT3G22320.1 \| Symbol: ATRPABC24.3 \| DNA-directed RNA polymerase, putative, similar to SP:P19388 DNA-directed RNA polymerase II 23 kDa polypeptide (EC 2.7.7.6) {Homo sapiens}; contains Pfam profiles PF03871: RNA polymerase Rpb5 N-terminal domain, PF01191: RNA polymerase Rpb5 C-terminal domain \| chr3:7890893-7892330 REVERSE \| Aliases: MCB17.20 | 10.0 | 8.2 | 1.8 | 7.8 | 0.11% | 7.5 |
| 1731 | AT3G49100.1 \| signal recognition particle 9 kDa protein, putative / SRP9, putative, similar to SP:P49458 Signal recognition particle 9 kDa protein (SRP9) {Homo sapiens}; contains Pfam PF05486: Signal recognition particle 9 kDa protein (SRP9) \| chr3:18207828-18209432 FORWARD \| Aliases: T2J13.60 | 6.2 | 3.3 | 2.9 | 7.8 | 0.11% | 7.3 |
| 1734 | AT5G44200.1 \| Symbol: CBP20 \| nuclear cap-binding protein, putative, similar to SP:P52298 20 kDa nuclear cap binding protein (CBP20) (NCBP interacting protein 1) {Homo sapiens}; non-consensus AT donor splice site at exon 4, AC acceptor splice site at exon 5; contains InterPro entry IPR000504: RNA-binding region RNP-1 (RNA recognition motif) (RRM) \| chr5:17819166-17821154 REVERSE \| Aliases: ATCBP20, CAP BINDING PROTEIN 20, MLN1.12, MLN1_12 | 9.2 | 7.2 | 2.0 | 7.8 | 0.11% | 7.5 |
| 1738 | AT4G33030.1 \| Symbol: SQD1 \| UDP-sulfoquinovose synthase / sulfite:UDP-glucose sulfotransferase / sulfolipid biosynthesis protein (SQD1), identical to gi:2736155 \| chr4:15935432-15937810 FORWARD \| Aliases: F4I10.6, UDP SULFOQUINOVOSE SYNTHASE | 8.3 | 5.9 | 2.4 | 7.8 | 0.11% | 7.4 |
| 1741 | AT3G61080.1 \| fructosamine kinase family protein, contains Pfam PF03881: Fructosamine kinase \| chr3:22618105-22620074 FORWARD \| Aliases: T27I15.170 | 6.0 | 3.3 | 2.7 | 7.8 | 0.11% | 6.7 |
| 1742 | AT5G03650.1 \| Symbol: SBE2.2 \| | 8.6 | 4.9 | 3.7 | 7.8 | 0.11% | 7.3 |
| 1744 | AT3G59820.2 \| similar to calcium-binding EF hand family protein [Arabidopsis thaliana] (TAIR:At1g65540.1); similar to OSJNBa0067K08.12 [Oryza sativa (japonica cultivar-group)] (GB:XP_473035.1) \| chr3:22109005-22112736 REVERSE \| Aliases: F24G16.90 | 7.5 | 4.3 | 3.2 | 7.8 | 0.11% | 7.0 |
| 1746 | AT1G73230.1 \| nascent polypeptide-associated complex (NAC) domain-containing protein, similar to SP:P20290 Transcription factor BTF3 (RNA polymerase B transcription factor 3) {Homo sapiens}; contains Pfam profile PF01849: NAC domain \| chr1:27543969-27545603 REVERSE \| Aliases: T18K17.10, T18K17_10 | 10.4 | 7.5 | 2.9 | 7.8 | 0.11% | 7.4 |
| 1747 | AT3G15260.2 \| protein phosphatase 2C, putative / PP2C, putative, similar to protein phosphatase type 2C GB:AAD17805 from (Lotus japonicus) \| chr3:5138703-5142651 FORWARD \| Aliases: K7L4.6 | 7.7 | 5.7 | 2.0 | 7.8 | 0.11% | 7.5 |
| 1748 | AT3G57610.1 \| adenylosuccinate synthetase (ADSS), identical to adenylosuccinate synthetase, chloroplast precursor (EC 6.3.4.4) (IMP-- aspartate ligase) (AdSS) (AMPSase) (Swiss-Prot:Q96529) (Arabidopsis thaliana) \| chr3:21345261-21347635 REVERSE \| Aliases: F15B8.200, F15B8_200 | 11.4 | 8.9 | 2.5 | 7.8 | 0.11% | 7.6 |
| 1749 | AT5G05340.1 \| peroxidase, putative, similar to peroxidase (Nicotiana tabacum) gi:5381253:dbj:BAA82306; similar to Peroxidase P7 (Brassica rapa (Turnip)) SWISS-PROT:P00434 \| chr5:1578952-1580876 REVERSE \| Aliases: K18I23.14, K18I23_14 | 5.8 | 2.1 | 3.7 | 7.8 | 0.11% | 7.5 |
| 1751 | AT5G64600.1 \| expressed protein, similar to axi 1 (Nicotiana tabacum) GI:559921; contains Pfam profile PF03138: Plant protein family \| chr5:25842393-25845580 FORWARD \| Aliases: MUB3.12, MUB3_12 | 7.0 | 4.7 | 2.3 | 7.8 | 0.11% | 7.2 |
| 1752 | AT4G36680.1 \| pentatricopeptide (PPR) repeat-containing protein, low similarity to fertility restorer (Petunia x hybrida) GI:22128587; contains Pfam profile PF01535: PPR repeat; identical to cDNA membrane-associated salt-inducible protein like GI:2632060 | 7.2 | 4.9 | 2.3 | 7.8 | 0.11% | 7.3 |
| 1753 | AT3G09735.1 \| DNA-binding S1FA family protein, contains Pfam profile: PF04689 DNA binding protein S1FA | 9.7 | 8.2 | 1.5 | 7.8 | 0.11% | 7.6 |
| 1754 | AT1G43560.1 \| thioredoxin family protein, contains Pfam profile: PF00085 Thioredoxin; similar to thioredoxin GI:142153 from (Synechococcus PCC6301) \| chr1:16400539-16402318 REVERSE \| Aliases: AT1G43565, T10P12.4, T10P12_4 | 6.5 | 3.2 | 3.4 | 7.8 | 0.11% | 6.9 |
| 1756 | AT5G49560.1 \| expressed protein, similar to SP:P40389 Rapid response to glucose protein 1 {Schizosaccharomyces pombe} \| chr5:20128187-20129011 REVERSE \| Aliases: K6M13.11, K6M13_11 | 6.8 | 3.7 | 3.1 | 7.8 | 0.11% | 6.7 |

| Rank | Description | Sync | Root | M | t | adj.q | B |
|---|---|---|---|---|---|---|---|
| 1757 | AT3G62940.2 \| OTU-like cysteine protease family protein, contains Pfam profile PF02338: OTU-like cysteine protease \| chr3:23273925-23275646 REVERSE \| Aliases: T20O10.40 | 8.6 | 6.9 | 1.7 | 7.8 | 0.11% | 7.5 |
| 1758 | AT4G26490.1 \| similar to hypothetical protein [Arabidopsis thaliana] (TAIR:At5g56050.1); similar to P0456E05.10 [Oryza sativa (japonica cultivar-group)] (GB:XP_463593.1) \| chr4:13380580-13381446 FORWARD \| Aliases: M3E9.80, M3E9_80 | 4.5 | 2.5 | 2.0 | 7.8 | 0.11% | 7.2 |
| 1759 | AT3G63250.2 \| Symbol: ATHMT 2/HMT 2 \| homocysteine S-methyltransferase 2 (HMT-2), 99.7% identical to homocysteine S-methyltransferase AtHMT-2 (GI:6685163) (Arabidopsis thaliana) \| chr3:23381313-23383591 REVERSE \| Aliases: ATHMT 2, F16M2.100, HMT 2 | 9.8 | 7.6 | 2.3 | 7.8 | 0.11% | 7.4 |
| 1762 | AT1G27400.1 \| 60S ribosomal protein L17 (RPL17A), similar to GB:P51413 from (Arabidopsis thaliana); similar to ESTs gb:L33542 and gb:AA660016 \| chr1:9515122-9516912 FORWARD \| Aliases: F17L21.19, F17L21_19 | 10.2 | 6.6 | 3.5 | 7.8 | 0.11% | 7.3 |
| 1763 | AT5G13070.1 \| MSF1-like family protein, similar to px19 (Gallus gallus) GI:969170; contains Pfam profile PF04707: MSF1-like conserved region \| chr5:4148219-4149215 FORWARD \| Aliases: T19L5.30, T19L5_30 | 8.2 | 5.3 | 2.9 | 7.8 | 0.11% | 7.3 |
| 1769 | AT3G63390.1 \| expressed protein \| chr3:23422141-23422943 REVERSE \| Aliases: MAA21.20 | 8.9 | 7.1 | 1.8 | 7.8 | 0.11% | 7.5 |
| 1771 | AT2G37120.1 \| DNA-binding S1FA family protein, contains Pfam profile: PF04689 DNA binding protein S1FA | 9.5 | 7.7 | 1.8 | 7.8 | 0.11% | 7.5 |
| 1772 | AT5G53400.1 \| nuclear movement family protein, contains Pfam profile: PF03593 nuclear movement protein | 10.0 | 6.7 | 3.3 | 7.8 | 0.11% | 7.3 |
| 1773 | AT2G14890.2 \| Symbol: AGP9 \| arabinogalactan-protein (AGP9), identical to gi:10880495:gb:AAG24277 \| chr2:6406703-6408064 FORWARD \| Aliases: ARABINOGALACTAN PROTEIN 9, T26I20.5, T26I20_5 | 9.3 | 7.0 | 2.3 | 7.7 | 0.11% | 7.3 |
| 1774 | AT1G19240.1 \| expressed protein \| chr1:6649089-6650411 REVERSE \| Aliases: T29M8.11, T29M8_11 | 8.3 | 6.3 | 2.0 | 7.7 | 0.11% | 7.3 |
| 1775 | AT5G23060.1 \| expressed protein \| chr5:7736593-7738518 REVERSE \| Aliases: MYJ24.5, MYJ24_5 | 6.2 | 3.2 | 2.9 | 7.7 | 0.11% | 6.7 |
| 1777 | AT1G76300.1 \| small nuclear ribonucleoprotein D3, putative / snRNP core protein D3, putative / Sm protein D3, putative, similar to SWISS-PROT:P43331 small nuclear ribonucleoprotein Sm D3 (snRNP core protein D3, Sm-D3) (Mouse) \| chr1:28630778-28632257 FORWARD \| Aliases: F15M4.20 | 7.3 | 4.9 | 2.4 | 7.7 | 0.11% | 7.3 |
| 1783 | AT5G62575.2 \| expressed protein \| chr5:25134749-25136118 FORWARD \| Aliases: None | 7.5 | 5.5 | 1.9 | 7.7 | 0.12% | 7.3 |
| 1784 | AT5G07340.1 \| calnexin, putative, identical to calnexin homolog 2 from Arabidopsis thaliana (SP:Q38798), strong similarity to calnexin homolog 1, Arabidopsis thaliana, EMBL:AT08315 (SP:P29402); contains Pfam profile PF00262 calreticulin family \| chr5:2317214-2319650 FORWARD \| Aliases: T2I1.50, T2I1_50 | 6.3 | 4.3 | 2.0 | 7.7 | 0.12% | 7.3 |
| 1785 | AT5G39600.1 \| expressed protein \| chr5:15871157-15872420 REVERSE \| Aliases: MIJ24.9, MIJ24_9 | 6.6 | 3.8 | 2.9 | 7.7 | 0.12% | 7.0 |
| 1787 | AT2G46450.1 \| Symbol: ATCNGC12 \| cyclic nucleotide-regulated ion channel, putative (CNGC12), similar to cyclic nucleotide and calmodulin-regulated ion channel (cngc3) GI:4581201 from (Arabidopsis thaliana) | 6.6 | 3.9 | 2.8 | 7.7 | 0.12% | 7.1 |
| 1789 | AT5G13630.1 \| Symbol: GUN5 \| magnesium-chelatase subunit chlH, chloroplast, putative / Mg-protoporphyrin IX chelatase, putative (CHLH), nearly identical to magnesium chelatase subunit GI:1154627 from (Arabidopsis thaliana); contains Pfam profile: PF02514 CobN/magnesium chelatase family protein \| chr5:4387337-4392230 REVERSE \| Aliases: CCH, CCH1, CHLH, CONDITIONAL CHLORINA, GENOMES UNCOUPLED 5, T6I14.12 | 9.6 | 6.2 | 3.4 | 7.7 | 0.12% | 7.4 |
| 1792 | AT4G34555.1 \| 40S ribosomal protein S25, putative \| chr4:16504235-16505477 REVERSE \| Aliases: None | 8.1 | 5.1 | 3.0 | 7.7 | 0.12% | 7.1 |
| 1794 | AT4G37380.1 \| pentatricopeptide (PPR) repeat-containing protein, contains Pfam profile PF01535: PPR repeat \| chr4:17572034-17573932 REVERSE \| Aliases: F6G17.30, F6G17_30 | 6.2 | 4.3 | 1.9 | 7.7 | 0.12% | 7.3 |
| 1795 | AT1G35140.1 \| Symbol: PHI 1 \| phosphate-responsive protein, putative, similar to phi-1 (phosphate-induced gene) (Nicotiana tabacum) GI:3759184; contains Pfam profile PF04674: Phosphate-induced protein 1 conserved region \| chr1:12851975-12853103 FORWARD \| Aliases: PHOSPHATE INDUCED 1, T32G9.32, T32G9_32 | 6.2 | 3.4 | 2.8 | 7.7 | 0.12% | 6.5 |
| 1797 | AT1G33030.1 \| O-methyltransferase family 2 protein, similar to caffeic acid 3-O-methyltransferase (SP:Q00763) (Populus tremuloides), catechol O-methyltransferase (GI:4808524)(Thalictrum tuberosum) \| chr1:11964756-11966256 REVERSE \| Aliases: F9L11.18, F9L11_18 | 5.7 | 2.7 | 3.0 | 7.7 | 0.12% | 7.0 |
| 1798 | AT3G09890.1 \| ankyrin repeat family protein, contains ankyrin repeats, Pfam:PF00023 \| chr3:3032564-3034448 FORWARD \| Aliases: F8A24.6 | 4.8 | 2.8 | 1.9 | 7.7 | 0.12% | 7.4 |
| 1800 | AT2G33570.1 \| expressed protein \| chr2:14224299-14226324 FORWARD \| Aliases: F4P9.34, F4P9_34 | 6.4 | 3.0 | 3.4 | 7.7 | 0.12% | 7.1 |

| Rank | Description | Sync | Root | M | t | adj.q | B |
|---|---|---|---|---|---|---|---|
| 1802 | AT3G53580.1 \| diaminopimelate epimerase family protein, contains Pfam profile PF01678: Diaminopimelate epimerase \| chr3:19875742-19878112 FORWARD \| Aliases: F4P12.280 | 10.9 | 9.5 | 1.3 | 7.7 | 0.12% | 7.5 |
| 1803 | AT5G65220.1 \| ribosomal protein L29 family protein, contains Pfam profile PF00831: ribosomal protein L29 \| chr5:26078455-26079888 FORWARD \| Aliases: MQN23.16, MQN23_16 | 6.6 | 4.7 | 1.9 | 7.7 | 0.12% | 7.3 |
| 1804 | AT3G60210.1 \| chloroplast chaperonin 10, putative, similar to chloroplast chaperonin 10 GI:14041813 from (Arabidopsis thaliana) \| chr3:22262219-22263800 REVERSE \| Aliases: F27H5.5 | 9.6 | 6.3 | 3.3 | 7.7 | 0.12% | 7.3 |
| 1806 | AT1G69570.1 \| Dof-type zinc finger domain-containing protein, nearly identical to H-protein promoter binding factor-2b (Arabidopsis thaliana) GI:3386548 \| chr1:26165191-26166927 REVERSE \| Aliases: F10D13.20, F10D13_20 | 6.2 | 3.1 | 3.2 | 7.7 | 0.12% | 6.5 |
| 1807 | AT2G21960.1 \| expressed protein \| chr2:9361999-9364499 FORWARD \| Aliases: F7D8.28, F7D8_28 | 7.8 | 5.4 | 2.4 | 7.7 | 0.12% | 7.2 |
| 1808 | AT5G09240.3 \| transcriptional coactivator p15 (PC4) family protein, similar to SP:P11031 Activated RNA polymerase II transcriptional coactivator p15 precursor (PC4) (p14) (Single-stranded DNA binding protein p9) {Mus musculus}; contains Pfam profile PF02229: Transcriptional Coactivator p15 (PC4) \| chr5:2873589-2874785 REVERSE \| Aliases: T2K12.3 | 5.1 | 3.0 | 2.1 | 7.7 | 0.12% | 7.1 |
| 1809 | AT5G25630.1 \| pentatricopeptide (PPR) repeat-containing protein, contains Pfam profile PF01535: PPR repeat \| chr5:8947392-8949602 FORWARD \| Aliases: T14C9.170, T14C9_170 | 8.1 | 5.7 | 2.3 | 7.7 | 0.12% | 7.3 |
| 1813 | AT4G39210.1 \| Symbol: APL3 \| glucose-1-phosphate adenylyltransferase large subunit 3 (APL3) / ADP-glucose pyrophosphorylase, identical to SP:P55231 \| chr4:18259603-18263411 FORWARD \| Aliases: T22F8.110, T22F8_110 | 6.2 | 3.1 | 3.1 | 7.7 | 0.12% | 6.5 |
| 1814 | AT5G46580.1 \| pentatricopeptide (PPR) repeat-containing protein, contains similarity to 67kD chloroplastic RNA-binding protein, P67.1 (Raphanus sativus) GI:9755886; contains Pfam profile PF01535: PPR repeat | 4.2 | 2.6 | 1.6 | 7.7 | 0.12% | 7.4 |
| 1815 | AT5G59480.2 \| haloacid dehalogenase-like hydrolase family protein, low similarity to SP:P53078 SSM1 protein {Saccharomyces cerevisiae}; contains InterPro accession IPR005834: Haloacid dehalogenase-like hydrolase \| chr5:23996649-23998793 REVERSE \| Aliases: F2O15.10, F2O15_10 | 8.9 | 4.3 | 4.6 | 7.7 | 0.12% | 7.1 |
| 1816 | AT4G28830.1 \| expressed protein \| chr4:14232452-14234005 FORWARD \| Aliases: F16A16.60, F16A16_60 | 8.8 | 6.5 | 2.4 | 7.7 | 0.12% | 7.1 |
| 1818 | AT3G01480.1 \| peptidyl-prolyl cis-trans isomerase, putative / cyclophilin, putative / rotamase, putative, similar to peptidyl-prolyl cis-trans isomerase, chloroplast precursor (40 kDa thylakoid lumen PPIase, 40 kDa thylakoid lumen rotamase) (Spinacia oleracea) SWISS-PROT:O49939 | 6.3 | 4.5 | 1.8 | 7.7 | 0.12% | 7.4 |
| 1820 | AT5G18800.2 \| NADH-ubiquinone oxidoreductase 19 kDa subunit (NDUFA8) family protein, contains Pfam profile: PF05850 NADH-ubiquinone oxidoreductase 19 kDa subunit \| chr5:6267067-6268616 FORWARD \| Aliases: F17K4.50, F17K4_50 | 12.4 | 11.0 | 1.4 | 7.7 | 0.12% | 7.3 |
| 1821 | AT3G44880.1 \| Symbol: ACD1 \| Rieske (2Fe-2S) domain-containing protein, similar to lethal leaf-spot 1 from Zea mays (gi:1935909); contains Pfam PF00355 Rieske (2Fe-2S) domain \| chr3:16394580-16397296 FORWARD \| Aliases: ACCELERATED CELL DEATH 1, F28D10.70, LLS1 | 5.9 | 3.9 | 2.0 | 7.7 | 0.12% | 7.2 |
| 1823 | AT3G15000.1 \| expressed protein, similar to DAG protein (required for chloroplast differentiation and palisade development) GB:Q38732 (Antirrhinum majus) \| chr3:5050271-5052439 FORWARD \| Aliases: K15M2.14 | 7.4 | 5.0 | 2.4 | 7.7 | 0.12% | 7.1 |
| 1825 | AT5G53420.2 \| similar to expressed protein [Arabidopsis thaliana] (TAIR:At4g27900.1); similar to expressed protein [Arabidopsis thaliana] (TAIR:At4g27900.2); similar to zinc finger CONSTANS-like protein [Oryza sativa (japonica cultivar-group)] (GB:BAD73395.1) \| chr5:21691615-21692959 FORWARD \| Aliases: MYN8.3, MYN8_3 | 8.7 | 6.6 | 2.0 | 7.7 | 0.12% | 7.3 |
| 1826 | AT1G20340.1 \| Symbol: DRT112 \| | 9.2 | 4.9 | 4.3 | 7.7 | 0.12% | 6.8 |
| 1827 | AT4G16146.1 \| expressed protein \| chr4:9142445-9144588 REVERSE \| Aliases: None | 5.7 | 3.3 | 2.4 | 7.7 | 0.12% | 7.1 |
| 1831 | AT5G62840.1 \| phosphoglycerate/bisphosphoglycerate mutase family protein, contains Pfam profile PF00300: phosphoglycerate mutase family \| chr5:25245451-25246909 REVERSE \| Aliases: MQB2.16, MQB2_16 | 5.5 | 2.9 | 2.6 | 7.6 | 0.12% | 6.5 |
| 1834 | AT3G53370.1 \| DNA-binding S1FA family protein, contains Pfam profile: PF04689 DNA binding protein S1FA | 7.1 | 3.9 | 3.2 | 7.6 | 0.12% | 6.5 |
| 1835 | AT5G06730.1 \| peroxidase, putative, similar to peroxidase (Arabidopsis thaliana) gi:1491617:emb:CAA68212 \| chr5:2079956-2081685 REVERSE \| Aliases: MPH15.9, MPH15_9 | 7.0 | 2.3 | 4.6 | 7.6 | 0.12% | 6.7 |
| 1837 | AT1G06460.1 \| Symbol: ACD32.1 \| 31.2 kDa small heat shock family protein / hsp20 family protein, contains Pfam profile: PF00011 Hsp20/alpha crystallin family | 5.5 | 2.9 | 2.6 | 7.6 | 0.12% | 7.2 |

| Rank | Description | Sync | Root | M | t | adj.q | B |
|---|---|---|---|---|---|---|---|
| 1840 | AT4G14070.1 \| AMP-binding protein, putative, similar to AMP-binding protein (gi:1617272) from Brassica napus; contains Pfam AMP-binding enzyme domain PF00501; identical to cDNA acyl-CoA synthetase-like protein GI:20799730 \| chr4:8111957-8118155 REVERSE \| Aliases: DL3075C, FCAALL.81 | 6.9 | 4.5 | 2.4 | 7.6 | 0.12% | 7.0 |
| 1842 | AT3G14600.1 \| 60S ribosomal protein L18A (RPL18aC), similar to GB:CAA08791 from (Podocoryne carnea) \| chr3:4910704-4912180 FORWARD \| Aliases: MIE1.10 | 10.3 | 7.7 | 2.6 | 7.6 | 0.12% | 7.3 |
| 1845 | AT2G37500.1 \| arginine biosynthesis protein ArgJ family, contains Pfam profile: PF01960 ArgJ family \| chr2:15746418-15749836 REVERSE \| Aliases: F3G5.29, F3G5_29 | 8.1 | 5.5 | 2.6 | 7.6 | 0.12% | 7.2 |
| 1847 | AT1G80750.1 \| 60S ribosomal protein L7 (RPL7A), similar to ribosomal protein L7 GB:AAA03081 GI:307388 from (Homo sapiens) \| chr1:30353715-30355456 FORWARD \| Aliases: F23A5.10, F23A5_10 | 8.3 | 5.2 | 3.2 | 7.6 | 0.12% | 7.0 |
| 1848 | AT4G09640.1 \| expressed protein, several hypothetical proteins - Arabidopsis thaliana \| chr4:6088214-6090885 REVERSE \| Aliases: T25P22.80, T25P22_80 | 5.8 | 3.8 | 2.0 | 7.6 | 0.12% | 7.1 |
| 1849 | AT4G03240.1 \| frataxin protein-related, contains weak similarity to Frataxin, mitochondrial precursor (Friedreich's ataxia protein) (Fxn) (Swiss-Prot:Q16595) (Homo sapiens) \| chr4:1423445-1424759 REVERSE \| Aliases: F4C21.17, F4C21_17 | 6.8 | 4.2 | 2.6 | 7.6 | 0.12% | 7.3 |
| 1850 | AT1G66530.1 \| arginyl-tRNA synthetase, putative / arginine--tRNA ligase, putative, similar to SP:P37880 Arginyl-tRNA synthetase (EC 6.1.1.19) (Arginine--tRNA ligase) (ArgRS) {Cricetulus longicaudatus}; contains Pfam profiles PF00750: arginyl-tRNA synthetase, PF03485: arginyl-tRNA synthetase N-terminal domain \| chr1:24822574-24826005 REVERSE \| Aliases: F28G11.14, F28G11_14 | 9.3 | 5.9 | 3.3 | 7.6 | 0.12% | 7.2 |
| 1851 | AT3G49320.1 \| expressed protein, contains Pfam profile PF03690: Uncharacterised protein family (UPF0160) \| chr3:18299232-18301643 REVERSE \| Aliases: F2K15.180 | 6.6 | 4.6 | 2.0 | 7.6 | 0.12% | 7.2 |
| 1855 | AT3G05630.1 \| Symbol: PLDP2 \| phospholipase D, putative (PLDP2), identical to SP:Q9M9W8 Phospholipase D p2 (EC 3.1.4.4) (AtPLDp2) (Phospholipase D2 PHOX and PX containing domain) (Phospholipase D zeta 2) (PLDzeta2) {Arabidopsis thaliana}; similar to phospholipase D GB:BAA24577 from (Rattus norvegicus); contains Pfam profile: PF00614 phospholipase D, PF00169 PH domain, PF00787 PX domain \| chr3:1635265-1640349 FORWARD \| Aliases: F18C1.10, F18C1_10, PLDZETA2 | 6.0 | 3.2 | 2.8 | 7.6 | 0.12% | 6.8 |
| 1859 | AT4G20150.1 \| expressed protein \| chr4:10888309-10889606 REVERSE \| Aliases: F1C12.70, F1C12_70 | 12.9 | 11.6 | 1.2 | 7.6 | 0.12% | 7.3 |
| 1861 | AT5G03740.1 \| Symbol: HD2C \| zinc finger (C2H2 type) family protein, contains Pfam domain, PF00096: Zinc finger, C2H2 type \| chr5:981896-984201 FORWARD \| Aliases: F17C15.160, F17C15_160, HISTONE DEACETYLASE 2C | 9.3 | 7.8 | 1.5 | 7.6 | 0.12% | 7.4 |
| 1865 | AT5G22880.1 \| histone H2B, putative, strong similarity to histone H2B-3 Lycopersicon esculentum GI:3021485, H2B Gossypium hirsutum SP:O22582; contains Pfam profile PF00125 Core histone H2A/H2B/H3/H4 | 8.5 | 4.5 | 4.0 | 7.6 | 0.12% | 6.9 |
| 1867 | AT2G19680.1 \| mitochondrial ATP synthase g subunit family protein, contains Pfam profile: PF04718 mitochondrial ATP synthase g subunit | 10.5 | 7.0 | 3.5 | 7.6 | 0.12% | 7.3 |
| 1869 | AT5G47930.1 \| 40S ribosomal protein S27 (RPS27D) \| chr5:19423403-19424624 REVERSE \| Aliases: K16F13.2, K16F13_2 | 12.1 | 10.2 | 1.9 | 7.6 | 0.13% | 7.3 |
| 1872 | AT3G18510.1 \| expressed protein \| chr3:6355670-6356159 FORWARD \| Aliases: MYF24.41 | 6.2 | 3.4 | 2.7 | 7.6 | 0.13% | 6.9 |
| 1873 | AT1G03310.2 \| Symbol: ATISA2/DBE1/ISA2 \| isoamylase, putative / starch debranching enzyme, putative, similar to isoamylase isoform 2 (Solanum tuberosum) GI:27728147, isoamylase from (Triticum aestivum) GI:17932898, (Hordeum vulgare) GI:21314275, (Oryza sativa) GI:3252794; contains Pfam profiles PF00128: Alpha amylase catalytic domain, PF02922: Isoamylase N-terminal domain; ESTs gb:H36690, gb:AA712462, gb:AA651230 and gb:N95932 come from this gene \| chr1:813493-816749 FORWARD \| Aliases: ATISA2, DBE1, DEBRANCHING ENZYME 1, F15K9.9, F15K9_9, ISA2 | 7.5 | 4.6 | 2.9 | 7.6 | 0.13% | 7.2 |
| 1874 | AT1G74240.1 \| mitochondrial substrate carrier family protein, contains Pfam profile: PF00153 mitochondrial carrier protein \| chr1:27921057-27923800 FORWARD \| Aliases: F1O17.9, F1O17_9 | 8.5 | 6.3 | 2.1 | 7.6 | 0.13% | 7.3 |
| 1875 | AT5G22650.2 \| Symbol: HD2B \| expressed protein, non-consensus AT donor splice site at exon 3, AC acceptor splice site at exon 4; \| chr5:7534067-7536276 FORWARD \| Aliases: ATHD2B, HD2, HDA4, HDT02, HDT2, HISTONE DEACETYLASE, HISTONE DEACETYLASE 2, HISTONE DEACETYLASE 2B, MDJ22.7, MDJ22_7 | 9.5 | 7.7 | 1.8 | 7.6 | 0.13% | 7.4 |
| 1876 | AT2G37870.1 \| protease inhibitor/seed storage/lipid transfer protein (LTP) family protein, contains Pfam protease inhibitor/seed storage/LTP family domain PF00234 \| chr2:15866317-15866985 FORWARD \| Aliases: T8P21.22, T8P21_22 | 10.2 | 6.6 | 3.7 | 7.6 | 0.13% | 7.2 |
| 1879 | AT4G21110.1 \| G10 family protein, contains Pfam profile: PF01125 G10 protein \| chr4:11267196-11268968 FORWARD \| Aliases: F7J7.50, F7J7_50 | 10.5 | 8.9 | 1.5 | 7.6 | 0.13% | 7.4 |

| Rank | Description | Sync | Root | M | t | adj.q | B |
|---|---|---|---|---|---|---|---|
| 1881 | AT1G50840.1 \| polI-like DNA polymerase, putative, similar to PolI-like DNA polymerase (Oryza sativa) GI:19912795 contains Pfam profiles PF01612: 3'-5' exonuclease, PF00476: DNA polymerase I family A | 7.6 | 5.2 | 2.4 | 7.6 | 0.13% | 6.8 |
| 1882 | AT3G28700.1 \| expressed protein, contains Pfam PF02636: Uncharacterized ACR, COG1565 \| chr3:10760802-10763351 FORWARD \| Aliases: MZN14.19 | 7.3 | 5.2 | 2.1 | 7.6 | 0.13% | 7.2 |
| 1885 | AT3G54120.1 \| reticulon family protein (RTNLB12), contains Pfam profile PF02453: Reticulon \| chr3:20051974-20053318 REVERSE \| Aliases: F24B22.80 | 6.7 | 3.7 | 3.0 | 7.6 | 0.13% | 6.9 |
| 1886 | AT5G58420.1 \| 40S ribosomal protein S4 (RPS4D), ribosomal protein S4, Arabidopsis thaliana, PIR:T48480 | 10.4 | 8.5 | 1.9 | 7.6 | 0.13% | 7.3 |
| 1890 | AT5G08100.2 \| L-asparaginase / L-asparagine amidohydrolase, identical to Swiss-Prot:P50287 L-asparaginase (EC 3.5.1.1) (L-asparagine amidohydrolase) (Arabidopsis thaliana) \| chr5:2593051-2594628 REVERSE \| Aliases: T22D6.40, T22D6_40 | 9.6 | 7.8 | 1.8 | 7.6 | 0.13% | 7.3 |
| 1891 | AT3G16650.1 \| PP1/PP2A phosphatases pleiotropic regulator 2 (PRL2), identical to SP:Q39190 PP1/PP2A phosphatases pleiotropic regulator PRL2 {Arabidopsis thaliana}, GB:Q39190 from (Arabidopsis thaliana); contains Pfam PF00400: WD domain, G-beta repeat (7 copies, 1 weak) \| chr3:5671087-5675330 FORWARD \| Aliases: MGL6.10 | 7.2 | 4.4 | 2.8 | 7.6 | 0.13% | 6.5 |
| 1892 | AT2G23670.1 \| expressed protein \| chr2:10070235-10070943 REVERSE \| Aliases: F26B6.28 | 5.6 | 3.6 | 2.0 | 7.6 | 0.13% | 7.0 |
| 1894 | AT5G02570.1 \| histone H2B, putative, similar to histone H2B-2 Lycopersicon esculentum GI:3021483, Gossypium hirsutum SP:O22582, Asparagus officinalis GI:563329; contains Pfam profile PF00125 Core histone H2A/H2B/H3/H4 \| chr5:576740-577138 REVERSE \| Aliases: T22P11.160, T22P11_160 | 7.5 | 3.5 | 4.0 | 7.6 | 0.13% | 6.6 |
| 1895 | AT5G20720.2 \| Symbol: CPN20 \| 20 kDa chaperonin, chloroplast (CPN21) (CHCPN10) (CPN20), identical to chloroplast 20 kDa chaperonin, chloroplast precursor (Protein Cpn21), chloroplast protein Cpn10, chloroplast chaperonin 10 (Ch-CPN10), SP:O65282 from (Arabidopsis thaliana); identical to cDNA chaperonin 20 GI:14587372 \| chr5:7014688-7016476 FORWARD \| Aliases: ATCPN21, CHAPERONIN 10, CHAPERONIN 20, CHCPN10, CHLOROPLAST CHAPERONIN 10, CPN10, CPN21, T1M15.120, T1M15_120 | 11.3 | 9.3 | 2.1 | 7.6 | 0.13% | 7.3 |
| 1897 | AT5G27670.1 \| histone H2A, putative, similar to histone H2A Lycopersicon esculentum SP:P25469, Pisum sativum SP:P25470, Petroselinum crispum SP:P19177; contains Pfam profile PF00125 Core histone H2A/H2B/H3/H4 \| chr5:9792579-9793458 REVERSE \| Aliases: T1G16.3 | 9.2 | 7.6 | 1.7 | 7.6 | 0.13% | 7.3 |
| 1899 | AT5G43970.1 \| Symbol: TOM22 V \| expressed protein \| chr5:17710034-17710630 FORWARD \| Aliases: ATTOM22 V, MRH10.8, MRH10_8, TOM22, TOM9 2 | 8.2 | 6.0 | 2.2 | 7.6 | 0.13% | 7.3 |
| 1900 | AT1G73110.1 \| ribulose bisphosphate carboxylase/oxygenase activase, putative / RuBisCO activase, putative, similar to ribulose bisphosphate carboxylase/oxygenase activase, chloroplast precursor (RuBisCO activase, RA) (Oryza sativa) SWISS-PROT:P93431 \| chr1:27497901-27500539 REVERSE \| Aliases: F3N23.32, F3N23_32 | 6.0 | 3.5 | 2.5 | 7.6 | 0.13% | 6.9 |
| 1901 | AT1G77670.1 \| aminotransferase class I and II family protein, similar to kynurenine aminotransferase /glutamine transaminase K GI:1030066 (Rattus norvegicus) \| chr1:29193795-29195869 REVERSE \| Aliases: T5M16.26, T5M16_26 | 8.5 | 4.7 | 3.8 | 7.6 | 0.13% | 6.7 |
| 1902 | AT3G50910.1 \| expressed protein \| chr3:18930494-18933219 FORWARD \| Aliases: F18B3.190 | 9.6 | 7.9 | 1.7 | 7.6 | 0.13% | 7.3 |
| 1903 | AT1G74010.1 \| strictosidine synthase family protein, similar to strictosidine synthase (Rauvolfia serpentina)(SP:P15324); contains strictosidine synthase domain PF03088 \| chr1:27835819-27837774 REVERSE \| Aliases: F2P9.12, F2P9_12 | 7.4 | 3.4 | 4.0 | 7.6 | 0.13% | 5.8 |
| 1905 | AT3G03150.1 \| expressed protein \| chr3:727561-729467 REVERSE \| Aliases: T17B22.16, T17B22_16 | 9.6 | 7.0 | 2.5 | 7.5 | 0.13% | 7.1 |
| 1907 | AT5G54380.1 \| protein kinase family protein, contains protein kinase domain, Pfam:PF00069 \| chr5:22094318-22097106 REVERSE \| Aliases: GA469.3, GA469_3 | 5.8 | 4.1 | 1.8 | 7.5 | 0.13% | 7.2 |
| 1908 | AT5G24840.1 \| expressed protein, contains Pfam profile PF02390: Putative methyltransferase \| chr5:8533790-8535046 REVERSE \| Aliases: F6A4.50, F6A4_50 | 7.9 | 6.0 | 1.9 | 7.5 | 0.13% | 7.1 |
| 1912 | AT4G17190.2 \| Symbol: FPS2 \| farnesyl pyrophosphate synthetase 2 (FPS2) / FPP synthetase 2 / farnesyl diphosphate synthase 2, identical to SP:Q43315 Farnesyl pyrophosphate synthetase 2 (FPP synthetase 2) (FPS 2) (Farnesyl diphosphate synthetase 2) (Includes: Dimethylallyltransferase (EC 2.5.1.1); Geranyltranstransferase (EC 2.5.1.10)) {Arabidopsis thaliana} \| chr4:9648552-9650857 REVERSE \| Aliases: DL4630C, FARNESYL DIPHOSPHATE SYNTHASE 2, FCAALL.91 | 6.7 | 3.7 | 3.1 | 7.5 | 0.13% | 6.6 |
| 1916 | AT1G10750.1 \| expressed protein, similar to gi 3128199 F4I1.5 putative proteinase from Arabidopsis thaliana BAC gb AC004521 \| chr1:3574808-3578475 REVERSE \| Aliases: T16B5.11, T16B5_11 | 6.4 | 3.5 | 2.9 | 7.5 | 0.13% | 6.3 |

| Rank | Description | Sync | Root | M | t | adj.q | B |
|---|---|---|---|---|---|---|---|
| 1920 | AT5G61310.3 \| similar to cytochrome c oxidase subunit Vc family protein / COX5C family protein [Arabidopsis thaliana] (TAIR:At2g47380.1); similar to cytochrome c oxidase subunit 5c [Helianthus annuus] (GB:AAL67939.1); contains InterPro domain Cytochrome c oxidase subunit Vc (InterPro:IPR008432) \| chr5:24670635-24672079 REVERSE \| Aliases: MFB13.16, MFB13_16 | 8.9 | 6.8 | 2.1 | 7.5 | 0.13% | 7.2 |
| 1921 | AT1G66890.1 \| expressed protein \| chr1:24961221-24961777 FORWARD \| Aliases: T4O24.4, T4O24_4 | 7.2 | 5.7 | 1.5 | 7.5 | 0.13% | 7.3 |
| 1922 | AT5G52220.1 \| expressed protein, ; expression supported by MPSS \| chr5:21224456-21224904 REVERSE \| Aliases: F17P19.12, F17P19_12 | 6.1 | 2.8 | 3.3 | 7.5 | 0.13% | 6.7 |
| 1925 | AT5G58490.1 \| cinnamoyl-CoA reductase family, similar to cinnamoyl-CoA reductase from Pinus taeda (GI:17978649), Eucalyptus gunnii (GI:2058311) \| chr5:23660248-23661824 FORWARD \| Aliases: MQJ2.6, MQJ2_6 | 9.6 | 6.3 | 3.2 | 7.5 | 0.13% | 7.2 |
| 1926 | AT1G74970.1 \| Symbol: RPS9 \| ribosomal protein S9 (RPS9), identical to ribosomal protein S9 (Arabidopsis thaliana) GI:5456946 | 5.9 | 2.8 | 3.0 | 7.5 | 0.13% | 6.6 |
| 1927 | AT1G77380.1 \| Symbol: AAP3 \| amino acid carrier, putative / amino acid permease, putative, strong similarity to amino acid carrier GI:3293031 from (Ricinus communis); contains Pfam profile PF01490: Transmembrane amino acid transporter protein; identical to cDNA AAP3 (Amino Acid Permease) GI:3970651 \| chr1:29079782-29082284 REVERSE \| Aliases: F2P24.9, F2P24_9 | 9.8 | 7.1 | 2.7 | 7.5 | 0.13% | 7.3 |
| 1929 | AT1G32160.1 \| expressed protein \| chr1:11568647-11570367 FORWARD \| Aliases: F3C3.6, F3C3_6 | 7.2 | 5.3 | 1.8 | 7.5 | 0.13% | 7.3 |
| 1930 | AT3G57000.1 \| nucleolar essential protein-related, contains weak similarity to Nucleolar essential protein 1 (Essential for mitotic growth 1) (Swiss-Prot:Q06287) (Saccharomyces cerevisiae) \| chr3:21103247-21105271 FORWARD \| Aliases: F24I3.80 | 7.8 | 5.7 | 2.1 | 7.5 | 0.13% | 7.2 |
| 1931 | AT5G27850.1 \| 60S ribosomal protein L18 (RPL18C), 60S ribosomal protein L18, Arabidopsis thaliana, SWISSPROT:RL18_ARATH \| chr5:9873160-9874602 FORWARD \| Aliases: F14I23.10, F14I23_10 | 10.9 | 9.1 | 1.8 | 7.5 | 0.13% | 7.3 |
| 1934 | AT5G05470.1 \| Symbol: EIF2 ALPHA \| eukaryotic translation initiation factor 2 subunit 1, putative / eIF-2A, putative / eIF-2-alpha, putative, similar to SWISS-PROT:P20459 eukaryotic translation initiation factor 2 alpha subunit (eIF-2- alpha) (Saccharomyces cerevisiae); identical to cDNA cohesin GI:6682280 \| chr5:1620459-1622777 FORWARD \| Aliases: ATEIF2 A2, EIF2 A2, K18I23.28, K18I23_28 | 5.8 | 3.9 | 2.0 | 7.5 | 0.13% | 7.3 |
| 1936 | AT2G36130.1 \| peptidyl-prolyl cis-trans isomerase, putative / cyclophilin, putative / rotamase, putative, contains Pfam domain, PF00160: peptidyl-prolyl cis-trans isomerase, cyclophilin-type | 8.2 | 5.6 | 2.6 | 7.5 | 0.13% | 7.1 |
| 1937 | AT5G06460.1 \| Symbol: ATUBA2 \| | 8.2 | 6.5 | 1.7 | 7.5 | 0.13% | 7.3 |
| 1938 | AT3G59140.1 \| Symbol: ATMRP14 \| ABC transporter family protein, putative multi resistance protein mrp - Arabidopsis thaliana, EMBL:ATMRPPROT \| chr3:21874496-21879678 REVERSE \| Aliases: F17J16.190 | 6.4 | 3.8 | 2.6 | 7.5 | 0.13% | 6.4 |
| 1940 | AT5G18660.1 \| Symbol: PCB2 \| Encodes a protein with 3,8-divinyl protochlorophyllide a 8-vinyl reductase activity. Mutants accumulate divinyl chlorophyll rather than monovinyl chlorophyll. \| chr5:6220783-6222230 REVERSE \| Aliases: DVR, PALE GREEN AND CHLOROPHYLL B REDUCED 2, T1A4.40, T1A4_40 | 6.3 | 3.4 | 2.8 | 7.5 | 0.13% | 6.3 |
| 1944 | AT4G29770.1 \| expressed protein \| chr4:14575846-14577645 FORWARD \| Aliases: F27B13.10, F27B13_10 | 6.6 | 3.1 | 3.5 | 7.5 | 0.13% | 5.8 |
| 1948 | AT5G10360.1 \| Symbol: EMB3010 \| 40S ribosomal protein S6 (RPS6B) \| chr5:3258475-3260228 REVERSE \| Aliases: EMB3010, EMBRYO DEFECTIVE 3010, F12B17.290, F12B17_290 | 10.7 | 9.2 | 1.5 | 7.5 | 0.13% | 7.2 |
| 1949 | AT5G24970.1 \| ABC1 family protein, contains Pfam domain, PF03109: ABC1 family \| chr5:8604339-8608820 REVERSE \| Aliases: F6A4.180, F6A4_180 | 6.9 | 4.6 | 2.2 | 7.5 | 0.13% | 7.1 |
| 1951 | AT4G11980.1 \| MutT/nudix family protein, low similarity to SP:P54570 ADP-ribose pyrophosphatase (EC 3.6.1.13) {Bacillus subtilis}; contains Pfam profile PF00293: NUDIX domain | 6.6 | 4.7 | 1.9 | 7.5 | 0.13% | 7.0 |
| 1953 | AT5G03940.1 \| Symbol: FFC \| signal recognition particle 54 kDa protein, chloroplast / 54 chloroplast protein / SRP54 (FFC), identical to Swiss-Prot:P37107 signal recognition particle 54 kDa protein, chloroplast precursor (SRP54) (54 chloroplast protein) (54CP) (FFC) (Arabidopsis thaliana) \| chr5:1059503-1063318 REVERSE \| Aliases: 54CP, CPSRP54, F8F6.150, F8F6_150, FIFTY FOUR CHLOROPLAST HOMOLOGUE, SIGNAL RECOGNITION PARTICLE 54 KDA SUBUNIT, SRP54CP | 8.0 | 5.1 | 2.9 | 7.5 | 0.13% | 7.2 |
| 1954 | AT1G06190.1 \| expressed protein \| chr1:1892272-1894137 REVERSE \| Aliases: F9P14.5, F9P14_5 | 5.0 | 3.5 | 1.5 | 7.5 | 0.13% | 7.2 |
| 1956 | AT5G67160.1 \| transferase family protein, similar to anthranilate N-hydroxycinnamoyl/benzoyltransferase, Dianthus caryophyllus (gi:2239091); contains Pfam transferase family domain PF002458 \| chr5:26814839-26816267 REVERSE \| Aliases: K21H1.12, K21H1_12 | 7.5 | 4.4 | 3.1 | 7.5 | 0.13% | 7.0 |

| Rank | Description | Sync | Root | M | t | adj.q | B |
|---|---|---|---|---|---|---|---|
| 1958 | AT1G72740.1 \| DNA-binding family protein / histone H1/H5 family protein, similar to DNA-binding protein PcMYB1 (Petroselinum crispum) GI:2224897; contains Pfam profiles PF00538: linker histone H1 and H5 family, PF00249: Myb-like DNA-binding domain \| chr1:27383956-27386586 REVERSE \| Aliases: F28P22.7, F28P22_7 | 6.7 | 3.9 | 2.9 | 7.5 | 0.13% | 7.2 |
| 1960 | AT5G56360.1 \| calmodulin-binding protein, similar to alpha glucosidase II beta subunit from GI:2104691 (Mus musculus) \| chr5:22840528-22845223 REVERSE \| Aliases: MCD7.9, MCD7_9 | 9.0 | 7.0 | 1.9 | 7.5 | 0.13% | 7.3 |
| 1961 | AT5G13510.1 \| ribosomal protein L10 family protein, ribosomal protein L10- Nicotiana tabacum, EMBL:AB010879 \| chr5:4341267-4342098 FORWARD \| Aliases: T6I14.40, T6I14_40 | 8.1 | 4.8 | 3.3 | 7.5 | 0.13% | 7.0 |
| 1962 | AT5G43330.1 \| malate dehydrogenase, cytosolic, putative, strong similarity to cytosolic malate dehydrogenase (EC 1.1.1.37) SP:O24047 {Mesembryanthemum crystallinum}, SP:O48905 {Medicago sativa}, (Prunus persica) GI:15982948; contains InterPro entry IPR001236: Lactate/malate dehydrogenase \| chr5:17407661-17409867 FORWARD \| Aliases: MWF20.2, MWF20_2 | 11.1 | 5.8 | 5.2 | 7.5 | 0.13% | 7.1 |
| 1964 | AT5G55560.1 \| protein kinase family protein, contains protein kinase domain, Pfam:PF00069 \| chr5:22523603-22525075 REVERSE \| Aliases: MWC10.1, MWC10_1 | 6.3 | 4.1 | 2.2 | 7.5 | 0.13% | 7.1 |
| 1965 | AT1G77540.1 \| expressed protein, similar to GB:AAD41433 \| chr1:29142570-29143103 REVERSE \| Aliases: T5M16.13, T5M16_13 | 8.9 | 6.1 | 2.8 | 7.5 | 0.13% | 6.9 |
| 1966 | AT3G28900.1 \| 60S ribosomal protein L34 (RPL34C), similar to 60S ribosomal protein L34 GB:P41098 (Nicotiana tabacum) \| chr3:10903924-10905686 REVERSE \| Aliases: MLD15.7 | 8.2 | 4.8 | 3.5 | 7.5 | 0.13% | 7.0 |
| 1967 | AT1G31970.1 \| DEAD/DEAH box helicase, putative, similar to p68 RNA helicase (Schizosaccharomyces pombe) GI:173419 \| chr1:11479846-11482870 FORWARD \| Aliases: F5M6.3 | 9.5 | 7.7 | 1.8 | 7.5 | 0.13% | 7.3 |
| 1968 | AT2G31200.1 \| Symbol: ADF6 \| actin-depolymerizing factor 6 (ADF6), identical to SP:Q9ZSK2 Actin-depolymerizing factor 6 (ADF-6) (AtADF6) {Arabidopsis thaliana} \| chr2:13301130-13302487 FORWARD \| Aliases: ACTIN DEPOLYMERIZING FACTOR 6, F16D14.4, F16D14_4 | 10.8 | 7.9 | 2.9 | 7.5 | 0.13% | 7.3 |
| 1971 | AT5G36170.3 \| Symbol: HCF109 \| | 7.3 | 5.7 | 1.6 | 7.5 | 0.14% | 7.2 |
| 1972 | AT1G75210.1 \| 5' nucleotidase family protein, contains Pfam profile: PF05761 5' nucleotidase family \| chr1:28227530-28232636 FORWARD \| Aliases: F22H5.7, F22H5_7 | 7.7 | 5.7 | 2.0 | 7.5 | 0.14% | 7.0 |
| 1974 | AT4G12410.1 \| auxin-responsive family protein, similar to GP:546362 small auxin up RNA {Arabidopsis thaliana}; auxin-induced protein 10A -Glycine max,PID:g255579 \| chr4:7342953-7343587 REVERSE \| Aliases: T1P17.3 | 7.9 | 5.1 | 2.8 | 7.4 | 0.14% | 6.9 |
| 1975 | AT4G34265.2 \| expressed protein \| chr4:16402284-16403827 FORWARD \| Aliases: None | 7.6 | 4.4 | 3.2 | 7.4 | 0.14% | 7.0 |
| 1976 | AT2G19760.1 \| Symbol: PRF1 \| profilin 1 (PRO1) (PFN1) (PRF1) / allergen Ara t 8, identical to profilin 1 (Allergen Ara t 8) SP:Q42449 GI:1353770 from (Arabidopsis thaliana) \| chr2:8523869-8525249 REVERSE \| Aliases: F6F22.21, F6F22_21, PFN1, PROFILIN 1 | 12.6 | 11.0 | 1.6 | 7.4 | 0.14% | 7.2 |
| 1977 | AT1G01170.2 \| similar to stress-related ozone-induced protein (OZI1) / stress-related ozone-responsive protein [Arabidopsis thaliana] (TAIR:At4g00860.1); similar to ozone-responsive stress-related protein-like [Oryza sativa (japonica cultivar-group)] (GB:NP_910318.1) \| chr1:73824-74731 REVERSE \| Aliases: F6F3.32 | 8.4 | 6.1 | 2.4 | 7.4 | 0.14% | 7.2 |
| 1981 | ATCG00800.1 \| Symbol: RPS3 \| encodes a chloroplast ribosomal protein S3, a constituent of the small subunit of the ribosomal complex \| chrC:82826-83482 REVERSE \| Aliases: RPS3 | 11.3 | 9.8 | 1.5 | 7.4 | 0.14% | 7.2 |
| 1983 | AT3G24030.1 \| hydroxyethylthiazole kinase family protein, contains Pfam profile: PF02110 hydroxyethylthiazole kinase family \| chr3:8679196-8680819 REVERSE \| Aliases: F14O13.24 | 7.8 | 5.7 | 2.1 | 7.4 | 0.14% | 7.0 |
| 1984 | AT2G29570.1 \| Symbol: PCNA2 \| proliferating cell nuclear antigen 2 (PCNA2), identical to SP:Q9ZW35 Proliferating cell nuclear antigen 2 (PCNA 2) {Arabidopsis thaliana}; nearly identical to SP:Q43124 Proliferating cell nuclear antigen (PCNA) {Brassica napus}; contains Pfam profiles PF00705: Proliferating cell nuclear antigen N-terminal domain, PF02747: Proliferating cell nuclear antigen C-terminal domain \| chr2:12656983-12658780 REVERSE \| Aliases: F16P2.5, F16P2_5, PROLIFERATING CELL NUCLEAR 2 | 8.2 | 5.1 | 3.1 | 7.4 | 0.14% | 7.0 |
| 1986 | AT3G05910.1 \| pectinacetylesterase, putative, similar to pectinacetylesterase precursor GI:1431629 from (Vigna radiata) \| chr3:1764301-1767581 REVERSE \| Aliases: F2O10.13 | 8.1 | 5.7 | 2.4 | 7.4 | 0.14% | 7.2 |

| Rank | Description | Sync | Root | M | t | adj.q | B |
|---|---|---|---|---|---|---|---|
| 1987 | AT2G45740.3 \| similar to peroxisomal biogenesis factor 11 family protein / PEX11 family protein [Arabidopsis thaliana] (TAIR:At3g61070.1); similar to peroxisomal biogenesis factor 11 protein-like [Oryza sativa (japonica cultivar-group)] (GB:XP_550574.1); contains InterPro domain Peroxisomal biogenesis factor 11 (InterPro:IPR008733) \| chr2:18846662-18848414 FORWARD \| Aliases: F4I18.28 | 6.8 | 3.9 | 2.9 | 7.4 | 0.14% | 6.8 |
| 1989 | AT4G37910.1 \| Symbol: MTHSC70 1 \| heat shock protein 70, mitochondrial, putative / HSP70, mitochondrial, putative, strong similarity to SP:Q01899 Heat shock 70 kDa protein, mitochondrial precursor {Phaseolus vulgaris} \| chr4:17825074-17828171 REVERSE \| Aliases: F20D10.30, F20D10_30, MTHSC70 1 | 10.2 | 7.7 | 2.5 | 7.4 | 0.14% | 7.2 |
| 1996 | AT5G11340.1 \| GCN5-related N-acetyltransferase (GNAT) family protein, low similarity to SP:Q03503 L-A virus GAG protein N-acetyltransferase (EC 2.3.1.-) {Saccharomyces cerevisiae}; contains Pfam profile PF00583: acetyltransferase, GNAT family \| chr5:3619156-3621232 FORWARD \| Aliases: F2I11.230, F2I11_230 | 9.7 | 7.6 | 2.1 | 7.4 | 0.14% | 7.1 |
| 1998 | AT2G47950.1 \| expressed protein \| chr2:19631293-19631879 FORWARD \| Aliases: T9J23.9, T9J23_9 | 6.1 | 3.4 | 2.7 | 7.4 | 0.14% | 6.9 |
| 1999 | AT1G72340.1 \| eukaryotic translation initiation factor 2B family protein / eIF-2B family protein, similar to SP:Q64270 Translation initiation factor eIF-2B alpha subunit {Rattus norvegicus}; contains Pfam profile PF01008: Initiation factor 2 subunit family \| chr1:27240543-27242274 FORWARD \| Aliases: T10D10.19, T10D10_19 | 9.0 | 7.4 | 1.5 | 7.4 | 0.14% | 7.1 |
| 2001 | AT1G06680.1 \| Symbol: PSBP \| photosystem II oxygen-evolving complex 23 (OEC23), JBC 14:211-238 (2002); identical to 23 kDa polypeptide of oxygen-evolving comlex (OEC) GB:CAA66785 GI:1769905 (Arabidopsis thaliana) \| chr1:2047878-2049417 FORWARD \| Aliases: F12K11.3, F12K11_3, OE23, OEE2, OXYGEN EVOLVING ENHANCER PROTEIN 2 | 10.5 | 6.7 | 3.8 | 7.4 | 0.14% | 7.0 |
| 2002 | AT5G08320.1 \| expressed protein, predicted proteins, Homo sapiens and Caenorhabditis elegans \| chr5:2677053-2678679 FORWARD \| Aliases: F8L15.50, F8L15_50 | 6.8 | 4.4 | 2.4 | 7.4 | 0.14% | 7.1 |
| 2003 | AT5G50930.1 \| expressed protein, similar to unknown protein (emb:CAB61034.1) \| chr5:20739295-20741543 FORWARD \| Aliases: K3K7.8, K3K7_8 | 6.7 | 3.5 | 3.2 | 7.4 | 0.14% | 5.8 |
| 2004 | AT5G21060.3 \| similar to bifunctional aspartate kinase/homoserine dehydrogenase, putative / AK-HSDH, putative [Arabidopsis thaliana] (TAIR:At4g19710.2); similar to COG0460: Homoserine dehydrogenase [Rubrobacter xylanophilus DSM 9941] (GB:ZP_00199840.1); contains InterPro domain Homoserine dehydrogenase (InterPro:IPR001342) \| chr5:7148980-7152866 REVERSE \| Aliases: T10F18.90, T10F18_90 | 7.9 | 5.0 | 2.9 | 7.4 | 0.14% | 6.8 |
| 2005 | AT3G14290.1 \| Symbol: PAE2 \| 20S proteasome alpha subunit E2 (PAE2), identical to 20S proteasome subunit PAE2 GB:AAC32061 from (Arabidopsis thaliana) | 10.2 | 8.9 | 1.4 | 7.4 | 0.14% | 7.2 |
| 2007 | AT3G27330.1 \| zinc finger (C3HC4-type RING finger) family protein, contains Pfam profiles: PF00097 zinc finger, C3HC4 type (RING finger), PF01697 Domain of unknown function \| chr3:10118187-10121882 REVERSE \| Aliases: K1G2.5 | 6.6 | 4.5 | 2.1 | 7.4 | 0.14% | 6.9 |
| 2008 | AT3G06450.1 \| anion exchange family protein, similar to putative Anion exchanger family members: GB:AAD39673, GB:AAD55295 (Arabidopsis thaliana) \| chr3:1975965-1980042 REVERSE \| Aliases: F24P17.6, F24P17_6 | 7.4 | 5.4 | 2.0 | 7.4 | 0.14% | 7.0 |
| 2012 | AT2G27690.1 \| Symbol: CYP94C1 \| cytochrome P450, putative, similar to Cytochrome P450 94A1 (P450-dependent fatty acid omega-hydroxylase) (SP:O81117) {Vicia sativa}; contains Pfam profile: PF00067 cytochrome P450; supported by cDNA: gi_13877668 \| chr2:11816383-11818292 FORWARD \| Aliases: F15K20.21, F15K20_21 | 5.6 | 3.6 | 2.0 | 7.4 | 0.15% | 6.8 |
| 2013 | AT2G34860.1 \| chaperone protein dnaJ-related, contains Pfam PF00684 : DnaJ central domain (4 repeats); similar to Chaperone protein dnaJ (Heat shock protein 40) (SP:Q9UXR9) {Methanosarcina thermophila} \| chr2:14715447-14717276 FORWARD \| Aliases: F19I3.9, F19I3_9 | 7.2 | 5.0 | 2.2 | 7.4 | 0.15% | 7.1 |
| 2014 | NA | 11.0 | 9.8 | 1.2 | 7.4 | 0.15% | 7.1 |
| 2015 | AT2G31725.1 \| expressed protein \| chr2:13493394-13494522 REVERSE \| Aliases: T9H9.25, T9H9_25 | 5.2 | 3.9 | 1.3 | 7.4 | 0.15% | 7.2 |
| 2016 | AT5G06060.1 \| tropinone reductase, putative / tropine dehydrogenase, putative, similar to tropinone reductase SP:P50165 from (Datura stramonium) \| chr5:1823845-1825895 REVERSE \| Aliases: K16F4.2, K16F4_2 | 8.1 | 6.2 | 1.9 | 7.4 | 0.15% | 7.0 |
| 2017 | AT4G01430.2 \| nodulin MtN21 family protein, similar to MtN21 GI:2598575 (root nodule development) from (Medicago truncatula) \| chr4:585609-588027 FORWARD \| Aliases: F3D13.4, F3D13_4 | 8.2 | 3.9 | 4.4 | 7.4 | 0.15% | 7.0 |
| 2018 | AT3G03980.1 \| short-chain dehydrogenase/reductase (SDR) family protein, similar to short-chain type dehydrogenase/reductase SP:Q08632 (Picea abies) \| chr3:1031749-1033214 FORWARD \| Aliases: T11I18.9, T11I18_9 | 9.3 | 5.6 | 3.6 | 7.4 | 0.15% | 6.5 |
| 2019 | AT2G38905.1 \| hydrophobic protein, putative / low temperature and salt responsive protein, putative, strong similarity to SP:Q9ZNQ7 Hydrophobic protein RCI2A (Low temperature and salt responsive protein LTI6A) {Arabidopsis thaliana}; contains Pfam profile PF01679: Uncharacterized protein family \| chr2:16251177-16251562 REVERSE \| Aliases: None | 6.6 | 2.9 | 3.7 | 7.4 | 0.15% | 6.5 |

| Rank | Description | Sync | Root | M | t | adj.q | B |
|---|---|---|---|---|---|---|---|
| 2020 | AT4G38020.1 \| tRNA/rRNA methyltransferase (SpoU) family protein, similar to SP:P18644 rRNA (adenosine-2'-O-)-methyltransferase (EC 2.1.1.66) {Streptomyces cyaneus}; contains Pfam profile PF00588: SpoU rRNA Methylase (RNA methyltransferase, TrmH) family \| chr4:17861520-17863054 FORWARD \| Aliases: F20D10.140, F20D10_140 | 6.6 | 4.3 | 2.2 | 7.4 | 0.15% | 6.7 |
| 2024 | AT1G67720.1 \| leucine-rich repeat family protein / protein kinase family protein, contains similarity to light repressible receptor protein kinase (Arabidopsis thaliana) gi:1321686:emb:CAA66376; contains Pfam doamins PF00069: Protein kinase domain and PF00560: Leucine Rich Repeat \| chr1:25390004-25394736 FORWARD \| Aliases: F12A21.30 | 6.5 | 4.7 | 1.8 | 7.3 | 0.15% | 7.1 |
| 2026 | AT1G59640.2 \| Symbol: ZCW32 \| basic helix-loop-helix (bHLH) family protein \| chr1:21913300-21914837 REVERSE \| Aliases: T30E16.21, T30E16_21, ZCW32 | 7.2 | 4.7 | 2.5 | 7.3 | 0.15% | 6.8 |
| 2028 | AT1G18500.1 \| 2-isopropylmalate synthase, putative, strong similarity to 2-isopropylmalate synthase (IMS1) (Arabidopsis thaliana) GI:12330687; contains Pfam profile PF00682: HMGL-like \| chr1:6369340-6373018 FORWARD \| Aliases: F15H18.3, F15H18_3 | 10.3 | 7.5 | 2.8 | 7.3 | 0.15% | 7.1 |
| 2030 | AT2G30920.1 \| Symbol: ATCOQ3 \| | 6.4 | 4.4 | 2.0 | 7.3 | 0.15% | 7.0 |
| 2032 | AT4G31360.1 \| expressed protein \| chr4:15221892-15223633 FORWARD \| Aliases: F8F16.180, F8F16_180 | 6.4 | 4.1 | 2.3 | 7.3 | 0.15% | 6.6 |
| 2036 | AT4G37940.1 \| Symbol: AGL21 \| MADS-box family protein, MADS-box protein AGL17 - Arabidopsis thaliana, PID:g862648; identical to cDNA MADS-box protein AGL21 GI:18478602 \| chr4:17835689-17838615 REVERSE \| Aliases: F20D10.60, F20D10_60 | 7.4 | 4.6 | 2.8 | 7.3 | 0.15% | 7.0 |
| 2037 | AT1G32550.1 \| ferredoxin family protein, similar to ferredoxin from Synechocystis sp. (GI:48019); contains Pfam profile PF00111 2Fe-2S iron-sulfur cluster binding domain \| chr1:11771746-11774158 REVERSE \| Aliases: T9G5.4, T9G5_4 | 6.3 | 4.5 | 1.8 | 7.3 | 0.15% | 6.9 |
| 2042 | AT1G74720.1 \| C2 domain-containing protein, contains INTERPRO:IPR000008 C2 domain \| chr1:28078834-28082417 FORWARD \| Aliases: F25A4.35, F25A4_35 | 7.1 | 4.6 | 2.5 | 7.3 | 0.15% | 6.7 |
| 2043 | AT1G76100.1 \| plastocyanin, identical to plastocyanin GI:1865683 from (Arabidopsis thaliana) \| chr1:28558881-28559651 REVERSE \| Aliases: T23E18.3, T23E18_3 | 5.1 | 2.4 | 2.6 | 7.3 | 0.15% | 6.5 |
| 2046 | AT1G48170.1 \| expressed protein \| chr1:17792455-17793762 FORWARD \| Aliases: F21D18.10 | 6.2 | 3.9 | 2.3 | 7.3 | 0.15% | 7.1 |
| 2048 | AT4G36580.1 \| AAA-type ATPase family protein, contains Pfam domain, PF00004: ATPase, AAA family ('A'TPases 'A'ssociated with diverse cellular 'A'ctivities) \| chr4:17257963-17260666 FORWARD \| Aliases: AP22.32, AP22_32 | 6.3 | 3.9 | 2.4 | 7.3 | 0.15% | 6.6 |
| 2050 | AT3G22230.1 \| 60S ribosomal protein L27 (RPL27B), similar to 60S RIBOSOMAL PROTEIN L27 GB:P41101 from (Solanum tuberosum) \| chr3:7843974-7844604 REVERSE \| Aliases: MKA23.22 | 9.2 | 6.8 | 2.4 | 7.3 | 0.15% | 7.0 |
| 2054 | AT1G46264.1 \| Symbol: AT HSFB4 \| heat shock transcription factor family protein, contains Pfam profile: PF00447 HSF-type DNA-binding domain \| chr1:17227282-17228720 FORWARD \| Aliases: F2G19.8, F2G19_8, HSFB4 | 4.7 | 2.9 | 1.8 | 7.3 | 0.15% | 7.0 |
| 2055 | AT1G53520.1 \| chalcone-flavanone isomerase-related, low similarity to GI:499036 (Vitis vinifera) \| chr1:19980083-19981623 REVERSE \| Aliases: F22G10.11 | 6.1 | 3.6 | 2.5 | 7.3 | 0.15% | 6.9 |
| 2061 | AT5G42060.1 \| expressed protein \| chr5:16835616-16836183 REVERSE \| Aliases: MJC20.17, MJC20_17 | 6.3 | 4.2 | 2.1 | 7.3 | 0.15% | 6.9 |
| 2062 | AT3G23990.1 \| Symbol: HSP60 \| chaperonin (CPN60) (HSP60), identical to SWISS-PROT:P29197- chaperonin CPN60, mitochondrial precursor (HSP60) (Arabidopsis thaliana) \| chr3:8668935-8672585 FORWARD \| Aliases: F14O13.1 | 10.4 | 6.9 | 3.5 | 7.3 | 0.15% | 6.9 |
| 2063 | AT1G26880.1 \| 60S ribosomal protein L34 (RPL34A), identical to GB:Q42351, location of EST 105E2T7, gb:T22624 \| chr1:9315370-9316716 REVERSE \| Aliases: T2P11.7, T2P11_7 | 10.4 | 7.9 | 2.5 | 7.3 | 0.15% | 7.0 |
| 2064 | AT2G24940.1 \| cytochrome b5 domain-containing protein, similar to SP:P70580 Membrane associated progesterone receptor component 1 {Rattus norvegicus}; contains Pfam profile PF00173: Heme/Steroid binding domain \| chr2:10616473-10619311 FORWARD \| Aliases: F27C12.14, F27C12_14 | 12.4 | 10.1 | 2.3 | 7.3 | 0.15% | 7.1 |
| 2065 | AT2G24945.1 \| expressed protein \| chr2:10616492-10619311 FORWARD \| Aliases: None | 12.4 | 10.1 | 2.3 | 7.3 | 0.15% | 7.1 |
| 2067 | AT2G40750.1 \| Symbol: WRKY54 \| | 5.8 | 4.5 | 1.3 | 7.3 | 0.15% | 7.1 |
| 2068 | AT2G41350.2 \| similar to hypothetical protein [Arabidopsis thaliana] (TAIR:At2g21980.1); similar to unnamed protein product [Mus musculus] (GB:BAC36526.1) \| chr2:17238428-17240536 FORWARD \| Aliases: F13H10.10, F13H10_10 | 7.8 | 5.9 | 1.9 | 7.3 | 0.15% | 7.1 |

| Rank | Description | Sync | Root | M | t | adj.q | B |
|---|---|---|---|---|---|---|---|
| 2069 | AT3G51510.1 \| expressed protein \| chr3:19120041-19121030 FORWARD \| Aliases: F26O13.150 | 6.8 | 3.9 | 2.9 | 7.3 | 0.15% | 6.3 |
| 2073 | AT2G40840.1 \| glycoside hydrolase family 77 protein, contains Pfam profiles PF02446: 4-alpha-glucanotransferase, PF00686: Starch binding domain; contains a non-consensus AT-AC intron between at intron 5 \| chr2:17052041-17058025 FORWARD \| Aliases: T20B5.4, T20B5_4 | 8.4 | 6.5 | 2.0 | 7.3 | 0.16% | 7.1 |
| 2074 | AT3G06850.2 \| Symbol: DIN3 \| branched chain alpha-keto acid dehydrogenase E2 subunit (din3), identical to branched chain alpha-keto acid dehydrogenase E2 subunit (din3) (Arabidopsis thaliana) GI:7021284 \| chr3:2157989-2160619 REVERSE \| Aliases: DARK INDUCIBLE 3, F3E22.1, LTA1 | 7.3 | 6.2 | 1.1 | 7.3 | 0.16% | 7.1 |
| 2078 | AT1G50110.1 \| branched-chain amino acid aminotransferase 6 / branched-chain amino acid transaminase 6 (BCAT6), contains Pfam profile: PF01063 aminotransferase class IV; identical to SP:Q9LPM9 Branched-chain amino acid aminotransferase 6 (EC 2.6.1.42) (Atbcat-6) {Arabidopsis thaliana} \| chr1:18561685-18563940 REVERSE \| Aliases: F2J10.4, F2J10_4 | 6.5 | 4.5 | 2.0 | 7.3 | 0.16% | 6.8 |
| 2081 | AT5G58110.1 \| expressed protein, predicted proteins, Homo sapiens and Drosophila melanogaster \| chr5:23532412-23533860 FORWARD \| Aliases: K21L19.11, K21L19_11 | 10.2 | 8.7 | 1.5 | 7.3 | 0.16% | 7.1 |
| 2083 | AT5G48630.1 \| cyclin family protein, similar to SP:P55168 Cyclin C {Gallus gallus}; contains Pfam profile PF00134: Cyclin, N-terminal domain \| chr5:19738776-19740460 REVERSE \| Aliases: K15N18.10, K15N18_10 | 6.5 | 3.9 | 2.6 | 7.3 | 0.16% | 7.0 |
| 2084 | AT2G14880.1 \| SWIB complex BAF60b domain-containing protein, contains Pfam profile PF02201: BAF60b domain of the SWIB complex \| chr2:6400414-6402020 REVERSE \| Aliases: T26I20.4, T26I20_4 | 6.7 | 3.6 | 3.0 | 7.3 | 0.16% | 6.5 |
| 2087 | AT5G11160.1 \| adenine phosphoribosyltransferase, putative, strong similarity to SP:Q42563 Adenine phosphoribosyltransferase 2 (EC 2.4.2.7) (APRT) {Arabidopsis thaliana}; contains Pfam profile PF00156: Phosphoribosyl transferase domain \| chr5:3550551-3552145 FORWARD \| Aliases: F2I11.50, F2I11_50 | 5.9 | 4.2 | 1.7 | 7.2 | 0.16% | 6.9 |
| 2088 | AT3G21420.1 \| oxidoreductase, 2OG-Fe(II) oxygenase family protein, similar to SP:Q9ZWQ9 Flavonol synthase (EC 1.14.11.-) {Citrus unshiu}; contains Pfam profile PF03171: oxidoreductase, 2OG-Fe(II) oxygenase family \| chr3:7541509-7543524 FORWARD \| Aliases: MHC9.10 | 8.8 | 5.7 | 3.0 | 7.2 | 0.16% | 6.9 |
| 2089 | AT2G24020.1 \| expressed protein, contains Pfam domain PF02575: Uncharacterized BCR, YbaB family COG0718 \| chr2:10224781-10226361 REVERSE \| Aliases: T29E15.22, T29E15_22 | 7.8 | 5.7 | 2.1 | 7.2 | 0.16% | 6.8 |
| 2092 | AT3G02540.2 \| ubiquitin family protein, contains Pfam profiles PF00240: Ubiquitin family, PF00627: UBA/TS-N domain; \| chr3:533461-536302 REVERSE \| Aliases: F16B3.17, F16B3_17 | 7.6 | 4.9 | 2.8 | 7.2 | 0.16% | 6.9 |
| 2093 | AT2G30580.1 \| zinc finger (C3HC4-type RING finger) family protein, contains Pfam profile: PF00097 zinc finger, C3HC4 type (RING finger) \| chr2:13033077-13037738 FORWARD \| Aliases: T6B20.7, T6B20_7 | 6.0 | 4.6 | 1.4 | 7.2 | 0.16% | 7.1 |
| 2096 | AT1G69380.1 \| expressed protein, predicated by genscan+ \| chr1:26085004-26087269 REVERSE \| Aliases: F23O10.4, F23O10_4 | 6.2 | 4.1 | 2.1 | 7.2 | 0.16% | 6.9 |
| 2098 | AT2G35605.1 \| SWIB complex BAF60b domain-containing protein, contains Pfam profile PF02201: BAF60b domain of the SWIB complex \| chr2:14953143-14954355 FORWARD \| Aliases: None | 7.8 | 4.2 | 3.6 | 7.2 | 0.16% | 6.6 |
| 2099 | AT4G03960.1 \| tyrosine specific protein phosphatase family protein, contains tyrosine specific protein phosphatases active site, PROSITE:PS00383 \| chr4:1887526-1889209 FORWARD \| Aliases: T24M8.4, T24M8_4 | 7.4 | 4.9 | 2.5 | 7.2 | 0.16% | 6.8 |
| 2101 | AT4G20170.1 \| expressed protein \| chr4:10896689-10899151 FORWARD \| Aliases: F1C12.90, F1C12_90 | 7.4 | 5.8 | 1.5 | 7.2 | 0.16% | 7.0 |
| 2103 | AT2G19670.1 \| protein arginine N-methyltransferase, putative, similar to protein arginine N-methyltransferase 1-variant 1 (Homo sapiens) GI:7453577 \| chr2:8506199-8508232 REVERSE \| Aliases: F6F22.30, F6F22_30 | 5.1 | 3.1 | 2.0 | 7.2 | 0.16% | 7.0 |
| 2104 | AT2G39930.1 \| Symbol: ATISA1/ISA1 \| isoamylase, putative / starch debranching enzyme, putative, similar to isoamylase from (Solanum tuberosum) GI:27728145, (Triticum aestivum) GI:17932898, (Hordeum vulgare) GI:21314275, (Oryza sativa) GI:3252794; contains Pfam profiles PF00128: Alpha amylase catalytic domain, PF02922: Isoamylase N-terminal domain.Mutants have reduced starch content and abnormally structured amylopectins and phytoglycogens. \| chr2:16673125-16680012 FORWARD \| Aliases: ATISA1, ISA1, T28M21.9, T28M21_9 | 7.2 | 4.9 | 2.3 | 7.2 | 0.16% | 6.8 |
| 2105 | AT5G26570.1 \| Symbol: PWD \| similar to starch excess protein (SEX1) [Arabidopsis thaliana] (TAIR:At1g10760.1); similar to starch associated protein R1 [Solanum tuberosum] (GB:AAK11735.1); contains InterPro domain Glycoside hydrolase, starch-binding domain (InterPro:IPR002044) \| chr5:9261583-9267732 FORWARD \| Aliases: ATGWD3, F9D12.1, F9D12_1, OK1, PHOSPHOGLUCAN WATER DIKINASE | 7.4 | 5.1 | 2.3 | 7.2 | 0.16% | 6.9 |
| 2106 | AT2G44310.1 \| calcium-binding EF hand family protein, contains INTERPRO:IPR002048 calcium-binding EF-hand domain \| chr2:18316288-18316902 FORWARD \| Aliases: F4I1.12 | 9.9 | 7.5 | 2.3 | 7.2 | 0.16% | 6.9 |

| Rank | Description | Sync | Root | M | t | adj.q | B |
|---|---|---|---|---|---|---|---|
| 2108 | AT5G12190.1 \| RNA recognition motif (RRM)-containing protein, similar to SP:P52298 20 kDa nuclear cap binding protein (NCBP 20 kDa subunit) (CBP20) (NCBP interacting protein 1) (NIP1) {Homo sapiens}; contains InterPro entry IPR000504: RNA-binding region RNP-1 (RNA recognition motif) (RRM) \| chr5:3940533-3941206 REVERSE \| Aliases: MXC9.15, MXC9_15 | 8.4 | 5.6 | 2.8 | 7.2 | 0.16% | 7.0 |
| 2111 | AT5G40970.1 \| expressed protein \| chr5:16431266-16431732 FORWARD \| Aliases: MEE6.4, MEE6_4 | 6.0 | 2.4 | 3.6 | 7.2 | 0.16% | 6.9 |
| 2114 | AT3G10620.1 \| diadenosine 5',5'''-P1,P4-tetraphosphate hydrolase, putative, similar to diadenosine 5',5'''-P1,P4-tetraphosphate hydrolase from (Lupinus angustifolius) GI:1888557, (Hordeum vulgare subsp. vulgare) GI:2564253; contains Pfam profile PF00293: NUDIX domain \| chr3:3320268-3322022 FORWARD \| Aliases: F13M14.9 | 7.6 | 5.0 | 2.6 | 7.2 | 0.16% | 6.5 |
| 2115 | AT1G75290.1 \| isoflavone reductase, putative, similar to SP:P52577 Isoflavone reductase homolog P3 (EC 1.3.1.-) {Arabidopsis thaliana}; contains Pfam profile PF02716: Isoflavone reductase \| chr1:28257496-28258947 FORWARD \| Aliases: F22H5.18, F22H5_18 | 6.3 | 3.4 | 2.9 | 7.2 | 0.16% | 6.4 |
| 2116 | AT2G23150.1 \| Symbol: NRAMP3 \| | 6.9 | 3.7 | 3.2 | 7.2 | 0.16% | 6.3 |
| 2117 | AT1G33040.1 \| nascent polypeptide-associated complex (NAC) domain-containing protein, similar to alpha-NAC, non-muscle form (Mus musculus) GI:1666690; contains Pfam profile PF01849: NAC domain \| chr1:11966479-11967823 FORWARD \| Aliases: F9L11.19, F9L11_19 | 10.6 | 7.1 | 3.5 | 7.2 | 0.16% | 7.0 |
| 2119 | AT3G06680.1 \| 60S ribosomal protein L29 (RPL29B), similar to 60S ribosomal protein L29 GB:P25886 from (Rattus norvegicus) \| chr3:2114408-2114885 REVERSE \| Aliases: T8E24.9 | 9.1 | 5.4 | 3.7 | 7.2 | 0.16% | 7.0 |
| 2120 | AT5G17840.1 \| chaperone protein dnaJ-related, similar to bundle sheath defective protein 2 (GI:4732091) (Zea mays) \| chr5:5895678-5897152 REVERSE \| Aliases: MVA3.190, MVA3_190 | 8.3 | 6.8 | 1.4 | 7.2 | 0.16% | 7.0 |
| 2121 | AT1G02150.1 \| pentatricopeptide (PPR) repeat-containing protein, low similiarity to DNA-binding protein (Triticum aestivum) GI:6958202; contains Pfam profile: PF01535 PPR repeat \| chr1:408622-410611 FORWARD \| Aliases: T6A9.13 | 6.5 | 5.5 | 1.0 | 7.2 | 0.16% | 7.0 |
| 2122 | AT5G47810.1 \| phosphofructokinase family protein, similar to phosphofructokinase (Amycolatopsis methanolica) GI:17432243; contains Pfam profile PF00365: Phosphofructokinase \| chr5:19373582-19375351 REVERSE \| Aliases: MCA23.13, MCA23_13 | 9.8 | 8.0 | 1.8 | 7.2 | 0.16% | 7.0 |
| 2126 | AT4G15790.1 \| expressed protein \| chr4:8982253-8984216 FORWARD \| Aliases: DL3935W, FCAALL.69 | 5.7 | 3.7 | 2.0 | 7.2 | 0.16% | 7.0 |
| 2127 | AT1G47960.1 \| Symbol: C/VIF1 \| | 9.8 | 7.4 | 2.4 | 7.2 | 0.16% | 7.0 |
| 2135 | AT3G16980.1 \| DNA-directed RNA polymerase II, putative, similar to SP:P36958 DNA-directed RNA polymerase II 15.1 kDa polypeptide (EC 2.7.7.6) {Drosophila melanogaster}; contains Pfam profile PF02150: RNA polymerases M/15 Kd subunit \| chr3:5794502-5795705 FORWARD \| Aliases: K14A17.16 | 5.7 | 2.9 | 2.8 | 7.2 | 0.16% | 6.5 |
| 2136 | AT4G35905.1 \| expressed protein, contains Pfam domain PF03966: Protein of unknown function (DUF343) \| chr4:17007031-17008157 REVERSE \| Aliases: None | 6.0 | 4.6 | 1.4 | 7.2 | 0.16% | 7.0 |
| 2137 | AT3G10530.1 \| transducin family protein / WD-40 repeat family protein, contains 2 WD-40 repeats (PF00400); BING4 (gi:3811380) {Mus musculus}; similar to hypothetical protein GB:P40055 (Saccharomyces cerevisiae) \| chr3:3286018-3288885 FORWARD \| Aliases: F13M14.19 | 6.7 | 4.4 | 2.3 | 7.2 | 0.16% | 6.8 |
| 2138 | AT5G49510.2 \| similar to unnamed protein product [Mus musculus] (GB:BAC37066.1); similar to PREDICTED: similar to von Hippel-Lindau binding protein 1 [Canis familiaris] (GB:XP_538210.1); similar to PFD3_MOUSE Prefoldin subunit 3 (Von Hippel-Lindau binding protein 1) (VHL binding protein-1) (VBP-1) (GB:P61759); similar to von Hippel-Lindau binding protein 1 [Homo sapiens] (GB:AAV38411.1); similar to von Hippel-Lindau binding protein 1 [synthetic construct] (GB:AAV38448.1); contains InterPro domain Putative prefoldin chaperone (InterPro:IPR004127) \| chr5:20097841-20100147 FORWARD \| Aliases: K6M13.5, K6M13_5 | 8.8 | 7.0 | 1.8 | 7.2 | 0.16% | 6.9 |
| 2147 | AT4G19400.1 \| expressed protein \| chr4:10580838-10582000 REVERSE \| Aliases: T5K18.180, T5K18_180 | 7.7 | 6.1 | 1.6 | 7.2 | 0.16% | 7.0 |
| 2149 | AT1G76520.2 \| auxin efflux carrier family protein, contains auxin efflux carrier domain, Pfam:PF03547 \| chr1:28720049-28722308 FORWARD \| Aliases: F14G6.12, F14G6_12 | 8.6 | 4.5 | 4.1 | 7.2 | 0.16% | 6.7 |

| Rank | Description | Sync | Root | M | t | adj.q | B |
|---|---|---|---|---|---|---|---|
| 2153 | AT5G56500.2 \| similar to chaperonin, putative [Arabidopsis thaliana] (TAIR:At1g26230.1); similar to RuBisCO subunit binding-protein beta subunit, chloroplast / 60 kDa chaperonin beta subunit / CPN-60 beta [Arabidopsis thaliana] (TAIR:At1g55490.2); similar to chaperonin, putative [Arabidopsis thaliana] (TAIR:At3g13470.1); similar to RuBisCO subunit binding-protein beta subunit, chloroplast / 60 kDa chaperonin beta subunit / CPN-60 beta [Arabidopsis thaliana] (TAIR:At1g55490.1); similar to putative RuBisCO subunit binding-protein beta subunit, chloroplast precursor [Oryza sativa (japonica cultivar-group)] (GB:XP_463795.1); similar to chaperonin [Secale cereale] (GB:CAA93139.1); similar to chaperonin 62.5K beta chain - rape (GB:PW0007); similar to putative chaperonin 60 beta precursor [Oryza sativa (japonica cultivar-group)] (GB:NP_910308.1); similar to chaperonin precursor [Pisum sativum] (GB:AAA66365.1); contains InterPro domain Chaperonin Cpn60 (InterPro:IPR001844); contains InterPro domain Chaperonin Cpn60/TCP-1 (InterPro:IPR002423) \| chr5:22891017-22894622 FORWARD \| Aliases: MCD7.27, MCD7_27 | 11.3 | 8.2 | 3.1 | 7.2 | 0.16% | 7.0 |
| 2155 | AT3G15080.1 \| exonuclease family protein, contains exonuclease domain, Pfam:PF00929 \| chr3:5074272-5076528 FORWARD \| Aliases: K15M2.23 | 6.6 | 4.4 | 2.2 | 7.2 | 0.16% | 7.0 |
| 2156 | AT1G74000.1 \| Symbol: SS3 \| similar to strictosidine synthase family protein [Arabidopsis thaliana] (TAIR:At1g74020.1); similar to strictosidine synthase (GB:AAB40594.1); contains InterPro domain Strictosidine synthase (InterPro:IPR004141) \| chr1:27832803-27835088 REVERSE \| Aliases: ATSS 3 STRICTOSIDINE SYNTHASE, F2P9.13, F2P9_13, STRICTOSIDINE SYNTHASE 3 | 6.1 | 3.6 | 2.5 | 7.2 | 0.16% | 6.5 |
| 2157 | AT1G71750.1 \| phosphoribosyltransferase family protein, similar to SP:Q02522 Hypoxanthine-guanine phosphoribosyltransferase (EC 2.4.2.8) (HGPRT) (HGPRTase) {Lactococcus lactis}; contains Pfam profile PF00156: Phosphoribosyl transferase domain \| chr1:26991465-26992822 REVERSE \| Aliases: F14O23.13, F14O23_13 | 5.6 | 3.7 | 1.9 | 7.2 | 0.16% | 7.0 |
| 2160 | AT5G57300.1 \| UbiE/COQ5 methyltransferase family protein, similar to ubiquinone biosynthesis methyltransferase COQ5 (Saccharomyces cerevisiae)(SP:P49017), ubiquinone/menaquinone biosynthesis methyltransferase ubiE (Escherichia coli)(SP:P27851); contains Pfam profile PF01209: methlytransferase, UbiE/COQ5 family \| chr5:23225809-23228316 REVERSE \| Aliases: MJB24.11, MJB24_11 | 9.1 | 6.6 | 2.5 | 7.2 | 0.16% | 6.9 |
| 2161 | AT1G34430.1 \| Symbol: EMB3003 \| dihydrolipoamide S-acetyltransferase, putative, similar to dihydrolipoamide S-acetyltransferase (LTA2) (Arabidopsis thaliana) GI:5881963; contains Pfam profiles PF00198: 2-oxo acid dehydrogenases acyltransferase (catalytic domain), PF00364: Biotin-requiring enzyme, PF02817: e3 binding domain \| chr1:12587730-12590120 REVERSE \| Aliases: EMB3003, EMBRYO DEFECTIVE 3003, F12K21.24, F12K21_24 | 9.5 | 6.7 | 2.8 | 7.2 | 0.16% | 6.9 |
| 2162 | AT1G20640.2 \| RWP-RK domain-containing protein, similar to nodule inception protein GI:6448579 from (Lotus japonicus); contains Pfam profile: PF02042 RWP-RK domain \| chr1:7154400-7158208 FORWARD \| Aliases: F5M15.4 | 8.0 | 5.6 | 2.5 | 7.2 | 0.16% | 6.6 |
| 2163 | AT5G08270.1 \| expressed protein \| chr5:2661748-2663483 FORWARD \| Aliases: F8L15.1, F8L15_1 | 4.5 | 3.6 | 1.0 | 7.2 | 0.16% | 7.0 |
| 2164 | AT3G54920.1 \| Symbol: PMR6 \| pectate lyase, putative / powdery mildew susceptibility protein (PMR6), identical to powdery mildew susceptibility protein (Arabidopsis thaliana) GI:22506901; similar to pectate lyase 2 GP:6606534 from (Musa acuminata) \| chr3:20356077-20359507 FORWARD \| Aliases: F28P10.100, POWDERY MILDEW RESISTANT 6 | 8.6 | 5.9 | 2.7 | 7.2 | 0.16% | 6.8 |
| 2165 | AT1G14450.1 \| expressed protein, contains similarity to cytochrome c oxidase subunit I GI:5678701 from (Loligo pealei) | 9.2 | 7.6 | 1.7 | 7.2 | 0.17% | 7.0 |
| 2166 | AT3G52390.2 \| tatD-related deoxyribonuclease family protein, similar to SP:P27859 Deoxyribonuclease tatD (EC 3.1.21.-) (DNAse tatD) {Escherichia coli}; contains Pfam profile PF01026: TatD related DNase \| chr3:19433692-19436161 REVERSE \| Aliases: F22O6.230 | 7.0 | 4.6 | 2.4 | 7.2 | 0.17% | 6.7 |
| 2167 | AT3G03920.1 \| Gar1 RNA-binding region family protein, contains Pfam profile PF04410: Gar1 protein RNA binding region \| chr3:1009005-1010477 REVERSE \| Aliases: F20H23.3, F20H23_3 | 9.1 | 6.5 | 2.6 | 7.2 | 0.17% | 6.8 |
| 2171 | AT5G01920.1 \| Symbol: STN8 \| protein kinase family protein, contains eukaryotic protein kinase domain, INTERPRO:IPR000719 \| chr5:359127-362901 FORWARD \| Aliases: T20L15.190, T20L15_190 | 7.8 | 6.3 | 1.5 | 7.2 | 0.17% | 7.0 |
| 2180 | AT1G47250.1 \| Symbol: PAF2 \| 20S proteasome alpha subunit F2 (PAF2) (PRC2B) (PRS1), identical to GB:AAC32063 from (Arabidopsis thaliana) (Genetics 149 (2), 677-692 (1998)); identical to cDNA proteasome subunit prc2b GI:2511585 \| chr1:17321617-17324100 FORWARD \| Aliases: 20S PROTEASOME SUBUNIT PAF2, F8G22.3, F8G22_3 | 9.5 | 6.2 | 3.3 | 7.1 | 0.17% | 6.6 |
| 2183 | AT2G34460.1 \| flavin reductase-related, low similarity to SP:P30043 Flavin reductase {Homo sapiens} \| chr2:14536687-14538037 FORWARD \| Aliases: F13P17.33 | 7.5 | 4.2 | 3.3 | 7.1 | 0.17% | 6.5 |
| 2185 | AT1G75670.2 \| expressed protein, similar to DNA-dependent RNA polymerase 19 kDa polypeptide (EC 2.7.7.6) (A43) (Swiss-Prot:O43036) (Schizosaccharomyces pombe) | 6.3 | 4.2 | 2.1 | 7.1 | 0.17% | 6.6 |
| 2186 | AT3G02650.1 \| pentatricopeptide (PPR) repeat-containing protein, contains Pfam profile PF01535: PPR repeat \| chr3:566278-570110 FORWARD \| Aliases: F16B3.28, F16B3_28 | 7.1 | 4.6 | 2.5 | 7.1 | 0.17% | 6.9 |

| Rank | Description | Sync | Root | M | t | adj.q | B |
|---|---|---|---|---|---|---|---|
| 2187 | AT1G01050.1 \| inorganic pyrophosphatase, putative (soluble) / pyrophosphate phospho-hydrolase, putative / PPase, putative, strong similarity to SP:Q43187 Soluble inorganic pyrophosphatase (EC 3.6.1.1) (Pyrophosphate phospho- hydrolase) (PPase) {Solanum tuberosum}; contains Pfam profile PF00719: inorganic pyrophosphatase \| chr1:31181-33148 REVERSE \| Aliases: T25K16.5, T25K16_5 | 10.6 | 8.4 | 2.2 | 7.1 | 0.17% | 7.0 |
| 2190 | AT3G50410.1 \| Symbol: OBP1 \| Dof-type zinc finger domain-containing protein \| chr3:18720729-18721724 FORWARD \| Aliases: F11C1.250, OBF BINDING PROTEIN | 6.3 | 3.1 | 3.2 | 7.1 | 0.17% | 5.9 |
| 2191 | AT1G74470.1 \| geranylgeranyl reductase, identical to geranylgeranyl reductase GB:Y14044 (Arabidopsis thaliana) (involvement: chlorophyll, the tocopherol and the phylloquinone pathways Eur J Biochem 1998 Jan 15;251(1-2):413-7) \| chr1:27994826-27996667 FORWARD \| Aliases: F1M20.15, F1M20_15 | 10.5 | 6.3 | 4.3 | 7.1 | 0.17% | 6.7 |
| 2193 | AT4G31780.2 \| Symbol: MGD1 \| 1,2-diacylglycerol 3-beta-galactosyltransferase, putative / monogalactosyldiacylglycerol synthase, putative / MGDG synthase, putative, similar to MGD synthase type A from Arabidopsis thaliana (gi:9927297), similar to monogalactosyldiacylglycerol synthase, Cucumis sativus, PID:g1805254 \| chr4:15374005-15377140 FORWARD \| Aliases: F11C18.2, MONOGALACTOSYLDIACYLGLYCEROL SYNTHASE, MONOGALACTOSYLDIACYLGLYCEROL SYNTHASE 1, MONOGALACTOSYLDIACYLGLYCEROL SYNTHASE A | 8.2 | 7.0 | 1.2 | 7.1 | 0.17% | 6.9 |
| 2195 | AT1G13810.1 \| expressed protein, ; expression supported by MPSS \| chr1:4734085-4735161 FORWARD \| Aliases: F16A14.30, F16A14_30 | 5.9 | 3.9 | 2.0 | 7.1 | 0.17% | 6.7 |
| 2198 | AT5G04900.1 \| short-chain dehydrogenase/reductase (SDR) family protein, contains INTERPRO family IPR002198 Short-chain dehydrogenase/reductase (SDR) superfamily \| chr5:1434816-1437326 FORWARD \| Aliases: MUG13.25, MUG13_25 | 5.1 | 3.2 | 2.0 | 7.1 | 0.17% | 6.6 |
| 2199 | AT3G17890.1 \| expressed protein \| chr3:6127047-6127880 FORWARD \| Aliases: MEB5.11 | 5.4 | 3.1 | 2.4 | 7.1 | 0.17% | 6.5 |
| 2200 | AT5G65280.1 \| lanthionine synthetase C-like family protein, contains Pfam domain, PF05147: Lanthionine synthetase C-like protein \| chr5:26103199-26105370 REVERSE \| Aliases: MQN23.23, MQN23_23 | 7.1 | 5.1 | 2.0 | 7.1 | 0.17% | 6.9 |
| 2201 | AT5G01210.1 \| transferase family protein, contains Pfam profile PF02458 transferase family \| chr5:84473-86274 FORWARD \| Aliases: F7J8.190, F7J8_190 | 11.1 | 9.0 | 2.1 | 7.1 | 0.17% | 6.9 |
| 2203 | AT4G34190.1 \| Symbol: SEP1 \| stress enhanced protein 1 (SEP1), identical to stress enhanced protein 1 (SEP1) GI:7384978 from (Arabidopsis thaliana) \| chr4:16372394-16373630 REVERSE \| Aliases: F10M10.5, SEP1, STRESS ENHANCED PROTEIN 1 | 7.0 | 5.2 | 1.8 | 7.1 | 0.17% | 6.9 |
| 2207 | AT5G44000.1 \| glutathione S-transferase C-terminal domain-containing protein, contains Pfam domain PF00043: Glutathione S-transferase, C-terminal domain \| chr5:17719357-17721178 REVERSE \| Aliases: MRH10.11, MRH10_11 | 8.8 | 6.6 | 2.2 | 7.1 | 0.17% | 6.8 |
| 2209 | AT3G61450.1 \| Symbol: SYP73 \| syntaxin 73 (SYP73), identical to syntaxin 73 (AtSYP73) (Swiss-Prot:Q94KK5) (Arabidopsis thaliana) \| chr3:22749369-22750959 FORWARD \| Aliases: ATSYP73, F2A19.50, SYNTAXIN OF PLANTS 73 | 5.3 | 3.2 | 2.1 | 7.1 | 0.17% | 6.6 |
| 2210 | AT2G40760.1 \| rhodanese-like domain-containing protein, contains rhodanese-like domain PF00581 | 7.8 | 6.2 | 1.6 | 7.1 | 0.17% | 6.9 |
| 2211 | AT3G56630.1 \| Symbol: CYP94D2 \| cytochrome P450, putative, cytochrome P450 CYP94A1 - Vicia sativa, PIR:T08014 | 6.6 | 3.9 | 2.6 | 7.1 | 0.17% | 6.1 |
| 2212 | AT3G07910.1 \| expressed protein \| chr3:2523163-2524171 REVERSE \| Aliases: F17A17.25 | 10.1 | 8.5 | 1.6 | 7.1 | 0.17% | 6.9 |
| 2213 | AT1G67440.1 \| Symbol: EMB1688 \| expressed protein, contains Pfam domain PF03193: Protein of unknown function, DUF258 \| chr1:25267467-25269382 REVERSE \| Aliases: EMB1688, EMBRYO DEFECTIVE 1688, T1F15.10, T1F15_10 | 6.5 | 4.0 | 2.4 | 7.1 | 0.17% | 6.7 |
| 2214 | AT3G04230.1 \| 40S ribosomal protein S16 (RPS16B), similar to 40S ribosomal protein S16 GB:AAD22696 (Arabidopsis thaliana) \| chr3:1112997-1113616 REVERSE \| Aliases: T6K12.15, T6K12_15 | 6.2 | 4.4 | 1.8 | 7.1 | 0.17% | 6.8 |
| 2218 | AT1G23100.1 \| 10 kDa chaperonin, putative, similar to 10 kDa chaperonin SP:P34893 from (Arabidopsis thaliana) \| chr1:8195769-8196464 FORWARD \| Aliases: T26J12.12, T26J12_12 | 5.7 | 3.7 | 2.0 | 7.1 | 0.17% | 6.7 |
| 2220 | AT4G00030.1 \| plastid-lipid associated protein PAP / fibrillin family protein, contains Pfam profile PF04755: PAP_fibrillin \| chr4:13527-14413 FORWARD \| Aliases: F6N15.13, F6N15_13 | 5.3 | 3.0 | 2.2 | 7.1 | 0.18% | 6.7 |
| 2227 | AT1G54160.1 \| CCAAT-binding transcription factor (CBF-B/NF-YA) family protein, contains Pfam profile: PF02045 CCAAT-binding transcription factor (CBF-B/NF-YA) subunit B \| chr1:20221003-20223028 REVERSE \| Aliases: F15I1.26, F15I1_26 | 6.7 | 3.9 | 2.7 | 7.1 | 0.18% | 6.3 |
| 2228 | AT5G60540.1 \| Symbol: EMB2407 \| SNO glutamine amidotransferase family protein, similar to pyridoxine synthesis protein PDX2 (Cercospora nicotianae) GI:9954418; contains Pfam profile PF01174: SNO glutamine amidotransferase family \| chr5:24353849-24356028 REVERSE \| Aliases: EMB2407, EMBRYO DEFECTIVE 2407, MUF9.1, MUF9_1 | 10.3 | 6.7 | 3.6 | 7.1 | 0.18% | 6.8 |
| 2229 | AT5G06670.1 \| kinesin motor protein-related \| chr5:2048244-2055020 REVERSE \| Aliases: F15M7.20, F15M7_20 | 6.1 | 4.8 | 1.3 | 7.1 | 0.18% | 6.9 |

| Rank | Description | Sync | Root | M | t | adj.q | B |
|---|---|---|---|---|---|---|---|
| 2231 | AT2G46820.2 \| Symbol: TMP14 \| expressed protein, similar to expressed protein [Arabidopsis thaliana] (TAIR:At1g52220.1); similar to hypothetical protein [Oryza sativa (japonica cultivar-group)] (GB:AAP54812.1) \| chr2:19250565-19252206 FORWARD \| Aliases: F19D11.10, THYLAKOID MEMBRANE PHOSPHOPROTEIN OF 14 KDA | 8.1 | 5.7 | 2.4 | 7.1 | 0.18% | 6.8 |
| 2232 | AT4G24050.1 \| short-chain dehydrogenase/reductase (SDR) family protein, contains INTERPRO family IPR002198 Short-chain dehydrogenase/reductase (SDR) superfamily \| chr4:12497077-12500138 FORWARD \| Aliases: T19F6.40, T19F6_40 | 5.3 | 3.9 | 1.4 | 7.1 | 0.18% | 6.9 |
| 2233 | AT4G25050.1 \| Symbol: ACP4 \| acyl carrier family protein / ACP family protein, similar to Acyl carrier protein, chloroplast precursor from {Spinacia oleracea} SP:P23235, {Casuarina glauca} SP:P93092; contains InterPro accession IPR003881: Isochorismatase \| chr4:12870077-12871228 FORWARD \| Aliases: F13M23.190, F24A6.4 | 7.1 | 3.5 | 3.7 | 7.1 | 0.18% | 6.3 |
| 2236 | AT3G53560.1 \| chloroplast lumen common family protein \| chr3:19870605-19872009 REVERSE \| Aliases: F4P12.260 | 6.7 | 4.6 | 2.1 | 7.0 | 0.18% | 6.8 |
| 2237 | AT1G44920.1 \| expressed protein \| chr1:16985344-16986849 REVERSE \| Aliases: T12C22.21, T12C22_21 | 6.7 | 4.2 | 2.5 | 7.0 | 0.18% | 6.2 |
| 2238 | AT3G06730.1 \| thioredoxin family protein, contains Pfam profile: PF00085 Thioredoxin \| chr3:2124240-2126148 FORWARD \| Aliases: F3E22.13 | 5.0 | 2.7 | 2.3 | 7.0 | 0.18% | 6.4 |
| 2239 | AT2G28740.1 \| Symbol: HIS4 \| histone H4, identical to histone H4 from Lycopersicon esculentum GI:297150, Lolium temulentum SP:P02308, Acropora formosa GI:455652, Citrus jambhiri GI:16797797 | 8.6 | 5.9 | 2.7 | 7.0 | 0.18% | 6.5 |
| 2241 | AT2G27960.1 \| Symbol: CKS1 \| cyclin-dependent kinase / CDK (CKS1), identical to Cks1 protein (Arabidopsis thaliana) gi:2274859:emb:CAA03859 \| chr2:11917803-11918703 REVERSE \| Aliases: CATALYTIC SUBUNIT OF CYCLIN DEPENDENT KINASE, CDK subunit 1, CKS1, CKS1AT, P10CKS1AT, SUC1, T1E2.12, T1E2_12 | 9.7 | 7.2 | 2.5 | 7.0 | 0.18% | 6.8 |
| 2242 | AT3G52750.1 \| Symbol: FTSZ2 2 \| chloroplast division protein, putative, strong similarity to plastid division protein FtsZ (Arabidopsis thaliana) GI:14195704, chloroplast division protein AtFtsZ2-1 (Arabidopsis thaliana) GI:15636809 \| chr3:19560639-19563992 REVERSE \| Aliases: F3C22.150 | 8.1 | 4.8 | 3.3 | 7.0 | 0.18% | 6.6 |
| 2243 | AT3G16520.3 \| UDP-glucoronosyl/UDP-glucosyl transferase family protein, contains Pfam profile: PF00201 UDP-glucoronosyl and UDP-glucosyl transferase \| chr3:5619134-5620879 REVERSE \| Aliases: MDC8.15 | 9.0 | 5.4 | 3.6 | 7.0 | 0.18% | 5.8 |
| 2244 | AT5G14600.1 \| expressed protein \| chr5:4706948-4709146 FORWARD \| Aliases: T15N1.90, T15N1_90 | 5.7 | 3.7 | 2.0 | 7.0 | 0.18% | 6.6 |
| 2245 | AT1G23750.1 \| DNA-binding protein-related, contains weak similarity to G-quartet DNA binding protein 3 (Tetrahymena thermophila) gi:4583503:gb:AAD25098 \| chr1:8400145-8401476 FORWARD \| Aliases: F5O8.30, F5O8_30 | 6.6 | 4.1 | 2.6 | 7.0 | 0.18% | 6.6 |
| 2247 | AT4G24280.1 \| Symbol: CPHSC70 1 \| heat shock protein 70, putative / HSP70, putative, strong similarity to heat shock protein 70 (Arabidopsis thaliana) GI:6746592; similar to heat shock 70 protein - Spinacia oleracea,PID:g2654208 \| chr4:12589998-12593640 FORWARD \| Aliases: CPHSC70 1, T22A6.110, T22A6_110 | 9.0 | 7.3 | 1.7 | 7.0 | 0.18% | 6.8 |
| 2250 | AT3G43920.1 \| Symbol: DCL3 \| ribonuclease III family protein, similar to RNA helicase/RNAseIII CAF protein (Arabidopsis thaliana) GI:6102610; contains Pfam profiles PF02170: PAZ domain, PF00636: RNase3 domain \| chr3:15764535-15771817 FORWARD \| Aliases: DICER LIKE 3, T15B3.60 | 5.3 | 3.7 | 1.6 | 7.0 | 0.18% | 6.7 |
| 2251 | AT3G02980.1 \| GCN5-related N-acetyltransferase (GNAT) family protein, contains Pfam profile: PF00583 acetyltransferase (GNAT) family \| chr3:670904-672222 REVERSE \| Aliases: F13E7.7, F13E7_7 | 4.6 | 3.4 | 1.2 | 7.0 | 0.18% | 6.9 |
| 2252 | AT1G70350.1 \| expressed protein \| chr1:26510777-26511932 REVERSE \| Aliases: F17O7.11, F17O7_11 | 6.0 | 3.1 | 2.8 | 7.0 | 0.18% | 6.6 |
| 2253 | AT1G07280.2 \| expressed protein, similar to expressed protein [Arabidopsis thaliana] (TAIR:At2g29670.1); similar to unknown protein [Oryza sativa (japonica cultivar-group)] (GB:AAU10760.1); contains domain SER_RICH (PS50324) \| chr1:2238109-2241172 FORWARD \| Aliases: F22G5.38, F22G5_38 | 6.6 | 4.0 | 2.5 | 7.0 | 0.18% | 6.7 |
| 2254 | AT3G29375.1 \| XH domain-containing protein, contains Pfam profile: PF03469: XH domain \| chr3:11281388-11283196 FORWARD \| Aliases: None | 5.3 | 3.1 | 2.2 | 7.0 | 0.18% | 6.4 |
| 2257 | AT2G19720.1 \| Symbol: RPS15AB \| 40S ribosomal protein S15A (RPS15aB) \| chr2:8516562-8518399 REVERSE \| Aliases: F6F22.25, F6F22_25, RPS15AB | 6.8 | 3.8 | 3.0 | 7.0 | 0.18% | 6.4 |
| 2259 | AT5G16290.2 \| acetolactate synthase small subunit, putative, similar to gi:5931761 from Nicotiana plumbaginifolia \| chr5:5333830-5337747 FORWARD \| Aliases: MQK4.1, MQK4_1 | 7.5 | 4.9 | 2.6 | 7.0 | 0.18% | 6.6 |
| 2261 | AT2G20890.1 \| Symbol: THF1 \| Chloroplast-localized Thylakoid formation1 gene product involved in vesicle-mediated formation of thylakoid membranes. Thf1 antisense lines contain abnormal chloroplasts early in leaf development (chloroplasts have loosely stacked thylakoid membranes). Expression was induced in the light and decreased under dark conditions. \| chr2:8994749-8996494 FORWARD \| Aliases: F5H14.14, F5H14_14, THF1, THYLAKOID FORMATION1 | 9.3 | 7.2 | 2.1 | 7.0 | 0.18% | 6.8 |
| 2268 | AT1G34210.1 \| Symbol: SERK2 \| somatic embryogenesis receptor-like kinase 2 (SERK2), nearly identical to somatic embryogenesis receptor-like kinase 2 (Arabidopsis thaliana) GI:14573457; contains Pfam domains PF00560: Leucine Rich Repeat and PF00069: Protein kinase domain; identical to cDNA somatic embryogenesis receptor-like kinase 2 (SERK2) GI:14573456 \| chr1:12458648-12463896 FORWARD \| Aliases: ATSERK2, F23M19.11, F23M19_11, SOMATIC EMBRYOGENESIS RECEPTOR LIKE KINASE 2 | 8.3 | 5.8 | 2.5 | 7.0 | 0.18% | 6.7 |

| Rank | Description | Sync | Root | M | t | adj.q | B |
|---|---|---|---|---|---|---|---|
| 2269 | AT4G23670.1 \| major latex protein-related / MLP-related, low similarity to major latex protein {Papaver somniferum}(GI:294060) contains Pfam profile PF00407: Pathogenesis-related protein Bet v I family \| chr4:12332570-12333814 REVERSE \| Aliases: F9D16.140, F9D16_140 | 6.2 | 2.7 | 3.5 | 7.0 | 0.18% | 6.3 |
| 2270 | AT5G62050.1 \| Symbol: OXA1 \| OXA1 protein (OXA1), identical to AtOXA1 (Arabidopsis thaliana) GI:6624207 \| chr5:24941000-24944538 REVERSE \| Aliases: ATOXA1, MTG10.19, MTG10_19, OXA1AT | 7.4 | 4.5 | 3.0 | 7.0 | 0.18% | 6.7 |
| 2272 | AT1G04710.1 \| acetyl-CoA C-acyltransferase, putative / 3-ketoacyl-CoA thiolase, putative, similar to 3-ketoacyl-CoA thiolase (E.C. 2.3.1.16) from (Arabidopsis thaliana) GI:2981616, (Cucumis sativus) GI:393707, (Cucurbita cv. Kurokawa Amakuri) GI:1694621; contains InterPro accession IPR002155: Thiolase | 8.1 | 5.1 | 2.9 | 7.0 | 0.19% | 5.9 |
| 2273 | AT3G56090.1 \| Symbol: ATFER3 \| | 7.5 | 3.5 | 4.0 | 7.0 | 0.19% | 5.9 |
| 2274 | AT2G41720.2 \| Symbol: EMB2654 \| pentatricopeptide (PPR) repeat-containing protein, contains Pfam profile PF01535: PPR repeat \| chr2:17410681-17413800 REVERSE \| Aliases: EMB2654, EMBRYO DEFECTIVE 2654, T11A7.18, T11A7_18 | 6.9 | 4.6 | 2.3 | 7.0 | 0.19% | 6.3 |
| 2276 | AT1G15790.2 \| expressed protein \| chr1:5438252-5440082 REVERSE \| Aliases: F7H2.13, F7H2_13 | 4.2 | 3.2 | 1.0 | 7.0 | 0.19% | 6.8 |
| 2277 | AT1G67560.1 \| lipoxygenase family protein, similar to 13-lipoxygenase GB:CAA65269 (Solanum tuberosum), gi:1654140 (Lycopersicon esculentum) \| chr1:25323562-25327927 FORWARD \| Aliases: F12B7.11, F12B7_11 | 7.6 | 5.8 | 1.7 | 7.0 | 0.19% | 6.8 |
| 2282 | AT4G28390.1 \| Symbol: AAC3 \| ADP, ATP carrier protein, mitochondrial, putative / ADP/ATP translocase, putative / adenine nucleotide translocator, putative, similar to mitochondrial ADP,ATP carrier protein SP:P12857 from (Zea mays) \| chr4:14040745-14043251 REVERSE \| Aliases: ADP/ATP CARRIER 3, ATAAC3, F20O9.60, F20O9_60 | 7.0 | 4.5 | 2.6 | 7.0 | 0.19% | 6.6 |
| 2283 | AT1G36320.1 \| expressed protein, similar to hypothetical protein GB:CAB37532 from (Arabidopsis thaliana), \| chr1:13673029-13675310 FORWARD \| Aliases: F7F23.4, F7F23_4 | 7.7 | 6.0 | 1.8 | 7.0 | 0.19% | 6.8 |
| 2286 | AT1G61550.1 \| S-locus protein kinase, putative, similar to receptor protein kinase (Ipomoea trifida) gi:836954:gb:AAC23542; contains S-locus glycoprotein family domain, Pfam:PF00954 \| chr1:22708531-22711491 REVERSE \| Aliases: T25B24.10, T25B24_10 | 4.7 | 2.8 | 2.0 | 7.0 | 0.19% | 6.5 |
| 2289 | AT2G26440.1 \| pectinesterase family protein, contains Pfam profile: PF01095 pectinesterase \| chr2:11254461-11256562 FORWARD \| Aliases: T9J22.11, T9J22_11 | 5.7 | 3.7 | 2.0 | 7.0 | 0.19% | 6.5 |
| 2290 | AT1G54690.1 \| histone H2A, putative, strong similarity to histone H2A GI:3204129 SP:O65759 from Cicer arietinum, Picea abies SP:P35063; contains Pfam profile PF00125 Core histone H2A/H2B/H3/H4 \| chr1:20417983-20418954 REVERSE \| Aliases: T22H22.12, T22H22_12 | 10.0 | 6.4 | 3.6 | 7.0 | 0.19% | 6.5 |
| 2293 | AT2G44920.2 \| thylakoid lumenal 15 kDa protein, chloroplast, identical to SP:O22160 Thylakoid lumenal 15 kDa protein, chloroplast precursor (p15) {Arabidopsis thaliana}; contains 8 pentapeptide repeats \| chr2:18531480-18533902 FORWARD \| Aliases: T13E15.7 | 5.1 | 3.2 | 1.9 | 7.0 | 0.19% | 6.6 |
| 2294 | AT5G52570.1 \| Symbol: BETA OHASE 2 \| beta-carotene hydroxylase, putative, similar to GI:1575296, beta-carotene hydroxylase \| chr5:21351630-21354206 REVERSE \| Aliases: BETA CAROTENE HYDROXYLASE 2, F6N7.5, F6N7_5 | 6.7 | 4.7 | 2.0 | 7.0 | 0.19% | 6.5 |
| 2296 | AT5G06000.1 \| Symbol: EIF3G2 \| | 6.3 | 4.2 | 2.1 | 7.0 | 0.19% | 6.4 |
| 2298 | AT2G37250.1 \| Symbol: ADK/ATPADK1 \| adenylate kinase family protein, contains Pfam profile: PF00406 adenylate kinase \| chr2:15649016-15650797 FORWARD \| Aliases: ADK, ATPADK1, F3G5.4, F3G5_4 | 9.1 | 7.4 | 1.7 | 7.0 | 0.19% | 6.8 |
| 2299 | AT2G27680.1 \| aldo/keto reductase family protein, contains Pfam profile PF00248: oxidoreductase, aldo/keto reductase family \| chr2:11810892-11813065 REVERSE \| Aliases: F15K20.22, F15K20_22 | 7.5 | 5.8 | 1.7 | 7.0 | 0.19% | 6.7 |
| 2301 | AT1G32990.1 \| Symbol: PRPL11 \| | 6.7 | 3.6 | 3.2 | 7.0 | 0.19% | 5.6 |
| 2302 | AT1G17650.1 \| similar to 6-phosphogluconate dehydrogenase NAD-binding domain-containing protein [Arabidopsis thaliana] (TAIR:At3g25530.1); similar to putative gamma hydroxybutyrate dehydrogenase [Oryza sativa (japonica cultivar-group)] (GB:NP_918497.1); contains InterPro domain 6-phosphogluconate dehydrogenase, NAD binding domain (InterPro:IPR006115) \| chr1:6069470-6072005 REVERSE \| Aliases: F11A6.12 | 6.0 | 3.6 | 2.4 | 7.0 | 0.19% | 6.2 |
| 2304 | AT1G09630.1 \| Symbol: ATRAB11C \| Ras-related GTP-binding protein, putative, similar to GTP-binding protein GI:1370146 from (Lotus japonicus) \| chr1:3118205-3119710 REVERSE \| Aliases: F21M12.2, F21M12_2, RAS RELATED SMALL GTPASE | 9.0 | 5.4 | 3.7 | 7.0 | 0.19% | 6.6 |
| 2305 | AT1G29390.2 \| Symbol: COR314 TM2 \| stress-responsive protein, putative, similar to cold acclimation WCOR413-like protein gamma form (Hordeum vulgare) gi:18449100:gb:AAL69988; similar to stress-regulated protein SAP1 (Xerophyta viscosa) gi:21360378:gb:AAM47505 \| chr1:10286279-10288089 REVERSE \| Aliases: F15D2.42 | 8.4 | 5.7 | 2.6 | 7.0 | 0.19% | 6.6 |

| Rank | Description | Sync | Root | M | t | adj.q | B |
|---|---|---|---|---|---|---|---|
| 2306 | AT1G07320.4 \| Symbol: RPL4 \| similar to ribosomal protein L4 family protein [Arabidopsis thaliana] (TAIR:At2g20060.1); similar to ribosomal protein L4 [Spinacia oleracea] (GB:CAA63651.1); contains InterPro domain Ribosomal protein L4/L1e (InterPro:IPR002136) \| chr1:2249133-2250490 FORWARD \| Aliases: F22G5.34, F22G5_34, RIBOSOMAL PROTEIN L4 | 7.2 | 5.3 | 1.9 | 7.0 | 0.19% | 6.5 |
| 2307 | AT4G33010.1 \| glycine dehydrogenase (decarboxylating), putative / glycine decarboxylase, putative / glycine cleavage system P-protein, putative, strong similarity to SP:P49361 Glycine dehydrogenase (decarboxylating) A, mitochondrial precursor (EC 1.4.4.2) {Flaveria pringlei}; contains Pfam profile PF02347: Glycine cleavage system P-protein \| chr4:15926673-15931335 REVERSE \| Aliases: F4I10.7 | 11.6 | 7.3 | 4.3 | 7.0 | 0.19% | 6.8 |
| 2309 | AT1G52300.1 \| 60S ribosomal protein L37 (RPL37B), similar to SP:Q43292 from (Arabidopsis thaliana) \| chr1:19478716-19479887 REVERSE \| Aliases: F19K6.12, F19K6_12 | 13.0 | 11.8 | 1.2 | 7.0 | 0.19% | 6.3 |
| 2311 | AT2G27530.2 \| 60S ribosomal protein L10A (RPL10aB) \| chr2:11770332-11771821 REVERSE \| Aliases: F10A12.22 | 10.7 | 8.3 | 2.4 | 7.0 | 0.19% | 6.7 |
| 2312 | AT1G62710.1 \| Symbol: BETA VPE \| vacuolar processing enzyme beta / beta-VPE, identical to SP:Q39044 Vacuolar processing enzyme, beta-isozyme precursor (EC 3.4.22.-) (Beta-VPE) {Arabidopsis thaliana} \| chr1:23227540-23230581 REVERSE \| Aliases: BETAVPE, F23N19.7, F23N19_7 | 7.2 | 5.3 | 2.0 | 7.0 | 0.19% | 6.5 |
| 2313 | AT1G50580.1 \| glycosyltransferase family protein, similar to UDP rhamnose: anthocyanidin-3-glucoside rhamnosyltransferase GB:CAA81057 GI:397567 from (Petunia x hybrida); contains Pfam profile: PF00201 UDP-glucoronosyl and UDP-glucosyl transferase | 6.0 | 4.4 | 1.5 | 7.0 | 0.19% | 6.7 |
| 2314 | AT3G16310.1 \| mitotic phosphoprotein N' end (MPPN) family protein, contains Pfam profile PF05172: MPPN (Mitotic PhosphoProtein N' end) (rrm-like) domain \| chr3:5526424-5528606 REVERSE \| Aliases: MYA6.11 | 8.7 | 6.2 | 2.4 | 6.9 | 0.19% | 6.8 |
| 2315 | AT1G70790.2 \| C2 domain-containing protein, similar to zinc finger and C2 domain protein GI:9957238 from (Arabidopsis thaliana) \| chr1:26704077-26706002 FORWARD \| Aliases: F15H11.4, F15H11_4 | 7.3 | 5.5 | 1.7 | 6.9 | 0.19% | 6.7 |
| 2316 | AT5G44520.1 \| ribose 5-phosphate isomerase-related, low similarity to SP:P47968 Ribose 5-phosphate isomerase (EC 5.3.1.6) (Phosphoriboisomerase) {Mus musculus} \| chr5:17951514-17953751 REVERSE \| Aliases: MFC16.20, MFC16_20 | 5.8 | 4.3 | 1.5 | 6.9 | 0.19% | 6.7 |
| 2319 | AT1G62750.1 \| elongation factor Tu family protein, similar to elongation factor G SP:P34811 (Glycine max (Soybean)) \| chr1:23237099-23240112 REVERSE \| Aliases: F23N19.11, F23N19_11 | 10.5 | 6.9 | 3.6 | 6.9 | 0.19% | 6.7 |
| 2320 | AT3G47650.1 \| bundle-sheath defective protein 2 family / bsd2 family, similar to bundle sheath defective protein 2 (Zea mays) GI:4732091 \| chr3:17580523-17581539 FORWARD \| Aliases: F1P2.200 | 7.8 | 5.2 | 2.7 | 6.9 | 0.19% | 6.2 |
| 2323 | AT3G13226.1 \| regulatory protein RecX family protein, contains Pfam PF02631: regulatory protein RecX \| chr3:4264311-4266369 REVERSE \| Aliases: None | 7.5 | 5.9 | 1.6 | 6.9 | 0.19% | 6.7 |
| 2324 | AT3G52910.1 \| Symbol: AtGRF4 \| | 7.6 | 4.8 | 2.8 | 6.9 | 0.19% | 6.0 |
| 2325 | AT5G16200.1 \| 50S ribosomal protein-related, contains weak similarity to Cyanelle 50S ribosomal protein L35. (Swiss-Prot:P14810) (Cyanophora paradoxa) | 5.5 | 3.8 | 1.7 | 6.9 | 0.19% | 6.7 |
| 2328 | AT3G60640.1 \| autophagy 8g (APG8g), identical to autophagy 8g (Arabidopsis thaliana) GI:19912163; contains Pfam profile PF02991: Microtubule associated protein 1A/1B, light chain 3; supporting cDNA gi:19912162:dbj:AB073181.1: \| chr3:22426816-22428133 FORWARD \| Aliases: T4C21.50 | 8.2 | 6.2 | 2.0 | 6.9 | 0.19% | 6.5 |
| 2332 | AT1G64185.1 \| lactoylglutathione lyase family protein / glyoxalase I family protein, contains Pfam domain PF00903: glyoxalase family protein \| chr1:23828177-23829447 FORWARD \| Aliases: None | 5.1 | 3.5 | 1.6 | 6.9 | 0.20% | 6.7 |
| 2335 | AT4G21480.1 \| glucose transporter, putative, similar to glucose transporter (Sugar carrier) STP1, Arabidopsis thaliana, SP:P23586; contains Pfam profile PF00083: major facilitator superfamily protein \| chr4:11433314-11435296 REVERSE \| Aliases: F18E5.100, F18E5_100 | 7.6 | 4.5 | 3.0 | 6.9 | 0.20% | 6.3 |
| 2337 | AT4G13200.1 \| expressed protein, hypothetical protein sll1769 - Synechocystis sp,PID:d1018406 \| chr4:7668146-7669197 REVERSE \| Aliases: F17N18.90, F17N18_90 | 6.3 | 5.2 | 1.1 | 6.9 | 0.20% | 6.7 |
| 2340 | AT2G27130.1 \| protease inhibitor/seed storage/lipid transfer protein (LTP) family protein, contains Pfam protease inhibitor/seed storage/LTP family domain PF00234 \| chr2:11602370-11603620 FORWARD \| Aliases: T20P8.18, T20P8_18 | 6.0 | 3.8 | 2.2 | 6.9 | 0.20% | 6.4 |
| 2341 | AT4G31930.1 \| mitochondrial glycoprotein family protein / MAM33 family protein, similar to SUAPRGA1 (Emericella nidulans) GI:6562379; contains Pfam profile PF02330: Mitochondrial glycoprotein \| chr4:15449667-15450842 REVERSE \| Aliases: F11C18.4 | 5.8 | 3.4 | 2.4 | 6.9 | 0.20% | 6.2 |

| Rank | Description | Sync | Root | M | t | adj.q | B |
|---|---|---|---|---|---|---|---|
| 2343 | AT4G18030.1 \| dehydration-responsive family protein, similar to early-responsive to dehydration stress ERD3 protein (Arabidopsis thaliana) GI:15320410; contains Pfam profile PF03141: Putative methyltransferase \| chr4:10012373-10015688 REVERSE \| Aliases: T6K21.210, T6K21_210 | 8.0 | 5.9 | 2.2 | 6.9 | 0.20% | 6.7 |
| 2344 | AT3G58100.1 \| glycosyl hydrolase family protein 17, similar to elicitor inducible chitinase Nt-SubE76 GI:11071974 from (Nicotiana tabacum); C-terminal homology only \| chr3:21526176-21527464 REVERSE \| Aliases: F9D24.10 | 6.7 | 4.4 | 2.3 | 6.9 | 0.20% | 6.3 |
| 2345 | AT3G20930.1 \| RNA recognition motif (RRM)-containing protein, contains Pfam profile: PF00076 RNA recognition motif \| chr3:7331731-7334034 FORWARD \| Aliases: MFD22.4 | 6.6 | 4.0 | 2.5 | 6.9 | 0.20% | 6.4 |
| 2346 | AT4G00490.1 \| beta-amylase, putative / 1,4-alpha-D-glucan maltohydrolase, putative, similar to beta-amylase from SP:O64407 (Vigna unguiculata); contains Pfam profile PF01373: Glycosyl hydrolase family 14 \| chr4:222348-225457 FORWARD \| Aliases: F6N23.1, F6N23_1 | 7.3 | 3.7 | 3.6 | 6.9 | 0.20% | 6.3 |
| 2347 | AT1G32470.1 \| glycine cleavage system H protein, mitochondrial, putative, similar to SP:P25855 Glycine cleavage system H protein 1, mitochondrial precursor {Arabidopsis thaliana}; contains Pfam profile PF01597: Glycine cleavage H-protein \| chr1:11739284-11740308 REVERSE \| Aliases: F5D14.25, F5D14_25 | 7.1 | 3.6 | 3.5 | 6.9 | 0.20% | 6.1 |
| 2348 | AT1G25260.1 \| acidic ribosomal protein P0-related, contains similarity to 60S acidic ribosomal protein GI:5815233 from (Homo sapiens) \| chr1:8853946-8855847 REVERSE \| Aliases: F4F7.35, F4F7_35 | 9.8 | 7.0 | 2.8 | 6.9 | 0.20% | 6.6 |
| 2349 | AT1G18640.2 \| Symbol: PSP \| 3-phosphoserine phosphatase (PSP), nearly identical to 3-phosphoserine phosphatase GI:3759177 from (Arabidopsis thaliana) | 9.7 | 7.7 | 2.0 | 6.9 | 0.20% | 6.7 |
| 2351 | AT4G34160.1 \| Symbol: CYCD3;1 \| cyclin delta-3 (CYCD3), identical to SP:P42753 Cyclin delta-3 {Arabidopsis thaliana} \| chr4:16357643-16359559 FORWARD \| Aliases: CYCD3, CYCD3;1, Cyclin D3;1, D3, F28A23.80, F28A23_80 | 5.2 | 2.7 | 2.5 | 6.9 | 0.20% | 6.4 |
| 2354 | AT5G56710.1 \| 60S ribosomal protein L31 (RPL31C) \| chr5:22961062-22962196 REVERSE \| Aliases: MIK19.16, MIK19_16 | 12.0 | 10.2 | 1.8 | 6.9 | 0.20% | 6.7 |
| 2355 | AT1G63960.1 \| hypothetical protein \| chr1:23741864-23742169 FORWARD \| Aliases: T12P18.2, T12P18_2 | 3.7 | 2.5 | 1.2 | 6.9 | 0.20% | 6.7 |
| 2359 | AT2G36145.1 \| expressed protein \| chr2:15175620-15176585 FORWARD \| Aliases: None | 5.3 | 2.8 | 2.5 | 6.9 | 0.20% | 6.0 |
| 2360 | AT3G24360.1 \| enoyl-CoA hydratase/isomerase family protein, similar to CHY1 (gi:8572760); contains Pfam profile PF00388 enoyl-CoA hydratase/isomerase family protein \| chr3:8839735-8842838 REVERSE \| Aliases: K7M2.21 | 4.7 | 3.2 | 1.5 | 6.9 | 0.20% | 6.6 |
| 2361 | AT2G27330.1 \| RNA recognition motif (RRM)-containing protein \| chr2:11702112-11703693 REVERSE \| Aliases: F12K2.9 | 6.3 | 4.8 | 1.5 | 6.9 | 0.20% | 6.7 |
| 2363 | AT4G32930.1 \| expressed protein, predicted protein, Caenorhabditis elegans, gb:Z70780 \| chr4:15898840-15900824 FORWARD \| Aliases: F26P21.50, F26P21_50 | 8.4 | 6.6 | 1.8 | 6.9 | 0.20% | 6.7 |
| 2364 | AT5G60230.2 \| Symbol: SEN2 \| tRNA-splicing endonuclease, putative, contains similarity to Swiss-Prot:P16658 tRNA-splicing endonuclease subunit SEN2 (tRNA-intron endonuclease) (Saccharomyces cerevisiae); contains Pfam domain PF01974: tRNA intron endonuclease, catalytic C-terminal domain \| chr5:24267317-24268821 REVERSE \| Aliases: ATSEN2, F15L12.18, F15L12_18, SPLICING ENDONUCLEASE 2 | 5.7 | 4.0 | 1.8 | 6.9 | 0.20% | 6.6 |
| 2365 | AT1G20830.1 \| expressed protein \| chr1:7242909-7244893 FORWARD \| Aliases: F2D10.35, F2D10_35 | 5.9 | 4.1 | 1.7 | 6.9 | 0.20% | 6.7 |
| 2366 | AT3G48420.1 \| haloacid dehalogenase-like hydrolase family protein, low similarity to SP:P95649 CbbY protein {Rhodobacter sphaeroides}; contains InterPro accession IPR005834: Haloacid dehalogenase-like hydrolase \| chr3:17940632-17942686 FORWARD \| Aliases: T29H11.1 | 6.7 | 3.2 | 3.5 | 6.9 | 0.20% | 6.4 |
| 2369 | AT1G12800.1 \| S1 RNA-binding domain-containing protein, contains Pfam domain, PF00575: S1 RNA binding domain \| chr1:4361549-4365314 REVERSE \| Aliases: F13K23.5, F13K23_5 | 6.9 | 4.2 | 2.8 | 6.9 | 0.20% | 6.4 |
| 2374 | AT5G50890.1 \| expressed protein \| chr5:20719910-20721461 FORWARD \| Aliases: K3K7.3, K3K7_3 | 7.5 | 4.9 | 2.6 | 6.9 | 0.20% | 6.4 |
| 2376 | AT1G16790.1 \| ribosomal protein-related, similar to Chloroplast 30S ribosomal protein S8 (SP:P21508) (Euglena gracilis) \| chr1:5744558-5745202 FORWARD \| Aliases: F17F16.10, F17F16_10 | 5.5 | 3.3 | 2.2 | 6.9 | 0.21% | 6.6 |
| 2377 | AT3G10050.1 \| Symbol: OMR1 \| threonine ammonia-lyase / threonine dehydratase / threonine deaminase (OMR1), identical to SP:Q9ZSS6 Threonine dehydratase biosynthetic, chloroplast precursor (EC 4.3.1.19, formerly EC 4.2.1.16) (Threonine deaminase) (TD) {Arabidopsis thaliana} \| chr3:3098730-3101766 REVERSE \| Aliases: L O METHYLTHREONINE RESISTANT 1, T22K18.12, THREONINE DEHYDRATASE/DEAMINASE | 6.5 | 3.6 | 2.9 | 6.9 | 0.21% | 6.6 |
| 2379 | AT5G23090.4 \| similar to TATA-binding protein-associated phosphoprotein Dr1 protein, putative [Arabidopsis thaliana] (TAIR:At5g08190.1); similar to repressor protein [Oryza sativa] (GB:AAL73485.1); contains InterPro domain Transcription factor CBF/NF-Y/archaeal histone (InterPro:IPR003958); contains InterPro domain Histone-fold/TFIID-TAF/NF-Y (InterPro:IPR007124) \| chr5:7749073-7750541 FORWARD \| Aliases: MYJ24.8, MYJ24_8 | 6.6 | 5.6 | 1.0 | 6.8 | 0.21% | 6.7 |

| Rank | Description | Sync | Root | M | t | adj.q | B |
|---|---|---|---|---|---|---|---|
| 2380 | AT5G07240.1 \| calmodulin-binding family protein, contains Pfam profile PF00612: IQ calmodulin-binding motif \| chr5:2271920-2274288 FORWARD \| Aliases: T28J14.180, T28J14_180 | 3.7 | 2.3 | 1.4 | 6.8 | 0.21% | 6.6 |
| 2383 | AT1G32580.1 \| plastid developmental protein DAG, putative, similar to DAG protein, chloroplast precursor (Garden snapdragon) SWISS-PROT:Q38732 | 6.2 | 3.9 | 2.3 | 6.8 | 0.21% | 6.5 |
| 2384 | AT4G01310.1 \| ribosomal protein L5 family protein, contains Pfam profiles PF00673: ribosomal L5P family C-terminus, PF00281: ribosomal protein L5 \| chr4:543970-545535 REVERSE \| Aliases: F2N1.5, F2N1_5 | 6.9 | 3.1 | 3.8 | 6.8 | 0.21% | 6.3 |
| 2385 | AT4G27652.1 \| expressed protein \| chr4:13809839-13810362 FORWARD \| Aliases: None | 7.7 | 4.7 | 3.0 | 6.8 | 0.21% | 6.5 |
| 2387 | AT4G37710.1 \| VQ motif-containing protein, contains PF05678: VQ motif \| chr4:17718122-17718736 FORWARD \| Aliases: T28I19.3 | 4.4 | 1.8 | 2.6 | 6.8 | 0.21% | 6.6 |
| 2389 | AT4G37280.1 \| MRG family protein, contains Pfam domain PF05712: MRG \| chr4:17546578-17549409 REVERSE \| Aliases: AP22.79, AP22_79 | 7.6 | 5.8 | 1.8 | 6.8 | 0.21% | 6.6 |
| 2391 | AT5G47650.1 \| MutT/nudix family protein, similar to Nucleoside diphosphate-linked moiety X motif 6 (Protein GFG) from {Xenopus laevis} SP:P13420, {Homo sapiens} SP:P53370; contains Pfam profile PF00293: NUDIX domain \| chr5:19326453-19329653 REVERSE \| Aliases: MNJ7.24, MNJ7_24 | 6.1 | 3.7 | 2.3 | 6.8 | 0.21% | 6.5 |
| 2393 | AT2G42770.1 \| peroxisomal membrane 22 kDa family protein, contains Mpv17 / PMP22 family domain, Pfam:PF04117 \| chr2:17805363-17807083 REVERSE \| Aliases: F7D19.23, F7D19_23 | 7.3 | 4.9 | 2.5 | 6.8 | 0.21% | 6.0 |
| 2395 | AT5G42680.1 \| expressed protein, contains Pfam profile PF04759: Protein of unknown function, DUF617 \| chr5:17131824-17132640 FORWARD \| Aliases: MJB21.5, MJB21_5 | 6.4 | 4.1 | 2.2 | 6.8 | 0.21% | 6.4 |
| 2399 | AT3G53880.1 \| aldo/keto reductase family protein, similar to chalcone reductase (Sesbania rostrata)(GI:2792155), and aldose reductase ALDRXV4 (Xerophyta viscosa)(GI:4539944), (Hordeum vulgare)(GI:728592) \| chr3:19964151-19966268 FORWARD \| Aliases: F5K20.180 | 6.7 | 5.2 | 1.4 | 6.8 | 0.21% | 6.7 |
| 2403 | AT3G49670.1 \| leucine-rich repeat transmembrane protein kinase, putative, CLAVATA1 receptor kinase, Arabidopsis thaliana, EMBL:ATU96879 \| chr3:18428553-18432091 FORWARD \| Aliases: T16K5.20 | 5.8 | 3.3 | 2.4 | 6.8 | 0.21% | 6.0 |
| 2404 | AT3G48425.1 \| endonuclease/exonuclease/phosphatase family protein, similar to SP:P51173:APEA_DICDI DNA-(apurinic or apyrimidinic site) lyase (EC 4.2.99.18)(Class II apurinic/apyrimidinic(AP)-endonuclease) {Dictyostelium discoideum}; contains Pfam profile PF03372: Endonuclease/Exonuclease/phosphatase family \| chr3:17942803-17945729 FORWARD \| Aliases: None | 5.2 | 3.8 | 1.4 | 6.8 | 0.21% | 6.6 |
| 2406 | AT5G35790.1 \| Symbol: G6PD1 \| Encodes a plastidic glucose-6-phosphate dehydrogenase that is sensitive to reduction by DTT and whose mRNA is more prevalent in developing organs but absent in the root. \| chr5:13973929-13976975 REVERSE \| Aliases: G6PD1, GLUCOSE 6 PHOSPHATE DEHYDROGENASE 1, MWP19.3 | 6.2 | 3.7 | 2.5 | 6.8 | 0.21% | 6.2 |
| 2407 | AT5G02830.1 \| pentatricopeptide (PPR) repeat-containing protein, contains Pfam profile PF01535: PPR repeat \| chr5:643609-648451 REVERSE \| Aliases: F9G14.140, F9G14_140 | 7.4 | 5.2 | 2.2 | 6.8 | 0.21% | 6.3 |
| 2409 | AT3G57810.2 \| OTU-like cysteine protease family protein, contains Pfam profile PF02338: OTU-like cysteine protease \| chr3:21426805-21428925 FORWARD \| Aliases: T10K17.20 | 6.9 | 5.1 | 1.8 | 6.8 | 0.21% | 6.5 |
| 2411 | AT2G21170.1 \| Symbol: TIM \| triosephosphate isomerase, chloroplast, putative, similar to Triosephosphate isomerase, chloroplast precursor: SP:P48496 from Spinacia oleracea, SP:P46225 from Secale cereale \| chr2:9077835-9080304 REVERSE \| Aliases: F26H11.7, F26H11_7, TRIOSEPHOSPHATE ISOMERASE | 11.3 | 9.3 | 2.0 | 6.8 | 0.21% | 6.6 |
| 2412 | AT3G24590.1 \| signal peptidase I family protein, contains Pfam profile: PF00461 signal peptidase I \| chr3:8970679-8972175 FORWARD \| Aliases: MOB24.17 | 8.5 | 7.4 | 1.1 | 6.8 | 0.21% | 6.6 |
| 2414 | AT3G18880.1 \| ribosomal protein S17 family protein, similar to 40S ribosomal protein S17 GB:Y08858 from (Nicotiana plumbaginifolia) \| chr3:6509584-6510631 FORWARD \| Aliases: MCB22.5 | 5.3 | 3.9 | 1.4 | 6.8 | 0.21% | 6.6 |
| 2416 | AT3G57010.1 \| strictosidine synthase family protein, similar to strictosidine synthase (Rauvolfia serpentina)(SP:P15324) \| chr3:21106633-21108430 REVERSE \| Aliases: F24I3.90 | 9.5 | 6.6 | 2.9 | 6.8 | 0.21% | 6.5 |
| 2417 | AT3G23570.1 \| dienelactone hydrolase family protein, similar to SP:Q9ZT66 Endo-1,3;1,4-beta-D-glucanase precursor (EC 3.2.1.-) {Zea mays}; contains Pfam profile: PF01738 dienelactone hydrolase family \| chr3:8457785-8459698 REVERSE \| Aliases: MDB19.5 | 10.3 | 6.6 | 3.7 | 6.8 | 0.21% | 6.2 |
| 2419 | AT1G41880.1 \| 60S ribosomal protein L35a (RPL35aB), identical to GB:CAB81600 from (Arabidopsis thaliana) \| chr1:15653564-15654932 REVERSE \| Aliases: F5A13.4, F5A13_4 | 11.5 | 9.5 | 2.0 | 6.8 | 0.21% | 6.4 |
| 2420 | AT3G55750.1 \| 60S ribosomal protein L35a (RPL35aD), ribosomal protein L35a.e.c15, Saccharomyces cerevisiae, PIR:S44069 | 11.5 | 9.5 | 2.0 | 6.8 | 0.21% | 6.4 |

| Rank | Description | Sync | Root | M | t | adj.q | B |
|---|---|---|---|---|---|---|---|
| 2421 | AT5G56590.1 \| glycosyl hydrolase family 17 protein, similar to elicitor inducible chitinase Nt-SubE76 GI:11071974 from (Nicotiana tabacum) \| chr5:22924549-22926715 FORWARD \| Aliases: MIK19.3, MIK19_3 | 7.1 | 5.4 | 1.7 | 6.8 | 0.21% | 6.6 |
| 2426 | AT4G25150.1 \| acid phosphatase, putative, similar to acid phosphatase-1(1); Apase-1(1) (Lycopersicon esculentum) GI:7705154, acid phosphatase (Glycine max) GI:3341443; contains Pfam profile PF03767: HAD superfamily (subfamily IIIB) phosphatase \| chr4:12901631-12903135 REVERSE \| Aliases: F24A6.12 | 5.9 | 3.2 | 2.7 | 6.8 | 0.22% | 6.1 |
| 2431 | AT5G54180.1 \| mitochondrial transcription termination factor-related / mTERF-related, contains Pfam profile PF02536: mTERF \| chr5:22005732-22007454 FORWARD \| Aliases: K18G13.5, K18G13_5 | 5.4 | 3.9 | 1.4 | 6.8 | 0.22% | 6.6 |
| 2432 | AT2G29500.1 \| 17.6 kDa class I small heat shock protein (HSP17.6B-CI), contains Pfam PF00011: Hsp20/alpha crystallin family; identified in Scharf, K-D., et al, Cell Stress & Chaperones (2001) 6: 225-237. \| chr2:12640180-12640882 REVERSE \| Aliases: F16P2.12, F16P2_12 | 7.4 | 4.7 | 2.8 | 6.8 | 0.22% | 6.1 |
| 2436 | AT2G25270.1 \| expressed protein \| chr2:10766828-10769797 FORWARD \| Aliases: T22F11.14, T22F11_14 | 7.1 | 4.6 | 2.5 | 6.8 | 0.22% | 6.2 |
| 2437 | AT3G62300.1 \| agenet domain-containing protein, contains Pfam PF05641: Agenet domain \| chr3:23065079-23068163 FORWARD \| Aliases: T17J13.260 | 6.6 | 4.9 | 1.7 | 6.8 | 0.22% | 6.5 |
| 2442 | AT2G01290.1 \| expressed protein \| chr2:148918-150381 REVERSE \| Aliases: F10A8.17, F10A8_17 | 6.2 | 3.8 | 2.4 | 6.8 | 0.22% | 6.0 |
| 2443 | AT2G41780.1 \| expressed protein \| chr2:17435176-17435890 FORWARD \| Aliases: T11A7.12, T11A7_12 | 8.3 | 7.3 | 1.1 | 6.8 | 0.22% | 6.6 |
| 2445 | AT2G20940.1 \| expressed protein \| chr2:9009113-9009782 REVERSE \| Aliases: F26H11.30, F26H11_30 | 6.5 | 3.5 | 3.1 | 6.8 | 0.22% | 6.1 |
| 2446 | AT1G79200.1 \| expressed protein \| chr1:29799281-29800933 FORWARD \| Aliases: YUP8H12R.41, YUP8H12R_41 | 4.9 | 3.4 | 1.6 | 6.8 | 0.22% | 6.5 |
| 2448 | AT4G39230.1 \| isoflavone reductase, putative, similar to allergenic isoflavone reductase-like protein Bet v 6.0102 (Betula pendula)(GI:10764491); contains Pfam profile PF02716: Isoflavone reductase \| chr4:18265879-18267718 REVERSE \| Aliases: T22F8.130, T22F8_130 | 7.3 | 3.3 | 4.0 | 6.8 | 0.22% | 6.2 |
| 2449 | AT4G16500.1 \| cysteine protease inhibitor family protein / cystatin family protein, similar to SP:Q06445 Cysteine proteinase inhibitor (Cystatin) {Vigna unguiculata}; contains Pfam profile PF00031: Cystatin domain \| chr4:9301413-9302045 REVERSE \| Aliases: DL4275C, FCAALL.171 | 11.2 | 10.0 | 1.2 | 6.8 | 0.22% | 6.4 |
| 2450 | AT2G01250.1 \| 60S ribosomal protein L7 (RPL7B) | 11.7 | 10.2 | 1.5 | 6.8 | 0.22% | 6.4 |
| 2451 | AT1G60870.1 \| expressed protein \| chr1:22413137-22414159 FORWARD \| Aliases: T7P1.2, T7P1_2 | 7.5 | 4.4 | 3.0 | 6.8 | 0.22% | 5.7 |
| 2452 | AT4G05400.2 \| expressed protein, similar to expressed protein [Arabidopsis thaliana] (TAIR:At4g21140.1); similar to unknown protein [Oryza sativa (japonica cultivar-group)] (GB:BAD73259.1) \| chr4:2741157-2743306 FORWARD \| Aliases: C6L9.80, C6L9_80 | 7.0 | 4.2 | 2.8 | 6.8 | 0.22% | 6.5 |
| 2453 | AT3G56070.1 \| Symbol: ROC2 \| peptidyl-prolyl cis-trans isomerase, putative / cyclophilin, putative / rotamase, putative, similar to peptidyl-prolyl cis-trans isomerase, PPIase (cyclophilin, cyclosporin A-binding protein) (Catharanthus roseus) SWISS-PROT:Q39613 \| chr3:20817728-20819071 REVERSE \| Aliases: F18O21.30, ROTAMASE CYCLOPHILIN 2, ROTAMASE CYP 2 | 7.4 | 4.6 | 2.8 | 6.8 | 0.22% | 6.4 |
| 2454 | AT4G35950.1 \| Symbol: ARAC6 \| Rac-like GTP-binding protein (ARAC6), identical to rac-like GTP binding protein Arac6 GI:3406757 from (Arabidopsis thaliana) \| chr4:17023840-17025866 REVERSE \| Aliases: RAC2, T19K4.80 | 8.2 | 6.7 | 1.5 | 6.8 | 0.22% | 6.6 |
| 2455 | AT3G58540.1 \| expressed protein \| chr3:21659570-21660364 FORWARD \| Aliases: F14P22.130 | 4.8 | 3.0 | 1.7 | 6.8 | 0.22% | 6.6 |
| 2458 | AT1G04430.1 \| dehydration-responsive protein-related, similar to early-responsive to dehydration stress ERD3 protein (Arabidopsis thaliana) GI:15320410; contains Pfam profile PF03141: Putative methyltransferase \| chr1:1198136-1201526 FORWARD \| Aliases: F19P19.11, F19P19_11 | 9.8 | 7.6 | 2.2 | 6.7 | 0.22% | 6.6 |
| 2460 | AT1G69390.1 \| chloroplast division protein, putative (MinE1), identical to chloroplast division protein homolog MinE1 GI:17511220 from (Arabidopsis thaliana) \| chr1:26088317-26090144 FORWARD \| Aliases: F23O10.25, F23O10_25 | 6.8 | 4.7 | 2.1 | 6.7 | 0.22% | 6.5 |
| 2463 | AT4G04870.1 \| CDP-alcohol phosphatidyltransferase family protein, similar to SP:Q07560 Cardiolipin synthetase (EC 2.7.8.-) {Saccharomyces cerevisiae; contains Pfam profile PF01066: CDP-alcohol phosphatidyltransferase \| chr4:2461211-2464141 REVERSE \| Aliases: T4B21.19, T4B21_19 | 8.5 | 6.7 | 1.8 | 6.7 | 0.22% | 6.5 |
| 2464 | AT5G52960.1 \| expressed protein, similar to unknown protein (pir :S77140) \| chr5:21494372-21495662 FORWARD \| Aliases: MNB8.2, MNB8_2 | 4.8 | 2.6 | 2.2 | 6.7 | 0.22% | 6.3 |
| 2465 | AT1G74530.3 \| expressed protein \| chr1:28013294-28015823 REVERSE \| Aliases: F1M20.21, F1M20_21 | 5.8 | 3.7 | 2.0 | 6.7 | 0.22% | 6.5 |
| 2466 | AT5G59740.1 \| UDP-galactose/UDP-glucose transporter-related, weak similarity to UDP-galactose/UDP-glucose transporter (Arabidopsis thaliana) GI:22651763 \| chr5:24087277-24090243 REVERSE \| Aliases: MTH12.11, MTH12_11 | 6.2 | 4.8 | 1.4 | 6.7 | 0.22% | 6.5 |

| Rank | Description | Sync | Root | M | t | adj.q | B |
|---|---|---|---|---|---|---|---|
| 2467 | AT5G08350.1 \| GRAM domain-containing protein / ABA-responsive protein-related, contains similarity to ABA-responsive protein in barley (GI:4103635) (Hordeum vulgare) (J. Exp. Bot. 50, 727-728 (1999); FH protein interacting protein FIP1, Arabidopsis thaliana, EMBL:AF174428; contains Pfam PF02893: GRAM domain \| chr5:2686246-2687250 REVERSE \| Aliases: F8L15.80, F8L15_80 | 7.1 | 4.8 | 2.3 | 6.7 | 0.22% | 6.3 |
| 2469 | AT5G06120.1 \| Ran-binding protein, putative, similar to SP:Q9UIA9 Ran-binding protein 16 {Homo sapiens}; contains Pfam profile PF03810: Importin-beta N-terminal domain \| chr5:1844079-1852602 FORWARD \| Aliases: K16F4.8, K16F4_8 | 7.7 | 5.5 | 2.2 | 6.7 | 0.22% | 6.5 |
| 2471 | AT2G21340.2 \| enhanced disease susceptibility protein, putative / salicylic acid induction deficient protein, putative, similar to enhanced disease susceptibility 5 (Arabidopsis thaliana) GI:16589070 \| chr2:9139638-9143524 FORWARD \| Aliases: F3K23.10, F3K23_10 | 6.4 | 5.3 | 1.1 | 6.7 | 0.22% | 6.5 |
| 2472 | AT1G08550.2 \| Symbol: NPQ1 \| similar to violaxanthin de-epoxidase-related [Arabidopsis thaliana] (TAIR:At2g21860.1); similar to violaxanthin de-epoxidase [Camellia sinensis] (GB:AAL67858.2) | 5.8 | 3.8 | 2.0 | 6.7 | 0.22% | 6.4 |
| 2474 | AT1G80600.1 \| acetylornithine aminotransferase, mitochondrial, putative / acetylornithine transaminase, putative / AOTA, putative / ACOAT, putative, similar to SP:O04866 Acetylornithine aminotransferase, mitochondrial precursor (EC 2.6.1.11) (ACOAT) (Acetylornithine transaminase) (AOTA) {Alnus glutinosa}; contains Pfam profile PF00202: aminotransferase, class III \| chr1:30303410-30305445 REVERSE \| Aliases: T21F11.7, T21F11_7 | 10.9 | 7.6 | 3.2 | 6.7 | 0.22% | 6.5 |
| 2476 | AT5G16980.1 \| NADP-dependent oxidoreductase, putative, strong similarity to probable NADP-dependent oxidoreductase (zeta-crystallin homolog) P1 (SP:Q39172)(gi:886428) and P2 (SP:Q39173)(gi:886430), Arabidopsis thaliana \| chr5:5579146-5580783 REVERSE \| Aliases: F2K13.130, F2K13_130 | 6.0 | 3.9 | 2.1 | 6.7 | 0.22% | 6.1 |
| 2477 | AT1G21560.1 \| expressed protein, similar to hypothetical protein [Arabidopsis thaliana] (TAIR:At4g01170.1); similar to unknown protein [Oryza sativa (japonica cultivar-group)] (GB:AAV24769.1) \| chr1:7554829-7557014 FORWARD \| Aliases: F24J8.19, F24J8_19 | 6.3 | 4.7 | 1.6 | 6.7 | 0.22% | 6.5 |
| 2478 | AT3G55600.1 \| expressed protein, predicted proteins, Arabidopsis thaliana \| chr3:20631661-20633449 FORWARD \| Aliases: F1I16.10 | 5.5 | 4.1 | 1.4 | 6.7 | 0.23% | 6.6 |
| 2479 | AT3G12490.2 \| similar to cysteine protease inhibitor, putative / cystatin, putative [Arabidopsis thaliana] (TAIR:At5g05110.1); similar to cysteine protease inhibitor CPI-1 [Brassica oleracea] (GB:AAL59842.1); contains InterPro domain Cystatin C/M (InterPro:IPR003243); contains InterPro domain Cysteine protease inhibitor (InterPro:IPR000010) | 12.7 | 10.6 | 2.1 | 6.7 | 0.23% | 6.5 |
| 2480 | AT5G14590.1 \| isocitrate dehydrogenase, putative / NADP+ isocitrate dehydrogenase, putative, strong similarity to isocitrate dehydrogenase (NADP+) (Nicotiana tabacum) GI:3021512; contains Pfam domain PF00180: dehydrogenase, isocitrate/isopropylmalate family \| chr5:4703301-4706738 REVERSE \| Aliases: T15N1.80, T15N1_80 | 10.3 | 8.1 | 2.2 | 6.7 | 0.23% | 6.5 |
| 2484 | AT2G44120.2 \| 60S ribosomal protein L7 (RPL7C) | 8.5 | 6.5 | 2.0 | 6.7 | 0.23% | 6.5 |
| 2488 | AT5G05180.2 \| expressed protein \| chr5:1536227-1538493 FORWARD \| Aliases: K2A11.5, K2A11_5 | 6.4 | 4.3 | 2.1 | 6.7 | 0.23% | 6.3 |
| 2489 | AT5G49280.1 \| hydroxyproline-rich glycoprotein family protein, contains proline-rich extensin domains, INTERPRO:IPR002965 \| chr5:19993820-19994675 REVERSE \| Aliases: K21P3.16, K21P3_16 | 5.4 | 3.5 | 1.9 | 6.7 | 0.23% | 6.4 |
| 2490 | AT2G20490.2 \| Symbol: NOP10 \| expressed protein, similar to hypothetical protein LOC548917 [Xenopus tropicalis] (GB:NP_001016163.1); contains InterPro domain Nucleolar RNA-binding protein Nop10p (InterPro:IPR007264) \| chr2:8838797-8839997 FORWARD \| Aliases: NOP10, T13C7.8, T13C7_8 | 7.3 | 4.2 | 3.1 | 6.7 | 0.23% | 6.2 |
| 2491 | AT2G29440.1 \| Symbol: ATGSTU6 \| glutathione S-transferase, putative \| chr2:12627161-12628224 REVERSE \| Aliases: F16P2.18, F16P2_18, GLUTATHIONE S TRANSFERASE 24, GST24 | 5.9 | 4.2 | 1.7 | 6.7 | 0.23% | 6.5 |
| 2493 | AT2G39820.1 \| eukaryotic translation initiation factor 6, putative / eIF-6, putative, similar to SP:O55135 Eukaryotic translation initiation factor 6 (eIF-6) (B4 integrin interactor) {Mus musculus}; contains Pfam profile PF01912: eIF-6 family \| chr2:16625355-16626767 FORWARD \| Aliases: T5I7.12, T5I7_12 | 5.3 | 3.3 | 2.1 | 6.7 | 0.23% | 6.1 |
| 2494 | AT3G09860.1 \| expressed protein \| chr3:3026125-3027213 FORWARD \| Aliases: F8A24.9 | 10.5 | 7.5 | 3.0 | 6.7 | 0.23% | 6.3 |
| 2495 | AT5G32450.1 \| RNA recognition motif (RRM)-containing protein, various predicted proteins, Arabidopsis thaliana and others \| chr5:12096848-12098803 FORWARD \| Aliases: F18O9.60, F18O9_60 | 9.3 | 8.5 | 0.9 | 6.7 | 0.23% | 6.4 |
| 2499 | AT4G35250.1 \| vestitone reductase-related, low similarity to vestitone reductase (Medicago sativa subsp. sativa) GI:973249 \| chr4:16771180-16773369 REVERSE \| Aliases: F23E12.190, F23E12_190 | 8.3 | 4.7 | 3.6 | 6.7 | 0.23% | 6.5 |
| 2500 | AT1G73530.1 \| RNA recognition motif (RRM)-containing protein, low similarity to SP:Q03251 Glycine-rich RNA-binding protein 8 (CCR1 protein) {Arabidopsis thaliana}; contains InterPro entry IPR000504: RNA-binding region RNP-1 (RNA recognition motif) (RRM) \| chr1:27647294-27649047 REVERSE \| Aliases: T9L24.48, T9L24_48 | 4.9 | 3.2 | 1.7 | 6.7 | 0.23% | 6.4 |

| Rank | Description | Sync | Root | M | t | adj.q | B |
|---|---|---|---|---|---|---|---|
| 2501 | AT1G78040.1 \| pollen Ole e 1 allergen and extensin family protein, contains Pfam domain, PF01190: Pollen proteins Ole e I family \| chr1:29350732-29352001 FORWARD \| Aliases: F28K19.26, F28K19_26 | 11.5 | 10.0 | 1.5 | 6.7 | 0.23% | 6.4 |
| 2503 | AT5G26710.1 \| glutamate-tRNA ligase, putative / glutamyl-tRNA synthetase, putatuve / GluRS, putative, identical to gi:3435196 \| chr5:9305600-9308774 FORWARD \| Aliases: None | 10.7 | 8.8 | 1.9 | 6.7 | 0.23% | 6.5 |
| 2506 | AT5G37780.1 \| Symbol: CAM1 \| calmodulin-1/4 (CAM1), identical to calmodulin 4 (Arabidopsis thaliana) GI:16223, SP:P25854 Calmodulin-1/4 {Arabidopsis thaliana} \| chr5:15021763-15023435 REVERSE \| Aliases: ACAM 1, CALMODULIN 1, CAM1, K22F20.20, K22F20_20, TCH1 | 11.9 | 9.5 | 2.4 | 6.7 | 0.23% | 6.4 |
| 2509 | AT2G32480.2 \| membrane-associated zinc metalloprotease, putative, similar to Hypothetical zinc metalloprotease All3971 (SP:Q8YQ64) (strain PCC 7120) {Anabaena sp.} Pfam PF00595: PDZ domain (Also known as DHR or GLGF); contains TIGRFAM TIGR00054: membrane-associated zinc metalloprotease, putative \| chr2:13795594-13797150 REVERSE \| Aliases: T26B15.4, T26B15_4 | 9.1 | 7.6 | 1.5 | 6.7 | 0.23% | 6.5 |
| 2510 | AT5G49010.1 \| DNA replication protein-related, similar to Sld5 (Xenopus laevis) GI:29365477; contains Pfam profile PF05916: Eukaryotic protein of unknown function (DUF873) \| chr5:19883690-19885367 FORWARD \| Aliases: K19E20.14, K19E20_14 | 7.3 | 4.3 | 3.0 | 6.7 | 0.23% | 6.2 |
| 2511 | AT5G27750.1 \| F-box family protein, contains F-box domain Pfam:PF00646 | 5.8 | 4.0 | 1.8 | 6.7 | 0.23% | 6.4 |
| 2512 | AT1G21190.1 \| small nuclear ribonucleoprotein, putative / snRNP, putative / Sm protein, putative, similar to SWISS-PROT:Q9Y4Z1 U6 snRNA-associated Sm-like protein LSm3 (MDS017) (Mouse) \| chr1:7419667-7420892 REVERSE \| Aliases: F16F4.12, F16F4_12 | 6.3 | 4.5 | 1.8 | 6.7 | 0.23% | 6.4 |
| 2514 | AT1G52930.1 \| brix domain-containing protein, contains Pfam domain, PF04427: Brix domain \| chr1:19714905-19716898 FORWARD \| Aliases: F14G24.20, F14G24_20 | 7.8 | 4.9 | 2.9 | 6.7 | 0.23% | 6.4 |
| 2518 | AT1G67250.1 \| proteasome maturation factor UMP1 family protein, contains Pfam profile PF05348: Proteasome maturation factor UMP1 \| chr1:25167305-25168715 REVERSE \| Aliases: F1N21.7 | 10.9 | 8.8 | 2.1 | 6.7 | 0.24% | 6.5 |
| 2524 | AT1G52890.1 \| Symbol: ANAC019 \| no apical meristem (NAM) family protein, contains Pfam PF02365: No apical meristem (NAM) domain; similar to NAM (no apical meristem) GB:CAA63101 from (Petunia x hybrida) \| chr1:19700672-19702140 REVERSE \| Aliases: ANAC019, F14G24.16, F14G24_16 | 4.9 | 3.4 | 1.5 | 6.7 | 0.24% | 6.4 |
| 2526 | AT5G19570.1 \| expressed protein \| chr5:6606702-6607205 FORWARD \| Aliases: T20D1.90, T20D1_90 | 7.6 | 5.8 | 1.8 | 6.7 | 0.24% | 6.5 |
| 2527 | AT3G22480.2 \| prefoldin-related KE2 family protein, similar to Swiss-Prot:Q9UHV9 prefoldin subunit 2 (Protein HSPC231) (Homo sapiens); contains Pfam domain, PF01920: KE2 family protein \| chr3:7968184-7969636 FORWARD \| Aliases: F16J14.4 | 8.4 | 5.7 | 2.7 | 6.7 | 0.24% | 6.4 |
| 2528 | AT1G51940.1 \| protein kinase family protein / peptidoglycan-binding LysM domain-containing protein, contains protein kinases ATP-binding region signature, PROSITE:PS00107 \| chr1:19299598-19302787 REVERSE \| Aliases: T14L22.13, T14L22_13 | 6.5 | 4.9 | 1.6 | 6.7 | 0.24% | 6.4 |
| 2530 | AT5G62440.1 \| expressed protein \| chr5:25089709-25091215 REVERSE \| Aliases: AT5G62450, K19B1.5, K19B1_5 | 7.4 | 6.2 | 1.2 | 6.7 | 0.24% | 6.4 |
| 2532 | AT4G11010.1 \| Symbol: NDPK3 \| nucleoside diphosphate kinase 3, mitochondrial (NDK3), identical to Nucleoside diphosphate kinase III, mitochondrial precursor (NDK III) (NDP kinase III) (NDPK III) (SP:O49203) (Arabidopsis thaliana); contains Pfam PF00334 : Nucleoside diphosphate kinase domain; | 10.7 | 8.5 | 2.2 | 6.6 | 0.24% | 6.5 |
| 2533 | AT2G46830.2 \| Symbol: CCA1 \| myb-related transcription factor (CCA1), identical to myb-related transcription factor (CCA1) GI:4090569 from (Arabidopsis thaliana) \| chr2:19253750-19255983 FORWARD \| Aliases: CIRCADIAN CLOCK ASSOCIATED 1, F19D11.11, MYB RELATED DNA BINDING PROTEIN | 7.6 | 4.3 | 3.3 | 6.6 | 0.24% | 6.4 |
| 2534 | AT1G48830.2 \| 40S ribosomal protein S7 (RPS7A), similar to 40S ribosomal protein S7 homolog GI:5532505 from (Brassica oleracea) \| chr1:18063304-18064925 REVERSE \| Aliases: F11I4.1, F11I4_1 | 12.2 | 10.5 | 1.6 | 6.6 | 0.24% | 6.2 |
| 2536 | AT4G15770.1 \| 60S ribosome subunit biogenesis protein, putative, contains similarity to 60S ribosome subunit biogenesis protein NIP7 (Swiss-Prot:Q08962) (Saccharomyces cerevisiae) \| chr4:8978009-8978877 FORWARD \| Aliases: DL3925W, FCAALL.377 | 9.0 | 8.2 | 0.8 | 6.6 | 0.24% | 6.4 |
| 2538 | AT1G75270.1 \| dehydroascorbate reductase, putative, similar to GI:6939839 from (Oryza sativa) \| chr1:28253736-28255010 REVERSE \| Aliases: F22H5.1, F22H5_1 | 9.9 | 7.7 | 2.2 | 6.6 | 0.24% | 6.5 |
| 2539 | AT1G20480.1 \| 4-coumarate--CoA ligase family protein / 4-coumaroyl-CoA synthase family protein, similar to SP:Q9S725 from Arabidopsis thaliana and SP:P17814 from Oryza sativa; contains Pfam AMP-binding enzyme domain PF00501 \| chr1:7094250-7097104 REVERSE \| Aliases: F5M15.29, F5M15_29 | 4.5 | 3.4 | 1.2 | 6.6 | 0.24% | 6.5 |
| 2541 | AT5G46160.2 \| ribosomal protein L14 family protein / huellenlos paralog (HLP), contains Pfam profile PF00238: Ribosomal protein L14p/L23e; identical to cDNA HUELLENLOS PARALOG (HLP) nuclear gene for mitochondrial product GU:18140859 \| chr5:18728582-18729734 REVERSE \| Aliases: MCL19.22, MCL19_22 | 9.4 | 7.7 | 1.6 | 6.6 | 0.24% | 6.5 |

| Rank | Description | Sync | Root | M | t | adj.q | B |
|---|---|---|---|---|---|---|---|
| 2543 | AT1G15310.1 \| Symbol: ATHSRP54A \| signal recognition particle 54 kDa protein 1 / SRP54 (SRP-54) (SRP-54A), identical to Swiss-Prot:P37106 signal recognition particle 54 kDa protein 1 (SRP54) (Arabidopsis thaliana) | 7.0 | 5.4 | 1.7 | 6.6 | 0.24% | 6.4 |
| 2548 | AT2G03420.1 \| expressed protein \| chr2:1034995-1035966 REVERSE \| Aliases: T4M8.15, T4M8_15 | 4.7 | 2.7 | 2.0 | 6.6 | 0.24% | 6.2 |
| 2549 | AT5G14710.1 \| expressed protein \| chr5:4746365-4747936 FORWARD \| Aliases: T9L3.10, T9L3_10 | 6.8 | 4.5 | 2.3 | 6.6 | 0.24% | 6.4 |
| 2550 | AT1G20630.1 \| Symbol: CAT1 \| catalase 1, identical to catalase 1 GI:2511725 from (Arabidopsis thaliana) \| chr1:7146720-7149967 FORWARD \| Aliases: CATALASE 1, F5M15.31, F5M15_31 | 8.4 | 4.7 | 3.7 | 6.6 | 0.24% | 6.4 |
| 2553 | AT2G28430.1 \| expressed protein \| chr2:12166639-12167528 REVERSE \| Aliases: T1B3.5, T1B3_5 | 8.5 | 6.0 | 2.5 | 6.6 | 0.24% | 6.3 |
| 2557 | AT5G63650.1 \| serine/threonine protein kinase, putative, similar to serine/threonine-protein kinase ASK2(Arabidopsis thaliana), SWISS-PROT:P43292; contains protein kinase domain, Pfam:PF00069 \| chr5:25498743-25500945 REVERSE \| Aliases: MBK5.13, MBK5_13 | 5.4 | 3.4 | 2.0 | 6.6 | 0.24% | 6.4 |
| 2558 | AT1G69935.1 \| expressed protein \| chr1:26345464-26346789 REVERSE \| Aliases: None | 6.1 | 4.3 | 1.8 | 6.6 | 0.24% | 6.2 |
| 2560 | AT5G55160.1 \| Symbol: SUM2 \| small ubiquitin-like modifier 2 (SUMO), similar to ubiquitin-like protein SMT3 SP:P55852 from (Arabidopsis thaliana); identical to cDNA small ubiquitin-like modifier 2 (SUMO) GI:22652843; contains Pfam profile PF00240: Ubiquitin family \| chr5:22400795-22402151 REVERSE \| Aliases: MCO15.11, MCO15_11, SMALL UBIQUITIN LIKE MODIFIER 2, SUMO 2, SUMO2 | 9.8 | 8.3 | 1.4 | 6.6 | 0.24% | 6.4 |
| 2561 | AT5G47070.1 \| protein kinase, putative, similar to protein kinase (Lophopyrum elongatum) gi:13022177:gb:AAK11674 \| chr5:19135770-19138136 REVERSE \| Aliases: K14A3.2, K14A3_2 | 6.1 | 3.7 | 2.4 | 6.6 | 0.24% | 6.4 |
| 2562 | AT1G79180.1 \| myb family transcription factor (MYB63), similar to myb-related protein GI:1370139 from (Lycopersicon esculentum) \| chr1:29791402-29792695 FORWARD \| Aliases: YUP8H12R.21, YUP8H12R_21 | 6.5 | 4.9 | 1.6 | 6.6 | 0.24% | 6.4 |
| 2563 | AT3G27360.1 \| histone H3, identical to histone H3 from Zea mays SP:P05203, Medicago sativa GI:166384, Encephalartos altensteinii SP:P08903, Pisum sativum SP:P02300; contains Pfam profile PF00125 Core histone H2A/H2B/H3/H4 \| chr3:10130520-10131174 REVERSE \| Aliases: K1G2.15 | 6.5 | 4.1 | 2.5 | 6.6 | 0.24% | 6.3 |
| 2564 | AT3G15352.1 \| Symbol: ATCOX17 \| cytochrome c oxidase copper chaperone-related, contains similarity to cytochrome c oxidase copper chaperone (Mus musculus) SWISS-PROT:P56394 \| chr3:5179507-5180672 FORWARD \| Aliases: COX17 | 7.4 | 5.6 | 1.8 | 6.6 | 0.24% | 6.4 |
| 2568 | AT2G41460.1 \| Symbol: ARP \| apurinic endonuclease-redox protein / DNA-(apurinic or apyrimidinic site) lyase, identical to apurinic endonuclease-redox protein SP: P45951 from (Arabidopsis thaliana) \| chr2:17292809-17296006 FORWARD \| Aliases: T26J13.5, T26J13_5 | 7.0 | 5.6 | 1.4 | 6.6 | 0.25% | 6.4 |
| 2570 | AT5G61330.1 \| rRNA processing protein-related, contains weak similarity to rRNA processing protein EBP2 (EBNA1-binding protein homolog) (Swiss-Prot:P36049) (Saccharomyces cerevisiae) | 7.7 | 6.5 | 1.2 | 6.6 | 0.25% | 6.4 |
| 2572 | AT5G59000.1 \| zinc finger (C3HC4-type RING finger) family protein, contains Pfam profile: PF00097 zinc finger, C3HC4 type (RING finger) ;contains PROSITE PS00190: Cytochrome c family heme-binding site signature \| chr5:23835980-23837463 FORWARD \| Aliases: K19M22.19, K19M22_19 | 5.1 | 3.2 | 1.9 | 6.6 | 0.25% | 6.2 |
| 2573 | AT3G04840.1 \| 40S ribosomal protein S3A (RPS3aA), similar to 40S ribosomal protein S3A (S phase specific protein GBIS289) GB:P49396 (Brassica rapa) \| chr3:1329699-1331581 FORWARD \| Aliases: T9J14.21, T9J14_21 | 10.4 | 8.2 | 2.2 | 6.6 | 0.25% | 6.4 |
| 2576 | AT3G25400.1 \| expressed protein \| chr3:9214469-9215516 FORWARD \| Aliases: MWL2.8 | 8.5 | 6.8 | 1.7 | 6.6 | 0.25% | 6.4 |
| 2577 | AT1G18040.1 \| Symbol: CDKD1;3 \| cell division protein kinase, putative, similar to cell division protein kinase 7 (Homo sapiens) SWISS-PROT:P50613 \| chr1:6206864-6209493 REVERSE \| Aliases: CDKD1;3, Cyclin dependent kinase D1;3, T10F20.5, T10F20_5 | 6.0 | 3.7 | 2.3 | 6.6 | 0.25% | 6.3 |
| 2578 | AT3G53400.1 \| expressed protein \| chr3:19809129-19811114 FORWARD \| Aliases: F4P12.100 | 7.2 | 5.2 | 2.0 | 6.6 | 0.25% | 6.2 |
| 2579 | AT5G44650.1 \| expressed protein \| chr5:18030376-18032630 FORWARD \| Aliases: K15C23.10, K15C23_10 | 7.5 | 5.9 | 1.6 | 6.6 | 0.25% | 6.4 |
| 2582 | AT2G25355.1 \| exonuclease-related, similar to Exosome complex exonuclease RRP40 (EC 3.1.13.-) (Ribosomal RNA processing protein 40) (p10) (CGI-102) (Swiss-Prot:Q9NQT5) (Homo sapiens) | 6.4 | 4.8 | 1.7 | 6.6 | 0.25% | 6.4 |
| 2583 | AT4G16265.1 \| DNA-directed RNA polymerase II, putative, similar to SP:P36958 DNA-directed RNA polymerase II 15.1 kDa polypeptide (EC 2.7.7.6) {Drosophila melanogaster}; contains Pfam profile PF02150: RNA polymerases M/15 Kd subunit \| chr4:9202371-9204086 REVERSE \| Aliases: None | 5.9 | 3.8 | 2.1 | 6.6 | 0.25% | 6.3 |

| Rank | Description | Sync | Root | M | t | adj.q | B |
|---|---|---|---|---|---|---|---|
| 2584 | AT5G50170.1 \| C2 domain-containing protein / GRAM domain-containing protein, low similarity to SP:P40748 Synaptotagmin III (SytIII) {Rattus norvegicus}; contains Pfam profiles PF00168: C2 domain, PF02893: GRAM domain \| chr5:20438299-20442812 FORWARD \| Aliases: K6A12.3, K6A12_3 | 5.5 | 3.6 | 2.0 | 6.6 | 0.25% | 6.3 |
| 2589 | AT5G10160.1 \| beta-hydroxyacyl-ACP dehydratase, putative, similar to beta-hydroxyacyl-ACP dehydratase from Toxoplasma gondii (GI:3850997); contains Pfam profile PF01377 Thioester dehydratase | 7.6 | 4.1 | 3.4 | 6.6 | 0.25% | 6.1 |
| 2590 | AT5G62290.1 \| nucleotide-sensitive chloride conductance regulator (ICln) family protein, contains PF03517: Nucleotide-sensitive chloride conductance regulator (ICln) \| chr5:25036713-25038357 FORWARD \| Aliases: MMI9.12, MMI9_12 | 5.8 | 4.7 | 1.2 | 6.6 | 0.25% | 6.4 |
| 2591 | AT5G58480.1 \| glycosyl hydrolase family 17 protein, similar to elicitor inducible chitinase Nt-SubE76 GI:11071974 from (Nicotiana tabacum) \| chr5:23658368-23660079 REVERSE \| Aliases: MQJ2.10, MQJ2_10 | 7.2 | 4.5 | 2.7 | 6.6 | 0.25% | 6.0 |
| 2593 | AT3G05190.1 \| aminotransferase class IV family protein, contains Pfam profile: PF01063 aminotransferase class IV \| chr3:1471403-1475790 FORWARD \| Aliases: T12H1.16, T12H1_16 | 5.8 | 3.7 | 2.2 | 6.6 | 0.25% | 6.0 |
| 2595 | AT5G61030.1 \| RNA-binding protein, putative, similar to RNA-binding protein from (Solanum tuberosum) GI:15822705, (Nicotiana tabacum) GI:15822703, (Nicotiana sylvestris) GI:624925; contains InterPro entry IPR000504: RNA-binding region RNP-1 (RNA recognition motif) (RRM) | 10.2 | 8.3 | 1.9 | 6.6 | 0.25% | 6.4 |
| 2596 | AT2G24820.1 \| Rieske (2Fe-2S) domain-containing protein, similar to Rieske iron-sulfur protein Tic55 from Pisum sativum (gi:2764524); contains Pfam PF00355 Rieske (2Fe-2S) domain \| chr2:10582058-10584553 FORWARD \| Aliases: F27C12.26, F27C12_26 | 7.0 | 4.4 | 2.6 | 6.6 | 0.25% | 6.3 |
| 2599 | AT5G66570.1 \| Symbol: PSBO 1 \| | 7.7 | 3.3 | 4.4 | 6.6 | 0.25% | 6.2 |
| 2600 | AT1G76570.1 \| chlorophyll A-B binding family protein, similar to chlorophyll A-B binding protein GB:P12470 (Nicotiana plumbaginifolia); contains Pfam profile: PF00504 Chlorophyll A-B binding proteins \| chr1:28734026-28735719 FORWARD \| Aliases: F14G6.17, F14G6_17 | 5.8 | 4.8 | 1.0 | 6.6 | 0.25% | 6.4 |
| 2601 | AT2G34160.1 \| expressed protein \| chr2:14433282-14434462 FORWARD \| Aliases: T14G11.28, T14G11_28 | 6.7 | 4.5 | 2.1 | 6.6 | 0.25% | 6.4 |
| 2608 | AT1G14320.1 \| 60S ribosomal protein L10 (RPL10A) / Wilm's tumor suppressor protein-related, similar to tumor suppressor GI:575354 from (Oryza sativa) \| chr1:4888209-4889656 FORWARD \| Aliases: F14L17.9, F14L17_9 | 13.1 | 12.0 | 1.1 | 6.6 | 0.25% | 5.9 |
| 2609 | AT3G22210.1 \| expressed protein \| chr3:7838791-7839474 REVERSE \| Aliases: MKA23.19 | 4.8 | 2.5 | 2.3 | 6.6 | 0.25% | 6.3 |
| 2610 | AT4G37110.1 \| expressed protein \| chr4:17484119-17486265 REVERSE \| Aliases: AP22.72, AP22_72 | 4.5 | 2.8 | 1.7 | 6.6 | 0.25% | 6.4 |
| 2611 | AT3G18080.1 \| glycosyl hydrolase family 1 protein, contains Pfam PF00232 : Glycosyl hydrolase family 1 domain; TIGRFAM TIGR01233: 6-phospho-beta-galactosidase; similar to beta-glucosidase BGQ60 precursor GB:A57512 (Hordeum vulgare); similar to beta-mannosidase enzyme (GI:17226270) (Lycopersicon esculentum) \| chr3:6191565-6194458 FORWARD \| Aliases: MRC8.20 | 6.8 | 3.9 | 2.9 | 6.6 | 0.25% | 6.3 |
| 2612 | AT4G21680.1 \| proton-dependent oligopeptide transport (POT) family protein, contains Pfam profile: PF00854 POT family \| chr4:11517043-11519777 REVERSE \| Aliases: F17L22.140, F17L22_140 | 6.6 | 4.1 | 2.5 | 6.6 | 0.25% | 5.9 |
| 2613 | AT5G27820.1 \| ribosomal protein L18 family protein, similar to SP:P52863 50S ribosomal protein L18 (Aeromonas proteolytica) {Vibrio proteolyticus} \| chr5:9859858-9861133 FORWARD \| Aliases: T1G16.150, T1G16_150 | 5.5 | 3.0 | 2.5 | 6.6 | 0.25% | 5.9 |
| 2614 | AT5G09590.1 \| Symbol: mtHSC70 2 \| heat shock protein 70 / HSP70 (HSC70-5), identical to heat shock protein 70 (Arabidopsis thaliana) GI:6746590 \| chr5:2975576-2978751 FORWARD \| Aliases: F17I14.220, F17I14_220, HEAT SHOCK PROTEIN 70, HSC70 5 | 11.0 | 8.4 | 2.7 | 6.6 | 0.25% | 6.4 |
| 2615 | AT2G21710.1 \| Symbol: EMB2219 \| mitochondrial transcription termination factor-related / mTERF-related, contains Pfam profile PF02536: mTERF \| chr2:9277966-9280498 FORWARD \| Aliases: EMB2219, EMBRYO DEFECTIVE 2219, F7D8.3, F7D8_3 | 3.6 | 2.4 | 1.1 | 6.6 | 0.25% | 6.4 |
| 2616 | AT2G39500.1 \| expressed protein \| chr2:16496151-16496666 FORWARD \| Aliases: F12L6.16, F12L6_16 | 6.8 | 4.8 | 2.0 | 6.6 | 0.26% | 6.3 |
| 2617 | AT1G31812.1 \| acyl-CoA binding protein / ACBP, identical to acyl-CoA-binding protein (ACBP) (Arabidopsis thaliana) SWISS-PROT:P57752 \| chr1:11410857-11412170 REVERSE \| Aliases: F5M6.27, F5M6_27 | 12.8 | 11.6 | 1.1 | 6.6 | 0.26% | 6.3 |
| 2619 | AT2G43030.1 \| ribosomal protein L3 family protein, contains Pfam profile PF00297: ribosomal protein L3 | 8.3 | 5.5 | 2.8 | 6.6 | 0.26% | 6.2 |
| 2620 | AT1G31860.1 \| Symbol: AT IE \| histidine biosynthesis bifunctional protein (HISIE), identical to histidine biosynthesis bifunctional protein hisIE, chloroplast (precursor) SP: O82768 from (Arabidopsis thaliana); identical to cDNA phosphoribosyl-ATP pyrophosphohydrolase GI:3461883 \| chr1:11434089-11436587 REVERSE \| Aliases: F5M6.13, F5M6_13, PHOSPHORIBOSYL ATP PYROPHOSPHOHYDROLASE | 7.0 | 5.4 | 1.6 | 6.6 | 0.26% | 6.4 |

| Rank | Description | Sync | Root | M | t | adj.q | B |
|---|---|---|---|---|---|---|---|
| 2621 | AT2G23360.1 \| transport protein-related, contains Pfam PF05911: Plant protein of unknown function (DUF869) profile; weak similarity to Intracellular protein transport protein USO1 (Swiss-Prot:P25386) (Saccharomyces cerevisiae) \| chr2:9956536-9959925 FORWARD \| Aliases: F26B6.1, F26B6_1 | 4.8 | 3.6 | 1.2 | 6.6 | 0.26% | 6.4 |
| 2622 | AT5G27390.1 \| expressed protein, CG6949 - Drosophila melanogaster, EMBL:AE003739 \| chr5:9671848-9676121 REVERSE \| Aliases: F21A20.100, F21A20_100 | 6.5 | 4.9 | 1.7 | 6.6 | 0.26% | 6.2 |
| 2624 | AT5G57290.1 \| 60S acidic ribosomal protein P3 (RPP3B) \| chr5:23224055-23225121 REVERSE \| Aliases: MJB24.10, MJB24_10 | 9.4 | 6.9 | 2.5 | 6.6 | 0.26% | 6.1 |
| 2625 | AT2G19740.1 \| 60S ribosomal protein L31 (RPL31A) \| chr2:8520599-8521601 FORWARD \| Aliases: F6F22.23, F6F22_23 | 8.9 | 6.1 | 2.8 | 6.6 | 0.26% | 6.2 |
| 2629 | AT4G22740.2 \| glycine-rich protein \| chr4:11942812-11945967 REVERSE \| Aliases: T12H17.1 | 10.3 | 8.0 | 2.3 | 6.5 | 0.26% | 6.4 |
| 2635 | AT4G12700.1 \| expressed protein \| chr4:7482463-7484402 REVERSE \| Aliases: T20K18.50, T20K18_50 | 5.1 | 3.9 | 1.2 | 6.5 | 0.26% | 6.4 |
| 2636 | AT5G61000.1 \| replication protein, putative, similar to replication protein A1 (Oryza sativa) GI:2258469; contains InterPro entry IPR004365: OB-fold nucleic acid binding domain \| chr5:24566804-24569947 REVERSE \| Aliases: MSL3.120, MSL3_120 | 6.7 | 4.0 | 2.7 | 6.5 | 0.26% | 6.0 |
| 2637 | AT3G23760.1 \| expressed protein \| chr3:8562836-8564677 REVERSE \| Aliases: MYM9.10 | 5.0 | 2.6 | 2.4 | 6.5 | 0.26% | 5.8 |
| 2638 | AT2G40430.1 \| expressed protein, identical to Protein At2g40430 (Swiss-Prot:O22892) (Arabidopsis thaliana); similar to Glioma tumor suppressor candidate region gene 2 protein (p60) (Swiss-Prot:Q9NZM5) (Homo sapiens) \| chr2:16886297-16889505 REVERSE \| Aliases: T2P4.22 | 9.6 | 8.3 | 1.3 | 6.5 | 0.26% | 6.4 |
| 2640 | AT1G70070.1 \| Symbol: PDE317 \| | 5.6 | 4.0 | 1.6 | 6.5 | 0.26% | 6.2 |
| 2641 | AT2G02500.1 \| Symbol: ATMEPCT/ISPD \| expressed protein, contains Pfam profile: PF01128 uncharacterized protein family UPF0007; identical to GP:12697583 2-C-methyl-D-erythritol 4-phosphate cytidyltransferase {Arabidopsis thaliana}; identical to cDNA 4-Diphosphocytidyl-2C-methyl-D-erythritol synthase (ISPD) GI:7385140 \| chr2:670877-673154 REVERSE \| Aliases: 2 C METHYL D ERYTHRITOL 4 PHOSPHATE CYTIDYLTRANSFERASE, 4 DIPHOSPHOCYTIDYL 2C METHYL D ERYTHRITOL SYNTHASE, T8K22.20, T8K22_20 | 5.4 | 3.9 | 1.6 | 6.5 | 0.26% | 6.3 |
| 2642 | AT4G14210.2 \| Symbol: PDS3 \| phytoene dehydrogenase, chloroplast / phytoene desaturase (PDS), identical to SP:Q07356 Phytoene dehydrogenase, chloroplast precursor (EC 1.14.99.-) (Phytoene desaturase){Arabidopsis thaliana}; high similarity to phytoene desaturase (Lycopersicon esculentum)(GI:19287) \| chr4:8190256-8195095 REVERSE \| Aliases: DL3145C, FCAALL.28, PDE226, PDS, PHYTOENE DESATURASE | 8.3 | 5.9 | 2.4 | 6.5 | 0.26% | 6.3 |
| 2643 | AT5G02790.1 \| In2-1 protein, putative, similar to In2-1, Zea mays, EMBL:X58573 | 10.1 | 6.3 | 3.8 | 6.5 | 0.26% | 6.2 |
| 2644 | AT4G29480.1 \| mitochondrial ATP synthase g subunit family protein, contains Pfam profile: PF04718 mitochondrial ATP synthase g subunit | 12.1 | 10.4 | 1.7 | 6.5 | 0.26% | 6.3 |
| 2645 | AT5G38510.1 \| rhomboid family protein, contains Pfam profile PF01694: Rhomboid family \| chr5:15435069-15437232 REVERSE \| Aliases: MBB18.4, MBB18_4 | 5.3 | 3.7 | 1.6 | 6.5 | 0.26% | 6.3 |
| 2648 | AT4G35450.4 \| Symbol: AKR2 \| ankyrin repeat family protein / AFT protein (AFT), contains ankyrin repeats, Pfam:PF00023; identical to cDNA AFT protein (AFT) GI:3478699 \| chr4:16839933-16842082 FORWARD \| Aliases: AFT, ANKYRIN REPEAT CONTAINING PROTEIN 2, F15J1.20, F15J1_20 | 11.0 | 8.6 | 2.4 | 6.5 | 0.26% | 6.4 |
| 2649 | AT3G62150.1 \| multidrug resistant (MDR) ABC transporter, putative, similar to multidrug-resistant protein CjMDR1 GI:14715462 from (Coptis japonica); contains Pfam profiles PF00005: ABC transporter, PF00664: ABC transporter transmembrane region \| chr3:23019730-23024554 REVERSE \| Aliases: T17J13.110 | 7.1 | 5.9 | 1.2 | 6.5 | 0.26% | 6.3 |
| 2651 | AT1G71350.1 \| eukaryotic translation initiation factor SUI1 family protein, weak similarity to SP:P41214 Ligatin (Hepatocellular carcinoma-associated antigen 56) {Homo sapiens}; contains Pfam profile PF01253: Translation initiation factor SUI1 \| chr1:26892070-26894893 REVERSE \| Aliases: F3I17.23, F3I17_23 | 8.5 | 6.5 | 2.0 | 6.5 | 0.26% | 6.3 |
| 2654 | AT2G38330.1 \| MATE efflux family protein, low similarity to enhanced disease susceptibility 5 (Arabidopsis thaliana) GI:16589070; contains Pfam profile PF01554: Uncharacterized membrane protein family \| chr2:16071602-16075369 FORWARD \| Aliases: T19C21.18, T19C21_18 | 6.8 | 5.2 | 1.7 | 6.5 | 0.26% | 6.3 |
| 2655 | AT2G21580.2 \| similar to 40S ribosomal protein S25 (RPS25E) [Arabidopsis thaliana] (TAIR:At4g39200.1); similar to PREDICTED P0562A06.14 gene product [Oryza sativa (japonica cultivar-group)] (GB:XP_507607.1); contains InterPro domain S25 ribosomal protein (InterPro:IPR004977) \| chr2:9243633-9244891 FORWARD \| Aliases: F2G1.15, F2G1_15 | 10.8 | 8.3 | 2.6 | 6.5 | 0.26% | 6.2 |
| 2656 | AT2G45730.1 \| eukaryotic initiation factor 3 gamma subunit family protein, contains Pfam profile PF04189: Eukaryotic initiation factor 3, gamma subunit \| chr2:18843459-18846234 REVERSE \| Aliases: F4I18.29 | 6.9 | 5.4 | 1.5 | 6.5 | 0.26% | 6.3 |
| 2658 | AT1G33790.1 \| jacalin lectin family protein, similar to myrosinase binding protein homolog GI:2997767 from (Arabidopsis thaliana); contains contains Pfam profile PF01419 jacalin-like lectin domain \| chr1:12256644-12260537 FORWARD \| Aliases: F14M2.9, F14M2_9 | 6.2 | 4.7 | 1.5 | 6.5 | 0.26% | 6.3 |

| Rank | Description | Sync | Root | M | t | adj.q | B |
|---|---|---|---|---|---|---|---|
| 2659 | AT1G64750.2 \| DSS1/SEM1 family protein, contains Pfam profile PF05160: DSS1/SEM1 family \| chr1:24056244-24057568 REVERSE \| Aliases: F13O11.6, F13O11_6 | 8.8 | 6.6 | 2.1 | 6.5 | 0.26% | 6.2 |
| 2662 | AT5G47190.1 \| ribosomal protein L19 family protein, similar to plastid ribosomal protein L19 precursor (Spinacia oleracea) gi:7582403:gb:AAF64312 \| chr5:19181475-19183334 REVERSE \| Aliases: MQL5.4, MQL5_4 | 6.1 | 4.1 | 2.0 | 6.5 | 0.26% | 6.1 |
| 2664 | AT1G16870.1 \| mitochondrial 28S ribosomal protein S29-related, contains weak similarity to Swiss-Prot:P51398 mitochondrial 28S ribosomal protein S29 (MRP-S29, Death-associated protein 3, DAP-3) (Homo sapiens) \| chr1:5770728-5773241 REVERSE \| Aliases: F17F16.14 | 8.0 | 5.4 | 2.6 | 6.5 | 0.26% | 6.2 |
| 2665 | AT5G13710.1 \| Symbol: SMT1 \| sterol 24-C-methyltransferase, putative, similar to SP:P25087 Sterol 24-C-methyltransferase, Delta(24)-sterol C- methyltransferase, Saccharomyces cerevisiae \| chr5:4423698-4427050 REVERSE \| Aliases: CEPHALOPOD, CPH, MSH12.18, MSH12_18, SAM:CYCLOARTENOL C24 METHYLTRANSFERASE, STEROL METHYLTRANSFERASE 1 | 9.3 | 7.2 | 2.1 | 6.5 | 0.26% | 6.3 |
| 2667 | AT4G24090.1 \| expressed protein \| chr4:12512746-12514591 FORWARD \| Aliases: T19F6.80, T19F6_80 | 5.3 | 3.8 | 1.5 | 6.5 | 0.26% | 6.3 |
| 2668 | AT4G09020.1 \| Symbol: ATISA3/ISA3 \| isoamylase, putative / starch debranching enzyme, putative, similar to isoamylase isoform 3 (Solanum tuberosum) GI:27728149, isoamylase (Oryza sativa) GI:3252794; contains Pfam profiles PF00128: Alpha amylase catalytic domain, PF02922: Isoamylase N-terminal domain \| chr4:5784073-5788997 FORWARD \| Aliases: ATISA3, F23J3.50, F23J3_50, ISA3 | 7.3 | 5.4 | 1.9 | 6.5 | 0.26% | 6.3 |
| 2669 | AT4G21090.3 \| similar to adrenodoxin-like ferredoxin 2 [Arabidopsis thaliana] (TAIR:At4g05450.1); similar to putative ferredoxin [Oryza sativa (japonica cultivar-group)] (GB:XP_476404.1); contains InterPro domain Adrenodoxin (InterPro:IPR001055); contains InterPro domain Ferredoxin (InterPro:IPR001041) \| chr4:11256470-11258598 REVERSE \| Aliases: F7J7.30, F7J7_30 | 7.2 | 5.7 | 1.5 | 6.5 | 0.26% | 6.3 |
| 2670 | AT5G50210.1 \| quinolinate synthetase A-related, contains weak similarity to Swiss-Prot:P11458 quinolinate synthetase A (Escherichia coli) \| chr5:20459873-20463217 FORWARD \| Aliases: K6A12.7, K6A12_7 | 8.1 | 5.5 | 2.6 | 6.5 | 0.27% | 6.1 |
| 2674 | AT4G37970.1 \| mannitol dehydrogenase, putative, similar to ELI3-2 (SP:Q02972), sinapyl alcohol dehydrogenase (Populus tremuloides) (gi:14279694); contains Pfam zinc-binding dehydrogenase domain PF00107 \| chr4:17849666-17852233 FORWARD \| Aliases: F20D10.90, F20D10_90 | 6.2 | 4.1 | 2.1 | 6.5 | 0.27% | 6.1 |
| 2676 | AT5G15200.1 \| 40S ribosomal protein S9 (RPS9B), 40S ribosomal protein S9, Chlamydomonas sp., EMBL:AU066528 \| chr5:4934886-4936384 REVERSE \| Aliases: F8M21.90, F8M21_90 | 11.6 | 10.1 | 1.5 | 6.5 | 0.27% | 6.3 |
| 2680 | AT4G31730.1 \| Symbol: GDU1 \| expressed protein \| chr4:15361097-15361801 FORWARD \| Aliases: F28M20.80, F28M20_80, GLUTAMINE DUMPER 1 | 6.9 | 5.2 | 1.7 | 6.5 | 0.27% | 6.3 |
| 2681 | AT5G14910.1 \| heavy-metal-associated domain-containing protein, Pfam profile PF00403: Heavy-metal-associated domain \| chr5:4823717-4825373 FORWARD \| Aliases: F2G14.30, F2G14_30 | 6.5 | 4.1 | 2.5 | 6.5 | 0.27% | 6.1 |
| 2682 | AT5G27700.1 \| 40S ribosomal protein S21 (RPS21C), ribosomal protein S21, Zea mays, PIR:T03945 \| chr5:9807323-9808480 REVERSE \| Aliases: T1G16.30, T1G16_30 | 11.2 | 8.9 | 2.3 | 6.5 | 0.27% | 6.3 |
| 2683 | AT2G32520.1 \| dienelactone hydrolase family protein, low similarity to dienelactone hydrolase (Pseudomonas resinovorans) GI:13094163; contains Pfam profile PF01738: Dienelactone hydrolase family \| chr2:13812596-13814613 REVERSE \| Aliases: T26B15.8, T26B15_8 | 11.3 | 9.4 | 1.9 | 6.5 | 0.27% | 6.2 |
| 2684 | AT3G50820.1 \| Symbol: PSBO 2/PSBO2 \| Encodes a protein which is an extrinsic subunit of photosystem II and which has been proposed to play a central role in stabilization of the catalytic manganese cluster. In <i>Arabidopsis thaliana</i> the PsbO proteins are encoded by two genes: <i>psbO1</i> and <i>psbO2</i>. PsbO2 is the minor isoform in the wild-type. | 6.3 | 4.3 | 2.0 | 6.5 | 0.27% | 6.2 |
| 2685 | AT3G24506.1 \| expressed protein \| chr3:8928177-8929336 REVERSE \| Aliases: None | 6.3 | 4.6 | 1.7 | 6.5 | 0.27% | 6.3 |
| 2687 | AT1G08980.1 \| Symbol: ATAMI1 \| | 6.8 | 5.2 | 1.7 | 6.5 | 0.27% | 6.2 |
| 2693 | AT5G41650.1 \| lactoylglutathione lyase family protein / glyoxalase I family protein, contains Pfam profile PF00903: glyoxalase family protein \| chr5:16672141-16673027 FORWARD \| Aliases: MBK23.15, MBK23_15 | 3.6 | 2.5 | 1.0 | 6.5 | 0.27% | 6.3 |
| 2694 | AT5G25475.2 \| expressed protein \| chr5:8867843-8869824 REVERSE \| Aliases: None | 6.3 | 4.1 | 2.1 | 6.5 | 0.27% | 6.2 |
| 2696 | AT5G08620.1 \| DEAD box RNA helicase (RH25), identical to RNA helicase (Arabidopsis thaliana) GI:3776023; contains Pfam profiles PF00270: DEAD/DEAH box helicase, PF00271: Helicase conserved C-terminal domain | 6.4 | 5.1 | 1.3 | 6.5 | 0.27% | 6.3 |

| Rank | Description | Sync | Root | M | t | adj.q | B |
|---|---|---|---|---|---|---|---|
| 2697 | AT4G02930.1 \| elongation factor Tu, putative / EF-Tu, putative, similar to mitochondrial elongation factor Tu (Arabidopsis thaliana) gi:1149571:emb:CAA61511 \| chr4:1295409-1298397 REVERSE \| Aliases: T4I9.19 | 10.0 | 7.1 | 2.9 | 6.5 | 0.27% | 6.1 |
| 2699 | AT1G16740.1 \| ribosomal protein L20 family protein, similar to ribosomal protein L20 GI:3603025 from (Guillardia theta) \| chr1:5727683-5729072 FORWARD \| Aliases: F19K19.15 | 11.0 | 9.0 | 2.0 | 6.5 | 0.27% | 6.3 |
| 2700 | AT2G18230.1 \| inorganic pyrophosphatase (soluble) (PPA) / pyrophosphate phospho-hydrolase / PPase, nearly identical to SP:P21216 Soluble inorganic pyrophosphatase (EC 3.6.1.1) (Pyrophosphate phospho- hydrolase) (PPase) {Arabidopsis thaliana} \| chr2:7939009-7941411 REVERSE \| Aliases: T30D6.26, T30D6_26 | 10.1 | 5.5 | 4.6 | 6.5 | 0.27% | 6.1 |
| 2701 | AT1G69530.3 \| Symbol: ATEXPA1 \| expansin, putative (EXP1), identical to expansin (At-EXP1) (Arabidopsis thaliana) GI:1041702; alpha-expansin gene family, PMID:11641069 \| chr1:26145499-26147159 FORWARD \| Aliases: ARABIDOPSIS THALIANA EXPANSIN A1, AT EXP1, ATEXP1, ATHEXP ALPHA 1.2, EXP1, F10D13.18, F10D13_18 | 7.9 | 2.2 | 5.7 | 6.5 | 0.27% | 5.8 |
| 2702 | AT3G16000.1 \| Symbol: MFP1 \| matrix-localized MAR DNA-binding protein-related, similar to matrix-localized MAR DNA binding protein MFP1 GI:1771158 from (Lycopersicon esculentum) \| chr3:5430895-5433823 REVERSE \| Aliases: MAR BINDING FILAMENT LIKE PROTEIN 1, MSL1.8 | 6.6 | 4.9 | 1.7 | 6.5 | 0.27% | 6.2 |
| 2704 | AT2G46390.1 \| expressed protein \| chr2:19049374-19050133 REVERSE \| Aliases: F11C10.8 | 11.6 | 10.4 | 1.2 | 6.5 | 0.27% | 6.2 |
| 2705 | AT4G14060.1 \| major latex protein-related / MLP-related, low similarity to major latex protein {Papaver somniferum}(GI:294060) ; contains Pfam profile PF00407: Pathogenesis-related protein Bet v I family \| chr4:8107332-8108302 FORWARD \| Aliases: DL3070W, FCAALL.193 | 5.8 | 3.0 | 2.8 | 6.5 | 0.27% | 5.7 |
| 2707 | AT1G01140.3 \| Symbol: CIPK9 \| CBL-interacting protein kinase 9 (CIPK9), identical to CBL-interacting protein kinase 9 (Arabidopsis thaliana) gi:13249117:gb:AAK16684; contains Pfam profiles PF00069: Protein kinase domain and PF03822: NAF domain; identical to cDNA CBL-interacting protein kinase 9 (CIPK9) GI:13249116 \| chr1:64167-67625 REVERSE \| Aliases: CBL INTERACTING PROTEIN KINASE 9, F6F3.28, PKS6 | 6.2 | 4.0 | 2.2 | 6.5 | 0.27% | 6.2 |
| 2708 | AT4G25700.2 \| Symbol: BETA OHASE 1 \| similar to beta-carotene hydroxylase, putative [Arabidopsis thaliana] (TAIR:At5g52570.1); similar to beta-carotene hydroxylase [Citrus unshiu] (GB:AAG10793.1); contains InterPro domain Carotene hydroxylase (InterPro:IPR005596); contains InterPro domain SUR2-type hydroxylase/desaturase, catalytic domain (InterPro:IPR006087) \| chr4:13094782-13095944 REVERSE \| Aliases: BETA HYDROXYLASE 1, L73G19.80, L73G19_80 | 8.0 | 6.3 | 1.7 | 6.5 | 0.27% | 6.3 |
| 2709 | AT3G13190.2 \| myosin heavy chain-related, contains weak similarity to Myosin II heavy chain, non muscle (Swiss-Prot:P05659) (Acanthamoeba castellanii) | 5.9 | 4.0 | 2.0 | 6.5 | 0.27% | 6.2 |
| 2710 | AT5G22875.2 \| expressed protein, similar to hypothetical protein [Ipomoea trifida] (GB:AAS79573.1) \| chr5:7650585-7651779 FORWARD \| Aliases: None | 4.7 | 3.1 | 1.6 | 6.5 | 0.27% | 6.3 |
| 2711 | AT3G20560.1 \| Symbol: ATPDIL5 3 \| Encodes a protein disulfide isomerase-like (PDIL) protein, a member of a multigene family within the thioredoxin (TRX) superfamily. | 6.7 | 4.8 | 1.9 | 6.5 | 0.27% | 6.2 |
| 2712 | AT1G22450.1 \| Symbol: COX6B \| cytochrome c oxidase subunit 6b, putative (COX6b), nearly identical to subunit 6b of cytochrome c oxidase (Arabidopsis thaliana) GI:6518353 \| chr1:7925303-7927267 FORWARD \| Aliases: ATCOX6B2, CYTOCHROME C OXIDASE 6B, F12K8.20, F12K8_20, SUBUNIT 6B OF CYTOCHROME C OXIDASE | 9.6 | 7.8 | 1.8 | 6.5 | 0.27% | 6.3 |
| 2713 | AT5G19700.1 \| MATE efflux protein-related, contains Pfam profile PF01554: Uncharacterized membrane protein family \| chr5:6660823-6662349 REVERSE \| Aliases: T29J13.120, T29J13_120 | 6.7 | 3.6 | 3.1 | 6.5 | 0.27% | 5.2 |
| 2716 | AT3G07670.1 \| SET domain-containing protein, similar to ribulose-1,5-bisphosphate carboxylase/oxygenase small subunit N-methyltransferase I (Spinacia oleracea) GI:3403236; contains Pfam profile PF00856: SET domain \| chr3:2451614-2454866 FORWARD \| Aliases: MLP3.12 | 5.8 | 3.3 | 2.5 | 6.5 | 0.28% | 6.2 |
| 2718 | AT2G20810.1 \| glycosyl transferase family 8 protein, contains Pfam profile: PF01501 glycosyl transferase family 8 \| chr2:8964874-8966861 FORWARD \| Aliases: F5H14.44 | 6.6 | 5.3 | 1.3 | 6.5 | 0.28% | 6.3 |
| 2719 | AT3G43980.1 \| 40S ribosomal protein S29 (RPS29A), ribosomal protein S29, rat, PIR:S30298 \| chr3:15789389-15790298 REVERSE \| Aliases: T15B3.120, T15B3_120 | 13.0 | 11.8 | 1.2 | 6.4 | 0.28% | 5.5 |
| 2720 | AT3G44010.1 \| 40S ribosomal protein S29 (RPS29B), ribosomal protein S29, rat, PIR:S30298 \| chr3:15810918-15812187 REVERSE \| Aliases: T15B3.150 | 13.0 | 11.8 | 1.2 | 6.4 | 0.28% | 5.5 |
| 2721 | AT4G39040.2 \| expressed protein, contains PF01985: Uncharacterised protein family \| chr4:18189006-18190550 REVERSE \| Aliases: F19H22.140, F19H22_140 | 8.4 | 6.6 | 1.8 | 6.4 | 0.28% | 6.2 |
| 2723 | AT2G40960.1 \| expressed protein \| chr2:17102600-17104527 FORWARD \| Aliases: T20B5.16, T20B5_16 | 3.7 | 2.6 | 1.2 | 6.4 | 0.28% | 6.3 |
| 2725 | AT1G56450.1 \| Symbol: PBG1 \| 20S proteasome beta subunit G1 (PBG1) (PRCH), identical to 20S proteasome beta subunit (PBG1) GI:3421123 (Arabidopsis thaliana); identical to cDNA proteasome subunit prch GI:2511597 \| chr1:21145558-21148065 FORWARD \| Aliases: F13N6.3, F13N6_3, PROTEASOME BETA SUBUNIT PBG1 | 10.8 | 8.0 | 2.8 | 6.4 | 0.28% | 6.2 |

| Rank | Description | Sync | Root | M | t | adj.q | B |
|---|---|---|---|---|---|---|---|
| 2726 | AT4G19460.1 \| glycosyl transferase family 1 protein, contains Pfam profile: PF00534 Glycosyl transferases group 1 \| chr4:10610320-10612016 REVERSE \| Aliases: F24J7.20, F24J7_20 | 6.4 | 5.0 | 1.4 | 6.4 | 0.28% | 6.3 |
| 2728 | AT2G21530.1 \| forkhead-associated domain-containing protein / FHA domain-containing protein \| chr2:9226404-9227666 FORWARD \| Aliases: F3K23.29, F3K23_29 | 5.0 | 2.6 | 2.4 | 6.4 | 0.28% | 5.6 |
| 2729 | AT4G27480.1 \| glycosyltransferase family 14 protein / core-2/I-branching enzyme family protein, contains Pfam profile: PF02485 Core-2/I-Branching enzyme \| chr4:13736841-13738921 REVERSE \| Aliases: F27G19.80, F27G19_80 | 5.7 | 4.0 | 1.8 | 6.4 | 0.28% | 6.1 |
| 2730 | AT4G39200.1 \| 40S ribosomal protein S25 (RPS25E), ribosomal protein S25, Lycopersicon esculentum, PIR2:S40089 \| chr4:18257330-18258670 FORWARD \| Aliases: T22F8.100, T22F8_100 | 11.3 | 9.6 | 1.7 | 6.4 | 0.28% | 6.2 |
| 2731 | AT4G11410.1 \| short-chain dehydrogenase/reductase (SDR) family protein, contains INTERPRO family IPR002198 Short-chain dehydrogenase/reductase (SDR) superfamily \| chr4:6944919-6947328 REVERSE \| Aliases: F25E4.30, F25E4_30 | 6.8 | 4.1 | 2.7 | 6.4 | 0.28% | 6.2 |
| 2732 | AT1G45170.1 \| expressed protein, similar to expressed protein [Arabidopsis thaliana] (TAIR:At5g42960.1); similar to expressed protein [Oryza sativa (japonica cultivar-group)] (GB:XP_470465.1) \| chr1:17098420-17100446 REVERSE \| Aliases: F27F5.25, F27F5_25 | 6.7 | 4.9 | 1.8 | 6.4 | 0.28% | 6.1 |
| 2733 | AT3G15110.1 \| expressed protein \| chr3:5084295-5085836 REVERSE \| Aliases: F4B12.2 | 4.1 | 2.8 | 1.3 | 6.4 | 0.28% | 6.2 |
| 2735 | AT5G59870.1 \| histone H2A, putative, similar to histone H2A Petroselinum crispum SP:P19177, Lycopersicon esculentum SP:P25469, Zea mays SP:P40280; contains Pfam profile PF00125 Core histone H2A/H2B/H3/H4 \| chr5:24132608-24133444 REVERSE \| Aliases: MMN10.22, MMN10_22 | 11.2 | 8.3 | 2.9 | 6.4 | 0.28% | 6.2 |
| 2736 | AT1G26740.1 \| expressed protein, similar to 50S ribosomal protein L32 (SP:P80339) {Thermus thermophilus} \| chr1:9244974-9246588 REVERSE \| Aliases: T24P13.27 | 7.3 | 4.4 | 2.9 | 6.4 | 0.28% | 6.0 |
| 2738 | AT3G02190.1 \| 60S ribosomal protein L39 (RPL39B), similar to ribosomal protein L39 GB:P51424 (Arabidopsis thaliana) \| chr3:405823-406408 REVERSE \| Aliases: F1C9.36 | 8.8 | 7.3 | 1.6 | 6.4 | 0.28% | 6.2 |
| 2739 | AT5G03880.1 \| expressed protein \| chr5:1038507-1041479 REVERSE \| Aliases: MED24.18, MED24_18 | 8.3 | 6.3 | 2.0 | 6.4 | 0.28% | 6.2 |
| 2741 | AT4G31700.1 \| Symbol: RPS6 \| 40S ribosomal protein S6 (RPS6A), ribosomal protein S6, Arabidopsis thaliana, PID:g2662469 \| chr4:15346083-15347731 REVERSE \| Aliases: F28M20.110, F28M20_110, RIBOSOMAL PROTEIN S6 | 11.4 | 9.5 | 1.9 | 6.4 | 0.28% | 6.2 |
| 2743 | AT1G09130.2 \| similar to ATP-dependent Clp protease proteolytic subunit (ClpR1) (nClpP5) [Arabidopsis thaliana] (TAIR:At1g49970.1); similar to COG0740: Protease subunit of ATP-dependent Clp proteases [Nostoc punctiforme PCC 73102] (GB:ZP_00108611.1); contains InterPro domain Clp protease (InterPro:IPR001907) \| chr1:2939928-2942269 REVERSE \| Aliases: F7G19.1, F7G19_1 | 8.9 | 5.6 | 3.3 | 6.4 | 0.28% | 6.0 |
| 2744 | AT5G65880.1 \| expressed protein \| chr5:26370262-26370900 FORWARD \| Aliases: K14B20.5, K14B20_5 | 8.0 | 6.0 | 2.0 | 6.4 | 0.28% | 6.2 |
| 2745 | AT1G54290.1 \| eukaryotic translation initiation factor SUI1, putative, similar to P:P32911 Protein translation factor SUI1 {Saccharomyces cerevisiae}; contains Pfam profile PF01253: Translation initiation factor SUI1 \| chr1:20272152-20273774 REVERSE \| Aliases: F20D21.11, F20D21_11 | 7.9 | 5.6 | 2.2 | 6.4 | 0.28% | 6.0 |
| 2746 | AT3G48510.1 \| expressed protein \| chr3:17978341-17979523 REVERSE \| Aliases: T8P19.20 | 6.7 | 4.2 | 2.5 | 6.4 | 0.28% | 5.9 |
| 2749 | AT4G08590.1 \| zinc finger (C3HC4-type RING finger) family protein, contains Pfam profiles PF00097: Zinc finger C3HC4 type (RING finger), PF02182: YDG/SRA domain \| chr4:5463892-5466653 REVERSE \| Aliases: T3F12.10 | 5.2 | 3.6 | 1.6 | 6.4 | 0.28% | 6.2 |
| 2750 | AT4G27490.1 \| 3' exoribonuclease family domain 1-containing protein, contains Pfam PF01138: 3' exoribonuclease family, domain 1 \| chr4:13739812-13741809 FORWARD \| Aliases: F27G19.90, F27G19_90 | 6.3 | 3.3 | 2.9 | 6.4 | 0.28% | 5.8 |
| 2751 | AT5G61930.1 \| Symbol: APO3 \| similar to expressed protein [Arabidopsis thaliana] (TAIR:At1g64810.1); similar to OSJNBa0008M17.9 [Oryza sativa (japonica cultivar-group)] (GB:XP_473885.1); contains InterPro domain Arabidopsis thaliana protein of unknown function DUF794 (InterPro:IPR008512) \| chr5:24883301-24885047 REVERSE \| Aliases: ACCUMULATION OF PHOTOSYSTEM ONE 3, K22G18.5, K22G18_5 | 7.0 | 5.3 | 1.7 | 6.4 | 0.28% | 6.1 |
| 2755 | AT5G39570.1 \| expressed protein \| chr5:15861187-15863406 FORWARD \| Aliases: MIJ24.6, MIJ24_6 | 13.4 | 12.6 | 0.8 | 6.4 | 0.28% | 6.1 |
| 2756 | AT1G18730.1 \| expressed protein \| chr1:6460590-6462212 FORWARD \| Aliases: F6A14.16, F6A14_16 | 6.0 | 3.9 | 2.1 | 6.4 | 0.28% | 6.1 |
| 2758 | AT1G77750.1 \| 30S ribosomal protein S13, chloroplast, putative, similar to putative 30S ribosomal protein S13, chloroplast precursor GB:P42732 (Arabidopsis thaliana) \| chr1:29235118-29236418 REVERSE \| Aliases: RPS13, T32E8.8, T32E8_8 | 7.4 | 3.7 | 3.7 | 6.4 | 0.28% | 5.8 |

| Rank | Description | Sync | Root | M | t | adj.q | B |
|---|---|---|---|---|---|---|---|
| 2759 | AT4G14680.1 \| Symbol: APS3 \| sulfate adenylyltransferase 3 / ATP-sulfurylase 3 (APS3), identical to ATP sulfurylase (APS3) (Arabidopsis thaliana) GI:1575327 \| chr4:8413286-8415349 REVERSE \| Aliases: DL3380C, FCAALL.128 | 9.0 | 5.6 | 3.4 | 6.4 | 0.29% | 6.2 |
| 2760 | AT1G09660.2 \| KH domain-containing quaking protein, putative, similar to GB:AAC67357 \| chr1:3127783-3131049 REVERSE \| Aliases: F21M12.5, F21M12_5 | 5.7 | 4.4 | 1.3 | 6.4 | 0.29% | 6.2 |
| 2761 | AT3G04000.1 \| short-chain dehydrogenase/reductase (SDR) family protein, similar to SP:Q08632 Short-chain type dehydrogenase/reductase (EC 1.-.-.-) {Picea abies}; contains Pfam:PF00106 oxidoreductase, short chain dehydrogenase/reductase family \| chr3:1035507-1036679 FORWARD \| Aliases: T11I18.11, T11I18_11 | 7.5 | 4.7 | 2.8 | 6.4 | 0.29% | 5.6 |
| 2762 | AT5G48030.1 \| Symbol: GFA2 \| DNAJ heat shock protein, mitochondrially targeted (GFA2), 99.8% identical to mitochondrially targeted DnaJ protein GFA2 (Arabidopsis thaliana) GI:21429604; contains Pfam profiles PF00226: DnaJ domain, PF01556: DnaJ C terminal region, PF00684: DnaJ central domain (4 repeats) \| chr5:19483304-19487128 REVERSE \| Aliases: GAMETOPHYTIC FACTOR 2, MDN11.11, MDN11_11 | 8.2 | 6.2 | 2.0 | 6.4 | 0.29% | 6.2 |
| 2763 | AT5G59910.1 \| histone H2B, nearly identical to histone H2B Arabidopsis thaliana GI:2407802; contains Pfam profile PF00125 Core histone H2A/H2B/H3/H4 \| chr5:24144354-24145161 FORWARD \| Aliases: MMN10.15, MMN10_15 | 10.6 | 9.3 | 1.3 | 6.4 | 0.29% | 6.2 |
| 2765 | AT4G32330.2 \| expressed protein \| chr4:15608918-15612291 FORWARD \| Aliases: F8B4.30 | 7.4 | 5.7 | 1.6 | 6.4 | 0.29% | 6.2 |
| 2770 | AT4G31820.1 \| phototropic-responsive NPH3 family protein, contains NPH3 family domain, Pfam:PF03000 \| chr4:15390656-15394436 REVERSE \| Aliases: F11C18.20, F11C18_20 | 6.5 | 4.3 | 2.2 | 6.4 | 0.29% | 6.0 |
| 2771 | AT4G21860.1 \| methionine sulfoxide reductase domain-containing protein / SelR domain-containing protein, low similarity to pilin-like transcription factor (Homo sapiens) GI:5059062, SP:P14930 Peptide methionine sulfoxide reductase msrA/msrB (EC 1.8.4.6) {Neisseria gonorrhoeae}; contains Pfam profile PF01641: SelR domain \| chr4:11600085-11601572 REVERSE \| Aliases: T8O5.70, T8O5_70 | 8.6 | 6.3 | 2.3 | 6.4 | 0.29% | 6.1 |
| 2773 | AT4G10480.1 \| nascent polypeptide associated complex alpha chain protein, putative / alpha-NAC, putative, similar to alpha-NAC, non-muscle form (Mus musculus) GI:1666690; contains Pfam profiles PF01849: NAC domain, PF00627: UBA/TS-N domain \| chr4:6477872-6479160 REVERSE \| Aliases: F7L13.60, F7L13_60 | 11.7 | 9.9 | 1.8 | 6.4 | 0.29% | 6.0 |
| 2776 | AT3G10860.1 \| ubiquinol-cytochrome C reductase complex ubiquinone-binding protein, putative / ubiquinol-cytochrome C reductase complex 8.2 kDa protein, putative, similar to ubiquinol--cytochrome c reductase GI:633687 from (Solanum tuberosum) \| chr3:3399741-3400732 FORWARD \| Aliases: T7M13.6 | 13.3 | 12.7 | 0.6 | 6.4 | 0.29% | 5.5 |
| 2778 | AT1G06510.1 \| expressed protein \| chr1:1992384-1993839 FORWARD \| Aliases: F12K11.16, F12K11_16 | 4.5 | 3.2 | 1.3 | 6.4 | 0.29% | 6.2 |
| 2779 | AT5G15520.1 \| 40S ribosomal protein S19 (RPS19B), 40S RIBOSOMAL PROTEIN S19 - Oryza sativa, SWISSPROT:RS19_ORYSA \| chr5:5037024-5038170 REVERSE \| Aliases: T20K14.130, T20K14_130 | 7.1 | 5.1 | 2.0 | 6.4 | 0.29% | 6.1 |
| 2785 | AT1G78690.1 \| phospholipid/glycerol acyltransferase family protein, similar to SP:Q16635 Tafazzin from Homo sapiens; contains Pfam profile: PF01553 Acyltransferase \| chr1:29601548-29603522 FORWARD \| Aliases: F9K20.27, F9K20_27 | 5.9 | 4.1 | 1.7 | 6.4 | 0.29% | 6.0 |
| 2787 | AT3G16780.1 \| 60S ribosomal protein L19 (RPL19B), similar to ribosomal protein L19 GB:CAA45090 from (Homo sapiens) \| chr3:5708931-5710415 FORWARD \| Aliases: MGL6.7 | 9.3 | 7.3 | 1.9 | 6.4 | 0.29% | 6.2 |
| 2791 | AT5G14050.1 \| transducin family protein / WD-40 repeat family protein, contains 4 WD-40 repeats (PF00400); similar to unknown protein (ref:NP_057085.1) \| chr5:4533292-4535303 FORWARD \| Aliases: MUA22.5, MUA22_5 | 8.1 | 6.8 | 1.3 | 6.4 | 0.29% | 6.2 |
| 2794 | AT5G02490.1 \| heat shock cognate 70 kDa protein 2 (HSC70-2) (HSP70-2), identical to SP:P22954 Heat shock cognate 70 kDa protein 2 (Hsc70.2) {Arabidopsis thaliana} \| chr5:550033-552643 REVERSE \| Aliases: T22P11.80, T22P11_80 | 8.1 | 6.0 | 2.1 | 6.4 | 0.29% | 5.8 |
| 2795 | AT1G09300.1 \| metallopeptidase M24 family protein, similar to SP:P15034 Xaa-Pro aminopeptidase (EC 3.4.11.9) (X-Pro aminopeptidase) (Aminopeptidase P II) (Aminoacylproline aminopeptidase) {Escherichia coli}; contains Pfam profiles PF00557: metallopeptidase family M24, PF05195: Aminopeptidase P, N-terminal domain \| chr1:3003928-3008215 FORWARD \| Aliases: T31J12.2, T31J12_2 | 6.0 | 4.8 | 1.2 | 6.4 | 0.29% | 6.2 |
| 2797 | AT3G02560.2 \| 40S ribosomal protein S7 (RPS7B), similar to ribosomal protein S7 GB:AAD26256 from (Secale cereale) \| chr3:541807-543341 FORWARD \| Aliases: F16B3.19, F16B3_19 | 10.4 | 8.4 | 2.1 | 6.4 | 0.29% | 6.1 |
| 2798 | AT5G38650.1 \| proteasome maturation factor UMP1 family protein, contains Pfam profile PF05348: Proteasome maturation factor UMP1 \| chr5:15488192-15490025 REVERSE \| Aliases: MBB18.20, MBB18_20 | 10.8 | 8.7 | 2.1 | 6.4 | 0.29% | 6.2 |
| 2799 | AT1G73790.1 \| expressed protein \| chr1:27748333-27749038 FORWARD \| Aliases: F25P22.21, F25P22_21 | 7.3 | 5.9 | 1.5 | 6.4 | 0.30% | 6.2 |

| Rank | Description | Sync | Root | M | t | adj.q | B |
|---|---|---|---|---|---|---|---|
| 2800 | AT3G53920.1 \| Symbol: SIGC \| RNA polymerase sigma subunit SigC (sigC) / sigma factor 3 (SIG3), identical to sigma factor SigC (Arabidopsis thaliana) GI:5478585, sigma factor 3 (SIG3) (Arabidopsis thaliana) GI:2353175, plastid RNA polymerase sigma-subunit (Arabidopsis thaliana) GI:2398853; contains Pfam profiles PF04545: Sigma-70, region 4, PF04539: Sigma-70 region 3, PF04542: Sigma-70 region 2 \| chr3:19971847-19974927 REVERSE \| Aliases: F5K20.220, RNA POLYMERASE SIGMA SUBUNIT, SIG3 | 6.7 | 4.9 | 1.8 | 6.4 | 0.30% | 6.2 |
| 2801 | AT1G69930.1 \| Symbol: ATGSTU11 \| glutathione S-transferase, putative, similar to glutathione transferase GB:CAA09188 (Alopecurus myosuroides) | 6.4 | 3.5 | 2.9 | 6.4 | 0.30% | 5.2 |
| 2804 | AT1G60080.1 \| 3' exoribonuclease family domain 1-containing protein, similar to SP:Q96B26 Exosome complex exonuclease RRP43 (EC 3.1.13.-) (Ribosomal RNA processing protein 43) {Homo sapiens}; contains Pfam profile PF01138: 3' exoribonuclease family, domain 1 \| chr1:22155895-22158246 REVERSE \| Aliases: T2K10.14, T2K10_14 | 6.1 | 4.6 | 1.5 | 6.4 | 0.30% | 6.1 |
| 2805 | AT3G53730.1 \| histone H4, identical to histone H4 from Lycopersicon esculentum GI:297150, Lolium temulentum SP:P02308, Acropora formosa GI:455652, Citrus jambhiri GI:16797797 | 12.2 | 10.3 | 1.9 | 6.4 | 0.30% | 6.1 |
| 2807 | AT3G21400.1 \| expressed protein \| chr3:7534639-7536515 REVERSE \| Aliases: MHC9.8 | 6.3 | 4.7 | 1.6 | 6.4 | 0.30% | 6.2 |
| 2808 | AT1G04070.1 \| Symbol: TOM22 I \| expressed protein, Contains similarity to hypothetical mitochondrial import receptor subunit gb Z98597 from S. pombe. ESTs gb:T45575 and gb:Z26435 and gb:AA394576 come from this gene \| chr1:1050552-1051185 REVERSE \| Aliases: ATTOM22 I, F20D22.15, TOM22 | 6.2 | 4.5 | 1.6 | 6.4 | 0.30% | 6.2 |
| 2809 | AT2G40060.1 \| expressed protein \| chr2:16733562-16735263 FORWARD \| Aliases: T28M21.22, T28M21_22 | 9.6 | 8.6 | 1.0 | 6.4 | 0.30% | 6.1 |
| 2811 | AT1G07010.1 \| calcineurin-like phosphoesterase family protein, contains Pfam profile: PF00149 calcineurin-like phosphoesterase | 6.2 | 4.3 | 1.9 | 6.3 | 0.30% | 6.0 |
| 2812 | AT3G19270.1 \| Symbol: CYP707A4 \| cytochrome P450 family protein, similar to Cytochrome P450 85 (SP:Q43147) {Lycopersicon esculentum}; | 6.9 | 3.7 | 3.2 | 6.3 | 0.30% | 5.9 |
| 2813 | AT3G20670.1 \| histone H2A, putative, strong similarity to histone H2A GB:AAF64418 GI:7595337 from Arabidopsis thaliana, Triticum aestivum GI:536892; contains Pfam profile PF00125 Core histone H2A/H2B/H3/H4 \| chr3:7229417-7230508 FORWARD \| Aliases: F3H11.6 | 8.8 | 5.9 | 3.0 | 6.3 | 0.30% | 6.1 |
| 2814 | AT4G18593.1 \| dual specificity protein phosphatase-related, contains weak similarity to Dual specificity protein phosphatase 12 (EC 3.1.3.48) (EC 3.1.3.16) (Dual-specificity tyrosine phosphatase YVH1) (Swiss-Prot:Q9UNI6) (Homo sapiens) \| chr4:10237583-10238847 FORWARD \| Aliases: None | 8.6 | 7.1 | 1.5 | 6.3 | 0.30% | 6.1 |
| 2816 | AT3G51530.1 \| F-box family protein, various predicted proteins, Arabidopsis thaliana; contains Pfam profile PF00646: F-box domain \| chr3:19123399-19125622 REVERSE \| Aliases: F26O13.170 | 7.2 | 5.3 | 1.9 | 6.3 | 0.30% | 6.1 |
| 2817 | AT4G20030.1 \| RNA recognition motif (RRM)-containing protein, low similarity to heterogeneous nuclear ribonucleoprotein G (Mus musculus) GI:5579009; contains InterPro entry IPR000504: RNA-binding region RNP-1 (RNA recognition motif) (RRM) | 4.7 | 3.1 | 1.6 | 6.3 | 0.30% | 6.1 |
| 2819 | AT2G16780.1 \| Symbol: MSI2 \| WD-40 repeat protein (MSI2), contains 5 WD-40 repeats (PF0400); identical to WD-40 repeat protein MSI2 (SP:O22468) (Arabidopsis thaliana) WD-40 repeats (PF0400); \| chr2:7288544-7290715 REVERSE \| Aliases: MSI02, MSI2, NFC02, NFC2, T24I21.19, T24I21_19 | 7.3 | 4.7 | 2.5 | 6.3 | 0.30% | 5.9 |
| 2820 | AT3G10970.2 \| haloacid dehalogenase-like hydrolase family protein, low similarity to genetic modifier (Zea mays) GI:10444400; contains InterPro accession IPR005834: Haloacid dehalogenase-like hydrolase \| chr3:3433078-3436284 REVERSE \| Aliases: F9F8.21 | 7.5 | 6.2 | 1.3 | 6.3 | 0.30% | 6.2 |
| 2822 | AT2G31240.1 \| tetratricopeptide repeat (TPR)-containing protein, contains Pfam profile PF00515: TPR Domain \| chr2:13324492-13326637 REVERSE \| Aliases: F16D14.8, F16D14_8 | 6.1 | 4.1 | 2.0 | 6.3 | 0.30% | 6.1 |
| 2825 | AT5G06050.1 \| dehydration-responsive protein-related, similar to early-responsive to dehydration stress ERD3 protein (Arabidopsis thaliana) GI:15320410; contains Pfam profile PF03141: Putative methyltransferase \| chr5:1820135-1823771 FORWARD \| Aliases: K18J17.25, K18J17_25 | 6.6 | 5.0 | 1.6 | 6.3 | 0.30% | 6.1 |
| 2827 | AT2G29590.1 \| thioesterase family protein, contains Pfam profile PF03061: thioesterase family protein \| chr2:12661533-12662525 REVERSE \| Aliases: F16P2.3, F16P2_3 | 7.4 | 6.1 | 1.2 | 6.3 | 0.30% | 6.1 |
| 2830 | AT3G54090.1 \| pfkB-type carbohydrate kinase family protein, contains Pfam profile: PF00294 pfkB family carbohydrate kinase \| chr3:20039102-20040964 FORWARD \| Aliases: F24B22.50 | 5.8 | 3.4 | 2.4 | 6.3 | 0.30% | 5.9 |
| 2831 | AT4G12580.1 \| expressed protein, predicted protein \| chr4:7449371-7449676 REVERSE \| Aliases: T1P17.170, T1P17_170 | 5.2 | 3.4 | 1.8 | 6.3 | 0.30% | 6.1 |
| 2833 | AT2G21090.1 \| pentatricopeptide (PPR) repeat-containing protein, contains INTERPRO:IPR002885 PPR repeats \| chr2:9052597-9054657 REVERSE \| Aliases: F26H11.15, F26H11_15 | 4.5 | 3.4 | 1.1 | 6.3 | 0.31% | 6.1 |
| 2834 | AT1G51380.1 \| eukaryotic translation initiation factor 4A, putative / eIF-4A, putative | 4.8 | 3.6 | 1.2 | 6.3 | 0.31% | 6.1 |

| Rank | Description | Sync | Root | M | t | adj.q | B |
|---|---|---|---|---|---|---|---|
| 2835 | AT5G52050.1 \| MATE efflux protein-related, contains Pfam profile PF01554: Uncharacterized membrane protein family \| chr5:21156088-21157869 FORWARD \| Aliases: MSG15.13, MSG15_13 | 6.7 | 3.3 | 3.3 | 6.3 | 0.31% | 5.3 |
| 2836 | AT4G20430.1 \| subtilase family protein, contains Pfam profile: PF00082 subtilase family \| chr4:11017667-11021116 REVERSE \| Aliases: F9F13.80, F9F13_80 | 4.7 | 3.5 | 1.3 | 6.3 | 0.31% | 6.1 |
| 2839 | AT5G15410.2 \| Symbol: DND1 \| cyclic nucleotide-regulated ion channel / cyclic nucleotide-gated channel (CNGC2), identical to cyclic nucleotide-gated cation channel GI:3894399 from (Arabidopsis thaliana) \| chr5:5003317-5006820 REVERSE \| Aliases: ATCNGC2, CNGC2, CYCLIC NUCLEOTIDE GATED CHANNEL 2, DEFENSE NO DEATH 1 | 5.3 | 3.2 | 2.1 | 6.3 | 0.31% | 5.7 |
| 2841 | AT1G67700.2 \| expressed protein \| chr1:25377897-25379693 FORWARD \| Aliases: F12A21.32 | 7.3 | 4.2 | 3.1 | 6.3 | 0.31% | 6.0 |
| 2842 | AT5G44730.2 \| haloacid dehalogenase-like hydrolase family protein, low similarity to SP:Q94915 Rhythmically expressed gene 2 protein (DREG-2) {Drosophila melanogaster}; contains InterPro accession IPR005834: Haloacid dehalogenase-like hydrolase \| chr5:18062758-18064135 REVERSE \| Aliases: K23L20.7, K23L20_7 | 6.5 | 4.5 | 2.0 | 6.3 | 0.31% | 6.1 |
| 2852 | AT3G53650.1 \| histone H2B, putative, similar to histone H2B from Lycopersicon esculentum, PIR:T06389 GI:3021483, Gossypium hirsutum SP:O22582, Capsicum annuum SP:O49118; contains Pfam profile PF00125 Core histone H2A/H2B/H3/H4 \| chr3:19900336-19900752 FORWARD \| Aliases: F4P12.350 | 7.1 | 4.0 | 3.1 | 6.3 | 0.31% | 5.8 |
| 2856 | AT3G12270.1 \| protein arginine N-methyltransferase family protein, similar to protein arginine N-methyltransferase 3 from {Rattus norvegicus} SP:O70467, {Homo sapiens} SP:O60678 \| chr3:3910661-3913261 FORWARD \| Aliases: F28J15.7 | 5.4 | 4.0 | 1.4 | 6.3 | 0.31% | 6.1 |
| 2857 | AT1G16920.1 \| Symbol: RAB11 \| Ras-related GTP-binding protein, putative, similar to GTP binding protein GI:218228 from (Vicia faba); identical to cDNA small GTP-binding protein (Rab11) GI:451859 \| chr1:5787323-5789242 REVERSE \| Aliases: ATRABA4B, F17F16.26, RAB11 | 8.3 | 5.5 | 2.8 | 6.3 | 0.31% | 5.9 |
| 2858 | AT2G05630.1 \| Symbol: atg8d \| autophagy 8d (APG8d), identical to autophagy 8d (Arabidopsis thaliana) GI:19912157; contains Pfam profile PF02991: Microtubule associated protein 1A/1B, light chain 3 \| chr2:2082983-2084879 REVERSE \| Aliases: T20G20.2, T20G20_2 | 8.8 | 7.2 | 1.6 | 6.3 | 0.31% | 6.1 |
| 2859 | AT3G26570.2 \| Symbol: PHT2;1 \| | 7.5 | 4.4 | 3.1 | 6.3 | 0.31% | 5.4 |
| 2860 | AT3G13580.3 \| 60S ribosomal protein L7 (RPL7D), similar to 60S ribosomal protein L7 GB:AAD14525 GI:4262232 from (Arabidopsis thaliana) | 10.3 | 8.7 | 1.5 | 6.3 | 0.31% | 6.1 |
| 2861 | AT1G02870.1 \| expressed protein \| chr1:637214-638677 REVERSE \| Aliases: F22D16.13, F22D16_13 | 6.4 | 3.1 | 3.3 | 6.3 | 0.31% | 6.0 |
| 2862 | AT4G00100.1 \| Symbol: ATRPS13A \| 40S ribosomal protein S13 (RPS13A), similar to ribosomal protein S13; PF00312 (View Sanger Pfam): ribosomal protein S15; identical to cDNA AtRPS13A mRNA for cytoplasmic ribosomal protein S13 GI:6521011 \| chr4:37096-38312 FORWARD \| Aliases: F6N15.7, F6N15_7, PFL2, POINTED FIRST LEAF 2, RIBOSOMAL PROTEIN S13, RIBOSOMAL PROTEIN S13A, RPS13 | 11.5 | 9.0 | 2.4 | 6.3 | 0.31% | 6.1 |
| 2863 | AT2G29530.1 \| Symbol: TIM10 \| mitochondrial import inner membrane translocase (TIM10), identical to mitochondrial import inner membrane translocase subunit Tim10 (Arabidopsis thaliana) Swiss-Prot:Q9ZW33; contains Pfam domain, PF02953: Tim10/DDP family zinc finger \| chr2:12647901-12649367 REVERSE \| Aliases: F16P2.9, F16P2_9, TIM10 | 9.3 | 6.0 | 3.3 | 6.3 | 0.31% | 5.9 |
| 2867 | AT3G43740.2 \| leucine-rich repeat family protein, contains leucine rich-repeat (LRR) domains Pfam:PF00560, INTERPRO:IPR001611; contains similarity to somatic embryogenesis receptor-like kinase 2 (Arabidopsis thaliana) gi:14573457:gb:AAK68073 \| chr3:15655114-15656433 FORWARD \| Aliases: T28A8.30 | 6.5 | 4.8 | 1.7 | 6.3 | 0.32% | 6.0 |
| 2871 | AT3G05570.1 \| expressed protein \| chr3:1616271-1616642 REVERSE \| Aliases: F18C1.16, F18C1_16 | 7.7 | 6.3 | 1.5 | 6.3 | 0.32% | 6.0 |
| 2873 | AT2G37860.3 \| Symbol: LCD1 \| similar to expressed protein [Arabidopsis thaliana] (TAIR:At5g22790.1); similar to chloroplast lumen common protein family-like protein [Oryza sativa (japonica cultivar-group)] (GB:XP_477219.1); contains domain GLY_RICH (PS50315) \| chr2:15863710-15865998 FORWARD \| Aliases: LOWER CELL DENSITY 1, T8P21.23, T8P21_23 | 8.2 | 4.8 | 3.4 | 6.3 | 0.32% | 6.0 |
| 2874 | AT3G29670.1 \| transferase family protein, similar to anthocyanin 5-aromatic acyltransferase from Gentiana triflora GI:4185599, malonyl CoA:anthocyanin 5-O-glucoside-6'''-O-malonyltransferase from Perilla frutescens GI:17980232, Salvia splendens GI:17980234; contains Pfam profile PF02458 transferase family \| chr3:11530351-11531829 FORWARD \| Aliases: MOD1.7 | 6.2 | 3.7 | 2.5 | 6.3 | 0.32% | 5.3 |
| 2876 | AT1G79490.1 \| Symbol: EMB2217 \| pentatricopeptide (PPR) repeat-containing protein, low similarity to fertility restorer (Petunia x hybrida) GI:22128587; contains Pfam profile PF01535: PPR repeat \| chr1:29905352-29908082 FORWARD \| Aliases: EMB2217, EMBRYO DEFECTIVE 2217, T8K14.9, T8K14_9 | 5.1 | 3.6 | 1.5 | 6.3 | 0.32% | 6.1 |
| 2878 | AT3G16500.1 \| Symbol: PAP1 \| auxin-responsive AUX/IAA family protein, similar to SP:O24408:AXII_ARATH Auxin-responsive protein IAA18 (Indoleacetic acid-induced protein 18) {Arabidopsis thaliana}; contains Pfam profile: PF02309: AUX/IAA family \| chr3:5612506-5614416 REVERSE \| Aliases: IAA26, MDC8.13, PAP1, PHYTOCHROME ASSOCIATED PROTEIN 1 | 7.0 | 5.2 | 1.8 | 6.3 | 0.32% | 6.0 |

| Rank | Description | Sync | Root | M | t | adj.q | B |
|---|---|---|---|---|---|---|---|
| 2881 | AT5G64990.1 \| Ras-related GTP-binding protein, putative, similar to GTP-binding protein GI:550072 from (Homo sapiens) \| chr5:25980788-25982018 REVERSE \| Aliases: MXK3.22, MXK3_22 | 5.5 | 3.2 | 2.3 | 6.3 | 0.32% | 5.8 |
| 2883 | AT2G41680.1 \| thioredoxin reductase, putative / NADPH-dependent thioredoxin reductase, putative, The last 2 exons encode thioredoxin. There is an EST match to exons 5-7, and the distance between exon 7 and exon 8 is only 90bp. It is unlikely this is two separate genes, but more likely a hybrid protein. \| chr2:17383233-17386122 REVERSE \| Aliases: T32G6.20, T32G6_20 | 7.4 | 6.0 | 1.5 | 6.3 | 0.32% | 6.1 |
| 2886 | AT5G16950.1 \| expressed protein \| chr5:5572579-5573073 FORWARD \| Aliases: F2K13.100, F2K13_100 | 7.6 | 6.2 | 1.4 | 6.3 | 0.32% | 6.1 |
| 2887 | AT4G25100.4 \| Symbol: FSD1 \| similar to superoxide dismutase (Fe), putative / iron superoxide dismutase, putative [Arabidopsis thaliana] (TAIR:At5g51100.1); similar to Fe-superoxide dismutase (GB:AAA32791.1); contains InterPro domain Manganese and iron superoxide dismutase (InterPro:IPR001189) \| chr4:12884310-12886537 REVERSE \| Aliases: F24A6.1, FE SUPEROXIDE DISMUTASE 1 | 8.7 | 4.4 | 4.3 | 6.3 | 0.32% | 5.6 |
| 2888 | AT1G12200.1 \| flavin-containing monooxygenase family protein / FMO family protein, low similarity to FMO2 from Homo sapiens (SP:Q99518); contains Pfam profile: PF00743 Flavin-binding monooxygenase-like \| chr1:4137531-4140026 FORWARD \| Aliases: T28K15.7, T28K15_7 | 9.8 | 7.5 | 2.3 | 6.3 | 0.32% | 6.1 |
| 2890 | AT3G62970.1 \| similar to zinc finger (C3HC4-type RING finger) family protein [Arabidopsis thaliana] (TAIR:At5g18650.1); similar to putative PGPD14 protein (pollen germination related protein) [Oryza sativa (japonica cultivar-group)] (GB:NP_916676.1); similar to zinc finger protein ZFP-like [Oryza sativa (japonica cultivar-group)] (GB:BAD87761.1); contains InterPro domain CHY zinc finger (InterPro:IPR008913); contains InterPro domain Zn-finger, RING (InterPro:IPR001841) \| chr3:23281609-23283895 FORWARD \| Aliases: T20O10.70 | 9.1 | 6.3 | 2.7 | 6.3 | 0.32% | 5.9 |
| 2891 | AT5G09960.1 \| expressed protein, similar to unknown protein (emb:CAB61744.1) \| chr5:3106321-3107830 FORWARD \| Aliases: MYH9.17, MYH9_17 | 8.2 | 5.2 | 3.0 | 6.3 | 0.32% | 6.0 |
| 2892 | AT3G61670.1 \| expressed protein, weak similarity to extra-large G-protein (Arabidopsis thaliana) GI:3201682 \| chr3:22829564-22832985 FORWARD \| Aliases: F15G16.60 | 5.9 | 4.6 | 1.3 | 6.3 | 0.32% | 6.1 |
| 2902 | AT5G24580.2 \| copper-binding family protein, similar to copper homeostasis factor gi:3168840 from Arabidopsis thaliana; contains Pfam profile PF00403: Heavy-metal-associated domain \| chr5:8410261-8412268 REVERSE \| Aliases: K18P6.11, K18P6_11 | 6.0 | 3.8 | 2.2 | 6.2 | 0.32% | 5.7 |
| 2906 | AT2G25870.1 \| haloacid dehalogenase-like hydrolase family protein, contains Pfam profiles PF00702: haloacid dehalogenase-like hydrolase, PF02130: Uncharacterized protein family UPF0054 \| chr2:11038193-11041532 FORWARD \| Aliases: AT2G25860, F17H15.10, F17H15_10 | 6.3 | 4.8 | 1.5 | 6.2 | 0.33% | 6.0 |
| 2907 | AT1G49245.1 \| expressed protein \| chr1:18222418-18222922 FORWARD \| Aliases: None | 4.3 | 3.1 | 1.2 | 6.2 | 0.33% | 6.0 |
| 2908 | AT1G33330.1 \| peptide chain release factor, putative, similar to peptide chain release factor 2 (Campylobacter jejuni) GI:6469929; contains Pfam profile PF00472: Peptidyl-tRNA hydrolase domain \| chr1:12084931-12086508 FORWARD \| Aliases: F10C21.2 | 5.9 | 4.5 | 1.4 | 6.2 | 0.33% | 6.0 |
| 2909 | AT1G76260.1 \| transducin family protein / WD-40 repeat family protein, contains 6 WD-40 repeats (PF00400); similar to retinoblastoma A associated protein; RbAp48 (GI:3309245) (Xenopus laevis) \| chr1:28615206-28618838 FORWARD \| Aliases: T23E18.19, T23E18_19 | 9.0 | 6.0 | 3.0 | 6.2 | 0.33% | 6.0 |
| 2910 | AT2G48020.2 \| sugar transporter, putative, similar to ERD6 protein {Arabidopsis thaliana} GI:3123712, sugar-porter family proteins 1 and 2 (Arabidopsis thaliana) GI:14585699, GI:14585701; contains Pfam profile PF00083: major facilitator superfamily protein \| chr2:19651325-19654250 FORWARD \| Aliases: T9J23.17 | 7.7 | 6.3 | 1.4 | 6.2 | 0.33% | 6.0 |
| 2913 | AT1G27530.1 \| expressed protein, Similar to gb:AF151884 CGI-126 protein from Homo sapiens. EST gb:Z18048 comes from this gene \| chr1:9562586-9563926 REVERSE \| Aliases: T17H3.3, T17H3_3 | 11.9 | 10.3 | 1.5 | 6.2 | 0.33% | 6.0 |
| 2914 | AT4G28210.1 \| Symbol: EMB1923 \| expressed protein \| chr4:13990626-13992084 FORWARD \| Aliases: EMB1923, EMBRYO DEFECTIVE 1923, F26K10.90, F26K10_90 | 7.0 | 4.2 | 2.7 | 6.2 | 0.33% | 5.5 |
| 2916 | AT4G18100.1 \| 60S ribosomal protein L32 (RPL32A), ribosomal protein L32, human, PIR1:R5HU32 \| chr4:10035497-10036552 REVERSE \| Aliases: F15J5.70, F15J5_70 | 9.5 | 6.2 | 3.2 | 6.2 | 0.33% | 5.9 |
| 2917 | AT5G46430.2 \| 60S ribosomal protein L32 (RPL32B) \| chr5:18850494-18851791 FORWARD \| Aliases: K11I1.2, K11I1_2 | 9.5 | 6.2 | 3.2 | 6.2 | 0.33% | 5.9 |
| 2918 | AT4G30430.1 \| senescence-associated family protein, similar to senescence-associated protein 5 (Hemerocallis hybrid cultivar) gi:3551954:gb:AAC34855 \| chr4:14878789-14880337 REVERSE \| Aliases: F17I23.230, F17I23_230 | 5.3 | 3.5 | 1.9 | 6.2 | 0.33% | 5.9 |
| 2919 | AT3G46430.1 \| expressed protein \| chr3:17098672-17099482 FORWARD \| Aliases: F18L15.150, F18L15_150 | 10.4 | 8.6 | 1.8 | 6.2 | 0.33% | 6.0 |
| 2920 | AT3G03900.1 \| adenylylsulfate kinase, putative, similar to adenylylsulfate kinase 1, chloroplast precursor (APS kinase, Adenosine-5'phosphosulfate kinase, ATP adenosine-5'- phosphosulfate 3'-phosphotransferase) (Arabidopsis thaliana) SWISS-PROT:Q43295 \| chr3:1002982-1004278 REVERSE \| Aliases: F20H23.5, F20H23_5 | 6.5 | 4.6 | 1.9 | 6.2 | 0.33% | 6.0 |

| Rank | Description | Sync | Root | M | t | adj.q | B |
|---|---|---|---|---|---|---|---|
| 2921 | AT4G31310.1 \| avirulence-responsive protein-related / avirulence induced gene (AIG) protein-related, similar to SP:P54121 AIG2 protein {Arabidopsis thaliana} \| chr4:15191331-15192602 FORWARD \| Aliases: F8F16.130, F8F16_130 | 6.4 | 3.7 | 2.7 | 6.2 | 0.33% | 5.9 |
| 2922 | AT2G27710.3 \| 60S acidic ribosomal protein P2 (RPP2B) \| chr2:11823868-11824944 FORWARD \| Aliases: F15K20.19, F15K20_19 | 10.1 | 7.6 | 2.5 | 6.2 | 0.33% | 6.0 |
| 2923 | AT5G10400.1 \| histone H3, identical to several histone H3 proteins, including Zea mays SP:P05203, Medicago sativa GI:166384, Encephalartos altensteinii SP:P08903, Pisum sativum SP:P02300; contains Pfam profile PF00125 Core histone H2A/H2B/H3/H4 \| chr5:3270290-3270953 REVERSE \| Aliases: F12B17.250 | 7.7 | 3.6 | 4.1 | 6.2 | 0.33% | 6.0 |
| 2924 | AT5G57170.1 \| macrophage migration inhibitory factor family protein / MIF family protein, contains Pfam profile: PF01187 Macrophage migration inhibitory factor(MIF) \| chr5:23179300-23180506 REVERSE \| Aliases: MUL3.12, MUL3_12 | 4.1 | 2.8 | 1.3 | 6.2 | 0.33% | 6.0 |
| 2926 | AT3G54210.1 \| ribosomal protein L17 family protein, contains Pfam profile: PF01196 ribosomal protein L17 \| chr3:20078525-20079413 REVERSE \| Aliases: F24B22.170 | 8.7 | 6.2 | 2.4 | 6.2 | 0.33% | 6.0 |
| 2928 | AT5G10700.1 \| expressed protein \| chr5:3378054-3378868 REVERSE \| Aliases: MAJ23.60, MAJ23_60 | 5.2 | 3.6 | 1.6 | 6.2 | 0.33% | 5.9 |
| 2929 | AT1G63020.1 \| Symbol: NRPD1a \| | 6.0 | 3.7 | 2.3 | 6.2 | 0.33% | 5.7 |
| 2930 | AT3G55605.1 \| mitochondrial glycoprotein family protein / MAM33 family protein, low similarity to SUAPRGA1 (Emericella nidulans) GI:6562379; contains Pfam profile PF02330: Mitochondrial glycoprotein | 7.1 | 5.5 | 1.6 | 6.2 | 0.33% | 5.9 |
| 2932 | AT5G14970.1 \| expressed protein \| chr5:4847281-4848868 FORWARD \| Aliases: F2G14.90, F2G14_90 | 8.3 | 6.9 | 1.4 | 6.2 | 0.33% | 6.0 |
| 2934 | AT2G30170.1 \| expressed protein \| chr2:12886757-12888575 REVERSE \| Aliases: T27E13.9, T27E13_9 | 6.5 | 4.8 | 1.7 | 6.2 | 0.33% | 6.0 |
| 2935 | AT4G26300.1 \| Symbol: EMB1027 \| arginyl-tRNA synthetase, putative / arginine--tRNA ligase, putative, similar to SP:P37880 Arginyl-tRNA synthetase (EC 6.1.1.19) (Arginine--tRNA ligase) (ArgRS) {Cricetulus longicaudatus}; contains Pfam profiles PF00750: arginyl-tRNA synthetase, PF03485: arginyl-tRNA synthetase N-terminal domain \| chr4:13307986-13313118 REVERSE \| Aliases: EMB1027, EMBRYO DEFECTIVE 1027, T25K17.110, T25K17_110 | 8.9 | 6.8 | 2.0 | 6.2 | 0.33% | 6.0 |
| 2939 | AT3G48730.1 \| glutamate-1-semialdehyde 2,1-aminomutase 2 (GSA 2) / glutamate-1-semialdehyde aminotransferase 2 (GSA-AT 2), identical to GSA2 (SP:Q42522) \| chr3:18060608-18062687 FORWARD \| Aliases: T8P19.240 | 8.9 | 5.2 | 3.6 | 6.2 | 0.34% | 5.7 |
| 2940 | AT3G13560.3 \| glycosyl hydrolase family 17 protein, similar to beta-1,3-glucanase GI:15150341 from (Camellia sinensis) | 6.3 | 4.6 | 1.8 | 6.2 | 0.34% | 5.9 |
| 2941 | AT5G45930.1 \| Symbol: CHLI2 \| magnesium-chelatase subunit chlI, chloroplast, putative / Mg-protoporphyrin IX chelatase, putative, similar to SP:P161127 from Arabidopsis thaliana, SP:P93162 from Glycine max, SP:O22436 from Nicotiana tabacum; non-consensus AA donor splice site at exon 1, TG acceptor splice site at exon 2 \| chr5:18645234-18646907 FORWARD \| Aliases: CHL I2, K15I22.13, K15I22_13, MAGNESIUM CHELETASE I2 | 4.3 | 3.0 | 1.3 | 6.2 | 0.34% | 6.0 |
| 2942 | AT2G19800.1 \| Symbol: MIOX2 \| expressed protein, similar to myo-inositol oxygenase (Sus scrofa) gi:17432544:gb:AAL39076 \| chr2:8537986-8540533 REVERSE \| Aliases: F6F22.17, F6F22_17, MYO INOSITOL OXYGENASE 2 | 9.3 | 6.4 | 2.9 | 6.2 | 0.34% | 5.8 |
| 2943 | AT1G80110.1 \| Symbol: ATPP2 B11 \| expressed protein, contains similarity to SKP1 interacting partner 3 (Arabidopsis thaliana) GI:10716951 \| chr1:30140274-30141848 FORWARD \| Aliases: ATPP2 B11, F18B13.19, F18B13_19 | 4.5 | 3.4 | 1.1 | 6.2 | 0.34% | 6.0 |
| 2944 | AT3G59760.3 \| Symbol: OASC \| cysteine synthase, mitochondrial, putative / O-acetylserine (thiol)-lyase, putative / O-acetylserine sulfhydrylase, putative, similar to SP:Q43725 Cysteine synthase, mitochondrial precursor (EC 4.2.99.8) (O- acetylserine sulfhydrylase) (O-acetylserine (Thiol)-lyase) {Arabidopsis thaliana} \| chr3:22083451-22086393 REVERSE \| Aliases: ATCS C, CSASE C, F24G16.30 | 10.5 | 7.6 | 2.9 | 6.2 | 0.34% | 6.0 |
| 2945 | AT1G60660.1 \| Symbol: B5 #5 \| cytochrome b5 domain-containing protein, contains InterPro accession IPR001199: Cytochrome b5 \| chr1:22346079-22346679 REVERSE \| Aliases: F8A5.18, F8A5_18 | 8.0 | 6.2 | 1.8 | 6.2 | 0.34% | 5.9 |
| 2946 | AT4G37040.1 \| Symbol: MAP1D \| metallopeptidase M24 family protein, similar to SP:O33343 Methionine aminopeptidase (EC 3.4.11.18) (Peptidase M) {Mycobacterium tuberculosis}; contains Pfam profile PF00557: metallopeptidase family M24 \| chr4:17455133-17457351 FORWARD \| Aliases: METHIONINE AMINOPEPTIDASE 1D | 6.7 | 4.4 | 2.2 | 6.2 | 0.34% | 6.0 |
| 2947 | AT2G22420.1 \| peroxidase 17 (PER17) (P17), identical to SP:Q9SJZ2 Peroxidase 17 precursor (EC 1.11.1.7) (Atperox P17) (ATP25a) {Arabidopsis thaliana} \| chr2:9520299-9521615 FORWARD \| Aliases: F14M13.18, F14M13_18 | 7.1 | 5.5 | 1.5 | 6.2 | 0.34% | 6.0 |
| 2948 | AT2G34640.1 \| expressed protein \| chr2:14588969-14591736 REVERSE \| Aliases: T31E10.2, T31E10_2 | 5.9 | 3.8 | 2.0 | 6.2 | 0.34% | 6.0 |
| 2952 | AT5G23070.1 \| thymidine kinase, putative, similar to thymidine kinase (Oryza sativa) SWISS-PROT:O81263 | 6.6 | 5.3 | 1.3 | 6.2 | 0.34% | 6.0 |

| Rank | Description | Sync | Root | M | t | adj.q | B |
|---|---|---|---|---|---|---|---|
| 2955 | AT5G15770.1 \| GCN5-related N-acetyltransferase (GNAT) family protein, similar to SP:O93806 Glucosamine-phosphate N-acetyltransferase (EC 2.3.1.4) (Phosphoglucosamine transacetylase) (Phosphoglucosamine acetylase) {Candida albicans}; contains Pfam profile PF00583: acetyltransferase, GNAT family | 5.2 | 3.4 | 1.8 | 6.2 | 0.34% | 5.9 |
| 2956 | AT1G67750.1 \| pectate lyase family protein, similar to pectate lyase GI:14289169 from (Salix gilgiana) | 6.1 | 3.3 | 2.7 | 6.2 | 0.34% | 5.2 |
| 2958 | AT3G07510.1 \| expressed protein \| chr3:2393697-2394681 REVERSE \| Aliases: F21O3.22 | 5.6 | 4.7 | 0.9 | 6.2 | 0.34% | 5.9 |
| 2961 | AT1G05620.1 \| inosine-uridine preferring nucleoside hydrolase family protein, similar to Chain A, Crystal Structure Of Nucleoside Hydrolase From Leishmania MajorGI:8569431; contains Pfam profile PF01156: Inosine-uridine preferring nucleoside hydrolase \| chr1:1679165-1681705 FORWARD \| Aliases: F3F20.7, F3F20_7 | 7.3 | 5.6 | 1.8 | 6.2 | 0.34% | 6.0 |
| 2962 | AT2G25570.1 \| expressed protein \| chr2:10892776-10894999 REVERSE \| Aliases: F3N11.2, F3N11_2 | 7.5 | 5.4 | 2.1 | 6.2 | 0.34% | 5.9 |
| 2964 | AT5G44785.2 \| expressed protein, contains Pfam PF05329: Protein of unknown function (DUF731) \| chr5:18087918-18090356 REVERSE \| Aliases: None | 6.4 | 4.7 | 1.7 | 6.2 | 0.34% | 6.0 |
| 2966 | AT5G62620.1 \| galactosyltransferase family protein, contains Pfam profile: PF01762 galactosyltransferase \| chr5:25153977-25157148 FORWARD \| Aliases: MRG21.3, MRG21_3 | 6.0 | 4.5 | 1.5 | 6.2 | 0.34% | 5.9 |
| 2967 | AT5G02450.1 \| 60S ribosomal protein L36 (RPL36C), 60S ribosomal protein L36, Arabidopsis thaliana, EMBL:AC004684 \| chr5:533119-534635 FORWARD \| Aliases: T22P11.40, T22P11_40 | 11.2 | 9.9 | 1.3 | 6.2 | 0.34% | 5.8 |
| 2968 | AT1G07790.1 \| histone H2B, putative, strong similarity to histone H2B Arabidopsis thaliana GI:2407802, Gossypium hirsutum SP:O22582, Lycopersicon esculentum GI:3021489, Capsicum annuum SP:O49118; contains Pfam profile PF00125 Core histone H2A/H2B/H3/H4 \| chr1:2412977-2413705 FORWARD \| Aliases: F24B9.10, F24B9_10 | 7.0 | 4.2 | 2.9 | 6.2 | 0.34% | 5.7 |
| 2969 | AT3G48440.1 \| zinc finger (CCCH-type) family protein, contains Pfam domain, PF00642: Zinc finger C-x8-C-x5-C-x3-H type (and similar) \| chr3:17952387-17954561 FORWARD \| Aliases: T29H11.40 | 4.9 | 3.2 | 1.7 | 6.2 | 0.34% | 5.9 |
| 2971 | AT1G12830.1 \| expressed protein \| chr1:4374248-4375168 REVERSE \| Aliases: F13K23.8, F13K23_8 | 7.5 | 4.7 | 2.8 | 6.2 | 0.35% | 5.9 |
| 2974 | AT5G08650.1 \| GTP-binding protein LepA, putative \| chr5:2806324-2813227 REVERSE \| Aliases: T2K12.1 | 5.5 | 3.8 | 1.7 | 6.2 | 0.35% | 6.0 |
| 2975 | AT4G17360.1 \| formyltetrahydrofolate deformylase, putative, similar to formyltetrahydrofolate deformylase (strain PCC 6803- Synechocystis sp.) SWISS-PROT:Q55135 \| chr4:9703187-9705480 REVERSE \| Aliases: DL4715C, FCAALL.417 | 4.1 | 3.0 | 1.2 | 6.2 | 0.35% | 6.0 |
| 2979 | AT3G22900.1 \| RNA polymerase Rpb7 N-terminal domain-containing protein, weak similarity to SP:P52433 DNA-directed RNA polymerase II 19 kDa polypeptide (EC 2.7.7.6) (RPB7) {Rattus norvegicus}; contains Pfam profile PF03876: RNA polymerase Rpb7, N-terminal domain \| chr3:8115184-8116260 FORWARD \| Aliases: F5N5.8 | 5.7 | 3.8 | 1.9 | 6.2 | 0.35% | 5.9 |
| 2980 | AT5G65010.2 \| Symbol: ASN2 \| asparagine synthetase 2 (ASN2), identical to asparagine synthetase (ASN2) (Arabidopsis thaliana) GI:3859536 \| chr5:25986416-25989801 FORWARD \| Aliases: ASPARAGINE SYNTHETASE 2, MXK3.25, MXK3_25 | 6.4 | 3.8 | 2.7 | 6.2 | 0.35% | 5.7 |
| 2981 | AT3G10840.1 \| hydrolase, alpha/beta fold family protein, low similarity to hydrolase (Terrabacter sp. DBF63) GI:14196240; contains Pfam profile: PF00561 alpha/beta hydrolase fold \| chr3:3391070-3393295 REVERSE \| Aliases: T7M13.8 | 5.8 | 3.7 | 2.1 | 6.2 | 0.35% | 5.7 |
| 2982 | AT5G41610.2 \| Symbol: ATCHX18 \| cation/hydrogen exchanger, putative (CHX18), monovalent cation:proton antiporter family 2 (CPA2) member, PMID:11500563 \| chr5:16655598-16658485 REVERSE \| Aliases: CHX18, MBK23.12, MBK23_12 | 6.7 | 4.4 | 2.3 | 6.2 | 0.35% | 5.4 |
| 2987 | AT2G27720.1 \| 60S acidic ribosomal protein P2 (RPP2A) \| chr2:11825568-11826655 FORWARD \| Aliases: F15K20.18, F15K20_18 | 11.7 | 10.4 | 1.3 | 6.1 | 0.35% | 5.8 |
| 2991 | AT1G74640.1 \| expressed protein, contains Pfam profile: PF00561 alpha/beta hydrolase fold \| chr1:28036424-28038434 FORWARD \| Aliases: F1M20.32, F1M20_32 | 8.1 | 5.8 | 2.3 | 6.1 | 0.35% | 5.9 |
| 2992 | AT3G55520.1 \| immunophilin, putative / FKBP-type peptidyl-prolyl cis-trans isomerase, putative, POSSIBLE PEPTIDYL-PROLYL CIS-TRANS ISOMERASE) (EC 5.2.1.8) (PPIASE) (ROTAMASE) SP:P30416(Mouse);P59 PROTEIN (HSP BINDING IMMUNOPHILIN), rabbit, SWISSPROT:P27124:FKB4_RABBIT \| chr3:20604691-20606324 FORWARD \| Aliases: T22E16.180 | 10.5 | 8.6 | 2.0 | 6.1 | 0.35% | 5.9 |
| 2993 | AT3G11510.1 \| 40S ribosomal protein S14 (RPS14B), similar to 40S ribosomal protein S14 GB:P19950 (Zea mays) \| chr3:3623463-3624945 REVERSE \| Aliases: F24K9.19 | 9.6 | 6.3 | 3.2 | 6.1 | 0.35% | 5.9 |

| Rank | Description | Sync | Root | M | t | adj.q | B |
|---|---|---|---|---|---|---|---|
| 2994 | AT1G29310.1 \| protein transport protein sec61, putative, similar to PfSec61 (Plasmodium falciparum) GI:3057044; contains Pfam profile PF00344: eubacterial secY protein \| chr1:10252292-10254676 FORWARD \| Aliases: F28N24.2, F28N24_2 | 5.7 | 3.9 | 1.8 | 6.1 | 0.35% | 5.9 |
| 2995 | AT4G30810.1 \| Symbol: SCPL29 \| | 9.1 | 7.1 | 1.9 | 6.1 | 0.35% | 5.9 |
| 2996 | AT3G23690.1 \| basic helix-loop-helix (bHLH) family protein, contains Pfam profile: PF00010 helix-loop-helix DNA-binding domain | 8.7 | 7.7 | 1.1 | 6.1 | 0.35% | 5.9 |
| 2997 | AT1G69030.1 \| similar to BSD domain-containing protein [Arabidopsis thaliana] (TAIR:At1g26300.1); contains InterPro domain BSD domain (InterPro:IPR005607) \| chr1:25950928-25952925 REVERSE \| Aliases: T6L1.21, T6L1_21 | 8.1 | 5.8 | 2.2 | 6.1 | 0.35% | 5.9 |
| 2998 | AT5G61830.1 \| short-chain dehydrogenase/reductase (SDR) family protein, contains INTERPRO family IPR002198 short chain dehydrogenase/reductase SDR family \| chr5:24854561-24856196 FORWARD \| Aliases: MAC9.11, MAC9_11 | 6.1 | 4.6 | 1.5 | 6.1 | 0.35% | 5.9 |
| 2999 | AT3G03160.1 \| expressed protein \| chr3:729775-730474 FORWARD \| Aliases: T17B22.15, T17B22_15 | 9.8 | 8.8 | 1.1 | 6.1 | 0.35% | 5.7 |
| 3000 | AT1G78790.1 \| expressed protein \| chr1:29628422-29629935 REVERSE \| Aliases: F9K20.17, F9K20_17 | 6.9 | 5.0 | 1.9 | 6.1 | 0.35% | 5.7 |
| 3001 | AT1G05070.1 \| expressed protein \| chr1:1457067-1458742 REVERSE \| Aliases: T7A14.6, T7A14_6 | 8.2 | 5.6 | 2.6 | 6.1 | 0.35% | 5.9 |
| 3002 | AT2G25100.1 \| ribonuclease HII family protein, contains Pfam profile: PF01351 ribonuclease HII | 5.3 | 3.8 | 1.5 | 6.1 | 0.35% | 5.9 |
| 3003 | AT5G59750.1 \| riboflavin biosynthesis protein, putative, similar to SP:P50855 Riboflavin biosynthesis protein ribA (Includes: GTP cyclohydrolase II (EC 3.5.4.25); 3,4-dihydroxy-2-butanone 4-phosphate synthase (DHBP synthase)) {Actinobacillus pleuropneumoniae}; contains Pfam profiles PF00925: GTP cyclohydrolase II, PF00926: 3,4-dihydroxy-2-butanone 4-phosphate synthase \| chr5:24090515-24092765 FORWARD \| Aliases: MTH12.13, MTH12_13 | 7.2 | 4.7 | 2.4 | 6.1 | 0.35% | 5.7 |
| 3004 | AT3G27340.2 \| similar to B1065G12.33 [Oryza sativa (japonica cultivar-group)] (GB:NP_915228.1) | 7.9 | 6.5 | 1.5 | 6.1 | 0.35% | 5.9 |
| 3010 | AT5G06980.2 \| expressed protein \| chr5:2167624-2169392 FORWARD \| Aliases: MOJ9.15, MOJ9_15 | 6.0 | 3.9 | 2.2 | 6.1 | 0.35% | 5.7 |
| 3011 | AT2G40360.1 \| transducin family protein / WD-40 repeat family protein, contains 4 WD-40 repeats (PF00400); similar to block of proliferation protein Bop1 (GI:1679772) (Mus musculus) \| chr2:16859958-16863768 REVERSE \| Aliases: T3G21.13, T3G21_13 | 9.2 | 7.9 | 1.3 | 6.1 | 0.35% | 5.9 |
| 3012 | AT2G42130.5 \| expressed protein, contains weak hit to Pfam PF04755: PAP_fibrillin \| chr2:17573301-17575182 FORWARD \| Aliases: T24P15.4, T24P15_4 | 5.3 | 3.4 | 1.9 | 6.1 | 0.35% | 5.9 |
| 3014 | AT4G21210.2 \| expressed protein, similar to expressed protein [Arabidopsis thaliana] (TAIR:At3g01200.1); similar to hypothetical protein GSU0450 [Geobacter sulfurreducens PCA] (GB:NP_951509.1); contains InterPro domain Protein of unknown function DUF299 (InterPro:IPR005177) \| chr4:11306955-11308752 FORWARD \| Aliases: F7J7.150, F7J7_150 | 5.0 | 3.5 | 1.6 | 6.1 | 0.35% | 5.9 |
| 3015 | AT5G67510.1 \| 60S ribosomal protein L26 (RPL26B) \| chr5:26955039-26955677 REVERSE \| Aliases: K9I9.7, K9I9_7 | 8.5 | 6.7 | 1.8 | 6.1 | 0.35% | 5.9 |
| 3016 | AT5G19030.3 \| RNA recognition motif (RRM)-containing protein, low similarity to Cold-inducible RNA-binding protein (Glycine-rich RNA-binding protein CIRP) from {Homo sapiens} SP:Q14011, {Rattus norvegicus} SP:Q61413,{Xenopus laevis} SP:O93235; contains InterPro entry IPR000504: RNA-binding region RNP-1 (RNA recognition motif) (RRM) \| chr5:6357436-6359078 REVERSE \| Aliases: T16G12.70, T16G12_70 | 5.0 | 3.8 | 1.2 | 6.1 | 0.35% | 5.9 |
| 3019 | AT3G09300.1 \| oxysterol-binding family protein, similar to SP:P22059 Oxysterol-binding protein 1 {Homo sapiens}; contains Pfam profile PF01237: Oxysterol-binding protein \| chr3:2857883-2860674 FORWARD \| Aliases: F3L24.17 | 10.6 | 9.2 | 1.4 | 6.1 | 0.36% | 5.9 |
| 3020 | AT3G47470.1 \| Symbol: CAB4 \| chlorophyll A-B binding protein 4, chloroplast / LHCI type III CAB-4 (CAB4), identical to SP:P27521 Chlorophyll A-B binding protein 4, chloroplast precursor (LHCI type III CAB-4) (LHCP) {Arabidopsis thaliana} | 10.1 | 6.5 | 3.6 | 6.1 | 0.36% | 5.7 |
| 3021 | AT3G13640.1 \| Symbol: ATRLI1 \| | 4.1 | 2.8 | 1.3 | 6.1 | 0.36% | 5.9 |
| 3022 | AT4G27130.1 \| eukaryotic translation initiation factor SUI1, putative, similar to SP:P32911 Protein translation factor SUI1 {Saccharomyces cerevisiae}; contains Pfam profile PF01253: Translation initiation factor SUI1 \| chr4:13604601-13606454 REVERSE \| Aliases: T24A18.80, T24A18_80 | 11.1 | 9.9 | 1.2 | 6.1 | 0.36% | 5.7 |
| 3023 | AT3G61640.1 \| Symbol: AGP20 \| arabinogalactan-protein (AGP20) | 6.3 | 4.2 | 2.1 | 6.1 | 0.36% | 5.9 |
| 3025 | AT1G31160.1 \| zinc-binding protein, putative / protein kinase C inhibitor, putative, similar to 14 kDa zinc-binding protein (Protein kinase C inhibitor, PKCI) (Zea mays) Swiss-Prot:P42856 \| chr1:11122640-11124133 REVERSE \| Aliases: F28K20.9, F28K20_9 | 6.7 | 3.5 | 3.2 | 6.1 | 0.36% | 5.8 |

| Rank | Description | Sync | Root | M | t | adj.q | B |
|---|---|---|---|---|---|---|---|
| 3026 | AT5G62430.1 \| Symbol: CDF1 \| Dof-type zinc finger domain-containing protein, similar to H-protein promoter binding factor-2a GI:3386546 from (Arabidopsis thaliana) \| chr5:25086321-25087403 REVERSE \| Aliases: CYCLING DOF FACTOR 1, K19B1.4, K19B1_4 | 5.2 | 3.6 | 1.7 | 6.1 | 0.36% | 5.9 |
| 3027 | AT3G47833.1 \| expressed protein \| chr3:17659618-17661125 FORWARD \| Aliases: None | 9.6 | 8.5 | 1.2 | 6.1 | 0.36% | 5.8 |
| 3032 | AT1G47260.1 \| Symbol: APFI \| bacterial transferase hexapeptide repeat-containing protein, contains Pfam profile PF00132: Bacterial transferase hexapeptide (four repeats) \| chr1:17323048-17325924 REVERSE \| Aliases: F8G22.2, F8G22_2 | 10.3 | 8.7 | 1.6 | 6.1 | 0.36% | 5.9 |
| 3033 | AT5G45010.1 \| DSS1/SEM1 family protein, contains Pfam profile PF05160: DSS1/SEM1 family \| chr5:18184471-18185601 REVERSE \| Aliases: K21C13.20, K21C13_20 | 10.6 | 8.9 | 1.7 | 6.1 | 0.36% | 5.9 |
| 3035 | AT4G12440.2 \| adenine phosphoribosyltransferase, putative, strong similarity to SP:P31166 Adenine phosphoribosyltransferase 1 (EC 2.4.2.7) (APRT) {Arabidopsis thaliana}; contains Pfam profile PF00156: Phosphoribosyl transferase domain \| chr4:7376265-7380148 FORWARD \| Aliases: T1P17.30, T1P17_30 | 5.9 | 4.5 | 1.5 | 6.1 | 0.36% | 5.9 |
| 3036 | AT2G43770.1 \| transducin family protein / WD-40 repeat family protein, contains 7 WD-40 repeats (PF00400); similar to U5 snRNP-specific 40 kDa protein (GI:3820594) (Homo sapiens) \| chr2:18141138-18143057 REVERSE \| Aliases: F18O19.12 | 8.2 | 5.4 | 2.8 | 6.1 | 0.36% | 5.9 |
| 3037 | AT4G11350.2 \| similar to fringe-related protein [Arabidopsis thaliana] (TAIR:At4g23490.1); similar to fringe-related protein-like [Oryza sativa (japonica cultivar-group)] (GB:BAD29388.1) \| chr4:6901677-6904220 FORWARD \| Aliases: F8L21.140, F8L21_140 | 5.1 | 3.6 | 1.5 | 6.1 | 0.36% | 5.9 |
| 3040 | AT1G49410.1 \| Symbol: TOM6 \| expressed protein \| chr1:18289409-18290570 REVERSE \| Aliases: F13F21.16, F13F21_16, TOM6 | 6.8 | 4.0 | 2.8 | 6.1 | 0.37% | 5.9 |
| 3041 | AT2G40460.1 \| proton-dependent oligopeptide transport (POT) family protein, contains Pfam profile: PF00854 POT family \| chr2:16903985-16908358 FORWARD \| Aliases: T2P4.19, T2P4_19 | 5.7 | 4.0 | 1.7 | 6.1 | 0.37% | 5.8 |
| 3042 | AT5G24280.1 \| expressed protein, ; expression supported by MPSS \| chr5:8251381-8261431 REVERSE \| Aliases: MOP9.10, MOP9_10 | 4.8 | 3.6 | 1.3 | 6.1 | 0.37% | 5.9 |
| 3043 | AT5G13720.1 \| expressed protein \| chr5:4427844-4429136 FORWARD \| Aliases: MSH12.19, MSH12_19 | 5.6 | 4.3 | 1.3 | 6.1 | 0.37% | 5.9 |
| 3044 | AT1G59840.2 \| expressed protein \| chr1:22030172-22033044 FORWARD \| Aliases: F23H11.16, F23H11_16 | 5.8 | 3.0 | 2.8 | 6.1 | 0.37% | 5.3 |
| 3051 | AT2G22600.1 \| KH domain-containing protein \| chr2:9603697-9606130 FORWARD \| Aliases: T9I22.4, T9I22_4 | 5.0 | 3.6 | 1.4 | 6.1 | 0.37% | 5.8 |
| 3054 | AT4G08790.1 \| nitrilase, putative, similar to nitrilase 1 (Mus musculus) GI:3228668; contains Pfam profile PF00795: hydrolase, carbon-nitrogen family \| chr4:5608237-5611212 REVERSE \| Aliases: T32A17.100, T32A17_100 | 5.5 | 4.2 | 1.3 | 6.1 | 0.37% | 5.9 |
| 3055 | AT3G46110.2 \| expressed protein \| chr3:16943097-16944627 FORWARD \| Aliases: F12M12.80 | 4.8 | 3.2 | 1.6 | 6.1 | 0.37% | 5.8 |
| 3057 | AT3G17040.1 \| Symbol: HCF107 \| | 5.2 | 3.9 | 1.3 | 6.1 | 0.37% | 5.8 |
| 3058 | AT5G01340.1 \| mitochondrial substrate carrier family protein, contains Pfam profile: PF00153 mitochondrial carrier protein \| chr5:143054-144719 REVERSE \| Aliases: T10O8.50, T10O8_50 | 9.7 | 6.9 | 2.8 | 6.1 | 0.37% | 5.7 |
| 3060 | ATCG00470.1 \| Symbol: ATPE \| ATPase epsilon subunit \| chrC:52265-52663 REVERSE \| Aliases: ATPE | 13.4 | 12.4 | 1.0 | 6.1 | 0.37% | 5.6 |
| 3061 | AT1G30080.1 \| glycosyl hydrolase family 17 protein, similar to beta-1,3-glucanase precursor GI:4097948 from (Oryza sativa) \| chr1:10550931-10553199 REVERSE \| Aliases: T1P2.13, T1P2_13 | 5.6 | 4.2 | 1.4 | 6.1 | 0.37% | 5.8 |
| 3062 | AT5G35530.1 \| 40S ribosomal protein S3 (RPS3C) | 11.8 | 10.6 | 1.1 | 6.1 | 0.37% | 5.7 |
| 3063 | AT3G14700.1 \| expressed protein \| chr3:4940325-4941405 FORWARD \| Aliases: MIE1.20 | 5.7 | 3.5 | 2.1 | 6.1 | 0.37% | 5.6 |
| 3064 | AT2G24970.1 \| expressed protein \| chr2:10627198-10628386 REVERSE \| Aliases: F27C12.11, F27C12_11 | 5.5 | 2.4 | 3.0 | 6.1 | 0.38% | 5.7 |
| 3066 | AT3G07630.2 \| prephenate dehydratase family protein, similar to P-protein: chorismate mutase, prephenate dehydratase GB:P43900 (Haemophilus influenzae) \| chr3:2435456-2437971 FORWARD \| Aliases: MLP3.8 | 6.2 | 4.4 | 1.9 | 6.1 | 0.38% | 5.9 |
| 3068 | AT3G61530.2 \| ketopantoate hydroxymethyltransferase family protein, similar to SP:Q9Y7B6 3-methyl-2-oxobutanoate hydroxymethyltransferase (EC 2.1.2.11) (Ketopantoate hydroxymethyltransferase) {Emericella nidulans}; contains Pfam profile PF02548: Ketopantoate hydroxymethyltransferase \| chr3:22782444-22784765 REVERSE \| Aliases: F2A19.130 | 7.1 | 3.6 | 3.6 | 6.1 | 0.38% | 5.5 |

| Rank | Description | Sync | Root | M | t | adj.q | B |
|---|---|---|---|---|---|---|---|
| 3070 | AT4G29590.1 \| expressed protein \| chr4:14512555-14514449 REVERSE \| Aliases: T16L4.100, T16L4_100 | 7.7 | 5.0 | 2.8 | 6.1 | 0.38% | 5.8 |
| 3072 | AT4G30840.1 \| WD-40 repeat protein family \| chr4:15017625-15019741 REVERSE \| Aliases: F6I18.250, F6I18_250 | 5.2 | 3.2 | 2.0 | 6.1 | 0.38% | 5.6 |
| 3073 | AT2G04795.1 \| expressed protein \| chr2:1682950-1683472 FORWARD \| Aliases: None | 8.0 | 6.5 | 1.5 | 6.1 | 0.38% | 5.8 |
| 3077 | AT2G04845.1 \| GCN5-related N-acetyltransferase (GNAT) family protein, contains Pfam profile PF00583: acetyltransferase, GNAT family \| chr2:1702220-1704000 REVERSE \| Aliases: None | 5.6 | 3.5 | 2.0 | 6.0 | 0.38% | 5.6 |
| 3078 | AT5G07900.1 \| mitochondrial transcription termination factor family protein / mTERF family protein, low similarity to SP:Q99551 Transcription termination factor, mitochondrial precursor (mTERF) {Homo sapiens}; contains Pfam profile PF02536: mTERF | 6.5 | 5.0 | 1.4 | 6.0 | 0.38% | 5.8 |
| 3080 | AT1G48560.1 \| expressed protein \| chr1:17956533-17958973 FORWARD \| Aliases: T1N15.18, T1N15_18 | 4.6 | 3.3 | 1.3 | 6.0 | 0.38% | 5.8 |
| 3081 | AT1G06690.1 \| aldo/keto reductase family protein, contains Pfam profile PF00248: oxidoreductase, aldo/keto reductase family \| chr1:2049601-2052059 REVERSE \| Aliases: F12K11.2, F12K11_2 | 6.8 | 5.0 | 1.8 | 6.0 | 0.38% | 5.8 |
| 3082 | AT1G31150.1 \| expressed protein, EST gb:Z33866 comes from this gene \| chr1:11120078-11122568 FORWARD \| Aliases: F28K20.8, F28K20_8 | 4.7 | 3.3 | 1.4 | 6.0 | 0.38% | 5.8 |
| 3084 | AT3G20230.1 \| 50S ribosomal protein L18 family, similar to ribosomal protein L18 (GI:29342325) (Enterococcus faecalis V583); similar to 50S ribosomal protein L18 (Swiss-Prot:O24704) (Synechococcus sp.) \| chr3:7054834-7056764 REVERSE \| Aliases: MAL21.25 | 5.3 | 3.2 | 2.1 | 6.0 | 0.39% | 5.7 |
| 3085 | AT4G26210.2 \| mitochondrial ATP synthase g subunit family protein, contains Pfam profile: PF04718 mitochondrial ATP synthase g subunit | 8.0 | 5.9 | 2.1 | 6.0 | 0.39% | 5.8 |
| 3090 | AT1G66620.1 \| seven in absentia (SINA) protein, putative, similar to SIAH1 protein (Brassica napus var. napus) GI:7657876; contains Pfam profile PF03145: Seven in absentia protein family \| chr1:24856469-24857707 REVERSE \| Aliases: T12I7.7, T12I7_7 | 6.8 | 3.8 | 3.0 | 6.0 | 0.39% | 5.6 |
| 3091 | AT1G63780.1 \| Symbol: IMP4 \| brix domain-containing protein, contains Pfam domain, PF04427: Brix domain \| chr1:23668536-23671015 REVERSE \| Aliases: F24D7.3 | 6.6 | 5.4 | 1.2 | 6.0 | 0.39% | 5.8 |
| 3092 | AT4G02400.1 \| U3 ribonucleoprotein (Utp) family protein, contains Pfam profile: PF04615 Utp14 protein \| chr4:1054243-1058236 FORWARD \| Aliases: T14P8.20, T14P8_20 | 6.6 | 3.8 | 2.8 | 6.0 | 0.39% | 5.7 |
| 3094 | AT2G23970.1 \| defense-related protein, putative, similar to defense-related protein (Brassica carinata) GI:14009290 \| chr2:10206778-10208161 FORWARD \| Aliases: T29E15.17, T29E15_17 | 4.8 | 3.1 | 1.7 | 6.0 | 0.39% | 5.7 |
| 3095 | AT4G24930.1 \| thylakoid lumenal 17.9 kDa protein, chloroplast, SP:Q9SW33;GI:17369630;PMID:11719511; similar to expressed protein gi:12321169 {Oryza sativa} \| chr4:12821233-12823752 REVERSE \| Aliases: F13M23.70, F13M23_70 | 5.2 | 3.2 | 2.0 | 6.0 | 0.39% | 5.5 |
| 3097 | AT5G03660.1 \| expressed protein, low similarity to outer surface protein F (Borrelia burgdorferi) GI:466482; contains Pfam profile PF04949: Family of unknown function (DUF662) \| chr5:937976-940057 FORWARD \| Aliases: F17C15.80, F17C15_80 | 9.1 | 6.5 | 2.6 | 6.0 | 0.39% | 5.8 |
| 3099 | AT1G07910.1 \| encodes a tRNA ligase that resembles the yeast RNA ligase in structure and function but very different in sequence. \| chr1:2446709-2454554 FORWARD \| Aliases: T6D22.1, T6D22_1 | 5.3 | 3.8 | 1.5 | 6.0 | 0.39% | 5.8 |
| 3100 | AT4G28250.1 \| Symbol: ATEXPB3 \| beta-expansin, putative (EXPB3), similar to soybean pollen allergen (cim1) protein - soybean, PIR2:S48032; beta-expansin gene family, PMID:11641069 \| chr4:14000044-14002047 REVERSE \| Aliases: ARABIDOPSIS THALIANA EXPANSIN B3, ATHEXP BETA 1.6, EXPB3, F26K10.130, F26K10_130 | 6.4 | 3.4 | 2.9 | 6.0 | 0.39% | 5.7 |
| 3101 | AT4G18260.1 \| cytochrome B561-related, related to cytochrome b-561 (GI:20345443) (Mus musculus); multidrug resistance protein, Homo sapiens, PIR2:S71841 \| chr4:10093535-10097514 REVERSE \| Aliases: T9A21.110, T9A21_110 | 5.0 | 3.7 | 1.3 | 6.0 | 0.40% | 5.8 |
| 3103 | AT1G68260.1 \| thioesterase family protein, contains Pfam profile: PF03061: thioesterase family protein | 5.5 | 3.3 | 2.2 | 6.0 | 0.40% | 5.7 |
| 3104 | AT1G23820.2 \| Symbol: SPDS1 \| spermidine synthase 1 (SPDSYN1) / putrescine aminopropyltransferase 1, identical to SP:Q9ZUB3 Spermidine synthase 1 (EC 2.5.1.16) (Putrescine aminopropyltransferase 1) (SPDSY 1) {Arabidopsis thaliana} \| chr1:8420276-8422928 FORWARD \| Aliases: F5O8.38, F5O8_38, SPERMIDINE SYNTHASE 1 | 12.2 | 10.5 | 1.7 | 6.0 | 0.40% | 5.5 |
| 3107 | AT3G03320.1 \| expressed protein, similar to hypothetical protein GB:BAA29429 (Pyrococcus horikoshii) \| chr3:781722-783471 FORWARD \| Aliases: T21P5.26, T21P5_26 | 5.6 | 3.7 | 1.8 | 6.0 | 0.40% | 5.7 |
| 3108 | AT3G53960.1 \| proton-dependent oligopeptide transport (POT) family protein, contains Pfam profile: PF00854 POT family \| chr3:19989100-19991912 REVERSE \| Aliases: F5K20.260 | 5.4 | 3.7 | 1.7 | 6.0 | 0.40% | 5.6 |

| Rank | Description | Sync | Root | M | t | adj.q | B |
|---|---|---|---|---|---|---|---|
| 3109 | AT5G64350.1 \| Symbol: FKBP12 \| | 13.2 | 12.2 | 1.0 | 6.0 | 0.40% | 5.6 |
| 3114 | AT3G18420.1 \| tetratricopeptide repeat (TPR)-containing protein, contains Pfam profile: PF00515 tetratricopeptide repeat domain (TPR domain) \| chr3:6324639-6325799 REVERSE \| Aliases: MYF24.14 | 7.4 | 4.9 | 2.5 | 6.0 | 0.40% | 5.7 |
| 3115 | AT5G24800.1 \| Symbol: BZO2H2 \| | 10.3 | 8.7 | 1.7 | 6.0 | 0.40% | 5.7 |
| 3116 | AT3G49160.1 \| pyruvate kinase family protein, similar to SP:Q92122 Pyruvate kinase, muscle isozyme (EC 2.7.1.40) (Cytosolic thyroid hormone binding protein) (CTHBP) {Xenopus laevis}; contains Pfam profile PF00224: Pyruvate kinase, barrel domain \| chr3:18232979-18235870 REVERSE \| Aliases: F2K15.20 | 5.3 | 3.2 | 2.1 | 6.0 | 0.40% | 5.7 |
| 3117 | AT3G50685.1 \| expressed protein \| chr3:18845200-18845815 REVERSE \| Aliases: None | 9.7 | 8.5 | 1.2 | 6.0 | 0.40% | 5.8 |
| 3118 | AT2G35820.1 \| expressed protein \| chr2:15057991-15059318 FORWARD \| Aliases: F11F19.27, F11F19_27 | 4.5 | 1.9 | 2.6 | 6.0 | 0.40% | 5.4 |
| 3119 | AT2G45200.1 \| Symbol: GOS12 \| Golgi SNARE 12 protein / Golgi SNAP receptor complex member 1, identical to Probable 28 kDa Golgi SNARE protein (Golgi SNAP receptor complex member 1) (SP:O22151) {Arabidopsis thaliana} \| chr2:18644582-18646858 REVERSE \| Aliases: ATGOS12, F4L23.29, GOLGI SNARE 12 | 9.3 | 7.5 | 1.8 | 6.0 | 0.40% | 5.8 |
| 3121 | AT2G01320.4 \| ABC transporter family protein \| chr2:154427-158181 REVERSE \| Aliases: F10A8.20, F10A8_20 | 5.1 | 3.5 | 1.6 | 6.0 | 0.40% | 5.7 |
| 3122 | AT3G53470.2 \| expressed protein, ribosomal protein S25, cytosolic, Arabidopsis thaliana, PIR:T08568 \| chr3:19833728-19834384 FORWARD \| Aliases: F4P12.170 | 5.8 | 3.9 | 1.9 | 6.0 | 0.40% | 5.6 |
| 3124 | AT1G22520.1 \| expressed protein, contains Pfam PF04418: Domain of unknown function (DUF543) \| chr1:7953011-7954391 REVERSE \| Aliases: F12K8.27, F12K8_27 | 7.6 | 5.7 | 1.9 | 6.0 | 0.40% | 5.8 |
| 3127 | AT4G39970.1 \| haloacid dehalogenase-like hydrolase family protein, low similarity to SP:P95649 CbbY protein {Rhodobacter sphaeroides} \| chr4:18536120-18538478 REVERSE \| Aliases: T5J17.140, T5J17_140 | 5.4 | 3.9 | 1.5 | 6.0 | 0.40% | 5.8 |
| 3130 | AT2G34300.2 \| similar to dehydration-responsive protein-related [Arabidopsis thaliana] (TAIR:At1g29470.1); similar to dehydration-responsive family protein [Arabidopsis thaliana] (TAIR:At2g40280.1); similar to dehydration-responsive protein-related [Arabidopsis thaliana] (TAIR:At5g64030.1); similar to dehydration-responsive protein-related [Arabidopsis thaliana] (TAIR:At3g51070.1); similar to OSJNBb0020J19.14 [Oryza sativa (japonica cultivar-group)] (GB:XP_474482.1); similar to ankyrin-like protein [Oryza sativa (japonica cultivar-group)] (GB:BAD82580.1); contains InterPro domain Putative methyltransferase DUF248 (InterPro:IPR004159) \| chr2:14480744-14484343 REVERSE \| Aliases: F13P17.14, F13P17_14 | 8.0 | 6.3 | 1.7 | 6.0 | 0.40% | 5.8 |
| 3132 | AT5G08540.1 \| expressed protein, similar to unknown protein (pir::T27191) \| chr5:2763824-2766156 FORWARD \| Aliases: MAH20.10, MAH20_10 | 7.3 | 4.8 | 2.5 | 6.0 | 0.40% | 5.8 |
| 3133 | AT5G17520.1 \| Symbol: RCP1 \| root cap 1 (RCP1), identical to GI:6137138 \| chr5:5772586-5775347 REVERSE \| Aliases: K3M16.90, K3M16_90, MALTOSE EXCESS 1, MEX1, ROOT CAP 1 | 7.1 | 4.4 | 2.7 | 6.0 | 0.40% | 5.4 |
| 3135 | AT4G15640.1 \| expressed protein \| chr4:8918983-8921928 REVERSE \| Aliases: DL3860C, FCAALL.350 | 6.5 | 3.7 | 2.8 | 6.0 | 0.41% | 5.6 |
| 3136 | AT4G17670.1 \| senescence-associated protein-related, similar to senescence-associated protein SAG102 (GI:22331931) (Arabidopsis thaliana); \| chr4:9833681-9834740 REVERSE \| Aliases: DL4870C, FCAALL.414 | 6.5 | 4.4 | 2.0 | 6.0 | 0.41% | 5.7 |
| 3141 | AT1G62810.1 \| copper amine oxidase, putative, similar to copper amine oxidase (Cicer arietinum) gi:3819099:emb:CAA08855 \| chr1:23261391-23265631 REVERSE \| Aliases: F23N19.18, F23N19_18 | 8.6 | 6.7 | 1.9 | 6.0 | 0.41% | 5.7 |
| 3144 | AT1G80480.1 \| PRLI-interacting factor L, putative, similar to PRLI-interacting factor L (Arabidopsis thaliana) GI:11139268; contains Pfam profile PF02492: Cobalamin synthesis protein/P47K \| chr1:30262968-30265462 REVERSE \| Aliases: T21F11.27, T21F11_27 | 7.7 | 5.9 | 1.8 | 6.0 | 0.41% | 5.8 |
| 3146 | AT3G51870.1 \| mitochondrial substrate carrier family protein, peroxisomal Ca-dependent solute carrier - Oryctolagus cuniculus, EMBL:AF004161 \| chr3:19254931-19257913 FORWARD \| Aliases: ATEM1.12 | 4.9 | 3.2 | 1.7 | 6.0 | 0.41% | 5.7 |
| 3148 | AT3G45740.1 \| hydrolase family protein / HAD-superfamily protein, contains TIGRfam domians TIGR01460: HAD-superfamily hydrolase, subfamily IIA and TIGR01456: HAD-superfamily subfamily IIA hydrolase, TIGR01456, CECR5 \| chr3:16801746-16804519 FORWARD \| Aliases: T6D9.70 | 6.0 | 4.4 | 1.6 | 6.0 | 0.41% | 5.7 |
| 3150 | AT3G08700.1 \| ubiquitin-conjugating enzyme, putative, strong similar to ubiquitin-conjugating enzymes E2-17 from (Arabidopsis thaliana) SP:P35134, SP:P35132, SP:P35133; contains Pfam profile PF00179: Ubiquitin-conjugating enzyme \| chr3:2643488-2644551 FORWARD \| Aliases: F17O14.17 | 3.9 | 3.1 | 0.7 | 6.0 | 0.41% | 5.5 |
| 3153 | AT4G27340.1 \| Met-10+ like family protein, ; contains Pfam profile PF02475: Met-10+ like-protein \| chr4:13687202-13690376 REVERSE \| Aliases: F27G19.9 | 5.2 | 4.0 | 1.3 | 6.0 | 0.41% | 5.7 |

| Rank | Description | Sync | Root | M | t | adj.q | B |
|---|---|---|---|---|---|---|---|
| 3155 | AT1G05410.2 \| similar to tropomyosin-related [Arabidopsis thaliana] (TAIR:At5g48160.1); similar to unknown protein [Oryza sativa (japonica cultivar-group)] (GB:XP_469730.1); contains domain CYS_RICH (PS50311) \| chr1:1585370-1587773 REVERSE \| Aliases: T25N20.6, T25N20_6 | 6.5 | 5.1 | 1.4 | 6.0 | 0.41% | 5.7 |
| 3156 | AT5G66460.1 \| (1-4)-beta-mannan endohydrolase, putative, similar to (1-4)-beta-mannan endohydrolase (Coffea arabica) GI:10178872; contains Pfam profile PF00150: Cellulase (glycosyl hydrolase family 5) \| chr5:26555621-26558262 REVERSE \| Aliases: K1F13.12, K1F13_12 | 5.7 | 2.6 | 3.1 | 6.0 | 0.41% | 5.5 |
| 3159 | AT2G02230.1 \| Symbol: ATPP2 B1 \| similar to F-box family protein / SKP1 interacting partner 3-related [Arabidopsis thaliana] (TAIR:At2g02240.1); similar to F-box family protein-like [Oryza sativa (japonica cultivar-group)] (GB:XP_468355.1); contains InterPro domain Cyclin-like F-box (InterPro:IPR001810) \| chr2:590222-591649 REVERSE \| Aliases: ATPP2 B1, T16F16.2, T16F16_2 | 9.4 | 5.5 | 3.9 | 5.9 | 0.42% | 5.6 |
| 3161 | AT4G31040.1 \| proton extrusion protein-related, contains weak similarity to Proton extrusion protein pcxA (Swiss-Prot:P75028) (Synechocystis sp.) \| chr4:15111491-15114132 REVERSE \| Aliases: F6I18.50, F6I18_50 | 7.1 | 6.1 | 1.0 | 5.9 | 0.42% | 5.7 |
| 3163 | AT2G18040.1 \| Symbol: PIN1AT \| | 9.8 | 7.6 | 2.3 | 5.9 | 0.42% | 5.7 |
| 3164 | AT2G45280.1 \| Symbol: ATRAD51C \| DNA repair family protein, contains similarity to Swiss-Prot:O43502 DNA repair protein RAD51 homolog 3 (Homo sapiens) \| chr2:18677083-18679351 FORWARD \| Aliases: F4L23.21, RAD51C | 4.9 | 3.4 | 1.5 | 5.9 | 0.42% | 5.7 |
| 3165 | AT1G26550.1 \| peptidyl-prolyl cis-trans isomerase PPIC-type family protein, similar to SP:Q9Y237 Peptidyl-prolyl cis-trans isomerase NIMA-interacting 4 (EC 5.2.1.8) (Rotamase Pin4) (PPIase Pin4) (Parvulin 14) {Homo sapiens}; contains Pfam profile PF00639: PPIC-type PPIASE domain \| chr1:9171119-9172926 FORWARD \| Aliases: T1K7.8, T1K7_8 | 11.4 | 10.6 | 0.8 | 5.9 | 0.42% | 5.5 |
| 3167 | AT5G20580.2 \| expressed protein, predicted protein, Arabidopsis thaliana \| chr5:6958619-6962768 REVERSE \| Aliases: F7C8.170, F7C8_170 | 5.6 | 3.9 | 1.7 | 5.9 | 0.42% | 5.7 |
| 3168 | AT5G64900.1 \| expressed protein \| chr5:25954396-25955302 FORWARD \| Aliases: MXK3.13, MXK3_13 | 5.6 | 4.1 | 1.4 | 5.9 | 0.42% | 5.7 |
| 3170 | AT1G59660.1 \| nucleoporin family protein, contains Pfam profiles: PF04096 nucleoporin autopeptidase, PF03093 nucleoporin FG repeat family \| chr1:21928358-21933089 FORWARD \| Aliases: T30E16.24, T30E16_24 | 5.9 | 4.1 | 1.7 | 5.9 | 0.42% | 5.5 |
| 3171 | AT2G40300.1 \| Symbol: ATFER4 \| | 5.8 | 3.3 | 2.5 | 5.9 | 0.42% | 5.4 |
| 3172 | AT2G17240.1 \| expressed protein \| chr2:7505214-7506288 FORWARD \| Aliases: T23A1.10, T23A1_10 | 6.8 | 3.5 | 3.3 | 5.9 | 0.42% | 5.6 |
| 3173 | AT3G51100.2 \| expressed protein \| chr3:18991762-18993241 REVERSE \| Aliases: F24M12.140 | 9.1 | 7.5 | 1.6 | 5.9 | 0.42% | 5.7 |
| 3174 | AT5G65420.1 \| Symbol: CYCD4;1 \| cyclin, putative, similar to cyclin D2.1 protein (Nicotiana tabacum) GI:4160298; contains Pfam profiles PF00134: Cyclin, N-terminal domain, PF02984: Cyclin, C-terminal domain \| chr5:26158699-26161219 REVERSE \| Aliases: CYCD4;1, Cyclin D4;1, MNA5.15, MNA5_15 | 5.9 | 4.9 | 1.0 | 5.9 | 0.42% | 5.7 |
| 3175 | AT4G19985.1 \| GCN5-related N-acetyltransferase (GNAT) family protein, contains Pfam profile PF00583: acetyltransferase, GNAT family \| chr4:10830318-10832104 REVERSE \| Aliases: None | 5.0 | 3.3 | 1.7 | 5.9 | 0.42% | 5.7 |
| 3179 | AT3G52730.1 \| ubiquinol-cytochrome C reductase UQCRX/QCR9-like family protein, contains Pfam profile: PF05365 ubiquinol-cytochrome C reductase, UQCRX/QCR9 like \| chr3:19553909-19555242 REVERSE \| Aliases: F3C22.130 | 11.9 | 10.8 | 1.0 | 5.9 | 0.42% | 5.5 |
| 3181 | AT5G41210.1 \| Symbol: ATGSTT1 \| glutathione S-transferase (GST10), identical to glutathione transferase AtGST 10 (Arabidopsis thaliana) GI:4049401 \| chr5:16509603-16511126 REVERSE \| Aliases: GST10, MEE6.28, MEE6_28 | 9.2 | 7.3 | 1.9 | 5.9 | 0.42% | 5.7 |
| 3184 | AT4G25910.1 \| Symbol: NFU3 \| nitrogen fixation protein, putative, nitrogen fixation protein nifU (SP:Q43885) (Anabaena sp.); contains Pfam profile: PF01106 NifU-like domain \| chr4:13163982-13165173 FORWARD \| Aliases: ATCNFU3, F20B18.20, F20B18_20 | 8.0 | 6.3 | 1.7 | 5.9 | 0.42% | 5.6 |
| 3185 | AT4G23820.1 \| glycoside hydrolase family 28 protein / polygalacturonase (pectinase) family protein, weak similarity to polygalacturonase PG1 (Glycine max) GI:5669846; contains PF00295: Glycosyl hydrolases family 28 | 5.6 | 3.9 | 1.7 | 5.9 | 0.42% | 5.6 |
| 3186 | AT1G68590.1 \| plastid-specific 30S ribosomal protein 3, putative / PSRP-3, putative, similar to SP:P82412 Plastid-specific 30S ribosomal protein 3, chloroplast precursor (PSRP-3) {Spinacia oleracea}; contains Pfam profile PF04839: Plastid and cyanobacterial ribosomal protein (PSRP-3 / Ycf65) \| chr1:25761072-25761890 REVERSE \| Aliases: F24J5.17, F24J5_17 | 5.8 | 3.4 | 2.4 | 5.9 | 0.42% | 5.4 |
| 3189 | AT2G19310.1 \| expressed protein \| chr2:8376876-8377488 REVERSE \| Aliases: F27F23.11, F27F23_11 | 6.0 | 4.1 | 1.9 | 5.9 | 0.42% | 5.7 |
| 3192 | AT3G29230.1 \| pentatricopeptide (PPR) repeat-containing protein, contains INTERPRO:IPR002885 PPR repeats \| chr3:11190040-11191842 FORWARD \| Aliases: MXO21.8 | 6.8 | 5.0 | 1.8 | 5.9 | 0.43% | 5.6 |

| Rank | Description | Sync | Root | M | t | adj.q | B |
|---|---|---|---|---|---|---|---|
| 3193 | AT2G46420.1 \| expressed protein \| chr2:19060759-19064163 FORWARD \| Aliases: F11C10.11 | 5.8 | 2.4 | 3.4 | 5.9 | 0.43% | 5.4 |
| 3194 | AT5G53190.1 \| nodulin MtN3 family protein, similar to MtN3 GI:1619602 (root nodule development) from (Medicago truncatula) \| chr5:21589643-21591510 REVERSE \| Aliases: MFH8.13, MFH8_13 | 5.8 | 3.7 | 2.2 | 5.9 | 0.43% | 5.6 |
| 3195 | AT5G08050.1 \| expressed protein, predicted protein, Arabidopsis thaliana \| chr5:2578370-2579164 FORWARD \| Aliases: T22D6.3 | 7.1 | 5.0 | 2.1 | 5.9 | 0.43% | 5.5 |
| 3196 | AT2G01640.1 \| expressed protein \| chr2:283003-284124 FORWARD \| Aliases: T8O11.19, T8O11_19 | 5.3 | 3.8 | 1.5 | 5.9 | 0.43% | 5.7 |
| 3197 | AT1G26665.2 \| expressed protein \| chr1:9214164-9215538 FORWARD \| Aliases: None | 8.6 | 6.9 | 1.6 | 5.9 | 0.43% | 5.7 |
| 3200 | AT5G24660.1 \| expressed protein \| chr5:8443282-8443837 REVERSE \| Aliases: MXC17.2, MXC17_2 | 11.9 | 8.8 | 3.1 | 5.9 | 0.43% | 5.7 |
| 3201 | AT4G29510.1 \| protein arginine N-methyltransferase, putative, similar to protein arginine N-methyltransferase 1-variant 2 (Homo sapiens) GI:7453575 \| chr4:14491631-14494007 FORWARD \| Aliases: T16L4.20, T16L4_20 | 8.2 | 5.5 | 2.6 | 5.9 | 0.43% | 5.7 |
| 3202 | AT5G57120.1 \| expressed protein, weak similarity to SP:Q14978 Nucleolar phosphoprotein p130 {Homo sapiens} \| chr5:23139766-23141739 REVERSE \| Aliases: MUL3.6, MUL3_6 | 9.7 | 8.2 | 1.5 | 5.9 | 0.43% | 5.6 |
| 3204 | AT3G08010.1 \| expressed protein \| chr3:2555988-2557511 FORWARD \| Aliases: F17A17.35 | 8.0 | 6.2 | 1.8 | 5.9 | 0.43% | 5.6 |
| 3207 | AT4G26400.2 \| zinc finger (C3HC4-type RING finger) family protein, contains Pfam domain, PF00097: Zinc finger, C3HC4 type (RING finger) \| chr4:13344857-13346583 REVERSE \| Aliases: M3E9.170, M3E9_170 | 8.2 | 6.8 | 1.4 | 5.9 | 0.43% | 5.7 |
| 3210 | AT3G24315.1 \| sec20 family protein, contains Pfam PF03908: Sec20 \| chr3:8820464-8822810 REVERSE \| Aliases: None | 9.3 | 7.9 | 1.4 | 5.9 | 0.43% | 5.7 |
| 3212 | AT5G45040.1 \| cytochrome c6 (ATC6), identical to c6 type cytochrome (Arabidopsis thaliana) gi:19571824:emb:CAD27418 \| chr5:18192200-18194110 REVERSE \| Aliases: K21C13.23, K21C13_23 | 4.7 | 2.3 | 2.3 | 5.9 | 0.43% | 5.4 |
| 3213 | AT5G40500.1 \| expressed protein \| chr5:16245062-16245839 FORWARD \| Aliases: MNF13.2, MNF13_2 | 4.4 | 3.0 | 1.4 | 5.9 | 0.43% | 5.7 |
| 3215 | AT5G10040.1 \| expressed protein \| chr5:3143724-3144158 FORWARD \| Aliases: T31P16.30, T31P16_30 | 6.3 | 4.1 | 2.2 | 5.9 | 0.43% | 5.6 |
| 3216 | AT1G15120.1 \| ubiquinol-cytochrome C reductase complex 7.8 kDa protein, putative / mitochondrial hinge protein, putative, similar to SP:P48504 Ubiquinol-cytochrome C reductase complex 7.8 kDa protein (EC 1.10.2.2) (Mitochondrial hinge protein) (CR7) {Solanum tuberosum}; contains Pfam profile PF02320: Ubiquinol-cytochrome C reductase hinge protein \| chr1:5202849-5204188 FORWARD \| Aliases: F9L1.5, F9L1_5 | 10.8 | 9.6 | 1.2 | 5.9 | 0.43% | 5.2 |
| 3217 | AT2G33735.1 \| DNAJ heat shock N-terminal domain-containing protein, similar to SP:P30725 Chaperone protein dnaJ Clostridium acetobutylicum; contains Pfam profile PF00226 DnaJ domain \| chr2:14275345-14276517 REVERSE \| Aliases: None | 4.9 | 3.3 | 1.6 | 5.9 | 0.43% | 5.7 |
| 3219 | AT4G13220.1 \| expressed protein \| chr4:7674295-7675303 REVERSE \| Aliases: F17N18.110, F17N18_110 | 5.3 | 3.7 | 1.6 | 5.9 | 0.43% | 5.6 |
| 3220 | AT5G38890.1 \| exoribonuclease-related, similar to SP:P53859 3'-5' exoribonuclease CSL4 (EC 3.1.13.-) {Saccharomyces cerevisiae} \| chr5:15586722-15588325 FORWARD \| Aliases: K15E6.10 | 6.1 | 4.3 | 1.8 | 5.9 | 0.43% | 5.5 |
| 3221 | AT5G14660.2 \| Symbol: PDF1B \| peptide deformylase, chloroplast / polypeptide deformylase 1B (PDF1B), identical to SP:Q9FUZ2 Peptide deformylase, chloroplast precursor (EC 3.5.1.88) (PDF) (Polypeptide deformylase) {Arabidopsis thaliana}; supporting cDNA gi:11320965:gb:AF269165.1:AF269165 \| chr5:4726736-4728749 REVERSE \| Aliases: DEF2, PEPTIDE DEFORMYLASE, PEPTIDE DEFORMYLASE 1B | 4.6 | 3.1 | 1.5 | 5.9 | 0.43% | 5.7 |
| 3222 | AT2G39725.2 \| complex 1 family protein / LVR family protein, contains Pfam PF05347: Complex 1 protein (LYR family) \| chr2:16576508-16577595 FORWARD \| Aliases: None | 7.1 | 4.6 | 2.5 | 5.9 | 0.43% | 5.6 |
| 3224 | AT2G05520.5 \| Symbol: GRP 3 \| similar to glycine-rich protein (GRP3S) [Arabidopsis thaliana] (TAIR:At2g05380.1); similar to glycine-rich protein [Citrus unshiu] (GB:BAA92155.1); contains domain GLY_RICH (PS50315) \| chr2:2026159-2027114 FORWARD \| Aliases: ATGRP 3, GLYCINE RICH PROTEIN 3, T20G20.13, T20G20_13 | 7.5 | 6.0 | 1.4 | 5.9 | 0.44% | 5.7 |
| 3225 | AT2G37190.1 \| 60S ribosomal protein L12 (RPL12A) \| chr2:15626486-15627198 REVERSE \| Aliases: T2N18.5, T2N18_5 | 9.1 | 6.9 | 2.2 | 5.9 | 0.44% | 5.6 |
| 3227 | AT3G20390.1 \| endoribonuclease L-PSP family protein, contains Pfam domain PF01042: Endoribonuclease L-PSP \| chr3:7109955-7111830 REVERSE \| Aliases: MQC12.17 | 11.6 | 9.8 | 1.8 | 5.9 | 0.44% | 5.6 |
| 3228 | AT4G30410.2 \| expressed protein, similar to cDNA bHLH transcription factor (bHLH eta gene) gi:32563007 | 5.5 | 3.6 | 1.9 | 5.9 | 0.44% | 5.5 |

| Rank | Description | Sync | Root | M | t | adj.q | B |
|---|---|---|---|---|---|---|---|
| 3229 | AT4G19150.1 \| ankyrin repeat family protein, contains ankyrin repeats, Pfam:PF00023 \| chr4:10471346-10472753 REVERSE \| Aliases: T18B16.120, T18B16_120 | 7.7 | 5.9 | 1.8 | 5.9 | 0.44% | 5.7 |
| 3230 | AT3G04260.1 \| SAP domain-containing protein, contains Pfam domain PF02037: SAP domain \| chr3:1123030-1127522 REVERSE \| Aliases: T6K12.12, T6K12_12 | 5.2 | 3.5 | 1.6 | 5.9 | 0.44% | 5.6 |
| 3232 | AT5G03850.1 \| 40S ribosomal protein S28 (RPS28B), ribosomal protein S28, Arabidopsis thaliana, EMBL:ATRP28A \| chr5:1028339-1029120 REVERSE \| Aliases: MED24.14 | 12.1 | 11.0 | 1.1 | 5.9 | 0.44% | 5.2 |
| 3233 | AT3G47490.2 \| HNH endonuclease domain-containing protein, contains Pfam domain PF01844: HNH endonuclease \| chr3:17509322-17510286 FORWARD \| Aliases: F1P2.40 | 6.3 | 4.9 | 1.4 | 5.9 | 0.44% | 5.6 |
| 3234 | AT2G01080.1 \| expressed protein \| chr2:77888-79333 FORWARD \| Aliases: F23H14.5, F23H14_5 | 5.6 | 4.7 | 0.9 | 5.9 | 0.44% | 5.6 |
| 3235 | AT2G27030.3 \| Symbol: CAM2 \| calmodulin-2/3/5 (CAM5) (TCH1), identical to calmodulin GI:474183 from (Arabidopsis thaliana), SP:P25069 Calmodulin-2/3/5 {Arabidopsis thaliana} | 9.0 | 6.5 | 2.4 | 5.9 | 0.44% | 5.6 |
| 3236 | AT1G16080.1 \| expressed protein \| chr1:5514311-5516012 FORWARD \| Aliases: T24D18.18, T24D18_18 | 6.0 | 4.3 | 1.7 | 5.9 | 0.44% | 5.6 |
| 3238 | AT3G52150.2 \| similar to 33 kDa ribonucleoprotein, chloroplast, putative / RNA-binding protein cp33, putative [Arabidopsis thaliana] (TAIR:At3g52380.1); similar to putative plastid-specific ribosomal protein 2 precursor [Oryza sativa (japonica cultivar-group)] (GB:XP_450482.1); contains InterPro domain Paraneoplastic encephalomyelitis antigen (InterPro:IPR002343); contains InterPro domain RNA-binding region RNP-1 (RNA recognition motif) (InterPro:IPR000504) | 7.0 | 3.2 | 3.8 | 5.9 | 0.44% | 5.4 |
| 3239 | AT4G28820.2 \| similar to PREDICTED: similar to thyroid hormone receptor interactor 3 [Canis familiaris] (GB:XP_537712.1); similar to ENSANGP00000021276 [Anopheles gambiae str. PEST] (GB:XP_320662.2); contains InterPro domain HIT Zn-finger (InterPro:IPR007529) \| chr4:14230704-14232348 REVERSE \| Aliases: F16A16.70, F16A16_70 | 4.6 | 2.8 | 1.8 | 5.9 | 0.44% | 5.6 |
| 3241 | AT3G53700.1 \| pentatricopeptide (PPR) repeat-containing protein, low similarity to fertility restorer (Petunia x hybrida) GI:22128587; contains Pfam profile PF01535: PPR repeat \| chr3:19911258-19913862 FORWARD \| Aliases: F5K20.2 | 5.4 | 4.0 | 1.4 | 5.9 | 0.44% | 5.6 |
| 3243 | AT1G52380.1 \| Ran-binding protein 1 domain-containing protein / RanBP1 domain-containing protein, weak similarity to SP:Q09717 Ran-specific GTPase-activating protein 1 (Ran binding protein 1) (RANBP1) (Spi1-binding protein) {Schizosaccharomyces pombe}; contains Pfam profile PF00638: RanBP1 domain \| chr1:19512654-19515207 FORWARD \| Aliases: F19K6.4, F19K6_4 | 8.9 | 7.6 | 1.3 | 5.9 | 0.44% | 5.6 |
| 3244 | AT3G18030.1 \| Symbol: ATHAL3A \| halotolerance protein (HAL3A), identical to GB:AAD51616 from (Arabidopsis thaliana); similar to Halotolerance protein Hal3b (SP:P94063)(Arabidopsis thaliana); contains Pfam PF02441: Flavoprotein \| chr3:6167690-6168954 REVERSE \| Aliases: ATHAL3, HAL3, HAL3A, MBG14.4 | 5.7 | 3.6 | 2.1 | 5.9 | 0.44% | 5.6 |
| 3245 | AT5G65990.1 \| amino acid transporter family protein, similar to proton/amino acid transporter 1 (Mus musculus) GI:21908024; contains Pfam profile PF01490: Transmembrane amino acid transporter protein \| chr5:26412108-26414108 FORWARD \| Aliases: K2A18.5, K2A18_5 | 6.9 | 4.5 | 2.4 | 5.9 | 0.44% | 5.5 |
| 3247 | AT3G52155.1 \| expressed protein \| chr3:19354673-19355827 FORWARD \| Aliases: None | 3.2 | 2.3 | 0.9 | 5.9 | 0.45% | 5.5 |
| 3248 | AT5G20180.2 \| ribosomal protein L36 family protein, contains Pfam profile: PF00444 ribosomal protein L36 \| chr5:6810966-6812148 FORWARD \| Aliases: F5O24.70, F5O24_70 | 10.3 | 8.8 | 1.5 | 5.9 | 0.45% | 5.6 |
| 3249 | AT2G40010.1 \| 60S acidic ribosomal protein P0 (RPP0A) \| chr2:16715457-16717526 REVERSE \| Aliases: T28M21.17, T28M21_17 | 6.6 | 2.6 | 4.0 | 5.9 | 0.45% | 5.5 |
| 3251 | AT2G47590.1 \| Symbol: PHR2 \| photolyase/blue light photoreceptor (PHR2), identical to photolyase/blue light photoreceptor PHR2 (Arabidopsis thaliana) GI:3319288; contains Pfam domain, PF00875: deoxyribodipyrimidine photolyase \| chr2:19528867-19531133 FORWARD \| Aliases: PHOTOLYASE/BLUE LIGHT RECEPTOR 2, T30B22.11 | 6.0 | 3.4 | 2.6 | 5.9 | 0.45% | 5.5 |
| 3253 | AT2G38730.1 \| peptidyl-prolyl cis-trans isomerase, putative / cyclophilin, putative / rotamase, putative, similar to cyclophilin (Homo sapiens) gi:3647230:gb:AAC60793 | 7.2 | 3.6 | 3.6 | 5.9 | 0.45% | 5.4 |
| 3259 | AT3G59780.1 \| expressed protein \| chr3:22097904-22101421 FORWARD \| Aliases: F24G16.50 | 8.0 | 6.0 | 2.1 | 5.9 | 0.45% | 5.4 |
| 3260 | AT5G13770.1 \| pentatricopeptide (PPR) repeat-containing protein, contains Pfam profile PF01535: PPR repeat \| chr5:4445426-4447839 FORWARD \| Aliases: MXE10.6, MXE10_6 | 7.9 | 4.6 | 3.4 | 5.8 | 0.45% | 5.6 |
| 3261 | AT1G25275.3 \| expressed protein \| chr1:8860659-8861349 FORWARD \| Aliases: None | 11.3 | 10.4 | 0.9 | 5.8 | 0.45% | 5.3 |

| Rank | Description | Sync | Root | M | t | adj.q | B |
|---|---|---|---|---|---|---|---|
| 3263 | AT2G27190.1 \| Symbol: PAP1 \| iron(III)-zinc(II) purple acid phosphatase (PAP12), identical to iron(III)-zinc(II) purple acid phosphatase (precursor) SP:Q38924 from (Arabidopsis thaliana) \| chr2:11628304-11630534 REVERSE \| Aliases: PURPLE ACID PHOSPHATASE 1, SECRETED PURPLE ACID PHOSPHATASE PRECURSOR, T22O13.4, T22O13_4 | 9.5 | 8.7 | 0.8 | 5.8 | 0.45% | 5.4 |
| 3264 | AT5G19150.2 \| carbohydrate kinase family, contains Pfam profile PF01256: Carbohydrate kinase \| chr5:6423307-6428456 REVERSE \| Aliases: T24G5.50, T24G5_50 | 8.1 | 7.2 | 1.0 | 5.8 | 0.45% | 5.6 |
| 3265 | AT2G19810.1 \| zinc finger (CCCH-type) family protein, contains Pfam domain, PF00642: Zinc finger C-x8-C-x5-C-x3-H type (and similar) \| chr2:8557395-8558940 FORWARD \| Aliases: F6F22.16, F6F22_16 | 6.3 | 5.2 | 1.0 | 5.8 | 0.45% | 5.6 |
| 3266 | AT1G07040.1 \| expressed protein \| chr1:2160944-2163302 REVERSE \| Aliases: F10K1.25, F10K1_25 | 9.4 | 6.9 | 2.5 | 5.8 | 0.45% | 5.6 |
| 3267 | AT3G20100.1 \| Symbol: CYP705A19 \| cytochrome P450 family protein, similar to Cytochrome P450 93A3 (P450 CP5) (SP:O81973) (Glycine max); contains Pfam profile: PF00067 cytochrome P450; supported by full-length cDNA: Ceres:149380. \| chr3:7019001-7020907 FORWARD \| Aliases: MAL21.14 | 7.6 | 6.6 | 1.0 | 5.8 | 0.45% | 5.6 |
| 3268 | AT5G23900.1 \| 60S ribosomal protein L13 (RPL13D) \| chr5:8064016-8065520 REVERSE \| Aliases: MRO11.6, MRO11_6 | 9.9 | 8.0 | 1.9 | 5.8 | 0.45% | 5.6 |
| 3269 | AT2G26990.1 \| Symbol: FUS12 \| COP9 signalosome complex subunit 2 / CSN complex subunit 2 (CSN2), proteasome, COP9-complex and eIF3-domain protein; identical to CSN complex subunit 2 (Arabidopsis thaliana) GI:18056655; identical to cDNA CSN complex subunit 2 (CSN2) GI:18056654 \| chr2:11526585-11529665 REVERSE \| Aliases: ATCSN2, CONSTITUTIVE PHOTOMORPHOGENIC 12, COP12, CSN2, FUSCA 12, T20P8.4, T20P8_4 | 9.8 | 7.8 | 2.0 | 5.8 | 0.45% | 5.6 |
| 3270 | AT1G15420.1 \| expressed protein \| chr1:5301608-5303372 REVERSE \| Aliases: None | 6.0 | 4.3 | 1.7 | 5.8 | 0.46% | 5.6 |
| 3274 | AT3G12210.2 \| expressed protein \| chr3:3894800-3896073 FORWARD \| Aliases: F28J15.5 | 6.9 | 4.9 | 2.0 | 5.8 | 0.46% | 5.5 |
| 3275 | AT5G62530.1 \| Symbol: ALDH12A1 \| delta-1-pyrroline-5-carboxylate dehydrogenase (P5CDH), identical to delta-1-pyrroline-5-carboxylate dehydrogenase precursor (Arabidopsis thaliana) gi:15383744:gb:AAK73756; identical to cDNA delta-1-pyrroline-5-carboxylate dehydrogenase precursor (P5CDH) nuclear gene for mitochondrial product GI:15383743; contains Pfam profile PF00171:aldehyde dehydrogenase (NAD) family protein \| chr5:25116229-25120476 REVERSE \| Aliases: ATP5CDH, K19B1.14, K19B1_14 | 7.7 | 5.0 | 2.6 | 5.8 | 0.46% | 5.5 |
| 3276 | AT4G34670.1 \| 40S ribosomal protein S3A (RPS3aB) \| chr4:16548651-16550453 FORWARD \| Aliases: T4L20.250, T4L20_250 | 11.4 | 9.9 | 1.5 | 5.8 | 0.46% | 5.4 |
| 3277 | AT3G26380.1 \| glycosyl hydrolase family protein 27 / alpha-galactosidase family protein / melibiase family protein, similar to alpha-galactosidase GI:10944326 from (Phanerochaete chrysosporium) \| chr3:9661302-9664535 FORWARD \| Aliases: F20C19.10 | 7.9 | 6.5 | 1.4 | 5.8 | 0.46% | 5.6 |
| 3279 | AT1G74880.1 \| Symbol: NDH O \| Encodes subunit NDH-O of NAD(P)H:plastoquinone dehydrogenase complex (Ndh complex) present in the thylakoid membrane of chloroplasts. This subunit is thought to be required for Ndh complex assembly. \| chr1:28133540-28134738 REVERSE \| Aliases: F25A4.15, F25A4_15, NDH O | 4.1 | 2.7 | 1.4 | 5.8 | 0.46% | 5.6 |
| 3280 | AT1G54220.2 \| similar to dihydrolipoamide S-acetyltransferase, putative [Arabidopsis thaliana] (TAIR:At3g13930.1); similar to dihydrolipoamide S-acetyltransferase [Zea mays] (GB:AAD46491.1); similar to unknown protein [Oryza sativa (japonica cultivar-group)] (GB:XP_477668.1); similar to putative dihydrolipoamide S-acetyltransferase [Oryza sativa (japonica cultivar-group)] (GB:XP_463813.1); contains InterPro domain Catalytic domain of components of various dehydrogenase complexes (InterPro:IPR001078); contains InterPro domain E3 binding domain (InterPro:IPR004167); contains InterPro domain Dihydrolipoamide acetyltransferase, long form (InterPro:IPR006257); contains InterPro domain 2-oxo acid dehydrogenase, lipoyl-binding site (InterPro:IPR003016); contains InterPro domain Biotin/lipoyl attachment (InterPro:IPR000089) \| chr1:20249704-20253977 REVERSE \| Aliases: F20D21.4, F20D21_4 | 6.1 | 4.5 | 1.5 | 5.8 | 0.46% | 5.6 |
| 3283 | AT5G48300.1 \| Symbol: ADG1 \| glucose-1-phosphate adenylyltransferase small subunit 1 (APS1) / ADP-glucose pyrophosphorylase (ADG1), identical to SP:P55228 \| chr5:19587472-19590030 FORWARD \| Aliases: ADP GLUCOSE PYROPHOSPHORYLASE SMALL SUBUNIT, ADP GLUCOSE PYROPHOSPHORYLASE SMALL SUBUNIT 1, APS1, K23F3.2, K23F3_2 | 8.3 | 5.4 | 2.9 | 5.8 | 0.46% | 5.6 |
| 3285 | AT2G17800.1 \| Symbol: ARAC1 \| Rac-like GTP-binding protein (ARAC1) (ATGP2), identical to Rac-like GTP-binding protein (ARAC1) SP:Q38902 from (Arabidopsis thaliana) \| chr2:7746954-7749237 FORWARD \| Aliases: ATGP2, T17A5.14, T17A5_14 | 7.4 | 5.6 | 1.7 | 5.8 | 0.46% | 5.6 |
| 3288 | AT1G51650.1 \| ATP synthase epsilon chain, mitochondrial, identical to ATP synthase epsilon chain, mitochondrial SP:Q96253 from (Arabidopsis thaliana) \| chr1:19156283-19157597 FORWARD \| Aliases: F19C24.25, F19C24_25 | 12.1 | 11.5 | 0.6 | 5.8 | 0.46% | 5.2 |
| 3291 | AT5G63400.2 \| Symbol: ADK1 \| similar to adenylate kinase, putative [Arabidopsis thaliana] (TAIR:At5g50370.1); similar to adenylate kinase-a [Oryza sativa] (GB:BAA01180.1); similar to adenylate kinase [Oryza sativa] (GB:BAA94761.1); contains InterPro domain Adenylate kinase, active site lid (InterPro:IPR007862); contains InterPro domain Adenylate kinase (InterPro:IPR000850) \| chr5:25410227-25412090 REVERSE \| Aliases: ADENYLATE KINASE 1, MLE2.3, MLE2_3 | 9.8 | 7.7 | 2.1 | 5.8 | 0.46% | 5.6 |
| 3292 | AT3G19810.1 \| expressed protein, contains Pfam PF02620: Uncharacterized ACR, COG1399 \| chr3:6877581-6879405 REVERSE \| Aliases: MPN9.5 | 5.5 | 4.2 | 1.3 | 5.8 | 0.46% | 5.6 |

| Rank | Description | Sync | Root | M | t | adj.q | B |
|---|---|---|---|---|---|---|---|
| 3293 | AT1G48570.1 \| zinc finger (Ran-binding) family protein, contains Pfam domain, PF00641: Zn-finger in Ran binding protein and others \| chr1:17958528-17961387 REVERSE \| Aliases: T1N15.19, T1N15_19 | 5.2 | 3.3 | 1.9 | 5.8 | 0.46% | 5.5 |
| 3294 | AT3G15690.2 \| biotin carboxyl carrier protein of acetyl-CoA carboxylase-related, contains weak similarity to Biotin carboxyl carrier protein of acetyl-CoA carboxylase, chloroplast precursor (BCCP) (Swiss-Prot:Q42533) (Arabidopsis thaliana) \| chr3:5316948-5319615 FORWARD \| Aliases: MSJ11.9 | 7.2 | 4.7 | 2.6 | 5.8 | 0.46% | 5.5 |
| 3295 | AT5G37010.1 \| expressed protein \| chr5:14637962-14640171 REVERSE \| Aliases: K15O15.3, K15O15_3 | 7.4 | 6.2 | 1.2 | 5.8 | 0.46% | 5.6 |
| 3296 | AT5G42765.1 \| expressed protein \| chr5:17166762-17168907 REVERSE \| Aliases: None | 5.3 | 4.1 | 1.1 | 5.8 | 0.46% | 5.6 |
| 3298 | AT4G33700.1 \| CBS domain-containing protein, contains Pfam profiles PF00571: CBS domain, PF01595: Domain of unknown function \| chr4:16176403-16179482 REVERSE \| Aliases: T16L1.190, T16L1_190 | 8.0 | 6.7 | 1.3 | 5.8 | 0.46% | 5.5 |
| 3300 | AT5G48240.1 \| expressed protein, similar to hypothetical protein [Oryza sativa (japonica cultivar-group)] (GB:AAX96070.1) \| chr5:19576469-19578464 FORWARD \| Aliases: MIF21.13, MIF21_13 | 8.7 | 6.6 | 2.1 | 5.8 | 0.46% | 5.5 |
| 3301 | AT4G21800.2 \| ATP-binding family protein, contains Pfam domain, PF03029: Conserved hypothetical ATP binding protein \| chr4:11572877-11575504 FORWARD \| Aliases: F17L22.260, F17L22_260 | 6.5 | 5.2 | 1.3 | 5.8 | 0.46% | 5.6 |
| 3303 | AT3G43540.2 \| expressed protein, hypothetical protein slr1699 - Synechocystis sp. (strain PCC 6803), PIR:S75306 \| chr3:15441747-15444175 FORWARD \| Aliases: T18D12.110 | 6.4 | 3.6 | 2.8 | 5.8 | 0.47% | 5.5 |
| 3305 | AT3G42660.1 \| transducin family protein / WD-40 repeat family protein, contains 5 WD-40 repeats (PF00400); AND-1 protein - Homo sapiens, EMBL:AJ006266 | 7.2 | 4.4 | 2.7 | 5.8 | 0.47% | 5.5 |
| 3306 | AT1G74560.2 \| similar to nucleosome assembly protein (NAP) family protein [Arabidopsis thaliana] (TAIR:At1g18800.1); similar to nucleosome/chromatin assembly factor A [Zea mays] (GB:AAK67146.1); contains InterPro domain Nucleosome assembly protein (NAP) (InterPro:IPR002164) \| chr1:28021245-28023702 REVERSE \| Aliases: F1M20.24, F1M20_24 | 6.3 | 4.1 | 2.1 | 5.8 | 0.47% | 5.5 |
| 3307 | AT5G52390.1 \| photoassimilate-responsive protein, putative, similar to PIR:S62698 photoassimilate-responsive protein precursor (clone PAR-1a) - common tobacco | 6.5 | 2.2 | 4.3 | 5.8 | 0.47% | 4.7 |
| 3308 | AT1G16640.1 \| transcriptional factor B3 family protein, low similarity to reproductive meristem protein 1 (Arabidopsis thaliana) GI:13604227; contains Pfam profile PF02362: B3 DNA binding domain \| chr1:5686596-5687693 FORWARD \| Aliases: F19K19.7, F19K19_7 | 4.0 | 2.4 | 1.6 | 5.8 | 0.47% | 5.6 |
| 3309 | AT1G70280.2 \| NHL repeat-containing protein, contains Pfam profile PF01436: NHL repeat | 5.0 | 3.1 | 1.9 | 5.8 | 0.47% | 5.6 |
| 3310 | AT1G77020.1 \| DNAJ heat shock N-terminal domain-containing protein, similar to SP:P39101 CAJ1 protein (Saccharomyces cerevisiae); contains Pfam profile PF00226 DnaJ domain \| chr1:28949775-28951761 REVERSE \| Aliases: F22K20.12, F22K20_12 | 6.0 | 4.8 | 1.3 | 5.8 | 0.47% | 5.6 |
| 3314 | AT3G06700.3 \| similar to 60S ribosomal protein L29 (RPL29B) [Arabidopsis thaliana] (TAIR:At3g06680.1); similar to P0475H04.11 [Oryza sativa (japonica cultivar-group)] (GB:NP_914175.1); contains InterPro domain Ribosomal L29e protein (InterPro:IPR002673) \| chr3:2117361-2117960 REVERSE \| Aliases: T8E24.8 | 11.1 | 9.0 | 2.2 | 5.8 | 0.47% | 5.5 |
| 3315 | AT5G51010.1 \| rubredoxin family protein, similar to SP:P04170 Rubredoxin (Rd) {Desulfovibrio desulfuricans}; contains Pfam profile PF00301: Rubredoxin | 6.8 | 5.0 | 1.8 | 5.8 | 0.47% | 5.5 |
| 3317 | AT1G26770.1 \| Symbol: ATEXPA10 \| expansin, putative (EXP10), similar to expansin At-EXP1 GI:1041702 from (Arabidopsis thaliana); alpha-expansin gene family, PMID:11641069 \| chr1:9259592-9261300 FORWARD \| Aliases: ARABIDOPSIS THALIANA EXPANSIN A10, AT EXP10, ATEXP10, ATHEXP ALPHA 1.1, EXP10, EXPANSIN 10, T24P13.15, T24P13_15 | 5.4 | 3.0 | 2.4 | 5.8 | 0.47% | 5.5 |
| 3318 | AT1G63660.2 \| GMP synthase (glutamine-hydrolyzing), putative / glutamine amidotransferase, putative, similar to SP:P38625 GMP synthase (glutamine-hydrolyzing) (EC 6.3.5.2) (Glutamine amidotransferase) (GMP synthetase) {Saccharomyces cerevisiae}; contains Pfam profile PF00117: glutamine amidotransferase class-I \| chr1:23607602-23610938 REVERSE \| Aliases: F24D7.15, F24D7_15 | 8.0 | 6.4 | 1.7 | 5.8 | 0.47% | 5.6 |
| 3319 | AT1G76405.2 \| expressed protein \| chr1:28666817-28668683 FORWARD \| Aliases: None | 6.3 | 4.1 | 2.3 | 5.8 | 0.47% | 5.5 |
| 3322 | AT1G26910.1 \| 60S ribosomal protein L10 (RPL10B), Nearly identical to ribosomal protein L10.e, Wilm's tumor suppressor homologue, gi:17682 (Z15157), however differences in sequence indicate this is a different member of the L10 family \| chr1:9321637-9322947 FORWARD \| Aliases: T2P11.10, T2P11_10 | 7.5 | 6.0 | 1.5 | 5.8 | 0.47% | 5.5 |
| 3323 | AT1G53645.1 \| hydroxyproline-rich glycoprotein family protein \| chr1:20029841-20032303 REVERSE \| Aliases: None | 7.2 | 5.5 | 1.8 | 5.8 | 0.47% | 5.6 |

| Rank | Description | Sync | Root | M | t | adj.q | B |
|---|---|---|---|---|---|---|---|
| 3326 | AT4G36430.1 \| peroxidase, putative, identical to peroxidase (Arabidopsis thaliana) gi:6822093:emb:CAB71009; identical to cDNA class III peroxidase ATP31, GI:17530561 | 6.9 | 2.7 | 4.1 | 5.8 | 0.47% | 5.2 |
| 3328 | AT5G10980.1 \| histone H3, identical to HISTONE H3.2, MINOR, Medicago sativa, SWISSPROT:P11105, histone H3 variant H3.3 Lycopersicon esculentum GI:1435157; contains Pfam profile PF00125 Core histone H2A/H2B/H3/H4 \| chr5:3472429-3473442 REVERSE \| Aliases: T30N20.250, T30N20_250 | 11.8 | 10.8 | 1.0 | 5.8 | 0.47% | 5.3 |
| 3334 | AT3G25660.1 \| glutamyl-tRNA(Gln) amidotransferase, putative, similar to SP:O06491 Glutamyl-tRNA(Gln) amidotransferase subunit A (EC 6.3.5.-) {Bacillus subtilis}; contains Pfam profile PF01425: Amidase \| chr3:9340678-9343441 REVERSE \| Aliases: T5M7.15, T5M7_15 | 7.1 | 6.1 | 1.1 | 5.8 | 0.47% | 5.5 |
| 3335 | AT3G01660.1 \| expressed protein, similar to putative protein GB:CAB45319 (Arabidopsis thaliana) \| chr3:245485-246439 FORWARD \| Aliases: F4P13.20, F4P13_20 | 4.5 | 2.8 | 1.7 | 5.8 | 0.47% | 5.4 |
| 3336 | AT2G16200.1 \| expressed protein \| chr2:7031806-7032186 FORWARD \| Aliases: F7H1.22, F7H1_22 | 3.3 | 2.3 | 1.0 | 5.8 | 0.47% | 5.5 |
| 3339 | AT5G22300.1 \| Symbol: NIT4 \| nitrilase 4 (NIT4), identical to SP:P46011 Nitrilase 4 (EC 3.5.5.1) {Arabidopsis thaliana} \| chr5:7379221-7381928 FORWARD \| Aliases: NITRILASE 4, T6G21.6 | 5.2 | 3.4 | 1.8 | 5.8 | 0.48% | 5.3 |
| 3341 | AT3G47590.1 \| esterase/lipase/thioesterase family protein, low similarity to cinnamoyl ester hydrolase CinI (Butyrivibrio fibrisolvens) GI:1622732; contains Interpro entry IPR000379 \| chr3:17546605-17548508 REVERSE \| Aliases: F1P2.140 | 8.2 | 6.8 | 1.4 | 5.8 | 0.48% | 5.5 |
| 3343 | AT4G11120.1 \| translation elongation factor Ts (EF-Ts), putative, similar to ethylene-responsive elongation factor EF-Ts precursor (Lycopersicon esculentum) GI:5669636; contains Pfam profile PF00889: Elongation factor TS \| chr4:6777800-6780162 FORWARD \| Aliases: T22B4.100, T22B4_100 | 6.6 | 5.1 | 1.5 | 5.8 | 0.48% | 5.5 |
| 3344 | AT1G08360.1 \| 60S ribosomal protein L10A (RPL10aA), similar to 60S ribosomal protein L10A GB:AAC73045 GI:3860277 from (Arabidopsis thaliana) \| chr1:2636027-2637912 FORWARD \| Aliases: T27G7.6, T27G7_6 | 11.4 | 9.6 | 1.8 | 5.8 | 0.48% | 5.5 |
| 3345 | AT4G29060.2 \| Symbol: EMB2726 \| similar to translation elongation factor Ts (EF-Ts), putative [Arabidopsis thaliana] (TAIR:At4g11120.1); similar to elongation factor TS [Synechocystis sp. PCC 6803] (GB:NP_441466.1); contains InterPro domain Elongation factor Ts (InterPro:IPR001816); contains InterPro domain Ubiquitin-associated domain (InterPro:IPR000449); contains InterPro domain RNA binding S1 (InterPro:IPR003029); contains InterPro domain Ribosomal protein S1 (InterPro:IPR000110) \| chr4:14317262-14321573 FORWARD \| Aliases: EMB2726, EMBRYO DEFECTIVE 2726, F19B15.90, F19B15_90 | 8.2 | 4.7 | 3.5 | 5.8 | 0.48% | 5.5 |
| 3346 | AT5G58970.2 \| Symbol: ATUCP2 \| | 8.1 | 5.7 | 2.4 | 5.8 | 0.48% | 5.5 |
| 3347 | AT1G18650.1 \| glycosyl hydrolase family protein 17, similar to beta-1,3-glucanase GI:15150341 from (Camellia sinensis); C-terminal homology only \| chr1:6418852-6420521 REVERSE \| Aliases: F25I16.1, F25I16_1 | 4.1 | 2.9 | 1.3 | 5.8 | 0.48% | 5.5 |
| 3350 | AT1G28140.1 \| expressed protein, contains similarity to cytochrome oxidase I GI:1289267 from (Xantholinus sp.) | 6.3 | 4.8 | 1.6 | 5.8 | 0.48% | 5.5 |
| 3352 | AT3G16480.1 \| Symbol: MPPALPHA \| mitochondrial processing peptidase alpha subunit, putative, similar to mitochondrial processing peptidase alpha subunit, mitochondrial precursor, Alpha-MPP (Ubiquinol-cytochrome C reductase subunit II) (Potato) SWISS-PROT:P29677 | 7.5 | 4.6 | 2.9 | 5.8 | 0.48% | 5.5 |
| 3354 | AT3G26900.1 \| shikimate kinase family protein, similar to shikimate kinase precursor GB:CAA45121 (Lycopersicon esculentum); contains Pfam shikimate kinase domain PF01202 \| chr3:9913449-9915715 REVERSE \| Aliases: MDJ14.24 | 7.0 | 5.4 | 1.6 | 5.8 | 0.49% | 5.5 |
| 3357 | AT4G21870.1 \| 26.5 kDa class P-related heat shock protein (HSP26.5-P), contains Pfam profile: PF00011 Hsp20/alpha crystallin family: identified in Scharf, K-D., et al,Cell Stress & Chaperones (2001) 6: 225-237. \| chr4:11603582-11604343 REVERSE \| Aliases: T8O5.80, T8O5_80 | 7.3 | 5.6 | 1.7 | 5.8 | 0.49% | 5.5 |
| 3358 | AT3G48540.1 \| cytidine/deoxycytidylate deaminase family protein, similar to SP:P32321 Deoxycytidylate deaminase (EC 3.5.4.12) (dCMP deaminase) {Homo sapiens}; contains Pfam profile PF00383: Cytidine and deoxycytidylate deaminase zinc-binding region \| chr3:18000783-18003000 REVERSE \| Aliases: T8P19.50 | 5.0 | 3.4 | 1.6 | 5.8 | 0.49% | 5.5 |
| 3361 | AT1G54050.1 \| 17.4 kDa class III heat shock protein (HSP17.4-CIII), contains Pfam profile: PF00011 Hsp20/alpha crystallin family; identified as class CIII in Scharf, K-D., et al,Cell Stress & Chaperones (2001) 6: 225-237. \| chr1:20183090-20183947 REVERSE \| Aliases: F15I1.13, F15I1_13 | 5.6 | 4.0 | 1.5 | 5.8 | 0.49% | 5.5 |
| 3362 | AT3G18850.3 \| Symbol: LPAT5 \| similar to phospholipid/glycerol acyltransferase family protein [Arabidopsis thaliana] (TAIR:At1g75020.2); similar to phospholipid/glycerol acyltransferase family protein [Arabidopsis thaliana] (TAIR:At1g75020.1); similar to putative acyl-CoA:1-acylglycerol-3-phosphate acyltransferase [Oryza sativa (japonica cultivar-group)] (GB:BAD53283.1); contains InterPro domain Phospholipid/glycerol acyltransferase (InterPro:IPR002123) \| chr3:6499421-6501345 REVERSE \| Aliases: LPAT5, MCB22.2 | 5.0 | 3.5 | 1.5 | 5.8 | 0.49% | 5.5 |
| 3363 | AT4G33510.1 \| Symbol: DHS2 \| 2-dehydro-3-deoxyphosphoheptonate aldolase 2 / 3-deoxy-D-arabino-heptulosonate 7-phosphate synthase 2 / DAHP synthetase 2 (DHS2), nearly identical to SP:Q00218 \| chr4:16116450-16118800 FORWARD \| Aliases: 3 DEOXY D ARABINO HEPTULOSONATE 7 PHOSPHATE SYNTHASE, F17M5.270, F17M5_270 | 10.2 | 8.8 | 1.4 | 5.8 | 0.49% | 5.5 |

| Rank | Description | Sync | Root | M | t | adj.q | B |
|---|---|---|---|---|---|---|---|
| 3365 | AT2G26680.1 \| expressed protein, similar to NLPE1 (GI:13022100) (Rhizobium etli); \| chr2:11350879-11352480 REVERSE \| Aliases: F18A8.5, F18A8_5 | 5.2 | 4.0 | 1.2 | 5.7 | 0.49% | 5.5 |
| 3366 | AT5G66000.1 \| expressed protein \| chr5:26413171-26414726 REVERSE \| Aliases: K2A18.6, K2A18_6 | 4.9 | 3.5 | 1.3 | 5.7 | 0.49% | 5.5 |
| 3368 | AT4G33350.1 \| chloroplast inner membrane import protein Tic22, putative, similar to Tic22 (Pisum sativum) gi:3769671:gb:AAC64606 \| chr4:16064552-16066740 REVERSE \| Aliases: F17M5.110, F17M5_110 | 6.2 | 4.5 | 1.7 | 5.7 | 0.49% | 5.5 |
| 3369 | AT1G26570.1 \| UDP-glucose 6-dehydrogenase, putative, strong similarity to SP:Q96558 UDP-glucose 6-dehydrogenase (EC 1.1.1.22) (UDP-Glc dehydrogenase) (UDP-GlcDH) (UDPGDH) {Glycine max}; contains Pfam profiles PF03721: UDP-glucose/GDP-mannose dehydrogenase family NAD binding domain, PF00984: UDP-glucose/GDP-mannose dehydrogenase family central domain, PF03720: UDP-glucose/GDP-mannose dehydrogenase family UDP binding domain \| chr1:9182191-9184380 FORWARD \| Aliases: T1K7.6, T1K7_6 | 5.0 | 3.7 | 1.2 | 5.7 | 0.49% | 5.5 |
| 3370 | AT4G21550.1 \| transcriptional factor B3 family protein, low similarity to SP:Q01593 Abscisic acid-insensitive protein 3 {Arabidopsis thaliana}, SP:P37398 Viviparous protein homolog {Oryza sativa}; contains Pfam profile PF02362: B3 DNA binding domain \| chr4:11463999-11468691 FORWARD \| Aliases: F18E5.170, F18E5_170 | 4.6 | 3.2 | 1.4 | 5.7 | 0.49% | 5.5 |
| 3372 | AT1G48580.1 \| expressed protein \| chr1:17961931-17964278 REVERSE \| Aliases: T1N15.20, T1N15_20 | 4.0 | 2.8 | 1.2 | 5.7 | 0.49% | 5.5 |
| 3373 | AT5G52470.1 \| Symbol: FIB1 \| fibrillarin 1 (FBR1) (FIB1) (SKIP7), identical to fibrillarin 1 GI:9965653 from (Arabidopsis thaliana); C-terminus identical to SKP1 interacting partner 7 GI:10716959 from (Arabidopsis thaliana); contains Pfam domain PF01269: Fibrillarin \| chr5:21311454-21313902 FORWARD \| Aliases: ATFBR1, ATFIB1, FBR1, FIBRILLARIN 1, K24M7.22, K24M7_22, SKIP7, SKP1 INTERACTING PARTNER 7 | 11.2 | 10.4 | 0.8 | 5.7 | 0.49% | 5.2 |
| 3374 | AT5G50160.1 \| ferric reductase-like transmembrane component family protein, contains Pfam profile PF01794: Ferric reductase like transmembrane component \| chr5:20432997-20435930 FORWARD \| Aliases: K6A12.2, K6A12_2 | 5.6 | 2.7 | 2.8 | 5.7 | 0.50% | 4.9 |
| 3376 | AT4G02230.1 \| 60S ribosomal protein L19 (RPL19C), similar to L19 from several species \| chr4:979229-980667 REVERSE \| Aliases: T2H3.3, T2H3_3 | 10.6 | 8.3 | 2.3 | 5.7 | 0.50% | 5.5 |
| 3379 | AT1G24490.1 \| Symbol: ARTEMIS \| 60 kDa inner membrane family protein, similar to chloroplast membrane protein (ALBINO3) (GI:3927828) (Arabidopsis thaliana) \| chr1:8678846-8685262 FORWARD \| Aliases: ARABIDOPSIS THALIANA ENVELOPE MEMBRANE INTEGRASE, F21J9.16 | 6.1 | 4.5 | 1.6 | 5.7 | 0.50% | 5.5 |
| 3380 | AT5G57330.1 \| aldose 1-epimerase family protein, contains Pfam profile PF01263 Aldose 1-epimerase \| chr5:23235440-23238315 FORWARD \| Aliases: MJB24.14, MJB24_14 | 9.0 | 6.7 | 2.4 | 5.7 | 0.50% | 5.5 |
| 3383 | AT5G28060.1 \| 40S ribosomal protein S24 (RPS24B), 40S ribosomal protein S19, Cyanophora paradoxa, EMBL:CPA245654 \| chr5:10069553-10070897 REVERSE \| Aliases: F15F15.130, F15F15_130 | 9.2 | 6.9 | 2.3 | 5.7 | 0.50% | 5.5 |
| 3384 | AT2G37600.1 \| 60S ribosomal protein L36 (RPL36A) \| chr2:15781323-15782420 REVERSE \| Aliases: F13M22.10, F13M22_10 | 6.2 | 3.7 | 2.5 | 5.7 | 0.50% | 5.5 |
| 3386 | AT1G62820.1 \| calmodulin, putative, similar to calmodulin SP:P04465 from (Trypanosoma brucei gambiense); contains INTERPRO:IPR002048 calcium-binding EF-hand domain \| chr1:23267336-23268014 REVERSE \| Aliases: F23N19.25, F23N19_25 | 6.9 | 5.4 | 1.5 | 5.7 | 0.50% | 5.5 |
| 3392 | AT5G51280.1 \| DEAD-box protein abstrakt, putative \| chr5:20858474-20861032 FORWARD \| Aliases: MWD22.23, MWD22_23 | 8.7 | 7.1 | 1.6 | 5.7 | 0.50% | 5.5 |
| 3399 | AT5G53070.1 \| ribosomal protein L9 family protein, contains similarity to ribosomal protein L9 | 4.6 | 2.9 | 1.6 | 5.7 | 0.50% | 5.4 |
| 3402 | AT4G24175.1 \| expressed protein \| chr4:12546979-12548968 FORWARD \| Aliases: None | 4.9 | 3.4 | 1.5 | 5.7 | 0.50% | 5.5 |
| 3404 | AT1G69920.1 \| Symbol: ATGSTU12 \| glutathione S-transferase, putative, similar to glutathione transferase GB:CAA09188 (Alopecurus myosuroides); supported by cDNA gi:15451157 gb:AY050343. \| chr1:26337913-26339206 REVERSE \| Aliases: T17F3.5, T17F3_5 | 5.5 | 3.0 | 2.5 | 5.7 | 0.50% | 4.8 |
| 3405 | AT5G16840.1 \| RNA recognition motif (RRM)-containing protein, predicted proteins - Arabidopsis thaliana \| chr5:5535923-5538253 FORWARD \| Aliases: F5E19.180, F5E19_180 | 9.5 | 7.2 | 2.2 | 5.7 | 0.50% | 5.5 |
| 3406 | AT2G35300.1 \| late embryogenesis abundant group 1 domain-containing protein / LEA group 1 domain-containing protein, contains Pfam domain, PF03760: Late embryogenesis abundant (LEA) group 1 \| chr2:14869980-14870466 REVERSE \| Aliases: T4C15.3, T4C15_3 | 3.3 | 2.4 | 0.9 | 5.7 | 0.50% | 5.3 |
| 3407 | AT1G09210.1 \| calreticulin 2 (CRT2), identical to SP:Q38858 Calreticulin 2 precursor {Arabidopsis thaliana} \| chr1:2972844-2976731 REVERSE \| Aliases: T12M4.8, T12M4_8 | 8.5 | 6.6 | 1.9 | 5.7 | 0.50% | 5.5 |
| 3408 | AT5G17560.1 \| BolA-like family protein, contains Pfam profile: PF01722 BolA-like protein \| chr5:5788456-5789925 FORWARD \| Aliases: K10A8.40, K10A8_40 | 6.7 | 5.2 | 1.5 | 5.7 | 0.50% | 5.5 |

| Rank | Description | Sync | Root | M | t | adj.q | B |
|---|---|---|---|---|---|---|---|
| 3410 | AT5G01230.2 \| FtsJ-like methyltransferase family protein, contains Pfam profile: PF01728 FtsJ-like methyltransferase \| chr5:92600-95685 REVERSE \| Aliases: F7J8.210, F7J8_210 | 6.0 | 4.9 | 1.2 | 5.7 | 0.50% | 5.4 |
| 3414 | AT4G28490.1 \| Symbol: HAESA \| leucine-rich repeat transmembrane protein kinase, putative \| chr4:14077863-14081177 FORWARD \| Aliases: F20O9.180, F20O9_180, RECEPTOR LIKE PROTEIN KINASE 5, RLK5 | 5.1 | 3.3 | 1.9 | 5.7 | 0.51% | 5.4 |
| 3415 | AT2G13560.1 \| malate oxidoreductase, putative, similar to NAD-dependent malic enzyme 62 kDa isoform, mitochondrial precursor (EC 1.1.1.39) (NAD-ME) (SP:P37221) {Solanum tuberosum} \| chr2:5657046-5662301 FORWARD \| Aliases: T10F5.10, T10F5_10 | 10.7 | 9.6 | 1.2 | 5.7 | 0.51% | 5.4 |
| 3416 | AT4G35840.1 \| zinc finger (C3HC4-type RING finger) family protein, contains a TG non-consensus donor splice site at exon 2; contains Pfam profile PF00097: Zinc finger, C3HC4 type (RING finger) \| chr4:16980965-16982368 FORWARD \| Aliases: F4B14.110, F4B14_110 | 8.6 | 6.3 | 2.4 | 5.7 | 0.51% | 5.5 |
| 3417 | AT2G01120.1 \| origin recognition complex subunit 4, putative (ORC4), similar to origin recognition complex subunit 4 (GI:15866782) (Zea mays) \| chr2:87317-88122 FORWARD \| Aliases: F23H14.9, F23H14_9 | 4.9 | 3.1 | 1.9 | 5.7 | 0.51% | 5.4 |
| 3418 | AT1G63980.2 \| similar to aldose 1-epimerase family protein [Arabidopsis thaliana] (TAIR:At5g15140.1); similar to CG11180-PA [Drosophila melanogaster] (GB:NP_611495.1); contains InterPro domain D111/G-patch domain (InterPro:IPR000467) \| chr1:23744225-23746965 FORWARD \| Aliases: F22C12.25, F22C12_25 | 9.2 | 7.4 | 1.8 | 5.7 | 0.51% | 5.5 |
| 3424 | AT1G06950.1 \| Symbol: ATTIC110/TIC110 \| Encodes a protein thought to be a part of the translocon at the chloroplast inner envelope. Involved in protein import into the chloroplast and chloroplast biogenesis. \| chr1:2130154-2135664 REVERSE \| Aliases: ATTIC110, F10K1.33, TIC110 | 8.4 | 5.9 | 2.6 | 5.7 | 0.51% | 5.5 |
| 3425 | AT3G04950.1 \| expressed protein, contains InterPro domain SEC-C motif (InterPro:IPR004027) \| chr3:1371711-1373483 FORWARD \| Aliases: T9J14.10, T9J14_10 | 3.4 | 2.2 | 1.3 | 5.7 | 0.51% | 5.5 |
| 3428 | AT4G25130.1 \| peptide methionine sulfoxide reductase, putative, strong similarity to SP:P54151 Peptide methionine sulfoxide reductase (EC 1.8.4.6) {Brassica napus}; contains Pfam profile PF01625: Peptide methionine sulfoxide reductase \| chr4:12898603-12900074 REVERSE \| Aliases: F24A6.2 | 9.9 | 6.8 | 3.2 | 5.7 | 0.51% | 5.4 |
| 3429 | AT4G18730.1 \| Symbol: RPL16B \| | 10.1 | 8.0 | 2.1 | 5.7 | 0.51% | 5.5 |
| 3430 | AT5G45775.2 \| 60S ribosomal protein L11 (RPL11D) \| chr5:18582292-18583785 REVERSE \| Aliases: None | 10.1 | 8.0 | 2.1 | 5.7 | 0.51% | 5.5 |
| 3431 | AT5G62630.1 \| expressed protein \| chr5:25160821-25163685 REVERSE \| Aliases: MRG21.5, MRG21_5 | 8.9 | 7.1 | 1.8 | 5.7 | 0.51% | 5.5 |
| 3432 | AT3G07440.1 \| expressed protein \| chr3:2381376-2383146 FORWARD \| Aliases: F21O3.15 | 5.2 | 3.5 | 1.7 | 5.7 | 0.51% | 5.3 |
| 3433 | AT5G38900.1 \| DSBA oxidoreductase family protein, contains Pfam profile: PF01323 DSBA-like thioredoxin domain \| chr5:15590735-15592848 REVERSE \| Aliases: K15E6.80, K15E6_80 | 6.1 | 4.1 | 2.0 | 5.7 | 0.51% | 5.3 |
| 3435 | NA | 9.7 | 4.3 | 5.3 | 5.7 | 0.51% | 5.2 |
| 3436 | AT4G11100.1 \| expressed protein \| chr4:6768611-6770180 FORWARD \| Aliases: T22B4.80, T22B4_80 | 3.4 | 2.5 | 0.8 | 5.7 | 0.51% | 5.4 |
| 3437 | AT2G20260.1 \| photosystem I reaction center subunit IV, chloroplast, putative / PSI-E, putative (PSAE2), identical to SP:Q9S714; similar to SP:P12354 Photosystem I reaction center subunit IV, chloroplast precursor (PSI-E) {Spinacia oleracea}; contains Pfam profile PF02427: Photosystem I reaction centre subunit IV / PsaE \| chr2:8743818-8744843 FORWARD \| Aliases: F11A3.19, F11A3_19 | 7.0 | 5.3 | 1.6 | 5.7 | 0.51% | 5.5 |
| 3438 | AT5G03455.1 \| Symbol: CDC25 \| rhodanese-like domain-containing protein, contains Rhodanese-like domain PF:00581 \| chr5:862558-864107 FORWARD \| Aliases: ARATH;CDC25 | 10.2 | 9.0 | 1.2 | 5.7 | 0.51% | 5.2 |
| 3440 | AT3G07170.1 \| sterile alpha motif (SAM) domain-containing protein, contains Pfam profile PF00536: SAM domain (Sterile alpha motif) \| chr3:2280207-2282344 FORWARD \| Aliases: T1B9.17 | 8.4 | 6.7 | 1.7 | 5.7 | 0.51% | 5.4 |
| 3441 | AT2G32830.1 \| Symbol: PHT5 \| inorganic phosphate transporter (PHT5), identical to inorganic phosphate transporter (PHT5) (Arabidopsis thaliana) GI:2780345 \| chr2:13934776-13936790 REVERSE \| Aliases: F24L7.2 | 5.6 | 3.3 | 2.3 | 5.7 | 0.51% | 4.9 |
| 3443 | AT5G18140.1 \| DNAJ heat shock N-terminal domain-containing protein, similar to DnaJ protein Tid-1 (Homo sapiens) GI:17066575; contains Pfam profile PF00226 DnaJ domain \| chr5:5998201-5999890 FORWARD \| Aliases: MRG7.10, MRG7_10 | 7.6 | 6.1 | 1.5 | 5.7 | 0.51% | 5.5 |
| 3444 | AT1G03820.1 \| expressed protein, similar to arabinogalactan-protein; AGP (GI:1087015) {Pyrus communis}; similar to Protein E6. (SP:Q01197) {Gossypium hirsutum} \| chr1:959927-960708 REVERSE \| Aliases: F21M11.27, F21M11_27 | 5.9 | 2.9 | 3.0 | 5.7 | 0.51% | 5.4 |

| Rank | Description | Sync | Root | M | t | adj.q | B |
|---|---|---|---|---|---|---|---|
| 3445 | AT3G01130.1 \| expressed protein \| chr3:44491-45597 REVERSE \| Aliases: T4P13.18, T4P13_18 | 12.3 | 11.2 | 1.1 | 5.7 | 0.51% | 4.6 |
| 3448 | AT5G17670.1 \| expressed protein \| chr5:5821062-5822738 FORWARD \| Aliases: MVA3.2, MVA3_2 | 7.4 | 4.9 | 2.5 | 5.7 | 0.51% | 5.3 |
| 3453 | AT5G26670.2 \| pectinacetylesterase, putative, similar to pectinacetylesterase precursor GI:1431629 from (Vigna radiata) \| chr5:9318283-9320990 FORWARD \| Aliases: None | 5.9 | 4.6 | 1.3 | 5.7 | 0.52% | 5.4 |
| 3454 | AT3G62810.1 \| complex 1 family protein / LVR family protein, contains Pfam PF05347: Complex 1 protein (LYR family) \| chr3:23238683-23239341 FORWARD \| Aliases: F26K9.240 | 10.5 | 7.4 | 3.1 | 5.7 | 0.52% | 5.4 |
| 3460 | AT1G07370.1 \| Symbol: PCNA1 \| proliferating cell nuclear antigen 1 (PCNA1), identical to SP:Q9M7Q7 Proliferating cellular nuclear antigen 1 (PCNA 1) {Arabidopsis thaliana}; nearly identical to SP:Q43124 Proliferating cell nuclear antigen (PCNA) {Brassica napus}; contains Pfam profiles PF00705: Proliferating cell nuclear antigen N-terminal domain, PF02747: Proliferating cell nuclear antigen C-terminal domain \| chr1:2263140-2264549 FORWARD \| Aliases: F22G5.29, F22G5_29, PROLIFERATING CELLULAR NUCLEAR ANTIGEN | 6.9 | 4.4 | 2.5 | 5.7 | 0.52% | 5.4 |
| 3462 | AT5G43430.2 \| expressed protein, similar to hypothetical protein DDB0204353 [Dictyostelium discoideum] (GB:EAL68168.1); contains InterPro domain Electron transfer flavoprotein beta-subunit (InterPro:IPR000049) \| chr5:17470826-17472918 FORWARD \| Aliases: MWF20.14, MWF20_14 | 6.4 | 4.4 | 2.1 | 5.7 | 0.52% | 5.4 |
| 3463 | AT1G11720.1 \| starch synthase, putative, strong similarity to soluble-starch-synthase (Solanum tuberosum) GI:1911166 \| chr1:3952511-3957044 FORWARD \| Aliases: F25C20.13, F25C20_13 | 5.1 | 3.6 | 1.5 | 5.7 | 0.52% | 5.4 |
| 3464 | AT5G46030.1 \| expressed protein, contains Pfam profile PF05129: Putative zinc binding domain (DUF701) \| chr5:18687356-18688566 FORWARD \| Aliases: MCL19.8, MCL19_8 | 9.6 | 8.4 | 1.2 | 5.7 | 0.52% | 5.4 |
| 3465 | AT1G12650.2 \| similar to similar to CG8461-PA [Apis mellifera] (GB:XP_394556.1); contains domain LYS_RICH (PS50318) \| chr1:4305958-4307957 FORWARD \| Aliases: T12C24.18, T12C24_18 | 8.2 | 6.2 | 2.0 | 5.7 | 0.52% | 5.4 |
| 3467 | AT4G23885.1 \| expressed protein \| chr4:12419404-12420477 FORWARD \| Aliases: None | 9.4 | 8.2 | 1.2 | 5.7 | 0.52% | 5.4 |
| 3468 | AT3G14790.1 \| Symbol: RHM3 \| NAD-dependent epimerase/dehydratase family protein, similar to dTDP-glucose 4,6-dehydratase from Aneurinibacillus thermoaerophilus GI:16357461, Saccharopolyspora spinosa GI:15077647, RmlB from Leptospira borgpetersenii GI:4234803; contains Pfam profile PF01370 NAD dependent epimerase/dehydratase family \| chr3:4964162-4967066 FORWARD \| Aliases: RHAMNOSE BIOSYNTHESIS 3, T21E2.5 | 7.0 | 5.5 | 1.5 | 5.7 | 0.52% | 5.4 |
| 3470 | AT3G11630.1 \| 2-cys peroxiredoxin, chloroplast (BAS1), identical to SP:Q96291 2-cys peroxiredoxin BAS1, chloroplast precursor {Arabidopsis thaliana}; contains Pfam profile: PF00578 AhpC/TSA (alkyl hydroperoxide reductase and thiol-specific antioxidant) family \| chr3:3672126-3674081 FORWARD \| Aliases: F24K9.28, F24K9_28, T19F11.3 | 10.5 | 7.1 | 3.3 | 5.7 | 0.52% | 5.4 |
| 3472 | AT4G37430.1 \| Symbol: CYP91A2 \| cytochrome P450 81F1 (CYP81F1) (CYP91A2), identical to cytochrome P450 81F1 (91A2) (SP:O65790) (Arabidopsis thaliana) | 5.2 | 4.2 | 1.1 | 5.7 | 0.52% | 5.4 |
| 3476 | AT3G07568.1 \| expressed protein \| chr3:2416249-2417796 REVERSE \| Aliases: None | 10.5 | 8.4 | 2.1 | 5.7 | 0.52% | 5.4 |
| 3479 | AT1G02780.1 \| Symbol: EMB2386 \| 60S ribosomal protein L19 (RPL19A), similar to ribosomal protein L19 GI:36127 from (Homo sapiens) \| chr1:607821-609435 REVERSE \| Aliases: EMB2386, EMBRYO DEFECTIVE 2386, T14P4.34 | 11.7 | 9.1 | 2.5 | 5.7 | 0.52% | 5.4 |
| 3480 | AT5G16040.1 \| regulator of chromosome condensation (RCC1) family protein, similar to UVB-resistance protein UVR8 (Arabidopsis thaliana) GI:5478530; contains Pfam profile PF00415: Regulator of chromosome condensation (RCC1) | 8.7 | 6.6 | 2.1 | 5.7 | 0.52% | 5.4 |
| 3481 | AT2G34050.1 \| expressed protein, contains Pfam PS00030: Eukaryotic putative RNA-binding region RNP-1 signature | 6.2 | 3.8 | 2.4 | 5.7 | 0.52% | 5.0 |
| 3483 | AT5G22440.1 \| 60S ribosomal protein L10A (RPL10aC) \| chr5:7435128-7436642 REVERSE \| Aliases: MWD9.24, MWD9_24 | 7.7 | 5.3 | 2.4 | 5.7 | 0.52% | 5.4 |
| 3484 | AT5G41960.1 \| expressed protein \| chr5:16806942-16808270 FORWARD \| Aliases: MJC20.6, MJC20_6 | 7.6 | 6.4 | 1.2 | 5.7 | 0.52% | 5.4 |
| 3485 | AT1G06380.1 \| ribosomal protein-related, similar to PBK1 protein (GI:3668141) {Homo sapiens}; weakly similar to 60S ribosomal protein L10a. (SP:Q963B6) (Fall armyworm) {Spodoptera frugiperda} \| chr1:1945123-1946075 FORWARD \| Aliases: T2D23.8, T2D23_8 | 5.1 | 3.8 | 1.3 | 5.7 | 0.53% | 5.4 |
| 3490 | AT5G42300.1 \| ubiquitin family protein, contains INTERPRO:IPR000626 ubiquitin domain \| chr5:16929561-16931158 REVERSE \| Aliases: K5J14.10, K5J14_10 | 13.3 | 12.7 | 0.6 | 5.7 | 0.53% | 4.9 |
| 3491 | AT5G43450.1 \| 2-oxoglutarate-dependent dioxygenase, putative, similar to 2A6 (GI:599622) and tomato ethylene synthesis regulatory protein E8 (SP:P10967) \| chr5:17474359-17476025 REVERSE \| Aliases: MWF20.16, MWF20_16 | 4.5 | 3.3 | 1.2 | 5.7 | 0.53% | 5.4 |

| Rank | Description | Sync | Root | M | t | adj.q | B |
|---|---|---|---|---|---|---|---|
| 3492 | AT1G69370.1 \| Symbol: CM3 \| chorismate mutase, putative, similar to gi:5732016 and SP:P42738; contains Pfam profile: PF01817: Chorismate mutase \| chr1:26083619-26085390 FORWARD \| Aliases: CHORISMATE MUTASE 3, CM 3, CM3, F10D13.6, F10D13_6 | 7.5 | 4.8 | 2.7 | 5.7 | 0.53% | 5.3 |
| 3495 | AT2G01870.1 \| expressed protein \| chr2:389469-390376 REVERSE \| Aliases: T23K3.6, T23K3_6 | 3.6 | 2.5 | 1.1 | 5.6 | 0.53% | 5.3 |
| 3496 | AT2G43190.3 \| ribonuclease P family protein, similar to Ribonuclease P protein subunit p29 (EC 3.1.26.5) (hPOP4) (Swiss-Prot:O95707) (Homo sapiens) \| chr2:17963066-17965118 FORWARD \| Aliases: F14B2.13 | 7.5 | 6.1 | 1.4 | 5.6 | 0.53% | 5.4 |
| 3499 | AT3G25120.1 \| mitochondrial import inner membrane translocase subunit Tim17/Tim22/Tim23 family protein, contains Pfam PF02466: Mitochondrial import inner membrane translocase subunit Tim17 \| chr3:9149551-9151112 REVERSE \| Aliases: MJL12.6 | 7.3 | 5.3 | 2.0 | 5.6 | 0.53% | 5.2 |
| 3500 | AT3G54430.1 \| lateral root primordium (LRP) protein-related, similar to lateral root primordium 1 (LRP1) (Arabidopsis thaliana) GI:882341; contains Pfam profile PF05142: Domain of unknown function (DUF702) \| chr3:20158079-20159105 REVERSE \| Aliases: T14E10.2 | 6.0 | 4.7 | 1.3 | 5.6 | 0.53% | 5.4 |
| 3502 | AT2G29450.1 \| Symbol: ATGSTU5 \| glutathione S-transferase (103-1A), identical to Swiss-Prot:P46421 glutathione S-transferase 103-1A (Arabidopsis thaliana) \| chr2:12631663-12632711 REVERSE \| Aliases: AT103 1A, ATGSTU1, F16P2.17, F16P2_17, GLUTATHIONE S TRANSFERASE | 6.0 | 4.0 | 2.0 | 5.6 | 0.53% | 5.4 |
| 3503 | AT1G18680.1 \| HNH endonuclease domain-containing protein, contains Pfam domain PF01844: HNH endonuclease \| chr1:6431930-6433437 REVERSE \| Aliases: F6A14.21, F6A14_21 | 6.0 | 4.9 | 1.1 | 5.6 | 0.53% | 5.4 |
| 3505 | AT2G26930.1 \| Symbol: ATCDPMEK \| 4-diphosphocytidyl-2-C-methyl-D-erythritol kinase (CMK) (CDPMEK), identical to 4-diphosphocytidyl-2-C-methyl-D-erythritol kinase, chloroplast precursor (CMK) (4-(cytidine-5'-diphospho)-2-C-methyl- D-erythritol kinase)(CDPMEK) (Arabidopsis thaliana) SWISS-PROT:O81014 \| chr2:11498299-11501324 REVERSE \| Aliases: 4 (CYTIDINE 5' PHOSPHO) 2 C METHYL D ERITHRITOL KINASE, 4 DIPHOSPHOCYTIDYL 2C METHYL D ERYTHRITOL KINASE, F12C20.3, F12C20_3, ISPE, PDE277, PIGMENT DEFECTIVE 277 | 5.2 | 3.6 | 1.5 | 5.6 | 0.53% | 5.4 |
| 3506 | AT3G01060.3 \| expressed protein \| chr3:16650-18847 REVERSE \| Aliases: T4P13.26, T4P13_26 | 4.8 | 3.8 | 1.0 | 5.6 | 0.53% | 5.4 |
| 3507 | AT5G13310.1 \| expressed protein \| chr5:4263496-4265340 FORWARD \| Aliases: T22N19.1 | 5.2 | 4.2 | 1.0 | 5.6 | 0.53% | 5.3 |
| 3509 | AT1G01070.2 \| nodulin MtN21 family protein, similar to MtN21 GI:2598575 (root nodule development) from (Medicago truncatula) \| chr1:38753-40927 REVERSE \| Aliases: T25K16.7, T25K16_7 | 9.3 | 7.0 | 2.2 | 5.6 | 0.54% | 5.3 |
| 3510 | AT3G13230.1 \| expressed protein \| chr3:4269985-4271211 FORWARD \| Aliases: MDC11.5 | 6.3 | 4.4 | 1.9 | 5.6 | 0.54% | 5.4 |
| 3511 | AT3G47560.2 \| esterase/lipase/thioesterase family protein, low similarity to cinnamoyl ester hydrolase CinI (Butyrivibrio fibrisolvens) GI:1622732; contains Interpro entry IPR000379 \| chr3:17536162-17537963 REVERSE \| Aliases: F1P2.110 | 7.1 | 4.7 | 2.3 | 5.6 | 0.54% | 5.3 |
| 3512 | AT2G36930.1 \| zinc finger (C2H2 type) family protein, contains Prosite PS00028: Zinc finger, C2H2 type, domain; weak similarity to Zinc finger protein T86 (Swiss-Prot:O00488) (Homo sapiens) \| chr2:15516981-15518584 REVERSE \| Aliases: T1J8.11, T1J8_11 | 9.0 | 7.0 | 2.0 | 5.6 | 0.54% | 5.4 |
| 3514 | AT3G09250.1 \| expressed protein \| chr3:2839007-2840871 FORWARD \| Aliases: F3L24.12 | 5.8 | 4.7 | 1.1 | 5.6 | 0.54% | 5.4 |
| 3516 | AT5G02820.1 \| Symbol: RHL2 \| DNA topoisomerase VIA (SPO11), identical to topoisomerase 6 subunit A (spo11) (Arabidopsis thaliana) GI:12331186 | 7.4 | 5.6 | 1.8 | 5.6 | 0.54% | 5.3 |
| 3518 | AT2G31410.1 \| expressed protein \| chr2:13399099-13399928 REVERSE \| Aliases: T28P16.10, T28P16_10 | 5.1 | 3.5 | 1.6 | 5.6 | 0.55% | 5.4 |
| 3519 | AT3G59540.1 \| 60S ribosomal protein L38 (RPL38B), 60S RIBOSOMAL PROTEIN L38 - Lycopersicon esculentum, EMBL:X69979 \| chr3:22006655-22007783 REVERSE \| Aliases: T16L24.90 | 10.3 | 7.7 | 2.6 | 5.6 | 0.55% | 5.4 |
| 3522 | AT2G15690.1 \| pentatricopeptide (PPR) repeat-containing protein, contains Pfam profile PF01535: PPR repeat \| chr2:6838077-6840859 REVERSE \| Aliases: F9O13.34 | 6.2 | 4.3 | 1.9 | 5.6 | 0.55% | 5.2 |
| 3523 | AT2G33250.1 \| expressed protein \| chr2:14104509-14105788 FORWARD \| Aliases: F25I18.1, F25I18_1 | 9.2 | 7.8 | 1.3 | 5.6 | 0.55% | 5.3 |
| 3524 | AT1G73430.1 \| sec34-like family protein, contains Pfam PF04136: Sec34-like family profile; similar to Conserved oligomeric Golgi complex component 3 (Vesicle docking protein SEC34 homolog) (p94) (Swiss-Prot:Q96JB2) (Homo sapiens) \| chr1:27607316-27614729 FORWARD \| Aliases: T9L24.37, T9L24_37 | 5.4 | 4.3 | 1.2 | 5.6 | 0.55% | 5.3 |
| 3525 | AT2G28450.1 \| zinc finger (CCCH-type) family protein, contains Pfam domain, PF00642: Zinc finger C-x8-C-x5-C-x3-H type (and similar) \| chr2:12169128-12172971 REVERSE \| Aliases: T1B3.3, T1B3_3 | 6.1 | 4.2 | 1.8 | 5.6 | 0.55% | 5.3 |

| Rank | Description | Sync | Root | M | t | adj.q | B |
|---|---|---|---|---|---|---|---|
| 3527 | AT5G45390.1 \| Symbol: CLPP4 \| ATP-dependent Clp protease proteolytic subunit (ClpP4), identical to nClpP4 GI:5360593 from (Arabidopsis thaliana) \| chr5:18413530-18415343 FORWARD \| Aliases: MFC19.6, MFC19_6, NCLPP3, NCLPP4 | 6.5 | 3.9 | 2.6 | 5.6 | 0.55% | 5.3 |
| 3534 | AT4G15110.1 \| Symbol: CYP97B3 \| cytochrome P450 97B3, putative (CYP97B3), identical to Cytochrome P450 97B3 (SP:O23365) (Arabidopsis thaliana) \| chr4:8629770-8633030 REVERSE \| Aliases: DL3600C, FCAALL.208 | 7.2 | 6.0 | 1.1 | 5.6 | 0.56% | 5.3 |
| 3537 | AT5G20740.1 \| invertase/pectin methylesterase inhibitor family protein, low similarity to pectinesterase from Arabidopsis thaliana SP:Q42534, Lycopersicon esculentum SP:Q43143; contains Pfam profile PF04043: Plant invertase/pectin methylesterase inhibitor \| chr5:7025688-7026534 REVERSE \| Aliases: T1M15.140, T1M15_140 | 5.8 | 2.7 | 3.0 | 5.6 | 0.56% | 4.4 |
| 3538 | AT2G37400.1 \| chloroplast lumen common family protein, very similar to GI:6729507 (At5g02590) and GI:7413648 (At3g53560) (Arabidopsis thaliana) \| chr2:15703234-15704481 REVERSE \| Aliases: F3G5.19, F3G5_19 | 6.0 | 3.8 | 2.2 | 5.6 | 0.56% | 5.3 |
| 3539 | AT3G20330.1 \| aspartate carabmoyltransferase, chloroplast / aspartate transcarbamylase / ATCase (PYRB), identical to SP:P49077 Aspartate carbamoyltransferase, chloroplast precursor (EC 2.1.3.2) (Aspartate transcarbamylase) (ATCase) {Arabidopsis thaliana} | 8.5 | 5.6 | 2.8 | 5.6 | 0.56% | 5.3 |
| 3543 | AT5G59180.1 \| DNA-directed RNA polymerase II, identical to Swiss-Prot:P38421 DNA-directed RNA polymerase II 19 kDa polypeptide (EC 2.7.7.6) (RNA polymerase II subunit 5) (Arabidopsis thaliana) \| chr5:23900643-23902432 FORWARD \| Aliases: MNC17.16, MNC17_16 | 7.8 | 4.8 | 3.0 | 5.6 | 0.56% | 5.3 |
| 3545 | AT2G33860.1 \| Symbol: ETT \| auxin-responsive factor (ARF3) / ETTIN protein (ETT), identical to ETTIN GB:AF007788 from (Arabidopsis thaliana) \| chr2:14332345-14335847 REVERSE \| Aliases: ARF3, AUXIN RESPONSE TRANSCRIPTION FACTOR 3, ETTIN, T1B8.30, T1B8_30 | 6.6 | 5.3 | 1.3 | 5.6 | 0.56% | 5.3 |
| 3546 | AT3G13620.1 \| amino acid permease family protein, weak similarity to SP:Q9WTR6 Cystine/glutamate transporter (Amino acid transport system xc-) {Mus musculus}; contains Pfam profile PF00324: Amino acid permease \| chr3:4450191-4452594 REVERSE \| Aliases: K20M4.6 | 7.0 | 5.1 | 1.9 | 5.6 | 0.56% | 5.3 |
| 3547 | AT5G24310.2 \| expressed protein, strong similarity to unknown protein (emb:CAB66408.1) | 8.4 | 6.6 | 1.7 | 5.6 | 0.56% | 5.3 |
| 3548 | AT3G27280.2 \| prohibitin, putative, strong similarity to prohibitin (Arabidopsis thaliana) GI:1946331; contains Pfam profile PF01145: SPFH domain / Band 7 family \| chr3:10078077-10079658 FORWARD \| Aliases: K17E12.10 | 6.5 | 5.4 | 1.2 | 5.6 | 0.56% | 5.3 |
| 3550 | AT5G58590.1 \| Symbol: RANBP1 \| | 11.3 | 8.9 | 2.4 | 5.6 | 0.56% | 5.3 |
| 3553 | AT1G54500.1 \| rubredoxin family protein, similar to SP:P00270 Rubredoxin (Rd) {Desulfovibrio gigas}; contains Pfam profile PF00301: Rubredoxin | 7.3 | 5.8 | 1.5 | 5.6 | 0.56% | 5.3 |
| 3556 | AT5G18440.1 \| expressed protein \| chr5:6113250-6115750 FORWARD \| Aliases: F20L16.160, F20L16_160 | 5.5 | 4.7 | 0.8 | 5.6 | 0.56% | 5.2 |
| 3558 | AT5G11460.1 \| senescence-associated protein-related, similar to senescence-associated protein SAG102 (GI:22331931) (Arabidopsis thaliana) \| chr5:3656812-3658932 REVERSE \| Aliases: F15N18.50, F15N18_50 | 6.0 | 4.6 | 1.4 | 5.6 | 0.56% | 5.3 |
| 3560 | AT2G37450.1 \| nodulin MtN21 family protein, similar to MtN21 GI:2598575 (root nodule development) from (Medicago truncatula) \| chr2:15729647-15732141 REVERSE \| Aliases: F3G5.24, F3G5_24 | 6.6 | 4.5 | 2.1 | 5.6 | 0.57% | 5.2 |
| 3563 | AT4G40045.1 \| expressed protein \| chr4:18559778-18560651 REVERSE \| Aliases: None | 6.7 | 5.3 | 1.4 | 5.6 | 0.57% | 5.3 |
| 3564 | AT2G33370.1 \| 60S ribosomal protein L23 (RPL23B) \| chr2:14150646-14151797 REVERSE \| Aliases: F4P9.14 | 8.5 | 6.7 | 1.8 | 5.6 | 0.57% | 5.3 |
| 3565 | AT3G55030.1 \| phosphatidylglycerolphosphate synthase, putative, similar to phosphatidylglycerolphosphate synthase GI:13365519 from (Arabidopsis thaliana); contains non-consensus CG acceptor splice site at exon 4 \| chr3:20407737-20409630 FORWARD \| Aliases: T15C9.30 | 7.1 | 5.5 | 1.6 | 5.6 | 0.57% | 5.3 |
| 3566 | AT5G10730.1 \| expressed protein \| chr5:3390615-3393107 REVERSE \| Aliases: MAJ23.90, MAJ23_90 | 8.6 | 6.6 | 2.1 | 5.6 | 0.57% | 5.3 |
| 3570 | AT3G25530.2 \| similar to 6-phosphogluconate dehydrogenase NAD-binding domain-containing protein [Arabidopsis thaliana] (TAIR:At1g17650.1); similar to oxidoreductase-like [Oryza sativa (japonica cultivar-group)] (GB:BAD45192.1); contains InterPro domain 3-hydroxyisobutyrate dehydrogenase (InterPro:IPR002204); contains InterPro domain 6-phosphogluconate dehydrogenase, NAD binding domain (InterPro:IPR006115) \| chr3:9273037-9274830 REVERSE \| Aliases: MWL2.23 | 11.7 | 10.2 | 1.5 | 5.6 | 0.57% | 5.0 |
| 3574 | AT3G53900.2 \| uracil phosphoribosyltransferase, putative / UMP pyrophosphorylase, putative / UPRTase, putative, similar to SP:P50926 Uracil phosphoribosyltransferase (EC 2.4.2.9) (UMP pyrophosphorylase) (UPRTase) {Lactococcus lactis}; contains Pfam profile PF00156: Phosphoribosyl transferase domain \| chr3:19967634-19970019 REVERSE \| Aliases: F5K20.200 | 5.7 | 3.8 | 1.9 | 5.6 | 0.57% | 5.3 |
| 3579 | AT5G42900.3 \| expressed protein, similar to expressed protein [Arabidopsis thaliana] (TAIR:At4g33980.1) \| chr5:17215358-17217007 REVERSE \| Aliases: MBD2.9, MBD2_9 | 5.7 | 4.1 | 1.6 | 5.6 | 0.57% | 5.3 |

| Rank | Description | Sync | Root | M | t | adj.q | B |
|---|---|---|---|---|---|---|---|
| 3580 | AT1G75330.1 \| Symbol: OTC \| ornithine carbamoyltransferase, chloroplast / ornithine transcarbamylase / OTCase (OTC), identical to SP:O50039 Ornithine carbamoyltransferase, chloroplast precursor (EC 2.1.3.3) (OTCase) (Ornithine transcarbamylase) {Arabidopsis thaliana} \| chr1:28269920-28272068 REVERSE \| Aliases: F1B16.13, F1B16_13, ORNITHINE CARBAMOYLTRANSFERASE | 11.3 | 9.1 | 2.2 | 5.6 | 0.57% | 5.3 |
| 3583 | AT1G26750.1 \| expressed protein \| chr1:9246774-9248261 FORWARD \| Aliases: T24P13.13, T24P13_13 | 7.5 | 5.4 | 2.0 | 5.6 | 0.57% | 5.2 |
| 3585 | AT3G21250.1 \| Symbol: ATMRP6 \| | 6.7 | 5.3 | 1.5 | 5.6 | 0.57% | 5.3 |
| 3587 | AT5G39850.1 \| 40S ribosomal protein S9 (RPS9C), 40S ribosomal protein S9 - Chlamydomonas sp.,EMBL:AU066528 \| chr5:15967250-15968653 FORWARD \| Aliases: MYH19.10, MYH19_10 | 8.2 | 6.1 | 2.1 | 5.6 | 0.57% | 5.2 |
| 3589 | AT2G34480.1 \| 60S ribosomal protein L18A (RPL18aB) \| chr2:14539787-14541280 REVERSE \| Aliases: F13P17.34 | 11.2 | 9.4 | 1.7 | 5.6 | 0.57% | 5.1 |
| 3590 | AT4G17720.1 \| RNA recognition motif (RRM)-containing protein \| chr4:9862459-9864676 REVERSE \| Aliases: DL4895C, FCAALL.73 | 7.0 | 4.8 | 2.2 | 5.6 | 0.57% | 5.3 |
| 3591 | AT5G58600.2 \| expressed protein, various predicted proteins, Arabidopsis thaliana and Oryza sativa \| chr5:23701017-23702941 REVERSE \| Aliases: MZN1.6, MZN1_6 | 5.4 | 3.7 | 1.7 | 5.6 | 0.58% | 5.2 |
| 3592 | AT3G13160.1 \| pentatricopeptide (PPR) repeat-containing protein, contains Pfam profile PF01535: PPR repeat \| chr3:4229978-4231293 REVERSE \| Aliases: MJG19.11 | 6.3 | 4.3 | 2.0 | 5.6 | 0.58% | 5.3 |
| 3593 | AT5G64780.1 \| expressed protein, similar to unknown protein (pir::T04031) \| chr5:25917785-25919351 REVERSE \| Aliases: MVP7.11, MVP7_11 | 3.9 | 2.8 | 1.1 | 5.6 | 0.58% | 5.3 |
| 3595 | AT5G12410.1 \| THUMP domain-containing protein, contains Pfam profile PF02926: THUMP domain \| chr5:4021816-4023989 REVERSE \| Aliases: None | 7.2 | 5.4 | 1.8 | 5.6 | 0.58% | 5.3 |
| 3596 | AT5G10320.3 \| expressed protein \| chr5:3244006-3246721 REVERSE \| Aliases: F18D22.90, F18D22_90 | 4.8 | 4.0 | 0.8 | 5.6 | 0.58% | 5.2 |
| 3599 | AT4G12340.1 \| expressed protein \| chr4:7321276-7322448 REVERSE \| Aliases: T4C9.180, T4C9_180 | 9.8 | 8.1 | 1.7 | 5.6 | 0.58% | 5.3 |
| 3602 | AT3G57220.1 \| UDP-GlcNAc:dolichol phosphate N-acetylglucosamine-1-phosphate transferase, putative, strong similarity to GI:5804772 \| chr3:21185438-21188080 FORWARD \| Aliases: F28O9.70, F28O9_70 | 6.6 | 4.6 | 2.0 | 5.6 | 0.58% | 5.2 |
| 3603 | AT2G02880.1 \| mucin-related, similar to putative mucin GI:18071389 (Oryza sativa) \| chr2:841260-843359 FORWARD \| Aliases: T17M13.5, T17M13_5 | 6.2 | 4.6 | 1.7 | 5.6 | 0.58% | 5.3 |
| 3605 | AT1G30040.2 \| similar to gibberellin 2-oxidase / GA2-oxidase (GA2OX3) [Arabidopsis thaliana] (TAIR:At2g34555.1); similar to GA 2-oxidase 2 [Nerium oleander] (GB:AAT92094.1); contains InterPro domain 2OG-Fe(II) oxygenase superfamily (InterPro:IPR005123) \| chr1:10537632-10539172 FORWARD \| Aliases: T1P2.6, T1P2_6 | 6.0 | 4.2 | 1.8 | 5.6 | 0.58% | 5.2 |
| 3608 | AT5G48570.1 \| peptidyl-prolyl cis-trans isomerase, putative / FK506-binding protein, putative, similar to rof1 (Arabidopsis thaliana) GI:1373396 \| chr5:19707768-19711071 REVERSE \| Aliases: K15N18.12, K15N18_12 | 10.2 | 8.2 | 2.0 | 5.6 | 0.58% | 5.3 |
| 3610 | AT5G58240.2 \| bis(5'-adenosyl)-triphosphatase, putative, similar to bis(5'-adenosyl)-triphosphatase (Diadenosine 5',5'''- P1,P3-triphosphate hydrolase, Dinucleosidetriphosphatase, AP3A hydrolase, AP3AASE, Fragile histidine triad protein) (Homo sapiens) Swiss-Prot:P49789 | 7.5 | 5.9 | 1.6 | 5.5 | 0.58% | 5.2 |
| 3611 | AT5G41685.1 \| mitochondrial import receptor subunit TOM7 / translocase of outer membrane 7 kDa subunit (TOM7.1), identical to SP:Q9ASY8 Mitochondrial import receptor subunit TOM7 (Translocase of outer membrane 7 kDa subunit) {Arabidopsis thaliana} \| chr5:16686314-16686950 REVERSE \| Aliases: None | 7.4 | 4.4 | 3.0 | 5.5 | 0.58% | 5.3 |
| 3614 | AT3G11250.1 \| 60S acidic ribosomal protein P0 (RPP0C), similar to 60S acidic ribosomal protein P0 GI:2088654 (Arabidopsis thaliana) \| chr3:3521366-3523006 FORWARD \| Aliases: F11B9.17 | 7.0 | 4.4 | 2.6 | 5.5 | 0.58% | 5.2 |
| 3616 | AT1G34350.1 \| expressed protein \| chr1:12534432-12536073 FORWARD \| Aliases: F7P12.4, F7P12_4 | 7.0 | 4.9 | 2.1 | 5.5 | 0.58% | 5.2 |
| 3617 | AT5G42760.1 \| expressed protein, similar to hypothetical protein [Streptomyces sp. KO-3988] (GB:BAD86809.1); contains InterPro domain Protein of unknown function DUF142 (InterPro:IPR003455) \| chr5:17166151-17167514 FORWARD \| Aliases: MJB21.14, MJB21_14 | 4.5 | 2.7 | 1.9 | 5.5 | 0.58% | 5.3 |
| 3618 | AT4G18170.1 \| Symbol: WRKY28 \| | 5.9 | 4.4 | 1.5 | 5.5 | 0.58% | 5.3 |
| 3619 | AT1G08540.1 \| Symbol: SIGB \| RNA polymerase sigma subunit SigB (sigB) / sigma factor 2 (SIG2), identical to sigma factor SigB (Arabidopsis thaliana) GI:5478446, plastid RNA polymerase sigma-subunit (Arabidopsis thaliana) GI:2879922, sigma factor (Arabidopsis thaliana) GI:2597831, sigma factor 2 (SIG2) (Arabidopsis thaliana) GI:2353173; contains Pfam profiles PF04545: Sigma-70, region 4, PF04539: Sigma-70 region 3, PF04542: Sigma-70 region 2 \| chr1:2703342-2706848 FORWARD \| Aliases: PUTATIVE PLASTID RNA POLYMERASE SIGMA SUBUNIT, SIG1, SIG2, SIGA, SIGMA FACTOR B, T27G7.22 | 5.7 | 4.2 | 1.5 | 5.5 | 0.58% | 5.3 |

| Rank | Description | Sync | Root | M | t | adj.q | B |
|---|---|---|---|---|---|---|---|
| 3620 | AT3G53450.1 \| hypothetical protein, contains Pfam profile PF03641: decarboxylase family protein \| chr3:19823955-19826408 REVERSE \| Aliases: F4P12.150 | 4.9 | 3.8 | 1.1 | 5.5 | 0.58% | 5.3 |
| 3621 | AT4G26370.2 \| antitermination NusB domain-containing protein, contains Pfam profile: PF01029 NusB family \| chr4:13333733-13336184 REVERSE \| Aliases: T25K17.4 | 3.8 | 2.6 | 1.2 | 5.5 | 0.58% | 5.3 |
| 3622 | AT1G20580.1 \| small nuclear ribonucleoprotein, putative / snRNP, putative / Sm protein, putative, similar to small nuclear ribonucleoprotein Sm D3 (snRNP core protein D3, Sm-D3) (Mus musculus) SWISS-PROT:P43331 \| chr1:7128878-7130631 FORWARD \| Aliases: F5M15.9, F5M15_9 | 9.2 | 7.7 | 1.6 | 5.5 | 0.58% | 5.3 |
| 3623 | AT5G66470.1 \| expressed protein \| chr5:26558965-26561585 REVERSE \| Aliases: K1F13.13, K1F13_13 | 5.1 | 3.6 | 1.5 | 5.5 | 0.58% | 5.2 |
| 3624 | AT4G24750.1 \| similar to rhodanese-like domain-containing protein [Arabidopsis thaliana] (TAIR:At3g08920.1); similar to rhodanese family protein-like [Oryza sativa (japonica cultivar-group)] (GB:XP_450481.1); contains InterPro domain Rhodanese-like (InterPro:IPR001763) \| chr4:12758224-12760892 REVERSE \| Aliases: F22K18.50, F22K18_50 | 6.7 | 5.4 | 1.3 | 5.5 | 0.58% | 5.3 |
| 3625 | AT1G76820.1 \| expressed protein \| chr1:28842467-28843745 FORWARD \| Aliases: F28O16.19, F28O16_19 | 7.9 | 5.0 | 2.9 | 5.5 | 0.58% | 5.2 |
| 3628 | AT4G19410.1 \| pectinacetylesterase, putative, similar to pectinacetylesterase precursor GI:1431629 from (Vigna radiata) \| chr4:10582032-10585769 REVERSE \| Aliases: T5K18.190, T5K18_190 | 11.5 | 9.7 | 1.8 | 5.5 | 0.59% | 5.2 |
| 3631 | AT4G17560.1 \| ribosomal protein L19 family protein, similar to plastid ribosomal protein L19 precursor (Spinacia oleracea) gi:7582403:gb:AAF64312 \| chr4:9780292-9782194 FORWARD \| Aliases: DL4815W, FCAALL.39 | 5.1 | 2.5 | 2.6 | 5.5 | 0.59% | 4.9 |
| 3633 | AT4G16370.1 \| Symbol: ATOPT3 \| | 9.4 | 6.4 | 2.9 | 5.5 | 0.59% | 5.3 |
| 3635 | AT3G52380.1 \| Symbol: CP33 \| 33 kDa ribonucleoprotein, chloroplast, putative / RNA-binding protein cp33, putative, similar to chloroplast RNA-binding protein (cp33) GB:BAA06523 (Arabidopsis thaliana) (Plant Mol. Biol. 27 (3), 529-539 (1995)); contains InterPro entry IPR000504: RNA-binding region RNP-1 (RNA recognition motif) (RRM) \| chr3:19432375-19434072 FORWARD \| Aliases: PDE322, PIGMENT DEFECTIVE 322, T25B15.18 | 8.2 | 6.5 | 1.7 | 5.5 | 0.59% | 5.2 |
| 3636 | AT4G37870.2 \| similar to phosphoenolpyruvate carboxykinase (ATP), putative / PEP carboxykinase, putative / PEPCK, putative [Arabidopsis thaliana] (TAIR:At5g65690.1); similar to phosphoenolpyruvate carboxykinase [Flaveria pringlei] (GB:BAB43909.1); similar to phosphoenolpyruvate carboxykinase [Zoysia japonica] (GB:BAD88616.1); similar to phosphoenolpyruvate carboxykinase [Flaveria trinervia] (GB:BAB43907.1); similar to PEPCK [Cucumis sativus] (GB:AAM00814.1); similar to phosphoenolpyruvate carboxykinase [Lycopersicon esculentum] (GB:AAG01894.2); contains InterPro domain Phosphoenolpyruvate carboxykinase (ATP) (InterPro:IPR001272); contains InterPro domain Phosphoenolpyruvate carboxykinase, N-terminal (InterPro:IPR008210) \| chr4:17802688-17806533 REVERSE \| Aliases: T28I19.150, T28I19_150 | 11.7 | 8.6 | 3.1 | 5.5 | 0.59% | 5.3 |
| 3638 | AT2G17230.1 \| phosphate-responsive 1 family protein, similar to phi-1 (phosphate-induced gene) (Nicotiana tabacum) GI:3759184; contains Pfam profile PF04674: Phosphate-induced protein 1 conserved region \| chr2:7501618-7503098 REVERSE \| Aliases: T23A1.9, T23A1_9 | 5.8 | 3.1 | 2.6 | 5.5 | 0.59% | 5.0 |
| 3639 | AT1G72190.1 \| oxidoreductase family protein, similar to D-3-phosphoglycerate dehydrogenase from Arabidopsis thaliana (SP:O04130), glyoxylate reductase from Homo sapiens (gi:6002730); contains Pfam D-isomer specific 2-hydroxyacid dehydrogenase, NAD binding domain PF02826 | 6.5 | 5.6 | 0.9 | 5.5 | 0.59% | 5.2 |
| 3641 | AT1G55890.1 \| pentatricopeptide (PPR) repeat-containing protein, contains Pfam profile PF01535: PPR repeat \| chr1:20905011-20906465 FORWARD \| Aliases: F14J16.14, F14J16_14 | 8.1 | 5.7 | 2.4 | 5.5 | 0.59% | 5.3 |
| 3643 | AT5G23310.1 \| Symbol: FSD3 \| superoxide dismutase (Fe) / iron superoxide dismutase 3 (FSD3), identical to iron superoxide dismutase 3 (Arabidopsis thaliana) gi:3273757:gb:AAC24834 \| chr5:7850550-7852535 FORWARD \| Aliases: FE SUPEROXIDE DISMUTASE 3, MKD15.17, MKD15_17 | 6.1 | 4.4 | 1.7 | 5.5 | 0.59% | 5.2 |
| 3644 | AT4G21140.1 \| expressed protein, ; expression supported by MPSS \| chr4:11277546-11278232 REVERSE \| Aliases: F7J7.80, F7J7_80 | 6.2 | 4.8 | 1.4 | 5.5 | 0.59% | 5.3 |
| 3646 | AT3G44380.1 \| expressed protein, predicted proteins, Arabidopsis thaliana \| chr3:16047170-16047867 REVERSE \| Aliases: T22K7.60 | 4.5 | 3.2 | 1.3 | 5.5 | 0.59% | 5.3 |
| 3649 | AT1G60850.3 \| Symbol: ATRPAC42 \| DNA-directed RNA polymerase, putative, identical to RNA polymerase subunit (Arabidopsis thaliana) GI:514322; contains Pfam profile PF01000: RNA polymerase Rpb3/RpoA insert domain \| chr1:22401581-22403834 REVERSE \| Aliases: AAC42, F23C21.3, F23C21_3, RNA POLYMERASE I SUBUNIT 42 | 6.6 | 4.7 | 1.9 | 5.5 | 0.59% | 5.3 |
| 3650 | AT1G32500.1 \| Symbol: ATNAP6 \| | 7.7 | 5.7 | 2.0 | 5.5 | 0.59% | 5.3 |
| 3652 | AT1G66580.1 \| 60S ribosomal protein L10 (RPL10C), contains Pfam profile: PF00826: Ribosomal L10 \| chr1:24842828-24844275 FORWARD \| Aliases: T12I7.3, T12I7_3 | 11.9 | 10.7 | 1.2 | 5.5 | 0.59% | 5.2 |

| Rank | Description | Sync | Root | M | t | adj.q | B |
|---|---|---|---|---|---|---|---|
| 3657 | AT4G33680.1 \| Symbol: AGD2 \| aminotransferase class I and II family protein, low similarity to Aromatic Aminotransferase from Pyrococcus horikoshii GP:14278621; contains Pfam profile PF00155 aminotransferase, classes I and II \| chr4:16171697-16174681 REVERSE \| Aliases: ABERRANT GROWTH AND DEATH 2, T16L1.170, T16L1_170 | 11.7 | 8.5 | 3.2 | 5.5 | 0.59% | 5.1 |
| 3659 | AT1G74270.1 \| 60S ribosomal protein L35a (RPL35aC), similar to ribosomal protein L33B GB:NP_014877 from (Saccharomyces cerevisiae) \| chr1:27931917-27933158 REVERSE \| Aliases: F1O17.6, F1O17_6 | 10.1 | 8.1 | 2.0 | 5.5 | 0.59% | 5.3 |
| 3660 | AT5G01350.1 \| expressed protein \| chr5:145635-147199 REVERSE \| Aliases: T10O8.60, T10O8_60 | 11.1 | 9.9 | 1.1 | 5.5 | 0.59% | 4.9 |
| 3661 | AT1G34770.2 \| MAGE-8 antigen-related, contains weak similarity to Swiss-Prot:P43361 melanoma-associated antigen 8 (MAGE-8 antigen) (Homo sapiens) \| chr1:12746598-12748538 FORWARD \| Aliases: F11O6.12 | 5.0 | 3.7 | 1.3 | 5.5 | 0.59% | 5.2 |
| 3663 | AT2G47020.2 \| peptide chain release factor, putative, similar to peptide chain release factor 1 (Escherichia coli) GI:147567; contains Pfam profiles PF00472: Peptidyl-tRNA hydrolase domain, PF03462: PCRF domain \| chr2:19326755-19329461 REVERSE \| Aliases: F14M4.15 | 3.4 | 2.7 | 0.7 | 5.5 | 0.60% | 5.1 |
| 3665 | AT5G56260.1 \| dimethylmenaquinone methyltransferase family protein, similar to bacterial S-adenosylmethionine:2-demethylmenaquinone methyltransferases; contains Pfam profile PF03737: Dimethylmenaquinone methyltransferase \| chr5:22792551-22794437 REVERSE \| Aliases: K24C1.7, K24C1_7 | 7.6 | 6.0 | 1.6 | 5.5 | 0.60% | 5.2 |
| 3666 | AT3G62060.1 \| pectinacetylesterase family protein, similar to pectinacetylesterase precursor GI:1431629 from (Vigna radiata); contains Pfam profile: PF03283 pectinacetylesterase \| chr3:22991031-22993919 FORWARD \| Aliases: T17J13.20 | 5.0 | 3.3 | 1.7 | 5.5 | 0.60% | 5.3 |
| 3668 | AT2G31170.1 \| tRNA synthetase class I (C) family protein, similar to cysteine-tRNA ligase (Escherichia coli) GI:41203; contains Pfam profile PF01406: tRNA synthetases class I (C) \| chr2:13289458-13292722 REVERSE \| Aliases: T16B12.16 | 3.1 | 2.4 | 0.6 | 5.5 | 0.60% | 5.0 |
| 3672 | AT5G42370.1 \| expressed protein \| chr5:16956367-16958953 REVERSE \| Aliases: MDH9.6, MDH9_6 | 6.0 | 4.0 | 2.0 | 5.5 | 0.60% | 5.1 |
| 3674 | AT5G58930.1 \| expressed protein, contains Pfam PF05340: Protein of unknown function (DUF740) \| chr5:23811541-23813687 REVERSE \| Aliases: K19M22.12, K19M22_12 | 9.8 | 9.2 | 0.7 | 5.5 | 0.60% | 5.1 |
| 3675 | AT5G41270.1 \| expressed protein \| chr5:16524937-16526107 FORWARD \| Aliases: K1O13.7, K1O13_7 | 4.3 | 3.4 | 0.9 | 5.5 | 0.60% | 5.2 |
| 3676 | AT4G32470.2 \| ubiquinol-cytochrome C reductase complex 14 kDa protein, putative, similar to SP:P48502 Ubiquinol-cytochrome C reductase complex 14 kDa protein (EC 1.10.2.2) (CR14) {Solanum tuberosum}; contains Pfam profile PF02271: Ubiquinol-cytochrome C reductase complex 14kD subunit \| chr4:15669225-15671200 REVERSE \| Aliases: F8B4.170, F8B4_170 | 12.1 | 10.7 | 1.4 | 5.5 | 0.60% | 5.0 |
| 3678 | AT5G14030.1 \| translocon-associated protein beta (TRAPB) family protein, low similarity to SP:P23438 Translocon-associated protein, beta subunit precursor (TRAP-beta) (Signal sequence receptor beta subunit) {Canis familiaris}; contains Pfam profile PF05753: Translocon-associated protein beta (TRAPB) \| chr5:4526813-4528411 FORWARD \| Aliases: MUA22.2, MUA22_2 | 10.2 | 8.9 | 1.3 | 5.5 | 0.60% | 5.2 |
| 3680 | AT4G01790.1 \| ribosomal protein L7Ae/L30e/S12e/Gadd45 family protein / ribonuclease P-related, similar to human RNaseP-associate protein P38, GenBank accession number U77664 \| chr4:769868-770728 FORWARD \| Aliases: T7B11.5 | 3.1 | 2.4 | 0.7 | 5.5 | 0.60% | 5.0 |
| 3681 | AT1G18060.1 \| expressed protein \| chr1:6211867-6213358 REVERSE \| Aliases: T10F20.7 | 6.9 | 5.8 | 1.2 | 5.5 | 0.60% | 5.2 |
| 3688 | AT2G35500.1 \| shikimate kinase-related, low similarity to shikimate kinase precursor from Lycopersicon esculentum (SP:Q00497) \| chr2:14921106-14923198 FORWARD \| Aliases: T32F12.12, T32F12_12 | 6.1 | 4.3 | 1.8 | 5.5 | 0.61% | 5.2 |
| 3689 | AT3G08990.1 \| yippee family protein, similar to qdgl-1 (Coturnix coturnix) GI:10441650, Yippee protein (Drosophila melanogaster) GI:5713279; contains Pfam profile PF03226: Yippee putative zinc-binding protein \| chr3:2743081-2744937 FORWARD \| Aliases: T16O11.5 | 5.9 | 4.5 | 1.4 | 5.5 | 0.61% | 5.2 |
| 3692 | AT1G64510.1 \| ribosomal protein S6 family protein, similar to plastid ribosomal protein S6 precursor GB:AAF64311 GI:7582401 from (Spinacia oleracea) \| chr1:23958466-23959914 REVERSE \| Aliases: F1N19.8, F1N19_8 | 6.6 | 3.4 | 3.1 | 5.5 | 0.61% | 5.0 |
| 3695 | AT5G20410.1 \| Symbol: MGD2 \| 1,2-diacylglycerol 3-beta-galactosyltransferase, putative / monogalactosyldiacylglycerol synthase, putative / MGDG synthase, putative, identical to monogalactosyldiacylglycerol synthase (gi:3367638) from Arabidopsis thaliana, similar to MGDG synthase type A (gi:9884651) from Glycine max \| chr5:6896679-6898785 FORWARD \| Aliases: ATMGD2, F5O24.300, F5O24_300 | 5.2 | 3.4 | 1.8 | 5.5 | 0.61% | 5.2 |
| 3700 | AT2G46580.1 \| pyridoxine 5'-phosphate oxidase-related, similar to pyridoxine 5'-phosphate oxidase (GI:3237304) (Rattus norvegicus); similar to pyridoxamine 5'-phosphate oxidase (GI:22294052) (Thermosynechococcus elongatus BP-1) \| chr2:19136313-19137667 REVERSE \| Aliases: F13A10.11 | 5.7 | 5.0 | 0.6 | 5.5 | 0.61% | 5.0 |

| Rank | Description | Sync | Root | M | t | adj.q | B |
|---|---|---|---|---|---|---|---|
| 3706 | AT1G50710.1 \| expressed protein \| chr1:18788177-18791470 REVERSE \| Aliases: F17J6.23, F17J6_23 | 6.0 | 4.6 | 1.4 | 5.5 | 0.62% | 5.2 |
| 3707 | AT1G48440.1 \| expressed protein \| chr1:17910669-17912191 FORWARD \| Aliases: T1N15.5, T1N15_5 | 11.0 | 9.8 | 1.2 | 5.5 | 0.62% | 4.9 |
| 3708 | AT1G55500.1 \| expressed protein, contains Pfam profile PF04146: YT521-B-like family \| chr1:20723241-20726399 FORWARD \| Aliases: T5A14.10, T5A14_10 | 5.5 | 4.5 | 1.1 | 5.5 | 0.62% | 5.2 |
| 3709 | AT5G46840.1 \| RNA recognition motif (RRM)-containing protein, contains InterPro entry IPR000504: RNA-binding region RNP-1 (RNA recognition motif) (RRM) \| chr5:19022828-19024993 FORWARD \| Aliases: MZA15.26 | 8.5 | 6.0 | 2.6 | 5.5 | 0.62% | 5.2 |
| 3710 | AT4G34710.2 \| Symbol: ADC2 \| arginine decarboxylase 2 (SPE2), identical to SP:O23141 Arginine decarboxylase 2 (EC 4.1.1.19) (ARGDC 2) (ADC 2) (ADC-N) {Arabidopsis thaliana} \| chr4:16560067-16562936 REVERSE \| Aliases: ARGININE DECARBOXYLASE, ARGININE DECARBOXYLASE 2, SPE2, T4L20.290, T4L20_290 | 9.4 | 7.7 | 1.7 | 5.5 | 0.62% | 5.2 |
| 3711 | NA | 12.0 | 10.7 | 1.3 | 5.5 | 0.62% | 4.7 |
| 3712 | AT5G27400.1 \| expressed protein, hypothetical proteins - different species \| chr5:9676351-9678506 FORWARD \| Aliases: F21A20.110, F21A20_110 | 7.4 | 5.8 | 1.7 | 5.5 | 0.62% | 5.2 |
| 3713 | AT3G27070.1 \| Symbol: TOM20 1 \| mitochondrial import receptor subunit TOM20-1 / translocase of outer membrane 20 kDa subunit 1 (TOM20-1), identical to mitochondrial import receptor subunit TOM20-1 SP:P82872 from (Arabidopsis thaliana) \| chr3:9984241-9985981 REVERSE \| Aliases: MOJ10.21, TRANSLOCASE OUTER MEMBRANE 20 1 | 5.0 | 3.9 | 1.0 | 5.5 | 0.62% | 5.2 |
| 3714 | AT2G14520.1 \| CBS domain-containing protein, contains Pfam profiles PF00571: CBS domain, PF01595: Domain of unknown function \| chr2:6189275-6191730 REVERSE \| Aliases: T13P21.10, T13P21_10 | 5.2 | 4.1 | 1.1 | 5.5 | 0.62% | 5.2 |
| 3718 | AT2G18330.1 \| AAA-type ATPase family protein, contains Pfam profile: PF00004 ATPase family associated with various cellular activities (AAA) \| chr2:7972739-7976187 FORWARD \| Aliases: T30D6.16, T30D6_16 | 6.8 | 5.0 | 1.7 | 5.5 | 0.62% | 5.2 |
| 3721 | AT1G10590.3 \| DNA-binding protein-related, contains weak similarity to G-quartet DNA binding protein 3 (Tetrahymena thermophila) gi:4583503:gb:AAD25098 \| chr1:3502051-3503410 REVERSE \| Aliases: F20B24.1 | 10.2 | 9.2 | 1.0 | 5.5 | 0.62% | 5.1 |
| 3722 | AT2G33820.1 \| mitochondrial substrate carrier family protein (BAC1), contains Pfam profile: PF00153 mitochondrial carrier protein \| chr2:14313369-14315369 REVERSE \| Aliases: T1B8.12, T1B8_12 | 5.9 | 5.1 | 0.9 | 5.5 | 0.62% | 5.1 |
| 3723 | AT2G34560.2 \| katanin, putative, similar to katanin p60 subunit (Strongylocentrotus purpuratus) GI:3098603; contains Pfam profile PF00004: ATPase AAA family \| chr2:14567277-14569924 FORWARD \| Aliases: T31E10.10, T31E10_10 | 8.4 | 6.5 | 1.9 | 5.5 | 0.62% | 5.2 |
| 3724 | ATCG00380.1 \| Symbol: RPS4 \| Chloroplast encoded ribosomal protein S4 | 11.7 | 9.2 | 2.6 | 5.5 | 0.62% | 5.1 |
| 3726 | AT4G37370.1 \| Symbol: CYP81D8 \| cytochrome P450, putative, similar to Cytochrome P450 91A1 (SP:Q9FG65 )(Arabidopsis thaliana); cytochrome P450, Glycyrrhiza echinata, AB001379 \| chr4:17569822-17571698 REVERSE \| Aliases: F6G17.20, F6G17_20 | 5.2 | 2.9 | 2.3 | 5.5 | 0.62% | 4.8 |
| 3727 | AT5G51110.1 \| expressed protein \| chr5:20795360-20796660 REVERSE \| Aliases: MWD22.5, MWD22_5 | 5.9 | 4.4 | 1.4 | 5.5 | 0.62% | 5.2 |
| 3728 | AT4G15000.1 \| 60S ribosomal protein L27 (RPL27C) \| chr4:8571829-8572485 FORWARD \| Aliases: DL3545W, FCAALL.99 | 10.4 | 8.0 | 2.4 | 5.5 | 0.62% | 5.2 |
| 3729 | AT3G24495.1 \| DNA mismatch repair protein MSH6-2 (MSH7), identical to SP:Q9SMV7 DNA mismatch repair protein MSH6-2 (AtMsh6-2) (MutS homolog 7) {Arabidopsis thaliana}; GC donor splice site at exon 11 | 4.8 | 3.3 | 1.5 | 5.5 | 0.62% | 5.2 |
| 3730 | AT1G79440.1 \| Symbol: ALDH5F1 \| succinate-semialdehyde dehydrogenase (SSADH1), similar to succinate-semialdehyde dehydrogenase (NADP+) (SSDH) (Escherichia coli) SWISS-PROT:P25526; identical to succinic semialdehyde dehydrogenase mRNA, nuclear gene encoding mitochondrial protein GI:6684441; contains TIGRfam profile TIGR01780:succinic semialdehyde dehydrogenase; contains Pfam profile PF00171: aldehyde dehydrogenase (NAD) family protein | 8.9 | 6.5 | 2.5 | 5.5 | 0.62% | 5.2 |
| 3731 | AT3G22110.1 \| Symbol: PAC1 \| 20S proteasome alpha subunit C (PAC1) (PRC9), identical to GB:AAC32057 from (Arabidopsis thaliana) (Genetics (1998) 149 (2), 677-692); identical to cDNA proteasome subunit prc9 GI:2511583 \| chr3:7792645-7794161 REVERSE \| Aliases: 20S PROTEASOME SUBUNIT PAC1, MKA23.2 | 11.4 | 9.5 | 1.9 | 5.5 | 0.62% | 5.0 |
| 3733 | AT5G08040.1 \| expressed protein \| chr5:2577203-2578120 REVERSE \| Aliases: T22D6.2 | 10.2 | 8.3 | 1.9 | 5.5 | 0.63% | 5.2 |
| 3737 | AT5G10810.1 \| Symbol: ATER \| enhancer of rudimentary protein, putative, identical to enhancer of rudimentary homolog SP:Q96319 from (Arabidopsis thaliana); contains Pfam profile: PF01133 enhancer of rudimentary \| chr5:3418667-3420636 REVERSE \| Aliases: ENHANCER OF RUDIMENTARY HOMOLOG ATER, T30N20.80, T30N20_80 | 8.6 | 6.3 | 2.4 | 5.5 | 0.63% | 5.2 |

| Rank | Description | Sync | Root | M | t | adj.q | B |
|---|---|---|---|---|---|---|---|
| 3738 | AT4G12130.1 \| similar to GA20785-PA [Drosophila pseudoobscura] (GB:EAL28126.1); contains InterPro domain Glycine cleavage T protein (aminomethyl transferase) (InterPro:IPR006222) \| chr4:7263593-7265806 FORWARD \| Aliases: F16J13.200, F16J13_200 | 6.4 | 4.7 | 1.7 | 5.5 | 0.63% | 5.0 |
| 3740 | AT1G11750.1 \| Symbol: CLPP6 \| ATP-dependent Clp protease proteolytic subunit (ClpP), identical to ATP-dependent Clp protease proteolytic subunit GI:2827888 from (Arabidopsis thaliana); contains Pfam profile PF00574: Clp protease; contains TIGRfam profile TIGR00493: ATP-dependent Clp protease, proteolytic subunit ClpP \| chr1:3967478-3969854 FORWARD \| Aliases: ATP DEPENDENT CLP PROTEASE PROTEOLYTIC SUBUNIT, F25C20.10, F25C20_10, NCLPP1, NCLPP6 | 6.6 | 4.5 | 2.2 | 5.5 | 0.63% | 5.1 |
| 3743 | AT1G16310.1 \| cation efflux family protein, contains cation efflux family protein domain, Pfam:PF01545 | 10.1 | 7.0 | 3.1 | 5.5 | 0.63% | 5.2 |
| 3745 | AT3G23330.1 \| pentatricopeptide (PPR) repeat-containing protein, contains INTERPRO:IPR002885 PPR repeats \| chr3:8347207-8349354 FORWARD \| Aliases: MLM24.6 | 4.9 | 3.3 | 1.6 | 5.4 | 0.63% | 5.2 |
| 3747 | AT3G20500.1 \| calcineurin-like phosphoesterase family protein, contains Pfam profile: PF00149 calcineurin-like phosphoesterase | 10.1 | 8.3 | 1.8 | 5.4 | 0.63% | 5.2 |
| 3748 | AT1G30290.1 \| pentatricopeptide (PPR) repeat-containing protein, contains Pfam profile PF01535: PPR repeat \| chr1:10670304-10672722 REVERSE \| Aliases: F12P21.10, F12P21_10 | 6.2 | 4.8 | 1.4 | 5.4 | 0.63% | 5.2 |
| 3750 | AT4G38100.1 \| expressed protein \| chr4:17886864-17888198 REVERSE \| Aliases: F20D10.220, F20D10_220 | 6.8 | 5.1 | 1.7 | 5.4 | 0.63% | 5.2 |
| 3751 | AT4G32540.1 \| Symbol: YUC \| flavin-containing monooxygenase / FMO (YUCCA), identical to gi:16555352 \| chr4:15700910-15702876 FORWARD \| Aliases: L23H3.20, L23H3_20, YUCCA | 5.4 | 3.7 | 1.7 | 5.4 | 0.63% | 5.1 |
| 3752 | AT3G61310.1 \| DNA-binding family protein, contains a AT hook motif (DNA binding motifs with a preference for A/T rich regions), Pfam:PF02178 \| chr3:22701167-22703797 REVERSE \| Aliases: T20K12.210 | 5.0 | 3.5 | 1.6 | 5.4 | 0.63% | 5.1 |
| 3754 | AT3G06140.1 \| zinc finger (C3HC4-type RING finger) family protein, contains Pfam profile: PF00097 Zinc finger, C3HC4 type (RING finger) \| chr3:1856688-1858856 REVERSE \| Aliases: F28L1.8, F28L1_8 | 4.2 | 2.7 | 1.4 | 5.4 | 0.63% | 5.2 |
| 3758 | AT3G16570.1 \| Symbol: RALFL23 \| rapid alkalinization factor (RALF) family protein, similar to RALF precursor (Nicotiana tabacum) GI:16566316 \| chr3:5644704-5645420 FORWARD \| Aliases: MGL6.3, RALF LIKE 23 | 6.6 | 5.1 | 1.6 | 5.4 | 0.63% | 5.2 |
| 3760 | AT2G47580.1 \| Symbol: U1A \| small nuclear ribonucleoprotein U1A / spliceosomal protein U1A / U1snRNP-specific protein, identical to GB:Z49991 U1snRNP-specific protein (Arabidopsis thaliana) | 8.0 | 5.6 | 2.4 | 5.4 | 0.63% | 5.1 |
| 3761 | AT5G40660.1 \| ATP12 protein-related, weak similarity to SP:P22135 ATP12 protein, mitochondrial precursor {Saccharomyces cerevisiae} \| chr5:16300739-16302554 REVERSE \| Aliases: MNF13.22, MNF13_22 | 8.1 | 5.9 | 2.2 | 5.4 | 0.63% | 5.1 |
| 3762 | AT4G34350.1 \| Symbol: CLB6 \| Arabidopsis ISPH is involved in the plastid nonmevalonate pathway of isoprenoid biosynthesis. It was shown to complement the lethal phenotype of E.coli ispH mutant and is therefore most likely encodes a protein with 4-hydroxy-3-methylbut-2-en-1-yl diphosphate reductase activity involved in the last step of mevalonate-independent isopentenyl biosynthesis. \| chr4:16428484-16431117 REVERSE \| Aliases: CHLOROPLAST BIOGENESIS, F10M10.120, F10M10_120, ISPH | 9.7 | 8.1 | 1.6 | 5.4 | 0.63% | 5.2 |
| 3763 | AT1G52870.2 \| peroxisomal membrane protein-related, contains weak similarity to Swiss-Prot:Q07066 22 kDa peroxisomal membrane protein (Rattus norvegicus) \| chr1:19689415-19691531 FORWARD \| Aliases: F14G24.14, F14G24_14 | 6.5 | 4.2 | 2.4 | 5.4 | 0.64% | 5.1 |
| 3764 | AT5G59610.1 \| DNAJ heat shock N-terminal domain-containing protein, similar to SP:Q9UXR9 Chaperone protein dnaJ (Heat shock protein 40 Methanosarcina thermophila, SP:Q9QYI6 DnaJ homolog subfamily B member 9 Mus musculus; contains Pfam profile PF00226 DnaJ domain | 4.8 | 3.5 | 1.3 | 5.4 | 0.64% | 5.1 |
| 3766 | AT5G04790.1 \| expressed protein \| chr5:1388156-1388320 REVERSE \| Aliases: MUK11.11 | 7.0 | 4.6 | 2.4 | 5.4 | 0.64% | 4.9 |
| 3770 | AT1G72040.1 \| deoxynucleoside kinase family, contains Pfam profile: PF01712 deoxynucleoside kinase \| chr1:27115559-27118004 REVERSE \| Aliases: F28P5.7, F28P5_7 | 6.9 | 4.6 | 2.2 | 5.4 | 0.64% | 5.1 |
| 3771 | AT2G01400.1 \| expressed protein \| chr2:174270-175616 FORWARD \| Aliases: F10A8.4 | 4.1 | 2.9 | 1.3 | 5.4 | 0.64% | 5.2 |
| 3773 | AT1G80030.3 \| DNAJ heat shock protein, putative, similar to SP:Q05646 Chaperone protein dnaJ {Erysipelothrix rhusiopathiae}; contains Pfam profiles PF00226: DnaJ domain, PF01556: DnaJ C terminal region, PF00684: DnaJ central domain (4 repeats) \| chr1:30109917-30113859 REVERSE \| Aliases: F18B13.12, F18B13_12 | 4.1 | 3.4 | 0.7 | 5.4 | 0.64% | 4.9 |
| 3774 | AT2G42540.2 \| Symbol: COR15A \| | 5.7 | 2.5 | 3.2 | 5.4 | 0.64% | 5.0 |
| 3775 | AT5G51545.1 \| expressed protein \| chr5:20953660-20954626 FORWARD \| Aliases: None | 6.1 | 4.4 | 1.6 | 5.4 | 0.64% | 5.1 |

| Rank | Description | Sync | Root | M | t | adj.q | B |
|---|---|---|---|---|---|---|---|
| 3776 | AT3G06740.1 \| zinc finger (GATA type) family protein \| chr3:2126425-2127473 FORWARD \| Aliases: F3E22.12 | 6.5 | 4.4 | 2.1 | 5.4 | 0.64% | 5.2 |
| 3778 | AT2G05790.1 \| glycosyl hydrolase family 17 protein, similar to elicitor inducible chitinase Nt-SubE76 GI:11071974 from (Nicotiana tabacum) \| chr2:2199389-2201480 FORWARD \| Aliases: T25M19.1, T25M19_1 | 7.7 | 5.7 | 2.0 | 5.4 | 0.64% | 5.1 |
| 3779 | AT1G31340.1 \| Symbol: RUB1 \| ubiquitin family protein, contains INTERPRO:IPR000626 ubiquitin domain \| chr1:11217865-11219437 REVERSE \| Aliases: ATRUB1, NEDD8, RELATED TO UBIQUITIN 1, T19E23.13, T19E23_13 | 9.5 | 6.6 | 2.9 | 5.4 | 0.64% | 5.1 |
| 3782 | AT3G49910.1 \| 60S ribosomal protein L26 (RPL26A), 60S RIBOSOMAL PROTEIN L26, Brassica rapa, EMBL:BRD495 \| chr3:18515241-18515952 FORWARD \| Aliases: F3A4.4 | 9.2 | 7.5 | 1.8 | 5.4 | 0.64% | 5.1 |
| 3783 | AT1G15730.1 \| PRLI-interacting factor L, putative, strong similarity to PRLI-interacting factor L GI:11139268 from (Arabidopsis thaliana); contains Pfam profile PF02492: Cobalamin synthesis protein/P47K \| chr1:5407023-5409979 REVERSE \| Aliases: F7H2.7, F7H2_7 | 7.2 | 5.8 | 1.4 | 5.4 | 0.64% | 5.1 |
| 3785 | AT2G22450.1 \| riboflavin biosynthesis protein, putative, similar to SP:P50855 Riboflavin biosynthesis protein ribA (Includes: GTP cyclohydrolase II (EC 3.5.4.25); 3,4-dihydroxy-2-butanone 4-phosphate synthase (DHBP synthase)) {Actinobacillus pleuropneumoniae}; contains Pfam profiles PF00925: GTP cyclohydrolase II, PF00926: 3,4-dihydroxy-2-butanone 4-phosphate synthase \| chr2:9537627-9539990 FORWARD \| Aliases: F14M13.15, F14M13_15 | 6.6 | 4.8 | 1.7 | 5.4 | 0.65% | 5.1 |
| 3787 | AT1G06570.1 \| Symbol: PDS1 \| 4-hydroxyphenylpyruvate dioxygenase (HPD), identical to 4-hydroxyphenylpyruvate dioxygenase (HPD) SP:P93836 (Arabidopsis thaliana (Mouse-ear cress)) \| chr1:2011885-2013544 REVERSE \| Aliases: 4 HYDROXYPHENYLPYRUVATE DIOXYGENASE, F12K11.9, F12K11_9, HPD, P HYDROXYPHENYLPYRUVATE DIOXYGENASE, PHYTOENE DESATURATION 1 | 9.5 | 6.9 | 2.7 | 5.4 | 0.65% | 5.1 |
| 3788 | AT5G35080.1 \| expressed protein \| chr5:13371503-13374047 REVERSE \| Aliases: F7N22.4, F7N22_4 | 7.2 | 5.7 | 1.5 | 5.4 | 0.65% | 5.1 |
| 3789 | AT1G79040.1 \| photosystem II 10 kDa polypeptide, identical to photosystem II 10 kDa polypeptide, chloroplast (precursor) SP:P27202 from (Arabidopsis thaliana); contains Pfam profile: PF04725 photosystem II 10 kDa polypeptide PsbR \| chr1:29740911-29741831 FORWARD \| Aliases: YUP8H12R.34, YUP8H12R_34 | 11.3 | 9.9 | 1.5 | 5.4 | 0.65% | 5.1 |
| 3791 | AT1G02700.1 \| expressed protein, similar to hypothetical protein GB:CAB80707 GI:7268598 from (Arabidopsis thaliana) \| chr1:588329-589480 FORWARD \| Aliases: T14P4.2, T14P4_2 | 4.5 | 3.0 | 1.5 | 5.4 | 0.65% | 5.1 |
| 3792 | AT1G70580.4 \| Symbol: AOAT2 \| similar to glutamate:glyoxylate aminotransferase 1 (GGT1) [Arabidopsis thaliana] (TAIR:At1g23310.1); similar to alanine aminotransferase [Oryza sativa (indica cultivar-group)] (GB:AAO84040.1); contains InterPro domain Aminotransferase, class I and II (InterPro:IPR004839); contains InterPro domain 1-aminocyclopropane-1-carboxylate synthase (InterPro:IPR001176) \| chr1:26616525-26619732 FORWARD \| Aliases: ALANINE 2 OXOGLUTARATE AMINOTRANSFERASE 2, F5A18.24, F5A18_24, GGT2, GLUTAMATE:GLYOXYLATE AMINOTRANSFERASE 2 | 7.6 | 5.4 | 2.2 | 5.4 | 0.65% | 5.1 |
| 3793 | AT5G42190.1 \| Symbol: ASK2 \| E3 ubiquitin ligase SCF complex subunit SKP1/ASK1 (At2) / UFO-binding protein (UIP2), E3 ubiquitin ligase; skp1b; identical to UIP2 GI:3719211 from (Arabidopsis thaliana); contains Pfam profiles PF01466: Skp1 family, dimerisation domain and PF03931:Skp1 family, tetramerisation domain; identical to cDNA UFO binding protein UIP2 mRNA, partial cds GI:3719210 \| chr5:16870883-16872793 REVERSE \| Aliases: ARABIDOPSIS SKP1 LIKE 2, MJC20.30, MJC20_30, SKP1B | 11.0 | 8.6 | 2.4 | 5.4 | 0.65% | 5.1 |
| 3794 | AT5G01370.1 \| expressed protein \| chr5:152529-154350 FORWARD \| Aliases: T10O8.80, T10O8_80 | 6.2 | 4.4 | 1.8 | 5.4 | 0.65% | 5.1 |
| 3797 | AT5G08180.1 \| ribosomal protein L7Ae/L30e/S12e/Gadd45 family protein, Similar to NHP2/L7Ae family proteins, see SWISSPROT:P32495 and PMID:2063628. \| chr5:2631658-2633449 REVERSE \| Aliases: T22D6.120, T22D6_120 | 7.2 | 5.8 | 1.4 | 5.4 | 0.65% | 5.1 |
| 3798 | AT1G51730.1 \| RWD domain-containing protein, contains Pfam profile PF05773: RWD domain; similar to GCN2 eIF2alpha kinase (GI:6066585) (Mus musculus); similar to GCN2beta (GI:10764163) (Mus musculus); similar to RING finger protein 25 (RING finger protein AO7) (Swiss-Prot:Q9QZR0) (Mus musculus); similar to RING finger protein 25 (Swiss-Prot:Q96BH1) (Homo sapiens) \| chr1:19190171-19192441 REVERSE \| Aliases: F19C24.6, F19C24_6 | 8.3 | 6.1 | 2.1 | 5.4 | 0.65% | 5.1 |
| 3799 | AT2G35190.1 \| Symbol: NPSN11 \| | 6.8 | 4.9 | 1.8 | 5.4 | 0.65% | 5.1 |
| 3800 | AT4G13750.1 \| expressed protein \| chr4:7978944-7987554 FORWARD \| Aliases: F18A5.140, F18A5_140 | 4.7 | 3.4 | 1.2 | 5.4 | 0.65% | 5.1 |
| 3801 | AT3G02520.1 \| Symbol: GRF7 \| 14-3-3 protein GF14 nu (GRF7), identical to 14-3-3 protein GF14 nu GI:1531631 from (Arabidopsis thaliana) \| chr3:526444-528320 REVERSE \| Aliases: F16B3.15, F16B3_15, GF14 NU | 9.5 | 6.4 | 3.1 | 5.4 | 0.65% | 5.1 |
| 3802 | AT1G03100.1 \| pentatricopeptide (PPR) repeat-containing protein, low similarity to CRP1 (Zea mays) GI:3289002; contains Pfam profile PF01535: PPR repeat \| chr1:744026-746407 REVERSE \| Aliases: F10O3.8, F10O3_8 | 5.3 | 4.1 | 1.2 | 5.4 | 0.65% | 5.1 |
| 3803 | AT3G61620.1 \| Symbol: RRP41 \| exonuclease RRP41 (RRP41), identical to exonuclease RRP41 (Arabidopsis thaliana) GI:6164938 \| chr3:22812212-22814083 REVERSE \| Aliases: EXONUCLEASE RRP41, F15G16.10 | 6.3 | 5.2 | 1.2 | 5.4 | 0.65% | 5.1 |

| Rank | Description | Sync | Root | M | t | adj.q | B |
|---|---|---|---|---|---|---|---|
| 3804 | AT5G17070.1 \| expressed protein \| chr5:5613371-5616141 REVERSE \| Aliases: F2K13.220, F2K13_220 | 6.0 | 3.3 | 2.8 | 5.4 | 0.65% | 5.1 |
| 3809 | AT2G30150.1 \| UDP-glucoronosyl/UDP-glucosyl transferase family protein, contains Pfam profile: PF00201 UDP-glucoronosyl and UDP-glucosyl transferase \| chr2:12881783-12883199 FORWARD \| Aliases: T27E13.11, T27E13_11 | 8.7 | 7.1 | 1.6 | 5.4 | 0.65% | 5.1 |
| 3810 | AT3G05900.1 \| neurofilament protein-related, similar to NF-180 (GI:632549) (Petromyzon marinus) similar to Neurofilament triplet H protein (200 kDa neurofilament protein) (Neurofilament heavy polypeptide) (NF-H) (Swiss-Prot:P12036) (Homo sapiens) | 6.9 | 5.2 | 1.7 | 5.4 | 0.65% | 5.1 |
| 3812 | AT1G78650.1 \| expressed protein, weak similarity to DNA polymerase delta subunit 3 (DNA polymerase delta subunit p66) (Swiss-Prot:Q15054) (Homo sapiens) \| chr1:29587822-29590534 FORWARD \| Aliases: T30F21.2, T30F21_2 | 4.7 | 3.3 | 1.4 | 5.4 | 0.65% | 5.1 |
| 3813 | AT1G31410.1 \| putrescine-binding periplasmic protein-related, similar to Chain A, Putrescine Receptor (Potf) (GI:3891734) (Escherichia coli); similar to Chain C, Putrescine Receptor (Potf) (GI:3891736) (Escherichia coli); similar to Putrescine-binding periplasmic protein precursor. (Swiss-Prot:P31133) (Escherichia coli) \| chr1:11246895-11249340 REVERSE \| Aliases: T8E3.6, T8E3_6 | 7.0 | 5.5 | 1.5 | 5.4 | 0.65% | 5.1 |
| 3814 | AT2G27760.1 \| Symbol: ATIPT2 \| | 6.9 | 5.5 | 1.5 | 5.4 | 0.65% | 5.1 |
| 3819 | AT3G47500.1 \| Dof-type zinc finger domain-containing protein, identical to H-protein promoter binding factor-2a GI:3386546 from (Arabidopsis thaliana) | 6.8 | 6.0 | 0.8 | 5.4 | 0.66% | 5.1 |
| 3822 | AT1G67850.2 \| expressed protein, contains Pfam profile PF05212: Protein of unknown function (DUF707) \| chr1:25441633-25445782 FORWARD \| Aliases: F12A21.2, F12A21_2 | 6.0 | 4.5 | 1.6 | 5.4 | 0.66% | 5.1 |
| 3823 | AT1G11870.3 \| seryl-tRNA synthetase, putative / serine--tRNA ligase, putative, similar to PIR:T03949 serine--tRNA ligase (EC 6.1.1.11) serS {Zea mays}; contains Pfam profiles PF00587: tRNA synthetase class II core domain (G, H, P, S and T), PF02403: Seryl-tRNA synthetase N-terminal domain \| chr1:4003845-4006737 FORWARD \| Aliases: F12F1.29, F12F1_29 | 4.9 | 3.4 | 1.5 | 5.4 | 0.66% | 5.1 |
| 3825 | AT5G12110.1 \| elongation factor 1B alpha-subunit 1 (eEF1Balpha1), identical to elongation factor 1B alpha-subunit (Arabidopsis thaliana) GI:6686819 \| chr5:3914482-3915936 FORWARD \| Aliases: MXC9.7, MXC9_7 | 7.6 | 6.5 | 1.1 | 5.4 | 0.66% | 5.1 |
| 3826 | AT4G37660.1 \| ribosomal protein L12 family protein, ribosomal protein L12, Liberobacter africanum, U09675 \| chr4:17695490-17696264 FORWARD \| Aliases: F19F18.150, F19F18_150 | 5.0 | 2.3 | 2.7 | 5.4 | 0.66% | 4.9 |
| 3828 | AT1G54830.3 \| CCAAT-box binding transcription factor Hap5a, putative, similar to heme activated protein GI:6289057 from (Arabidopsis thaliana) GI:14577940 CCAAT-binding protein subunit HAP5 {Hypocrea jecorina} similar to Transcription factor GB:CAA74053 GI:2398533 from (Arabidopsis thaliana) similarity to transcription factor Hap5a similar to transcription factor Hap5a (Arabidopsis thaliana)(GI:6523090) | 8.2 | 6.8 | 1.4 | 5.4 | 0.66% | 5.1 |
| 3829 | AT5G05200.1 \| ABC1 family protein, contains Pfam domain, PF03109: ABC1 family \| chr5:1543606-1547138 REVERSE \| Aliases: K2A11.7, K2A11_7 | 9.5 | 8.5 | 1.0 | 5.4 | 0.66% | 5.0 |
| 3831 | AT5G07320.1 \| mitochondrial substrate carrier family protein, similar to peroxisomal Ca-dependent solute carrier (Oryctolagus cuniculus) GI:2352427 (mitochondrial carrier superfamily); contains INTERPRO:IPR001993 Mitochondrial substrate carrier family, INTERPRO:IPR002048 calcium-binding EF-hand domain | 6.4 | 4.6 | 1.7 | 5.4 | 0.66% | 5.1 |
| 3833 | AT4G17010.1 \| expressed protein \| chr4:9575831-9577922 FORWARD \| Aliases: DL4535W, FCAALL.229 | 6.7 | 4.1 | 2.5 | 5.4 | 0.66% | 5.1 |
| 3834 | AT1G10522.1 \| expressed protein \| chr1:3469637-3471880 FORWARD \| Aliases: None | 4.8 | 3.5 | 1.4 | 5.4 | 0.66% | 5.1 |
| 3836 | AT3G25140.1 \| Symbol: QUA1 \| glycosyl transferase family 8 protein, contains Pfam profile: PF01501 glycosyl transferase family 8 \| chr3:9154718-9156851 FORWARD \| Aliases: MJL12.8, QUASIMODO1 | 11.2 | 10.3 | 0.9 | 5.4 | 0.66% | 4.9 |
| 3837 | AT2G26400.1 \| acireductone dioxygenase (ARD/ARD') family protein, similar to iron-deficiency induced gene (Hordeum vulgare) GI:14522834, SIPL (Homo sapiens) GI:16551383; contains Pfam profile PF03079: ARD/ARD' family \| chr2:11238925-11240352 REVERSE \| Aliases: T9J22.7, T9J22_7 | 6.7 | 4.9 | 1.8 | 5.4 | 0.66% | 5.0 |
| 3840 | AT1G54110.1 \| cation exchanger, putative (CAX10), Ca2+:Cation Antiporter (CaCA) Family member PMID:11500563 \| chr1:20203086-20205363 FORWARD \| Aliases: F15I1.20 | 5.5 | 4.3 | 1.2 | 5.4 | 0.66% | 5.1 |
| 3843 | AT2G38090.1 \| myb family transcription factor, contains Pfam profile: PF00249 myb-like DNA-binding domain \| chr2:15951789-15954163 FORWARD \| Aliases: F16M14.2, F16M14_2 | 6.1 | 4.9 | 1.2 | 5.4 | 0.66% | 5.1 |
| 3844 | AT5G08580.1 \| calcium-binding EF hand family protein, contains INTERPRO:IPR002048 calcium-binding EF-hand domain \| chr5:2780513-2783067 REVERSE \| Aliases: MAH20.14, MAH20_14 | 6.8 | 5.1 | 1.7 | 5.4 | 0.66% | 5.1 |
| 3847 | AT1G20816.1 \| expressed protein \| chr1:7233757-7235117 FORWARD \| Aliases: F2D10.33, F2D10_33 | 4.9 | 2.5 | 2.5 | 5.4 | 0.66% | 4.8 |

| Rank | Description | Sync | Root | M | t | adj.q | B |
|---|---|---|---|---|---|---|---|
| 3848 | AT1G02370.1 \| pentatricopeptide (PPR) repeat-containing protein, low similarity to DNA-binding protein (Triticum aestivum) GI:6958202; contains Pfam profile PF01535: PPR repeat \| chr1:474500-476511 FORWARD \| Aliases: T6A9.6, T6A9_6 | 5.5 | 3.8 | 1.7 | 5.4 | 0.66% | 5.1 |
| 3849 | AT1G24360.1 \| 3-oxoacyl-(acyl-carrier protein) reductase, chloroplast / 3-ketoacyl-acyl carrier protein reductase, identical to 3-oxoacyl-(acyl-carrier protein) reductase SP:P33207 from (Arabidopsis thaliana) \| chr1:8640725-8643467 FORWARD \| Aliases: F21J9.2, F21J9.34, F21J9_34 | 11.0 | 9.8 | 1.2 | 5.4 | 0.66% | 5.0 |
| 3852 | AT3G15460.1 \| brix domain-containing protein, contains Pfam domain, PF04427: Brix domain \| chr3:5213343-5216268 REVERSE \| Aliases: MJK13.12 | 6.7 | 5.1 | 1.6 | 5.4 | 0.67% | 5.1 |
| 3853 | AT1G54580.1 \| Symbol: ACP2 \| acyl carrier protein, chloroplast, putative / ACP, putative, strong similarity to SP:P25701 Acyl carrier protein 2, chloroplast precursor (ACP) {Arabidopsis thaliana}; contains InterPro accession IPR003881: Isochorismatase \| chr1:20393100-20394682 FORWARD \| Aliases: ACYL CARRIER PROTEIN 2, T22H22.3, T22H22_3 | 12.3 | 11.2 | 1.1 | 5.4 | 0.67% | 5.0 |
| 3854 | AT1G54630.2 \| Symbol: ACP3 \| similar to acyl carrier protein, chloroplast, putative / ACP, putative [Arabidopsis thaliana] (TAIR:At1g54580.1); similar to acyl carrier protein [Brassica napus] (GB:CAA34248.1); contains InterPro domain Phosphopantetheine-binding domain (InterPro:IPR006163); contains InterPro domain Acyl carrier protein (ACP) (InterPro:IPR003231) \| chr1:20405044-20406671 REVERSE \| Aliases: ACYL CARRIER PROTEIN 3, T22H22.7, T22H22_7 | 12.3 | 11.2 | 1.1 | 5.4 | 0.67% | 5.0 |
| 3855 | AT2G28590.1 \| protein kinase family protein, contains protein kinase domain, Pfam:PF00069 \| chr2:12256912-12258745 FORWARD \| Aliases: T8O18.12, T8O18_12 | 5.3 | 4.0 | 1.2 | 5.4 | 0.67% | 5.1 |
| 3856 | AT4G31990.3 \| Symbol: ASP5 \| similar to aspartate aminotransferase, cytoplasmic isozyme 1 / transaminase A (ASP2) [Arabidopsis thaliana] (TAIR:At5g19550.1); similar to Asp aminotransferase (GB:1908424A); similar to aspartate transaminase (EC 2.6.1.1) AAT5 precursor - soybean (GB:S33528); similar to aspartate aminotransferase [Lotus corniculatus] (GB:AAC12674.1); similar to aspartate aminotransferase [Phaseolus vulgaris] (GB:AAN76499.1); similar to aspartate aminotransferase isozyme 5 [Glycine max] (GB:AAB26677.2); contains InterPro domain Aminotransferase, class I and II (InterPro:IPR004839); contains InterPro domain Aminotransferases class-I pyridoxal-phosphate-binding site (InterPro:IPR004838); contains InterPro domain Aspartate/other aminotransferase (InterPro:IPR000796) \| chr4:15470767-15473859 REVERSE \| Aliases: AAT3, ASPARTATE AMINOTRANSFERASE, ASPARTATE AMINOTRANSFERASE 5, ASPARTATE AMINOTRANSFERASE DEFICIENT 3, F11C18.15 | 11.0 | 9.3 | 1.7 | 5.4 | 0.67% | 5.1 |
| 3857 | AT1G17470.1 \| Symbol: ATDRG1 \| | 9.6 | 8.0 | 1.6 | 5.4 | 0.67% | 5.1 |
| 3859 | AT1G64490.1 \| expressed protein \| chr1:23955801-23956376 FORWARD \| Aliases: F1N19.6, F1N19_6 | 8.0 | 6.0 | 1.9 | 5.4 | 0.67% | 5.1 |
| 3861 | AT4G27440.2 \| Symbol: PORB \| similar to protochlorophyllide reductase A, chloroplast / PCR A / NADPH-protochlorophyllide oxidoreductase A (PORA) [Arabidopsis thaliana] (TAIR:At5g54190.1); similar to NADPH-protochlorophyllide oxidoreductase [Cucumis sativus] (GB:BAA21089.1); similar to protochlorophyllide reductase (EC 1.3.1.33) precursor - garden pea (GB:S20941); similar to protochlorophyllide reductase [Pisum sativum] (GB:CAA44786.1); similar to NADPH:protochlorophyllide oxidoreductase [Nicotiana tabacum] (GB:BAB93003.1); similar to NADPH:protochlorophyllide oxidoreductase [Daucus carota] (GB:AAF20949.1); contains InterPro domain Glucose/ribitol dehydrogenase (InterPro:IPR002347); contains InterPro domain Light-dependent protochlorophyllide reductase (InterPro:IPR005979); contains InterPro domain Short-chain dehydrogenase/reductase SDR (InterPro:IPR002198) | 6.8 | 4.7 | 2.2 | 5.4 | 0.67% | 5.1 |
| 3863 | AT3G21750.1 \| UDP-glucoronosyl/UDP-glucosyl transferase family protein, contains Pfam profile: PF00201 UDP-glucoronosyl and UDP-glucosyl transferase \| chr3:7664352-7666202 FORWARD \| Aliases: MSD21.8 | 5.4 | 3.4 | 2.1 | 5.4 | 0.67% | 4.7 |
| 3865 | AT3G06950.1 \| tRNA pseudouridine synthase family protein, similar to SP:P07649 tRNA pseudouridine synthase A (EC 4.2.1.70) (Uracil hydrolyase) {Escherichia coli}; contains Pfam profile PF01416: tRNA pseudouridine synthase \| chr3:2192805-2194278 FORWARD \| Aliases: F17A9.10 | 5.9 | 4.6 | 1.3 | 5.4 | 0.67% | 5.1 |
| 3868 | AT4G19350.1 \| Symbol: EMB3006 \| expressed protein \| chr4:10562034-10563768 REVERSE \| Aliases: EMB3006, EMBRYO DEFECTIVE 3006, T5K18.130, T5K18_130 | 6.6 | 5.0 | 1.6 | 5.4 | 0.68% | 5.1 |
| 3873 | AT1G31330.1 \| photosystem I reaction center subunit III family protein, contains Pfam profile: PF02507: photosystem I reaction center subunit III \| chr1:11214805-11215992 REVERSE \| Aliases: T19E23.12, T19E23_12 | 7.3 | 5.8 | 1.5 | 5.4 | 0.68% | 5.0 |
| 3878 | AT3G10670.1 \| Symbol: ATNAP7 \| | 9.3 | 6.6 | 2.8 | 5.4 | 0.68% | 5.1 |
| 3879 | AT5G15930.1 \| Symbol: PAM1 \| plant adhesion molecule 1 (PAM1), identical to plant adhesion molecule 1 (Arabidopsis thaliana) GI:3511223; contains Pfam profile PF00566: TBC domain \| chr5:5199749-5202452 FORWARD \| Aliases: F1N13.70, F1N13_70, PLANT ADHESION MOLECULE 1 | 7.5 | 6.1 | 1.4 | 5.4 | 0.68% | 5.1 |
| 3880 | AT2G36660.1 \| Symbol: PAB7 \| polyadenylate-binding protein, putative / PABP, putative. Member of the class III family of PABP proteins. \| chr2:15368400-15371477 REVERSE \| Aliases: F13K3.6, F13K3_6, PAB7, POLY(A) BINDING PROTEIN 7 | 4.5 | 3.4 | 1.1 | 5.4 | 0.68% | 5.1 |
| 3881 | AT5G16400.1 \| thioredoxin, putative, similar to SP:P29450 Thioredoxin F-type, chloroplast precursor (TRX-F) {Pisum sativum}; contains Pfam profile: PF00085 Thioredoxin \| chr5:5363664-5365319 REVERSE \| Aliases: MQK4.13, MQK4_13 | 6.7 | 5.2 | 1.5 | 5.4 | 0.68% | 5.1 |
| 3882 | AT3G20680.1 \| expressed protein \| chr3:7230078-7231200 REVERSE \| Aliases: F3H11.7 | 4.4 | 3.3 | 1.1 | 5.4 | 0.68% | 5.1 |

| Rank | Description | Sync | Root | M | t | adj.q | B |
|---|---|---|---|---|---|---|---|
| 3883 | AT2G30970.1 \| Symbol: ASP1 \| aspartate aminotransferase, mitochondrial / transaminase A (ASP1), identical to SP:P46643 Aspartate aminotransferase, mitochondrial precursor (EC 2.6.1.1) (Transaminase A) {Arabidopsis thaliana} \| chr2:13185926-13188984 FORWARD \| Aliases: ASPARTATE AMINOTRANSFERASE 1, F7F1.18, F7F1_18 | 10.7 | 6.3 | 4.3 | 5.4 | 0.68% | 5.1 |
| 3884 | AT3G54560.1 \| histone H2A.F/Z, identical to GI:2407800 | 8.0 | 5.4 | 2.6 | 5.4 | 0.68% | 5.1 |
| 3885 | AT5G42240.1 \| Symbol: SCPL42 \| | 4.5 | 3.3 | 1.2 | 5.4 | 0.68% | 5.1 |
| 3887 | AT1G31020.1 \| thioredoxin o (TRXO2), similar to thioredoxin 2 from Saccharomyces cerevisiae GI:173050, 3'-end of protein contains similarity to thioredoxins; contains Pfam profile: PF00085 Thioredoxin; identical to cDNA thioredoxin o (TRXO2) GI:15081458 | 6.4 | 5.2 | 1.3 | 5.3 | 0.69% | 5.0 |
| 3891 | AT2G28930.3 \| Symbol: APK1B \| similar to protein kinase (APK1a) [Arabidopsis thaliana] (TAIR:At1g07570.2); similar to protein kinase (APK1a) [Arabidopsis thaliana] (TAIR:At1g07570.1); similar to putative protein kinase [Oryza sativa] (GB:XP_493889.1); similar to putative protein kinase [Oryza sativa (japonica cultivar-group)] (GB:NP_910058.1); contains InterPro domain Serine/threonine protein kinase, active site (InterPro:IPR008271); contains InterPro domain Tyrosine protein kinase (InterPro:IPR001245); contains InterPro domain Serine/threonine protein kinase (InterPro:IPR002290); contains InterPro domain Protein kinase (InterPro:IPR000719) \| chr2:12431381-12434189 FORWARD \| Aliases: T9I4.1, T9I4_1 | 6.5 | 5.0 | 1.6 | 5.3 | 0.69% | 5.1 |
| 3892 | AT1G23310.2 \| Symbol: GGT1 \| similar to glutamate:glyoxylate aminotransferase 2 (GGT2) [Arabidopsis thaliana] (TAIR:At1g70580.1); similar to glutamate:glyoxylate aminotransferase 2 (GGT2) [Arabidopsis thaliana] (TAIR:At1g70580.2); similar to alanine aminotransferase [Oryza sativa (indica cultivar-group)] (GB:AAO84040.1); contains InterPro domain 1-aminocyclopropane-1-carboxylate synthase (InterPro:IPR001176) \| chr1:8268393-8271922 REVERSE \| Aliases: ALANINE 2 OXOGLUTARATE AMINOTRANSFERASE 1, AOAT1, F26F24.16, F26F24_16, GLUTAMATE:GLYOXYLATE AMINOTRANSFERASE | 10.3 | 9.0 | 1.3 | 5.3 | 0.69% | 5.0 |
| 3896 | AT3G62910.1 \| Symbol: APG3 \| peptide chain release factor, putative, similar to peptide chain release factor 1 (Escherichia coli) GI:147567; contains Pfam profiles PF00472: Peptidyl-tRNA hydrolase domain, PF03462: PCRF domain \| chr3:23268530-23271402 REVERSE \| Aliases: ALBINO AND PALE GREEN, T20O10.10 | 5.0 | 3.6 | 1.4 | 5.3 | 0.69% | 5.1 |
| 3897 | AT1G08280.1 \| glycosyl transferase family 29 protein / sialyltransferase family protein, contains Pfam profile: PF00777 sialyltransferase (Glycosyltransferase family 29) \| chr1:2608405-2609850 FORWARD \| Aliases: T23G18.14, T23G18_14 | 5.9 | 4.0 | 2.0 | 5.3 | 0.69% | 5.0 |
| 3902 | AT1G54770.1 \| expressed protein \| chr1:20438329-20439675 FORWARD \| Aliases: T22H22.18, T22H22_18 | 4.9 | 3.6 | 1.3 | 5.3 | 0.69% | 5.1 |
| 3905 | AT4G14965.1 \| cytochrome b5 domain-containing protein, similar to SP:O15173 Membrane associated progesterone receptor component 2 (Steroid receptor protein DG6) {Homo sapiens}; contains Pfam profile PF00173: Heme/Steroid binding domain \| chr4:8551240-8553549 FORWARD \| Aliases: None | 7.3 | 6.3 | 1.0 | 5.3 | 0.69% | 5.0 |
| 3906 | AT3G55970.1 \| oxidoreductase, 2OG-Fe(II) oxygenase family protein, similar to leucoanthocyanidin dioxygenase, Malus domestica, SP:P51091; contains Pfam profile PF03171: oxidoreductase, 2OG-Fe(II) oxygenase family \| chr3:20777718-20780303 REVERSE \| Aliases: F27K19.150 | 3.6 | 2.5 | 1.1 | 5.3 | 0.69% | 5.0 |
| 3908 | AT1G54570.1 \| esterase/lipase/thioesterase family protein, contains Interpro entry IPR000379 \| chr1:20384145-20388688 REVERSE \| Aliases: T22H22.2, T22H22_2 | 7.1 | 5.8 | 1.3 | 5.3 | 0.69% | 5.0 |
| 3909 | AT5G04140.2 \| Symbol: GLU1 \| glutamate synthase (GLU1) / ferredoxin-dependent glutamate synthase (Fd-GOGAT 1), identical to ferredoxin-dependent glutamate synthase precursor (Arabidopsis thaliana) GI:3869251 \| chr5:1130032-1138439 FORWARD \| Aliases: F21E1.60, F21E1_60, FERREDOXIN DEPENDENT GLUAMATE SYNTHASE 1, GLS1, GLUAMATE SYNTHASE 1, GLUTAMATE SYNTHASE | 6.3 | 4.5 | 1.8 | 5.3 | 0.69% | 5.0 |
| 3912 | AT1G48630.1 \| guanine nucleotide-binding family protein / activated protein kinase C receptor, putative / RACK, putative, contains 7 WD-40 repeats (PF00400); very similar to guanine nucleotide-binding protein; activated protein kinase C receptor; RACK1 (GI:9294068) {Arabidopsis thaliana}; similar to WD-40 repeat auxin-dependent protein ARCA (SP:O24456) (Arabidopsis thaliana); \| chr1:17985448-17986995 REVERSE \| Aliases: F11I4.18, F11I4_18 | 10.8 | 8.9 | 1.9 | 5.3 | 0.70% | 5.0 |
| 3913 | AT3G26420.1 \| Symbol: ARRZ 1A \| Zinc finger-containing glycine-rich RNA-binding protein. Cold-inducible. Contributes to the enhancement of freezing tolerance. \| chr3:9672754-9677242 FORWARD \| Aliases: ARRZ 1A, F20C19.15 | 7.1 | 6.1 | 1.0 | 5.3 | 0.70% | 4.9 |
| 3914 | AT3G26430.1 \| GDSL-motif lipase/hydrolase family protein, similar to early nodulin ENOD8 (Medicago sativa) GI:304037; contains InterPro Entry IPR001087 Lipolytic enzyme, G-D-S-L family \| chr3:9672754-9677164 FORWARD \| Aliases: F20C19.19 | 7.1 | 6.1 | 1.0 | 5.3 | 0.70% | 4.9 |
| 3916 | AT3G17940.1 \| aldose 1-epimerase family protein, similar to ALDOSE 1-EPIMERASE PRECURSOR GB:P05149 (SP:P05149) from (Acinetobacter calcoaceticus); contains Pfam profile PF01263 Aldose 1-epimerase \| chr3:6143525-6145324 REVERSE \| Aliases: MEB5.16 | 9.7 | 8.6 | 1.0 | 5.3 | 0.70% | 4.9 |
| 3918 | AT1G12320.1 \| expressed protein \| chr1:4189353-4190340 FORWARD \| Aliases: F5O11.5, F5O11_5 | 6.7 | 5.3 | 1.4 | 5.3 | 0.70% | 5.0 |
| 3919 | AT3G19590.1 \| WD-40 repeat family protein / mitotic checkpoint protein, putative, contains 5 WD-40 repeats (PF00400) (1 weak); similar to testis mitotic checkpoint protein BUB3 (GB:AAC28439,SP:O43684)(Homo sapiens) | 6.2 | 4.8 | 1.4 | 5.3 | 0.70% | 5.0 |

| Rank | Description | Sync | Root | M | t | adj.q | B |
|---|---|---|---|---|---|---|---|
| 3921 | AT1G26230.1 \| chaperonin, putative, similar to SWISS-PROT:P08927- RuBisCO subunit binding-protein beta subunit, chloroplast precursor (60 kDa chaperonin beta subunit, CPN-60 beta) (Pisum sativum); contains Pfam:PF00118 domain, TCP-1/cpn60 chaperonin family \| chr1:9072375-9075266 REVERSE \| Aliases: F28B23.11, F28B23_11 | 4.5 | 3.1 | 1.4 | 5.3 | 0.70% | 5.0 |
| 3923 | AT4G05050.2 \| Symbol: UBQ11 \| similar to polyubiquitin (UBQ14) [Arabidopsis thaliana] (TAIR:At4g02890.1); similar to polyubiquitin (UBQ3) [Arabidopsis thaliana] (TAIR:At5g03240.2); similar to polyubiquitin (UBQ4) [Arabidopsis thaliana] (TAIR:At5g20620.1); similar to polyubiquitin (UBQ14) [Arabidopsis thaliana] (TAIR:At4g02890.2); similar to polyubiquitin (UBQ14) [Arabidopsis thaliana] (TAIR:At4g02890.3); similar to ubiquitin-like protein [Phaseolus vulgaris] (GB:AAB36545.1); similar to polyubiquitin [Fragaria x ananassa] (GB:AAB68045.1); similar to pentameric polyubiquitin (GB:AAA34124.1); contains InterPro domain Ubiquitin domain (InterPro:IPR000626) \| chr4:2588002-2589137 REVERSE \| Aliases: C17L7.6, UBIQUITIN 11 | 13.5 | 12.4 | 1.1 | 5.3 | 0.70% | 4.4 |
| 3924 | AT1G07440.2 \| similar to short-chain dehydrogenase/reductase (SDR) family protein [Arabidopsis thaliana] (TAIR:At2g29340.2); similar to short-chain dehydrogenase/reductase (SDR) family protein [Arabidopsis thaliana] (TAIR:At2g29340.1); similar to putative pfam00106, adh_short, short chain dehydrogenase [Oryza sativa (japonica cultivar-group)] (GB:NP_912375.1); contains InterPro domain Glucose/ribitol dehydrogenase (InterPro:IPR002347); contains InterPro domain Short-chain dehydrogenase/reductase SDR (InterPro:IPR002198) \| chr1:2286146-2287727 REVERSE \| Aliases: F22G5.39, F22G5_39 | 5.6 | 4.0 | 1.7 | 5.3 | 0.70% | 5.0 |
| 3925 | AT2G22475.2 \| GRAM domain-containing protein / ABA-responsive protein-related, similar to ABA-responsive protein (Hordeum vulgare) GI:4103635; contains Pfam profile PF02893: GRAM domain \| chr2:9548481-9552089 FORWARD \| Aliases: None | 7.5 | 6.2 | 1.3 | 5.3 | 0.70% | 5.0 |
| 3927 | AT3G45590.1 \| Symbol: ATSEN1 \| | 7.7 | 6.3 | 1.4 | 5.3 | 0.70% | 5.0 |
| 3929 | AT1G60230.1 \| radical SAM domain-containing protein, contains Pfam profile PF04055: radical SAM domain protein \| chr1:22215749-22218089 REVERSE \| Aliases: T13D8.12, T13D8_12 | 4.9 | 3.4 | 1.5 | 5.3 | 0.71% | 5.0 |
| 3930 | AT1G18710.1 \| myb family transcription factor (MYB47), contains Pfam profile: PF00249 myb-like DNA-binding domain \| chr1:6450586-6453106 FORWARD \| Aliases: F6A14.18, F6A14_18 | 4.3 | 2.8 | 1.5 | 5.3 | 0.71% | 5.0 |
| 3931 | AT4G14800.1 \| Symbol: PBD2 \| 20S proteasome beta subunit D2 (PBD2) (PRCGA), identical to SP:O24633 Proteasome subunit beta type 2-2 (EC 3.4.25.1) (20S proteasome alpha subunit D2) {Arabidopsis thaliana}, cDNA proteasome subunit prcga GI:2511571 \| chr4:8500283-8502224 FORWARD \| Aliases: 20S PROTEASOME BETA SUBUNIT 2, 20S PROTEASOME BETA SUBUNIT PBD2, DL3440W, FCAALL.135 | 10.9 | 8.6 | 2.4 | 5.3 | 0.71% | 5.0 |
| 3932 | AT1G75280.1 \| isoflavone reductase, putative, identical to SP:P52577 Isoflavone reductase homolog P3 (EC 1.3.1.-) {Arabidopsis thaliana}; contains Pfam profile PF02716: isoflavone reductase. Involved in response to oxidative stress. \| chr1:28255622-28257280 FORWARD \| Aliases: F22H5.17, F22H5_17, P3 | 11.7 | 8.2 | 3.6 | 5.3 | 0.71% | 5.0 |
| 3933 | AT3G52960.1 \| peroxiredoxin type 2, putative, similar to type 2 peroxiredoxin (Brassica rapa subsp. pekinensis) GI:4928472; contains Pfam profile: PF00578 AhpC/TSA (alkyl hydroperoxide reductase and thiol-specific antioxidant) family \| chr3:19650646-19651597 FORWARD \| Aliases: F8J2.130 | 8.3 | 6.7 | 1.6 | 5.3 | 0.71% | 5.0 |
| 3934 | AT5G17990.1 \| Symbol: TRP1 \| anthranilate phosphoribosyltransferase, identical to anthranilate phosphoribosyltransferase, chloroplast precursor (EC 2.4.2.18) SP:Q02166 from (Arabidopsis thaliana) \| chr5:5957215-5959906 FORWARD \| Aliases: MCM23.6, MCM23_6, PAT1, PHOSPHORIBOSYLANTHRANILATE TRANSFERASE, PHOSPHORIBOSYLANTHRANILATE TRANSFERASE 1, TRYPTOPHAN BIOSYNTHESIS 1 | 9.6 | 7.2 | 2.4 | 5.3 | 0.71% | 5.0 |
| 3935 | AT3G02220.1 \| expressed protein \| chr3:412090-413611 REVERSE \| Aliases: F14P3.13, F14P3_13 | 7.9 | 5.9 | 2.0 | 5.3 | 0.71% | 5.0 |
| 3937 | AT2G39730.3 \| Symbol: RCA \| ribulose bisphosphate carboxylase/oxygenase activase / RuBisCO activase, identical to SWISS-PROT:P10896 ribulose bisphosphate carboxylase/oxygenase activase, chloroplast precursor (RuBisCO activase, RA)(Arabidopsis thaliana) \| chr2:16577824-16580597 REVERSE \| Aliases: RUBISCO ACTIVASE, T5I7.18 | 8.8 | 5.1 | 3.7 | 5.3 | 0.71% | 5.0 |
| 3939 | AT3G57520.3 \| alkaline alpha galactosidase, putative, similar to alkaline alpha galactosidase II (Cucumis melo) GI:29838631; contains Pfam profile PF05691: Raffinose synthase or seed imbibition protein Sip1 | 7.0 | 5.1 | 1.9 | 5.3 | 0.71% | 5.0 |
| 3941 | AT4G00370.1 \| Symbol: ANTR2 \| sugar transporter family protein, contains Pfam profile PF00083: major facilitator superfamily protein \| chr4:161539-166357 REVERSE \| Aliases: A_IG005I10.NN, A_IG005I10_NN, F5I10.7, F5I10_7 | 7.6 | 5.4 | 2.2 | 5.3 | 0.71% | 4.9 |
| 3942 | AT5G14450.1 \| GDSL-motif lipase/hydrolase family protein, similar to early nodulin ENOD8 (Medicago sativa) GI:304037, elicitor-induced glycoprotein iEP4 (Daucus carota) GI:1911765, pollen-expressed coil protein (Medicago sativa) GI:1110502; contains InterPro Entry IPR001087 Lipolytic enzyme, G-D-S-L family \| chr5:4658444-4660178 FORWARD \| Aliases: F18O22.240, F18O22_240 | 4.5 | 3.6 | 0.9 | 5.3 | 0.71% | 5.0 |
| 3946 | AT5G07330.1 \| expressed protein \| chr5:2315981-2316875 FORWARD \| Aliases: T2I1.40, T2I1_40 | 4.2 | 2.3 | 1.9 | 5.3 | 0.71% | 5.0 |
| 3948 | AT2G17670.2 \| pentatricopeptide (PPR) repeat-containing protein, contains Pfam profile PF01535: PPR repeat \| chr2:7681433-7683239 FORWARD \| Aliases: T17A5.11, T17A5_11 | 7.0 | 5.8 | 1.2 | 5.3 | 0.71% | 5.0 |

| Rank | Description | Sync | Root | M | t | adj.q | B |
|---|---|---|---|---|---|---|---|
| 3951 | AT1G53290.1 \| galactosyltransferase family protein, contains Pfam profile: PF01762 galactosyltransferase ;contains similarity to Avr9 elicitor response protein GI:4138265 from (Nicotiana tabacum) \| chr1:19874967-19877158 FORWARD \| Aliases: F12M16.19, F12M16_19 | 8.8 | 6.8 | 2.0 | 5.3 | 0.71% | 5.0 |
| 3954 | AT5G20230.1 \| Symbol: ATBCB \| plastocyanin-like domain-containing protein \| chr5:6826508-6827601 FORWARD \| Aliases: ARABIDOPSIS BLUE COPPER BINDING PROTEIN, BCB, F5O24.120, F5O24_120 | 5.3 | 2.6 | 2.7 | 5.3 | 0.72% | 5.0 |
| 3956 | AT4G17610.1 \| tRNA/rRNA methyltransferase (SpoU) family protein, similar to TAR RNA loop binding protein (Homo sapiens) GI:1184692; contains Pfam profile PF00588: SpoU rRNA Methylase (RNA methyltransferase, TrmH) family \| chr4:9805665-9814923 REVERSE \| Aliases: DL4840C, FCAALL.71 | 7.2 | 5.8 | 1.4 | 5.3 | 0.72% | 5.0 |
| 3960 | AT1G04760.1 \| Symbol: ATVAMP726 \| synaptobrevin family protein, similar to Vesicle-associated membrane protein 722 (AtVAMP722) Synaptobrevin-related protein 1 (SP:P47192) {Arabidopsis thaliana} \| chr1:1334759-1336069 FORWARD \| Aliases: F13M7.25, F13M7_25, VAMP726 | 4.6 | 3.5 | 1.1 | 5.3 | 0.72% | 5.0 |
| 3962 | AT4G14930.1 \| acid phosphatase survival protein SurE, putative, similar to Swiss-Prot:P36664 acid phosphatase surE (EC 3.1.3.2) (Stationary-phase survival protein surE) (Escherichia coli O157:H7); contains Pfam domain PF01975: Survival protein SurE \| chr4:8538582-8541895 FORWARD \| Aliases: DL3505W, FCAALL.200 | 6.9 | 4.8 | 2.1 | 5.3 | 0.72% | 5.0 |
| 3963 | AT4G33780.1 \| expressed protein \| chr4:16201833-16203643 REVERSE \| Aliases: T16L1.270, T16L1_270 | 8.5 | 6.9 | 1.6 | 5.3 | 0.72% | 5.0 |
| 3966 | AT5G58330.3 \| malate dehydrogenase (NADP), chloroplast, putative, strong similiarity to chloroplast NADP-dependent malate dehydrogenase (EC 1.1.1.82) SP:O48902 {Medicago sativa}, SP:P21528 {Pisum sativum}, SP:Q05145 {Mesembryanthemum crystallinum}, SP:P46489 {Flaveria bidentis}, (Flaveria trinervia) GI:726334, SP:P17606I {Sorghum bicolor}; contains InterPro entry IPR001236: Lactate/malate dehydrogenase \| chr5:23596427-23599521 REVERSE \| Aliases: MCK7.20, MCK7_20 | 8.1 | 5.5 | 2.6 | 5.3 | 0.72% | 4.9 |
| 3970 | ATCG00070.1 \| Symbol: PSBK \| PSII K protein \| chrC:7017-7202 FORWARD \| Aliases: PSBK | 5.2 | 3.2 | 2.0 | 5.3 | 0.72% | 5.0 |
| 3971 | AT2G24790.2 \| zinc finger (B-box type) family protein \| chr2:10573977-10574702 FORWARD \| Aliases: F27A10.10 | 7.0 | 5.4 | 1.6 | 5.3 | 0.73% | 5.0 |
| 3972 | AT3G57880.1 \| C2 domain-containing protein, contains INTERPRO:IPR000008 C2 domain \| chr3:21441993-21445363 REVERSE \| Aliases: T10K17.90 | 5.5 | 4.4 | 1.2 | 5.3 | 0.73% | 5.0 |
| 3973 | AT4G20280.1 \| transcription initiation factor IID (TFIID) 28 kDa subunit (TAFII-28) family protein, similar to SP:Q15544 Transcription initiation factor TFIID 28 kDa subunit (TAFII-28) (TAFII28) (TFIID subunit p30-beta) {Homo sapiens}; contains Pfam profile PF04719: hTAFII28-like protein conserved region \| chr4:10953557-10954983 FORWARD \| Aliases: F1C12.195, F1C12_195 | 7.4 | 6.3 | 1.1 | 5.3 | 0.73% | 5.0 |
| 3974 | AT5G19350.1 \| RNA-binding protein 45 (RBP45), putative | 8.9 | 7.9 | 1.0 | 5.3 | 0.73% | 4.9 |
| 3975 | AT1G66670.1 \| Symbol: CLPP3 \| ATP-dependent Clp protease proteolytic subunit (ClpP3), identical to ATP-dependent Clp protease (nClpP3) GI:5360591 (Arabidopsis thaliana) \| chr1:24867448-24869363 REVERSE \| Aliases: F4N21.19, F4N21_19, NCLPP3, NCLPP4 | 8.8 | 7.2 | 1.7 | 5.3 | 0.73% | 5.0 |
| 3976 | AT1G07770.2 \| Symbol: RPS15A \| | 11.3 | 10.5 | 0.9 | 5.3 | 0.73% | 4.8 |
| 3977 | AT1G07170.1 \| expressed protein, contains Pfam domain PF03660: Uncharacterised protein family (UPF0123) \| chr1:2200133-2201139 FORWARD \| Aliases: F10K1.35 | 9.1 | 8.1 | 1.0 | 5.3 | 0.73% | 4.9 |
| 3978 | AT1G54250.1 \| Symbol: ATRPABC16.5 \| DNA-directed RNA polymerase I, II, and III, putative, similar to SP:P52434 DNA-directed RNA polymerases I, II, and III 17.1 kDa polypeptide (EC 2.7.7.6) (RPB17) (RPB8) {Homo sapiens}; contains Pfam profile PF03870: RNA polymerase Rpb8 \| chr1:20258605-20260220 FORWARD \| Aliases: F20D21.7, F20D21_7, RNA POLYMERASE I, II AND III 16.5 KDA SUBUNIT | 7.1 | 5.4 | 1.7 | 5.3 | 0.73% | 4.9 |
| 3979 | AT3G59600.1 \| DNA-directed RNA polymerase I, II, and III, putative, similar to SP:P52434 DNA-directed RNA polymerases I, II, and III 17.1 kDa polypeptide (EC 2.7.7.6) (RPB17) (RPB8) {Homo sapiens}; contains Pfam profile PF03870: RNA polymerase Rpb8 \| chr3:22027362-22029073 FORWARD \| Aliases: T16L24.150 | 7.1 | 5.4 | 1.7 | 5.3 | 0.73% | 4.9 |
| 3980 | AT3G19820.2 \| Symbol: DWF1 \| cell elongation protein / DWARF1 / DIMINUTO (DIM), identical to GB:S71189 (SP:Q39085) from (Arabidopsis thaliana); contains Pfam FAD binding domain PF01565 \| chr3:6879624-6882265 REVERSE \| Aliases: CABBAGE 1, CBB1, DIM, DIM1, DIMINUTIA, DIMINUTO 1, DW1, DWARF 1, EVE1, MPN9.6 | 9.7 | 7.0 | 2.6 | 5.3 | 0.73% | 5.0 |
| 3981 | AT1G72550.2 \| tRNA synthetase beta subunit family protein, contains Pfam profiles: PF03484 phenylalanine-tRNA synthetase, B5 domain, PF03483 B3/4 domain; an isoform contains a non-consensus TG acceptor splice site at a terminal exon. \| chr1:27323413-27327677 REVERSE \| Aliases: F28P22.26, F28P22_26 | 8.9 | 7.0 | 1.9 | 5.3 | 0.73% | 5.0 |
| 3985 | AT2G39510.1 \| nodulin MtN21 family protein, similar to MtN21 GI:2598575 (root nodule development) from (Medicago truncatula) \| chr2:16498221-16500210 REVERSE \| Aliases: F12L6.17, F12L6_17 | 5.9 | 4.0 | 1.9 | 5.3 | 0.74% | 4.7 |

| Rank | Description | Sync | Root | M | t | adj.q | B |
|---|---|---|---|---|---|---|---|
| 3986 | AT3G55620.1 \| Symbol: EMB1624 \| eukaryotic translation initiation factor 6, putative / eIF-6, putative, similar to SP:O55135 Eukaryotic translation initiation factor 6 (eIF-6) (B4 integrin interactor) {Mus musculus}; contains Pfam profile PF01912: eIF-6 family \| chr3:20645182-20647485 FORWARD \| Aliases: EMB1624, EMBRYO DEFECTIVE 1624, F1I16.30 | 10.1 | 8.2 | 1.9 | 5.3 | 0.74% | 4.9 |
| 3988 | AT1G02140.1 \| mago nashi family protein, similar to Mago Nashi, Genbank Accession Number U03559; contains Pfam PF02792: Mago nashi protein domain \| chr1:403195-404439 REVERSE \| Aliases: T7I23.7, T7I23_7 | 9.7 | 6.9 | 2.7 | 5.3 | 0.74% | 5.0 |
| 3991 | AT5G13120.1 \| peptidyl-prolyl cis-trans isomerase cyclophilin-type family protein, contains Pfam domain, PF00160: peptidyl-prolyl cis-trans isomerase, cyclophilin-type \| chr5:4162506-4164787 REVERSE \| Aliases: T19L5.80, T19L5_80 | 9.3 | 8.1 | 1.2 | 5.3 | 0.74% | 4.7 |
| 3992 | AT2G02170.1 \| remorin family protein, contains Pfam domain, PF03763: Remorin, C-terminal region \| chr2:556492-558797 REVERSE \| Aliases: F5O4.6, F5O4_6 | 5.4 | 4.1 | 1.3 | 5.3 | 0.74% | 5.0 |
| 3993 | AT2G45600.1 \| expressed protein, low similarity to PrMC3 (Pinus radiata) GI:5487873 \| chr2:18796662-18798026 FORWARD \| Aliases: F17K2.13 | 4.0 | 2.6 | 1.4 | 5.3 | 0.74% | 5.0 |
| 3996 | AT3G01920.1 \| yrdC family protein, contains Pfam PF01300: yrdC domain; similar to Protein yciO. (Swiss-Prot:P45847) (Shigella flexneri); similar to Chain A, Crystal Structure Of E. Coli Ycio Length (GI:27573707) (Escherichia coli) | 5.7 | 4.2 | 1.4 | 5.3 | 0.74% | 5.0 |
| 3998 | AT4G17370.1 \| oxidoreductase family protein, weak similarity to SP:P26935 Myo-inositol 2-dehydrogenase (EC 1.1.1.18). {Bacillus subtilis}; contains Pfam profiles PF01408: Oxidoreductase family NAD-binding Rossmann fold, PF02894: Oxidoreductase family C-terminal alpha/beta domain \| chr4:9706070-9708273 FORWARD \| Aliases: DL4720W, FCAALL.420 | 8.1 | 7.2 | 0.9 | 5.3 | 0.74% | 4.9 |
| 3999 | AT1G07220.1 \| expressed protein \| chr1:2217072-2219378 REVERSE \| Aliases: F10K1.7, F10K1_7 | 5.3 | 3.7 | 1.6 | 5.3 | 0.74% | 5.0 |
| 4001 | AT2G16060.1 \| Symbol: AHB1 \| non-symbiotic hemoglobin 1 (HB1) (GLB1), identical to SP:O24520 Non-symbiotic hemoglobin 1 (Hb1) (ARAth GLB1) {Arabidopsis thaliana} \| chr2:6989703-6990713 REVERSE \| Aliases: ARATH GLB1, F7H1.8, F7H1_8, GLB1, HEMOGLOBIN, NSHB1 | 10.3 | 9.1 | 1.3 | 5.3 | 0.75% | 5.0 |
| 4002 | AT3G47450.2 \| expressed protein \| chr3:17494053-17497332 REVERSE \| Aliases: T21L8.200 | 5.5 | 3.7 | 1.8 | 5.3 | 0.75% | 4.8 |
| 4005 | AT5G12890.1 \| UDP-glucoronosyl/UDP-glucosyl transferase family protein, contains Pfam profile: PF00201 UDP-glucoronosyl and UDP-glucosyl transferase \| chr5:4069580-4071230 REVERSE \| Aliases: T24H18.60, T24H18_60 | 6.9 | 5.8 | 1.1 | 5.3 | 0.75% | 4.8 |
| 4006 | AT2G43500.1 \| RWP-RK domain-containing protein, low similarity to nodule inception protein (Lotus japonicus) GI:6448579; contains Pfam profile: PF02042 RWP-RK domain \| chr2:18069792-18073527 FORWARD \| Aliases: T1O24.24 | 5.7 | 4.3 | 1.4 | 5.3 | 0.75% | 5.0 |
| 4010 | AT3G54110.1 \| Symbol: ATPUMP1 \| plant uncoupling mitochondrial protein (PUMP), identical to plant uncoupling mitochondrial protein (Arabidopsis thaliana) GI:3115108 \| chr3:20049670-20052179 FORWARD \| Aliases: F24B22.70, PUMP, UCP, UCP2, UNCOUPLING PROTEIN, UNCOUPLING PROTEIN PUMP2 | 10.9 | 8.6 | 2.3 | 5.3 | 0.75% | 4.9 |
| 4011 | AT5G06700.1 \| expressed protein, strong similarity to unknown protein (emb:CAB82953.1) | 8.4 | 6.7 | 1.7 | 5.3 | 0.75% | 4.9 |
| 4012 | AT1G53000.1 \| cytidylyltransferase family, contains Pfam profile: PF02348 cytidylyltransferase | 7.7 | 5.3 | 2.4 | 5.3 | 0.75% | 4.9 |
| 4015 | AT1G44835.2 \| similar to 25.7 kDa protein [Cicer arietinum] (GB:CAB81546.1); contains InterPro domain YbaK/prolyl-tRNA synthetase associated region (InterPro:IPR007214) \| chr1:16942246-16944949 FORWARD \| Aliases: T12C22.11, T12C22_11 | 5.9 | 4.7 | 1.2 | 5.2 | 0.75% | 4.9 |
| 4019 | AT4G15520.1 \| tRNA/rRNA methyltransferase (SpoU) family protein, similar to SP:P19396 tRNA (Guanosine-2'-O-)-methyltransferase (EC 2.1.1.34) {Escherichia coli O157:H7}; contains Pfam profile PF00588: SpoU rRNA Methylase (RNA methyltransferase, TrmH) family; contains non_consensus donor splice site TA at exon 4 \| chr4:8862813-8864616 FORWARD \| Aliases: DL3800W, FCAALL.322 | 6.5 | 4.9 | 1.6 | 5.2 | 0.75% | 4.9 |
| 4021 | AT3G56290.1 \| expressed protein \| chr3:20889518-20890547 REVERSE \| Aliases: F18O21.250 | 5.8 | 4.3 | 1.6 | 5.2 | 0.75% | 4.9 |
| 4023 | AT5G13470.1 \| expressed protein \| chr5:4318565-4319834 FORWARD \| Aliases: T6I14.1 | 5.0 | 3.9 | 1.1 | 5.2 | 0.75% | 4.9 |
| 4024 | AT5G16440.1 \| Symbol: IPP1 \| similar to MutT/nudix family protein [Arabidopsis thaliana] (TAIR:At1g79690.1); similar to isopentenyl pyrophosphate:dimethyllallyl pyrophosphate isomerase [Adonis palaestina] (GB:AAF29973.1); similar to isopentenyl pyrophosphate isomerase [Pueraria montana var. lobata] (GB:AAQ84167.1); contains InterPro domain Isopentenyl-diphosphate delta-isomerase (InterPro:IPR002667); contains InterPro domain NUDIX hydrolase (InterPro:IPR000086) \| chr5:5371743-5373738 FORWARD \| Aliases: IDI1, ISOPENTENYL DIPHOSPHATE ISOMERASE, MQK4.17, MQK4_17 | 7.0 | 3.6 | 3.5 | 5.2 | 0.75% | 4.9 |
| 4026 | AT3G20240.1 \| mitochondrial substrate carrier family protein, contains Pfam profile: PF00153 mitochondrial carrier proteins \| chr3:7057023-7058897 FORWARD \| Aliases: MAL21.26 | 6.0 | 4.3 | 1.6 | 5.2 | 0.75% | 4.9 |

| Rank | Description | Sync | Root | M | t | adj.q | B |
|---|---|---|---|---|---|---|---|
| 4027 | AT4G32770.1 \| Symbol: VTE1 \| tocopherol cyclase, chloroplast / vitamin E deficient 1 (VTE1) / sucrose export defective 1 (SXD1), identical to SP:Q94FY7 Tocopherol cyclase, chloroplast precursor (Vitamin E deficient 1) (Sucrose export defective 1) {Arabidopsis thaliana} | 6.1 | 4.2 | 1.8 | 5.2 | 0.76% | 4.9 |
| 4029 | AT5G48500.1 \| expressed protein, similar to unknown protein (gb:AAD28645.1) \| chr5:19670217-19671470 FORWARD \| Aliases: MJE7.14, MJE7_14 | 5.0 | 3.8 | 1.2 | 5.2 | 0.76% | 4.9 |
| 4031 | AT2G43560.1 \| immunophilin / FKBP-type peptidyl-prolyl cis-trans isomerase family protein, identical to Probable FKBP-type peptidyl-prolyl cis-trans isomerase 2, chloroplast precursor (Ppiase) (Rotamase) (SP:O22870)(Arabidopsis thaliana); contains Pfam PF00254: peptidyl-prolyl cis-trans isomerase, FKBP-type \| chr2:18080566-18082514 REVERSE \| Aliases: T1O24.30 | 6.6 | 4.6 | 2.0 | 5.2 | 0.76% | 4.8 |
| 4032 | AT2G39350.1 \| ABC transporter family protein \| chr2:16436774-16439635 REVERSE \| Aliases: T16B24.1, T16B24_1 | 6.0 | 4.2 | 1.9 | 5.2 | 0.76% | 4.9 |
| 4035 | AT1G76120.2 \| tRNA pseudouridine synthase family protein, similar to SP:Q9Y606 tRNA pseudouridine synthase A (EC 4.2.1.70) (Uracil hydrolyase) {Homo sapiens}; contains Pfam profile PF01416: tRNA pseudouridine synthase \| chr1:28563568-28565330 REVERSE \| Aliases: T23E18.5, T23E18_5 | 4.5 | 3.5 | 1.0 | 5.2 | 0.76% | 4.9 |
| 4037 | AT5G49840.1 \| ATP-dependent Clp protease ATP-binding subunit ClpX, putative, similar to CLP protease regulatory subunit CLPX GI:2674203 from (Arabidopsis thaliana); non-consensus splice donor GC at exon 4; non-consensus splice donor AA at exon 7 \| chr5:20272469-20276261 FORWARD \| Aliases: K21G20.5, K21G20_5 | 5.7 | 4.6 | 1.1 | 5.2 | 0.76% | 4.9 |
| 4038 | AT1G67040.1 \| expressed protein, ; expression supported by MPSS \| chr1:25022768-25025585 REVERSE \| Aliases: F1O19.10, F1O19_10 | 4.1 | 3.0 | 1.1 | 5.2 | 0.76% | 4.9 |
| 4039 | AT2G30330.1 \| GCN5L1 family protein, similar to GCN5-like protein 1 (RT14 protein) (Swiss-Prot:P78537) (Homo sapiens) \| chr2:12935844-12936814 REVERSE \| Aliases: T9D9.14, T9D9_14 | 4.4 | 3.5 | 0.9 | 5.2 | 0.76% | 4.8 |
| 4040 | AT1G21065.1 \| expressed protein \| chr1:7374199-7375778 FORWARD \| Aliases: None | 8.8 | 7.5 | 1.4 | 5.2 | 0.76% | 4.7 |
| 4041 | AT4G08170.2 \| inositol 1,3,4-trisphosphate 5/6-kinase family protein, similar to inositol phosphate kinase (GI:27549256) (Zea mays); similar to inositol 1,3,4-trisphosphate 5/6-kinase (GI:3396079) (Arabidopsis thaliana) \| chr4:5163396-5167176 REVERSE \| Aliases: T12G13.10, T12G13_10 | 5.2 | 4.2 | 0.9 | 5.2 | 0.77% | 4.9 |
| 4043 | AT1G78780.2 \| pathogenesis-related family protein, contains similarity to pathogen-related protein (GI:499073) (Swiss-Prot:P16273) (Hordeum vulgare) \| chr1:29626237-29627338 REVERSE \| Aliases: F9K20.18, F9K20_18 | 6.3 | 4.3 | 2.0 | 5.2 | 0.77% | 4.9 |
| 4044 | AT5G47210.1 \| nuclear RNA-binding protein, putative, similar to nuclear RNA binding protein GI:6492264 from (Arabidopsis thaliana) \| chr5:19186249-19188446 REVERSE \| Aliases: MQL5.6, MQL5_6 | 11.7 | 10.6 | 1.2 | 5.2 | 0.77% | 4.5 |
| 4045 | AT2G34357.1 \| expressed protein \| chr2:14506315-14512806 FORWARD \| Aliases: None | 6.3 | 4.6 | 1.8 | 5.2 | 0.77% | 4.9 |
| 4051 | AT2G02400.1 \| cinnamoyl-CoA reductase family, similar to cinnamoyl-CoA reductase from Pinus taeda (GI:17978649), Eucalyptus gunnii (GI:2058311) \| chr2:631266-632574 REVERSE \| Aliases: T16F16.19, T16F16_19 | 7.6 | 5.8 | 1.7 | 5.2 | 0.77% | 4.9 |
| 4052 | AT4G32260.1 \| ATP synthase family, contains Pfam profile: PF00430 ATP synthase B/B' CF(0); identical to cDNA chloroplast ATP synthase beta chain precursor (atpG) GI:5730140 \| chr4:15573643-15574743 REVERSE \| Aliases: F10M6.100, F10M6_100 | 7.5 | 4.3 | 3.2 | 5.2 | 0.77% | 4.9 |
| 4053 | AT4G29140.1 \| MATE efflux protein-related, several hypothetical proteins - Arabidopsis thaliana; contains Pfam profile PF01554: Uncharacterized membrane protein family \| chr4:14368925-14370898 FORWARD \| Aliases: F19B15.170, F19B15_170 | 8.5 | 6.7 | 1.8 | 5.2 | 0.78% | 4.8 |
| 4055 | AT2G20860.1 \| Symbol: LIP1 \| lipoic acid synthase (LIP1), identical to gi:3928758 contains Pfam profile PF04055: radical SAM domain protein \| chr2:8986660-8988286 FORWARD \| Aliases: F5H14.17, F5H14_17, LIPOIC ACID SYNTHASE | 10.6 | 8.3 | 2.3 | 5.2 | 0.78% | 4.8 |
| 4057 | AT3G24090.1 \| glucosamine--fructose-6-phosphate aminotransferase (isomerizing), putative / hexosephosphate aminotransferase, putative / glucosamine-6-phosphate synthase, putative / D-fructose-6-phosphate amidotransferase, putative / GLCN6P synthase, putative, similar to SP:O94808 Glucosamine--fructose-6-phosphate aminotransferase (isomerizing) 2 (EC 2.6.1.16) (Hexosephosphate aminotransferase 2) (D-fructose-6- phosphate amidotransferase 2) {Homo sapiens}; contains Pfam profiles PF00310: Glutamine amidotransferases class-II, PF01380:SIS domain \| chr3:8697845-8700978 REVERSE \| Aliases: F14O13.29 | 4.2 | 3.2 | 1.1 | 5.2 | 0.78% | 4.9 |
| 4058 | AT5G38720.1 \| expressed protein, predicted protein, Drosophila melanogaster \| chr5:15525477-15527789 REVERSE \| Aliases: MKD10.4, MKD10_4 | 7.3 | 5.4 | 1.9 | 5.2 | 0.78% | 4.9 |
| 4064 | AT1G15440.2 \| transducin family protein / WD-40 repeat family protein, Strong similarity to gb X95263 Periodic tryptophan protein 2 gene (PWP2) from Homo sapiens and contains 6 WD40, G-beta repeat domains \| chr1:5306056-5309509 REVERSE \| Aliases: F9L1.40, F9L1_40 | 6.9 | 5.8 | 1.0 | 5.2 | 0.78% | 4.8 |
| 4066 | AT5G53860.3 \| Symbol: EMB2737 \| expressed protein \| chr5:21882494-21886068 FORWARD \| Aliases: EMB2737, EMBRYO DEFECTIVE 2737, K6O8.3 | 6.9 | 5.6 | 1.3 | 5.2 | 0.79% | 4.9 |

| Rank | Description | Sync | Root | M | t | adj.q | B |
|---|---|---|---|---|---|---|---|
| 4069 | AT3G18110.1 \| Symbol: EMB1270 \| pentatricopeptide (PPR) repeat-containing protein, contains Pfam profile: PF01535 PPR repeat \| chr3:6204810-6209697 REVERSE \| Aliases: EMB1270, EMBRYO DEFECTIVE 1270, MRC8.9 | 6.2 | 4.4 | 1.8 | 5.2 | 0.79% | 4.8 |
| 4070 | AT5G44680.1 \| methyladenine glycosylase family protein, similar to SP:P05100 DNA-3-methyladenine glycosylase I (EC 3.2.2.20) (3-methyladenine-DNA glycosylase I, constitutive) {Escherichia coli}; contains Pfam profile PF03352: Methyladenine glycosylase \| chr5:18041442-18043172 REVERSE \| Aliases: K23L20.1, K23L20_1 | 3.7 | 2.7 | 1.0 | 5.2 | 0.79% | 4.9 |
| 4073 | AT3G55140.2 \| pectate lyase family protein, similar to pollen allergen Amb a 1.3 SP:P27761 from (Ambrosia artemisiifolia) \| chr3:20449760-20451404 FORWARD \| Aliases: T26I12.20 | 7.4 | 5.9 | 1.5 | 5.2 | 0.79% | 4.9 |
| 4075 | AT1G69840.4 \| band 7 family protein, strong similarity to hypersensitive-induced response protein (Zea mays) GI:7716466; contains Pfam profile PF01145: SPFH domain / Band 7 family \| chr1:26297331-26299298 REVERSE \| Aliases: T17F3.13, T17F3_13 | 7.5 | 4.2 | 3.3 | 5.2 | 0.79% | 4.8 |
| 4079 | AT1G13060.1 \| Symbol: PBE1 \| 20S proteasome beta subunit E1 (PBE1) (PRCE), identical to GB:O23717; identical to cDNA proteasome subunit prce GI:2511595 \| chr1:4452269-4454872 FORWARD \| Aliases: 20S PROTEASOME BETA SUBUNIT PBE1, F3F19.8, F3F19_8 | 12.2 | 10.5 | 1.7 | 5.2 | 0.79% | 4.7 |
| 4080 | AT3G26340.1 \| 20S proteasome beta subunit E, putative, very strong similarity to SP:O23717 Proteasome subunit beta type 5 precursor (EC 3.4.25.1) (20S proteasome subunit E) (Proteasome epsilon chain) {Arabidopsis thaliana} | 12.2 | 10.5 | 1.7 | 5.2 | 0.79% | 4.7 |
| 4084 | AT5G22740.1 \| Symbol: ATCSLA02 \| glycosyl transferase family 2 protein, similar to beta-(1-3)-glucosyl transferase GB:AAC62210 GI:3687658 from (Bradyrhizobium japonicum), cellulose synthase from Agrobacterium tumeficiens (gi:710492) and Agrobacterium radiobacter (gi:710493); contains Pfam glycosyl transferase, group 2 family protein domain PF00535 | 9.7 | 7.1 | 2.6 | 5.2 | 0.80% | 4.9 |
| 4087 | AT1G63610.2 \| expressed protein \| chr1:23587190-23589386 REVERSE \| Aliases: F2K11.3, F2K11_3 | 6.5 | 4.4 | 2.1 | 5.2 | 0.81% | 4.9 |
| 4088 | AT1G48420.1 \| desulfhydrase family, similar to similar to D-cysteine desulfhydrase (EC 4.4.1.15). (Swiss-Prot:P59329) (Escherichia coli O6); contains TIGRFAM TIGR01275: pyridoxal phosphate-dependent enzymes, D-cysteine desulfhydrase family profile \| chr1:17900267-17902472 REVERSE \| Aliases: T1N15.3, T1N15_3 | 11.6 | 10.1 | 1.4 | 5.2 | 0.81% | 4.6 |
| 4089 | AT5G48910.1 \| pentatricopeptide (PPR) repeat-containing protein, contains Pfam profile PF01535: PPR repeat \| chr5:19850098-19852160 REVERSE \| Aliases: K19E20.2, K19E20_2 | 5.6 | 4.6 | 1.0 | 5.2 | 0.81% | 4.8 |
| 4093 | AT1G15140.3 \| oxidoreductase NAD-binding domain-containing protein, Contains Pfam profile PF00175: Oxidoreductase NAD-binding domain; ESTs gb:H76345 and gb:AA651465 come from this gene \| chr1:5210277-5212344 REVERSE \| Aliases: F9L1.8, F9L1_8 | 6.9 | 5.3 | 1.6 | 5.2 | 0.81% | 4.9 |
| 4095 | AT3G02630.1 \| acyl-(acyl-carrier-protein) desaturase, putative / stearoyl-ACP desaturase, putative, similar to Acyl-(acyl-carrier protein) desaturase from Sesamum indicum GI:575942, Cucumis sativus SP:P32061, Ricinus communis SP:P22337; contains Pfam profile PF03405 Fatty acid desaturase \| chr3:562038-564877 FORWARD \| Aliases: F16B3.26, F16B3_26 | 9.8 | 6.2 | 3.5 | 5.2 | 0.81% | 4.9 |
| 4096 | AT1G78580.1 \| Symbol: ATTPS1 \| | 6.9 | 5.5 | 1.4 | 5.2 | 0.81% | 4.8 |
| 4100 | AT4G01570.1 \| pentatricopeptide (PPR) repeat-containing protein, contains Pfam profile PF01535: PPR repeat \| chr4:679472-681940 FORWARD \| Aliases: T15B16.21, T15B16_21 | 4.6 | 3.8 | 0.9 | 5.2 | 0.81% | 4.8 |
| 4101 | AT2G45520.1 \| expressed protein \| chr2:18761830-18763362 REVERSE \| Aliases: F17K2.5 | 8.6 | 7.4 | 1.2 | 5.2 | 0.81% | 4.9 |
| 4102 | AT3G19360.1 \| zinc finger (CCCH-type) family protein, contains Pfam domain, PF00642: Zinc finger C-x8-C-x5-C-x3-H type (and similar) \| chr3:6707270-6709034 FORWARD \| Aliases: MLD14.9 | 5.8 | 4.4 | 1.4 | 5.2 | 0.81% | 4.9 |
| 4104 | AT5G45020.1 \| expressed protein \| chr5:18185909-18187843 FORWARD \| Aliases: K21C13.21, K21C13_21 | 5.6 | 4.8 | 0.8 | 5.2 | 0.81% | 4.8 |
| 4107 | AT4G32720.2 \| similar to La domain-containing protein [Arabidopsis thaliana] (TAIR:At1g79880.1); similar to putative RNA recognition motif (RRM)-containing protein [Oryza sativa (japonica cultivar-group)] (GB:XP_466667.1); contains InterPro domain RNA-binding protein Lupus LaI (InterPro:IPR006630); contains InterPro domain Lupus La protein (InterPro:IPR002344); contains InterPro domain RNA-binding region RNP-1 (RNA recognition motif) (InterPro:IPR000504) \| chr4:15787218-15789982 FORWARD \| Aliases: F4D11.80, F4D11_80 | 7.7 | 6.4 | 1.4 | 5.2 | 0.81% | 4.9 |
| 4112 | AT3G13980.1 \| expressed protein \| chr3:4619045-4620269 REVERSE \| Aliases: MDC16.10 | 4.2 | 3.3 | 0.9 | 5.2 | 0.81% | 4.8 |
| 4113 | AT4G29735.1 \| expressed protein, contains Pfam domain PF05251: Uncharacterised protein family (UPF0197) \| chr4:14562645-14564384 REVERSE \| Aliases: None | 10.7 | 8.9 | 1.8 | 5.2 | 0.82% | 4.8 |

| Rank | Description | Sync | Root | M | t | adj.q | B |
|---|---|---|---|---|---|---|---|
| 4114 | AT2G45790.1 \| eukaryotic phosphomannomutase family protein, contains Pfam profile: PF03332 eukaryotic phosphomannomutase \| chr2:18862853-18865442 FORWARD \| Aliases: F4I18.23 | 10.3 | 8.7 | 1.6 | 5.2 | 0.82% | 4.8 |
| 4115 | AT1G56170.1 \| Symbol: HAP5B \| transcription factor, putative, similar to Transcription factor GB:CAA74053 GI:2398533 from (Arabidopsis thaliana) similarity to transcription factor Hap5a similar to transcription factor Hap5a (Arabidopsis thaliana)(GI:6523090) \| chr1:21028429-21029548 FORWARD \| Aliases: ATHAP5B, F14G9.21 | 6.6 | 3.3 | 3.2 | 5.2 | 0.82% | 4.7 |
| 4116 | AT1G78490.1 \| Symbol: CYP708A3 \| cytochrome P450 family protein, similar to Cytochrome P450 90A1 (SP:Q42569) (Arabidopsis thaliana) \| chr1:29533169-29535558 FORWARD \| Aliases: T30F21.17, T30F21_17 | 5.0 | 3.6 | 1.4 | 5.2 | 0.82% | 4.8 |
| 4117 | AT3G01910.2 \| Symbol: SOX \| similar to nitrate reductase 1 (NR1) [Arabidopsis thaliana] (TAIR:At1g77760.1); similar to B Chain B, Sulfite Oxidase From Chicken Liver (GB:1SOX); contains InterPro domain Eukaryotic molybdopterin oxidoreductase (InterPro:IPR008335); contains InterPro domain Mo-co oxidoreductase dimerisation domain (InterPro:IPR005066); contains InterPro domain Oxidoreductase, molybdopterin binding (InterPro:IPR000572) \| chr3:314703-316719 REVERSE \| Aliases: F28J7.38, F28J7_38, SULFITE OXIDASE | 11.4 | 10.5 | 1.0 | 5.2 | 0.82% | 4.6 |
| 4119 | AT3G47520.1 \| Symbol: MDH \| malate dehydrogenase (NAD), chloroplast (MDH), identical to chloroplast NAD-malate dehydrogenase (Arabidopsis thaliana) GI:3256066; contains InterPro entry IPR001236: Lactate/malate dehydrogenase; contains Pfam profiles PF00056: lactate/malate dehydrogenase, NAD binding domain and PF02866: lactate/malate dehydrogenase, alpha/beta C-terminal domain \| chr3:17524259-17526026 FORWARD \| Aliases: F1P2.70, MALATE DEHYDROGENASE | 10.2 | 8.5 | 1.7 | 5.2 | 0.82% | 4.8 |
| 4121 | AT3G59670.1 \| expressed protein, similar to expressed protein [Arabidopsis thaliana] (TAIR:At4g37440.1); similar to unknown protein [Oryza sativa (japonica cultivar-group)] (GB:AAV32239.1) \| chr3:22050454-22053637 FORWARD \| Aliases: T16L24.220 | 5.4 | 3.9 | 1.4 | 5.2 | 0.82% | 4.8 |
| 4123 | AT4G08685.1 \| Symbol: SAH7 \| pollen Ole e 1 allergen and extensin family protein, contains Pfam domain, PF01190: Pollen proteins Ole e I family \| chr4:5550426-5551816 FORWARD \| Aliases: None | 9.1 | 6.1 | 2.9 | 5.2 | 0.83% | 4.8 |
| 4124 | AT1G74440.1 \| expressed protein, similar to YGL010w-like protein GB:AAC32136 (Picea mariana) \| chr1:27980163-27984065 REVERSE \| Aliases: F1M20.12, F1M20_12 | 4.6 | 3.7 | 0.9 | 5.2 | 0.83% | 4.8 |
| 4125 | AT4G33400.1 \| dem protein-related / defective embryo and meristems protein-related, identical to dem GI:2190419 from (Lycopersicon esculentum) | 7.3 | 4.7 | 2.6 | 5.2 | 0.83% | 4.8 |
| 4127 | AT2G34520.1 \| Symbol: RPS14 \| ribosomal protein S14 mitochondrial family protein, identical to ribosomal protein S14 {Arabidopsis thaliana} NCBI_gi:4583554 \| chr2:14554863-14555837 REVERSE \| Aliases: T31E10.14, T31E10_14 | 8.2 | 6.4 | 1.9 | 5.1 | 0.83% | 4.8 |
| 4128 | AT1G08200.1 \| Symbol: AXS2 \| expressed protein \| chr1:2573857-2576709 REVERSE \| Aliases: T23G18.6, T23G18_6, UDP D APIOSE/UDP D XYLOSE SYNTHASE 2 | 10.4 | 8.3 | 2.1 | 5.1 | 0.83% | 4.7 |
| 4129 | AT2G27860.1 \| Symbol: AXS1 \| expressed protein \| chr2:11871470-11873975 REVERSE \| Aliases: F15K20.4, F15K20_4, UDP D APIOSE/UDP D XYLOSE SYNTHASE 1 | 10.4 | 8.3 | 2.1 | 5.1 | 0.83% | 4.7 |
| 4130 | AT3G54400.1 \| aspartyl protease family protein, contains Pfam profile: PF00026 eukaryotic aspartyl protease \| chr3:20151036-20153620 REVERSE \| Aliases: T14E10.1 | 6.3 | 3.6 | 2.7 | 5.1 | 0.83% | 4.8 |
| 4131 | AT4G34360.1 \| protease-related, similar to PIR:I46078 endothelin converting enzyme, Bos primigenius taurus \| chr4:16431945-16433962 FORWARD \| Aliases: F10M10.130, F10M10_130 | 3.9 | 2.8 | 1.1 | 5.1 | 0.83% | 4.8 |
| 4133 | AT2G30540.1 \| glutaredoxin family protein, contains INTERPRO Domain IPR002109, Glutaredoxin (thioltransferase) | 5.3 | 2.8 | 2.5 | 5.1 | 0.83% | 4.8 |
| 4134 | AT4G32590.4 \| similar to ferredoxin-related [Arabidopsis thaliana] (TAIR:At3g16250.1); similar to 2Fe-2S iron-sulfur cluster protein-like [Oryza sativa (japonica cultivar-group)] (GB:XP_478158.1) \| chr4:15721385-15722875 FORWARD \| Aliases: F4D11.210, F4D11_210 | 5.5 | 4.6 | 0.9 | 5.1 | 0.83% | 4.8 |
| 4135 | AT5G16150.3 \| hexose transporter, putative, strong similarity to hexose transporter (Arabidopsis thaliana) GI:8347250; contains Pfam profile PF00083: major facilitator superfamily protein \| chr5:5272690-5275818 FORWARD \| Aliases: T21H19.70, T21H19_70 | 9.8 | 8.6 | 1.2 | 5.1 | 0.83% | 4.8 |
| 4136 | AT5G15320.1 \| expressed protein, predicted protein, Arabidopsis thaliana \| chr5:4977541-4978897 FORWARD \| Aliases: F8M21.210, F8M21_210 | 10.3 | 9.3 | 1.0 | 5.1 | 0.83% | 4.7 |
| 4137 | AT1G67950.4 \| RNA recognition motif (RRM)-containing protein \| chr1:25482030-25483839 REVERSE \| Aliases: T23K23.20, T23K23_20 | 5.7 | 4.3 | 1.5 | 5.1 | 0.83% | 4.8 |
| 4139 | AT3G15990.1 \| Symbol: SULTR3;4 \| sulfate transporter, putative, similar to sulfate transporter (Arabidopsis thaliana) GI:2285885; contains Pfam profiles PF00916: Sulfate transporter family, PF01740: STAS domain \| chr3:5426993-5430885 FORWARD \| Aliases: MSL1.24, sulfate transporter | 4.3 | 3.5 | 0.8 | 5.1 | 0.83% | 4.7 |
| 4140 | AT5G48470.1 \| expressed protein \| chr5:19658987-19661588 FORWARD \| Aliases: MJE7.11, MJE7_11 | 5.1 | 4.1 | 1.0 | 5.1 | 0.83% | 4.8 |
| 4142 | AT1G03680.1 \| Symbol: ATHM1 \| thioredoxin M-type 1, chloroplast (TRX-M1), nearly identical to SP:O48737 Thioredoxin M-type 1, chloroplast precursor (TRX-M1) {Arabidopsis thaliana}; similar to ESTs gb:T13714, gb:H76398, gb:N37762, gb:AA042639, gb:T21104, emb:Z30901 \| chr1:916845-918001 REVERSE \| Aliases: None | 8.4 | 5.6 | 2.8 | 5.1 | 0.83% | 4.8 |

| Rank | Description | Sync | Root | M | t | adj.q | B |
|---|---|---|---|---|---|---|---|
| 4145 | AT2G39020.1 \| GCN5-related N-acetyltransferase (GNAT) family protein, similar to SP:Q9SMB8 Tyramine N-feruloyltransferase 4/11 (EC 2.3.1.110) (Hydroxycinnamoyl-CoA: tyramine N-hydroxycinnamoyltransferase) {Nicotiana tabacum}; contains Pfam profile PF00583: acetyltransferase, GNAT family \| chr2:16302393-16303406 FORWARD \| Aliases: T7F6.19, T7F6_19 | 5.8 | 4.0 | 1.8 | 5.1 | 0.84% | 4.7 |
| 4146 | AT1G65230.1 \| expressed protein \| chr1:24232813-24234565 FORWARD \| Aliases: T23K8.14, T23K8_14 | 3.6 | 2.5 | 1.1 | 5.1 | 0.84% | 4.8 |
| 4148 | AT5G25580.1 \| expressed protein \| chr5:8903508-8906314 FORWARD \| Aliases: T14C9.120, T14C9_120 | 4.9 | 2.7 | 2.2 | 5.1 | 0.84% | 4.7 |
| 4151 | AT5G47570.1 \| expressed protein \| chr5:19309839-19311962 REVERSE \| Aliases: MNJ7.16, MNJ7_16 | 12.2 | 11.0 | 1.2 | 5.1 | 0.84% | 4.6 |
| 4152 | AT3G23580.1 \| Symbol: RNR2A \| ribonucleoside-diphosphate reductase small chain / ribonucleotide reductase, nearly identical to SP:P50651 Ribonucleoside-diphosphate reductase small chain (EC 1.17.4.1) (Ribonucleotide reductase) (R2 subunit) {Arabidopsis thaliana} \| chr3:8460059-8462822 FORWARD \| Aliases: MDB19.1, R2, RNR2 | 7.9 | 6.6 | 1.3 | 5.1 | 0.84% | 4.8 |
| 4154 | AT5G54510.1 \| Symbol: DFL1 \| encodes an IAA-amido synthase that conjugates Ala, Asp, Phe, and Trp to auxin. Lines overexpressing this gene accumulate IAA-ASP and are hypersensitive to several auxins. \| chr5:22148319-22150904 REVERSE \| Aliases: DWARF IN LIGHT 1, F24B18.13, F24B18_13, GH3.6 | 8.1 | 5.3 | 2.7 | 5.1 | 0.84% | 4.8 |
| 4155 | AT5G44400.1 \| FAD-binding domain-containing protein, similar to SP:P30986 reticuline oxidase precursor (Berberine-bridge-forming enzyme) (BBE) (Tetrahydroprotoberberine synthase) (Eschscholzia californica); contains PF01565 FAD binding domain \| chr5:17903421-17905707 REVERSE \| Aliases: K9L2.20, K9L2_20 | 4.6 | 2.8 | 1.8 | 5.1 | 0.84% | 4.8 |
| 4156 | AT5G28840.1 \| NAD-dependent epimerase/dehydratase family protein, similar to sugar epimerase BlmG from Streptomyces verticillus GI:9937230; contains Pfam profile PF01370 NAD dependent epimerase/dehydratase family \| chr5:10862177-10864906 REVERSE \| Aliases: F7P1.20, F7P1_20 | 8.5 | 6.1 | 2.4 | 5.1 | 0.84% | 4.8 |
| 4159 | AT3G52300.2 \| similar to putative mitochondrial F0 ATP synthase D chain [Oryza sativa (japonica cultivar-group)] (GB:XP_482965.1) \| chr3:19407543-19409355 FORWARD \| Aliases: T25B15.70 | 13.2 | 11.6 | 1.6 | 5.1 | 0.84% | 4.3 |
| 4160 | AT3G20420.1 \| ribonuclease III family protein, similar to CAF protein (RNA helicase/RNAseIII) (Arabidopsis thaliana) GI:6102610; contains Pfam profiles: PF00636 RNase3 domain, PF00035 Double-stranded RNA binding motif \| chr3:7119288-7120925 REVERSE \| Aliases: MQC12.21 | 4.2 | 3.1 | 1.1 | 5.1 | 0.84% | 4.8 |
| 4161 | AT3G57050.3 \| Symbol: CBL \| cystathionine beta-lyase, chloroplast / beta-cystathionase / cysteine lyase (CBL), identical to SP:P53780 Cystathionine beta-lyase, chloroplast precursor (EC 4.4.1.8) (CBL) (Beta-cystathionase) (Cysteine lyase) {Arabidopsis thaliana} | 10.5 | 9.2 | 1.3 | 5.1 | 0.84% | 4.7 |
| 4162 | AT3G02690.1 \| integral membrane family protein, similar to PecM protein (GI:5852331) {Vogesella indigofera} and PecM protein (SP:P42194) (Erwinia chrysanthemi) \| chr3:579593-582136 FORWARD \| Aliases: F16B3.32, F16B3_32 | 7.0 | 5.4 | 1.6 | 5.1 | 0.84% | 4.8 |
| 4164 | AT5G11060.1 \| Symbol: KNAT4 \| homeobox protein knotted-1 like 4 (KNAT4), identical to homeobox protein knotted-1 like 4 ( KNAT4) SP:P48001 from (Arabidopsis thaliana) \| chr5:3510119-3513306 FORWARD \| Aliases: T5K6.50, T5K6_50 | 5.4 | 4.2 | 1.2 | 5.1 | 0.84% | 4.8 |
| 4168 | AT2G25880.1 \| Encodes a member of a family of Ser/Thr kinases whose activities peak during cell division. Transcripts are abundant in tissues rich in dividing cells like roots and flowers but are low or absent in fully expanded leaves and stems. | 6.3 | 4.7 | 1.6 | 5.1 | 0.85% | 4.8 |
| 4171 | AT2G30320.1 \| tRNA pseudouridine synthase family protein, similar to pseudouridine synthase (Schizosaccharomyces pombe) GI:6469502; contains Pfam profile PF01416: tRNA pseudouridine synthase | 5.7 | 3.9 | 1.8 | 5.1 | 0.85% | 4.8 |
| 4173 | AT1G71770.1 \| Symbol: PAB5 \| polyadenylate-binding protein 5 (PABP5), identical to GB:Q05196 from (Arabidopsis thaliana) \| chr1:26994170-26997109 REVERSE \| Aliases: F14O23.15, F14O23_15, POLY(A) BINDING PROTEIN | 4.6 | 3.2 | 1.4 | 5.1 | 0.85% | 4.8 |
| 4174 | AT3G55430.1 \| glycosyl hydrolase family 17 protein / beta-1,3-glucanase, putative, similar to beta-1,3 glucanase GI:7414433 from (Pisum sativum); contains Pfam profile PF00332: Glycosyl hydrolases family 17 | 8.6 | 7.0 | 1.7 | 5.1 | 0.85% | 4.8 |
| 4176 | AT2G14660.1 \| expressed protein, contains Pfam PF04543: Family of unknown function (DUF589); similar to cThy28kD (GI:995778) (Gallus gallus); similar to thymocyte protein mThy28 (GI:23978374) (Mus musculus) | 4.7 | 3.6 | 1.1 | 5.1 | 0.85% | 4.7 |
| 4178 | AT5G18250.1 \| expressed protein, similar to unknown protein (dbj:BAA90342.1) \| chr5:6033706-6035382 FORWARD \| Aliases: MRG7.21, MRG7_21 | 5.1 | 3.5 | 1.6 | 5.1 | 0.86% | 4.8 |
| 4179 | AT5G06410.1 \| DNAJ heat shock N-terminal domain-containing protein, similar to SP:P36540 Chaperone protein hscB (HSC20) {Escherichia coli O157:H7}; contains Pfam profile PF00226 DnaJ domain \| chr5:1959537-1961159 REVERSE \| Aliases: MHF15.7, MHF15_7 | 4.5 | 3.2 | 1.3 | 5.1 | 0.86% | 4.7 |
| 4180 | AT4G11060.1 \| single-strand-binding family protein, contains Pfam domain PF00436: Single-strand binding protein family | 6.4 | 3.7 | 2.7 | 5.1 | 0.86% | 4.6 |

| Rank | Description | Sync | Root | M | t | adj.q | B |
|---|---|---|---|---|---|---|---|
| 4181 | AT4G34030.1 \| Symbol: MCCB \| methylcrotonyl-CoA carboxylase beta chain, mitochondrial / 3-methylcrotonyl-CoA carboxylase 2 (MCCB), identical to SP:Q9LDD8 Methylcrotonyl-CoA carboxylase beta chain, mitochondrial precursor (EC 6.4.1.4) (3-Methylcrotonyl-CoA carboxylase 2) (MCCase beta subunit) (3-methylcrotonyl-CoA:carbon dioxide ligase beta subunit) {Arabidopsis thaliana}; contains Pfam profile: PF01039 carboxyl transferase domain \| chr4:16301198-16304031 FORWARD \| Aliases: 3 METHYLCROTONYL COA CARBOXYLASE, F28A23.210, F28A23_210 | 10.3 | 9.5 | 0.9 | 5.1 | 0.86% | 4.5 |
| 4185 | AT5G25570.2 \| expressed protein \| chr5:8901758-8903219 FORWARD \| Aliases: T14C9.110, T14C9_110 | 3.9 | 2.4 | 1.6 | 5.1 | 0.86% | 4.8 |
| 4188 | AT1G14345.1 \| expressed protein, contains one transmembrane domain \| chr1:4899141-4899974 FORWARD \| Aliases: None | 4.1 | 3.2 | 0.9 | 5.1 | 0.86% | 4.6 |
| 4189 | AT2G32440.1 \| Symbol: KAO2 \| ent-kaurenoic acid hydroxylase, putative / cytochrome P450, putative, identical to ent-kaurenoic acid hydroxylase / cytochrome P450 CYP88A (GI:13021856) (Arabidopsis thaliana); similar to ent-kaurenoic acid hydroxylase (Arabidopsis thaliana) GI:13021853 \| chr2:13782665-13785079 FORWARD \| Aliases: CYP88A4, ENT KAURENOIC ACID HYDROXYLASE 2, T32F6.4, T32F6_4 | 4.6 | 3.4 | 1.1 | 5.1 | 0.86% | 4.8 |
| 4190 | AT5G49210.2 \| expressed protein \| chr5:19967069-19968910 FORWARD \| Aliases: K21P3.8, K21P3_8 | 6.7 | 5.5 | 1.2 | 5.1 | 0.86% | 4.8 |
| 4192 | AT1G69510.3 \| expressed protein \| chr1:26130049-26131596 FORWARD \| Aliases: F10D13.16, F10D13_16 | 11.2 | 9.9 | 1.3 | 5.1 | 0.86% | 4.4 |
| 4195 | AT2G23560.1 \| hydrolase, alpha/beta fold family protein, similar to ethylene-induced esterase (Citrus sinensis) GI:14279437, polyneuridine aldehyde esterase (Rauvolfia serpentina) GI:6651393; contains Pfam profile PF00561: hydrolase, alpha/beta fold family \| chr2:10036416-10037362 REVERSE \| Aliases: F26B6.21, F26B6_21 | 8.1 | 6.6 | 1.5 | 5.1 | 0.86% | 4.8 |
| 4196 | AT3G12650.1 \| expressed protein \| chr3:4017837-4018638 REVERSE \| Aliases: T2E22.4 | 8.9 | 8.0 | 0.9 | 5.1 | 0.86% | 4.7 |
| 4199 | AT5G64310.1 \| Symbol: AGP1 \| arabinogalactan-protein (AGP1), identical to gi:3883120 gb:AAC77823 \| chr5:25739159-25739872 FORWARD \| Aliases: ARABINOGALACTAN PROTEIN 1, MSJ1.15, MSJ1_15 | 9.8 | 7.9 | 1.9 | 5.1 | 0.86% | 4.8 |
| 4200 | AT5G46020.1 \| expressed protein \| chr5:18680104-18682054 REVERSE \| Aliases: MCL19.6, MCL19_6 | 10.6 | 9.4 | 1.2 | 5.1 | 0.86% | 4.7 |
| 4202 | AT5G53650.1 \| expressed protein \| chr5:21808403-21809666 FORWARD \| Aliases: MNC6.19, MNC6_19 | 8.4 | 7.0 | 1.4 | 5.1 | 0.86% | 4.6 |
| 4203 | AT4G10760.1 \| Symbol: EMB1706 \| methyltransferase MT-A70, putative, similar to (N6-adenosine)-methyltransferase (Mus musculus) GI:10179948, m6A methyltransferase (MT-A70) (Homo sapiens) GI:2460037; contains Pfam profile PF05063: MT-A70 (S-adenosylmethionine-binding subunit of human mRNA:m6A methyl-transferase (MTase)) \| chr4:6619480-6623347 REVERSE \| Aliases: EMB1706, EMBRYO DEFECTIVE 1706, T12H20.6, T12H20_6 | 8.2 | 5.4 | 2.7 | 5.1 | 0.87% | 4.7 |
| 4204 | AT2G46240.1 \| IQ domain-containing protein / BAG domain-containing protein, contains Pfam profiles PF00612: IQ calmodulin-binding motif, PF02179: BAG (Apoptosis regulator Bcl-2 protein) domain \| chr2:18993234-18997020 FORWARD \| Aliases: T3F17.11 | 4.8 | 3.9 | 0.9 | 5.1 | 0.87% | 4.6 |
| 4205 | AT5G43440.1 \| 2-oxoglutarate-dependent dioxygenase, putative, similar to 2A6 (GI:599622) and tomato ethylene synthesis regulatory protein E8 (SP:P10967) \| chr5:17472461-17473885 REVERSE \| Aliases: MWF20.15, MWF20_15 | 5.0 | 3.7 | 1.3 | 5.1 | 0.87% | 4.8 |
| 4209 | AT4G02060.1 \| Symbol: PRL \| prolifera protein (PRL) / DNA replication licensing factor Mcm7 (MCM7), identical to DNA replication licensing factor Mcm7 SP:P43299 PROLIFERA protein {Arabidopsis thaliana}; contains Pfam profile PF00493: MCM2/3/5 family \| chr4:901388-905590 FORWARD \| Aliases: MCM7, PROLIFERA, T10M13.7, T10M13_7 | 5.5 | 3.4 | 2.1 | 5.1 | 0.87% | 4.8 |
| 4210 | AT3G03590.1 \| SWIB complex BAF60b domain-containing protein, contains Pfam profile PF02201: BAF60b domain of the SWIB complex \| chr3:865190-867015 REVERSE \| Aliases: T12J13.13, T12J13_13 | 4.0 | 3.1 | 0.9 | 5.1 | 0.87% | 4.6 |
| 4211 | AT5G46420.1 \| 16S rRNA processing protein RimM family, contains weak similarity to Swiss-Prot:O74933 UDP-N-acetylglucosamine pyrophosphorylase (Candida albicans); contains Pfam profiles PF01782: 16S rRNA processing protein RimM, PF05239: PRC-barrel domain \| chr5:18847182-18850180 FORWARD \| Aliases: K11I1.1, K11I1_1 | 6.3 | 5.1 | 1.2 | 5.1 | 0.87% | 4.7 |
| 4212 | AT1G56670.1 \| GDSL-motif lipase/hydrolase family protein, similarity to early early nodulin ENOD8 (Medicago sativa) GI:304037, lanatoside 15'-O-acetylesterase (Digitalis lanata) GI:3688284, elicitor-induced glycoprotein iEP4 (Daucus carota) GI:1911765; contains InterPro Entry IPR001087 Lipolytic enzyme, G-D-S-L family \| chr1:21245350-21247712 FORWARD \| Aliases: F25P12.90, F25P12_90 | 4.9 | 4.0 | 0.9 | 5.1 | 0.87% | 4.6 |
| 4215 | AT2G22860.1 \| Symbol: ATPSK2 \| | 10.0 | 8.9 | 1.2 | 5.1 | 0.87% | 4.7 |
| 4216 | AT2G22870.1 \| Symbol: EMB2001 \| expressed protein \| chr2:9746537-9748586 FORWARD \| Aliases: EMB2001, EMBRYO DEFECTIVE 2001, T20K9.8, T20K9_8 | 6.3 | 5.0 | 1.4 | 5.1 | 0.87% | 4.8 |

| Rank | Description | Sync | Root | M | t | adj.q | B |
|---|---|---|---|---|---|---|---|
| 4219 | AT5G16310.1 \| ubiquitin carboxyl-terminal hydrolase family 1 protein, similar to 26S proteasome regulatory complex subunit p37A (Drosophila melanogaster) GI:6434962; contains Pfam profile PF01088: Ubiquitin carboxyl-terminal hydrolase, family 1 \| chr5:5342062-5344286 REVERSE \| Aliases: MQK4.3, MQK4_3 | 5.1 | 4.0 | 1.1 | 5.1 | 0.87% | 4.8 |
| 4220 | AT2G47250.1 \| RNA helicase, putative, similar to SP:P53131 Pre-mRNA splicing factor RNA helicase PRP43 (Helicase JA1) {Saccharomyces cerevisiae}; contains Pfam profiles PF04408: Helicase associated domain (HA2), PF00271: Helicase conserved C-terminal domain \| chr2:19406765-19410107 REVERSE \| Aliases: T8I13.9 | 7.7 | 6.5 | 1.2 | 5.1 | 0.87% | 4.8 |
| 4221 | AT2G16700.1 \| Symbol: ADF5 \| actin-depolymerizing factor 5 (ADF5), identical to SP:Q9ZNT3 Actin-depolymerizing factor 5 (ADF-5) (AtADF5) {Arabidopsis thaliana} \| chr2:7251704-7252823 FORWARD \| Aliases: ACTIN DEPOLYMERIZING FACTOR 5, T24I21.11, T24I21_11 | 7.3 | 6.4 | 0.9 | 5.1 | 0.88% | 4.7 |
| 4222 | AT1G19640.1 \| Symbol: JMT \| S-adenosyl-L-methionine:jasmonic acid carboxyl methyltransferase (JMT), nearly identical to gi:13676829 \| chr1:6788876-6791837 REVERSE \| Aliases: F14P1.3, F14P1_3, JASMONIC ACID CARBOXYL METHYLTRANSFERASE | 4.7 | 2.9 | 1.9 | 5.1 | 0.88% | 4.6 |
| 4225 | AT3G04760.1 \| pentatricopeptide (PPR) repeat-containing protein, contains Pfam profile PF01535: PPR repeat \| chr3:1303581-1305884 REVERSE \| Aliases: F7O18.25, F7O18_25 | 5.1 | 3.1 | 2.0 | 5.1 | 0.88% | 4.6 |
| 4226 | AT4G30700.1 \| pentatricopeptide (PPR) repeat-containing protein, contains INTERPRO:IPR002885 PPR repeats \| chr4:14962499-14965020 REVERSE \| Aliases: T10C21.50, T10C21_50 | 3.9 | 2.8 | 1.1 | 5.1 | 0.88% | 4.7 |
| 4229 | AT5G43260.1 \| chaperone protein dnaJ-related, similar to Chaperone protein dnaJ (SP:Q9WZV3) (Thermotoga maritima) \| chr5:17374615-17375310 REVERSE \| Aliases: MNL12.8, MNL12_8 | 8.7 | 7.4 | 1.3 | 5.1 | 0.88% | 4.7 |
| 4230 | AT4G26430.1 \| Symbol: CSN6B \| COP9 signalosome subunit 6 / CSN subunit 6 (CSN6B), identical to COP9 signalosome subunit 6 (Arabidopsis thaliana) GI:17940314, CSN complex subunit 6B (Arabidopsis thaliana) GI:18056667; contains Pfam profile PF01398: Mov34/MPN/PAD-1 family; supporting cDNA gi:17940313:gb:AF434762.1:AF434762; identical to cDNA CSN complex subunit 6B (CSN6B) GI:18056666 \| chr4:13355000-13357610 FORWARD \| Aliases: M3E9.140, M3E9_140 | 5.5 | 4.1 | 1.4 | 5.1 | 0.88% | 4.7 |
| 4231 | AT2G43280.1 \| far-red impaired responsive family protein / FAR1 family protein, weak similarity to far-red impaired response protein (Arabidopsis thaliana) GI:5764395; contains Pfam profile PF03101: FAR1 family \| chr2:17996775-17998099 FORWARD \| Aliases: F14B2.32 | 5.5 | 4.1 | 1.4 | 5.1 | 0.88% | 4.7 |
| 4232 | AT4G02220.1 \| zinc finger (MYND type) family protein / programmed cell death 2 C-terminal domain-containing protein, similar to SP:Q16342 Programmed cell death protein 2 (Zinc finger protein Rp-8) {Homo sapiens}; contains Pfam profiles PF01753: MYND finger, PF04194: Programmed cell death protein 2, C-terminal putative domain | 5.8 | 4.6 | 1.2 | 5.1 | 0.88% | 4.7 |
| 4234 | AT3G53130.1 \| Symbol: LUT1 \| cytochrome P450 family protein, similar to Cytochrome P450 97B2 (SP:048921) (Glycine max) \| chr3:19703749-19708520 FORWARD \| Aliases: CYP97C1, LUTEIN DEFICIENT 1, T4D2.60 | 4.8 | 3.9 | 1.0 | 5.1 | 0.88% | 4.7 |
| 4235 | AT5G02740.2 \| expressed protein \| chr5:616492-618595 FORWARD \| Aliases: F9G14.50, F9G14_50 | 6.1 | 4.2 | 1.9 | 5.1 | 0.89% | 4.7 |
| 4236 | AT4G34870.1 \| Symbol: ROC5 \| peptidyl-prolyl cis-trans isomerase / cyclophilin (CYP1) / rotamase, identical to cyclophilin (CYP1) gi:992643:gb:AAA75512; similar to peptidyl-prolyl cis-trans isomerase, PPIase (cyclophilin, cyclosporin A-binding protein) (Catharanthus roseus) SWISS-PROT:Q39613 \| chr4:16614332-16615318 FORWARD \| Aliases: ATCYP1, CYCLOPHILIN, F11I11.110, F11I11_110, ROTAMASE CYP | 10.6 | 7.9 | 2.7 | 5.1 | 0.89% | 4.7 |
| 4239 | AT2G47400.1 \| Symbol: CP12 1 \| | 9.2 | 7.7 | 1.5 | 5.1 | 0.89% | 4.7 |
| 4241 | AT5G36950.1 \| DegP protease, putative, contains similarity to DegP2 protease GI:13172275 from (Arabidopsis thaliana) \| chr5:14612211-14615570 FORWARD \| Aliases: MLF18.70, MLF18_70 | 5.9 | 4.4 | 1.5 | 5.1 | 0.89% | 4.7 |
| 4243 | AT5G47550.1 \| cysteine protease inhibitor, putative / cystatin, putative, similar to SP:P09229 Cysteine proteinase inhibitor-I (Oryzacystatin-I) {Oryza sativa}; contains Pfam profile PF00031: Cystatin domain \| chr5:19303676-19304221 REVERSE \| Aliases: MNJ7.14, MNJ7_14 | 6.7 | 5.0 | 1.7 | 5.1 | 0.89% | 4.7 |
| 4244 | AT5G62650.1 \| expressed protein \| chr5:25169851-25173154 REVERSE \| Aliases: MRG21.7, MRG21_7 | 7.0 | 5.9 | 1.1 | 5.1 | 0.89% | 4.7 |
| 4245 | AT1G71430.1 \| expressed protein \| chr1:26924102-26924801 FORWARD \| Aliases: F3I17.24, F3I17_24 | 6.8 | 4.9 | 1.9 | 5.1 | 0.89% | 4.6 |
| 4246 | AT4G33100.1 \| expressed protein, contains Pfam PF05254: Uncharacterised protein family (UPF0203) \| chr4:15971177-15972492 FORWARD \| Aliases: F4I10.30, F4I10_30 | 7.1 | 6.0 | 1.1 | 5.1 | 0.90% | 4.7 |
| 4248 | AT5G17000.1 \| NADP-dependent oxidoreductase, putative, strong similarity to probable NADP-dependent oxidoreductase (zeta-crystallin homolog) P1 (SP:Q39172)(gi:886428) and P2 (SP:Q39173)(gi:886430), Arabidopsis thaliana \| chr5:5584804-5587066 REVERSE \| Aliases: F2K13.150, F2K13_150 | 8.6 | 6.9 | 1.7 | 5.1 | 0.90% | 4.7 |

| Rank | Description | Sync | Root | M | t | adj.q | B |
|---|---|---|---|---|---|---|---|
| 4249 | AT1G06730.1 \| pfkB-type carbohydrate kinase family protein, contains Pfam profile: PF00294 pfkB family carbohydrate kinase \| chr1:2067528-2070445 FORWARD \| Aliases: F4H5.18, F4H5_18 | 5.1 | 3.9 | 1.2 | 5.1 | 0.90% | 4.7 |
| 4250 | AT1G27900.1 \| RNA helicase, putative, similar to SP:Q14562 ATP-dependent helicase DDX8 (RNA helicase HRH1) (DEAH-box protein 8) {Homo sapiens}; contains Pfam profiles PF04408: Helicase associated domain (HA2), PF00271: Helicase conserved C-terminal domain \| chr1:9715440-9720548 REVERSE \| Aliases: F13K9.28, F13K9_28 | 7.2 | 5.5 | 1.7 | 5.1 | 0.90% | 4.7 |
| 4251 | AT5G23440.1 \| ferredoxin-thioredoxin reductase, putative, similar to ferredoxin-thioredoxin reductase, variable chain (FTR-V, Ferredoxin- thioredoxin reductase subunit A, FTR-A) (Zea mays) SWISS-PROT:P80680 | 4.5 | 3.4 | 1.0 | 5.1 | 0.90% | 4.7 |
| 4252 | AT4G31985.1 \| 60S ribosomal protein L39 (RPL39C) \| chr4:15469901-15470565 FORWARD \| Aliases: None | 10.8 | 8.9 | 1.9 | 5.1 | 0.90% | 4.4 |
| 4253 | AT5G04000.1 \| expressed protein \| chr5:1079102-1079839 REVERSE \| Aliases: F8F6.210, F8F6_210 | 5.2 | 3.0 | 2.2 | 5.1 | 0.90% | 4.7 |
| 4254 | AT1G15260.1 \| expressed protein, EST gb:N65467 comes from this gene \| chr1:5249755-5250568 FORWARD \| Aliases: F9L1.20, F9L1_20 | 4.1 | 2.6 | 1.5 | 5.1 | 0.90% | 4.7 |
| 4256 | AT1G27680.1 \| Symbol: APL2 \| glucose-1-phosphate adenylyltransferase large subunit 2 (APL2) / ADP-glucose pyrophosphorylase, identical to SP:P55230 \| chr1:9631272-9634584 FORWARD \| Aliases: APL2, T22C5.13, T22C5_13 | 5.2 | 3.9 | 1.3 | 5.1 | 0.90% | 4.7 |
| 4257 | AT3G24890.1 \| Symbol: ATVAMP728 \| synaptobrevin-related, similar to SYNAPTOBREVIN-RELATED PROTEIN GB:P47192 from (Arabidopsis thaliana) (Proc. Natl. Acad. Sci. U.S.A. (1992) 89(9), 3894-3898); contains Pfam profile PF00957: synaptobrevin | 4.2 | 2.7 | 1.6 | 5.1 | 0.90% | 4.7 |
| 4258 | AT5G41760.2 \| similar to nucleotide-sugar transporter family protein [Arabidopsis thaliana] (TAIR:At3g59360.2); similar to nucleotide-sugar transporter family protein [Arabidopsis thaliana] (TAIR:At3g59360.1); similar to putative CMP-sialic acid transporter [Oryza sativa (japonica cultivar-group)] (GB:XP_478801.1); contains InterPro domain UDP-galactose transporter (InterPro:IPR004689); contains InterPro domain Nucleotide-sugar transporter (InterPro:IPR007271) \| chr5:16724121-16727137 FORWARD \| Aliases: K16L22.3, K16L22_3 | 6.3 | 4.6 | 1.7 | 5.1 | 0.90% | 4.7 |
| 4260 | AT1G27050.1 \| similar to homeobox-leucine zipper protein 5 (HAT5) / HD-ZIP protein 5 / HD-ZIP protein (HB-1) [Arabidopsis thaliana] (TAIR:At3g01470.1); similar to homeodomain leucine zipper protein HDZ2 [Phaseolus vulgaris] (GB:AAK84886.1); similar to homeodomain leucine zipper protein 16 [Oryza sativa (japonica cultivar-group)] (GB:AAS68137.1); similar to Hox16 [Oryza sativa (japonica cultivar-group)] (GB:AAS83417.1); contains InterPro domain Leucine zipper, homeobox-associated (InterPro:IPR003106); contains InterPro domain Helix-turn-helix motif, lambda-like repressor (InterPro:IPR000047); contains InterPro domain Homeobox (InterPro:IPR001356); contains InterPro domain RNA-binding region RNP-1 (RNA recognition motif) (InterPro:IPR000504) | 4.4 | 3.5 | 1.0 | 5.1 | 0.91% | 4.7 |
| 4261 | AT5G55740.1 \| pentatricopeptide (PPR) repeat-containing protein, contains INTERPRO:IPR002885 PPR repeats \| chr5:22579167-22581659 REVERSE \| Aliases: MDF20.18, MDF20_18 | 3.8 | 2.6 | 1.2 | 5.1 | 0.91% | 4.7 |
| 4268 | AT3G49730.1 \| pentatricopeptide (PPR) repeat-containing protein, contains Pfam profile PF01535: PPR repeat \| chr3:18453260-18458631 REVERSE \| Aliases: T16K5.80 | 6.9 | 5.3 | 1.7 | 5.0 | 0.91% | 4.7 |
| 4270 | AT3G47250.3 \| expressed protein, contains Pfam profile PF03140: Plant protein of unknown function \| chr3:17410862-17412705 REVERSE \| Aliases: F13I12.300 | 3.7 | 2.5 | 1.2 | 5.0 | 0.91% | 4.7 |
| 4271 | AT1G72520.1 \| lipoxygenase, putative, similar to lipoxygenase gi:1495804 (Solanum tuberosum), gi:1654140 (Lycopersicon esculentum), GB:CAB56692 (Arabidopsis thaliana) | 4.9 | 3.5 | 1.5 | 5.0 | 0.91% | 4.7 |
| 4273 | AT3G11964.1 \| S1 RNA-binding domain-containing protein, similar to SP:Q05022 rRNA biogenesis protein RRP5 {Saccharomyces cerevisiae}; contains Pfam profile PF00575: S1 RNA binding domain \| chr3:3793963-3806632 REVERSE \| Aliases: MEC18.13 | 6.2 | 4.3 | 1.9 | 5.0 | 0.91% | 4.6 |
| 4274 | AT5G54750.1 \| transport protein particle (TRAPP) component Bet3, putative, similar to SP:P36149 Transport protein particle 22 kDa subunit (TRAPP 22 kDa subunit) {Saccharomyces cerevisiae}; contains Pfam profile PF04051: Transport protein particle (TRAPP) component, Bet3 | 9.0 | 7.3 | 1.6 | 5.0 | 0.91% | 4.6 |
| 4275 | AT3G03600.1 \| Symbol: RPS2 \| ribosomal protein S2, mitochondrial (RPS2), identical to SP:Q9GCB9 Mitochondrial ribosomal protein S2 {Arabidopsis thaliana}; contains Pfam profile PF00318: ribosomal protein S2 \| chr3:867669-868585 REVERSE \| Aliases: RIBOSOMAL PROTEIN S2, T12J13.12, T12J13_12 | 6.7 | 5.2 | 1.5 | 5.0 | 0.91% | 4.7 |
| 4276 | AT5G47435.2 \| formyltetrahydrofolate deformylase, putative, similar to formyltetrahydrofolate deformylase (strain PCC 6803- Synechocystis sp.) SWISS-PROT:Q55135 \| chr5:19258930-19261181 FORWARD \| Aliases: None | 11.0 | 9.9 | 1.1 | 5.0 | 0.91% | 4.4 |
| 4277 | AT1G04340.1 \| lesion inducing protein-related, similar to ORF, able to induce HR-like lesions (Nicotiana tabacum) \| chr1:1163096-1164850 REVERSE \| Aliases: F19P19.23, F19P19_23 | 7.1 | 5.7 | 1.5 | 5.0 | 0.91% | 4.7 |

| Rank | Description | Sync | Root | M | t | adj.q | B |
|---|---|---|---|---|---|---|---|
| 4280 | AT1G09150.1 \| pseudouridine synthase and archaeosine transglycosylase (PUA) domain-containing protein, similar to MCT-1 (putative oncogene) (Homo sapiens) GI:6177738; contains Pfam profile PF01472: PUA domain \| chr1:2946264-2949161 FORWARD \| Aliases: T12M4.18, T12M4_18 | 7.7 | 5.1 | 2.5 | 5.0 | 0.91% | 4.7 |
| 4281 | AT5G63890.2 \| Symbol: ATHDH \| histidinol dehydrogenase, putative / HDH, putative, strong similarity to SP:P24226 Histidinol dehydrogenase, chloroplast precursor (EC 1.1.1.23) (HDH) {Brassica oleracea var.capitata}; contains Pfam profile PF00815: histidinol dehydrogenase \| chr5:25582657-25585421 REVERSE \| Aliases: HISTIDINOL DEHYDROGENASE, MGI19.9, MGI19_9 | 9.3 | 7.3 | 2.0 | 5.0 | 0.91% | 4.7 |
| 4283 | AT2G38550.1 \| expressed protein, contains Pfam profile PF03647: Uncharacterised protein family (UPF0136) \| chr2:16139092-16141493 FORWARD \| Aliases: T6A23.25, T6A23_25 | 9.4 | 6.9 | 2.5 | 5.0 | 0.91% | 4.7 |
| 4284 | AT5G40930.1 \| Symbol: TOM20 4 \| mitochondrial import receptor subunit TOM20-4 / translocase of outer membrane 20 kDa subunit 4, identical to mitochondrial import receptor subunit TOM20-4 SP:P82805 from (Arabidopsis thaliana) \| chr5:16421688-16423224 FORWARD \| Aliases: MMG1.2, MMG1_2, TOM20, TRANSLOCASE OF OUTER MEMBRANE 20 4 | 10.2 | 8.5 | 1.7 | 5.0 | 0.91% | 4.5 |
| 4285 | AT1G62180.1 \| Symbol: APR2 \| 5'-adenylylsulfate reductase 2, chloroplast (APR2) (APSR) / adenosine 5'-phosphosulfate 5'-adenylylsulfate (APS) sulfotransferase 2 / 3'-phosphoadenosine-5'-phosphosulfate (PAPS) reductase homolog 43 (PRH-43), identical to SP:P92981 5'-adenylylsulfate reductase 2, chloroplast precursor (EC 1.8.4.9) (Adenosine 5'-phosphosulfate 5'-adenylylsulfate sulfotransferase 2) (APS sulfotransferase 2) (Thioredoxin independent APS reductase 2) (3'-phosphoadenosine-5'-phosphosulfate reductase homolog 43) (PAPS reductase homolog 43) (Prh-43) {Arabidopsis thaliana}; identical to cDNA PAPS reductase homolog (PRH43) GI:1710115 | 9.8 | 8.4 | 1.5 | 5.0 | 0.91% | 4.7 |
| 4286 | AT1G53030.1 \| cytochrome c oxidase copper chaperone family protein, contains Pfam domian, PF05051: Cytochrome C oxidase copper chaperone (COX17) \| chr1:19763721-19765110 REVERSE \| Aliases: F8L10.24, F8L10_24 | 6.3 | 4.6 | 1.7 | 5.0 | 0.92% | 4.7 |
| 4287 | AT5G13480.1 \| Symbol: FY \| similar to WD-40 repeat family protein [Arabidopsis thaliana] (TAIR:At5g67320.1); similar to putative FY protein [Oryza sativa (japonica cultivar-group)] (GB:BAD87887.1); contains InterPro domain G-protein beta WD-40 repeat (InterPro:IPR001680) \| chr5:4326587-4331641 REVERSE \| Aliases: FY, T6I14.10, T6I14_10 | 6.2 | 5.1 | 1.1 | 5.0 | 0.92% | 4.7 |
| 4288 | AT3G60910.1 \| expressed protein, low similarity to PIR:I46078 endothelin converting enzyme from Bos primigenius taurus | 7.8 | 7.0 | 0.7 | 5.0 | 0.92% | 4.5 |
| 4291 | AT4G28230.1 \| expressed protein \| chr4:13995793-13997313 REVERSE \| Aliases: F26K10.110, F26K10_110 | 7.7 | 5.8 | 2.0 | 5.0 | 0.92% | 4.7 |
| 4292 | AT5G10180.1 \| Symbol: AST68 \| sulfate transporter, identical to sulfate transporter (Arabidopsis thaliana) GI:2114104 | 7.1 | 4.7 | 2.3 | 5.0 | 0.92% | 4.7 |
| 4295 | AT1G77060.1 \| mutase family protein, similar to carboxyvinyl-carboxyphosphonate phosphorylmutase GB:O49290 from (Arabidopsis thaliana); similar to carboxyphosphonoenolpyruvate mutase (GI:47149) (Streptomyces hygroscopicus); contains Prosite PS00161: Isocitrate lyase signature \| chr1:28956579-28958374 REVERSE \| Aliases: F22K20.14, F22K20_14 | 4.9 | 3.0 | 1.9 | 5.0 | 0.92% | 4.7 |
| 4296 | AT3G55040.1 \| In2-1 protein, putative, similar to In2-1 protein, Zea mays, P49248 \| chr3:20409695-20411282 REVERSE \| Aliases: T15C9.60 | 5.2 | 4.2 | 1.1 | 5.0 | 0.92% | 4.7 |
| 4299 | AT4G16770.1 \| oxidoreductase, 2OG-Fe(II) oxygenase family protein, low similarity to flavonol synthase from Petunia hybrida (SP:Q07512), Citrus unshiu (GI:4126403); contains Pfam domain PF03171, 2OG-Fe(II) oxygenase superfamily; non-consensus GG acceptor splice site at exon 8 \| chr4:9434376-9437187 REVERSE \| Aliases: DL4410C, FCAALL.233 | 6.5 | 5.1 | 1.4 | 5.0 | 0.92% | 4.7 |
| 4300 | AT5G28490.1 \| expressed protein, contains Pfam profile PF04852: Protein of unknown function (DUF640) \| chr5:10454545-10455117 REVERSE \| Aliases: F24J2.30, F24J2_30 | 3.5 | 2.7 | 0.9 | 5.0 | 0.92% | 4.6 |
| 4301 | AT3G48490.1 \| expressed protein \| chr3:17972274-17972890 FORWARD \| Aliases: T29H11.260 | 4.3 | 3.1 | 1.2 | 5.0 | 0.93% | 4.7 |
| 4302 | AT5G37980.1 \| NADP-dependent oxidoreductase, putative, similar to probable NADP-dependent oxidoreductase (zeta-crystallin homolog) P1 (SP:Q39172)(gi:886428) and P2 (SP:Q39173)(gi:886430), Arabidopsis thaliana \| chr5:15148590-15150315 FORWARD \| Aliases: K18L3.140, K18L3_140 | 5.7 | 4.7 | 1.1 | 5.0 | 0.93% | 4.6 |
| 4303 | AT3G23250.2 \| similar to myb family transcription factor [Arabidopsis thaliana] (TAIR:At1g06180.1); similar to myb-related transcription factor LBM4 [Nicotiana tabacum] (GB:BAA88224.1); contains InterPro domain Myb DNA-binding domain (InterPro:IPR001005) | 5.5 | 3.7 | 1.8 | 5.0 | 0.93% | 4.7 |
| 4304 | AT5G23590.2 \| similar to DNAJ heat shock protein, mitochondrially targeted (GFA2) [Arabidopsis thaliana] (TAIR:At5g48030.1); similar to PREDICTED: similar to hypothetical protein FLJ10634 [Canis familiaris] (GB:XP_535435.1); contains InterPro domain Heat shock protein DnaJ (InterPro:IPR003095); contains InterPro domain Heat shock protein DnaJ, N-terminal (InterPro:IPR001623) \| chr5:7953532-7955625 REVERSE \| Aliases: MQM1.14, MQM1_14 | 9.4 | 7.7 | 1.6 | 5.0 | 0.93% | 4.7 |
| 4306 | AT4G32610.1 \| mitochondrial glycoprotein family protein / MAM33 family protein, low similarity to SP:P40513 Mitochondrial acidic protein MAM33, mitochondrial precursor {Saccharomyces cerevisiae}; contains Pfam profile PF02330: Mitochondrial glycoprotein \| chr4:15726279-15730362 REVERSE \| Aliases: F4D11.190, F4D11_190 | 6.9 | 4.6 | 2.2 | 5.0 | 0.93% | 4.6 |

| Rank | Description | Sync | Root | M | t | adj.q | B |
|---|---|---|---|---|---|---|---|
| 4309 | AT4G33500.1 \| protein phosphatase 2C-related / PP2C-related, YHR077c (NMD2,IFS1) protein -Saccharomyces cerevisiae,PID:g555939 | 6.3 | 5.1 | 1.2 | 5.0 | 0.93% | 4.7 |
| 4310 | AT1G44790.1 \| ChaC-like family protein, contains Pfam profile: PF04752 ChaC-like protein \| chr1:16914594-16916104 REVERSE \| Aliases: T12C22.6, T12C22_6 | 6.7 | 4.4 | 2.2 | 5.0 | 0.93% | 4.7 |
| 4311 | AT5G58710.1 \| Symbol: ROC7 \| peptidyl-prolyl cis-trans isomerase, putative / cyclophilin, putative / rotamase, putative (ROC7), similar to cyclophilin (Arabidopsis thaliana) gi:2443755:gb:AAB71401 \| chr5:23735018-23736975 FORWARD \| Aliases: CYCLOPHILIN, MZN1.23, MZN1_23 | 10.7 | 9.1 | 1.5 | 5.0 | 0.93% | 4.5 |
| 4313 | AT1G53840.1 \| Symbol: ATPME1 \| | 5.4 | 3.3 | 2.1 | 5.0 | 0.93% | 4.6 |
| 4315 | AT5G66680.1 \| Symbol: DGL1 \| dolichyl-diphosphooligosaccharide-protein glycosyltransferase 48kDa subunit family protein, similar to SP:Q05052 Dolichyl-diphosphooligosaccharide--protein glycosyltransferase 48 kDa subunit precursor (EC 2.4.1.119) (Oligosaccharyl transferase 48 kDa subunit) {Canis familiaris}; contains Pfam profile PF03345: Dolichyl-diphosphooligosaccharide-protein glycosyltransferase 48kD subunit \| chr5:26634770-26637893 REVERSE \| Aliases: MSN2.7, MSN2_7 | 10.6 | 9.5 | 1.1 | 5.0 | 0.93% | 4.5 |
| 4316 | AT5G66240.2 \| transducin family protein / WD-40 repeat family protein, contains Pfam PF00400: WD domain, G-beta repeat (4 copies, 1 weak); similar to Will die slowly protein. {Drosophila melanogaster} (SP:Q9V3J8) {Drosophila melanogaster} | 8.3 | 7.3 | 1.0 | 5.0 | 0.94% | 4.5 |
| 4319 | AT5G10695.1 \| expressed protein \| chr5:3377664-3378222 FORWARD \| Aliases: None | 9.3 | 8.0 | 1.3 | 5.0 | 0.94% | 4.6 |
| 4323 | AT3G22680.1 \| expressed protein \| chr3:8019736-8020515 FORWARD \| Aliases: MWI23.5 | 7.3 | 5.4 | 1.9 | 5.0 | 0.94% | 4.7 |
| 4324 | AT5G53060.1 \| KH domain-containing protein \| chr5:21532252-21535663 FORWARD \| Aliases: MNB8.12, MNB8_12 | 5.3 | 4.1 | 1.2 | 5.0 | 0.94% | 4.6 |
| 4326 | AT1G70730.1 \| phosphoglucomutase, cytoplasmic, putative / glucose phosphomutase, putative, strong similarity to SP:P93804 Phosphoglucomutase, cytoplasmic 1 (EC 5.4.2.2) (Glucose phosphomutase 1) (PGM 1) {Zea mays}; contains InterPro accession IPR006352: Phosphoglucosamine mutase \| chr1:26672527-26676418 REVERSE \| Aliases: F5A18.9, F5A18_9 | 10.7 | 8.5 | 2.2 | 5.0 | 0.94% | 4.6 |
| 4327 | AT1G27480.1 \| lecithin:cholesterol acyltransferase family protein / LACT family protein, similar to LCAT-like lysophospholipase (LLPL) (Homo sapiens) GI:4589720; contains Pfam profile PF02450: Lecithin:cholesterol acyltransferase (phosphatidylcholine-sterol acyltransferase) \| chr1:9544501-9546272 FORWARD \| Aliases: F17L21.27, F17L21_27 | 4.6 | 3.2 | 1.4 | 5.0 | 0.94% | 4.6 |
| 4329 | AT3G13845.1 \| expressed protein \| chr3:4557333-4558586 REVERSE \| Aliases: None | 9.9 | 8.7 | 1.2 | 5.0 | 0.94% | 4.5 |
| 4332 | AT2G44690.1 \| Symbol: ARAC9 \| Rac-like GTP-binding protein (ARAC9), identical to rac-like protein ARAC9 GI:5381419 from (Arabidopsis thaliana) \| chr2:18436339-18437879 FORWARD \| Aliases: F16B22.18 | 4.9 | 4.0 | 0.9 | 5.0 | 0.94% | 4.6 |
| 4333 | AT4G17420.1 \| expressed protein, contains Pfam domain, PF01987: Protein of unknown function \| chr4:9722451-9724652 REVERSE \| Aliases: DL4745C, FCAALL.389 | 7.5 | 6.3 | 1.2 | 5.0 | 0.94% | 4.7 |
| 4334 | AT4G34410.1 \| encodes a member of the ERF (ethylene response factor) subfamily B-3 of ERF/AP2 transcription factor family. The protein contains one AP2 domain. There are 18 members in this subfamily including ATERF-1, ATERF-2, AND ATERF-5. \| chr4:16451880-16453264 FORWARD \| Aliases: F10M10.180, F10M10_180 | 4.1 | 2.6 | 1.5 | 5.0 | 0.94% | 4.7 |
| 4335 | AT3G11050.1 \| Symbol: ATFER2 \| | 6.4 | 4.4 | 2.0 | 5.0 | 0.94% | 4.4 |
| 4336 | AT1G67830.1 \| GDSL-motif lipase/hydrolase family protein, similar to early nodulin ENOD8 (Medicago sativa) GI:304037, elicitor-induced glycoprotein iEP4 (Daucus carota) GI:1911765, lanatoside 15'-O-acetylesterase (Digitalis lanata) GI:3688284; contains Pfam profile PF00657: Lipase/Acylhydrolase with GDSL-like motif \| chr1:25434992-25436635 REVERSE \| Aliases: F12A21.4, F12A21_4 | 5.6 | 4.5 | 1.1 | 5.0 | 0.94% | 4.7 |
| 4338 | AT3G14220.1 \| GDSL-motif lipase/hydrolase family protein, similar to myrosinase-associated proteins GI:1769968, GI:1769970 from (Brassica napus); contains InterPro Entry IPR001087 Lipolytic enzyme, G-D-S-L family; contains 1 predicted transmembrane domain; \| chr3:4732900-4734616 FORWARD \| Aliases: MLE3.1 | 8.3 | 7.2 | 1.1 | 5.0 | 0.94% | 4.7 |
| 4341 | AT1G64105.1 \| Symbol: ANAC027 \| no apical meristem (NAM) family protein, contains Pfam PF02365: No apical meristem (NAM) domain \| chr1:23798713-23799305 FORWARD \| Aliases: ANAC027 | 8.4 | 6.7 | 1.7 | 5.0 | 0.95% | 4.7 |
| 4342 | AT5G64680.2 \| expressed protein \| chr5:25871408-25872710 FORWARD \| Aliases: MUB3.20, MUB3_20 | 5.8 | 4.6 | 1.2 | 5.0 | 0.95% | 4.6 |
| 4344 | AT3G15590.1 \| DNA-binding protein, putative, similar to DNA-binding protein (Triticum aestivum) GI:6958202; contains Pfam profile: PF01535 PPR repeat \| chr3:5275408-5277994 REVERSE \| Aliases: MQD17.5 | 10.4 | 9.6 | 0.8 | 5.0 | 0.95% | 4.3 |
| 4347 | AT4G39710.1 \| immunophilin, putative / FKBP-type peptidyl-prolyl cis-trans isomerase, putative, similar to FK506 binding protein 1 (GP:21535744) (Arabidopsis thaliana) \| chr4:18427033-18428353 REVERSE \| Aliases: T19P19.100, T19P19_100 | 4.0 | 2.6 | 1.3 | 5.0 | 0.95% | 4.7 |

| Rank | Description | Sync | Root | M | t | adj.q | B |
|---|---|---|---|---|---|---|---|
| 4349 | AT3G30300.1 \| expressed protein, contains Pfam PF03138: Plant protein family. The function of this family of plant proteins is unknown; previously annotated as 'auxin-independent growth promoter -related' based on similarity to axi 1 protein (GB:X80301) (GI:559920) from (Nicotiana tabacum), which, due to scienitific fraud was retracted. Retraction in: Schell J. EMBO J 1999 May 17;18(10):2908. PMID:10400497. \| chr3:11923677-11926884 REVERSE \| Aliases: T6J22.5 | 6.2 | 5.0 | 1.2 | 5.0 | 0.95% | 4.6 |
| 4350 | AT2G06010.1 \| Symbol: ORG4 \| expressed protein \| chr2:2336815-2338899 FORWARD \| Aliases: F5K7.23, F5K7_23, OBP3 RESPONSIVE GENE 4 | 5.0 | 4.0 | 0.9 | 5.0 | 0.96% | 4.6 |
| 4351 | AT1G16780.1 \| Symbol: AVPL1 \| vacuolar-type H+-translocating inorganic pyrophosphatase, putative, similar to vacuolar-type H+-translocating inorganic pyrophosphatase GI:6901676 from (Arabidopsis thaliana) \| chr1:5739292-5743562 REVERSE \| Aliases: AVPL1, F17F16.2, F17F16_2, VACUOLAR PYROPHOSPHATASE LIKE PROTEIN 1 | 5.9 | 4.4 | 1.5 | 5.0 | 0.96% | 4.6 |
| 4354 | AT3G02080.1 \| 40S ribosomal protein S19 (RPS19A), similar to 40S ribosomal protein S19 GB:P40978 (Oryza sativa) \| chr3:363918-365255 REVERSE \| Aliases: F1C9.13, F1C9_13 | 10.7 | 9.2 | 1.5 | 5.0 | 0.96% | 4.6 |
| 4355 | AT4G24700.1 \| expressed protein \| chr4:12744632-12745323 REVERSE \| Aliases: F22K18.100, F22K18_100 | 3.4 | 2.5 | 0.8 | 5.0 | 0.96% | 4.5 |
| 4356 | AT1G53240.1 \| malate dehydrogenase (NAD), mitochondrial, identical to mitochondrial NAD-dependent malate dehydrogenase GI:3929649 SP:Q9ZP06 from (Arabidopsis thaliana); contains InterPro entry IPR001236: Lactate/malate dehydrogenase \| chr1:19858283-19860605 REVERSE \| Aliases: F12M16.14, F12M16_14 | 11.3 | 9.0 | 2.3 | 5.0 | 0.96% | 4.5 |
| 4357 | AT3G56490.1 \| zinc-binding protein, putative / protein kinase C inhibitor, putative, similar to 14 kDa zinc-binding protein (Protein kinase C inhibitor, PKCI) (Zea mays) Swiss-Prot:P42856 \| chr3:20952481-20954381 FORWARD \| Aliases: T5P19.140 | 8.0 | 5.8 | 2.1 | 5.0 | 0.96% | 4.6 |
| 4358 | AT3G51140.1 \| expressed protein \| chr3:19008748-19010883 FORWARD \| Aliases: F24M12.180 | 7.1 | 4.8 | 2.4 | 5.0 | 0.96% | 4.6 |
| 4361 | AT1G06720.1 \| expressed protein, contains Pfam domain, PF04950: Protein of unknown function (DUF663) \| chr1:2060443-2067322 REVERSE \| Aliases: F4H5.19, F4H5_19 | 7.0 | 4.4 | 2.6 | 5.0 | 0.97% | 4.6 |
| 4363 | AT3G18750.2 \| Symbol: WNK6 \| similar to protein kinase family protein [Arabidopsis thaliana] (TAIR:At1g49160.2); similar to protein kinase family protein [Arabidopsis thaliana] (TAIR:At1g49160.1); similar to putative MAP kinase [Oryza sativa (japonica cultivar-group)] (GB:BAD27820.1); contains InterPro domain Serine/threonine protein kinase, active site (InterPro:IPR008271); contains InterPro domain Tyrosine protein kinase (InterPro:IPR001245); contains InterPro domain Serine/threonine protein kinase (InterPro:IPR002290); contains InterPro domain Protein kinase (InterPro:IPR000719) \| chr3:6454116-6457503 REVERSE \| Aliases: MVE11.20, ZIK5 | 4.0 | 2.4 | 1.6 | 5.0 | 0.97% | 4.6 |
| 4364 | AT5G24490.1 \| 30S ribosomal protein, putative, similar to SP:P19954 Plastid-specific 30S ribosomal protein 1, chloroplast precursor (CS-S5) (CS5) (S22) (Ribosomal protein 1) (PSRP-1) {Spinacia oleracea}; contains Pfam profile PF02482: Sigma 54 modulation protein / S30EA ribosomal protein \| chr5:8365624-8367429 FORWARD \| Aliases: T31K7.7, T31K7_7 | 8.7 | 7.4 | 1.3 | 5.0 | 0.97% | 4.6 |
| 4367 | AT1G50920.1 \| GTP-binding protein-related, similar to GTP-binding protein SP:Q99ME9 from (Mus musculus) \| chr1:18873807-18876598 FORWARD \| Aliases: F8A12.14, F8A12_14 | 9.1 | 6.3 | 2.7 | 5.0 | 0.97% | 4.6 |
| 4368 | AT2G25840.3 \| tRNA synthetase class I (W and Y) family protein, contains Pfam profile: PF00579 tRNA synthetases class I (W and Y) \| chr2:11028983-11032448 FORWARD \| Aliases: F17H15.13, F17H15_13 | 5.4 | 4.0 | 1.4 | 5.0 | 0.97% | 4.6 |
| 4369 | AT3G56510.2 \| similar to unnamed protein product [Yarrowia lipolytica CLIB99] (GB:CAG82708.1) \| chr3:20954907-20957640 REVERSE \| Aliases: T5P19.160 | 9.3 | 8.2 | 1.1 | 5.0 | 0.97% | 4.6 |
| 4372 | AT1G18850.1 \| expressed protein \| chr1:6504797-6506412 REVERSE \| Aliases: F6A14.6, F6A14_6 | 5.5 | 3.9 | 1.6 | 5.0 | 0.97% | 4.6 |
| 4374 | AT2G36835.1 \| expressed protein \| chr2:15456686-15458505 REVERSE \| Aliases: None | 7.7 | 5.4 | 2.4 | 5.0 | 0.97% | 4.6 |
| 4375 | AT5G13610.1 \| expressed protein \| chr5:4383016-4385032 FORWARD \| Aliases: T6I14.13 | 7.3 | 5.9 | 1.4 | 5.0 | 0.98% | 4.6 |
| 4377 | AT1G11420.1 \| agenet domain-containing protein, contains Pfam PF05641: Agenet domain \| chr1:3844581-3846872 REVERSE \| Aliases: T23J18.9, T23J18_9 | 5.5 | 3.3 | 2.2 | 5.0 | 0.98% | 4.6 |
| 4378 | AT3G48570.1 \| protein transport protein SEC61 gamma subunit, putative, similar to Swiss-Prot:Q19967 protein transport protein SEC61 gamma subunit (Caenorhabditis elegans) \| chr3:18014898-18015959 REVERSE \| Aliases: T8P19.80 | 9.0 | 7.1 | 1.9 | 5.0 | 0.98% | 4.5 |
| 4379 | AT5G06130.2 \| chaperone protein dnaJ-related, similar to unknown protein (pir::T00468); contains Pfam PF00684 : DnaJ central domain (4 repeats) \| chr5:1853450-1855873 REVERSE \| Aliases: K16F4.10, K16F4_10 | 8.0 | 6.0 | 2.0 | 5.0 | 0.98% | 4.6 |
| 4381 | AT3G53540.1 \| expressed protein \| chr3:19857478-19861946 REVERSE \| Aliases: F4P12.240 | 5.5 | 4.6 | 1.0 | 5.0 | 0.98% | 4.5 |

| Rank | Description | Sync | Root | M | t | adj.q | B |
|---|---|---|---|---|---|---|---|
| 4382 | AT1G14720.1 \| Symbol: XTR2 \| xyloglucan:xyloglucosyl transferase / xyloglucan endotransglycosylase / endo-xyloglucan transferase (XTR2), identical to endoxyloglucan transferase (Arabidopsis thaliana) GI:5533311 \| chr1:5066631-5068629 REVERSE \| Aliases: ENDOXYLOGLUCAN TRANSFERASE A2, EXGT A2, F10B6.12, F10B6_12, XYLOGLUCAN ENDOTRANSGLYCOSYLASE 2, XYLOGLUCAN ENDOTRANSGLYCOSYLASE RELATED 2, XYLOGLUCAN ENDOTRANSGLYCOSYLASE RELATED PROTEIN | 8.9 | 7.7 | 1.2 | 5.0 | 0.98% | 4.6 |
| 4384 | AT5G05160.1 \| leucine-rich repeat transmembrane protein kinase, putative \| chr5:1528001-1530063 FORWARD \| Aliases: K2A11.3, K2A11_3 | 9.4 | 8.3 | 1.1 | 5.0 | 0.98% | 4.5 |
| 4386 | AT5G61810.2 \| similar to mitochondrial substrate carrier family protein [Arabidopsis thaliana] (TAIR:At5g07320.1); similar to putative small calcium-binding mitochondrial carrier 2 [Oryza sativa (japonica cultivar-group)] (GB:BAD35532.1); contains InterPro domain Mitochondrial substrate carrier (InterPro:IPR001993); contains InterPro domain Mitochondrial carrier protein (InterPro:IPR002067) \| chr5:24848341-24851096 REVERSE \| Aliases: MAC9.1, MAC9_1 | 6.1 | 5.0 | 1.1 | 5.0 | 0.98% | 4.6 |
| 4387 | AT2G20480.1 \| expressed protein \| chr2:8837658-8838579 REVERSE \| Aliases: T13C7.7, T13C7_7 | 3.3 | 2.4 | 0.9 | 5.0 | 0.99% | 4.3 |
| 4389 | AT3G13570.1 \| Symbol: SCL30a \| | 6.5 | 5.0 | 1.5 | 5.0 | 0.99% | 4.6 |
| 4390 | AT5G10010.1 \| expressed protein \| chr5:3127907-3131721 FORWARD \| Aliases: T31P16.7, T31P16_7 | 5.2 | 3.9 | 1.3 | 5.0 | 0.99% | 4.6 |
| 4391 | AT2G03350.1 \| expressed protein, contains Pfam profile PF04398: Protein of unknown function, DUF538 \| chr2:1019576-1021183 REVERSE \| Aliases: T4M8.22, T4M8_22 | 6.1 | 4.8 | 1.3 | 5.0 | 0.99% | 4.6 |
| 4393 | AT1G02160.1 \| expressed protein \| chr1:410785-411590 FORWARD \| Aliases: T6A9.18 | 10.5 | 9.4 | 1.0 | 5.0 | 0.99% | 4.3 |
| 4394 | AT1G47400.1 \| expressed protein \| chr1:17388157-17388626 FORWARD \| Aliases: T3F24.21, T3F24_21 | 4.1 | 3.0 | 1.1 | 5.0 | 0.99% | 4.6 |
| 4396 | AT3G29810.1 \| phytochelatin synthetase family protein / COBRA cell expansion protein COBL2, similar to phytochelatin synthetase (Hordeum vulgare subsp. vulgare) GI:29570314; identified in Roudier, et al, Plant Phys. (2002) 130:538-548 (PMID:12376623); contains Pfam profile PF04833: Phytochelatin synthetase-like conserved region \| chr3:11730596-11732718 FORWARD \| Aliases: K17E7.12 | 8.2 | 6.5 | 1.7 | 5.0 | 0.99% | 4.6 |
| 4397 | AT5G23300.1 \| Symbol: PYRD \| dihydroorotate dehydrogenase, mitochondrial / dihydroorotate oxidase / DHOdehase (PYRD), nearly identical to SP:P32746 Dihydroorotate dehydrogenase, mitochondrial precursor (EC 1.3.3.1) (Dihydroorotate oxidase) (DHOdehase) {Arabidopsis thaliana}; identical to cDNA pyrD mRNA for dihydroorotate dehydrogenase GI:16448 \| chr5:7847593-7850407 REVERSE \| Aliases: DIHYDROOROTATE DEHYDROGENASE, MKD15.16, MKD15_16 | 6.4 | 4.7 | 1.7 | 5.0 | 0.99% | 4.6 |
| 4399 | AT5G20500.1 \| glutaredoxin, putative, similar to glutaredoxin (Populus tremula x Populus tremuloides) gi:19548658:gb:AAL90750 \| chr5:6938600-6940269 FORWARD \| Aliases: F7C8.90, F7C8_90 | 9.8 | 8.0 | 1.9 | 5.0 | 0.99% | 4.6 |
| 4401 | AT3G60320.1 \| Symbol: P9L \| expressed protein, contains Pfam profiles: PF04782: protein of unknown function (DUF632), PF04783: protein of unknown function (DUF630) \| chr3:22302861-22306346 REVERSE \| Aliases: F27H5.110, P9L | 7.8 | 6.5 | 1.3 | 5.0 | 1.00% | 4.6 |
| 4402 | AT3G57800.2 \| basic helix-loop-helix (bHLH) family protein, contains Pfam profile: PF00010 helix-loop-helix DNA-binding domain; supported by full-length cDNA gi:20127059 \| chr3:21418786-21422698 REVERSE \| Aliases: AT3G57795, T10K17.10 | 4.6 | 3.9 | 0.7 | 5.0 | 1.00% | 4.4 |
| 4403 | AT4G10450.1 \| 60S ribosomal protein L9 (RPL90D), ribosomal protein L9, cytosolic - garden pea, PIR2:S19978 \| chr4:6462949-6464521 REVERSE \| Aliases: F7L13.30, F7L13_30 | 5.8 | 4.4 | 1.4 | 5.0 | 1.00% | 4.6 |
| 4405 | AT3G09350.1 \| armadillo/beta-catenin repeat family protein, contains Pfam profile: PF00514 armadillo/beta-catenin-like repeat \| chr3:2871051-2873318 FORWARD \| Aliases: F3L24.22 | 5.2 | 3.1 | 2.1 | 4.9 | 1.00% | 4.6 |
| 4406 | AT1G44575.2 \| Symbol: NPQ4 \| photosystem II 22kDa protein, chloroplast / CP22 (PSBS), identical to photosystem II 22 kDa protein, chloroplast (precursor) SP:Q9XF91 from (Arabidopsis thaliana); contains Pfam profile PF00504: Chlorophyll A-B binding protein \| chr1:16874170-16875818 FORWARD \| Aliases: NONPHOTOCHEMICAL QUENCHING, PSBS, T18F15.3, T18F15_3 | 6.4 | 4.6 | 1.8 | 4.9 | 1.00% | 4.6 |
| 4409 | AT4G28320.1 \| glycosyl hydrolase family 5 protein / cellulase family protein, mannan endo-1,4-beta-mannosidase (EC 3.2.1.78) - tomato, PIR2:T04323 \| chr4:14018189-14020223 REVERSE \| Aliases: F26K10.200, F26K10_200 | 3.4 | 2.7 | 0.7 | 4.9 | 1.00% | 4.2 |
| 4412 | AT2G19780.1 \| leucine-rich repeat family protein / extensin family protein, contains leucine rich-repeat (LRR) domains Pfam:PF00560, INTERPRO:IPR001611; similar to leucine-rich repeat/extensin 1 (GI:13809918) (Arabidopsis thaliana); contains similarity to disease resistance protein (Lycopersicon esculentum) gi:3894383:gb:AAC78591 \| chr2:8529760-8531156 REVERSE \| Aliases: F6F22.19, F6F22_19 | 4.7 | 3.4 | 1.4 | 4.9 | 1.01% | 4.6 |
| 4413 | AT3G18490.1 \| aspartyl protease family protein, contains Pfam domain, PF00026: eukaryotic aspartyl protease \| chr3:6348761-6350674 REVERSE \| Aliases: MYF24.39 | 9.4 | 7.9 | 1.5 | 4.9 | 1.01% | 4.6 |

| Rank | Description | Sync | Root | M | t | adj.q | B |
|---|---|---|---|---|---|---|---|
| 4414 | AT3G06483.1 \| Symbol: PDK \| similar to mitochondrial pyruvate dehydrogenase kinase isoform 1 [Glycine max] (GB:AAT02655.1); contains InterPro domain Bacterial sensor protein, C-terminal (InterPro:IPR004358); contains InterPro domain ATP-binding region, ATPase-like (InterPro:IPR003594) | 5.8 | 4.2 | 1.6 | 4.9 | 1.01% | 4.6 |
| 4416 | AT5G65810.1 \| expressed protein, similar to unknown protein (emb CAB66910.1) \| chr5:26354895-26356963 REVERSE \| Aliases: K22J17.2, K22J17_2 | 7.9 | 5.8 | 2.1 | 4.9 | 1.01% | 4.6 |
| 4420 | AT1G65500.1 \| expressed protein \| chr1:24361530-24362279 FORWARD \| Aliases: F5I14.4, F5I14_4 | 5.1 | 2.6 | 2.5 | 4.9 | 1.01% | 4.4 |
| 4421 | AT2G14860.1 \| peroxisomal membrane protein 22 kDa, putative, similar to 22 kDa peroxisomal membrane protein {Mus musculus} SWISS-PROT:P42925, NCBI_gi:454833 \| chr2:6394902-6396645 REVERSE \| Aliases: T26I20.2, T26I20_2 | 3.8 | 2.5 | 1.3 | 4.9 | 1.01% | 4.6 |
| 4422 | AT2G47170.1 \| Symbol: ARF1 \| Gene encoding ADP-ribosylation factor and similar to other ARFs and ARF-like proteins. Members of this family are known to be essential for vesicle coating and uncoating and functions in GTP-binding. The gene is shown to play a role in cell division, cell expansion and cellulose production using antisense construct. \| chr2:19373694-19375870 FORWARD \| Aliases: ADP RIBOSYLATION FACTOR, ADP RIBOSYLATION FACTOR 1, T8I13.1 | 12.1 | 10.6 | 1.6 | 4.9 | 1.01% | 4.3 |
| 4424 | AT5G66780.1 \| expressed protein \| chr5:26680799-26681629 FORWARD \| Aliases: MUD21.2, MUD21_2 | 4.0 | 2.4 | 1.6 | 4.9 | 1.01% | 4.5 |
| 4426 | AT2G40550.1 \| expressed protein \| chr2:16941914-16945213 FORWARD \| Aliases: T2P4.10, T2P4_10 | 6.2 | 3.5 | 2.7 | 4.9 | 1.01% | 4.5 |
| 4428 | AT2G47790.1 \| transducin family protein / WD-40 repeat family protein, similar to WD-repeat protein 5 (SP:Q9UGP9) (Homo sapiens); The first 3 exons are identical to that of GB:AJ224957. This gene appears to be a truncated version of that in GB:AJ224957; contains 4 WD-40 repeats (PF00400) \| chr2:19577641-19580086 REVERSE \| Aliases: F17A22.18 | 8.8 | 6.8 | 2.0 | 4.9 | 1.01% | 4.6 |
| 4429 | AT4G32240.1 \| expressed protein \| chr4:15568306-15569194 FORWARD \| Aliases: F10M6.120, F10M6_120 | 11.2 | 10.4 | 0.8 | 4.9 | 1.01% | 3.9 |
| 4430 | AT2G18740.1 \| small nuclear ribonucleoprotein E, putative / snRNP-E, putative / Sm protein E, putative, similar to SWISS-PROT:P08578 small nuclear ribonucleoprotein E (snRNP-E) (Sm protein E, Sm-E, SmE) (Chicken) \| chr2:8130303-8132086 FORWARD \| Aliases: MSF3.12, MSF3_12 | 5.7 | 2.5 | 3.2 | 4.9 | 1.01% | 4.6 |
| 4432 | AT3G22290.1 \| expressed protein \| chr3:7881672-7885549 FORWARD \| Aliases: AT3G22280, MCB17.1 | 7.9 | 7.2 | 0.7 | 4.9 | 1.01% | 4.4 |
| 4433 | AT2G38740.1 \| haloacid dehalogenase-like hydrolase family protein, similar to SP:P71447 Beta-phosphoglucomutase (EC 5.4.2.6) {Lactococcus lactis}; contains InterPro accession IPR005834: Haloacid dehalogenase-like hydrolase | 8.3 | 6.6 | 1.8 | 4.9 | 1.01% | 4.5 |
| 4434 | AT4G13860.1 \| glycine-rich RNA-binding protein, putative, similar to Glycine-rich RNA-binding protein 2, mitochondrial precursor (AtGRP2) (Swiss-Prot:Q9SVM8) (Arabidopsis thaliana) ; contains InterPro entry IPR000504: RNA-binding region RNP-1 (RNA recognition motif) (RRM) \| chr4:8022597-8023158 FORWARD \| Aliases: F18A5.250, F18A5_250 | 7.1 | 5.8 | 1.3 | 4.9 | 1.01% | 4.6 |
| 4437 | AT1G72680.1 \| cinnamyl-alcohol dehydrogenase, putative, similar to cinnamyl-alcohol dehydrogenase GB:AAC35846 (Medicago sativa), SP:Q08350 (Picea abies) \| chr1:27362894-27364678 REVERSE \| Aliases: F28P22.13, F28P22_13 | 9.7 | 6.4 | 3.3 | 4.9 | 1.02% | 4.5 |
| 4438 | AT2G16570.1 \| Symbol: ATASE \| amidophosphoribosyltransferase / glutamine phosphoribosylpyrophosphate amidotransferase / phosphoribosyldiphosphate 5-amidotransferase, identical to amidophosphoribosyltransferase (Arabidopsis thaliana) GI:469193; identical to cDNA amidophosphoribosyltransferase GI:469192 \| chr2:7187227-7189275 REVERSE \| Aliases: ATASE1, F1P15.5, F1P15_5, GLN PHOSPHORIBOSYL PYROPHOSPHATE AMIDOTRANSFERASE 1 | 8.0 | 6.4 | 1.6 | 4.9 | 1.02% | 4.6 |
| 4440 | AT5G02710.1 \| expressed protein, contains Pfam PF03692: Uncharacterised protein family (UPF0153) \| chr5:612606-614418 FORWARD \| Aliases: F9G14.8 | 3.5 | 2.4 | 1.1 | 4.9 | 1.02% | 4.5 |
| 4441 | AT3G06040.2 \| ribosomal protein L12 family protein, contains similarity to 50S ribosomal protein L12-C, chloroplast precursor GB:P36212 from (Arabidopsis thaliana) \| chr3:1824382-1825634 REVERSE \| Aliases: F24F17.2, F24F17_2 | 5.6 | 3.7 | 1.9 | 4.9 | 1.02% | 4.6 |
| 4442 | AT5G19940.1 \| plastid-lipid associated protein PAP-related / fibrillin-related, weak hit to Pfam profile PF04755: PAP_fibrillin \| chr5:6739626-6740984 FORWARD \| Aliases: F28I16.90, F28I16_90 | 5.3 | 3.4 | 1.8 | 4.9 | 1.02% | 4.6 |
| 4443 | AT1G80280.1 \| hydrolase, alpha/beta fold family protein, contains Pfam profile: PF00561 alpha/beta hydrolase fold \| chr1:30188433-30191478 REVERSE \| Aliases: F5I6.3, F5I6_3 | 3.8 | 2.4 | 1.3 | 4.9 | 1.02% | 4.5 |
| 4444 | AT2G28260.1 \| Symbol: ATCNGC15 \| cyclic nucleotide-regulated ion channel, putative (CNGC15), similar to cyclic nucleotide and calmodulin-regulated ion channel (cngc6) GI:4581207 from (Arabidopsis thaliana) | 5.7 | 4.9 | 0.9 | 4.9 | 1.02% | 4.5 |
| 4446 | AT1G21350.3 \| expressed protein \| chr1:7476930-7479021 REVERSE \| Aliases: F24J8.1, F24J8_1 | 4.3 | 3.2 | 1.1 | 4.9 | 1.02% | 4.6 |

| Rank | Description | Sync | Root | M | t | adj.q | B |
|---|---|---|---|---|---|---|---|
| 4449 | AT5G60960.1 \| pentatricopeptide (PPR) repeat-containing protein, contains Pfam profile PF01535: PPR repeat \| chr5:24545476-24547237 REVERSE \| Aliases: MSL3.8, MSL3_8 | 8.5 | 5.8 | 2.8 | 4.9 | 1.02% | 4.6 |
| 4450 | AT4G25730.1 \| FtsJ-like methyltransferase family protein, contains Pfam profile: PF01728 FtsJ-like methyltransferase \| chr4:13102688-13106789 REVERSE \| Aliases: F14M19.10, F14M19_10 | 5.5 | 4.4 | 1.0 | 4.9 | 1.02% | 4.5 |
| 4451 | AT3G18390.1 \| Symbol: EMB1865 \| expressed protein, contains Pfam domain, PF04581: Protein of unknown function (DUF578) \| chr3:6313491-6317977 FORWARD \| Aliases: EMB1865, EMBRYO DEFECTIVE 1865, MYF24.11 | 5.2 | 4.1 | 1.2 | 4.9 | 1.02% | 4.5 |
| 4453 | AT1G73010.1 \| expressed protein, similar to phosphatase, orphan 1 (GI:20196841) (Mus musculus), (GI:20196839) (Homo sapiens); contains TIGRFAM TIGR01489: 2,3-diketo-5-methylthio-1-phosphopentane phosphatase profile; contains TIGRFAM TIGR01488: HAD-superfamily hydrolase, subfamily IB (PSPase-like) \| chr1:27468233-27469917 REVERSE \| Aliases: F3N23.21, F3N23_21 | 5.8 | 4.3 | 1.5 | 4.9 | 1.02% | 4.6 |
| 4454 | AT5G35360.2 \| Symbol: CAC2 \| similar to methylcrotonyl-CoA carboxylase alpha chain, mitochondrial / 3-methylcrotonyl-CoA carboxylase 1 (MCCA) [Arabidopsis thaliana] (TAIR:At1g03090.2); similar to biotin carboxylase precursor [Glycine max] (GB:AAC23573.1); similar to biotin carboxylase precursor [Glycine max] (GB:AAC02267.1); similar to acetyl-CoA carboxylase [Glycine max] (GB:AAF80469.1); similar to biotin carboxylase subunit (GB:AAC41659.1); contains InterPro domain Carbamoyl-phosphate synthetase large chain, N-terminal (InterPro:IPR005481); contains InterPro domain Acetyl-CoA carboxylase, biotin carboxylase (InterPro:IPR004549); contains InterPro domain Biotin carboxylase, C-terminal (InterPro:IPR005482); contains InterPro domain Carbamoyl-phosphate synthase L chain, ATP-binding (InterPro:IPR005479) \| chr5:13601388-13606337 FORWARD \| Aliases: ACETYL COA CARBOXYLASE, T26D22.8, T26D22_8 | 10.8 | 9.7 | 1.0 | 4.9 | 1.02% | 4.2 |
| 4457 | AT5G40480.1 \| Symbol: EMB3012 \| expressed protein, ; expression supported by MPSS \| chr5:16230589-16241210 FORWARD \| Aliases: EMB3012, EMBRYO DEFECTIVE 3012, K21I16.30, K21I16_30 | 6.3 | 5.0 | 1.3 | 4.9 | 1.02% | 4.6 |
| 4459 | AT3G25560.2 \| protein kinase family protein, contains Prosite:PS00108: Serine/Threonine protein kinases active-site signature and PS00107: Protein kinases ATP-binding region signature \| chr3:9280750-9283924 REVERSE \| Aliases: MWL2.18 | 6.2 | 5.4 | 0.8 | 4.9 | 1.02% | 4.4 |
| 4462 | AT1G11120.1 \| expressed protein \| chr1:3715229-3719681 FORWARD \| Aliases: T19D16.22, T19D16_22 | 5.3 | 3.7 | 1.6 | 4.9 | 1.03% | 4.6 |
| 4466 | AT4G39800.1 \| Symbol: MI 1 P SYNTHASE \| inositol-3-phosphate synthase isozyme 1 / myo-inositol-1-phosphate synthase 1 / MI-1-P synthase 1 / IPS 1, identical to SP:P42801 Inositol-3-phosphate synthase isozyme 1 (EC 5.5.1.4) (Myo-inositol-1- phosphate synthase 1) (MI-1-P synthase 1) (IPS 1) {Arabidopsis thaliana} \| chr4:18469342-18471958 REVERSE \| Aliases: MYO INOSITOL 1 PHOSPHATE SYNTHASE, T19P19.190, T19P19_190, T5J17.3 | 6.1 | 4.4 | 1.7 | 4.9 | 1.03% | 4.5 |
| 4469 | AT5G46740.1 \| Symbol: UBP21 \| ubiquitin-specific protease 21 (UBP21), identical to ubiquitin-specific protease 21 GI:11993482 (Arabidopsis thaliana) \| chr5:18982498-18985969 REVERSE \| Aliases: MZA15.16, MZA15_16, UBIQUITIN SPECIFIC PROTEASE 21 | 4.3 | 3.1 | 1.2 | 4.9 | 1.03% | 4.4 |
| 4470 | AT2G15290.1 \| expressed protein \| chr2:6649408-6651117 REVERSE \| Aliases: F27O10.6, F27O10_6 | 8.8 | 6.7 | 2.1 | 4.9 | 1.03% | 4.6 |
| 4471 | AT5G10390.1 \| histone H3, identical to histone H3 from Zea mays SP:P05203, Medicago sativa GI:166384, Encephalartos altensteinii SP:P08903, Pisum sativum SP:P02300; contains Pfam profile PF00125 Core histone H2A/H2B/H3/H4 \| chr5:3268848-3269551 REVERSE \| Aliases: F12B17.260 | 6.3 | 3.4 | 2.9 | 4.9 | 1.03% | 4.4 |
| 4473 | AT1G56050.1 \| GTP-binding protein-related, similar to GTP-binding protein GI:10176676 from (Bacillus halodurans) \| chr1:20967384-20970006 FORWARD \| Aliases: T6H22.14, T6H22_14 | 4.8 | 3.2 | 1.6 | 4.9 | 1.04% | 4.5 |
| 4475 | AT1G76130.1 \| Symbol: ATAMY2 \| | 4.5 | 3.3 | 1.2 | 4.9 | 1.04% | 4.5 |
| 4478 | AT3G47930.1 \| Symbol: ATGLDH \| | 8.4 | 6.6 | 1.8 | 4.9 | 1.04% | 4.5 |
| 4479 | AT5G19110.1 \| extracellular dermal glycoprotein-related / EDGP-related, similar to extracellular dermal glycoprotein EDGP precursor (Daucus carota) GI:285741 \| chr5:6411563-6413172 REVERSE \| Aliases: T24G5.10, T24G5_10 | 5.8 | 3.6 | 2.2 | 4.9 | 1.04% | 4.4 |
| 4485 | AT2G29550.1 \| Symbol: TUB7 \| tubulin beta-7 chain (TUB7), identical to GB:M84704 SP:P29515 Tubulin beta-7 chain {Arabidopsis thaliana} \| chr2:12651124-12653114 REVERSE \| Aliases: F16P2.7, F16P2_7 | 7.7 | 5.0 | 2.7 | 4.9 | 1.05% | 4.5 |
| 4487 | AT4G35490.1 \| ribosomal protein L11 family protein, several ribosomal proteins L11 \| chr4:16855907-16856679 FORWARD \| Aliases: F15J1.60, F15J1_60 | 9.5 | 7.1 | 2.4 | 4.9 | 1.05% | 4.5 |
| 4492 | AT1G67370.1 \| Symbol: ASY1 \| meiotic asynaptic mutant 1 (ASY1), identical to meiotic asynaptic mutant 1 (Arabidopsis thaliana) GI:7939627; contains Pfam profiles PF02301: DNA-binding HORMA domain, PF04433: SWIRM domain \| chr1:25242780-25247500 REVERSE \| Aliases: ASY1, ASYNAPTIC 1, F1N21.19 | 4.1 | 3.1 | 1.0 | 4.9 | 1.05% | 4.5 |
| 4494 | AT5G59380.1 \| Symbol: MBD6 \| methyl-CpG-binding domain-containing protein, similar to testis specific methyl-CpG binding protein MBD2 (Homo sapiens) GI:3800801; contains Pfam profile PF01429: Methyl-CpG binding domain \| chr5:23969487-23970933 FORWARD \| Aliases: F2O15.4, F2O15_4 | 6.3 | 5.0 | 1.3 | 4.9 | 1.06% | 4.5 |

| Rank | Description | Sync | Root | M | t | adj.q | B |
|---|---|---|---|---|---|---|---|
| 4495 | AT5G55920.1 \| nucleolar protein, putative, similar to SP:P46087 Proliferating-cell nucleolar antigen p120 (Proliferation-associated nucleolar protein p120) {Homo sapiens}, SP:P40991 Nucleolar protein NOP2 {Saccharomyces cerevisiae}; contains Pfam profile PF01189: NOL1/NOP2/sun family \| chr5:22662709-22666673 REVERSE \| Aliases: None | 9.5 | 8.2 | 1.3 | 4.9 | 1.06% | 4.5 |
| 4496 | AT5G23130.1 \| peptidoglycan-binding LysM domain-containing protein, contains Pfam profile PF01476: LysM domain | 3.1 | 2.5 | 0.7 | 4.9 | 1.06% | 4.2 |
| 4497 | AT1G12250.1 \| thylakoid lumenal protein-related, weak similarity to SP:O22160 Thylakoid lumenal 15 kDa protein, chloroplast precursor (p15) {Arabidopsis thaliana}; contains Pfam profile PF00805: Pentapeptide repeats (8 copies) \| chr1:4159268-4161453 FORWARD \| Aliases: T28K15.2, T28K15_2 | 5.6 | 4.9 | 0.7 | 4.9 | 1.06% | 4.3 |
| 4498 | AT1G18250.2 \| Symbol: ATLP 1 \| | 5.2 | 3.6 | 1.6 | 4.9 | 1.06% | 4.4 |
| 4499 | AT3G26090.1 \| Symbol: RGS1 \| expressed protein \| chr3:9533985-9536866 FORWARD \| Aliases: ATRGS1, MPE11.28, REGULATOR OF G PROTEIN SIGNALING 1 | 7.9 | 6.6 | 1.3 | 4.9 | 1.06% | 4.5 |
| 4500 | AT4G14150.1 \| Symbol: PAKRP1 \| | 6.5 | 4.1 | 2.5 | 4.9 | 1.06% | 4.5 |
| 4501 | AT4G27520.1 \| plastocyanin-like domain-containing protein, similar to PIR:JC7196 phytocyanin-related protein Pn14 {Ipomoea nil}; contains Pfam profile PF02298: Plastocyanin-like domain \| chr4:13750446-13751911 REVERSE \| Aliases: T29A15.10, T29A15_10 | 5.1 | 3.5 | 1.6 | 4.9 | 1.06% | 4.5 |
| 4502 | AT1G68430.1 \| expressed protein \| chr1:25658568-25659107 REVERSE \| Aliases: T2E12.2, T2E12_2 | 4.3 | 3.2 | 1.1 | 4.9 | 1.06% | 4.5 |
| 4507 | AT5G39740.1 \| 60S ribosomal protein L5 (RPL5B), ribosomal protein L5, rice \| chr5:15920551-15922688 FORWARD \| Aliases: MKM21.30, MKM21_30 | 8.8 | 5.6 | 3.2 | 4.9 | 1.07% | 4.5 |
| 4508 | AT4G30935.1 \| WRKY family transcription factor, contains Pfam profile: PF03106 | 6.5 | 5.4 | 1.0 | 4.9 | 1.07% | 4.5 |
| 4510 | AT4G20930.1 \| 3-hydroxyisobutyrate dehydrogenase, putative, similar to SP:P29266 3-hydroxyisobutyrate dehydrogenase, mitochondrial precursor (EC 1.1.1.31) {Rattus norvegicus}; contains Pfam profile PF03446: NAD binding domain of 6-phosphogluconate dehydrogenase \| chr4:11198417-11201084 REVERSE \| Aliases: T13K14.90, T13K14_90 | 4.3 | 3.4 | 0.9 | 4.9 | 1.07% | 4.4 |
| 4511 | AT1G16750.1 \| expressed protein, contains Pfam profile PF04784: Protein of unknown function, DUF547 \| chr1:5729090-5731863 REVERSE \| Aliases: F19K19.16 | 7.0 | 4.8 | 2.2 | 4.9 | 1.07% | 4.3 |
| 4512 | AT4G34960.1 \| peptidyl-prolyl cis-trans isomerase, putative / cyclophilin, putative / rotamase, putative, similar to cyclophilin (Arabidopsis thaliana) gi:2443755:gb:AAB71401 | 5.2 | 4.0 | 1.2 | 4.9 | 1.07% | 4.5 |
| 4513 | AT4G17520.1 \| nuclear RNA-binding protein, putative, similar to nuclear RNA binding protein GI:6492264 from (Arabidopsis thaliana) \| chr4:9771343-9773605 FORWARD \| Aliases: DL4795W, FCAALL.142 | 10.2 | 8.8 | 1.4 | 4.9 | 1.07% | 4.4 |
| 4514 | AT5G37360.1 \| expressed protein \| chr5:14822459-14825454 REVERSE \| Aliases: MNJ8.18, MNJ8_18 | 6.5 | 5.4 | 1.1 | 4.9 | 1.07% | 4.5 |
| 4515 | AT5G25800.1 \| exonuclease family protein, contains exonuclease domain, Pfam:PF00929 \| chr5:8979625-8982876 REVERSE \| Aliases: F18A17.50, F18A17_50 | 4.7 | 3.7 | 1.0 | 4.9 | 1.07% | 4.5 |
| 4517 | AT3G59280.1 \| Symbol: TXR1 \| signaling molecule-related, contains similarity to mitochondria-associated granulocyte macrophage CSF signaling molecule, mitochondrial precursor (CGI-136) (Mus musculus) SWISS-PROT:Q9CQV1 \| chr3:21919890-21921699 REVERSE \| Aliases: F25L23.140, THAXTOMIN A RESISTANT 1 | 10.5 | 9.1 | 1.4 | 4.9 | 1.07% | 4.2 |
| 4518 | AT4G20760.1 \| short-chain dehydrogenase/reductase (SDR) family protein, contains Pfam profile: PF00106 short chain dehydrogenase \| chr4:11129026-11130781 FORWARD \| Aliases: AT4G20750, F21C20.110, F21C20_110 | 5.6 | 4.6 | 1.0 | 4.9 | 1.07% | 4.5 |
| 4520 | AT1G75110.1 \| expressed protein \| chr1:28197776-28199594 REVERSE \| Aliases: F9E10.4, F9E10_4 | 3.8 | 2.9 | 0.9 | 4.9 | 1.07% | 4.4 |
| 4522 | AT5G50790.1 \| nodulin MtN3 family protein, similar to MtN3 GI:1619602 (root nodule development) from (Medicago truncatula) \| chr5:20673479-20675225 REVERSE \| Aliases: MFB16.26, MFB16_26 | 6.0 | 4.3 | 1.7 | 4.9 | 1.07% | 4.4 |
| 4524 | AT3G57030.1 \| strictosidine synthase family protein, similar to strictosidine synthase (Rauvolfia serpentina)(SP:P15324) \| chr3:21112436-21114272 REVERSE \| Aliases: F24I3.110 | 8.3 | 6.4 | 1.8 | 4.9 | 1.07% | 4.5 |
| 4525 | AT5G19260.1 \| expressed protein, various predicted proteins \| chr5:6479402-6480448 REVERSE \| Aliases: F7K24.10, F7K24_10 | 4.8 | 3.5 | 1.3 | 4.9 | 1.07% | 4.4 |
| 4528 | AT4G21940.1 \| Symbol: CPK15 \| calcium-dependent protein kinase, putative / CDPK, putative, similar to calcium-dependent protein kinase (Nicotiana tabacum) gi:3283996:gb:AAC25423 \| chr4:11640819-11643653 FORWARD \| Aliases: F1N20.5 | 3.6 | 2.8 | 0.8 | 4.9 | 1.07% | 4.3 |

| Rank | Description | Sync | Root | M | t | adj.q | B |
|---|---|---|---|---|---|---|---|
| 4529 | AT5G20220.1 \| zinc knuckle (CCHC-type) family protein, contains Pfam domain, PF00098: Zinc knuckle \| chr5:6821992-6825983 FORWARD \| Aliases: F5O24.110, F5O24_110 | 4.7 | 4.0 | 0.8 | 4.9 | 1.07% | 4.4 |
| 4531 | AT1G65290.1 \| acyl carrier family protein / ACP family protein, similar to SP:P53665 Acyl carrier protein, mitochondrial precursor (ACP) (NADH-ubiquinone oxidoreductase 9.6 kDa subunit) (MtACP-1) {Arabidopsis thaliana}; contains InterPro accession IPR003881: Isochorismatase \| chr1:24252515-24254065 REVERSE \| Aliases: T8F5.6, T8F5_6 | 10.6 | 9.1 | 1.5 | 4.9 | 1.08% | 4.4 |
| 4532 | AT3G13360.1 \| expressed protein \| chr3:4338366-4340403 REVERSE \| Aliases: MDC11.20 | 8.0 | 6.2 | 1.8 | 4.9 | 1.08% | 4.5 |
| 4534 | AT1G61160.1 \| expressed protein \| chr1:22548684-22550022 REVERSE \| Aliases: F11P17.19, F11P17_19 | 4.7 | 3.6 | 1.1 | 4.9 | 1.08% | 4.5 |
| 4536 | AT4G33670.1 \| L-galactose dehydrogenase (L-GalDH), identical to L-galactose dehydrogenase (Arabidopsis thaliana) GI:16555790; similar to L-fucose dehydrogenase (Pseudomonas sp.) GI:829054; contains Pfam profile PF00248: oxidoreductase, aldo/keto reductase family | 8.5 | 6.9 | 1.6 | 4.9 | 1.08% | 4.5 |
| 4537 | AT5G40040.1 \| 60S acidic ribosomal protein P2 (RPP2E), acidic ribosomal protein P2, Parthenium argentatum,SWISSPROT:RLA2_PARAR | 5.5 | 3.8 | 1.7 | 4.9 | 1.08% | 4.4 |
| 4539 | AT5G06360.1 \| ribosomal protein S8e family protein, contains Pfam profile PF01201: Ribosomal protein S8e \| chr5:1944772-1946725 FORWARD \| Aliases: MHF15.12, MHF15_12 | 9.2 | 6.9 | 2.3 | 4.9 | 1.08% | 4.5 |
| 4540 | AT4G24190.2 \| Symbol: SHD \| shepherd protein (SHD) / clavata formation protein, putative, nearly identical to SHEPHERD (Arabidopsis thaliana) GI:19570872; contains Pfam profiles PF02518: ATPase, histidine kinase-, DNA gyrase B-, and HSP90-like domain protein, PF00183: Hsp90 protein \| chr4:12551731-12555915 REVERSE \| Aliases: SHEPHERD, T22A6.20, T22A6_20 | 11.6 | 10.0 | 1.6 | 4.9 | 1.09% | 4.3 |
| 4542 | AT2G24765.1 \| ADP-ribosylation factor 3 (ARF3), identical to GP:453191 ADP-ribosylation factor 3 {Arabidopsis thaliana}; contains domain PF00025: ADP-ribosylation factor family \| chr2:10569805-10572274 FORWARD \| Aliases: F27A10.8 | 11.2 | 10.2 | 0.9 | 4.9 | 1.09% | 4.3 |
| 4544 | AT2G41600.2 \| expressed protein \| chr2:17352455-17353793 REVERSE \| Aliases: T32G6.12, T32G6_12 | 5.7 | 4.2 | 1.5 | 4.9 | 1.10% | 4.5 |
| 4545 | AT4G16410.1 \| expressed protein, contains Pfam PF05421: Protein of unknown function (DUF751) \| chr4:9261708-9262614 REVERSE \| Aliases: DL4233C, FCAALL.144 | 6.5 | 4.8 | 1.7 | 4.9 | 1.10% | 4.5 |
| 4547 | AT1G21600.2 \| similar to putative tonoplast membrane integral protein [Oryza sativa (japonica cultivar-group)] (GB:BAD81369.1) | 4.0 | 2.9 | 1.1 | 4.9 | 1.10% | 4.2 |
| 4550 | AT3G05330.1 \| cyclin family, low similarity to microtubule-binding protein TANGLED1 (Zea mays) GI:11228986; contains Pfam profile PF00134: Cyclin, N-terminal domain \| chr3:1518950-1521465 REVERSE \| Aliases: T12H1.30, T12H1_30 | 5.4 | 3.0 | 2.4 | 4.9 | 1.10% | 4.0 |
| 4551 | AT5G38290.2 \| peptidyl-tRNA hydrolase family protein, contains Pfam profile PF01195: peptidyl-tRNA hydrolase \| chr5:15320610-15322776 FORWARD \| Aliases: MSI17.2, MSI17_2 | 4.1 | 3.2 | 0.9 | 4.9 | 1.10% | 4.4 |
| 4552 | AT1G05055.1 \| basic transcription factor 2, 44kD subunit-related, contains weak similarity to Swiss-Prot:Q13888 TFIIH basal transcription factor complex p44 subunit (Basic transcription factor 2 44 kDa subunit, BTF2-p44, General transcription factor IIH polypeptide 2) (Homo sapiens) \| chr1:1448718-1450855 REVERSE \| Aliases: AT1G05050 | 6.4 | 5.3 | 1.1 | 4.8 | 1.10% | 4.4 |
| 4553 | AT3G14880.1 \| DNA-binding protein-related, low similarity to tumor-related protein (Nicotiana glauca x Nicotiana langsdorffii) GI:688423, SP:O24160 TGACG-sequence specific DNA-binding protein TGA-2.1 {Nicotiana tabacum} \| chr3:5006572-5007696 FORWARD \| Aliases: K15M2.2 | 4.5 | 3.5 | 1.0 | 4.8 | 1.10% | 4.4 |
| 4554 | AT5G63040.2 \| expressed protein, similar to unknown protein (emb:CAB81845.1) \| chr5:25305372-25307638 FORWARD \| Aliases: MJH22.10, MJH22_10 | 3.9 | 3.0 | 0.9 | 4.8 | 1.10% | 4.4 |
| 4555 | AT1G30630.1 \| coatomer protein epsilon subunit family protein / COPE family protein, similar to SP:O14579 Coatomer epsilon subunit (Epsilon-coat protein) (Epsilon-COP) from Homo sapiens, SP:Q60445 from Cricetulus griseus; ESTs gb:Z17908, gb:AA728673, gb:N96555, gb:H76335, gb:AA712463, gb:W43247, gb:T45611, gb:T21160, gb:T14119 and AI100483 come from this gene \| chr1:10858296-10860243 REVERSE \| Aliases: T5I8.8, T5I8_8 | 10.8 | 8.8 | 2.0 | 4.8 | 1.10% | 4.2 |
| 4557 | AT1G68680.1 \| expressed protein \| chr1:25789169-25790587 FORWARD \| Aliases: F24J5.21, F24J5_21 | 7.8 | 6.3 | 1.6 | 4.8 | 1.11% | 4.5 |
| 4558 | AT1G50950.1 \| thioredoxin-related, contains weak hit to Pfam PF00085: Thioredoxin; contains 2 predicted transmembrane domains \| chr1:18883988-18887893 FORWARD \| Aliases: F8A12.17, F8A12_17 | 3.7 | 2.6 | 1.0 | 4.8 | 1.11% | 4.4 |
| 4559 | AT5G16720.1 \| expressed protein, contains Pfam profile PF04576: Protein of unknown function, DUF593 \| chr5:5494231-5496813 FORWARD \| Aliases: F5E19.60, F5E19_60 | 4.3 | 3.5 | 0.8 | 4.8 | 1.11% | 4.3 |

| Rank | Description | Sync | Root | M | t | adj.q | B |
|---|---|---|---|---|---|---|---|
| 4560 | AT2G29980.2 \| Symbol: FAD3 \| omega-3 fatty acid desaturase, endoplasmic reticulum (FAD3), identical to SP:48623 \| chr2:12788668-12792114 REVERSE \| Aliases: F23F1.10, F23F1_10, FATTY ACID DESATURASE 3, OMEGA 3 FATTY ACID DESATURASE, ENDOPLASMIC RETICULUM18:2 DESATURASE (OMEGA 3 FATTY ACID DESATURASE) | 8.1 | 5.0 | 3.1 | 4.8 | 1.11% | 4.4 |
| 4561 | AT1G68360.1 \| zinc finger protein-related, similar to zinc finger protein GI:790683 from (Arabidopsis thaliana) \| chr1:25625341-25626075 REVERSE \| Aliases: T22E19.1, T22E19_1 | 7.3 | 5.5 | 1.8 | 4.8 | 1.11% | 4.4 |
| 4562 | AT5G15510.1 \| expressed protein \| chr5:5034123-5036798 FORWARD \| Aliases: T20K14.120, T20K14_120 | 5.6 | 4.3 | 1.2 | 4.8 | 1.11% | 4.5 |
| 4564 | AT1G62780.1 \| expressed protein \| chr1:23252844-23254770 REVERSE \| Aliases: F23N19.15, F23N19_15 | 4.1 | 2.5 | 1.6 | 4.8 | 1.11% | 4.5 |
| 4566 | AT1G55300.2 \| similar to TATA-binding protein-associated factor TAFII55-like [Oryza sativa (japonica cultivar-group)] (GB:BAD68329.1); contains InterPro domain TAFII55 protein conserved region (InterPro:IPR006751) \| chr1:20631690-20633359 REVERSE \| Aliases: F7A10.3, F7A10_3 | 5.1 | 4.0 | 1.1 | 4.8 | 1.11% | 4.5 |
| 4567 | AT1G48200.1 \| expressed protein \| chr1:17800524-17802006 REVERSE \| Aliases: F21D18.7, F21D18_7 | 5.5 | 3.4 | 2.1 | 4.8 | 1.11% | 4.4 |
| 4568 | AT5G02500.1 \| Symbol: HSC70 1 \| heat shock cognate 70 kDa protein 1 (HSC70-1) (HSP70-1), identical to SP:P22953 Heat shock cognate 70 kDa protein 1 (Hsc70.1) {Arabidopsis thaliana} \| chr5:553743-556437 REVERSE \| Aliases: AT HSC70 1, HSC70, HSP70 1, T22P11.90, T22P11_90 | 11.1 | 10.2 | 0.9 | 4.8 | 1.11% | 4.1 |
| 4569 | AT4G34260.1 \| expressed protein \| chr4:16398119-16401814 FORWARD \| Aliases: F10M10.30, F10M10_30 | 6.0 | 4.8 | 1.2 | 4.8 | 1.11% | 4.5 |
| 4570 | AT1G73880.1 \| UDP-glucoronosyl/UDP-glucosyl transferase family protein, contains Pfam profile: PF00201 UDP-glucoronosyl and UDP-glucosyl transferase \| chr1:27788642-27790465 FORWARD \| Aliases: F2P9.25, F2P9_25 | 10.4 | 7.4 | 3.0 | 4.8 | 1.11% | 4.5 |
| 4572 | AT5G05000.3 \| Symbol: OEP34 \| translocate of chloroplast 34 (TOC34) / GTP-binding protein (OEP34), contains Pfam PF04548: AIG1 family;contains TIGRFAM TIGR00991: GTP-binding protein and TIGR00231: small GTP-binding protein domain; 99.7% identical to atToc34 protein (GI:11557975) (Arabidopsis thaliana); similar to Chain A, | 9.0 | 7.7 | 1.3 | 4.8 | 1.11% | 4.5 |
| 4573 | AT4G30020.1 \| subtilase family protein, contains similarity to meiotic serine proteinase TMP GI:6468325 from (Lycopersicon esculentum) | 6.7 | 5.6 | 1.1 | 4.8 | 1.11% | 4.4 |
| 4580 | AT3G58180.1 \| PBS lyase HEAT-like repeat-containing protein, contains Pfam profile: PF03130: PBS lyase HEAT-like repeat; contains alternative donor splice site TT at exon 2,hypothetical protein C14A4.1 - Caenorhabditis elegans, PIR:T19243 | 7.6 | 6.1 | 1.5 | 4.8 | 1.12% | 4.4 |
| 4583 | AT1G28570.2 \| GDSL-motif lipase, putative, similar to lipase (Arabidopsis thaliana) GI:1145627; contains InterPro Entry IPR001087 Lipolytic enzyme, G-D-S-L family \| chr1:10041627-10044136 REVERSE \| Aliases: F1K23.19, F1K23_19 | 8.1 | 6.2 | 1.9 | 4.8 | 1.13% | 4.4 |
| 4584 | AT3G09470.2 \| expressed protein \| chr3:2911521-2914505 REVERSE \| Aliases: AT3G09460, F11F8.4 | 6.5 | 4.7 | 1.8 | 4.8 | 1.13% | 4.3 |
| 4586 | AT1G41830.1 \| Symbol: SKS6 \| multi-copper oxidase type I family protein, similar to pollen-specific BP10 protein (SP:Q00624)(Brassica napus); contains Pfam profile: PF00394 Multicopper oxidase \| chr1:15606058-15610302 REVERSE \| Aliases: F5A13.5, F5A13_5, SKS6 | 8.8 | 6.7 | 2.1 | 4.8 | 1.13% | 4.4 |
| 4588 | AT5G50960.1 \| nucleotide-binding family protein, similar to Nucleotide-binding protein 1 (NBP 1) (SP:Q9R060) (Mus musculus); contains Pfam PF00991 : ParA family ATPase \| chr5:20751403-20753281 FORWARD \| Aliases: K3K7.12, K3K7_12 | 7.4 | 6.2 | 1.2 | 4.8 | 1.13% | 4.4 |
| 4589 | AT2G22260.1 \| oxidoreductase, 2OG-Fe(II) oxygenase family protein, contains Pfam profile PF03171: oxidoreductase, 2OG-Fe(II) oxygenase family \| chr2:9468205-9470264 FORWARD \| Aliases: T26C19.8, T26C19_8 | 4.7 | 3.5 | 1.2 | 4.8 | 1.13% | 4.4 |
| 4590 | AT3G09630.2 \| similar to 60S ribosomal protein L4/L1 (RPL4D) [Arabidopsis thaliana] (TAIR:At5g02870.1); similar to PREDICTED OJ1014_E09.28 gene product [Oryza sativa (japonica cultivar-group)] (GB:XP_507356.1); contains InterPro domain Ribosomal protein L4/L1e (InterPro:IPR002136) \| chr3:2953742-2955753 FORWARD \| Aliases: F11F8.22 | 12.3 | 11.2 | 1.2 | 4.8 | 1.13% | 4.0 |
| 4593 | AT1G52280.1 \| Ras-related GTP-binding protein, putative, similar to RAB7D GI:1370187 from (Lotus japonicus) (Plant J. 11 (2), 237-250 (1997)); contains Pfam PF00071: Ras family \| chr1:19471638-19473255 REVERSE \| Aliases: F19K6.10, F19K6_10 | 7.4 | 5.9 | 1.4 | 4.8 | 1.13% | 4.3 |
| 4595 | AT2G31060.2 \| similar to elongation factor family protein [Arabidopsis thaliana] (TAIR:At5g13650.2); similar to putative GTP-binding protein [Oryza sativa (japonica cultivar-group)] (GB:NP_916146.1); contains InterPro domain Small GTP-binding protein domain (InterPro:IPR005225); contains InterPro domain Elongation factor G, C-terminal (InterPro:IPR000640); contains InterPro domain Elongation factor, GTP-binding (InterPro:IPR000795); contains InterPro domain GTP-binding protein TypA (InterPro:IPR006298); contains InterPro domain Elongation factor Tu, domain 2 (InterPro:IPR004161) \| chr2:13220275-13225662 REVERSE \| Aliases: T16B12.13, T16B12_13 | 5.5 | 3.9 | 1.6 | 4.8 | 1.13% | 4.4 |

| Rank | Description | Sync | Root | M | t | adj.q | B |
|---|---|---|---|---|---|---|---|
| 4596 | AT4G39150.1 \| DNAJ heat shock N-terminal domain-containing protein, similar to SP:P39101 CAJ1 protein, Saccharomyces cerevisiae, PIR2:S48085; contains Pfam profile PF00226 DnaJ domain \| chr4:18233499-18236058 REVERSE \| Aliases: T22F8.50, T22F8_50 | 8.1 | 7.0 | 1.0 | 4.8 | 1.14% | 4.3 |
| 4597 | AT3G62880.2 \| similar to mitochondrial import inner membrane translocase subunit Tim17/Tim22/Tim23 family protein [Arabidopsis thaliana] (TAIR:At2g28900.1); similar to B1146F03.20 [Oryza sativa (japonica cultivar-group)] (GB:NP_914110.1); contains InterPro domain Mitochondrial import inner membrane translocase, subunit Tim17/22 (InterPro:IPR003397) | 8.5 | 6.8 | 1.7 | 4.8 | 1.14% | 4.4 |
| 4598 | AT1G17560.1 \| Symbol: HLL \| ribosomal protein L14 family protein, similar to GB:Z98756 from (Mycobacterium leprae) \| chr1:6037457-6038685 REVERSE \| Aliases: F1L3.27, F1L3_27, HUELLENLOS | 7.0 | 4.5 | 2.5 | 4.8 | 1.14% | 4.3 |
| 4599 | AT1G08450.2 \| Symbol: CRT3 \| calreticulin 3 (CRT3), identical to similar to SP:O04153 Calreticulin 3 precursor {Arabidopsis thaliana} | 8.7 | 7.7 | 1.0 | 4.8 | 1.14% | 4.2 |
| 4600 | AT4G37780.1 \| myb family transcription factor (MYB87), identical to AtMYB87 R2R3-MYB transcription factor GI:2832559 from (Arabidopsis thaliana); contains Pfam profile: PF00249 myb-like DNA-binding domain \| chr4:17758237-17759493 REVERSE \| Aliases: T28I19.60, T28I19_60 | 4.1 | 2.5 | 1.6 | 4.8 | 1.14% | 4.4 |
| 4602 | AT4G09030.1 \| Symbol: AGP10 \| arabinogalactan-protein (AGP10), identical to gi:10880497:gb:AAG24278; supported by Ceres cDNA 265772 \| chr4:5792204-5792787 FORWARD \| Aliases: F23J3.60, F23J3_60 | 8.4 | 6.2 | 2.2 | 4.8 | 1.14% | 4.4 |
| 4603 | AT5G66550.1 \| Maf family protein, contains Pfam domain PF02545: Maf-like protein \| chr5:26576856-26578404 REVERSE \| Aliases: K1F13.22, K1F13_22 | 5.4 | 4.3 | 1.1 | 4.8 | 1.14% | 4.4 |
| 4605 | AT5G40950.1 \| 50S ribosomal protein L27, chloroplast, putative (RPL27), identical to SP:Q9FLN4 ribosomal protein L27, chloroplast precursor {Arabidopsis thaliana}; similar to SP:P30155 50S ribosomal protein L27, chloroplast precursor (CL27) {Nicotiana tabacum} \| chr5:16428037-16429284 FORWARD \| Aliases: MMG1.7 | 7.1 | 4.9 | 2.2 | 4.8 | 1.14% | 4.4 |
| 4608 | AT3G03060.1 \| AAA-type ATPase family protein, contains a ATP/GTP-binding site motif A (P-loop), PROSITE:PS00017 \| chr3:692195-695431 FORWARD \| Aliases: T17B22.25, T17B22_25 | 4.9 | 3.7 | 1.2 | 4.8 | 1.14% | 4.4 |
| 4611 | AT5G48480.1 \| expressed protein \| chr5:19661901-19663103 FORWARD \| Aliases: MJE7.12, MJE7_12 | 7.9 | 6.1 | 1.9 | 4.8 | 1.15% | 4.4 |
| 4613 | AT3G26710.1 \| expressed protein \| chr3:9814763-9815987 FORWARD \| Aliases: MLJ15.10 | 6.5 | 5.3 | 1.2 | 4.8 | 1.15% | 4.4 |
| 4617 | AT1G06630.1 \| F-box family protein, contains F-box domain Pfam:PF00646 | 4.4 | 3.6 | 0.8 | 4.8 | 1.15% | 4.3 |
| 4620 | AT2G20820.2 \| expressed protein, . \| chr2:8971430-8972505 FORWARD \| Aliases: F5H14.21, F5H14_21 | 11.5 | 10.2 | 1.3 | 4.8 | 1.15% | 4.3 |
| 4621 | AT4G34760.1 \| auxin-responsive family protein, auxin-induced protein X15, Glycine max, PIR2:JQ1097 \| chr4:16582188-16582884 REVERSE \| Aliases: F11I11.11 | 4.4 | 2.9 | 1.5 | 4.8 | 1.15% | 4.3 |
| 4622 | AT2G29960.1 \| Symbol: CYP5 \| peptidyl-prolyl cis-trans isomerase / cyclophilin (CYP5) / rotamase, identical to cyclophilin (Arabidopsis thaliana) gi:2443755:gb:AAB71401 \| chr2:12776134-12777656 REVERSE \| Aliases: ATCYP5, F23F1.12, F23F1_12 | 9.4 | 8.1 | 1.3 | 4.8 | 1.15% | 4.3 |
| 4625 | AT5G64010.1 \| expressed protein \| chr5:25635445-25637405 REVERSE \| Aliases: MBM17.11, MBM17_11 | 7.1 | 5.9 | 1.3 | 4.8 | 1.16% | 4.4 |
| 4627 | AT5G54930.2 \| similar to AT hook motif-containing protein [Arabidopsis thaliana] (TAIR:At5g52890.1); contains InterPro domain HMG-I and HMG-Y DNA-binding domain (A+T-hook) (InterPro:IPR000637) \| chr5:22322959-22324787 REVERSE \| Aliases: MBG8.20, MBG8_20 | 7.6 | 6.4 | 1.2 | 4.8 | 1.16% | 4.4 |
| 4628 | AT5G06340.1 \| diadenosine 5',5'''-P1,P4-tetraphosphate hydrolase, putative, similar to diadenosine 5',5'''-P1,P4-tetraphosphate hydrolase from (Lupinus angustifolius) GI:1888557, (Hordeum vulgare subsp. vulgare) GI:2564253; contains Pfam profile PF00293: NUDIX domain \| chr5:1936407-1938112 FORWARD \| Aliases: MHF15.14, MHF15_14 | 8.9 | 6.2 | 2.7 | 4.8 | 1.16% | 4.4 |
| 4629 | AT3G56900.1 \| aladin-related / adracalin-related, weak similarity to SP:Q9NRG9 Aladin (Adracalin) (GL003) {Homo sapiens}; non-consensus AT-AC splice sites at intron 6 | 5.9 | 4.2 | 1.8 | 4.8 | 1.16% | 4.4 |
| 4633 | AT1G13220.2 \| nuclear matrix constituent protein-related, similar to nuclear matrix constituent protein 1 (NMCP1) (Daucus carota) GI:2190187 \| chr1:4515696-4520281 FORWARD \| Aliases: F3F19.25, F3F19_25 | 5.8 | 4.5 | 1.4 | 4.8 | 1.17% | 4.4 |
| 4635 | AT5G27430.1 \| signal peptidase subunit family protein, contains Pfam profile: PF04573 signal peptidase subunit \| chr5:9687390-9689366 FORWARD \| Aliases: F21A20.140, F21A20_140 | 5.6 | 3.9 | 1.6 | 4.8 | 1.17% | 4.3 |
| 4637 | AT1G63640.2 \| kinesin motor protein-related, C-terminal region is similar to C-term region of kinesin motor protein GB:AAB51397 (Mus musculus); contains Pfam profile: PF00225 Kinesin motor domain \| chr1:23592730-23599438 REVERSE \| Aliases: F2K11.1, F2K11_1 | 5.5 | 4.2 | 1.3 | 4.8 | 1.17% | 4.4 |

| Rank | Description | Sync | Root | M | t | adj.q | B |
|---|---|---|---|---|---|---|---|
| 4640 | AT5G06240.1 \| Symbol: EMB2735 \| expressed protein \| chr5:1888314-1889697 FORWARD \| Aliases: EMB2735, EMBRYO DEFECTIVE 2735, MBL20.12, MBL20_12 | 6.1 | 5.2 | 0.9 | 4.8 | 1.17% | 4.3 |
| 4641 | AT3G50980.1 \| dehydrin, putative, similar to dehydrin Xero 1 (Arabidopsis thaliana) SWISS-PROT:P25863 \| chr3:18952957-18953846 FORWARD \| Aliases: F24M12.20 | 5.5 | 4.0 | 1.5 | 4.8 | 1.17% | 4.4 |
| 4643 | AT4G37000.1 \| Symbol: ACD2 \| accelerated cell death 2 (ACD2), identical to accelerated cell death 2 (ACD2) GI:12484129 from (Arabidopsis thaliana) \| chr4:17442606-17443844 FORWARD \| Aliases: ACCELERATED CELL DEATH 2, AP22.36, AP22_36 | 6.5 | 4.8 | 1.7 | 4.8 | 1.17% | 4.4 |
| 4645 | AT1G58220.1 \| myb family transcription factor, contains Pfam profile: PF00249: Myb-like DNA-binding domain \| chr1:21561908-21566630 REVERSE \| Aliases: F16M22.4, F16M22_4 | 5.3 | 4.1 | 1.2 | 4.8 | 1.18% | 4.4 |
| 4646 | AT1G03860.3 \| Symbol: ATPHB2 \| | 8.1 | 5.4 | 2.7 | 4.8 | 1.18% | 4.4 |
| 4648 | AT3G43520.1 \| expressed protein, contains Pfam profile PF03647: Uncharacterised protein family (UPF0136) \| chr3:15417012-15418896 FORWARD \| Aliases: T18D12.90 | 9.9 | 8.3 | 1.6 | 4.8 | 1.18% | 4.3 |
| 4649 | AT3G61110.1 \| Symbol: ARS27A \| | 11.3 | 9.3 | 2.0 | 4.8 | 1.18% | 4.0 |
| 4650 | AT5G38110.1 \| ASF1-like anti-silencing family protein, similar to SP:P32447 Anti-silencing protein 1 {Saccharomyces cerevisiae}; contains Pfam profile PF04729: Anti-silencing protein, ASF1-like \| chr5:15225702-15227445 FORWARD \| Aliases: F16F17.110, F16F17_110 | 6.9 | 4.8 | 2.1 | 4.8 | 1.18% | 4.4 |
| 4651 | AT1G77230.1 \| tetratricopeptide repeat (TPR)-containing protein, contains Pfam profile PF00515 TPR Domain \| chr1:29021023-29022858 FORWARD \| Aliases: T14N5.9, T14N5_9 | 5.6 | 3.9 | 1.7 | 4.8 | 1.18% | 4.4 |
| 4653 | AT5G09460.1 \| expressed protein \| chr5:2944438-2945418 REVERSE \| Aliases: T5E8.260, T5E8_260 | 5.4 | 3.8 | 1.6 | 4.8 | 1.18% | 4.4 |
| 4655 | AT1G64810.2 \| Symbol: APO1 \| expressed protein, similar to expressed protein [Arabidopsis thaliana] (TAIR:At5g57930.1); similar to expressed protein [Arabidopsis thaliana] (TAIR:At5g57930.2); similar to unknown protein [Oryza sativa (japonica cultivar-group)] (GB:XP_467639.1); contains InterPro domain Arabidopsis thaliana protein of unknown function DUF794 (InterPro:IPR008512) \| chr1:24090402-24092201 FORWARD \| Aliases: ACCUMULATION OF PHOTOSYSTEM ONE 1, F13O11.11, F13O11_11 | 5.5 | 4.3 | 1.2 | 4.8 | 1.18% | 4.4 |
| 4656 | AT5G16240.1 \| acyl-(acyl-carrier-protein) desaturase, putative / stearoyl-ACP desaturase, putative, similar to Acyl-(acyl-carrier protein) desaturase from Sesamum indicum GI:575942, Cucumis sativus SP:P32061, Ricinus communis SP:P22337; contains Pfam profile PF03405 Fatty acid desaturase \| chr5:5306810-5309935 FORWARD \| Aliases: T21H19.160, T21H19_160 | 4.9 | 3.8 | 1.1 | 4.8 | 1.18% | 4.4 |
| 4657 | AT1G49380.1 \| cytochrome c biogenesis protein family, contains Pfam PF05140: ResB-like family; similar to CCS1 (GI:2749939) (Chlamydomonas reinhardtii); similar to Ccs1 (GI:6137102) (Synechococcus sp. PCC 6301) \| chr1:18280375-18283196 FORWARD \| Aliases: F13F21.19, F13F21_19 | 4.0 | 2.7 | 1.4 | 4.8 | 1.18% | 4.4 |
| 4658 | AT3G17210.1 \| stable protein 1-related, similar to stable protein 1 (GI:13445204) (Populus tremula) PMID:12376651; similar to pop3 peptide GB:AAC26526 from (Populus balsamifera subsp. trichocarpa X Populus deltoides) \| chr3:5882290-5883206 FORWARD \| Aliases: MGD8.2 | 9.2 | 7.9 | 1.3 | 4.8 | 1.18% | 4.3 |
| 4662 | AT3G28200.1 \| peroxidase, putative, similar to peroxidase ATP26a GB:CAA72487 GI:1890317 (Arabidopsis thaliana) | 5.7 | 4.8 | 1.0 | 4.8 | 1.18% | 4.3 |
| 4666 | AT1G66840.1 \| expressed protein, contains Pfam profile PF05701: Plant protein of unknown function (DUF827); expression supported by MPSS \| chr1:24937661-24939779 REVERSE \| Aliases: F4N21.22, F4N21_22 | 3.9 | 2.9 | 0.9 | 4.8 | 1.18% | 4.3 |
| 4668 | AT5G13730.1 \| Symbol: SIGD \| RNA polymerase sigma subunit SigD (sigD) / sigma-like factor (SIG4), identical to RNA polymerase sigma subunit SigD (Arabidopsis thaliana) GI:4972296, sigma-like factor (Arabidopsis thaliana) GI:3983260; contains Pfam profiles PF04545: Sigma-70, region 4, PF04539: Sigma-70 region 3, PF04542: Sigma-70 region 2 \| chr5:4428700-4430837 REVERSE \| Aliases: MSH12.20, MSH12_20, SIG4, SIGMA LIKE FACTOR 4 | 4.5 | 2.7 | 1.8 | 4.8 | 1.18% | 4.4 |
| 4669 | AT5G45350.1 \| proline-rich family protein, contains proline rich extensin domains, INTERPRO:IPR002965 \| chr5:18399064-18400926 REVERSE \| Aliases: MFC19.1, MFC19_1 | 9.0 | 6.9 | 2.2 | 4.8 | 1.18% | 4.4 |
| 4670 | AT5G24020.1 \| Symbol: MIND \| septum site-determining protein (MIND), identical to MinD (Arabidopsis thaliana) GI:6759277; contains Pfam PF00991 : ParA family ATPase \| chr5:8116683-8118033 FORWARD \| Aliases: MZF18.10, MZF18_10 | 5.8 | 4.9 | 0.9 | 4.8 | 1.18% | 4.3 |
| 4671 | AT1G31780.1 \| similar to Component of oligomeric golgi complex 6 [Xenopus tropicalis] (GB:AAH75361.1) \| chr1:11391210-11394729 FORWARD \| Aliases: F5M6.21, F5M6_21 | 6.8 | 4.8 | 2.0 | 4.8 | 1.19% | 4.4 |

| Rank | Description | Sync | Root | M | t | adj.q | B |
|---|---|---|---|---|---|---|---|
| 4674 | AT2G42680.1 \| ethylene-responsive transcriptional coactivator, putative, similar to ethylene-responsive transcriptional coactivator (Lycopersicon esculentum) gi:5669634:gb:AAD46402 \| chr2:17781980-17783387 FORWARD \| Aliases: F14N22.5, F14N22_5 | 12.3 | 11.2 | 1.1 | 4.8 | 1.19% | 3.7 |
| 4675 | AT2G42720.1 \| F-box family protein, contains F-box domain Pfam:PF00646 | 4.0 | 3.2 | 0.9 | 4.8 | 1.19% | 4.3 |
| 4676 | AT5G51050.1 \| mitochondrial substrate carrier family protein, similar to peroxisomal Ca-dependent solute carrier (Oryctolagus cuniculus) GI:2352427; contains INTERPRO:IPR001993 Mitochondrial substrate carrier family, INTERPRO:IPR002048 calcium-binding EF-hand domain \| chr5:20770607-20772940 FORWARD \| Aliases: K3K7.23, K3K7_23 | 7.0 | 5.9 | 1.2 | 4.8 | 1.19% | 4.3 |
| 4678 | AT3G16050.1 \| Symbol: A37 \| stress-responsive protein, putative, similar to ethylene-inducible protein HEVER (Hevea brasiliensis) SWISS-PROT:Q39963; contains Pfam domain, PF01680: SOR/SNZ family \| chr3:5443957-5445335 REVERSE \| Aliases: MSL1.3 | 8.4 | 6.4 | 2.0 | 4.8 | 1.19% | 4.4 |
| 4679 | AT3G18980.1 \| F-box family protein, contains Pfam:PF00646 F-box domain ; contains TIGRFAM TIGR01640: F-box protein interaction domain ; contains TIGRFAM TIGR01640: F-box protein interaction domain \| chr3:6546469-6548068 FORWARD \| Aliases: K13E13.9 | 5.0 | 3.9 | 1.0 | 4.8 | 1.19% | 4.4 |
| 4680 | AT5G03440.2 \| expressed protein \| chr5:856871-858404 REVERSE \| Aliases: F12E4.200, F12E4_200 | 5.8 | 4.4 | 1.4 | 4.8 | 1.19% | 4.3 |
| 4682 | AT4G21510.1 \| F-box family protein, contains Pfam PF00646: F-box domain; similar to SKP1 interacting partner 2 (SKIP2) TIGR_Ath1:At5g67250 \| chr4:11445868-11447175 REVERSE \| Aliases: F18E5.130, F18E5_130 | 4.8 | 4.0 | 0.8 | 4.8 | 1.19% | 4.2 |
| 4685 | AT2G33430.1 \| plastid developmental protein DAG, putative, similar to DAG protein, chloroplast precursor (Garden snapdragon) SWISS-PROT:Q38732 | 6.3 | 5.1 | 1.2 | 4.8 | 1.19% | 4.4 |
| 4688 | AT1G61800.1 \| glucose-6-phosphate/phosphate translocator, putative, similar to glucose-6-phosphate/phosphate-translocator precursor GI:2997591 from (Pisum sativum) \| chr1:22828105-22830411 FORWARD \| Aliases: T13M11.18, T13M11_18 | 4.5 | 3.4 | 1.1 | 4.8 | 1.19% | 4.4 |
| 4690 | AT3G07880.1 \| Rho GDP-dissociation inhibitor family protein, similar to SP:P52565 Rho GDP-dissociation inhibitor 1 (Rho GDI 1) (Rho-GDI alpha) {Homo sapiens}; contains Pfam profile PF02115: RHO protein GDP dissociation inhibitor \| chr3:2513977-2515828 FORWARD \| Aliases: F17A17.22 | 8.9 | 7.4 | 1.5 | 4.8 | 1.20% | 4.2 |
| 4691 | AT4G27230.1 \| histone H2A, putative, strong similarity to histone H2A Arabidopsis thaliana GI:7595337, Triticum aestivum GI:536892, Picea abies SP:P35063; contains Pfam profile PF00125 Core histone H2A/H2B/H3/H4 \| chr4:13637345-13638425 REVERSE \| Aliases: M4I22.40, M4I22_40 | 8.9 | 6.4 | 2.5 | 4.8 | 1.20% | 4.4 |
| 4692 | AT5G19440.1 \| similar to Eucalyptus gunnii alcohol dehydrogenase of unknown physiological function (GI:1143445), apple tree, PIR:T16995; NOT a cinnamyl-alcohol dehydrogenase \| chr5:6556422-6558344 FORWARD \| Aliases: F7K24.190, F7K24_190 | 10.7 | 8.4 | 2.3 | 4.8 | 1.20% | 4.3 |
| 4693 | AT4G04620.2 \| autophagy 8b (APG8b), identical to autophagy 8b (Arabidopsis thaliana) GI:19912153; contains Pfam profile PF02991: Microtubule associated protein 1A/1B, light chain 3 \| chr4:2328506-2330134 REVERSE \| Aliases: F4H6.14, F4H6_14 | 7.9 | 6.5 | 1.4 | 4.8 | 1.20% | 4.2 |
| 4694 | AT4G15850.1 \| Symbol: ATRH1 \| DEAD/DEAH box helicase, putative, similar to D-E-A-D box protein (Drosophila melanogaster) GI:499204; contains Pfam profiles PF00270: DEAD/DEAH box helicase, PF00271: Helicase conserved C-terminal domain \| chr4:9001344-9004749 FORWARD \| Aliases: DL3965W, FCAALL.401 | 3.9 | 2.9 | 0.9 | 4.8 | 1.20% | 4.3 |
| 4695 | AT4G39280.1 \| phenylalanyl-tRNA synthetase, putative / phenylalanine--tRNA ligase, putative, similar to SP:Q9Y285 Phenylalanyl-tRNA synthetase alpha chain (EC 6.1.1.20) (Phenylalanine- -tRNA ligase alpha chain) (PheRS) {Homo sapiens}; contains Pfam profile PF01409: tRNA synthetases class II core domain (F) \| chr4:18281456-18284843 REVERSE \| Aliases: T22F8.180, T22F8_180 | 9.2 | 6.9 | 2.3 | 4.8 | 1.20% | 4.4 |
| 4696 | AT1G03070.1 \| expressed protein, low similarity to N-methyl-D-aspartate receptor-associated protein (Drosophila melanogaster) GI:567104; contains Pfam profile PF01027: Uncharacterized protein family UPF0005 | 5.8 | 4.4 | 1.5 | 4.8 | 1.20% | 4.4 |
| 4697 | AT1G24240.1 \| ribosomal protein L19 family protein, similar to plastid ribosomal protein L19 precursor (Spinacia oleracea) gi:7582403:gb:AAF64312 \| chr1:8586342-8588070 FORWARD \| Aliases: F3I6.17, F3I6_17 | 4.8 | 3.9 | 0.9 | 4.8 | 1.20% | 4.3 |
| 4698 | AT3G07540.1 \| formin homology 2 domain-containing protein / FH2 domain-containing protein, contains formin homology 2 domain, Pfam:PF02181 \| chr3:2404515-2407547 REVERSE \| Aliases: F21O3.25 | 6.1 | 4.7 | 1.4 | 4.8 | 1.20% | 4.4 |
| 4699 | AT2G14460.1 \| expressed protein \| chr2:6161575-6162086 REVERSE \| Aliases: T13P21.16, T13P21_16 | 7.1 | 4.6 | 2.5 | 4.8 | 1.20% | 4.3 |
| 4701 | AT2G29760.1 \| pentatricopeptide (PPR) repeat-containing protein, contains Pfam profile PF01535: PPR repeat \| chr2:12719961-12722177 FORWARD \| Aliases: T27A16.14, T27A16_14 | 4.7 | 3.9 | 0.8 | 4.8 | 1.20% | 4.2 |
| 4703 | AT4G31720.2 \| Symbol: TAFII15 \| transcription initiation factor IID (TFIID) 23-30kDa subunit (TAF2H) family protein, contains Pfam profile: PF03540 transcription initiation factor TFIID 23-30kDa \| chr4:15353958-15356352 REVERSE \| Aliases: F28M20.90, F28M20_90 | 9.5 | 7.7 | 1.8 | 4.8 | 1.21% | 4.4 |

| Rank | Description | Sync | Root | M | t | adj.q | B |
|---|---|---|---|---|---|---|---|
| 4704 | AT3G58670.1 \| expressed protein \| chr3:21714367-21717027 REVERSE \| Aliases: T20N10.20 | 4.8 | 3.6 | 1.2 | 4.8 | 1.21% | 4.3 |
| 4706 | AT3G16770.1 \| Symbol: ATEBP/RAP2.3 \| Encodes a member of the ERF (ethylene response factor) subfamily B-2 of ERF/AP2 transcription factor family (RAP2.3). The protein contains one AP2 domain. There are 5 members in this subfamily including RAP2.2 AND RAP2.12. \| chr3:5705721-5707029 FORWARD \| Aliases: ATEBP, MGL6.1, RAP2.3, RELATED TO AP2 3 | 11.0 | 9.2 | 1.8 | 4.8 | 1.21% | 4.1 |
| 4707 | AT3G19480.1 \| D-3-phosphoglycerate dehydrogenase, putative / 3-PGDH, putative, similar to SP:O04130 from (Arabidopsis thaliana) \| chr3:6752537-6754791 FORWARD \| Aliases: MLD14.34 | 4.1 | 2.8 | 1.3 | 4.7 | 1.21% | 4.4 |
| 4708 | AT3G62720.2 \| similar to galactosyl transferase GMA12/MNN10 family protein [Arabidopsis thaliana] (TAIR:At4g02500.1); similar to beta-6-xylosyltransferase [Gossypium raimondii] (GB:CAI11451.1); similar to beta-6-xylosyltransferase [Pinus taeda] (GB:CAI11450.1); contains InterPro domain Galactosyl transferase (InterPro:IPR008630) \| chr3:23212132-23213889 FORWARD \| Aliases: F26K9.150 | 7.2 | 5.9 | 1.3 | 4.7 | 1.21% | 4.4 |
| 4710 | AT5G16480.1 \| tyrosine specific protein phosphatase family protein, contains tyrosine specific protein phosphatases active site, PROSITE:PS00383 \| chr5:5381116-5382900 REVERSE \| Aliases: MQK4.21, MQK4_21 | 6.3 | 4.8 | 1.6 | 4.7 | 1.22% | 4.4 |
| 4711 | AT1G80620.1 \| ribosomal protein S15 family protein, similar to ribosomal protein S15 GB:AAD36415 from (Thermotoga maritima) \| chr1:30310256-30312308 FORWARD \| Aliases: None | 5.3 | 3.9 | 1.5 | 4.7 | 1.22% | 4.3 |
| 4713 | AT1G43670.1 \| fructose-1,6-bisphosphatase, putative / D-fructose-1,6-bisphosphate 1-phosphohydrolase, putative / FBPase, putative, very strong similarity to SP:P46267 Fructose-1,6-bisphosphatase, cytosolic (EC 3.1.3.11) (D-fructose-1,6- bisphosphate 1-phosphohydrolase) (FBPase) {Brassica napus}; contains Pfam profile PF00316: fructose-1,6-bisphosphatase \| chr1:16470547-16472937 FORWARD \| Aliases: F2J6.2, F2J6_2 | 7.1 | 4.6 | 2.6 | 4.7 | 1.22% | 4.3 |
| 4714 | AT4G14910.1 \| Symbol: IGPD \| imidazoleglycerol-phosphate dehydratase, putative, similar to SP:P34047 Imidazoleglycerol-phosphate dehydratase 1 (EC 4.2.1.19) (IGPD 1) {Arabidopsis thaliana}; contains Pfam profile PF00475: imidazoleglycerol-phosphate dehydratase \| chr4:8528163-8530110 REVERSE \| Aliases: DL3495C, FCAALL.36, IMIDAZOLEGLYCEROLPHOSPHATE DEHYDRATASE | 5.5 | 4.0 | 1.5 | 4.7 | 1.22% | 4.3 |
| 4715 | AT3G57785.1 \| expressed protein \| chr3:21415551-21416194 REVERSE \| Aliases: None | 6.5 | 4.4 | 2.0 | 4.7 | 1.22% | 4.3 |
| 4716 | AT3G08630.1 \| expressed protein \| chr3:2620838-2622226 FORWARD \| Aliases: F17O14.10 | 7.0 | 4.0 | 3.0 | 4.7 | 1.22% | 4.3 |
| 4717 | AT3G16190.1 \| isochorismatase hydrolase family protein, low similarity to SP:P32400 N-carbamoylsarcosine amidase (EC 3.5.1.59) (N-carbamoylsarcosine amidohydrolase) {Arthrobacter sp}; contains Pfam profile PF00857: isochorismatase family protein \| chr3:5489707-5491254 REVERSE \| Aliases: MYA6.5 | 7.8 | 6.6 | 1.2 | 4.7 | 1.22% | 4.3 |
| 4718 | AT1G26660.1 \| c-myc binding protein, putative / prefoldin, putative, similar to Swiss-Prot:Q99471 prefoldin subunit 5 (C-myc binding protein Mm-1) (Myc modulator 1) (Homo sapiens) \| chr1:9211727-9213961 FORWARD \| Aliases: T24P13.26 | 7.7 | 5.8 | 1.9 | 4.7 | 1.23% | 4.3 |
| 4720 | AT5G49520.1 \| Symbol: WRKY48 \| | 4.2 | 2.3 | 1.9 | 4.7 | 1.23% | 4.3 |
| 4722 | AT1G02110.1 \| proline-rich family protein, contains proline-rich domain, INTERPRO:IPR000694 \| chr1:392939-395554 FORWARD \| Aliases: T7I23.5, T7I23_5 | 5.6 | 4.4 | 1.2 | 4.7 | 1.23% | 4.3 |
| 4723 | AT5G58260.1 \| Encodes subunit NDH-N of NAD(P)H:plastoquinone dehydrogenase complex (Ndh complex) present in the thylakoid membrane of chloroplasts. This subunit is thought to be required for Ndh complex assembly. \| chr5:23578108-23579167 REVERSE \| Aliases: MCK7.13, MCK7_13, NDH N | 4.0 | 3.3 | 0.7 | 4.7 | 1.23% | 4.1 |
| 4724 | AT4G29670.2 \| thioredoxin family protein, contains Pfam profile PF00085: Thioredoxin \| chr4:14535613-14537244 REVERSE \| Aliases: T16L4.180, T16L4_180 | 7.3 | 6.2 | 1.1 | 4.7 | 1.23% | 4.3 |
| 4725 | AT1G15370.1 \| expressed protein \| chr1:5287057-5288506 FORWARD \| Aliases: F9L1.32, F9L1_32 | 8.8 | 7.5 | 1.3 | 4.7 | 1.23% | 4.2 |
| 4726 | AT1G30240.1 \| similar to proline-, glutamic acid-, leucine-rich protein 1 [Mus musculus] (GB:NP_083507.2) \| chr1:10641083-10645919 REVERSE \| Aliases: F12P21.4, F12P21_4 | 5.6 | 4.3 | 1.3 | 4.7 | 1.23% | 4.3 |
| 4727 | AT3G10940.1 \| protein phosphatase-related, similar to protein phosphatase PTPKIS1 protein (GI:11595504) (Arabidopsis thaliana) | 6.6 | 5.6 | 1.0 | 4.7 | 1.23% | 4.3 |
| 4730 | AT2G03270.1 \| DNA-binding protein, putative, similar to Swiss-Prot:Q60560 DNA-binding protein SMUBP-2 (Immunoglobulin MU binding protein 2) (SMUBP-2) (Insulin II gene enhancer-binding protein)(RIPE3B-binding complex 3B2 P110 subunit) (RIP-1)(Mesocricetus auratus); identical to putative helicase (atpc-2 gene) cDNA NCBI_gi:11191230 \| chr2:993780-996125 FORWARD \| Aliases: T4M8.30, T4M8_30 | 6.7 | 5.2 | 1.5 | 4.7 | 1.23% | 4.3 |

| Rank | Description | Sync | Root | M | t | adj.q | B |
|---|---|---|---|---|---|---|---|
| 4732 | AT3G46210.4 \| similar to hypothetical protein [Arabidopsis thaliana] (TAIR:At2g07110.1); similar to putative exosome component [Oryza sativa (japonica cultivar-group)] (GB:XP_470449.1); contains InterPro domain 3' exoribonuclease (InterPro:IPR001247) \| chr3:16987739-16989877 REVERSE \| Aliases: F12M12.180 | 5.6 | 4.3 | 1.2 | 4.7 | 1.23% | 4.3 |
| 4733 | AT1G02680.1 \| transcription initiation factor IID (TFIID) 18 kDa subunit (TAFII-18) family protein, similar to transcription factor TFIID (SP:Q15543) (Homo sapiens); contains Pfam PF02269: Transcription initiation factor IID, 18kD subunit | 5.5 | 4.3 | 1.3 | 4.7 | 1.23% | 4.3 |
| 4735 | AT1G22800.1 \| expressed protein, similar to Biotin synthesis protein bioC. {Serratia marcescens} (SP:P36571); ESTs gb:Z34075, gb:Z34835 and gb:AA404888 come from this gene \| chr1:8071924-8074346 FORWARD \| Aliases: T22J18.3, T22J18_3 | 7.1 | 5.7 | 1.4 | 4.7 | 1.23% | 4.3 |
| 4739 | AT4G05520.2 \| calcium-binding EF hand family protein, similar to EH-domain containing protein 1 from {Mus musculus} SP:Q9WVK4, {Homo sapiens} SP:Q9H4M9, receptor-mediated endocytosis 1 from (Caenorhabditis elegans) GI:13487775, GI:13487777, GI:13487779; contains INTERPRO:IPR002048 calcium-binding EF-hand domain \| chr4:2804448-2807926 FORWARD \| Aliases: T1J24.10, T1J24_10 | 6.0 | 4.3 | 1.7 | 4.7 | 1.23% | 4.3 |
| 4741 | AT2G36885.2 \| expressed protein \| chr2:15489090-15490785 REVERSE \| Aliases: None | 7.2 | 5.0 | 2.2 | 4.7 | 1.23% | 4.3 |
| 4743 | AT3G27740.1 \| Symbol: CARA \| carbamoyl-phosphate synthase (glutamine-hydrolyzing) (CARA) / glutamine-dependent carbamoyl-phosphate synthase small subunit, identical to carbamoyl phosphate synthetase small subunit GI:2462781 (Arabidopsis thaliana) \| chr3:10282498-10285103 REVERSE \| Aliases: CARBAMOYL PHOSPHATE SYNTHETASE A, CARBAMOYL PHOSPHATE SYNTHETASE SMALL SUBUNIT, MGF10.14 | 8.2 | 6.0 | 2.3 | 4.7 | 1.23% | 4.3 |
| 4750 | AT3G27160.1 \| Symbol: GHS1 \| ribosomal protein S21 family protein, contains Pfam profile: PF01165 ribosomal protein S21 \| chr3:10018713-10020327 FORWARD \| Aliases: GLUCOSE HYPERSENSITIVE 1, MYF5.3 | 6.9 | 6.0 | 0.9 | 4.7 | 1.23% | 4.3 |
| 4754 | AT1G58160.1 \| jacalin lectin family protein, similar to myrosinase-binding protein homolog (Arabidopsis thaliana) GI:2997767; contains Pfam profile PF01419 jacalin-like lectin domain | 3.8 | 3.0 | 0.8 | 4.7 | 1.24% | 4.1 |
| 4760 | AT5G22290.1 \| Symbol: ANAC089 \| no apical meristem (NAM) family protein, contains Pfam PF02365: No apical meristem (NAM) domain \| chr5:7375926-7377626 REVERSE \| Aliases: ANAC089, T6G21.9 | 8.8 | 6.9 | 1.9 | 4.7 | 1.25% | 4.3 |
| 4763 | AT1G69780.1 \| Symbol: ATHB13 \| | 4.0 | 3.1 | 0.9 | 4.7 | 1.25% | 4.2 |
| 4765 | AT1G55130.1 \| endomembrane protein 70, putative, similar to multispanning membrane protein GI:2276460 from (Homo sapiens) \| chr1:20573321-20576060 FORWARD \| Aliases: T7N22.7, T7N22_7 | 5.8 | 4.4 | 1.4 | 4.7 | 1.25% | 4.3 |
| 4766 | AT5G11910.1 \| esterase/lipase/thioesterase family protein, low similarity to cinnamoyl ester hydrolase CinI (Butyrivibrio fibrisolvens) GI:1622732; contains Interpro entry IPR000379 \| chr5:3836338-3838214 REVERSE \| Aliases: F14F18.80, F14F18_80 | 8.2 | 7.1 | 1.2 | 4.7 | 1.25% | 4.3 |
| 4767 | AT5G39890.1 \| expressed protein \| chr5:15991621-15993498 FORWARD \| Aliases: MYH19.8, MYH19_8 | 6.8 | 4.7 | 2.1 | 4.7 | 1.25% | 4.3 |
| 4771 | AT5G25940.1 \| early nodulin-related, contains weak similarity to Swiss-Prot:Q02921 early nodulin 93 (N-93) (Glycine max) \| chr5:9053704-9055217 REVERSE \| Aliases: T1N24.3, T1N24_3 | 9.8 | 8.2 | 1.6 | 4.7 | 1.26% | 4.3 |
| 4772 | AT5G37055.1 \| zinc finger (HIT type) family protein, contains Pfam profile: PF04438 HIT zinc finger \| chr5:14658781-14659670 REVERSE \| Aliases: None | 5.1 | 4.3 | 0.8 | 4.7 | 1.26% | 4.2 |
| 4773 | AT3G01370.1 \| expressed protein, contains Pfam domain, PF04581: Protein of unknown function (DUF578) \| chr3:139018-143699 FORWARD \| Aliases: T13O15.1, T13O15_1 | 5.6 | 4.3 | 1.3 | 4.7 | 1.26% | 4.3 |
| 4775 | AT5G05680.1 \| nuclear pore complex protein-related, contains weak similarity to Nuclear pore complex protein Nup88 (Nucleoporin Nup88) (88 kDa nuclear pore complex protein) (Swiss-Prot:Q99567) (Homo sapiens) \| chr5:1698271-1702616 FORWARD \| Aliases: MJJ3.8, MJJ3_8 | 5.7 | 4.8 | 0.9 | 4.7 | 1.26% | 4.1 |
| 4777 | AT5G42330.1 \| expressed protein \| chr5:16943887-16944716 FORWARD \| Aliases: MDH9.2, MDH9_2 | 7.2 | 4.9 | 2.3 | 4.7 | 1.26% | 4.0 |
| 4781 | AT1G07980.1 \| histone-like transcription factor (CBF/NF-Y) family protein, contains Pfam profile PF00808: Histone-like transcription factor (CBF/NF-Y) and archaeal histone; similar to Chromatin accessibility complex protein 1 (CHRAC-1) (CHRAC-15) (HuCHRAC15) (DNA polymerase epsilon subunit p15) (SP:Q9NRG0) {Homo sapiens} \| chr1:2473162-2474924 REVERSE \| Aliases: T6D22.7, T6D22_7 | 6.4 | 5.3 | 1.1 | 4.7 | 1.26% | 4.3 |
| 4783 | AT4G28025.1 \| expressed protein \| chr4:13935641-13937388 REVERSE \| Aliases: None | 8.5 | 7.5 | 0.9 | 4.7 | 1.26% | 4.3 |
| 4786 | AT1G61870.1 \| pentatricopeptide (PPR) repeat-containing protein, contains Pfam profile PF01535: PPR repeat \| chr1:22868669-22870280 REVERSE \| Aliases: F8K4.8, F8K4_8 | 7.1 | 5.2 | 1.9 | 4.7 | 1.27% | 4.3 |
| 4788 | AT2G40020.2 \| expressed protein \| chr2:16718064-16720148 FORWARD \| Aliases: T28M21.18, T28M21_18 | 4.6 | 3.4 | 1.2 | 4.7 | 1.27% | 4.3 |

| Rank | Description | Sync | Root | M | t | adj.q | B |
|---|---|---|---|---|---|---|---|
| 4789 | AT1G04985.1 \| expressed protein \| chr1:1416342-1418438 REVERSE \| Aliases: F13M7.2, F13M7_2 | 7.8 | 6.6 | 1.2 | 4.7 | 1.27% | 4.3 |
| 4792 | AT3G05130.1 \| expressed protein, ; expression supported by MPSS \| chr3:1433481-1435385 REVERSE \| Aliases: T12H1.9, T12H1_9 | 5.0 | 3.7 | 1.3 | 4.7 | 1.27% | 4.3 |
| 4794 | AT5G07720.1 \| galactosyl transferase GMA12/MNN10 family protein, very low similarity to alpha-1,2-galactosyltransferase, Schizosaccharomyces pombe (SP:Q09174) | 4.4 | 2.9 | 1.5 | 4.7 | 1.27% | 4.2 |
| 4795 | AT4G01050.1 \| hydroxyproline-rich glycoprotein family protein, contains a rhodanese homology domain. \| chr4:455768-458510 FORWARD \| Aliases: F2N1.31, F2N1_31 | 6.1 | 3.8 | 2.3 | 4.7 | 1.27% | 4.3 |
| 4797 | AT1G78020.1 \| senescence-associated protein-related, similar to senescence-associated protein SAG102 (GI:22331931) (Arabidopsis thaliana) \| chr1:29343587-29344649 FORWARD \| Aliases: F28K19.24, F28K19_24 | 5.5 | 4.0 | 1.5 | 4.7 | 1.27% | 4.3 |
| 4798 | AT5G45920.1 \| similar to GDSL-motif lipase/hydrolase family protein [Arabidopsis thaliana] (TAIR:At5g62930.1); similar to OSJNBa0038J17.19 [Oryza sativa (japonica cultivar-group)] (GB:NP_913321.1); contains InterPro domain Lipolytic enzyme, G-D-S-L family (InterPro:IPR001087) \| chr5:18639992-18642012 FORWARD \| Aliases: K15I22.12, K15I22_12 | 5.3 | 4.1 | 1.2 | 4.7 | 1.27% | 4.3 |
| 4799 | AT5G63000.1 \| expressed protein \| chr5:25297238-25298759 REVERSE \| Aliases: MJH22.6, MJH22_6 | 10.4 | 9.0 | 1.4 | 4.7 | 1.28% | 4.2 |
| 4806 | AT1G52260.1 \| Symbol: ATPDIL1 5 \| Encodes a protein disulfide isomerase-like (PDIL) protein, a member of a multigene family within the thioredoxin (TRX) superfamily. | 6.0 | 4.7 | 1.3 | 4.7 | 1.28% | 4.3 |
| 4807 | AT2G40935.2 \| expressed protein, low similarity to PGPS/D12 (Petunia x hybrida) GI:4105794; contains Pfam profile PF04749: Protein of unknown function, DUF614 | 8.1 | 6.9 | 1.1 | 4.7 | 1.28% | 4.3 |
| 4808 | AT1G30960.1 \| GTP-binding protein (ERG), identical to GTP-binding protein ERG SP:O82653 from (Arabidopsis thaliana) \| chr1:11037635-11039982 REVERSE \| Aliases: F17F8.15 | 4.9 | 3.4 | 1.5 | 4.7 | 1.28% | 4.3 |
| 4811 | AT3G47800.1 \| aldose 1-epimerase family protein, similar to ALDOSE 1-EPIMERASE PRECURSOR GB:P05149 (SP:P05149) from (Acinetobacter calcoaceticus); contains Pfam profile PF01263 Aldose 1-epimerase \| chr3:17645795-17648595 FORWARD \| Aliases: T23J7.130 | 5.0 | 3.2 | 1.9 | 4.7 | 1.29% | 4.1 |
| 4816 | AT1G09680.1 \| pentatricopeptide (PPR) repeat-containing protein, contains Pfam profile PF01535: PPR repeat \| chr1:3134109-3135932 REVERSE \| Aliases: F21M12.7, F21M12_7 | 5.4 | 4.2 | 1.2 | 4.7 | 1.29% | 4.2 |
| 4817 | AT2G35160.1 \| Symbol: SUVH5 \| SET domain-containing protein (SUVH5), identical to SUVH5 (Arabidopsis thaliana) GI:13517751; contains Pfam profiles PF00856: SET domain, PF05033: Pre-SET motif, PF02182: YDG/SRA domain; identical to cDNA SUVH5 (SUVH5) GI:13517750 | 4.0 | 3.2 | 0.7 | 4.7 | 1.29% | 3.9 |
| 4818 | AT1G26270.1 \| phosphatidylinositol 3- and 4-kinase family protein, similar to phosphatidylinositol 4-kinase type-II beta (Homo sapiens) GI:20159767; contains Pfam profile PF00454: Phosphatidylinositol 3- and 4-kinase \| chr1:9089511-9092444 REVERSE \| Aliases: F28B23.7, F28B23_7 | 10.9 | 8.2 | 2.6 | 4.7 | 1.29% | 4.3 |
| 4819 | AT3G03020.1 \| expressed protein \| chr3:680951-682006 FORWARD \| Aliases: F13E7.3, F13E7_3 | 3.9 | 3.0 | 0.9 | 4.7 | 1.29% | 4.2 |
| 4821 | AT1G67350.1 \| expressed protein, contains similarity to MHC class II antigen GI:9502037 from (Aotus nancymaae) | 11.8 | 11.2 | 0.6 | 4.7 | 1.30% | 3.5 |
| 4822 | AT5G57590.1 \| Symbol: BIO1 \| aminotransferase class III family protein, low similarity to 7,8-diaminopelargonic acid aminotransferase BioA (Mesorhizobium loti) GI:12044306; contains Pfam profile PF00202: aminotransferase, class III \| chr5:23335734-23338765 REVERSE \| Aliases: BIOTIN AUXOTROPH 1, MUA2.17, MUA2_17 | 6.2 | 4.6 | 1.5 | 4.7 | 1.30% | 4.2 |
| 4824 | AT4G26840.1 \| Symbol: SUM1 \| ubiquitin-like protein (SMT3), identical to Ubiquitin-like protein SMT3 SP:P55852 from(Arabidopsis thaliana); identical to cDNA SMT3 protein GI:1707371 \| chr4:13497334-13498733 FORWARD \| Aliases: F10M23.180, F10M23_180, SMALL UBIQUITIN LIKE MODIFIER 1, SUMO 1, SUMO1 | 9.3 | 7.9 | 1.3 | 4.7 | 1.30% | 4.0 |
| 4828 | AT4G10750.1 \| HpcH/HpaI aldolase family protein, similar to 2,4-dihydroxydec-2-ene-1,10-dioic acid aldolase (Sphingopyxis macrogoltabida) GI:23330203; contains Pfam profile PF03328: HpcH/HpaI aldolase family \| chr4:6618672-6619935 FORWARD \| Aliases: T12H20.10, T12H20_10 | 8.7 | 7.3 | 1.4 | 4.7 | 1.30% | 4.2 |
| 4829 | AT4G33760.1 \| tRNA synthetase class II (D, K and N) family protein, similar to SP:P36419 Aspartyl-tRNA synthetase (EC 6.1.1.12) (Aspartate--tRNA ligase) (AspRS) {Thermus thermophilus}; contains Pfam profile PF00152: tRNA synthetases class II (D, K and N) | 5.9 | 4.5 | 1.4 | 4.7 | 1.30% | 4.3 |
| 4830 | AT4G17390.1 \| 60S ribosomal protein L15 (RPL15B) \| chr4:9714225-9715624 REVERSE \| Aliases: DL4730C, FCAALL.32 | 10.9 | 9.9 | 1.0 | 4.7 | 1.30% | 4.0 |
| 4835 | AT5G42970.1 \| Symbol: COP8 \| COP9 signalosome complex subunit 4 / CSN complex subunit 4 (CSN4) (COP8) (FUS4), FUSCA4, COP8, CSN4; identical to CSN complex subunit 4 (Arabidopsis thaliana) GI:18056659, COP8 (Arabidopsis thaliana) GI:5802627; contains Pfam profile PF01399: PCI domain; identical to cDNA CSN complex subunit 4 (CSN4) GI:18056658 \| chr5:17254413-17257961 REVERSE \| Aliases: ATS4, CONSTITUTIVE PHOTOMORPHOGENIC 14, CONSTITUTIVE PHOTOMORPHOGENIC 8, COP14, CSN4, EMB134, EMBRYO DEFECTIVE 134, FUS4, FUS8, FUSCA 4, FUSCA 8, MBD2.17, MBD2_17 | 5.2 | 4.0 | 1.2 | 4.7 | 1.31% | 4.1 |

| Rank | Description | Sync | Root | M | t | adj.q | B |
|---|---|---|---|---|---|---|---|
| 4836 | AT1G69740.1 \| porphobilinogen synthase, putative / delta-aminolevulinic acid dehydratase, putative, similar to delta-aminolevulinic acid dehydratase (Alad) GI:493019 (SP:P43210) from Glycine max, SP:P24493 from Spinacia oleracea, SP:P30124 from Pisum sativum \| chr1:26235499-26238626 FORWARD \| Aliases: T6C23.6, T6C23_6 | 8.0 | 6.9 | 1.1 | 4.7 | 1.31% | 4.2 |
| 4837 | AT4G10500.1 \| oxidoreductase, 2OG-Fe(II) oxygenase family protein, similar to hyoscyamine 6 beta-hydroxylase (Atropa belladona)(GI:4996123); contains PF03171 2OG-Fe(II) oxygenase superfamily domain \| chr4:6491085-6492442 FORWARD \| Aliases: F7L13.80, F7L13_80 | 2.7 | 2.3 | 0.4 | 4.7 | 1.31% | 3.8 |
| 4838 | AT3G23070.1 \| expressed protein, contains Pfam domain, PF04581: Protein of unknown function (DUF578) \| chr3:8203476-8208003 FORWARD \| Aliases: MXC7.10 | 4.8 | 3.8 | 1.1 | 4.7 | 1.31% | 4.2 |
| 4841 | AT5G07090.2 \| similar to 40S ribosomal protein S4 (RPS4D) [Arabidopsis thaliana] (TAIR:At5g58420.1); similar to ribosomal protein S4 [Solanum tuberosum] (GB:CAA54095.1); contains InterPro domain RNA-binding S4 (InterPro:IPR002942); contains InterPro domain KOW (Kyrpides, Ouzounis, Woese) motif (InterPro:IPR006646); contains InterPro domain Ribosomal protein S4E (InterPro:IPR000876); contains InterPro domain KOW (InterPro:IPR005824) \| chr5:2202398-2204079 FORWARD \| Aliases: T28J14.30 | 9.1 | 7.0 | 2.1 | 4.7 | 1.31% | 4.3 |
| 4842 | AT3G48500.1 \| Symbol: PDE312 \| | 5.4 | 4.1 | 1.3 | 4.7 | 1.31% | 4.2 |
| 4843 | AT1G07210.1 \| 30S ribosomal protein S18 family, contains Pfam profile: PF01084 ribosomal protein S18; similar to 30S ribosomal protein S18 (SP:P80382) {Thermus thermophilus} \| chr1:2215235-2217391 FORWARD \| Aliases: F10K1.8, F10K1_8 | 5.8 | 4.5 | 1.3 | 4.7 | 1.31% | 4.2 |
| 4845 | AT5G49030.2 \| similar to isoleucyl-tRNA synthetase, putative / isoleucine--tRNA ligase, putative [Arabidopsis thaliana] (TAIR:At4g10320.1); similar to COG0060: Isoleucyl-tRNA synthetase [Anabaena variabilis ATCC 29413] (GB:ZP_00158029.2); similar to COG0060: Isoleucyl-tRNA synthetase [Crocosphaera watsonii WH 8501] (GB:ZP_00178195.1); similar to COG0060: Isoleucyl-tRNA synthetase [Trichodesmium erythraeum IMS101] (GB:ZP_00327549.1); similar to COG0060: Isoleucyl-tRNA synthetase [Nostoc punctiforme PCC 73102] (GB:ZP_00106717.1); similar to isoleucyl-tRNA synthetase [Nostoc sp. PCC 7120] (GB:BAB73030.1); contains InterPro domain Aminoacyl-tRNA synthetase, class I (InterPro:IPR001412); contains InterPro domain Isoleucyl-tRNA synthetase, class Ia (InterPro:IPR002301); contains InterPro domain Aminoacyl-tRNA synthetase, class Ia (InterPro:IPR002300) \| chr5:19893392-19899688 REVERSE \| Aliases: K19E20.18, K19E20_18 | 7.4 | 6.1 | 1.2 | 4.7 | 1.31% | 4.2 |
| 4846 | AT4G27060.1 \| Symbol: TOR1 \| expressed protein \| chr4:13581414-13585161 REVERSE \| Aliases: CN, CONVOLUTA, SPIRAL 2, SPR2, T24A18.10, T24A18_10, TORTIFOLIA 1 | 7.0 | 6.0 | 1.0 | 4.7 | 1.31% | 4.2 |
| 4847 | AT3G25230.1 \| Symbol: ROF1 \| peptidyl-prolyl cis-trans isomerase / FK506-binding protein (ROF1), identical to rotamase FKBP (ROF1) GB:U49453 (Arabidopsis thaliana) (Mol. Gen. Genet. 252 (5), 510-517 (1996)) \| chr3:9189444-9192642 FORWARD \| Aliases: MJL12.21, ROTAMASE FKBP 1 | 10.1 | 7.9 | 2.2 | 4.7 | 1.32% | 4.2 |
| 4849 | AT5G62540.1 \| ubiquitin-conjugating enzyme 3 (UBC3), E2; identical to gi:431261, SP:P42746 \| chr5:25121160-25122845 FORWARD \| Aliases: K19B1.15, K19B1_15 | 8.9 | 7.3 | 1.6 | 4.7 | 1.32% | 4.2 |
| 4850 | AT1G48950.1 \| expressed protein, similar to expressed protein [Arabidopsis thaliana] (TAIR:At1g17210.1); similar to hypothetical protein [Oryza sativa (japonica cultivar-group)] (GB:BAD52672.1) \| chr1:18110565-18113905 FORWARD \| Aliases: F27K7.14 | 6.2 | 4.9 | 1.2 | 4.7 | 1.32% | 4.2 |
| 4851 | AT2G40660.1 \| tRNA-binding region domain-containing protein, similar to SP:Q12904 Multisynthetase complex auxiliary component p43 (Contains: Endothelial-monocyte activating polypeptide II (EMAP-II) (Small inducible cytokine subfamily E member 1)) {Homo sapiens}; contains Pfam profile PF01588: Putative tRNA binding domain \| chr2:16973011-16976075 FORWARD \| Aliases: T7D17.16, T7D17_16 | 8.4 | 7.0 | 1.4 | 4.7 | 1.32% | 4.2 |
| 4852 | AT2G33590.1 \| cinnamoyl-CoA reductase family, similar to cinnamoyl-CoA reductase from Pinus taeda (GI:17978649), Eucalyptus gunnii (GI:2058311) \| chr2:14231344-14233678 FORWARD \| Aliases: F4P9.36, F4P9_36 | 8.7 | 6.5 | 2.2 | 4.7 | 1.32% | 4.2 |
| 4854 | AT2G22610.1 \| kinesin motor protein-related \| chr2:9606630-9611827 FORWARD \| Aliases: T9I22.5, T9I22_5 | 4.6 | 3.3 | 1.3 | 4.7 | 1.32% | 4.2 |
| 4857 | AT5G52300.2 \| Symbol: RD29B \| similar to stress-responsive protein-related [Arabidopsis thaliana] (TAIR:At4g25580.1); similar to cor78 (GB:AAA32776.1) \| chr5:21254282-21256755 FORWARD \| Aliases: K24M7.3, K24M7_3, LTI65, RESPONSIVE TO DESSICATION 29B | 6.7 | 5.2 | 1.6 | 4.7 | 1.33% | 4.2 |
| 4858 | AT2G47680.1 \| zinc finger (CCCH type) helicase family protein, similar to SP:Q28141 ATP-dependent RNA helicase A (Nuclear DNA helicase II) (DEAD-box protein 9) {Bos taurus}; contains Pfam profiles PF00271: Helicase conserved C-terminal domain, PF00642: Zinc finger C-x8-C-x5-C-x3-H type (and similar) \| chr2:19552898-19557975 REVERSE \| Aliases: F17A22.7 | 3.7 | 2.9 | 0.8 | 4.7 | 1.33% | 4.0 |
| 4859 | AT3G21580.2 \| expressed protein \| chr3:7601975-7603727 REVERSE \| Aliases: MIL23.15 | 5.1 | 4.1 | 1.0 | 4.7 | 1.33% | 4.2 |
| 4861 | AT1G52670.1 \| biotin/lipoyl attachment domain-containing protein, similar to SP:Q06881 Biotin carboxyl carrier protein of acetyl-CoA carboxylase (BCCP) {Anabaena sp.}; contains Pfam profile PF00364: Biotin-requiring enzyme | 6.3 | 4.1 | 2.2 | 4.7 | 1.33% | 4.2 |

| Rank | Description | Sync | Root | M | t | adj.q | B |
|---|---|---|---|---|---|---|---|
| 4862 | AT1G52290.1 \| protein kinase family protein, contains Pfam PF00069: Protein kinase domain \| chr1:19473733-19476031 REVERSE \| Aliases: F19K6.9, F19K6_9 | 4.3 | 3.3 | 1.0 | 4.7 | 1.33% | 4.2 |
| 4864 | AT1G49670.1 \| ARP protein (REF), identical to ARP protein GB:CAA89858 GI:886434 from (Arabidopsis thaliana); contains Pfam profile PF00107: oxidoreductase, zinc-binding dehydrogenase family \| chr1:18384930-18389775 REVERSE \| Aliases: F14J22.10, F14J22_10 | 10.4 | 9.0 | 1.4 | 4.7 | 1.33% | 4.1 |
| 4865 | AT4G27654.1 \| expressed protein \| chr4:13811651-13812132 FORWARD \| Aliases: None | 6.0 | 3.3 | 2.8 | 4.6 | 1.34% | 4.2 |
| 4866 | AT5G28500.1 \| expressed protein, predicted proteins, Arabidopsis thaliana and Synechocystis sp. \| chr5:10477775-10479388 FORWARD \| Aliases: T26D3.4, T26D3_4 | 8.0 | 6.2 | 1.7 | 4.6 | 1.34% | 4.2 |
| 4867 | AT2G32770.3 \| purple acid phosphatase (PAP13), identical to purple acid phosphatase (PAP13) (Arabidopsis thaliana) GI:20257489; contains Pfam profile PF00149: Ser/Thr protein phosphatase \| chr2:13902915-13905354 FORWARD \| Aliases: F24L7.9, F24L7_9 | 3.1 | 2.5 | 0.6 | 4.6 | 1.34% | 3.9 |
| 4868 | AT4G13520.1 \| expressed protein \| chr4:7864390-7864920 FORWARD \| Aliases: T6G15.70, T6G15_70 | 10.9 | 9.8 | 1.1 | 4.6 | 1.34% | 3.7 |
| 4869 | AT4G22380.1 \| ribosomal protein L7Ae/L30e/S12e/Gadd45 family protein, Similar to NHP2/L7Ae family proteins, see SWISSPROT:P32495 and PMID:2063628. \| chr4:11812053-11812934 REVERSE \| Aliases: F7K2.1 | 5.5 | 3.8 | 1.7 | 4.6 | 1.34% | 4.2 |
| 4872 | AT4G17970.1 \| expressed protein, contains Pfam profile PF01027: Uncharacterized protein family UPF0005 | 4.8 | 3.2 | 1.6 | 4.6 | 1.34% | 4.1 |
| 4874 | AT1G51390.2 \| Symbol: NFU5 \| similar to nitrogen fixation NifU-like family protein [Arabidopsis thaliana] (TAIR:At3g20970.1); similar to hypothetical protein UM04972.1 [Ustilago maydis 521] (GB:EAK85802.1); contains InterPro domain Nitrogen-fixing NifU, C-terminal (InterPro:IPR001075) | 4.5 | 3.8 | 0.8 | 4.6 | 1.34% | 4.0 |
| 4878 | AT3G26460.1 \| major latex protein-related / MLP-related, low similarity to major latex protein {Papaver somniferum}(GI:294060) contains Pfam profile PF00407: Pathogenesis-related protein Bet v I family \| chr3:9685110-9685894 REVERSE \| Aliases: F20C19.18 | 4.6 | 2.8 | 1.8 | 4.6 | 1.34% | 4.2 |
| 4879 | AT2G21830.1 \| DC1 domain-containing protein, contains Pfam profilePF03107: DC1 domain \| chr2:9310793-9312977 FORWARD \| Aliases: F7D8.15, F7D8_15 | 6.4 | 5.2 | 1.1 | 4.6 | 1.35% | 4.2 |
| 4880 | AT4G14660.1 \| RNA polymerase Rpb7 N-terminal domain-containing protein, similar to SP:P52433 DNA-directed RNA polymerase II 19 kDa polypeptide (EC 2.7.7.6) (RPB7) {Rattus norvegicus}; contains Pfam profile PF03876: RNA polymerase Rpb7, N-terminal domain \| chr4:8406330-8407042 REVERSE \| Aliases: DL3370C, FCAALL.283 | 5.1 | 4.3 | 0.8 | 4.6 | 1.35% | 4.0 |
| 4881 | AT2G29180.1 \| expressed protein \| chr2:12550111-12551306 FORWARD \| Aliases: F16P2.44, F16P2_44 | 6.3 | 4.5 | 1.7 | 4.6 | 1.35% | 4.1 |
| 4887 | AT2G42120.2 \| DNA polymerase delta small subunit-related, similar to DNA polymerase delta small subunit SP:Q9LRE5 from (Oryza sativa) \| chr2:17570254-17573110 REVERSE \| Aliases: T24P15.3, T24P15_3 | 4.9 | 3.7 | 1.2 | 4.6 | 1.35% | 4.2 |
| 4888 | AT1G11840.5 \| Symbol: ATGLX1 \| | 11.8 | 10.3 | 1.6 | 4.6 | 1.35% | 4.0 |
| 4889 | AT3G55080.2 \| similar to SET domain-containing protein [Arabidopsis thaliana] (TAIR:At3g07670.1); similar to putative ribulose-1,5 bisphosphate carboxylase/oxygenase large subunit N-methyltransferase, chloroplast precursor [Oryza sativa (japonica cultivar-group)] (GB:BAD28364.1) \| chr3:20422380-20426896 REVERSE \| Aliases: T15C9.90 | 5.4 | 4.2 | 1.2 | 4.6 | 1.35% | 4.2 |
| 4890 | AT5G20590.1 \| expressed protein, various predicted proteins, Arabidopsis thaliana \| chr5:6963440-6966607 FORWARD \| Aliases: F7C8.180, F7C8_180 | 7.0 | 6.0 | 1.1 | 4.6 | 1.35% | 4.2 |
| 4891 | AT1G48380.1 \| Symbol: RHL1 \| root hair initiation protein root hairless 1 (RHL1) \| chr1:17881081-17882938 REVERSE \| Aliases: F11A17.7, F11A17_7, ROOT HAIRLESS 1 | 6.3 | 5.1 | 1.2 | 4.6 | 1.35% | 4.2 |
| 4895 | AT3G24320.1 \| Symbol: CHM \| DNA mismatch repair MutS family (MSH1), low similarity to SP:Q56239 DNA mismatch repair protein mutS {Thermus aquaticus}; contains Pfam profiles PF05190: MutS family domain IV, PF01624: MutS domain I, PF01541: Endo/excinuclease amino terminal domain | 3.7 | 2.7 | 1.1 | 4.6 | 1.36% | 4.2 |
| 4897 | AT5G20130.1 \| expressed protein \| chr5:6797445-6798901 FORWARD \| Aliases: F5O24.20, F5O24_20 | 6.5 | 5.4 | 1.0 | 4.6 | 1.36% | 4.2 |
| 4899 | AT3G17350.1 \| expressed protein \| chr3:5934100-5935042 FORWARD \| Aliases: MGD8.19 | 5.1 | 3.7 | 1.4 | 4.6 | 1.36% | 4.2 |
| 4902 | AT4G23940.1 \| FtsH protease, putative, contains similarity to zinc dependent protease GI:7650138 from (Arabidopsis thaliana) \| chr4:12437118-12441978 FORWARD \| Aliases: T32A16.110, T32A16_110 | 4.7 | 3.1 | 1.7 | 4.6 | 1.36% | 4.2 |
| 4903 | AT5G46280.1 \| DNA replication licensing factor, putative, similar to SP:Q43704 DNA replication licensing factor MCM3 homolog (Replication origin activator) (ROA protein) {Zea mays}; contains Pfam profile PF00493: MCM2/3/5 family \| chr5:18786924-18790885 REVERSE \| Aliases: MPL12.6, MPL12_6 | 6.5 | 4.4 | 2.2 | 4.6 | 1.36% | 4.2 |

| Rank | Description | Sync | Root | M | t | adj.q | B |
|---|---|---|---|---|---|---|---|
| 4904 | AT3G15360.1 \| Symbol: ATHM4 \| thioredoxin M-type 4, chloroplast (TRX-M4), nearly identical to SP:Q9SEU6 Thioredoxin M-type 4, chloroplast precursor (TRX-M4) {Arabidopsis thaliana} \| chr3:5188395-5189704 FORWARD \| Aliases: MJK13.20 | 8.3 | 6.7 | 1.6 | 4.6 | 1.36% | 4.1 |
| 4908 | AT4G27820.1 \| glycosyl hydrolase family 1 protein, contains Pfam PF00232 : Glycosyl hydrolase family 1 domain; TIGRFAM TIGR01233: 6-phospho-beta-galactosidase; similar to hydroxyisourate hydrolase (GI:19569603) (Glycine max) \| chr4:13857764-13860577 REVERSE \| Aliases: T27E11.60, T27E11_60 | 3.9 | 3.1 | 0.8 | 4.6 | 1.37% | 4.1 |
| 4909 | AT1G03687.2 \| DTW domain-containing protein, contains Pfam PF03942: DTW domain | 5.1 | 4.0 | 1.1 | 4.6 | 1.37% | 4.2 |
| 4911 | AT3G10640.2 \| SNF7 family protein, contains Pfam domain, PF03357: SNF7 family \| chr3:3323315-3325051 REVERSE \| Aliases: F13M14.7 | 8.2 | 6.9 | 1.3 | 4.6 | 1.37% | 4.1 |
| 4912 | AT2G16595.1 \| similar to putative SSR alpha subunit [Oryza sativa (japonica cultivar-group)] (GB:BAD53577.1); contains InterPro domain Translocon-associated protein (TRAP), alpha subunit (InterPro:IPR005595) \| chr2:7205209-7207379 FORWARD \| Aliases: None | 6.4 | 5.2 | 1.2 | 4.6 | 1.37% | 4.1 |
| 4915 | AT3G23620.1 \| brix domain-containing protein, contains Pfam domain, PF04427: Brix domain \| chr3:8480076-8482164 FORWARD \| Aliases: MDB19.11 | 6.5 | 3.7 | 2.7 | 4.6 | 1.37% | 4.1 |
| 4917 | AT4G28220.1 \| NADH dehydrogenase-related, similar to 64 kDa mitochondrial NADH dehydrogenase (Neurospora crassa) GI:4753821, alternative NADH-dehydrogenase (Yarrowia lipolytica) GI:3718005; contains Pfam profile PF00070: Pyridine nucleotide-disulphide oxidoreductase \| chr4:13993003-13998353 FORWARD \| Aliases: F26K10.100, F26K10_100 | 5.6 | 4.6 | 1.0 | 4.6 | 1.38% | 4.2 |
| 4920 | AT1G63260.3 \| similar to senescence-associated family protein [Arabidopsis thaliana] (TAIR:At3g45600.1); similar to senescence-associated protein DH [Zea mays] (GB:AAV31120.1); contains InterPro domain CD9/CD37/CD63 antigen (InterPro:IPR000301) \| chr1:23470425-23472855 REVERSE \| Aliases: F9N12.12, F9N12_12 | 5.0 | 4.2 | 0.8 | 4.6 | 1.38% | 4.1 |
| 4921 | AT2G34510.1 \| expressed protein, contains Pfam profile PF04862: Protein of unknown function, DUF642 \| chr2:14551076-14553886 REVERSE \| Aliases: T31E10.15, T31E10_15 | 5.0 | 3.6 | 1.4 | 4.6 | 1.38% | 4.2 |
| 4922 | AT1G13330.1 \| expressed protein, similar to nuclear receptor coactivator GT198 (GI:16506273) {Rattus norvegicus}; similar to TBP-1 interacting protein (GI:7328534) (Homo sapiens) \| chr1:4567932-4570565 FORWARD \| Aliases: T6J4.9, T6J4_9 | 4.3 | 3.4 | 0.9 | 4.6 | 1.38% | 4.1 |
| 4923 | AT5G67130.1 \| expressed protein \| chr5:26808592-26811253 FORWARD \| Aliases: K21H1.9, K21H1_9 | 6.6 | 5.5 | 1.0 | 4.6 | 1.38% | 4.1 |
| 4924 | AT2G43130.1 \| Symbol: ARA4 \| Ras-related protein (ARA-4) / small GTP-binding protein, putative, identical to SP:P28187 Ras-related protein ARA-4 {Arabidopsis thaliana} \| chr2:17936731-17937998 REVERSE \| Aliases: ARA 4, F14B2.27, F14B2.7, F14B2_27 | 3.7 | 2.7 | 1.0 | 4.6 | 1.38% | 4.0 |
| 4927 | AT2G16800.1 \| high-affinity nickel-transport family protein, contains Pfam domain, PF03824: High-affinity nickel-transport protein \| chr2:7292820-7294447 FORWARD \| Aliases: T24I21.21, T24I21_21 | 9.3 | 8.4 | 0.9 | 4.6 | 1.39% | 4.0 |
| 4928 | AT1G14410.1 \| DNA-binding protein-related, similar to DNA-binding protein p24 GI:9651810 from (Solanum tuberosum) \| chr1:4929118-4930888 REVERSE \| Aliases: F14L17.18, F14L17_18 | 6.1 | 4.7 | 1.4 | 4.6 | 1.39% | 4.2 |
| 4933 | AT4G12060.1 \| Clp amino terminal domain-containing protein, contains Pfam profile: PF02861 Clp amino terminal domain \| chr4:7228001-7229935 REVERSE \| Aliases: F16J13.130, F16J13_130 | 8.9 | 7.4 | 1.5 | 4.6 | 1.39% | 4.1 |
| 4934 | AT1G75990.1 \| 26S proteasome regulatory subunit S3, putative (RPN3), similar to 26S proteasome regulatory subunit S3 SP:P93768 (Nicotiana tabacum (Common tobacco)) \| chr1:28528160-28530437 REVERSE \| Aliases: T4O12.21, T4O12_21 | 6.5 | 4.4 | 2.1 | 4.6 | 1.39% | 4.1 |
| 4935 | AT5G38660.1 \| Symbol: APE1 \| expressed protein, similar to unknown protein (pir::S75762) \| chr5:15490363-15492765 REVERSE \| Aliases: ACCLIMATION OF PHOTOSYNTHESIS TO ENVIRONMENT, MBB18.21, MBB18_21 | 9.6 | 7.4 | 2.2 | 4.6 | 1.39% | 4.2 |
| 4936 | AT2G28800.2 \| Symbol: ALB3 \| chloroplast membrane protein (ALBINO3), Oxa1p homolog {PMID:11148275}; identical to chloroplast membrane protein ALBINO3 (Arabidopsis thaliana) GI:2209332 \| chr2:12364233-12366329 REVERSE \| Aliases: ABL3, ALBINA 3, ALBINO 3, CHLOROPLAST MEMBRANE PROTEIN ALB3, F8N16.9, F8N16_9 | 8.1 | 6.3 | 1.9 | 4.6 | 1.39% | 4.2 |
| 4937 | AT3G46040.1 \| 40S ribosomal protein S15A (RPS15aD), cytoplasmic ribosomal protein S15a, Arabidopsis thaliana, EMBL:ATAF1412 \| chr3:16925554-16926744 FORWARD \| Aliases: F12M12.10 | 10.4 | 9.2 | 1.1 | 4.6 | 1.39% | 3.9 |
| 4938 | AT5G27630.1 \| acyl-CoA binding family protein, similar to RING finger rngB protein, cytosolic - Dictyostelium discoideum, PIR:S68824; contains Pfam profiles PF01344: Kelch motif, PF00887: Acyl CoA binding protein (ACBP) \| chr5:9776026-9781530 FORWARD \| Aliases: F15A18.90, F15A18_90 | 4.6 | 3.4 | 1.2 | 4.6 | 1.39% | 4.1 |

| Rank | Description | Sync | Root | M | t | adj.q | B |
|---|---|---|---|---|---|---|---|
| 4940 | AT2G36240.1 \| similar to pentatricopeptide (PPR) repeat-containing protein [Arabidopsis thaliana] (TAIR:At1g09900.1); similar to putative pentatricopeptide repeat protein [Oryza sativa (japonica cultivar-group)] (GB:NP_909693.1); contains InterPro domain PPR repeat (InterPro:IPR002885); contains InterPro domain Proline-rich region (InterPro:IPR000694) \| chr2:15202732-15204310 FORWARD \| Aliases: F2H17.15, F2H17_15 | 8.5 | 6.8 | 1.7 | 4.6 | 1.39% | 4.1 |
| 4942 | AT1G52550.1 \| expressed protein \| chr1:19576596-19577699 REVERSE \| Aliases: F6D8.23, F6D8_23 | 3.8 | 2.8 | 1.0 | 4.6 | 1.39% | 4.1 |
| 4944 | AT5G16750.1 \| Symbol: TOZ \| transducin family protein / WD-40 repeat family protein, contains 8 WD-40 repeats (PF00400); similar to transducin homolog sazD - Homo sapiens, EMBL:U02609 \| chr5:5504349-5509345 REVERSE \| Aliases: F5E19.90, F5E19_90, TORMOZ, TORMOZEMBRYO DEFECTIVE | 5.4 | 4.2 | 1.1 | 4.6 | 1.39% | 4.1 |
| 4946 | AT1G77490.1 \| L-ascorbate peroxidase, thylakoid-bound (tAPX), identical to thylakoid-bound ascorbate peroxidase GB:CAA67426 (Arabidopsis thaliana) \| chr1:29122504-29125109 FORWARD \| Aliases: T5M16.8, T5M16_8 | 4.8 | 3.3 | 1.5 | 4.6 | 1.39% | 4.2 |
| 4948 | AT5G59850.1 \| 40S ribosomal protein S15A (RPS15aF), cytoplasmic ribosomal protein S15a, Arabidopsis thaliana, EMBL:ATAF1412 \| chr5:24129426-24130836 REVERSE \| Aliases: MMN10.16 | 9.2 | 7.0 | 2.2 | 4.6 | 1.39% | 4.2 |
| 4950 | AT4G22220.1 \| Symbol: ISU1 \| iron-sulfur cluster assembly complex protein, putative, similar to iron-sulfur cluster assembly complex ISCU1 (GI:11545705) (Homo sapiens); nifU protein homolog YPL135w (GI:15619823) (Saccharomyces cerevisiae) PIR2:S69049 \| chr4:11759173-11760943 REVERSE \| Aliases: ATISU1, T10I14.50, T10I14_50 | 6.7 | 3.9 | 2.8 | 4.6 | 1.39% | 4.2 |
| 4951 | AT3G05430.1 \| PWWP domain-containing protein, contains Pfam profile:PF00855 PWWP domain \| chr3:1567631-1571078 FORWARD \| Aliases: F22F7.12, F22F7_12 | 3.9 | 3.4 | 0.5 | 4.6 | 1.39% | 3.8 |
| 4952 | AT2G40280.1 \| dehydration-responsive family protein, similar to early-responsive to dehydration stress ERD3 protein (Arabidopsis thaliana) GI:15320410; contains Pfam profile PF03141: Putative methyltransferase \| chr2:16832578-16835476 REVERSE \| Aliases: T7M7.24 | 7.4 | 6.1 | 1.3 | 4.6 | 1.40% | 4.2 |
| 4953 | AT2G27840.2 \| Symbol: HDT4 \| histone deacetylase-related / HD-related, similar to nucleolar histone deacetylase HD2-p39 (Zea mays) GI:2257756; contains non-consensus donor splice site AT at exon2 and acceptor splice site AC at exon3. \| chr2:11869605-11870963 FORWARD \| Aliases: F15K20.6, F15K20_6, HDA13, HDT04, HISTONE DEACETYLASE | 6.3 | 4.1 | 2.3 | 4.6 | 1.40% | 4.2 |
| 4955 | AT3G51010.1 \| expressed protein \| chr3:18957681-18959137 REVERSE \| Aliases: F24M12.50 | 7.1 | 5.2 | 1.9 | 4.6 | 1.40% | 4.2 |
| 4956 | AT5G05610.2 \| PHD finger family protein, contains Pfam domain, PF00628: PHD-finger \| chr5:1676941-1679105 REVERSE \| Aliases: MOP10.15, MOP10_15 | 6.0 | 4.5 | 1.5 | 4.6 | 1.40% | 4.1 |
| 4957 | AT3G60830.1 \| Symbol: ATARP7 \| | 7.2 | 6.1 | 1.2 | 4.6 | 1.40% | 4.1 |
| 4959 | AT1G07500.1 \| expressed protein, ; expression supported by MPSS \| chr1:2304763-2305028 REVERSE \| Aliases: F22G5.11, F22G5_11 | 4.9 | 3.4 | 1.5 | 4.6 | 1.40% | 4.1 |
| 4962 | AT3G53430.1 \| 60S ribosomal protein L12 (RPL12B), 60S RIBOSOMAL PROTEIN L12, Prunus armeniaca, SWISSPROT:RL12_PRUAR \| chr3:19820665-19821447 REVERSE \| Aliases: F4P12.130 | 10.2 | 8.2 | 2.0 | 4.6 | 1.40% | 4.2 |
| 4965 | AT3G58140.1 \| phenylalanyl-tRNA synthetase class IIc family protein, similar to phenylalanine-tRNA synthetase (Homo sapiens) GI:3983103; contains Pfam profile PF01409: tRNA synthetases class II core domain (F) \| chr3:21540708-21543549 REVERSE \| Aliases: F9D24.50 | 7.3 | 6.2 | 1.1 | 4.6 | 1.41% | 4.0 |
| 4966 | AT4G32810.1 \| Symbol: CCD8 \| Encodes a protein with similarity to carotenoid cleaving deoxygenases. Involved in the production of a graft transmissable signal to suppress axillary branching. Mutants have increased axillary branches. Expressed primarily in root apex. \| chr4:15828232-15831481 FORWARD \| Aliases: CAROTENOID CLEAVAGE DIOXYGENASE 8, MAX4, T16I18.20, T16I18_20 | 4.3 | 2.3 | 2.0 | 4.6 | 1.41% | 4.2 |
| 4969 | AT5G59460.1 \| scarecrow-like transcription factor 11 (SCL11), identical to cDNA scarecrow-like 11 (SCL11) mRNA, partial cds gi:4580526 | 5.1 | 3.7 | 1.3 | 4.6 | 1.41% | 4.0 |
| 4970 | AT5G27990.1 \| expressed protein, predicted proteins, Saccharomyces cerevisiae and Schizosaccharomyces pombe \| chr5:10017052-10018445 FORWARD \| Aliases: F15F15.60, F15F15_60 | 6.7 | 5.5 | 1.2 | 4.6 | 1.41% | 4.0 |
| 4971 | AT5G13270.1 \| pentatricopeptide (PPR) repeat-containing protein, contains INTERPRO:IPR002885 PPR repeats \| chr5:4246957-4249215 REVERSE \| Aliases: T31B5.90, T31B5_90 | 3.0 | 2.5 | 0.5 | 4.6 | 1.41% | 3.7 |
| 4974 | AT3G21360.1 \| expressed protein \| chr3:7522810-7524586 FORWARD \| Aliases: MHC9.4 | 6.5 | 5.4 | 1.1 | 4.6 | 1.42% | 4.1 |
| 4975 | AT4G02280.1 \| sucrose synthase, putative / sucrose-UDP glucosyltransferase, putative, strong similarity to sucrose synthase GI:6682841 from (Citrus unshiu) \| chr4:994927-998963 FORWARD \| Aliases: T2H3.8, T2H3_8 | 5.4 | 4.2 | 1.2 | 4.6 | 1.42% | 4.2 |

| Rank | Description | Sync | Root | M | t | adj.q | B |
|---|---|---|---|---|---|---|---|
| 4976 | AT3G58200.1 \| meprin and TRAF homology domain-containing protein / MATH domain-containing protein, similar to ubiquitin-specific protease 12 (Arabidopsis thaliana) GI:11993471; contains Pfam profile PF00917: MATH domain \| chr3:21570870-21572429 REVERSE \| Aliases: F9D24.110 | 5.6 | 3.2 | 2.4 | 4.6 | 1.42% | 4.1 |
| 4977 | AT1G26150.1 \| similar to protein kinase family protein [Arabidopsis thaliana] (TAIR:At5g38560.1); similar to putative receptor protein kinase PERK1 [Oryza sativa (japonica cultivar-group)] (GB:BAD87028.1); contains InterPro domain Serine/threonine protein kinase, active site (InterPro:IPR008271); contains InterPro domain Tyrosine protein kinase (InterPro:IPR001245); contains InterPro domain Serine/threonine protein kinase (InterPro:IPR002290); contains InterPro domain Protein kinase (InterPro:IPR000719); contains InterPro domain Proline-rich region (InterPro:IPR000694) \| chr1:9039615-9043275 REVERSE \| Aliases: F28B23.17, F28B23_17 | 6.8 | 5.6 | 1.2 | 4.6 | 1.42% | 4.1 |
| 4981 | AT1G08970.4 \| Symbol: HAP5C \| CCAAT-box binding transcription factor Hap5a, putative \| chr1:2882546-2884285 FORWARD \| Aliases: F7G19.16, F7G19_16, HAP5C, HEME ACTIVATED PROTEIN 5C | 9.4 | 7.1 | 2.3 | 4.6 | 1.42% | 4.1 |
| 4983 | AT4G14100.1 \| expressed protein \| chr4:8120715-8122406 FORWARD \| Aliases: DL3085W, FCAALL.191 | 4.9 | 3.7 | 1.2 | 4.6 | 1.43% | 4.1 |
| 4984 | AT2G07050.1 \| Symbol: CAS1 \| cycloartenol synthase (CAS1) / 2,3-epoxysqualene--cycloartenol cyclase / (S)-2,3-epoxysqualene mutase, identical to cycloartenol synthase (SP:P38605 : GI:452446) (PMID:7505443) | 8.4 | 7.0 | 1.4 | 4.6 | 1.43% | 4.1 |
| 4992 | AT5G47970.1 \| nitrogen regulation family protein, strong similarity to unknown protein (emb:CAB87804.1) ; contains Pfam domain PF01207: Dihydrouridine synthase (Dus); similar to (SP:P45672) NIFR3-like protein (SP:P45672) (Azospirillum brasilense) | 4.7 | 3.9 | 0.9 | 4.6 | 1.43% | 4.0 |
| 4998 | AT1G29700.1 \| expressed protein \| chr1:10384967-10387427 REVERSE \| Aliases: F15D2.25 | 4.2 | 3.0 | 1.1 | 4.6 | 1.44% | 4.0 |
| 5000 | AT2G37420.1 \| kinesin motor protein-related \| chr2:15707410-15712422 FORWARD \| Aliases: F3G5.21, F3G5_21 | 4.9 | 3.0 | 1.8 | 4.6 | 1.44% | 4.1 |
| 5003 | AT5G48460.1 \| fimbrin-like protein, putative, strong similarity to fimbrin-like protein AtFim2 (Arabidopsis thaliana) GI:2737926; contains Pfam profile PF00307: Calponin homology (CH) domain | 6.1 | 5.4 | 0.7 | 4.6 | 1.44% | 3.9 |
| 5008 | AT2G33180.1 \| expressed protein \| chr2:14073545-14074544 FORWARD \| Aliases: F25I18.8, F25I18_8 | 3.8 | 2.6 | 1.2 | 4.6 | 1.45% | 4.1 |
| 5011 | AT2G17250.1 \| expressed protein, weak similarity to Ribosome biogenesis protein MAK21 (Swiss-Prot:Q12176) (Saccharomyces cerevisiae) \| chr2:7506559-7510207 FORWARD \| Aliases: T23A1.11, T23A1_11 | 7.1 | 5.9 | 1.1 | 4.6 | 1.45% | 4.0 |
| 5012 | AT5G42130.1 \| mitochondrial substrate carrier family protein, contains Pfam profile: PF00153 mitochondrial carrier protein \| chr5:16852527-16854067 REVERSE \| Aliases: MJC20.24, MJC20_24 | 6.2 | 5.0 | 1.2 | 4.6 | 1.45% | 4.1 |
| 5013 | AT3G60880.2 \| dihydrodipicolinate synthase 1 (DHDPS1) (DHDPS) (DHPS1), identical to SP:Q9LZX6 Dihydrodipicolinate synthase 1, chloroplast precursor (EC 4.2.1.52) (DHDPS 1) {Arabidopsis thaliana} \| chr3:22505827-22507672 FORWARD \| Aliases: T4C21.290 | 7.6 | 5.3 | 2.3 | 4.6 | 1.45% | 4.1 |
| 5014 | AT1G28480.1 \| glutaredoxin family protein, contains INTERPRO Domain IPR002109, Glutaredoxin (thioltransferase) | 8.1 | 7.0 | 1.1 | 4.6 | 1.45% | 4.0 |
| 5016 | AT3G04830.2 \| expressed protein \| chr3:1326255-1329370 FORWARD \| Aliases: T9J14.22, T9J14_22 | 8.7 | 6.5 | 2.2 | 4.6 | 1.45% | 4.1 |
| 5017 | AT4G22530.1 \| embryo-abundant protein-related, similar to embryo-abundant protein (Picea glauca) GI:1350531 \| chr4:11859015-11860159 REVERSE \| Aliases: F7K2.110, F7K2_110 | 6.8 | 5.5 | 1.3 | 4.6 | 1.46% | 4.1 |
| 5018 | AT1G30825.1 \| Symbol: ARPC2A/DIS2 \| actin-related protein 2/3 complex 34kDa subunit family / arp2/3 complex 34kDa subunit family, contains Pfam PF04045: Arp2/3 complex, 34kD subunit p34-Arc; similar to ARP2/3 complex 34 kDa subunit (P34-ARC) (Swiss-Prot:O96623) (Dictyostelium discoideum); similar to ARP2/3 complex 34 kDa subunit (P34-ARC) (Actin-related protein 2/3 complex subunit 2) (Swiss-Prot:O15144) (Homo sapiens) | 6.1 | 4.9 | 1.2 | 4.6 | 1.46% | 4.1 |
| 5019 | AT3G03950.3 \| similar to YT521-B-like family protein [Arabidopsis thaliana] (TAIR:At5g61020.2); similar to YT521-B-like family protein [Arabidopsis thaliana] (TAIR:At5g61020.1); similar to high-glucose-regulated protein 8-like [Oryza sativa (japonica cultivar-group)] (GB:XP_476753.1); contains InterPro domain YT521-B-like protein (InterPro:IPR007275) \| chr3:1021267-1024016 FORWARD \| Aliases: T11I18.6, T11I18_6 | 7.8 | 5.6 | 2.2 | 4.6 | 1.46% | 4.1 |
| 5021 | AT2G45640.1 \| sin3 associated polypeptide p18 family protein, similar to Sin3 associated polypeptide p18 (2HOR0202) (Swiss-Prot:O00422) (Homo sapiens) | 7.5 | 6.7 | 0.8 | 4.6 | 1.46% | 4.0 |
| 5022 | AT3G13490.1 \| tRNA synthetase class II (D, K and N) family protein, similar to SP:Q9RHV9 Lysyl-tRNA synthetase (EC 6.1.1.6) (Lysine--tRNA ligase) {Bacillus stearothermophilus}; contains Pfam profile: PF00152 tRNA synthetases class II (D, K and N) | 5.2 | 4.3 | 1.0 | 4.6 | 1.46% | 3.9 |

| Rank | Description | Sync | Root | M | t | adj.q | B |
|---|---|---|---|---|---|---|---|
| 5024 | AT1G29900.1 \| Symbol: CARB \| carbamoyl-phosphate synthase family protein, similar to carbamoylphosphate synthetase GI:6552726 from (Medicago sativa); contains Pfam profiles PF02786: Carbamoyl-phosphate synthase L chain ATP binding domain, PF00289: Carbamoyl-phosphate synthase L chain N-terminal domain, PF02787: Carbamoyl-phosphate synthetase large chain oligomerisation domain \| chr1:10468038-10472195 FORWARD \| Aliases: CARBAMOYL PHOSPHATE SYNTHETASE B, CARBAMOYL PHOSPHATE SYNTHETASE LARGE SUBUNIT, F1N18.6, F1N18_6 | 9.0 | 6.8 | 2.3 | 4.6 | 1.46% | 4.1 |
| 5025 | AT5G62810.1 \| Symbol: PEX14 \| peroxisomal protein (PEX14), identical to PEX14 (Arabidopsis thaliana) GI:11094252; contains Pfam profile PF04695: Peroxisomal membrane anchor protein (Pex14p) conserved region; supporting cDNA gi:11094253:dbj:AB037539.1: \| chr5:25237391-25241015 FORWARD \| Aliases: ATPEX14, MQB2.13, MQB2_13, PED2, PEROXISOME DEFECTIVE 2 | 7.3 | 6.2 | 1.2 | 4.6 | 1.46% | 4.0 |
| 5026 | AT5G64580.1 \| AAA-type ATPase family protein, similar to zinc dependent protease (Arabidopsis thaliana) GI:7650138; contains Pfam profile PF00004: ATPase AAA family \| chr5:25834318-25838691 REVERSE \| Aliases: MUB3.10, MUB3_10 | 4.9 | 3.5 | 1.4 | 4.6 | 1.46% | 4.1 |
| 5027 | AT3G54050.1 \| fructose-1,6-bisphosphatase, putative / D-fructose-1,6-bisphosphate 1-phosphohydrolase, putative / FBPase, putative, strong similarity to fructose-1,6-bisphosphatase (Brassica napus) GI:289367; identical to SP:P25851 Fructose-1,6-bisphosphatase, chloroplast precursor (EC 3.1.3.11) (D-fructose-1,6-bisphosphate 1-phosphohydrolase) (FBPase) {Arabidopsis thaliana}; contains Pfam profile PF00316: fructose-1,6-bisphosphatase \| chr3:20027891-20029726 FORWARD \| Aliases: F24B22.10 | 3.6 | 2.8 | 0.8 | 4.6 | 1.46% | 4.0 |
| 5028 | AT2G32500.1 \| expressed protein \| chr2:13802337-13804259 REVERSE \| Aliases: T26B15.6, T26B15_6 | 4.3 | 3.1 | 1.2 | 4.6 | 1.46% | 4.1 |
| 5029 | AT2G37890.1 \| mitochondrial substrate carrier family protein, contains Pfam profile: PF00153 mitochondrial carrier protein \| chr2:15868766-15871087 REVERSE \| Aliases: T8P21.20, T8P21_20 | 5.2 | 4.1 | 1.2 | 4.6 | 1.46% | 4.1 |
| 5031 | AT3G09650.1 \| Symbol: HCF152 \| | 7.2 | 5.7 | 1.5 | 4.6 | 1.47% | 4.1 |
| 5034 | AT1G69230.2 \| expressed protein \| chr1:26029981-26031371 REVERSE \| Aliases: F4N2.18 | 4.5 | 3.1 | 1.4 | 4.6 | 1.47% | 4.1 |
| 5036 | AT1G48460.1 \| expressed protein \| chr1:17915014-17916968 FORWARD \| Aliases: T1N15.7, T1N15_7 | 5.0 | 3.8 | 1.2 | 4.6 | 1.47% | 4.1 |
| 5037 | AT3G02490.1 \| pentatricopeptide (PPR) repeat-containing protein, contains Pfam profile PF01535: PPR repeat \| chr3:513532-515932 FORWARD \| Aliases: F16B3.12, F16B3_12 | 7.0 | 5.8 | 1.2 | 4.6 | 1.47% | 4.0 |
| 5038 | AT1G69020.1 \| prolyl oligopeptidase family protein, similar to SP:Q59536 Protease II (EC 3.4.21.83) (Oligopeptidase B) {Moraxella lacunata}; contains Pfam profiles PF00326: prolyl oligopeptidase family, PF02897: Prolyl oligopeptidase, N-terminal beta-propeller domain; contains non-consensus GA donor splice site at intron 5 \| chr1:25947549-25950860 REVERSE \| Aliases: T6L1.20, T6L1_20 | 4.4 | 3.6 | 0.8 | 4.5 | 1.47% | 4.0 |
| 5043 | AT4G38890.1 \| dihydrouridine synthase family protein, contains Pfam domain, PF01207: Dihydrouridine synthase (Dus) \| chr4:18135903-18139094 REVERSE \| Aliases: F19H22.4 | 9.0 | 7.9 | 1.1 | 4.5 | 1.48% | 4.1 |
| 5044 | AT5G37770.1 \| Symbol: TCH2 \| touch-responsive protein / calmodulin-related protein 2, touch-induced (TCH2), identical to calmodulin-related protein 2,touch-induced SP:P25070 from (Arabidopsis thaliana) \| chr5:15016084-15016849 REVERSE \| Aliases: K22F20.10, K22F20_10, TOUCH 2 | 5.3 | 3.3 | 2.0 | 4.5 | 1.48% | 4.1 |
| 5046 | AT4G39880.1 \| ribosomal protein L23 family protein, contains Pfam profile PF00276: ribosomal protein L23 \| chr4:18504531-18505332 FORWARD \| Aliases: T5J17.50, T5J17_50 | 6.6 | 4.7 | 1.9 | 4.5 | 1.48% | 4.1 |
| 5047 | AT2G35340.1 \| RNA helicase, putative, similar to ATP-dependent RNA helicase #3 (Homo sapiens) GI:3107913; contains Pfam profiles PF04408: Helicase associated domain (HA2), PF00271: Helicase conserved C-terminal domain \| chr2:14879807-14886694 FORWARD \| Aliases: T32F12.28, T32F12_28 | 3.3 | 2.9 | 0.5 | 4.5 | 1.48% | 3.6 |
| 5049 | AT5G57850.1 \| aminotransferase class IV family protein, contains Pfam profile: PF01063 aminotransferase class IV \| chr5:23452528-23454552 REVERSE \| Aliases: MTI20.10, MTI20_10 | 5.8 | 4.6 | 1.2 | 4.5 | 1.49% | 4.1 |
| 5050 | AT5G63530.1 \| Symbol: ATFP3 \| copper chaperone (CCH)-related, low similarity to copper homeostasis factor (GI:3168840); nearly identical to farnesylated protein ATFP3 (GI:4097547); contains Pfam profile PF00403: Heavy-metal-associated domain \| chr5:25450469-25453060 FORWARD \| Aliases: MLE2.16, MLE2_16 | 7.5 | 4.5 | 3.0 | 4.5 | 1.49% | 4.1 |
| 5051 | AT1G64355.2 \| expressed protein, similar to hypothetical protein Tery02003428 [Trichodesmium erythraeum IMS101] (GB:ZP_00326379.1) \| chr1:23889765-23890875 FORWARD \| Aliases: None | 3.8 | 2.9 | 0.8 | 4.5 | 1.49% | 4.0 |
| 5052 | AT3G62220.1 \| serine/threonine protein kinase, putative, similar to Pto kinase interactor 1 (Pti1)(Lycopersicon esculentum) gi:3668069:gb:AAC61805 \| chr3:23040075-23042130 REVERSE \| Aliases: T17J13.180 | 5.9 | 4.7 | 1.2 | 4.5 | 1.49% | 4.0 |
| 5057 | AT5G27740.1 \| expressed protein \| chr5:9823744-9827068 FORWARD \| Aliases: T1G16.70, T1G16_70 | 5.2 | 4.0 | 1.2 | 4.5 | 1.50% | 4.1 |

| Rank | Description | Sync | Root | M | t | adj.q | B |
|---|---|---|---|---|---|---|---|
| 5059 | AT3G06030.1 \| Symbol: ANP3 \| NPK1-related protein kinase, putative (ANP3), similar to protein kinase (Nicotiana tabacum) gi:456309:dbj:BAA05648; identical to cDNA NPK1-related protein kinase 3 GI:2342426 \| chr3:1818749-1822846 REVERSE \| Aliases: F24F17.1, F24F17_1, MAPKKK12 | 5.0 | 3.8 | 1.1 | 4.5 | 1.50% | 4.1 |
| 5061 | AT3G09690.2 \| similar to hydrolase, alpha/beta fold family protein [Arabidopsis thaliana] (TAIR:At5g02970.1); similar to putative alpha/beta hydrolase [Oryza sativa (japonica cultivar-group)] (GB:AAP54670.1); similar to hypothetical protein [Oryza sativa (japonica cultivar-group)] (GB:AAM92284.1); contains InterPro domain Alpha/beta hydrolase fold (InterPro:IPR000073) \| chr3:2972228-2974848 FORWARD \| Aliases: F11F8.28 | 5.4 | 4.6 | 0.8 | 4.5 | 1.50% | 4.0 |
| 5062 | AT1G55520.2 \| Symbol: TBP2 \| transcription initiation factor IID-2 (TFIID-2) / TATA-box factor 2 / TATA sequence-binding protein 2 (TBP2), identical to Swiss-Prot:P28148:TF22_ARATH Transcription initiation factor TFIID-2 (TATA-box factor 2) (TATA sequence-binding protein 2) (TBP-2) (Arabidopsis thaliana) \| chr1:20729392-20731756 REVERSE \| Aliases: ATTBP2, T5A14.8, T5A14_8 | 6.4 | 4.9 | 1.5 | 4.5 | 1.50% | 4.1 |
| 5063 | AT1G05720.1 \| selenoprotein family protein, contains Prosite PS00190: Cytochrome c family heme-binding site signature; similar to 15 kDa selenoprotein (GI:12314088) {Homo sapiens} \| chr1:1717461-1718852 REVERSE \| Aliases: F3F20.17, F3F20_17 | 9.0 | 7.8 | 1.3 | 4.5 | 1.50% | 4.0 |
| 5065 | AT3G49640.1 \| nitrogen regulation family protein, similar to NITROGEN REGULATION PROTEIN NIFR3 (SP:Q08111) (Rhodobacter capsulatus); contains Pfam domain PF01207: Dihydrouridine synthase (Dus) \| chr3:18411764-18415791 REVERSE \| Aliases: T9C5.230 | 5.9 | 4.4 | 1.5 | 4.5 | 1.50% | 4.1 |
| 5066 | AT5G54900.1 \| Symbol: ATRBP45A \| RNA-binding protein 45 (RBP45), putative, contains similarity to polyadenylate-binding protein 5 \| chr5:22312609-22315572 FORWARD \| Aliases: ATRBP45A, MBG8.17, MBG8_17 | 10.5 | 9.2 | 1.4 | 4.5 | 1.50% | 3.9 |
| 5067 | AT3G18720.1 \| F-box family protein, contains Pfam PF00646: F-box domain \| chr3:6444373-6445765 REVERSE \| Aliases: MVE11.8 | 2.7 | 2.3 | 0.4 | 4.5 | 1.50% | 3.5 |
| 5069 | AT3G17465.1 \| Symbol: RPL3P \| ribosomal protein L3 family protein \| chr3:5977823-5979578 REVERSE \| Aliases: MKP6.7, RIBOSOMAL PROTEIN L3 PLASTID | 6.7 | 5.1 | 1.6 | 4.5 | 1.50% | 4.1 |
| 5074 | AT4G15830.1 \| expressed protein \| chr4:8995126-8996614 REVERSE \| Aliases: DL3955C, FCAALL.419 | 8.2 | 6.7 | 1.5 | 4.5 | 1.51% | 4.0 |
| 5077 | AT1G20910.1 \| ARID/BRIGHT DNA-binding domain-containing protein, low similarity to Chain A, Human Mrf-2 Domain (Homo sapiens) GI:14278238; contains Pfam profile PF01388: ARID/BRIGHT DNA binding domain \| chr1:7276932-7281127 REVERSE \| Aliases: F9H16.11, F9H16_11 | 5.6 | 4.5 | 1.1 | 4.5 | 1.52% | 4.0 |
| 5078 | AT2G24440.1 \| expressed protein \| chr2:10398169-10400326 FORWARD \| Aliases: T28I24.17, T28I24_17 | 5.3 | 3.9 | 1.3 | 4.5 | 1.52% | 4.1 |
| 5080 | AT5G49410.2 \| expressed protein, similar to expressed protein [Arabidopsis thaliana] (TAIR:At1g73940.1); similar to unknown protein [Oryza sativa (japonica cultivar-group)] (GB:XP_470431.1) \| chr5:20049713-20051152 FORWARD \| Aliases: K7J8.8, K7J8_8 | 3.1 | 2.6 | 0.5 | 4.5 | 1.52% | 3.6 |
| 5083 | AT5G22790.1 \| expressed protein \| chr5:7599149-7601693 REVERSE \| Aliases: K8E10.2, K8E10_2 | 7.4 | 6.2 | 1.3 | 4.5 | 1.52% | 4.1 |
| 5084 | AT1G04610.1 \| flavin-containing monooxygenase / FMO (YUCCA3), identical to gi:16555356 | 6.5 | 4.5 | 2.0 | 4.5 | 1.52% | 4.1 |
| 5089 | AT3G49080.1 \| ribosomal protein S9 family protein, contains Pfam profile PF00380: ribosomal protein S9 | 6.8 | 5.8 | 1.0 | 4.5 | 1.52% | 4.0 |
| 5092 | AT1G27385.1 \| expressed protein \| chr1:9510925-9512847 REVERSE \| Aliases: None | 7.4 | 6.4 | 1.0 | 4.5 | 1.53% | 4.1 |
| 5093 | AT2G23090.1 \| expressed protein \| chr2:9836530-9837490 REVERSE \| Aliases: F21P24.15 | 12.0 | 10.9 | 1.1 | 4.5 | 1.53% | 3.6 |
| 5094 | AT3G17160.1 \| expressed protein \| chr3:5852158-5853694 FORWARD \| Aliases: K14A17.24 | 8.4 | 6.7 | 1.7 | 4.5 | 1.53% | 4.0 |
| 5097 | AT3G13120.1 \| 30S ribosomal protein S10, chloroplast, putative, similar to 30S ribosomal protein S10 GB:P02364 (Escherichia coli) (est matches suggest the N-terminal extension) \| chr3:4219790-4221637 REVERSE \| Aliases: MJG19.7 | 6.1 | 4.2 | 1.9 | 4.5 | 1.53% | 4.0 |
| 5099 | AT2G31890.1 \| expressed protein \| chr2:13563222-13566844 REVERSE \| Aliases: F20M17.7, F20M17_7 | 6.7 | 5.7 | 1.0 | 4.5 | 1.53% | 4.0 |
| 5100 | AT4G34270.1 \| TIP41-like family protein, contains Pfam PF04176: TIP41-like family; identical to cDNA putative cytoskeletal protein mRNA, partial cds GI:5031529 \| chr4:16403848-16406210 REVERSE \| Aliases: F10M10.40, F10M10_40 | 7.0 | 6.4 | 0.6 | 4.5 | 1.53% | 3.6 |
| 5101 | AT1G65980.2 \| Symbol: TPX1 \| similar to peroxiredoxin type 2, putative [Arabidopsis thaliana] (TAIR:At1g65970.1); similar to thioredoxin peroxidase 1 [Lycopersicon esculentum] (GB:AAP34571.1); similar to thioredoxin-dependent peroxidase [Plantago major] (GB:CAH58634.1); contains InterPro domain Alkyl hydroperoxide reductase/ Thiol specific antioxidant/ Mal allergen (InterPro:IPR000866) \| chr1:24562969-24564599 REVERSE \| Aliases: F12P19.14, F12P19_14, PEROXIREDOXIN TPX1 | 12.4 | 11.9 | 0.6 | 4.5 | 1.54% | 3.6 |
| 5102 | AT1G08880.1 \| histone H2A, putative, Strong similarity to histone H2A Cicer arietinum SP:O65759, Picea abies SP:P35063; contains Pfam profile PF00125 Core histone H2A/H2B/H3/H4; ESTs gb:ATTS3874,gb:T46627,gb:T14194 come from this gene \| chr1:2846956-2847784 REVERSE \| Aliases: F7G19.24, F7G19_24 | 8.6 | 5.8 | 2.8 | 4.5 | 1.54% | 4.1 |

| Rank | Description | Sync | Root | M | t | adj.q | B |
|---|---|---|---|---|---|---|---|
| 5103 | AT2G47420.1 \| dimethyladenosine transferase, putative, similar to SP:P41819 Dimethyladenosine transferase (EC 2.1.1.-) (S-adenosylmethionine-6-N', N'-adenosyl (rRNA) dimethyltransferase) {Saccharomyces cerevisiae}; contains Pfam profile PF00398: ribosomal RNA adenine dimethylase family protein \| chr2:19464555-19465951 FORWARD \| Aliases: T30B22.28 | 6.6 | 5.2 | 1.5 | 4.5 | 1.54% | 4.0 |
| 5105 | AT5G04600.1 \| RNA recognition motif (RRM)-containing protein, contains InterPro entry IPR000504: RNA-binding region RNP-1 (RNA recognition motif) (RRM) \| chr5:1323641-1325614 FORWARD \| Aliases: T1E3.1 | 7.2 | 5.7 | 1.5 | 4.5 | 1.54% | 4.1 |
| 5106 | AT1G03130.1 \| photosystem I reaction center subunit II, chloroplast, putative / photosystem I 20 kDa subunit, putative / PSI-D, putative (PSAD2), similar to SP:P12353 Photosystem I reaction center subunit II, chloroplast precursor (Photosystem I 20 kDa subunit) (PSI-D) {Spinacia oleracea}; contains Pfam profile PF02531: PsaD | 6.2 | 4.7 | 1.5 | 4.5 | 1.54% | 4.0 |
| 5109 | AT5G53170.1 \| Symbol: FTSH11 \| | 6.9 | 4.6 | 2.4 | 4.5 | 1.55% | 4.0 |
| 5110 | AT3G16290.1 \| Symbol: EMB2083 \| FtsH protease, putative, contains similarity to cell division protein FtsH GI:1652085 from (Synechocystis sp. PCC 6803) \| chr3:5521193-5525001 REVERSE \| Aliases: EMB2083, EMBRYO DEFECTIVE 2083, MYA6.12 | 5.7 | 4.9 | 0.8 | 4.5 | 1.55% | 3.8 |
| 5111 | AT1G31817.1 \| chloroplast 30S ribosomal protein S11, putative, contains Pfam profile: PF00411: Ribosomal protein S11 \| chr1:11414730-11416418 REVERSE \| Aliases: F5M6.25, F5M6_25 | 5.9 | 3.9 | 2.0 | 4.5 | 1.55% | 4.1 |
| 5112 | AT5G05690.2 \| Symbol: CPD \| similar to steroid 22-alpha-hydroxylase (CYP90B1) (DWF4) [Arabidopsis thaliana] (TAIR:At3g50660.1); similar to cytochrome P450 [Nicotiana tabacum] (GB:CAD27417.1); contains InterPro domain E-class P450, group I (InterPro:IPR002401); contains InterPro domain Cytochrome P450 (InterPro:IPR001128) \| chr5:1702689-1706788 REVERSE \| Aliases: CABBAGE 3, CBB3, CONSTITUTIVE PHOTOMORPHOGENIC DWARF, CYP90, CYP90A, CYP90A1, CYTOCHROME P450, CYTOCHROME P450 MONOOXYGENASE, DWARF 3, DWF3, MJJ3.9, MJJ3_9 | 6.3 | 5.0 | 1.3 | 4.5 | 1.55% | 4.0 |
| 5113 | AT5G56510.1 \| pumilio/Puf RNA-binding domain-containing protein, contains similarity to RNA-binding protein \| chr5:22898947-22901068 FORWARD \| Aliases: MKN22.2, MKN22_2 | 3.3 | 2.6 | 0.7 | 4.5 | 1.55% | 3.8 |
| 5119 | AT5G11520.1 \| Symbol: ASP3 \| aspartate aminotransferase, chloroplast / transaminase A (ASP3) (YLS4), identical to SP:P46644 Aspartate aminotransferase, chloroplast precursor (EC 2.6.1.1) (Transaminase A) {Arabidopsis thaliana}; identical to cDNA YLS4 mRNA for aspartate aminotransferase (ASP3), partial cds GI:13122285 \| chr5:3685090-3687765 REVERSE \| Aliases: ASPARTATE AMINOTRANSFERASE 3, ASPARTATE AMINOTRANSFERASE, CHLOROPLAST PRECURSOR, F15N18.110, F15N18_110, YELLOW LEAF SPECIFIC GENE 4, YLS4 | 11.7 | 9.7 | 1.9 | 4.5 | 1.56% | 3.8 |
| 5120 | AT1G01725.1 \| expressed protein \| chr1:269794-270775 REVERSE \| Aliases: None | 8.1 | 6.5 | 1.6 | 4.5 | 1.56% | 4.0 |
| 5121 | AT5G67180.1 \| AP2 domain-containing transcription factor, putative, similar to (SP:P47927) Floral homeotic protein APETALA2. (Mouse-ear cress) {Arabidopsis thaliana} \| chr5:26819233-26821245 REVERSE \| Aliases: K21H1.22, K21H1_22 | 3.8 | 3.0 | 0.8 | 4.5 | 1.56% | 3.8 |
| 5122 | AT4G09730.1 \| DEAD/DEAH box helicase, putative, RNA helicase -Mus musculus,PIR2:I84741 | 4.0 | 2.9 | 1.0 | 4.5 | 1.56% | 3.9 |
| 5125 | AT3G55380.1 \| ubiquitin-conjugating enzyme 14 (UBC14), E2; UbcAT3; identical to gi:2129757, S46656 \| chr3:20542396-20544150 FORWARD \| Aliases: T22E16.40 | 7.1 | 5.6 | 1.4 | 4.5 | 1.57% | 4.0 |
| 5126 | AT1G66330.2 \| senescence-associated family protein, similar to senescence-associated protein (GI:12836895) (Ipomoea batatas) \| chr1:24733292-24736125 REVERSE \| Aliases: T27F4.8, T27F4_8 | 5.8 | 4.1 | 1.7 | 4.5 | 1.57% | 4.0 |
| 5127 | AT2G44525.1 \| expressed protein \| chr2:18389172-18390587 FORWARD \| Aliases: None | 8.4 | 6.9 | 1.5 | 4.5 | 1.57% | 4.0 |
| 5128 | AT2G18710.1 \| preprotein translocase secY subunit, chloroplast (CpSecY), Identical to SP:Q38885 Preprotein translocase secY subunit, chloroplast precursor (CpSecY) {Arabidopsis thaliana} \| chr2:8119166-8121559 REVERSE \| Aliases: MSF3.9, MSF3_9 | 7.2 | 4.6 | 2.6 | 4.5 | 1.57% | 4.0 |
| 5129 | AT4G33540.1 \| metallo-beta-lactamase family protein \| chr4:16130876-16133963 FORWARD \| Aliases: T16L1.30, T16L1_30 | 8.5 | 6.4 | 2.0 | 4.5 | 1.57% | 4.0 |
| 5130 | AT2G39310.1 \| jacalin lectin family protein, similar to myrosinase-binding protein homolog (Arabidopsis thaliana) GI:2997767; contains Pfam profile PF01419 jacalin-like lectin domain | 4.5 | 3.3 | 1.3 | 4.5 | 1.57% | 4.0 |
| 5133 | AT4G15940.1 \| fumarylacetoacetate hydrolase family protein, contains Pfam domain, PF01557: fumarylacetoacetate hydrolase family protein \| chr4:9038304-9040758 FORWARD \| Aliases: DL4011W, FCAALL.203 | 9.5 | 7.9 | 1.6 | 4.5 | 1.58% | 3.9 |
| 5139 | AT1G17130.1 \| cell cycle control protein-related, contains similarity to Swiss-Prot:Q9P7C5 cell cycle control protein cwf16 (Schizosaccharomyces pombe) \| chr1:5854141-5856968 FORWARD \| Aliases: F20D23.18, F20D23_18 | 6.8 | 5.1 | 1.8 | 4.5 | 1.58% | 4.0 |

| Rank | Description | Sync | Root | M | t | adj.q | B |
|---|---|---|---|---|---|---|---|
| 5140 | AT1G45474.2 \| Symbol: LHCA5 \| chlorophyll A-B binding protein, putative (LHCA5), identical to Lhca5 protein (Arabidopsis thaliana) GI:4741942; contains Pfam profile: PF00504 chlorophyll A-B binding protein; similar to light-harvesting complex protein GI:22752 from (Pinus sylvestris) \| chr1:17181742-17183246 FORWARD \| Aliases: F2G19.4, F2G19_4 | 6.3 | 4.6 | 1.7 | 4.5 | 1.58% | 4.0 |
| 5145 | AT5G02280.1 \| synbindin, putative, similar to Swiss-Prot:Q9ES56 synbindin (TRS23 homolog) (Mus musculus) \| chr5:469324-470792 FORWARD \| Aliases: T1E22.40, T1E22_40 | 7.7 | 6.4 | 1.3 | 4.5 | 1.58% | 4.0 |
| 5148 | AT1G79460.1 \| Symbol: GA2 \| ent-kaurene synthase / ent-kaurene synthetase B (KS) (GA2), identical to GI:3056725 (PMID:9536043); formerly called ent-kaurene synthetase B \| chr1:29895285-29899428 FORWARD \| Aliases: ATKS, ENT KAURENE SYNTHASE, GA REQUIRING 2, KS, T8K14.12, T8K14_12 | 5.3 | 4.5 | 0.8 | 4.5 | 1.58% | 3.8 |
| 5150 | AT2G47640.3 \| small nuclear ribonucleoprotein D2, putative / snRNP core protein D2, putative / Sm protein D2, putative, similar to small nuclear ribonucleoprotein Sm D2 (snRNP core protein D2) (Sm-D2) (Mus musculus) SWISS-PROT:P43330 \| chr2:19544275-19545669 FORWARD \| Aliases: F17A22.3 | 10.2 | 9.2 | 1.0 | 4.5 | 1.59% | 3.8 |
| 5151 | ATCG01090.1 \| Symbol: NDHI \| Encodes subunit of the chloroplast NAD(P)H dehydrogenase complex | 8.8 | 7.1 | 1.7 | 4.5 | 1.59% | 4.0 |
| 5152 | AT1G06130.2 \| hydroxyacylglutathione hydrolase, putative / glyoxalase II, putative, similar to glyoxalase II isozyme GB:AAC49865 GI:2570338 from (Arabidopsis thaliana) \| chr1:1857766-1860697 REVERSE \| Aliases: T21E18.18, T21E18_18 | 5.1 | 3.5 | 1.6 | 4.5 | 1.59% | 4.0 |
| 5153 | AT4G01710.1 \| Symbol: CRK \| actin polymerization factor protein-related, similar to human ARP2/3 complex 16 kd subunit, GenBank accession number O15511 likely functions to control the polymerization of actin \| chr4:735753-736604 FORWARD \| Aliases: ARPC5, CROOKED, T15B16.22, T15B16_22 | 6.9 | 5.4 | 1.6 | 4.5 | 1.59% | 4.0 |
| 5154 | AT4G33520.3 \| Symbol: PAA1 \| metal-transporting P-type ATPase, putative (PAA1), nearly identical to gi:2668492; contains Pfam heavy-metal-associated domain PF00403 \| chr4:16118899-16126153 FORWARD \| Aliases: F17M5.280, F17M5_280 | 6.7 | 5.7 | 1.0 | 4.5 | 1.59% | 3.9 |
| 5155 | AT1G69200.1 \| pfkB-type carbohydrate kinase family protein, contains Pfam profile: PF00294 pfkB family carbohydrate kinase \| chr1:26019687-26022528 FORWARD \| Aliases: F4N2.16, F4N2_16 | 4.0 | 2.8 | 1.2 | 4.5 | 1.59% | 4.0 |
| 5156 | AT5G60250.1 \| zinc finger (C3HC4-type RING finger) family protein, contains a Prosite:PS00518 Zinc finger, C3HC4 type (RING finger), signature and Pfam domain PF01485: IBR domain \| chr5:24269384-24272066 FORWARD \| Aliases: F15L12.13, F15L12_13 | 4.0 | 3.2 | 0.8 | 4.5 | 1.59% | 3.8 |
| 5157 | AT3G15970.1 \| Ran-binding protein 1 domain-containing protein / RanBP1 domain-containing protein, similar to Ran binding protein (Homo sapiens) GI:624232; contains Pfam profile PF00638: RanBP1 domain \| chr3:5408803-5411114 REVERSE \| Aliases: MSL1.1 | 6.5 | 5.9 | 0.6 | 4.5 | 1.60% | 3.7 |
| 5158 | AT4G28660.1 \| photosystem II reaction centre W (PsbW) family protein, contains Pfam profile: PF03912 photosystem II reaction centre W protein, PsbW \| chr4:14149922-14151103 FORWARD \| Aliases: T5F17.110, T5F17_110 | 6.0 | 3.9 | 2.1 | 4.5 | 1.60% | 4.0 |
| 5159 | AT1G09910.1 \| expressed protein \| chr1:3220037-3224543 REVERSE \| Aliases: F21M12.30, F21M12_30 | 7.2 | 6.2 | 1.0 | 4.5 | 1.60% | 3.9 |
| 5164 | AT3G27230.1 \| expressed protein \| chr3:10055166-10056928 FORWARD \| Aliases: K17E12.5 | 7.7 | 6.1 | 1.6 | 4.5 | 1.60% | 4.0 |
| 5165 | AT3G57090.1 \| expressed protein \| chr3:21139509-21141167 FORWARD \| Aliases: F24I3.170 | 9.0 | 7.4 | 1.6 | 4.5 | 1.60% | 3.9 |
| 5166 | AT2G27020.1 \| Symbol: PAG1 \| 20S proteasome alpha subunit G (PAG1) (PRC8), identical to proteasome subunit alpha type 3 SP:O23715, GI:12644056 from (Arabidopsis thaliana); identical to cDNA proteasome subunit prc8 GI:2511591 \| chr2:11535437-11538054 REVERSE \| Aliases: 20S PROTEASOME SUBUNIT PAG1, T20P8.7, T20P8_7 | 10.7 | 7.8 | 2.9 | 4.5 | 1.60% | 3.9 |
| 5168 | AT3G14450.1 \| Symbol: CID9 \| RNA-binding protein, putative, contains Pfam profile: PF00076 RNA recognition motif. (a.k.a. RRM, RBD, or RNP domain) (2 copies). Contains PAM PABC binding domain. \| chr3:4849795-4851608 FORWARD \| Aliases: CID9, MOA2.5 | 3.6 | 3.1 | 0.6 | 4.5 | 1.60% | 3.6 |
| 5171 | AT5G13850.1 \| nascent polypeptide-associated complex (NAC) domain-containing protein, similar to alpha-NAC, non-muscle form (Mus musculus) GI:1666690; contains Pfam profile PF01849: NAC domain \| chr5:4471315-4472857 FORWARD \| Aliases: MAC12.19, MAC12_19 | 8.4 | 7.0 | 1.5 | 4.5 | 1.61% | 4.0 |
| 5173 | AT2G25920.1 \| expressed protein \| chr2:11061369-11063120 REVERSE \| Aliases: F17H15.5, F17H15_5 | 5.2 | 4.2 | 1.0 | 4.5 | 1.61% | 3.9 |
| 5175 | AT5G67440.1 \| phototropic-responsive NPH3 family protein, contains NPH3 family domain, Pfam:PF03000 \| chr5:26929933-26933306 REVERSE \| Aliases: K8K14.18, K8K14_18 | 5.0 | 4.1 | 0.9 | 4.5 | 1.61% | 3.8 |
| 5177 | AT1G35160.1 \| Symbol: GRF4 \| 14-3-3 protein GF14 phi (GRF4), identical to GF14 protein phi chain GI:1493805, SP:P46077 from (Arabidopsis thaliana) \| chr1:12867159-12868771 FORWARD \| Aliases: GF14 PHI, GF14 PROTEIN PHI CHAIN, GRF4, T32G9.30, T32G9_30 | 11.3 | 6.7 | 4.6 | 4.5 | 1.61% | 4.0 |
| 5178 | AT4G09000.1 \| Symbol: GRF1 \| 14-3-3-like protein GF14 chi / general regulatory factor 1 (GRF1), identical to 14-3-3 protein GF14 chi chain GI:1702986, SP:P42643 from (Arabidopsis thaliana) | 11.3 | 6.7 | 4.6 | 4.5 | 1.61% | 4.0 |

| Rank | Description | Sync | Root | M | t | adj.q | B |
|---|---|---|---|---|---|---|---|
| 5180 | AT2G03670.1 \| Symbol: CDC48B \| | 4.3 | 2.9 | 1.4 | 4.5 | 1.61% | 3.9 |
| 5183 | AT3G60380.1 \| expressed protein \| chr3:22327888-22330241 REVERSE \| Aliases: T8B10.40 | 7.1 | 5.7 | 1.5 | 4.5 | 1.61% | 3.9 |
| 5185 | AT1G24290.1 \| AAA-type ATPase family protein, similar to Werner helicase interacting protein (Homo sapiens) GI:14349166; contains Pfam profiles PF00004: ATPase family associated with various cellular activities (AAA), PF00627: UBA/TS-N domain; contains ATP/GTP-binding site motif A (P-loop) \| chr1:8612261-8614266 REVERSE \| Aliases: F3I6.23, F3I6_23 | 3.2 | 2.5 | 0.7 | 4.5 | 1.61% | 3.7 |
| 5187 | AT3G60820.2 \| Symbol: PBF1 \| similar to 20S proteasome beta subunit C1 (PBC1) (PRCT) [Arabidopsis thaliana] (TAIR:At1g21720.1); similar to beta 6 subunit of 20S proteasome [Oryza sativa (japonica cultivar-group)] (GB:BAA28276.1); contains InterPro domain Proteasome B-type subunit (InterPro:IPR000243); contains InterPro domain Multispecific proteasome protease (InterPro:IPR001353) \| chr3:22482730-22484904 REVERSE \| Aliases: 20S PROTEASOME BETA SUBUNIT PBF1, T4C21.230 | 10.3 | 8.2 | 2.1 | 4.5 | 1.61% | 4.0 |
| 5189 | AT3G05500.1 \| rubber elongation factor (REF) family protein, contains Pfam profile: PF05755 rubber elongation factor protein (REF) \| chr3:1593440-1595021 FORWARD \| Aliases: F22F7.5, F22F7_5 | 8.2 | 6.2 | 2.0 | 4.5 | 1.61% | 3.9 |
| 5191 | AT1G56560.1 \| beta-fructofuranosidase, putative / invertase, putative / saccharase, putative / beta-fructosidase, putative, similar to neutral invertase (Daucus carota) GI:4200165; contains Pfam profile PF04853: Plant neutral invertase \| chr1:21196164-21198777 FORWARD \| Aliases: F25P12.99, F25P12_99 | 7.2 | 5.8 | 1.4 | 4.5 | 1.62% | 4.0 |
| 5193 | AT2G39050.1 \| hydroxyproline-rich glycoprotein family protein, contains QXW lectin repeat domain, Pfam:PF00652 | 7.3 | 6.0 | 1.3 | 4.5 | 1.62% | 4.0 |
| 5194 | AT2G22240.2 \| inositol-3-phosphate synthase isozyme 2 / myo-inositol-1-phosphate synthase 2 / MI-1-P synthase 2 / IPS 2, identical to SP:Q38862 Myo-inositol-1-phosphate synthase isozyme 2 (EC 5.5.1.4) (MI-1-P synthase 2) (IPS 2) {Arabidopsis thaliana} \| chr2:9458255-9461159 REVERSE \| Aliases: T26C19.10, T26C19_10 | 5.3 | 3.8 | 1.5 | 4.5 | 1.62% | 4.0 |
| 5197 | AT5G06480.1 \| MD-2-related lipid recognition domain-containing protein / ML domain-containing protein, contains Pfam profile PF02221: ML domain \| chr5:1976087-1977291 REVERSE \| Aliases: F15M7.1, F15M7_1 | 3.5 | 2.7 | 0.8 | 4.5 | 1.62% | 3.8 |
| 5198 | AT3G57560.1 \| aspartate/glutamate/uridylate kinase family protein, similar to acetylglutamate kinase from Porphyra purpurea (SP:P31595), Campylobacter jejuni (GI:6650364) contains Pfam profile PF00696: Amino acid kinase family \| chr3:21321802-21323289 REVERSE \| Aliases: T8H10.160 | 6.7 | 5.1 | 1.7 | 4.5 | 1.62% | 4.0 |
| 5199 | AT2G18030.2 \| peptide methionine sulfoxide reductase family protein, similar to SP:P08761 Ecdysone-induced protein 28/29 kDa {Drosophila melanogaster}; contains Pfam profile PF01625: Peptide methionine sulfoxide reductase \| chr2:7847149-7848736 FORWARD \| Aliases: T27K22.10, T27K22_10 | 6.4 | 4.7 | 1.7 | 4.5 | 1.62% | 4.0 |
| 5201 | AT5G04590.1 \| Symbol: SIR \| sulfite reductase / ferredoxin (SIR), identical to sulfite reductase (Arabidopsis thaliana) GI:804953, GI:2584721 | 9.0 | 6.6 | 2.4 | 4.5 | 1.62% | 4.0 |
| 5202 | AT5G56580.1 \| Symbol: ATMKK6 \| | 4.2 | 3.0 | 1.2 | 4.5 | 1.62% | 3.9 |
| 5203 | AT3G62000.1 \| O-methyltransferase family 3 protein, several O-methyltransferases - different species; contains Pfam 01596 O-methyltransferase domain \| chr3:22970176-22972184 REVERSE \| Aliases: F21F14.170 | 5.6 | 4.6 | 0.9 | 4.5 | 1.62% | 4.0 |
| 5204 | AT2G31320.1 \| poly (ADP-ribose) polymerase, putative / NAD(+) ADP-ribosyltransferase, putative / poly(ADP-ribose) synthetase, putative, similar to poly(ADP)-ribose polymerase (Zea mays) GI:3928871 ; contains Pfam profiles PF00644: Poly(ADP-ribose) polymerase catalytic domain, PF00645: Poly(ADP-ribose) polymerase and DNA-Ligase Zn-finger region, PF02877: Poly(ADP-ribose) polymerase, regulatory domain, PF00533: BRCA1 C Terminus (BRCT) domain \| chr2:13360898-13366805 REVERSE \| Aliases: F16D14.16, F16D14_16 | 5.4 | 4.3 | 1.1 | 4.5 | 1.62% | 4.0 |
| 5208 | AT3G23805.1 \| Symbol: RALFL24 \| rapid alkalinization factor (RALF) family protein, similar to RALF precursor (Nicotiana tabacum) GI:16566316 \| chr3:8586389-8587063 FORWARD \| Aliases: RALF LIKE 24 | 6.1 | 4.9 | 1.2 | 4.4 | 1.63% | 3.9 |
| 5210 | AT5G51960.1 \| expressed protein \| chr5:21127929-21128574 FORWARD \| Aliases: MSG15.4, MSG15_4 | 6.3 | 4.6 | 1.6 | 4.4 | 1.63% | 3.9 |
| 5212 | AT1G47720.1 \| expressed protein, contains Pfam PF05329: Protein of unknown function (DUF731) \| chr1:17563795-17565323 REVERSE \| Aliases: T2E6.21 | 5.6 | 4.4 | 1.2 | 4.4 | 1.64% | 4.0 |
| 5213 | AT3G52940.2 \| Symbol: FK \| C-14 sterol reductase / delta(14)-sterol reductase / FACKEL (FK), identical to gi:9082182 \| chr3:19641176-19644261 REVERSE \| Aliases: ELL1, EXTRA LONG LIFESPAN 1, F8J2.111, FACKEL, HYD2 | 6.9 | 5.8 | 1.2 | 4.4 | 1.64% | 3.9 |
| 5215 | AT1G14830.1 \| Symbol: ADL1C \| dynamin-like protein C (DL1C), nearly identical to dynamin-like protein C (Arabidopsis thaliana) GI:19569772 \| chr1:5107416-5111665 REVERSE \| Aliases: ADL1C, ADL5, DYNAMIN LIKE PROTEIN 5, F10B6.23, F10B6_23 | 8.6 | 6.4 | 2.2 | 4.4 | 1.64% | 4.0 |

| Rank | Description | Sync | Root | M | t | adj.q | B |
|---|---|---|---|---|---|---|---|
| 5218 | AT5G63750.1 \| IBR domain-containing protein, contains a Prosite:PS00518 Zinc finger, C3HC4 type (RING finger), signature and Pfam:PF01485 IBR domain \| chr5:25530220-25532164 REVERSE \| Aliases: MBK5.23, MBK5_23 | 4.9 | 3.9 | 1.0 | 4.4 | 1.65% | 3.9 |
| 5219 | AT2G36485.1 \| expressed protein \| chr2:15314035-15314869 REVERSE \| Aliases: None | 3.9 | 2.8 | 1.1 | 4.4 | 1.65% | 3.9 |
| 5221 | AT3G12120.1 \| Symbol: FAD2 \| omega-6 fatty acid desaturase, endoplasmic reticulum (FAD2) / delta-12 desaturase, identical to omega-6 fatty acid desaturase, endoplasmic reticulum (FAD2) SP:P46313 (Arabidopsis thaliana (Mouse-ear cress)) (Plant Cell 6:147-158(1994)) \| chr3:3860291-3863036 REVERSE \| Aliases: DELTA 12 DESATURASE, FATTY ACID DESATURASE 2, T21B14.6 | 11.7 | 9.6 | 2.2 | 4.4 | 1.65% | 3.6 |
| 5223 | AT2G45440.1 \| Symbol: DHDPS2 \| | 7.7 | 4.7 | 3.1 | 4.4 | 1.65% | 3.9 |
| 5224 | AT4G29070.2 \| expressed protein \| chr4:14321895-14323313 FORWARD \| Aliases: F19B15.100, F19B15_100 | 6.2 | 5.0 | 1.2 | 4.4 | 1.65% | 4.0 |
| 5225 | AT5G55210.1 \| expressed protein, similar to unknown protein (pir::T04913) \| chr5:22413756-22414614 REVERSE \| Aliases: MCO15.16, MCO15_16 | 4.4 | 3.2 | 1.2 | 4.4 | 1.65% | 3.9 |
| 5226 | AT5G16120.1 \| hydrolase, alpha/beta fold family protein, similar to monoglyceride lipase from (Homo sapiens) GI:14594904, (Mus musculus) GI:2632162; contains Pfam profile PF00561: hydrolase, alpha/beta fold family \| chr5:5265957-5267982 FORWARD \| Aliases: T21H19.40, T21H19_40 | 7.7 | 6.8 | 0.8 | 4.4 | 1.65% | 3.8 |
| 5227 | AT3G13180.1 \| NOL1/NOP2/sun family protein / antitermination NusB domain-containing protein, low similarity to SP:P36929 SUN protein (FMU protein) {Escherichia coli}; contains Pfam profiles PF01189: NOL1/NOP2/sun family, PF01029: NusB family \| chr3:4236024-4240016 REVERSE \| Aliases: MJG19.14 | 4.4 | 3.6 | 0.9 | 4.4 | 1.65% | 3.9 |
| 5231 | AT5G38730.1 \| pentatricopeptide (PPR) repeat-containing protein, contains Pfam profile PF01535: PPR repeat \| chr5:15528131-15529921 FORWARD \| Aliases: MKD10.5, MKD10_5 | 5.0 | 4.0 | 1.0 | 4.4 | 1.66% | 3.8 |
| 5232 | AT4G35650.1 \| isocitrate dehydrogenase, putative / NAD+ isocitrate dehydrogenase, putative, strong similarity to NAD+ dependent isocitrate dehydrogenase subunit 1 (Arabidopsis thaliana) GI:1766046 \| chr4:16908524-16910195 FORWARD \| Aliases: F8D20.160, F8D20_160 | 6.2 | 3.9 | 2.3 | 4.4 | 1.66% | 3.9 |
| 5233 | AT2G34710.1 \| Symbol: PHB \| homeobox-leucine zipper transcription factor (HB-14), identical to homeodomain transcription factor (ATHB-14)GP:3132474 GB:Y11122 (Arabidopsis thaliana); | 6.8 | 5.9 | 0.9 | 4.4 | 1.66% | 3.9 |
| 5234 | AT3G02920.1 \| replication protein-related, similar to replication protein A 30kDa (Oryza sativa (japonica cultivar-group)) GI:13516746; contains InterPro entry IPR004365: OB-fold nucleic acid binding domain | 6.4 | 4.9 | 1.5 | 4.4 | 1.66% | 3.9 |
| 5238 | AT5G10620.1 \| expressed protein, contains Pfam profile PF02590: Uncharacterized ACR, COG1576 \| chr5:3355467-3356616 REVERSE \| Aliases: F12B17.30, F12B17_30 | 5.1 | 3.7 | 1.3 | 4.4 | 1.67% | 4.0 |
| 5240 | AT4G10620.1 \| expressed protein \| chr4:6564275-6566500 FORWARD \| Aliases: T4F9.80, T4F9_80 | 6.9 | 5.9 | 1.0 | 4.4 | 1.67% | 3.9 |
| 5241 | AT1G02560.1 \| Symbol: CLPP5 \| ATP-dependent Clp protease proteolytic subunit (ClpP1), identical to nClpP1 GB:BAA82065 GI:5360579 from (Arabidopsis thaliana); contains Pfam profile PF00574: Clp protease; contains TIGRfam profile TIGR00493: ATP-dependent Clp protease, proteolytic subunit ClpP \| chr1:537888-540109 FORWARD \| Aliases: CLP PROTEASE 1 PROTEOLYTIC SUBUNIT, NCLPP1, NCLPP5, NUCLEAR ENCODED CLP PROTEASE 1, T14P4.12, T14P4_12 | 9.4 | 7.2 | 2.2 | 4.4 | 1.67% | 3.9 |
| 5242 | AT1G01380.1 \| Symbol: ETC1 \| myb family transcription factor, similar to myb homolog (CPC) GI:2346966 from (Arabidopsis thaliana) \| chr1:147267-147935 FORWARD \| Aliases: ENHANCER OF TRY AND CPC 1, F6F3.18, F6F3_18 | 5.8 | 4.6 | 1.2 | 4.4 | 1.67% | 3.9 |
| 5243 | AT1G77480.2 \| nucellin protein, putative, similar to nucellin GB:AAB96882 GI:2290202 (Hordeum vulgare) (nucellin: similar to aspartic protease and its specific expression in nucellar cells during degeneration) \| chr1:29119452-29122232 REVERSE \| Aliases: T5M16.7, T5M16_7 | 5.1 | 3.9 | 1.2 | 4.4 | 1.67% | 3.8 |
| 5244 | AT4G05330.1 \| zinc finger and C2 domain protein, putative, similar to zinc finger and C2 domain protein GI:9957238 from (Arabidopsis thaliana) | 4.8 | 3.6 | 1.2 | 4.4 | 1.67% | 4.0 |
| 5245 | AT5G43640.1 \| 40S ribosomal protein S15 (RPS15E) \| chr5:17548529-17549597 FORWARD \| Aliases: K9D7.14, K9D7_14 | 3.0 | 2.7 | 0.3 | 4.4 | 1.67% | 3.1 |
| 5246 | AT4G33905.1 \| peroxisomal membrane protein 22 kDa, putative, similar to 22 kDa peroxisomal membrane protein PMP22 (Mus musculus) gi:10954089:gb:AAG25724 \| chr4:16253820-16255617 REVERSE \| Aliases: None | 3.5 | 2.8 | 0.7 | 4.4 | 1.67% | 3.8 |
| 5248 | AT5G50460.1 \| protein transport protein SEC61 gamma subunit, putative, similar to Swiss-Prot:Q19967 protein transport protein SEC61 gamma subunit (Caenorhabditis elegans) \| chr5:20568663-20569792 REVERSE \| Aliases: MBA10.8 | 10.0 | 7.9 | 2.1 | 4.4 | 1.67% | 3.9 |
| 5249 | AT4G21865.1 \| expressed protein \| chr4:11601917-11603287 FORWARD \| Aliases: None | 5.7 | 3.6 | 2.1 | 4.4 | 1.67% | 3.7 |

| Rank | Description | Sync | Root | M | t | adj.q | B |
|---|---|---|---|---|---|---|---|
| 5251 | AT5G51100.1 \| Symbol: FSD2 \| superoxide dismutase (Fe), putative / iron superoxide dismutase, putative, similar to Fe-superoxide dismutase precursor (Medicago sativa) gi:16974682:gb:AAL32441 \| chr5:20790522-20792898 REVERSE \| Aliases: FE SUPEROXIDE DISMUTASE 2, MWD22.4, MWD22_4 | 4.5 | 3.0 | 1.4 | 4.4 | 1.68% | 3.9 |
| 5254 | AT5G61770.3 \| brix domain-containing protein, contains Pfam domain, PF04427: Brix domain \| chr5:24832863-24834869 FORWARD \| Aliases: MAC9.8, MAC9_8 | 5.8 | 4.2 | 1.6 | 4.4 | 1.68% | 4.0 |
| 5255 | AT2G29360.1 \| tropinone reductase, putative / tropine dehydrogenase, putative, similar to tropinone reductase SP:P50165 from (Datura stramonium) \| chr2:12610902-12612312 FORWARD \| Aliases: F16P2.26, F16P2_26 | 3.4 | 2.6 | 0.7 | 4.4 | 1.68% | 3.7 |
| 5256 | AT3G02660.1 \| tRNA synthetase class I (W and Y) family protein, similar to SP:P00952 Tyrosyl-tRNA synthetase (EC 6.1.1.1) (Tyrosine--tRNA ligase) (TyrRS) {Bacillus stearothermophilus}; contains Pfam profiles PF00579: tRNA synthetases class I (W and Y), PF01479: S4 domain \| chr3:570153-572341 REVERSE \| Aliases: F16B3.29, F16B3_29 | 4.7 | 3.8 | 0.9 | 4.4 | 1.68% | 3.8 |
| 5257 | AT1G15710.1 \| prephenate dehydrogenase family protein, contains Pfam profile: PF02153 prephenate dehydrogenase | 7.1 | 4.3 | 2.7 | 4.4 | 1.68% | 3.9 |
| 5258 | AT3G51260.2 \| Symbol: PAD1 \| similar to 20S proteasome alpha subunit D2 (PAD2) (PRS1) (PRC6) [Arabidopsis thaliana] (TAIR:At5g66140.1); similar to proteasome alpha subunit [Oryza sativa (japonica cultivar-group)] (GB:XP_483663.1); similar to proteasome alpha subunit [Lycopersicon esculentum] (GB:CAA74725.1); contains InterPro domain Proteasome subunit, A-type (InterPro:IPR000426); contains InterPro domain Multispecific proteasome protease (InterPro:IPR001353) \| chr3:19041974-19044043 FORWARD \| Aliases: 20S PROTEASOME ALPHA SUBUNIT PAD1, F24M12.300 | 9.2 | 6.2 | 3.0 | 4.4 | 1.68% | 3.9 |
| 5259 | AT1G45000.1 \| 26S proteasome regulatory complex subunit p42D, putative, similar to 26S proteasome regulatory complex subunit p42D (Drosophila melanogaster) gi:6434958:gb:AAF08391 \| chr1:17011584-17014326 FORWARD \| Aliases: F27F5.8, F27F5_8 | 10.3 | 9.4 | 1.0 | 4.4 | 1.68% | 3.8 |
| 5260 | AT1G67660.1 \| expressed protein \| chr1:25367198-25368892 FORWARD \| Aliases: F12A21.19, F12A21_19 | 5.1 | 4.1 | 1.0 | 4.4 | 1.68% | 3.9 |
| 5262 | AT4G26760.1 \| microtubule associated protein (MAP65/ASE1) family protein, low similarity to protein regulating cytokinesis 1 (PRC1) (Homo sapiens) GI:2865521; contains Pfam profile PF03999: Microtubule associated protein (MAP65/ASE1 family) \| chr4:13478598-13481757 REVERSE \| Aliases: F10M23.100, F10M23_100 | 4.4 | 3.2 | 1.2 | 4.4 | 1.68% | 3.8 |
| 5263 | AT4G16510.1 \| YbaK/prolyl-tRNA synthetase-related, contains weak hit to Pfam profile PF04073: YbaK/prolyl-tRNA synthetases associated domain \| chr4:9302821-9305070 FORWARD \| Aliases: DL4280W, FCAALL.85 | 5.1 | 4.4 | 0.7 | 4.4 | 1.68% | 3.7 |
| 5264 | AT1G55170.1 \| expressed protein \| chr1:20583990-20585642 FORWARD \| Aliases: T7N22.12, T7N22_12 | 6.8 | 5.6 | 1.2 | 4.4 | 1.68% | 3.9 |
| 5265 | AT3G25520.1 \| Symbol: ATL5 \| 60S ribosomal protein L5, similar to 60S ribosomal protein L5 GB:P49625 from (Oryza sativa) \| chr3:9270524-9272626 REVERSE \| Aliases: A. THALIANA RIBOSOMAL PROTEIN L5, MWL2.17, RIBOSOMAL PROTEIN L5 | 10.1 | 7.7 | 2.4 | 4.4 | 1.68% | 4.0 |
| 5266 | AT1G71450.1 \| encodes a member of the DREB subfamily A-4 of ERF/AP2 transcription factor family. The protein contains one AP2 domain. There are 17 members in this subfamily including TINY. \| chr1:26930750-26931618 FORWARD \| Aliases: F26A9.17 | 3.1 | 2.5 | 0.6 | 4.4 | 1.68% | 3.7 |
| 5267 | AT1G10660.4 \| expressed protein \| chr1:3532474-3535132 FORWARD \| Aliases: F20B24.10, F20B24_10 | 6.8 | 4.5 | 2.2 | 4.4 | 1.69% | 3.9 |
| 5269 | AT2G43550.1 \| Encodes a defensin-like (DEFL) family protein. \| chr2:18080311-18080982 FORWARD \| Aliases: T1O24.29 | 4.2 | 3.2 | 1.0 | 4.4 | 1.69% | 3.9 |
| 5270 | AT1G65650.1 \| ubiquitin carboxyl-terminal hydrolase family 1 protein, similar to 26S proteasome regulatory complex subunit p37A (Drosophila melanogaster) GI:6434962; contains Pfam profile PF01088: Ubiquitin carboxyl-terminal hydrolase, family 1 \| chr1:24418678-24421372 REVERSE \| Aliases: F1E22.3 | 8.2 | 6.1 | 2.0 | 4.4 | 1.69% | 3.9 |
| 5272 | AT1G47200.1 \| MFP1 attachment factor, putative, contains similarity to MFP1 attachment factor 1 GI:7546725 from (Lycopersicon esculentum) similar to MFP1 attachment factor 1 (Glycine max) gi:7546729:gb:AAF63659 \| chr1:17300471-17301257 REVERSE \| Aliases: F2G19.18, F2G19_18 | 5.0 | 3.6 | 1.5 | 4.4 | 1.69% | 3.9 |
| 5273 | AT2G17270.1 \| mitochondrial substrate carrier family protein, contains Pfam profile: PF00153 mitochondrial carrier protein \| chr2:7517182-7519469 FORWARD \| Aliases: F5J6.3, F5J6_3 | 4.9 | 3.8 | 1.1 | 4.4 | 1.69% | 3.9 |
| 5274 | AT5G55050.1 \| GDSL-motif lipase/hydrolase family protein, similar to family II lipases EXL3 GI:15054386, EXL1 GI:15054382, EXL2 GI:15054384 from (Arabidopsis thaliana); contains Pfam profile PF00657: GDSL-like Lipase/Acylhydrolase | 5.0 | 3.6 | 1.4 | 4.4 | 1.69% | 3.9 |
| 5275 | AT5G47600.1 \| heat shock protein-related, contains weak similarity to Heat shock 22 kDa protein, mitochondrial precursor (Swiss-Prot:P46254) (Pisum sativum) \| chr5:19317008-19317703 REVERSE \| Aliases: MNJ7.19, MNJ7_19 | 3.9 | 2.3 | 1.6 | 4.4 | 1.69% | 3.9 |

| Rank | Description | Sync | Root | M | t | adj.q | B |
|---|---|---|---|---|---|---|---|
| 5278 | AT5G50100.1 \| expressed protein, contains Pfam domain PF04134: Protein of unknown function, DUF393 \| chr5:20389079-20390667 FORWARD \| Aliases: MPF21.11, MPF21_11 | 5.8 | 4.7 | 1.0 | 4.4 | 1.70% | 3.9 |
| 5279 | AT5G53920.1 \| ribosomal protein L11 methyltransferase-related, similar to ribosomal protein L11 methyltransferase; PrmA (Escherichia coli) GI:455655 \| chr5:21909350-21911493 FORWARD \| Aliases: K19P17.9, K19P17_9 | 2.8 | 2.3 | 0.5 | 4.4 | 1.71% | 3.4 |
| 5280 | AT3G56710.1 \| Symbol: SIB1 \| sigA-binding protein, identical to SigA binding protein (Arabidopsis thaliana) gi:6980074:gb:AAF34713; contains Pfam PF05678: VQ motif \| chr3:21017729-21018544 REVERSE \| Aliases: SIGMA FACTOR BINDING PROTEIN 1, T8M16.40 | 4.1 | 3.2 | 0.9 | 4.4 | 1.71% | 3.9 |
| 5281 | AT1G20330.1 \| Symbol: SMT2 \| S-adenosyl-methionine-sterol-C-methyltransferase, identical to sterol-C-methyltransferase GI:1061040 from (Arabidopsis thaliana) | 7.0 | 5.1 | 1.9 | 4.4 | 1.71% | 3.9 |
| 5286 | AT1G65260.1 \| PspA/IM30 family protein, contains Pfam PF04012: PspA/IM30 family profile; similar to Membrane-associated 30 kDa protein, chloroplast precursor (M30) (Swiss-Prot:Q03943) (Pisum sativum); similar to phage shock protein A (GI:28806161) (Vibrio parahaemolyticus); similar to Phage shock protein A. (Swiss-Prot:P23853) (Shigella flexneri) \| chr1:24239923-24244322 FORWARD \| Aliases: T8F5.2, T8F5_2 | 7.5 | 5.4 | 2.1 | 4.4 | 1.72% | 3.9 |
| 5287 | AT1G32190.1 \| expressed protein \| chr1:11592935-11595962 REVERSE \| Aliases: F3C3.3, F3C3_3 | 4.4 | 3.7 | 0.7 | 4.4 | 1.72% | 3.7 |
| 5289 | AT5G25470.2 \| expressed protein \| chr5:8865732-8867688 REVERSE \| Aliases: T14C9.20, T14C9_20 | 5.0 | 3.5 | 1.5 | 4.4 | 1.72% | 3.9 |
| 5290 | AT5G14500.1 \| aldose 1-epimerase family protein, similar to apospory-associated protein C, Chlamydomonas reinhardtii, EMBL:AF195243 Pfam profile PF01263: Aldose 1-epimerase \| chr5:4674236-4676768 REVERSE \| Aliases: T15N1.5 | 4.7 | 3.6 | 1.1 | 4.4 | 1.72% | 3.9 |
| 5291 | AT5G57390.1 \| similar to ovule development protein, putative [Arabidopsis thaliana] (TAIR:At1g51190.1); similar to OSJNBb0022F16.3 [Oryza sativa (japonica cultivar-group)] (GB:XP_474162.1); contains InterPro domain Pathogenesis-related transcriptional factor and ERF (InterPro:IPR001471) \| chr5:23270685-23273676 REVERSE \| Aliases: MSF19.5, MSF19_5 | 5.0 | 3.9 | 1.1 | 4.4 | 1.72% | 3.9 |
| 5292 | AT3G02950.1 \| expressed protein \| chr3:665365-666910 REVERSE \| Aliases: F13E7.10, F13E7_10 | 4.5 | 3.3 | 1.2 | 4.4 | 1.72% | 3.8 |
| 5293 | AT2G18940.1 \| pentatricopeptide (PPR) repeat-containing protein, contains Pfam profile: PF01535 PPR repeat \| chr2:8210812-8213477 REVERSE \| Aliases: F19F24.14, F19F24_14 | 5.1 | 3.8 | 1.4 | 4.4 | 1.72% | 3.9 |
| 5296 | AT2G05840.2 \| Symbol: PAA2 \| similar to 20S proteasome alpha subunit A1 (PAA1) (PRC1) [Arabidopsis thaliana] (TAIR:At5g35590.1); similar to proteasome IOTA subunit [Glycine max] (GB:AAC28135.1); contains InterPro domain Proteasome subunit, A-type (InterPro:IPR000426); contains InterPro domain Multispecific proteasome protease (InterPro:IPR001353) \| chr2:2234089-2236287 FORWARD \| Aliases: 20S PROTEASOME SUBUNIT PAA2, T6P5.4, T6P5_4 | 11.3 | 10.4 | 0.9 | 4.4 | 1.72% | 3.4 |
| 5297 | AT5G35590.1 \| Symbol: PAA1 \| 20S proteasome alpha subunit A1 (PAA1) (PRC1), identical to proteasome subunit alpha type 6-1 SP:O81146 GI:12643647 from (Arabidopsis thaliana); identical to cDNA proteasome subunit prc1 GI:2511587 \| chr5:13782400-13785047 REVERSE \| Aliases: 20S PROTEASOME SUBUNIT PAA1, K2K18.4, K2K18_4 | 11.3 | 10.4 | 0.9 | 4.4 | 1.72% | 3.4 |
| 5300 | AT5G42790.1 \| Symbol: PAF1 \| 20S proteasome alpha subunit F1 (PAF1), (gb:AAC32062.1) \| chr5:17176278-17178298 REVERSE \| Aliases: 20S PROTEASOME SUBUNIT PAF1, MJB21.17, MJB21_17, PAF1 | 10.1 | 8.1 | 2.1 | 4.4 | 1.73% | 3.8 |
| 5304 | AT1G69800.1 \| CBS domain-containing protein, low similarity to SP:Q9UGI9 5'-AMP-activated protein kinase, gamma-3 subunit (AMPK gamma-3 chain) (AMPK gamma3) {Homo sapiens}; contains Pfam profile PF00571: CBS domain | 7.2 | 6.3 | 0.9 | 4.4 | 1.73% | 3.8 |
| 5305 | AT5G05740.2 \| peptidase M50 family protein / sterol-regulatory element binding protein (SREBP) site 2 protease family protein, contains Pfam PF02163: Sterol-regulatory element binding protein (SREBP) site 2 protease \| chr5:1723837-1726860 REVERSE \| Aliases: ATEGY2, MJJ3.15, MJJ3_15 | 6.5 | 4.7 | 1.7 | 4.4 | 1.73% | 3.8 |
| 5306 | AT1G57820.2 \| zinc finger (C3HC4-type RING finger) family protein, low similarity to nuclear protein np95 (Mus musculus) GI:4220590; contains Pfam profiles PF02182: YDG/SRA domain, PF00097: Zinc finger, C3HC4 type (RING finger), PF00628: PHD-finger \| chr1:21417876-21421602 REVERSE \| Aliases: F12K22.14, F12K22_14 | 5.6 | 4.3 | 1.3 | 4.4 | 1.73% | 3.9 |
| 5308 | AT3G58680.1 \| ethylene-responsive transcriptional coactivator, putative, similar to ethylene-responsive transcriptional coactivator (Lycopersicon esculentum) gi:5669634:gb:AAD46402 \| chr3:21718253-21719881 FORWARD \| Aliases: T20N10.30, T20N10_30 | 10.5 | 8.9 | 1.6 | 4.4 | 1.73% | 3.7 |
| 5309 | AT5G12140.1 \| Symbol: ATCYS1 \| | 12.0 | 10.8 | 1.1 | 4.4 | 1.73% | 3.6 |
| 5310 | AT4G34740.1 \| Symbol: ATASE2 \| | 4.4 | 3.5 | 0.9 | 4.4 | 1.73% | 3.8 |

| Rank | Description | Sync | Root | M | t | adj.q | B |
|---|---|---|---|---|---|---|---|
| 5311 | AT1G20540.1 \| transducin family protein / WD-40 repeat family protein, contains 6 WD-40 repeats (PF00400); similar to Rbap46 polypeptide (GI:9454362) (Gallus gallus) \| chr1:7112611-7115810 FORWARD \| Aliases: F5M15.14, F5M15_14 | 8.8 | 7.5 | 1.3 | 4.4 | 1.73% | 3.8 |
| 5312 | AT3G04860.1 \| expressed protein \| chr3:1339114-1340629 REVERSE \| Aliases: T9J14.19, T9J14_19 | 5.7 | 4.5 | 1.2 | 4.4 | 1.73% | 3.9 |
| 5313 | AT1G79050.1 \| DNA repair protein recA, identical to DNA repair protein recA, chloroplast (Precursor) SP:Q39199 from (Arabidopsis thaliana) ;contains Pfam profile: PF00154 recA bacterial DNA recombination protein \| chr1:29741854-29745049 REVERSE \| Aliases: YUP8H12R.33, YUP8H12R_33 | 8.7 | 7.0 | 1.7 | 4.4 | 1.73% | 3.9 |
| 5316 | AT4G19130.1 \| replication protein-related, similar to replication protein A 70kDa (Oryza sativa) GI:13536993; contains Pfam profile PF00098: Zinc knuckle \| chr4:10466488-10468071 REVERSE \| Aliases: T18B16.100, T18B16_100 | 4.7 | 3.8 | 1.0 | 4.4 | 1.74% | 3.9 |
| 5317 | AT3G25290.2 \| similar to auxin-responsive protein, putative [Arabidopsis thaliana] (TAIR:At4g12980.1); similar to putative auxin-induced protein [Oryza sativa (japonica cultivar-group)] (GB:XP_483411.1); contains InterPro domain Cytochrome b561 / ferric reductase transmembrane (InterPro:IPR006593); contains InterPro domain Protein of unknown function DUF568 (InterPro:IPR007613); contains InterPro domain DOMON domain (InterPro:IPR005018) \| chr3:9210178-9211824 FORWARD \| Aliases: MJL12.25 | 6.7 | 5.1 | 1.6 | 4.4 | 1.74% | 3.9 |
| 5318 | AT3G53220.1 \| thioredoxin family protein, low similarity to SP:P29451 Thioredoxin (Rhesus macaque) {Macaca mulatta}; contains Pfam profile: PF00085 Thioredoxin \| chr3:19732972-19733680 FORWARD \| Aliases: T4D2.150 | 3.3 | 2.7 | 0.6 | 4.4 | 1.74% | 3.5 |
| 5322 | AT1G09160.2 \| protein phosphatase 2C-related / PP2C-related, similar to GB:AAC16260 \| chr1:2952773-2955606 REVERSE \| Aliases: T12M4.15, T12M4_15 | 6.4 | 5.3 | 1.1 | 4.4 | 1.74% | 3.9 |
| 5323 | AT1G74020.1 \| Symbol: SS2 \| strictosidine synthase family protein, similar to strictosidine synthase (Rauvolfia serpentina)(SP:P15324); contains strictosidine synthase domain PF03088 \| chr1:27838792-27840970 REVERSE \| Aliases: ATSS 2 STRICTOSIDINE SYNTHASE, F2P9.11, F2P9_11, STRICTOSIDINE SYNTHASE 2 | 11.2 | 10.4 | 0.8 | 4.4 | 1.74% | 3.6 |
| 5325 | AT2G35490.1 \| plastid-lipid associated protein PAP, putative, similar to plastid-lipid associated protein PAP3 (Brassica rapa) GI:14248552; contains Pfam profile PF04755: PAP_fibrillin \| chr2:14919223-14920956 REVERSE \| Aliases: T32F12.13, T32F12_13 | 8.3 | 6.8 | 1.5 | 4.4 | 1.74% | 3.9 |
| 5326 | AT5G60730.1 \| anion-transporting ATPase family protein, low similarity to SP:O43681 Arsenical pump-driving ATPase (EC 3.6.3.16) (Arsenite-translocating ATPase) (Arsenical resistance ATPase) (Arsenite-transporting ATPase) (ARSA) (ASNA-I) {Homo sapiens}; contains Pfam profile PF02374: Anion-transporting ATPase \| chr5:24439961-24442718 FORWARD \| Aliases: MUP24.8, MUP24_8 | 3.7 | 3.0 | 0.7 | 4.4 | 1.75% | 3.7 |
| 5327 | AT2G40780.1 \| hypothetical protein \| chr2:17028953-17029826 FORWARD \| Aliases: T7D17.4, T7D17_4 | 4.4 | 3.5 | 0.9 | 4.4 | 1.75% | 3.8 |
| 5328 | AT5G52440.1 \| Symbol: HCF106 \| | 7.0 | 5.3 | 1.7 | 4.4 | 1.75% | 3.9 |
| 5329 | AT1G31920.1 \| pentatricopeptide (PPR) repeat-containing protein, contains Pfam profile PF01535: PPR repeat \| chr1:11461835-11463782 REVERSE \| Aliases: F5M6.8, F5M6_8 | 3.7 | 2.7 | 1.0 | 4.4 | 1.75% | 3.7 |
| 5333 | AT1G18090.2 \| exonuclease, putative, similar to Swiss-Prot:P53695 exonuclease I (EXO I) (Schizosaccharomyces pombe) \| chr1:6224497-6228233 FORWARD \| Aliases: T10F20.10 | 3.8 | 3.0 | 0.8 | 4.4 | 1.76% | 3.8 |
| 5334 | AT1G12280.1 \| disease resistance protein (CC-NBS-LRR class), putative, domain signature CC-NBS-LRR exists, suggestive of a disease resistance protein. | 3.1 | 2.4 | 0.7 | 4.4 | 1.76% | 3.6 |
| 5335 | AT1G80860.2 \| expressed protein \| chr1:30393156-30394194 REVERSE \| Aliases: F23A5.21, F23A5_21 | 7.1 | 6.1 | 1.1 | 4.4 | 1.76% | 3.8 |
| 5340 | AT5G53360.1 \| seven in absentia (SINA) family protein, low similarity to siah-1A protein (Mus musculus) GI:297035; contains Pfam profile PF03145: Seven in absentia protein family \| chr5:21665186-21666801 FORWARD \| Aliases: K19E1.16, K19E1_16 | 8.2 | 7.4 | 0.8 | 4.4 | 1.76% | 3.7 |
| 5344 | AT1G69830.1 \| Symbol: ATAMY3 \| | 7.1 | 5.0 | 2.2 | 4.4 | 1.76% | 3.9 |
| 5345 | AT1G11790.2 \| similar to prephenate dehydratase family protein [Arabidopsis thaliana] (TAIR:At3g07630.2); similar to prephenate dehydratase family protein [Arabidopsis thaliana] (TAIR:At3g07630.1); similar to putative prephenate dehydratase [Oryza sativa (japonica cultivar-group)] (GB:XP_479626.1); contains InterPro domain Prephenate dehydratase (InterPro:IPR001086) \| chr1:3981247-3985214 FORWARD \| Aliases: F25C20.4, F25C20_4 | 6.6 | 5.5 | 1.1 | 4.4 | 1.77% | 3.8 |
| 5347 | AT2G27450.2 \| Symbol: NLP1 \| carbon-nitrogen hydrolase family protein, low similarity to beta-alanine synthase (Drosophila melanogaster) GI:14334063; contains Pfam profile PF00795: hydrolase, carbon-nitrogen family \| chr2:11744510-11746676 REVERSE \| Aliases: ATNLP1, CPA, F10A12.13, F10A12_13, N CARBAMOYLPUTRESCINE AMIDOHYDROLASE, NITRILASE LIKE PROTEIN 1 | 10.6 | 8.7 | 1.9 | 4.4 | 1.77% | 3.7 |

| Rank | Description | Sync | Root | M | t | adj.q | B |
|---|---|---|---|---|---|---|---|
| 5349 | AT2G37020.1 \| translin family protein, similar to SP:Q62348 Translin {Mus musculus}; contains Pfam profile PF01997: Translin family \| chr2:15553609-15556116 FORWARD \| Aliases: T1J8.20, T1J8_20 | 6.0 | 4.3 | 1.8 | 4.4 | 1.77% | 3.9 |
| 5354 | AT1G42480.1 \| expressed protein \| chr1:15938670-15941301 FORWARD \| Aliases: T8D8.4, T8D8_4 | 6.1 | 5.2 | 0.8 | 4.4 | 1.77% | 3.6 |
| 5355 | AT5G51220.1 \| ubiquinol-cytochrome C chaperone family protein, contains Pfam PF03981: Ubiquinol-cytochrome C chaperone | 7.9 | 6.6 | 1.3 | 4.4 | 1.78% | 3.8 |
| 5356 | AT2G47720.1 \| expressed protein \| chr2:19563694-19564209 FORWARD \| Aliases: F17A22.11 | 4.4 | 3.4 | 1.0 | 4.4 | 1.78% | 3.8 |
| 5358 | AT1G26460.1 \| pentatricopeptide (PPR) repeat-containing protein, contains Pfam profile PF01535: PPR repeat \| chr1:9151719-9154727 FORWARD \| Aliases: T1K7.17, T1K7_17 | 7.5 | 4.8 | 2.7 | 4.4 | 1.78% | 3.9 |
| 5360 | AT2G31380.1 \| Symbol: STH \| zinc finger (B-box type) family protein / salt tolerance-like protein (STH), contains Pfam profile PF00643: B-box zinc finger; identical to cDNA B-box zinc finger protein STH GI:12698721, SP:Q9SID1 Salt tolerance-like protein (Arabidopsis thaliana) \| chr2:13389030-13390834 FORWARD \| Aliases: STO HOMOLOG, T28P16.13, T28P16_13 | 3.9 | 2.7 | 1.2 | 4.4 | 1.78% | 3.8 |
| 5365 | AT3G17970.1 \| chloroplast outer membrane translocon subunit, putative, similar to Toc64 (Pisum sativum) GI:7453538; contains Pfam profile PF00515 TPR Domain \| chr3:6147968-6151981 FORWARD \| Aliases: AT3G17960, MEB5.19 | 5.0 | 4.4 | 0.6 | 4.4 | 1.79% | 3.6 |
| 5366 | AT1G68830.1 \| STN7 protein kinase; required for state transitions, phosphorylation of the major antenna complex (LHCII) between PSII and PSI, and light adaptation \| chr1:25876119-25879169 REVERSE \| Aliases: STN7, T6L1.2, T6L1_2 | 5.5 | 4.7 | 0.8 | 4.4 | 1.79% | 3.7 |
| 5367 | AT4G22730.1 \| leucine-rich repeat transmembrane protein kinase, putative, leucine rich repeat receptor-like kinase, Oryza sativa, PATCHX:E267533 \| chr4:11941395-11943750 FORWARD \| Aliases: T12H17.120, T12H17_120 | 3.6 | 2.9 | 0.7 | 4.4 | 1.79% | 3.7 |
| 5368 | AT5G64640.1 \| pectinesterase family protein, contains Pfam profile: PF01095 pectinesterase \| chr5:25853953-25856279 FORWARD \| Aliases: MUB3.16, MUB3_16 | 4.6 | 3.7 | 0.9 | 4.4 | 1.79% | 3.8 |
| 5370 | AT3G62800.2 \| double-stranded RNA-binding domain (DsRBD)-containing protein, weak similarity to SP:P19525 Interferon-induced, double-stranded RNA-activated protein kinase (EC 2.7.1.-) {Homo sapiens}; contains Pfam profile PF00035: Double-stranded RNA binding motif \| chr3:23236567-23238487 REVERSE \| Aliases: F26K9.230 | 6.4 | 5.5 | 0.9 | 4.4 | 1.79% | 3.6 |
| 5376 | AT3G53690.1 \| zinc finger (C3HC4-type RING finger) family protein, contains a Zinc finger, C3HC4 type (RING finger) signature, PROSITE:PS00518 and a IBR domain, Pfam:PF01485 \| chr3:19909794-19911043 REVERSE \| Aliases: F5K20.1 | 6.0 | 4.8 | 1.2 | 4.4 | 1.79% | 3.9 |
| 5377 | AT4G16690.1 \| esterase/lipase/thioesterase family protein, similar to ethylene-induced esterase (Citrus sinensis) GI:14279437, polyneuridine aldehyde esterase (Rauvolfia serpentina) GI:6651393, SP:Q40708 PIR7A protein {Oryza sativa}; contains Interpro entry IPR000379 \| chr4:9392169-9393491 REVERSE \| Aliases: DL4370C, FCAALL.12 | 3.4 | 2.7 | 0.7 | 4.4 | 1.79% | 3.7 |
| 5379 | AT1G67180.1 \| zinc finger (C3HC4-type RING finger) family protein / BRCT domain-containing protein, contains Pfam domains PF00533: BRCA1 C Terminus (BRCT) domain and PF00097: Zinc finger, C3HC4 type (RING finger) \| chr1:25133762-25135832 FORWARD \| Aliases: F5A8.9, F5A8_9 | 2.5 | 2.1 | 0.4 | 4.4 | 1.79% | 3.0 |
| 5381 | AT3G06200.1 \| guanylate kinase, putative, similar to guanylate kinase (GMP kinase) (Bacillus halodurans) Swiss-Prot:Q9K9Y2; contains Pfam profile: PF00625 guanylate kinase \| chr3:1877600-1879244 FORWARD \| Aliases: F28L1.14, F28L1_14 | 5.9 | 4.8 | 1.1 | 4.4 | 1.80% | 3.8 |
| 5382 | AT4G18750.1 \| pentatricopeptide (PPR) repeat-containing protein, contains INTERPRO:IPR002885 PPR repeats \| chr4:10304861-10307476 FORWARD \| Aliases: F28A21.160, F28A21_160 | 3.1 | 2.6 | 0.5 | 4.4 | 1.80% | 3.3 |
| 5383 | AT1G11890.1 \| Symbol: SEC22 \| vesicle transport protein SEC22, putative, identified as SEC22 by Raikhel, NV, et al. in Plant Physiol. 124: 1558-69 (2000); similar to vesicle trafficking protein gb:U91538 from Mus musculus; ESTs gb:F15494 and gb:F14097 come from this gene \| chr1:4010778-4013122 FORWARD \| Aliases: ATSEC22, F12F1.27, F12F1_27 | 7.6 | 6.2 | 1.4 | 4.4 | 1.80% | 3.8 |
| 5384 | AT5G27110.1 \| pentatricopeptide (PPR) repeat-containing protein, contains INTERPRO:IPR002885 PPR repeats \| chr5:9538575-9540650 REVERSE \| Aliases: T21B4.20, T21B4_20 | 5.5 | 4.4 | 1.0 | 4.4 | 1.80% | 3.8 |
| 5386 | AT5G39660.2 \| Dof-type zinc finger domain-containing protein, similar to H-protein promoter binding factor-2a GI:3386546 from (Arabidopsis thaliana) \| chr5:15895954-15898272 FORWARD \| Aliases: MIJ24.16, MIJ24_16 | 6.0 | 5.0 | 1.0 | 4.4 | 1.80% | 3.8 |

| Rank | Description | Sync | Root | M | t | adj.q | B |
|---|---|---|---|---|---|---|---|
| 5387 | AT1G17180.1 \| Symbol: ATGSTU25 \| glutathione S-transferase, putative, Second of three repeated putative glutathione transferases. 72% identical to glutathione transferase (Arabidopsis thaliana) (gi:4006934). Location of ests 191A10T7 (gb:R90188) and 171N13T7 (gb:R65532) \| chr1:5872142-5873079 FORWARD \| Aliases: F20D23.12, F20D23_12 | 5.2 | 3.6 | 1.5 | 4.4 | 1.80% | 3.8 |
| 5388 | AT2G17560.2 \| Symbol: HMGB4 \| similar to high mobility group protein delta (HMGdelta) / HMG protein delta [Arabidopsis thaliana] (TAIR:At4g35570.1); similar to HMG-1 like protein gene [Glycine max] (GB:CAA41200.1); similar to high mobility group protein [Solanum tuberosum] (GB:CAA05365.1); contains InterPro domain HMG1/2 (high mobility group) box (InterPro:IPR000910) \| chr2:7649247-7650749 REVERSE \| Aliases: HIGH MOBILITY GROUP B 4, HMG GAMMA, MJB20.12, MJB20_12, NFD04, NFD4 | 7.7 | 5.5 | 2.2 | 4.4 | 1.80% | 3.9 |
| 5391 | AT2G33620.3 \| DNA-binding family protein / AT-hook protein 1 (AHP1), identical to AT-hook protein 1 (Arabidopsis thaliana) gi:2598227:emb:CAA10857 \| chr2:14241325-14244634 FORWARD \| Aliases: F4P9.39, F4P9_39 | 6.9 | 5.3 | 1.6 | 4.3 | 1.81% | 3.8 |
| 5395 | AT3G10090.1 \| 40S ribosomal protein S28 (RPS28A), similar to ribosomal protein S28 GB:P34789 (Arabidopsis thaliana) \| chr3:3108815-3110022 REVERSE \| Aliases: T22K18.8 | 10.8 | 8.7 | 2.1 | 4.3 | 1.81% | 3.5 |
| 5396 | AT3G54690.1 \| sugar isomerase (SIS) domain-containing protein / CBS domain-containing protein, similar to SP:Q47334 Polysialic acid capsule expression protein kpsF {Escherichia coli}; contains Pfam profiles PF01380: sugar isomerase (SIS) domain, PF00571: CBS domain \| chr3:20257517-20259002 FORWARD \| Aliases: T5N23.50 | 5.5 | 4.1 | 1.4 | 4.3 | 1.81% | 3.8 |
| 5406 | AT4G30440.1 \| Symbol: GAE1 \| NAD-dependent epimerase/dehydratase family protein, similar to nucleotide sugar epimerase from Vibrio vulnificus GI:3093975 (PID:g3093975), WbnF (Escherichia coli) GI:5739472, CAPI protein {Staphylococcus aureus} SP:P39858; contains Pfam profile PF01370 NAD dependent epimerase/dehydratase family | 7.9 | 6.8 | 1.1 | 4.3 | 1.81% | 3.7 |
| 5409 | AT5G53470.1 \| Symbol: ACBP1 \| acyl-CoA binding protein, putative / ACBP, putative, similar to acyl-CoA binding protein 2 (Arabidopsis thaliana) gi:12039034:gb:AAG46057 \| chr5:21727577-21729836 FORWARD \| Aliases: ACBP, ACYL COA BINDING PROTEIN, MYN8.8, MYN8_8 | 5.2 | 3.6 | 1.6 | 4.3 | 1.82% | 3.9 |
| 5415 | AT3G28480.1 \| oxidoreductase, 2OG-Fe(II) oxygenase family protein, similar to prolyl 4-hydroxylase, alpha subunit, from Gallus gallus (GI:212530), Rattus norvegicus (GI:474940), Mus musculus (SP:Q60715); contains PF03171 2OG-Fe(II) oxygenase superfamily domain \| chr3:10677275-10679525 REVERSE \| Aliases: MFJ20.16 | 8.4 | 6.9 | 1.5 | 4.3 | 1.83% | 3.8 |
| 5416 | AT4G04180.1 \| AAA-type ATPase family protein, contains Pfam domain, PF00004: ATPase, AAA family \| chr4:2020434-2023990 FORWARD \| Aliases: T27D20.13, T27D20_13 | 4.7 | 3.7 | 1.0 | 4.3 | 1.83% | 3.8 |
| 5417 | AT5G64940.2 \| Symbol: ATATH13 \| ABC1 family protein, contains Pfam domain, PF03109: ABC1 family \| chr5:25966199-25971048 FORWARD \| Aliases: MXK3.17, MXK3_17 | 5.5 | 3.3 | 2.2 | 4.3 | 1.83% | 3.8 |
| 5418 | AT5G51830.1 \| pfkB-type carbohydrate kinase family protein, contains Pfam profile: PF00294 pfkB family carbohydrate kinase \| chr5:21086811-21088961 FORWARD \| Aliases: MIO24.3, MIO24_3 | 5.5 | 3.9 | 1.7 | 4.3 | 1.83% | 3.8 |
| 5419 | AT1G12410.1 \| Symbol: CLPR2 \| ATP-dependent Clp protease proteolytic subunit (ClpP2), identical to nClpP2 GI:5360589 from (Arabidopsis thaliana) \| chr1:4223035-4225112 FORWARD \| Aliases: CLPP2, F5O11.13, F5O11_13, NCLPP2 | 9.0 | 7.4 | 1.6 | 4.3 | 1.83% | 3.8 |
| 5420 | AT5G27290.2 \| similar to expressed protein [Arabidopsis thaliana] (TAIR:At1g54680.1); similar to similar to Oryza sativa chromosome 3, OSJNBa0077G22.24 [Oryza sativa (japonica cultivar-group)] (GB:NP_918819.1) \| chr5:9617978-9620536 REVERSE \| Aliases: F21A20.3 | 4.1 | 3.4 | 0.7 | 4.3 | 1.83% | 3.7 |
| 5422 | AT2G35790.1 \| expressed protein \| chr2:15047758-15049201 FORWARD \| Aliases: T20F21.20 | 6.9 | 5.3 | 1.6 | 4.3 | 1.83% | 3.8 |
| 5423 | AT2G42450.1 \| lipase class 3 family protein, similar to calmodulin-binding heat-shock protein CaMBP (Nicotiana tabacum) GI:1087073; contains Pfam profile PF01764: Lipase, PF03893: Lipase 3 N-terminal region | 4.9 | 3.7 | 1.2 | 4.3 | 1.83% | 3.8 |
| 5425 | AT3G26740.1 \| light responsive protein-related, similar to light regulated protein precursor SP:Q03200 (Oryza sativa) (Plant Mol. Biol. 22 (1), 165-170 (1993)), ccr protein GB:S52663 (Citrus X paradisi) (Plant Mol. Biol. 26 (1), 165-173 (1994)) \| chr3:9829039-9829917 FORWARD \| Aliases: MLJ15.9 | 3.4 | 2.4 | 1.0 | 4.3 | 1.83% | 3.7 |
| 5428 | AT1G72920.1 \| disease resistance protein (TIR-NBS class), putative, domain signature TIR-NBS exists, suggestive of a disease resistance protein. \| chr1:27441608-27442709 FORWARD \| Aliases: F3N23.12, F3N23_12 | 5.4 | 3.7 | 1.7 | 4.3 | 1.84% | 3.8 |
| 5429 | AT2G46210.1 \| delta-8 sphingolipid desaturase, putative, similar to delta-8 sphingolipid desaturase GI:3819708 from (Brassica napus) \| chr2:18984417-18986074 FORWARD \| Aliases: T3F17.14 | 4.8 | 3.6 | 1.3 | 4.3 | 1.84% | 3.8 |
| 5430 | AT1G04190.1 \| tetratricopeptide repeat (TPR)-containing protein, low similarity to protein antigen LmSTI1 (Leishmania major) GI:1698880; contains Pfam profile PF00515 TPR Domain; EST gb:Z47802 and gb:Z48402 come from this gene \| chr1:1106427-1108707 REVERSE \| Aliases: F20D22.4, F20D22_4 | 8.0 | 6.4 | 1.6 | 4.3 | 1.84% | 3.8 |

| Rank | Description | Sync | Root | M | t | adj.q | B |
|---|---|---|---|---|---|---|---|
| 5431 | AT1G22570.1 \| proton-dependent oligopeptide transport (POT) family protein, contains Pfam profile: PF00854 POT family \| chr1:7976609-7978562 REVERSE \| Aliases: F12K8.8, F12K8_8 | 6.2 | 5.0 | 1.2 | 4.3 | 1.84% | 3.8 |
| 5432 | AT2G33220.1 \| expressed protein \| chr2:14085948-14087213 FORWARD \| Aliases: F25I18.4, F25I18_4 | 10.1 | 9.0 | 1.2 | 4.3 | 1.84% | 3.7 |
| 5434 | AT4G32340.1 \| expressed protein \| chr4:15612597-15614259 REVERSE \| Aliases: F8B4.40 | 6.8 | 5.4 | 1.4 | 4.3 | 1.84% | 3.8 |
| 5436 | AT3G17090.2 \| similar to protein phosphatase 2C family protein / PP2C family protein [Arabidopsis thaliana] (TAIR:At4g38520.2); similar to protein phosphatase 2C family protein / PP2C family protein [Arabidopsis thaliana] (TAIR:At4g38520.1); similar to protein phosphatase 2c-like protein [Thellungiella halophila] (GB:AAM19705.1); contains InterPro domain Protein phosphatase 2C-like (InterPro:IPR001932) \| chr3:5826809-5829627 FORWARD \| Aliases: K14A17.4 | 10.4 | 9.3 | 1.1 | 4.3 | 1.84% | 3.7 |
| 5438 | AT3G25620.1 \| ABC transporter family protein, similar to GB:AAC61893 from (Bactrocera tryoni) (Insect Mol. Biol. 6 (4), 343-356 (1997)) \| chr3:9317311-9320805 REVERSE \| Aliases: T5M7.11 | 4.7 | 3.7 | 1.0 | 4.3 | 1.85% | 3.8 |
| 5440 | AT4G36360.2 \| Symbol: BGAL3 \| beta-galactosidase, putative / lactase, putative, similar to beta-galactosidase precursor SP:P48980 from (Lycopersicon esculentum) \| chr4:17176345-17181245 REVERSE \| Aliases: F23E13.200, F23E13_200 | 6.2 | 4.4 | 1.8 | 4.3 | 1.85% | 3.8 |
| 5442 | AT4G15660.1 \| glutaredoxin family protein, contains INTERPRO Domain IPR002109, Glutaredoxin (thioltransferase) | 3.1 | 2.7 | 0.4 | 4.3 | 1.85% | 3.2 |
| 5443 | AT5G52380.1 \| zinc knuckle (CCHC-type) family protein, contains Pfam domain, PF00098: Zinc knuckle \| chr5:21278579-21280364 REVERSE \| Aliases: K24M7.12, K24M7_12 | 6.0 | 4.8 | 1.2 | 4.3 | 1.85% | 3.7 |
| 5445 | AT5G64160.1 \| expressed protein \| chr5:25688156-25689689 FORWARD \| Aliases: MHJ24.14, MHJ24_14 | 7.4 | 5.7 | 1.6 | 4.3 | 1.85% | 3.8 |
| 5446 | AT5G42670.1 \| agenet domain-containing protein, contains Pfam PF05641: Agenet domain \| chr5:17125490-17127463 FORWARD \| Aliases: MJB21.4, MJB21_4 | 6.3 | 4.9 | 1.4 | 4.3 | 1.85% | 3.8 |
| 5448 | AT4G22930.1 \| Symbol: PYR4 \| dihydroorotase, mitochondrial / DHOase (PYR4), identical to SP:O04904 Dihydroorotase, mitochondrial precursor (EC 3.5.2.3) (DHOase) {Arabidopsis thaliana} \| chr4:12018906-12021602 FORWARD \| Aliases: DIHYDROOROTASE, F7H19.110, F7H19_110 | 7.4 | 6.0 | 1.4 | 4.3 | 1.86% | 3.8 |
| 5450 | AT1G30530.1 \| UDP-glucoronosyl/UDP-glucosyl transferase family protein, contains Pfam profile: PF00201 UDP-glucoronosyl and UDP-glucosyl transferase \| chr1:10814641-10816565 FORWARD \| Aliases: F26G16.15, F26G16_15 | 5.6 | 4.0 | 1.6 | 4.3 | 1.86% | 3.8 |
| 5451 | AT2G46100.1 \| expressed protein \| chr2:18960353-18962069 FORWARD \| Aliases: T3F17.25 | 9.2 | 7.8 | 1.3 | 4.3 | 1.86% | 3.8 |
| 5455 | AT4G31200.3 \| SWAP (Suppressor-of-White-APricot)/surp domain-containing protein, related to DAN26 (Homo sapiens) gi:1770394:emb:CAA69591 \| chr4:15161642-15164914 REVERSE \| Aliases: F8F16.10, F8F16_10 | 5.8 | 5.0 | 0.8 | 4.3 | 1.86% | 3.6 |
| 5457 | AT3G09150.3 \| Symbol: HY2 \| phytochromobilin:ferredoxin oxidoreductase, chloroplast / phytochromobilin synthase (HY2), identical to SP:Q9SR43 Phytochromobilin:ferredoxin oxidoreductase, chloroplast precursor (EC 1.3.7.4) (Phytochromobilin synthase) (PFB synthase) (PPhiB synthase) {Arabidopsis thaliana}; identical to cDNA for phytochromobilin synthase HY2 protein, GI:13359272 \| chr3:2803615-2805569 FORWARD \| Aliases: ELONGATED HYPOCOTYL 2, F3L24.1, GENOMES UNCOUPLED 3, GUN3, PHYTOCHROMOBILIN SYNTHASE | 4.9 | 3.7 | 1.3 | 4.3 | 1.86% | 3.8 |
| 5458 | AT2G04230.1 \| F-box family protein, contains F-box domain Pfam:PF00646 | 4.2 | 3.2 | 1.0 | 4.3 | 1.86% | 3.7 |
| 5460 | AT5G65250.1 \| expressed protein \| chr5:26095759-26097424 REVERSE \| Aliases: MQN23.20, MQN23_20 | 6.1 | 4.4 | 1.6 | 4.3 | 1.87% | 3.8 |
| 5461 | AT5G39840.1 \| ATP-dependent RNA helicase, mitochondrial, putative, similar to mitochondrial RNA helicase (Arabidopsis thaliana) GI:5823579; contains Pfam profile PF00271: Helicase conserved C-terminal domain \| chr5:15963997-15966414 FORWARD \| Aliases: K13H13.20, K13H13_20 | 2.9 | 2.3 | 0.6 | 4.3 | 1.87% | 3.5 |
| 5463 | AT4G05530.1 \| short-chain dehydrogenase/reductase (SDR) family protein, similar to peroxisomal short-chain alcohol dehydrogenase GI:4105190 from (Homo sapiens) \| chr4:2816392-2818362 FORWARD \| Aliases: T1J24.9, T1J24_9 | 9.0 | 6.8 | 2.2 | 4.3 | 1.87% | 3.8 |
| 5465 | AT1G74430.1 \| Symbol: MYB95 \| myb family transcription factor (MYB95), contains Pfam profile: PF00249 myb-like DNA-binding domain \| chr1:27978842-27981095 FORWARD \| Aliases: F1M20.11, F1M20_11, MYB95 | 3.4 | 2.4 | 1.0 | 4.3 | 1.87% | 3.6 |
| 5466 | AT1G70480.2 \| expressed protein, similar to expressed protein [Arabidopsis thaliana] (TAIR:At1g23560.1); contains InterPro domain Protein of unknown function DUF220 (InterPro:IPR003863) \| chr1:26565722-26567472 FORWARD \| Aliases: F24J13.5, F24J13_5 | 9.2 | 7.2 | 2.0 | 4.3 | 1.87% | 3.8 |
| 5467 | AT1G15480.1 \| DNA-binding protein, putative, similar to DNA-binding protein (Triticum aestivum) GI:6958202; contains Pfam profile PF01535: PPR repeat \| chr1:5318208-5320589 FORWARD \| Aliases: F9L1.43, F9L1_43 | 4.7 | 3.7 | 1.0 | 4.3 | 1.87% | 3.8 |

| Rank | Description | Sync | Root | M | t | adj.q | B |
|---|---|---|---|---|---|---|---|
| 5469 | AT4G25020.1 \| KOW domain-containing protein / D111/G-patch domain-containing protein, contains Pfam profiles PF01585: G-patch domain, PF00467: KOW motif \| chr4:12860644-12861985 REVERSE \| Aliases: F13M23.160, F13M23_160 | 6.5 | 5.0 | 1.5 | 4.3 | 1.87% | 3.8 |
| 5470 | AT5G65760.1 \| serine carboxypeptidase S28 family protein, similar to SP:P42785 Lysosomal Pro-X carboxypeptidase precursor (EC 3.4.16.2) (Prolylcarboxypeptidase) (PRCP) (Proline carboxypeptidase) {Homo sapiens}; contains Pfam profile PF05577: Serine carboxypeptidase S28 | 10.3 | 8.8 | 1.5 | 4.3 | 1.88% | 3.6 |
| 5471 | AT2G04030.2 \| Symbol: CR88 \| heat shock protein, putative, strong similarity to heat shock protein (Arabidopsis thaliana) GI:1906830; contains Pfam profiles PF02518: ATPase, histidine kinase-, DNA gyrase B-, and HSP90-like domain protein, PF00183: Hsp90 protein | 6.7 | 4.8 | 1.9 | 4.3 | 1.88% | 3.8 |
| 5472 | AT2G42530.1 \| cold-responsive protein / cold-regulated protein (cor15b), nearly identical to cold-regulated gene cor15b (Arabidopsis thaliana) GI:456016; contains Pfam profile PF02987: Late embryogenesis abundant protein \| chr2:17716114-17717023 REVERSE \| Aliases: F14N22.20, F14N22_20 | 5.8 | 4.0 | 1.8 | 4.3 | 1.88% | 3.8 |
| 5473 | AT5G55070.1 \| 2-oxoacid dehydrogenase family protein, similar to SP:Q01205 Dihydrolipoamide succinyltransferase component of 2-oxoglutarate dehydrogenase complex, mitochondrial precursor (EC 2.3.1.61) {Rattus norvegicus}; contains Pfam profiles PF00198: 2-oxo acid dehydrogenases acyltransferase (catalytic domain), PF00364: Biotin-requiring enzyme \| chr5:22364490-22368043 FORWARD \| Aliases: MCO15.2, MCO15_2 | 9.6 | 7.3 | 2.3 | 4.3 | 1.88% | 3.8 |
| 5475 | AT5G08400.2 \| expressed protein, similar to expressed protein [Arabidopsis thaliana] (TAIR:At4g29400.1); similar to unknown protein [Oryza sativa (japonica cultivar-group)] (GB:BAD87659.1) \| chr5:2706459-2708946 REVERSE \| Aliases: F8L15.130, F8L15_130 | 6.2 | 4.8 | 1.5 | 4.3 | 1.88% | 3.8 |
| 5477 | AT3G45770.2 \| oxidoreductase, zinc-binding dehydrogenase family protein, similar to nuclear receptor binding factor-1 NRBF-1 - Rattus norvegicus, EMBL:AB015724; contains Pfam zinc-binding dehydrogenase domain PF00107 \| chr3:16816547-16818780 REVERSE \| Aliases: F16L2.2 | 9.8 | 7.9 | 1.9 | 4.3 | 1.88% | 3.7 |
| 5482 | AT5G43810.1 \| Symbol: ZLL \| pinhead protein (PINHEAD) / zwille protein (ZWILLE), identical to SP:Q9XGW1 PINHEAD protein (ZWILLE protein) {Arabidopsis thaliana}; contains Pfam profiles PF02171: Piwi domain, PF02170: PAZ domain \| chr5:17628199-17634024 FORWARD \| Aliases: MQD19.17, MQD19_17, PINHEAD, PNH, ZWILLE | 5.4 | 3.5 | 1.9 | 4.3 | 1.89% | 3.8 |
| 5485 | AT5G03370.1 \| acylphosphatase family, contains Pfam PF00708: Acylphosphatase \| chr5:825572-827033 REVERSE \| Aliases: F12E4.110, F12E4_110 | 5.4 | 4.5 | 0.8 | 4.3 | 1.89% | 3.7 |
| 5486 | AT5G53490.2 \| thylakoid lumenal 17.4 kDa protein, chloroplast, identical to SP:P81760 Thylakoid lumenal 17.4 kDa protein, chloroplast precursor (P17.4) {Arabidopsis thaliana} | 4.3 | 3.3 | 1.0 | 4.3 | 1.90% | 3.7 |
| 5488 | AT1G49170.1 \| expressed protein \| chr1:18186934-18188102 REVERSE \| Aliases: F27J15.6, F27J15_6 | 3.7 | 2.6 | 1.1 | 4.3 | 1.90% | 3.6 |
| 5490 | AT5G27710.1 \| expressed protein \| chr5:9812992-9815424 FORWARD \| Aliases: T1G16.40, T1G16_40 | 5.1 | 4.0 | 1.1 | 4.3 | 1.90% | 3.7 |
| 5491 | AT3G07200.1 \| zinc finger (C3HC4-type RING finger) family protein, contains Pfam PF00097: Zinc finger, C3HC4 type (RING finger) \| chr3:2290773-2292498 FORWARD \| Aliases: T1B9.13 | 7.0 | 5.7 | 1.4 | 4.3 | 1.90% | 3.8 |
| 5492 | AT4G14330.1 \| phragmoplast-associated kinesin-related protein 2 (PAKRP2), identical to cDNA phragmoplast-associated kinesin-related protein 2 (PAKRP2) GI:16973450 \| chr4:8244190-8247437 FORWARD \| Aliases: DL3205W, FCAALL.179 | 3.5 | 2.6 | 0.9 | 4.3 | 1.90% | 3.6 |
| 5494 | AT5G64980.1 \| expressed protein \| chr5:25978001-25980461 REVERSE \| Aliases: MXK3.21, MXK3_21 | 3.8 | 2.9 | 0.9 | 4.3 | 1.90% | 3.8 |
| 5495 | AT1G51110.1 \| plastid-lipid associated protein PAP / fibrillin family protein, similar to plastid-lipid associated protein PAP2 (Brassica rapa) GI:14248550; contains Pfam profile PF04755: PAP_fibrillin \| chr1:18939016-18941327 FORWARD \| Aliases: AT1G51115, F23H24.16, F23H24_16 | 4.2 | 3.2 | 1.0 | 4.3 | 1.90% | 3.6 |
| 5496 | AT1G16490.1 \| myb family transcription factor (MYB58), contains PFAM profile: myb DNA binding domain PF00249 \| chr1:5629648-5630988 REVERSE \| Aliases: F3O9.29, F3O9_29 | 4.1 | 3.0 | 1.0 | 4.3 | 1.91% | 3.7 |
| 5497 | AT2G42210.4 \| expressed protein, similar to hypothetical protein [Oryza sativa (japonica cultivar-group)] (GB:XP_450480.1); contains InterPro domain Mitochondrial import inner membrane translocase, subunit Tim17/22 (InterPro:IPR003397) \| chr2:17597291-17598930 FORWARD \| Aliases: T24P15.12, T24P15_12 | 11.2 | 10.0 | 1.2 | 4.3 | 1.91% | 3.5 |
| 5498 | AT3G56760.1 \| calcium-dependent protein kinase, putative / CDPK, putative, similar to calcium/calmodulin-dependent protein kinase CaMK3 (Nicotiana tabacum) gi:16904226:gb:AAL30820 \| chr3:21031537-21034735 REVERSE \| Aliases: T8M16.90 | 6.6 | 5.7 | 0.9 | 4.3 | 1.91% | 3.8 |
| 5500 | AT1G32060.1 \| phosphoribulokinase (PRK) / phosphopentokinase, nearly identical to SP:P25697 Phosphoribulokinase, chloroplast precursor (EC 2.7.1.19) (Phosphopentokinase) (PRKASE) (PRK) {Arabidopsis thaliana} \| chr1:11532530-11534619 FORWARD \| Aliases: T12O21.4, T12O21_4 | 4.0 | 3.0 | 1.0 | 4.3 | 1.91% | 3.7 |
| 5502 | AT4G34990.1 \| myb family transcription factor (MYB32), similar to myb DNA-binding protein GI:19052 from (Hordeum vulgare) \| chr4:16661334-16662372 REVERSE \| Aliases: M4E13.50, M4E13_50 | 6.3 | 5.3 | 1.0 | 4.3 | 1.91% | 3.8 |

| Rank | Description | Sync | Root | M | t | adj.q | B |
|---|---|---|---|---|---|---|---|
| 5503 | AT1G60690.1 \| aldo/keto reductase family protein, contains Pfam profile PF00248: oxidoreductase, aldo/keto reductase family \| chr1:22353557-22355333 REVERSE \| Aliases: F8A5.21, F8A5_21 | 6.8 | 5.9 | 0.9 | 4.3 | 1.91% | 3.7 |
| 5504 | AT1G30620.2 \| Symbol: MUR4 \| similar to NAD-dependent epimerase/dehydratase family protein [Arabidopsis thaliana] (TAIR:At5g44480.1); similar to NAD-dependent epimerase/dehydratase family protein [Arabidopsis thaliana] (TAIR:At4g20460.1); similar to UDP-galactose 4-epimerase-like protein [Oryza sativa (japonica cultivar-group)] (GB:BAC24804.1); similar to putative UDP-glucose 4-epimerase [Oryza sativa (japonica cultivar-group)] (GB:XP_479925.1); similar to putative UDP-glucose 4-epimerase [Oryza sativa (japonica cultivar-group)] (GB:XP_476598.1); similar to OSJNBa0093O08.14 [Oryza sativa (japonica cultivar-group)] (GB:XP_473906.1); similar to PREDICTED P0582D05.120 gene product [Oryza sativa (japonica cultivar-group)] (GB:XP_507116.1); contains InterPro domain NAD-dependent epimerase/dehydratase (InterPro:IPR001509); contains InterPro domain UDP-glucose 4-epimerase (InterPro:IPR005886) \| chr1:10854587-10858196 FORWARD \| Aliases: MURUS 4, T5I8.7, T5I8_7, UXE1 | 6.1 | 4.8 | 1.3 | 4.3 | 1.92% | 3.8 |
| 5505 | AT1G78560.1 \| bile acid:sodium symporter family protein, low similarity to SP:Q12908 Ileal sodium/bile acid cotransporter {Homo sapiens}; contains Pfam profile PF01758: Sodium Bile acid symporter family \| chr1:29551498-29553717 REVERSE \| Aliases: T30F21.11, T30F21_11 | 6.1 | 5.0 | 1.1 | 4.3 | 1.93% | 3.7 |
| 5506 | AT5G65860.2 \| expressed protein, similar to hypothetical protein SPAC26A3.17c - fission yeast (Schizosaccharomyces pombe) (GB:T38405); contains InterPro domain Ankyrin (InterPro:IPR002110) \| chr5:26364860-26366282 REVERSE \| Aliases: K14B20.3, K14B20_3 | 6.3 | 5.3 | 1.0 | 4.3 | 1.93% | 3.7 |
| 5510 | AT1G69250.2 \| nuclear transport factor 2 (NTF2) family protein / RNA recognition motif (RRM)-containing protein, contains Pfam profiles PF02136: Nuclear transport factor 2 (NTF2) domain, PF00076: RNA recognition motif (a.k.a. RRM, RBD, or RNP domain) \| chr1:26036558-26039167 FORWARD \| Aliases: F4N2.20 | 5.2 | 3.7 | 1.6 | 4.3 | 1.93% | 3.6 |
| 5512 | AT1G48450.2 \| similar to expressed protein [Arabidopsis thaliana] (TAIR:At3g17800.1); similar to unknown [Brassica napus] (GB:AAK30572.1); contains InterPro domain Protein of unknown function DUF760 (InterPro:IPR008479) \| chr1:17912171-17914113 REVERSE \| Aliases: T1N15.6, T1N15_6 | 9.3 | 8.3 | 1.0 | 4.3 | 1.93% | 3.6 |
| 5514 | AT2G17900.1 \| Symbol: SDG37 \| zinc finger (MYND type) family protein / SET domain-containing protein, contains Pfam profiles PF00856: SET domain, PF01753: MYND zinc finger (ZnF) domain; confirmed by cDNA (Paola Vittorioso--accession not assigned yet (paola.vittorioso@uniroma1.it) \| chr2:7780410-7783765 REVERSE \| Aliases: SET DOMAIN GROUP 37, T13L16.8, T13L16_8 | 4.7 | 3.7 | 0.9 | 4.3 | 1.93% | 3.6 |
| 5518 | AT4G26410.1 \| expressed protein \| chr4:13346757-13349025 FORWARD \| Aliases: M3E9.160, M3E9_160 | 9.3 | 8.1 | 1.2 | 4.3 | 1.95% | 3.6 |
| 5520 | AT1G50700.1 \| Symbol: CPK33 \| calcium-dependent protein kinase, putative / CDPK, putative, similar to calmodulin-domain protein kinase CDPK isoform 9 (Arabidopsis thaliana) gi:1399265:gb:AAB03242 \| chr1:18785882-18788053 FORWARD \| Aliases: F17J6.22, F17J6_22 | 5.0 | 3.9 | 1.0 | 4.3 | 1.95% | 3.7 |
| 5521 | AT1G49650.1 \| cell death associated protein-related, similar to PrMC3 (Pinus radiata) GI:5487873; weak similarity to cell death associated protein (Nicotiana tabacum) GI:7417008, hsr203J (Nicotiana tabacum) GI:22830761 \| chr1:18380923-18382302 REVERSE \| Aliases: F14J22.21, F14J22_21 | 5.7 | 4.7 | 1.0 | 4.3 | 1.95% | 3.7 |
| 5524 | AT1G66070.1 \| translation initiation factor-related, similar to Eukaryotic translation initiation factor 3 subunit 1 (eIF-3 alpha) (eIF3 p35) (eIF3j) (Swiss-Prot:O75822) (Homo sapiens) \| chr1:24599549-24602181 REVERSE \| Aliases: F15E12.10, F15E12_10 | 5.3 | 4.4 | 0.9 | 4.3 | 1.95% | 3.6 |
| 5526 | AT1G43860.1 \| expressed protein \| chr1:16624508-16626909 REVERSE \| Aliases: F28H19.11, F28H19_11 | 7.6 | 6.6 | 0.9 | 4.3 | 1.96% | 3.7 |
| 5527 | AT4G33060.1 \| peptidyl-prolyl cis-trans isomerase cyclophilin-type family protein, contains Pfam domain, PF00160: peptidyl-prolyl cis-trans isomerase, cyclophilin-type \| chr4:15948507-15952172 FORWARD \| Aliases: F4I10.3 | 6.6 | 4.9 | 1.7 | 4.3 | 1.96% | 3.7 |
| 5528 | AT1G75040.1 \| Symbol: PR5 \| pathogenesis-related protein 5 (PR-5), identical to SP:P28493 Pathogenesis-related protein 5 precursor (PR-5) {Arabidopsis thaliana}; contains Pfam profile: PF00314 Thaumatin family \| chr1:28181364-28182601 FORWARD \| Aliases: PATHOGENESIS RELATED GENE 5, PATHOGENESIS RELATED PROTEIN 5 | 4.2 | 3.1 | 1.1 | 4.3 | 1.96% | 3.7 |
| 5529 | AT1G14310.1 \| haloacid dehalogenase-like hydrolase family protein, contains InterPro accession IPR005834: Haloacid dehalogenase-like hydrolase | 7.8 | 6.9 | 1.0 | 4.3 | 1.96% | 3.7 |
| 5531 | AT1G42960.1 \| expressed protein localized to the inner membrane of the chloroplast. \| chr1:16128235-16129727 FORWARD \| Aliases: F13A11.2, F13A11_2 | 10.2 | 9.1 | 1.1 | 4.3 | 1.96% | 3.5 |
| 5532 | AT1G12230.1 \| transaldolase, putative, similar to Swiss-Prot:P30148 transaldolase B (EC 2.2.1.2) (Escherichia coli O157:H7) \| chr1:4148032-4151023 FORWARD \| Aliases: T28K15.4, T28K15_4 | 10.6 | 8.3 | 2.3 | 4.3 | 1.96% | 3.7 |
| 5536 | AT2G33850.1 \| expressed protein, contains 1 transmembrane domain; similar to Protein E6 (Swiss-Prot:Q01197) (Gossypium hirsutum) \| chr2:14328138-14329125 REVERSE \| Aliases: T1B8.15, T1B8_15 | 3.4 | 2.3 | 1.1 | 4.3 | 1.96% | 3.8 |
| 5538 | AT5G55940.1 \| Symbol: EMB2731 \| expressed protein, contains Pfam PF03665: Uncharacterised protein family (UPF0172) \| chr5:22672658-22675274 REVERSE \| Aliases: EMB2731, EMBRYO DEFECTIVE 2731, MYN21.5, MYN21_5 | 6.9 | 5.7 | 1.2 | 4.3 | 1.97% | 3.7 |

| Rank | Description | Sync | Root | M | t | adj.q | B |
|---|---|---|---|---|---|---|---|
| 5541 | AT1G19140.2 \| expressed protein \| chr1:6610774-6612458 REVERSE \| Aliases: F14D16.30, F14D16_30 | 8.1 | 6.5 | 1.7 | 4.3 | 1.97% | 3.7 |
| 5543 | AT4G39300.2 \| expressed protein \| chr4:18286787-18288332 FORWARD \| Aliases: T22F8.210, T22F8_210 | 4.7 | 3.1 | 1.6 | 4.3 | 1.97% | 3.7 |
| 5544 | AT1G23550.1 \| Symbol: SRO2 \| Encodes a protein with similarity to RCD1 but without the WWE domain. The protein does have a PARP signature upstream of the C-terminal protein interaction domain. The PARP signature may bind NAD+ and attach the ADP-ribose-moiety from NAD+ to the target molecule. Its presence suggests a role for the protein in ADP ribosylation. \| chr1:8350901-8352221 FORWARD \| Aliases: F28C11.18, SIMILAR TO RCD ONE 2, SIMILAR TO RCD ONE 2, SRO2 | 6.0 | 4.9 | 1.1 | 4.3 | 1.97% | 3.7 |
| 5545 | AT2G40240.1 \| pentatricopeptide (PPR) repeat-containing protein, contains INTERPRO:IPR002885 PPR repeats \| chr2:16818104-16819306 FORWARD \| Aliases: T7M7.22 | 3.7 | 3.1 | 0.6 | 4.3 | 1.98% | 3.4 |
| 5548 | AT2G06255.1 \| expressed protein \| chr2:2457570-2459808 FORWARD \| Aliases: None | 3.6 | 3.1 | 0.5 | 4.3 | 1.98% | 3.4 |
| 5554 | AT5G48580.1 \| Symbol: FKBP15 2 \| FK506-binding protein 2-2 (FKBP15-2) / immunophilin / peptidyl-prolyl cis-trans isomerase / rotamase, identical to SP:Q38936: FK506-binding protein 2-2 precursor (EC 5.2.1.8); \| chr5:19713155-19714551 REVERSE \| Aliases: IMMUNOPHILIN, K15N18.5, K15N18_5 | 8.3 | 6.8 | 1.5 | 4.3 | 1.99% | 3.7 |
| 5555 | AT1G02816.1 \| expressed protein, contains Pfam profile PF04398: Protein of unknown function, DUF538 \| chr1:621461-622469 FORWARD \| Aliases: None | 6.5 | 4.7 | 1.8 | 4.3 | 1.99% | 3.7 |
| 5557 | AT2G45530.1 \| zinc finger (C3HC4-type RING finger) family protein, contains Pfam profile: PF00097 zinc finger, C3HC4 type (RING finger) \| chr2:18763552-18765002 FORWARD \| Aliases: F17K2.6 | 7.7 | 6.7 | 1.0 | 4.3 | 2.00% | 3.7 |
| 5558 | AT4G26520.1 \| fructose-bisphosphate aldolase, cytoplasmic, identical to SP:P22197 Fructose-bisphosphate aldolase, cytoplasmic isozyme (EC 4.1.2.13) {Arabidopsis thaliana} \| chr4:13388683-13390381 FORWARD \| Aliases: M3E9.50, M3E9_50 | 2.8 | 2.3 | 0.5 | 4.3 | 2.00% | 3.3 |
| 5559 | AT4G30930.1 \| Symbol: WRKY32 \| | 7.2 | 5.4 | 1.9 | 4.3 | 2.00% | 3.7 |
| 5563 | AT1G20920.1 \| DEAD box RNA helicase, putative, similar to RNA helicase (Rattus norvegicus) GI:897915; contains Pfam profiles PF00270: DEAD/DEAH box helicase, PF00271: Helicase conserved C-terminal domain \| chr1:7285103-7288831 FORWARD \| Aliases: F9H16.10, F9H16_10 | 6.0 | 5.3 | 0.7 | 4.3 | 2.00% | 3.6 |
| 5564 | AT2G29490.1 \| Symbol: ATGSTU1 \| glutathione S-transferase, putative, similar to glutathione S-transferase 103-1A (Arabidopsis thaliana) SWISS-PROT:P46421 \| chr2:12638556-12639550 REVERSE \| Aliases: F16P2.13, F16P2_13, GLUTATHIONE S TRANSFERASE 19, GST19 | 5.8 | 4.0 | 1.7 | 4.3 | 2.00% | 3.6 |
| 5565 | AT2G36970.1 \| UDP-glucoronosyl/UDP-glucosyl transferase family protein, contains Pfam profile: PF00201 UDP-glucoronosyl and UDP-glucosyl transferase \| chr2:15536085-15537828 FORWARD \| Aliases: T1J8.15, T1J8_15 | 6.1 | 4.5 | 1.6 | 4.2 | 2.01% | 3.7 |
| 5566 | AT4G04020.1 \| plastid-lipid associated protein PAP, putative / fibrillin, putative, strong similarity to plastid-lipid associated proteins PAP1 GI:14248554, PAP2 GI:14248556 from (Brassica rapa), fibrillin (Brassica napus) GI:4139097; contains Pfam profile PF04755: PAP_fibrillin \| chr4:1932149-1933948 FORWARD \| Aliases: T24H24.16, T24H24_16 | 8.0 | 4.0 | 4.0 | 4.2 | 2.01% | 3.1 |
| 5567 | AT4G22240.1 \| plastid-lipid associated protein PAP, putative, similar to plastid-lipid associated proteins PAP2 (Brassica rapa) GI:14248550 GI:14248556; contains Pfam profile PF04755: PAP_fibrillin \| chr4:11765815-11767316 REVERSE \| Aliases: T10I14.70, T10I14_70 | 8.0 | 4.0 | 4.0 | 4.2 | 2.01% | 3.1 |
| 5569 | AT2G32580.1 \| expressed protein \| chr2:13834740-13836386 FORWARD \| Aliases: T26B15.14, T26B15_14 | 8.6 | 6.1 | 2.6 | 4.2 | 2.01% | 3.7 |
| 5570 | AT3G51750.2 \| expressed protein \| chr3:19207482-19208313 REVERSE \| Aliases: T18N14.130 | 4.4 | 2.9 | 1.5 | 4.2 | 2.01% | 3.6 |
| 5571 | AT1G29395.1 \| Symbol: COR414 TM1 \| stress-responsive protein, putative, similar to cold acclimation WCOR413-like protein gamma form (Hordeum vulgare) gi:18449100:gb:AAL69988; similar to stress-regulated protein SAP1 (Xerophyta viscosa) gi:21360378:gb:AAM47505 \| chr1:10288236-10289579 REVERSE \| Aliases: None | 4.2 | 3.0 | 1.2 | 4.2 | 2.01% | 3.7 |
| 5573 | AT1G60550.1 \| naphthoate synthase, putative / dihydroxynaphthoic acid synthetase, putative / DHNA synthetase, putative, contains similarity to MENB from Escherichia coli (SP:P27290), Bacillus subtilis (SP:P23966); contains Pfam profile PF00378 enoyl-CoA hydratase/isomerase family protein \| chr1:22309496-22311838 REVERSE \| Aliases: F8A5.9, F8A5_9 | 6.1 | 4.1 | 2.0 | 4.2 | 2.01% | 3.7 |
| 5575 | AT2G19470.1 \| casein kinase, putative, similar to casein kinase I, delta isoform (Arabidopsis thaliana) SWISS-PROT:P42158; contains protein kinase domain, Pfam:PF00069 \| chr2:8440697-8443625 REVERSE \| Aliases: F3P11.7, F3P11_7 | 6.0 | 5.2 | 0.8 | 4.2 | 2.01% | 3.5 |
| 5576 | AT2G23610.1 \| esterase, putative, similar to ethylene-induced esterase (Citrus sinensis) GI:14279437, polyneuridine aldehyde esterase (Rauvolfia serpentina) GI:6651393; contains Pfam profile PF00561: hydrolase, alpha/beta fold family \| chr2:10051259-10053612 REVERSE \| Aliases: F26B6.26, F26B6_26 | 9.2 | 6.4 | 2.8 | 4.2 | 2.01% | 3.7 |
| 5579 | AT3G01640.1 \| GHMP kinase family protein, contains GHMP kinases putative ATP-binding protein domain, Pfam:PF00288 \| chr3:239388-241450 FORWARD \| Aliases: F4P13.18, F4P13_18 | 6.4 | 5.4 | 1.0 | 4.2 | 2.01% | 3.7 |

| Rank | Description | Sync | Root | M | t | adj.q | B |
|---|---|---|---|---|---|---|---|
| 5581 | AT5G65390.1 \| Symbol: AGP7 \| arabinogalactan-protein (AGP7) \| chr5:26145810-26146564 REVERSE \| Aliases: MNA5.12, MNA5_12 | 6.9 | 5.7 | 1.2 | 4.2 | 2.01% | 3.7 |
| 5586 | AT2G25790.1 \| leucine-rich repeat transmembrane protein kinase, putative \| chr2:11007677-11011313 FORWARD \| Aliases: F17H15.18, F17H15_18 | 7.6 | 6.4 | 1.3 | 4.2 | 2.01% | 3.6 |
| 5588 | AT2G01140.1 \| fructose-bisphosphate aldolase, putative, similar to plastidic aldolase NPALDP1 from Nicotiana paniculata (GI:4827251); contains Pfam profile PF00274 Fructose-bisphosphate aldolase class-I \| chr2:94810-96634 REVERSE \| Aliases: F10A8.2, F10A8_2 | 11.3 | 9.8 | 1.5 | 4.2 | 2.02% | 3.2 |
| 5593 | AT5G51040.2 \| expressed protein, contains InterPro domain Protein of unknown function DUF339 (InterPro:IPR005631) \| chr5:20767886-20769250 FORWARD \| Aliases: K3K7.22, K3K7_22 | 10.1 | 9.1 | 1.0 | 4.2 | 2.02% | 3.5 |
| 5596 | AT3G13930.1 \| dihydrolipoamide S-acetyltransferase, putative, similar to dihydrolipoamide S-acetyltransferase (Zea mays) GI:5669871; contains Pfam profiles PF00198: 2-oxo acid dehydrogenases acyltransferase (catalytic domain), PF00364: Biotin-requiring enzyme, PF02817: e3 binding domain \| chr3:4595883-4600476 FORWARD \| Aliases: MDC16.18 | 11.4 | 10.8 | 0.7 | 4.2 | 2.03% | 3.1 |
| 5597 | AT5G43750.1 \| expressed protein \| chr5:17597193-17598449 REVERSE \| Aliases: MQD19.9, MQD19_9 | 4.5 | 2.9 | 1.6 | 4.2 | 2.03% | 3.7 |
| 5598 | AT4G04830.1 \| methionine sulfoxide reductase domain-containing protein / SeIR domain-containing protein, low similarity to pilin-like transcription factor (Homo sapiens) GI:5059062, SP:P14930 Peptide methionine sulfoxide reductase msrA/msrB (EC 1.8.4.6) {Neisseria gonorrhoeae}; contains Pfam profile PF01641: SelR domain \| chr4:2445857-2447082 FORWARD \| Aliases: T4B21.5, T4B21_5 | 12.6 | 12.1 | 0.5 | 4.2 | 2.03% | 3.2 |
| 5599 | AT3G60360.1 \| expressed protein \| chr3:22323285-22325375 REVERSE \| Aliases: T8B10.20 | 6.4 | 4.7 | 1.7 | 4.2 | 2.03% | 3.7 |
| 5601 | AT5G21150.1 \| PAZ domain-containing protein / piwi domain-containing protein, similar to SP:O04379 Argonaute protein (AGO1) {Arabidopsis thaliana}; contains Pfam profile: PF02170 PAZ (Piwi Argonaut and Zwille), PF02171 Piwi domain \| chr5:7192242-7198327 FORWARD \| Aliases: T10F18.180, T10F18_180 | 2.6 | 2.1 | 0.4 | 4.2 | 2.04% | 3.1 |
| 5602 | AT4G17890.2 \| Symbol: UBP20 \| similar to human Rev interacting-like family protein / hRIP family protein [Arabidopsis thaliana] (TAIR:At5g46750.1); similar to putative zinc finger protein [Oryza sativa (japonica cultivar-group)] (GB:AAO66537.1); contains InterPro domain Human Rev interacting-like protein (hRIP) (InterPro:IPR001164) | 8.1 | 6.5 | 1.6 | 4.2 | 2.04% | 3.7 |
| 5604 | AT3G62450.1 \| expressed protein \| chr3:23109120-23109606 FORWARD \| Aliases: T12C14.150 | 9.1 | 8.3 | 0.8 | 4.2 | 2.04% | 3.5 |
| 5605 | AT1G14460.1 \| DNA polymerase-related, weak similarity to DNA polymerase III holoenzyme tau subunit (Thermus thermophilus) GI:2583049 \| chr1:4948743-4952922 REVERSE \| Aliases: F14L17.24, F14L17_24 | 5.2 | 4.5 | 0.7 | 4.2 | 2.04% | 3.5 |
| 5606 | AT4G16800.1 \| enoyl-CoA hydratase, putative, similar to AU-binding protein/Enoyl-CoA hydratase (Homo sapiens) GI:780241, (Mus musculus)GI:6840920; contains Pfam profile PF00378 enoyl-CoA hydratase/isomerase family protein \| chr4:9454835-9456328 REVERSE \| Aliases: DL4425C, FCAALL.21 | 7.2 | 5.3 | 1.9 | 4.2 | 2.04% | 3.6 |
| 5607 | AT2G18910.1 \| hydroxyproline-rich glycoprotein family protein \| chr2:8199790-8201420 REVERSE \| Aliases: F19F24.21 | 6.4 | 5.0 | 1.4 | 4.2 | 2.04% | 3.7 |
| 5608 | AT1G14490.1 \| DNA-binding protein-related, contains similarity to DNA-binding proteins | 4.2 | 3.4 | 0.8 | 4.2 | 2.04% | 3.6 |
| 5609 | AT5G40770.1 \| Symbol: ATPHB3 \| | 9.2 | 7.4 | 1.8 | 4.2 | 2.04% | 3.7 |
| 5610 | AT5G09990.1 \| expressed protein \| chr5:3124569-3125073 FORWARD \| Aliases: MYH9.1 | 3.3 | 2.4 | 0.9 | 4.2 | 2.04% | 3.3 |
| 5612 | AT2G25830.1 \| YebC-related, contains Pfam profile PF01709: Domain of unknown function; contains TIGRFAM TIGR01033: conserved hypothetical protein; similar to YebC (GI:5771432) (Legionella pneumophila) \| chr2:11025846-11028768 REVERSE \| Aliases: F17H15.14, F17H15_14 | 6.5 | 4.5 | 2.1 | 4.2 | 2.04% | 3.7 |
| 5614 | AT1G14710.2 \| hydroxyproline-rich glycoprotein family protein, contains proline-rich extensin domains, INTERPRO:IPR002965 \| chr1:5061861-5065097 REVERSE \| Aliases: F10B6.11, F10B6_11 | 6.1 | 4.7 | 1.4 | 4.2 | 2.05% | 3.6 |
| 5619 | AT2G39970.1 \| peroxisomal membrane protein (PMP36), identical to 36kDa-peroxisomal membrane protein (PMP36) GI:15146342 from (Arabidopsis thaliana) \| chr2:16690910-16693740 REVERSE \| Aliases: T28M21.13, T28M21_13 | 8.3 | 7.2 | 1.1 | 4.2 | 2.06% | 3.6 |
| 5623 | AT4G27540.1 \| prenylated rab acceptor (PRA1) protein-related, contains Pfam profile PF03208: PRA1 family protein \| chr4:13753219-13754751 REVERSE \| Aliases: T29A15.30, T29A15_30 | 5.1 | 4.2 | 0.9 | 4.2 | 2.06% | 3.6 |
| 5625 | AT4G27720.1 \| expressed protein, contains Pfam PF05631: Protein of unknown function (DUF791) \| chr4:13831126-13833820 FORWARD \| Aliases: T29A15.210, T29A15_210 | 6.2 | 4.4 | 1.8 | 4.2 | 2.06% | 3.7 |
| 5626 | AT2G39450.1 \| cation efflux family protein, contains cation efflux family protein domain, Pfam:PF01545 | 7.5 | 5.7 | 1.9 | 4.2 | 2.06% | 3.7 |

| Rank | Description | Sync | Root | M | t | adj.q | B |
|---|---|---|---|---|---|---|---|
| 5627 | AT5G13650.2 \| elongation factor family protein, contains Pfam profiles: PF00009 elongation factor Tu GTP binding domain,PF00679 elongation factor G C-terminus, PF03144 elongation factor Tu domain 2 | 5.9 | 4.1 | 1.8 | 4.2 | 2.06% | 3.7 |
| 5628 | AT5G16470.1 \| zinc finger (C2H2 type) family protein, contains Pfam profile: PF00096 zinc finger, C2H2 type \| chr5:5379419-5380043 FORWARD \| Aliases: MQK4.20, MQK4_20 | 10.4 | 9.2 | 1.2 | 4.2 | 2.07% | 3.5 |
| 5629 | AT4G13140.1 \| expressed protein \| chr4:7650322-7651141 REVERSE \| Aliases: F17N18.30, F17N18_30 | 8.3 | 7.0 | 1.3 | 4.2 | 2.07% | 3.7 |
| 5631 | AT2G33740.2 \| Symbol: CUTA \| copper-binding protein (CUTA), identical to copper-binding protein CUTA GI:12963361 from (Arabidopsis thaliana); contains Pfam profile: PF03091 CutA1 divalent ion tolerance protein \| chr2:14276657-14278752 FORWARD \| Aliases: T1B8.5, T1B8_5 | 8.5 | 7.3 | 1.2 | 4.2 | 2.07% | 3.7 |
| 5633 | AT3G61980.1 \| serine protease inhibitor, Kazal-type family protein, contains Pfam domain PF00050: Kazal-type serine protease inhibitor domain \| chr3:22967114-22967858 REVERSE \| Aliases: F21F14.150 | 7.3 | 6.2 | 1.1 | 4.2 | 2.07% | 3.6 |
| 5636 | AT4G23100.2 \| Symbol: RML1 \| similar to gamma-glutamylcysteine synthetase [Brassica juncea] (GB:CAA06613.1); similar to glutamate-cysteine ligase [Brassica juncea] (GB:CAD91714.1); similar to gamma-glutamylcysteine synthetase precursor [Pisum sativum] (GB:AAF22137.1); similar to gamma-glutamylcysteine synthetase [Lotus corniculatus var. japonicus] (GB:AAO27827.1); similar to glutamate-cysteine ligase [Brassica juncea] (GB:CAD91713.1); contains InterPro domain Glutamate--cysteine ligase, plant (InterPro:IPR006336) \| chr4:12103172-12107310 REVERSE \| Aliases: CAD2, CADMIUM SENSITIVE 2, F7H19.290, F7H19_290, GAMMA GLUTAMYL CYSTEINE SYNTHETASE, GAMMA GLUTAMYL CYSTEINE SYNTHETASE 1, GAMMA GLUTAMYLCYSTEINE SYNTHETASE, GSH1, ROOT MERISTEMLESS 1 | 11.1 | 8.4 | 2.7 | 4.2 | 2.07% | 3.7 |
| 5637 | AT1G25420.3 \| expressed protein, contains Pfam profile: PF03398 eukaryotic protein of unknown function, DUF292 \| chr1:8916086-8918175 FORWARD \| Aliases: F2J7.16, F2J7_16 | 7.9 | 6.7 | 1.2 | 4.2 | 2.07% | 3.6 |
| 5638 | AT5G46110.3 \| Symbol: APE2 \| similar to glucose-6-phosphate/phosphate translocator, putative [Arabidopsis thaliana] (TAIR:At5g54800.1); similar to triose phosphate/phosphate translocator precursor [Mesembryanthemum crystallinum] (GB:AAF86906.1); contains InterPro domain Tpt phosphate/phosphoenolpyruvate translocator (InterPro:IPR004696) \| chr5:18714477-18717883 FORWARD \| Aliases: ACCLIMATION OF PHOTOSYNTHESIS TO ENVIRONMENT, MCL19.16, MCL19_16, TPT | 6.0 | 3.8 | 2.2 | 4.2 | 2.07% | 3.7 |
| 5639 | AT5G40990.1 \| GDSL-motif lipase/hydrolase family protein, similar to lipase (Arabidopsis thaliana) GI:1145627; contains Pfam profile PF00657: Lipase/Acylhydrolase with GDSL-like motif \| chr5:16436148-16437628 FORWARD \| Aliases: MEE6.6, MEE6_6 | 3.8 | 2.2 | 1.6 | 4.2 | 2.08% | 3.6 |
| 5640 | AT2G29480.1 \| Symbol: ATGSTU2 \| glutathione S-transferase, putative, similar to Glutathione S-Transferase (Arabidopsis thaliana) gi:940381:16226389:gb:AF428387. \| chr2:12637459-12638309 REVERSE \| Aliases: F16P2.14, F16P2_14, GLUTATHIONE S TRANSFERASE 20, GST20 | 4.5 | 3.7 | 0.8 | 4.2 | 2.08% | 3.6 |
| 5641 | AT3G23210.1 \| basic helix-loop-helix (bHLH) family protein, similar to hypothetical protein GB:CAB10220 from (Arabidopsis thaliana) \| chr3:8283097-8285087 REVERSE \| Aliases: K14B15.12 | 5.9 | 5.3 | 0.6 | 4.2 | 2.08% | 3.4 |
| 5642 | AT4G31830.1 \| expressed protein \| chr4:15400610-15401138 REVERSE \| Aliases: F11C18.30, F11C18_30 | 2.9 | 2.5 | 0.4 | 4.2 | 2.08% | 3.1 |
| 5645 | AT4G30480.3 \| expressed protein \| chr4:14897352-14899127 FORWARD \| Aliases: F17I23.180, F17I23_180 | 9.1 | 7.3 | 1.9 | 4.2 | 2.08% | 3.5 |
| 5646 | AT1G23790.1 \| expressed protein \| chr1:8409952-8412030 FORWARD \| Aliases: F5O8.34, F5O8_34 | 3.6 | 2.8 | 0.8 | 4.2 | 2.08% | 3.5 |
| 5649 | AT1G80170.1 \| polygalacturonase, putative / pectinase, putative, similar to polygalacturonase GI:7381227 from (Lycopersicon esculentum); contains Pfam profile PF00295: Glycosyl hydrolases family 28 (polygalacturonases) \| chr1:30158300-30160584 REVERSE \| Aliases: F18B13.25, F18B13_25 | 5.5 | 4.4 | 1.1 | 4.2 | 2.09% | 3.6 |
| 5650 | AT5G01470.1 \| expressed protein \| chr5:190927-192610 FORWARD \| Aliases: T10O8.180, T10O8_180 | 5.1 | 4.0 | 1.1 | 4.2 | 2.09% | 3.7 |
| 5654 | AT5G39710.1 \| Symbol: EMB2745 \| pentatricopeptide (PPR) repeat-containing protein, contains Pfam profile PF01535: PPR repeat \| chr5:15912957-15915200 FORWARD \| Aliases: EMB2745, EMBRYO DEFECTIVE 2745, MIJ24.190, MIJ24_190 | 3.5 | 2.9 | 0.6 | 4.2 | 2.10% | 3.4 |
| 5655 | AT5G63580.1 \| flavonol synthase, putative, similar to SP:Q96330; contains PF03171 2OG-Fe(II) oxygenase superfamily \| chr5:25471956-25473702 FORWARD \| Aliases: MBK5.4, MBK5_4 | 2.7 | 2.2 | 0.5 | 4.2 | 2.10% | 3.0 |
| 5657 | AT3G04600.2 \| tRNA synthetase class I (W and Y) family protein, contains Pfam profile: PF00579 tRNA synthetases class I (W and Y) \| chr3:1242993-1246184 FORWARD \| Aliases: F7O18.7, F7O18_7 | 5.9 | 5.0 | 0.9 | 4.2 | 2.10% | 3.6 |
| 5658 | AT1G76710.2 \| SET domain-containing protein (ASHH1), low similarity to huntingtin interacting protein 1 (Homo sapiens) GI:12697196; contains Pfam profile PF00856: SET domain; identical to cDNA ASH1-like protein 1 (ASHH1) partial cds GI:15488417 \| chr1:28794623-28797475 REVERSE \| Aliases: F28O16.8, F28O16_8 | 6.0 | 4.9 | 1.1 | 4.2 | 2.10% | 3.6 |

| Rank | Description | Sync | Root | M | t | adj.q | B |
|---|---|---|---|---|---|---|---|
| 5659 | AT1G12775.1 \| similar to helicase domain-containing protein / pentatricopeptide (PPR) repeat-containing protein [Arabidopsis thaliana] (TAIR:At1g12700.1); similar to pentatricopeptide (PPR) repeat-containing protein [Arabidopsis thaliana] (TAIR:At1g12300.1); similar to pentatricopeptide (PPR) repeat-containing protein [Arabidopsis thaliana] (TAIR:At1g12620.1); similar to pentatricopeptide (PPR) repeat-containing protein [Arabidopsis thaliana] (TAIR:At1g62590.1); similar to pentatricopeptide (PPR) repeat-containing protein [Arabidopsis thaliana] (TAIR:At3g22470.1); similar to fertility restorer homologue A [Raphanus sativus] (GB:CAD80166.1); contains InterPro domain PPR repeat (InterPro:IPR002885) \| chr1:4353904-4355927 FORWARD \| Aliases: None | 4.5 | 3.7 | 0.8 | 4.2 | 2.10% | 3.5 |
| 5660 | AT4G17960.1 \| expressed protein \| chr4:9970859-9972468 FORWARD \| Aliases: T6K21.140, T6K21_140 | 5.9 | 4.3 | 1.6 | 4.2 | 2.10% | 3.6 |
| 5663 | AT5G08300.1 \| succinyl-CoA ligase (GDP-forming) alpha-chain, mitochondrial, putative / succinyl-CoA synthetase, alpha chain, putative / SCS-alpha, putative, identical to SP:P53586 Succinyl-CoA ligase (GDP-forming) alpha-chain, mitochondrial precursor (EC 6.2.1.4) (Succinyl-CoA synthetase, alpha chain) (SCS-alpha) {Arabidopsis thaliana}; strong similarity to SP:P13086 Succinyl-CoA ligase (GDP-forming) alpha-chain, mitochondrial precursor {Rattus norvegicus}; contains Pfam profiles PF00549: CoA-ligase, PF02629: CoA binding domain \| chr5:2667471-2669918 FORWARD \| Aliases: F8L15.30, F8L15_30 | 12.1 | 10.3 | 1.9 | 4.2 | 2.11% | 3.6 |
| 5665 | AT3G60660.1 \| expressed protein \| chr3:22432385-22434452 FORWARD \| Aliases: T4C21.70 | 3.1 | 2.4 | 0.7 | 4.2 | 2.11% | 3.4 |
| 5666 | AT2G23390.1 \| expressed protein \| chr2:9967452-9970699 REVERSE \| Aliases: F26B6.4, F26B6_4 | 6.2 | 5.1 | 1.1 | 4.2 | 2.11% | 3.6 |
| 5667 | AT3G27520.1 \| expressed protein \| chr3:10193659-10194736 FORWARD \| Aliases: MMJ24.7 | 3.1 | 2.6 | 0.5 | 4.2 | 2.11% | 3.3 |
| 5668 | AT3G22880.1 \| Symbol: ATDMC1 \| | 8.4 | 6.3 | 2.0 | 4.2 | 2.11% | 3.6 |
| 5670 | AT3G27930.1 \| expressed protein \| chr3:10370536-10373673 REVERSE \| Aliases: K24A2.1 | 6.5 | 5.8 | 0.7 | 4.2 | 2.12% | 3.5 |
| 5672 | AT4G35920.3 \| expressed protein, contains Pfam profile PF04749: Protein of unknown function, DUF614; isoform contains AT-acceptor splice site at intron 8 \| chr4:17011791-17014821 REVERSE \| Aliases: T19K4.50 | 8.2 | 6.5 | 1.7 | 4.2 | 2.12% | 3.5 |
| 5674 | AT1G11940.1 \| expressed protein, contains Pfam profile PF03267: Arabidopsis protein of unknown function, DUF266 \| chr1:4031578-4034076 REVERSE \| Aliases: F12F1.19, F12F1_19 | 4.9 | 3.8 | 1.1 | 4.2 | 2.12% | 3.6 |
| 5675 | AT3G22780.1 \| Symbol: TSO1 \| CXC domain protein (TSO1), identical to CXC domain protein TSO1 (Arabidopsis thaliana) GI:7767425 \| chr3:8048809-8052819 FORWARD \| Aliases: MWI23.15 | 7.0 | 5.5 | 1.5 | 4.2 | 2.12% | 3.5 |
| 5681 | ATCG00080.1 \| Symbol: PSBI \| PSII I protein \| chrC:7583-7693 FORWARD \| Aliases: PSBI | 10.1 | 7.3 | 2.8 | 4.2 | 2.13% | 3.4 |
| 5682 | AT3G63090.1 \| expressed protein \| chr3:23324403-23325964 FORWARD \| Aliases: T20O10.190 | 4.7 | 3.8 | 0.9 | 4.2 | 2.13% | 3.6 |
| 5683 | AT5G65350.1 \| histone H3, nearly identical to histone H3 from Zea mays SP:P05203, Medicago sativa GI:166384, Encephalartos altensteinii SP:P08903, Pisum sativum SP:P02300; contains Pfam profile PF00125 Core histone H2A/H2B/H3/H4 \| chr5:26136437-26137051 REVERSE \| Aliases: MNA5.8, MNA5_8 | 2.9 | 2.4 | 0.5 | 4.2 | 2.13% | 3.2 |
| 5685 | AT1G59890.1 \| paired amphipathic helix repeat-containing protein, similar to transcription co-repressor Sin3 (Xenopus laevis) GI:4960210; contains Pfam profile PF02671: Paired amphipathic helix repeat \| chr1:22047991-22054726 FORWARD \| Aliases: F23H11.20, F23H11_20 | 5.2 | 4.0 | 1.2 | 4.2 | 2.13% | 3.7 |
| 5687 | AT1G44890.1 \| expressed protein \| chr1:16965584-16967406 FORWARD \| Aliases: T12C22.18, T12C22_18 | 7.0 | 5.7 | 1.3 | 4.2 | 2.13% | 3.6 |
| 5689 | AT1G19010.2 \| expressed protein \| chr1:6565774-6567515 FORWARD \| Aliases: F14D16.16, F14D16_16 | 4.4 | 3.6 | 0.9 | 4.2 | 2.13% | 3.6 |
| 5691 | AT5G13970.1 \| expressed protein \| chr5:4506234-4507807 REVERSE \| Aliases: MAC12.6, MAC12_6 | 7.6 | 6.4 | 1.2 | 4.2 | 2.14% | 3.4 |
| 5692 | AT1G30490.1 \| Symbol: PHV \| homeobox-leucine zipper transcription factor (HB-9), identical to HD-Zip protein GB:CAA71854 GI:2145358 from (Arabidopsis thaliana) \| chr1:10796117-10800885 REVERSE \| Aliases: ATHB9, F26G16.11, F26G16_11, PHAVOLUTA | 6.9 | 4.9 | 2.0 | 4.2 | 2.14% | 3.7 |
| 5693 | AT3G26360.1 \| ribosomal protein-related, similar to SP:Q9Z3S4 30S ribosomal protein S21 {Rhizobium meliloti} \| chr3:9656519-9657610 REVERSE \| Aliases: F20C19.8 | 7.4 | 6.3 | 1.1 | 4.2 | 2.14% | 3.6 |
| 5695 | AT2G25950.1 \| expressed protein \| chr2:11075729-11077738 FORWARD \| Aliases: F17H15.2 | 9.4 | 7.3 | 2.0 | 4.2 | 2.14% | 3.7 |
| 5696 | AT3G22550.1 \| senescence-associated protein-related, similar to senescence-associated protein SAG102 (GI:22331931) (Arabidopsis thaliana) \| chr3:7991653-7993461 REVERSE \| Aliases: F16J14.11 | 4.8 | 3.9 | 0.9 | 4.2 | 2.14% | 3.6 |
| 5698 | AT1G76170.1 \| expressed protein, contains Pfam profile PF01171: PP-loop family \| chr1:28589073-28591119 REVERSE \| Aliases: T23E18.11, T23E18_11 | 4.1 | 3.4 | 0.7 | 4.2 | 2.14% | 3.4 |

| Rank | Description | Sync | Root | M | t | adj.q | B |
|---|---|---|---|---|---|---|---|
| 5699 | AT4G34700.1 \| complex 1 family protein / LVR family protein, contains Pfam PF05347: Complex 1 protein (LYR family) \| chr4:16556850-16558851 FORWARD \| Aliases: T4L20.280, T4L20_280 | 11.7 | 10.4 | 1.3 | 4.2 | 2.14% | 3.1 |
| 5701 | AT1G10510.1 \| Symbol: EMB2004 \| leucine-rich repeat family protein, similar to ribonuclease inhibitor (GI:164639) (Sus scrofa (pig)); contains Pfam PF00560: Leucine Rich Repeat domains \| chr1:3461640-3465787 FORWARD \| Aliases: EMB2004, EMBRYO DEFECTIVE 2004, T10O24.12, T10O24_12 | 6.8 | 5.9 | 0.9 | 4.2 | 2.14% | 3.5 |
| 5702 | AT4G33250.1 \| Symbol: EIF3K \| eukaryotic translation initiation factor 3 subunit 11 / eIF-3 p25 / eIF3k (TIF3K1), identical to Swiss-Prot:Q9SZA3 eukaryotic translation initiation factor 3 subunit 11 (eIF-3 p25) (eIF3k) (Arabidopsis thaliana); identical to cDNA initiation factor 3k GI:12407752 | 10.2 | 8.3 | 1.9 | 4.2 | 2.14% | 3.5 |
| 5703 | AT2G39080.1 \| expressed protein \| chr2:16316907-16319587 REVERSE \| Aliases: T7F6.25, T7F6_25 | 6.4 | 5.9 | 0.6 | 4.2 | 2.14% | 3.3 |
| 5705 | AT1G19920.1 \| Symbol: APS2 \| sulfate adenylyltransferase 2 / ATP-sulfurylase 2 (ASA1) (MET3-1) (APS2), identical to ATP sulfurylase (APS2) (Arabidopsis thaliana) GI:1575324 \| chr1:6914604-6916745 REVERSE \| Aliases: ASA1, ATP SULFURYLASE PRECURSOR, F6F9.2, F6F9_2 | 9.3 | 6.8 | 2.5 | 4.2 | 2.15% | 3.6 |
| 5706 | AT5G45590.1 \| expressed protein \| chr5:18501734-18503505 REVERSE \| Aliases: K2N11.6, K2N11_6 | 4.8 | 3.2 | 1.7 | 4.2 | 2.15% | 3.6 |
| 5707 | AT2G34750.1 \| RNA polymerase I specific transcription initiation factor RRN3 family protein, contains Pfam PF05327: RNA polymerase I specific transcription initiation factor RRN3; similar to RRN3 (GI:7670100) (Homo sapiens) similar to RNA polymerase I specific transcription initiation factor RRN3 (Swiss-Prot:P36070) (Saccharomyces cerevisiae) \| chr2:14667387-14671345 REVERSE \| Aliases: T29F13.4, T29F13_4 | 9.0 | 7.9 | 1.1 | 4.2 | 2.15% | 3.6 |
| 5708 | AT5G48485.1 \| Symbol: DIR1 \| protease inhibitor/seed storage/lipid transfer protein (LTP) family protein, contains Pfam protease inhibitor/seed storage/LTP family domain PF00234 \| chr5:19663458-19664028 REVERSE \| Aliases: DEFECTIVE IN INDUCED RESISTANCE 1 | 3.5 | 3.0 | 0.6 | 4.2 | 2.15% | 3.1 |
| 5709 | AT2G33600.1 \| cinnamoyl-CoA reductase family, similar to cinnamoyl-CoA reductase from Pinus taeda (GI:17978649), Eucalyptus gunnii (GI:2058311) \| chr2:14233842-14235787 FORWARD \| Aliases: F4P9.37, F4P9_37 | 5.2 | 3.9 | 1.3 | 4.2 | 2.15% | 3.6 |
| 5712 | AT3G11580.2 \| DNA-binding protein, putative, similar to DNA-binding proteins from (Arabidopsis thaliana) RAV1 GI:3868857 and RAV2 GI:3868859, AP2 domain containing protein RAP2.8 (Arabidopsis thaliana) GI:2281641; contains Pfam profile PF02362: B3 DNA binding domain \| chr3:3650546-3651323 REVERSE \| Aliases: F24K9.25 | 5.3 | 3.7 | 1.6 | 4.2 | 2.15% | 3.6 |
| 5714 | AT3G21780.1 \| UDP-glucoronosyl/UDP-glucosyl transferase family protein, contains Pfam profile: PF00201 UDP-glucoronosyl and UDP-glucosyl transferase \| chr3:7675058-7676353 REVERSE \| Aliases: MSD21.11 | 3.6 | 2.5 | 1.1 | 4.2 | 2.16% | 3.6 |
| 5715 | AT1G05060.1 \| expressed protein \| chr1:1451057-1452871 REVERSE \| Aliases: T7A14.7, T7A14_7 | 5.8 | 4.0 | 1.7 | 4.2 | 2.16% | 3.7 |
| 5716 | AT1G30880.1 \| expressed protein, similar to ESTs gb:R30049 and gb:T46176 \| chr1:10993215-10994100 REVERSE \| Aliases: F17F8.25 | 8.7 | 6.9 | 1.8 | 4.2 | 2.16% | 3.7 |
| 5717 | AT2G34090.2 \| expressed protein, similar to GP:1301832:gnl:PID:e239862:Z71287 | 3.8 | 3.0 | 0.8 | 4.2 | 2.16% | 3.5 |
| 5723 | AT1G21720.1 \| Symbol: PBC1 \| 20S proteasome beta subunit C1 (PBC1) (PRCT), almost identical to GB:AAC32069 from (Arabidopsis thaliana), EST gb:T76747 comes from this gene; identical to cDNA proteasome subunit prct GI:2511567 \| chr1:7626272-7628251 FORWARD \| Aliases: 20S PROTEASOME BETA SUBUNIT PBC1, F8K7.15, F8K7_15 | 10.0 | 6.1 | 3.9 | 4.2 | 2.16% | 3.6 |
| 5725 | AT2G44210.2 \| similar to expressed protein [Arabidopsis thaliana] (TAIR:At1g55360.1); similar to putative DD1A protein [Oryza sativa (japonica cultivar-group)] (GB:XP_477068.1); contains InterPro domain Protein of unknown function DUF239 (InterPro:IPR004314) \| chr2:18287699-18289952 FORWARD \| Aliases: F4I1.2 | 5.1 | 3.6 | 1.4 | 4.2 | 2.16% | 3.6 |
| 5727 | AT2G39960.1 \| microsomal signal peptidase 25 kDa subunit, putative (SPC25), identical to Probable microsomal signal peptidase 25 kDa subunit (EC 3.4.-.-) (SPase 25 kDa subunit) (SPC25) (Swiss-Prot:P58684) (Arabidopsis thaliana); contains non-consensus AT-AC splice sites; contains 1 transmembrane domain; \| chr2:16688609-16690718 REVERSE \| Aliases: T28M21.12, T28M21_12 | 8.2 | 6.9 | 1.3 | 4.2 | 2.16% | 3.6 |
| 5732 | AT3G20060.1 \| Symbol: UBC19 \| ubiquitin-conjugating enzyme 19 (UBC19), nearly identical to ubiquitin-conjugating enzyme UBC19 (Arabidopsis thaliana) GI:22530865; contains Pfam profile PF00179: Ubiquitin-conjugating enzyme | 7.3 | 6.1 | 1.2 | 4.2 | 2.16% | 3.5 |
| 5734 | AT1G61640.2 \| ABC1 family protein, contains Pfam domain, PF03109: ABC1 family \| chr1:22750066-22752849 REVERSE \| Aliases: T25B24.1, T25B24_1 | 4.4 | 3.5 | 0.9 | 4.2 | 2.17% | 3.5 |
| 5735 | AT5G43850.1 \| acireductone dioxygenase (ARD/ARD') family protein, similar to iron-deficiency induced gene (Hordeum vulgare) GI:14522834, SIPL (Homo sapiens) GI:16551383; contains Pfam profile PF03079: ARD/ARD' family \| chr5:17644412-17646385 REVERSE \| Aliases: MQD19.21, MQD19_21 | 9.8 | 9.0 | 0.9 | 4.2 | 2.17% | 3.3 |
| 5736 | AT2G27040.1 \| Symbol: AGO4 \| PAZ domain-containing protein / piwi domain-containing protein, similar to SP:Q9QZ81 Eukaryotic translation initiation factor 2C 2 {Rattus norvegicus}; contains Pfam profiles PF02171: Piwi domain, PF02170: PAZ domain | 6.5 | 5.0 | 1.5 | 4.2 | 2.17% | 3.6 |

| Rank | Description | Sync | Root | M | t | adj.q | B |
|---|---|---|---|---|---|---|---|
| 5737 | AT2G31370.5 \| similar to bZIP transcription factor, putative (bZIP69) [Arabidopsis thaliana] (TAIR:At1g06070.1); similar to RF2a [Oryza sativa] (GB:AAC49832.1); contains InterPro domain Basic-leucine zipper (bZIP) transcription factor (InterPro:IPR004827) \| chr2:13386110-13388600 FORWARD \| Aliases: T28P16.14, T28P16_14 | 6.6 | 5.0 | 1.6 | 4.2 | 2.17% | 3.6 |
| 5738 | AT1G56350.1 \| peptide chain release factor, putative, similar to SP:P28353 Peptide chain release factor 2 (RF-2). {Salmonella typhi}; contains Pfam profiles PF00472: Peptidyl-tRNA hydrolase domain, PF03462: PCRF domain \| chr1:21098443-21101491 FORWARD \| Aliases: F13N6.6, F13N6_6 | 5.7 | 4.7 | 1.0 | 4.2 | 2.17% | 3.6 |
| 5739 | AT1G28510.1 \| expressed protein \| chr1:10023875-10026281 FORWARD \| Aliases: F3M18.5, F3M18_5 | 6.4 | 5.1 | 1.3 | 4.2 | 2.18% | 3.5 |
| 5742 | AT3G03070.1 \| NADH-ubiquinone oxidoreductase-related, contains weak similarity to NADH-ubiquinone oxidoreductase 13 kDa-A subunit, mitochondrial precursor (EC 1.6.5.3) (EC 1.6.99.3) (Complex I-13KD-A) (CI-13KD-A) (Swiss-Prot:P23934) (Bos taurus) \| chr3:696306-698163 REVERSE \| Aliases: T17B22.24, T17B22_24 | 8.9 | 7.7 | 1.3 | 4.2 | 2.18% | 3.6 |
| 5743 | AT5G20550.1 \| oxidoreductase, 2OG-Fe(II) oxygenase family protein, similar to leucoanthocyanidin dioxygenase (Malus domestica)(SP:P51091), flavonol synthase (Petunia x hybrida)(GI:311658); contains PF03171 2OG-Fe(II) oxygenase superfamily domain \| chr5:6952419-6953883 REVERSE \| Aliases: F7C8.140, F7C8_140 | 7.6 | 5.9 | 1.7 | 4.2 | 2.18% | 3.6 |
| 5745 | AT2G40490.1 \| uroporphyrinogen decarboxylase, putative / UPD, putative, similar to uroporphyrinogen decarboxylase (chloroplast) from Nicotiana tabacum (SP:Q42967), Hordeum vulgare (SP:Q42855), Zea mays (SP:O81220); contains Pfam domain uroporphyrinogen decarboxylase (URO-D) PF01208 \| chr2:16919940-16922255 FORWARD \| Aliases: T2P4.16, T2P4_16 | 5.3 | 4.4 | 0.9 | 4.2 | 2.19% | 3.4 |
| 5747 | AT3G62310.1 \| RNA helicase, putative, similar to SP:P53131 Pre-mRNA splicing factor RNA helicase PRP43 (Helicase JA1) {Saccharomyces cerevisiae}; contains Pfam profiles PF04408: Helicase associated domain (HA2), PF00271: Helicase conserved C-terminal domain \| chr3:23068221-23071665 REVERSE \| Aliases: T12C14.10 | 7.4 | 6.2 | 1.2 | 4.2 | 2.19% | 3.5 |
| 5748 | AT3G12260.1 \| complex 1 family protein / LVR family protein, contains Pfam PF05347: Complex 1 protein (LYR family) \| chr3:3908947-3910403 REVERSE \| Aliases: F28J15.12 | 10.5 | 8.2 | 2.3 | 4.2 | 2.20% | 3.5 |
| 5753 | AT2G24050.1 \| MIF4G domain-containing protein / MA3 domain-containing protein, similar to SP:Q03387 Eukaryotic initiation factor (iso)4F subunit P82-34 (eIF-(iso)4F P82-34) {Triticum aestivum}; contains Pfam profiles PF02854: MIF4G domain, PF02847: MA3 domain \| chr2:10232305-10235718 REVERSE \| Aliases: T29E15.25, T29E15_25 | 6.7 | 5.8 | 0.9 | 4.2 | 2.21% | 3.4 |
| 5755 | AT5G01410.1 \| stress-responsive protein, putative, similar to ethylene-inducible protein HEVER (Hevea brasiliensis) SWISS-PROT:Q39963 \| chr5:172317-173613 REVERSE \| Aliases: T10O8.120, T10O8_120 | 10.1 | 7.9 | 2.2 | 4.2 | 2.21% | 3.6 |
| 5759 | AT4G25550.1 \| expressed protein \| chr4:13048386-13051178 FORWARD \| Aliases: M7J2.80, M7J2_80 | 7.3 | 5.4 | 1.9 | 4.2 | 2.21% | 3.6 |
| 5760 | AT1G03475.1 \| Symbol: LIN2 \| coproporphyrinogen III oxidase, putative / coproporphyrinogenase, putative / coprogen oxidase, putative, similar to coproporphyrinogen III oxidase, chloroplast (precursor) from Glycine max (SP:P35055), Nicotiana tabacum (SP:Q42946), Hordeum vulgare (SP:Q42840), ESTs gb:AA586260 and dbj:D48620; contains Pfam domain coproporphyrinogen III oxidase, aerobic (PF01218) \| chr1:869051-871212 REVERSE \| Aliases: COPROPORPHYRINOGEN III OXIDASE, F21B7.10, LESION INITIATION 2 | 5.3 | 3.9 | 1.4 | 4.2 | 2.21% | 3.6 |
| 5761 | AT4G09900.1 \| hydrolase, alpha/beta fold family protein, similar to ethylene-induced esterase (Citrus sinensis) GI:14279437, polyneuridine aldehyde esterase (Rauvolfia serpentina) GI:6651393, SP:Q40708 PIR7A protein {Oryza sativa}; contains Pfam profile PF00561: hydrolase, alpha/beta fold family \| chr4:6221661-6224130 REVERSE \| Aliases: AT4G09910, T5L19.30, T5L19_30 | 3.5 | 2.8 | 0.7 | 4.2 | 2.21% | 3.3 |
| 5762 | AT3G03630.1 \| Symbol: CS26 \| cysteine synthase, chloroplast, putative / O-acetylserine (thiol)-lyase, putative / O-acetylserine sulfhydrylase, putative, identical to SP:O22682 Probable cysteine synthase, chloroplast precursor {Arabidopsis thaliana}, similar to SP:P31300 Cysteine synthase, chloroplast precursor {Capsicum annuum} \| chr3:877951-880453 REVERSE \| Aliases: O ACETYLSERINE (THIOL) LYASE, T12J13.9, T12J13_9 | 4.5 | 3.5 | 1.0 | 4.2 | 2.21% | 3.5 |
| 5763 | AT5G59980.1 \| RNase P subunit p30 family protein, contains Pfam PF01876: RNase P subunit p30 \| chr5:24164433-24166953 FORWARD \| Aliases: MMN10.23, MMN10_23 | 3.3 | 2.6 | 0.7 | 4.2 | 2.21% | 3.5 |
| 5764 | AT1G74070.1 \| peptidyl-prolyl cis-trans isomerase cyclophilin-type family protein, contains Pfam domain, PF00160: peptidyl-prolyl cis-trans isomerase, cyclophilin-type \| chr1:27855149-27856637 REVERSE \| Aliases: F2P9.6, F2P9_6 | 4.4 | 3.3 | 1.1 | 4.2 | 2.22% | 3.6 |
| 5765 | AT3G61770.1 \| expressed protein, contains Pfam domain, PF02681: Uncharacterized BCR, COG1963 \| chr3:22875518-22877380 FORWARD \| Aliases: F21F14.5 | 5.0 | 4.0 | 1.1 | 4.2 | 2.22% | 3.6 |
| 5766 | AT5G20890.1 \| chaperonin, putative, similar to SWISS-PROT:P78371- T-complex protein 1, beta subunit (TCP-1-beta) (Homo sapiens); contains Pfam:PF00118 domain, TCP-1/cpn60 chaperonin family \| chr5:7086655-7090083 REVERSE \| Aliases: F22D1.60, F22D1_60 | 9.7 | 8.6 | 1.1 | 4.2 | 2.22% | 3.3 |

| Rank | Description | Sync | Root | M | t | adj.q | B |
|---|---|---|---|---|---|---|---|
| 5769 | AT5G04910.1 \| expressed protein, ; expression supported by MPSS \| chr5:1437696-1438771 FORWARD \| Aliases: MUG13.24, MUG13_24 | 8.9 | 7.9 | 1.0 | 4.2 | 2.22% | 3.3 |
| 5770 | AT3G06530.1 \| BAP28-related, similar to Protein BAP28 (Swiss-Prot:Q9H583) (Homo sapiens) \| chr3:2022602-2033643 FORWARD \| Aliases: F5E6.14, F5E6_14 | 4.4 | 3.7 | 0.7 | 4.2 | 2.22% | 3.4 |
| 5772 | AT3G04890.1 \| expressed protein \| chr3:1347652-1349205 FORWARD \| Aliases: T9J14.16, T9J14_16 | 6.7 | 5.9 | 0.8 | 4.1 | 2.22% | 3.5 |
| 5776 | AT3G22660.1 \| rRNA processing protein-related, contains weak similarity to rRNA processing protein EBP2 (EBNA1-binding protein homolog) (Swiss-Prot:P36049) (Saccharomyces cerevisiae) | 7.1 | 6.3 | 0.8 | 4.1 | 2.23% | 3.4 |
| 5784 | AT5G38480.2 \| Symbol: GRF3 \| similar to 14-3-3 protein GF14 nu (GRF7) [Arabidopsis thaliana] (TAIR:At3g02520.1); similar to 14-3-3 e-1 protein [Nicotiana tabacum] (GB:BAD12176.1); similar to 14-3-3 e-2 protein [Nicotiana tabacum] (GB:BAD12177.1); contains InterPro domain 14-3-3 protein (InterPro:IPR000308) \| chr5:15426927-15428746 FORWARD \| Aliases: GENERAL REGULATORY FACTOR 3, GF14PSI ISOFORM, MXI10.21, MXI10_21, RCI1 | 9.2 | 7.0 | 2.2 | 4.1 | 2.25% | 3.5 |
| 5785 | AT5G25900.1 \| Symbol: GA3 \| ent-kaurene oxidase, putative (GA3) / cytochrome P450, identical to GA3 (Arabidopsis thaliana) GI:3342249; similar to ent-kaurene oxidase (Cucurbita maxima) GI:11934675; contains Pfam profile PF00067: Cytochrome P450 \| chr5:9036046-9038399 FORWARD \| Aliases: CYP701A3, ENT KAURENE OXIDASE, GA REQUIRING 3, T1N24.23, T1N24_23 | 7.3 | 6.4 | 0.8 | 4.1 | 2.25% | 3.5 |
| 5786 | AT3G18500.1 \| similar to endonuclease/exonuclease/phosphatase family protein [Arabidopsis thaliana] (TAIR:At1g73875.1); similar to endonuclease/exonuclease/phosphatase family protein-like [Oryza sativa (japonica cultivar-group)] (GB:BAD52532.1); contains InterPro domain Endonuclease/exonuclease/phosphatase family (InterPro:IPR005135) \| chr3:6352544-6355446 FORWARD \| Aliases: MYF24.23 | 4.6 | 3.5 | 1.1 | 4.1 | 2.25% | 3.5 |
| 5788 | AT4G31120.2 \| Skb1 methyltransferase family protein, contains Pfam profile: PF05185 Skb1 methyltransferase \| chr4:15132125-15136624 REVERSE \| Aliases: F6E21.40, F6E21_40 | 7.2 | 6.4 | 0.9 | 4.1 | 2.25% | 3.4 |
| 5789 | AT3G19240.1 \| expressed protein \| chr3:6664256-6666564 FORWARD \| Aliases: MVI11.21 | 9.6 | 8.3 | 1.3 | 4.1 | 2.25% | 3.6 |
| 5790 | AT1G56340.2 \| Symbol: CRT1 \| similar to calreticulin 2 (CRT2) [Arabidopsis thaliana] (TAIR:At1g09210.1); similar to calreticulin [Beta vulgaris subsp. vulgaris] (GB:CAA05161.1); similar to calreticulin [Nicotiana plumbaginifolia] (GB:CAA95999.1); similar to calreticulin (GB:AAA80652.1); similar to calcium-binding protein calreticulin [Prunus armeniaca] (GB:AAD32207.1); similar to CRTC_RICCO Calreticulin precursor (GB:P93508); contains InterPro domain Calreticulin (InterPro:IPR001580) \| chr1:21093545-21096322 REVERSE \| Aliases: CALRETICULIN 1, F14G9.5, F14G9_5 | 10.3 | 9.0 | 1.3 | 4.1 | 2.25% | 3.6 |
| 5791 | AT5G47455.6 \| expressed protein \| chr5:19267950-19269591 FORWARD \| Aliases: None | 4.5 | 3.3 | 1.1 | 4.1 | 2.26% | 3.5 |
| 5792 | AT4G31460.1 \| ribosomal protein L28 family protein, ribosomal protein YmL14 precursor, mitochondrial - Saccharomyces cerevisiae,PIR2:S50921 \| chr4:15259475-15261197 REVERSE \| Aliases: F3L17.30, F3L17_30 | 6.2 | 4.8 | 1.4 | 4.1 | 2.26% | 3.5 |
| 5795 | AT5G63700.1 \| zinc finger (C3HC4 type RING finger) family protein, contains Pfam PF03126: Plus-3 domain; contains Pfam PF02201: BAF60b domain of the SWIB complex; contains Pfam PF00628: PHD-finger domain; contains Prosite Zinc finger, C3HC4 type (RING finger), signature; similar to CPRF interacting protein (GI:9588690) (Petroselinum crispum) \| chr5:25511160-25514858 REVERSE \| Aliases: MBK5.18, MBK5_18 | 5.8 | 4.4 | 1.3 | 4.1 | 2.26% | 3.5 |
| 5798 | AT3G12860.1 \| nucleolar protein Nop56, putative, similar to XNop56 protein (Xenopus laevis) GI:14799394; contains Pfam profile PF01798: Putative snoRNA binding domain | 6.4 | 5.1 | 1.3 | 4.1 | 2.27% | 3.5 |
| 5799 | AT1G26330.1 \| expressed protein \| chr1:9107612-9113507 FORWARD \| Aliases: F28B23.2, F28B23_2 | 4.1 | 2.9 | 1.3 | 4.1 | 2.27% | 3.6 |
| 5800 | AT3G57190.1 \| peptide chain release factor, putative, similar to SP:P07012 Peptide chain release factor 2 (RF-2) {Escherichia coli}; contains Pfam profile PF03462: PCRF domain \| chr3:21177382-21179355 FORWARD \| Aliases: F28O9.40 | 4.0 | 3.2 | 0.8 | 4.1 | 2.27% | 3.5 |
| 5801 | AT2G26380.1 \| disease resistance protein-related / LRR protein-related, contains leucine rich-repeat domains Pfam:PF00560, INTERPRO:IPR001611; similar to Hcr2-2A (Lycopersicon pimpinellifolium) gi:3894389:gb:AAC78594 \| chr2:11233693-11235135 REVERSE \| Aliases: T9J22.5, T9J22_5 | 3.3 | 2.6 | 0.7 | 4.1 | 2.27% | 3.4 |
| 5802 | AT1G35550.1 \| elongation factor Tu C-terminal domain-containing protein, similar to SP:P13905 Elongation factor 1-alpha (EF-1-alpha) {Arabidopsis thaliana}; contains Pfam profile PF03143: Elongation factor Tu C-terminal domain \| chr1:13112484-13113014 FORWARD \| Aliases: F15O4.37 | 6.1 | 5.0 | 1.1 | 4.1 | 2.28% | 3.6 |
| 5803 | AT2G37540.1 \| short-chain dehydrogenase/reductase (SDR) family protein, contains INTERPRO family IPR002198 Short-chain dehydrogenase/reductase (SDR) superfamily \| chr2:15758653-15761045 REVERSE \| Aliases: F13M22.4, F13M22_4 | 3.2 | 2.4 | 0.9 | 4.1 | 2.28% | 3.2 |

| Rank | Description | Sync | Root | M | t | adj.q | B |
|---|---|---|---|---|---|---|---|
| 5805 | AT4G12740.1 \| adenine-DNA glycosylase-related / MYH-related, similar to MYH (GI:18845094) (Rattus norvegicus); similar to adenine-DNA glycosylase (GI:12656850) (Mus musculus); contains TIGRFAM profile TIGR01084: A/G-specific adenine glycosylase (hits below the trusted cutoff) \| chr4:7494575-7497593 REVERSE \| Aliases: T20K18.90, T20K18_90 | 3.9 | 3.2 | 0.7 | 4.1 | 2.28% | 3.4 |
| 5807 | AT1G14260.2 \| zinc finger (C3HC4-type RING finger) family protein, contains Pfam domain, PF00097: Zinc finger, C3HC4 type (RING finger) \| chr1:4872900-4874821 FORWARD \| Aliases: F14L17.2, F14L17_2 | 3.1 | 2.5 | 0.6 | 4.1 | 2.29% | 3.3 |
| 5809 | AT1G48240.1 \| Symbol: ATNPSN12 \| novel plant SNARE 12 (NPSN12), identical to Novel plant SNARE 12 (AtNPSN12) (Swiss-Prot:Q9LNH6) (Arabidopsis thaliana) \| chr1:17812676-17815563 REVERSE \| Aliases: F21D18.4, NPSN12 | 6.7 | 4.9 | 1.8 | 4.1 | 2.30% | 3.5 |
| 5811 | AT4G01080.1 \| expressed protein \| chr4:466391-468293 REVERSE \| Aliases: F2N1.14, F2N1_14 | 3.8 | 2.9 | 0.9 | 4.1 | 2.30% | 3.5 |
| 5812 | AT5G42890.1 \| sterol carrier protein 2 (SCP-2) family protein, similar to sterol carrier protein-2 (Homo sapiens) GI:432973; contains Pfam profile PF02036: SCP-2 sterol transfer family \| chr5:17211500-17213260 REVERSE \| Aliases: MBD2.8, MBD2_8 | 9.9 | 8.7 | 1.1 | 4.1 | 2.30% | 3.4 |
| 5814 | AT3G20000.1 \| Symbol: TOM40 \| porin family protein, low similarity to haymaker protein (Mus musculus) GI:17834089, mitochondrial outer membrane protein MOM35 (Mus musculus) GI:6650562; contains Pfam profile PF01459: Eukaryotic porin \| chr3:6967596-6970571 FORWARD \| Aliases: MZE19.5 | 8.6 | 7.0 | 1.5 | 4.1 | 2.30% | 3.5 |
| 5819 | AT2G44640.1 \| expressed protein \| chr2:18424319-18426472 FORWARD \| Aliases: F16B22.13 | 7.6 | 5.3 | 2.3 | 4.1 | 2.31% | 3.6 |
| 5820 | AT5G11310.1 \| pentatricopeptide (PPR) repeat-containing protein, contains Pfam profile PF01535: PPR repeat \| chr5:3606403-3608608 FORWARD \| Aliases: F2I11.200, F2I11_200 | 3.4 | 2.8 | 0.6 | 4.1 | 2.31% | 3.1 |
| 5821 | AT2G22370.1 \| expressed protein \| chr2:9507895-9509269 REVERSE \| Aliases: F14M13.23, F14M13_23 | 6.8 | 5.7 | 1.2 | 4.1 | 2.31% | 3.4 |
| 5822 | AT5G16780.1 \| SART-1 family protein, contains Pfam domain, PF03343: SART-1 family \| chr5:5517528-5522261 REVERSE \| Aliases: F5E19.120, F5E19_120 | 8.2 | 6.2 | 2.0 | 4.1 | 2.31% | 3.6 |
| 5824 | AT3G22790.1 \| kinase interacting family protein, similar to kinase interacting protein 1 (GI:13936326) (Petunia integrifolia) \| chr3:8052453-8057895 REVERSE \| Aliases: MWI23.16 | 4.1 | 3.4 | 0.7 | 4.1 | 2.31% | 3.4 |
| 5827 | AT4G18040.1 \| Symbol: EIF4E \| eukaryotic translation initiation factor 4E 1 / eIF-4E1 / mRNA cap-binding protein 1 (EIF4E1), identical to SP:O23252 Eukaryotic translation initiation factor 4E (eIF-4E) (eIF4E) (mRNA cap-binding protein) (eIF-4F 25 kDa subunit) (eIF-4F P26 subunit) {Arabidopsis thaliana} \| chr4:10016583-10018198 REVERSE \| Aliases: AT.EIF4E1, CUCUMOVIRUS MULTIPLICATION 1, CUM1, EUKARYOTIC TRANSLATION INITATION FACTOR 4E, F15J5.10, F15J5_10, LSP1 | 10.3 | 8.1 | 2.2 | 4.1 | 2.32% | 3.5 |
| 5828 | AT3G60440.1 \| similar to expressed protein [Arabidopsis thaliana] (TAIR:At3g60450.1); similar to COG0406: Fructose-2,6-bisphosphatase [Trichodesmium erythraeum IMS101] (GB:ZP_00325866.1) \| chr3:22348768-22350421 FORWARD \| Aliases: T8B10.100 | 4.6 | 3.6 | 0.9 | 4.1 | 2.32% | 3.5 |
| 5829 | AT1G19960.1 \| expressed protein \| chr1:6928074-6928268 FORWARD \| Aliases: T20H2.25, T20H2_25 | 6.7 | 4.6 | 2.1 | 4.1 | 2.32% | 3.6 |
| 5830 | AT4G11175.1 \| translation initiation factor IF-1, chloroplast, putative, similar to Swiss-Prot:P08698 translation initiation factor IF-1, chloroplast (Spinacia oleracea); contains Pfam profile PF00575: S1 RNA binding domain \| chr4:6818089-6819038 FORWARD \| Aliases: T22B4.1, T22B4_1 | 4.3 | 3.5 | 0.8 | 4.1 | 2.32% | 3.2 |
| 5831 | AT3G13440.1 \| expressed protein \| chr3:4377104-4379582 FORWARD \| Aliases: MRP15.19 | 7.3 | 6.2 | 1.1 | 4.1 | 2.32% | 3.5 |
| 5833 | AT1G53850.1 \| Symbol: PAE1 \| 20S proteasome alpha subunit E1 (PAE1), identical to 20S proteasome subunit PAE1 GI:3421087 from (Arabidopsis thaliana) \| chr1:20107622-20109663 REVERSE \| Aliases: 20S PROTEASOME SUBUNIT PAE1, T18A20.8, T18A20_8 | 9.0 | 7.7 | 1.3 | 4.1 | 2.32% | 3.4 |
| 5835 | AT1G54350.1 \| ABC transporter family protein, similar to hypothetical ABC transporter ATP-binding protein GI:9955395 from (Microcystis aeruginosa) \| chr1:20290549-20294068 FORWARD \| Aliases: F20D21.17, F20D21_17 | 6.3 | 5.5 | 0.8 | 4.1 | 2.33% | 3.3 |
| 5837 | AT2G41530.1 \| esterase, putative, similar to SP:P10768 Esterase D (EC 3.1.1.1) {Homo sapiens}; contains Pfam profile: PF00756 putative esterase \| chr2:17330573-17332689 REVERSE \| Aliases: T32G6.5, T32G6_5 | 10.4 | 8.9 | 1.5 | 4.1 | 2.33% | 3.4 |
| 5841 | AT2G18850.1 \| similar to SET domain-containing protein [Arabidopsis thaliana] (TAIR:At3g07670.1); similar to hypothetical protein DDB0218318 [Dictyostelium discoideum] (GB:EAL66753.1); contains InterPro domain Nuclear protein SET (InterPro:IPR001214) \| chr2:8167816-8170771 FORWARD \| Aliases: MSF3.23, MSF3_23 | 4.0 | 3.4 | 0.6 | 4.1 | 2.34% | 3.2 |

| Rank | Description | Sync | Root | M | t | adj.q | B |
|---|---|---|---|---|---|---|---|
| 5842 | AT1G18800.1 \| nucleosome assembly protein (NAP) family protein, similar to SP:Q01105:SET protein (HLA-DR associated protein II) (PHAPII) (Phosphatase 2A inhibitor I2PP2A) {Homo sapiens}; contains Pfam profile PF00956: Nucleosome assembly protein (NAP) \| chr1:6481104-6483587 REVERSE \| Aliases: F6A14.10, F6A14_10 | 3.6 | 2.5 | 1.1 | 4.1 | 2.34% | 3.3 |
| 5843 | AT5G08380.1 \| alpha-galactosidase, putative / melibiase, putative / alpha-D-galactoside galactohydrolase, putative, similar to alpha-galactosidase SP:Q42656 from (Coffea arabica); contains Pfam profile PF02065: Melibiase \| chr5:2694678-2697688 REVERSE \| Aliases: F8L15.110, F8L15_110 | 9.0 | 7.6 | 1.5 | 4.1 | 2.34% | 3.5 |
| 5844 | AT3G21140.1 \| expressed protein \| chr3:7409463-7412231 REVERSE \| Aliases: MSA6.18 | 6.3 | 5.1 | 1.2 | 4.1 | 2.35% | 3.5 |
| 5846 | AT2G03680.1 \| Symbol: SPR1 \| expressed protein, Alternative splicing exists based on EST evidence \| chr2:1120797-1122004 FORWARD \| Aliases: F19B11.13, F19B11_13, SKU6, SPIRAL1 | 11.8 | 10.7 | 1.1 | 4.1 | 2.35% | 2.9 |
| 5847 | AT3G56730.1 \| expressed protein, contains Pfam profile PF04396: Protein of unknown function, DUF537 \| chr3:21025166-21026053 FORWARD \| Aliases: T8M16.60 | 8.0 | 6.7 | 1.2 | 4.1 | 2.35% | 3.4 |
| 5848 | AT1G26340.1 \| Symbol: B5 #6 \| cytochrome b5, putative, similar to cytochrome b5 GB:BAA74839 GI:4240120 from (Arabidopsis thaliana) \| chr1:9113887-9114958 FORWARD \| Aliases: F28B23.1 | 4.5 | 4.0 | 0.5 | 4.1 | 2.35% | 2.9 |
| 5849 | AT4G33865.1 \| 40S ribosomal protein S29 (RPS29C) \| chr4:16233214-16234201 REVERSE \| Aliases: None | 12.4 | 11.8 | 0.6 | 4.1 | 2.35% | 3.0 |
| 5852 | AT5G47520.1 \| Ras-related GTP-binding protein, putative, similar to GTP-binding protein RAB11J GI:1370160 from (Lotus japonicus) \| chr5:19294588-19295593 REVERSE \| Aliases: MNJ7.11, MNJ7_11 | 7.2 | 6.2 | 1.1 | 4.1 | 2.36% | 3.4 |
| 5855 | AT1G68660.1 \| expressed protein \| chr1:25781514-25782824 REVERSE \| Aliases: F24J5.10, F24J5_10 | 8.8 | 7.6 | 1.2 | 4.1 | 2.36% | 3.5 |
| 5858 | AT5G07590.1 \| WD-40 repeat protein family, contains 3 WD-40 repeats (PF00400); similarity to WD-repeat protein 8 (WDR8)(SP:Q9P2S5) (HOMO SAPIENS) \| chr5:2401621-2403855 REVERSE \| Aliases: MBK20.2, MBK20_2 | 4.7 | 4.0 | 0.7 | 4.1 | 2.37% | 3.3 |
| 5859 | AT1G01470.1 \| Symbol: LEA14 \| late embryogenesis abundant protein, putative / LEA protein, putative, similar to SP:P46518 Late embryogenesis abundant protein Lea14-A {Gossypium hirsutum}; contains Pfam profile PF03168: Late embryogenesis abundant protein \| chr1:172146-172923 REVERSE \| Aliases: F22L4.3, F22L4_3, LATE EMBRYOGENESIS ABUNDANT 14, LIGHT STRESS REGULATED 3, LSR3 | 12.3 | 10.4 | 2.0 | 4.1 | 2.37% | 3.4 |
| 5860 | AT1G05140.1 \| membrane-associated zinc metalloprotease, putative, similar to Hypothetical zinc metalloprotease All3971 (SP:Q8YQ64) (strain PCC 7120) {Anabaena sp.}; Similar to Synechocystis hypothetical protein (gb:D90908);contains Pfam PF00595: PDZ domain (Also known as DHR or GLGF); contains TIGRFAM TIGR00054: membrane-associated zinc metalloprotease, putative \| chr1:1482547-1484049 FORWARD \| Aliases: YUP8H12.25, YUP8H12_25 | 8.2 | 7.3 | 0.8 | 4.1 | 2.37% | 3.2 |
| 5862 | AT5G45600.1 \| Symbol: GAS41 \| YEATS family protein, contains Pfam domain PF03366: YEATS family \| chr5:18505129-18507096 FORWARD \| Aliases: K2N11.8, K2N11_8 | 7.9 | 6.4 | 1.5 | 4.1 | 2.37% | 3.5 |
| 5865 | AT2G30695.2 \| expressed protein \| chr2:13086184-13088427 REVERSE \| Aliases: None | 4.4 | 3.3 | 1.1 | 4.1 | 2.38% | 3.5 |
| 5866 | AT5G64380.1 \| fructose-1,6-bisphosphatase family protein, similar to SP:P22418 Fructose-1,6-bisphosphatase, chloroplast precursor (EC 3.1.3.11) (D-fructose-1,6-bisphosphate 1-phosphohydrolase) (FBPase) {Spinacia oleracea}; contains Pfam profile PF00316: fructose-1,6-bisphosphatase \| chr5:25758475-25760424 FORWARD \| Aliases: MSJ1.22, MSJ1_22 | 4.8 | 3.7 | 1.0 | 4.1 | 2.38% | 3.5 |
| 5870 | AT5G65490.1 \| expressed protein, similar to unknown protein (dbj BAA75199.1) \| chr5:26194728-26197542 REVERSE \| Aliases: K19O4.5, K19O4_5 | 6.3 | 5.2 | 1.2 | 4.1 | 2.38% | 3.5 |
| 5873 | AT5G16010.1 \| 3-oxo-5-alpha-steroid 4-dehydrogenase family protein / steroid 5-alpha-reductase family protein, similar to steroid 5alpha-reductase - Rattus norvegicus, PIR:A34239 (SP:24008); contains Pfam 3-oxo-5-alpha-steroid 4-dehydrogenase domain PF02544 \| chr5:5227927-5229119 FORWARD \| Aliases: F1N13.150, F1N13_150 | 8.6 | 6.6 | 2.0 | 4.1 | 2.39% | 3.5 |
| 5875 | AT1G05385.1 \| photosystem II 11 kDa protein-related, similar to Photosystem II 11 kDa protein precursor (SP:P74367)(strain PCC 6803) {Synechocystis sp.} \| chr1:1582673-1583717 REVERSE \| Aliases: None | 5.2 | 4.4 | 0.8 | 4.1 | 2.39% | 3.4 |
| 5876 | AT1G05430.1 \| expressed protein \| chr1:1594056-1595817 REVERSE \| Aliases: T25N20.8, T25N20_8 | 7.3 | 5.8 | 1.5 | 4.1 | 2.39% | 3.5 |
| 5879 | AT5G59830.2 \| expressed protein \| chr5:24122304-24124406 FORWARD \| Aliases: MMN10.5, MMN10_5 | 8.0 | 7.3 | 0.7 | 4.1 | 2.40% | 3.3 |
| 5880 | AT1G12940.1 \| Symbol: ATNRT2.5 \| high-affinity nitrate transporter, putative, similar to trans-membrane nitrate transporter protein AtNRT2:1 (Arabidopsis thaliana) GI:3747058, high-affinity nitrate transporter ACH1 (Arabidopsis thaliana) GI:3608362 \| chr1:4416403-4418588 FORWARD \| Aliases: F13K23.20, F13K23_20 | 4.8 | 3.4 | 1.4 | 4.1 | 2.40% | 3.5 |

| Rank | Description | Sync | Root | M | t | adj.q | B |
|---|---|---|---|---|---|---|---|
| 5883 | AT1G75340.1 \| zinc finger (CCCH-type) family protein, weak similarity to Nucleoporin NUP42 (Nuclear pore protein NUP42) (Swiss-Prot:P49686) (Saccharomyces cerevisiae); contains Pfam profile PF00642: Zinc finger C-x8-C-x5-C-x3-H type (and similar) \| chr1:28272631-28275609 REVERSE \| Aliases: F1B16.12, F1B16_12 | 4.7 | 3.8 | 0.9 | 4.1 | 2.41% | 3.4 |
| 5886 | AT5G65450.1 \| ubiquitin carboxyl-terminal hydrolase family protein / zinc finger (MYND type) family protein, similar to ubiquitin-specific protease 16 (UBP16) (Arabidopsis thaliana) GI:11993477; contains Pfam profiles PF00443: Ubiquitin carboxyl-terminal hydrolase, PF01753: MYND finger | 6.9 | 5.7 | 1.1 | 4.1 | 2.42% | 3.5 |
| 5890 | AT4G19230.2 \| Symbol: CYP707A1 \| cytochrome P450 family protein, cytochrome P450, Arabidopsis thaliana; supported by cDNA: gi_15293092_gb_AY050980.1_ | 5.5 | 4.2 | 1.3 | 4.1 | 2.42% | 3.4 |
| 5891 | AT1G10480.1 \| Symbol: ZFP5 \| zinc finger (C2H2 type) family protein, contains Pfam profile: PF00096 zinc finger, C2H2 type \| chr1:3449692-3451227 FORWARD \| Aliases: T10O24.9, T10O24_9, ZFP5, ZINC FINGER PROTEIN 5 | 5.1 | 4.3 | 0.9 | 4.1 | 2.42% | 3.3 |
| 5893 | AT1G14990.1 \| expressed protein \| chr1:5167147-5168238 REVERSE \| Aliases: T15D22.3, T15D22_3 | 7.1 | 5.7 | 1.3 | 4.1 | 2.42% | 3.4 |
| 5895 | AT1G31870.2 \| similar to splicing factor PWI domain-containing protein [Arabidopsis thaliana] (TAIR:At2g29210.1); similar to unknown protein [Oryza sativa (japonica cultivar-group)] (GB:XP_480359.1); contains InterPro domain Bipartite nuclear localization signal (InterPro:IPR001472) \| chr1:11436703-11440089 FORWARD \| Aliases: F5M6.12, F5M6_12 | 8.8 | 7.4 | 1.4 | 4.1 | 2.43% | 3.5 |
| 5896 | AT1G05590.1 \| glycosyl hydrolase family 20 protein, similar to beta-hexosaminidase precursor SP:P43077 from (Candida albicans) | 5.4 | 4.5 | 0.9 | 4.1 | 2.43% | 3.4 |
| 5897 | AT1G68560.1 \| Symbol: XYL1 \| alpha-xylosidase (XYL1), identical to alpha-xylosidase precursor GB:AAD05539 GI:4163997 from (Arabidopsis thaliana); contains Pfam profile PF01055: Glycosyl hydrolases family 31; identical to cDNA alpha-xylosidase precursor (XYL1) partial cds GI:4163996 \| chr1:25737845-25741658 REVERSE \| Aliases: F24J5.20, F24J5_20 | 3.9 | 2.5 | 1.4 | 4.1 | 2.43% | 3.5 |
| 5899 | AT5G10910.1 \| mraW methylase family protein, contains Pfam profile PF01795: MraW methylase family \| chr5:3439554-3441583 FORWARD \| Aliases: T30N20.180, T30N20_180 | 5.6 | 4.3 | 1.4 | 4.1 | 2.43% | 3.4 |
| 5902 | AT3G23710.1 \| chloroplast inner membrane import protein Tic22, putative, similar to Tic22 (Pisum sativum) gi:3769671:gb:AAC64606; contains Pfam domain PF04278: Tic22-like family \| chr3:8534039-8536381 FORWARD \| Aliases: MYM9.5 | 5.8 | 4.5 | 1.3 | 4.1 | 2.43% | 3.5 |
| 5904 | AT1G27730.1 \| Symbol: ZAT10 \| zinc finger (C2H2 type) family protein (ZAT10) / salt-tolerance zinc finger protein (STZ), identical to salt-tolerance zinc finger protein GB:CAA64820 GI:1565227 from (Arabidopsis thaliana); contains Pfam domain, PF00096: Zinc finger, C2H2 type; identical to cDNA | 7.7 | 6.5 | 1.2 | 4.1 | 2.43% | 3.5 |
| 5905 | AT5G16790.1 \| expressed protein \| chr5:5522499-5523914 REVERSE \| Aliases: F5E19.130, F5E19_130 | 3.8 | 3.1 | 0.6 | 4.1 | 2.43% | 3.3 |
| 5906 | AT5G42070.1 \| expressed protein, similar to unknown protein (dbj:BAA92898.1) \| chr5:16836215-16837423 REVERSE \| Aliases: MJC20.18, MJC20_18 | 4.9 | 3.4 | 1.4 | 4.1 | 2.44% | 3.5 |
| 5908 | AT4G17180.1 \| glycosyl hydrolase family 17 protein, similar to 3-glucanase GI:18483232 from (Sorghum bicolor) \| chr4:9646518-9648306 FORWARD \| Aliases: DL4625W, FCAALL.368 | 4.6 | 3.7 | 0.9 | 4.1 | 2.44% | 3.4 |
| 5909 | AT1G09700.1 \| Symbol: HYL1 \| double-stranded RNA-binding domain (DsRBD)-containing protein, contains Pfam profile PF00035: Double-stranded RNA binding motif; supporting cDNA gi:12247456:gb:AF276440.1:AF276440 \| chr1:3137769-3140236 REVERSE \| Aliases: F21M12.9, F21M12_9, HYPONASTIC LEAVES 1 | 5.2 | 4.1 | 1.1 | 4.1 | 2.44% | 3.5 |
| 5910 | AT1G74210.1 \| glycerophosphoryl diester phosphodiesterase family protein, low similarity to glycerophosphodiester phosphodiesterase (Borrelia hermsii) GI:1399038; contains Pfam profile PF03009: Glycerophosphoryl diester phosphodiesterase family \| chr1:27913979-27916602 FORWARD \| Aliases: F1O17.12, F1O17_12 | 10.1 | 8.7 | 1.3 | 4.1 | 2.44% | 3.4 |
| 5912 | AT3G11710.1 \| lysyl-tRNA synthetase, putative / lysine--tRNA ligase, putative, similar to SP:Q43776 Lysyl-tRNA synthetase (EC 6.1.1.6) (Lysine--tRNA ligase) {Lycopersicon esculentum}; contains Pfam profile PF00152: tRNA synthetases class II (D, K and N) \| chr3:3702179-3705707 REVERSE \| Aliases: T19F11.11, T19F11_11 | 10.1 | 9.1 | 0.9 | 4.1 | 2.44% | 3.4 |
| 5913 | AT1G49840.1 \| expressed protein, contains Pfam profile PF04788: Protein of unknown function (DUF620) \| chr1:18455738-18458026 FORWARD \| Aliases: F10F5.2, F10F5_2 | 5.1 | 4.0 | 1.1 | 4.1 | 2.44% | 3.5 |
| 5917 | AT5G20570.1 \| Symbol: RBX1 \| ring-box protein-related, similar to ring-box protein 1 GI:4769004 from (Homo sapiens) \| chr5:6956662-6958313 REVERSE \| Aliases: F7C8.160, F7C8_160, HRT1, ROC1 | 10.2 | 9.2 | 1.1 | 4.1 | 2.45% | 3.3 |
| 5920 | AT3G56620.1 \| integral membrane family protein / nodulin MtN21-related, similar to MtN21 GI:2598575 (root nodule development) from (Medicago truncatula) \| chr3:20983482-20985585 REVERSE \| Aliases: T5P19.270 | 6.6 | 5.4 | 1.3 | 4.1 | 2.45% | 3.5 |

| Rank | Description | Sync | Root | M | t | adj.q | B |
|---|---|---|---|---|---|---|---|
| 5922 | AT1G34380.1 \| 5'-3' exonuclease family protein, similar to DNA polymerase type I (Rhodothermus sp. 'ITI 518') gi:4090935:gb:AAC98908 ; contains Pfam profile PF02739: 5'-3' exonuclease, N-terminal resolvase-like domain \| chr1:12552633-12554298 REVERSE \| Aliases: F7P12.3, F7P12_3 | 4.3 | 3.1 | 1.2 | 4.1 | 2.45% | 3.4 |
| 5923 | AT3G24200.2 \| similar to putative oxygenase [Oryza sativa (japonica cultivar-group)] (GB:XP_470364.1); contains InterPro domain Flavoprotein monooxygenase (InterPro:IPR000733); contains InterPro domain NAD-binding site (InterPro:IPR000205); contains InterPro domain Aromatic-ring hydroxylase (InterPro:IPR003042); contains InterPro domain UbiH/COQ6 monooxygenase (InterPro:IPR000689) \| chr3:8747838-8751376 REVERSE \| Aliases: MUJ8.10 | 9.8 | 8.4 | 1.4 | 4.1 | 2.45% | 3.3 |
| 5924 | AT2G15490.2 \| UDP-glucoronosyl/UDP-glucosyl transferase family protein, contains Pfam profile: PF00201 UDP-glucoronosyl and UDP-glucosyl transferase \| chr2:6768717-6770556 FORWARD \| Aliases: F9O13.4 | 4.7 | 3.5 | 1.2 | 4.1 | 2.46% | 3.4 |
| 5926 | AT2G04700.1 \| ferredoxin thioredoxin reductase catalytic beta chain family protein, contains Pfam profile: PF02943 ferredoxin thioredoxin reductase catalytic beta \| chr2:1646805-1648554 FORWARD \| Aliases: F28I8.26, F28I8_26 | 7.2 | 6.3 | 0.9 | 4.1 | 2.46% | 3.2 |
| 5929 | AT3G25780.1 \| Symbol: AOC2 \| allene oxide cyclase, putative / early-responsive to dehydration protein, putative / ERD protein, putative, similar to allene oxide cyclase GI:8977961 from (Lycopersicon esculentum); similar to early-responsive to dehydration (ERD12) protein (GI:15320414); contains Pfam profile PF06351: Allene oxide cyclase \| chr3:9410527-9411803 FORWARD \| Aliases: ALLENE OXIDE CYCLASE 2, K13N2.12 | 6.8 | 5.6 | 1.2 | 4.1 | 2.46% | 3.4 |
| 5932 | AT2G24860.1 \| chaperone protein dnaJ-related, similar to Tsi1-interacting protein TSIP1 (GI:4337001) (Nicotiana tabacum) \| chr2:10593854-10595408 REVERSE \| Aliases: F27C12.22, F27C12_22 | 6.4 | 5.8 | 0.6 | 4.1 | 2.46% | 3.0 |
| 5933 | AT4G36530.2 \| hydrolase, alpha/beta fold family protein, low similarity to hydrolase (Sphingomonas sp.) GI:3426124; contains Pfam profile PF00561: hydrolase, alpha/beta fold family \| chr4:17239961-17241938 REVERSE \| Aliases: AP22.96, AP22_96 | 8.1 | 7.5 | 0.6 | 4.1 | 2.46% | 3.3 |
| 5935 | AT5G26940.4 \| exonuclease family protein, contains exonuclease domain, Pfam:PF00929 \| chr5:9480597-9482772 FORWARD \| Aliases: F2P16.200, F2P16_200 | 7.3 | 6.0 | 1.3 | 4.1 | 2.46% | 3.5 |
| 5937 | AT1G17070.1 \| D111/G-patch domain-containing protein, Similar to SP:Q9ERA6 Tuftelin-interacting protein 11 {Mus musculus}; contains Pfam profile PF01585: G-patch domain \| chr1:5837583-5840261 FORWARD \| Aliases: F20D23.23, F20D23_23 | 5.7 | 4.5 | 1.2 | 4.1 | 2.46% | 3.5 |
| 5938 | AT2G11890.2 \| expressed protein, contains InterPro domain Adenylate cyclase (InterPro:IPR008172) \| chr2:4809865-4811457 FORWARD \| Aliases: None | 6.8 | 5.6 | 1.2 | 4.1 | 2.46% | 3.5 |
| 5939 | AT2G22425.1 \| expressed protein, weak similarity to Swiss-Prot:Q9Y6A9 microsomal signal peptidase 12 kDa subunit (SPase 12 kDa subunit, SPC12, HSPC033) (Homo sapiens) | 10.7 | 10.2 | 0.5 | 4.1 | 2.46% | 2.8 |
| 5941 | AT2G47450.1 \| Symbol: CAO \| chloroplast signal recognition particle component (CAO), nearly identical to CAO (Arabidopsis thaliana) GI:4102582 \| chr2:19479751-19481384 FORWARD \| Aliases: CHAOS, CHLOROPHYLL A/B BINDING PROTEIN HARVESTING ORGANELLE SPECIFIC, CPSRP43, T30B22.25 | 9.2 | 7.7 | 1.4 | 4.1 | 2.47% | 3.4 |
| 5943 | AT1G17160.1 \| pfkB-type carbohydrate kinase family protein, contains Pfam profile: PF00294 pfkB family carbohydrate kinase \| chr1:5867639-5869323 FORWARD \| Aliases: F20D23.14, F20D23_14 | 9.1 | 8.0 | 1.1 | 4.1 | 2.48% | 3.3 |
| 5944 | AT1G07880.2 \| Symbol: ATMPK13 \| similar to mitogen-activated protein kinase, putative / MAPK, putative (MPK6) [Arabidopsis thaliana] (TAIR:At2g43790.1); similar to NRK1 MAPK [Nicotiana tabacum] (GB:BAB32406.1); similar to p43Nft6 serine/threonine protein kinase [Nicotiana tabacum] (GB:CAA58760.1); contains InterPro domain Serine/threonine protein kinase, active site (InterPro:IPR008271); contains InterPro domain MAP kinase (InterPro:IPR003527); contains InterPro domain Tyrosine protein kinase (InterPro:IPR001245); contains InterPro domain Serine/threonine protein kinase (InterPro:IPR002290); contains InterPro domain Protein kinase (InterPro:IPR000719) \| chr1:2434056-2435742 REVERSE \| Aliases: F24B9.3, F24B9_3 | 3.9 | 3.2 | 0.7 | 4.1 | 2.48% | 3.2 |
| 5945 | AT2G41500.1 \| WD-40 repeat family protein / small nuclear ribonucleoprotein Prp4p-related, similar to U4/U6 small nuclear ribonucleoprotein hPrp4 (GP:2708305) {Homo sapiens}; contains Pfam PF00400: WD domain, G-beta repeat (7 copies):19877698:gb:AU238529.1:AU238529 \| chr2:17311209-17314504 REVERSE \| Aliases: T26J13.9, T26J13_9 | 6.8 | 6.0 | 0.7 | 4.1 | 2.48% | 3.3 |
| 5946 | AT5G52190.1 \| sugar isomerase (SIS) domain-containing protein, similar to 6-phospho-3-hexuloisomerase (Mycobacterium gastri) GI:6899860; contains Pfam profile PF01380: SIS domain \| chr5:21218956-21219769 REVERSE \| Aliases: F17P19.9, F17P19_9 | 4.8 | 3.4 | 1.3 | 4.1 | 2.48% | 3.5 |
| 5947 | AT1G51440.1 \| lipase class 3 family protein, similar to DEFECTIVE IN ANTHER DEHISCENCE1 (Arabidopsis thaliana) GI:16215706, lipase (Dianthus caryophyllus) GI:4103627; contains Pfam profile PF01764: Lipase \| chr1:19074585-19076362 FORWARD \| Aliases: F5D21.19, F5D21_19 | 3.5 | 2.8 | 0.7 | 4.0 | 2.48% | 3.3 |
| 5948 | AT5G11980.1 \| conserved oligomeric Golgi complex component-related / COG complex component-related, similar to SP:Q96MW5 Conserved oligomeric Golgi complex component 8 {Homo sapiens}; contains Pfam profile PF04124: Dor1-like family \| chr5:3868653-3872270 REVERSE \| Aliases: F14F18.150, F14F18_150 | 6.2 | 5.1 | 1.1 | 4.0 | 2.49% | 3.4 |

| Rank | Description | Sync | Root | M | t | adj.q | B |
|---|---|---|---|---|---|---|---|
| 5952 | AT1G71220.1 \| UDP-glucose:glycoprotein glucosyltransferase, putative, similar to UDP-glucose:glycoprotein glucosyltransferase precursor GB:Q09332 (SP:Q09332) from Drosophila melanogaster, (gi:7670746) and (gi:11346464) from Homo sapiens \| chr1:26845326-26855583 FORWARD \| Aliases: F3I17.13, F3I17_13 | 6.7 | 5.5 | 1.2 | 4.0 | 2.49% | 3.4 |
| 5953 | AT1G19980.1 \| cytomatrix protein-related, contains weak similarity to CAST1 (Rattus norvegicus) gi:22138113:gb:AAL07517 \| chr1:6933022-6934993 FORWARD \| Aliases: T20H2.28, T20H2_28 | 4.9 | 4.1 | 0.7 | 4.0 | 2.49% | 3.4 |
| 5955 | AT5G57030.1 \| Symbol: LUT2 \| lycopene epsilon cyclase, identical to lycopene epsilon cyclase (GI:1399181) \| chr5:23094491-23097262 FORWARD \| Aliases: LUTEIN DEFICIENT 2, LYCOPENE EPSILON CYCLASE, MHM17.16, MHM17_16 | 4.9 | 4.0 | 0.9 | 4.0 | 2.49% | 3.4 |
| 5957 | AT2G21510.1 \| DNAJ heat shock N-terminal domain-containing protein, similar to SP:P39101 CAJ1 protein, Saccharomyces cerevisiae; contains Pfam profile PF00226 DnaJ domain \| chr2:9217735-9220138 REVERSE \| Aliases: F3K23.27, F3K23_27 | 3.6 | 2.7 | 0.9 | 4.0 | 2.50% | 3.4 |
| 5959 | AT5G55990.1 \| Symbol: CBL2 \| calcineurin B-like protein 2 (CBL2), identical to calcineurin B-like protein 2 GI:3309084 from (Arabidopsis thaliana) \| chr5:22688678-22691081 FORWARD \| Aliases: ATCBL2, MDA7.3, MDA7_3 | 7.5 | 6.4 | 1.1 | 4.0 | 2.50% | 3.3 |
| 5960 | AT1G15940.1 \| expressed protein, similar To androgen-induced prostate proliferative shutoff associated protein (GI:4559410) (Homo sapiens) \| chr1:5473584-5478246 FORWARD \| Aliases: T24D18.4, T24D18_4 | 5.9 | 4.9 | 1.0 | 4.0 | 2.50% | 3.3 |
| 5961 | AT3G02820.1 \| zinc knuckle (CCHC-type) family protein, contains Pfam domain, PF00098: Zinc knuckle \| chr3:611543-613390 FORWARD \| Aliases: F13E7.24, F13E7_24 | 4.2 | 3.2 | 1.0 | 4.0 | 2.50% | 3.3 |
| 5962 | AT2G35760.1 \| integral membrane family protein, contains TIGRFAM TIGR01569 : plant integral membrane protein TIGR01569; contains Pfam PF04535 : Domain of unknown function (DUF588) \| chr2:15039002-15040384 FORWARD \| Aliases: T20F21.5, T20F21_5 | 7.1 | 5.9 | 1.2 | 4.0 | 2.51% | 3.4 |
| 5965 | AT2G38720.1 \| microtubule associated protein (MAP65/ASE1) family protein, low similarity to myosin (Schistosoma japonicum) GI:3941320; contains Pfam profile PF03999: Microtubule associated protein (MAP65/ASE1 family) \| chr2:16195125-16199180 FORWARD \| Aliases: T6A23.8, T6A23_8 | 4.8 | 3.1 | 1.7 | 4.0 | 2.52% | 3.4 |
| 5967 | AT1G28390.1 \| protein kinase family protein, contains protein kinase domain, Pfam:PF00069 \| chr1:9966614-9968272 REVERSE \| Aliases: F3M18.17, F3M18_17 | 5.6 | 4.8 | 0.8 | 4.0 | 2.52% | 3.4 |
| 5968 | AT3G07150.1 \| hypothetical protein \| chr3:2263812-2264582 REVERSE \| Aliases: T1B9.19 | 3.5 | 2.7 | 0.8 | 4.0 | 2.53% | 3.3 |
| 5970 | AT2G33210.1 \| chaperonin, putative, similar to SWISS-PROT:Q05046- chaperonin CPN60-2, mitochondrial precursor (HSP60-2) (Cucurbita maxima); contains Pfam:PF00118 domain, TCP-1/cpn60 chaperonin family \| chr2:14081986-14085797 REVERSE \| Aliases: F25I18.5, F25I18_5 | 9.2 | 7.8 | 1.4 | 4.0 | 2.53% | 3.4 |
| 5972 | AT5G17210.2 \| expressed protein \| chr5:5656435-5658149 FORWARD \| Aliases: MKP11.6, MKP11_6 | 7.2 | 5.4 | 1.8 | 4.0 | 2.53% | 3.4 |
| 5976 | AT1G24310.1 \| expressed protein \| chr1:8624050-8626394 FORWARD \| Aliases: F3I6.25, F3I6_25 | 4.2 | 3.6 | 0.6 | 4.0 | 2.53% | 3.2 |
| 5977 | AT1G06500.2 \| expressed protein \| chr1:1989485-1990727 FORWARD \| Aliases: F12K11.25, F12K11_25 | 7.4 | 6.1 | 1.3 | 4.0 | 2.53% | 3.4 |
| 5981 | AT5G35400.1 \| tRNA pseudouridine synthase family protein, weak similarity to SP:P07649 tRNA pseudouridine synthase A (EC 4.2.1.70) (Uracil hydrolyase) {Escherichia coli}; contains Pfam profile PF01416: tRNA pseudouridine synthase | 5.2 | 4.4 | 0.9 | 4.0 | 2.54% | 3.3 |
| 5982 | AT5G23400.1 \| disease resistance family protein / LRR family protein, similar to disease resistance protein (Lycopersicon esculentum) gi:3894383:gb:AAC78591; contains leucine rich-repeat domain Pfam:PF00560, INTERPRO:IPR001611 \| chr5:7880338-7882637 FORWARD \| Aliases: K19M13.1, K19M13_1 | 4.6 | 3.5 | 1.1 | 4.0 | 2.54% | 3.4 |
| 5984 | AT4G31300.2 \| Symbol: PBA1 \| similar to 20S proteasome beta subunit E1 (PBE1) (PRCE) [Arabidopsis thaliana] (TAIR:At1g13060.1); similar to PREDICTED OJ1079_F11.26 gene product [Oryza sativa (japonica cultivar-group)] (GB:XP_507536.1); contains InterPro domain Proteasome B-type subunit (InterPro:IPR000243); contains InterPro domain Multispecific proteasome protease (InterPro:IPR001353) \| chr4:15188769-15191159 FORWARD \| Aliases: 20S PROTEASOME SUBUNIT PBA1, F8F16.120, F8F16_120 | 11.0 | 9.2 | 1.8 | 4.0 | 2.54% | 3.3 |
| 5985 | AT1G23280.1 \| MAK16 protein-related, contains similarity to MAK16 protein SP:P10962 from (Saccharomyces cerevisiae) \| chr1:8260854-8262639 REVERSE \| Aliases: F26F24.12, F26F24_12 | 5.7 | 3.7 | 2.0 | 4.0 | 2.54% | 3.4 |
| 5986 | AT5G46700.1 \| senescence-associated protein, putative, similar to senescence-associated protein 5 (Hemerocallis hybrid cultivar) gi:3551954:gb:AAC34855 \| chr5:18968188-18969995 FORWARD \| Aliases: MZA15.11, MZA15_11 | 6.9 | 5.4 | 1.5 | 4.0 | 2.54% | 3.4 |
| 5987 | AT5G12020.1 \| 17.6 kDa | 4.3 | 3.4 | 0.9 | 4.0 | 2.54% | 3.4 |

| Rank | Description | Sync | Root | M | t | adj.q | B |
|---|---|---|---|---|---|---|---|
| 5988 | AT3G21690.1 \| MATE efflux family protein, similar to ripening regulated protein DDTFR18 (Lycopersicon esculentum) GI:12231296; contains Pfam profile: PF01554 MatE uncharacterized membrane protein family \| chr3:7638672-7642212 FORWARD \| Aliases: MIL23.25 | 4.7 | 4.0 | 0.7 | 4.0 | 2.55% | 3.1 |
| 5990 | AT1G44900.1 \| DNA replication licensing factor, putative, similar to DNA replication licensing factor MCM2 from {Xenopus laevis} SP:P55861, SP:P49736 {Homo sapiens}; contains Pfam profile PF00493: MCM2/3/5 family \| chr1:16972701-16977030 FORWARD \| Aliases: T12C22.19, T12C22_19 | 4.1 | 2.5 | 1.6 | 4.0 | 2.55% | 3.5 |
| 5991 | AT1G10500.1 \| Symbol: ATCPISCA \| Involved in chloroplast Fe-S cluster | 7.8 | 6.7 | 1.0 | 4.0 | 2.55% | 3.4 |
| 5995 | AT1G17220.1 \| translation initiation factor IF-2, chloroplast, putative, similar to SP:P57997:IF2C_PHAVU Translation initiation factor IF-2, chloroplast precursor (PvIF2cp) {Phaseolus vulgaris} \| chr1:5885076-5890410 FORWARD \| Aliases: F20D23.8, F20D23_8 | 8.4 | 7.2 | 1.2 | 4.0 | 2.56% | 3.2 |
| 5996 | AT4G32690.1 \| Symbol: GLB3 \| 2-on-2 hemoglobin (GLB3), identical to 2-on-2 hemoglobin (GLB3) GI:14165163 from (Arabidopsis thaliana) \| chr4:15765436-15766990 FORWARD \| Aliases: F4D11.1 | 7.2 | 5.3 | 1.9 | 4.0 | 2.56% | 3.4 |
| 5998 | AT1G07840.3 \| similar to Sas10/U3 ribonucleoprotein (Utp) family protein [Arabidopsis thaliana] (TAIR:At2g43650.1); similar to P0456E05.27 [Oryza sativa (japonica cultivar-group)] (GB:XP_463608.1) \| chr1:2424051-2426673 FORWARD \| Aliases: F24B9.6, F24B9_6 | 5.2 | 4.2 | 1.0 | 4.0 | 2.57% | 3.3 |
| 6001 | AT5G55220.1 \| trigger factor type chaperone family protein, contains Pfam profiles PF05697: Bacterial trigger factor protein (TF), PF05698: Bacterial trigger factor protein (TF) C-terminus, PF00254: peptidyl-prolyl cis-trans isomerase, FKBP-type \| chr5:22414842-22418062 FORWARD \| Aliases: MCO15.17, MCO15_17 | 3.5 | 2.7 | 0.8 | 4.0 | 2.57% | 3.2 |
| 6002 | AT2G45100.1 \| transcription factor IIB (TFIIB) family protein, contains Pfam domain, PF00382: Transcription factor TFIIB repeat \| chr2:18602423-18605163 REVERSE \| Aliases: T14P1.9 | 5.7 | 4.4 | 1.3 | 4.0 | 2.57% | 3.4 |
| 6003 | AT4G02820.1 \| pentatricopeptide (PPR) repeat-containing protein, low similarity to DNA-binding protein (Triticum aestivum) GI:6958202; contains Pfam profile PF01535: PPR repeat \| chr4:1258568-1260665 FORWARD \| Aliases: T5J8.14, T5J8_14 | 3.8 | 3.1 | 0.7 | 4.0 | 2.57% | 3.1 |
| 6004 | AT4G30650.1 \| hydrophobic protein, putative / low temperature and salt responsive protein, putative, similar to SP:Q9ZNQ7 Hydrophobic protein RCI2A (Low temperature and salt responsive protein LTI6A) {Arabidopsis thaliana}; contains Pfam profile PF01679: Uncharacterized protein family \| chr4:14954326-14954974 FORWARD \| Aliases: F17I23.10, F17I23_10 | 7.8 | 6.4 | 1.5 | 4.0 | 2.57% | 3.3 |
| 6006 | AT5G27830.2 \| expressed protein \| chr5:9861293-9862900 FORWARD \| Aliases: T1G16.160, T1G16_160 | 7.9 | 6.1 | 1.8 | 4.0 | 2.57% | 3.3 |
| 6007 | AT1G26220.1 \| GCN5-related N-acetyltransferase (GNAT) family protein, low similarity to SP:P09453 Ribosomal-protein-alanine acetyltransferase (EC 2.3.1.128) {Escherichia coli}; contains Pfam profile PF00583: acetyltransferase, GNAT family | 3.1 | 2.5 | 0.7 | 4.0 | 2.57% | 3.1 |
| 6008 | AT1G26160.1 \| metal-dependent phosphohydrolase HD domain-containing protein, contains Pfam profile PF01966: HD domain \| chr1:9044428-9046966 REVERSE \| Aliases: F28B23.16, F28B23_16 | 6.3 | 4.8 | 1.6 | 4.0 | 2.57% | 3.4 |
| 6009 | AT5G51200.1 \| expressed protein \| chr5:20822152-20836818 FORWARD \| Aliases: MWD22.15, MWD22_15 | 6.3 | 5.0 | 1.3 | 4.0 | 2.57% | 3.3 |
| 6010 | AT5G15450.1 \| heat shock protein 100, putative / HSP100, putative / heat shock protein clpB, putative / HSP100/ClpB, putative, similar to HSP100/ClpB GI:9651530 (Phaseolus lunatus) \| chr5:5014402-5018350 REVERSE \| Aliases: T20K14.60, T20K14_60 | 9.0 | 6.1 | 2.9 | 4.0 | 2.57% | 3.3 |
| 6011 | AT5G01940.1 \| eukaryotic translation initiation factor 2B family protein / eIF-2B family protein, similar to SP:P41035 Eukaryotic translation initiation factor 2 subunit (eIF-2-beta) {Oryctolagus cuniculus}; contains Pfam profile PF01873: Domain found in IF2B/IF5 \| chr5:363591-364766 REVERSE \| Aliases: T20L15.210, T20L15_210 | 5.4 | 4.5 | 0.8 | 4.0 | 2.57% | 3.3 |
| 6012 | AT2G21790.1 \| Symbol: R1 \| ribonucleoside-diphosphate reductase small chain, putative / ribonucleotide reductase, putative, similar to ribonucleotide reductase GI:4151068 from (Nicotiana tabacum) \| chr2:9300435-9304922 FORWARD \| Aliases: F7D8.11, F7D8_11, R1 | 6.5 | 4.8 | 1.7 | 4.0 | 2.58% | 3.4 |
| 6014 | AT5G12940.1 \| leucine-rich repeat family protein, contains leucine rich-repeat domains Pfam:PF00560, INTERPRO:IPR001611 \| chr5:4087715-4089007 FORWARD \| Aliases: T24H18.110, T24H18_110 | 4.4 | 3.3 | 1.1 | 4.0 | 2.58% | 3.4 |
| 6017 | AT3G60540.2 \| sec61beta family protein, similar to SP:P52870 Protein transport protein SEC61 beta 1 subunit {Saccharomyces cerevisiae}; contains Pfam profile PF03911: Sec61beta family \| chr3:22385938-22386840 REVERSE \| Aliases: T8B10.200 | 6.7 | 5.4 | 1.3 | 4.0 | 2.59% | 3.4 |
| 6018 | AT1G03300.1 \| agenet domain-containing protein, contains Pfam PF05641: Agenet domain \| chr1:811033-813086 REVERSE \| Aliases: F15K9.10, F15K9_10 | 4.0 | 3.4 | 0.7 | 4.0 | 2.59% | 3.2 |

| Rank | Description | Sync | Root | M | t | adj.q | B |
|---|---|---|---|---|---|---|---|
| 6019 | AT5G20060.3 \| similar to phospholipase/carboxylesterase family protein [Arabidopsis thaliana] (TAIR:At1g52700.1); similar to biostress-resistance-related protein [Triticum aestivum] (GB:AAM29178.1); contains InterPro domain Phospholipase/Carboxylesterase (InterPro:IPR003140) \| chr5:6776277-6779616 FORWARD \| Aliases: F28I16.210, F28I16_210 | 9.0 | 7.5 | 1.4 | 4.0 | 2.59% | 3.4 |
| 6020 | AT1G07970.1 \| expressed protein \| chr1:2469288-2472620 REVERSE \| Aliases: T6D22.6, T6D22_6 | 5.2 | 4.4 | 0.8 | 4.0 | 2.59% | 3.3 |
| 6023 | AT3G54900.1 \| Symbol: CXIP1 \| CAX-interacting protein 1 (CAXIP1), identical to cDNA CAXIP1 protein (CAXIP1) GI:27752304, CAXIP1 protein (Arabidopsis thaliana) GI:27752305 \| chr3:20352566-20353371 REVERSE \| Aliases: CAX INTERACTING PROTEIN 1, F28P10.120 | 5.0 | 3.6 | 1.4 | 4.0 | 2.59% | 3.4 |
| 6026 | AT5G57440.1 \| Symbol: GS1 \| haloacid dehalogenase-like hydrolase family protein, similar to SP:Q08623 GS1 protein {Homo sapiens}; contains InterPro accession IPR005834: Haloacid dehalogenase-like hydrolase | 5.1 | 3.3 | 1.9 | 4.0 | 2.60% | 3.4 |
| 6029 | AT3G58470.1 \| expressed protein, several hypothetical proteins - Saccharomyces cerevisiae \| chr3:21637648-21639546 REVERSE \| Aliases: F14P22.60 | 6.2 | 4.3 | 1.9 | 4.0 | 2.60% | 3.3 |
| 6030 | AT3G11210.1 \| GDSL-motif lipase/hydrolase family protein, contains Pfam profile PF00657: Lipase/Acylhydrolase with GDSL-like motif \| chr3:3510669-3513066 REVERSE \| Aliases: F11B9.13 | 4.3 | 3.1 | 1.2 | 4.0 | 2.61% | 3.4 |
| 6031 | AT1G26560.1 \| glycosyl hydrolase family 1 protein, contains Pfam PF00232 : Glycosyl hydrolase family 1 domain; TIGRFAM TIGR01233: 6-phospho-beta-galactosidase; similar to amygdalin hydrolase isoform AH I precursor (GI:16757966) (Prunus serotina) | 4.7 | 3.9 | 0.8 | 4.0 | 2.61% | 3.2 |
| 6032 | AT5G27620.1 \| Symbol: CYCH;1 \| | 4.6 | 3.5 | 1.2 | 4.0 | 2.61% | 3.3 |
| 6035 | AT1G72530.1 \| plastid developmental protein DAG, putative, similar to DAG protein, chloroplast precursor (required for chloroplast differentiation) GB:Q38732 (Antirrhinum majus) \| chr1:27316661-27317599 FORWARD \| Aliases: F28P22.28, F28P22_28 | 4.1 | 3.4 | 0.7 | 4.0 | 2.61% | 3.3 |
| 6037 | AT2G37330.1 \| Symbol: ALS3 \| expressed protein, and genefinder \| chr2:15676217-15677744 FORWARD \| Aliases: ALUMINUM SENSITIVE 3, F3G5.12, F3G5_12 | 6.7 | 5.4 | 1.3 | 4.0 | 2.62% | 3.4 |
| 6041 | AT5G20140.1 \| SOUL heme-binding family protein, contains PFam profile PF04832: SOUL heme-binding protein \| chr5:6798834-6800905 REVERSE \| Aliases: F5O24.30, F5O24_30 | 5.1 | 4.4 | 0.7 | 4.0 | 2.62% | 3.3 |
| 6042 | AT5G62930.1 \| GDSL-motif lipase/hydrolase family protein, similar to SP:P41734 Isoamyl acetate-hydrolyzing esterase (EC 3.1.-.-) {Saccharomyces cerevisiae}; contains Pfam profile PF00657: Lipase/Acylhydrolase with GDSL-like motif \| chr5:25272114-25273843 FORWARD \| Aliases: MQB2.25, MQB2_25 | 7.8 | 6.0 | 1.8 | 4.0 | 2.63% | 3.4 |
| 6043 | AT3G15640.1 \| cytochrome c oxidase family protein, contains Pfam domain, PF01215: Cytochrome c oxidase subunit Vb \| chr3:5299117-5301752 FORWARD \| Aliases: MSJ11.5 | 10.0 | 9.1 | 1.0 | 4.0 | 2.63% | 3.2 |
| 6045 | AT1G72090.1 \| radical SAM domain-containing protein / TRAM domain-containing protein, contains Pfam profiles PF00919: UPF0004 family protein, PF04055: radical SAM domain protein, PF01938: TRAM domain \| chr1:27127237-27130557 FORWARD \| Aliases: F28P5.4, F28P5_4 | 8.4 | 7.3 | 1.1 | 4.0 | 2.63% | 3.2 |
| 6046 | AT3G50520.1 \| phosphoglycerate/bisphosphoglycerate mutase family protein, similar to SP:P00950 Phosphoglycerate mutase 1 (EC 5.4.2.1) {Saccharomyces cerevisiae}; contains Pfam profile PF00300: phosphoglycerate mutase family \| chr3:18759010-18760552 FORWARD \| Aliases: T20E23.120 | 6.9 | 5.4 | 1.5 | 4.0 | 2.63% | 3.3 |
| 6048 | AT1G20770.1 \| expressed protein \| chr1:7215016-7216693 REVERSE \| Aliases: F2D10.26, F2D10_26 | 7.5 | 5.5 | 2.0 | 4.0 | 2.64% | 3.4 |
| 6051 | AT1G69210.1 \| expressed protein \| chr1:26021765-26023721 REVERSE \| Aliases: F4N2.17, F4N2_17 | 3.8 | 3.1 | 0.7 | 4.0 | 2.64% | 3.1 |
| 6052 | AT4G17310.2 \| expressed protein, similar to expressed protein [Arabidopsis thaliana] (TAIR:At5g47455.3) \| chr4:9685190-9686940 REVERSE \| Aliases: DL4690C, FCAALL.398 | 4.5 | 3.7 | 0.8 | 4.0 | 2.64% | 3.2 |
| 6054 | AT1G04020.2 \| Symbol: ATBRCA1 \| zinc finger (C3HC4-type RING finger) family protein / BRCT domain-containing protein, contains Pfam domain, PF00533: BRCA1 C Terminus (BRCT) domain \| chr1:1036563-1040223 FORWARD \| Aliases: ATBRCA1, F21M11.4, F21M11_4 | 5.6 | 4.1 | 1.5 | 4.0 | 2.65% | 3.4 |
| 6055 | AT2G47840.1 \| tic20 protein-related, similar to Tic20 (GI:3769673) (Pisum sativum) \| chr2:19601257-19602071 REVERSE \| Aliases: F17A22.23 | 7.8 | 7.2 | 0.7 | 4.0 | 2.65% | 3.1 |
| 6059 | AT5G02370.1 \| kinesin motor protein-related, kinesin, Xenopus laevis, EMBL:XLA249840 \| chr5:503406-506922 FORWARD \| Aliases: T1E22.130, T1E22_130 | 4.7 | 3.7 | 1.0 | 4.0 | 2.65% | 3.2 |
| 6063 | AT4G39460.1 \| mitochondrial substrate carrier family protein \| chr4:18355987-18358887 REVERSE \| Aliases: F23K16.90, F23K16_90 | 6.2 | 4.5 | 1.7 | 4.0 | 2.66% | 3.4 |
| 6064 | AT5G15070.1 \| expressed protein \| chr5:4876518-4885824 FORWARD \| Aliases: F2G14.190, F2G14_190 | 4.4 | 3.5 | 0.9 | 4.0 | 2.66% | 3.2 |

| Rank | Description | Sync | Root | M | t | adj.q | B |
|---|---|---|---|---|---|---|---|
| 6072 | AT1G70310.1 \| Symbol: SPDS2 \| spermidine synthase 2 (SPDSYN2) / putrescine aminopropyltransferase 2, identical to SP:O48661 Spermidine synthase 2 (EC 2.5.1.16) (Putrescine aminopropyltransferase 2) (SPDSY 2) {Arabidopsis thaliana} \| chr1:26488969-26491076 REVERSE \| Aliases: F17O7.16, F17O7_16, SPERMIDINE SYNTHASE 2 | 11.8 | 10.6 | 1.1 | 4.0 | 2.67% | 3.0 |
| 6073 | AT4G33000.2 \| Symbol: CBL10 \| calcineurin B-like protein 10 (CBL10), identical to calcineurin B-like protein 10 (Arabidopsis thaliana) GI:29150248 \| chr4:15924759-15926631 FORWARD \| Aliases: ATCBL10, F26P21.120, F26P21_120 | 3.5 | 2.7 | 0.8 | 4.0 | 2.67% | 3.2 |
| 6074 | AT3G56330.1 \| N2,N2-dimethylguanosine tRNA methyltransferase family protein, low similarity to SP:Q9P804 N(2),N(2)-dimethylguanosine tRNA methyltransferase (EC 2.1.1.32) (tRNA(guanine-26,N(2)-N(2)) methyltransferase) {Schizosaccharomyces pombe}; contains Pfam profile PF02005: N2,N2-dimethylguanosine tRNA methyltransferase \| chr3:20901229-20902923 FORWARD \| Aliases: T5P19.2 | 4.0 | 3.3 | 0.7 | 4.0 | 2.68% | 3.1 |
| 6077 | AT3G63490.2 \| ribosomal protein L1 family protein, ribosomal protein L1, S.oleracea, EMBL:SORPL1 \| chr3:23455190-23457820 FORWARD \| Aliases: MAA21.120 | 5.9 | 4.8 | 1.1 | 4.0 | 2.68% | 3.3 |
| 6078 | AT5G09270.2 \| expressed protein \| chr5:2878885-2880817 FORWARD \| Aliases: T2K12.7 | 6.8 | 5.9 | 0.8 | 4.0 | 2.68% | 3.2 |
| 6082 | AT3G22845.1 \| emp24/gp25L/p24 protein-related, contains weak similarity to transmembrane protein (GI:1212965) (Homo sapiens) \| chr3:8087314-8088754 FORWARD \| Aliases: F5N5.1 | 8.9 | 7.7 | 1.2 | 4.0 | 2.69% | 3.2 |
| 6083 | AT5G02160.1 \| expressed protein \| chr5:426352-427252 FORWARD \| Aliases: T7H20.210, T7H20_210 | 3.4 | 2.6 | 0.8 | 4.0 | 2.69% | 3.2 |
| 6085 | AT1G62690.1 \| expressed protein \| chr1:23214109-23215054 REVERSE \| Aliases: F23N19.23, F23N19_23 | 4.0 | 3.1 | 0.8 | 4.0 | 2.69% | 3.2 |
| 6086 | AT4G30520.1 \| leucine-rich repeat family protein / protein kinase family protein, contains Pfam domains PF00560: Leucine Rich Repeat and PF00069: Protein kinase domain \| chr4:14908006-14911313 REVERSE \| Aliases: F17I23.140, F17I23_140 | 6.1 | 5.0 | 1.0 | 4.0 | 2.69% | 3.3 |
| 6087 | AT5G56020.1 \| expressed protein \| chr5:22701715-22703595 FORWARD \| Aliases: MDA7.6, MDA7_6 | 7.0 | 5.8 | 1.2 | 4.0 | 2.69% | 3.3 |
| 6088 | AT1G67690.1 \| peptidase M3 family protein / thimet oligopeptidase family protein, similar to SP:P42676 Neurolysin, mitochondrial precursor (EC 3.4.24.16) (Mitochondrial oligopeptidase M) {Rattus norvegicus}; contains Pfam profile PF01432: Peptidase family M3 \| chr1:25372749-25377393 FORWARD \| Aliases: F12A21.16 | 3.3 | 2.7 | 0.6 | 4.0 | 2.69% | 3.1 |
| 6090 | AT1G16460.3 \| Symbol: ATRDH2 \| | 9.4 | 7.6 | 1.7 | 4.0 | 2.70% | 3.2 |
| 6093 | AT1G73240.1 \| expressed protein \| chr1:27545848-27548581 REVERSE \| Aliases: T18K17.9, T18K17_9 | 3.9 | 2.9 | 1.1 | 4.0 | 2.70% | 3.2 |
| 6096 | AT1G80720.1 \| mitochondrial glycoprotein family protein / MAM33 family protein, similar to SUAPRGA1 (Emericella nidulans) GI:6562379; contains Pfam profile PF02330: Mitochondrial glycoprotein \| chr1:30341030-30342450 FORWARD \| Aliases: F23A5.7, F23A5_7 | 3.6 | 2.7 | 0.9 | 4.0 | 2.71% | 3.2 |
| 6098 | AT1G16700.1 \| NADH-ubiquinone oxidoreductase 23 kDa subunit, mitochondrial, putative, very strong similarity to SP:Q42599 NADH-ubiquinone oxidoreductase 23 kDa subunit, mitochondrial precursor (EC 1.6.5.3) (EC 1.6.99.3) (Complex I-23KD) (CI-23KD) (Complex I- 28.5KD) (CI-28.5KD) {Arabidopsis thaliana}; contains Pfam profile PF00037: iron-sulfur cluster-binding protein \| chr1:5709718-5711889 FORWARD \| Aliases: F19K19.1, F19K19_1 | 10.0 | 6.9 | 3.1 | 4.0 | 2.71% | 3.2 |
| 6099 | AT1G79010.1 \| NADH-ubiquinone oxidoreductase 23 kDa subunit, mitochondrial (TYKY), identical to SP:Q42599 NADH-ubiquinone oxidoreductase 23 kDa subunit, mitochondrial precursor (EC 1.6.5.3) (EC 1.6.99.3) (Complex I-23KD) (CI-23KD) (Complex I- 28.5KD) (CI-28.5KD) {Arabidopsis thaliana} \| chr1:29729814-29731910 REVERSE \| Aliases: YUP8H12R.37, YUP8H12R_37 | 10.0 | 6.9 | 3.1 | 4.0 | 2.71% | 3.2 |
| 6101 | AT1G66080.1 \| expressed protein \| chr1:24603881-24604820 REVERSE \| Aliases: F15E12.12, F15E12_12 | 3.6 | 2.9 | 0.7 | 4.0 | 2.71% | 3.2 |
| 6104 | AT2G34650.1 \| Symbol: PID \| protein kinase PINOID (PID), identical to protein kinase PINOID (Arabidopsis thaliana) gi:7208442:gb:AAF40202; contains protein kinase domain, Pfam:PF00069 \| chr2:14596851-14598867 REVERSE \| Aliases: ABR, ABRUPTUS, PINOID, PROTEIN KINASE PINOID, T31E10.1, T31E10_1 | 5.6 | 4.4 | 1.1 | 4.0 | 2.71% | 3.4 |
| 6105 | AT5G41870.1 \| glycoside hydrolase family 28 protein / polygalacturonase (pectinase) family protein, weak similarity to polygalacturonase PG1 (Glycine max) GI:5669846; contains PF00295: Glycosyl hydrolases family 28 | 4.2 | 3.6 | 0.7 | 4.0 | 2.72% | 3.2 |
| 6106 | AT4G33410.1 \| signal peptide peptidase family protein, contains Pfam domain PF04258: Membrane protein of unknown function (DUF435) \| chr4:16080879-16083240 FORWARD \| Aliases: F17M5.170, F17M5_170 | 8.4 | 7.4 | 1.0 | 4.0 | 2.72% | 3.3 |
| 6107 | AT5G21105.1 \| L-ascorbate oxidase, putative, similar to L-ascorbate oxidase from {Nicotiana tabacum} SP:Q40588, {Cucurbita pepo var. melopepo} SP:P37064; contains Pfam profile PF00394: Multicopper oxidase; supported by cDNA gi_15215753_gb_AY050406.1_; A false intron was added between exons 4 and 5 to circumvent the single nucleotide insertion in this BAC which, otherwise, causes a frameshift. \| chr5:7172777-7177749 FORWARD \| Aliases: None | 7.7 | 5.0 | 2.7 | 4.0 | 2.72% | 3.4 |

| Rank | Description | Sync | Root | M | t | adj.q | B |
|---|---|---|---|---|---|---|---|
| 6112 | AT1G45230.2 \| similar to expressed protein [Arabidopsis thaliana] (TAIR:At3g46630.1); similar to DCL protein [Coffea arabica] (GB:CAD12248.1) \| chr1:17171979-17173901 REVERSE \| Aliases: None | 3.9 | 2.4 | 1.5 | 4.0 | 2.73% | 3.4 |
| 6113 | AT1G17460.1 \| Symbol: TRFL3 \| myb family transcription factor, contains Pfam PF00249: Myb-like DNA-binding domain \| chr1:5998813-6002709 FORWARD \| Aliases: F28G4.5, TRF LIKE 3 | 3.9 | 3.2 | 0.7 | 4.0 | 2.73% | 3.1 |
| 6115 | AT2G24090.1 \| ribosomal protein L35 family protein, contains Pfam profile PF01632: ribosomal protein L35 \| chr2:10249056-10250693 FORWARD \| Aliases: T29E15.29, T29E15_29 | 4.7 | 2.5 | 2.2 | 4.0 | 2.73% | 3.4 |
| 6117 | AT3G56430.1 \| expressed protein, unknown protein At2g40800 - Arabidopsis thaliana, EMBL:AC007660 \| chr3:20934579-20936788 REVERSE \| Aliases: T5P19.80 | 5.1 | 4.3 | 0.8 | 4.0 | 2.74% | 3.2 |
| 6119 | AT5G14530.1 \| transducin family protein / WD-40 repeat family protein, similar to Will die slowly protein (SP:Q9V3J8) (Drosophila melanogaster) ; contains Pfam PF00400: WD domain, G-beta repeat (4 copies, 1 weak) \| chr5:4684623-4686912 REVERSE \| Aliases: T15N1.20, T15N1_20 | 5.8 | 4.6 | 1.2 | 4.0 | 2.74% | 3.4 |
| 6120 | AT2G22490.1 \| Symbol: CYCD2;1 \| cyclin delta-2 (CYCD2), identical to SP:P42752 Cyclin delta-2 {Arabidopsis thaliana} \| chr2:9560883-9563221 REVERSE \| Aliases: CYCD2;1, Cyclin D2;1, F14M13.11, F14M13_11 | 7.2 | 5.9 | 1.2 | 4.0 | 2.74% | 3.3 |
| 6122 | AT3G07140.2 \| GPI transamidase component Gpi16 subunit family protein, similar to phosphatidyl inositol glycan class T (GI:14456615) (Homo sapiens); contains Pfam profile PF04113: Gpi16 subunit, GPI transamidase component \| chr3:2261203-2263740 FORWARD \| Aliases: T1B9.20 | 6.7 | 5.0 | 1.7 | 4.0 | 2.74% | 3.4 |
| 6124 | AT2G39920.1 \| acid phosphatase class B family protein, weak similarity to pod storage protein (Phaseolus vulgaris GI:2627233 SP:P10743 STEM 31 kDa glycoprotein precursor (Vegetative storage protein B) {Glycine max}; contains Pfam profile PF03767: HAD superfamily (subfamily IIIB) phosphatase \| chr2:16670102-16671616 REVERSE \| Aliases: T28M21.8, T28M21_8 | 3.0 | 2.4 | 0.6 | 4.0 | 2.75% | 3.0 |
| 6125 | AT5G66540.1 \| expressed protein, ; supported by full-Length cDNA gi:12057175 from (Arabidopsis thaliana) \| chr5:26573576-26576653 REVERSE \| Aliases: K1F13.21, K1F13_21 | 4.0 | 3.3 | 0.7 | 4.0 | 2.75% | 3.1 |
| 6126 | AT1G16530.1 \| LOB domain protein 3 / lateral organ boundaries domain protein 3 (LBD3), identical to SP:Q9SA51 LOB domain protein 3 {Arabidopsis thaliana}; identical to ASYMMETRIC LEAVES2-like protein 9 (Arabidopsis thaliana) GI:19918989 \| chr1:5651463-5652832 REVERSE \| Aliases: F3O9.33, F3O9_33 | 4.4 | 3.3 | 1.1 | 4.0 | 2.75% | 3.3 |
| 6128 | AT2G17300.1 \| expressed protein \| chr2:7529547-7530018 REVERSE \| Aliases: F5J6.6, F5J6_6 | 3.9 | 3.3 | 0.6 | 4.0 | 2.75% | 3.1 |
| 6129 | AT4G26110.1 \| nucleosome assembly protein (NAP), putative, similar to nucleosome assembly protein 1 (Glycine max) GI:1161252; contains Pfam profile PF00956: Nucleosome assembly protein (NAP) | 7.1 | 6.1 | 0.9 | 4.0 | 2.76% | 3.1 |
| 6130 | AT1G16900.1 \| curculin-like (mannose-binding) lectin family protein, very low similarity to Ser Thr protein kinase GI:2598067 from (Zea mays); contains Pfam lectin (probable mannose binding) domain PF01453 but not the protein kinase domain of the Z. mays protein | 4.8 | 3.8 | 1.0 | 4.0 | 2.76% | 3.2 |
| 6131 | AT5G03680.1 \| trihelix DNA-binding protein, putative, similar to DNA-binding protein DF1 (Pisum sativum) GI:13646986 \| chr5:957857-960759 FORWARD \| Aliases: F17C15.100, F17C15_100 | 4.1 | 3.3 | 0.8 | 4.0 | 2.76% | 3.2 |
| 6133 | AT1G12180.1 \| expressed protein \| chr1:4130419-4131135 FORWARD \| Aliases: T28K15.15, T28K15_15 | 4.5 | 3.0 | 1.5 | 4.0 | 2.76% | 3.3 |
| 6134 | AT5G64840.1 \| Symbol: ATGCN5 \| | 8.2 | 6.4 | 1.7 | 4.0 | 2.76% | 3.2 |
| 6135 | AT3G13674.1 \| expressed protein \| chr3:4474784-4475888 REVERSE \| Aliases: MMM17.23 | 3.3 | 2.4 | 0.9 | 4.0 | 2.76% | 3.2 |
| 6136 | AT4G16566.1 \| histidine triad family protein / HIT family protein, contains Pfam domain, PF01230: HIT family (histidine triad protein family) \| chr4:9334648-9335980 REVERSE \| Aliases: None | 6.8 | 5.6 | 1.3 | 4.0 | 2.77% | 3.3 |
| 6139 | AT1G13370.1 \| histone H3, putative, strong similarity to Histone H3.2, minor Medicago sativa SP:P11105, histone H3 Rubus idaeus GI:10732809; contains Pfam profile PF00125 Core histone H2A/H2B/H3/H4 \| chr1:4587777-4588610 REVERSE \| Aliases: T6J4.12, T6J4_12 | 4.0 | 2.4 | 1.6 | 3.9 | 2.77% | 3.3 |
| 6141 | AT5G20380.1 \| transporter-related, low similarity to vesicular glutamate transporter 3 (Rattus norvegicus) GI:21685382 | 4.8 | 3.8 | 1.0 | 3.9 | 2.78% | 3.2 |
| 6142 | AT2G22900.1 \| galactosyl transferase GMA12/MNN10 family protein, very low similarity to alpha-1,2-galactosyltransferase, Schizosaccharomyces pombe (SP:Q09174) | 7.3 | 6.0 | 1.3 | 3.9 | 2.78% | 3.1 |
| 6143 | AT2G27290.1 \| expressed protein \| chr2:11685478-11686870 REVERSE \| Aliases: F12K2.13, F12K2_13 | 5.1 | 4.2 | 0.8 | 3.9 | 2.78% | 3.3 |

| Rank | Description | Sync | Root | M | t | adj.q | B |
|---|---|---|---|---|---|---|---|
| 6144 | AT3G56040.1 \| expressed protein \| chr3:20803052-20808551 REVERSE \| Aliases: F27K19.220 | 4.4 | 3.6 | 0.9 | 3.9 | 2.78% | 3.2 |
| 6145 | AT1G08640.1 \| expressed protein \| chr1:2748430-2751281 REVERSE \| Aliases: F22O13.12, F22O13_12 | 6.5 | 5.2 | 1.4 | 3.9 | 2.78% | 3.4 |
| 6148 | AT2G43530.1 \| Encodes a defensin-like (DEFL) family protein. \| chr2:18077163-18077710 FORWARD \| Aliases: T1O24.27 | 3.1 | 2.4 | 0.6 | 3.9 | 2.79% | 3.1 |
| 6151 | AT5G01790.1 \| expressed protein, similar to expressed protein [Arabidopsis thaliana] (TAIR:At2g40475.1); similar to unknown protein [Oryza sativa (japonica cultivar-group)] (GB:XP_475660.1) \| chr5:304894-305693 REVERSE \| Aliases: T20L15.60, T20L15_60 | 3.5 | 2.7 | 0.8 | 3.9 | 2.79% | 3.2 |
| 6152 | AT4G25370.1 \| Clp amino terminal domain-containing protein, contains Pfam profile: PF02861 Clp amino terminal domain \| chr4:12972718-12974880 FORWARD \| Aliases: T30C3.40, T30C3_40 | 7.0 | 6.1 | 1.0 | 3.9 | 2.79% | 3.0 |
| 6153 | AT2G43690.1 \| lectin protein kinase, putative, similar to receptor-like kinase LECRK1 (Arabidopsis thaliana) gi:2150023:gb:AAB58725 \| chr2:18119666-18121660 FORWARD \| Aliases: F18O19.20 | 3.2 | 2.7 | 0.5 | 3.9 | 2.79% | 2.9 |
| 6154 | AT5G18290.1 \| major intrinsic protein-related / MIP-related, contains weak similarity to Pfam profile: MIP PF00230; annotated based on segmental duplication \| chr5:6055030-6056702 REVERSE \| Aliases: F20L16.1 | 5.1 | 3.9 | 1.3 | 3.9 | 2.80% | 3.3 |
| 6155 | AT1G74870.1 \| expressed protein, contains similarity to hypothetical proteins \| chr1:28130754-28131936 FORWARD \| Aliases: F25A4.16, F25A4_16 | 4.7 | 3.7 | 1.0 | 3.9 | 2.80% | 3.3 |
| 6156 | AT1G08220.1 \| expressed protein, similar to hypothetical protein [Dictyostelium discoideum] (GB:AAO52382.1); contains InterPro domain ATP10 (InterPro:IPR007849) \| chr1:2581019-2583319 REVERSE \| Aliases: T23G18.8, T23G18_8 | 5.7 | 4.3 | 1.4 | 3.9 | 2.80% | 3.3 |
| 6157 | AT5G24600.1 \| expressed protein, contains Pfam profile PF04654: Protein of unknown function, DUF599 \| chr5:8421632-8423483 REVERSE \| Aliases: K18P6.13, K18P6_13 | 4.8 | 3.8 | 1.0 | 3.9 | 2.80% | 3.3 |
| 6159 | AT4G34540.1 \| isoflavone reductase family protein, similar to phenylcoumaran benzylic ether reductase homolog Fi1 (Forsythia x intermedia)(GI:7578895); contains isoflavone reductase domain PF02716 \| chr4:16498396-16502179 FORWARD \| Aliases: T4L20.120, T4L20_120 | 4.8 | 3.2 | 1.6 | 3.9 | 2.80% | 3.3 |
| 6161 | AT5G20290.1 \| 40S ribosomal protein S8 (RPS8A), ribosomal protein S8 - Zea mays, PIR:T04088 \| chr5:6851483-6853065 REVERSE \| Aliases: F5O24.180, F5O24_180 | 11.9 | 10.9 | 1.0 | 3.9 | 2.81% | 3.1 |
| 6164 | AT3G29575.3 \| similar to expressed protein [Arabidopsis thaliana] (TAIR:At1g13740.1); similar to ABRC5 [Helianthus annuus] (GB:AAR06258.1) \| chr3:11384710-11386443 REVERSE \| Aliases: MWE13.5 | 6.9 | 5.9 | 1.0 | 3.9 | 2.81% | 3.2 |
| 6165 | AT5G57345.1 \| expressed protein \| chr5:23246652-23247861 REVERSE \| Aliases: None | 4.6 | 3.9 | 0.8 | 3.9 | 2.81% | 3.2 |
| 6166 | AT1G63470.1 \| DNA-binding family protein, contains a AT hook motif (DNA binding motifs with a preference for A/T rich regions), Pfam:PF02178 \| chr1:23540212-23542885 REVERSE \| Aliases: F2K11.28, F2K11_28 | 7.3 | 6.0 | 1.2 | 3.9 | 2.82% | 3.2 |
| 6169 | AT4G03180.1 \| expressed protein \| chr4:1403201-1405570 FORWARD \| Aliases: F4C21.10, F4C21_10 | 6.7 | 6.0 | 0.7 | 3.9 | 2.82% | 3.1 |
| 6173 | AT3G46940.1 \| deoxyuridine 5'-triphosphate nucleotidohydrolase family, contains Pfam profile: PF00692 deoxyuridine 5'-triphosphate nucleotidohydrolase | 6.6 | 5.3 | 1.3 | 3.9 | 2.82% | 3.3 |
| 6176 | AT5G18100.1 \| Symbol: CSD3 \| superoxide dismutase (Cu-Zn) / copper/zinc superoxide dismutase (CSD3), identical to copper/zinc superoxide dismutase GI:3273755 | 7.5 | 6.6 | 0.9 | 3.9 | 2.83% | 3.1 |
| 6178 | AT5G11070.1 \| expressed protein \| chr5:3516218-3516996 REVERSE \| Aliases: T5K6.60, T5K6_60 | 7.1 | 5.7 | 1.4 | 3.9 | 2.83% | 3.3 |
| 6180 | AT5G06910.1 \| Symbol: ATJ6 \| DNAJ heat shock protein, putative (J6), identical to DnaJ homologue (Arabidopsis thaliana) GI:2689720; contains Pfam profile PF00226 DnaJ domain | 7.5 | 5.8 | 1.7 | 3.9 | 2.83% | 3.3 |
| 6181 | AT1G80910.1 \| expressed protein \| chr1:30405628-30408934 FORWARD \| Aliases: F23A5.27, F23A5_27 | 6.0 | 5.0 | 1.0 | 3.9 | 2.85% | 3.2 |
| 6182 | AT5G41340.1 \| Symbol: UBC4 \| ubiquitin-conjugating enzyme 4 (UBC4), E2; identical to gi:431265, SP:P42748 \| chr5:16555351-16557358 REVERSE \| Aliases: ATUBC4, MYC6.5, MYC6_5, UBIQUITIN CONJUGATING ENZYME 4 | 8.1 | 6.2 | 2.0 | 3.9 | 2.85% | 3.2 |
| 6183 | AT4G28190.1 \| Symbol: ULT1 \| expressed protein \| chr4:13985476-13987267 FORWARD \| Aliases: F26K10.70, F26K10_70, ULT, ULTRAPETALA, ULTRAPETALA1 | 7.3 | 6.0 | 1.3 | 3.9 | 2.85% | 3.2 |

| Rank | Description | Sync | Root | M | t | adj.q | B |
|---|---|---|---|---|---|---|---|
| 6186 | AT5G61240.1 \| similar to leucine-rich repeat family protein [Arabidopsis thaliana] (TAIR:At1g13910.1); similar to Hcr2-0B [Lycopersicon esculentum] (GB:AAC78593.1); similar to putative leucine-rich repeat resistance protein [Solanum demissum] (GB:AAT38740.1); contains InterPro domain Leucine-rich repeat, typical subtype (InterPro:IPR003591); contains InterPro domain Leucine-rich repeat, plant specific (InterPro:IPR007090); contains InterPro domain Leucine-rich repeat (InterPro:IPR001611) \| chr5:24646613-24649812 FORWARD \| Aliases: MFB13.23, MFB13_23 | 7.6 | 6.0 | 1.6 | 3.9 | 2.85% | 3.3 |
| 6188 | AT3G16250.1 \| ferredoxin-related, contains Pfam profile: PF00111 2Fe-2S iron-sulfur cluster binding domains \| chr3:5506268-5508420 REVERSE \| Aliases: MYA6.6 | 2.8 | 2.2 | 0.6 | 3.9 | 2.86% | 2.9 |
| 6190 | AT5G62210.1 \| embryo-specific protein-related, contains weak similarity to embryo-specific protein 3 (GI:3335171) (Arabidopsis thaliana) \| chr5:25003553-25004715 REVERSE \| Aliases: MMI9.4, MMI9_4 | 4.3 | 3.3 | 0.9 | 3.9 | 2.86% | 3.1 |
| 6191 | AT5G66710.1 \| protein kinase, putative, similar to protein kinase ATN1 GP:1054633 (Arabidopsis thaliana) \| chr5:26653835-26655790 FORWARD \| Aliases: MSN2.10, MSN2_10 | 4.0 | 3.2 | 0.8 | 3.9 | 2.86% | 3.1 |
| 6193 | AT3G09820.2 \| Symbol: ADK1 \| adenosine kinase 1 (ADK1) / adenosine 5'-phosphotransferase 1, identical to adenosine kinase 1 /adenosine 5'-phosphotransferase 1 SP:Q9SF85 from (Arabidopsis thaliana) \| chr3:3012099-3014949 FORWARD \| Aliases: ADENOSINE KINASE 1, F8A24.13 | 11.3 | 9.3 | 2.0 | 3.9 | 2.86% | 3.3 |
| 6194 | AT5G47830.2 \| expressed protein \| chr5:19390124-19391557 REVERSE \| Aliases: MCA23.17, MCA23_17 | 5.8 | 5.0 | 0.8 | 3.9 | 2.86% | 3.2 |
| 6198 | AT5G40650.1 \| Symbol: SDH2 2 \| | 9.5 | 7.9 | 1.6 | 3.9 | 2.87% | 3.1 |
| 6200 | AT2G27970.1 \| Symbol: CKS2 \| cyclin-dependent kinase, putative / CDK, putative, similar to Cks1 protein (Arabidopsis thaliana) gi:2274859:emb:CAA03859 \| chr2:11919295-11920348 REVERSE \| Aliases: CDK subunit 2, CKS2, T1E2.11, T1E2_11 | 9.1 | 7.4 | 1.6 | 3.9 | 2.87% | 3.2 |
| 6201 | AT1G62830.1 \| amine oxidase family protein / SWIRM domain-containing protein, contains Pfam profile: PF01593 Flavin containing amine oxidase \| chr1:23268155-23270867 REVERSE \| Aliases: F23N19.21, F23N19_21 | 5.0 | 4.4 | 0.6 | 3.9 | 2.87% | 3.0 |
| 6203 | AT5G23570.1 \| Symbol: SGS3 \| XS domain-containing protein / XS zinc finger domain-containing protein-related, contains Pfam profiles PF03468: XS domain, weak hit to PF03470: XS zinc finger domain \| chr5:7943127-7946053 FORWARD \| Aliases: MQM1.17, MQM1_17, SUPPRESSOR OF GENE SILENCING 3 | 4.3 | 2.9 | 1.4 | 3.9 | 2.88% | 3.2 |
| 6206 | AT5G67070.1 \| Symbol: RALFL34 \| rapid alkalinization factor (RALF) family protein, similar to RALF precursor (Nicotiana tabacum) GI:16566316 \| chr5:26785321-26786072 FORWARD \| Aliases: K21H1.3, K21H1_3, RALF LIKE 34 | 4.8 | 3.7 | 1.1 | 3.9 | 2.89% | 3.3 |
| 6207 | AT5G13290.2 \| protein kinase family protein, contains protein kinase domain, Pfam:PF00069 \| chr5:4252846-4254435 REVERSE \| Aliases: T31B5.110, T31B5_110 | 5.9 | 5.0 | 0.8 | 3.9 | 2.89% | 3.2 |
| 6209 | AT3G63190.1 \| ribosome recycling factor, chloroplast, putative / ribosome releasing factor, chloroplast, putative, similar to SP:P82231 Ribosome recycling factor, chloroplast precursor (Ribosome releasing factor, chloroplast) (RRF) (CpFrr) (RRFHCP) {Spinacia oleracea}; contains Pfam profile PF01765: ribosome recycling factor | 5.6 | 4.3 | 1.3 | 3.9 | 2.89% | 3.3 |
| 6210 | AT1G77810.2 \| galactosyltransferase family protein, contains Pfam profile PF01762: Galactosyltransferase \| chr1:29265515-29267895 REVERSE \| Aliases: T32E8.14 | 6.4 | 5.5 | 0.9 | 3.9 | 2.89% | 3.2 |
| 6212 | AT4G37510.1 \| ribonuclease III family protein, contains Pfam profile PF00636 RNase3 domain \| chr4:17626098-17628941 REVERSE \| Aliases: F6G17.160, F6G17_160 | 5.5 | 4.2 | 1.2 | 3.9 | 2.89% | 3.3 |
| 6213 | AT4G16450.1 \| expressed protein \| chr4:9280104-9280740 FORWARD \| Aliases: DL4250W, FCAALL.388 | 10.6 | 9.8 | 0.7 | 3.9 | 2.90% | 2.9 |
| 6216 | AT3G62790.1 \| NADH-ubiquinone oxidoreductase-related, contains weak similarity to NADH-ubiquinone oxidoreductase 15 kDa subunit (EC 1.6.5.3) (EC 1.6.99.3) (Complex I-15 kDa) (CI-15 kDa) (Swiss-Prot:O43920) (Homo sapiens) \| chr3:23234059-23235283 REVERSE \| Aliases: F26K9.220 | 10.8 | 10.3 | 0.5 | 3.9 | 2.90% | 2.8 |
| 6217 | AT2G35150.1 \| phosphate-responsive 1 family protein, similar to phi-1 (phosphate-induced gene) (Nicotiana tabacum) GI:3759184; contains Pfam profile PF04674: Phosphate-induced protein 1 conserved region \| chr2:14824126-14825290 REVERSE \| Aliases: T4C15.11 | 3.5 | 2.5 | 1.0 | 3.9 | 2.90% | 3.2 |
| 6218 | AT1G14270.2 \| CAAX amino terminal protease family protein, contains Pfam profile PF02517: CAAX amino terminal protease family \| chr1:4874870-4877221 REVERSE \| Aliases: F14L17.3, F14L17_3 | 5.5 | 4.7 | 0.8 | 3.9 | 2.90% | 3.1 |
| 6219 | AT3G56870.1 \| expressed protein \| chr3:21063452-21066569 FORWARD \| Aliases: T8M16.200 | 3.7 | 2.7 | 1.0 | 3.9 | 2.91% | 3.3 |

| Rank | Description | Sync | Root | M | t | adj.q | B |
|---|---|---|---|---|---|---|---|
| 6220 | AT1G08260.1 \| Symbol: EMB2284 \| DNA-directed DNA polymerase epsilon catalytic subunit, putative, similar to SP:Q07864 DNA polymerase epsilon, catalytic subunit A (EC 2.7.7.7) (DNA polymerase II subunit A) {Homo sapiens}; contains Pfam profiles: PF03175 DNA polymerase type B, organellar and viral, PF00136 DNA polymerase family B, PF03104 DNA polymerase family B, exonuclease domain \| chr1:2590941-2606889 FORWARD \| Aliases: EMB2284, EMBRYO DEFECTIVE 2284, T23G18.21, T23G18_21 | 4.9 | 4.2 | 0.7 | 3.9 | 2.91% | 3.1 |
| 6222 | AT1G47870.1 \| Symbol: E2FC \| E2F transcription factor-2 (E2F2) / transcription factor E2Fc (E2Fc), identical to transcription factor E2Fc (Arabidopsis thaliana) GI:19578311; contains Pfam profile PF02319: Transcription factor E2F/dimerisation partner; identical to cDNA E2F transcription factor-2 E2F2 GI:10443850 \| chr1:17637228-17640210 FORWARD \| Aliases: ATE2F2, E2FC, E2FC TRANSCRIPTION FACTOR, T2E6.2, T2E6_2, TRANSCRIPTION FACTOR 2 E2F2 | 6.4 | 5.6 | 0.8 | 3.9 | 2.91% | 3.2 |
| 6224 | AT2G24390.1 \| similar to avirulence-responsive protein-related / avirulence induced gene (AIG) protein-related [Arabidopsis thaliana] (TAIR:At4g31310.1); similar to avrRpt2-induced protein 2-like [Oryza sativa (japonica cultivar-group)] (GB:XP_464100.1) \| chr2:10380811-10382032 FORWARD \| Aliases: T28I24.12, T28I24_12 | 3.6 | 3.0 | 0.6 | 3.9 | 2.91% | 3.0 |
| 6225 | AT3G24440.1 \| fibronectin type III domain-containing protein, contains Pfam profile PF00041: Fibronectin type III domain \| chr3:8876034-8878178 REVERSE \| Aliases: MXP5.1 | 5.6 | 4.6 | 1.0 | 3.9 | 2.91% | 3.1 |
| 6226 | AT2G22740.2 \| Symbol: SUVH6 \| SET domain-containing protein (SUVH6), identical to SUVH6 (Arabidopsis thaliana) GI:13517753; contains Pfam profiles PF00856: SET domain, PF05033: Pre-SET motif, PF02182: YDG/SRA domain \| chr2:9669718-9674061 REVERSE \| Aliases: T9I22.18, T9I22_18 | 6.9 | 6.0 | 0.8 | 3.9 | 2.91% | 3.1 |
| 6232 | AT5G60860.1 \| Ras-related GTP-binding protein, putative, similar to GTP-binding protein GI:303742 from (Pisum sativum) | 5.0 | 3.8 | 1.2 | 3.9 | 2.92% | 3.2 |
| 6233 | AT5G01390.1 \| DNAJ heat shock protein, putative, similar to SP:Q9QYJ3 DnaJ homolog subfamily B member 1 (Heat shock 40 kDa protein 1) {Mus musculus}; contains Pfam profile PF00226: DnaJ domain \| chr5:160263-162270 REVERSE \| Aliases: T10O8.100, T10O8_100 | 5.3 | 4.3 | 1.0 | 3.9 | 2.93% | 3.2 |
| 6235 | AT3G58790.1 \| glycosyl transferase family 8 protein, contains Pfam profile: PF01501 glycosyl transferase family 8; general stress protein gspA, Bacillus subtilis, PIR:S16423 \| chr3:21753048-21757301 REVERSE \| Aliases: T20N10.140 | 4.7 | 3.9 | 0.9 | 3.9 | 2.93% | 3.1 |
| 6236 | AT1G77200.1 \| encodes a member of the DREB subfamily A-4 of ERF/AP2 transcription factor family. The protein contains one AP2 domain. There are 17 members in this subfamily including TINY. \| chr1:29009251-29009985 REVERSE \| Aliases: T14N5.6, T14N5_6 | 3.0 | 2.3 | 0.7 | 3.9 | 2.93% | 3.1 |
| 6237 | AT1G05160.1 \| Symbol: CYP88A3 \| ent-kaurenoic acid hydroxylase (KAO1) / cytochrome P450 88A3, putative (CYP88A3), identical to Cytochrome P450 88A3 (SP:O23051) (Arabidopsis thaliana); nearly identical to ent-kaurenoic acid hydroxylase (KAO1) GI:13021852 from (Arabidopsis thaliana) \| chr1:1487377-1490946 REVERSE \| Aliases: ATKAO1, ENT KAURENOIC ACID HYDROXYLASE 1, KAO1, YUP8H12.23, YUP8H12_23 | 4.7 | 3.9 | 0.8 | 3.9 | 2.93% | 3.1 |
| 6238 | AT1G72660.3 \| similar to developmentally regulated GTP-binding protein (DRG1) [Arabidopsis thaliana] (TAIR:At1g17470.1); similar to developmentally regulated GTP binding protein [Pisum sativum] (GB:AAB67829.1); contains InterPro domain Small GTP-binding protein domain (InterPro:IPR005225); contains InterPro domain TGS domain (InterPro:IPR004095); contains InterPro domain GTP1/OBG domain (InterPro:IPR006074); contains InterPro domain GTP1/OBG (InterPro:IPR006073); contains InterPro domain GTP1/OBG sub-domain (InterPro:IPR006169) \| chr1:27357542-27360332 FORWARD \| Aliases: F28P22.15, F28P22_15 | 5.2 | 4.1 | 1.2 | 3.9 | 2.93% | 3.2 |
| 6241 | AT4G31580.1 \| Symbol: SRZ 22 \| | 7.4 | 6.2 | 1.2 | 3.9 | 2.93% | 3.3 |
| 6243 | AT1G10840.2 \| Symbol: TIF3H1 \| | 8.7 | 7.5 | 1.2 | 3.9 | 2.93% | 3.2 |
| 6245 | AT5G42850.2 \| expressed protein \| chr5:17199108-17199807 REVERSE \| Aliases: MBD2.4, MBD2_4 | 7.7 | 6.6 | 1.1 | 3.9 | 2.93% | 3.0 |
| 6251 | AT3G08640.1 \| alphavirus core protein family, contains Pfam profile: PF00944 alphavirus core protein \| chr3:2622886-2624314 FORWARD \| Aliases: F17O14.11 | 8.3 | 6.1 | 2.2 | 3.9 | 2.95% | 3.3 |
| 6252 | AT3G08920.1 \| rhodanese-like domain-containing protein, contains rhodanese-like domain PF:00581 \| chr3:2712242-2713272 FORWARD \| Aliases: T16O11.14 | 5.0 | 4.0 | 1.0 | 3.9 | 2.95% | 3.2 |
| 6254 | AT3G61740.1 \| PHD finger family protein (ATX3), contains Pfam domains PF00628: PHD-finger and PF00855: PWWP domain; identical to cDNA trithorax 3 (ATX3) partial cds GI:15217142 \| chr3:22861810-22867947 REVERSE \| Aliases: F21F14.13 | 4.7 | 3.8 | 0.8 | 3.9 | 2.95% | 3.2 |
| 6256 | AT1G74920.1 \| Symbol: ALDH10A8 \| betaine-aldehyde dehydrogenase, putative, identical to betaine-aldehyde dehydrogenase, chloroplast precursor (BADH) (Arabidopsis thaliana) SWISS-PROT:Q9S795; strong similarity to betaine aldehyde dehydrogenase (Amaranthus hypochondriacus) GI:2388710 \| chr1:28142686-28146405 REVERSE \| Aliases: F25A4.11, F25A4_11 | 10.9 | 9.5 | 1.4 | 3.9 | 2.95% | 3.1 |
| 6258 | AT5G51720.1 \| expressed protein \| chr5:21026822-21027571 FORWARD \| Aliases: MIO24.14, MIO24_14 | 6.1 | 4.1 | 2.0 | 3.9 | 2.96% | 3.3 |

| Rank | Description | Sync | Root | M | t | adj.q | B |
|---|---|---|---|---|---|---|---|
| 6259 | AT2G24490.1 \| replication protein, putative, similar to replication protein A 30kDa (Oryza sativa (japonica cultivar-group)) GI:13516746; contains InterPro entry IPR004365: OB-fold nucleic acid binding domain \| chr2:10405484-10407568 REVERSE \| Aliases: T28I24.22, T28I24_22 | 5.9 | 4.6 | 1.2 | 3.9 | 2.96% | 3.3 |
| 6260 | AT3G11400.1 \| Symbol: EIF3G1 \| | 8.9 | 7.3 | 1.6 | 3.9 | 2.96% | 3.2 |
| 6261 | AT5G48090.1 \| expressed protein, ; expression supported by MPSS \| chr5:19502460-19505609 FORWARD \| Aliases: MDN11.17, MDN11_17 | 3.0 | 2.5 | 0.4 | 3.9 | 2.96% | 2.7 |
| 6262 | AT1G09180.1 \| Symbol: ATSAR1/ATSARA1A \| GTP-binding protein, putative, strong similarity to SP:Q01474 GTP-binding protein SAR1B and SP:O04834 GTP-binding protein SAR1A (Arabidopsis thaliana) \| chr1:2965025-2965974 FORWARD \| Aliases: ATSAR1, ATSARA1A, T12M4.12, T12M4_12 | 4.6 | 3.8 | 0.7 | 3.9 | 2.96% | 3.0 |
| 6263 | AT3G22930.1 \| calmodulin, putative, strong similarity to calmodulin 8 GI:5825600 from (Arabidopsis thaliana); contains INTERPRO:IPR002048 calcium-binding EF-hand domain \| chr3:8124090-8125938 REVERSE \| Aliases: F5N5.10 | 3.5 | 2.2 | 1.3 | 3.9 | 2.96% | 2.9 |
| 6264 | AT3G61220.1 \| short-chain dehydrogenase/reductase (SDR) family protein, similar to carbonyl reductase GI:1049108 from (Mus musculus) \| chr3:22673962-22675468 FORWARD \| Aliases: T20K12.120 | 10.2 | 9.2 | 1.1 | 3.9 | 2.96% | 3.0 |
| 6265 | AT3G52570.1 \| expressed protein, contains Interpro entry IPR000379 \| chr3:19512108-19513977 FORWARD \| Aliases: F3C22.3 | 7.1 | 6.2 | 0.9 | 3.9 | 2.97% | 3.0 |
| 6269 | AT3G57150.1 \| Symbol: NAP57 \| dyskerin, putative / nucleolar protein NAP57, putative, similar to SP:P40615 Dyskerin (Nucleolar protein NAP57) {Rattus norvegicus}; contains Pfam profiles PF01509: TruB family pseudouridylate synthase (N terminal domain), PF01472: PUA domain; supporting cDNA gi:8901185:gb:AF234984.2:AF234984 \| chr3:21164952-21166988 REVERSE \| Aliases: ATNAP57, F24I3.230 | 10.7 | 9.4 | 1.4 | 3.9 | 2.97% | 2.9 |
| 6271 | AT3G05750.2 \| similar to expressed protein [Arabidopsis thaliana] (TAIR:At5g26910.1); similar to expressed protein [Arabidopsis thaliana] (TAIR:At3g58650.1); similar to serine-threonine rich antigen [Staphylococcus aureus] (GB:AAL58470.1) | 2.8 | 2.4 | 0.5 | 3.9 | 2.97% | 2.7 |
| 6272 | AT4G34200.1 \| D-3-phosphoglycerate dehydrogenase, putative / 3-PGDH, putative, similar to phosphoglycerate dehydrogenase, Arabidopsis thaliana, SP:O04130 \| chr4:16373850-16376626 REVERSE \| Aliases: F10M10.7 | 8.7 | 6.0 | 2.7 | 3.9 | 2.97% | 3.3 |
| 6273 | AT4G04940.1 \| transducin family protein / WD-40 repeat family protein, contains seven G-protein beta WD-40 repeats \| chr4:2511026-2517191 REVERSE \| Aliases: T1J1.5, T1J1_5 | 4.4 | 3.5 | 0.9 | 3.9 | 2.97% | 3.2 |
| 6276 | AT4G28450.1 \| transducin family protein / WD-40 repeat family protein, SOF1 (involved in rRNA processing) protein-yeast \| chr4:14061465-14064679 REVERSE \| Aliases: F20O9.130, F20O9_130 | 5.6 | 4.6 | 1.0 | 3.9 | 2.97% | 3.1 |
| 6278 | AT3G62920.1 \| expressed protein \| chr3:23271692-23272268 FORWARD \| Aliases: T20O10.20 | 4.9 | 4.1 | 0.7 | 3.9 | 2.98% | 3.1 |
| 6280 | AT1G31290.1 \| PAZ domain-containing protein / piwi domain-containing protein, contains Pfam profiles PF02170: PAZ domain, PF02171: Piwi domain | 4.5 | 3.2 | 1.3 | 3.9 | 2.98% | 3.2 |
| 6282 | AT2G35155.1 \| expressed protein \| chr2:14826073-14829044 REVERSE \| Aliases: None | 5.6 | 4.4 | 1.2 | 3.9 | 2.99% | 3.2 |
| 6283 | AT1G28100.3 \| expressed protein \| chr1:9801069-9804413 FORWARD \| Aliases: F13K9.27, F13K9_27 | 8.3 | 7.2 | 1.1 | 3.9 | 2.99% | 3.1 |
| 6284 | AT3G62140.1 \| similar to P0426D06.11 [Oryza sativa (japonica cultivar-group)] (GB:NP_908670.1) | 8.3 | 7.1 | 1.3 | 3.9 | 2.99% | 3.1 |
| 6287 | AT1G19330.1 \| expressed protein \| chr1:6680558-6683498 REVERSE \| Aliases: F18O14.5, F18O14_5 | 6.7 | 5.5 | 1.2 | 3.9 | 3.00% | 3.3 |
| 6288 | AT4G04190.2 \| expressed protein \| chr4:2024576-2026415 FORWARD \| Aliases: T27D20.12, T27D20_12 | 5.0 | 4.0 | 1.0 | 3.9 | 3.00% | 3.1 |
| 6291 | AT5G50840.2 \| expressed protein \| chr5:20703046-20706144 FORWARD \| Aliases: K7B16.6, K7B16_6 | 7.4 | 5.8 | 1.6 | 3.9 | 3.01% | 3.1 |
| 6292 | AT3G13910.1 \| expressed protein \| chr3:4590926-4591490 REVERSE \| Aliases: MDC16.3 | 6.7 | 5.9 | 0.9 | 3.9 | 3.02% | 3.2 |
| 6294 | AT1G78450.1 \| SOUL heme-binding family protein, weak similarity to SOUL protein (Mus musculus) GI:4886906; contains Pfam profile PF04832: SOUL heme-binding protein \| chr1:29521882-29522826 REVERSE \| Aliases: F3F9.4 | 4.1 | 2.7 | 1.4 | 3.9 | 3.03% | 3.3 |
| 6295 | AT1G28530.2 \| expressed protein \| chr1:10031983-10035485 REVERSE \| Aliases: F3M18.3 | 4.2 | 3.5 | 0.7 | 3.9 | 3.03% | 3.1 |
| 6297 | AT3G61150.1 \| homeobox-leucine zipper family protein / homeodomain GLABRA2 like protein 1 (HD-GL2-1), similar to Anthocyaninless2 (ANL2) (GP:5702094) Arabidopsis thaliana, EMBL:AF077335 \| chr3:22641585-22645950 FORWARD \| Aliases: T20K12.50 | 4.8 | 3.7 | 1.1 | 3.9 | 3.04% | 3.2 |

| Rank | Description | Sync | Root | M | t | adj.q | B |
|---|---|---|---|---|---|---|---|
| 6298 | AT1G63150.1 \| pentatricopeptide (PPR) repeat-containing protein, contains Pfam profile PF01535: PPR repeat \| chr1:23423061-23425244 FORWARD \| Aliases: F16M19.13, F16M19_13 | 3.1 | 2.6 | 0.4 | 3.9 | 3.04% | 2.7 |
| 6299 | AT5G46640.1 \| DNA-binding family protein, contains a AT hook motif (DNA binding motifs with a preference for A/T rich regions), Pfam:PF02178 \| chr5:18941712-18943802 FORWARD \| Aliases: MZA15.3, MZA15_3 | 5.0 | 4.1 | 1.0 | 3.9 | 3.04% | 3.2 |
| 6300 | AT4G19830.1 \| immunophilin / FKBP-type peptidyl-prolyl cis-trans isomerase family protein, similar to 70 kDa peptidylprolyl isomerase (Peptidylprolyl cis-trans isomerase) (PPiase) (Rotamase) (SP:Q43207) (Triticum aestivum); FKBP-type peptidyl-prolyl cis-trans isomerase,Synechocystis sp., PIR2:S75144; contains Pfam PF00254: peptidyl-prolyl cis-trans isomerase, FKBP-type \| chr4:10772441-10774033 REVERSE \| Aliases: T16H5.190, T16H5_190 | 5.5 | 4.5 | 1.0 | 3.9 | 3.04% | 3.2 |
| 6301 | AT1G29710.1 \| pentatricopeptide (PPR) repeat-containing protein, contains Pfam profile PF01535: PPR repeat \| chr1:10387659-10389086 FORWARD \| Aliases: T3M22.4, T3M22_4 | 3.3 | 2.9 | 0.4 | 3.9 | 3.04% | 2.6 |
| 6302 | AT1G74260.1 \| AIR synthase-related family protein, contains Pfam profiles: PF00586 AIR synthase related protein, N-terminal domain, PF02769 AIR synthase related protein, C-terminal domain \| chr1:27926494-27931107 REVERSE \| Aliases: F1O17.7, F1O17_7 | 7.7 | 6.8 | 1.0 | 3.9 | 3.04% | 3.0 |
| 6303 | AT4G24370.1 \| expressed protein \| chr4:12611038-12612141 REVERSE \| Aliases: T22A6.200, T22A6_200 | 4.4 | 3.3 | 1.1 | 3.9 | 3.05% | 3.1 |
| 6304 | AT5G38150.1 \| expressed protein \| chr5:15240346-15242165 REVERSE \| Aliases: MXA21.20, MXA21_20 | 4.5 | 3.3 | 1.2 | 3.9 | 3.05% | 3.2 |
| 6306 | AT5G65900.1 \| DEAD/DEAH box helicase, putative, contains Pfam profiles | 4.6 | 3.4 | 1.1 | 3.9 | 3.05% | 3.1 |
| 6308 | AT3G17120.2 \| expressed protein \| chr3:5841323-5843491 FORWARD \| Aliases: K14A17.19 | 5.4 | 4.1 | 1.3 | 3.9 | 3.05% | 3.3 |
| 6309 | AT5G44170.1 \| expressed protein, low similarity to SP:P40389 Rapid response to glucose protein 1 {Schizosaccharomyces pombe} \| chr5:17797605-17798510 FORWARD \| Aliases: MLN1.9, MLN1_9 | 4.0 | 3.6 | 0.4 | 3.9 | 3.06% | 2.5 |
| 6310 | AT5G26880.1 \| tRNA/rRNA methyltransferase (SpoU) family protein, low similarity to tRNA (Gm18) methyltransferase (Thermus thermophilus) GI:11079208; contains Pfam profile PF00588: SpoU rRNA Methylase (RNA methyltransferase, TrmH) family \| chr5:9457700-9459233 REVERSE \| Aliases: F2P16.25, F2P16_25 | 10.1 | 9.5 | 0.6 | 3.9 | 3.06% | 3.0 |
| 6311 | ATCG00750.1 \| Symbol: RPS11 \| 30S chloroplast ribosomal protein S11 \| chrC:78960-79376 REVERSE \| Aliases: RPS11 | 8.2 | 6.1 | 2.1 | 3.9 | 3.07% | 3.2 |
| 6313 | AT5G19140.2 \| expressed protein, similar to expressed protein [Arabidopsis thaliana] (TAIR:At5g43830.1); similar to hypothetical protein ARG10 - mung bean (GB:T07820) | 7.0 | 5.3 | 1.7 | 3.9 | 3.09% | 3.1 |
| 6315 | AT5G41190.1 \| expressed protein, ; expression supported by MPSS \| chr5:16504856-16507240 REVERSE \| Aliases: MEE6.26, MEE6_26 | 6.5 | 5.6 | 0.9 | 3.9 | 3.09% | 3.1 |
| 6316 | AT4G35730.1 \| expressed protein, similar to expressed protein [Arabidopsis thaliana] (TAIR:At1g34220.2); similar to unknown protein [Oryza sativa (japonica cultivar-group)] (GB:XP_480332.1); contains InterPro domain Eukaryotic protein of unknown function DUF292 (InterPro:IPR005061) \| chr4:16931044-16933361 FORWARD \| Aliases: F8D20.240, F8D20_240 | 4.5 | 3.7 | 0.8 | 3.9 | 3.09% | 3.1 |
| 6317 | AT4G22260.1 \| Symbol: IM \| alternative oxidase, putative / immutans protein (IM), identical to IMMUTANS from Arabidopsis thaliana (gi:4138855); contains Pfam profile PF01786 alternative oxidase \| chr4:11769768-11772526 REVERSE \| Aliases: IM1, IMMUTANS, T10I14.90, T10I14_90 | 6.7 | 5.4 | 1.3 | 3.9 | 3.09% | 3.1 |
| 6318 | AT1G06650.2 \| 2-oxoglutarate-dependent dioxygenase, putative, similar to 2A6 (GI:599622) and tomato ethylene synthesis regulatory protein E8 (SP:P10967); contains Pfam profile: PF00671 Iron/Ascorbate oxidoreductase family \| chr1:2035838-2037362 FORWARD \| Aliases: F12K11.26, F12K11_26 | 6.4 | 5.3 | 1.2 | 3.9 | 3.10% | 3.2 |
| 6320 | AT4G19540.1 \| expressed protein \| chr4:10657393-10659243 FORWARD \| Aliases: F24J7.100, F24J7_100 | 6.0 | 5.2 | 0.8 | 3.9 | 3.10% | 3.1 |
| 6321 | AT3G21300.1 \| RNA methyltransferase family protein, contains TIGRfam TIGR00479: RNA methyltransferase, TrmA family \| chr3:7493445-7496295 REVERSE \| Aliases: MXL8.18 | 3.0 | 2.5 | 0.5 | 3.9 | 3.10% | 2.7 |
| 6322 | AT5G63420.1 \| Symbol: EMB2746 \| metallo-beta-lactamase family protein | 4.5 | 3.8 | 0.7 | 3.9 | 3.11% | 3.0 |
| 6323 | AT5G61040.1 \| expressed protein \| chr5:24579559-24582185 REVERSE \| Aliases: MAF19.5, MAF19_5 | 4.8 | 4.2 | 0.6 | 3.9 | 3.11% | 2.7 |
| 6325 | AT1G62020.1 \| coatomer protein complex, subunit alpha, putative, contains Pfam PF00400: WD domain, G-beta repeat; similar to Coatomer alpha subunit (Alpha-coat protein) (Alpha-COP) (HEPCOP) (HEP-COP) (SP:P53621) (Homo sapiens) \| chr1:22923479-22927689 FORWARD \| Aliases: F8K4.21, F8K4_21 | 8.3 | 6.9 | 1.4 | 3.8 | 3.11% | 3.1 |

| Rank | Description | Sync | Root | M | t | adj.q | B |
|---|---|---|---|---|---|---|---|
| 6329 | AT4G38740.1 \| Symbol: ROC1 \| peptidyl-prolyl cis-trans isomerase / cyclophilin / rotamase / cyclosporin A-binding protein (ROC1), identical to SP:P34790 Peptidyl-prolyl cis-trans isomerase (EC 5.2.1.8) (PPIase) (Rotamase) (Cyclophilin) (Cyclosporin A-binding protein) {Arabidopsis thaliana} \| chr4:18083389-18084245 REVERSE \| Aliases: CYCLOPHILIN, T9A14.20, T9A14_20 | 9.9 | 6.7 | 3.2 | 3.8 | 3.11% | 3.2 |
| 6330 | AT1G54150.1 \| zinc finger (C3HC4-type RING finger) family protein, contains Pfam profile: PF00097 zinc finger, C3HC4 type (RING finger) \| chr1:20219101-20221365 FORWARD \| Aliases: F15I1.25, F15I1_25 | 6.6 | 5.9 | 0.6 | 3.8 | 3.11% | 2.8 |
| 6332 | AT5G16140.1 \| peptidyl-tRNA hydrolase family protein, contains Pfam profile PF01195: peptidyl-tRNA hydrolase \| chr5:5270311-5271520 REVERSE \| Aliases: T21H19.60, T21H19_60 | 4.1 | 3.4 | 0.8 | 3.8 | 3.12% | 3.1 |
| 6333 | AT3G51500.1 \| expressed protein, predicted protein, Oryza sativa, EMBL:AP000367 \| chr3:19119113-19119866 FORWARD \| Aliases: F26O13.140 | 4.3 | 3.4 | 0.9 | 3.8 | 3.12% | 3.1 |
| 6335 | AT1G64220.1 \| preprotein translocase-related, similar to TOM7 protein (Solanum tuberosum) GI:3319774 \| chr1:23836751-23836984 REVERSE \| Aliases: F22C12.30, F22C12_30 | 2.9 | 2.5 | 0.4 | 3.8 | 3.13% | 2.6 |
| 6336 | AT3G55760.2 \| expressed protein \| chr3:20711414-20714035 FORWARD \| Aliases: F1I16.170 | 6.7 | 5.0 | 1.7 | 3.8 | 3.13% | 3.1 |
| 6337 | AT5G66140.1 \| Symbol: PAD2 \| 20S proteasome alpha subunit D2 (PAD2) (PRS1) (PRC6), identical to SP:O24616 Proteasome subunit alpha type 7-2 (EC 3.4.25.1) (20S proteasome alpha subunit D2) {Arabidopsis thaliana} \| chr5:26454396-26455947 REVERSE \| Aliases: 20S PROTEASOME SUBUNIT PAD2, K2A18.22, K2A18_22 | 9.2 | 7.1 | 2.1 | 3.8 | 3.13% | 3.1 |
| 6340 | AT4G33925.1 \| expressed protein \| chr4:16263242-16264229 FORWARD \| Aliases: None | 5.4 | 4.2 | 1.2 | 3.8 | 3.13% | 3.2 |
| 6341 | AT5G16620.2 \| Symbol: PDE120 \| | 5.4 | 4.0 | 1.4 | 3.8 | 3.13% | 3.2 |
| 6342 | AT5G61300.1 \| expressed protein \| chr5:24668131-24670002 REVERSE \| Aliases: MFB13.7, MFB13_7 | 5.1 | 4.3 | 0.8 | 3.8 | 3.14% | 2.9 |
| 6344 | AT3G15770.2 \| expressed protein, similar to expressed protein [Arabidopsis thaliana] (TAIR:At5g25360.1); similar to hypothetical protein [Picea mariana] (GB:AAC32109.1) \| chr3:5340081-5341346 FORWARD \| Aliases: MSJ11.17 | 5.9 | 4.9 | 1.1 | 3.8 | 3.14% | 3.0 |
| 6346 | AT2G45660.1 \| Symbol: AGL20 \| MADS-box protein (AGL20) \| chr2:18814612-18818094 REVERSE \| Aliases: AGAMOUS LIKE 20, AGL20, F17K2.19, MADS BOX PROTEIN AGL20, SOC1, SUPPRESSOR OF OVEREXPRESSION OF CO 1 | 5.3 | 4.6 | 0.6 | 3.8 | 3.15% | 2.9 |
| 6348 | AT5G02150.2 \| similar to armadillo/beta-catenin repeat family protein [Arabidopsis thaliana] (TAIR:At3g09350.1); similar to Zgc:55259 protein [Danio rerio] (GB:AAH49402.1) \| chr5:424290-426045 REVERSE \| Aliases: T7H20.200, T7H20_200 | 5.3 | 4.4 | 0.9 | 3.8 | 3.15% | 3.1 |
| 6349 | AT3G24100.1 \| four F5 family protein / 4F5 family protein, contains Pfam PF04419: 4F5 protein family \| chr3:8703613-8704235 FORWARD \| Aliases: MUJ8.7 | 7.9 | 7.0 | 0.9 | 3.8 | 3.15% | 3.1 |
| 6350 | AT1G30380.1 \| photosystem I reaction center subunit psaK, chloroplast, putative / photosystem I subunit X, putative / PSI-K, putative (PSAK), identical to SP:Q9SUI5; strong similarity to SP:P36886 Photosystem I reaction center subunit psaK, chloroplast precursor (Photosystem I subunit X) (PSI-K) (Light-harvesting complex I 7 kDa protein){Hordeum vulgare}; contains Pfam profile PF01241: Photosystem I psaG / psaK | 9.1 | 8.0 | 1.1 | 3.8 | 3.15% | 3.1 |
| 6351 | AT4G14600.1 \| expressed protein \| chr4:8376396-8378331 FORWARD \| Aliases: DL3340W, FCAALL.126 | 5.4 | 4.2 | 1.2 | 3.8 | 3.16% | 3.1 |
| 6355 | AT1G76650.1 \| calcium-binding EF hand family protein, similar to regulator of gene silencing calmodulin-related protein GI:12963415 from (Nicotiana tabacum); contains INTERPRO:IPR002048 calcium-binding EF-hand domain \| chr1:28771644-28772411 REVERSE \| Aliases: F28O16.2, F28O16_2 | 6.1 | 4.8 | 1.3 | 3.8 | 3.16% | 3.2 |
| 6356 | ATCG00720.1 \| Symbol: PETB \| Encodes the cytochrome b(6) subunit of the cytochrome b6f complex. \| chrC:74841-76292 FORWARD \| Aliases: PETB | 8.5 | 6.7 | 1.8 | 3.8 | 3.16% | 3.2 |
| 6358 | AT2G38670.1 \| ethanolamine-phosphate cytidylyltransferase, putative / phosphorylethanolamine transferase, putative / CTP:phosphoethanolamine cytidylyltransferase, putative, similar to SP:Q99447 Ethanolamine-phosphate cytidylyltransferase (EC 2.7.7.14) {Homo sapiens}; contains Pfam profile PF01467: Cytidylyltransferase \| chr2:16175945-16178996 FORWARD \| Aliases: T6A23.13, T6A23_13 | 7.6 | 6.0 | 1.6 | 3.8 | 3.16% | 3.1 |
| 6361 | AT1G66660.1 \| seven in absentia (SINA) protein, putative, similar to SIAH2 protein (Brassica napus var. napus) GI:7657878; contains Pfam profile PF03145: Seven in absentia protein family \| chr1:24865539-24867031 REVERSE \| Aliases: F4N21.20, F4N21_20 | 4.7 | 4.0 | 0.8 | 3.8 | 3.16% | 3.0 |
| 6364 | AT2G40820.1 \| proline-rich family protein, contains proline rich extensin domains, INTERPRO:IPR002965 \| chr2:17041814-17046552 REVERSE \| Aliases: T20B5.2, T20B5_2 | 4.7 | 4.0 | 0.8 | 3.8 | 3.17% | 3.1 |

| Rank | Description | Sync | Root | M | t | adj.q | B |
|---|---|---|---|---|---|---|---|
| 6365 | AT5G39900.1 \| similar to GTP-binding protein LepA, putative [Arabidopsis thaliana] (TAIR:At5g08650.1); similar to hypothetical protein LOC231279 isoform a [Mus musculus] (GB:NP_766299.1); similar to PREDICTED: similar to Hypothetical protein FLJ13220 [Canis familiaris] (GB:XP_539246.1); similar to PREDICTED: similar to expressed sequence AA407526 isoform a [Rattus norvegicus] (GB:XP_223381.3); similar to unnamed protein product [Homo sapiens] (GB:BAB14507.1); similar to Hypothetical protein FLJ13220 [Homo sapiens] (GB:AAH36768.1); contains InterPro domain GTP-binding protein LepA (InterPro:IPR006297); contains InterPro domain Small GTP-binding protein domain (InterPro:IPR005225); contains InterPro domain Elongation factor G, C-terminal (InterPro:IPR000640); contains InterPro domain Elongation factor, GTP-binding (InterPro:IPR000795) \| chr5:15993775-15996229 REVERSE \| Aliases: MYH19.60, MYH19_60 | 3.6 | 3.1 | 0.5 | 3.8 | 3.17% | 2.5 |
| 6366 | AT5G50370.1 \| adenylate kinase, putative, similar to adenylate kinase (ATP-AMP transphosphorylase) (Arabidopsis thaliana) SWISS-PROT:O82514 \| chr5:20526385-20527926 REVERSE \| Aliases: MXI22.8, MXI22_8 | 8.9 | 6.4 | 2.6 | 3.8 | 3.17% | 3.2 |
| 6367 | AT5G60800.1 \| heavy-metal-associated domain-containing protein, similar to farnesylated protein ATFP3 (GI:4097547); contains Pfam profile PF00403: Heavy-metal-associated domain \| chr5:24478115-24479760 REVERSE \| Aliases: MAE1.5, MAE1_5 | 8.0 | 5.9 | 2.1 | 3.8 | 3.17% | 3.2 |
| 6370 | AT5G63150.1 \| expressed protein \| chr5:25347426-25348070 FORWARD \| Aliases: MDC12.12, MDC12_12 | 5.5 | 4.2 | 1.2 | 3.8 | 3.18% | 3.1 |
| 6371 | AT5G16930.1 \| AAA-type ATPase family protein, contains Pfam domain, PF00004: ATPase, AAA family \| chr5:5568396-5571958 FORWARD \| Aliases: F2K13.80, F2K13_80 | 3.1 | 2.5 | 0.5 | 3.8 | 3.18% | 2.7 |
| 6372 | AT2G34140.1 \| Dof-type zinc finger domain-containing protein \| chr2:14421120-14421805 REVERSE \| Aliases: T14G11.26, T14G11_26 | 6.6 | 5.8 | 0.7 | 3.8 | 3.18% | 3.0 |
| 6373 | AT5G55000.2 \| Symbol: FIP2 \| potassium channel tetramerisation domain-containing protein / pentapeptide repeat-containing protein, contains Pfam profiles PF02214: K+ channel tetramerisation domain, PF00805: Pentapeptide repeats (8 copies) \| chr5:22335536-22338973 FORWARD \| Aliases: MBG8.27, MBG8_27 | 9.5 | 7.4 | 2.1 | 3.8 | 3.18% | 3.1 |
| 6376 | AT4G02580.1 \| NADH-ubiquinone oxidoreductase 24 kDa subunit, putative, similar to NADH-ubiquinone oxidoreductase 24 kDa subunit, mitochondrial precursor (EC 1.6.5.3) (EC 1.6.99.3) (Polypeptide II) (Swiss-Prot:P04394) (Bos taurus) \| chr4:1134494-1137156 FORWARD \| Aliases: T10P11.14, T10P11_14 | 9.9 | 7.2 | 2.7 | 3.8 | 3.19% | 3.2 |
| 6377 | AT5G13100.1 \| expressed protein \| chr5:4156663-4158539 FORWARD \| Aliases: T19L5.60, T19L5_60 | 5.8 | 4.8 | 1.0 | 3.8 | 3.19% | 3.1 |
| 6380 | AT5G01380.1 \| expressed protein \| chr5:155638-157521 REVERSE \| Aliases: T10O8.90, T10O8_90 | 4.8 | 3.3 | 1.6 | 3.8 | 3.19% | 3.2 |
| 6382 | AT3G57180.1 \| expressed protein \| chr3:21174524-21177009 REVERSE \| Aliases: F28O9.30 | 5.6 | 3.9 | 1.7 | 3.8 | 3.20% | 3.2 |
| 6383 | AT1G15960.1 \| Symbol: NRAMP6 \| | 3.0 | 2.5 | 0.5 | 3.8 | 3.20% | 2.5 |
| 6384 | AT4G34370.1 \| IBR domain-containing protein, similar to SP:Q94981 Ariadne-1 protein (Ari-1) {Drosophila melanogaster}; contains Pfam profile PF01485: IBR domain \| chr4:16434337-16437313 FORWARD \| Aliases: F10M10.140, F10M10_140 | 7.3 | 5.8 | 1.5 | 3.8 | 3.20% | 3.2 |
| 6385 | AT4G00860.1 \| Symbol: ATOZI1 \| | 11.8 | 10.8 | 1.0 | 3.8 | 3.20% | 2.6 |
| 6386 | AT3G51310.1 \| vacuolar protein sorting-associated protein 35 family protein / VPS35 family protein, similar to vacuolar protein sorting 35 (Mus musculus) GI:11875394; contains Pfam profile PF03635: Vacuolar protein sorting-associated protein 35 \| chr3:19055502-19060300 REVERSE \| Aliases: F24M12.350 | 8.9 | 8.1 | 0.8 | 3.8 | 3.20% | 2.9 |
| 6388 | AT2G42690.1 \| lipase, putative, similar to lipase (Dianthus caryophyllus) GI:4103627; contains Pfam profile PF01764: Lipase \| chr2:17783318-17784794 REVERSE \| Aliases: F14N22.2 | 6.5 | 4.9 | 1.6 | 3.8 | 3.20% | 3.2 |
| 6389 | AT2G44520.1 \| UbiA prenyltransferase family protein, similar to SP:Q12887 Protoheme IX farnesyltransferase, mitochondrial precursor (EC 2.5.1.-) (Heme O synthase) {Homo sapiens}, SP:P21592 COX10 {Saccharomyces cerevisiae} \| chr2:18386651-18389018 FORWARD \| Aliases: F4I1.50, F4I1_50 | 8.4 | 6.5 | 1.9 | 3.8 | 3.21% | 3.1 |
| 6390 | AT3G26085.1 \| CAAX amino terminal protease family protein, contains Pfam profile PF02517 CAAX amino terminal protease family protein \| chr3:9532006-9533850 FORWARD \| Aliases: None | 5.6 | 4.9 | 0.7 | 3.8 | 3.21% | 3.0 |
| 6393 | AT1G61380.1 \| S-locus protein kinase, putative, similar to KI domain interacting kinase 1 (Zea mays) gi:2735017:gb:AAB93834; contains S-locus glycoprotein family domain, Pfam:PF00954 | 4.5 | 3.7 | 0.8 | 3.8 | 3.21% | 3.0 |
| 6394 | AT5G43170.1 \| Symbol: AZF3 \| zinc finger (C2H2 type) protein 3 (AZF3), identical to Cys2/His2-type zinc finger protein 3 (Arabidopsis thaliana) gi:6009889:dbj:BAA85109 \| chr5:17348173-17348874 REVERSE \| Aliases: ARABIDOPSIS ZINC FINGER PROTEIN 3, MMG4.21, MMG4_21 | 8.4 | 7.6 | 0.8 | 3.8 | 3.22% | 3.0 |
| 6396 | AT1G01800.1 \| short-chain dehydrogenase/reductase (SDR) family protein, similar to carbonyl reductase GI:1049108 from (Mus musculus) \| chr1:293342-295040 FORWARD \| Aliases: T1N6.22, T1N6_22 | 11.6 | 10.0 | 1.5 | 3.8 | 3.23% | 2.9 |
| 6398 | AT4G26700.3 \| Symbol: ATFIM1 \| | 6.4 | 5.2 | 1.3 | 3.8 | 3.23% | 3.2 |

| Rank | Description | Sync | Root | M | t | adj.q | B |
|---|---|---|---|---|---|---|---|
| 6401 | AT3G14750.1 \| expressed protein, weak similarity to Septation ring formation regulator (Swiss-Prot:O34894) (Bacillus subtilis) | 7.5 | 6.7 | 0.8 | 3.8 | 3.23% | 2.8 |
| 6402 | AT5G55200.1 \| co-chaperone grpE protein, putative, similar to chaperone GrpE type 2 (Nicotiana tabacum) GI:3851640; contains Pfam profile PF01025: co-chaperone GrpE \| chr5:22411860-22413777 FORWARD \| Aliases: MCO15.15, MCO15_15 | 8.9 | 7.8 | 1.1 | 3.8 | 3.23% | 2.9 |
| 6405 | AT1G67320.1 \| DNA primase, large subunit family, contains Pfam profile PF04104: Eukaryotic-type DNA primase, large subunit; similar to DNA primase large subunit (EC 2.7.7.-) (DNA primase 58 kDa subunit) (p58) (Swiss-Prot:P49643) (Homo sapiens) \| chr1:25209055-25212635 REVERSE \| Aliases: F1N21.14 | 5.8 | 4.5 | 1.3 | 3.8 | 3.24% | 3.2 |
| 6407 | AT4G28330.1 \| expressed protein, similar to hypothetical protein [Arabidopsis thaliana] (TAIR:At4g28340.1) \| chr4:14022393-14022993 REVERSE \| Aliases: F20O9.10, F20O9_10 | 6.6 | 5.2 | 1.4 | 3.8 | 3.24% | 3.2 |
| 6408 | AT2G16390.1 \| Symbol: DRD1 \| SNF2 domain-containing protein / helicase domain-containing protein, low similarity to RAD54 (Drosophila melanogaster) GI:1765914; contains Pfam profiles PF00271: Helicase conserved C-terminal domain, PF00176: SNF2 family N-terminal domain \| chr2:7104720-7108178 FORWARD \| Aliases: CHR35, F16F14.11, F16F14_11 | 3.4 | 2.9 | 0.5 | 3.8 | 3.24% | 2.7 |
| 6415 | AT1G48520.3 \| Symbol: GATB \| glutamyl-tRNA(Gln) amidotransferase B family protein, contains Pfam profiles: PF02934 PET112 family, N terminal region, PF02637 GatB/Yqey domain, PF01162 PET112 family, C terminal region \| chr1:17943756-17946490 FORWARD \| Aliases: GLU ADT SUBUNIT B, GLU TRNA(GLN) AMIDOTRANSFERASE SUBUNIT B | 5.1 | 4.3 | 0.8 | 3.8 | 3.24% | 3.1 |
| 6418 | AT5G50250.1 \| 31 kDa ribonucleoprotein, chloroplast, putative / RNA-binding protein RNP-T, putative / RNA-binding protein 1/2/3, putative / RNA-binding protein cp31, putative, similar to SP:Q04836 31 kDa ribonucleoprotein, chloroplast precursor (RNA-binding protein RNP-T) (1/2/3) (AtRBP33) (cp31) {Arabidopsis thaliana}; contains InterPro entry IPR000504: RNA-binding region RNP-1 (RNA recognition motif) (RRM) \| chr5:20469713-20471202 REVERSE \| Aliases: K6A12.11, K6A12_11 | 7.1 | 6.2 | 0.9 | 3.8 | 3.25% | 2.9 |
| 6421 | AT2G39830.1 \| LIM domain-containing protein, contains Pfam profile PF00412: LIM domain | 5.0 | 4.3 | 0.7 | 3.8 | 3.26% | 3.0 |
| 6423 | AT3G25990.1 \| DNA-binding protein GT-1-related, similar to GT-1 GI:598073 from (Arabidopsis thaliana) \| chr3:9505922-9507940 REVERSE \| Aliases: MPE11.20 | 3.5 | 2.9 | 0.7 | 3.8 | 3.26% | 2.9 |
| 6425 | AT1G47420.1 \| expressed protein, identical to hypothetical protein GB:AAD46040 GI:5668814 from (Arabidopsis thaliana) \| chr1:17397958-17399693 REVERSE \| Aliases: T3F24.12 | 12.5 | 10.4 | 2.1 | 3.8 | 3.27% | 3.0 |
| 6427 | AT1G15230.1 \| expressed protein, ESTs gb:R30529, gb:Z48463, gb:Z48467, gb:AA597369 and gb:AA394772 come from this gene | 6.3 | 5.3 | 0.9 | 3.8 | 3.27% | 3.0 |
| 6428 | AT1G28540.1 \| expressed protein \| chr1:10035650-10036927 FORWARD \| Aliases: F3M18.29 | 7.6 | 6.6 | 1.0 | 3.8 | 3.27% | 2.9 |
| 6429 | AT5G18820.1 \| Symbol: EMB3007 \| chaperonin, putative, similar to SWISS-PROT:P08926- RuBisCO subunit binding-protein alpha subunit, chloroplast precursor (60 kDa chaperonin alpha subunit, CPN-60 alpha)(Pisum sativum); contains Pfam:PF00118 domain, TCP-1/cpn60 chaperonin family \| chr5:6271551-6274155 FORWARD \| Aliases: EMB3007, EMBRYO DEFECTIVE 3007, F17K4.70, F17K4_70 | 3.4 | 2.7 | 0.6 | 3.8 | 3.28% | 2.8 |
| 6430 | AT2G41110.1 \| Symbol: ATCAL4 \| | 9.0 | 6.9 | 2.2 | 3.8 | 3.28% | 3.1 |
| 6431 | AT1G52570.1 \| Symbol: PLDALPHA2 \| phospholipase D alpha 2 / PLD alpha 2 (PLDALPHA2) (PLD2) / choline phosphatase 2, identical to phospholipase D alpha 2 ( PLD alpha 2) SP:Q9SSQ9 from (Arabidopsis thaliana) | 3.1 | 2.7 | 0.4 | 3.8 | 3.28% | 2.6 |
| 6432 | AT5G54970.1 \| expressed protein \| chr5:22330436-22330990 FORWARD \| Aliases: MBG8.24, MBG8_24 | 6.6 | 4.0 | 2.5 | 3.8 | 3.28% | 3.2 |
| 6435 | AT4G28200.1 \| expressed protein \| chr4:13987608-13990456 REVERSE \| Aliases: F26K10.80, F26K10_80 | 6.6 | 5.5 | 1.1 | 3.8 | 3.29% | 2.9 |
| 6436 | AT1G01970.1 \| pentatricopeptide (PPR) repeat-containing protein, low similarity to 67 kD chloroplastic RNA-binding protein RSP67.2 (Raphanus sativus) GI:9755888; contains Pfam profile PF01535: PPR repeat \| chr1:338292-340074 FORWARD \| Aliases: F22M8.10, F22M8_10 | 6.1 | 5.2 | 1.0 | 3.8 | 3.29% | 3.1 |
| 6437 | AT4G18820.1 \| expressed protein \| chr4:10330382-10334279 FORWARD \| Aliases: F28A21.230, F28A21_230 | 5.5 | 4.6 | 1.0 | 3.8 | 3.29% | 3.0 |
| 6442 | AT5G44420.1 \| Symbol: PDF1.2 \| | 3.0 | 2.5 | 0.5 | 3.8 | 3.31% | 2.8 |
| 6443 | AT5G46920.1 \| intron maturase, type II family protein, similar to SP:Q57005 Group II intron-encoded protein ltrA {Lactococcus lactis subsp}; contains Pfam profiles PF00078: Reverse transcriptase (RNA-dependent DNA polymerase), PF01348: Type II intron maturase \| chr5:19070855-19073234 FORWARD \| Aliases: MQD22.6, MQD22_6 | 5.8 | 4.9 | 0.9 | 3.8 | 3.31% | 3.0 |
| 6446 | ATCG00120.1 \| Symbol: ATPA \| ATPase alpha subunit \| chrC:9938-11461 REVERSE \| Aliases: ATPA | 6.9 | 5.1 | 1.8 | 3.8 | 3.31% | 3.1 |

| Rank | Description | Sync | Root | M | t | adj.q | B |
|---|---|---|---|---|---|---|---|
| 6447 | AT1G30520.1 \| acyl-activating enzyme 14 (AAE14), identical to acyl-activating enzyme 14 (Arabidopsis thaliana); similar to SP:Q42524 4-coumarate--CoA ligase 1 (EC 6.2.1.12) (4-coumaroyl-CoA synthase 1) {Arabidopsis thaliana}; contains Pfam profile PF00501: AMP-binding enzyme; identical to cDNA acyl-activating enzyme 14 (At1g30520) GI:29893263 \| chr1:10810966-10813603 FORWARD \| Aliases: F26G16.14, F26G16_14 | 6.2 | 5.3 | 0.9 | 3.8 | 3.31% | 3.0 |
| 6448 | AT5G07660.1 \| structural maintenance of chromosomes (SMC) family protein, similar to SMC-like protein (MIM) (Arabidopsis thaliana) GI:5880614; contains Pfam profile PF02463: RecF/RecN/SMC N terminal domain \| chr5:2422840-2429913 FORWARD \| Aliases: MBK20.10, MBK20_10 | 4.1 | 3.3 | 0.8 | 3.8 | 3.31% | 3.0 |
| 6450 | AT5G55140.1 \| ribosomal protein L30 family protein, contains similarity to 50S ribosomal protein L30 \| chr5:22398262-22399254 FORWARD \| Aliases: MCO15.9, MCO15_9 | 6.2 | 4.7 | 1.5 | 3.8 | 3.31% | 3.1 |
| 6452 | AT2G28060.1 \| protein kinase-related, similar to GAL83 protein (Solanum tuberosum) GI:5702015; contains Pfam profile PF04739: 5'-AMP-activated protein kinase, beta subunit, complex-interacting region; supporting cDNA gi:22652763:gb:AF491295.1: \| chr2:11957063-11959279 REVERSE \| Aliases: F24D13.15, F24D13_15 | 7.0 | 6.2 | 0.8 | 3.8 | 3.31% | 2.9 |
| 6454 | AT3G05280.1 \| integral membrane Yip1 family protein, contains Pfam domain, PF04893: Yip1 domain \| chr3:1503764-1505648 REVERSE \| Aliases: T12H1.25, T12H1_25 | 7.2 | 5.7 | 1.5 | 3.8 | 3.32% | 3.1 |
| 6455 | AT1G31170.1 \| parB-like nuclease domain-containing protein, contains Pfam profile PF02195: ParB-like nuclease domain \| chr1:11133470-11134386 FORWARD \| Aliases: F28K20.12, F28K20_12 | 7.9 | 6.9 | 1.0 | 3.8 | 3.32% | 3.1 |
| 6456 | AT1G30480.1 \| Symbol: DRT111 \| | 7.3 | 6.1 | 1.1 | 3.8 | 3.32% | 3.0 |
| 6457 | AT3G29330.1 \| expressed protein \| chr3:11258939-11260290 REVERSE \| Aliases: MUO10.3 | 4.4 | 3.8 | 0.6 | 3.8 | 3.33% | 2.9 |
| 6459 | AT3G62630.1 \| expressed protein \| chr3:23174723-23176171 REVERSE \| Aliases: F26K9.60 | 3.4 | 2.9 | 0.6 | 3.8 | 3.33% | 2.8 |
| 6460 | AT4G09760.3 \| choline kinase, putative, similar to GmCK2p choline kinase gi:1438881:gb:AAC49375 \| chr4:6148755-6151380 REVERSE \| Aliases: F17A8.110, F17A8_110 | 5.9 | 4.8 | 1.1 | 3.8 | 3.33% | 3.1 |
| 6464 | AT1G24330.1 \| armadillo/beta-catenin repeat family protein / U-box domain-containing family protein, contains Pfam domain, PF00514: Armadillo/beta-catenin-like repeats and Pfam, PF04564: U-box domain \| chr1:8631768-8634824 FORWARD \| Aliases: F3I6.27, F3I6_27 | 3.6 | 2.9 | 0.7 | 3.8 | 3.33% | 3.0 |
| 6465 | AT3G05520.1 \| F-actin capping protein alpha subunit family protein, contains Pfam profile: PF01267 F-actin capping protein alpha subunit \| chr3:1598556-1601306 FORWARD \| Aliases: F22F7.25 | 7.6 | 6.7 | 0.9 | 3.8 | 3.33% | 2.8 |
| 6466 | AT1G08110.4 \| similar to lactoylglutathione lyase, putative / glyoxalase I, putative [Arabidopsis thaliana] (TAIR:At1g67280.1); similar to Glyoxalase I [Cicer arietinum] (GB:CAA12028.1); contains InterPro domain Glyoxalase I (InterPro:IPR004361); contains InterPro domain Glyoxalase/Bleomycin resistance protein/dioxygenase domain (InterPro:IPR004360) \| chr1:2535368-2537928 FORWARD \| Aliases: T6D22.20, T6D22_20 | 10.0 | 7.8 | 2.2 | 3.8 | 3.33% | 3.1 |
| 6467 | AT5G18770.1 \| F-box family protein, contains F-box domain Pfam:PF00646 | 3.1 | 2.6 | 0.5 | 3.8 | 3.33% | 2.6 |
| 6469 | AT2G45270.1 \| glycoprotease M22 family protein, similar to SP:P36175 O-sialoglycoprotein endopeptidase (EC 3.4.24.57) (Glycoprotease) {Pasteurella haemolytica}; contains Pfam profile PF00814: Glycoprotease family \| chr2:18673622-18676947 FORWARD \| Aliases: F4L23.22 | 5.8 | 4.5 | 1.3 | 3.8 | 3.33% | 3.1 |
| 6471 | AT2G19460.1 \| expressed protein \| chr2:8438564-8439187 FORWARD \| Aliases: F27F23.23 | 4.0 | 2.7 | 1.3 | 3.8 | 3.33% | 3.0 |
| 6472 | AT1G18600.1 \| rhomboid family protein, contains Pfam profile PF01694: Rhomboid family \| chr1:6400396-6402770 FORWARD \| Aliases: F25I16.6, F25I16_6 | 8.2 | 5.7 | 2.5 | 3.8 | 3.34% | 2.9 |
| 6474 | AT3G12380.1 \| Symbol: ATARP5 \| | 6.9 | 6.2 | 0.6 | 3.8 | 3.34% | 2.8 |
| 6475 | AT5G08570.1 \| pyruvate kinase, putative, similar to pyruvate kinase, cytosolic isozyme (Glycine max) SWISS-PROT:Q42806 | 9.4 | 7.9 | 1.6 | 3.8 | 3.34% | 2.9 |
| 6476 | AT5G42000.1 \| ORMDL family protein, contains Pfam domain PF04061: ORMDL family | 5.6 | 4.1 | 1.5 | 3.8 | 3.34% | 3.0 |
| 6477 | AT1G34360.1 \| translation initiation factor 3 (IF-3) family protein, low similarity to Translation initiation factor IF-3 from (subsp. Schizaphis graminum) {Buchnera aphidicola} SP:P46243, {Salmonella typhimurium} SP:P33321; contains Pfam profiles PF05198: Translation initiation factor IF-3 N-terminal domain, PF00707: Translation initiation factor IF-3 C-terminal domain \| chr1:12542867-12546150 FORWARD \| Aliases: F7P12.8, F7P12_8 | 4.1 | 3.5 | 0.5 | 3.8 | 3.34% | 2.7 |
| 6478 | AT3G06490.1 \| Symbol: MYB108 \| | 4.0 | 3.2 | 0.8 | 3.8 | 3.34% | 2.9 |

| Rank | Description | Sync | Root | M | t | adj.q | B |
|---|---|---|---|---|---|---|---|
| 6480 | AT5G09650.1 \| inorganic pyrophosphatase family protein, similar to SP:Q15181 Inorganic pyrophosphatase (EC 3.6.1.1) (Pyrophosphate {Homo sapiens}; contains Pfam profile PF00719: inorganic pyrophosphatase \| chr5:2991156-2993214 REVERSE \| Aliases: F17I14.160, F17I14_160 | 9.8 | 8.5 | 1.4 | 3.8 | 3.35% | 3.0 |
| 6481 | AT5G51970.2 \| sorbitol dehydrogenase, putative / L-iditol 2-dehydrogenase, putative, similar to NAD-dependent sorbitol dehydrogenase from Malus x domestica (gi:4519539) \| chr5:21128820-21130629 FORWARD \| Aliases: MSG15.7, MSG15_7 | 12.1 | 10.9 | 1.2 | 3.8 | 3.35% | 2.9 |
| 6482 | AT1G67620.1 \| expressed protein, contains Pfam domain PF02410: Domain of unknown function DUF143 \| chr1:25344239-25345605 FORWARD \| Aliases: F12B7.17, F12B7_17 | 4.9 | 3.3 | 1.5 | 3.8 | 3.35% | 3.1 |
| 6484 | AT4G17695.1 \| myb family transcription factor (KAN3), contains Pfam profile: PF00249 myb-like DNA-binding domain; identical to cDNA GARP-like putative transcription factor KANADI3 (KAN3) GI:15723596 \| chr4:9848142-9850717 REVERSE \| Aliases: None | 3.5 | 2.9 | 0.6 | 3.8 | 3.36% | 2.8 |
| 6485 | AT5G10460.1 \| haloacid dehalogenase-like hydrolase family protein, contains InterPro accession IPR005834: Haloacid dehalogenase-like hydrolase | 6.7 | 5.3 | 1.4 | 3.8 | 3.37% | 3.0 |
| 6486 | AT4G11600.1 \| Symbol: ATGPX6 \| | 11.1 | 9.8 | 1.3 | 3.8 | 3.37% | 2.8 |
| 6488 | AT1G28490.2 \| Symbol: SYP61 \| similar to syntaxin 52 (SYP52) [Arabidopsis thaliana] (TAIR:At1g79590.1); similar to putative syntaxin 6 [Oryza sativa (japonica cultivar-group)] (GB:BAD87234.1); contains InterPro domain Target SNARE coiled-coil domain (InterPro:IPR000727) \| chr1:10016419-10018011 FORWARD \| Aliases: ATSYP61, F3M18.7, F3M18_7, OSM1, SYNTAXIN OF PLANTS 61 | 8.0 | 7.0 | 1.0 | 3.8 | 3.38% | 3.0 |
| 6489 | AT5G06400.1 \| pentatricopeptide (PPR) repeat-containing protein, contains Pfam profile PF01535: PPR repeat \| chr5:1955960-1959052 FORWARD \| Aliases: MHF15.8, MHF15_8 | 3.1 | 2.7 | 0.4 | 3.8 | 3.38% | 2.5 |
| 6492 | AT3G28500.1 \| 60S acidic ribosomal protein P2 (RPP2C), similar to acidic ribosomal protein P2b (rpp2b) GB:U62753 GI:2431770 from (Zea mays) \| chr3:10683334-10683968 FORWARD \| Aliases: T20D4.1 | 6.1 | 4.9 | 1.2 | 3.8 | 3.38% | 3.1 |
| 6493 | AT4G32840.1 \| phosphofructokinase family protein, similar to phosphofructokinase (Amycolatopsis methanolica) GI:17432243; contains Pfam profile PF00365: Phosphofructokinase \| chr4:15844784-15848445 REVERSE \| Aliases: T16I18.50, T16I18_50 | 5.0 | 4.2 | 0.9 | 3.8 | 3.38% | 2.7 |
| 6496 | AT1G27970.1 \| nuclear transport factor 2 (NTF2), putative, similar to Swiss-Prot:P33331 nuclear transport factor 2 (NTF-2) (Nuclear transport factor P10) (Saccharomyces cerevisiae) \| chr1:9746798-9748006 FORWARD \| Aliases: F13K9.26, F13K9_26 | 9.1 | 6.2 | 2.9 | 3.8 | 3.38% | 3.1 |
| 6498 | AT4G39620.1 \| Symbol: EMB2453 \| pentatricopeptide (PPR) repeat-containing protein, contains Pfam profile PF01535: PPR repeat \| chr4:18395288-18397572 FORWARD \| Aliases: EMB2453, EMBRYO DEFECTIVE 2453, F23K16.250, F23K16_250 | 5.2 | 4.4 | 0.7 | 3.8 | 3.39% | 2.7 |
| 6500 | AT2G26080.1 \| glycine dehydrogenase (decarboxylating), putative / glycine decarboxylase, putative / glycine cleavage system P-protein, putative, strong similarity to SP:P26969 Glycine dehydrogenase (decarboxylating), mitochondrial precursor (EC 1.4.4.2) {Pisum sativum}; contains Pfam profile PF02347: Glycine cleavage system P-protein \| chr2:11116098-11120906 REVERSE \| Aliases: T19L18.11, T19L18_11 | 10.4 | 8.8 | 1.7 | 3.8 | 3.39% | 2.9 |
| 6501 | AT2G17630.1 \| phosphoserine aminotransferase, putative, similar to Phosphoserine aminotransferase, chloroplast precursor (PSAT) (SP:Q96255) (Arabidopsis thaliana); contains TIGRFAM TIGR01364: phosphoserine aminotransferase; contains Pfam PF00266: aminotransferase, class V \| chr2:7673667-7675138 FORWARD \| Aliases: T19E12.3, T19E12_3 | 7.8 | 6.2 | 1.7 | 3.8 | 3.39% | 3.1 |
| 6502 | AT1G30330.2 \| Symbol: ARF6 \| similar to auxin-responsive factor (ARF8) [Arabidopsis thaliana] (TAIR:At5g37020.1); similar to auxin response factor 6b [Oryza sativa] (GB:BAB85915.1); similar to OSJNBb0004A17.5 [Oryza sativa (japonica cultivar-group)] (GB:XP_474307.1); similar to auxin response factor 4 [Cucumis sativus] (GB:BAD19064.1); similar to putative auxin response factor [Oryza sativa (japonica cultivar-group)] (GB:BAD45924.1); similar to putative auxin response transcription factor(ARF6) [Oryza sativa (japonica cultivar-group)] (GB:XP_464221.1); contains InterPro domain Transcriptional factor B3 (InterPro:IPR003340) \| chr1:10685804-10690018 REVERSE \| Aliases: ARF6, AUXIN RESPONSE FACTOR 6, T4K22.6, T4K22_6 | 5.5 | 4.6 | 0.9 | 3.8 | 3.40% | 3.1 |
| 6503 | AT4G35910.1 \| expressed protein \| chr4:17008584-17011454 REVERSE \| Aliases: T19K4.40 | 6.4 | 5.2 | 1.2 | 3.8 | 3.40% | 3.0 |
| 6504 | AT1G20870.1 \| expressed protein \| chr1:7259091-7260764 REVERSE \| Aliases: F9H16.15, F9H16_15 | 4.7 | 3.8 | 1.0 | 3.8 | 3.40% | 3.1 |
| 6512 | AT3G59110.1 \| protein kinase family protein, contains protein kinase domain, Pfam:PF00069 \| chr3:21865697-21869181 FORWARD \| Aliases: F17J16.160 | 4.8 | 4.1 | 0.7 | 3.8 | 3.43% | 2.8 |
| 6513 | AT4G25630.1 \| Symbol: FIB2 \| fibrillarin 2 (FIB2), identical to fibrillarin 2 GI:9965655 from (Arabidopsis thaliana) \| chr4:13074142-13076432 FORWARD \| Aliases: ATFIB2, FIBRILLARIN 2, L73G19.10, L73G19_10 | 8.1 | 6.9 | 1.2 | 3.8 | 3.44% | 3.0 |
| 6516 | AT3G22520.1 \| expressed protein \| chr3:7974769-7977597 FORWARD \| Aliases: F16J14.8 | 5.1 | 4.1 | 1.1 | 3.8 | 3.45% | 3.0 |

| Rank | Description | Sync | Root | M | t | adj.q | B |
|---|---|---|---|---|---|---|---|
| 6518 | AT3G55510.1 \| expressed protein \| chr3:20589963-20593382 FORWARD \| Aliases: T22E16.170 | 4.7 | 3.4 | 1.3 | 3.8 | 3.45% | 3.1 |
| 6523 | AT3G04400.1 \| Symbol: EMB2171 \| 60S ribosomal protein L23 (RPL23C), similar to ribosomal protein L17 GB:AAA34113.1 from (Nicotiana tabacum) \| chr3:1167282-1168663 FORWARD \| Aliases: EMB2171, EMBRYO DEFECTIVE 2171, T27C4.4, T27C4_4 | 10.9 | 10.1 | 0.8 | 3.8 | 3.47% | 2.9 |
| 6524 | AT5G40600.1 \| Symbol: EMB1875 \| expressed protein \| chr5:16272272-16273993 REVERSE \| Aliases: EMB1875, EMBRYO DEFECTIVE 1875, MNF13.13, MNF13_13 | 3.7 | 3.1 | 0.5 | 3.8 | 3.47% | 2.7 |
| 6526 | AT1G72940.1 \| disease resistance protein (TIR-NBS class), putative, domain signature TIR-NBS exists, suggestive of a disease resistance protein. \| chr1:27445899-27447517 FORWARD \| Aliases: F3N23.14, F3N23_14 | 4.6 | 3.9 | 0.7 | 3.8 | 3.47% | 2.9 |
| 6527 | AT1G01990.1 \| expressed protein \| chr1:343166-344319 REVERSE \| Aliases: F22M8.12, F22M8_12 | 5.5 | 5.0 | 0.5 | 3.8 | 3.47% | 2.6 |
| 6528 | AT3G03250.1 \| Symbol: UGP \| Is thought to encodes a cytosolic UDP-glucose pyrophosphorylase with strong similarity to UTP--glucose-1-phosphate uridylyltransferase (SwissProt P19595, EC 2.7.7.9, UDP-glucose pyrophosphorylase <i>Solanum tuberosum</i>). \| chr3:749486-754166 REVERSE \| Aliases: T17B22.6, T17B22_6, UGP, UGPASE | 9.7 | 7.6 | 2.1 | 3.8 | 3.47% | 3.0 |
| 6531 | AT2G47610.1 \| 60S ribosomal protein L7A (RPL7aA) \| chr2:19536860-19538725 FORWARD \| Aliases: T30B22.8 | 9.6 | 7.8 | 1.8 | 3.7 | 3.49% | 2.9 |
| 6535 | AT1G71850.1 \| expressed protein, ; expression supported by MPSS \| chr1:27028537-27029949 REVERSE \| Aliases: F14O23.23, F14O23_23 | 4.9 | 4.0 | 0.9 | 3.7 | 3.51% | 3.0 |
| 6536 | AT4G22000.1 \| expressed protein \| chr4:11659650-11660916 REVERSE \| Aliases: F1N20.100, F1N20_100 | 9.2 | 7.8 | 1.4 | 3.7 | 3.51% | 3.0 |
| 6539 | AT4G17430.1 \| expressed protein, weak similarity to CigA protein (Mucor circinelloides) GI:9717020 \| chr4:9724852-9726840 REVERSE \| Aliases: DL4750C, FCAALL.1 | 4.9 | 4.2 | 0.7 | 3.7 | 3.51% | 2.9 |
| 6540 | AT5G66440.1 \| expressed protein \| chr5:26547461-26548323 REVERSE \| Aliases: K1F13.9, K1F13_9 | 9.1 | 7.5 | 1.6 | 3.7 | 3.52% | 3.0 |
| 6542 | AT5G59140.1 \| SKP1 family protein, similar to elongin C, Drosophila melanogaster, GI:2780365 PIR:JC5794; contains Pfam profile PF01466: Skp1 family, dimerisation domain \| chr5:23891676-23892910 REVERSE \| Aliases: MNC17.5, MNC17_5 | 9.2 | 8.1 | 1.2 | 3.7 | 3.52% | 2.8 |
| 6543 | AT4G37080.2 \| expressed protein, contains Pfam profile PF04784: Protein of unknown function, DUF547 \| chr4:17473266-17476946 FORWARD \| Aliases: AP22.58, AP22_58 | 4.5 | 3.8 | 0.7 | 3.7 | 3.53% | 2.8 |
| 6545 | AT2G39700.1 \| Symbol: ATEXPA4 \| expansin, putative (EXP4), similar to alpha-expansin 6 precursor GI:16923359 from (Cucumis sativus); alpha-expansin gene family, PMID:11641069 \| chr2:16550910-16552662 REVERSE \| Aliases: ARABIDOPSIS THALIANA EXPANSIN A4, ATEXP4, ATHEXP ALPHA 1.6, F17A14.7, F17A14_7 | 6.6 | 2.3 | 4.2 | 3.7 | 3.53% | 3.1 |
| 6547 | AT4G00570.1 \| malate oxidoreductase, putative, similar to NAD-dependent malic enzyme 59 kDa isoform, mitochondrial precursor (EC 1.1.1.39) (NAD-ME) (SP:P37225) {Solanum tuberosum} \| chr4:242516-246736 REVERSE \| Aliases: F6N23.16, F6N23_16 | 8.7 | 7.4 | 1.3 | 3.7 | 3.53% | 3.0 |
| 6548 | AT3G59480.1 \| pfkB-type carbohydrate kinase family protein, contains Pfam profile: PF00294 pfkB family carbohydrate kinase \| chr3:21993998-21995572 FORWARD \| Aliases: T16L24.30 | 4.9 | 3.7 | 1.2 | 3.7 | 3.53% | 3.1 |
| 6550 | AT3G08730.1 \| Symbol: ATPK1 \| serine/threonine protein kinase (PK1) (PK6), identical to serine/threonine-protein kinase AtPK1/AtPK6 (ribosomal-protein S6 kinase ATPK6) (Arabidopsis thaliana) SWISS-PROT:P42818 \| chr3:2651453-2654189 REVERSE \| Aliases: ATPK6, F17O14.20, PROTEIN SERINE KINASE | 9.0 | 8.1 | 0.9 | 3.7 | 3.54% | 2.9 |
| 6551 | AT1G14680.1 \| expressed protein \| chr1:5040856-5041728 FORWARD \| Aliases: F10B6.4, F10B6_4 | 4.2 | 2.9 | 1.4 | 3.7 | 3.55% | 2.9 |
| 6552 | AT5G18280.1 \| Symbol: ATAPY2 \| | 5.4 | 4.7 | 0.7 | 3.7 | 3.55% | 2.7 |
| 6555 | AT4G17060.1 \| expressed protein \| chr4:9593548-9595268 REVERSE \| Aliases: None | 5.9 | 5.0 | 0.9 | 3.7 | 3.55% | 2.9 |
| 6557 | AT5G06870.1 \| Symbol: PGIP2 \| polygalacturonase inhibiting protein 2 (PGIP2), identical to polygalacturonase inhibiting protein 2 (PGIP2) (Arabidopsis thaliana) gi:7800201:gb:AAF69828; contains leucine rich-repeat (LRR) domains Pfam:PF00560, INTERPRO:IPR001611 \| chr5:2133919-2135162 FORWARD \| Aliases: MOJ9.4, MOJ9_4, POLYGALACTURONASE INHIBITING PROTEIN 2 | 3.2 | 2.5 | 0.7 | 3.7 | 3.55% | 2.8 |
| 6558 | AT1G03040.1 \| basic helix-loop-helix (bHLH) family protein, component of the pyruvate dehydrogenase complex E3, contains PF:00010 helix-loop-helix DNA-binding domain. ESTs gb:T45640 and gb:T22783 come from this gene \| chr1:703906-706631 REVERSE \| Aliases: F10O3.14, F10O3_14 | 7.5 | 5.4 | 2.1 | 3.7 | 3.55% | 3.0 |
| 6559 | AT3G15710.1 \| signal peptidase, putative, similar to SP:P13679 Microsomal signal peptidase 21 kDa subunit (EC 3.4.-.-) {Canis familiaris}; contains Pfam profile: PF00461 signal peptidase I \| chr3:5323365-5324915 REVERSE \| Aliases: MSJ11.11 | 8.3 | 7.2 | 1.0 | 3.7 | 3.55% | 2.9 |

| Rank | Description | Sync | Root | M | t | adj.q | B |
|---|---|---|---|---|---|---|---|
| 6562 | AT1G66980.1 \| protein kinase family protein / glycerophosphoryl diester phosphodiesterase family protein, similar to leaf rust resistance kinase Lr10 GI:1680685 from (Triticum aestivum); contains Pfam profiles PF03009: Glycerophosphoryl diester phosphodiesterase family, PF00069: Protein kinase domain \| chr1:25000972-25005624 REVERSE \| Aliases: F1O19.6, F1O19_6 | 3.3 | 2.8 | 0.6 | 3.7 | 3.55% | 2.7 |
| 6563 | AT3G15980.3 \| coatomer protein complex, subunit beta 2 (beta prime), putative, contains 7 WD-40 repeats (PF00400) (1 weak); identical to coatomer protein complex, beta prime (beta'-COP) protein {Arabidopsis thaliana} (GI:9294445); similar to Coatomer beta' subunit (Beta'-coat protein) (Beta'-COP) (p102) (SP:P35606) (Homo sapiens) \| chr3:5411678-5418451 REVERSE \| Aliases: MSL1.4 | 6.2 | 4.7 | 1.5 | 3.7 | 3.56% | 3.0 |
| 6564 | AT1G78210.1 \| hydrolase, alpha/beta fold family protein, low similarity to hydrolases from Rhodococcus sp. EtbD2 GI:3273241, EtbD1 GI:3273239; contains Pfam profile PF00561: hydrolase, alpha/beta fold family \| chr1:29427611-29429268 REVERSE \| Aliases: T11I11.15, T11I11_15 | 6.9 | 6.2 | 0.8 | 3.7 | 3.56% | 2.9 |
| 6566 | AT4G05450.1 \| adrenodoxin-like ferredoxin 2, similar to SP:P46656 Adrenodoxin, mitochondrial precursor (Adrenal ferredoxin) from Mus musculus, SP:P10109 Adrenodoxin, mitochondrial precursor (Hepatoredoxin) from Homo sapiens, SP:P29330 Adrenodoxin from Ovis aries; contains Pfam profile: PF00111 2Fe-2S iron-sulfur cluster binding domains; identical to cDNA GI: 28192430 \| chr4:2758653-2760895 FORWARD \| Aliases: C6L9.130, C6L9_130 | 5.4 | 4.5 | 0.8 | 3.7 | 3.56% | 2.9 |
| 6567 | AT2G20340.1 \| tyrosine decarboxylase, putative, similar to tyrosine/dopa decarboxylase (Papaver somniferum) GI:3282527, SP:Q06087 Tyrosine decarboxylase 3 (EC 4.1.1.25) {Petroselinum crispum}; contains Pfam profile PF00282: Pyridoxal-dependent decarboxylase conserved domain \| chr2:8786816-8789853 FORWARD \| Aliases: F11A3.11, F11A3_11 | 3.5 | 2.6 | 1.0 | 3.7 | 3.56% | 2.9 |
| 6568 | AT1G29990.1 \| prefoldin, putative, similar to Swiss-Prot:O15212 prefoldin subunit 6 (Protein Ke2) (Homo sapiens) \| chr1:10507577-10509029 FORWARD \| Aliases: T1P2.3, T1P2_3 | 8.9 | 7.2 | 1.6 | 3.7 | 3.57% | 3.0 |
| 6569 | AT4G10250.1 \| Symbol: ATHSP22.0 \| 22.0 kDa ER small heat shock protein (HSP22.0-ER), identical to endomembrane-localized small heat shock protein GI:511795 from (Arabidopsis thaliana) \| chr4:6370339-6371286 FORWARD \| Aliases: ATHSP22.0, T9A4.7 | 3.2 | 2.6 | 0.6 | 3.7 | 3.57% | 2.8 |
| 6570 | AT1G61340.1 \| F-box family protein, contains Pfam PF00646: F-box domain; similar to late embryogenesis abundant protein GI:1350540 from (Picea glauca) | 6.4 | 5.2 | 1.3 | 3.7 | 3.57% | 3.0 |
| 6572 | AT3G46460.1 \| ubiquitin-conjugating enzyme 13 (UBC13), E2; identical to gi:992706 \| chr3:17106886-17108437 REVERSE \| Aliases: F18L15.180 | 7.5 | 6.2 | 1.3 | 3.7 | 3.57% | 3.0 |
| 6574 | AT1G29850.2 \| double-stranded DNA-binding family protein, contains Pfam profile: PF01984 double-stranded DNA-binding domain \| chr1:10447747-10449930 FORWARD \| Aliases: F1N18.11, F1N18_11 | 11.6 | 10.3 | 1.2 | 3.7 | 3.58% | 2.6 |
| 6575 | AT3G59000.2 \| similar to F-box family protein [Arabidopsis thaliana] (TAIR:At3g58930.1); similar to unknown protein [Oryza sativa (japonica cultivar-group)] (GB:BAD54099.1); contains InterPro domain Cyclin-like F-box (InterPro:IPR001810); contains InterPro domain FBD (InterPro:IPR006566) \| chr3:21810253-21812720 FORWARD \| Aliases: F17J16.50 | 6.1 | 5.4 | 0.7 | 3.7 | 3.58% | 2.9 |
| 6576 | AT4G24530.1 \| expressed protein, contains Pfam PF03138: Plant protein family. The function of this family of plant proteins is unknown; previously annotated as 'PsRT17-1 like protein' based on similarity to PsRT17-1 (GP:1778376) (Pisum sativum) which was based upon similarity to axi 1 protein (GB:X80301) (GI:559920) from (Nicotiana tabacum), which, due to scienitific fraud was retracted. Retraction in: Schell J. EMBO J 1999 May 17;18(10):2908. PMID:10400497. \| chr4:12667368-12670023 REVERSE \| Aliases: F22K18.270, F22K18_270 | 5.8 | 5.0 | 0.8 | 3.7 | 3.58% | 2.9 |
| 6577 | AT4G39690.1 \| expressed protein \| chr4:18417664-18421965 FORWARD \| Aliases: T19P19.80, T19P19_80 | 7.6 | 5.9 | 1.7 | 3.7 | 3.58% | 3.0 |
| 6578 | AT2G46230.1 \| expressed protein, contains Pfam profile: PF04900 protein of unknown function, DUF652 \| chr2:18991045-18993032 REVERSE \| Aliases: T3F17.12 | 8.2 | 7.0 | 1.2 | 3.7 | 3.58% | 2.9 |
| 6579 | AT3G22670.1 \| pentatricopeptide (PPR) repeat-containing protein, contains Pfam profile PF01535: PPR repeat \| chr3:8017778-8019466 REVERSE \| Aliases: MWI23.4 | 5.2 | 4.0 | 1.2 | 3.7 | 3.58% | 3.0 |
| 6580 | AT2G42300.2 \| similar to basic helix-loop-helix (bHLH) family protein [Arabidopsis thaliana] (TAIR:At3g57800.2); similar to bHLH transcription factor GBOF-1 [Tulipa gesneriana] (GB:AAD56411.1); similar to hypothetical protein [Oryza sativa (japonica cultivar-group)] (GB:XP_469986.1) \| chr2:17628235-17631916 FORWARD \| Aliases: MHK10.2, MHK10_2 | 4.8 | 3.6 | 1.2 | 3.7 | 3.58% | 3.0 |
| 6582 | AT1G79210.1 \| 20S proteasome alpha subunit B, putative, nearly identical to SP:O23708 Proteasome subunit alpha type 2 (EC 3.4.25.1) (20S proteasome alpha subunit B) {Arabidopsis thaliana} and to At1g16470 \| chr1:29800987-29803624 REVERSE \| Aliases: YUP8H12R.19, YUP8H12R_19 | 7.7 | 6.2 | 1.6 | 3.7 | 3.58% | 3.0 |
| 6584 | AT1G19525.1 \| pentatricopeptide (PPR) repeat-containing protein, contains Pfam profile PF01535: PPR repeat \| chr1:6761511-6762769 FORWARD \| Aliases: None | 6.3 | 5.1 | 1.2 | 3.7 | 3.59% | 2.9 |

| Rank | Description | Sync | Root | M | t | adj.q | B |
|---|---|---|---|---|---|---|---|
| 6585 | AT4G02290.1 \| glycosyl hydrolase family 9 protein, similar to endo-1,4-beta glucanase; ATCEL2 GI:3132891 from (Arabidopsis thaliana) \| chr4:1002446-1005202 REVERSE \| Aliases: T2H3.5, T2H3_5 | 5.4 | 3.8 | 1.5 | 3.7 | 3.59% | 3.1 |
| 6586 | AT5G67590.1 \| Symbol: FRO1 \| NADH-ubiquinone oxidoreductase-related, contains weak similarity to NADH-ubiquinone oxidoreductase 21 kDa subunit, mitochondrial precursor (EC 1.6.5.3) (EC 1.6.99.3) (Complex I-21KD) (CI-21KD). (Swiss-Prot:P25711) (Neurospora crassa); contains Pfam PF04800: ETC complex I subunit conserved region | 10.5 | 8.8 | 1.7 | 3.7 | 3.59% | 2.9 |
| 6588 | AT2G38170.3 \| Symbol: CAX1 \| calcium exchanger (CAX1), identical to high affinity calcium antiporter CAX1 (Arabidopsis thaliana) gi:9256741:gb:AAB05913, except a possible frameshift at base 58008. Sequence has been confirmed with 5 sequencing reads.; Ca2+:Cation Antiporter (CaCA) Family member PMID:11500563 \| chr2:15997127-16000354 REVERSE \| Aliases: ATCAX1, F16M14.10, F16M14_10, HIGH AFFINITY CALCIUM ANTIPORTER CAX1, RARE COLD INDUCIBLE4, RCI4 | 7.7 | 6.5 | 1.2 | 3.7 | 3.59% | 3.0 |
| 6592 | AT5G55290.1 \| ATP synthase subunit H family protein, contains weak similarity to Vacuolar ATP synthase subunit H (EC 3.6.3.14) (V-ATPase H subunit) (Vacuolar proton pump H subunit) (V-ATPase M9.2 subunit) (V-ATPase 9.2 kDa membrane accessory protein) (Swiss-Prot:P81103) (Bos taurus); contains Pfam profile PF05493: ATP synthase subunit H \| chr5:22440263-22441312 REVERSE \| Aliases: MCO15.24, MCO15_24 | 10.1 | 9.5 | 0.6 | 3.7 | 3.60% | 2.6 |
| 6595 | AT5G12080.2 \| mechanosensitive ion channel domain-containing protein / MS ion channel domain-containing protein, contains Pfam profile PF00924: Mechanosensitive ion channel \| chr5:3898048-3901521 REVERSE \| Aliases: F14F18.230, F14F18_230 | 8.7 | 7.1 | 1.6 | 3.7 | 3.60% | 3.0 |
| 6598 | AT1G03320.1 \| expressed protein \| chr1:817027-817767 FORWARD \| Aliases: F15K9.8, F15K9_8 | 2.6 | 2.2 | 0.4 | 3.7 | 3.60% | 2.4 |
| 6599 | AT3G02770.1 \| dimethylmenaquinone methyltransferase family protein, similar to bacterial S-adenosylmethionine:2-demethylmenaquinone methyltransferases; contains Pfam profile PF03737: Dimethylmenaquinone methyltransferase \| chr3:600900-602218 REVERSE \| Aliases: F13E7.29, F13E7_29 | 9.6 | 8.4 | 1.1 | 3.7 | 3.60% | 3.0 |
| 6601 | AT5G37510.2 \| Symbol: EMB1467 \| NADH-ubiquinone dehydrogenase, mitochondrial, putative, similar to NADH-ubiquinone oxidoreductase 75 kDa subunit, mitochondrial from Solanum tuberosum (SP:Q43644) \| chr5:14914640-14917877 FORWARD \| Aliases: EMB1467, EMBRYO DEFECTIVE 1467, MPA22.5, MPA22_5 | 11.1 | 8.7 | 2.4 | 3.7 | 3.60% | 2.9 |
| 6603 | AT4G31560.1 \| expressed protein \| chr4:15295207-15296233 FORWARD \| Aliases: F3L17.130, F3L17_130 | 5.6 | 4.5 | 1.1 | 3.7 | 3.61% | 3.1 |
| 6604 | AT5G58790.1 \| expressed protein, ; expression supported by MPSS \| chr5:23762232-23763018 FORWARD \| Aliases: MZN1.28 | 4.9 | 4.0 | 0.9 | 3.7 | 3.61% | 2.7 |
| 6605 | AT3G02530.1 \| chaperonin, putative, similar to SWISS-PROT:P80317- T-complex protein 1, zeta subunit (TCP-1-zeta) (Mus musculus); contains Pfam:PF00118 domain, TCP-1/cpn60 chaperonin family \| chr3:528581-532589 REVERSE \| Aliases: F16B3.16, F16B3_16 | 8.4 | 6.4 | 2.0 | 3.7 | 3.62% | 2.9 |
| 6606 | AT4G23493.1 \| expressed protein \| chr4:12256192-12257049 REVERSE \| Aliases: None | 6.6 | 5.4 | 1.2 | 3.7 | 3.62% | 2.9 |
| 6609 | AT1G61990.1 \| mitochondrial transcription termination factor-related / mTERF-related, contains Pfam profile PF02536: mTERF \| chr1:22914870-22916600 FORWARD \| Aliases: F8K4.18, F8K4_18 | 5.1 | 3.7 | 1.4 | 3.7 | 3.63% | 3.0 |
| 6610 | AT1G15950.1 \| Symbol: CCR1 \| cinnamoyl-CoA reductase, putative, nearly identical to CCR1 (GI:12034897), similar to cinnamoyl CoA reductase GI:2058310 from (Eucalyptus gunnii) \| chr1:5478748-5482159 FORWARD \| Aliases: CINNAMOYL COA REDUCTASE, CINNAMOYL COA REDUCTASE 1, IRREGULAR XYLEM 4, IRX4, T24D18.5, T24D18_5 | 10.9 | 9.3 | 1.7 | 3.7 | 3.63% | 2.6 |
| 6611 | AT3G04340.1 \| Symbol: EMB2458 \| FtsH protease family protein, similar to chloroplast FtsH protease (Arabidopsis thaliana) GI:1483215; contains Pfam profiles PF01434: Peptidase family M41, PF00004: ATPase AAA family \| chr3:1146807-1152455 REVERSE \| Aliases: EMB2458, EMBRYO DEFECTIVE 2458, T6K12.4, T6K12_4 | 4.5 | 3.8 | 0.7 | 3.7 | 3.63% | 2.9 |
| 6612 | AT1G53200.2 \| expressed protein \| chr1:19844044-19847071 REVERSE \| Aliases: F12M16.11, F12M16_11 | 4.1 | 3.4 | 0.7 | 3.7 | 3.63% | 2.8 |
| 6613 | AT5G03300.1 \| Symbol: ADK2 \| adenosine kinase 2 (ADK2), contains Pfam profile: PF00294 pfkB family carbohydrate kinase; identical to cDNA adenosine kinase 2 (ADK2) GI:12017763 \| chr5:796441-799153 FORWARD \| Aliases: ADENOSINE KINASE 2, F12E4.30, F12E4_30 | 11.0 | 9.8 | 1.1 | 3.7 | 3.63% | 2.8 |
| 6620 | AT3G19280.1 \| Symbol: FUT11 \| glycoprotein 3-alpha-L-fucosyltransferase A / FucTA / core alpha-(1,3)-fucosyltransferase (FUT11), identical to Glycoprotein 3-alpha-L-fucosyltransferase A (Core alpha-(1,3)-fucosyltransferase)(Fuc-T C3) (FucTA) (FucT1) (AtFUT11)(SP:Q9LJK1) from (Arabidopsis thaliana); contains Pfam profile PF00852: Fucosyl transferase; identical to cDNA alpha1,3-fucosyltransferase (FucTA) GI:13992482 \| chr3:6681303-6683898 REVERSE \| Aliases: ATFUT11, FUCT1, FUCTA, MVI11.20 | 4.5 | 4.0 | 0.5 | 3.7 | 3.65% | 2.6 |
| 6626 | AT1G04980.1 \| Symbol: ATPDIL2 2 \| Encodes a protein disulfide isomerase-like (PDIL) protein, a member of a multigene family within the thioredoxin (TRX) superfamily. | 6.0 | 5.2 | 0.9 | 3.7 | 3.66% | 2.9 |
| 6628 | AT5G36120.1 \| YGGT family protein, contains Pfam profile PF02325: YGGT family (unknown function) \| chr5:14215818-14216473 REVERSE \| Aliases: MAB16.6, MAB16_6 | 4.2 | 3.6 | 0.6 | 3.7 | 3.66% | 2.8 |

| Rank | Description | Sync | Root | M | t | adj.q | B |
|---|---|---|---|---|---|---|---|
| 6631 | AT3G56940.1 \| Symbol: AT103 \| dicarboxylate diiron protein, putative (Crd1), similar to leucine-containing zipper protein At103 GP:6911864; contains Pfam profile PF05447: Copper response defect 1 (CRD1) \| chr3:21087524-21089571 FORWARD \| Aliases: CHL27, CRD1, DICARBOXYLATE DIIRON 1, T8M16.1 | 6.2 | 5.1 | 1.1 | 3.7 | 3.66% | 3.0 |
| 6632 | AT1G12780.1 \| Encodes a UDP-glucose epimerase that catalyzes the interconversion of the sugar nucleotides UDP-glucose UDP-galactose via a UDP-4-keto-hexose intermediate. \| chr1:4355924-4358326 REVERSE \| Aliases: F13K23.3, F13K23_3 | 8.3 | 6.6 | 1.7 | 3.7 | 3.67% | 2.9 |
| 6633 | AT3G45100.2 \| Symbol: SETH2 \| N-acetylglucosaminyl-phosphatidylinositol biosynthetic protein, putative, similar to PIG-A from Mus musculus (gi:577723(, Homo sapiens (SP:P37287); contains Pfam glycosyl transferase, group 1 family protein domain PF00534 \| chr3:16515445-16517988 FORWARD \| Aliases: T14D3.40 | 7.8 | 6.8 | 1.0 | 3.7 | 3.67% | 2.9 |
| 6634 | AT1G76160.1 \| Symbol: SKS5 \| multi-copper oxidase type I family protein, similar to pollen-specific BP10 protein (SP:Q00624)(Brassica napus); contains Pfam profile: PF00394 Multicopper oxidase \| chr1:28582836-28586006 REVERSE \| Aliases: SKS5, T23E18.10, T23E18_10 | 8.3 | 6.7 | 1.6 | 3.7 | 3.68% | 2.9 |
| 6635 | AT1G45332.1 \| mitochondrial elongation factor, putative, similar to mitochondrial elongation factor GI:3917 from (Saccharomyces cerevisiae) \| chr1:17174795-17179261 REVERSE \| Aliases: AT1G45261, F2G19.2, F2G19_2 | 9.3 | 6.8 | 2.6 | 3.7 | 3.68% | 3.0 |
| 6636 | AT2G45030.1 \| mitochondrial elongation factor, putative, similar to SP:P25039 Elongation factor G 1, mitochondrial precursor (mEF-G-1) {Saccharomyces cerevisiae}; contains Pfam profiles PF00009: Elongation factor Tu GTP binding domain, PF03764: Elongation factor G domain IV, PF00679: Elongation factor G C-terminus \| chr2:18579486-18584045 FORWARD \| Aliases: T14P1.16 | 9.3 | 6.8 | 2.6 | 3.7 | 3.68% | 3.0 |
| 6639 | AT5G25170.1 \| expressed protein \| chr5:8693187-8695098 FORWARD \| Aliases: F21J6.101, F21J6_101 | 5.9 | 4.7 | 1.1 | 3.7 | 3.69% | 2.8 |
| 6640 | AT5G53760.2 \| Symbol: MLO11 \| similar to seven transmembrane MLO family protein / MLO-like protein 14 (MLO14) [Arabidopsis thaliana] (TAIR:At1g26700.1); similar to seven transmembrane protein Mlo9 [Zea mays] (GB:AAK38345.1); contains InterPro domain Mlo-related protein (InterPro:IPR004326) \| chr5:21839760-21843677 FORWARD \| Aliases: MGN6.12, MGN6_12 | 6.1 | 5.1 | 1.0 | 3.7 | 3.69% | 3.0 |
| 6641 | AT5G56290.1 \| Symbol: PEX5 \| peroxisomal targeting signal type 1 receptor (PEX5), identical to GI:3603353; contains Pfam profile PF00515 TPR Domain \| chr5:22803755-22809345 FORWARD \| Aliases: MXK23.3, MXK23_3 | 8.9 | 7.5 | 1.3 | 3.7 | 3.69% | 2.9 |
| 6642 | AT4G27240.1 \| zinc finger (C2H2 type) family protein, contains zinc finger, C2H2 type, domain, PROSITE:PS00028 | 5.5 | 3.8 | 1.7 | 3.7 | 3.69% | 3.0 |
| 6643 | AT1G63770.2 \| peptidase M1 family protein, similar to SP:P04825 Aminopeptidase N (EC 3.4.11.2) (Alpha-aminoacylpeptide hydrolase) {Escherichia coli}; contains Pfam profile PF01433: Peptidase family M1 \| chr1:23661169-23667906 REVERSE \| Aliases: F24D7.4, F24D7_4 | 10.7 | 9.5 | 1.3 | 3.7 | 3.70% | 2.7 |
| 6645 | AT5G58290.1 \| Symbol: RPT3 \| 26S proteasome AAA-ATPase subunit (RPT3), identical to 26S proteasome AAA-ATPase subunit RPT3 GI:6652882 from (Arabidopsis thaliana) \| chr5:23586304-23588556 FORWARD \| Aliases: 26S PROTEASOME AAA ATPASE SUBUNIT RPT3, MCK7.16, MCK7_16 | 10.6 | 8.7 | 2.0 | 3.7 | 3.71% | 2.7 |
| 6646 | AT3G15380.1 \| choline transporter-related, contains weak similarity to CD92 protein (Homo sapiens) gi:16945323:emb:CAC82175 \| chr3:5193235-5196596 FORWARD \| Aliases: MJK13.4 | 4.8 | 4.0 | 0.9 | 3.7 | 3.71% | 2.9 |
| 6648 | AT4G12980.1 \| auxin-responsive protein, putative, similar to auxin-induced protein AIR12 GI:11357190 (Arabidopsis thaliana) \| chr4:7589392-7591099 REVERSE \| Aliases: F25G13.70, F25G13_70 | 5.0 | 4.3 | 0.7 | 3.7 | 3.71% | 2.8 |
| 6650 | AT3G45230.1 \| hydroxyproline-rich glycoprotein family protein, contains proline-rich extensin domains, INTERPRO:IPR002965; related to vegetative cell wall protein gp1 (Chlamydomonas reinhardtii) gi:12018147:gb:AAG45420 \| chr3:16580036-16580812 FORWARD \| Aliases: T14D3.170 | 5.8 | 5.0 | 0.8 | 3.7 | 3.71% | 2.8 |
| 6651 | AT2G46470.1 \| OXA1 protein, putative, similar to Oxa1 protein (Arabidopsis thaliana) GI:10176922 \| chr2:19079385-19082195 FORWARD \| Aliases: F11C10.16 | 5.2 | 4.2 | 1.0 | 3.7 | 3.72% | 3.0 |
| 6656 | AT3G18890.1 \| expressed protein, similar to UV-B and ozone similarly regulated protein 1 UOS1 (Pisum sativum) GI:20339364 \| chr3:6511031-6514975 FORWARD \| Aliases: MCB22.6 | 2.8 | 2.3 | 0.5 | 3.7 | 3.73% | 2.6 |
| 6658 | AT4G13640.2 \| similar to myb family transcription factor [Arabidopsis thaliana] (TAIR:At3g24120.2); similar to transfactor-like [Oryza sativa (japonica cultivar-group)] (GB:XP_481813.1); contains InterPro domain Myb DNA-binding domain (InterPro:IPR001005); contains InterPro domain Myb-like DNA-binding domain, SHAQKYF class (InterPro:IPR006447) \| chr4:7936602-7938626 REVERSE \| Aliases: F18A5.30, F18A5_30 | 6.1 | 5.3 | 0.8 | 3.7 | 3.74% | 2.7 |
| 6659 | AT3G52860.1 \| expressed protein \| chr3:19602826-19603743 REVERSE \| Aliases: F8J2.30 | 4.0 | 3.2 | 0.8 | 3.7 | 3.74% | 2.8 |
| 6660 | AT5G58575.1 \| expressed protein \| chr5:23691244-23692462 FORWARD \| Aliases: None | 4.9 | 3.7 | 1.3 | 3.7 | 3.74% | 3.0 |

| Rank | Description | Sync | Root | M | t | adj.q | B |
|---|---|---|---|---|---|---|---|
| 6661 | AT1G11530.1 \| Encodes a single cysteine active site thioredoxin-related protein, similar to thioredoxin H-type from Arabidopsis thaliana SP:P29448, Nicotiana tabacum SP:Q07090; contains Pfam profile: PF00085 Thioredoxin; \| chr1:3874437-3875484 FORWARD \| Aliases: T23J18.19, T23J18_19 | 6.3 | 5.6 | 0.7 | 3.7 | 3.75% | 2.7 |
| 6664 | AT1G20230.1 \| pentatricopeptide (PPR) repeat-containing protein, contains Pfam profile PF01535: PPR repeat \| chr1:7009561-7011843 FORWARD \| Aliases: T20H2.1, T20H2_1 | 3.9 | 3.1 | 0.8 | 3.7 | 3.75% | 2.9 |
| 6665 | AT1G27390.1 \| Symbol: TOM20 2 \| mitochondrial import receptor subunit TOM20-2 (TOM20-2), identical to mitochondrial import receptor subunit TOM20-2 SP:P82873 from (Arabidopsis thaliana) \| chr1:9513076-9514988 REVERSE \| Aliases: F17L21.18, F17L21_18, TRANSLOCASE OUTER MEMBRANE 20 2 | 5.8 | 4.9 | 0.9 | 3.7 | 3.76% | 2.8 |
| 6673 | AT3G14650.1 \| Symbol: CYP72A11 \| cytochrome P450, putative, similar to GB:Q05047 from (Catharanthus roseus) \| chr3:4922138-4924695 FORWARD \| Aliases: MIE1.15 | 4.8 | 3.7 | 1.0 | 3.7 | 3.77% | 2.8 |
| 6678 | AT4G37490.1 \| Symbol: CYC1 \| G2/mitotic-specific cyclin (CYC1) / B-like cyclin (CYC1), identical to SP:P30183 G2/mitotic-specific cyclin (B-like cyclin) {Arabidopsis thaliana} \| chr4:17621880-17624279 REVERSE \| Aliases: CYCB1, CYCB1;1, CYCLIN 1, Cyclin B1;1, F6G17.140, F6G17_140 | 4.3 | 3.7 | 0.7 | 3.7 | 3.79% | 2.7 |
| 6679 | AT1G63050.1 \| membrane bound O-acyl transferase (MBOAT) family protein, low similarity to porcupine from (Xenopus laevis) GI:6714514, GI:6714520, GI:6714518, GI:6714516; contains Pfam profile PF03062: MBOAT family \| chr1:23379549-23381927 REVERSE \| Aliases: F16M19.14, F16M19_14 | 7.4 | 6.1 | 1.3 | 3.7 | 3.79% | 2.8 |
| 6681 | AT1G27460.1 \| Symbol: NPGR1 \| calmodulin-binding protein, similar to calmodulin-binding protein MPCBP (Zea mays) GI:10086260; contains Pfam profile PF00515: TPR Domain \| chr1:9534519-9537548 FORWARD \| Aliases: F17L21.25, F17L21_25, NO POLLEN GERMINATION RELATED 1 | 4.3 | 3.2 | 1.2 | 3.7 | 3.80% | 3.0 |
| 6682 | AT1G31850.3 \| dehydration-responsive protein, putative, strong similarity to early-responsive to dehydration stress ERD3 protein (Arabidopsis thaliana) GI:15320410; contains Pfam profile PF03141: Putative methyltransferase | 7.8 | 6.4 | 1.4 | 3.7 | 3.81% | 2.8 |
| 6683 | AT2G25300.1 \| similar to galactosyltransferase family protein [Arabidopsis thaliana] (TAIR:At4g32120.1); similar to beta 1,3-glycosyltransferase-like protein I [Lycopersicon esculentum] (GB:CAD30015.1); contains InterPro domain Glycosyl transferase, family 31 (InterPro:IPR002659) \| chr2:10778773-10781373 REVERSE \| Aliases: T22F11.11, T22F11_11 | 7.0 | 5.9 | 1.1 | 3.7 | 3.81% | 2.9 |
| 6685 | AT5G59440.2 \| thymidylate kinase family protein, similar to thymidylate kinase GI:291900 \| chr5:23988461-23990097 FORWARD \| Aliases: F2O15.12, F2O15_12 | 6.1 | 4.9 | 1.2 | 3.7 | 3.81% | 3.0 |
| 6689 | AT3G25740.1 \| Symbol: MAP1B \| metallopeptidase M24 family protein, similar to SP:O33343 Methionine aminopeptidase (EC 3.4.11.18) (Peptidase M) {Mycobacterium tuberculosis}; contains Pfam profile PF00557: metallopeptidase family M24 \| chr3:9399023-9400999 FORWARD \| Aliases: K13N2.17, MAP1C, METHIONINE AMINOPEPTIDASE 1C | 4.7 | 3.8 | 0.8 | 3.7 | 3.81% | 2.7 |
| 6690 | AT1G35340.3 \| ATP-dependent protease La (LON) domain-containing protein, contains Pfam profile PF02190: ATP-dependent protease La (LON) domain | 7.5 | 6.8 | 0.8 | 3.7 | 3.81% | 2.8 |
| 6692 | AT5G27600.1 \| Symbol: LACS7 \| AMP-binding protein, putative, similar to AMP-binding protein (MF39P) gi:1617274 from Brassica napus, long-chain-fatty-acid--CoA ligase - Brassica napus, EMBL:Z72152; contains Pfam AMP-binding enzyme domain PF00501 \| chr5:9742619-9747008 FORWARD \| Aliases: F15A18.60, F15A18_60, LONG CHAIN ACYL COA SYNTHETASE 7 | 6.0 | 5.0 | 1.0 | 3.7 | 3.82% | 2.8 |
| 6695 | AT5G55540.1 \| expressed protein \| chr5:22513422-22517769 REVERSE \| Aliases: MTE17.26, MTE17_26 | 6.2 | 5.4 | 0.8 | 3.7 | 3.83% | 2.8 |
| 6696 | AT1G34270.1 \| exostosin family protein, contains Pfam profile: PF03016 Exostosin family \| chr1:12492389-12494522 REVERSE \| Aliases: F23M19.7, F23M19_7 | 4.4 | 3.7 | 0.7 | 3.7 | 3.83% | 2.8 |
| 6701 | AT4G28590.1 \| expressed protein \| chr4:14125286-14126841 REVERSE \| Aliases: T5F17.40, T5F17_40 | 3.0 | 2.6 | 0.5 | 3.7 | 3.83% | 2.4 |
| 6702 | AT5G15810.1 \| N2,N2-dimethylguanosine tRNA methyltransferase family protein, similar to SP:Q9P804 N(2),N(2)-dimethylguanosine tRNA methyltransferase (EC 2.1.1.32) (tRNA(guanine-26,N(2)-N(2)) methyltransferase) {Schizosaccharomyces pombe}; contains Pfam profile PF02005: N2,N2-dimethylguanosine tRNA methyltransferase \| chr5:5157801-5161361 FORWARD \| Aliases: F14F8.190, F14F8_190 | 7.0 | 6.1 | 0.9 | 3.7 | 3.84% | 2.8 |
| 6707 | AT2G17260.1 \| Symbol: GLR2 \| glutamate receptor family protein (GLR3.1) (GLR2), identical to putative glutamate receptor GLR2 (Arabidopsis thaliana) gi:4185740:gb:AAD09174; plant glutamate receptor family, PMID:11379626 \| chr2:7511838-7515476 FORWARD \| Aliases: ATGLR2, ATGLR3.1, F5J6.2, F5J6_2, GLR3.1, GLUTAMATE RECEPTOR 2 | 4.8 | 4.0 | 0.8 | 3.7 | 3.84% | 2.8 |
| 6709 | AT1G07410.1 \| Ras-related GTP-binding protein, putative, similar to GTP-binding protein RAB11C GI:1370146 from (Lotus japonicus) \| chr1:2276267-2277151 FORWARD \| Aliases: F22G5.24, F22G5_24 | 3.8 | 3.0 | 0.8 | 3.7 | 3.84% | 2.7 |
| 6710 | AT4G32920.1 \| glycine-rich protein \| chr4:15887578-15896009 REVERSE \| Aliases: F26P21.40, F26P21_40 | 4.3 | 3.6 | 0.7 | 3.7 | 3.85% | 2.8 |

| Rank | Description | Sync | Root | M | t | adj.q | B |
|---|---|---|---|---|---|---|---|
| 6711 | AT1G48470.1 \| Symbol: GLN1;5 \| | 5.7 | 4.3 | 1.3 | 3.7 | 3.85% | 3.0 |
| 6714 | AT1G48230.1 \| phosphate translocator-related, low similarity to phosphoenolpyruvate/phosphate translocator precursor (Mesembryanthemum crystallinum) GI:9295275, SP:P52178 Triose phosphate/phosphate translocator, non-green plastid, chloroplast precursor (CTPT) {Brassica oleracea} \| chr1:17809932-17812553 FORWARD \| Aliases: F21D18.5 | 6.1 | 5.2 | 0.8 | 3.7 | 3.85% | 2.7 |
| 6715 | AT4G19610.1 \| RNA recognition motif (RRM)-containing protein, contains InterPro entry IPR000504: RNA-binding region RNP-1 (RNA recognition motif) (RRM) \| chr4:10677675-10681633 FORWARD \| Aliases: F24J7.162, F24J7_162 | 7.0 | 6.0 | 1.0 | 3.7 | 3.85% | 2.7 |
| 6717 | AT1G18490.1 \| expressed protein \| chr1:6367061-6368842 FORWARD \| Aliases: F15H18.26, F15H18_26 | 7.9 | 6.7 | 1.2 | 3.7 | 3.86% | 2.8 |
| 6718 | AT5G02550.1 \| expressed protein \| chr5:573052-573556 REVERSE \| Aliases: T22P11.140, T22P11_140 | 4.1 | 3.0 | 1.1 | 3.7 | 3.86% | 2.7 |
| 6720 | AT5G04360.1 \| pullulanase, putative / starch debranching enzyme, putative, similar to pullulanase (Spinacia oleracea) GI:634093 (EC 3.2.1.41); contains Pfam profiles PF00128: Alpha amylase catalytic domain, PF02922: Isoamylase N-terminal domain \| chr5:1221531-1228612 FORWARD \| Aliases: T19N18.90, T19N18_90 | 4.5 | 3.1 | 1.3 | 3.7 | 3.87% | 2.9 |
| 6722 | AT4G14695.1 \| expressed protein, contains Pfam domain, PF03650: Uncharacterized protein family (UPF0041) \| chr4:8419828-8420871 FORWARD \| Aliases: None | 3.1 | 2.4 | 0.7 | 3.7 | 3.87% | 2.3 |
| 6726 | AT1G03590.1 \| protein phosphatase 2C family protein / PP2C family protein, similar to protein phosphatase-2C; PP2C (GI:3643088) (Mesembryanthemum crystallinum); contains Pfam PF00481 : Protein phosphatase 2C domain; \| chr1:894254-896439 REVERSE \| Aliases: F21B7.20 | 6.0 | 5.3 | 0.7 | 3.7 | 3.88% | 2.7 |
| 6727 | AT2G34620.1 \| mitochondrial transcription termination factor-related / mTERF-related, contains Pfam profile PF02536: mTERF \| chr2:14584275-14585367 FORWARD \| Aliases: T31E10.4, T31E10_4 | 2.8 | 2.4 | 0.4 | 3.7 | 3.88% | 2.3 |
| 6730 | AT5G58500.1 \| expressed protein, contains Pfam profile PF04852: Protein of unknown function (DUF640) \| chr5:23662761-23663309 REVERSE \| Aliases: MQJ2.11, MQJ2_11 | 5.3 | 4.6 | 0.7 | 3.7 | 3.88% | 2.7 |
| 6731 | AT5G49650.2 \| xylulose kinase, putative, similar to D-xylulokinase (Pichia stipitis) gi:8100400:gb:AAF72328 \| chr5:20170007-20172994 FORWARD \| Aliases: MNI5.3, MNI5_3 | 8.0 | 6.4 | 1.7 | 3.7 | 3.88% | 2.9 |
| 6734 | AT2G41550.1 \| expressed protein \| chr2:17335617-17338211 REVERSE \| Aliases: T32G6.7, T32G6_7 | 5.2 | 4.3 | 0.9 | 3.7 | 3.89% | 2.9 |
| 6737 | AT1G78995.1 \| expressed protein \| chr1:29719972-29721004 REVERSE \| Aliases: None | 3.9 | 3.2 | 0.7 | 3.7 | 3.89% | 2.6 |
| 6738 | AT1G10990.2 \| expressed protein, and genscan \| chr1:3670278-3671187 REVERSE \| Aliases: T19D16.27, T19D16_27 | 4.4 | 3.7 | 0.7 | 3.7 | 3.89% | 2.5 |
| 6739 | AT5G03390.1 \| expressed protein, contains Pfam profile PF03478: Protein of unknown function (DUF295) \| chr5:834987-836750 FORWARD \| Aliases: F12E4.130, F12E4_130 | 3.2 | 2.4 | 0.8 | 3.7 | 3.89% | 2.8 |
| 6740 | AT3G56010.1 \| expressed protein \| chr3:20799670-20800903 FORWARD \| Aliases: F27K19.190 | 5.1 | 4.5 | 0.7 | 3.7 | 3.89% | 2.8 |
| 6741 | AT2G01710.1 \| DNAJ heat shock N-terminal domain-containing protein, simlar to AHM1 (Triticum aestivum) GI:6691467; contains Pfam profile PF00226: DnaJ domain \| chr2:315725-316992 FORWARD \| Aliases: T8O11.12, T8O11_12 | 5.0 | 4.3 | 0.7 | 3.7 | 3.90% | 2.6 |
| 6743 | AT2G41040.1 \| methyltransferase-related, eak similarity to C5-O-methyltransferase (GI:5921167) (Streptomyces avermitilis); weak similarity to Probable menaquinone biosynthesis methyltransferase (EC 2.1.1.-) (gerC2 protein homolog) (Swiss-Prot:P49016) (Lactococcus lactis) \| chr2:17128454-17130260 FORWARD \| Aliases: T3K9.19, T3K9_19 | 4.7 | 3.2 | 1.5 | 3.6 | 3.90% | 3.0 |
| 6745 | AT1G63220.1 \| C2 domain-containing protein, similar to phloem protein RPP16 (Oryza sativa (japonica cultivar-group)) GI:21998839; contains Pfam profile PF00168: C2 domain \| chr1:23452584-23454106 FORWARD \| Aliases: F9N12.16, F9N12_16 | 7.4 | 5.6 | 1.9 | 3.6 | 3.90% | 2.9 |
| 6746 | AT3G11670.2 \| Symbol: DGD1 \| digalactosyldiacylglycerol synthase 1 (DGD1) / MGDG:MGDG galactosyltransferase / galactolipid galactosyltransferase, identical to digalactosyldiacylglycerol synthase (DGD1) GI:5354158 (Arabidopsis thaliana) \| chr3:3681612-3684552 REVERSE \| Aliases: DIGALACTOSYL DIACYLGLYCEROL DEFICIENT 1, DIGALACTOSYLDIACYLGLYCEROL SYNTHASE 1, T19F11.7 | 6.0 | 4.7 | 1.3 | 3.6 | 3.91% | 2.8 |
| 6749 | AT1G62730.1 \| expressed protein \| chr1:23232724-23234401 REVERSE \| Aliases: F23N19.9, F23N19_9 | 5.3 | 4.2 | 1.1 | 3.6 | 3.92% | 2.8 |
| 6751 | AT5G05670.2 \| expressed protein \| chr5:1695731-1698105 REVERSE \| Aliases: MJJ3.7, MJJ3_7 | 5.5 | 4.4 | 1.1 | 3.6 | 3.92% | 2.9 |

| Rank | Description | Sync | Root | M | t | adj.q | B |
|---|---|---|---|---|---|---|---|
| 6753 | AT5G42990.1 \| ubiquitin-conjugating enzyme 18 (UBC18), E2; identical to gi:2801448 \| chr5:17261219-17263182 REVERSE \| Aliases: MBD2.19, MBD2_19 | 6.9 | 6.0 | 0.9 | 3.6 | 3.93% | 2.8 |
| 6754 | AT4G18520.1 \| similar to pentatricopeptide (PPR) repeat-containing protein [Arabidopsis thaliana] (TAIR:At3g09040.1); similar to PPR986-12 [Physcomitrella patens] (GB:BAD67155.1); contains InterPro domain PPR repeat (InterPro:IPR002885) \| chr4:10215132-10217114 REVERSE \| Aliases: F28J12.180, F28J12_180 | 3.7 | 3.1 | 0.7 | 3.6 | 3.93% | 2.8 |
| 6755 | AT5G65650.1 \| expressed protein \| chr5:26256728-26258741 FORWARD \| Aliases: K21L13.17, K21L13_17 | 5.5 | 4.3 | 1.2 | 3.6 | 3.93% | 2.8 |
| 6757 | AT3G56120.1 \| Met-10+ like family protein, non-consensus TT donor splice site at exon 4 ; contains Pfam profile PF02475: Met-10+ like-protein \| chr3:20834129-20837522 FORWARD \| Aliases: F18O21.80 | 6.7 | 5.3 | 1.4 | 3.6 | 3.94% | 2.9 |
| 6760 | AT3G63160.1 \| expressed protein \| chr3:23344488-23345009 REVERSE \| Aliases: F16M2.10 | 4.9 | 4.1 | 0.8 | 3.6 | 3.94% | 2.7 |
| 6762 | AT5G11100.1 \| C2 domain-containing protein, similar to Ca2+-dependent lipid-binding protein (CLB1) GI:2789434 from (Lycopersicon esculentum) \| chr5:3532403-3535222 FORWARD \| Aliases: T5K6.90, T5K6_90 | 5.5 | 4.6 | 0.9 | 3.6 | 3.94% | 2.9 |
| 6763 | AT5G15550.2 \| transducin family protein / WD-40 repeat family protein, similar to YTM1 - Homo sapiens, EMBL:AF242546; contains Pfam PF00400: WD domain, G-beta repeat (7 copies,1 weak); \| chr5:5058839-5062090 REVERSE \| Aliases: T20K14.160, T20K14_160 | 6.9 | 5.8 | 1.1 | 3.6 | 3.94% | 2.9 |
| 6765 | AT1G01090.1 \| Symbol: PDH E1 ALPHA \| pyruvate dehydrogenase E1 component alpha subunit, chloroplast, identical to pyruvate dehydrogenase E1 alpha subunit GB:AAB86803 GI:2454182 from (Arabidopsis thaliana); identical to cDNA pyruvate dehydrogenase E1 alpha subunit mRNA, nuclear gene encoding plastid protein GI:2454181 \| chr1:47485-49279 REVERSE \| Aliases: PYRUVATE DEHYDROGENASE E1 ALPHA, T25K16.8, T25K16_8 | 10.8 | 9.5 | 1.4 | 3.6 | 3.95% | 2.7 |
| 6766 | AT5G53080.1 \| kinesin light chain-related, low similarity to kinesin light chain from (Plectonema boryanum) GI:2645229, (Loligo pealei) GI:403179; contains Pfam profile PF00515: TPR Domain \| chr5:21537739-21540492 FORWARD \| Aliases: MNB8.14, MNB8_14 | 3.7 | 3.2 | 0.5 | 3.6 | 3.95% | 2.6 |
| 6768 | AT2G30960.1 \| expressed protein \| chr2:13183781-13185405 REVERSE \| Aliases: F7F1.17, F7F1_17 | 7.4 | 5.9 | 1.6 | 3.6 | 3.95% | 2.9 |
| 6770 | AT2G18410.2 \| expressed protein \| chr2:7997847-7999856 FORWARD \| Aliases: T30D6.8, T30D6_8 | 4.4 | 4.0 | 0.4 | 3.6 | 3.96% | 2.4 |
| 6771 | AT1G13700.1 \| glucosamine/galactosamine-6-phosphate isomerase family protein, similar to SP:O95336 6-phosphogluconolactonase (EC 3.1.1.31) (6PGL) {Homo sapiens}; contains Pfam profile PF01182: Glucosamine-6-phosphate isomerase/6-phosphogluconolactonase \| chr1:4693959-4695979 REVERSE \| Aliases: F21F23.14, F21F23_14 | 3.8 | 3.0 | 0.9 | 3.6 | 3.96% | 2.6 |
| 6772 | AT1G10770.1 \| invertase/pectin methylesterase inhibitor family protein, contains Pfam profile PF04043: Plant invertase/pectin methylesterase inhibitor | 4.6 | 3.8 | 0.8 | 3.6 | 3.96% | 2.8 |
| 6773 | AT4G34290.1 \| SWIB complex BAF60b domain-containing protein, contains Pfam profile PF02201: BAF60b domain of the SWIB complex \| chr4:16410836-16412389 FORWARD \| Aliases: F10M10.60, F10M10_60 | 4.0 | 2.7 | 1.2 | 3.6 | 3.97% | 2.8 |
| 6775 | AT2G06030.1 \| expressed protein \| chr2:2352158-2353770 REVERSE \| Aliases: F5K7.21, F5K7_21 | 3.9 | 3.4 | 0.5 | 3.6 | 3.98% | 2.6 |
| 6776 | AT5G16715.1 \| Symbol: EMB2247 \| tRNA synthetase class I (I, L, M and V) family protein, similar to SP:P11931 Valyl-tRNA synthetase (EC 6.1.1.9) (Valine--tRNA ligase) (VALRS) {Bacillus stearothermophilus}; contains Pfam profile PF00133: tRNA synthetases class I (I, L, M and V) \| chr5:5485356-5493407 FORWARD \| Aliases: EMB2247, EMBRYO DEFECTIVE 2247 | 5.0 | 4.0 | 1.0 | 3.6 | 3.98% | 2.9 |
| 6782 | AT5G57500.1 \| expressed protein \| chr5:23303463-23304797 REVERSE \| Aliases: MUA2.7, MUA2_7 | 2.8 | 2.4 | 0.5 | 3.6 | 3.99% | 2.4 |
| 6786 | AT5G67150.1 \| transferase family protein, similar to anthranilate N-hydroxycinnamoyl/benzoyltransferase, Dianthus caryophyllus (gi:2239091); contains Pfam transferase family domain PF002458 \| chr5:26812997-26814538 REVERSE \| Aliases: K21H1.11, K21H1_11 | 8.0 | 6.7 | 1.2 | 3.6 | 4.00% | 2.8 |
| 6787 | AT2G30260.1 \| Symbol: U2B" \| small nuclear ribonucleoprotein U2B, putative / spliceosomal protein, putative, similar to spliceosomal protein (Solanum tuberosum) GI:169589 \| chr2:12912482-12914586 REVERSE \| Aliases: T9D9.7, T9D9_7, U2B" | 7.2 | 6.1 | 1.1 | 3.6 | 4.00% | 2.8 |
| 6788 | AT1G64970.1 \| Symbol: G TMT \| expressed protein \| chr1:24137050-24139723 REVERSE \| Aliases: F13O11.27, F13O11_27, GAMMA TOCOPHEROL METHYLTRANSFERASE, TMT1, VITAMIN E DEFICIENT 4, VTE4 | 4.1 | 3.3 | 0.8 | 3.6 | 4.01% | 2.8 |
| 6791 | AT2G43090.1 \| aconitase C-terminal domain-containing protein, contains Pfam profile PF00694: Aconitase C-terminal domain \| chr2:17926004-17927506 FORWARD \| Aliases: MFL8.15 | 8.6 | 5.2 | 3.4 | 3.6 | 4.01% | 2.9 |
| 6793 | AT1G21410.1 \| F-box family protein, similar to SKP1 interacting partner 2 (SKIP2) TIGR_Ath1:At5g67250 | 7.4 | 6.4 | 1.1 | 3.6 | 4.01% | 2.8 |

| Rank | Description | Sync | Root | M | t | adj.q | B |
|---|---|---|---|---|---|---|---|
| 6795 | AT5G63135.1 \| expressed protein \| chr5:25342205-25343705 REVERSE \| Aliases: None | 3.6 | 2.3 | 1.3 | 3.6 | 4.01% | 2.7 |
| 6798 | AT3G55330.1 \| photosystem II reaction center PsbP family protein, contains Pfam profile PF01789: PsbP \| chr3:20524870-20526328 REVERSE \| Aliases: T26I12.210 | 5.0 | 4.0 | 1.1 | 3.6 | 4.02% | 2.9 |
| 6804 | AT3G12610.1 \| Symbol: DRT100 \| | 4.0 | 3.0 | 1.1 | 3.6 | 4.03% | 2.9 |
| 6806 | AT3G51780.1 \| BAG domain-containing protein, low similarity to SP:Q99933 BAG-family molecular chaperone regulator-1 {Homo sapiens}; contains Pfam profile PF02179: BAG (Apoptosis regulator Bcl-2 protein) domain \| chr3:19217745-19219260 REVERSE \| Aliases: ATEM1.3 | 7.5 | 6.5 | 1.1 | 3.6 | 4.03% | 2.7 |
| 6807 | AT5G62800.1 \| seven in absentia (SINA) family protein, similar to SIAH1 protein (Brassica napus var. napus) GI:7657876; contains Pfam profile PF03145: Seven in absentia protein family \| chr5:25235889-25237193 FORWARD \| Aliases: MQB2.12, MQB2_12 | 3.9 | 3.0 | 0.9 | 3.6 | 4.03% | 2.8 |
| 6808 | AT4G39660.1 \| Symbol: AGT2 \| alanine--glyoxylate aminotransferase, putative / beta-alanine-pyruvate aminotransferase, putative / AGT, putative, similar to SP:Q64565 Alanine--glyoxylate aminotransferase 2, mitochondrial precursor (EC 2.6.1.44) (AGT 2) (Beta-alanine-pyruvate aminotransferase) {Rattus norvegicus}; contains Pfam profile PF00202: aminotransferase, class III \| chr4:18406767-18409466 FORWARD \| Aliases: ALANINE:GLYOXYLATE AMINOTRANSFERASE 2, T19P19.50, T19P19_50 | 9.4 | 8.1 | 1.3 | 3.6 | 4.03% | 2.8 |
| 6812 | AT4G10090.1 \| expressed protein \| chr4:6305439-6307514 FORWARD \| Aliases: F28M11.10, F28M11_10 | 4.6 | 4.0 | 0.5 | 3.6 | 4.05% | 2.5 |
| 6813 | AT4G38700.1 \| disease resistance-responsive family protein, related to disease resistance response protein 206-d (Pisum sativum) gi:508844:gb:AAB18669G \| chr4:18076453-18077171 REVERSE \| Aliases: T9A14.4 | 4.6 | 3.8 | 0.8 | 3.6 | 4.05% | 2.7 |
| 6816 | AT4G27450.1 \| expressed protein, similar to auxin down-regulated protein ARG10 (Vigna radiata) GI:2970051, wali7 (aluminum-induced protein) (Triticum aestivum) GI:451193 \| chr4:13727493-13728892 REVERSE \| Aliases: F27G19.50, F27G19_50 | 9.3 | 8.2 | 1.1 | 3.6 | 4.05% | 2.9 |
| 6817 | AT2G16270.1 \| expressed protein, and genefinder; expression supported by MPSS | 4.3 | 3.7 | 0.5 | 3.6 | 4.05% | 2.6 |
| 6821 | AT5G08280.1 \| hydroxymethylbilane synthase / porphobilinogen deaminase, chloroplast / pre-uroporphyrinogen synthase, identical to SP:Q43316 \| chr5:2663191-2665793 REVERSE \| Aliases: F8L15.10, F8L15_10 | 5.8 | 4.1 | 1.6 | 3.6 | 4.07% | 2.9 |
| 6822 | AT5G02470.3 \| Symbol: DPA \| DP-2 transcription factor, putative (DPA), similar to Swiss-Prot:Q14188 transcription factor DP-2 (E2F dimerization partner 2) (Homo sapiens) \| chr5:542356-544500 REVERSE \| Aliases: DPA TRANSCRIPTION FACTOR, T22P11.60, T22P11_60 | 5.5 | 4.5 | 1.0 | 3.6 | 4.07% | 2.7 |
| 6823 | AT4G11960.1 \| expressed protein, hypothetical protein F7H19.70 - Arabidopsis thaliana, PID:e1310057 \| chr4:7174880-7177752 REVERSE \| Aliases: F16J13.30, F16J13_30 | 6.8 | 5.7 | 1.1 | 3.6 | 4.07% | 2.8 |
| 6824 | AT2G02560.2 \| Symbol: CAND1 \| similar to TBP-interacting protein 120A [Rattus norvegicus] (GB:NP_446456.1); similar to PREDICTED: TBP-interacting protein [Mus musculus] (GB:XP_125901.5); similar to TIP120 protein [Homo sapiens] (GB:AAH50341.1); similar to mKIAA0829 protein [Mus musculus] (GB:BAC98035.1); similar to hypothetical protein [Homo sapiens] (GB:CAB66744.1); contains InterPro domain TBP-interacting 120 (InterPro:IPR007723) \| chr2:689787-697595 FORWARD \| Aliases: ATCAND1, ETA2, T8K22.14, T8K22_14, TIP120 | 6.7 | 5.3 | 1.4 | 3.6 | 4.08% | 2.8 |
| 6827 | AT5G52910.1 \| Symbol: ATIM \| timeless family protein, contains Pfam domains PF05029: Timeless protein C terminal region and PF04821: Timeless protein | 4.3 | 3.7 | 0.6 | 3.6 | 4.08% | 2.6 |
| 6828 | AT2G42600.2 \| Symbol: ATPPC2 \| | 8.1 | 7.2 | 0.9 | 3.6 | 4.10% | 2.6 |
| 6829 | AT5G52450.1 \| MATE efflux protein-related, strong similarity to unknown protein (pir::T02324); contains Pfam profile PF01554 Uncharacterized membrane protein family \| chr5:21306125-21309088 REVERSE \| Aliases: K24M7.20, K24M7_20 | 7.2 | 5.9 | 1.2 | 3.6 | 4.10% | 2.8 |
| 6830 | ATCG00710.1 \| Symbol: PSBH \| Encodes a 8 kD phosphoprotein that is a component of the photosystem II oxygen evolving core. Its exact molecular function has not been determined but it may play a role in mediating electron transfer between the secondary quinone acceptors, QA and QB, associated with the acceptor side of PSII. \| chrC:74485-74706 FORWARD \| Aliases: PSBH | 6.4 | 5.0 | 1.4 | 3.6 | 4.10% | 2.7 |
| 6832 | AT2G27775.2 \| expressed protein \| chr2:11849812-11850370 REVERSE \| Aliases: None | 4.7 | 3.4 | 1.2 | 3.6 | 4.11% | 2.5 |
| 6834 | AT4G08280.1 \| expressed protein, hypothetical protein ssr1391 - Synechocystis sp. (strain PCC 6803),PIR2:S75571 \| chr4:5230323-5232128 FORWARD \| Aliases: T12G13.120, T12G13_120 | 5.8 | 4.2 | 1.5 | 3.6 | 4.11% | 2.8 |
| 6835 | AT4G28020.1 \| expressed protein, contains Pfam PF01980: Uncharacterised protein family | 5.0 | 4.4 | 0.6 | 3.6 | 4.11% | 2.7 |
| 6836 | AT5G16070.1 \| chaperonin, putative, similar to SWISS-PROT:P80317 T-complex protein 1, zeta subunit (TCP-1-zeta) (Mus musculus); contains Pfam:PF00118 domain, TCP-1/cpn60 chaperonin family \| chr5:5247357-5251182 REVERSE \| Aliases: F1N13.210, F1N13_210 | 9.2 | 7.3 | 1.8 | 3.6 | 4.11% | 2.9 |

| Rank | Description | Sync | Root | M | t | adj.q | B |
|---|---|---|---|---|---|---|---|
| 6837 | AT3G11770.1 \| expressed protein \| chr3:3721334-3722652 REVERSE \| Aliases: F26K24.6 | 8.3 | 7.5 | 0.8 | 3.6 | 4.11% | 2.7 |
| 6839 | AT1G28410.1 \| similar to myosin heavy chain-related [Arabidopsis thaliana] (TAIR:At4g31340.1); similar to putative stress related-like protein interactor [Oryza sativa (japonica cultivar-group)] (GB:XP_483357.1); similar to unknown [Oryza sativa (japonica cultivar-group)] (GB:AAO72581.1) \| chr1:9976168-9979253 REVERSE \| Aliases: F3M18.15, F3M18_15 | 4.8 | 3.8 | 1.0 | 3.6 | 4.12% | 2.6 |
| 6843 | AT1G68790.1 \| expressed protein \| chr1:25838372-25842982 REVERSE \| Aliases: F14K14.10, F14K14_10 | 9.5 | 7.5 | 2.0 | 3.6 | 4.13% | 2.7 |
| 6845 | AT4G01510.1 \| Symbol: ARV2 \| similar to transmembrane ARV1-like family protein [Arabidopsis thaliana] (TAIR:At1g01020.1); similar to hypothetical protein DDB0188786 [Dictyostelium discoideum] (GB:EAL62332.1); contains InterPro domain Arv1-like protein (InterPro:IPR007290) \| chr4:642733-644336 FORWARD \| Aliases: ARV2, F11O4.16, F11O4_16 | 5.2 | 4.3 | 0.9 | 3.6 | 4.13% | 2.7 |
| 6846 | AT4G20910.1 \| Symbol: HEN1 \| double-stranded RNA binding protein-related / DsRBD protein-related, contains weak similarity to Pfam profile PF00035: Double-stranded RNA binding motif | 3.6 | 3.0 | 0.6 | 3.6 | 4.13% | 2.6 |
| 6850 | AT5G60190.1 \| Ulp1 protease family protein, low similarity to sentrin/SUMO-specific protease (Homo sapiens) GI:6906859; contains Pfam profile PF02902: Ulp1 protease family, C-terminal catalytic domain \| chr5:24251780-24253381 FORWARD \| Aliases: F15L12.8, F15L12_8 | 3.5 | 3.0 | 0.6 | 3.6 | 4.14% | 2.6 |
| 6851 | AT3G59550.1 \| Symbol: SYN3 \| cohesion family protein SYN3 (SYN3), nearly identical to cohesion family protein SYN3 (Arabidopsis thaliana) GI:12006362; supporting cDNA gi:12006361:gb:AF281155.1:AF281155 \| chr3:22008031-22011655 FORWARD \| Aliases: T16L24.100 | 4.2 | 3.0 | 1.2 | 3.6 | 4.14% | 2.9 |
| 6853 | AT1G63210.1 \| expressed protein, contains Pfam profile: PF04283 protein of unknown function (DUF439) \| chr1:23447353-23451019 FORWARD \| Aliases: F16M19.22, F16M19_22 | 3.5 | 2.9 | 0.6 | 3.6 | 4.14% | 2.5 |
| 6854 | AT3G46440.2 \| Symbol: UXS5 \| similar to NAD-dependent epimerase/dehydratase family protein [Arabidopsis thaliana] (TAIR:At2g28760.2); similar to NAD-dependent epimerase/dehydratase family protein [Arabidopsis thaliana] (TAIR:At2g28760.1); similar to UDP-glucuronic acid decarboxylase (UXS3) [Arabidopsis thaliana] (TAIR:At5g59290.1); similar to UDP-D-glucuronate carboxy-lyase [Pisum sativum] (GB:BAB40967.1); contains InterPro domain NAD-dependent epimerase/dehydratase (InterPro:IPR001509) \| chr3:17100030-17102811 REVERSE \| Aliases: F18L15.160 | 11.0 | 8.5 | 2.5 | 3.6 | 4.14% | 2.8 |
| 6855 | AT3G03420.1 \| Ku70-binding family protein, similar to Ku70-binding protein GB:AAD31085 GI:4867999 (Homo sapiens); contains Prosite PS00142: Neutral zinc metallopeptidases, zinc-binding region signature \| chr3:812455-814034 FORWARD \| Aliases: T21P5.16, T21P5_16 | 6.2 | 5.0 | 1.3 | 3.6 | 4.14% | 2.8 |
| 6858 | AT1G20370.1 \| tRNA pseudouridine synthase family protein, similar to SP:Q9WU56 tRNA pseudouridine synthase A (EC 4.2.1.70) (Uracil hydrolyase) {Mus musculus}; contains Pfam profile PF01416: tRNA pseudouridine synthase \| chr1:7051567-7053631 REVERSE \| Aliases: F14O10.3, F14O10_3 | 4.0 | 3.4 | 0.6 | 3.6 | 4.15% | 2.5 |
| 6859 | AT1G50900.1 \| expressed protein \| chr1:18869874-18870872 FORWARD \| Aliases: F8A12.12, F8A12_12 | 4.3 | 2.9 | 1.3 | 3.6 | 4.15% | 2.8 |
| 6861 | AT4G29830.1 \| Symbol: VIP3 \| transducin family protein / WD-40 repeat family protein, contains 7 WD-40 repeats (PF00400); G protein beta subunit-like protein, Schistosoma mansoni, gb:U30261 | 7.7 | 6.8 | 0.9 | 3.6 | 4.15% | 2.8 |
| 6862 | AT2G45340.1 \| leucine-rich repeat transmembrane protein kinase, putative \| chr2:18698796-18701776 FORWARD \| Aliases: F4L23.15 | 4.0 | 3.2 | 0.9 | 3.6 | 4.15% | 2.7 |
| 6864 | AT3G51450.1 \| strictosidine synthase family protein, similar to hemomucin (Drosophila melanogaster)(GI:1280434), strictosidine synthase (Rauvolfia serpentina)(SP:P15324) \| chr3:19101947-19103482 FORWARD \| Aliases: F26O13.90 | 5.9 | 5.2 | 0.7 | 3.6 | 4.16% | 2.6 |
| 6868 | AT3G47630.2 \| similar to Hypothetical protein CBG04116 [Caenorhabditis briggsae] (GB:CAE60498.1) \| chr3:17574031-17576414 REVERSE \| Aliases: F1P2.180 | 4.9 | 4.2 | 0.7 | 3.6 | 4.17% | 2.7 |
| 6871 | AT1G18630.1 \| glycine-rich RNA-binding protein, putative, similar to glycine-rich RNA-binding protein from {Sorghum bicolor} SP:Q99070, GI:1778373 from (Pisum sativum); contains InterPro entry IPR000504: RNA-binding region RNP-1 (RNA recognition motif) (RRM) \| chr1:6414717-6416445 FORWARD \| Aliases: F25I16.4, F25I16_4 | 5.8 | 5.2 | 0.7 | 3.6 | 4.18% | 2.6 |
| 6872 | AT4G35230.1 \| protein kinase family protein, contains protein kinase domain, Pfam:PF00069 \| chr4:16755123-16758096 REVERSE \| Aliases: F23E12.210, F23E12_210 | 9.6 | 8.8 | 0.8 | 3.6 | 4.18% | 2.5 |
| 6873 | AT5G25590.1 \| expressed protein, contains Pfam profile PF04783: Protein of unknown function (DUF630) \| chr5:8906601-8909923 REVERSE \| Aliases: T14C9.130, T14C9_130 | 3.7 | 3.2 | 0.5 | 3.6 | 4.18% | 2.4 |
| 6874 | AT1G19800.3 \| Symbol: TGD1 \| expressed protein, contains Pfam PF02405: Domain of unknown function DUF140; similar to TOLUENE TOLERANCE PROTEIN TTG2B {Brucella melitensis 16M} (GI:17982923) \| chr1:6846363-6848087 FORWARD \| Aliases: F14P1.27, F14P1_27, TRIGALACTOSYLDIACYLGLYCEROL 1 | 6.5 | 5.0 | 1.5 | 3.6 | 4.19% | 2.6 |

| Rank | Description | Sync | Root | M | t | adj.q | B |
|---|---|---|---|---|---|---|---|
| 6875 | AT2G19900.1 \| malate oxidoreductase, putative, similar to NADP-dependent malic enzyme (EC 1.1.1.40) (NADP-ME) (SP:P51615) {Vitis vinifera} \| chr2:8598981-8602535 REVERSE \| Aliases: F6F22.7, F6F22_7 | 6.0 | 4.7 | 1.3 | 3.6 | 4.19% | 2.9 |
| 6878 | AT5G15760.1 \| plastid-specific 30S ribosomal protein 3, putative / PSRP-3, putative, similar to SP:P82412 Plastid-specific 30S ribosomal protein 3, chloroplast precursor (PSRP-3) {Spinacia oleracea}; contains Pfam profile PF04839: Plastid and cyanobacterial ribosomal protein (PSRP-3 / Ycf65) \| chr5:5143056-5144056 FORWARD \| Aliases: F14F8.140, F14F8_140 | 2.4 | 2.0 | 0.4 | 3.6 | 4.19% | 2.2 |
| 6879 | AT3G17310.2 \| methyltransferase family protein, similar to cytosine methyltransferase (GI:7658293) (Arabidopsis thaliana) \| chr3:5909007-5913248 REVERSE \| Aliases: MGD8.15 | 5.0 | 4.1 | 0.9 | 3.6 | 4.20% | 2.8 |
| 6880 | AT5G23080.2 \| expressed protein, similar to expressed protein [Arabidopsis thaliana] (TAIR:At5g53800.1); similar to hypothetical protein [Homo sapiens] (GB:CAD39124.1); contains InterPro domain SWAP/Surp (InterPro:IPR000061) \| chr5:7742648-7748931 REVERSE \| Aliases: MYJ24.7, MYJ24_7 | 7.0 | 5.1 | 2.0 | 3.6 | 4.20% | 2.9 |
| 6883 | AT1G77920.1 \| bZIP family transcription factor, contains Pfam profile: PF00170 bZIP transcription factor \| chr1:29303299-29305757 FORWARD \| Aliases: F28K19.13, F28K19_13 | 7.7 | 7.0 | 0.7 | 3.6 | 4.22% | 2.7 |
| 6885 | AT4G26200.1 \| Symbol: ACS7 \| 1-aminocyclopropane-1-carboxylate synthase, putative / ACC synthase, putative, similar to ACC synthase from Malus x domestica, GI:1658062 (U73816), Pyrus pyrifolia GI:4586411 \| chr4:13275316-13277197 FORWARD \| Aliases: T25K17.10, T25K17_10 | 5.5 | 4.3 | 1.2 | 3.6 | 4.22% | 2.9 |
| 6889 | AT5G57960.1 \| GTP-binding family protein, similar to SP:P25519 GTP-binding protein hflX {Escherichia coli} \| chr5:23480024-23483726 REVERSE \| Aliases: MTI20.22, MTI20_22 | 4.6 | 4.0 | 0.7 | 3.6 | 4.23% | 2.6 |
| 6893 | AT5G12910.1 \| histone H3, putative, similar to histone H3 from Mus musculus GI:51301, Gallus gallus GI:211859, Medicago sativa GI:166384, Pisum sativum SP:P02300; contains Pfam profile PF00125 Core histone H2A/H2B/H3/H4 \| chr5:4077165-4077560 FORWARD \| Aliases: T24H18.80, T24H18_80 | 2.8 | 2.3 | 0.5 | 3.6 | 4.23% | 2.4 |
| 6897 | AT5G41620.1 \| expressed protein, weak similarity to microtubule binding protein D-CLIP-190 (GI:2773363) (Drosophila melanogaster); weak similarity to Synaptonemal complex protein 1 (SCP-1 protein) (Swiss-Prot:Q15431) (Homo sapiens); weak similarity to DNA double-strand break repair rad50 ATPase. (Swiss-Prot:P58301) (Pyrococcus furiosus) \| chr5:16663666-16666196 FORWARD \| Aliases: MBK23.17, MBK23_17 | 6.5 | 5.3 | 1.1 | 3.6 | 4.24% | 2.7 |
| 6898 | AT3G23670.2 \| similar to phragmoplast-associated kinesin-related protein (PAKRP1) [Arabidopsis thaliana] (TAIR:At4g14150.1); similar to OSJNBa0038P21.12 [Oryza sativa (japonica cultivar-group)] (GB:CAE05519.1); contains InterPro domain Kinesin, motor region (InterPro:IPR001752) | 4.7 | 3.6 | 1.1 | 3.6 | 4.24% | 2.7 |
| 6899 | AT4G10890.1 \| expressed protein \| chr4:6688829-6693754 FORWARD \| Aliases: None | 4.2 | 3.5 | 0.7 | 3.6 | 4.24% | 2.5 |
| 6901 | AT5G15840.2 \| Symbol: CO \| similar to zinc finger protein CONSTANS-LIKE 1 (COL1) [Arabidopsis thaliana] (TAIR:At5g15850.1); similar to constans [Brassica napus] (GB:AAC27694.1); contains InterPro domain Zn-finger, CONSTANS type (InterPro:IPR002926); contains InterPro domain Zn-finger, B-box (InterPro:IPR000315) \| chr5:5171186-5172738 REVERSE \| Aliases: CONSTANS, F14F8.220, F14F8_220, FG | 3.3 | 2.8 | 0.4 | 3.6 | 4.24% | 2.2 |
| 6903 | AT1G01060.3 \| Symbol: LHY \| similar to myb-related transcription factor (CCA1) [Arabidopsis thaliana] (TAIR:At2g46830.1); similar to LHY protein [Phaseolus vulgaris] (GB:CAD12767.2); contains InterPro domain Myb DNA-binding domain (InterPro:IPR001005); contains InterPro domain Myb-like DNA-binding domain, SHAQKYF class (InterPro:IPR006447) | 4.1 | 3.0 | 1.1 | 3.6 | 4.25% | 2.7 |
| 6904 | AT4G08240.2 \| expressed protein \| chr4:5194672-5196758 FORWARD \| Aliases: T12G13.80, T12G13_80 | 5.6 | 4.3 | 1.4 | 3.6 | 4.26% | 2.7 |
| 6905 | AT1G19950.1 \| abscisic acid-responsive HVA22 family protein, weak similarity to SP:Q00765 Polyposis locus protein 1 (TB2 protein) {Homo sapiens}; contains Pfam profile PF03134: TB2/DP1, HVA22 family | 3.1 | 2.7 | 0.4 | 3.6 | 4.26% | 2.3 |
| 6906 | AT5G15910.1 \| dehydrogenase-related, low similarity to SP:Q9R1J0 NAD(P)-dependent steroid dehydrogenase (EC 1.1.1.-) {Mus musculus} \| chr5:5193140-5195399 FORWARD \| Aliases: F1N13.50, F1N13_50 | 8.9 | 8.3 | 0.6 | 3.6 | 4.26% | 2.4 |
| 6907 | AT4G16140.1 \| proline-rich family protein, contains proline-rich extensin domains, INTERPRO:IPR002965 \| chr4:9129058-9129853 FORWARD \| Aliases: DL4110W, FCAALL.289 | 3.5 | 2.8 | 0.7 | 3.6 | 4.27% | 2.7 |
| 6909 | AT3G11745.1 \| expressed protein \| chr3:3713769-3714585 REVERSE \| Aliases: None | 4.1 | 3.4 | 0.7 | 3.6 | 4.27% | 2.6 |
| 6910 | AT1G48140.1 \| dolichol-phosphate mannosyltransferase-related, contains weak similarity to Swiss-Prot:Q9P2X0 dolichol-phosphate mannosyltransferase subunit 3 (Dolichol-phosphate mannose synthase subunit 3, Dolichyl-phosphate beta-D- mannosyltransferase subunit 3, Mannose-P-dolichol synthase subunit 3, MPD synthase subunit 3, DPM synthase complex subunit 3, Prostin 1 (Homo sapiens) | 8.7 | 7.8 | 0.8 | 3.6 | 4.27% | 2.6 |

| Rank | Description | Sync | Root | M | t | adj.q | B |
|---|---|---|---|---|---|---|---|
| 6911 | AT5G53000.1 \| Symbol: TAP46 \| protein phosphatase 2A-associated 46 kDa protein / PP2A regulatory subunit (TAP46), identical to PP2A regulatory subunit (46 kDa protein phosphatase 2A-associated protein) (TAP46) (Arabidopsis thaliana) GI:5107033; contains Pfam profile PF04177: TAP42-like family | 7.5 | 6.2 | 1.2 | 3.6 | 4.27% | 2.7 |
| 6915 | AT3G46580.1 \| Symbol: MBD5 \| methyl-CpG-binding domain-containing protein, weak similarity to SP:P51608 Methyl-CpG-binding protein 2 (MeCP-2 protein) (MeCP2) {Homo sapiens}; contains Pfam profile PF01429: Methyl-CpG binding domain \| chr3:17159327-17160574 FORWARD \| Aliases: F12A12.100 | 5.2 | 4.2 | 1.0 | 3.6 | 4.29% | 2.8 |
| 6917 | AT4G27700.1 \| rhodanese-like domain-containing protein, contains rhodanese-like domain PF00581 | 6.0 | 5.4 | 0.7 | 3.6 | 4.30% | 2.7 |
| 6918 | AT1G09470.1 \| expressed protein, ; expression supported by MPSS \| chr1:3055393-3056933 REVERSE \| Aliases: F14J9.13, F14J9_13 | 4.4 | 3.3 | 1.1 | 3.6 | 4.30% | 2.8 |
| 6919 | AT1G69070.1 \| expressed protein \| chr1:25970975-25975052 REVERSE \| Aliases: F4N2.4, F4N2_4 | 4.2 | 3.6 | 0.6 | 3.6 | 4.30% | 2.5 |
| 6921 | AT3G07640.1 \| expressed protein \| chr3:2438144-2439847 FORWARD \| Aliases: MLP3.9 | 6.3 | 4.8 | 1.4 | 3.6 | 4.30% | 2.8 |
| 6922 | AT1G74850.1 \| pentatricopeptide (PPR) repeat-containing protein, contains Pfam profile PF01535: PPR repeat \| chr1:28122510-28125975 REVERSE \| Aliases: F25A4.18, F25A4_18 | 4.3 | 3.8 | 0.5 | 3.6 | 4.30% | 2.5 |
| 6925 | AT1G72370.2 \| Symbol: P40 \| similar to 40S ribosomal protein SA (RPSaB) [Arabidopsis thaliana] (TAIR:At3g04770.2); similar to RSSA_DAUCA 40S ribosomal protein SA (p40) (GB:O80377); contains InterPro domain Ribosomal protein S2, eukaryotic and archaeal form (InterPro:IPR005707); contains InterPro domain Ribosomal protein S2 (InterPro:IPR001865) \| chr1:27246607-27248589 REVERSE \| Aliases: AP40, P40, RP40, RPSAA, T10D10.16, T10D10_16 | 10.7 | 8.8 | 1.9 | 3.6 | 4.32% | 2.7 |
| 6926 | AT1G75010.1 \| similar to phosphatidylinositol-4-phosphate 5-kinase family protein [Arabidopsis thaliana] (TAIR:At1g60890.1); similar to putative phosphatidylinositol-4-phosphate 5-kinase [Bacteroides thetaiotaomicron VPI-5482] (GB:AAO75854.1); contains InterPro domain MORN motif (InterPro:IPR003409) \| chr1:28168501-28173705 REVERSE \| Aliases: F25A4.3, F25A4_3 | 5.1 | 4.1 | 1.0 | 3.6 | 4.32% | 2.8 |
| 6927 | AT3G60740.1 \| Symbol: TTN1 \| tubulin folding cofactor D, identical to tubulin folding cofactor D GI:20514263 from (Arabidopsis thaliana) \| chr3:22458244-22464339 REVERSE \| Aliases: CHAMPIGNON, CHO, EMB133, EMBRYO DEFECTIVE 133, T4C21.150, TFC D, TITAN 1, TTN1, TUBULIN FOLDING COFACTOR D | 4.2 | 3.8 | 0.4 | 3.6 | 4.32% | 2.1 |
| 6929 | AT1G02800.1 \| Symbol: ATCEL2 \| | 4.7 | 3.7 | 1.0 | 3.6 | 4.33% | 2.8 |
| 6930 | AT3G12930.1 \| expressed protein, contains Pfam domain PF02410: Domain of unknown function DUF143 \| chr3:4128414-4129963 FORWARD \| Aliases: MGH6.4 | 4.4 | 3.6 | 0.8 | 3.6 | 4.33% | 2.8 |
| 6932 | AT2G01760.1 \| Symbol: ARR14 \| two-component responsive regulator family protein / response regulator family protein, similar to response regulator 9 (GI:14189890) (Zea mays); similar to ARR1 protein (GI:4210449) (Arabidopsis thaliana); contains Pfam profile: PF00072 response regulator receiver domain | 3.3 | 2.7 | 0.5 | 3.6 | 4.33% | 2.5 |
| 6933 | AT1G62040.1 \| autophagy 8c (APG8c), identical to autophagy 8c (Arabidopsis thaliana) GI:19912155; contains Pfam profile PF02991: Microtubule associated protein 1A/1B, light chain 3 \| chr1:22936566-22938088 FORWARD \| Aliases: F8K4.23, F8K4_23 | 9.0 | 8.2 | 0.8 | 3.6 | 4.33% | 2.6 |
| 6934 | AT1G74960.2 \| Symbol: FAB1 \| 3-ketoacyl-ACP synthase, putative, similar to 3-ketoacyl-ACP synthase (Cuphea pulcherrima) gi:3800747:gb:AAC68860; identical to cDNA beta-ketoacyl-ACP synthetase 2 nuclear gene for plastid product GI:14582700 | 7.7 | 7.0 | 0.7 | 3.6 | 4.33% | 2.6 |
| 6936 | AT1G74370.1 \| zinc finger (C3HC4-type RING finger) family protein, contains Pfam profile: PF00097 zinc finger, C3HC4 type (RING finger) \| chr1:27961323-27963018 REVERSE \| Aliases: F1M20.5, F1M20_5 | 6.3 | 5.5 | 0.8 | 3.6 | 4.34% | 2.7 |
| 6937 | AT1G18360.1 \| hydrolase, alpha/beta fold family protein, similar to monoglyceride lipase from (Homo sapiens) GI:14594904, (Mus musculus) GI:2632162,(Rattus norvegicus) GI:19697886; contains Pfam profile PF00561: hydrolase, alpha/beta fold family \| chr1:6316688-6319420 REVERSE \| Aliases: F15H18.13, F15H18_13 | 5.2 | 4.1 | 1.2 | 3.6 | 4.34% | 2.7 |
| 6939 | AT5G39730.1 \| avirulence-responsive protein-related / avirulence induced gene (AIG) protein-related, similar to SP:P54121 AIG2 protein {Arabidopsis thaliana} \| chr5:15918907-15920057 FORWARD \| Aliases: MKM21.20, MKM21_20 | 7.7 | 5.1 | 2.7 | 3.6 | 4.34% | 2.8 |
| 6940 | AT4G38680.1 \| Symbol: GRP2 \| cold-shock DNA-binding family protein, contains Pfam domains PF00313: 'Cold-shock' DNA-binding domain and PF00098: Zinc knuckle \| chr4:18071878-18073176 REVERSE \| Aliases: GRP 2, T9A14.9 | 6.2 | 5.2 | 1.0 | 3.6 | 4.34% | 2.7 |
| 6941 | AT5G17610.1 \| expressed protein \| chr5:5803964-5805254 FORWARD \| Aliases: K10A8.90, K10A8_90 | 7.7 | 7.1 | 0.6 | 3.6 | 4.35% | 2.5 |
| 6942 | AT4G39630.1 \| expressed protein \| chr4:18397589-18399381 REVERSE \| Aliases: F23K16.7 | 4.6 | 3.8 | 0.9 | 3.6 | 4.35% | 2.7 |
| 6943 | AT1G61065.1 \| expressed protein \| chr1:22493909-22495126 REVERSE \| Aliases: None | 7.4 | 6.4 | 1.0 | 3.6 | 4.35% | 2.7 |

| Rank | Description | Sync | Root | M | t | adj.q | B |
|---|---|---|---|---|---|---|---|
| 6946 | AT5G57860.3 \| ubiquitin family protein, contains Pfam profile PF00240: Ubiquitin family \| chr5:23454871-23456538 FORWARD \| Aliases: MTI20.11, MTI20_11 | 5.5 | 4.4 | 1.2 | 3.6 | 4.35% | 2.4 |
| 6949 | AT1G49880.1 \| Erv1/Alr family protein, similar to SP:Q63042 Augmenter of liver regeneration {Rattus norvegicus}; contains Pfam profile PF04777: Erv1 / Alr family \| chr1:18467685-18469745 FORWARD \| Aliases: F10F5.3, F10F5_3 | 3.3 | 2.9 | 0.4 | 3.6 | 4.35% | 2.1 |
| 6951 | AT1G20823.1 \| zinc finger (C3HC4-type RING finger) family protein, contains Pfam domain, PF00097: Zinc finger, C3HC4 type (RING finger) \| chr1:7238789-7239658 FORWARD \| Aliases: F2D10.34, F2D10_34 | 7.7 | 6.4 | 1.2 | 3.6 | 4.36% | 2.8 |
| 6953 | AT5G40960.1 \| expressed protein \| chr5:16429973-16430179 REVERSE \| Aliases: MEE6.3, MEE6_3 | 8.7 | 7.4 | 1.3 | 3.6 | 4.36% | 2.7 |
| 6954 | AT3G48150.1 \| Symbol: APC8 \| cell division cycle family protein / CDC family protein, similar to cell division cycle protein 23 (Homo sapiens) GI:3283051, anaphase-promoting complex subunit 8 (Homo sapiens) GI:6180017; contains Pfam profile PF00515: TPR Domain \| chr3:17790641-17793630 REVERSE \| Aliases: CDC23, T24C20.30 | 6.5 | 5.7 | 0.9 | 3.6 | 4.36% | 2.6 |
| 6955 | AT3G62620.1 \| sucrose-phosphatase-related, contains weak similarity to sucrose-phosphatase (Arabidopsis thaliana) gi:11127757:gb:AAG31075 \| chr3:23170779-23172830 FORWARD \| Aliases: F26K9.50 | 3.9 | 3.3 | 0.6 | 3.6 | 4.36% | 2.5 |
| 6956 | AT3G15020.1 \| malate dehydrogenase (NAD), mitochondrial, putative, similar to mitochondrial NAD-dependent malate dehydrogenase GB:CAA10320 SP:Q9ZP06 (Arabidopsis thaliana); contains InterPro entry IPR001236: Lactate/malate dehydrogenase \| chr3:5056083-5058255 FORWARD \| Aliases: K15M2.16 | 9.7 | 7.9 | 1.7 | 3.6 | 4.36% | 2.7 |
| 6958 | AT3G53620.1 \| inorganic pyrophosphatase, putative (soluble) / pyrophosphate phospho-hydrolase, putative / PPase, putative, similar to magnesium dependent soluble inorganic pyrophosphatase (Solanum tuberosum) GI:2706450; contains Pfam profile PF00719: inorganic pyrophosphatase \| chr3:19891482-19894648 FORWARD \| Aliases: F4P12.320 | 7.1 | 5.1 | 2.0 | 3.6 | 4.37% | 2.8 |
| 6959 | AT5G64620.1 \| Symbol: C/VIF2 \| | 4.7 | 3.8 | 0.9 | 3.6 | 4.38% | 2.8 |
| 6960 | AT1G27310.1 \| nuclear transport factor 2 (NTF2), putative, similar to Swiss-Prot:P33331 nuclear transport factor 2 (NTF-2) (Nuclear transport factor P10) (Saccharomyces cerevisiae) \| chr1:9484338-9485883 REVERSE \| Aliases: F17L21.10, F17L21_10 | 7.0 | 4.7 | 2.3 | 3.5 | 4.38% | 2.8 |
| 6961 | AT2G30160.1 \| mitochondrial substrate carrier family protein, contains Pfam profile: PF00153 mitochondrial carrier protein \| chr2:12884915-12886726 FORWARD \| Aliases: T27E13.10, T27E13_10 | 5.4 | 4.4 | 1.1 | 3.5 | 4.39% | 2.8 |
| 6963 | AT3G27430.2 \| Symbol: PBB1 \| 20S proteasome beta subunit B (PBB1), identical to 20S proteasome beta subunit PBB1 (PBB1) GB:AAC32066 (Arabidopsis thaliana) (Genetics 149 (2), 677-692 (1998)); contains Pfam profile: PF00227 proteasome A-type and B-type; \| chr3:10153754-10156579 FORWARD \| Aliases: 20S PROTEASOME BETA SUBUNIT PBB1, K1G2.26 | 9.3 | 6.4 | 2.8 | 3.5 | 4.39% | 2.8 |
| 6965 | AT4G33650.1 \| Symbol: ADL2 \| dynamin-like protein 2a (ADL2a), identical to dynamin like protein 2a (ADL2a) (Arabidopsis thaliana) GI:19032337; supported by cDNA gi:19032336 \| chr4:16160938-16166932 FORWARD \| Aliases: ADL2A, ARABIDOPSIS DYNAMIN LIKE 2, T16L1.140, T16L1_140 | 6.4 | 5.4 | 1.0 | 3.5 | 4.40% | 2.6 |
| 6967 | AT1G68410.2 \| similar to protein phosphatase 2C-related / PP2C-related [Arabidopsis thaliana] (TAIR:At1g09160.2); similar to protein phosphatase 2C-related / PP2C-related [Arabidopsis thaliana] (TAIR:At1g09160.1); similar to protein phosphatase 2C-like [Oryza sativa (japonica cultivar-group)] (GB:XP_479610.1); contains InterPro domain Protein phosphatase 2C-like (InterPro:IPR001932) \| chr1:25653662-25656682 REVERSE \| Aliases: T2E12.9, T2E12_9 | 6.8 | 6.0 | 0.8 | 3.5 | 4.40% | 2.7 |
| 6968 | AT3G18870.1 \| mitochondrial transcription termination factor-related / mTERF-related, contains Pfam profile PF02536: mTERF \| chr3:6508226-6509349 REVERSE \| Aliases: MCB22.4 | 2.9 | 2.5 | 0.4 | 3.5 | 4.40% | 2.3 |
| 6969 | AT1G14060.1 \| expressed protein \| chr1:4817141-4818017 REVERSE \| Aliases: None | 5.8 | 4.9 | 0.9 | 3.5 | 4.40% | 2.8 |
| 6971 | AT1G16280.1 \| DEAD/DEAH box helicase, putative, similar to gb:L13612 DEAD-box protein (dbp45A) from Drosophila melanogaster and is a member of PF:00270 DEAD/DEAH box helicase family \| chr1:5568476-5570481 REVERSE \| Aliases: F3O9.8, F3O9_8 | 4.6 | 3.5 | 1.1 | 3.5 | 4.41% | 2.6 |
| 6973 | AT2G17265.1 \| Symbol: HSK \| homoserine kinase (HSK), identical to homoserine kinase (Arabidopsis thaliana) gi:4927412:gb:AAD33097 \| chr2:7515670-7516837 FORWARD \| Aliases: F5J6.24, F5J6_24, HOMOSERINE KINASE | 9.2 | 7.0 | 2.2 | 3.5 | 4.41% | 2.7 |
| 6975 | AT1G55510.1 \| Symbol: BCDH BETA1 \| 2-oxoisovalerate dehydrogenase, putative / 3-methyl-2-oxobutanoate dehydrogenase, putative / branched-chain alpha-keto acid dehydrogenase E1 beta subunit, putative, strong similarity to branched chain alpha-keto acid dehydrogenase E1 beta subunit (Arabidopsis thaliana) GI:7021286; contains Pfam profiles PF02779: Transketolase, pyridine binding domain, PF02780: Transketolase, C-terminal domain \| chr1:20727016-20729284 FORWARD \| Aliases: BRANCHED CHAIN ALPHA KETO ACID DECARBOXYLASE E1 BETA SUBUNIT, T5A14.9, T5A14_9 | 6.9 | 6.0 | 1.0 | 3.5 | 4.41% | 2.6 |

| Rank | Description | Sync | Root | M | t | adj.q | B |
|---|---|---|---|---|---|---|---|
| 6976 | AT2G44620.1 \| Symbol: mtACP 1 \| acyl carrier protein, mitochondrial / ACP / NADH-ubiquinone oxidoreductase 9.6 kDa subunit, identical to SP:P53665 Acyl carrier protein, mitochondrial precursor (ACP) (NADH-ubiquinone oxidoreductase 9.6 kDa subunit) (MtACP-1) {Arabidopsis thaliana}; identical to cDNA acyl carrier protein precursor GI:468265 \| chr2:18421359-18422310 FORWARD \| Aliases: F16B22.11, F16B22.28, F16B22_28, MITOCHONDRIAL ACYL CARRIER PROTEIN 1 | 9.0 | 7.3 | 1.6 | 3.5 | 4.41% | 2.7 |
| 6981 | AT2G03780.1 \| translin family protein, similar to SP:Q99598 Translin-associated protein X (Translin-associated factor X) {Homo sapiens}; contains Pfam profile PF01997: Translin family \| chr2:1152507-1154284 REVERSE \| Aliases: F19B11.23, F19B11_23 | 4.0 | 3.5 | 0.5 | 3.5 | 4.42% | 2.3 |
| 6985 | AT5G24650.1 \| mitochondrial import inner membrane translocase subunit Tim17/Tim22/Tim23 family protein, contains Pfam PF02466: Mitochondrial import inner membrane translocase subunit Tim17 \| chr5:8437088-8439107 FORWARD \| Aliases: K18P6.19, K18P6_19 | 7.5 | 5.6 | 1.9 | 3.5 | 4.44% | 2.7 |
| 6986 | AT5G51600.1 \| Symbol: PLE \| microtubule associated protein (MAP65/ASE1) family protein, low similarity to SP:P50275 Anaphase spindle elongation protein {Saccharomyces cerevisiae}, protein regulating cytokinesis 1 (PRC1) (Homo sapiens) GI:2865521; contains Pfam profile PF03999: Microtubule associated protein (MAP65/ASE1 family) \| chr5:20978137-20981306 REVERSE \| Aliases: ATMAP65 3, K17N15.15, K17N15_15, PLEIADE | 4.2 | 3.4 | 0.8 | 3.5 | 4.44% | 2.7 |
| 6987 | AT1G79150.1 \| expressed protein, ; expression supported by MPSS \| chr1:29779996-29782767 REVERSE \| Aliases: YUP8H12R.24, YUP8H12R_24 | 7.2 | 5.8 | 1.5 | 3.5 | 4.44% | 2.5 |
| 6988 | AT3G16175.1 \| thioesterase family protein, contains Pfam profile PF03061: thioesterase family protein \| chr3:5480470-5482048 FORWARD \| Aliases: None | 3.4 | 2.7 | 0.7 | 3.5 | 4.44% | 2.4 |
| 6989 | AT3G46230.1 \| Symbol: ATHSP17.4 \| 17.4 kDa class I heat shock protein (HSP17.4-CI), identical to 17.4 kDa class I heat shock protein SP:P19036 from (Arabidopsis thaliana) \| chr3:16994886-16995826 REVERSE \| Aliases: F12M12.200, SMALL HEAT SHOCK PROTEIN 17.4 | 5.8 | 4.8 | 1.0 | 3.5 | 4.44% | 2.7 |
| 6990 | AT4G31210.1 \| DNA topoisomerase family protein, similar to DNA Topoisomerase I (SP:Q9X3X7) {Zymomonas mobilis} | 4.6 | 3.7 | 0.9 | 3.5 | 4.45% | 2.7 |
| 6991 | AT2G07690.1 \| minichromosome maintenance family protein / MCM family protein, similar to SP:P55862 DNA replication licensing factor MCM5 (CDC46 homolog) {Xenopus laevis}; contains Pfam profile PF00493: MCM2/3/5 family \| chr2:3523243-3527428 REVERSE \| Aliases: T12J2.1 | 5.3 | 4.7 | 0.6 | 3.5 | 4.45% | 2.5 |
| 6992 | AT1G08270.2 \| expressed protein, low similarity to SP:P46467 SKD1 protein (Vacuolar sorting protein 4b) {Mus musculus} | 3.8 | 3.4 | 0.4 | 3.5 | 4.45% | 2.1 |
| 6993 | AT1G20700.1 \| homeobox-leucine zipper family protein, strong similarity to homeodomain protein PALE-2 (GI:20152544) (Arabidopsis thaliana); contains Pfam profile: PF00046 homeobox domain \| chr1:7182548-7183999 FORWARD \| Aliases: F2D10.19, F2D10_19 | 4.9 | 4.0 | 0.9 | 3.5 | 4.45% | 2.7 |
| 6999 | AT3G59580.2 \| similar to RWP-RK domain-containing protein [Arabidopsis thaliana] (TAIR:At2g43500.1); similar to OSJNBa0067K08.5 [Oryza sativa (japonica cultivar-group)] (GB:XP_473028.1); contains InterPro domain Plant regulator RWP-RK (InterPro:IPR003035); contains InterPro domain Octicosapeptide/Phox/Bem1p (InterPro:IPR000270) \| chr3:22020190-22023743 FORWARD \| Aliases: T16L24.130 | 3.7 | 3.0 | 0.7 | 3.5 | 4.46% | 2.6 |
| 7000 | AT1G16480.1 \| pentatricopeptide (PPR) repeat-containing protein, contains Pfam profile PF01535: PPR repeat \| chr1:5625696-5628562 REVERSE \| Aliases: F3O9.28, F3O9_28 | 3.3 | 2.8 | 0.5 | 3.5 | 4.47% | 2.3 |
| 7001 | AT5G18950.1 \| pentatricopeptide (PPR) repeat-containing protein, contains Pfam profile PF01535: PPR repeat \| chr5:6328521-6329972 REVERSE \| Aliases: F17K4.200, F17K4_200 | 6.2 | 5.3 | 0.8 | 3.5 | 4.47% | 2.5 |
| 7003 | AT3G06810.1 \| acyl-CoA dehydrogenase-related, low similarity to acyl-CoA dehydrogenase (Acinetobacter sp. NCIMB9871) GI:14587418; contains Pfam profiles PF01636: Phosphotransferase enzyme family, PF00441: Acyl-CoA dehydrogenase C-terminal domain, PF02770: Acyl-CoA dehydrogenase middle domain \| chr3:2146398-2150837 FORWARD \| Aliases: AT3G06800, F3E22.5 | 4.5 | 3.7 | 0.8 | 3.5 | 4.48% | 2.5 |
| 7005 | AT2G41840.1 \| 40S ribosomal protein S2 (RPS2C) | 10.5 | 9.2 | 1.2 | 3.5 | 4.49% | 2.5 |
| 7006 | AT4G38050.1 \| similar to xanthine/uracil permease family protein [Arabidopsis thaliana] (TAIR:At2g27810.1); similar to PREDICTED P0477A12.37 gene product [Oryza sativa (japonica cultivar-group)] (GB:XP_506346.1); contains InterPro domain Xanthine/uracil/vitamin C permease family (InterPro:IPR006043) \| chr4:17869374-17872476 REVERSE \| Aliases: F20D10.170, F20D10_170 | 4.3 | 3.5 | 0.8 | 3.5 | 4.49% | 2.6 |
| 7007 | AT3G20050.1 \| Symbol: ATTCP 1 \| T-complex protein 1 alpha subunit / TCP-1-alpha / chaperonin (CCT1), identical to SWISS-PROT:P28769- T-complex protein 1, alpha subunit (TCP-1-alpha) (Arabidopsis thaliana) \| chr3:6998260-7002407 REVERSE \| Aliases: MAL21.5, MAL21_5 | 9.3 | 7.5 | 1.9 | 3.5 | 4.49% | 2.7 |
| 7008 | AT2G46610.2 \| arginine/serine-rich splicing factor, putative, similar to SP:P92964 Arginine/serine-rich splicing factor RSP31 {Arabidopsis thaliana} \| chr2:19143672-19145751 REVERSE \| Aliases: F13A10.14 | 6.5 | 5.1 | 1.4 | 3.5 | 4.50% | 2.8 |
| 7011 | AT5G10190.1 \| transporter-related, low similarity to spinster membrane proteins from (Drosophila melanogaster) GI:12003974, GI:12003976, GI:12003972, GI:12003970; contains Pfam profile PF00083: major facilitator superfamily protein \| chr5:3199134-3201364 FORWARD \| Aliases: F18D22.5 | 5.7 | 4.3 | 1.4 | 3.5 | 4.50% | 2.6 |

| Rank | Description | Sync | Root | M | t | adj.q | B |
|---|---|---|---|---|---|---|---|
| 7013 | AT2G17390.1 \| ankyrin repeat family protein, contains ankyrin repeats, Pfam:PF00023 \| chr2:7562732-7565120 FORWARD \| Aliases: F5J6.15, F5J6_15 | 11.1 | 10.4 | 0.7 | 3.5 | 4.50% | 2.1 |
| 7014 | AT2G21290.1 \| expressed protein \| chr2:9119937-9120411 FORWARD \| Aliases: F3K23.5, F3K23_5 | 10.8 | 10.1 | 0.7 | 3.5 | 4.50% | 2.1 |
| 7015 | AT5G15050.1 \| glycosyltransferase family 14 protein / core-2/I-branching enzyme family protein, contains Pfam profile: PF02485 Core-2/I-Branching enzyme \| chr5:4871642-4873601 REVERSE \| Aliases: F2G14.170, F2G14_170 | 5.6 | 4.6 | 0.9 | 3.5 | 4.51% | 2.6 |
| 7016 | AT5G61510.1 \| NADP-dependent oxidoreductase, putative, similar to zeta-crystallin homolog TED2 from Zinnia elegans (gi:531096); contains Pfam zinc-binding dehydrogenase domain PF00107 \| chr5:24754171-24756224 REVERSE \| Aliases: K11J9.5, K11J9_5 | 10.1 | 8.8 | 1.3 | 3.5 | 4.51% | 2.6 |
| 7017 | AT1G61770.1 \| DNAJ heat shock N-terminal domain-containing protein, similar to SP:Q9UBS4 DnaJ homolog subfamily B member 11 precursor Homo sapiens; contains Pfam profile PF00226 DnaJ domain \| chr1:22813830-22816272 FORWARD \| Aliases: T13M11.13, T13M11_13 | 8.0 | 7.4 | 0.7 | 3.5 | 4.51% | 2.5 |
| 7018 | AT2G38880.6 \| Symbol: HAP3A \| similar to CCAAT-box binding transcription factor subunit B (NF-YB) (HAP3 ) (AHAP3) family [Arabidopsis thaliana] (TAIR:At4g14540.1); similar to CAAT-box DNA binding protein subunit B (NF-YB) [Zea mays] (GB:CAA42234.1); contains InterPro domain Transcription factor CBF/NF-Y/archaeal histone (InterPro:IPR003958); contains InterPro domain Histone-like transcription factor/archaeal histone/topoisomerase (InterPro:IPR003957); contains InterPro domain Histone-fold/TFIID-TAF/NF-Y (InterPro:IPR007124); contains InterPro domain Histone-like transcription factor CBF/NF-Y/archaeal histone, subunit A (InterPro:IPR003956) \| chr2:16245570-16247833 FORWARD \| Aliases: ATHAP3, HAP3, T7F6.5, T7F6_5 | 7.1 | 6.2 | 0.9 | 3.5 | 4.51% | 2.6 |
| 7019 | AT1G77470.1 \| replication factor C 36 kDA, putative, similar to SWISS-PROT:P40937 activator 1 36 kDa subunit (Replication factor C 36 kDa subunit, A1 36 kDa subunit, RF-C 36 kDa subunit, RFC36) (Homo sapiens) \| chr1:29116842-29119329 REVERSE \| Aliases: T5M16.6, T5M16_6 | 5.8 | 5.1 | 0.7 | 3.5 | 4.51% | 2.5 |
| 7020 | AT3G04680.2 \| pre-mRNA cleavage complex family protein, similar to Pre-mRNA cleavage complex II protein Clp1 (Swiss-Prot:Q92989) (Homo sapiens) \| chr3:1269859-1272922 REVERSE \| Aliases: F7O18.15, F7O18_15 | 6.6 | 5.8 | 0.8 | 3.5 | 4.51% | 2.7 |
| 7021 | AT5G38450.1 \| Symbol: CYP735A1 \| cytochrome P450 family protein, similar to cytochrome P450 72A1 (SP:Q05047) (Catharanthus roseus) \| chr5:15410984-15414509 REVERSE \| Aliases: MXI10.18, MXI10_18 | 3.3 | 2.9 | 0.4 | 3.5 | 4.51% | 2.2 |
| 7023 | AT3G24570.1 \| peroxisomal membrane 22 kDa family protein, contains Mpv17 / PMP22 family domain, Pfam:PF04117 \| chr3:8966451-8968662 REVERSE \| Aliases: MOB24.15 | 7.5 | 6.4 | 1.0 | 3.5 | 4.52% | 2.5 |
| 7028 | AT5G20810.2 \| similar to auxin-responsive protein-related [Arabidopsis thaliana] (TAIR:At3g43120.1); similar to auxin-induced protein-like [Oryza sativa (japonica cultivar-group)] (GB:BAD36439.1); contains InterPro domain Auxin responsive SAUR protein (InterPro:IPR003676) \| chr5:7043900-7045714 FORWARD \| Aliases: T1M15.210, T1M15_210 | 3.1 | 2.7 | 0.4 | 3.5 | 4.52% | 2.2 |
| 7029 | AT1G71140.1 \| MATE efflux family protein, similar to ripening regulated protein DDTFR18 (Lycopersicon esculentum) GI:12231296; contains Pfam profile PF01554: Uncharacterized membrane protein family \| chr1:26828424-26830410 FORWARD \| Aliases: F23N20.13, F23N20_13 | 4.4 | 3.6 | 0.8 | 3.5 | 4.53% | 2.6 |
| 7031 | AT5G65270.1 \| Ras-related GTP-binding family protein, similar to GTP-binding protein RAB11A GI:1370142 from (Lotus japonicus); contains Pfam profile: PF00071 Ras family \| chr5:26100602-26101940 FORWARD \| Aliases: MQN23.22, MQN23_22 | 9.3 | 7.3 | 2.0 | 3.5 | 4.53% | 2.6 |
| 7034 | AT2G29580.1 \| zinc finger (CCCH-type) family protein / RNA recognition motif (RRM)-containing protein, similar to SP:O59800 Cell cycle control protein cwf5 {Schizosaccharomyces pombe}; contains Pfam profile: PF00076 RNA recognition motif (aka RRM, RBD, or RNP domain) \| chr2:12659000-12661413 FORWARD \| Aliases: F16P2.4, F16P2_4 | 4.1 | 3.4 | 0.7 | 3.5 | 4.53% | 2.5 |
| 7036 | AT5G43690.1 \| sulfotransferase family protein, similar to steroid sulfotransferase 3 (Brassica napus) GI:3420008, steroid sulfotransferase 1 (Brassica napus) GI:3420004; contains Pfam profile PF00685: Sulfotransferase domain \| chr5:17563500-17564561 FORWARD \| Aliases: MQD19.2, MQD19_2 | 5.0 | 4.0 | 1.0 | 3.5 | 4.53% | 2.7 |
| 7038 | AT3G14890.2 \| phosphoesterase, identical to phosphoesterase (Arabidopsis thaliana) GI:21630064; contains Pfam profile PF00645: Poly(ADP-ribose) polymerase and DNA-Ligase Zn-finger region \| chr3:5008828-5013272 FORWARD \| Aliases: K15M2.3 | 5.4 | 3.9 | 1.4 | 3.5 | 4.53% | 2.8 |
| 7039 | AT5G62200.1 \| embryo-specific protein-related, contains weak similarity to embryo-specific protein 3 (GI:3335171) (Arabidopsis thaliana) \| chr5:25001494-25002900 REVERSE \| Aliases: MMI9.3, MMI9_3 | 9.2 | 7.3 | 1.9 | 3.5 | 4.54% | 2.7 |
| 7040 | AT3G52230.1 \| expressed protein \| chr3:19382265-19383653 FORWARD \| Aliases: T25B15.4 | 7.8 | 5.0 | 2.8 | 3.5 | 4.54% | 2.7 |
| 7041 | AT3G20520.1 \| glycerophosphoryl diester phosphodiesterase family protein, contains Pfam PF03009 : Glycerophosphoryl diester phosphodiesterase family; similar to glycerophosphodiester phosphodiesterase (GI:1399038) (Borrelia hermsii) \| chr3:7162834-7165865 FORWARD \| Aliases: K10D20.6 | 3.5 | 3.0 | 0.5 | 3.5 | 4.54% | 2.4 |

| Rank | Description | Sync | Root | M | t | adj.q | B |
|---|---|---|---|---|---|---|---|
| 7044 | AT1G23070.1 \| expressed protein, contains Pfam profile PF03619: Domain of unknown function \| chr1:8174000-8175747 REVERSE \| Aliases: T26J12.15, T26J12_15 | 3.8 | 3.1 | 0.7 | 3.5 | 4.54% | 2.4 |
| 7045 | AT5G48730.1 \| pentatricopeptide (PPR) repeat-containing protein, contains Pfam profile PF01535: PPR repeat \| chr5:19780378-19782362 FORWARD \| Aliases: K24G6.6, K24G6_6 | 4.8 | 3.8 | 1.0 | 3.5 | 4.54% | 2.7 |
| 7046 | AT3G27050.1 \| expressed protein \| chr3:9979396-9980901 REVERSE \| Aliases: MOJ10.12 | 8.4 | 7.4 | 1.0 | 3.5 | 4.54% | 2.5 |
| 7047 | AT1G07640.3 \| Symbol: OBP2 \| similar to Dof-type zinc finger domain-containing protein [Arabidopsis thaliana] (TAIR:At2g28810.1); similar to putative DNA-binding protein [Oryza sativa (japonica cultivar-group)] (GB:XP_470142.1); contains InterPro domain Zn-finger, Dof type (InterPro:IPR003851) \| chr1:2354351-2356224 REVERSE \| Aliases: F24B9.30, F24B9_30, ZINC FINGER PROTEIN OBP2 | 6.3 | 5.5 | 0.8 | 3.5 | 4.54% | 2.7 |
| 7049 | AT2G34450.2 \| similar to high mobility group protein beta1 (HMGbeta1) / HMG protein beta1 [Arabidopsis thaliana] (TAIR:At1g20693.1); similar to high mobility group protein beta2 (HMGbeta2) / HMG protein beta2 [Arabidopsis thaliana] (TAIR:At1g20696.1); similar to OSJNBb0063G05.2 [Oryza sativa (japonica cultivar-group)] (GB:NP_916804.1); contains InterPro domain HMG1/2 (high mobility group) box (InterPro:IPR000910) \| chr2:14534815-14536571 REVERSE \| Aliases: F13P17.25 | 3.7 | 2.8 | 0.9 | 3.5 | 4.54% | 2.6 |
| 7050 | AT3G25470.1 \| bacterial hemolysin-related, similar to hemolysine GB:AAD36643 from (Thermotoga maritima), contains Pfam profile: PF01479 S4 domain \| chr3:9234178-9236450 FORWARD \| Aliases: MWL2.14 | 9.2 | 8.8 | 0.4 | 3.5 | 4.54% | 2.1 |
| 7051 | AT3G61490.2 \| glycoside hydrolase family 28 protein / polygalacturonase (pectinase) family protein, weak similarity to SP:P27644 Polygalacturonase (EC 3.2.1.15) (Pectinase) {Agrobacterium tumefaciens}; contains PF00295: Glycosyl hydrolases family 28 \| chr3:22769363-22771167 FORWARD \| Aliases: F2A19.90 | 5.6 | 5.1 | 0.5 | 3.5 | 4.54% | 2.3 |
| 7052 | AT1G43910.1 \| AAA-type ATPase family protein, contains Pfam profile: ATPase family PF00004 \| chr1:16658044-16660096 REVERSE \| Aliases: F9C16.7, F9C16_7 | 7.6 | 6.0 | 1.6 | 3.5 | 4.55% | 2.6 |
| 7053 | AT2G48120.1 \| Symbol: PAC \| pale cress protein (PAC), identical to PAC (Arabidopsis thaliana) gi:1483213:emb:CAA65334 | 3.3 | 2.7 | 0.5 | 3.5 | 4.55% | 2.4 |
| 7055 | AT1G49400.1 \| Symbol: EMB1129 \| ribosomal protein S17 family protein, similar to 40S ribosomal protein S17 GI:1620985 from (Nicotiana plumbaginifolia) \| chr1:18286708-18287870 FORWARD \| Aliases: EMB1129, EMBRYO DEFECTIVE 1129, F13F21.17, F13F21_17 | 5.6 | 3.4 | 2.1 | 3.5 | 4.55% | 2.7 |
| 7056 | AT1G07570.2 \| Symbol: APK1A \| protein kinase (APK1a), identical to Protein kinase APK1A from (Arabidopsis thaliana) SWISS-PROT:Q06548 | 4.7 | 4.0 | 0.7 | 3.5 | 4.55% | 2.6 |
| 7057 | AT4G02485.1 \| oxidoreductase, 2OG-Fe(II) oxygenase family protein, contains Pfam domain PF03171, 2OG-Fe(II) oxygenase superfamily \| chr4:1092681-1094595 FORWARD \| Aliases: None | 3.8 | 3.3 | 0.5 | 3.5 | 4.55% | 2.4 |
| 7058 | AT2G18780.1 \| F-box family protein, contains F-box domain Pfam:PF00646 | 3.9 | 2.8 | 1.1 | 3.5 | 4.55% | 2.7 |
| 7059 | AT3G03890.2 \| expressed protein \| chr3:999474-1002040 REVERSE \| Aliases: F20H23.6, F20H23_6 | 8.3 | 6.8 | 1.5 | 3.5 | 4.55% | 2.6 |
| 7064 | AT1G14030.1 \| ribulose-1,5 bisphosphate carboxylase oxygenase large subunit N-methyltransferase, putative, strong similarity to SP:P94026 Ribulose-1,5 bisphosphate carboxylase/oxygenase large subunit N- methyltransferase, chloroplast precursor (EC 2.1.1.127) ((Ribulose- bisphosphate-carboxylase)-lysine N-methyltransferase) {Nicotiana tabacum}; contains Pfam profile PF00856: SET domain; Rare GC intron splice site at 49572 is inferred from protein alignment and is not confirmed experimentally \| chr1:4805303-4807514 REVERSE \| Aliases: F7A19.12, F7A19_12 | 4.4 | 3.7 | 0.7 | 3.5 | 4.56% | 2.6 |
| 7067 | AT4G20440.3 \| Symbol: SMB \| similar to small nuclear ribonucleoprotein associated protein B, putative / snRNP-B, putative / Sm protein B, putative [Arabidopsis thaliana] (TAIR:At5g44500.1); similar to small nuclear ribonucleoprotein B' [Macropus eugenii] (GB:AAF40115.1); similar to small nuclear ribonucleoprotein-associated protein N - rat (GB:A35448); similar to small nuclear ribonucleoprotein polypeptide B/B' isoform B' [Bos taurus] (GB:AAX46330.1); contains InterPro domain Proline-rich region (InterPro:IPR000694); contains InterPro domain Small nuclear ribonucleoprotein (Sm protein) (InterPro:IPR001163) | 5.1 | 3.5 | 1.6 | 3.5 | 4.58% | 2.7 |
| 7069 | AT5G60450.1 \| Symbol: ARF4 \| auxin-responsive factor (ARF4), contains Pfam profile: PF02362 B3 DNA binding domain; identical to cDNA auxin response factor 4 (ARF4) GI:4102597 \| chr5:24325549-24329924 REVERSE \| Aliases: AUXIN RESPONSE FACTOR 4, MUF9.7, MUF9_7 | 5.6 | 4.8 | 0.8 | 3.5 | 4.59% | 2.7 |
| 7072 | AT3G15520.1 \| peptidyl-prolyl cis-trans isomerase TLP38, chloroplast / thylakoid lumen PPIase of 38 kDa / cyclophilin / rotamase, cyclophylin-type; identical to SP:P82869 Thylakoid lumenal 38 kDa protein, chloroplast precursor (P38) {Arabidopsis thaliana} \| chr3:5248936-5252439 REVERSE \| Aliases: MJK13.18 | 4.3 | 3.6 | 0.7 | 3.5 | 4.60% | 2.5 |
| 7073 | AT5G66380.1 \| Encodes a folate transporter that is located in the chloroplast envelope and is able to mediate exogenous folate uptake when expressed in E. coli. However, this is not the sole folate transporter for chloroplasts as null mutants of this gene have no discernable phenotype when grown under folate-sufficient conditions and contained wild-type levels of folates in leaves. \| chr5:26530750-26532891 REVERSE \| Aliases: K1F13.3, K1F13_3 | 7.4 | 6.4 | 1.0 | 3.5 | 4.60% | 2.6 |

| Rank | Description | Sync | Root | M | t | adj.q | B |
|---|---|---|---|---|---|---|---|
| 7076 | AT5G38470.1 \| DNA repair protein RAD23, putative, similar to DNA repair by nucleotide excision (NER) RAD23 protein, isoform I GI:1914683 from (Daucus carota) \| chr5:15421872-15424941 FORWARD \| Aliases: MXI10.20, MXI10_20 | 8.5 | 6.4 | 2.0 | 3.5 | 4.61% | 2.7 |
| 7078 | AT1G57770.1 \| amine oxidase family, contains similarity to carotenoid isomerase (Lycopersicon esculentum) GI:19550437, phytoene dehydrogenase (PDH1) GI:433144 from (Cercospora nicotianae); contains Pfam profile PF01593 amine oxidase, flavin-containing \| chr1:21398861-21401945 FORWARD \| Aliases: F12K22.18 | 4.2 | 3.0 | 1.1 | 3.5 | 4.61% | 2.7 |
| 7080 | AT5G08415.1 \| lipoic acid synthase family protein, similar to lipoic acid synthase from Arabidopsis thaliana (gi:3928758), from Mus musculus (gi:14669826) Pfam profile PF04055: radical SAM domain protein \| chr5:2710820-2713133 REVERSE \| Aliases: None | 7.7 | 6.3 | 1.4 | 3.5 | 4.62% | 2.6 |
| 7081 | AT5G40550.1 \| expressed protein \| chr5:16258333-16260609 FORWARD \| Aliases: MNF13.7, MNF13_7 | 3.8 | 3.1 | 0.7 | 3.5 | 4.62% | 2.4 |
| 7084 | AT1G77510.1 \| Symbol: ATPDIL1 2 \| Encodes a protein disulfide isomerase-like (PDIL) protein, a member of a multigene family within the thioredoxin (TRX) superfamily. | 8.4 | 6.6 | 1.8 | 3.5 | 4.62% | 2.6 |
| 7086 | AT5G56150.2 \| ubiquitin-conjugating enzyme, putative, strong similarity to ubiquitin-conjugating enzyme UBC2 (Mesembryanthemum crystallinum) GI:5762457, UBC4 (Pisum sativum) GI:456568; contains Pfam profile PF00179: Ubiquitin-conjugating enzyme \| chr5:22746408-22748409 FORWARD \| Aliases: MDA7.21, MDA7_21 | 11.3 | 10.6 | 0.7 | 3.5 | 4.63% | 2.1 |
| 7087 | AT1G61900.2 \| expressed protein, contains similarity to glutamic acid/alanine-rich protein GI:6707830 from (Trypanosoma congolense) \| chr1:22885880-22888718 REVERSE \| Aliases: F8K4.10, F8K4_10 | 5.0 | 4.4 | 0.6 | 3.5 | 4.63% | 2.5 |
| 7091 | AT3G60420.1 \| expressed protein \| chr3:22345406-22346760 FORWARD \| Aliases: T8B10.80 | 3.1 | 2.0 | 1.1 | 3.5 | 4.63% | 2.5 |
| 7092 | AT2G06050.2 \| Symbol: OPR3 \| 12-oxophytodienoate reductase (OPR3) / delayed dehiscence1 (DDE1), nearly identical to DELAYED DEHISCENCE1 (GI:7688991) and to OPR3 (GI:10242314); contains Pfam profile PF00724:oxidoreductase, FAD/FMN-binding; identical to cDNA OPDA-reductase homolog GI:5059114 \| chr2:2359112-2362124 REVERSE \| Aliases: 12 OXO PHYTODIENOATE REDUCTASE, 12 OXOPHYTODIENOATE 10,11 REDUCTASE, F5K7.19, F5K7_19 | 10.2 | 8.7 | 1.5 | 3.5 | 4.63% | 2.6 |
| 7095 | AT1G79220.1 \| mitochondrial transcription termination factor family protein / mTERF family protein, weak similarity to mtDBP protein (Paracentrotus lividus) GI:4584695; contains Pfam profile PF02536: mTERF \| chr1:29803828-29805418 FORWARD \| Aliases: YUP8H12R.18, YUP8H12R_18 | 3.9 | 3.3 | 0.6 | 3.5 | 4.64% | 2.5 |
| 7096 | AT1G06790.2 \| RNA polymerase Rpb7 N-terminal domain-containing protein, similar to SP:P35718 DNA-directed RNA polymerase III 25 kDa polypeptide (EC 2.7.7.6) {Saccharomyces cerevisiae}; contains Pfam profile PF03876: RNA polymerase Rpb7, N-terminal domain | 5.7 | 4.6 | 1.0 | 3.5 | 4.64% | 2.7 |
| 7099 | AT5G14960.1 \| Symbol: DEL2/E2FD/E2L1 \| | 3.2 | 2.8 | 0.4 | 3.5 | 4.64% | 2.1 |
| 7101 | AT1G47830.1 \| clathrin coat assembly protein, putative, similar to clathrin coat assembly protein AP17 GB:CAA65533 GI:2959358 from (Zea mays); contains Pfam profile: PF01217 clathrin adaptor complex small chain \| chr1:17615538-17617355 REVERSE \| Aliases: T2E6.6, T2E6_6 | 7.8 | 7.0 | 0.8 | 3.5 | 4.64% | 2.7 |
| 7102 | AT3G49850.1 \| myb family transcription factor, contains Pfam profile: PF00249 myb-like DNA-binding domain \| chr3:18500030-18501981 FORWARD \| Aliases: T16K5.200 | 5.1 | 4.1 | 1.0 | 3.5 | 4.65% | 2.7 |
| 7103 | AT3G08890.2 \| expressed protein, contains Pfam profile PF04398: Protein of unknown function, DUF538 \| chr3:2706642-2708753 FORWARD \| Aliases: T16O11.17 | 4.4 | 3.8 | 0.6 | 3.5 | 4.65% | 2.4 |
| 7106 | AT4G09570.1 \| Symbol: CPK4 \| calcium-dependent protein kinase, putative / CDPK, putative, similar to calcium-dependent protein kinase (Arabidopsis thaliana) gi:604881:dbj:BAA04830; contains protein kinase domain, Pfam:PF00069; contains EF hand domain (calcium-binding EF-hand), Pfam:PF00036, INTERPRO:IPR002048 \| chr4:6049518-6052332 FORWARD \| Aliases: T25P22.10, T25P22_10 | 9.2 | 8.3 | 0.9 | 3.5 | 4.65% | 2.4 |
| 7107 | AT3G01610.1 \| Symbol: CDC48C \| | 6.3 | 5.4 | 0.8 | 3.5 | 4.65% | 2.4 |
| 7109 | AT2G44050.1 \| Symbol: COS1 \| 6,7-dimethyl-8-ribityllumazine synthase / DMRL synthase / lumazine synthase / riboflavin synthase, identical to 6,7-dimethyl-8-ribityllumazine synthase, chloroplast (precursor) SP:O80575 from (Arabidopsis thaliana) \| chr2:18231342-18233236 FORWARD \| Aliases: COI1 SUPPRESSOR1, F6E13.18, F6E13.41, F6E13_41 | 6.4 | 5.0 | 1.4 | 3.5 | 4.66% | 2.7 |
| 7111 | AT1G79080.1 \| pentatricopeptide (PPR) repeat-containing protein, contains Pfam profile PF01535: PPR repeat \| chr1:29751865-29753837 REVERSE \| Aliases: YUP8H12R.30, YUP8H12R_30 | 4.3 | 3.2 | 1.1 | 3.5 | 4.66% | 2.7 |
| 7113 | AT3G22425.2 \| imidazoleglycerol-phosphate dehydratase 1 (IGPD1), identical to SP:P34047 Imidazoleglycerol-phosphate dehydratase 1 (EC 4.2.1.19) (IGPD 1) {Arabidopsis thaliana}; supporting cDNA gi:437212:gb:U02689.1:ATU02689 \| chr3:7951062-7953230 FORWARD \| Aliases: None | 5.8 | 4.0 | 1.8 | 3.5 | 4.66% | 2.6 |

| Rank | Description | Sync | Root | M | t | adj.q | B |
|---|---|---|---|---|---|---|---|
| 7114 | AT3G11830.1 \| chaperonin, putative, similar to SWISS-PROT:P80313 T-complex protein 1, eta subunit (TCP-1-eta) (Mus musculus); contains Pfam:PF00118 domain, TCP-1/cpn60 chaperonin family \| chr3:3732670-3736377 FORWARD \| Aliases: F26K24.12 | 7.9 | 6.6 | 1.4 | 3.5 | 4.67% | 2.7 |
| 7115 | AT5G07880.1 \| Symbol: SNAP29 \| | 3.4 | 2.7 | 0.7 | 3.5 | 4.68% | 2.6 |
| 7117 | AT5G03690.2 \| fructose-bisphosphate aldolase, putative, similar to PIR:S65073 fructose-bisphosphate aldolase (EC 4.1.2.13) isoenzyme C-1, cytosolic (Oryza sativa); contains Pfam profile PF00274 Fructose-bisphosphate aldolase class-I \| chr5:963147-964988 REVERSE \| Aliases: F17C15.110, F17C15_110 | 4.4 | 3.8 | 0.6 | 3.5 | 4.68% | 2.5 |
| 7118 | AT2G30060.1 \| Ran-binding protein 1b (RanBP1b), nearly identical to atranbp1b (Arabidopsis thaliana) GI:2058284 \| chr2:12834036-12835996 FORWARD \| Aliases: T27E13.20, T27E13_20 | 7.7 | 6.9 | 0.9 | 3.5 | 4.68% | 2.3 |
| 7119 | AT4G00010.1 \| expressed protein \| chr4:3894-5908 REVERSE \| Aliases: T15P10.10, T15P10_10 | 4.2 | 3.2 | 0.9 | 3.5 | 4.69% | 2.7 |
| 7120 | AT5G53540.1 \| MSP1 protein, putative / intramitochondrial sorting protein, putative, similar to Swiss-Prot:P28737 MSP1 protein (TAT-binding homolog 4) (Saccharomyces cerevisiae); contains Pfam domain, PF00004: ATPase, AAA family \| chr5:21766512-21768463 REVERSE \| Aliases: MNC6.8, MNC6_8 | 8.7 | 8.1 | 0.6 | 3.5 | 4.70% | 2.4 |
| 7122 | AT1G10740.2 \| expressed protein \| chr1:3568191-3570761 REVERSE \| Aliases: T16B5.12, T16B5_12 | 5.8 | 4.9 | 1.0 | 3.5 | 4.71% | 2.6 |
| 7125 | AT5G05820.1 \| phosphate translocator-related, low similarity to phosphoenolpyruvate/phosphate translocator precursor (Mesembryanthemum crystallinum) GI:9295275, glucose-6-phosphate/phosphate-translocator precursor (Zea mays) GI:2997589; contains Pfam profile PF00892: Integral membrane protein \| chr5:1751689-1754179 REVERSE \| Aliases: MJJ3.24, MJJ3_24 | 5.5 | 4.7 | 0.9 | 3.5 | 4.71% | 2.6 |
| 7126 | AT5G49100.1 \| expressed protein \| chr5:19914596-19916340 REVERSE \| Aliases: K20J1.7, K20J1_7 | 6.7 | 4.9 | 1.8 | 3.5 | 4.72% | 2.8 |
| 7127 | AT5G50740.1 \| copper chaperone (CCH)-related, low similarity to copper homeostasis factor (GI:3168840)(PMID: 9701579); similar to farnesylated protein ATFP3 (GI:4097547); contains Pfam profile PF00403: Heavy-metal-associated domain \| chr5:20654374-20655086 REVERSE \| Aliases: MFB16.14, MFB16_14 | 4.2 | 3.4 | 0.8 | 3.5 | 4.72% | 2.5 |
| 7128 | AT1G63830.2 \| proline-rich family protein, contains proline-rich extensin domains, INTERPRO:IPR002965; contains 1 predicted transmembrane domain \| chr1:23689014-23690985 FORWARD \| Aliases: T12P18.15, T12P18_15 | 7.8 | 6.1 | 1.8 | 3.5 | 4.72% | 2.7 |
| 7129 | AT4G24810.2 \| similar to ABC1 family protein [Arabidopsis thaliana] (TAIR:At5g50330.1); similar to ABC transporter-like protein [Oryza sativa (japonica cultivar-group)] (GB:NP_915227.1); contains InterPro domain ABC1 protein (InterPro:IPR004147) \| chr4:12786637-12789723 REVERSE \| Aliases: F6I7.20, F6I7_20 | 4.1 | 3.3 | 0.7 | 3.5 | 4.73% | 2.6 |
| 7130 | AT1G14290.1 \| acid phosphatase, putative, similar to acid phosphatase (Lupinus albus) GI:5360721; contains Pfam profile PF01598 sterol desaturase \| chr1:4880046-4881947 REVERSE \| Aliases: F14L17.5, F14L17_5 | 4.4 | 3.4 | 1.1 | 3.5 | 4.73% | 2.6 |
| 7131 | AT3G63130.1 \| Symbol: RANGAP1 \| RAN GTPase activating protein 1 (RanGAP1), contains Pfam PF00560: Leucine Rich Repeat domains; identical to RAN GTPase activating protein 1 (GI:6708466)(Arabidopsis thaliana) \| chr3:23335596-23337888 FORWARD \| Aliases: RAN GTPASE ACTIVATING PROTEIN 1, T20O10.230 | 3.7 | 3.1 | 0.7 | 3.5 | 4.74% | 2.4 |
| 7133 | AT1G73250.1 \| Symbol: ATFX/GER1 \| encodes a bifunctional 3, 5-epimerase-4-reductase in L-fucose synthesis and converts GDP-D-mannose to GDP-L-fucose in vitro along with MUR1 (GDP-D-mannose 4,6-dehydratase). It is expressed in all tissues examined, but most abundantly in roots and flowers. \| chr1:27548667-27550085 REVERSE \| Aliases: ATFX, GDP 4 KETO 6 DEOXY D MANNOSE 3,5 EPIMERASE 4 REDUCTASE, GER1, T18K17.8, T18K17_8 | 6.1 | 5.5 | 0.6 | 3.5 | 4.74% | 2.3 |
| 7134 | AT1G54270.1 \| Symbol: EIF4A 2 \| eukaryotic translation initiation factor 4A-2 / eIF-4A-2, similar to eukaryotic translation initiation factor 4A GI:19696 from (Nicotiana plumbaginifolia) \| chr1:20263359-20265933 FORWARD \| Aliases: F20D21.9, F20D21_9 | 9.7 | 8.6 | 1.1 | 3.5 | 4.74% | 2.5 |
| 7135 | AT3G06660.1 \| PAPA-1-like family protein / zinc finger (HIT type) family protein, contains Pfam domains, PF04795: PAPA-1-like conserved region and PF04438: HIT zinc finger \| chr3:2102477-2104912 FORWARD \| Aliases: T8E24.3 | 6.0 | 4.8 | 1.2 | 3.5 | 4.74% | 2.6 |
| 7136 | AT5G58920.1 \| expressed protein \| chr5:23810355-23811471 FORWARD \| Aliases: K19M22.6, K19M22_6 | 7.0 | 5.9 | 1.0 | 3.5 | 4.74% | 2.6 |
| 7138 | ATCG00810.1 \| Symbol: RPL22 \| encodes a chloroplast ribosomal protein L22, a constituent of the large subunit of the ribosomal complex \| chrC:83467-83949 REVERSE \| Aliases: RPL22 | 9.4 | 6.8 | 2.5 | 3.5 | 4.75% | 2.4 |
| 7141 | AT4G29870.1 \| expressed protein, predicted protein, Arabidopsis thaliana, PIR2:T01282 \| chr4:14606007-14606680 FORWARD \| Aliases: F27B13.110, F27B13_110 | 6.1 | 5.3 | 0.8 | 3.5 | 4.76% | 2.6 |
| 7142 | AT3G51880.2 \| Symbol: HMGB1 \| high mobility group protein alpha (HMGalpha) / HMG protein alpha, nearly identical to HMG protein (HMGalpha) (Arabidopsis thaliana) GI:2832357; contains Pfam profile PF00505: HMG (high mobility group) box \| chr3:19257980-19260072 REVERSE \| Aliases: ATEM1.13, HIGH MOBILITY GROUP B 1, HMG ALPHA, NFD1 | 8.8 | 7.8 | 1.0 | 3.5 | 4.76% | 2.5 |

| Rank | Description | Sync | Root | M | t | adj.q | B |
|---|---|---|---|---|---|---|---|
| 7145 | AT3G58830.1 \| haloacid dehalogenase (HAD) superfamily protein, contains TIGRfam TIGR01668: HAD superfamily (subfamily IIIA) phosphatase, TIGR01668 and TIGR01662: hydrolase, HAD-superfamily, subfamily IIIA \| chr3:21766033-21767615 REVERSE \| Aliases: T20N10.180 | 4.9 | 4.0 | 0.9 | 3.5 | 4.76% | 2.7 |
| 7146 | AT2G35320.1 \| Symbol: ATEYA \| homologue of the animal Eys Absent genes. encodes a tyrosine-specific phosphatase. the protein sequence lacks the cys-containing signature of the classical tyrosine phosphatases. belongs to the aspartate-based phosphatases. The enzyme activity is strictly metal-dependent. \| chr2:14873911-14875939 REVERSE \| Aliases: ATEYA, T4C15.1, T4C15_1 | 6.1 | 5.4 | 0.7 | 3.5 | 4.76% | 2.5 |
| 7147 | AT5G13410.1 \| immunophilin / FKBP-type peptidyl-prolyl cis-trans isomerase family protein, SP:Q9LYR5; similar to FK506-binding protein (Peptidyl-prolyl cis-trans isomerase) (PPiase) (Rotamase) (SP:P25138) ({Neisseria meningitidis); peptidyl-prolyl cis-trans isomerase, Spodoptera frugiperda, EMBL:SF15038; contains Pfam PF00254: peptidyl-prolyl cis-trans isomerase, FKBP-type \| chr5:4299744-4301770 REVERSE \| Aliases: T22N19.60, T22N19_60 | 6.8 | 5.5 | 1.3 | 3.5 | 4.77% | 2.6 |
| 7148 | AT1G54680.1 \| expressed protein \| chr1:20416721-20418149 FORWARD \| Aliases: T22H22.11, T22H22_11 | 8.9 | 7.7 | 1.2 | 3.5 | 4.77% | 2.5 |
| 7149 | AT2G23950.1 \| leucine-rich repeat family protein / protein kinase family protein, contains Pfam domains PF00560: Leucine Rich Repeat and PF00069: Protein kinase domain \| chr2:10194171-10197365 REVERSE \| Aliases: T29E15.15, T29E15_15 | 4.6 | 3.5 | 1.1 | 3.5 | 4.77% | 2.7 |
| 7153 | AT1G21910.1 \| encodes a member of the DREB subfamily A-5 of ERF/AP2 transcription factor family. The protein contains one AP2 domain. There are 15 members in this subfamily including RAP2.1, RAP2.9 and RAP2.10. \| chr1:7696525-7697688 FORWARD \| Aliases: T26F17.14, T26F17_14 | 4.5 | 3.5 | 1.0 | 3.5 | 4.78% | 2.7 |
| 7154 | AT1G79560.1 \| Symbol: EMB1047/FTSH12 \| | 5.0 | 3.8 | 1.2 | 3.5 | 4.78% | 2.5 |
| 7155 | AT3G14900.1 \| expressed protein \| chr3:5013299-5015284 REVERSE \| Aliases: K15M2.4 | 5.0 | 3.3 | 1.7 | 3.5 | 4.78% | 2.6 |
| 7156 | ATCG00670.1 \| Symbol: CLPP1 \| Encodes the only ClpP (caseinolytic protease) encoded within the plastid genome. Contains a highly conserved catalytic triad of Ser-type proteases (Ser-His-Asp). Part of the 350 kDa chloroplast Clp complex. The name reflects nomenclature described in Adam et. al (2001). | 9.5 | 7.9 | 1.6 | 3.5 | 4.78% | 2.6 |
| 7158 | AT4G18580.2 \| expressed protein \| chr4:10234421-10236028 REVERSE \| Aliases: F28J12.240, F28J12_240 | 7.5 | 5.8 | 1.7 | 3.5 | 4.79% | 2.6 |
| 7159 | AT1G80070.1 \| Symbol: SUS2 \| splicing factor, putative, strong similarity to splicing factor Prp8 (Homo sapiens) GI:3661610; contains Pfam profile PF01398: Mov34/MPN/PAD-1 family \| chr1:30122945-30132704 FORWARD \| Aliases: ABNORMAL SUSPENSOR 2, EMB14, EMB158, EMB177, EMB33, EMBRYO DEFECTIVE 14, EMBRYO DEFECTIVE 177, EMBRYO DEFECTIVE 33, F18B13.15, F18B13_15 | 8.7 | 7.5 | 1.2 | 3.5 | 4.79% | 2.6 |
| 7160 | AT5G24390.1 \| RabGAP/TBC domain-containing protein, similar to GTPase activating protein (Yarrowia lipolytica) GI:2370595; contains Pfam profile PF00566: TBC domain \| chr5:8326003-8329466 REVERSE \| Aliases: K16H17.10, K16H17_10 | 4.6 | 4.0 | 0.6 | 3.5 | 4.79% | 2.4 |
| 7164 | AT5G44670.1 \| expressed protein, contains Pfam:PF01697 Domain of unknown function \| chr5:18036132-18038612 REVERSE \| Aliases: K15C23.12, K15C23_12 | 7.0 | 6.0 | 1.0 | 3.5 | 4.81% | 2.6 |
| 7167 | AT1G61620.1 \| expressed protein, contains Pfam profile: PF01363 FYVE zinc finger \| chr1:22741208-22743095 FORWARD \| Aliases: T25B24.3, T25B24_3 | 8.3 | 7.1 | 1.3 | 3.5 | 4.83% | 2.6 |
| 7168 | AT2G02810.1 \| UDP-galactose/UDP-glucose transporter, contains transmembrane domains; identical to UDP-galactose/UDP-glucose transporter (GI:22651763) (Arabidopsis thaliana) similar to UGTrel1 (GI:1669564) (Rattus rattus); identical to cDNA UDP-galactose/UDP-glucose transporter GI:22651762 \| chr2:801529-803435 FORWARD \| Aliases: T20F6.5, T20F6_5 | 5.2 | 4.6 | 0.6 | 3.5 | 4.83% | 2.2 |
| 7169 | AT3G25580.1 \| thioredoxin-related, contains weak similarity to thioredoxin (Swiss-Prot:O17486) (Echinococcus granulosus) \| chr3:9293725-9295584 FORWARD \| Aliases: MWL2.25 | 5.1 | 4.2 | 0.9 | 3.5 | 4.84% | 2.7 |
| 7170 | AT5G63570.1 \| Symbol: GSA1 \| glutamate-1-semialdehyde 2,1-aminomutase 1 (GSA 1) / glutamate-1-semialdehyde aminotransferase 1 (GSA-AT 1), identical to GSA 1 (SP:P42799) \| chr5:25469103-25471067 FORWARD \| Aliases: GLUTAMATE 1 SEMIALDEHYDE 2,1 AMINOMUTASE, MBK5.3, MBK5_3 | 8.3 | 7.1 | 1.2 | 3.5 | 4.84% | 2.6 |
| 7172 | AT1G21060.1 \| expressed protein, contains Pfam profile PF04784: Protein of unknown function, DUF547 \| chr1:7371788-7374093 FORWARD \| Aliases: T22I11.12, T22I11_12 | 3.9 | 3.1 | 0.8 | 3.5 | 4.85% | 2.5 |
| 7173 | AT1G72750.1 \| Symbol: ATTIM23 2 \| mitochondrial import inner membrane translocase subunit Tim17/Tim22/Tim23 family protein, contains Pfam PF02466: Mitochondrial import inner membrane translocase subunit Tim17 \| chr1:27387027-27388186 FORWARD \| Aliases: F28P22.6, F28P22_6 | 6.1 | 4.4 | 1.7 | 3.5 | 4.85% | 2.7 |
| 7176 | AT3G17380.1 \| meprin and TRAF homology domain-containing protein / MATH domain-containing protein, similar to ubiquitin-specific protease 12 (Arabidopsis thaliana) GI:11993471; contains Pfam profile PF00917: MATH domain \| chr3:5950206-5953699 FORWARD \| Aliases: MGD8.22 | 7.4 | 5.4 | 2.0 | 3.5 | 4.85% | 2.6 |
| 7177 | AT5G10710.2 \| expressed protein \| chr5:3379145-3382780 FORWARD \| Aliases: MAJ23.70, MAJ23_70 | 8.2 | 7.4 | 0.8 | 3.5 | 4.85% | 2.4 |

| Rank | Description | Sync | Root | M | t | adj.q | B |
|---|---|---|---|---|---|---|---|
| 7179 | AT3G48580.1 \| xyloglucan:xyloglucosyl transferase, putative / xyloglucan endotransglycosylase, putative / endo-xyloglucan transferase, putative, similar to endoxyloglucan transferase EXGT-A4 GI:5139002 from (Arabidopsis thaliana) \| chr3:18018166-18019472 FORWARD \| Aliases: T8P19.90 | 3.1 | 2.5 | 0.6 | 3.5 | 4.85% | 2.4 |
| 7180 | AT3G27060.1 \| ribonucleoside-diphosphate reductase small chain, putative / ribonucleotide reductase, putative, similar to ribonucleotide reductase R2 (Nicotiana tabacum) GI:1044912; contains Pfam profile PF00268: Ribonucleotide reductase, small chain \| chr3:9981103-9982532 REVERSE \| Aliases: MOJ10.15 | 7.4 | 5.9 | 1.5 | 3.5 | 4.85% | 2.7 |
| 7183 | AT3G10370.1 \| mitochondrial FAD-dependent glycerol-3-phosphate dehydrogenase. possibly involved in storage lipid catabolism and glycerol assimilation, and in glycerol-3-phosphate shuttle which transports reducing power from cytosol to mitochondrion. \| chr3:3216374-3219275 FORWARD \| Aliases: F14P13.3 | 6.8 | 5.8 | 1.0 | 3.5 | 4.87% | 2.5 |
| 7185 | AT1G76020.1 \| expressed protein \| chr1:28535786-28537218 REVERSE \| Aliases: T4O12.23, T4O12_23 | 4.2 | 3.3 | 0.9 | 3.5 | 4.87% | 2.4 |
| 7186 | AT4G12840.1 \| expressed protein, contains Pfam profile PF05212: Protein of unknown function (DUF707) \| chr4:7533618-7535830 REVERSE \| Aliases: T20K18.190, T20K18_190 | 4.9 | 4.3 | 0.6 | 3.5 | 4.88% | 2.3 |
| 7188 | AT5G09810.1 \| Symbol: ACT7 \| actin 7 (ACT7) / actin 2, identical to SP:P53492 Actin 7 (Actin-2) {Arabidopsis thaliana} \| chr5:3052167-3054615 FORWARD \| Aliases: ACTIN_2, MYH9.2, MYH9_2 | 11.1 | 9.4 | 1.7 | 3.5 | 4.89% | 2.4 |
| 7189 | ATCG00420.1 \| Symbol: NDHJ \| NADH dehydrogenase subunit \| chrC:48677-49153 REVERSE \| Aliases: NDHJ | 6.3 | 4.2 | 2.1 | 3.5 | 4.89% | 2.7 |
| 7190 | AT1G12120.1 \| expressed protein, contains Pfam domain PF05904: Plant protein of unknown function (DUF863) \| chr1:4114769-4117592 REVERSE \| Aliases: T28K15.14, T28K15_14 | 7.7 | 6.7 | 1.0 | 3.5 | 4.89% | 2.4 |
| 7196 | AT1G74690.1 \| calmodulin-binding family protein, low similarity to SF16 protein (Helianthus annuus) GI:560150; contains Pfam profile PF00612: IQ calmodulin-binding motif \| chr1:28064275-28068286 REVERSE \| Aliases: F25A4.33, F25A4_33 | 6.6 | 5.4 | 1.1 | 3.5 | 4.91% | 2.7 |
| 7197 | AT2G21590.2 \| Symbol: APL4 \| similar to glucose-1-phosphate adenylyltransferase large subunit 2 (APL2) / ADP-glucose pyrophosphorylase [Arabidopsis thaliana] (TAIR:At1g27680.1); similar to glucose-1-phosphate adenylyltransferase large subunit 3 (APL3) / ADP-glucose pyrophosphorylase [Arabidopsis thaliana] (TAIR:At4g39210.1); similar to ADP-glucose pyrophosphorylase large subunit 1 [Lycopersicon esculentum] (GB:AAC49941.1); similar to ADP-glucose pyrophosphorylase large subunit PvAGPL1 [Phaseolus vulgaris] (GB:BAC66692.1); similar to ADP-glucose pyrophosphorylase large subunit [Solanum tuberosum] (GB:CAA43490.1); similar to ADP-glucose pyrophosphorylase large subunit [Citrus unshiu] (GB:AAD56042.1); similar to ADP-glucose pyrophosphorylase large subunit 1 [Citrullus lanatus] (GB:AAB91467.1); contains InterPro domain Nucleotidyl transferase (InterPro:IPR005835); contains InterPro domain ADP-glucose pyrophosphorylase (InterPro:IPR005836) \| chr2:9245827-9249520 FORWARD \| Aliases: F2G1.14, F2G1_14 | 3.0 | 2.5 | 0.5 | 3.4 | 4.92% | 2.2 |
| 7198 | AT1G64680.1 \| expressed protein \| chr1:24039684-24041064 FORWARD \| Aliases: F1N19.25, F1N19_25 | 5.2 | 4.3 | 0.9 | 3.4 | 4.92% | 2.6 |
| 7199 | AT5G67080.1 \| Symbol: MAPKKK19 \| protein kinase family protein, contains protein kinase domain, Pfam:PF00069 \| chr5:26789952-26790986 FORWARD \| Aliases: K21H1.4, K21H1_4 | 3.7 | 3.0 | 0.8 | 3.4 | 4.92% | 2.4 |
| 7200 | AT1G20010.1 \| Symbol: TUB5 \| tubulin beta-5 chain (TUB5), nearly identical to SP:P29513 Tubulin beta-5 chain {Arabidopsis thaliana} \| chr1:6937786-6940573 REVERSE \| Aliases: T20H2.21, T20H2_21 | 6.2 | 5.2 | 1.0 | 3.4 | 4.92% | 2.4 |
| 7201 | AT5G18620.2 \| DNA-dependent ATPase, putative, similar to DNA-dependent ATPase SNF2H (Mus musculus) GI:14028669; contains Pfam profiles PF00271: Helicase conserved C-terminal domain, PF00176: SNF2 family N-terminal domain, PF00249: Myb-like DNA-binding domain \| chr5:6195919-6202168 REVERSE \| Aliases: T1A4.2 | 7.9 | 6.2 | 1.7 | 3.4 | 4.92% | 2.6 |
| 7202 | AT4G15510.3 \| similar to photosystem II reaction center PsbP family protein [Arabidopsis thaliana] (TAIR:At3g55330.1); similar to photosystem II oxygen-evolving complex 23K protein PsbP homolog [Thermosynechococcus elongatus BP-1] (GB:NP_682865.1); contains InterPro domain Photosystem II reaction center protein PsbP (InterPro:IPR002683) \| chr4:8860717-8862656 FORWARD \| Aliases: DL3795W, FCAALL.88 | 3.5 | 2.7 | 0.8 | 3.4 | 4.93% | 2.5 |
| 7203 | AT1G76790.1 \| O-methyltransferase family 2 protein, similar to caffeic acid O-methyltransferase (Catharanthus roseus)(GI:18025321), catechol O-methyltransferase GB:CAA55358 (Vanilla planifolia) \| chr1:28827080-28828567 REVERSE \| Aliases: F28O16.16, F28O16_16 | 9.4 | 8.2 | 1.2 | 3.4 | 4.93% | 2.3 |
| 7205 | AT5G15350.1 \| plastocyanin-like domain-containing protein, contains plastocyanin-like domain Pfam:PF02298 \| chr5:4984962-4986221 REVERSE \| Aliases: F8M21.240, F8M21_240 | 8.5 | 6.8 | 1.8 | 3.4 | 4.93% | 2.6 |
| 7206 | AT5G11930.1 \| glutaredoxin family protein, contains INTERPRO Domain IPR002109, Glutaredoxin (thioltransferase) | 5.3 | 4.4 | 0.8 | 3.4 | 4.93% | 2.6 |
| 7210 | AT3G04940.1 \| Symbol: ATCYSD1 \| cysteine synthase, putative / O-acetylserine (thiol)-lyase, putative / O-acetylserine sulfhydrylase, putative, similar to O-acetylserine(thiol) lyase (EC 4.2.99.8) (Brassica juncea) GI:2245144; contains Pfam profile PF00291: Pyridoxal-phosphate dependent enzyme | 6.7 | 5.9 | 0.8 | 3.4 | 4.95% | 2.5 |

| Rank | Description | Sync | Root | M | t | adj.q | B |
|---|---|---|---|---|---|---|---|
| 7211 | AT1G32920.1 \| expressed protein \| chr1:11928787-11929281 FORWARD \| Aliases: F9L11.25, F9L11_25 | 7.9 | 6.3 | 1.6 | 3.4 | 4.95% | 2.6 |
| 7212 | AT1G14120.1 \| 2-oxoglutarate-dependent dioxygenase, putative, similar to adventitious rooting related oxygenase ARRO-1 from Malus x domestica, gi:3492806; contains Pfam domain PF03171, 2OG-Fe(II) oxygenase superfamily \| chr1:4833516-4835083 REVERSE \| Aliases: F7A19.20, F7A19_20 | 2.9 | 2.5 | 0.4 | 3.4 | 4.95% | 2.1 |
| 7213 | AT5G57370.1 \| expressed protein, low similarity to nucleic acid binding protein (Homo sapiens) GI:431953 \| chr5:23261960-23263639 FORWARD \| Aliases: MSF19.3, MSF19_3 | 4.3 | 3.6 | 0.7 | 3.4 | 4.95% | 2.5 |
| 7214 | AT1G54030.1 \| GDSL-motif lipase, putative, similar to myrosinase-associated proteins GI:1769968, GI:1769970, GI:1216391, GI:1216389 from (Brassica napus); contains Pfam profile PF00657: GDSL-like Lipase/Acylhydrolase \| chr1:20171302-20173268 FORWARD \| Aliases: F15I1.11, F15I1_11 | 7.8 | 5.9 | 1.9 | 3.4 | 4.96% | 2.6 |
| 7218 | AT2G17360.1 \| 40S ribosomal protein S4 (RPS4A), contains ribosomal protein S4 signature from residues 8 to 22 \| chr2:7553567-7555395 FORWARD \| Aliases: F5J6.12, F5J6_12 | 9.3 | 7.6 | 1.7 | 3.4 | 4.97% | 2.5 |
| 7220 | AT3G19515.1 \| expressed protein \| chr3:6766674-6769032 REVERSE \| Aliases: MLD14.38 | 8.3 | 6.9 | 1.4 | 3.4 | 4.98% | 2.4 |
| 7223 | AT4G35130.1 \| pentatricopeptide (PPR) repeat-containing protein, contains Pfam profile PF01535: PPR repeat \| chr4:16721089-16723503 REVERSE \| Aliases: T12J5.8 | 3.8 | 3.4 | 0.4 | 3.4 | 4.98% | 1.9 |
| 7225 | AT5G18920.1 \| expressed protein \| chr5:6310066-6310848 FORWARD \| Aliases: F17K4.170, F17K4_170 | 4.0 | 3.3 | 0.7 | 3.4 | 4.99% | 2.6 |
| 7227 | AT1G03445.1 \| Symbol: BSU1 \| similar to kelch repeat-containing serine/threonine phosphoesterase family protein [Arabidopsis thaliana] (TAIR:At4g03080.1); similar to protein serine/threonine phosphatase, putative [Plasmodium berghei] (GB:CAH95465.1); similar to protein serine/threonine phosphatase alpha [Plasmodium yoelii yoelii] (GB:EAA18849.1); contains InterPro domain Kelch repeat (InterPro:IPR006652); contains InterPro domain Metallo-phosphoesterase (InterPro:IPR004843); contains InterPro domain Serine/threonine-specific protein phosphatase and bis(5-nucleosyl)-tetraphosphatase (InterPro:IPR006186) \| chr1:854409-859701 REVERSE \| Aliases: F21B7.7 | 4.3 | 3.4 | 0.9 | 3.4 | 5.00% | 2.6 |
| 7228 | AT5G13450.2 \| similar to F1-ATP synthase delta subunit [Ipomoea batatas] (GB:BAA77508.1); similar to oligomycin sensitivity conferring protein [Silene latifolia] (GB:AAN38066.1); contains InterPro domain H+-transporting two-sector ATPase, delta (OSCP) subunit (InterPro:IPR000711) \| chr5:4310317-4312015 REVERSE \| Aliases: T22N19.100, T22N19_100 | 11.0 | 9.5 | 1.5 | 3.4 | 5.00% | 2.5 |
| 7230 | AT5G63060.1 \| similar to SEC14 cytosolic factor family protein / phosphoglyceride transfer family protein [Arabidopsis thaliana] (TAIR:At4g08690.1); similar to sec14 like protein [Oryza sativa (japonica cultivar-group)] (GB:XP_463685.1); contains InterPro domain Cellular retinaldehyde-binding)/triple function, C-terminal (InterPro:IPR001251) \| chr5:25311788-25314075 REVERSE \| Aliases: MDC12.2, MDC12_2 | 4.1 | 3.4 | 0.6 | 3.4 | 5.00% | 2.3 |
| 7231 | AT5G66760.1 \| Symbol: SDH1 1 \| | 10.5 | 9.1 | 1.4 | 3.4 | 5.00% | 2.5 |

| Rank | Description | Sync | Root | M | t | adj.q | B |
|---|---|---|---|---|---|---|---|
| 1 | AT1G61590.1 \| protein kinase, putative, contains protein kinase domain, Pfam:PF00069; similar to serine/threonine protein kinase gi:1066501:gb:AAA81538 \| chr1:22727166-22729739 REVERSE \| Aliases: T25B24.6, T25B24_6 | 2.6 | 11.6 | -9.0 | -57.3 | 1.3E-7 | 17.6 |
| 2 | AT5G59090.3 \| similar to subtilase family protein [Arabidopsis thaliana] (TAIR:At5g58820.1); similar to subtilase family protein [Arabidopsis thaliana] (TAIR:At5g59100.1); similar to subtilase family protein [Arabidopsis thaliana] (TAIR:At5g59130.1); similar to subtilase family protein [Arabidopsis thaliana] (TAIR:At5g58840.1); similar to subtilase family protein [Arabidopsis thaliana] (TAIR:At5g59120.1); similar to pre-pro-cucumisin [Cucumis melo] (GB:BAA06905.1); contains InterPro domain Protease-associated PA (InterPro:IPR003137); contains InterPro domain Subtilase serine protease (InterPro:IPR000209) \| chr5:23869131-23872501 REVERSE \| Aliases: K18B18.5, K18B18_5 | 2.8 | 12.1 | -9.3 | -56.8 | 1.3E-7 | 18.4 |
| 3 | AT1G05260.1 \| Symbol: RCI3 \| peroxidase 3 (PER3) (P3) / rare cold-inducible protein (RCI3A) (PRC), identical to SP:O23044 Peroxidase 3 precursor (EC 1.11.1.7) (Atperox P3) (Rare cold inducible protein) (RCI3A) (ATPRC) {Arabidopsis thalliana} \| chr1:1529767-1531438 FORWARD \| Aliases: PEROXIDASE PRECURSOR, RARE COLD INDUCIBLE GENE 3, RCI3A, YUP8H12.13, YUP8H12_13 | 2.9 | 11.9 | -9.0 | -44.1 | 3.1E-7 | 14.8 |
| 4 | AT3G25930.1 \| universal stress protein (USP) family protein, contains Pfam PF00582: universal stress protein family \| chr3:9494082-9495154 REVERSE \| Aliases: MPE11.10 | 3.1 | 10.9 | -7.8 | -44.0 | 3.1E-7 | 16.2 |
| 5 | AT1G43160.1 \| Symbol: RAP2.6 \| | 2.2 | 11.3 | -9.1 | -43.7 | 3.1E-7 | 15.2 |
| 6 | AT5G44020.1 \| acid phosphatase class B family protein, similar to SP:P15490 STEM 28 kDa glycoprotein precursor (Vegetative storage protein A) {Glycine max}, acid phosphatase (Glycine max) GI:3341443; contains Pfam profile PF03767: HAD superfamily (subfamily IIIB) phosphatase \| chr5:17729638-17731563 FORWARD \| Aliases: MRH10.13, MRH10_13 | 3.3 | 11.0 | -7.7 | -42.9 | 3.1E-7 | 14.9 |
| 7 | AT1G09090.2 \| Symbol: ATRBOHB \| respiratory burst oxidase protein B (RbohB) / NADPH oxidase, identical to respiratory burst oxidase protein B from Arabidopsis thaliana (gi:3242783) \| chr1:2932741-2936588 FORWARD \| Aliases: ATRBOHB, F7G19.4, F7G19_4, RESPIRATORY BURST OXIDASE HOMOLOG B | 1.9 | 12.0 | -10.0 | -42.9 | 3.1E-7 | 14.8 |
| 8 | AT2G16750.1 \| protein kinase family protein, contains Pfam domain, PF00069: Protein kinase domain \| chr2:7278094-7281775 FORWARD \| Aliases: T24I21.16, T24I21_16 | 2.5 | 10.9 | -8.4 | -42.8 | 3.1E-7 | 17.7 |
| 9 | AT2G32270.1 \| Symbol: ZIP3 \| zinc transporter (ZIP3), identical to zinc transporter (Arabidopsis thaliana) gi:3252870:gb:AAC24199; member of the Zinc (Zn2+)-Iron (Fe2+) permease (ZIP) family, PMID:11500563 | 3.4 | 12.7 | -9.3 | -42.2 | 3.1E-7 | 13.3 |
| 10 | AT1G74760.1 \| zinc finger (C3HC4-type RING finger) family protein, contains Pfam domain PF00097: Zinc finger, C3HC4 type (RING finger) | 2.7 | 10.9 | -8.2 | -41.8 | 3.1E-7 | 14.5 |
| 11 | AT3G61430.1 \| Symbol: PIP1A \| plasma membrane intrinsic protein 1A (PIP1A) / aquaporin PIP1.1 (PIP1.1) (AQ1), identical to plasma membrane intrinsic protein 1A SP:P43285 from (Arabidopsis thaliana) | 4.5 | 15.3 | -10.8 | -41.0 | 3.3E-7 | 12.1 |
| 12 | AT1G08320.3 \| similar to bZIP family transcription factor [Arabidopsis thaliana] (TAIR:At5g06839.1); similar to basic leucine zipper protein [Zea mays] (GB:AAC39351.1); similar to bZIP transcription factor [Oryza sativa (japonica cultivar-group)] (GB:AAX94845.1); contains InterPro domain Basic-leucine zipper (bZIP) transcription factor (InterPro:IPR004827) | 3.2 | 10.5 | -7.3 | -38.9 | 4.7E-7 | 15.1 |
| 13 | AT3G01190.1 \| peroxidase 27 (PER27) (P27) (PRXR7), identical to SP:Q43735 Peroxidase 27 precursor (EC 1.11.1.7) (Atperox P27) (PRXR7) (ATP12a) {Arabidopsis thaliana} \| chr3:67065-68543 REVERSE \| Aliases: T4P13.12, T4P13_12 | 3.5 | 12.4 | -8.9 | -36.6 | 7.0E-7 | 13.4 |
| 14 | AT3G48920.1 \| myb family transcription factor (MYB45), similar to MybHv33 GI:456214 from (Hordeum vulgare); contains PFAM profile: myb DNA binding domain PF00249 \| chr3:18150377-18151546 FORWARD \| Aliases: T2J13.240 | 2.4 | 9.7 | -7.3 | -35.8 | 7.8E-7 | 14.0 |
| 15 | AT1G18100.1 \| Symbol: E12A11 \| | 2.3 | 9.8 | -7.4 | -35.4 | 8.1E-7 | 13.4 |
| 16 | AT5G47450.1 \| Symbol: AtTIP2;3 \| major intrinsic family protein / MIP family protein, contains Pfam profile: MIP PF00230 \| chr5:19265476-19266731 REVERSE \| Aliases: DELTA TIP3, MNJ7.4, MNJ7_4, TIP2;3 | 2.8 | 12.3 | -9.4 | -34.0 | 1.0E-6 | 12.7 |
| 17 | AT3G53420.2 \| Symbol: PIP2A \| similar to plasma membrane intrinsic protein 2B (PIP2B) / aquaporin PIP2.2 (PIP2.2) [Arabidopsis thaliana] (TAIR:At2g37170.1); similar to Plasma membrane aquaporin (PAQ2) [Raphanus sativus] (GB:BAA32778.1); contains InterPro domain MIP family (InterPro:IPR000425) | 4.3 | 13.6 | -9.2 | -33.4 | 1.1E-6 | 13.0 |
| 19 | AT4G05200.1 \| protein kinase family protein, contains Prosite:PS00108: Serine/Threonine protein kinases active-site signature \| chr4:2679721-2682307 REVERSE \| Aliases: C17L7.120, C17L7_120 | 2.9 | 10.6 | -7.7 | -32.2 | 1.4E-6 | 12.8 |
| 20 | AT4G20110.1 \| vacuolar sorting receptor, putative, similar to BP-80 vacuolar sorting receptor (Pisum sativum) GI:1737222; identical to vacuolar sorting receptor-like protein (GI:2827665) (Arabidopsis thaliana) \| chr4:10875299-10878740 FORWARD \| Aliases: F1C12.2 | 3.5 | 12.0 | -8.5 | -32.0 | 1.4E-6 | 13.7 |

| Rank | Description | Sync | Root | M | t | adj.q | B |
|---|---|---|---|---|---|---|---|
| 21 | AT3G22600.1 \| protease inhibitor/seed storage/lipid transfer protein (LTP) family protein, contains Pfam protease inhibitor/seed storage/LTP family domain PF00234 \| chr3:8006521-8007478 REVERSE \| Aliases: F16J14.16 | 4.2 | 12.7 | -8.5 | -31.7 | 1.4E-6 | 13.2 |
| 22 | AT3G53480.1 \| ABC transporter family protein, PDR5-like ABC transporter, Spirodela polyrrhiza, EMBL:Z70524 \| chr3:19836285-19842755 FORWARD \| Aliases: F4P12.180 | 3.4 | 11.1 | -7.7 | -31.1 | 1.6E-6 | 14.7 |
| 24 | AT2G28160.1 \| basic helix-loop-helix (bHLH) family protein \| chr2:12011785-12013199 FORWARD \| Aliases: F24D13.5, F24D13_5 | 2.8 | 10.8 | -8.1 | -29.8 | 1.9E-6 | 12.9 |
| 25 | AT1G01620.1 \| Symbol: PIP1C \| plasma membrane intrinsic protein 1C (PIP1C) / aquaporin PIP1.3 (PIP1.3) / transmembrane protein B (TMPB), identical to plasma membrane intrinsic protein 1c SP:Q08733 from (Arabidopsis thaliana) \| chr1:225722-227302 REVERSE \| Aliases: F22L4.16, F22L4_16, PIP1;3, TMP B | 3.6 | 12.2 | -8.6 | -29.8 | 1.9E-6 | 13.7 |
| 27 | AT5G64100.1 \| peroxidase, putative, identical to peroxidase ATP3a (Arabidopsis thaliana) gi:1546698:emb:CAA67340 \| chr5:25667867-25669349 REVERSE \| Aliases: MHJ24.8, MHJ24_8 | 3.2 | 11.8 | -8.6 | -29.7 | 1.9E-6 | 11.6 |
| 28 | AT4G15390.1 \| transferase family protein, similar to alcohol acyltransferase (Fragaria x ananassa)(GI:10121328)(PMID:10810141), deacetylvindoline 4-O-acetyltransferase (Catharanthus roseus)(GI:4091808)(PMID:9681034) \| chr4:8792812-8794293 REVERSE \| Aliases: DL3740C, FCAALL.282 | 3.7 | 13.5 | -9.8 | -29.6 | 1.9E-6 | 10.2 |
| 29 | AT1G48930.1 \| endo-1,4-beta-glucanase, putative / cellulase, putative, similar to endo-beta-1,4-glucanase GI:4972236 from (Fragaria x ananassa) \| chr1:18105311-18108329 REVERSE \| Aliases: F27K7.5 | 2.8 | 9.2 | -6.4 | -29.3 | 2.0E-6 | 12.9 |
| 30 | AT3G11340.1 \| UDP-glucoronosyl/UDP-glucosyl transferase family protein, contains Pfam profile: PF00201 UDP-glucoronosyl and UDP-glucosyl transferase \| chr3:3556713-3558275 FORWARD \| Aliases: F11B9.23 | 3.0 | 10.5 | -7.4 | -28.8 | 2.1E-6 | 12.0 |
| 31 | AT1G72200.1 \| zinc finger (C3HC4-type RING finger) family protein, contains Pfam profile: PF00097: Zinc finger, C3HC4 type (RING finger) \| chr1:27173457-27174983 REVERSE \| Aliases: T9N14.11, T9N14_11 | 3.0 | 10.9 | -7.9 | -28.7 | 2.1E-6 | 13.8 |
| 32 | AT4G11530.1 \| protein kinase family protein, contains Pfam PF00069: Protein kinase domain \| chr4:6985617-6989593 FORWARD \| Aliases: F25E4.150, F25E4_150 | 3.0 | 8.7 | -5.7 | -28.6 | 2.1E-6 | 13.4 |
| 33 | AT5G44480.1 \| Symbol: DUR \| NAD-dependent epimerase/dehydratase family protein, similar to SP:P55180 UDP-glucose 4-epimerase (EC 5.1.3.2) from Bacillus subtilis, GI:3021357 UDP-galactose 4-epimerase from Cyamopsis tetragonoloba; contains Pfam profile PF01370 NAD dependent epimerase/dehydratase family | 3.6 | 11.3 | -7.7 | -28.3 | 2.3E-6 | 13.1 |
| 34 | AT5G09530.1 \| hydroxyproline-rich glycoprotein family protein, contains proline-rich extensin domains, INTERPRO:IPR002965 \| chr5:2959735-2961272 REVERSE \| Aliases: T5E8.2 | 3.2 | 10.4 | -7.2 | -28.2 | 2.3E-6 | 14.9 |
| 35 | AT5G63600.2 \| similar to flavonol synthase 1 (FLS1) [Arabidopsis thaliana] (TAIR:At5g08640.1); similar to FLS_MATIN Flavonol synthase/flavanone 3-hydroxylase (FLS) (GB:O04395); contains InterPro domain 2OG-Fe(II) oxygenase superfamily (InterPro:IPR005123) \| chr5:25477910-25479684 REVERSE \| Aliases: MBK5.7, MBK5_7 | 4.4 | 13.0 | -8.6 | -28.0 | 2.3E-6 | 12.1 |
| 39 | AT5G07680.2 \| Symbol: ANAC079/ANAC080 \| no apical meristem (NAM) family protein, contains Pfam PF02365 : No apical meristem (NAM) protein; similar to cup-shaped cotyledon CUC2 (GI:1944132) (Arabidopsis thaliana) \| chr5:2435984-2437500 FORWARD \| Aliases: ANAC079, ANAC080, MBK20.13, MBK20_13 | 2.8 | 8.6 | -5.7 | -27.3 | 2.6E-6 | 14.2 |
| 41 | AT4G16260.1 \| glycosyl hydrolase family 17 protein, similar to glucan endo-1,3-beta-glucosidase, basic vacuolar isoform precursor SP:P52407 from (Hevea brasiliensis) \| chr4:9200025-9201544 REVERSE \| Aliases: DL4170C, FCAALL.386 | 3.8 | 11.4 | -7.6 | -27.0 | 2.6E-6 | 12.5 |
| 42 | AT4G02270.1 \| pollen Ole e 1 allergen and extensin family protein, contains Pfam domain, PF01190: Pollen proteins Ole e I family \| chr4:992175-993031 REVERSE \| Aliases: T2H3.4, T2H3_4 | 2.5 | 11.2 | -8.7 | -27.0 | 2.6E-6 | 10.3 |
| 43 | AT5G67400.1 \| peroxidase 73 (PER73) (P73) (PRXR11), identical to SP:Q43873 Peroxidase 73 precursor (EC 1.11.1.7) (Atperox P73) (PRXR11) (ATP10a) {Arabidopsis thaliana} \| chr5:26912082-26913714 FORWARD \| Aliases: K8K14.13, K8K14_13 | 2.9 | 11.0 | -8.1 | -26.9 | 2.7E-6 | 12.4 |
| 44 | AT5G63590.1 \| flavonol synthase, putative, similar to SP:Q96330 Flavonol synthase 1 (EC 1.14.11.-) (FLS 1) {Arabidopsis thaliana}; contains Pfam profile PF03171: oxidoreductase, 2OG-Fe(II) oxygenase family \| chr5:25474219-25475696 REVERSE \| Aliases: MBK5.5, MBK5_5 | 4.4 | 10.8 | -6.5 | -26.8 | 2.7E-6 | 12.4 |
| 45 | AT2G26820.1 \| Symbol: ATPP2 A3 \| avirulence-responsive family protein / avirulence induced gene (AIG1) family protein, similar to SP:P54120 AIG1 protein {Arabidopsis thaliana}; contains Pfam profile PF04548: AIG1 family \| chr2:11444886-11449277 FORWARD \| Aliases: ATPP2 A3, F12C20.14, F12C20_14 | 3.1 | 10.2 | -7.2 | -26.7 | 2.7E-6 | 12.7 |
| 46 | AT4G23400.1 \| Symbol: PIP1;5/PIP1D \| major intrinsic family protein / MIP family protein, contains Pfam profile: MIP PF00230 \| chr4:12220753-12222380 FORWARD \| Aliases: F16G20.100, F16G20_100, PCR55, PIP1;5, PIP1D | 3.4 | 12.4 | -9.0 | -26.6 | 2.7E-6 | 12.6 |

| Rank | Description | Sync | Root | M | t | adj.q | B |
|---|---|---|---|---|---|---|---|
| 48 | AT5G19890.1 \| peroxidase, putative, identical to peroxidase (Arabidopsis thaliana) gi:1403134:emb:CAA67092 \| chr5:6724117-6725925 REVERSE \| Aliases: F28I16.40, F28I16_40 | 3.4 | 11.9 | -8.5 | -26.5 | 2.7E-6 | 13.6 |
| 49 | AT4G11290.1 \| peroxidase, putative, identical to peroxidase ATP19a (Arabidopsis thaliana) gi:1546692:emb:CAA67337 \| chr4:6869959-6871657 FORWARD \| Aliases: F8L21.80, F8L21_80 | 2.0 | 9.0 | -7.0 | -25.9 | 3.1E-6 | 12.3 |
| 50 | AT3G26520.1 \| Symbol: TIP2 \| tonoplast intrinsic protein, putative, similar to tonoplast intrinsic protein GI:5081419 from (Brassica napus) \| chr3:9723680-9725052 REVERSE \| Aliases: GAMMA TIP2, MFE16.17, SALT STRESS INDUCIBLE PROTEIN, SITIP, TIP1;2, TONOPLAST INTRINSIC PROTEIN 2 | 3.9 | 12.4 | -8.5 | -25.6 | 3.3E-6 | 11.9 |
| 52 | AT3G29035.1 \| Symbol: ANAC059 \| no apical meristem (NAM) family protein, contains Pfam PF02365 : No apical meristem (NAM) protein; similar to cup-shaped cotyledon CUC2 (GI:1944132) (Arabidopsis thaliana) \| chr3:11035069-11036467 FORWARD \| Aliases: ANAC059, MRI12.1 | 3.4 | 10.6 | -7.2 | -25.5 | 3.3E-6 | 12.1 |
| 53 | AT5G26280.1 \| meprin and TRAF homology domain-containing protein / MATH domain-containing protein, low similarity to ubiquitin-specific protease 12 (Arabidopsis thaliana) GI:11993471; contains Pfam profile PF00917: MATH domain \| chr5:9208715-9210575 FORWARD \| Aliases: F9D12.7 | 4.5 | 11.4 | -6.9 | -25.2 | 3.6E-6 | 13.4 |
| 54 | AT1G10970.1 \| Symbol: ZIP4 \| metal transporter, putative (ZIP4), similar to Zn and Cd transporter ZNT1 (Thlaspi caerulescens) gi:7381054:gb:AAF61374; member of the Zinc (Zn2+)-Iron (Fe2+) permease (ZIP) family, PMID:11500563 \| chr1:3665089-3667139 REVERSE \| Aliases: T19D16.11, T19D16_11, ZRT, IRT LIKE PROTEIN | 4.8 | 11.4 | -6.5 | -25.2 | 3.6E-6 | 11.8 |
| 55 | AT5G19970.1 \| expressed protein, ; expression supported by MPSS \| chr5:6747552-6748643 FORWARD \| Aliases: F28I16.120, F28I16_120 | 3.3 | 10.2 | -6.9 | -25.1 | 3.6E-6 | 11.5 |
| 56 | AT1G49500.1 \| expressed protein \| chr1:18325476-18325952 REVERSE \| Aliases: F13F21.6, F13F21_6 | 4.4 | 10.9 | -6.5 | -25.0 | 3.7E-6 | 12.8 |
| 57 | AT4G17340.1 \| Symbol: DELTA TIP2/TIP2;2 \| major intrinsic family protein / MIP family protein, contains Pfam profile: MIP PF00230 \| chr4:9699265-9700421 FORWARD \| Aliases: DELTA TIP2, DL4705W, FCAALL.412, TIP2;2 | 5.3 | 13.7 | -8.4 | -24.8 | 3.8E-6 | 10.2 |
| 58 | AT5G44380.1 \| FAD-binding domain-containing protein, similar to SP:P30986 reticuline oxidase precursor (Berberine-bridge-forming enzyme) (BBE) (Tetrahydroprotoberberine synthase) (Eschscholzia californica); contains PF01565 FAD binding domain \| chr5:17895949-17898666 REVERSE \| Aliases: K9L2.18, K9L2_18 | 3.7 | 11.0 | -7.3 | -24.8 | 3.8E-6 | 13.5 |
| 59 | AT2G36100.1 \| integral membrane family protein, contains TIGRFAM TIGR01569 : plant integral membrane protein TIGR01569; contains Pfam PF04535 : Domain of unknown function (DUF588) \| chr2:15166823-15167801 REVERSE \| Aliases: F9C22.3, F9C22_3 | 4.4 | 11.1 | -6.7 | -24.7 | 3.9E-6 | 11.2 |
| 60 | AT4G25820.1 \| Symbol: XTR9 \| xyloglucan:xyloglucosyl transferase / xyloglucan endotransglycosylase / endo-xyloglucan transferase (XTR9), identical to xyloglucan endotransglycosylase GI:4218963 from (Arabidopsis thaliana) \| chr4:13130306-13131797 FORWARD \| Aliases: F14M19.100, F14M19_100, XYLOGLUCAN ENDOTRANSGLYCOSYLASE 9 | 4.1 | 11.4 | -7.2 | -24.6 | 3.9E-6 | 12.9 |
| 61 | AT1G13300.1 \| myb family transcription factor, contains Pfam domain, PF00249: Myb-like DNA-binding domain \| chr1:4556889-4558705 FORWARD \| Aliases: T6J4.6, T6J4_6 | 5.5 | 11.7 | -6.2 | -24.5 | 3.9E-6 | 12.4 |
| 62 | AT5G54040.1 \| DC1 domain-containing protein, contains Pfam profile PF03107: DC1 domain | 2.6 | 11.5 | -8.9 | -24.3 | 4.2E-6 | 15.0 |
| 63 | AT3G61390.2 \| U-box domain-containing protein, several hypothetical proteins - Arabidopsis thaliana \| chr3:22727328-22729348 FORWARD \| Aliases: F2A19.1 | 2.6 | 9.2 | -6.6 | -24.2 | 4.2E-6 | 14.7 |
| 64 | AT1G44050.1 \| DC1 domain-containing protein, contains Pfam profile PF03107: DC1 domain | 2.4 | 9.2 | -6.8 | -24.1 | 4.4E-6 | 14.6 |
| 65 | AT5G56870.1 \| beta-galactosidase, putative / lactase, putative, similar to beta-galactosidase precursor GI:3869280 from (Carica papaya) \| chr5:23021422-23025875 FORWARD \| Aliases: MPI10.3, MPI10_3 | 4.3 | 12.0 | -7.7 | -24.0 | 4.4E-6 | 10.7 |
| 67 | AT1G13110.1 \| Symbol: CYP71B7 \| cytochrome P450 71B7 (CYP71B7), identical to (SP:Q96514) cytochrome P450 71B7 (Arabidopsis thaliana); | 2.9 | 9.9 | -6.9 | -24.0 | 4.4E-6 | 12.0 |
| 69 | AT4G32650.3 \| Symbol: ATKC1 \| similar to inward rectifying potassium channel (KAT2) [Arabidopsis thaliana] (TAIR:At4g18290.1); similar to potassium channel [Daucus carota] (GB:CAB62555.1); contains InterPro domain Cation channel, non-ligand gated (InterPro:IPR005820); contains InterPro domain EAG/ELK/ERG potassium channel (InterPro:IPR003938); contains InterPro domain Ion transport protein (InterPro:IPR005821); contains InterPro domain Cyclic nucleotide-binding domain (InterPro:IPR000595) | 2.8 | 10.0 | -7.3 | -23.4 | 5.0E-6 | 12.1 |
| 70 | AT5G66390.1 \| peroxidase 72 (PER72) (P72) (PRXR8), identical to SP:Q9FJZ9 Peroxidase 72 precursor (EC 1.11.1.7) (Atperox P72) (PRXR8) (ATP6a) {Arabidopsis thaliana} \| chr5:26533142-26534610 REVERSE \| Aliases: K1F13.4, K1F13_4 | 2.9 | 11.0 | -8.0 | -23.4 | 5.1E-6 | 13.1 |

| Rank | Description | Sync | Root | M | t | adj.q | B |
|---|---|---|---|---|---|---|---|
| 71 | AT1G60960.1 \| Symbol: IRT3 \| metal transporter, putative (IRT3), identical to putative metal transporter IRT3 (Arabidopsis thaliana) gi:17385796:gb:AAL38438; similar to iron-regulated transporter 1 (Lycopersicon esculentum) gi:9716481:gb:AAF97509; member of the Zinc (Zn2+)-Iron (Fe2+) permease (ZIP) family, PMID:11500563 \| chr1:22448988-22450810 REVERSE \| Aliases: T7P1.10, T7P1_10 | 8.0 | 12.2 | -4.2 | -23.3 | 5.1E-6 | 13.6 |
| 72 | AT4G30170.1 \| peroxidase, putative, identical to peroxidase ATP8a (Arabidopsis thaliana) gi:1546706:emb:CAA67361 \| chr4:14762847-14764633 FORWARD \| Aliases: F9N11.20, F9N11_20 | 2.4 | 10.0 | -7.6 | -23.2 | 5.3E-6 | 11.9 |
| 73 | AT5G52790.1 \| CBS domain-containing protein-related, contains Pfam profile PF01595: Domain of unknown function, weak hit to PF00571: CBS domain \| chr5:21408943-21411585 REVERSE \| Aliases: F6N7.28, F6N7_28 | 3.0 | 10.7 | -7.6 | -23.0 | 5.6E-6 | 10.4 |
| 74 | AT2G35585.1 \| expressed protein \| chr2:14943745-14945042 FORWARD \| Aliases: None | 2.8 | 9.4 | -6.7 | -22.6 | 6.4E-6 | 12.0 |
| 75 | AT1G30750.1 \| expressed protein \| chr1:10904889-10905691 REVERSE \| Aliases: T5I8.20 | 2.6 | 9.4 | -6.8 | -22.5 | 6.5E-6 | 11.0 |
| 79 | AT4G00700.1 \| C2 domain-containing protein, contains INTERPRO:IPR000008 C2 domain \| chr4:286051-289514 FORWARD \| Aliases: F6N23.8, F6N23_8 | 3.3 | 9.7 | -6.4 | -22.2 | 6.9E-6 | 11.0 |
| 80 | AT1G13080.2 \| Symbol: CYP71B2 \| cytochrome P450 family protein, identical to gb:D78605 cytochrome P450 monooxygenase from Arabidopsis thaliana and is a member of the PF:00067 Cytochrome P450 family. ESTs gb:Z18072, gb:Z35218 and gb:T43466 come from this gene \| chr1:4459164-4460938 FORWARD \| Aliases: CYTOCHROME P450 71B2, CYTOCHROME P450 MONOOXYGENASE 71B2, F3F19.10, F3F19_10 | 2.9 | 10.7 | -7.7 | -22.1 | 7.0E-6 | 11.4 |
| 81 | AT3G21260.2 \| glycolipid transfer protein-related, contains weak similarity to Glycolipid transfer protein (GLTP) (Swiss-Prot:P17403) (Sus scrofa) \| chr3:7464026-7465100 REVERSE \| Aliases: MXL8.12 | 3.3 | 10.3 | -7.0 | -21.8 | 7.6E-6 | 12.6 |
| 82 | AT4G05100.1 \| myb family transcription factor (MYB74), contains Pfam profile: PF00249 myb-like DNA-binding domain; identical to cDNA putative transcription factor (MYB74) mRNA, partial cds GI:3941505 \| chr4:2618452-2619885 FORWARD \| Aliases: C17L7.20, C17L7_20 | 2.8 | 8.7 | -6.0 | -21.7 | 7.9E-6 | 12.8 |
| 83 | AT5G65210.5 \| similar to bZIP family transcription factor (OBF4) [Arabidopsis thaliana] (TAIR:At5g10030.1); similar to TGACG-motif-binding protein (GB:AAA75414.1); contains InterPro domain Basic-leucine zipper (bZIP) transcription factor (InterPro:IPR004827) \| chr5:26075057-26078330 FORWARD \| Aliases: MQN23.15, MQN23_15 | 3.8 | 10.1 | -6.3 | -21.7 | 7.9E-6 | 12.4 |
| 86 | AT5G58350.1 \| Symbol: WNK4 \| protein kinase family protein, contains protein kinase domain, Pfam:PF00069 \| chr5:23602401-23605027 FORWARD \| Aliases: MCK7.22, MCK7_22, ZIK2 | 3.6 | 9.2 | -5.6 | -21.5 | 8.2E-6 | 13.3 |
| 87 | AT3G62680.1 \| Symbol: PRP3 \| proline-rich family protein, contains proline-rich region, INTERPRO:IPR000694 \| chr3:23193698-23194970 FORWARD \| Aliases: ATPRP3, F26K9.110, PROLINE RICH PROTEIN 3 | 5.0 | 11.0 | -6.0 | -21.4 | 8.2E-6 | 11.8 |
| 88 | AT2G01880.1 \| purple acid phosphatase (PAP7), identical to purple acid phosphatase (PAP7) GI:20257476 from (Arabidopsis thaliana); contains Pfam profile: PF00149 calcineurin-like phosphoesterase \| chr2:391422-393999 REVERSE \| Aliases: T23K3.7, T23K3_7 | 2.8 | 9.9 | -7.1 | -21.4 | 8.4E-6 | 11.7 |
| 89 | AT2G29330.1 \| tropinone reductase, putative / tropine dehydrogenase, putative, similar to tropinone reductase SP:P50165 from (Datura stramonium) \| chr2:12601667-12603407 FORWARD \| Aliases: F16P2.29, F16P2_29 | 2.1 | 7.9 | -5.8 | -21.2 | 8.5E-6 | 13.0 |
| 91 | AT4G13580.1 \| disease resistance-responsive family protein, contains similarity to pathogenesis-related protein (Pisum sativum) gi:4585273:gb:AAD25355 | 3.7 | 11.7 | -8.0 | -21.2 | 8.5E-6 | 9.5 |
| 92 | AT2G22170.1 \| lipid-associated family protein, contains PLAT/LH2 (Polycystin-1, Lipoxygenase, Alpha-Toxin/Lipoxygenase homology) domain Pfam:PF01477 \| chr2:9433929-9434891 REVERSE \| Aliases: T26C19.17, T26C19_17 | 3.8 | 11.5 | -7.7 | -21.1 | 8.5E-6 | 10.0 |
| 94 | AT3G20960.1 \| Symbol: CYP705A33 \| cytochrome P450 family protein, similar to Cytochrome P450 93A1 (SP:Q42798) | 2.8 | 8.5 | -5.7 | -21.1 | 8.5E-6 | 12.2 |
| 95 | AT1G20380.1 \| prolyl oligopeptidase, putative / prolyl endopeptidase, putative / post-proline cleaving enzyme, putative, similar to SP:P48147 Prolyl endopeptidase (EC 3.4.21.26) (Post-proline cleaving enzyme) {Homo sapiens}; contains Pfam profiles PF00326: prolyl oligopeptidase family, PF02897: Prolyl oligopeptidase, N-terminal beta-propeller domain \| chr1:7061655-7065168 FORWARD \| Aliases: F14O10.2, F14O10_2 | 4.8 | 10.7 | -5.9 | -21.1 | 8.5E-6 | 10.8 |

| Rank | Description | Sync | Root | M | t | adj.q | B |
|---|---|---|---|---|---|---|---|
| 97 | AT1G05570.1 \| Symbol: CALS1 \| similar to glycosyl transferase family 48 protein [Arabidopsis thaliana] (TAIR:At5g13000.1); similar to glycosyl transferase family 48 protein [Arabidopsis thaliana] (TAIR:At2g31960.1); similar to glycosyl transferase family 48 protein [Arabidopsis thaliana] (TAIR:At1g06490.1); similar to glycosyl transferase family 48 protein [Arabidopsis thaliana] (TAIR:At2g13680.1); similar to glycosyl transferase family 48 protein [Arabidopsis thaliana] (TAIR:At3g59100.1); similar to putative callose synthase 1 catalytic subunit [Oryza sativa (japonica cultivar-group)] (GB:BAD72533.1); similar to Putative glucan synthase [Oryza sativa (japonica cultivar-group)] (GB:NP_912480.1); similar to OJ1029_F04.4 [Oryza sativa (japonica cultivar-group)] (GB:NP_918100.1); similar to putative beta-1,3-glucan synthase [Nicotiana alata] (GB:AAK49452.2); similar to putative callose synthase 1 catalytic subunit [Oryza sativa (japonica cultivar-group)] (GB:BAD62105.1); contains InterPro domain Glycosyl transferase, family 48 (InterPro:IPR003440) \| chr1:1647719-1658685 REVERSE \| Aliases: ATGSL06, ATGSL6, CALLOSE SYNTHASE 1, F3F20.1, F3F20_1, GLUCAN SYNTHASE LIKE 6, GSL06, GSL6 | 6.2 | 11.9 | -5.7 | -21.0 | 8.8E-6 | 11.1 |
| 98 | AT4G24130.1 \| expressed protein, contains Pfam profile PF04398: Protein of unknown function, DUF538 \| chr4:12527785-12528602 FORWARD \| Aliases: T19F6.120, T19F6_120 | 3.5 | 10.5 | -7.0 | -20.9 | 8.8E-6 | 11.7 |
| 100 | AT1G77330.1 \| 1-aminocyclopropane-1-carboxylate oxidase, putative / ACC oxidase, putative, similar to 1-aminocyclopropane-1-carboxylate oxidase GI:3386565 from (Sorghum bicolor) \| chr1:29067884-29069431 REVERSE \| Aliases: F2P24.4, F2P24_4 | 6.0 | 12.0 | -6.0 | -20.7 | 9.4E-6 | 11.2 |
| 101 | AT2G32150.1 \| haloacid dehalogenase-like hydrolase family protein, contains InterPro accession IPR005834: Haloacid dehalogenase-like hydrolase | 5.2 | 11.0 | -5.8 | -20.7 | 9.5E-6 | 11.3 |
| 102 | AT4G28890.1 \| zinc finger (C3HC4-type RING finger) family protein, contains Pfam profile: PF00097 zinc finger, C3HC4 type (RING finger) \| chr4:14256339-14257726 REVERSE \| Aliases: F25O24.10, F25O24_10 | 3.3 | 10.5 | -7.3 | -20.6 | 9.6E-6 | 11.9 |
| 103 | AT4G33020.1 \| Symbol: ZIP9 \| metal transporter, putative (ZIP9), identical to putative metal transporter ZIP9 (Arabidopsis thaliana) gi:17385790:gb:AAL38435; similar to Zn and Cd transporter ZNT1 (Thlaspi caerulescens) gi:7381054:gb:AAF61374; member of the Zinc (Zn2+)-Iron (Fe2+) permease (ZIP) family, PMID:11500563 | 2.9 | 10.5 | -7.7 | -20.5 | 1.0E-5 | 9.8 |
| 105 | AT2G45960.1 \| Symbol: PIP1B \| plasma membrane intrinsic protein 1B (PIP1B) / aquaporin PIP1.2 (PIP1.2) / transmembrane protein A (TMPA), identical to plasma membrane intrinsic protein 1B SP:Q06611 from (Arabidopsis thaliana) \| chr2:18917384-18919035 FORWARD \| Aliases: ATHH2, F4I18.6, PIP1;2, PIP1B, TMP A | 6.0 | 13.4 | -7.4 | -20.4 | 1.0E-5 | 10.4 |
| 107 | AT3G13610.1 \| oxidoreductase, 2OG-Fe(II) oxygenase family protein, similar to desacetoxyvindoline 4-hydroxylase (Catharanthus roseus)(GI:1916643), flavonol synthase 1 (SP:Q96330); contains PF03171 2OG-Fe(II) oxygenase superfamily domain \| chr3:4449455-4451184 FORWARD \| Aliases: K20M4.9 | 3.0 | 10.0 | -6.9 | -20.3 | 1.0E-5 | 10.7 |
| 108 | AT1G30870.1 \| cationic peroxidase, putative, similar to cationic peroxidase (gi:1232069); similar to EST gb:AI100412 \| chr1:10991466-10993004 FORWARD \| Aliases: T17H7.19 | 3.1 | 10.6 | -7.4 | -20.3 | 1.0E-5 | 12.2 |
| 109 | AT4G15340.1 \| Symbol: ATPEN1 \| | 2.3 | 8.6 | -6.3 | -20.3 | 1.0E-5 | 11.7 |
| 110 | AT5G01520.2 \| zinc finger (C3HC4-type RING finger) family protein, similar to MTD2 (Medicago truncatula) GI:9294812; contains Pfam profile PF00097: Zinc finger, C3HC4 type (RING finger) \| chr5:206431-208610 FORWARD \| Aliases: F7A7.40, F7A7_40 | 4.4 | 9.7 | -5.2 | -20.2 | 1.1E-5 | 11.2 |
| 111 | AT1G62660.1 \| beta-fructosidase (BFRUCT3) / beta-fructofuranosidase / invertase, vacuolar, identical to beta-fructosidase GB:CAA67560 GI:1429209 (Arabidopsis thaliana); supported by full-length cDNA GI:14517549; identical to cDNA | 3.0 | 10.7 | -7.7 | -20.1 | 1.1E-5 | 12.3 |
| 112 | AT3G29410.1 \| terpene synthase/cyclase family protein, similar to terpene synthase GB:CAA72074 from (Arabidopsis thaliana), contains Pfam profile: PF01397 terpene synthase family \| chr3:11303672-11306361 REVERSE \| Aliases: MUO10.2 | 2.8 | 9.7 | -6.8 | -20.0 | 1.1E-5 | 9.9 |
| 113 | AT5G43580.1 \| protease inhibitor, putative, similar to SP:P19873 Inhibitor of trypsin and hageman factor (CMTI-V) {Cucurbita maxima}; contains Pfam profile PF00280: Potato inhibitor I family | 3.5 | 9.8 | -6.3 | -20.0 | 1.1E-5 | 11.3 |
| 114 | AT5G48290.2 \| similar to heavy-metal-associated domain-containing protein [Arabidopsis thaliana] (TAIR:At3g07600.1); similar to OSJNBa0016O02.17 [Oryza sativa (japonica cultivar-group)] (GB:XP_472812.1); contains InterPro domain Proline-rich region (InterPro:IPR000694) \| chr5:19586122-19587025 FORWARD \| Aliases: K23F3.1, K23F3_1 | 2.3 | 9.5 | -7.1 | -20.0 | 1.1E-5 | 11.7 |
| 115 | AT5G04730.1 \| expressed protein, ; expression supported by MPSS \| chr5:1364102-1365067 REVERSE \| Aliases: MUK11.5 | 2.4 | 8.3 | -5.9 | -20.0 | 1.1E-5 | 11.6 |
| 116 | AT2G28110.1 \| Encodes a protein with similarity to glycosyl transferase family 47 proteins that is involved in secondary cell wall biosynthesis. Mutants have irregular xylem formation, reduced cellulose levels and plants are smaller than normal siblings. | 3.1 | 9.9 | -6.8 | -19.9 | 1.1E-5 | 10.4 |
| 117 | AT3G48360.1 \| speckle-type POZ | 3.3 | 9.7 | -6.4 | -19.8 | 1.1E-5 | 10.9 |

| Rank | Description | Sync | Root | M | t | adj.q | B |
|---|---|---|---|---|---|---|---|
| 118 | AT1G08930.2 \| Symbol: ERD6 \| similar to sugar transporter, putative [Arabidopsis thaliana] (TAIR:At1g08920.1); similar to integral membrane protein [Beta vulgaris] (GB:AAB53155.1); contains InterPro domain Sugar transporter superfamily (InterPro:IPR005829); contains InterPro domain Major facilitator superfamily (MFS) (InterPro:IPR007114); contains InterPro domain General substrate transporter (InterPro:IPR005828); contains InterPro domain Sugar transporter (InterPro:IPR003663) \| chr1:2873468-2877273 FORWARD \| Aliases: ERD6, F7G19.19, F7G19_19 | 4.3 | 10.1 | -5.7 | -19.8 | 1.2E-5 | 12.4 |
| 119 | AT4G35060.1 \| heavy-metal-associated domain-containing protein / copper chaperone (CCH)-related, low similarity to copper homeostasis factor (GI:3168840)(PMID:9701579); similar to farnesylated proteins GMFP7 (Glycine max)(GI:4097573) and ATFP6 (GI:4097553); contains heavy-metal-associated domain PF00403 \| chr4:16685725-16686708 REVERSE \| Aliases: T12J5.12 | 3.3 | 9.8 | -6.6 | -19.8 | 1.2E-5 | 10.9 |
| 121 | AT3G14850.2 \| expressed protein, similar to expressed protein [Arabidopsis thaliana] (TAIR:At1g29050.1); similar to unknown protein [Oryza sativa (japonica cultivar-group)] (GB:BAD73054.1); similar to unknown protein [Oryza sativa (japonica cultivar-group)] (GB:BAD73055.1); contains InterPro domain Protein of unknown function DUF231 (InterPro:IPR004253) \| chr3:4995604-4997700 FORWARD \| Aliases: T21E2.11 | 3.4 | 9.6 | -6.3 | -19.5 | 1.2E-5 | 10.0 |
| 122 | AT2G37130.1 \| peroxidase 21 (PER21) (P21) (PRXR5), identical to SP:Q42580 Peroxidase 21 precursor (EC 1.11.1.7) (Atperox P21) (PRXR5) (ATP2a/ATP2b) {Arabidopsis thaliana} \| chr2:15605000-15607137 REVERSE \| Aliases: T2N18.11, T2N18_11 | 4.0 | 12.1 | -8.1 | -19.4 | 1.3E-5 | 12.6 |
| 123 | AT2G36380.1 \| ABC transporter family protein, related to multi drug resistance proteins and P-glycoproteins \| chr2:15264662-15270766 FORWARD \| Aliases: F1O11.1, F1O11_1 | 6.1 | 10.9 | -4.7 | -19.4 | 1.3E-5 | 12.3 |
| 125 | AT4G17215.1 \| expressed protein \| chr4:9655848-9656588 FORWARD \| Aliases: None | 3.8 | 9.8 | -6.0 | -19.3 | 1.3E-5 | 11.5 |
| 126 | AT2G37750.1 \| expressed protein \| chr2:15838067-15838636 FORWARD \| Aliases: T8P21.37 | 3.8 | 10.8 | -7.0 | -19.3 | 1.3E-5 | 9.5 |
| 127 | AT3G43800.1 \| Symbol: ATGSTU27 \| glutathione S-transferase, putative, glutathione transferase, papaya, PIR:T09781 \| chr3:15671846-15672912 FORWARD \| Aliases: T28A8.90 | 5.1 | 10.6 | -5.5 | -19.3 | 1.3E-5 | 12.4 |
| 128 | AT2G21045.1 \| similar to senescence-associated family protein [Arabidopsis thaliana] (TAIR:At5g66170.1); similar to Ntdin [Nicotiana tabacum] (GB:BAA88985.2); contains InterPro domain Rhodanese-like (InterPro:IPR001763) \| chr2:9034933-9036101 FORWARD \| Aliases: None | 4.5 | 11.2 | -6.7 | -19.2 | 1.3E-5 | 10.0 |
| 129 | AT3G14680.1 \| Symbol: CYP72A14 \| cytochrome P450, putative, similar to GB:Q05047 from (Catharanthus roseus) \| chr3:4934428-4936570 FORWARD \| Aliases: MIE1.1 | 3.4 | 10.1 | -6.6 | -19.2 | 1.3E-5 | 10.4 |
| 130 | AT4G34580.1 \| similar to SEC14 cytosolic factor, putative / phosphoglyceride transfer protein, putative [Arabidopsis thaliana] (TAIR:At4g39180.1); similar to SEC14 cytosolic factor family protein / phosphoglyceride transfer family protein [Arabidopsis thaliana] (TAIR:At2g16380.1); similar to SEC14 cytosolic factor, putative / phosphoglyceride transfer protein, putative [Arabidopsis thaliana] (TAIR:At2g21540.1); similar to putative hosphatidylinositol/phophatidylcholine transfer protein [Oryza sativa (japonica cultivar-group)] (GB:XP_464026.1); similar to putative SEC14 cytosolic factor [Oryza sativa (japonica cultivar-group)] (GB:XP_465384.1); contains InterPro domain Cellular retinaldehyde-binding)/triple function, C-terminal (InterPro:IPR001251); contains InterPro domain Cellular retinaldehyde binding/alpha-tocopherol transport (InterPro:IPR001071); contains InterPro domain Cellular retinaldehyde-binding/triple function, N-terminal (InterPro:IPR008273) \| chr4:16515332-16519539 FORWARD \| Aliases: T4L20.160, T4L20_160 | 3.2 | 10.4 | -7.2 | -19.2 | 1.3E-5 | 10.4 |
| 131 | AT4G18510.1 \| Symbol: CLE2 \| Clavata3 / ESR-Related-2 (CLE2), CLAVATA3/ESR-Related-2 (CLE2) \| chr4:10211880-10212412 FORWARD \| Aliases: CLAVATA3/ESR RELATED, F28J12.170, F28J12_170 | 2.9 | 9.5 | -6.6 | -19.1 | 1.4E-5 | 10.5 |
| 132 | AT4G26470.1 \| calcium-binding EF hand family protein, low similarity to SP:P06787 Calmodulin {Saccharomyces cerevisiae}; contains INTERPRO:IPR002048 calcium-binding EF-hand domain \| chr4:13371078-13372351 FORWARD \| Aliases: M3E9.100, M3E9_100 | 3.2 | 9.3 | -6.1 | -18.8 | 1.6E-5 | 10.6 |
| 133 | AT4G36860.2 \| LIM domain-containing protein, low similarity to LIM-domain protein (Branchiostoma floridae) GI:3360516, DRAL/Slim3/FHL2 (Homo sapiens) GI:7209525; contains Pfam profile PF00412: LIM domain \| chr4:17358376-17361872 REVERSE \| Aliases: AP22.33, AP22_33 | 5.9 | 11.3 | -5.4 | -18.7 | 1.6E-5 | 11.7 |
| 136 | AT3G16560.1 \| protein phosphatase 2C-related / PP2C-related, contains protein phosphatase 2C domain \| chr3:5635602-5638488 REVERSE \| Aliases: MDC8.3 | 3.4 | 8.8 | -5.5 | -18.6 | 1.6E-5 | 10.2 |
| 138 | AT5G63410.1 \| leucine-rich repeat transmembrane protein kinase, putative, contains similarity to receptor-like protein kinase \| chr5:25412211-25415379 REVERSE \| Aliases: MLE2.4, MLE2_4 | 4.1 | 9.4 | -5.3 | -18.6 | 1.6E-5 | 10.9 |
| 139 | AT3G45600.1 \| senescence-associated family protein, similar to senescence-associated protein 5 (Hemerocallis hybrid cultivar) gi:3551954:gb:AAC34855 \| chr3:16744740-16746936 REVERSE \| Aliases: F9K21.180 | 4.2 | 9.6 | -5.4 | -18.5 | 1.6E-5 | 11.4 |
| 141 | AT3G47730.1 \| Symbol: ATATH1 \| | 4.9 | 10.4 | -5.5 | -18.5 | 1.7E-5 | 11.5 |

| Rank | Description | Sync | Root | M | t | adj.q | B |
|---|---|---|---|---|---|---|---|
| 142 | AT2G28670.1 \| disease resistance-responsive family protein / fibroin-related, contains similarity to silk fibroin heavy chain (Bombyx mori) gi:765323:gb:AAB31861; contains disease resistance response protien domain Pfam:FP03018 \| chr2:12307336-12308859 REVERSE \| Aliases: T8O18.4, T8O18_4 | 6.1 | 11.8 | -5.7 | -18.5 | 1.7E-5 | 10.3 |
| 143 | AT5G50400.1 \| calcineurin-like phosphoesterase family protein, contains Pfam profile: PF00149 calcineurin-like phosphoesterase | 9.1 | 12.0 | -2.9 | -18.5 | 1.7E-5 | 13.4 |
| 144 | AT5G27350.1 \| Symbol: SFP1 \| sugar-porter family protein 1 (SFP1), identical to sugar-porter family protein 1 (Arabidopsis thaliana) GI:14585699 \| chr5:9648670-9654429 FORWARD \| Aliases: F21A20.60, F21A20_60, sugar porter family protein 1 | 3.6 | 9.0 | -5.5 | -18.4 | 1.7E-5 | 11.8 |
| 145 | AT1G01750.1 \| actin-depolymerizing factor, putative, strong similarity to SP:P30175 Actin-depolymerizing factor (ADF) {Lilium longiflorum}; contains Pfam profile PF00241: Cofilin/tropomyosin-type actin-binding protein \| chr1:275366-276310 FORWARD \| Aliases: T1N6.16, T1N6_16 | 3.1 | 8.4 | -5.2 | -18.3 | 1.7E-5 | 10.4 |
| 147 | AT1G08090.1 \| Symbol: ATNRT2:1 \| high-affinity nitrate transporter (ACH1), identical to trans-membrane nitrate transporter protein AtNRT2:1 (Arabidopsis thaliana) GI:3747058, high-affinity nitrate transporter ACH1 (Arabidopsis thaliana) GI:3608362 \| chr1:2524073-2526161 FORWARD \| Aliases: ACH1, ATNRT2.1, LATERAL ROOT INITIATION 1, LIN1, NITRATE TRANSPORTER 1, NRT2, NRT2;1AT, T6D22.17, T6D22_17, TRANS MEMBRANE NITRATE TRANSPORTER PROTEIN ATNRT2:1 | 6.4 | 13.3 | -6.9 | -18.2 | 1.8E-5 | 9.5 |
| 148 | AT3G24503.1 \| Symbol: ALDH2C4 \| aldehyde dehydrogenase (ALDH1a), identical to aldehyde dehydrogenase ALDH1a (Arabidopsis thaliana) gi:20530143:gb:AAM27004 \| chr3:8919567-8923074 REVERSE \| Aliases: ALDH1A, REDUCED EPIDERMAL FLUORESCENCE1, REF1 | 6.7 | 11.4 | -4.8 | -18.0 | 1.9E-5 | 11.7 |
| 150 | AT5G41040.2 \| transferase family protein, similar to hypersensitivity-related gene product HSR201 - Nicotiana tabacum, EMBL:X95343; contains Pfam transferase family domain PF00248 \| chr5:16448619-16450533 FORWARD \| Aliases: MEE6.11, MEE6_11 | 2.8 | 9.4 | -6.6 | -18.0 | 2.0E-5 | 9.4 |
| 152 | AT5G03570.1 \| iron-responsive transporter-related, similar to iron-regulated transporter IREG1 (Mus musculus) GI:7264727 \| chr5:904488-906663 FORWARD \| Aliases: F12E4.370, F12E4_370 | 3.6 | 10.8 | -7.2 | -18.0 | 2.0E-5 | 9.5 |
| 153 | AT3G44990.1 \| Symbol: XTR8 \| xyloglucan:xyloglucosyl transferase, putative / xyloglucan endotransglycosylase, putative / endo-xyloglucan transferase, putative \| chr3:16457960-16459749 REVERSE \| Aliases: ATXTR8, F14D17.60 | 6.4 | 12.1 | -5.7 | -17.9 | 2.0E-5 | 11.9 |
| 156 | AT1G43020.2 \| similar to expressed protein [Arabidopsis thaliana] (TAIR:At1g21060.1); similar to putative ternary complex factor MIP1 [Antirrhinum majus] (GB:AAO59425.1); contains domain SER_RICH (PS50324) \| chr1:16157843-16159570 FORWARD \| Aliases: F13A11.7, F13A11_7 | 2.4 | 8.5 | -6.1 | -17.8 | 2.1E-5 | 9.9 |
| 157 | AT5G13900.1 \| protease inhibitor/seed storage/lipid transfer protein (LTP) family protein, contains Pfam protease inhibitor/seed storage/LTP family domain PF00234 \| chr5:4481234-4481879 REVERSE \| Aliases: MAC12.14, MAC12_14 | 2.7 | 8.5 | -5.8 | -17.7 | 2.1E-5 | 9.4 |
| 159 | AT2G39200.1 \| Symbol: MLO12 \| seven transmembrane MLO family protein / MLO-like protein 12 (MLO12), identical to SP:O80961 MLO-like protein 12 (AtMlo12) {Arabidopsis thaliana}, membrane protein Mlo12 (Arabidopsis thaliana) gi:14091594:gb:AAK53805; similar to MLO protein SWISS-PROT:P93766, NCBI_gi:1877221 (Hordeum vulgare)(Barley) \| chr2:16363237-16366891 REVERSE \| Aliases: T16B24.16, T16B24_16 | 2.8 | 7.7 | -5.0 | -17.6 | 2.2E-5 | 10.6 |
| 160 | AT4G12470.1 \| protease inhibitor/seed storage/lipid transfer protein (LTP) family protein, similar to pEARLI 1 (Accession No. L43080): an Arabidopsis member of a conserved gene family (PGF95-099), Plant Physiol. 109 (4), 1497 (1995); contains Pfam protease inhibitor/seed storage/LTP family domain PF00234 \| chr4:7401106-7401904 REVERSE \| Aliases: T1P17.60, T1P17_60 | 2.8 | 8.4 | -5.6 | -17.6 | 2.2E-5 | 9.7 |
| 161 | AT5G65530.1 \| protein kinase, putative, contains protein kinase domain, Pfam:PF00069 \| chr5:26207823-26210192 REVERSE \| Aliases: K21L13.3, K21L13_3 | 3.3 | 9.7 | -6.4 | -17.6 | 2.2E-5 | 10.3 |
| 163 | AT2G44380.1 \| DC1 domain-containing protein, highly similar to GP:2435515:AF024504; contains Pfam profile PF03107: DC1 domain \| chr2:18330666-18331504 REVERSE \| Aliases: F4I1.19 | 3.2 | 9.8 | -6.5 | -17.4 | 2.4E-5 | 9.1 |
| 165 | AT2G14100.1 \| Symbol: CYP705A13 \| cytochrome P450 family protein, contains Pfam profile PF00067: Cytochrome P450 \| chr2:5941638-5943453 REVERSE \| Aliases: T22C12.3, T22C12_3 | 3.5 | 9.3 | -5.8 | -17.3 | 2.4E-5 | 9.7 |
| 167 | AT1G78080.1 \| Symbol: RAP2.4 \| | 3.8 | 8.9 | -5.1 | -17.3 | 2.5E-5 | 11.1 |
| 170 | AT5G04950.1 \| nicotianamine synthase, putative, similar to nicotianamine synthase (Lycopersicon esculentum)(GI:4753801), nicotianamine synthase 2 (Hordeum vulgare)(GI:4894912) \| chr5:1457646-1458891 REVERSE \| Aliases: MUG13.19, MUG13_19 | 3.1 | 9.9 | -6.7 | -17.2 | 2.5E-5 | 8.5 |
| 171 | AT3G04720.1 \| Symbol: PR4 \| hevein-like protein (HEL), identical to SP:P43082 Hevein-like protein precursor {Arabidopsis thaliana}; similar to SP:P09762 Wound-induced protein WIN2 precursor {Solanum tuberosum}; contains Pfam profile PF00187: Chitin recognition protein \| chr3:1285573-1286568 REVERSE \| Aliases: F7O18.21, F7O18_21, HEL, HEVEIN LIKE, PATHOGENESIS RELATED 4, PR 4 | 6.8 | 12.3 | -5.6 | -17.2 | 2.5E-5 | 9.6 |

| Rank | Description | Sync | Root | M | t | adj.q | B |
|---|---|---|---|---|---|---|---|
| 172 | AT1G15670.1 \| kelch repeat-containing F-box family protein, similar to SP:Q9ER30 Kelch-related protein 1 (Sarcosin) {Rattus norvegicus}; contains Pfam profiles PF01344: Kelch motif, PF00646: F-box domain \| chr1:5389947-5391485 FORWARD \| Aliases: F7H2.1, F7H2_1 | 3.7 | 9.8 | -6.2 | -17.2 | 2.5E-5 | 10.5 |
| 173 | AT2G48130.1 \| protease inhibitor/seed storage/lipid transfer protein (LTP) family protein, contains Pfam protease inhibitor/seed storage/LTP family domain PF00234 \| chr2:19692138-19693063 REVERSE \| Aliases: F11L15.3 | 2.9 | 9.1 | -6.2 | -17.2 | 2.5E-5 | 9.5 |
| 174 | AT1G70300.1 \| Symbol: KUP6 \| potassium transporter, putative, similar to potassium transporter HAK2p (Mesembryanthemum crystallinum) gi:14091471:gb:AAK53759; KUP/HAK/KT Transporter family member, PMID:11500563; contains Pfam profile PF02705: K+ potassium transporter \| chr1:26481543-26485293 REVERSE \| Aliases: F17O7.17, F17O7_17 | 5.7 | 9.6 | -4.0 | -17.2 | 2.5E-5 | 11.5 |
| 177 | AT1G49570.1 \| peroxidase, putative, identical to peroxidase ATP5a (Arabidopsis thaliana) gi:1546702:emb:CAA67341; similar to peroxidase SWISS-PROT:P80679 from (Armoracia rusticana) \| chr1:18350704-18352619 FORWARD \| Aliases: F14J22.19, F14J22_19 | 3.1 | 9.8 | -6.7 | -17.0 | 2.6E-5 | 12.3 |
| 178 | AT2G45920.1 \| U-box domain-containing protein, contains Pfam profile PF04564: U-box domain \| chr2:18906288-18908376 FORWARD \| Aliases: F4I18.10 | 4.3 | 9.9 | -5.6 | -17.0 | 2.7E-5 | 11.6 |
| 179 | AT5G59930.1 \| DC1 domain-containing protein / UV-B light-insensitive protein, putative, similar to ULI3 (UV-B light insensitive) (Arabidopsis thaliana) GI:17225050; contains Pfam profile PF03107: DC1 domain | 2.6 | 8.2 | -5.6 | -16.9 | 2.7E-5 | 11.0 |
| 180 | AT1G51860.1 \| leucine-rich repeat protein kinase, putative, similar to light repressible receptor protein kinase (Arabidopsis thaliana) gi:1321686:emb:CAA66376; contains leucine rich repeat (LRR) domains, Pfam:PF00560; contains protein kinase domain, Pfam:PF00069 \| chr1:19261303-19265148 REVERSE \| Aliases: T14L22.7, T14L22_7 | 2.7 | 9.0 | -6.4 | -16.9 | 2.7E-5 | 9.3 |
| 181 | AT1G54370.1 \| sodium proton exchanger, putative (NHX5), contains similarity to Na+/H+ antiporter GI:1655701 from (Xenopus laevis); Member of The Monovalent Cation:Proton Antiporter (CPA1) Family, PMID:11500563; contains non-consensus AT-AC splice sites at introns 3 and 10 \| chr1:20296818-20300796 REVERSE \| Aliases: F20D21.19, F20D21_19 | 3.1 | 8.2 | -5.0 | -16.9 | 2.7E-5 | 10.2 |
| 182 | AT1G16060.2 \| ovule development protein, putative, similar to ovule development protein AINTEGUMENTA (GI:1209099)(Arabidopsis thaliana) \| chr1:5508537-5511729 FORWARD \| Aliases: T24D18.16, T24D18_16 | 3.3 | 7.9 | -4.5 | -16.9 | 2.7E-5 | 11.6 |
| 183 | AT2G28470.1 \| Symbol: BGAL8 \| beta-galactosidase, putative / lactase, putative, similar to Beta-galactosidase precursor SP:P48980 from (Lycopersicon esculentum) \| chr2:12175930-12180647 REVERSE \| Aliases: T17D12.3, T17D12_3 | 6.6 | 11.7 | -5.1 | -16.9 | 2.7E-5 | 11.7 |
| 184 | AT5G61340.1 \| expressed protein \| chr5:24679230-24680343 REVERSE \| Aliases: MFB13.11, MFB13_11 | 2.9 | 8.8 | -5.9 | -16.8 | 2.7E-5 | 10.7 |
| 185 | AT5G35190.1 \| proline-rich extensin-like family protein, contains proline-rich extensin domains, INTERPRO:IPR002965 \| chr5:13451411-13452397 FORWARD \| Aliases: T25C13.70, T25C13_70 | 3.6 | 10.0 | -6.4 | -16.8 | 2.7E-5 | 10.3 |
| 186 | AT5G05500.1 \| pollen Ole e 1 allergen and extensin family protein, contains Pfam domain, PF01190: Pollen proteins Ole e I family \| chr5:1629668-1630397 FORWARD \| Aliases: MOP10.4, MOP10_4 | 2.6 | 9.0 | -6.4 | -16.8 | 2.8E-5 | 10.7 |
| 188 | AT1G68440.1 \| expressed protein \| chr1:25661696-25662936 FORWARD \| Aliases: T2E12.1, T2E12_1 | 4.4 | 9.9 | -5.5 | -16.7 | 2.8E-5 | 10.6 |
| 191 | AT5G47740.1 \| similar to protein kinase family protein / U-box domain-containing protein [Arabidopsis thaliana] (TAIR:At2g45910.1); similar to putative protein kinase [Oryza sativa (japonica cultivar-group)] (GB:AAP55019.1) \| chr5:19355737-19357636 FORWARD \| Aliases: MCA23.6, MCA23_6 | 2.8 | 8.5 | -5.7 | -16.7 | 2.8E-5 | 10.2 |
| 192 | AT2G03730.2 \| Symbol: ACR5 \| similar to ACT domain containing protein (ACR4) [Arabidopsis thaliana] (TAIR:At1g69040.2); similar to putative ACT domain repeat protein [Oryza sativa (japonica cultivar-group)] (GB:NP_909842.1); contains InterPro domain Amino acid-binding ACT (InterPro:IPR002912) | 4.6 | 8.8 | -4.2 | -16.7 | 2.8E-5 | 10.8 |
| 196 | AT1G20440.1 \| Symbol: COR47 \| dehydrin (COR47), identical to dehydrin COR47 (Cold-induced COR47 protein) (Arabidopsis thaliana) SWISS-PROT:P31168 \| chr1:7084399-7085762 REVERSE \| Aliases: F5M15.22, F5M15_22, RD17 | 8.0 | 12.6 | -4.5 | -16.7 | 2.8E-5 | 11.3 |
| 197 | AT1G71400.1 \| disease resistance family protein / LRR family protein, contains leucine rich-repeat domains Pfam:PF00560, INTERPRO:IPR001611; similar to Hcr2-5D (Lycopersicon esculentum) gi:3894393:gb:AAC78596 \| chr1:26913497-26916259 FORWARD \| Aliases: F3I17.30, F3I17_30 | 5.3 | 9.0 | -3.7 | -16.7 | 2.8E-5 | 11.7 |
| 198 | AT1G71697.1 \| Symbol: ATCK1 \| choline kinase, putative, similar to GmCK2p choline kinase gi:1438881:gb:AAC49375 \| chr1:26974885-26977126 FORWARD \| Aliases: CHOLINE KINASE, CK, F14O23.8, F14O23_8 | 4.5 | 9.8 | -5.3 | -16.7 | 2.8E-5 | 11.5 |
| 204 | AT3G23160.1 \| expressed protein, contains Pfam domain PF05003: protein of unknown function (DUF668); expression supported by MPSS \| chr3:8260066-8261634 REVERSE \| Aliases: K14B15.5 | 4.5 | 8.8 | -4.3 | -16.6 | 2.8E-5 | 11.1 |

| Rank | Description | Sync | Root | M | t | adj.q | B |
|---|---|---|---|---|---|---|---|
| 207 | AT3G58810.2 \| Symbol: MTPA2 \| zinc transporter, putative, similar to zinc transporter 4; ZnT4 (Mus musculus) gi:2582990:gb:AAB82593; similar to zinc transporter ZAT (Arabidopsis thaliana) gi:4206640:gb:AAD11757; member of the cation diffusion facilitator (CDF) family, or cation efflux (CE) family, PMID:11500563 \| chr3:21760943-21762983 FORWARD \| Aliases: ATMTPA2, T20N10.160 | 4.4 | 10.8 | -6.4 | -16.5 | 2.9E-5 | 10.4 |
| 210 | AT3G21710.2 \| expressed protein \| chr3:7648366-7649792 FORWARD \| Aliases: MSD21.2 | 5.4 | 10.9 | -5.5 | -16.3 | 3.0E-5 | 9.2 |
| 211 | AT5G04960.1 \| pectinesterase family protein, contains Pfam profile: PF01095 pectinesterase \| chr5:1461911-1463970 FORWARD \| Aliases: MUG13.18, MUG13_18 | 2.9 | 9.8 | -6.9 | -16.3 | 3.1E-5 | 10.3 |
| 214 | AT3G54590.1 \| Symbol: ATHRGP1 \| proline-rich extensin-like family protein, contains proline-rich extensin domains, INTERPRO:IPR002965 | 3.0 | 11.2 | -8.2 | -16.1 | 3.3E-5 | 8.4 |
| 215 | AT5G59680.1 \| leucine-rich repeat protein kinase, putative, similar to light repressible receptor protein kinase (Arabidopsis thaliana) gi:1321686:emb:CAA66376; contains leucine rich repeat (LRR) domains, Pfam:PF00560; contains protein kinase domain, Pfam:PF00069 \| chr5:24064018-24068027 FORWARD \| Aliases: MTH12.14, MTH12_14 | 3.8 | 8.8 | -5.0 | -16.1 | 3.4E-5 | 10.0 |
| 216 | AT5G58900.1 \| myb family transcription factor, contains Pfam profile: PF00249 myb-like DNA-binding domain \| chr5:23800349-23802560 REVERSE \| Aliases: K19M22.10, K19M22_10 | 6.9 | 10.5 | -3.6 | -16.1 | 3.4E-5 | 11.2 |
| 217 | AT3G14470.1 \| disease resistance protein (NBS-LRR class), putative, domain signature NBS-LRR exists, suggestive of a disease resistance protein. \| chr3:4857947-4861254 FORWARD \| Aliases: MOA2.9 | 2.9 | 7.2 | -4.3 | -16.1 | 3.4E-5 | 10.8 |
| 218 | AT4G03510.2 \| Symbol: RMA1 \| zinc finger (C3HC4-type RING finger) family protein (RMA1), identical to RING zinc finger protein RMA1 gi:3164222 \| chr4:1557766-1559374 REVERSE \| Aliases: F9H3.14, F9H3_14 | 4.0 | 8.6 | -4.6 | -16.0 | 3.4E-5 | 10.6 |
| 219 | AT1G29020.1 \| calcium-binding EF hand family protein, contains INTERPRO:IPR002048 calcium-binding EF-hand domain \| chr1:10120483-10127054 REVERSE \| Aliases: F28N24.34 | 3.2 | 8.0 | -4.8 | -16.0 | 3.5E-5 | 9.8 |
| 220 | AT3G48530.1 \| CBS domain-containing protein, low similarity to SP:Q9UGI9 5'-AMP-activated protein kinase, gamma-3 subunit (AMPK gamma-3 chain) (AMPK gamma3) {Homo sapiens}; contains Pfam profile PF00571: CBS domain | 6.4 | 11.3 | -4.9 | -16.0 | 3.5E-5 | 11.2 |
| 221 | AT2G01890.2 \| Symbol: PAP8 \| purple acid phosphatase, putative, contains Pfam profile: PF00149 calcineurin-like phosphoesterase; identical to purple acid phosphatase (GI:20257479) (Arabidopsis thaliana); \| chr2:396808-399190 REVERSE \| Aliases: PAP8, PURPLE ACID PHOSPHATASE PRECURSOR, T23K3.8, T23K3_8 | 3.2 | 8.9 | -5.7 | -15.9 | 3.6E-5 | 9.5 |
| 224 | AT5G58860.1 \| Symbol: CYP86A1 \| cytochrome P450 86A1 (CYP86) (CYP86A1) / CYPLXXXVI / P450-dependent fatty acid omega-hydroxylase, identical to Cytochrome P450 86A1 (CYPLXXXVI) (P450-dependent fatty acid omega-hydroxylase) (SP:P48422) (Arabidopsis thaliana) \| chr5:23783040-23785275 REVERSE \| Aliases: CYP86, K19M22.14, K19M22_14 | 2.7 | 8.3 | -5.6 | -15.8 | 3.7E-5 | 10.2 |
| 225 | AT1G64405.1 \| expressed protein \| chr1:23927395-23927999 FORWARD \| Aliases: None | 3.2 | 7.9 | -4.7 | -15.8 | 3.7E-5 | 11.3 |
| 226 | AT3G16800.3 \| similar to protein phosphatase 2C, putative / PP2C, putative [Arabidopsis thaliana] (TAIR:At3g05640.1); similar to protein phosphatase 2C, putative / PP2C, putative [Arabidopsis thaliana] (TAIR:At3g05640.2); similar to putative protein phosphatase 2C [Oryza sativa (japonica cultivar-group)] (GB:XP_483187.1); contains InterPro domain Protein phosphatase 2C-like (InterPro:IPR001932) \| chr3:5721026-5723187 FORWARD \| Aliases: K20I9.2 | 5.0 | 9.1 | -4.1 | -15.8 | 3.7E-5 | 10.8 |
| 230 | AT3G54420.1 \| Symbol: ATEP3 \| class IV chitinase (CHIV), almost identical to class IV chitinase from GI:2597826 (Arabidopsis thaliana) \| chr3:20156888-20158041 FORWARD \| Aliases: ATCHITIV, CHIV, T14E10.4 | 4.5 | 10.8 | -6.3 | -15.7 | 3.9E-5 | 11.0 |
| 231 | AT4G27410.2 \| Symbol: RD26 \| no apical meristem (NAM) family protein (RD26), contains Pfam PF02365: No apical meristem (NAM) domain; Arabidopsis thaliana nap gene,PID:e1234813; identical to cDNA RD26 mRNA for NAM-like protein GI:15375403 | 7.2 | 11.8 | -4.6 | -15.7 | 3.9E-5 | 10.2 |
| 232 | AT2G23810.1 \| similar to senescence-associated family protein [Arabidopsis thaliana] (TAIR:At4g30430.1); similar to senescence-associated protein DH [Zea mays] (GB:AAV31120.1); contains InterPro domain CD9/CD37/CD63 antigen (InterPro:IPR000301) \| chr2:10142685-10144531 REVERSE \| Aliases: F27L4.1, F27L4_1 | 7.7 | 11.0 | -3.3 | -15.6 | 3.9E-5 | 11.5 |
| 234 | AT1G05300.2 \| Symbol: ZIP5 \| metal transporter, putative (ZIP5), identical to putative metal transporter ZIP5 (Arabidopsis thaliana) gi:17385784:gb:AAL38432; similar to zinc transporter protein ZIP1 (Glycine max) gi:15418778:gb:AAK37761; member of the Zinc (Zn2+)-Iron (Fe2+) permease (ZIP) family, PMID:11500563 \| chr1:1545215-1547797 REVERSE \| Aliases: YUP8H12.8, YUP8H12_8 | 4.0 | 10.3 | -6.3 | -15.5 | 4.0E-5 | 9.0 |
| 235 | AT2G21100.1 \| disease resistance-responsive protein-related / dirigent protein-related, similar to dirigent protein (Thuja plicata) gi:6694699:gb:AAF25360; similar to disease resistance response protein 206-d (Pisum sativum) gi:508844:gb:AAB18669 | 3.7 | 8.7 | -5.1 | -15.5 | 4.0E-5 | 9.6 |

| Rank | Description | Sync | Root | M | t | adj.q | B |
|---|---|---|---|---|---|---|---|
| 236 | AT4G38950.1 \| kinesin motor family protein, similar to AtNACK1 kinesin-like protein (GI:19979627) (Arabidopsis thaliana); similar to kinesin-like protein NACK1 (GI:19570247) (Nicotiana tabacum) \| chr4:18154466-18158455 REVERSE \| Aliases: F19H22.50, F19H22_50 | 3.9 | 8.8 | -4.9 | -15.5 | 4.0E-5 | 11.6 |
| 237 | AT3G26100.2 \| regulator of chromosome condensation (RCC1) family protein, weak similarity to UVB-resistance protein UVR8 (Arabidopsis thaliana) GI:5478530; contains Pfam profile PF00415: Regulator of chromosome condensation (RCC1) | 6.6 | 11.0 | -4.4 | -15.5 | 4.0E-5 | 10.7 |
| 238 | AT5G58010.1 \| basic helix-loop-helix (bHLH) family protein, bHLH transcription factor GBOF-1, Tulipa gesneriana, EMBL:AF185269; contains Pfam profile PF00010: Helix-loop-helix DNA-binding domain \| chr5:23500771-23502115 REVERSE \| Aliases: F2C19.2, F2C19_2 | 4.0 | 9.7 | -5.7 | -15.5 | 4.0E-5 | 9.3 |
| 239 | AT1G73260.1 \| trypsin and protease inhibitor family protein / Kunitz family protein, similar to trypsin inhibitor propeptide (Brassica oleracea) GI:841208; contains Pfam profile PF00197: Trypsin and protease inhibitor \| chr1:27550935-27551851 REVERSE \| Aliases: T18K17.7, T18K17_7 | 5.3 | 11.1 | -5.8 | -15.5 | 4.1E-5 | 8.9 |
| 245 | AT3G61410.1 \| similar to protein kinase family protein / U-box domain-containing protein [Arabidopsis thaliana] (TAIR:At2g45910.1); similar to putative protein kinase [Oryza sativa (japonica cultivar-group)] (GB:AAP51741.1) \| chr3:22732013-22733626 FORWARD \| Aliases: F2A19.10 | 3.5 | 8.2 | -4.7 | -15.4 | 4.2E-5 | 10.6 |
| 246 | AT1G76990.4 \| Symbol: ACR3 \| similar to ACT domain containing protein (ACR4) [Arabidopsis thaliana] (TAIR:At1g69040.2); similar to ACT domain containing protein (ACR4) [Arabidopsis thaliana] (TAIR:At1g69040.1); similar to putative ACT domain containing protein, ACR4, with alternative splicing forms [Oryza sativa (japonica cultivar-group)] (GB:XP_468663.1); contains InterPro domain Amino acid-binding ACT (InterPro:IPR002912) \| chr1:28937056-28940444 FORWARD \| Aliases: F22K20.9, F22K20_9 | 4.9 | 9.5 | -4.6 | -15.4 | 4.2E-5 | 10.9 |
| 247 | AT3G46900.1 \| Symbol: COPT2 \| copper transporter, putative, similar to SP:Q39065 Copper transporter 1 (COPT1) {Arabidopsis thaliana}; contains Pfam profile PF04145: Ctr copper transporter family \| chr3:17279860-17280495 FORWARD \| Aliases: T6H20.70 | 2.7 | 8.5 | -5.7 | -15.3 | 4.2E-5 | 9.2 |
| 248 | AT1G18910.1 \| zinc finger (C3HC4-type RING finger) family protein, contains Pfam domain PF00097: Zinc finger, C3HC4 type (RING finger) | 3.8 | 8.8 | -5.0 | -15.3 | 4.2E-5 | 10.0 |
| 249 | AT5G42180.1 \| peroxidase 64 (PER64) (P64) (PRXR4), identical to SP:Q43872 Peroxidase 64 precursor (EC 1.11.1.7) (Atperox P64) (PRXR4) (ATP17a) {Arabidopsis thaliana} \| chr5:16869860-16871448 FORWARD \| Aliases: MJC20.29, MJC20_29 | 3.1 | 10.6 | -7.4 | -15.3 | 4.2E-5 | 8.8 |
| 251 | AT3G61930.1 \| expressed protein \| chr3:22946485-22947156 FORWARD \| Aliases: F21F14.100 | 4.2 | 10.6 | -6.4 | -15.3 | 4.3E-5 | 9.2 |
| 254 | AT3G27170.1 \| Symbol: CLC B \| chloride channel protein (CLC-b), identical to CLC-b chloride channel protein GB:CAA96058 from (Arabidopsis thaliana) (J. Biol. Chem. 271 (52), 33632-33638 (1996)) \| chr3:10025254-10028435 FORWARD \| Aliases: ATCLC B, MYF5.4 | 6.7 | 11.2 | -4.5 | -15.2 | 4.3E-5 | 10.9 |
| 256 | AT1G78000.2 \| Symbol: SULTR1;2 \| sulfate transporter (Sultr1;2), identical to sulfate transporter Sultr1;2 (Arabidopsis thaliana) GI:7768660; contaisn Pfam profiles PF00916: Sulfate transporter family and PF01740: STAS domain; contains TIGRfam profile TIGR00815: sulfate permease \| chr1:29334738-29338040 FORWARD \| Aliases: F28K19.22, F28K19_22, SEL1, SELENATE RESISTANT 1, SULFATE TRANSPORTER, SULFATE TRANSPORTER 1;2 | 7.2 | 11.4 | -4.2 | -15.2 | 4.3E-5 | 10.6 |
| 257 | AT3G61940.1 \| Symbol: MTPA1 \| zinc transporter, putative, similar to zinc transporter ZAT (Arabidopsis thaliana) gi:4206640:gb:AAD11757; similar to zinc transporter ZnT-2 (Rattus norvegicus) gi:1256378:gb:AAB02775; member of the cation diffusion facilitator (CDF) family, or cation efflux (CE) family, PMID:11500563 \| chr3:22948145-22949549 FORWARD \| Aliases: ATMTPA1, F21F14.110 | 3.9 | 10.5 | -6.6 | -15.2 | 4.5E-5 | 8.7 |
| 258 | AT2G26650.1 \| Symbol: AKT1 \| potassium channel protein 1 (AKT1), identical to AKT1 (Arabidopsis thaliana) gi:563112:gb:AAA96810; member of the 1 pore, 6 transmembrane (1P/6TM- Shaker-type) K+ channel family, PMID:11500563 | 5.7 | 9.0 | -3.3 | -15.2 | 4.5E-5 | 11.0 |
| 260 | AT5G25770.2 \| expressed protein \| chr5:8969219-8972049 REVERSE \| Aliases: F18A17.20, F18A17_20 | 9.7 | 11.7 | -2.1 | -15.1 | 4.5E-5 | 12.3 |
| 261 | AT4G21410.1 \| protein kinase family protein, contains Pfam PF00069: Protein kinase domain \| chr4:11402325-11405067 REVERSE \| Aliases: F18E5.30 | 5.5 | 9.9 | -4.4 | -15.1 | 4.6E-5 | 10.3 |
| 262 | AT4G20390.1 \| integral membrane family protein, contains TIGRFAM TIGR01569 : plant integral membrane protein TIGR01569; contains Pfam PF04535 : Domain of unknown function (DUF588) \| chr4:11007046-11008075 FORWARD \| Aliases: F9F13.40, F9F13_40 | 4.3 | 9.1 | -4.8 | -15.1 | 4.6E-5 | 10.1 |
| 263 | AT1G24440.1 \| expressed protein, similar to MTD2 (Medicago truncatula) GI:9294812 \| chr1:8662007-8663952 FORWARD \| Aliases: F21J9.10 | 6.5 | 9.5 | -2.9 | -15.0 | 4.6E-5 | 11.3 |
| 264 | AT1G78680.1 \| gamma-glutamyl hydrolase (GGH1) / gamma-Glu-X carboxypeptidase / conjugase, identical to SP:O65355 Gamma-glutamyl hydrolase precursor (EC 3.4.19.9) (Gamma-Glu-X carboxypeptidase) (Conjugase) (GH) {Arabidopsis thaliana} \| chr1:29598758-29601163 FORWARD \| Aliases: F9K20.28, F9K20_28 | 5.3 | 9.8 | -4.5 | -15.0 | 4.6E-5 | 10.5 |
| 265 | AT5G42250.1 \| alcohol dehydrogenase, putative, similar to alcohol dehydrogenase ADH GI:7705214 from (Lycopersicon esculentum); contains Pfam zinc-binding dehydrogenase domain PF00107 | 4.1 | 10.5 | -6.5 | -15.0 | 4.7E-5 | 8.9 |
| 266 | AT2G20030.1 \| zinc finger (C3HC4-type RING finger) family protein, similar to RING-H2 zinc finger protein ATL6 (Arabidopsis thaliana) GI:4928403; contains Pfam profile PF00097: Zinc finger, C3HC4 type (RING finger) | 3.5 | 9.0 | -5.6 | -15.0 | 4.7E-5 | 9.5 |

| Rank | Description | Sync | Root | M | t | adj.q | B |
|---|---|---|---|---|---|---|---|
| 268 | AT2G41540.3 \| similar to NAD-dependent glycerol-3-phosphate dehydrogenase family protein [Arabidopsis thaliana] (TAIR:At3g07690.1); similar to putative glycerol-3-phosphate dehydrogenase [Oryza sativa (japonica cultivar-group)] (GB:NP_915827.1); similar to putative glycerol-3-phosphate dehydrogenase [Oryza sativa (japonica cultivar-group)] (GB:AAU44049.1); contains InterPro domain NAD-dependent glycerol-3-phosphate dehydrogenase (InterPro:IPR006168) \| chr2:17333206-17336863 FORWARD \| Aliases: T32G6.6, T32G6_6 | 5.2 | 9.2 | -4.0 | -15.0 | 4.7E-5 | 10.7 |
| 270 | AT1G67480.1 \| kelch repeat-containing F-box family protein, similar to SKP1 interacting partner 6 (Arabidopsis thaliana) GI:10716957; contains Pfam profiles PF01344: Kelch motif, PF00646: F-box domain \| chr1:25279958-25282381 FORWARD \| Aliases: T1F15.5, T1F15_5 | 7.3 | 9.5 | -2.3 | -15.0 | 4.7E-5 | 12.0 |
| 271 | AT5G12170.1 \| expressed protein, similar to unknown protein (pir::T09909) \| chr5:3933503-3934962 FORWARD \| Aliases: MXC9.13, MXC9_13 | 6.3 | 10.4 | -4.1 | -15.0 | 4.7E-5 | 10.1 |
| 272 | AT2G20880.1 \| AP2 domain-containing transcription factor, putative, similar to AP2 domain containing protein RAP2.4 (Arabidopsis thaliana) GI:2281633 \| chr2:8993054-8994344 FORWARD \| Aliases: F5H14.15, F5H14_15 | 2.7 | 8.3 | -5.6 | -15.0 | 4.7E-5 | 8.4 |
| 273 | AT1G80180.1 \| expressed protein \| chr1:30161718-30162476 REVERSE \| Aliases: F18B13.26, F18B13_26 | 6.7 | 11.2 | -4.4 | -15.0 | 4.7E-5 | 10.3 |
| 274 | AT3G50260.1 \| encodes a member of the DREB subfamily A-5 of ERF/AP2 transcription factor family. The protein contains one AP2 domain. There are 16 members in this subfamily including RAP2.1, RAP2.9 and RAP2.10. \| chr3:18645558-18646380 FORWARD \| Aliases: F11C1.100 | 4.7 | 10.8 | -6.1 | -14.9 | 4.8E-5 | 10.6 |
| 275 | AT2G28550.2 \| Symbol: TOE1 \| AP2 domain-containing transcription factor RAP2.7 (RAP2.7), nearly identical to AP2 domain transcription factor RAP2.7 (GI:2281639) (Arabidopsis thaliana) \| chr2:12233168-12235620 REVERSE \| Aliases: RAP2.7, T17D12.11, T17D12_11 | 4.4 | 8.7 | -4.3 | -14.9 | 4.8E-5 | 10.7 |
| 276 | AT3G08040.1 \| Symbol: FRD3 \| MATE efflux family protein, low similarity to enhanced disease susceptibility 5 (Arabidopsis thaliana) GI:16589070; contains TIGRfam profile: TIGR00797: MATE efflux family protein, Pfam profile PF01554 Uncharacterized membrane protein family \| chr3:2566283-2569505 REVERSE \| Aliases: FERRIC REDUCTASE DEFECTIVE 3, MAN1, MANGANESE ACCUMULATOR 1, T8G24.8 | 4.2 | 8.9 | -4.7 | -14.8 | 4.9E-5 | 10.0 |
| 278 | AT3G01260.1 \| aldose 1-epimerase family protein, similar to non-cell-autonomous protein pathway2, plasmodesmal receptor (Nicotiana tabacum) GI:15824567; contains Pfam profile PF01263: Aldose 1-epimerase \| chr3:80183-81958 REVERSE \| Aliases: T4P13.5, T4P13_5 | 3.1 | 8.5 | -5.4 | -14.8 | 5.0E-5 | 10.0 |
| 279 | AT1G27030.1 \| expressed protein \| chr1:9381790-9383970 FORWARD \| Aliases: T7N9.9, T7N9_9 | 5.2 | 9.5 | -4.3 | -14.8 | 5.0E-5 | 11.0 |
| 283 | AT4G26010.1 \| peroxidase, putative, peroxidase ATP13a - Arabidopsis thaliana, PID:e264765; identical to cDNA class III peroxidase ATP35, GI:17530565 \| chr4:13200602-13201950 FORWARD \| Aliases: F20B18.120, F20B18_120 | 3.3 | 10.5 | -7.2 | -14.7 | 5.2E-5 | 10.7 |
| 285 | AT1G52200.1 \| expressed protein, similar to PGPS/D12 (Petunia x hybrida) GI:4105794; contains Pfam profile PF04749: Protein of unknown function, DUF614 \| chr1:19445744-19447094 REVERSE \| Aliases: F9I5.19, F9I5_19 | 3.1 | 9.6 | -6.5 | -14.7 | 5.2E-5 | 8.7 |
| 286 | AT1G30700.1 \| FAD-binding domain-containing protein, similar to SP:P30986 reticuline oxidase precursor (Berberine-bridge-forming enzyme) (BBE) (Tetrahydroprotoberberine synthase) (Eschscholzia californica); contains PF01565 FAD binding domain \| chr1:10892552-10894554 FORWARD \| Aliases: T5I8.15, T5I8_15 | 2.9 | 8.2 | -5.3 | -14.6 | 5.4E-5 | 11.2 |
| 287 | AT1G02900.1 \| Symbol: RALFL1 \| | 6.2 | 11.1 | -4.9 | -14.6 | 5.4E-5 | 9.7 |
| 288 | AT1G03850.2 \| glutaredoxin family protein, contains INTERPRO Domain IPR002109, Glutaredoxin (thioltransferase) | 4.5 | 10.0 | -5.5 | -14.5 | 5.6E-5 | 8.8 |
| 290 | AT1G17340.1 \| phosphoinositide phosphatase family protein, contains similarity to phosphoinositide phosphatase SAC1 (Rattus norvegicus) gi:11095248:gb:AAG29810; contains Pfam domain, PF02383: SacI homology domain; identical to cDNA SAC domain protein 5 (SAC5) GI:31415726 \| chr1:5933893-5938754 FORWARD \| Aliases: F28G4.21, F28G4_21 | 2.8 | 5.6 | -2.7 | -14.5 | 5.7E-5 | 11.6 |
| 291 | AT1G56010.2 \| Symbol: NAC1 \| transcription activator NAC1 (NAC1), contains Pfam PF02365: No apical meristem (NAM) domain; identical to NAC1 GB:AAF21437 GI:6649236 from (Arabidopsis thaliana) \| chr1:20950236-20952906 REVERSE \| Aliases: ANAC021, ANAC022, F14J16.32, NAC1 | 2.9 | 7.7 | -4.8 | -14.4 | 5.9E-5 | 9.3 |
| 292 | AT5G49770.1 \| leucine-rich repeat transmembrane protein kinase, putative \| chr5:20240086-20244493 FORWARD \| Aliases: K2I5.14, K2I5_14 | 2.5 | 8.9 | -6.5 | -14.4 | 5.9E-5 | 9.8 |
| 294 | AT4G13770.1 \| Symbol: CYP83A1 \| cytochrome P450 family protein \| chr4:7990481-7992305 REVERSE \| Aliases: CYTOCHROME P450 83A1, CYTOCHROME P450 MONOOXYGENASE, F18A5.160, F18A5_160, REF2 | 3.2 | 9.7 | -6.5 | -14.4 | 5.9E-5 | 10.1 |
| 299 | AT5G60660.1 \| Symbol: PIP2;4/PIP2F \| major intrinsic family protein / MIP family protein, similar to mipC protein GI:1657948 from (Mesembryanthemum crystallinum) \| chr5:24392686-24394215 REVERSE \| Aliases: MUP24.9, MUP24_9, PIP2;4, PIP2F | 3.7 | 10.3 | -6.7 | -14.3 | 6.1E-5 | 8.7 |
| 302 | AT3G19030.1 \| expressed protein, contains similarity to phosphoserine aminotransferase GB:P19689 from (Yersinia enterocolitica) \| chr3:6563985-6564430 REVERSE \| Aliases: K13E13.14 | 3.5 | 9.5 | -6.0 | -14.2 | 6.3E-5 | 9.5 |

| Rank | Description | Sync | Root | M | t | adj.q | B |
|---|---|---|---|---|---|---|---|
| 303 | AT3G53980.2 \| protease inhibitor/seed storage/lipid transfer protein (LTP) family protein, contains Pfam protease inhibitor/seed storage/LTP family domain PF00234 \| chr3:19998559-19999237 REVERSE \| Aliases: F5K20.280 | 6.4 | 12.0 | -5.7 | -14.2 | 6.3E-5 | 10.7 |
| 307 | AT1G23020.1 \| ferric-chelate reductase, putative, similar to ferric-chelate reductase (FRO1) (Pisum sativum) GI:15341529; contains Pfam profile PF01794: Ferric reductase like transmembrane component | 6.3 | 10.8 | -4.5 | -14.2 | 6.4E-5 | 10.0 |
| 308 | AT4G39675.1 \| expressed protein \| chr4:18413769-18414232 FORWARD \| Aliases: None | 5.6 | 11.4 | -5.7 | -14.2 | 6.4E-5 | 11.3 |
| 311 | AT5G66490.1 \| expressed protein \| chr5:26564453-26564994 REVERSE \| Aliases: K1F13.15, K1F13_15 | 4.5 | 9.6 | -5.1 | -14.1 | 6.4E-5 | 10.5 |
| 312 | AT5G23190.1 \| Symbol: CYP86B1 \| cytochrome P450 family protein, contains Pfam profile: PF00067 cytochrome P450 \| chr5:7803144-7805818 REVERSE \| Aliases: MKD15.5, MKD15_5 | 2.1 | 5.3 | -3.2 | -14.1 | 6.5E-5 | 10.8 |
| 314 | AT5G17820.1 \| peroxidase 57 (PER57) (P57) (PRXR10), identical to SP:Q43729 Peroxidase 57 precursor (EC 1.11.1.7) (Atperox P57) (PRXR10) (ATP13a) {Arabidopsis thaliana} \| chr5:5887908-5890164 REVERSE \| Aliases: MVA3.170, MVA3_170 | 2.9 | 9.8 | -6.9 | -14.0 | 6.7E-5 | 8.8 |
| 316 | AT1G55850.1 \| Symbol: ATCSLE1 \| cellulose synthase family protein, similar to cellulose synthase catalytic subunit (gi:13925881) from Nicotiana alata, cellulose synthase-5 (gi:9622882) from Zea mays \| chr1:20880365-20883146 FORWARD \| Aliases: CSLE1, F14J16.9, F14J16_9 | 7.3 | 10.9 | -3.5 | -14.0 | 6.7E-5 | 10.8 |
| 317 | AT1G08190.1 \| vacuolar assembly protein, putative (VPS41), 99.8% identical to Vacuolar assembly protein VPS41 homolog (SP:P93043) (Arabidopsis thaliana); similar to vacuolar assembly protein vps41 GI:1835787 from (Lycopersicon esculentum) | 5.5 | 9.0 | -3.6 | -14.0 | 6.7E-5 | 10.6 |
| 318 | AT2G48140.1 \| protease inhibitor/seed storage/lipid transfer protein (LTP) family protein, similar to pEARLI 1 (Accession No. L43080): an Arabidopsis member of a conserved gene family (PGF95-099), Plant Physiol. 109 (4), 1497 (1995); contains Pfam protease inhibitor/seed storage/LTP family domain PF00234 \| chr2:19693479-19694734 FORWARD \| Aliases: F11L15.4 | 3.2 | 9.5 | -6.4 | -14.0 | 6.7E-5 | 7.7 |
| 319 | AT2G20960.1 \| Symbol: PEARLI4 \| expressed protein, pEARLI 4 gene product (Arabidopsis thaliana) GI:871782 \| chr2:9013729-9016844 FORWARD \| Aliases: F26H11.28, F26H11_28, PEARLI4 | 4.3 | 7.4 | -3.1 | -14.0 | 6.7E-5 | 10.6 |
| 320 | AT1G34040.1 \| alliinase family protein, contains Pfam profiles: PF04864 allinase C-terminal domain, PF04863 alliinase EGF-like domain \| chr1:12374413-12376159 FORWARD \| Aliases: F12G12.14, F12G12_14 | 2.6 | 7.2 | -4.7 | -14.0 | 6.7E-5 | 9.1 |
| 321 | AT1G34060.1 \| alliinase family protein, contains Pfam profiles: PF04864 allinase C-terminal domain, PF04863 alliinase EGF-like domain \| chr1:12396399-12398348 REVERSE \| Aliases: F12G12.12, F12G12_12 | 2.6 | 7.2 | -4.7 | -14.0 | 6.7E-5 | 9.1 |
| 322 | AT1G32450.1 \| proton-dependent oligopeptide transport (POT) family protein, contains Pfam profile: PF00854 POT family \| chr1:11715130-11719935 REVERSE \| Aliases: F5D14.23, F5D14_23 | 6.5 | 10.3 | -3.9 | -14.0 | 6.7E-5 | 10.1 |
| 324 | AT2G44490.1 \| glycosyl hydrolase family 1 protein, contains Pfam PF00232 : Glycosyl hydrolase family 1 domain; TIGRFAM TIGR01233: 6-phospho-beta-galactosidase; similar to anther-specific protein ATA27 (GI:2746341) (Arabidopsis thaliana) \| chr2:18371859-18374801 FORWARD \| Aliases: F4I1.30 | 5.3 | 10.5 | -5.2 | -14.0 | 6.7E-5 | 9.9 |
| 325 | AT3G59660.1 \| C2 domain-containing protein / GRAM domain-containing protein, low similarity to GLUT4 vesicle protein (Rattus norvegicus) GI:4193489; contains Pfam profiles PF00168: C2 domain, PF02893: GRAM domain \| chr3:22045155-22049634 REVERSE \| Aliases: T16L24.210 | 5.6 | 9.2 | -3.5 | -13.9 | 6.9E-5 | 10.4 |
| 327 | AT4G19160.3 \| expressed protein \| chr4:10477307-10480150 FORWARD \| Aliases: T18B16.130, T18B16_130 | 5.7 | 10.4 | -4.7 | -13.9 | 6.9E-5 | 10.3 |
| 328 | AT4G20460.1 \| NAD-dependent epimerase/dehydratase family protein, similar to UDP-galactose 4-epimerase from Cyamopsis tetragonoloba GI:3021357 (EMBL:AJ005082), Bacillus subtilis SP:P55180; contains Pfam profile PF01370 NAD dependent epimerase/dehydratase family \| chr4:11029747-11031583 REVERSE \| Aliases: F9F13.110, F9F13_110 | 4.7 | 9.0 | -4.3 | -13.9 | 7.1E-5 | 10.0 |
| 329 | AT5G59780.3 \| Symbol: MYB59 \| myb family transcription factor (MYB59), contains PFAM profile: myb DNA binding domain PF00249 \| chr5:24099423-24100612 REVERSE \| Aliases: MTH12.19, MTH12_19 | 6.2 | 11.9 | -5.7 | -13.9 | 7.1E-5 | 10.1 |
| 332 | AT2G25160.1 \| Symbol: CYP82F1 \| cytochrome P450, putative, similar to cytochrome p450(CYP82C1p) GI:2739004 from (Glycine max) | 2.6 | 7.2 | -4.6 | -13.8 | 7.2E-5 | 8.8 |
| 333 | AT2G01530.1 \| major latex protein-related / MLP-related, low similarity to major latex protein {Papaver somniferum}(GI:20810) \| chr2:239719-240492 FORWARD \| Aliases: F2I9.15, F2I9_15 | 6.0 | 11.3 | -5.3 | -13.8 | 7.3E-5 | 8.4 |
| 334 | AT3G48520.1 \| Symbol: CYP94B3 \| cytochrome P450 family protein, similar to Cytochrome P450 94A1 (P450-dependent fatty acid omega-hydroxylase) (SP:O81117) {Vicia sativa}; contains Pfam profile: PF00067 cytochrome P450 \| chr3:17985962-17987655 REVERSE \| Aliases: T8P19.30 | 5.4 | 9.2 | -3.7 | -13.8 | 7.3E-5 | 10.3 |

| Rank | Description | Sync | Root | M | t | adj.q | B |
|---|---|---|---|---|---|---|---|
| 335 | AT1G67730.1 \| b-keto acyl reductase, putative (GLOSSY8), similar to b-keto acyl reductase GI:2586127 from (Hordeum vulgare) \| chr1:25395123-25397273 FORWARD \| Aliases: F12A21.14, F12A21_14 | 8.8 | 11.5 | -2.7 | -13.8 | 7.3E-5 | 11.4 |
| 337 | AT3G02850.1 \| Symbol: SKOR \| stelar K+ outward rectifier (SKOR) / potassium channel protein, identical to SKOR (Arabidopsis thaliana) gi:3810676:emb:CAA11280; member of the 1 pore, 6 transmembrane (1P/6TM) Shaker K+ channel family, PMID:11500563 | 2.8 | 8.5 | -5.7 | -13.7 | 7.5E-5 | 7.9 |
| 340 | AT5G10820.1 \| integral membrane transporter family protein, contains 11 transmembrane domains; similar to folate/methotrexate transporter FT1 (GI:5813863) {Leishmania donovani}; lignostilbene-alpha,beta-dioxygenase gene, Synechococcus PCC7942, EMBL:AF055873 \| chr5:3420838-3423222 REVERSE \| Aliases: T30N20.90, T30N20_90 | 7.5 | 10.2 | -2.7 | -13.7 | 7.5E-5 | 10.9 |
| 344 | AT3G61380.1 \| expressed protein \| chr3:22719459-22722254 REVERSE \| Aliases: F2A19.4 | 4.6 | 7.8 | -3.2 | -13.6 | 7.9E-5 | 10.8 |
| 345 | AT5G27920.1 \| F-box family protein, contains similarity to leucine-rich repeats containing F-box protein FBL3 GI:5919219 from (Homo sapiens) \| chr5:9941909-9944782 REVERSE \| Aliases: F15F15.2 | 6.1 | 8.6 | -2.5 | -13.6 | 7.9E-5 | 11.3 |
| 346 | AT3G14050.1 \| Symbol: RSH2 \| RelA/SpoT protein, putative (RSH2), nearly identical to RelA/SpoT homolog RSH2 (Arabidopsis thaliana) GI:7141306; contains Pfam profiles PF01966: HD domain, PF04607: Region found in RelA / SpoT proteins \| chr3:4650768-4653921 REVERSE \| Aliases: AT RSH2, MDC16.19, RELA SPOT HOMOLOG | 6.8 | 10.4 | -3.5 | -13.6 | 7.9E-5 | 10.2 |
| 347 | AT2G47890.2 \| zinc finger (B-box type) family protein \| chr2:19615066-19616702 FORWARD \| Aliases: F17A22.28 | 6.1 | 10.1 | -4.0 | -13.6 | 7.9E-5 | 9.9 |
| 348 | AT4G23700.1 \| Symbol: ATCHX17 \| cation/hydrogen exchanger, putative (CHX17), similar to Na+/H+-exchanging protein slr1595 - Synechocystis sp., EMBL:D90902; monovalent cation:proton antiporter family 2 (CPA2) member, PMID:11500563 | 3.7 | 8.9 | -5.2 | -13.5 | 8.1E-5 | 9.7 |
| 349 | AT5G07220.1 \| BAG domain-containing protein, contains Pfam:PF02179 BAG domain \| chr5:2265257-2266968 REVERSE \| Aliases: T28J14.160, T28J14_160 | 5.4 | 9.5 | -4.0 | -13.5 | 8.3E-5 | 10.0 |
| 350 | AT4G02380.1 \| Symbol: SAG21 \| late embryogenesis abundant 3 family protein / LEA3 family protein, similar to several small proteins (~100 aa) that are induced by heat, auxin, ethylene and wounding such as Phaseolus aureus indole-3-acetic acid induced protein ARG (SW:32292); contains Pfam profile PF03242: Late embryogenesis abundant protein | 11.1 | 12.7 | -1.5 | -13.5 | 8.3E-5 | 11.7 |
| 352 | AT1G12110.1 \| Symbol: NRT1.1 \| | 7.8 | 11.4 | -3.6 | -13.5 | 8.3E-5 | 10.7 |
| 353 | AT3G08760.1 \| Symbol: ATSIK \| protein kinase family protein, contains protein kinase domain, Pfam:PF00069 \| chr3:2657924-2660833 REVERSE \| Aliases: F17O14.23 | 4.5 | 8.0 | -3.6 | -13.5 | 8.3E-5 | 10.7 |
| 357 | AT5G49990.1 \| xanthine/uracil permease family protein, similar to permease 1 (Zea mays) GI:7844006; contains Pfam profile: PF00860 Xanthine/uracil permeases family \| chr5:20354867-20359077 REVERSE \| Aliases: K9P8.13, K9P8_13 | 3.8 | 8.4 | -4.6 | -13.4 | 8.6E-5 | 10.4 |
| 358 | AT3G57790.1 \| glycoside hydrolase family 28 protein / polygalacturonase (pectinase) family protein, weak similarity to SP:P15922 Exo-poly-alpha-D-galacturonosidase precursor (EC 3.2.1.82) (Exo-PG) {Erwinia chrysanthemi}; contains PF00295: Glycosyl hydrolases family 28 \| chr3:21416364-21418065 REVERSE \| Aliases: F15B8.20 | 5.6 | 9.1 | -3.5 | -13.4 | 8.6E-5 | 9.9 |
| 360 | AT1G70780.1 \| expressed protein \| chr1:26698845-26700041 REVERSE \| Aliases: F5A18.4, F5A18_4 | 10.2 | 11.8 | -1.5 | -13.4 | 8.7E-5 | 11.6 |
| 361 | AT5G57740.1 \| Symbol: XBAT32 \| | 3.2 | 5.9 | -2.7 | -13.3 | 8.8E-5 | 10.6 |
| 362 | AT3G56980.1 \| Symbol: ORG3 \| basic helix-loop-helix (bHLH) family protein \| chr3:21097525-21098473 REVERSE \| Aliases: T8M16.8 | 4.8 | 9.8 | -5.0 | -13.3 | 8.9E-5 | 9.0 |
| 363 | AT3G62040.1 \| similar to haloacid dehalogenase-like hydrolase family protein [Arabidopsis thaliana] (TAIR:At5g59490.1); similar to expressed protein [Oryza sativa (japonica cultivar-group)] (GB:XP_470321.1); contains InterPro domain HAD-superfamily hydrolase, subfamily IA, variant 3 (InterPro:IPR006402); contains InterPro domain Haloacid dehalogenase-like hydrolase (InterPro:IPR005834) \| chr3:22986251-22987558 REVERSE \| Aliases: F21F14.210 | 3.8 | 9.6 | -5.8 | -13.3 | 8.9E-5 | 8.7 |
| 364 | AT3G55150.1 \| exocyst subunit EXO70 family protein, contains Pfam domain PF03081: Exo70 exocyst complex subunit; tomato leucine zipper-containing protein, Lycopersicon esculentum, PIR:S21495 | 3.0 | 7.0 | -4.0 | -13.3 | 8.9E-5 | 9.3 |
| 367 | AT2G16720.1 \| Symbol: MYB7 \| myb family transcription factor, contains Pfam profile: PF00249 myb-like DNA-binding domain \| chr2:7262671-7263643 REVERSE \| Aliases: ATMYB7, T24I21.13, T24I21_13 | 6.6 | 9.5 | -2.9 | -13.3 | 8.9E-5 | 10.5 |
| 370 | AT1G03660.1 \| expressed protein, contains 2 predicted transmembrane domains; expression supported by MPSS \| chr1:911435-911758 REVERSE \| Aliases: F21B7.26, F21B7_26 | 3.8 | 8.0 | -4.2 | -13.2 | 9.3E-5 | 9.6 |

| Rank | Description | Sync | Root | M | t | adj.q | B |
|---|---|---|---|---|---|---|---|
| 371 | AT3G22620.1 \| protease inhibitor/seed storage/lipid transfer protein (LTP) family protein, similar to pEARLI 1 (Accession No. L43080): an Arabidopsis member of a conserved gene family (PGF95-099), Plant Physiol. 109 (4), 1497 (1995); contains Pfam protease inhibitor/seed storage/LTP family domain PF00234 \| chr3:8008541-8010024 FORWARD \| Aliases: F16J14.17 | 3.7 | 9.0 | -5.3 | -13.2 | 9.3E-5 | 9.2 |
| 372 | AT2G45830.2 \| expressed protein \| chr2:18873135-18875616 FORWARD \| Aliases: F4I18.19 | 4.6 | 9.0 | -4.4 | -13.2 | 9.4E-5 | 10.3 |
| 374 | AT3G25190.1 \| nodulin, putative, similar to nodulin 21 (N-21) (Glycine max) SWISS-PROT:P16313 \| chr3:9175694-9177013 FORWARD \| Aliases: MJL12.26 | 4.8 | 9.7 | -4.9 | -13.2 | 9.4E-5 | 9.1 |
| 378 | AT5G24530.1 \| oxidoreductase, 2OG-Fe(II) oxygenase family protein, similar to flavanone 3-hydroxylase (Persea americana)(GI:727410); contains PF03171 2OG-Fe(II) oxygenase superfamily domain \| chr5:8378836-8383404 FORWARD \| Aliases: K18P6.6, K18P6_6 | 3.9 | 8.8 | -4.9 | -13.1 | 9.6E-5 | 9.4 |
| 382 | AT3G20015.1 \| similar to aspartyl protease family protein [Arabidopsis thaliana] (TAIR:At3g18490.1); similar to putative aspartic proteinase nepenthesin I [Oryza sativa (japonica cultivar-group)] (GB:NP_909181.1); contains InterPro domain Aspartic protease A1, pepsin (InterPro:IPR001461) \| chr3:6978483-6980374 REVERSE \| Aliases: MZE19.7 | 3.8 | 8.6 | -4.8 | -13.0 | 9.9E-5 | 9.0 |
| 384 | AT2G28890.1 \| protein phosphatase 2C family protein / PP2C family protein, similar to protein phosphatase-2c (GI:3608412) (Mesembryanthemum crystallinum); contains Pfam PF00481 : Protein phosphatase 2C domain \| chr2:12412673-12415312 REVERSE \| Aliases: F8N16.18, F8N16_18 | 8.4 | 10.6 | -2.2 | -13.0 | 1.0E-4 | 10.9 |
| 385 | AT5G44610.1 \| DREPP plasma membrane polypeptide-related, contains Pfam profile: PF05558 DREPP plasma membrane polypeptide \| chr5:18010792-18011895 REVERSE \| Aliases: K15C23.5, K15C23_5 | 5.8 | 11.8 | -5.9 | -13.0 | 1.0E-4 | 8.9 |
| 392 | AT1G64780.1 \| Symbol: ATAMT1;2 \| ammonium transporter 1, member 2 (AMT1.2), nearly identical to SP:Q9ZPJ8 Ammonium transporter 1, member 2 (AtAMT1;2) {Arabidopsis thaliana} \| chr1:24064433-24066293 REVERSE \| Aliases: None | 2.8 | 8.4 | -5.6 | -13.0 | 1.0E-4 | 8.1 |
| 394 | AT5G56080.1 \| nicotianamine synthase, putative, similar to nicotianamine synthase (Lycopersicon esculentum)(GI:4753801), nicotianamine synthase 2 (Hordeum vulgare)(GI:4894912) \| chr5:22728628-22729590 REVERSE \| Aliases: MDA7.14, MDA7_14 | 2.6 | 8.5 | -5.9 | -12.9 | 1.0E-4 | 7.2 |
| 395 | AT4G02090.1 \| expressed protein \| chr4:923100-923916 FORWARD \| Aliases: T10M13.10, T10M13_10 | 3.7 | 8.8 | -5.1 | -12.9 | 1.0E-4 | 9.1 |
| 397 | AT4G20140.1 \| leucine-rich repeat transmembrane protein kinase, putative, Cf-2.2, Lycopersicon pimpinellifolium, PIR:T10515 \| chr4:10884207-10888280 FORWARD \| Aliases: F1C12.60, F1C12_60 | 3.7 | 8.1 | -4.4 | -12.9 | 1.0E-4 | 9.4 |
| 399 | AT3G52190.1 \| transducin family protein / WD-40 repeat family protein, similar to St12p protein (GI:166878) {Arabidopsis thaliana}; contains Pfam profile: PF00400 WD domain, G-beta repeat \| chr3:19364950-19368036 REVERSE \| Aliases: T25B15.3 | 5.8 | 8.9 | -3.1 | -12.9 | 1.0E-4 | 10.4 |
| 400 | AT2G27920.3 \| Symbol: SCPL51 \| | 3.7 | 8.2 | -4.4 | -12.9 | 1.0E-4 | 9.2 |
| 401 | AT1G05010.1 \| Symbol: EFE \| 1-aminocyclopropane-1-carboxylate oxidase / ACC oxidase / ethylene-forming enzyme (ACO) (EAT1), Identical to 1-aminocyclopropane-1-carboxylate oxidase (ACC oxidase) gb:X66719 (EAT1). ESTs gb:T43073, gb:T5714, gb:R90435, gb:R44023, gb:AA597926, gb:AI099676, gb:AA650810 and gb:29725 come from this gene \| chr1:1431189-1432857 REVERSE \| Aliases: 1 AMINOCYCLOPROPANE 1 CARBOXYLATE OXIDASE, EAT1, ETHYLENE FORMING ENZYME, T7A14.12, T7A14_12 | 4.0 | 8.0 | -4.0 | -12.9 | 1.1E-4 | 9.5 |
| 402 | AT1G44160.1 \| DNAJ chaperone C-terminal domain-containing protein, contains Pfam profile PF01556: DnaJ C terminal region \| chr1:16797269-16798856 FORWARD \| Aliases: T7O23.16, T7O23_16 | 3.3 | 8.5 | -5.2 | -12.8 | 1.1E-4 | 8.0 |
| 403 | AT1G77000.1 \| F-box family protein, similar to GP:21554029: F-box protein AtFBL5 from (Arabidopsis thaliana); similar to F-box protein FBL2 GI:6063090 from (Homo sapiens) \| chr1:28945726-28947490 FORWARD \| Aliases: F22K20.10, F22K20_10 | 6.1 | 10.2 | -4.1 | -12.8 | 1.1E-4 | 10.9 |
| 404 | AT2G17710.1 \| expressed protein \| chr2:7700678-7701828 FORWARD \| Aliases: T17A5.17, T17A5_17 | 6.1 | 11.2 | -5.1 | -12.8 | 1.1E-4 | 9.5 |
| 409 | AT4G30450.1 \| glycine-rich protein \| chr4:14886033-14886681 REVERSE \| Aliases: F17I23.210, F17I23_210 | 8.4 | 10.8 | -2.4 | -12.8 | 1.1E-4 | 11.0 |
| 412 | AT1G73920.2 \| lipase family protein, similar to lipase GB:CAA74737 (SP:O46108) from (Drosophila melanogaster) \| chr1:27794537-27798575 FORWARD \| Aliases: F2P9.21, F2P9_21 | 6.1 | 9.8 | -3.8 | -12.7 | 1.1E-4 | 9.8 |
| 414 | AT2G21880.1 \| Ras-related GTP-binding protein, putative, similar to Ras family GTP-binding protein SP:Q43463 from (Glycine max) \| chr2:9331713-9333401 REVERSE \| Aliases: F7D8.20, F7D8_20 | 3.1 | 6.2 | -3.1 | -12.7 | 1.1E-4 | 10.1 |
| 416 | AT1G54970.1 \| Symbol: ATPRP1 \| | 3.0 | 9.0 | -6.1 | -12.7 | 1.1E-4 | 8.8 |

| Rank | Description | Sync | Root | M | t | adj.q | B |
|---|---|---|---|---|---|---|---|
| 418 | AT1G63440.1 \| copper-exporting ATPase, putative / responsive-to-antagonist 1, putative / copper-transporting ATPase, putative, similar to ATP dependent copper transporter SP:Q9S7J8 (Arabidopsis thaliana) \| chr1:23531320-23534774 FORWARD \| Aliases: F2K11.18, F2K11_18 | 7.4 | 9.6 | -2.2 | -12.7 | 1.1E-4 | 10.7 |
| 419 | AT3G50300.1 \| transferase family protein, similar to anthranilate N-hydroxycinnamoyl/benzoyltransferase, Dianthus caryophyllus (gi:2239091); contains Pfam transferase family domain PF002458 \| chr3:18654937-18656388 FORWARD \| Aliases: F11C1.140 | 5.9 | 9.9 | -4.0 | -12.6 | 1.1E-4 | 9.1 |
| 420 | AT1G49580.1 \| calcium-dependent protein kinase, putative / CDPK, putative, similar to calcium/calmodulin-dependent protein kinase CaMK3 (Nicotiana tabacum) gi:16904226:gb:AAL30820; contains protein kinase domain, Pfam:PF00069; contains serine/threonine protein kinase domain, INTERPRO:IPR002290 | 4.5 | 8.2 | -3.7 | -12.6 | 1.1E-4 | 9.8 |
| 421 | AT2G19060.1 \| GDSL-motif lipase/hydrolase family protein, similar to family II lipases EXL6 GI:15054390, EXL1 GI:15054382, EXL2 GI:15054384 from (Arabidopsis thaliana); contains Pfam profile PF00657: GDSL-like Lipase/Acylhydrolase | 3.6 | 8.0 | -4.5 | -12.6 | 1.1E-4 | 9.0 |
| 424 | AT5G58770.1 \| dehydrodolichyl diphosphate synthase, putative / DEDOL-PP synthase, putative, similar to GI:796076 \| chr5:23751602-23752860 FORWARD \| Aliases: MZN1.21, MZN1_21 | 2.6 | 7.7 | -5.1 | -12.6 | 1.1E-4 | 8.0 |
| 425 | AT5G23020.1 \| Symbol: MAM L \| 2-isopropylmalate synthase 2 (IMS2), identical to 2-isopropylmalate synthase (IMS2) (Arabidopsis thaliana) GI:12330689 \| chr5:7718206-7721842 FORWARD \| Aliases: 2 ISOPROPYLMALATE SYNTHASE 2, IMS2, METHYLTHIOALKYMALATE SYNTHASE LIKE, MYJ24.1, MYJ24_1 | 9.8 | 11.1 | -1.3 | -12.6 | 1.2E-4 | 11.2 |
| 427 | AT1G56320.1 \| expressed protein \| chr1:21089213-21090124 REVERSE \| Aliases: F14G9.7, F14G9_7 | 4.1 | 9.3 | -5.2 | -12.6 | 1.2E-4 | 8.9 |
| 430 | AT1G71010.1 \| phosphatidylinositol-4-phosphate 5-kinase family protein, low similarity to phosphatidylinositol 3,5-kinase (Candida albicans) GI:14571648; contains Pfam profile PF01504: Phosphatidylinositol-4-phosphate 5-Kinase \| chr1:26786501-26792630 FORWARD \| Aliases: F23N20.21 | 8.1 | 10.3 | -2.3 | -12.6 | 1.2E-4 | 10.8 |
| 431 | AT1G28130.2 \| Symbol: GH3.17 \| | 4.2 | 6.7 | -2.4 | -12.6 | 1.2E-4 | 10.6 |
| 432 | AT3G51440.1 \| strictosidine synthase family protein, similar to hemomucin (Drosophila melanogaster)(GI:1280434), strictosidine synthase (Rauvolfia serpentina)(SP:P15324); contains strictosidine synthase domain PF03088 \| chr3:19100060-19101618 FORWARD \| Aliases: F26O13.80 | 5.5 | 9.0 | -3.5 | -12.6 | 1.2E-4 | 10.1 |
| 437 | AT4G17080.1 \| MORN (Membrane Occupation and Recognition Nexus) repeat-containing protein /phosphatidylinositol-4-phosphate 5-kinase-related, low similarity to phosphatidylinositol-4-phosphate 5-kinase AtPIP5K1 (Arabidopsis thaliana) GI:3702691; contains Pfam profile PF02493: MORN repeat \| chr4:9601587-9603831 FORWARD \| Aliases: DL4570W, FCAALL.347 | 5.4 | 9.5 | -4.1 | -12.5 | 1.2E-4 | 10.3 |
| 442 | AT2G40140.2 \| similar to zinc finger (CCCH-type) family protein [Arabidopsis thaliana] (TAIR:At3g55980.1); similar to putative finger transcription factor [Oryza sativa (japonica cultivar-group)] (GB:AAU10743.1); contains InterPro domain Zn-finger, C-x8-C-x5-C-x3-H type (InterPro:IPR000571); contains InterPro domain Ankyrin (InterPro:IPR002110) \| chr2:16779294-16781735 FORWARD \| Aliases: T7M7.3, T7M7_3 | 5.6 | 8.4 | -2.9 | -12.5 | 1.2E-4 | 10.4 |
| 443 | AT3G17790.1 \| Symbol: ATACP5 \| | 3.6 | 8.7 | -5.1 | -12.5 | 1.2E-4 | 9.3 |
| 448 | AT1G68070.1 \| zinc finger (C3HC4-type RING finger) family protein, very low similarity to RING-H2 finger protein RHG1a (Arabidopsis thaliana) GI:3822225; contains Pfam profile: PF00097 zinc finger, C3HC4 type (RING finger) | 5.5 | 7.2 | -1.6 | -12.4 | 1.2E-4 | 10.9 |
| 450 | AT2G02960.5 \| zinc finger (C3HC4-type RING finger) family protein, contains Pfam profile PF00097: Zinc finger, C3HC4 type (RING finger); contains PROSITE PS00190: Cytochrome c family heme-binding site signature \| chr2:862086-864358 REVERSE \| Aliases: T17M13.13, T17M13_13 | 6.3 | 9.9 | -3.6 | -12.4 | 1.2E-4 | 10.2 |
| 451 | AT5G10520.1 \| protein kinase family protein, contains protein kinase domain, INTERPRO:IPR000719 \| chr5:3320379-3322911 REVERSE \| Aliases: F12B17.130, F12B17_130 | 3.3 | 7.9 | -4.5 | -12.4 | 1.2E-4 | 8.8 |
| 453 | AT1G12750.1 \| rhomboid family protein, contains PFAM domain PF01694, Rhomboid family \| chr1:4344983-4348325 REVERSE \| Aliases: T12C24.28, T12C24_28 | 4.8 | 8.5 | -3.6 | -12.4 | 1.3E-4 | 10.4 |
| 456 | AT4G11650.1 \| Symbol: ATOSM34 \| osmotin-like protein (OSM34), nearly identical to SP:P50700:OSL3_ARATH Osmotin-like protein OSM34 precursor {Arabidopsis thaliana}; contains Pfam profile PF00314: Thaumatin family \| chr4:7024850-7026140 REVERSE \| Aliases: T5C23.80, T5C23_80 | 4.3 | 9.4 | -5.2 | -12.3 | 1.3E-4 | 9.1 |
| 458 | AT4G02050.1 \| sugar transporter, putative, similar to SP:Q10710 Sugar carrier protein A {Ricinus communis}, glucose transporter (Saccharum hybrid cultivar H65-7052) GI:347855; contains Pfam profile PF00083: major facilitator superfamily protein \| chr4:898307-900199 REVERSE \| Aliases: T10M13.6, T10M13_6 | 6.2 | 9.3 | -3.2 | -12.3 | 1.3E-4 | 10.2 |
| 460 | AT4G23050.2 \| protein kinase, putative, similar to MAP3K delta-1 protein kinase (Arabidopsis thaliana) gi:2253010:emb:CAA74591; contains Pfam PF00069 Protein kinase domain and PF00989 PAS domain \| chr4:12080071-12084267 FORWARD \| Aliases: F7H19.240, F7H19_240 | 7.1 | 9.6 | -2.5 | -12.3 | 1.3E-4 | 10.3 |
| 461 | AT3G20340.1 \| expressed protein \| chr3:7092906-7093428 REVERSE \| Aliases: MQC12.8 | 2.4 | 7.8 | -5.3 | -12.2 | 1.3E-4 | 10.1 |

| Rank | Description | Sync | Root | M | t | adj.q | B |
|---|---|---|---|---|---|---|---|
| 464 | AT1G69810.1 \| Symbol: WRKY36 \| | 3.2 | 7.5 | -4.3 | -12.2 | 1.3E-4 | 9.6 |
| 465 | AT2G29995.1 \| expressed protein \| chr2:12803987-12806496 REVERSE \| Aliases: None | 4.7 | 9.4 | -4.6 | -12.2 | 1.4E-4 | 9.2 |
| 467 | AT1G44800.1 \| nodulin MtN21 family protein, similar to MtN21 (Medicago truncatula) GI:2598575; contains Pfam profile PF00892: Integral membrane protein \| chr1:16916449-16919436 REVERSE \| Aliases: T12C22.7, T12C22_7 | 5.7 | 10.7 | -5.1 | -12.2 | 1.4E-4 | 8.6 |
| 470 | AT1G15130.1 \| hydroxyproline-rich glycoprotein family protein \| chr1:5205997-5209910 REVERSE \| Aliases: F9L1.7, F9L1_7 | 4.1 | 7.3 | -3.2 | -12.2 | 1.4E-4 | 10.2 |
| 471 | AT1G01610.1 \| Symbol: ATGPAT4/GPAT4 \| Encodes a protein with glycerol-3-phosphate acyltransferase activity. \| chr1:221691-224315 REVERSE \| Aliases: ATGPAT4, F22L4.15, F22L4_15, GPAT4 | 2.4 | 6.6 | -4.2 | -12.2 | 1.4E-4 | 8.7 |
| 474 | AT3G49210.1 \| expressed protein \| chr3:18249033-18252208 REVERSE \| Aliases: F2K15.70 | 4.0 | 7.8 | -3.7 | -12.2 | 1.4E-4 | 9.3 |
| 476 | AT1G49860.1 \| Symbol: ATGSTF14 \| glutathione S-transferase, putative, similar to GI:860955 from (Hyoscyamus muticus) (Plant Physiol. 109 (1), 253-260 (1995)) | 3.0 | 8.2 | -5.1 | -12.1 | 1.4E-4 | 8.1 |
| 477 | AT5G02480.1 \| expressed protein, p \| chr5:547561-549910 FORWARD \| Aliases: T22P11.70, T22P11_70 | 7.6 | 9.7 | -2.1 | -12.1 | 1.4E-4 | 10.7 |
| 478 | AT3G04060.1 \| Symbol: ANAC046 \| no apical meristem (NAM) family protein, contains Pfam PF02365 : No apical meristem (NAM) protein; similar to cup-shaped cotyledon CUC2 (GI:1944132) (Arabidopsis thaliana) \| chr3:1053373-1055170 REVERSE \| Aliases: ANAC046, T11I18.17, T11I18_17 | 3.0 | 7.8 | -4.8 | -12.1 | 1.4E-4 | 8.7 |
| 481 | AT2G20630.2 \| protein phosphatase 2C, putative / PP2C, putative \| chr2:8903993-8906916 REVERSE \| Aliases: F23N11.5, F23N11_5 | 6.1 | 9.0 | -2.8 | -12.1 | 1.4E-4 | 10.4 |
| 482 | AT5G40690.1 \| expressed protein \| chr5:16307939-16308811 FORWARD \| Aliases: MNF13.25, MNF13_25 | 3.3 | 7.1 | -3.8 | -12.1 | 1.4E-4 | 8.9 |
| 483 | AT1G21920.1 \| MORN (Membrane Occupation and Recognition Nexus) repeat-containing protein /phosphatidylinositol-4-phosphate 5-kinase-related, similar to phosphatidylinositol-4-phosphate 5-kinase AtPIP5K1 (Arabidopsis thaliana) GI:3702691; contains Pfam profile PF02493: MORN repeat \| chr1:7704244-7706129 REVERSE \| Aliases: T26F17.15, T26F17_15 | 8.4 | 12.0 | -3.5 | -12.1 | 1.4E-4 | 10.5 |
| 486 | AT1G64400.1 \| long-chain-fatty-acid--CoA ligase, putative / long-chain acyl-CoA synthetase, putative, similar to GI:1617270 (MF7P) from (Brassica napus) \| chr1:23919261-23923446 REVERSE \| Aliases: F15H21.7, F15H21_7 | 3.0 | 7.2 | -4.2 | -12.1 | 1.4E-4 | 8.6 |
| 487 | AT3G15630.1 \| expressed protein \| chr3:5296854-5297606 REVERSE \| Aliases: MSJ11.3 | 3.8 | 7.7 | -4.0 | -12.1 | 1.4E-4 | 9.3 |
| 488 | AT1G72700.1 \| haloacid dehalogenase-like hydrolase family protein, similar to Potential phospholipid-transporting ATPase (EC 3.6.3.1) from Homo sapiens (SP:Q9Y2Q0, SP:O43520); contains InterPro accession IPR005834: Haloacid dehalogenase-like hydrolase \| chr1:27370572-27375395 FORWARD \| Aliases: F28P22.11, F28P22_11 | 4.3 | 7.7 | -3.4 | -12.1 | 1.4E-4 | 9.4 |
| 490 | AT1G29180.1 \| DC1 domain-containing protein, contains Pfam profile PF03107: DC1 domain | 3.2 | 7.8 | -4.5 | -12.1 | 1.4E-4 | 9.2 |
| 491 | AT1G21000.1 \| zinc-binding family protein, similar to zinc-binding protein (Pisum sativum) GI:16117799; contains Pfam profile PF04640 : Protein of unknown function, DUF597 \| chr1:7337740-7339441 FORWARD \| Aliases: F9H16.1, F9H16_1 | 6.4 | 9.1 | -2.7 | -12.0 | 1.4E-4 | 10.4 |
| 492 | AT4G05070.1 \| expressed protein \| chr4:2593596-2594117 REVERSE \| Aliases: C17L7.2 | 8.3 | 11.2 | -2.8 | -12.0 | 1.4E-4 | 10.3 |
| 494 | AT4G38830.1 \| protein kinase family protein, contains Pfam domain PF00069: Protein kinase domain \| chr4:18122320-18124937 FORWARD \| Aliases: T9A14.110, T9A14_110 | 3.7 | 8.2 | -4.5 | -12.0 | 1.4E-4 | 8.4 |
| 498 | AT1G49230.1 \| zinc finger (C3HC4-type RING finger) family protein, contains Pfam domain, PF00097: Zinc finger, C3HC4 type (RING finger) \| chr1:18212952-18213765 FORWARD \| Aliases: F27J15.2, F27J15_2 | 3.8 | 6.9 | -3.1 | -12.0 | 1.5E-4 | 9.9 |
| 499 | AT1G78340.1 \| Symbol: ATGSTU22 \| glutathione S-transferase, putative, similar to glutathione transferase GI:2853219 from (Carica papaya) \| chr1:29477785-29478756 REVERSE \| Aliases: F3F9.13, F3F9_13 | 6.1 | 10.8 | -4.7 | -12.0 | 1.5E-4 | 9.6 |
| 500 | AT3G14660.1 \| Symbol: CYP72A13 \| cytochrome P450, putative, similar to GB:Q05047 from (Catharanthus roseus) \| chr3:4924784-4927441 FORWARD \| Aliases: MIE1.16 | 6.8 | 9.5 | -2.7 | -12.0 | 1.5E-4 | 10.4 |
| 501 | AT5G18780.1 \| F-box family protein, contains F-box domain Pfam:PF00646 | 6.6 | 9.8 | -3.2 | -12.0 | 1.5E-4 | 9.9 |
| 502 | AT5G45070.1 \| Symbol: ATPP2 A8 \| disease resistance protein (TIR class), putative, domain signature TIR exists, suggestive of a disease resistance protein. \| chr5:18205126-18206589 REVERSE \| Aliases: ATPP2 A8, K17O22.3, K17O22_3 | 3.6 | 8.4 | -4.9 | -12.0 | 1.5E-4 | 7.6 |

| Rank | Description | Sync | Root | M | t | adj.q | B |
|---|---|---|---|---|---|---|---|
| 503 | AT5G37260.1 \| myb family transcription factor, contains Pfam profile: PF00249 myb-like DNA-binding domain \| chr5:14768574-14770202 REVERSE \| Aliases: MNJ8.4, MNJ8_4 | 3.8 | 8.7 | -4.9 | -11.9 | 1.5E-4 | 9.4 |
| 507 | AT4G03500.1 \| ankyrin repeat family protein, contains ankyrin repeats, Pfam:PF00023 \| chr4:1553090-1556569 FORWARD \| Aliases: F9H3.13, F9H3_13 | 3.4 | 7.4 | -4.0 | -11.9 | 1.5E-4 | 8.4 |
| 509 | AT4G40090.1 \| Symbol: AGP3 \| arabinogalactan-protein (AGP3), InDels between the genome sequence and the cDNA prevent identical translations from being annotated; the basis for this is under investigation. | 2.0 | 7.3 | -5.4 | -11.9 | 1.5E-4 | 7.2 |
| 513 | AT1G66150.1 \| Symbol: TMK1 \| leucine-rich repeat protein kinase, putative (TMK1), identical to protein kinase TMK1 gi:166888:gb:AAA32876, SP:P43298 Putative receptor protein kinase TMK1 precursor (EC 2.7.1.-) {Arabidopsis thaliana} \| chr1:24635072-24638412 FORWARD \| Aliases: F15E12.4, F15E12_4, TRANSMEMBRANE KINASE 1 | 3.0 | 5.9 | -2.9 | -11.9 | 1.5E-4 | 9.5 |
| 515 | AT5G12420.1 \| expressed protein \| chr5:4024315-4026742 REVERSE \| Aliases: None | 2.5 | 6.6 | -4.1 | -11.9 | 0.02% | 8.5 |
| 516 | AT4G30670.1 \| expressed protein \| chr4:14957371-14957968 REVERSE \| Aliases: T10C21.20, T10C21_20 | 2.9 | 8.8 | -5.9 | -11.8 | 0.02% | 8.8 |
| 517 | AT1G67920.1 \| expressed protein \| chr1:25477207-25477803 FORWARD \| Aliases: T23K23.23, T23K23_23 | 4.4 | 9.4 | -5.0 | -11.8 | 0.02% | 9.5 |
| 518 | AT2G39410.2 \| hydrolase, alpha/beta fold family protein, similar to monoglyceride lipase from (Homo sapiens) GI:14594904, (Mus musculus) GI:2632162; contains Pfam profile PF00561: hydrolase, alpha/beta fold family \| chr2:16462647-16465295 FORWARD \| Aliases: F12L6.7, F12L6_7 | 3.0 | 6.5 | -3.5 | -11.8 | 0.02% | 8.9 |
| 519 | AT4G15380.1 \| Symbol: CYP705A4 \| cytochrome P450 family protein, similar to CYTOCHROME P450 93A3 (P450 CP5) (SP:O81973) (Glycine max) \| chr4:8788739-8790422 FORWARD \| Aliases: DL3735W, FCAALL.280 | 3.1 | 7.0 | -3.9 | -11.8 | 0.02% | 8.9 |
| 520 | AT3G13760.1 \| DC1 domain-containing protein, contains Pfam profile PF03107: DC1 domain | 4.9 | 8.2 | -3.3 | -11.8 | 0.02% | 9.2 |
| 521 | AT5G55170.1 \| Symbol: SUM3 \| small ubiquitin-like modifier 3 (SUMO), similar to SP:O13351 Ubiquitin-like protein smt3/pmt3 {Schizosaccharomyces pombe}; identical to cDNA small ubiquitin-like modifier 3 (SUMO) GI:22652845 \| chr5:22402694-22403297 REVERSE \| Aliases: MCO15.12, MCO15_12, SMALL UBIQUITIN LIKE MODIFIER 3, SUMO 3, SUMO3 | 4.4 | 8.7 | -4.3 | -11.8 | 0.02% | 9.0 |
| 522 | AT5G57530.1 \| xyloglucan:xyloglucosyl transferase, putative / xyloglucan endotransglycosylase, putative / endo-xyloglucan transferase, putative, similar to xyloglucan endotransglycosylase XTR9 GI:4218963 from (Arabidopsis thaliana) \| chr5:23317547-23318831 REVERSE \| Aliases: MUA2.10, MUA2_10 | 2.5 | 8.3 | -5.8 | -11.8 | 0.02% | 8.1 |
| 523 | AT1G54320.1 \| LEM3 (ligand-effect modulator 3) family protein / CDC50 family protein, Similar to GI:11994416; GI:4966357; GI:4835763; GI:9757735 from (Arabidopsis thaliana) \| chr1:20279202-20281624 REVERSE \| Aliases: F20D21.14, F20D21_14 | 10.2 | 11.9 | -1.7 | -11.8 | 0.02% | 10.7 |
| 525 | AT1G54730.3 \| sugar transporter, putative, similar to ERD6 protein (Arabidopsis thaliana) GI:3123712, sugar-porter family proteins 1 and 2 (Arabidopsis thaliana) GI:14585699, GI:14585701; contains Pfam profile PF00083: major facilitator superfamily protein \| chr1:20428077-20433790 FORWARD \| Aliases: T22H22.15, T22H22_15 | 3.4 | 6.3 | -2.9 | -11.8 | 0.02% | 10.0 |
| 526 | AT4G29800.1 \| patatin-related, low similarity to patatin precursor (Solanum brevidens)(GI:563125); contains Patatin domain PF01743 \| chr4:14590837-14592542 REVERSE \| Aliases: F27B13.40, F27B13_40 | 4.9 | 10.3 | -5.4 | -11.8 | 0.02% | 7.7 |
| 529 | AT2G45430.1 \| DNA-binding protein-related, contains Pfam domain PF03479: Domain of unknown function (DUF296), found in AT-hook motifs Pfam:PF02178 \| chr2:18734635-18736009 FORWARD \| Aliases: F4L23.6 | 5.3 | 8.5 | -3.1 | -11.8 | 0.02% | 9.8 |
| 532 | AT4G21390.1 \| S-locus lectin protein kinase family protein, contains Pfam profiles: PF00954 S-locus glycoprotein family, PF00069 protein kinase domain, PF01453 lectin (probable mannose binding) \| chr4:11394368-11397594 REVERSE \| Aliases: T6K22.120, T6K22_120 | 4.5 | 8.6 | -4.1 | -11.7 | 0.02% | 10.0 |
| 533 | AT1G68850.1 \| peroxidase, putative, identical to peroxidase ATP23a GB:CAA70035 (Arabidopsis thaliana) \| chr1:25887279-25888896 REVERSE \| Aliases: T6L1.4, T6L1_4 | 3.1 | 6.1 | -3.0 | -11.7 | 0.02% | 9.6 |
| 539 | AT1G80440.1 \| kelch repeat-containing F-box family protein, similar to SP:Q9ER30 Kelch-related protein 1 (Sarcosin) {Rattus norvegicus}; contains Pfam profiles PF01344: Kelch motif, PF00646: F-box domain \| chr1:30246499-30247956 FORWARD \| Aliases: T21F11.23, T21F11_23 | 5.0 | 9.5 | -4.5 | -11.7 | 0.02% | 9.3 |
| 540 | AT3G04070.1 \| Symbol: ANAC047 \| no apical meristem (NAM) family protein, contains Pfam PF02365: No apical meristem (NAM) domain; similar to NAM GB:CAA63101 (Petunia x hybrida) \| chr3:1061533-1063101 REVERSE \| Aliases: ANAC047, T11I18.18, T11I18_18 | 4.8 | 8.3 | -3.5 | -11.7 | 0.02% | 9.4 |
| 541 | AT1G55590.1 \| F-box family protein, ; similar to SKP1 interacting partner 2 (SKIP2) TIGR_Ath1:At5g67250 \| chr1:20772986-20775537 REVERSE \| Aliases: F20N2.2 | 5.4 | 7.8 | -2.3 | -11.7 | 0.02% | 9.9 |

| Rank | Description | Sync | Root | M | t | adj.q | B |
|---|---|---|---|---|---|---|---|
| 546 | AT4G36380.1 \| Symbol: ROT3 \| cytochrome P450 90C1 (CYP90C1) / rotundifolia3 (ROT3), identical | 3.3 | 8.2 | -4.9 | -11.6 | 0.02% | 7.5 |
| 547 | AT5G56540.1 \| Symbol: AGP14 \| arabinogalactan-protein (AGP14), identical to gi:10880505:gb:AAG24282 \| chr5:22910377-22910868 FORWARD \| Aliases: ARABINOGALACTAN PROTEIN 14, MKN22.5, MKN22_5 | 5.5 | 11.9 | -6.3 | -11.6 | 0.02% | 8.8 |
| 548 | AT2G06925.1 \| phospholipase A2 family protein, similar to secretory low molecular weight phospholipase A2 beta (Arabidopsis thaliana) GI:25992715; contains INTERPRO domain IPR001211 phospholipase A2 | 4.0 | 7.3 | -3.3 | -11.6 | 0.02% | 9.1 |
| 549 | AT3G12740.1 \| LEM3 (ligand-effect modulator 3) family protein / CDC50 family protein, Similar to GI:4585976; GI:4966357; GI:4835763; GI:9757735 from (Arabidopsis thaliana) \| chr3:4049522-4052050 FORWARD \| Aliases: MBK21.12 | 9.5 | 11.3 | -1.8 | -11.6 | 0.02% | 10.5 |
| 552 | AT2G30840.1 \| 2-oxoglutarate-dependent dioxygenase, putative, similar to 2A6 (GI:599622) and tomato ethylene synthesis regulatory protein E8 (SP:P10967) \| chr2:13142507-13143926 REVERSE \| Aliases: F7F1.5, F7F1_5 | 3.1 | 7.5 | -4.4 | -11.6 | 0.02% | 8.0 |
| 556 | AT2G25090.1 \| Symbol: CIPK16 \| | 3.0 | 8.4 | -5.4 | -11.6 | 0.02% | 7.2 |
| 557 | AT3G29250.1 \| short-chain dehydrogenase/reductase (SDR) family protein, similar to 3-beta-hydroxysteroiddehydrogenase GI:15983819 from (Digitalis lanata); contains Pfam profile: PF00106 short chain dehydrogenase \| chr3:11195004-11200309 REVERSE \| Aliases: MXO21.10 | 3.1 | 8.1 | -5.0 | -11.6 | 0.02% | 9.3 |
| 558 | AT1G64460.1 \| phosphatidylinositol 3- and 4-kinase family protein, contains Pfam profile PF00454: Phosphatidylinositol 3- and 4-kinase | 3.7 | 7.3 | -3.6 | -11.6 | 0.02% | 9.6 |
| 559 | AT3G48970.1 \| copper-binding family protein, similar to copper homeostasis factor gi:3168840 from Arabidopsis thaliana; contains Pfam heavy-metal-associated domain PF00403 \| chr3:18163273-18164360 REVERSE \| Aliases: T2J13.190 | 3.2 | 7.5 | -4.3 | -11.5 | 0.02% | 8.8 |
| 564 | AT5G25440.1 \| protein kinase family protein, contains protein kinase domain, Pfam:PF00069 \| chr5:8854842-8856761 REVERSE \| Aliases: F18G18.180, F18G18_180 | 3.3 | 5.2 | -1.9 | -11.5 | 0.02% | 10.2 |
| 565 | AT1G60140.1 \| Symbol: ATTPS10 \| glycosyl transferase family 20 protein / trehalose-phosphatase family protein, contains Pfam profile: PF02358 trehalose-phosphatase \| chr1:22180599-22184495 REVERSE \| Aliases: T13D8.4, T13D8_4, TPS10 | 4.9 | 9.0 | -4.1 | -11.5 | 0.02% | 9.6 |
| 566 | AT3G09240.1 \| protein kinase-related, low similarity to protein kinase GI:166809; contains Pfam profile: Eukaryotic protein kinase domain \| chr3:2835674-2837962 REVERSE \| Aliases: F3L24.11 | 2.3 | 7.4 | -5.1 | -11.5 | 0.02% | 7.1 |
| 568 | AT4G32950.1 \| protein phosphatase 2C, putative / PP2C, putative, phosphoprotein phosphatase, Arabidopsis thaliana, PIR2:S55457 | 5.9 | 10.0 | -4.1 | -11.5 | 0.02% | 8.8 |
| 569 | AT3G10500.1 \| Symbol: ANAC053 \| no apical meristem (NAM) family protein, similar to to NAC2 (GI:645671) (Arabidopsis thaliana); contains Pfam PF02365: No apical meristem (NAM) protein \| chr3:3271617-3274035 FORWARD \| Aliases: ANAC053, F13M14.22 | 7.2 | 10.5 | -3.3 | -11.5 | 0.02% | 9.4 |
| 570 | AT3G45710.1 \| proton-dependent oligopeptide transport (POT) family protein, contains Pfam profile: PF00854 POT family \| chr3:16793629-16795720 FORWARD \| Aliases: T6D9.40 | 3.1 | 7.4 | -4.3 | -11.5 | 0.02% | 9.4 |
| 573 | AT4G39940.1 \| Symbol: AKN2 \| adenylylsulfate kinase 2 (AKN2), identical to adenylylsulfate kinase 2, chloroplast precursor (APS kinase, Adenosine-5'phosphosulfate kinase, ATP adenosine-5'- phosphosulfate 3'-phosphotransferase) (Arabidopsis thaliana) SWISS-PROT:O49196 \| chr4:18519711-18521562 FORWARD \| Aliases: ADENOSINE 5' PHOSPHOSULFATE KINASE, T5J17.110, T5J17_110 | 3.0 | 6.4 | -3.5 | -11.5 | 0.02% | 8.7 |
| 574 | AT1G55940.1 \| Symbol: CYP708A1 \| cytochrome P450, putative, similar to SP:Q42569 from (Arabidopsis thaliana) \| chr1:20926133-20929284 REVERSE \| Aliases: F14J16.21, F14J16_21 | 2.6 | 6.7 | -4.1 | -11.4 | 0.02% | 9.0 |
| 576 | AT5G24410.1 \| glucosamine/galactosamine-6-phosphate isomerase-related, contains weak similarity to Swiss-Prot:O95336 6-phosphogluconolactonase (EC 3.1.1.31) (6PGL) (Homo sapiens) \| chr5:8332476-8333987 REVERSE \| Aliases: K16H17.12, K16H17_12 | 3.1 | 7.4 | -4.3 | -11.4 | 0.02% | 7.9 |
| 577 | AT3G23090.1 \| expressed protein \| chr3:8214318-8217087 REVERSE \| Aliases: MXC7.13 | 4.8 | 7.9 | -3.1 | -11.4 | 0.02% | 9.8 |
| 578 | AT3G57040.1 \| Symbol: ARR9 \| two-component responsive regulator / response reactor 4 (RR4), identical to responce reactor4 GI:3273202 from (Arabidopsis thaliana); contains Pfam profile: PF00072 response regulator receiver domain \| chr3:21120593-21122411 FORWARD \| Aliases: ATRR4, F24I3.120, F24I3_120, RESPONSE REACTOR 4 | 4.1 | 7.9 | -3.9 | -11.4 | 0.02% | 9.4 |
| 580 | AT2G23760.3 \| Symbol: BLH4 \| similar to BEL1-like homeobox 2 protein (BLH2) [Arabidopsis thaliana] (TAIR:At4g36870.1); similar to BEL1-related homeotic protein 13 [Solanum tuberosum] (GB:AAN03623.1); contains InterPro domain POX (InterPro:IPR006563); contains InterPro domain Homeobox (InterPro:IPR001356) \| chr2:10114789-10120085 REVERSE \| Aliases: BEL1 like homeobox 4, F27L4.6, F27L4_6 | 2.7 | 6.9 | -4.2 | -11.4 | 0.02% | 8.5 |

| Rank | Description | Sync | Root | M | t | adj.q | B |
|---|---|---|---|---|---|---|---|
| 581 | AT1G19270.1 \| ubiquitin interaction motif-containing protein / LIM domain-containing protein, weak similarity to LIM-homeobox protein (Mus musculus) GI:2149584, Hic-5 (Mus musculus) GI:664955; contains Pfam profiles PF02809: Ubiquitin interaction motif, PF00412: LIM domain \| chr1:6662492-6666019 FORWARD \| Aliases: T29M8.14, T29M8_14 | 4.7 | 6.6 | -1.9 | -11.4 | 0.02% | 10.1 |
| 582 | AT2G22620.1 \| expressed protein \| chr2:9611881-9617675 REVERSE \| Aliases: T9I22.6, T9I22_6 | 3.2 | 6.7 | -3.5 | -11.4 | 0.02% | 9.6 |
| 583 | AT2G45750.1 \| dehydration-responsive family protein, similar to early-responsive to dehydration stress ERD3 protein (Arabidopsis thaliana) GI:15320410; contains Pfam profile PF03141: Putative methyltransferase \| chr2:18849613-18852541 FORWARD \| Aliases: F4I18.27 | 3.9 | 7.5 | -3.6 | -11.4 | 0.02% | 8.8 |
| 585 | AT1G01120.1 \| Symbol: KCS1 \| fatty acid elongase 3-ketoacyl-CoA synthase 1 (KCS1), nearly identical to GB:AAC99312 GI:4091810 from (Arabidopsis thaliana) \| chr1:57269-59167 REVERSE \| Aliases: T25K16.11, T25K16_11 | 4.2 | 8.1 | -3.9 | -11.4 | 0.02% | 8.3 |
| 586 | AT5G40730.1 \| Symbol: AGP24 \| arabinogalactan-protein (AGP24) | 9.5 | 12.7 | -3.2 | -11.4 | 0.02% | 10.3 |
| 587 | AT1G08340.1 \| rac GTPase activating protein, putative, similar to rac GTPase activating protein 1 GI:3695059 from (Lotus japonicus); contains Pfam profile PF00620: RhoGAP domain \| chr1:2631065-2632666 FORWARD \| Aliases: T23G18.20, T23G18_20 | 2.9 | 7.4 | -4.5 | -11.4 | 0.02% | 7.9 |
| 588 | AT1G06110.1 \| F-box family protein, contains similarity to F-box protein FBX3 GI:6103643 from (Homo sapiens) ; similar to SKP1 interacting partner 2 (SKIP2) TIGR_Ath1:At5g67250 \| chr1:1853076-1855021 REVERSE \| Aliases: T21E18.16, T21E18_16 | 6.2 | 8.5 | -2.3 | -11.4 | 0.02% | 9.8 |
| 590 | AT5G40890.2 \| Symbol: ATCLC A \| similar to chloride channel protein (CLC-c) [Arabidopsis thaliana] (TAIR:At5g49890.1); similar to chloride channel-like (CLC) protein, putative [Arabidopsis thaliana] (TAIR:At5g33280.1); similar to chloride channel protein (CLC-b) [Arabidopsis thaliana] (TAIR:At3g27170.1); similar to chloride channel [Oryza sativa (japonica cultivar-group)] (GB:XP_466225.1); similar to chloride channel [Oryza sativa (japonica cultivar-group)] (GB:BAB97268.1); similar to chloride channel [Oryza sativa (japonica cultivar-group)] (GB:BAB97267.1); similar to chloride channel Stclc1 [Solanum tuberosum] (GB:CAA71369.1); similar to C1C-Nt1 [Nicotiana tabacum] (GB:CAA64829.1); contains InterPro domain Cl- channel, voltage gated (InterPro:IPR001807); contains InterPro domain Chloride channel plant CLC (InterPro:IPR002251); contains InterPro domain CBS domain (InterPro:IPR000644) \| chr5:16398542-16401547 REVERSE \| Aliases: CHLORIDE CHANNEL A, CLC A, CLCA, MHK7.12, MHK7_12 | 6.3 | 9.2 | -2.9 | -11.4 | 0.02% | 9.5 |
| 591 | AT3G19710.1 \| branched-chain amino acid aminotransferase, putative / branched-chain amino acid transaminase, putative (BCAT4), similar to branched-chain amino acid transaminase 6 (Arabidopsis thaliana) GI:13810195; contains Pfam profile: PF01063 aminotransferase class IV \| chr3:6846996-6849503 REVERSE \| Aliases: MMB12.20 | 3.3 | 8.4 | -5.0 | -11.4 | 0.02% | 8.2 |
| 592 | AT3G06420.1 \| autophagy 8h (APG8h), identical to autophagy 8h (Arabidopsis thaliana) GI:19912165; contains Pfam profile PF02991: Microtubule associated protein 1A/1B, light chain 3; supporting cDNA gi:19912164:dbj:AB073182.1: \| chr3:1954996-1956399 REVERSE \| Aliases: F24P17.11, F24P17_11 | 6.3 | 10.0 | -3.7 | -11.4 | 0.02% | 9.4 |
| 593 | AT2G23540.1 \| GDSL-motif lipase/hydrolase family protein, similar to family II lipase EXL1 GI:15054382 from (Arabidopsis thaliana); contains Pfam profile PF00657: GDSL-like Lipase/Acylhydrolase \| chr2:10031433-10033265 FORWARD \| Aliases: F26B6.19, F26B6_19 | 3.5 | 8.1 | -4.6 | -11.4 | 0.02% | 7.9 |
| 595 | AT3G05200.1 \| Symbol: ATL6 \| zinc finger (C3HC4-type RING finger) family protein (ATL6), contains Pfam profile: PF00097: Zinc finger, C3HC4 type (RING finger) \| chr3:1476988-1478736 FORWARD \| Aliases: T12H1.17, T12H1_17 | 6.5 | 9.2 | -2.7 | -11.3 | 0.02% | 9.6 |
| 596 | AT1G07610.1 \| Symbol: MT1C \| metallothionein-like protein 1C (MT-1C), identical to Metallothionein-like protein 1C (MT-1C). (SP:Q38804) (Arabidopsis thaliana) \| chr1:2341539-2342120 FORWARD \| Aliases: F22G5.40 | 11.3 | 14.1 | -2.7 | -11.3 | 0.02% | 10.3 |
| 597 | AT4G38540.1 \| monooxygenase, putative (MO2), identical to GI:3426064 \| chr4:18023173-18025021 FORWARD \| Aliases: F20M13.100, F20M13_100 | 7.4 | 9.8 | -2.4 | -11.3 | 0.02% | 9.9 |
| 598 | AT5G24270.1 \| Symbol: SOS3 \| calcineurin B-like protein, putative / calcium sensor homolog (SOS3), identical to calcium sensor homolog (Arabidopsis thaliana) GI:3309575; similar to calcineurin B-like protein 8 (GI:15866276) (Arabidopsis thaliana) \| chr5:8238784-8240182 REVERSE \| Aliases: CALCINEURIN B LIKE PROTEIN 4, CBL4, MOP9.19, MOP9_19, SALT OVERLY SENSITIVE 3 | 3.8 | 7.8 | -3.9 | -11.3 | 0.02% | 8.2 |
| 600 | AT5G53460.1 \| Symbol: GLT1 \| glutamate synthase (NADH), chloroplast, putative, similar to SP:Q03460 Glutamate synthase (NADH), chloroplast precursor (EC 1.4.1.14) (NADH- GOGAT) {Medicago sativa} \| chr5:21717744-21726855 FORWARD \| Aliases: MYN8.7, MYN8_7, NADH GOGAT | 7.9 | 10.6 | -2.7 | -11.3 | 0.02% | 10.0 |
| 601 | AT3G48450.1 \| nitrate-responsive NOI protein, putative, similar to nitrate-induced NOI protein (Zea mays) GI:2642213 \| chr3:17955017-17955873 REVERSE \| Aliases: T29H11.30 | 3.9 | 6.9 | -3.0 | -11.3 | 0.02% | 9.4 |
| 605 | AT4G16350.1 \| Symbol: CBL6 \| calcineurin B-like protein 6 (CBL6), identical to calcineurin B-like protein 6 (GI:11065943) (Arabidopsis thaliana) \| chr4:9242522-9243686 REVERSE \| Aliases: CALCINEURIN B LIKE PROTEIN 6, DL4205C, FCAALL.89, SCABP2, SOS3 LIKE CALCIUM BINDING PROTEIN 2 | 3.3 | 7.2 | -3.9 | -11.3 | 0.02% | 8.4 |

| Rank | Description | Sync | Root | M | t | adj.q | B |
|---|---|---|---|---|---|---|---|
| 609 | AT4G20260.4 \| similar to DREPP plasma membrane polypeptide-related [Arabidopsis thaliana] (TAIR:At5g44610.1); similar to DREPP2 protein [Nicotiana tabacum] (GB:CAB91552.1); contains InterPro domain DREPP plasma membrane polypeptide (InterPro:IPR008469) \| chr4:10940749-10943512 FORWARD \| Aliases: F1C12.180, F1C12_180 | 10.2 | 12.2 | -2.0 | -11.2 | 0.02% | 10.2 |
| 613 | AT3G49960.1 \| peroxidase, putative, identical to peroxidase ATP21a (Arabidopsis thaliana) gi:1546696:emb:CAA67339 \| chr3:18535069-18536672 REVERSE \| Aliases: F3A4.40 | 3.0 | 8.0 | -5.0 | -11.2 | 0.02% | 9.8 |
| 615 | AT1G69450.1 \| early-responsive to dehydration protein-related / ERD protein-related, low similarity to ERD4 protein (early-responsive to dehydration stress) (Arabidopsis thaliana) GI:15375406; contains Pfam profile PF02714: Domain of unknown function DUF221 \| chr1:26110783-26113444 REVERSE \| Aliases: F10D13.27, F10D13_27 | 5.4 | 8.7 | -3.3 | -11.2 | 0.02% | 8.7 |
| 617 | AT3G25790.1 \| myb family transcription factor, contains Pfam domain, PF00249: Myb-like DNA-binding domain \| chr3:9414364-9416240 FORWARD \| Aliases: K13N2.13 | 5.2 | 10.1 | -4.9 | -11.2 | 0.02% | 8.4 |
| 619 | AT4G18640.1 \| leucine-rich repeat transmembrane protein kinase, putative \| chr4:10259695-10263775 FORWARD \| Aliases: F28A21.50, F28A21_50 | 3.9 | 7.4 | -3.5 | -11.1 | 0.02% | 9.4 |
| 620 | AT4G08160.2 \| similar to glycosyl hydrolase family 10 protein / carbohydrate-binding domain-containing protein [Arabidopsis thaliana] (TAIR:At1g10050.1); similar to glycosyl hydrolase family 10 protein / carbohydrate-binding domain-containing protein [Arabidopsis thaliana] (TAIR:At1g58370.1); similar to putative 1,4-beta-D xylan xylanohydrolase [Oryza sativa (japonica cultivar-group)] (GB:XP_477949.1); similar to putative xylan xylanohydrolase isoenzyme [Oryza sativa (japonica cultivar-group)] (GB:XP_469240.1); contains InterPro domain Glycoside hydrolase, family 10 (InterPro:IPR001000); contains InterPro domain Carbohydrate-binding, CenC-like (InterPro:IPR003305) \| chr4:5159031-5162771 REVERSE \| Aliases: F9M13.1, F9M13_1 | 3.2 | 8.0 | -4.8 | -11.1 | 0.02% | 7.4 |
| 626 | AT1G08500.1 \| plastocyanin-like domain-containing protein \| chr1:2689057-2690048 FORWARD \| Aliases: T27G7.18, T27G7_18 | 3.9 | 8.0 | -4.1 | -11.1 | 0.02% | 8.2 |
| 627 | AT3G44510.1 \| expressed protein \| chr3:16119094-16120345 REVERSE \| Aliases: F14L2.60 | 3.0 | 6.5 | -3.5 | -11.1 | 0.02% | 8.3 |
| 628 | AT5G24080.1 \| protein kinase family protein, contains protein kinase domain, Pfam:PF00069 \| chr5:8139256-8141125 REVERSE \| Aliases: MZF18.3, MZF18_3 | 3.0 | 6.9 | -3.9 | -11.1 | 0.02% | 8.3 |
| 634 | AT2G16970.1 \| expressed protein, ; expression supported by MPSS \| chr2:7376631-7382610 FORWARD \| Aliases: F12A24.15, F12A24_15 | 3.9 | 7.2 | -3.3 | -11.1 | 0.02% | 8.8 |
| 635 | AT1G30370.1 \| lipase class 3 family protein, similar to DEFECTIVE IN ANTHER DEHISCENCE1 (Arabidopsis thaliana) GI:16215706; contains Pfam profile: PF01764: Lipase \| chr1:10719151-10720740 REVERSE \| Aliases: T4K22.3, T4K22_3 | 3.5 | 7.7 | -4.3 | -11.1 | 0.02% | 7.8 |
| 636 | AT5G39970.1 \| expressed protein, low similarity to up-regulated by thyroid hormone in tadpoles; expressed specifically in the tail and only at metamorphosis; membrane bound or extracellular protein; C-terminal basic region (Xenopus laevis) GI:1234787 \| chr5:16015253-16018850 FORWARD \| Aliases: MYH19.17, MYH19_17 | 4.3 | 7.7 | -3.4 | -11.0 | 0.02% | 9.3 |
| 637 | AT3G62860.1 \| esterase/lipase/thioesterase family protein, similar to monoglyceride lipase from (Homo sapiens) GI:14594904, (Mus musculus) GI:2632162; contains Interpro entry IPR000379 \| chr3:23250421-23253226 REVERSE \| Aliases: F26K9.290 | 4.9 | 7.4 | -2.5 | -11.0 | 0.02% | 9.3 |
| 638 | AT2G23030.1 \| protein kinase, putative, similar to protein kinase 3 (Glycine max) GP:310582:gb:AAB68961 \| chr2:9810582-9813759 REVERSE \| Aliases: F21P24.9, F21P24_9 | 5.3 | 9.1 | -3.8 | -11.0 | 0.02% | 9.0 |
| 639 | AT2G47000.1 \| multidrug resistant (MDR) ABC transporter, putative, similar to multidrug-resistant protein CjMDR1 (Coptis japonica) GI:14715462, MDR-like p-glycoprotein (Arabidopsis thaliana) GI:24324262; contains Pfam profiles PF00005: ABC transporter, PF00664: ABC transporter transmembrane region \| chr2:19316937-19321819 REVERSE \| Aliases: F14M4.17 | 4.8 | 8.4 | -3.6 | -11.0 | 0.02% | 9.3 |
| 645 | AT4G30120.1 \| Symbol: HMA3 \| ATPase E1-E2 type family protein / heavy-metal-associated domain-containing protein, similar to cadmium efflux ATPase (Streptococcus thermophilus) GI:22416341; contains Pfam profile PF00122: E1-E2 ATPase \| chr4:14730407-14733508 REVERSE \| Aliases: F6G3.150, F6G3_150 | 3.7 | 7.7 | -4.0 | -11.0 | 0.02% | 8.1 |
| 646 | AT1G03210.1 \| phenazine biosynthesis PhzC/PhzF family protein, contains Pfam profile: PF02567 phenazine biosynthesis-like protein \| chr1:782948-784240 FORWARD \| Aliases: F15K9.19, F15K9_19 | 7.7 | 10.4 | -2.8 | -11.0 | 0.02% | 9.3 |
| 648 | AT1G32230.2 \| Symbol: RCD1 \| WWE domain-containing protein / ceo protein, putative (CEO), contains Pfam domain, PF02825: WWE domain; identical to cDNA for ceo protein (ceo gene) GI:11044956 | 7.0 | 10.4 | -3.4 | -11.0 | 0.02% | 9.4 |
| 649 | AT1G05320.3 \| similar to expressed protein [Arabidopsis thaliana] (TAIR:At2g32240.1); similar to unnamed protein product [Candida glabrata CBS138] (GB:CAG58365.1) \| chr1:1553607-1557769 FORWARD \| Aliases: YUP8H12.6, YUP8H12_6 | 5.5 | 8.8 | -3.3 | -11.0 | 0.02% | 8.8 |

| Rank | Description | Sync | Root | M | t | adj.q | B |
|---|---|---|---|---|---|---|---|
| 650 | AT2G03720.1 \| universal stress protein (USP) family protein, contains Pfam profile PF00582: universal stress protein family \| chr2:1132167-1133376 FORWARD \| Aliases: F19B11.17, F19B11_17 | 2.9 | 7.8 | -4.9 | -11.0 | 0.02% | 7.7 |
| 652 | AT1G21120.1 \| O-methyltransferase, putative, similar to GI:2781394 \| chr1:7395221-7396738 REVERSE \| Aliases: T22I11.5, T22I11_5 | 4.0 | 7.4 | -3.3 | -11.0 | 0.02% | 9.6 |
| 653 | AT3G32040.1 \| geranylgeranyl pyrophosphate synthase, putative / GGPP synthetase, putative / farnesyltranstransferase, putative, identical to gi:413730; similar to GB:P34802 from (Arabidopsis thaliana) (Plant Physiol. 104 (4), 1469-1470 (1994)) \| chr3:13051535-13052722 REVERSE \| Aliases: F1M23.7 | 4.6 | 8.4 | -3.8 | -11.0 | 0.02% | 9.1 |
| 654 | AT1G07380.1 \| ceramidase family protein, contains similarity to mitochondrial ceramidase (Homo sapiens) gi:9246993:gb:AAF86240 | 5.5 | 7.9 | -2.4 | -11.0 | 0.02% | 9.6 |
| 656 | AT3G06380.1 \| F-box family protein / tubby family protein, similar to phosphodiesterase (GI:467578) (Mus musculus); similar to C-terminal half of tubby protein (A mutation in the tub gene causes maturity-onset obesity, insulin resistance, and sensory deficits) (GB:P50586) (GI:6730158)(Mus musculus); contains Pfam PF00646: F-box domain and Pfam PF01167: Tub family; similar to Tubby protein homolog (Swiss-Prot:O88808) (Rattus norvegicus) \| chr3:1936130-1938227 FORWARD \| Aliases: F24P17.15, F24P17_15 | 4.6 | 7.0 | -2.4 | -11.0 | 0.02% | 9.8 |
| 657 | AT1G22440.1 \| alcohol dehydrogenase, putative, similar to alcohol dehydrogenase ADH GI:7705214 from (Lycopersicon esculentum); contains Pfam zinc-binding dehydrogenase domain PF00107 | 6.8 | 10.3 | -3.5 | -11.0 | 0.02% | 9.7 |
| 659 | AT3G53150.1 \| UDP-glucoronosyl/UDP-glucosyl transferase family protein, contains Pfam profile: PF00201 UDP-glucoronosyl and UDP-glucosyl transferase \| chr3:19708714-19710237 REVERSE \| Aliases: T4D2.80 | 3.4 | 7.9 | -4.5 | -10.9 | 0.02% | 8.3 |
| 660 | AT5G66815.1 \| expressed protein \| chr5:26694591-26695091 FORWARD \| Aliases: None | 2.8 | 5.7 | -3.0 | -10.9 | 0.02% | 9.3 |
| 665 | AT3G12700.1 \| aspartyl protease family protein, contains Pfam PF00026: Eukaryotic aspartyl protease; similar to CND41, chloroplast nucleoid DNA binding protein (GI:2541876) (Nicotiana tabacum) \| chr3:4037077-4039141 FORWARD \| Aliases: MBK21.7 | 5.3 | 8.1 | -2.8 | -10.9 | 0.02% | 9.1 |
| 668 | AT1G20160.2 \| similar to subtilase family protein [Arabidopsis thaliana] (TAIR:At1g20150.1); similar to putative subtilisin precursor [Glycine max] (GB:CAB87247.1); similar to subtilisin-like protein [Glycine max] (GB:AAK53589.1); similar to subtilisin-like protein [Picea abies] (GB:BAA13135.1); contains InterPro domain Protease-associated PA (InterPro:IPR003137); contains InterPro domain Subtilase serine protease (InterPro:IPR000209) | 5.9 | 9.4 | -3.6 | -10.9 | 0.02% | 9.5 |
| 670 | AT2G40080.1 \| Symbol: ELF4 \| expressed protein \| chr2:16741372-16741990 REVERSE \| Aliases: T28M21.24, T28M21_24 | 7.4 | 10.9 | -3.5 | -10.9 | 0.02% | 9.4 |
| 672 | AT4G31240.2 \| expressed protein \| chr4:15176461-15178514 REVERSE \| Aliases: F8F16.60, F8F16_60 | 4.1 | 6.8 | -2.7 | -10.9 | 0.02% | 9.2 |
| 673 | AT3G04810.2 \| similar to protein kinase family protein [Arabidopsis thaliana] (TAIR:At1g54510.1); similar to protein kinase, putative [Arabidopsis thaliana] (TAIR:At5g28290.1); similar to putative LSTK-1-like kinase [Oryza sativa (japonica cultivar-group)] (GB:AAR01739.1); similar to LSTK-1-like kinase [Lycopersicon esculentum] (GB:AAL04423.1); contains InterPro domain Serine/threonine protein kinase, active site (InterPro:IPR008271); contains InterPro domain Tyrosine protein kinase (InterPro:IPR001245); contains InterPro domain Serine/threonine protein kinase (InterPro:IPR002290); contains InterPro domain Protein kinase (InterPro:IPR000719) \| chr3:1317266-1321300 FORWARD \| Aliases: T9J14.24, T9J14_24 | 4.3 | 8.9 | -4.6 | -10.9 | 0.02% | 8.8 |
| 675 | AT3G45640.1 \| Symbol: ATMPK3 \| | 5.5 | 9.7 | -4.2 | -10.9 | 0.02% | 9.8 |
| 677 | AT2G27000.1 \| Symbol: CYP705A8 \| cytochrome P450 family protein \| chr2:11530382-11532173 REVERSE \| Aliases: T20P8.5, T20P8_5 | 4.3 | 7.8 | -3.5 | -10.9 | 0.02% | 9.3 |
| 680 | AT1G27980.1 \| pyridoxal-dependent decarboxylase family protein, similar to sphingosine-1-phosphate lyase (Homo sapiens) GI:10129683; contains Pfam profile PF00282: Pyridoxal-dependent decarboxylase conserved domain | 7.8 | 10.2 | -2.5 | -10.8 | 0.02% | 9.6 |
| 681 | AT3G01420.1 \| Symbol: ALPHA DOX1 \| pathogen-responsive alpha-dioxygenase, putative, similar to pathogen-inducible alpha-dioxygenase (Nicotiana attenuata) GI:12539609; contains Pfam profile PF03098: Animal haem peroxidase \| chr3:159473-162844 REVERSE \| Aliases: ALPHA DIOXYGENASE 1, T13O15.6, T13O15_6 | 3.9 | 8.5 | -4.6 | -10.8 | 0.02% | 7.2 |
| 687 | AT1G74460.1 \| GDSL-motif lipase/hydrolase family protein, similar to family II lipases EXL3 GI:15054386, EXL1 GI:15054382, EXL2 GI:15054384 from (Arabidopsis thaliana); contains Pfam profile: PF00657 lipase/acylhydrolase with GDSL-like motif \| chr1:27991622-27993462 REVERSE \| Aliases: F1M20.14, F1M20_14 | 4.0 | 7.3 | -3.3 | -10.8 | 0.02% | 8.6 |
| 689 | AT5G16900.1 \| leucine-rich repeat protein kinase, putative, similar to light repressible receptor protein kinase (Arabidopsis thaliana) gi:1321686:emb:CAA66376; contains leucine rich repeat (LRR) domains, Pfam:PF00560; contains protein kinase domain, Pfam:PF00069 \| chr5:5555257-5559718 FORWARD \| Aliases: F2K13.50, F2K13_50 | 3.3 | 5.6 | -2.3 | -10.8 | 0.02% | 9.4 |
| 691 | AT1G04220.1 \| beta-ketoacyl-CoA synthase, putative, Strong similarity to beta-keto-Coa synthase gb:U37088 from Simmondsia chinensis, GI:4091810 \| chr1:1119359-1122525 REVERSE \| Aliases: F20D22.1, F20D22_1 | 2.7 | 8.7 | -6.0 | -10.8 | 0.02% | 6.7 |

| Rank | Description | Sync | Root | M | t | adj.q | B |
|---|---|---|---|---|---|---|---|
| 693 | AT3G16460.2 \| similar to jacalin lectin family protein [Arabidopsis thaliana] (TAIR:At1g52040.1); similar to myrosinase-binding protein [Brassica napus] (GB:AAC08048.1); contains InterPro domain Jacalin-related lectin (InterPro:IPR001229) | 6.9 | 9.9 | -3.1 | -10.8 | 0.02% | 9.5 |
| 694 | AT5G07360.1 \| amidase family protein, low similarity to enantiomerase-selective amidase (Rhodococcus sp.) GI:152052; contains Pfam profile PF01425: Amidase \| chr5:2326709-2330137 REVERSE \| Aliases: T2I1.70, T2I1_70 | 5.4 | 8.4 | -3.0 | -10.8 | 0.02% | 9.2 |
| 697 | AT3G22830.1 \| Symbol: AT HSFA6B \| heat shock transcription factor family protein, contains Pfam profile: PF00447 HSF-type DNA-binding domain \| chr3:8078827-8081058 FORWARD \| Aliases: HSFA6B, MWI23.20 | 5.0 | 9.1 | -4.1 | -10.7 | 0.02% | 8.7 |
| 698 | AT4G31500.1 \| Symbol: CYP83B1 \| cytochrome P450 83B1 (CYP83B1), Identical to Cytochrome P450 (SP:O65782 )(Arabidopsis thaliana) \| chr4:15273477-15275316 REVERSE \| Aliases: ALTERED TRYPTOPHAN REGULATION 4, ATR4, CYTOCHROME P450 MONOOXYGENASE 83B1, F3L17.70, F3L17_70, RED LIGHT ELONGATED 1, RED1, RNT1, SUPERROOT 2, SUR2 | 6.0 | 10.1 | -4.1 | -10.7 | 0.02% | 8.9 |
| 699 | AT1G55920.1 \| Symbol: AtSerat2;1 \| serine O-acetyltransferase, putative, identical to GI:608677 from (Arabidopsis thaliana) \| chr1:20915781-20917112 FORWARD \| Aliases: F14J16.18, F14J16_18, SAT1, SAT5, SERINE ACETYLTRANSFERASE, SERINE ACETYLTRANSFERASE 1 | 6.7 | 10.0 | -3.3 | -10.7 | 0.02% | 8.7 |
| 700 | AT5G44910.1 \| Toll-Interleukin-Resistance (TIR) domain-containing protein, domain signature TIR exists, suggestive of a disease resistance protein. \| chr5:18154581-18155462 FORWARD \| Aliases: K21C13.9, K21C13_9 | 3.3 | 7.6 | -4.3 | -10.7 | 0.02% | 8.3 |
| 701 | AT5G14880.1 \| potassium transporter, putative, similar to potassium transporter HAK2p (Mesembryanthemum crystallinum) gi:14091471:gb:AAK53759; KUP/HAK/KT Transporter family member, PMID:11500563; contains Pfam profile PF02705: K+ potassium transporter \| chr5:4814247-4817670 FORWARD \| Aliases: T9L3.180, T9L3_180 | 4.6 | 6.8 | -2.2 | -10.7 | 0.02% | 9.4 |
| 706 | AT4G32060.1 \| calcium-binding EF hand family protein, contains INTERPRO:IPR002048 calcium-binding EF-hand domain \| chr4:15501084-15503599 FORWARD \| Aliases: F10N7.140, F10N7_140 | 9.2 | 11.5 | -2.3 | -10.7 | 0.02% | 9.8 |
| 707 | AT1G54120.1 \| expressed protein \| chr1:20210688-20211208 FORWARD \| Aliases: F15I1.22, F15I1_22 | 4.2 | 8.4 | -4.3 | -10.7 | 0.03% | 8.0 |
| 710 | AT1G70460.1 \| protein kinase, putative, contains Pfam PF00069: Protein kinase domain \| chr1:26559511-26562947 FORWARD \| Aliases: F24J13.3, F24J13_3 | 3.5 | 7.2 | -3.7 | -10.7 | 0.03% | 8.0 |
| 712 | AT2G38470.1 \| Symbol: WRKY33 \| | 5.1 | 10.3 | -5.1 | -10.7 | 0.03% | 9.0 |
| 713 | AT3G04010.1 \| glycosyl hydrolase family 17 protein, similar to beta-1,3-glucanase GB:S12402 (Nicotiana sp), GB:CAA03908 (Citrus sinensis), GB:S44364 (Lycopersicon esculentum) | 3.7 | 8.6 | -4.9 | -10.7 | 0.03% | 6.8 |
| 714 | AT1G11670.1 \| MATE efflux family protein, similar to ripening regulated protein DDTFR18 (Lycopersicon esculentum) GI:12231296; contains Pfam profile PF01554: Uncharacterized membrane protein family; EST gb:W43487 comes from this gene \| chr1:3928182-3931576 REVERSE \| Aliases: F25C20.18, F25C20_18 | 3.9 | 7.3 | -3.4 | -10.7 | 0.03% | 8.6 |
| 715 | AT5G64930.1 \| Symbol: CPR5 \| CPR5 protein, putative, Constitutive expressor of Pathogensis Related genes 5 (cpr5); regulator of disease resistance and senescence (Plant J. (2001)26(4)409-420. \| chr5:25962938-25965565 REVERSE \| Aliases: CONSTITUTIVE EXPRESSION OF PR GENES 5, HYS1, MXK3.16, MXK3_16 | 6.6 | 8.6 | -2.0 | -10.7 | 0.03% | 9.7 |
| 717 | AT3G60330.1 \| ATPase, plasma membrane-type, putative / proton pump, putative, similar to P-type H(+)-transporting ATPase from Nicotiana plumbaginifolia (SP:Q08435, SP:Q08436), Lycopersicon esculentum (GI:5901757, SP:P22180), Solanum tuberosum (GI:435003); contains InterPro accession IPR001757: ATPase, E1-E2 type \| chr3:22309738-22314484 FORWARD \| Aliases: T8B10.1 | 3.2 | 8.6 | -5.4 | -10.7 | 0.03% | 7.9 |
| 719 | AT4G33720.1 \| pathogenesis-related protein, putative, similar to SP:P33154 Pathogenesis-related protein 1 precursor (PR-1) {Arabidopsis thaliana}; contains Pfam profile PF00188: SCP-like extracellular protein \| chr4:16182755-16183536 FORWARD \| Aliases: T16L1.210, T16L1_210 | 3.8 | 9.2 | -5.4 | -10.7 | 0.03% | 7.0 |
| 721 | AT1G67870.1 \| glycine-rich protein, contains non-consensus GG donor splice site at exon2; modeled to est match. \| chr1:25453030-25454675 REVERSE \| Aliases: T23K23.28, T23K23_28 | 2.7 | 7.0 | -4.3 | -10.7 | 0.03% | 7.7 |
| 722 | AT1G17090.1 \| expressed protein, Pfam \| chr1:5842209-5842875 REVERSE \| Aliases: F20D23.31, F20D23_31 | 3.5 | 7.7 | -4.2 | -10.6 | 0.03% | 8.4 |
| 723 | AT1G30900.1 \| vacuolar sorting receptor, putative, similar to BP-80 vacuolar sorting receptor (Pisum sativum) GI:1737222 \| chr1:10997256-11000524 FORWARD \| Aliases: F17F8.23 | 5.6 | 9.7 | -4.1 | -10.6 | 0.03% | 8.7 |
| 726 | AT2G47160.1 \| Symbol: BOR1 \| anion exchange family protein, contains some similarity to SWISS-PROT:P04919 anion transport protein (anion exchange protein 1) (Mouse) {Mus musculus} \| chr2:19364556-19368213 REVERSE \| Aliases: REQUIRES HIGH BORON 1, T3D7.3 | 3.2 | 7.9 | -4.7 | -10.6 | 0.03% | 9.2 |

| Rank | Description | Sync | Root | M | t | adj.q | B |
|---|---|---|---|---|---|---|---|
| 727 | AT5G23010.1 \| Symbol: MAM1 \| 2-isopropylmalate synthase 3 (IMS3), identical to 2-isopropylmalate synthase (IMS3) (Arabidopsis thaliana) GI:15983745; identical to cDNA 2-isopropylmalate synthase (IMS3) GI:15983744 | 3.6 | 8.3 | -4.7 | -10.6 | 0.03% | 8.0 |
| 730 | AT3G50740.1 \| UDP-glucoronosyl/UDP-glucosyl transferase family protein, contains Pfam profile: PF00201 UDP-glucoronosyl and UDP-glucosyl transferase \| chr3:18866142-18867865 REVERSE \| Aliases: F18B3.20 | 3.1 | 9.4 | -6.2 | -10.6 | 0.03% | 8.1 |
| 733 | AT4G29950.2 \| microtubule-associated protein, identical to microtubule-associated protein GI:5032258 from (Arabidopsis thaliana); similar to TBC1 domain family member 5 (Swiss-Prot:Q92609) (Homo sapiens); contains Pfam profile PF00566: TBC domain | 5.7 | 9.0 | -3.3 | -10.6 | 0.03% | 8.8 |
| 738 | AT2G28710.1 \| zinc finger (C2H2 type) family protein, contains Pfam domain, PF00096: Zinc finger, C2H2 type \| chr2:12330051-12330521 FORWARD \| Aliases: T11P11.2, T11P11_2 | 3.7 | 7.5 | -3.8 | -10.6 | 0.03% | 8.0 |
| 740 | AT5G14120.1 \| nodulin family protein, similar to nodulin-like protein (Arabidopsis thaliana) GI:3329368, nodule-specific protein Nlj70 (Lotus japonicus) GI:3329366 \| chr5:4555984-4559116 FORWARD \| Aliases: MUA22.12, MUA22_12 | 6.9 | 10.1 | -3.2 | -10.6 | 0.03% | 9.1 |
| 741 | AT3G01290.1 \| band 7 family protein, similar to hypersensitive-induced response protein (Zea mays) GI:7716470; contains Pfam profile PF01145: SPFH domain / Band 7 family \| chr3:88064-89429 REVERSE \| Aliases: T22N4.8, T22N4_8 | 3.7 | 7.8 | -4.2 | -10.5 | 0.03% | 7.8 |
| 743 | AT5G61250.2 \| glycosyl hydrolase family 79 N-terminal domain-containing protein, similar to beta-glucuronidase GI:8918740 from (Scutellaria baicalensis) \| chr5:24649210-24652505 REVERSE \| Aliases: MFB13.2, MFB13_2 | 4.8 | 10.1 | -5.3 | -10.5 | 0.03% | 9.0 |
| 747 | AT4G12080.1 \| DNA-binding family protein, contains a AT hook motif (DNA binding motifs with a preference for A/T rich regions), Pfam:PF02178 \| chr4:7239213-7241488 FORWARD \| Aliases: F16J13.150, F16J13_150 | 7.4 | 10.8 | -3.3 | -10.5 | 0.03% | 9.4 |
| 751 | AT4G37010.2 \| similar to caltractin / centrin [Arabidopsis thaliana] (TAIR:At3g50360.1); similar to centrin [Nicotiana tabacum] (GB:AAF07221.1); contains InterPro domain Calcium-binding EF-hand (InterPro:IPR002048); contains InterPro domain Flagellar calcium-binding protein (calflagin) (InterPro:IPR003299) \| chr4:17444303-17445609 FORWARD \| Aliases: AP22.11, AP22_11 | 3.2 | 7.3 | -4.1 | -10.5 | 0.03% | 7.9 |
| 753 | AT2G39110.1 \| protein kinase, putative, similar to protein kinase (Lophopyrum elongatum) gi:13022177:gb:AAK11674 \| chr2:16326742-16328755 FORWARD \| Aliases: T7F6.28, T7F6_28 | 4.9 | 8.2 | -3.3 | -10.5 | 0.03% | 8.2 |
| 755 | AT2G42850.1 \| Symbol: CYP718 \| | 3.3 | 8.1 | -4.8 | -10.5 | 0.03% | 7.5 |
| 757 | AT4G23270.1 \| protein kinase family protein, contains Pfam PF00069: Protein kinase domain \| chr4:12171113-12173935 FORWARD \| Aliases: F21P8.160, F21P8_160 | 6.8 | 9.0 | -2.2 | -10.5 | 0.03% | 9.6 |
| 759 | AT2G31260.1 \| Symbol: APG9 \| autophagy 9 (APG9), identical to autophagy 9 protein GI:19912149 from (Arabidopsis thaliana) \| chr2:13329098-13334477 REVERSE \| Aliases: ATAPG9, AUTOPHAGY 9, F16D14.10, F16D14_10 | 3.8 | 6.2 | -2.4 | -10.5 | 0.03% | 9.4 |
| 765 | AT5G67420.1 \| LOB domain protein 37 / lateral organ boundaries domain protein 37 (LBD37), identical to LOB DOMAIN 37 (Arabidopsis thaliana) GI:17227170 \| chr5:26921515-26923252 REVERSE \| Aliases: K8K14.16, K8K14_16 | 4.4 | 8.9 | -4.5 | -10.5 | 0.03% | 8.7 |
| 766 | AT3G21630.1 \| protein kinase family protein, contains protein kinase domain, Pfam:PF00069 \| chr3:7615416-7618588 REVERSE \| Aliases: MIL23.20 | 7.7 | 9.8 | -2.1 | -10.4 | 0.03% | 9.6 |
| 768 | AT3G54040.1 \| photoassimilate-responsive protein-related, contains weak similarity to mRNA inducible by sucrose and salicylic acid expressed in sugar-accumulating tobacco plants (GI:871487) (Nicotiana tabacum) \| chr3:20024739-20026415 REVERSE \| Aliases: F5K20.340 | 4.0 | 9.1 | -5.1 | -10.4 | 0.03% | 8.5 |
| 769 | AT1G72300.1 \| leucine-rich repeat transmembrane protein kinase, putative, similar to GI:3641252 from (Malus x domestica) (Plant Mol. Biol. 40 (6), 945-957 (1999)) \| chr1:27221162-27224738 REVERSE \| Aliases: T9N14.20, T9N14_20 | 5.6 | 8.8 | -3.2 | -10.4 | 0.03% | 9.3 |
| 770 | AT4G14410.2 \| basic helix-loop-helix (bHLH) family protein \| chr4:8300072-8301663 FORWARD \| Aliases: DL3245W, FCAALL.201 | 8.3 | 10.1 | -1.7 | -10.4 | 0.03% | 9.7 |
| 771 | AT5G04890.1 \| Symbol: RTM2 \| small heat shock-like protein (RTM2), similar to 17.9 kDa heat-shock protein (Helianthus annuus) GI:11990130; contains Pfam profile PF00011: Hsp20/alpha crystallin family; supporting cDNA gi:7407072:gb:AF208051.1:AF208051; identical to cDNA small heat shock-like protein (RTM2) GI:7407072, small heat shock-like protein (Arabidopsis thaliana) GI:7407073 \| chr5:1427174-1428455 FORWARD \| Aliases: MUK11.22, MUK11_22, RESTRICTED TEV MOVEMENT 2 | 5.3 | 10.3 | -5.1 | -10.4 | 0.03% | 8.1 |
| 773 | AT5G47040.1 \| Lon protease homolog 1, mitochondrial (LON), identical to Lon protease homolog 1 | 5.5 | 8.9 | -3.4 | -10.4 | 0.03% | 9.1 |
| 776 | AT5G43780.1 \| Symbol: APS4 \| sulfate adenylyltransferase 4 / ATP-sulfurylase 4 (APS4), identical to ATP sulfurylase precursor (APS4) (Arabidopsis thaliana) GI:4633131 \| chr5:17606418-17608762 REVERSE \| Aliases: MQD19.13, MQD19_13 | 7.3 | 11.7 | -4.5 | -10.4 | 0.03% | 8.6 |

| Rank | Description | Sync | Root | M | t | adj.q | B |
|---|---|---|---|---|---|---|---|
| 779 | AT2G28250.2 \| similar to protein kinase family protein [Arabidopsis thaliana] (TAIR:At1g10620.1); similar to putative receptor protein kinase PERK1 [Oryza sativa (japonica cultivar-group)] (GB:XP_463824.1); contains InterPro domain Serine/threonine protein kinase, active site (InterPro:IPR008271); contains InterPro domain Tyrosine protein kinase (InterPro:IPR001245); contains InterPro domain Serine/threonine protein kinase (InterPro:IPR002290); contains InterPro domain Protein kinase (InterPro:IPR000719) \| chr2:12050559-12053614 FORWARD \| Aliases: T3B23.8, T3B23_8 | 4.0 | 8.1 | -4.1 | -10.4 | 0.03% | 8.4 |
| 780 | AT1G68710.1 \| haloacid dehalogenase-like hydrolase family protein, similar to Potential phospholipid-transporting ATPase (EC 3.6.3.1) from {Mus musculus} SP:P98200, {Bos taurus} SP:Q29449, {Homo sapiens} SP:O43520; contains InterPro accession IPR005834: Haloacid dehalogenase-like hydrolase \| chr1:25796870-25801638 REVERSE \| Aliases: F24J5.6, F24J5_6 | 4.2 | 8.4 | -4.2 | -10.4 | 0.03% | 8.9 |
| 782 | AT3G20460.1 \| sugar transporter, putative, similar to ERD6 protein (Arabidopsis thaliana) GI:3123712, sugar-porter family proteins 1 and 2 (Arabidopsis thaliana) GI:14585699, GI:14585701; contains Pfam profile PF00083: major facilitator superfamily protein \| chr3:7135056-7139478 FORWARD \| Aliases: MQC12.5 | 3.5 | 7.1 | -3.6 | -10.4 | 0.03% | 8.1 |
| 783 | AT3G07720.1 \| kelch repeat-containing protein, similar to epithiospecifier (GI:16118838) (Arabidopsis thaliana); contains Pfam PF01344: Kelch motif (5 repeats) \| chr3:2465371-2467239 FORWARD \| Aliases: MLP3.17, MLP3_17 | 7.5 | 11.1 | -3.7 | -10.4 | 0.03% | 8.7 |
| 784 | AT5G20250.3 \| Symbol: DIN10 \| similar to alkaline alpha galactosidase, putative [Arabidopsis thaliana] (TAIR:At3g57520.2); similar to alkaline alpha galactosidase, putative [Arabidopsis thaliana] (TAIR:At3g57520.3); similar to alkaline alpha galactosidase, putative [Arabidopsis thaliana] (TAIR:At1g55740.1); similar to alkaline alpha galactosidase, putative [Arabidopsis thaliana] (TAIR:At3g57520.1); similar to putative alkaline alpha-galactosidase seed imbibition protein [Oryza sativa (japonica cultivar-group)] (GB:XP_483143.1); similar to Sip1 protein - barley (GB:S27762); similar to putative imbibition protein [Brassica oleracea] (GB:CAA55893.1); similar to putative raffinose synthase or seed imbibition protein [Oryza sativa (japonica cultivar-group)] (GB:AAT77910.1); similar to alkaline alpha galactosidase II [Cucumis melo] (GB:AAM75140.1); contains InterPro domain Raffinose synthase (InterPro:IPR008811) \| chr5:6833680-6836790 FORWARD \| Aliases: DARK INDUCIBLE 10, F5O24.140, F5O24_140 | 7.8 | 10.4 | -2.7 | -10.3 | 0.03% | 9.3 |
| 792 | AT1G14040.1 \| similar to EXS family protein / ERD1/XPR1/SYG1 family protein [Arabidopsis thaliana] (TAIR:At2g03250.1); similar to EXS family protein / ERD1/XPR1/SYG1 family protein [Arabidopsis thaliana] (TAIR:At2g03260.1); similar to EXS family protein / ERD1/XPR1/SYG1 family protein [Arabidopsis thaliana] (TAIR:At2g03240.1); similar to EXS family protein / ERD1/XPR1/SYG1 family protein [Arabidopsis thaliana] (TAIR:At1g35350.1); similar to EXS family protein / ERD1/XPR1/SYG1 family protein [Arabidopsis thaliana] (TAIR:At1g26730.1); similar to putative PHO1-like protein [Oryza sativa (japonica cultivar-group)] (GB:XP_549873.1); contains InterPro domain SPX, N-terminal (InterPro:IPR004331); contains InterPro domain EXS, C-terminal (InterPro:IPR004342) \| chr1:4810485-4814540 FORWARD \| Aliases: F7A19.13, F7A19_13 | 6.3 | 8.7 | -2.3 | -10.3 | 0.03% | 9.4 |
| 793 | AT5G41990.1 \| Symbol: WNK8 \| protein kinase family protein, contains protein kinase domain, Pfam:PF00069 \| chr5:16811805-16815234 REVERSE \| Aliases: MJC20.9, MJC20_9 | 7.5 | 10.2 | -2.7 | -10.3 | 0.03% | 9.3 |
| 796 | AT2G16980.2 \| expressed protein \| chr2:7383431-7388176 FORWARD \| Aliases: F12A24.16, F12A24_16 | 4.6 | 8.6 | -4.0 | -10.3 | 0.03% | 8.7 |
| 800 | AT5G02880.1 \| Symbol: UPL4 \| HECT-domain-containing protein / ubiquitin-transferase family protein / armadillo/beta-catenin-like repeat-containing protein, similar to SP:Q14669 Thyroid receptor interacting protein 12 (TRIP12) {Homo sapiens}; contains Pfam profiles PF00632: HECT-domain (ubiquitin-transferase), PF00514: Armadillo/beta-catenin-like repeat \| chr5:662641-669105 FORWARD \| Aliases: F9G14.190, F9G14_190 | 6.0 | 8.5 | -2.5 | -10.3 | 0.03% | 9.0 |
| 802 | AT5G49760.1 \| leucine-rich repeat family protein / protein kinase family protein, contains Pfam domains PF00560: Leucine Rich Repeat and PF00069: Protein kinase domain \| chr5:20233598-20238525 FORWARD \| Aliases: K2I5.13, K2I5_13 | 5.6 | 8.9 | -3.3 | -10.3 | 0.03% | 8.8 |
| 803 | AT1G61820.3 \| glycosyl hydrolase family 1 protein, contains Pfam PF00232 : Glycosyl hydrolase family 1 domain; TIGRFAM TIGR01233: 6-phospho-beta-galactosidase; similar to beta-mannosidase enzyme (GI:17226270) (Lycopersicon esculentum) \| chr1:22840043-22842280 FORWARD \| Aliases: F8K4.3, F8K4_3 | 5.5 | 9.0 | -3.5 | -10.2 | 0.03% | 8.1 |
| 808 | AT1G09970.2 \| leucine-rich repeat transmembrane protein kinase, putative, Similar to A. thaliana receptor-like protein kinase (gb:RLK5_ARATH). ESTs gb:ATTS0475,gb:ATTS4362 come from this gene isoform contains a TG acceptor site at intron. | 5.7 | 9.4 | -3.7 | -10.2 | 0.03% | 9.2 |
| 810 | AT5G45800.1 \| leucine-rich repeat transmembrane protein kinase, putative \| chr5:18592809-18596324 REVERSE \| Aliases: MRA19.24, MRA19_24 | 4.1 | 7.8 | -3.7 | -10.2 | 0.03% | 9.1 |
| 813 | AT1G54540.1 \| expressed protein \| chr1:20371369-20372088 REVERSE \| Aliases: F20D21.35, F20D21_35 | 3.5 | 7.0 | -3.5 | -10.2 | 0.03% | 7.8 |
| 819 | AT5G25810.1 \| Symbol: TNY \| encodes a member of the DREB subfamily A-4 of ERF/AP2 transcription factor family (TINY). The protein contains one AP2 domain. There are 17 members in this subfamily including TINY. Ectopic or overexpression of this gene in a Ds tagged line has reduced cell expansion. The expression of this gene is induced by ethylene and light and appears to stimulate cytokinin biosynthesis. \| chr5:8986774-8987790 REVERSE \| Aliases: F18A17.60, F18A17_60, TINY, TNY | 2.6 | 6.8 | -4.2 | -10.2 | 0.03% | 7.0 |
| 822 | AT1G62200.1 \| proton-dependent oligopeptide transport (POT) family protein, contains Pfam profile: PF00854 POT family ; contains non-consensus GA donor site at intron 4 \| chr1:22985701-22988024 REVERSE \| Aliases: F19K23.13, F19K23_13 | 7.0 | 9.1 | -2.1 | -10.2 | 0.03% | 9.4 |

| Rank | Description | Sync | Root | M | t | adj.q | B |
|---|---|---|---|---|---|---|---|
| 826 | AT3G22750.1 \| protein kinase, putative, similar to protein kinase ATMRK1 (Arabidopsis thaliana) gi:2351097:dbj:BAA22079 \| chr3:8037223-8039910 REVERSE \| Aliases: MWI23.12 | 5.0 | 7.5 | -2.4 | -10.2 | 0.03% | 8.8 |
| 828 | AT4G38430.1 \| member of KPP-like gene family, homolog of KPP (kinase partner protein) gene in tomato; expressed protein, contains Pfam profile PF03759: Domain of unknown function (DUF315) \| chr4:17986461-17988801 FORWARD \| Aliases: F22I13.200, F22I13_200, KINASE PARTNER PROTEIN LIKE, KPP LIKE | 3.6 | 6.3 | -2.8 | -10.2 | 0.03% | 8.8 |
| 829 | AT3G02240.1 \| expressed protein \| chr3:418522-419158 FORWARD \| Aliases: F14P3.11, F14P3_11 | 3.2 | 6.8 | -3.7 | -10.1 | 0.03% | 8.0 |
| 833 | AT5G09690.3 \| magnesium transporter CorA-like family protein (MRS2-7), weak similarity to SP:Q01926 RNA splicing protein MRS2, mitochondrial precursor {Saccharomyces cerevisiae}; contains Pfam profile PF01544: CorA-like Mg2+ transporter protein \| chr5:3000810-3003501 REVERSE \| Aliases: F17I14.120, F17I14_120 | 3.9 | 6.5 | -2.6 | -10.1 | 0.03% | 8.6 |
| 837 | AT5G45500.1 \| expressed protein, weak similarity to resistance complex protein I2C-2 (Lycopersicon esculentum) GI:2258317 \| chr5:18449685-18451475 REVERSE \| Aliases: MFC19.17, MFC19_17 | 6.2 | 9.2 | -3.0 | -10.1 | 0.03% | 9.1 |
| 838 | AT3G12900.1 \| oxidoreductase, 2OG-Fe(II) oxygenase family protein, similar to SP:P10967 1-aminocyclopropane-1-carboxylate oxidase homolog (Protein E8) {Lycopersicon esculentum}, desacetoxyvindoline-4-hydroxylase (Catharanthus roseus) GI:2352812; contains Pfam profile PF03171: oxidoreductase, 2OG-Fe(II) oxygenase family \| chr3:4104583-4106119 FORWARD \| Aliases: MJM20.4 | 3.4 | 8.8 | -5.4 | -10.1 | 0.03% | 7.9 |
| 840 | AT3G10910.1 \| zinc finger (C3HC4-type RING finger) family protein, contains Pfam domain, PF00097: Zinc finger, C3HC4 type (RING finger) \| chr3:3412916-3414024 REVERSE \| Aliases: T7M13.1 | 2.4 | 5.9 | -3.5 | -10.1 | 0.03% | 7.7 |
| 841 | AT4G18780.1 \| Symbol: CESA8 \| cellulose synthase, catalytic subunit (IRX1), nearly identical to gi:12836997 \| chr4:10312665-10316797 REVERSE \| Aliases: ATCESA8, CELLULASE SYNTHASE 8, F28A21.190, F28A21_190, IRREGULAR XYLEM 1, IRX1, LEAF WILTING 2, LEW2 | 3.6 | 7.8 | -4.2 | -10.1 | 0.03% | 7.9 |
| 842 | AT4G24160.2 \| hydrolase, alpha/beta fold family protein, contains Pfam profile PF00561: hydrolase, alpha/beta fold family \| chr4:12539787-12542499 FORWARD \| Aliases: T19F6.150, T19F6_150 | 8.3 | 10.2 | -1.8 | -10.1 | 0.03% | 9.3 |
| 845 | AT5G17600.1 \| zinc finger (C3HC4-type RING finger) family protein, contains Pfam profile: PF00097 zinc finger, C3HC4 type (RING finger) \| chr5:5799907-5801149 REVERSE \| Aliases: K10A8.80, K10A8_80 | 3.1 | 5.5 | -2.4 | -10.1 | 0.03% | 8.9 |
| 846 | AT1G20450.2 \| Symbol: ERD10 \| dehydrin (ERD10), identical to dehydrin ERD10 (Low-temperature-induced protein LTI45) (Arabidopsis thaliana) SWISS-PROT:P42759 \| chr1:7087993-7089596 REVERSE \| Aliases: ER10, ERD10, F5M15.21, F5M15_21, LTI45 | 10.3 | 12.8 | -2.5 | -10.1 | 0.03% | 9.5 |
| 847 | AT5G59520.1 \| Symbol: ZIP2 \| zinc transporter (ZIP2), identical to zinc transporter ZIP2 (Arabidopsis thaliana) gi:3252868:gb:AAC24198; member of the Zinc (Zn2+)-Iron (Fe2+) permease (ZIP) family, PMID:11500563 \| chr5:24008479-24010024 REVERSE \| Aliases: F2O15.25, F2O15_25, ZIP2 | 5.1 | 7.6 | -2.5 | -10.1 | 0.03% | 8.8 |
| 849 | AT1G23870.1 \| Symbol: ATTPS9 \| | 3.3 | 8.2 | -4.9 | -10.1 | 0.03% | 8.6 |
| 850 | AT2G26510.1 \| Symbol: PDE135 \| | 7.2 | 9.2 | -2.0 | -10.1 | 0.03% | 9.3 |
| 851 | AT1G65610.1 \| endo-1,4-beta-glucanase, putative / cellulase, putative, similar to endo-1,4-beta-glucanase GI:2065530 from (Lycopersicon esculentum) \| chr1:24395342-24399023 REVERSE \| Aliases: F5I14.14, F5I14_14 | 3.4 | 7.0 | -3.6 | -10.1 | 0.03% | 8.1 |
| 852 | AT2G35380.2 \| similar to peroxidase, putative [Arabidopsis thaliana] (TAIR:At1g44970.1); similar to peroxidase prx15 precursor [Spinacia oleracea] (GB:AAF63027.1); contains InterPro domain Haem peroxidase (InterPro:IPR002016); contains InterPro domain Plant peroxidase (InterPro:IPR000823) \| chr2:14899728-14901072 FORWARD \| Aliases: T32F12.24, T32F12_24 | 2.7 | 6.5 | -3.8 | -10.1 | 0.03% | 7.6 |
| 861 | AT1G44770.1 \| expressed protein \| chr1:16909979-16911903 REVERSE \| Aliases: T12C22.4, T12C22_4 | 8.4 | 10.1 | -1.7 | -10.0 | 0.03% | 9.4 |
| 863 | AT1G75130.1 \| Symbol: CYP721A1 \| cytochrome P450 family protein, similar to Cytochrome P450 72A1 (SP:Q05047) (Catharanthus roseus) \| chr1:28203636-28205611 REVERSE \| Aliases: F22H5.19 | 5.3 | 7.0 | -1.7 | -10.0 | 0.03% | 9.3 |
| 865 | AT3G26690.2 \| MutT/nudix family protein, similar to SP:Q09790 Diadenosine 5',5'''-P1,P6-hexaphosphate hydrolase (EC 3.6.1.-) (Ap6A hydrolase) {Schizosaccharomyces pombe}; contains Pfam profile PF00293: NUDIX domain | 8.4 | 11.2 | -2.8 | -10.0 | 0.03% | 9.4 |
| 868 | AT5G17640.1 \| expressed protein \| chr5:5810189-5813286 REVERSE \| Aliases: K10A8.120, K10A8_120 | 5.1 | 8.1 | -3.0 | -10.0 | 0.03% | 8.8 |

| Rank | Description | Sync | Root | M | t | adj.q | B |
|---|---|---|---|---|---|---|---|
| 869 | AT2G01900.1 \| endonuclease/exonuclease/phosphatase family protein, similar to inositol polyphosphate 5-phosphatase I (GI:10444261) and II (GI:10444263) (Arabidopsis thaliana); contains Pfam profile PF03372: Endonuclease/Exonuclease/phosphatase family \| chr2:405917-409013 FORWARD \| Aliases: T23K3.9, T23K3_9 | 2.4 | 7.6 | -5.1 | -10.0 | 0.03% | 7.8 |
| 872 | AT1G22500.1 \| zinc finger (C3HC4-type RING finger) family protein, contains Pfam profile: PF00097: Zinc finger, C3HC4 type (RING finger) \| chr1:7949465-7950845 FORWARD \| Aliases: F12K8.15, F12K8_15 | 2.9 | 8.9 | -5.9 | -10.0 | 0.03% | 8.5 |
| 874 | AT5G50200.3 \| Symbol: WR3 \| expressed protein, similar to unknown protein (pir::T05562) isoform contains a non-consensus AT acceptor splice site at intron 1 \| chr5:20453406-20454952 FORWARD \| Aliases: K6A12.6, K6A12_6 | 8.1 | 12.0 | -3.8 | -10.0 | 0.03% | 9.2 |
| 875 | AT5G58780.1 \| dehydrodolichyl diphosphate synthase, putative / DEDOL-PP synthase, putative, similar to GI:796076 \| chr5:23752855-23754561 REVERSE \| Aliases: MZN1.33 | 2.9 | 6.6 | -3.7 | -10.0 | 0.03% | 7.8 |
| 877 | AT3G51540.1 \| expressed protein, mucin 5AC, Homo sapiens, PIR:S53363 \| chr3:19126321-19128189 FORWARD \| Aliases: F26O13.180 | 3.0 | 5.9 | -2.8 | -10.0 | 0.03% | 8.3 |
| 881 | AT1G07560.1 \| leucine-rich repeat protein kinase, putative, similar to light repressible receptor protein kinase (Arabidopsis thaliana) gi:1321686:emb:CAA66376; contains leucine rich repeat (LRR) domains, Pfam:PF00560; contains protein kinase domain, Pfam:PF00069 \| chr1:2327317-2331093 FORWARD \| Aliases: F22G5.6, F22G5_6 | 3.4 | 7.0 | -3.6 | -10.0 | 0.03% | 7.9 |
| 882 | AT3G20450.1 \| expressed protein \| chr3:7130668-7131280 REVERSE \| Aliases: MQC12.24 | 4.1 | 6.9 | -2.8 | -10.0 | 0.04% | 8.6 |
| 884 | AT5G10580.2 \| expressed protein, contains Pfam profile PF04654: Protein of unknown function, DUF599 \| chr5:3347438-3350072 FORWARD \| Aliases: F12B17.70, F12B17_70 | 5.0 | 8.5 | -3.5 | -9.9 | 0.04% | 7.7 |
| 886 | AT2G31730.1 \| ethylene-responsive protein, putative, similar to ethylene-inducible ER33 protein (Lycopersicon esculentum) gi:5669656:gb:AAD46413 \| chr2:13494769-13495670 REVERSE \| Aliases: T9H9.27 | 4.0 | 6.9 | -2.9 | -9.9 | 0.04% | 8.8 |
| 887 | AT3G50660.1 \| Symbol: DWF4 \| steroid 22-alpha-hydroxylase (CYP90B1) (DWF4), identical to gi:2935342 \| chr3:18825122-18828214 REVERSE \| Aliases: CLAM, CLM, CYP90B1, DWARF 4, DWF4, STEROID 22 ALPHA HYDROXYLASE, T3A5.40 | 7.3 | 9.6 | -2.3 | -9.9 | 0.04% | 9.2 |
| 888 | AT5G64410.1 \| Symbol: ATOPT4 \| | 4.2 | 7.9 | -3.7 | -9.9 | 0.04% | 7.7 |
| 890 | AT3G17770.1 \| dihydroxyacetone kinase family protein, contains Pfam domains, PF02733: DAK1 domain and PF02734: DAK2 domain \| chr3:6081677-6085963 REVERSE \| Aliases: MIG5.7 | 6.3 | 8.9 | -2.6 | -9.9 | 0.04% | 9.0 |
| 898 | AT1G20070.1 \| expressed protein \| chr1:6958001-6959114 REVERSE \| Aliases: T20H2.15, T20H2_15 | 5.4 | 9.5 | -4.1 | -9.9 | 0.04% | 7.9 |
| 899 | AT3G05920.1 \| heavy-metal-associated domain-containing protein, contains Pfam profile PF00403: Heavy-metal-associated domain \| chr3:1768796-1769592 REVERSE \| Aliases: F2O10.12, F2O10_12 | 2.9 | 9.8 | -6.9 | -9.9 | 0.04% | 8.3 |
| 900 | AT1G09560.1 \| Symbol: GLP5 \| germin-like protein (GLP4) (GLP5), identical to Arabidopsis germin-like protein subfamily 2 member 1 (SP:P94014); Location of EST 180L10T7, gi:906417 \| chr1:3093841-3094864 FORWARD \| Aliases: F14J9.22, F14J9_22, GERMIN LIKE PROTEIN 5, GLP5 | 8.5 | 12.2 | -3.7 | -9.9 | 0.04% | 8.3 |
| 903 | AT2G39360.1 \| protein kinase family protein, contains protein kinase domain, Pfam:PF00069 \| chr2:16444550-16447232 REVERSE \| Aliases: F12L6.2, F12L6_2 | 4.4 | 8.2 | -3.7 | -9.9 | 0.04% | 8.2 |
| 906 | AT5G13180.1 \| Symbol: ANAC083 \| no apical meristem (NAM) family protein, contains Pfam PF02365: No apical meristem (NAM) domain; hypothetical protein SENU5, senescence up-regulated - Lycopersicon esculentum, EMBL:Z75524 \| chr5:4196579-4197851 FORWARD \| Aliases: ANAC083, T19L5.140, T19L5_140 | 4.1 | 9.7 | -5.6 | -9.9 | 0.04% | 8.3 |
| 912 | AT4G38470.1 \| protein kinase family protein, similar to protein kinase (gi:170047) from Glycine max; contains Pfam protein kinase domain PF00069 \| chr4:17999426-18003675 FORWARD \| Aliases: F20M13.30, F20M13_30 | 6.5 | 9.4 | -2.9 | -9.8 | 0.04% | 9.1 |
| 913 | AT1G59520.3 \| Symbol: CW7 \| expressed protein (CW7) \| chr1:21865049-21869855 FORWARD \| Aliases: CW7, T30E16.4, T30E16_4 | 5.5 | 7.9 | -2.3 | -9.8 | 0.04% | 9.1 |
| 915 | AT3G14350.3 \| leucine-rich repeat transmembrane protein kinase, putative, similar to leucine-rich repeat transmembrane protein kinase 1 GB:AAC27894 from (Zea mays) \| chr3:4782764-4787174 REVERSE \| Aliases: MLN21.15 | 5.5 | 8.8 | -3.3 | -9.8 | 0.04% | 8.8 |
| 916 | AT2G45720.2 \| similar to armadillo/beta-catenin repeat family protein [Arabidopsis thaliana] (TAIR:At1g01830.1); similar to arm repeat containing protein-like [Oryza sativa (japonica cultivar-group)] (GB:XP_478704.1); contains InterPro domain Armadillo repeat (InterPro:IPR000225) \| chr2:18841223-18843448 FORWARD \| Aliases: F4I18.30 | 7.0 | 9.0 | -2.1 | -9.8 | 0.04% | 9.1 |

| Rank | Description | Sync | Root | M | t | adj.q | B |
|---|---|---|---|---|---|---|---|
| 917 | AT3G18170.1 \| expressed protein, contains Pfam domain, PF04577: Protein of unknown function (DUF563) \| chr3:6228348-6229668 FORWARD \| Aliases: MRC8.17 | 4.6 | 9.6 | -5.0 | -9.8 | 0.04% | 7.0 |
| 918 | AT1G67330.1 \| expressed protein, contains Pfam profile PF04669: Protein of unknown function (DUF579) \| chr1:25217515-25218834 FORWARD \| Aliases: F1N21.15 | 3.9 | 9.0 | -5.1 | -9.8 | 0.04% | 8.5 |
| 921 | AT5G27930.2 \| protein phosphatase 2C, putative / PP2C, putative, protein phosphatase-2C, Mesembryanthemum crystallinum, EMBL:AF075581 \| chr5:9958102-9961170 REVERSE \| Aliases: F15F15.3 | 4.6 | 7.0 | -2.4 | -9.8 | 0.04% | 8.8 |
| 924 | AT4G33300.2 \| Symbol: ADR1 L1 \| similar to disease resistance protein (NBS-LRR class), putative [Arabidopsis thaliana] (TAIR:At5g47280.1); similar to disease resistance protein (CC-NBS-LRR class), putative [Arabidopsis thaliana] (TAIR:At5g04720.1); similar to disease resistance protein (CC-NBS-LRR class), putative [Arabidopsis thaliana] (TAIR:At1g33560.1); similar to disease resistance protein-like protein MsR1 [Medicago sativa] (GB:AAN62760.1); contains InterPro domain NB-ARC domain (InterPro:IPR002182); contains InterPro domain Disease resistance protein (InterPro:IPR000767); contains InterPro domain Leucine-rich repeat (InterPro:IPR001611) \| chr4:16050233-16056530 REVERSE \| Aliases: ADR1 L1, ADR1 LIKE 1, F17M5.60, F17M5_60 | 7.4 | 9.8 | -2.5 | -9.8 | 0.04% | 8.9 |
| 925 | AT4G13130.1 \| DC1 domain-containing protein, contains Pfam protein PF03107 DC1 domain \| chr4:7647385-7649827 FORWARD \| Aliases: F17N18.20, F17N18_20 | 3.2 | 6.5 | -3.4 | -9.8 | 0.04% | 8.4 |
| 926 | AT2G04240.2 \| zinc finger (C3HC4-type RING finger) family protein, contains Pfam profile: PF00097 zinc finger, C3HC4 type (RING finger) \| chr2:1461627-1462934 REVERSE \| Aliases: T23O15.13, T23O15_13 | 5.9 | 8.7 | -2.8 | -9.8 | 0.04% | 8.9 |
| 932 | AT3G21510.1 \| Symbol: AHP1 \| two-component phosphorelay mediator 3 (HP3), identical to ATHP3 (Arabidopsis thaliana) GI:4156245 \| chr3:7578181-7579603 REVERSE \| Aliases: ATHP3, HISTIDINE CONTAINING PHOSPHOTRANSMITTER 3, MIL23.7 | 3.9 | 7.4 | -3.5 | -9.8 | 0.04% | 8.1 |
| 933 | AT3G62270.1 \| anion exchange family protein, contains similarity to anion exchanger 3, cardiac splice form - Rattus norvegicus, PIR:A42497 \| chr3:23053259-23057023 REVERSE \| Aliases: T17J13.230 | 3.4 | 7.2 | -3.8 | -9.8 | 0.04% | 8.5 |
| 934 | AT3G60520.1 \| expressed protein \| chr3:22372505-22373554 REVERSE \| Aliases: T8B10.180 | 3.2 | 5.2 | -2.0 | -9.8 | 0.04% | 9.0 |
| 936 | AT3G07900.1 \| expressed protein, contains Pfam PF03138: Plant protein family. The function of this family of plant proteins is unknown; previously annotated as 'auxin-independent growth promoter -related' based on similarity to axi 1 protein (GB:X80301) (GI:559920) from (Nicotiana tabacum), which, due to scienitific fraud was retracted. Retraction in: Schell J. EMBO J 1999 May 17;18(10):2908. PMID:10400497. \| chr3:2520780-2523114 FORWARD \| Aliases: F17A17.24 | 3.6 | 7.0 | -3.4 | -9.7 | 0.04% | 7.5 |
| 937 | AT3G51330.1 \| aspartyl protease family protein, contains Eukaryotic and viral aspartyl proteases active site, PROSITE:PS00141 \| chr3:19064347-19067203 REVERSE \| Aliases: F24M12.370, F24M12_370 | 2.6 | 7.2 | -4.6 | -9.7 | 0.04% | 7.0 |
| 939 | AT5G48560.1 \| basic helix-loop-helix (bHLH) family protein, contains Pfam profile: PF00010 helix-loop-helix DNA-binding domain | 3.4 | 7.2 | -3.8 | -9.7 | 0.04% | 8.6 |
| 943 | AT4G30320.1 \| allergen V5/Tpx-1-related family protein, similar to SP:Q40374 Pathogenesis-related protein PR-1 precursor {Medicago truncatula}; contains Pfam profile PF00188: SCP-like extracellular protein \| chr4:14835839-14836324 FORWARD \| Aliases: F17I23.340, F17I23_340 | 3.5 | 7.7 | -4.3 | -9.7 | 0.04% | 6.9 |
| 947 | AT2G24240.1 \| potassium channel tetramerisation domain-containing protein, contains Pfam profile PF02214: K+ channel tetramerisation domain \| chr2:10317728-10319408 FORWARD \| Aliases: F27D4.15, F27D4_15 | 5.4 | 9.2 | -3.8 | -9.7 | 0.04% | 7.8 |
| 949 | AT5G10030.1 \| Symbol: TGA4 \| bZIP family transcription factor | 4.5 | 7.6 | -3.1 | -9.7 | 0.04% | 8.6 |
| 952 | AT5G39610.1 \| Symbol: ANAC092 \| no apical meristem (NAM) family protein, contains Pfam PF02365 : No apical meristem (NAM) protein; similar to cup-shaped cotyledon CUC2 (GI:1944132) (Arabidopsis thaliana) \| chr5:15875628-15877011 REVERSE \| Aliases: ANAC092, MIJ24.11, MIJ24_11 | 2.1 | 6.2 | -4.0 | -9.7 | 0.04% | 7.8 |
| 954 | AT4G26220.1 \| caffeoyl-CoA 3-O-methyltransferase, putative, similar to caffeoyl-CoA O-methyltransferase (GI:1622926)(Nicotiana tabacum), GI:2960356 (Populus balsamifera subsp. trichocarpa), AF036095 (Pinus taeda) \| chr4:13283667-13285332 FORWARD \| Aliases: T25K17.30, T25K17_30 | 3.4 | 7.8 | -4.5 | -9.7 | 0.04% | 6.4 |
| 959 | AT1G76890.2 \| Symbol: GT2 \| trihelix DNA-binding protein / GT-2 factor (GT2), identical to GT2 factor (Arabidopsis thaliana) GI:416490, GI:2664202 (DNA binding factor GT-2 from Arabidopsis) | 3.7 | 8.2 | -4.5 | -9.7 | 0.04% | 7.3 |
| 961 | AT5G05960.1 \| protease inhibitor/seed storage/lipid transfer protein (LTP) family protein, contains Pfam protease inhibitor/seed storage/LTP family domain PF00234 \| chr5:1790232-1790834 FORWARD \| Aliases: K18J17.13, K18J17_13 | 5.0 | 8.8 | -3.8 | -9.6 | 0.04% | 7.2 |
| 965 | AT3G25640.1 \| expressed protein, contains Pfam profile PF04759: Protein of unknown function, DUF617 \| chr3:9334956-9336041 FORWARD \| Aliases: T5M7.9 | 3.8 | 6.9 | -3.1 | -9.6 | 0.04% | 8.4 |

| Rank | Description | Sync | Root | M | t | adj.q | B |
|---|---|---|---|---|---|---|---|
| 966 | AT1G34370.3 \| similar to zinc finger (C2H2 type) family protein [Arabidopsis thaliana] (TAIR:At5g22890.1); similar to putative zinc finger protein [Oryza sativa (japonica cultivar-group)] (GB:XP_470361.1); contains InterPro domain Zn-finger, C2H2 type (InterPro:IPR007087) \| chr1:12550455-12552677 FORWARD \| Aliases: F7P12.7, F7P12_7 | 8.7 | 11.1 | -2.4 | -9.6 | 0.04% | 9.0 |
| 968 | AT3G22570.1 \| protease inhibitor/seed storage/lipid transfer protein (LTP) family protein, contains Pfam protease inhibitor/seed storage/LTP family domain PF00234 \| chr3:8000522-8001245 REVERSE \| Aliases: F16J14.13 | 4.2 | 7.8 | -3.6 | -9.6 | 0.04% | 7.6 |
| 969 | AT1G02810.1 \| pectinesterase family protein, contains Pfam profile: PF01095 pectinesterase \| chr1:618270-620480 FORWARD \| Aliases: F22D16.20, F22D16_20 | 2.5 | 5.6 | -3.0 | -9.6 | 0.04% | 8.1 |
| 972 | AT1G73390.3 \| expressed protein \| chr1:27594477-27598803 REVERSE \| Aliases: T9L24.40, T9L24_40 | 4.9 | 7.8 | -3.0 | -9.6 | 0.04% | 8.3 |
| 974 | AT5G01060.1 \| protein kinase family protein, contains eukaryotic protein kinase domain, INTERPRO:IPR000719 \| chr5:22739-24846 FORWARD \| Aliases: F7J8.40, F7J8_40 | 3.8 | 7.0 | -3.2 | -9.6 | 0.04% | 8.1 |
| 977 | AT1G07730.2 \| disease resistance-responsive family protein, contains Pfam PF03018: Plant disease resistance response protein \| chr1:2397524-2398693 REVERSE \| Aliases: None | 4.7 | 8.9 | -4.2 | -9.6 | 0.04% | 7.3 |
| 979 | AT5G62150.1 \| peptidoglycan-binding LysM domain-containing protein, contains Pfam profile PF01476: LysM domain | 5.1 | 7.8 | -2.6 | -9.6 | 0.04% | 8.2 |
| 981 | AT5G66040.2 \| senescence-associated family protein, almost identical to ketoconazole resistant protein GI:928938 from (Arabidopsis thaliana) full-length cDNA: Ceres:101608. \| chr5:26427756-26428553 FORWARD \| Aliases: K2A18.11, K2A18_11 | 6.5 | 9.6 | -3.1 | -9.6 | 0.04% | 8.4 |
| 982 | AT4G38810.2 \| calcium-binding EF hand family protein, contains INTERPRO:IPR002048 calcium-binding EF-hand domain \| chr4:18115277-18119071 REVERSE \| Aliases: T9A14.90, T9A14_90 | 5.7 | 7.9 | -2.2 | -9.6 | 0.04% | 8.6 |
| 984 | AT2G34830.1 \| Symbol: WRKY35 \| | 3.4 | 6.8 | -3.4 | -9.6 | 0.04% | 7.8 |
| 986 | AT5G66690.1 \| UDP-glucoronosyl/UDP-glucosyl transferase family protein, contains Pfam profile: PF00201 UDP-glucoronosyl and UDP-glucosyl transferase \| chr5:26642306-26644019 FORWARD \| Aliases: MSN2.8, MSN2_8 | 5.5 | 9.5 | -4.0 | -9.6 | 0.04% | 8.2 |
| 988 | AT3G14060.1 \| expressed protein \| chr3:4657053-4657608 REVERSE \| Aliases: MDC16.20 | 4.0 | 7.1 | -3.0 | -9.6 | 0.04% | 8.2 |
| 991 | AT1G56430.1 \| nicotianamine synthase, putative, similar to nicotianamine synthase (Lycopersicon esculentum)(GI:4753801), nicotianamine synthase 2 (Hordeum vulgare)(GI:4894912) \| chr1:21140609-21141752 FORWARD \| Aliases: F13N6.10, F13N6_10 | 4.7 | 7.3 | -2.6 | -9.6 | 0.04% | 8.3 |
| 992 | AT1G07000.1 \| exocyst subunit EXO70 family protein, similar to leucine zipper protein GI:10177020 from (Arabidopsis thaliana) contains Pfam domain PF03081: Exo70 exocyst complex subunit; \| chr1:2150193-2152324 REVERSE \| Aliases: F10K1.28, F10K1_28 | 4.6 | 7.6 | -3.0 | -9.6 | 0.04% | 8.5 |
| 993 | AT5G19730.1 \| pectinesterase family protein, contains Pfam profile: PF01095 pectinesterase \| chr5:6670460-6673284 FORWARD \| Aliases: T29J13.150, T29J13_150 | 4.1 | 8.5 | -4.4 | -9.6 | 0.04% | 7.6 |
| 997 | AT1G25230.1 \| purple acid phosphatase family protein, contains Pfam profile: PF00149 calcineurin-like phosphoesterase; similar to purple acid phosphatase (GI:20257479) (Arabidopsis thaliana) | 3.2 | 7.1 | -4.0 | -9.6 | 0.04% | 7.6 |
| 998 | AT3G27200.1 \| plastocyanin-like domain-containing protein, contains similarity to uclacyanin I GI:3399767 GB:AAC32038 from (Arabidopsis thaliana) \| chr3:10044759-10045608 REVERSE \| Aliases: K17E12.2 | 4.0 | 8.5 | -4.5 | -9.5 | 0.04% | 6.7 |
| 1000 | AT3G22460.1 \| cysteine synthase, putative / O-acetylserine (thiol)-lyase, putative / O-acetylserine sulfhydrylase, putative, nearly identical over 185 amino acids to SP:P47998 Cysteine synthase (EC 4.2.99.8) (O-acetylserine sulfhydrylase) (O-acetylserine (Thiol)-lyase) {Arabidopsis thaliana} \| chr3:7963544-7965914 FORWARD \| Aliases: F16J14.18 | 5.6 | 8.6 | -3.0 | -9.5 | 0.04% | 8.5 |
| 1001 | AT1G33320.1 \| cystathionine gamma-synthase, chloroplast, putative / O-succinylhomoserine (Thiol)-lyase, putative, strong similarity to SP:P55217 Cystathionine gamma-synthase, chloroplast precursor (EC 4.2.99.9) (CGS) (O-succinylhomoserine (Thiol)-lyase) {Arabidopsis thaliana}; contains Pfam profile PF01053: Cys/Met metabolism PLP-dependent enzyme \| chr1:12080989-12083442 FORWARD \| Aliases: F10C21.1 | 3.1 | 6.2 | -3.1 | -9.5 | 0.04% | 7.7 |
| 1007 | AT1G70590.1 \| F-box family protein, contains Pfam PF00646: F-box domain; similar to SKP1 interacting partner 2 (SKIP2) TIGR_Ath1:At5g67250 \| chr1:26621984-26623946 FORWARD \| Aliases: F24J13.16, F24J13_16 | 7.6 | 9.7 | -2.1 | -9.5 | 0.04% | 8.8 |

| Rank | Description | Sync | Root | M | t | adj.q | B |
|---|---|---|---|---|---|---|---|
| 1009 | AT1G63840.1 \| zinc finger (C3HC4-type RING finger) family protein, similar to RING-H2 finger protein RHA1a (GI:3790554) (Arabidopsis thaliana)' similar to BRH1 RING finger protein (Arabidopsis thaliana) GI:4689366; contains Pfam profile PF00097: Zinc finger, C3HC4 type (RING finger) \| chr1:23693360-23694470 REVERSE \| Aliases: T12P18.14, T12P18_14 | 7.2 | 9.7 | -2.5 | -9.5 | 0.04% | 8.9 |
| 1010 | AT2G28940.2 \| protein kinase family protein, contains protein kinase domain, Pfam:PF00069 \| chr2:12433348-12435762 REVERSE \| Aliases: T9I4.2, T9I4_2 | 3.7 | 6.8 | -3.1 | -9.5 | 0.04% | 7.9 |
| 1011 | AT3G20250.1 \| pumilio/Puf RNA-binding domain-containing protein, contains Pfam profile: PF00806 Pumilio-family RNA binding domains (aka PUM-HD, Pumilio homology domain) (8 copies at C-terminus) \| chr3:7058964-7063246 REVERSE \| Aliases: MAL21.29 | 6.6 | 9.2 | -2.6 | -9.5 | 0.04% | 8.8 |
| 1014 | AT4G28040.4 \| similar to nodulin MtN21 family protein [Arabidopsis thaliana] (TAIR:At4g30420.1); similar to putative MtN21 [Oryza sativa (japonica cultivar-group)] (GB:BAD33609.1); contains InterPro domain Protein of unknown function DUF6 (InterPro:IPR000620) \| chr4:13940352-13942829 FORWARD \| Aliases: T13J8.150, T13J8_150 | 3.5 | 8.1 | -4.6 | -9.5 | 0.04% | 6.7 |
| 1016 | AT4G15610.1 \| integral membrane family protein, contains TIGRFAM TIGR01569 : plant integral membrane protein TIGR01569; contains Pfam PF04535 : Domain of unknown function (DUF588) \| chr4:8909098-8910906 FORWARD \| Aliases: DL3845W, FCAALL.139 | 5.0 | 7.2 | -2.2 | -9.5 | 0.04% | 8.5 |
| 1017 | AT1G69080.2 \| universal stress protein (USP) family protein, contains Pfam profile PF00582: universal stress protein family \| chr1:25975580-25976920 REVERSE \| Aliases: F4N2.5, F4N2_5 | 3.6 | 7.2 | -3.6 | -9.5 | 0.04% | 7.1 |
| 1019 | AT3G26470.1 \| expressed protein \| chr3:9687335-9688879 FORWARD \| Aliases: F20C19.20 | 3.1 | 7.4 | -4.2 | -9.5 | 0.04% | 6.7 |
| 1022 | AT2G03450.1 \| purple acid phosphatase (PAP9), identical to purple acid phosphatase (Arabidopsis thaliana) GI:20257481; contains Pfam profile: PF00149 calcineurin-like phosphoesterase; contains metallo-phosphoesterase motif (PS50185) \| chr2:1041385-1043664 FORWARD \| Aliases: T4M8.12, T4M8_12 | 5.6 | 7.3 | -1.8 | -9.5 | 0.04% | 9.0 |
| 1023 | AT1G07240.1 \| UDP-glucoronosyl/UDP-glucosyl transferase family protein, contains Pfam profile: PF00201 UDP-glucoronosyl and UDP-glucosyl transferase \| chr1:2223690-2225447 FORWARD \| Aliases: F10K1.5, F10K1_5 | 5.3 | 7.6 | -2.3 | -9.5 | 0.04% | 8.6 |
| 1026 | AT3G53780.2 \| rhomboid family protein, contains PFAM domain PF01694, Rhomboid family \| chr3:19935546-19938004 REVERSE \| Aliases: F5K20.80 | 8.0 | 10.1 | -2.1 | -9.5 | 0.04% | 8.8 |
| 1027 | AT3G26720.1 \| glycosyl hydrolase family 38 protein, similar to lysosomal alpha-mannosidase GI:3522867 from (Homo sapiens) \| chr3:9817891-9824423 FORWARD \| Aliases: MLJ15.19 | 7.8 | 10.9 | -3.1 | -9.5 | 0.04% | 8.6 |
| 1029 | AT4G38080.1 \| hydroxyproline-rich glycoprotein family protein, contains proline-rich extensin domains, INTERPRO:IPR002965; Common family member: At2g22510 (Arabidopsis thaliana) \| chr4:17882958-17883606 FORWARD \| Aliases: F20D10.200, F20D10_200 | 2.8 | 8.7 | -5.9 | -9.5 | 0.04% | 8.7 |
| 1031 | AT4G04900.1 \| Symbol: RIC10 \| p21-rho-binding domain-containing protein, contains Pfam PF00786: P21-Rho-binding domain \| chr4:2486216-2487777 REVERSE \| Aliases: ROP INTERACTIVE CRIB MOTIF CONTAINING PROTEIN 10, T1J1.7, T1J1_7 | 3.6 | 6.8 | -3.2 | -9.5 | 0.04% | 8.5 |
| 1032 | AT1G27380.2 \| Symbol: RIC2 \| p21-rho-binding domain-containing protein, contains Pfam PF00786: P21-Rho-binding domain | 3.0 | 5.5 | -2.5 | -9.4 | 0.04% | 8.6 |
| 1038 | AT5G44920.2 \| Toll-Interleukin-Resistance (TIR) domain-containing protein, domain signature TIR exists, suggestive of a disease resistance protein. \| chr5:18156002-18157410 FORWARD \| Aliases: K21C13.10, K21C13_10 | 6.4 | 9.3 | -2.9 | -9.4 | 0.04% | 8.6 |
| 1040 | AT1G68670.1 \| myb family transcription factor, contains Pfam domain, PF00249: Myb-like DNA-binding domain \| chr1:25785745-25787737 FORWARD \| Aliases: F24J5.9, F24J5_9 | 6.4 | 8.1 | -1.7 | -9.4 | 0.04% | 8.8 |
| 1043 | AT2G39530.1 \| integral membrane protein, putative, contains 3 transmembrane domains; contains Pfam PF04535 : Domain of unknown function (DUF588); similar to putative ethylene responsive element binding protein (GI:22135858) (Arabidopsis thaliana) \| chr2:16505564-16506549 REVERSE \| Aliases: F12L6.19, F12L6_19 | 3.6 | 6.3 | -2.6 | -9.4 | 0.05% | 8.4 |
| 1045 | AT2G45400.1 \| dihydroflavonol 4-reductase family / dihydrokaempferol 4-reductase family, similar to dihydroflavonol 4-reductase (SP:P51102), vestitone reductase (Medicago sativa, GI:973249) \| chr2:18710903-18713319 REVERSE \| Aliases: F4L23.9 | 4.2 | 9.2 | -5.0 | -9.4 | 0.05% | 7.8 |
| 1046 | AT1G27290.1 \| expressed protein \| chr1:9481546-9482899 FORWARD \| Aliases: F17L21.8, F17L21_8 | 6.9 | 9.1 | -2.2 | -9.4 | 0.05% | 8.9 |

| Rank | Description | Sync | Root | M | t | adj.q | B |
|---|---|---|---|---|---|---|---|
| 1051 | AT2G47460.1 \| Symbol: MYB12 \| MYB12 belongs to subgroup 7 of the R2R3-MYB family. It strongly activates the promoters of chalcone synthase (CHS), flavanone 3-hydroxylase (F3H), flavonol synthase (FLS) and - to a lesser extent - chalcone flavanone isomerase (CHI), but cannot activate the promoters of flavonoid-3'hydroxylase (F3'H) and dihydroflavonol 4-reductase (DF). The activation requires a functional MYB recognition element (MRE). Results from the myb12-1f allele indicate that an activation domain might be present in the C-terminus. Overexpression or knock-out plants do not show any obvious phenotype under greenhouse conditions. Young myb12-ko seedlings contain reduced amounts of flavonoids (quercetin and kaempferol), while seedlings as well as leaves of MYB12-OX plants displayed an increased flavonoid content. They did not show any significant difference in anthocyanin content. Expression of CHS and FLS shows a clear correlation to MYB12 expression levels. CHI and F3H show increased transcript levels in the MYB12-OX lines, but no differences in the knock-out. Even in the absence of functional MYB12, flavonol biosynthesis is not completely absent, suggesting functional redundancy. \| chr2:19483407-19486538 FORWARD \| Aliases: MYB12, T30B22.24 | 3.2 | 7.0 | -3.8 | -9.4 | 0.05% | 7.5 |
| 1056 | AT5G07080.1 \| transferase family protein, similar to 10-deacetylbaccatin III-10-O-acetyl transferase - Taxus cuspidata, AF193765, EMBL:AF193765; contains Pfam transferase family domain PF00248 \| chr5:2200333-2202111 FORWARD \| Aliases: T28J14.20, T28J14_20 | 3.1 | 8.1 | -5.0 | -9.4 | 0.05% | 7.9 |
| 1057 | AT4G38620.1 \| Symbol: MYB4 \| myb family transcription factor (MYB4), contains Pfam profile: PF00249 myb-like DNA-binding domain \| chr4:18053545-18054993 FORWARD \| Aliases: ATMYB4, T9A14.11 | 6.5 | 8.8 | -2.3 | -9.4 | 0.05% | 8.5 |
| 1058 | AT5G25640.1 \| expressed protein, ; expression supported by MPSS \| chr5:8949852-8950334 REVERSE \| Aliases: T14C9.180, T14C9_180 | 5.2 | 7.3 | -2.1 | -9.4 | 0.05% | 8.7 |
| 1059 | AT3G07570.1 \| membrane protein, putative, similar to membrane protein SDR2 (GI:1747306) (Mus musculus) | 6.8 | 9.4 | -2.6 | -9.4 | 0.05% | 8.4 |
| 1061 | AT3G18560.1 \| expressed protein \| chr3:6393916-6394783 FORWARD \| Aliases: K24M9.5 | 4.2 | 6.9 | -2.7 | -9.4 | 0.05% | 7.9 |
| 1062 | AT5G53250.1 \| arabinogalactan-protein, putative (AGP22), similar to arabinogalactan protein (Arabidopsis thaliana) gi:10880509:gb:AAG24284 \| chr5:21620846-21621457 FORWARD \| Aliases: K19E1.5, K19E1_5 | 5.9 | 9.8 | -4.0 | -9.4 | 0.05% | 8.3 |
| 1067 | AT2G01450.4 \| Symbol: ATMPK17 \| similar to mitogen-activated protein kinase, putative / MAPK, putative (MPK16) [Arabidopsis thaliana] (TAIR:At5g19010.1); similar to mitogen-activated protein kinase, putative / MAPK, putative (MPK8) [Arabidopsis thaliana] (TAIR:At1g18150.1); similar to mitogen-activated protein kinase, putative / MAPK, putative (MPK9) [Arabidopsis thaliana] (TAIR:At3g18040.1); similar to mitogen-activated protein kinase, putative / MAPK, putative (MPK8) [Arabidopsis thaliana] (TAIR:At1g18150.2); similar to putative MAPK protein kinase [Triticum aestivum] (GB:AAX20166.1); similar to putative MAP kinase [Oryza sativa (japonica cultivar-group)] (GB:BAD53616.1); similar to putative MAP kinase [Hordeum vulgare subsp. vulgare] (GB:CAD42638.1); similar to blast and wounding induced mitogen-activated protein kinase [Oryza sativa] (GB:AAD52659.1); similar to putative MAPK protein kinase [Triticum aestivum] (GB:AAX20165.1); contains InterPro domain Tyrosine protein kinase (InterPro:IPR001245); contains InterPro domain Serine/threonine protein kinase (InterPro:IPR002290); contains InterPro domain Protein kinase (InterPro:IPR000719) \| chr2:199510-203125 REVERSE \| Aliases: F2I9.7, F2I9_7 | 7.5 | 11.2 | -3.7 | -9.3 | 0.05% | 8.2 |
| 1070 | AT1G07870.1 \| protein kinase family protein, contains protein kinase domain, Pfam:PF00069 \| chr1:2429696-2432018 REVERSE \| Aliases: F24B9.4, F24B9_4 | 6.8 | 9.7 | -2.9 | -9.3 | 0.05% | 8.4 |
| 1071 | AT1G11540.1 \| expressed protein, contains Pfam profile: PF01925 domain of unknown function DUF81 \| chr1:3875160-3877155 REVERSE \| Aliases: T23J18.20, T23J18_20 | 5.0 | 7.1 | -2.2 | -9.3 | 0.05% | 8.5 |
| 1075 | AT5G53450.3 \| Symbol: ORG1 \| expressed protein, contains InterPro domain Serine/threonine protein kinase (InterPro:IPR002290); contains InterPro domain Protein kinase (InterPro:IPR000719) \| chr5:21705999-21709677 FORWARD \| Aliases: MYN8.6, MYN8_6 | 7.6 | 9.5 | -1.9 | -9.3 | 0.05% | 8.8 |
| 1078 | AT3G14280.1 \| expressed protein \| chr3:4760984-4762402 REVERSE \| Aliases: MLN21.7 | 4.3 | 8.7 | -4.4 | -9.3 | 0.05% | 7.3 |
| 1080 | AT2G36830.1 \| Symbol: GAMMA TIP \| major intrinsic family protein / MIP family protein, contains Pfam profile: MIP PF00230 \| chr2:15452505-15453653 FORWARD \| Aliases: GAMMA TIP1, GAMMA TONOPLAST INTRINSIC PROTEIN, T1J8.1, T1J8_1, TIP1;1 | 9.1 | 12.6 | -3.6 | -9.3 | 0.05% | 8.4 |
| 1083 | AT4G36920.1 \| Symbol: AP2 \| floral homeotic protein APETALA2 (AP2), Identical to (SP:P47927) Floral homeotic protein APETALA2. (Mouse-ear cress) {Arabidopsis thaliana} \| chr4:17400844-17403329 FORWARD \| Aliases: AP22.49, AP22_49, APETALA 2, APETALA2, FL1, FLO2, FLORAL HOMEOTIC PROTEIN APETALA 2, FLOWER1 | 5.1 | 7.6 | -2.5 | -9.3 | 0.05% | 8.3 |
| 1084 | AT4G39350.1 \| Symbol: CESA2 \| cellulose synthase, catalytic subunit (Ath-A), identical to gi:2827141 \| chr4:18296903-18302186 FORWARD \| Aliases: ATH A, CELLULASE SYNTHASE 2, CELLULOSE SYNTHASE, T22F8.250, T22F8_250 | 6.5 | 9.1 | -2.6 | -9.3 | 0.05% | 8.6 |
| 1085 | AT5G62420.1 \| aldo/keto reductase family protein, similar to chalcone reductase (Sesbania rostrata)(GI:2792155); contains Pfam profile PF00248: oxidoreductase, aldo/keto reductase family \| chr5:25082061-25083337 FORWARD \| Aliases: K19B1.2, K19B1_2 | 3.2 | 8.1 | -5.0 | -9.3 | 0.05% | 8.2 |
| 1086 | AT5G35200.1 \| epsin N-terminal homology (ENTH) domain-containing protein, contains Pfam PF01417: ENTH domain. ENTH (Epsin N-terminal homology) domain; similar to clathrin assembly protein short form, Rattus norvegicus, EMBL:AF041373;similar to Chain A, Calm-N N-Terminal Domain Of Clathrin Assembly Lymphoid Myeloid Leukaemia Protein, Pi(4,5)p2 Complex (GP:13399999) {Homo sapiens} \| chr5:13479392-13483281 REVERSE \| Aliases: T25C13.80, T25C13_80 | 6.2 | 9.1 | -2.9 | -9.3 | 0.05% | 8.8 |

| Rank | Description | Sync | Root | M | t | adj.q | B |
|---|---|---|---|---|---|---|---|
| 1090 | AT4G36670.1 \| mannitol transporter, putative, similar to mannitol transporter (Apium graveolens var. dulce) GI:12004316; contains Pfam profile PF00083: major facilitator superfamily protein \| chr4:17287503-17289594 REVERSE \| Aliases: AP22.86, AP22_86 | 4.8 | 8.5 | -3.8 | -9.3 | 0.05% | 8.1 |
| 1092 | AT2G29900.1 \| presenilin family protein, similar to presenilin (Drosophila melanogaster) GI:2062395; contains Pfam profile PF01080: Presenilin | 5.8 | 7.2 | -1.4 | -9.3 | 0.05% | 8.8 |
| 1093 | AT3G23200.1 \| expressed protein \| chr3:8281370-8282785 FORWARD \| Aliases: K14B15.11 | 6.2 | 9.0 | -2.8 | -9.3 | 0.05% | 8.0 |
| 1094 | AT2G34940.1 \| vacuolar sorting receptor, putative, similar to BP-80 vacuolar sorting receptor (Pisum sativum) GI:1737222 \| chr2:14747550-14750496 FORWARD \| Aliases: F19I3.17, F19I3_17 | 3.4 | 7.5 | -4.1 | -9.3 | 0.05% | 6.8 |
| 1097 | AT4G08930.1 \| Symbol: ATAPRL6 \| Encodes a protein disulfide isomerase-like (PDIL) protein, a member of a multigene family within the thioredoxin (TRX) superfamily. This protein also belongs to the adenosine 5'-phosphosulfate reductase-like (APRL) group. \| chr4:5729189-5730858 FORWARD \| Aliases: APR LIKE 6, ATAPRL6, T3H13.4, T3H13_4 | 5.5 | 8.5 | -3.0 | -9.3 | 0.05% | 8.2 |
| 1098 | AT2G25590.1 \| agenet domain-containing protein, contains Pfam PF05641: Agenet domain \| chr2:10898373-10900850 FORWARD \| Aliases: F3N11.4, F3N11_4 | 6.6 | 9.4 | -2.8 | -9.3 | 0.05% | 8.4 |
| 1099 | AT1G62300.1 \| Symbol: WRKY6 \| WRKY family transcription factor, similar to putative DNA-binding protein GI:7268215 from (Arabidopsis thaliana) \| chr1:23020348-23022944 REVERSE \| Aliases: F19K23.22, F19K23_22 | 5.1 | 8.6 | -3.5 | -9.3 | 0.05% | 8.3 |
| 1100 | AT1G20110.1 \| zinc finger (FYVE type) family protein, contains Pfam profile: PF01363 FYVE zinc finger \| chr1:6971476-6974812 FORWARD \| Aliases: T20H2.10, T20H2_10 | 8.8 | 10.7 | -1.9 | -9.3 | 0.05% | 8.8 |
| 1101 | AT2G40110.2 \| yippee family protein, similar to mdgl-1 (Mus musculus) GI:10441648, Yippee protein (Drosophila melanogaster) GI:5713279; contains Pfam profile PF03226: Yippee putative zinc-binding protein \| chr2:16755018-16756590 FORWARD \| Aliases: F27I1.3, F27I1_3 | 6.8 | 10.1 | -3.3 | -9.2 | 0.05% | 8.4 |
| 1104 | AT3G23880.1 \| F-box family protein, contains F-box domain Pfam:PF00646 | 3.5 | 7.4 | -3.9 | -9.2 | 0.05% | 7.6 |
| 1105 | AT5G66250.3 \| kinectin-related, contains weak similarity to kinectin (GI:3766232) (Vulpes vulpes) \| chr5:26485761-26487170 FORWARD \| Aliases: K1L20.3, K1L20_3 | 6.6 | 8.9 | -2.3 | -9.2 | 0.05% | 8.6 |
| 1110 | AT4G29220.1 \| phosphofructokinase family protein, similar to phosphofructokinase (Amycolatopsis methanolica) GI:17432243; contains Pfam profile PF00365: Phosphofructokinase \| chr4:14403041-14406511 REVERSE \| Aliases: F17A13.40, F17A13_40 | 4.6 | 8.8 | -4.1 | -9.2 | 0.05% | 7.7 |
| 1115 | AT3G27190.1 \| uracil phosphoribosyltransferase, putative / UMP pyrophosphorylase, putative / UPRTase, putative, similar to SP:O65583 Uracil phosphoribosyltransferase (EC 2.4.2.9) (UMP pyrophosphorylase) (UPRTase) {Arabidopsis thaliana}; contains Pfam profile PF00485: Phosphoribulokinase / Uridine kinase family \| chr3:10040535-10044254 REVERSE \| Aliases: K17E12.1 | 5.7 | 8.3 | -2.6 | -9.2 | 0.05% | 8.4 |
| 1116 | AT5G50760.1 \| auxin-responsive family protein, similar to auxin-induced protein TGSAUR22 (GI:10185820) (Tulipa gesneriana) \| chr5:20661887-20662695 FORWARD \| Aliases: MFB16.16, MFB16_16 | 2.7 | 6.6 | -4.0 | -9.2 | 0.05% | 6.6 |
| 1119 | AT4G31550.3 \| Symbol: WRKY11 \| | 4.0 | 6.3 | -2.3 | -9.2 | 0.05% | 8.3 |
| 1123 | AT1G58370.1 \| Symbol: RXF12 \| glycosyl hydrolase family 10 protein / carbohydrate-binding domain-containing protein, similar to (1,4)-beta-xylan endohydrolase GI:5306060 from (Triticum aestivum) ; contains Pfam profiles PF00331: Glycosyl hydrolase family 10, PF02018: Carbohydrate binding domain \| chr1:21688399-21691986 FORWARD \| Aliases: F9K23.10, RXF12 | 4.1 | 7.4 | -3.2 | -9.2 | 0.05% | 7.9 |
| 1126 | AT1G30130.2 \| expressed protein, similar to hypothetical protein GI:1469227 from (Brassica oleracea) \| chr1:10587587-10589606 FORWARD \| Aliases: T2H7.7, T2H7_7 | 5.4 | 8.5 | -3.2 | -9.2 | 0.05% | 8.4 |
| 1136 | AT3G11550.1 \| integral membrane family protein, similar to unknown protein GB:AAD26967 (Arabidopsis thaliana); contains TIGRFAM TIGR01569 : plant integral membrane protein TIGR01569; contains Pfam PF04535 : Domain of unknown function (DUF588) \| chr3:3638201-3639203 FORWARD \| Aliases: F24K9.22 | 3.2 | 6.8 | -3.7 | -9.1 | 0.05% | 7.0 |
| 1139 | AT1G30400.2 \| Symbol: ATMRP1 \| | 6.0 | 9.0 | -3.0 | -9.1 | 0.05% | 8.4 |
| 1140 | AT1G21360.1 \| expressed protein \| chr1:7481353-7483425 FORWARD \| Aliases: F24J8.2, F24J8_2 | 2.6 | 7.8 | -5.2 | -9.1 | 0.05% | 5.5 |
| 1142 | AT1G56230.1 \| expressed protein \| chr1:21049699-21052690 REVERSE \| Aliases: F14G9.16, F14G9_16 | 6.8 | 9.9 | -3.0 | -9.1 | 0.05% | 8.5 |
| 1144 | AT5G45480.1 \| expressed protein, contains Pfam domain, PF04578: Protein of unknown function, DUF594 \| chr5:18443331-18446317 REVERSE \| Aliases: MFC19.15, MFC19_15 | 2.8 | 5.9 | -3.1 | -9.1 | 0.05% | 7.6 |

| Rank | Description | Sync | Root | M | t | adj.q | B |
|---|---|---|---|---|---|---|---|
| 1146 | AT1G22190.1 \| AP2 domain-containing transcription factor, putative, similar to AP2 domain containing protein RAP2.4 GI:2281633 from (Arabidopsis thaliana) \| chr1:7835771-7837277 FORWARD \| Aliases: F16L1.8, F16L1_8 | 4.6 | 7.9 | -3.3 | -9.1 | 0.05% | 7.5 |
| 1147 | AT5G64240.2 \| latex-abundant family protein (AMC3) / caspase family protein, contains similarity to latex-abundant protein (Hevea brasiliensis) gb:AAD13216; contains Pfam profile PF00656: ICE-like protease (caspase) p20 domain \| chr5:25712846-25715046 FORWARD \| Aliases: MSJ1.8, MSJ1_8 | 3.1 | 6.6 | -3.4 | -9.1 | 0.05% | 7.4 |
| 1148 | AT3G52360.1 \| expressed protein \| chr3:19423098-19423896 FORWARD \| Aliases: T25B15.130 | 6.8 | 9.6 | -2.8 | -9.1 | 0.05% | 8.6 |
| 1149 | AT5G55910.1 \| protein kinase, putative, contains protein kinase domain, Pfam:PF00069 \| chr5:22657070-22659528 REVERSE \| Aliases: MYN21.2, MYN21_2 | 5.3 | 7.7 | -2.4 | -9.1 | 0.05% | 8.5 |
| 1150 | AT1G75820.1 \| Symbol: CLV1 \| CLAVATA1 receptor kinase (CLV1), identical to receptor kinase (CLV1) GB:AAB58929 GI:2160756 (Arabidopsis thaliana) \| chr1:28467123-28470632 REVERSE \| Aliases: CLAVATA 1, CLV1 RECEPTOR KINASE PRECURSOR, FAS3, FASCIATA 3, FLO5, FLOWER DEVELOPMENT 5, T4O12.5, T4O12_5 | 4.2 | 7.4 | -3.1 | -9.1 | 0.05% | 7.3 |
| 1151 | AT5G44110.2 \| Symbol: POP1 \| similar to ABC transporter family protein [Arabidopsis thaliana] (TAIR:At1g03905.1); similar to hypothetical protein UM03153.1 [Ustilago maydis 521] (GB:EAK84383.1); contains InterPro domain ABC transporter (InterPro:IPR003439); contains InterPro domain ATP/GTP-binding site motif A (P-loop) (InterPro:IPR001687) | 9.0 | 11.5 | -2.5 | -9.1 | 0.05% | 8.6 |
| 1153 | AT4G18700.1 \| Symbol: CIPK12 \| | 8.5 | 10.2 | -1.8 | -9.1 | 0.05% | 8.7 |
| 1155 | AT4G15330.1 \| Symbol: CYP705A1 \| cytochrome P450 family protein \| chr4:8751391-8753134 REVERSE \| Aliases: DL3710C, FCAALL.270 | 3.3 | 7.8 | -4.5 | -9.1 | 0.05% | 6.3 |
| 1157 | AT3G07130.1 \| serine/threonine protein phosphatase family protein, contains similarity to purple acid phosphatase (Arabidopsis thaliana) gi:20257489:gb:AAM15914 \| chr3:2255716-2258011 REVERSE \| Aliases: T1B9.21 | 7.3 | 9.4 | -2.1 | -9.1 | 0.05% | 8.5 |
| 1161 | AT5G59450.1 \| scarecrow-like transcription factor 11 (SCL11), scarecrow-like 11, Arabidopsis thaliana, EMBL:AF036307 \| chr5:23991894-23994017 FORWARD \| Aliases: F2O15.5, F2O15_5 | 4.1 | 5.6 | -1.6 | -9.1 | 0.05% | 8.6 |
| 1162 | AT4G10380.1 \| major intrinsic family protein / MIP family protein, contains Pfam profile: MIP PF00230 \| chr4:6431235-6434818 REVERSE \| Aliases: F7L13.6 | 5.4 | 7.6 | -2.2 | -9.1 | 0.05% | 8.6 |
| 1163 | AT4G02410.1 \| lectin protein kinase family protein, contains Pfam domains, PF00069: Protein kinase domain, PF00139: Legume lectins beta domain and PF00138: Legume lectins alpha domain \| chr4:1059889-1062153 REVERSE \| Aliases: T14P8.3, T14P8_3 | 5.2 | 8.0 | -2.8 | -9.1 | 0.05% | 8.2 |
| 1165 | AT3G14270.1 \| phosphatidylinositol-4-phosphate 5-kinase family protein, similar to SP:Q9Z1T6 FYVE finger-containing phosphoinositide kinase (EC 2.7.1.68) (1-phosphatidylinositol-4-phosphate kinase) (PIP5K) (PtdIns(4)P-5-kinase) {Mus musculus}; contains Pfam profiles PF01504: Phosphatidylinositol-4-phosphate 5-Kinase, PF01363: FYVE zinc finger, PF00118: TCP-1/cpn60 chaperonin family \| chr3:4753932-4761460 FORWARD \| Aliases: MLN21.6 | 6.4 | 9.5 | -3.1 | -9.1 | 0.05% | 8.5 |
| 1166 | AT1G04440.1 \| casein kinase, putative, similar to casein kinase I (Arabidopsis thaliana) gi:1103318:emb:CAA55395; contains protein kinase domain, Pfam:PF00069 | 5.6 | 9.3 | -3.7 | -9.1 | 0.05% | 8.2 |
| 1167 | AT3G52450.1 \| U-box domain-containing protein, similar to immediate-early fungal elicitor protein CMPG1 (Petroselinum crispum) GI:14582200; contains Pfam profile PF04564: U-box domain \| chr3:19451717-19453361 REVERSE \| Aliases: F22O6.170 | 7.0 | 9.4 | -2.4 | -9.1 | 0.05% | 8.5 |
| 1171 | AT4G13020.3 \| Symbol: MHK \| similar to protein kinase, putative [Arabidopsis thaliana] (TAIR:At4g19110.1); similar to protein kinase, putative [Arabidopsis thaliana] (TAIR:At4g19110.2); similar to putative Cdc2-related protein kinase CRK2 [Beta vulgaris] (GB:CAB90209.1); contains InterPro domain Serine/threonine protein kinase, active site (InterPro:IPR008271); contains InterPro domain Tyrosine protein kinase (InterPro:IPR001245); contains InterPro domain Serine/threonine protein kinase (InterPro:IPR002290); contains InterPro domain Protein kinase (InterPro:IPR000719) \| chr4:7603823-7607152 FORWARD \| Aliases: F25G13.110, F25G13_110, PROTEIN KINASE | 5.5 | 8.1 | -2.5 | -9.0 | 0.05% | 8.3 |
| 1172 | AT4G02075.1 \| Symbol: PIT1 \| zinc finger (C3HC4-type RING finger) family protein, contains InterPro Entry IPR001841 Zn-finger, RING \| chr4:913419-916476 REVERSE \| Aliases: PITCHOUN 1 | 2.8 | 8.0 | -5.2 | -9.0 | 0.05% | 8.1 |
| 1175 | AT5G23030.1 \| senescence-associated family protein, similar to senescence-associated protein 5 (Hemerocallis hybrid cultivar) gi:3551954:gb:AAC34855 \| chr5:7726791-7727712 FORWARD \| Aliases: MYJ24.2, MYJ24_2 | 2.2 | 4.5 | -2.2 | -9.0 | 0.05% | 8.4 |
| 1180 | AT3G47160.1 \| expressed protein, similar to MTD2 (Medicago truncatula) GI:9294812; supporting cDNA gi:25083983:gb:BT002136.1: \| chr3:17370976-17372990 REVERSE \| Aliases: F13I12.210 | 6.4 | 9.1 | -2.8 | -9.0 | 0.06% | 8.1 |

| Rank | Description | Sync | Root | M | t | adj.q | B |
|---|---|---|---|---|---|---|---|
| 1185 | AT4G34500.1 \| protein kinase family protein, contains protein kinase domain, Pfam:PF00069; contains serine/threonine protein kinase domain, INTERPRO:IPR002290 | 4.9 | 7.1 | -2.2 | -9.0 | 0.06% | 8.2 |
| 1188 | AT4G27290.1 \| S-locus protein kinase, putative, similar to S-receptor kinase gi:392557:gb:AAA62232; contains Pfam domains PF00954: S-locus glycoprotein family, PF00069: Protein kinase domain and PF01453: Lectin (probable mannose binding) \| chr4:13666287-13669208 FORWARD \| Aliases: M4I22.100, M4I22_100 | 2.3 | 3.7 | -1.3 | -9.0 | 0.06% | 8.6 |
| 1189 | AT3G07690.1 \| similar to NAD-dependent glycerol-3-phosphate dehydrogenase family protein [Arabidopsis thaliana] (TAIR:At2g41540.1); similar to NAD-dependent glycerol-3-phosphate dehydrogenase family protein [Arabidopsis thaliana] (TAIR:At2g41540.2); similar to putative glycerol-3-phosphate dehydrogenase [Oryza sativa (japonica cultivar-group)] (GB:NP_915827.1); similar to putative glycerol-3-phosphate dehydrogenase [Oryza sativa (japonica cultivar-group)] (GB:AAU44049.1); contains InterPro domain NAD-dependent glycerol-3-phosphate dehydrogenase (InterPro:IPR006168) \| chr3:2457114-2459700 FORWARD \| Aliases: MLP3.14 | 5.8 | 8.0 | -2.2 | -9.0 | 0.06% | 8.4 |
| 1190 | AT1G32700.2 \| zinc-binding family protein, similar to zinc-binding protein (Pisum sativum) GI:16117799; contains Pfam profile PF04640 : Protein of unknown function, DUF597 \| chr1:11827447-11829739 FORWARD \| Aliases: F6N18.8, F6N18_8 | 5.9 | 9.2 | -3.3 | -9.0 | 0.06% | 8.2 |
| 1192 | AT5G03280.1 \| Symbol: EIN2 \| ethylene-insensitive 2 (EIN2), identical to EIN2 (Arabidopsis thaliana) gi:5231113:gb:AAD41076; member of the natural resistance-associated macrophage protein (NRAMP) metal transporter family, PMID:11500563; metal transport capacity has not been shown, PMID:11500563, PMID:1038174 | 4.4 | 8.3 | -4.0 | -9.0 | 0.06% | 8.5 |
| 1193 | AT1G07600.1 \| Symbol: MT1A \| metallothionein-like protein 1A (MT-1A) (MT-Q) (MT-2), identical to Metallothionein-like protein 1A (MT-1A) (MT-Q) (MT-2) SP:P43392 from (Arabidopsis thaliana) \| chr1:2338901-2339318 REVERSE \| Aliases: ATMT 2, ATMT Q, F22G5.2, LIGHT STRESS REGULATED 4, LSR4, METALLOTHIONEIN 1A, MT Q, MT1 | 12.5 | 14.3 | -1.8 | -9.0 | 0.06% | 8.4 |
| 1194 | AT4G02390.1 \| Symbol: APP \| poly (ADP-ribose) polymerase / NAD(+) ADP-ribosyltransferase / poly(ADP-ribose) synthetase (APP), identical to SP:Q11207 Poly (ADP-ribose) polymerase (EC 2.4.2.30) (PARP) (ADPRT) (NAD(+) ADP- ribosyltransferase) (Poly(ADP-ribose) synthetase) {Arabidopsis thaliana} | 4.9 | 6.7 | -1.7 | -9.0 | 0.06% | 8.5 |
| 1196 | AT1G22930.1 \| T-complex protein 11, contains Pfam PF05794: T-complex protein 11 \| chr1:8117386-8122116 FORWARD \| Aliases: F19G10.11, F19G10_11 | 8.0 | 10.6 | -2.6 | -9.0 | 0.06% | 8.2 |
| 1199 | AT2G44110.2 \| Symbol: MLO15 \| seven transmembrane MLO family protein / MLO-like protein 15 (MLO15), identical to MLO-like protein 15 (AtMlo15) SP:O80580 from (Arabidopsis thaliana); similar to MLO protein SWISS-PROT:P93766, NCBI_gi:1877221 (Hordeum vulgare)(Barley) \| chr2:18252433-18255405 REVERSE \| Aliases: F6E13.24 | 2.9 | 6.9 | -4.0 | -9.0 | 0.06% | 6.3 |
| 1200 | AT5G18460.1 \| expressed protein, predicted proteins, Arabidopsis thaliana Pfam profile PF03080: Arabidopsis proteins of unknown function \| chr5:6123048-6126723 FORWARD \| Aliases: F20L16.180, F20L16_180 | 2.7 | 5.3 | -2.5 | -9.0 | 0.06% | 8.0 |
| 1201 | AT5G07250.1 \| rhomboid family protein, contains PFAM domain PF01694, Rhomboid family \| chr5:2273555-2275991 REVERSE \| Aliases: T28J14.190, T28J14_190 | 5.4 | 7.9 | -2.5 | -9.0 | 0.06% | 8.4 |
| 1203 | AT1G34760.1 \| Symbol: GRF11 \| 14-3-3 protein GF14 omicron (GRF11), identical to SP:Q9S9Z8, 14-3-3-like protein GF14 omicron (General regulatory factor 11){Arabidopsis thaliana} \| chr1:12743826-12745581 REVERSE \| Aliases: 14 3 3 PROTEIN GF14OMICRON, F11O6.13, GF14 OMICRON | 3.5 | 8.1 | -4.7 | -9.0 | 0.06% | 8.0 |
| 1205 | AT1G17430.1 \| hydrolase, alpha/beta fold family protein, low similarity to SP:Q02104 Lipase 1 precursor (EC 3.1.1.3) (Triacylglycerol lipase) {Psychrobacter immobilis}, contains Pfam profile PF00561: hydrolase, alpha/beta fold family \| chr1:5982260-5984004 FORWARD \| Aliases: F28G4.9 | 6.8 | 9.3 | -2.4 | -9.0 | 0.06% | 8.3 |
| 1207 | AT1G70290.1 \| Symbol: ATTPS8 \| | 6.3 | 8.6 | -2.2 | -9.0 | 0.06% | 8.5 |
| 1210 | AT4G18430.1 \| Ras-related GTP-binding protein, putative, similar to GTP-binding protein GI:303742 from (Pisum sativum) | 3.0 | 5.7 | -2.7 | -9.0 | 0.06% | 7.7 |
| 1211 | AT5G61820.1 \| expressed protein, MtN19, Medicago truncatula, EMBL:MTY15367 \| chr5:24851544-24853917 REVERSE \| Aliases: MAC9.6, MAC9_6 | 10.2 | 11.8 | -1.6 | -9.0 | 0.06% | 8.6 |
| 1213 | AT2G17440.1 \| leucine-rich repeat family protein, contains Pfam PF00560: Leucine Rich Repeats \| chr2:7578222-7580838 FORWARD \| Aliases: F5J6.20, F5J6_20 | 4.0 | 6.9 | -3.0 | -9.0 | 0.06% | 8.3 |
| 1215 | AT3G18830.1 \| Symbol: ATPLT5 \| | 6.4 | 8.1 | -1.7 | -8.9 | 0.06% | 8.6 |
| 1223 | AT5G67520.1 \| adenylylsulfate kinase, putative, similar to adenylylsulfate kinase, chloroplast precursor (APS kinase, Adenosine-5'phosphosulfate kinase, ATP adenosine-5'- phosphosulfate 3'-phosphotransferase) (Catharanthus roseus) SWISS-PROT:O49204 \| chr5:26956385-26958172 FORWARD \| Aliases: K9I9.8, K9I9_8 | 5.3 | 7.7 | -2.4 | -8.9 | 0.06% | 8.0 |
| 1224 | AT4G23850.1 \| long-chain-fatty-acid--CoA ligase / long-chain acyl-CoA synthetase, nearly identical to acyl-CoA synthetase (MF7P) from Brassica napus (gi:1617270) \| chr4:12403477-12408343 REVERSE \| Aliases: T32A16.20, T32A16_20 | 8.3 | 10.9 | -2.5 | -8.9 | 0.06% | 8.5 |

| Rank | Description | Sync | Root | M | t | adj.q | B |
|---|---|---|---|---|---|---|---|
| 1225 | AT3G06650.1 \| Symbol: ACLB 1 \| | 5.9 | 8.7 | -2.8 | -8.9 | 0.06% | 8.2 |
| 1226 | AT2G04430.1 \| similar to MutT/nudix family protein [Arabidopsis thaliana] (TAIR:At2g04450.1); similar to OSJNBa0079A21.19 [Oryza sativa (japonica cultivar-group)] (GB:XP_473408.1); contains InterPro domain Anti-sense to fibroblast growth factor protein GFG (InterPro:IPR003293); contains InterPro domain NUDIX hydrolase (InterPro:IPR000086) \| chr2:1538898-1541091 FORWARD \| Aliases: T1O3.16, T1O3_16 | 4.3 | 6.7 | -2.4 | -8.9 | 0.06% | 8.2 |
| 1227 | AT1G44100.1 \| Symbol: AAP5 \| amino acid permease 5, putative (AAP5), nearly identical to amino acid permease (AAP5) GI:608673 from (Arabidopsis thaliana) \| chr1:16766845-16769973 REVERSE \| Aliases: T7O23.19, T7O23_19 | 5.2 | 7.8 | -2.7 | -8.9 | 0.06% | 8.1 |
| 1228 | AT3G01470.1 \| Symbol: ATHB 1 \| | 8.6 | 11.5 | -2.9 | -8.9 | 0.06% | 8.3 |
| 1232 | AT3G56230.1 \| speckle-type POZ protein-related, contains Pfam PF00651 : BTB/POZ domain; similar to Speckle-type POZ protein (SP:O43791) (Homo sapiens) | 2.5 | 5.2 | -2.7 | -8.9 | 0.06% | 7.7 |
| 1234 | AT2G37090.1 \| Symbol: IXR9 \| Encodes a protein with similarity to glycosyltransferases. Mutants have irregular xylem phenotype suggesting a role in secondary cell wall biosynthesis. | 4.0 | 7.2 | -3.2 | -8.9 | 0.06% | 7.3 |
| 1237 | AT5G15600.1 \| expressed protein \| chr5:5078203-5079094 FORWARD \| Aliases: T20K14.210, T20K14_210 | 7.1 | 9.6 | -2.5 | -8.9 | 0.06% | 7.9 |
| 1240 | AT2G22800.1 \| Symbol: HAT9 \| homeobox-leucine zipper protein 9 (HAT9) / HD-ZIP protein 9, identical to GB:U09341 \| chr2:9711796-9713230 REVERSE \| Aliases: T30L20.6, TRANSCRIPTION FACTOR HAT9 | 2.5 | 7.6 | -5.1 | -8.9 | 0.06% | 7.7 |
| 1241 | AT3G24520.1 \| Symbol: AT HSFC1 \| heat shock transcription factor family protein, contains Pfam profile: PF00447 HSF-type DNA-binding domain \| chr3:8941073-8942881 FORWARD \| Aliases: HSFC1, MOB24.9 | 6.1 | 9.5 | -3.4 | -8.9 | 0.06% | 8.3 |
| 1242 | AT3G19390.1 \| cysteine proteinase, putative / thiol protease, putative, contains similarity to cysteine proteinase RD21A (thiol protease) GI:435619, SP:P43297 from (Arabidopsis thaliana) \| chr3:6722995-6724957 FORWARD \| Aliases: MLD14.3 | 6.0 | 8.2 | -2.2 | -8.9 | 0.06% | 8.3 |
| 1244 | AT3G55850.2 \| Symbol: LAF3.1 \| | 4.6 | 6.6 | -2.1 | -8.9 | 0.06% | 8.3 |
| 1245 | AT3G19660.1 \| expressed protein \| chr3:6825527-6826012 REVERSE \| Aliases: MMB12.13 | 5.6 | 8.2 | -2.6 | -8.8 | 0.06% | 8.2 |
| 1251 | AT5G34940.3 \| glycosyl hydrolase family 79 N-terminal domain-containing protein, similar to beta-glucuronidase precursor (Scutellaria baicalensis) GI:8918740; contains Pfam profile PF03662: Glycosyl hydrolase family 79, N-terminal domain \| chr5:13253059-13255948 REVERSE \| Aliases: T2L5.6 | 7.7 | 10.2 | -2.5 | -8.8 | 0.06% | 8.4 |
| 1257 | AT3G20370.1 \| meprin and TRAF homology domain-containing protein / MATH domain-containing protein, similar to ubiquitin-specific protease 12 (Arabidopsis thaliana) GI:11993471; contains Pfam profile PF00917: MATH domain \| chr3:7105377-7107231 FORWARD \| Aliases: MQC12.15 | 4.4 | 7.6 | -3.2 | -8.8 | 0.06% | 7.6 |
| 1259 | AT1G26440.4 \| Symbol: ATUPS5 \| | 3.8 | 7.4 | -3.6 | -8.8 | 0.06% | 7.3 |
| 1268 | AT3G57020.2 \| similar to strictosidine synthase family protein [Arabidopsis thaliana] (TAIR:At3g57010.1); similar to putative strictosidine synthase [Lycopersicon esculentum] (GB:AAF75751.1); contains InterPro domain Strictosidine synthase (InterPro:IPR004141) \| chr3:21109294-21111338 REVERSE \| Aliases: F24I3.100 | 4.3 | 9.3 | -5.0 | -8.8 | 0.06% | 7.9 |
| 1270 | AT2G47130.1 \| short-chain dehydrogenase/reductase (SDR) family protein, similar to 3-beta-hydroxysteroiddehydrogenase GI:15983819 from (Digitalis lanata) \| chr2:19356606-19357591 REVERSE \| Aliases: F14M4.4 | 4.4 | 6.2 | -1.8 | -8.8 | 0.06% | 8.3 |
| 1272 | AT4G17280.1 \| auxin-responsive family protein, similar to auxin-induced protein AIR12 (GI:11357190) (Arabidopsis thaliana) \| chr4:9678541-9680401 REVERSE \| Aliases: DL4675C, FCAALL.393 | 3.3 | 6.0 | -2.7 | -8.8 | 0.06% | 7.8 |
| 1277 | AT5G62480.2 \| Symbol: ATGSTU9 \| glutathione S-transferase, putative \| chr5:25105944-25106819 REVERSE \| Aliases: GLUTATHIONE S TRANSFERASE 14, GLUTATHIONE S TRANSFERASE 14B, GLUTATHIONE S TRANSFERASE TAU 9, GST14, GST14B, K19B1.9, K19B1_9 | 4.7 | 7.7 | -3.1 | -8.8 | 0.06% | 7.1 |
| 1279 | AT5G49900.1 \| expressed protein, contains Pfam domain PF04685: Protein of unknown function, DUF608 \| chr5:20314349-20319572 REVERSE \| Aliases: K9P8.4, K9P8_4 | 8.0 | 9.7 | -1.7 | -8.8 | 0.06% | 8.4 |

| Rank | Description | Sync | Root | M | t | adj.q | B |
|---|---|---|---|---|---|---|---|
| 1280 | AT5G58700.2 \| similar to phosphoinositide-specific phospholipase C family protein [Arabidopsis thaliana] (TAIR:At2g40116.1); similar to phosphoinositide-specific phospholipase C (PLC2) [Arabidopsis thaliana] (TAIR:At3g08510.1); similar to 1-phosphatidylinositol-4,5-bisphosphate phosphodiesterase [Nicotiana rustica] (GB:CAA72681.1); similar to phospholipase C [Pisum sativum] (GB:CAA75546.2); contains InterPro domain Phosphoinositide-specific phospholipase C (PLC) (InterPro:IPR001192); contains InterPro domain C2 domain (InterPro:IPR000008); contains InterPro domain Phosphatidylinositol-specific phospholipase C, X domain (InterPro:IPR000909); contains InterPro domain Phosphatidylinositol-specific phospholipase C, Y domain (InterPro:IPR001711) | 7.7 | 9.4 | -1.7 | -8.8 | 0.06% | 8.3 |
| 1283 | AT1G02400.1 \| gibberellin 2-oxidase, putative / GA2-oxidase, putative, similar to GA2ox2 (GI:4678368); similar to dioxygenase GI:1666096 from (Marah macrocarpus); contains PF03171 2OG-Fe(II) oxygenase superfamily domain \| chr1:486802-489577 FORWARD \| Aliases: T6A9.9, T6A9_9 | 3.2 | 6.8 | -3.6 | -8.8 | 0.06% | 7.8 |
| 1284 | AT3G21430.1 \| expressed protein \| chr3:7544634-7550830 REVERSE \| Aliases: MHC9.11 | 5.4 | 7.6 | -2.1 | -8.8 | 0.06% | 8.4 |
| 1286 | AT3G11410.1 \| protein phosphatase 2C, putative / PP2C, putative, identical to protein phosphatase 2C (PP2C) GB:P49598 (Arabidopsis thaliana); contains Pfam profile PF00481: Protein phosphatase 2C; identical to cDNA protein phosphatase 2C GI:633027 \| chr3:3583889-3585796 REVERSE \| Aliases: F24K9.8 | 5.9 | 8.0 | -2.1 | -8.8 | 0.06% | 8.2 |
| 1287 | AT1G61740.1 \| expressed protein, contains Pfam profile: PF01925 domain of unknown function DUF81; identical to cDNA hypothetical protein, partial GI:4079631 \| chr1:22801734-22804738 REVERSE \| Aliases: T13M11.10, T13M11_10 | 5.2 | 8.3 | -3.2 | -8.7 | 0.06% | 7.8 |
| 1288 | AT2G23450.2 \| protein kinase family protein, contains protein kinase domain, Pfam:PF00069 \| chr2:9995834-9998739 REVERSE \| Aliases: F26B6.10, F26B6_10 | 4.9 | 7.3 | -2.5 | -8.7 | 0.06% | 7.7 |
| 1289 | AT3G54220.1 \| Symbol: SCR \| scarecrow transcription factor, putative, nearly identical to SCARECROW (Arabidopsis thaliana) GI:1497987 | 3.3 | 6.1 | -2.8 | -8.7 | 0.06% | 7.9 |
| 1293 | AT4G19670.1 \| zinc finger (C3HC4-type RING finger) family protein, contains a Prosite:PS00518 Zinc finger, C3HC4 type (RING finger), signature and Pfam domain PF01485: IBR domain \| chr4:10699251-10701462 REVERSE \| Aliases: T16H5.30, T16H5_30 | 6.1 | 9.1 | -3.0 | -8.7 | 0.06% | 7.7 |
| 1296 | AT1G24320.1 \| alpha-glucosidase, putative, similar to alpha-glucosidase I from GI:16506680 (Arabidopsis thaliana) \| chr1:8626316-8630960 REVERSE \| Aliases: F3I6.26, F3I6_26 | 3.3 | 6.6 | -3.3 | -8.7 | 0.06% | 7.1 |
| 1297 | AT4G22270.1 \| expressed protein \| chr4:11773340-11775984 FORWARD \| Aliases: T10I14.100, T10I14_100 | 4.1 | 6.1 | -2.0 | -8.7 | 0.06% | 8.1 |
| 1298 | AT4G22780.1 \| Symbol: ACR7 \| ACT domain-containing protein (ACR7), low similarity to uridylyltransferase SP:P56884 from Rhizobium meliloti; contains Pfam ACT domain PF01842 \| chr4:11968287-11971158 REVERSE \| Aliases: T12H17.170, T12H17_170 | 3.3 | 5.8 | -2.6 | -8.7 | 0.06% | 7.8 |
| 1299 | AT4G20860.1 \| FAD-binding domain-containing protein, simlar to SP:P30986 reticuline oxidase precursor (Berberine-bridge-forming enzyme) (BBE) (Tetrahydroprotoberberine synthase) (Eschscholzia californica); contains PF01565 FAD binding domain \| chr4:11172633-11174478 FORWARD \| Aliases: T13K14.20, T13K14_20 | 4.4 | 9.2 | -4.7 | -8.7 | 0.06% | 7.7 |
| 1300 | AT2G39430.1 \| disease resistance-responsive protein-related / dirigent protein-related, contains similarity to disease resistance response protein 206-d (Pisum sativum) gi:508844:gb:AAB18669; contains similarity to dirigent protein (Thuja plicata) gi:6694709:gb:AAF25365 | 3.3 | 8.0 | -4.7 | -8.7 | 0.06% | 7.6 |
| 1301 | AT3G23870.1 \| permease-related, low similarity to purine permease (Arabidopsis thaliana) GI:7620007; contains 9 predicted transmembrane domains; contains Pfam PF05653: Protein of unknown function (DUF803); identified as COG0697, Permeases of the drug/metabolite transporter (DMT) superfamily \| chr3:8619199-8621914 FORWARD \| Aliases: F14O13.5 | 3.5 | 5.5 | -2.0 | -8.7 | 0.06% | 8.2 |
| 1302 | AT2G24550.1 \| expressed protein, weak similarity to MTD1 (Medicago truncatula) GI:9294810 \| chr2:10434936-10436301 REVERSE \| Aliases: F25P17.15, F25P17_15 | 5.8 | 9.8 | -4.0 | -8.7 | 0.06% | 8.2 |
| 1303 | AT4G36610.1 \| hydrolase, alpha/beta fold family protein, low similarity to 2-hydroxy-6-oxo-7-methylocta-2,4-dienoate hydrolase (Pseudomonas fluorescens) GI:1871461; contains Pfam profile PF00561: hydrolase, alpha/beta fold family \| chr4:17265396-17267320 REVERSE \| Aliases: AP22.94, AP22_94 | 3.9 | 6.8 | -2.9 | -8.7 | 0.06% | 7.8 |
| 1304 | AT5G54840.2 \| GTP-binding family protein, similar to SP:P87027 Septum-promoting GTP-binding protein 1 (GTPase spg1)(Sid3 protein) {Schizosaccharomyces pombe} \| chr5:22293856-22295643 REVERSE \| Aliases: MBG8.10, MBG8_10 | 6.7 | 9.6 | -2.8 | -8.7 | 0.06% | 8.4 |
| 1305 | AT1G23040.1 \| hydroxyproline-rich glycoprotein family protein, contains proline-rich domains, INTERPRO:IPR000694 \| chr1:8164948-8165851 FORWARD \| Aliases: F19G10.19 | 6.3 | 8.5 | -2.2 | -8.7 | 0.06% | 8.0 |
| 1307 | AT1G67340.1 \| zinc finger (MYND type) family protein / F-box family protein \| chr1:25233928-25235503 FORWARD \| Aliases: F1N21.16 | 4.1 | 6.5 | -2.5 | -8.7 | 0.07% | 8.2 |
| 1308 | AT2G30040.1 \| Symbol: MAPKKK14 \| protein kinase family protein, contains protein kinase domain, Pfam:PF00069 \| chr2:12828787-12830246 FORWARD \| Aliases: F23F1.4, F23F1_4 | 4.9 | 7.5 | -2.6 | -8.7 | 0.07% | 7.9 |

| Rank | Description | Sync | Root | M | t | adj.q | B |
|---|---|---|---|---|---|---|---|
| 1309 | AT3G58710.2 \| Symbol: WRKY69 \| | 5.8 | 9.0 | -3.2 | -8.7 | 0.07% | 8.1 |
| 1313 | AT1G05575.1 \| expressed protein \| chr1:1661690-1662186 REVERSE \| Aliases: None | 2.4 | 6.6 | -4.2 | -8.7 | 0.07% | 8.2 |
| 1314 | AT5G62390.1 \| calmodulin-binding family protein, contains IQ calmodulin-binding motif, Pfam:PF00612 \| chr5:25069275-25071515 REVERSE \| Aliases: MMI9.22, MMI9_22 | 7.0 | 10.1 | -3.1 | -8.7 | 0.07% | 8.1 |
| 1315 | AT1G29280.1 \| Symbol: WRKY65 \| | 4.5 | 9.0 | -4.5 | -8.7 | 0.07% | 6.8 |
| 1317 | AT5G06570.2 \| expressed protein, similar to PrMC3 (Pinus radiata) GI:5487873 \| chr5:2007601-2011042 REVERSE \| Aliases: F15M7.10, F15M7_10 | 3.4 | 7.8 | -4.4 | -8.7 | 0.07% | 7.6 |
| 1318 | AT5G40390.1 \| raffinose synthase family protein, similar to galactinol-raffinose galactosyltransferase (Vigna angularis) GI:6634701, seed imbibition protein GB:AAA32975 GI:167100 from (Hordeum vulgare); contains Pfam profile PF05691: Raffinose synthase or seed imbibition protein Sip1 \| chr5:16178743-16182585 FORWARD \| Aliases: MPO12.100, MPO12_100 | 5.8 | 7.8 | -2.0 | -8.7 | 0.07% | 8.0 |
| 1319 | AT2G26760.1 \| Symbol: CYCB1;4 \| cyclin, putative, similar to CYCB1-1 protein (Petunia x hybrida) GI:6093215, B-type cyclin (Nicotiana tabacum) GI:849074; contains Pfam profiles PF00134: Cyclin, N-terminal domain, PF02984: Cyclin, C-terminal domain \| chr2:11408407-11410505 FORWARD \| Aliases: CYC3, CYCB1;4, CYCLIN 3, CYCLIN B1;4, F18A8.13, F18A8_13 | 3.2 | 6.3 | -3.1 | -8.7 | 0.07% | 8.1 |
| 1320 | AT5G01360.2 \| expressed protein, similar to expressed protein [Arabidopsis thaliana] (TAIR:At3g55990.1); similar to unknown protein [Oryza sativa (japonica cultivar-group)] (GB:AAV43889.1) \| chr5:147947-149366 REVERSE \| Aliases: T10O8.70, T10O8_70 | 3.6 | 7.8 | -4.2 | -8.7 | 0.07% | 7.0 |
| 1321 | AT2G01850.1 \| Symbol: EXGT A3 \| xyloglucan:xyloglucosyl transferase / xyloglucan endotransglycosylase / endo-xyloglucan transferase (EXGT-A3), identical to endoxyloglucan transferase (Arabidopsis thaliana) GI:5533313 \| chr2:385222-387343 FORWARD \| Aliases: ATXTH27, T23K3.4, T23K3_4, XTH27 | 6.9 | 10.2 | -3.3 | -8.6 | 0.07% | 8.1 |
| 1322 | AT5G44030.1 \| Symbol: CESA4 \| cellulose synthase, catalytic subunit (IRX5), nearly identical to cellulose synthase (Arabidopsis thaliana) GI:27462651; contains Pfam profile PF03552: Cellulose synthase \| chr5:17731872-17737318 FORWARD \| Aliases: CELLULASE SYNTHASE 4, IRREGULAR XYLEM5, IRX5, MRH10.14, MRH10_14 | 3.0 | 6.8 | -3.8 | -8.6 | 0.07% | 6.5 |
| 1323 | AT5G02420.1 \| expressed protein \| chr5:523387-524074 FORWARD \| Aliases: T22P11.10, T22P11_10 | 3.0 | 6.6 | -3.7 | -8.6 | 0.07% | 7.9 |
| 1328 | AT1G21310.1 \| Symbol: ATEXT3 \| | 4.3 | 11.0 | -6.7 | -8.6 | 0.07% | 6.4 |
| 1329 | AT5G61430.1 \| Symbol: ANAC100 \| no apical meristem (NAM) family protein, PF02365 : No apical meristem (NAM) protein; similar to cup-shaped cotyledon CUC2 (GI:1944132) (Arabidopsis thaliana) | 3.3 | 4.5 | -1.2 | -8.6 | 0.07% | 8.3 |
| 1330 | AT1G71090.1 \| auxin efflux carrier family protein, contains auxin efflux carrier domain, Pfam:PF03547 \| chr1:26815874-26817792 FORWARD \| Aliases: F23N20.8, F23N20_8 | 7.0 | 9.4 | -2.4 | -8.6 | 0.07% | 8.0 |
| 1332 | AT1G31050.1 \| expressed protein \| chr1:11075634-11076180 REVERSE \| Aliases: F17F8.3 | 5.7 | 9.9 | -4.2 | -8.6 | 0.07% | 7.1 |
| 1333 | AT4G22460.1 \| protease inhibitor/seed storage/lipid transfer protein (LTP) family protein, contains Pfam profile: PF00234 protease inhibitor/seed storage/LTP family \| chr4:11839172-11839573 REVERSE \| Aliases: F7K2.40, F7K2_40 | 3.0 | 4.8 | -1.9 | -8.6 | 0.07% | 8.2 |
| 1334 | AT5G15290.1 \| integral membrane family protein, contains TIGRFAM TIGR01569 : plant integral membrane protein TIGR01569; contains Pfam PF04535 : Domain of unknown function (DUF588) contains plant integral membrane protein domain, TIGR01569 | 2.4 | 5.8 | -3.4 | -8.6 | 0.07% | 6.8 |
| 1341 | AT5G43910.3 \| pfkB-type carbohydrate kinase family protein, contains Pfam profile: PF00294 pfkB family carbohydrate kinase \| chr5:17684634-17687466 REVERSE \| Aliases: F6B6.5, F6B6_5 | 4.3 | 6.9 | -2.6 | -8.6 | 0.07% | 7.5 |
| 1343 | AT5G11020.1 \| protein kinase family protein, contains protein kinase domain, Pfam:PF00069 \| chr5:3486440-3488381 REVERSE \| Aliases: None | 3.3 | 6.5 | -3.2 | -8.6 | 0.07% | 8.0 |
| 1345 | AT1G33800.1 \| expressed protein, contains Pfam profile PF04669: Protein of unknown function (DUF579) \| chr1:12261399-12262697 FORWARD \| Aliases: F14M2.8, F14M2_8 | 2.8 | 6.7 | -3.9 | -8.6 | 0.07% | 6.6 |
| 1346 | AT5G22850.1 \| aspartyl protease family protein, contains Pfam profile: PF00026 eukaryotic aspartyl protease \| chr5:7633440-7636570 REVERSE \| Aliases: MRN17.8, MRN17_8 | 7.1 | 8.5 | -1.4 | -8.6 | 0.07% | 8.3 |
| 1350 | AT5G13930.1 \| Symbol: CHS \| chalcone synthase / naringenin-chalcone synthase, identical to SP:P13114 \| chr5:4488692-4490266 FORWARD \| Aliases: ATCHS, CHALCONE SYNTHASE, CHALCONE/STILBENE SYNTHASE, MAC12.28, MAC12_28, NARINGENIN CHALCONE SYNTHASE, TESTA 4 PROTEIN, TRANSPARENT TESTA 4, TT4 | 6.0 | 9.4 | -3.4 | -8.6 | 0.07% | 7.9 |

| Rank | Description | Sync | Root | M | t | adj.q | B |
|---|---|---|---|---|---|---|---|
| 1352 | AT1G35350.1 \| similar to EXS family protein / ERD1/XPR1/SYG1 family protein [Arabidopsis thaliana] (TAIR:At2g03250.1); similar to EXS family protein / ERD1/XPR1/SYG1 family protein [Arabidopsis thaliana] (TAIR:At2g03240.1); similar to EXS family protein / ERD1/XPR1/SYG1 family protein [Arabidopsis thaliana] (TAIR:At1g14040.1); similar to EXS family protein / ERD1/XPR1/SYG1 family protein [Arabidopsis thaliana] (TAIR:At4g25350.1); similar to EXS family protein / ERD1/XPR1/SYG1 family protein [Arabidopsis thaliana] (TAIR:At1g26730.1); similar to putative xenotropic and polytropic murine retrovirus receptor [Oryza sativa (japonica cultivar-group)] (GB:BAD46522.1); contains InterPro domain SPX, N-terminal (InterPro:IPR004331); contains InterPro domain EXS, C-terminal (InterPro:IPR004342) \| chr1:12981229-12984561 REVERSE \| Aliases: T9I1.12, T9I1_12 | 3.8 | 7.5 | -3.7 | -8.6 | 0.07% | 7.2 |
| 1356 | AT4G14130.1 \| Symbol: XTR7 \| xyloglucan:xyloglucosyl transferase, putative / xyloglucan endotransglycosylase, putative / endo-xyloglucan transferase, putative (XTR7), almost identical to xyloglucan endotransglycosylase-related protein XTR7 GI:1244760 from (Arabidopsis thaliana), one amino acid difference \| chr4:8137047-8138276 REVERSE \| Aliases: DL3105C, FCAALL.173, XYLOGLUCAN ENDOTRANSGLYCOSYLASE 7 | 5.9 | 9.4 | -3.5 | -8.6 | 0.07% | 8.1 |
| 1357 | AT1G69325.1 \| remorin family protein, contains Pfam domain, PF03763: Remorin, C-terminal region \| chr1:26066868-26067399 FORWARD \| Aliases: None | 3.0 | 8.7 | -5.7 | -8.6 | 0.07% | 6.6 |
| 1358 | AT3G51550.1 \| protein kinase family protein, contains protein kinase domain, Pfam:PF00069 \| chr3:19128563-19131840 REVERSE \| Aliases: F26O13.190 | 5.2 | 8.0 | -2.8 | -8.6 | 0.07% | 8.0 |
| 1361 | AT3G01690.1 \| expressed protein \| chr3:256562-258554 FORWARD \| Aliases: F4P13.23, F4P13_23 | 9.9 | 11.6 | -1.6 | -8.6 | 0.07% | 8.2 |
| 1362 | AT1G25400.1 \| expressed protein, similar to unknown protein GI:6714347 from (Arabidopsis thaliana) \| chr1:8911117-8912385 REVERSE \| Aliases: F2J7.13, F2J7_13 | 4.4 | 6.7 | -2.2 | -8.6 | 0.07% | 7.9 |
| 1363 | AT2G42350.1 \| zinc finger (C3HC4-type RING finger) family protein, contains Pfam domain, PF00097: Zinc finger, C3HC4 type (RING finger) \| chr2:17646323-17646976 FORWARD \| Aliases: MHK10.7, MHK10_7 | 4.1 | 8.4 | -4.3 | -8.6 | 0.07% | 6.0 |
| 1364 | AT3G49350.1 \| RabGAP/TBC domain-containing protein, similar to GTPase activating protein (Yarrowia lipolytica) GI:2370595; contains Pfam profile PF00566: TBC domain \| chr3:18308178-18311174 REVERSE \| Aliases: F2K15.210 | 5.7 | 7.7 | -2.0 | -8.5 | 0.07% | 7.9 |
| 1365 | AT4G22110.2 \| alcohol dehydrogenase, putative, similar to alcohol dehydrogenase ADH GI:7705214 from (Lycopersicon esculentum); contains Pfam zinc-binding dehydrogenase domain PF00107 | 3.5 | 6.2 | -2.7 | -8.5 | 0.07% | 7.6 |
| 1366 | AT4G26080.1 \| Symbol: ABI1 \| protein phosphatase 2C ABI1 / PP2C ABI1 / abscisic acid-insensitive 1 (ABI1), nearly identical to SP:P49597 Protein phosphatase 2C ABI1 (EC 3.1.3.16) (PP2C) (Abscisic acid- insensitive 1) {Arabidopsis thaliana} \| chr4:13219970-13222293 REVERSE \| Aliases: ABA INSENSITIVE 1, ABSCISIC ACID INSENSITIVE 1, F20B18.190, F20B18_190, PROTEIN PHOSPHATASE 2C ABI1 | 6.7 | 9.5 | -2.8 | -8.5 | 0.07% | 8.2 |
| 1369 | AT4G29500.1 \| prolidase-related, contains similarity to prolidase GI:3114966 from (Suberites domuncula) \| chr4:14490217-14491495 FORWARD \| Aliases: T16L4.10, T16L4_10 | 7.8 | 9.4 | -1.6 | -8.5 | 0.07% | 8.2 |
| 1371 | AT2G41970.1 \| protein kinase, putative, similar to Pto kinase interactor 1 (serine/threonine protein kinase) (Lycopersicon esculentum) gi:3668069:gb:AAC61805 \| chr2:17527595-17529722 REVERSE \| Aliases: T6D20.14, T6D20_14 | 2.5 | 5.9 | -3.3 | -8.5 | 0.07% | 7.2 |
| 1372 | AT2G37460.1 \| nodulin MtN21 family protein, similar to MtN21 GI:2598575 (root nodule development) from (Medicago truncatula) \| chr2:15733527-15736176 REVERSE \| Aliases: F3G5.25, F3G5_25 | 4.2 | 7.2 | -2.9 | -8.5 | 0.07% | 7.2 |
| 1374 | AT4G31875.1 \| expressed protein \| chr4:15411707-15412295 REVERSE \| Aliases: None | 3.3 | 5.9 | -2.6 | -8.5 | 0.07% | 7.5 |
| 1375 | AT1G72160.1 \| SEC14 cytosolic factor family protein / phosphoglyceride transfer family protein, similar to GI:807956 from (Saccharomyces cerevisiae)similar to polyphosphoinositide binding protein Ssh2p (GI:2739046) {Glycine max}; contains Pfam PF00650 : CRAL/TRIO domain; contains Pfam PF03765 : CRAL/TRIO, N-terminus \| chr1:27157243-27159326 REVERSE \| Aliases: T9N14.8, T9N14_8 | 6.5 | 9.9 | -3.5 | -8.5 | 0.07% | 7.6 |
| 1378 | AT5G04470.1 \| expressed protein \| chr5:1266692-1267417 REVERSE \| Aliases: T19N18.8 | 6.7 | 8.9 | -2.2 | -8.5 | 0.07% | 8.1 |
| 1380 | AT3G59700.1 \| Symbol: ATHLECRK \| lectin protein kinase, putative, similar to receptor lectin kinase 3 (Arabidopsis thaliana) gi:4100060:gb:AAD00733 \| chr3:22063110-22065252 FORWARD \| Aliases: ARABIDOPSIS THALIANA LECTIN RECEPTOR KINASE, LECRK1, T16L24.250 | 3.5 | 6.8 | -3.2 | -8.5 | 0.07% | 7.8 |
| 1381 | AT3G26810.1 \| transport inhibitor response protein, putative, E3 ubiquitin ligase SCF complex F-box subunit; similar to transport inhibitor response 1 GI:2352492 from (Arabidopsis thaliana) \| chr3:9869082-9871877 FORWARD \| Aliases: MDJ14.9 | 3.2 | 4.7 | -1.5 | -8.5 | 0.07% | 8.2 |
| 1382 | AT3G06500.1 \| beta-fructofuranosidase, putative / invertase, putative / saccharase, putative / beta-fructosidase, putative, similar to neutral invertase (Daucus carota) GI:4200165; contains Pfam profile PF04853: Plant neutral invertase \| chr3:2012085-2015711 FORWARD \| Aliases: F5E6.17, F5E6_17 | 6.2 | 8.0 | -1.9 | -8.5 | 0.07% | 8.2 |

| Rank | Description | Sync | Root | M | t | adj.q | B |
|---|---|---|---|---|---|---|---|
| 1383 | AT5G18630.3 \| lipase class 3 family protein, low similarity to Triacylglycerol Acylhydrolase (E.C.3.1.1.3) (Rhizomucor miehei) GI:230348; contains Pfam profile PF01764: Lipase \| chr5:6202635-6205108 FORWARD \| Aliases: T1A4.10, T1A4_10 | 4.9 | 7.2 | -2.4 | -8.5 | 0.07% | 7.9 |
| 1387 | AT1G02340.1 \| Symbol: HFR1 \| long hypocotyl in far-red 1 (HFR1) / reduced phytochrome signaling (REP1) / basic helix-loop-helix FBI1 protein (FBI1) / reduced sensitivity to far-red light (RSF1) / bHLH protein 26 (BHLH026) (BHLH26), identical to SP:Q9FE22 Long hypocotyl in far-red 1 (bHLH-like protein HFR1) (Reduced phytochrome signaling) (Basic helix-loop-helix FBI1 protein) (Reduced sensitivity to far-red light) (Arabidopsis thaliana) \| chr1:465718-467844 REVERSE \| Aliases: FBI1, LONG HYPOCOTYL IN FAR RED, REDUCED PHYTOCHROME SIGNALING 1, REDUCED SENSITIVITY TO FAR RED LIGHT 1, REP1, RSF1, T6A9.4, T6A9_4 | 2.7 | 4.8 | -2.1 | -8.5 | 0.07% | 7.8 |
| 1388 | AT5G43180.1 \| expressed protein, contains Pfam profile PF04654: Protein of unknown function, DUF599 \| chr5:17353566-17355080 REVERSE \| Aliases: K24F5.2, K24F5_2 | 4.6 | 8.2 | -3.6 | -8.5 | 0.07% | 7.4 |
| 1391 | AT1G01660.1 \| U-box domain-containing protein \| chr1:240057-242608 REVERSE \| Aliases: T1N6.4, T1N6_4 | 2.8 | 4.9 | -2.0 | -8.5 | 0.07% | 7.9 |
| 1392 | AT5G52060.1 \| BAG domain-containing protein, contains Pfam:PF02179 BAG domain \| chr5:21169454-21170919 REVERSE \| Aliases: MSG15.15, MSG15_15 | 7.1 | 9.8 | -2.7 | -8.5 | 0.07% | 7.9 |
| 1395 | AT3G15430.2 \| regulator of chromosome condensation (RCC1) family protein, low similarity to UVB-resistance protein UVR8 (Arabidopsis thaliana) GI:5478530; contains Pfam profile PF00415: Regulator of chromosome condensation (RCC1) \| chr3:5208766-5211752 FORWARD \| Aliases: MJK13.9 | 6.7 | 8.5 | -1.8 | -8.5 | 0.07% | 8.1 |
| 1396 | AT4G26140.2 \| Symbol: BGAL12 \| | 2.6 | 5.7 | -3.1 | -8.5 | 0.07% | 7.3 |
| 1397 | AT3G08650.2 \| metal transporter family protein, contains ZIP Zinc transporter domain, Pfam:PF02535 \| chr3:2624467-2627376 REVERSE \| Aliases: F17O14.12 | 8.0 | 9.9 | -1.9 | -8.5 | 0.07% | 8.2 |
| 1399 | AT4G30350.1 \| heat shock protein-related, contains similarity to heat shock protein 101 (Triticum aestivum) gi:6013196:gb:AAF01280 \| chr4:14847746-14851149 FORWARD \| Aliases: F17I23.310, F17I23_310 | 5.2 | 9.4 | -4.2 | -8.5 | 0.07% | 7.5 |
| 1400 | AT5G40510.1 \| expressed protein \| chr5:16246416-16248326 FORWARD \| Aliases: MNF13.5, MNF13_5 | 3.7 | 6.9 | -3.2 | -8.5 | 0.07% | 7.5 |
| 1401 | AT2G21850.1 \| similar to CHP-rich zinc finger protein, putative [Arabidopsis thaliana] (TAIR:At2g21840.1); similar to DC1 domain-containing protein [Arabidopsis thaliana] (TAIR:At2g21830.1); similar to CHP-rich zinc finger protein-like [Oryza sativa (japonica cultivar-group)] (GB:XP_479125.1); contains InterPro domain Zn-finger-like, PHD finger (InterPro:IPR001965); contains InterPro domain Protein kinase C, phorbol ester/diacylglycerol binding (InterPro:IPR002219); contains InterPro domain DC1 domain (InterPro:IPR004146) \| chr2:9322298-9325318 FORWARD \| Aliases: F7D8.17, F7D8_17 | 2.9 | 6.2 | -3.3 | -8.5 | 0.07% | 6.8 |
| 1404 | AT4G36790.1 \| transporter-related, low similarity to spinster membrane proteins from (Drosophila melanogaster) GI:12003976, GI:12003972, GI:12003974, GI:12003970; contains Pfam profile PF00083: major facilitator superfamily protein \| chr4:17336177-17338376 FORWARD \| Aliases: AP22.91, AP22_91 | 6.0 | 7.4 | -1.4 | -8.5 | 0.08% | 8.2 |
| 1408 | AT3G27110.2 \| peptidase M48 family protein, contains Pfam domain, PF01435: Peptidase family M48 \| chr3:9999133-10001412 FORWARD \| Aliases: MOJ10.19 | 4.1 | 7.1 | -3.0 | -8.4 | 0.08% | 7.2 |
| 1409 | AT5G03730.2 \| Symbol: CTR1 \| serine/threonine protein kinase (CTR1), identical to serine/threonine-protein kinase CTR1 (Arabidopsis thaliana) SWISS-PROT:Q05609 \| chr5:974507-979848 REVERSE \| Aliases: CONSTITUTIVE TRIPLE RESPONSE 1, F17C15.150, F17C15_150, SERINE/THREONINE PROTEIN KINASE CTR1, SIS1 | 5.3 | 6.8 | -1.5 | -8.4 | 0.08% | 8.1 |
| 1410 | AT1G02260.1 \| transmembrane protein, putative, similar to P protein (Melanocyte-specific transporter protein) (SP:Q04671) {Homo sapiens}; contains 8 transmembrane domains \| chr1:440441-442877 REVERSE \| Aliases: T6A9.21 | 4.5 | 7.9 | -3.4 | -8.4 | 0.08% | 7.4 |
| 1414 | AT1G80570.3 \| F-box family protein (FBL14), contains similarity to F-box protein FBL2 GI:6063090 from (Homo sapiens) \| chr1:30295255-30297310 FORWARD \| Aliases: T21F11.10, T21F11_10 | 3.7 | 6.3 | -2.6 | -8.4 | 0.08% | 7.4 |
| 1415 | AT1G43790.1 \| expressed protein \| chr1:16578335-16578857 FORWARD \| Aliases: F28H19.19, F28H19_19 | 4.8 | 8.1 | -3.3 | -8.4 | 0.08% | 7.2 |
| 1417 | AT3G16350.1 \| myb family transcription factor, ; contains Pfam profile: PF00249 Myb-like DNA-binding domain \| chr3:5547724-5549666 FORWARD \| Aliases: T2O4.10 | 5.2 | 7.1 | -1.9 | -8.4 | 0.08% | 8.0 |
| 1419 | AT2G46690.1 \| auxin-responsive family protein, similar to indole-3-acetic acid induced protein ARG7 (SP:P32295) (Phaseolus aureus) \| chr2:19187800-19188537 FORWARD \| Aliases: T3A4.7 | 8.6 | 10.5 | -1.9 | -8.4 | 0.08% | 8.1 |
| 1422 | AT1G79320.1 \| latex abundant protein, putative (AMC5) / caspase family protein, similar to latex-abundant protein (Hevea brasiliensis) gb:AAD13216; contains Pfam domain, PF00656: ICE-like protease (caspase) p20 domain \| chr1:29841579-29842801 FORWARD \| Aliases: YUP8H12R.6, YUP8H12R_6 | 3.3 | 6.5 | -3.2 | -8.4 | 0.08% | 6.6 |

| Rank | Description | Sync | Root | M | t | adj.q | B |
|---|---|---|---|---|---|---|---|
| 1428 | AT3G44190.1 \| pyridine nucleotide-disulphide oxidoreductase family protein, low similarity to dihydrolipoamide dehydrogenase from Clostridium magnum (GI:472330); contains Pfam profile PF00070 Pyridine nucleotide-disulphide oxidoreductase domain \| chr3:15912784-15914595 REVERSE \| Aliases: F26G5.140 | 7.4 | 9.8 | -2.4 | -8.4 | 0.08% | 8.0 |
| 1432 | AT4G25390.2 \| protein kinase family protein, contains protein kinase domain, Pfam:PF00069 \| chr4:12977351-12979578 FORWARD \| Aliases: T30C3.60, T30C3_60 | 5.2 | 8.3 | -3.0 | -8.4 | 0.08% | 7.8 |
| 1433 | AT3G46130.2 \| Symbol: MYB111 \| | 7.3 | 9.8 | -2.5 | -8.4 | 0.08% | 7.8 |
| 1434 | AT5G43560.2 \| meprin and TRAF homology domain-containing protein / MATH domain-containing protein, weak similarity to ubiquitin-specific protease 12 (Arabidopsis thaliana) GI:11993471; contains Pfam profile PF00917: MATH domain \| chr5:17517656-17522873 FORWARD \| Aliases: K9D7.6, K9D7_6 | 8.3 | 9.9 | -1.6 | -8.4 | 0.08% | 8.1 |
| 1435 | AT5G09520.1 \| hydroxyproline-rich glycoprotein family protein, contains proline-rich extensin domains, INTERPRO:IPR002965 \| chr5:2958293-2958857 REVERSE \| Aliases: T5E8.1 | 2.8 | 4.6 | -1.8 | -8.4 | 0.08% | 7.8 |
| 1436 | AT5G22270.1 \| expressed protein, similar to unknown protein (gb AAF02129.1) \| chr5:7372236-7373105 REVERSE \| Aliases: T6G21.5 | 4.2 | 9.8 | -5.6 | -8.4 | 0.08% | 7.6 |
| 1437 | AT5G48430.1 \| expressed protein, low similarity to extracellular dermal glycoprotein EDGP precursor (Daucus carota) GI:285741, SP:P13917 Basic 7S globulin precursor {Glycine max}; expression supported by MPSS \| chr5:19645118-19646338 REVERSE \| Aliases: MJE7.6, MJE7_6 | 3.8 | 8.7 | -5.0 | -8.4 | 0.08% | 7.5 |
| 1440 | AT1G02220.1 \| Symbol: ANAC003 \| no apical meristem (NAM) family protein, similar to NAC domain protein NAC2 (GI:15148914) {Phaseolus vulgaris}; similar to NAC domain protein NAC2 (GI:21554255) {Arabidopsis thaliana}; contains Pfam PF02365 : No apical meristem (NAM) protein \| chr1:428902-430567 REVERSE \| Aliases: ANAC003, T6A9.17 | 3.1 | 7.0 | -3.9 | -8.4 | 0.08% | 7.5 |
| 1441 | AT4G24480.1 \| serine/threonine protein kinase, putative, similar to serine/threonine-protein kinase CTR1 (Arabidopsis thaliana) SWISS-PROT:Q05609 \| chr4:12649997-12654994 FORWARD \| Aliases: T22A6.310, T22A6_310 | 4.6 | 6.5 | -1.9 | -8.4 | 0.08% | 8.0 |
| 1444 | AT3G13720.1 \| prenylated rab acceptor (PRA1) family protein, contains Pfam profile PF03208: Prenylated rab acceptor (PRA1) \| chr3:4495026-4495963 REVERSE \| Aliases: MMM17.14 | 3.7 | 6.3 | -2.7 | -8.4 | 0.08% | 7.3 |
| 1446 | AT3G06390.1 \| integral membrane family protein, similar to hypothetical protein GB:CAB10339 (Arabidopsis thaliana); contains TIGRFAM TIGR01569 : plant integral membrane protein TIGR01569; contains Pfam PF04535 : Domain of unknown function (DUF588) \| chr3:1938738-1939785 REVERSE \| Aliases: F24P17.14, F24P17_14 | 3.8 | 7.1 | -3.3 | -8.3 | 0.08% | 6.8 |
| 1450 | AT5G51060.1 \| Symbol: RHD2 \| respiratory burst oxidase protein C (RbohC) / NADPH oxidase, nearly identical to respiratory burst oxidase protein C from Arabidopsis thaliana (gi:3242785) \| chr5:20774510-20779657 REVERSE \| Aliases: A. THALIANA RESPIRATORY BURST OXIDASE HOMOLOG C, ATRBOHC, K3K7.25, K3K7_25, RBOHC, ROOT HAIR DEFECTIVE 2 | 5.7 | 8.8 | -3.1 | -8.3 | 0.08% | 7.7 |
| 1451 | AT3G60070.1 \| lactose permease-related, contains weak similarity to Lactose permease (Lactose-proton symport) (Lactose transport protein). (Swiss-Prot:O33814) (Staphylococcus xylosus) \| chr3:22194220-22197305 REVERSE \| Aliases: T2O9.50 | 7.6 | 8.8 | -1.2 | -8.3 | 0.08% | 8.1 |
| 1452 | AT4G05150.1 \| octicosapeptide/Phox/Bem1p (PB1) domain-containing protein, various predicted proteins contains Pfam profile PF00564: PB1 domain | 5.6 | 8.8 | -3.2 | -8.3 | 0.08% | 7.6 |
| 1457 | AT2G35930.1 \| U-box domain-containing protein, similar to immediate-early fungal elicitor protein CMPG1 (Petroselinum crispum) GI:14582200; contains Pfam profile PF04564: U-box domain \| chr2:15089934-15091552 REVERSE \| Aliases: F11F19.16, F11F19_16 | 5.5 | 8.7 | -3.2 | -8.3 | 0.08% | 7.7 |
| 1459 | AT3G28550.1 \| proline-rich extensin-like family protein, contains proline-rich extensin domains, INTERPRO:IPR002965 \| chr3:10701944-10705166 REVERSE \| Aliases: MZN14.2 | 2.7 | 8.7 | -6.0 | -8.3 | 0.08% | 8.0 |
| 1464 | AT1G56720.2 \| protein kinase family protein, contains protein kinase domain, Pfam:PF00069 \| chr1:21267093-21270033 REVERSE \| Aliases: F25P12.84, F25P12_84 | 3.6 | 6.0 | -2.4 | -8.3 | 0.08% | 7.9 |
| 1465 | AT5G15440.1 \| circadian clock coupling factor-related, similar to circadian clock coupling factor ZGT (Nicotiana tabacum) GI:14210079 \| chr5:5012073-5014649 FORWARD \| Aliases: T20K14.50, T20K14_50 | 6.4 | 7.5 | -1.1 | -8.3 | 0.08% | 8.0 |
| 1468 | AT4G33790.1 \| acyl CoA reductase, putative, similar to acyl CoA reductase (Simmondsia chinensis) GI:5020215; contains Pfam profile PF03015: Male sterility protein; No start codon identified, may contain anomalous splicing at 5' end. \| chr4:16204159-16207947 REVERSE \| Aliases: T16L1.280 | 2.8 | 5.5 | -2.8 | -8.3 | 0.08% | 7.4 |
| 1469 | AT3G56360.1 \| expressed protein, unknown protein 110K5.12 - Sorghum bicolor, TREMBL:AF124045_5 | 9.1 | 11.4 | -2.2 | -8.3 | 0.08% | 7.8 |

| Rank | Description | Sync | Root | M | t | adj.q | B |
|---|---|---|---|---|---|---|---|
| 1475 | AT5G26820.1 \| ferroportin-related, low similarity to ferroportin1 (Danio rerio) GI:7109245 \| chr5:9433622-9437103 FORWARD \| Aliases: F2P16.6, F2P16_6 | 7.8 | 9.3 | -1.5 | -8.3 | 0.08% | 8.0 |
| 1477 | AT4G35600.1 \| Symbol: CONNEXIN 32 \| protein kinase family protein, contains protein kinase domain, Pfam:PF00069 \| chr4:16896242-16898881 FORWARD \| Aliases: F8D20.110, F8D20_110 | 4.8 | 7.2 | -2.4 | -8.3 | 0.08% | 7.9 |
| 1483 | AT3G47210.1 \| expressed protein, contains Pfam profile PF03140: Plant protein of unknown function \| chr3:17396298-17398229 FORWARD \| Aliases: F13I12.260 | 5.1 | 7.4 | -2.2 | -8.3 | 0.08% | 7.5 |
| 1484 | AT3G16030.1 \| lectin protein kinase family protein, contains Pfam domains PF00069: Protein kinase domain and PF01453: Lectin (probable mannose binding) \| chr3:5439615-5442808 FORWARD \| Aliases: MSL1.2 | 2.6 | 4.3 | -1.7 | -8.3 | 0.08% | 7.9 |
| 1492 | AT3G58990.1 \| aconitase C-terminal domain-containing protein, contains Pfam profile PF00694: Aconitase C-terminal domain \| chr3:21808035-21809280 REVERSE \| Aliases: F17J16.40 | 3.9 | 6.9 | -3.0 | -8.3 | 0.08% | 6.8 |
| 1493 | AT2G19130.1 \| S-locus lectin protein kinase family protein, contains Pfam domains PF00954: S-locus glycoprotein family, PF00069: Protein kinase domain and PF01453: Lectin (probable mannose binding) \| chr2:8300815-8303357 FORWARD \| Aliases: T20K24.15, T20K24_15 | 5.2 | 8.4 | -3.2 | -8.3 | 0.08% | 7.5 |
| 1494 | AT5G20050.1 \| protein kinase family protein, contains protein kinase domain, Pfam:PF00069 \| chr5:6774304-6775847 FORWARD \| Aliases: F28I16.200, F28I16_200 | 7.6 | 9.9 | -2.4 | -8.3 | 0.08% | 8.0 |
| 1496 | AT4G36970.1 \| remorin family protein, contains Pfam domain, PF03763: Remorin, C-terminal region \| chr4:17429739-17431680 REVERSE \| Aliases: AP22.30, AP22_30 | 5.5 | 7.8 | -2.3 | -8.2 | 0.08% | 7.8 |
| 1498 | AT2G21550.1 \| bifunctional dihydrofolate reductase-thymidylate synthase, putative / DHFR-TS, putative, similar to THY-1 (SP: Q05762) and THY-2 (SP:Q05763) from Arabidopsis thaliana; contains Pfam profiles PF00303 thymidylate synthase and PF00186 dihydrofolate reductase \| chr2:9234289-9237269 FORWARD \| Aliases: F2G1.18, F2G1_18 | 3.6 | 6.0 | -2.4 | -8.2 | 0.09% | 7.5 |
| 1499 | AT1G68150.1 \| Symbol: WRKY9 \| WRKY family transcription factor, similar to DNA-binding protein ABF2 GI:1159879 from (Avena fatua) \| chr1:25547633-25549380 FORWARD \| Aliases: ATWRKY9, T22E19.22, T22E19_22, WRKY9 | 3.1 | 7.2 | -4.1 | -8.2 | 0.09% | 7.8 |
| 1501 | AT5G05400.1 \| disease resistance protein (CC-NBS-LRR class), putative, domain signature CC-NBS-LRR exists, suggestive of a disease resistance protein. | 3.4 | 5.8 | -2.5 | -8.2 | 0.09% | 7.2 |
| 1502 | AT2G27740.1 \| expressed protein, contains Pfam profile PF04949: Family of unknown function (DUF662) \| chr2:11829855-11830808 FORWARD \| Aliases: F15K20.16, F15K20_16 | 3.6 | 6.1 | -2.5 | -8.2 | 0.09% | 7.7 |
| 1504 | AT3G29780.1 \| Symbol: RALFL27 \| expressed protein \| chr3:11674220-11674668 FORWARD \| Aliases: RALF LIKE 27, T26G12.18 | 3.0 | 7.1 | -4.1 | -8.2 | 0.09% | 6.1 |
| 1506 | AT1G18160.1 \| protein kinase family protein, contains Pfam domain, PF00069: Protein kinase domain \| chr1:6248786-6254032 FORWARD \| Aliases: T10F20.16 | 7.0 | 9.1 | -2.2 | -8.2 | 0.09% | 7.7 |
| 1507 | AT5G13580.1 \| ABC transporter family protein \| chr5:4370695-4373204 FORWARD \| Aliases: T6I14.7 | 3.5 | 5.6 | -2.1 | -8.2 | 0.09% | 7.6 |
| 1509 | AT3G60130.2 \| similar to glycosyl hydrolase family 1 protein [Arabidopsis thaliana] (TAIR:At5g44640.1); similar to glycosyl hydrolase family 1 protein [Arabidopsis thaliana] (TAIR:At2g44450.1); similar to glycosyl hydrolase family 1 protein [Arabidopsis thaliana] (TAIR:At5g42260.1); similar to putative prunasin hydrolase precursor [Prunus serotina] (GB:AAL07490.1); similar to putative prunasin hydrolase isoform PH-L1 precursor [Prunus serotina] (GB:AAF34651.2); similar to prunasin hydrolase isoform PH B precursor [Prunus serotina] (GB:AAL39079.1); similar to prunasin hydrolase isoform PH B precursor [Prunus serotina] (GB:AAL06338.1); contains InterPro domain Glycoside hydrolase, family 1 (InterPro:IPR001360) \| chr3:22221409-22224792 FORWARD \| Aliases: T2O9.110 | 3.7 | 6.8 | -3.1 | -8.2 | 0.09% | 7.1 |
| 1510 | AT3G56170.1 \| Symbol: CAN \| Ca(2+)-dependent nuclease, identical to Ca(2+)-dependent nuclease (Arabidopsis thaliana) GI:7684292; supporting cDNA gi:7684291:dbj:D84226.1: \| chr3:20853556-20855437 FORWARD \| Aliases: CA 2+ DEPENDENT NUCLEASE, F18O21.130, F18O21_130 | 5.4 | 8.3 | -2.9 | -8.2 | 0.09% | 7.7 |
| 1513 | AT1G74790.1 \| expressed protein, contains similarity to hedgehog-interacting protein GI:4868122 from (Mus musculus) \| chr1:28102471-28105557 FORWARD \| Aliases: F25A4.24, F25A4_24 | 5.3 | 7.2 | -1.9 | -8.2 | 0.09% | 7.7 |
| 1515 | AT4G19690.2 \| Symbol: IRT1 \| similar to metal transporter, putative (ZIP8) [Arabidopsis thaliana] (TAIR:At5g45105.1); similar to iron-regulated transporter 1 [Lycopersicon esculentum] (GB:AAD30548.1); contains InterPro domain ZIP zinc/iron transport (InterPro:IPR004698); contains InterPro domain Zinc transporter ZIP (InterPro:IPR003689) \| chr4:10707456-10709025 FORWARD \| Aliases: T16H5.50, T16H5_50 | 2.7 | 7.0 | -4.3 | -8.2 | 0.09% | 6.2 |

| Rank | Description | Sync | Root | M | t | adj.q | B |
|---|---|---|---|---|---|---|---|
| 1516 | AT1G50420.1 \| Symbol: SCL3 \| scarecrow-like transcription factor 3 (SCL3), identical to GB:AAD24404 GI:4580515 from (Arabidopsis thaliana) (Plant J. 18 (1), 111-119 (1999)) \| chr1:18681598-18683740 REVERSE \| Aliases: F11F12.22, F11F12_22 | 5.8 | 8.0 | -2.2 | -8.2 | 0.09% | 7.8 |
| 1517 | AT2G01670.1 \| MutT/nudix family protein, similar to SP:Q09790 Diadenosine 5',5'''-P1,P6-hexaphosphate hydrolase (EC 3.6.1.-) (Ap6A hydrolase) {Schizosaccharomyces pombe}; contains Pfam profile PF00293: NUDIX domain | 4.4 | 7.5 | -3.2 | -8.2 | 0.09% | 7.3 |
| 1520 | AT4G25560.1 \| myb family transcription factor (MYB18), contains PFAM profile: Myb DNA binding domain PF00249 \| chr4:13052564-13053627 FORWARD \| Aliases: M7J2.70, M7J2_70 | 3.1 | 4.7 | -1.6 | -8.2 | 0.09% | 7.9 |
| 1524 | AT4G00360.1 \| Symbol: CYP86A2 \| cytochrome P450, putative \| chr4:160768-163002 FORWARD \| Aliases: A_IG005I10.21, A_IG005I10_21, F5I10.21, F5I10_21 | 4.0 | 6.9 | -2.9 | -8.2 | 0.09% | 7.4 |
| 1525 | AT5G05410.2 \| Symbol: DREB2A \| | 6.5 | 9.9 | -3.5 | -8.2 | 0.09% | 7.5 |
| 1526 | AT3G56930.1 \| zinc finger (DHHC type) family protein, low similarity to Golgi-specific DHHC zinc figer protein (Mus musculus) GI:21728103; contains Pfam profile PF01529: DHHC zinc finger domain \| chr3:21084474-21087293 FORWARD \| Aliases: T8M16.5 | 3.2 | 5.2 | -2.0 | -8.2 | 0.09% | 7.6 |
| 1527 | AT5G14690.1 \| expressed protein, predicted protein, Arabidopsis thaliana \| chr5:4733802-4734550 REVERSE \| Aliases: T9L3.3 | 3.2 | 6.0 | -2.8 | -8.2 | 0.09% | 7.5 |
| 1528 | AT2G35000.1 \| zinc finger (C3HC4-type RING finger) family protein, contains Pfam profile: PF00097: Zinc finger, C3HC4 type (RING finger) \| chr2:14758653-14760398 REVERSE \| Aliases: F19I3.23, F19I3_23 | 2.4 | 6.0 | -3.6 | -8.2 | 0.09% | 7.2 |
| 1529 | AT3G04520.1 \| threonine aldolase family protein, similar to L-allo-threonine aldolase SP:O07051 from (Aeromonas jandaei) \| chr3:1217248-1219765 REVERSE \| Aliases: None | 6.6 | 8.3 | -1.7 | -8.2 | 0.09% | 7.8 |
| 1530 | AT3G26890.3 \| expressed protein, similar to expressed protein [Arabidopsis thaliana] (TAIR:At5g41110.1); similar to unknown protein [Oryza sativa (japonica cultivar-group)] (GB:XP_483533.1) \| chr3:9908499-9912853 REVERSE \| Aliases: MDJ14.23 | 8.7 | 9.5 | -0.8 | -8.2 | 0.09% | 7.8 |
| 1534 | AT5G18490.1 \| expressed protein \| chr5:6134154-6136767 REVERSE \| Aliases: T28N17.2 | 4.5 | 6.7 | -2.1 | -8.2 | 0.09% | 7.5 |
| 1535 | AT5G16250.1 \| expressed protein \| chr5:5310100-5310954 REVERSE \| Aliases: T21H19.170, T21H19_170 | 3.9 | 6.0 | -2.1 | -8.2 | 0.09% | 7.5 |
| 1537 | AT3G63200.1 \| patatin-related, low similarity to patatin-like latex protein allergen Hev b 7 - Hevea brasiliensis, EMBL:AF113546; contains patatin domain PF01734 \| chr3:23356877-23358539 FORWARD \| Aliases: F16M2.50 | 5.4 | 8.6 | -3.2 | -8.1 | 0.09% | 7.5 |
| 1538 | AT2G17450.1 \| Symbol: RHA3A \| zinc finger (C3HC4-type RING finger) family protein, contains Pfam domain, PF00097: Zinc finger, C3HC4 type (RING finger) \| chr2:7583556-7584382 REVERSE \| Aliases: F5J6.22, RING H2 FINGER PROTEIN RHA3A | 6.8 | 8.6 | -1.8 | -8.1 | 0.09% | 7.8 |
| 1540 | AT5G62720.2 \| integral membrane HPP family protein, contains Pfam domain, PF04982: HPP family \| chr5:25209086-25212181 FORWARD \| Aliases: MQB2.4, MQB2_4 | 7.3 | 9.4 | -2.1 | -8.1 | 0.09% | 7.6 |
| 1541 | AT1G80310.1 \| expressed protein \| chr1:30199510-30201446 FORWARD \| Aliases: F5I6.6, F5I6_6 | 6.9 | 8.9 | -2.0 | -8.1 | 0.09% | 7.8 |
| 1542 | AT4G10390.1 \| protein kinase family protein, contains protein kinase domain, Pfam:PF00069 \| chr4:6441689-6443385 REVERSE \| Aliases: F7L13.4 | 4.2 | 6.3 | -2.1 | -8.1 | 0.09% | 7.6 |
| 1544 | AT3G14940.1 \| Symbol: ATPPC3 \| | 7.4 | 11.2 | -3.8 | -8.1 | 0.09% | 7.7 |
| 1547 | AT2G27050.1 \| Symbol: EIL1 \| ethylene-insensitive3-like1 (EIL1), identical to ethylene-insensitive3-like1 GI:2224927 from (Arabidopsis thaliana) \| chr2:11552873-11555371 FORWARD \| Aliases: ETHYLENE INSENSITIVE3 LIKE 1, T20P8.10, T20P8_10 | 6.0 | 8.0 | -2.0 | -8.1 | 0.09% | 7.8 |
| 1549 | AT3G09540.1 \| pectate lyase family protein, simliar to style development-specific protein 9612 SP:P24396 from (Lycopersicon esculentum) \| chr3:2928875-2931234 REVERSE \| Aliases: F11F8.12 | 3.8 | 6.0 | -2.2 | -8.1 | 0.09% | 7.5 |
| 1554 | AT3G46620.1 \| zinc finger (C3HC4-type RING finger) family protein, contains Pfam domain, PF00097: Zinc finger, C3HC4 type (RING finger) \| chr3:17189709-17191052 REVERSE \| Aliases: F12A12.140 | 7.5 | 9.1 | -1.7 | -8.1 | 0.09% | 7.8 |
| 1555 | AT2G31990.1 \| exostosin family protein, contains Pfam profile: PF03016 | 3.9 | 6.6 | -2.6 | -8.1 | 0.09% | 7.4 |
| 1561 | AT3G44540.2 \| similar to acyl CoA reductase, putative / male-sterility protein, putative [Arabidopsis thaliana] (TAIR:At5g22500.1); similar to putative fatty acyl coA reductase [Oryza sativa (japonica cultivar-group)] (GB:XP_481392.1); similar to putative fatty acyl coA reductase [Oryza sativa (japonica cultivar-group)] (GB:BAD31294.1); contains InterPro domain Male sterility protein (InterPro:IPR004262) \| chr3:16135008-16138869 FORWARD \| Aliases: F14L2.90 | 2.8 | 4.7 | -1.9 | -8.1 | 0.09% | 7.6 |

| Rank | Description | Sync | Root | M | t | adj.q | B |
|---|---|---|---|---|---|---|---|
| 1569 | AT5G45510.1 \| leucine-rich repeat family protein, contains leucine rich repeat (LRR) domains, Pfam:PF00560 \| chr5:18461884-18466673 FORWARD \| Aliases: MFC19.18, MFC19_18 | 5.8 | 8.4 | -2.6 | -8.1 | 0.09% | 7.6 |
| 1576 | AT5G56795.1 \| Symbol: MT1B \| one of the five metallothioneins (MTs) genes identified in Arabidopsis. MTs are cysteine-rich proteins required for heavy metal tolerance. The MT1b gene, however, is indicated to be inactive. \| chr5:22988283-22990379 REVERSE \| Aliases: None | 12.4 | 14.3 | -1.9 | -8.1 | 0.09% | 7.3 |
| 1578 | AT5G06300.1 \| expressed protein, similar to hypothetical protein [Arabidopsis thaliana] (TAIR:At3g53450.1); similar to unknown protein [Oryza sativa (japonica cultivar-group)] (GB:AAP54293.1); contains InterPro domain Conserved hypothetical protein 730 (InterPro:IPR005269) \| chr5:1921745-1925349 REVERSE \| Aliases: MHF15.18, MHF15_18 | 3.8 | 6.6 | -2.8 | -8.1 | 0.09% | 7.2 |
| 1579 | AT1G50590.1 \| pirin, putative, similar to SP:O00625 Pirin {Homo sapiens}; contains Pfam profile PF02678: Pirin | 3.7 | 6.2 | -2.4 | -8.1 | 0.09% | 7.6 |
| 1585 | AT1G49390.1 \| oxidoreductase, 2OG-Fe(II) oxygenase family protein, similar to flavonol synthase GI:311658 from (Petunia hybrida), leucoanthocyanidin dioxygenase (Malus domestica)(SP:P51091); contains PF03171 2OG-Fe(II) oxygenase superfamily domain \| chr1:18283268-18284646 FORWARD \| Aliases: F13F21.18, F13F21_18 | 3.3 | 6.3 | -2.9 | -8.1 | 0.09% | 6.9 |
| 1586 | AT5G13980.3 \| similar to glycosyl hydrolase family 38 protein [Arabidopsis thaliana] (TAIR:At3g26720.1); similar to glycosyl hydrolase family 38 protein [Arabidopsis thaliana] (TAIR:At5g66150.1); similar to MA2B1_MACFA Lysosomal alpha-mannosidase precursor (Mannosidase, alpha B) (Lysosomal acid alpha-mannosidase) (Laman) (Mannosidase alpha class 2B member 1) (GB:Q60HE9); similar to hypothetical protein [Pongo pygmaeus] (GB:CAH91346.1); similar to unnamed protein product [Tetraodon nigroviridis] (GB:CAG12505.1); similar to mannosidase, alpha, class 2B, member 1 precursor variant [Homo sapiens] (GB:BAD93158.1); similar to hypothetical protein LOC541519 [Danio rerio] (GB:NP_001014354.1); contains InterPro domain Glycoside hydrolase, family 38 (InterPro:IPR000602) \| chr5:4508498-4514427 FORWARD \| Aliases: MAC12.38, MAC12_38 | 7.0 | 8.9 | -1.8 | -8.1 | 0.09% | 7.7 |
| 1587 | AT1G10380.1 \| expressed protein \| chr1:3400607-3402325 FORWARD \| Aliases: F14N23.27 | 3.5 | 7.0 | -3.5 | -8.1 | 0.10% | 6.7 |
| 1588 | AT1G62980.1 \| Symbol: ATEXPA18 \| expansin, putative (EXP18), identical to SWISS-PROT:Q9LQ07 alpha-expansin 18 precursor (At-EXP18)(Arabidopsis thaliana); alpha-expansin gene family, PMID:11641069 \| chr1:23335341-23336773 FORWARD \| Aliases: ARABIDOPSIS THALIANA EXPANSIN A18, ATEXP18, ATHEXP ALPHA 1.25, EXP18, F16P17.14, F16P17_14 | 3.1 | 7.2 | -4.1 | -8.1 | 0.10% | 6.5 |
| 1590 | AT1G36160.1 \| Symbol: ACC1 \| similar to acetyl-CoA carboxylase 2 (ACC2) [Arabidopsis thaliana] (TAIR:At1g36180.1); similar to putative acetyl-CoA carboxylase [Oryza sativa (japonica cultivar-group)] (GB:AAP53321.1); similar to acetyl-CoA carboxylase [Medicago sativa] (GB:AAB42144.1); similar to acetyl-CoA carboxylase [Brassica napus] (GB:CAC19875.1); similar to acetyl-CoA carboxylase [Brassica napus] (GB:CAC19876.1); similar to acetyl CoA carboxylase (GB:AAA75528.1); contains InterPro domain Carboxyl transferase (InterPro:IPR000022); contains InterPro domain Carbamoyl-phosphate synthetase large chain, N-terminal (InterPro:IPR005481); contains InterPro domain Biotin carboxylase, C-terminal (InterPro:IPR005482); contains InterPro domain Biotin/lipoyl attachment (InterPro:IPR000089); contains InterPro domain Carbamoyl-phosphate synthase L chain, ATP-binding (InterPro:IPR005479) \| chr1:13535377-13545220 FORWARD \| Aliases: ACETYL COA CARBOXYLASE, ACETYL COENZYME A CARBOXYLASE 1, AT ACC1, EMB22, EMBRYO DEFECTIVE 22, F15C21.1, GK, GREEN BLIMP, GURKE, PAS3, PASTICCINO 3 | 6.8 | 9.4 | -2.6 | -8.1 | 0.10% | 7.6 |
| 1591 | AT3G51300.1 \| Symbol: ROP1 \| Rac-like GTP-binding protein (ARAC11) / Rho-like GTP-binding protein (ROP1), identical to GTP binding protein Rop1At (Arabidopsis thaliana) GI:2558666, rac-like GTP binding protein Arac11 (Arabidopsis thaliana) GI:3603426 \| chr3:19053866-19055330 FORWARD \| Aliases: F24M12.340, ROP1AT | 2.8 | 4.6 | -1.8 | -8.1 | 0.10% | 7.7 |
| 1592 | AT2G48030.1 \| endonuclease/exonuclease/phosphatase family protein, contains Pfam profile PF03372: Endonuclease/Exonuclease/phosphatase family \| chr2:19654597-19657218 REVERSE \| Aliases: T9J23.18 | 4.5 | 6.4 | -1.9 | -8.0 | 0.10% | 7.6 |
| 1593 | AT1G79670.2 \| Symbol: RFO1 \| wall-associated kinase, putative, similar to wall-associated kinase 4 GI:3355308 from (Arabidopsis thaliana); isoform contains non-consensus AT-acceptor splice site. \| chr1:29981780-29984230 REVERSE \| Aliases: F20B17.27, F20B17_27, RESISTANCE TO FUSARIUM OXYSPORUM 1, WAKL22 | 5.1 | 7.2 | -2.1 | -8.0 | 0.10% | 7.7 |
| 1594 | AT4G35380.1 \| guanine nucleotide exchange family protein, similar to guanine nucleotide exchange factor (Homo sapiens) GI:5456754; contains Pfam profile PF01369: Sec7 domain \| chr4:16819888-16826066 FORWARD \| Aliases: F23E12.60, F23E12_60 | 3.7 | 7.5 | -3.8 | -8.0 | 0.10% | 7.5 |
| 1597 | AT4G01720.1 \| Symbol: WRKY47 \| | 2.7 | 5.1 | -2.3 | -8.0 | 0.10% | 7.3 |
| 1598 | AT3G07310.1 \| expressed protein \| chr3:2327824-2329787 REVERSE \| Aliases: T1B9.2 | 4.7 | 6.9 | -2.2 | -8.0 | 0.10% | 7.6 |
| 1599 | AT4G36010.2 \| similar to pathogenesis-related thaumatin family protein [Arabidopsis thaliana] (TAIR:At2g17860.1); similar to putative thaumatin-like protein [Oryza sativa (japonica cultivar-group)] (GB:AAP52110.1); contains InterPro domain Thaumatin, pathogenesis-related (InterPro:IPR001938) \| chr4:17039066-17041134 REVERSE \| Aliases: T19K4.140, T19K4_140 | 4.3 | 6.7 | -2.4 | -8.0 | 0.10% | 7.3 |
| 1600 | AT4G24960.1 \| Symbol: ATHVA22D \| ABA-responsive protein (HVA22d), identical to AtHVA22d (Arabidopsis thaliana) GI:4884938 \| chr4:12827947-12829249 FORWARD \| Aliases: F13M23.100, F13M23_100 | 4.9 | 8.6 | -3.8 | -8.0 | 0.10% | 7.2 |

| Rank | Description | Sync | Root | M | t | adj.q | B |
|---|---|---|---|---|---|---|---|
| 1605 | AT5G60280.1 \| lectin protein kinase family protein, contains Pfam domains, PF00138: Legume lectins alpha domain, PF00139: Legume lectins beta domain, and PF00069: Protein kinase domain \| chr5:24277789-24279762 FORWARD \| Aliases: F15L12.12, F15L12_12 | 2.6 | 5.0 | -2.4 | -8.0 | 0.10% | 7.1 |
| 1606 | AT3G57410.1 \| Symbol: VLN3 \| villin 3 (VLN3), nearly identical to villin 3 (VLN3) (Arabidopsis thaliana) GI:3415117 \| chr3:21254113-21262077 REVERSE \| Aliases: F28O9.1, VILLIN 3 | 4.0 | 6.4 | -2.4 | -8.0 | 0.10% | 7.4 |
| 1607 | AT2G26730.1 \| leucine-rich repeat transmembrane protein kinase, putative \| chr2:11395485-11398719 FORWARD \| Aliases: F18A8.10, F18A8_10 | 4.3 | 6.3 | -2.0 | -8.0 | 0.10% | 7.5 |
| 1608 | AT1G49100.1 \| leucine-rich repeat protein kinase, putative, similar to light repressible receptor protein kinase (Arabidopsis thaliana) gi:1321686:emb:CAA66376; contains leucine rich repeat (LRR) domains, Pfam:PF00560; contains protein kinase domain, Pfam:PF00069 \| chr1:18169815-18173773 REVERSE \| Aliases: F27J15.13, F27J15_13 | 3.1 | 5.7 | -2.6 | -8.0 | 0.10% | 7.2 |
| 1609 | AT1G11310.1 \| Symbol: MLO2 \| seven transmembrane MLO family protein / MLO-like protein 2 (MLO2), idenctical to membrane protein Mlo2 (Arabidopsis thaliana) gi:14091574:gb:AAK53795; similar to Mlo (Hordeum vulgare subsp. vulgare) gi:1877221:emb:CAB06083 SWISS-PROT:P93766 \| chr1:3800747-3804144 REVERSE \| Aliases: T28P6.4, T28P6_4 | 6.0 | 8.6 | -2.6 | -8.0 | 0.10% | 7.6 |
| 1610 | AT5G03380.2 \| similar to heavy-metal-associated domain-containing protein [Arabidopsis thaliana] (TAIR:At2g36950.1); similar to GMFP5 [Glycine max] (GB:AAD09514.1); contains InterPro domain Heavy metal binding (InterPro:IPR006191); contains InterPro domain Heavy metal transport/detoxification protein (InterPro:IPR006121) \| chr5:832103-834284 REVERSE \| Aliases: F12E4.120, F12E4_120 | 8.6 | 12.3 | -3.7 | -8.0 | 0.10% | 7.4 |
| 1611 | AT5G06800.1 \| myb family transcription factor, contains Pfam profile: PF00249 myb-like DNA-binding domain \| chr5:2103172-2105899 FORWARD \| Aliases: MPH15.16, MPH15_16 | 2.9 | 6.2 | -3.3 | -8.0 | 0.10% | 7.1 |
| 1615 | AT3G06410.1 \| similar to zinc finger (CCCH-type) family protein [Arabidopsis thaliana] (TAIR:At5g18550.1); similar to putative floral homeotic protein HUA1 [Oryza sativa (japonica cultivar-group)] (GB:BAD81402.1); contains InterPro domain Zn-finger, C-x8-C-x5-C-x3-H type (InterPro:IPR000571) \| chr3:1947310-1949775 REVERSE \| Aliases: F24P17.12, F24P17_12 | 6.9 | 9.4 | -2.5 | -8.0 | 0.10% | 7.7 |
| 1626 | AT1G64280.1 \| Symbol: NPR1 \| regulatory protein (NPR1), identical to regulatory protein NPR1 (nonexpresser of PR genes 1, NPR1; noninducible immunity 1, Nim1; salicylic acid insensitive 1, Sai1) (Arabidopsis thaliana) SWISS-PROT:P93002 \| chr1:23856411-23859229 REVERSE \| Aliases: F15H21.6, F15H21_6, NIM1, NON INDUCIBLE IMMUNITY 1, NONEXPRESSER OF PR GENES 1, NPR1, REGULATORY PROTEIN NPR1, SAI1, SALICYLIC ACID INSENSITIVE 1 | 5.0 | 7.7 | -2.6 | -8.0 | 0.10% | 7.3 |
| 1628 | AT3G09490.1 \| chloroplast lumen common family protein, 2 TPR domains; similar to chloroplast lumen proteins (GI:4056493 (F3G5.19)(At2g37400)) and (GI:7413648 (T22P11.180),(At5g02590)) (Arabidopsis thaliana); + \| chr3:2915565-2916679 FORWARD \| Aliases: F11F8.6 | 4.2 | 6.7 | -2.5 | -8.0 | 0.10% | 7.2 |
| 1631 | AT4G33666.1 \| expressed protein \| chr4:16169051-16169538 FORWARD \| Aliases: None | 3.1 | 6.5 | -3.4 | -8.0 | 0.10% | 6.6 |
| 1634 | AT4G23450.1 \| zinc finger (C3HC4-type RING finger) family protein, contains Pfam profile: PF00097 zinc finger, C3HC4 type \| chr4:12240837-12242716 REVERSE \| Aliases: F16G20.150, F16G20_150 | 3.7 | 5.3 | -1.6 | -8.0 | 0.10% | 7.6 |
| 1635 | AT5G11790.1 \| Ndr family protein, similar to SP:O23969 Pollen specific protein SF21 {Helianthus annuus}; contains Pfam profile PF03096: Ndr family \| chr5:3799551-3802793 FORWARD \| Aliases: T22P22.180, T22P22_180 | 5.4 | 7.3 | -1.9 | -8.0 | 0.10% | 7.5 |
| 1636 | AT3G03000.1 \| calmodulin, putative, similar to calmodulin SP:P04352 from (Chlamydomonas reinhardtii); contains Pfam profile: PF00036 EF hand (4 copies) \| chr3:677247-678091 FORWARD \| Aliases: F13E7.5, F13E7_5 | 2.9 | 4.9 | -2.0 | -8.0 | 0.10% | 7.5 |
| 1637 | AT5G54080.2 \| Symbol: HGO \| homogentisate 1,2-dioxygenase / homogentisicase/homogentisate oxygenase / homogentisic acid oxidase (HGO), identical to SP:Q9ZRA2 Homogentisate 1,2-dioxygenase (EC 1.13.11.5) (Homogentisicase) (Homogentisate oxygenase) (Homogentisic acid oxidase) {Arabidopsis thaliana}; contains Pfam profile PF04209: homogentisate 1,2-dioxygenase \| chr5:21963095-21966139 FORWARD \| Aliases: HOMOGENTISATE 1,2 DIOXYGENASE, MJP23.6, MJP23_6 | 8.2 | 9.8 | -1.6 | -8.0 | 0.10% | 7.7 |
| 1639 | AT4G30420.1 \| nodulin MtN21 family protein, similar to MtN21 (Medicago truncatula) GI:2598575; contains Pfam profile PF00892: Integral membrane protein \| chr4:14877075-14878920 FORWARD \| Aliases: F17I23.240, F17I23_240 | 4.4 | 7.6 | -3.2 | -8.0 | 0.10% | 7.1 |
| 1641 | AT1G27520.1 \| glycoside hydrolase family 47 protein, Similar to gb:U04299 mannosyl-oligosaccharide alpha-1,2-mannosidase from Mus musculus. ESTs gb:R84145 and gb:AA394707 come from this gene | 5.3 | 7.1 | -1.8 | -8.0 | 0.10% | 7.7 |
| 1642 | AT2G22470.1 \| Symbol: AGP2 \| arabinogalactan-protein (AGP2), identical to gi:3883122:gb:AAC77824; supported by cDNA gi:3883121:gb:AF082299 \| chr2:9545277-9545959 REVERSE \| Aliases: ARABINOGALACTAN PROTEIN 2, F14M13.13, F14M13_13 | 6.9 | 8.8 | -1.9 | -8.0 | 0.10% | 7.7 |

| Rank | Description | Sync | Root | M | t | adj.q | B |
|---|---|---|---|---|---|---|---|
| 1645 | AT5G66170.2 \| similar to senescence-associated family protein [Arabidopsis thaliana] (TAIR:At2g17850.1); similar to A Chain A, Solution Structure Of At5g66040, A Putative Protein From Arabidosis Thaliana (GB:1TQ1); contains InterPro domain Rhodanese-like (InterPro:IPR001763) \| chr5:26464874-26465953 FORWARD \| Aliases: K2A18.25, K2A18_25 | 7.4 | 9.7 | -2.3 | -8.0 | 0.10% | 7.6 |
| 1646 | AT4G13030.2 \| expressed protein \| chr4:7607264-7609840 FORWARD \| Aliases: F25G13.120, F25G13_120 | 5.8 | 7.8 | -2.0 | -8.0 | 0.10% | 7.4 |
| 1650 | AT4G00430.2 \| Symbol: TMP C \| plasma membrane intrinsic protein, putative, identical to transmembrane protein GI:535780 from (Arabidopsis thaliana); very strong similarity to SP:Q08733 Plasma membrane intrinsic protein 1C (Transmembrane protein B) (TMP-B) {Arabidopsis thaliana}; contains Pfam profile PF00230: Major intrinsic protein; \| chr4:185854-187617 REVERSE \| Aliases: A_IG005I10.2, A_IG005I10_2, F5I10.2, F5I10_2, PIP1;4, PIP1E | 5.0 | 10.1 | -5.1 | -8.0 | 0.10% | 6.5 |
| 1651 | AT5G27100.1 \| Symbol: ATGLR2.1 \| glutamate receptor family protein (GLR2.1) (GLR3), plant glutamate receptor family, PMID:11379626 \| chr5:9535108-9538336 REVERSE \| Aliases: GLR2.1, T21B4.10, T21B4_10 | 3.4 | 6.1 | -2.7 | -8.0 | 0.10% | 7.2 |
| 1652 | AT3G15300.1 \| VQ motif-containing protein, contains PF05678: VQ motif \| chr3:5147412-5148217 REVERSE \| Aliases: K7L4.10 | 6.8 | 8.8 | -2.0 | -8.0 | 0.10% | 7.3 |
| 1656 | AT1G16150.1 \| Symbol: WAKL4 \| wall-associated kinase, putative, contains similarity to wall-associated kinase 4 GI:3355308 from (Arabidopsis thaliana) \| chr1:5532409-5534871 FORWARD \| Aliases: T24D18.23, T24D18_23, WALL ASSOCIATED KINASE LIKE 4 | 3.6 | 5.8 | -2.2 | -7.9 | 0.10% | 7.3 |
| 1658 | AT3G27470.1 \| expressed protein, contains Pfam profile PF05212: Protein of unknown function (DUF707) \| chr3:10164286-10168111 FORWARD \| Aliases: K1G2.18 | 3.6 | 5.8 | -2.1 | -7.9 | 0.10% | 7.5 |
| 1659 | AT4G40060.1 \| Symbol: ATHB16 \| | 4.9 | 7.2 | -2.3 | -7.9 | 0.10% | 7.4 |
| 1660 | AT1G44170.2 \| Symbol: ALDH3H1 \| aldehyde dehydrogenase, putative (ALDH), similar to aldehyde dehydrogenase ALDH (Craterostigma plantagineum) gi:17065918:emb:CAC84900 | 6.5 | 10.0 | -3.5 | -7.9 | 0.10% | 7.5 |
| 1661 | AT1G20850.1 \| Symbol: XCP2 \| cysteine endopeptidase, papain-type (XCP2), identical to papain-type cysteine endopeptidase XCP2 GI:6708183 from (Arabidopsis thaliana) \| chr1:7252173-7253716 FORWARD \| Aliases: F9H16.17, F9H16_17, PAPAIN TYPE CYSTEINE ENDOPEPTIDASE XCP2, XYLEM CYSTEINE PEPTIDASE 2 | 5.1 | 10.4 | -5.3 | -7.9 | 0.10% | 7.0 |
| 1663 | AT5G25280.2 \| serine-rich protein-related, contains some similarity to serine-rich proteins \| chr5:8773795-8776003 FORWARD \| Aliases: F18G18.20, F18G18_20 | 5.3 | 8.4 | -3.1 | -7.9 | 0.10% | 7.2 |
| 1667 | AT5G43760.1 \| beta-ketoacyl-CoA synthase, putative, similar to beta-ketoacyl-CoA synthase (Simmondsia chinensis)(GI:1045614) \| chr5:17602990-17605824 FORWARD \| Aliases: MQD19.11, MQD19_11 | 3.7 | 5.8 | -2.0 | -7.9 | 0.10% | 7.6 |
| 1668 | AT1G08310.1 \| esterase/lipase/thioesterase family protein, contains Interpro entry IPR000379 \| chr1:2618683-2620408 FORWARD \| Aliases: T23G18.18, T23G18_18 | 3.6 | 6.7 | -3.2 | -7.9 | 0.10% | 7.3 |
| 1672 | AT2G21540.2 \| similar to SEC14 cytosolic factor, putative / phosphoglyceride transfer protein, putative [Arabidopsis thaliana] (TAIR:At4g34580.1); similar to SEC14 cytosolic factor, putative / phosphoglyceride transfer protein, putative [Arabidopsis thaliana] (TAIR:At4g39180.1); similar to SEC14 cytosolic factor, putative / phosphoglyceride transfer protein, putative [Arabidopsis thaliana] (TAIR:At4g36490.1); similar to SEC14 cytosolic factor, putative / phosphoglyceride transfer protein, putative [Arabidopsis thaliana] (TAIR:At2g18180.1); similar to putative SEC14 cytosolic factor [Oryza sativa (japonica cultivar-group)] (GB:XP_465384.1); contains InterPro domain Cellular retinaldehyde-binding)/triple function, C-terminal (InterPro:IPR001251); contains InterPro domain Cellular retinaldehyde binding/alpha-tocopherol transport (InterPro:IPR001071); contains InterPro domain Cellular retinaldehyde-binding/triple function, N-terminal (InterPro:IPR008273) \| chr2:9227629-9231359 REVERSE \| Aliases: F3K23.30 | 3.2 | 5.9 | -2.6 | -7.9 | 0.10% | 7.2 |
| 1677 | AT5G40020.1 \| pathogenesis-related thaumatin family protein, similar to SP:P50699 Thaumatin-like protein precursor {Arabidopsis thaliana}, pathogenesis-related group 5 protein (Brassica rapa) GI:2749943; contains Pfam profile PF00314: Thaumatin family \| chr5:16039990-16041544 REVERSE \| Aliases: MYH19.180, MYH19_180 | 3.8 | 7.3 | -3.4 | -7.9 | 0.10% | 6.2 |
| 1682 | AT4G27600.1 \| pfkB-type carbohydrate kinase family protein, contains Pfam profile: PF00294 pfkB family carbohydrate kinase \| chr4:13782521-13785118 REVERSE \| Aliases: T29A15.90, T29A15_90 | 5.4 | 6.8 | -1.5 | -7.9 | 0.10% | 7.7 |
| 1683 | AT2G46370.2 \| Symbol: JAR1 \| auxin-responsive GH3 family protein, similar to auxin-responsive GH3 product (Glycine max) GI:18591; contains Pfam profile PF03321: GH3 auxin-responsive promoter \| chr2:19040863-19043732 FORWARD \| Aliases: F11C10.6, FAR RED INSENSITIVE 219, FIN219, JAR, JASMONATE RESISTANT 1 | 7.1 | 9.0 | -1.9 | -7.9 | 0.10% | 7.7 |
| 1684 | AT2G25680.1 \| expressed protein, ; expression supported by MPSS \| chr2:10940139-10941615 REVERSE \| Aliases: F3N11.13, F3N11_13 | 4.8 | 7.9 | -3.2 | -7.9 | 0.10% | 6.7 |
| 1688 | AT5G37690.1 \| GDSL-motif lipase/hydrolase family protein, similar to family II lipase EXL3 (GI:15054386), EXL1 (GI:15054382), EXL2 (GI:15054384) (Arabidopsis thaliana) \| chr5:14990583-14993399 REVERSE \| Aliases: AT5G37700, K12B20.140, K12B20_140 | 4.2 | 7.0 | -2.8 | -7.9 | 0.10% | 6.8 |

| Rank | Description | Sync | Root | M | t | adj.q | B |
|---|---|---|---|---|---|---|---|
| 1689 | AT2G41705.1 \| camphor resistance CrcB family protein, contains Pfam profile PF02537: CrcB-like protein | 4.5 | 6.6 | -2.1 | -7.9 | 0.10% | 7.4 |
| 1692 | AT5G66420.2 \| similar to transcriptional regulator [Clostridium tetani E88] (GB:NP_782382.1) \| chr5:26538900-26542307 REVERSE \| Aliases: K1F13.7, K1F13_7 | 7.7 | 9.7 | -2.0 | -7.9 | 0.11% | 7.4 |
| 1697 | AT4G29340.1 \| Symbol: PRF4 \| profilin 3 (PRO3) (PFN3), identical to profilin 3 SP:Q38904 GI:1353765 from (Arabidopsis thaliana) \| chr4:14447653-14448704 FORWARD \| Aliases: F17A13.160, F17A13_160, PROFILIN 4 | 3.2 | 5.8 | -2.6 | -7.9 | 0.11% | 7.2 |
| 1700 | AT3G27580.2 \| Symbol: ATPK7 \| similar to protein kinase, putative [Arabidopsis thaliana] (TAIR:At5g47750.1); similar to protein kinase C (EC 2.7.1.-) homolog - kidney bean (GB:A30311); contains InterPro domain Serine/threonine protein kinase, active site (InterPro:IPR008271); contains InterPro domain Tyrosine protein kinase (InterPro:IPR001245); contains InterPro domain Serine/threonine protein kinase (InterPro:IPR002290); contains InterPro domain Protein kinase (InterPro:IPR000719) \| chr3:10218664-10221213 REVERSE \| Aliases: MMJ24.13 | 2.7 | 5.2 | -2.4 | -7.9 | 0.11% | 7.1 |
| 1705 | AT2G45220.1 \| pectinesterase family protein, contains Pfam profile: PF01095 pectinesterase \| chr2:18651160-18653521 REVERSE \| Aliases: F4L23.27 | 3.6 | 8.8 | -5.2 | -7.9 | 0.11% | 7.0 |
| 1707 | AT5G25350.1 \| Symbol: EBF2 \| F-box family protein, contains Pfam PF00646: F-box domain and Pfam PF00560: Leucine Rich Repeat (6 copies); similar to F-box protein FBL6 (GI:4432860) (Homo sapiens) \| chr5:8794845-8797015 REVERSE \| Aliases: EIN3 BINDING F BOX PROTEIN 2, F18G18.90, F18G18_90 | 3.1 | 6.9 | -3.7 | -7.9 | 0.11% | 7.1 |
| 1711 | AT4G28530.1 \| Symbol: ANAC074 \| no apical meristem (NAM) family protein, contains Pfam PF02365: No apical meristem (NAM) domain; No apical meristem gene (NAM), required for pattern formation in embryos and flowers-Petunia hybrida, PATCHX:E205713 | 3.5 | 5.2 | -1.7 | -7.9 | 0.11% | 7.5 |
| 1713 | AT4G40010.1 \| serine/threonine protein kinase, putative, similar to serine-threonine protein kinase (Triticum aestivum) gi:2055374:gb:AAB58348 | 4.7 | 9.2 | -4.6 | -7.8 | 0.11% | 6.6 |
| 1714 | AT1G29270.1 \| expressed protein \| chr1:10230305-10231128 FORWARD \| Aliases: F28N24.5, F28N24_5 | 2.5 | 6.4 | -4.0 | -7.8 | 0.11% | 6.6 |
| 1717 | AT1G33840.1 \| hypothetical protein, contains Pfam profile PF04525: Protein of unknown function (DUF567) \| chr1:12283842-12285330 REVERSE \| Aliases: F14M2.3, F14M2_3 | 2.7 | 5.3 | -2.6 | -7.8 | 0.11% | 7.1 |
| 1719 | AT4G32140.1 \| expressed protein \| chr4:15522905-15525433 REVERSE \| Aliases: F10N7.50, F10N7_50 | 5.5 | 7.5 | -1.9 | -7.8 | 0.11% | 7.5 |
| 1720 | AT1G12290.1 \| disease resistance protein (CC-NBS-LRR class), putative, domain signature CC-NBS-LRR exists, suggestive of a disease resistance protein. | 2.6 | 3.9 | -1.3 | -7.8 | 0.11% | 7.6 |
| 1722 | AT5G64260.1 \| Symbol: MSJ1.10 \| phosphate-responsive protein, putative, similar to phi-1 (phosphate-induced gene) (Nicotiana tabacum) GI:3759184; contains Pfam profile PF04674: Phosphate-induced protein 1 conserved region | 6.7 | 10.3 | -3.5 | -7.8 | 0.11% | 6.9 |
| 1723 | AT1G12740.1 \| Symbol: CYP87A2 \| cytochrome P450 family protein, similar to Cytochrome P450 90A1 (SP:Q42569) (Arabidopsis thaliana) \| chr1:4342460-4344615 FORWARD \| Aliases: T12C24.27, T12C24_27 | 1.9 | 5.7 | -3.8 | -7.8 | 0.11% | 6.8 |
| 1726 | AT4G04960.1 \| lectin protein kinase, putative, similar to receptor lectin kinase 3 (Arabidopsis thaliana) gi:4100060:gb:AAD00733; contains protein kinase domain, Pfam:PF00069; contains legume lectins alpha and beta domains, Pfam:PF00138 and Pfam:PF00139 \| chr4:2533054-2535356 FORWARD \| Aliases: T32N4.9, T32N4_9 | 5.0 | 7.2 | -2.1 | -7.8 | 0.11% | 7.5 |
| 1727 | AT5G63930.1 \| leucine-rich repeat transmembrane protein kinase, putative \| chr5:25600232-25603725 FORWARD \| Aliases: MBM17.3, MBM17_3 | 6.3 | 8.1 | -1.8 | -7.8 | 0.11% | 7.5 |
| 1730 | AT2G30360.1 \| Symbol: CIPK11 \| | 7.1 | 10.2 | -3.1 | -7.8 | 0.11% | 7.5 |
| 1732 | AT3G11280.2 \| myb family transcription factor, contains Pfam profile: PF00249 Myb-like DNA-binding domain \| chr3:3533261-3534502 REVERSE \| Aliases: F11B9.25 | 7.9 | 10.0 | -2.1 | -7.8 | 0.11% | 7.6 |
| 1733 | AT4G37790.1 \| Symbol: HAT22 \| homeobox-leucine zipper protein 22 (HAT22) / HD-ZIP protein 22, identical to homeobox-leucine zipper protein HAT22 (HD-ZIP protein 22) (SP:P46604) (Arabidopsis thaliana) \| chr4:17768130-17769600 FORWARD \| Aliases: HOMEOBOX PROTEIN, T28I19.70, T28I19_70 | 4.4 | 8.6 | -4.2 | -7.8 | 0.11% | 7.1 |
| 1735 | AT2G01650.1 \| Symbol: pux2 \| zinc finger (C2H2 type) family protein, contains Pfam domain, PF00096: Zinc finger, C2H2 type \| chr2:284671-286492 REVERSE \| Aliases: PLANT UBX DOMAIN CONTAINING PROTEIN 2, T8O11.18, T8O11_18 | 7.3 | 9.1 | -1.8 | -7.8 | 0.11% | 7.6 |
| 1736 | AT1G62380.1 \| Symbol: ACO2 \| 1-aminocyclopropane-1-carboxylate oxidase, putative / ACC oxidase, putative, nearly identical to ACC oxidase (ACC ox1) GI:587086 from (Brassica oleracea) \| chr1:23085927-23087918 FORWARD \| Aliases: ACC OXIDASE, ACC OXIDASE 2, ATACO2, F24O1.40, F24O1_40 | 8.7 | 12.1 | -3.3 | -7.8 | 0.11% | 7.5 |
| 1737 | AT2G05260.1 \| lipase class 3 family protein, contains Pfam profile PF01764: Lipase \| chr2:1918332-1920039 FORWARD \| Aliases: F5G3.16, F5G3_16 | 6.4 | 8.0 | -1.6 | -7.8 | 0.11% | 7.5 |

| Rank | Description | Sync | Root | M | t | adj.q | B |
|---|---|---|---|---|---|---|---|
| 1739 | AT4G19680.2 \| Symbol: IRT2 \| similar to iron-responsive transporter (IRT1) [Arabidopsis thaliana] (TAIR:At4g19690.2); similar to iron-regulated transporter 1 [Lycopersicon esculentum] (GB:AAD30548.1); contains InterPro domain ZIP zinc/iron transport (InterPro:IPR004698); contains InterPro domain Zinc transporter ZIP (InterPro:IPR003689) \| chr4:10703386-10704811 FORWARD \| Aliases: T16H5.40, T16H5_40 | 3.1 | 8.4 | -5.3 | -7.8 | 0.11% | 5.9 |
| 1740 | AT3G54770.2 \| similar to RNA recognition motif (RRM)-containing protein [Arabidopsis thaliana] (TAIR:At1g20880.1); similar to putative RRM-containing protein [Oryza sativa (japonica cultivar-group)] (GB:BAD37252.1); contains InterPro domain RNA-binding region RNP-1 (RNA recognition motif) (InterPro:IPR000504) \| chr3:20284762-20286802 REVERSE \| Aliases: T5N23.130 | 2.4 | 3.6 | -1.2 | -7.8 | 0.11% | 7.6 |
| 1743 | AT5G53550.1 \| transporter, putative, similar to iron-phytosiderophore transporter protein yellow stripe 1 (Zea mays) GI:10770865; contains Pfam profile PF03169: OPT oligopeptide transporter protein \| chr5:21773307-21776002 FORWARD \| Aliases: MNC6.9, MNC6_9 | 4.7 | 7.6 | -2.9 | -7.8 | 0.11% | 7.1 |
| 1745 | AT3G45410.1 \| lectin protein kinase family protein, contains Pfam profiles: PF00069 protein kinase domain, PF00138 legume lectins alpha domain, PF00139 legume lectins beta domain \| chr3:16664884-16666998 REVERSE \| Aliases: F18N11.170 | 3.6 | 7.6 | -3.9 | -7.8 | 0.11% | 6.4 |
| 1750 | AT1G70690.1 \| kinase-related, contains Pfam PF01657:Domain of unknown function; similar to receptor-like protein kinase 4 GI:13506745 (Arabidopsis thaliana) \| chr1:26655676-26657287 FORWARD \| Aliases: F5A18.13, F5A18_13 | 3.6 | 7.3 | -3.7 | -7.8 | 0.11% | 6.7 |
| 1755 | AT5G63970.2 \| similar to copine-related [Arabidopsis thaliana] (TAIR:At3g01650.1); similar to P0401G10.23 [Oryza sativa (japonica cultivar-group)] (GB:NP_914244.1); contains InterPro domain von Willebrand factor, type A (InterPro:IPR002035) \| chr5:25623997-25626213 REVERSE \| Aliases: MBM17.7, MBM17_7 | 8.6 | 9.7 | -1.2 | -7.8 | 0.11% | 7.5 |
| 1760 | AT5G19930.1 \| integral membrane family protein, contains Pfam domain PF01940: Integral membrane protein \| chr5:6737585-6739350 REVERSE \| Aliases: F28I16.80, F28I16_80 | 6.9 | 9.6 | -2.7 | -7.8 | 0.11% | 7.4 |
| 1761 | AT3G14225.1 \| Symbol: EMB1474 \| GDSL-motif lipase/hydrolase family protein, contains Pfam profile PF00657: Lipase/Acylhydrolase with GDSL-like motif \| chr3:4734623-4736000 REVERSE \| Aliases: EMB1474, EMBRYO DEFECTIVE 1474, MLE3.2 | 3.3 | 5.7 | -2.3 | -7.8 | 0.11% | 6.9 |
| 1764 | AT5G65090.1 \| endonuclease/exonuclease/phosphatase family protein, similar to inositol polyphosphate 5-phosphatase I (GI:10444261) and II (GI:10444263) (Arabidopsis thaliana); contains Pfam profile PF03372: Endonuclease/Exonuclease/phosphatase family \| chr5:26021422-26024077 FORWARD \| Aliases: MQN23.2, MQN23_2 | 2.6 | 5.7 | -3.2 | -7.8 | 0.11% | 6.5 |
| 1765 | AT4G16750.1 \| encodes a member of the DREB subfamily A-4 of ERF/AP2 transcription factor family. The protein contains one AP2 domain. There are 17 members in this subfamily including TINY. \| chr4:9421143-9421682 REVERSE \| Aliases: DL4400C, FCAALL.19 | 2.8 | 3.9 | -1.0 | -7.8 | 0.11% | 7.5 |
| 1766 | AT2G02780.1 \| leucine-rich repeat transmembrane protein kinase, putative \| chr2:781846-784645 REVERSE \| Aliases: T20F6.8, T20F6_8 | 2.9 | 4.3 | -1.4 | -7.8 | 0.11% | 7.5 |
| 1767 | AT3G22240.1 \| expressed protein \| chr3:7863684-7864586 REVERSE \| Aliases: MMP21.2 | 9.3 | 11.8 | -2.5 | -7.8 | 0.11% | 7.3 |
| 1768 | AT1G22740.1 \| Symbol: RAB7 \| Ras-related protein (RAB7) / AtRab75 / small GTP-binding protein, putative, identical to SP:O04157 Ras-related protein Rab7 (AtRab75) (Arabidopsis thaliana) \| chr1:8049089-8050697 FORWARD \| Aliases: T22J18.9, T22J18_9 | 4.3 | 5.9 | -1.6 | -7.8 | 0.11% | 7.5 |
| 1770 | AT1G72630.1 \| expressed protein \| chr1:27347932-27349238 FORWARD \| Aliases: F28P22.18, F28P22_18 | 6.0 | 7.4 | -1.4 | -7.8 | 0.11% | 7.5 |
| 1776 | AT5G02560.1 \| histone H2A, putative, similar to histone H2A from Pisum sativum SP:P25470, Zea mays SP:P40280, Petroselinum crispum SP:P19177; contains Pfam profile PF00125 Core histone H2A/H2B/H3/H4 | 7.7 | 8.8 | -1.1 | -7.7 | 0.11% | 7.5 |
| 1778 | AT3G14340.1 \| expressed protein \| chr3:4782764-4783144 FORWARD \| Aliases: MLN21.13 | 5.2 | 7.6 | -2.5 | -7.7 | 0.11% | 7.0 |
| 1779 | AT3G26440.2 \| expressed protein, similar to expressed protein [Arabidopsis thaliana] (TAIR:At1g13000.1); similar to unknown protein [Oryza sativa (japonica cultivar-group)] (GB:AAU44134.1); contains InterPro domain Protein of unknown function DUF707 (InterPro:IPR007877) | 4.6 | 8.6 | -4.0 | -7.7 | 0.11% | 6.7 |
| 1780 | AT1G80380.2 \| phosphoribulokinase/uridine kinase-related \| chr1:30222157-30224863 FORWARD \| Aliases: F5I6.13, F5I6_13 | 7.8 | 9.8 | -2.0 | -7.7 | 0.11% | 7.2 |
| 1781 | AT3G03380.1 \| DegP protease, putative, contains similarity to degP GI:2623992 from (Bradyrhizobium japonicum) \| chr3:799622-808529 FORWARD \| Aliases: AT3G03390, T21P5.20, T21P5_20 | 5.0 | 6.4 | -1.5 | -7.7 | 0.11% | 7.5 |
| 1782 | AT3G07490.1 \| calcium-binding protein, putative, similar to calcium-binding protein GI:6580549 from (Lotus japonicus) \| chr3:2391195-2391656 FORWARD \| Aliases: F21O3.20 | 3.0 | 5.9 | -2.9 | -7.7 | 0.12% | 6.8 |
| 1786 | AT1G45688.2 \| expressed protein \| chr1:17193844-17195598 FORWARD \| Aliases: F2G19.25, F2G19_25 | 5.8 | 7.8 | -2.0 | -7.7 | 0.12% | 7.3 |

| Rank | Description | Sync | Root | M | t | adj.q | B |
|---|---|---|---|---|---|---|---|
| 1788 | AT2G41220.1 \| Symbol: GLU2 \| glutamate synthase, chloroplast (GLU2) / ferredoxin-dependent glutamate synthase (Fd-GOGAT 2), identical to SP:Q9T0P4 Ferredoxin-dependent glutamate synthase 2, chloroplast precursor (EC 1.4.7.1) (Fd-GOGAT 2) {Arabidopsis thaliana} \| chr2:17185012-17195829 FORWARD \| Aliases: F13H10.23, F13H10_23, GLUTAMATE SYNTHASE PRECURSOR | 8.7 | 10.8 | -2.1 | -7.7 | 0.12% | 7.5 |
| 1790 | AT2G26980.5 \| Symbol: CIPK3 \| similar to protein kinase family protein / NAF domain-containing protein [Arabidopsis thaliana] (TAIR:At5g21326.1); similar to serine/threonine kinase [Sorghum bicolor] (GB:CAA73067.1); similar to serine/threonine kinase [Sorghum bicolor] (GB:CAA73068.1); contains InterPro domain Serine/threonine protein kinase, active site (InterPro:IPR008271); contains InterPro domain Tyrosine protein kinase (InterPro:IPR001245); contains InterPro domain NAF domain (InterPro:IPR004041); contains InterPro domain Serine/threonine protein kinase (InterPro:IPR002290); contains InterPro domain Protein kinase (InterPro:IPR000719) \| chr2:11521781-11525401 REVERSE \| Aliases: CBL INTERACTING PROTEIN KINASE 3, T20P8.3, T20P8_3 | 3.4 | 5.5 | -2.1 | -7.7 | 0.12% | 7.3 |
| 1791 | AT3G47780.1 \| Symbol: ATATH6 \| | 6.4 | 8.5 | -2.1 | -7.7 | 0.12% | 7.3 |
| 1793 | AT2G46260.1 \| BTB/POZ domain-containing protein, contains Pfam PF00651: BTB/POZ domain; contains Interpro IPR000210/ PS50097: BTBB/POZ domain; similar to POZ/BTB containing-protein AtPOB1 (GI:12006855) (Arabidopsis thaliana); similar to actinfilin (GI:21667852) (Rattus norvegicus) \| chr2:19003041-19005706 FORWARD \| Aliases: T3F17.9 | 6.0 | 7.6 | -1.6 | -7.7 | 0.12% | 7.5 |
| 1796 | AT2G22660.2 \| similar to glycine-rich protein [Arabidopsis thaliana] (TAIR:At4g37900.1); similar to hypothetical protein [Sorghum bicolor] (GB:AAD27577.1) \| chr2:9634488-9638161 FORWARD \| Aliases: T9I22.10, T9I22_10 | 8.2 | 11.6 | -3.3 | -7.7 | 0.12% | 6.8 |
| 1799 | AT1G51420.1 \| sucrose-phosphatase, putative, similar to sucrose-phosphatase (SPP1) (Arabidopsis thaliana) GI:11127757; contains Pfam profile PF05116: Sucrose-6F-phosphate phosphohydrolase \| chr1:19068520-19070372 REVERSE \| Aliases: F5D21.9, F5D21_9 | 4.4 | 7.6 | -3.2 | -7.7 | 0.12% | 7.4 |
| 1801 | AT5G60020.1 \| laccase, putative / diphenol oxidase, putative, similar to laccase LAC2-4, Liriodendron tulipifera, EMBL:LTU73106 (GI:1621467) \| chr5:24185222-24187688 FORWARD \| Aliases: MMN10.20, MMN10_20 | 3.7 | 6.8 | -3.0 | -7.7 | 0.12% | 7.0 |
| 1805 | AT3G17920.1 \| leucine-rich repeat family protein, contains leucine rich repeat (LRR) domains, Pfam:PF00560 \| chr3:6137261-6142029 FORWARD \| Aliases: MEB5.14 | 5.4 | 7.0 | -1.6 | -7.7 | 0.12% | 7.5 |
| 1810 | AT2G28960.1 \| leucine-rich repeat protein kinase, putative, similar to light repressible receptor protein kinase (Arabidopsis thaliana) gi:1321686:emb:CAA66376; contains leucine rich repeat (LRR) domains, Pfam:PF00560; contains protein kinase domain, Pfam:PF00069 \| chr2:12444991-12449424 REVERSE \| Aliases: T9I4.4, T9I4_4 | 2.2 | 4.1 | -1.9 | -7.7 | 0.12% | 7.3 |
| 1811 | AT1G20900.1 \| Symbol: ESC \| DNA-binding protein-related, contains Pfam domain PF03479: Domain of unknown function (DUF296), found in AT-hook motifs Pfam:PF02178 \| chr1:7272545-7274061 FORWARD \| Aliases: ESC, ESCAROLA, F9H16.12, F9H16_12 | 5.3 | 7.3 | -1.9 | -7.7 | 0.12% | 7.2 |
| 1812 | AT1G80920.1 \| Symbol: J8 \| DNAJ heat shock N-terminal domain-containing protein, similar to SP:Q05646 Chaperone protein dnaJ Erysipelothrix rhusiopathiae, SP:P45555 Chaperone protein dnaJ (HSP40) Staphylococcus aureus; contains Pfam profile PF00226 DnaJ domain \| chr1:30408536-30409577 REVERSE \| Aliases: F23A5.28, F23A5_28, J8 | 6.7 | 9.0 | -2.3 | -7.7 | 0.12% | 7.0 |
| 1817 | AT4G21230.1 \| protein kinase family protein, contains Pfam domain, PF00069: Protein kinase domain \| chr4:11319196-11321689 REVERSE \| Aliases: F7J7.170, F7J7_170 | 3.3 | 6.9 | -3.6 | -7.7 | 0.12% | 6.2 |
| 1819 | AT4G32440.2 \| agenet domain-containing protein, contains Pfam PF05641: Agenet domain \| chr4:15656691-15659754 FORWARD \| Aliases: F8B4.140, F8B4_140 | 4.9 | 6.4 | -1.5 | -7.7 | 0.12% | 7.4 |
| 1822 | AT4G12030.2 \| bile acid:sodium symporter family protein, low similarity to SP:Q12908 Ileal sodium/bile acid cotransporter {Homo sapiens}; contains Pfam profile PF01758: Sodium Bile acid symporter family \| chr4:7210913-7212830 FORWARD \| Aliases: F16J13.100, F16J13_100 | 3.5 | 6.1 | -2.5 | -7.7 | 0.12% | 7.1 |
| 1824 | AT3G09270.1 \| Symbol: ATGSTU8 \| glutathione S-transferase, putative, similar to glutathione transferase GB:CAA71784 (Glycine max) \| chr3:2848295-2849293 REVERSE \| Aliases: F3L24.14 | 5.3 | 8.6 | -3.3 | -7.7 | 0.12% | 7.1 |
| 1828 | AT1G77280.1 \| protein kinase family protein, contains Pfam domain, PF00069: Protein kinase domain \| chr1:29036362-29040776 REVERSE \| Aliases: T14N5.13, T14N5_13 | 4.2 | 8.0 | -3.7 | -7.7 | 0.12% | 7.0 |
| 1829 | AT1G04250.1 \| Symbol: AXR3 \| auxin-responsive protein / indoleacetic acid-induced protein 17 (IAA17), Identical to SP:P93830 Auxin-responsive protein IAA17 (Indoleacetic acid-induced protein 17) {Arabidopsis thaliana}; ESTs gb:H36782 and gb:F14074 come from this gene \| chr1:1136257-1138582 FORWARD \| Aliases: AUXIN RESISTANT 3, F19P19.31, F19P19_31, IAA17 | 4.9 | 8.1 | -3.3 | -7.7 | 0.12% | 6.7 |
| 1830 | AT5G01740.1 \| expressed protein, wound-inducible protein wun1 protein - Solanum tuberosum, PIR:JQ0398 \| chr5:280719-281442 FORWARD \| Aliases: T20L15.10, T20L15_10 | 4.2 | 6.7 | -2.5 | -7.7 | 0.12% | 7.0 |

| Rank | Description | Sync | Root | M | t | adj.q | B |
|---|---|---|---|---|---|---|---|
| 1832 | AT5G10480.1 \| Symbol: PAS2 \| protein tyrosine phosphatase-like protein, putative (PAS2), identical to PEPINO/PASTICCINO2 protein GI:24411193, GI:24575153 from (Arabidopsis thaliana); contains Pfam:04387: protein tyrosine phosphatase-like protein | 5.4 | 8.2 | -2.8 | -7.6 | 0.12% | 7.3 |
| 1833 | AT3G12640.1 \| RNA recognition motif (RRM)-containing protein, contains InterPro entry IPR000504: RNA-binding region RNP-1 (RNA recognition motif) (RRM) \| chr3:4014462-4017851 FORWARD \| Aliases: T2E22.5 | 5.5 | 7.6 | -2.1 | -7.6 | 0.12% | 7.4 |
| 1836 | AT3G62660.1 \| glycosyl transferase family 8 protein, low similarity to glycosyl transferase lgtC - Neisseria gonorrhoeae, EMBL:AF208062; contains Pfam glycosyl transferase family 8 domain PF01501 \| chr3:23183782-23185737 FORWARD \| Aliases: F26K9.90 | 5.7 | 6.9 | -1.2 | -7.6 | 0.12% | 7.4 |
| 1838 | AT1G51850.1 \| leucine-rich repeat protein kinase, putative, similar to light repressible receptor protein kinase (Arabidopsis thaliana) gi:1321686:emb:CAA66376 \| chr1:19256516-19260452 REVERSE \| Aliases: T14L22.6, T14L22_6 | 2.5 | 4.7 | -2.2 | -7.6 | 0.12% | 6.8 |
| 1839 | AT4G03560.1 \| Symbol: ATTPC1 \| | 7.8 | 9.8 | -2.0 | -7.6 | 0.12% | 7.4 |
| 1841 | AT1G55020.1 \| Symbol: LOX1 \| lipoxygenase (LOX1), identical to SP:Q06327 \| chr1:20529375-20533940 FORWARD \| Aliases: LIPOXYGENASE | 5.0 | 8.1 | -3.1 | -7.6 | 0.12% | 7.2 |
| 1843 | AT5G43030.1 \| DC1 domain-containing protein, contains Pfam profile PF03107: DC1 domain | 2.3 | 3.8 | -1.5 | -7.6 | 0.12% | 7.4 |
| 1844 | AT5G04150.1 \| basic helix-loop-helix (bHLH) family protein, contains Pfam profile: PF00010 helix-loop-helix DNA-binding domain; PMID: 12679534 | 3.6 | 5.0 | -1.4 | -7.6 | 0.12% | 7.4 |
| 1846 | AT5G60710.1 \| zinc finger (C3HC4-type RING finger) family protein, low similarity to SP:P97280 Inter-alpha-trypsin inhibitor heavy chain H3 precursor {Mesocricetus auratus}; contains Pfam profiles PF00097: Zinc finger, C3HC4 type (RING finger), PF00092: von Willebrand factor type A domain \| chr5:24427944-24432294 REVERSE \| Aliases: MUP24.2, MUP24_2 | 3.7 | 5.7 | -2.0 | -7.6 | 0.12% | 7.4 |
| 1852 | AT2G24610.1 \| Symbol: ATCNGC14 \| cyclic nucleotide-regulated ion channel, putative (CNGC14), similar to cyclic nucleotide and calmodulin-regulated ion channel (GI:4581205) (Arabidopsis thaliana) \| chr2:10464124-10467587 FORWARD \| Aliases: CNGC14, F25P17.9, F25P17_9 | 2.7 | 5.0 | -2.3 | -7.6 | 0.12% | 7.3 |
| 1853 | AT1G05700.1 \| leucine-rich repeat protein kinase, putative, similar to light repressible receptor protein kinase, gi:2129635; contains leucine rich repeat (LRR) domains, Pfam:PF00560; contains protein kinase domain, Pfam:PF00069 \| chr1:1709795-1713244 FORWARD \| Aliases: F3F20.15, F3F20_15 | 2.8 | 5.6 | -2.9 | -7.6 | 0.12% | 6.5 |
| 1854 | AT3G14430.1 \| expressed protein \| chr3:4824204-4824844 REVERSE \| Aliases: MOA2.3 | 9.2 | 10.0 | -0.8 | -7.6 | 0.12% | 7.2 |
| 1856 | AT1G23720.1 \| proline-rich extensin-like family protein, contains proline-rich extensin domains, INTERPRO:IPR002965 \| chr1:8388644-8391529 FORWARD \| Aliases: F5O8.27, F5O8_27 | 3.0 | 9.0 | -5.9 | -7.6 | 0.12% | 6.1 |
| 1857 | AT1G74770.1 \| expressed protein \| chr1:28094772-28098495 REVERSE \| Aliases: F25A4.26, F25A4_26 | 3.0 | 8.7 | -5.7 | -7.6 | 0.12% | 7.1 |
| 1858 | AT5G26010.1 \| similar to protein phosphatase 2C, putative / PP2C, putative [Arabidopsis thaliana] (TAIR:At4g32950.1); similar to putative protein phosphatase 2C [Oryza sativa (japonica cultivar-group)] (GB:XP_465582.1); contains InterPro domain Protein phosphatase 2C-like (InterPro:IPR001932) \| chr5:9085396-9087375 REVERSE \| Aliases: T1N24.8, T1N24_8 | 4.5 | 8.0 | -3.5 | -7.6 | 0.12% | 6.8 |
| 1860 | AT4G25220.1 \| transporter, putative, similar to glycerol-3-phosphate transporter (glycerol 3-phosphate permease) (Homo sapiens) GI:7543982 \| chr4:12920937-12922895 FORWARD \| Aliases: F24A6.60, F24A6_60 | 2.6 | 6.1 | -3.5 | -7.6 | 0.12% | 7.1 |
| 1862 | AT1G52690.2 \| late embryogenesis abundant protein, putative / LEA protein, putative, similar to SP:P13934 Late embryogenesis abundant protein 76 (LEA 76) {Brassica napus}; contains Pfam profile PF02987: Late embryogenesis abundant protein | 3.1 | 6.9 | -3.9 | -7.6 | 0.12% | 7.2 |
| 1863 | AT1G78420.1 \| expressed protein \| chr1:29509426-29512379 FORWARD \| Aliases: F3F9.7, F3F9_7 | 6.2 | 8.0 | -1.8 | -7.6 | 0.12% | 7.2 |
| 1864 | AT4G03340.1 \| glycosyltransferase family 14 protein / core-2/I-branching enzyme family protein, contains Pfam profile: PF02485 Core-2/I-Branching enzyme \| chr4:1467749-1470062 REVERSE \| Aliases: F4C21.27, F4C21_27 | 4.3 | 6.1 | -1.8 | -7.6 | 0.12% | 7.3 |
| 1866 | AT5G49080.1 \| proline-rich extensin-like family protein, contains proline-rich extensin domains, INTERPRO:IPR002965 \| chr5:19908010-19910035 REVERSE \| Aliases: K20J1.5, K20J1_5 | 4.1 | 10.6 | -6.6 | -7.6 | 0.12% | 6.2 |
| 1868 | AT3G11320.1 \| similar to phosphate translocator-related [Arabidopsis thaliana] (TAIR:At5g05820.1); similar to Putative phosphate/phosphoenolpyruvate translocator protein [Oryza sativa (japonica cultivar-group)] (GB:XP_470662.1) \| chr3:3546652-3548862 REVERSE \| Aliases: F11B9.28 | 4.4 | 6.4 | -2.0 | -7.6 | 0.12% | 7.2 |
| 1870 | AT5G43230.1 \| hypothetical protein \| chr5:17367665-17369975 FORWARD \| Aliases: MNL12.5, MNL12_5 | 2.6 | 3.6 | -1.0 | -7.6 | 0.13% | 7.4 |
| 1871 | AT1G31720.1 \| expressed protein \| chr1:11356083-11357062 FORWARD \| Aliases: F27M3.8, F27M3_8 | 2.8 | 6.7 | -3.8 | -7.6 | 0.13% | 5.4 |

| Rank | Description | Sync | Root | M | t | adj.q | B |
|---|---|---|---|---|---|---|---|
| 1877 | AT3G07870.1 \| F-box family protein, contains F-box domain Pfam:PF00646 | 5.6 | 6.8 | -1.2 | -7.6 | 0.13% | 7.4 |
| 1878 | AT1G18390.1 \| protein kinase family protein, contains protein kinase domain, Pfam:PF00069 \| chr1:6327456-6329928 FORWARD \| Aliases: F15H18.25, F15H18_25 | 4.2 | 5.7 | -1.5 | -7.6 | 0.13% | 7.4 |
| 1880 | AT5G07270.1 \| ankyrin repeat family protein, contains ankyrin repeats, Pfam:PF00023 \| chr5:2280672-2283594 FORWARD \| Aliases: T28J14.210, T28J14_210 | 3.4 | 5.4 | -2.0 | -7.6 | 0.13% | 7.2 |
| 1883 | AT2G27370.1 \| integral membrane family protein, contains TIGRFAM TIGR01569 : plant integral membrane protein TIGR01569; contains Pfam PF04535 : Domain of unknown function (DUF588) \| chr2:11715532-11716983 REVERSE \| Aliases: F12K2.5, F12K2_5 | 2.6 | 6.2 | -3.6 | -7.6 | 0.13% | 6.1 |
| 1884 | AT5G25820.1 \| exostosin family protein, contains Pfam profile: PF03016 exostosin family \| chr5:8997186-9000318 REVERSE \| Aliases: F18A17.70, F18A17_70 | 3.9 | 6.6 | -2.6 | -7.6 | 0.13% | 6.6 |
| 1887 | AT4G37470.1 \| hydrolase, alpha/beta fold family protein, low similarity to SP:Q59093 3-oxoadipate enol-lactonase I (EC 3.1.1.24) (Enol-lactone hydrolase I) (Beta-ketoadipate enol-lactone hydrolase I) {Acinetobacter calcoaceticus}; contains Pfam profile PF00561: hydrolase, alpha/beta fold family \| chr4:17616653-17618452 REVERSE \| Aliases: F6G17.120, F6G17_120 | 9.0 | 10.4 | -1.4 | -7.6 | 0.13% | 7.4 |
| 1888 | AT3G57330.1 \| calcium-transporting ATPase, plasma membrane-type, putative / Ca2+-ATPase, putative (ACA11), identical to SP:Q9M2L4:ACAB_ARATH Potential calcium-transporting ATPase 11, plasma membrane-type (EC 3.6.3.8) (Ca(2+)-ATPase isoform 11) {Arabidopsis thaliana}; strong similarity to calmodulin-stimulated calcium-ATPase (Brassica oleracea) GI:1805654 Belongs to plant 2B ATPase??s with an N-terminal autoinhibitor. \| chr3:21222455-21227353 REVERSE \| Aliases: F28O9.180 | 6.4 | 7.6 | -1.3 | -7.6 | 0.13% | 7.3 |
| 1889 | AT2G38860.2 \| Symbol: YLS5 \| proteaseI (pfpI)-like protein (YLS5), contains Pfam profile PF01965: DJ-1/PfpI family; supporting cDNA gi:13122287:dbj:AB047808.1:; identical to proteaseI (pfpI)-like protein (Arabidopsis thaliana) GI:13122288, cDNA proteaseI (pfpI)-like protein GI:13122287 \| chr2:16240522-16242309 REVERSE \| Aliases: T7F6.3, T7F6_3 | 8.4 | 10.1 | -1.7 | -7.6 | 0.13% | 7.3 |
| 1893 | AT2G37060.3 \| similar to CCAAT-box binding transcription factor, putative [Arabidopsis thaliana] (TAIR:At3g53340.1); similar to CAAT-box DNA binding protein subunit B (NF-YB) [Zea mays] (GB:CAA42234.1); contains InterPro domain Transcription factor CBF/NF-Y/archaeal histone (InterPro:IPR003958); contains InterPro domain Histone-like transcription factor/archaeal histone/topoisomerase (InterPro:IPR003957); contains InterPro domain Histone-fold/TFIID-TAF/NF-Y (InterPro:IPR007124); contains InterPro domain Histone-like transcription factor CBF/NF-Y/archaeal histone, subunit A (InterPro:IPR003956) \| chr2:15583103-15584956 FORWARD \| Aliases: T2N18.18, T2N18_18 | 3.8 | 6.1 | -2.3 | -7.6 | 0.13% | 7.0 |
| 1896 | AT2G18800.1 \| xyloglucan:xyloglucosyl transferase, putative / xyloglucan endotransglycosylase, putative / endo-xyloglucan transferase, putative, similar to xyloglucan endotransglycosylase TCH4 GI:886116 from (Arabidopsis thaliana) | 2.9 | 4.7 | -1.8 | -7.6 | 0.13% | 7.2 |
| 1898 | AT3G59940.1 \| kelch repeat-containing F-box family protein, contains Pfam profiles PF01344: Kelch motif, PF00646: F-box domain | 3.5 | 6.5 | -2.9 | -7.6 | 0.13% | 6.6 |
| 1904 | AT1G53940.1 \| GDSL-motif lipase/hydrolase family protein, similar to lipase GI:1145627 from (Arabidopsis thaliana); contains Pfam profile PF00657: Lipase/Acylhydrolase with GDSL-like motif \| chr1:20146947-20149448 FORWARD \| Aliases: T18A20.17 | 2.8 | 6.4 | -3.6 | -7.5 | 0.13% | 6.3 |
| 1906 | AT1G66450.1 \| DC1 domain-containing protein, contains Pfam profile PF03107: DC1 domain | 3.4 | 5.9 | -2.4 | -7.5 | 0.13% | 7.2 |
| 1909 | AT4G22770.1 \| DNA-binding family protein, contains a AT hook motif (DNA binding motifs with a preference for A/T rich regions), Pfam:PF02178 \| chr4:11963639-11965695 REVERSE \| Aliases: T12H17.160, T12H17_160 | 4.8 | 6.9 | -2.0 | -7.5 | 0.13% | 7.1 |
| 1910 | AT5G01830.1 \| armadillo/beta-catenin repeat family protein / U-box domain-containing protein, contains Pfam domain, PF00514: Armadillo/beta-catenin-like repeats and Pfam, PF04564: U-box domain \| chr5:320692-323165 FORWARD \| Aliases: T20L15.100, T20L15_100 | 5.0 | 8.3 | -3.2 | -7.5 | 0.13% | 7.2 |
| 1911 | AT1G72220.1 \| zinc finger (C3HC4-type RING finger) family protein, contains Pfam profile: PF00097 zinc finger, C3HC4 type (RING finger); similar to GI:4928397 from (Arabidopsis thaliana) (Plant Mol. Biol. 40 (4), 579-590 (1999)) \| chr1:27187943-27189492 REVERSE \| Aliases: T9N14.22, T9N14_22 | 4.9 | 7.6 | -2.7 | -7.5 | 0.13% | 6.8 |
| 1913 | AT5G42010.1 \| WD-40 repeat family protein, contains Pfam PF00400: WD domain, G-beta repeat; similar to WD-repeat protein 5 (WD repeat protein BIG-3) (SP: Q9UGP9) (Homo sapiens) \| chr5:16819508-16822241 FORWARD \| Aliases: MJC20.11, MJC20_11 | 3.8 | 5.2 | -1.4 | -7.5 | 0.13% | 7.3 |

| Rank | Description | Sync | Root | M | t | adj.q | B |
|---|---|---|---|---|---|---|---|
| 1914 | AT2G32390.2 \| Symbol: ATGLR3.5 \| similar to glutamate receptor family protein (GLR3.6) [Arabidopsis thaliana] (TAIR:At3g51480.1); similar to glutamate receptor family protein (GLR3.3) [Arabidopsis thaliana] (TAIR:At1g42540.1); similar to glutamate receptor family protein (GLR3.2) (GLUR2) [Arabidopsis thaliana] (TAIR:At4g35290.2); similar to glutamate receptor family protein (GLR3.4) [Arabidopsis thaliana] (TAIR:At1g05200.1); similar to glutamate receptor family protein (GLR3.2) (GLUR2) [Arabidopsis thaliana] (TAIR:At4g35290.1); similar to ligand gated channel-like protein [Brassica napus] (GB:AAF21901.1); similar to glutamate receptor, ionotropic kainate 5 precursor-like protein [Oryza sativa (japonica cultivar-group)] (GB:NP_919189.1); similar to putative ionotropic glutamate receptor ortholog GLR6 [Oryza sativa (japonica cultivar-group)] (GB:BAD45881.1); similar to putative ionotropic glutamate receptor homolog GLR4 [Oryza sativa (japonica cultivar-group)] (GB:XP_478449.1); similar to OSJNBa0013K16.8 [Oryza sativa (japonica cultivar-group)] (GB:XP_473671.1); contains InterPro domain Extracellular ligand-binding receptor (InterPro:IPR001828); contains InterPro domain Ionotropic glutamate receptor (InterPro:IPR001320); contains InterPro domain G-protein coupled receptors family 3 (Metabotropic glutamate receptor-like) (InterPro:IPR000337) \| chr2:13755545-13758834 REVERSE \| Aliases: GLR3.5, GLR6, GLUTAMATE RECEPTOR 6, T32F6.9, T32F6_9 | 4.0 | 5.5 | -1.5 | -7.5 | 0.13% | 7.3 |
| 1915 | AT2G24570.1 \| Symbol: WRKY17 \| | 3.8 | 6.4 | -2.6 | -7.5 | 0.13% | 7.1 |
| 1917 | AT4G15920.1 \| nodulin MtN3 family protein, similar to MtN3 GI:1619602 (root nodule development) from (Medicago truncatula) \| chr4:9030531-9033409 REVERSE \| Aliases: DL4000C, FCAALL.237 | 6.3 | 7.9 | -1.6 | -7.5 | 0.13% | 7.2 |
| 1918 | AT1G36070.1 \| WD-40 repeat family protein, contains 2 WD-40 repeats (PF0400);similar to guanine nucleotide-binding protein beta subunit GPBA (SP:P36408) (Dictyostelium discoideum (Slime mold)); similar to katanin p80 (WD40-containing) subunit B 1 (GI:12655011) (Homo sapiens) \| chr1:13468123-13471888 REVERSE \| Aliases: F5J5.6, F5J5_6 | 3.0 | 4.2 | -1.3 | -7.5 | 0.13% | 7.3 |
| 1919 | AT4G32070.1 \| octicosapeptide/Phox/Bem1p (PB1) domain-containing protein / tetratricopeptide repeat (TPR)-containing protein, similar to SP:Q99614 Tetratricopeptide repeat protein 1 {Homo sapiens}; contains Pfam profiles PF00564: PB1 domain, PF00515: TPR Domain \| chr4:15504668-15507103 REVERSE \| Aliases: F10N7.120, F10N7_120 | 3.7 | 6.7 | -3.0 | -7.5 | 0.13% | 7.2 |
| 1923 | AT5G40850.1 \| Symbol: UPM1 \| urophorphyrin III methylase (UPM1), identical to urophorphyrin III methylase (GI:1146165) (Arabidopsis thaliana); similar to s-adenosyl-L-methionine-dependent uroporphyrinogen III methyltransferase (GI:1490606) (Arabidopsis thaliana); similar to Diphthine synthase (Diphtamide biosynthesis methyltransferase) (DPH5) (SP:P32469) (Saccharomyces cerevisiae); contains Pfam PF00590 : Tetrapyrrole (Corrin/Porphyrin) Methylases domain; contains TIGRFAM PF00590: Tetrapyrrole (Corrin/Porphyrin) Methylases \| chr5:16383742-16386327 FORWARD \| Aliases: MHK7.8, MHK7_8, UROPHORPHYRIN METHYLASE 1 | 7.6 | 9.8 | -2.1 | -7.5 | 0.13% | 7.3 |
| 1924 | AT1G34510.1 \| peroxidase, putative, similar to peroxidase ATP13a GB:CAA67312 from (Arabidopsis thaliana) \| chr1:12615711-12617010 REVERSE \| Aliases: F12K21.18, F12K21_18 | 2.5 | 6.4 | -3.9 | -7.5 | 0.13% | 6.5 |
| 1928 | AT1G65840.1 \| amine oxidase family protein, similar to polyamine oxidase SP:O64411 (Zea mays); contains Pfam profile PF01593 amine oxidase, flavin-containing \| chr1:24493447-24496687 FORWARD \| Aliases: F1E22.18, F1E22_18 | 7.6 | 10.2 | -2.6 | -7.5 | 0.13% | 7.0 |
| 1932 | AT4G19880.3 \| similar to expressed protein [Arabidopsis thaliana] (TAIR:At5g45020.1); similar to putative transferase [Escherichia coli O157:H7 EDL933] (GB:AAG58235.1) \| chr4:10784401-10786433 REVERSE \| Aliases: T16H5.240, T16H5_240 | 9.1 | 10.4 | -1.2 | -7.5 | 0.13% | 7.2 |
| 1933 | AT2G01170.2 \| amino acid permease family protein, weak similarity to GABA permease (Emericella nidulans) GI:4972245; contains Pfam profile PF00324: Amino acid permease \| chr2:102210-104514 REVERSE \| Aliases: F10A8.5, F10A8_5 | 5.6 | 8.2 | -2.7 | -7.5 | 0.13% | 6.8 |
| 1935 | AT4G40050.1 \| expressed protein \| chr4:18560854-18563832 REVERSE \| Aliases: T5J17.220, T5J17_220 | 3.3 | 5.6 | -2.3 | -7.5 | 0.13% | 7.2 |
| 1939 | AT2G34680.1 \| Symbol: AIR9 \| similar to leucine-rich repeat family protein [Arabidopsis thaliana] (TAIR:At1g78230.1); similar to putative leucine rich repeat protein [Oryza sativa (japonica cultivar-group)] (GB:XP_476635.1); contains InterPro domain Leucine-rich repeat (InterPro:IPR001611) \| chr2:14623701-14636208 REVERSE \| Aliases: T29F13.11, T29F13_11 | 7.7 | 9.5 | -1.8 | -7.5 | 0.13% | 7.3 |
| 1941 | AT4G19040.1 \| pleckstrin homology (PH) domain-containing protein / lipid-binding START domain-containing protein, contains Pfam profiles PF01852: START domain, PF00169: PH domain \| chr4:10431532-10437436 REVERSE \| Aliases: F13C5.210, F13C5_210 | 7.3 | 8.7 | -1.4 | -7.5 | 0.13% | 7.3 |
| 1942 | AT4G09110.1 \| zinc finger (C3HC4-type RING finger) family protein, contains Pfam profile: PF00097: Zinc finger, C3HC4 type (RING finger) \| chr4:5812485-5813393 FORWARD \| Aliases: T8A17.5 | 2.7 | 7.8 | -5.1 | -7.5 | 0.13% | 7.3 |
| 1943 | AT5G02270.1 \| Symbol: ATNAP9 \| | 10.4 | 12.0 | -1.6 | -7.5 | 0.13% | 7.1 |
| 1945 | AT4G31710.1 \| Symbol: ATGLR2.4 \| glutamate receptor family protein (GLR2.4), plant glutamate receptor family, PMID:11379626 \| chr4:15349127-15352968 FORWARD \| Aliases: F28M20.100, F28M20_100, GLR2.4 | 4.0 | 6.8 | -2.8 | -7.5 | 0.13% | 6.6 |

| Rank | Description | Sync | Root | M | t | adj.q | B |
|---|---|---|---|---|---|---|---|
| 1946 | AT5G58940.1 \| Symbol: CRCK1 \| similar to protein kinase family protein [Arabidopsis thaliana] (TAIR:At4g00330.1); similar to P0529H11.30 [Oryza sativa (japonica cultivar-group)] (GB:NP_915524.1); contains InterPro domain Serine/threonine protein kinase, active site (InterPro:IPR008271); contains InterPro domain Tyrosine protein kinase (InterPro:IPR001245); contains InterPro domain Serine/threonine protein kinase (InterPro:IPR002290); contains InterPro domain Protein kinase (InterPro:IPR000719) \| chr5:23815812-23818049 FORWARD \| Aliases: CALMODULIN BINDING RECEPTOR LIKE CYTOPLASMIC KINASE 1, K19M22.13, K19M22_13 | 3.5 | 5.9 | -2.4 | -7.5 | 0.13% | 7.0 |
| 1947 | AT2G34070.1 \| expressed protein \| chr2:14394505-14397309 REVERSE \| Aliases: T14G11.19, T14G11_19 | 5.1 | 6.6 | -1.5 | -7.5 | 0.13% | 7.2 |
| 1950 | AT3G05490.1 \| Symbol: RALFL22 \| rapid alkalinization factor (RALF) family protein, similar to RALF precursor (Nicotiana tabacum) GI:16566316 \| chr3:1591283-1591989 FORWARD \| Aliases: F22F7.6, F22F7_6, RALF LIKE 22 | 6.8 | 9.3 | -2.5 | -7.5 | 0.13% | 7.2 |
| 1952 | AT5G63370.1 \| protein kinase family protein, contains protein kinase domain, Pfam:PF00069 \| chr5:25401132-25404450 REVERSE \| Aliases: K9H21.10, K9H21_10 | 5.7 | 7.7 | -2.0 | -7.5 | 0.13% | 7.2 |
| 1955 | AT4G20450.1 \| leucine-rich repeat protein kinase, putative, similar to light repressible receptor protein kinase (Arabidopsis thaliana) gi:1321686:emb:CAA66376; contains leucine rich repeat (LRR) domains, Pfam:PF00560; contains protein kinase domain, Pfam:PF00069 \| chr4:11024065-11029019 REVERSE \| Aliases: F9F13.100, F9F13_100 | 2.6 | 5.2 | -2.7 | -7.5 | 0.13% | 6.6 |
| 1957 | AT3G57380.1 \| expressed protein, contains Pfam domain, PF04577: Protein of unknown function (DUF563) \| chr3:21240779-21242706 FORWARD \| Aliases: F28O9.230 | 4.1 | 6.1 | -2.0 | -7.5 | 0.13% | 7.1 |
| 1959 | AT1G12560.1 \| Symbol: ATEXPA7 \| expansin, putative (EXP7), similar to expansin GI:2828241 from (Brassica napus); alpha-expansin gene family, PMID:11641069 \| chr1:4276555-4277691 FORWARD \| Aliases: ARABIDOPSIS THALIANA EXPANSIN A7, ATEXP7, ATHEXP ALPHA 1.26, EXP7, F5O11.30, F5O11_30 | 3.1 | 8.3 | -5.2 | -7.5 | 0.13% | 7.0 |
| 1963 | AT4G03110.2 \| RNA-binding protein, putative, similar to Etr-1 (Danio rerio) GI:7670536, BRUNO-like 6 RNA-binding protein (Homo sapiens) GI:15341327, CUG-BP and ETR-3 like factor 3 (Homo sapiens) GI:12746392; contains InterPro entry IPR000504: RNA-binding region RNP-1 (RNA recognition motif) (RRM) \| chr4:1376306-1379398 REVERSE \| Aliases: F4C21.3 | 4.1 | 6.9 | -2.8 | -7.5 | 0.13% | 7.2 |
| 1969 | AT1G22360.2 \| Symbol: AT2 \| similar to UDP-glucoronosyl/UDP-glucosyl transferase family protein [Arabidopsis thaliana] (TAIR:At1g22380.1); similar to UDP-glucoronosyl/UDP-glucosyl transferase family protein [Arabidopsis thaliana] (TAIR:At1g22340.1); similar to UDP-glucoronosyl/UDP-glucosyl transferase family protein [Arabidopsis thaliana] (TAIR:At1g22400.1); similar to UDP-glucoronosyl/UDP-glucosyl transferase family protein [Arabidopsis thaliana] (TAIR:At1g22370.2); similar to glycosyltransferase NTGT5a [Nicotiana tabacum] (GB:BAD93689.1); contains InterPro domain UDP-glucoronosyl/UDP-glucosyl transferase (InterPro:IPR002213) \| chr1:7894847-7897580 REVERSE \| Aliases: None | 5.5 | 9.5 | -4.0 | -7.5 | 0.13% | 6.5 |
| 1970 | AT5G58690.1 \| phosphoinositide-specific phospholipase C family protein, contains Pfam profile: PF00388 phosphatidylinositol-specific phospholipase C \| chr5:23726755-23729357 REVERSE \| Aliases: MZN1.15, MZN1_15 | 6.7 | 10.4 | -3.7 | -7.5 | 0.14% | 7.1 |
| 1973 | AT3G51830.1 \| Symbol: ATG5 \| phosphoinositide phosphatase family protein, contains similarity to phosphoinositide phosphatase SAC1 (Rattus norvegicus) gi:11095248:gb:AAG29810; contains Pfam domain, PF02383: SacI homology domain ; Contains nonconsensus AT/AA splice site at intron 7 \| chr3:19231126-19236257 FORWARD \| Aliases: ATEM1.8, TRANSMEMBRANE PROTEIN G5P | 5.4 | 8.1 | -2.7 | -7.5 | 0.14% | 7.2 |
| 1978 | AT1G01340.1 \| Symbol: ATCNGC10 \| cyclic nucleotide-regulated ion channel (CNGC10) (ACBK1), almost identical to CaM-regulated potassium ion channel (ACBK1) GI:8515883 from (Arabidopsis thaliana); contains Pfam domain, PF00520: Ion transport protein \| chr1:132332-135322 REVERSE \| Aliases: ACBK1, CNGC10, F6F3.13, F6F3_13 | 4.4 | 7.8 | -3.4 | -7.4 | 0.14% | 7.0 |
| 1979 | AT5G18650.1 \| zinc finger (C3HC4-type RING finger) family protein, contains Pfam domain PF00097: Zinc finger, C3HC4 type (RING finger) | 7.5 | 9.9 | -2.3 | -7.4 | 0.14% | 7.2 |
| 1980 | AT5G27150.1 \| Symbol: NHX1 \| sodium proton exchanger / Na+/H+ antiporter (NHX1), identical to Na+/H+ exchanger (Arabidopsis thaliana) gi:6650177:gb:AAF21755 and sodium proton exchanger Nhx1 (Arabidopsis thaliana) gi:4324597:gb:AAD16946; Member of The Monovalent Cation:Proton Antiporter (CPA1) Family, PMID:11500563 \| chr5:9553441-9557516 FORWARD \| Aliases: AT NHX1, ATNHX, ATNHX1, NA(+)/H(+) ANTIPORTER, NA+/H+ EXCHANGER, T21B4.60, T21B4_60 | 7.0 | 9.6 | -2.6 | -7.4 | 0.14% | 7.0 |
| 1982 | AT1G73640.1 \| Ras-related GTP-binding family protein, contains Pfam profile: PF00071 ras family Pfam profile: PF00071 Ras family \| chr1:27690653-27691788 FORWARD \| Aliases: F25P22.5, F25P22_5 | 4.8 | 7.4 | -2.6 | -7.4 | 0.14% | 7.1 |
| 1985 | AT3G14090.1 \| exocyst subunit EXO70 family protein, contains Pfam domain PF03081: Exo70 exocyst complex subunit; \| chr3:4669260-4671560 REVERSE \| Aliases: MAG2.5 | 4.2 | 6.0 | -1.7 | -7.4 | 0.14% | 7.1 |
| 1988 | AT4G38550.1 \| expressed protein \| chr4:18025653-18028794 FORWARD \| Aliases: F20M13.110, F20M13_110 | 4.2 | 7.8 | -3.6 | -7.4 | 0.14% | 7.0 |
| 1990 | AT1G13420.1 \| sulfotransferase family protein, similar to steroid sulfotransferase 1 GI:3420004 from (Brassica napus); contains Pfam profile PF00685: Sulfotransferase domain \| chr1:4604838-4606050 FORWARD \| Aliases: T6J4.16, T6J4_16 | 2.9 | 4.9 | -2.1 | -7.4 | 0.14% | 7.0 |

| Rank | Description | Sync | Root | M | t | adj.q | B |
|---|---|---|---|---|---|---|---|
| 1991 | AT2G25010.1 \| expressed protein \| chr2:10638649-10640736 FORWARD \| Aliases: F27C12.7, F27C12_7 | 4.0 | 6.9 | -3.0 | -7.4 | 0.14% | 6.8 |
| 1992 | AT3G28050.1 \| nodulin MtN21 family protein, similar to MtN21 (Medicago truncatula) GI:2598575; contains Pfam profile PF00892: Integral membrane protein \| chr3:10444054-10446622 FORWARD \| Aliases: MMG15.22 | 5.1 | 8.6 | -3.5 | -7.4 | 0.14% | 6.9 |
| 1993 | AT1G19450.1 \| integral membrane protein, putative / sugar transporter family protein, similar to GB:U43629 GI:1209756 integral membrane protein from (Beta vulgaris); contains Pfam profile PF00083: major facilitator superfamily protein; contains TIGRfam TIGR00879: Sugar transporter \| chr1:6731425-6734873 REVERSE \| Aliases: F18O14.22, F18O14_22 | 7.9 | 10.5 | -2.6 | -7.4 | 0.14% | 7.2 |
| 1994 | AT2G20670.1 \| expressed protein, contains Pfam profile PF04720: Protein of unknown function (DUF506) \| chr2:8918941-8920615 REVERSE \| Aliases: F23N11.1 | 3.3 | 4.9 | -1.6 | -7.4 | 0.14% | 7.2 |
| 1995 | AT2G17720.1 \| oxidoreductase, 2OG-Fe(II) oxygenase family protein, similar to prolyl 4-hydroxylase, alpha subunit, from Gallus gallus (GI:212530), Rattus norvegicus (GI:474940), Mus musculus (SP:Q60715); contains PF03171 2OG-Fe(II) oxygenase superfamily domain \| chr2:7704418-7706636 FORWARD \| Aliases: T17A5.10, T17A5_10 | 7.7 | 9.5 | -1.8 | -7.4 | 0.14% | 7.2 |
| 1997 | AT1G27740.1 \| basic helix-loop-helix (bHLH) family protein, contains Pfam profile: PF00010 helix-loop-helix DNA-binding domain | 2.8 | 6.1 | -3.3 | -7.4 | 0.14% | 6.0 |
| 2000 | AT3G07070.1 \| protein kinase family protein, contains protein kinase domain, Pfam:PF00069 \| chr3:2237964-2240080 FORWARD \| Aliases: F17A9.25 | 2.4 | 4.3 | -1.9 | -7.4 | 0.14% | 7.0 |
| 2006 | AT5G06330.1 \| hairpin-responsive protein, putative (HIN1), similar to harpin-induced protein hin1 ( GI:1619321) (Nicotiana tabacum) \| chr5:1934740-1935645 REVERSE \| Aliases: MHF15.15, MHF15_15 | 3.2 | 5.1 | -1.9 | -7.4 | 0.14% | 6.9 |
| 2009 | AT5G13080.1 \| Symbol: WRKY75 \| | 3.8 | 6.7 | -2.9 | -7.4 | 0.14% | 7.1 |
| 2010 | AT1G48260.1 \| Symbol: CIPK17 \| | 3.5 | 5.9 | -2.4 | -7.4 | 0.14% | 6.7 |
| 2011 | AT1G51880.1 \| leucine-rich repeat protein kinase, putative, similar to light repressible receptor protein kinase (Arabidopsis thaliana) gi:1321686:emb:CAA66376; contains leucine rich repeat (LRR) domains, Pfam:PF00560; contains protein kinase domain, Pfam:PF00069 \| chr1:19273862-19277737 REVERSE \| Aliases: T14L22.9, T14L22_9 | 3.0 | 5.2 | -2.2 | -7.4 | 0.14% | 6.9 |
| 2021 | AT1G68940.1 \| armadillo/beta-catenin repeat protein-related / U-box domain-containing protein, ; contains Pfam profile PF04564: U-box domain \| chr1:25925664-25929037 REVERSE \| Aliases: T6L1.12, T6L1_12 | 4.0 | 6.7 | -2.7 | -7.4 | 0.15% | 6.4 |
| 2022 | AT3G09960.1 \| calcineurin-like phosphoesterase family protein, contains Pfam profile: PF00149 calcineurin-like phosphoesterase | 3.5 | 5.3 | -1.8 | -7.4 | 0.15% | 7.0 |
| 2023 | AT5G49710.3 \| expressed protein, similar to unknown protein (pir::T05575) \| chr5:20212653-20214579 FORWARD \| Aliases: K2I5.7, K2I5_7 | 5.0 | 7.6 | -2.6 | -7.3 | 0.15% | 6.4 |
| 2025 | AT3G28690.2 \| similar to protein kinase, putative [Arabidopsis thaliana] (TAIR:At5g15080.1); similar to putative protein kinase [Oryza sativa (japonica cultivar-group)] (GB:NP_917446.1); similar to serine/threonine protein kinase [Aster tripolium] (GB:BAC57958.1); contains InterPro domain Serine/threonine protein kinase, active site (InterPro:IPR008271); contains InterPro domain Tyrosine protein kinase (InterPro:IPR001245); contains InterPro domain Serine/threonine protein kinase (InterPro:IPR002290); contains InterPro domain Protein kinase (InterPro:IPR000719) \| chr3:10756744-10759105 FORWARD \| Aliases: MZN14.22 | 4.2 | 7.5 | -3.2 | -7.3 | 0.15% | 6.9 |
| 2027 | AT1G17960.1 \| threonyl-tRNA synthetase, putative / threonine--tRNA ligase, putative, similar to SP:O04630 Threonyl-tRNA synthetase, mitochondrial precursor (EC 6.1.1.3) (Threonine--tRNA ligase) (ThrRS) {Arabidopsis thaliana}; contains Pfam profiles PF00587: tRNA synthetase class II core domain (G, H, P, S and T), PF03129: Anticodon binding domain, PF02824: TGS domain \| chr1:6181008-6183730 REVERSE \| Aliases: F2H15.18, F2H15_18 | 3.5 | 5.2 | -1.7 | -7.3 | 0.15% | 7.0 |
| 2029 | AT5G07780.1 \| formin homology 2 domain-containing protein / FH2 domain-containing protein, contains formin homology 2 domain, Pfam:PF02181 \| chr5:2479708-2482834 FORWARD \| Aliases: MXM12.2, MXM12_2 | 2.8 | 5.4 | -2.6 | -7.3 | 0.15% | 6.8 |
| 2031 | AT3G55720.1 \| expressed protein, contains Pfam profile PF04788: Protein of unknown function (DUF620) \| chr3:20690799-20692426 FORWARD \| Aliases: F1I16.130 | 2.7 | 4.9 | -2.2 | -7.3 | 0.15% | 6.7 |
| 2033 | AT1G72850.1 \| disease resistance protein (TIR-NBS class), putative, domain signature TIR-NBS exists, suggestive of a disease resistance protein. \| chr1:27417632-27418996 FORWARD \| Aliases: F3N23.5, F3N23_5 | 3.3 | 5.7 | -2.4 | -7.3 | 0.15% | 6.9 |
| 2034 | AT4G33150.2 \| Symbol: LKR \| lysine-ketoglutarate reductase/saccharopine dehydrogenase bifunctional enzyme, identical to lysine-ketoglutarate reductase/saccharopine dehydrogenase GI:2052508 from (Arabidopsis thaliana) \| chr4:15985201-15991541 REVERSE \| Aliases: F4I10.80, F4I10_80, LYSINE KETOGLUTARATE REDUCTASE/SACCHAROPINE DEHYDROGENASE, SACCHAROPINE DEHYDROGENASE | 7.5 | 9.5 | -2.0 | -7.3 | 0.15% | 7.1 |

| Rank | Description | Sync | Root | M | t | adj.q | B |
|---|---|---|---|---|---|---|---|
| 2035 | AT5G06640.1 \| proline-rich extensin-like family protein, contains proline-rich extensin domains, INTERPRO:IPR002965 \| chr5:2039860-2041929 REVERSE \| Aliases: F15M7.17, F15M7_17 | 3.2 | 9.1 | -5.9 | -7.3 | 0.15% | 6.4 |
| 2038 | AT5G20820.1 \| auxin-responsive protein-related, similar to auxin-induced protein TGSAUR21 (GI:10185818) Tulipa gesneriana) \| chr5:7046753-7047398 REVERSE \| Aliases: T1M15.220, T1M15_220 | 4.3 | 8.0 | -3.7 | -7.3 | 0.15% | 6.0 |
| 2039 | AT5G18640.1 \| lipase class 3 family protein, low similarity to Triacylglycerol Acylhydrolase (E.C.3.1.1.3) (Rhizomucor miehei) GI:230348; contains Pfam profile PF01764: Lipase \| chr5:6213249-6215496 FORWARD \| Aliases: T1A4.20, T1A4_20 | 4.9 | 7.5 | -2.5 | -7.3 | 0.15% | 6.7 |
| 2040 | AT2G40000.1 \| expressed protein \| chr2:16707588-16709106 REVERSE \| Aliases: T28M21.16, T28M21_16 | 8.2 | 10.3 | -2.1 | -7.3 | 0.15% | 7.1 |
| 2041 | AT2G46750.1 \| FAD-binding domain-containing protein, low similarity to SP:P58710 L-gulonolactone oxidase (EC 1.1.3.8) {Mus musculus}; contains Pfam profile PF01565: FAD binding domain \| chr2:19215390-19218028 REVERSE \| Aliases: F19D11.3 | 3.6 | 5.8 | -2.2 | -7.3 | 0.15% | 6.7 |
| 2044 | AT5G42590.1 \| Symbol: CYP71A16 \| cytochrome P450 71A16, putative (CYP71A16), Identical to Cytochrome P450 71A16 (SP:Q9FH66) (Arabidopsis thaliana) \| chr5:17048375-17050924 REVERSE \| Aliases: K16E1.6, K16E1_6 | 3.0 | 5.2 | -2.2 | -7.3 | 0.15% | 6.7 |
| 2045 | AT5G22410.1 \| peroxidase, putative, identical to peroxidase ATP14a (Arabidopsis thaliana) gi:1546690:emb:CAA67335 \| chr5:7426328-7427967 FORWARD \| Aliases: MWD9.21, MWD9_21 | 2.9 | 5.4 | -2.4 | -7.3 | 0.15% | 7.0 |
| 2047 | AT2G37950.1 \| zinc finger (C3HC4-type RING finger) family protein, contains Pfam profile PF00097: Zinc finger, C3HC4 type (RING finger); contains PROSITE PS00190: Cytochrome c family heme-binding site signature \| chr2:15889566-15891057 REVERSE \| Aliases: T8P21.14, T8P21_14 | 3.5 | 7.3 | -3.8 | -7.3 | 0.15% | 5.8 |
| 2049 | AT3G50400.1 \| GDSL-motif lipase/hydrolase family protein, similar to family II lipase EXL3 (GI:15054386), EXL1 (GI:15054382), EXL2 (GI:15054384) (Arabidopsis thaliana); contains Pfam profile PF00657: Lipase/Acylhydrolase with GDSL-like motif \| chr3:18715746-18717551 FORWARD \| Aliases: F11C1.240 | 2.2 | 5.5 | -3.3 | -7.3 | 0.15% | 6.4 |
| 2051 | AT1G10900.1 \| phosphatidylinositol-4-phosphate 5-kinase family protein, similar to phosphatidylinositol-4-phosphate 5-kinase AtPIP5K1 (Arabidopsis thaliana) GI:3702691; contains Pfam profiles PF01504: Phosphatidylinositol-4-phosphate 5-Kinase, PF02493: MORN repeat \| chr1:3632274-3637141 REVERSE \| Aliases: T19D16.18, T19D16_18 | 5.2 | 6.8 | -1.7 | -7.3 | 0.15% | 7.1 |
| 2052 | AT2G43210.2 \| UBX domain-containing protein, contains Pfam profile PF00789: UBX domain | 7.3 | 9.1 | -1.8 | -7.3 | 0.15% | 7.0 |
| 2053 | AT3G23540.2 \| expressed protein, similar to expressed protein [Arabidopsis thaliana] (TAIR:At4g14290.1); similar to unknown protein [Oryza sativa (japonica cultivar-group)] (GB:XP_464484.1) \| chr3:8444945-8448077 FORWARD \| Aliases: MDB19.2 | 4.8 | 6.3 | -1.6 | -7.3 | 0.15% | 7.0 |
| 2056 | AT3G45900.1 \| expressed protein \| chr3:16885387-16887007 REVERSE \| Aliases: F16L2.110 | 5.6 | 6.8 | -1.3 | -7.3 | 0.15% | 7.1 |
| 2057 | AT1G76900.2 \| F-box family protein / tubby family protein, similar to Tubby protein (SP:P50586) {Mus musculus}; similar to Chain A, C-Terminal Domain Of Mouse Brain Tubby Protein (GI:6730158) (Mus musculus); similar to Tubby related protein 1 (Tubby-like protein 1) (Swiss-Prot:O00294) (Homo sapiens); similar to phosphodiesterase (GI:467578) (Mus musculus); contains Pfam profile: PF01167: Tub family; contains Pfam PF00646: F-box domain | 5.8 | 8.0 | -2.2 | -7.3 | 0.15% | 6.9 |
| 2058 | AT3G13690.1 \| protein kinase family protein, contains protein kinase domain, Pfam:PF00069 \| chr3:4485799-4490238 FORWARD \| Aliases: MMM17.11 | 3.6 | 5.9 | -2.2 | -7.3 | 0.15% | 7.1 |
| 2059 | AT4G35560.1 \| expressed protein \| chr4:16881567-16887024 FORWARD \| Aliases: F8D20.70, F8D20_70 | 5.4 | 8.1 | -2.7 | -7.3 | 0.15% | 6.9 |
| 2060 | AT3G55910.1 \| expressed protein, PA26, p53 regulated PA26-T3 nuclear protein, Homo sapiens, EMBL:AF033121 \| chr3:20753878-20754458 FORWARD \| Aliases: F27K19.90 | 3.8 | 5.9 | -2.1 | -7.3 | 0.15% | 6.8 |
| 2066 | AT4G08690.2 \| similar to SEC14 cytosolic factor family protein / phosphoglyceride transfer family protein [Arabidopsis thaliana] (TAIR:At1g22180.2); similar to putative sec14 like protein [Oryza sativa (japonica cultivar-group)] (GB:BAD81256.1); similar to unnamed protein product [Oryza sativa (japonica cultivar-group)] (GB:NP_912885.1); contains InterPro domain Cellular retinaldehyde-binding)/triple function, C-terminal (InterPro:IPR001251); contains InterPro domain Cellular retinaldehyde-binding/triple function, N-terminal (InterPro:IPR008273) \| chr4:5550937-5552953 REVERSE \| Aliases: T32A17.1 | 5.2 | 6.4 | -1.2 | -7.3 | 0.15% | 7.1 |
| 2070 | AT4G11360.1 \| Symbol: RHA1B \| zinc finger (C3HC4-type RING finger) family protein (RHA1b), identical to RING-H2 finger protein RHA1b (Arabidopsis thaliana) GI:3790567 \| chr4:6906009-6906754 FORWARD \| Aliases: F8L21.150, F8L21_150, RING H2 FINGER PROTEIN RHA1B | 4.7 | 7.0 | -2.3 | -7.3 | 0.15% | 7.0 |
| 2071 | AT1G60490.1 \| Symbol: ATVPS34 \| phosphatidylinositol 3-kinase (PI3K), identical to SP:P42339 Phosphatidylinositol 3-kinase (EC 2.7.1.137) (PI3-kinase) (PtdIns-3-kinase) (PI3K) {Arabidopsis thaliana} \| chr1:22289249-22293966 REVERSE \| Aliases: F8A5.4, F8A5_4, PHOSPHATIDYLINOSITOL 3 KINASE | 6.2 | 7.8 | -1.6 | -7.3 | 0.15% | 7.1 |

| Rank | Description | Sync | Root | M | t | adj.q | B |
|---|---|---|---|---|---|---|---|
| 2072 | AT1G44970.1 \| peroxidase, putative, similar to peroxidase GI:993004 from (Mercurialis annua) \| chr1:17004652-17006124 FORWARD \| Aliases: F27F5.6, F27F5_6 | 2.4 | 4.9 | -2.5 | -7.3 | 0.15% | 6.4 |
| 2075 | AT5G37930.1 \| seven in absentia (SINA) family protein, similar to SIAH1 protein (Brassica napus var. napus) GI:7657876; contains Pfam profile PF03145: Seven in absentia protein family \| chr5:15119430-15120899 REVERSE \| Aliases: K18L3.13, K18L3_13 | 6.1 | 7.7 | -1.6 | -7.3 | 0.16% | 7.0 |
| 2076 | AT5G60890.1 \| Symbol: ATR1 \| receptor-like protein kinase (ATR1) (MYB34), identical to receptor-like protein kinase(ATR1) GI:3150037 from (Arabidopsis thaliana); contains PFAM profile: myb DNA binding domain PF00249; identical to cDNA putative transcription factor (MYB34) mRNA, partial cds GI:3941443 | 3.2 | 6.4 | -3.2 | -7.3 | 0.16% | 6.6 |
| 2077 | AT1G23140.1 \| C2 domain-containing protein, similar to zinc finger and C2 domain protein GI:9957238 from (Arabidopsis thaliana) \| chr1:8202351-8203201 REVERSE \| Aliases: T26J12.9, T26J12_9 | 5.8 | 7.9 | -2.1 | -7.3 | 0.16% | 6.8 |
| 2079 | AT2G35170.1 \| MORN (Membrane Occupation and Recognition Nexus) repeat-containing protein /phosphatidylinositol-4-phosphate 5-kinase-related, low similarity to phosphatidylinositol-4-phosphate 5-kinase AtPIP5K1 (Arabidopsis thaliana) GI:3702691; contains Pfam profile PF02493: MORN repeat \| chr2:14834542-14836881 FORWARD \| Aliases: T4C15.16, T4C15_16 | 4.6 | 7.0 | -2.5 | -7.3 | 0.16% | 7.1 |
| 2080 | AT4G10360.2 \| expressed protein, similar to expressed protein [Arabidopsis thaliana] (TAIR:At1g31300.1); similar to unknown protein [Oryza sativa (japonica cultivar-group)] (GB:XP_475446.1); contains InterPro domain TRAM, LAG1 and CLN8 homology (InterPro:IPR006634) \| chr4:6420465-6422376 FORWARD \| Aliases: F24G24.160, F24G24_160 | 5.4 | 7.7 | -2.3 | -7.3 | 0.16% | 6.8 |
| 2082 | AT5G24880.1 \| expressed protein, ; expression supported by MPSS \| chr5:8552746-8554077 FORWARD \| Aliases: F6A4.90, F6A4_90 | 3.5 | 8.5 | -5.0 | -7.3 | 0.16% | 6.5 |
| 2085 | AT3G26290.1 \| Symbol: CYP71B26 \| cytochrome P450 71B26, putative (CYP71B26), identical to cytochrome P450 71B26 (SP:Q9LTL0) (Arabidopsis thaliana); contains Pfam profile: PF00067 cytochrome P450 \| chr3:9633919-9635703 REVERSE \| Aliases: MTC11.20 | 4.0 | 6.5 | -2.5 | -7.3 | 0.16% | 6.5 |
| 2086 | AT5G19790.1 \| Symbol: RAP2.11 \| encodes a member of the ERF (ethylene response factor) subfamily B-6 of ERF/AP2 transcription factor family (RAP2.11). The protein contains one AP2 domain. There are 12 members in this subfamily including RAP2.11. | 4.3 | 6.3 | -2.0 | -7.3 | 0.16% | 6.8 |
| 2090 | AT3G43230.1 \| zinc finger (FYVE type) family protein, contains Pfam domain PF01363: FYVE zinc finger \| chr3:15218355-15220813 FORWARD \| Aliases: F7K15.80 | 8.2 | 9.7 | -1.5 | -7.2 | 0.16% | 7.1 |
| 2091 | AT1G48690.1 \| auxin-responsive GH3 family protein, similar to auxin-responsive GH3 product (Glycine max) GI:18591; contains Pfam profile PF03321: GH3 auxin-responsive promoter \| chr1:18012773-18013636 REVERSE \| Aliases: F11I4.13, F11I4_13 | 2.5 | 7.1 | -4.6 | -7.2 | 0.16% | 6.2 |
| 2094 | AT2G19150.1 \| pectinesterase family protein, contains Pfam profile: PF01095 pectinesterase \| chr2:8312182-8314526 FORWARD \| Aliases: T20K24.17, T20K24_1 7 | 3.3 | 6.6 | -3.3 | -7.2 | 0.16% | 6.1 |
| 2095 | AT1G58410.1 \| disease resistance protein (CC-NBS-LRR class), putative, domain signature CC-NBS-LRR exists, suggestive of a disease resistance protein. | 2.9 | 5.1 | -2.2 | -7.2 | 0.16% | 6.8 |
| 2097 | AT4G30200.3 \| expressed protein, contains weak similarities to Pfam profiles: PF00041 Fibronectin type III domain, PF00628 PHD-finger; supporting cDNA gi:11177136:dbj:AB050977.1: \| chr4:14786639-14790479 REVERSE \| Aliases: F9N11.50, F9N11_50 | 5.3 | 7.6 | -2.3 | -7.2 | 0.16% | 7.0 |
| 2100 | AT2G26410.1 \| calmodulin-binding family protein, similar to SF16 protein (Helianthus annuus) GI:560150; contains Pfam profile PF00612: IQ calmodulin-binding motif \| chr2:11241729-11243829 REVERSE \| Aliases: T9J22.8, T9J22_8 | 3.3 | 6.6 | -3.4 | -7.2 | 0.16% | 6.9 |
| 2102 | AT4G39870.1 \| expressed protein, hypothetical protein, Schizosaccharomyces cerevisae, Z99168 \| chr4:18501889-18504422 FORWARD \| Aliases: T5J17.40, T5J17_40 | 5.1 | 6.7 | -1.6 | -7.2 | 0.16% | 7.0 |
| 2107 | AT1G54710.1 \| expressed protein, contains 3 WD-40 repeats (PF00400) (1 weak) submitForm(); \| chr1:20420437-20424702 REVERSE \| Aliases: T22H22.14, T22H22_14 | 6.2 | 7.9 | -1.7 | -7.2 | 0.16% | 7.0 |
| 2109 | AT5G67430.1 \| GCN5-related N-acetyltransferase (GNAT) family protein, contains Pfam profile PF00583: acetyltransferase, GNAT family \| chr5:26927562-26929327 FORWARD \| Aliases: K8K14.17, K8K14_17 | 4.0 | 7.1 | -3.1 | -7.2 | 0.16% | 6.0 |
| 2110 | AT4G08770.1 \| peroxidase, putative, identical to class III peroxidase ATP38 (Arabidopsis thaliana) gi:17530568:gb:AAL40851; similar to peroxidase C2 precursor (Armoracia rusticana) SWISS-PROT: P17179; identical to cDNA class III peroxidase ATP38 GI:17530567 \| chr4:5598112-5600309 REVERSE \| Aliases: T32A17.80, T32A17_80 | 5.9 | 9.5 | -3.6 | -7.2 | 0.16% | 6.9 |
| 2112 | AT1G75500.1 \| nodulin MtN21 family protein, similar to MtN21 GB:CAA75575 GI:2598575 from (Medicago truncatula) (Mol. Plant Microbe Interact. 9 (4), 233-242 (1996)); contains Pfam profile PF00892: Integral membrane protein \| chr1:28341453-28343821 REVERSE \| Aliases: F1B16.19 | 3.8 | 7.7 | -3.8 | -7.2 | 0.16% | 6.4 |

| Rank | Description | Sync | Root | M | t | adj.q | B |
|---|---|---|---|---|---|---|---|
| 2113 | AT5G19530.1 \| Symbol: ACL5 \| spermine/spermidine synthase family protein, similar to SP:P09158 Spermidine synthase (EC 2.5.1.16) (Putrescine aminopropyltransferase) {Escherichia coli}; contains Pfam profile PF01564: Spermine/spermidine synthase \| chr5:6588960-6591183 REVERSE \| Aliases: ACAULIS 5, SPERMINE SYNTHASE, T20D1.50, T20D1_50 | 3.1 | 5.0 | -1.9 | -7.2 | 0.16% | 6.7 |
| 2118 | AT2G24150.1 \| expressed protein, contains Pfam profile PF03006: Uncharacterised protein family (Hly-III / UPF0073) \| chr2:10272564-10274466 REVERSE \| Aliases: F27D4.6, F27D4_6 | 4.6 | 8.4 | -3.7 | -7.2 | 0.16% | 6.6 |
| 2123 | AT5G15830.1 \| bZIP transcription factor family protein, similar to common plant regulatory factor 7 GI:9650828 from (Petroselinum crispum); contains Pfam profile: PF00170 bZIP transcription factor \| chr5:5168546-5169364 FORWARD \| Aliases: F14F8.210, F14F8_210 | 3.8 | 6.4 | -2.6 | -7.2 | 0.16% | 6.8 |
| 2124 | AT2G44790.1 \| Symbol: UCC2 \| uclacyanin II, strong similarity to uclacyanin II GI:3399769 from (Arabidopsis thaliana); contains Pfam profile PF02298: Plastocyanin-like domain; identical to cDNA uclacyanin II GI:3399768 \| chr2:18468981-18470384 REVERSE \| Aliases: F16B22.32, UCLACYANIN 2 | 5.6 | 11.2 | -5.6 | -7.2 | 0.16% | 6.7 |
| 2125 | AT3G01860.2 \| expressed protein \| chr3:303725-304473 FORWARD \| Aliases: F28J7.19, F28J7_19 | 5.5 | 10.2 | -4.8 | -7.2 | 0.16% | 6.4 |
| 2128 | AT1G66440.1 \| DC1 domain-containing protein, contains Pfam protein PF03107 DC1 domain \| chr1:24785834-24788014 FORWARD \| Aliases: F28G11.16, F28G11_16 | 3.5 | 6.3 | -2.8 | -7.2 | 0.16% | 6.5 |
| 2129 | AT4G22070.1 \| Symbol: WRKY31 \| | 2.0 | 2.9 | -0.9 | -7.2 | 0.16% | 7.0 |
| 2130 | AT5G24870.2 \| zinc finger (C3HC4-type RING finger) family protein, similar to Pfam domain, PF00097: Zinc finger, C3HC4 type (RING finger) \| chr5:8544788-8547116 REVERSE \| Aliases: F6A4.80, F6A4_80 | 4.6 | 7.0 | -2.4 | -7.2 | 0.16% | 6.7 |
| 2131 | AT1G59910.1 \| formin homology 2 domain-containing protein / FH2 domain-containing protein, contains formin homology 2 domain, Pfam:PF02128 \| chr1:22057616-22060717 REVERSE \| Aliases: F23H11.22, F23H11_22 | 6.0 | 8.4 | -2.4 | -7.2 | 0.16% | 6.8 |
| 2132 | AT4G12550.1 \| Symbol: AIR1 \| protease inhibitor/seed storage/lipid transfer protein (LTP) family protein, similar to pEARLI 1 (Accession No. L43080): an Arabidopsis member of a conserved gene family (PGF95-099), Plant Physiol. 109 (4), 1497 (1995); contains Pfam protease inhibitor/seed storage/LTP family domain PF00234; identical to cDNA AIR1 mRNA, partial cds GI:3695016 | 2.8 | 6.5 | -3.7 | -7.2 | 0.16% | 5.9 |
| 2133 | AT3G51390.1 \| zinc finger (DHHC type) family protein, contains Pfam profile PF01529: DHHC zinc finger domain \| chr3:19086555-19089096 FORWARD \| Aliases: F26O13.30 | 7.7 | 9.1 | -1.4 | -7.2 | 0.16% | 7.0 |
| 2134 | AT1G07630.1 \| protein phosphatase 2C family protein / PP2C family protein, similar to protein phosphatase-2c (GI:3608412) (Mesembryanthemum crystallinum); contains Pfam PF00481 : Protein phosphatase 2C domain \| chr1:2349094-2351564 FORWARD \| Aliases: F24B9.31, F24B9_31 | 5.0 | 7.1 | -2.2 | -7.2 | 0.16% | 6.7 |
| 2139 | AT5G39580.2 \| similar to peroxidase, putative [Arabidopsis thaliana] (TAIR:At5g64120.1); similar to peroxidase precursor [Lycopersicon esculentum] (GB:CAA64413.1); contains InterPro domain Haem peroxidase (InterPro:IPR002016); contains InterPro domain Plant peroxidase (InterPro:IPR000823) \| chr5:15864306-15866336 REVERSE \| Aliases: MIJ24.50, MIJ24_50 | 2.5 | 4.0 | -1.4 | -7.2 | 0.16% | 7.0 |
| 2140 | AT1G08670.1 \| epsin N-terminal homology (ENTH) domain-containing protein / clathrin assembly protein-related, contains Pfam PF01417: ENTH domain. ENTH (Epsin N-terminal homology) domain;similar to epsin 2b (GI:3894397) (Homo sapiens) \| chr1:2760349-2761554 REVERSE \| Aliases: F22O13.15, F22O13_15 | 3.4 | 8.8 | -5.4 | -7.2 | 0.16% | 6.4 |
| 2141 | AT2G29340.2 \| short-chain dehydrogenase/reductase (SDR) family protein, similar to tropinone reductase-I GI:424160 from (Datura stramonium) \| chr2:12604194-12605496 FORWARD \| Aliases: F16P2.28, F16P2_28 | 3.3 | 4.7 | -1.4 | -7.2 | 0.16% | 7.0 |
| 2142 | AT5G56980.1 \| expressed protein, non-consensus CG donor splice site at exon 1, GA donor splice site at exon 3 \| chr5:23073306-23074968 REVERSE \| Aliases: MHM17.10, MHM17_10 | 3.6 | 5.9 | -2.3 | -7.2 | 0.16% | 6.9 |
| 2143 | AT1G78320.1 \| Symbol: ATGSTU23 \| glutathione S-transferase, putative, similar to glutathione transferase GI:2853219 from (Carica papaya) \| chr1:29472333-29473267 REVERSE \| Aliases: F3F9.14, F3F9_14 | 3.4 | 5.0 | -1.7 | -7.2 | 0.16% | 6.9 |
| 2144 | AT4G32850.8 \| Symbol: nPAP \| similar to nucleotidyltransferase family protein [Arabidopsis thaliana] (TAIR:At1g17980.1); similar to nucleotidyltransferase family protein [Arabidopsis thaliana] (TAIR:At2g25850.1); similar to nucleotidyltransferase family protein [Arabidopsis thaliana] (TAIR:At2g25850.2); similar to putative polynucleotide adenylyltransferase [Oryza sativa (indica cultivar-group)] (GB:AAW68018.1); similar to putative polynucleotide adenylyltransferase [Oryza sativa (indica cultivar-group)] (GB:AAW68015.1); similar to putative poly(A) polymerase [Oryza sativa (japonica cultivar-group)] (GB:XP_464757.1); contains InterPro domain Poly(A) polymerase, predicted RNA-binding domain (InterPro:IPR007010); contains InterPro domain Poly(A) polymerase, central region (InterPro:IPR007012); contains InterPro domain PAP/25A core domain (InterPro:IPR001201); contains InterPro domain DNA polymerase, beta-like region (InterPro:IPR002934) \| chr4:15849776-15854464 FORWARD \| Aliases: NUCLEAR POLY(A) POLYMERASE, T16I18.60, T16I18_60 | 6.4 | 7.9 | -1.5 | -7.2 | 0.16% | 7.0 |

| Rank | Description | Sync | Root | M | t | adj.q | B |
|---|---|---|---|---|---|---|---|
| 2145 | AT5G01980.1 \| zinc finger (C3HC4-type RING finger) family protein, contains Pfam profile: PF00097 zinc finger, C3HC4 type (RING finger) \| chr5:375242-377205 FORWARD \| Aliases: T7H20.30, T7H20_30 | 5.9 | 7.8 | -1.9 | -7.2 | 0.16% | 6.8 |
| 2146 | AT4G28260.1 \| expressed protein \| chr4:14004491-14006976 FORWARD \| Aliases: F26K10.140, F26K10_140 | 8.4 | 11.0 | -2.6 | -7.2 | 0.16% | 7.0 |
| 2148 | AT5G43520.1 \| DC1 domain-containing protein, contains Pfam profile PF03107: DC1 domain | 2.7 | 5.7 | -3.0 | -7.2 | 0.16% | 6.1 |
| 2150 | AT2G16760.1 \| expressed protein \| chr2:7282783-7284314 FORWARD \| Aliases: T24I21.17, T24I21_17 | 4.9 | 6.2 | -1.4 | -7.2 | 0.16% | 7.0 |
| 2151 | AT5G64740.1 \| Symbol: CESA6 \| cellulose synthase, catalytic subunit, putative, similar to gi:2827141 cellulose synthase catalytic subunit (Ath-A), Arabidopsis thaliana \| chr5:25898292-25903920 FORWARD \| Aliases: CELLULASE SYNTHASE 6, E112, ISOXABEN RESISTANT 2, IXR2, MVP7.7, MVP7_7, PRC1, PROCUSTE 1 | 7.3 | 9.5 | -2.2 | -7.2 | 0.16% | 7.0 |
| 2152 | AT4G12070.1 \| expressed protein \| chr4:7231816-7234495 FORWARD \| Aliases: F16J13.140, F16J13_140 | 4.7 | 6.2 | -1.5 | -7.2 | 0.16% | 7.0 |
| 2154 | AT4G05170.1 \| similar to ethylene-responsive protein-related [Arabidopsis thaliana] (TAIR:At4g21340.1); similar to bHLH transcription factor-like protein [Oryza sativa (japonica cultivar-group)] (GB:BAD53363.1); contains InterPro domain Basic helix-loop-helix dimerization domain bHLH (InterPro:IPR001092) \| chr4:2667988-2669362 REVERSE \| Aliases: C17L7.90, C17L7_90 | 2.3 | 5.8 | -3.5 | -7.2 | 0.16% | 5.9 |
| 2158 | AT3G18990.1 \| Symbol: VRN1 \| transcriptional factor B3 family protein, contains Pfam profile PF02362: B3 DNA binding domain \| chr3:6548875-6551847 REVERSE \| Aliases: K13E13.10, REDUCED VERNALIZATION RESPONSE 1, REM39 | 6.0 | 7.8 | -1.8 | -7.2 | 0.16% | 7.0 |
| 2159 | AT3G10740.1 \| Symbol: ASD1 \| glycosyl hydrolase family protein 51, similar to arabinoxylan arabinofuranohydrolase isoenzyme AXAH-II from GI:13398414 (Hordeum vulgare) \| chr3:3360893-3365368 REVERSE \| Aliases: T7M13.18 | 7.1 | 10.4 | -3.2 | -7.2 | 0.16% | 6.8 |
| 2168 | AT3G48760.1 \| zinc finger (DHHC type) family protein, contains Pfam profile PF01529: DHHC zinc finger domain \| chr3:18086817-18089276 FORWARD \| Aliases: T21J18.30 | 6.6 | 8.9 | -2.2 | -7.2 | 0.17% | 6.9 |
| 2169 | AT2G46600.1 \| calcium-binding protein, putative, similar to EF-hand Ca2+-binding protein CCD1 (Triticum aestivum) GI:9255753 \| chr2:19143090-19143643 FORWARD \| Aliases: F13A10.13 | 7.3 | 9.5 | -2.1 | -7.2 | 0.17% | 7.0 |
| 2170 | AT3G12360.1 \| ankyrin repeat family protein, contains ankyrin repeat domains, Pfam:PF00023 \| chr3:3934085-3936701 FORWARD \| Aliases: T2E22.31 | 8.5 | 9.8 | -1.3 | -7.2 | 0.17% | 6.9 |
| 2172 | AT1G35580.3 \| similar to beta-fructofuranosidase, putative / invertase, putative / saccharase, putative / beta-fructosidase, putative [Arabidopsis thaliana] (TAIR:At4g09510.1); similar to beta-fructofuranosidase, putative / invertase, putative / saccharase, putative / beta-fructosidase, putative [Arabidopsis thaliana] (TAIR:At1g22650.1); similar to beta-fructofuranosidase, putative / invertase, putative / saccharase, putative / beta-fructosidase, putative [Arabidopsis thaliana] (TAIR:At4g09510.2); similar to beta-fructofuranosidase, putative / invertase, putative / saccharase, putative / beta-fructosidase, putative [Arabidopsis thaliana] (TAIR:At1g72000.1); similar to beta-fructofuranosidase, putative / invertase, putative / saccharase, putative / beta-fructosidase, putative [Arabidopsis thaliana] (TAIR:At4g34860.1); similar to invertase, putative [Oryza sativa (japonica cultivar-group)] (GB:AAX95795.1); similar to putative alkaline/neutral invertase [Oryza sativa (japonica cultivar-group)] (GB:XP_466154.1); similar to OSJNBa0084A10.15 [Oryza sativa (japonica cultivar-group)] (GB:XP_472554.1); contains InterPro domain Plant neutral invertase (InterPro:IPR006937) \| chr1:13122855-13125241 REVERSE \| Aliases: F15O4.33 | 4.7 | 8.3 | -3.6 | -7.2 | 0.17% | 6.8 |
| 2173 | AT1G12030.1 \| expressed protein, contains Pfam profile PF04720: Protein of unknown function (DUF506) \| chr1:4064749-4066317 REVERSE \| Aliases: F12F1.10, F12F1_10 | 4.1 | 6.2 | -2.0 | -7.2 | 0.17% | 6.9 |
| 2174 | AT5G45080.1 \| Symbol: ATPP2 A6 \| disease resistance protein-related, weak similarity to disease resistance protein rps4-RLD (Arabidopsis thaliana) GI:5823585; contains Pfam profile PF01582: TIR domain \| chr5:18208802-18210434 REVERSE \| Aliases: ATPP2 A6, K17O22.4, K17O22_4 | 3.6 | 6.4 | -2.8 | -7.2 | 0.17% | 6.3 |
| 2175 | AT4G39950.1 \| Symbol: CYP79B2 \| cytochrome P450 79B2, putative (CYP79B2), identical to cytochrome P450 (79B2) SP:O81346 from (Arabidopsis thaliana) \| chr4:18525240-18527573 FORWARD \| Aliases: T5J17.120, T5J17_120 | 4.0 | 6.5 | -2.5 | -7.1 | 0.17% | 6.4 |
| 2176 | AT1G29760.1 \| expressed protein \| chr1:10422316-10424312 FORWARD \| Aliases: F1N18.18, F1N18_18 | 6.5 | 8.8 | -2.3 | -7.1 | 0.17% | 6.8 |
| 2177 | AT5G08240.1 \| expressed protein \| chr5:2650838-2652387 REVERSE \| Aliases: F8L15.11 | 3.1 | 7.3 | -4.2 | -7.1 | 0.17% | 6.1 |
| 2178 | AT1G71190.1 \| Symbol: SAG18 \| expressed protein \| chr1:26837072-26838689 REVERSE \| Aliases: F23N20.18, F23N20_18, TTN4 | 6.6 | 8.1 | -1.5 | -7.1 | 0.17% | 7.0 |
| 2179 | AT3G63010.1 \| expressed protein, similar to PrMC3 (Pinus radiata) GI:5487873 \| chr3:23300400-23302459 FORWARD \| Aliases: T20O10.110 | 3.9 | 5.2 | -1.2 | -7.1 | 0.17% | 7.0 |
| 2181 | AT5G17880.1 \| disease resistance protein (TIR-NBS-LRR class), putative, domain signature TIR-NBS-LRR exists, suggestive of a disease resistance protein. \| chr5:5908876-5913101 REVERSE \| Aliases: MPI7.20, MPI7_20 | 3.8 | 8.1 | -4.3 | -7.1 | 0.17% | 6.2 |

| Rank | Description | Sync | Root | M | t | adj.q | B |
|---|---|---|---|---|---|---|---|
| 2182 | AT1G63010.2 \| similar to SPX (SYG1/Pho81/XPR1) domain-containing protein [Arabidopsis thaliana] (TAIR:At4g22990.1); similar to SPX (SYG1/Pho81/XPR1) domain-containing protein [Arabidopsis thaliana] (TAIR:At4g11810.1); similar to OSJNBa0019K04.6 [Oryza sativa (japonica cultivar-group)] (GB:XP_473572.1); similar to SPX (SYG1/Pho81/XPR1) domain-containing protein-like [Oryza sativa (japonica cultivar-group)] (GB:BAD29367.1); similar to hypothetical protein [Oryza sativa (japonica cultivar-group)] (GB:BAD46701.1); contains InterPro domain SPX, N-terminal (InterPro:IPR004331) \| chr1:23351382-23356310 REVERSE \| Aliases: F16P17.18, F16P17_18 | 7.5 | 8.9 | -1.4 | -7.1 | 0.17% | 6.9 |
| 2184 | AT3G23800.1 \| selenium-binding family protein, contains Pfam profile: PF05694 56kDa selenium binding protein (SBP56) \| chr3:8581661-8583842 FORWARD \| Aliases: MYM9.16 | 7.0 | 10.2 | -3.2 | -7.1 | 0.17% | 6.7 |
| 2188 | AT1G62800.2 \| Symbol: ASP4 \| aspartate aminotransferase, cytoplasmic isozyme 2 / transaminase A (ASP4), identical to aspartate aminotransferase, cytoplasmic isozyme 2 SP:P46646 (Arabidopsis thaliana) \| chr1:23257368-23261128 REVERSE \| Aliases: ASPARTATE AMINOTRANSFERASE 4, F23N19.17, F23N19_17 | 7.2 | 8.9 | -1.7 | -7.1 | 0.17% | 6.9 |
| 2189 | AT5G18680.1 \| F-box family protein / tubby family protein, similar to phosphodiesterase (GI:467578) (Mus musculus); similar to Chain A, C-Terminal Domain Of Mouse Brain Tubby Protein (GI:6730158) (Mus musculus); contains Pfam PF00646: F-box domain and Pfam PF01167: Tub family \| chr5:6228167-6230539 REVERSE \| Aliases: T1A4.60, T1A4_60 | 7.5 | 10.6 | -3.1 | -7.1 | 0.17% | 6.9 |
| 2192 | AT5G10280.1 \| Symbol: MYB92 \| myb family transcription factor (MYB92), contains PFAM profile myb DNA binding domain PF00249; identical to cDNA putative transcription factor (MYB92) GI:3941523 \| chr5:3232570-3234211 FORWARD \| Aliases: F18D22.50, F18D22_50 | 2.5 | 6.2 | -3.6 | -7.1 | 0.17% | 6.4 |
| 2194 | AT1G08800.1 \| expressed protein, weak similarity to SP:Q02455 Myosin-like protein MLP1 {Saccharomyces cerevisiae}; contains Pfam profile PF04576: Protein of unknown function, DUF593 \| chr1:2813064-2817642 REVERSE \| Aliases: F22O13.29, F22O13_29 | 3.3 | 6.2 | -2.9 | -7.1 | 0.17% | 6.7 |
| 2196 | AT5G44290.3 \| similar to protein kinase family protein [Arabidopsis thaliana] (TAIR:At1g03740.1); similar to putative CRK1 protein [Oryza sativa (japonica cultivar-group)] (GB:XP_479750.1); similar to putative CRK1 protein [Oryza sativa (japonica cultivar-group)] (GB:BAD89473.1); contains InterPro domain Serine/threonine protein kinase, active site (InterPro:IPR008271); contains InterPro domain Tyrosine protein kinase (InterPro:IPR001245); contains InterPro domain Serine/threonine protein kinase (InterPro:IPR002290); contains InterPro domain Protein kinase (InterPro:IPR000719) \| chr5:17857651-17860824 REVERSE \| Aliases: K9L2.5, K9L2_5 | 5.2 | 7.1 | -1.8 | -7.1 | 0.17% | 6.9 |
| 2197 | AT3G23000.1 \| Symbol: CIPK7 \| CBL-interacting protein kinase 7 (CIPK7), identical to CBL-interacting protein kinase 7 (Arabidopsis thaliana) gi:13249113:gb:AAK16682; contains Pfam profiles PF00069: Protein kinase domain and PF03822: NAF domain; identical to cDNA CBL-interacting protein kinase 7 (CIPK7) GI:13249112 \| chr3:8172604-8174138 FORWARD \| Aliases: ATSR2, ATSRPK1, CBL INTERACTING PROTEIN KINASE 7, MXC7.3, PKS7 | 7.7 | 10.9 | -3.2 | -7.1 | 0.17% | 6.9 |
| 2202 | AT2G42430.1 \| Symbol: LBD16 \| LOB domain protein 16 / lateral organ boundaries domain protein 16 (LBD16), identical to LOB DOMAIN 16 (Arabidopsis thaliana) GI:17227162 \| chr2:17670522-17672370 FORWARD \| Aliases: MHK10.15, MHK10_15 | 4.5 | 6.2 | -1.7 | -7.1 | 0.17% | 6.9 |
| 2204 | AT2G03500.1 \| myb family transcription factor, contains Pfam profile: PF00249 myb-like DNA-binding domain \| chr2:1059707-1062407 FORWARD \| Aliases: T4M8.7, T4M8_7 | 2.9 | 4.1 | -1.2 | -7.1 | 0.17% | 6.9 |
| 2205 | AT1G66020.1 \| terpene synthase/cyclase family protein, contains Pfam profile: PF01397: Terpene synthase family | 3.4 | 5.7 | -2.4 | -7.1 | 0.17% | 6.6 |
| 2206 | AT5G48380.1 \| leucine-rich repeat family protein / protein kinase family protein, contains protein kinase domain, Pfam:PF00069; contains leucine-rich repeats, Pfam:PF00560 \| chr5:19621315-19624235 REVERSE \| Aliases: K23F3.10 | 5.2 | 7.5 | -2.3 | -7.1 | 0.17% | 6.6 |
| 2208 | AT1G14070.1 \| Symbol: FUT7 \| xyloglucan fucosyltransferase, putative (FUT7), nearly identical to SP:Q9XI81 Probable fucosyltransferase 7 (EC 2.4.1.-) (AtFUT7) {Arabidopsis thaliana}; similar to xyloglucan fucosyltransferase GI:5231145 from (Arabidopsis thaliana) \| chr1:4818545-4820147 FORWARD \| Aliases: F7A19.15, F7A19_15 | 2.5 | 4.1 | -1.6 | -7.1 | 0.17% | 6.8 |
| 2215 | AT1G72120.1 \| proton-dependent oligopeptide transport (POT) family protein, contains Pfam profile: PF00854 POT family \| chr1:27135795-27140051 FORWARD \| Aliases: F28P5.2, F28P5_2 | 2.5 | 4.3 | -1.8 | -7.1 | 0.17% | 6.8 |
| 2216 | AT2G02710.3 \| PAC motif-containing protein, similar to nonphototropic hypocotyl 1 (Zea mays) GI:2687358; contains Pfam profile PF00785: PAC motif \| chr2:758696-760815 REVERSE \| Aliases: T20F6.15, T20F6_15 | 8.5 | 10.6 | -2.2 | -7.1 | 0.17% | 6.8 |
| 2217 | AT1G78960.1 \| Symbol: ATLUP2 \| | 4.1 | 6.1 | -1.9 | -7.1 | 0.17% | 6.8 |
| 2219 | AT1G71740.1 \| expressed protein \| chr1:26990907-26991299 FORWARD \| Aliases: F14O23.12, F14O23_12 | 3.9 | 6.8 | -2.8 | -7.1 | 0.18% | 5.9 |
| 2221 | AT3G21550.1 \| expressed protein, contains Pfam profile PF05078: Protein of unknown function (DUF679) \| chr3:7591489-7592328 REVERSE \| Aliases: MIL23.12 | 3.4 | 6.0 | -2.6 | -7.1 | 0.18% | 6.4 |
| 2222 | AT2G42890.2 \| Symbol: MEI2 \| RNA recognition motif (RRM)-containing protein \| chr2:17857639-17861607 FORWARD \| Aliases: F7D19.11, F7D19_11 | 5.7 | 8.3 | -2.6 | -7.1 | 0.18% | 6.5 |

| Rank | Description | Sync | Root | M | t | adj.q | B |
|---|---|---|---|---|---|---|---|
| 2223 | AT5G46520.1 \| disease resistance protein (TIR-NBS-LRR class), putative, domain signature TIR-NBS-LRR exists, suggestive of a disease resistance protein. \| chr5:18884953-18889533 FORWARD \| Aliases: K11I1.11, K11I1_11 | 3.1 | 5.8 | -2.8 | -7.1 | 0.18% | 6.5 |
| 2224 | AT2G36310.1 \| inosine-uridine preferring nucleoside hydrolase family protein, similar to Chain A, Crystal Structure Of Nucleoside Hydrolase From Leishmania MajorGI:8569431; contains Pfam profile PF01156: Inosine-uridine preferring nucleoside hydrolase \| chr2:15231576-15233951 REVERSE \| Aliases: F2H17.8, F2H17_8 | 7.4 | 9.0 | -1.6 | -7.1 | 0.18% | 6.9 |
| 2225 | AT4G21400.1 \| protein kinase family protein, contains Pfam domain PF00069: Protein kinase domain \| chr4:11399142-11401720 REVERSE \| Aliases: F18E5.20 | 5.1 | 7.7 | -2.6 | -7.1 | 0.18% | 6.9 |
| 2226 | AT5G04460.1 \| expressed protein \| chr5:1260010-1263846 FORWARD \| Aliases: T19N18.3 | 3.9 | 5.4 | -1.6 | -7.1 | 0.18% | 6.8 |
| 2230 | AT3G21600.2 \| senescence/dehydration-associated protein-related, similar to senescence-associated protein 12 (Hemerocallis hybrid cultivar) gi:3551958:gb:AAC34857; similar to early-responsive to dehydration stress ERD7 protein (Arabidopsis thaliana) gi:15320412:dbj:BAB63916 \| chr3:7606552-7608371 REVERSE \| Aliases: MIL23.17 | 3.1 | 4.7 | -1.6 | -7.1 | 0.18% | 6.9 |
| 2234 | AT5G02780.1 \| In2-1 protein, putative, similar to In2-1 (Zea mays) EMBL:X58573 | 5.7 | 9.0 | -3.4 | -7.1 | 0.18% | 6.7 |
| 2235 | AT2G26890.1 \| Symbol: GRV2 \| DNAJ heat shock N-terminal domain-containing protein, contains Pfam profile PF00226: DnaJ domain | 6.7 | 8.6 | -1.9 | -7.0 | 0.18% | 6.9 |
| 2240 | AT3G26610.1 \| polygalacturonase, putative / pectinase, putative, similar to polygalacturonase (PG1) GI:5669846, (PG2) GI:5669848 (Glycine max); contains PF00295: Glycosyl hydrolases family 28 (polygalacturonases) \| chr3:9778197-9781736 FORWARD \| Aliases: MFE16.14 | 3.3 | 5.6 | -2.2 | -7.0 | 0.18% | 6.4 |
| 2246 | AT4G00040.1 \| chalcone and stilbene synthase family protein, similar to chalcone synthase homolog PrChS1, Pinus radiata, gb:U90341; similar to anther-specific protein (Nicotiana sylvestris)(GI:2326774), YY2 protein (Oryza sativa)(GI:2645170) \| chr4:14627-16079 FORWARD \| Aliases: F6N15.12, F6N15_12 | 6.2 | 8.5 | -2.3 | -7.0 | 0.18% | 6.7 |
| 2248 | AT5G09480.1 \| hydroxyproline-rich glycoprotein family protein, contains proline-rich extensin domains, INTERPRO:IPR002965; Common family members At5g09530, At5g09520, At1g44222 (Arabidopsis thaliana) \| chr5:2950915-2951694 REVERSE \| Aliases: T5E8.280, T5E8_280 | 3.1 | 6.9 | -3.7 | -7.0 | 0.18% | 6.7 |
| 2249 | AT2G36320.1 \| zinc finger (AN1-like) family protein, contains Pfam domain, PF01428: AN1-like Zinc finger \| chr2:15235867-15237575 FORWARD \| Aliases: F2H17.7, F2H17_7 | 6.9 | 9.8 | -2.8 | -7.0 | 0.18% | 6.5 |
| 2255 | AT4G12910.1 \| Symbol: SCPL20 \| | 4.6 | 7.1 | -2.5 | -7.0 | 0.18% | 6.7 |
| 2256 | AT1G68100.1 \| Symbol: IAR1 \| IAA-alanine resistance protein 1, putative, similar to IAA-alanine resistance protein 1 (Arabidopsis thaliana) SWISS-PROT:Q9M647; contains ZIP Zinc transporter domain, Pfam:PF02535; identical to cDNA IAA-alanine resistance protein 1 mRNA GI:6942042 | 5.9 | 7.4 | -1.4 | -7.0 | 0.18% | 6.8 |
| 2258 | AT5G54730.1 \| expressed protein \| chr5:22251015-22254030 REVERSE \| Aliases: K5F14.9, K5F14_9 | 6.5 | 8.5 | -2.0 | -7.0 | 0.18% | 6.8 |
| 2260 | AT5G40450.1 \| expressed protein \| chr5:16202027-16212756 REVERSE \| Aliases: MPO12.160, MPO12_160 | 9.0 | 11.2 | -2.2 | -7.0 | 0.18% | 6.8 |
| 2262 | AT4G00680.1 \| actin-depolymerizing factor, putative, strong similarity to SP:P30175 Actin-depolymerizing factor (ADF) {Lilium longiflorum}; contains Pfam profile PF00241: Cofilin/tropomyosin-type actin-binding protein \| chr4:279603-280699 REVERSE \| Aliases: F6N23.12, F6N23_12 | 5.7 | 8.6 | -2.9 | -7.0 | 0.18% | 6.7 |
| 2263 | AT5G11090.1 \| serine-rich protein-related, contains some similarity to serine-rich proteins \| chr5:3524681-3525847 FORWARD \| Aliases: T5K6.80, T5K6_80 | 5.0 | 7.5 | -2.5 | -7.0 | 0.18% | 6.7 |
| 2264 | AT5G05440.1 \| expressed protein, low similarity to cytokinin-specific binding protein (Vigna radiata) GI:4190976 \| chr5:1609251-1610447 FORWARD \| Aliases: K18I23.25, K18I23_25 | 7.1 | 9.4 | -2.3 | -7.0 | 0.18% | 6.6 |
| 2265 | AT5G65910.1 \| BSD domain-containing protein, contains Pfam profile PF03909: BSD domain | 5.5 | 7.2 | -1.7 | -7.0 | 0.18% | 6.8 |
| 2266 | AT4G19860.1 \| lecithin:cholesterol acyltransferase family protein / LACT family protein, similar to lysosomal phospholipase A2 (Mus musculus) GI:18699602; contains Pfam profile PF02450: Lecithin:cholesterol acyltransferase (phosphatidylcholine-sterol acyltransferase) \| chr4:10777522-10780373 REVERSE \| Aliases: T16H5.220, T16H5_220 | 8.9 | 11.4 | -2.5 | -7.0 | 0.18% | 6.8 |
| 2267 | AT3G60690.1 \| auxin-responsive family protein, similar to auxin-induced protein SAUR-AC1 (GP:546362) (PIR:T06084)(Arabidopsis thaliana) PIR:T06084 \| chr3:22446096-22446924 FORWARD \| Aliases: T4C21.100 | 5.3 | 7.5 | -2.2 | -7.0 | 0.18% | 6.8 |

| Rank | Description | Sync | Root | M | t | adj.q | B |
|---|---|---|---|---|---|---|---|
| 2271 | AT3G22120.1 \| Symbol: CWLP \| protease inhibitor/seed storage/lipid transfer protein (LTP) family protein, similar to SP:Q00451:PRF1_LYCES 36.4 kDa proline-rich protein Lycopersicon esculentum, proline-rich cell wall protein (Medicago sativa) GI:3818416; contains Pfam profile PF00234 Protease inhibitor/seed storage/LTP family \| chr3:7794964-7796317 REVERSE \| Aliases: CELL WALL PLASMA MEMBRANE LINKER PROTEIN, MKA23.6 | 2.6 | 3.9 | -1.3 | -7.0 | 0.19% | 6.8 |
| 2275 | AT5G57540.1 \| xyloglucan:xyloglucosyl transferase, putative / xyloglucan endotransglycosylase, putative / endo-xyloglucan transferase, putative, similar to xyloglucan endotransglycosylase (XTR9) GI:4218963 from (Arabidopsis thaliana) \| chr5:23320222-23321275 REVERSE \| Aliases: MUA2.11, MUA2_11 | 3.1 | 5.7 | -2.7 | -7.0 | 0.19% | 5.9 |
| 2278 | AT4G14940.1 \| Symbol: ATAO1 \| copper amine oxidase, putative, highly similar to copper amine oxidase (Arabidopsis thaliana) gi:2654118:gb:AAB87690 \| chr4:8541875-8544300 FORWARD \| Aliases: DL3510W, FCAALL.145 | 2.8 | 4.9 | -2.1 | -7.0 | 0.19% | 6.5 |
| 2279 | AT1G30410.1 \| Symbol: ATMRP13 \| ATP-binding cassette transport protein, putative, similar to MgATP-energized glutathione S-conjugate pump (Arabidopsis thaliana) GI:2909781; contains Pfam profiles PF00005: ABC transporter, PF00664: ABC transporter transmembrane region \| chr1:10739339-10747305 FORWARD \| Aliases: T4K22.13, T4K22_13 | 4.8 | 8.1 | -3.3 | -7.0 | 0.19% | 6.3 |
| 2280 | AT1G30420.1 \| Symbol: ATMRP12 \| ATP-binding cassette transport protein, putative, contains Pfam profiles PF00005: ABC transporter, PF00664: ABC transporter transmembrane region \| chr1:10748798-10756298 FORWARD \| Aliases: T4K22.1, T4K22_1 | 4.8 | 8.1 | -3.3 | -7.0 | 0.19% | 6.3 |
| 2281 | AT5G42840.1 \| DC1 domain-containing protein, contains Pfam profile PF03107: DC1 domain | 2.6 | 4.1 | -1.5 | -7.0 | 0.19% | 6.8 |
| 2284 | AT4G36030.1 \| armadillo/beta-catenin repeat family protein, contains Pfam profile: PF00514 armadillo/beta-catenin-like repeat \| chr4:17044948-17047318 REVERSE \| Aliases: T19K4.160, T19K4_160 | 2.8 | 4.8 | -2.0 | -7.0 | 0.19% | 6.6 |
| 2285 | AT3G25250.1 \| Symbol: AGC2 1 \| | 4.2 | 5.9 | -1.6 | -7.0 | 0.19% | 6.8 |
| 2287 | AT5G62680.1 \| proton-dependent oligopeptide transport (POT) family protein, contains Pfam profile: PF00854 POT family \| chr5:25182656-25185169 REVERSE \| Aliases: MRG21.10, MRG21_10 | 3.1 | 6.4 | -3.3 | -7.0 | 0.19% | 6.5 |
| 2288 | AT5G05310.3 \| expressed protein \| chr5:1571272-1574580 FORWARD \| Aliases: K18I23.11, K18I23_11 | 4.8 | 6.2 | -1.4 | -7.0 | 0.19% | 6.8 |
| 2291 | AT4G24140.1 \| hydrolase, alpha/beta fold family protein, low similarity to 2-hydroxy-6-oxo-7-methylocta-2,4-dienoate hydrolase (Pseudomonas putida) GI:2822275, hydroxymuconic semialdehyde hydrolase, Pseudomonas stutzeri, AF039534; contains Pfam profile PF00561: hydrolase, alpha/beta fold family \| chr4:12529885-12533803 REVERSE \| Aliases: T19F6.130, T19F6_130 | 2.5 | 5.6 | -3.0 | -7.0 | 0.19% | 6.5 |
| 2292 | AT5G25930.1 \| leucine-rich repeat family protein / protein kinase family protein, contains similarity to Swiss-Prot:P47735 receptor-like protein kinase 5 precursor (Arabidopsis thaliana); contains Pfam domains PF00560: Leucine Rich Repeat and PF00069: Protein kinase domain \| chr5:9050797-9053981 FORWARD \| Aliases: F18A17.4 | 5.6 | 7.9 | -2.3 | -7.0 | 0.19% | 6.7 |
| 2295 | AT1G79030.1 \| DNAJ heat shock N-terminal domain-containing protein / S-locus protein, putative, similar to S-locus protein 5 (GI:6069485) (Brassica rapa); contains Pfam profile PF00226 DnaJ domain \| chr1:29735807-29738463 REVERSE \| Aliases: YUP8H12R.35, YUP8H12R_35 | 4.6 | 6.9 | -2.3 | -7.0 | 0.19% | 6.5 |
| 2297 | AT5G26310.1 \| UDP-glucoronosyl/UDP-glucosyl transferase family protein, contains Pfam profile: PF00201 UDP-glucoronosyl and UDP-glucosyl transferase \| chr5:9234688-9236388 FORWARD \| Aliases: F9D12.4, F9D12_4 | 3.4 | 7.0 | -3.6 | -7.0 | 0.19% | 6.6 |
| 2300 | AT1G30850.1 \| expressed protein \| chr1:10985097-10985999 FORWARD \| Aliases: T17H7.17, T17H7_17 | 2.6 | 4.1 | -1.5 | -7.0 | 0.19% | 6.8 |
| 2303 | AT1G11210.1 \| expressed protein, contains similarity to cotton fiber expressed protein 1 (Gossypium hirsutum) gi:3264828:gb:AAC33276 \| chr1:3755677-3756998 REVERSE \| Aliases: T28P6.13, T28P6_13 | 4.1 | 6.9 | -2.8 | -7.0 | 0.19% | 6.7 |
| 2308 | AT2G42010.1 \| Symbol: PLDBETA1 \| phospholipase D beta 1 / PLD beta 1 (PLDBETA1), identical to SP:P93733 Phospholipase D beta 1 (EC 3.1.4.4) (AtPLDbeta1) (PLD beta 1) (PLDbeta) {Arabidopsis thaliana}; contains Pfam profiles: PF00614 phospholipase D.active site motif, PF00168 C2 domain \| chr2:17539891-17545476 REVERSE \| Aliases: PHOSPHOLIPASE D, PHOSPHOLIPASE D BETA 1, PLDBETA, T6D20.10, T6D20_10 | 5.1 | 6.2 | -1.1 | -7.0 | 0.19% | 6.8 |
| 2310 | AT1G64480.1 \| Symbol: CBL8 \| calcineurin B-like protein 8 (CBL8), identical to calcineurin B-like protein 8 (GI:15866276) (Arabidopsis thaliana); similar to CALCINEURIN B SUBUNIT GB:P25296 from (Saccharomyces cerevisiae) \| chr1:23951691-23953414 REVERSE \| Aliases: CALCINEURIN B LIKE PROTEIN 5, CBL8, F1N19.5, F1N19_5 | 2.5 | 4.7 | -2.2 | -7.0 | 0.19% | 6.3 |
| 2317 | AT1G11920.1 \| pectate lyase family protein, similar to pectate lyase GI:14289169 from (Salix gilgiana) | 2.7 | 4.5 | -1.8 | -6.9 | 0.19% | 6.8 |
| 2318 | AT5G19240.1 \| expressed protein \| chr5:6470131-6471064 FORWARD \| Aliases: T24G5.140, T24G5_140 | 2.8 | 7.0 | -4.2 | -6.9 | 0.19% | 6.3 |

| Rank | Description | Sync | Root | M | t | adj.q | B |
|---|---|---|---|---|---|---|---|
| 2321 | AT2G03200.1 \| aspartyl protease family protein, contains Pfam domain, PF00026: eukaryotic aspartyl protease \| chr2:966448-967915 REVERSE \| Aliases: T18E12.13, T18E12_13 | 2.1 | 5.9 | -3.7 | -6.9 | 0.19% | 6.5 |
| 2322 | AT1G66930.1 \| serine/threonine protein kinase family protein, contains Pfam PF00069: Protein kinase domain \| chr1:24974186-24976732 FORWARD \| Aliases: T4O24.2 | 2.8 | 4.7 | -1.9 | -6.9 | 0.19% | 6.5 |
| 2326 | AT5G41680.2 \| protein kinase family protein, contains protein kinase domain, Pfam:PF00069; similar to receptor-like protein kinase (GI:4008006) (Arabidopsis thaliana); similar to receptor-like kinase RHG1 (GI:21239380) (GI:21239382) (Glycine max) | 3.4 | 6.1 | -2.7 | -6.9 | 0.19% | 6.6 |
| 2327 | AT1G53590.1 \| C2 domain-containing protein \| chr1:20000118-20003933 FORWARD \| Aliases: F22G10.28 | 5.1 | 6.9 | -1.8 | -6.9 | 0.19% | 6.7 |
| 2329 | AT1G18150.2 \| Symbol: ATMPK8 \| | 5.7 | 7.6 | -1.9 | -6.9 | 0.20% | 6.7 |
| 2330 | AT2G18370.1 \| protease inhibitor/seed storage/lipid transfer protein (LTP) family protein, similar to lipid-transfer protein (Nicotiana glauca) GI:6782436; contains Pfam protease inhibitor/seed storage/LTP family domain PF00234 \| chr2:7987711-7988826 FORWARD \| Aliases: T30D6.12, T30D6_12 | 3.0 | 4.8 | -1.8 | -6.9 | 0.20% | 6.7 |
| 2331 | AT1G49320.1 \| BURP domain-containing protein, similarity to SP:Q08298 Dehydration-responsive protein RD22 precursor {Arabidopsis thaliana}; contains Pfam profile PF03181: BURP domain | 3.9 | 6.3 | -2.4 | -6.9 | 0.20% | 6.2 |
| 2333 | AT4G23510.1 \| disease resistance protein (TIR class), putative, domain signature TIR exists, suggestive of a disease resistance protein. \| chr4:12267526-12270553 REVERSE \| Aliases: F16G20.210, F16G20_210 | 2.6 | 4.8 | -2.2 | -6.9 | 0.20% | 6.3 |
| 2334 | AT4G29900.1 \| Symbol: ACA10 \| calcium-transporting ATPase, plasma membrane-type, putative / Ca2+-ATPase, putative (ACA10), identical to SP:Q9SZR1 Potential calcium-transporting ATPase 10, plasma membrane-type (EC 3.6.3.8) (Ca(2+)-ATPase isoform 10) {Arabidopsis thaliana}; similar to SP:Q9LF79 Calcium-transporting ATPase 8, plasma membrane-type (EC 3.6.3.8) (Ca(2+)-ATPase isoform 8) {Arabidopsis thaliana} \| chr4:14610889-14618781 REVERSE \| Aliases: ATACA10, AUTOINHIBITED CA(2+) ATPASE, F27B13.140, F27B13_140 | 6.7 | 8.5 | -1.8 | -6.9 | 0.20% | 6.7 |
| 2336 | AT4G33730.1 \| pathogenesis-related protein, putative, similar to SP:P33154 Pathogenesis-related protein 1 precursor (PR-1) {Arabidopsis thaliana}; contains Pfam profile PF00188: SCP-like extracellular protein \| chr4:16185101-16185619 FORWARD \| Aliases: T16L1.220, T16L1_220 | 3.0 | 5.7 | -2.7 | -6.9 | 0.20% | 6.0 |
| 2338 | AT5G56340.1 \| zinc finger (C3HC4-type RING finger) family protein, contains Pfam domain, PF00097: Zinc finger, C3HC4 type (RING finger) \| chr5:22834937-22836883 FORWARD \| Aliases: MCD7.7, MCD7_7 | 5.2 | 6.7 | -1.4 | -6.9 | 0.20% | 6.7 |
| 2339 | AT3G26300.1 \| Symbol: CYP71B34 \| cytochrome P450 family protein, contains Pfam profile: PF00067 cytochrome P450 \| chr3:9640436-9642103 REVERSE \| Aliases: F20C19.2 | 3.3 | 6.4 | -3.1 | -6.9 | 0.20% | 6.0 |
| 2342 | AT1G18470.2 \| zinc finger (C3HC4-type RING finger) family protein, contains Pfam profile: PF00097 zinc finger, C3HC4 type \| chr1:6356126-6360159 REVERSE \| Aliases: F15H18.5, F15H18_5 | 6.2 | 8.3 | -2.1 | -6.9 | 0.20% | 6.6 |
| 2350 | AT1G30510.3 \| ferredoxin--NADP(+) reductase, putative / adrenodoxin reductase, putative, strong similarity to SP:P41345 Ferredoxin--NADP reductase, root isozyme, chloroplast precursor (EC 1.18.1.2) (FNR) {Oryza sativa}, ferredoxin-NADP reductase precursor (Zea mays) GI:500751 \| chr1:10806968-10809144 REVERSE \| Aliases: F26G16.13, F26G16_13 | 9.5 | 11.5 | -2.0 | -6.9 | 0.20% | 6.7 |
| 2352 | AT3G57420.1 \| expressed protein, contains Pfam domain PF03385: Protein of unknown function, DUF288 \| chr3:21263396-21266253 REVERSE \| Aliases: T8H10.20 | 3.5 | 5.3 | -1.9 | -6.9 | 0.20% | 6.7 |
| 2353 | AT5G13150.1 \| exocyst subunit EXO70 family protein, leucine zipper-containing protein - Lycopersicon esculentum, EMBL:Z12127 contains Pfam domain PF03081: Exo70 exocyst complex subunit; \| chr5:4172972-4174933 REVERSE \| Aliases: T19L5.110, T19L5_110 | 4.0 | 6.0 | -2.0 | -6.9 | 0.20% | 6.6 |
| 2356 | AT5G14540.1 \| proline-rich family protein, contains proline rich extensin domains, INTERPRO:IPR002965 \| chr5:4687150-4689677 REVERSE \| Aliases: T15N1.30, T15N1_30 | 4.7 | 7.5 | -2.8 | -6.9 | 0.20% | 6.6 |
| 2357 | AT4G25360.2 \| similar to leaf senescence protein-related (YLS7 ) [Arabidopsis thaliana] (TAIR:At5g51640.1); similar to leaf senescence protein-like [Oryza sativa (japonica cultivar-group)] (GB:BAD68439.1); contains InterPro domain Protein of unknown function DUF231 (InterPro:IPR004253) | 6.2 | 7.9 | -1.7 | -6.9 | 0.20% | 6.7 |
| 2358 | AT4G15400.1 \| transferase family protein, similar to deacetylvindoline 4-O-acetyltransferase (Catharanthus roseus)(GI:4091808)(PMID:9681034), benzylalcohol acetyltransferase (Clarkia breweri)(GI:6166336)(PMID:10588064) \| chr4:8811928-8813478 REVERSE \| Aliases: DL3745C, FCAALL.284 | 2.7 | 4.8 | -2.2 | -6.9 | 0.20% | 6.3 |
| 2362 | AT2G01150.1 \| Symbol: RHA2B \| zinc finger (C3HC4-type RING finger) family protein, contains Pfam profile: PF00097 zinc finger, C3HC4 type (RING finger) \| chr2:100648-101494 FORWARD \| Aliases: F10A8.3, F10A8_3, RING H2 FINGER PROTEIN 2B, RING H2 FINGER PROTEIN RHA2B | 5.3 | 7.2 | -1.9 | -6.9 | 0.20% | 6.6 |

| Rank | Description | Sync | Root | M | t | adj.q | B |
|---|---|---|---|---|---|---|---|
| 2367 | AT2G40160.1 \| expressed protein \| chr2:16784468-16786486 FORWARD \| Aliases: T7M7.25, T7M7_25 | 3.4 | 6.2 | -2.7 | -6.9 | 0.20% | 6.1 |
| 2368 | AT1G67300.2 \| hexose transporter, putative, similar to hexose transporters from Solanum tuberosum (GI:8347246), Nicotiana tabacum (GI:8347244), Arabidopsis thaliana (GI:8347250); contains Pfam profile PF00083: major facilitator superfamily protein \| chr1:25197367-25200594 REVERSE \| Aliases: F1N21.12 | 6.8 | 7.8 | -1.0 | -6.9 | 0.20% | 6.6 |
| 2370 | AT1G70330.1 \| Symbol: ENT1,AT \| equilibrative nucleoside transporter family protein, contains similarity to SWISS-PROT:Q14542 equilibrative nucleoside transporter 2 (Equilibrative nitrobenzylmercaptopurine riboside-insensitive nucleoside transporter, Equilibrative NBMPR-insensitive nucleoside transporter, Nucleoside transporter, ei-type, 36 kDa nucleolar protein HNP36, Hydrophobic nucleolar protein, 36 kDa, Delayed-early response protein 12) (Homo sapiens) \| chr1:26506572-26508282 FORWARD \| Aliases: EQUILIBRATIVE NUCLEOTIDE TRANSPORTER 1 | 7.0 | 10.5 | -3.4 | -6.9 | 0.20% | 6.6 |
| 2371 | AT2G30860.1 \| Symbol: ATGSTF9 \| glutathione S-transferase, putative, identical to GB:Y12295 \| chr2:13146074-13147322 FORWARD \| Aliases: ATGSTF7, F7F1.7, F7F1_7, GLUTTR | 8.4 | 12.0 | -3.7 | -6.9 | 0.20% | 6.5 |
| 2372 | AT1G47530.1 \| ripening-responsive protein, putative, similar to ripening regulated protein DDTFR18 (Lycopersicon esculentum) GI:12231296; contains Pfam profile PF01554: Uncharacterized membrane protein family \| chr1:17454120-17456861 FORWARD \| Aliases: F16N3.20, F16N3_20 | 4.8 | 6.4 | -1.6 | -6.9 | 0.20% | 6.6 |
| 2373 | AT3G07940.1 \| zinc finger and C2 domain protein, putative, similar to zinc finger and C2 domain protein GI:9957238 from (Arabidopsis thaliana);contains Pfam profile: PF01412 Putative GTP-ase activating protein for Arf \| chr3:2529245-2531539 FORWARD \| Aliases: F17A17.28 | 4.8 | 7.1 | -2.3 | -6.9 | 0.20% | 6.5 |
| 2375 | AT2G44500.2 \| expressed protein, contains Pfam PF03138: Plant protein family. The function of this family of plant proteins is unknown; previously annotated as 'axi 1 protein from Nicotiana tabacum -related' based on similarity to axi 1 protein (GB:X80301) (GI:559920) from (Nicotiana tabacum), which, due to scienitific fraud was retracted. Retraction in: Schell J. EMBO J 1999 May 17;18(10):2908. PMID:10400497. | 3.1 | 7.1 | -4.0 | -6.9 | 0.21% | 6.4 |
| 2378 | AT1G79380.1 \| copine-related, low similarity to SP:Q99829 Copine I {Homo sapiens} \| chr1:29865384-29868528 FORWARD \| Aliases: T8K14.20, T8K14_20 | 6.5 | 9.7 | -3.2 | -6.9 | 0.21% | 6.5 |
| 2381 | AT2G01570.1 \| Symbol: RGA1 \| gibberellin response modulator (RGA1) / gibberellin-responsive modulator, identical to GB:Y11336, member of SCARECROW family \| chr2:255248-257549 REVERSE \| Aliases: F2I9.19, F2I9_19, REPRESSOR OF GA1 3, REPRESSOR OF GA1 3 1, RGA, RGA1 | 4.0 | 5.5 | -1.5 | -6.8 | 0.21% | 6.7 |
| 2382 | AT4G17030.1 \| Symbol: ATEXLB1 \| expansin-related, identical to SWISS-PROT:O23547 expansin-related protein 1 precursor (At-EXPR1)(Arabidopsis thaliana); related to expansins, http://www.bio.psu.edu/expansins/ \| chr4:9581605-9583309 REVERSE \| Aliases: ARABIDOPSIS THALIANA EXPANSIN LIKE B1, AT EXPR, ATEXPR1, ATHEXP BETA 3.1, DL4545C, EXPR, FCAALL.341 | 3.1 | 5.3 | -2.2 | -6.8 | 0.21% | 6.3 |
| 2386 | AT5G23920.1 \| expressed protein \| chr5:8073227-8074173 REVERSE \| Aliases: MRO11.4, MRO11_4 | 6.5 | 8.6 | -2.0 | -6.8 | 0.21% | 6.6 |
| 2388 | AT1G27920.1 \| microtubule associated protein (MAP65/ASE1) family protein, low similarity to protein regulating cytokinesis 1 (PRC1) (Homo sapiens) GI:2865521; contains Pfam profile PF03999: Microtubule associated protein (MAP65/ASE1 family) \| chr1:9726935-9729835 FORWARD \| Aliases: F13K9.3, F13K9_3 | 2.9 | 5.8 | -2.9 | -6.8 | 0.21% | 6.4 |
| 2390 | AT2G36890.1 \| myb family transcription factor (MYB38), contains Pfam profile: PF00249 myb-like DNA-binding domain \| chr2:15492858-15494356 FORWARD \| Aliases: T1J8.7, T1J8_7 | 2.6 | 5.4 | -2.8 | -6.8 | 0.21% | 6.3 |
| 2392 | AT3G51360.1 \| aspartyl protease family protein, contains Eukaryotic and viral aspartyl proteases active site, PROSITE:PS00141 \| chr3:19075273-19077539 REVERSE \| Aliases: F26O13.1 | 2.3 | 4.6 | -2.3 | -6.8 | 0.21% | 6.4 |
| 2394 | AT5G28630.1 \| glycine-rich protein \| chr5:10636635-10637253 FORWARD \| Aliases: F4I4.10, F4I4_10 | 3.4 | 9.0 | -5.6 | -6.8 | 0.21% | 6.5 |
| 2396 | AT4G37445.1 \| expressed protein \| chr4:17604088-17604926 REVERSE \| Aliases: None | 3.0 | 4.7 | -1.7 | -6.8 | 0.21% | 6.6 |
| 2397 | AT5G06839.1 \| bZIP family transcription factor, contains Pfam profile: PF00170 bZIP transcription factor \| chr5:2120528-2126455 FORWARD \| Aliases: None | 2.9 | 6.3 | -3.3 | -6.8 | 0.21% | 6.0 |
| 2398 | AT5G15180.1 \| peroxidase, putative, similar to peroxidase ATP12a (Arabidopsis thaliana) gi:1429217:emb:CAA67311 \| chr5:4930522-4932345 FORWARD \| Aliases: F8M21.70, F8M21_70 | 3.0 | 6.9 | -3.9 | -6.8 | 0.21% | 6.6 |
| 2400 | AT2G01980.1 \| Symbol: SOS1 \| sodium proton exchanger, putative (NHX7) (SOS1), identical to putative Na+/H+ antiporter SOS1 (Arabidopsis thaliana) gi:8515714:gb:AAF76139; Member of The Monovalent Cation:Proton Antiporter (CPA1) Family, PMID:11500563 \| chr2:457009-463325 FORWARD \| Aliases: ATSOS1, F14H20.5, F14H20_5, SALT OVERLY SENSITIVE 1 | 5.6 | 7.1 | -1.5 | -6.8 | 0.21% | 6.6 |
| 2401 | AT4G28370.1 \| zinc finger (C3HC4-type RING finger) family protein, contains Pfam PF00097: Zinc finger, C3HC4 type (RING finger) \| chr4:14035616-14041672 FORWARD \| Aliases: F20O9.50, F20O9_50 | 3.4 | 4.8 | -1.4 | -6.8 | 0.21% | 6.6 |

| Rank | Description | Sync | Root | M | t | adj.q | B |
|---|---|---|---|---|---|---|---|
| 2402 | AT2G30010.1 \| expressed protein \| chr2:12812801-12816462 FORWARD \| Aliases: F23F1.7, F23F1_7 | 3.5 | 4.8 | -1.3 | -6.8 | 0.21% | 6.6 |
| 2405 | AT5G25110.1 \| Symbol: CIPK25 \| | 5.5 | 9.2 | -3.7 | -6.8 | 0.21% | 6.4 |
| 2408 | AT5G58787.2 \| similar to expressed protein [Arabidopsis thaliana] (TAIR:At3g47160.1); similar to zinc finger protein family-like [Oryza sativa (japonica cultivar-group)] (GB:XP_479418.1); contains InterPro domain Zn-finger, RING (InterPro:IPR001841) \| chr5:23759676-23761845 FORWARD \| Aliases: None | 6.8 | 8.2 | -1.5 | -6.8 | 0.21% | 6.6 |
| 2410 | AT3G08720.2 \| Symbol: ATPK19 \| | 8.5 | 9.8 | -1.3 | -6.8 | 0.21% | 6.6 |
| 2413 | AT1G52910.1 \| expressed protein \| chr1:19711729-19712991 FORWARD \| Aliases: F14G24.18, F14G24_18 | 4.5 | 6.4 | -2.0 | -6.8 | 0.21% | 6.5 |
| 2415 | AT3G10680.1 \| heat shock protein-related, contains weak similarity to Pfam profile PF00011: Hsp20/alpha crystallin family \| chr3:3337778-3339350 FORWARD \| Aliases: T7M13.24 | 3.7 | 5.2 | -1.5 | -6.8 | 0.21% | 6.6 |
| 2418 | AT1G61360.1 \| S-locus lectin protein kinase family protein, contains protein kinase domain, Pfam:PF00069; contains S-locus glycoprotein family domain, Pfam:PF00954 \| chr1:22641393-22644681 REVERSE \| Aliases: T1F9.15, T1F9_15 | 6.3 | 8.7 | -2.3 | -6.8 | 0.21% | 6.6 |
| 2422 | AT3G59950.2 \| similar to autophagy 4a (APG4a) [Arabidopsis thaliana] (TAIR:At2g44140.1); similar to putative autophagy protein (with alternative splicing) [Oryza sativa (japonica cultivar-group)] (GB:XP_470812.1); similar to putative autophagy protein (with alternative splicing) [Oryza sativa (japonica cultivar-group)] (GB:XP_470811.1); contains InterPro domain Peptidase family C54 (InterPro:IPR005078) \| chr3:22155328-22158609 REVERSE \| Aliases: F24G16.220 | 6.1 | 8.0 | -1.9 | -6.8 | 0.21% | 6.6 |
| 2423 | AT1G51790.1 \| leucine-rich repeat protein kinase, putative, smilar to light repressible receptor protein kinase (Arabidopsis thaliana) gi:1321686:emb:CAA66376; contains leucine rich repeat (LRR) domains, Pfam:PF00560; contains protein kinase domain, Pfam:PF00069 | 2.4 | 6.0 | -3.7 | -6.8 | 0.21% | 6.5 |
| 2424 | AT3G22970.2 \| expressed protein, similar to expressed protein [Arabidopsis thaliana] (TAIR:At4g14620.1); similar to expressed protein [Oryza sativa (japonica cultivar-group)] (GB:AAT76408.1); contains InterPro domain Uncharacterized plant-specific domain 01615 (InterPro:IPR006502) \| chr3:8152045-8154113 FORWARD \| Aliases: F5N5.17 | 5.9 | 8.8 | -2.9 | -6.8 | 0.21% | 6.5 |
| 2425 | AT4G35190.1 \| expressed protein, contains Pfam profile PF03641: decarboxylase family protein \| chr4:16746604-16748266 FORWARD \| Aliases: T12J5.60, T12J5_60 | 4.6 | 6.0 | -1.4 | -6.8 | 0.22% | 6.6 |
| 2427 | AT2G24540.1 \| kelch repeat-containing F-box family protein, similar to SKP1 interacting partner 4 (Arabidopsis thaliana) GI:10716953; contains Pfam profiles PF01344: Kelch motif, PF00646: F-box domain \| chr2:10433422-10434674 FORWARD \| Aliases: F25P17.16, F25P17_16 | 5.5 | 7.0 | -1.5 | -6.8 | 0.22% | 6.6 |
| 2428 | AT5G47530.1 \| auxin-responsive protein, putative, similar to auxin-induced protein AIR12 (GI:11357190) (Arabidopsis thaliana); similar to stromal cell derived factor receptor 2 (GI:20381292) (Mus musculus) \| chr5:19298601-19300288 FORWARD \| Aliases: MNJ7.12, MNJ7_12 | 2.9 | 4.5 | -1.6 | -6.8 | 0.22% | 6.5 |
| 2429 | AT2G22920.2 \| Symbol: SCPL12 \| | 3.6 | 5.9 | -2.3 | -6.8 | 0.22% | 6.5 |
| 2430 | AT3G62390.1 \| expressed protein, several hypothetical proteins - Arabidopsis thaliana \| chr3:23097996-23100166 REVERSE \| Aliases: T12C14.90 | 4.3 | 6.0 | -1.7 | -6.8 | 0.22% | 6.6 |
| 2433 | AT1G47128.1 \| cysteine proteinase (RD21A) / thiol protease, identical to SP:P43297 Cysteine proteinase RD21A precursor (EC 3.4.22.-) {Arabidopsis thaliana}, thiol protease RD21A SP:P43297 from (Arabidopsis thaliana) \| chr1:17285265-17288110 REVERSE \| Aliases: F2G19.31, F2G19_31 | 9.2 | 11.7 | -2.6 | -6.8 | 0.22% | 6.6 |
| 2434 | AT3G51280.1 \| male sterility MS5, putative, similar to male sterility MS5 (Arabidopsis thaliana) GI:3859112; contains Pfam profile PF00515 TPR Domain \| chr3:19048142-19049930 FORWARD \| Aliases: F24M12.320 | 3.6 | 5.1 | -1.5 | -6.8 | 0.22% | 6.6 |
| 2435 | AT2G24360.1 \| serine/threonine/tyrosine kinase, putative, similar to serine/threonine/tyrosine kinase (Arachis hypogaea) gi:13124865:gb:AAK11734 \| chr2:10371531-10373971 REVERSE \| Aliases: T28I24.9, T28I24_9 | 8.7 | 10.0 | -1.3 | -6.8 | 0.22% | 6.6 |
| 2438 | AT3G17510.2 \| Symbol: CIPK1 \| CBL-interacting protein kinase 1 (CIPK1), identical to CBL-interacting protein kinase 1 (Arabidopsis thaliana) gi:11066952:gb:AAG28776; contains Pfam profiles PF00069: Protein kinase domain and PF03822: NAF domain; identical to cDNA CBL-interacting protein kinase 1 (CIPK1) GI:11066951 \| chr3:5988997-5991287 REVERSE \| Aliases: CBL INTERACTING PROTEIN KINASE 1, MKP6.20 | 5.0 | 8.5 | -3.4 | -6.8 | 0.22% | 6.6 |
| 2439 | AT5G36940.1 \| Symbol: CAT3 \| amino acid permease family protein, similar to SP:Q09143 High-affinity cationic amino acid transporter-1 (CAT-1) {Mus musculus}; contains Pfam profile PF00324: Amino acid permease \| chr5:14607424-14611614 FORWARD \| Aliases: CATIONIC AMINO ACID TRANSPORTER 3, MLF18.60, MLF18_60 | 6.0 | 7.8 | -1.8 | -6.8 | 0.22% | 6.5 |
| 2440 | AT1G66200.2 \| Symbol: ATGSR2 \| | 9.0 | 12.5 | -3.5 | -6.8 | 0.22% | 6.4 |
| 2441 | AT1G07530.1 \| scarecrow-like transcription factor 14 (SCL14), identical to GB:AAD24412 from (Arabidopsis thaliana) (Plant J. 18 (1), 111-119 (1999)) \| chr1:2313579-2316425 REVERSE \| Aliases: F22G5.9, F22G5_9 | 4.3 | 5.7 | -1.3 | -6.8 | 0.22% | 6.6 |

| Rank | Description | Sync | Root | M | t | adj.q | B |
|---|---|---|---|---|---|---|---|
| 2444 | AT4G33050.3 \| similar to calmodulin-binding family protein [Arabidopsis thaliana] (TAIR:At2g26190.1); similar to putative calmodulin-binding protein [Oryza sativa (japonica cultivar-group)] (GB:BAD45140.1); contains InterPro domain IQ calmodulin-binding region (InterPro:IPR000048) \| chr4:15944325-15947029 REVERSE \| Aliases: F4I10.2 | 3.5 | 6.1 | -2.5 | -6.8 | 0.22% | 6.4 |
| 2447 | AT5G59490.1 \| haloacid dehalogenase-like hydrolase family protein, low similarity to SP:P53078 SSM1 protein {Saccharomyces cerevisiae}; contains InterPro accession IPR005834: Haloacid dehalogenase-like hydrolase \| chr5:23999977-24001645 REVERSE \| Aliases: F2O15.11, F2O15_11 | 3.5 | 6.2 | -2.6 | -6.8 | 0.22% | 6.2 |
| 2456 | AT1G16860.1 \| merozoite surface protein-related, contains weak similarity to merozoite surface protein (Plasmodium falciparum) gi:12043655:gb:AAG47601 | 4.2 | 5.7 | -1.5 | -6.8 | 0.22% | 6.5 |
| 2457 | AT3G22540.1 \| expressed protein \| chr3:7984097-7984634 REVERSE \| Aliases: F16J14.10 | 4.7 | 7.6 | -3.0 | -6.8 | 0.22% | 6.1 |
| 2459 | AT1G75850.1 \| similar to vacuolar protein sorting-associated protein 35 family protein / VPS35 family protein [Arabidopsis thaliana] (TAIR:At3g51310.1); similar to vacuolar protein sorting-associated protein 35 family protein / VPS35 family protein [Arabidopsis thaliana] (TAIR:At2g17790.1); similar to novel protein simlar to human and mouse vacuolar protein sorting 35 (yeast) (VPS35) [Danio rerio] (GB:CAE49237.1); contains InterPro domain Vacuolar protein sorting-associated protein 35 (InterPro:IPR005378) | 4.8 | 7.0 | -2.3 | -6.7 | 0.22% | 6.5 |
| 2461 | AT2G22430.1 \| Symbol: ATHB6 \| homeobox-leucine zipper protein 6 (HB-6) / HD-ZIP transcription factor 6, identical to homeobox-leucine zipper protein ATHB-6 (HD-ZIP protein ATHB-6) (SP:P46668) (Arabidopsis thaliana) \| chr2:9533175-9534910 REVERSE \| Aliases: F14M13.17, F14M13_17 | 6.0 | 8.3 | -2.3 | -6.7 | 0.22% | 6.4 |
| 2462 | AT4G03330.1 \| Symbol: SYP123 \| | 3.7 | 5.9 | -2.2 | -6.7 | 0.22% | 6.3 |
| 2468 | AT3G26210.1 \| Symbol: CYP71B23 \| cytochrome P450 71B23, putative (CYP71B23), Identical to Cytochrome P450 71B23 (SP:Q9LTM0)(Arabidopsis thaliana);contains Pfam profile: PF00067 cytochrome P450 \| chr3:9594382-9596470 REVERSE \| Aliases: MTC11.12 | 2.9 | 4.6 | -1.7 | -6.7 | 0.22% | 6.5 |
| 2470 | AT5G53320.1 \| leucine-rich repeat transmembrane protein kinase, putative \| chr5:21653295-21655622 REVERSE \| Aliases: K19E1.12, K19E1_12 | 6.0 | 7.5 | -1.4 | -6.7 | 0.22% | 6.5 |
| 2473 | AT5G17890.1 \| LIM domain-containing protein / disease resistance protein-related, low similarity to disease resistance protein RPP4 (Arabidopsis thaliana) GI:20270890; contains Pfam profiles PF00412: LIM domain, PF00931: NB-ARC domain, PF00560: Leucine Rich Repeat \| chr5:5916972-5923350 FORWARD \| Aliases: MPI7.6, MPI7_6 | 3.1 | 5.2 | -2.1 | -6.7 | 0.22% | 6.5 |
| 2475 | AT3G09410.3 \| pectinacetylesterase family protein, similar to pectinacetylesterase precursor GB:CAA67728 (Vigna radiata); contains Pfam profile: PF03283 pectinacetylesterase \| chr3:2897644-2900990 REVERSE \| Aliases: F3L24.30 | 3.3 | 4.6 | -1.2 | -6.7 | 0.22% | 6.6 |
| 2481 | AT3G07420.1 \| Symbol: NS2 \| asparaginyl-tRNA synthetase 2, cytoplasmic / asparagine-tRNA ligase 2 (SYNC2), nearly identical to SP:Q9SW95; HMM hit: tRNA synthetases class II \| chr3:2373983-2376748 REVERSE \| Aliases: ATNS2, F21O3.13, SYNC2, SYNC2_ARATH | 3.9 | 6.4 | -2.4 | -6.7 | 0.23% | 6.1 |
| 2482 | AT2G46340.1 \| Symbol: SPA1 \| phytochrome A supressor spa1 (SPA1), identical to phytochrome A supressor spa1 (GI:4809171) (Arabidopsis thaliana); contains 8 WD-40 repeats (Pfam PF00400) (1 weak) \| chr2:19029417-19034440 REVERSE \| Aliases: AT2G46350, F11C10.3, PHYTOCHROME A SUPRESSOR 1, PHYTOCHROME A SUPRESSOR SPA1, SUPPRESSOR OF PHYA 105 1 | 4.2 | 5.3 | -1.0 | -6.7 | 0.23% | 6.5 |
| 2483 | AT1G12090.1 \| Symbol: ELP \| protease inhibitor/seed storage/lipid transfer protein (LTP) family protein, similar to 14 kDa polypeptide (Catharanthus roseus) GI:407410; contains Pfam protease inhibitor/seed storage/LTP family domain PF00234 \| chr1:4089845-4090723 REVERSE \| Aliases: EXTENSIN LIKE PROTEIN, F12F1.3, F12F1_3 | 2.5 | 4.2 | -1.7 | -6.7 | 0.23% | 6.4 |
| 2485 | AT5G24910.1 \| Symbol: CYP714A1 \| cytochrome P450 family protein, similar to Cytochrome P450 72A1 (SP:Q05047) (Catharanthus roseus); similar to fatty acid omega-hydroxylase cytochrome P450 4A11 - Homo sapiens, PIR:I53015; supported by cDNA: gi_16604323_gb_AY058060.1_ \| chr5:8567584-8570361 REVERSE \| Aliases: F6A4.120, F6A4_120 | 3.2 | 5.4 | -2.2 | -6.7 | 0.23% | 6.5 |
| 2486 | AT4G09990.1 \| expressed protein, contains Pfam profile PF04669: Protein of unknown function (DUF579) \| chr4:6258877-6260061 REVERSE \| Aliases: T5L19.120, T5L19_120 | 3.0 | 5.3 | -2.3 | -6.7 | 0.23% | 6.3 |
| 2487 | AT5G65480.1 \| expressed protein \| chr5:26193247-26195140 FORWARD \| Aliases: MNA5.18 | 9.4 | 11.1 | -1.7 | -6.7 | 0.23% | 6.5 |
| 2492 | AT2G26290.1 \| protein kinase, putative, similar to auxin-regulated dual specificity cytosolic kinase (Lycopersicon esculentum) gi:14484938:gb:AAK62821 | 3.2 | 5.9 | -2.7 | -6.7 | 0.23% | 6.3 |
| 2496 | AT5G40860.1 \| hypothetical protein \| chr5:16388659-16389213 FORWARD \| Aliases: MHK7.9, MHK7_9 | 2.9 | 5.4 | -2.6 | -6.7 | 0.23% | 6.1 |
| 2497 | AT1G29340.1 \| armadillo/beta-catenin repeat family protein / U-box domain-containing protein, contains Pfam domain, PF00514: Armadillo/beta-catenin-like repeats and Pfam, PF04564: U-box domain \| chr1:10264381-10266804 FORWARD \| Aliases: F15D2.34, F15D2_34 | 6.8 | 8.3 | -1.5 | -6.7 | 0.23% | 6.5 |

| Rank | Description | Sync | Root | M | t | adj.q | B |
|---|---|---|---|---|---|---|---|
| 2498 | AT1G50520.1 \| Symbol: CYP705A27 \| cytochrome P450 family protein, similar to CYTOCHROME P450 93A3 GB:O81973 from (Glycine max) \| chr1:18723046-18724887 FORWARD \| Aliases: F11F12.13, F11F12_13 | 3.6 | 5.2 | -1.7 | -6.7 | 0.23% | 6.4 |
| 2502 | AT4G17800.1 \| DNA-binding protein-related, contains Pfam domain PF03479: Domain of unknown function (DUF296), found in AT-hook motifs Pfam:PF02178 \| chr4:9895303-9896681 REVERSE \| Aliases: DL4935C, FCAALL.129 | 4.4 | 6.0 | -1.7 | -6.7 | 0.23% | 6.5 |
| 2504 | AT4G18550.1 \| lipase class 3 family protein, similar to lipase (Dianthus caryophyllus) GI:4103627; contains Pfam profile PF01764: Lipase \| chr4:10225017-10226873 REVERSE \| Aliases: F28J12.210, F28J12_210 | 2.7 | 4.4 | -1.8 | -6.7 | 0.23% | 6.4 |
| 2505 | AT5G42690.1 \| expressed protein, contains Pfam profile PF04784: Protein of unknown function, DUF547; expression supported by MPSS \| chr5:17133858-17136351 REVERSE \| Aliases: MJB21.6, MJB21_6 | 3.8 | 5.6 | -1.8 | -6.7 | 0.23% | 6.5 |
| 2507 | AT2G39420.1 \| esterase/lipase/thioesterase family protein, similar to monoglyceride lipase from (Homo sapiens) GI:14594904, (Mus musculus) GI:2632162; contains Interpro entry IPR000379 \| chr2:16467442-16470111 FORWARD \| Aliases: F12L6.8, F12L6_8 | 6.5 | 9.0 | -2.5 | -6.7 | 0.23% | 6.4 |
| 2508 | AT1G22640.1 \| Symbol: MYB3 \| myb family transcription factor (MYB4), similar to myb-related protein GI:1020155 from (Arabidopsis thaliana) \| chr1:8006186-8007417 FORWARD \| Aliases: ATMYB3, T22J18.19, T22J18_19, TRANSCRIPTION FACTOR MYB3 | 5.7 | 7.1 | -1.4 | -6.7 | 0.23% | 6.5 |
| 2513 | AT4G34000.4 \| Symbol: ABF3 \| similar to ABA-responsive element-binding protein 2 (AREB2) [Arabidopsis thaliana] (TAIR:At3g19290.1); similar to AREB-like protein [Lycopersicon esculentum] (GB:AAS20434.1); contains InterPro domain Basic-leucine zipper (bZIP) transcription factor (InterPro:IPR004827) \| chr4:16295436-16298248 FORWARD \| Aliases: ABSCISIC ACID RESPONSIVE ELEMENTS BINDING FACTOR 3, F17I5.200 | 4.4 | 7.1 | -2.8 | -6.7 | 0.23% | 6.3 |
| 2515 | AT3G11420.1 \| fringe-related protein, similar to hypothetical protein GB:AAC23643 (Arabidopsis thaliana) + weak similarity to Fringe (Schistocerca gregaria)(GI:6573138);Fringe encodes an extracellular protein that regulates Notch signalling. \| chr3:3591481-3594539 FORWARD \| Aliases: F24K9.9 | 4.7 | 6.0 | -1.3 | -6.7 | 0.23% | 6.5 |
| 2516 | AT4G32600.1 \| zinc finger (C3HC4-type RING finger) family protein, low similarity to RING finger protein 38 (Homo sapiens) GI:21666412; contains Pfam profile PF00097: Zinc finger, C3HC4 type (RING finger) \| chr4:15723468-15726105 FORWARD \| Aliases: F4D11.200, F4D11_200 | 6.8 | 8.1 | -1.3 | -6.7 | 0.23% | 6.5 |
| 2517 | AT4G12330.1 \| Symbol: CYP706A7 \| cytochrome P450 family protein, contains Pfam profile:PF00067 cytochrome p450 \| chr4:7317558-7319737 REVERSE \| Aliases: T4C9.170, T4C9_170 | 2.5 | 6.6 | -4.1 | -6.7 | 0.24% | 5.9 |
| 2519 | AT1G10340.2 \| ankyrin repeat family protein, contains ankyrin repeat domains, Pfam:PF00023 \| chr1:3390324-3392526 REVERSE \| Aliases: F14N23.22, F14N23_22 | 3.3 | 4.8 | -1.5 | -6.7 | 0.24% | 6.5 |
| 2520 | AT2G04680.1 \| DC1 domain-containing protein, contains Pfam profile PF03107: DC1 domain | 2.8 | 4.6 | -1.8 | -6.7 | 0.24% | 6.4 |
| 2521 | AT1G23560.1 \| expressed protein, contains Pfam profile PF02713: Domain of unknown function DUF220 \| chr1:8352604-8354251 REVERSE \| Aliases: F28C11.32 | 4.8 | 7.0 | -2.2 | -6.7 | 0.24% | 6.4 |
| 2522 | AT5G07810.1 \| SNF2 domain-containing protein / helicase domain-containing protein / HNH endonuclease domain-containing protein, similar to HepA-related protein HARP (Homo sapiens) GI:6693791; contains Pfam profiles PF00271: Helicase conserved C-terminal domain, PF00176: SNF2 family N-terminal domain, PF01844: HNH endonuclease \| chr5:2491284-2498485 REVERSE \| Aliases: MXM12.5, MXM12_5 | 2.6 | 3.8 | -1.2 | -6.7 | 0.24% | 6.5 |
| 2523 | AT3G49360.1 \| glucosamine/galactosamine-6-phosphate isomerase family protein, similar to SP:O95336 6-phosphogluconolactonase (EC 3.1.1.31) (6PGL) {Homo sapiens}; contains Pfam profile PF01182: Glucosamine-6-phosphate isomerase/6-phosphogluconolactonase \| chr3:18314031-18315383 REVERSE \| Aliases: F2K15.220 | 5.1 | 7.3 | -2.2 | -6.7 | 0.24% | 6.4 |
| 2525 | AT5G59260.1 \| lectin protein kinase, putative, similar to receptor lectin kinase 3 (Arabidopsis thaliana) gi:4100060:gb:AAD00733; contains protein kinase domain, Pfam:PF00069; contains legume lectins alpha and beta domains, Pfam:PF00138 and Pfam:PF00139 \| chr5:23925127-23927151 REVERSE \| Aliases: MNC17.17, MNC17_17 | 3.3 | 5.4 | -2.1 | -6.7 | 0.24% | 6.2 |
| 2529 | AT2G45890.1 \| member of KPP-like gene family, homolog of KPP (kinase partner protein) gene in tomato; expressed protein, contains Pfam profile PF03759: Domain of unknown function (DUF315) \| chr2:18890546-18892639 FORWARD \| Aliases: F4I18.13, KINASE PARTNER PROTEIN LIKE, KPP LIKE | 1.7 | 2.6 | -0.9 | -6.7 | 0.24% | 6.4 |
| 2531 | AT1G59870.1 \| ABC transporter family protein, similar to PDR5-like ABC transporter GI:1514643 from (Spirodela polyrhiza) \| chr1:22038171-22043703 FORWARD \| Aliases: F23H11.19, F23H11_19 | 6.3 | 9.8 | -3.5 | -6.7 | 0.24% | 6.4 |

| Rank | Description | Sync | Root | M | t | adj.q | B |
|---|---|---|---|---|---|---|---|
| 2535 | AT4G32180.2 \| similar to eukaryotic pantothenate kinase family protein [Arabidopsis thaliana] (TAIR:At1g60440.1); similar to pantothenate kinase 4 [Homo sapiens] (GB:CAI20410.1); similar to pantothenate kinase 4 variant [Homo sapiens] (GB:BAD97266.1); similar to hypothetical protein [Homo sapiens] (GB:CAC09438.1); contains InterPro domain Eukaryotic pantothenate kinase (InterPro:IPR004567) \| chr4:15537565-15543910 REVERSE \| Aliases: F10M6.180, F10M6_180 | 7.3 | 8.9 | -1.6 | -6.6 | 0.24% | 6.5 |
| 2537 | AT5G44130.1 \| fasciclin-like arabinogalactan-protein, putative, similar to gi_13377784_gb_AAK20861 \| chr5:17778317-17779230 FORWARD \| Aliases: MLN1.5, MLN1_5 | 4.7 | 6.9 | -2.1 | -6.6 | 0.24% | 6.3 |
| 2540 | AT3G20040.1 \| similar to hexokinase, putative [Arabidopsis thaliana] (TAIR:At1g50460.1); similar to putative hexokinase [Oryza sativa (japonica cultivar-group)] (GB:XP_463697.1); contains InterPro domain Hexokinase (InterPro:IPR001312) \| chr3:6994900-6998177 FORWARD \| Aliases: MAL21.4 | 4.3 | 6.2 | -1.9 | -6.6 | 0.24% | 6.4 |
| 2542 | AT3G48990.1 \| AMP-dependent synthetase and ligase family protein, similar to peroxisomal-coenzyme A synthetase (FAT2) (gi:586339) from Saccharomyces cerevisiae; contains Pfam AMP-binding enzyme domain PF00501; identical to cDNA; identical to cDNA adenosine monophosphate binding protein 3 AMPBP3 (AMPBP3)GI:20799714 \| chr3:18169732-18172334 REVERSE \| Aliases: T2J13.170 | 11.2 | 12.3 | -1.2 | -6.6 | 0.24% | 6.3 |
| 2544 | AT5G45370.3 \| nodulin-related / integral membrane family protein, contains Pfam profile:PF00892 integral membrane protein DUF6 | 4.4 | 6.1 | -1.7 | -6.6 | 0.24% | 6.4 |
| 2545 | AT3G49760.1 \| bZIP transcription factor family protein, similar to bZIP transcription factor GI:1769891 from (Arabidopsis thaliana) \| chr3:18466333-18467109 REVERSE \| Aliases: T16K5.110 | 2.6 | 5.3 | -2.7 | -6.6 | 0.24% | 6.0 |
| 2546 | AT3G24110.1 \| calcium-binding EF hand family protein, contains Pfam profile: PF00036 EF hand, similar to calcium-modulated proteins \| chr3:8704436-8705420 REVERSE \| Aliases: MUJ8.1 | 4.3 | 6.0 | -1.6 | -6.6 | 0.24% | 6.4 |
| 2547 | AT3G16180.1 \| proton-dependent oligopeptide transport (POT) family protein, contains Pfam profile: PF00854 POT family \| chr3:5481331-5485100 REVERSE \| Aliases: MSL1.22 | 4.9 | 6.9 | -2.0 | -6.6 | 0.24% | 6.4 |
| 2551 | AT3G14840.2 \| leucine-rich repeat family protein / protein kinase family protein, contains Pfam domains PF00560: Leucine Rich Repeat and PF00069: Protein kinase domain; contains 2 predicted transmembrane domains \| chr3:4988278-4994065 FORWARD \| Aliases: T21E2.2 | 6.8 | 8.6 | -1.8 | -6.6 | 0.24% | 6.5 |
| 2552 | AT2G25980.1 \| jacalin lectin family protein, similar to myrosinase-binding protein homolog (Arabidopsis thaliana) GI:2997767; contains Pfam profile PF01419 jacalin-like lectin domain | 3.1 | 4.5 | -1.3 | -6.6 | 0.24% | 6.5 |
| 2554 | AT3G27150.1 \| kelch repeat-containing F-box family protein, contains Pfam:PF01344 Kelch motif, Pfam:PF00646 F-box domain \| chr3:10010679-10012878 REVERSE \| Aliases: MYF5.2 | 6.1 | 8.5 | -2.3 | -6.6 | 0.24% | 6.4 |
| 2555 | AT5G20350.1 \| Symbol: TIP1 \| similar to ankyrin repeat family protein [Arabidopsis thaliana] (TAIR:At2g14250.1); similar to putative ankyrin repeat-containing protein [Oryza sativa (japonica cultivar-group)] (GB:XP_464354.1); contains InterPro domain Zn-finger, DHHC type (InterPro:IPR001594); contains InterPro domain Ankyrin (InterPro:IPR002110) \| chr5:6876591-6881272 FORWARD \| Aliases: F5O24.240, F5O24_240, TIP GROWTH DEFECTIVE 1 | 8.2 | 9.3 | -1.2 | -6.6 | 0.24% | 6.4 |
| 2556 | AT5G17420.1 \| Symbol: IRX3 \| cellulose synthase, catalytic subunit (IRX3), identical to gi:5230423 \| chr5:5736294-5741454 REVERSE \| Aliases: ATCESA7, CELLULASE SYNTHASE 7, CELLULOSE SYNTHASE CATALYTIC SUBUNIT, CESA7, IRREGULAR XYLEM 3, T10B6.80, T10B6_80 | 3.3 | 5.6 | -2.3 | -6.6 | 0.24% | 5.9 |
| 2559 | AT5G14340.1 \| myb family transcription factor (MYB40), contains Pfam profile: PF00249 myb-like DNA-binding domain \| chr5:4623370-4624850 FORWARD \| Aliases: F18O22.130, F18O22_130 | 4.6 | 6.2 | -1.6 | -6.6 | 0.24% | 6.3 |
| 2565 | AT3G04910.2 \| Symbol: WNK1 \| similar to protein kinase family protein [Arabidopsis thaliana] (TAIR:At5g28080.1); similar to mitogen activated protein kinase kinase [Oryza sativa] (GB:AAC32599.1); contains InterPro domain Serine/threonine protein kinase, active site (InterPro:IPR008271); contains InterPro domain Tyrosine protein kinase (InterPro:IPR001245); contains InterPro domain Serine/threonine protein kinase (InterPro:IPR002290); contains InterPro domain Protein kinase (InterPro:IPR000719) \| chr3:1354641-1358225 FORWARD \| Aliases: T9J14.14, T9J14_14, WITH NO LYSINE (K) 1, ZIK4 | 5.9 | 7.8 | -1.8 | -6.6 | 0.24% | 6.4 |
| 2566 | AT3G59090.3 \| similar to transmembrane protein-related (TOM1) [Arabidopsis thaliana] (TAIR:At4g21790.1); similar to P0046E05.19 [Oryza sativa (japonica cultivar-group)] (GB:NP_916134.1) \| chr3:21850175-21853732 FORWARD \| Aliases: F17J16.140 | 3.9 | 5.5 | -1.6 | -6.6 | 0.24% | 6.4 |
| 2567 | AT5G37740.1 \| C2 domain-containing protein, similar to zinc finger and C2 domain protein GI:9957238 from (Arabidopsis thaliana) \| chr5:15009295-15010901 FORWARD \| Aliases: K12B20.22, K12B20_22 | 5.9 | 8.7 | -2.8 | -6.6 | 0.24% | 6.4 |
| 2569 | AT4G15290.1 \| Symbol: ATCSLB05 \| cellulose synthase family protein, similar to Zea mays cellulose synthase-5 (gi:9622882), -4 (gi:9622880) \| chr4:8721490-8726669 REVERSE \| Aliases: ATCSLB5, CSLB05, DL3690C, FCAALL.256 | 4.5 | 6.8 | -2.3 | -6.6 | 0.25% | 6.1 |

| Rank | Description | Sync | Root | M | t | adj.q | B |
|---|---|---|---|---|---|---|---|
| 2571 | AT4G10790.1 \| UBX domain-containing protein, low similarity to SP:Q9UNN5 FAS-associated factor 1 (FAF1 protein) {Homo sapiens}; contains Pfam profile PF00789: UBX domain \| chr4:6640609-6643058 REVERSE \| Aliases: T12H20.9, T12H20_9 | 6.8 | 8.5 | -1.8 | -6.6 | 0.25% | 6.4 |
| 2574 | AT1G13260.1 \| Symbol: RAV1 \| DNA-binding protein RAV1 (RAV1), identical to SP:Q9ZWM9 DNA-binding protein RAV1 {Arabidopsis thaliana}, RAV1 GI:3868857 from (Arabidopsis thaliana) \| chr1:4542165-4543739 FORWARD \| Aliases: RAV1, T6J4.2, T6J4_2 | 7.2 | 8.8 | -1.7 | -6.6 | 0.25% | 6.3 |
| 2575 | AT5G54700.1 \| ankyrin repeat family protein, contains ankyrin repeat domains, Pfam:PF00023 \| chr5:22240322-22242735 REVERSE \| Aliases: K5F14.4, K5F14_4 | 2.9 | 5.0 | -2.0 | -6.6 | 0.25% | 6.3 |
| 2580 | AT1G77740.1 \| 1-phosphatidylinositol-4-phosphate 5-kinase, putative / PIP kinase, putative / PtdIns(4)P-5-kinase, putative / diphosphoinositide kinase, putative, strong similarity to phosphatidylinositol-4-phosphate 5-kinase AtPIP5K1 (Arabidopsis thaliana) GI:3702691; contains Pfam profiles PF01504: Phosphatidylinositol-4-phosphate 5-Kinase, PF02493: MORN repeat \| chr1:29225432-29228755 FORWARD \| Aliases: T32E8.7, T32E8_7 | 4.5 | 6.2 | -1.7 | -6.6 | 0.25% | 6.4 |
| 2581 | AT5G62900.1 \| expressed protein \| chr5:25266023-25267103 FORWARD \| Aliases: MQB2.22, MQB2_22 | 5.4 | 7.7 | -2.3 | -6.6 | 0.25% | 6.3 |
| 2585 | AT2G17760.1 \| aspartyl protease family protein, contains Eukaryotic and viral aspartyl proteases active site, PROSITE:PS00141 \| chr2:7720396-7723546 FORWARD \| Aliases: T17A5.8, T17A5_8 | 6.2 | 8.4 | -2.2 | -6.6 | 0.25% | 6.4 |
| 2586 | AT4G25900.1 \| aldose 1-epimerase family protein, similar to apospory-associated protein C; APOC (Chlamydomonas reinhardtii) GI:6970044 Pfam profile PF01263: Aldose 1-epimerase \| chr4:13161388-13163607 FORWARD \| Aliases: F14M19.180, F14M19_180 | 6.9 | 9.2 | -2.3 | -6.6 | 0.25% | 6.3 |
| 2587 | AT2G36690.1 \| oxidoreductase, 2OG-Fe(II) oxygenase family protein, similar to IDS3 (Hordeum vulgare)(GI:4514655), leucoanthocyanidin dioxygenase (SP:P51091)(Malus domestica); contains PF03171 2OG-Fe(II) oxygenase superfamily domain \| chr2:15387009-15389066 FORWARD \| Aliases: F13K3.9, F13K3_9 | 2.8 | 4.2 | -1.4 | -6.6 | 0.25% | 6.4 |
| 2588 | AT3G30390.2 \| similar to amino acid transporter family protein [Arabidopsis thaliana] (TAIR:At5g38820.1); similar to hypothetical protein [Oryza sativa (japonica cultivar-group)] (GB:NP_910699.1); similar to putative system A transporter isoform 2 [Oryza sativa (japonica cultivar-group)] (GB:BAD37809.1); contains InterPro domain Amino acid/polyamine transporter, family II (InterPro:IPR002422); contains InterPro domain Aromatic amino acid permease (InterPro:IPR002091) \| chr3:11979316-11982100 REVERSE \| Aliases: T6J22.19 | 5.9 | 10.3 | -4.5 | -6.6 | 0.25% | 6.3 |
| 2592 | AT2G48080.1 \| oxidoreductase, 2OG-Fe(II) oxygenase family protein, contains Pfam domain PF03171 2OG-Fe(II) oxygenase superfamily \| chr2:19672060-19674162 REVERSE \| Aliases: T9J23.23 | 3.2 | 6.4 | -3.2 | -6.6 | 0.25% | 5.9 |
| 2594 | AT1G70450.1 \| protein kinase family protein, contains protein kinase domain, Pfam:PF00069 \| chr1:26556239-26558100 FORWARD \| Aliases: F24J13.2, F24J13_2 | 3.7 | 7.8 | -4.1 | -6.6 | 0.25% | 6.1 |
| 2597 | AT2G19590.1 \| Symbol: ACO1 \| 1-aminocyclopropane-1-carboxylate oxidase, putative / ACC oxidase, putative, similar to ACC oxidase (Cucumis melo)(GI:1183898) \| chr2:8483048-8484539 REVERSE \| Aliases: ACC OXIDASE 1, ACO1, F3P11.19, F3P11_19 | 5.6 | 8.0 | -2.4 | -6.6 | 0.25% | 6.2 |
| 2598 | AT1G15110.1 \| phosphatidyl serine synthase family protein, contains Pfam profile: PF03034 phosphatidyl serine synthase | 4.6 | 6.5 | -1.9 | -6.6 | 0.25% | 6.4 |
| 2602 | AT1G69600.1 \| zinc finger homeobox family protein / ZF-HD homeobox family protein \| chr1:26186133-26186861 FORWARD \| Aliases: F24J1.29, F24J1_29 | 4.4 | 6.2 | -1.8 | -6.6 | 0.25% | 6.2 |
| 2603 | AT1G47480.1 \| expressed protein, similar to PrMC3 (Pinus radiata) GI:5487873 \| chr1:17420019-17421872 FORWARD \| Aliases: F16N3.25, F16N3_25 | 2.7 | 5.3 | -2.5 | -6.6 | 0.25% | 6.2 |
| 2604 | AT5G01490.1 \| Symbol: CAX4 \| cation exchanger, putative (CAX4), identical to cation/proton antiporter (Arabidopsis thaliana) gi:15426028:gb:AAK97656; similar to high affinity calcium antiporter CAX1 (Arabidopsis thaliana) gi:9256741:gb:AAB05913; Ca2+:Cation Antiporter (CaCA) Family member PMID:11500563 \| chr5:195588-198523 FORWARD \| Aliases: ATCAX4, F7A7.10, F7A7_10 | 4.1 | 6.3 | -2.3 | -6.6 | 0.25% | 6.3 |
| 2605 | AT1G12150.1 \| expressed protein, contains Pfam profile PF05701: Plant protein of unknown function (DUF827); expression supported by MPSS \| chr1:4123528-4125326 REVERSE \| Aliases: T28K15.11, T28K15_11 | 2.9 | 5.0 | -2.1 | -6.6 | 0.25% | 6.3 |
| 2606 | AT5G20270.1 \| expressed protein, contains Pfam domain, PF03006: Uncharacterised protein family (Hly-III / UPF0073) \| chr5:6840835-6842860 REVERSE \| Aliases: F5O24.160, F5O24_160 | 3.0 | 5.5 | -2.5 | -6.6 | 0.25% | 5.9 |
| 2607 | AT5G14330.1 \| expressed protein \| chr5:4619800-4620554 REVERSE \| Aliases: F18O22.120, F18O22_120 | 3.1 | 6.9 | -3.8 | -6.6 | 0.25% | 4.6 |
| 2618 | AT5G38820.1 \| amino acid transporter family protein, low similarity to N system amino acids transporter NAT-1 (Mus musculus) GI:7406950; contains Pfam profile PF01490: Transmembrane amino acid transporter protein \| chr5:15560711-15562412 FORWARD \| Aliases: K15E6.7 | 4.9 | 7.9 | -3.0 | -6.6 | 0.26% | 5.8 |

| Rank | Description | Sync | Root | M | t | adj.q | B |
|---|---|---|---|---|---|---|---|
| 2623 | AT2G30740.1 \| serine/threonine protein kinase, putative, similar to Pto kinase interactor 1 (Pti1)(Lycopersicon esculentum) gi:3668069:gb:AAC61805 \| chr2:13103434-13105671 FORWARD \| Aliases: T11J7.13, T11J7_13 | 5.1 | 7.8 | -2.7 | -6.6 | 0.26% | 6.0 |
| 2626 | AT5G07820.1 \| expressed protein \| chr5:2498750-2500547 REVERSE \| Aliases: F13G24.20, F13G24_20 | 3.2 | 5.8 | -2.6 | -6.6 | 0.26% | 6.1 |
| 2627 | AT4G15740.1 \| C2 domain-containing protein, similar to cold-regulated gene SRC2 (Glycine max) GI:2055230; contains Pfam profile PF00168: C2 domain \| chr4:8964743-8966316 REVERSE \| Aliases: DL3910C, FCAALL.373 | 3.6 | 5.8 | -2.2 | -6.5 | 0.26% | 5.9 |
| 2628 | AT5G51630.2 \| disease resistance protein (TIR-NBS-LRR class), putative, domain signature TIR-NBS-LRR exists, suggestive of a disease resistance protein. \| chr5:20986847-20991926 FORWARD \| Aliases: K17N15.18, K17N15_18 | 3.2 | 5.7 | -2.5 | -6.5 | 0.26% | 6.3 |
| 2630 | AT1G43710.1 \| Symbol: EMB1075 \| serine decarboxylase, identical to serine decarboxylase (Arabidopsis thaliana) GI:15011302; contains Pfam profile PF00282: Pyridoxal-dependent decarboxylase conserved domain | 9.4 | 10.7 | -1.4 | -6.5 | 0.26% | 6.3 |
| 2631 | AT3G58350.1 \| meprin and TRAF homology domain-containing protein / MATH domain-containing protein, similar to ubiquitin-specific protease 12 (Arabidopsis thaliana) GI:11993471; contains Pfam profile PF00917: MATH domain \| chr3:21602429-21603939 REVERSE \| Aliases: F9D24.260 | 3.0 | 5.0 | -2.0 | -6.5 | 0.26% | 6.1 |
| 2632 | AT1G11440.1 \| expressed protein \| chr1:3849360-3850662 FORWARD \| Aliases: T23J18.11, T23J18_11 | 4.9 | 6.7 | -1.8 | -6.5 | 0.26% | 6.3 |
| 2633 | AT1G74360.1 \| leucine-rich repeat transmembrane protein kinase, putative, similar to brassinosteroid insensitive 1 GB:AAC49810 (putative receptor protein kinase); contains Pfam profiles: PF00560 Leucine Rich Repeat (17 repeats), PF00069 Eukaryotic protein kinase domain \| chr1:27957892-27962176 FORWARD \| Aliases: F1M20.4, F1M20_4 | 3.5 | 5.8 | -2.3 | -6.5 | 0.26% | 6.2 |
| 2634 | AT1G51800.1 \| leucine-rich repeat protein kinase, putative, similar to light repressible receptor protein kinase (Arabidopsis thaliana) gi:1321686:emb:CAA66376; contains leucine rich repeat (LRR) domains, Pfam:PF00560; contains protein kinase domain, Pfam:PF00069 \| chr1:19217817-19221639 FORWARD \| Aliases: F19C24.3, F19C24_3 | 3.1 | 5.7 | -2.6 | -6.5 | 0.26% | 6.1 |
| 2639 | AT1G70170.1 \| Symbol: MMP \| matrixin family protein, similar to SP:P29136 Metalloendoproteinase 1 precursor (EC 3.4.24.-) (SMEP1) {Glycine max}; contains InterPro accession IPR001818: Matrixin \| chr1:26427581-26428985 FORWARD \| Aliases: F20P5.11, F20P5_11, MATRIX METALLOPROTEINASE | 3.1 | 4.6 | -1.5 | -6.5 | 0.26% | 6.3 |
| 2646 | AT5G54050.1 \| DC1 domain-containing protein \| chr5:21950849-21953221 REVERSE \| Aliases: K19P17.23, K19P17_23 | 2.9 | 4.7 | -1.8 | -6.5 | 0.26% | 6.4 |
| 2647 | AT5G55250.1 \| S-adenosyl-L-methionine:carboxyl methyltransferase family protein, similar to SAM:jasmonic acid carboxyl methyltransferase (JMT)(GI:13676829) and to SAM:benzoic acid carboxyl methyltransferase (BAMT)(GI:9789277)(Antirrhinum majus) \| chr5:22424775-22428170 REVERSE \| Aliases: MCO15.20, MCO15_20 | 2.8 | 5.5 | -2.7 | -6.5 | 0.26% | 6.0 |
| 2650 | AT3G03190.1 \| Symbol: ATGSTF11 \| glutathione S-transferase, putative, identical to glutathione S-transferase GB:AAB09584 from (Arabidopsis thaliana) \| chr3:735035-735994 FORWARD \| Aliases: ATGSTF6, GLUTATHIONE S TRANSFERASE, GLUTATHIONE S TRANSFERASE F11, T17B22.12, T17B22_12 | 3.3 | 5.4 | -2.1 | -6.5 | 0.26% | 6.2 |
| 2652 | AT5G49890.1 \| Symbol: CLC C \| chloride channel protein (CLC-c), identical to gi:1742956 \| chr5:20305346-20309576 REVERSE \| Aliases: ATCLC C, CHLORIDE CHANNEL C, K9P8.3, K9P8_3 | 3.8 | 6.4 | -2.6 | -6.5 | 0.26% | 6.3 |
| 2653 | AT5G64250.2 \| 2-nitropropane dioxygenase family / NPD family, contains Pfam profile PF03060: oxidoreductase, 2-nitropropane dioxygenase (NPD) family \| chr5:25714450-25716438 REVERSE \| Aliases: MSJ1.9, MSJ1_9 | 9.2 | 10.6 | -1.4 | -6.5 | 0.26% | 6.2 |
| 2657 | AT5G59820.1 \| Symbol: RHL41 \| zinc finger (C2H2 type) family protein (ZAT12), identical to zinc finger protein ZAT12 (Arabidopsis thaliana) gi:1418325:emb:CAA67232 \| chr5:24120198-24121013 FORWARD \| Aliases: MMN10.11, MMN10_11, RESPONSIVE TO HIGH LIGHT 41, ZAT12 | 5.9 | 8.1 | -2.3 | -6.5 | 0.26% | 6.1 |
| 2660 | AT4G00670.1 \| similar to DNA-binding protein, putative [Arabidopsis thaliana] (TAIR:At2g45820.1); similar to OSJNBb0039L24.13 [Oryza sativa (japonica cultivar-group)] (GB:XP_473296.1); contains InterPro domain Remorin, C-terminal region (InterPro:IPR005516) \| chr4:278335-279184 REVERSE \| Aliases: F6N23.13, F6N23_13 | 4.2 | 6.8 | -2.6 | -6.5 | 0.26% | 5.9 |
| 2661 | AT2G43020.1 \| amine oxidase family protein, similar to polyamine oxidase SP:O64411 (Zea mays); contains Pfam profile PF01593 amine oxidase, flavin-containing \| chr2:17898580-17901712 FORWARD \| Aliases: MFL8.12, MFL8_12 | 7.1 | 8.8 | -1.7 | -6.5 | 0.26% | 6.3 |
| 2663 | AT2G47060.4 \| similar to serine/threonine protein kinase, putative [Arabidopsis thaliana] (TAIR:At3g62220.1); similar to Pto kinase interactor 1 [Lycopersicon esculentum] (GB:AAC61805.1); contains InterPro domain Serine/threonine protein kinase, active site (InterPro:IPR008271); contains InterPro domain Tyrosine protein kinase (InterPro:IPR001245); contains InterPro domain Serine/threonine protein kinase (InterPro:IPR002290); contains InterPro domain Protein kinase (InterPro:IPR000719) \| chr2:19339974-19342041 REVERSE \| Aliases: F14M4.11 | 5.5 | 7.2 | -1.6 | -6.5 | 0.26% | 6.3 |

| Rank | Description | Sync | Root | M | t | adj.q | B |
|---|---|---|---|---|---|---|---|
| 2666 | AT5G65110.2 \| Symbol: ACX2 \| similar to acyl-CoA oxidase, putative [Arabidopsis thaliana] (TAIR:At1g06310.1); similar to acyl-CoA oxidase homolog [Phalaenopsis sp. 'True Lady'] (GB:AAB67883.1); similar to acyl-CoA oxidase [Phalaenopsis cv. 'True Lady'] (GB:AAR00586.1); similar to acyl CoA oxidase homolog [Cucurbita sp. cv. Kurokawa Amakuri] (GB:AAC15870.1); contains InterPro domain Acyl-CoA dehydrogenase, C-terminal (InterPro:IPR006090) \| chr5:26026861-26029745 REVERSE \| Aliases: ACYL COA OXIDASE 2, ATACX2, MQN23.4, MQN23_4 | 9.2 | 10.8 | -1.7 | -6.5 | 0.26% | 6.3 |
| 2671 | AT5G13990.1 \| exocyst subunit EXO70 family protein, contains Pfam domain PF03081: Exo70 exocyst complex subunit; \| chr5:4514570-4516894 REVERSE \| Aliases: MAC12.17, MAC12_17 | 2.9 | 4.2 | -1.4 | -6.5 | 0.27% | 6.3 |
| 2672 | AT2G31960.1 \| Symbol: ATGSL03 \| glycosyl transferase family 48 protein, contains Pfam profile: PF02364 1,3-beta-glucan synthase; contains non-consensus splice aite AC at exon 33 \| chr2:13596622-13607143 FORWARD \| Aliases: ATGSL3, F22D22.29, F22D22_29, GLUCAN SYNTHASE LIKE 3, GSL03 | 5.8 | 7.7 | -1.9 | -6.5 | 0.27% | 6.3 |
| 2673 | AT1G13970.1 \| expressed protein \| chr1:4779520-4782531 REVERSE \| Aliases: F16A14.19 | 3.5 | 6.3 | -2.9 | -6.5 | 0.27% | 5.8 |
| 2675 | AT1G80340.1 \| Symbol: GA4H \| gibberellin 3-beta-dioxygenase / gibberellin 3 beta-hydroxylase (GA4H), nearly identical to gibberellin 3 beta-hydroxylase GI:3982753 GB:AAC83647 (Arabidopsis thaliana) | 2.7 | 3.7 | -1.0 | -6.5 | 0.27% | 6.3 |
| 2677 | AT4G33090.1 \| Symbol: APM1 \| aminopeptidase M, similar to SP:Q11011 Puromycin-sensitive aminopeptidase (EC 3.4.11.-) (PSA) {Mus musculus}; contains Pfam profile PF01433: Peptidase family M1 | 8.9 | 10.9 | -2.0 | -6.5 | 0.27% | 6.3 |
| 2678 | AT4G37540.1 \| LOB domain protein 39 / lateral organ boundaries domain protein 39 (LBD39), identical to SP:Q9SZE8 LOB domain protein 39 {Arabidopsis thaliana} \| chr4:17639527-17640651 REVERSE \| Aliases: F19F18.30, F19F18_30 | 4.7 | 8.3 | -3.6 | -6.5 | 0.27% | 6.2 |
| 2679 | AT4G37340.1 \| Symbol: CYP81D3 \| cytochrome P450 family protein, Similar to Cytochrome P450 91A1 (SP:Q9FG65) (Arabidopsis thaliana); \| chr4:17564845-17566719 REVERSE \| Aliases: F6G17.1 | 2.8 | 4.2 | -1.4 | -6.5 | 0.27% | 6.3 |
| 2686 | AT2G21410.1 \| vacuolar proton ATPase, putative, similar to vacuolar proton ATPase 100-kDa subunit from Dictyostelium discoideum P:1384136:gb:AAB49621 \| chr2:9169700-9175532 FORWARD \| Aliases: F3K23.17, F3K23_17 | 6.8 | 7.8 | -0.9 | -6.5 | 0.27% | 6.2 |
| 2688 | AT5G25520.2 \| transcription elongation factor-related, contains weak similarity to transcription elongation factors \| chr5:8885091-8889623 FORWARD \| Aliases: T14C9.60, T14C9_60 | 5.1 | 7.1 | -2.0 | -6.5 | 0.27% | 6.3 |
| 2689 | AT1G30650.1 \| Symbol: WRKY14 \| | 2.4 | 5.0 | -2.7 | -6.5 | 0.27% | 6.0 |
| 2690 | AT1G25390.1 \| protein kinase family protein, contains protein kinase domain, Pfam:PF00069 \| chr1:8906439-8908841 REVERSE \| Aliases: F2J7.14, F2J7_14 | 5.2 | 7.0 | -1.8 | -6.5 | 0.27% | 6.1 |
| 2691 | AT2G30210.1 \| laccase, putative / diphenol oxidase, putative, similar to laccase (Populus balsamifera subsp. trichocarpa)(GI:3805960) \| chr2:12894523-12896904 REVERSE \| Aliases: T9D9.2 | 3.8 | 7.2 | -3.4 | -6.5 | 0.27% | 5.9 |
| 2692 | AT1G58440.1 \| Symbol: XF1 \| squalene monooxygenase, putative / squalene epoxidase, putative, similar to SP:O65404 (SE 1,1), SP:O65402 (SE 1,2) 6566341 dbj AB008021.1 AB008021 \| chr1:21717507-21720955 REVERSE \| Aliases: F9K23.3, F9K23_3, XF1 | 4.1 | 5.7 | -1.6 | -6.5 | 0.27% | 6.2 |
| 2695 | AT5G14730.1 \| expressed protein \| chr5:4756221-4757660 FORWARD \| Aliases: T9L3.30, T9L3_30 | 3.6 | 5.5 | -1.9 | -6.5 | 0.27% | 6.2 |
| 2698 | AT4G36520.1 \| trichohyalin-related, low similarity to SP:Q07283 Trichohyalin {Homo sapiens} \| chr4:17230592-17235438 REVERSE \| Aliases: AP22.19, AP22_19 | 4.0 | 6.7 | -2.7 | -6.5 | 0.27% | 6.0 |
| 2703 | AT3G62020.2 \| Symbol: GLP10 \| germin-like protein (GLP10), identical to germin-like protein subfamily 2 member 4 (SP:Q9M263) \| chr3:22982333-22983214 REVERSE \| Aliases: F21F14.190, GERMIN LIKE PROTEIN 10 | 3.7 | 6.3 | -2.6 | -6.5 | 0.27% | 6.0 |
| 2706 | AT2G39550.1 \| Symbol: ATGGT IB \| geranylgeranyl transferase type I beta subunit (GGT-IB), nearly identical to GI:11878247 \| chr2:16508701-16511382 FORWARD \| Aliases: GERANYLGERANYLTRANSFERASE I BETA SUBUNIT | 6.8 | 8.8 | -2.0 | -6.5 | 0.27% | 6.2 |
| 2714 | AT3G52480.1 \| expressed protein \| chr3:19463232-19464140 REVERSE \| Aliases: F22O6.140 | 3.3 | 4.9 | -1.6 | -6.5 | 0.27% | 6.1 |
| 2715 | AT1G59580.2 \| Symbol: ATMPK2 \| | 3.9 | 5.3 | -1.4 | -6.5 | 0.28% | 6.2 |
| 2717 | AT3G20570.1 \| plastocyanin-like domain-containing protein \| chr3:7186312-7187527 REVERSE \| Aliases: K10D20.11 | 4.2 | 6.0 | -1.8 | -6.5 | 0.28% | 6.1 |
| 2722 | AT3G04420.1 \| Symbol: ANAC048 \| no apical meristem (NAM) family protein, similar to NAC1 (GI:7716952) {Medicago truncatula}; contains Pfam PF02365 : No apical meristem (NAM) protein \| chr3:1172752-1174473 FORWARD \| Aliases: ANAC048, T27C4.6, T27C4_6 | 3.6 | 5.2 | -1.6 | -6.4 | 0.28% | 6.2 |

| Rank | Description | Sync | Root | M | t | adj.q | B |
|---|---|---|---|---|---|---|---|
| 2724 | AT3G05050.1 \| protein kinase family protein, contains Pfam domain, PF00069: Protein kinase domain \| chr3:1408619-1411807 REVERSE \| Aliases: T12H1.1, T12H1_1 | 5.1 | 6.5 | -1.4 | -6.4 | 0.28% | 6.2 |
| 2727 | AT3G21770.1 \| peroxidase 30 (PER30) (P30) (PRXR9), identical to SP:Q9LSY7 Peroxidase 30 precursor (EC 1.11.1.7) (Atperox P30) (PRXR9) (ATP7a) {Arabidopsis thaliana} \| chr3:7673283-7674846 FORWARD \| Aliases: MSD21.10 | 8.3 | 9.9 | -1.6 | -6.4 | 0.28% | 6.3 |
| 2734 | AT5G26270.1 \| expressed protein, ; expression supported by MPSS \| chr5:9206511-9207515 FORWARD \| Aliases: T19G15.120, T19G15_120 | 3.4 | 4.5 | -1.1 | -6.4 | 0.28% | 6.2 |
| 2737 | AT4G18650.1 \| transcription factor-related, contains weak similarity to TGACG-sequence specific DNA-binding protein TGA-2.1 (TGA2.1) (Swiss-Prot:O24160) (Nicotiana tabacum) \| chr4:10263980-10265450 REVERSE \| Aliases: F28A21.60, F28A21_60 | 4.1 | 7.1 | -3.0 | -6.4 | 0.28% | 5.2 |
| 2740 | AT1G27090.1 \| glycine-rich protein \| chr1:9403898-9406243 REVERSE \| Aliases: T7N9.15, T7N9_15 | 5.4 | 7.5 | -2.1 | -6.4 | 0.28% | 6.2 |
| 2742 | AT5G67480.2 \| TAZ zinc finger family protein / BTB/POZ domain-containing protein, contains Pfam PF00651 : BTB/POZ domain; contains Pfam PF02135 : TAZ zinc finger; simiar to Chain A, Taz2 Domain Of The Transcriptional Adaptor Protein Cbp (GI:11514507) (Homo sapiens); similar to (SP:Q09472) E1A-associated protein p300 (SP:Q09472) (Homo sapiens); similar to histone acetyltransferase HAC4 (GI:21105785) (Arabidopsis thaliana) \| chr5:26948166-26949982 REVERSE \| Aliases: K9I9.4, K9I9_4 | 5.3 | 7.0 | -1.7 | -6.4 | 0.28% | 6.2 |
| 2747 | AT1G80360.1 \| aminotransferase class I and II family protein, low similarity to GI:14278621 Aromatic Aminotransferase from Pyrococcus horikoshii \| chr1:30213391-30215579 REVERSE \| Aliases: F5I6.11, F5I6_11 | 8.1 | 9.9 | -1.8 | -6.4 | 0.28% | 6.2 |
| 2748 | AT3G14560.1 \| expressed protein \| chr3:4889079-4890166 FORWARD \| Aliases: MIE1.6 | 3.7 | 6.8 | -3.2 | -6.4 | 0.28% | 5.9 |
| 2752 | AT5G47950.1 \| transferase family protein, similar to deacetylvindoline 4-O-acetyltransferase (Catharanthus roseus)(GI:4091808)(PMID:9681034), acetyl-CoA:benzylalcohol acetyltranferase (Clarkia concinna)(GI:6166328)(PMID:10588064) | 3.8 | 6.1 | -2.3 | -6.4 | 0.28% | 6.0 |
| 2753 | AT2G31790.1 \| UDP-glucoronosyl/UDP-glucosyl transferase family protein, contains Pfam profile: PF00201 UDP-glucoronosyl and UDP-glucosyl transferase \| chr2:13525288-13527441 FORWARD \| Aliases: F20M17.17, F20M17_17 | 6.1 | 7.8 | -1.6 | -6.4 | 0.28% | 6.2 |
| 2754 | AT5G52120.1 \| Symbol: ATPP2 A14 \| F-box family protein / SKP1 interacting partner 3-related, contains Pfam profile PF00646: F-box domain \| chr5:21193659-21197536 REVERSE \| Aliases: ATPP2 A14, MSG15.5, MSG15_5 | 4.8 | 6.2 | -1.4 | -6.4 | 0.28% | 6.2 |
| 2757 | AT5G02350.1 \| DC1 domain-containing protein, contains Pfam profile PF03107: DC1 domain | 2.7 | 4.6 | -1.9 | -6.4 | 0.28% | 6.2 |
| 2764 | AT2G18690.1 \| expressed protein \| chr2:8104504-8105909 FORWARD \| Aliases: MSF3.7, MSF3_7 | 4.9 | 6.7 | -1.7 | -6.4 | 0.29% | 6.2 |
| 2766 | AT4G35350.2 \| Symbol: XCP1 \| cysteine endopeptidase, papain-type (XCP1), identical to papain-type cysteine endopeptidase XCP1 GI:6708181 from (Arabidopsis thaliana) \| chr4:16810487-16812009 FORWARD \| Aliases: F23E12.90, F23E12_90, XYLEM CYSTEINE PEPTIDASE 1 | 3.6 | 8.4 | -4.9 | -6.4 | 0.29% | 6.1 |
| 2767 | AT5G55970.2 \| zinc finger (C3HC4-type RING finger) family protein, contains Pfam profile: PF00097 zinc finger, C3HC4 type (RING finger) \| chr5:22684612-22686771 FORWARD \| Aliases: MDA7.1, MDA7_1 | 3.7 | 5.7 | -2.0 | -6.4 | 0.29% | 6.1 |
| 2768 | AT1G54090.1 \| exocyst subunit EXO70 family protein, contains Pfam domain PF03081: Exo70 exocyst complex subunit \| chr1:20193235-20195568 FORWARD \| Aliases: F15I1.17, F15I1_17 | 3.3 | 5.0 | -1.6 | -6.4 | 0.29% | 6.2 |
| 2769 | AT5G03170.1 \| Symbol: FLA11 \| fasciclin-like arabinogalactan-protein (FLA11) \| chr5:752775-753656 REVERSE \| Aliases: F15A17.200, F15A17_200 | 3.2 | 5.4 | -2.2 | -6.4 | 0.29% | 6.1 |
| 2772 | AT5G49720.1 \| Symbol: KOR1 \| endo-1,4-beta-glucanase KORRIGAN (KOR) / cellulase (OR16pep), identical to endo-1,4-beta-D-glucanase KORRIGAN (Arabidopsis thaliana) GI:3978258; similar to endo-1,4-beta-D-glucanase; cellulase GI:5689613 from (Brassica napus); identical to cDNA cellulase (OR16pep) | 7.6 | 10.4 | -2.8 | -6.4 | 0.29% | 6.2 |
| 2774 | AT5G57785.1 \| expressed protein \| chr5:23426122-23426648 REVERSE \| Aliases: None | 3.3 | 5.8 | -2.5 | -6.4 | 0.29% | 5.6 |
| 2775 | AT5G57910.1 \| expressed protein, similar to unknown protein (emb:CAB79781.1) \| chr5:23468375-23469828 REVERSE \| Aliases: MTI20.17, MTI20_17 | 5.5 | 6.9 | -1.4 | -6.4 | 0.29% | 6.2 |
| 2777 | AT3G29630.1 \| glycosyltransferase family protein, contains Pfam profile: PF00201 UDP-glucoronosyl and UDP-glucosyl transferase | 3.3 | 5.6 | -2.3 | -6.4 | 0.29% | 5.8 |
| 2780 | AT4G21850.2 \| methionine sulfoxide reductase domain-containing protein / SeIR domain-containing protein, low similarity to pilin-like transcription factor (Homo sapiens) GI:5059062, SP:P14930 Peptide methionine sulfoxide reductase msrA/msrB (EC 1.8.4.6) {Neisseria gonorrhoeae}; contains Pfam profile PF01641: SeIR domain \| chr4:11591128-11592250 REVERSE \| Aliases: T8O5.60, T8O5_60 | 4.6 | 7.2 | -2.6 | -6.4 | 0.29% | 6.0 |

| Rank | Description | Sync | Root | M | t | adj.q | B |
|---|---|---|---|---|---|---|---|
| 2781 | AT2G34500.1 \| Symbol: CYP710A1 \| cytochrome P450 family protein, similar to Cytochrome P450 61 (C-22 sterol desaturase) (SP:P54781) {Saccharomyces cerevisiae} \| chr2:14546558-14548485 REVERSE \| Aliases: F13P17.35 | 2.9 | 4.9 | -2.1 | -6.4 | 0.29% | 5.9 |
| 2782 | AT1G73120.1 \| expressed protein \| chr1:27500847-27501611 REVERSE \| Aliases: T18K17.22, T18K17_22 | 3.9 | 7.4 | -3.6 | -6.4 | 0.29% | 5.9 |
| 2783 | AT5G45810.1 \| Symbol: CIPK19 \| | 4.3 | 6.1 | -1.8 | -6.4 | 0.29% | 6.1 |
| 2784 | AT1G03370.1 \| C2 domain-containing protein / GRAM domain-containing protein, contains Pfam profiles PF00168: C2 domain; contains PF02893: GRAM domain; similar to Chain A, Crystal Structure Of Synaptotagmin Iii C2aC2B Length(GI:6980525); similar to Synaptotagmin III (SytIII) (Swiss-Prot:P40748) (Rattus norvegicus) \| chr1:827182-835361 FORWARD \| Aliases: F15K9.2, F15K9_2 | 4.6 | 6.7 | -2.1 | -6.4 | 0.29% | 6.1 |
| 2786 | AT1G19540.1 \| isoflavone reductase, putative, similar to SP:P52577; contains isoflavone reductase domain PF02716 \| chr1:6765662-6767332 FORWARD \| Aliases: F18O14.30, F18O14_30 | 3.4 | 4.5 | -1.1 | -6.4 | 0.29% | 6.2 |
| 2788 | AT5G63780.1 \| zinc finger (C3HC4-type RING finger) family protein, contains Pfam profile: PF00097 zinc finger, C3HC4 type (RING finger) \| chr5:25541071-25544452 FORWARD \| Aliases: MBK5.26, MBK5_26 | 7.3 | 8.7 | -1.4 | -6.4 | 0.29% | 6.2 |
| 2789 | AT1G79270.1 \| expressed protein, contains Pfam profile PF04146: YT521-B-like family \| chr1:29820399-29824585 FORWARD \| Aliases: YUP8H12R.13, YUP8H12R_13 | 6.3 | 8.9 | -2.6 | -6.4 | 0.29% | 6.1 |
| 2790 | AT5G09880.1 \| RNA recognition motif (RRM)-containing protein \| chr5:3081458-3085255 REVERSE \| Aliases: MYH9.9, MYH9_9 | 6.5 | 8.5 | -2.0 | -6.4 | 0.29% | 6.2 |
| 2792 | AT5G08640.1 \| Symbol: FLS \| flavonol synthase 1 (FLS1), identical to SP:Q96330; contains PF03171 2OG-Fe(II) oxygenase superfamily \| chr5:2803959-2805448 FORWARD \| Aliases: FLAVONOL SYNTHASE, T2K12.5 | 3.9 | 6.3 | -2.4 | -6.4 | 0.29% | 6.1 |
| 2793 | AT1G70850.3 \| similar to Bet v I allergen family protein [Arabidopsis thaliana] (TAIR:At1g70830.1); similar to Bet v I allergen family protein [Arabidopsis thaliana] (TAIR:At1g70830.2); similar to Csf-2 [Cucumis sativus] (GB:BAA83470.1); contains InterPro domain Bet v I allergen (InterPro:IPR000916) \| chr1:26718528-26720445 REVERSE \| Aliases: F15H11.10, F15H11_10 | 4.7 | 6.3 | -1.6 | -6.4 | 0.29% | 6.2 |
| 2796 | AT4G09100.1 \| zinc finger (C3HC4-type RING finger) family protein, contains Pfam profile: PF00097 zinc finger, C3HC4 type (RING finger) \| chr4:5811253-5811651 FORWARD \| Aliases: T8A17.4 | 3.3 | 5.4 | -2.0 | -6.4 | 0.29% | 5.9 |
| 2802 | AT4G38390.1 \| expressed protein, contains Pfam PF03138: Plant protein family. The function of this family of plant proteins is unknown; previously annotated as 'growth regulator protein -related' based on similarity to axi 1 protein (GB:X80301) (GI:559920) from (Nicotiana tabacum), which, due to scienitific fraud was retracted. Retraction in: Schell J. EMBO J 1999 May 17;18(10):2908. PMID:10400497. \| chr4:17975770-17978374 FORWARD \| Aliases: F22I13.160, F22I13_160 | 2.8 | 5.1 | -2.3 | -6.4 | 0.30% | 6.0 |
| 2803 | AT3G52900.1 \| expressed protein, contains Pfam profile PF04949: Family of unknown function (DUF662) \| chr3:19624098-19625005 FORWARD \| Aliases: F8J2.70 | 3.6 | 6.8 | -3.1 | -6.4 | 0.30% | 5.5 |
| 2806 | AT4G28270.1 \| zinc finger (C3HC4-type RING finger) family protein, contains Pfam profile: PF00097 zinc finger, C3HC4 type (RING finger) \| chr4:14007545-14009025 REVERSE \| Aliases: F26K10.150, F26K10_150 | 6.4 | 7.5 | -1.1 | -6.4 | 0.30% | 6.2 |
| 2810 | AT2G42280.2 \| basic helix-loop-helix (bHLH) family protein \| chr2:17618361-17620565 REVERSE \| Aliases: T24P15.19, T24P15_19 | 3.0 | 4.7 | -1.6 | -6.3 | 0.30% | 6.1 |
| 2815 | AT5G59940.1 \| DC1 domain-containing protein / UV-B light-insensitive protein, putative, similar to ULI3 (UV-B light insensitive) (Arabidopsis thaliana) GI:17225050; contains Pfam profile PF03107: DC1 domain | 3.7 | 5.8 | -2.1 | -6.3 | 0.30% | 6.0 |
| 2818 | AT1G11190.1 \| Symbol: BFN1 \| bifunctional nuclease (BFN1), identical to bifunctional nuclease bfn1 (Arabidopsis thaliana) gi:4099831:gb:AAD00693 \| chr1:3750066-3752780 REVERSE \| Aliases: BIFUNCTIONAL NUCLEASE I, T28P6.14, T28P6_14 | 3.9 | 5.7 | -1.8 | -6.3 | 0.30% | 6.1 |
| 2821 | AT4G01850.1 \| S-adenosylmethionine synthetase 2 (SAM2), identical to S-adenosylmethionine synthetase 2 (Methionine adenosyltransferase 2, AdoMet synthetase 2) (Arabidopsis thaliana) SWISS-PROT:P17562 \| chr4:796097-798285 REVERSE \| Aliases: T7B11.11, T7B11_11 | 10.1 | 11.5 | -1.3 | -6.3 | 0.30% | 6.1 |
| 2823 | AT5G53850.3 \| haloacid dehalogenase-like hydrolase family protein, low similarity to enolase-phosphatase E-1 enzyme (Klebsiella oxytoca) GI:401712; contains InterPro accession IPR005834: Haloacid dehalogenase-like hydrolase \| chr5:21878223-21882133 REVERSE \| Aliases: K19P17.1, K19P17_1 | 9.2 | 10.5 | -1.2 | -6.3 | 0.30% | 6.1 |
| 2824 | AT2G26250.1 \| Symbol: FDH \| beta-ketoacyl-CoA synthase family (FIDDLEHEAD) (FDH), identical to GB:AJ010713 (fiddlehead protein) \| chr2:11177496-11180220 REVERSE \| Aliases: FIDDLEHEAD, T1D16.11, T1D16_11 | 2.7 | 4.7 | -2.0 | -6.3 | 0.30% | 5.8 |

| Rank | Description | Sync | Root | M | t | adj.q | B |
|---|---|---|---|---|---|---|---|
| 2826 | AT1G24170.1 \| glycosyl transferase family 8 protein, contains Pfam profile: PF01501 glycosyl transferase family 8 \| chr1:8557205-8558821 REVERSE \| Aliases: F3I6.10, F3I6_10 | 7.3 | 8.1 | -0.8 | -6.3 | 0.30% | 6.0 |
| 2828 | AT1G08680.3 \| Symbol: ZIGA4 \| similar to human Rev interacting-like protein-related / hRIP protein-related [Arabidopsis thaliana] (TAIR:At4g13350.2); similar to human Rev interacting-like protein-related / hRIP protein-related [Arabidopsis thaliana] (TAIR:At4g13350.1); similar to human Rev interacting-like protein-like [Oryza sativa (japonica cultivar-group)] (GB:XP_464581.1); similar to ZIGA2 protein-like [Oryza sativa (japonica cultivar-group)] (GB:XP_464580.1); contains InterPro domain Human Rev interacting-like protein (hRIP) (InterPro:IPR001164) \| chr1:2762646-2768955 FORWARD \| Aliases: AT1G08690, F22O13.16, F22O13_16, ZIGA4 | 3.9 | 4.8 | -1.0 | -6.3 | 0.30% | 6.1 |
| 2829 | AT5G25560.1 \| zinc finger (C3HC4-type RING finger) family protein, contains Pfam domain PF00097: Zinc finger, C3HC4 type (RING finger) | 7.2 | 8.8 | -1.6 | -6.3 | 0.30% | 6.1 |
| 2832 | AT1G63430.1 \| leucine-rich repeat transmembrane protein kinase, putative, contains Pfam profiles: PF00069 Eukaryotic protein kinase domain, PF00560 Leucine Rich Repeat; contains 1 predicted transmembrane domain \| chr1:23526273-23530435 FORWARD \| Aliases: F2K11.19, F2K11_19 | 5.9 | 7.7 | -1.8 | -6.3 | 0.30% | 6.1 |
| 2837 | AT5G23220.1 \| isochorismatase hydrolase family protein, low similarity to SP:P45743Isochorismatase (EC 3.3.2.1) (2,3 dihydro-2,3 dihydroxybenzoate synthase) (Superoxide-inducible protein) (SOI1) {Bacillus subtilis}; contains Pfam profile PF00857: isochorismatase family protein \| chr5:7819819-7820679 REVERSE \| Aliases: MKD15.8, MKD15_8 | 2.7 | 5.3 | -2.7 | -6.3 | 0.31% | 5.3 |
| 2838 | AT2G24170.1 \| endomembrane protein 70, putative, similar to MURA transposase of maize Mutator transposon \| chr2:10281224-10284066 REVERSE \| Aliases: F27D4.8, F27D4_8 | 5.1 | 6.6 | -1.5 | -6.3 | 0.31% | 6.1 |
| 2840 | AT4G25710.1 \| kelch repeat-containing F-box family protein, similar to SKP1 interacting partner 6 (Arabidopsis thaliana) GI:10716957; contains Pfam profiles PF01344: Kelch motif, PF00646: F-box domain \| chr4:13098150-13099540 REVERSE \| Aliases: L73G19.90, L73G19_90 | 6.1 | 7.5 | -1.4 | -6.3 | 0.31% | 6.1 |
| 2843 | AT1G33990.1 \| hydrolase, alpha/beta fold family protein, similar to polyneuridine aldehyde esterase GI:6651393 from (Rauvolfia serpentina), SP:Q40708 PIR7A protein {Oryza sativa}, ethylene-induced esterase (Citrus sinensis) GI:14279437; contains Pfam profile PF00561: hydrolase, alpha/beta fold family \| chr1:12355548-12358237 FORWARD \| Aliases: F12G12.19, F12G12_19 | 7.9 | 9.6 | -1.6 | -6.3 | 0.31% | 6.1 |
| 2844 | AT1G33560.1 \| Symbol: ADR1 \| disease resistance protein (CC-NBS-LRR class), putative, domain signature CC-NBS-LRR exists, suggestive of a disease resistance protein. | 3.6 | 5.1 | -1.5 | -6.3 | 0.31% | 6.1 |
| 2845 | AT1G70230.1 \| expressed protein \| chr1:26453983-26455608 FORWARD \| Aliases: F20P5.5, F20P5_5 | 4.2 | 6.6 | -2.4 | -6.3 | 0.31% | 5.9 |
| 2846 | AT3G01720.1 \| expressed protein \| chr3:262258-265912 REVERSE \| Aliases: F4P13.27 | 7.6 | 9.8 | -2.2 | -6.3 | 0.31% | 6.1 |
| 2847 | AT2G15830.1 \| expressed protein \| chr2:6901200-6901633 FORWARD \| Aliases: F19G14.17, F19G14_17 | 3.2 | 7.2 | -4.0 | -6.3 | 0.31% | 4.8 |
| 2848 | AT1G22880.2 \| similar to glycosyl hydrolase family 9 protein [Arabidopsis thaliana] (TAIR:At1g71380.1); similar to endo-1,4-beta-glucanase [Malus x domestica] (GB:AAQ55294.1); similar to basic cellulase [Citrus sinensis] (GB:AAB65156.1); contains InterPro domain Glycoside hydrolase, family 9 (InterPro:IPR001701) \| chr1:8095491-8097698 FORWARD \| Aliases: F19G10.16, F19G10_16 | 2.7 | 3.8 | -1.1 | -6.3 | 0.31% | 6.1 |
| 2849 | AT3G45040.1 \| phosphatidate cytidylyltransferase family protein, weak similarity to SP:P20048 Dolichol kinase (EC 2.7.1.108) {Saccharomyces cerevisiae}; contains Pfam profile: PF01148 phosphatidate cytidylyltransferase \| chr3:16483647-16486738 REVERSE \| Aliases: F14D17.110 | 7.0 | 8.2 | -1.2 | -6.3 | 0.31% | 6.1 |
| 2850 | AT1G08990.1 \| glycogenin glucosyltransferase (glycogenin)-related, low similarity to glycogenin-1 from Mus musculus (SP:Q9R062), Rattus norvegicus (SP:O08730), Oryctolagus cuniculus (SP:P13280) \| chr1:2888927-2890962 FORWARD \| Aliases: F7G19.14, F7G19_14 | 2.4 | 4.4 | -2.0 | -6.3 | 0.31% | 5.7 |
| 2851 | AT2G15790.1 \| Symbol: SQN \| peptidyl-prolyl cis-trans isomerase / cyclophilin-40 (CYP40) / rotamase, identical to cyclophilin-40 (Arabidopsis thaliana) GI:13442983; supporting cDNA gi:13442982:gb:AY026065.1: \| chr2:6884857-6887980 REVERSE \| Aliases: CYCLOPHILIN 40, CYP40, F19G14.21, F19G14_21, SQUINT | 3.8 | 6.8 | -3.0 | -6.3 | 0.31% | 6.1 |
| 2853 | AT1G74510.2 \| kelch repeat-containing F-box family protein, contains Pfam:PF01344 Kelch motif, Pfam:PF00646 F-box domain \| chr1:28009212-28011400 FORWARD \| Aliases: F1M20.19, F1M20_19 | 6.3 | 7.7 | -1.4 | -6.3 | 0.31% | 6.0 |
| 2854 | AT3G46370.1 \| leucine-rich repeat protein kinase, putative, similar to light repressible receptor protein kinase (Arabidopsis thalian) gi:1321686:emb:CAA66376; contains leucine rich repeat (LRR) domains, Pfam:PF00560; contains protein kinase domain, Pfam:PF00069 | 2.7 | 4.1 | -1.4 | -6.3 | 0.31% | 6.1 |
| 2855 | AT1G23120.1 \| major latex protein-related / MLP-related, low similarity to major latex protein {Papaver somniferum}(GI:294060); Location of EST gb:T88564 ; contains Pfam profile PF00407: Pathogenesis-related protein Bet v I family | 5.4 | 7.0 | -1.6 | -6.3 | 0.31% | 6.1 |
| 2864 | AT1G34300.1 \| lectin protein kinase family protein, contains Pfam domains, PF01453: Lectin (probable mannose binding) and PF00069: Protein kinase domain \| chr1:12503384-12506026 FORWARD \| Aliases: F23M19.5, F23M19_5 | 6.5 | 7.7 | -1.2 | -6.3 | 0.31% | 6.1 |

| Rank | Description | Sync | Root | M | t | adj.q | B |
|---|---|---|---|---|---|---|---|
| 2865 | AT5G11650.1 \| hydrolase, alpha/beta fold family protein, contains Pfam profile PF00561: hydrolase, alpha/beta fold family; low similarity to monoglyceride lipase from (Homo sapiens) GI:14594904, (Mus musculus) GI:2632162 \| chr5:3744981-3747126 FORWARD \| Aliases: T22P22.40, T22P22_40 | 4.4 | 6.8 | -2.5 | -6.3 | 0.31% | 6.0 |
| 2866 | AT4G00240.1 \| Symbol: PLDBETA2 \| phospholipase D beta 2 / PLD beta 2 (PLDBETA2) / PLDdelta1, identical to SP:O23078 Phospholipase D beta 2 (EC 3.1.4.4) (AtPLDbeta2) (PLD beta 2) (PLDdelta1) (Arabidopsis thaliana); contains Pfam profiles: PF00614 phospholipase D.active site motif, PF00168 C2 domain \| chr4:106380-110718 REVERSE \| Aliases: F5I10.13 | 6.1 | 7.7 | -1.6 | -6.3 | 0.32% | 6.1 |
| 2868 | AT5G13550.1 \| Symbol: SULTR4;1 \| sulfate transporter family protein, similar to sulfate transporter (Arabidopsis thaliana) GI:3777483; contains Pfam profiles PF00916: Sulfate transporter family, PF01740: STAS domain \| chr5:4355279-4359555 REVERSE \| Aliases: None | 4.9 | 6.0 | -1.1 | -6.3 | 0.32% | 6.1 |
| 2869 | AT5G02620.1 \| ankyrin repeat family protein, contains ankyrin repeat domains, Pfam:PF00023 \| chr5:589525-591675 FORWARD \| Aliases: T22P11.210, T22P11_210 | 4.4 | 5.5 | -1.1 | -6.3 | 0.32% | 6.1 |
| 2870 | AT2G36910.1 \| Symbol: ATPGP1 \| | 8.6 | 10.1 | -1.5 | -6.3 | 0.32% | 6.1 |
| 2872 | AT3G05210.1 \| Symbol: ERCC1 \| nucleotide repair protein, putative, similar to nucleotide repair protein (Lilium longiflorum) GI:3297891, SP:P07992 DNA excision repair protein ERCC-1 {Homo sapiens}; contains Pfam profile PF00633: Helix-hairpin-helix motif \| chr3:1479560-1482048 FORWARD \| Aliases: T12H1.18, T12H1_18 | 5.4 | 6.4 | -1.1 | -6.3 | 0.32% | 6.0 |
| 2875 | AT3G11690.1 \| expressed protein \| chr3:3694994-3696484 FORWARD \| Aliases: T19F11.9 | 4.2 | 5.7 | -1.5 | -6.3 | 0.32% | 6.0 |
| 2877 | AT2G44180.1 \| Symbol: MAP2A \| methionyl aminopeptidase, putative / methionine aminopeptidase, putative / peptidase M, putative, similar to SP:P50579 Methionine aminopeptidase 2 (EC 3.4.11.18) (MetAP 2) {Homo sapiens}; contains Pfam profile PF00557: metallopeptidase family M24 \| chr2:18276351-18278919 REVERSE \| Aliases: F6E13.31, METHIONINE AMINOPEPTIDASE 2A | 6.5 | 7.7 | -1.1 | -6.3 | 0.32% | 6.1 |
| 2879 | AT3G57630.2 \| exostosin family protein, contains Pfam profile: PF03016 | 5.2 | 6.8 | -1.7 | -6.3 | 0.32% | 6.0 |
| 2880 | AT3G53100.1 \| GDSL-motif lipase/hydrolase family protein, similar to family II lipase EXL3 (GI:15054386), EXL1 (GI:15054382) (Arabidopsis thaliana), SP:P40603 Anther-specific proline-rich protein APG {Brassica napus}; contains InterPro Entry IPR001087 Lipolytic enzyme, G-D-S-L family \| chr3:19695725-19697575 REVERSE \| Aliases: T4D2.30 | 2.2 | 4.4 | -2.2 | -6.3 | 0.32% | 6.1 |
| 2882 | AT2G19110.1 \| Symbol: HMA4 \| ATPase E1-E2 type family protein / haloacid dehalogenase-like hydrolase family protein / heavy-metal-associated domain-containing protein, similar to cadmium efflux pump protein from Geobacillus stearothermophilus (GI:16753175), cadmium resistance protein B from Staphylococcus aureus (GI:14020985); T20K24.13 has been merged with T20K24.12 per suggestion of Dr. Kristian Axelsen (axe@biobase.dk) \| chr2:8285965-8293527 FORWARD \| Aliases: T20K24.12, T20K24_12 | 7.9 | 10.2 | -2.3 | -6.3 | 0.32% | 6.0 |
| 2884 | AT3G18660.2 \| similar to glycogenin glucosyltransferase (glycogenin)-related [Arabidopsis thaliana] (TAIR:At1g77130.1); similar to P0018C10.26 [Oryza sativa (japonica cultivar-group)] (GB:NP_915047.1); similar to glycogenin-like protein [Oryza sativa (japonica cultivar-group)] (GB:BAD81673.1); similar to secondary cell wall-related glycosyltransferase family 8 [Populus tremula x Populus tremuloides] (GB:AAX33316.1); similar to putative glycogenin [Oryza sativa (japonica cultivar-group)] (GB:XP_475247.1); contains InterPro domain Glycosyl transferase, family 8 (InterPro:IPR002495) \| chr3:6419037-6421965 REVERSE \| Aliases: MVE11.2 | 2.3 | 4.0 | -1.7 | -6.3 | 0.32% | 6.1 |
| 2885 | AT3G05890.1 \| Symbol: RCI2B \| hydrophobic protein (RCI2B) / low temperature and salt responsive protein (LTI6B), identical to SP:Q9ZNS6 Hydrophobic protein RCI2B (Low temperature and salt responsive protein LTI6B) {Arabidopsis thaliana} \| chr3:1757637-1758498 REVERSE \| Aliases: F2O10.15, F2O10_15, RARE COLD INDUCIBLE 2B | 5.3 | 9.4 | -4.1 | -6.3 | 0.32% | 6.0 |
| 2889 | AT1G31440.1 \| SH3 domain-containing protein 1 (SH3P1), nearly identical to SH3 domain-containing protein 1 (Arabidopsis thaliana) GI:16974676; contains Pfam profile PF00018: SH3 domain \| chr1:11255817-11258685 REVERSE \| Aliases: T8E3.10, T8E3_10 | 5.6 | 7.0 | -1.4 | -6.3 | 0.32% | 6.1 |
| 2893 | AT4G11300.1 \| expressed protein \| chr4:6872182-6873537 REVERSE \| Aliases: F8L21.90, F8L21_90 | 4.2 | 6.3 | -2.1 | -6.3 | 0.32% | 6.0 |
| 2894 | AT3G52400.1 \| Symbol: SYP122 \| | 5.2 | 7.5 | -2.2 | -6.2 | 0.32% | 6.0 |
| 2895 | AT3G05160.1 \| sugar transporter, putative, similar to sugar-porter family proteins 1 and 2 (Arabidopsis thaliana) GI:14585699, GI:14585701; contains Pfam profile PF00083: major facilitator superfamily protein \| chr3:1453112-1457155 REVERSE \| Aliases: T12H1.12 | 6.9 | 8.4 | -1.5 | -6.2 | 0.32% | 6.1 |
| 2896 | AT3G05165.3 \| similar to sugar-porter family protein 2 (SFP2) [Arabidopsis thaliana] (TAIR:At5g27360.1); similar to sugar transporter, putative [Arabidopsis thaliana] (TAIR:At3g05160.1); similar to sugar-porter family protein 1 (SFP1) [Arabidopsis thaliana] (TAIR:At5g27350.1); similar to putative integral membrane protein [Oryza sativa (japonica cultivar-group)] (GB:AAU10669.1); contains InterPro domain Major facilitator superfamily (MFS) (InterPro:IPR007114); contains InterPro domain General substrate transporter (InterPro:IPR005828); contains InterPro domain Sugar transporter (InterPro:IPR003663) \| chr3:1458142-1462922 REVERSE \| Aliases: None | 6.9 | 8.4 | -1.5 | -6.2 | 0.32% | 6.1 |

| Rank | Description | Sync | Root | M | t | adj.q | B |
|---|---|---|---|---|---|---|---|
| 2897 | AT1G58100.1 \| TCP family transcription factor, putative, similar to auxin-induced basic helix-loop-helix transcription factor GI:5731257 from (Gossypium hirsutum) \| chr1:21516112-21517592 REVERSE \| Aliases: T15M6.11 | 6.1 | 7.5 | -1.3 | -6.2 | 0.32% | 6.0 |
| 2898 | AT1G13210.1 \| haloacid dehalogenase-like hydrolase family protein, similar to Potential phospholipid-transporting ATPase (EC 3.6.3.1) (Chromaffin granule ATPase) from {Homo sapiens} SP:Q9Y2Q0, {Mus musculus} SP:P98200, {Bos taurus} SP:Q29449; contains InterPro accession IPR005834: Haloacid dehalogenase-like hydrolase; ESTs gb:T45045 and gb:AA394473 come from this gene \| chr1:4508960-4513935 REVERSE \| Aliases: F3F19.24, F3F19_24 | 3.3 | 4.4 | -1.1 | -6.2 | 0.32% | 6.1 |
| 2899 | AT4G13660.1 \| pinoresinol-lariciresinol reductase, putative, similar to pinoresinol-lariciresinol reductase TH1 (Tsuga heterophylla)(GI:7578915); contains isoflavone reductase domain PF02716 | 8.7 | 11.0 | -2.3 | -6.2 | 0.32% | 6.0 |
| 2900 | AT2G39340.1 \| SAC3/GANP family protein, contains Pfam profile: PF03399 SAC3/GANP family \| chr2:16430719-16437679 FORWARD \| Aliases: T16B24.2, T16B24_2 | 5.0 | 6.2 | -1.2 | -6.2 | 0.32% | 6.0 |
| 2901 | AT1G79620.1 \| leucine-rich repeat transmembrane protein kinase, putative, similar to receptor protein kinase GI:1389566 from (Arabidopsis thaliana) \| chr1:29962455-29967198 REVERSE \| Aliases: F20B17.5, F20B17_5 | 3.4 | 5.6 | -2.2 | -6.2 | 0.32% | 5.9 |
| 2903 | AT2G27310.1 \| F-box family protein, contains Pfam PF00646: F-box domain;; similar to SKP1 interacting partner 2 (SKIP2) TIGR_Ath1:At5g67250 \| chr2:11690940-11692045 REVERSE \| Aliases: F12K2.11, F12K2_11 | 5.5 | 8.2 | -2.6 | -6.2 | 0.32% | 6.0 |
| 2904 | AT1G03090.2 \| Symbol: MCCA \| methylcrotonyl-CoA carboxylase alpha chain, mitochondrial / 3-methylcrotonyl-CoA carboxylase 1 (MCCA), nearly identical to SP:Q42523 Methylcrotonyl-CoA carboxylase alpha chain, mitochondrial precursor (EC 6.4.1.4) (3-Methylcrotonyl-CoA carboxylase 1) (MCCase alpha subunit) (3-methylcrotonyl-CoA:carbon dioxide ligase alpha subunit) {Arabidopsis thaliana} \| chr1:739687-744184 FORWARD \| Aliases: None | 8.5 | 9.4 | -1.0 | -6.2 | 0.32% | 6.0 |
| 2905 | AT1G15740.1 \| leucine-rich repeat family protein \| chr1:5410929-5415297 FORWARD \| Aliases: F7H2.8, F7H2_8 | 6.6 | 9.7 | -3.0 | -6.2 | 0.32% | 6.0 |
| 2911 | AT5G54020.1 \| expressed protein \| chr5:21939090-21940760 REVERSE \| Aliases: K19P17.19, K19P17_19 | 2.8 | 5.8 | -3.0 | -6.2 | 0.33% | 5.6 |
| 2912 | AT4G39780.1 \| encodes a member of the DREB subfamily A-6 of ERF/AP2 transcription factor family. The protein contains one AP2 domain. There are 8 members in this subfamily including RAP2.4. \| chr4:18457951-18459174 REVERSE \| Aliases: T19P19.170, T19P19_170 | 4.0 | 6.0 | -2.0 | -6.2 | 0.33% | 5.8 |
| 2915 | AT3G01540.4 \| Symbol: DRH1 \| similar to DEAD box RNA helicase, putative [Arabidopsis thaliana] (TAIR:At5g14610.1); similar to DEAD box RNA helicase, putative [Arabidopsis thaliana] (TAIR:At3g06480.1); similar to putative p68 RNA helicase [Oryza sativa (japonica cultivar-group)] (GB:XP_550286.1); similar to putative RNA helicase, DRH1 [Oryza sativa (japonica cultivar-group)] (GB:XP_462826.1); similar to putative RNA helicase, DRH1 [Oryza sativa (japonica cultivar-group)] (GB:NP_918275.1); similar to P72 DEAD box protein [Pisum sativum] (GB:AAF04377.1); similar to putative ATP-dependent RNA helicase DB10 [Oryza sativa (japonica cultivar-group)] (GB:BAD88050.1); contains InterPro domain WW/Rsp5/WWP domain (InterPro:IPR001202); contains InterPro domain ATP-dependent helicase, DEAD-box (InterPro:IPR000629); contains InterPro domain Helicase, C-terminal (InterPro:IPR001650); contains InterPro domain DEAD/DEAH box helicase (InterPro:IPR001410) \| chr3:212525-216678 REVERSE \| Aliases: ATDRH1, F4P13.9, F4P13_9, RNA HELICASE DRH1 | 9.2 | 10.7 | -1.4 | -6.2 | 0.33% | 6.0 |
| 2925 | AT2G26830.1 \| Symbol: EMB1187 \| choline/ethanolamine kinase family protein, similar to SP:Q9HBU6 Ethanolamine kinase (EC 2.7.1.82) (EKI){Homo sapiens}, GmCK3p (Glycine max) GI:1438883; contains Pfam profile PF01633: Choline/ethanolamine kinase \| chr2:11450639-11454184 FORWARD \| Aliases: EMB1187, EMBRYO DEFECTIVE 1187, F12C20.13, F12C20_13 | 5.6 | 7.9 | -2.3 | -6.2 | 0.33% | 6.0 |
| 2927 | AT3G45390.1 \| lectin protein kinase family protein, contains Protein kinases ATP-binding region signature, Prosite:PS00107 and Legume lectins beta-chain signature, Prosite:PS00307 \| chr3:16658906-16660959 REVERSE \| Aliases: F18N11.150 | 2.7 | 3.9 | -1.2 | -6.2 | 0.33% | 6.0 |
| 2931 | AT5G37500.1 \| Symbol: GORK \| guard cell outward rectifying K+ channel (GORK), identical to guard cell outward rectifying K+ channel (Arabidopsis thaliana) gi:11414742:emb:CAC17380; member of the 1 pore, 6 transmembrane (1P/6TM) Shaker K+ channel family, PMID:11500563 \| chr5:14906923-14912134 REVERSE \| Aliases: GATED OUTWARDLY RECTIFYING K+ CHANNEL, MPA22.4, MPA22_4 | 3.5 | 5.2 | -1.7 | -6.2 | 0.33% | 6.0 |
| 2933 | AT3G52370.1 \| beta-Ig-H3 domain-containing protein / fasciclin domain-containing protein, weak similarity to osteoblast specific factor 2 (Homo sapiens) GI:393319; contains Pfam profile PF02469: Fasciclin domain; supporting cDNA gi:26450295:dbj:AK117608.1: \| chr3:19428468-19430760 FORWARD \| Aliases: T25B15.140 | 6.1 | 8.8 | -2.7 | -6.2 | 0.33% | 6.0 |
| 2936 | AT4G31170.3 \| similar to serine/threonine/tyrosine kinase, putative [Arabidopsis thaliana] (TAIR:At2g24360.1); similar to OSJNBa0060N03.16 [Oryza sativa (japonica cultivar-group)] (GB:XP_473833.1); contains InterPro domain Serine/threonine protein kinase, active site (InterPro:IPR008271); contains InterPro domain Tyrosine protein kinase (InterPro:IPR001245); contains InterPro domain Serine/threonine protein kinase (InterPro:IPR002290); contains InterPro domain Protein kinase (InterPro:IPR000719) \| chr4:15153188-15155644 REVERSE \| Aliases: F6E21.90, F6E21_90 | 7.9 | 9.7 | -1.8 | -6.2 | 0.33% | 6.0 |
| 2937 | AT5G64500.1 \| membrane protein-related, contains weak similarity to spinster type IV (GI:12003976) (Drosophila melanogaster) \| chr5:25797608-25800880 FORWARD \| Aliases: T12B11.9, T12B11_9 | 8.5 | 9.9 | -1.4 | -6.2 | 0.33% | 6.0 |

| Rank | Description | Sync | Root | M | t | adj.q | B |
|---|---|---|---|---|---|---|---|
| 2938 | AT2G32260.1 \| cholinephosphate cytidylyltransferase, putative / phosphorylcholine transferase, putative / CTP:phosphocholine cytidylyltransferase, putative, strong similarity to CTP:phosphocholine cytidylyltransferase (Brassica napus) GI:1418125; contains Pfam profile PF01467: Cytidylyltransferase \| chr2:13704593-13707718 FORWARD \| Aliases: T32F6.22, T32F6_22 | 7.7 | 9.7 | -2.0 | -6.2 | 0.34% | 6.0 |
| 2949 | AT1G18860.1 \| Symbol: WRKY61 \| | 2.6 | 4.2 | -1.6 | -6.2 | 0.34% | 6.0 |
| 2950 | AT3G18400.1 \| Symbol: ANAC058 \| no apical meristem (NAM) family protein, contains Pfam PF02365: No apical meristem (NAM) domain; similar to GP:1279640 NAM {Petunia x hybrida} \| chr3:6318751-6320599 REVERSE \| Aliases: ANAC058, MYF24.12 | 2.8 | 4.5 | -1.8 | -6.2 | 0.34% | 6.0 |
| 2951 | AT4G24740.2 \| Symbol: AFC2 \| protein kinase (AFC2), identical to protein kinase AFC2 (Arabidopsis thaliana) SWISS-PROT:P51567 | 6.8 | 8.3 | -1.5 | -6.2 | 0.34% | 6.0 |
| 2953 | AT1G50030.1 \| Symbol: TOR \| target of rapamycin protein (TOR), identical to pTOR (Arabidopsis thaliana) GI:12002902; contains Pfam profiles PF00454 Phosphatidylinositol 3- and 4-kinase, PF02259 FAT domain, PF02260 FATC domain \| chr1:18526109-18543663 REVERSE \| Aliases: F2J10.19, F2J10_19, TARGET OF RAPAMYCIN, TOR | 5.9 | 7.5 | -1.5 | -6.2 | 0.34% | 6.0 |
| 2954 | AT1G07310.1 \| C2 domain-containing protein, contains similarity to shock protein SRC2 (Glycine max) gi:2055230:dbj:BAA19769 ; contains Pfam profile PF00168:C2 domain \| chr1:2247508-2248960 REVERSE \| Aliases: F22G5.35, F22G5_35 | 4.3 | 5.7 | -1.4 | -6.2 | 0.34% | 6.0 |
| 2957 | AT1G16670.1 \| protein kinase family protein, contains protein kinase domain, Pfam:PF00069; similar to receptor-like serine/threonine kinase GI:2465923 from (Arabidopsis thaliana) \| chr1:5697332-5699762 FORWARD \| Aliases: F19K19.4, F19K19_4 | 5.9 | 7.5 | -1.5 | -6.2 | 0.34% | 6.0 |
| 2959 | AT1G13250.1 \| glycosyl transferase family 8 protein, contains Pfam profile: PF01501 Glycosyl transferase family 8 \| chr1:4528620-4530060 REVERSE \| Aliases: T6J4.1, T6J4_1 | 3.3 | 4.2 | -0.9 | -6.2 | 0.34% | 5.9 |
| 2960 | AT4G18340.1 \| glycosyl hydrolase family 17 protein, similar to elicitor inducible chitinase Nt-SubE76 GI:11071974 from (Nicotiana tabacum) \| chr4:10130153-10132072 REVERSE \| Aliases: T9A21.190, T9A21_190 | 6.5 | 7.8 | -1.3 | -6.2 | 0.34% | 6.0 |
| 2963 | AT3G62420.1 \| bZIP transcription factor family protein, similar to common plant regulatory factor 6 GI:9650826 from (Petroselinum crispum) \| chr3:23102820-23103260 REVERSE \| Aliases: T12C14.120 | 9.1 | 10.2 | -1.2 | -6.2 | 0.34% | 5.9 |
| 2965 | AT2G27010.1 \| Symbol: CYP705A9 \| cytochrome P450 family protein, similar to Cytochrome P450 93A1 (SP:Q42798) {Glycine max}; \| chr2:11533246-11534932 REVERSE \| Aliases: T20P8.6, T20P8_6 | 3.9 | 6.9 | -3.0 | -6.2 | 0.34% | 5.9 |
| 2970 | AT3G50950.2 \| disease resistance protein (CC-NBS-LRR class), putative, domain signature CC-NBS-LRR exists, suggestive of a disease resistance protein. | 4.6 | 6.4 | -1.8 | -6.2 | 0.34% | 5.9 |
| 2972 | AT4G05190.1 \| Symbol: ATK5 \| kinesin-like protein A, putative, kinesin like protein A, Arabidopsis thaliana, gb:Q07970 \| chr4:2675136-2679480 FORWARD \| Aliases: ARABIDOPSIS THALIANA KINESIN 5, C17L7.110, C17L7_110 | 2.3 | 3.3 | -0.9 | -6.2 | 0.35% | 5.9 |
| 2973 | AT1G71820.1 \| expressed protein \| chr1:27013307-27020770 FORWARD \| Aliases: F14O23.20, F14O23_20 | 4.3 | 5.7 | -1.4 | -6.2 | 0.35% | 5.9 |
| 2976 | AT2G20900.4 \| similar to diacylglycerol kinase accessory domain-containing protein [Arabidopsis thaliana] (TAIR:At4g28130.1); similar to putative diacylglycerol kinase [Oryza sativa (japonica cultivar-group)] (GB:XP_480362.1); similar to calmodulin-binding diacylglycerol kinase [Lycopersicon esculentum] (GB:AAG23128.1); similar to diacylglycerol kinase variant A [Lycopersicon esculentum] (GB:AAG23130.1); similar to diacylglycerol kinase [Lycopersicon esculentum] (GB:AAG23129.1); similar to OSJNBa0043L09.15 [Oryza sativa (japonica cultivar-group)] (GB:XP_474019.1); contains InterPro domain Diacylglycerol kinase, catalytic domain (InterPro:IPR001206); contains InterPro domain Diacylglycerol kinase accessory domain (presumed) (InterPro:IPR000756) \| chr2:8996356-9000066 REVERSE \| Aliases: F5H14.13, F5H14_13 | 4.7 | 6.2 | -1.5 | -6.2 | 0.35% | 5.9 |
| 2977 | AT1G44110.1 \| Symbol: CYCA1;1 \| cyclin, putative, similar to mitotic cyclin a2-type (Glycine max) GI:857397, cyclin A-like protein (Nicotiana tabacum) GI:1064927; contains Pfam profiles PF00134: Cyclin, N-terminal domain, PF02984: Cyclin, C-terminal domain \| chr1:16777361-16779679 REVERSE \| Aliases: CYCA1;1, Cyclin A1;1, T7O23.18, T7O23_18 | 3.4 | 4.9 | -1.5 | -6.2 | 0.35% | 5.9 |
| 2978 | AT1G45976.1 \| Symbol: SBP1 \| expressed protein \| chr1:17201791-17203173 FORWARD \| Aliases: F2G19.22, F2G19_22, S RIBONUCLEASE BINDING PROTEIN 1 | 7.4 | 8.8 | -1.4 | -6.2 | 0.35% | 5.9 |
| 2983 | AT3G59690.1 \| calmodulin-binding family protein, similar to SF16 protein (Helianthus annuus) GI:560150; contains Pfam profile PF00612: IQ calmodulin-binding motif \| chr3:22059731-22062650 FORWARD \| Aliases: T16L24.240 | 4.7 | 6.2 | -1.5 | -6.2 | 0.35% | 6.0 |
| 2984 | AT5G19120.1 \| expressed protein, low similarity to extracellular dermal glycoprotein EDGP precursor (Daucus carota) GI:285741, SP:P13917 Basic 7S globulin precursor {Glycine max} \| chr5:6414564-6415807 FORWARD \| Aliases: T24G5.20, T24G5_20 | 6.5 | 8.7 | -2.2 | -6.2 | 0.35% | 5.9 |

| Rank | Description | Sync | Root | M | t | adj.q | B |
|---|---|---|---|---|---|---|---|
| 2985 | AT1G78050.1 \| phosphoglycerate/bisphosphoglycerate mutase family protein, similar to SP:P31217 Phosphoglycerate mutase 1 (EC 5.4.2.1) {Escherichia coli O157:H7}; contains Pfam profile PF00300: phosphoglycerate mutase family \| chr1:29352475-29355175 FORWARD \| Aliases: F28K19.30, F28K19_30 | 7.6 | 9.2 | -1.6 | -6.2 | 0.35% | 6.0 |
| 2986 | AT1G78120.1 \| tetratricopeptide repeat (TPR)-containing protein, low similarity to SP:Q99615 DnaJ homolog subfamily C member 7 (Tetratricopeptide repeat protein 2) {Homo sapiens}; contains Pfam profile PF00515: TPR Domain \| chr1:29398914-29400896 REVERSE \| Aliases: T11I11.6, T11I11_6 | 5.4 | 6.6 | -1.3 | -6.1 | 0.35% | 5.9 |
| 2988 | AT4G20270.1 \| leucine-rich repeat transmembrane protein kinase, putative, CLAVATA1 receptor kinase, Arabidopsis th., PATX:G2160756 \| chr4:10949671-10953089 FORWARD \| Aliases: F1C12.190, F1C12_190 | 7.0 | 8.2 | -1.1 | -6.1 | 0.35% | 5.9 |
| 2989 | AT2G26480.1 \| UDP-glucoronosyl/UDP-glucosyl transferase family protein, contains Pfam profile: PF00201 UDP-glucoronosyl and UDP-glucosyl transferase \| chr2:11271041-11272762 FORWARD \| Aliases: T9J22.15, T9J22_15 | 2.8 | 4.5 | -1.7 | -6.1 | 0.35% | 5.9 |
| 2990 | AT3G13590.1 \| DC1 domain-containing protein, contains Pfam protein PF03107 DC1 domain \| chr3:4435323-4437341 REVERSE \| Aliases: K20M4.3 | 3.1 | 4.2 | -1.1 | -6.1 | 0.35% | 5.9 |
| 3005 | AT1G17170.1 \| Symbol: ATGSTU24 \| glutathione S-transferase, putative, One of three repeated putative glutathione transferases. 72% identical to glutathione transferase (Arabidopsis thaliana) (gi:4006934) \| chr1:5869839-5870833 FORWARD \| Aliases: F20D23.13, F20D23_13 | 4.9 | 6.5 | -1.6 | -6.1 | 0.35% | 5.9 |
| 3006 | AT1G19530.1 \| expressed protein \| chr1:6763906-6764944 FORWARD \| Aliases: F18O14.29, F18O14_29 | 5.8 | 8.8 | -3.0 | -6.1 | 0.35% | 5.8 |
| 3007 | AT4G28300.2 \| hydroxyproline-rich glycoprotein family protein \| chr4:14014679-14017074 FORWARD \| Aliases: F26K10.180, F26K10_180 | 3.8 | 6.0 | -2.1 | -6.1 | 0.35% | 5.7 |
| 3008 | AT4G26050.1 \| leucine-rich repeat family protein, contains Pfam PF00560: Leucine Rich Repeat domains; \| chr4:13210468-13213443 FORWARD \| Aliases: F20B18.160, F20B18_160 | 2.8 | 4.6 | -1.8 | -6.1 | 0.35% | 5.9 |
| 3009 | AT4G27435.1 \| expressed protein \| chr4:13723824-13725143 FORWARD \| Aliases: None | 2.8 | 4.2 | -1.4 | -6.1 | 0.35% | 5.9 |
| 3013 | AT4G38060.2 \| expressed protein \| chr4:17873003-17874801 REVERSE \| Aliases: F20D10.180, F20D10_180 | 7.1 | 9.8 | -2.7 | -6.1 | 0.35% | 5.9 |
| 3017 | AT5G44240.1 \| haloacid dehalogenase-like hydrolase family protein, similar to Potential phospholipid-transporting ATPase (EC 3.6.3.1) from {Mus musculus} SP:P70704, {Bos taurus} SP:Q29449; contains InterPro accession IPR005834: Haloacid dehalogenase-like hydrolase \| chr5:17834846-17840933 FORWARD \| Aliases: MLN1.17, MLN1_17 | 6.0 | 7.7 | -1.7 | -6.1 | 0.35% | 5.9 |
| 3018 | AT1G13830.1 \| beta-1,3-glucanase-related, similar to beta-1,3-glucanase-like protein (GI:14279169) (Olea europaea) similar to Glucan endo-1,3-beta-glucosidase precursor (EC 3.2.1.39) ((1-3)-beta- glucan endohydrolase) ((1-3)-beta-glucanase) (Beta-1,3- endoglucanase) (Swiss-Prot:P52409) (Triticum aestivum) \| chr1:4739877-4741029 REVERSE \| Aliases: F16A14.5, F16A14_5 | 2.5 | 5.3 | -2.8 | -6.1 | 0.36% | 5.4 |
| 3024 | AT2G02630.1 \| DC1 domain-containing protein, contains Pfam profile PF03107: DC1 domain | 2.8 | 3.9 | -1.1 | -6.1 | 0.36% | 5.9 |
| 3028 | AT5G35370.1 \| similar to lectin protein kinase family protein [Arabidopsis thaliana] (TAIR:At4g32300.1); similar to putative S-receptor kinase, homolog precursor [Oryza sativa (japonica cultivar-group)] (GB:BAD38273.1); contains InterPro domain Serine/threonine protein kinase, active site (InterPro:IPR008271); contains InterPro domain Tyrosine protein kinase (InterPro:IPR001245); contains InterPro domain Serine/threonine protein kinase (InterPro:IPR002290); contains InterPro domain Curculin-like (mannose-binding) lectin (InterPro:IPR001480); contains InterPro domain Protein kinase (InterPro:IPR000719) \| chr5:13605794-13608501 REVERSE \| Aliases: T26D22.12, T26D22_12 | 5.3 | 7.1 | -1.8 | -6.1 | 0.36% | 5.9 |
| 3029 | AT1G53270.1 \| ABC transporter family protein, contains similarity to ABC transporter GI:10280532 from (Homo sapiens) \| chr1:19866546-19868318 FORWARD \| Aliases: F12M16.17, F12M16_17 | 2.5 | 3.6 | -1.1 | -6.1 | 0.36% | 5.9 |
| 3030 | AT2G15620.1 \| Symbol: NIR1 \| ferredoxin--nitrite reductase, putative, strong similarity to ferredoxin--nitrite reductase (Nicotiana tabacum) GI:19893; contains Pfam profiles PF03460: Nitrite/Sulfite reductase ferredoxin-like half domain, PF01077: Nitrite and sulphite reductase 4Fe-4S domain \| chr2:6817558-6820021 FORWARD \| Aliases: ATHNIR, F9O13.17, F9O13_17, NIR, NITRITE REDUCTASE | 8.3 | 10.9 | -2.6 | -6.1 | 0.36% | 5.9 |
| 3031 | AT4G38520.2 \| protein phosphatase 2C family protein / PP2C family protein, similar to Ser/Thr protein phosphatase 2C (PP2C6) (GI:15020818) (Arabidopsis thaliana); similar to protein phosphatase 2C (GI:3608412) (Mesembryanthemum crystallinum); contains Pfam PF00481 : Protein phosphatase 2C domain; \| chr4:18015730-18018267 REVERSE \| Aliases: F20M13.80, F20M13_80 | 6.2 | 7.9 | -1.7 | -6.1 | 0.36% | 5.9 |
| 3034 | AT1G06160.1 \| encodes a member of the ERF (ethylene response factor) subfamily B-3 of ERF/AP2 transcription factor family. The protein contains one AP2 domain. There are 18 members in this subfamily including ATERF-1, ATERF-2, AND ATERF-5. \| chr1:1883003-1883933 FORWARD \| Aliases: F9P14.2, F9P14_2 | 3.6 | 5.7 | -2.1 | -6.1 | 0.36% | 5.6 |
| 3038 | AT5G35580.1 \| protein kinase, putative, similar to auxin-regulated dual specificity cytosolic kinase (Lycopersicon esculentum) gi:14484938:gb:AAK62821 | 6.1 | 8.0 | -1.9 | -6.1 | 0.37% | 5.9 |

| Rank | Description | Sync | Root | M | t | adj.q | B |
|---|---|---|---|---|---|---|---|
| 3039 | AT5G48250.1 \| zinc finger (B-box type) family protein, contains similarity to CONSTANS homologs \| chr5:19578545-19580948 REVERSE \| Aliases: MIF21.14, MIF21_14 | 3.8 | 4.7 | -0.9 | -6.1 | 0.37% | 5.8 |
| 3045 | AT1G53180.1 \| expressed protein \| chr1:19835109-19836510 FORWARD \| Aliases: F12M16.8, F12M16_8 | 2.5 | 4.7 | -2.3 | -6.1 | 0.37% | 5.8 |
| 3046 | AT5G17250.1 \| phosphatidylinositolglycan class O (PIG-O) family protein, similar to Pig-o (Mus musculus) GI:8099973 \| chr5:5668902-5673518 REVERSE \| Aliases: MKP11.10, MKP11_10 | 3.4 | 4.4 | -1.0 | -6.1 | 0.37% | 5.8 |
| 3047 | AT5G54230.1 \| Symbol: MYB49 \| myb family transcription factor (MYB49), contains Pfam profile: PF00249 myb-like DNA-binding domain \| chr5:22033476-22035159 REVERSE \| Aliases: MDK4.5, MDK4_5 | 2.1 | 6.9 | -4.7 | -6.1 | 0.37% | 5.4 |
| 3048 | AT1G50200.1 \| Symbol: ALATS \| aminoacyl-tRNA synthetase family protein, contains Pfam profiles: PF01411 tRNA synthetases class II (A), PF02272 DHHA1 domain \| chr1:18594882-18602149 REVERSE \| Aliases: ACD, ALANYL TRNA SYNTHETASE, ALARS, F14I3.17, F14I3_17 | 9.9 | 10.9 | -1.0 | -6.1 | 0.37% | 5.8 |
| 3049 | AT5G05250.1 \| expressed protein \| chr5:1557038-1557999 REVERSE \| Aliases: K18I23.5, K18I23_5 | 8.1 | 10.6 | -2.5 | -6.1 | 0.37% | 5.9 |
| 3050 | AT5G60570.1 \| kelch repeat-containing F-box family protein, contains Pfam:PF01344 Kelch motif, Pfam:PF00646 F-box domain \| chr5:24365939-24367120 FORWARD \| Aliases: MUF9.20, MUF9_20 | 5.6 | 7.9 | -2.3 | -6.1 | 0.37% | 5.9 |
| 3052 | AT1G64610.2 \| WD-40 repeat family protein, contains Pfam PF00400: WD domain, G-beta repeat; similar to WD-repeat protein 5 (WD repeat protein BIG-3) (SP: Q9UGP9) (Homo sapiens) \| chr1:24002388-24004991 REVERSE \| Aliases: F1N19.18, F1N19_18 | 4.4 | 5.1 | -0.7 | -6.1 | 0.37% | 5.6 |
| 3053 | AT4G33950.1 \| Symbol: OST1 \| protein kinase, putative, similar to abscisic acid-activated protein kinase (Vicia faba) gi:6739629:gb:AAF27340; contains protein kinase domain, Pfam:PF00069 \| chr4:16272324-16274815 FORWARD \| Aliases: F17I5.140, F17I5_140, OPEN STOMATA 1 | 5.1 | 7.3 | -2.3 | -6.1 | 0.37% | 5.9 |
| 3056 | AT4G17900.1 \| zinc-binding family protein, similar to zinc-binding protein (Pisum sativum) GI:16117799; contains Pfam profile PF04640 : Protein of unknown function, DUF597 \| chr4:9945974-9948091 FORWARD \| Aliases: T6K21.80, T6K21_80 | 4.8 | 7.6 | -2.8 | -6.1 | 0.37% | 5.4 |
| 3059 | AT3G20130.1 \| Symbol: CYP705A22 \| cytochrome P450 family protein, contains Pfam profile: PF00067 cytochrome P450 \| chr3:7026935-7028842 FORWARD \| Aliases: MAL21.17 | 2.4 | 3.3 | -0.9 | -6.1 | 0.37% | 5.8 |
| 3065 | AT1G09170.1 \| kinesin motor protein-related, similar to GB:AAB61066 \| chr1:2956591-2962209 REVERSE \| Aliases: T12M4.14, T12M4_14 | 2.7 | 4.1 | -1.4 | -6.1 | 0.38% | 5.8 |
| 3067 | AT1G70000.1 \| DNA-binding family protein, contains Pfam domains, PF00249: Myb-like DNA-binding domain and PF00098: Zinc knuckle \| chr1:26366941-26368362 REVERSE \| Aliases: F20P5.26, F20P5_26 | 4.5 | 6.1 | -1.6 | -6.1 | 0.38% | 5.9 |
| 3069 | AT2G36090.1 \| F-box family protein, contains Pfam PF00646: F-box domain; similar to SKP1 interacting partner 2 (SKIP2) TIGR_Ath1:At5g67250 \| chr2:15165710-15166663 FORWARD \| Aliases: F9C22.2, F9C22_2 | 6.1 | 8.8 | -2.7 | -6.1 | 0.38% | 5.7 |
| 3071 | AT3G28450.1 \| leucine-rich repeat transmembrane protein kinase, putative, similar to receptor kinase GB:AAD02501 from (Arabidopsis thaliana) \| chr3:10668499-10670614 FORWARD \| Aliases: MFJ20.14 | 6.1 | 7.8 | -1.7 | -6.1 | 0.38% | 5.8 |
| 3074 | AT2G36650.1 \| expressed protein \| chr2:15366994-15368407 FORWARD \| Aliases: F13K3.5, F13K3_5 | 3.7 | 6.0 | -2.3 | -6.1 | 0.38% | 5.7 |
| 3075 | AT2G42060.1 \| CHP-rich zinc finger protein, putative \| chr2:17555268-17556078 FORWARD \| Aliases: T6D20.5, T6D20_5 | 2.3 | 4.0 | -1.7 | -6.0 | 0.38% | 5.8 |
| 3076 | AT4G01910.1 \| DC1 domain-containing protein, contains Pfam profile PF03107: DC1 domain | 5.0 | 6.6 | -1.6 | -6.0 | 0.38% | 5.8 |
| 3079 | AT5G60170.1 \| RNA recognition motif (RRM)-containing protein, low similarity to transcriptional repressor Not4-Np (Homo sapiens) GI:6856207; contains INTERPRO:IPR000504 RNA-binding region RNP-1 (RNA recognition motif) \| chr5:24245715-24250095 FORWARD \| Aliases: F15L12.6, F15L12_6 | 3.9 | 4.8 | -0.9 | -6.0 | 0.38% | 5.7 |
| 3083 | AT2G41100.3 \| Symbol: TCH3 \| similar to calmodulin-2/3/5 (CAM2) (CAL1) [Arabidopsis thaliana] (TAIR:At2g41110.1); similar to calmodulin-2/3/5 (CAM3) [Arabidopsis thaliana] (TAIR:At3g56800.1); similar to calmodulin-2/3/5 (CAM5) (TCH1) [Arabidopsis thaliana] (TAIR:At2g27030.1); similar to calmodulin-2/3/5 (CAM5) (TCH1) [Arabidopsis thaliana] (TAIR:At2g27030.3); similar to calmodulin-7 (CAM7) [Arabidopsis thaliana] (TAIR:At3g43810.1); similar to CALM_PATSP Calmodulin (CaM) (GB:P02595); contains InterPro domain Calcium-binding EF-hand (InterPro:IPR002048); contains InterPro domain Flagellar calcium-binding protein (calflagin) (InterPro:IPR003299) \| chr2:17145157-17146690 FORWARD \| Aliases: ATCAL5, CALMODULIN LIKE 5, T3K9.13, T3K9_13, TOUCH 3 | 5.5 | 7.5 | -2.0 | -6.0 | 0.39% | 5.8 |
| 3086 | AT4G33240.1 \| phosphatidylinositol-4-phosphate 5-kinase family protein, similar to SP:Q9Z1T6 FYVE finger-containing phosphoinositide kinase (EC 2.7.1.68) (1-phosphatidylinositol-4-phosphate kinase) (PIP5K) (PtdIns(4)P-5-kinase) {Mus musculus}; contains Pfam profiles PF01504: Phosphatidylinositol-4-phosphate 5-Kinase, PF01363: FYVE zinc finger, PF00118: TCP-1/cpn60 chaperonin family \| chr4:16029209-16037282 REVERSE \| Aliases: F4I10.170, F4I10_170 | 4.4 | 5.7 | -1.3 | -6.0 | 0.39% | 5.8 |

| Rank | Description | Sync | Root | M | t | adj.q | B |
|---|---|---|---|---|---|---|---|
| 3087 | AT2G20650.2 \| zinc finger (C3HC4-type RING finger) family protein \| chr2:8910718-8914363 REVERSE \| Aliases: F23N11.3 | 4.4 | 5.9 | -1.5 | -6.0 | 0.39% | 5.8 |
| 3088 | AT4G30110.1 \| Symbol: HMA2 \| ATPase E1-E2 type family protein / haloacid dehalogenase-like hydrolase family protein / heavy-metal-associated domain-containing protein, similar to cadmium efflux pump protein from Geobacillus stearothermophilus (GI:16753175), cadmium resistance protein B from Staphylococcus aureus (GI:14020985) \| chr4:14720259-14724583 REVERSE \| Aliases: F6G3.140, F6G3_140 | 4.7 | 6.8 | -2.1 | -6.0 | 0.39% | 5.8 |
| 3089 | AT4G35500.2 \| protein kinase family protein, contains eukaryotic protein kinase domain, INTERPRO:IPR000719 \| chr4:16857358-16859608 FORWARD \| Aliases: F8D20.10, F8D20_10 | 4.5 | 6.1 | -1.6 | -6.0 | 0.39% | 5.8 |
| 3093 | AT5G52830.1 \| Symbol: WRKY27 \| | 4.3 | 6.7 | -2.4 | -6.0 | 0.39% | 5.8 |
| 3096 | AT5G26200.1 \| mitochondrial substrate carrier family protein, contains Pfam profile: PF00153 mitochondrial carrier protein \| chr5:9157145-9158780 FORWARD \| Aliases: F9D12.12 | 4.8 | 6.9 | -2.1 | -6.0 | 0.39% | 5.4 |
| 3098 | AT5G38770.1 \| hypothetical protein \| chr5:15543344-15543637 FORWARD \| Aliases: MKD10.70, MKD10_70 | 2.7 | 3.6 | -0.9 | -6.0 | 0.39% | 5.7 |
| 3102 | AT4G12760.1 \| expressed protein, ; expression supported by MPSS \| chr4:7504873-7506088 FORWARD \| Aliases: T20K18.110, T20K18_110 | 7.2 | 8.6 | -1.4 | -6.0 | 0.40% | 5.8 |
| 3105 | AT5G08560.1 \| transducin family protein / WD-40 repeat family protein, contains 7 WD-40 repeats (PF00400); similar to will die slowly protein (WDS) (SP:Q9V3J8) (Drosophila melanogaster) \| chr5:2770875-2774673 REVERSE \| Aliases: MAH20.12, MAH20_12 | 9.0 | 10.1 | -1.1 | -6.0 | 0.40% | 5.7 |
| 3106 | AT3G13100.1 \| Symbol: ATMRP7 \| | 3.7 | 6.2 | -2.5 | -6.0 | 0.40% | 5.7 |
| 3110 | AT5G03320.1 \| protein kinase, putative, similar to serine/threonine-protein kinase NAK (Arabidopsis thaliana) SWISS-PROT:P43293 \| chr5:802055-804397 FORWARD \| Aliases: F12E4.50, F12E4_50 | 4.9 | 6.7 | -1.8 | -6.0 | 0.40% | 5.8 |
| 3111 | AT3G46930.1 \| protein kinase family protein, contains protein kinase domain, Pfam:PF00069 \| chr3:17296628-17299017 FORWARD \| Aliases: F13I12.1 | 4.3 | 6.4 | -2.1 | -6.0 | 0.40% | 5.8 |
| 3112 | AT5G10210.1 \| expressed protein \| chr5:3206237-3206830 FORWARD \| Aliases: F18D22.2 | 3.9 | 6.2 | -2.3 | -6.0 | 0.40% | 5.8 |
| 3113 | AT3G06330.2 \| similar to zinc finger (C3HC4-type RING finger) family protein [Arabidopsis thaliana] (TAIR:At5g18760.1); similar to zinc finger (C3HC4-type RING finger) protein-like [Oryza sativa (japonica cultivar-group)] (GB:XP_464222.1); contains InterPro domain Zn-finger, RING (InterPro:IPR001841) \| chr3:1917246-1919931 FORWARD \| Aliases: F24P17.21, F24P17_21 | 4.5 | 6.4 | -2.0 | -6.0 | 0.40% | 5.8 |
| 3120 | AT2G38320.1 \| expressed protein \| chr2:16062367-16065058 FORWARD \| Aliases: T19C21.19, T19C21_19 | 3.1 | 5.3 | -2.2 | -6.0 | 0.40% | 5.5 |
| 3123 | AT2G28320.1 \| pleckstrin homology (PH) domain-containing protein / lipid-binding START domain-containing protein, contains Pfam profiles PF01852: START domain, PF00169: PH domain \| chr2:12101823-12106721 FORWARD \| Aliases: T1B3.16, T1B3_16 | 6.5 | 7.8 | -1.3 | -6.0 | 0.40% | 5.8 |
| 3125 | AT4G26060.1 \| expressed protein \| chr4:13214755-13216470 REVERSE \| Aliases: F20B18.170, F20B18_170 | 8.0 | 9.4 | -1.4 | -6.0 | 0.40% | 5.8 |
| 3126 | AT1G55450.1 \| embryo-abundant protein-related, similar to embryo-abundant protein GI:1350531 from (Picea glauca) \| chr1:20708593-20710556 REVERSE \| Aliases: T5A14.14, T5A14_14 | 5.5 | 7.2 | -1.7 | -6.0 | 0.40% | 5.8 |
| 3128 | AT5G44700.1 \| leucine-rich repeat transmembrane protein kinase, putative \| chr5:18050091-18054121 REVERSE \| Aliases: K23L20.3, K23L20_3 | 2.1 | 3.0 | -0.9 | -6.0 | 0.40% | 5.5 |
| 3129 | AT5G51300.3 \| similar to KH domain-containing quaking protein, putative [Arabidopsis thaliana] (TAIR:At1g09660.1); similar to related to branch point bridging protein (MSL5) [Neurospora crassa] (GB:CAD36971.1); contains InterPro domain KH domain (InterPro:IPR004087); contains InterPro domain Zn-finger, CCHC type (InterPro:IPR001878); contains InterPro domain RNA-binding region RNP-1 (RNA recognition motif) (InterPro:IPR000504) \| chr5:20866213-20869556 REVERSE \| Aliases: MWD22.25, MWD22_25 | 3.9 | 6.5 | -2.5 | -6.0 | 0.40% | 5.8 |
| 3131 | AT5G27420.1 \| zinc finger (C3HC4-type RING finger) family protein, similar to RING-H2 zinc finger protein ATL6 (Arabidopsis thaliana) gi:4928403:gb:AAD33584.1:AF132016_1(4928403); contains Pfam domain, PF00097: Zinc finger, C3HC4 type (RING finger) \| chr5:9684075-9685564 FORWARD \| Aliases: F21A20.130, F21A20_130 | 3.8 | 5.4 | -1.5 | -6.0 | 0.40% | 5.7 |
| 3134 | AT1G15550.1 \| Symbol: GA4 \| gibberellin 3-beta-dioxygenase / gibberellin 3 beta-hydroxylase (GA4), identical to gibberellin 3 beta-hydroxylase (GI:2160454) \| chr1:5344473-5346161 REVERSE \| Aliases: ATGA3OX1, GA REQUIRING 4, GA4, GIBBERELLIN 3 BETA HYDROXYLASE, T16N11.6, T16N11_6 | 7.3 | 8.7 | -1.4 | -6.0 | 0.40% | 5.8 |
| 3137 | AT3G27350.1 \| expressed protein \| chr3:10127272-10129192 FORWARD \| Aliases: K1G2.6 | 6.0 | 7.3 | -1.4 | -6.0 | 0.41% | 5.8 |

| Rank | Description | Sync | Root | M | t | adj.q | B |
|---|---|---|---|---|---|---|---|
| 3138 | AT2G28990.1 \| leucine-rich repeat protein kinase, putative, similar to light repressible receptor protein kinase (Arabidopsis thaliana) gi:1321686:emb:CAA66376; contains leucine rich repeat (LRR) domains, Pfam:PF00560; contains protein kinase domain, Pfam:PF00069 \| chr2:12462132-12466618 FORWARD \| Aliases: T9I4.7, T9I4_7 | 2.7 | 4.1 | -1.4 | -6.0 | 0.41% | 5.7 |
| 3139 | AT5G27840.2 \| Symbol: TOPP8 \| serine/threonine protein phosphatase PP1 isozyme 8 (TOPP8), identical to SP:O82734 Serine/threonine protein phosphatase PP1 isozyme 8 (EC 3.1.3.16) {Arabidopsis thaliana} \| chr5:9862928-9865033 REVERSE \| Aliases: T1G16.170, T1G16_170 | 5.5 | 6.9 | -1.4 | -6.0 | 0.41% | 5.8 |
| 3140 | AT3G20410.1 \| Symbol: CPK9 \| calmodulin-domain protein kinase isoform 9 (CPK9), identical to calmodulin-domain protein kinase CDPK isoform 9 (Arabidopsis thaliana) gi:1399265:gb:AAB03242 \| chr3:7116207-7119127 FORWARD \| Aliases: CALMODULIN DOMAIN PROTEIN KINASE 9, MQC12.23 | 5.6 | 7.1 | -1.5 | -6.0 | 0.41% | 5.8 |
| 3142 | AT4G34138.1 \| UDP-glucoronosyl/UDP-glucosyl transferase family protein, contains Pfam profile: PF00201 UDP-glucoronosyl and UDP-glucosyl transferase \| chr4:16348110-16349986 REVERSE \| Aliases: None | 5.8 | 8.3 | -2.5 | -6.0 | 0.41% | 5.8 |
| 3143 | AT1G73040.1 \| jacalin lectin family protein, similar to jacalin Artocarpus heterophyllus GI:289160, SP:P18674 Agglutinin alpha chain (MPA) Maclura pomifera, SP:P18670 Agglutinin alpha chain (Jacalin alpha chain) Artocarpus integrifolia; contains Pfam profile PF01419 jacalin-like lectin domain \| chr1:27478236-27479623 REVERSE \| Aliases: F3N23.24, F3N23_24 | 2.9 | 3.9 | -1.0 | -6.0 | 0.41% | 5.7 |
| 3145 | AT5G66810.1 \| expressed protein, similar to unknown protein (gb:AAB71479.1:) \| chr5:26690152-26693821 FORWARD \| Aliases: MUD21.5, MUD21_5 | 4.1 | 5.5 | -1.3 | -6.0 | 0.41% | 5.8 |
| 3147 | AT2G35270.1 \| DNA-binding protein-related, contains Pfam domain PF03479: Domain of unknown function (DUF296), found in AT-hook motifs Pfam:PF02178 \| chr2:14864100-14864957 FORWARD \| Aliases: T4C15.6, T4C15_6 | 4.3 | 7.0 | -2.7 | -6.0 | 0.41% | 5.7 |
| 3149 | AT4G37050.1 \| patatin, putative, similar to patatin-like latex allergen (Hevea brasiliensis)(PMID:10589016); contains patatin domain PF01734 \| chr4:17457129-17459613 REVERSE \| Aliases: AP22.16, AP22_16 | 3.9 | 5.6 | -1.7 | -6.0 | 0.41% | 5.6 |
| 3151 | AT5G62600.1 \| transportin-SR-related, contains weak similarity to transportin-SR (GI:5052414) (Homo sapiens) \| chr5:25140537-25149893 REVERSE \| Aliases: K19B1.1, K19B1_1 | 5.8 | 7.6 | -1.8 | -6.0 | 0.41% | 5.7 |
| 3152 | AT3G49370.1 \| calcium-dependent protein kinase, putative / CDPK, putative, similar to calcium/calmodulin-dependent protein kinase CaMK1 (Nicotiana tabacum) gi:16904222:gb:AAL30818 \| chr3:18315727-18318891 REVERSE \| Aliases: F2K15.230 | 3.1 | 4.6 | -1.4 | -6.0 | 0.41% | 5.7 |
| 3154 | AT2G46740.1 \| FAD-binding domain-containing protein, strong similarity to At1g32300, At5g56490, At2g46750, At2g46760; contains PF01565: FAD binding domain \| chr2:19212227-19214525 REVERSE \| Aliases: F19D11.2 | 2.9 | 4.9 | -1.9 | -6.0 | 0.41% | 5.5 |
| 3157 | AT2G37040.1 \| Symbol: PAL1 \| phenylalanine ammonia-lyase 1 (PAL1), nearly identical to SP:P35510 \| chr2:15564455-15567442 REVERSE \| Aliases: CI0004, T1J8.22, T1J8_22 | 8.4 | 11.0 | -2.5 | -6.0 | 0.41% | 5.7 |
| 3158 | AT1G61370.1 \| S-locus lectin protein kinase family protein, contains Pfam domains, PF00954: S-locus glycoprotein family, PF00069: Protein kinase domain, and PF01453: Lectin (probable mannose binding) \| chr1:22645761-22648812 REVERSE \| Aliases: T1F9.14, T1F9_14 | 5.6 | 7.0 | -1.4 | -5.9 | 0.41% | 5.7 |
| 3160 | AT1G58030.1 \| Symbol: CAT2 \| amino acid permease family protein, similar to SP:P18581 Low-affinity cationic amino acid transporter-2 (CAT-2) (T-cell early activation protein) {Mus musculus}; contains Pfam profile PF00324: Amino acid permease \| chr1:21467671-21472085 REVERSE \| Aliases: CATIONIC AMINO ACID TRANSPORTER 2 | 7.3 | 8.8 | -1.6 | -5.9 | 0.42% | 5.7 |
| 3162 | AT4G37460.1 \| tetratricopeptide repeat (TPR)-containing protein, contains Pfam profile PF00515: TPR Domain \| chr4:17608371-17615770 REVERSE \| Aliases: F6G17.110, F6G17_110 | 7.0 | 8.5 | -1.5 | -5.9 | 0.42% | 5.7 |
| 3166 | AT2G33830.2 \| dormancy/auxin associated family protein, contains Pfam profile: PF05564 dormancy/auxin associated protein \| chr2:14316600-14317417 REVERSE \| Aliases: T1B8.13, T1B8_13 | 7.7 | 9.9 | -2.3 | -5.9 | 0.42% | 5.7 |
| 3169 | AT5G18270.2 \| Symbol: ANAC087 \| no apical meristem (NAM) family protein, contains Pfam PF02365 : No apical meristem (NAM) protein; similar to cup-shaped cotyledon CUC2 (GI:1944132) (Arabidopsis thaliana) \| chr5:6040921-6042940 REVERSE \| Aliases: ANAC087, MRG7.23, MRG7_23 | 3.5 | 5.4 | -1.9 | -5.9 | 0.42% | 5.5 |
| 3176 | AT1G17145.1 \| expressed protein \| chr1:5860112-5862672 REVERSE \| Aliases: None | 6.6 | 7.9 | -1.3 | -5.9 | 0.42% | 5.7 |
| 3177 | AT3G63210.1 \| Symbol: MARD1 \| expressed protein, identical to senescence-associated protein SAG102 (GI::22331931) (Arabidopsis thaliana) (unpublished); contains Pfam profile PF04570: Protein of unknown function (DUF581) \| chr3:23364590-23366135 REVERSE \| Aliases: F16M2.60, MEDIATOR OF ABA REGULATED DORMANCY 1 | 2.7 | 6.2 | -3.6 | -5.9 | 0.42% | 5.7 |

| Rank | Description | Sync | Root | M | t | adj.q | B |
|---|---|---|---|---|---|---|---|
| 3178 | AT2G15480.1 \| similar to UDP-glucoronosyl/UDP-glucosyl transferase family protein [Arabidopsis thaliana] (TAIR:At4g34131.1); similar to UDP-glucoronosyl/UDP-glucosyl transferase family protein [Arabidopsis thaliana] (TAIR:At4g34135.1); similar to immediate-early salicylate-induced glucosyltransferase (GB:AAB36653.1); similar to betanidin-5-O-glucosyltransferase [Dorotheanthus bellidiformis] (GB:CAB56231.1); similar to phenylpropanoid:glucosyltransferase 1 [Nicotiana tabacum] (GB:AAK28303.1); contains InterPro domain UDP-glucoronosyl/UDP-glucosyl transferase (InterPro:IPR002213) \| chr2:6765763-6767715 FORWARD \| Aliases: F9O13.3 | 5.1 | 7.1 | -1.9 | -5.9 | 0.42% | 5.7 |
| 3180 | AT3G46690.1 \| UDP-glucoronosyl/UDP-glucosyl transferase family protein, contains Pfam profile: PF00201 UDP-glucoronosyl and UDP-glucosyl transferase \| chr3:17208614-17210322 REVERSE \| Aliases: T6H20.280 | 3.9 | 6.1 | -2.2 | -5.9 | 0.42% | 5.5 |
| 3182 | AT2G32300.1 \| Symbol: UCC1 \| uclacyanin I, identical to uclacyanin I GI:3399767 from (Arabidopsis thaliana); contains Pfam profile PF02298: Plastocyanin-like domain; identical to cDNA uclacyanin I GI:3399766 \| chr2:13729504-13730667 FORWARD \| Aliases: UCLACYANIN 1 | 3.4 | 5.4 | -2.0 | -5.9 | 0.42% | 5.4 |
| 3183 | AT4G39270.2 \| leucine-rich repeat transmembrane protein kinase, putative, receptor protein kinase erecta, Arabidopsis thaliana | 4.5 | 9.2 | -4.7 | -5.9 | 0.42% | 5.6 |
| 3187 | AT1G60190.1 \| armadillo/beta-catenin repeat family protein / U-box domain-containing protein, contains Pfam domain, PF00514: Armadillo/beta-catenin-like repeats and Pfam, PF04564: U-box domain \| chr1:22202068-22204343 FORWARD \| Aliases: T13D8.8, T13D8_8 | 6.5 | 7.8 | -1.3 | -5.9 | 0.42% | 5.7 |
| 3188 | AT5G52310.1 \| Symbol: COR78 \| low-temperature-responsive protein 78 (LTI78) / desiccation-responsive protein 29A (RD29A) \| chr5:21258075-21260839 FORWARD \| Aliases: COLD REGULATED 78, K24M7.4, K24M7_4, LTI140, LTI78, RD29A, RESPONSIVE TO DESSICATION 29A | 10.6 | 12.7 | -2.0 | -5.9 | 0.42% | 5.5 |
| 3190 | AT5G24030.1 \| C4-dicarboxylate transporter/malic acid transport family protein, weak similarity to SP:P25396 Tellurite resistance protein tehA. {Escherichia coli}; contains Pfam profile PF03595: C4-dicarboxylate transporter/malic acid transport protein \| chr5:8118320-8122755 REVERSE \| Aliases: MZF18.9, MZF18_9 | 3.6 | 5.2 | -1.7 | -5.9 | 0.42% | 5.7 |
| 3191 | AT5G51640.1 \| Symbol: YLS7 \| leaf senescence protein-related (YLS7 ), annotation temporarily based on supporting cDNA gi:13122291:dbj:AB047810.1:; identical to cDNA YLS7 leaf-senescence-related protein GI:13122291 | 6.7 | 8.0 | -1.4 | -5.9 | 0.42% | 5.7 |
| 3198 | AT3G18200.2 \| similar to nodulin MtN21 family protein [Arabidopsis thaliana] (TAIR:At1g75500.1); similar to putative nodulin MtN21 [Oryza sativa (japonica cultivar-group)] (GB:XP_463858.1); contains InterPro domain Protein of unknown function DUF6 (InterPro:IPR000620) \| chr3:6234312-6236097 REVERSE \| Aliases: MRC8.19 | 3.8 | 5.7 | -1.9 | -5.9 | 0.43% | 5.6 |
| 3199 | AT2G27660.1 \| DC1 domain-containing protein, contains Pfam profile PF03107: DC1 domain | 3.8 | 7.2 | -3.3 | -5.9 | 0.43% | 5.5 |
| 3203 | AT3G26330.1 \| Symbol: CYP71B37 \| similar to cytochrome P450 family protein [Arabidopsis thaliana] (TAIR:At3g26300.1); similar to cytochrome P450 family protein [Arabidopsis thaliana] (TAIR:At3g26310.1); similar to cytochrome P450 71B26, putative (CYP71B26) [Arabidopsis thaliana] (TAIR:At3g26290.1); similar to cytochrome P450 family protein [Arabidopsis thaliana] (TAIR:At2g02580.1); similar to cytochrome P450 71B10 [Arabidopsis thaliana] (TAIR:At5g57260.1); similar to cytochrome P450 [Citrus sinensis] (GB:AAL24049.1); contains InterPro domain E-class P450, group I (InterPro:IPR002401); contains InterPro domain Cytochrome P450 (InterPro:IPR001128) | 4.8 | 6.4 | -1.6 | -5.9 | 0.43% | 5.7 |
| 3205 | AT1G13100.1 \| Symbol: CYP71B29 \| cytochrome P450 71B29, putative (CYP71B29), strong similarity to gb:X97864 cytochrome P450 and identical to Cytochrome P450 71B29 (SP:Q9SAE4)(Arabidopsis thaliana);PF:00067 Cytochrome P450 family \| chr1:4463922-4465536 FORWARD \| Aliases: F3F19.12, F3F19_12 | 2.7 | 4.4 | -1.7 | -5.9 | 0.43% | 5.6 |
| 3206 | AT5G16660.1 \| expressed protein \| chr5:5465490-5467066 REVERSE \| Aliases: MTG13.11, MTG13_11 | 8.2 | 9.4 | -1.2 | -5.9 | 0.43% | 5.7 |
| 3208 | AT5G45940.1 \| MutT/nudix family protein, contains Pfam profile PF00293: NUDIX domain \| chr5:18646279-18647884 REVERSE \| Aliases: K15I22.14, K15I22_14 | 4.8 | 6.2 | -1.4 | -5.9 | 0.43% | 5.7 |
| 3209 | AT4G32020.1 \| expressed protein, NuLL \| chr4:15487950-15489456 FORWARD \| Aliases: F10N7.170, F10N7_170 | 2.4 | 6.2 | -3.8 | -5.9 | 0.43% | 5.6 |
| 3211 | AT4G30960.1 \| Symbol: CIPK6 \| CBL-interacting protein kinase 6 (CIPK6), identical to CBL-interacting protein kinase 6 (Arabidopsis thaliana) gi:9280634:gb:AAF86505 \| chr4:15067059-15069016 FORWARD \| Aliases: CBL INTERACTING PROTEIN KINASE 6, F6I18.130, F6I18_130 | 7.2 | 9.5 | -2.3 | -5.9 | 0.43% | 5.7 |
| 3214 | AT4G21980.1 \| Symbol: APG8A \| autophagy 8a (APG8a), identical to autophagy 8a (Arabidopsis thaliana) GI:19912151; contains Pfam profile PF02991: Microtubule associated protein 1A/1B, light chain 3 \| chr4:11655742-11656980 FORWARD \| Aliases: ATG8A, F1N20.80, F1N20_80 | 7.1 | 8.9 | -1.8 | -5.9 | 0.43% | 5.7 |
| 3218 | AT1G01240.3 \| expressed protein \| chr1:99894-101834 FORWARD \| Aliases: F633.5, F633_5, F6F3.27 | 6.7 | 8.1 | -1.4 | -5.9 | 0.43% | 5.6 |
| 3223 | AT3G12920.1 \| expressed protein \| chr3:4121890-4123503 REVERSE \| Aliases: MGH6.3 | 9.8 | 10.5 | -0.8 | -5.9 | 0.43% | 5.6 |
| 3226 | AT5G19410.1 \| ABC transporter family protein, white membrane transporter, Bactrocera tryoni, EMBL:U97104 \| chr5:6545239-6547113 REVERSE \| Aliases: F7K24.160, F7K24_160 | 2.7 | 4.7 | -2.0 | -5.9 | 0.44% | 5.5 |

| Rank | Description | Sync | Root | M | t | adj.q | B |
|---|---|---|---|---|---|---|---|
| 3231 | AT2G42520.1 \| DEAD box RNA helicase, putative, similar to SP:O00571 DEAD-box protein 3 (Helicase-like protein 2) {Homo sapiens}, DEAD box RNA helicase DDX3 (Homo sapiens) GI:3523150; contains Pfam profiles PF00270: DEAD/DEAH box helicase, PF00271: Helicase conserved C-terminal domain \| chr2:17711913-17716025 FORWARD \| Aliases: F14N22.21, F14N22_21 | 5.4 | 6.2 | -0.9 | -5.9 | 0.44% | 5.5 |
| 3237 | AT1G23480.3 \| Symbol: ATCSLA03 \| similar to glycosyl transferase family 2 protein [Arabidopsis thaliana] (TAIR:At5g03760.1); similar to glycosyl transferase family 2 protein [Arabidopsis thaliana] (TAIR:At1g24070.1); similar to glycosyl transferase family 2 protein [Arabidopsis thaliana] (TAIR:At3g56000.1); similar to glycosyl transferase family 2 protein [Arabidopsis thaliana] (TAIR:At4g13410.1); similar to glycosyl transferase family 2 protein [Arabidopsis thaliana] (TAIR:At5g16190.1); similar to glycosyltransferase 10 [Ipomoea trifida] (GB:AAQ62572.1); similar to glycosyltransferase 5 [Ipomoea trifida] (GB:AAQ62571.1); similar to glycosyltransferase 1 [Ipomoea trifida] (GB:AAQ62570.1); similar to TPA: cellulose synthase-like A1 [Oryza sativa (japonica cultivar-group)] (GB:DAA01743.1); contains InterPro domain Glycosyl transferase, family 2 (InterPro:IPR001173) \| chr1:8333685-8336374 FORWARD \| Aliases: ATCSLA3, CSLA03, F28C11.11 | 3.3 | 4.9 | -1.6 | -5.9 | 0.44% | 5.6 |
| 3240 | AT5G12120.1 \| ubiquitin-associated (UBA)/TS-N domain-containing protein, contains Pfam profile PF00627: UBA/TS-N domain \| chr5:3916049-3918660 REVERSE \| Aliases: MXC9.8, MXC9_8 | 5.8 | 7.0 | -1.2 | -5.9 | 0.44% | 5.7 |
| 3242 | AT3G09020.1 \| alpha 1,4-glycosyltransferase family protein / glycosyltransferase sugar-binding DXD motif-containing protein, low similarity to alpha-1,4-N-acetylglucosaminyltransferase, Homo sapiens (gi:5726306), Gb3 synthase, Rattus norvegicus (gi:9082162) ; contains Pfam profiles PF04572: Alpha 1,4-glycosyltransferase conserved region, PF04488: Glycosyltransferase sugar-binding region containing DXD motif \| chr3:2753312-2754626 FORWARD \| Aliases: T16O11.2 | 2.3 | 5.1 | -2.8 | -5.9 | 0.44% | 5.4 |
| 3246 | AT4G11850.1 \| Symbol: PLDGAMMA1 \| phospholipase D gamma 1 / PLD gamma 1 (PLDGAMMA1), identical to phospholipase D gamma 1 SP:Q9T053 from (Arabidopsis thaliana) \| chr4:7129117-7133194 REVERSE \| Aliases: T26M18.60, T26M18_60 | 4.5 | 5.2 | -0.7 | -5.9 | 0.44% | 5.5 |
| 3250 | AT3G52430.1 \| Symbol: PAD4 \| phytoalexin-deficient 4 protein (PAD4), identical to phytoalexin-deficient 4 protein (Arabidopsis thaliana) GI:6457331; contains Pfam profile PF01764: Lipase \| chr3:19442349-19445381 FORWARD \| Aliases: F22O6.190, PAD4, PHYTOALEXIN DEFICIENT 4, PHYTOALEXIN DEFICIENT 4 | 4.7 | 6.5 | -1.8 | -5.9 | 0.45% | 5.6 |
| 3252 | AT2G41560.1 \| Symbol: ACA4 \| calcium-transporting ATPase 4, plasma membrane-type / Ca2+-ATPase, isoform 4 (ACA4), identical to SP:O22218 Calcium-transporting ATPase 4, plasma membrane-type (EC 3.6.3.8) (Ca(2+)-ATPase isoform 4) {Arabidopsis thaliana} Belongs to plant 2B ATPase??s with an N-terminal autoinhibitor. \| chr2:17339162-17344276 REVERSE \| Aliases: AUTO INHIBITED CA(2+) ATPASE, ISOFORM 4, T32G6.8, T32G6_8 | 5.2 | 6.5 | -1.3 | -5.9 | 0.45% | 5.6 |
| 3254 | AT5G46250.2 \| RNA recognition motif (RRM)-containing protein, contains similarity to RNA-binding protein; contains InterPro entry IPR000504: RNA-binding region RNP-1 (RNA recognition motif) (RRM) \| chr5:18772535-18775505 FORWARD \| Aliases: MPL12.3, MPL12_3 | 10.2 | 11.1 | -0.9 | -5.9 | 0.45% | 5.5 |
| 3255 | AT4G13390.1 \| proline-rich extensin-like family protein, contains proline-rich extensin domains, INTERPRO:IPR002965 \| chr4:7783814-7785322 FORWARD \| Aliases: T9E8.130, T9E8_130 | 2.9 | 6.0 | -3.1 | -5.9 | 0.45% | 4.6 |
| 3256 | AT2G21520.1 \| similar to SEC14 cytosolic factor, putative / phosphoglyceride transfer protein, putative [Arabidopsis thaliana] (TAIR:At4g39170.1); similar to putative hosphatidylinositol/phophatidylcholine transfer protein [Oryza sativa (japonica cultivar-group)] (GB:XP_464026.1); contains InterPro domain Cellular retinaldehyde-binding)/triple function, C-terminal (InterPro:IPR001251); contains InterPro domain Cellular retinaldehyde binding/alpha-tocopherol transport (InterPro:IPR001071); contains InterPro domain Cellular retinaldehyde-binding/triple function, N-terminal (InterPro:IPR008273) | 7.2 | 9.3 | -2.2 | -5.9 | 0.45% | 5.6 |
| 3257 | AT4G16640.1 \| matrix metalloproteinase, putative, metalloproteinase (Arabidopsis thaliana) GI:3128477; contains InterPro accession IPR001818: Matrixin | 2.8 | 3.9 | -1.0 | -5.9 | 0.45% | 5.6 |
| 3258 | AT1G33050.2 \| expressed protein \| chr1:11968897-11971001 REVERSE \| Aliases: T9L6.12, T9L6_12 | 5.5 | 7.3 | -1.8 | -5.9 | 0.45% | 5.6 |
| 3262 | AT3G13050.1 \| transporter-related, low similarity to apical organic cation transporter (Sus scrofa) GI:2062135, SP:Q02563 Synaptic vesicle protein 2 (SV2) {Rattus norvegicus}; contains Pfam profile PF00083: major facilitator superfamily protein \| chr3:4176518-4179073 FORWARD \| Aliases: MGH6.18 | 4.7 | 6.0 | -1.3 | -5.8 | 0.45% | 5.6 |
| 3271 | AT5G57150.3 \| basic helix-loop-helix (bHLH) family protein, contains Pfam profile: PF00010 helix-loop-helix DNA-binding domain | 5.1 | 7.3 | -2.2 | -5.8 | 0.46% | 5.6 |
| 3272 | AT3G20650.2 \| mRNA capping enzyme family protein, contains Pfam PF03291: mRNA capping enzyme, large subunit \| chr3:7220887-7224034 REVERSE \| Aliases: F3H11.3 | 3.7 | 4.8 | -1.1 | -5.8 | 0.46% | 5.6 |
| 3273 | AT1G09430.1 \| Symbol: ACLA 3 \| | 8.7 | 10.3 | -1.6 | -5.8 | 0.46% | 5.5 |
| 3278 | AT4G23010.1 \| UDP-galactose transporter-related, contains weak similarity to UDP-galactose transporter related isozyme 1 (GI:1669562) (Mus musculus) \| chr4:12060179-12062827 REVERSE \| Aliases: F7H19.200, F7H19_200 | 6.2 | 8.4 | -2.2 | -5.8 | 0.46% | 5.6 |
| 3281 | AT1G63310.1 \| expressed protein, similar to unknown protein GB:AAC69930 from (Arabidopsis thaliana) \| chr1:23489982-23490750 REVERSE \| Aliases: F9N12.7, F9N12_7 | 2.5 | 4.9 | -2.4 | -5.8 | 0.46% | 5.0 |

| Rank | Description | Sync | Root | M | t | adj.q | B |
|---|---|---|---|---|---|---|---|
| 3282 | AT1G73270.1 \| Symbol: SCPL6 \| serine carboxypeptidase S10 family protein, similar to serine carboxypeptidase I precursor (SP:P07519) (Hordeum vulgare), glucose acyltransferase GB:AAD01263 (Solanum berthaultii); contains Pfam profile: PF00450 Serine carboxypeptidase; \| chr1:27553067-27556178 REVERSE \| Aliases: SCPL6, T18K17.6, T18K17_6 | 2.8 | 3.8 | -0.9 | -5.8 | 0.46% | 5.6 |
| 3284 | AT1G22540.1 \| proton-dependent oligopeptide transport (POT) family protein, contains Pfam profile: PF00854 POT family \| chr1:7964031-7966425 FORWARD \| Aliases: F12K8.12, F12K8_12 | 5.7 | 6.9 | -1.2 | -5.8 | 0.46% | 5.6 |
| 3286 | AT1G14080.1 \| Symbol: FUT6 \| xyloglucan fucosyltransferase, putative (FUT6), nearly identical to SP:Q9XI80 Probable fucosyltransferase 6 (EC 2.4.1.-) (AtFUT6) {Arabidopsis thaliana}; similar to xyloglucan fucosyltransferase GI:5231145 from (Arabidopsis thaliana) \| chr1:4822525-4824411 FORWARD \| Aliases: ATFUT6, F7A19.16, F7A19_16 | 2.6 | 4.1 | -1.5 | -5.8 | 0.46% | 5.5 |
| 3287 | AT2G33700.1 \| protein phosphatase 2C, putative / PP2C, putative, contains PF00481: Protein phosphatase 2C domain; similar to protein phosphatase-2C (PP2C) (GI:3643085) (Mesembryanthemum crystallinum) \| chr2:14260500-14263069 FORWARD \| Aliases: T1B8.2, T1B8_2 | 5.6 | 6.7 | -1.2 | -5.8 | 0.46% | 5.6 |
| 3289 | AT5G67090.1 \| subtilase family protein, contains similarity to subtilisin-like protease ag12 GI:757522 from (Alnus glutinosa) \| chr5:26791337-26793547 REVERSE \| Aliases: K21H1.5, K21H1_5 | 3.0 | 4.1 | -1.1 | -5.8 | 0.46% | 5.6 |
| 3290 | AT1G15100.1 \| Symbol: RHA2A \| zinc finger (C3HC4-type RING finger) family protein, contains Pfam profile: PF00097 zinc finger, C3HC4 type (RING finger) \| chr1:5193421-5194198 REVERSE \| Aliases: RING H2 FINGER PROTEIN RHA2A | 6.3 | 8.3 | -2.0 | -5.8 | 0.46% | 5.6 |
| 3297 | AT3G22260.2 \| OTU-like cysteine protease family protein, contains Pfam profile PF02338: OTU-like cysteine protease \| chr3:7871015-7873572 FORWARD \| Aliases: MMP21.4 | 4.9 | 6.3 | -1.4 | -5.8 | 0.46% | 5.6 |
| 3299 | AT2G46620.1 \| AAA-type ATPase family protein, contains Pfam profile: ATPase family PF00004 \| chr2:19145871-19147817 REVERSE \| Aliases: F13A10.15 | 3.7 | 5.0 | -1.3 | -5.8 | 0.46% | 5.6 |
| 3302 | AT5G63790.1 \| Symbol: ANAC102 \| no apical meristem (NAM) family protein, contains Pfam PF02365: No apical meristem (NAM) domain; contains similarity to NAC-domain protein \| chr5:25543735-25545239 REVERSE \| Aliases: ANAC102, MBK5.27, MBK5_27 | 6.7 | 9.0 | -2.2 | -5.8 | 0.47% | 5.6 |
| 3304 | AT1G76920.1 \| F-box family protein (FBX3), contains similarity to stamina pistilloidia GI:4101570, the pea ortholog of Fim and UFO from (Pisum sativum) \| chr1:28896956-28898570 FORWARD \| Aliases: F22K20.2, F22K20_2 | 5.1 | 6.7 | -1.6 | -5.8 | 0.47% | 5.6 |
| 3311 | AT5G10740.1 \| protein phosphatase 2C-related / PP2C-related, protein phosphatase 2C, alfalfa, PIR:T09640 \| chr5:3393570-3396177 REVERSE \| Aliases: MAJ23.3 | 6.9 | 8.0 | -1.1 | -5.8 | 0.47% | 5.5 |
| 3312 | AT1G15400.2 \| expressed protein, ESTs gb:H37295 and gb:R64895 come from this gene \| chr1:5296220-5296897 REVERSE \| Aliases: F9L1.35, F9L1_35 | 8.4 | 9.7 | -1.4 | -5.8 | 0.47% | 5.6 |
| 3313 | AT1G80050.1 \| Symbol: APT2 \| adenine phosphoribosyltransferase 2 (APT2), identical to SP:Q42563 Adenine phosphoribosyltransferase 2 (EC 2.4.2.7) (APRT) {Arabidopsis thaliana} \| chr1:30116407-30118217 REVERSE \| Aliases: ADENINE PHOSPHORIBOSYL TRANSFERASE 2, ATAPT2, F18B13.14, F18B13_14 | 5.0 | 6.4 | -1.4 | -5.8 | 0.47% | 5.6 |
| 3316 | AT5G47635.1 \| expressed protein \| chr5:19324280-19325121 REVERSE \| Aliases: None | 3.0 | 4.6 | -1.6 | -5.8 | 0.47% | 5.5 |
| 3320 | AT1G74660.1 \| zinc finger homeobox family protein / ZF-HD homeobox family protein, contains Pfam PF04770: ZF-HD protein dimerisation region; contains TIGRFAM TIGR01566: ZF-HD homeobox protein Cys/His-rich dimerization domain; similar to ZF-HD homeobox protein (GI:13374061) (Flaveria bidentis) \| chr1:28051237-28051788 REVERSE \| Aliases: F1M20.34, F1M20_34 | 5.1 | 7.4 | -2.3 | -5.8 | 0.47% | 5.6 |
| 3321 | AT4G35030.1 \| protein kinase family protein, contains Pfam domain, PF00069: Protein kinase domain \| chr4:16676134-16677541 FORWARD \| Aliases: M4E13.90, M4E13_90 | 3.1 | 7.1 | -4.0 | -5.8 | 0.47% | 3.9 |
| 3324 | AT5G07390.1 \| Symbol: ATRBOHA \| respiratory burst oxidase protein A (RbohA) / NADPH oxidase, identical to respiratory burst oxidase protein A from Arabidopsis thaliana (gi:3242781) \| chr5:2335896-2339914 REVERSE \| Aliases: ATRBOHA, RESPIRATORY BURST OXIDASE HOMOLOG A, T2I1.100, T2I1_100 | 3.9 | 6.6 | -2.7 | -5.8 | 0.47% | 5.4 |
| 3325 | AT3G04350.1 \| expressed protein \| chr3:1153734-1156783 REVERSE \| Aliases: T6K12.3, T6K12_3 | 5.0 | 6.2 | -1.2 | -5.8 | 0.47% | 5.5 |
| 3327 | AT5G40090.1 \| disease resistance protein-related, contains Pfam domain, PF00931: NB-ARC domain, a novel signalling motif found in plant resistance gene products \| chr5:16059147-16060680 REVERSE \| Aliases: MUD12.70, MUD12_70 | 2.7 | 3.6 | -0.9 | -5.8 | 0.47% | 5.5 |
| 3329 | AT4G34390.1 \| extra-large guanine nucleotide binding protein, putative / G-protein, putative, similar to extra-large G-protein (XLG) (Arabidopsis thaliana) GI:3201680; contains Pfam profile PF00503: G-protein alpha subunit | 4.1 | 5.6 | -1.5 | -5.8 | 0.47% | 5.6 |

| Rank | Description | Sync | Root | M | t | adj.q | B |
|---|---|---|---|---|---|---|---|
| 3330 | AT1G64190.1 \| 6-phosphogluconate dehydrogenase family protein, contains Pfam profiles: PF00393 6-phosphogluconate dehydrogenase C-terminal domain, PF03446 NAD binding domain of 6-phosphogluconate \| chr1:23828991-23830799 REVERSE \| Aliases: F22C12.5, F22C12_5 | 11.3 | 12.2 | -0.9 | -5.8 | 0.47% | 5.3 |
| 3331 | AT5G52260.1 \| myb family transcription factor (MYB19), contains PFAM profile: Myb DNA binding domain PF00249 \| chr5:21237391-21238506 FORWARD \| Aliases: F17P19.16, F17P19_16 | 2.5 | 3.5 | -1.0 | -5.8 | 0.47% | 5.5 |
| 3332 | AT2G22330.1 \| Symbol: CYP79B3 \| cytochrome P450, putative, similar to cytochrome P450 79B2 (SP:O81346) (Arabidopsis thaliana) | 3.6 | 4.8 | -1.1 | -5.8 | 0.47% | 5.6 |
| 3333 | AT2G42320.2 \| expressed protein, similar to expressed protein [Arabidopsis thaliana] (TAIR:At3g57780.1); similar to unknown protein [Oryza sativa (japonica cultivar-group)] (GB:XP_483236.1) \| chr2:17635041-17637870 FORWARD \| Aliases: MHK10.4, MHK10_4 | 3.8 | 5.4 | -1.5 | -5.8 | 0.47% | 5.5 |
| 3337 | AT1G50410.1 \| SNF2 domain-containing protein / helicase domain-containing protein / RING finger domain-containing protein, similar to transcription factor RUSH-1alpha (Oryctolagus cuniculus) GI:1655930; contains Pfam profiles PF00271: Helicase conserved C-terminal domain, PF00176: SNF2 family N-terminal domain, PF00097: Zinc finger, C3HC4 type (RING finger) \| chr1:18675831-18681224 FORWARD \| Aliases: F11F12.23, F11F12_23 | 5.4 | 6.9 | -1.5 | -5.8 | 0.47% | 5.6 |
| 3338 | AT3G03040.1 \| F-box family protein, contains F-box domain Pfam:PF00646 | 4.2 | 5.4 | -1.2 | -5.8 | 0.48% | 5.5 |
| 3340 | AT4G29700.1 \| type I phosphodiesterase/nucleotide pyrophosphatase family protein, similar to SP:P22413 Ectonucleotide pyrophosphatase/phosphodiesterase 1 (Plasma-cell membrane glycoprotein PC-1) (Includes: Alkaline phosphodiesterase I (EC 3.1.4.1); Nucleotide pyrophosphatase (EC 3.6.1.9) {Homo sapiens}; contains Pfam profile PF01663: Type I phosphodiesterase / nucleotide pyrophosphatase \| chr4:14543463-14545144 REVERSE \| Aliases: T16L4.210, T16L4_210 | 2.8 | 4.6 | -1.8 | -5.8 | 0.48% | 5.6 |
| 3342 | AT3G53230.1 \| cell division cycle protein 48, putative / CDC48, putative, very strong similarity to SP:P54609 Cell division cycle protein 48 homolog {Arabidopsis thaliana}; contains Pfam profiles PF00004: ATPase AAA family, PF02359: Cell division protein 48 (CDC48) N-terminal domain \| chr3:19734353-19737650 FORWARD \| Aliases: T4D2.160 | 6.6 | 8.3 | -1.7 | -5.8 | 0.48% | 5.5 |
| 3348 | AT3G15050.1 \| calmodulin-binding family protein, similar to SF16 protein (Helianthus annuus) GI:560150; contains Pfam profile PF00612: IQ calmodulin-binding motif \| chr3:5066886-5067967 REVERSE \| Aliases: K15M2.19 | 2.9 | 3.8 | -0.9 | -5.8 | 0.48% | 5.5 |
| 3349 | AT5G61650.1 \| Symbol: CYCP4;2 \| cyclin family protein, similar to cyclin 2 (Trypanosoma brucei) GI:7339572, cyclin 6 (Trypanosoma cruzi) GI:12005317; contains Pfam profile PF00134: Cyclin, N-terminal domain \| chr5:24795491-24796245 FORWARD \| Aliases: CYCP4, K11J9.17, K11J9_17 | 3.0 | 5.0 | -2.0 | -5.8 | 0.48% | 5.4 |
| 3351 | AT4G14270.2 \| similar to dehydration-induced protein (ERD15) [Arabidopsis thaliana] (TAIR:At2g41430.2); similar to dehydration-induced protein (ERD15) [Arabidopsis thaliana] (TAIR:At2g41430.3); similar to dehydration-induced protein (ERD15) [Arabidopsis thaliana] (TAIR:At2g41430.4); similar to dehydration-induced protein (ERD15) [Arabidopsis thaliana] (TAIR:At2g41430.1); similar to early response to dehydration 15-like protein [Pseudotsuga menziesii var. menziesii] (GB:AAV92278.1); similar to early response to dehydration 15-like protein [Pseudotsuga menziesii var. menziesii] (GB:AAV92296.1) | 7.8 | 9.3 | -1.5 | -5.8 | 0.48% | 5.5 |
| 3353 | AT5G35180.1 \| expressed protein \| chr5:13441648-13450248 FORWARD \| Aliases: T25C13.60, T25C13_60 | 6.7 | 9.0 | -2.3 | -5.8 | 0.49% | 5.5 |
| 3355 | AT2G41660.1 \| expressed protein, contains Pfam profile PF04759: Protein of unknown function, DUF617 \| chr2:17374900-17376267 FORWARD \| Aliases: T32G6.18, T32G6_18 | 3.6 | 7.1 | -3.6 | -5.8 | 0.49% | 5.3 |
| 3356 | AT5G05600.1 \| oxidoreductase, 2OG-Fe(II) oxygenase family protein, similar to flavonol synthase (Citrus unshiu)(gi:4126403), leucoanthocyanidin dioxygenase (Daucus carota)(gi:5924383); contains PF03171 2OG-Fe(II) oxygenase superfamily domain \| chr5:1672121-1674740 FORWARD \| Aliases: MOP10.14, MOP10_14 | 4.4 | 6.8 | -2.4 | -5.8 | 0.49% | 5.5 |
| 3359 | AT2G17590.1 \| DC1 domain-containing protein, contains Pfam profile PF03107: DC1 domain | 2.3 | 3.2 | -0.8 | -5.8 | 0.49% | 5.4 |
| 3360 | AT2G39010.1 \| Symbol: PIP2;6/PIP2E \| aquaporin, putative, similar to plasma membrane aquaporin 2b GI:7209560 from (Raphanus sativus) \| chr2:16298555-16301112 FORWARD \| Aliases: PIP2;6, PIP2E, T7F6.18, T7F6_18 | 2.6 | 4.5 | -1.9 | -5.8 | 0.49% | 5.3 |
| 3364 | AT4G31450.1 \| zinc finger (C3HC4-type RING finger) family protein, similar to Pfam domain, PF00097: Zinc finger, C3HC4 type (RING finger) \| chr4:15255634-15258818 REVERSE \| Aliases: F3L17.20, F3L17_20 | 4.8 | 6.3 | -1.4 | -5.7 | 0.49% | 5.5 |
| 3367 | AT5G24670.1 \| similar to novel protein [Danio rerio] (GB:CAD43470.1); contains InterPro domain Cytidine/deoxycytidylate deaminase, zinc-binding region (InterPro:IPR002125) \| chr5:8448100-8451221 FORWARD \| Aliases: MXC17.5, MXC17_5 | 6.0 | 8.1 | -2.1 | -5.7 | 0.49% | 5.5 |
| 3371 | AT1G50560.1 \| Symbol: CYP705A25 \| cytochrome P450, putative, similar to CYTOCHROME P450 93A3 (P450 CP5) (SP:O81973) (Glycine max) \| chr1:18727875-18731215 FORWARD \| Aliases: F11F12.12, F11F12_12 | 2.5 | 5.6 | -3.1 | -5.7 | 0.49% | 5.2 |

| Rank | Description | Sync | Root | M | t | adj.q | B |
|---|---|---|---|---|---|---|---|
| 3375 | AT1G73330.1 \| Symbol: ATDR4 \| protease inhibitor, putative (DR4), identical to Dr4 GI:469114 from (Arabidopsis thaliana); contains Pfam profile PF00197: Trypsin and protease inhibitor \| chr1:27572379-27573378 REVERSE \| Aliases: T9L24.45, T9L24_45 | 4.9 | 6.8 | -2.0 | -5.7 | 0.50% | 5.5 |
| 3377 | AT3G07600.1 \| heavy-metal-associated domain-containing protein, identical to residues 23 to 179 of farnesylated protein ATFP4 (putative metal-binding protein) GB:AAD09508 (Arabidopsis thaliana); contains Pfam profile PF00403: Heavy-metal-associated domain \| chr3:2424123-2425028 REVERSE \| Aliases: MLP3.5 | 3.2 | 5.6 | -2.4 | -5.7 | 0.50% | 5.5 |
| 3378 | AT1G13590.1 \| Symbol: ATPSK1 \| | 4.9 | 7.9 | -3.0 | -5.7 | 0.50% | 5.2 |
| 3381 | AT2G01180.2 \| Symbol: ATPAP1 \| | 4.8 | 6.2 | -1.4 | -5.7 | 0.50% | 5.5 |
| 3382 | AT2G22080.1 \| expressed protein \| chr2:9393621-9395263 FORWARD \| Aliases: T16B14.7, T16B14_7 | 8.0 | 10.3 | -2.3 | -5.7 | 0.50% | 5.5 |
| 3385 | AT5G52510.1 \| scarecrow-like transcription factor 8 (SCL8) \| chr5:21324275-21326528 FORWARD \| Aliases: T4M5.2, T4M5_2 | 5.4 | 7.2 | -1.8 | -5.7 | 0.50% | 5.5 |
| 3387 | AT4G36400.2 \| FAD linked oxidase family protein, low similarity to SP:Q12627 from Kluyveromyces lactis and SP:P32891 from Saccharomyces cerevisiae; contains Pfam FAD linked oxidases, C-terminal domain PF02913, Pfam FAD binding domain PF01565 \| chr4:17197252-17200924 FORWARD \| Aliases: AP22.14, AP22_14 | 3.9 | 5.1 | -1.2 | -5.7 | 0.50% | 5.5 |
| 3388 | AT5G51160.1 \| ankyrin repeat family protein, contains ankyrin repeats, Pfam:PF00023 \| chr5:20809506-20811000 FORWARD \| Aliases: MWD22.10, MWD22_10 | 3.7 | 6.7 | -3.0 | -5.7 | 0.50% | 5.5 |
| 3389 | AT5G46340.1 \| O-acetyltransferase-related, similar to O-acetyltransferase (Homo sapiens) GI:17016934 \| chr5:18813153-18817321 REVERSE \| Aliases: MPL12.14, MPL12_14 | 4.3 | 5.9 | -1.5 | -5.7 | 0.50% | 5.5 |
| 3390 | AT5G44070.1 \| Symbol: CAD1 \| phytochelatin synthase 1 (PCS1), identical to phytochelatin synthase (Arabidopsis thaliana) gi:18254401:gb:AAL66747; identical to cDNA phytochelatin synthase, GI:18254400 \| chr5:17751985-17755066 FORWARD \| Aliases: ARA8, ATPCS1, CADMIUM SENSITIVE 1, MRH10.18, MRH10_18, PCS1, PHYTOCHELATIN SYNTHASE | 7.9 | 9.1 | -1.2 | -5.7 | 0.50% | 5.4 |
| 3391 | AT3G58640.2 \| protein kinase family protein, contains eukaryotic protein kinase domain, INTERPRO:IPR000719 \| chr3:21697718-21704818 REVERSE \| Aliases: F14P22.230 | 6.1 | 7.0 | -0.8 | -5.7 | 0.50% | 5.4 |
| 3393 | AT4G27710.1 \| Symbol: CYP709B3 \| cytochrome P450 family protein, contains Pfam profile: PF00067 cytochrome P450 \| chr4:13828468-13830602 FORWARD \| Aliases: T29A15.200, T29A15_200 | 2.9 | 4.7 | -1.8 | -5.7 | 0.50% | 5.5 |
| 3394 | AT1G45545.1 \| expressed protein, contains Pfam profile PF05701: Plant protein of unknown function (DUF827) \| chr1:17183055-17185397 REVERSE \| Aliases: F2G19.5, F2G19_5 | 3.1 | 5.7 | -2.7 | -5.7 | 0.50% | 5.1 |
| 3395 | AT1G20980.1 \| Symbol: SPL14 \| SPL1-Related2 protein (SPL1R2), strong similarity to SPL1-Related2 protein (Arabidopsis thaliana) GI:6006427; contains Pfam profile PF03110: SBP domain | 6.3 | 7.7 | -1.5 | -5.7 | 0.50% | 5.5 |
| 3396 | AT2G20790.3 \| expressed protein, weak similarity to clathrin-adaptor medium chain apm 3 (GI:12000359) (Dictyostelium discoideum); weak similarity to Adapter-related protein complex 3 mu 2 subunit (Clathrin coat assembly protein AP47 homolog 2) (Golgi adaptor AP-1 47 kDa protein homolog 2) (HA1 47 kDa subunit homolog 2) (P47B) (Swiss-Prot:P53678) (Rattus norvegicus) \| chr2:8957607-8959772 REVERSE \| Aliases: F5H14.24, F5H14_24 | 5.7 | 6.6 | -0.9 | -5.7 | 0.50% | 5.4 |
| 3397 | AT1G76590.1 \| zinc-binding family protein, similar to zinc-binding protein (Pisum sativum) GI:16117799; contains Pfam profile PF04640 : Protein of unknown function, DUF597 \| chr1:28745585-28747101 FORWARD \| Aliases: F14G6.19, F14G6_19 | 3.7 | 6.4 | -2.7 | -5.7 | 0.50% | 5.3 |
| 3398 | AT4G09420.1 \| disease resistance protein (TIR-NBS class), putative, domain signature TIR-NBS exists, suggestive of a disease resistance protein. \| chr4:5962208-5963788 REVERSE \| Aliases: T15G18.160, T15G18_160 | 3.7 | 5.4 | -1.7 | -5.7 | 0.50% | 5.5 |
| 3400 | AT4G13510.1 \| Symbol: AMT1 \| ammonium transporter 1, member 1 (AMT1.1), identical to SP:P54144 High affinity ammonium transporter (AtAMT1;1) {Arabidopsis thaliana} \| chr4:7858179-7859914 FORWARD \| Aliases: AMMONIUM TRANSPORT 1, ATAMT1, T6G15.60, T6G15_60 | 5.4 | 8.5 | -3.1 | -5.7 | 0.50% | 5.5 |
| 3401 | AT3G47040.1 \| glycosyl hydrolase family 3 protein, beta-D-glucan exohydrolase, Nicotiana tabacum, TREMBL:AB017502_1 \| chr3:17335216-17338090 REVERSE \| Aliases: F13I12.90 | 4.7 | 8.0 | -3.3 | -5.7 | 0.50% | 5.4 |
| 3403 | AT1G47670.1 \| amino acid transporter family protein, similar to lysine and histidine specific transporter GI:2576361 from (Arabidopsis thaliana); contains Pfam profile PF01490: Transmembrane amino acid transporter protein \| chr1:17539107-17541973 REVERSE \| Aliases: F16N3.4, F16N3_4 | 5.0 | 5.9 | -1.0 | -5.7 | 0.50% | 5.4 |
| 3409 | AT5G59590.1 \| UDP-glucoronosyl/UDP-glucosyl transferase family protein, contains Pfam profile: PF00201 UDP-glucoronosyl and UDP-glucosyl transferase \| chr5:24026209-24027875 REVERSE \| Aliases: F2O15.19, F2O15_19 | 3.5 | 6.1 | -2.5 | -5.7 | 0.50% | 5.3 |

| Rank | Description | Sync | Root | M | t | adj.q | B |
|---|---|---|---|---|---|---|---|
| 3411 | AT1G62570.1 \| flavin-containing monooxygenase family protein / FMO family protein, low similarity to flavin-containing monooxygenase FMO3 (Rattus norvegicus) GI:12006730; contains Pfam profile PF00743: Flavin-binding monooxygenase-like \| chr1:23172801-23175770 FORWARD \| Aliases: T3P18.13, T3P18_13 | 4.4 | 8.1 | -3.7 | -5.7 | 0.50% | 5.5 |
| 3412 | AT2G26570.1 \| expressed protein, contains Pfam profile PF05701: Plant protein of unknown function (DUF827); weak similarity to merozoite surface protein 3 alpha (GI:27596802) (Plasmodium vivax) \| chr2:11306222-11309269 FORWARD \| Aliases: T9J22.24, T9J22_24 | 6.6 | 8.6 | -2.0 | -5.7 | 0.51% | 5.5 |
| 3413 | AT3G12540.1 \| similar to expressed protein [Arabidopsis thaliana] (TAIR:At2g39690.1); similar to putative ternary complex factor [Oryza sativa (japonica cultivar-group)] (GB:XP_550141.1); contains InterPro domain Protein of unknown function DUF547 (InterPro:IPR006869) \| chr3:3975253-3977319 FORWARD \| Aliases: T2E22.15 | 2.8 | 4.1 | -1.2 | -5.7 | 0.51% | 5.5 |
| 3419 | AT4G17110.1 \| expressed protein, ; expression supported by MPSS \| chr4:9613629-9615510 REVERSE \| Aliases: DL4590C, FCAALL.359 | 5.5 | 7.7 | -2.2 | -5.7 | 0.51% | 5.4 |
| 3420 | AT2G23320.2 \| Symbol: WRKY15 \| | 5.5 | 6.9 | -1.5 | -5.7 | 0.51% | 5.5 |
| 3421 | AT5G36260.1 \| aspartyl protease family protein, contains Pfam profile: PF00026 eukaryotic aspartyl protease \| chr5:14302185-14305563 REVERSE \| Aliases: T30G6.12, T30G6_12 | 2.8 | 4.0 | -1.2 | -5.7 | 0.51% | 5.5 |
| 3422 | AT1G70500.1 \| polygalacturonase, putative / pectinase, putative, similar to polygalacturonase (Cucumis sativus) GI:6624205; contains Pfam profile PF00295: Glycosyl hydrolases family 28 (polygalacturonases) | 3.0 | 4.2 | -1.2 | -5.7 | 0.51% | 5.4 |
| 3423 | AT2G27300.1 \| Symbol: ANAC040 \| no apical meristem (NAM) family protein, contains Pfam PF02365: No apical meristem (NAM) domain; \| chr2:11687495-11689033 REVERSE \| Aliases: ANAC040, F12K2.12, F12K2_12 | 2.4 | 3.0 | -0.6 | -5.7 | 0.51% | 5.2 |
| 3426 | AT5G16800.2 \| GCN5-related N-acetyltransferase (GNAT) family protein, very low similarity to SP:P39909 Spermine/spermidine acetyltransferase (EC 2.3.1.57) {Bacillus subtilis}; contains Pfam profile PF00583: acetyltransferase, GNAT family \| chr5:5524317-5526566 REVERSE \| Aliases: F5E19.140, F5E19_140 | 6.5 | 8.2 | -1.7 | -5.7 | 0.51% | 5.5 |
| 3427 | AT4G17230.1 \| scarecrow-like transcription factor 13 (SCL13) \| chr4:9660996-9663782 REVERSE \| Aliases: DL4650C, FCAALL.225 | 4.1 | 6.0 | -1.8 | -5.7 | 0.51% | 5.4 |
| 3434 | AT3G19340.1 \| expressed protein \| chr3:6701279-6704232 REVERSE \| Aliases: MLD14.7 | 7.2 | 8.7 | -1.4 | -5.7 | 0.51% | 5.5 |
| 3439 | AT1G09740.1 \| ethylene-responsive protein, putative, similar to ER6 protein (Lycopersicon esculentum) GI:5669654; contains Pfam profile PF00582: universal stress protein family \| chr1:3154534-3156252 FORWARD \| Aliases: F21M12.12, F21M12_12 | 6.2 | 9.4 | -3.2 | -5.7 | 0.51% | 5.4 |
| 3442 | AT1G27300.1 \| expressed protein \| chr1:9483250-9484576 FORWARD \| Aliases: F17L21.9, F17L21_9 | 6.9 | 8.2 | -1.3 | -5.7 | 0.51% | 5.5 |
| 3446 | AT3G03180.1 \| Got1-like family protein, contains Pfam profile: PF04178 Got1-like family \| chr3:733394-734434 FORWARD \| Aliases: T17B22.13, T17B22_13 | 5.8 | 6.6 | -0.8 | -5.7 | 0.51% | 5.4 |
| 3447 | AT1G51890.1 \| leucine-rich repeat protein kinase, putative, similar to light repressible receptor protein kinase (Arabidopsis thaliana) gi:1321686:emb:CAA66376; contains leucine rich repeat (LRR) domains, Pfam:PF00560; contains protein kinase domain, Pfam:PF00069 \| chr1:19278471-19282197 REVERSE \| Aliases: T14L22.10, T14L22_10 | 2.7 | 4.8 | -2.1 | -5.7 | 0.51% | 5.4 |
| 3449 | AT5G66010.1 \| heterogeneous nuclear ribonucleoprotein, putative / hnRNP, putative, similar to Heterogeneous nuclear ribonucleoprotein SP:P55795, SP:P31943, SP:P52597 {Homo sapiens}; contains InterPro entry IPR000504: RNA-binding region RNP-1 (RNA recognition motif) (RRM) domain | 5.4 | 6.6 | -1.2 | -5.7 | 0.51% | 5.4 |
| 3450 | AT2G32280.1 \| expressed protein \| chr2:13720137-13721086 FORWARD \| Aliases: T32F6.20, T32F6_20 | 3.5 | 4.7 | -1.2 | -5.7 | 0.51% | 5.4 |
| 3451 | AT2G39940.1 \| Symbol: COI1 \| coronatine-insensitive 1 / COI1 (FBL2), E3 ubiquitin ligase SCF complex F-box subunit; identical to LRR-containing F-box protein GI:3158394 from (Arabidopsis thaliana) \| chr2:16679040-16682826 REVERSE \| Aliases: CORONATINE INSENSITIVE 1, T28M21.10, T28M21_10 | 5.2 | 6.1 | -0.9 | -5.7 | 0.51% | 5.2 |
| 3452 | AT2G47850.1 \| zinc finger (CCCH-type) family protein, contains Pfam domain, PF00642: Zinc finger C-x8-C-x5-C-x3-H type (and similar) \| chr2:19602743-19605651 FORWARD \| Aliases: F17A22.24 | 5.9 | 7.0 | -1.1 | -5.7 | 0.51% | 5.4 |
| 3455 | AT1G02500.2 \| Symbol: SAM1 \| S-adenosylmethionine synthetase 1 (SAM1), identical to S-adenosylmethionine synthetase 1 (Methionine adenosyltransferase 1, AdoMet synthetase 1) (Arabidopsis thaliana) SWISS-PROT:P23686 \| chr1:518254-520437 FORWARD \| Aliases: SAM 1, T14P4.17, T14P4_17 | 7.5 | 10.6 | -3.1 | -5.7 | 0.52% | 5.3 |
| 3456 | AT4G10770.1 \| Symbol: ATOPT7 \| | 3.0 | 4.2 | -1.3 | -5.7 | 0.52% | 5.4 |
| 3457 | AT3G46340.1 \| leucine-rich repeat protein kinase, putative, similar to light repressible receptor protein kinase (Arabidopsis thaliana) gi:1321686:emb:CAA66376; contains leucine rich repeat (LRR) domains, Pfam:PF00560; contains protein kinase domain, Pfam:PF00069 \| chr3:17037643-17042827 FORWARD \| Aliases: F18L15.60 | 2.7 | 5.6 | -2.9 | -5.7 | 0.52% | 5.4 |

| Rank | Description | Sync | Root | M | t | adj.q | B |
|---|---|---|---|---|---|---|---|
| 3458 | AT4G02180.1 \| DC1 domain-containing protein, contains Pfam profile PF03107: DC1 domain | 3.4 | 5.5 | -2.1 | -5.7 | 0.52% | 5.4 |
| 3459 | AT3G61180.1 \| zinc finger (C3HC4-type RING finger) family protein, low similarity to RNF6 protein (Mus musculus) GI:20530241; contains Pfam profile PF00097: Zinc finger, C3HC4 type (RING finger) \| chr3:22656541-22658642 FORWARD \| Aliases: T20K12.80 | 5.4 | 7.4 | -2.0 | -5.7 | 0.52% | 5.4 |
| 3461 | AT1G07990.1 \| SIT4 phosphatase-associated family protein, contains Pfam profile: PF04499 SIT4 phosphatase-associated protein \| chr1:2477155-2483135 FORWARD \| Aliases: T6D22.8, T6D22_8 | 3.8 | 5.3 | -1.6 | -5.7 | 0.52% | 5.4 |
| 3466 | AT4G14650.1 \| similar to expressed protein [Arabidopsis thaliana] (TAIR:At2g22795.1); similar to mature parasite-infected erythrocyte surface antigen [Plasmodium falciparum] (GB:AAC13303.1); similar to GH09355p [Drosophila melanogaster] (GB:AAL68190.1); contains domain GLU_RICH (PS50313) \| chr4:8400569-8402906 REVERSE \| Aliases: DL3365C, FCAALL.281 | 2.6 | 4.0 | -1.4 | -5.7 | 0.52% | 5.4 |
| 3469 | AT5G45840.1 \| leucine-rich repeat transmembrane protein kinase, putative, and genscan+ \| chr5:18611307-18614448 REVERSE \| Aliases: K15I22.4, K15I22_4 | 2.4 | 3.5 | -1.1 | -5.7 | 0.52% | 5.4 |
| 3471 | AT1G08050.1 \| zinc finger (C3HC4-type RING finger) family protein, low similarity to SP:P79263 Inter-alpha-trypsin inhibitor heavy chain H4 precursor {Sus scrofa}; contains Pfam profiles PF00097: Zinc finger, C3HC4 type (RING finger), PF00092: von Willebrand factor type A domain \| chr1:2498833-2501388 REVERSE \| Aliases: T6D22.13, T6D22_13 | 5.3 | 7.4 | -2.0 | -5.7 | 0.52% | 5.4 |
| 3473 | AT1G27210.1 \| expressed protein \| chr1:9455455-9458013 REVERSE \| Aliases: T7N9.27, T7N9_27 | 4.5 | 6.8 | -2.3 | -5.7 | 0.52% | 5.4 |
| 3474 | AT1G61860.1 \| protein kinase, putative, similar to protein kinase GI:9294282 from (Arabidopsis thaliana) \| chr1:22866524-22868284 REVERSE \| Aliases: F8K4.7, F8K4_7 | 2.6 | 3.7 | -1.1 | -5.7 | 0.52% | 5.4 |
| 3475 | AT4G36410.1 \| Symbol: UBC17 \| ubiquitin-conjugating enzyme 17 (UBC17), E2; identical to gi:2801446 \| chr4:17201930-17202988 FORWARD \| Aliases: AP22.89, AP22_89, UBIQUITIN CONJUGATING ENZYME 17 | 3.8 | 5.3 | -1.5 | -5.7 | 0.52% | 5.4 |
| 3477 | AT5G44210.1 \| Symbol: ATERF 9/ATERF9/ERF9 \| encodes a member of the ERF (ethylene response factor) subfamily B-1 of ERF/AP2 transcription factor family (ATERF-9). The protein contains one AP2 domain. There are 15 members in this subfamily including ATERF-3, ATERF-4, ATERF-7, and leafy petiole. \| chr5:17823699-17824760 FORWARD \| Aliases: ATERF 9, ATERF9, ERF9, MLN1.14, MLN1_14 | 3.4 | 5.5 | -2.1 | -5.7 | 0.52% | 5.4 |
| 3478 | AT1G13750.1 \| calcineurin-like phosphoesterase family protein, contains Pfam profile: PF00149 calcineurin-like phosphoesterase | 5.3 | 6.6 | -1.3 | -5.7 | 0.52% | 5.4 |
| 3482 | AT5G01820.1 \| Symbol: ATSR1 \| CBL-interacting protein kinase 14 (CIPK14), identical to CBL-interacting protein kinase 14 (Arabidopsis thaliana) gi:13249127:gb:AAK16689; contains Pfam profiles PF00069: Protein kinase domain and PF03822: NAF domain; identical to cDNA CBL-interacting protein kinase 14 (CIPK14) GI:13249126 \| chr5:313190-314997 REVERSE \| Aliases: CBL INTERACTING PROTEIN KINASE 14, CIPK14, SERINE/THREONINE PROTEIN KINASE 1, T20L15.90, T20L15_90 | 6.8 | 9.0 | -2.2 | -5.7 | 0.52% | 5.4 |
| 3486 | AT1G02360.1 \| chitinase, putative, similar to chitinase precursor GI:5880845 from (Petroselinum crispum) \| chr1:471990-473140 REVERSE \| Aliases: T6A9.5, T6A9_5 | 5.1 | 8.7 | -3.6 | -5.7 | 0.53% | 5.3 |
| 3487 | AT2G36290.1 \| hydrolase, alpha/beta fold family protein, low similarity to 2-hydroxy-6-oxo-6-phenylhexa-2,4-dienoate hydrolase (Rhodococcus sp. RHA1) GI:8978311; contains Pfam profile PF00561: hydrolase, alpha/beta fold family \| chr2:15215706-15217918 REVERSE \| Aliases: F2H17.10, F2H17_10 | 8.7 | 10.4 | -1.8 | -5.7 | 0.53% | 5.2 |
| 3488 | AT5G47730.1 \| SEC14 cytosolic factor, putative / polyphosphoinositide-binding protein, putative, similar to polyphosphoinositide binding protein SEC14 homolog Ssh1p (GI:2739044) {Glycine max} \| chr5:19351523-19354327 REVERSE \| Aliases: MCA23.5, MCA23_5 | 4.5 | 6.5 | -2.0 | -5.7 | 0.53% | 5.4 |
| 3489 | AT2G45420.1 \| LOB domain protein 18 / lateral organ boundaries domain protein 18 (LBD18), identical to LOB DOMAIN 18 (Arabidopsis thaliana) GI:17227164; supported by full-length cDNA gi:17227163 \| chr2:18725422-18727803 FORWARD \| Aliases: F4L23.7 | 2.1 | 2.6 | -0.5 | -5.7 | 0.53% | 5.0 |
| 3493 | AT2G28120.1 \| nodulin family protein, similar to nodulin-like protein (Arabidopsis thaliana) GI:3329368, nodule-specific protein Nlj70 (Lotus japonicus) GI:3329366 \| chr2:11992764-11994815 FORWARD \| Aliases: F24D13.9, F24D13_9 | 4.5 | 5.7 | -1.2 | -5.7 | 0.53% | 5.4 |
| 3494 | AT5G63190.2 \| MA3 domain-containing protein, low similarity to programmed cell death 4 protein (Gallus gallus) GI:12958564; contains Pfam profile PF02847: MA3 domain \| chr5:25362822-25366022 FORWARD \| Aliases: MDC12.16, MDC12_16 | 6.6 | 8.5 | -1.9 | -5.7 | 0.53% | 5.4 |
| 3497 | AT5G62165.3 \| similar to MADS-box protein (AGL20) [Arabidopsis thaliana] (TAIR:At2g45660.1); similar to transcription factor SaMADS A (GB:AAB41526.1); contains InterPro domain Transcription factor, MADS-box (InterPro:IPR002100); contains InterPro domain Transcription factor, K-box (InterPro:IPR002487) | 4.6 | 6.5 | -1.9 | -5.6 | 0.53% | 5.4 |

| Rank | Description | Sync | Root | M | t | adj.q | B |
|---|---|---|---|---|---|---|---|
| 3498 | AT3G61060.2 \| Symbol: ATPP2 A13 \| F-box family protein / lectin-related, low similarity to PP2 lectin polypeptide (Cucurbita maxima) GI:410437; contains Pfam profile PF00646: F-box domain \| chr3:22613885-22615553 FORWARD \| Aliases: ATPP2 A13, T27I15.150 | 4.8 | 7.2 | -2.4 | -5.6 | 0.53% | 5.3 |
| 3501 | AT1G15380.2 \| similar to lactoylglutathione lyase family protein / glyoxalase I family protein [Arabidopsis thaliana] (TAIR:At1g80160.1); similar to hypothetical protein [Oryza sativa (japonica cultivar-group)] (GB:NP_916470.1); contains InterPro domain Glyoxalase I (InterPro:IPR004361); contains InterPro domain Glyoxalase/Bleomycin resistance protein/dioxygenase domain (InterPro:IPR004360) \| chr1:5290742-5292529 FORWARD \| Aliases: F9L1.33, F9L1_33 | 5.3 | 7.4 | -2.1 | -5.6 | 0.53% | 5.1 |
| 3504 | AT5G05700.1 \| Symbol: ATE1 \| arginine-tRNA-protein transferase 1 / arginyltransferase 1 / arginyl-tRNA-protein transferase 1 (ATE1), identical to SP:Q9ZT48 Arginine-tRNA-protein transferase 1 (EC 2.3.2.8) (R-transferase 1) (Arginyltransferase 1) (Arginyl-tRNA--protein transferase 1) {Arabidopsis thaliana} \| chr5:1712671-1715700 REVERSE \| Aliases: ARGININE TRNA PROTEIN TRANSFERASE 1, ATATE1, DELAYED LEAF SENESCENCE 1, DLS1, MJJ3.1, MJJ3_1 | 5.8 | 7.4 | -1.6 | -5.6 | 0.53% | 5.4 |
| 3508 | AT4G18425.1 \| expressed protein, contains Pfam profile PF05078: Protein of unknown function (DUF679) \| chr4:10181345-10182151 REVERSE \| Aliases: None | 2.9 | 6.1 | -3.2 | -5.6 | 0.53% | 5.1 |
| 3513 | AT2G42150.1 \| DNA-binding bromodomain-containing protein, contains Pfam domains, PF00439: Bromodomain and PF00249: Myb-like DNA-binding domain | 4.0 | 5.8 | -1.8 | -5.6 | 0.54% | 5.4 |
| 3515 | AT2G02610.1 \| DC1 domain-containing protein, contains Pfam profile PF03107: DC1 domain \| chr2:710711-712818 FORWARD \| Aliases: T8K22.9, T8K22_9 | 2.8 | 4.2 | -1.4 | -5.6 | 0.54% | 5.3 |
| 3517 | AT5G40780.2 \| Symbol: LHT1 \| lysine and histidine specific transporter, putative, strong similarity to lysine and histidine specific transporter GI:2576361 from (Arabidopsis thaliana); contains Pfam profile PF01490: Transmembrane amino acid transporter protein \| chr5:16340910-16344502 FORWARD \| Aliases: K1B16.3, K1B16_3, LYSINE AND HISTIDINE SPECIFIC TRANSPORTER | 9.4 | 11.2 | -1.8 | -5.6 | 0.54% | 5.4 |
| 3520 | AT3G61710.3 \| expressed protein, similar to expressed protein [Oryza sativa (japonica cultivar-group)] (GB:AAU90282.1); contains InterPro domain Autophagy protein Apg6 (InterPro:IPR007243) \| chr3:22850290-22853407 REVERSE \| Aliases: F21F14.6 | 6.6 | 7.8 | -1.2 | -5.6 | 0.55% | 5.3 |
| 3521 | AT3G19580.1 \| Symbol: AZF2 \| zinc finger (C2H2 type) protein 2 (AZF2), identical to Cys2/His2-type zinc finger protein 2 (Arabidopsis thaliana) gi:6009885:dbj:BAA85107 \| chr3:6803172-6804239 REVERSE \| Aliases: ARABIDOPSIS ZINC FINGER PROTEIN 2, MMB12.27 | 5.0 | 6.4 | -1.5 | -5.6 | 0.55% | 5.3 |
| 3526 | AT3G10710.1 \| pectinesterase family protein, contains similarity to pectinesterase GB:AAB57671 (Citrus sinensis); contains Pfam profile: PF01095 pectinesterase \| chr3:3352294-3354242 FORWARD \| Aliases: T7M13.21 | 4.0 | 6.8 | -2.8 | -5.6 | 0.55% | 5.0 |
| 3528 | AT3G05990.1 \| leucine-rich repeat family protein, contains leucine rich-repeat (LRR) domains Pfam:PF00560, INTERPRO:IPR001611; contains similarity to leaf senescence-associated receptor-like protein kinase (Phaseolus vulgaris) gi:9837280:gb:AAG00510 \| chr3:1796871-1799876 REVERSE \| Aliases: F2O10.5, F2O10_5 | 7.0 | 8.4 | -1.4 | -5.6 | 0.55% | 5.3 |
| 3529 | AT1G26930.1 \| kelch repeat-containing F-box family protein, contains Pfam:PF01344 Kelch motif, Pfam:PF00646 F-box domain \| chr1:9336044-9337574 REVERSE \| Aliases: T2P11.12, T2P11_12 | 7.7 | 9.4 | -1.7 | -5.6 | 0.55% | 5.3 |
| 3530 | AT1G69526.2 \| similar to UbiE/COQ5 methyltransferase family protein [Arabidopsis thaliana] (TAIR:At1g69523.1); similar to COG0500: SAM-dependent methyltransferases [Nostoc punctiforme PCC 73102] (GB:ZP_00110216.1); contains InterPro domain Generic methyltransferase (InterPro:IPR001601); contains InterPro domain SAM (and some other nucleotide) binding motif (InterPro:IPR000051); contains InterPro domain UbiE/COQ5 methyltransferase (InterPro:IPR004033) | 2.9 | 4.8 | -1.9 | -5.6 | 0.55% | 5.3 |
| 3531 | AT1G31470.1 \| nodulin-related, weak similarity to nodule-specific protein Nlj70 (Lotus japonicus) GI:3329366 \| chr1:11262918-11264817 REVERSE \| Aliases: T8E3.15, T8E3_15 | 6.0 | 7.2 | -1.3 | -5.6 | 0.55% | 5.3 |
| 3532 | AT4G36500.1 \| expressed protein \| chr4:17225915-17226613 REVERSE \| Aliases: AP22.75, AP22_75 | 3.7 | 5.5 | -1.8 | -5.6 | 0.56% | 5.3 |
| 3533 | AT2G20010.1 \| expressed protein \| chr2:8644828-8648555 REVERSE \| Aliases: T2G17.19, T2G17_19 | 5.0 | 7.1 | -2.2 | -5.6 | 0.56% | 5.3 |
| 3535 | AT5G03545.1 \| expressed protein, No ATG start, annotated according to PMID:11123795 \| chr5:894705-895399 FORWARD \| Aliases: None | 8.2 | 11.8 | -3.6 | -5.6 | 0.56% | 4.2 |
| 3536 | AT5G64000.1 \| Symbol: SAL2 \| 3'(2'),5'-bisphosphate nucleotidase, putative / inositol polyphosphate 1-phosphatase, putative, similar to SP:Q42546 3'(2'),5'-bisphosphate nucleotidase (EC 3.1.3.7) {Arabidopsis thaliana}; contains Pfam profile PF00459: Inositol monophosphatase family \| chr5:25633864-25635850 FORWARD \| Aliases: ATSAL2, MBM17.10, MBM17_10 | 3.4 | 4.5 | -1.1 | -5.6 | 0.56% | 5.3 |
| 3540 | AT5G58300.1 \| leucine-rich repeat transmembrane protein kinase, putative \| chr5:23589105-23592587 FORWARD \| Aliases: MCK7.17, MCK7_17 | 4.4 | 6.3 | -1.9 | -5.6 | 0.56% | 5.3 |
| 3541 | AT5G51310.1 \| gibberellin 20-oxidase-related, low similarity to GI:9791186, GI:1109695 | 2.9 | 4.1 | -1.3 | -5.6 | 0.56% | 5.3 |

| Rank | Description | Sync | Root | M | t | adj.q | B |
|---|---|---|---|---|---|---|---|
| 3542 | AT3G27210.1 \| expressed protein \| chr3:10046423-10048580 REVERSE \| Aliases: K17E12.3 | 4.2 | 5.6 | -1.4 | -5.6 | 0.56% | 5.3 |
| 3544 | AT5G05320.1 \| monooxygenase, putative (MO3), similar to GI:3426064; identical to cDNA monooxygenase 3, partial GI:3426065 \| chr5:1575007-1576732 REVERSE \| Aliases: K18I23.12, K18I23_12 | 6.4 | 7.7 | -1.3 | -5.6 | 0.56% | 5.3 |
| 3549 | AT5G58200.1 \| expressed protein \| chr5:23566860-23569278 FORWARD \| Aliases: MCK7.7, MCK7_7 | 7.4 | 9.0 | -1.6 | -5.6 | 0.56% | 5.3 |
| 3551 | AT5G67450.1 \| Symbol: AZF1 \| zinc finger (C2H2 type) protein 1 (AZF1), identical to Cys2/His2-type zinc finger protein 1 (Arabidopsis thaliana) gi:6009887:dbj:BAA85108 \| chr5:26936019-26937175 REVERSE \| Aliases: ARABIDOPSIS ZINC FINGER PROTEIN 1, K8K14.19, K8K14_19 | 3.2 | 5.7 | -2.5 | -5.6 | 0.56% | 5.3 |
| 3552 | AT5G48310.1 \| expressed protein \| chr5:19592187-19597588 REVERSE \| Aliases: K23F3.3, K23F3_3 | 5.8 | 6.9 | -1.1 | -5.6 | 0.56% | 5.3 |
| 3554 | AT5G56180.2 \| Symbol: ATARP8 \| | 4.9 | 5.8 | -0.8 | -5.6 | 0.56% | 5.2 |
| 3555 | AT3G09920.2 \| similar to phosphatidylinositol-4-phosphate 5-kinase family protein [Arabidopsis thaliana] (TAIR:At1g60890.1); similar to phosphatidylinositol-4-phosphate 5-kinase family protein [Arabidopsis thaliana] (TAIR:At1g10900.1); similar to phosphatidylinositol-4-phosphate 5-kinase [Oryza sativa (japonica cultivar-group)] (GB:CAD27794.1); similar to Putative Phosphatidylinositol-4-phosphate 5-Kinase [Oryza sativa (japonica cultivar-group)] (GB:AAX95508.1); contains InterPro domain Phosphatidylinositol-4-phosphate 5-kinase (InterPro:IPR002498); contains InterPro domain MORN motif (InterPro:IPR003409) \| chr3:3040308-3044772 REVERSE \| Aliases: F8A24.2 | 5.1 | 6.8 | -1.7 | -5.6 | 0.56% | 5.3 |
| 3557 | AT1G49960.2 \| xanthine/uracil permease family protein, contains Pfam profile: PF00860 permease family \| chr1:18502308-18505554 FORWARD \| Aliases: F2J10.15, F2J10_15 | 3.4 | 5.2 | -1.8 | -5.6 | 0.56% | 5.3 |
| 3559 | AT2G15390.2 \| Symbol: FUT4 \| xyloglucan fucosyltransferase, putative (FUT4), identical to SP:Q9SJP2 Probable fucosyltransferase 4 (EC 2.4.1.-) (AtFUT4) {Arabidopsis thaliana}; similar to SP:Q9SWH5 Galactoside 2-alpha-L-fucosyltransferase (EC 2.4.1.69) (Xyloglucan alpha-(1,2)-fucosyltransferase) (AtFUT1) {Arabidopsis thaliana} \| chr2:6716343-6718117 REVERSE \| Aliases: ATFUT4, F26H6.9, F26H6_9 | 3.2 | 5.4 | -2.2 | -5.6 | 0.56% | 5.2 |
| 3561 | AT5G49270.1 \| phytochelatin synthetase-related, contains Pfam PF04833: Phytochelatin synthetase-like conserved region \| chr5:19989138-19991670 REVERSE \| Aliases: K21P3.15, K21P3_15 | 3.7 | 5.8 | -2.2 | -5.6 | 0.57% | 5.1 |
| 3562 | AT3G56240.1 \| Symbol: CCH \| copper homeostasis factor / copper chaperone (CCH) (ATX1), identical to gi:3168840 Pfam profile PF00403: Heavy-metal-associated domain \| chr3:20874236-20875470 REVERSE \| Aliases: ATX1, COPPER CHAPERONE, COPPER HOMEOSTASIS FACTOR, F18O21.200 | 9.1 | 10.7 | -1.6 | -5.6 | 0.57% | 5.3 |
| 3567 | AT3G51520.1 \| diacylglycerol acyltransferase family, contains Pfam PF03982: Diacylglycerol acyltransferase \| chr3:19121628-19123739 FORWARD \| Aliases: F26O13.160 | 6.2 | 8.7 | -2.5 | -5.6 | 0.57% | 5.3 |
| 3568 | AT5G25370.1 \| Symbol: PLDALPHA3 \| phospholipase D, putative (PLDZETA), identical to phospholipase D zeta SP:P58766 from (Arabidopsis thaliana); similar to phospholipase D (Lycopersicon esculentum) GI:12060550; contains Pfam profile PF00614: Phospholipase D. Active site motif \| chr5:8804243-8807550 REVERSE \| Aliases: F18G18.110, F18G18_110, PLD ZETA 1, PLDZETA | 3.1 | 4.3 | -1.2 | -5.6 | 0.57% | 5.3 |
| 3569 | AT2G16430.2 \| purple acid phosphatase (PAP10), identical to purple acid phosphatase (PAP10) GI:20257482 from (Arabidopsis thaliana) \| chr2:7127501-7129901 REVERSE \| Aliases: F16F14.7, F16F14_7 | 6.1 | 8.8 | -2.7 | -5.6 | 0.57% | 5.3 |
| 3571 | AT1G80870.1 \| protein kinase family protein, contains eukaryotic protein kinase domain, INTERPRO:IPR000719 \| chr1:30397025-30399275 FORWARD \| Aliases: F23A5.23, F23A5_23 | 5.5 | 7.3 | -1.8 | -5.6 | 0.57% | 5.3 |
| 3572 | AT3G12870.1 \| expressed protein \| chr3:4094424-4095044 REVERSE \| Aliases: MJM20.1 | 5.0 | 7.7 | -2.8 | -5.6 | 0.57% | 5.0 |
| 3573 | AT1G26360.1 \| hydrolase, alpha/beta fold family protein, similar to SP:Q40708 PIR7A protein {Oryza sativa}, ethylene-induced esterase (Citrus sinensis) GI:14279437, polyneuridine aldehyde esterase (Rauvolfia serpentina) GI:6651393; contains Pfam profile PF00561: alpha/beta hydrolase fold \| chr1:9118935-9121204 REVERSE \| Aliases: T1K7.26, T1K7_26 | 2.0 | 2.5 | -0.5 | -5.6 | 0.57% | 4.8 |
| 3575 | AT1G22750.3 \| expressed protein, similar to unknown protein [Oryza sativa (japonica cultivar-group)] (GB:BAD68607.1) \| chr1:8050851-8052893 FORWARD \| Aliases: T22J18.8, T22J18_8 | 5.3 | 7.1 | -1.8 | -5.6 | 0.57% | 5.3 |
| 3576 | AT1G78550.1 \| oxidoreductase, 2OG-Fe(II) oxygenase family protein, similar to naringenin,2-oxoglutarate 3-dioxygenase (flavonone-3-hydroxylase) (SP:Q06942)(Malus domestica); contains PF03171 2OG-Fe(II) oxygenase superfamily domain | 5.7 | 7.0 | -1.4 | -5.6 | 0.57% | 5.3 |

| Rank | Description | Sync | Root | M | t | adj.q | B |
|---|---|---|---|---|---|---|---|
| 3577 | AT2G38400.1 \| Symbol: AGT3 \| alanine--glyoxylate aminotransferase, putative / beta-alanine-pyruvate aminotransferase, putative / AGT, putative, similar to SP:Q64565 Alanine--glyoxylate aminotransferase 2, mitochondrial precursor (EC 2.6.1.44) (AGT 2) (Beta-alanine-pyruvate aminotransferase) {Rattus norvegicus}; contains Pfam profile PF00202: aminotransferase, class III \| chr2:16090769-16093427 FORWARD \| Aliases: ALANINE:GLYOXYLATE AMINOTRANSFERASE 2 HOMOLOG, ALANINE:GLYOXYLATE AMINOTRANSFERASE 3, T19C21.11, T19C21_11 | 7.4 | 9.2 | -1.7 | -5.6 | 0.57% | 5.3 |
| 3578 | AT4G31410.2 \| expressed protein \| chr4:15243743-15245767 FORWARD \| Aliases: F3L17.5 | 6.0 | 7.0 | -1.1 | -5.6 | 0.57% | 5.3 |
| 3581 | AT4G29720.1 \| amine oxidase family protein, similar to polyamine oxidase isoform-2 (Homo sapiens) GI:16554963; contains Pfam profile PF01593: amine oxidase, flavin-containing | 3.8 | 5.0 | -1.2 | -5.6 | 0.57% | 5.3 |
| 3582 | AT5G23660.1 \| Symbol: MTN3 \| nodulin MtN3 family protein, similar to MtN3 GI:1619602 (root nodule development) from (Medicago truncatula) \| chr5:7971654-7973928 REVERSE \| Aliases: MQM1.8, MQM1_8 | 3.8 | 6.5 | -2.8 | -5.6 | 0.57% | 5.1 |
| 3584 | AT5G47390.1 \| myb family transcription factor, contains Pfam profile: PF00249 myb-like DNA-binding domain \| chr5:19244017-19246085 FORWARD \| Aliases: MQL5.25, MQL5_25 | 5.4 | 7.7 | -2.3 | -5.6 | 0.57% | 5.3 |
| 3586 | AT2G01460.1 \| phosphoribulokinase/uridine kinase family protein, contains Pfam profile PF00485: Phosphoribulokinase / Uridine kinase family \| chr2:206135-210974 FORWARD \| Aliases: F2I9.8, F2I9_8 | 3.3 | 4.2 | -0.9 | -5.6 | 0.57% | 5.2 |
| 3588 | AT5G06610.1 \| expressed protein, contains Pfam profile PF04788: Protein of unknown function (DUF620) \| chr5:2029898-2033724 FORWARD \| Aliases: F15M7.14, F15M7_14 | 5.1 | 7.2 | -2.0 | -5.6 | 0.57% | 5.3 |
| 3594 | AT1G24030.1 \| protein kinase family protein, contains protein kinase domain, Pfam:PF00069; similar to protein kinase APK1A, SWISS-PROT:Q06548 (Arabidopsis thaliana) \| chr1:8503242-8505449 FORWARD \| Aliases: T23E23.18, T23E23_18 | 3.6 | 4.8 | -1.2 | -5.6 | 0.58% | 5.3 |
| 3597 | AT1G52760.1 \| esterase/lipase/thioesterase family protein, low similarity to monoglyceride lipase from (Homo sapiens) GI:14594904, (Mus musculus) GI:2632162; contains Interpro entry IPR000379 \| chr1:19655013-19656411 FORWARD \| Aliases: F14G24.3, F14G24_3 | 9.2 | 11.0 | -1.9 | -5.6 | 0.58% | 5.2 |
| 3598 | AT2G25940.1 \| Symbol: ALPHA VPE \| vacuolar processing enzyme alpha / alpha-VPE, identical to SP:P49047 Vacuolar processing enzyme, alpha-isozyme precursor (EC 3.4.22.-) (Alpha-VPE) {Arabidopsis thaliana} \| chr2:11069798-11073106 REVERSE \| Aliases: ALPHAVPE, F17H15.4 | 4.9 | 7.3 | -2.4 | -5.6 | 0.58% | 5.2 |
| 3600 | AT5G54650.2 \| formin homology 2 domain-containing protein / FH2 domain-containing protein, contains formin homology 2 domain, Pfam:PF02181 \| chr5:22214855-22219249 REVERSE \| Aliases: MRB17.15, MRB17_15 | 5.2 | 6.6 | -1.4 | -5.6 | 0.58% | 5.3 |
| 3601 | AT3G23150.1 \| Symbol: ETR2 \| ethylene receptor, putative (ETR2), similar to putative ethylene receptor; ETR2 (Arabidopsis thaliana) gi:3687654:gb:AAC62208. \| chr3:8254670-8257742 FORWARD \| Aliases: ETHYLENE RESPONSE 2, ETR2, K14B15.9 | 5.5 | 7.4 | -1.9 | -5.6 | 0.58% | 5.3 |
| 3604 | AT2G34810.1 \| FAD-binding domain-containing protein, similar to SP:P30986 reticuline oxidase precursor (Berberine-bridge-forming enzyme) (BBE) (Tetrahydroprotoberberine synthase) (Eschscholzia californica); contains PF01565 FAD binding domain \| chr2:14692335-14694141 FORWARD \| Aliases: F19I3.4, F19I3_4 | 2.7 | 4.0 | -1.3 | -5.6 | 0.58% | 5.3 |
| 3606 | AT4G22212.1 \| Encodes a defensin-like (DEFL) family protein. \| chr4:11753998-11754700 FORWARD \| Aliases: None | 10.0 | 11.4 | -1.4 | -5.6 | 0.58% | 5.1 |
| 3607 | AT1G31120.1 \| Symbol: KUP10 \| potassium transporter family protein, similar to HAK2 (Hordeum vulgare) GI:7108599, potassium transporter (Arabidopsis thaliana) gi:2654088:gb:AAB87687; KUP/HAK/KT Transporter family member, PMID:11500563; contains Pfam profile PF02705: K+ potassium transporter \| chr1:11104282-11108012 REVERSE \| Aliases: F28K20.5, F28K20_5 | 4.6 | 6.0 | -1.4 | -5.6 | 0.58% | 5.3 |
| 3609 | AT2G47860.2 \| phototropic-responsive NPH3 family protein, contains NPH3 family domain, Pfam:PF03000 \| chr2:19607018-19609262 FORWARD \| Aliases: F17A22.25 | 2.6 | 3.7 | -1.1 | -5.6 | 0.58% | 5.2 |
| 3612 | AT3G19290.1 \| Symbol: ABF4 \| ABA-responsive element-binding protein 2 (AREB2), almost identical (one amino acid) to GB:AAF27182 from (Arabidopsis thaliana); contains Pfam profile PF00170:bZIP transcription factor; identical to cDNA abscisic acid responsive elements-binding factor (ABRE) mRNA, partial cds GI:6739282 \| chr3:6687146-6690129 FORWARD \| Aliases: ABA RESPONSIVE ELEMENT BINDING PROTEIN 2, ABRE BINDING FACTOR 4, AREB2, MVI11.7 | 4.6 | 6.6 | -1.9 | -5.5 | 0.58% | 5.3 |
| 3613 | AT1G01650.2 \| similar to protease-associated (PA) domain-containing protein [Arabidopsis thaliana] (TAIR:At1g63690.2); similar to growth-on protein GRO10 [Euphorbia esula] (GB:AAL14629.1); contains InterPro domain Presenilin signal peptide peptidase (InterPro:IPR006639); contains InterPro domain Membrane protein of unknown function DUF435 (InterPro:IPR007369) \| chr1:232841-237817 REVERSE \| Aliases: T1N6.3, T1N6_3 | 7.1 | 9.3 | -2.2 | -5.5 | 0.58% | 5.3 |
| 3615 | AT2G36950.1 \| heavy-metal-associated domain-containing protein, nearly identical to farnesylated protein ATFP2 (GI:4097545) Pfam profile PF00403: Heavy-metal-associated domain \| chr2:15522212-15524010 FORWARD \| Aliases: T1J8.13, T1J8_13 | 9.6 | 11.6 | -2.0 | -5.5 | 0.58% | 5.3 |

| Rank | Description | Sync | Root | M | t | adj.q | B |
|---|---|---|---|---|---|---|---|
| 3626 | AT1G27150.1 \| expressed protein \| chr1:9429044-9432235 FORWARD \| Aliases: T7N9.21, T7N9_21 | 9.0 | 9.9 | -0.8 | -5.5 | 0.58% | 5.1 |
| 3627 | AT3G23190.1 \| lesion inducing protein-related, similar to ORF, able to induce HR-like lesions (Nicotiana tabacum) \| chr3:8279339-8280919 FORWARD \| Aliases: K14B15.15 | 3.7 | 7.7 | -4.0 | -5.5 | 0.58% | 5.2 |
| 3629 | AT5G39590.1 \| expressed protein \| chr5:15868639-15871194 FORWARD \| Aliases: MIJ24.8, MIJ24_8 | 7.2 | 9.7 | -2.5 | -5.5 | 0.59% | 5.3 |
| 3630 | AT1G09250.1 \| expressed protein \| chr1:2989380-2990352 FORWARD \| Aliases: T12M4.4, T12M4_4 | 4.2 | 5.7 | -1.4 | -5.5 | 0.59% | 5.3 |
| 3632 | AT1G19230.1 \| respiratory burst oxidase protein E (RbohE) / NADPH oxidase, nearly identical to respiratory burst oxidase protein E GI:3242787 (gi:3242787) from (Arabidopsis thaliana) | 2.9 | 4.6 | -1.7 | -5.5 | 0.59% | 5.2 |
| 3634 | AT1G26580.1 \| expressed protein, similar to putative MYB family transcription factor GB:AAD17429 GI:4335752 from (Arabidopsis thaliana) \| chr1:9184593-9187492 FORWARD \| Aliases: T1K7.5, T1K7_5 | 5.6 | 8.3 | -2.7 | -5.5 | 0.59% | 5.3 |
| 3637 | AT1G73980.1 \| phosphoribulokinase/uridine kinase family protein, weak similarity to SP:Q59190 Uridine kinase (EC 2.7.1.48) (Uridine monophosphokinase) (Cytidine monophosphokinase) {Borrelia burgdorferi}; contains Pfam profile PF00485: Phosphoribulokinase / Uridine kinase family | 7.6 | 9.1 | -1.5 | -5.5 | 0.59% | 5.3 |
| 3640 | AT1G24280.1 \| Symbol: G6PD3 \| Encodes a plastidic glucose-6-phosphate dehydrogenase that is sensitive to reduction by DTT and whose mRNA is most highly expressed in root. \| chr1:8609434-8612569 FORWARD \| Aliases: F3I6.22, F3I6_22, G6PD3, GLUCOSE 6 PHOSPHATE DEHYDROGENASE 3 | 8.7 | 9.8 | -1.1 | -5.5 | 0.59% | 5.2 |
| 3642 | AT3G20080.3 \| Symbol: CYP705A15 \| cytochrome P450 family protein, similar to Cytochrome P450 93A1 (SP:Q42798) | 3.6 | 4.8 | -1.2 | -5.5 | 0.59% | 5.3 |
| 3645 | AT1G56145.1 \| leucine-rich repeat family protein / protein kinase family protein, contains Pfam profiles: PF00069: Eukaryotic protein kinase domain, multiple PF00560: Leucine Rich Repeat \| chr1:21011758-21017648 REVERSE \| Aliases: None | 3.8 | 6.0 | -2.2 | -5.5 | 0.59% | 5.1 |
| 3647 | AT2G16790.1 \| shikimate kinase family protein, similar to thermosensitive glucokinase from Escherichia coli (SP:P39208) (SP:P46859); contains Pfam profile PF01202: shikimate kinase \| chr2:7291039-7292319 REVERSE \| Aliases: T24I21.20, T24I21_20 | 6.9 | 9.1 | -2.1 | -5.5 | 0.59% | 5.3 |
| 3648 | AT5G60950.1 \| phytochelatin synthetase-related, contains Pfam profile PF04833: Phytochelatin synthetase-like conserved region \| chr5:24544383-24545232 REVERSE \| Aliases: MSL3.7, MSL3_7 | 4.2 | 6.5 | -2.3 | -5.5 | 0.59% | 5.2 |
| 3651 | AT3G09260.1 \| Symbol: PYK10 \| glycosyl hydrolase family 1 protein, contains Pfam PF00232 : Glycosyl hydrolase family 1 domain; TIGRFAM TIGR01233: 6-phospho-beta-galactosidase; almost identical to beta-glucosidase GI:1732570 from (Arabidopsis thaliana); similar to beta-glucosidase 1 (GI:12043529) (Arabidopsis thaliana) \| chr3:2840486-2843784 REVERSE \| Aliases: F3L24.13, PSR3.1 | 6.5 | 10.8 | -4.3 | -5.5 | 0.59% | 5.2 |
| 3653 | AT1G10350.1 \| DNAJ heat shock protein, putative, similar to SP:Q9QYJ3 DnaJ homolog subfamily B member 1 (Heat shock 40 kDa protein 1) {Mus musculus}; contains Pfam profile PF00226: DnaJ domain \| chr1:3393409-3395057 REVERSE \| Aliases: F14N23.23, F14N23_23 | 6.2 | 7.4 | -1.2 | -5.5 | 0.59% | 5.3 |
| 3654 | AT1G19250.1 \| flavin-containing monooxygenase family protein / FMO family protein, low similarity to SP:P97501 Dimethylaniline monooxygenase (N-oxide forming) 3 (EC 1.14.13.8) (Hepatic flavin-containing monooxygenase 3) (FMO 3) {Mus musculus}; contains Pfam profile PF00743: Flavin-binding monooxygenase-like \| chr1:6650520-6653069 REVERSE \| Aliases: T29M8.12, T29M8_12 | 2.4 | 3.2 | -0.8 | -5.5 | 0.59% | 5.1 |
| 3655 | AT2G41120.1 \| expressed protein \| chr2:17147973-17149497 REVERSE \| Aliases: T3K9.11, T3K9_11 | 3.7 | 4.9 | -1.2 | -5.5 | 0.59% | 5.3 |
| 3656 | AT1G07740.1 \| pentatricopeptide (PPR) repeat-containing protein, contains Pfam profile PF01535: PPR repeat; possible frameshift prevents accurate annotation of the gene product \| chr1:2397439-2400505 REVERSE \| Aliases: AT1G07730, F24B9.15, F24B9_15 | 4.3 | 5.7 | -1.5 | -5.5 | 0.59% | 5.3 |
| 3658 | AT3G45190.1 \| SIT4 phosphatase-associated family protein, contains Pfam profile: PF04499 SIT4 phosphatase-associated protein \| chr3:16552979-16559146 REVERSE \| Aliases: T14D3.130 | 2.9 | 3.7 | -0.7 | -5.5 | 0.59% | 5.0 |
| 3662 | AT1G70470.1 \| expressed protein \| chr1:26563022-26563697 REVERSE \| Aliases: F24J13.4, F24J13_4 | 2.8 | 6.1 | -3.3 | -5.5 | 0.60% | 5.2 |
| 3664 | AT3G15040.1 \| expressed protein, contains Pfam profile PF04520: Protein of unknown function, DUF584 \| chr3:5064711-5066697 REVERSE \| Aliases: K15M2.18 | 6.0 | 7.0 | -1.0 | -5.5 | 0.60% | 5.2 |
| 3667 | AT4G39670.1 \| expressed protein \| chr4:18410166-18410994 FORWARD \| Aliases: T19P19.60, T19P19_60 | 3.9 | 6.0 | -2.1 | -5.5 | 0.60% | 5.2 |
| 3669 | AT1G28320.1 \| protease-related, similar to Protease degS (Precursor) (SP:P44947) (Haemophilus influenzae); similar to DegP protease precursor (GI:2565436) (Arabidopsis thaliana) \| chr1:9920494-9924475 REVERSE \| Aliases: F3H9.3, F3H9_3 | 5.6 | 7.1 | -1.4 | -5.5 | 0.60% | 5.2 |

| Rank | Description | Sync | Root | M | t | adj.q | B |
|---|---|---|---|---|---|---|---|
| 3670 | AT5G05570.1 \| transducin family protein / WD-40 repeat family protein, similar to unknown protein (pir::T04661); contains Pfam PF00400: WD domain, G-beta repeat (4 copies, 2 weak):8683726:gb:AV524198.1:AV524198 \| chr5:1656567-1663930 FORWARD \| Aliases: MOP10.11, MOP10_11 | 6.0 | 8.1 | -2.1 | -5.5 | 0.60% | 5.2 |
| 3671 | AT1G05940.1 \| Symbol: CAT9 \| amino acid permease family protein, low similarity to SP:P30823 High-affinity cationic amino acid transporter-1 (CAT-1) {Rattus norvegicus}; contains Pfam profile PF00324: Amino acid permease \| chr1:1801177-1803975 REVERSE \| Aliases: CATIONIC AMINO ACID TRANSPORTER 9, T21E18.1, T21E18_1 | 7.0 | 8.1 | -1.1 | -5.5 | 0.60% | 5.2 |
| 3673 | AT4G23410.1 \| senescence-associated family protein, similar to senescence-associated protein 5 (Hemerocallis hybrid cultivar) gi:3551954:gb:AAC34855 \| chr4:12223963-12225235 FORWARD \| Aliases: F16G20.110, F16G20_110 | 3.5 | 5.1 | -1.5 | -5.5 | 0.60% | 5.2 |
| 3677 | AT1G14960.1 \| major latex protein-related / MLP-related, low similarity to major latex protein {Papaver somniferum}(GI:20810) contains Pfam profile PF00407: Pathogenesis-related protein Bet v I family \| chr1:5159194-5160076 REVERSE \| Aliases: F10B6.39, F10B6_39 | 3.2 | 6.8 | -3.6 | -5.5 | 0.60% | 5.2 |
| 3679 | AT3G50810.1 \| integral membrane protein, putative, contains 4 transmembrane domains; contains Pfam PF04535 : Domain of unknown function (DUF588); similar to putative ethylene responsive element binding protein (GI:22135858) (Arabidopsis thaliana) \| chr3:18898595-18900849 REVERSE \| Aliases: F18B3.90 | 5.9 | 7.2 | -1.3 | -5.5 | 0.60% | 5.2 |
| 3682 | AT5G12010.1 \| expressed protein \| chr5:3877813-3879540 REVERSE \| Aliases: F14F18.180, F14F18_180 | 7.9 | 9.3 | -1.4 | -5.5 | 0.60% | 5.0 |
| 3683 | AT4G27500.1 \| Symbol: PPI1 \| expressed protein, non-consensus GA donor splice site at exon 6 \| chr4:13743178-13745906 FORWARD \| Aliases: F27G19.100, F27G19_100, PROTON PUMP INTERACTOR 1 | 10.0 | 11.3 | -1.3 | -5.5 | 0.61% | 5.0 |
| 3684 | AT2G15970.1 \| Symbol: COR413 PM1 \| cold-acclimation protein, putative (FL3-5A3), similar to cold acclimation WCOR413-like protein gamma form (Hordeum vulgare) gi:18449100:gb:AAL69988; similar to stress-regulated protein SAP1 (Xerophyta viscosa) gi:21360378:gb:AAM47505; identical to cDNA cold acclimation protein WCOR413-like protein alpha form GI:10121840, cold acclimation protein homolog (Arabidopsis thaliana) GI:11127595 \| chr2:6957170-6958274 FORWARD \| Aliases: ATCOR413 PM1, COLD ACCLIMATION PROTEIN WCOR413 LIKE PROTEIN ALPHA FORM, F19G14.3, F19G14_3, FL3 5A3, WCOR413, WCOR413 LIKE | 4.7 | 7.8 | -3.1 | -5.5 | 0.61% | 5.1 |
| 3685 | AT5G61260.1 \| chromosome scaffold protein-related, contains weak similarity to chromosome scaffold protein p85 (Moneuplotes crassus) gi:25990101:gb:AAN75020 \| chr5:24654335-24655825 FORWARD \| Aliases: MFB13.3, MFB13_3 | 2.7 | 6.4 | -3.7 | -5.5 | 0.61% | 4.9 |
| 3686 | AT5G16520.1 \| expressed protein \| chr5:5394656-5397458 REVERSE \| Aliases: MQK4.27, MQK4_27 | 4.6 | 6.1 | -1.5 | -5.5 | 0.61% | 5.2 |
| 3687 | AT1G70410.3 \| carbonic anhydrase, putative / carbonate dehydratase, putative, similar to SP:P42737 Carbonic anhydrase 2 (EC 4.2.1.1) (Carbonate dehydratase 2) {Arabidopsis thaliana}; contains Pfam profile PF00484: Carbonic anhydrase \| chr1:26537589-26540336 REVERSE \| Aliases: F17O7.5, F17O7_5 | 7.8 | 9.4 | -1.6 | -5.5 | 0.61% | 5.2 |
| 3690 | AT5G05880.1 \| UDP-glucoronosyl/UDP-glucosyl transferase family protein, contains Pfam profile: PF00201 UDP-glucoronosyl and UDP-glucosyl transferase \| chr5:1769649-1771516 FORWARD \| Aliases: K18J17.3, K18J17_3 | 3.8 | 5.0 | -1.2 | -5.5 | 0.61% | 5.2 |
| 3691 | AT3G61880.1 \| Symbol: CYP78A9 \| cytochrome P450, putative, similar to cytochrome p450 SP:O48927 from (Arabidopsis thaliana) \| chr3:22916843-22918933 REVERSE \| Aliases: CYTOCHROME P450 78A9, F21F14.50 | 3.8 | 5.7 | -1.9 | -5.5 | 0.61% | 5.1 |
| 3693 | AT1G71530.2 \| protein kinase family protein, contains Serine/Threonine protein kinases active-site signature, PROSITE:PS00108 \| chr1:26943229-26946614 FORWARD \| Aliases: F26A9.10 | 3.8 | 5.7 | -2.0 | -5.5 | 0.61% | 5.2 |
| 3694 | AT1G62510.1 \| protease inhibitor/seed storage/lipid transfer protein (LTP) family protein, contains Pfam protease inhibitor/seed storage/LTP family domain PF00234 \| chr1:23140036-23140856 REVERSE \| Aliases: T3P18.7, T3P18_7 | 1.9 | 2.8 | -0.9 | -5.5 | 0.61% | 5.1 |
| 3696 | AT4G13530.2 \| expressed protein, predicted protein, Arabidopsis thaliana \| chr4:7866780-7868716 FORWARD \| Aliases: T6G15.80, T6G15_80 | 8.4 | 9.9 | -1.6 | -5.5 | 0.61% | 5.2 |
| 3697 | AT5G15630.1 \| Symbol: COBL4/IRX6 \| Encodes a member of the COBRA family, similar to phytochelatin synthetase. Involved in secondary cell wall biosynthesis. Mutants make smaller plants with reduced levels of cellulose and cell wall sugars. \| chr5:5084708-5086795 FORWARD \| Aliases: COBL4, COBRA LIKE4, F14F8.10, F14F8_10, IRX6 | 3.3 | 5.4 | -2.0 | -5.5 | 0.61% | 5.2 |
| 3698 | AT3G10660.1 \| Symbol: CPK2 \| calcium-dependent protein kinase isoform 2 (CPK2), identical to calcium-dependent protein kinase isoform 2 (Arabidopsis thaliana) gi:9837343:gb:AAG00535; contains protein kinase domain, Pfam:PF00069; contains EF hand domain (calcium-binding EF-hand), Pfam:PF00036, INTERPRO:IPR002048 \| chr3:3331403-3334273 REVERSE \| Aliases: ATCPK2, CALMODULIN DOMAIN PROTEIN KINASE CDPK ISOFORM 2, F13M14.5 | 3.7 | 4.9 | -1.2 | -5.5 | 0.61% | 5.2 |
| 3699 | AT3G06620.1 \| protein kinase family protein, contains Pfam domain, PF00069: Protein kinase domain \| chr3:2062212-2067422 REVERSE \| Aliases: F5E6.5, F5E6_5 | 2.7 | 3.2 | -0.5 | -5.5 | 0.61% | 4.8 |

| Rank | Description | Sync | Root | M | t | adj.q | B |
|---|---|---|---|---|---|---|---|
| 3701 | AT1G03550.1 \| secretory carrier membrane protein (SCAMP) family protein, contains Pfam domain, PF04144: SCAMP family \| chr1:885623-887956 REVERSE \| Aliases: F21B7.17, F21B7_17 | 4.4 | 6.4 | -2.0 | -5.5 | 0.61% | 5.2 |
| 3702 | AT5G19600.1 \| Symbol: SULTR3;5 \| sulfate transporter, putative, similar to sulfate transporter (Arabidopsis thaliana) GI:2285885; contains Pfam profiles PF00916: Sulfate transporter family, PF01740: STAS domain; supporting cDNA gi:14141683:dbj:AB061739.1: \| chr5:6612823-6616999 REVERSE \| Aliases: T29J13.20, T29J13_20, sulfate transporter 3;5 | 2.9 | 4.1 | -1.2 | -5.5 | 0.62% | 5.2 |
| 3703 | AT2G47930.1 \| hydroxyproline-rich glycoprotein family protein, contains proline-rich extensin domains, INTERPRO:IPR002965 \| chr2:19624045-19624751 REVERSE \| Aliases: T9J23.8, T9J23_8 | 3.0 | 4.6 | -1.6 | -5.5 | 0.62% | 5.2 |
| 3704 | AT5G27000.1 \| Symbol: ATK4 \| kinesin motor protein-related, non-consensus AT donor splice site at exon 12; non-consensus AC acceptor splice site at exon 13 \| chr5:9498055-9503137 FORWARD \| Aliases: ARABIDOPSIS THALIANA KINESIN 4, F2P16.12, F2P16_12, KATD, KINESIN LIKE PROTEIN D | 3.3 | 4.2 | -0.9 | -5.5 | 0.62% | 5.2 |
| 3705 | AT5G20730.3 \| Symbol: NPH4 \| auxin-responsive factor (ARF7), identical to auxin response factor 7 GI:4104929 from (Arabidopsis thaliana) \| chr5:7016472-7022115 REVERSE \| Aliases: ARF7, AUXIN RESPONSE FACTOR 7, AUXIN REGULATED TRANSCRIPTIONAL ACTIVATOR 7, AUXIN RESPONSIVE TRANSCRIPTIONAL ACTIVATOR 7, BIP, BIPOSTO, IAA21, MASSUGU1, MSG1, NON PHOTOTROPHIC HYPOCOTYL, T1M15.130, T1M15_130, TIR5, TRANSPORT INHIBITOR RESPONSE 5 | 8.2 | 9.1 | -0.8 | -5.5 | 0.62% | 5.1 |
| 3715 | AT2G20520.1 \| Symbol: FLA6 \| fasciclin-like arabinogalactan-protein (FLA6), identical to gi:13377780_gb_AAK20859 \| chr2:8847744-8848542 FORWARD \| Aliases: T13C7.11, T13C7_11, fasciclin like arabinogalactan protein 6 | 3.0 | 5.3 | -2.2 | -5.5 | 0.62% | 5.2 |
| 3716 | AT3G09940.1 \| monodehydroascorbate reductase, putative, similar to monodehydroascorbate reductase (NADH) GB:JU0182 (Cucumis sativus) \| chr3:3056384-3059153 REVERSE \| Aliases: F8A24.20 | 3.3 | 4.6 | -1.3 | -5.5 | 0.62% | 5.2 |
| 3717 | AT1G48300.1 \| expressed protein \| chr1:17851024-17852466 FORWARD \| Aliases: F11A17.15, F11A17_15 | 9.1 | 11.7 | -2.6 | -5.5 | 0.62% | 5.1 |
| 3719 | AT2G28210.1 \| carbonic anhydrase family protein, similar to storage protein (dioscorin) (Dioscorea cayenensis) GI:433463; contains Pfam profile PF00194: Eukaryotic-type carbonic anhydrase \| chr2:12037074-12039497 FORWARD \| Aliases: T3B23.12, T3B23_12 | 2.1 | 4.7 | -2.6 | -5.5 | 0.62% | 5.1 |
| 3720 | AT1G02310.1 \| glycosyl hydrolase family protein 5 / cellulase family protein / (1-4)-beta-mannan endohydrolase, putative, similar to (1-4)-beta-mannan endohydrolase precursor GI:9836826 from (Lycopersicon esculentum) \| chr1:458133-460696 REVERSE \| Aliases: T6A9.1, T6A9_1 | 3.9 | 5.0 | -1.1 | -5.5 | 0.62% | 5.2 |
| 3725 | AT3G25610.1 \| haloacid dehalogenase-like hydrolase family protein, similar to Potential phospholipid-transporting ATPase (EC 3.6.3.1) from Mus musculus (SP:P98200, SP:P70704), {Bos taurus} SP:Q29449; contains InterPro accession IPR005834: Haloacid dehalogenase-like hydrolase \| chr3:9310179-9314590 REVERSE \| Aliases: T5M7.10 | 4.7 | 6.1 | -1.4 | -5.5 | 0.62% | 5.2 |
| 3732 | AT3G47420.1 \| glycerol-3-phosphate transporter, putative / glycerol 3-phosphate permease, putative, similar to cAMP inducible 2 protein (Mus musculus) GI:4580997, glycerol-3-phosphate transporter (glycerol 3-phosphate permease) (Homo sapiens) GI:7543982; contains Pfam profile PF00083: major facilitator superfamily protein \| chr3:17483327-17486198 REVERSE \| Aliases: T21L8.170 | 4.9 | 8.2 | -3.4 | -5.5 | 0.63% | 5.2 |
| 3734 | AT4G25350.1 \| EXS family protein / ERD1/XPR1/SYG1 family protein, similar to PHO1 protein (Arabidopsis thaliana) GI:20069032; contains Pfam profiles PF03105: SPX domain, PF03124: EXS family \| chr4:12963392-12966458 REVERSE \| Aliases: T30C3.10, T30C3_10 | 2.7 | 3.4 | -0.7 | -5.5 | 0.63% | 4.9 |
| 3735 | AT3G18450.1 \| expressed protein, similar to PGPS/D12 (Petunia x hybrida) GI:4105794; contains Pfam profile PF04749: Protein of unknown function, DUF614 \| chr3:6331040-6332124 REVERSE \| Aliases: MYF24.17 | 1.9 | 3.4 | -1.4 | -5.5 | 0.63% | 5.2 |
| 3736 | AT3G55090.1 \| ABC transporter family protein, ATP-binding cassette-sub-family G-member 2, Mus musculus, EMBL:AF140218 \| chr3:20427319-20429481 REVERSE \| Aliases: T15C9.80 | 3.2 | 4.7 | -1.5 | -5.5 | 0.63% | 5.2 |
| 3739 | AT1G77130.1 \| glycogenin glucosyltransferase (glycogenin)-related, contains similarity to glycogenin-1 from Mus musculus (SP:Q9R062), Rattus norvegicus (SP:O08730), Homo sapiens (SP:P46976) \| chr1:28983788-28986185 REVERSE \| Aliases: T14N5.1, T14N5_1 | 6.1 | 8.7 | -2.6 | -5.5 | 0.63% | 5.2 |
| 3741 | AT5G44750.1 \| Symbol: REV1 \| similar to UMUC-like DNA repair family protein [Arabidopsis thaliana] (TAIR:At1g49980.1); similar to PREDICTED: similar to deoxycytidyl transferase [Rattus norvegicus] (GB:XP_237071.3); contains InterPro domain UMUC-like DNA-repair protein (InterPro:IPR001126); contains InterPro domain BRCT (InterPro:IPR001357) | 3.6 | 4.8 | -1.2 | -5.5 | 0.63% | 5.1 |
| 3742 | AT5G03150.1 \| zinc finger (C2H2 type) family protein, contains Pfam domain, PF00096: Zinc finger, C2H2 type \| chr5:745643-749002 FORWARD \| Aliases: F15A17.180, F15A17_180 | 3.1 | 6.3 | -3.2 | -5.5 | 0.63% | 5.2 |
| 3744 | AT2G26640.1 \| beta-ketoacyl-CoA synthase, putative, similar to beta-ketoacyl-CoA synthase (Simmondsia chinensis)(GI:1045614) \| chr2:11336948-11339528 FORWARD \| Aliases: F18A8.1, F18A8_1 | 3.8 | 4.9 | -1.0 | -5.4 | 0.63% | 5.2 |

| Rank | Description | Sync | Root | M | t | adj.q | B |
|---|---|---|---|---|---|---|---|
| 3746 | AT5G54130.1 \| calcium-binding EF hand family protein, contains INTERPRO:IPR002048 calcium-binding EF-hand domain \| chr5:21981074-21982505 FORWARD \| Aliases: MJP23.11, MJP23_11 | 4.0 | 5.7 | -1.7 | -5.4 | 0.63% | 5.1 |
| 3749 | AT2G32610.1 \| Symbol: ATCSLB01 \| cellulose synthase family protein, similar to Zea mays cellulose synthase-3 (gi:9622878), -2 (gi:9622876), -1 (gi:9622874) \| chr2:13843311-13846590 FORWARD \| Aliases: ATCSLB1, CSLB01, T26B15.17, T26B15_17 | 3.2 | 4.6 | -1.4 | -5.4 | 0.63% | 5.2 |
| 3753 | AT5G38350.1 \| disease resistance protein (NBS-LRR class), putative, domain signature NBS-LRR exists, suggestive of a disease resistance protein. \| chr5:15345889-15348758 FORWARD \| Aliases: MXI10.1, MXI10_1 | 2.2 | 4.7 | -2.5 | -5.4 | 0.63% | 5.2 |
| 3755 | AT5G60270.1 \| lectin protein kinase family protein, contains Pfam domains, PF00069: Protein kinase domain, PF00139: Legume lectins beta domain, and PF00138: Legume lectins alpha domain \| chr5:24274987-24276993 FORWARD \| Aliases: F15L12.9, F15L12_9 | 2.7 | 3.2 | -0.5 | -5.4 | 0.63% | 4.8 |
| 3756 | AT5G60920.1 \| Symbol: COB \| phytochelatin synthetase, putative / COBRA cell expansion protein COB, putative, similar to phytochelatin synthetase GI:29570314; similar to GB:AAK56072; identified in Roudier, et al, Plant Phys. (2002) 130:538-548 (PMID:12376623); identical to cDNA putative phytochelatin synthetase GI:3559804; contains Pfam profile PF04833: Phytochelatin synthetase-like conserved region \| chr5:24528422-24531354 REVERSE \| Aliases: COBRA, MSL3.40, MSL3_40 | 5.5 | 8.1 | -2.6 | -5.4 | 0.63% | 5.2 |
| 3757 | AT4G10310.1 \| Symbol: HKT1 \| sodium transporter (HKT1), identical to sodium transporter AtHKT1 (Arabidopsis thaliana) gi:7716474:gb:AAF68393 \| chr4:6391980-6395803 FORWARD \| Aliases: ATHKT1, HIGH AFFINITY K+ TRANSPORTER 1, HKT1, T9A4.5 | 2.2 | 2.9 | -0.7 | -5.4 | 0.63% | 5.0 |
| 3759 | AT2G35610.1 \| expressed protein \| chr2:14954444-14958326 REVERSE \| Aliases: T20F21.19, T20F21_19 | 5.2 | 6.8 | -1.6 | -5.4 | 0.63% | 5.2 |
| 3765 | AT4G33580.2 \| similar to carbonic anhydrase family protein / carbonate dehydratase family protein [Arabidopsis thaliana] (TAIR:At1g58180.2); similar to carbonate dehydratase (EC 4.2.1.1) precursor, chloroplast - Flaveria linearis (GB:S61883); similar to chloroplast carbonic anhydrase precursor [Thlaspi caerulescens] (GB:AAS65454.1); contains InterPro domain Carbonic anhydrase, prokaryotic (InterPro:IPR001765) \| chr4:16138362-16141626 FORWARD \| Aliases: T16L1.70, T16L1_70 | 4.3 | 5.5 | -1.2 | -5.4 | 0.64% | 5.1 |
| 3767 | AT2G28970.1 \| leucine-rich repeat protein kinase, putative, similar to light repressible receptor protein kinase (Arabidopsis thaliana) gi:1321686:emb:CAA66376; contains leucine rich repeat (LRR) domains, Pfam:PF00560; contains protein kinase domain, Pfam:PF00069 \| chr2:12450996-12455240 FORWARD \| Aliases: T9I4.5, T9I4_5 | 2.6 | 4.6 | -2.0 | -5.4 | 0.64% | 5.1 |
| 3768 | AT1G55265.1 \| expressed protein, contains Pfam profile PF04398: Protein of unknown function, DUF538 \| chr1:20620980-20621668 FORWARD \| Aliases: None | 4.3 | 5.5 | -1.2 | -5.4 | 0.64% | 5.2 |
| 3769 | AT1G78240.1 \| dehydration-responsive protein-related, similar to early-responsive to dehydration stress ERD3 protein (Arabidopsis thaliana) GI:15320410; contains Pfam profile PF03141: Putative methyltransferase \| chr1:29437722-29441377 REVERSE \| Aliases: F3F9.21, F3F9_21 | 6.1 | 7.6 | -1.5 | -5.4 | 0.64% | 5.2 |
| 3772 | AT1G73370.1 \| sucrose synthase, putative / sucrose-UDP glucosyltransferase, putative, similar to sucrose synthase GI:6682841 from (Citrus unshiu) \| chr1:27588140-27591987 REVERSE \| Aliases: T9L24.42, T9L24_42 | 4.7 | 6.5 | -1.8 | -5.4 | 0.64% | 5.1 |
| 3777 | AT1G77500.1 \| expressed protein, contains Pfam domains, PF04782: Protein of unknown function (DUF632) and PF04783: Protein of unknown function (DUF630) \| chr1:29126383-29130085 FORWARD \| Aliases: T5M16.9, T5M16_9 | 3.5 | 4.2 | -0.7 | -5.4 | 0.64% | 5.0 |
| 3780 | AT5G24260.1 \| prolyl oligopeptidase family protein, similar to dipeptidyl peptidase IV (Stenotrophomonas maltophilia) GI:1753197; contains Pfam profiles PF00326: prolyl oligopeptidase family, PF00930: Dipeptidyl peptidase IV (DPP IV) N-terminal region \| chr5:8234726-8238246 REVERSE \| Aliases: MOP9.8, MOP9_8 | 6.5 | 7.5 | -1.1 | -5.4 | 0.64% | 5.1 |
| 3781 | AT5G35750.1 \| Symbol: AHK2 \| histidine kinase (AHK2), identical to histidine kinase AHK2 (Arabidopsis thaliana) gi:13537196:dbj:BAB40774 \| chr5:13928601-13933567 REVERSE \| Aliases: ARABIDOPSIS HISTIDINE KINASE 2, MXH1.16, MXH1_16 | 4.0 | 5.7 | -1.7 | -5.4 | 0.64% | 5.1 |
| 3784 | AT4G15475.1 \| F-box family protein (FBL4), 99.7% identical to F-box protein family, AtFBL4 (GP:21536497) (Arabidopsis thaliana); similar to grr1 GI:2407790 from (Glycine max) | 5.8 | 7.2 | -1.4 | -5.4 | 0.64% | 5.2 |
| 3786 | AT4G02540.1 \| DC1 domain-containing protein, contains Pfam profile PF03107: DC1 domain | 6.8 | 9.3 | -2.5 | -5.4 | 0.65% | 5.1 |
| 3790 | AT5G47560.1 \| Symbol: ATSDAT/ATTDT \| Encodes a tonoplast malate/fumarate transporter. | 2.9 | 4.4 | -1.5 | -5.4 | 0.65% | 5.1 |
| 3795 | AT5G53010.1 \| calcium-transporting ATPase, putative \| chr5:21505716-21513763 REVERSE \| Aliases: MNB8.7, MNB8_7 | 2.8 | 4.3 | -1.5 | -5.4 | 0.65% | 5.1 |
| 3796 | AT2G18550.1 \| Symbol: HB 2 \| homeobox-leucine zipper family protein, similar to CRHB6 (GI:3868839) (Ceratopteris richardii); contains Pfam PF00046: Homeobox domain \| chr2:8056745-8058295 REVERSE \| Aliases: F24H14.10, F24H14_10, HB 2 | 3.2 | 4.2 | -1.0 | -5.4 | 0.65% | 5.1 |

| Rank | Description | Sync | Root | M | t | adj.q | B |
|---|---|---|---|---|---|---|---|
| 3805 | AT5G46050.1 \| proton-dependent oligopeptide transport (POT) family protein, contains Pfam profile: PF00854 POT family \| chr5:18692262-18696373 REVERSE \| Aliases: MCL19.10, MCL19_10 | 2.9 | 4.3 | -1.4 | -5.4 | 0.65% | 5.1 |
| 3806 | AT3G63330.2 \| similar to Snf1-related protein kinase, putative [Arabidopsis thaliana] (TAIR:At5g39440.1); similar to protein kinase [Cryptosporidium hominis] (GB:EAL35993.1); contains InterPro domain Serine/threonine protein kinase (InterPro:IPR002290); contains InterPro domain Protein kinase (InterPro:IPR000719) \| chr3:23402603-23405738 REVERSE \| Aliases: MAA21.2 | 3.8 | 4.9 | -1.1 | -5.4 | 0.65% | 5.1 |
| 3807 | AT2G28305.1 \| expressed protein, contains Pfam profile PF03641: decarboxylase family protein \| chr2:12088190-12091741 FORWARD \| Aliases: T1B3.18 | 2.6 | 3.1 | -0.6 | -5.4 | 0.65% | 4.8 |
| 3808 | AT1G12950.1 \| MATE efflux family protein, similar to ripening regulated protein DDTFR18 (Lycopersicon esculentum) GI:12231296; contains Pfam profile PF01554: MatE \| chr1:4419768-4422632 FORWARD \| Aliases: F13K23.21, F13K23_21 | 3.1 | 4.4 | -1.3 | -5.4 | 0.65% | 5.1 |
| 3811 | AT4G14280.1 \| expressed protein \| chr4:8222513-8225069 FORWARD \| Aliases: DL3180W, FCAALL.154 | 2.4 | 3.2 | -0.8 | -5.4 | 0.65% | 5.0 |
| 3815 | AT5G13750.3 \| similar to sugar transporter family protein [Arabidopsis thaliana] (TAIR:At5g13740.1); similar to putative major facilitator superfamily antiporter [Oryza sativa (japonica cultivar-group)] (GB:BAD81140.1); contains InterPro domain Major facilitator superfamily (MFS) (InterPro:IPR007114); contains InterPro domain Tetracycline resistance protein (InterPro:IPR001958) \| chr5:4438203-4441570 FORWARD \| Aliases: MXE10.2, MXE10_2 | 6.1 | 7.3 | -1.2 | -5.4 | 0.65% | 5.1 |
| 3816 | AT5G13110.1 \| Symbol: G6PD2 \| Encodes a plastidic glucose-6-phosphate dehydrogenase that is sensitive to reduction by DTT and whose mRNA is most highly expressed in root. \| chr5:4158814-4161823 FORWARD \| Aliases: G6PD2, GLUCOSE 6 PHOSPHATE DEHYDROGENASE 2, T19L5.70, T19L5_70 | 9.1 | 10.6 | -1.5 | -5.4 | 0.66% | 5.1 |
| 3817 | AT3G02880.1 \| leucine-rich repeat transmembrane protein kinase, putative, contains Pfam profiles: PF00069 Eukaryotic protein kinase domain, PF00560 Leucine Rich Repeat (5 copies) \| chr3:634660-637289 FORWARD \| Aliases: F13E7.17, F13E7_17 | 6.3 | 8.6 | -2.3 | -5.4 | 0.66% | 5.1 |
| 3818 | AT5G62580.1 \| Symbol: VEX1 \| Encodes a protein that is expressed in vegetative cells of pollen. \| chr5:25137089-25139752 FORWARD \| Aliases: K19B1.19, K19B1_19, VEGETATIVE CELL EXPRESSED1, VEX1 | 5.2 | 6.3 | -1.1 | -5.4 | 0.66% | 5.1 |
| 3820 | AT1G80350.1 \| Symbol: ERH3 \| katanin 1 (KTN1), identical to katanin 1 (KTN1) (Arabidopsis thaliana) GI:14133602 \| chr1:30210216-30213097 REVERSE \| Aliases: AAA1, ATKTN1, BOT1, BOTERO 1, CAD ATPASE, ECTOPIC ROOT HAIR 3, F5I6.10, F5I6_10, FAT ROOT, FRA2, FRAGILE FIBER 2, FRC2, FTR, FURCA2, KATANIN 1, KTN1, LUE1 | 6.0 | 7.8 | -1.8 | -5.4 | 0.66% | 5.1 |
| 3821 | AT4G19840.1 \| Symbol: ATPP2 A1 \| lectin-related, similar to PP2 lectin polypeptide (Cucurbita maxima) GI:410437 \| chr4:10774286-10775911 FORWARD \| Aliases: T16H5.200, T16H5_200 | 4.4 | 5.7 | -1.3 | -5.4 | 0.66% | 5.1 |
| 3824 | AT4G28770.1 \| expressed protein \| chr4:14211328-14213677 REVERSE \| Aliases: F16A16.120, F16A16_120 | 7.5 | 9.2 | -1.7 | -5.4 | 0.66% | 5.0 |
| 3827 | AT1G68820.1 \| membrane protein, putative, contains 7 transmembrane domains; similar to inhibitor of apoptosis-2 IAP-2 (GI:20043383) (Mamestra configurata nucleopolyhedrovirus) \| chr1:25869337-25872648 FORWARD \| Aliases: F14K14.7, F14K14_7 | 5.7 | 7.2 | -1.5 | -5.4 | 0.66% | 5.1 |
| 3830 | AT4G31840.1 \| plastocyanin-like domain-containing protein \| chr4:15401646-15402623 FORWARD \| Aliases: F11C18.40, F11C18_40 | 4.2 | 5.8 | -1.5 | -5.4 | 0.66% | 5.1 |
| 3832 | AT2G38800.1 \| calmodulin-binding protein-related, contains similarity to potato calmodulin-binding protein PCBP GI:17933110 from (Solanum tuberosum) \| chr2:16224077-16226237 FORWARD \| Aliases: F13I13.3, F13I13_3 | 8.2 | 10.0 | -1.8 | -5.4 | 0.66% | 5.1 |
| 3835 | AT1G05450.2 \| protease inhibitor/seed storage/lipid transfer protein (LTP)-related, similar to geranyl diphosphate synthase large subunit (Mentha x piperita) GI:6449052 \| chr1:1599955-1601624 FORWARD \| Aliases: T25N20.10, T25N20_10 | 2.7 | 3.4 | -0.7 | -5.4 | 0.66% | 4.7 |
| 3838 | AT4G23690.1 \| disease resistance-responsive family protein / dirigent family protein, similar to disease resistance response protein 206-d (Pisum sativum) gi:508844:gb:AAB18669; similar to dirigent protein (Forsythia x intermedia) gi:6694693:gb:AAF25357 \| chr4:12338881-12339757 REVERSE \| Aliases: F9D16.160, F9D16_160 | 5.0 | 8.0 | -3.0 | -5.4 | 0.66% | 4.7 |
| 3839 | AT4G21310.1 \| expressed protein, predicted protein, Arabidopsis thaliana \| chr4:11339058-11340080 REVERSE \| Aliases: T6K22.40, T6K22_40 | 4.1 | 5.6 | -1.5 | -5.4 | 0.66% | 5.1 |
| 3841 | AT2G45170.2 \| autophagy 8e (APG8e), identical to autophagy 8e (Arabidopsis thaliana) GI:19912159; contains Pfam profile PF02991: Microtubule associated protein 1A/1B, light chain 3 \| chr2:18631456-18632773 FORWARD \| Aliases: T14P1.2 | 7.0 | 8.8 | -1.8 | -5.4 | 0.66% | 5.1 |
| 3842 | AT3G53340.1 \| CCAAT-box binding transcription factor, putative, similar to CAAT-box DNA binding protein subunit B (NF-YB) (SP:P25209) (GI:22380) (Zea mays); contains Pfam PF00808 : Histone-like transcription factor (CBF/NF-Y) and archaeal histone | 6.0 | 7.6 | -1.5 | -5.4 | 0.66% | 5.1 |

| Rank | Description | Sync | Root | M | t | adj.q | B |
|---|---|---|---|---|---|---|---|
| 3845 | AT1G65960.1 \| Symbol: GAD2 \| similar to glutamate decarboxylase 1 (GAD 1) [Arabidopsis thaliana] (TAIR:At5g17330.1); similar to glutamate decarboxylase [Nicotiana tabacum] (GB:AAM48129.1); similar to DCE_PETHY Glutamate decarboxylase (GAD) (GB:Q07346); contains InterPro domain Pyridoxal-dependent decarboxylase (InterPro:IPR002129) \| chr1:24558084-24561314 FORWARD \| Aliases: F12P19.12, F12P19_12, GLUTAMATE DECARBOXYLASE 2 | 7.5 | 9.4 | -1.9 | -5.4 | 0.66% | 5.1 |
| 3846 | AT4G24170.1 \| kinesin motor family protein, contains Pfam domain, PF00225: Kinesin motor domain \| chr4:12543216-12546815 FORWARD \| Aliases: T19F6.1 | 2.3 | 3.2 | -0.9 | -5.4 | 0.66% | 4.9 |
| 3850 | AT3G27560.1 \| Symbol: ATN1 \| protein kinase (ATN1), almost identical (1 amino acid difference) to protein kinase ATN1 (Arabidopsis thaliana) gi:1054633:emb:CAA63387 \| chr3:10211668-10214241 REVERSE \| Aliases: ATN, MMJ24.11 | 7.4 | 8.7 | -1.3 | -5.4 | 0.67% | 5.1 |
| 3851 | AT5G56230.1 \| prenylated rab acceptor (PRA1) family protein, contains Pfam profile PF03208: Prenylated rab acceptor (PRA1) \| chr5:22775994-22776597 REVERSE \| Aliases: K24C1.4, K24C1_4 | 4.2 | 5.4 | -1.3 | -5.4 | 0.67% | 5.1 |
| 3858 | AT3G23590.1 \| expressed protein \| chr3:8467456-8473626 FORWARD \| Aliases: MDB19.7 | 5.8 | 6.9 | -1.1 | -5.4 | 0.67% | 5.0 |
| 3860 | AT1G11050.1 \| protein kinase family protein, contains protein kinase domain, Pfam:PF00069 \| chr1:3681888-3684169 FORWARD \| Aliases: T19D16.6, T19D16_6 | 4.2 | 5.9 | -1.7 | -5.4 | 0.67% | 5.1 |
| 3862 | AT3G09010.1 \| protein kinase family protein, contains protein kinase domain, Pfam:PF00069 \| chr3:2749958-2752281 FORWARD \| Aliases: T16O11.3 | 3.8 | 4.8 | -1.0 | -5.4 | 0.67% | 5.1 |
| 3864 | AT2G16900.1 \| expressed protein \| chr2:7330697-7333303 REVERSE \| Aliases: F12A24.8, F12A24_8 | 5.6 | 6.7 | -1.1 | -5.4 | 0.67% | 5.0 |
| 3866 | AT1G06000.1 \| UDP-glucoronosyl/UDP-glucosyl transferase family protein, contains similarity to UDPG glucosyltransferase GB:AAB62270 GI:2232354 from (Solanum berthaultii) \| chr1:1820307-1821892 REVERSE \| Aliases: T21E18.5, T21E18_5 | 6.8 | 8.2 | -1.4 | -5.4 | 0.67% | 5.0 |
| 3867 | AT2G38410.1 \| VHS domain-containing protein / GAT domain-containing protein, weak similarity to hepatocyte growth factor-regulated tyrosine kinase substrate HRS isoform 2 (Homo sapiens) GI:9022389; contains Pfam profiles PF00790: VHS domain, PF03127: GAT domain \| chr2:16093708-16097286 REVERSE \| Aliases: T19C21.10, T19C21_10 | 3.7 | 5.0 | -1.3 | -5.4 | 0.67% | 5.0 |
| 3869 | AT2G22930.1 \| glycosyltransferase family protein, contains Pfam profile: PF00201 UDP-glucoronosyl and UDP-glucosyl transferase | 3.0 | 4.2 | -1.2 | -5.4 | 0.68% | 5.1 |
| 3870 | AT5G48060.1 \| C2 domain-containing protein, contains INTERPRO:IPR000008 C2 domain \| chr5:19492522-19496228 FORWARD \| Aliases: MDN11.14, MDN11_14 | 3.1 | 4.1 | -1.0 | -5.4 | 0.68% | 5.1 |
| 3871 | AT1G33080.2 \| MATE efflux family protein, similar to ripening regulated protein DDTFR18 (Lycopersicon esculentum) GI:12231296; contains Pfam profile PF01554: Uncharacterized membrane protein family \| chr1:11985699-11990391 FORWARD \| Aliases: T9L6.14, T9L6_14 | 3.0 | 4.1 | -1.1 | -5.4 | 0.68% | 5.1 |
| 3872 | AT5G50900.1 \| armadillo/beta-catenin repeat family protein, armadillo/beta-catenin-like repeats, Pfam:PF00514 \| chr5:20722030-20724004 REVERSE \| Aliases: K3K7.4, K3K7_4 | 8.1 | 9.2 | -1.1 | -5.4 | 0.68% | 5.0 |
| 3874 | AT3G52740.1 \| expressed protein \| chr3:19557352-19557942 REVERSE \| Aliases: F3C22.140 | 7.0 | 8.2 | -1.2 | -5.4 | 0.68% | 5.1 |
| 3875 | AT1G49470.1 \| expressed protein, contains Pfam profile PF04819: Family of unknown function (DUF716) (Plant viral-response family) \| chr1:18314162-18315302 FORWARD \| Aliases: F13F21.10, F13F21_10 | 6.4 | 7.4 | -1.0 | -5.4 | 0.68% | 5.0 |
| 3876 | AT2G47630.1 \| esterase/lipase/thioesterase family protein, similar to monoglyceride lipase from (Homo sapiens) GI:14594904, (Mus musculus) GI:2632162; contains Interpro entry IPR000379 \| chr2:19541573-19544073 FORWARD \| Aliases: F17A22.2 | 6.9 | 9.0 | -2.1 | -5.4 | 0.68% | 5.0 |
| 3877 | AT1G73750.1 \| expressed protein, contains Pfam profile: PF00561 alpha/beta hydrolase fold \| chr1:27740291-27742740 FORWARD \| Aliases: F25P22.17, F25P22_17 | 4.5 | 6.1 | -1.7 | -5.4 | 0.68% | 5.1 |
| 3886 | AT1G58110.2 \| similar to bZIP family transcription factor [Arabidopsis thaliana] (TAIR:At1g35490.1); similar to putative transcription activator RF2a [Oryza sativa (japonica cultivar-group)] (GB:BAD87301.1); contains InterPro domain Basic-leucine zipper (bZIP) transcription factor (InterPro:IPR004827) | 3.4 | 5.5 | -2.0 | -5.3 | 0.68% | 5.1 |
| 3888 | AT1G31710.1 \| copper amine oxidase, putative, similar to copper amine oxidase (Lens culinaris) gi:15451834:gb:AAB34918 | 3.9 | 4.8 | -0.9 | -5.3 | 0.69% | 5.0 |
| 3889 | AT5G40340.1 \| PWWP domain-containing protein, KED, Nicotiana tabacum, EMBL:AB009883 \| chr5:16148882-16151908 REVERSE \| Aliases: MPO12.6, MPO12_6 | 4.0 | 7.1 | -3.1 | -5.3 | 0.69% | 5.0 |
| 3890 | AT1G74940.1 \| senescence-associated protein-related, similar to senescence-associated protein SAG102 (GI:22331931) (Arabidopsis thaliana) \| chr1:28149720-28150839 FORWARD \| Aliases: F25A4.9, F25A4_9 | 8.0 | 9.4 | -1.5 | -5.3 | 0.69% | 5.0 |

| Rank | Description | Sync | Root | M | t | adj.q | B |
|---|---|---|---|---|---|---|---|
| 3893 | AT4G33430.1 \| Symbol: BAK1 \| brassinosteroid insensitive 1-associated receptor kinase 1 (BAK1) / somatic embryogenesis receptor-like kinase 3 (SERK3), identical to SP:Q94F62 BRASSINOSTEROID INSENSITIVE 1-associated receptor kinase 1 precursor (EC 2.7.1.37) (BRI1-associated receptor kinase 1) (Somatic embryogenesis receptor-like kinase 3) {Arabidopsis thaliana}; contains Pfam domains PF00560: Leucine Rich Repeat and PF00069: Protein kinase domain; identical to cDNA somatic embryogenesis receptor-like kinase 3 (SERK3) GI:14573458 | 6.4 | 8.7 | -2.4 | -5.3 | 0.69% | 5.1 |
| 3894 | AT2G40230.1 \| transferase family protein, similar to taxadienol acetyl transferase from Taxus cuspidata (gi:6978038); contains Pfam transferase family domain PF002458 | 3.8 | 5.1 | -1.3 | -5.3 | 0.69% | 5.0 |
| 3895 | AT5G54500.1 \| Symbol: FQR1 \| quinone reductase, putative, similar to 1,4-benzoquinone reductase (Phanerochaete chrysosporium)(GI:4454993); contains flavodoxin domain PF00258 | 9.0 | 10.3 | -1.3 | -5.3 | 0.69% | 5.0 |
| 3898 | AT1G71980.1 \| protease-associated zinc finger (C3HC4-type RING finger) family protein, identical to ReMembR-H2 protein JR702 (Arabidopsis thaliana) gi:6942149:gb:AAF32326; contains Pfam domain, PF02225: protease-associated (PA) domain and Pfam domain, PF00097: Zinc finger, C3HC4 type (RING finger); identical to cDNA ReMembR-H2 protein JR702 mRNA, partial cds GI:6942148 \| chr1:27101628-27103801 FORWARD \| Aliases: F17M19.13, F17M19_13 | 5.3 | 7.1 | -1.8 | -5.3 | 0.69% | 5.1 |
| 3899 | AT3G03560.1 \| expressed protein \| chr3:853976-856493 REVERSE \| Aliases: T21P5.2 | 4.0 | 5.0 | -1.0 | -5.3 | 0.69% | 5.0 |
| 3900 | AT1G63720.1 \| expressed protein, similar to putative protein GB:CAA18164 (Arabidopsis thaliana) \| chr1:23639519-23641178 REVERSE \| Aliases: F24D7.9, F24D7_9 | 3.8 | 4.6 | -0.8 | -5.3 | 0.69% | 5.0 |
| 3901 | AT2G27550.1 \| Symbol: ATC \| centroradialis protein, putative (CEN), strong similarity to SP:Q41261 CENTRORADIALIS protein {Antirrhinum majus}; contains Pfam profile PF01161: Phosphatidylethanolamine-binding protein \| chr2:11780330-11781758 FORWARD \| Aliases: ARABIDOPSIS THALIANA CENTRORADIALIS, ATC, F10A12.20 | 3.5 | 6.9 | -3.4 | -5.3 | 0.69% | 5.0 |
| 3903 | AT1G18570.1 \| Symbol: MYB51 \| myb family transcription factor (MYB51), contains PFAM profile: PF00249 | 3.0 | 4.5 | -1.5 | -5.3 | 0.69% | 5.1 |
| 3904 | AT5G04340.1 \| Symbol: C2H2 \| zinc finger (C2H2 type) family protein, contains Pfam domain, PF00096: Zinc finger, C2H2 type \| chr5:1216132-1217107 REVERSE \| Aliases: C2H2 ZINC FINGER TRANSCRIPTION FACTOR, T19N18.70, T19N18_70 | 4.0 | 6.2 | -2.2 | -5.3 | 0.69% | 4.9 |
| 3907 | AT1G58180.3 \| similar to carbonic anhydrase family protein / carbonate dehydratase family protein [Arabidopsis thaliana] (TAIR:At4g33580.1); similar to carbonic anhydrase 2 [Flaveria bidentis] (GB:AAO17573.1); contains InterPro domain Carbonic anhydrase, prokaryotic (InterPro:IPR001765) \| chr1:21541600-21543579 REVERSE \| Aliases: T15M6.18 | 5.1 | 6.5 | -1.3 | -5.3 | 0.69% | 5.0 |
| 3910 | AT2G31880.1 \| leucine-rich repeat transmembrane protein kinase, putative \| chr2:13561973-13564054 FORWARD \| Aliases: F20M17.8, F20M17_8 | 5.0 | 6.7 | -1.7 | -5.3 | 0.69% | 5.0 |
| 3911 | AT1G04970.2 \| lipid-binding serum glycoprotein family protein, low similarity to SP:P17213 Bactericidal permeability-increasing protein precursor (BPI) {Homo sapiens}; contains Pfam profile PF02886: LBP / BPI / CETP family, C-terminal domain \| chr1:1411889-1413625 FORWARD \| Aliases: F13M7.4, F13M7_4 | 7.3 | 9.3 | -2.0 | -5.3 | 0.70% | 5.0 |
| 3915 | AT3G20150.1 \| kinesin motor family protein, contains Pfam domain, PF00225: Kinesin motor domain \| chr3:7031418-7036505 FORWARD \| Aliases: MAL21.18 | 2.8 | 3.9 | -1.1 | -5.3 | 0.70% | 5.0 |
| 3917 | AT3G27390.1 \| expressed protein \| chr3:10134450-10137730 REVERSE \| Aliases: K1G2.10 | 3.6 | 5.1 | -1.6 | -5.3 | 0.70% | 5.0 |
| 3920 | AT5G20610.1 \| expressed protein \| chr5:6968692-6972997 FORWARD \| Aliases: T1M15.10, T1M15_10 | 4.9 | 6.9 | -2.0 | -5.3 | 0.70% | 5.0 |
| 3922 | AT1G03840.2 \| similar to zinc finger (C2H2 type) family protein [Arabidopsis thaliana] (TAIR:At5g44160.1); similar to zinc finger protein-like [Oryza sativa (japonica cultivar-group)] (GB:NP_913610.1); contains InterPro domain Zn-finger, C2H2 type (InterPro:IPR007087) \| chr1:967520-970057 REVERSE \| Aliases: F21M11.25, F21M11_25 | 2.6 | 3.3 | -0.8 | -5.3 | 0.70% | 4.9 |
| 3926 | AT4G33960.1 \| expressed protein \| chr4:16277371-16277784 FORWARD \| Aliases: F17I5.150, F17I5_150 | 5.1 | 7.3 | -2.2 | -5.3 | 0.70% | 4.9 |
| 3928 | AT1G49430.1 \| Symbol: LACS2 \| long-chain-fatty-acid--CoA ligase / long-chain acyl-CoA synthetase, nearly identical to acyl CoA synthetase (MF45P) GI:1617268 from (Brassica napus) \| chr1:18294712-18299493 FORWARD \| Aliases: F13F21.14, F13F21_14, LONG CHAIN ACYL COA SYNTHETASE 2 | 3.0 | 4.5 | -1.5 | -5.3 | 0.70% | 5.0 |
| 3936 | AT3G21560.1 \| UDP-glucosyltransferase, putative, similar to UDP-glucose:sinapate glucosyltransferase GI:9794913 from (Brassica napus) \| chr3:7595812-7597583 FORWARD \| Aliases: MIL23.13 | 5.1 | 6.0 | -0.9 | -5.3 | 0.71% | 4.9 |
| 3938 | AT1G32100.1 \| pinoresinol-lariciresinol reductase, putative, similar to pinoresinol-lariciresinol reductase TH1 (Tsuga heterophylla)(GI:7578915); contains isoflavone reductase domain PF02716 | 5.9 | 9.1 | -3.2 | -5.3 | 0.71% | 5.0 |

| Rank | Description | Sync | Root | M | t | adj.q | B |
|---|---|---|---|---|---|---|---|
| 3940 | AT5G58720.1 \| PRLI-interacting factor, putative, similar to PRLI-interacting factor N (Arabidopsis thaliana) GI:11139276; contains Pfam profile PF01713: Smr domain \| chr5:23736607-23740363 REVERSE \| Aliases: MZN1.26, MZN1_26 | 5.0 | 6.6 | -1.6 | -5.3 | 0.71% | 5.0 |
| 3943 | AT5G60120.1 \| Symbol: TOE2 \| AP2 domain-containing transcription factor, putative, Similar to Floral homeotic protein APETALA2 protein (SP:P47927) (Arabidopsis thaliana); homolog HAP2, Hyacinthus orientalis, EMBL:AF134116 | 3.2 | 5.8 | -2.6 | -5.3 | 0.71% | 4.8 |
| 3944 | AT5G05140.1 \| transcription elongation factor-related, low similarity to transcription elongation factor TFIIS.h (Mus musculus) GI:3288547, elongin A (Mus musculus) GI:6009624 \| chr5:1519914-1522668 FORWARD \| Aliases: MUG13.28 | 4.0 | 5.7 | -1.7 | -5.3 | 0.71% | 5.0 |
| 3945 | AT4G19660.1 \| Symbol: NPR4 \| ankyrin repeat family protein / BTB/POZ domain-containing protein, contains Pfam domain, PF00023: Ankyrin repeat and Pfam domain, PF00651: BTB/POZ domain \| chr4:10696276-10698253 REVERSE \| Aliases: NPR1 LIKE PROTEIN 4, T16H5.20, T16H5_20 | 4.3 | 5.9 | -1.7 | -5.3 | 0.71% | 5.0 |
| 3947 | AT1G09155.1 \| Symbol: ATPP2 B15 \| SKP1 interacting partner 3-related, low similarity to SKP1 interacting partner 3 (Arabidopsis thaliana) GI:10716951 | 2.7 | 6.3 | -3.6 | -5.3 | 0.71% | 4.8 |
| 3949 | AT1G75190.1 \| expressed protein \| chr1:28223043-28223888 FORWARD \| Aliases: F22H5.8, F22H5_8 | 3.3 | 5.9 | -2.6 | -5.3 | 0.71% | 5.0 |
| 3950 | AT1G14370.1 \| Symbol: APK2A \| protein kinase (APK2a), identical to protein kinase APK2a GI:2852447 from (Arabidopsis thaliana) \| chr1:4915662-4918303 FORWARD \| Aliases: F14L17.14, F14L17_14, PROTEIN KINASE 2A | 5.0 | 6.4 | -1.4 | -5.3 | 0.71% | 5.0 |
| 3952 | AT1G74100.1 \| sulfotransferase family protein, similar to SP:P52837 Flavonol 4'-sulfotransferase (EC 2.8.2.-) (F4-ST) {Flaveria chloraefolia}; contains Pfam profile PF00685: Sulfotransferase domain \| chr1:27868006-27869355 REVERSE \| Aliases: F2P9.3, F2P9_3 | 6.6 | 8.7 | -2.1 | -5.3 | 0.71% | 5.0 |
| 3953 | AT1G17860.1 \| trypsin and protease inhibitor family protein / Kunitz family protein, similar to LeMir (miraculin homolog) GI:2654440 from (Lycopersicon esculentum), tumor-related protein (Nicotiana tabacum) GI:1762933; contains Pfam profile PF00197: Trypsin and protease inhibitor \| chr1:6149294-6150031 FORWARD \| Aliases: F2H15.9, F2H15_9 | 6.8 | 10.0 | -3.1 | -5.3 | 0.72% | 5.0 |
| 3955 | AT1G76360.1 \| protein kinase, putative, similar to protein kinase APK1B, SWISS-PROT:P46573; contains protein kinase domain, Pfam:PF00069 \| chr1:28647254-28651489 REVERSE \| Aliases: F15M4.14, F15M4_14 | 3.9 | 5.1 | -1.3 | -5.3 | 0.72% | 5.0 |
| 3957 | AT4G15500.1 \| UDP-glucoronosyl/UDP-glucosyl transferase family protein, contains Pfam profile: PF00201 UDP-glucoronosyl and UDP-glucosyl transferase \| chr4:8857093-8858520 REVERSE \| Aliases: DL3790C, FCAALL.307 | 4.4 | 7.2 | -2.8 | -5.3 | 0.72% | 4.9 |
| 3958 | AT1G49050.1 \| aspartyl protease family protein, contains Pfam PF00026: Eukaryotic aspartyl protease; contains similarity to nucellin GI:2290203 from (Hordeum vulgare) | 6.8 | 9.2 | -2.3 | -5.3 | 0.72% | 5.0 |
| 3959 | AT1G30640.1 \| protein kinase, putative, contains protein kinase domain, Pfam:PF00069 \| chr1:10861279-10864682 FORWARD \| Aliases: T5I8.9, T5I8_9 | 5.2 | 6.8 | -1.6 | -5.3 | 0.72% | 5.0 |
| 3961 | AT2G14900.1 \| gibberellin-regulated family protein, similar to SP:P46690 Gibberellin-regulated protein 4 precursor {Arabidopsis thaliana} GASA4; contains Pfam profile PF02704: Gibberellin regulated protein \| chr2:6411259-6412412 FORWARD \| Aliases: T26I20.6, T26I20_6 | 3.7 | 6.1 | -2.4 | -5.3 | 0.72% | 4.8 |
| 3964 | AT4G07820.1 \| pathogenesis-related protein, putative, similar to SP:P33154 Pathogenesis-related protein 1 precursor (PR-1) {Arabidopsis thaliana}; contains Pfam profile PF00188: SCP-like extracellular protein \| chr4:4636861-4637348 REVERSE \| Aliases: F5K24.6, F5K24_6 | 4.0 | 5.5 | -1.5 | -5.3 | 0.72% | 5.0 |
| 3965 | AT5G28020.5 \| Symbol: ATCYSD2 \| similar to cysteine synthase, putative / O-acetylserine (thiol)-lyase, putative / O-acetylserine sulfhydrylase, putative [Arabidopsis thaliana] (TAIR:At5g28030.1); similar to cysteine synthase, putative / O-acetylserine (thiol)-lyase, putative / O-acetylserine sulfhydrylase, putative [Arabidopsis thaliana] (TAIR:At5g28030.2); similar to cysteine synthase [Nicotiana plumbaginifolia] (GB:AAR18402.1); contains InterPro domain Pyridoxal-5'-phosphate-dependent enzyme, beta family (InterPro:IPR001926) \| chr5:10026191-10027872 REVERSE \| Aliases: CYSTEINE SYNTHASE, F15F15.90, F15F15_90 | 4.1 | 6.2 | -2.1 | -5.3 | 0.72% | 5.0 |
| 3967 | AT5G02010.1 \| member of KPP-like gene family, homolog of KPP (kinase partner protein) gene in tomato; expressed protein, contains Pfam profile PF03759: Domain of unknown function (DUF315) \| chr5:383150-385847 FORWARD \| Aliases: KINASE PARTNER PROTEIN LIKE, KPP LIKE, T7H20.60, T7H20_60 | 3.1 | 4.0 | -0.9 | -5.3 | 0.72% | 4.9 |
| 3968 | AT4G24240.1 \| Symbol: WRKY7 \| WRKY family transcription factor, contains Pfam profile: PF03106 WRKY DNA -binding domain \| chr4:12571776-12573735 FORWARD \| Aliases: ATWRKY7, T22A6.70, T22A6_70 | 3.6 | 4.7 | -1.1 | -5.3 | 0.72% | 5.0 |
| 3969 | AT2G36810.1 \| expressed protein \| chr2:15432818-15443960 REVERSE \| Aliases: F13K3.21, F13K3_21 | 3.4 | 5.7 | -2.4 | -5.3 | 0.72% | 5.0 |
| 3982 | AT1G25280.2 \| F-box family protein / tubby family protein, similar to Tubby protein homolog (SP:O88808) (Rattus norvegicus); contains Pfam PF00646: F-box domain and Pfam PF01167: Tub family; | 7.0 | 8.1 | -1.1 | -5.3 | 0.73% | 4.9 |
| 3983 | AT2G34910.1 \| expressed protein \| chr2:14733979-14735126 FORWARD \| Aliases: F19I3.14, F19I3_14 | 3.4 | 5.7 | -2.4 | -5.3 | 0.73% | 4.6 |

| Rank | Description | Sync | Root | M | t | adj.q | B |
|---|---|---|---|---|---|---|---|
| 3984 | AT5G65630.1 \| DNA-binding bromodomain-containing protein, similar to 5.9 kb fsh membrane protein (Drosophila melanogaster) GI:157455; contains Pfam profile PF00439: Bromodomain \| chr5:26243058-26245675 REVERSE \| Aliases: K21L13.15, K21L13_15 | 4.9 | 6.6 | -1.6 | -5.3 | 0.73% | 4.9 |
| 3987 | AT2G31090.1 \| expressed protein \| chr2:13263215-13263788 FORWARD \| Aliases: T16B12.10, T16B12_10 | 9.6 | 10.8 | -1.2 | -5.3 | 0.74% | 4.8 |
| 3989 | AT2G42760.1 \| expressed protein \| chr2:17803061-17804308 REVERSE \| Aliases: F7D19.24, F7D19_24 | 8.2 | 9.9 | -1.7 | -5.3 | 0.74% | 4.9 |
| 3990 | AT5G23530.1 \| expressed protein, contains similarity to PrMC3 (Pinus radiata) GI:5487873 \| chr5:7932969-7934439 REVERSE \| Aliases: MQM1.21, MQM1_21 | 3.1 | 4.4 | -1.2 | -5.3 | 0.74% | 5.0 |
| 3994 | AT3G27270.1 \| expressed protein \| chr3:10074517-10075397 FORWARD \| Aliases: K17E12.9 | 4.4 | 5.7 | -1.3 | -5.3 | 0.74% | 5.0 |
| 3995 | AT2G28780.1 \| expressed protein \| chr2:12347111-12350498 REVERSE \| Aliases: F8N16.7, F8N16_7 | 3.6 | 4.9 | -1.4 | -5.3 | 0.74% | 5.0 |
| 3997 | AT1G60010.1 \| expressed protein \| chr1:22099025-22100242 REVERSE \| Aliases: T2K10.6, T2K10_6 | 4.7 | 6.7 | -1.9 | -5.3 | 0.74% | 4.9 |
| 4000 | AT2G19710.1 \| expressed protein, contains Pfam profile: PF03398 eukaryotic protein of unknown function, DUF292 \| chr2:8513216-8517163 FORWARD \| Aliases: F6F22.26, F6F22_26 | 4.0 | 5.0 | -1.0 | -5.3 | 0.75% | 4.9 |
| 4003 | AT5G57670.1 \| protein kinase family protein, contains protein kinase domain, Pfam:PF00069 \| chr5:23377626-23379690 REVERSE \| Aliases: MRI1.2, MRI1_2 | 2.1 | 2.4 | -0.4 | -5.3 | 0.75% | 4.3 |
| 4004 | AT2G20740.3 \| expressed protein \| chr2:8943759-8944591 FORWARD \| Aliases: AT2G20730, F5H14.29, F5H14_29 | 5.7 | 6.9 | -1.1 | -5.3 | 0.75% | 4.9 |
| 4007 | AT5G45380.1 \| sodium:solute symporter family protein, contains Pfam profile: PF00474 sodium:solute symporter family \| chr5:18408351-18413053 FORWARD \| Aliases: MFC19.5, MFC19_5 | 4.7 | 6.5 | -1.8 | -5.3 | 0.75% | 4.9 |
| 4008 | AT2G13650.2 \| Symbol: GONST1 \| | 5.7 | 8.1 | -2.4 | -5.3 | 0.75% | 5.0 |
| 4009 | AT2G42490.1 \| copper amine oxidase, putative, similar to copper methylamine oxidase precursor (MAOXII) (Arthrobacter sp.) SWISS-PROT:Q07123 \| chr2:17698497-17702926 REVERSE \| Aliases: MHK10.21, MHK10_21 | 8.6 | 10.1 | -1.4 | -5.3 | 0.75% | 4.8 |
| 4013 | AT2G17500.4 \| similar to auxin efflux carrier family protein [Arabidopsis thaliana] (TAIR:At5g65980.1); similar to unknown protein [Oryza sativa (japonica cultivar-group)] (GB:AAW56872.1); contains InterPro domain Auxin Efflux Carrier (InterPro:IPR004776) \| chr2:7613997-7616360 FORWARD \| Aliases: MJB20.6, MJB20_6 | 3.6 | 5.6 | -1.9 | -5.3 | 0.75% | 4.9 |
| 4014 | AT1G12680.1 \| protein kinase family protein, contains protein kinase domain, Pfam:PF00069 \| chr1:4319884-4322943 REVERSE \| Aliases: T12C24.32, T12C24_32 | 5.3 | 6.6 | -1.3 | -5.2 | 0.75% | 4.9 |
| 4016 | AT2G35730.1 \| heavy-metal-associated domain-containing protein, contains PS1047 Heavy-metal-associated domain contains Pfam profile PF00403: Heavy-metal-associated domain \| chr2:15027579-15028635 REVERSE \| Aliases: T20F21.3 | 2.4 | 6.0 | -3.5 | -5.2 | 0.75% | 4.9 |
| 4017 | AT3G16920.1 \| glycoside hydrolase family 19 protein, similar to class I chitinase GI:7798670 from (Arabis microphylla) | 2.8 | 6.9 | -4.1 | -5.2 | 0.75% | 4.9 |
| 4018 | AT2G28350.1 \| Symbol: ARF10 \| auxin-responsive factor (ARF10), similar to auxin response factor 10 GI:6165644 from (Arabidopsis thaliana); identical to cDNA auxin response factor 10 (ARF10) mRNA, partial cds GI:6165643 \| chr2:12121408-12123914 FORWARD \| Aliases: ARF10, AUXIN RESPONSE FACTOR 10, T1B3.13, T1B3_13 | 6.2 | 7.3 | -1.1 | -5.2 | 0.75% | 4.8 |
| 4020 | AT3G11370.1 \| DC1 domain-containing protein, contains Pfam protein PF03107 DC1 domain \| chr3:3561105-3562886 FORWARD \| Aliases: F11B9.29 | 2.1 | 4.6 | -2.5 | -5.2 | 0.75% | 4.9 |
| 4022 | AT1G19770.1 \| Symbol: ATPUP14 \| purine permease-related, low similarity to purine permease (Arabidopsis thaliana) GI:7620007; contains Pfam profile PF03151: Domain of unknown function, DUF250 \| chr1:6832341-6833693 FORWARD \| Aliases: F14P1.13, F14P1_13 | 7.7 | 9.0 | -1.3 | -5.2 | 0.75% | 4.8 |
| 4025 | AT3G13730.1 \| Symbol: CYP90D1 \| Encodes a cytochrome P-450 gene that is involved in brassinosteroid biosynthesis, most likely in the conversion step of teasterone (TE) to 3-dehydroteasterone (3DT), and/or 6-deoxoteasterone (6-deoxoTE) to 6-deoxo-3-dehydroteasterone (6-deoxo3DT); or the conversion of cathasterone (CT) to TE, and/or 6-deoxocathasterone (6-deoxoCT) to 6-deoxoTE. Member of the CYP90C CYP450 family. Similar to Cytochrome P450 90C1 (ROT3). \| chr3:4497983-4500927 REVERSE \| Aliases: MMM17.20 | 5.5 | 6.7 | -1.2 | -5.2 | 0.75% | 4.9 |
| 4028 | AT3G53510.1 \| ABC transporter family protein, breast cancer resistance protein (BCRP), Homo sapiens, EMBL:AF098951 \| chr3:19848191-19850840 REVERSE \| Aliases: F4P12.210 | 2.5 | 3.5 | -1.0 | -5.2 | 0.76% | 4.9 |
| 4030 | AT5G16600.1 \| Symbol: MYB43 \| myb family transcription factor (MYB43), contains PFAM profile: myb DNA binding domain PF00249 \| chr5:5438294-5440248 FORWARD \| Aliases: MTG13.12, MTG13_12 | 3.2 | 5.8 | -2.7 | -5.2 | 0.76% | 4.9 |

| Rank | Description | Sync | Root | M | t | adj.q | B |
|---|---|---|---|---|---|---|---|
| 4033 | AT5G60720.1 \| expressed protein, contains Pfam profile PF04784: Protein of unknown function, DUF547 \| chr5:24435455-24439460 REVERSE \| Aliases: MUP24.14, MUP24_14 | 3.2 | 5.1 | -1.9 | -5.2 | 0.76% | 4.9 |
| 4034 | AT4G11190.1 \| disease resistance-responsive family protein / dirigent family protein, similar to dirigent protein (Forsythia x intermedia) gi:6694693:gb:AAF25357; similar to disease resistance response protein 206-d (Pisum sativum) gi:508844:gb:AAB18669 \| chr4:6826673-6827379 FORWARD \| Aliases: T22B4.170, T22B4_170 | 2.9 | 3.9 | -1.0 | -5.2 | 0.76% | 4.9 |
| 4036 | AT1G18940.1 \| nodulin family protein, similar to nodulin-like protein (Arabidopsis thaliana) GI:3329368, nodule-specific protein Nlj70 (Lotus japonicus) GI:3329366 \| chr1:6543843-6545583 FORWARD \| Aliases: F14D16.8, F14D16_8 | 3.7 | 5.8 | -2.1 | -5.2 | 0.76% | 4.9 |
| 4042 | AT2G45210.1 \| auxin-responsive protein-related, weakly similar to small auxin up RNA (GI:546362) {Arabidopsis thaliana} \| chr2:18648640-18649778 FORWARD \| Aliases: F4L23.28 | 4.7 | 6.3 | -1.6 | -5.2 | 0.77% | 4.9 |
| 4046 | AT5G49830.1 \| expressed protein \| chr5:20267712-20272215 REVERSE \| Aliases: K21G20.4, K21G20_4 | 4.1 | 5.0 | -0.9 | -5.2 | 0.77% | 4.9 |
| 4047 | AT4G38220.2 \| aminoacylase, putative / N-acyl-L-amino-acid amidohydrolase, putative, similar to aminoacylase-1 (N-acyl-L-amino-acid amidohydrolase, ACY-1)(Homo sapiens) SWISS-PROT:Q03154 \| chr4:17925174-17927091 FORWARD \| Aliases: F20D10.340, F20D10_340 | 9.4 | 10.7 | -1.3 | -5.2 | 0.77% | 4.9 |
| 4048 | AT1G10090.1 \| expressed protein \| chr1:3290059-3296181 REVERSE \| Aliases: T27I1.11, T27I1_11 | 4.4 | 5.1 | -0.7 | -5.2 | 0.77% | 4.8 |
| 4049 | AT4G23496.1 \| expressed protein \| chr4:12257689-12258421 REVERSE \| Aliases: None | 3.4 | 4.3 | -0.9 | -5.2 | 0.77% | 4.8 |
| 4050 | AT1G52250.1 \| dynein light chain type 1 family protein, similar to SP:O02414 Dynein light chain LC6, flagellar outer arm {Anthocidaris crassispina}; contains Pfam profile PF01221: Dynein light chain type 1 \| chr1:19462342-19462904 REVERSE \| Aliases: F19K6.18 | 3.0 | 4.2 | -1.1 | -5.2 | 0.77% | 4.8 |
| 4054 | AT2G35770.1 \| Symbol: SCPL28 \| | 2.1 | 3.8 | -1.7 | -5.2 | 0.78% | 4.9 |
| 4056 | AT5G62000.3 \| Symbol: ARF2 \| transcriptional factor B3 family protein / auxin-responsive factor, putative (ARF1), contains Pfam profile: PF02362 B3 DNA binding domain; identical to cDNA ARF1 (auxin response factor) binding protein GI:2245393 \| chr5:24927608-24932431 FORWARD \| Aliases: ARF1 BINDING PROTEIN, ARF1 BP, AT5G62010, AUXIN RESPONSE FACTOR 2, HLS1 SUPPRESSOR, HSS, MTG10.1, MTG10_1 | 4.3 | 5.2 | -0.8 | -5.2 | 0.78% | 4.8 |
| 4059 | AT5G43880.1 \| expressed protein \| chr5:17657202-17660817 REVERSE \| Aliases: F6B6.2, F6B6_2 | 2.8 | 3.7 | -0.9 | -5.2 | 0.78% | 4.7 |
| 4060 | AT3G09320.1 \| zinc finger (DHHC type) family protein, similar to Golgi-specific DHHC zinc figer protein (Mus musculus) GI:21728103; contains Pfam profile PF01529: DHHC zinc finger domain \| chr3:2861749-2864251 REVERSE \| Aliases: F3L24.19 | 5.4 | 6.9 | -1.5 | -5.2 | 0.78% | 4.9 |
| 4061 | AT5G39320.1 \| UDP-glucose 6-dehydrogenase, putative, very strong similarity to SP:Q96558 UDP-glucose 6-dehydrogenase (EC 1.1.1.22) (UDP-Glc dehydrogenase) (UDP-GlcDH) (UDPGDH) {Glycine max}; contains Pfam profiles PF03721: UDP-glucose/GDP-mannose dehydrogenase family NAD binding domain, PF00984: UDP-glucose/GDP-mannose dehydrogenase family central domain, PF03720: UDP-glucose/GDP-mannose dehydrogenase family UDP binding domain | 4.3 | 6.3 | -2.0 | -5.2 | 0.78% | 4.9 |
| 4062 | AT4G33940.1 \| zinc finger (C3HC4-type RING finger) family protein, contains Pfam profile: PF00097 zinc finger, C3HC4 type (RING finger) \| chr4:16266035-16267786 FORWARD \| Aliases: F17I5.1 | 5.8 | 6.9 | -1.1 | -5.2 | 0.78% | 4.9 |
| 4063 | AT4G26320.1 \| Symbol: AGP13 \| arabinogalactan-protein (AGP13), identical to gi:10880503:gb:AAG24281 \| chr4:13317109-13317479 REVERSE \| Aliases: ARABINOGALACTAN PROTEIN 13, T25K17.130, T25K17_130 | 6.1 | 8.4 | -2.3 | -5.2 | 0.78% | 4.9 |
| 4065 | AT1G14780.1 \| expressed protein \| chr1:5090985-5094147 FORWARD \| Aliases: F10B6.18, F10B6_18 | 3.5 | 5.5 | -2.0 | -5.2 | 0.78% | 4.9 |
| 4067 | AT5G15790.2 \| zinc finger (C3HC4-type RING finger) family protein, contains Pfam profile: PF00097 zinc finger, C3HC4 type (RING finger) \| chr5:5148765-5151412 FORWARD \| Aliases: F14F8.170, F14F8_170 | 2.9 | 3.6 | -0.7 | -5.2 | 0.79% | 4.6 |
| 4068 | AT1G79450.2 \| LEM3 (ligand-effect modulator 3) family protein / CDC50 family protein, similar to GI:4966357; GI:4585976; GI:11994416; GI:9757735 from (Arabidopsis thaliana) \| chr1:29892582-29895072 FORWARD \| Aliases: T8K14.13, T8K14_13 | 3.6 | 6.0 | -2.4 | -5.2 | 0.79% | 4.9 |
| 4071 | AT3G46590.3 \| Symbol: TRFL1 \| similar to telomere repeat-binding protein 1 (TRP1) [Arabidopsis thaliana] (TAIR:At5g59430.2); similar to telomere repeat-binding protein 1 (TRP1) [Arabidopsis thaliana] (TAIR:At5g59430.1); similar to BPF-1 [Petroselinum crispum] (GB:CAA48413.1) \| chr3:17163760-17167212 FORWARD \| Aliases: F12A12.110, TRF LIKE 1 | 4.7 | 6.5 | -1.8 | -5.2 | 0.79% | 4.9 |
| 4072 | AT2G32430.1 \| galactosyltransferase family protein, contains Pfam profile: PF01762 galactosyltransferase \| chr2:13778373-13781579 FORWARD \| Aliases: T32F6.5, T32F6_5 | 5.4 | 6.5 | -1.1 | -5.2 | 0.79% | 4.8 |

| Rank | Description | Sync | Root | M | t | adj.q | B |
|---|---|---|---|---|---|---|---|
| 4074 | AT3G45840.1 \| similar to DC1 domain-containing protein / UV-B light-insensitive protein, putative [Arabidopsis thaliana] (TAIR:At5g59940.1); contains InterPro domain Zn-finger-like, PHD finger (InterPro:IPR001965); contains InterPro domain Cytochrome c heme-binding site (InterPro:IPR000345); contains InterPro domain DC1 domain (InterPro:IPR004146) | 3.0 | 7.3 | -4.2 | -5.2 | 0.79% | 4.9 |
| 4076 | NA | 3.7 | 7.1 | -3.4 | -5.2 | 0.79% | 4.9 |
| 4077 | AT2G39380.1 \| exocyst subunit EXO70 family protein, contains Pfam domain PF03081: Exo70 exocyst complex subunit; \| chr2:16454084-16456161 REVERSE \| Aliases: F12L6.4, F12L6_4 | 3.3 | 4.9 | -1.6 | -5.2 | 0.79% | 4.9 |
| 4078 | AT4G34600.1 \| expressed protein \| chr4:16529230-16530191 FORWARD \| Aliases: T4L20.180, T4L20_180 | 6.6 | 8.1 | -1.5 | -5.2 | 0.79% | 4.8 |
| 4081 | AT3G50280.1 \| transferase family protein, similar to anthranilate N-hydroxycinnamoyl/benzoyltransferase, Dianthus caryophyllus, PIR:T10717 (gi:2239091); contains Pfam transferase family domain PF002458 \| chr3:18648836-18650167 FORWARD \| Aliases: F11C1.120 | 3.4 | 4.2 | -0.9 | -5.2 | 0.79% | 4.8 |
| 4082 | AT1G53140.1 \| dynamin family protein, low similarity to dynamin-like protein E (Arabidopsis thaliana) GI:19423872; contains Pfam profile PF00350: Dynamin family \| chr1:19802906-19806262 FORWARD \| Aliases: F8L10.1, F8L10_1 | 5.3 | 6.0 | -0.7 | -5.2 | 0.79% | 4.6 |
| 4083 | AT2G27830.1 \| expressed protein \| chr2:11867295-11868552 FORWARD \| Aliases: F15K20.7, F15K20_7 | 3.7 | 5.8 | -2.1 | -5.2 | 0.79% | 4.7 |
| 4085 | AT4G36990.1 \| Symbol: HSF4 \| heat shock factor protein 4 (HSF4) / heat shock transcription factor 4 (HSTF4), identical to heat shock transcription factor 4 (HSF4) SP:Q96320 from (Arabidopsis thaliana) \| chr4:17440287-17442145 FORWARD \| Aliases: AP22.9, AP22_9, AT HSFB1, ATHSF4, HEAT SHOCK FACTOR 4, HEAT SHOCK TRANSCRIPTION FACTOR 4, HSFB1 | 8.6 | 11.3 | -2.7 | -5.2 | 0.80% | 4.8 |
| 4086 | AT5G41150.2 \| Symbol: UVH1 \| similar to ERCC4_CRIGR DNA repair endonuclease XPF (DNA excision repair protein ERCC-4) (GB:Q9QYM7); similar to ERCC4 [Cricetulus griseus] (GB:BAA89229.1) \| chr5:16491281-16494179 REVERSE \| Aliases: ATRAD1, MEE6.22, MEE6_22, RAD1, REPAIR ENDONUCLEASE, ULTRAVIOLET HYPERSENSITIVE 1 | 3.4 | 4.4 | -0.9 | -5.2 | 0.80% | 4.8 |
| 4090 | AT1G49660.1 \| expressed protein \| chr1:18382365-18383467 REVERSE \| Aliases: F14J22.11, F14J22_11 | 7.1 | 9.5 | -2.4 | -5.2 | 0.81% | 4.8 |
| 4091 | AT3G59900.1 \| expressed protein, hypothetical protein At2g44080 - Arabidopsis thaliana, EMBL:AC004005 \| chr3:22140703-22141434 FORWARD \| Aliases: F24G16.170 | 6.1 | 8.7 | -2.6 | -5.2 | 0.81% | 4.9 |
| 4092 | AT1G54740.1 \| expressed protein, similar to expressed protein [Arabidopsis thaliana] (TAIR:At1g22110.1); similar to hypothetical protein [Plasmodium falciparum 3D7] (GB:NP_704767.1) \| chr1:20434478-20435653 FORWARD \| Aliases: T22H22.16, T22H22_16 | 2.2 | 3.6 | -1.4 | -5.2 | 0.81% | 4.9 |
| 4094 | AT2G13820.2 \| protease inhibitor/seed storage/lipid transfer protein (LTP) family protein, contains Pfam protease inhibitor/seed storage/LTP family domain PF00234 \| chr2:5782333-5783371 REVERSE \| Aliases: F17L24.13, F17L24_13 | 6.3 | 7.5 | -1.2 | -5.2 | 0.81% | 4.9 |
| 4097 | AT5G20990.1 \| Symbol: CHL6/CNX/CNX1/SIR4 \| Involved in molybdenum cofactor (Moco) biosynthesis, inserting Mo into Molybdopterin. sir loss-of-function mutants are resistant to sirtinol, a modulator of auxin signaling. \| chr5:7128477-7133459 REVERSE \| Aliases: CHL6, CHLORATE RESISTANT 6, CNX, CNX1, COFACTOR OF NR/CHL6, MOLYBDENUM COFACTOR BIOSYNTHESIS ENZYME CNX1, SIR4, T10F18.20, T10F18_20 | 8.2 | 9.1 | -0.9 | -5.2 | 0.81% | 4.7 |
| 4098 | AT1G03380.1 \| expressed protein \| chr1:835825-840891 FORWARD \| Aliases: F21B7.1 | 5.0 | 6.3 | -1.3 | -5.2 | 0.81% | 4.8 |
| 4099 | AT2G41160.1 \| ubiquitin-associated (UBA)/TS-N domain-containing protein, contains Pfam profile PF00627: UBA/TS-N domain \| chr2:17163107-17165936 REVERSE \| Aliases: T3K9.7, T3K9_7 | 4.2 | 5.3 | -1.2 | -5.2 | 0.81% | 4.8 |
| 4103 | AT5G47620.3 \| heterogeneous nuclear ribonucleoprotein, putative / hnRNP, putative \| chr5:19319496-19321316 REVERSE \| Aliases: MNJ7.21, MNJ7_21 | 9.9 | 10.8 | -0.9 | -5.2 | 0.81% | 4.7 |
| 4105 | AT5G06850.1 \| C2 domain-containing protein, contains INTERPRO:IPR000008 C2 domain \| chr5:2126976-2129325 REVERSE \| Aliases: MOJ9.2, MOJ9_2 | 3.9 | 5.3 | -1.4 | -5.2 | 0.81% | 4.9 |
| 4106 | AT2G33990.1 \| similar to calmodulin-binding family protein [Arabidopsis thaliana] (TAIR:At3g15050.1); similar to putative SF16 protein [Oryza sativa (japonica cultivar-group)] (GB:BAD73780.1); contains InterPro domain IQ calmodulin-binding region (InterPro:IPR000048) \| chr2:14367355-14369257 REVERSE \| Aliases: T14G11.11, T14G11_11 | 4.7 | 6.0 | -1.3 | -5.2 | 0.81% | 4.8 |
| 4108 | AT1G02950.3 \| Symbol: ATGSTF4 \| similar to glutathione S-transferase, putative [Arabidopsis thaliana] (TAIR:At1g02940.1); similar to glutathione S-transferase 1 [Brassica juncea] (GB:AAP58391.1); contains InterPro domain Glutathione S-transferase, N-terminal (InterPro:IPR004045); contains InterPro domain Glutathione S-transferase, C-terminal (InterPro:IPR004046) \| chr1:665152-666451 REVERSE \| Aliases: F22D16.5, F22D16_5, GLUTATHIONE S TRANSFERASE 31, GST31 | 2.5 | 3.3 | -0.8 | -5.2 | 0.81% | 4.7 |
| 4109 | AT1G35780.1 \| expressed protein \| chr1:13278486-13281472 REVERSE \| Aliases: F14D7.8, F14D7_8 | 5.5 | 6.6 | -1.1 | -5.2 | 0.81% | 4.8 |

| Rank | Description | Sync | Root | M | t | adj.q | B |
|---|---|---|---|---|---|---|---|
| 4110 | AT1G75390.1 \| bZIP transcription factor family protein, contains Pfam profile: PF00170 bZIP transcription factor \| chr1:28295426-28296669 FORWARD \| Aliases: F1B16.8, F1B16_8 | 7.4 | 8.9 | -1.5 | -5.2 | 0.81% | 4.8 |
| 4111 | AT1G01420.1 \| UDP-glucoronosyl/UDP-glucosyl transferase family protein, contains Pfam profile: PF00201 UDP-glucoronosyl and UDP-glucosyl transferase \| chr1:154566-156011 REVERSE \| Aliases: F6F3.22, F6F3_22 | 3.4 | 4.1 | -0.8 | -5.2 | 0.81% | 4.7 |
| 4118 | AT3G61630.1 \| AP2 domain-containing transcription factor, putative, transcription factor Pti6 - Lycopersicon esculentum, PIR:T07728 \| chr3:22816155-22817499 FORWARD \| Aliases: F15G16.20 | 2.3 | 3.0 | -0.8 | -5.2 | 0.82% | 4.7 |
| 4120 | AT1G74080.1 \| Symbol: MYB122 \| | 2.7 | 4.0 | -1.3 | -5.2 | 0.82% | 4.8 |
| 4122 | AT5G48110.1 \| terpene synthase/cyclase family protein \| chr5:19518383-19521583 REVERSE \| Aliases: MDN11.20, MDN11_20 | 2.8 | 4.3 | -1.5 | -5.2 | 0.82% | 4.8 |
| 4126 | AT4G29380.1 \| protein kinase family protein / WD-40 repeat family protein, contains Pfam PF00400: WD domain, G-beta repeat; contains Pfam PF00069: Protein kinase domain; contains PF02985: HEAT repeat; similar to adaptor protein (GI:1817584) (Homo sapiens); similar to VPS15 protein (GI:6103009) (Pichia pastoris) \| chr4:14458828-14464865 FORWARD \| Aliases: F17A13.200, F17A13_200 | 6.1 | 7.3 | -1.1 | -5.1 | 0.83% | 4.8 |
| 4132 | AT4G18120.1 \| RNA recognition motif (RRM)-containing protein, Mei2-like protein, Arabidopsis thaliana, gb:D86122 \| chr4:10038890-10042023 FORWARD \| Aliases: F15J5.90, F15J5_90 | 5.8 | 7.5 | -1.6 | -5.1 | 0.83% | 4.7 |
| 4138 | AT1G29970.1 \| expressed protein, similar to GI:3128228 from (Arabidopsis thaliana) (Nature 402 (6763), 761-768 (1999)) | 6.7 | 7.5 | -0.8 | -5.1 | 0.83% | 4.7 |
| 4141 | AT3G20940.1 \| Symbol: CYP705A30 \| cytochrome P450 family protein, similar to Cytochrome P450 93A3 (P450 CP5) (SP:O81973) (Glycine max); similar to cytochrome P450 (SP:H71417) (Arabidopsis thaliana) \| chr3:7339723-7341656 FORWARD \| Aliases: MFD22.8 | 3.0 | 3.8 | -0.8 | -5.1 | 0.83% | 4.7 |
| 4143 | AT4G28550.1 \| RabGAP/TBC domain-containing protein, similar to SP:P09379 GTPase-activating protein GYP7 (Fragment) {Yarrowia lipolytica}; contains Pfam profile PF00566: TBC domain \| chr4:14110890-14113754 FORWARD \| Aliases: F20O9.250, F20O9_250 | 3.8 | 4.6 | -0.8 | -5.1 | 0.83% | 4.6 |
| 4144 | AT1G70210.1 \| Symbol: CYCD1;1 \| cyclin delta-1 (CYCD1), nearly identical to SP:P42751 Cyclin delta-1 {Arabidopsis thaliana} \| chr1:26443560-26446492 FORWARD \| Aliases: CYCD1;1, Cyclin D1;1, F20P5.7, F20P5_7 | 2.8 | 3.4 | -0.6 | -5.1 | 0.84% | 4.6 |
| 4147 | AT4G08620.1 \| Symbol: SULTR1;1 \| sulfate transporter, identical to sulfate transporter (Arabidopsis thaliana) GI:3777483 \| chr4:5500310-5506262 FORWARD \| Aliases: SULTR1.2 | 3.3 | 5.0 | -1.6 | -5.1 | 0.84% | 4.8 |
| 4149 | AT5G65930.2 \| Symbol: ZWI \| kinesin-like calmodulin-binding protein (ZWICHEL), identical to kinesin-like protein GI:2224925 from (Arabidopsis thaliana) \| chr5:26387427-26393746 REVERSE \| Aliases: K14B20.10, K14B20_10, KCBP, KINESIN LIKE CALMODULIN BINDING PROTEIN, PKCBP, ZWICHEL | 4.6 | 6.2 | -1.5 | -5.1 | 0.84% | 4.8 |
| 4150 | AT5G49610.1 \| F-box family protein, contains Pfam PF00646: F-box domain; contains TIGRFAM TIGR01640: F-box protein interaction domain; similar to unknown protein (gb:AAF30317.1) \| chr5:20148500-20149955 REVERSE \| Aliases: K6M13.17, K6M13_17 | 4.5 | 5.7 | -1.2 | -5.1 | 0.84% | 4.8 |
| 4153 | AT3G49870.1 \| Symbol: ATARLA1C \| ADP-ribosylation factor, putative, similar to ADP-ribosylation factor-like protein 1 (SP:P40616) (Homo sapiens); ARF3 ADP-RIBOSYLATION FACTOR,GP:453191 Arabidopsis thaliana; contains domain PF00025: ADP-ribosylation factor family \| chr3:18503435-18505124 REVERSE \| Aliases: ATARLA1C, T16K5.220 | 9.0 | 10.1 | -1.2 | -5.1 | 0.84% | 4.8 |
| 4157 | AT4G32410.1 \| Symbol: CESA1 \| cellulose synthase, catalytic subunit, putative, similar to cellulose synthase-1 (gi:9622874) and -2 (gi:9622876) from Zea mays | 6.6 | 7.9 | -1.3 | -5.1 | 0.84% | 4.7 |
| 4158 | AT3G51660.1 \| macrophage migration inhibitory factor family protein / MIF family protein, contains Pfam profile: PF01187 Macrophage migration inhibitory factor family(MIF) \| chr3:19175042-19176151 REVERSE \| Aliases: T18N14.40 | 2.9 | 4.9 | -2.0 | -5.1 | 0.84% | 4.7 |
| 4163 | AT1G21050.1 \| expressed protein, contains Pfam profile PF04759: Protein of unknown function, DUF617 \| chr1:7366764-7367667 FORWARD \| Aliases: T22I11.13, T22I11_13 | 4.4 | 6.4 | -2.0 | -5.1 | 0.84% | 4.8 |
| 4165 | AT1G62320.1 \| early-responsive to dehydration protein-related / ERD protein-related, similar to ERD4 protein (early-responsive to dehydration stress) (Arabidopsis thaliana) GI:15375406; contains Pfam profile PF02714: Domain of unknown function DUF221 \| chr1:23045333-23048520 REVERSE \| Aliases: F24O1.4, F24O1_4 | 2.4 | 3.1 | -0.7 | -5.1 | 0.85% | 4.5 |
| 4166 | AT4G14630.1 \| Symbol: GLP9 \| germin-like protein (GLP9), identical to germin-like protein subfamily 1 member 8 (SP:Q9LEA7) \| chr4:8392915-8393802 FORWARD \| Aliases: DL3355W, FCAALL.278, GERMIN LIKE PROTEIN 9 | 2.6 | 4.0 | -1.4 | -5.1 | 0.85% | 4.7 |
| 4167 | AT5G43040.1 \| DC1 domain-containing protein, contains Pfam profile PF03107: DC1 domain | 3.1 | 3.7 | -0.7 | -5.1 | 0.85% | 4.5 |

| Rank | Description | Sync | Root | M | t | adj.q | B |
|---|---|---|---|---|---|---|---|
| 4169 | AT5G62570.1 \| calmodulin-binding protein, similar to calmodulin-binding protein TCB60 GI:1698548 from (Nicotiana tabacum) \| chr5:25131617-25134193 FORWARD \| Aliases: K19B1.18, K19B1_18 | 6.2 | 7.3 | -1.2 | -5.1 | 0.85% | 4.8 |
| 4170 | AT5G25270.1 \| expressed protein \| chr5:8757786-8762005 REVERSE \| Aliases: F21J6.3 | 4.3 | 7.2 | -2.9 | -5.1 | 0.85% | 4.7 |
| 4172 | AT5G40470.1 \| expressed protein \| chr5:16225237-16227125 REVERSE \| Aliases: K21I16.4, K21I16_4 | 5.5 | 7.2 | -1.8 | -5.1 | 0.85% | 4.8 |
| 4175 | AT3G12955.1 \| auxin-responsive protein-related, similar to indole-3-acetic acid induced protein arg7 (SP:P32295) (Vigna radiata) \| chr3:4135507-4136150 REVERSE \| Aliases: None | 3.1 | 4.0 | -1.0 | -5.1 | 0.85% | 4.7 |
| 4177 | AT1G42990.1 \| Symbol: ATBZIP60 \| AtbZIP60 consists of a bZIP DNA binding domain followed by a putative transmembrane domain. GFP fusions containing the first 260 amino acids (AtbZIP60deltaC) are nuclear-localized. AtbZIP60 is upregulated by the addition of tunicamycin (ER stress response inductor), DTT (inhibitor of disulfide bond formation) and azetin-2-carboxylate (proline analog perturbing protein structure). It is hypothesized that upon ER stress the protein is proteolyzed and the soluble part translocalized into the nucleus. AtbZIP60deltaC can activate the promoters of the ER chaperones BiP1, BiP2 and BiP3 and CNX1 and CNX2 via binding to the ER stress response element (ERSE) and the plant unfolded protein response element(P-UPRE). It can also activate its own transcription. \| chr1:16138292-16139903 REVERSE \| Aliases: ATBZIP60, F13A11.5, F13A11_5 | 8.5 | 9.7 | -1.2 | -5.1 | 0.86% | 4.7 |
| 4182 | AT4G30360.1 \| Symbol: ATCNGC17 \| cyclic nucleotide-regulated ion channel, putative (CNGC17), similar to cyclic nucleotide and calmodulin-regulated ion channel cngc5 GI:4581205 from (Arabidopsis thaliana) \| chr4:14854820-14858001 REVERSE \| Aliases: CNGC17, F17I23.300, F17I23_300 | 4.5 | 5.5 | -1.0 | -5.1 | 0.86% | 4.7 |
| 4183 | AT1G49310.1 \| expressed protein \| chr1:18241531-18242030 REVERSE \| Aliases: F13F21.30, F13F21_30 | 4.4 | 7.3 | -2.9 | -5.1 | 0.86% | 4.8 |
| 4184 | AT5G06265.1 \| hyaluronan mediated motility receptor-related, contains weak similarity to hyaluronan mediated motility receptor (Intracellular hyaluronic acid binding protein) (Receptor for hyaluronan-mediated motility) (CD168 antigen) (Swiss-Prot:O75330) (Homo sapiens) \| chr5:1905293-1908168 FORWARD \| Aliases: None | 5.8 | 7.0 | -1.2 | -5.1 | 0.86% | 4.7 |
| 4186 | AT5G01960.1 \| zinc finger (C3HC4-type RING finger) family protein, contains Pfam profile: PF00097 zinc finger, C3HC4 type (RING finger) \| chr5:370425-373314 FORWARD \| Aliases: T7H20.10, T7H20_10 | 6.5 | 7.6 | -1.1 | -5.1 | 0.86% | 4.8 |
| 4187 | AT3G11430.1 \| Symbol: ATGPAT5/GPAT5 \| Encodes a protein with glycerol-3-phosphate acyltransferase activity. \| chr3:3595771-3597958 FORWARD \| Aliases: ATGPAT5, F24K9.10, GPAT5 | 3.2 | 4.4 | -1.2 | -5.1 | 0.86% | 4.8 |
| 4191 | AT5G65640.1 \| basic helix-loop-helix (bHLH) family protein \| chr5:26254285-26256310 FORWARD \| Aliases: K21L13.16, K21L13_16 | 3.9 | 5.4 | -1.5 | -5.1 | 0.86% | 4.8 |
| 4193 | AT2G29750.1 \| UDP-glucoronosyl/UDP-glucosyl transferase family protein, contains Pfam profile: PF00201 UDP-glucoronosyl and UDP-glucosyl transferase \| chr2:12716804-12718773 FORWARD \| Aliases: T27A16.15, T27A16_15 | 3.7 | 5.6 | -1.9 | -5.1 | 0.86% | 4.8 |
| 4194 | AT1G55430.1 \| DC1 domain-containing protein, contains Pfam profile PF03107: DC1 domain | 2.8 | 3.4 | -0.6 | -5.1 | 0.86% | 4.5 |
| 4197 | AT5G18670.1 \| Symbol: BMY3 \| beta-amylase, putative (BMY3) / 1,4-alpha-D-glucan maltohydrolase, putative, almost identical to beta-amylase BMY3 GI:15149457 from (Arabidopsis thaliana); identical to cDNA putative beta-amylase BMY3 (BMY3) GI:15149456 \| chr5:6225958-6228134 FORWARD \| Aliases: T1A4.50, T1A4_50 | 6.9 | 7.8 | -0.9 | -5.1 | 0.86% | 4.7 |
| 4198 | AT3G62160.1 \| transferase family protein, low similarity to Taxus cuspidata transferases: 10-deacetylbaccatin III-10-O-acetyl transferase GI:6746554, taxadienol acetyl transferase GI:6978038, 2-debenzoyl-7,13-diacetylbaccatin III-2-O-benzoyl transferase GI:11559716; contains Pfam profile PF02458 transferase family \| chr3:23025183-23026814 REVERSE \| Aliases: T17J13.120 | 3.1 | 4.7 | -1.6 | -5.1 | 0.86% | 4.7 |
| 4201 | AT5G46780.2 \| VQ motif-containing protein, contains PF05678: VQ motif \| chr5:18996204-18997283 FORWARD \| Aliases: MZA15.20, MZA15_20 | 5.6 | 8.0 | -2.4 | -5.1 | 0.86% | 4.8 |
| 4206 | AT3G27610.1 \| expressed protein \| chr3:10226779-10229428 REVERSE \| Aliases: MGF10.2 | 4.9 | 6.9 | -1.9 | -5.1 | 0.87% | 4.8 |
| 4207 | AT5G07580.1 \| encodes a member of the ERF (ethylene response factor) subfamily B-3 of ERF/AP2 transcription factor family. The protein contains one AP2 domain. There are 18 members in this subfamily including ATERF-1, ATERF-2, AND ATERF-5. \| chr5:2399505-2400602 FORWARD \| Aliases: MBK20.1 | 5.9 | 6.9 | -1.0 | -5.1 | 0.87% | 4.7 |
| 4208 | AT4G16190.1 \| cysteine proteinase, putative, contains similarity to papain-like cysteine proteinase isoform I GI:7381219 from (Ipomoea batatas) \| chr4:9171482-9173120 FORWARD \| Aliases: DL4135W, FCAALL.298 | 6.8 | 9.8 | -3.0 | -5.1 | 0.87% | 4.7 |
| 4213 | AT1G71170.1 \| 6-phosphogluconate dehydrogenase NAD-binding domain-containing protein, contains Pfam profile: PF03446 NAD binding domain of 6-phosphogluconate \| chr1:26834331-26835761 FORWARD \| Aliases: F23N20.16, F23N20_16 | 7.8 | 8.8 | -1.1 | -5.1 | 0.87% | 4.5 |

| Rank | Description | Sync | Root | M | t | adj.q | B |
|---|---|---|---|---|---|---|---|
| 4214 | AT3G16857.2 \| Symbol: ARR1 \| two-component responsive regulator family protein / response regulator family protein, contains Pfam profile: PF00072 response regulator receiver domain; similar to ARR1 protein GB:BAA74528 from (Arabidopsis thaliana) (Plant Cell Physiol. (1998) 39 (11), 1232-1239) \| chr3:5755930-5759376 FORWARD \| Aliases: ARABIDOPSIS RESPONSE REGULATOR 1, MUH15.5 | 4.2 | 5.2 | -1.0 | -5.1 | 0.87% | 4.7 |
| 4217 | AT5G04120.1 \| phosphoglycerate/bisphosphoglycerate mutase family protein, low similarity to SP:P36623 Phosphoglycerate mutase (EC 5.4.2.1) {Schizosaccharomyces pombe}; contains Pfam profile PF00300: phosphoglycerate mutase family \| chr5:1120024-1121776 FORWARD \| Aliases: F21E1.40, F21E1_40 | 2.7 | 3.9 | -1.2 | -5.1 | 0.87% | 4.7 |
| 4218 | AT1G58070.1 \| expressed protein \| chr1:21506570-21507739 FORWARD \| Aliases: T15M6.8, T15M6_8 | 2.9 | 4.3 | -1.3 | -5.1 | 0.87% | 4.8 |
| 4223 | AT2G46225.1 \| Symbol: ABI1L1 \| | 4.0 | 5.1 | -1.1 | -5.1 | 0.88% | 4.8 |
| 4224 | AT1G12080.2 \| expressed protein \| chr1:4084203-4085041 FORWARD \| Aliases: F12F1.4, F12F1_4 | 4.0 | 7.1 | -3.2 | -5.1 | 0.88% | 4.5 |
| 4227 | AT4G30850.2 \| expressed protein, contains Pfam domain, PF03006: Uncharacterised protein family (Hly-III / UPF0073) \| chr4:15020188-15022451 REVERSE \| Aliases: F6I18.240, F6I18_240 | 7.5 | 8.7 | -1.2 | -5.1 | 0.88% | 4.7 |
| 4228 | AT4G02120.1 \| CTP synthase, putative / UTP--ammonia ligase, putative, similar to SP:P17812 CTP synthase (EC 6.3.4.2) (UTP--ammonia ligase) {Homo sapiens}; contains Pfam profile PF00117: glutamine amidotransferase class-I \| chr4:940841-944284 FORWARD \| Aliases: T10M13.13, T10M13_13 | 4.9 | 7.2 | -2.3 | -5.1 | 0.88% | 4.7 |
| 4233 | AT1G48600.2 \| phosphoethanolamine N-methyltransferase 2, putative (NMT2), very similar to :PEM2_ARATH Putative phosphoethanolamine N-methyltransferase 2 (EC 2.1.1.103) (SP:Q944H0){Arabidopsis thaliana}; very similar to Halotolerance protein Hal3b (SP:P94063)(Arabidopsis thaliana); to similar to GB:AAF61950 from (Spinacia oleracea) \| chr1:17969539-17973005 FORWARD \| Aliases: T1N15.23, T1N15_23 | 11.5 | 12.3 | -0.7 | -5.1 | 0.88% | 4.3 |
| 4237 | AT5G26240.1 \| Symbol: CLC D \| chloride channel protein (CLC-d), identical to CLC-d chloride channel protein (Arabidopsis thaliana) GI:1742959 | 3.6 | 5.1 | -1.5 | -5.1 | 0.89% | 4.7 |
| 4238 | AT3G19100.1 \| calcium-dependent protein kinase, putative / CDPK, putative, similar to calcium/calmodulin-dependent protein kinase CaMK3 (Nicotiana tabacum) gi:16904226:gb:AAL30820; contains protein kinase domain, Pfam:PF00069; contains serine/threonine protein kinase domain, INTERPRO:IPR002290 | 4.5 | 6.0 | -1.5 | -5.1 | 0.89% | 4.7 |
| 4240 | AT3G05220.2 \| heavy-metal-associated domain-containing protein, similar to farnesylated protein 1 (GI:23304411) {Hordeum vulgare subsp. spontaneum}; contains Pfam profile PF00403: Heavy-metal-associated domain \| chr3:1489425-1491320 FORWARD \| Aliases: T12H1.19, T12H1_19 | 9.5 | 10.5 | -1.0 | -5.1 | 0.89% | 4.3 |
| 4242 | AT4G17785.1 \| myb family transcription factor (MYB39), contains Pfam profile: PF00249 myb-like DNA-binding domain \| chr4:9881648-9883445 REVERSE \| Aliases: None | 2.3 | 2.8 | -0.5 | -5.1 | 0.89% | 4.1 |
| 4247 | AT5G13810.1 \| glutaredoxin family protein \| chr5:4455354-4457022 FORWARD \| Aliases: MAC12.24, MAC12_24 | 6.4 | 7.7 | -1.4 | -5.1 | 0.90% | 4.6 |
| 4255 | AT1G52240.1 \| member of KPP-like gene family, homologs of KPP (kinase partner protein) gene in tomato. | 2.8 | 4.1 | -1.3 | -5.1 | 0.90% | 4.7 |
| 4259 | AT1G50290.1 \| expressed protein \| chr1:18631227-18632102 REVERSE \| Aliases: F14I3.24, F14I3_24 | 3.1 | 4.1 | -1.0 | -5.1 | 0.91% | 4.7 |
| 4262 | AT5G60760.1 \| 2-phosphoglycerate kinase-related, contains weak similarity to 2-phosphoglycerate kinase (GI:467751) (Methanothermus fervidus) \| chr5:24455497-24459513 FORWARD \| Aliases: MAE1.1, MAE1_1 | 4.6 | 7.7 | -3.0 | -5.1 | 0.91% | 4.7 |
| 4263 | AT1G75920.1 \| family II extracellular lipase 5 (EXL5), EXL5 (PMID:11431566); similar to anter-specific proline-rich protein (APG) SP:P40602 (Arabidopsis thaliana (Mouse-ear cress)) \| chr1:28509252-28510684 FORWARD \| Aliases: T4O12.14, T4O12_14 | 3.8 | 5.2 | -1.3 | -5.0 | 0.91% | 4.7 |
| 4264 | AT5G01610.1 \| expressed protein, contains Pfam profile PF04398: Protein of unknown function, DUF538 \| chr5:231042-232198 FORWARD \| Aliases: F7A7.130, F7A7_130 | 4.1 | 5.7 | -1.6 | -5.0 | 0.91% | 4.7 |
| 4265 | AT2G19410.1 \| protein kinase family protein, contains Pfam profile: PF00069 Eukaryotic protein kinase domain \| chr2:8411983-8416094 REVERSE \| Aliases: F27F23.21, F27F23_21 | 2.6 | 3.5 | -0.9 | -5.0 | 0.91% | 4.6 |
| 4266 | AT5G48740.1 \| leucine-rich repeat family protein / protein kinase family protein, contains Pfam domains PF00560: Leucine Rich Repeat and PF00069: Protein kinase domain \| chr5:19781910-19786595 REVERSE \| Aliases: K24G6.7, K24G6_7 | 3.4 | 4.2 | -0.8 | -5.0 | 0.91% | 4.6 |
| 4267 | AT5G52640.1 \| Symbol: HSP81 1 \| heat shock protein 81-1 (HSP81-1) / heat shock protein 83 (HSP83), nearly identical to SP:P27323 Heat shock protein 81-1 (HSP81-1) (Heat shock protein 83) {Arabidopsis thaliana}; contains Pfam profiles PF02518: ATPase, histidine kinase-, DNA gyrase B-, and HSP90-like domain protein, PF00183: Hsp90 protein \| chr5:21369660-21372587 FORWARD \| Aliases: ATHS83, F6N7.13, F6N7_13, HEAT SHOCK PROTEIN 81 1, HEAT SHOCK PROTEIN 83, HSP81 1, HSP81.1, HSP83 | 6.9 | 8.3 | -1.5 | -5.0 | 0.91% | 4.7 |
| 4269 | AT2G44010.1 \| expressed protein \| chr2:18223760-18224514 FORWARD \| Aliases: F6E13.14 | 3.5 | 7.9 | -4.3 | -5.0 | 0.91% | 4.6 |

| Rank | Description | Sync | Root | M | t | adj.q | B |
|---|---|---|---|---|---|---|---|
| 4272 | AT5G18970.1 \| AWPM-19-like membrane family protein, contains Pfam profile: PF05512 AWPM-19-like family | 2.9 | 5.1 | -2.2 | -5.0 | 0.91% | 4.6 |
| 4278 | AT1G07690.1 \| expressed protein \| chr1:2378073-2378997 FORWARD \| Aliases: F24B9.22, F24B9_22 | 3.1 | 3.8 | -0.7 | -5.0 | 0.91% | 4.4 |
| 4279 | AT1G01190.1 \| Symbol: CYP78A8 \| cytochrome P450, putative, similar to cytochrome P450 SP:O48927 from (Glycine max) \| chr1:83045-84864 REVERSE \| Aliases: F6F3.25 | 3.6 | 5.1 | -1.5 | -5.0 | 0.91% | 4.7 |
| 4282 | AT1G75160.1 \| expressed protein, contains Pfam profile PF04788: Protein of unknown function (DUF620) \| chr1:28213098-28215465 FORWARD \| Aliases: F22H5.11, F22H5_11 | 2.1 | 2.6 | -0.5 | -5.0 | 0.91% | 4.3 |
| 4289 | AT3G19970.1 \| expressed protein \| chr3:6958654-6961466 FORWARD \| Aliases: MZE19.2 | 4.3 | 5.5 | -1.2 | -5.0 | 0.92% | 4.7 |
| 4290 | AT3G16220.1 \| expressed protein, similar to CGI-18 protein GB:AAD27727 (Homo sapiens) \| chr3:5497620-5499241 FORWARD \| Aliases: MYA6.3 | 5.2 | 6.7 | -1.6 | -5.0 | 0.92% | 4.6 |
| 4293 | AT2G29620.1 \| expressed protein \| chr2:12670277-12672880 REVERSE \| Aliases: T27A16.28, T27A16_28 | 2.5 | 3.2 | -0.7 | -5.0 | 0.92% | 4.4 |
| 4294 | AT4G00940.1 \| Dof-type zinc finger domain-containing protein, similar to DNA-binding protein DAG1/BBFa GI:4581965 (Arabidopsis thaliana) \| chr4:403320-404234 REVERSE \| Aliases: A_TM018A10.25, A_TM018A10_25, T18A10.24, T18A10_24 | 3.3 | 5.2 | -1.9 | -5.0 | 0.92% | 4.6 |
| 4297 | AT3G56310.2 \| alpha-galactosidase, putative / melibiase, putative / alpha-D-galactoside galactohydrolase, putative, similar to alpha-galactosidase SP:Q42656 from (Coffea arabica) \| chr3:20893806-20897302 FORWARD \| Aliases: F18O21.270 | 10.9 | 11.9 | -1.0 | -5.0 | 0.92% | 4.5 |
| 4298 | AT4G16563.1 \| aspartyl protease family protein, contains Pfam profile: PF00026 eukaryotic aspartyl protease \| chr4:9329679-9331545 REVERSE \| Aliases: None | 3.7 | 5.7 | -2.0 | -5.0 | 0.92% | 4.6 |
| 4305 | AT5G53980.1 \| homeobox-leucine zipper family protein, contains Pfam PF00046: Homeobox domain; similar to homeobox protein PpHB5 (GI:7415622) (Physcomitrella patens) \| chr5:21931271-21931965 FORWARD \| Aliases: K19P17.15, K19P17_15 | 2.7 | 3.8 | -1.1 | -5.0 | 0.93% | 4.6 |
| 4307 | AT1G75620.1 \| glyoxal oxidase-related, contains similarity to glyoxal oxidase precursor (Phanerochaete chrysosporium) gi:1050302:gb:AAA87594 \| chr1:28398440-28400257 REVERSE \| Aliases: F10A5.18, F10A5_18 | 2.8 | 3.8 | -1.0 | -5.0 | 0.93% | 4.7 |
| 4308 | AT2G47140.1 \| short-chain dehydrogenase/reductase (SDR) family protein, similar to 3-beta-hydroxysteroiddehydrogenase GI:15983819 from (Digitalis lanata) \| chr2:19357810-19359158 REVERSE \| Aliases: F14M4.3 | 3.1 | 4.0 | -0.8 | -5.0 | 0.93% | 4.6 |
| 4312 | AT3G16340.1 \| ABC transporter family protein, similar to PDR5-like ABC transporter GI:1514643 from (Spirodela polyrhiza); contains Pfam profile: PF00005 ABC transporter \| chr3:5539903-5546410 FORWARD \| Aliases: MYA6.19 | 5.4 | 7.1 | -1.7 | -5.0 | 0.93% | 4.7 |
| 4314 | AT1G69240.1 \| hydrolase, alpha/beta fold family protein, low similarity to SP:Q40708 PIR7A protein {Oryza sativa}, polyneuridine aldehyde esterase GI:6651393 from (Rauvolfia serpentina), ethylene-induced esterase (Citrus sinensis) GI:14279437; contains Pfam profile PF00561: hydrolase, alpha/beta fold family \| chr1:26031639-26033897 REVERSE \| Aliases: F4N2.19, F4N2_19 | 2.3 | 3.2 | -0.8 | -5.0 | 0.93% | 4.6 |
| 4317 | AT5G35560.1 \| DENN (AEX-3) domain-containing protein, contains Pfam domain PF02141: DENN (AEX-3) domain; this cDNA may contain an anomalously spliced intron...will require further examination. \| chr5:13759339-13765493 REVERSE \| Aliases: MOK9.21, MOK9_21 | 5.1 | 6.5 | -1.4 | -5.0 | 0.94% | 4.7 |
| 4318 | AT1G14200.1 \| zinc finger (C3HC4-type RING finger) family protein, contains Pfam profile: PF00097 zinc finger, C3HC4 type (RING finger) \| chr1:4854379-4855195 REVERSE \| Aliases: F7A19.29, F7A19_29 | 5.5 | 6.9 | -1.4 | -5.0 | 0.94% | 4.6 |
| 4320 | AT1G51170.1 \| protein kinase family protein \| chr1:18957126-18958560 REVERSE \| Aliases: F23H24.1 | 4.3 | 6.0 | -1.7 | -5.0 | 0.94% | 4.7 |
| 4321 | AT2G35910.1 \| zinc finger (C3HC4-type RING finger) family protein, low similarity to RING-H2 zinc finger protein ATL6 (Arabidopsis thaliana) GI:4928403; contains Pfam profile PF00097: Zinc finger, C3HC4 type (RING finger) \| chr2:15080011-15081160 REVERSE \| Aliases: F11F19.18, F11F19_18 | 2.8 | 3.8 | -1.1 | -5.0 | 0.94% | 4.6 |
| 4322 | AT5G06200.1 \| integral membrane family protein, similar to unknown protein (gb:AAF00668.1); contains TIGRFAM TIGR01569 : plant integral membrane protein TIGR01569; contains Pfam PF04535 : Domain of unknown function (DUF588) \| chr5:1877334-1878117 FORWARD \| Aliases: MBL20.8, MBL20_8 | 2.6 | 4.3 | -1.7 | -5.0 | 0.94% | 4.6 |
| 4325 | AT5G58520.1 \| protein kinase family protein, contains protein kinase domain, Pfam:PF00069 \| chr5:23672476-23675388 FORWARD \| Aliases: MQJ2.14, MQJ2_14 | 5.9 | 7.2 | -1.2 | -5.0 | 0.94% | 4.7 |
| 4328 | AT3G48020.1 \| expressed protein \| chr3:17735578-17735985 FORWARD \| Aliases: T17F15.110 | 4.3 | 5.6 | -1.3 | -5.0 | 0.94% | 4.6 |

| Rank | Description | Sync | Root | M | t | adj.q | B |
|---|---|---|---|---|---|---|---|
| 4330 | AT4G33160.1 \| F-box family protein (FBX13), contains similarity to fimbriata GI:547307 from (Antirrhinum majus) \| chr4:15994240-15995590 FORWARD \| Aliases: F4I10.90, F4I10_90 | 4.8 | 5.5 | -0.7 | -5.0 | 0.94% | 4.4 |
| 4331 | AT1G32740.1 \| expressed protein \| chr1:11844857-11846482 FORWARD \| Aliases: F6N18.12, F6N18_12 | 4.5 | 6.8 | -2.3 | -5.0 | 0.94% | 4.7 |
| 4337 | AT3G45700.1 \| proton-dependent oligopeptide transport (POT) family protein, contains Pfam profile: PF00854 POT family \| chr3:16789698-16792183 FORWARD \| Aliases: T6D9.30 | 4.0 | 5.6 | -1.6 | -5.0 | 0.94% | 4.7 |
| 4339 | AT5G08250.1 \| cytochrome P450 family protein, contains Pfam profile: PF00067 cytochrome P450 \| chr5:2653581-2655727 REVERSE \| Aliases: F8L15.12 | 2.4 | 3.5 | -1.1 | -5.0 | 0.94% | 4.5 |
| 4340 | AT1G11080.1 \| Symbol: SCPL31 \| | 2.5 | 3.9 | -1.4 | -5.0 | 0.94% | 4.7 |
| 4343 | AT5G41810.2 \| expressed protein \| chr5:16755290-16756891 FORWARD \| Aliases: K16L22.9, K16L22_9 | 3.4 | 5.1 | -1.7 | -5.0 | 0.95% | 4.6 |
| 4345 | AT1G70530.1 \| protein kinase family protein, contains Pfam domain, PF00069: Protein kinase domain \| chr1:26592413-26595042 REVERSE \| Aliases: F24J13.10, F24J13_10 | 4.7 | 6.1 | -1.4 | -5.0 | 0.95% | 4.6 |
| 4346 | AT5G20300.3 \| similar to chloroplast outer membrane protein, putative [Arabidopsis thaliana] (TAIR:At3g16620.1); similar to chloroplast Toc125 [Physcomitrella patens] (GB:AAS47583.1) | 6.6 | 8.2 | -1.6 | -5.0 | 0.95% | 4.5 |
| 4348 | AT3G43430.1 \| zinc finger (C3HC4-type RING finger) family protein, contains Pfam profile: PF00097 zinc finger, C3HC4 type (RING finger) \| chr3:15365547-15366275 REVERSE \| Aliases: T5C2.130 | 4.4 | 6.0 | -1.6 | -5.0 | 0.95% | 4.6 |
| 4352 | AT5G09620.1 \| octicosapeptide/Phox/Bem1p (PB1) domain-containing protein, predicted proteins, Arabidopsis thaliana and Drosophila melanogaster contains Pfam profile PF00564: PB1 domain \| chr5:2983450-2985436 REVERSE \| Aliases: F17I14.190, F17I14_190 | 7.3 | 8.3 | -0.9 | -5.0 | 0.96% | 4.4 |
| 4353 | AT2G31820.1 \| ankyrin repeat family protein, contains ankyrin repeat domains, Pfam:PF00023 \| chr2:13537427-13539639 FORWARD \| Aliases: F20M17.14, F20M17_14 | 7.9 | 8.7 | -0.8 | -5.0 | 0.96% | 4.3 |
| 4359 | AT5G19050.1 \| expressed protein \| chr5:6369269-6371901 FORWARD \| Aliases: T16G12.90, T16G12_90 | 6.0 | 7.7 | -1.8 | -5.0 | 0.96% | 4.6 |
| 4360 | AT4G03260.1 \| leucine-rich repeat family protein, contains leucine rich repeat (LRR) domains, Pfam:PF00560 \| chr4:1428171-1431663 FORWARD \| Aliases: F4C21.19, F4C21_19 | 8.0 | 9.2 | -1.1 | -5.0 | 0.96% | 4.4 |
| 4362 | AT1G29120.4 \| similar to expressed protein [Arabidopsis thaliana] (TAIR:At4g25770.1); similar to putative serine esterase [Oryza sativa (japonica cultivar-group)] (GB:AAT77089.1); similar to hypothetical protein [Oryza sativa (japonica cultivar-group)] (GB:XP_468171.1); contains InterPro domain Esterase/lipase/thioesterase (InterPro:IPR000379) \| chr1:10174268-10178475 FORWARD \| Aliases: AT1G29130, F28N24.29, F28N24_29 | 5.7 | 7.0 | -1.3 | -5.0 | 0.97% | 4.6 |
| 4365 | AT1G65580.1 \| endonuclease/exonuclease/phosphatase family protein, similar to inositol polyphosphate 5-phosphatase II isoform (GI:15418718) (Mus musculus); contains 6 (5 weak) Pfam: Pf00400 WD domain, G-beta repeats and Pfam PF03372: Endonuclease/Exonuclease/phosphatase family \| chr1:24380977-24387742 REVERSE \| Aliases: F5I14.11, F5I14_11 | 8.6 | 9.9 | -1.3 | -5.0 | 0.97% | 4.4 |
| 4366 | AT3G19190.1 \| expressed protein \| chr3:6639717-6648242 FORWARD \| Aliases: MVI11.11 | 4.6 | 6.2 | -1.6 | -5.0 | 0.97% | 4.6 |
| 4370 | AT1G73290.1 \| Symbol: SCPL5 \| serine carboxypeptidase S10 family protein, similar to serine carboxypeptidase I precursor (SP:P07519) (Hordeum vulgare); glucose acyltransferase GB:AAD01263 (Solanum berthaultii); contains Pfam profile: PF00450 Serine carboxypeptidase; \| chr1:27560058-27562434 REVERSE \| Aliases: SCPL5, T18K17.4, T18K17_4 | 2.2 | 2.6 | -0.3 | -5.0 | 0.97% | 3.8 |
| 4371 | AT1G49720.1 \| Symbol: ABF1 \| ABA-responsive element-binding protein / abscisic acid responsive elements-binding factor (ABRE), identical to abscisic acid responsive elements-binding factor GB:AAF27179 GI:6739274 from (Arabidopsis thaliana); identical to cDNA abscisic acid responsive elements-binding factor (ABRE) mRNA, complete cds GI:6739273 \| chr1:18403760-18406735 FORWARD \| Aliases: ABSCISIC ACID RESPONSIVE ELEMENT BINDING FACTOR 1, F14J22.7, F14J22_7 | 5.0 | 6.3 | -1.4 | -5.0 | 0.97% | 4.6 |
| 4373 | AT1G08590.1 \| similar to CLV1-like leucine rich repeat transmembrane receptor-like protein kinase (Ipomoea nil) (U77888) \| chr1:2718781-2722224 FORWARD \| Aliases: F22O13.7, F22O13_7 | 3.4 | 4.4 | -1.0 | -5.0 | 0.97% | 4.6 |
| 4376 | AT2G43820.1 \| UDP-glucoronosyl/UDP-glucosyl transferase family protein, contains Pfam profile: PF00201 UDP-glucoronosyl and UDP-glucosyl transferase \| chr2:18159304-18160985 FORWARD \| Aliases: F18O19.7 | 4.4 | 7.0 | -2.6 | -5.0 | 0.98% | 4.6 |
| 4380 | AT5G63080.1 \| transcription factor jumonji (jmjC) domain-containing protein, contains Pfam domain PF02373: jmjC domain | 5.5 | 6.8 | -1.3 | -5.0 | 0.98% | 4.6 |
| 4383 | AT3G47770.1 \| Symbol: ATATH5 \| | 2.8 | 3.6 | -0.8 | -5.0 | 0.98% | 4.4 |

| Rank | Description | Sync | Root | M | t | adj.q | B |
|---|---|---|---|---|---|---|---|
| 4385 | AT3G52800.1 \| zinc finger (AN1-like) family protein, contains Pfam domain, PF01428: AN1-like Zinc finger \| chr3:19580289-19581660 FORWARD \| Aliases: F3C22.200 | 6.2 | 7.8 | -1.6 | -5.0 | 0.98% | 4.6 |
| 4388 | AT1G80240.1 \| expressed protein, contains Pfam profile PF04862: Protein of unknown function, DUF642 \| chr1:30176249-30177718 REVERSE \| Aliases: F18B13.30, F18B13_30 | 2.8 | 3.8 | -1.1 | -5.0 | 0.99% | 4.6 |
| 4392 | AT1G08040.2 \| similar to expressed protein [Arabidopsis thaliana] (TAIR:At2g28310.2); similar to expressed protein [Arabidopsis thaliana] (TAIR:At2g28310.1); similar to lysine ketoglutarate reductase trans-splicing related 1-like [Oryza sativa (japonica cultivar-group)] (GB:BAD87361.1); contains InterPro domain Protein of unknown function DUF707 (InterPro:IPR007877) \| chr1:2495339-2498638 REVERSE \| Aliases: T6D22.12, T6D22_12 | 6.8 | 8.0 | -1.2 | -5.0 | 0.99% | 4.6 |
| 4395 | AT3G46730.1 \| disease resistance protein (CC-NBS class), putative, domain signature CC-NBS exists, suggestive of a disease resistance protein. | 2.3 | 3.3 | -1.0 | -5.0 | 0.99% | 4.5 |
| 4398 | AT3G47180.1 \| zinc finger (C3HC4-type RING finger) family protein, contains Pfam domain, PF00097: Zinc finger, C3HC4 type (RING finger) \| chr3:17383785-17384438 REVERSE \| Aliases: F13I12.230 | 3.6 | 4.7 | -1.1 | -5.0 | 0.99% | 4.6 |
| 4400 | AT1G79430.2 \| Symbol: APL \| myb family transcription factor-related \| chr1:29882121-29884097 REVERSE \| Aliases: ALTERED PHLOEM DEVELOPMENT, T8K14.15, T8K14_15, WDY, WOODY | 3.0 | 4.3 | -1.3 | -5.0 | 1.00% | 4.6 |
| 4404 | AT3G10550.1 \| expressed protein \| chr3:3292904-3296874 REVERSE \| Aliases: F13M14.17 | 5.6 | 6.9 | -1.3 | -4.9 | 1.00% | 4.5 |
| 4407 | AT5G65660.1 \| hydroxyproline-rich glycoprotein family protein \| chr5:26262475-26263605 REVERSE \| Aliases: K21L13.18, K21L13_18 | 9.6 | 10.8 | -1.2 | -4.9 | 1.00% | 4.6 |
| 4408 | AT3G07990.1 \| Symbol: SCPL27 \| | 2.8 | 3.6 | -0.8 | -4.9 | 1.00% | 4.5 |
| 4410 | AT2G26670.1 \| Symbol: HY1 \| heme oxygenase 1 (HO1) (HY1), identical to plastid heme oxygenase (HY1) (Arabidopsis thaliana) GI:4877362, heme oxygenase 1 (Arabidopsis thaliana) GI:4530591 GB:AF132475; annotation updated per Seth J. Davis at University of Wisconsin-Madison \| chr2:11348762-11350650 FORWARD \| Aliases: ATHO1, ELONGATED HYPOCOTYL 1, F18A8.4, F18A8_4, GENOMES UNCOUPLED 2, GUN2, HEME OXYGENASE, HEME OXYGENASE 1, HO1, PLASTID HEME OXYGENASE, TED 4 | 8.0 | 10.0 | -2.0 | -4.9 | 1.00% | 4.6 |
| 4411 | AT3G11490.1 \| rac GTPase activating protein, putative, similar to rac GTPase activating protein 1 GB:AAC62624 GI:3695059 (Lotus japonicus); contains Pfam profile PF00620: RhoGAP domain \| chr3:3617529-3619573 REVERSE \| Aliases: F24K9.16 | 5.2 | 6.7 | -1.5 | -4.9 | 1.00% | 4.6 |
| 4415 | AT5G23720.3 \| Symbol: PHS1 \| similar to dual specificity protein phosphatase (DsPTP1) [Arabidopsis thaliana] (TAIR:At3g23610.1); similar to B1146B04.18 [Oryza sativa (japonica cultivar-group)] (GB:NP_908883.1); similar to P0551A11.24 [Oryza sativa (japonica cultivar-group)] (GB:NP_908585.1); similar to putative PROPYZAMIDE-HTPERSENSITIVE 1 [Oryza sativa (japonica cultivar-group)] (GB:BAD52745.1); contains InterPro domain Tyrosine specific protein phosphatase and dual specificity protein phosphatase (InterPro:IPR000387); contains InterPro domain Dual specificity protein phosphatase (InterPro:IPR000340) \| chr5:7998477-8002808 FORWARD \| Aliases: MQM1.1, MQM1_1, PROPYZAMIDE HYPERSENSITIVE 1 | 6.7 | 7.8 | -1.1 | -4.9 | 1.01% | 4.6 |
| 4417 | AT3G03520.1 \| phosphoesterase family protein, low similarity to SP:P95246 Phospholipase C 2 precursor (EC 3.1.4.3) {Mycobacterium tuberculosis}; contains Pfam profile PF04185: Phosphoesterase family | 4.0 | 5.5 | -1.5 | -4.9 | 1.01% | 4.6 |
| 4418 | AT4G37610.1 \| TAZ zinc finger family protein / BTB/POZ domain-containing protein, contains Pfam PF00651 : BTB/POZ domain; contains Pfam PF02135 : TAZ zinc finger; similar to Speckle-type POZ protein (SP:O43791) (Homo sapiens) \| chr4:17670475-17672334 REVERSE \| Aliases: F19F18.100, F19F18_100 | 7.3 | 8.8 | -1.5 | -4.9 | 1.01% | 4.5 |
| 4419 | AT3G49590.2 \| expressed protein \| chr3:18388545-18391269 FORWARD \| Aliases: T9C5.180 | 5.3 | 6.9 | -1.5 | -4.9 | 1.01% | 4.6 |
| 4423 | AT2G35330.1 \| zinc finger (C3HC4-type RING finger) protein-related, contains weak hit to Pfam profile: PF00097 zinc finger, C3HC4 type (RING finger); weak similarity to RING finger protein 8 (Swiss-Prot:O76064) (Homo sapiens) \| chr2:14876068-14879019 FORWARD \| Aliases: T32F12.29, T32F12_29 | 5.0 | 6.8 | -1.8 | -4.9 | 1.01% | 4.6 |
| 4425 | AT5G54860.1 \| integral membrane transporter family protein, contains 10 transmembrane domains; contains Pfam PF03092: BT1 family; contains TIGRFAMS TIGR00788: folate/biopterin transporter; similar to high affinity folic acid/methotrexate transporter 5 (GI:21898554) (Leishmania tarentolae) \| chr5:22301855-22304432 FORWARD \| Aliases: MBG8.12, MBG8_12 | 5.2 | 6.7 | -1.5 | -4.9 | 1.01% | 4.6 |
| 4427 | AT3G01730.1 \| expressed protein \| chr3:266565-266994 FORWARD \| Aliases: F4P13.28 | 3.2 | 5.0 | -1.8 | -4.9 | 1.01% | 4.6 |
| 4431 | AT1G34120.3 \| Symbol: IP5PI \| inositol polyphosphate 5-phosphatase I (IP5PI), nearly identical to inositol polyphosphate 5-phosphatase I (Arabidopsis thaliana) GI:10444261 \| chr1:12426756-12429688 FORWARD \| Aliases: F12G12.6, F12G12_6, INOSITOL POLYPHOSPHATE 5 PHOSPHATASE AT5P1, INOSITOL POLYPHOSPHATE 5 PHOSPHATASE I | 4.8 | 6.8 | -2.0 | -4.9 | 1.01% | 4.6 |
| 4435 | AT5G19040.1 \| Symbol: ATIPT5 \| | 3.9 | 5.5 | -1.6 | -4.9 | 1.01% | 4.6 |

| Rank | Description | Sync | Root | M | t | adj.q | B |
|---|---|---|---|---|---|---|---|
| 4436 | AT5G03550.1 \| hypothetical protein \| chr5:895812-896144 REVERSE \| Aliases: F12E4.340, F12E4_340 | 2.4 | 3.0 | -0.7 | -4.9 | 1.01% | 4.2 |
| 4439 | AT1G28290.1 \| pollen Ole e 1 allergen and extensin family protein, similar to arabinogalactan protein (Daucus carota) GI:11322245; contains Pfam profile PF01190: Pollen proteins Ole e I family \| chr1:9889112-9890980 REVERSE \| Aliases: F3H9.6, F3H9_6 | 5.4 | 6.9 | -1.5 | -4.9 | 1.02% | 4.6 |
| 4445 | AT5G37070.1 \| expressed protein, contains Pfam profile PF04398: Protein of unknown function, DUF538 \| chr5:14668321-14669377 FORWARD \| Aliases: MJG14.7, MJG14_7 | 6.8 | 7.6 | -0.8 | -4.9 | 1.02% | 4.5 |
| 4447 | AT3G05620.1 \| pectinesterase family protein, contains Pfam profile: PF01095 pectinesterase \| chr3:1629664-1631772 REVERSE \| Aliases: F18C1.11, F18C1_11 | 2.8 | 4.7 | -1.8 | -4.9 | 1.02% | 4.4 |
| 4448 | AT3G52460.1 \| hydroxyproline-rich glycoprotein family protein, contains proline-rich extensin domains, INTERPRO:IPR002965 \| chr3:19457948-19458850 FORWARD \| Aliases: F22O6.160 | 5.1 | 6.0 | -0.9 | -4.9 | 1.02% | 4.4 |
| 4452 | AT5G61350.1 \| protein kinase family protein, contains protein kinase domain, Pfam:PF00069 \| chr5:24685199-24687727 FORWARD \| Aliases: MFB13.1, MFB13_1 | 2.6 | 3.8 | -1.2 | -4.9 | 1.02% | 4.6 |
| 4455 | AT3G03360.1 \| F-box family protein, low similarity to ribosomal RNA apurinic site specific lyase (Triticum aestivum) GI:6505722; contains F-box domain Pfam:PF00646 \| chr3:795225-796925 FORWARD \| Aliases: T21P5.22, T21P5_22 | 5.4 | 6.9 | -1.5 | -4.9 | 1.02% | 4.5 |
| 4456 | AT1G48670.1 \| auxin-responsive GH3 family protein, similar to auxin-responsive GH3 product (Glycine max) GI:18591; contains Pfam profile PF03321: GH3 auxin-responsive promoter \| chr1:18002614-18004868 REVERSE \| Aliases: F11I4.14, F11I4_14 | 2.6 | 5.1 | -2.6 | -4.9 | 1.02% | 4.6 |
| 4458 | AT2G23980.1 \| Symbol: ATCNGC6 \| cyclic nucleotide-regulated ion channel / cyclic nucleotide-gated channel (CNGC6), identical to cyclic nucleotide and calmodulin-regulated ion channel (cngc6) GI:4581207 from (Arabidopsis thaliana) | 6.7 | 7.8 | -1.2 | -4.9 | 1.02% | 4.5 |
| 4460 | AT1G16370.1 \| transporter-related, low similarity to organic cation transporter OCTN1 from (Homo sapiens) GI:2605501, (Mus musculus) GI:4126605, (Rattus norvegicus) GI:5679326; contains Pfam profile PF00083: major facilitator superfamily protein \| chr1:5596688-5598441 FORWARD \| Aliases: F3O9.17, F3O9_17 | 4.9 | 5.8 | -0.9 | -4.9 | 1.02% | 4.5 |
| 4461 | AT5G43930.3 \| similar to transducin family protein / WD-40 repeat family protein [Arabidopsis thaliana] (TAIR:At1g04140.1); similar to transducin family protein / WD-40 repeat family protein [Arabidopsis thaliana] (TAIR:At1g04140.2); similar to transducin family protein-like [Oryza sativa (japonica cultivar-group)] (GB:BAD33212.1); contains InterPro domain G-protein beta WD-40 repeat (InterPro:IPR001680) \| chr5:17694050-17698309 FORWARD \| Aliases: MRH10.2, MRH10_2 | 4.2 | 5.4 | -1.2 | -4.9 | 1.03% | 4.5 |
| 4463 | AT1G08315.1 \| armadillo/beta-catenin repeat family protein, contains Pfam profile: PF00514 armadillo/beta-catenin-like repeat \| chr1:2620334-2621542 REVERSE \| Aliases: None | 6.0 | 7.0 | -1.0 | -4.9 | 1.03% | 4.5 |
| 4464 | AT4G37150.1 \| esterase, putative, similar to ethylene-induced esterase (Citrus sinensis) GI:14279437, polyneuridine aldehyde esterase (Rauvolfia serpentina) GI:6651393; contains Pfam profile PF00561: hydrolase, alpha/beta fold family \| chr4:17492872-17494101 REVERSE \| Aliases: AP22.78, AP22_78 | 2.9 | 3.8 | -0.9 | -4.9 | 1.03% | 4.4 |
| 4465 | AT4G31250.1 \| leucine-rich repeat transmembrane protein kinase, putative, receptor kinase, Petunia inflata, Patchx:G498278 \| chr4:15178939-15181757 REVERSE \| Aliases: F8F16.70, F8F16_70 | 3.8 | 5.2 | -1.4 | -4.9 | 1.03% | 4.6 |
| 4467 | AT4G30190.1 \| Symbol: AHA2 \| ATPase 2, plasma membrane-type, putative / proton pump 2, putative / proton-exporting ATPase, putative, strong similarity to SP:P19456 ATPase 2, plasma membrane-type (EC 3.6.3.6) (Proton pump 2) {Arabidopsis thaliana}; contains InterPro accession IPR001757: ATPase, E1-E2 type; contains Pfam profile PF00690: Cation transporter/ATPase, N-terminus \| chr4:14770505-14776059 REVERSE \| Aliases: F9N11.40, F9N11_40, P TYPE H(+) ATPASE ISOFORM 2, PMA2 | 10.9 | 12.2 | -1.2 | -4.9 | 1.03% | 4.3 |
| 4468 | AT5G24230.1 \| expressed protein \| chr5:8228895-8230967 FORWARD \| Aliases: MOP9.4, MOP9_4 | 2.4 | 3.3 | -1.0 | -4.9 | 1.03% | 4.5 |
| 4472 | AT1G73660.1 \| protein kinase family protein, contains Pfam profile: PF00069 eukaryotic protein kinase domain \| chr1:27695554-27700872 REVERSE \| Aliases: F25P22.8, F25P22_8 | 4.1 | 5.0 | -0.9 | -4.9 | 1.03% | 4.5 |
| 4474 | AT4G28500.1 \| Symbol: ANAC073 \| no apical meristem (NAM) family protein, contains Pfam PF02365: No apical meristem (NAM) domain; \| chr4:14083020-14084272 FORWARD \| Aliases: ANAC073, F20O9.190, F20O9_190 | 3.7 | 5.0 | -1.3 | -4.9 | 1.04% | 4.5 |
| 4476 | AT5G47980.1 \| transferase family protein, similar to alcohol acyltransferase (Fragaria x ananassa)(GI:10121328)(PMID:10810141), deacetylvindoline 4-O-acetyltransferase (Catharanthus roseus)(GI:4091808)(PMID:9681034) \| chr5:19446114-19447702 FORWARD \| Aliases: MDN11.1 | 3.3 | 4.5 | -1.1 | -4.9 | 1.04% | 4.5 |
| 4477 | AT1G53700.1 \| protein kinase, putative, similar to cucumber protein kinase CsPK3 (Cucumis sativus) gi:7416109:dbj:BAA93704 \| chr1:20052254-20053783 FORWARD \| Aliases: F22G10.21, F22G10_21 | 3.5 | 4.4 | -1.0 | -4.9 | 1.04% | 4.5 |

| Rank | Description | Sync | Root | M | t | adj.q | B |
|---|---|---|---|---|---|---|---|
| 4480 | AT2G18730.1 \| diacylglycerol kinase, putative, contains INTERPRO domain, IPR001206, DAG-kinase catalytic domain \| chr2:8125912-8129003 FORWARD \| Aliases: MSF3.11, MSF3_11 | 4.6 | 5.5 | -0.8 | -4.9 | 1.04% | 4.4 |
| 4481 | AT3G24450.1 \| copper-binding family protein, similar to copper homeostasis factor gi:3168840 from Arabidopsis thaliana; contains Pfam profile PF00403: Heavy-metal-associated domain \| chr3:8880075-8881729 REVERSE \| Aliases: MXP5.2 | 3.3 | 5.5 | -2.2 | -4.9 | 1.04% | 4.5 |
| 4482 | AT4G38250.1 \| amino acid transporter family protein, low similarity to lysosomal amino acid transporter 1 (Rattus norvegicus) GI:14571904; contains Pfam profile PF01490: Transmembrane amino acid transporter protein | 7.0 | 8.6 | -1.7 | -4.9 | 1.04% | 4.5 |
| 4483 | AT4G16920.1 \| disease resistance protein (TIR-NBS-LRR class), putative, domain signature TIR-NBS-LRR exists, suggestive of a disease resistance protein. \| chr4:9519192-9525710 REVERSE \| Aliases: DL4490C, FCAALL.311 | 3.7 | 5.7 | -1.9 | -4.9 | 1.05% | 4.5 |
| 4484 | AT5G56140.1 \| KH domain-containing protein \| chr5:22742688-22745569 FORWARD \| Aliases: MDA7.20, MDA7_20 | 4.0 | 5.6 | -1.6 | -4.9 | 1.05% | 4.5 |
| 4486 | AT1G58080.1 \| Symbol: ATATP PRT1 \| ATP phosphoribosyl transferase 1 (ATP-PRT1), identical to ATP phosphoribosyl transferase GI:6683617 from (Arabidopsis thaliana) \| chr1:21507979-21511118 REVERSE \| Aliases: ATP PHOSPHORIBOSYL TRANSFERASE, T15M6.9, T15M6_9 | 7.2 | 9.0 | -1.9 | -4.9 | 1.05% | 4.5 |
| 4488 | AT4G39070.1 \| zinc finger (B-box type) family protein, salt-tolerance protein - Arabidopsis thaliana, PID:e224078 \| chr4:18204864-18206768 REVERSE \| Aliases: F19H22.170, F19H22_170 | 3.2 | 4.3 | -1.1 | -4.9 | 1.05% | 4.5 |
| 4489 | AT3G12980.1 \| histone acetyltransferase 5 (HAC5), identical to HAC5 (GI:21105780) (Arabidopsis thaliana); similar to CREB-binding protein GB:S39162 from (Homo sapiens) \| chr3:4146926-4154902 FORWARD \| Aliases: MGH6.20 | 4.5 | 5.4 | -0.9 | -4.9 | 1.05% | 4.4 |
| 4490 | AT1G67050.1 \| expressed protein \| chr1:25032290-25033451 REVERSE \| Aliases: F1O19.11, F1O19_11 | 4.2 | 6.3 | -2.1 | -4.9 | 1.05% | 4.5 |
| 4491 | AT1G19440.1 \| very-long-chain fatty acid condensing enzyme, putative, similar to GB:AAD37122 from (Arabidopsis thaliana) \| chr1:6728975-6730922 FORWARD \| Aliases: F18O14.21, F18O14_21 | 3.2 | 4.0 | -0.8 | -4.9 | 1.05% | 4.4 |
| 4493 | AT3G05580.1 \| serine/threonine protein phosphatase, putative, similar to serine/threonine protein phosphatase PP1 isozyme 8 SP:O82734 from (Arabidopsis thaliana) \| chr3:1617853-1619995 REVERSE \| Aliases: F18C1.15, F18C1_15 | 5.4 | 6.9 | -1.5 | -4.9 | 1.05% | 4.5 |
| 4503 | AT2G15310.1 \| Symbol: ATARFB1A \| Gene encoding ADP-ribosylation factor and similar to ADP-ribosylation factor (GI:861205) (Chlamydomonas reinhardtii), other ARFs and ARF-like proteins. Members of this family are known to be essential for vesicle coating and uncoating and functions in GTP-binding. \| chr2:6660874-6662583 FORWARD \| Aliases: ATARFB1A, F27O10.4, F27O10_4 | 3.0 | 4.1 | -1.1 | -4.9 | 1.06% | 4.5 |
| 4504 | AT5G58730.1 \| pfkB-type carbohydrate kinase family protein, contains Pfam profile: PF00294 pfkB family carbohydrate kinase \| chr5:23740634-23742691 REVERSE \| Aliases: MZN1.17, MZN1_17 | 7.0 | 8.2 | -1.3 | -4.9 | 1.06% | 4.5 |
| 4505 | AT2G22680.1 \| zinc finger (C3HC4-type RING finger) family protein, contains Pfam profiles PF00097: Zinc finger, C3HC4 type (RING finger), PF00092: von Willebrand factor type A domain | 4.3 | 6.0 | -1.7 | -4.9 | 1.07% | 4.5 |
| 4506 | AT4G00755.2 \| F-box family protein, ; similar to SKP1 interacting partner 2 (SKIP2) TIGR_Ath1:At5g67250 \| chr4:324994-326964 FORWARD \| Aliases: None | 5.5 | 6.3 | -0.7 | -4.9 | 1.07% | 4.3 |
| 4509 | AT1G47655.1 \| Dof-type zinc finger domain-containing protein \| chr1:17527805-17528746 FORWARD \| Aliases: None | 3.9 | 5.1 | -1.1 | -4.9 | 1.07% | 4.5 |
| 4516 | AT3G52820.1 \| purple acid phosphatase (PAP22), identical to purple acid phosphatase (PAP22)GI:20257494 from (Arabidopsis thaliana) \| chr3:19584922-19587955 REVERSE \| Aliases: F3C22.220 | 2.4 | 3.3 | -0.8 | -4.9 | 1.07% | 4.4 |
| 4519 | AT5G05840.1 \| expressed protein, contains Pfam profile PF04788: Protein of unknown function (DUF620); expression supported by MPSS \| chr5:1757269-1760459 REVERSE \| Aliases: MJJ3.26, MJJ3_26 | 3.7 | 4.5 | -0.8 | -4.9 | 1.07% | 4.2 |
| 4521 | AT3G46760.1 \| protein kinase family protein, contains protein kinase domain, Pfam:PF00069 \| chr3:17233012-17234025 FORWARD \| Aliases: T6H20.210 | 2.7 | 3.9 | -1.1 | -4.9 | 1.07% | 4.5 |
| 4523 | AT3G45870.1 \| integral membrane family protein / nodulin MtN21-related, simlar to MtN21 GI:2598575 (root nodule development) Medicago truncatula, EMBL:MTY15293 \| chr3:16878187-16879911 FORWARD \| Aliases: F16L2.80 | 3.1 | 3.9 | -0.8 | -4.9 | 1.07% | 4.3 |
| 4526 | AT3G13430.2 \| similar to zinc finger (C3HC4-type RING finger) family protein [Arabidopsis thaliana] (TAIR:At1g55530.1); similar to hypothetical protein [Oryza sativa (japonica cultivar-group)] (GB:AAT77283.1); contains InterPro domain Zn-finger, RING (InterPro:IPR001841) \| chr3:4367514-4368886 FORWARD \| Aliases: MRP15.6 | 3.3 | 4.2 | -0.9 | -4.9 | 1.07% | 4.5 |

| Rank | Description | Sync | Root | M | t | adj.q | B |
|---|---|---|---|---|---|---|---|
| 4527 | AT5G64905.1 \| expressed protein \| chr5:25956800-25957285 FORWARD \| Aliases: None | 3.0 | 4.1 | -1.1 | -4.9 | 1.07% | 4.4 |
| 4530 | AT1G59940.1 \| Symbol: ARR3 \| two-component responsive regulator / response regulator 3 (ARR3), identical to response regulator 3 GI:3953595 from (Arabidopsis thaliana) \| chr1:22069282-22070638 REVERSE \| Aliases: F23H11.25, F23H11_25, RESPONSE REGULATOR 3 | 4.0 | 4.9 | -1.0 | -4.9 | 1.08% | 4.5 |
| 4533 | AT4G01070.1 \| the glycosyltransferase (UGT72B1) is involved in metabolizing xenobiotica (chloroaniline and chlorophenole). Comparison between wild type and knock-out mutant demonstrates the central role of this gene for metabolizing chloroaniline but significantly less for chlorophenole. The glucosyltransferase preferred UDP-xylose over UDP-glucose indicating its (additional) functioning as a xylosyltransferase in planta \| chr4:461592-463449 REVERSE \| Aliases: F2N1.15, F2N1_15, GT72B1 | 5.2 | 7.6 | -2.4 | -4.9 | 1.08% | 4.5 |
| 4535 | AT2G29320.1 \| tropinone reductase, putative / tropine dehydrogenase, putative, similar to tropinone reductase SP:P50165 from (Datura stramonium) \| chr2:12599225-12600763 FORWARD \| Aliases: F16P2.30, F16P2_30 | 3.5 | 4.9 | -1.4 | -4.9 | 1.08% | 4.5 |
| 4538 | AT5G49450.1 \| bZIP family transcription factor, similar to bZIP transcription factor GI:1769891 from (Arabidopsis thaliana) \| chr5:20069055-20069961 FORWARD \| Aliases: K7J8.13, K7J8_13 | 5.7 | 7.8 | -2.1 | -4.9 | 1.08% | 4.5 |
| 4541 | AT5G12880.1 \| proline-rich family protein, similar to hydroxyproline-rich glycoprotein gi:169345:gb:AAA33763 \| chr5:4068522-4068965 REVERSE \| Aliases: T24H18.50, T24H18_50 | 3.2 | 7.1 | -3.9 | -4.9 | 1.09% | 4.5 |
| 4543 | AT2G17130.2 \| Symbol: IDH2 \| isocitrate dehydrogenase subunit 2 / NAD+ isocitrate dehydrogenase subunit 2, nearly identical to NAD+ dependent isocitrate dehydrogenase subunit 2 (Arabidopsis thaliana) GI:1766048 \| chr2:7467896-7469636 REVERSE \| Aliases: F6P23.14, F6P23_14, ISOCITRATE DEHYDROGENASE SUBUNIT 2, NAD+ DEPENDENT ISOCITRATE DEHYDROGENASE SUBUNIT 2 | 9.2 | 10.7 | -1.5 | -4.9 | 1.09% | 4.4 |
| 4546 | AT5G40230.1 \| nodulin-related, low similarity to MtN21 (Medicago truncatula) GI:2598575; contains Pfam profile PF00892: Integral membrane protein \| chr5:16096876-16098963 REVERSE \| Aliases: MSN9.130, MSN9_130 | 3.7 | 4.6 | -0.8 | -4.9 | 1.10% | 4.4 |
| 4548 | AT1G34750.1 \| protein phosphatase 2C, putative / PP2C, putative, similar to GB:AAD17805 from (Lotus japonicus) (Proc. Natl. Acad. Sci. U.S.A. 96 (4), 1738-1743 (1999)) \| chr1:12736073-12737705 REVERSE \| Aliases: F11O6.14 | 4.5 | 5.8 | -1.3 | -4.9 | 1.10% | 4.5 |
| 4549 | AT4G08410.1 \| proline-rich extensin-like family protein, contains proline-rich extensin domains, INTERPRO:IPR002965; \| chr4:5339083-5341206 REVERSE \| Aliases: T28D5.100, T28D5_100 | 3.6 | 8.4 | -4.8 | -4.9 | 1.10% | 3.9 |
| 4556 | AT1G12880.1 \| MutT/nudix family protein, similar to diphosphoinositol polyphosphate phosphohydrolase (Homo sapiens) GI:3978224; contains Pfam profile PF00293: NUDIX domain \| chr1:4389749-4391149 FORWARD \| Aliases: F13K23.14, F13K23_14 | 3.7 | 7.1 | -3.4 | -4.8 | 1.10% | 4.4 |
| 4563 | AT1G78100.1 \| F-box family protein, contains F-box domain Pfam:PF00646 | 7.1 | 8.4 | -1.3 | -4.8 | 1.11% | 4.3 |
| 4565 | AT3G54870.1 \| armadillo/beta-catenin repeat family protein / kinesin motor family protein, kinesin, Syncephalastrum racemosum, AJ225894 \| chr3:20341784-20347695 FORWARD \| Aliases: F28P10.150 | 3.4 | 4.5 | -1.0 | -4.8 | 1.11% | 4.3 |
| 4571 | AT3G04370.2 \| similar to 33 kDa secretory protein-related [Arabidopsis thaliana] (TAIR:At1g04520.1); similar to 33 kDa secretory protein-like [Oryza sativa (japonica cultivar-group)] (GB:BAD46165.1); contains InterPro domain Protein of unknown function DUF26 (InterPro:IPR002902) \| chr3:1159429-1161132 FORWARD \| Aliases: T27C4.1, T27C4_1 | 3.6 | 4.4 | -0.8 | -4.8 | 1.11% | 4.3 |
| 4574 | AT3G09830.2 \| protein kinase, putative, similar to protein kinase (Lophopyrum elongatum) gi:13022177:gb:AAK11674 \| chr3:3016545-3018988 FORWARD \| Aliases: F8A24.12 | 4.8 | 6.1 | -1.3 | -4.8 | 1.12% | 4.4 |
| 4575 | AT3G55700.1 \| UDP-glucoronosyl/UDP-glucosyl transferase family protein, glucuronosyl transferase homolog, Lycopersicon esculentum, PIR:S39507 ;contains Pfam profile: PF00201 UDP-glucoronosyl and UDP-glucosyl transferase \| chr3:20682094-20684351 FORWARD \| Aliases: F1I16.110 | 2.8 | 3.7 | -0.9 | -4.8 | 1.12% | 4.3 |
| 4576 | AT5G56720.1 \| malate dehydrogenase, cytosolic, putative, similar to cytosolic malate dehydrogenase from Mesembryanthemum crystallinum (SP:O24047), Medicago sativa (SP:O48905), Prunus persica (GI:15982948); contains InterPro entry IPR001236: Lactate/malate dehydrogenase \| chr5:22962763-22963944 FORWARD \| Aliases: MIK19.17, MIK19_17 | 3.7 | 4.9 | -1.3 | -4.8 | 1.12% | 4.5 |
| 4577 | AT4G29190.1 \| zinc finger (CCCH-type) family protein, contains Pfam domain, PF00642: Zinc finger C-x8-C-x5-C-x3-H type (and similar) \| chr4:14391944-14393379 REVERSE \| Aliases: F17A13.10, F17A13_10 | 5.8 | 7.1 | -1.3 | -4.8 | 1.12% | 4.3 |
| 4578 | AT4G36040.1 \| DNAJ heat shock N-terminal domain-containing protein (J11), identical to dnaJ heat shock protein J11 (Arabidopsis thaliana) GI:9843641; contains Pfam profile PF00226 DnaJ domain \| chr4:17049174-17050277 REVERSE \| Aliases: T19K4.170, T19K4_170 | 5.4 | 7.4 | -2.0 | -4.8 | 1.12% | 4.4 |

| Rank | Description | Sync | Root | M | t | adj.q | B |
|---|---|---|---|---|---|---|---|
| 4579 | AT5G27450.2 \| mevalonate kinase (MK), identical to mevalonate kinase (Arabidopsis thaliana) SWISS-PROT:P46086 \| chr5:9690745-9693227 FORWARD \| Aliases: F21A20.160, F21A20_160 | 6.9 | 8.3 | -1.4 | -4.8 | 1.12% | 4.5 |
| 4581 | AT4G11670.1 \| expressed protein, contains Pfam PF05664: Protein of unknown function (DUF810) \| chr4:7044395-7053085 REVERSE \| Aliases: T5C23.100, T5C23_100 | 5.1 | 6.4 | -1.3 | -4.8 | 1.12% | 4.4 |
| 4582 | AT5G09290.1 \| 3'(2'),5'-bisphosphate nucleotidase, putative / inositol polyphosphate 1-phosphatase, putative, similar to SP:Q42546 3'(2'),5'-bisphosphate nucleotidase (EC 3.1.3.7) {Arabidopsis thaliana}; contains Pfam profile PF00459: Inositol monophosphatase family \| chr5:2882429-2884099 FORWARD \| Aliases: T5E8.90, T5E8_90 | 2.9 | 4.0 | -1.1 | -4.8 | 1.12% | 4.4 |
| 4585 | AT4G12480.1 \| Symbol: PEARLI 1 1 \| protease inhibitor/seed storage/lipid transfer protein (LTP) family protein, identical to pEARLI 1 (Accession No. L43080): an Arabidopsis member of a conserved gene family (PGF95-099), Plant Physiol. 109 (4), 1497 (1995); contains Pfam protease inhibitor/seed storage/LTP family domain PF00234 \| chr4:7406102-7406934 REVERSE \| Aliases: T1P17.70, T1P17_70 | 2.3 | 3.4 | -1.1 | -4.8 | 1.13% | 4.4 |
| 4587 | AT4G21810.1 \| Der1-like family protein / degradation in the ER-like family protein, contains Pfam profile: PF04511 Der1-like family \| chr4:11575271-11577165 REVERSE \| Aliases: T8O5.20, T8O5_20 | 6.0 | 7.4 | -1.4 | -4.8 | 1.13% | 4.3 |
| 4591 | AT1G42470.1 \| patched family protein, similar to SP:O15118 Niemann-Pick C1 protein precursor from Homo sapiens (GB:AAB63982) (GI:2276463); contains Pfam profile PF02460 Patched family \| chr1:15929427-15937648 FORWARD \| Aliases: F7F22.1, F7F22_1 | 7.3 | 8.7 | -1.4 | -4.8 | 1.13% | 4.3 |
| 4592 | AT3G06440.1 \| galactosyltransferase family protein, contains Pfam profile: PF01762 galactosyltransferase \| chr3:1972628-1975760 REVERSE \| Aliases: F24P17.7, F24P17_7 | 4.4 | 5.2 | -0.8 | -4.8 | 1.13% | 4.3 |
| 4594 | AT3G53710.2 \| similar to arabidopsis pde1 suppressor 1 protein (ASP1) [Arabidopsis thaliana] (TAIR:At2g37550.1); similar to CG4237-PA [Drosophila melanogaster] (GB:NP_524040.2); similar to putative ARF1 GTPase activating protein [Drosophila melanogaster] (GB:AAB64300.1); contains InterPro domain Human Rev interacting-like protein (hRIP) (InterPro:IPR001164) \| chr3:19914291-19916494 REVERSE \| Aliases: F5K20.10 | 7.0 | 7.8 | -0.8 | -4.8 | 1.13% | 4.2 |
| 4601 | AT2G19450.1 \| Symbol: TAG1 \| diacylglycerol O-acyltransferase / acyl CoA:diacylglycerol acyltransferase (DGAT), identical to gi:5050913, gi:6625553 \| chr2:8433288-8436818 FORWARD \| Aliases: ABX45, AS11, ATDGAT, DGAT1, DIACYLGLYCEROL ACYLTRANSFERASE, F27F23.24, RDS1, REGULATION OF DESATURASES IN SEEDS 1, TRIACYLGLYCEROL BIOSYNTHESIS DEFECT 1 | 6.8 | 8.0 | -1.2 | -4.8 | 1.14% | 4.4 |
| 4604 | AT2G44370.1 \| DC1 domain-containing protein, highly similar to GP:2435515:AF024504 \| chr2:18329070-18330197 REVERSE \| Aliases: F4I1.18 | 2.7 | 4.1 | -1.4 | -4.8 | 1.14% | 4.4 |
| 4606 | AT2G44260.2 \| expressed protein \| chr2:18302914-18304943 FORWARD \| Aliases: F4I1.7 | 3.0 | 3.9 | -0.9 | -4.8 | 1.14% | 4.4 |
| 4607 | AT2G07360.1 \| SH3 domain-containing protein, contains Pfam profile PF00018: SH3 domain | 4.4 | 5.0 | -0.6 | -4.8 | 1.14% | 4.1 |
| 4609 | AT1G75760.1 \| ER lumen protein retaining receptor family protein, similar to SP:P33946 ER lumen protein retaining receptor 1 (KDEL receptor 1) {Bos taurus}; contains Pfam profile PF00810: ER lumen protein retaining receptor \| chr1:28450377-28452293 REVERSE \| Aliases: F10A5.5, F10A5_5 | 4.3 | 5.4 | -1.2 | -4.8 | 1.14% | 4.4 |
| 4610 | AT5G23340.1 \| expressed protein \| chr5:7856195-7859280 FORWARD \| Aliases: MKD15.20, MKD15_20 | 3.7 | 5.5 | -1.8 | -4.8 | 1.15% | 4.4 |
| 4612 | AT1G06620.1 \| 2-oxoglutarate-dependent dioxygenase, putative, similar to 2A6 (GI:599622) and tomato ethylene synthesis regulatory protein E8 (SP:P10967); contains Pfam profile: PF00671 Iron/Ascorbate oxidoreductase family \| chr1:2025600-2027270 FORWARD \| Aliases: F12K11.24, F12K11_24 | 2.6 | 3.5 | -0.9 | -4.8 | 1.15% | 4.4 |
| 4614 | AT1G28580.2 \| GDSL-motif lipase, putative, similar to lipase (Arabidopsis thaliana) GI:1145627; contains InterPro Entry IPR001087 Lipolytic enzyme, G-D-S-L family \| chr1:10044323-10046413 REVERSE \| Aliases: F1K23.18, F1K23_18 | 8.9 | 10.1 | -1.2 | -4.8 | 1.15% | 4.2 |
| 4615 | AT1G67310.1 \| similar to calmodulin-binding protein [Arabidopsis thaliana] (TAIR:At5g64220.1); similar to calmodulin-binding transcription activator [Brassica napus] (GB:AAM10969.1); contains InterPro domain IQ calmodulin-binding region (InterPro:IPR000048); contains InterPro domain CG-1 domain (InterPro:IPR005559); contains InterPro domain Ankyrin (InterPro:IPR002110) \| chr1:25201845-25206789 REVERSE \| Aliases: F1N21.13 | 4.9 | 6.7 | -1.9 | -4.8 | 1.15% | 4.4 |
| 4616 | AT5G54980.1 \| integral membrane family protein, similar to unknown protein (gb:AAD50013.1); contains TIGRFAM TIGR01569 : plant integral membrane protein TIGR01569; contains Pfam PF04535 : Domain of unknown function (DUF588) \| chr5:22332925-22333827 FORWARD \| Aliases: MBG8.25, MBG8_25 | 5.8 | 7.0 | -1.2 | -4.8 | 1.15% | 4.4 |
| 4618 | AT2G19210.1 \| leucine-rich repeat protein kinase, putative, similar to light repressible receptor protein kinase (Arabidopsis thaliana) gi:1321686:emb:CAA66376; contains leucine rich repeat (LRR) domains, Pfam:PF00560; contains protein kinase domain, Pfam:PF00069 \| chr2:8342721-8346389 REVERSE \| Aliases: F27F23.1, F27F23_1 | 3.1 | 4.4 | -1.3 | -4.8 | 1.15% | 4.4 |
| 4619 | AT3G11820.2 \| Symbol: SYP121 \| | 6.0 | 7.6 | -1.6 | -4.8 | 1.15% | 4.4 |

| Rank | Description | Sync | Root | M | t | adj.q | B |
|---|---|---|---|---|---|---|---|
| 4623 | AT1G21210.1 \| Symbol: WAK4 \| wall-associated kinase 4 | 2.8 | 3.4 | -0.6 | -4.8 | 1.16% | 4.1 |
| 4624 | AT4G13940.3 \| Symbol: HOG1 \| similar to adenosylhomocysteinase, putative / S-adenosyl-L-homocysteine hydrolase, putative / AdoHcyase, putative [Arabidopsis thaliana] (TAIR:At3g23810.1); similar to adenosylhomocysteinase (EC 3.3.1.1) - wheat (GB:T06764); similar to S-adenosyl-L-homocystein hydrolase [Nicotiana sylvestris] (GB:BAA03709.1); similar to adenosylhomocysteinase [Medicago truncatula] (GB:AAO89238.1); similar to S-adenosyl-L-homocysteinase [Lupinus luteus] (GB:AAD56048.1); similar to wheat adenosylhomocysteinase-like protein [Oryza sativa (japonica cultivar-group)] (GB:AAO72664.1); contains InterPro domain S-adenosyl-L-homocysteine hydrolase (InterPro:IPR000043) \| chr4:8054856-8057176 FORWARD \| Aliases: DL3010W, EMB1395, EMBRYO DEFECTIVE 1395, FCAALL.35, HOMOLOGY DEPENDENT GENE SILENCING 1, S ADENOSYL L HOMOCYSTEINE HYDROLASE, SAHH, SAHH1 | 9.3 | 11.8 | -2.4 | -4.8 | 1.16% | 4.4 |
| 4626 | AT4G21790.1 \| Symbol: TOM1 \| transmembrane protein-related (TOM1), contains some similarity to transmembrane protein TOM3 GI:15425641 from (Arabidopsis thaliana); identical to cDNA TOM1 GI:9967414 \| chr4:11569793-11572561 FORWARD \| Aliases: F17L22.250, F17L22_250, TOBAMOVIRUS MULTIPLICATION 1 | 7.4 | 8.5 | -1.1 | -4.8 | 1.16% | 4.3 |
| 4630 | AT5G24890.1 \| expressed protein, weak similarity to MTD1 (Medicago truncatula) GI:9294810 \| chr5:8558207-8559409 REVERSE \| Aliases: F6A4.100, F6A4_100 | 5.5 | 7.9 | -2.4 | -4.8 | 1.16% | 4.4 |
| 4631 | AT2G35060.2 \| Symbol: KUP11 \| similar to potassium transporter family protein [Arabidopsis thaliana] (TAIR:At1g31120.1); similar to potassium transporter (KUP1) [Arabidopsis thaliana] (TAIR:At2g30070.1); similar to potassium transporter family protein [Arabidopsis thaliana] (TAIR:At1g60160.1); similar to potassium transporter family protein [Arabidopsis thaliana] (TAIR:At4g19960.1); similar to potassium transporter (KUP3) [Arabidopsis thaliana] (TAIR:At3g02050.1); similar to putative potasium transporter [Oryza sativa (japonica cultivar-group)] (GB:CAD21002.1); similar to OSJNBa0070C17.23 [Oryza sativa (japonica cultivar-group)] (GB:XP_473875.1); similar to putative potassium transporter [Oryza sativa (japonica cultivar-group)] (GB:BAD46101.1); similar to POT9_ARATH Putative potassium transporter 9 (AtPOT9) (GB:O49423); similar to putative potassium transporter [Oryza sativa (japonica cultivar-group)] (GB:XP_479449.1); contains InterPro domain K+ potassium transporter (InterPro:IPR003855) \| chr2:14782142-14785949 REVERSE \| Aliases: F19I3.29, F19I3_29 | 4.2 | 5.0 | -0.8 | -4.8 | 1.16% | 4.2 |
| 4632 | AT3G54010.2 \| Symbol: PAS1 \| peptidyl-prolyl cis-trans isomerase, putative / FK506-binding protein, putative / pasticcino 1-D (PAS1-D), nearly identical to pasticcino 1-D (Arabidopsis thaliana) GI:3080740 \| chr3:20011867-20016161 FORWARD \| Aliases: F5K20.310, PASTICCINO 1 | 9.1 | 10.2 | -1.1 | -4.8 | 1.16% | 4.2 |
| 4634 | AT4G23880.1 \| expressed protein \| chr4:12415729-12416460 REVERSE \| Aliases: T32A16.50, T32A16_50 | 3.5 | 4.5 | -1.0 | -4.8 | 1.17% | 4.4 |
| 4636 | AT3G14010.1 \| Symbol: CID4 \| hydroxyproline-rich glycoprotein family protein, similar to Mrs16p (GI:2737884) (Saccharomyces cerevisiae); weak similarity to ataxin-2 related protein (GI:1679686) (Homo sapiens). Included in a family of CTC interacting domain proteins found to interact with PAB2. \| chr3:4636373-4640977 FORWARD \| Aliases: CID4, MDC16.14 | 5.9 | 8.3 | -2.4 | -4.8 | 1.17% | 4.4 |
| 4638 | AT1G70100.3 \| expressed protein \| chr1:26406746-26409751 FORWARD \| Aliases: F20P5.17, F20P5_17 | 2.7 | 3.0 | -0.3 | -4.8 | 1.17% | 3.6 |
| 4639 | AT5G42870.1 \| lipin family protein, contains Pfam profile: PF04571 lipin, N-terminal conserved region \| chr5:17202691-17206909 REVERSE \| Aliases: MBD2.6, MBD2_6 | 3.2 | 4.9 | -1.7 | -4.8 | 1.17% | 4.3 |
| 4642 | AT3G02340.1 \| zinc finger (C3HC4-type RING finger) family protein, contains Pfam profile: PF00097 Zinc finger, C3HC4 type (RING finger) \| chr3:477010-478449 FORWARD \| Aliases: F11A12.3, F11A12_3 | 6.6 | 7.4 | -0.8 | -4.8 | 1.17% | 4.0 |
| 4644 | AT1G45130.1 \| beta-galactosidase, putative / lactase, putative, similar to beta-galactosidase (Lycopersicon esculentum) GI:7939619, beta-galactosidase BG1 GI:15081596 from (Vitis vinifera); contains Pfam profile PF01301: Glycosyl hydrolases family 35 \| chr1:17067692-17071702 FORWARD \| Aliases: F27F5.20, F27F5_20 | 9.3 | 10.5 | -1.2 | -4.8 | 1.17% | 4.2 |
| 4647 | AT1G04400.2 \| Symbol: CRY2 \| cryptochrome 2 apoprotein (CRY2) / blue light photoreceptor (PHH1), 97% identical to photolysase (PHH1) | 6.8 | 8.3 | -1.5 | -4.8 | 1.18% | 4.3 |
| 4652 | AT4G35580.1 \| no apical meristem (NAM) family protein, similar to TIP (Arabidopsis thaliana) GI:9408601; contains Pfam profile PF02365: No apical meristem (NAM) protein \| chr4:16888410-16890772 REVERSE \| Aliases: F8D20.90, F8D20_90 | 5.1 | 6.3 | -1.2 | -4.8 | 1.18% | 4.4 |
| 4654 | AT3G53180.1 \| glutamine synthetase, putative, similar to glutamine synthetase (glutamate--ammonia ligase) (Bacillus subtilis) SWISS-PROT:P12425 \| chr3:19717872-19722393 FORWARD \| Aliases: T4D2.110 | 5.6 | 6.9 | -1.2 | -4.8 | 1.18% | 4.3 |
| 4659 | AT3G33530.1 \| transducin family protein / WD-40 repeat family protein, similar to WD-repeat protein 11 (SP:Q9BZH6) {Homo sapiens}; contains Pfam PF00400: WD domain, G-beta repeat (4 copies, 3 weak):2763187:gb:T46504.1:T46504 \| chr3:14096340-14104656 FORWARD \| Aliases: T4P3.9 | 5.4 | 6.3 | -1.0 | -4.8 | 1.18% | 4.3 |
| 4660 | AT4G20210.1 \| terpene synthase/cyclase family protein, (+)-delta-cadinene synthase isozyme XC14, Gossypiumarboreum, PIR2:S68366 \| chr4:10913623-10918292 REVERSE \| Aliases: F1C12.130, F1C12_130 | 3.5 | 4.6 | -1.1 | -4.8 | 1.18% | 4.3 |
| 4661 | AT1G27320.1 \| Symbol: AHK3 \| histidine kinase (AHK3), identical to histidine kinase AHK3 (Arabidopsis thaliana) gi:13537198:dbj:BAB40775 \| chr1:9487767-9492280 FORWARD \| Aliases: ARABIDOPSIS HISTIDINE KINASE 3, F17L21.11, F17L21_11 | 3.7 | 5.1 | -1.4 | -4.8 | 1.18% | 4.4 |

| Rank | Description | Sync | Root | M | t | adj.q | B |
|---|---|---|---|---|---|---|---|
| 4663 | AT5G40590.1 \| DC1 domain-containing protein, predicted protein, Arabidopsis thaliana \| chr5:16268665-16269546 REVERSE \| Aliases: MNF13.11, MNF13_11 | 2.7 | 5.2 | -2.6 | -4.8 | 1.18% | 4.2 |
| 4664 | AT1G03870.1 \| Symbol: FLA9 \| fasciclin-like arabinogalactan-protein (FLA9), identical to gi_13377784_gb_AAK20861 \| chr1:982508-983539 REVERSE \| Aliases: F21M11.20, F21M11_20, fasciclin like arabinogalactan protein 9 | 4.7 | 5.8 | -1.2 | -4.8 | 1.18% | 4.4 |
| 4665 | AT5G42830.1 \| transferase family protein, similar to anthranilate N-hydroxycinnamoyl/benzoyltransferase, Dianthus caryophyllus (gi:2239091); contains Pfam transferase family domain PF002458 \| chr5:17193612-17195246 FORWARD \| Aliases: MBD2.2, MBD2_2 | 5.3 | 6.5 | -1.2 | -4.8 | 1.18% | 4.4 |
| 4667 | AT4G37180.2 \| myb family transcription factor, contains Pfam domain, PF00249: Myb-like DNA-binding domain \| chr4:17504558-17506361 FORWARD \| Aliases: AP22.100, AP22_100 | 3.3 | 4.2 | -0.9 | -4.8 | 1.18% | 4.3 |
| 4672 | AT4G01920.1 \| DC1 domain-containing protein, similar to A. thaliana CHP-rich proteins \| chr4:828890-831200 REVERSE \| Aliases: T7B11.18, T7B11_18 | 2.9 | 3.7 | -0.8 | -4.8 | 1.19% | 4.3 |
| 4673 | AT5G60300.2 \| lectin protein kinase family protein, contains Pfam domains, PF00069: Protein kinase domain and PF00139: Legume lectins beta domain \| chr5:24281848-24284436 FORWARD \| Aliases: F15L12.17, F15L12_17 | 5.9 | 7.2 | -1.4 | -4.8 | 1.19% | 4.4 |
| 4677 | AT5G43380.3 \| Symbol: TOPP6 \| similar to serine/threonine protein phosphatase PP1 isozyme 4 (TOPP4) / phosphoprotein phosphatase 1 [Arabidopsis thaliana] (TAIR:At2g39840.1); similar to protein phosphatase 1, catalytic beta subunit [Medicago sativa] (GB:CAA05491.1); contains InterPro domain Metallo-phosphoesterase (InterPro:IPR004843); contains InterPro domain Serine/threonine-specific protein phosphatase and bis(5-nucleosyl)-tetraphosphatase (InterPro:IPR006186) \| chr5:17437047-17439210 REVERSE \| Aliases: None | 2.6 | 3.1 | -0.5 | -4.8 | 1.19% | 3.9 |
| 4681 | AT1G21780.1 \| BTB/POZ domain-containing protein. Contains similarity to gb:AJ000644 SPOP (speckle-type POZ protein) from Homo sapiens and contains a PF:00651 BTB/POZ domain. ESTs gb:T75841, gb:R89974, gb:R30221, gb:N96386, gb:T76457, gb:AI100013 and gb:T76456 come from this gene;supported by full-length. Interacts with CUL3A and CUL3B. \| chr1:7652431-7654003 FORWARD \| Aliases: F8K7.22, F8K7_22 | 4.0 | 5.2 | -1.2 | -4.8 | 1.19% | 4.3 |
| 4683 | AT1G64060.1 \| Symbol: ATRBOH F F \| respiratory burst oxidase protein F (RbohF) (RbohAp108) / NADPH oxidase, identical to cytochrome b245 beta chain homolog RbohAp108 (GI:2654868), respiratory burst oxidase protein F (gi:3242456), from Arabidopsis thaliana \| chr1:23773437-23780594 FORWARD \| Aliases: ATRBOH F, CYTOCHROME B245 BETA CHAIN HOMOLOG RBOHAP108, F22C12.18, F22C12_18, RBOHAP108 | 5.5 | 7.3 | -1.8 | -4.8 | 1.19% | 4.4 |
| 4684 | AT5G44550.1 \| integral membrane family protein, similar to unknown protein (pir::T10581) This family of plant proteins contains a domain that may have a catalytic activity. It has a conserved arginine and aspartate that could form an active site. These proteins are predicted to contain 3 or 4 transmembrane helices; contains TIGRFAM TIGR01569 : plant integral membrane protein TIGR01569; contains Pfam PF04535 : Domain of unknown function (DUF588) \| chr5:17959102-17960435 REVERSE \| Aliases: MFC16.23, MFC16_23 | 2.3 | 3.6 | -1.3 | -4.8 | 1.19% | 4.4 |
| 4686 | AT3G54380.2 \| SAC3/GANP family protein, contains Pfam profile: PF03399 SAC3/GANP family \| chr3:20144872-20147769 REVERSE \| Aliases: T14E10.7 | 5.6 | 6.4 | -0.7 | -4.8 | 1.19% | 3.9 |
| 4687 | AT1G75640.1 \| leucine-rich repeat family protein / protein kinase family protein, contains protein kinase domain, Pfam:PF00069; contains leucine-rich repeats, Pfam:PF00560 \| chr1:28407261-28410683 REVERSE \| Aliases: F10A5.16, F10A5_16 | 3.1 | 3.9 | -0.8 | -4.8 | 1.19% | 4.2 |
| 4689 | AT5G04840.1 \| bZIP protein \| chr5:1405306-1407935 FORWARD \| Aliases: MUK11.16, MUK11_16 | 3.9 | 5.0 | -1.2 | -4.8 | 1.20% | 4.4 |
| 4700 | AT5G05980.2 \| Symbol: ATDFB \| similar to dihydrofolate synthetase/folylpolyglutamate synthetase (DHFS/FPGS4) [Arabidopsis thaliana] (TAIR:At3g55630.3); similar to folylpoly-gammaglutamate synthetase precursor [Cricetulus griseus] (GB:AAK69546.1); contains InterPro domain Folylpolyglutamate synthetase (InterPro:IPR001645) \| chr5:1799491-1804296 REVERSE \| Aliases: A. THALIANA DHFS FPGS HOMOLOG B, K18J17.17, K18J17_17 | 8.6 | 9.5 | -0.9 | -4.8 | 1.20% | 4.3 |
| 4702 | AT1G61260.1 \| expressed protein, contains similarity to cotton fiber expressed protein 1 (Gossypium hirsutum) gi:3264828:gb:AAC33276 \| chr1:22597130-22598734 REVERSE \| Aliases: F11P17.2, F11P17_2 | 4.2 | 6.7 | -2.4 | -4.8 | 1.21% | 4.4 |
| 4705 | AT1G03700.1 \| integral membrane family protein, contains TIGRFAM TIGR01569 : plant integral membrane protein TIGR01569; contains Pfam PF04535 : Domain of unknown function (DUF588) \| chr1:920950-921901 FORWARD \| Aliases: F21B7.30 | 2.6 | 4.6 | -1.9 | -4.8 | 1.21% | 4.4 |
| 4709 | AT2G17060.1 \| disease resistance protein (TIR-NBS-LRR class), putative, domain signature TIR-NBS-LRR exists, suggestive of a disease resistance protein. \| chr2:7429493-7433959 FORWARD \| Aliases: F6P23.21, F6P23_21 | 2.2 | 3.0 | -0.8 | -4.7 | 1.21% | 4.2 |
| 4712 | AT3G53410.1 \| zinc finger (C3HC4-type RING finger) family protein, contains Pfam domain: PF00097: Zinc finger, C3HC4 type (RING finger) \| chr3:19811930-19813298 REVERSE \| Aliases: F4P12.110 | 5.8 | 6.7 | -0.9 | -4.7 | 1.22% | 4.2 |

| Rank | Description | Sync | Root | M | t | adj.q | B |
|---|---|---|---|---|---|---|---|
| 4719 | AT3G06540.1 \| GDP dissociation inhibitor family protein / Rab GTPase activator family protein, similar to SP:P26374 Rab proteins geranylgeranyltransferase component A 2 (Rab escort protein 2) {Homo sapiens}; contains Pfam profile PF00996: GDP dissociation inhibitor \| chr3:2035010-2037794 REVERSE \| Aliases: F5E6.13, F5E6_13 | 4.9 | 5.9 | -1.0 | -4.7 | 1.23% | 4.3 |
| 4721 | AT1G67060.1 \| expressed protein \| chr1:25040279-25043029 FORWARD \| Aliases: F1O19.19, F1O19_19 | 3.4 | 4.7 | -1.3 | -4.7 | 1.23% | 4.3 |
| 4728 | AT5G03470.1 \| Symbol: ATB' ALPHA \| serine/threonine protein phosphatase 2A (PP2A) regulatory subunit B' (B'alpha), similar to SWISS-PROT:Q28653 serine/threonine protein phosphatase 2A, 56 kDa regulatory subunit, delta isoform (PP2A, B subunit, B' delta isoform, PP2A, B subunit, B56 delta isoform, PP2A, B subunit, PR61 delta isoform, PP2A, B subunit, R5 delta isoform, PP2A, B subunit, B'-gamma) (Oryctolagus cuniculus); contains Pfam domain, PF01603: Protein phosphatase 2A regulatory B subunit (B56 family) | 5.9 | 7.3 | -1.4 | -4.7 | 1.23% | 4.2 |
| 4729 | AT5G47990.1 \| Symbol: CYP705A5 \| cytochrome P450 family protein, similar to Cytochrome P450 93A3 (P450 CP5) (SP:O81973) (Glycine max); \| chr5:19452053-19453915 FORWARD \| Aliases: MDN11.4, MDN11_4 | 2.6 | 3.3 | -0.7 | -4.7 | 1.23% | 4.1 |
| 4731 | AT5G10550.1 \| DNA-binding bromodomain-containing protein, low similarity to kinase (Gallus gallus) GI:1370092; contains Pfam profile PF00439: Bromodomain \| chr5:3332856-3335233 REVERSE \| Aliases: F12B17.100, F12B17_100 | 5.8 | 7.1 | -1.3 | -4.7 | 1.23% | 4.3 |
| 4734 | AT1G75540.1 \| zinc finger (B-box type) family protein, similar to zinc finger protein GB:BAA33202 GI:3618312 from (Oryza sativa) \| chr1:28369720-28371313 FORWARD \| Aliases: F10A5.24, F10A5_24 | 6.4 | 8.4 | -2.0 | -4.7 | 1.23% | 4.3 |
| 4736 | AT2G26710.1 \| Symbol: BAS1 \| Encodes a member of the cytochrome p450 family. Involved in brassinolide metabolism. Mediates response to a variety of light signals including hypocotyl elongation and cotyledon expansion. \| chr2:11387584-11390690 FORWARD \| Aliases: BAS1, CYP72B1, CYP734A1, F18A8.8, F18A8_8, PHYB ACTIVATION TAGGED SUPPRESSOR 1 | 2.4 | 3.3 | -0.9 | -4.7 | 1.23% | 4.1 |
| 4737 | AT2G47830.2 \| cation efflux family protein / metal tolerance protein, putative (MTPc1), member of the cation diffusion facilitator (CDF) family, or cation efflux (CE) family, PMID:11500563 \| chr2:19597926-19601067 REVERSE \| Aliases: F17A22.22 | 4.6 | 5.9 | -1.3 | -4.7 | 1.23% | 4.3 |
| 4738 | AT5G20680.1 \| expressed protein, predicted proteins, Arabidopsis thaliana \| chr5:6998105-7001912 FORWARD \| Aliases: T1M15.80, T1M15_80 | 5.0 | 5.5 | -0.6 | -4.7 | 1.23% | 4.0 |
| 4740 | AT1G19110.1 \| inter-alpha-trypsin inhibitor heavy chain-related, similar to SP:Q61704 Inter-alpha-trypsin inhibitor heavy chain H3 precursor {Mus musculus}; contains Pfam profile PF00092: von Willebrand factor type A domain \| chr1:6602117-6605899 FORWARD \| Aliases: F14D16.26, F14D16_26 | 8.6 | 9.4 | -0.8 | -4.7 | 1.23% | 4.2 |
| 4742 | AT1G17190.1 \| Symbol: ATGSTU26 \| glutathione S-transferase, putative, One of three repeated glutathione transferases. 65% identical to glutathione transferase (Arabidopsis thaliana) (gi:4006934). Location of est 141C5T7 (gb:T46669); supported by fl cDNA gi:14326476gb:AF385691. | 2.9 | 4.7 | -1.8 | -4.7 | 1.23% | 4.2 |
| 4744 | AT4G32290.1 \| expressed protein, contains Pfam profile PF03267: Arabidopsis protein of unknown function, DUF266 \| chr4:15589680-15591170 REVERSE \| Aliases: F10M6.70, F10M6_70 | 4.5 | 5.7 | -1.2 | -4.7 | 1.23% | 4.3 |
| 4745 | AT5G65970.1 \| Symbol: MLO10 \| seven transmembrane MLO family protein / MLO-like protein 10 (MLO10), identical to membrane protein Mlo10 (Arabidopsis thaliana) gi:14091590:gb:AAK53803; similar to MLO protein SWISS-PROT:P93766, NCBI_gi:1877221 (Hordeum vulgare)(Barley) \| chr5:26404654-26407678 REVERSE \| Aliases: K2A18.3, K2A18_3 | 5.9 | 7.1 | -1.2 | -4.7 | 1.23% | 4.3 |
| 4746 | AT5G15890.1 \| expressed protein \| chr5:5187690-5189351 REVERSE \| Aliases: F1N13.30, F1N13_30 | 2.6 | 3.6 | -0.9 | -4.7 | 1.23% | 4.3 |
| 4747 | AT5G62890.3 \| similar to xanthine/uracil permease family protein [Arabidopsis thaliana] (TAIR:At1g10540.1); similar to xanthine/uracil permease family protein [Arabidopsis thaliana] (TAIR:At1g60030.1); similar to xanthine/uracil permease family protein [Arabidopsis thaliana] (TAIR:At5g49990.1); similar to putative permease 1 [Lycopersicon esculentum] (GB:AAX95758.1); similar to putative permease [Oryza sativa (japonica cultivar-group)] (GB:XP_482444.1); similar to putative permease 1 [Oryza sativa (japonica cultivar-group)] (GB:XP_450798.1); similar to putative permease 1 [Oryza sativa (japonica cultivar-group)] (GB:XP_467723.1); similar to putative permease [Oryza sativa (japonica cultivar-group)] (GB:NP_910042.1); contains InterPro domain Xanthine/uracil/vitamin C permease family (InterPro:IPR006043) \| chr5:25260477-25264068 FORWARD \| Aliases: MQB2.190, MQB2_190 | 5.3 | 8.0 | -2.7 | -4.7 | 1.23% | 4.3 |
| 4748 | AT5G38610.1 \| invertase/pectin methylesterase inhibitor family protein, low similarity to pistil-specific gene sts15 (Solanum tuberosum) GI:1616628; contains Pfam profile PF04043: Plant invertase/pectin methylesterase inhibitor \| chr5:15478075-15478674 REVERSE \| Aliases: MBB18.16, MBB18_16 | 4.0 | 5.3 | -1.3 | -4.7 | 1.23% | 4.3 |
| 4749 | AT4G04990.1 \| expressed protein, contains Pfam domain PF05553: Cotton fiber expressed protein \| chr4:2555088-2557044 FORWARD \| Aliases: C17L7.3 | 3.0 | 4.3 | -1.2 | -4.7 | 1.23% | 4.3 |
| 4751 | AT1G12040.1 \| Symbol: LRX1 \| leucine-rich repeat family protein / extensin family protein (LRX1), similar to extensin-like protein (Lycopersicon esculentum) gi:5917664:gb:AAD55979; contains leucine-rich repeats, Pfam:PF00560; contains proline rich extensin domains, INTERPRO:IPR002965 \| chr1:4070122-4072565 FORWARD \| Aliases: F12F1.9, F12F1_9, LEUCINE RICH REPEAT/EXTENSIN 1 | 3.0 | 6.7 | -3.7 | -4.7 | 1.23% | 4.3 |

| Rank | Description | Sync | Root | M | t | adj.q | B |
|---|---|---|---|---|---|---|---|
| 4752 | AT4G31910.1 \| transferase family protein, low similarity to anthranilate N-hydroxycinnamoyl/benzoyltransferase Dianthus caryophyllus GI:3288180, 10-deacetylbaccatin III-10-O-acetyl transferase Taxus cuspidata GI:6746554; contains Pfam profile PF02458 transferase family \| chr4:15441088-15443906 FORWARD \| Aliases: F11C18.110, F11C18_110 | 6.6 | 8.6 | -2.0 | -4.7 | 1.24% | 4.3 |
| 4753 | AT1G71695.1 \| peroxidase 12 (PER12) (P12) (PRXR6), identical to SP:Q96520 Peroxidase 12 precursor (EC 1.11.1.7) (Atperox P12) (PRXR6) (ATP4a) {Arabidopsis thaliana} \| chr1:26967967-26970350 FORWARD \| Aliases: F14O23.6, F14O23_6 | 3.7 | 6.6 | -2.9 | -4.7 | 1.24% | 4.3 |
| 4755 | AT3G14460.1 \| disease resistance protein (NBS-LRR class), putative, domain signature NBS-LRR exists, suggestive of a disease resistance protein. \| chr3:4851997-4856271 REVERSE \| Aliases: MOA2.7 | 2.8 | 3.9 | -1.0 | -4.7 | 1.24% | 4.3 |
| 4756 | AT4G10550.1 \| subtilase family protein, contains similarity to subtilisin-like protease AIR3 GI:4218991 from (Arabidopsis thaliana) \| chr4:6516578-6519763 REVERSE \| Aliases: T4F9.10, T4F9_10 | 3.5 | 4.7 | -1.2 | -4.7 | 1.24% | 4.3 |
| 4757 | AT5G24430.1 \| calcium-dependent protein kinase, putative / CDPK, putative, similar to calcium/calmodulin-dependent protein kinase CaMK1 (Nicotiana tabacum) gi:16904222:gb:AAL30818 \| chr5:8339147-8343104 REVERSE \| Aliases: K16H17.14, K16H17_14 | 6.3 | 7.2 | -0.8 | -4.7 | 1.24% | 4.3 |
| 4758 | AT2G02860.2 \| Symbol: SUT2 \| sucrose transporter / sucrose-proton symporter (SUC3), identical to sucrose transporter (Arabidopsis thaliana) GI:8052190; similar to sucrose transporters from (Oryza sativa (japonica cultivar-group)) GI:2723471, (Zea mays) GI:5771354, (Triticum aestivum) GI:19548165; contains Pfam profile PF00083: major facilitator superfamily protein \| chr2:828351-832454 REVERSE \| Aliases: ATSUC3, SUC3, T17M13.3, T17M13_3 | 5.4 | 6.5 | -1.1 | -4.7 | 1.24% | 4.3 |
| 4759 | AT4G16520.2 \| autophagy 8f (APG8f), identical to autophagy 8f (Arabidopsis thaliana) GI:19912161; contains Pfam profile PF02991: Microtubule associated protein 1A/1B, light chain 3 \| chr4:9306708-9308465 REVERSE \| Aliases: DL4285C, FCAALL.383 | 11.5 | 12.2 | -0.7 | -4.7 | 1.25% | 3.9 |
| 4761 | AT1G60440.1 \| eukaryotic pantothenate kinase family protein, similar to pantothenate kinase GI:4191500 from (Aspergillus nidulans); contains Pfam profile PF03630: Fumble \| chr1:22270175-22272906 REVERSE \| Aliases: T13D8.31, T13D8_31 | 8.7 | 9.2 | -0.6 | -4.7 | 1.25% | 3.8 |
| 4762 | AT3G17420.1 \| Symbol: GPK1 \| protein kinase family protein, contains protein kinase domain, Pfam:PF00069; contains serine/threonine protein kinase domain, INTERPRO:IPR002290 | 6.0 | 7.8 | -1.8 | -4.7 | 1.25% | 4.3 |
| 4764 | AT5G20110.1 \| dynein light chain, putative, similar to SP:O02414 Dynein light chain LC6, flagellar outer arm {Anthocidaris crassispina}; contains Pfam profile PF01221: Dynein light chain type 1 \| chr5:6791489-6793348 REVERSE \| Aliases: F28I16.260, F28I16_260 | 4.2 | 6.8 | -2.6 | -4.7 | 1.25% | 4.3 |
| 4768 | AT2G23430.1 \| Symbol: ICK1 \| kip-related protein 1 (KRP1) / cyclin-dependent kinase inhibitor 1 (ICK1), identical to cyclin-dependent kinase inhibitor (ICK1) (Arabidopsis thaliana) GI:3550262, GI:2052502 \| chr2:9983826-9985040 REVERSE \| Aliases: CYCLIN DEPENDENT KINASE INHIBITOR PROTEIN, F26B6.8, F26B6_8, KIP RELATED PROTEIN 1, KRP1, Kip related protein 1 | 4.7 | 6.8 | -2.1 | -4.7 | 1.25% | 4.3 |
| 4769 | AT5G12370.3 \| similar to Brain secretory protein SEC10P [Homo sapiens] (GB:AAH41126.1) | 5.1 | 5.8 | -0.7 | -4.7 | 1.25% | 4.1 |
| 4770 | AT3G27820.1 \| monodehydroascorbate reductase, putative, similar to cytosolic monodehydroascorbate reductase GB:BAA77214 (Oryza sativa) | 7.8 | 8.8 | -1.0 | -4.7 | 1.25% | 4.1 |
| 4774 | AT5G06630.1 \| proline-rich extensin-like family protein, contains proline-rich extensin domains, INTERPRO:IPR002965 \| chr5:2036362-2037844 REVERSE \| Aliases: F15M7.16, F15M7_16 | 3.5 | 8.2 | -4.7 | -4.7 | 1.26% | 4.0 |
| 4776 | AT3G20860.1 \| protein kinase family protein, contains protein kinase domain, Pfam:PF00069; contains serine/threonine protein kinase domain, INTERPRO:IPR002290 | 3.6 | 6.6 | -2.9 | -4.7 | 1.26% | 4.2 |
| 4778 | AT4G38350.1 \| patched family protein, similar to SP:O15118 Niemann-Pick C1 protein precursor from Homo sapiens (PID:g2276463); contains Pfam profile PF02460 Patched family \| chr4:17958318-17966411 REVERSE \| Aliases: F22I13.120, F22I13_120 | 6.5 | 7.9 | -1.3 | -4.7 | 1.26% | 4.2 |
| 4779 | AT1G28050.1 \| zinc finger (B-box type) family protein \| chr1:9775515-9777797 REVERSE \| Aliases: F13K9.15, F13K9_15 | 4.9 | 5.9 | -0.9 | -4.7 | 1.26% | 4.2 |
| 4780 | AT4G27860.1 \| integral membrane family protein, contains Pfam PF01988: Integral membrane protein \| chr4:13873803-13876532 FORWARD \| Aliases: T27E11.100, T27E11_100 | 2.5 | 3.3 | -0.7 | -4.7 | 1.26% | 4.2 |
| 4782 | AT3G56410.2 \| expressed protein \| chr3:20927151-20932185 REVERSE \| Aliases: T5P19.60 | 3.5 | 5.1 | -1.7 | -4.7 | 1.26% | 4.3 |
| 4784 | AT3G01350.1 \| proton-dependent oligopeptide transport (POT) family protein, contains Pfam profile: PF00854 POT family \| chr3:135031-137467 FORWARD \| Aliases: T13O15.11 | 4.1 | 5.2 | -1.1 | -4.7 | 1.26% | 4.3 |
| 4785 | AT2G01580.1 \| expressed protein, and genefinder \| chr2:265043-265904 REVERSE \| Aliases: F2I9.20, F2I9_20 | 5.0 | 6.8 | -1.8 | -4.7 | 1.27% | 4.3 |

| Rank | Description | Sync | Root | M | t | adj.q | B |
|---|---|---|---|---|---|---|---|
| 4787 | AT5G58510.1 \| expressed protein, KIAA0066, Homo sapiens, EMBL:HSORFKG1O \| chr5:23665379-23671778 REVERSE \| Aliases: MQJ2.13, MQJ2_13 | 5.5 | 6.5 | -1.1 | -4.7 | 1.27% | 4.2 |
| 4790 | AT5G07110.1 \| prenylated rab acceptor (PRA1) family protein, weak similarity to prenylated Rab acceptor 1 (PRA1) (Homo sapiens) GI:4877285; contains Pfam profile PF03208: Prenylated rab acceptor (PRA1) \| chr5:2206001-2207153 FORWARD \| Aliases: T28J14.50 | 4.8 | 6.1 | -1.3 | -4.7 | 1.27% | 4.3 |
| 4791 | AT4G24690.1 \| ubiquitin-associated (UBA)/TS-N domain-containing protein / octicosapeptide/Phox/Bemp1 (PB1) domain-containing protein, contains Pfam profiles PF00627: Ubiquitin-associated (UBA)/TS-N domain, PF00569: Zinc finger ZZ type domain, PF00564: PB1 domain | 8.0 | 9.4 | -1.5 | -4.7 | 1.27% | 4.2 |
| 4793 | AT3G52060.2 \| expressed protein, contains Pfam profile PF03267: Arabidopsis protein of unknown function, DUF266 \| chr3:19321511-19323264 REVERSE \| Aliases: F4F15.170 | 4.8 | 6.4 | -1.6 | -4.7 | 1.27% | 4.2 |
| 4796 | AT5G60490.1 \| Symbol: FLA12 \| fasciclin-like arabinogalactan-protein (FLA12) \| chr5:24342953-24343928 REVERSE \| Aliases: MUF9.12, MUF9_12 | 3.7 | 4.5 | -0.8 | -4.7 | 1.27% | 4.1 |
| 4800 | AT2G17050.1 \| disease resistance protein (TIR-NBS-LRR class), putative, domain signature TIR-NBS-LRR exists, suggestive of a disease resistance protein. \| chr2:7417917-7422692 REVERSE \| Aliases: F6P23.20, F6P23_20 | 2.5 | 3.3 | -0.8 | -4.7 | 1.28% | 4.1 |
| 4801 | AT2G22090.2 \| UBP1 interacting protein 1a (UBA1a), nearly identical to UBP1 interacting protein 1a (Arabidopsis thaliana) GI:19574236; contains InterPro entry IPR000504: RNA-binding region RNP-1 (RNA recognition motif) (RRM); based on cDNA of partial mRNA for UBP1 interacting protein 1a (uba1a) GI:19574235 \| chr2:9396080-9397586 REVERSE \| Aliases: T16B14.6, T16B14_6 | 5.1 | 6.2 | -1.1 | -4.7 | 1.28% | 4.3 |
| 4802 | AT4G05320.5 \| Symbol: UBQ10 \| polyubiquitin (UBQ10) (SEN3), senescence-associated protein; identical to GI:870791 \| chr4:2718168-2720130 FORWARD \| Aliases: C17L7.240, C17L7_240, POLYUBIQUITIN 10 | 13.1 | 13.6 | -0.5 | -4.7 | 1.28% | 3.2 |
| 4803 | AT3G11520.1 \| Symbol: CYCB1;3 \| cyclin, putative (CYC2), similar to cyclin (Arabidopsis thaliana) GI:1360646; contains Pfam profiles PF00134: Cyclin, N-terminal domain, PF02984: Cyclin, C-terminal domain; identical to cDNA cyclin box (cyc2) partial cds GI:456019 \| chr3:3625330-3627236 REVERSE \| Aliases: CYC2, CYCLIN B1;3, F24K9.20 | 3.6 | 5.2 | -1.6 | -4.7 | 1.28% | 4.3 |
| 4804 | AT1G24100.1 \| UDP-glucoronosyl/UDP-glucosyl transferase family protein, contains Pfam profile: PF00201 UDP-glucoronosyl and UDP-glucosyl transferase \| chr1:8525424-8527076 REVERSE \| Aliases: F3I6.2, F3I6_2 | 7.2 | 9.2 | -1.9 | -4.7 | 1.28% | 4.1 |
| 4805 | AT3G12100.2 \| similar to cation efflux family protein [Arabidopsis thaliana] (TAIR:At2g04620.1); similar to cation efflux protein, putative [Entamoeba histolytica HM-1:IMSS] (GB:EAL49993.1); contains InterPro domain Cation efflux protein (InterPro:IPR002524) \| chr3:3854521-3857325 REVERSE \| Aliases: T21B14.8 | 4.7 | 5.9 | -1.2 | -4.7 | 1.28% | 4.2 |
| 4809 | AT5G16490.1 \| Symbol: RIC4 \| p21-rho-binding domain-containing protein, contains Pfam PF00786: P21-Rho-binding domain | 3.2 | 5.0 | -1.8 | -4.7 | 1.28% | 4.3 |
| 4810 | AT3G58060.1 \| cation efflux family protein / metal tolerance protein, putative (MTPc3), member of the cation diffusion facilitator (CDF) family, or cation efflux (CE) family, PMID:11500563 \| chr3:21508755-21510653 REVERSE \| Aliases: T10K17.270 | 3.1 | 4.8 | -1.7 | -4.7 | 1.29% | 4.2 |
| 4812 | AT3G26510.5 \| similar to octicosapeptide/Phox/Bem1p (PB1) domain-containing protein [Arabidopsis thaliana] (TAIR:At1g70640.1); similar to PB1 domain, putative [Oryza sativa (japonica cultivar-group)] (GB:AAX96261.1); contains InterPro domain Octicosapeptide/Phox/Bem1p (InterPro:IPR000270) \| chr3:9711574-9713897 REVERSE \| Aliases: MFE16.2 | 4.4 | 7.7 | -3.3 | -4.7 | 1.29% | 4.3 |
| 4813 | AT4G35040.1 \| bZIP transcription factor family protein, contains Pfam profile: PF00170 bZIP transcription factor \| chr4:16680419-16682098 FORWARD \| Aliases: M4E13.100, M4E13_100 | 8.2 | 9.4 | -1.2 | -4.7 | 1.29% | 4.1 |
| 4814 | AT3G03210.1 \| expressed protein \| chr3:740777-742316 FORWARD \| Aliases: T17B22.10, T17B22_10 | 5.9 | 7.2 | -1.3 | -4.7 | 1.29% | 4.2 |
| 4815 | AT5G01710.1 \| expressed protein \| chr5:263516-265613 REVERSE \| Aliases: F7A7.230, F7A7_230 | 8.8 | 9.6 | -0.9 | -4.7 | 1.29% | 4.0 |
| 4820 | AT1G13600.1 \| bZIP transcription factor family protein, contains Pfam profile: PF00170 bZIP transcription factor \| chr1:4650784-4651466 REVERSE \| Aliases: F21F23.3 | 3.6 | 5.0 | -1.3 | -4.7 | 1.30% | 4.3 |
| 4823 | AT3G51570.1 \| disease resistance protein (TIR-NBS-LRR class), putative, domain signature TIR-NBS-LRR exists, suggestive of a disease resistance protein. \| chr3:19137337-19141435 FORWARD \| Aliases: T18N14.4 | 2.3 | 3.1 | -0.8 | -4.7 | 1.30% | 4.1 |
| 4825 | AT4G25835.1 \| AAA-type ATPase family protein, contains Pfam profile: PF00004 ATPase family \| chr4:13136116-13137739 FORWARD \| Aliases: None | 3.1 | 5.7 | -2.6 | -4.7 | 1.30% | 4.3 |
| 4826 | AT5G24360.1 \| protein kinase family protein / Ire1 homolog-1 (IRE1-1), identical to Ire1 homolog-1 (Arabidopsis thaliana) GI:15277137; cDNA Ire1 homolog-1 GI:15277136; \| chr5:8316446-8319980 FORWARD \| Aliases: K16H17.7, K16H17_7 | 6.7 | 7.4 | -0.8 | -4.7 | 1.30% | 4.1 |
| 4827 | AT4G38640.1 \| choline transporter-related, contains weak similarity to CD92 protein (Homo sapiens) gi:16945323:emb:CAC82175 \| chr4:18059647-18062283 REVERSE \| Aliases: T9A14.3 | 5.4 | 6.9 | -1.5 | -4.7 | 1.30% | 4.2 |

| Rank | Description | Sync | Root | M | t | adj.q | B |
|---|---|---|---|---|---|---|---|
| 4831 | AT2G25060.1 \| plastocyanin-like domain-containing protein \| chr2:10669331-10670177 FORWARD \| Aliases: F13D4.2 | 3.8 | 5.0 | -1.2 | -4.7 | 1.30% | 4.3 |
| 4832 | AT5G23210.2 \| Symbol: SCPL34 \| | 2.5 | 3.2 | -0.7 | -4.7 | 1.30% | 4.1 |
| 4833 | AT1G79330.1 \| Symbol: AMC6/ATMCP2B \| Metacaspase AtMCPb2/AMC6. Caspase family protein. Arginine/lysine-specific cysteine protease activity. Induces apoptosis in yeast. Contains Pfam domain, PF00656: ICE-like protease (caspase) p20 domain. \| chr1:29843546-29845030 FORWARD \| Aliases: AMC6, ATMCP2B, YUP8H12R.5, YUP8H12R_5 | 2.4 | 3.2 | -0.7 | -4.7 | 1.31% | 4.1 |
| 4834 | AT1G23440.2 \| pyrrolidone-carboxylate peptidase family protein, similar to Pyrrolidone-carboxylate peptidase (Swiss-Prot:O58321) (Pyrococcus horikoshii); contains Prosite PS00141: Eukaryotic and viral aspartyl proteases active site \| chr1:8321694-8323359 FORWARD \| Aliases: F28C11.26, F28C11_26 | 7.1 | 8.6 | -1.5 | -4.7 | 1.31% | 4.2 |
| 4839 | AT3G08850.1 \| Encodes one of two Arabidopsis RAPTOR/KOG1 homologs. RAPTOR proteins are binding partners of the target of rapamycin kinase that is present in all eukaryotes and play a central role in the stimulation of cell growth and metabolism in response to nutrients. Mutants show embryo lethal phenotype which occurs at pre-globular stage. May interact with TOR kinase in a rapamycin like signaling pathway. | 4.0 | 5.2 | -1.2 | -4.7 | 1.31% | 4.2 |
| 4840 | AT5G02020.2 \| expressed protein \| chr5:386079-388120 REVERSE \| Aliases: T7H20.70, T7H20_70 | 5.5 | 7.0 | -1.5 | -4.7 | 1.31% | 4.3 |
| 4844 | AT5G66100.1 \| La domain-containing protein, similar to SP:P40796 La protein homolog (La ribonucleoprotein) (La autoantigen homolog) {Drosophila melanogaster}; contains Pfam profile PF05383: La domain \| chr5:26444715-26447232 FORWARD \| Aliases: K2A18.18, K2A18_18 | 3.9 | 4.6 | -0.6 | -4.7 | 1.31% | 4.0 |
| 4848 | AT1G17120.1 \| Symbol: CAT8 \| amino acid permease family protein, low similarity to SP:O43246 Cationic amino acid transporter-4 (CAT-4) {Homo sapiens}; contains Pfam profile PF00324: Amino acid permease \| chr1:5851946-5853948 FORWARD \| Aliases: CATIONIC AMINO ACID TRANSPORTER 8, F20D23.19, F20D23_19 | 5.6 | 6.5 | -0.9 | -4.7 | 1.32% | 4.1 |
| 4853 | AT4G13420.1 \| Symbol: HAK5 \| potassium transporter (HAK5), identical to K+ transporter HAK5 (Arabidopsis thaliana) gi:7108597:gb:AAF36490; similar to high-affinity potassium transporter AtKUP1p (Arabidopsis thaliana) gi:2688979:gb:AAB88901; KUP/HAK/KT Transporter family member, PMID:11500563 \| chr4:7796846-7802247 REVERSE \| Aliases: T9E8.160, T9E8_160 | 2.7 | 3.8 | -1.1 | -4.7 | 1.32% | 4.2 |
| 4855 | AT5G54790.1 \| expressed protein \| chr5:22272356-22272715 REVERSE \| Aliases: MBG8.5, MBG8_5 | 2.7 | 3.5 | -0.8 | -4.7 | 1.32% | 4.0 |
| 4856 | AT4G39740.1 \| electron transport SCO1/SenC family protein, similar to SP:P23833 SCO1 protein, mitochondrial precursor {Saccharomyces cerevisiae}; contains Pfam profile PF02630: SCO1/SenC \| chr4:18435288-18437118 REVERSE \| Aliases: T19P19.130, T19P19_130 | 4.1 | 4.8 | -0.8 | -4.7 | 1.33% | 4.1 |
| 4860 | AT1G48500.1 \| expressed protein, ; expression supported by MPSS \| chr1:17936665-17938101 FORWARD \| Aliases: T1N15.11, T1N15_11 | 3.2 | 4.6 | -1.4 | -4.7 | 1.33% | 4.2 |
| 4863 | AT1G31350.1 \| F-box family protein, similar to hypothetical protein GB:AAF24593 GI:6692128 from (Arabidopsis thaliana) ; similar to SKP1 interacting partner 2 (SKIP2) TIGR_Ath1:At5g67250 \| chr1:11221321-11222764 REVERSE \| Aliases: T19E23.14, T19E23_14 | 4.9 | 6.5 | -1.7 | -4.7 | 1.33% | 4.2 |
| 4870 | AT4G33420.1 \| peroxidase, putative, identical to class III peroxidase ATP32 (Arabidopsis thaliana) gi:17530547:gb:AAL40837; identical to cDNA class III peroxidase ATP32 GI:17530546 | 5.7 | 8.3 | -2.6 | -4.6 | 1.34% | 4.2 |
| 4871 | AT2G35710.3 \| glycogenin glucosyltransferase (glycogenin)-related, low similarity to glycogenin-2 from Homo sapiens (SP:O15488) \| chr2:15020656-15021226 REVERSE \| Aliases: T20F21.27 | 4.0 | 4.6 | -0.6 | -4.6 | 1.34% | 3.8 |
| 4873 | AT5G05460.1 \| similar to glycosyl hydrolase family 85 protein [Arabidopsis thaliana] (TAIR:At3g11040.1); similar to PREDICTED: similar to RIKEN cDNA D230014K01 [Rattus norvegicus] (GB:XP_221141.2); contains InterPro domain Glycoside hydrolase, family 85 (InterPro:IPR005201) \| chr5:1615616-1618903 FORWARD \| Aliases: K18I23.27, K18I23_27 | 5.2 | 5.9 | -0.7 | -4.6 | 1.34% | 4.0 |
| 4875 | AT1G43700.1 \| Symbol: VIP1 \| VirE2-interacting protein (VIP1), identical to VirE2-interacting protein VIP1 GB:AAF37279 GI:7258340 from (Arabidopsis thaliana) | 7.5 | 9.0 | -1.4 | -4.6 | 1.34% | 4.2 |
| 4876 | AT1G63530.1 \| expressed protein \| chr1:23567317-23568899 FORWARD \| Aliases: F2K11.11, F2K11_11 | 2.6 | 3.0 | -0.4 | -4.6 | 1.34% | 3.7 |
| 4877 | AT2G14095.1 \| expressed protein \| chr2:5939477-5940873 REVERSE \| Aliases: None | 3.6 | 5.1 | -1.5 | -4.6 | 1.34% | 4.2 |
| 4882 | AT5G26660.1 \| Symbol: ATMYB4 \| | 2.6 | 3.5 | -0.9 | -4.6 | 1.35% | 4.1 |
| 4883 | AT1G68450.1 \| VQ motif-containing protein, contains PF05678: VQ motif \| chr1:25665164-25665902 REVERSE \| Aliases: T2E12.4 | 4.0 | 6.8 | -2.7 | -4.6 | 1.35% | 4.1 |
| 4884 | AT3G24240.1 \| leucine-rich repeat transmembrane protein kinase, putative, similar to CLV1 receptor kinase GB:AAB58929 from (Arabidopsis thaliana) \| chr3:8780558-8784157 FORWARD \| Aliases: K13K6.1 | 4.8 | 5.8 | -1.0 | -4.6 | 1.35% | 4.2 |
| 4885 | AT5G15730.1 \| serine/threonine protein kinase, putative, similar to protein-serine/threonine kinase (Nicotiana tabacum) gi:505146:dbj:BAA06538 | 5.1 | 6.1 | -0.9 | -4.6 | 1.35% | 4.1 |

| Rank | Description | Sync | Root | M | t | adj.q | B |
|---|---|---|---|---|---|---|---|
| 4886 | AT1G20840.1 \| transporter-related, low similarity to D-xylose proton-symporter (Lactobacillus brevis) GI:2895856; contains Pfam profile PF00083: major facilitator superfamily protein | 4.3 | 5.1 | -0.8 | -4.6 | 1.35% | 4.1 |
| 4892 | AT2G26190.1 \| calmodulin-binding family protein, contains Pfam profile PF00612: IQ calmodulin-binding motif \| chr2:11154717-11157588 REVERSE \| Aliases: T1D16.17, T1D16_17 | 5.2 | 8.6 | -3.4 | -4.6 | 1.35% | 4.2 |
| 4893 | AT1G49180.1 \| protein kinase family protein, contains eukaryotic protein kinase domain, INTERPRO:IPR000719 \| chr1:18188322-18191197 REVERSE \| Aliases: F27J15.5, F27J15_5 | 2.8 | 3.8 | -0.9 | -4.6 | 1.36% | 4.2 |
| 4894 | AT5G64110.1 \| peroxidase, putative, similar to peroxidase ATP3a (Arabidopsis thaliana) gi:1546698:emb:CAA67340 | 2.3 | 2.8 | -0.5 | -4.6 | 1.36% | 3.6 |
| 4896 | AT3G47050.1 \| glycosyl hydrolase family 3 protein, beta-D-glucan exohydrolase, Nicotiana tabacum, TREMBL:AB017502_1 \| chr3:17339020-17341874 REVERSE \| Aliases: F13I12.100 | 3.4 | 4.5 | -1.1 | -4.6 | 1.36% | 4.1 |
| 4898 | AT3G58750.1 \| citrate synthase, glyoxysomal, putative, strong similarity to SP:P49299 Citrate synthase, glyoxysomal precursor {Cucurbita maxima}; contains Pfam profile PF00285: Citrate synthase \| chr3:21735291-21738667 REVERSE \| Aliases: T20N10.100 | 5.8 | 7.4 | -1.6 | -4.6 | 1.36% | 4.2 |
| 4900 | AT5G25890.1 \| Symbol: IAA28 \| auxin-responsive protein / indoleacetic acid-induced protein 28 (IAA28), identical to SP:Q9XFM0:AXIS_ARATH Auxin-responsive protein IAA28 (Indoleacetic acid-induced protein 28) {Arabidopsis thaliana} | 5.7 | 6.9 | -1.3 | -4.6 | 1.36% | 4.2 |
| 4901 | AT1G75180.3 \| expressed protein \| chr1:28219134-28222046 REVERSE \| Aliases: F22H5.9, F22H5_9 | 4.8 | 5.7 | -0.9 | -4.6 | 1.36% | 3.9 |
| 4905 | AT1G21390.1 \| Symbol: EMB2170 \| expressed protein \| chr1:7489140-7490726 REVERSE \| Aliases: EMB2170, EMBRYO DEFECTIVE 2170, F24J8.30, F24J8_30 | 4.0 | 5.3 | -1.3 | -4.6 | 1.37% | 4.2 |
| 4906 | AT1G68000.1 \| Symbol: ATPIS1 \| | 5.2 | 7.6 | -2.4 | -4.6 | 1.37% | 4.2 |
| 4907 | AT1G64000.1 \| Symbol: WRKY56 \| | 2.4 | 2.9 | -0.5 | -4.6 | 1.37% | 3.5 |
| 4910 | AT3G55260.1 \| glycosyl hydrolase family 20 protein, similar to beta-hexosaminidase A SP:P13723 from (Dictyostelium discoideum) | 7.1 | 8.5 | -1.4 | -4.6 | 1.37% | 4.2 |
| 4913 | AT1G16970.1 \| Symbol: KU70 \| Ku70-like protein, identical to Ku70-like protein GI:12006424 from (Arabidopsis thaliana); contains Pfam profiles PF03731: Ku70/Ku80 N-terminal alpha/beta domain, PF02735: Ku70/Ku80 beta-barrel domain, PF03730: Ku70/Ku80 C-terminal arm, and PF02037: SAP domain; contains TIGRfam profile TIGR00578: ATP-dependent DNA helicase ii, 70 kDa subunit \| chr1:5800904-5805767 REVERSE \| Aliases: F6I1.2, F6I1_2, KU70 LIKE PROTEIN | 4.6 | 5.5 | -0.9 | -4.6 | 1.37% | 4.1 |
| 4914 | AT3G14770.1 \| nodulin MtN3 family protein, similar to MtN3 GI:1619602 (root nodule development) from (Medicago truncatula) \| chr3:4957363-4959709 REVERSE \| Aliases: T21E2.6 | 2.5 | 3.3 | -0.8 | -4.6 | 1.37% | 4.1 |
| 4916 | AT5G04080.1 \| expressed protein \| chr5:1104706-1105389 FORWARD \| Aliases: F21E1.3 | 3.0 | 4.7 | -1.7 | -4.6 | 1.37% | 4.2 |
| 4918 | AT5G14130.1 \| peroxidase, putative, identical to peroxidase ATP20a (Arabidopsis thaliana) gi:1546694:emb:CAA67338 \| chr5:4558101-4560069 REVERSE \| Aliases: MUA22.13, MUA22_13 | 3.5 | 6.2 | -2.7 | -4.6 | 1.38% | 4.1 |
| 4919 | AT3G45330.1 \| lectin protein kinase family protein, contains Serine/Threonine protein kinases active-site signature, Prosite:PS00108; contains Pfam profiles PF00069: Protein kinase domain, PF00139: Legume lectins beta domain, PF00138: Legume lectins alpha domain | 2.8 | 5.1 | -2.4 | -4.6 | 1.38% | 4.2 |
| 4925 | AT1G72230.1 \| plastocyanin-like domain-containing protein, similar to blue copper protein SP:Q41001 from (Pisum sativum) \| chr1:27191728-27192906 FORWARD \| Aliases: T9N14.17, T9N14_17 | 5.8 | 8.3 | -2.5 | -4.6 | 1.38% | 4.2 |
| 4926 | AT5G51070.1 \| Symbol: ERD1 \| ATP-dependent Clp protease ATP-binding subunit (ClpD), (ERD1), SAG15/ERD1; identical to ERD1 protein GI:497629, SP:P42762 from (Arabidopsis thaliana); contains Pfam profile PF02861: Clp amino terminal domain \| chr5:20781541-20785907 FORWARD \| Aliases: CLPD, EARLY RESPONSIVE TO DEHYDRATION 1, K3K7.27, K3K7_27 | 6.8 | 8.3 | -1.5 | -4.6 | 1.39% | 4.2 |
| 4929 | AT2G17940.1 \| expressed protein, contains Pfam PF05701: Plant protein of unknown function (DUF827) \| chr2:7809907-7811029 FORWARD \| Aliases: T27K22.19, T27K22_19 | 3.3 | 4.1 | -0.8 | -4.6 | 1.39% | 4.0 |
| 4930 | AT2G45910.1 \| protein kinase family protein / U-box domain-containing protein, contains Pfam profiles PF00069 Eukaryotic protein kinase domain, PF04564: U-box domain; supported by tandem duplication of (GI:3386604) (TIGR_Ath1:At2g45920) (Arabidopsis thaliana) \| chr2:18901148-18905601 FORWARD \| Aliases: F4I18.11 | 3.5 | 4.7 | -1.2 | -4.6 | 1.39% | 4.0 |
| 4931 | AT4G18270.1 \| Symbol: ATTRANS11 \| glycosyl transferase family 4 protein, contains Pfam profile PF00953: Glycosyl transferase \| chr4:10099375-10101908 REVERSE \| Aliases: T9A21.120, T9A21_120, TRANSLOCASE I | 5.3 | 6.8 | -1.5 | -4.6 | 1.39% | 4.2 |

| Rank | Description | Sync | Root | M | t | adj.q | B |
|---|---|---|---|---|---|---|---|
| 4932 | AT5G47580.1 \| expressed protein, strong similarity to unknown protein (pir::E71441) \| chr5:19312072-19314644 REVERSE \| Aliases: MNJ7.17, MNJ7_17 | 5.5 | 6.5 | -0.9 | -4.6 | 1.39% | 4.1 |
| 4939 | AT3G60860.1 \| guanine nucleotide exchange family protein, similar to guanine nucleotide exchange factor, Homo sapiens, GI:5456754; contains Pfam profile PF01369: Sec7 domain \| chr3:22495779-22502555 FORWARD \| Aliases: T4C21.270 | 7.8 | 9.3 | -1.5 | -4.6 | 1.39% | 4.1 |
| 4941 | AT1G56220.3 \| dormancy/auxin associated family protein, similar to Auxin-repressed 12.5 kDa protein (Swiss-Prot:Q05349) (Fragaria ananassa); similar to auxin-repressed protein (GI:927034) (Fragaria x ananassa); similar to dormancy-associated protein (GI:2605887) (Pisum sativum) | 6.0 | 8.0 | -2.1 | -4.6 | 1.39% | 4.1 |
| 4943 | AT5G41180.1 \| leucine-rich repeat protein kinase, putative, contains leucine rich repeat (LRR) domains, Pfam:PF00560; contains protein kinase domain, Pfam:PF00069 \| chr5:16501099-16504654 FORWARD \| Aliases: MEE6.25, MEE6_25 | 4.6 | 5.6 | -1.1 | -4.6 | 1.39% | 4.1 |
| 4945 | AT1G32120.1 \| expressed protein, contains Pfam profile PF04819: Family of unknown function (DUF716) (Plant viral-response family) \| chr1:11552906-11558757 FORWARD \| Aliases: F3C3.9, F3C3_9 | 3.4 | 4.6 | -1.3 | -4.6 | 1.39% | 4.1 |
| 4947 | AT3G51890.1 \| expressed protein, protein At2g40060 - Arabidopsis thaliana, EMBL:AF002109 \| chr3:19260457-19261942 REVERSE \| Aliases: ATEM1.14 | 6.6 | 8.2 | -1.5 | -4.6 | 1.39% | 4.2 |
| 4949 | AT1G05805.1 \| basic helix-loop-helix (bHLH) family protein \| chr1:1744773-1747692 FORWARD \| Aliases: None | 4.2 | 5.1 | -0.9 | -4.6 | 1.39% | 4.1 |
| 4954 | AT1G06780.1 \| glycosyl transferase family 8 protein, contains Pfam profile: PF01501 glycosyl transferase family 8 \| chr1:2083423-2086961 FORWARD \| Aliases: F4H5.13, F4H5_13 | 9.2 | 10.9 | -1.7 | -4.6 | 1.40% | 3.9 |
| 4958 | AT5G07130.1 \| laccase, putative / diphenol oxidase, putative, similar to laccase (Populus balsamifera subsp. trichocarpa)(GI:3805960) \| chr5:2210570-2212621 FORWARD \| Aliases: T28J14.70, T28J14_70 | 4.0 | 5.4 | -1.4 | -4.6 | 1.40% | 4.1 |
| 4960 | AT2G01930.2 \| expressed protein \| chr2:427116-428682 REVERSE \| Aliases: F23I14.2, F23I14_2 | 4.8 | 6.0 | -1.2 | -4.6 | 1.40% | 4.0 |
| 4961 | AT3G20810.3 \| similar to transcription factor jumonji (jmjC) domain-containing protein [Arabidopsis thaliana] (TAIR:At5g19840.1); similar to hypothetical protein LOC77035 [Mus musculus] (GB:NP_084118.1); contains InterPro domain Transcription factor jumonji, jmjC (InterPro:IPR003347) \| chr3:7275712-7278326 FORWARD \| Aliases: MOE17.12 | 4.8 | 6.0 | -1.2 | -4.6 | 1.40% | 4.2 |
| 4963 | AT4G03030.1 \| kelch repeat-containing F-box family protein, contains Pfam profiles PF01344: Kelch motif, PF00646: F-box domain | 7.3 | 8.7 | -1.4 | -4.6 | 1.41% | 4.1 |
| 4964 | AT4G08180.3 \| oxysterol-binding family protein, similar to SP:Q969R2 Oxysterol-binding protein 2 {Homo sapiens}; contains Pfam profiles PF00169: PH domain, PF01237: Oxysterol-binding protein | 3.8 | 5.7 | -1.9 | -4.6 | 1.41% | 4.2 |
| 4967 | AT1G59850.1 \| expressed protein \| chr1:22032174-22033872 REVERSE \| Aliases: F23H11.17, F23H11_17 | 3.0 | 4.0 | -0.9 | -4.6 | 1.41% | 4.1 |
| 4968 | AT5G54940.2 \| eukaryotic translation initiation factor SUI1, putative, similar to SP:P32911 Protein translation factor SUI1 {Saccharomyces cerevisiae}; contains Pfam profile PF01253: Translation initiation factor SUI1 \| chr5:22325497-22326633 REVERSE \| Aliases: MBG8.21, MBG8_21 | 10.6 | 11.3 | -0.7 | -4.6 | 1.41% | 3.8 |
| 4972 | AT4G26790.2 \| GDSL-motif lipase/hydrolase family protein, similar to family II lipase EXL3 (GI:15054386), EXL1 (GI:15054382), EXL2 (GI:15054384) (Arabidopsis thaliana); contains Pfam profile PF00657: Lipase/Acylhydrolase with GDSL-like motif \| chr4:13487712-13489382 FORWARD \| Aliases: F10M23.130, F10M23_130 | 2.7 | 3.8 | -1.1 | -4.6 | 1.41% | 4.1 |
| 4973 | AT1G50630.2 \| similar to expressed protein [Arabidopsis thaliana] (TAIR:At3g20300.1); similar to OSJNBa0064H22.7 [Oryza sativa (japonica cultivar-group)] (GB:XP_462655.1) \| chr1:18755124-18757337 REVERSE \| Aliases: F11F12.5, F11F12_5 | 4.2 | 5.7 | -1.5 | -4.6 | 1.42% | 4.2 |
| 4978 | AT2G29660.1 \| zinc finger (C2H2 type) family protein, contains zinc finger, C2H2 type, domain, PROSITE:PS00028 | 6.2 | 7.3 | -1.0 | -4.6 | 1.42% | 4.0 |
| 4979 | AT3G01670.1 \| expressed protein \| chr3:247241-250477 FORWARD \| Aliases: F4P13.21, F4P13_21 | 3.0 | 4.4 | -1.3 | -4.6 | 1.42% | 4.2 |
| 4980 | AT1G03730.1 \| expressed protein, similar to ESTs gb:AA605440 and gb:H37232 \| chr1:930828-931895 REVERSE \| Aliases: F21B7.33 | 6.0 | 6.9 | -0.9 | -4.6 | 1.42% | 4.0 |
| 4982 | AT1G08520.1 \| Symbol: PDE166 \| | 7.1 | 7.9 | -0.7 | -4.6 | 1.43% | 3.9 |
| 4985 | AT2G46560.1 \| transducin family protein / WD-40 repeat family protein, similar to CPY (GI:3096961) {Chironomus thummi}; contains Pfam PF00400: WD domain, G-beta repeat (8 copies, 3 weak):9780477:gb:BE522499.1:BE522499 \| chr2:19122443-19132928 REVERSE \| Aliases: F13A10.9 | 6.5 | 7.9 | -1.4 | -4.6 | 1.43% | 4.1 |
| 4986 | AT2G42390.1 \| protein kinase C substrate, heavy chain-related, similar to Protein kinase C substrate, 80 kDa protein, heavy chain (PKCSH) (80K-H protein) (Swiss-Prot:P14314) (Homo sapiens); contains 1 transmembrane domain; | 7.1 | 7.9 | -0.8 | -4.6 | 1.43% | 4.1 |

| Rank | Description | Sync | Root | M | t | adj.q | B |
|---|---|---|---|---|---|---|---|
| 4987 | AT5G11000.1 \| expressed protein \| chr5:3479030-3480426 REVERSE \| Aliases: T30N20.270, T30N20_270 | 7.2 | 8.8 | -1.7 | -4.6 | 1.43% | 4.0 |
| 4988 | AT3G11780.1 \| MD-2-related lipid recognition domain-containing protein / ML domain-containing protein, weak similarity to phosphatidylglycerol/phosphatidylinositol transfer protein (Aspergillus oryzae) GI:10178615; contains Pfam profile PF02221: ML domain \| chr3:3724153-3725595 REVERSE \| Aliases: F26K24.7 | 6.2 | 8.3 | -2.1 | -4.6 | 1.43% | 4.1 |
| 4989 | AT4G24020.1 \| RWP-RK domain-containing protein, similar to nodule inception protein (Lotus japonicus) GI:6448579; contains Pfam profile: PF02042 RWP-RK domain | 4.5 | 5.9 | -1.4 | -4.6 | 1.43% | 4.1 |
| 4990 | AT3G54790.1 \| armadillo/beta-catenin repeat family protein / U-box domain-containing protein, contains Pfam domain, PF00514: Armadillo/beta-catenin-like repeats and Pfam, PF04564: U-box domain \| chr3:20291469-20296104 REVERSE \| Aliases: T5N23.150 | 4.3 | 5.7 | -1.4 | -4.6 | 1.43% | 4.1 |
| 4991 | AT1G57570.1 \| jacalin lectin family protein, similar to myrosinase-binding protein homolog (Arabidopsis thaliana) GI:2997767; contains Pfam profile PF01419 jacalin-like lectin domain | 3.9 | 5.1 | -1.2 | -4.6 | 1.43% | 3.9 |
| 4993 | AT5G04410.1 \| Symbol: NAC2 \| no apical meristem (NAM) family protein, contains Pfam PF02365: No apical meristem (NAM) protein; supporting cDNA gi:6456750:gb:AF201456.1:AF201456 \| chr5:1243759-1247015 FORWARD \| Aliases: ANAC078, T19N18.11 | 8.3 | 9.3 | -1.0 | -4.6 | 1.43% | 4.0 |
| 4994 | AT5G44160.1 \| zinc finger (C2H2 type) family protein, contains Pfam domain, PF00096: Zinc finger, C2H2 type \| chr5:17790168-17792913 FORWARD \| Aliases: MLN1.8, MLN1_8 | 3.5 | 3.9 | -0.4 | -4.6 | 1.43% | 3.6 |
| 4995 | AT4G33330.1 \| similar to glycogenin glucosyltransferase (glycogenin)-related [Arabidopsis thaliana] (TAIR:At3g18660.1); similar to P0018C10.26 [Oryza sativa (japonica cultivar-group)] (GB:NP_915047.1); similar to glycogenin-like protein [Oryza sativa (japonica cultivar-group)] (GB:BAD81673.1); similar to putative glycogenin [Oryza sativa (japonica cultivar-group)] (GB:XP_475247.1); similar to secondary cell wall-related glycosyltransferase family 8 [Populus tremula x Populus tremuloides] (GB:AAX33317.1); contains InterPro domain Glycosyl transferase, family 8 (InterPro:IPR002495) \| chr4:16060016-16063074 REVERSE \| Aliases: F17M5.90, F17M5_90 | 3.4 | 4.6 | -1.2 | -4.6 | 1.43% | 3.9 |
| 4996 | AT1G22480.1 \| plastocyanin-like domain-containing protein \| chr1:7934158-7935371 REVERSE \| Aliases: F12K8.17, F12K8_17 | 2.7 | 3.4 | -0.8 | -4.6 | 1.43% | 4.0 |
| 4997 | AT5G11350.1 \| endonuclease/exonuclease/phosphatase family protein, contains Pfam profile PF03372: Endonuclease/Exonuclease/phosphatase family \| chr5:3621570-3625646 FORWARD \| Aliases: F2I11.240, F2I11_240 | 5.6 | 6.4 | -0.8 | -4.6 | 1.43% | 3.8 |
| 4999 | AT1G75410.1 \| Symbol: BLH3 \| BEL1-like homeodomain 3 protein (BLH3), identical to BEL1-like homeodomain 3 (GI:13877515) (Arabidopsis thaliana) \| chr1:28303453-28306232 REVERSE \| Aliases: BEL1 like homeodomain 3, F1B16.6, F1B16_6 | 4.8 | 6.6 | -1.8 | -4.6 | 1.44% | 4.1 |
| 5001 | AT5G12900.1 \| expressed protein \| chr5:4071922-4074622 REVERSE \| Aliases: T24H18.70, T24H18_70 | 3.9 | 4.8 | -0.9 | -4.6 | 1.44% | 3.9 |
| 5002 | AT5G63130.1 \| octicosapeptide/Phox/Bem1p (PB1) domain-containing protein, contains Pfam profile PF00564: PB1 domain \| chr5:25340335-25341498 FORWARD \| Aliases: MDC12.9, MDC12_9 | 3.4 | 4.7 | -1.3 | -4.6 | 1.44% | 4.1 |
| 5004 | AT5G10290.1 \| leucine-rich repeat family protein / protein kinase family protein, contains Pfam domains PF00560: Leucine Rich Repeat and PF00069: Protein kinase domain \| chr5:3235296-3238720 REVERSE \| Aliases: F18D22.60, F18D22_60 | 7.6 | 8.9 | -1.4 | -4.6 | 1.44% | 3.8 |
| 5005 | AT1G09570.1 \| Symbol: PHYA \| phytochrome A (PHYA), identical to SP:P14712 Phytochrome A {Arabidopsis thaliana} \| chr1:3095258-3100327 REVERSE \| Aliases: ELONGATED HYPOCOTYL 8, F14J9.23, F14J9_23, FAR RED ELONGATED 1, FHY2, FRE1, HY8, PHYTOCHROME 2, PHYTOCHROME A | 6.9 | 8.2 | -1.3 | -4.6 | 1.44% | 4.1 |
| 5006 | AT5G61380.1 \| Symbol: TOC1 \| ABI3-interacting protein 1 (AIP1), identical to pseudo-response regulator 1 GI:7576354 from (Arabidopsis thaliana); timing of CAB expression 1 protein (TOC1) GI:9247019; contains Pfam profile PF00072: Response regulator receiver domain; identical to cDNA ABI3-interacting protein 1 (aip1 gene) GI:6996312 \| chr5:24692313-24695776 FORWARD \| Aliases: APRR1, MFB13.13, MFB13_13, PRR1, PSEUDO RESPONSE REGULATOR 1, TIMING OF CAB EXPRESSION 1, TIMING OF CAB1 1 | 5.3 | 6.4 | -1.1 | -4.6 | 1.44% | 4.0 |
| 5007 | AT1G49450.1 \| transducin family protein / WD-40 repeat family protein, contains 7 WD-40 repeats (PF00400); similar to En/Spm-like transposon protein GI:2739374 from (Arabidopsis thaliana); no characterized homologs | 3.5 | 4.6 | -1.1 | -4.6 | 1.45% | 4.1 |
| 5009 | AT2G02350.1 \| Symbol: SKIP3 \| SKP1 interacting partner 3 (SKIP3), nearly identical to SKP1 interacting partner 3 GI:10716951 from (Arabidopsis thaliana) \| chr2:617799-619090 REVERSE \| Aliases: ATPP2 B9, SKP1 INTERACTING PARTNER 3, T16F16.14, T16F16_14 | 4.6 | 5.7 | -1.1 | -4.6 | 1.45% | 3.9 |
| 5010 | AT3G13040.2 \| myb family transcription factor, contains Pfam profile: PF00249 myb-like DNA-binding domain \| chr3:4172149-4175255 REVERSE \| Aliases: MGH6.22 | 8.5 | 9.7 | -1.2 | -4.6 | 1.45% | 4.0 |

| Rank | Description | Sync | Root | M | t | adj.q | B |
|---|---|---|---|---|---|---|---|
| 5015 | AT4G35480.1 \| Symbol: RHA3B \| zinc finger (C3HC4-type RING finger) family protein, contains Pfam domain PF00097: Zinc finger, C3HC4 type (RING finger) \| chr4:16852079-16852952 REVERSE \| Aliases: F15J1.50, F15J1_50, RING H2 FINGER PROTEIN RHA3B | 5.0 | 6.9 | -1.9 | -4.6 | 1.45% | 4.1 |
| 5020 | AT3G59370.1 \| expressed protein, hypothetical protein F12F1.4 - Arabidopsis thaliana, EMBL:AC002131 \| chr3:21952849-21953509 REVERSE \| Aliases: F25L23.230 | 2.3 | 5.5 | -3.2 | -4.6 | 1.46% | 4.1 |
| 5023 | AT4G38090.2 \| expressed protein, contains Pfam PF01205: Uncharacterized protein family UPF0029 | 6.3 | 7.5 | -1.2 | -4.6 | 1.46% | 4.1 |
| 5030 | AT1G08350.1 \| endomembrane protein 70 family protein, KNOLLE; similar to putative endosomal protein GB:AAD20090 GI:4406780 from (Arabidopsis thaliana) | 4.7 | 5.4 | -0.7 | -4.6 | 1.47% | 3.9 |
| 5032 | AT3G13790.1 \| beta-fructosidase (BFRUCT1) / beta-fructofuranosidase / cell wall invertase, identical to beta-fructofuranosidase GI:402740 from (Arabidopsis thaliana) \| chr3:4532918-4535865 REVERSE \| Aliases: MMM17.26 | 3.3 | 7.0 | -3.7 | -4.6 | 1.47% | 4.1 |
| 5033 | AT3G10760.1 \| myb family transcription factor, contains Pfam profile: PF00249 myb-like DNA-binding domain \| chr3:3369585-3371232 FORWARD \| Aliases: T7M13.16 | 3.5 | 4.9 | -1.4 | -4.6 | 1.47% | 4.1 |
| 5035 | AT1G68580.2 \| agenet domain-containing protein / bromo-adjacent homology (BAH) domain-containing protein, contains Pfam profile PF01426: BAH domain and PF05641: Agenet domain | 4.3 | 5.8 | -1.5 | -4.6 | 1.47% | 4.0 |
| 5039 | AT4G01330.1 \| protein kinase family protein, contains protein kinase domain, Pfam:PF00069; contains serine/threonine protein kinase domain, INTERPRO:IPR002290 | 4.0 | 4.8 | -0.8 | -4.5 | 1.47% | 4.0 |
| 5040 | AT5G52070.1 \| agenet domain-containing protein, contains Pfam PF05641: Agenet domain \| chr5:21178537-21182251 FORWARD \| Aliases: MSG15.17, MSG15_17 | 3.2 | 5.4 | -2.2 | -4.5 | 1.47% | 4.1 |
| 5041 | AT1G32170.1 \| Symbol: XTR4 \| xyloglucan:xyloglucosyl transferase, putative / xyloglucan endotransglycosylase, putative / endo-xyloglucan transferase, putative (XTR4), identical to N-terminal partial sequence of xyloglucan endotransglycosylase-related protein XTR4 GI:1244754 from (Arabidopsis thaliana); similar to endoxyloglucan transferase (Arabidopsis thaliana) GI:5533311 \| chr1:11575301-11577907 FORWARD \| Aliases: F3C3.5, F3C3_5, XYLOGLUCAN ENDOTRANSGLYCOSYLASE 4, XYLOGLUCAN ENDOTRANSGLYCOSYLASE RELATED PROTEIN | 6.1 | 8.0 | -1.9 | -4.5 | 1.48% | 4.1 |
| 5042 | AT4G40080.1 \| epsin N-terminal homology (ENTH) domain-containing protein / clathrin assembly protein-related, contains Pfam PF01417: ENTH domain. ENTH (Epsin N-terminal homology) domain; similar to CLATHRIN COAT ASSEMBLY PROTEIN AP180 - Mus musculus, SWISSPROT:Q61548 \| chr4:18579308-18580711 FORWARD \| Aliases: T5J17.250, T5J17_250 | 4.7 | 6.1 | -1.4 | -4.5 | 1.48% | 4.1 |
| 5045 | AT4G29230.1 \| Symbol: ANAC075 \| no apical meristem (NAM) family protein, similar to NAM family proteins GP:12751304:, GP:6223650:, GP:9758909 - Arabidopsis thaliana,PIR2:T04621 \| chr4:14410344-14414962 FORWARD \| Aliases: ANAC075, F17A13.50, F17A13_50 | 2.7 | 3.4 | -0.7 | -4.5 | 1.48% | 3.8 |
| 5048 | AT2G27440.1 \| rac GTPase activating protein, putative, similar to rac GTPase activating protein 3 (Lotus japonicus) GI:3695063; contains Pfam profiles PF00620: RhoGAP domain, PF00786: P21-Rho-binding domain | 2.7 | 3.3 | -0.6 | -4.5 | 1.49% | 3.8 |
| 5053 | AT1G77770.2 \| expressed protein \| chr1:29251099-29252921 REVERSE \| Aliases: T32E8.10, T32E8_10 | 6.4 | 7.6 | -1.2 | -4.5 | 1.49% | 3.9 |
| 5054 | AT1G03010.1 \| phototropic-responsive NPH3 family protein, contains NPH3 family domain, Pfam:PF03000 \| chr1:693480-696382 FORWARD \| Aliases: F10O3.17, F10O3_17 | 2.3 | 2.8 | -0.6 | -4.5 | 1.49% | 3.7 |
| 5055 | AT3G49690.1 \| myb family transcription factor, contains PFAM profile: myb DNA binding domain PF00249 \| chr3:18438821-18440186 FORWARD \| Aliases: T16K5.40 | 5.5 | 7.3 | -1.9 | -4.5 | 1.50% | 4.1 |
| 5056 | AT2G23200.1 \| protein kinase family protein, contains protein kinase domain, Pfam:PF00069 \| chr2:9886356-9888988 FORWARD \| Aliases: T20D16.17, T20D16_17 | 3.7 | 4.6 | -0.8 | -4.5 | 1.50% | 4.0 |
| 5058 | AT5G01930.1 \| (1-4)-beta-mannan endohydrolase, putative, similar to (1-4)-beta-mannan endohydrolase (Coffea arabica) GI:10178872; contains Pfam profile PF00150: Cellulase (glycosyl hydrolase family 5) \| chr5:361085-362895 REVERSE \| Aliases: T20L15.200, T20L15_200 | 3.2 | 5.8 | -2.6 | -4.5 | 1.50% | 4.1 |
| 5060 | AT2G28690.1 \| expressed protein \| chr2:12313967-12315705 REVERSE \| Aliases: T8O18.2, T8O18_2 | 2.9 | 3.9 | -1.0 | -4.5 | 1.50% | 3.9 |
| 5064 | AT3G61300.1 \| C2 domain-containing protein, anthranilate phosphoribosyltransferase (fragment) - Pisum sativum, PIR:T06460 \| chr3:22698545-22701978 FORWARD \| Aliases: T20K12.200 | 4.3 | 7.1 | -2.8 | -4.5 | 1.50% | 4.0 |

| Rank | Description | Sync | Root | M | t | adj.q | B |
|---|---|---|---|---|---|---|---|
| 5068 | AT4G19110.2 \| protein kinase, putative, contains protein kinase domain, Pfam:PF00069 \| chr4:10454540-10459309 REVERSE \| Aliases: T18B16.80, T18B16_80 | 6.0 | 8.4 | -2.4 | -4.5 | 1.50% | 4.1 |
| 5070 | AT1G72840.1 \| disease resistance protein (TIR-NBS-LRR class), putative, domain signature TIR-NBS-LRR exists, suggestive of a disease resistance protein. \| chr1:27412963-27417284 REVERSE \| Aliases: F3N23.4, F3N23_4 | 2.5 | 3.1 | -0.6 | -4.5 | 1.50% | 3.8 |
| 5071 | AT1G07520.1 \| scarecrow transcription factor family protein, similar to GB:AAD24412 from (Arabidopsis thaliana) (Plant J. 18 (1), 111-119 (1999)); contains Pfam profile: PF03514 GRAS family transcription factor \| chr1:2309561-2311802 REVERSE \| Aliases: F22G5.41, F22G5_41 | 3.5 | 4.2 | -0.7 | -4.5 | 1.51% | 3.7 |
| 5072 | AT3G53090.2 \| Symbol: UPL7 \| similar to HECT-domain-containing protein / ubiquitin-transferase family protein [Arabidopsis thaliana] (TAIR:At3g17205.1); similar to putative ubiquitin protein ligase, 5'-partial [Oryza sativa (japonica cultivar-group)] (GB:AAP68382.1); contains InterPro domain IQ calmodulin-binding region (InterPro:IPR000048); contains InterPro domain HECT domain (Ubiquitin-protein ligase) (InterPro:IPR000569) \| chr3:19690186-19696338 FORWARD \| Aliases: T4D2.20 | 3.7 | 5.3 | -1.6 | -4.5 | 1.51% | 4.0 |
| 5073 | AT2G25000.1 \| Symbol: WRKY60 \| | 3.4 | 4.2 | -0.8 | -4.5 | 1.51% | 4.0 |
| 5075 | AT1G78670.1 \| gamma-glutamyl hydrolase, putative / gamma-Glu-X carboxypeptidase, putative / conjugase, putative, similar to gamma glutamyl hydrolase GI:1679658 SP:P93164 from (Glycine max) \| chr1:29595848-29598392 FORWARD \| Aliases: F9K20.29, F9K20_29 | 8.5 | 10.0 | -1.5 | -4.5 | 1.51% | 4.1 |
| 5076 | AT2G37050.3 \| similar to leucine-rich repeat family protein / protein kinase family protein [Arabidopsis thaliana] (TAIR:At1g67720.1); similar to putative light repressible receptor protein kinase [Oryza sativa (japonica cultivar-group)] (GB:BAD87040.1); contains InterPro domain Serine/threonine protein kinase, active site (InterPro:IPR008271); contains InterPro domain Tyrosine protein kinase (InterPro:IPR001245); contains InterPro domain Serine/threonine protein kinase (InterPro:IPR002290); contains InterPro domain Protein kinase (InterPro:IPR000719); contains InterPro domain Leucine-rich repeat (InterPro:IPR001611) \| chr2:15576065-15580805 FORWARD \| Aliases: T2N18.19, T2N18_19 | 5.6 | 7.4 | -1.8 | -4.5 | 1.51% | 4.0 |
| 5079 | AT1G78090.1 \| Symbol: ATTPPB \| | 4.3 | 5.9 | -1.6 | -4.5 | 1.52% | 4.1 |
| 5081 | AT3G47740.1 \| Symbol: ATATH2 \| | 2.2 | 3.2 | -1.0 | -4.5 | 1.52% | 4.0 |
| 5082 | AT5G11570.1 \| proton-dependent oligopeptide transport (POT) family protein, contains Pfam profile: PF00854 POT family \| chr5:3715944-3718277 REVERSE \| Aliases: F15N18.160, F15N18_160 | 2.7 | 3.5 | -0.9 | -4.5 | 1.52% | 4.0 |
| 5085 | AT3G58550.1 \| protease inhibitor/seed storage/lipid transfer protein (LTP) family protein, contains Pfam protease inhibitor/seed storage/LTP family domain \| chr3:21660341-21661264 REVERSE \| Aliases: F14P22.140 | 4.0 | 5.5 | -1.5 | -4.5 | 1.52% | 3.9 |
| 5086 | AT1G50660.1 \| expressed protein, similar to liver stage antigen-1 (GI:510184) (Plasmodium falciparum); similar to Myosin II heavy chain, non muscle (Swiss-Prot:P08799) (Dictyostelium discoideum); similar to liver stage antigen (GI:9916) (Plasmodium falciparum); similar to Kinesin-like protein KLPA (Swiss-Prot:P28739) (Emericella nidulans) \| chr1:18774605-18778354 FORWARD \| Aliases: F17J6.18, F17J6_18 | 4.1 | 5.3 | -1.2 | -4.5 | 1.52% | 4.1 |
| 5087 | AT2G47540.1 \| pollen Ole e 1 allergen and extensin family protein, contains Pfam domain, PF01190: Pollen proteins Ole e I family \| chr2:19512948-19513578 FORWARD \| Aliases: T30B22.16 | 2.4 | 3.1 | -0.7 | -4.5 | 1.52% | 3.9 |
| 5088 | AT5G59700.1 \| protein kinase, putative, similar to receptor-like protein kinase (Catharanthus roseus) gi:1644291:emb:CAA97692 \| chr5:24069611-24072651 REVERSE \| Aliases: MTH12.1, MTH12_1 | 4.3 | 5.2 | -0.9 | -4.5 | 1.52% | 4.0 |
| 5090 | AT5G61590.1 \| encodes a member of the ERF (ethylene response factor) subfamily B-3 of ERF/AP2 transcription factor family. The protein contains one AP2 domain. There are 18 members in this subfamily including ATERF-1, ATERF-2, AND ATERF-5. \| chr5:24781664-24782550 REVERSE \| Aliases: K11J9.4, K11J9_4 | 6.1 | 7.9 | -1.8 | -4.5 | 1.52% | 4.0 |
| 5091 | AT1G24530.1 \| transducin family protein / WD-40 repeat family protein, similar to Vegetatible incompatibility protein HET-E-1 (SP:Q00808) {Podospora anserina}; contains 7 WD-40 repeats (PF00400) \| chr1:8693214-8694692 FORWARD \| Aliases: F21J9.19 | 5.4 | 6.4 | -1.0 | -4.5 | 1.53% | 4.0 |
| 5095 | AT2G37820.1 \| DC1 domain-containing protein, contains Pfam profile PF03107: DC1 domain | 2.8 | 3.8 | -1.1 | -4.5 | 1.53% | 4.0 |
| 5096 | AT1G68740.1 \| EXS family protein / ERD1/XPR1/SYG1 family protein, similar to PHO1 protein (Arabidopsis thaliana) GI:20069032; contains Pfam profiles PF03105: SPX domain, PF03124: EXS family \| chr1:25816112-25820320 REVERSE \| Aliases: F24J5.2, F24J5_2 | 5.3 | 6.3 | -1.0 | -4.5 | 1.53% | 4.0 |
| 5098 | ATMG00110.1 \| Symbol: CCB206 \| | 3.4 | 5.3 | -1.9 | -4.5 | 1.53% | 4.0 |
| 5104 | AT1G74680.1 \| exostosin family protein, contains Pfam profile: PF03016 Exostosin family \| chr1:28062849-28064872 FORWARD \| Aliases: F25A4.34, F25A4_34 | 7.2 | 7.9 | -0.7 | -4.5 | 1.54% | 3.9 |

| Rank | Description | Sync | Root | M | t | adj.q | B |
|---|---|---|---|---|---|---|---|
| 5107 | AT4G14010.1 \| Symbol: RALFL32 \| rapid alkalinization factor (RALF) family protein, similar to RALF precursor (Nicotiana tabacum) GI:16566316 \| chr4:8092832-8093377 REVERSE \| Aliases: DL3045C, FCAALL.140, RALF LIKE 32 | 8.6 | 9.8 | -1.1 | -4.5 | 1.54% | 3.8 |
| 5108 | AT5G01700.2 \| similar to protein phosphatase 2C family protein / PP2C family protein [Arabidopsis thaliana] (TAIR:At3g02750.1); similar to putative protein phosphatase 2C [Oryza sativa (japonica cultivar-group)] (GB:XP_478745.1); similar to putative protein phosphatase 2C [Oryza sativa (japonica cultivar-group)] (GB:XP_465582.1); contains InterPro domain Protein phosphatase 2C-like (InterPro:IPR001932) \| chr5:260745-262579 REVERSE \| Aliases: F7A7.220, F7A7_220 | 3.5 | 4.6 | -1.1 | -4.5 | 1.54% | 4.0 |
| 5114 | AT1G59970.1 \| matrixin family protein, similar to SP:P29136 Metalloendoproteinase 1 precursor (EC 3.4.24.-) (SMEP1) {Glycine max}; contains InterPro accession IPR001818: Matrixin \| chr1:22077199-22078514 FORWARD \| Aliases: F23H11.28 | 5.7 | 6.4 | -0.7 | -4.5 | 1.55% | 3.8 |
| 5115 | AT4G23980.2 \| Symbol: ARF9 \| similar to transcriptional factor B3 family protein / auxin-responsive factor AUX/IAA-related [Arabidopsis thaliana] (TAIR:At2g46530.1); similar to ARFO_ARATH Putative auxin response factor 15 (GB:Q9LQE3); contains InterPro domain Transcriptional factor B3 (InterPro:IPR003340); contains InterPro domain AUX/IAA protein (InterPro:IPR003311) \| chr4:12451208-12455277 FORWARD \| Aliases: AUXIN RESPONSE FACTOR 9, T32A16.150, T32A16_150 | 4.0 | 6.0 | -2.1 | -4.5 | 1.55% | 4.1 |
| 5116 | AT3G53350.3 \| myosin heavy chain-related, low similarity to filamin-interacting protein S-FILIP (Rattus norvegicus) GI:21392397, nonmuscle heavy chain myosin II-A (Mus musculus) GI:17978023 \| chr3:19791053-19792719 REVERSE \| Aliases: F4P12.50 | 4.7 | 6.3 | -1.5 | -4.5 | 1.55% | 4.0 |
| 5117 | AT5G50140.1 \| ankyrin repeat family protein, contains ankyrin repeat domains, Pfam:PF00023 \| chr5:20413082-20415423 FORWARD \| Aliases: MPF21.16, MPF21_16 | 3.0 | 4.3 | -1.3 | -4.5 | 1.55% | 4.0 |
| 5118 | AT3G19370.1 \| expressed protein \| chr3:6710797-6713656 FORWARD \| Aliases: MLD14.10 | 6.4 | 8.3 | -2.0 | -4.5 | 1.56% | 4.0 |
| 5123 | AT4G14440.1 \| enoyl-CoA hydratase/isomerase family protein, low similarity to PhaB (Pseudomonas putida) GI:3253198, SP:P31551 Carnitine racemase {Escherichia coli}; contains Pfam profile PF00378 enoyl-CoA hydratase/isomerase family protein \| chr4:8306741-8307749 REVERSE \| Aliases: DL3260C, FCAALL.212 | 3.9 | 5.4 | -1.4 | -4.5 | 1.57% | 4.0 |
| 5124 | AT5G24380.1 \| Symbol: YSL2 \| similar to transporter, putative [Arabidopsis thaliana] (TAIR:At5g53550.1); similar to oligopeptide transporter OPT family protein [Arabidopsis thaliana] (TAIR:At5g41000.1); similar to oligopeptide transporter OPT family protein [Arabidopsis thaliana] (TAIR:At1g65730.1); similar to oligopeptide transporter OPT family protein [Arabidopsis thaliana] (TAIR:At3g17650.1); similar to transporter, putative [Arabidopsis thaliana] (TAIR:At4g24120.1); similar to OSJNBb0103I08.15 [Oryza sativa (japonica cultivar-group)] (GB:XP_473374.1); similar to putative iron-phytosiderophore transporter protein yellow stripe 1 [Oryza sativa (japonica cultivar-group)] (GB:XP_467066.1); similar to iron transport protein 1 [Oryza sativa] (GB:AAS49493.1); similar to hypothetical protein [Oryza sativa (japonica cultivar-group)] (GB:BAD90812.1); similar to iron-phytosiderophore transporter protein yellow stripe 1 [Zea mays] (GB:AAG17016.2); contains InterPro domain Oligopeptide transporter OPT superfamily (InterPro:IPR004813) \| chr5:8323898-8326708 FORWARD \| Aliases: ATYSL2, K16H17.9, K16H17_9, YELLOW STRIPE LIKE 2 | 6.8 | 8.1 | -1.3 | -4.5 | 1.57% | 4.0 |
| 5131 | AT5G61900.3 \| Symbol: BON1 \| copine BONZAI1 (BON1), nearly identical to BONZAI1 (Arabidopsis thaliana) GI:15487382; contains Pfam profile PF00168: C2 domain \| chr5:24872887-24876453 REVERSE \| Aliases: BON, BONSAI, BONZAI1, COPINE 1, CPN1, K22G18.2, K22G18_2 | 6.3 | 7.3 | -1.0 | -4.5 | 1.57% | 3.9 |
| 5132 | AT3G28960.1 \| amino acid transporter family protein, low similarity to vesicular inhibitory amino acid transporter (Mus musculus) GI:2826776; contains INTERPRO:IPR002422 amino acid/polyamine transporter, family II \| chr3:10985482-10987004 REVERSE \| Aliases: K5K13.9 | 2.2 | 2.6 | -0.4 | -4.5 | 1.58% | 3.5 |
| 5134 | AT4G37900.1 \| glycine-rich protein \| chr4:17821731-17824439 REVERSE \| Aliases: F20D10.20, F20D10_20 | 5.0 | 6.3 | -1.3 | -4.5 | 1.58% | 4.0 |
| 5135 | AT1G75370.1 \| SEC14 cytosolic factor, putative / phosphatidylinositol transfer-like protein, putative, similar to phosphatidylinositol transfer-like protein III (GI:14486705) (Lotus japonicus); contains Pfam PF00650 : CRAL/TRIO domain; contains Pfam PF03765 : CRAL/TRIO, N-terminu \| chr1:28280105-28283559 REVERSE \| Aliases: F1B16.10, F1B16_10 | 5.6 | 6.4 | -0.8 | -4.5 | 1.58% | 3.9 |
| 5136 | AT2G21120.1 \| expressed protein \| chr2:9058829-9061694 REVERSE \| Aliases: F26H11.12, F26H11_12 | 5.5 | 6.9 | -1.4 | -4.5 | 1.58% | 4.0 |
| 5137 | AT3G15450.3 \| similar to expressed protein [Arabidopsis thaliana] (TAIR:At4g27450.1); similar to unknown [Asparagus officinalis] (GB:CAA54526.1) \| chr3:5213004-5214126 FORWARD \| Aliases: MJK13.11 | 6.9 | 8.0 | -1.1 | -4.5 | 1.58% | 4.0 |
| 5138 | AT5G58750.1 \| wound-responsive protein-related, similar to induced upon wounding stress (Arabidopsis thaliana) GI:1483218 \| chr5:23746014-23747348 FORWARD \| Aliases: MZN1.13, MZN1_13 | 3.0 | 4.6 | -1.6 | -4.5 | 1.58% | 4.0 |
| 5141 | AT3G28210.1 \| Symbol: PMZ \| zinc finger (AN1-like) family protein, contains Pfam profile: PF01428 AN1-like zinc finger \| chr3:10521758-10522769 FORWARD \| Aliases: T19D11.2 | 3.5 | 4.6 | -1.0 | -4.5 | 1.58% | 4.0 |
| 5142 | AT5G03030.1 \| DNAJ heat shock N-terminal domain-containing protein, contains Pfam profile PF00226 DnaJ domain; DNAJ domain-containing protein, Homo sapiens, EMBL:AF126743 \| chr5:708341-709701 REVERSE \| Aliases: F15A17.60, F15A17_60 | 8.0 | 9.0 | -1.0 | -4.5 | 1.58% | 3.9 |

| Rank | Description | Sync | Root | M | t | adj.q | B |
|---|---|---|---|---|---|---|---|
| 5143 | AT3G48080.1 \| lipase class 3 family protein / disease resistance protein-related, similar to disease resistance protein/lipase homolog EDS1 GI:4454567; contains Pfam profile PF01764: Lipase \| chr3:17763923-17766175 REVERSE \| Aliases: T17F15.50 | 2.5 | 3.3 | -0.8 | -4.5 | 1.58% | 3.9 |
| 5144 | AT5G49660.1 \| leucine-rich repeat transmembrane protein kinase, putative, contains leucine rich repeat (LRR) domains, Pfam:PF00560; contains protein kinase domain, Pfam:PF00069 \| chr5:20178432-20181955 REVERSE \| Aliases: MNI5.4, MNI5_4 | 6.3 | 7.3 | -1.0 | -4.5 | 1.58% | 3.9 |
| 5146 | AT3G15800.1 \| glycosyl hydrolase family 17 protein, similar to elicitor inducible chitinase Nt-SubE76 GI:11071974 from (Nicotiana tabacum) \| chr3:5345058-5346995 FORWARD \| Aliases: MSJ11.20 | 2.4 | 3.1 | -0.7 | -4.5 | 1.58% | 3.7 |
| 5147 | AT1G59590.1 \| Symbol: ZCF37 \| expressed protein \| chr1:21891417-21892457 FORWARD \| Aliases: T30E16.15, T30E16_15, ZCF37 | 4.0 | 5.7 | -1.7 | -4.5 | 1.58% | 4.0 |
| 5149 | AT5G37370.2 \| pre-mRNA splicing factor PRP38 family protein (SRL1), contains Pfam profile PF03371: PRP38 family \| chr5:14829915-14833750 REVERSE \| Aliases: MNJ8.19, MNJ8_19 | 6.4 | 7.9 | -1.5 | -4.5 | 1.59% | 3.9 |
| 5160 | AT5G03555.1 \| permease, cytosine/purines, uracil, thiamine, allantoin family protein, contains Pfam PF02133: permease, cytosine/purines, uracil, thiamine, allantoin family \| chr5:898240-900158 REVERSE \| Aliases: None | 5.0 | 6.9 | -1.9 | -4.5 | 1.60% | 4.0 |
| 5161 | AT1G35260.1 \| Bet v I allergen family protein, similar to Csf-2 (Cucumis sativus)(GI:5762258)(J Am Soc Hortic Sci 124, 136-139 (1999)) contains Pfam profile PF00407: Pathogenesis-related protein Bet v I family \| chr1:12936827-12937765 REVERSE \| Aliases: T9I1.17, T9I1_17 | 3.6 | 5.3 | -1.7 | -4.5 | 1.60% | 4.0 |
| 5162 | AT3G28130.2 \| similar to nodulin MtN21 family protein [Arabidopsis thaliana] (TAIR:At3g28100.1); similar to putative nodulin MtN21 protein [Oryza sativa (japonica cultivar-group)] (GB:XP_475475.1); contains InterPro domain Protein of unknown function DUF6 (InterPro:IPR000620) \| chr3:10466753-10470148 FORWARD \| Aliases: MMG15.31 | 3.1 | 4.3 | -1.2 | -4.5 | 1.60% | 4.0 |
| 5163 | AT5G65790.1 \| Symbol: MYB68 \| myb family transcription factor (MYB68), identical to putative transcription factor (MYB68) GI:3941493 from (Arabidopsis thaliana); contains Pfam profile: PF00249 myb-like DNA-binding domain \| chr5:26340174-26341804 FORWARD \| Aliases: MPA24.14, MPA24_14 | 3.9 | 5.9 | -1.9 | -4.5 | 1.60% | 4.0 |
| 5167 | AT3G49790.1 \| expressed protein, predicted protein, Arabidopsis thaliana \| chr3:18479036-18480748 REVERSE \| Aliases: T16K5.140 | 3.3 | 4.4 | -1.1 | -4.5 | 1.60% | 3.7 |
| 5169 | AT1G79600.1 \| ABC1 family protein, contains Pfam domain, PF03109: ABC1 family \| chr1:29954839-29957583 REVERSE \| Aliases: F20B17.3, F20B17_3 | 6.0 | 7.1 | -1.0 | -4.5 | 1.60% | 3.9 |
| 5170 | AT3G10250.2 \| expressed protein \| chr3:3167956-3170908 REVERSE \| Aliases: F14P13.15 | 3.4 | 5.2 | -1.7 | -4.5 | 1.60% | 4.0 |
| 5172 | AT5G61600.1 \| encodes a member of the ERF (ethylene response factor) subfamily B-3 of ERF/AP2 transcription factor family. The protein contains one AP2 domain. There are 18 members in this subfamily including ATERF-1, ATERF-2, AND ATERF-5. \| chr5:24783612-24784656 REVERSE \| Aliases: K11J9.13, K11J9_13 | 5.9 | 7.6 | -1.7 | -4.5 | 1.61% | 4.0 |
| 5174 | AT3G57570.1 \| expressed protein \| chr3:21323475-21331276 FORWARD \| Aliases: T8H10.170 | 3.5 | 4.2 | -0.7 | -4.5 | 1.61% | 3.8 |
| 5176 | AT1G25550.1 \| myb family transcription factor, contains Pfam domain, PF00249: Myb-like DNA-binding domain \| chr1:8976525-8978080 FORWARD \| Aliases: F2J7.21, F2J7_21 | 7.0 | 7.9 | -1.0 | -4.5 | 1.61% | 3.7 |
| 5179 | AT4G30280.1 \| Symbol: ATXTH18 \| putative xyloglucan endotransglycosylase/hydrolase, expressed in the mature or basal regions of both the main and lateral roots, but not in the tip of these roots where cell division occurs. \| chr4:14825814-14827053 REVERSE \| Aliases: ATXTH18, F17I23.380, F17I23_380, XYLOGLUCAN ENDOTRANSGLUCOSYLASE/HYDROLASE 18 | 3.3 | 5.0 | -1.7 | -4.5 | 1.61% | 3.9 |
| 5181 | AT3G46220.1 \| expressed protein \| chr3:16990060-16995776 FORWARD \| Aliases: F12M12.190 | 6.5 | 7.6 | -1.2 | -4.5 | 1.61% | 4.0 |
| 5182 | AT5G02890.1 \| transferase family protein, contains Pfam profile PF02458: Transferase family \| chr5:670063-671467 REVERSE \| Aliases: F9G14.200, F9G14_200 | 3.0 | 4.0 | -1.0 | -4.5 | 1.61% | 3.9 |
| 5184 | AT5G58375.1 \| expressed protein \| chr5:23613500-23614186 FORWARD \| Aliases: None | 9.2 | 10.8 | -1.6 | -4.5 | 1.61% | 4.0 |
| 5186 | AT5G13090.1 \| expressed protein, predicted proteins - Arabidopsis thaliana \| chr5:4153589-4154675 REVERSE \| Aliases: T19L5.50, T19L5_50 | 5.5 | 6.5 | -1.0 | -4.5 | 1.61% | 3.9 |
| 5188 | AT3G50970.1 \| Symbol: XERO2 \| dehydrin xero2 (XERO2) / low-temperature-induced protein LTI30 (LTI30), identical to dehydrin Xero 2 (Low-temperature-induced protein LTI30) (Arabidopsis thaliana) SWISS-PROT:P42758 \| chr3:18951758-18952535 FORWARD \| Aliases: F24M12.10 | 8.6 | 10.8 | -2.2 | -4.5 | 1.61% | 4.0 |
| 5190 | AT5G55500.1 \| Symbol: AtXylT \| | 5.4 | 6.3 | -0.9 | -4.5 | 1.61% | 3.9 |

| Rank | Description | Sync | Root | M | t | adj.q | B |
|---|---|---|---|---|---|---|---|
| 5192 | AT5G11710.1 \| epsin N-terminal homology (ENTH) domain-containing protein / clathrin assembly protein-related, contains Pfam PF01417: ENTH domain. ENTH (Epsin N-terminal homology) domain; similar to Af10-protein (GI:1724114) (Avena fatua); similar to clathrin assembly protein AP180 (GI:6492344) (Xenopus laevis); Af10-protein, Avena fatua, EMBL:U80041 \| chr5:3772865-3776663 FORWARD \| Aliases: T22P22.100, T22P22_100 | 5.9 | 6.6 | -0.7 | -4.5 | 1.62% | 3.8 |
| 5195 | AT1G19835.1 \| expressed protein, contains Pfam PF05911: Plant protein of unknown function (DUF869) \| chr1:6855943-6860323 REVERSE \| Aliases: F14P1.24, F14P1_24 | 9.0 | 9.9 | -1.0 | -4.5 | 1.62% | 3.8 |
| 5196 | AT5G66650.1 \| expressed protein, contains Pfam domain, PF04678: Protein of unknown function, DUF607 \| chr5:26620229-26621602 REVERSE \| Aliases: MSN2.3, MSN2_3 | 3.5 | 4.5 | -1.1 | -4.5 | 1.62% | 4.0 |
| 5200 | AT3G21340.1 \| leucine-rich repeat protein kinase, putative, similar to light repressible receptor protein kinase (Arabidopsis thaliana) gi:1321686:emb:CAA66376; contains leucine rich repeat (LRR) domains, Pfam:PF00560; contains protein kinase domain, Pfam:PF00069 \| chr3:7511793-7515943 REVERSE \| Aliases: MHC9.2 | 2.5 | 3.0 | -0.6 | -4.5 | 1.62% | 3.7 |
| 5205 | AT5G22920.1 \| zinc finger (C3HC4-type RING finger) family protein, contains Pfam profiles:PF05495 CHY zinc finger, PF00097 zinc finger, C3HC4 type (RING finger) \| chr5:7664994-7667268 FORWARD \| Aliases: MRN17.15, MRN17_15 | 7.8 | 9.1 | -1.3 | -4.5 | 1.63% | 3.8 |
| 5206 | AT2G01600.1 \| epsin N-terminal homology (ENTH) domain-containing protein, contains Pfam PF01417: ENTH domain. ENTH (Epsin N-terminal homology) domain; similar to clathrin assembly protein AP180 (GI:6492344) (Xenopus laevis) \| chr2:268817-272609 FORWARD \| Aliases: F2I9.22, F2I9_22 | 4.7 | 6.5 | -1.8 | -4.5 | 1.63% | 4.0 |
| 5207 | AT2G23300.1 \| leucine-rich repeat transmembrane protein kinase, putative \| chr2:9921688-9924210 FORWARD \| Aliases: T20D16.7, T20D16_7 | 3.4 | 5.0 | -1.6 | -4.4 | 1.63% | 4.0 |
| 5209 | AT5G59530.1 \| 2-oxoglutarate-dependent dioxygenase, putative, similar to 2A6 (GI:599622) and tomato ethylene synthesis regulatory protein E8 (SP:P10967); 1-aminocyclopropane-1-carboxylate oxidase kidney bean, PIR:T10818 \| chr5:24011410-24012941 REVERSE \| Aliases: F2O15.26, F2O15_26 | 2.7 | 3.5 | -0.7 | -4.4 | 1.63% | 3.8 |
| 5211 | AT3G53260.1 \| Symbol: PAL2 \| phenylalanine ammonia-lyase 2 (PAL2), nearly identical to SP:P45724 \| chr3:19755029-19757758 REVERSE \| Aliases: T4D2.190 | 8.1 | 10.4 | -2.3 | -4.4 | 1.64% | 3.8 |
| 5214 | AT2G30250.1 \| Symbol: WRKY25 \| | 3.3 | 4.5 | -1.3 | -4.4 | 1.64% | 4.0 |
| 5216 | AT3G16690.1 \| nodulin MtN3 family protein, contains Pfam PF03083 MtN3/saliva family \| chr3:5684386-5686496 REVERSE \| Aliases: MGL6.16 | 3.3 | 4.7 | -1.4 | -4.4 | 1.64% | 3.9 |
| 5217 | AT3G06670.1 \| expressed protein \| chr3:2105465-2113421 REVERSE \| Aliases: T8E24.10 | 3.7 | 4.6 | -0.9 | -4.4 | 1.65% | 3.9 |
| 5220 | AT3G17340.1 \| importin-related, contains Pfam profile PF03810: Importin-beta N-terminal domain | 2.8 | 3.5 | -0.7 | -4.4 | 1.65% | 3.8 |
| 5222 | AT1G60610.2 \| expressed protein \| chr1:22331577-22333941 REVERSE \| Aliases: F8A5.13, F8A5_13 | 6.3 | 7.2 | -0.9 | -4.4 | 1.65% | 3.7 |
| 5228 | AT1G17060.1 \| Symbol: CYP72C1 \| cytochrome P450, putative, 41% identical to Cytochrome P450 (Catharanthus roseus) (gi:404690) | 3.0 | 3.6 | -0.6 | -4.4 | 1.65% | 3.7 |
| 5229 | AT4G22640.1 \| expressed protein, various predicted proteins, Arabidopsis thaliana \| chr4:11911611-11912019 FORWARD \| Aliases: T12H17.30 | 3.9 | 5.8 | -1.9 | -4.4 | 1.65% | 3.9 |
| 5230 | AT5G57160.1 \| Symbol: ATLIG4 \| | 6.1 | 7.3 | -1.2 | -4.4 | 1.65% | 3.8 |
| 5235 | AT1G34420.1 \| leucine-rich repeat family protein / protein kinase family protein, contains leucine rich repeat (LRR) domains, Pfam:PF00560; contains protein kinase domain, Pfam:PF00069 \| chr1:12584565-12587548 FORWARD \| Aliases: F12K21.25, F12K21_25 | 2.9 | 4.0 | -1.1 | -4.4 | 1.66% | 3.9 |
| 5236 | AT5G47230.1 \| Symbol: ATERF 5/ATERF5 \| | 3.8 | 5.2 | -1.5 | -4.4 | 1.67% | 4.0 |
| 5237 | AT3G59050.1 \| amine oxidase family protein, similar to polyamine oxidase (EC 1.5.3.11) precursor - Zea mays (SP:O64411); contains Pfam profile PF01593 amine oxidase, flavin-containing \| chr3:21835673-21838486 REVERSE \| Aliases: F17J16.100 | 6.2 | 7.5 | -1.3 | -4.4 | 1.67% | 3.9 |
| 5239 | AT1G80010.1 \| far-red impaired responsive protein, putative, similar to far-red impaired response protein FAR1 (Arabidopsis thaliana) gi:5764395:gb:AAD51282 \| chr1:30102649-30104739 FORWARD \| Aliases: F18B13.10, F18B13_10 | 4.9 | 5.6 | -0.7 | -4.4 | 1.67% | 3.7 |
| 5247 | AT1G13920.1 \| remorin family protein, contains Pfam domain, PF03763: Remorin, C-terminal region \| chr1:4758185-4759751 FORWARD \| Aliases: F16A14.13 | 2.8 | 3.8 | -0.9 | -4.4 | 1.67% | 3.9 |
| 5250 | AT5G56220.1 \| expressed protein \| chr5:22772097-22775146 FORWARD \| Aliases: K24C1.3, K24C1_3 | 5.0 | 5.6 | -0.6 | -4.4 | 1.67% | 3.7 |

| Rank | Description | Sync | Root | M | t | adj.q | B |
|---|---|---|---|---|---|---|---|
| 5252 | AT2G38840.1 \| guanylate-binding family protein, similar to SP:Q01514 Interferon-induced guanylate-binding protein 1 (Guanine nucleotide-binding protein 1) (Interferon-gamma inducible protein MAG-1) {Mus musculus}; contains Pfam profile PF02263: Guanylate-binding protein, N-terminal domain \| chr2:16234407-16239434 FORWARD \| Aliases: F13I13.7, F13I13_7 | 5.0 | 5.9 | -0.8 | -4.4 | 1.68% | 3.5 |
| 5253 | AT2G30340.1 \| LOB domain protein 13 / lateral organ boundaries domain protein 13 (LBD13), identical to LOB DOMAIN 13 (Arabidopsis thaliana) GI:17227158 SP:Q9AT61 \| chr2:12938321-12939659 REVERSE \| Aliases: T9D9.15, T9D9_15 | 2.2 | 3.2 | -1.1 | -4.4 | 1.68% | 3.8 |
| 5261 | AT3G16230.1 \| expressed protein, similar to ASC-1 complex subunit P50 (GI:12061189) (Homo sapiens) \| chr3:5500562-5503549 FORWARD \| Aliases: MYA6.4 | 7.5 | 8.4 | -0.8 | -4.4 | 1.68% | 3.9 |
| 5268 | AT1G32640.1 \| Symbol: ATMYC2 \| | 6.1 | 7.4 | -1.3 | -4.4 | 1.69% | 3.9 |
| 5271 | AT1G27430.1 \| GYF domain-containing protein, contains Pfam profile: PF02213 GYF domain | 4.7 | 5.8 | -1.0 | -4.4 | 1.69% | 3.8 |
| 5276 | AT1G04240.1 \| Symbol: SHY2 \| auxin-responsive protein / indoleacetic acid-induced protein 3 (IAA3), identical to SP:Q38822 Auxin-responsive protein IAA3 (Indoleacetic acid-induced protein 3) {Arabidopsis thaliana}; EST gb:T04296 comes from this gene \| chr1:1128187-1129414 REVERSE \| Aliases: F19P19.32, F19P19_32, IAA3, SHORT HYPOCOTYL 2 | 3.6 | 5.0 | -1.5 | -4.4 | 1.70% | 3.9 |
| 5277 | AT1G13195.1 \| zinc finger (C3HC4-type RING finger) family protein, similar to MTD2 (Medicago truncatula) GI:9294812; contains Pfam profile PF00097: Zinc finger, C3HC4 type (RING finger) \| chr1:4501081-4503588 REVERSE \| Aliases: None | 5.9 | 7.2 | -1.3 | -4.4 | 1.70% | 3.9 |
| 5282 | AT4G08040.1 \| Symbol: ACS11 \| 1-aminocyclopropane-1-carboxylate synthase, putative / ACC synthase, putative, similar to ACC synthase from Malus sylvestris (SP:P37821), Solanum tuberosum (GI:520914) | 2.8 | 3.8 | -1.0 | -4.4 | 1.72% | 3.9 |
| 5283 | AT1G07650.1 \| leucine-rich repeat transmembrane protein kinase, putative, similar to GB:AAC50043 from (Arabidopsis thaliana) (Plant Mol. Biol. 37 (4), 587-596 (1998)) | 4.7 | 5.6 | -0.9 | -4.4 | 1.72% | 3.8 |
| 5284 | AT1G77700.1 \| pathogenesis-related thaumatin family protein, similar to thaumatin-like protein (Arabidopsis thaliana) GI:2435406; contains Pfam profile PF00314: Thaumatin family \| chr1:29209641-29211111 FORWARD \| Aliases: T32E8.3, T32E8_3 | 2.4 | 3.2 | -0.8 | -4.4 | 1.72% | 3.7 |
| 5285 | AT3G02460.2 \| plant adhesion molecule, putative, strong similarity to plant adhesion molecule 1 (Arabidopsis thaliana) GI:3511223; contains Pfam profile PF00566: TBC domain \| chr3:505441-508410 FORWARD \| Aliases: F16B3.9, F16B3_9 | 5.5 | 6.3 | -0.8 | -4.4 | 1.72% | 3.7 |
| 5288 | AT5G49870.1 \| jacalin lectin family protein, similar to myrosinase-binding protein homolog (Arabidopsis thaliana) GI:2997767; contains Pfam profile PF01419 jacalin-like lectin domain | 3.1 | 3.8 | -0.6 | -4.4 | 1.72% | 3.5 |
| 5294 | AT4G00230.1 \| Symbol: XSP1 \| subtilisin-like serine endopeptidase (XSP1), identical to subtilisin-type serine endopeptidase XSP1 GI:6708179 from (Arabidopsis thaliana) \| chr4:93923-97449 FORWARD \| Aliases: F6N15.3, F6N15_3, SUBTILISIN TYPE SERINE ENDOPEPTIDASE XSP1, XYLEM SERINE PEPTIDASE 1 | 3.8 | 5.3 | -1.5 | -4.4 | 1.72% | 3.9 |
| 5295 | AT1G19180.2 \| expressed protein \| chr1:6622105-6623611 FORWARD \| Aliases: T29M8.5, T29M8_5 | 5.8 | 7.5 | -1.8 | -4.4 | 1.72% | 3.9 |
| 5298 | AT5G14550.2 \| expressed protein, contains Pfam profile PF03267: Arabidopsis protein of unknown function, DUF266 \| chr5:4691381-4693735 REVERSE \| Aliases: T15N1.40, T15N1_40 | 5.2 | 6.0 | -0.8 | -4.4 | 1.73% | 3.8 |
| 5299 | AT2G47760.3 \| expressed protein, similar to PREDICTED: hypothetical protein XP_535823 [Canis familiaris] (GB:XP_535823.1); contains InterPro domain ALG3 (InterPro:IPR007873) \| chr2:19572552-19575335 REVERSE \| Aliases: F17A22.15 | 6.6 | 7.7 | -1.0 | -4.4 | 1.73% | 3.8 |
| 5301 | AT5G25240.1 \| expressed protein \| chr5:8746608-8747200 REVERSE \| Aliases: F21J6.107, F21J6_107 | 4.9 | 6.5 | -1.6 | -4.4 | 1.73% | 3.8 |
| 5302 | AT4G29180.1 \| leucine-rich repeat protein kinase, putative, similar to light repressible receptor protein kinase (Arabidopsis thaliana) gi:1321686:emb:CAA66376; contains leucine rich repeat (LRR) domains, Pfam:PF00560; contains protein kinase domain, Pfam:PF00069 \| chr4:14385599-14389695 FORWARD \| Aliases: F19B15.210, F19B15_210 | 2.8 | 4.1 | -1.3 | -4.4 | 1.73% | 3.9 |
| 5303 | AT1G08100.1 \| Symbol: ATNRT2.2 \| high-affinity nitrate transporter (ACH2), identical to high-affinity nitrate transporter ACH2 (Arabidopsis thaliana) GI:3608364 \| chr1:2527167-2529117 REVERSE \| Aliases: ACH2, NITRATE TRANSPORTER 2, NRT2;2AT, T6D22.18, T6D22_18 | 3.2 | 4.3 | -1.1 | -4.4 | 1.73% | 3.9 |
| 5307 | AT1G55330.1 \| Symbol: AGP21 \| arabinogalactan-protein (AGP21) | 9.2 | 10.7 | -1.6 | -4.4 | 1.73% | 3.5 |
| 5314 | AT2G04880.2 \| Symbol: ZAP1 \| WRKY family transcription factor (ZAP1), identical to ZAP1 GI:1064883 from (Arabidopsis thaliana); contains Pfam profile: PF03106 WRKY DNA -binding domain \| chr2:1717890-1720971 FORWARD \| Aliases: ATWRKY1, F1O13.1, F1O13_1, WRKY1, ZAP1 | 6.6 | 7.6 | -1.0 | -4.4 | 1.73% | 3.8 |

| Rank | Description | Sync | Root | M | t | adj.q | B |
|---|---|---|---|---|---|---|---|
| 5315 | AT5G43190.1 \| F-box family protein (FBX6), contains similarity to unusual floral organs (UFO) GI:4376159 from (Arabidopsis thaliana) \| chr5:17357367-17358740 REVERSE \| Aliases: MNL12.1, MNL12_1 | 5.5 | 6.1 | -0.6 | -4.4 | 1.74% | 3.6 |
| 5319 | AT5G49460.1 \| Symbol: ACLB 2 \| | 5.8 | 7.7 | -1.9 | -4.4 | 1.74% | 3.8 |
| 5320 | AT1G75400.1 \| expressed protein \| chr1:28300580-28303347 FORWARD \| Aliases: F1B16.7, F1B16_7 | 3.6 | 4.8 | -1.3 | -4.4 | 1.74% | 3.9 |
| 5321 | AT5G58540.3 \| protein kinase family protein, contains Pfam domain, PF00069: Protein kinase domain \| chr5:23680362-23682905 FORWARD \| Aliases: MQJ2.16, MQJ2_16 | 3.1 | 3.8 | -0.8 | -4.4 | 1.74% | 3.8 |
| 5324 | AT1G53430.1 \| leucine-rich repeat family protein / protein kinase family protein, contains Pfam domains PF00560: Leucine Rich Repeat and PF00069: Protein kinase domain \| chr1:19938835-19944756 FORWARD \| Aliases: T3F20.25, T3F20_25 | 4.9 | 6.8 | -1.9 | -4.4 | 1.74% | 3.9 |
| 5330 | AT5G43100.1 \| aspartyl protease family protein, low similarity to CND41, chloroplast nucleoid DNA binding protein (Nicotiana tabacum) GI:2541876; contains Pfam profile PF00026: Eukaryotic aspartyl protease \| chr5:17316351-17320286 FORWARD \| Aliases: MMG4.12, MMG4_12 | 6.5 | 7.7 | -1.1 | -4.4 | 1.75% | 3.9 |
| 5331 | AT3G15890.1 \| protein kinase family protein, contains protein kinase domain, Pfam:PF00069 \| chr3:5374254-5376281 FORWARD \| Aliases: MVC8.1 | 4.7 | 5.8 | -1.1 | -4.4 | 1.75% | 3.7 |
| 5332 | AT1G80450.1 \| VQ motif-containing protein, contains PF05678: VQ motif \| chr1:30248847-30249580 REVERSE \| Aliases: T21F11.22, T21F11_22 | 2.0 | 2.4 | -0.5 | -4.4 | 1.76% | 3.0 |
| 5336 | AT3G30720.1 \| expressed protein \| chr3:12351626-12352205 FORWARD \| Aliases: F21A17.18 | 3.4 | 4.9 | -1.5 | -4.4 | 1.76% | 3.9 |
| 5337 | AT2G18440.1 \| Symbol: GUT15 \| expressed protein \| chr2:8002811-8004000 REVERSE \| Aliases: GENE WITH UNSTABLE TRANSCRIPT 15, GUT15, T30D6.5, T30D6_5 | 8.1 | 9.8 | -1.7 | -4.4 | 1.76% | 3.8 |
| 5338 | AT1G78990.1 \| transferase family protein, low similarity to acetyl CoA: benzylalcohol acetyltransferase Clarkia breweri GI:3170250, GI:6166336, Clarkia concinna GI:6166326, anthranilate N-hydroxycinnamoyl/benzoyltransferase Dianthus caryophyllus GI:2239091; contains Pfam profile PF02458 transferase family \| chr1:29718424-29719862 REVERSE \| Aliases: YUP8H12R.39, YUP8H12R_39 | 2.6 | 3.5 | -0.9 | -4.4 | 1.76% | 3.8 |
| 5339 | AT2G27360.1 \| lipase, putative, similar to lipase (Arabidopsis thaliana) GI:1145627; contains Pfam profile PF00657: Lipase/Acylhydrolase with GDSL-like motif \| chr2:11713276-11715120 FORWARD \| Aliases: F12K2.6, F12K2_6 | 4.3 | 5.6 | -1.4 | -4.4 | 1.76% | 3.9 |
| 5341 | AT5G60410.4 \| Symbol: ATSIZ1 \| | 4.5 | 5.1 | -0.6 | -4.4 | 1.76% | 3.6 |
| 5342 | AT2G45970.1 \| Symbol: CYP86A8 \| Encodes a member of the CYP86A subfamily of cytochrome p450 genes. \| chr2:18919333-18921812 REVERSE \| Aliases: CYP86A6, F4I18.5, LACERATA, LCR | 2.8 | 3.5 | -0.7 | -4.4 | 1.76% | 3.7 |
| 5343 | AT4G01650.2 \| expressed protein \| chr4:704407-706965 REVERSE \| Aliases: T15B16.3, T15B16_3 | 3.5 | 4.4 | -0.9 | -4.4 | 1.76% | 3.8 |
| 5346 | AT1G12550.1 \| oxidoreductase family protein, similar to glyoxylate reductase from Homo sapiens (gi:6002730); contains Pfam D-isomer specific 2-hydroxyacid dehydrogenase, NAD binding domain PF02826 \| chr1:4274647-4275829 FORWARD \| Aliases: F5O11.29, F5O11_29 | 5.8 | 7.2 | -1.4 | -4.4 | 1.77% | 3.8 |
| 5348 | AT4G08320.2 \| similar to serine/threonine protein phosphatase, putative [Arabidopsis thaliana] (TAIR:At2g42810.1); similar to CAA30373.1 protein [Oryza sativa] (GB:CAB53476.1); contains InterPro domain TPR repeat (InterPro:IPR001440) \| chr4:5252808-5255516 FORWARD \| Aliases: T28D5.10, T28D5_10 | 6.0 | 7.0 | -1.0 | -4.4 | 1.77% | 3.7 |
| 5350 | AT3G62650.2 \| expressed protein, putative mitochondrial carrier protein At2g47490 - Arabidopsis thaliana, EMBL:AC002535 \| chr3:23179037-23180163 FORWARD \| Aliases: F26K9.80 | 8.1 | 9.2 | -1.1 | -4.4 | 1.77% | 3.8 |
| 5351 | AT2G43220.1 \| DC1 domain-containing protein, contains Pfam profile PF03107: DC1 domain | 4.3 | 5.2 | -0.9 | -4.4 | 1.77% | 3.6 |
| 5352 | AT2G42750.1 \| DNAJ heat shock N-terminal domain-containing protein, low similarity to GFA2 (Arabidopsis thaliana) GI:21429604; contains Pfam profile PF00226: DnaJ domain \| chr2:17800384-17802757 FORWARD \| Aliases: F7D19.25, F7D19_25 | 5.3 | 6.5 | -1.2 | -4.4 | 1.77% | 3.9 |
| 5353 | AT4G32040.1 \| Symbol: KNAT5 \| homeobox protein knotted-1 like 5 (KNAT5) / homeodomain containing protein 1 (H1), identical to homeobox protein knotted-1 like 5 (KNAT5) SP:P48002 from (Arabidopsis thaliana) | 4.9 | 6.5 | -1.6 | -4.4 | 1.77% | 3.9 |
| 5357 | AT4G00520.1 \| acyl-CoA thioesterase family protein, contains Pfam profile: PF02551 acyl-CoA thioesterase \| chr4:229163-231699 FORWARD \| Aliases: F6N23.3, F6N23_3 | 3.1 | 4.0 | -0.8 | -4.4 | 1.78% | 3.6 |
| 5359 | AT4G16830.1 \| nuclear RNA-binding protein (RGGA), identical to nuclear RNA binding protein GI:6492264 from (Arabidopsis thaliana) \| chr4:9470458-9472613 FORWARD \| Aliases: DL4440W, FCAALL.13 | 9.1 | 10.4 | -1.3 | -4.4 | 1.78% | 3.8 |

| Rank | Description | Sync | Root | M | t | adj.q | B |
|---|---|---|---|---|---|---|---|
| 5361 | AT1G17310.1 \| MADS-box protein (AGL100), similar to transcription factor GB:BAA25245 GI:2981610 from (Ceratopteris richardii); contains Pfam profile PF00319: SRF-type transcription factor (DNA-binding and dimerisation domain) \| chr1:5928007-5928660 REVERSE \| Aliases: T13M22.2, T13M22_2 | 3.1 | 4.1 | -1.0 | -4.4 | 1.78% | 3.8 |
| 5362 | AT1G10050.1 \| glycosyl hydrolase family 10 protein / carbohydrate-binding domain-containing protein, similar to GB:AAD27896 to endoxylanases gi:1255238 (Thermoanaerobacterium thermosulfurigenes), gi:1813595 (Hordeum vulgare) and others ; contains Pfam profiles PF00331: Glycosyl hydrolase family 10, PF02018: Carbohydrate binding domain \| chr1:3279272-3283484 FORWARD \| Aliases: T27I1.7, T27I1_7 | 4.5 | 5.4 | -1.0 | -4.4 | 1.78% | 3.7 |
| 5363 | AT2G02070.1 \| zinc finger (C2H2 type) family protein, contains Pfam domain, PF00096: Zinc finger, C2H2 type \| chr2:505486-509424 FORWARD \| Aliases: F5O4.16, F5O4_16 | 3.0 | 4.1 | -1.0 | -4.4 | 1.79% | 3.8 |
| 5364 | AT2G17430.1 \| seven transmembrane MLO family protein / MLO-like protein 7 (MLO7), identical to membrane protein Mlo7 (Arabidopsis thaliana) gi:14091584:gb:AAK53800; similar to MLO protein SWISS-PROT:P93766, NCBI_gi:1877221 (Hordeum vulgare)(Barley) \| chr2:7575100-7577906 FORWARD \| Aliases: F5J6.19, F5J6_19 | 2.7 | 3.4 | -0.7 | -4.4 | 1.79% | 3.5 |
| 5369 | AT1G02460.1 \| glycoside hydrolase family 28 protein / polygalacturonase (pectinase) family protein, similar to polygalacturonase PG1 GI:5669846, PG2 GI:5669848 from (Glycine max); contains PF00295: Glycosyl hydrolases family 28 (polygalacturonases) \| chr1:504673-507204 REVERSE \| Aliases: T6A9.22 | 4.0 | 4.7 | -0.7 | -4.4 | 1.79% | 3.7 |
| 5371 | AT5G19330.2 \| armadillo/beta-catenin repeat family protein / BTB/POZ domain-containing protein, contains armadillo/beta-catenin-like repeats, Pfam:PF00514 and a BTB/POZ domain, Pfam:PF00651 \| chr5:6508302-6512746 REVERSE \| Aliases: F7K24.80, F7K24_80 | 6.0 | 6.8 | -0.8 | -4.4 | 1.79% | 3.7 |
| 5372 | AT2G17420.1 \| Symbol: NTRA \| similar to thioredoxin reductase 1 / NADPH-dependent thioredoxin reductase 1 (NTR1) [Arabidopsis thaliana] (TAIR:At4g35460.1); similar to NADPH-thioredoxin reductase [Triticum aestivum] (GB:CAD19162.1); contains InterPro domain Thioredoxin reductase (InterPro:IPR005982); contains InterPro domain Pyridine nucleotide-disulphide oxidoreductase, class I (InterPro:IPR001100); contains InterPro domain FAD-dependent pyridine nucleotide-disulphide oxidoreductase (InterPro:IPR001327); contains InterPro domain Pyridine nucleotide-disulphide oxidoreductase, class-II, active site (InterPro:IPR008255); contains InterPro domain Adrenodoxin reductase (InterPro:IPR000759); contains InterPro domain Pyridine nucleotide-disulphide oxidoreductase, class-II (InterPro:IPR000103) \| chr2:7571427-7573621 FORWARD \| Aliases: ATNTRA, F5J6.18, F5J6_18 | 12.3 | 12.9 | -0.5 | -4.4 | 1.79% | 3.0 |
| 5373 | AT4G35460.1 \| Symbol: NTR1 \| thioredoxin reductase 1 / NADPH-dependent thioredoxin reductase 1 (NTR1), identical to SP:Q39243 | 12.3 | 12.9 | -0.5 | -4.4 | 1.79% | 3.0 |
| 5374 | AT2G32800.1 \| protein kinase family protein, contains dual protein kinase domains, Pfam:PF00069 \| chr2:13923529-13926629 FORWARD \| Aliases: F24L7.6, F24L7_6 | 4.7 | 6.2 | -1.4 | -4.4 | 1.79% | 3.8 |
| 5375 | AT2G29130.1 \| laccase, putative / diphenol oxidase, putative, similar to laccase (Liriodendron tulipifera)(GI:1621467) | 2.9 | 3.5 | -0.6 | -4.4 | 1.79% | 3.5 |
| 5378 | AT5G64880.1 \| expressed protein \| chr5:25949516-25951373 FORWARD \| Aliases: MXK3.11, MXK3_11 | 4.7 | 5.8 | -1.1 | -4.4 | 1.79% | 3.8 |
| 5380 | AT1G17250.1 \| leucine-rich repeat family protein, contains leucine rich-repeat (LRR) domains Pfam:PF00560, INTERPRO:IPR001611; similar to Hcr2-0B (Lycopersicon esculentum) gi:3894387:gb:AAC78593 \| chr1:5901162-5903432 REVERSE \| Aliases: F20D23.5, F20D23_5 | 2.6 | 3.4 | -0.8 | -4.4 | 1.80% | 3.7 |
| 5385 | AT1G19880.1 \| regulator of chromosome condensation (RCC1) family protein, low similarity to UVB-resistance protein UVR8 (Arabidopsis thaliana) GI:5478530; contains Pfam profile PF00415: Regulator of chromosome condensation (RCC1) \| chr1:6900406-6904060 REVERSE \| Aliases: F6F9.7, F6F9_7 | 4.4 | 5.4 | -1.0 | -4.4 | 1.80% | 3.8 |
| 5389 | AT5G24313.1 \| expressed protein \| chr5:8276788-8277405 REVERSE \| Aliases: None | 2.7 | 3.7 | -1.0 | -4.4 | 1.80% | 3.7 |
| 5390 | AT1G73630.1 \| calcium-binding protein, putative, similar to calcium binding protein GI:14589311 from (Sesbania rostrata); contains Pfam profile: PF00036 EF hand (4 copies) \| chr1:27688397-27689114 FORWARD \| Aliases: F25P22.4, F25P22_4 | 2.6 | 3.3 | -0.7 | -4.3 | 1.81% | 3.7 |
| 5392 | AT1G05900.2 \| endonuclease-related, similar to endonuclease III (Homo sapiens) GI:1753174; contains Pfam profile PF00633: Helix-hairpin-helix motif \| chr1:1786871-1789673 FORWARD \| Aliases: T20M3.18, T20M3_18 | 3.6 | 4.2 | -0.7 | -4.3 | 1.81% | 3.6 |
| 5393 | AT1G08510.1 \| Symbol: FATB \| acyl-(acyl carrier protein) thioesterase / acyl-ACP thioesterase / oleoyl-(acyl-carrier protein) hydrolase / S-acyl fatty acid synthase thioesterase, identical to acyl-(acyl carrier protein) thioesterase (Arabidopsis thaliana) GI:804948 \| chr1:2691083-2694380 REVERSE \| Aliases: T27G7.19, T27G7_19 | 8.5 | 10.5 | -2.1 | -4.3 | 1.81% | 3.7 |
| 5394 | AT3G12500.1 \| Symbol: ATHCHIB \| basic endochitinase, identical to basic endochitinase precursor SP:P19171 from (Arabidopsis thaliana) | 5.7 | 8.4 | -2.7 | -4.3 | 1.81% | 3.8 |
| 5397 | AT3G24740.2 \| expressed protein, similar to expressed protein [Arabidopsis thaliana] (TAIR:At3g25910.1); similar to unknown protein [Oryza sativa (japonica cultivar-group)] (GB:XP_468005.1) \| chr3:9032404-9033790 FORWARD \| Aliases: K7P8.3 | 4.1 | 5.1 | -1.0 | -4.3 | 1.81% | 3.8 |
| 5398 | AT1G78130.1 \| transporter-related, low similarity to spinster type III (Drosophila melanogaster) GI:12003974; contains Pfam profile: PF00083 major facilitator superfamily protein \| chr1:29405033-29406961 FORWARD \| Aliases: T11I11.7, T11I11_7 | 3.6 | 5.6 | -2.0 | -4.3 | 1.81% | 3.8 |

| Rank | Description | Sync | Root | M | t | adj.q | B |
|---|---|---|---|---|---|---|---|
| 5399 | AT1G65310.1 \| Symbol: ATXTH17 \| putative xyloglucan endotransglycosylase/hydrolase, expressed in the mature or basal regions of both the main and lateral roots, but not in the tip of these roots where cell division occurs. \| chr1:24260925-24262136 FORWARD \| Aliases: ATXTH17, T8F5.9, T8F5_9, XYLOGLUCAN ENDOTRANSGLUCOSYLASE/HYDROLASE 17 | 2.6 | 3.9 | -1.2 | -4.3 | 1.81% | 3.9 |
| 5400 | AT2G20780.1 \| mannitol transporter, putative, similar to mannitol transporter (Apium graveolens var. dulce) GI:12004316; contains Pfam profile PF00083: major facilitator superfamily protein | 5.1 | 5.8 | -0.7 | -4.3 | 1.81% | 3.7 |
| 5401 | AT3G23030.1 \| Symbol: IAA2 \| auxin-responsive protein / indoleacetic acid-induced protein 2 (IAA2), identical to SP:P49678 Auxin-responsive protein IAA2 (Indoleacetic acid-induced protein 2) {Arabidopsis thaliana} | 4.2 | 5.1 | -0.9 | -4.3 | 1.81% | 3.7 |
| 5402 | AT5G47020.1 \| glycine-rich protein, strong similarity to unknown protein (emb:CAB87688.1) \| chr5:19099232-19107244 FORWARD \| Aliases: MQD22.16, MQD22_16 | 8.0 | 9.1 | -1.0 | -4.3 | 1.81% | 3.6 |
| 5403 | AT3G13782.1 \| nucleosome assembly protein (NAP) family protein, similar to nucleosome assembly protein 1 (Glycine max) GI:1161252; contains Pfam profile PF00956: Nucleosome assembly protein (NAP) \| chr3:4526667-4528405 FORWARD \| Aliases: MMM17.24 | 3.4 | 5.9 | -2.4 | -4.3 | 1.81% | 3.9 |
| 5404 | AT1G06800.2 \| lipase class 3 family protein, similar to DEFECTIVE IN ANTHER DEHISCENCE1 (Arabidopsis thaliana) GI:16215706; contains Pfam profile PF01764: Lipase \| chr1:2090040-2091685 REVERSE \| Aliases: F4H5.11, F4H5_11 | 3.6 | 4.4 | -0.8 | -4.3 | 1.81% | 3.6 |
| 5405 | AT3G07810.2 \| heterogeneous nuclear ribonucleoprotein, putative / hnRNP, putative, contains Pfam profile: PF00076 RNA recognition motif. (a.k.a. RRM, RBD, or RNP domain) \| chr3:2492299-2495756 FORWARD \| Aliases: F17A17.15 | 4.8 | 5.8 | -1.0 | -4.3 | 1.81% | 3.8 |
| 5407 | AT2G35940.3 \| Symbol: BLH1 \| similar to BEL1-like homeobox 4 protein (BLH4) [Arabidopsis thaliana] (TAIR:At2g23760.1); similar to BEL1-like homeobox 4 protein (BLH4) [Arabidopsis thaliana] (TAIR:At2g23760.2); similar to bell-like homeodomain protein 2 [Lycopersicon esculentum] (GB:AAP47025.1); contains InterPro domain POX (InterPro:IPR006563); contains InterPro domain Homeobox (InterPro:IPR001356) \| chr2:15095911-15099027 REVERSE \| Aliases: BEL1 like homeodomain 1, F11F19.15, F11F19_15 | 5.0 | 6.0 | -1.0 | -4.3 | 1.82% | 3.7 |
| 5408 | AT3G54950.1 \| patatin-related, low similarity to patatin (GI:169500)(Solanum tuberosum); contains Patatin domain PF01734 \| chr3:20369873-20372210 REVERSE \| Aliases: T15C9.3 | 3.7 | 4.7 | -1.0 | -4.3 | 1.82% | 3.8 |
| 5410 | AT5G59960.1 \| expressed protein \| chr5:24159240-24162559 REVERSE \| Aliases: MMN10.21, MMN10_21 | 5.4 | 6.6 | -1.3 | -4.3 | 1.82% | 3.8 |
| 5411 | AT1G64200.1 \| Symbol: VHA E3 \| | 5.8 | 7.1 | -1.2 | -4.3 | 1.82% | 3.8 |
| 5412 | AT4G25090.1 \| respiratory burst oxidase, putative / NADPH oxidase, putative, similar to respiratory burst oxidase protein A from Arabidopsis thaliana, gb:AF055353 (gi:3242781), protein D (gi:3242789); contains Pfam profile PF01794 Ferric reductase like transmembrane component \| chr4:12878785-12883609 REVERSE \| Aliases: F24A6.3 | 3.3 | 4.4 | -1.1 | -4.3 | 1.82% | 3.8 |
| 5413 | AT2G40260.1 \| myb family transcription factor, contains Pfam profile: PF00249 myb-like DNA-binding domain \| chr2:16823896-16825551 REVERSE \| Aliases: T7M7.13 | 3.1 | 3.8 | -0.7 | -4.3 | 1.82% | 3.6 |
| 5414 | AT3G16090.1 \| zinc finger (C3HC4-type RING finger) family protein, contains Pfam profile: PF00097 zinc finger, C3HC4 type (RING finger) \| chr3:5456150-5458972 FORWARD \| Aliases: MSL1.13 | 3.5 | 4.1 | -0.6 | -4.3 | 1.82% | 3.5 |
| 5421 | AT3G61460.1 \| Symbol: BRH1 \| zinc finger (C3HC4-type RING finger) family protein (BRH1), identical to BRH1 RING finger protein (Arabidopsis thaliana) GI:4689366; identical to cDNA BRH1 RING finger protein, GI:4689365 \| chr3:22752486-22753259 REVERSE \| Aliases: BRASSINOSTEROID RESPONSIVE RING H2, F2A19.60 | 4.8 | 5.9 | -1.1 | -4.3 | 1.83% | 3.8 |
| 5424 | AT1G01430.1 \| expressed protein, similar to hypothetical protein GB:CAB80917 GI:7267605 from (Arabidopsis thaliana) \| chr1:156801-158655 REVERSE \| Aliases: F6F3.23, F6F3_23 | 4.2 | 4.9 | -0.7 | -4.3 | 1.83% | 3.7 |
| 5426 | AT5G49570.1 \| transglutaminase-like family protein, low similarity to peptide:N-glycanase PNGase (Mus musculus) GI:8347622; contains Pfam profile PF01841: Transglutaminase-like superfamily \| chr5:20129085-20133411 REVERSE \| Aliases: K6M13.12, K6M13_12 | 5.2 | 6.9 | -1.7 | -4.3 | 1.83% | 3.8 |
| 5427 | AT2G36080.2 \| DNA-binding protein, putative, strong similarity to DNA-binding proteins from (Arabidopsis thaliana) RAV1 GI:3868857, RAV2 GI:3868859; contains Pfam profile PF02362: B3 DNA binding domain \| chr2:15157630-15158654 REVERSE \| Aliases: F9C22.1 | 2.7 | 3.3 | -0.6 | -4.3 | 1.83% | 3.5 |
| 5433 | AT1G73860.1 \| kinesin motor protein-related, similar to kinesin-C GB:AAF04841 from (Strongylocentrotus purpuratus) \| chr1:27774647-27780518 REVERSE \| Aliases: F2P9.27, F2P9_27 | 3.0 | 3.7 | -0.8 | -4.3 | 1.84% | 3.6 |
| 5435 | AT3G25950.1 \| expressed protein \| chr3:9497593-9498348 FORWARD \| Aliases: MPE11.12 | 4.6 | 5.2 | -0.6 | -4.3 | 1.84% | 3.5 |

| Rank | Description | Sync | Root | M | t | adj.q | B |
|---|---|---|---|---|---|---|---|
| 5437 | AT1G15640.1 \| expressed protein \| chr1:5379072-5380022 FORWARD \| Aliases: T16N11.15, T16N11_15 | 3.8 | 4.9 | -1.1 | -4.3 | 1.84% | 3.8 |
| 5439 | AT2G37480.2 \| expressed protein \| chr2:15744961-15746926 FORWARD \| Aliases: F3G5.27, F3G5_27 | 4.9 | 5.8 | -1.0 | -4.3 | 1.85% | 3.6 |
| 5441 | AT2G25850.3 \| similar to nucleotidyltransferase family protein [Arabidopsis thaliana] (TAIR:At4g32850.5); similar to nucleotidyltransferase family protein [Arabidopsis thaliana] (TAIR:At4g32850.1); similar to nucleotidyltransferase family protein [Arabidopsis thaliana] (TAIR:At4g32850.3); similar to nucleotidyltransferase family protein [Arabidopsis thaliana] (TAIR:At4g32850.2); similar to nucleotidyltransferase family protein [Arabidopsis thaliana] (TAIR:At4g32850.6); similar to putative polynucleotide adenylyltransferase [Oryza sativa (indica cultivar-group)] (GB:AAW68018.1); similar to putative polynucleotide adenylyltransferase [Oryza sativa (indica cultivar-group)] (GB:AAW68015.1); similar to putative poly(A) polymerase [Oryza sativa (japonica cultivar-group)] (GB:XP_464757.1); contains InterPro domain Poly(A) polymerase, predicted RNA-binding domain (InterPro:IPR007010); contains InterPro domain Poly(A) polymerase, central region (InterPro:IPR007012); contains InterPro domain PAP/25A core domain (InterPro:IPR001201); contains InterPro domain DNA polymerase, beta-like region (InterPro:IPR002934) \| chr2:11032588-11037705 REVERSE \| Aliases: F17H15.12, F17H15_12 | 5.3 | 8.3 | -3.0 | -4.3 | 1.85% | 3.8 |
| 5444 | AT1G48790.1 \| mov34 family protein, similar to AMSH (Homo sapiens) GI:4098124; contains Pfam profile PF01398: Mov34/MPN/PAD-1 family \| chr1:18047378-18051325 REVERSE \| Aliases: F11I4.4, F11I4_4 | 8.0 | 9.2 | -1.2 | -4.3 | 1.85% | 3.8 |
| 5447 | AT2G47070.1 \| Symbol: SPL1 \| squamosa promoter-binding protein-like 1 (SPL1), identical to squamosa promoter binding protein-like 1 (Arabidopsis thaliana) GI:5931655; contains Pfam profile PF03110: SBP domain \| chr2:19343767-19347939 FORWARD \| Aliases: F14M4.10, SQUAMOSA PROMOTER BINDING PROTEIN LIKE 1 | 5.1 | 5.6 | -0.5 | -4.3 | 1.86% | 3.5 |
| 5449 | AT5G14000.1 \| Symbol: ANAC084 \| no apical meristem (NAM) family protein, contains Pfam PF02365: No apical meristem (NAM) domain; \| chr5:4518010-4519302 FORWARD \| Aliases: ANAC084, MAC12.3, MAC12_3 | 3.4 | 4.6 | -1.3 | -4.3 | 1.86% | 3.8 |
| 5452 | AT4G16400.1 \| expressed protein, similar to expressed protein [Arabidopsis thaliana] (TAIR:At3g13175.1) \| chr4:9260587-9263019 FORWARD \| Aliases: DL4230W, FCAALL.356 | 4.3 | 5.8 | -1.5 | -4.3 | 1.86% | 3.8 |
| 5453 | AT4G31670.1 \| ubiquitin carboxyl-terminal hydrolase family protein / zinc finger (MYND type) family protein, similar to ubiquitin-specific protease 15 (UBP15) (Arabidopsis thaliana) GI:11993475; contains Pfam profiles PF00443: Ubiquitin carboxyl-terminal hydrolase, PF01753: MYND finger | 5.4 | 6.4 | -1.0 | -4.3 | 1.86% | 3.7 |
| 5454 | AT2G47500.1 \| kinesin motor protein-related \| chr2:19500065-19505226 FORWARD \| Aliases: T30B22.20 | 3.9 | 4.9 | -1.0 | -4.3 | 1.86% | 3.8 |
| 5456 | AT4G17830.1 \| peptidase M20/M25/M40 family protein, similar to acetylornithine deacetylase (Acetylornithinase, AO; N-acetylornithinase, NAO) (Dictyostelium discoideum) SWISS-PROT:P54638 \| chr4:9915893-9918256 FORWARD \| Aliases: T6K21.10 | 6.0 | 7.3 | -1.3 | -4.3 | 1.86% | 3.8 |
| 5459 | AT3G59220.1 \| Symbol: PRN \| pirin, putative, similar to SP:O00625 Pirin {Homo sapiens}; contains Pfam profile PF02678: Pirin \| chr3:21905111-21906600 FORWARD \| Aliases: ATPIRIN1, F25L23.80, PIRIN, PRN1 | 3.4 | 4.2 | -0.8 | -4.3 | 1.87% | 3.6 |
| 5462 | AT4G38260.1 \| expressed protein, contains Pfam PF05742: Protein of unknown function (DUF833) \| chr4:17936342-17938266 REVERSE \| Aliases: F22I13.30, F22I13_30 | 5.6 | 7.8 | -2.2 | -4.3 | 1.87% | 3.8 |
| 5464 | AT3G63300.2 \| expressed protein \| chr3:23395963-23398817 FORWARD \| Aliases: MAA21.9 | 4.0 | 5.4 | -1.4 | -4.3 | 1.87% | 3.8 |
| 5468 | AT5G19000.1 \| Symbol: ATBPM1 \| | 6.6 | 8.0 | -1.4 | -4.3 | 1.87% | 3.8 |
| 5474 | AT5G40540.1 \| protein kinase, putative, similar to protein kinase ATN1 (Arabidopsis thaliana) gi:1054633:emb:CAA63387 \| chr5:16254609-16256818 FORWARD \| Aliases: MNF13.60, MNF13_60 | 6.8 | 7.8 | -1.0 | -4.3 | 1.88% | 3.8 |
| 5476 | AT2G28570.1 \| expressed protein \| chr2:12248847-12249806 FORWARD \| Aliases: T17D12.13, T17D12_13 | 2.7 | 4.8 | -2.1 | -4.3 | 1.88% | 3.8 |
| 5478 | AT3G25570.1 \| adenosylmethionine decarboxylase family protein, contains Pfam profile: PF01536 adenosylmethionine decarboxylase | 3.4 | 4.7 | -1.4 | -4.3 | 1.88% | 3.7 |
| 5479 | AT1G29520.1 \| AWPM-19-like membrane family protein, contains Pfam profile: PF05512 AWPM-19-like family | 2.5 | 3.5 | -0.9 | -4.3 | 1.88% | 3.8 |
| 5480 | AT1G20030.2 \| pathogenesis-related thaumatin family protein, similar to receptor serine/threonine kinase PR5K (Arabidopsis thaliana) GI:1235680; contains Pfam profile PF00314: Thaumatin family \| chr1:6945416-6947335 FORWARD \| Aliases: T20H2.19, T20H2_19 | 3.4 | 4.6 | -1.2 | -4.3 | 1.89% | 3.7 |
| 5481 | AT5G48070.1 \| Symbol: ATXTH20 \| putative xyloglucan endotransglycosylase/hydrolase, expressed primarily in the stele of mature non-elongating regions of both the main and the lateral root. Is expressed in lateral root primordia but expression ceases after lateral root begins to grow. | 3.2 | 4.2 | -1.0 | -4.3 | 1.89% | 3.8 |
| 5483 | AT3G52830.1 \| expressed protein, MobA, Pseudomonas alcaligenes, EMBL:PAU88088 \| chr3:19588983-19589243 FORWARD \| Aliases: F8J2.1 | 2.7 | 3.4 | -0.7 | -4.3 | 1.89% | 2.9 |

| Rank | Description | Sync | Root | M | t | adj.q | B |
|---|---|---|---|---|---|---|---|
| 5484 | AT4G08290.2 \| nodulin MtN21 family protein, similar to MtN21 GI:2598575 (root nodule development) from (Medicago truncatula) \| chr4:5238924-5241027 FORWARD \| Aliases: T12G13.130, T12G13_130 | 3.6 | 6.9 | -3.2 | -4.3 | 1.89% | 3.8 |
| 5487 | AT1G54170.1 \| Symbol: CID3 \| ataxin-2-related, similar to SCA2 (GI:1770390) (Homo sapiens); similar to ataxin-2 (GI:3005020) (Mus musculus). Member of a family of PAM2 motif containing proteins. \| chr1:20224727-20229613 REVERSE \| Aliases: CID3, F15I1.27, F15I1_27 | 3.8 | 4.6 | -0.8 | -4.3 | 1.90% | 3.6 |
| 5489 | AT5G62520.2 \| Symbol: SRO5 \| expressed protein \| chr5:25115253-25117133 FORWARD \| Aliases: K19B1.13, K19B1_13, SIMILAR TO RCD ONE 5, SIMILAR TO RCD ONE 5, SRO5 | 4.7 | 6.2 | -1.5 | -4.3 | 1.90% | 3.8 |
| 5493 | AT3G48860.2 \| expressed protein \| chr3:18128471-18133015 FORWARD \| Aliases: T21J18.130 | 6.8 | 8.2 | -1.4 | -4.3 | 1.90% | 3.7 |
| 5499 | AT1G53390.1 \| ABC transporter family protein, similar to ATP-binding cassette, sub-family G, member 2 (Placenta-specific ATP- binding cassette transporter) (Breast cancer resistance protein) SP:Q9UNQ0 from (Homo sapiens) \| chr1:19921715-19927475 FORWARD \| Aliases: F12M16.28, F12M16_28 | 5.2 | 5.9 | -0.8 | -4.3 | 1.91% | 3.7 |
| 5501 | AT1G71970.1 \| expressed protein \| chr1:27096690-27097689 FORWARD \| Aliases: F17M19.12, F17M19_12 | 4.6 | 5.7 | -1.1 | -4.3 | 1.91% | 3.8 |
| 5507 | AT2G43050.1 \| pectinesterase family protein, contains Pfam profile: PF01095 pectinesterase \| chr2:17909563-17911520 FORWARD \| Aliases: ATPMEPCRD, MFL8.9 | 3.1 | 4.5 | -1.4 | -4.3 | 1.93% | 3.7 |
| 5508 | AT3G11600.1 \| expressed protein, weak similarity to B-type cyclin (GI:849074) (Nicotiana tabacum) \| chr3:3667235-3667985 FORWARD \| Aliases: F24K9.6 | 3.3 | 4.8 | -1.6 | -4.3 | 1.93% | 3.7 |
| 5509 | AT1G77660.1 \| MORN (Membrane Occupation and Recognition Nexus) repeat-containing protein /phosphatidylinositol-4-phosphate 5-kinase-related, low similarity to phosphatidylinositol-4-phosphate 5-kinase AtPIP5K1 (Arabidopsis thaliana) GI:3702691; contains Pfam profile PF02493: MORN repeat \| chr1:29190967-29192394 REVERSE \| Aliases: T5M16.25, T5M16_25 | 3.4 | 4.3 | -0.9 | -4.3 | 1.93% | 3.6 |
| 5511 | AT5G03140.1 \| lectin protein kinase family protein, contains Pfam domains, PF00138: Legume lectins alpha domain, PF00139: Legume lectins beta domain and PF00069: Protein kinase domain \| chr5:737589-740015 REVERSE \| Aliases: F15A17.170, F15A17_170 | 6.4 | 8.0 | -1.6 | -4.3 | 1.93% | 3.8 |
| 5513 | AT4G00630.1 \| Symbol: KEA2 \| K+ efflux antiporter, putative (KEA2), Monovalent cation:proton antiporter family 2 (CPA2 family) member, PMID:11500563; similar to SWISS-PROT:SPP03819 Glutathione-regulated potassium-efflux system protein kefC (K(+)/H(+) antiporter) (Escherichia coli) \| chr4:261655-265958 REVERSE \| Aliases: ATKEA2, F6N23.15, F6N23_15 | 8.6 | 9.6 | -1.0 | -4.3 | 1.93% | 3.5 |
| 5515 | AT5G10650.2 \| similar to zinc finger (C3HC4-type RING finger) family protein [Arabidopsis thaliana] (TAIR:At5g24870.1); similar to zinc finger (C3HC4-type RING finger) family protein [Arabidopsis thaliana] (TAIR:At5g24870.2); similar to putative RING-H2 finger protein RHG1a [Oryza sativa (japonica cultivar-group)] (GB:BAD38048.1); contains InterPro domain Zn-finger, RING (InterPro:IPR001841) \| chr5:3365018-3367604 REVERSE \| Aliases: MAJ23.10, MAJ23_10 | 5.2 | 6.2 | -1.0 | -4.3 | 1.93% | 3.7 |
| 5516 | AT4G18465.1 \| RNA helicase, putative, similar to SP:Q14562 ATP-dependent helicase DDX8 (RNA helicase HRH1) (DEAH-box protein 8) {Homo sapiens}; contains Pfam profiles PF04408: Helicase associated domain (HA2), PF00271: Helicase conserved C-terminal domain \| chr4:10197067-10201622 FORWARD \| Aliases: None | 3.8 | 4.8 | -0.9 | -4.3 | 1.94% | 3.7 |
| 5517 | AT2G35736.1 \| expressed protein, ; expression supported by MPSS \| chr2:15030163-15030333 REVERSE \| Aliases: None | 5.4 | 6.6 | -1.2 | -4.3 | 1.94% | 3.8 |
| 5519 | AT2G23340.1 \| encodes a member of the DREB subfamily A-5 of ERF/AP2 transcription factor family. The protein contains one AP2 domain. There are 16 members in this subfamily including RAP2.1, RAP2.9 and RAP2.10. \| chr2:9945079-9945953 FORWARD \| Aliases: T20D16.3, T20D16_3 | 9.6 | 11.4 | -1.9 | -4.3 | 1.95% | 3.4 |
| 5522 | AT2G27390.1 \| proline-rich family protein, contains proline-rich extensin domains, INTERPRO:IPR002965 \| chr2:11727562-11727966 REVERSE \| Aliases: F12K2.3, F12K2_3 | 2.4 | 2.9 | -0.6 | -4.3 | 1.95% | 3.4 |
| 5523 | AT1G66880.1 \| serine/threonine protein kinase family protein, contains protein kinase domain, Pfam:PF00069; contains serine/threonine protein kinase domain, INTERPRO:IPR002290 \| chr1:24950591-24959274 FORWARD \| Aliases: F4N21.1, F4N21_1 | 4.8 | 6.1 | -1.3 | -4.3 | 1.95% | 3.6 |
| 5525 | AT4G28850.1 \| xyloglucan:xyloglucosyl transferase, putative / xyloglucan endotransglycosylase, putative / endo-xyloglucan transferase, putative, contains similarity to xyloglucan endotransglycosylase XET2 GI:8886867 from (Asparagus officinalis) \| chr4:14244301-14245963 FORWARD \| Aliases: F16A16.40, F16A16_40 | 2.0 | 2.7 | -0.7 | -4.3 | 1.96% | 3.6 |
| 5530 | AT4G25830.1 \| integral membrane family protein, contains TIGRFAM TIGR01569 : plant integral membrane protein TIGR01569; contains Pfam PF04535 : Domain of unknown function (DUF588) \| chr4:13133645-13135033 FORWARD \| Aliases: F14M19.9 | 2.8 | 3.3 | -0.5 | -4.3 | 1.96% | 3.2 |
| 5533 | AT4G36150.1 \| disease resistance protein (TIR-NBS-LRR class), putative, domain signature TIR-NBS-LRR exists, suggestive of a disease resistance protein. \| chr4:17104779-17108714 FORWARD \| Aliases: F23E13.40, F23E13_40 | 4.1 | 4.9 | -0.8 | -4.3 | 1.96% | 3.6 |

| Rank | Description | Sync | Root | M | t | adj.q | B |
|---|---|---|---|---|---|---|---|
| 5534 | AT5G49950.1 \| embryogenesis-associated protein-related, contains weak similarity to Embryogenesis-associated protein EMB8 (Swiss-Prot:Q40863) (Picea glauca) \| chr5:20336442-20340842 REVERSE \| Aliases: K9P8.9, K9P8_9 | 7.2 | 7.9 | -0.7 | -4.3 | 1.96% | 3.6 |
| 5535 | AT1G74840.1 \| myb family transcription factor, similar to myb-related transcription activator GI:9279717 from (Arabidopsis thaliana) \| chr1:28119558-28121066 REVERSE \| Aliases: F25A4.19, F25A4_19 | 8.9 | 10.2 | -1.3 | -4.3 | 1.96% | 3.5 |
| 5537 | AT5G42400.1 \| SET domain-containing protein (TXR7), contains Pfam profile PF00856: SET domain \| chr5:16971697-16977899 REVERSE \| Aliases: MDH9.9, MDH9_9 | 5.0 | 5.9 | -1.0 | -4.3 | 1.97% | 3.6 |
| 5539 | AT4G17130.1 \| expressed protein \| chr4:9624275-9627621 REVERSE \| Aliases: DL4600C, FCAALL.361 | 4.0 | 6.3 | -2.3 | -4.3 | 1.97% | 3.7 |
| 5540 | AT5G58220.3 \| similar to putative transthyretin, having alternative splicing products [Oryza sativa (japonica cultivar-group)] (GB:AAR06357.1); contains InterPro domain Transthyretin (InterPro:IPR000895) \| chr5:23571153-23573092 REVERSE \| Aliases: MCK7.9, MCK7_9 | 7.7 | 8.7 | -1.1 | -4.3 | 1.97% | 3.7 |
| 5542 | AT3G18260.1 \| reticulon family protein (RTNLB9), weak similarity to RTN2-C (Homo sapiens) GI:3435090; contains Pfam profile PF02453: Reticulon | 2.8 | 3.6 | -0.7 | -4.3 | 1.97% | 3.4 |
| 5546 | AT4G00355.4 \| similar to expressed protein [Arabidopsis thaliana] (TAIR:At2g45980.1); similar to OSJNBa0019G23.5 [Oryza sativa (japonica cultivar-group)] (GB:XP_474580.1) \| chr4:154721-156376 FORWARD \| Aliases: None | 6.7 | 8.4 | -1.7 | -4.3 | 1.98% | 3.7 |
| 5547 | AT5G41940.1 \| RabGAP/TBC domain-containing protein, similar to GTPase activating protein (Yarrowia lipolytica) GI:2370595; contains Pfam profile PF00566: TBC domain \| chr5:16799224-16802804 FORWARD \| Aliases: MJC20.4, MJC20_4 | 7.1 | 7.9 | -0.7 | -4.3 | 1.98% | 3.5 |
| 5549 | AT4G17490.1 \| Symbol: ATERF6 \| | 4.2 | 6.3 | -2.1 | -4.3 | 1.98% | 3.8 |
| 5550 | AT4G11230.1 \| respiratory burst oxidase, putative / NADPH oxidase, putative, similar to respiratory burst oxidase homolog F (gi:3242456), RbohAp108 (gi:2654868) from Arabidopsis thaliana, respiratory burst oxidase homolog (GI:16549087) from Solanum tuberosum; contains Pfam profile PF01794 Ferric reductase like transmembrane component \| chr4:6840487-6845587 REVERSE \| Aliases: F8L21.20, F8L21_20 | 2.3 | 3.1 | -0.8 | -4.3 | 1.98% | 3.5 |
| 5551 | AT1G55640.1 \| prenylated rab acceptor (PRA1) family protein, weak similarity to prenylated Rab acceptor 1 (PRA1) (Homo sapiens) GI:4877285; contains Pfam profile PF03208: Prenylated rab acceptor (PRA1) \| chr1:20797437-20798000 FORWARD \| Aliases: F20N2.7 | 3.2 | 4.2 | -0.9 | -4.3 | 1.98% | 3.6 |
| 5552 | AT5G46410.1 \| NLI interacting factor (NIF) family protein, contains Pfam profile PF03031: NLI interacting factor \| chr5:18842330-18846448 FORWARD \| Aliases: MPL12.21, MPL12_21 | 5.1 | 6.0 | -0.9 | -4.3 | 1.98% | 3.7 |
| 5553 | AT3G02640.1 \| expressed protein \| chr3:564891-565715 REVERSE \| Aliases: F16B3.27, F16B3_27 | 3.7 | 4.4 | -0.7 | -4.3 | 1.98% | 3.5 |
| 5556 | AT5G20170.1 \| expressed protein \| chr5:6807392-6810800 REVERSE \| Aliases: F5O24.60, F5O24_60 | 6.1 | 6.9 | -0.8 | -4.3 | 2.00% | 3.3 |
| 5560 | AT5G27410.1 \| aminotransferase class IV family protein, contains Pfam profile: PF01063 aminotransferase class IV \| chr5:9678795-9682943 FORWARD \| Aliases: F21A20.120, F21A20_120 | 4.4 | 5.3 | -0.9 | -4.3 | 2.00% | 3.6 |
| 5561 | AT2G45690.1 \| Symbol: SSE1 \| shrunken seed protein (SSE1), identical to shrunken seed protein (Arabidopsis thaliana) GI:4837733; annotation based on supporting cDNA gi:4837732:gb:AF085354.1:AF085354 \| chr2:18830298-18832796 REVERSE \| Aliases: F17K2.22, PEX16, SHRUNKEN SEED 1, SSE | 4.9 | 6.1 | -1.1 | -4.3 | 2.00% | 3.6 |
| 5562 | AT3G57530.1 \| Symbol: CPK32 \| calcium-dependent protein kinase, putative / CDPK, putative, similar to calmodulin-domain protein kinase CDPK isoform 7 (Arabidopsis thaliana) gi:1399277:gb:AAB03247 \| chr3:21307531-21310568 REVERSE \| Aliases: T8H10.130 | 6.2 | 7.7 | -1.5 | -4.3 | 2.00% | 3.7 |
| 5568 | AT5G06270.1 \| expressed protein \| chr5:1912551-1913675 FORWARD \| Aliases: MHF15.21, MHF15_21 | 3.5 | 4.4 | -0.9 | -4.2 | 2.01% | 3.6 |
| 5572 | AT3G05710.2 \| Symbol: SYP43 \| syntaxin, putative, similar to syntaxin of plants 42 (Arabidopsis thaliana) GI:5059352 \| chr3:1685009-1687543 FORWARD \| Aliases: ATSYP43, F18C1.2, F18C1_2 | 4.3 | 5.6 | -1.4 | -4.2 | 2.01% | 3.7 |
| 5574 | AT3G54990.1 \| Symbol: SMZ \| similar to AP2 domain-containing transcription factor, putative [Arabidopsis thaliana] (TAIR:At2g39250.1); similar to PHAP2B protein [Petunia x hybrida] (GB:AAD39440.1); contains InterPro domain Pathogenesis-related transcriptional factor and ERF (InterPro:IPR001471) \| chr3:20384695-20387499 REVERSE \| Aliases: SCHLAFMUTZE, T15C9.6 | 4.5 | 6.0 | -1.5 | -4.2 | 2.01% | 3.7 |
| 5577 | AT2G20625.1 \| expressed protein \| chr2:8903235-8904299 REVERSE \| Aliases: F23N11.15, F23N11_15 | 2.9 | 4.2 | -1.3 | -4.2 | 2.01% | 3.7 |
| 5578 | AT3G22380.1 \| expressed protein \| chr3:7913126-7919491 FORWARD \| Aliases: None | 5.9 | 7.1 | -1.1 | -4.2 | 2.01% | 3.7 |
| 5580 | AT2G48040.1 \| expressed protein \| chr2:19658266-19659835 REVERSE \| Aliases: T9J23.19 | 5.8 | 7.1 | -1.2 | -4.2 | 2.01% | 3.7 |

| Rank | Description | Sync | Root | M | t | adj.q | B |
|---|---|---|---|---|---|---|---|
| 5582 | AT1G53510.1 \| Symbol: ATMPK18 \| similar to mitogen-activated protein kinase, putative / MAPK, putative (MPK19) [Arabidopsis thaliana] (TAIR:At3g14720.1); similar to mitogen-activated protein kinase, putative / MAPK, putative (MPK16) [Arabidopsis thaliana] (TAIR:At5g19010.1); similar to mitogen-activated protein kinase, putative / MAPK, putative (MPK20) [Arabidopsis thaliana] (TAIR:At2g42880.1); similar to putative mitogen-activated protein kinase, putative / MAPK, putative (MPK9) [Arabidopsis thaliana] (TAIR:At3g18040.1); similar to mitogen-activated protein kinase, putative / MAPK, putative (MPK8) [Arabidopsis thaliana] (TAIR:At1g18150.2); similar to MAP kinase-like protein [Oryza sativa (japonica cultivar-group)] (GB:NP_917813.1); similar to putative mitogen-activated protein kinase [Oryza sativa (japonica cultivar-group)] (GB:NP_916793.1); similar to putative mitogen-activated protein kinase wjumk1 [Oryza sativa (japonica cultivar-group)] (GB:CAD54742.1); similar to MAPK6 [Oryza sativa (japonica cultivar-group)] (GB:AAR11478.1); similar to mitogen-activated protein kinase 7-like [Oryza sativa (japonica cultivar-group)] (GB:BAD61401.1); contains InterPro domain Tyrosine protein kinase (InterPro:IPR001245); contains InterPro domain Serine/threonine protein kinase (InterPro:IPR002290); contains InterPro domain Protein kinase (InterPro:IPR000719) \| chr1:19974332-19978061 REVERSE \| Aliases: F22G10.12 | 3.3 | 6.1 | -2.8 | -4.2 | 2.01% | 3.7 |
| 5583 | AT4G32940.1 \| Symbol: GAMMA VPE \| vacuolar processing enzyme gamma / gamma-VPE, nearly identical to SP:Q39119 Vacuolar processing enzyme, gamma-isozyme precursor (EC 3.4.22.-) (Gamma-VPE) {Arabidopsis thaliana} \| chr4:15900273-15903271 REVERSE \| Aliases: F26P21.60, F26P21_60, GAMMAVPE | 10.5 | 11.8 | -1.3 | -4.2 | 2.01% | 3.5 |
| 5584 | AT1G67030.1 \| Symbol: ZFP6 \| zinc finger (C2H2 type) family protein (ZFP6), identical to zinc finger protein, ZFP6 gi:790683:gb:AAA87302; contains Pfam domain, PF00096: Zinc finger, C2H2 type \| chr1:25020206-25021175 FORWARD \| Aliases: F1O19.8, F1O19_8, ZFP6, ZINC FINGER PROTEIN 6 | 5.8 | 6.5 | -0.7 | -4.2 | 2.01% | 3.6 |
| 5585 | AT1G05790.1 \| lipase class 3 family protein, contains Pfam profile PF01764: Lipase \| chr1:1733038-1737484 FORWARD \| Aliases: T20M3.5, T20M3_5 | 6.3 | 7.6 | -1.3 | -4.2 | 2.01% | 3.7 |
| 5587 | AT5G37580.1 \| expressed protein \| chr5:14944543-14946800 REVERSE \| Aliases: K12B20.4, K12B20_4 | 5.3 | 6.4 | -1.0 | -4.2 | 2.02% | 3.7 |
| 5589 | AT1G23530.1 \| expressed protein \| chr1:8345824-8346637 FORWARD \| Aliases: F28C11.16 | 3.0 | 3.7 | -0.7 | -4.2 | 2.02% | 3.5 |
| 5590 | AT1G77180.2 \| similar to RNA recognition motif (RRM)-containing protein [Arabidopsis thaliana] (TAIR:At2g16940.1); similar to WD-40 repeat family protein [Arabidopsis thaliana] (TAIR:At5g67320.1); similar to GAMYB-binding protein [Hordeum vulgare subsp. vulgare] (GB:AAO25542.1); contains InterPro domain SKIP/SNW domain (InterPro:IPR004015) \| chr1:29004502-29007019 REVERSE \| Aliases: T14N5.5, T14N5_5 | 9.2 | 10.1 | -0.9 | -4.2 | 2.02% | 3.4 |
| 5591 | AT1G01570.1 \| fringe-related protein, + similar to hypothetical protein GB:AAC23643 (Arabidopsis thaliana) + weak similarity to Fringe (Schistocerca gregaria)(GI:6573138);Fringe encodes an extracellular protein that regulates Notch signalling. \| chr1:205176-207435 FORWARD \| Aliases: F22L4.11, F22L4_11 | 4.4 | 5.4 | -1.0 | -4.2 | 2.02% | 3.6 |
| 5592 | AT1G59820.1 \| haloacid dehalogenase-like hydrolase family protein, similar to Potential phospholipid-transporting ATPase (EC 3.6.3.1) from Mus musculus (SP:P70704), {Bos taurus} SP:Q29449; contains InterPro accession IPR005834: Haloacid dehalogenase-like hydrolase \| chr1:22014993-22023959 FORWARD \| Aliases: F23H11.14, F23H11_14 | 5.0 | 5.8 | -0.8 | -4.2 | 2.02% | 3.6 |
| 5594 | AT2G42980.1 \| aspartyl protease family protein, contains pfam profile: PF00026 eukaryotic aspartyl protease \| chr2:17882082-17883665 REVERSE \| Aliases: F23E6.3, F23E6_3 | 2.6 | 3.1 | -0.5 | -4.2 | 2.02% | 3.1 |
| 5595 | AT1G75020.2 \| Symbol: LPAT4 \| phospholipid/glycerol acyltransferase family protein, contains Pfam profile: PF01553 Acyltransferase \| chr1:28174786-28177087 FORWARD \| Aliases: F25A4.2, F25A4_2, LPAT4 | 6.3 | 8.1 | -1.7 | -4.2 | 2.02% | 3.7 |
| 5600 | AT1G26820.1 \| Symbol: RNS3 \| ribonuclease 3 (RNS3), identical to ribonuclease SP:P42815 Ribonuclease 3 precursor (EC 3.1.27.1) {Arabidopsis thaliana} | 3.8 | 5.3 | -1.6 | -4.2 | 2.04% | 3.7 |
| 5603 | AT1G54790.2 \| GDSL-motif lipase/hydrolase family protein, similar to early nodulin ENOD8 (Medicago sativa) GI:304037, elicitor-induced glycoprotein iEP4 (Daucus carota) GI:1911765, lanatoside 15'-O-acetylesterase (Digitalis lanata) GI:3688284; contains InterPro Entry IPR001087 Lipolytic enzyme, G-D-S-L family | 3.1 | 4.8 | -1.7 | -4.2 | 2.04% | 3.7 |
| 5611 | AT2G46700.1 \| calcium-dependent protein kinase, putative / CDPK, putative, similar to calcium/calmodulin-dependent protein kinase homolog MCK1 (Zea mays) gi:1839597:gb:AAB47181 \| chr2:19189794-19193648 REVERSE \| Aliases: T3A4.8 | 5.5 | 6.2 | -0.8 | -4.2 | 2.04% | 3.6 |
| 5613 | AT5G08370.2 \| similar to alpha-galactosidase, putative / melibiase, putative / alpha-D-galactoside galactohydrolase, putative [Arabidopsis thaliana] (TAIR:At5g08380.1); similar to alpha galactosidase [Coffea arabica] (GB:CAI47559.1); contains InterPro domain Glycoside hydrolase, clan GH-D (InterPro:IPR000111); contains InterPro domain Glycoside hydrolase, family 27 (InterPro:IPR002241) \| chr5:2690560-2693551 REVERSE \| Aliases: F8L15.100, F8L15_100 | 5.4 | 6.9 | -1.6 | -4.2 | 2.05% | 3.7 |
| 5615 | AT2G46940.1 \| expressed protein \| chr2:19293846-19294650 REVERSE \| Aliases: F14M4.23 | 3.3 | 4.5 | -1.2 | -4.2 | 2.05% | 3.7 |
| 5616 | AT1G14740.1 \| expressed protein \| chr1:5075208-5077786 REVERSE \| Aliases: F10B6.14, F10B6_14 | 4.6 | 5.7 | -1.1 | -4.2 | 2.05% | 3.4 |
| 5617 | AT3G46500.1 \| oxidoreductase, 2OG-Fe(II) oxygenase family protein, similar to gibberellin 20-oxidase from A. thaliana (gi:1109699), N. tabacum (GI:3402332); contains Pfam profile: PF03171 oxidoreductase, 2OG-Fe(II) oxygenase family \| chr3:17131465-17133730 FORWARD \| Aliases: F12A12.20 | 2.2 | 3.4 | -1.2 | -4.2 | 2.05% | 3.7 |
| 5618 | AT1G48960.1 \| universal stress protein (USP) family protein, contains Pfam PF00582: universal stress protein family; similar to hypothetical protein GI:7770340 from (Arabidopsis thaliana) \| chr1:18116154-18117284 FORWARD \| Aliases: F27K7.3 | 5.2 | 6.9 | -1.8 | -4.2 | 2.05% | 3.7 |

| Rank | Description | Sync | Root | M | t | adj.q | B |
|---|---|---|---|---|---|---|---|
| 5620 | AT5G12050.1 \| expressed protein \| chr5:3890105-3891514 FORWARD \| Aliases: F14F18.220, F14F18_220 | 2.3 | 2.7 | -0.4 | -4.2 | 2.06% | 2.9 |
| 5621 | AT4G28540.1 \| casein kinase, putative, similar to casein kinase I (Arabidopsis thaliana) gi:1103318:emb:CAA55395; contains protein kinase domain, Pfam:PF00069 | 7.0 | 8.1 | -1.1 | -4.2 | 2.06% | 3.7 |
| 5622 | AT3G01680.1 \| expressed protein \| chr3:251988-255520 FORWARD \| Aliases: F4P13.22, F4P13_22 | 3.4 | 5.9 | -2.5 | -4.2 | 2.06% | 3.7 |
| 5624 | AT5G59430.2 \| telomere repeat-binding protein 1 (TRP1), identical to telomere repeat-binding protein TRP1 (Arabidopsis thaliana) GI:5459298 \| chr5:23984541-23988171 FORWARD \| Aliases: F2O15.20, F2O15_20 | 2.6 | 3.3 | -0.7 | -4.2 | 2.06% | 3.4 |
| 5630 | AT4G18050.1 \| ABC transporter family protein, contains Pfam profile: PF00005 ABC transporter; similar to multidrug-resistant protein CjMDR1 GI:14715462 from (Coptis japonica) | 2.7 | 3.4 | -0.7 | -4.2 | 2.07% | 3.4 |
| 5632 | AT5G63560.1 \| transferase family protein, similar to hypersensitivity-related gene product HSR201 - Nicotiana tabacum, EMBL:X95343; contains Pfam transferase family domain PF00248 \| chr5:25466707-25468640 FORWARD \| Aliases: MBK5.2, MBK5_2 | 4.1 | 4.9 | -0.8 | -4.2 | 2.07% | 3.6 |
| 5634 | AT1G74590.1 \| Symbol: ATGSTU10 \| glutathione S-transferase, putative, similar to putative glutathione S-transferase GB:CAA10060 (Arabidopsis thaliana); contains Pfam profile: PF00043 Glutathione S-transferases \| chr1:28027288-28028387 REVERSE \| Aliases: F1M20.27, F1M20_27 | 3.5 | 5.3 | -1.8 | -4.2 | 2.07% | 3.7 |
| 5635 | AT1G16440.1 \| protein kinase, putative, similar to viroid symptom modulation protein (Lycopersicon esculentum) gi:7672777:gb:AAF66637 \| chr1:5616259-5617666 FORWARD \| Aliases: F3O9.24, F3O9_24 | 2.7 | 3.3 | -0.6 | -4.2 | 2.07% | 3.4 |
| 5643 | AT4G11840.1 \| Symbol: PLDGAMMA3 \| phospholipase D gamma 3 / PLD gamma 3 (PLDGAMMA3), identical to phospholipase D gamma 3 sp:Q9T052 from (Arabidopsis thaliana) \| chr4:7121959-7125876 REVERSE \| Aliases: T26M18.50, T26M18_50 | 5.2 | 6.9 | -1.7 | -4.2 | 2.08% | 3.6 |
| 5644 | AT3G52290.1 \| calmodulin-binding family protein, similar to SF16 protein (Helianthus annuus) GI:560150; contains Pfam profile PF00612: IQ calmodulin-binding motif \| chr3:19404797-19407381 FORWARD \| Aliases: T25B15.60 | 3.4 | 4.3 | -0.9 | -4.2 | 2.08% | 3.4 |
| 5647 | AT2G43840.2 \| UDP-glucoronosyl/UDP-glucosyl transferase family protein, contains Pfam profile: PF00201 UDP-glucoronosyl and UDP-glucosyl transferase \| chr2:18164715-18166252 FORWARD \| Aliases: F18O19.5 | 5.7 | 7.0 | -1.2 | -4.2 | 2.09% | 3.4 |
| 5648 | AT5G24470.1 \| Symbol: APRR5 \| pseudo-response regulator 5 (APRR5), identical to pseudo-response regulator 5 GI:10281006 from (Arabidopsis thaliana) \| chr5:8355985-8358876 REVERSE \| Aliases: PRR5, PSEUDO RESPONSE REGULATOR 5, T31K7.5, T31K7_5 | 5.0 | 5.8 | -0.8 | -4.2 | 2.09% | 3.6 |
| 5651 | AT1G57600.1 \| membrane bound O-acyl transferase (MBOAT) family protein, low similarity to skinny hedgehog (Drosophila melanogaster) GI:15420842; contains Pfam profile PF03062: MBOAT family \| chr1:21334255-21338392 REVERSE \| Aliases: T8L23.7, T8L23_7 | 6.0 | 7.1 | -1.1 | -4.2 | 2.10% | 3.6 |
| 5652 | AT1G23760.1 \| Symbol: JP630 \| BURP domain-containing protein / polygalacturonase, putative, similar to polygalacturonase isoenzyme 1 beta subunit (Lycopersicon esculentum) GI:170480; contains Pfam profile PF03181: BURP domain \| chr1:8402035-8404280 FORWARD \| Aliases: F5O8.31, F5O8_31, JP630 | 4.2 | 6.2 | -2.0 | -4.2 | 2.10% | 3.7 |
| 5653 | AT4G14760.1 \| M protein repeat-containing protein, contains Pfam profile: PF02370 M protein repeat \| chr4:8475964-8481089 FORWARD \| Aliases: DL3420W, FCAALL.305 | 6.1 | 7.4 | -1.3 | -4.2 | 2.10% | 3.7 |
| 5656 | AT5G50820.1 \| Symbol: ANAC097 \| no apical meristem (NAM) family protein, contains Pfam PF02365: No apical meristem (NAM) domain; similar to unknown protein (pir::T07182) \| chr5:20696459-20697351 FORWARD \| Aliases: ANAC097, K7B16.4, K7B16_4 | 3.8 | 4.6 | -0.8 | -4.2 | 2.10% | 3.5 |
| 5661 | AT1G79700.1 \| ovule development protein, putative, similar to ovule development protein AINTEGUMENTA (GI:1209099) (Arabidopsis thaliana) \| chr1:29995164-29998617 REVERSE \| Aliases: F20B17.12, F20B17_12 | 3.0 | 4.2 | -1.2 | -4.2 | 2.10% | 3.6 |
| 5662 | AT1G49040.2 \| Symbol: SCD1 \| stomatal cytokinesis defective / SCD1 protein (SCD1), contains Pfam PF02141: DENN (AEX-3) domain; contains Pfam PF00400: WD domain, G-beta repeat (8 copies); identical to stomatal cytokinesis defective (Arabidopsis thaliana) GI:19743728; supporting cDNA gi:19743727:gb:AY082605.1:; PMID 12874123 \| chr1:18145470-18152601 REVERSE \| Aliases: F27J15.17, F27J15_17, STOMATAL CYTOKINESIS DEFECTIVE 1 | 7.4 | 8.1 | -0.7 | -4.2 | 2.11% | 3.4 |
| 5664 | AT1G22280.2 \| protein phosphatase 2C, putative / PP2C, putative, similar to protein phosphatase type 2C GI:4336436 from (Lotus japonicus) \| chr1:7873985-7875867 FORWARD \| Aliases: T16E15.10, T16E15_10 | 6.8 | 7.8 | -1.1 | -4.2 | 2.11% | 3.5 |
| 5669 | AT3G28850.1 \| glutaredoxin family protein \| chr3:10849906-10851359 FORWARD \| Aliases: T19N8.15 | 6.4 | 7.5 | -1.1 | -4.2 | 2.11% | 3.5 |
| 5671 | AT4G01450.3 \| nodulin MtN21 family protein, similar to MtN21 GI:2598575 (root nodule development) from (Medicago truncatula) \| chr4:608456-610705 FORWARD \| Aliases: F11O4.14, F11O4_14 | 7.5 | 8.5 | -0.9 | -4.2 | 2.12% | 3.4 |

| Rank | Description | Sync | Root | M | t | adj.q | B |
|---|---|---|---|---|---|---|---|
| 5673 | AT1G73280.1 \| Symbol: SCPL3 \| serine carboxypeptidase S10 family protein, similar to serine carboxypeptidase I precursor (SP:P07519) (Hordeum vulgare); glucose acyltransferase GB:AAD01263 (Solanum berthaultii); contains Pfam profile: PF00450 Serine carboxypeptidase; \| chr1:27556631-27558983 REVERSE \| Aliases: SCPL3, T18K17.5, T18K17_5 | 2.8 | 3.4 | -0.7 | -4.2 | 2.12% | 3.4 |
| 5676 | AT1G76970.1 \| VHS domain-containing protein / GAT domain-containing protein, weak similarity to HGF-regulated tyrosine kinase substrate (Mus musculus) GI:1089781; contains Pfam profiles PF00790: VHS domain, PF03127: GAT domain \| chr1:28927560-28930298 REVERSE \| Aliases: F22K20.7, F22K20_7 | 3.2 | 4.6 | -1.3 | -4.2 | 2.12% | 3.6 |
| 5677 | AT4G13100.2 \| zinc finger (C3HC4-type RING finger) family protein, contains Pfam profile: PF00097 zinc finger, C3HC4 type (RING finger) \| chr4:7636435-7639034 REVERSE \| Aliases: F25G13.190, F25G13_190 | 3.7 | 4.6 | -0.9 | -4.2 | 2.12% | 3.5 |
| 5678 | AT5G48175.1 \| expressed protein, contains weak similarity to thioglucoside glucohydrolase (GI:5524767) (Arabidopsis thaliana) \| chr5:19556422-19557007 FORWARD \| Aliases: None | 3.0 | 4.1 | -1.1 | -4.2 | 2.12% | 3.4 |
| 5679 | AT5G36870.1 \| Symbol: ATGSL09 \| glycosyl transferase family 48 protein, contains Pfam profile: PF02364 1,3-beta-glucan synthase \| chr5:14535546-14551160 FORWARD \| Aliases: ATGSL9, F5H8.14, F5H8_14, GLUCAN SYNTHASE LIKE 9, GSL09 | 4.9 | 7.2 | -2.3 | -4.2 | 2.13% | 3.7 |
| 5680 | AT1G13990.1 \| expressed protein \| chr1:4794761-4796594 FORWARD \| Aliases: F7A19.8, F7A19_8 | 6.6 | 7.7 | -1.1 | -4.2 | 2.13% | 3.6 |
| 5684 | AT1G57560.1 \| myb family transcription factor (MYB50), similar to DNA-binding protein GI:19058 from (Hordeum vulgare) \| chr1:21320493-21321729 FORWARD \| Aliases: T8L23.3, T8L23_3 | 3.4 | 4.7 | -1.4 | -4.2 | 2.13% | 3.6 |
| 5686 | AT4G18140.2 \| similar to NLI interacting factor (NIF) family protein [Arabidopsis thaliana] (TAIR:At5g46410.1) | 4.2 | 5.0 | -0.8 | -4.2 | 2.13% | 3.6 |
| 5688 | AT2G25810.1 \| tonoplast intrinsic protein, putative, similar to tonoplast intrinsic protein GI:4584429 from (Nicotiana tabacum) \| chr2:11019679-11021071 FORWARD \| Aliases: F17H15.16, F17H15_16 | 2.7 | 3.6 | -0.9 | -4.2 | 2.13% | 3.5 |
| 5690 | AT5G06930.1 \| expressed protein \| chr5:2145140-2148040 FORWARD \| Aliases: MOJ9.10, MOJ9_10 | 3.4 | 4.3 | -0.9 | -4.2 | 2.14% | 3.6 |
| 5694 | AT1G73310.1 \| Symbol: SCPL4 \| serine carboxypeptidase S10 family protein, similar to serine carboxypeptidase I precursor (SP:P07519) (Hordeum vulgare); glucose acyltransferase GB:AAD01263 (Solanum berthaultii); contains Pfam profile: PF00450 Serine carboxypeptidase \| chr1:27566476-27568838 REVERSE \| Aliases: SCPL4, T18K17.2, T18K17_2 | 3.0 | 3.9 | -0.9 | -4.2 | 2.14% | 3.5 |
| 5697 | AT3G46600.2 \| scarecrow transcription factor family protein, scarecrow-like 11 - Arabidopsis thaliana, EMBL:AF036307 \| chr3:17168929-17170950 FORWARD \| Aliases: F12A12.120 | 5.1 | 5.7 | -0.6 | -4.2 | 2.14% | 3.4 |
| 5700 | AT5G51680.1 \| hydroxyproline-rich glycoprotein family protein, contains proline-rich extensin domains, INTERPRO:IPR002965; similar to hydroxyproline-rich Glycoprotein (HRGP) (Zea mays) gi:4007865:emb:CAA10387 \| chr5:21014546-21016067 FORWARD \| Aliases: MIO24.19, MIO24_19 | 2.8 | 3.7 | -0.9 | -4.2 | 2.14% | 3.6 |
| 5704 | AT4G12010.1 \| disease resistance protein (TIR-NBS-LRR class), putative, domain signature TIR-NBS-LRR exists, suggestive of a disease resistance protein. \| chr4:7197205-7201387 REVERSE \| Aliases: F16J13.80, F16J13_80 | 3.1 | 4.0 | -0.9 | -4.2 | 2.15% | 3.6 |
| 5710 | AT5G46040.1 \| proton-dependent oligopeptide transport (POT) family protein, contains Pfam profile: PF00854 POT family \| chr5:18688624-18690778 REVERSE \| Aliases: MCL19.9, MCL19_9 | 2.7 | 3.3 | -0.7 | -4.2 | 2.15% | 3.4 |
| 5711 | AT5G16210.1 \| HEAT repeat-containing protein, contains Pfam profile PF02985: HEAT repeat \| chr5:5290870-5297977 REVERSE \| Aliases: T21H19.130, T21H19_130 | 5.4 | 6.1 | -0.7 | -4.2 | 2.15% | 3.5 |
| 5713 | AT1G09790.1 \| phytochelatin synthetase-related, contains Pfam profile PF04833: Phytochelatin synthetase-like conserved region \| chr1:3168484-3170821 REVERSE \| Aliases: F21M12.17, F21M12_17 | 3.7 | 5.0 | -1.3 | -4.2 | 2.16% | 3.5 |
| 5718 | AT5G56610.2 \| similar to dual specificity protein phosphatase family protein [Arabidopsis thaliana] (TAIR:At2g35680.1); similar to putative PTEN-like phosphatase [Oryza sativa (japonica cultivar-group)] (GB:BAD86944.1); contains InterPro domain Tyrosine specific protein phosphatase and dual specificity protein phosphatase (InterPro:IPR000387); contains InterPro domain Dual specificity protein phosphatase (InterPro:IPR000340) \| chr5:22933957-22935564 FORWARD \| Aliases: MIK19.6, MIK19_6 | 6.2 | 7.5 | -1.3 | -4.2 | 2.16% | 3.6 |
| 5719 | AT1G68795.1 \| Symbol: CLE12 \| CLE12, CLAVATA3/ESR-Related 12 \| chr1:25844518-25845315 REVERSE \| Aliases: CLAVATA3/ESR RELATED 12 | 2.9 | 4.0 | -1.0 | -4.2 | 2.16% | 3.3 |
| 5720 | AT3G21700.3 \| expressed protein \| chr3:7644482-7646362 FORWARD \| Aliases: MSD21.1 | 3.1 | 3.9 | -0.9 | -4.2 | 2.16% | 3.4 |
| 5721 | AT5G66880.1 \| serine/threonine protein kinase, putative, similar to serine-threonine protein kinase (Triticum aestivum) gi:2055374:gb:AAB58348 | 6.1 | 7.9 | -1.7 | -4.2 | 2.16% | 3.6 |

| Rank | Description | Sync | Root | M | t | adj.q | B |
|---|---|---|---|---|---|---|---|
| 5722 | AT1G24560.1 \| expressed protein \| chr1:8702372-8705835 FORWARD \| Aliases: F21J9.22 | 8.0 | 9.1 | -1.1 | -4.2 | 2.16% | 3.6 |
| 5724 | AT5G15130.1 \| Symbol: WRKY72 \| | 2.5 | 3.7 | -1.2 | -4.2 | 2.16% | 3.6 |
| 5726 | AT5G66280.1 \| Symbol: GMD1 \| GDP-D-mannose 4,6-dehydratase, putative, strong similarity to GDP-D-mannose-4,6-dehydratase (Arabidopsis thaliana) GI:1764100 \| chr5:26493392-26495344 FORWARD \| Aliases: GDP D MANNOSE 4,6 DEHYDRATASE 1, K1L20.6, K1L20_6 | 4.1 | 4.9 | -0.9 | -4.2 | 2.16% | 3.5 |
| 5728 | AT1G29690.1 \| Symbol: CAD1 \| Encodes a protein containing a domain with significant homology to the MACPF (membrane attack complex and perforin) domain of complements and perforin proteins that are involved in innate immunity in animals. Transgenic <i>cad1-1</i> mutant plants show lesions seen in the hypersensitive response, as well as a spontaneous activation of expression of pathogenesis-related genes and leading to a 32-fold increase in salicylic acid (SA). CAD1 is postulated to act as a negative regulator controlling SA-mediated pathway of programmed cell death in plant immunity. \| chr1:10379012-10382109 REVERSE \| Aliases: CAD1, F15D2.24, F15D2_24 | 3.7 | 4.5 | -0.8 | -4.2 | 2.16% | 3.5 |
| 5729 | AT3G09290.1 \| zinc finger (C2H2 type) family protein, ; contains Pfam profile: PF00096 Zinc finger, C2H2 type \| chr3:2856147-2856665 FORWARD \| Aliases: F3L24.16 | 3.2 | 4.9 | -1.7 | -4.2 | 2.16% | 3.6 |
| 5730 | AT3G03370.1 \| expressed protein \| chr3:797126-798480 REVERSE \| Aliases: T21P5.21, T21P5_21 | 2.7 | 3.3 | -0.6 | -4.2 | 2.16% | 3.3 |
| 5731 | AT5G65260.1 \| polyadenylate-binding protein family protein / PABP family protein, low similarity to poly(A)-binding protein II (Drosophila melanogaster) GI:6007612; contains InterPro entry IPR000504: RNA-binding region RNP-1 (RNA recognition motif) (RRM) domain \| chr5:26097457-26100243 REVERSE \| Aliases: MQN23.21, MQN23_21 | 7.7 | 9.2 | -1.5 | -4.2 | 2.16% | 3.5 |
| 5733 | AT5G26600.2 \| expressed protein, weak similarity to SP:P18549 Isopenicillin N epimerase (EC 5.-.- ) {Streptomyces clavuligerus} \| chr5:9377405-9379800 FORWARD \| Aliases: None | 7.3 | 8.0 | -0.7 | -4.2 | 2.17% | 3.4 |
| 5740 | AT4G12720.3 \| MutT/nudix family protein, similar to SP:P53370 Nucleoside diphosphate-linked moiety X motif 6 {Homo sapiens}; contains Pfam profile PF00293: NUDIX domain \| chr4:7487540-7490073 FORWARD \| Aliases: T20K18.70, T20K18_70 | 9.0 | 9.5 | -0.5 | -4.2 | 2.18% | 3.0 |
| 5741 | AT1G34630.1 \| expressed protein \| chr1:12685178-12687710 FORWARD \| Aliases: F12K21.3 | 5.6 | 7.2 | -1.6 | -4.2 | 2.18% | 3.6 |
| 5744 | AT1G17140.2 \| tropomyosin-related, similar to Tropomyosin 1. (Baker's yeast)(SP:P17536) {Saccharomyces cerevisiae} \| chr1:5856257-5858525 REVERSE \| Aliases: F20D23.32 | 3.1 | 4.8 | -1.8 | -4.2 | 2.18% | 3.6 |
| 5746 | AT3G01810.1 \| expressed protein \| chr3:288580-292762 FORWARD \| Aliases: F28J7.14, F28J7_14 | 4.9 | 6.5 | -1.6 | -4.2 | 2.19% | 3.6 |
| 5749 | AT3G01175.1 \| expressed protein \| chr3:59478-60852 FORWARD \| Aliases: None | 2.2 | 2.6 | -0.3 | -4.2 | 2.20% | 2.7 |
| 5750 | AT3G23340.1 \| casein kinase, putative, similar to casein kinase I (Arabidopsis thaliana) gi:1197461:emb:CAA55396 \| chr3:8350810-8353966 FORWARD \| Aliases: MLM24.21 | 5.6 | 6.3 | -0.8 | -4.2 | 2.20% | 3.4 |
| 5751 | AT5G45090.1 \| Symbol: ATPP2 A7 \| lectin-related, low similarity to PP2 lectin polypeptide (Cucurbita maxima) GI:410437; contains Pfam profile PF01582: TIR domain \| chr5:18219980-18222227 REVERSE \| Aliases: ATPP2 A7, K17O22.8, K17O22_8 | 3.9 | 5.3 | -1.4 | -4.2 | 2.21% | 3.6 |
| 5752 | AT2G40330.1 \| Bet v I allergen family protein, contains Pfam profile PF00407: Pathogenesis-related protein Bet v I family \| chr2:16851942-16853012 REVERSE \| Aliases: T7M7.15 | 4.2 | 5.6 | -1.3 | -4.2 | 2.21% | 3.6 |
| 5754 | AT1G28680.1 \| transferase family protein, similar to elicitor inducible gene product EIG-I24 (Nicotiana tabacum) (gi:10798748); contains Pfam transferase family domain PF00248 \| chr1:10078175-10080015 FORWARD \| Aliases: F1K23.12, F1K23_12 | 9.1 | 10.3 | -1.3 | -4.2 | 2.21% | 3.3 |
| 5756 | AT5G55860.1 \| expressed protein, contains Pfam profile PF05701: Plant protein of unknown function (DUF827); expression supported by MPSS \| chr5:22627372-22629392 FORWARD \| Aliases: MWJ3.4, MWJ3_4 | 5.7 | 7.3 | -1.5 | -4.2 | 2.21% | 3.6 |
| 5757 | AT3G44660.1 \| histone deacetylase-related / HD-related, similar to SP:O09106 Histone deacetylase 1 (HD1) {Mus musculus} \| chr3:16225951-16226772 REVERSE \| Aliases: T18B22.60 | 8.4 | 9.1 | -0.7 | -4.2 | 2.21% | 3.5 |
| 5758 | AT3G23050.2 \| Symbol: IAA7 \| auxin-responsive protein / indoleacetic acid-induced protein 7 (IAA7), identical to SP:Q38825:AXI7_ARATH Auxin-responsive protein IAA7 (Indoleacetic acid-induced protein 7) \| chr3:8194718-8196521 FORWARD \| Aliases: AUXIN RESISTANT 2, AXR2, MXC7.8 | 5.7 | 7.1 | -1.4 | -4.2 | 2.21% | 3.6 |

| Rank | Description | Sync | Root | M | t | adj.q | B |
|---|---|---|---|---|---|---|---|
| 5767 | AT1G79840.1 \| Symbol: GL2 \| homeobox-leucine zipper protein 10 (HB-10) / HD-ZIP transcription factor 10 / homeobox protein (GLABRA2), identical to homeobox protein (GLABRA2) (homeobox-leucine zipper protein ATHB-10) (HD-ZIP protein ATHB-10) GB:P46607 (Arabidopsis thaliana) \| chr1:30042308-30045984 FORWARD \| Aliases: F19K16.20, F19K16_20, GLABRA 2, HOMEOBOX PROTEIN GLABRA 2 | 2.9 | 3.7 | -0.8 | -4.2 | 2.22% | 3.5 |
| 5768 | AT5G24165.1 \| expressed protein \| chr5:8188563-8189279 FORWARD \| Aliases: None | 6.6 | 7.9 | -1.3 | -4.2 | 2.22% | 3.5 |
| 5771 | AT5G62550.1 \| expressed protein \| chr5:25122864-25125177 REVERSE \| Aliases: K19B1.16, K19B1_16 | 4.2 | 6.4 | -2.1 | -4.1 | 2.22% | 3.6 |
| 5773 | AT2G39690.2 \| similar to expressed protein [Arabidopsis thaliana] (TAIR:At3g12540.1); similar to putative ternary complex factor [Oryza sativa (japonica cultivar-group)] (GB:XP_550141.1); contains InterPro domain Protein of unknown function DUF547 (InterPro:IPR006869) \| chr2:16548104-16550456 FORWARD \| Aliases: F17A14.6, F17A14_6 | 2.2 | 2.9 | -0.7 | -4.1 | 2.22% | 3.3 |
| 5774 | AT4G01740.1 \| DC1 domain-containing protein, similar to T15B16.6 similar to A. thaliana CHP-rich hypothetical proteins encoded by T10M13, GenBank accession number AF001308 \| chr4:753422-755456 FORWARD \| Aliases: T15B16.10, T15B16_10 | 3.3 | 5.4 | -2.1 | -4.1 | 2.23% | 3.6 |
| 5775 | AT3G50930.1 \| AAA-type ATPase family protein, contains Pfam profile: ATPase family PF00004 \| chr3:18940507-18942820 FORWARD \| Aliases: F18B3.210 | 3.3 | 3.7 | -0.4 | -4.1 | 2.23% | 2.9 |
| 5777 | AT5G17850.1 \| cation exchanger, putative (CAX8), similar to sodium/calcium exchanger protein (Mus musculus) gi:13925661:gb:AAK49407; Ca2+:Cation Antiporter (CaCA) Family member PMID:11500563 | 6.8 | 7.8 | -1.0 | -4.1 | 2.23% | 3.6 |
| 5778 | AT1G28330.3 \| Symbol: DRM1 \| dormancy-associated protein, putative (DRM1), identical to dormancy-associated protein (Arabidopsis thaliana) GI:2995990; similar to dormancy-associated protein GI:2605887 from (Pisum sativum); contains Pfam profile PF05564: Dormancy/auxin associated protein \| chr1:9933896-9935251 REVERSE \| Aliases: DORMANCY ASSOCIATED PROTEIN 1, DRM1, F3H9.1, F3H9_1 | 5.6 | 7.7 | -2.1 | -4.1 | 2.23% | 3.5 |
| 5779 | AT2G25520.1 \| phosphate translocator-related, low similarity to SP:P52178 Triose phosphate/phosphate translocator, non-green plastid, chloroplast precursor (CTPT) {Brassica oleracea}, phosphoenolpyruvate/phosphate translocator precursor (Mesembryanthemum crystallinum) GI:9295275 \| chr2:10867906-10869435 FORWARD \| Aliases: F13B15.18, F13B15_18 | 7.0 | 8.3 | -1.4 | -4.1 | 2.24% | 3.5 |
| 5780 | AT5G45970.1 \| Symbol: ARAC2 \| Rac-like GTP-binding protein (ARAC2), identical to RAC-like GTP binding protein ARAC2 SP:Q38903 | 2.8 | 3.5 | -0.7 | -4.1 | 2.24% | 3.4 |
| 5781 | AT5G35980.2 \| similar to protein kinase family protein [Arabidopsis thaliana] (TAIR:At2g40120.1); similar to putative protein kinase YakA [Oryza sativa (japonica cultivar-group)] (GB:XP_467340.1); contains InterPro domain Serine/threonine protein kinase, active site (InterPro:IPR008271); contains InterPro domain Tyrosine protein kinase (InterPro:IPR001245); contains InterPro domain Serine/threonine protein kinase (InterPro:IPR002290); contains InterPro domain Protein kinase (InterPro:IPR000719) \| chr5:14145214-14151682 FORWARD \| Aliases: MEE13.9, MEE13_9 | 5.1 | 6.1 | -1.0 | -4.1 | 2.24% | 3.3 |
| 5782 | AT4G23870.1 \| expressed protein, predicted proteins, Arabidopsis thaliana \| chr4:12414240-12415059 FORWARD \| Aliases: T32A16.40, T32A16_40 | 3.6 | 4.9 | -1.3 | -4.1 | 2.24% | 3.5 |
| 5783 | AT5G02640.1 \| expressed protein, predicted proteins, Arabidopsis thaliana and Oryza sativa \| chr5:594177-594865 REVERSE \| Aliases: T22P11.230, T22P11_230 | 3.8 | 4.5 | -0.6 | -4.1 | 2.24% | 3.3 |
| 5787 | AT3G45970.1 \| Symbol: ATEXLA1 \| expansin family protein (EXPL1), similar to cim1 induced allergen, Glycine max, EMBL:U03860; expansin-like gene, PMID:11641069, www.bio.psu.edu/expansins \| chr3:16907151-16908293 FORWARD \| Aliases: ARABIDOPSIS THALIANA EXPANSIN LIKE A1, ATEXPL1, ATHEXP BETA 2.1, EXPL1, F16L2.180, F16L2_180 | 10.0 | 10.7 | -0.8 | -4.1 | 2.25% | 3.4 |
| 5793 | AT2G37940.1 \| expressed protein \| chr2:15883878-15886733 FORWARD \| Aliases: T8P21.15, T8P21_15 | 5.6 | 6.6 | -0.9 | -4.1 | 2.26% | 3.4 |
| 5794 | AT1G44750.3 \| Symbol: ATPUP11 \| similar to purine permease family protein [Arabidopsis thaliana] (TAIR:At4g08700.1); similar to putative purine permease [Oryza sativa (japonica cultivar-group)] (GB:XP_467223.1); contains InterPro domain Protein of unknown function DUF250 (InterPro:IPR004853) \| chr1:16896163-16897905 FORWARD \| Aliases: T12C22.2, T12C22_2 | 6.5 | 7.8 | -1.3 | -4.1 | 2.26% | 3.5 |
| 5796 | AT1G67510.1 \| leucine-rich repeat family protein, contains protein kinase domain, Pfam:PF00069; contains leucine-rich repeats, Pfam:PF00560 \| chr1:25301140-25303847 REVERSE \| Aliases: T1F15.2, T1F15_2 | 3.5 | 4.6 | -1.1 | -4.1 | 2.26% | 3.5 |
| 5797 | AT1G16540.1 \| Symbol: ABA3/LOS5/SIR3 \| | 3.3 | 4.2 | -0.9 | -4.1 | 2.27% | 3.4 |
| 5804 | AT3G51470.1 \| protein phosphatase 2C, putative / PP2C, putative, protein phosphatase-2C, Mesembryanthemum crystallinum, EMBL:AF075580 \| chr3:19108766-19110254 REVERSE \| Aliases: F26O13.110 | 3.2 | 3.8 | -0.6 | -4.1 | 2.28% | 3.3 |

| Rank | Description | Sync | Root | M | t | adj.q | B |
|---|---|---|---|---|---|---|---|
| 5806 | AT2G17530.1 \| protein kinase family protein, identical to SRPK2 (Arabidopsis thaliana) gi:9843645:emb:CAC03676; contains protein kinase domain, Pfam:PF00069 \| chr2:7633478-7636231 FORWARD \| Aliases: MJB20.9, MJB20_9 | 4.8 | 6.5 | -1.7 | -4.1 | 2.29% | 3.5 |
| 5808 | AT3G51350.1 \| aspartyl protease family protein, contains Eukaryotic and viral aspartyl proteases active site, PROSITE:PS00141 \| chr3:19071301-19074489 REVERSE \| Aliases: F26O13.3 | 2.9 | 4.3 | -1.5 | -4.1 | 2.29% | 3.5 |
| 5810 | AT5G37450.1 \| leucine-rich repeat transmembrane protein kinase, putative \| chr5:14870031-14874328 REVERSE \| Aliases: T25O11.15, T25O11_15 | 2.7 | 3.3 | -0.6 | -4.1 | 2.30% | 3.3 |
| 5813 | AT1G47240.1 \| Symbol: NRAMP2 \| | 6.6 | 7.7 | -1.1 | -4.1 | 2.30% | 3.5 |
| 5815 | AT1G72870.1 \| disease resistance protein (TIR-NBS class), putative, domain signature TIR-NBS exists, suggestive of a disease resistance protein. \| chr1:27424739-27426761 FORWARD \| Aliases: F3N23.7, F3N23_7 | 2.9 | 3.6 | -0.7 | -4.1 | 2.30% | 3.3 |
| 5816 | AT1G17970.1 \| zinc finger (C3HC4-type RING finger) family protein, similar to Pfam domain, PF00097: Zinc finger, C3HC4 type (RING finger) \| chr1:6184897-6187465 FORWARD \| Aliases: F2H15.19, F2H15_19 | 2.8 | 3.7 | -0.9 | -4.1 | 2.30% | 3.5 |
| 5817 | AT1G53920.1 \| GDSL-motif lipase/hydrolase family protein, similar to Anther-specific proline-rich proteins SP:P40603 SP:P40602 from {Arabidopsis thaliana}; contains Pfam profile PF00657: GDSL-like Lipase/Acylhydrolase \| chr1:20141380-20143305 FORWARD \| Aliases: T18A20.15, T18A20_15 | 3.7 | 4.3 | -0.6 | -4.1 | 2.31% | 3.2 |
| 5818 | AT4G02370.1 \| expressed protein, contains Pfam profile PF04398: Protein of unknown function, DUF538 \| chr4:1042338-1043206 FORWARD \| Aliases: T14P8.18, T14P8_18 | 8.0 | 9.7 | -1.7 | -4.1 | 2.31% | 3.5 |
| 5823 | AT5G03540.1 \| exocyst subunit EXO70 family protein, contains Pfam domain PF03081: Exo70 exocyst complex subunit \| chr5:889565-894155 FORWARD \| Aliases: F12E4.330, F12E4_330 | 6.6 | 8.2 | -1.6 | -4.1 | 2.31% | 3.6 |
| 5825 | AT3G15940.1 \| glycosyl transferase family 1 protein, contains Pfam profile:PF00534 Glycosyl transferases group 1 \| chr3:5393519-5396765 REVERSE \| Aliases: MVC8.8 | 6.0 | 7.0 | -1.0 | -4.1 | 2.32% | 3.4 |
| 5826 | AT2G01260.3 \| expressed protein, similar to expressed protein [Arabidopsis thaliana] (TAIR:At1g15030.1); similar to hypothetical protein [Oryza sativa (japonica cultivar-group)] (GB:XP_477257.1); contains InterPro domain Protein of unknown function DUF789 (InterPro:IPR008507) \| chr2:135243-137802 REVERSE \| Aliases: F10A8.14, F10A8_14 | 3.9 | 5.5 | -1.5 | -4.1 | 2.32% | 3.4 |
| 5832 | AT2G41790.1 \| peptidase M16 family protein / insulinase family protein, contains Pfam domain, PF05193: Peptidase M16 inactive domain; similar to insulin-degrading enzyme (Insulysin, Insulinase, Insulin protease) (Mouse) SWISS-PROT:Q9JHR7 \| chr2:17436372-17443188 REVERSE \| Aliases: T11A7.11, T11A7_11 | 6.5 | 7.7 | -1.2 | -4.1 | 2.32% | 3.5 |
| 5834 | AT5G60850.1 \| Symbol: OBP4 \| Dof-type zinc finger domain-containing protein, similar to zinc finger protein OBP4 gi:5059396 from (Arabidopsis thaliana); EMBL:AF155817 \| chr5:24497675-24499094 FORWARD \| Aliases: MAE1.2, MAE1_2, ZINC FINGER PROTEIN OBP4 | 4.6 | 5.4 | -0.9 | -4.1 | 2.32% | 3.5 |
| 5836 | AT2G18240.2 \| RER1 protein, putative, similar to SP:O48671 RER1B protein (AtRER1B) {Arabidopsis thaliana}, SP:O48670 RER1A protein (AtRER1A) {Arabidopsis thaliana}; contains Pfam profile PF03248: Rer1 family \| chr2:7942367-7943803 FORWARD \| Aliases: T30D6.25 | 4.4 | 5.1 | -0.7 | -4.1 | 2.33% | 3.4 |
| 5838 | AT2G31940.1 \| expressed protein \| chr2:13587677-13588189 FORWARD \| Aliases: F20M17.26 | 2.7 | 3.5 | -0.8 | -4.1 | 2.33% | 3.2 |
| 5839 | AT4G17500.1 \| Symbol: ATERF 1 \| encodes a member of the ERF (ethylene response factor) subfamily B-3 of ERF/AP2 transcription factor family (ATERF-1). The protein contains one AP2 domain. There are 18 members in this subfamily including ATERF-1, ATERF-2, AND ATERF-5. \| chr4:9759337-9760353 FORWARD \| Aliases: DL4785W, ETHYLENE RESPONSIVE ELEMENT BINDING FACTOR 1, FCAALL.123 | 5.8 | 6.8 | -1.0 | -4.1 | 2.34% | 3.5 |
| 5840 | AT1G64380.1 \| encodes a member of the DREB subfamily A-6 of ERF/AP2 transcription factor family. The protein contains one AP2 domain. There are 8 members in this subfamily including RAP2.4. \| chr1:23894309-23895836 REVERSE \| Aliases: F15H21.12, F15H21_12 | 2.4 | 3.7 | -1.3 | -4.1 | 2.34% | 3.5 |
| 5845 | AT1G33780.1 \| expressed protein, similar to At3g29240 (Arabidopsis thaliana); contains Pfam profile PF02622: Uncharacterized ACR, COG1678 \| chr1:12244565-12246168 REVERSE \| Aliases: F14M2.10, F14M2_10 | 7.2 | 8.0 | -0.9 | -4.1 | 2.35% | 3.4 |
| 5850 | AT1G51720.1 \| glutamate dehydrogenase, putative, similar to NADP-specific glutatamate dehydrogenase (NADP-GDH) SP:P28724 (Giardia lamblia (Giardia intestinalis)) \| chr1:19184440-19190200 FORWARD \| Aliases: F19C24.7, F19C24_7 | 8.1 | 8.7 | -0.7 | -4.1 | 2.36% | 3.2 |
| 5851 | AT3G04530.1 \| Symbol: PPCK2 \| phosphoenolpyruvate carboxylase kinase 2 (PPCK2), phosphoenolpyruvate carboxylase kinase 2 (Arabidopsis thaliana) gi:13877128:gb:AAK43710; contains protein kinase domain, Pfam:PF00069 | 4.3 | 6.0 | -1.7 | -4.1 | 2.36% | 3.5 |

| Rank | Description | Sync | Root | M | t | adj.q | B |
|---|---|---|---|---|---|---|---|
| 5853 | AT4G01550.1 \| Symbol: ANAC069 \| no apical meristem (NAM) family protein, similar to NAC1 (GI:7716952) {Medicago truncatula}; contains Pfam PF02365 : No apical meristem (NAM) protein \| chr4:673868-676392 REVERSE \| Aliases: ANAC069, F11O4.5, F11O4_5 | 2.2 | 3.1 | -0.8 | -4.1 | 2.36% | 3.5 |
| 5854 | AT1G34260.1 \| phosphatidylinositol-4-phosphate 5-kinase family protein, low similarity to SP:Q9Z1T6 FYVE finger-containing phosphoinositide kinase (EC 2.7.1.68) (1-phosphatidylinositol-4-phosphate kinase) (PIP5K) (PtdIns(4)P-5-kinase) {Mus musculus}; contains Pfam profile PF01504: Phosphatidylinositol-4-phosphate 5-Kinase \| chr1:12485945-12492126 FORWARD \| Aliases: F23M19.8, F23M19_8 | 4.9 | 5.8 | -1.0 | -4.1 | 2.36% | 3.5 |
| 5856 | AT5G25160.1 \| Symbol: ZFP3 \| zinc finger (C2H2 type) family protein (ZFP3), identical to zinc finger protein, ZFP3 gi:790677:gb:AAA87299; contains Pfam domain, PF00096: Zinc finger, C2H2 type \| chr5:8687473-8688418 FORWARD \| Aliases: F21J6.6, F21J6_6, ZFP3, ZINC FINGER PROTEIN 3 | 5.5 | 6.3 | -0.8 | -4.1 | 2.36% | 3.4 |
| 5857 | AT1G69040.2 \| Symbol: ACR4 \| ACT domain containing protein (ACR4), low similarity to uridylyltransferase (Gluconacetobacter diazotrophicus) GI:17226253; contains Pfam profile PF01842: ACT domain \| chr1:25961482-25963970 FORWARD \| Aliases: ACT REPEAT 4, F4N2.2, F4N2_2 | 4.6 | 5.3 | -0.6 | -4.1 | 2.37% | 3.3 |
| 5861 | AT3G24670.1 \| pectate lyase family protein, similar to pectate lyase GP:14531296 from (Fragaria x ananassa) \| chr3:9006212-9008808 REVERSE \| Aliases: MSD24.10 | 5.3 | 7.2 | -1.9 | -4.1 | 2.37% | 3.5 |
| 5863 | AT5G19800.1 \| hydroxyproline-rich glycoprotein family protein, similar to extensin (Catharanthus roseus) gi:1486263:dbj:BAA13175; contains proline-rich extensin domains, INTERPRO:IPR002965 \| chr5:6690650-6690940 FORWARD \| Aliases: T29J13.220, T29J13_220 | 3.8 | 5.9 | -2.2 | -4.1 | 2.37% | 3.5 |
| 5864 | AT5G39020.1 \| protein kinase family protein, contains protein kinase domain, Pfam:PF00069 \| chr5:15634147-15636588 FORWARD \| Aliases: MXF12.30, MXF12_30 | 2.7 | 3.9 | -1.2 | -4.1 | 2.38% | 3.4 |
| 5867 | AT2G20950.4 \| expressed protein \| chr2:9010220-9013019 FORWARD \| Aliases: F26H11.29 | 5.7 | 8.0 | -2.2 | -4.1 | 2.38% | 3.5 |
| 5868 | AT3G10080.1 \| germin-like protein, putative, similar to germin-like protein 2 (Oryza sativa) GI:2655287 \| chr3:3107281-3108182 REVERSE \| Aliases: T22K18.9 | 3.7 | 4.6 | -1.0 | -4.1 | 2.38% | 3.5 |
| 5869 | AT1G04330.1 \| expressed protein, EST gb:H76414 comes from this gene \| chr1:1161310-1161885 REVERSE \| Aliases: F19P19.24, F19P19_24 | 2.5 | 3.1 | -0.6 | -4.1 | 2.38% | 3.2 |
| 5871 | AT2G29000.1 \| leucine-rich repeat family protein / protein kinase family protein, contains Pfam domains PF00560: Leucine Rich Repeat and PF00069: Protein kinase domain \| chr2:12467858-12472114 FORWARD \| Aliases: T9I4.8, T9I4_8 | 2.6 | 3.2 | -0.6 | -4.1 | 2.39% | 3.0 |
| 5872 | AT4G39400.1 \| Symbol: BRI1 \| brassinosteroid insensitive 1 (BRI1), identical to GI:2392895 \| chr4:18324655-18328820 FORWARD \| Aliases: BIN1, BRASSINOSTEROID INSENSITIVE 1, CABBAGE 2, CBB2, DWARF 2, DWF2, F23K16.30, F23K16_30 | 5.5 | 6.2 | -0.7 | -4.1 | 2.39% | 3.3 |
| 5874 | AT3G62780.1 \| C2 domain-containing protein, contains similarity to shock protein SRC2 (Glycine max) gi:2055230:dbj:BAA19769 ; contains Pfam profile PF00168:C2 domain \| chr3:23233004-23233916 REVERSE \| Aliases: F26K9.210 | 2.6 | 3.5 | -0.9 | -4.1 | 2.39% | 3.1 |
| 5877 | AT3G61690.1 \| expressed protein \| chr3:22839324-22845011 FORWARD \| Aliases: F15G16.80 | 5.3 | 6.2 | -0.9 | -4.1 | 2.40% | 3.4 |
| 5878 | AT3G51630.1 \| Symbol: WNK5 \| protein kinase family protein, contains Pfam profile: PF00069 protein kinase domain \| chr3:19159886-19163172 FORWARD \| Aliases: T18N14.10, ZIK1 | 6.5 | 7.2 | -0.7 | -4.1 | 2.40% | 3.3 |
| 5881 | AT4G16447.1 \| expressed protein \| chr4:9278809-9279411 FORWARD \| Aliases: None | 3.4 | 6.1 | -2.7 | -4.1 | 2.40% | 3.5 |
| 5882 | AT2G43250.1 \| expressed protein, and genefinder \| chr2:17984305-17986685 FORWARD \| Aliases: F14B2.19 | 4.1 | 4.9 | -0.8 | -4.1 | 2.40% | 3.2 |
| 5884 | AT3G61190.1 \| Symbol: BAP1 \| BON1-associated protein 1 (BAP1), identical to BON1-associated protein 1 (Arabidopsis thaliana) GI:15487384; contains Pfam profile PF00168: C2 domain; supporting cDNA gi:15487383:gb:AY045765.1: \| chr3:22661502-22662424 REVERSE \| Aliases: BON ASSOCIATION PROTEIN 1, T20K12.90 | 4.8 | 6.2 | -1.5 | -4.1 | 2.41% | 3.5 |
| 5885 | AT4G10510.1 \| subtilase family protein, contains similarity to subtilase; SP1 GI:9957714 from (Oryza sativa) \| chr4:6495951-6499006 FORWARD \| Aliases: F7L13.90, F7L13_90 | 2.6 | 3.2 | -0.7 | -4.1 | 2.41% | 3.3 |
| 5887 | AT1G35290.1 \| thioesterase family protein, contains Pfam profile PF03061: thioesterase family protein \| chr1:12947522-12948819 REVERSE \| Aliases: T9I1.6, T9I1_6 | 2.5 | 3.5 | -1.0 | -4.1 | 2.42% | 3.5 |
| 5888 | AT1G02000.1 \| Symbol: GAE2 \| NAD-dependent epimerase/dehydratase family protein, similar to nucleotide sugar epimerase from Vibrio vulnificus GI:3093975 (PID:g3093975), WbnF (Escherichia coli) GI:5739472, CAPI protein {Staphylococcus aureus} SP:P39858; contains Pfam profile PF01370 NAD dependent epimerase/dehydratase family | 5.7 | 6.7 | -1.0 | -4.1 | 2.42% | 3.2 |

| Rank | Description | Sync | Root | M | t | adj.q | B |
|---|---|---|---|---|---|---|---|
| 5889 | AT1G26970.1 \| protein kinase, putative, similar to protein kinase (Arabidopsis thaliana) gi:2852449:dbj:BAA24695; contains protein kinase domain, Pfam:PF00069 | 3.3 | 4.2 | -0.9 | -4.1 | 2.42% | 3.4 |
| 5892 | AT3G53810.1 \| lectin protein kinase, putative, similar to receptor lectin kinase 3 (Arabidopsis thaliana) gi:4100060:gb:AAD00733; contains protein kinase domain, Pfam:PF00069; contains legume lectins alpha and beta domains, Pfam:PF00138 and Pfam:PF00139 \| chr3:19943970-19946212 REVERSE \| Aliases: F5K20.110 | 4.2 | 5.1 | -0.9 | -4.1 | 2.42% | 3.3 |
| 5894 | AT5G09850.1 \| transcription elongation factor-related, low similarity to SP:P10712 Transcription elongation factor S-II (Transcription elongation factor A) {Mus musculus} \| chr5:3063252-3065890 REVERSE \| Aliases: MYH9.6, MYH9_6 | 5.2 | 6.7 | -1.5 | -4.1 | 2.42% | 3.4 |
| 5898 | AT4G28650.1 \| leucine-rich repeat transmembrane protein kinase, putative, receptor-like protein kinase 5, Arabidopsis thaliana, PIR1:S27756 \| chr4:14143990-14147543 REVERSE \| Aliases: T5F17.100, T5F17_100 | 2.9 | 3.4 | -0.5 | -4.1 | 2.43% | 3.2 |
| 5900 | AT4G24250.1 \| Symbol: MLO13 \| seven transmembrane MLO family protein / MLO-like protein 13 (MLO13), identical to membrane protein Mlo13 (Arabidopsis thaliana) gi:14091596:gb:AAK53806; similar to MLO protein SWISS-PROT:P93766, NCBI_gi:1877221 (Hordeum vulgare)(Barley) \| chr4:12574822-12577593 REVERSE \| Aliases: T22A6.80, T22A6_80 | 2.6 | 3.5 | -0.9 | -4.1 | 2.43% | 3.2 |
| 5901 | AT5G03640.1 \| protein kinase family protein, contains serine/threonine protein kinase domain, INTERPRO:IPR002290 \| chr5:927914-930780 FORWARD \| Aliases: F17C15.60, F17C15_60 | 2.7 | 3.3 | -0.6 | -4.1 | 2.43% | 3.3 |
| 5903 | AT2G36880.1 \| S-adenosylmethionine synthetase, putative, similar to S-adenosylmethionine synthetase 3 (Methionine adenosyltransferase 3, AdoMet synthetase 3) (Lycopersicon esculentum) SWISS-PROT:P43282 \| chr2:15486445-15488486 REVERSE \| Aliases: T1J8.6, T1J8_6 | 7.8 | 10.4 | -2.6 | -4.1 | 2.43% | 3.5 |
| 5907 | AT3G44550.1 \| acyl CoA reductase, putative, similar to acyl CoA reductase (Simmondsia chinensis) GI:5020215; contains Pfam profile PF03015: Male sterility protein \| chr3:16149004-16151325 FORWARD \| Aliases: F14L2.100 | 3.0 | 4.4 | -1.3 | -4.1 | 2.44% | 3.4 |
| 5911 | AT5G14260.3 \| SET domain-containing protein, low similarity to ribulose-1,5-bisphosphate carboxylase/oxygenase small subunit N-methyltransferase I (Spinacia oleracea) GI:3403236; contains Pfam profile PF00856: SET domain \| chr5:4600999-4604450 FORWARD \| Aliases: F18O22.50, F18O22_50 | 6.6 | 7.9 | -1.2 | -4.1 | 2.44% | 3.4 |
| 5914 | AT5G45110.1 \| ankyrin repeat family protein / BTB/POZ domain-containing protein, contains Pfam domain, PF00023: Ankyrin repeat and Pfam domain, PF00651: BTB/POZ domain \| chr5:18246137-18248749 FORWARD \| Aliases: K17O22.11, K17O22_11 | 6.1 | 7.2 | -1.0 | -4.1 | 2.44% | 3.3 |
| 5915 | AT2G25930.1 \| Symbol: ELF3 \| hydroxyproline-rich glycoprotein family protein, identical to cDNA nematode responsive protein GI:2213418 \| chr2:11066113-11070402 FORWARD \| Aliases: EARLY FLOWERING 3, F17H15.25, PYK20 | 6.5 | 7.9 | -1.3 | -4.1 | 2.45% | 3.4 |
| 5916 | AT2G18360.1 \| hydrolase, alpha/beta fold family protein, low similarity to SP:P24640:LIP3_MORSP Lipase 3 precursor (EC 3.1.1.3) (Triacylglycerol lipase) {Moraxella sp}; contains Pfam profile PF00561: hydrolase, alpha/beta fold family \| chr2:7983794-7986459 REVERSE \| Aliases: T30D6.13, T30D6_13 | 3.3 | 4.1 | -0.8 | -4.1 | 2.45% | 3.4 |
| 5918 | AT3G53670.1 \| expressed protein \| chr3:19901399-19903380 FORWARD \| Aliases: AT3G53660, F4P12.370 | 4.9 | 5.5 | -0.6 | -4.1 | 2.45% | 3.1 |
| 5919 | AT1G53660.1 \| phosphate translocator-related, low similarity to SP:P52178 Triose phosphate/phosphate translocator, non-green plastid, chloroplast precursor (CTPT) {Brassica oleracea}, phosphoenolpyruvate/phosphate translocator precursor (Mesembryanthemum crystallinum) GI:9295275; contains 8 predicted transmembrane domains \| chr1:20036950-20039614 FORWARD \| Aliases: F22G10.26, F22G10_26 | 3.0 | 3.5 | -0.6 | -4.1 | 2.45% | 3.2 |
| 5921 | AT1G21570.1 \| zinc finger (CCCH-type) family protein, contains Pfam domain, PF00642: Zinc finger C-x8-C-x5-C-x3-H type (and similar) \| chr1:7557251-7560272 REVERSE \| Aliases: F24J8.21, F24J8_21 | 8.9 | 10.4 | -1.5 | -4.1 | 2.45% | 3.4 |
| 5925 | AT1G76700.1 \| DNAJ heat shock N-terminal domain-containing protein, similar to SP:P39101 CAJ1 protein, Saccharomyces cerevisiae; contains Pfam profile PF00226 DnaJ domain \| chr1:28784670-28788142 REVERSE \| Aliases: F28O16.7, F28O16_7 | 3.8 | 4.8 | -1.0 | -4.1 | 2.46% | 3.4 |
| 5927 | AT5G27320.1 \| expressed protein, similar to PrMC3 (Pinus radiata) GI:5487873 \| chr5:9629095-9631213 FORWARD \| Aliases: F21A20.30, F21A20_30 | 4.2 | 5.2 | -1.0 | -4.1 | 2.46% | 3.3 |
| 5928 | AT2G04780.2 \| Symbol: FLA7 \| fasciclin-like arabinogalactan-protein (FLA7), identical to gi_13377782_gb_AAK20860 \| chr2:1676996-1678452 FORWARD \| Aliases: F28I8.18, F28I8_18, fasciclin like arabinogalactan protein 7 | 6.9 | 8.6 | -1.7 | -4.1 | 2.46% | 3.5 |
| 5930 | AT5G41000.1 \| oligopeptide transporter OPT family protein, contains Pfam profile PF03169: OPT oligopeptide transporter protein | 4.0 | 5.1 | -1.1 | -4.1 | 2.46% | 3.4 |
| 5931 | AT5G54690.1 \| glycosyl transferase family 8 protein, contains Pfam profile: PF01501 glycosyl transferase family 8 \| chr5:22236661-22239066 REVERSE \| Aliases: K5F14.3, K5F14_3 | 3.2 | 5.7 | -2.6 | -4.1 | 2.46% | 3.4 |

| Rank | Description | Sync | Root | M | t | adj.q | B |
|---|---|---|---|---|---|---|---|
| 5934 | AT1G15800.1 \| expressed protein, similar to expressed protein [Arabidopsis thaliana] (TAIR:At1g80610.1) \| chr1:5441077-5443653 REVERSE \| Aliases: F7H2.14, F7H2_14 | 4.8 | 6.4 | -1.6 | -4.1 | 2.46% | 3.5 |
| 5936 | AT3G57480.1 \| zinc finger (C2H2 type, AN1-like) family protein, contains Pfam domain, PF00096: Zinc finger, C2H2 type; contains Pfam domain, PF01428: AN1-like Zinc finger \| chr3:21288917-21290331 REVERSE \| Aliases: T8H10.80 | 5.6 | 6.3 | -0.7 | -4.1 | 2.46% | 3.2 |
| 5940 | AT3G10340.1 \| phenylalanine ammonia-lyase, putative, similar to phenylalanine ammonia-lyase GB:S48726 (Petroselinum crispum) \| chr3:3204018-3208027 FORWARD \| Aliases: F14P13.6 | 4.8 | 6.2 | -1.4 | -4.1 | 2.47% | 3.5 |
| 5942 | AT5G67340.1 \| armadillo/beta-catenin repeat family protein / U-box domain-containing protein, contains Pfam domain, PF00514: Armadillo/beta-catenin-like repeats and Pfam, PF04564: U-box domain \| chr5:26881782-26884825 FORWARD \| Aliases: K8K14.6, K8K14_6 | 3.7 | 4.6 | -0.9 | -4.1 | 2.47% | 3.4 |
| 5949 | AT2G32080.2 \| Symbol: PUR ALPHA 1 \| PUR alpha-1 protein, identical to PUR alpha-1 GI:5081612 from (Arabidopsis thaliana); contains Pfam profile: PF04845 PurA ssDNA and RNA-binding protein \| chr2:13649314-13651243 REVERSE \| Aliases: F22D22.17, F22D22_17, PUR ALPHA 1 | 4.4 | 5.7 | -1.4 | -4.0 | 2.49% | 3.4 |
| 5950 | AT1G22335.1 \| expressed protein, ; expression supported by MPSS \| chr1:7888300-7889230 FORWARD \| Aliases: None | 2.7 | 3.6 | -0.9 | -4.0 | 2.49% | 3.4 |
| 5951 | AT3G01400.1 \| armadillo/beta-catenin repeat family protein, contains Pfam profile: PF00514 armadillo/beta-catenin-like repeats (4 copies) \| chr3:151769-153224 FORWARD \| Aliases: T13O15.4, T13O15_4 | 7.0 | 7.8 | -0.8 | -4.0 | 2.49% | 3.3 |
| 5954 | AT1G29230.1 \| Symbol: CIPK18 \| | 3.9 | 4.9 | -1.0 | -4.0 | 2.49% | 3.4 |
| 5956 | AT1G73410.1 \| Symbol: MYB54 \| myb family transcription factor (MYB54), identical to putative transcription factor (MYB54) GI:3941471 from (Arabidopsis thaliana) \| chr1:27605293-27606978 FORWARD \| Aliases: ATMYB54, BW54, MYB54, T9L24.38, T9L24_38 | 2.4 | 2.9 | -0.4 | -4.0 | 2.50% | 2.8 |
| 5958 | AT5G38700.1 \| similar to expressed protein [Arabidopsis thaliana] (TAIR:At4g02170.1); similar to disease resistance gene [Pinus sylvestris] (GB:AAR36911.1) \| chr5:15506825-15507468 FORWARD \| Aliases: MBB18.26, MBB18_26 | 2.9 | 3.7 | -0.8 | -4.0 | 2.50% | 3.3 |
| 5963 | AT2G38300.1 \| similar to myb family transcription factor [Arabidopsis thaliana] (TAIR:At2g40260.1); similar to myb family transcription factor-like [Oryza sativa (japonica cultivar-group)] (GB:BAD28879.1); contains InterPro domain Myb DNA-binding domain (InterPro:IPR001005); contains InterPro domain Myb-like DNA-binding domain, SHAQKYF class (InterPro:IPR006447) \| chr2:16051038-16053070 REVERSE \| Aliases: F16M14.25 | 2.4 | 2.7 | -0.3 | -4.0 | 2.51% | 2.6 |
| 5964 | AT4G15415.2 \| serine/threonine protein phosphatase 2A (PP2A) regulatory subunit B' (B'gamma), identical to B' regulatory subunit of PP2A (Arabidopsis thaliana) GI:2160694; similar to SWISS-PROT:Q28653 serine/threonine protein phosphatase 2A, 56 kDa regulatory subunit, delta isoform (PP2A, B subunit, B' delta isoform, PP2A, B subunit, B56 delta isoform, PP2A, B subunit, PR61 delta isoform, PP2A, B subunit, R5 delta isoform, PP2A, B subunit, B'-gamma) (Oryctolagus cuniculus); contains Pfam domain, PF01603: Protein phosphatase 2A regulatory B subunit (B56 family) | 3.1 | 3.9 | -0.7 | -4.0 | 2.52% | 3.3 |
| 5966 | AT5G52670.1 \| heavy-metal-associated domain-containing protein, contains Pfam profile PF00403: Heavy-metal-associated domain \| chr5:21380954-21381460 FORWARD \| Aliases: F6N7.16, F6N7_16 | 2.9 | 4.2 | -1.4 | -4.0 | 2.52% | 3.4 |
| 5969 | AT4G04750.1 \| similar to sugar transporter family protein [Arabidopsis thaliana] (TAIR:At4g04760.1); similar to integral membrane protein [Beta vulgaris] (GB:AAB53155.1); contains InterPro domain Sugar transporter superfamily (InterPro:IPR005829); contains InterPro domain Major facilitator superfamily (MFS) (InterPro:IPR007114); contains InterPro domain General substrate transporter (InterPro:IPR005828); contains InterPro domain Sugar transporter (InterPro:IPR003663) \| chr4:2417886-2422622 FORWARD \| Aliases: T4B21.10, T4B21_10 | 2.6 | 3.4 | -0.8 | -4.0 | 2.53% | 3.3 |
| 5971 | AT5G14700.1 \| cinnamoyl-CoA reductase-related, similar to cinnamoyl-CoA reductase from Pinus taeda (GI:17978649), Saccharum officinarum (GI:3341511) \| chr5:4740255-4743449 REVERSE \| Aliases: T9L3.2 | 3.9 | 5.2 | -1.3 | -4.0 | 2.53% | 3.3 |
| 5973 | AT4G05590.2 \| similar to expressed protein [Arabidopsis thaliana] (TAIR:At4g22310.1); similar to light induced protein like protein [Oryza sativa (japonica cultivar-group)] (GB:XP_477903.1); contains InterPro domain Protein of unknown function UPF0041 (InterPro:IPR005336) \| chr4:2907169-2908532 FORWARD \| Aliases: F6H8.10, F6H8_10 | 4.7 | 5.8 | -1.1 | -4.0 | 2.53% | 3.4 |
| 5974 | AT2G43850.2 \| ankyrin protein kinase, putative (APK1), similar to ankyrin-kinase (Medicago truncatula) gi:18700701:gb:AAL78674;contains Pfam profile PF00069: Protein kinase domain; contains Pfam profile PF00023: Ankyrin repeat \| chr2:18166259-18169121 REVERSE \| Aliases: F18O19.4 | 4.2 | 5.2 | -0.9 | -4.0 | 2.53% | 3.4 |
| 5975 | AT4G04460.1 \| aspartyl protease family protein, contains Pfam profiles: PF00026 eukaryotic aspartyl protease, PF03489 surfactant protein B, PF05184 saposin-like type B, region 1 \| chr4:2224549-2227872 FORWARD \| Aliases: T26N6.7, T26N6_7 | 2.8 | 3.8 | -1.0 | -4.0 | 2.53% | 3.3 |
| 5978 | AT1G66260.1 \| RNA and export factor-binding protein, putative, similar to GI:7159943 from (Mus musculus) (RNA 6 (4), 638-650 (2000)) \| chr1:24699303-24702093 REVERSE \| Aliases: T6J19.1, T6J19_1 | 9.8 | 10.6 | -0.8 | -4.0 | 2.53% | 3.2 |

| Rank | Description | Sync | Root | M | t | adj.q | B |
|---|---|---|---|---|---|---|---|
| 5979 | AT1G64590.1 \| short-chain dehydrogenase/reductase (SDR) family protein, contains INTERPRO family IPR002198 short-chain dehydrogenase/reductase (SDR) superfamily \| chr1:23997196-23999172 FORWARD \| Aliases: F1N19.16, F1N19_16 | 5.8 | 7.5 | -1.8 | -4.0 | 2.53% | 3.5 |
| 5980 | AT2G35680.1 \| dual specificity protein phosphatase family protein, contains Pfam profile: PF00782 dual specificity phosphatase, catalytic domain \| chr2:15003792-15006164 REVERSE \| Aliases: T20F21.13, T20F21_13 | 7.0 | 8.5 | -1.5 | -4.0 | 2.53% | 3.4 |
| 5983 | AT1G74300.1 \| esterase/lipase/thioesterase family protein, low similarity to 2-hydroxy-6-oxo-6-phenylhexa-2,4-dienoate hydrolase (Rhodococcus sp. RHA1) GI:8978311; contains Interpro entry IPR000379 \| chr1:27938959-27940650 FORWARD \| Aliases: F1O17.3, F1O17_3 | 4.4 | 5.2 | -0.8 | -4.0 | 2.54% | 3.3 |
| 5989 | AT2G30520.2 \| Symbol: RPT2 \| similar to phototropic-responsive NPH3 family protein [Arabidopsis thaliana] (TAIR:At5g48800.1); similar to Hypothetical protein [Oryza sativa (japonica cultivar-group)] (GB:AAN77296.1); contains InterPro domain NPH3 (InterPro:IPR004249) \| chr2:13009819-13012672 REVERSE \| Aliases: AT2G30510, ROOT PHOTOTROPISM 2, RPT2, T6B20.13, T6B20_13 | 3.0 | 3.3 | -0.4 | -4.0 | 2.55% | 2.7 |
| 5992 | AT4G12690.1 \| expressed protein \| chr4:7480804-7481867 FORWARD \| Aliases: T20K18.40, T20K18_40 | 3.5 | 4.2 | -0.7 | -4.0 | 2.55% | 3.2 |
| 5993 | AT2G17975.1 \| zinc finger (Ran-binding) family protein, contains Pfam domain, PF00641: Zn-finger in Ran binding protein and others \| chr2:7829320-7831052 REVERSE \| Aliases: T27K22.20 | 4.0 | 5.0 | -1.0 | -4.0 | 2.55% | 3.2 |
| 5994 | AT1G36640.1 \| expressed protein \| chr1:13854388-13854899 REVERSE \| Aliases: T15P17.13, T15P17_13 | 2.6 | 5.5 | -2.9 | -4.0 | 2.55% | 3.5 |
| 5997 | AT2G22980.2 \| Symbol: SCPL13 \| | 2.9 | 3.6 | -0.7 | -4.0 | 2.56% | 2.9 |
| 5999 | AT5G19560.1 \| member of KPP-like gene family, homolog of KPP (kinase partner protein) gene in tomato; expressed protein, contains Pfam profile PF03759: Domain of unknown function (DUF315) \| chr5:6603293-6606132 FORWARD \| Aliases: KINASE PARTNER PROTEIN LIKE, KPP LIKE, T20D1.80, T20D1_80 | 2.9 | 4.4 | -1.5 | -4.0 | 2.57% | 3.4 |
| 6000 | AT5G35960.1 \| protein kinase, putative, contains protein kinase domain, Pfam:PF00069 \| chr5:14125754-14127766 REVERSE \| Aliases: MEE13.6, MEE13_6 | 3.0 | 3.6 | -0.6 | -4.0 | 2.57% | 2.9 |
| 6005 | AT1G12820.1 \| Symbol: IPS1 \| transport inhibitor response protein, putative, E3 ubiquitin ligase SCF complex F-box subunit; similar to transport inhibitor response 1 GI:2352492 from (Arabidopsis thaliana) \| chr1:4368548-4371291 REVERSE \| Aliases: F13K23.7, F13K23_7 | 6.4 | 7.3 | -0.8 | -4.0 | 2.57% | 3.3 |
| 6013 | AT3G49840.1 \| proline-rich family protein, contains proline rich extensin domains, INTERPRO:IPR002965 \| chr3:18495527-18499283 FORWARD \| Aliases: T16K5.190 | 3.1 | 3.6 | -0.5 | -4.0 | 2.58% | 2.9 |
| 6015 | AT3G50630.1 \| Symbol: ICK2 \| kip-related protein 2 (KRP2) / cyclin-dependent kinase inhibitor 2 (ICK2) / cdc2a-interacting protein, identical to Cdc2a-interacting protein (ICK2) (cyclin-dependent kinase inhibitor) (Arabidopsis thaliana) GI:7160669 \| chr3:18811382-18812568 FORWARD \| Aliases: KRP2, Kip related protein 2, T3A5.10 | 5.3 | 6.6 | -1.3 | -4.0 | 2.58% | 3.3 |
| 6016 | AT4G00460.1 \| member of KPP-like gene family, homolog of KPP (kinase partner protein) gene in tomato; expressed protein, contains Pfam profile PF03759: Domain of unknown function (DUF315) \| chr4:211085-213697 REVERSE \| Aliases: A_IG005I10.1, A_IG005I10_1, F6N23.18, KINASE PARTNER PROTEIN LIKE, KPP LIKE | 3.2 | 3.9 | -0.7 | -4.0 | 2.58% | 3.1 |
| 6021 | AT3G49050.1 \| lipase class 3 family protein / calmodulin-binding heat-shock protein, putative, calmodulin-binding heat-shock protein, Nicotiana tabacum, PIR:T04107 \| chr3:18191953-18194737 FORWARD \| Aliases: T2J13.110 | 4.8 | 6.3 | -1.5 | -4.0 | 2.59% | 3.3 |
| 6022 | AT4G37890.2 \| zinc finger (C3HC4-type RING finger) family protein, contains Pfam profiles PF00097: Zinc finger, C3HC4 type (RING finger), PF00092: von Willebrand factor type A domain | 5.1 | 6.1 | -1.1 | -4.0 | 2.59% | 3.3 |
| 6024 | AT5G65500.1 \| protein kinase family protein, contains Pfam profile: PF00069 Eukaryotic protein kinase domain \| chr5:26198319-26201223 REVERSE \| Aliases: K19O4.3, K19O4_3 | 3.3 | 3.8 | -0.5 | -4.0 | 2.59% | 2.8 |
| 6025 | AT1G13910.1 \| leucine-rich repeat family protein, contains leucine rich-repeat (LRR) domains Pfam:PF00560, INTERPRO:IPR001611; contains similarity to Hcr2-0A (Lycopersicon esculentum) gi:3894385:gb:AAC78592 | 4.2 | 5.1 | -0.9 | -4.0 | 2.59% | 3.4 |
| 6027 | AT3G43220.1 \| phosphoinositide phosphatase family protein, contains similarity to phosphoinositide phosphatase SAC1 (Rattus norvegicus) gi:11095248:gb:AAG29810; contains Pfam domain, PF02383: SacI homology domain; identical to SAC domain protein 3 (SAC3) GI:31415722 \| chr3:15207851-15214050 REVERSE \| Aliases: F7K15.70 | 6.2 | 6.9 | -0.7 | -4.0 | 2.60% | 3.1 |
| 6028 | AT1G64210.1 \| leucine-rich repeat transmembrane protein kinase, putative, contains 1 predicted transmembrane domain; similar to receptor-like protein kinase (GI:4008006) (Arabidopsis thaliana); similar to receptor-like kinase RHG1 (GI:21239382) (Glycine max); similar to receptor-like protein kinase 3 (GI:13506810) (Lycopersicon esculentum) \| chr1:23834696-23836526 FORWARD \| Aliases: F22C12.3, F22C12_3 | 3.1 | 4.0 | -0.8 | -4.0 | 2.60% | 2.9 |

| Rank | Description | Sync | Root | M | t | adj.q | B |
|---|---|---|---|---|---|---|---|
| 6033 | AT3G54580.1 \| proline-rich extensin-like family protein, contains proline-rich extensin domains, INTERPRO:IPR002965 \| chr3:20211666-20214760 REVERSE \| Aliases: T14E10.150 | 3.0 | 4.7 | -1.7 | -4.0 | 2.61% | 3.3 |
| 6034 | AT2G18170.1 \| Symbol: ATMPK7 \| | 2.7 | 4.0 | -1.3 | -4.0 | 2.61% | 3.2 |
| 6036 | AT5G40010.1 \| AAA-type ATPase family protein, contains Pfam profile: ATPase family PF00004 \| chr5:16037446-16038990 REVERSE \| Aliases: MYH19.21, MYH19_21 | 3.1 | 4.3 | -1.2 | -4.0 | 2.61% | 3.3 |
| 6038 | AT1G33055.1 \| expressed protein \| chr1:11972220-11972570 REVERSE \| Aliases: None | 5.4 | 8.5 | -3.1 | -4.0 | 2.62% | 3.4 |
| 6039 | AT1G20100.1 \| expressed protein \| chr1:6969098-6970947 REVERSE \| Aliases: T20H2.11, T20H2_11 | 7.5 | 9.4 | -1.8 | -4.0 | 2.62% | 3.4 |
| 6040 | AT2G02850.1 \| Symbol: ARPN \| plastocyanin-like domain-containing protein / plantacyanin, putative, similar to plantacyanin GI:3395754 from (Spinacia oleracea) \| chr2:826542-827785 REVERSE \| Aliases: PLANTACYANIN, T17M13.2, T17M13_2 | 4.1 | 5.7 | -1.6 | -4.0 | 2.62% | 3.4 |
| 6044 | AT5G05860.1 \| Symbol: UGT76C2 \| UDP-glucoronosyl/UDP-glucosyl transferase family protein, contains Pfam profile: PF00201 UDP-glucoronosyl and UDP-glucosyl transferase \| chr5:1765508-1767456 FORWARD \| Aliases: MJJ3.28, MJJ3_28 | 5.4 | 6.6 | -1.2 | -4.0 | 2.63% | 3.4 |
| 6047 | AT5G57630.1 \| Symbol: CIPK21 \| | 4.3 | 5.7 | -1.4 | -4.0 | 2.64% | 3.4 |
| 6049 | AT1G14550.1 \| anionic peroxidase, putative, similar to anionic peroxidase GI:170202 from (Nicotiana sylvestris) \| chr1:4979023-4980319 FORWARD \| Aliases: F14L17.33, F14L17_33 | 2.7 | 3.6 | -0.8 | -4.0 | 2.64% | 3.3 |
| 6050 | AT2G46920.2 \| Symbol: POL \| protein phosphatase 2C family protein / PP2C family protein, similar to protein phosphatase-2c (GI:3608412) (Mesembryanthemum crystallinum); contains Pfam PF00481 : Protein phosphatase 2C domain \| chr2:19285056-19288283 REVERSE \| Aliases: F14M4.25, POLTERGEIST | 6.7 | 7.9 | -1.2 | -4.0 | 2.64% | 3.4 |
| 6053 | AT5G63800.1 \| Symbol: BGAL6 \| glycosyl hydrolase family 35 protein, similar to beta-galactosidase GI:7939621 from (Lycopersicon esculentum); contains Pfam profile PF01301: Glycosyl hydrolases family 35 \| chr5:25547359-25553062 FORWARD \| Aliases: MBK5.28, MBK5_28 | 3.6 | 4.5 | -0.9 | -4.0 | 2.65% | 3.3 |
| 6056 | AT1G75810.1 \| expressed protein \| chr1:28465326-28466007 FORWARD \| Aliases: T4O12.4, T4O12_4 | 6.0 | 7.1 | -1.1 | -4.0 | 2.65% | 3.3 |
| 6057 | AT5G01240.2 \| amino acid permease, putative, strong similarity to AUX1 GI:1531758 from (Arabidopsis thaliana); contains Pfam profile PF01490: Transmembrane amino acid transporter protein \| chr5:98532-101830 FORWARD \| Aliases: F7J8.220, F7J8_220 | 4.7 | 5.4 | -0.7 | -4.0 | 2.65% | 3.1 |
| 6058 | AT5G66070.2 \| similar to zinc finger (C3HC4-type RING finger) family protein [Arabidopsis thaliana] (TAIR:At2g17730.1); similar to putative zinc finger protein [Oryza sativa] (GB:AAL79729.1); contains InterPro domain Zn-finger, RING (InterPro:IPR001841) \| chr5:26439081-26440458 FORWARD \| Aliases: K2A18.15, K2A18_15 | 5.1 | 6.4 | -1.3 | -4.0 | 2.65% | 3.4 |
| 6060 | AT1G51640.1 \| exocyst subunit EXO70 family protein, contains Pfam domain PF03081: Exo70 exocyst complex subunit contains Pfam domain PF03081: Exo70 exocyst complex subunit; \| chr1:19153429-19155520 REVERSE \| Aliases: F19C24.13, F19C24_13 | 2.5 | 3.3 | -0.8 | -4.0 | 2.65% | 3.1 |
| 6061 | AT5G03510.1 \| zinc finger (C2H2 type) family protein, contains Pfam domain, PF00096: Zinc finger, C2H2 type \| chr5:880224-881252 FORWARD \| Aliases: F12E4.290, F12E4_290 | 3.2 | 4.2 | -1.0 | -4.0 | 2.66% | 3.0 |
| 6062 | AT1G34670.1 \| myb family transcription factor, similar to myb-related protein mixta GI:485867 from (Antirrhinum majus) | 2.8 | 3.9 | -1.1 | -4.0 | 2.66% | 3.3 |
| 6065 | AT1G18270.1 \| ketose-bisphosphate aldolase class-II family protein, low similarity to KbaY (tagatose-1,6-bisphosphate aldolase) (Escherichia coli) GI:8895753; contains Pfam profile PF01116: Fructose-bisphosphate aldolase class-II \| chr1:6283405-6293821 REVERSE \| Aliases: T10O22.24, T10O22_24 | 7.9 | 8.9 | -1.1 | -4.0 | 2.66% | 3.4 |
| 6066 | AT2G19570.1 \| Symbol: CDA1 \| cytidine deaminase (CDD) / cytidine aminohydrolase, identical to cytidine deaminase (CDD) (Arabidopsis thaliana) GI:3046700 \| chr2:8477460-8478726 REVERSE \| Aliases: AT CDA1, CYTIDINE DEAMINASE 1, DESZ, F3P11.17, F3P11_17 | 7.3 | 8.2 | -0.9 | -4.0 | 2.66% | 3.2 |
| 6067 | AT4G17220.1 \| Symbol: ATMAP70 5 \| Encodes a microtubule associated protein (MAP70-5). Expressed in all tissues. \| chr4:9656849-9659474 REVERSE \| Aliases: ATMAP70 5, DL4645C, FCAALL.385 | 3.5 | 5.9 | -2.5 | -4.0 | 2.66% | 3.4 |
| 6068 | AT4G30560.1 \| Symbol: ATCNGC9 \| cyclic nucleotide-regulated ion channel, putative, similar to cyclic nucleotide and calmodulin-regulated ion channel cngc6 GI:4581207 from (Arabidopsis thaliana) \| chr4:14926980-14929687 REVERSE \| Aliases: CNGC9, CYCLIC NUCLEOTIDE GATED CHANNEL 9, F17I23.100, F17I23_100 | 4.7 | 5.6 | -0.9 | -4.0 | 2.66% | 3.2 |
| 6069 | AT1G55810.3 \| uracil phosphoribosyltransferase, putative / UMP pyrophosphorylase, putative / UPRTase, putative, similar to SP:O65583 Uracil phosphoribosyltransferase (EC 2.4.2.9) (UMP pyrophosphorylase) (UPRTase) {Arabidopsis thaliana}; contains Pfam profile PF00485: Phosphoribulokinase / Uridine kinase family \| chr1:20864294-20867932 FORWARD \| Aliases: F20N2.21, F20N2_21 | 5.3 | 6.1 | -0.8 | -4.0 | 2.66% | 3.0 |

| Rank | Description | Sync | Root | M | t | adj.q | B |
|---|---|---|---|---|---|---|---|
| 6070 | AT4G37060.1 \| patatin, putative, similar to patatin-like latex allergen (Hevea brasiliensis)(PMID:10589016); contains patatin domain PF01734 \| chr4:17461567-17463728 REVERSE \| Aliases: AP22.93, AP22_93 | 2.9 | 4.3 | -1.4 | -4.0 | 2.66% | 3.3 |
| 6071 | AT4G37070.3 \| similar to patatin, putative [Arabidopsis thaliana] (TAIR:At4g37060.1); similar to putative patatin homolog [Oryza sativa (japonica cultivar-group)] (GB:BAD38550.1); contains InterPro domain Patatin (InterPro:IPR002641) \| chr4:17464797-17467103 REVERSE \| Aliases: AP22.83, AP22_83 | 2.9 | 4.3 | -1.4 | -4.0 | 2.66% | 3.3 |
| 6075 | AT4G35540.1 \| expressed protein, transcription factor IIIB chain BRF1, Saccharomycescerevisiae, PIR2:A44072 \| chr4:16873840-16875603 FORWARD \| Aliases: F8D20.50, F8D20_50 | 5.7 | 7.0 | -1.3 | -4.0 | 2.68% | 3.3 |
| 6076 | AT3G01560.1 \| proline-rich family protein, contains proline rich extensin domains, INTERPRO:IPR002965 \| chr3:219589-221886 FORWARD \| Aliases: F4P13.11, F4P13_11 | 5.2 | 6.0 | -0.8 | -4.0 | 2.68% | 3.3 |
| 6079 | AT1G15890.1 \| disease resistance protein (CC-NBS-LRR class), putative, domain signature CC-NBS-LRR exists, suggestive of a disease resistance protein. | 3.3 | 4.5 | -1.2 | -4.0 | 2.68% | 3.2 |
| 6080 | AT2G40940.1 \| Symbol: ERS1 \| ethylene response sensor / ethylene-responsive sensor (ERS), identical to ethylene response sensor (ERS) (Arabidopsis thaliana) GI:1046225 \| chr2:17091434-17094367 REVERSE \| Aliases: ERS, ETHYLENE RESPONSE SENSOR 1, T20B5.14, T20B5_14 | 6.4 | 8.0 | -1.6 | -4.0 | 2.69% | 3.4 |
| 6081 | AT2G26240.1 \| expressed protein, contains Pfam profile PF03647: Uncharacterised protein family (UPF0136) \| chr2:11174335-11175535 REVERSE \| Aliases: T1D16.12, T1D16_12 | 9.2 | 9.9 | -0.7 | -4.0 | 2.69% | 3.2 |
| 6084 | AT1G62280.1 \| C4-dicarboxylate transporter/malic acid transport family protein, contains Pfam profile PF03595: C4-dicarboxylate transporter/malic acid transport protein \| chr1:23010812-23012250 REVERSE \| Aliases: F19K23.20, F19K23_20 | 3.2 | 3.7 | -0.4 | -4.0 | 2.69% | 2.7 |
| 6089 | AT4G23470.3 \| similar to proline-rich family protein [Arabidopsis thaliana] (TAIR:At1g63830.2); similar to proline-rich family protein [Arabidopsis thaliana] (TAIR:At1g63830.1); similar to Unknown protein [Oryza sativa (japonica cultivar-group)] (GB:XP_468591.1); contains InterPro domain Proline-rich region (InterPro:IPR000694) \| chr4:12249081-12251406 FORWARD \| Aliases: F16G20.170, F16G20_170 | 5.4 | 7.3 | -1.9 | -4.0 | 2.69% | 3.3 |
| 6091 | AT4G09950.1 \| avirulence-responsive family protein / avirulence induced gene (AIG1) family protein, similar to AIG1 protein SP:P54120 (Arabidopsis thaliana); contains Pfam PF04548: AIG1 family; \| chr4:6234759-6236309 FORWARD \| Aliases: T5L19.80, T5L19_80 | 2.9 | 3.7 | -0.8 | -4.0 | 2.70% | 3.3 |
| 6092 | AT3G03870.2 \| expressed protein, predicted using genefinder \| chr3:995224-997417 FORWARD \| Aliases: F20H23.8, F20H23_8 | 4.9 | 5.7 | -0.8 | -4.0 | 2.70% | 3.3 |
| 6094 | AT2G29410.1 \| Symbol: MTPB1 \| zinc transporter, putative, similar to zinc transporter ZAT (Arabidopsis thaliana) gi:4206640:gb:AAD11757; similar to zinc transporter ZnT-2 (Rattus norvegicus) gi:1256378:gb:AAB02775; member of the cation diffusion facilitator (CDF) family, or cation efflux (CE) family, PMID:11500563 \| chr2:12623652-12625064 FORWARD \| Aliases: ATMTPB1, F16P2.21, F16P2_21 | 2.9 | 3.8 | -0.9 | -4.0 | 2.70% | 3.2 |
| 6095 | AT1G19940.1 \| glycosyl hydrolase family 9 protein, similar to endo-beta-1,4-D-glucanase GI:4165132 from (Lycopersicon esculentum) \| chr1:6918182-6920368 REVERSE \| Aliases: F6F9.1, F6F9_1 | 2.7 | 3.2 | -0.5 | -4.0 | 2.70% | 2.6 |
| 6097 | AT3G51860.1 \| Symbol: CAX3 \| cation exchanger, putative (CAX3), similar to high affinity calcium antiporter CAX1 (Arabidopsis thaliana) gi:9256741:gb:AAB05913; Ca2+:Cation Antiporter (CaCA) Family member PMID:11500563; non-consensus AT-acceptor splice site at intron 1 \| chr3:19250404-19253856 FORWARD \| Aliases: ATCAX3, ATEM1.11, ATHCX1, CALCIUM/PROTON EXCHANGER CAX1 LIKE PROTEIN, CAX1 LIKE, CAX3 | 3.7 | 4.6 | -0.9 | -4.0 | 2.71% | 3.3 |
| 6100 | AT3G51740.1 \| Symbol: IMK2 \| leucine-rich repeat transmembrane protein kinase, putative, brassinosteroid-insensitive protein BRI1 - Arabidopsis thaliana, PIR:T09356 \| chr3:19200028-19203028 FORWARD \| Aliases: INFLORESCENCE MERISTEM RECEPTOR LIKE KINASE 2, T18N14.120 | 2.4 | 2.9 | -0.5 | -4.0 | 2.71% | 2.9 |
| 6102 | AT2G16740.1 \| ubiquitin-conjugating enzyme, putative, strong similarity to SP:P35133 Ubiquitin-conjugating enzyme E2-17 kDa 10 (EC 6.3.2.19) (Ubiquitin- protein ligase 10) (Ubiquitin carrier protein 10) {Arabidopsis thaliana}; contains Pfam profile PF00179: Ubiquitin-conjugating enzyme | 5.7 | 6.9 | -1.3 | -4.0 | 2.71% | 3.4 |
| 6103 | AT5G19950.3 \| expressed protein \| chr5:6741111-6743775 REVERSE \| Aliases: F28I16.100, F28I16_100 | 3.4 | 4.2 | -0.9 | -4.0 | 2.71% | 3.2 |
| 6108 | AT5G14510.1 \| armadillo/beta-catenin repeat family protein, contains Pfam profile: PF00514 armadillo/beta-catenin-like repeat \| chr5:4677976-4679197 REVERSE \| Aliases: T15N1.2 | 3.3 | 4.7 | -1.4 | -4.0 | 2.72% | 3.4 |
| 6109 | AT1G63580.1 \| protein kinase-related \| chr1:23580996-23581917 FORWARD \| Aliases: F2K11.6, F2K11_6 | 2.6 | 3.1 | -0.5 | -4.0 | 2.72% | 3.0 |
| 6110 | AT1G27620.1 \| transferase family protein, similar to hypersensitivity-related gene product HSR201 - Nicotiana tabacum, EMBL:X95343; contains Pfam transferase family domain PF00248 \| chr1:9608406-9610529 FORWARD \| Aliases: T22C5.6 | 4.1 | 5.2 | -1.2 | -4.0 | 2.72% | 3.3 |
| 6111 | AT5G43550.1 \| expressed protein \| chr5:17515120-17515491 FORWARD \| Aliases: K9D7.5, K9D7_5 | 2.3 | 2.6 | -0.4 | -4.0 | 2.72% | 2.5 |

| Rank | Description | Sync | Root | M | t | adj.q | B |
|---|---|---|---|---|---|---|---|
| 6114 | AT5G03670.1 \| expressed protein \| chr5:947145-950170 FORWARD \| Aliases: F17C15.90, F17C15_90 | 2.4 | 2.9 | -0.5 | -4.0 | 2.73% | 2.8 |
| 6116 | AT3G43930.2 \| similar to expressed protein [Arabidopsis thaliana] (TAIR:At2g26270.1); similar to unknown [Oryza sativa (japonica cultivar-group)] (GB:AAO72659.1); contains InterPro domain BRCT (InterPro:IPR001357) \| chr3:15771885-15774317 REVERSE \| Aliases: T15B3.70 | 2.6 | 3.1 | -0.5 | -4.0 | 2.73% | 2.8 |
| 6118 | AT1G72950.1 \| disease resistance protein (TIR-NBS class), putative, domain signature TIR-NBS exists, suggestive of a disease resistance protein. \| chr1:27448246-27449641 FORWARD \| Aliases: F3N23.15, F3N23_15 | 3.7 | 4.3 | -0.6 | -4.0 | 2.74% | 3.0 |
| 6121 | AT5G56100.1 \| glycine-rich protein / oleosin, contains Pfam PF01277: Oleosin domain \| chr5:22734341-22734977 FORWARD \| Aliases: MDA7.16, MDA7_16 | 7.2 | 9.3 | -2.1 | -4.0 | 2.74% | 3.3 |
| 6123 | AT5G18470.1 \| curculin-like (mannose-binding) lectin family protein, contains Pfam profile: PF01453 lectin (probable mannose binding) \| chr5:6127799-6129679 FORWARD \| Aliases: T28N17.6 | 4.2 | 5.1 | -0.9 | -4.0 | 2.74% | 3.2 |
| 6127 | AT2G44940.1 \| encodes a member of the DREB subfamily A-4 of ERF/AP2 transcription factor family. The protein contains one AP2 domain. There are 17 members in this subfamily including TINY. \| chr2:18544332-18545488 FORWARD \| Aliases: T13E15.25 | 4.6 | 5.7 | -1.1 | -4.0 | 2.75% | 3.3 |
| 6132 | AT2G27500.3 \| similar to glycosyl hydrolase family 17 protein [Arabidopsis thaliana] (TAIR:At1g32860.1); similar to putative beta-1,3-glucanase [Oryza sativa (japonica cultivar-group)] (GB:NP_915593.1); similar to putative elicitor inducible beta-1,3-glucanase NtEIG-E76 [Oryza sativa (japonica cultivar-group)] (GB:BAD82640.1); similar to OSJNBa0073L04.8 [Oryza sativa (japonica cultivar-group)] (GB:XP_472401.1); contains InterPro domain Glycoside hydrolase, family 17 (InterPro:IPR000490) \| chr2:11759248-11760845 REVERSE \| Aliases: F10A12.18, F10A12_18 | 4.5 | 5.5 | -1.0 | -4.0 | 2.76% | 3.2 |
| 6137 | AT2G23510.1 \| transferase family protein, low similarity to EIG-I24 from Nicotiana tabacum (gi:10798748), 10-deacetylbaccatin III-10-O-acetyl transferase from Taxus cuspidata (gi:6746554); contains Pfam transferase family domain PF02458 \| chr2:10018597-10020613 REVERSE \| Aliases: F26B6.16, F26B6_16 | 2.7 | 3.5 | -0.8 | -4.0 | 2.77% | 3.1 |
| 6138 | AT4G15230.1 \| ABC transporter family protein, similar to pleiotropic drug resistance like protein (Nicotiana tabacum) GI:20522008, PDR5-like ABC transporter (Spirodela polyrhiza) GI:1514643; contains Pfam profile PF00005: ABC transporter \| chr4:8680182-8686880 FORWARD \| Aliases: DL3660W, FCAALL.241 | 3.1 | 3.8 | -0.7 | -4.0 | 2.77% | 3.0 |
| 6140 | AT3G62700.1 \| Symbol: ATMRP10 \| glutathione-conjugate transporter, putative, similar to glutathione-conjugate transporter AtMRP4 GI:2959767 from (Arabidopsis thaliana) \| chr3:23201243-23206738 REVERSE \| Aliases: F26K9.130 | 7.4 | 8.9 | -1.5 | -3.9 | 2.78% | 3.2 |
| 6146 | AT1G16180.1 \| TMS membrane family protein / tumour differentially expressed (TDE) family protein, contains Pfam domain, PF03348: TMS membrane protein/tumour differentially expressed protein (TDE) \| chr1:5540899-5542896 FORWARD \| Aliases: T24D18.26, T24D18_26 | 9.4 | 11.2 | -1.8 | -3.9 | 2.78% | 3.1 |
| 6147 | AT5G38790.1 \| expressed protein, predicted protein, Arabidopsis thaliana; expression supported by MPSS | 3.0 | 6.8 | -3.8 | -3.9 | 2.79% | 3.3 |
| 6149 | AT1G51620.1 \| protein kinase family protein, contains protein kinase domain, Pfam:PF00069 \| chr1:19143798-19145455 FORWARD \| Aliases: F19C24.15, F19C24_15 | 2.4 | 2.7 | -0.4 | -3.9 | 2.79% | 2.6 |
| 6150 | AT1G76560.1 \| CP12 domain-containing protein, contains Pfam domain PF02672: CP12 domain \| chr1:28733147-28733747 FORWARD \| Aliases: F14G6.16, F14G6_16 | 6.9 | 8.3 | -1.4 | -3.9 | 2.79% | 3.3 |
| 6158 | AT5G04720.1 \| Symbol: ADR1 L2 \| disease resistance protein (CC-NBS-LRR class), putative, domain signature CC-NBS-LRR exists, suggestive of a disease resistance protein. \| chr5:1360713-1363885 FORWARD \| Aliases: ADR1 L2, ADR1 LIKE 2, MUK11.4 | 7.1 | 8.5 | -1.4 | -3.9 | 2.80% | 3.3 |
| 6160 | AT5G57360.1 \| Symbol: ZTL \| F-box family protein / LOV kelch protein 1 (LKP1), E3 ubiquitin ligase SCF complex F-box subunit; identical to clock-associated PAS protein ZTL; ZEITLUPE GI:7839456, LOV kelch protein 1 GI:11610573, Adagio 1 GI:13487068 from (Arabidopsis thaliana); contains Pfam profile PF01344: Kelch motif; identical to cDNA Adagio 1 (ADO1) GI:13487067; identical to cDNA LKP1 mRNA for LOV kelch protein 1, GI:11610572 \| chr5:23258653-23261777 FORWARD \| Aliases: ADO1, FKF1 LIKE PROTEIN 2, FKL2, LKP1, LOV KELCH PROTEIN 1, MSF19.2, MSF19_2, ZEITLUPE, ZTL | 3.9 | 4.6 | -0.7 | -3.9 | 2.81% | 3.0 |
| 6162 | AT1G30090.1 \| kelch repeat-containing F-box family protein, similar to SP:O95198 Kelch-like protein 2 (Actin-binding protein Mayven) {Homo sapiens}; contains Pfam profiles PF01344: Kelch motif, PF00646: F-box domain \| chr1:10559545-10561407 REVERSE \| Aliases: T2H7.11, T2H7_11 | 7.2 | 8.6 | -1.4 | -3.9 | 2.81% | 3.2 |
| 6163 | AT5G28770.3 \| Symbol: BZO2H3 \| | 4.1 | 5.4 | -1.3 | -3.9 | 2.81% | 3.3 |
| 6167 | AT3G20090.1 \| Symbol: CYP705A18 \| cytochrome P450 family protein, similar to Cytochrome P450 93A3 (P450 CP5) (SP:O81973) {Glycine max}; contains Pfam profile: PF00067 cytochrome P450 \| chr3:7015803-7018366 FORWARD \| Aliases: MAL21.13 | 3.2 | 4.0 | -0.8 | -3.9 | 2.82% | 3.0 |
| 6168 | AT1G72470.1 \| exocyst subunit EXO70 family protein, contains Pfam domain PF03081: Exo70 exocyst complex subunit; \| chr1:27287355-27289557 FORWARD \| Aliases: T10D10.6, T10D10_6 | 4.4 | 5.2 | -0.8 | -3.9 | 2.82% | 3.1 |

| Rank | Description | Sync | Root | M | t | adj.q | B |
|---|---|---|---|---|---|---|---|
| 6170 | AT4G36160.1 \| Symbol: ANAC076 \| no apical meristem (NAM) family protein, similar to NAC2 (GI:6456751) | 2.8 | 3.2 | -0.4 | -3.9 | 2.82% | 2.5 |
| 6171 | AT1G55190.1 \| prenylated rab acceptor (PRA1) family protein, contains Pfam profile PF03208: Prenylated rab acceptor (PRA1) \| chr1:20592064-20592866 FORWARD \| Aliases: F7A10.20, F7A10_20 | 5.4 | 6.2 | -0.8 | -3.9 | 2.82% | 3.2 |
| 6172 | AT4G36980.1 \| expressed protein \| chr4:17433639-17437057 REVERSE \| Aliases: AP22.35, AP22_35 | 6.6 | 8.4 | -1.9 | -3.9 | 2.82% | 3.2 |
| 6174 | AT3G54820.1 \| Symbol: PIP2;5/PIP2D \| aquaporin, putative, similar to plasma membrane aquaporin GI:3551133 from (Raphanus sativus) \| chr3:20312999-20314988 FORWARD \| Aliases: F28P10.200, PIP2;5, PIP2D | 4.0 | 5.2 | -1.2 | -3.9 | 2.83% | 3.3 |
| 6175 | AT3G10320.1 \| expressed protein, contains Pfam domain, PF04577: Protein of unknown function (DUF563) \| chr3:3195319-3197351 REVERSE \| Aliases: F14P13.8 | 3.2 | 4.2 | -0.9 | -3.9 | 2.83% | 3.2 |
| 6177 | AT2G44420.1 \| protein N-terminal asparagine amidohydrolase family protein, similar to Protein N-terminal asparagine amidohydrolase (EC 3.5.1.-) (Protein NH2-terminal asparagine deamidase) (NTN-amidase) (PNAD) (Protein NH2- terminal asparagine amidohydrolase) (PNAA) (Swiss-Prot:Q64311) (Mus musculus) \| chr2:18337549-18339985 FORWARD \| Aliases: F4I1.23 | 3.6 | 4.3 | -0.7 | -3.9 | 2.83% | 3.1 |
| 6179 | AT3G60840.1 \| microtubule associated protein (MAP65/ASE1) family protein, low similarity to protein regulating cytokinesis 1 (PRC1) (Homo sapiens) GI:2865521; contains Pfam profile PF03999: Microtubule associated protein (MAP65/ASE1 family) \| chr3:22488548-22491438 REVERSE \| Aliases: T4C21.250 | 3.9 | 4.6 | -0.7 | -3.9 | 2.83% | 3.1 |
| 6184 | AT1G16160.1 \| Symbol: WAKL5 \| protein kinase family protein, contains similarity to wall-associated kinase 4 GI:3355308 from (Arabidopsis thaliana) \| chr1:5535967-5538263 FORWARD \| Aliases: T24D18.24, T24D18_24, WALL ASSOCIATED KINASE LIKE 5 | 2.8 | 3.3 | -0.5 | -3.9 | 2.85% | 2.9 |
| 6185 | AT3G46960.1 \| similar to HUA enhancer 2 (HEN2) / DExH-box RNA helicase, putative [Arabidopsis thaliana] (TAIR:At2g06990.1); similar to superkiller viralicidic activity 2-like (S. cerevisiae) [Homo sapiens] (GB:CAI41730.1); similar to superkiller viralicidic activity 2-like (S. cerevisiae) [Homo sapiens] (GB:CAI41863.1); similar to similar to putative DEAD/DEAH box helicase [Schizosaccharomyces pombe] [Dictyostelium discoideum] (GB:AAO51181.1); similar to superkiller viralicidic activity 2-like [Rattus norvegicus] (GB:NP_998724.1); similar to superkiller viralicidic activity 2-like [Mus musculus] (GB:NP_067312.1); contains InterPro domain Helicase, C-terminal (InterPro:IPR001650); contains InterPro domain DEAD/DEAH box helicase (InterPro:IPR001410) \| chr3:17301878-17309052 REVERSE \| Aliases: F13I12.10 | 7.2 | 7.7 | -0.5 | -3.9 | 2.85% | 2.9 |
| 6187 | AT1G71710.1 \| inositol polyphosphate 5-phosphatase, putative, similar to inositol polyphosphate 5-phosphatase I (Arabidopsis thaliana) GI:10444261 \| chr1:26977229-26980493 REVERSE \| Aliases: F14O23.9, F14O23_9 | 3.7 | 4.8 | -1.1 | -3.9 | 2.85% | 3.2 |
| 6189 | AT2G43160.4 \| similar to epsin N-terminal homology (ENTH) domain-containing protein [Arabidopsis thaliana] (TAIR:At3g59290.1); similar to OSJNBa0083N12.8 [Oryza sativa (japonica cultivar-group)] (GB:XP_473754.1); contains InterPro domain Epsin N-terminal homology (InterPro:IPR001026) \| chr2:17955139-17960798 FORWARD \| Aliases: F14B2.10 | 5.6 | 6.3 | -0.7 | -3.9 | 2.86% | 3.1 |
| 6192 | AT4G32450.1 \| pentatricopeptide (PPR) repeat-containing protein, contains Pfam profile PF01535: PPR repeat \| chr4:15661098-15662711 FORWARD \| Aliases: F8B4.150, F8B4_150 | 3.4 | 5.8 | -2.5 | -3.9 | 2.86% | 3.2 |
| 6195 | AT5G24290.2 \| integral membrane family protein, contains Pfam domain PF01988: Integral membrane protein \| chr5:8263135-8265658 REVERSE \| Aliases: MOP9.11, MOP9_11 | 3.8 | 4.6 | -0.8 | -3.9 | 2.87% | 3.1 |
| 6196 | AT1G09920.1 \| TRAF-type zinc finger-related, contains Pfam PF02176: TRAF-type zinc finger; similar to TNF receptor associated factor 4 (GI:7274404) (Mus musculus) (GI:7274404); similar to Cysteine rich motif Associated to Ring and Traf domains protein (mCART1)(GI:1041446) (Mus musculus) \| chr1:3224686-3227068 REVERSE \| Aliases: F21M12.31, F21M12_31 | 6.1 | 7.1 | -1.0 | -3.9 | 2.87% | 3.3 |
| 6197 | AT1G79410.1 \| transporter-related, low similarity to organic anion transporter 3 (Rattus norvegicus) GI:5545293; contains Pfam profile PF00083: major facilitator superfamily protein \| chr1:29872773-29874526 REVERSE \| Aliases: T8K14.17, T8K14_17 | 3.8 | 6.1 | -2.3 | -3.9 | 2.87% | 3.3 |
| 6199 | AT5G44820.1 \| expressed protein, similar to unknown protein (pir::T04881) \| chr5:18112940-18114812 REVERSE \| Aliases: K23L20.17, K23L20_17 | 2.7 | 2.9 | -0.3 | -3.9 | 2.87% | 2.4 |
| 6202 | AT1G14540.1 \| anionic peroxidase, putative, similar to lignin forming anionic peroxidase (Nicotiana sylvestris) SWISS-PROT: Q02200 \| chr1:4974062-4975595 REVERSE \| Aliases: F14L17.32, F14L17_32 | 3.1 | 3.7 | -0.5 | -3.9 | 2.87% | 2.9 |
| 6204 | AT3G51920.1 \| Symbol: CAM9 \| calmodulin-9 (CAM9), identical to calmodulin 9 GI:5825602 from (Arabidopsis thaliana); contains Pfam profile PF00036: EF hand \| chr3:19279026-19280366 REVERSE \| Aliases: CALMODULIN 9, F4F15.30 | 9.4 | 10.2 | -0.8 | -3.9 | 2.88% | 3.1 |
| 6205 | AT5G60930.1 \| chromosome-associated kinesin, putative, microtubule-associated motor KIF4 , Mus musculus, PIR:A54803 \| chr5:24532624-24539737 REVERSE \| Aliases: MSL3.50, MSL3_50 | 3.9 | 4.8 | -0.8 | -3.9 | 2.88% | 3.0 |

| Rank | Description | Sync | Root | M | t | adj.q | B |
|---|---|---|---|---|---|---|---|
| 6208 | AT5G41090.1 \| Symbol: ANAC095 \| no apical meristem (NAM) family protein, contains Pfam PF02365: No apical meristem (NAM) domain; similar to unknown protein (emb:CAB88055.1) \| chr5:16462992-16463765 FORWARD \| Aliases: ANAC095, MEE6.16, MEE6_16 | 2.4 | 2.7 | -0.3 | -3.9 | 2.89% | 2.5 |
| 6211 | AT1G56160.1 \| myb family transcription factor (MYB72), contains Pfam profile: PF00249 myb-like DNA-binding domain; identical to cDNA putative transcription factor (MYB72) mRNA, partial cds GI:3941501 \| chr1:21026049-21027252 FORWARD \| Aliases: F14G9.22 | 3.0 | 4.8 | -1.8 | -3.9 | 2.89% | 3.2 |
| 6214 | AT3G26000.1 \| F-box family protein, contains Pfam PF00646: F-box domain; similar to SKP1 interacting partner 2 (SKIP2) TIGR_Ath1:At5g67250 \| chr3:9508015-9510495 REVERSE \| Aliases: MPE11.17 | 6.4 | 7.2 | -0.7 | -3.9 | 2.90% | 2.9 |
| 6215 | AT5G25130.1 \| Symbol: CYP71B12 \| cytochrome P450 family protein, CYTOCHROME P450 71B1 - Thlaspi arvense, EMBL:L24438 \| chr5:8668302-8670107 FORWARD \| Aliases: F21J6.2 | 3.2 | 3.9 | -0.7 | -3.9 | 2.90% | 3.1 |
| 6221 | AT3G55660.1 \| member of KPP-like gene family, homolog of KPP (kinase partner protein) gene in tomato; expressed protein, contains Pfam profile PF03759: Domain of unknown function (DUF315) \| chr3:20659921-20662367 REVERSE \| Aliases: F1I16.70, KINASE PARTNER PROTEIN LIKE, KPP LIKE | 3.4 | 3.9 | -0.5 | -3.9 | 2.91% | 2.8 |
| 6223 | AT4G01440.1 \| nodulin MtN21 family protein, similar to MtN21 GI:2598575 (root nodule development) from (Medicago truncatula) \| chr4:596399-598718 FORWARD \| Aliases: F3D13.3, F3D13_3 | 4.3 | 5.6 | -1.3 | -3.9 | 2.91% | 3.2 |
| 6227 | AT1G66680.1 \| Symbol: AR401 \| S locus-linked protein, putative, similar to S locus-linked protein SLL2 (Brassica napus) GI:1518113 \| chr1:24869758-24872638 REVERSE \| Aliases: AR401, F4N21.18, F4N21_18 | 7.9 | 9.2 | -1.2 | -3.9 | 2.92% | 3.1 |
| 6228 | AT4G29890.1 \| choline monooxygenase, putative (CMO-like), similar to Choline monooxygenase, chloroplast precursor (EC 1.14.15.7) from {Amaranthus tricolor} SP:Q93XE1, {Beta vulgaris} SP:O22553; contains Pfam Rieske (2Fe-2S) domain PF00355 | 5.0 | 6.3 | -1.4 | -3.9 | 2.92% | 3.3 |
| 6229 | AT5G50260.1 \| cysteine proteinase, putative, similar to cysteine endopeptidase precursor CysEP GI:2944446 from (Ricinus communis) \| chr5:20472543-20474255 FORWARD \| Aliases: K6A12.12, K6A12_12 | 2.7 | 3.4 | -0.6 | -3.9 | 2.92% | 3.0 |
| 6230 | AT3G44490.1 \| histone deacetylase-related / HD-related, similar to SP:O09106 Histone deacetylase 1 (HD1) {Mus musculus} \| chr3:16108466-16109373 FORWARD \| Aliases: F14L2.40 | 6.3 | 6.9 | -0.6 | -3.9 | 2.92% | 3.0 |
| 6231 | AT5G55620.1 \| expressed protein, similar to unknown protein (gb:AAF04428.1) \| chr5:22547430-22548165 FORWARD \| Aliases: MDF20.6, MDF20_6 | 2.6 | 3.0 | -0.3 | -3.9 | 2.92% | 2.5 |
| 6234 | AT3G05120.1 \| expressed protein, low similarity to PrMC3 (Pinus radiata) GI:5487873 \| chr3:1430483-1432784 FORWARD \| Aliases: T12H1.8, T12H1_8 | 5.2 | 6.0 | -0.7 | -3.9 | 2.93% | 3.1 |
| 6239 | AT5G08480.2 \| VQ motif-containing protein, contains PF05678: VQ motif \| chr5:2743621-2745112 FORWARD \| Aliases: F8L15.9 | 2.1 | 2.5 | -0.4 | -3.9 | 2.93% | 2.6 |
| 6240 | AT1G54130.1 \| Symbol: RSH3 \| RelA/SpoT protein, putative (RSH3), identical to RSH3 (RelA/SpoT homolog) GI:7141308 from (Arabidopsis thaliana); contains Pfam profiles PF01966: HD domain, PF04607: Region found in RelA / SpoT proteins \| chr1:20214540-20217720 FORWARD \| Aliases: AT RSH3, F15I1.23, F15I1_23, RELA/SPOT HOMOLOG 3, RSH3 | 7.9 | 9.0 | -1.1 | -3.9 | 2.93% | 3.1 |
| 6242 | AT1G15460.1 \| anion exchange family protein, member of the PF:00955 Anion exchanger family \| chr1:5309996-5313424 REVERSE \| Aliases: F9L1.41, F9L1_41 | 2.4 | 2.9 | -0.5 | -3.9 | 2.93% | 2.8 |
| 6244 | AT5G44190.1 \| Symbol: GLK2 \| myb family transcription factor (GLK2), contains Pfam profile: PF00249 myb-like DNA-binding domain \| chr5:17815596-17818050 FORWARD \| Aliases: ATGLK2, GOLDEN2 LIKE 2, GPRI2, MLN1.11, MLN1_11 | 3.5 | 4.2 | -0.6 | -3.9 | 2.93% | 3.0 |
| 6246 | AT3G26030.1 \| Symbol: ATB' DELTA \| serine/threonine protein phosphatase 2A (PP2A) regulatory subunit B', putative, similar to SWISS-PROT:Q28653 serine/threonine protein phosphatase 2A, 56 kDa regulatory subunit, delta isoform (PP2A, B subunit, B' delta isoform, PP2A, B subunit, B56 delta isoform, PP2A, B subunit, PR61 delta isoform, PP2A, B subunit, R5 delta isoform, PP2A, B subunit, B'-gamma) (Oryctolagus cuniculus); contains Pfam domain, PF01603: Protein phosphatase 2A regulatory B subunit (B56 family) | 5.5 | 6.6 | -1.1 | -3.9 | 2.93% | 3.1 |
| 6247 | AT5G65160.1 \| tetratricopeptide repeat (TPR)-containing protein, contains Pfam profile PF00515 TPR Domain \| chr5:26048419-26051040 REVERSE \| Aliases: MQN23.9, MQN23_9 | 4.2 | 5.1 | -0.9 | -3.9 | 2.93% | 3.1 |
| 6248 | AT2G42030.1 \| zinc finger (C3HC4-type RING finger) family protein, contains Pfam profile: PF00097 zinc finger, C3HC4 type (RING finger) \| chr2:17545986-17548044 REVERSE \| Aliases: T6D20.8, T6D20_8 | 6.5 | 7.3 | -0.8 | -3.9 | 2.94% | 3.0 |
| 6249 | AT1G15280.2 \| glycine-rich protein \| chr1:5252332-5256746 FORWARD \| Aliases: F9L1.22, F9L1_22 | 3.6 | 4.2 | -0.6 | -3.9 | 2.94% | 3.0 |

| Rank | Description | Sync | Root | M | t | adj.q | B |
|---|---|---|---|---|---|---|---|
| 6250 | AT1G68230.1 \| reticulon family protein (RTNLB14), contains Pfam profile PF02453: Reticulon \| chr1:25575848-25576584 FORWARD \| Aliases: T22E19.14, T22E19_14 | 3.2 | 3.9 | -0.8 | -3.9 | 2.95% | 3.2 |
| 6253 | AT1G77990.1 \| Symbol: AST56 \| similar to sulfate transporter [Arabidopsis thaliana] (TAIR:At5g10180.1); similar to low affinity sulphate transporter [Stylosanthes hamata] (GB:CAA57831.1); similar to Putative sulfate transporter [Oryza sativa (japonica cultivar-group)] (GB:AAN59769.1); similar to Putative sulfate transporter [Oryza sativa (japonica cultivar-group)] (GB:AAN59770.1); similar to sulfate permease [Brassica juncea] (GB:CAA11413.1); contains InterPro domain Sulphate transporter (InterPro:IPR001902); contains InterPro domain Sulfate transporter/antisigma-factor antagonist STAS (InterPro:IPR002645) | 3.6 | 5.0 | -1.4 | -3.9 | 2.95% | 3.3 |
| 6255 | AT5G07100.2 \| Symbol: WRKY26 \| | 3.1 | 4.6 | -1.4 | -3.9 | 2.95% | 3.0 |
| 6257 | AT1G53300.1 \| thioredoxin family protein, contains Pfam profiles PF00085: Thioredoxin, PF00515: TPR Domain; similar to tetratricopeptide repeat protein 2 (GI:7248701) (Drosophila melanogaster); similar to DnaJ homolog subfamily C member 7 (Tetratricopeptide repeat protein 2) (TPR repeat protein 2) (Swiss-Prot:Q99615) (Homo sapiens) \| chr1:19883090-19886043 FORWARD \| Aliases: F12M16.20, F12M16_20 | 2.7 | 3.4 | -0.7 | -3.9 | 2.96% | 3.0 |
| 6266 | AT3G25655.1 \| expressed protein \| chr3:9338769-9339302 REVERSE \| Aliases: None | 3.0 | 4.3 | -1.3 | -3.9 | 2.97% | 3.2 |
| 6267 | AT4G17910.1 \| zinc finger (C3HC4-type RING finger) family protein / pentatricopeptide (PPR) repeat-containing protein, contains Pfam domains PF01535: PPR repeat and PF00097: Zinc finger, C3HC4 type (RING finger) \| chr4:9948866-9960416 REVERSE \| Aliases: T6K21.90, T6K21_90 | 3.1 | 3.6 | -0.4 | -3.9 | 2.97% | 2.6 |
| 6268 | AT5G17040.1 \| UDP-glucoronosyl/UDP-glucosyl transferase family protein, similar to UDP glucose:flavonoid 3-o-glucosyltransferase GI:13620861 from (Vitis vinifera); contains Pfam profile: PF00201 UDP-glucoronosyl and UDP-glucosyl transferase \| chr5:5605287-5606973 REVERSE \| Aliases: F2K13.190, F2K13_190 | 3.4 | 4.3 | -0.9 | -3.9 | 2.97% | 3.1 |
| 6270 | AT5G23390.1 \| expressed protein, contains Pfam profile: PF04842 plant protein of unknown function (DUF639) \| chr5:7870516-7874508 REVERSE \| Aliases: T32G24.2, T32G24_2 | 5.9 | 7.6 | -1.8 | -3.9 | 2.97% | 3.2 |
| 6274 | AT3G49940.1 \| LOB domain protein 38 / lateral organ boundaries domain protein 38 (LBD38), identical to SP:Q9SN23 LOB domain protein 38 {Arabidopsis thaliana} \| chr3:18525328-18526460 FORWARD \| Aliases: F3A4.20 | 7.1 | 8.4 | -1.3 | -3.9 | 2.97% | 3.1 |
| 6275 | AT2G21180.1 \| expressed protein \| chr2:9081741-9082461 REVERSE \| Aliases: F26H11.6, F26H11_6 | 6.1 | 6.8 | -0.7 | -3.9 | 2.97% | 3.0 |
| 6277 | AT1G61080.1 \| proline-rich family protein \| chr1:22496859-22500684 REVERSE \| Aliases: T7P1.21, T7P1_21 | 2.6 | 3.2 | -0.6 | -3.9 | 2.98% | 2.9 |
| 6279 | AT3G54860.1 \| Symbol: ATVPS33 \| vacuolar protein sorting protein, putative, similar to Swiss-Prot:Q63615 vacuolar protein sorting 33A (r-vps33a) (Rattus norvegicus); contains Pfam domain, PF00995: Sec1 family \| chr3:20335068-20340828 REVERSE \| Aliases: F28P10.160, VPS33 | 4.1 | 5.5 | -1.5 | -3.9 | 2.98% | 3.2 |
| 6281 | AT1G51280.2 \| similar to Toll-Interleukin-Resistance (TIR) domain-containing protein [Arabidopsis thaliana] (TAIR:At5g45000.1); similar to R 1 protein [Glycine max] (GB:AAO23076.1); contains InterPro domain TIR domain (InterPro:IPR000157) | 3.4 | 4.7 | -1.3 | -3.9 | 2.99% | 3.1 |
| 6285 | AT3G27100.1 \| expressed protein \| chr3:9995832-9997249 REVERSE \| Aliases: MOJ10.18 | 7.9 | 9.1 | -1.2 | -3.9 | 3.00% | 3.2 |
| 6286 | AT4G27260.1 \| Symbol: GH3.5 \| encodes an IAA-amido synthase that conjugates Asp and other amino acids to auxin in vitro. Lines carrying insertions in this gene are hypersensitive to auxin. \| chr4:13653624-13656210 FORWARD \| Aliases: GH3.5, M4I22.70, M4I22_70 | 4.4 | 5.0 | -0.6 | -3.9 | 3.00% | 3.0 |
| 6289 | AT3G07780.1 \| expressed protein \| chr3:2484476-2487030 FORWARD \| Aliases: MLP3.23 | 8.3 | 9.7 | -1.4 | -3.9 | 3.01% | 3.0 |
| 6290 | AT2G43535.1 \| Encodes a defensin-like (DEFL) family protein. \| chr2:18078050-18078739 FORWARD \| Aliases: None | 4.2 | 6.1 | -1.9 | -3.9 | 3.01% | 3.2 |
| 6293 | AT4G31750.1 \| protein phosphatase 2C, putative / PP2C, putative, protein phosphatase 2C, Medicago sativa, PID:g2582800 | 8.2 | 8.9 | -0.8 | -3.9 | 3.02% | 2.9 |
| 6296 | AT1G72180.1 \| leucine-rich repeat transmembrane protein kinase, putative, similar to GI:3641252 from (Malus x domestica) (Plant Mol. Biol. 40 (6), 945-957 (1999)) \| chr1:27166482-27171092 FORWARD \| Aliases: T9N14.3, T9N14_3 | 4.4 | 5.3 | -0.9 | -3.9 | 3.04% | 3.1 |
| 6305 | AT3G52240.1 \| expressed protein \| chr3:19383880-19386858 REVERSE \| Aliases: T25B15.10 | 4.4 | 5.0 | -0.6 | -3.9 | 3.05% | 3.0 |
| 6307 | AT4G38230.1 \| Symbol: CPK26 \| calcium-dependent protein kinase, putative / CDPK, putative, calmodulin-domain protein kinase CDPK isoform 6 (Arabidopsis thaliana) gi:1399275:gb:AAB03246; contains protein kinase domain, Pfam:PF00069; contains EF hand domain (calcium-binding EF-hand), Pfam:PF00036, INTERPRO:IPR002048 \| chr4:17928671-17931176 REVERSE \| Aliases: F20D10.350, F20D10_350 | 2.8 | 3.3 | -0.5 | -3.9 | 3.05% | 2.7 |
| 6312 | AT3G58050.1 \| expressed protein \| chr3:21503516-21508134 FORWARD \| Aliases: T10K17.260 | 4.5 | 5.1 | -0.6 | -3.9 | 3.08% | 2.9 |
| 6314 | AT1G80830.1 \| Symbol: NRAMP1 \| | 9.2 | 10.5 | -1.3 | -3.9 | 3.09% | 2.7 |

| Rank | Description | Sync | Root | M | t | adj.q | B |
|---|---|---|---|---|---|---|---|
| 6319 | AT5G01870.1 \| lipid transfer protein, putative, similar to lipid transfer protein 6 from Arabidopsis thaliana (gi:8571927); contains Pfam protease inhibitor/seed storage/LTP family domain PF00234 \| chr5:337174-337812 FORWARD \| Aliases: T20L15.140, T20L15_140 | 3.3 | 5.2 | -1.9 | -3.9 | 3.10% | 3.2 |
| 6324 | AT5G16880.3 \| VHS domain-containing protein / GAT domain-containing protein, weak similarity to hepatocyte growth factor-regulated tyrosine kinase substrate (Homo sapiens) GI:2731383; contains Pfam profiles PF00790: VHS domain, PF03127: GAT domain \| chr5:5548993-5551466 FORWARD \| Aliases: F2K13.30, F2K13_30 | 7.7 | 9.3 | -1.6 | -3.8 | 3.11% | 3.1 |
| 6326 | AT3G03690.1 \| glycosyltransferase family 14 protein / core-2/I-branching enzyme family protein, contains Pfam profile: PF02485 Core-2/I-Branching enzyme \| chr3:911245-913684 REVERSE \| Aliases: T12J13.3, T12J13_3 | 3.0 | 3.5 | -0.5 | -3.8 | 3.11% | 2.8 |
| 6327 | AT4G25880.3 \| similar to pumilio/Puf RNA-binding domain-containing protein [Arabidopsis thaliana] (TAIR:At3g20250.1); similar to pumilio domain-containing protein PPD1 [Populus tremula x Populus tremuloides] (GB:AAF71823.1); contains InterPro domain Pumilio/Puf RNA-binding (InterPro:IPR001313) | 5.3 | 6.4 | -1.1 | -3.8 | 3.11% | 3.1 |
| 6328 | AT5G18590.2 \| kelch repeat-containing protein, identical to RanGAP1 interacting protein (GI:21950739) (Arabidopsis thaliana); similar to Tip elongation aberrant protein 1 (Cell polarity protein tea1) (SP:P87061) (Schizosaccharomyces pombe); contains Pfam PF01344: Kelch motif (5 repeats) \| chr5:6177967-6182708 REVERSE \| Aliases: T28N17.70, T28N17_70 | 7.1 | 8.0 | -0.8 | -3.8 | 3.11% | 2.8 |
| 6331 | AT1G61130.1 \| serine carboxypeptidase S10 family protein, similar to Serine carboxypeptidase II-3 precursor (SP:P52711) (CP-MII.3. (Hordeum vulgare) \| chr1:22532689-22535449 REVERSE \| Aliases: F11P17.14, F11P17_14, SCPL32 | 3.0 | 3.9 | -1.0 | -3.8 | 3.11% | 3.0 |
| 6334 | AT4G31320.1 \| auxin-responsive protein, putative / small auxin up RNA (SAUR_C), similar to auxin-induced protein TGSAUR22 (GI:10185820) (Tulipa gesnerian); similar to auxin-induced protein 15A (SP:P33081) from (Glycine max) \| chr4:15193999-15194568 REVERSE \| Aliases: F8F16.140, F8F16_140 | 2.8 | 3.8 | -1.0 | -3.8 | 3.12% | 3.0 |
| 6338 | AT3G52070.2 \| expressed protein \| chr3:19323731-19324444 FORWARD \| Aliases: F4F15.180 | 4.9 | 6.4 | -1.4 | -3.8 | 3.13% | 3.2 |
| 6339 | AT2G18650.1 \| zinc finger (C3HC4-type RING finger) family protein, similar to RING-H2 finger protein RHX1a (Arabidopsis thaliana) GI:3790591; contains Pfam profile PF00097: Zinc finger, C3HC4 type (RING finger) \| chr2:8093942-8095213 REVERSE \| Aliases: MSF3.3, MSF3_3 | 2.9 | 3.6 | -0.8 | -3.8 | 3.13% | 2.9 |
| 6343 | AT5G36270.1 \| dehydroascorbate reductase, putative, similar to dehydroascorbate reductase {Spinacia oleracea} gi:10952511 gb:AF195783, PMID:11148269 \| chr5:14310161-14310814 FORWARD \| Aliases: T30G6.13, T30G6_13 | 4.2 | 5.3 | -1.1 | -3.8 | 3.14% | 3.2 |
| 6345 | AT1G79310.1 \| latex-abundant protein, putative (AMC4) / caspase family protein, similar to latex-abundant protein (Hevea brasiliensis) gb:AAD13216; contains Pfam profile PF00656: ICE-like protease (caspase) p20 domain \| chr1:29838879-29840438 FORWARD \| Aliases: YUP8H12R.7, YUP8H12R_7 | 3.0 | 3.5 | -0.4 | -3.8 | 3.14% | 2.7 |
| 6347 | AT5G18830.2 \| Symbol: SPL7 \| squamosa promoter-binding protein-like 7 (SPL7), identical to squamosa promoter binding protein-like 7 (Arabidopsis thaliana) GI:5931635; contains Pfam profile PF03110: SBP domain \| chr5:6276024-6280683 FORWARD \| Aliases: F17K4.80, F17K4_80, SQUAMOSA PROMOTER BINDING PROTEIN LIKE 7 | 4.2 | 5.2 | -1.0 | -3.8 | 3.15% | 3.1 |
| 6352 | AT1G61560.1 \| Symbol: MLO6 \| seven transmembrane MLO family protein / MLO-like protein 6 (MLO6), idenctical to membrane protein Mlo6 (Arabidopsis thaliana) gi:14091582:gb:AAK53799; similar to MLO protein SWISS-PROT:P93766, NCBI_gi:1877221 (Hordeum vulgare)(Barley); contains Pfam profile | 2.6 | 3.1 | -0.4 | -3.8 | 3.16% | 2.7 |
| 6353 | AT3G47980.1 \| integral membrane HPP family protein, contains Pfam domain, PF04982: HPP family \| chr3:17721462-17723786 FORWARD \| Aliases: T17F15.150 | 3.9 | 4.5 | -0.6 | -3.8 | 3.16% | 2.8 |
| 6354 | AT2G25710.2 \| Symbol: HCS1 \| holocarboxylase synthetase 1 (HCS1), identical to holocarboxylase synthetase 1 (hcs1) (Arabidopsis thaliana) GI:19698365 | 5.2 | 6.8 | -1.6 | -3.8 | 3.16% | 3.2 |
| 6357 | AT2G25735.1 \| expressed protein \| chr2:10982138-10982837 REVERSE \| Aliases: None | 2.8 | 3.7 | -0.9 | -3.8 | 3.16% | 3.1 |
| 6359 | AT5G10930.1 \| Symbol: CIPK5 \| CBL-interacting protein kinase 5 (CIPK5), identical to CBL-interacting protein kinase 5 GP:9280632:gb:AAF86504 (Arabidopsis thaliana) \| chr5:3445367-3447115 REVERSE \| Aliases: CBL INTERACTING PROTEIN KINASE 5, T30N20.200, T30N20_200 | 5.4 | 6.3 | -0.9 | -3.8 | 3.16% | 2.9 |
| 6360 | AT1G07260.1 \| UDP-glucoronosyl/UDP-glucosyl transferase family protein, contains Pfam profile: PF00201 UDP-glucoronosyl and UDP-glucosyl transferase \| chr1:2227593-2229318 REVERSE \| Aliases: F10K1.3, F10K1_3 | 5.1 | 6.0 | -0.9 | -3.8 | 3.16% | 3.0 |
| 6362 | AT3G09850.1 \| D111/G-patch domain-containing protein, contains Pfam profile PF01585: G-patch domain \| chr3:3023216-3025856 REVERSE \| Aliases: F8A24.10 | 8.3 | 8.9 | -0.7 | -3.8 | 3.17% | 3.0 |
| 6363 | AT5G14650.1 \| polygalacturonase, putative / pectinase, putative, similar to polygalacturonase PG1 GP:5669846, PG2 GI:5669848 from (Glycine max); contains PF00295: Glycosyl hydrolases family 28 (polygalacturonases) \| chr5:4724453-4726516 FORWARD \| Aliases: T15N1.140, T15N1_140 | 3.0 | 3.6 | -0.6 | -3.8 | 3.17% | 2.8 |

| Rank | Description | Sync | Root | M | t | adj.q | B |
|---|---|---|---|---|---|---|---|
| 6368 | AT1G32690.1 \| expressed protein, similar to hypothetical protein GB:AAC61817 GI:3668085 from (Arabidopsis thaliana) \| chr1:11820888-11821690 REVERSE \| Aliases: F6N18.23, F6N18_23 | 5.7 | 6.7 | -1.0 | -3.8 | 3.18% | 3.1 |
| 6369 | AT5G10470.1 \| kinesin motor protein-related, TH65 protein, Arabidopsis thaliana, EMBL:AJ001729; contains Pfam profile PF00225: Kinesin motor domain \| chr5:3290122-3297249 REVERSE \| Aliases: F12B17.180, F12B17_180 | 3.7 | 5.0 | -1.4 | -3.8 | 3.18% | 3.1 |
| 6374 | AT1G79860.1 \| member of KPP-like gene family, homolog of KPP (kinase partner protein) gene in tomato; expressed protein, contains Pfam profile PF03759: Domain of unknown function (DUF315) \| chr1:30046992-30049068 REVERSE \| Aliases: F19K16.18, F19K16_18, KINASE PARTNER PROTEIN LIKE, KPP LIKE | 2.5 | 3.8 | -1.3 | -3.8 | 3.19% | 3.2 |
| 6375 | AT2G31350.2 \| hydroxyacylglutathione hydrolase, putative / glyoxalase II, putative, similar to glyoxalase II isozyme (Arabidopsis thaliana) gi:2570338:gb:AAC49865 \| chr2:13375370-13378070 FORWARD \| Aliases: T28P16.16, T28P16_16 | 9.1 | 10.0 | -0.9 | -3.8 | 3.19% | 2.9 |
| 6378 | AT1G22070.1 \| Symbol: TGA3 \| bZIP family transcription factor (TGA3), identical to transcription factor GI:304113 from (Arabidopsis thaliana) | 6.9 | 7.8 | -0.9 | -3.8 | 3.19% | 2.9 |
| 6379 | AT5G54660.1 \| heat shock protein-related, contains weak similarity to 17.6 kDa class I heat shock protein (HSP 17.6) (Swiss-Prot:P13853) (Arabidopsis thaliana) \| chr5:22221131-22222378 FORWARD \| Aliases: MRB17.16, MRB17_16 | 6.6 | 7.6 | -1.0 | -3.8 | 3.19% | 2.9 |
| 6381 | AT5G04040.1 \| patatin-related, contains Patatin domain PF01734 | 6.0 | 7.0 | -0.9 | -3.8 | 3.20% | 3.1 |
| 6387 | AT1G10800.1 \| expressed protein \| chr1:3598159-3598592 REVERSE \| Aliases: T16B5.6 | 5.1 | 5.8 | -0.7 | -3.8 | 3.20% | 2.9 |
| 6391 | AT1G78260.2 \| RNA recognition motif (RRM)-containing protein, similar to RNA recognition motif-containing protein SEB-4 GI:8895698 from (Xenopus laevis); contains InterPro entry IPR000504: RNA-binding region RNP-1 (RNA recognition motif) (RRM) \| chr1:29451878-29455297 FORWARD \| Aliases: F3F9.20, F3F9_20 | 3.7 | 5.1 | -1.4 | -3.8 | 3.21% | 3.2 |
| 6392 | AT3G62980.1 \| Symbol: TIR1 \| transport inhibitor response 1 (TIR1) (FBL1), E3 ubiquitin ligase SCF complex F-box subunit; identical to transport inhibitor response 1 GI:2352492 from (Arabidopsis thaliana) \| chr3:23284091-23287350 REVERSE \| Aliases: T20O10.80, TRANSPORT INHIBITOR RESPONSE 1 | 5.2 | 5.9 | -0.7 | -3.8 | 3.21% | 2.9 |
| 6395 | AT1G17240.1 \| leucine-rich repeat family protein, contains leucine rich-repeat (LRR) domains Pfam:PF00560, INTERPRO:IPR001611; similar to Hcr2-0B (Lycopersicon esculentum) gi:3894387:gb:AAC78593 \| chr1:5896409-5898710 REVERSE \| Aliases: F20D23.6, F20D23_6 | 2.8 | 3.5 | -0.7 | -3.8 | 3.22% | 2.8 |
| 6397 | AT2G41180.1 \| sigA-binding protein-related, low similarity to SigA binding protein (Arabidopsis thaliana) GI:6980074; contains Pfam domain PF05678: VQ motif \| chr2:17172269-17173103 FORWARD \| Aliases: T3K9.5, T3K9_5 | 3.9 | 4.7 | -0.8 | -3.8 | 3.23% | 3.0 |
| 6399 | AT3G42640.1 \| ATPase, plasma membrane-type, putative / proton pump, putative, strong similarity to P-type H+-ATPase from (Lycopersicon esculentum) GI:1621440, (Solanum tuberosum) GI:435001, SP:Q03194 {Nicotiana plumbaginifolia}; contains InterPro accession IPR001757: ATPase, E1-E2 type | 3.9 | 5.3 | -1.4 | -3.8 | 3.23% | 3.1 |
| 6400 | AT1G30990.1 \| major latex protein-related / MLP-related, low similarity to major latex protein {Papaver somniferum}(GI:20810); similar to ESTs gb:W43264, gb:H36765, gb:R30617, gb:AA042095, and emb:Z27272 ; contains Pfam profile PF00407: Pathogenesis-related protein Bet v I family \| chr1:11052111-11053044 FORWARD \| Aliases: F17F8.9 | 2.6 | 3.3 | -0.7 | -3.8 | 3.23% | 2.9 |
| 6403 | AT4G25110.2 \| similar to latex-abundant family protein (AMC1) / caspase family protein [Arabidopsis thaliana] (TAIR:At1g02170.1); similar to putative metacaspase [Oryza sativa (japonica cultivar-group)] (GB:AAR06365.1); contains InterPro domain Zn-finger, LSD1 type (InterPro:IPR005735); contains InterPro domain Proline-rich region (InterPro:IPR000694); contains InterPro domain Caspase-1, p20 (InterPro:IPR001309) \| chr4:12887528-12890271 REVERSE \| Aliases: F24A6.7 | 2.8 | 3.5 | -0.7 | -3.8 | 3.24% | 2.9 |
| 6404 | AT2G22310.1 \| Symbol: ATUBP4 \| | 6.2 | 7.0 | -0.8 | -3.8 | 3.24% | 3.0 |
| 6406 | AT1G48840.1 \| expressed protein, contains Pfam profile: PF04842 plant protein of unknown function (DUF639) \| chr1:18065203-18068948 FORWARD \| Aliases: T24P22.6, T24P22_6 | 4.5 | 5.3 | -0.8 | -3.8 | 3.24% | 3.0 |
| 6409 | AT5G12870.1 \| myb family transcription factor (MYB46), contains PFAM profile: myb DNA binding domain PF00249 \| chr5:4062727-4064995 REVERSE \| Aliases: T24H18.40, T24H18_40 | 2.6 | 3.2 | -0.6 | -3.8 | 3.24% | 2.8 |
| 6410 | NA | 11.3 | 12.0 | -0.7 | -3.8 | 3.24% | 1.9 |
| 6411 | AT2G40180.1 \| protein phosphatase 2C, putative / PP2C, putative, contains PF00481: Protein phosphatase 2C domain; identical to protein phosphatase 2C (GI:4587992) (Arabidopsis thaliana) \| chr2:16789600-16791225 FORWARD \| Aliases: ATHPP2C5, T7M7.17 | 3.2 | 4.1 | -0.9 | -3.8 | 3.24% | 2.8 |
| 6412 | AT4G20820.1 \| FAD-binding domain-containing protein, similar to SP:P30986 reticuline oxidase precursor (Berberine-bridge-forming enzyme) (BBE) (Tetrahydroprotoberberine synthase) (Eschscholzia californica); contains PF01565 FAD binding domain \| chr4:11150171-11151769 FORWARD \| Aliases: F21C20.170, F21C20_170 | 3.0 | 3.7 | -0.7 | -3.8 | 3.24% | 2.9 |

| Rank | Description | Sync | Root | M | t | adj.q | B |
|---|---|---|---|---|---|---|---|
| 6413 | AT4G27430.1 \| Symbol: CIP7 \| COP1-interacting protein 7 (CIP7), identical to COP1-Interacting Protein 7 (CIP7) GI:3327868 from (Arabidopsis thaliana) | 4.4 | 5.4 | -0.9 | -3.8 | 3.24% | 3.0 |
| 6414 | AT1G48110.1 \| expressed protein, contains Pfam profile PF04146: YT521-B-like family \| chr1:17772787-17777120 REVERSE \| Aliases: F21D18.17, F21D18_17 | 4.6 | 5.4 | -0.8 | -3.8 | 3.24% | 2.9 |
| 6416 | AT2G18160.1 \| Symbol: GBF5 \| bZIP transcription factor family protein, contains a bZIP transcription factor basic domain signature (PDOC00036) \| chr2:7905094-7906324 REVERSE \| Aliases: F8D23.6, F8D23_6, TRANSCRIPTION FACTOR GBF5 | 8.1 | 9.6 | -1.5 | -3.8 | 3.24% | 2.7 |
| 6417 | AT5G06750.1 \| protein phosphatase 2C family protein / PP2C family protein, similar to Ser/Thr protein phosphatase 2C (PP2C6) (GI:15020818) (Arabidopsis thaliana); similar to protein phosphatase 2C (GI:3608412) (Mesembryanthemum crystallinum); contains Pfam PF00481 : Protein phosphatase 2C domain; \| chr5:2086267-2088529 REVERSE \| Aliases: MPH15.11, MPH15_11 | 8.2 | 9.2 | -1.0 | -3.8 | 3.25% | 3.0 |
| 6419 | AT2G35870.1 \| expressed protein \| chr2:15068888-15069190 REVERSE \| Aliases: F11F19.22, F11F19_22 | 2.9 | 3.4 | -0.5 | -3.8 | 3.26% | 2.6 |
| 6420 | AT3G50190.1 \| expressed protein, similar to expressed protein [Arabidopsis thaliana] (TAIR:At3g50130.1); similar to expressed protein [Arabidopsis thaliana] (TAIR:At3g50140.1); similar to hypothetical protein [Oryza sativa (japonica cultivar-group)] (GB:XP_481746.1); contains InterPro domain Plant protein of unknown function (InterPro:IPR004158); contains InterPro domain Proline-rich region (InterPro:IPR000694) | 2.8 | 4.1 | -1.3 | -3.8 | 3.26% | 3.0 |
| 6422 | AT3G09220.1 \| laccase family protein / diphenol oxidase family protein, similar to laccase (Pinus taeda)(GI:13661201), laccase GB:CAA74105 (Populus balsamifera subsp. trichocarpa); contains Pfam profile: Multicopper oxidases \| chr3:2827245-2830548 REVERSE \| Aliases: F3L24.9 | 4.1 | 5.2 | -1.1 | -3.8 | 3.26% | 3.1 |
| 6424 | AT1G53340.1 \| DC1 domain-containing protein, contains Pfam protein PF03107 DC1 domain \| chr1:19902596-19904599 REVERSE \| Aliases: F12M16.24, F12M16_24 | 4.4 | 6.4 | -2.0 | -3.8 | 3.27% | 3.1 |
| 6426 | AT3G29760.1 \| NLI interacting factor (NIF) family protein, contains Pfam profile PF03031: NLI interacting factor \| chr3:11591984-11594312 REVERSE \| Aliases: MOD1.21 | 4.5 | 5.4 | -0.9 | -3.8 | 3.27% | 3.0 |
| 6433 | AT5G55590.1 \| pectinesterase family protein, contains Pfam profile: PF01095 pectinesterase \| chr5:22537137-22538925 FORWARD \| Aliases: MDF20.3, MDF20_3 | 3.3 | 4.6 | -1.2 | -3.8 | 3.29% | 3.1 |
| 6434 | AT3G52120.1 \| SWAP (Suppressor-of-White-APricot)/surp domain-containing protein / D111/G-patch domain-containing protein, contains Pfam profiles PF01585: G-patch domain, PF01805: Surp module | 3.9 | 4.5 | -0.6 | -3.8 | 3.29% | 2.9 |
| 6438 | AT1G10560.1 \| armadillo/beta-catenin repeat family protein / U-box domain-containing protein, contains Pfam domain, PF00514: Armadillo/beta-catenin-like repeats and Pfam, PF04564: U-box domain \| chr1:3484581-3486966 FORWARD \| Aliases: T10O24.19, T10O24_19 | 4.7 | 5.8 | -1.1 | -3.8 | 3.30% | 3.0 |
| 6439 | AT1G75220.1 \| integral membrane protein, putative, strong similarity to integral membrane protein GI:1209756 from (Beta vulgaris); contains Pfam profile PF00083: major facilitator superfamily protein \| chr1:28232851-28236492 REVERSE \| Aliases: F22H5.6, F22H5_6 | 6.5 | 8.5 | -1.9 | -3.8 | 3.30% | 3.1 |
| 6440 | AT1G59650.1 \| Symbol: CW14 \| expressed protein, ; supporting cDNA gi:6520232:dbj:AB028233.1: \| chr1:21923410-21926588 FORWARD \| Aliases: CW14, T30E16.22, T30E16_22 | 7.4 | 8.0 | -0.6 | -3.8 | 3.30% | 2.9 |
| 6441 | AT2G38790.1 \| expressed protein \| chr2:16221269-16222342 REVERSE \| Aliases: F13I13.2 | 4.0 | 5.0 | -1.0 | -3.8 | 3.31% | 3.0 |
| 6444 | AT1G56020.1 \| expressed protein \| chr1:20955811-20957077 FORWARD \| Aliases: F14J16.33 | 3.6 | 4.5 | -0.9 | -3.8 | 3.31% | 3.1 |
| 6445 | AT4G12020.1 \| Symbol: WRKY19 \| | 3.4 | 5.5 | -2.1 | -3.8 | 3.31% | 3.0 |
| 6449 | AT2G34180.1 \| Symbol: CIPK13 \| | 2.8 | 3.5 | -0.7 | -3.8 | 3.31% | 3.0 |
| 6451 | AT1G72900.1 \| disease resistance protein (TIR-NBS class), putative, domain signature TIR-NBS exists, suggestive of a disease resistance protein. \| chr1:27435226-27437443 FORWARD \| Aliases: F3N23.10, F3N23_10 | 4.1 | 5.7 | -1.5 | -3.8 | 3.31% | 3.0 |
| 6453 | AT2G31750.1 \| UDP-glucoronosyl/UDP-glucosyl transferase family protein, contains Pfam profile: PF00201 UDP-glucoronosyl and UDP-glucosyl transferase \| chr2:13504310-13507763 FORWARD \| Aliases: F20M17.21, F20M17_21 | 3.7 | 4.8 | -1.1 | -3.8 | 3.32% | 3.1 |
| 6458 | AT3G53990.2 \| universal stress protein (USP) family protein, contains Pfam PF00582: universal stress protein family \| chr3:20000315-20002084 REVERSE \| Aliases: F5K20.290, F5K20_290 | 6.3 | 8.2 | -1.9 | -3.8 | 3.33% | 2.9 |
| 6461 | AT1G66180.1 \| aspartyl protease family protein, contains Pfam PF00026: Eukaryotic aspartyl protease profile; similar to CND41, chloroplast nucleoid DNA binding protein (GI:2541876) (Nicotiana tabacum) \| chr1:24650851-24652476 FORWARD \| Aliases: F15E12.7, F15E12_7 | 6.9 | 7.8 | -0.8 | -3.8 | 3.33% | 2.6 |

| Rank | Description | Sync | Root | M | t | adj.q | B |
|---|---|---|---|---|---|---|---|
| 6462 | AT1G53680.1 \| Symbol: ATGSTU28 \| glutathione S-transferase, putative, similar to GI:2853219 from (Carica papaya) \| chr1:20042026-20042785 FORWARD \| Aliases: F22G10.22, F22G10_22 | 3.6 | 4.7 | -1.1 | -3.8 | 3.33% | 2.9 |
| 6463 | AT5G65380.1 \| ripening-responsive protein, putative, similar to ripening regulated protein DDTFR18 (Lycopersicon esculentum) GI:12231296; contains Pfam profile PF01554: Uncharacterized membrane protein family \| chr5:26140215-26143665 REVERSE \| Aliases: MNA5.11, MNA5_11 | 3.6 | 4.7 | -1.1 | -3.8 | 3.33% | 3.1 |
| 6468 | AT1G69790.1 \| protein kinase, putative, similar to protein kinase APK1A (Arabidopsis thaliana) SWISS-PROT:Q06548 \| chr1:26270422-26272646 FORWARD \| Aliases: T6C23.1, T6C23_1 | 3.7 | 5.4 | -1.6 | -3.8 | 3.33% | 3.1 |
| 6470 | AT3G24050.1 \| GATA transcription factor 1 (GATA-1), identical to GATA transcription factor 1 (AtGATA-1) GB:Y13648 (Arabidopsis thaliana) \| chr3:8685929-8687684 FORWARD \| Aliases: F14O13.7 | 4.5 | 6.5 | -2.0 | -3.8 | 3.33% | 3.1 |
| 6473 | AT1G06640.2 \| 2-oxoglutarate-dependent dioxygenase, putative, similar to 2A6 (GI:599622) and tomato ethylene synthesis regulatory protein E8 (SP:P10967); contains Pfam profile: PF00671 Iron/Ascorbate oxidoreductase family \| chr1:2032344-2034013 FORWARD \| Aliases: F12K11.27, F12K11_27 | 10.7 | 11.6 | -0.9 | -3.8 | 3.34% | 2.5 |
| 6479 | AT1G53910.2 \| Symbol: RAP2.12 \| similar to AP2 domain-containing protein RAP2.2 (RAP2.2) [Arabidopsis thaliana] (TAIR:At3g14230.2); similar to AP2 domain-containing protein RAP2.2 (RAP2.2) [Arabidopsis thaliana] (TAIR:At3g14230.3); similar to AP2 domain-containing protein RAP2.2 (RAP2.2) [Arabidopsis thaliana] (TAIR:At3g14230.1); similar to ethylene transcription factor [Fagus sylvatica] (GB:CAE54591.1); contains InterPro domain Pathogenesis-related transcriptional factor and ERF (InterPro:IPR001471) \| chr1:20138781-20140609 FORWARD \| Aliases: RAP2.12, T18A20.14, T18A20_14 | 4.6 | 6.7 | -2.2 | -3.8 | 3.35% | 3.1 |
| 6483 | AT5G11700.1 \| glycine-rich protein, predicted protein, Arabidopsis thaliana \| chr5:3762467-3771124 REVERSE \| Aliases: T22P22.90, T22P22_90 | 3.6 | 4.6 | -1.0 | -3.8 | 3.36% | 3.0 |
| 6487 | AT5G53430.1 \| Symbol: SDG29 \| PHD finger family protein / SET domain-containing protein (TX5), contains Pfam domain, PF00628: PHD-finger and PF00856: SET domain; identical to cDNA trithorax 5 (TX5) partial cds GI:16118406 \| chr5:21694372-21700720 FORWARD \| Aliases: MYN8.4, MYN8_4, SET DOMAIN GROUP 29 | 3.9 | 4.6 | -0.7 | -3.8 | 3.38% | 2.9 |
| 6490 | AT3G48940.1 \| remorin family protein, contains Pfam domain, PF03766: Remorin, N-terminal region and Pfam domain, PF03763: Remorin, C-terminal region \| chr3:18153848-18155148 REVERSE \| Aliases: T2J13.220 | 2.9 | 3.6 | -0.7 | -3.8 | 3.38% | 2.9 |
| 6491 | AT5G49780.1 \| leucine-rich repeat transmembrane protein kinase, putative \| chr5:20245813-20250321 FORWARD \| Aliases: K2I5.15, K2I5_15 | 3.5 | 4.6 | -1.1 | -3.8 | 3.38% | 3.0 |
| 6494 | AT5G45650.1 \| subtilase family protein, contains Pfam domain, PF00082: Subtilase family; contains Pfam domain, PF02225: protease associated (PA) domain \| chr5:18530658-18536095 REVERSE \| Aliases: MRA19.5, MRA19_5 | 2.6 | 3.3 | -0.7 | -3.8 | 3.38% | 2.9 |
| 6495 | AT3G50500.1 \| Symbol: SPK 2 2 \| protein kinase, putative, similar to abscisic acid-activated protein kinase (Vicia faba) gi:6739629:gb:AAF27340 \| chr3:18752571-18755054 REVERSE \| Aliases: PROTEIN KINASE, T20E23.100 | 6.2 | 8.2 | -2.1 | -3.8 | 3.38% | 3.0 |
| 6497 | AT3G20310.1 \| Symbol: ATERF 7/ATERF7 \| | 6.0 | 7.4 | -1.4 | -3.8 | 3.39% | 3.0 |
| 6499 | AT3G19450.1 \| Symbol: CAD4 \| cinnamyl-alcohol dehydrogenase (CAD), identical to SP:P48523 Cinnamyl-alcohol dehydrogenase (EC 1.1.1.195) (CAD) (Arabidopsis thaliana) \| chr3:6744769-6747220 FORWARD \| Aliases: ATCAD4, CAD, CAD C, CINNAMYL ALCOHOL DEHYDROGENASE 4, MLD14.30 | 8.2 | 10.1 | -1.9 | -3.8 | 3.39% | 3.1 |
| 6505 | AT5G27950.1 \| kinesin motor protein-related, kinesin heavy chain-like protein, potato, PIR:T07397 \| chr5:9984055-9987994 FORWARD \| Aliases: F15F15.20, F15F15_20 | 3.1 | 4.2 | -1.1 | -3.8 | 3.41% | 2.8 |
| 6506 | AT1G21200.1 \| expressed protein \| chr1:7421206-7423132 FORWARD \| Aliases: F16F4.11, F16F4_11 | 3.1 | 4.6 | -1.5 | -3.8 | 3.41% | 3.0 |
| 6507 | AT1G02730.1 \| Symbol: ATCSLD5 \| cellulose synthase family protein, similar to cellulose synthase catalytic subunit (gi:13925881) from Nicotiana alata, cellulose synthase-4 (gi:9622880) from Zea mays \| chr1:594590-598657 REVERSE \| Aliases: CSLD5, T14P4.29 | 4.5 | 5.2 | -0.7 | -3.8 | 3.42% | 2.9 |
| 6508 | AT3G26744.4 \| Symbol: ICE1 \| similar to basic helix-loop-helix (bHLH) family protein / F-box family protein [Arabidopsis thaliana] (TAIR:At1g12860.1); similar to transcription factor ICE1-like [Oryza sativa (japonica cultivar-group)] (GB:BAD88203.1); similar to OSJNBa0093F16.27 [Oryza sativa (japonica cultivar-group)] (GB:NP_914885.1) \| chr3:9834843-9836454 REVERSE \| Aliases: INDUCER OF CBF EXPRESSION 1, MDJ14.8 | 6.6 | 7.2 | -0.6 | -3.8 | 3.42% | 2.8 |
| 6509 | AT2G02870.3 \| similar to kelch repeat-containing F-box family protein [Arabidopsis thaliana] (TAIR:At1g14330.1); similar to kelch repeat-containing F-box-like [Oryza sativa (japonica cultivar-group)] (GB:XP_467797.1); contains InterPro domain Kelch motif (InterPro:IPR006651); contains InterPro domain Cyclin-like F-box (InterPro:IPR001810); contains InterPro domain Kelch repeat (InterPro:IPR006652) \| chr2:838035-840152 FORWARD \| Aliases: T17M13.4, T17M13_4 | 6.5 | 7.6 | -1.2 | -3.8 | 3.42% | 2.8 |
| 6510 | AT5G33390.1 \| glycine-rich protein, similar to nuclear antigen EBNA-1 (GI:3342234) {Cercopithecine herpesvirus 15} \| chr5:12679019-12680050 FORWARD \| Aliases: None | 5.4 | 6.5 | -1.0 | -3.8 | 3.43% | 3.1 |

| Rank | Description | Sync | Root | M | t | adj.q | B |
|---|---|---|---|---|---|---|---|
| 6511 | AT2G46550.1 \| expressed protein \| chr2:19119061-19121224 REVERSE \| Aliases: F13A10.8 | 6.8 | 7.8 | -0.9 | -3.8 | 3.43% | 2.9 |
| 6514 | AT1G49240.1 \| Symbol: ACT8 \| actin 8 (ACT8), identical to SP:Q96293 Actin 8 {Arabidopsis thaliana}; nearly identical to SP:Q96292 Actin 2 (Arabidopsis thaliana) GI:1669387, and to At3g18780 | 7.4 | 9.5 | -2.2 | -3.8 | 3.44% | 3.1 |
| 6515 | AT5G17300.1 \| myb family transcription factor, similar to CCA1 (Arabidopsis thaliana) GI:4090569; contains Pfam profile PF00249: Myb-like DNA-binding domain \| chr5:5690229-5692904 REVERSE \| Aliases: MKP11.15, MKP11_15 | 4.0 | 4.6 | -0.6 | -3.8 | 3.45% | 2.7 |
| 6517 | AT2G28460.1 \| DC1 domain-containing protein, contains Pfam profile PF03107: DC1 domain | 3.2 | 4.8 | -1.6 | -3.8 | 3.45% | 3.1 |
| 6519 | AT5G16910.1 \| Symbol: ATCSLD2 \| cellulose synthase family protein, similar to gi:2827143 cellulose synthase catalytic subunit, Arabidopsis thaliana, gi:9622886 cellulose synthase-7 from Zea mays \| chr5:5561682-5565583 FORWARD \| Aliases: CSLD2, F2K13.60, F2K13_60 | 6.4 | 7.2 | -0.8 | -3.8 | 3.45% | 2.9 |
| 6520 | AT3G14690.1 \| Symbol: CYP72A15 \| cytochrome P450, putative, similar to GB:Q05047 from (Catharanthus roseus) \| chr3:4937386-4939472 FORWARD \| Aliases: MIE1.19 | 7.0 | 8.8 | -1.8 | -3.8 | 3.46% | 3.0 |
| 6521 | AT5G17450.2 \| heavy-metal-associated domain-containing protein / copper chaperone (CCH)-related, similar to copper homeostasis factor (Arabidopsis thaliana)(GI:3168840), and farnesylated proteins GMFP7 (Glycine max)(GI:4097573), ATFP7 (GI:4097555), and ATFP6 (GI:4097553); contains heavy-metal-associated domain PF00403 \| chr5:5755350-5756880 FORWARD \| Aliases: K3M16.20, K3M16_20 | 2.8 | 3.8 | -1.0 | -3.8 | 3.46% | 2.8 |
| 6522 | AT5G04020.1 \| calmodulin-binding protein-related (PICBP), contains similarity to potato calmodulin-binding protein PCBP GI:17933110 from (Solanum tuberosum) \| chr5:1081610-1086545 REVERSE \| Aliases: F8F6.230, F8F6_230 | 6.0 | 6.8 | -0.8 | -3.8 | 3.47% | 2.9 |
| 6525 | AT2G21210.1 \| auxin-responsive protein, putative, similar to small auxin-up regulated protein SAUR (GI:3043536) (Raphanus sativus) \| chr2:9092291-9092957 REVERSE \| Aliases: F26H11.3, F26H11_3 | 2.5 | 2.8 | -0.3 | -3.8 | 3.47% | 2.3 |
| 6529 | AT3G01150.1 \| Symbol: PTB \| polypyrimidine tract-binding protein, putative / heterogeneous nuclear ribonucleoprotein, putative, similar to Polypyrimidine tract-binding protein 1 (PTB) (Heterogeneous nuclear ribonucleoprotein I) (hnRNP I) from {Sus scrofa} SP:Q29099, {Mus musculus} SP:P17225; contains InterPro entry IPR000504: RNA-binding region RNP-1 (RNA recognition motif) (RRM) \| chr3:51644-54513 FORWARD \| Aliases: POLYPYRIMIDINE TRACT BINDING, T4P13.16, T4P13_16 | 4.8 | 6.1 | -1.3 | -3.8 | 3.47% | 3.0 |
| 6530 | AT1G69340.1 \| appr-1-p processing enzyme family protein, contains Pfam domain PF01661: Appr-1-p processing enzyme family \| chr1:26069419-26073484 FORWARD \| Aliases: F10D13.28, F10D13_28 | 6.4 | 7.5 | -1.0 | -3.7 | 3.48% | 2.8 |
| 6532 | AT3G12560.1 \| Symbol: TRFL9 \| telomeric DNA-binding protein, putative, similar to telomeric DNA-binding protein 1 (Arabidopsis thaliana) gi:13641340:gb:AAK31590 \| chr3:3981991-3985557 REVERSE \| Aliases: T2E22.13, TRF LIKE 9 | 4.9 | 5.9 | -0.9 | -3.7 | 3.49% | 2.9 |
| 6533 | AT1G25240.1 \| epsin N-terminal homology (ENTH) domain-containing protein / clathrin assembly protein-related, contains Pfam PF01417: ENTH domain. ENTH (Epsin N-terminal homology) domain; similar to clathrin assembly protein AP180 (GI:6492344) (Xenopus laevis) \| chr1:8845218-8846480 FORWARD \| Aliases: F4F7.37, F4F7_37 | 2.5 | 3.1 | -0.6 | -3.7 | 3.49% | 2.7 |
| 6534 | AT3G26700.1 \| protein kinase family protein, contains protein kinase domain, Pfam:PF00069 \| chr3:9811187-9813901 FORWARD \| Aliases: MLJ15.17 | 3.1 | 3.7 | -0.6 | -3.7 | 3.49% | 2.8 |
| 6537 | AT2G37300.1 \| expressed protein, and genefinder \| chr2:15669678-15670524 REVERSE \| Aliases: F3G5.9, F3G5_9 | 2.9 | 3.5 | -0.5 | -3.7 | 3.51% | 2.7 |
| 6538 | AT1G61810.2 \| glycosyl hydrolase family 1 protein, contains Pfam PF00232 : Glycosyl hydrolase family 1 domain; TIGRFAM TIGR01233: 6-phospho-beta-galactosidase; similar to beta-glucosidase (GI:3820531) (Pinus contorta); similar to beta-glucosidase GI:804655 from (Hordeum vulgare) \| chr1:22833698-22834606 FORWARD \| Aliases: T13M11.19, T13M11_19 | 3.7 | 4.6 | -0.9 | -3.7 | 3.51% | 2.9 |
| 6541 | AT5G56620.1 \| Symbol: ANAC099 \| no apical meristem (NAM) family protein, contains Pfam PF02365: No apical meristem (NAM) domain; \| chr5:22935747-22938365 REVERSE \| Aliases: ANAC099, MIK19.7, MIK19_7 | 2.8 | 3.2 | -0.4 | -3.7 | 3.52% | 2.2 |
| 6544 | AT4G25940.1 \| epsin N-terminal homology (ENTH) domain-containing protein, contains Pfam PF01417: ENTH domain. ENTH (Epsin N-terminal homology) domain; similar to Chain B, Crystal Structure Of N-Terminal Domain Of Drosophila Ap180 (GP:13399617) (Drosophila melanogaster); supporting cDNA gi:20465326:gb:AY096427.1: \| chr4:13169650-13172842 REVERSE \| Aliases: F20B18.50, F20B18_50 | 2.4 | 3.0 | -0.6 | -3.7 | 3.53% | 2.6 |
| 6546 | AT5G15680.1 \| expressed protein \| chr5:5101191-5110796 REVERSE \| Aliases: F14F8.60, F14F8_60 | 3.8 | 4.3 | -0.6 | -3.7 | 3.53% | 2.7 |
| 6549 | AT4G01680.2 \| Symbol: MYB55 \| similar to myb family transcription factor (MYB61) [Arabidopsis thaliana] (TAIR:At1g09540.1); similar to MYB2 [Dendrobium sp. XMW-2002-2] (GB:AAO49411.1); contains InterPro domain Myb DNA-binding domain (InterPro:IPR001005) | 2.3 | 2.7 | -0.4 | -3.7 | 3.54% | 2.4 |

| Rank | Description | Sync | Root | M | t | adj.q | B |
|---|---|---|---|---|---|---|---|
| 6553 | AT3G44700.1 \| expressed protein, contains Pfam profile PF03140: Plant protein of unknown function; expression supported by MPSS \| chr3:16247330-16248723 REVERSE \| Aliases: T18B22.100 | 2.2 | 2.6 | -0.5 | -3.7 | 3.55% | 2.2 |
| 6554 | AT5G01550.1 \| lectin protein kinase, putative, similar to receptor lectin kinase 3 (Arabidopsis thaliana) gi:4100060:gb:AAD00733; contains protein kinase domain, Pfam:PF00069; contains legume lectins alpha and beta domains, Pfam:PF00138 and Pfam:PF00139 \| chr5:214516-216582 REVERSE \| Aliases: F7A7.70, F7A7_70 | 3.3 | 4.3 | -1.0 | -3.7 | 3.55% | 3.0 |
| 6556 | AT1G80320.1 \| oxidoreductase, 2OG-Fe(II) oxygenase family protein, similar to GS-AOP loci (GI:16118889, GI:16118887, GI:16118891, GI:16118893); contains PF03171 2OG-Fe(II) oxygenase superfamily domain \| chr1:30201674-30202788 FORWARD \| Aliases: F5I6.7, F5I6_7 | 2.6 | 4.0 | -1.4 | -3.7 | 3.55% | 2.8 |
| 6560 | AT4G28350.1 \| lectin protein kinase family protein, contains Pfam domains, PF00138: Legume lectins alpha domain, PF00139: Legume lectins beta domain and PF00069: Protein kinase domain \| chr4:14026583-14028628 FORWARD \| Aliases: F20O9.40, F20O9_40 | 3.5 | 4.7 | -1.3 | -3.7 | 3.55% | 2.9 |
| 6561 | AT5G18500.2 \| similar to protein kinase family protein [Arabidopsis thaliana] (TAIR:At3g17420.1); similar to putative kinase [Oryza sativa (japonica cultivar-group)] (GB:AAP54446.1); contains InterPro domain Serine/threonine protein kinase, active site (InterPro:IPR008271); contains InterPro domain Tyrosine protein kinase (InterPro:IPR001245); contains InterPro domain Serine/threonine protein kinase (InterPro:IPR002290); contains InterPro domain Protein kinase (InterPro:IPR000719) \| chr5:6138440-6141632 FORWARD \| Aliases: T28N17.1 | 4.8 | 6.5 | -1.7 | -3.7 | 3.55% | 3.0 |
| 6565 | AT5G40170.1 \| disease resistance family protein, contains leucine rich-repeat (LRR) domains Pfam:PF00560, INTERPRO:IPR001611; similar to Cf-4 (Lycopersicon hirsutum) gi:2808683:emb:CAA05268 \| chr5:16082319-16084802 REVERSE \| Aliases: MSN9.70, MSN9_70 | 4.7 | 5.6 | -0.9 | -3.7 | 3.56% | 3.0 |
| 6571 | AT3G58780.1 \| Symbol: SHP1 \| agamous-like MADS box protein AGL1 / shatterproof 1 (AGL1) (SHP1), identical to SP:P29381 Agamous-like MADS box protein AGL1 (Protein Shatterproof 1) {Arabidopsis thaliana} \| chr3:21749437-21752884 FORWARD \| Aliases: AGL1, SHATTERPROOF 1, T20N10.130 | 3.8 | 4.5 | -0.7 | -3.7 | 3.57% | 2.9 |
| 6573 | AT2G39720.1 \| Symbol: RHC2A \| zinc finger (C3HC4-type RING finger) family protein, contains Pfam domain, PF00097: Zinc finger, C3HC4 type (RING finger) \| chr2:16574287-16575900 REVERSE \| Aliases: RING H2 FINGER PROTEIN RHC2A, T5I7.2, T5I7_2 | 5.9 | 6.6 | -0.6 | -3.7 | 3.57% | 2.8 |
| 6581 | AT1G65180.1 \| DC1 domain-containing protein, contains Pfam profile PF03107: DC1 domain | 2.5 | 3.1 | -0.7 | -3.7 | 3.58% | 2.7 |
| 6583 | AT2G34610.1 \| expressed protein \| chr2:14582471-14583596 FORWARD \| Aliases: T31E10.5, T31E10_5 | 3.8 | 4.4 | -0.6 | -3.7 | 3.58% | 2.6 |
| 6587 | AT5G61550.1 \| protein kinase family protein, contains Pfam profile: PF00069 Eukaryotic protein kinase domain; protein kinase 1, PnPK1, Populus nigra, EMBL:AB041503 \| chr5:24765328-24768980 FORWARD \| Aliases: K11J9.12, K11J9_12 | 2.5 | 3.2 | -0.7 | -3.7 | 3.59% | 2.7 |
| 6589 | AT1G04360.1 \| zinc finger (C3HC4-type RING finger) family protein, contains Pfam profile: PF00097 zinc finger, C3HC4 type (RING finger) \| chr1:1167456-1168877 REVERSE \| Aliases: F19P19.21, F19P19_21 | 2.7 | 3.6 | -0.9 | -3.7 | 3.60% | 2.9 |
| 6590 | AT3G04030.2 \| myb family transcription factor, contains Pfam profile: PF00249 myb-like DNA-binding domain \| chr3:1042889-1044685 REVERSE \| Aliases: T11I18.14, T11I18_14 | 2.4 | 2.9 | -0.5 | -3.7 | 3.60% | 2.7 |
| 6591 | AT5G44580.1 \| expressed protein \| chr5:17996538-17997634 FORWARD \| Aliases: K15C23.2, K15C23_2 | 2.7 | 3.3 | -0.6 | -3.7 | 3.60% | 2.5 |
| 6593 | AT1G08180.1 \| expressed protein \| chr1:2564738-2565073 REVERSE \| Aliases: T23G18.4, T23G18_4 | 3.0 | 4.0 | -1.0 | -3.7 | 3.60% | 2.8 |
| 6594 | AT2G33480.1 \| Symbol: ANAC041 \| no apical meristem (NAM) family protein, contains Pfam PF02365: No apical meristem (NAM) domain; \| chr2:14188283-14189478 FORWARD \| Aliases: ANAC041, F4P9.25, F4P9_25 | 7.2 | 9.4 | -2.2 | -3.7 | 3.60% | 2.9 |
| 6596 | AT1G64170.1 \| Symbol: ATCHX16 \| cation/hydrogen exchanger, putative (CHX16), monovalent cation:proton antiporter family 2 (CPA2) member, PMID:11500563 \| chr1:23818839-23821956 REVERSE \| Aliases: CHX16, F22C12.7, F22C12_7 | 4.3 | 5.1 | -0.8 | -3.7 | 3.60% | 2.9 |
| 6597 | AT1G18140.1 \| laccase family protein / diphenol oxidase family protein, similar to high-pI laccase (LAC2-1) GI:1621460 from (Liriodendron tulipifera) \| chr1:6238837-6241429 REVERSE \| Aliases: T10F20.14 | 3.1 | 3.6 | -0.6 | -3.7 | 3.60% | 2.7 |
| 6600 | AT5G51500.1 \| pectinesterase family protein, contains Pfam profile: PF01095 pectinesterase \| chr5:20935155-20937064 REVERSE \| Aliases: K17N15.5, K17N15_5 | 2.5 | 3.0 | -0.5 | -3.7 | 3.60% | 2.5 |
| 6602 | AT2G42270.1 \| U5 small nuclear ribonucleoprotein helicase, putative \| chr2:17611408-17618411 FORWARD \| Aliases: T24P15.18, T24P15_18 | 5.7 | 6.8 | -1.2 | -3.7 | 3.61% | 2.7 |
| 6607 | AT4G31000.1 \| calmodulin-binding protein, similar to calmodulin-binding protein TCB60 GI:1698548 from (Nicotiana tabacum); contains non-consensus donor splice site AT at exon 4; supported by cDNA gi:17065559 \| chr4:15103165-15105985 FORWARD \| Aliases: F6I18.90, F6I18_90 | 3.4 | 4.5 | -1.1 | -3.7 | 3.62% | 2.7 |

| Rank | Description | Sync | Root | M | t | adj.q | B |
|---|---|---|---|---|---|---|---|
| 6608 | AT1G13450.2 \| similar to DNA-binding protein GT-1-related [Arabidopsis thaliana] (TAIR:At3g25990.1); similar to DNA-binding protein [Nicotiana tabacum] (GB:AAA34085.1); similar to DNA-binding protein B2F - common tobacco (fragment) (GB:PQ0446); contains InterPro domain Myb DNA-binding domain (InterPro:IPR001005) \| chr1:4612728-4615350 REVERSE \| Aliases: T6J4.18, T6J4_18 | 6.2 | 7.2 | -1.0 | -3.7 | 3.62% | 2.9 |
| 6614 | AT2G38500.1 \| expressed protein \| chr2:16124505-16126177 REVERSE \| Aliases: T6A23.30 | 4.2 | 4.7 | -0.5 | -3.7 | 3.63% | 2.5 |
| 6615 | AT3G46810.1 \| DC1 domain-containing protein, contains Pfam profile PF03107: DC1 domain | 2.8 | 3.5 | -0.8 | -3.7 | 3.63% | 2.6 |
| 6616 | AT4G36140.1 \| disease resistance protein (TIR-NBS-LRR class), putative, domain signature TIR-NBS-LRR exists, suggestive of a disease resistance protein. \| chr4:17098959-17104482 REVERSE \| Aliases: F23E13.30, F23E13_30 | 3.6 | 5.6 | -2.0 | -3.7 | 3.64% | 2.9 |
| 6617 | AT3G56130.3 \| similar to biotin/lipoyl attachment domain-containing protein [Arabidopsis thaliana] (TAIR:At1g52670.1); similar to acetyl-CoA carboxylasesubunit (biotin carboxyl carrier subunit) [Geobacillus kaustophilus HTA426] (GB:YP_148253.1); contains InterPro domain Biotin/lipoyl attachment (InterPro:IPR000089) \| chr3:20837637-20840249 FORWARD \| Aliases: F18O21.90 | 4.9 | 6.4 | -1.5 | -3.7 | 3.64% | 3.0 |
| 6618 | AT2G11520.1 \| Symbol: CRCK3 \| protein kinase family protein, contains protein kinase domain, Pfam:PF00069 \| chr2:4625743-4628658 FORWARD \| Aliases: F14P14.15, F14P14_15 | 5.7 | 6.5 | -0.8 | -3.7 | 3.64% | 2.7 |
| 6619 | AT1G26650.1 \| expressed protein \| chr1:9210076-9211547 FORWARD \| Aliases: T24P13.3, T24P13_3 | 5.5 | 6.4 | -0.9 | -3.7 | 3.65% | 2.8 |
| 6621 | AT2G31180.1 \| myb family transcription factor (MYB14), similar to myb-related transcription factor GI:1370140 from (Lycopersicon esculentum) \| chr2:13293798-13295252 REVERSE \| Aliases: F16D14.2 | 4.1 | 4.8 | -0.7 | -3.7 | 3.66% | 2.7 |
| 6622 | AT4G15420.1 \| PRLI-interacting factor K, nearly identical to PRLI-interacting factor K (Arabidopsis thaliana) GI:11139266; contains Pfam profiles PF03152: Ubiquitin fusion degradation protein UFD1, PF00096: Zinc finger, C2H2 type \| chr4:8823725-8826096 FORWARD \| Aliases: DL3755W, FCAALL.127 | 7.1 | 8.1 | -1.0 | -3.7 | 3.66% | 2.8 |
| 6623 | AT5G24620.1 \| thaumatin-like protein, putative, similar to thaumatin-like protein (Arabidopsis thaliana) GI:2435406; contains Pfam profile PF00314: Thaumatin family \| chr5:8430771-8432417 FORWARD \| Aliases: K18P6.16, K18P6_16 | 4.7 | 6.2 | -1.5 | -3.7 | 3.66% | 3.0 |
| 6624 | AT1G13310.1 \| expressed protein \| chr1:4561067-4562295 REVERSE \| Aliases: T6J4.7, T6J4_7 | 2.6 | 3.1 | -0.5 | -3.7 | 3.66% | 2.4 |
| 6625 | AT4G16850.1 \| expressed protein \| chr4:9480100-9481757 FORWARD \| Aliases: DL4450W, FCAALL.194 | 5.0 | 5.5 | -0.5 | -3.7 | 3.66% | 2.5 |
| 6627 | AT4G36060.2 \| basic helix-loop-helix (bHLH) family protein, contains Pfam profile: PF00010 helix-loop-helix DNA-binding domain | 2.5 | 2.9 | -0.4 | -3.7 | 3.66% | 2.5 |
| 6629 | AT2G17990.1 \| expressed protein \| chr2:7834004-7835836 FORWARD \| Aliases: T27K22.14, T27K22_14 | 8.6 | 9.5 | -0.9 | -3.7 | 3.66% | 2.8 |
| 6630 | AT4G20780.1 \| calcium-binding protein, putative, similar to SP:Q09011 Calcium-binding protein CAST {Solanum tuberosum}; contains INTERPRO:IPR002048 calcium-binding EF-hand domain \| chr4:11133197-11133981 REVERSE \| Aliases: F21C20.130, F21C20_130 | 3.2 | 4.0 | -0.8 | -3.7 | 3.66% | 2.8 |
| 6637 | AT3G61900.1 \| auxin-responsive family protein, similar to auxin-induced protein SAUR-AC1 (GI:546362) (PIR:T06084) (Arabidopsis thaliana) \| chr3:22936788-22937354 FORWARD \| Aliases: F21F14.70 | 3.4 | 4.5 | -1.1 | -3.7 | 3.68% | 2.9 |
| 6638 | AT3G20770.1 \| Symbol: EIN3 \| ethylene-insensitive 3 (EIN3), identical to ethylene-insensitive3 GI:2224933 from (Arabidopsis thaliana) | 7.1 | 8.5 | -1.4 | -3.7 | 3.68% | 2.9 |
| 6644 | AT5G51890.1 \| similar to peroxidase 64 (PER64) (P64) (PRXR4) [Arabidopsis thaliana] (TAIR:At5g42180.1); similar to cationic peroxidase [Zinnia elegans] (GB:BAD93164.1); contains InterPro domain Haem peroxidase (InterPro:IPR002016); contains InterPro domain Plant peroxidase (InterPro:IPR000823) | 3.8 | 6.8 | -2.9 | -3.7 | 3.71% | 3.0 |
| 6647 | AT1G02170.1 \| Symbol: LOL3 \| Metacaspase AtMCP1b. Arginine/lysine-specific cysteine protease activity. Induces apoptosis in yeast. Contains Pfam profile PF00656: ICE-like protease (caspase) p20 domain \| chr1:411883-413932 FORWARD \| Aliases: AMC1, ATMCPB1, LSD ONE LIKE 3, MCP1B, T6A9.24 | 7.3 | 8.2 | -0.9 | -3.7 | 3.71% | 2.9 |
| 6649 | AT1G63910.1 \| Symbol: ATMYB103 \| myb family transcription factor (MYB103), contains Pfam profile: PF00249 myb-like DNA-binding domain | 2.3 | 2.7 | -0.4 | -3.7 | 3.71% | 2.3 |
| 6652 | AT2G18640.1 \| Symbol: GGPS4 \| geranylgeranyl pyrophosphate synthase, putative / GGPP synthetase, putative / farnesyltranstransferase, putative, similar to gi:1944371; contains GB:L22347 \| chr2:8090822-8091970 REVERSE \| Aliases: F24H14.2, GERANYLGERANYL PYROPHOSPHATE SYNTHASE 4 | 2.6 | 3.0 | -0.4 | -3.7 | 3.72% | 2.3 |
| 6653 | AT2G20320.1 \| DENN (AEX-3) domain-containing protein, contains Pfam domain PF02141: DENN (AEX-3) domain \| chr2:8774187-8779217 FORWARD \| Aliases: F11A3.13, F11A3_13 | 5.2 | 6.0 | -0.8 | -3.7 | 3.73% | 2.8 |
| 6654 | AT5G42630.1 \| myb family transcription factor (KAN4), contains Pfam profile: PF00249 myb-like DNA-binding domain; identical to | 2.2 | 2.6 | -0.4 | -3.7 | 3.73% | 2.3 |

| Rank | Description | Sync | Root | M | t | adj.q | B |
|---|---|---|---|---|---|---|---|
| 6655 | AT1G25540.1 \| Symbol: PFT1 \| phytochrome and flowering time regulatory protein (PFT1), PMID: 12815435 | 5.4 | 6.2 | -0.8 | -3.7 | 3.73% | 2.8 |
| 6657 | AT3G55940.1 \| phosphoinositide-specific phospholipase C, putative, similar to phosphoinositide specific phospholipase C GI:857374 from (Arabidopsis thaliana) \| chr3:20758766-20761163 FORWARD \| Aliases: F27K19.120 | 3.1 | 3.7 | -0.6 | -3.7 | 3.73% | 2.7 |
| 6662 | AT1G65690.1 \| harpin-induced protein-related / HIN1-related / harpin-responsive protein-related, similar to hin1 homolog (GI:13122296) (Arabidopsis thaliana); similar to hin1 (GI:22830759) (Nicotiana tabacum); contains 1 transmembrane domain; \| chr1:24435096-24436616 REVERSE \| Aliases: F1E22.7, F1E22_7 | 3.3 | 3.9 | -0.5 | -3.7 | 3.75% | 2.6 |
| 6663 | AT3G10800.1 \| bZIP transcription factor family protein, contains Pfam profile: PF00170 bZIP transcription factor; contains similarity to TGACG-sequence specific DNA-binding protein TGA-1B (HSBF) GB:P14233 (Nicotiana tabacum) \| chr3:3379259-3381618 FORWARD \| Aliases: T7M13.12 | 5.9 | 6.9 | -1.0 | -3.7 | 3.75% | 2.9 |
| 6666 | AT1G74410.1 \| zinc finger (C3HC4-type RING finger) family protein, contains Pfam profile: PF00097 zinc finger, C3HC4 type (RING finger) \| chr1:27969215-27971563 FORWARD \| Aliases: F1M20.9, F1M20_9 | 5.9 | 6.6 | -0.7 | -3.7 | 3.76% | 2.9 |
| 6667 | AT4G39640.2 \| gamma-glutamyltranspeptidase family protein, similar to SP:P19440 Gamma-glutamyltranspeptidase 1 precursor (EC 2.3.2.2) (Gamma-glutamyltransferase 1) (CD224 antigen) {Homo sapiens}; contains Pfam profilePF01019: Gamma-glutamyltranspeptidase | 3.8 | 4.4 | -0.7 | -3.7 | 3.77% | 2.7 |
| 6668 | AT2G27480.1 \| similar to calcium-binding EF hand family protein [Arabidopsis thaliana] (TAIR:At5g04170.1); similar to fiber protein Fb1 [Gossypium barbadense] (GB:AAN77144.1); contains InterPro domain Calcium-binding EF-hand (InterPro:IPR002048) \| chr2:11753798-11755551 FORWARD \| Aliases: F10A12.16, F10A12_16 | 4.0 | 5.1 | -1.1 | -3.7 | 3.77% | 2.9 |
| 6669 | AT3G51580.1 \| expressed protein \| chr3:19141813-19144259 REVERSE \| Aliases: T18N14.6 | 6.4 | 7.0 | -0.6 | -3.7 | 3.77% | 2.7 |
| 6670 | AT4G31330.1 \| expressed protein, contains Pfam profile PF04654: Protein of unknown function, DUF599 \| chr4:15202302-15203437 FORWARD \| Aliases: F8F16.150, F8F16_150 | 5.4 | 6.5 | -1.0 | -3.7 | 3.77% | 2.8 |
| 6671 | AT3G02040.1 \| Symbol: SRG3 \| glycerophosphoryl diester phosphodiesterase family protein, contains Pfam profile PF03009: Glycerophosphoryl diester phosphodiesterase family \| chr3:348174-350006 REVERSE \| Aliases: F1C9.18, F1C9_18, SENESCENCE RELATED GENE 3 | 5.3 | 6.1 | -0.7 | -3.7 | 3.77% | 2.9 |
| 6672 | AT4G26770.1 \| phosphatidate cytidylyltransferase, putative / CDP-diglyceride synthetase, putative, similar to CDP-diacylglycerol synthetase GI:2182104 from (Solanum tuberosum) \| chr4:13482082-13484846 FORWARD \| Aliases: F10M23.110, F10M23_110 | 2.6 | 3.0 | -0.5 | -3.7 | 3.77% | 2.4 |
| 6674 | AT5G65320.1 \| basic helix-loop-helix (bHLH) family protein, contains similarity to bHLH DNA-binding protein \| chr5:26124395-26125952 REVERSE \| Aliases: MNA5.5, MNA5_5 | 3.7 | 4.1 | -0.5 | -3.7 | 3.78% | 2.5 |
| 6675 | AT5G66950.1 \| expressed protein \| chr5:26750559-26753372 FORWARD \| Aliases: K8A10.2, K8A10_2 | 6.1 | 7.2 | -1.2 | -3.7 | 3.78% | 2.9 |
| 6676 | AT3G02300.1 \| regulator of chromosome condensation (RCC1) family protein, weak similarity to UVB-resistance protein UVR8 (Arabidopsis thaliana) GI:5478530; contains Pfam profile PF00415: Regulator of chromosome condensation (RCC1) | 6.3 | 7.2 | -1.0 | -3.7 | 3.79% | 2.8 |
| 6677 | AT3G13090.1 \| Symbol: ATMRP8 \| | 3.8 | 4.5 | -0.7 | -3.7 | 3.79% | 2.6 |
| 6680 | AT3G61010.1 \| glycosyl hydrolase family protein 85, hypothetical protein F9F8.14 - Arabidopsis thaliana, EMBL:AC009991 | 4.1 | 5.1 | -1.0 | -3.7 | 3.80% | 2.8 |
| 6684 | AT5G65460.1 \| kinesin motor protein-related, contains similarity to kinesin heavy chain \| chr5:26179057-26186227 REVERSE \| Aliases: MNA5.20, MNA5_20 | 3.4 | 4.2 | -0.8 | -3.7 | 3.81% | 2.6 |
| 6686 | AT1G11570.1 \| nuclear transport factor 2 (NTF2), putative, similar to Swiss-Prot:P33331 nuclear transport factor 2 (NTF-2) (Nuclear transport factor P10) (Saccharomyces cerevisiae) \| chr1:3883280-3883663 FORWARD \| Aliases: T23J18.32, T23J18_32 | 2.1 | 2.6 | -0.5 | -3.7 | 3.81% | 2.5 |
| 6687 | AT1G79630.3 \| similar to protein phosphatase 2C family protein / PP2C family protein [Arabidopsis thaliana] (TAIR:At1g16220.1); similar to putative protein phosphatase 2C [Oryza sativa (japonica cultivar-group)] (GB:XP_465582.1); contains InterPro domain Protein phosphatase 2C-like (InterPro:IPR001932) \| chr1:29967538-29970688 REVERSE \| Aliases: F20B17.6, F20B17_6 | 4.4 | 5.1 | -0.7 | -3.7 | 3.81% | 2.5 |
| 6688 | AT4G34640.1 \| Symbol: SQS1 \| farnesyl-diphosphate farnesyltransferase 1 / squalene synthase 1 (SQS1), identical to SP:P53799 Farnesyl-diphosphate farnesyltransferase (EC 2.5.1.21) (Squalene synthetase) (SQS) (SS) (FPP:FPP farnesyltransferase) {Arabidopsis thaliana}; non-consensus GC donor splice site at exon 3, squalene synthase 2 (SQS2), Arabidopsis thaliana \| chr4:16538287-16541913 FORWARD \| Aliases: ERG9, SQUALENE SYNTHASE, SQUALENE SYNTHASE 1, T4L20.220, T4L20_220 | 8.0 | 9.1 | -1.1 | -3.7 | 3.81% | 2.8 |
| 6691 | AT2G15300.1 \| leucine-rich repeat transmembrane protein kinase, putative \| chr2:6656712-6659092 FORWARD \| Aliases: F27O10.5, F27O10_5 | 3.0 | 3.5 | -0.5 | -3.7 | 3.81% | 2.5 |

| Rank | Description | Sync | Root | M | t | adj.q | B |
|---|---|---|---|---|---|---|---|
| 6693 | AT5G01990.1 \| auxin efflux carrier family protein, contains auxin efflux carrier domain, Pfam:PF03547 \| chr5:377228-379908 REVERSE \| Aliases: T7H20.40, T7H20_40 | 5.2 | 5.9 | -0.7 | -3.7 | 3.82% | 2.7 |
| 6694 | AT1G23830.1 \| expressed protein \| chr1:8422949-8424077 REVERSE \| Aliases: F5O8.39, F5O8_39 | 4.9 | 6.0 | -1.1 | -3.7 | 3.83% | 2.9 |
| 6697 | AT2G38600.1 \| acid phosphatase class B family protein, similar to acid phosphatase (Glycine max) GI:3341443; contains Pfam profile PF03767: HAD superfamily (subfamily IIIB) phosphatase \| chr2:16152985-16153935 FORWARD \| Aliases: T6A23.20, T6A23_20 | 4.3 | 5.5 | -1.2 | -3.7 | 3.83% | 3.0 |
| 6698 | AT4G39330.2 \| similar to mannitol dehydrogenase, putative [Arabidopsis thaliana] (TAIR:At2g21730.1); similar to mannitol dehydrogenase, putative [Arabidopsis thaliana] (TAIR:At2g21890.1); similar to putative sinapyl alcohol dehydrogenase [Populus tremula x Populus tremuloides] (GB:AAM95578.1); contains InterPro domain Zinc-containing alcohol dehydrogenase superfamily (InterPro:IPR002085) | 3.5 | 4.2 | -0.7 | -3.7 | 3.83% | 2.6 |
| 6699 | AT4G24560.1 \| Symbol: UBP16 \| ubiquitin-specific protease 16, putative (UBP16), similar to ubiquitin-specific protease 16 GI:11993477 (Arabidopsis thaliana) \| chr4:12679220-12684949 REVERSE \| Aliases: F22K18.240, F22K18_240, UBIQUITIN SPECIFIC PROTEASE 16 | 4.8 | 6.0 | -1.2 | -3.7 | 3.83% | 2.9 |
| 6700 | AT5G64120.1 \| peroxidase, putative, identical to peroxidase (Arabidopsis thaliana) gi:1483222:emb:CAA67551 \| chr5:25676532-25678228 REVERSE \| Aliases: MHJ24.10, MHJ24_10 | 3.3 | 5.4 | -2.0 | -3.7 | 3.83% | 2.9 |
| 6703 | AT5G40910.1 \| disease resistance protein (TIR-NBS-LRR class), putative, domain signature TIR-NBS-LRR exists, suggestive of a disease resistance protein. Non-consensus TT donor splice site at exon 1 \| chr5:16412735-16416357 FORWARD \| Aliases: MHK7.14, MHK7_14 | 4.3 | 6.4 | -2.0 | -3.7 | 3.84% | 2.8 |
| 6704 | AT4G34100.1 \| zinc finger (C3HC4-type RING finger) family protein, contains Pfam profile: PF00097 zinc finger, C3HC4 type (RING finger) \| chr4:16330595-16334869 FORWARD \| Aliases: F28A23.140, F28A23_140 | 7.4 | 8.4 | -1.0 | -3.7 | 3.84% | 2.7 |
| 6705 | AT5G51770.1 \| protein kinase family protein, contains protein kinase domain, Pfam:PF00069 \| chr5:21048256-21050220 FORWARD \| Aliases: MIO24.10, MIO24_10 | 5.1 | 6.3 | -1.2 | -3.7 | 3.84% | 2.9 |
| 6706 | AT3G27320.2 \| similar to expressed protein [Arabidopsis thaliana] (TAIR:At5g14310.1); similar to putative esterase [Oryza sativa (japonica cultivar-group)] (GB:XP_469930.1) \| chr3:10091302-10094028 FORWARD \| Aliases: K17E12.14 | 3.7 | 4.3 | -0.6 | -3.7 | 3.84% | 2.5 |
| 6708 | AT3G21620.1 \| early-responsive to dehydration protein-related / ERD protein-related, similar to ERD4 protein (early-responsive to dehydration stress) (Arabidopsis thaliana) GI:15375406; contains Pfam profile PF02714: Domain of unknown function DUF221 \| chr3:7611051-7614048 REVERSE \| Aliases: MIL23.19 | 2.6 | 3.0 | -0.4 | -3.7 | 3.84% | 2.4 |
| 6712 | AT1G53210.1 \| sodium/calcium exchanger family protein / calcium-binding EF hand family protein, contains Pfam profiles: PF01699 sodium/calcium exchanger protein, PF00036 EF hand \| chr1:19848300-19851504 FORWARD \| Aliases: F12M16.12, F12M16_12 | 9.5 | 10.7 | -1.2 | -3.7 | 3.85% | 2.7 |
| 6713 | AT5G28040.1 \| expressed protein, contains Pfam profile: PF04504 protein of unknown function, DUF573 \| chr5:10037273-10039005 REVERSE \| Aliases: F15F15.110, F15F15_110 | 5.9 | 7.1 | -1.2 | -3.7 | 3.85% | 2.7 |
| 6716 | AT1G29400.2 \| Symbol: AML5 \| RNA recognition motif (RRM)-containing protein, similar to GI:6650523 from (Arabidopsis thaliana) \| chr1:10290026-10294508 REVERSE \| Aliases: ARABIDOPSIS MEI2 LIKE PROTEIN 5, F15D2.30, F15D2_30 | 3.4 | 4.4 | -1.0 | -3.7 | 3.86% | 2.7 |
| 6719 | AT1G68840.1 \| Symbol: RAV2 \| DNA-binding protein RAV2 (RAV2) / AP2 domain-containing protein RAP2.8, identical to RAV2 GI:3868859 from (Arabidopsis thaliana), AP2 domain containing protein RAP2.8 (Arabidopsis thaliana) GI:2281641; contains Pfam profile: PF00847 AP2-domain \| chr1:25883990-25885399 FORWARD \| Aliases: RAP2.8, RAV2, T6L1.3, T6L1_3 | 4.7 | 5.8 | -1.0 | -3.7 | 3.86% | 2.7 |
| 6721 | AT5G43730.1 \| disease resistance protein (CC-NBS-LRR class), putative, domain signature CC-NBS-LRR exists, suggestive of a disease resistance protein. | 2.9 | 4.9 | -2.1 | -3.7 | 3.87% | 2.7 |
| 6723 | AT1G05710.4 \| ethylene-responsive protein, putative, similar to ethylene-inducible ER33 protein (Lycopersicon esculentum) gi:5669656:gb:AAD46413; identical to bHLH transcription factor (bHLH-alpha gene) \| chr1:1715779-1717243 FORWARD \| Aliases: F3F20.16, F3F20_16 | 6.2 | 7.1 | -0.9 | -3.7 | 3.87% | 2.8 |
| 6724 | AT5G37550.1 \| expressed protein \| chr5:14936131-14937022 REVERSE \| Aliases: MPA22.10, MPA22_10 | 5.7 | 6.9 | -1.1 | -3.7 | 3.88% | 2.9 |
| 6725 | AT2G33630.1 \| 3-beta hydroxysteroid dehydrogenase/isomerase family protein, contains Pfam profile PF01073 3-beta hydroxysteroid dehydrogenase/isomerase domain; similar to NAD(P)-dependent cholesterol dehydrogenase (Nocardia sp.) (GI:216809) \| chr2:14243467-14245866 REVERSE \| Aliases: F4P9.40, F4P9_40 | 7.7 | 9.6 | -1.9 | -3.7 | 3.88% | 2.9 |
| 6728 | AT1G72500.1 \| inter-alpha-trypsin inhibitor heavy chain-related, low similarity to Inter-alpha-inhibitor H4 heavy chain (Rattus norvegicus) GI:2292988; contains Pfam profile PF00092: von Willebrand factor type A domain \| chr1:27298998-27302218 REVERSE \| Aliases: T10D10.3, T10D10_3 | 9.2 | 10.2 | -1.0 | -3.7 | 3.88% | 2.7 |

| Rank | Description | Sync | Root | M | t | adj.q | B |
|---|---|---|---|---|---|---|---|
| 6729 | AT2G46650.1 \| Symbol: B5 #1 \| cytochrome b5, putative, similar to cytochome b5 GI:2695711 from (Olea europaea) \| chr2:19158723-19159663 FORWARD \| Aliases: T3A4.3, T3A4_3 | 4.5 | 6.5 | -2.0 | -3.7 | 3.88% | 3.0 |
| 6732 | AT4G33610.1 \| glycine-rich protein \| chr4:16146264-16146996 FORWARD \| Aliases: T16L1.100, T16L1_100 | 3.7 | 4.6 | -0.9 | -3.7 | 3.88% | 2.7 |
| 6733 | AT4G36480.2 \| Symbol: ATLCB1 \| | 4.4 | 5.4 | -1.0 | -3.7 | 3.88% | 2.8 |
| 6735 | AT4G21350.1 \| U-box domain-containing protein, similar to immediate-early fungal elicitor protein CMPG1 (Petroselinum crispum) GI:14582198; contains Pfam profile PF04564: U-box domain \| chr4:11356154-11357278 REVERSE \| Aliases: T6K22.80, T6K22_80 | 4.7 | 5.6 | -1.0 | -3.7 | 3.89% | 2.9 |
| 6736 | AT2G39580.1 \| expressed protein \| chr2:16517503-16524151 FORWARD \| Aliases: F12L6.24, F12L6_24 | 3.6 | 4.9 | -1.3 | -3.7 | 3.89% | 2.7 |
| 6742 | AT2G47270.1 \| expressed protein \| chr2:19418630-19419221 REVERSE \| Aliases: T8I13.11 | 2.6 | 3.3 | -0.7 | -3.7 | 3.90% | 2.5 |
| 6744 | AT1G52660.1 \| disease resistance protein, putative, similar to NBS/LRR disease resistance protein GI:9758302 from (Arabidopsis thaliana) \| chr1:19617143-19618354 FORWARD \| Aliases: F6D8.12, F6D8_12 | 3.5 | 4.2 | -0.7 | -3.6 | 3.90% | 2.6 |
| 6747 | AT2G02310.1 \| Symbol: ATPP2 B6 \| F-box family protein / SKP1 interacting partner 3-related, contains similarity to SKP1 interacting partner 3 GI:10716951 from (Arabidopsis thaliana) | 2.6 | 3.8 | -1.1 | -3.6 | 3.91% | 2.8 |
| 6748 | AT2G35800.1 \| mitochondrial substrate carrier family protein, contains INTERPRO:IPR001993 Mitochondrial substrate carrier family, INTERPRO:IPR002048 calcium-binding EF-hand domain \| chr2:15051124-15055682 FORWARD \| Aliases: F11F19.29, F11F19_29 | 5.1 | 5.7 | -0.6 | -3.6 | 3.91% | 2.7 |
| 6750 | AT4G19030.1 \| Symbol: NLM1 \| major intrinsic family protein / MIP family protein, contains Pfam profile: MIP PF00230; identical to cDNA NLM1 protein GI:2677613 \| chr4:10421543-10423498 REVERSE \| Aliases: AT NLM1, F13C5.200, F13C5_200 | 2.6 | 3.0 | -0.4 | -3.6 | 3.92% | 2.3 |
| 6752 | AT1G12640.1 \| membrane bound O-acyl transferase (MBOAT) family protein, low similarity to porcupine from (Xenopus laevis) GI:6714514, GI:6714520, GI:6714518, GI:6714516; contains Pfam profile PF03062: MBOAT family \| chr1:4303315-4305786 REVERSE \| Aliases: T12C24.17, T12C24_17 | 7.1 | 9.0 | -1.9 | -3.6 | 3.92% | 2.8 |
| 6756 | AT3G48090.2 \| Symbol: EDS1 \| similar to lipase class 3 family protein / disease resistance protein-related [Arabidopsis thaliana] (TAIR:At3g48080.1); similar to EDS1-like protein [Nicotiana benthamiana] (GB:AAL85347.1) \| chr3:17766359-17768742 REVERSE \| Aliases: EDS1 PROTEIN, ENHANCED DISEASE SUSCEPTIBILITY 1, T17F15.40 | 3.7 | 4.5 | -0.7 | -3.6 | 3.93% | 2.7 |
| 6758 | AT3G12480.1 \| transcription factor, putative \| chr3:3957844-3960479 FORWARD \| Aliases: T2E22.20 | 8.2 | 9.1 | -0.9 | -3.6 | 3.94% | 2.5 |
| 6759 | AT1G65510.1 \| expressed protein \| chr1:24363116-24363532 FORWARD \| Aliases: F5I14.24, F5I14_24 | 3.0 | 4.7 | -1.7 | -3.6 | 3.94% | 2.9 |
| 6761 | AT2G30990.2 \| similar to expressed protein [Arabidopsis thaliana] (TAIR:At1g29240.1); similar to B1131G08.12 [Oryza sativa (japonica cultivar-group)] (GB:NP_916200.1); contains InterPro domain Protein of unknown function DUF688 (InterPro:IPR007789) \| chr2:13194660-13197496 REVERSE \| Aliases: F7F1.20, F7F1_20 | 5.3 | 6.2 | -0.8 | -3.6 | 3.94% | 2.7 |
| 6764 | AT5G45250.1 \| Symbol: RPS4 \| disease resistance protein (TIR-NBS-LRR class), putative (RPS4), domain signature TIR-NBS-LRR exists, suggestive of a disease resistance protein. Identical to RPS4 (GI:11357255). False intron created at intron 2 to escape a frameshift in the BAC sequence. \| chr5:18339141-18343249 REVERSE \| Aliases: DISEASE RESISTANCE PROTEIN RPS4, K9E15.1, K9E15_1, RESISTANT TO P. SYRINGAE 4 | 5.7 | 7.5 | -1.8 | -3.6 | 3.95% | 2.8 |
| 6767 | AT3G20110.1 \| Symbol: CYP705A20 \| cytochrome P450 family protein, similar to Cytochrome P450 93A1 (SP:Q42798) | 2.7 | 3.1 | -0.4 | -3.6 | 3.95% | 2.3 |
| 6769 | AT1G28240.1 \| expressed protein \| chr1:9868303-9872198 REVERSE \| Aliases: F3H9.11, F3H9_11 | 4.0 | 4.8 | -0.8 | -3.6 | 3.96% | 2.5 |
| 6774 | AT5G50450.1 \| zinc finger (MYND type) family protein, contains Pfam profile PF01753: MYND finger \| chr5:20560536-20561979 REVERSE \| Aliases: MXI22.17, MXI22_17 | 4.2 | 5.3 | -1.1 | -3.6 | 3.98% | 2.8 |
| 6777 | AT3G04640.1 \| glycine-rich protein, predicted proteins, Arabidopsis thaliana \| chr3:1260912-1261689 REVERSE \| Aliases: F7O18.12, F7O18_12 | 5.5 | 6.8 | -1.3 | -3.6 | 3.98% | 2.9 |
| 6778 | AT5G15110.1 \| pectate lyase family protein, similar to pectate lyase P59 SP:P15722 from (Lycopersicon esculentum) \| chr5:4895969-4897685 FORWARD \| Aliases: F2G14.230, F2G14_230 | 2.3 | 2.8 | -0.5 | -3.6 | 3.98% | 2.2 |
| 6779 | AT5G38380.2 \| expressed protein \| chr5:15362890-15365657 FORWARD \| Aliases: MXI10.5, MXI10_5 | 4.8 | 5.4 | -0.7 | -3.6 | 3.98% | 2.7 |

| Rank | Description | Sync | Root | M | t | adj.q | B |
|---|---|---|---|---|---|---|---|
| 6780 | AT4G33080.2 \| similar to protein kinase, putative [Arabidopsis thaliana] (TAIR:At2g19400.1); similar to protein kinase [Raphanus sativus] (GB:BAC76895.1); similar to putative serine/threonine kinase 38 [Oryza sativa (japonica cultivar-group)] (GB:BAD72247.1); similar to unnamed protein product [Oryza sativa (japonica cultivar-group)] (GB:NP_914515.1); similar to protein kinase [Spinacia oleracea] (GB:CAA82991.1); contains InterPro domain Serine/threonine protein kinase, active site (InterPro:IPR008271); contains InterPro domain Tyrosine protein kinase (InterPro:IPR001245); contains InterPro domain Serine/threonine protein kinase (InterPro:IPR002290); contains InterPro domain Protein kinase (InterPro:IPR000719) \| chr4:15959965-15963980 FORWARD \| Aliases: F4I10.10, F4I10_10 | 4.9 | 5.8 | -0.9 | -3.6 | 3.99% | 2.7 |
| 6781 | AT1G33440.1 \| proton-dependent oligopeptide transport (POT) family protein, contains Pfam profile: PF00854 POT family \| chr1:12127454-12130369 REVERSE \| Aliases: F10C21.11, F10C21_11 | 3.0 | 3.5 | -0.6 | -3.6 | 3.99% | 2.6 |
| 6783 | AT1G60890.1 \| phosphatidylinositol-4-phosphate 5-kinase family protein, similar to phosphatidylinositol-4-phosphate 5-kinase AtPIP5K1 (Arabidopsis thaliana) GI:3702691; contains Pfam profiles PF01504: Phosphatidylinositol-4-phosphate 5-Kinase, PF02493: MORN repeat \| chr1:22415911-22420898 REVERSE \| Aliases: T7P1.4, T7P1_4 | 4.2 | 5.4 | -1.2 | -3.6 | 3.99% | 2.9 |
| 6784 | AT3G21890.1 \| zinc finger (B-box type) family protein, contains Pfam profile: PF01760 CONSTANS family zinc finger \| chr3:7709187-7709740 REVERSE \| Aliases: MZN24.1 | 5.4 | 7.0 | -1.6 | -3.6 | 3.99% | 2.9 |
| 6785 | AT1G15210.1 \| ABC transporter family protein, Similar to gb:Z70524 GI:1514643 PDR5-like ABC transporter from Spirodela polyrrhiza and is a member of the PF:00005 ABC transporter family. ESTs gb:N97039 and gb:T43169 come from this gene \| chr1:5231343-5236568 REVERSE \| Aliases: F9L1.15, F9L1_15 | 6.5 | 7.6 | -1.1 | -3.6 | 4.00% | 2.8 |
| 6789 | AT4G00400.1 \| phospholipid/glycerol acyltransferase family protein \| chr4:174072-176837 REVERSE \| Aliases: A_IG005I10.4, A_IG005I10_4, F5I10.4, F5I10_4 | 2.9 | 3.3 | -0.4 | -3.6 | 4.01% | 2.3 |
| 6790 | AT2G18970.1 \| expressed protein \| chr2:8236198-8236837 FORWARD \| Aliases: F19F24.17, F19F24_17 | 3.4 | 4.3 | -0.9 | -3.6 | 4.01% | 2.6 |
| 6792 | AT4G34150.1 \| C2 domain-containing protein, similar to calcium-dependent protein kinase (Dunaliella tertiolecta) GI:6644464; contains Pfam profile PF00168: C2 domain \| chr4:16354978-16357297 FORWARD \| Aliases: F28A23.90, F28A23_90 | 3.4 | 4.3 | -0.8 | -3.6 | 4.01% | 2.8 |
| 6794 | AT1G52180.1 \| major intrinsic family protein / MIP family protein, contains Pfam profile: MIP PF00230 \| chr1:19428613-19429597 REVERSE \| Aliases: F9I5.3, F9I5_3 | 3.0 | 3.8 | -0.8 | -3.6 | 4.01% | 2.8 |
| 6796 | AT3G50690.1 \| leucine-rich repeat family protein \| chr3:18845948-18848078 REVERSE \| Aliases: T3A5.70 | 7.7 | 9.2 | -1.5 | -3.6 | 4.02% | 2.7 |
| 6797 | AT1G51140.1 \| basic helix-loop-helix (bHLH) family protein, contains Pfam profile: PF00010 helix-loop-helix DNA-binding domain | 5.2 | 6.2 | -1.0 | -3.6 | 4.02% | 2.8 |
| 6799 | AT3G25855.1 \| expressed protein \| chr3:9460658-9461532 REVERSE \| Aliases: None | 2.4 | 2.7 | -0.3 | -3.6 | 4.02% | 2.0 |
| 6800 | AT5G23810.2 \| Symbol: AAP7 \| similar to amino acid carrier, putative / amino acid permease, putative [Arabidopsis thaliana] (TAIR:At1g77380.1); similar to putative amino acid transport protein AAP2 [Oryza sativa (japonica cultivar-group)] (GB:AAL87189.1); contains InterPro domain Amino acid/polyamine transporter, family II (InterPro:IPR002422) \| chr5:8028381-8030166 FORWARD \| Aliases: MRO11.15, MRO11_15 | 4.1 | 5.2 | -1.1 | -3.6 | 4.02% | 2.8 |
| 6801 | AT1G61930.1 \| expressed protein, contains Pfam profile PF04520: Protein of unknown function, DUF584 \| chr1:22896766-22897635 FORWARD \| Aliases: F8K4.12, F8K4_12 | 5.8 | 7.1 | -1.3 | -3.6 | 4.03% | 2.8 |
| 6802 | AT3G59120.1 \| DC1 domain-containing protein, contains Pfam profile PF03107: DC1 domain | 2.8 | 3.6 | -0.7 | -3.6 | 4.03% | 2.6 |
| 6803 | AT1G03470.2 \| similar to kinase interacting family protein [Arabidopsis thaliana] (TAIR:At2g47920.1); similar to putative centromere protein [Oryza sativa (japonica cultivar-group)] (GB:AAP53815.1); contains domain GLU_RICH (PS50313) \| chr1:865915-867674 REVERSE \| Aliases: F21B7.9 | 5.1 | 6.5 | -1.3 | -3.6 | 4.03% | 2.9 |
| 6805 | AT3G45290.1 \| Symbol: MLO3 \| seven transmembrane MLO family protein / MLO-like protein 3 (MLO3), membrane protein Mlo3 (Arabidopsis thaliana) gi:14091576:gb:AAK53796; similar to MLO protein SWISS-PROT:P93766, NCBI_gi:1877221 (Hordeum vulgare)(Barley) \| chr3:16628529-16632355 FORWARD \| Aliases: F18N11.50 | 2.4 | 2.8 | -0.4 | -3.6 | 4.03% | 2.1 |
| 6809 | AT4G26890.1 \| Symbol: MAPKKK16 \| protein kinase family protein, contains protein kinase domain, Pfam:PF00069 \| chr4:13511845-13513412 FORWARD \| Aliases: F10M23.230, F10M23_230 | 3.7 | 4.8 | -1.0 | -3.6 | 4.03% | 2.9 |
| 6810 | AT2G01410.1 \| expressed protein \| chr2:174777-176398 REVERSE \| Aliases: F10A8.30 | 6.5 | 7.1 | -0.6 | -3.6 | 4.04% | 2.3 |
| 6811 | AT1G24600.1 \| expressed protein \| chr1:8720295-8720707 FORWARD \| Aliases: None | 3.5 | 4.3 | -0.8 | -3.6 | 4.04% | 2.7 |

| Rank | Description | Sync | Root | M | t | adj.q | B |
|---|---|---|---|---|---|---|---|
| 6814 | AT4G02650.1 \| epsin N-terminal homology (ENTH) domain-containing protein / clathrin assembly protein-related, contains Pfam PF01417: ENTH domain. ENTH (Epsin N-terminal homology) domain; similar to Chain A, Calm-N N-Terminal Domain Of Clathrin Assembly Lymphoid Myeloid Leukaemia Protein, Pi(4,5)p2 Complex (GP:13399999) {Homo sapiens}; supporting cDNA gi:26451912:dbj:AK118440.1: \| chr4:1156453-1158653 FORWARD \| Aliases: T10P11.8, T10P11_8 | 4.9 | 6.6 | -1.7 | -3.6 | 4.05% | 2.9 |
| 6815 | AT5G49320.1 \| expressed protein \| chr5:20012251-20013440 REVERSE \| Aliases: K21P3.22, K21P3_22 | 4.7 | 5.5 | -0.9 | -3.6 | 4.05% | 2.7 |
| 6818 | AT2G23770.1 \| protein kinase family protein / peptidoglycan-binding LysM domain-containing protein, contains Pfam domains, PF00069: Protein kinase domain and PF01476: LysM domain \| chr2:10127322-10129160 REVERSE \| Aliases: F27L4.5, F27L4_5 | 2.4 | 3.0 | -0.7 | -3.6 | 4.06% | 2.6 |
| 6819 | AT3G17609.3 \| similar to bZIP protein HY5 (HY5) [Arabidopsis thaliana] (TAIR:At5g11260.1); similar to putative bZIP protein HY5 [Oryza sativa (japonica cultivar-group)] (GB:BAD32844.1); contains InterPro domain Basic-leucine zipper (bZIP) transcription factor (InterPro:IPR004827) \| chr3:6023957-6024699 FORWARD \| Aliases: None | 4.1 | 5.5 | -1.4 | -3.6 | 4.06% | 2.8 |
| 6820 | AT3G16940.1 \| calmodulin-binding protein, similar to anther ethylene-upregulated protein ER1 GI:11612392 from (Nicotiana tabacum); contains Pfam profile: PF00612 IQ calmodulin-binding motif (3 copies) \| chr3:5781781-5785991 FORWARD \| Aliases: K14A17.29 | 5.6 | 7.0 | -1.4 | -3.6 | 4.06% | 2.6 |
| 6825 | AT3G16440.1 \| Symbol: ATMLP 300B \| jacalin lectin family protein, contains Pfam profile: PF01419 jacalin-like lectin domain; similar to myrosinase-binding protein homolog (Arabidopsis thaliana) GI:2997767 \| chr3:5585950-5587753 FORWARD \| Aliases: MYROSINASE BINDING PROTEIN LIKE PROTEIN 300B, T2O4.19 | 3.1 | 3.7 | -0.6 | -3.6 | 4.08% | 2.6 |
| 6826 | AT3G14205.1 \| phosphoinositide phosphatase family protein, contains similarity to phosphoinositide phosphatase SAC1 (Rattus norvegicus) gi:11095248:gb:AAG29810; contains Pfam domain, PF02383: SacI homology domain; identical to SAC domain protein 2 (SAC2) GI:31415720 \| chr3:4715755-4720958 REVERSE \| Aliases: None | 6.0 | 7.7 | -1.7 | -3.6 | 4.08% | 2.7 |
| 6831 | AT5G20150.1 \| SPX (SYG1/Pho81/XPR1) domain-containing protein, similar to PHO1 protein (Arabidopsis thaliana) GI:20069032; contains Pfam profile PF03105: SPX domain \| chr5:6802374-6803627 FORWARD \| Aliases: F5O24.40, F5O24_40 | 4.7 | 5.5 | -0.8 | -3.6 | 4.11% | 2.8 |
| 6833 | AT2G27140.1 \| heat shock family protein, contains similarity to Swiss-Prot:P27397 18.0 kDa class I heat shock protein (Daucus carota) \| chr2:11605457-11606400 REVERSE \| Aliases: T20P8.19, T20P8_19 | 3.0 | 3.5 | -0.5 | -3.6 | 4.11% | 2.6 |
| 6838 | AT1G07590.1 \| pentatricopeptide (PPR) repeat-containing protein, low similarity to DNA-binding protein (Triticum aestivum) GI:6958202; contains Pfam profile PF01535: PPR repeat \| chr1:2336520-2339388 REVERSE \| Aliases: F22G5.3, F22G5_3 | 8.3 | 9.0 | -0.7 | -3.6 | 4.11% | 2.6 |
| 6840 | AT2G44150.1 \| SET domain-containing protein (ASHH3), low similarity to huntingtin interacting protein 1 (Homo sapiens) GI:12697196; contains Pfam profile PF00856: SET domain; identical to cDNA ASH1-like protein 3 (ASHH3) partial cds GI:15488419 \| chr2:18265686-18268512 FORWARD \| Aliases: F6E13.28 | 3.9 | 4.6 | -0.7 | -3.6 | 4.12% | 2.6 |
| 6841 | AT4G32760.1 \| similar to VHS domain-containing protein / GAT domain-containing protein [Arabidopsis thaliana] (TAIR:At3g08790.1); similar to putative VHS domain-containing protein [Oryza sativa (japonica cultivar-group)] (GB:XP_464916.1); contains InterPro domain GAT domain (InterPro:IPR004152); contains InterPro domain VHS (InterPro:IPR002014) \| chr4:15799144-15804180 FORWARD \| Aliases: F4D11.40, F4D11_40 | 6.0 | 7.1 | -1.1 | -3.6 | 4.12% | 2.7 |
| 6842 | AT2G17890.1 \| Symbol: CPK16 \| calcium-dependent protein kinase family protein / CDPK family protein, contains Pfam domains, PF00069: Protein kinase domain and PF00036: EF hand \| chr2:7776967-7779709 REVERSE \| Aliases: T13L16.9, T13L16_9 | 2.7 | 3.1 | -0.5 | -3.6 | 4.13% | 2.4 |
| 6844 | AT2G33070.2 \| similar to jacalin lectin family protein [Arabidopsis thaliana] (TAIR:At3g16400.1); similar to jacalin lectin family protein [Arabidopsis thaliana] (TAIR:At3g16390.1); similar to jacalin lectin family protein [Arabidopsis thaliana] (TAIR:At3g16410.1); similar to putative D-protein [Oryza sativa (japonica cultivar-group)] (GB:XP_450269.1); contains InterPro domain Jacalin-related lectin (InterPro:IPR001229); contains InterPro domain Kelch repeat (InterPro:IPR006652) \| chr2:14036264-14038146 REVERSE \| Aliases: F25I18.19, F25I18_19 | 3.7 | 4.4 | -0.7 | -3.6 | 4.13% | 2.5 |
| 6847 | AT1G27190.1 \| leucine-rich repeat transmembrane protein kinase, putative, similar to CLV1 receptor kinase GB:AAB58929 GI:2160756 from (Arabidopsis thaliana) \| chr1:9446644-9448715 REVERSE \| Aliases: T7N9.25, T7N9_25 | 4.7 | 5.4 | -0.6 | -3.6 | 4.13% | 2.7 |
| 6848 | AT2G39440.1 \| expressed protein \| chr2:16471796-16477549 REVERSE \| Aliases: F12L6.10, F12L6_10 | 3.8 | 4.3 | -0.5 | -3.6 | 4.13% | 2.5 |
| 6849 | AT3G43940.1 \| expressed protein \| chr3:15775252-15776358 REVERSE \| Aliases: T15B3.80 | 3.2 | 3.8 | -0.6 | -3.6 | 4.14% | 2.5 |
| 6852 | AT2G18060.1 \| Symbol: ANAC037 \| no apical meristem (NAM) family protein, similar to NAC2 (GI:6456751) | 2.9 | 3.6 | -0.7 | -3.6 | 4.14% | 2.7 |
| 6856 | AT1G70810.1 \| C2 domain-containing protein, similar to zinc finger and C2 domain protein GI:9957238 from (Arabidopsis thaliana) \| chr1:26707792-26708898 FORWARD \| Aliases: F15H11.6, F15H11_6 | 4.2 | 5.1 | -0.9 | -3.6 | 4.15% | 2.8 |

| Rank | Description | Sync | Root | M | t | adj.q | B |
|---|---|---|---|---|---|---|---|
| 6857 | AT4G30400.1 \| zinc finger (C3HC4-type RING finger) family protein, similar to RING-H2 finger protein RHX1a (Arabidopsis thaliana) GI:3790591; contains Pfam profile PF00097: Zinc finger, C3HC4 type (RING finger) \| chr4:14866952-14868634 FORWARD \| Aliases: F17I23.260, F17I23_260 | 4.3 | 4.8 | -0.5 | -3.6 | 4.15% | 2.4 |
| 6860 | AT5G48850.1 \| male sterility MS5 family protein, similar to male sterility MS5 (Arabidopsis thaliana) GI:3859112; contains Pfam profile PF00515 TPR Domain \| chr5:19822663-19824985 REVERSE \| Aliases: K24G6.19, K24G6_19 | 5.3 | 7.0 | -1.7 | -3.6 | 4.15% | 2.9 |
| 6863 | AT1G05760.1 \| Symbol: RTM1 \| jacalin lectin family protein (RTM1), identical to gi:6503088 (GB:AAF14583) from (Arabidopsis thaliana) (Proc. Natl. Acad. Sci. U.S.A. 97 (1), 489-494 (2000)); contains Pfam profile PF01419 jacalin-like lectin domain | 3.8 | 4.9 | -1.1 | -3.6 | 4.15% | 2.9 |
| 6865 | AT1G48640.1 \| lysine and histidine specific transporter, putative, similar to lysine and histidine specific transporter GI:2576361 from (Arabidopsis thaliana); contains Pfam profile PF01490: Transmembrane amino acid transporter protein \| chr1:17990026-17992659 FORWARD \| Aliases: F11I4.17, F11I4_17 | 3.7 | 5.2 | -1.5 | -3.6 | 4.16% | 2.8 |
| 6866 | AT3G12140.3 \| similar to emsy N terminus domain-containing protein / ENT domain-containing protein [Arabidopsis thaliana] (TAIR:At5g06780.1); similar to emsy N terminus domain-containing protein-like [Oryza sativa (japonica cultivar-group)] (GB:XP_450476.1); contains InterPro domain ENT domain (InterPro:IPR005491) \| chr3:3869075-3871707 REVERSE \| Aliases: T21B14.4 | 5.7 | 6.6 | -0.9 | -3.6 | 4.17% | 2.7 |
| 6867 | AT4G15840.1 \| expressed protein \| chr4:8997042-9001045 FORWARD \| Aliases: DL3960W, FCAALL.399 | 6.3 | 7.0 | -0.7 | -3.6 | 4.17% | 2.5 |
| 6869 | AT4G32750.1 \| expressed protein \| chr4:15795990-15798245 REVERSE \| Aliases: F4D11.50, F4D11_50 | 4.7 | 5.3 | -0.6 | -3.6 | 4.17% | 2.6 |
| 6870 | AT5G03860.1 \| malate synthase, putative, strong similarity to glyoxysomal malate synthase from Brassica napus (SP:P13244) \| chr5:1032103-1034569 REVERSE \| Aliases: MED24.5 | 3.0 | 3.5 | -0.5 | -3.6 | 4.18% | 2.4 |
| 6876 | AT1G03520.1 \| glycosyltransferase family 14 protein / core-2/I-branching enzyme family protein, contains Pfam profile PF02485: Core-2/I-Branching enzyme \| chr1:877863-880159 REVERSE \| Aliases: F21B7.14 | 5.4 | 6.1 | -0.6 | -3.6 | 4.19% | 2.6 |
| 6877 | AT3G53590.1 \| leucine-rich repeat transmembrane protein kinase, putative, CLV1 receptor kinase, Arabidopsis thaliana, EMBL:ATU96879 \| chr3:19878357-19882629 REVERSE \| Aliases: F4P12.290 | 3.1 | 4.2 | -1.1 | -3.6 | 4.19% | 2.7 |
| 6881 | AT2G43570.1 \| chitinase, putative, similar to chitinase class IV GI:722272 from (Brassica napus) \| chr2:18083301-18084539 REVERSE \| Aliases: F18O19.32 | 3.9 | 5.0 | -1.2 | -3.6 | 4.22% | 2.8 |
| 6882 | AT2G37840.2 \| protein kinase family protein, contains protein kinase domain, Pfam:PF00069 \| chr2:15858968-15863379 FORWARD \| Aliases: AT2G37850, T8P21.25, T8P21_25 | 4.6 | 5.7 | -1.1 | -3.6 | 4.22% | 2.6 |
| 6884 | AT1G34320.1 \| expressed protein, contains Pfam domain PF05003: protein of unknown function (DUF668) \| chr1:12520691-12524341 FORWARD \| Aliases: F23M19.3, F23M19_3 | 4.2 | 5.1 | -0.9 | -3.6 | 4.22% | 2.8 |
| 6886 | AT5G54030.1 \| DC1 domain-containing protein, contains Pfam profile PF03107: DC1 domain | 2.8 | 3.3 | -0.5 | -3.6 | 4.23% | 2.2 |
| 6887 | AT2G28200.1 \| similar to zinc finger (C2H2 type) family protein [Arabidopsis thaliana] (TAIR:At5g04390.1); similar to putative zinc finger protein [Pisum sativum] (GB:CAA60828.1); contains InterPro domain Zn-finger, C2H2 type (InterPro:IPR007087) | 5.7 | 6.6 | -0.9 | -3.6 | 4.23% | 2.7 |
| 6888 | AT4G34690.1 \| expressed protein \| chr4:16555468-16556102 REVERSE \| Aliases: T4L20.270, T4L20_270 | 2.6 | 3.0 | -0.5 | -3.6 | 4.23% | 2.3 |
| 6890 | AT1G53100.1 \| similar to glycosyltransferase family 14 protein / core-2/I-branching enzyme family protein [Arabidopsis thaliana] (TAIR:At3g15350.1); similar to glycosyltransferase family 14 protein / core-2/I-branching enzyme family protein [Arabidopsis thaliana] (TAIR:At3g15350.2); similar to BGGP Beta-1-3-galactosyl-O-glycosyl-glycoprotein [Triticum aestivum] (GB:CAH10194.1); similar to putative N-acetylglucosaminyltransferase [Hordeum vulgare subsp. vulgare] (GB:AAV49991.1); similar to glycosylation enzyme-like [Oryza sativa (japonica cultivar-group)] (GB:BAD73208.1); contains InterPro domain Glycosyl transferase, family 14 (InterPro:IPR003406) \| chr1:19790624-19792637 REVERSE \| Aliases: F8L10.4, F8L10_4 | 3.1 | 3.7 | -0.6 | -3.6 | 4.23% | 2.5 |
| 6891 | AT2G05320.1 \| beta-1,2-N-acetylglucosaminyltransferase II, identical to beta-1,2-N-acetylglucosaminyltransferase II GI:10183645 from (Arabidopsis thaliana) \| chr2:1936013-1937461 FORWARD \| Aliases: F5G3.22, F5G3_22 | 4.8 | 5.6 | -0.8 | -3.6 | 4.23% | 2.6 |
| 6892 | AT2G36960.2 \| Symbol: TKI1 \| myb family transcription factor, contains Pfam profile: PF00249 myb-like DNA-binding domain \| chr2:15530027-15534556 FORWARD \| Aliases: T1J8.14, T1J8_14 | 5.1 | 5.7 | -0.7 | -3.6 | 4.23% | 2.6 |
| 6894 | AT2G43680.2 \| calmodulin-binding family protein, similar to SF16 protein (Helianthus annuus) GI:560150; contains Pfam profile PF00612: IQ calmodulin-binding motif \| chr2:18115428-18118904 FORWARD \| Aliases: F18O19.21 | 5.8 | 7.0 | -1.2 | -3.6 | 4.23% | 2.7 |

| Rank | Description | Sync | Root | M | t | adj.q | B |
|---|---|---|---|---|---|---|---|
| 6895 | AT1G33700.2 \| similar to expressed protein [Arabidopsis thaliana] (TAIR:At5g49900.1); similar to expressed protein [Arabidopsis thaliana] (TAIR:At4g10060.1); similar to expressed protein [Arabidopsis thaliana] (TAIR:At3g24180.1); similar to unknown protein [Oryza sativa (japonica cultivar-group)] (GB:AAP54244.1); similar to At5g49900 [Oryza sativa (japonica cultivar-group)] (GB:AAX95400.1); contains InterPro domain Protein of unknown function DUF608 (InterPro:IPR006775) \| chr1:12207208-12214162 REVERSE \| Aliases: F14M2.16, F14M2_16 | 9.1 | 9.6 | -0.5 | -3.6 | 4.24% | 2.2 |
| 6896 | AT2G41140.1 \| Symbol: CRK1 \| calcium-dependent protein kinase, putative / CDPK, putative, similar to calcium/calmodulin-dependent protein kinase CaMK3 (Nicotiana tabacum) gi:16904226:gb:AAL30820 \| chr2:17157465-17160768 FORWARD \| Aliases: CDPK RELATED KINASE 1, T3K9.9, T3K9_9 | 5.4 | 6.3 | -0.9 | -3.6 | 4.24% | 2.7 |
| 6900 | AT2G37360.1 \| ABC transporter family protein \| chr2:15680634-15682999 REVERSE \| Aliases: F3G5.15, F3G5_15 | 2.4 | 3.2 | -0.8 | -3.6 | 4.24% | 2.7 |
| 6902 | NA | 12.5 | 13.1 | -0.5 | -3.6 | 4.25% | 1.7 |
| 6908 | AT1G55390.1 \| DC1 domain-containing protein, similar to hypothetical protein GI:4204272 from (Arabidopsis thaliana) contains weak PHD zinc finger motifs contains weak PHD zinc finger motifs DC1 domain, a divergent protein kinase C domain of unknown function. \| chr1:20684542-20686709 REVERSE \| Aliases: T18I3.3, T18I3_3 | 3.0 | 3.7 | -0.6 | -3.6 | 4.27% | 2.5 |
| 6912 | AT1G07250.1 \| UDP-glucoronosyl/UDP-glucosyl transferase family protein, similar to UDP-glucose glucosyltransferase GI:453245 from (Manihot esculenta) \| chr1:2225899-2227565 FORWARD \| Aliases: F10K1.4, F10K1_4 | 8.2 | 9.0 | -0.8 | -3.6 | 4.27% | 2.3 |
| 6913 | AT5G05190.1 \| expressed protein, similar to unknown protein (emb:CAB88044.1) \| chr5:1541522-1544365 FORWARD \| Aliases: K2A11.6, K2A11_6 | 4.7 | 5.3 | -0.6 | -3.6 | 4.27% | 2.6 |
| 6914 | AT1G27670.1 \| expressed protein \| chr1:9627559-9628483 REVERSE \| Aliases: T22C5.12, T22C5_12 | 2.8 | 3.3 | -0.5 | -3.6 | 4.29% | 2.4 |
| 6916 | AT3G19930.1 \| Symbol: STP4 \| sugar transport protein (STP4), identical to GB:S25009 GI:16524 from (Arabidopsis thaliana) \| chr3:6934780-6937120 FORWARD \| Aliases: MPN9.19, SUGAR TRANSPORTER | 6.2 | 8.2 | -2.0 | -3.6 | 4.30% | 2.7 |
| 6920 | AT1G48480.1 \| Symbol: RKL1 \| leucine-rich repeat transmembrane protein kinase, putative, contains similarity to many predicted protein kinases \| chr1:17922059-17924653 FORWARD \| Aliases: T1N15.9, T1N15_9 | 2.9 | 3.3 | -0.4 | -3.6 | 4.30% | 2.3 |
| 6923 | AT3G49930.1 \| zinc finger (C2H2 type) family protein, contains Pfam profile: PF00096 zinc finger, C2H2 type \| chr3:18521231-18521948 FORWARD \| Aliases: F3A4.10 | 3.7 | 4.5 | -0.8 | -3.6 | 4.31% | 2.7 |
| 6924 | AT3G17611.2 \| rhomboid family protein / zinc finger protein-related, contains Pfam profiles PF01694: Rhomboid family, PF00641: Zn-finger in Ran binding protein and others \| chr3:6024919-6026449 FORWARD \| Aliases: None | 5.3 | 6.6 | -1.4 | -3.6 | 4.31% | 2.7 |
| 6928 | AT4G30630.1 \| expressed protein \| chr4:14950644-14952382 FORWARD \| Aliases: F17I23.30, F17I23_30 | 4.4 | 5.6 | -1.2 | -3.6 | 4.33% | 2.6 |
| 6931 | AT1G47540.2 \| trypsin inhibitor, putative, similar to SP:P26780 Trypsin inhibitor 2 precursor (MTI-2) {Sinapis alba} \| chr1:17457974-17458596 REVERSE \| Aliases: F16N3.19, F16N3_19 | 2.8 | 3.3 | -0.5 | -3.6 | 4.33% | 2.1 |
| 6935 | AT3G08490.1 \| expressed protein \| chr3:2574111-2575131 REVERSE \| Aliases: T8G24.9 | 3.4 | 4.2 | -0.8 | -3.6 | 4.33% | 2.7 |
| 6938 | AT2G25510.1 \| expressed protein \| chr2:10863505-10864234 FORWARD \| Aliases: F13B15.17, F13B15_17 | 2.6 | 3.1 | -0.5 | -3.6 | 4.34% | 2.0 |
| 6944 | AT1G80690.1 \| expressed protein \| chr1:30333961-30335695 REVERSE \| Aliases: F23A5.4, F23A5_4 | 5.3 | 6.1 | -0.8 | -3.6 | 4.35% | 2.6 |
| 6945 | AT4G09130.1 \| zinc finger (C3HC4-type RING finger) family protein, contains Pfam profile: PF00097: Zinc finger, C3HC4 type (RING finger) \| chr4:5815846-5816919 FORWARD \| Aliases: T8A17.9 | 2.3 | 2.5 | -0.3 | -3.6 | 4.35% | 1.9 |
| 6947 | AT5G03040.1 \| calmodulin-binding family protein, similar to SF16 protein (Helianthus annuus) GI:560150; contains Pfam profile PF00612: IQ calmodulin-binding motif \| chr5:710100-712787 REVERSE \| Aliases: F15A17.70, F15A17_70 | 4.2 | 5.2 | -1.0 | -3.6 | 4.35% | 2.7 |
| 6948 | AT4G34140.1 \| D111/G-patch domain-containing protein, contains Pfam PF01585: G-patch domain \| chr4:16350736-16354258 FORWARD \| Aliases: F28A23.100, F28A23_100 | 3.5 | 4.8 | -1.3 | -3.6 | 4.35% | 2.7 |
| 6950 | AT1G20880.1 \| RNA recognition motif (RRM)-containing protein, similar to RRM-containing protein SEB-4 (Xenopus laevis) GI:8895698; contains InterPro entry IPR000504: RNA-binding region RNP-1 (RNA recognition motif) (RRM); is the location of EST 197B1T7 , gb:AA597386 \| chr1:7262032-7265427 REVERSE \| Aliases: F9H16.14, F9H16_14 | 2.9 | 3.5 | -0.6 | -3.6 | 4.36% | 2.5 |
| 6952 | AT1G48430.1 \| dihydroxyacetone kinase family protein, similar to dihydroxyacetone kinases; contains Pfam profiles PF02733: DAK1 domain, PF02734: DAK2 domain \| chr1:17906383-17910461 REVERSE \| Aliases: T1N15.4, T1N15_4 | 6.0 | 7.8 | -1.8 | -3.6 | 4.36% | 2.8 |

| Rank | Description | Sync | Root | M | t | adj.q | B |
|---|---|---|---|---|---|---|---|
| 6957 | AT3G56100.1 \| Symbol: MRLK \| leucine-rich repeat transmembrane protein kinase, putative, hypothetical proteins - Arabidopsis thaliana | 3.3 | 4.0 | -0.7 | -3.6 | 4.37% | 2.6 |
| 6962 | AT5G51390.1 \| expressed protein, similar to unknown protein (gb:AAB68039.1) \| chr5:20895193-20895690 REVERSE \| Aliases: MFG13.10, MFG13_10 | 3.2 | 4.4 | -1.2 | -3.5 | 4.39% | 2.5 |
| 6964 | AT5G20790.1 \| expressed protein, predicted protein, Arabidopsis thaliana \| chr5:7039913-7040656 REVERSE \| Aliases: T1M15.190, T1M15_190 | 4.2 | 5.3 | -1.1 | -3.5 | 4.39% | 2.8 |
| 6966 | AT3G54230.1 \| similar to RNA recognition motif (RRM)-containing protein [Arabidopsis thaliana] (TAIR:At2g16940.1); similar to putative RNA-binding protein 10 [Oryza sativa (japonica cultivar-group)] (GB:XP_464248.1); contains InterPro domain D111/G-patch domain (InterPro:IPR000467); contains InterPro domain RNA-binding region RNP-1 (RNA recognition motif) (InterPro:IPR000504); contains InterPro domain Zn-finger, Ran-binding (InterPro:IPR001876) \| chr3:20084082-20091242 FORWARD \| Aliases: F24B22.190 | 4.7 | 5.7 | -0.9 | -3.5 | 4.40% | 2.6 |
| 6970 | AT5G16320.1 \| Symbol: FRL1 \| expressed protein \| chr5:5344505-5346022 FORWARD \| Aliases: FRIGIDA LIKE 1, MQK4.4, MQK4_4 | 4.0 | 4.5 | -0.6 | -3.5 | 4.40% | 2.3 |
| 6972 | AT3G25410.1 \| bile acid:sodium symporter family protein, low similarity to SP:Q14973 Sodium/bile acid cotransporter (Na(+)/bile acid cotransporter) {Homo sapiens}; contains Pfam profile PF01758: Sodium Bile acid symporter family \| chr3:9215576-9217727 REVERSE \| Aliases: MWL2.2 | 5.3 | 5.9 | -0.7 | -3.5 | 4.41% | 2.5 |
| 6974 | AT1G21370.2 \| expressed protein \| chr1:7483772-7486311 FORWARD \| Aliases: F24J8.26, F24J8_26 | 6.9 | 7.7 | -0.8 | -3.5 | 4.41% | 2.5 |
| 6977 | AT3G06170.1 \| TMS membrane family protein / tumour differentially expressed (TDE) family protein, contains Pfam domain, PF03348: TMS membrane protein/tumour differentially expressed protein (TDE) \| chr3:1867518-1869944 FORWARD \| Aliases: F28L1.11, F28L1_11 | 5.2 | 7.2 | -2.0 | -3.5 | 4.42% | 2.7 |
| 6978 | AT1G65850.1 \| disease resistance protein (TIR-NBS-LRR class), putative, domain signature TIR-NBS-LRR exists, suggestive of a disease resistance protein. \| chr1:24498397-24502148 FORWARD \| Aliases: F12P19.1, F12P19_1 | 2.5 | 2.9 | -0.4 | -3.5 | 4.42% | 2.2 |
| 6979 | AT5G51420.1 \| long-chain-alcohol O-fatty-acyltransferase family protein / wax synthase family protein, contains similarity to wax synthase wax synthase - Simmondsia chinensis, PID:g5020219 similar to wax synthase (gi:5020219) from Simmondsia chinensis \| chr5:20902645-20903952 REVERSE \| Aliases: MFG13.13, MFG13_13 | 3.3 | 3.9 | -0.6 | -3.5 | 4.42% | 2.4 |
| 6980 | AT5G62380.1 \| Symbol: ANAC101 \| no apical meristem (NAM) family protein, contains Pfam PF02365: No apical meristem (NAM) domain; OsNAC7, Oryza sativa, EMBL:AB028186 \| chr5:25067910-25069084 FORWARD \| Aliases: ANAC101, MMI9.6, MMI9_6 | 2.5 | 2.7 | -0.3 | -3.5 | 4.42% | 1.7 |
| 6982 | AT1G63820.1 \| expressed protein, similar to hypothetical protein [Arabidopsis thaliana] (TAIR:At5g41380.1); similar to 'unknown protein, contains CCT motif, PF06203' [Oryza sativa (japonica cultivar-group)] (GB:XP_475389.1) \| chr1:23686085-23688030 REVERSE \| Aliases: T12P18.16, T12P18_16 | 3.4 | 4.1 | -0.7 | -3.5 | 4.43% | 2.5 |
| 6983 | AT4G16143.1 \| importin alpha-2, putative (IMPA-2), similar to importin alpha 2 (Capsicum annuum) GI:13752562; contains Pfam profiles PF01749: Importin beta binding domain, PF00514: Armadillo/beta-catenin-like repeat; non-consensus GG donor splice site at exon 1 and 6; CT acceptor splice site at exon 2 | 3.2 | 3.5 | -0.3 | -3.5 | 4.43% | 1.8 |
| 6984 | AT4G35985.1 \| senescence/dehydration-associated protein-related, similar to senescence-associated protein 12 (Hemerocallis hybrid cultivar) gi:3551958:gb:AAC34857; similar to early-responsive to dehydration stress ERD7 protein (Arabidopsis thaliana) gi:15320412:dbj:BAB63916 \| chr4:17032271-17033865 REVERSE \| Aliases: None | 9.6 | 10.5 | -0.9 | -3.5 | 4.43% | 2.3 |
| 6994 | AT4G26180.1 \| mitochondrial substrate carrier family protein, contains Pfam profile: PF00153 mitochondrial carrier protein \| chr4:13260272-13262044 REVERSE \| Aliases: T25K17.6 | 4.4 | 5.0 | -0.6 | -3.5 | 4.46% | 2.3 |
| 6995 | AT1G70570.1 \| anthranilate phosphoribosyltransferase, putative, similar to anthranilate phosphoribosyltransferase (EC 2.4.2.18) SP:O66576 from (Aquifex aeolicus) \| chr1:26612290-26615947 FORWARD \| Aliases: F24J13.14, F24J13_14 | 6.8 | 7.9 | -1.1 | -3.5 | 4.46% | 2.6 |
| 6996 | AT3G02070.1 \| OTU-like cysteine protease family protein, contains Pfam profile PF02338: OTU-like cysteine protease \| chr3:360928-363336 FORWARD \| Aliases: F1C9.14, F1C9_14 | 4.8 | 5.7 | -0.9 | -3.5 | 4.46% | 2.8 |
| 6997 | AT4G24990.1 \| Symbol: ATGP4 \| ubiquitin family protein, contains INTERPRO:IPR000626 ubiquitin domain \| chr4:12849719-12851643 REVERSE \| Aliases: F13M23.130, F13M23_130 | 9.1 | 10.1 | -1.0 | -3.5 | 4.46% | 2.5 |
| 6998 | AT1G30140.1 \| expressed protein, similar to hypothetical protein [Arabidopsis thaliana] (TAIR:At5g27260.1) \| chr1:10598611-10599680 FORWARD \| Aliases: T2H7.6, T2H7_6 | 3.1 | 3.5 | -0.4 | -3.5 | 4.46% | 2.1 |
| 7002 | AT1G04300.1 \| meprin and TRAF homology domain-containing protein / MATH domain-containing protein, weak similarity to ubiquitin-specific protease 12 (Arabidopsis thaliana) GI:11993471;contains Pfam PF00917: Meprin And TRAF-Homology (MATH) domain \| chr1:1148586-1154597 REVERSE \| Aliases: F19P19.26, F19P19_26 | 6.2 | 7.2 | -1.0 | -3.5 | 4.48% | 2.3 |

| Rank | Description | Sync | Root | M | t | adj.q | B |
|---|---|---|---|---|---|---|---|
| 7004 | AT4G08380.1 \| proline-rich extensin-like family protein, contains proline-rich extensin domains, INTERPRO:IPR002965 \| chr4:5311090-5312403 REVERSE \| Aliases: T28D5.70, T28D5_70 | 3.2 | 4.2 | -1.0 | -3.5 | 4.48% | 2.7 |
| 7009 | AT5G12430.1 \| DNAJ heat shock N-terminal domain-containing protein, similarity to TETRATRICOPEPTIDE REPEAT PROTEIN 2 , human, SWISSPROT:TTC2_HUMAN; contains Pfam profiles PF00226: DnaJ domain, PF00515: TPR Domain \| chr5:4027980-4034087 REVERSE \| Aliases: None | 2.9 | 3.5 | -0.6 | -3.5 | 4.50% | 2.4 |
| 7010 | AT3G22160.1 \| VQ motif-containing protein, contains PF05678: VQ motif \| chr3:7817864-7818992 REVERSE \| Aliases: MKA23.7 | 3.6 | 4.1 | -0.5 | -3.5 | 4.50% | 2.1 |
| 7012 | AT4G36900.1 \| Symbol: RAP2.10 \| encodes a member of the DREB subfamily A-5 of ERF/AP2 transcription factor family (RAP2.10). The protein contains one AP2 domain. There are 16 members in this subfamily including RAP2.9 and RAP2.1. | 6.4 | 7.5 | -1.1 | -3.5 | 4.50% | 2.6 |
| 7022 | AT3G18280.1 \| protease inhibitor/seed storage/lipid transfer protein (LTP) family protein, similar to TED4 (Zinnia elegans) GI:493721; contains Pfam protease inhibitor/seed storage/LTP family domain PF00234 | 9.3 | 10.6 | -1.3 | -3.5 | 4.52% | 2.2 |
| 7024 | AT4G33490.1 \| similar to nucellin protein, putative [Arabidopsis thaliana] (TAIR:At1g44130.1); similar to nucellin-like aspartic protease [Oryza sativa (japonica cultivar-group)] (GB:AAY23257.1); contains InterPro domain Aspartic protease A1, pepsin (InterPro:IPR001461) | 4.2 | 5.0 | -0.8 | -3.5 | 4.52% | 2.6 |
| 7025 | AT1G63500.1 \| protein kinase-related, low similarity to protein kinase (Arabidopsis thaliana); contains Pfam profile: PF00069 Eukaryotic protein kinase domain | 3.5 | 4.1 | -0.6 | -3.5 | 4.52% | 2.5 |
| 7026 | AT2G18680.1 \| expressed protein \| chr2:8101976-8102861 FORWARD \| Aliases: MSF3.6, MSF3_6 | 3.4 | 4.2 | -0.8 | -3.5 | 4.52% | 2.5 |
| 7027 | AT4G07960.1 \| Symbol: ATCSLC12 \| glycosyl transferase family 2 protein, similar to cellulose synthase from Agrobacterium tumeficiens (gi:710492) and Agrobacterium radiobacter (gi:710493); contains Pfam glycosyl transferase, group 2 family protein domain PF00535 | 3.1 | 3.9 | -0.8 | -3.5 | 4.52% | 2.6 |
| 7030 | AT4G30870.1 \| similar to repair endonuclease family protein [Arabidopsis thaliana] (TAIR:At5g39770.1); similar to unnamed protein product [Debaryomyces hansenii CBS767] (GB:CAG90230.1); contains InterPro domain ERCC4 domain (InterPro:IPR006166) \| chr4:15028592-15032467 FORWARD \| Aliases: F6I18.220, F6I18_220 | 3.8 | 5.0 | -1.2 | -3.5 | 4.53% | 2.8 |
| 7032 | AT5G45280.2 \| pectinacetylesterase, putative, similar to pectinacetylesterase precursor GI:1431629 from (Vigna radiata) \| chr5:18362822-18366919 FORWARD \| Aliases: K9E15.6, K9E15_6 | 5.1 | 5.8 | -0.6 | -3.5 | 4.53% | 2.5 |
| 7033 | AT2G47350.2 \| PAPA-1-like family protein / zinc finger (HIT type) family protein, contains Pfam domains, PF04795: PAPA-1-like conserved region and PF04438: HIT zinc finger \| chr2:19440861-19444319 FORWARD \| Aliases: T8I13.19 | 4.8 | 5.5 | -0.7 | -3.5 | 4.53% | 2.3 |
| 7035 | AT2G03760.1 \| Symbol: ST \| steroid sulfotransferase, putative, strong similarity to steroid sulfotransferases from (Brassica napus) GI:3420008, GI:3420004, GI:3420006; contains Pfam profile PF00685: Sulfotransferase domain \| chr2:1149334-1150660 REVERSE \| Aliases: F19B11.21, F19B11_21, RAR047, SULFOTRANSFERASE, SULFURTRANSFERASE | 3.7 | 4.6 | -0.8 | -3.5 | 4.53% | 2.7 |
| 7037 | AT5G54670.1 \| Symbol: ATK3 \| kinesin-like protein C (KATC) \| chr5:22226698-22231360 FORWARD \| Aliases: ARABIDOPSIS THALIANA KINESIN 3, KATC, KINESIN LIKE PROTEIN C, MRB17.18 | 4.4 | 5.5 | -1.2 | -3.5 | 4.53% | 2.6 |
| 7042 | AT3G21270.1 \| Symbol: ADOF2 \| Dof-type zinc finger domain-containing protein (ADOF2), identical to Dof zinc finger protein ADOF2 GI:3608263 from (Arabidopsis thaliana); identical to cDNA adof2 mRNA for Dof zinc finger protein GI:3608262; contains Pfam profile PF02701: Dof domain, zinc finger \| chr3:7474685-7475762 FORWARD \| Aliases: MXL8.14 | 6.0 | 7.1 | -1.1 | -3.5 | 4.54% | 2.5 |
| 7043 | AT3G49880.1 \| glycosyl hydrolase family protein 43, contains similarity to xylanase GI:2645416 from (Caldicellulosiruptor saccharolyticus) \| chr3:18507234-18509698 FORWARD \| Aliases: T16K5.230 | 4.4 | 5.1 | -0.7 | -3.5 | 4.54% | 2.6 |
| 7048 | AT1G15410.1 \| aspartate-glutamate racemase family, contains Pfam profile PF:01177 Aspartate-glutamate racemase family; contains TIGRFAM TIGR00035: aspartate racemase; similar to aspartate racemase (GI:5458794){Pyrococcus abyssi} \| chr1:5298939-5300898 FORWARD \| Aliases: F9L1.36, F9L1_36 | 5.2 | 5.9 | -0.7 | -3.5 | 4.54% | 2.5 |
| 7054 | AT1G76460.1 \| RNA recognition motif (RRM)-containing protein, low similarity to RRM-containing protein SEB-4 (Xenopus laevis) GI:8895698; contains Pfam profile: PF00076 RNA recognition motif. (a.k.a. RRM, RBD, or RNP domain) \| chr1:28691023-28694015 REVERSE \| Aliases: F15M4.25 | 3.1 | 4.1 | -1.0 | -3.5 | 4.55% | 2.6 |
| 7060 | AT4G34590.1 \| Symbol: GBF6 \| bZIP transcription factor family protein, similar to common plant regulatory factor 7 GI:9650828 from (Petroselinum crispum) \| chr4:16521866-16523235 FORWARD \| Aliases: T4L20.170, T4L20_170 | 7.0 | 8.6 | -1.7 | -3.5 | 4.55% | 2.7 |
| 7061 | AT4G30640.1 \| F-box family protein (FBL19), contains similarity to SKP1 interacting partner 1 GI:10716947 from (Arabidopsis thaliana) \| chr4:14952676-14953688 FORWARD \| Aliases: F17I23.20, F17I23_20 | 2.9 | 3.5 | -0.6 | -3.5 | 4.55% | 2.4 |

| Rank | Description | Sync | Root | M | t | adj.q | B |
|---|---|---|---|---|---|---|---|
| 7062 | AT2G35890.1 \| Symbol: CPK25 \| calcium-dependent protein kinase, putative / CDPK, putative, similar to calcium-dependent protein kinase, isoform AK1 (CDPK). (Arabidopsis thaliana) SWISS-PROT:Q06850; contains protein kinase domain, Pfam:PF00069; contains EF hand domain (calcium-binding EF-hand), Pfam:PF00036, INTERPRO:IPR002048 \| chr2:15074254-15076215 REVERSE \| Aliases: F11F19.20, F11F19_20 | 2.9 | 3.4 | -0.4 | -3.5 | 4.55% | 1.9 |
| 7063 | AT5G18760.1 \| zinc finger (C3HC4-type RING finger) family protein, predicted proteins, Arabidopsis thaliana ; contains Pfam profile PF00097: Zinc finger, C3HC4 type (RING finger) \| chr5:6258300-6260882 REVERSE \| Aliases: F17K4.10, F17K4_10 | 3.8 | 4.2 | -0.5 | -3.5 | 4.56% | 2.3 |
| 7065 | AT5G59320.1 \| Symbol: LTP3 \| lipid transfer protein 3 (LTP3), identical to lipid transfer protein 3 from Arabidopsis thaliana (gi:8571921); contains Pfam protease inhibitor/seed storage/LTP family domain PF00234 \| chr5:23946197-23946965 FORWARD \| Aliases: LIPID TRANSFER PROTEIN 3, MNC17.10, MNC17_10 | 2.8 | 3.7 | -0.9 | -3.5 | 4.57% | 2.2 |
| 7066 | AT3G26760.1 \| short-chain dehydrogenase/reductase (SDR) family protein, similar to sex determination protein tasselseed 2 SP:P50160 from (Zea mays) \| chr3:9844828-9846413 FORWARD \| Aliases: MDJ14.5 | 4.3 | 4.9 | -0.6 | -3.5 | 4.57% | 2.5 |
| 7068 | AT3G58650.1 \| expressed protein \| chr3:21707223-21709828 REVERSE \| Aliases: F14P22.240 | 2.9 | 3.2 | -0.3 | -3.5 | 4.58% | 1.9 |
| 7070 | AT3G56500.1 \| serine-rich protein-related, contains some similarity to serine-rich proteins \| chr3:20954617-20954949 FORWARD \| Aliases: T5P19.150 | 2.7 | 3.3 | -0.6 | -3.5 | 4.60% | 2.4 |
| 7071 | AT3G12730.1 \| myb family transcription factor, contains Pfam profile: PF00249 myb-like DNA-binding domain \| chr3:4047079-4048621 REVERSE \| Aliases: MBK21.11 | 2.2 | 2.5 | -0.3 | -3.5 | 4.60% | 2.1 |
| 7074 | AT3G15950.2 \| similar to caldesmon-related [Arabidopsis thaliana] (TAIR:At1g52410.2); similar to caldesmon-related [Arabidopsis thaliana] (TAIR:At1g52410.1); similar to hypothetical protein FG09227.1 [Gibberella zeae PH-1] (GB:EAA76462.1) | 3.5 | 4.4 | -1.0 | -3.5 | 4.60% | 2.4 |
| 7075 | AT5G11250.1 \| disease resistance protein (TIR-NBS-LRR class), putative, domain signature TIR-NBS-LRR exists, suggestive of a disease resistance protein. \| chr5:3587979-3591961 REVERSE \| Aliases: F2I11.140, F2I11_140 | 3.1 | 3.5 | -0.4 | -3.5 | 4.60% | 2.1 |
| 7077 | AT3G60030.1 \| squamosa promoter-binding protein-like 12 (SPL12), identical to squamosa promoter binding protein-like 12 (Arabidopsis thaliana) GI:6006395; contains Pfam profiles PF03110: SBP domain, PF00023: Ankyrin repeat | 6.4 | 7.8 | -1.3 | -3.5 | 4.61% | 2.6 |
| 7079 | AT1G02860.1 \| SPX (SYG1/Pho81/XPR1) domain-containing protein / zinc finger (C3HC4-type RING finger) protein-related, weak similarity to tripartite motif protein TRIM13 (Mus musculus) GI:12407427, gpStaf50 (Homo sapiens) GI:899300; contains Pfam profiles PF03105: SPX domain, PF00097: Zinc finger, C3HC4 type (RING finger) \| chr1:635144-637262 FORWARD \| Aliases: F22D16.14, F22D16_14 | 6.5 | 7.7 | -1.2 | -3.5 | 4.62% | 2.4 |
| 7082 | AT5G01800.1 \| saposin B domain-containing protein, contains Pfam profiles: PF00026 eukaryotic aspartyl protease, PF03489 surfactant protein B, PF05184 saposin-like type B, region 1 \| chr5:306967-308905 FORWARD \| Aliases: T20L15.70, T20L15_70 | 6.4 | 8.0 | -1.6 | -3.5 | 4.62% | 2.7 |
| 7083 | AT1G70660.1 \| ubiquitin-conjugating enzyme family protein, similar to TRAF6-regulated IKK activator 1 beta Uev1A (Homo sapiens) GI:10880969; contains Pfam profile PF00179: Ubiquitin-conjugating enzyme \| chr1:26644258-26645810 FORWARD \| Aliases: F5A18.16, F5A18_16 | 8.1 | 9.1 | -1.0 | -3.5 | 4.62% | 2.7 |
| 7085 | AT3G47060.1 \| Symbol: FTSH7 \| encodes an FtsH protease that is localized to the chloroplast \| chr3:17343970-17347951 FORWARD \| Aliases: F13I12.110, FTSH7 | 4.8 | 5.7 | -0.8 | -3.5 | 4.62% | 2.6 |
| 7088 | AT5G54780.1 \| similar to RabGAP/TBC domain-containing protein [Arabidopsis thaliana] (TAIR:At4g27100.1); similar to putative GTPase activating protein [Oryza sativa (japonica cultivar-group)] (GB:XP_468334.1); contains InterPro domain RabGAP/TBC domain (InterPro:IPR000195) \| chr5:22265673-22269089 REVERSE \| Aliases: MBG8.4, MBG8_4 | 3.7 | 4.3 | -0.6 | -3.5 | 4.63% | 2.5 |
| 7089 | AT1G66800.1 \| similar to Eucalyptus gunnii alcohol dehydrogenase of unknown physiological function (GI:1143445), apple tree, PIR:T16995; NOT a cinnamyl-alcohol dehydrogenase \| chr1:24928476-24930028 FORWARD \| Aliases: F4N21.7, F4N21_7 | 3.1 | 4.0 | -0.9 | -3.5 | 4.63% | 2.6 |
| 7090 | AT5G59100.1 \| subtilase family protein, contains similarity to prepro-cucumisin GI:807698 from (Cucumis melo) \| chr5:23876120-23879355 REVERSE \| Aliases: K18B18.7, K18B18_7 | 2.9 | 3.2 | -0.3 | -3.5 | 4.63% | 1.8 |
| 7093 | AT5G42440.1 \| protein kinase family protein, contains protein kinase domain, Pfam:PF00069 \| chr5:16990532-16991802 REVERSE \| Aliases: MDH9.13, MDH9_13 | 3.6 | 4.1 | -0.5 | -3.5 | 4.64% | 2.2 |
| 7094 | AT2G23700.1 \| expressed protein, contains Pfam profile PF04784: Protein of unknown function, DUF547 \| chr2:10083449-10087312 REVERSE \| Aliases: F27L4.12, F27L4_12 | 3.7 | 4.9 | -1.1 | -3.5 | 4.64% | 2.4 |
| 7097 | AT1G06840.1 \| leucine-rich repeat transmembrane protein kinase, putative, similar to receptor protein kinase GB:BAA11869 GI:1389566 from (Arabidopsis thaliana) \| chr1:2097746-2103478 REVERSE \| Aliases: F4H5.8, F4H5_8 | 4.5 | 5.3 | -0.8 | -3.5 | 4.64% | 2.3 |

| Rank | Description | Sync | Root | M | t | adj.q | B |
|---|---|---|---|---|---|---|---|
| 7098 | AT1G56660.1 \| expressed protein \| chr1:21241553-21244223 FORWARD \| Aliases: F25P12.91, F25P12_91 | 6.4 | 8.5 | -2.0 | -3.5 | 4.64% | 2.6 |
| 7100 | AT2G46330.2 \| Symbol: AGP16 \| arabinogalactan-protein (AGP16), identical to gi:10880509:gb:AAG24284 \| chr2:19025581-19026328 REVERSE \| Aliases: ARABINOGALACTAN PROTEIN 16, F11C10.2 | 9.0 | 10.2 | -1.2 | -3.5 | 4.64% | 2.6 |
| 7104 | AT2G45820.1 \| DNA-binding protein, putative, identical to DNA-binding protein gi:601843:gb:AAA57124 (Arabidopsis thaliana); contains Pfam domain, PF03766: Remorin, N-terminal region; contains Pfam domain, PF03763: Remorin, C-terminal region \| chr2:18870032-18871735 REVERSE \| Aliases: F4I18.20 | 9.5 | 10.3 | -0.8 | -3.5 | 4.65% | 2.5 |
| 7105 | AT2G24130.1 \| leucine-rich repeat transmembrane protein kinase, putative \| chr2:10265228-10268300 FORWARD \| Aliases: F27D4.4, F27D4_4 | 3.1 | 3.6 | -0.5 | -3.5 | 4.65% | 2.0 |
| 7108 | AT1G24620.1 \| polcalcin, putative / calcium-binding pollen allergen, putative, similar to polcalcin Jun o 2 (calcium-binding pollen allergen Jun o 2) SP:O64943 from (Juniperus oxycedrus) \| chr1:8723698-8724445 REVERSE \| Aliases: F21J9.28 | 3.3 | 3.8 | -0.5 | -3.5 | 4.66% | 2.2 |
| 7110 | AT5G57970.2 \| similar to methyladenine glycosylase family protein [Arabidopsis thaliana] (TAIR:At1g80850.1); similar to OSJNBa0029H02.18 [Oryza sativa (japonica cultivar-group)] (GB:XP_473062.1); contains InterPro domain Methyladenine glycosylase (InterPro:IPR005019) \| chr5:23484171-23486241 FORWARD \| Aliases: MTI20.23, MTI20_23 | 5.5 | 6.9 | -1.4 | -3.5 | 4.66% | 2.7 |
| 7112 | AT1G14380.3 \| similar to calmodulin-binding family protein [Arabidopsis thaliana] (TAIR:At2g02790.1); similar to 'unknown protein, contains IQ calmodulin-binding motif' [Oryza sativa (japonica cultivar-group)] (GB:XP_475770.1); contains InterPro domain IQ calmodulin-binding region (InterPro:IPR000048) | 5.6 | 6.4 | -0.7 | -3.5 | 4.66% | 2.4 |
| 7116 | AT2G25730.1 \| expressed protein \| chr2:10963380-10979807 REVERSE \| Aliases: F3N11.1, F3N11_1 | 7.2 | 7.9 | -0.7 | -3.5 | 4.68% | 2.3 |
| 7121 | AT3G47990.1 \| zinc finger (C3HC4-type RING finger) family protein, contains Pfam profile: PF00097 zinc finger, C3HC4 type (RING finger) \| chr3:17724129-17727293 REVERSE \| Aliases: T17F15.140 | 6.3 | 8.0 | -1.6 | -3.5 | 4.71% | 2.6 |
| 7123 | AT3G47400.1 \| pectinesterase family protein, similar to pectinesterase (EC 3.1.1.11) from Vitis vinifera GI:15081598, Lycopersicon esculentum SP:Q43143 SP:P14280; contains Pfam profile PF01095 pectinesterase \| chr3:17476575-17479103 FORWARD \| Aliases: T21L8.150 | 3.0 | 3.7 | -0.7 | -3.5 | 4.71% | 2.4 |
| 7124 | AT3G18670.1 \| ankyrin repeat family protein, contains ankyrin repeats, Pfam domain PF00023 \| chr3:6424141-6426477 REVERSE \| Aliases: MVE11.3 | 3.1 | 3.6 | -0.5 | -3.5 | 4.71% | 2.2 |
| 7132 | AT5G24170.1 \| expressed protein \| chr5:8189489-8191394 REVERSE \| Aliases: K12G2.6, K12G2_6 | 4.3 | 5.0 | -0.7 | -3.5 | 4.74% | 2.5 |
| 7137 | AT5G28080.2 \| Symbol: WNK9 \| similar to protein kinase family protein [Arabidopsis thaliana] (TAIR:At3g04910.1); similar to mitogen activated protein kinase kinase [Oryza sativa] (GB:AAC32599.1); contains InterPro domain Serine/threonine protein kinase, active site (InterPro:IPR008271); contains InterPro domain Tyrosine protein kinase (InterPro:IPR001245); contains InterPro domain Serine/threonine protein kinase (InterPro:IPR002290); contains InterPro domain Protein kinase (InterPro:IPR000719) \| chr5:10090074-10092406 REVERSE \| Aliases: T24G3.10, T24G3_10 | 3.8 | 4.6 | -0.8 | -3.5 | 4.75% | 2.4 |
| 7139 | AT5G11210.1 \| Symbol: ATGLR2.5 \| glutamate receptor family protein (GLR2.5), plant glutamate receptor family, PMID:11379626 \| chr5:3571215-3574538 REVERSE \| Aliases: F2I11.100, F2I11_100, GLR2.5 | 2.4 | 2.8 | -0.4 | -3.5 | 4.75% | 1.9 |
| 7140 | AT2G25620.1 \| protein phosphatase 2C, putative / PP2C, putative \| chr2:10910021-10912323 REVERSE \| Aliases: F3N11.7, F3N11_7 | 4.5 | 5.5 | -1.0 | -3.5 | 4.76% | 2.6 |
| 7143 | AT5G36840.1 \| expressed protein, similar to At3g24380, At5g35010, At3g42740, At4g05290, At2g14770, At3g43390, At2g05560, At4g08880, At1g34730, At1g27790 \| chr5:14525317-14527433 REVERSE \| Aliases: F5H8.10, F5H8_10 | 2.5 | 3.1 | -0.6 | -3.5 | 4.76% | 2.3 |
| 7144 | AT5G04980.1 \| endonuclease/exonuclease/phosphatase family protein, contains similarity to inositol polyphosphate 5-phosphatase I (GI:10444261) and II (GI:10444263) (Arabidopsis thaliana); contains Pfam profile PF03372: Endonuclease/Exonuclease/phosphatase family | 3.5 | 4.1 | -0.6 | -3.5 | 4.76% | 2.3 |
| 7150 | AT3G03570.1 \| expressed protein, similar to hypothetical protein GB:CAB38918 (Arabidopsis thaliana) \| chr3:856903-860476 FORWARD \| Aliases: T12J13.15, T12J13_15 | 3.7 | 4.3 | -0.6 | -3.5 | 4.78% | 2.5 |
| 7151 | AT4G24220.1 \| Symbol: VEP1 \| expressed protein, protein induced upon wounding - Arabidopsis thaliana, PID:e257749 \| chr4:12564955-12567274 FORWARD \| Aliases: AWI31, T22A6.50, T22A6_50, VEIN PATTERNING | 7.6 | 8.8 | -1.3 | -3.5 | 4.78% | 2.6 |
| 7152 | AT2G27590.1 \| expressed protein \| chr2:11784714-11786928 REVERSE \| Aliases: F10A12.26, F10A12_26 | 4.9 | 5.6 | -0.7 | -3.5 | 4.78% | 2.4 |
| 7157 | AT2G14210.1 \| Symbol: ANR1 \| MADS-box protein (ANR1), identical to ANR1, MADS-box protein (Arabidopsis thaliana) GI:2959320 \| chr2:6025640-6030945 FORWARD \| Aliases: F15N24.5, F15N24_5 | 2.5 | 3.1 | -0.5 | -3.5 | 4.79% | 2.1 |
| 7161 | AT5G58620.1 \| zinc finger (CCCH-type) family protein, contains Pfam domain, PF00642: Zinc finger C-x8-C-x5-C-x3-H type (and similar) and Pfam domain, PF00023: Ankyrin repeat \| chr5:23710566-23713448 FORWARD \| Aliases: MZN1.16, MZN1_16 | 3.7 | 5.0 | -1.3 | -3.5 | 4.80% | 2.6 |

| Rank | Description | Sync | Root | M | t | adj.q | B |
|---|---|---|---|---|---|---|---|
| 7162 | AT1G61670.1 \| expressed protein, similar to membrane protein PTM1 precursor isolog GI:1931644 from (Arabidopsis thaliana) \| chr1:22773694-22776091 FORWARD \| Aliases: T13M11.2, T13M11_2 | 6.3 | 7.4 | -1.2 | -3.5 | 4.80% | 2.7 |
| 7163 | AT4G18070.4 \| expressed protein, similar to expressed protein [Arabidopsis thaliana] (TAIR:At1g29530.1); similar to hypothetical protein DDB0183896 [Dictyostelium discoideum] (GB:EAL61706.1); contains domain SER_RICH (PS50324) \| chr4:10030709-10032947 FORWARD \| Aliases: F15J5.40, F15J5_40 | 6.2 | 7.4 | -1.2 | -3.5 | 4.80% | 2.4 |
| 7165 | AT1G70140.1 \| Symbol: ATFH8 \| Encodes a group I formin. Binds to F-actin barbed ends. Has severing actin filaments activity. Binds profilin. Involved in the initiation and tip growth of root hairs through regulation of actin cytoskeleton. \| chr1:26416351-26418893 REVERSE \| Aliases: ATFH8, F20P5.14, F20P5_14, FORMIN 8 | 6.6 | 7.5 | -0.9 | -3.5 | 4.82% | 2.6 |
| 7166 | AT1G17580.1 \| Symbol: MYA1 \| myosin, putative, similar to myosin GI:433663 from (Arabidopsis thaliana) \| chr1:6039296-6049549 FORWARD \| Aliases: ATMYA1, F1L3.28, F1L3_28 | 5.7 | 6.5 | -0.8 | -3.5 | 4.83% | 2.6 |
| 7171 | AT3G49530.1 \| Symbol: ANAC062 \| no apical meristem (NAM) family protein, contains Pfam PF02365: No apical meristem (NAM) domain; similar to NAC2 - Arabidopsis thaliana, EMBL:AF201456 \| chr3:18373429-18375898 REVERSE \| Aliases: ANAC062, T9C5.120 | 6.9 | 7.6 | -0.8 | -3.5 | 4.84% | 2.5 |
| 7174 | AT4G37520.1 \| peroxidase 50 (PER50) (P50) (PRXR2), identical to SP:Q43731 Peroxidase 50 precursor (EC 1.11.1.7) (Atperox P50) (PRXR2) (ATP9a)) {Arabidopsis thaliana} \| chr4:17631556-17633243 FORWARD \| Aliases: F19F18.10, F19F18_10 | 3.3 | 6.2 | -2.9 | -3.5 | 4.85% | 2.5 |
| 7175 | AT4G37530.1 \| peroxidase, putative, similar to peroxidase (Arabidopsis thaliana) gi:1402906:emb:CAA66958; identical to Pfam profile PF00141: Peroxidase; identical to cDNA peroxidase ATP37 GI:18874553 \| chr4:17634778-17636282 FORWARD \| Aliases: F19F18.20, F19F18_20 | 3.3 | 6.2 | -2.9 | -3.5 | 4.85% | 2.5 |
| 7178 | AT4G14020.1 \| rapid alkalinization factor (RALF) family protein \| chr4:8095210-8095731 REVERSE \| Aliases: DL3050C, FCAALL.138 | 4.1 | 5.2 | -1.1 | -3.5 | 4.85% | 2.7 |
| 7181 | AT3G06780.1 \| glycine-rich protein \| chr3:2143494-2144527 FORWARD \| Aliases: F3E22.8 | 4.7 | 5.2 | -0.5 | -3.5 | 4.85% | 2.3 |
| 7182 | AT5G01620.2 \| expressed protein, several hypothetical proteins - Arabidopsis thaliana \| chr5:232732-234878 FORWARD \| Aliases: F7A7.140, F7A7_140 | 6.7 | 7.8 | -1.0 | -3.5 | 4.86% | 2.4 |
| 7184 | AT4G09670.1 \| oxidoreductase family protein, similar to AX110P (Daucus carota) GI:285739; contains Pfam profiles PF01408: Oxidoreductase family NAD-binding Rossmann fold, PF02894: Oxidoreductase family C-terminal alpha/beta domain | 5.6 | 6.9 | -1.3 | -3.5 | 4.87% | 2.5 |
| 7187 | AT4G00900.1 \| Symbol: ECA2 \| calcium-transporting ATPase 2, endoplasmic reticulum-type (ECA2), nearly identical to SP:O23087 Calcium-transporting ATPase 2, endoplasmic reticulum-type (EC 3.6.3.8) {Arabidopsis thaliana}; contains InterPro Accession IPR006069: Cation transporting ATPase | 4.9 | 6.8 | -1.8 | -3.5 | 4.88% | 2.5 |
| 7191 | AT5G45710.2 \| Symbol: AT HSFA4C \| heat shock transcription factor family protein, contains Pfam profile: PF00447 HSF-type DNA-binding domain \| chr5:18558096-18560090 FORWARD \| Aliases: HSFA4C, MRA19.11, MRA19_11, RHA1 | 4.7 | 5.6 | -0.9 | -3.5 | 4.89% | 2.7 |
| 7192 | AT1G07680.1 \| hypothetical protein \| chr1:2376059-2376990 FORWARD \| Aliases: F24B9.23, F24B9_23 | 3.5 | 4.3 | -0.9 | -3.5 | 4.90% | 2.5 |
| 7193 | AT2G16660.1 \| nodulin family protein, similar to nodulin-like protein (Arabidopsis thaliana) GI:3329368, nodule-specific protein Nlj70 (Lotus japonicus) GI:3329366 \| chr2:7225788-7228707 REVERSE \| Aliases: T24I21.7, T24I21_7 | 7.6 | 9.7 | -2.1 | -3.5 | 4.90% | 2.5 |
| 7194 | AT5G54490.1 \| Symbol: PBP1 \| calcium-binding EF-hand protein, putative, similar to EF-hand Ca2+-binding protein CCD1 (Triticum aestivum) GI:9255753; contains INTERPRO:IPR002048 calcium-binding EF-hand domain \| chr5:22138630-22139157 FORWARD \| Aliases: F24B18.11, F24B18_11, PID BINDING PROTEIN 1, PINOID BINDING PROTEIN 1 | 3.6 | 4.7 | -1.1 | -3.5 | 4.91% | 2.6 |
| 7195 | AT4G25160.1 \| protein kinase family protein, contains Pfam profile: PF00069 Eukaryotic protein kinase domain \| chr4:12903308-12907348 REVERSE \| Aliases: F24A6.13 | 3.2 | 3.9 | -0.6 | -3.5 | 4.91% | 2.4 |
| 7204 | AT1G47410.1 \| expressed protein, identical to hypothetical protein GB:AAD46041 GI:5668815 from (Arabidopsis thaliana) \| chr1:17397258-17397948 FORWARD \| Aliases: T3F24.15 | 5.5 | 6.8 | -1.3 | -3.4 | 4.93% | 2.7 |
| 7207 | AT2G43590.1 \| chitinase, putative, similar to basic endochitinase CHB4 precursor SP:Q06209 from (Brassica napus) \| chr2:18088408-18089826 REVERSE \| Aliases: F18O19.30 | 4.3 | 4.8 | -0.5 | -3.4 | 4.94% | 2.3 |
| 7208 | AT5G24240.1 \| phosphatidylinositol 3- and 4-kinase family protein / ubiquitin family protein, contains INTERPRO:IPR000626 ubiquitin domain, Pfam profile PF00454: Phosphatidylinositol 3- and 4-kinase \| chr5:8231113-8232928 REVERSE \| Aliases: MOP9.5, MOP9_5 | 3.6 | 4.1 | -0.6 | -3.4 | 4.95% | 2.2 |
| 7209 | AT5G57070.1 \| hydroxyproline-rich glycoprotein family protein, Common family members: At5g26070, At5g19800, At1g72790 (Arabidopsis thaliana) \| chr5:23113400-23115677 FORWARD \| Aliases: MUL3.1, MUL3_1 | 2.5 | 3.3 | -0.8 | -3.4 | 4.95% | 2.4 |

| Rank | Description | Sync | Root | M | t | adj.q | B |
|---|---|---|---|---|---|---|---|
| 7215 | AT4G19990.1 \| similar to far-red impaired responsive protein, putative [Arabidopsis thaliana] (TAIR:At3g22170.1); similar to putative far-red impaired response protein [Oryza sativa (japonica cultivar-group)] (GB:AAP50996.1); similar to putative FAR1 protein [Oryza sativa (japonica cultivar-group)] (GB:AAT78829.1); contains InterPro domain SWIM Zn-finger (InterPro:IPR007527); contains InterPro domain Zn-finger, PMZ type (InterPro:IPR006564); contains InterPro domain FAR1 (InterPro:IPR004330) \| chr4:10832858-10835710 FORWARD \| Aliases: F18F4.90, F18F4_90 | 4.8 | 5.8 | -1.0 | -3.4 | 4.96% | 2.7 |
| 7216 | AT1G66400.1 \| calmodulin-related protein, putative, similar to calmodulin-related protein 2, touch-induced from SP:P25070 (Arabidopsis thaliana); contains Pfam profile: PF00036 EF hand (4 copies) \| chr1:24774238-24775034 REVERSE \| Aliases: T27F4.15, T27F4_15 | 4.1 | 4.9 | -0.8 | -3.4 | 4.97% | 2.6 |
| 7217 | AT1G65040.2 \| similar to zinc finger (C3HC4-type RING finger) family protein [Arabidopsis thaliana] (TAIR:At3g16090.1); similar to hypothetical protein [Homo sapiens] (GB:CAD38937.1); similar to PREDICTED: similar to synoviolin 1 isoform a [Pan troglodytes] (GB:XP_522059.1); similar to Syvn1 protein [Mus musculus] (GB:AAH46829.1); similar to Synoviolin1 [Homo sapiens] (GB:BAC57449.1); similar to Synoviolin 1, isoform b [Homo sapiens] (GB:AAH30530.1); contains InterPro domain Zn-finger, RING (InterPro:IPR001841) \| chr1:24163497-24167291 REVERSE \| Aliases: F16G16.3, F16G16_3 | 4.3 | 5.5 | -1.2 | -3.4 | 4.97% | 2.7 |
| 7219 | AT3G02470.2 \| Symbol: SAMDC \| similar to adenosylmethionine decarboxylase family protein [Arabidopsis thaliana] (TAIR:At5g15950.1); similar to S-adenosyl-L-methionine decarboxylase [Brassica juncea] (GB:AAF20160.1); contains InterPro domain S-adenosylmethionine decarboxylase (InterPro:IPR001985) \| chr3:509428-511583 FORWARD \| Aliases: ADOMETDC, F16B3.10, F16B3_10, S ADENOSYLMETHIONINE DECARBOXYLASE | 11.8 | 12.4 | -0.6 | -3.4 | 4.97% | 1.8 |
| 7221 | AT5G53110.1 \| expressed protein \| chr5:21546248-21546970 FORWARD \| Aliases: MFH8.3, MFH8_3 | 3.0 | 3.7 | -0.7 | -3.4 | 4.98% | 2.4 |
| 7222 | AT1G11160.1 \| WD-40 repeat family protein / katanin p80 subunit, putative, similar to contains 6 WD-40 repeats (PF00400); katanin p80 subunit (GI:3005601) (Strongylocentrotus purpuratus) \| chr1:3733925-3739703 FORWARD \| Aliases: T28P6.17, T28P6_17 | 2.7 | 3.1 | -0.4 | -3.4 | 4.98% | 2.1 |
| 7224 | AT1G05030.1 \| hexose transporter, putative, similar to hexose transporters from Nicotiana tabacum (GI:8347244), Solanum tuberosum (GI:8347246), Arabidopsis thaliana (GI:8347250); contains Pfam profile PF00083: major facilitator superfamily protein \| chr1:1438151-1441416 REVERSE \| Aliases: T7A14.10, T7A14_10 | 5.6 | 6.8 | -1.2 | -3.4 | 4.98% | 2.5 |
| 7226 | AT4G38240.2 \| Symbol: CGL1 \| alpha-1,3-mannosyl-glycoprotein beta-1,2-N-acetylglucosaminyltransferase, putative, similar to N-acetylglucosaminyltransferase I from Arabidopsis thaliana (gi:5139335); contains AT-AC non-consensus splice sites at intron 13 \| chr4:17931564-17935319 REVERSE \| Aliases: CGL, COMPLEX GLYCAN LESS, F20D10.360, F20D10_360 | 4.0 | 5.0 | -1.0 | -3.4 | 4.99% | 2.3 |
| 7229 | AT4G35090.2 \| Symbol: CAT2 \| similar to catalase 3 (SEN2) [Arabidopsis thaliana] (TAIR:At1g20620.2); similar to catalase 1 [Arabidopsis thaliana] (TAIR:At1g20630.1); similar to catalase 3 (SEN2) [Arabidopsis thaliana] (TAIR:At1g20620.1); similar to catalase [Raphanus sativus] (GB:AAF71742.1); similar to catalase [Raphanus sativus] (GB:AAB86582.2); similar to catalase [Brassica juncea] (GB:AAD17935.1); similar to catalase [Brassica juncea] (GB:AAD17936.1); similar to catalase [Brassica juncea] (GB:AAD17934.1); contains InterPro domain Catalase (InterPro:IPR002226) \| chr4:16700347-16703291 REVERSE \| Aliases: CATALASE, CATALASE 2, T12J5.2 | 8.3 | 10.6 | -2.4 | -3.4 | 5.00% | 2.6 |

| Rank | Description | Sync | Root | M | t | adj.q | B |
|---|---|---|---|---|---|---|---|
| 7232 | AT1G04550.2 \| Symbol: IAA12 \| auxin-responsive protein / indoleacetic acid-induced protein 12 (IAA12), identical to SP:Q38830 Auxin-responsive protein IAA12 (Indoleacetic acid-induced protein 12) {Arabidopsis thaliana} \| chr1:1240413-1242119 FORWARD \| Aliases: AUXIN INDUCED PROTEIN 12, BDL, BODENLOS, T1G11.20, T1G11_20 | 5.5 | 4.6 | 0.9 | 3.4 | 5.0% | 2.4 |
| 7233 | AT5G22890.1 \| zinc finger (C2H2 type) family protein, contains Pfam domain PF00096: Zinc finger, C2H2 type \| chr5:7653264-7654727 REVERSE \| Aliases: MRN17.12, MRN17_12 | 2.9 | 3.3 | -0.4 | -3.4 | 5.0% | 2.0 |
| 7234 | AT1G49160.2 \| Symbol: WNK7 \| protein kinase family protein, contains protein kinase domain, Pfam:PF00069 \| chr1:18182972-18185535 REVERSE \| Aliases: F27J15.7, F27J15_7 | 5.0 | 6.3 | -1.3 | -3.4 | 5.0% | 2.5 |
| 7235 | AT1G23880.1 \| NHL repeat-containing protein, contains Pfam profile PF01436: NHL repeat | 3.8 | 3.4 | 0.4 | 3.4 | 5.0% | 2.1 |
| 7236 | AT1G61000.1 \| Nuf2 family protein, contains Pfam PF03800: Nuf2 family domain; similar to Myosin-like protein NUF2 (Nuclear filament-containing protein 2) (Nuclear division protein nuf2) (Swiss-Prot:Q10173) (Schizosaccharomyces pombe) \| chr1:22469433-22477711 REVERSE \| Aliases: T7P1.14, T7P1_14 | 3.7 | 4.3 | -0.6 | -3.4 | 5.0% | 2.2 |
| 7237 | AT5G12220.1 \| las1-like family protein, similar to Las1p (Saccharomyces cerevisiae) GI:495504; contains Pfam profile PF04031: Las1-like \| chr5:3950156-3953086 FORWARD \| Aliases: MXC9.18, MXC9_18 | 4.0 | 3.3 | 0.6 | 3.4 | 5.0% | 2.3 |
| 7238 | AT2G05160.1 \| zinc finger (CCCH-type) family protein / RNA recognition motif (RRM)-containing protein, contains InterPro entry IPR000504: RNA-binding region RNP-1 (RNA recognition motif) (RRM) \| chr2:1859008-1860928 REVERSE \| Aliases: F5G3.6, F5G3_6 | 2.1 | 2.5 | -0.4 | -3.4 | 5.0% | 1.8 |
| 7239 | AT4G39080.1 \| vacuolar proton ATPase, putative, similar to Swiss-Prot:Q93050 vacuolar proton translocating ATPase 116 kDa subunit A isoform 1 (Clathrin-coated vesicle/synaptic vesicle proton pump 116 kDa subunit, Vacuolar proton pump subunit 1, Vacuolar adenosine triphosphatase subunit Ac116) (Homo sapiens) \| chr4:18209423-18216343 FORWARD \| Aliases: F19H22.180, F19H22_180 | 6.3 | 7.9 | -1.5 | -3.4 | 5.0% | 2.6 |
| 7240 | AT5G38030.1 \| MATE efflux family protein, similar to ripening regulated protein DDTFR18 (Lycopersicon esculentum) GI:12231296; contains Pfam profile PF01554: Uncharacterized membrane protein family; putative multidrug efflux protein NorM - Vibrio parahaemolyticus, EMBL:AB010463 | 3.3 | 4.0 | -0.7 | -3.4 | 5.0% | 2.5 |
| 7241 | AT1G11820.1 \| similar to glycosyl hydrolase family 17 protein / beta-1,3-glucanase, putative [Arabidopsis thaliana] (TAIR:At2g01630.1); similar to E131_ARATH Putative glucan endo-1,3-beta-glucosidase 1 precursor ((1->3)-beta-glucan endohydrolase) ((1->3)-beta-glucanase) (Beta-1,3-endoglucanase) (Beta-1,3-glucanase) (GB:O65399); contains InterPro domain Glycoside hydrolase, family 17 (InterPro:IPR000490) | 5.7 | 4.6 | 1.1 | 3.4 | 5.0% | 2.7 |
| 7242 | AT5G44780.1 \| expressed protein, low similarity to SP:Q38732 DAG protein, chloroplast precursor {Antirrhinum majus} \| chr5:18085327-18087868 FORWARD \| Aliases: K23L20.12, K23L20_12 | 3.8 | 3.0 | 0.8 | 3.4 | 5.0% | 2.4 |
| 7243 | AT4G38580.1 \| Symbol: ATFP6 \| heavy-metal-associated domain-containing protein / copper chaperone (CCH)-related, low similarity to copper homeostasis factor (PMID:9701579)(GI:3168840); nearly identical to farnesylated protein TFP6 (GI:4097553); contains Heavy-metal-associated domain PF00403 \| chr4:18034445-18035879 FORWARD \| Aliases: F20M13.140, F20M13_140, FARNESYLATED PROTEIN 6 | 7.3 | 5.7 | 1.6 | 3.4 | 5.0% | 2.6 |
| 7244 | AT2G43900.1 \| endonuclease/exonuclease/phosphatase family protein, belongs to Pfam:PF03372: Endonuclease/Exonuclease/phosphatase family; contains 3 WD-40 repeats (PF00400);similar to Type II inositol-1,4,5-trisphosphate 5-phosphatase EC 3.1.3.56 (5PTASE) (INPP5B) (SP:P32019) (Homo sapiens) \| chr2:18185756-18190899 REVERSE \| Aliases: F6E13.3 | 4.8 | 4.0 | 0.8 | 3.4 | 5.0% | 2.5 |
| 7245 | AT1G71930.1 \| Symbol: ANAC030 \| no apical meristem (NAM) family protein, similar to NAM GB:CAA63101 from (Petunia x hybrida) \| chr1:27079802-27081619 FORWARD \| Aliases: ANAC030, F17M19.8, F17M19_8 | 2.4 | 2.8 | -0.4 | -3.4 | 5.0% | 2.1 |
| 7246 | AT1G71440.1 \| Symbol: PFI \| tubulin folding cofactor E / Pfifferling (PFI), almost identical to tubulin folding cofactor E (Pfifferling; PFI) GI:20514267 from (Arabidopsis thaliana); identical to cDNA tubulin folding cofactor E, GI:20514266 \| chr1:26924125-26928048 REVERSE \| Aliases: F3I17.21, F3I17_21, PFIFFERLING, TFC E, TUBULIN FOLDING COFACTOR E | 6.6 | 5.8 | 0.9 | 3.4 | 5.0% | 2.4 |
| 7247 | AT3G18165.1 \| expressed protein, similar to DAM1 (GI:3985930) (Homo sapiens); contains Pfam profile PF05700: Breast carcinoma amplified sequence 2 (BCAS2) \| chr3:6223194-6225378 FORWARD \| Aliases: None | 5.5 | 4.5 | 1.0 | 3.4 | 5.0% | 2.5 |
| 7248 | AT1G51580.1 \| KH domain-containing protein \| chr1:19129251-19131827 FORWARD \| Aliases: F5D21.23, F5D21_23 | 6.4 | 5.5 | 0.9 | 3.4 | 5.0% | 2.6 |
| 7249 | AT5G50310.1 \| kelch repeat-containing protein, similar to Kelch repeats protein 3 (SP:Q08979) (Saccharomyces cerevisiae); contains Pfam PF01344: Kelch motif (6 repeats) \| chr5:20491713-20496641 FORWARD \| Aliases: MXI22.1, MXI22_1 | 6.9 | 5.9 | 1.1 | 3.4 | 5.0% | 2.4 |
| 7250 | AT1G01300.1 \| aspartyl protease family protein, contains Pfam domain, PF00026: eukaryotic aspartyl protease \| chr1:116943-118764 FORWARD \| Aliases: F6F3.10, F6F3_10 | 6.2 | 4.4 | 1.9 | 3.4 | 5.0% | 2.7 |

| Rank | Description | Sync | Root | M | t | adj.q | B |
|---|---|---|---|---|---|---|---|
| 7251 | AT5G54390.1 \| Symbol: AHL \| inositol monophosphatase family protein, similar to SP:Q42546 3'(2'),5'-bisphosphate nucleotidase (EC 3.1.3.7) {Arabidopsis thaliana}; contains Pfam profile PF00459: Inositol monophosphatase family; supporting cDNA gi:1354509:gb:U55205.1:ATU55205 \| chr5:22103224-22105243 FORWARD \| Aliases: ATAHL, F24B18.1, F24B18_1, HAL2 LIKE | 7.3 | 5.7 | 1.6 | 3.4 | 5.0% | 2.5 |
| 7252 | AT5G25510.1 \| serine/threonine protein phosphatase 2A (PP2A) regulatory subunit B', putative, similar to SWISS-PROT:Q28653 serine/threonine protein phosphatase 2A, 56 kDa regulatory subunit, delta isoform (PP2A, B subunit, B' delta isoform, PP2A, B subunit, B56 delta isoform, PP2A, B subunit, PR61 delta isoform, PP2A, B subunit, R5 delta isoform, PP2A, B subunit, B'-gamma) (Oryctolagus cuniculus); contains Pfam domain, PF01603: Protein phosphatase 2A regulatory B subunit (B56 family) \| chr5:8882471-8884837 REVERSE \| Aliases: T14C9.50, T14C9_50 | 5.1 | 4.3 | 0.8 | 3.4 | 5.1% | 2.5 |
| 7253 | AT1G16470.2 \| Symbol: PAB1 \| similar to 20S proteasome alpha subunit D2 (PAD2) (PRS1) (PRC6) [Arabidopsis thaliana] (TAIR:At5g66140.1); similar to proteasome subunit alpha type 2 [Oryza sativa (japonica cultivar-group)] (GB:AAT78811.1); contains InterPro domain Proteasome subunit, A-type (InterPro:IPR000426); contains InterPro domain Multispecific proteasome protease (InterPro:IPR001353) \| chr1:5622832-5625637 FORWARD \| Aliases: F3O9.27, F3O9_27, PROTEASOME SUBUNIT PAB1 | 9.2 | 7.6 | 1.6 | 3.4 | 5.1% | 2.6 |
| 7254 | AT2G38080.1 \| Symbol: IRX12 \| Encodes a protein with similarity to laccase that may be involved in cell wall biosynthesis. Mutants have a mild irregular xylem phenotype. \| chr2:15941540-15944694 FORWARD \| Aliases: F16M14.1, F16M14_1, IRX12 | 2.8 | 5.0 | -2.2 | -3.4 | 5.1% | 2.5 |
| 7255 | AT3G01280.1 \| porin, putative, similar to SP:P42055 34 kDa outer mitochondrial membrane protein porin (Voltage-dependent anion-selective channel protein) (VDAC) {Solanum tuberosum}; contains Pfam profile PF01459: Eukaryotic porin \| chr3:85619-87865 FORWARD \| Aliases: T22N4.9, T22N4_9 | 11.2 | 9.1 | 2.1 | 3.4 | 5.1% | 2.5 |
| 7256 | AT5G28510.1 \| glycosyl hydrolase family 1 protein, contains Pfam PF00232 : Glycosyl hydrolase family 1 domain; TIGRFAM TIGR01233: 6-phospho-beta-galactosidase; similar to beta-glucosidase 1 (GI:12043529) (Arabidopsis thaliana) \| chr5:10481045-10484026 REVERSE \| Aliases: T26D3.6, T26D3_6 | 3.1 | 3.4 | -0.3 | -3.4 | 5.1% | 2.0 |
| 7257 | AT1G06470.2 \| phosphate translocator-related, low similarity to SP:P52178 Triose phosphate/phosphate translocator, non-green plastid, chloroplast precursor (CTPT) {Brassica oleracea}, phosphoenolpyruvate/phosphate translocator precursor (Mesembryanthemum crystallinum) GI:9295275 \| chr1:1969751-1973755 FORWARD \| Aliases: F12K11.18, F12K11_18 | 5.6 | 6.4 | -0.8 | -3.4 | 5.1% | 2.4 |
| 7258 | AT5G14370.1 \| expressed protein \| chr5:4631894-4633716 REVERSE \| Aliases: F18O22.160, F18O22_160 | 3.5 | 2.8 | 0.7 | 3.4 | 5.1% | 2.5 |
| 7259 | AT1G49700.1 \| expressed protein, ; expression supported by MPSS \| chr1:18392460-18393976 REVERSE \| Aliases: F14J22.9, F14J22_9 | 3.4 | 2.8 | 0.5 | 3.4 | 5.1% | 2.2 |
| 7260 | AT5G49550.1 \| expressed protein \| chr5:20123879-20124417 REVERSE \| Aliases: K6M13.19 | 7.6 | 6.9 | 0.6 | 3.4 | 5.1% | 2.4 |
| 7261 | AT1G61040.1 \| plus-3 domain-containing protein, contains Pfam profile PF03126: Plus-3 domain \| chr1:22486872-22489634 FORWARD \| Aliases: T7P1.17, T7P1_17 | 6.2 | 8.0 | -1.8 | -3.4 | 5.1% | 2.3 |
| 7262 | AT5G59510.1 \| expressed protein \| chr5:24007300-24007922 FORWARD \| Aliases: F2O15.21, F2O15_21 | 2.3 | 2.5 | -0.3 | -3.4 | 5.1% | 1.7 |
| 7263 | AT5G22700.1 \| F-box family protein, contains F-box domain Pfam:PF00646 | 3.5 | 4.3 | -0.9 | -3.4 | 5.1% | 2.1 |
| 7264 | AT5G40150.1 \| peroxidase, putative, identical to peroxidase ATP26a {Arabidopsis thaliana} GP:1890317:emb:CAA72487 \| chr5:16076737-16078271 REVERSE \| Aliases: MSN9.50, MSN9_50 | 3.9 | 3.5 | 0.5 | 3.4 | 5.1% | 2.1 |
| 7265 | AT4G13010.1 \| oxidoreductase, zinc-binding dehydrogenase family protein, low similarity to probable NADP-dependent oxidoreductase (zeta-crystallin homolog) P1 (SP:Q39172)(gi:886428) and P2 (SP:Q39173)(gi:886430); contains Pfam profile PF00107: oxidoreductase, zinc-binding dehydrogenase family \| chr4:7600548-7602726 FORWARD \| Aliases: F25G13.100, F25G13_100 | 10.1 | 8.8 | 1.3 | 3.4 | 5.1% | 2.5 |
| 7266 | AT3G46720.1 \| UDP-glucoronosyl/UDP-glucosyl transferase family protein, contains Pfam profile: PF00201 UDP-glucoronosyl and UDP-glucosyl transferase \| chr3:17221840-17223333 REVERSE \| Aliases: T6H20.250 | 4.1 | 4.7 | -0.6 | -3.4 | 5.1% | 2.1 |
| 7267 | AT5G48770.1 \| disease resistance protein (TIR-NBS-LRR class), putative, domain signature TIR-NBS-LRR exists, suggestive of a disease resistance protein. \| chr5:19790503-19794468 REVERSE \| Aliases: K24G6.10, K24G6_10 | 2.2 | 2.6 | -0.4 | -3.4 | 5.1% | 1.8 |
| 7268 | ATCG00480.1 \| Symbol: ATPB \| chloroplast-encoded gene for beta subunit of ATP synthase \| chrC:52660-54156 REVERSE \| Aliases: ATPB | 9.4 | 7.6 | 1.7 | 3.4 | 5.1% | 2.5 |
| 7269 | AT5G05370.1 \| ubiquinol-cytochrome C reductase complex ubiquinone-binding protein, putative / ubiquinol-cytochrome C reductase complex 8.2 kDa protein, putative, strong similarity to SP:P46269 Ubiquinol-cytochrome C reductase complex ubiquinone-binding protein QP-C (EC 1.10.2.2) (Ubiquinol-cytochrome C reductase complex 8.2 kDa protein) {Solanum tuberosum} | 11.0 | 10.5 | 0.4 | 3.4 | 5.1% | 2.1 |
| 7270 | AT5G57840.1 \| transferase family protein, similar to anthranilate N-hydroxycinnamoyl/benzoyltransferase from Dianthus caryophyllus (gi:2239091) \| chr5:23450030-23452458 REVERSE \| Aliases: MTI20.9, MTI20_9 | 4.8 | 5.8 | -1.0 | -3.4 | 5.1% | 2.5 |

| Rank | Description | Sync | Root | M | t | adj.q | B |
|---|---|---|---|---|---|---|---|
| 7271 | AT4G35310.1 \| Symbol: CPK5 \| calcium-dependent protein kinase, putative / CDPK, putative, similar to calmodulin-domain protein kinase CDPK isoform 6 (Arabidopsis thaliana) gi:1399275:gb:AAB03246; contains protein kinase domain, Pfam:PF00069; contains EF hand domain (calcium-binding EF-hand), Pfam:PF00036, INTERPRO:IPR002048 | 6.0 | 6.7 | -0.7 | -3.4 | 5.1% | 2.3 |
| 7272 | AT4G26500.1 \| Symbol: EMB1374 \| BolA-like family protein / Fe-S metabolism associated domain-containing protein, similar to SufE protein (Erwinia chrysanthemi) GI:11342550; contains Pfam profiles PF02657: Fe-S metabolism associated domain, PF01722: BolA-like protein \| chr4:13382206-13383649 REVERSE \| Aliases: EMB1374, EMBRYO DEFECTIVE 1374, M3E9.70, M3E9_70 | 6.6 | 5.9 | 0.7 | 3.4 | 5.1% | 2.5 |
| 7273 | AT3G22170.1 \| Symbol: FHY3 \| far-red impaired responsive protein, putative, similar to far-red impaired response protein FAR1 (Arabidopsis thaliana) gi:5764395:gb:AAD51282; contains Pfam:PF03101 domain: FAR1 family \| chr3:7822025-7825891 REVERSE \| Aliases: MKA23.12 | 5.3 | 7.0 | -1.7 | -3.4 | 5.1% | 2.7 |
| 7274 | AT3G14410.1 \| transporter-related, low similarity to SP:Q96A29 GDP-fucose transporter 1 {Homo sapiens}, phosphoenolpyruvate/phosphate translocator precursor (Mesembryanthemum crystallinum) GI:9295275; contains 10 predicted transmembrane domains; \| chr3:4815833-4817987 REVERSE \| Aliases: MLN21.19 | 8.0 | 8.8 | -0.8 | -3.4 | 5.1% | 2.2 |
| 7275 | AT1G55240.1 \| expressed protein, contains Pfam profile PF04819: Family of unknown function (DUF716) (Plant viral-response family) \| chr1:20608666-20610066 FORWARD \| Aliases: F7A10.18, F7A10_18 | 2.9 | 3.3 | -0.4 | -3.4 | 5.1% | 2.0 |
| 7276 | AT4G22720.2 \| glycoprotease M22 family protein, similar to sialylglycoprotease (Haemophilus ducreyi) GI:6942294; contains Pfam profile PF00814: Glycoprotease family \| chr4:11937075-11938977 FORWARD \| Aliases: T12H17.110, T12H17_110 | 7.5 | 6.9 | 0.6 | 3.4 | 5.1% | 2.3 |
| 7277 | AT1G79975.2 \| expressed protein \| chr1:30089065-30090457 FORWARD \| Aliases: None | 5.2 | 6.0 | -0.8 | -3.4 | 5.1% | 2.5 |
| 7278 | AT2G02990.1 \| Symbol: RNS1 \| ribonuclease 1 (RNS1), identical to ribonuclease SP:P42813 Ribonuclease 1 precursor (EC 3.1.27.1) {Arabidopsis thaliana}, GI:561998 from (Arabidopsis thaliana) \| chr2:873505-874810 FORWARD \| Aliases: RIBONUCLEASE 1, S LIKE RIBONUCLEASE 1, S LIKE RNASE 1, T17M13.16, T17M13_16 | 3.1 | 3.6 | -0.5 | -3.4 | 5.1% | 2.0 |
| 7279 | AT5G20870.1 \| glycosyl hydrolase family 17 protein, similar to elicitor inducible chitinase Nt-SubE76 GI:11071974 (Nicotiana tabacum) \| chr5:7079977-7081771 REVERSE \| Aliases: F22D1.40, F22D1_40 | 3.5 | 4.1 | -0.5 | -3.4 | 5.1% | 2.3 |
| 7280 | AT1G02820.1 \| late embryogenesis abundant 3 family protein / LEA3 family protein, similar to late embryogenis abundant protein 5 GI:2981167 from (Nicotiana tabacum); contains Pfam profile PF03242: Late embryogenesis abundant protein \| chr1:623782-624474 REVERSE \| Aliases: F22D16.18, F22D16_18 | 3.9 | 3.2 | 0.6 | 3.4 | 5.1% | 2.3 |
| 7281 | AT1G65520.1 \| enoyl-CoA hydratase/isomerase family protein, low similarity to enoyl-CoA hydratase (Escherichia coli) GI:2764828, carnitine racemase SP:P31551 (Escherichia coli); contains Pfam profile PF00378 enoyl-CoA hydratase/isomerase family protein \| chr1:24364705-24365556 REVERSE \| Aliases: F5I14.5, F5I14_5 | 4.9 | 5.6 | -0.6 | -3.4 | 5.1% | 2.4 |
| 7282 | AT2G36350.1 \| protein kinase, putative, similar to protein kinase KIPK (KCBP-interacting protein kinase) (Arabidopsis thaliana) gi:7716430:gb:AAF68383 | 4.5 | 3.7 | 0.7 | 3.4 | 5.1% | 2.5 |
| 7283 | AT4G32150.1 \| Symbol: VAMP7C \| | 7.1 | 7.8 | -0.7 | -3.4 | 5.1% | 2.4 |
| 7284 | AT5G38070.1 \| zinc finger (C3HC4-type RING finger) family protein, contains InterPro Entry IPR001841 Zn-finger, RING; contains PROSITE PS00190: Cytochrome c family heme-binding site signature \| chr5:15207484-15208919 FORWARD \| Aliases: F16F17.8, F16F17_8 | 2.8 | 3.4 | -0.6 | -3.4 | 5.1% | 2.4 |
| 7285 | AT2G41880.1 \| Symbol: GK 1 \| guanylate kinase 1 (GK-1), identical to guanylate kinase (GK-1) (Arabidopsis thaliana) gi:7861795:gb:AAF70408 \| chr2:17481713-17484301 FORWARD \| Aliases: AGK1, GUANYLATE KINASE 1, T11A7.23 | 6.2 | 7.4 | -1.2 | -3.4 | 5.1% | 2.5 |
| 7286 | AT1G28380.1 \| expressed protein \| chr1:9963579-9966306 FORWARD \| Aliases: F3M18.18, F3M18_18 | 5.3 | 6.1 | -0.8 | -3.4 | 5.1% | 2.4 |
| 7287 | AT3G47680.1 \| expressed protein, contains similarity to hypothetical proteins of (Arabidopsis thaliana) \| chr3:17588468-17589376 REVERSE \| Aliases: T23J7.10 | 3.9 | 5.5 | -1.6 | -3.4 | 5.2% | 2.6 |
| 7288 | AT3G08510.2 \| Symbol: ATPLC2 \| | 5.2 | 6.4 | -1.2 | -3.4 | 5.2% | 2.4 |
[truncated: 580,265 more chars]
