# Supplementary material for: The transcriptome of syncytia induced by the cyst nematode Heterodera schachtii in Arabidopsis roots
Source: Plant J. 2008 Dec 9;57(5):771–84. doi: 10.1111/j.1365-313X.2008.03727.x (PMC2667683; doi:10.1111/j.1365-313X.2008.03727.x)
Supplement: Supporting Information [file TableS7.pdf]

List of affected genes, confidential preview.

Statistics are testing for differential expression (two-sided non-null).

Ranks refer to this test.

Results are grouped by

Differentially expressed with *q*<5%	*M*>0	Up-regulation
Differentially expressed with *q*<5%	*M*<0	Down-regulation
Non-differentially expressed, *q* of 5% or higher		Insignificant

Orthogonal contrasts examined:

		up	*down*	*non-diff*
15 dpi Syncytium vs 5 dpi Syncytium	**... Table 3 & Table S3**	page 2	page 3	page 4

Contact: D. Kreil
http://bioinf.boku.ac.at/

Rank	Description	15.dpi	5.dpi	M	t	adj.q	B
2	AT3G15840.3 | expressed protein | chr3:5356657-5358506 REVERSE | Aliases: MSJ11.24, MSJ11_24	6.4	2.3	4.0	14.9	0.65%	7.4
3	AT4G39675.1 | expressed protein | chr4:18413769-18414232 FORWARD | Aliases: None	8.8	2.4	6.5	12.4	1.93%	6.1
4	AT5G54940.2 | eukaryotic translation initiation factor SUI1, putative, similar to SP:P32911 Protein translation factor SUI1 {Saccharomyces cerevisiae}; contains Pfam profile PF01253: Translation initiation factor SUI1 | chr5:22325497-22326633 REVERSE | Aliases: MBG8.21, MBG8_21	11.7	9.6	2.1	11.1	3.43%	6.3
5	AT5G50790.1 | nodulin MtN3 family protein, similar to MtN3 GI:1619602 (root nodule development) from (Medicago truncatula) | chr5:20673479-20675225 REVERSE | Aliases: MFB16.26, MFB16_26	8.4	3.7	4.7	10.6	3.46%	3.3
6	AT5G10040.1 | expressed protein | chr5:3143724-3144158 FORWARD | Aliases: T31P16.30, T31P16_30	8.9	3.8	5.1	10.5	3.46%	4.4
7	AT1G08430.1 | expressed protein, contains Pfam profile PF01027: Uncharacterized protein family UPF0005	9.1	5.4	3.7	10.1	3.67%	3.8
9	AT1G27730.1 | Symbol: ZAT10 | zinc finger (C2H2 type) family protein (ZAT10) / salt-tolerance zinc finger protein (STZ), identical to salt-tolerance zinc finger protein GB:CAA64820 GI:1565227 from (Arabidopsis thaliana); contains Pfam domain, PF00096: Zinc finger, C2H2 type; identical to cDNA	9.6	5.8	3.7	9.8	3.67%	4.4
10	AT4G33560.1 | expressed protein | chr4:16135403-16135917 FORWARD | Aliases: T16L1.50, T16L1_50	8.6	4.9	3.7	9.7	3.67%	4.0
11	AT5G65870.1 | Symbol: ATPSK5 |	7.1	3.6	3.5	9.6	3.67%	4.4
12	AT1G62480.1 | vacuolar calcium-binding protein-related, contains weak similarity to vacuolar calcium binding protein (Raphanus sativus) gi:9049359:dbj:BAA99394 | chr1:23132370-23133424 FORWARD | Aliases: T3P18.4, T3P18_4	10.6	7.0	3.6	9.5	3.67%	3.6
13	AT3G19680.1 | expressed protein | chr3:6840287-6842326 FORWARD | Aliases: MMB12.17	7.5	5.3	2.2	8.7	4.98%	4.4
14	AT5G10695.1 | expressed protein | chr5:3377664-3378222 FORWARD | Aliases: None	10.7	7.8	2.9	8.7	4.98%	4.2
16	AT3G03910.1 | glutamate dehydrogenase, putative, similar to glutamate dehydrogenase 1 (GDH 1) (Arabidopsis thaliana) SWISS-PROT:Q43314	9.8	5.5	4.3	8.6	4.98%	2.0
17	AT1G10010.1 | Symbol: AAP8 | amino acid permease, putative, similar to amino acid permease I GI:22641 from (Arabidopsis thaliana); GC splice site at position 1256 is predicted from alignment and not confirmed experimentally | chr1:3265978-3268728 FORWARD | Aliases: T27I1.3, T27I1_3	8.6	5.4	3.2	8.5	4.98%	3.3
18	AT2G18050.2 | Symbol: HIS1 3 |	6.3	3.5	2.9	8.5	4.98%	3.8
19	AT3G48740.1 | nodulin MtN3 family protein, similar to MtN3 GI:1619602 (root nodule development) from (Medicago truncatula) | chr3:18063492-18065738 REVERSE | Aliases: T21J18.1	9.8	4.4	5.4	8.4	4.98%	1.9
20	AT2G22860.1 | Symbol: ATPSK2 |	11.2	8.8	2.5	8.4	4.98%	4.1
21	AT2G17710.1 | expressed protein | chr2:7700678-7701828 FORWARD | Aliases: T17A5.17, T17A5_17	8.3	3.9	4.4	8.4	4.98%	3.1
22	AT1G74880.1 | Symbol: NDH O | Encodes subunit NDH-O of NAD(P)H:plastoquinone dehydrogenase complex (Ndh complex) present in the thylakoid membrane of chloroplasts. This subunit is thought to be required for Ndh complex assembly. | chr1:28133540-28134738 REVERSE | Aliases: F25A4.15, F25A4_15, NDH O	5.4	2.8	2.6	8.4	4.98%	3.6

Rank	Description	15.dpi	5.dpi	M	t	adj.q	B
1	AT3G60140.1 | Symbol: DIN2 | glycosyl hydrolase family 1 protein, contains Pfam PF00232 : Glycosyl hydrolase family 1 domain; TIGRFAM TIGR01233: 6-phospho-beta-galactosidase; similar to Cyanogenic Beta-Glucosidase (GI:1311386)(pdb:1CBG) (Trifolium Repens); identical beta-glucosidase GI:10834547 | chr3:22227648-22231792 FORWARD | Aliases: DARK INDUCIBLE 2, SENESCENCE RELATED GENE 2, SRG2, T2O9.120, T2O9_120	3.0	8.6	-5.6	-20.2	0.12%	7.3
8	AT5G05340.1 | peroxidase, putative, similar to peroxidase (Nicotiana tabacum) gi:5381253:dbj:BAA82306; similar to Peroxidase P7 (Brassica rapa (Turnip)) SWISS-PROT:P00434 | chr5:1578952-1580876 REVERSE | Aliases: K18I23.14, K18I23_14	2.8	8.8	-6.0	-9.8	3.67%	4.9
15	AT3G50300.1 | transferase family protein, similar to anthranilate N-hydroxycinnamoyl/benzoyltransferase, Dianthus caryophyllus (gi:2239091); contains Pfam transferase family domain PF002458 | chr3:18654937-18656388 FORWARD | Aliases: F11C1.140	4.1	7.7	-3.5	-8.6	4.98%	3.0

Rank	Description	15.dpi	5.dpi	M	t	adj.q	B
23	AT4G27450.1 | expressed protein, similar to auxin down-regulated protein ARG10 (Vigna radiata) GI:2970051, wali7 (aluminum-induced protein) (Triticum aestivum) GI:451193 | chr4:13727493-13728892 REVERSE | Aliases: F27G19.50, F27G19_50	10.9	7.7	3.2	8.3	5.3%	3.4
24	AT3G14780.1 | similar to glycosyl transferase family 48 protein [Arabidopsis thaliana] (TAIR:At3g14570.1); similar to putative callose synthase 1 catalytic subunit [Oryza sativa (japonica cultivar-group)] (GB:XP_468556.1) | chr3:4961813-4963513 REVERSE | Aliases: T21E2.4	6.7	4.6	2.1	8.3	5.3%	4.0
25	AT2G35290.1 | expressed protein | chr2:14868491-14869108 REVERSE | Aliases: T4C15.4, T4C15_4	7.8	2.9	4.9	8.1	5.3%	3.4
26	AT4G34410.1 | encodes a member of the ERF (ethylene response factor) subfamily B-3 of ERF/AP2 transcription factor family. The protein contains one AP2 domain. There are 18 members in this subfamily including ATERF-1, ATERF-2, AND ATERF-5. | chr4:16451880-16453264 FORWARD | Aliases: F10M10.180, F10M10_180	5.7	2.5	3.2	8.1	5.3%	3.5
27	AT4G33985.1 | expressed protein | chr4:16288117-16288918 REVERSE | Aliases: None	9.0	5.6	3.4	8.1	5.3%	2.8
28	AT4G18340.1 | glycosyl hydrolase family 17 protein, similar to elicitor inducible chitinase Nt-SubE76 GI:11071974 from (Nicotiana tabacum) | chr4:10130153-10132072 REVERSE | Aliases: T9A21.190, T9A21_190	7.6	5.3	2.2	8.0	5.3%	3.4
29	AT2G41480.1 | peroxidase, putative, similar to peroxidase (Spinacia oleracea) gi:1781334:emb:CAA71494 | chr2:17303890-17305805 REVERSE | Aliases: T26J13.7, T26J13_7	7.3	4.1	3.2	8.0	5.3%	2.6
30	AT3G45970.1 | Symbol: ATEXLA1 | expansin family protein (EXPL1), similar to cim1 induced allergen, Glycine max, EMBL:U03860; expansin-like gene, PMID:11641069, www.bio.psu.edu/expansins | chr3:16907151-16908293 FORWARD | Aliases: ARABIDOPSIS THALIANA EXPANSIN LIKE A1, ATEXPL1, ATHEXP BETA 2.1, EXPL1, F16L2.180, F16L2_180	10.9	9.1	1.9	7.9	5.3%	3.8
31	AT1G61340.1 | F-box family protein, contains Pfam PF00646: F-box domain; similar to late embryogenesis abundant protein GI:1350540 from (Picea glauca)	8.2	4.7	3.5	7.9	5.3%	3.2
32	AT4G10270.1 | wound-responsive family protein, similar to wound induced protein (GI:19320) (Lycopersicon esculentum) | chr4:6374730-6375206 FORWARD | Aliases: T9A4.6	10.9	6.6	4.3	7.9	5.3%	2.5
33	AT1G07500.1 | expressed protein, ; expression supported by MPSS | chr1:2304763-2305028 REVERSE | Aliases: F22G5.11, F22G5_11	6.6	3.2	3.4	7.9	5.3%	3.7
34	AT5G38710.1 | proline oxidase, putative / osmotic stress-responsive proline dehydrogenase, putative, similar to proline oxidase, mitochondrial precursor (Osmotic stress- induced proline dehydrogenase) (Arabidopsis thaliana) SWISS-PROT:P92983 | chr5:15518570-15521129 FORWARD | Aliases: MKD10.10, MKD10_10	9.0	5.6	3.4	7.8	5.3%	2.5
35	AT1G32450.1 | proton-dependent oligopeptide transport (POT) family protein, contains Pfam profile: PF00854 POT family | chr1:11715130-11719935 REVERSE | Aliases: F5D14.23, F5D14_23	7.9	5.1	2.8	7.8	5.3%	3.0
36	AT1G18250.2 | Symbol: ATLP 1 |	3.5	6.9	-3.3	-7.7	5.6%	2.3
37	AT1G20823.1 | zinc finger (C3HC4-type RING finger) family protein, contains Pfam domain, PF00097: Zinc finger, C3HC4 type (RING finger) | chr1:7238789-7239658 FORWARD | Aliases: F2D10.34, F2D10_34	9.4	6.0	3.4	7.5	6.8%	2.8
38	AT2G27240.1 | expressed protein, contains Pfam profile PF01027: Uncharacterized protein family UPF0005	9.6	6.0	3.6	7.5	7.0%	1.9
39	AT3G14660.1 | Symbol: CYP72A13 | cytochrome P450, putative, similar to GB:Q05047 from (Catharanthus roseus) | chr3:4924784-4927441 FORWARD | Aliases: MIE1.16	7.9	5.8	2.1	7.3	8.3%	3.2
40	AT4G37710.1 | VQ motif-containing protein, contains PF05678: VQ motif | chr4:17718122-17718736 FORWARD | Aliases: T28I19.3	6.2	2.6	3.6	7.2	8.5%	3.0
41	AT4G02290.1 | glycosyl hydrolase family 9 protein, similar to endo-1,4-beta glucanase; ATCEL2 GI:3132891 from (Arabidopsis thaliana) | chr4:1002446-1005202 REVERSE | Aliases: T2H3.5, T2H3_5	3.5	7.3	-3.8	-7.2	8.5%	2.4
42	AT2G11810.1 | Symbol: MGDC | 1,2-diacylglycerol 3-beta-galactosyltransferase, putative / monogalactosyldiacylglycerol synthase, putative / MGDG synthase, putative, identical to monogalactosyldiacylglycerol synthase type C (gi:9927295) from Arabidopsis thaliana, similar to MGDG synthase type A (gi:9884651) from Glycine max | chr2:4750448-4753859 FORWARD | Aliases: ATMGD3, F7E22.4, F7E22_4, MGD3, MONOGALACTOSYLDIACYLGLYCEROL SYNTHASE	5.4	3.2	2.2	7.1	8.5%	2.9
43	AT2G40610.1 | Symbol: ATEXPA8 | expansin, putative (EXP8), similar to expansin 2 GI:7025493 from (Zinnia elegans); alpha-expansin gene family, PMID:11641069 | chr2:16955941-16957635 REVERSE | Aliases: ARABIDOPSIS THALIANA EXPANSIN A8, ATEXP8, ATHEXP ALPHA 1.11, EXP8, T2P4.4, T2P4_4	10.0	6.3	3.7	7.1	8.5%	2.1

Rank	Description	15.dpi	5.dpi	M	t	adj.q	B
44	AT1G48600.2 | phosphoethanolamine N-methyltransferase 2, putative (NMT2), very similar to :PEM2_ARATH Putative phosphoethanolamine N-methyltransferase 2 (EC 2.1.1.103) (SP:Q944H0){Arabidopsis thaliana}; very similar to Halotolerance protein Hal3b (SP:P94063)(Arabidopsis thaliana); to similar to GB:AAF61950 from (Spinacia oleracea) | chr1:17969539-17973005 FORWARD | Aliases: T1N15.23, T1N15_23	10.9	12.2	-1.3	-7.1	8.5%	3.2
45	AT2G38870.1 | protease inhibitor, putative, similar to SP:P24076 Glu S.griseus protease inhibitor (BGIA) {Momordica charantia}; contains Pfam profile PF00280: Potato inhibitor I family | chr2:16243459-16244358 REVERSE | Aliases: T7F6.4, T7F6_4	7.3	11.1	-3.8	-7.1	8.5%	2.8
46	AT2G41640.1 | expressed protein, contains Pfam domain, PF04577: Protein of unknown function (DUF563) | chr2:17367574-17369613 FORWARD | Aliases: T32G6.16, T32G6_16	8.1	4.8	3.3	7.0	9.2%	2.7
47	AT3G53450.1 | hypothetical protein, contains Pfam profile PF03641: decarboxylase family protein | chr3:19823955-19826408 REVERSE | Aliases: F4P12.150	5.8	4.0	1.8	6.9	9.6%	2.9
48	AT2G40000.1 | expressed protein | chr2:16707588-16709106 REVERSE | Aliases: T28M21.16, T28M21_16	9.5	7.0	2.5	6.9	10.0%	2.7
49	AT2G41540.3 | similar to NAD-dependent glycerol-3-phosphate dehydrogenase family protein [Arabidopsis thaliana] (TAIR:At3g07690.1); similar to putative glycerol-3-phosphate dehydrogenase [Oryza sativa (japonica cultivar-group)] (GB:NP_915827.1); similar to putative glycerol-3-phosphate dehydrogenase [Oryza sativa (japonica cultivar-group)] (GB:AAU44049.1); contains InterPro domain NAD-dependent glycerol-3-phosphate dehydrogenase (InterPro:IPR006168) | chr2:17333206-17336863 FORWARD | Aliases: T32G6.6, T32G6_6	4.0	6.3	-2.3	-6.8	10.7%	2.5
50	AT1G79180.1 | myb family transcription factor (MYB63), similar to myb-related protein GI:1370139 from (Lycopersicon esculentum) | chr1:29791402-29792695 FORWARD | Aliases: YUP8H12R.21, YUP8H12R_21	5.5	7.5	-2.1	-6.8	10.8%	2.5
51	AT4G34160.1 | Symbol: CYCD3;1 | cyclin delta-3 (CYCD3), identical to SP:P42753 Cyclin delta-3 {Arabidopsis thaliana} | chr4:16357643-16359559 FORWARD | Aliases: CYCD3, CYCD3;1, Cyclin D3;1, D3, F28A23.80, F28A23_80	3.7	6.8	-3.2	-6.7	10.8%	2.2
52	AT1G69880.1 | thioredoxin, putative, similar to SP:Q38879 Thioredoxin H-type 2 (TRX-H-2) {Arabidopsis thaliana}; contains Pfam profile: PF00085 Thioredoxin | chr1:26325142-26326656 FORWARD | Aliases: T17F3.9, T17F3_9	9.0	5.3	3.7	6.7	10.8%	1.6
53	AT1G06460.1 | Symbol: ACD32.1 | 31.2 kDa small heat shock family protein / hsp20 family protein, contains Pfam profile: PF00011 Hsp20/alpha crystallin family	7.0	4.0	3.0	6.7	11.0%	2.4
54	AT3G16150.1 | L-asparaginase, putative / L-asparagine amidohydrolase, putative, similar to Swiss-Prot:P30364 L-asparaginase (EC 3.5.1.1) (L-asparagine amidohydrolase) (Lupinus angustifolius)	10.2	6.7	3.5	6.6	11.4%	1.8
55	AT4G25010.1 | nodulin MtN3 family protein, similar to MtN3 GI:1619602 (root nodule development) from (Medicago truncatula) | chr4:12854640-12856361 REVERSE | Aliases: F13M23.150, F13M23_150	9.8	6.2	3.6	6.6	11.4%	1.0
56	AT4G27652.1 | expressed protein | chr4:13809839-13810362 FORWARD | Aliases: None	9.6	5.8	3.7	6.6	11.4%	2.2
57	AT1G02900.1 | Symbol: RALFL1 |	4.8	7.6	-2.8	-6.6	11.4%	1.9
58	AT3G02800.1 | similar to tyrosine specific protein phosphatase family protein [Arabidopsis thaliana] (TAIR:At5g16480.1); similar to putative tyrosine specific protein phosphatase protein [Oryza sativa (japonica cultivar-group)] (GB:XP_450177.1); contains InterPro domain Putative tyrosine phosphatase (InterPro:IPR004861) | chr3:606478-607893 REVERSE | Aliases: F13E7.26, F13E7_26	6.5	4.9	1.6	6.6	11.4%	2.5
59	AT4G30270.1 | Symbol: MERI5B |	8.9	5.8	3.1	6.5	11.4%	1.7
60	AT1G76650.1 | calcium-binding EF hand family protein, similar to regulator of gene silencing calmodulin-related protein GI:12963415 from (Nicotiana tabacum); contains INTERPRO:IPR002048 calcium-binding EF-hand domain | chr1:28771644-28772411 REVERSE | Aliases: F28O16.2, F28O16_2	7.5	4.7	2.8	6.5	11.4%	2.2
61	AT5G17350.1 | expressed protein | chr5:5718774-5719610 FORWARD | Aliases: MKP11.31, MKP11_31	6.2	3.7	2.4	6.5	11.4%	2.0
62	AT1G64700.1 | expressed protein | chr1:24043473-24044258 REVERSE | Aliases: F13O11.2, F13O11_2	7.9	6.1	1.8	6.4	12.6%	2.5
63	AT1G80840.1 | Symbol: WRKY40 |	8.7	5.5	3.2	6.3	13.4%	2.0
64	AT3G30775.1 | Symbol: ERD5 | proline oxidase, mitochondrial / osmotic stress-responsive proline dehydrogenase (POX) (PRO1) (ERD5), nearly identical to SP:P92983 Proline oxidase, mitochondrial precursor (EC 1.5.3.-) (Osmotic stress- induced proline dehydrogenase) (Arabidopsis thaliana); identical to cDNA proline oxidase precursor GI:1817543	11.1	8.9	2.2	6.3	13.6%	2.0

Rank	Description	15.dpi	5.dpi	M	t	adj.q	B
65	AT5G66110.1 | similar to heavy-metal-associated domain-containing protein / copper chaperone (CCH)-related [Arabidopsis thaliana] (TAIR:At4g38580.1); similar to Putative atfp6-like protein [Oryza sativa (japonica cultivar-group)] (GB:XP_470243.1); contains InterPro domain Heavy metal binding (InterPro:IPR006191); contains InterPro domain Heavy metal transport/detoxification protein (InterPro:IPR006121) | chr5:26447428-26448138 FORWARD | Aliases: K2A18.19, K2A18_19	9.6	7.4	2.2	6.3	13.7%	2.3
66	AT1G18980.1 | germin-like protein, putative, similar to germin-like protein subfamily T member 1 (SP:P92995); contains PS00725 germin family signature	6.4	10.7	-4.3	-6.3	13.7%	1.6
67	AT5G45340.2 | Symbol: CYP707A3 | cytochrome P450 family protein, similar to SP:Q42569:C901_ARATH Cytochrome P450 90A1 (SP:Q42569) (Arabidopsis thaliana); contains Pfam profile: PF00067: Cytochrome P450 | chr5:18385907-18388218 REVERSE | Aliases: K9E15.12, K9E15_12	8.8	6.5	2.3	6.3	13.7%	2.2
68	AT4G00880.1 | auxin-responsive family protein, similar to small auxin up RNA (GI:546362) {Arabidopsis thaliana} | chr4:366373-367274 REVERSE | Aliases: A_TM018A10.6, A_TM018A10_6, T18A10.18, T18A10_18	6.7	4.9	1.8	6.2	13.7%	2.1
69	AT2G27080.2 | harpin-induced protein-related / HIN1-related / harpin-responsive protein-related, contains 1 transmembrane domain; similar to hin1 homolog (GI:13122296) (Arabidopsis thaliana); similar to hin1 (GI:22830759) (Nicotiana tabacum) | chr2:11571011-11574582 FORWARD | Aliases: T20P8.13, T20P8_13	7.2	5.5	1.7	6.2	14.1%	2.2
70	AT4G02850.1 | phenazine biosynthesis PhzC/PhzF family protein, contains Pfam profile: PF02567 phenazine biosynthesis-like protein | chr4:1266463-1268612 REVERSE | Aliases: T5J8.17, T5J8_17	3.6	5.9	-2.3	-6.2	14.2%	2.0
71	AT1G22160.1 | senescence-associated protein-related, similar to senescence-associated protein SAG102 (GI:22331931) (Arabidopsis thaliana) | chr1:7823138-7823960 FORWARD | Aliases: F2E2.23, F2E2_23	5.8	3.6	2.2	6.2	14.3%	2.1
72	AT2G01530.1 | major latex protein-related / MLP-related, low similarity to major latex protein {Papaver somniferum}(GI:20810) | chr2:239719-240492 FORWARD | Aliases: F2I9.15, F2I9_15	4.5	7.5	-3.1	-6.2	14.3%	1.1
73	AT5G26220.1 | ChaC-like family protein, contains Pfam profile: PF04752 ChaC-like protein | chr5:9162972-9164680 REVERSE | Aliases: T19G15.70, T19G15_70	6.0	9.6	-3.6	-6.1	14.4%	1.4
74	AT2G40880.1 | Symbol: FL3 27 |	9.1	7.0	2.1	6.1	14.5%	2.0
75	AT1G43800.1 | acyl-(acyl-carrier-protein) desaturase, putative / stearoyl-ACP desaturase, putative, similar to Acyl-(acyl-carrier protein) desaturase from Lupinus luteus GI:4704824, Asclepias syriaca GI:1762436, Ricinus communis SP:P22337; contains Pfam profile PF03405 Fatty acid desaturase | chr1:16580056-16582162 FORWARD | Aliases: F28H19.7, F28H19_7	5.8	2.5	3.3	6.1	14.5%	2.1
76	AT5G08350.1 | GRAM domain-containing protein / ABA-responsive protein-related, contains similarity to ABA-responsive protein in barley (GI:4103635) (Hordeum vulgare) (J. Exp. Bot. 50, 727-728 (1999); FH protein interacting protein FIP1, Arabidopsis thaliana, EMBL:AF174428; contains Pfam PF02893: GRAM domain | chr5:2686246-2687250 REVERSE | Aliases: F8L15.80, F8L15_80	8.4	5.8	2.7	6.1	14.5%	1.7
77	AT5G42900.3 | expressed protein, similar to expressed protein [Arabidopsis thaliana] (TAIR:At4g33980.1) | chr5:17215358-17217007 REVERSE | Aliases: MBD2.9, MBD2_9	6.8	4.6	2.2	6.1	14.5%	2.0
78	AT1G67810.1 | Fe-S metabolism associated domain-containing protein, contains Pfam PF02657: Fe-S metabolism associated domain | chr1:25430151-25430968 FORWARD | Aliases: F12A21.6, F12A21_6	7.9	6.1	1.8	6.1	14.5%	2.1
79	AT3G14430.1 | expressed protein | chr3:4824204-4824844 REVERSE | Aliases: MOA2.3	9.6	8.7	0.9	6.1	14.5%	2.0
80	AT2G35780.1 | Symbol: SCPL26 |	6.9	8.3	-1.3	-6.1	14.5%	2.1
81	AT5G43150.1 | expressed protein | chr5:17342246-17343126 FORWARD | Aliases: MMG4.18, MMG4_18	11.6	10.8	0.8	6.0	14.5%	2.0
82	AT4G08950.1 | phosphate-responsive protein, putative (EXO), similar to phi-1 (phosphate-induced gene) (Nicotiana tabacum) GI:3759184; contains Pfam profile PF04674: Phosphate-induced protein 1 conserved region | chr4:5740294-5741521 FORWARD | Aliases: T3H13.3, T3H13_3	9.0	6.0	3.0	6.0	14.5%	1.1
83	AT1G28480.1 | glutaredoxin family protein, contains INTERPRO Domain IPR002109, Glutaredoxin (thioltransferase)	9.0	7.2	1.8	6.0	14.5%	2.1
84	AT1G17380.1 | expressed protein | chr1:5955481-5957205 REVERSE | Aliases: F28G4.16	8.2	5.4	2.8	6.0	14.6%	1.5
85	AT2G47550.1 | pectinesterase family protein, contains Pfam profile: PF01095 pectinesterase | chr2:19516050-19519205 FORWARD | Aliases: T30B22.15	2.5	4.8	-2.3	-6.0	14.6%	1.8
86	AT5G40980.1 | expressed protein | chr5:16432076-16432448 REVERSE | Aliases: MEE6.5, MEE6_5	6.8	4.2	2.6	6.0	14.6%	2.0
87	AT2G40750.1 | Symbol: WRKY54 |	6.5	5.1	1.3	6.0	14.7%	2.0

Rank	Description	15.dpi	5.dpi	M	t	adj.q	B
88	AT1G07370.1 | Symbol: PCNA1 | proliferating cell nuclear antigen 1 (PCNA1), identical to SP:Q9M7Q7 Proliferating cellular nuclear antigen 1 (PCNA 1) {Arabidopsis thaliana}; nearly identical to SP:Q43124 Proliferating cell nuclear antigen (PCNA) {Brassica napus}; contains Pfam profiles PF00705: Proliferating cell nuclear antigen N-terminal domain, PF02747: Proliferating cell nuclear antigen C-terminal domain | chr1:2263140-2264549 FORWARD | Aliases: F22G5.29, F22G5_29, PROLIFERATING CELLULAR NUCLEAR ANTIGEN	5.2	8.6	-3.4	-6.0	14.7%	1.6
89	AT2G30750.1 | Symbol: CYP71A12 | cytochrome P450 71A12, putative (CYP71A12), Identical to Cytochrome P450 (SP:O49340) (Arabidopsis thaliana); contains Pfam profile: PF00067 cytochrome P450 | chr2:13106475-13108490 REVERSE | Aliases: T11J7.14, T11J7_14	3.1	5.1	-2.0	-5.9	14.7%	1.7
90	AT5G67080.1 | Symbol: MAPKKK19 | protein kinase family protein, contains protein kinase domain, Pfam:PF00069 | chr5:26789952-26790986 FORWARD | Aliases: K21H1.4, K21H1_4	4.6	2.9	1.7	5.9	14.7%	2.0
91	AT4G23690.1 | disease resistance-responsive family protein / dirigent family protein, similar to disease resistance response protein 206-d (Pisum sativum) gi:508844:gb:AAB18669; similar to dirigent protein (Forsythia x intermedia) gi:6694693:gb:AAF25357 | chr4:12338881-12339757 REVERSE | Aliases: F9D16.160, F9D16_160	2.8	7.1	-4.3	-5.9	14.7%	0.8
92	AT2G24040.1 | hydrophobic protein, putative / low temperature and salt responsive protein, putative, similar to SP:Q9ZNQ7 Hydrophobic protein RCI2A (Low temperature and salt responsive protein LTI6A) {Arabidopsis thaliana}; contains Pfam profile PF01679: Uncharacterized protein family | chr2:10231057-10231686 FORWARD | Aliases: T29E15.24, T29E15_24	8.2	6.7	1.4	5.9	15.3%	2.0
93	AT1G61800.1 | glucose-6-phosphate/phosphate translocator, putative, similar to glucose-6-phosphate/phosphate-translocator precursor GI:2997591 from (Pisum sativum) | chr1:22828105-22830411 FORWARD | Aliases: T13M11.18, T13M11_18	5.3	3.6	1.7	5.9	15.3%	1.7
94	AT2G21820.1 | expressed protein | chr2:9309965-9310327 REVERSE | Aliases: F7D8.14, F7D8_14	10.3	8.2	2.1	5.8	15.3%	1.7
95	AT4G20260.4 | similar to DREPP plasma membrane polypeptide-related [Arabidopsis thaliana] (TAIR:At5g44610.1); similar to DREPP2 protein [Nicotiana tabacum] (GB:CAB91552.1); contains InterPro domain DREPP plasma membrane polypeptide (InterPro:IPR008469) | chr4:10940749-10943512 FORWARD | Aliases: F1C12.180, F1C12_180	10.9	9.5	1.4	5.8	15.3%	1.9
96	AT3G53190.1 | pectate lyase family protein, similar to pectate lyase 2 GP:6606534 from (Musa acuminata) | chr3:19725019-19728568 FORWARD | Aliases: T4D2.120	6.2	7.8	-1.6	-5.8	15.3%	1.9
97	AT2G17850.1 | senescence-associated family protein, contains similarity to ketoconazole resistant protein GI:928938 from (Arabidopsis thaliana) and din1 GI:2190012 from (Raphanus sativus);contains a thiosulfate sulfurtransferase domain | chr2:7767087-7767791 REVERSE | Aliases: T13L16.13, T13L16_13	6.6	3.0	3.6	5.8	15.3%	1.7
98	AT5G47240.1 | MutT/nudix family protein, similar to SP:P53370 Nucleoside diphosphate-linked moiety X motif 6 {Homo sapiens}; contains Pfam profile PF00293: NUDIX domain | chr5:19200944-19202871 FORWARD | Aliases: MQL5.10, MQL5_10	6.5	4.4	2.1	5.8	15.3%	1.8
99	AT4G33420.1 | peroxidase, putative, identical to class III peroxidase ATP32 (Arabidopsis thaliana) gi:17530547:gb:AAL40837; identical to cDNA class III peroxidase ATP32 GI:17530546	7.8	3.6	4.2	5.8	15.3%	1.6
100	AT4G29780.1 | expressed protein | chr4:14579710-14581706 FORWARD | Aliases: F27B13.20, F27B13_20	7.4	5.2	2.2	5.8	15.8%	1.7
101	AT5G07580.1 | encodes a member of the ERF (ethylene response factor) subfamily B-3 of ERF/AP2 transcription factor family. The protein contains one AP2 domain. There are 18 members in this subfamily including ATERF-1, ATERF-2, AND ATERF-5. | chr5:2399505-2400602 FORWARD | Aliases: MBK20.1	6.6	5.2	1.4	5.8	15.8%	1.8
102	AT3G04890.1 | expressed protein | chr3:1347652-1349205 FORWARD | Aliases: T9J14.16, T9J14_16	7.4	6.0	1.5	5.8	15.8%	1.8
103	AT2G47950.1 | expressed protein | chr2:19631293-19631879 FORWARD | Aliases: T9J23.9, T9J23_9	7.4	4.8	2.7	5.7	16.3%	1.5
104	AT5G42200.1 | zinc finger (C3HC4-type RING finger) family protein, contains Pfam profile: PF00097 zinc finger, C3HC4 type (RING finger) | chr5:16877695-16878423 FORWARD | Aliases: MJC20.31, MJC20_31	6.7	4.3	2.4	5.7	16.5%	1.7
105	AT4G24570.1 | mitochondrial substrate carrier family protein, contains Pfam profile: PF00153 mitochondrial carrier protein | chr4:12686469-12687660 FORWARD | Aliases: F22K18.230, F22K18_230	8.0	5.8	2.2	5.7	16.5%	1.7
106	AT4G27654.1 | expressed protein | chr4:13811651-13812132 FORWARD | Aliases: None	8.2	3.8	4.4	5.7	16.6%	1.7
107	AT1G28290.1 | pollen Ole e 1 allergen and extensin family protein, similar to arabinogalactan protein (Daucus carota) GI:11322245; contains Pfam profile PF01190: Pollen proteins Ole e I family | chr1:9889112-9890980 REVERSE | Aliases: F3H9.6, F3H9_6	4.3	6.5	-2.3	-5.7	16.6%	1.4

Rank	Description	15.dpi	5.dpi	M	t	adj.q	B
108	AT5G07840.1 | ankyrin repeat family protein, contains ankyrin repeats, Pfam:PF00023 | chr5:2506657-2508374 REVERSE | Aliases: F13G24.40	7.2	6.3	1.0	5.7	16.6%	1.7
109	AT3G08720.2 | Symbol: ATPK19 |	9.2	7.8	1.4	5.7	16.6%	1.7
110	AT5G59820.1 | Symbol: RHL41 | zinc finger (C2H2 type) family protein (ZAT12), identical to zinc finger protein ZAT12 (Arabidopsis thaliana) gi:1418325:emb:CAA67232 | chr5:24120198-24121013 FORWARD | Aliases: MMN10.11, MMN10_11, RESPONSIVE TO HIGH LIGHT 41, ZAT12	7.1	4.6	2.5	5.7	16.6%	1.3
111	AT1G67320.1 | DNA primase, large subunit family, contains Pfam profile PF04104: Eukaryotic-type DNA primase, large subunit; similar to DNA primase large subunit (EC 2.7.7.-) (DNA primase 58 kDa subunit) (p58) (Swiss-Prot:P49643) (Homo sapiens) | chr1:25209055-25212635 REVERSE | Aliases: F1N21.14	4.6	7.1	-2.5	-5.7	16.6%	1.2
112	AT4G02060.1 | Symbol: PRL | prolifera protein (PRL) / DNA replication licensing factor Mcm7 (MCM7), identical to DNA replication licensing factor Mcm7 SP:P43299 PROLIFERA protein {Arabidopsis thaliana}; contains Pfam profile PF00493: MCM2/3/5 family | chr4:901388-905590 FORWARD | Aliases: MCM7, PROLIFERA, T10M13.7, T10M13_7	4.0	7.0	-3.0	-5.6	17.0%	1.4
113	AT2G01520.1 | major latex protein-related / MLP-related, low similarity to major latex protein {Papaver somniferum}(GI:169000) contains Pfam profile PF00407: Pathogenesis-related protein Bet v I family | chr2:235928-237130 FORWARD | Aliases: F2I9.14, F2I9_14	7.3	9.4	-2.0	-5.6	17.1%	1.5
114	AT1G47410.1 | expressed protein, identical to hypothetical protein GB:AAD46041 GI:5668815 from (Arabidopsis thaliana) | chr1:17397258-17397948 FORWARD | Aliases: T3F24.15	4.1	6.8	-2.7	-5.6	17.1%	1.2
115	AT5G03545.1 | expressed protein, No ATG start, annotated according to PMID:11123795 | chr5:894705-895399 FORWARD | Aliases: None	10.5	5.8	4.7	5.6	17.1%	-0.3
116	AT4G37490.1 | Symbol: CYC1 | G2/mitotic-specific cyclin (CYC1) / B-like cyclin (CYC1), identical to SP:P30183 G2/mitotic-specific cyclin (B-like cyclin) {Arabidopsis thaliana} | chr4:17621880-17624279 REVERSE | Aliases: CYCB1, CYCB1;1, CYCLIN 1, Cyclin B1;1, F6G17.140, F6G17_140	5.0	3.7	1.3	5.6	17.1%	1.6
117	AT2G19670.1 | protein arginine N-methyltransferase, putative, similar to protein arginine N-methyltransferase 1-variant 1 (Homo sapiens) GI:7453577 | chr2:8506199-8508232 REVERSE | Aliases: F6F22.30, F6F22_30	4.1	6.2	-2.0	-5.6	17.2%	1.5
118	AT5G19890.1 | peroxidase, putative, identical to peroxidase (Arabidopsis thaliana) gi:1403134:emb:CAA67092 | chr5:6724117-6725925 REVERSE | Aliases: F28I16.40, F28I16_40	4.5	2.2	2.3	5.5	18.5%	1.5
119	AT3G44260.1 | CCR4-NOT transcription complex protein, putative, similar to SWISS-PROT:Q9UFF9 CCR4-NOT transcription complex, subunit 8 (CAF1-like protein, CALIFp) (Homo sapiens) | chr3:15963032-15964071 REVERSE | Aliases: T10D17.50	6.0	4.3	1.6	5.5	18.8%	1.5
120	AT3G48540.1 | cytidine/deoxycytidylate deaminase family protein, similar to SP:P32321 Deoxycytidylate deaminase (EC 3.5.4.12) (dCMP deaminase) {Homo sapiens}; contains Pfam profile PF00383: Cytidine and deoxycytidylate deaminase zinc-binding region | chr3:18000783-18003000 REVERSE | Aliases: T8P19.50	4.0	6.0	-2.0	-5.5	18.8%	1.3
121	AT5G20150.1 | SPX (SYG1/Pho81/XPR1) domain-containing protein, similar to PHO1 protein (Arabidopsis thaliana) GI:20069032; contains Pfam profile PF03105: SPX domain | chr5:6802374-6803627 FORWARD | Aliases: F5O24.40, F5O24_40	5.5	3.8	1.7	5.5	18.8%	1.4
122	AT5G11930.1 | glutaredoxin family protein, contains INTERPRO Domain IPR002109, Glutaredoxin (thioltransferase)	6.1	4.4	1.7	5.5	18.9%	1.4
123	AT2G43620.1 | chitinase, putative, similar to basic endochitinase CHB4 precursor SP:Q06209 from (Brassica napus) | chr2:18100847-18102102 REVERSE | Aliases: F18O19.27	2.7	4.4	-1.7	-5.4	19.3%	1.5
124	AT1G72430.1 | auxin-responsive protein-related, similar to auxin-induced protein TGSAUR22 (GI:10185820) (Tulipa gesneriana) | chr1:27268573-27269292 REVERSE | Aliases: T10D10.10, T10D10_10	7.4	5.4	1.9	5.4	19.8%	1.3
125	AT5G61000.1 | replication protein, putative, similar to replication protein A1 (Oryza sativa) GI:2258469; contains InterPro entry IPR004365: OB-fold nucleic acid binding domain | chr5:24566804-24569947 REVERSE | Aliases: MSL3.120, MSL3_120	5.3	8.2	-2.9	-5.4	19.8%	1.0
126	AT1G78000.2 | Symbol: SULTR1;2 | sulfate transporter (Sultr1;2), identical to sulfate transporter Sultr1;2 (Arabidopsis thaliana) GI:7768660; contaisn Pfam profiles PF00916: Sulfate transporter family and PF01740: STAS domain; contains TIGRfam profile TIGR00815: sulfate permease | chr1:29334738-29338040 FORWARD | Aliases: F28K19.22, F28K19_22, SEL1, SELENATE RESISTANT 1, SULFATE TRANSPORTER, SULFATE TRANSPORTER 1;2	6.3	8.2	-1.9	-5.4	19.8%	1.2
127	AT1G75390.1 | bZIP transcription factor family protein, contains Pfam profile: PF00170 bZIP transcription factor | chr1:28295426-28296669 FORWARD | Aliases: F1B16.8, F1B16_8	8.4	6.3	2.0	5.4	19.8%	1.4
128	AT4G37240.1 | expressed protein | chr4:17524455-17525153 FORWARD | Aliases: AP22.53, AP22_53	5.4	3.7	1.7	5.4	19.8%	1.3

Rank	Description	15.dpi	5.dpi	M	t	adj.q	B
129	AT5G41880.1 | DNA primase small subunit family, contains Pfam profile: PF01896 DNA primase small subunit | chr5:16778299-16781011 FORWARD | Aliases: K16L22.17, K16L22_17	4.3	6.8	-2.5	-5.4	19.8%	1.1
130	AT1G20920.1 | DEAD box RNA helicase, putative, similar to RNA helicase (Rattus norvegicus) GI:897915; contains Pfam profiles PF00270: DEAD/DEAH box helicase, PF00271: Helicase conserved C-terminal domain | chr1:7285103-7288831 FORWARD | Aliases: F9H16.10, F9H16_10	6.6	5.5	1.1	5.4	19.8%	1.4
131	AT2G38860.2 | Symbol: YLS5 | proteaseI (pfpI)-like protein (YLS5), contains Pfam profile PF01965: DJ-1/PfpI family; supporting cDNA gi:13122287:dbj:AB047808.1:; identical to proteaseI (pfpI)-like protein (Arabidopsis thaliana) GI:13122288, cDNA proteaseI (pfpI)-like protein GI:13122287 | chr2:16240522-16242309 REVERSE | Aliases: T7F6.3, T7F6_3	7.6	9.2	-1.6	-5.4	19.8%	1.4
132	AT5G63600.2 | similar to flavonol synthase 1 (FLS1) [Arabidopsis thaliana] (TAIR:At5g08640.1); similar to FLS_MATIN Flavonol synthase/flavanone 3-hydroxylase (FLS) (GB:O04395); contains InterPro domain 2OG-Fe(II) oxygenase superfamily (InterPro:IPR005123) | chr5:25477910-25479684 REVERSE | Aliases: MBK5.7, MBK5_7	3.4	5.5	-2.1	-5.4	19.8%	1.2
133	AT5G63590.1 | flavonol synthase, putative, similar to SP:Q96330 Flavonol synthase 1 (EC 1.14.11.-) (FLS 1) {Arabidopsis thaliana}; contains Pfam profile PF03171: oxidoreductase, 2OG-Fe(II) oxygenase family | chr5:25474219-25475696 REVERSE | Aliases: MBK5.5, MBK5_5	3.5	5.2	-1.7	-5.3	19.8%	1.2
134	AT1G63640.2 | kinesin motor protein-related, C-terminal region is similar to C-term region of kinesin motor protein GB:AAB51397 (Mus musculus); contains Pfam profile: PF00225 Kinesin motor domain | chr1:23592730-23599438 REVERSE | Aliases: F2K11.1, F2K11_1	4.5	6.4	-1.9	-5.3	19.8%	1.1
135	AT5G43260.1 | chaperone protein dnaJ-related, similar to Chaperone protein dnaJ (SP:Q9WZV3) (Thermotoga maritima) | chr5:17374615-17375310 REVERSE | Aliases: MNL12.8, MNL12_8	9.6	7.8	1.8	5.3	19.8%	1.3
136	AT5G08020.1 | replication protein, putative, similar to replication protein A1 (Oryza sativa) GI:2258469; contains InterPro entry IPR004365: OB-fold nucleic acid binding domain | chr5:2572108-2574880 FORWARD | Aliases: F13G24.220, F13G24_220	3.2	5.0	-1.8	-5.3	19.8%	1.1
137	AT5G42290.1 | transcription activator-related, contains weak similarity to beta-cell E-box transcription activator 1 gi:529197:gb:AAA65702 | chr5:16928928-16929332 FORWARD | Aliases: K5J14.9, K5J14_9	6.7	4.7	2.1	5.3	19.8%	1.0
138	AT5G50800.1 | nodulin MtN3 family protein, similar to MtN3 GI:1619602 (root nodule development) from (Medicago truncatula) | chr5:20682303-20684530 REVERSE | Aliases: K7B16.1, K7B16_1	11.3	8.3	3.0	5.3	20.3%	1.1
139	AT2G28470.1 | Symbol: BGAL8 | beta-galactosidase, putative / lactase, putative, similar to Beta-galactosidase precursor SP:P48980 from (Lycopersicon esculentum) | chr2:12175930-12180647 REVERSE | Aliases: T17D12.3, T17D12_3	5.6	7.6	-2.1	-5.3	20.3%	1.3
140	AT5G12170.1 | expressed protein, similar to unknown protein (pir::T09909) | chr5:3933503-3934962 FORWARD | Aliases: MXC9.13, MXC9_13	7.2	5.3	1.9	5.3	20.3%	1.0
141	AT2G22170.1 | lipid-associated family protein, contains PLAT/LH2 (Polycystin-1, Lipoxygenase, Alpha-Toxin/Lipoxygenase homology) domain Pfam:PF01477 | chr2:9433929-9434891 REVERSE | Aliases: T26C19.17, T26C19_17	2.5	5.0	-2.5	-5.3	20.3%	0.8
142	AT5G10010.1 | expressed protein | chr5:3127907-3131721 FORWARD | Aliases: T31P16.7, T31P16_7	4.3	6.0	-1.8	-5.3	20.3%	1.2
143	AT1G52880.1 | Symbol: NAM | no apical meristem (NAM) family protein, contains Pfam PF02365: No apical meristem (NAM) domain; similar to NAM (no apical meristem) GB:CAA63101 from (Petunia x hybrida); identical to cDNA NAC domain protein GI:4325285 | chr1:19692625-19694210 REVERSE | Aliases: ANAC018, ATNAM, F14G24.15, F14G24_15, NAC DOMAIN PROTEIN NAM	5.4	3.7	1.6	5.3	20.3%	1.1
144	AT4G04830.1 | methionine sulfoxide reductase domain-containing protein / SeIR domain-containing protein, low similarity to pilin-like transcription factor (Homo sapiens) GI:5059062, SP:P14930 Peptide methionine sulfoxide reductase msrA/msrB (EC 1.8.4.6) {Neisseria gonorrhoeae}; contains Pfam profile PF01641: SelR domain | chr4:2445857-2447082 FORWARD | Aliases: T4B21.5, T4B21_5	12.2	13.0	-0.8	-5.3	20.3%	1.1
145	AT4G38970.2 | fructose-bisphosphate aldolase, putative, strong similarity to plastidic fructose-bisphosphate aldolase (EC 4.1.2.13) from Nicotiana paniculata (NPALDP1) (GI:4827251), Oryza sativa, PIR2:T02057 (SP:Q40677) | chr4:18163490-18165734 REVERSE | Aliases: F19H22.70, F19H22_70	10.7	9.1	1.6	5.3	20.3%	1.2
146	AT1G22570.1 | proton-dependent oligopeptide transport (POT) family protein, contains Pfam profile: PF00854 POT family | chr1:7976609-7978562 REVERSE | Aliases: F12K8.8, F12K8_8	7.1	5.2	1.9	5.3	20.4%	1.1
147	AT5G16570.1 | Symbol: GLN1;4 |	10.4	8.9	1.5	5.2	20.5%	1.2
148	AT2G44460.1 | glycosyl hydrolase family 1 protein, contains Pfam PF00232 : Glycosyl hydrolase family 1 domain; TIGRFAM TIGR01233: 6-phospho-beta-galactosidase; similar to beta-glucosidase 1 (GI:12043529) (Arabidopsis thaliana) | chr2:18353576-18357042 FORWARD | Aliases: F4I1.27	7.5	11.6	-4.2	-5.2	20.5%	0.9

Rank	Description	15.dpi	5.dpi	M	t	adj.q	B
149	AT5G16940.1 | expressed protein, contains Pfam profile PF04828: Protein of unknown function (DUF636) | chr5:5571554-5572393 REVERSE | Aliases: F2K13.90, F2K13_90	6.2	4.2	2.0	5.2	20.6%	1.2
150	AT2G39350.1 | ABC transporter family protein | chr2:16436774-16439635 REVERSE | Aliases: T16B24.1, T16B24_1	4.8	7.2	-2.4	-5.2	20.6%	0.9
151	AT1G70780.1 | expressed protein | chr1:26698845-26700041 REVERSE | Aliases: F5A18.4, F5A18_4	10.6	9.8	0.8	5.2	20.7%	1.0
152	AT5G11840.1 | expressed protein, predicted proteins in Synechococcus ,Cyanophora and other organisms | chr5:3813611-3814806 REVERSE | Aliases: F14F18.10, F14F18_10	6.2	4.6	1.6	5.2	20.8%	1.1
153	AT1G70590.1 | F-box family protein, contains Pfam PF00646: F-box domain; similar to SKP1 interacting partner 2 (SKIP2) TIGR_Ath1:At5g67250 | chr1:26621984-26623946 FORWARD | Aliases: F24J13.16, F24J13_16	8.3	6.8	1.5	5.2	20.8%	1.2
154	AT1G12940.1 | Symbol: ATNRT2.5 | high-affinity nitrate transporter, putative, similar to trans-membrane nitrate transporter protein AtNRT2:1 (Arabidopsis thaliana) GI:3747058, high-affinity nitrate transporter ACH1 (Arabidopsis thaliana) GI:3608362 | chr1:4416403-4418588 FORWARD | Aliases: F13K23.20, F13K23_20	5.9	3.6	2.2	5.2	20.9%	0.7
155	AT5G42650.1 | Symbol: AOS | allene oxide synthase (AOS) / hydroperoxide dehydrase / cytochrome P450 74A (CYP74A), identical to Allene oxide synthase, chloroplast precursor (Hydroperoxide dehydrase) (Cytochrome P450 74A) (SP:Q96242) {Arabidopsis thaliana} | chr5:17114823-17116623 REVERSE | Aliases: ALLENE OXIDASE SYNTHASE, ALLENE OXIDE SYNTHASE, CYP74A	6.4	5.3	1.1	5.2	20.9%	1.1
156	AT1G18970.1 | Symbol: GLP4 | germin-like protein (GLP1) (GLP4), identical to germin-like protein subfamily T member 1 (SP:P92995) | chr1:6554559-6555331 REVERSE | Aliases: F14D16.12, F14D16_12, GERMIN LIKE PROTEIN 4	3.3	5.3	-1.9	-5.2	21.0%	1.0
157	AT5G43450.1 | 2-oxoglutarate-dependent dioxygenase, putative, similar to 2A6 (GI:599622) and tomato ethylene synthesis regulatory protein E8 (SP:P10967) | chr5:17474359-17476025 REVERSE | Aliases: MWF20.16, MWF20_16	5.2	3.8	1.4	5.2	21.2%	1.1
158	AT1G50240.1 | armadillo/beta-catenin repeat family protein, contains Pfam profile: PF00514 armadillo/beta-catenin-like repeat | chr1:18613094-18617830 FORWARD | Aliases: F14I3.25, F14I3_25	4.3	5.7	-1.4	-5.2	21.2%	1.1
159	AT1G15550.1 | Symbol: GA4 | gibberellin 3-beta-dioxygenase / gibberellin 3 beta-hydroxylase (GA4), identical to gibberellin 3 beta-hydroxylase (GI:2160454) | chr1:5344473-5346161 REVERSE | Aliases: ATGA3OX1, GA REQUIRING 4, GA4, GIBBERELLIN 3 BETA HYDROXYLASE, T16N11.6, T16N11_6	6.5	8.1	-1.6	-5.2	21.4%	1.1
160	AT5G34940.3 | glycosyl hydrolase family 79 N-terminal domain-containing protein, similar to beta-glucuronidase precursor (Scutellaria baicalensis) GI:8918740; contains Pfam profile PF03662: Glycosyl hydrolase family 79, N-terminal domain | chr5:13253059-13255948 REVERSE | Aliases: T2L5.6	6.8	8.6	-1.9	-5.1	21.4%	1.1
161	AT3G62720.2 | similar to galactosyl transferase GMA12/MNN10 family protein [Arabidopsis thaliana] (TAIR:At4g02500.1); similar to beta-6-xylosyltransferase [Gossypium raimondii] (GB:CAI11451.1); similar to beta-6-xylosyltransferase [Pinus taeda] (GB:CAI11450.1); contains InterPro domain Galactosyl transferase (InterPro:IPR008630) | chr3:23212132-23213889 FORWARD | Aliases: F26K9.150	8.2	6.3	1.9	5.1	21.6%	1.0
162	AT2G17150.2 | similar to RWP-RK domain-containing protein [Arabidopsis thaliana] (TAIR:At4g35270.1); similar to Putataive nodule inception protein [Oryza sativa (japonica cultivar-group)] (GB:XP_470173.1) | chr2:7475164-7478340 REVERSE | Aliases: F6P23.15, F6P23_15	6.5	5.4	1.1	5.1	21.8%	1.1
163	AT1G16705.2 | similar to TAZ zinc finger family protein / zinc finger (ZZ type) family protein [Arabidopsis thaliana] (TAIR:At1g16710.1) | chr1:5712185-5713745 FORWARD | Aliases: None	4.1	3.0	1.1	5.1	21.8%	1.1
164	AT4G39630.1 | expressed protein | chr4:18397589-18399381 REVERSE | Aliases: F23K16.7	3.8	5.4	-1.6	-5.1	22.0%	1.0
165	AT4G29740.2 | Symbol: CKX4 | FAD-binding domain-containing protein / cytokinin oxidase family protein, similar to cytokinin oxidase, Zea mays (gi:3882018) (gi:3441978) | chr4:14566271-14569338 FORWARD | Aliases: CYTOKININ OXIDASE 4, T16L4.250, T16L4_250	5.0	3.5	1.5	5.1	22.0%	1.1
166	AT5G43170.1 | Symbol: AZF3 | zinc finger (C2H2 type) protein 3 (AZF3), identical to Cys2/His2-type zinc finger protein 3 (Arabidopsis thaliana) gi:6009889:dbj:BAA85109 | chr5:17348173-17348874 REVERSE | Aliases: ARABIDOPSIS ZINC FINGER PROTEIN 3, MMG4.21, MMG4_21	9.1	7.7	1.4	5.1	22.0%	1.1
167	AT4G10120.2 | similar to sucrose-phosphate synthase, putative [Arabidopsis thaliana] (TAIR:At1g04920.1); similar to sucrose-phosphate synthase, putative [Arabidopsis thaliana] (TAIR:At5g20280.1); similar to sucrose-phosphate synthase, putative [Arabidopsis thaliana] (TAIR:At5g11110.1); similar to sucrose-phosphate synthase [Craterostigma plantagineum] (GB:CAA72491.1); similar to sucrose-phosphate synthase [Triticum aestivum] (GB:AAQ14552.1); similar to sucrose phosphate synthase [Oryza sativa (japonica cultivar-group)] (GB:BAD87626.1); similar to Sucrose-Phosphate Synthase [Saccharum officinarum] (GB:BAA19241.1); similar to sucrose-phosphate synthase (EC 2.4.1.14) - maize (GB:JQ1329); contains InterPro domain Glycosyl transferase, group 1 (InterPro:IPR001296) | chr4:6314784-6319932 FORWARD | Aliases: F28M11.40, F28M11_40	4.7	3.0	1.7	5.1	22.2%	1.1

Rank	Description	15.dpi	5.dpi	M	t	adj.q	B
168	AT1G20440.1 | Symbol: COR47 | dehydrin (COR47), identical to dehydrin COR47 (Cold-induced COR47 protein) (Arabidopsis thaliana) SWISS-PROT:P31168 | chr1:7084399-7085762 REVERSE | Aliases: F5M15.22, F5M15_22, RD17	8.9	7.2	1.8	5.1	22.6%	1.0
169	AT2G19590.1 | Symbol: ACO1 | 1-aminocyclopropane-1-carboxylate oxidase, putative / ACC oxidase, putative, similar to ACC oxidase (Cucumis melo)(GI:1183898) | chr2:8483048-8484539 REVERSE | Aliases: ACC OXIDASE 1, ACO1, F3P11.19, F3P11_19	6.8	4.4	2.4	5.1	22.6%	0.8
170	AT1G67180.1 | zinc finger (C3HC4-type RING finger) family protein / BRCT domain-containing protein, contains Pfam domains PF00533: BRCA1 C Terminus (BRCT) domain and PF00097: Zinc finger, C3HC4 type (RING finger) | chr1:25133762-25135832 FORWARD | Aliases: F5A8.9, F5A8_9	2.2	2.8	-0.6	-5.0	22.8%	0.6
171	AT3G03270.2 | universal stress protein (USP) family protein / early nodulin ENOD18 family protein, contains Pfam profile PF00582: universal stress protein family; similar to early nodulin ENOD18 (GI:11602747) (Vicia faba) | chr3:761991-763089 REVERSE | Aliases: T17B22.4, T17B22_4	9.2	5.6	3.6	5.0	22.9%	0.6
172	AT2G30930.1 | expressed protein | chr2:13169517-13170473 FORWARD | Aliases: F7F1.14, F7F1_14	10.0	7.7	2.3	5.0	22.9%	0.9
173	AT1G26840.1 | similar to putative origin recognition complex subunit 6 (ORC6) containing protein [Solanum demissum] (GB:AAT39295.1); contains InterPro domain Origin recognition complex subunit 6 (InterPro:IPR008721) | chr1:9298809-9300686 FORWARD | Aliases: T2P11.3, T2P11_3	4.6	6.7	-2.1	-5.0	22.9%	0.8
174	AT3G11930.4 | similar to universal stress protein (USP) family protein [Arabidopsis thaliana] (TAIR:At3g58450.1); similar to putative ethylene-responsive protein [Oryza sativa (japonica cultivar-group)] (GB:AAP53941.1); contains InterPro domain Usp domain (InterPro:IPR006016); contains InterPro domain Universal stress protein (Usp) (InterPro:IPR006015) | chr3:3776265-3777700 FORWARD | Aliases: MEC18.3	10.7	9.1	1.5	5.0	22.9%	1.0
175	AT1G19770.1 | Symbol: ATPUP14 | purine permease-related, low similarity to purine permease (Arabidopsis thaliana) GI:7620007; contains Pfam profile PF03151: Domain of unknown function, DUF250 | chr1:6832341-6833693 FORWARD | Aliases: F14P1.13, F14P1_13	8.5	7.0	1.6	5.0	22.9%	1.0
176	AT3G18250.1 | expressed protein, ; expression supported by MPSS | chr3:6258183-6258413 REVERSE | Aliases: MIE15.4	4.0	5.3	-1.3	-5.0	23.0%	0.9
177	AT1G52890.1 | Symbol: ANAC019 | no apical meristem (NAM) family protein, contains Pfam PF02365: No apical meristem (NAM) domain; similar to NAM (no apical meristem) GB:CAA63101 from (Petunia x hybrida) | chr1:19700672-19702140 REVERSE | Aliases: ANAC019, F14G24.16, F14G24_16	5.7	4.2	1.5	5.0	23.0%	0.9
178	AT2G37640.1 | Symbol: ATEXPA3 | expansin, putative (EXP3), identical to Alpha-expansin 3 precursor (At-EXP3)(Arabidopsis thaliana) SWISS-PROT:O80932; alpha-expansin gene family, PMID:11641069 | chr2:15794783-15796931 REVERSE | Aliases: ARABIDOPSIS THALIANA EXPANSIN A3, ATEXP3, ATHEXP ALPHA 1.9, F13M22.14, F13M22_14	8.4	5.8	2.6	5.0	23.3%	0.5
179	AT1G04020.2 | Symbol: ATBRCA1 | zinc finger (C3HC4-type RING finger) family protein / BRCT domain-containing protein, contains Pfam domain, PF00533: BRCA1 C Terminus (BRCT) domain | chr1:1036563-1040223 FORWARD | Aliases: ATBRCA1, F21M11.4, F21M11_4	4.4	6.8	-2.4	-5.0	23.3%	0.7
180	AT5G41080.2 | glycerophosphoryl diester phosphodiesterase family protein, weak similarity to SP:P37965 Glycerophosphoryl diester phosphodiesterase (EC 3.1.4.46) {Bacillus subtilis}; contains Pfam profile PF03009: Glycerophosphoryl diester phosphodiesterase family | chr5:16459036-16461213 FORWARD | Aliases: MEE6.15, MEE6_15	5.3	3.7	1.5	5.0	23.3%	0.9
181	AT4G13530.2 | expressed protein, predicted protein, Arabidopsis thaliana | chr4:7866780-7868716 FORWARD | Aliases: T6G15.80, T6G15_80	9.3	7.5	1.8	5.0	23.3%	0.9
182	AT2G02560.2 | Symbol: CAND1 | similar to TBP-interacting protein 120A [Rattus norvegicus] (GB:NP_446456.1); similar to PREDICTED: TBP-interacting protein [Mus musculus] (GB:XP_125901.5); similar to TIP120 protein [Homo sapiens] (GB:AAH50341.1); similar to mKIAA0829 protein [Mus musculus] (GB:BAC98035.1); similar to hypothetical protein [Homo sapiens] (GB:CAB66744.1); contains InterPro domain TBP-interacting 120 (InterPro:IPR007723) | chr2:689787-697595 FORWARD | Aliases: ATCAND1, ETA2, T8K22.14, T8K22_14, TIP120	5.4	7.9	-2.5	-5.0	23.3%	0.9
183	AT5G43500.2 | Symbol: ATARP9 |	3.3	4.4	-1.0	-5.0	23.3%	0.9
184	AT5G43440.1 | 2-oxoglutarate-dependent dioxygenase, putative, similar to 2A6 (GI:599622) and tomato ethylene synthesis regulatory protein E8 (SP:P10967) | chr5:17472461-17473885 REVERSE | Aliases: MWF20.15, MWF20_15	5.8	4.1	1.7	5.0	23.3%	0.8
185	AT5G35670.1 | calmodulin-binding family protein, contains IQ calmodulin-binding motif, Pfam:PF00612 | chr5:13873345-13875756 FORWARD | Aliases: MXH1.1, MXH1_1	2.7	4.2	-1.5	-5.0	23.4%	0.9
186	AT1G50440.2 | zinc finger (C3HC4-type RING finger) family protein, contains Pfam profile: PF00097 zinc finger, C3HC4 type (RING finger) | chr1:18689479-18691575 FORWARD | Aliases: F11F12.20, F11F12_20	6.6	5.4	1.2	4.9	23.6%	0.9

Rank	Description	15.dpi	5.dpi	M	t	adj.q	B
187	AT2G29890.2 | Symbol: VLN1 | similar to villin, putative [Arabidopsis thaliana] (TAIR:At5g57320.1); similar to villin, putative [Arabidopsis thaliana] (TAIR:At4g30160.1); similar to putative villin [Oryza sativa (japonica cultivar-group)] (GB:XP_480904.1); similar to OSJNBa0041A02.24 [Oryza sativa (japonica cultivar-group)] (GB:XP_473786.1); similar to actin filament bundling protein P-115-ABP [Lilium longiflorum] (GB:BAC77209.1); contains InterPro domain Gelsolin (InterPro:IPR007122); contains InterPro domain Gelsolin region (InterPro:IPR007123) | chr2:12751227-12756663 FORWARD | Aliases: ATVLN1, F6K5.2, F6K5_2, VILLIN 1	3.7	5.3	-1.7	-4.9	23.6%	0.9
188	AT1G78290.2 | serine/threonine protein kinase, putative, similar to serine-threonine protein kinase (Triticum aestivum) gi:2055374:gb:AAB58348	5.8	4.5	1.3	4.9	23.6%	0.9
189	AT3G05220.2 | heavy-metal-associated domain-containing protein, similar to farnesylated protein 1 (GI:23304411) {Hordeum vulgare subsp. spontaneum}; contains Pfam profile PF00403: Heavy-metal-associated domain | chr3:1489425-1491320 FORWARD | Aliases: T12H1.19, T12H1_19	10.1	8.9	1.2	4.9	23.6%	0.7
190	AT2G06850.1 | Symbol: EXGT A1 | xyloglucan:xyloglucosyl transferase / xyloglucan endotransglycosylase / endo-xyloglucan transferase (EXT) (EXGT-A1), identical to endo-xyloglucan transferase (ext) GI:469484 and endoxyloglucan transferase (EXGT-A1) GI:5533309 from (Arabidopsis thaliana)	5.7	7.5	-1.8	-4.9	23.6%	0.8
191	AT4G29520.1 | expressed protein | chr4:14493920-14495735 REVERSE | Aliases: T16L4.30, T16L4_30	5.1	7.0	-1.9	-4.9	23.6%	0.8
192	AT3G51310.1 | vacuolar protein sorting-associated protein 35 family protein / VPS35 family protein, similar to vacuolar protein sorting 35 (Mus musculus) GI:11875394; contains Pfam profile PF03635: Vacuolar protein sorting-associated protein 35 | chr3:19055502-19060300 REVERSE | Aliases: F24M12.350	8.2	9.5	-1.3	-4.9	23.6%	0.8
193	AT1G71450.1 | encodes a member of the DREB subfamily A-4 of ERF/AP2 transcription factor family. The protein contains one AP2 domain. There are 17 members in this subfamily including TINY. | chr1:26930750-26931618 FORWARD | Aliases: F26A9.17	3.6	2.7	0.9	4.9	23.7%	0.8
194	AT5G66400.2 | Symbol: RAB18 | similar to dehydrin, putative [Arabidopsis thaliana] (TAIR:At3g50980.1); similar to putative dehydrin [Helianthus hirsutus] (GB:CAC80706.1); contains InterPro domain Eggshell protein (InterPro:IPR002952); contains InterPro domain Dehydrin (InterPro:IPR000167) | chr5:26535503-26536468 REVERSE | Aliases: ARABIDOPSIS THALIANA DROUGHT INDUCED 8, ATDI8, K1F13.5, K1F13_5, RESPONSIVE TO ABA 18	6.3	3.9	2.4	4.9	23.7%	0.6
195	AT1G07280.2 | expressed protein, similar to expressed protein [Arabidopsis thaliana] (TAIR:At2g29670.1); similar to unknown protein [Oryza sativa (japonica cultivar-group)] (GB:AAU10760.1); contains domain SER_RICH (PS50324) | chr1:2238109-2241172 FORWARD | Aliases: F22G5.38, F22G5_38	7.7	5.4	2.3	4.9	23.7%	0.8
196	AT2G02130.1 | Symbol: LCR68/PDF2.3 | plant defensin-fusion protein, putative (PDF2.3), plant defensin protein family member, personal communication, Bart Thomma (Bart.Thomma@agr.kuleuven.ac.be) | chr2:540017-540615 FORWARD | Aliases: F5O4.10, F5O4_10, LCR68, Low molecular weight cysteine rich 68, PDF2.3	12.9	11.9	0.9	4.9	23.7%	0.5
197	AT2G24490.1 | replication protein, putative, similar to replication protein A 30kDa (Oryza sativa (japonica cultivar-group)) GI:13516746; contains InterPro entry IPR004365: OB-fold nucleic acid binding domain | chr2:10405484-10407568 REVERSE | Aliases: T28I24.22, T28I24_22	4.8	6.9	-2.0	-4.9	23.7%	0.7
198	AT1G12180.1 | expressed protein | chr1:4130419-4131135 FORWARD | Aliases: T28K15.15, T28K15_15	3.3	5.7	-2.4	-4.9	23.7%	0.8
199	AT5G13520.1 | peptidase M1 family protein, similar to SP:P09960 Leukotriene A-4 hydrolase (EC 3.3.2.6) (LTA-4 hydrolase) {Homo sapiens}; contains Pfam profile PF01433: Peptidase family M1 | chr5:4341543-4344674 REVERSE | Aliases: T6I14.50, T6I14_50	8.2	10.5	-2.3	-4.9	24.5%	0.7
200	AT2G28760.2 | NAD-dependent epimerase/dehydratase family protein, similar to UDP-glucuronic acid decarboxylase Uxs1p from Filobasidiella neoformans GI:14318327; contains Pfam profile PF01370 NAD dependent epimerase/dehydratase family | chr2:12343268-12346104 REVERSE | Aliases: F8N16.5, F8N16_5	6.2	7.7	-1.5	-4.9	24.6%	0.8
201	AT5G38110.1 | ASF1-like anti-silencing family protein, similar to SP:P32447 Anti-silencing protein 1 {Saccharomyces cerevisiae}; contains Pfam profile PF04729: Anti-silencing protein, ASF1-like | chr5:15225702-15227445 FORWARD | Aliases: F16F17.110, F16F17_110	5.5	8.2	-2.7	-4.9	24.8%	0.5
202	AT1G30500.2 | CCAAT-binding transcription factor (CBF-B/NF-YA) family protein, contains Pfam profile: PF02045 CCAAT-binding transcription factor (CBF-B/NF-YA)	8.5	6.8	1.6	4.8	24.8%	0.6
203	AT1G06650.2 | 2-oxoglutarate-dependent dioxygenase, putative, similar to 2A6 (GI:599622) and tomato ethylene synthesis regulatory protein E8 (SP:P10967); contains Pfam profile: PF00671 Iron/Ascorbate oxidoreductase family | chr1:2035838-2037362 FORWARD | Aliases: F12K11.26, F12K11_26	7.4	5.5	1.9	4.8	24.8%	0.7
204	AT4G08850.2 | leucine-rich repeat family protein / protein kinase family protein, contains Pfam domains PF00560: Leucine Rich Repeat and PF00069: Protein kinase domain | chr4:5637335-5640633 REVERSE | Aliases: T32A17.160, T32A17_160	9.6	8.2	1.4	4.8	24.8%	0.7
205	AT5G57560.1 | Symbol: TCH4 | xyloglucan:xyloglucosyl transferase / xyloglucan endotransglycosylase / endo-xyloglucan transferase (TCH4), identical to xyloglucan endotransglycosylase TCH4 protein GI:886116 | chr5:23324356-23325507 REVERSE | Aliases: MUA2.13, MUA2_13, TOUCH 4, XTH22, XYLOGLUCAN ENDOTRANSGLYCOSYLASE	10.0	7.1	2.9	4.8	25.1%	0.5
206	AT4G31120.2 | Skb1 methyltransferase family protein, contains Pfam profile: PF05185 Skb1 methyltransferase | chr4:15132125-15136624 REVERSE | Aliases: F6E21.40, F6E21_40	6.6	7.9	-1.3	-4.8	25.1%	0.7

Rank	Description	15.dpi	5.dpi	M	t	adj.q	B
207	AT1G05210.1 | expressed protein | chr1:1510138-1511678 FORWARD | Aliases: YUP8H12.18, YUP8H12_18	4.8	5.8	-1.0	-4.8	25.6%	0.7
208	AT4G18470.1 | Symbol: SNI1 | negative regulator of systemic acquired resistance (SNI1), identical to negative regulator of systemic acquired resistance SNI1 (Arabidopsis thaliana) gi:5758933:gb:AAD50900 | chr4:10192920-10195673 FORWARD | Aliases: F28J12.3, F28J12_3, SUPPRESSOR OF NPR1 1, INDUCIBLE 1	2.4	3.0	-0.6	-4.8	25.6%	0.4
209	AT3G15300.1 | VQ motif-containing protein, contains PF05678: VQ motif | chr3:5147412-5148217 REVERSE | Aliases: K7L4.10	6.0	7.5	-1.6	-4.8	25.7%	0.6
210	AT2G24860.1 | chaperone protein dnaJ-related, similar to Tsi1-interacting protein TSIP1 (GI:4337001) (Nicotiana tabacum) | chr2:10593854-10595408 REVERSE | Aliases: F27C12.22, F27C12_22	6.9	6.0	0.9	4.8	25.7%	0.5
211	AT2G47440.1 | DNAJ heat shock N-terminal domain-containing protein, contains Pfam profiles PF00226: DnaJ domain, PF00515: TPR Domain; similar to GP:2104534:AF001308 (T10M13.11) | chr2:19476789-19478956 FORWARD | Aliases: T30B22.26	8.4	6.8	1.5	4.8	25.8%	0.7
212	AT4G16146.1 | expressed protein | chr4:9142445-9144588 REVERSE | Aliases: None	6.7	4.8	1.9	4.8	26.1%	0.6
213	AT1G77450.1 | Symbol: ANAC032 | no apical meristem (NAM) family protein, contains Pfam PF02365: No apical meristem (NAM) domain; similar to GRAB1 protein GB:CAA09371, a novel member of the NAC domain family | chr1:29104848-29106155 FORWARD | Aliases: ANAC032, T5M16.4, T5M16_4	9.4	7.9	1.5	4.8	26.1%	0.6
214	AT3G25600.1 | calmodulin, putative, similar to calmodulin GI:239841 from (Paramecium tetraurelia) | chr3:9308491-9309199 FORWARD | Aliases: T5M7.6	8.0	6.0	2.0	4.8	26.1%	0.7
215	AT1G43650.1 | integral membrane family protein / nodulin MtN21-related, similar to MtN21 GI:2598575 (root nodule development) from (Medicago truncatula)similar to MtN21 GI:2598575 (root nodule development) from (Medicago truncatula) | chr1:16446301-16449268 REVERSE | Aliases: F2J6.1, F2J6_1	7.1	5.0	2.1	4.8	26.1%	0.6
216	AT1G27100.1 | expressed protein, contains Pfam profile: PF04601 protein of unknown function (DUF569 | chr1:9407112-9411159 REVERSE | Aliases: T7N9.16, T7N9_16	6.4	5.2	1.2	4.7	26.6%	0.6
217	AT4G31290.1 | ChaC-like family protein, contains Pfam profile: PF04752 ChaC-like protein | chr4:15185997-15188490 FORWARD | Aliases: F8F16.110, F8F16_110	5.1	6.9	-1.8	-4.7	26.9%	0.6
218	AT1G64590.1 | short-chain dehydrogenase/reductase (SDR) family protein, contains INTERPRO family IPR002198 short-chain dehydrogenase/reductase (SDR) superfamily | chr1:23997196-23999172 FORWARD | Aliases: F1N19.16, F1N19_16	7.1	4.4	2.7	4.7	27.4%	0.4
219	AT3G05130.1 | expressed protein, ; expression supported by MPSS | chr3:1433481-1435385 REVERSE | Aliases: T12H1.9, T12H1_9	4.2	5.9	-1.7	-4.7	27.7%	0.6
220	AT3G59350.3 | similar to serine/threonine protein kinase, putative [Arabidopsis thaliana] (TAIR:At2g43230.1); similar to salt-inducible putative protein serine/threonine/tyrosine kinase [Zea mays] (GB:AAU11815.1); contains InterPro domain Tyrosine protein kinase, active site (InterPro:IPR008266); contains InterPro domain Tyrosine protein kinase (InterPro:IPR001245); contains InterPro domain Serine/threonine protein kinase (InterPro:IPR002290); contains InterPro domain Protein kinase (InterPro:IPR000719) | chr3:21943689-21946131 FORWARD | Aliases: F25L23.210	6.4	4.7	1.7	4.7	27.7%	0.6
221	AT4G11650.1 | Symbol: ATOSM34 | osmotin-like protein (OSM34), nearly identical to SP:P50700:OSL3_ARATH Osmotin-like protein OSM34 precursor {Arabidopsis thaliana}; contains Pfam profile PF00314: Thaumatin family | chr4:7024850-7026140 REVERSE | Aliases: T5C23.80, T5C23_80	3.0	5.5	-2.5	-4.7	27.7%	0.4
222	AT4G08780.1 | peroxidase, putative, similar to peroxidase isozyme (Armoracia rusticana) gi:217932:dbj:BAA14143	3.8	5.7	-1.9	-4.7	27.7%	0.6
223	AT5G67480.2 | TAZ zinc finger family protein / BTB/POZ domain-containing protein, contains Pfam PF00651 : BTB/POZ domain; contains Pfam PF02135 : TAZ zinc finger; simiar to Chain A, Taz2 Domain Of The Transcriptional Adaptor Protein Cbp (GI:11514507) (Homo sapiens); similar to (SP:Q09472) E1A-associated protein p300 (SP:Q09472) (Homo sapiens); similar to histone acetyltransferase HAC4 (GI:21105785) (Arabidopsis thaliana) | chr5:26948166-26949982 REVERSE | Aliases: K9I9.4, K9I9_4	6.1	4.5	1.6	4.7	27.7%	0.5
224	AT1G26150.1 | similar to protein kinase family protein [Arabidopsis thaliana] (TAIR:At5g38560.1); similar to putative receptor protein kinase PERK1 [Oryza sativa (japonica cultivar-group)] (GB:BAD87028.1); contains InterPro domain Serine/threonine protein kinase, active site (InterPro:IPR008271); contains InterPro domain Tyrosine protein kinase (InterPro:IPR001245); contains InterPro domain Serine/threonine protein kinase (InterPro:IPR002290); contains InterPro domain Protein kinase (InterPro:IPR000719); contains InterPro domain Proline-rich region (InterPro:IPR000694) | chr1:9039615-9043275 REVERSE | Aliases: F28B23.17, F28B23_17	6.0	7.6	-1.5	-4.7	27.7%	0.6
225	AT1G71692.1 | Symbol: AGL12 | MADS-box protein (AGL12), identical to GB:AAC49085 GI:862650 from (Arabidopsis thaliana) (Plant Cell 7 (8), 1259-1269 (1995)) | chr1:26956307-26958789 REVERSE | Aliases: AGAMOUS LIKE 12, F14O23.5, F14O23_5	7.8	6.1	1.7	4.7	27.7%	0.6

Rank	Description	15.dpi	5.dpi	M	t	adj.q	B
226	AT5G20250.3 | Symbol: DIN10 | similar to alkaline alpha galactosidase, putative [Arabidopsis thaliana] (TAIR:At3g57520.2); similar to alkaline alpha galactosidase, putative [Arabidopsis thaliana] (TAIR:At3g57520.3); similar to alkaline alpha galactosidase, putative [Arabidopsis thaliana] (TAIR:At1g55740.1); similar to alkaline alpha galactosidase, putative [Arabidopsis thaliana] (TAIR:At3g57520.1); similar to putative alkaline alpha-galactosidase seed imbibition protein [Oryza sativa (japonica cultivar-group)] (GB:XP_483143.1); similar to Sip1 protein - barley (GB:S27762); similar to putative imbibition protein [Brassica oleracea] (GB:CAA55893.1); similar to putative raffinose synthase or seed imbibition protein [Oryza sativa (japonica cultivar-group)] (GB:AAT77910.1); similar to alkaline alpha galactosidase II [Cucumis melo] (GB:AAM75140.1); contains InterPro domain Raffinose synthase (InterPro:IPR008811) | chr5:6833680-6836790 FORWARD | Aliases: DARK INDUCIBLE 10, F5O24.140, F5O24_140	8.6	7.0	1.6	4.7	27.7%	0.6
227	AT4G02380.1 | Symbol: SAG21 | late embryogenesis abundant 3 family protein / LEA3 family protein, similar to several small proteins (~100 aa) that are induced by heat, auxin, ethylene and wounding such as Phaseolus aureus indole-3-acetic acid induced protein ARG (SW:32292); contains Pfam profile PF03242: Late embryogenesis abundant protein	11.5	10.8	0.7	4.7	27.7%	0.1
228	AT2G30080.1 | Symbol: ZIP6 | metal transporter, putative (ZIP6), identical to putative metal transporter ZIP6 (Arabidopsis thaliana) gi:17385786:gb:AAL38433; similar to zinc transporter protein ZIP1 (Glycine max) gi:15418778:gb:AAK37761; member of the Zinc (Zn2+)-Iron (Fe2+) permease (ZIP) family, PMID:11500563 | chr2:12845807-12847189 REVERSE | Aliases: T27E13.18, T27E13_18	8.0	5.6	2.3	4.7	27.7%	0.5
229	AT3G21890.1 | zinc finger (B-box type) family protein, contains Pfam profile: PF01760 CONSTANS family zinc finger | chr3:7709187-7709740 REVERSE | Aliases: MZN24.1	6.7	4.0	2.6	4.7	27.7%	0.4
230	AT4G15800.1 | Symbol: RALFL33 | rapid alkalinization factor (RALF) family protein, similar to RALF precursor (Nicotiana tabacum) GI:16566316 | chr4:8984866-8985413 FORWARD | Aliases: DL3940C, FCAALL.206, RALF LIKE 33	7.8	6.4	1.4	4.7	27.7%	0.5
231	AT5G12030.1 | Symbol: AT HSP17.6A | 17.7 kDa class II heat shock protein 17.6A (HSP17.7-CII), identical to heat shock protein 17.6A GI:3256075 from (Arabidopsis thaliana) | chr5:3884108-3884738 REVERSE | Aliases: F14F18.200, F14F18_200, HSP17.6	4.6	3.4	1.2	4.7	27.7%	0.6
232	AT1G54260.1 | histone H1/H5 family protein, contains Pfam domain, PF00538: linker histone H1 and H5 family | chr1:20261007-20262843 FORWARD | Aliases: F20D21.8, F20D21_8	6.9	5.2	1.7	4.6	28.0%	0.5
233	AT1G47960.1 | Symbol: C/VIF1 |	10.8	8.8	2.0	4.6	28.0%	0.5
234	AT4G14770.1 | tesmin/TSO1-like CXC domain-containing protein, similar to CXC domain containing TSO1-like protein 1 (SOL1) (Arabidopsis thaliana) GI:7767427, CXC domain protein TSO1 (Arabidopsis thaliana) GI:7767425; contains Pfam profile PF03638: Tesmin/TSO1-like CXC domain | chr4:8481369-8484820 REVERSE | Aliases: DL3425C, FCAALL.306	4.5	5.9	-1.4	-4.6	28.2%	0.5
235	AT4G32940.1 | Symbol: GAMMA VPE | vacuolar processing enzyme gamma / gamma-VPE, nearly identical to SP:Q39119 Vacuolar processing enzyme, gamma-isozyme precursor (EC 3.4.22.-) (Gamma-VPE) {Arabidopsis thaliana} | chr4:15900273-15903271 REVERSE | Aliases: F26P21.60, F26P21_60, GAMMAVPE	11.4	9.6	1.8	4.6	28.2%	0.5
236	AT1G19530.1 | expressed protein | chr1:6763906-6764944 FORWARD | Aliases: F18O14.29, F18O14_29	7.3	4.4	2.9	4.6	28.2%	0.4
237	AT5G53090.1 | similar to oxidoreductase, forever young (FEY3) [Arabidopsis thaliana] (TAIR:At4g27760.1); similar to forever young oxidoreductase [Lycopersicon esculentum] (GB:AAL60068.1); contains InterPro domain Glucose/ribitol dehydrogenase (InterPro:IPR002347); contains InterPro domain Short-chain dehydrogenase/reductase SDR (InterPro:IPR002198)	3.8	4.8	-1.1	-4.6	28.3%	0.5
238	AT5G54855.1 | pollen Ole e 1 allergen and extensin family protein, contains Pfam domain, PF01190: Pollen proteins Ole e I family | chr5:22300026-22301613 FORWARD | Aliases: None	10.4	9.6	0.8	4.6	28.3%	0.2
239	AT2G34710.1 | Symbol: PHB | homeobox-leucine zipper transcription factor (HB-14), identical to homeodomain transcription factor (ATHB-14)GP:3132474 GB:Y11122 (Arabidopsis thaliana);	6.2	7.4	-1.3	-4.6	28.3%	0.5
240	AT3G27640.1 | transducin family protein / WD-40 repeat family protein, contains seven WD-40 G-protein beta repeats; similar to RA-regulated nuclear matrix-associated protein (GI:14161320) {Homo sapiens} | chr3:10233485-10236858 FORWARD | Aliases: MGF10.5	3.1	4.1	-1.0	-4.6	28.3%	0.4
241	AT4G16690.1 | esterase/lipase/thioesterase family protein, similar to ethylene-induced esterase (Citrus sinensis) GI:14279437, polyneuridine aldehyde esterase (Rauvolfia serpentina) GI:6651393, SP:Q40708 PIR7A protein {Oryza sativa}; contains Interpro entry IPR000379 | chr4:9392169-9393491 REVERSE | Aliases: DL4370C, FCAALL.12	3.8	2.9	1.0	4.6	28.3%	0.5
242	AT2G35300.1 | late embryogenesis abundant group 1 domain-containing protein / LEA group 1 domain-containing protein, contains Pfam domain, PF03760: Late embryogenesis abundant (LEA) group 1 | chr2:14869980-14870466 REVERSE | Aliases: T4C15.3, T4C15_3	3.8	2.8	1.0	4.6	28.3%	0.3

Rank	Description	15.dpi	5.dpi	M	t	adj.q	B
243	AT2G21330.3 | similar to fructose-bisphosphate aldolase, putative [Arabidopsis thaliana] (TAIR:At4g38970.1); similar to plastidic aldolase NPALDP1 [Nicotiana paniculata] (GB:BAA77604.1); similar to latex plastidic aldolase-like protein [Hevea brasiliensis] (GB:AAM46780.1); contains InterPro domain Fructose-bisphosphate aldolase, class-I (InterPro:IPR000741) | chr2:9135228-9137293 REVERSE | Aliases: F3K23.9, F3K23_9	10.7	9.2	1.6	4.6	28.3%	0.4
244	AT5G66985.1 | expressed protein | chr5:26760505-26761034 FORWARD | Aliases: None	7.2	4.4	2.7	4.6	28.3%	0.4
245	AT4G24050.1 | short-chain dehydrogenase/reductase (SDR) family protein, contains INTERPRO family IPR002198 Short-chain dehydrogenase/reductase (SDR) superfamily | chr4:12497077-12500138 FORWARD | Aliases: T19F6.40, T19F6_40	5.9	4.7	1.2	4.6	28.6%	0.4
246	AT2G23360.1 | transport protein-related, contains Pfam PF05911: Plant protein of unknown function (DUF869) profile; weak similarity to Intracellular protein transport protein USO1 (Swiss-Prot:P25386) (Saccharomyces cerevisiae) | chr2:9956536-9959925 FORWARD | Aliases: F26B6.1, F26B6_1	4.3	5.4	-1.1	-4.6	28.6%	0.5
247	AT3G22600.1 | protease inhibitor/seed storage/lipid transfer protein (LTP) family protein, contains Pfam protease inhibitor/seed storage/LTP family domain PF00234 | chr3:8006521-8007478 REVERSE | Aliases: F16J14.16	3.4	5.0	-1.6	-4.6	28.6%	0.4
248	AT3G23810.1 | Symbol: SAHH2 | adenosylhomocysteinase, putative / S-adenosyl-L-homocysteine hydrolase, putative / AdoHcyase, putative, strong similarity to SP:P50248:SAHH_TOBAC Adenosylhomocysteinase (EC 3.3.1.1) (S-adenosyl-L-homocysteine hydrolase) (AdoHcyase) {Nicotiana sylvestris}; contains Pfam profile PF00670: S-adenosyl-L-homocysteine hydrolase, NAD binding domain | chr3:8587671-8589720 REVERSE | Aliases: MYM9.17, SAHH2	3.9	7.3	-3.5	-4.6	28.6%	0.3
249	AT5G14650.1 | polygalacturonase, putative / pectinase, putative, similar to polygalacturonase PG1 GP:5669846, PG2 GI:5669848 from (Glycine max); contains PF00295: Glycosyl hydrolases family 28 (polygalacturonases) | chr5:4724453-4726516 FORWARD | Aliases: T15N1.140, T15N1_140	2.6	3.4	-0.9	-4.6	28.6%	0.3
250	AT5G44400.1 | FAD-binding domain-containing protein, similar to SP:P30986 reticuline oxidase precursor (Berberine-bridge-forming enzyme) (BBE) (Tetrahydroprotoberberine synthase) (Eschscholzia californica); contains PF01565 FAD binding domain | chr5:17903421-17905707 REVERSE | Aliases: K9L2.20, K9L2_20	3.6	5.6	-2.0	-4.6	28.6%	0.3
251	AT1G14710.2 | hydroxyproline-rich glycoprotein family protein, contains proline-rich extensin domains, INTERPRO:IPR002965 | chr1:5061861-5065097 REVERSE | Aliases: F10B6.11, F10B6_11	5.2	7.1	-1.9	-4.6	28.6%	0.4
252	AT2G29570.1 | Symbol: PCNA2 | proliferating cell nuclear antigen 2 (PCNA2), identical to SP:Q9ZW35 Proliferating cell nuclear antigen 2 (PCNA 2) {Arabidopsis thaliana}; nearly identical to SP:Q43124 Proliferating cell nuclear antigen (PCNA) {Brassica napus}; contains Pfam profiles PF00705: Proliferating cell nuclear antigen N-terminal domain, PF02747: Proliferating cell nuclear antigen C-terminal domain | chr2:12656983-12658780 REVERSE | Aliases: F16P2.5, F16P2_5, PROLIFERATING CELL NUCLEAR 2	7.0	9.4	-2.4	-4.6	28.6%	0.4
253	AT5G19140.2 | expressed protein, similar to expressed protein [Arabidopsis thaliana] (TAIR:At5g43830.1); similar to hypothetical protein ARG10 - mung bean (GB:T07820)	8.3	5.7	2.6	4.6	28.6%	0.4
254	AT3G43190.1 | sucrose synthase, putative / sucrose-UDP glucosyltransferase, putative, strong similarity to SP:P49040 Sucrose synthase (EC 2.4.1.13) (Sucrose-UDP glucosyltransferase) {Arabidopsis thaliana} (SUS1) | chr3:15190055-15194977 REVERSE | Aliases: F7K15.40	6.0	4.2	1.9	4.6	28.6%	0.3
255	AT4G13750.1 | expressed protein | chr4:7978944-7987554 FORWARD | Aliases: F18A5.140, F18A5_140	4.0	5.3	-1.4	-4.6	28.6%	0.4
256	AT4G27040.1 | expressed protein | chr4:13572444-13575544 REVERSE | Aliases: F10M23.380, F10M23_380	9.3	8.2	1.1	4.6	28.9%	0.4
257	AT5G51190.1 | encodes a member of the ERF (ethylene response factor) subfamily B-3 of ERF/AP2 transcription factor family. The protein contains one AP2 domain. There are 18 members in this subfamily including ATERF-1, ATERF-2, AND ATERF-5. | chr5:20817810-20818642 REVERSE | Aliases: MWD22.13, MWD22_13	6.3	4.7	1.6	4.5	29.1%	0.4
258	AT3G20260.1 | expressed protein | chr3:7064080-7066504 REVERSE | Aliases: MQC12.1	2.9	3.9	-1.1	-4.5	29.1%	0.4
259	AT4G38770.1 | Symbol: PRP4 | proline-rich family protein (PRP4), similar to proline-rich protein (Arabidopsis thaliana) gi:6782442:gb:AAF28388; contains proline-rich extensin domains, INTERPRO:IPR002965 | chr4:18096803-18098558 REVERSE | Aliases: ATPRP4, PROLINE RICH PROTEIN 4, T9A14.50, T9A14_50	3.0	4.0	-1.0	-4.5	29.1%	0.4
260	AT5G11070.1 | expressed protein | chr5:3516218-3516996 REVERSE | Aliases: T5K6.60, T5K6_60	8.1	6.1	2.0	4.5	29.1%	0.3
261	AT4G33720.1 | pathogenesis-related protein, putative, similar to SP:P33154 Pathogenesis-related protein 1 precursor (PR-1) {Arabidopsis thaliana}; contains Pfam profile PF00188: SCP-like extracellular protein | chr4:16182755-16183536 FORWARD | Aliases: T16L1.210, T16L1_210	2.4	5.3	-2.9	-4.5	29.2%	-0.3
262	AT1G05000.1 | tyrosine specific protein phosphatase family protein, contains tyrosine specific protein phosphatases active site, PROSITE:PS00383 | chr1:1425592-1428781 FORWARD | Aliases: T7A14.14, T7A14_14	5.3	4.0	1.3	4.5	29.2%	0.4

Rank	Description	15.dpi	5.dpi	M	t	adj.q	B
263	AT3G26740.1 | light responsive protein-related, similar to light regulated protein precursor SP:Q03200 (Oryza sativa) (Plant Mol. Biol. 22 (1), 165-170 (1993)), ccr protein GB:S52663 (Citrus X paradisi) (Plant Mol. Biol. 26 (1), 165-173 (1994)) | chr3:9829039-9829917 FORWARD | Aliases: MLJ15.9	4.1	2.8	1.3	4.5	29.2%	0.4
264	AT5G11160.1 | adenine phosphoribosyltransferase, putative, strong similarity to SP:Q42563 Adenine phosphoribosyltransferase 2 (EC 2.4.2.7) (APRT) {Arabidopsis thaliana}; contains Pfam profile PF00156: Phosphoribosyl transferase domain | chr5:3550551-3552145 FORWARD | Aliases: F2I11.50, F2I11_50	6.6	5.2	1.4	4.5	29.4%	0.3
265	AT5G64900.1 | expressed protein | chr5:25954396-25955302 FORWARD | Aliases: MXK3.13, MXK3_13	6.3	4.9	1.4	4.5	29.4%	0.4
266	AT4G34710.2 | Symbol: ADC2 | arginine decarboxylase 2 (SPE2), identical to SP:O23141 Arginine decarboxylase 2 (EC 4.1.1.19) (ARGDC 2) (ADC 2) (ADC-N) {Arabidopsis thaliana} | chr4:16560067-16562936 REVERSE | Aliases: ARGININE DECARBOXYLASE, ARGININE DECARBOXYLASE 2, SPE2, T4L20.290, T4L20_290	8.5	10.3	-1.8	-4.5	29.4%	0.4
267	AT1G65500.1 | expressed protein | chr1:24361530-24362279 FORWARD | Aliases: F5I14.4, F5I14_4	3.7	6.6	-2.9	-4.5	29.6%	-0.0
268	AT3G15780.1 | expressed protein | chr3:5341433-5342564 FORWARD | Aliases: MSJ11.18	8.6	6.8	1.9	4.5	29.8%	0.3
269	AT3G61450.1 | Symbol: SYP73 | syntaxin 73 (SYP73), identical to syntaxin 73 (AtSYP73) (Swiss-Prot:Q94KK5) (Arabidopsis thaliana) | chr3:22749369-22750959 FORWARD | Aliases: ATSYP73, F2A19.50, SYNTAXIN OF PLANTS 73	6.2	4.4	1.7	4.5	29.8%	0.2
270	AT3G15210.1 | Symbol: ATERF 4/ATERF4/ERF4/RAP2.5 | encodes a member of the ERF (ethylene response factor) subfamily B-1 of ERF/AP2 transcription factor family (ATERF-4). The protein contains one AP2 domain. There are 15 members in this subfamily including ATERF-3, ATERF-4, ATERF-7, and leafy petiole.	6.0	4.6	1.4	4.5	30.1%	0.3
271	AT1G21910.1 | encodes a member of the DREB subfamily A-5 of ERF/AP2 transcription factor family. The protein contains one AP2 domain. There are 15 members in this subfamily including RAP2.1, RAP2.9 and RAP2.10. | chr1:7696525-7697688 FORWARD | Aliases: T26F17.14, T26F17_14	5.3	3.7	1.6	4.5	30.1%	0.3
272	AT2G27690.1 | Symbol: CYP94C1 | cytochrome P450, putative, similar to Cytochrome P450 94A1 (P450-dependent fatty acid omega-hydroxylase) (SP:O81117) {Vicia sativa}; contains Pfam profile: PF00067 cytochrome P450; supported by cDNA: gi_13877668 | chr2:11816383-11818292 FORWARD | Aliases: F15K20.21, F15K20_21	6.4	4.8	1.6	4.5	30.1%	0.2
273	AT2G36200.1 | kinesin motor protein-related | chr2:15186802-15192293 REVERSE | Aliases: F2H17.19, F2H17_19	4.1	5.5	-1.4	-4.5	30.3%	0.3
274	AT2G43290.1 | Symbol: MSS3 | calmodulin-like protein (MSS3), identical to calmodulin-like MSS3 from GI:9965747 (Arabidopsis thaliana)	4.8	3.7	1.2	4.5	30.3%	0.3
275	AT3G55500.1 | Symbol: ATEXPA16 | expansin, putative (EXP16), similar to expansin GI:2828241 from (Brassica napus); alpha-expansin gene family, PMID:11641069 | chr3:20586052-20587125 REVERSE | Aliases: ARABIDOPSIS THALIANA EXPANSIN A16, ATEXP16, ATHEXP ALPHA 1.7, EXP16, T22E16.160	8.5	6.0	2.5	4.5	30.3%	-0.3
276	AT1G19960.1 | expressed protein | chr1:6928074-6928268 FORWARD | Aliases: T20H2.25, T20H2_25	8.2	5.2	3.0	4.5	30.3%	0.1
277	AT3G55980.1 | zinc finger (CCCH-type) family protein, contains Pfam domain, PF00642: Zinc finger C-x8-C-x5-C-x3-H type (and similar) and Pfam domain, PF00023: Ankyrin repeat | chr3:20787274-20789821 FORWARD | Aliases: F27K19.160	5.5	4.4	1.1	4.5	30.3%	0.3
278	AT1G09470.1 | expressed protein, ; expression supported by MPSS | chr1:3055393-3056933 REVERSE | Aliases: F14J9.13, F14J9_13	3.5	5.3	-1.8	-4.5	30.3%	0.3
279	AT1G58160.1 | jacalin lectin family protein, similar to myrosinase-binding protein homolog (Arabidopsis thaliana) GI:2997767; contains Pfam profile PF01419 jacalin-like lectin domain	4.3	3.3	1.0	4.5	30.3%	0.2
280	AT1G21100.1 | O-methyltransferase, putative, similar to GI:2781394 | chr1:7386828-7388417 REVERSE | Aliases: T22I11.7, T22I11_7	4.1	3.1	1.0	4.4	30.3%	-0.0
281	AT1G69770.1 | Symbol: CMT3 | chromomethylase 3 (CMT3), nearly identical to chromomethylase CMT3 (Arabidopsis thaliana) GI:14583092, GI:14647157 | chr1:26251990-26257248 REVERSE | Aliases: CHROMOMETHYLASE 3, T6C23.3, T6C23_3	3.7	5.4	-1.7	-4.4	30.4%	0.3
282	AT3G51920.1 | Symbol: CAM9 | calmodulin-9 (CAM9), identical to calmodulin 9 GI:5825602 from (Arabidopsis thaliana); contains Pfam profile PF00036: EF hand | chr3:19279026-19280366 REVERSE | Aliases: CALMODULIN 9, F4F15.30	10.0	8.9	1.1	4.4	30.8%	0.3
283	AT1G70900.1 | expressed protein | chr1:26735523-26737537 FORWARD | Aliases: F15H11.13, F15H11_13	6.7	5.3	1.4	4.4	31.4%	0.2
284	AT3G56360.1 | expressed protein, unknown protein 110K5.12 - Sorghum bicolor, TREMBL:AF124045_5	9.9	8.3	1.5	4.4	31.4%	0.2
285	AT5G04310.1 | pectate lyase family protein, similar to pectate lyase GP:14531296 from (Fragaria x ananassa) | chr5:1203204-1207353 REVERSE | Aliases: T19N18.40, T19N18_40	8.6	7.3	1.2	4.4	31.4%	0.2
286	AT1G19730.1 | Symbol: ATTRX4 |	6.5	5.4	1.0	4.4	31.4%	0.2

Rank	Description	15.dpi	5.dpi	M	t	adj.q	B
287	AT1G06000.1 | UDP-glucoronosyl/UDP-glucosyl transferase family protein, contains similarity to UDPG glucosyltransferase GB:AAB62270 GI:2232354 from (Solanum berthaultii) | chr1:1820307-1821892 REVERSE | Aliases: T21E18.5, T21E18_5	6.0	7.5	-1.5	-4.4	31.4%	0.2
288	AT5G62350.1 | invertase/pectin methylesterase inhibitor family protein / DC 1.2 homolog (FL5-2I22), similar to SP:Q42534 Pectinesterase 2 precursor (EC 3.1.1.11) (Pectin methylesterase 2) (PE 2) {Arabidopsis thaliana}; contains Pfam profile PF04043: Plant invertase/pectin methylesterase inhibitor; FL5-2I22 mRNA for DC 1.2 homolog, partial cds GI:11127598 | chr5:25054652-25055588 FORWARD | Aliases: MMI9.21, MMI9_21	8.2	9.6	-1.4	-4.4	31.4%	0.2
289	AT3G52180.2 | similar to protein phosphatase-related [Arabidopsis thaliana] (TAIR:At3g10940.1); similar to unknown protein [Oryza sativa (japonica cultivar-group)] (GB:XP_482104.1); contains InterPro domain Tyrosine specific protein phosphatase and dual specificity protein phosphatase (InterPro:IPR000387); contains InterPro domain Dual specificity protein phosphatase (InterPro:IPR000340)	9.9	8.4	1.5	4.4	31.4%	0.2
290	AT2G38465.1 | expressed protein | chr2:16113341-16113978 REVERSE | Aliases: None	7.2	5.9	1.3	4.4	31.4%	0.2
291	AT5G66710.1 | protein kinase, putative, similar to protein kinase ATN1 GP:1054633 (Arabidopsis thaliana) | chr5:26653835-26655790 FORWARD | Aliases: MSN2.10, MSN2_10	3.5	4.5	-1.1	-4.4	31.4%	0.2
292	AT2G22420.1 | peroxidase 17 (PER17) (P17), identical to SP:Q9SJZ2 Peroxidase 17 precursor (EC 1.11.1.7) (Atperox P17) (ATP25a) {Arabidopsis thaliana} | chr2:9520299-9521615 FORWARD | Aliases: F14M13.18, F14M13_18	6.4	7.7	-1.4	-4.4	31.4%	0.1
293	AT5G05960.1 | protease inhibitor/seed storage/lipid transfer protein (LTP) family protein, contains Pfam protease inhibitor/seed storage/LTP family domain PF00234 | chr5:1790232-1790834 FORWARD | Aliases: K18J17.13, K18J17_13	3.9	6.1	-2.2	-4.4	31.4%	-0.2
294	AT2G31110.1 | expressed protein | chr2:13265406-13266629 REVERSE | Aliases: T16B12.8, T16B12_8	7.5	6.4	1.2	4.4	31.4%	0.2
295	AT5G23240.1 | DNAJ heat shock N-terminal domain-containing protein, low similarity to SP:O34136 Chaperone protein dnaJ (40 kDa heat shock chaperone protein) (HSP40) {Deinococcus proteolyticus}; contains Pfam profile PF00226: DnaJ domain | chr5:7826737-7828727 REVERSE | Aliases: MKD15.10, MKD15_10	6.0	4.0	2.0	4.4	31.4%	0.2
296	AT5G24800.1 | Symbol: BZO2H2 |	11.1	9.5	1.6	4.4	31.4%	0.2
297	AT5G62630.1 | expressed protein | chr5:25160821-25163685 REVERSE | Aliases: MRG21.5, MRG21_5	8.0	9.8	-1.8	-4.4	31.8%	0.2
298	AT3G22890.1 | Symbol: APS1 | sulfate adenylyltransferase 1 / ATP-sulfurylase 1 (APS1), nearly identical to ATP sulfurylase (APS1) (Arabidopsis thaliana) GI:6606509 | chr3:8112730-8114996 FORWARD | Aliases: F5N5.7	7.6	10.3	-2.7	-4.4	31.8%	0.2
299	AT3G27220.1 | kelch repeat-containing protein, contains Pfam PF01344: Kelch motif (4 repeats); contains Prosite PS00334: Myb DNA-binding domain repeat signature 2; similar to Male enhanced Antigen-1 (peas) (GI:20513270) (Mus musculus) | chr3:10052849-10054915 REVERSE | Aliases: K17E12.4	6.7	4.3	2.4	4.4	31.8%	0.1
300	AT1G72230.1 | plastocyanin-like domain-containing protein, similar to blue copper protein SP:Q41001 from (Pisum sativum) | chr1:27191728-27192906 FORWARD | Aliases: T9N14.17, T9N14_17	4.3	7.3	-3.0	-4.4	31.8%	-0.0
301	AT3G08030.2 | expressed protein, contains Pfam profile PF04862: Protein of unknown function, DUF642 | chr3:2564159-2566008 FORWARD | Aliases: F17A17.37	6.9	9.6	-2.7	-4.4	31.8%	0.0
302	AT5G13200.1 | GRAM domain-containing protein / ABA-responsive protein-related, similar to ABA-responsive protein (Hordeum vulgare) GI:4103635; contains Pfam profile PF02893: GRAM domain | chr5:4207007-4208292 FORWARD | Aliases: T19L5.4	5.0	3.9	1.1	4.4	31.8%	0.2
303	AT2G01400.1 | expressed protein | chr2:174270-175616 FORWARD | Aliases: F10A8.4	4.8	3.5	1.3	4.4	31.8%	0.2
304	AT4G27280.1 | calcium-binding EF hand family protein, similar to EF-hand Ca2+-binding protein CCD1 (Triticum aestivum) GI:9255753; contains INTERPRO:IPR002048 calcium-binding EF-hand domain | chr4:13663582-13664246 REVERSE | Aliases: M4I22.90, M4I22_90	8.9	7.5	1.4	4.4	31.8%	0.1
305	AT5G53420.2 | similar to expressed protein [Arabidopsis thaliana] (TAIR:At4g27900.1); similar to expressed protein [Arabidopsis thaliana] (TAIR:At4g27900.2); similar to zinc finger CONSTANS-like protein [Oryza sativa (japonica cultivar-group)] (GB:BAD73395.1) | chr5:21691615-21692959 FORWARD | Aliases: MYN8.3, MYN8_3	9.4	7.9	1.5	4.3	31.9%	0.2
306	AT1G18740.1 | expressed protein | chr1:6463985-6466445 FORWARD | Aliases: F6A14.15, F6A14_15	4.7	3.5	1.3	4.3	32.2%	0.2
307	AT5G65280.1 | lanthionine synthetase C-like family protein, contains Pfam domain, PF05147: Lanthionine synthetase C-like protein | chr5:26103199-26105370 REVERSE | Aliases: MQN23.23, MQN23_23	7.9	6.3	1.6	4.3	32.2%	0.1

Rank	Description	15.dpi	5.dpi	M	t	adj.q	B
308	AT4G25790.1 | allergen V5/Tpx-1-related family protein, similar to SP:Q40374 Pathogenesis-related protein PR-1 precursor {Medicago truncatula}; contains Pfam profile PF00188: SCP-like extracellular protein | chr4:13122257-13123299 REVERSE | Aliases: F14M19.70, F14M19_70	8.5	6.8	1.7	4.3	32.2%	0.1
309	AT5G58150.1 | leucine-rich repeat transmembrane protein kinase, putative | chr5:23547307-23550066 REVERSE | Aliases: MCK7.2, MCK7_2	5.2	6.2	-0.9	-4.3	32.2%	-0.0
310	AT1G48520.3 | Symbol: GATB | glutamyl-tRNA(Gln) amidotransferase B family protein, contains Pfam profiles: PF02934 PET112 family, N terminal region, PF02637 GatB/Yqey domain, PF01162 PET112 family, C terminal region | chr1:17943756-17946490 FORWARD | Aliases: GLU ADT SUBUNIT B, GLU TRNA(GLN) AMIDOTRANSFERASE SUBUNIT B	4.6	5.7	-1.2	-4.3	32.2%	0.1
311	AT5G54080.2 | Symbol: HGO | homogentisate 1,2-dioxygenase / homogentisicase/homogentisate oxygenase / homogentisic acid oxidase (HGO), identical to SP:Q9ZRA2 Homogentisate 1,2-dioxygenase (EC 1.13.11.5) (Homogentisicase) (Homogentisate oxygenase) (Homogentisic acid oxidase) {Arabidopsis thaliana}; contains Pfam profile PF04209: homogentisate 1,2-dioxygenase | chr5:21963095-21966139 FORWARD | Aliases: HOMOGENTISATE 1,2 DIOXYGENASE, MJP23.6, MJP23_6	8.7	7.6	1.1	4.3	32.2%	-0.0
312	AT5G53250.1 | arabinogalactan-protein, putative (AGP22), similar to arabinogalactan protein (Arabidopsis thaliana) gi:10880509:gb:AAG24284 | chr5:21620846-21621457 FORWARD | Aliases: K19E1.5, K19E1_5	7.1	4.7	2.4	4.3	32.2%	0.1
313	AT3G24495.1 | DNA mismatch repair protein MSH6-2 (MSH7), identical to SP:Q9SMV7 DNA mismatch repair protein MSH6-2 (AtMsh6-2) (MutS homolog 7) {Arabidopsis thaliana}; GC donor splice site at exon 11	4.0	5.5	-1.5	-4.3	32.2%	0.1
314	AT3G54810.2 | zinc finger (GATA type) family protein, GATA transcription factor 3, Arabidopsis thaliana, Y13650 | chr3:20307246-20309669 FORWARD | Aliases: T5N23.3	5.3	3.9	1.3	4.3	32.2%	0.1
315	AT4G34600.1 | expressed protein | chr4:16529230-16530191 FORWARD | Aliases: T4L20.180, T4L20_180	7.4	5.8	1.6	4.3	32.2%	0.1
316	AT2G02120.1 | Symbol: LCR70/PDF2.1 | plant defensin-fusion protein, putative (PDF2.1), plant defensin protein family member, personal communication, Bart Thomma (Bart.Thomma@agr.kuleuven.ac.be); contains a gamma-thionin family signature (PDOC00725) | chr2:538254-539054 FORWARD | Aliases: F5O4.11, F5O4_11, LCR70, Low molecular weight cysteine rich 70, PDF2.1	11.7	10.4	1.3	4.3	32.2%	0.1
317	AT3G22240.1 | expressed protein | chr3:7863684-7864586 REVERSE | Aliases: MMP21.2	8.4	10.2	-1.8	-4.3	32.2%	0.1
318	AT3G55040.1 | In2-1 protein, putative, similar to In2-1 protein, Zea mays, P49248 | chr3:20409695-20411282 REVERSE | Aliases: T15C9.60	5.8	4.6	1.2	4.3	32.2%	0.1
319	AT2G43510.1 | Symbol: ATTI1 | Encodes a defensin-like (DEFL) family protein. | chr2:18074251-18074877 FORWARD | Aliases: T1O24.25, TRYPSIN INHIBITOR	3.6	4.8	-1.2	-4.3	32.2%	0.1
320	AT1G61160.1 | expressed protein | chr1:22548684-22550022 REVERSE | Aliases: F11P17.19, F11P17_19	5.3	4.0	1.2	4.3	32.2%	0.1
321	AT5G56580.1 | Symbol: ATMKK6 |	3.5	5.0	-1.5	-4.3	32.6%	0.1
322	AT2G41010.1 | VQ motif-containing protein, contains PF05678: VQ motif | chr2:17120944-17121882 REVERSE | Aliases: T3K9.22, T3K9_22	7.3	5.7	1.6	4.3	32.6%	0.1
323	AT3G25130.1 | expressed protein | chr3:9152411-9153878 FORWARD | Aliases: MJL12.7	3.0	4.7	-1.7	-4.3	32.7%	0.1
324	AT2G45730.1 | eukaryotic initiation factor 3 gamma subunit family protein, contains Pfam profile PF04189: Eukaryotic initiation factor 3, gamma subunit | chr2:18843459-18846234 REVERSE | Aliases: F4I18.29	6.3	7.6	-1.3	-4.3	32.7%	0.1
325	AT3G15500.1 | Symbol: ATNAC3 |	4.8	3.8	1.1	4.3	32.7%	0.1
326	AT2G31400.1 | pentatricopeptide (PPR) repeat-containing protein, low similarity to fertility restorer (Petunia x hybrida) GI:22128587, post-transcriptional control of chloroplast gene expression CRP1 (Zea mays) GI:3289002; contains Pfam profile PF01535: PPR repeat | chr2:13394081-13397783 REVERSE | Aliases: T28P16.11, T28P16_11	9.0	10.1	-1.1	-4.3	32.8%	-0.0
327	AT3G42660.1 | transducin family protein / WD-40 repeat family protein, contains 5 WD-40 repeats (PF00400); AND-1 protein - Homo sapiens, EMBL:AJ006266	5.9	8.5	-2.6	-4.3	33.0%	-0.0
328	AT3G53900.2 | uracil phosphoribosyltransferase, putative / UMP pyrophosphorylase, putative / UPRTase, putative, similar to SP:P50926 Uracil phosphoribosyltransferase (EC 2.4.2.9) (UMP pyrophosphorylase) (UPRTase) {Lactococcus lactis}; contains Pfam profile PF00156: Phosphoribosyl transferase domain | chr3:19967634-19970019 REVERSE | Aliases: F5K20.200	4.8	6.7	-1.9	-4.3	33.1%	0.0
329	AT1G15660.1 | expressed protein, similar to CENPCA protein (GI:11863170) {Zea mays} | chr1:5381165-5385249 FORWARD | Aliases: T16N11.16, T16N11_16	5.7	7.3	-1.7	-4.3	33.3%	0.0

Rank	Description	15.dpi	5.dpi	M	t	adj.q	B
330	AT4G13660.1 | pinoresinol-lariciresinol reductase, putative, similar to pinoresinol-lariciresinol reductase TH1 (Tsuga heterophylla)(GI:7578915); contains isoflavone reductase domain PF02716	7.7	9.7	-2.0	-4.3	33.3%	0.0
331	AT1G71880.1 | Symbol: SUC1 | sucrose transporter / sucrose-proton symporter (SUC1), identical to sucrose-proton symporter SUC1 (Arabidopsis thaliana) GI:407094 | chr1:27057842-27060001 FORWARD | Aliases: ATSUC1, F17M19.3, F17M19_3, SUCROSE H+ SYMPORTER	6.3	5.1	1.1	4.2	33.6%	0.0
332	AT4G19370.1 | expressed protein | chr4:10566274-10567546 REVERSE | Aliases: T5K18.150, T5K18_150	5.2	4.0	1.2	4.2	33.6%	0.0
333	AT4G35800.1 | Symbol: NRPB1 | DNA-directed RNA polymerase II largest subunit (RPB205) (RPII) (RPB1), nearly identical to P:P18616 DNA-directed RNA polymerase II largest subunit (EC 2.7.7.6) {Arabidopsis thaliana} | chr4:16960661-16967896 REVERSE | Aliases: F4B14.70, F4B14_70, RNA POLYMERASE II LARGE SUBUNIT, RNA_POL_II_LS, RNA_POL_II_LSRNA_POL_II_LS, RPB1	3.4	4.6	-1.2	-4.2	33.6%	0.0
334	AT5G11780.1 | expressed protein | chr5:3793929-3796142 FORWARD | Aliases: T22P22.170, T22P22_170	2.9	3.7	-0.8	-4.2	34.2%	-0.1
335	AT1G68585.1 | expressed protein | chr1:25760521-25761532 FORWARD | Aliases: None	8.1	6.8	1.3	4.2	34.2%	0.0
336	AT1G75030.1 | Symbol: ATLP 3 |	3.7	5.3	-1.6	-4.2	34.6%	-0.0
337	AT5G65420.1 | Symbol: CYCD4;1 | cyclin, putative, similar to cyclin D2.1 protein (Nicotiana tabacum) GI:4160298; contains Pfam profiles PF00134: Cyclin, N-terminal domain, PF02984: Cyclin, C-terminal domain | chr5:26158699-26161219 REVERSE | Aliases: CYCD4;1, Cyclin D4;1, MNA5.15, MNA5_15	5.4	6.3	-0.9	-4.2	34.8%	-0.0
338	AT5G40990.1 | GDSL-motif lipase/hydrolase family protein, similar to lipase (Arabidopsis thaliana) GI:1145627; contains Pfam profile PF00657: Lipase/Acylhydrolase with GDSL-like motif | chr5:16436148-16437628 FORWARD | Aliases: MEE6.6, MEE6_6	2.8	4.9	-2.1	-4.2	34.9%	-0.4
339	AT5G04760.1 | myb family transcription factor, contains Pfam profile: PF00249 myb-like DNA-binding domain | chr5:1373531-1374736 REVERSE | Aliases: MUK11.7	7.5	6.0	1.5	4.2	35.0%	-0.0
340	AT2G37790.1 | aldo/keto reductase family protein, similar to chalcone reductase (Sesbania rostrata)(GI:2792155), and aldose reductase ALDRXV4 (Xerophyta viscosa)(GI:4539944),	7.7	9.5	-1.7	-4.2	35.0%	-0.0
341	AT4G25170.1 | expressed protein | chr4:12909585-12911590 FORWARD | Aliases: F24A6.10, F24A6_10	7.6	5.7	1.9	4.2	35.1%	-0.0
342	AT1G76570.1 | chlorophyll A-B binding family protein, similar to chlorophyll A-B binding protein GB:P12470 (Nicotiana plumbaginifolia); contains Pfam profile: PF00504 Chlorophyll A-B binding proteins | chr1:28734026-28735719 FORWARD | Aliases: F14G6.17, F14G6_17	6.2	5.4	0.9	4.2	35.1%	-0.1
343	AT3G02550.1 | LOB domain protein 41 / lateral organ boundaries domain protein 41 (LBD41), identical to LOB DOMAIN 41 (Arabidopsis thaliana) GI:17227172 | chr3:536512-538005 REVERSE | Aliases: F16B3.18, F16B3_18	8.8	6.6	2.2	4.2	35.3%	-0.0
344	AT5G53460.1 | Symbol: GLT1 | glutamate synthase (NADH), chloroplast, putative, similar to SP:Q03460 Glutamate synthase (NADH), chloroplast precursor (EC 1.4.1.14) (NADH- GOGAT) {Medicago sativa} | chr5:21717744-21726855 FORWARD | Aliases: MYN8.7, MYN8_7, NADH GOGAT	7.3	8.5	-1.3	-4.2	35.3%	-0.1
345	AT3G61790.1 | seven in absentia (SINA) family protein, similar to siah-1A protein (Mus musculus) GI:297035; contains Pfam profile PF03145: Seven in absentia protein family | chr3:22882768-22884665 REVERSE | Aliases: F21F14.2	6.7	5.3	1.4	4.2	35.3%	-0.0
346	AT5G18130.2 | expressed protein, similar to unknown protein (gb:AAF00631.1) | chr5:5995482-5997400 FORWARD | Aliases: MRG7.9, MRG7_9	6.3	5.2	1.1	4.2	35.3%	-0.1
347	AT3G50980.1 | dehydrin, putative, similar to dehydrin Xero 1 (Arabidopsis thaliana) SWISS-PROT:P25863 | chr3:18952957-18953846 FORWARD | Aliases: F24M12.20	6.3	4.6	1.7	4.2	35.3%	-0.1
348	AT1G18730.1 | expressed protein | chr1:6460590-6462212 FORWARD | Aliases: F6A14.16, F6A14_16	6.9	5.1	1.7	4.2	35.5%	-0.1
349	AT3G55470.1 | C2 domain-containing protein, similar to phloem protein GI:4164539 from (Cucurbita maxima) | chr3:20575222-20577665 FORWARD | Aliases: T22E16.130	5.7	4.2	1.4	4.2	35.6%	-0.1
350	AT1G73780.1 | protease inhibitor/seed storage/lipid transfer protein (LTP) family protein, contains Pfam profile: PF00234 protease inhibitor/seed storage/LTP family | chr1:27747533-27747829 FORWARD | Aliases: F25P22.20, F25P22_20	2.1	2.4	-0.3	-4.2	35.9%	-0.9
351	AT2G39310.1 | jacalin lectin family protein, similar to myrosinase-binding protein homolog (Arabidopsis thaliana) GI:2997767; contains Pfam profile PF01419 jacalin-like lectin domain	3.8	5.3	-1.5	-4.2	36.0%	-0.1

Rank	Description	15.dpi	5.dpi	M	t	adj.q	B
352	AT2G22470.1 | Symbol: AGP2 | arabinogalactan-protein (AGP2), identical to gi:3883122:gb:AAC77824; supported by cDNA gi:3883121:gb:AF082299 | chr2:9545277-9545959 REVERSE | Aliases: ARABINOGALACTAN PROTEIN 2, F14M13.13, F14M13_13	7.5	6.3	1.3	4.2	36.1%	-0.1
353	AT1G08090.1 | Symbol: ATNRT2:1 | high-affinity nitrate transporter (ACH1), identical to trans-membrane nitrate transporter protein AtNRT2:1 (Arabidopsis thaliana) GI:3747058, high-affinity nitrate transporter ACH1 (Arabidopsis thaliana) GI:3608362 | chr1:2524073-2526161 FORWARD | Aliases: ACH1, ATNRT2.1, LATERAL ROOT INITIATION 1, LIN1, NITRATE TRANSPORTER 1, NRT2, NRT2;1AT, T6D22.17, T6D22_17, TRANS MEMBRANE NITRATE TRANSPORTER PROTEIN ATNRT2:1	5.4	7.4	-2.0	-4.2	36.1%	-0.3
354	AT1G76600.1 | expressed protein | chr1:28751782-28752739 FORWARD | Aliases: F14G6.20, F14G6_20	6.8	5.7	1.2	4.2	36.1%	-0.1
355	AT1G29060.1 | expressed protein | chr1:10147679-10149667 FORWARD | Aliases: F28N24.23, F28N24_23	5.9	4.7	1.2	4.1	36.1%	-0.1
356	AT1G01990.1 | expressed protein | chr1:343166-344319 REVERSE | Aliases: F22M8.12, F22M8_12	5.9	5.2	0.7	4.1	36.1%	-0.3
357	AT2G20290.1 | Symbol: XIG | myosin, putative, similar to myosin (GI:499047) (Arabidopsis thaliana) | chr2:8750356-8758959 REVERSE | Aliases: ATXIG, F11A3.16, F11A3_16	3.4	4.7	-1.4	-4.1	36.1%	-0.1
358	AT1G78490.1 | Symbol: CYP708A3 | cytochrome P450 family protein, similar to Cytochrome P450 90A1 (SP:Q42569) (Arabidopsis thaliana) | chr1:29533169-29535558 FORWARD | Aliases: T30F21.17, T30F21_17	5.7	4.2	1.4	4.1	36.1%	-0.1
359	AT1G73500.1 | Symbol: ATMKK9 |	7.3	6.2	1.1	4.1	36.1%	-0.1
360	AT4G36430.1 | peroxidase, putative, identical to peroxidase (Arabidopsis thaliana) gi:6822093:emb:CAB71009; identical to cDNA class III peroxidase ATP31, GI:17530561	5.0	8.8	-3.8	-4.1	36.2%	-0.4
361	AT3G22790.1 | kinase interacting family protein, similar to kinase interacting protein 1 (GI:13936326) (Petunia integrifolia) | chr3:8052453-8057895 REVERSE | Aliases: MWI23.16	3.7	4.6	-0.9	-4.1	36.2%	-0.1
362	AT5G49160.1 | Symbol: MET1 | DNA (cytosine-5-)-methyltransferase (ATHIM), identical to SP:P34881 DNA (cytosine-5)-methyltransferase AthI (EC 2.1.1.37) {Arabidopsis thaliana}	5.2	7.2	-2.0	-4.1	36.2%	-0.1
363	AT3G23170.1 | expressed protein | chr3:8267949-8268742 REVERSE | Aliases: K14B15.6	5.4	3.8	1.6	4.1	36.4%	-0.1
364	AT1G32900.1 | starch synthase, putative, similar to starch synthase SP:Q42857 from (Ipomoea batatas) | chr1:11920371-11923746 REVERSE | Aliases: F9L11.8, F9L11_8	9.2	7.5	1.7	4.1	36.7%	-0.1
365	AT1G04610.1 | flavin-containing monooxygenase / FMO (YUCCA3), identical to gi:16555356	5.3	7.7	-2.4	-4.1	36.8%	-0.2
366	AT1G18090.2 | exonuclease, putative, similar to Swiss-Prot:P53695 exonuclease I (EXO I) (Schizosaccharomyces pombe) | chr1:6224497-6228233 FORWARD | Aliases: T10F20.10	3.3	4.3	-1.0	-4.1	36.8%	-0.2
367	AT3G01970.1 | Symbol: WRKY45 |	6.1	4.6	1.5	4.1	36.8%	-0.1
368	AT5G49650.2 | xylulose kinase, putative, similar to D-xylulokinase (Pichia stipitis) gi:8100400:gb:AAF72328 | chr5:20170007-20172994 FORWARD | Aliases: MNI5.3, MNI5_3	6.8	9.3	-2.4	-4.1	36.8%	-0.1
369	AT1G15380.2 | similar to lactoylglutathione lyase family protein / glyoxalase I family protein [Arabidopsis thaliana] (TAIR:At1g80160.1); similar to hypothetical protein [Oryza sativa (japonica cultivar-group)] (GB:NP_916470.1); contains InterPro domain Glyoxalase I (InterPro:IPR004361); contains InterPro domain Glyoxalase/Bleomycin resistance protein/dioxygenase domain (InterPro:IPR004360) | chr1:5290742-5292529 FORWARD | Aliases: F9L1.33, F9L1_33	6.3	4.3	2.0	4.1	36.9%	-0.3
370	AT3G24660.1 | Symbol: TMKL1 | leucine-rich repeat transmembrane protein kinase, putative, identical to putative kinase-like protein TMKL1 precursor GB:P33543 from (Arabidopsis thaliana), (Plant Mol. Biol. 23 (2), 415-421 (1993)) | chr3:9003583-9005950 FORWARD | Aliases: MSD24.6, TRANSMEMBRANE KINASE LIKE 1	5.4	6.1	-0.7	-4.1	37.0%	-0.3
371	AT2G15580.1 | zinc finger (C3HC4-type RING finger) family protein, contains Pfam profile: PF00097 zinc finger, C3HC4 type (RING finger) | chr2:6804723-6806021 FORWARD | Aliases: F9O13.13	7.0	5.4	1.5	4.1	37.1%	-0.2
372	AT1G67940.1 | Symbol: ATNAP3 |	8.7	7.8	0.9	4.1	37.1%	-0.3
373	AT4G16190.1 | cysteine proteinase, putative, contains similarity to papain-like cysteine proteinase isoform I GI:7381219 from (Ipomoea batatas) | chr4:9171482-9173120 FORWARD | Aliases: DL4135W, FCAALL.298	8.4	5.3	3.1	4.1	37.1%	-0.3

Rank	Description	15.dpi	5.dpi	M	t	adj.q	B
374	AT1G42990.1 | Symbol: ATBZIP60 | AtbZIP60 consists of a bZIP DNA binding domain followed by a putative transmembrane domain. GFP fusions containing the first 260 amino acids (AtbZIP60deltaC) are nuclear-localized. AtbZIP60 is upregulated by the addition of tunicamycin (ER stress response inductor), DTT (inhibitor of disulfide bond formation) and azetin-2-carboxylate (proline analog perturbing protein structure). It is hypothesized that upon ER stress the protein is proteolyzed and the soluble part translocalized into the nucleus. AtbZIP60deltaC can activate the promoters of the ER chaperones BiP1, BiP2 and BiP3 and CNX1 and CNX2 via binding to the ER stress response element (ERSE) and the plant unfolded protein response element(P-UPRE). It can also activate its own transcription. | chr1:16138292-16139903 REVERSE | Aliases: ATBZIP60, F13A11.5, F13A11_5	9.1	7.9	1.2	4.1	37.1%	-0.2
375	AT5G53320.1 | leucine-rich repeat transmembrane protein kinase, putative | chr5:21653295-21655622 REVERSE | Aliases: K19E1.12, K19E1_12	5.5	6.6	-1.1	-4.1	37.3%	-0.2
376	AT1G56300.1 | DNAJ heat shock N-terminal domain-containing protein, similar to SP:Q9QYI7 DnaJ homolog subfamily B member 8 Mus musculus; contains Pfam profile: PF00226: DnaJ domain | chr1:21082485-21083991 REVERSE | Aliases: F14G9.9, F14G9_9	8.1	6.7	1.3	4.1	37.3%	-0.2
377	AT1G77720.1 | protein kinase family protein, contains protein kinase domain, Pfam:PF00069 | chr1:29215415-29218973 FORWARD | Aliases: T32E8.5, T32E8_5	2.5	3.0	-0.5	-4.1	37.4%	-0.6
378	AT1G16490.1 | myb family transcription factor (MYB58), contains PFAM profile: myb DNA binding domain PF00249 | chr1:5629648-5630988 REVERSE | Aliases: F3O9.29, F3O9_29	3.4	4.7	-1.2	-4.1	37.5%	-0.2
379	AT1G72450.1 | expressed protein | chr1:27277727-27279950 REVERSE | Aliases: T10D10.8, T10D10_8	7.1	5.5	1.7	4.1	37.9%	-0.3
380	AT2G07690.1 | minichromosome maintenance family protein / MCM family protein, similar to SP:P55862 DNA replication licensing factor MCM5 (CDC46 homolog) {Xenopus laevis}; contains Pfam profile PF00493: MCM2/3/5 family | chr2:3523243-3527428 REVERSE | Aliases: T12J2.1	4.9	5.7	-0.9	-4.1	37.9%	-0.3
381	AT2G38720.1 | microtubule associated protein (MAP65/ASE1) family protein, low similarity to myosin (Schistosoma japonicum) GI:3941320; contains Pfam profile PF03999: Microtubule associated protein (MAP65/ASE1 family) | chr2:16195125-16199180 FORWARD | Aliases: T6A23.8, T6A23_8	3.7	5.9	-2.1	-4.1	38.3%	-0.4
382	AT1G27590.1 | expressed protein, similar to hypothetical protein GB:AAD45997 GI:5668770 from (Arabidopsis thaliana) | chr1:9592149-9593949 FORWARD | Aliases: T22C5.2	3.6	2.9	0.7	4.1	38.4%	-0.3
383	AT3G59550.1 | Symbol: SYN3 | cohesion family protein SYN3 (SYN3), nearly identical to cohesion family protein SYN3 (Arabidopsis thaliana) GI:12006362; supporting cDNA gi:12006361:gb:AF281155.1:AF281155 | chr3:22008031-22011655 FORWARD | Aliases: T16L24.100	3.3	5.1	-1.8	-4.0	38.5%	-0.3
384	AT3G05490.1 | Symbol: RALFL22 | rapid alkalinization factor (RALF) family protein, similar to RALF precursor (Nicotiana tabacum) GI:16566316 | chr3:1591283-1591989 FORWARD | Aliases: F22F7.6, F22F7_6, RALF LIKE 22	7.7	5.9	1.7	4.0	38.5%	-0.2
385	AT3G18940.1 | clast3-related, contains Prosite PS00626: Regulator of chromosome condensation (RCC1) signature 2; similar to Clast3 protein (GI:9857631) (Mus musculus)	5.7	7.0	-1.2	-4.0	38.5%	-0.2
386	AT4G27800.3 | protein phosphatase 2C PPH1 / PP2C PPH1 (PPH1), identical to SP:P49599:P2C3_ARATH Protein phosphatase 2C PPH1 (EC 3.1.3.16) (PP2C) {Arabidopsis thaliana}; similar to protein phosphatase-2C; PP2C (GI:3643090) (Mesembryanthemum crystallinum) | chr4:13851876-13854197 REVERSE | Aliases: T27E11.40, T27E11_40	9.2	8.3	0.9	4.0	38.5%	-0.3
387	AT4G19830.1 | immunophilin / FKBP-type peptidyl-prolyl cis-trans isomerase family protein, similar to 70 kDa peptidylprolyl isomerase (Peptidylprolyl cis-trans isomerase) (PPiase) (Rotamase) (SP:Q43207) (Triticum aestivum); FKBP-type peptidyl-prolyl cis-trans isomerase,Synechocystis sp., PIR2:S75144; contains Pfam PF00254: peptidyl-prolyl cis-trans isomerase, FKBP-type | chr4:10772441-10774033 REVERSE | Aliases: T16H5.190, T16H5_190	6.1	4.8	1.3	4.0	38.7%	-0.2
388	AT5G05470.1 | Symbol: EIF2 ALPHA | eukaryotic translation initiation factor 2 subunit 1, putative / eIF-2A, putative / eIF-2-alpha, putative, similar to SWISS-PROT:P20459 eukaryotic translation initiation factor 2 alpha subunit (eIF-2- alpha) (Saccharomyces cerevisiae); identical to cDNA cohesin GI:6682280 | chr5:1620459-1622777 FORWARD | Aliases: ATEIF2 A2, EIF2 A2, K18I23.28, K18I23_28	5.1	6.5	-1.4	-4.0	38.7%	-0.3
389	AT4G12580.1 | expressed protein, predicted protein | chr4:7449371-7449676 REVERSE | Aliases: T1P17.170, T1P17_170	5.9	4.5	1.5	4.0	39.1%	-0.2
390	AT1G04730.1 | AAA-type ATPase family protein, contains Pfam domain, PF00004: ATPase, AAA family ('A'TPases 'A'ssociated with diverse cellular 'A'ctivities) | chr1:1325384-1331085 REVERSE | Aliases: T1G11.3, T1G11_3	3.2	4.1	-0.9	-4.0	39.4%	-0.3
391	AT4G30430.1 | senescence-associated family protein, similar to senescence-associated protein 5 (Hemerocallis hybrid cultivar) gi:3551954:gb:AAC34855 | chr4:14878789-14880337 REVERSE | Aliases: F17I23.230, F17I23_230	6.1	4.6	1.6	4.0	39.4%	-0.3
392	AT5G27280.1 | zinc finger (DNL type) family protein, contains Pfam profile PF05180: DNL zinc finger | chr5:9617489-9618484 FORWARD | Aliases: F21A20.2	6.7	5.9	0.9	4.0	39.4%	-0.3

Rank	Description	15.dpi	5.dpi	M	t	adj.q	B
393	AT5G07660.1 | structural maintenance of chromosomes (SMC) family protein, similar to SMC-like protein (MIM) (Arabidopsis thaliana) GI:5880614; contains Pfam profile PF02463: RecF/RecN/SMC N terminal domain | chr5:2422840-2429913 FORWARD | Aliases: MBK20.10, MBK20_10	3.5	4.6	-1.1	-4.0	39.4%	-0.3
394	AT2G15000.2 | expressed protein | chr2:6488440-6489469 REVERSE | Aliases: T15J14.4, T15J14_4	5.8	7.5	-1.7	-4.0	39.4%	-0.3
395	AT2G21790.1 | Symbol: R1 | ribonucleoside-diphosphate reductase small chain, putative / ribonucleotide reductase, putative, similar to ribonucleotide reductase GI:4151068 from (Nicotiana tabacum) | chr2:9300435-9304922 FORWARD | Aliases: F7D8.11, F7D8_11, R1	5.4	7.6	-2.1	-4.0	39.6%	-0.3
396	AT5G20220.1 | zinc knuckle (CCHC-type) family protein, contains Pfam domain, PF00098: Zinc knuckle | chr5:6821992-6825983 FORWARD | Aliases: F5O24.110, F5O24_110	5.1	4.3	0.8	4.0	39.7%	-0.3
397	AT3G11230.1 | yippee family protein, similar to qdgl-1 (Coturnix coturnix) GI:10441650, Yippee protein (Homo sapiens) GI:5713281; contains Pfam profile PF03226: Yippee putative zinc-binding protein | chr3:3516583-3518377 FORWARD | Aliases: F11B9.15	9.4	8.8	0.6	4.0	39.7%	-0.5
398	AT3G14450.1 | Symbol: CID9 | RNA-binding protein, putative, contains Pfam profile: PF00076 RNA recognition motif. (a.k.a. RRM, RBD, or RNP domain) (2 copies). Contains PAM PABC binding domain. | chr3:4849795-4851608 FORWARD | Aliases: CID9, MOA2.5	4.0	3.3	0.7	4.0	39.7%	-0.6
399	AT1G52550.1 | expressed protein | chr1:19576596-19577699 REVERSE | Aliases: F6D8.23, F6D8_23	4.4	3.2	1.2	4.0	40.3%	-0.3
400	AT5G52910.1 | Symbol: ATIM | timeless family protein, contains Pfam domains PF05029: Timeless protein C terminal region and PF04821: Timeless protein	3.8	4.7	-0.8	-4.0	40.4%	-0.4
401	AT4G01250.1 | Symbol: WRKY22 |	4.6	3.2	1.3	4.0	40.4%	-0.3
402	AT5G40960.1 | expressed protein | chr5:16429973-16430179 REVERSE | Aliases: MEE6.3, MEE6_3	9.6	7.7	1.9	4.0	40.4%	-0.3
403	AT1G07135.1 | glycine-rich protein | chr1:2189927-2190623 REVERSE | Aliases: None	8.9	5.8	3.1	4.0	40.4%	-0.6
404	AT5G18620.2 | DNA-dependent ATPase, putative, similar to DNA-dependent ATPase SNF2H (Mus musculus) GI:14028669; contains Pfam profiles PF00271: Helicase conserved C-terminal domain, PF00176: SNF2 family N-terminal domain, PF00249: Myb-like DNA-binding domain | chr5:6195919-6202168 REVERSE | Aliases: T1A4.2	6.7	9.2	-2.5	-4.0	40.4%	-0.3
405	AT2G18600.1 | RUB1-conjugating enzyme, putative, strong similarity to gi:6635457 RUB1 conjugating enzyme (Arabidopsis thaliana); contains Pfam profile PF00179: Ubiquitin-conjugating enzyme | chr2:8080282-8082030 REVERSE | Aliases: F24H14.5, F24H14_5	8.3	6.9	1.4	4.0	40.4%	-0.3
406	AT3G59480.1 | pfkB-type carbohydrate kinase family protein, contains Pfam profile: PF00294 pfkB family carbohydrate kinase | chr3:21993998-21995572 FORWARD | Aliases: T16L24.30	4.1	5.8	-1.7	-4.0	40.4%	-0.3
407	AT5G37990.1 | similar to S-adenosyl-L-methionine:carboxyl methyltransferase family protein [Arabidopsis thaliana] (TAIR:At5g37970.1); similar to AtPP homologue [Brassica napus] (GB:CAC80637.1); contains InterPro domain SAM dependent carboxyl methyltransferase (InterPro:IPR005299) | chr5:15150967-15152730 REVERSE | Aliases: K18L3.150, K18L3_150	3.4	4.5	-1.2	-4.0	40.4%	-0.3
408	AT5G16480.1 | tyrosine specific protein phosphatase family protein, contains tyrosine specific protein phosphatases active site, PROSITE:PS00383 | chr5:5381116-5382900 REVERSE | Aliases: MQK4.21, MQK4_21	7.2	5.5	1.7	4.0	40.4%	-0.3
409	AT1G64660.1 | Cys/Met metabolism pyridoxal-phosphate-dependent enzyme family protein, similar to SP:P13254 Methionine gamma-lyase (EC 4.4.1.11) (L-methioninase) {Pseudomonas putida}; contains Pfam profile PF01053: Cys/Met metabolism PLP-dependent enzyme | chr1:24032581-24034443 FORWARD | Aliases: F1N19.23, F1N19_23	4.7	6.3	-1.6	-4.0	40.4%	-0.3
410	AT2G48010.1 | Symbol: RKF3 | serine/threonine protein kinase (RFK3), identical to receptor-like serine/threonine kinase (Arabidopsis thaliana) gi:2465927:gb:AAC50045	8.0	6.9	1.1	4.0	40.4%	-0.5
411	AT3G27360.1 | histone H3, identical to histone H3 from Zea mays SP:P05203, Medicago sativa GI:166384, Encephalartos altensteinii SP:P08903, Pisum sativum SP:P02300; contains Pfam profile PF00125 Core histone H2A/H2B/H3/H4 | chr3:10130520-10131174 REVERSE | Aliases: K1G2.15	5.6	7.5	-1.9	-4.0	40.5%	-0.3
412	AT1G19020.1 | expressed protein | chr1:6568064-6568569 FORWARD | Aliases: F14D16.17, F14D16_17	7.6	6.2	1.5	4.0	41.1%	-0.4
413	AT1G77230.1 | tetratricopeptide repeat (TPR)-containing protein, contains Pfam profile PF00515 TPR Domain | chr1:29021023-29022858 FORWARD | Aliases: T14N5.9, T14N5_9	6.5	4.7	1.8	3.9	41.2%	-0.4
414	AT4G33940.1 | zinc finger (C3HC4-type RING finger) family protein, contains Pfam profile: PF00097 zinc finger, C3HC4 type (RING finger) | chr4:16266035-16267786 FORWARD | Aliases: F17I5.1	6.4	5.3	1.1	3.9	41.2%	-0.3

Rank	Description	15.dpi	5.dpi	M	t	adj.q	B
415	AT3G48110.1 | Symbol: EDD1 | similar to glycyl-tRNA synthetase alpha chain and beta chain [Tropheryma whipplei str. Twist] (GB:AAO44597.1); similar to probable glycyl-tRNA synthetase [Parachlamydia sp. UWE25] (GB:YP_007692.1); similar to putative glycine--tRNA ligase [Oryza sativa (japonica cultivar-group)] (GB:XP_476283.1); similar to glycyl-tRNA synthetase [Tropheryma whipplei TW08/27] (GB:NP_789201.1); contains InterPro domain Glycyl-tRNA synthetase, beta subunit (InterPro:IPR002311); contains InterPro domain Heterodimeric glycyl-transfer RNA synthetase (InterPro:IPR006194); contains InterPro domain Glycyl-tRNA synthetase, alpha subunit (InterPro:IPR002310) | chr3:17773986-17782131 FORWARD | Aliases: EDD, EMBRYO DEFECTIVE DEVELOPMENT 1, GLYCYL TRNA SYNTHETASE, T17F15.20	4.3	5.6	-1.3	-3.9	41.3%	-0.3
416	AT4G32240.1 | expressed protein | chr4:15568306-15569194 FORWARD | Aliases: F10M6.120, F10M6_120	11.7	10.8	0.8	3.9	41.4%	-0.9
417	AT1G27120.1 | galactosyltransferase family protein, contains Pfam profile:PF01762 galactosyltransferase | chr1:9421164-9424054 FORWARD | Aliases: T7N9.18, T7N9_18	5.1	7.0	-1.9	-3.9	41.4%	-0.5
418	AT1G18030.1 | protein phosphatase 2C, putative / PP2C, putative, contains similarity to protein phosphatase 2C GI:3777604 from (Rattus norvegicus) | chr1:6204336-6206981 FORWARD | Aliases: T10F20.4, T10F20_4	5.5	4.8	0.7	3.9	41.5%	-0.5
419	AT1G29050.1 | expressed protein, similar to hypothetical protein GB:AAB67625 GI:2342727 from (Arabidopsis thaliana) | chr1:10136217-10139194 REVERSE | Aliases: F28N24.24, F28N24_24	8.0	7.1	0.9	3.9	41.5%	-0.5
420	AT4G18910.1 | aquaglyceroporin / NOD26-like major intrinsic protein 2 (NLM2), contains Pfam profile: MIP PF00230; similar to SP:P08995 {Glycine max} Nodulin-26 (N-26); identical to cDNA aquaglyceroporin (nlm2 gene) GI:11071655, aquaglyceroporin (Arabidopsis thaliana) GI:11071656 | chr4:10366070-10368392 FORWARD | Aliases: F13C5.80, F13C5_80	4.6	6.8	-2.1	-3.9	41.6%	-0.4
421	AT5G46280.1 | DNA replication licensing factor, putative, similar to SP:Q43704 DNA replication licensing factor MCM3 homolog (Replication origin activator) (ROA protein) {Zea mays}; contains Pfam profile PF00493: MCM2/3/5 family | chr5:18786924-18790885 REVERSE | Aliases: MPL12.6, MPL12_6	5.3	7.7	-2.4	-3.9	41.6%	-0.4
422	AT2G01120.1 | origin recognition complex subunit 4, putative (ORC4), similar to origin recognition complex subunit 4 (GI:15866782) (Zea mays) | chr2:87317-88122 FORWARD | Aliases: F23H14.9, F23H14_9	4.1	5.8	-1.6	-3.9	42.0%	-0.4
423	AT1G20070.1 | expressed protein | chr1:6958001-6959114 REVERSE | Aliases: T20H2.15, T20H2_15	6.4	4.3	2.1	3.9	42.5%	-0.5
424	AT2G38640.1 | expressed protein, contains Pfam profile PF04525: Protein of unknown function (DUF567) | chr2:16164682-16165632 REVERSE | Aliases: T6A23.16, T6A23_16	5.7	4.2	1.5	3.9	42.5%	-0.4
425	AT5G59400.2 | expressed protein, predicted protein, Arabidopsis thaliana | chr5:23974971-23976858 FORWARD | Aliases: F2O15.8, F2O15_8	5.1	4.1	1.1	3.9	42.5%	-0.4
426	AT1G26730.1 | EXS family protein / ERD1/XPR1/SYG1 family protein, similar to PHO1 protein (Arabidopsis thaliana) GI:20069032; contains Pfam profiles PF03105: SPX domain, PF03124: EXS family	7.9	6.4	1.5	3.9	42.5%	-0.4
427	AT4G16520.2 | autophagy 8f (APG8f), identical to autophagy 8f (Arabidopsis thaliana) GI:19912161; contains Pfam profile PF02991: Microtubule associated protein 1A/1B, light chain 3 | chr4:9306708-9308465 REVERSE | Aliases: DL4285C, FCAALL.383	11.9	11.2	0.7	3.9	42.5%	-0.7
428	AT3G04980.1 | DNAJ heat shock N-terminal domain-containing protein, contains Pfam profile PF00226 DnaJ domain | chr3:1378690-1382187 REVERSE | Aliases: T9J14.7, T9J14_7	3.5	4.6	-1.1	-3.9	42.5%	-0.4
429	AT2G01110.1 | Symbol: APG2 | thylakoid membrane formation protein / cpTatC (APG2), identical to thylakoid membrane formation protein (cpTatC) GI:15004994 from (Arabidopsis thaliana); contains Pfam profile PF00902:	5.7	4.4	1.3	3.9	42.5%	-0.4
430	AT1G16240.2 | Symbol: SYP51 | similar to syntaxin 52 (SYP52) [Arabidopsis thaliana] (TAIR:At1g79590.1); similar to putative syntaxin of plants 52 [Oryza sativa (japonica cultivar-group)] (GB:XP_463898.1); contains InterPro domain Target SNARE coiled-coil domain (InterPro:IPR000727) | chr1:5555001-5556797 REVERSE | Aliases: ATSYP51, F3O9.4, F3O9_4, SYNTAXIN OF PLANTS 51	10.5	9.7	0.8	3.9	42.5%	-0.6
431	AT1G45474.2 | Symbol: LHCA5 | chlorophyll A-B binding protein, putative (LHCA5), identical to Lhca5 protein (Arabidopsis thaliana) GI:4741942; contains Pfam profile: PF00504 chlorophyll A-B binding protein; similar to light-harvesting complex protein GI:22752 from (Pinus sylvestris) | chr1:17181742-17183246 FORWARD | Aliases: F2G19.4, F2G19_4	7.3	5.3	2.0	3.9	42.8%	-0.4
432	AT3G12080.1 | Symbol: EMB2738 | GTP-binding family protein, contains Pfam domain, PF01926: GTPase of unknown function	5.8	7.0	-1.2	-3.9	42.8%	-0.4
433	AT4G00010.1 | expressed protein | chr4:3894-5908 REVERSE | Aliases: T15P10.10, T15P10_10	3.5	4.8	-1.3	-3.9	42.8%	-0.4
434	AT5G62410.1 | Symbol: SMC2 | SMC2-like condensin, putative (SMC2) (TITAN3), very strong similarity to SMC2-like condensin (TITAN3) (Arabidopsis thaliana) GI:14279543; contains Pfam profiles PF02483: SMC family C-terminal domain, PF02463: RecF/RecN/SMC N terminal domain	4.7	6.3	-1.6	-3.9	43.2%	-0.5

Rank	Description	15.dpi	5.dpi	M	t	adj.q	B
435	AT4G26760.1 | microtubule associated protein (MAP65/ASE1) family protein, low similarity to protein regulating cytokinesis 1 (PRC1) (Homo sapiens) GI:2865521; contains Pfam profile PF03999: Microtubule associated protein (MAP65/ASE1 family) | chr4:13478598-13481757 REVERSE | Aliases: F10M23.100, F10M23_100	3.7	5.1	-1.4	-3.9	43.2%	-0.5
436	AT5G05110.1 | cysteine protease inhibitor, putative / cystatin, putative, similar to cysteine proteinase inhibitor (Glycine max) GI:1944342; contains Pfam profile PF00031: Cystatin domain | chr5:1507420-1508868 REVERSE | Aliases: MUG13.3, MUG13_3	8.4	5.6	2.8	3.9	43.6%	-0.4
437	AT1G01670.1 | U-box domain-containing protein | chr1:242844-245988 REVERSE | Aliases: T1N6.28, T1N6_28	5.4	4.1	1.3	3.9	43.6%	-0.5
438	AT3G54430.1 | lateral root primordium (LRP) protein-related, similar to lateral root primordium 1 (LRP1) (Arabidopsis thaliana) GI:882341; contains Pfam profile PF05142: Domain of unknown function (DUF702) | chr3:20158079-20159105 REVERSE | Aliases: T14E10.2	6.5	5.4	1.1	3.9	43.6%	-0.4
439	AT1G63660.2 | GMP synthase (glutamine-hydrolyzing), putative / glutamine amidotransferase, putative, similar to SP:P38625 GMP synthase (glutamine-hydrolyzing) (EC 6.3.5.2) (Glutamine amidotransferase) (GMP synthetase) {Saccharomyces cerevisiae}; contains Pfam profile PF00117: glutamine amidotransferase class-I | chr1:23607602-23610938 REVERSE | Aliases: F24D7.15, F24D7_15	7.3	8.7	-1.4	-3.9	43.6%	-0.4
440	AT5G56030.1 | Symbol: HSP81 2 | heat shock protein 81-2 (HSP81-2), nearly identical to SP:P55737 Heat shock protein 81-2 (HSP81-2) {Arabidopsis thaliana} | chr5:22704058-22706876 FORWARD | Aliases: HEAT SHOCK PROTEIN 81 2, MDA7.7, MDA7_7	9.3	10.2	-0.9	-3.9	43.6%	-0.6
441	AT5G23380.1 | expressed protein | chr5:7866745-7870135 FORWARD | Aliases: T32G24.1, T32G24_1	4.2	3.4	0.8	3.9	43.6%	-0.6
442	AT1G16500.1 | expressed protein | chr1:5639025-5640166 FORWARD | Aliases: F3O9.30, F3O9_30	6.7	5.3	1.5	3.9	43.7%	-0.5
443	AT1G20640.2 | RWP-RK domain-containing protein, similar to nodule inception protein GI:6448579 from (Lotus japonicus); contains Pfam profile: PF02042 RWP-RK domain | chr1:7154400-7158208 FORWARD | Aliases: F5M15.4	8.9	7.2	1.7	3.9	43.7%	-0.5
444	AT5G22250.1 | CCR4-NOT transcription complex protein, putative, similar to SWISS-PROT:Q60809 CCR4-NOT transcription complex, subunit 7 (CCR4-associated factor 1, (CAF1) (Mus musculus) | chr5:7365535-7366781 REVERSE | Aliases: T6G21.2	4.8	4.2	0.7	3.9	43.7%	-0.7
445	AT5G67520.1 | adenylylsulfate kinase, putative, similar to adenylylsulfate kinase, chloroplast precursor (APS kinase, Adenosine-5'phosphosulfate kinase, ATP adenosine-5'- phosphosulfate 3'-phosphotransferase) (Catharanthus roseus) SWISS-PROT:O49204 | chr5:26956385-26958172 FORWARD | Aliases: K9I9.8, K9I9_8	6.0	4.6	1.3	3.9	43.8%	-0.5
446	AT5G15530.1 | Symbol: BCCP2 | biotin carboxyl carrier protein 2 (BCCP2), identical to biotin carboxyl carrier protein isoform 2 (Arabidopsis thaliana) gi:8886869:gb:AAF80592	4.1	5.6	-1.5	-3.9	43.8%	-0.5
447	AT1G31290.1 | PAZ domain-containing protein / piwi domain-containing protein, contains Pfam profiles PF02170: PAZ domain, PF02171: Piwi domain	5.3	3.7	1.6	3.8	43.9%	-0.5
448	AT4G11480.1 | protein kinase family protein, contains Pfam PF00069: Protein kinase domain | chr4:6971403-6973794 FORWARD | Aliases: F25E4.100, F25E4_100	2.8	3.5	-0.7	-3.8	43.9%	-0.6
449	AT5G24090.1 | acidic endochitinase (CHIB1), identical to SP:P19172 Acidic endochitinase precursor (EC 3.2.1.14) {Arabidopsis thaliana}	7.8	6.8	1.0	3.8	43.9%	-0.6
450	AT3G54750.2 | expressed protein | chr3:20275683-20279726 REVERSE | Aliases: T5N23.110	5.5	7.7	-2.2	-3.8	44.1%	-0.5
451	AT4G11960.1 | expressed protein, hypothetical protein F7H19.70 - Arabidopsis thaliana, PID:e1310057 | chr4:7174880-7177752 REVERSE | Aliases: F16J13.30, F16J13_30	7.5	6.0	1.5	3.8	44.3%	-0.5
452	AT4G23670.1 | major latex protein-related / MLP-related, low similarity to major latex protein {Papaver somniferum}(GI:294060) contains Pfam profile PF00407: Pathogenesis-related protein Bet v I family | chr4:12332570-12333814 REVERSE | Aliases: F9D16.140, F9D16_140	4.9	7.4	-2.5	-3.8	44.3%	-0.6
453	AT1G57820.2 | zinc finger (C3HC4-type RING finger) family protein, low similarity to nuclear protein np95 (Mus musculus) GI:4220590; contains Pfam profiles PF02182: YDG/SRA domain, PF00097: Zinc finger, C3HC4 type (RING finger), PF00628: PHD-finger | chr1:21417876-21421602 REVERSE | Aliases: F12K22.14, F12K22_14	4.8	6.3	-1.5	-3.8	44.3%	-0.5
454	AT3G02480.1 | ABA-responsive protein-related, similar to ABA-inducible protein (Fagus sylvatica) GI:3901016, cold-induced protein kin1 (Brassica napus) GI:167146 | chr3:512321-513034 FORWARD | Aliases: F16B3.11, F16B3_11	7.2	10.0	-2.8	-3.8	44.3%	-0.5
455	AT1G70790.2 | C2 domain-containing protein, similar to zinc finger and C2 domain protein GI:9957238 from (Arabidopsis thaliana) | chr1:26704077-26706002 FORWARD | Aliases: F15H11.4, F15H11_4	7.9	6.6	1.2	3.8	44.3%	-0.5

Rank	Description	15.dpi	5.dpi	M	t	adj.q	B
456	AT2G34770.1 | Symbol: FAH1 | fatty acid hydroxylase (FAH1), identical to fatty acid hydroxylase Fah1p GB:AF021804 GI:2736147 from (Arabidopsis thaliana) | chr2:14672746-14675329 FORWARD | Aliases: FAH1P, FATTY ACID HYDROXYLASE 1, T29F13.2, T29F13_2	8.7	6.6	2.1	3.8	44.6%	-0.5
457	AT4G30460.1 | glycine-rich protein | chr4:14888986-14889808 REVERSE | Aliases: F17I23.200, F17I23_200	7.3	5.4	1.9	3.8	44.6%	-0.5
458	AT5G49520.1 | Symbol: WRKY48 |	5.2	3.2	2.0	3.8	44.7%	-0.5
459	AT5G45920.1 | similar to GDSL-motif lipase/hydrolase family protein [Arabidopsis thaliana] (TAIR:At5g62930.1); similar to OSJNBa0038J17.19 [Oryza sativa (japonica cultivar-group)] (GB:NP_913321.1); contains InterPro domain Lipolytic enzyme, G-D-S-L family (InterPro:IPR001087) | chr5:18639992-18642012 FORWARD | Aliases: K15I22.12, K15I22_12	5.9	4.6	1.2	3.8	44.7%	-0.5
460	AT1G75290.1 | isoflavone reductase, putative, similar to SP:P52577 Isoflavone reductase homolog P3 (EC 1.3.1.-) {Arabidopsis thaliana}; contains Pfam profile PF02716: Isoflavone reductase | chr1:28257496-28258947 FORWARD | Aliases: F22H5.18, F22H5_18	5.3	7.3	-2.0	-3.8	44.7%	-0.6
461	AT2G22880.1 | VQ motif-containing protein, contains PF05678: VQ motif | chr2:9748199-9748543 REVERSE | Aliases: T20K9.9, T20K9_9	4.5	3.2	1.2	3.8	44.7%	-0.5
462	AT5G62430.1 | Symbol: CDF1 | Dof-type zinc finger domain-containing protein, similar to H-protein promoter binding factor-2a GI:3386546 from (Arabidopsis thaliana) | chr5:25086321-25087403 REVERSE | Aliases: CYCLING DOF FACTOR 1, K19B1.4, K19B1_4	5.9	4.6	1.3	3.8	44.7%	-0.5
463	AT4G39800.1 | Symbol: MI 1 P SYNTHASE | inositol-3-phosphate synthase isozyme 1 / myo-inositol-1-phosphate synthase 1 / MI-1-P synthase 1 / IPS 1, identical to SP:P42801 Inositol-3-phosphate synthase isozyme 1 (EC 5.5.1.4) (Myo-inositol-1- phosphate synthase 1) (MI-1-P synthase 1) (IPS 1) {Arabidopsis thaliana} | chr4:18469342-18471958 REVERSE | Aliases: MYO INOSITOL 1 PHOSPHATE SYNTHASE, T19P19.190, T19P19_190, T5J17.3	5.2	7.0	-1.7	-3.8	44.7%	-0.5
464	AT4G18280.1 | glycine-rich cell wall protein-related, glycine-rich protein 1.0 precursor, Phaseolus vulgaris, PIR1:S01821 | chr4:10104007-10104735 FORWARD | Aliases: T9A21.130, T9A21_130	11.9	10.3	1.5	3.8	44.7%	-0.5
465	AT2G04410.1 | expressed protein | chr2:1533838-1535165 REVERSE | Aliases: T1O3.18, T1O3_18	8.2	7.0	1.2	3.8	44.8%	-0.5
466	AT2G04780.2 | Symbol: FLA7 | fasciclin-like arabinogalactan-protein (FLA7), identical to gi_13377782_gb_AAK20860 | chr2:1676996-1678452 FORWARD | Aliases: F28I8.18, F28I8_18, fasciclin like arabinogalactan protein 7	5.9	7.9	-2.0	-3.8	44.8%	-0.5
467	AT5G52750.1 | heavy-metal-associated domain-containing protein, Pfam profile PF00403: Heavy-metal-associated domain | chr5:21401269-21402150 FORWARD | Aliases: F6N7.24, F6N7_24	2.8	3.4	-0.6	-3.8	44.8%	-0.8
468	AT2G39830.1 | LIM domain-containing protein, contains Pfam profile PF00412: LIM domain	5.5	4.6	0.9	3.8	44.8%	-0.6
469	AT1G52590.1 | expressed protein | chr1:19592678-19593942 REVERSE | Aliases: F6D8.19, F6D8_19	7.6	6.4	1.2	3.8	44.8%	-0.5
470	AT1G05160.1 | Symbol: CYP88A3 | ent-kaurenoic acid hydroxylase (KAO1) / cytochrome P450 88A3, putative (CYP88A3), identical to Cytochrome P450 88A3 (SP:O23051) (Arabidopsis thaliana); nearly identical to ent-kaurenoic acid hydroxylase (KAO1) GI:13021852 from (Arabidopsis thaliana) | chr1:1487377-1490946 REVERSE | Aliases: ATKAO1, ENT KAURENOIC ACID HYDROXYLASE 1, KAO1, YUP8H12.23, YUP8H12_23	5.3	4.2	1.1	3.8	44.8%	-0.6
471	AT3G48520.1 | Symbol: CYP94B3 | cytochrome P450 family protein, similar to Cytochrome P450 94A1 (P450-dependent fatty acid omega-hydroxylase) (SP:O81117) {Vicia sativa}; contains Pfam profile: PF00067 cytochrome P450 | chr3:17985962-17987655 REVERSE | Aliases: T8P19.30	6.1	4.7	1.3	3.8	44.8%	-0.5
472	AT3G01060.3 | expressed protein | chr3:16650-18847 REVERSE | Aliases: T4P13.26, T4P13_26	5.3	4.4	0.9	3.8	44.9%	-0.6
473	AT5G53360.1 | seven in absentia (SINA) family protein, low similarity to siah-1A protein (Mus musculus) GI:297035; contains Pfam profile PF03145: Seven in absentia protein family | chr5:21665186-21666801 FORWARD | Aliases: K19E1.16, K19E1_16	8.7	7.8	0.9	3.8	44.9%	-0.6
474	AT1G78020.1 | senescence-associated protein-related, similar to senescence-associated protein SAG102 (GI:22331931) (Arabidopsis thaliana) | chr1:29343587-29344649 FORWARD | Aliases: F28K19.24, F28K19_24	6.3	4.7	1.5	3.8	44.9%	-0.5
475	AT5G05410.2 | Symbol: DREB2A |	7.5	5.4	2.1	3.8	45.3%	-0.6
476	AT2G45560.2 | Symbol: CYP76C1 | cytochrome P450 family protein | chr2:18784299-18785448 REVERSE | Aliases: F17K2.9	3.1	2.6	0.5	3.8	45.3%	-1.0
477	AT2G47720.1 | expressed protein | chr2:19563694-19564209 FORWARD | Aliases: F17A22.11	4.9	3.9	1.1	3.8	45.4%	-0.6
478	AT5G37070.1 | expressed protein, contains Pfam profile PF04398: Protein of unknown function, DUF538 | chr5:14668321-14669377 FORWARD | Aliases: MJG14.7, MJG14_7	7.2	6.4	0.8	3.8	45.4%	-0.6

Rank	Description	15.dpi	5.dpi	M	t	adj.q	B
479	AT4G28270.1 | zinc finger (C3HC4-type RING finger) family protein, contains Pfam profile: PF00097 zinc finger, C3HC4 type (RING finger) | chr4:14007545-14009025 REVERSE | Aliases: F26K10.150, F26K10_150	6.8	6.0	0.9	3.8	45.4%	-0.6
480	AT1G62690.1 | expressed protein | chr1:23214109-23215054 REVERSE | Aliases: F23N19.23, F23N19_23	4.5	3.5	1.0	3.8	45.4%	-0.6
481	AT2G20800.1 | pyridine nucleotide-disulphide oxidoreductase family protein, similar to GI:3718005 alternative NADH-dehydrogenase {Yarrowia lipolytica} ; contains Pfam profile PF00070: Pyridine nucleotide-disulphide oxidoreductase | chr2:8960333-8962780 REVERSE | Aliases: F5H14.23, F5H14_23	3.0	4.7	-1.7	-3.8	45.4%	-0.6
482	AT1G62990.1 | Symbol: KNAT7 | homeodomain transcription factor (KNAT7), contains Pfam profiles: PF03789 ELK domain, PF03790 KNOX1 domain, PF03791 KNOX2 domain; similar to homeobox protein HD1 SP:P46606 from (Brassica napus); identical to cDNA homeodomain transcription factor KNAT7 (KNAT7) GI:11878229 | chr1:23341030-23344353 FORWARD | Aliases: F16P17.16, F16P17_16, IXR11, TRANSCRIPTION FACTOR KNAT7	6.2	4.9	1.4	3.8	45.4%	-0.6
483	AT5G17850.1 | cation exchanger, putative (CAX8), similar to sodium/calcium exchanger protein (Mus musculus) gi:13925661:gb:AAK49407; Ca2+:Cation Antiporter (CaCA) Family member PMID:11500563	7.4	6.2	1.2	3.8	45.4%	-0.6
484	AT1G08080.1 | carbonic anhydrase family protein, similar to storage protein (dioscorin) (Dioscorea cayenensis) GI:433463; contains Pfam profile PF00194: Eukaryotic-type carbonic anhydrase | chr1:2516988-2518543 REVERSE | Aliases: T6D22.16, T6D22_16	2.7	3.9	-1.2	-3.8	45.5%	-0.6
485	AT2G37560.2 | Symbol: ATORC2 |	3.3	4.4	-1.1	-3.8	45.5%	-0.6
486	AT2G44480.1 | glycosyl hydrolase family 1 protein, contains Pfam PF00232 : Glycosyl hydrolase family 1 domain; TIGRFAM TIGR01233: 6-phospho-beta-galactosidase; similar to amygdalin hydrolase isoform AH I precursor (GI:16757966) (Prunus serotina) | chr2:18366810-18370164 FORWARD | Aliases: F4I1.29	3.1	4.6	-1.5	-3.8	45.5%	-0.6
487	AT5G25160.1 | Symbol: ZFP3 | zinc finger (C2H2 type) family protein (ZFP3), identical to zinc finger protein, ZFP3 gi:790677:gb:AAA87299; contains Pfam domain, PF00096: Zinc finger, C2H2 type | chr5:8687473-8688418 FORWARD | Aliases: F21J6.6, F21J6_6, ZFP3, ZINC FINGER PROTEIN 3	5.9	5.0	0.9	3.8	45.5%	-0.6
488	AT5G40630.1 | ubiquitin family protein, contains INTERPRO:IPR000626 ubiquitin domain | chr5:16288368-16289698 REVERSE | Aliases: MNF13.18, MNF13_18	4.7	3.8	0.9	3.8	45.5%	-0.7
489	AT3G24310.1 | Symbol: MYB305 |	9.0	7.4	1.6	3.8	45.6%	-0.6
490	AT5G13980.3 | similar to glycosyl hydrolase family 38 protein [Arabidopsis thaliana] (TAIR:At3g26720.1); similar to glycosyl hydrolase family 38 protein [Arabidopsis thaliana] (TAIR:At5g66150.1); similar to MA2B1_MACFA Lysosomal alpha-mannosidase precursor (Mannosidase, alpha B) (Lysosomal acid alpha-mannosidase) (Laman) (Mannosidase alpha class 2B member 1) (GB:Q60HE9); similar to hypothetical protein [Pongo pygmaeus] (GB:CAH91346.1); similar to unnamed protein product [Tetraodon nigroviridis] (GB:CAG12505.1); similar to mannosidase, alpha, class 2B, member 1 precursor variant [Homo sapiens] (GB:BAD93158.1); similar to hypothetical protein LOC541519 [Danio rerio] (GB:NP_001014354.1); contains InterPro domain Glycoside hydrolase, family 38 (InterPro:IPR000602) | chr5:4508498-4514427 FORWARD | Aliases: MAC12.38, MAC12_38	6.5	7.6	-1.1	-3.8	45.6%	-0.6
491	AT5G13720.1 | expressed protein | chr5:4427844-4429136 FORWARD | Aliases: MSH12.19, MSH12_19	6.1	5.1	1.0	3.8	45.6%	-0.6
492	AT5G58900.1 | myb family transcription factor, contains Pfam profile: PF00249 myb-like DNA-binding domain | chr5:23800349-23802560 REVERSE | Aliases: K19M22.10, K19M22_10	7.4	6.4	1.1	3.8	45.6%	-0.6
493	AT3G46620.1 | zinc finger (C3HC4-type RING finger) family protein, contains Pfam domain, PF00097: Zinc finger, C3HC4 type (RING finger) | chr3:17189709-17191052 REVERSE | Aliases: F12A12.140	8.0	7.0	1.0	3.8	45.6%	-0.7
494	AT5G06760.1 | late embryogenesis abundant group 1 domain-containing protein / LEA group 1 domain-containing protein, low similarity to SP:P46515 11 kDa late embryogenesis abundant protein (DS11) {Helianthus annuus}; contains Pfam profile PF03760: Late embryogenesis abundant (LEA) group 1 | chr5:2089493-2090431 REVERSE | Aliases: MPH15.12, MPH15_12	6.4	4.6	1.8	3.8	45.6%	-0.6
495	AT3G12500.1 | Symbol: ATHCHIB | basic endochitinase, identical to basic endochitinase precursor SP:P19171 from (Arabidopsis thaliana)	4.2	7.2	-3.0	-3.8	45.6%	-0.6
496	AT4G16660.1 | heat shock protein 70, putative / HSP70, putative | chr4:9376773-9381529 FORWARD | Aliases: DL4355W, FCAALL.64	4.4	6.6	-2.1	-3.8	45.6%	-0.6
497	AT2G28450.1 | zinc finger (CCCH-type) family protein, contains Pfam domain, PF00642: Zinc finger C-x8-C-x5-C-x3-H type (and similar) | chr2:12169128-12172971 REVERSE | Aliases: T1B3.3, T1B3_3	5.3	6.9	-1.6	-3.7	45.6%	-0.6
498	AT5G62500.1 | Symbol: ATEB1B |	4.0	5.7	-1.7	-3.7	45.6%	-0.6
499	AT2G01180.2 | Symbol: ATPAP1 |	5.4	4.2	1.2	3.7	45.6%	-0.6

Rank	Description	15.dpi	5.dpi	M	t	adj.q	B
500	AT5G65300.1 | expressed protein | chr5:26112373-26112997 REVERSE | Aliases: MNA5.2, MNA5_2	6.1	4.9	1.2	3.7	45.6%	-0.6
501	AT5G37550.1 | expressed protein | chr5:14936131-14937022 REVERSE | Aliases: MPA22.10, MPA22_10	6.5	5.0	1.5	3.7	45.6%	-0.6
502	AT4G01270.1 | zinc finger (C3HC4-type RING finger) family protein, contains Pfam profile: PF00097 zinc finger, C3HC4 type (RING finger) | chr4:532351-534891 FORWARD | Aliases: F2N1.19, F2N1_19	3.1	3.8	-0.7	-3.7	45.8%	-0.7
503	AT3G21250.1 | Symbol: ATMRP6 |	6.1	7.4	-1.3	-3.7	45.8%	-0.6
504	AT1G28410.1 | similar to myosin heavy chain-related [Arabidopsis thaliana] (TAIR:At4g31340.1); similar to putative stress related-like protein interactor [Oryza sativa (japonica cultivar-group)] (GB:XP_483357.1); similar to unknown [Oryza sativa (japonica cultivar-group)] (GB:AAO72581.1) | chr1:9976168-9979253 REVERSE | Aliases: F3M18.15, F3M18_15	5.5	4.1	1.4	3.7	45.8%	-0.7
505	AT1G32460.1 | expressed protein | chr1:11738190-11739306 FORWARD | Aliases: F5D14.24, F5D14_24	11.5	10.2	1.3	3.7	46.1%	-0.8
506	AT3G12630.1 | zinc finger (AN1-like) family protein, contains Pfam domain, PF01428: AN1-like Zinc finger | chr3:4012615-4013590 FORWARD | Aliases: T2E22.6	9.9	8.3	1.6	3.7	46.1%	-0.6
507	AT3G16470.2 | Symbol: JR1 | similar to jacalin lectin family protein [Arabidopsis thaliana] (TAIR:At1g52040.1); similar to myrosinase binding protein [Brassica napus] (GB:CAA70587.1); contains InterPro domain Jacalin-related lectin (InterPro:IPR001229) | chr3:5595902-5598033 REVERSE | Aliases: T2O4.6	5.3	7.0	-1.6	-3.7	46.3%	-0.6
508	AT3G25780.1 | Symbol: AOC2 | allene oxide cyclase, putative / early-responsive to dehydration protein, putative / ERD protein, putative, similar to allene oxide cyclase GI:8977961 from (Lycopersicon esculentum); similar to early-responsive to dehydration (ERD12) protein (GI:15320414); contains Pfam profile PF06351: Allene oxide cyclase | chr3:9410527-9411803 FORWARD | Aliases: ALLENE OXIDE CYCLASE 2, K13N2.12	7.6	6.1	1.4	3.7	46.3%	-0.7
509	AT5G26731.1 | expressed protein | chr5:9295577-9296383 FORWARD | Aliases: None	7.6	6.4	1.2	3.7	46.8%	-0.7
510	AT3G13930.1 | dihydrolipoamide S-acetyltransferase, putative, similar to dihydrolipoamide S-acetyltransferase (Zea mays) GI:5669871; contains Pfam profiles PF00198: 2-oxo acid dehydrogenases acyltransferase (catalytic domain), PF00364: Biotin-requiring enzyme, PF02817: e3 binding domain | chr3:4595883-4600476 FORWARD | Aliases: MDC16.18	11.1	11.8	-0.8	-3.7	46.8%	-1.1
511	AT2G37040.1 | Symbol: PAL1 | phenylalanine ammonia-lyase 1 (PAL1), nearly identical to SP:P35510 | chr2:15564455-15567442 REVERSE | Aliases: CI0004, T1J8.22, T1J8_22	7.4	9.5	-2.0	-3.7	46.8%	-0.8
512	AT1G63090.1 | Symbol: ATPP2 A11 | F-box family protein / SKP1 interacting partner 3-related, contains Pfam profile PF00646: F-box domain | chr1:23394728-23396502 REVERSE | Aliases: ATPP2 A11, F16M19.16, F16M19_16	6.2	4.5	1.8	3.7	46.8%	-0.6
513	AT1G10180.1 | expressed protein | chr1:3338305-3341573 REVERSE | Aliases: F14N23.6, F14N23_6	7.0	8.3	-1.3	-3.7	46.9%	-0.7
514	AT5G45020.1 | expressed protein | chr5:18185909-18187843 FORWARD | Aliases: K21C13.21, K21C13_21	5.2	6.0	-0.8	-3.7	47.2%	-0.8
515	AT1G03070.1 | expressed protein, low similarity to N-methyl-D-aspartate receptor-associated protein (Drosophila melanogaster) GI:567104; contains Pfam profile PF01027: Uncharacterized protein family UPF0005	6.6	5.1	1.5	3.7	47.2%	-0.7
516	AT3G59580.2 | similar to RWP-RK domain-containing protein [Arabidopsis thaliana] (TAIR:At2g43500.1); similar to OSJNBa0067K08.5 [Oryza sativa (japonica cultivar-group)] (GB:XP_473028.1); contains InterPro domain Plant regulator RWP-RK (InterPro:IPR003035); contains InterPro domain Octicosapeptide/Phox/Bem1p (InterPro:IPR000270) | chr3:22020190-22023743 FORWARD | Aliases: T16L24.130	4.2	3.2	1.0	3.7	48.1%	-0.7
517	AT3G23800.1 | selenium-binding family protein, contains Pfam profile: PF05694 56kDa selenium binding protein (SBP56) | chr3:8581661-8583842 FORWARD | Aliases: MYM9.16	5.9	8.1	-2.1	-3.7	48.1%	-0.7
518	AT5G05200.1 | ABC1 family protein, contains Pfam domain, PF03109: ABC1 family | chr5:1543606-1547138 REVERSE | Aliases: K2A11.7, K2A11_7	9.9	9.0	0.9	3.7	48.1%	-0.8
519	AT1G33410.1 | expressed protein | chr1:12109600-12118808 REVERSE | Aliases: F10C21.8, F10C21_8	5.6	6.4	-0.8	-3.7	48.1%	-0.8
520	AT1G16220.1 | protein phosphatase 2C family protein / PP2C family protein, similar to protein phosphatase-2C; PP2C (GI:3643088) (Mesembryanthemum crystallinum); contains Pfam PF00481 : Protein phosphatase 2C domain; | chr1:5548647-5550547 FORWARD | Aliases: F3O9.3, F3O9_3	4.5	5.2	-0.7	-3.7	48.1%	-0.9
521	AT1G24170.1 | glycosyl transferase family 8 protein, contains Pfam profile: PF01501 glycosyl transferase family 8 | chr1:8557205-8558821 REVERSE | Aliases: F3I6.10, F3I6_10	7.6	6.9	0.6	3.7	48.1%	-1.0

Rank	Description	15.dpi	5.dpi	M	t	adj.q	B
522	AT2G19460.1 | expressed protein | chr2:8438564-8439187 FORWARD | Aliases: F27F23.23	4.8	3.2	1.6	3.7	48.1%	-0.7
523	AT2G39570.1 | ACT domain-containing protein, contains Pfam ACT domain PF01842 | chr2:16514974-16517276 FORWARD | Aliases: F12L6.23, F12L6_23	7.1	5.4	1.7	3.7	48.3%	-0.7
524	AT4G34360.1 | protease-related, similar to PIR:I46078 endothelin converting enzyme, Bos primigenius taurus | chr4:16431945-16433962 FORWARD | Aliases: F10M10.130, F10M10_130	3.4	4.4	-1.0	-3.7	48.3%	-0.8
525	AT1G73010.1 | expressed protein, similar to phosphatase, orphan 1 (GI:20196841) (Mus musculus), (GI:20196839) (Homo sapiens); contains TIGRFAM TIGR01489: 2,3-diketo-5-methylthio-1-phosphopentane phosphatase profile; contains TIGRFAM TIGR01488: HAD-superfamily hydrolase, subfamily IB (PSPase-like) | chr1:27468233-27469917 REVERSE | Aliases: F3N23.21, F3N23_21	6.6	5.1	1.4	3.7	48.3%	-0.7
526	AT1G66040.1 | zinc finger (C3HC4-type RING finger) family protein, contains zinc finger, C3HC4 type (RING finger), signature, PROSITE:PS00518 | chr1:24587483-24590344 REVERSE | Aliases: F15E12.8, F15E12_8	3.4	4.5	-1.0	-3.7	48.6%	-0.7
527	AT1G66050.1 | zinc finger (C3HC4-type RING finger) family protein, contains zinc finger, C3HC4 type (RING finger), signature, PROSITE:PS00518 | chr1:24593197-24596279 FORWARD | Aliases: F15E12.5, F15E12_5	3.4	4.5	-1.0	-3.7	48.6%	-0.7
528	AT5G39550.1 | zinc finger (C3HC4-type RING finger) family protein, contains zinc finger, C3HC4 type (RING finger), signature, PROSITE:PS00518 | chr5:15854636-15857731 REVERSE | Aliases: MIJ24.3, MIJ24_3	3.4	4.5	-1.0	-3.7	48.6%	-0.7
529	AT4G38340.1 | RWP-RK domain-containing protein, similar to nodule inception protein GI:6448579 from (Lotus japonicus); contains Pfam profile: PF02042 RWP-RK domain | chr4:17954704-17957822 FORWARD | Aliases: F22I13.110, F22I13_110	4.4	3.5	0.9	3.7	48.6%	-0.8
530	AT4G29670.2 | thioredoxin family protein, contains Pfam profile PF00085: Thioredoxin | chr4:14535613-14537244 REVERSE | Aliases: T16L4.180, T16L4_180	7.9	6.8	1.1	3.7	48.6%	-0.7
531	AT1G44575.2 | Symbol: NPQ4 | photosystem II 22kDa protein, chloroplast / CP22 (PSBS), identical to photosystem II 22 kDa protein, chloroplast (precursor) SP:Q9XF91 from (Arabidopsis thaliana); contains Pfam profile PF00504: Chlorophyll A-B binding protein | chr1:16874170-16875818 FORWARD | Aliases: NONPHOTOCHEMICAL QUENCHING, PSBS, T18F15.3, T18F15_3	7.2	5.5	1.7	3.7	48.6%	-0.7
532	AT2G27130.1 | protease inhibitor/seed storage/lipid transfer protein (LTP) family protein, contains Pfam protease inhibitor/seed storage/LTP family domain PF00234 | chr2:11602370-11603620 FORWARD | Aliases: T20P8.18, T20P8_18	5.3	6.8	-1.5	-3.7	48.6%	-0.7
533	AT2G29670.1 | expressed protein | chr2:12689221-12692164 REVERSE | Aliases: T27A16.23, T27A16_23	9.5	7.9	1.6	3.7	48.7%	-0.8
534	AT3G25470.1 | bacterial hemolysin-related, similar to hemolysine GB:AAD36643 from (Thermotoga maritima), contains Pfam profile: PF01479 S4 domain | chr3:9234178-9236450 FORWARD | Aliases: MWL2.14	9.0	9.5	-0.5	-3.7	48.9%	-1.1
535	AT2G29440.1 | Symbol: ATGSTU6 | glutathione S-transferase, putative | chr2:12627161-12628224 REVERSE | Aliases: F16P2.18, F16P2_18, GLUTATHIONE S TRANSFERASE 24, GST24	5.3	6.5	-1.2	-3.7	48.9%	-0.8
536	AT1G71380.1 | glycosyl hydrolase family 9 protein, similar to beta-glucanase GB:AAB72171 | chr1:26903446-26905451 REVERSE | Aliases: F3I17.16, F3I17_16	2.5	3.0	-0.5	-3.6	49.0%	-1.2
537	AT5G01670.2 | aldose reductase, putative, similar to aldose reductase (Hordeum vulgare)(GI:728592), aldose reductase ALDRXV4 (Xerophyta viscosa)(GI:4539944) | chr5:251975-253957 FORWARD | Aliases: F7A7.190, F7A7_190	7.6	6.3	1.2	3.6	49.0%	-0.7
538	AT3G62420.1 | bZIP transcription factor family protein, similar to common plant regulatory factor 6 GI:9650826 from (Petroselinum crispum) | chr3:23102820-23103260 REVERSE | Aliases: T12C14.120	9.5	8.6	0.9	3.6	49.0%	-0.9
539	AT5G47640.1 | CCAAT-box binding transcription factor subunit B (NF-YB) (HAP3) (AHAP3) family (Hap3b), similar to CAAT-box DNA binding protein subunit B (NF-YB) (SP:P25209) (GI:22380) (Zea mays); contains Pfam PF00808 : Histone-like transcription factor (CBF/NF-Y) and archaeal histone; identical to cDNA transcription factor Hap3b (Hap3b) mRNA, partial cds GI:9965734 | chr5:19326453-19327494 FORWARD | Aliases: MNJ7.23, MNJ7_23	6.7	5.7	1.0	3.6	49.1%	-0.8
540	AT1G65900.1 | expressed protein | chr1:24519509-24522111 REVERSE | Aliases: F12P19.7, F12P19_7	3.5	4.2	-0.8	-3.6	49.2%	-0.9
541	AT1G15010.1 | expressed protein | chr1:5171202-5171737 FORWARD | Aliases: T15D22.5, T15D22_5	3.2	2.4	0.8	3.6	49.2%	-0.9
542	AT2G34140.1 | Dof-type zinc finger domain-containing protein | chr2:14421120-14421805 REVERSE | Aliases: T14G11.26, T14G11_26	7.0	6.1	0.9	3.6	49.4%	-0.8

Rank	Description	15.dpi	5.dpi	M	t	adj.q	B
543	AT1G07440.2 | similar to short-chain dehydrogenase/reductase (SDR) family protein [Arabidopsis thaliana] (TAIR:At2g29340.2); similar to short-chain dehydrogenase/reductase (SDR) family protein [Arabidopsis thaliana] (TAIR:At2g29340.1); similar to putative pfam00106, adh_short, short chain dehydrogenase [Oryza sativa (japonica cultivar-group)] (GB:NP_912375.1); contains InterPro domain Glucose/ribitol dehydrogenase (InterPro:IPR002347); contains InterPro domain Short-chain dehydrogenase/reductase SDR (InterPro:IPR002198) | chr1:2286146-2287727 REVERSE | Aliases: F22G5.39, F22G5_39	6.4	4.9	1.5	3.6	49.9%	-0.8
544	AT3G02660.1 | tRNA synthetase class I (W and Y) family protein, similar to SP:P00952 Tyrosyl-tRNA synthetase (EC 6.1.1.1) (Tyrosine--tRNA ligase) (TyrRS) {Bacillus stearothermophilus}; contains Pfam profiles PF00579: tRNA synthetases class I (W and Y), PF01479: S4 domain | chr3:570153-572341 REVERSE | Aliases: F16B3.29, F16B3_29	4.2	5.2	-0.9	-3.6	50.6%	-0.9
545	AT1G44800.1 | nodulin MtN21 family protein, similar to MtN21 (Medicago truncatula) GI:2598575; contains Pfam profile PF00892: Integral membrane protein | chr1:16916449-16919436 REVERSE | Aliases: T12C22.7, T12C22_7	6.6	4.7	1.9	3.6	50.6%	-0.8
546	AT1G09070.1 | C2 domain-containing protein / src2-like protein, putative, similar to cold-regulated gene SRC2 (Glycine max) GI:2055230; contains Pfam profile PF00168: C2 domain; identical to cDNA src2-like protein GI:3426059 | chr1:2927706-2928971 FORWARD | Aliases: F7G19.6, F7G19_6	7.3	4.5	2.8	3.6	50.7%	-0.8
547	AT1G63470.1 | DNA-binding family protein, contains a AT hook motif (DNA binding motifs with a preference for A/T rich regions), Pfam:PF02178 | chr1:23540212-23542885 REVERSE | Aliases: F2K11.28, F2K11_28	6.5	8.0	-1.4	-3.6	51.2%	-0.8
548	AT2G07170.1 | similar to expressed protein [Arabidopsis thaliana] (TAIR:At4g27060.1); similar to potyviral helper component protease-interacting protein 2 [Solanum tuberosum subsp. andigena] (GB:CAD45375.1); contains InterPro domain Arabidopsis thaliana protein of unknown function DUF813 (InterPro:IPR008531) | chr2:2975562-2978817 FORWARD | Aliases: T25N22.13, T25N22_13	3.4	4.1	-0.7	-3.6	51.2%	-1.0
549	AT5G01230.2 | FtsJ-like methyltransferase family protein, contains Pfam profile: PF01728 FtsJ-like methyltransferase | chr5:92600-95685 REVERSE | Aliases: F7J8.210, F7J8_210	5.6	6.5	-0.9	-3.6	51.3%	-0.9
550	AT3G59710.1 | short-chain dehydrogenase/reductase (SDR) family protein, contains INTERPRO family IPR002198 short chain dehydrogenase/reductase SDR family | chr3:22065914-22068133 REVERSE | Aliases: T16L24.260	2.7	3.3	-0.6	-3.6	51.3%	-1.0
551	AT3G12740.1 | LEM3 (ligand-effect modulator 3) family protein / CDC50 family protein, Similar to GI:4585976; GI:4966357; GI:4835763; GI:9757735 from (Arabidopsis thaliana) | chr3:4049522-4052050 FORWARD | Aliases: MBK21.12	9.9	9.1	0.7	3.6	51.3%	-1.1
552	AT1G17180.1 | Symbol: ATGSTU25 | glutathione S-transferase, putative, Second of three repeated putative glutathione transferases. 72% identical to glutathione transferase (Arabidopsis thaliana) (gi:4006934). Location of ests 191A10T7 (gb:R90188) and 171N13T7 (gb:R65532) | chr1:5872142-5873079 FORWARD | Aliases: F20D23.12, F20D23_12	4.3	6.0	-1.6	-3.6	51.3%	-0.9
553	AT4G09800.1 | Symbol: RPS18C |	10.8	11.5	-0.6	-3.6	51.3%	-1.4
554	AT2G16200.1 | expressed protein | chr2:7031806-7032186 FORWARD | Aliases: F7H1.22, F7H1_22	3.7	2.9	0.8	3.6	51.3%	-1.0
555	AT5G10580.2 | expressed protein, contains Pfam profile PF04654: Protein of unknown function, DUF599 | chr5:3347438-3350072 FORWARD | Aliases: F12B17.70, F12B17_70	5.8	4.2	1.6	3.6	51.3%	-0.9
556	AT2G47910.2 | expressed protein | chr2:19621768-19622839 FORWARD | Aliases: F17A22.30, F17A22.40, F17A22_30, F17A22_40	4.4	3.7	0.7	3.6	51.3%	-1.0
557	AT5G60780.1 | Symbol: ATNRT2.3 | high-affinity nitrate transporter, putative, similar to trans-membrane nitrate transporter protein AtNRT2:1 (Arabidopsis thaliana) GI:3747058, high-affinity nitrate transporter ACH1 (Arabidopsis thaliana) GI:3608362 | chr5:24468065-24469982 FORWARD | Aliases: MAE1.4, MAE1_4	5.4	3.4	2.0	3.6	51.3%	-0.9
558	AT5G25880.1 | malate oxidoreductase, putative, similar to NADP-dependent malic enzyme (EC 1.1.1.40) (NADP-ME) (SP:P12628) {Phaseolus vulgaris}	3.4	4.4	-1.0	-3.6	51.3%	-0.9
559	AT3G26460.1 | major latex protein-related / MLP-related, low similarity to major latex protein {Papaver somniferum}(GI:294060) contains Pfam profile PF00407: Pathogenesis-related protein Bet v I family | chr3:9685110-9685894 REVERSE | Aliases: F20C19.18	3.7	5.5	-1.8	-3.6	51.6%	-0.8
560	AT3G18280.1 | protease inhibitor/seed storage/lipid transfer protein (LTP) family protein, similar to TED4 (Zinnia elegans) GI:493721; contains Pfam protease inhibitor/seed storage/LTP family domain PF00234	8.4	10.1	-1.7	-3.6	51.6%	-1.2
561	AT5G43780.1 | Symbol: APS4 | sulfate adenylyltransferase 4 / ATP-sulfurylase 4 (APS4), identical to ATP sulfurylase precursor (APS4) (Arabidopsis thaliana) GI:4633131 | chr5:17606418-17608762 REVERSE | Aliases: MQD19.13, MQD19_13	6.3	8.2	-2.0	-3.6	51.6%	-0.8
562	AT3G01330.1 | Symbol: DEL3 | transcription factor, putative / E2F-like repressor E2L2 (E2L2), identical to E2F-like repressor E2L2 (Arabidopsis thaliana) GI:20502506	3.6	4.2	-0.6	-3.6	51.6%	-1.0

Rank	Description	15.dpi	5.dpi	M	t	adj.q	B
563	AT5G22840.1 | protein kinase family protein, contains protein kinase domain, Pfam:PF00069 | chr5:7630720-7633226 REVERSE | Aliases: MRN17.7, MRN17_7	4.1	5.3	-1.2	-3.6	51.7%	-0.8
564	AT3G20670.1 | histone H2A, putative, strong similarity to histone H2A GB:AAF64418 GI:7595337 from Arabidopsis thaliana, Triticum aestivum GI:536892; contains Pfam profile PF00125 Core histone H2A/H2B/H3/H4 | chr3:7229417-7230508 FORWARD | Aliases: F3H11.6	7.8	9.9	-2.2	-3.6	51.7%	-0.8
565	AT3G52800.1 | zinc finger (AN1-like) family protein, contains Pfam domain, PF01428: AN1-like Zinc finger | chr3:19580289-19581660 FORWARD | Aliases: F3C22.200	6.9	5.4	1.5	3.6	51.7%	-0.8
566	AT5G54280.1 | Symbol: ATM2 | myosin heavy chain, putative, similar to myosin (Arabidopsis thaliana) gi:499045:emb:CAA84065 | chr5:22055583-22062468 REVERSE | Aliases: ARABIDOPSIS THALIANA MYOSIN 4, ATATM2, ATM4, ATMYOS1, MDK4.10, MDK4_10, MYOSIN 4	4.4	5.8	-1.4	-3.6	51.7%	-0.9
567	AT5G63030.1 | glutaredoxin, putative, similar to glutaredoxin (Ricinus communis) gi:1732424:emb:CAA89699 | chr5:25303511-25304906 FORWARD | Aliases: MJH22.9, MJH22_9	10.2	9.3	0.8	3.6	51.7%	-1.0
568	AT1G10070.3 | similar to branched-chain amino acid aminotransferase 5 / branched-chain amino acid transaminase 5 (BCAT5) [Arabidopsis thaliana] (TAIR:At5g65780.1); similar to branched-chain amino acid aminotransferase [Solanum tuberosum] (GB:AAF07192.1); contains InterPro domain Aminotransferase, class IV (InterPro:IPR001544); contains InterPro domain Branched-chain amino acid aminotransferase II (InterPro:IPR005786) | chr1:3288089-3290841 FORWARD | Aliases: T27I1.9, T27I1_9	5.5	3.8	1.8	3.6	51.7%	-0.8
569	AT2G21560.1 | expressed protein, contains weak similarity to reticulocyte-binding protein 2 homolog A (Plasmodium falciparum) gi:9754767:gb:AAF98066 | chr2:9237348-9238588 REVERSE | Aliases: F2G1.17, F2G1_17	11.2	9.3	1.9	3.6	51.7%	-0.9
570	AT5G06050.1 | dehydration-responsive protein-related, similar to early-responsive to dehydration stress ERD3 protein (Arabidopsis thaliana) GI:15320410; contains Pfam profile PF03141: Putative methyltransferase | chr5:1820135-1823771 FORWARD | Aliases: K18J17.25, K18J17_25	6.0	7.2	-1.2	-3.6	51.9%	-0.8
571	AT1G56110.1 | Symbol: NOP56 | nucleolar protein Nop56, putative, similar to XNop56 protein (Xenopus laevis) GI:14799394; contains Pfam profile PF01798: Putative snoRNA binding domain | chr1:20988055-20990614 REVERSE | Aliases: NOP56 LIKE PROTEIN, T6H22.10, T6H22_10	7.5	9.0	-1.5	-3.6	51.9%	-0.8
572	AT5G04490.1 | phosphatidate cytidylyltransferase family protein, contains Pfam profile: PF01148 phosphatidate cytidylyltransferase | chr5:1279761-1281670 FORWARD | Aliases: T32M21.90, T32M21_90	5.6	4.4	1.2	3.6	51.9%	-0.8
573	AT1G70710.1 | Symbol: CEL1 | endo-1,4-beta-glucanase (EGASE) / cellulase, identical to endo-1,4-beta-glucanase GB:CAA67157 GI:2440035 from (Arabidopsis thaliana) | chr1:26662794-26666662 REVERSE | Aliases: F5A18.11, F5A18_11	3.5	5.6	-2.1	-3.6	51.9%	-0.9
574	AT5G19875.1 | expressed protein | chr5:6718343-6718997 FORWARD | Aliases: None	7.4	6.2	1.2	3.6	51.9%	-0.9
575	AT4G20430.1 | subtilase family protein, contains Pfam profile: PF00082 subtilase family | chr4:11017667-11021116 REVERSE | Aliases: F9F13.80, F9F13_80	4.2	5.2	-0.9	-3.6	52.1%	-0.9
576	AT2G33830.2 | dormancy/auxin associated family protein, contains Pfam profile: PF05564 dormancy/auxin associated protein | chr2:14316600-14317417 REVERSE | Aliases: T1B8.13, T1B8_13	8.6	6.8	1.8	3.6	52.1%	-0.9
577	AT4G19540.1 | expressed protein | chr4:10657393-10659243 FORWARD | Aliases: F24J7.100, F24J7_100	5.5	6.5	-0.9	-3.6	52.1%	-0.9
578	AT3G06035.1 | expressed protein | chr3:1823105-1824188 REVERSE | Aliases: None	8.3	9.4	-1.1	-3.6	52.1%	-0.9
579	AT5G06980.2 | expressed protein | chr5:2167624-2169392 FORWARD | Aliases: MOJ9.15, MOJ9_15	6.8	5.2	1.6	3.6	52.2%	-0.9
580	AT2G41820.1 | leucine-rich repeat transmembrane protein kinase, putative | chr2:17453822-17457149 FORWARD | Aliases: T11A7.8, T11A7_8	5.1	5.8	-0.7	-3.6	52.4%	-1.0
581	AT5G64710.2 | expressed protein, contains Pfam profile PF04396: Protein of unknown function, DUF537 | chr5:25887071-25889455 FORWARD | Aliases: MVP7.3, MVP7_3	3.0	3.6	-0.6	-3.5	52.8%	-1.1
582	AT4G17670.1 | senescence-associated protein-related, similar to senescence-associated protein SAG102 (GI:22331931) (Arabidopsis thaliana); | chr4:9833681-9834740 REVERSE | Aliases: DL4870C, FCAALL.414	7.2	5.7	1.6	3.5	52.8%	-0.9
583	AT4G27990.1 | YGGT family protein, contains Pfam profile PF02325: YGGT family (unknown function) | chr4:13923779-13924698 REVERSE | Aliases: T13J8.100, T13J8_100	6.2	4.5	1.7	3.5	53.1%	-0.9

Rank	Description	15.dpi	5.dpi	M	t	adj.q	B
584	AT3G19580.1 | Symbol: AZF2 | zinc finger (C2H2 type) protein 2 (AZF2), identical to Cys2/His2-type zinc finger protein 2 (Arabidopsis thaliana) gi:6009885:dbj:BAA85107 | chr3:6803172-6804239 REVERSE | Aliases: ARABIDOPSIS ZINC FINGER PROTEIN 2, MMB12.27	5.6	4.4	1.2	3.5	53.2%	-0.9
585	AT3G15450.3 | similar to expressed protein [Arabidopsis thaliana] (TAIR:At4g27450.1); similar to unknown [Asparagus officinalis] (GB:CAA54526.1) | chr3:5213004-5214126 FORWARD | Aliases: MJK13.11	7.5	6.3	1.1	3.5	53.2%	-0.9
586	AT2G33850.1 | expressed protein, contains 1 transmembrane domain; similar to Protein E6 (Swiss-Prot:Q01197) (Gossypium hirsutum) | chr2:14328138-14329125 REVERSE | Aliases: T1B8.15, T1B8_15	2.8	4.0	-1.2	-3.5	53.2%	-0.9
587	AT2G19770.1 | profilin 4 (PRO4) (PFN4), identical to profilin 4 SP:Q38905 GI:1353768 from (Arabidopsis thaliana) | chr2:8526720-8528274 REVERSE | Aliases: F6F22.20, F6F22_20	4.5	3.2	1.3	3.5	53.2%	-0.9
588	AT4G36040.1 | DNAJ heat shock N-terminal domain-containing protein (J11), identical to dnaJ heat shock protein J11 (Arabidopsis thaliana) GI:9843641; contains Pfam profile PF00226 DnaJ domain | chr4:17049174-17050277 REVERSE | Aliases: T19K4.170, T19K4_170	6.3	4.5	1.9	3.5	53.2%	-0.9
589	AT5G64670.1 | ribosomal protein L15 family protein | chr5:25869493-25871215 REVERSE | Aliases: MUB3.19, MUB3_19	7.7	8.6	-0.9	-3.5	53.2%	-0.9
590	AT4G01700.1 | chitinase, putative, similar to peanut type II chitinase GI:1237025 from (Arachis hypogaea) | chr4:732010-733510 REVERSE | Aliases: T15B16.5, T15B16_5	6.0	9.2	-3.1	-3.5	53.2%	-0.9
591	AT5G28150.1 | expressed protein | chr5:10135475-10136981 FORWARD | Aliases: T24G3.80, T24G3_80	6.5	7.3	-0.8	-3.5	53.2%	-1.1
592	AT1G80110.1 | Symbol: ATPP2 B11 | expressed protein, contains similarity to SKP1 interacting partner 3 (Arabidopsis thaliana) GI:10716951 | chr1:30140274-30141848 FORWARD | Aliases: ATPP2 B11, F18B13.19, F18B13_19	4.9	4.1	0.8	3.5	53.2%	-1.0
593	AT2G38480.1 | integral membrane protein, putative, contains 4 transmembrane domains; contains plant integral membrane protein domain, TIGR01569 and PF04535;: Domain of unknown function (DUF588); At3g16300, At1g45222 both share this domain structure; distantly related to GP:14030504 salicylic acid-induced fragment 1 protein {Gossypium hirsutum}; similar to putative ethylene responsive element binding protein (GI:22135858) (Arabidopsis thaliana) | chr2:16117342-16118875 REVERSE | Aliases: T19C21.23	4.6	3.8	0.8	3.5	53.2%	-1.0
594	AT3G13520.1 | Symbol: AGP12 | arabinogalactan-protein (AGP12), identical to gi:10880501:gb:AAG24280 | chr3:4408932-4409447 FORWARD | Aliases: ARABINOGALACTAN PROTEIN 12, MRP15.18	12.4	11.6	0.8	3.5	53.3%	-1.5
595	AT4G24110.1 | expressed protein | chr4:12519942-12520823 REVERSE | Aliases: T19F6.100, T19F6_100	8.9	5.2	3.7	3.5	53.3%	-1.0
596	AT2G30040.1 | Symbol: MAPKKK14 | protein kinase family protein, contains protein kinase domain, Pfam:PF00069 | chr2:12828787-12830246 FORWARD | Aliases: F23F1.4, F23F1_4	5.6	4.2	1.3	3.5	53.3%	-0.9
597	AT3G62550.1 | universal stress protein (USP) family protein, similar to ER6 protein (Lycopersicon esculentum) GI:5669654; contains Pfam profile PF00582: universal stress protein family | chr3:23146960-23148146 FORWARD | Aliases: T12C14.250	5.0	3.6	1.4	3.5	53.3%	-0.9
598	AT1G48470.1 | Symbol: GLN1;5 |	6.5	4.8	1.7	3.5	53.3%	-0.9
599	AT1G74790.1 | expressed protein, contains similarity to hedgehog-interacting protein GI:4868122 from (Mus musculus) | chr1:28102471-28105557 FORWARD | Aliases: F25A4.24, F25A4_24	4.8	5.8	-1.0	-3.5	53.3%	-0.9
600	AT3G47500.1 | Dof-type zinc finger domain-containing protein, identical to H-protein promoter binding factor-2a GI:3386546 from (Arabidopsis thaliana)	7.2	6.5	0.7	3.5	53.7%	-1.0
601	AT2G20920.1 | expressed protein | chr2:9005767-9007013 FORWARD | Aliases: F5H14.11, F5H14_11	9.5	8.6	0.9	3.5	53.7%	-1.1
602	AT1G20950.1 | pyrophosphate--fructose-6-phosphate 1-phosphotransferase-related / pyrophosphate-dependent 6-phosphofructose-1-kinase-related, similar to pyrophosphate--fructose 6-phosphate 1-phosphotransferase alpha subunit SP:Q41140 from (Ricinus communis) | chr1:7297228-7301404 REVERSE | Aliases: F9H16.6, F9H16_6	6.0	8.5	-2.6	-3.5	53.7%	-0.9
603	AT4G12070.1 | expressed protein | chr4:7231816-7234495 FORWARD | Aliases: F16J13.140, F16J13_140	5.1	4.2	0.9	3.5	53.9%	-1.0
604	AT5G65070.1 | Symbol: MAF4 | MADS-box protein (MAF4), contains Pfam profile PF00319: SRF-type transcription factor (DNA-binding and dimerisation domain); contains Pfam profile PF01486: K-box region	3.3	2.7	0.6	3.5	53.9%	-1.2

Rank	Description	15.dpi	5.dpi	M	t	adj.q	B
605	AT1G28370.1 | Symbol: ATERF11/ERF11 | encodes a member of the ERF (ethylene response factor) subfamily B-1 of ERF/AP2 transcription factor family. The protein contains one AP2 domain. There are 15 members in this subfamily including ATERF-3, ATERF-4, ATERF-7, and leafy petiole. | chr1:9955955-9956926 REVERSE | Aliases: ATERF11, ERF11, F3M18.20, F3M18_20	5.7	4.1	1.6	3.5	54.0%	-1.0
606	AT3G60490.1 | encodes a member of the DREB subfamily A-4 of ERF/AP2 transcription factor family. The protein contains one AP2 domain. There are 17 members in this subfamily including TINY. | chr3:22360502-22361346 FORWARD | Aliases: T8B10.150	4.4	3.4	1.0	3.5	54.0%	-1.0
607	AT1G26830.1 | Symbol: ATCUL3/ATCUL3A/CUL3/CUL3A | Cullin, putative, similar to Cullin homolog 3 (CUL-3) SP:Q13618, GI:3639052 from (Homo sapiens); contains Pfam profile PF00888: Cullin family. Interacts with other components of E3 ligase complex suggesting it functions in RUB-modification. Forms complexes with BTB domain proteins forming a novel class of E3-based ubiquitin protein-ligase complexes. Mutant is early flowering and has a reduced sensitivity to far-red light. cul3a/cul3b homozygous/heterozygous plants are embryo lethal. | chr1:9295766-9298525 FORWARD | Aliases: ATCUL3, ATCUL3A, CUL3, CUL3A, T24P13.25	7.1	8.4	-1.3	-3.5	54.0%	-0.9
608	AT5G13170.1 | nodulin MtN3 family protein, similar to MtN3 GI:1619602 (root nodule development) from (Medicago truncatula); identical to cDNA senescence-associated protein (SAG29) mRNA, partial cds GI:4426938 | chr5:4181045-4183309 REVERSE | Aliases: T19L5.130, T19L5_130	6.0	4.3	1.7	3.5	54.0%	-0.9
609	AT3G29170.1 | expressed protein, contains Pfam PF05915: Eukaryotic protein of unknown function (DUF872) | chr3:11137243-11139086 REVERSE | Aliases: MXE2.17	7.6	6.8	0.9	3.5	54.0%	-1.0
610	AT3G02980.1 | GCN5-related N-acetyltransferase (GNAT) family protein, contains Pfam profile: PF00583 acetyltransferase (GNAT) family | chr3:670904-672222 REVERSE | Aliases: F13E7.7, F13E7_7	5.0	4.3	0.8	3.5	54.0%	-1.0
611	AT1G26770.1 | Symbol: ATEXPA10 | expansin, putative (EXP10), similar to expansin At-EXP1 GI:1041702 from (Arabidopsis thaliana); alpha-expansin gene family, PMID:11641069 | chr1:9259592-9261300 FORWARD | Aliases: ARABIDOPSIS THALIANA EXPANSIN A10, AT EXP10, ATEXP10, ATHEXP ALPHA 1.1, EXP10, EXPANSIN 10, T24P13.15, T24P13_15	6.4	4.5	1.9	3.5	54.0%	-0.9
612	AT5G08330.1 | TCP family transcription factor, putative, similar to PCF1 (GI:2580438) and PCF2 ((GI:2580440) Oryza sativa) ; auxin-induced basic helix-loop-helix transcription factor, Gossypium hirsutum, EMBL:AF165924 | chr5:2680745-2681814 FORWARD | Aliases: F8L15.60, F8L15_60	5.1	4.2	0.9	3.5	54.1%	-1.0
613	AT4G16980.1 | arabinogalactan-protein family, similar to arabinogalactan protein (Arabidopsis thaliana) gi:10880495:gb:AAG24277; contains proline-rich extensin domains, INTERPRO:IPR002965 | chr4:9556997-9557831 FORWARD | Aliases: DL4520C, FCAALL.321	2.9	3.9	-1.0	-3.5	54.3%	-1.0
614	AT1G11790.2 | similar to prephenate dehydratase family protein [Arabidopsis thaliana] (TAIR:At3g07630.2); similar to prephenate dehydratase family protein [Arabidopsis thaliana] (TAIR:At3g07630.1); similar to putative prephenate dehydratase [Oryza sativa (japonica cultivar-group)] (GB:XP_479626.1); contains InterPro domain Prephenate dehydratase (InterPro:IPR001086) | chr1:3981247-3985214 FORWARD | Aliases: F25C20.4, F25C20_4	6.0	7.1	-1.1	-3.5	54.3%	-1.0
615	AT5G23750.2 | remorin family protein, contains Pfam domain, PF03766: Remorin, N-terminal region; contains Pfam domain, PF03763: Remorin, C-terminal region | chr5:8009812-8011614 REVERSE | Aliases: MRO11.21, MRO11_21	6.4	5.1	1.4	3.5	54.3%	-0.9
616	AT1G69380.1 | expressed protein, predicated by genscan+ | chr1:26085004-26087269 REVERSE | Aliases: F23O10.4, F23O10_4	5.5	6.8	-1.3	-3.5	54.3%	-0.9
617	AT4G20380.6 | Symbol: LSD1 | similar to zinc finger protein, putative [Arabidopsis thaliana] (TAIR:At1g32540.1); similar to zinc finger protein, putative [Arabidopsis thaliana] (TAIR:At1g32540.2); similar to zinc finger protein LSD2 [Brassica oleracea] (GB:AAL50982.1); contains InterPro domain Zn-finger, LSD1 type (InterPro:IPR005735) | chr4:11004558-11006799 FORWARD | Aliases: F9F13.30, F9F13_30, LESION SIMULATING DISEASE	8.4	7.3	1.1	3.5	54.3%	-1.0
618	AT5G18850.1 | expressed protein | chr5:6286862-6287592 FORWARD | Aliases: F17K4.100, F17K4_100	7.0	6.1	0.9	3.5	54.3%	-1.0
619	AT2G17340.1 | pantothenate kinase-related, contains Pfam domain, PF01937: Protein of unknown function; supported by tandem duplication of pantothenate kinase -related protein (TIGR_Ath1:At2g17320) (Arabidopsis thaliana) | chr2:7548494-7551196 REVERSE | Aliases: F5J6.10, F5J6_10	7.8	8.7	-0.9	-3.5	54.4%	-1.1
620	AT1G61890.1 | MATE efflux family protein, similar to ripening regulated protein DDTFR18 (Lycopersicon esculentum) GI:12231296; contains Pfam profile PF01554: Uncharacterized membrane protein family | chr1:22871438-22874945 REVERSE | Aliases: F8K4.9, F8K4_9	7.1	5.5	1.6	3.5	54.6%	-1.0
621	AT1G35140.1 | Symbol: PHI 1 | phosphate-responsive protein, putative, similar to phi-1 (phosphate-induced gene) (Nicotiana tabacum) GI:3759184; contains Pfam profile PF04674: Phosphate-induced protein 1 conserved region | chr1:12851975-12853103 FORWARD | Aliases: PHOSPHATE INDUCED 1, T32G9.32, T32G9_32	7.0	5.4	1.6	3.5	54.7%	-1.1
622	AT1G43910.1 | AAA-type ATPase family protein, contains Pfam profile: ATPase family PF00004 | chr1:16658044-16660096 REVERSE | Aliases: F9C16.7, F9C16_7	8.6	6.6	2.0	3.5	54.7%	-1.0
623	AT2G05120.1 | expressed protein | chr2:1841906-1846855 REVERSE | Aliases: F5G3.2, F5G3_2	3.2	3.9	-0.7	-3.5	54.7%	-1.2

Rank	Description	15.dpi	5.dpi	M	t	adj.q	B
624	AT3G57770.1 | protein kinase, putative, contains protein kinase domain, Pfam:PF00069 | chr3:21408519-21409830 FORWARD | Aliases: F15B8.40	4.5	3.1	1.4	3.5	54.8%	-1.0
625	AT3G62050.1 | expressed protein, contains Pfam profile PF04396: Protein of unknown function, DUF537 | chr3:22989221-22989994 FORWARD | Aliases: T17J13.10	4.8	5.9	-1.1	-3.5	54.8%	-1.0
626	AT2G04795.1 | expressed protein | chr2:1682950-1683472 FORWARD | Aliases: None	8.5	7.4	1.1	3.5	54.9%	-1.0
627	AT2G26400.1 | acireductone dioxygenase (ARD/ARD') family protein, similar to iron-deficiency induced gene (Hordeum vulgare) GI:14522834, SIPL (Homo sapiens) GI:16551383; contains Pfam profile PF03079: ARD/ARD' family | chr2:11238925-11240352 REVERSE | Aliases: T9J22.7, T9J22_7	7.4	5.9	1.5	3.5	54.9%	-1.0
628	AT4G17340.1 | Symbol: DELTA TIP2/TIP2;2 | major intrinsic family protein / MIP family protein, contains Pfam profile: MIP PF00230 | chr4:9699265-9700421 FORWARD | Aliases: DELTA TIP2, DL4705W, FCAALL.412, TIP2;2	6.1	4.5	1.5	3.5	54.9%	-1.0
629	AT1G68600.1 | expressed protein, contains Pfam profile PF01027: Uncharacterized protein family UPF0005	3.6	3.0	0.6	3.5	54.9%	-1.3
630	AT3G47620.1 | TCP family transcription factor, putative, auxin-induced basic helix-loop-helix transcription factor - Gossypium hirsutum, EMBL:AF165924	8.0	7.1	1.0	3.5	55.1%	-1.0
631	AT1G05340.1 | expressed protein | chr1:1558849-1559757 REVERSE | Aliases: YUP8H12.4, YUP8H12_4	5.8	3.4	2.5	3.5	55.2%	-1.0
632	AT1G23880.1 | NHL repeat-containing protein, contains Pfam profile PF01436: NHL repeat	3.6	4.1	-0.6	-3.5	55.4%	-1.3
633	AT3G27540.1 | glycosyl transferase family 17 protein, low similarity to beta-1,4-mannosyl-glycoprotein beta-1,4-N-acetylglucosaminyltransferase (EC 2.4.1.144) from Mus musculus (SP:Q10470), Rattus norvegicus (SP:Q02527), Homo sapiens (SP:Q09327) ; contains Pfam profile PF04724 :Glycosyltransferase family 17 | chr3:10206545-10208543 FORWARD | Aliases: MMJ24.9	4.1	3.3	0.8	3.5	55.5%	-1.1
634	AT4G21870.1 | 26.5 kDa class P-related heat shock protein (HSP26.5-P), contains Pfam profile: PF00011 Hsp20/alpha crystallin family: identified in Scharf, K-D., et al,Cell Stress & Chaperones (2001) 6: 225-237. | chr4:11603582-11604343 REVERSE | Aliases: T8O5.80, T8O5_80	8.0	6.7	1.3	3.5	55.5%	-1.0
635	AT3G14650.1 | Symbol: CYP72A11 | cytochrome P450, putative, similar to GB:Q05047 from (Catharanthus roseus) | chr3:4922138-4924695 FORWARD | Aliases: MIE1.15	5.4	4.1	1.2	3.5	55.5%	-1.0
636	AT3G06480.1 | DEAD box RNA helicase, putative, similar to RNA helicase DRH1 (Arabidopsis thaliana) GI:3149952; contains Pfam profiles PF00270: DEAD/DEAH box helicase, PF00271: Helicase conserved C-terminal domain, PF00397: WW domain | chr3:1985461-1990159 REVERSE | Aliases: F24P17.2, F24P17_2	5.6	4.2	1.4	3.5	55.5%	-1.0
637	AT1G59540.1 | Symbol: ZCF125 |	2.7	3.2	-0.6	-3.4	55.5%	-1.3
638	AT3G19900.1 | expressed protein | chr3:6922574-6925029 REVERSE | Aliases: MPN9.15	6.0	4.9	1.2	3.4	55.5%	-1.0
639	AT3G54560.1 | histone H2A.F/Z, identical to GI:2407800	6.9	9.1	-2.2	-3.4	55.6%	-1.0
640	AT4G36710.1 | scarecrow transcription factor family protein | chr4:17305762-17307647 FORWARD | Aliases: AP22.56, AP22_56	8.2	6.6	1.6	3.4	55.6%	-1.0
641	AT5G48850.1 | male sterility MS5 family protein, similar to male sterility MS5 (Arabidopsis thaliana) GI:3859112; contains Pfam profile PF00515 TPR Domain | chr5:19822663-19824985 REVERSE | Aliases: K24G6.19, K24G6_19	4.2	6.3	-2.1	-3.4	55.6%	-1.0
642	AT4G39650.1 | similar to gamma-glutamyltranspeptidase family protein [Arabidopsis thaliana] (TAIR:At4g39640.2); similar to gamma-glutamyltranspeptidase family protein [Arabidopsis thaliana] (TAIR:At4g39640.1); similar to gamma-glutamyl transferase [Raphanus sativus] (GB:BAC45233.1); similar to OSJNBa0036B21.10 [Oryza sativa (japonica cultivar-group)] (GB:XP_472730.1); similar to gamma-glutamyltransferase [Allium cepa] (GB:AAL61611.2); contains InterPro domain Gamma-glutamyltranspeptidase (InterPro:IPR000101) | chr4:18403554-18406019 FORWARD | Aliases: T19P19.40, T19P19_40	4.1	3.4	0.7	3.4	55.6%	-1.1
643	AT1G32920.1 | expressed protein | chr1:11928787-11929281 FORWARD | Aliases: F9L11.25, F9L11_25	9.0	6.9	2.1	3.4	56.1%	-1.0
644	AT2G27290.1 | expressed protein | chr2:11685478-11686870 REVERSE | Aliases: F12K2.13, F12K2_13	5.5	4.6	0.9	3.4	56.1%	-1.0
645	AT3G57880.1 | C2 domain-containing protein, contains INTERPRO:IPR000008 C2 domain | chr3:21441993-21445363 REVERSE | Aliases: T10K17.90	6.0	5.1	1.0	3.4	56.1%	-1.0
646	AT1G01780.1 | LIM domain-containing protein, similar to PGPS/D1 (Petunia x hybrida) GI:4105772, LIM domain protein PLIM1 (Nicotiana tabacum) GI:5932418; contains Pfam profile PF00412: LIM domain | chr1:282772-284082 FORWARD | Aliases: T1N6.19, T1N6_19	5.8	5.0	0.8	3.4	56.1%	-1.2
647	AT5G01350.1 | expressed protein | chr5:145635-147199 REVERSE | Aliases: T10O8.60, T10O8_60	11.5	10.6	0.9	3.4	56.1%	-1.5

Rank	Description	15.dpi	5.dpi	M	t	adj.q	B
648	AT3G03320.1 | expressed protein, similar to hypothetical protein GB:BAA29429 (Pyrococcus horikoshii) | chr3:781722-783471 FORWARD | Aliases: T21P5.26, T21P5_26	4.9	6.2	-1.4	-3.4	56.1%	-1.0
649	AT5G25360.1 | expressed protein | chr5:8799713-8802649 REVERSE | Aliases: F18G18.100, F18G18_100	7.7	5.9	1.8	3.4	56.1%	-1.0
650	AT2G16500.1 | Symbol: ADC1 | arginine decarboxylase 1 (SPE1) (ARGDC), identical to SP:Q9SI64 Arginine decarboxylase 1 (EC 4.1.1.19) (ARGDC 1) (ADC 1) (ADC-O) {Arabidopsis thaliana} | chr2:7157704-7160421 REVERSE | Aliases: ARGDC, ARGDC1, ARGININE DECARBOXYLASE, ARGININE DECARBOXYLASE 1, F1P15.12, F1P15_12	7.7	9.1	-1.4	-3.4	56.1%	-1.0
651	AT1G80050.1 | Symbol: APT2 | adenine phosphoribosyltransferase 2 (APT2), identical to SP:Q42563 Adenine phosphoribosyltransferase 2 (EC 2.4.2.7) (APRT) {Arabidopsis thaliana} | chr1:30116407-30118217 REVERSE | Aliases: ADENINE PHOSPHORIBOSYL TRANSFERASE 2, ATAPT2, F18B13.14, F18B13_14	4.4	5.5	-1.1	-3.4	56.1%	-1.1
652	AT3G04640.1 | glycine-rich protein, predicted proteins, Arabidopsis thaliana | chr3:1260912-1261689 REVERSE | Aliases: F7O18.12, F7O18_12	6.3	4.7	1.5	3.4	56.5%	-1.0
653	AT3G60070.1 | lactose permease-related, contains weak similarity to Lactose permease (Lactose-proton symport) (Lactose transport protein). (Swiss-Prot:O33814) (Staphylococcus xylosus) | chr3:22194220-22197305 REVERSE | Aliases: T2O9.50	7.9	7.3	0.6	3.4	56.5%	-1.2
654	AT3G19740.1 | similar to AAA-type ATPase family protein [Arabidopsis thaliana] (TAIR:At1g50140.1); similar to spastin-like [Oryza sativa (japonica cultivar-group)] (GB:BAD37292.1); contains InterPro domain AAA ATPase (InterPro:IPR003593); contains InterPro domain AAA ATPase, central region (InterPro:IPR003959) | chr3:6855843-6859040 REVERSE | Aliases: MMB12.22	9.2	9.9	-0.7	-3.4	56.6%	-1.2
655	AT1G65060.2 | Symbol: 4CL3 | 4-coumarate--CoA ligase 3 / 4-coumaroyl-CoA synthase 3 (4CL3), identical to SP:Q9S777 4-coumarate--CoA ligase 3 (EC 6.2.1.12) (4CL 3) (4-coumaroyl-CoA synthase 3) {Arabidopsis thaliana} | chr1:24170890-24175165 REVERSE | Aliases: 4 COUMARATE:COA LIGASE, F16G16.6, F16G16_6	4.5	6.0	-1.4	-3.4	56.6%	-1.0
656	AT5G61330.1 | rRNA processing protein-related, contains weak similarity to rRNA processing protein EBP2 (EBNA1-binding protein homolog) (Swiss-Prot:P36049) (Saccharomyces cerevisiae)	8.1	7.3	0.8	3.4	56.6%	-1.2
657	AT5G10180.1 | Symbol: AST68 | sulfate transporter, identical to sulfate transporter (Arabidopsis thaliana) GI:2114104	6.0	8.1	-2.1	-3.4	56.7%	-1.0
658	AT3G47510.1 | expressed protein | chr3:17522084-17522600 FORWARD | Aliases: F1P2.60	8.8	8.0	0.8	3.4	56.7%	-1.3
659	AT1G02300.1 | cathepsin B-like cysteine protease, putative, similar to cathepsin B-like cysteine proteinase GI:609175 from (Nicotiana rustica)	6.9	5.5	1.4	3.4	56.7%	-1.1
660	AT2G30990.2 | similar to expressed protein [Arabidopsis thaliana] (TAIR:At1g29240.1); similar to B1131G08.12 [Oryza sativa (japonica cultivar-group)] (GB:NP_916200.1); contains InterPro domain Protein of unknown function DUF688 (InterPro:IPR007789) | chr2:13194660-13197496 REVERSE | Aliases: F7F1.20, F7F1_20	5.8	4.8	1.0	3.4	56.7%	-1.2
661	AT1G14685.3 | expressed protein | chr1:5042583-5044289 FORWARD | Aliases: F10B6.5, F10B6_5	7.0	6.2	0.8	3.4	56.7%	-1.2
662	AT5G05730.1 | Symbol: ASA1 | anthranilate synthase, alpha subunit, component I-1 (ASA1), identical to SP:P32068 | chr5:1719574-1722904 REVERSE | Aliases: A METHYL TRYPTOPHAN RESISTANT 1, AMT1, ANTHRANILATE SYNTHASE ALPHA SUBUNIT, ANTHRANILATE SYNTHASE ALPHA SUBUNIT 1, MJJ3.14, MJJ3_14, TRP5, TRYPTOPHAN BIOSYNTHESIS 5, WEI2	10.4	11.1	-0.7	-3.4	56.7%	-1.5
663	AT4G30190.1 | Symbol: AHA2 | ATPase 2, plasma membrane-type, putative / proton pump 2, putative / proton-exporting ATPase, putative, strong similarity to SP:P19456 ATPase 2, plasma membrane-type (EC 3.6.3.6) (Proton pump 2) {Arabidopsis thaliana}; contains InterPro accession IPR001757: ATPase, E1-E2 type; contains Pfam profile PF00690: Cation transporter/ATPase, N-terminus | chr4:14770505-14776059 REVERSE | Aliases: F9N11.40, F9N11_40, P TYPE H(+) ATPASE ISOFORM 2, PMA2	10.4	11.5	-1.1	-3.4	57.0%	-1.4
664	AT3G60420.1 | expressed protein | chr3:22345406-22346760 FORWARD | Aliases: T8B10.80	3.8	2.4	1.3	3.4	57.1%	-1.2
665	AT1G10780.1 | F-box family protein, ; similar to SKP1 interacting partner 2 (SKIP2) TIGR_Ath1:At5g67250 | chr1:3592747-3595078 REVERSE | Aliases: T16B5.8	3.3	3.9	-0.6	-3.4	57.1%	-1.4
666	AT4G30850.2 | expressed protein, contains Pfam domain, PF03006: Uncharacterised protein family (Hly-III / UPF0073) | chr4:15020188-15022451 REVERSE | Aliases: F6I18.240, F6I18_240	6.9	8.0	-1.1	-3.4	57.1%	-1.1
667	AT1G43700.1 | Symbol: VIP1 | VirE2-interacting protein (VIP1), identical to VirE2-interacting protein VIP1 GB:AAF37279 GI:7258340 from (Arabidopsis thaliana)	8.2	6.8	1.4	3.4	57.1%	-1.1
668	AT4G09010.1 | L-ascorbate peroxidase, chloroplast, putative, identical to SP:P82281:TL29_ARATH (EC 1.11.1.11) {Arabidopsis thaliana}; ascorbate peroxidase - Spinacia oleracea, (gi:1669585); contains Pfam domain PF00141: Peroxidase; contains TIGRfam domain TIGR01409: Tat (twin-arginine translocation) pathway signal sequence; identical to ascorbate peroxidase APX4 (AT4g09010) mRNA, partial cds GI:31980499 | chr4:5777080-5779418 REVERSE | Aliases: F23J3.40, F23J3_40	8.4	9.7	-1.4	-3.4	57.4%	-1.1

Rank	Description	15.dpi	5.dpi	M	t	adj.q	B
669	AT1G11360.2 | universal stress protein (USP) family protein, contains Pfam PF00582: universal stress protein family domain; similar to ethylene-responsive ER6 protein (GI:5669654) (Lycopersicon esculentum) | chr1:3821529-3823053 REVERSE | Aliases: T23J18.35, T23J18_35	10.1	9.0	1.0	3.4	57.4%	-1.5
670	AT5G54970.1 | expressed protein | chr5:22330436-22330990 FORWARD | Aliases: MBG8.24, MBG8_24	5.1	8.0	-2.9	-3.4	57.5%	-1.1
671	AT3G10420.2 | sporulation protein-related, similar to hypothetical proteins: GB:P51281 (Chloroplast Porphyra purpurea), GB:BAA16982 (Synechocystis sp), GB:P49540 (Odontella sinensis), GB:AAB82669 (Chloroplast Cyanidium caldarium); similar to stage III sporulation protein AA (GI:18145497) (Clostridium perfringens str. 13); similar to stage III sporulation protein AA (mutants block sporulation after engulfment) (GI:22777578) (Oceanobacillus iheyensis) | chr3:3239185-3242542 FORWARD | Aliases: F13M14.30	5.3	4.2	1.1	3.4	57.6%	-1.1
672	AT4G17350.1 | expressed protein | chr4:9701144-9703838 FORWARD | Aliases: DL4710W, FCAALL.415	8.0	7.1	0.8	3.4	57.8%	-1.2
673	AT3G05020.1 | Symbol: ACP1 | acyl carrier protein 1, chloroplast (ACP-1), identical to SP:P11829 Acyl carrier protein 1, chloroplast precursor (ACP) {Arabidopsis thaliana} | chr3:1391658-1392960 REVERSE | Aliases: ACP, ACYL CARRIER PROTEIN, T9J14.3, T9J14_3	6.8	8.6	-1.8	-3.4	57.8%	-1.1
674	AT3G21770.1 | peroxidase 30 (PER30) (P30) (PRXR9), identical to SP:Q9LSY7 Peroxidase 30 precursor (EC 1.11.1.7) (Atperox P30) (PRXR9) (ATP7a) {Arabidopsis thaliana} | chr3:7673283-7674846 FORWARD | Aliases: MSD21.10	7.7	8.8	-1.1	-3.4	57.8%	-1.1
675	AT5G24260.1 | prolyl oligopeptidase family protein, similar to dipeptidyl peptidase IV (Stenotrophomonas maltophilia) GI:1753197; contains Pfam profiles PF00326: prolyl oligopeptidase family, PF00930: Dipeptidyl peptidase IV (DPP IV) N-terminal region | chr5:8234726-8238246 REVERSE | Aliases: MOP9.8, MOP9_8	6.0	6.9	-0.8	-3.4	58.0%	-1.2
676	ATMG01370.1 | Symbol: ORF111D | hypothetical protein | chrM:360717-361052 REVERSE | Aliases: ORF111D	5.2	8.3	-3.1	-3.4	58.3%	-1.1
677	AT2G02020.1 | proton-dependent oligopeptide transport (POT) family protein, contains Pfam profile: PF00854 POT family | chr2:479100-481184 FORWARD | Aliases: F14H20.9, F14H20_9	5.5	4.5	1.1	3.4	58.3%	-1.1
678	AT3G55480.2 | adaptin family protein, similar to AP-3 complex beta3A subunit, Homo sapiens, SP:O00203; contains Pfam profile: PF01602 Adaptin N terminal region | chr3:20577226-20582227 REVERSE | Aliases: T22E16.140	4.9	6.2	-1.3	-3.4	58.3%	-1.1
679	AT2G21180.1 | expressed protein | chr2:9081741-9082461 REVERSE | Aliases: F26H11.6, F26H11_6	6.5	5.7	0.8	3.4	58.3%	-1.2
680	AT5G03760.1 | Symbol: ATCSLA09 | glycosyl transferase family 2 protein, similar to beta-(1-3)-glucosyl transferase GB:AAC62210 GI:3687658 from (Bradyrhizobium japonicum), cellulose synthase from Agrobacterium tumeficiens (gi:710492) and Agrobacterium radiobacter (gi:710493); contains Pfam glycosyl transferase, group 2 family protein domain PF00535	3.8	4.7	-0.9	-3.4	58.3%	-1.1
681	AT4G21560.3 | vacuolar protein sorting-associated protein 28 family protein / VPS28 family protein, contains similarity to Swiss-Prot:Q02767 vacuolar protein sorting-associated protein VPS28 (Saccharomyces cerevisiae) | chr4:11468773-11470490 REVERSE | Aliases: F17L22.20	6.5	5.7	0.8	3.4	58.3%	-1.4
682	AT1G45130.1 | beta-galactosidase, putative / lactase, putative, similar to beta-galactosidase (Lycopersicon esculentum) GI:7939619, beta-galactosidase BG1 GI:15081596 from (Vitis vinifera); contains Pfam profile PF01301: Glycosyl hydrolases family 35 | chr1:17067692-17071702 FORWARD | Aliases: F27F5.20, F27F5_20	8.8	9.8	-1.0	-3.4	58.3%	-1.3
683	AT1G73540.1 | MutT/nudix family protein, low similarity to SP:Q09790 Diadenosine 5',5'''-P1,P6-hexaphosphate hydrolase (EC 3.6.1.-) (Ap6A hydrolase) {Schizosaccharomyces pombe}; contains Pfam profile PF00293: NUDIX domain | chr1:27649322-27650359 REVERSE | Aliases: T9L24.30, T9L24_30	6.0	5.1	1.0	3.4	58.4%	-1.2
684	AT2G46550.1 | expressed protein | chr2:19119061-19121224 REVERSE | Aliases: F13A10.8	7.4	6.3	1.1	3.4	58.7%	-1.2
685	AT3G07180.2 | GPI transamidase component PIG-S-related, similar to GPI transamidase component PIG-S (Phosphatidylinositol-glycan biosynthesis, class S protein) (Swiss-Prot:Q96S52) (Homo sapiens) | chr3:2282094-2285519 REVERSE | Aliases: T1B9.16	7.3	8.0	-0.7	-3.4	58.9%	-1.3
686	AT4G22730.1 | leucine-rich repeat transmembrane protein kinase, putative, leucine rich repeat receptor-like kinase, Oryza sativa, PATCHX:E267533 | chr4:11941395-11943750 FORWARD | Aliases: T12H17.120, T12H17_120	3.2	4.0	-0.7	-3.4	58.9%	-1.3
687	AT5G08410.1 | ferredoxin-thioredoxin reductase, putative, similar to ferredoxin-thioredoxin reductase, variable chain (FTR-V, Ferredoxin- thioredoxin reductase subunit A, FTR-A) (Zea mays) SWISS-PROT:P80680	5.8	4.9	0.8	3.4	58.9%	-1.3
688	AT5G10540.1 | peptidase M3 family protein / thimet oligopeptidase family protein, similar to SP:P27237 Oligopeptidase A (EC 3.4.24.70) {Salmonella typhimurium}; contains Pfam profile PF01432: Peptidase family M3 | chr5:3328093-3334972 FORWARD | Aliases: F12B17.110, F12B17_110	7.8	9.6	-1.8	-3.4	58.9%	-1.1
689	AT4G30900.1 | expressed protein | chr4:15039514-15042352 FORWARD | Aliases: F6I18.190, F6I18_190	8.1	6.9	1.2	3.4	58.9%	-1.2

Rank	Description	15.dpi	5.dpi	M	t	adj.q	B
690	AT3G20720.1 | expressed protein | chr3:7239914-7247011 FORWARD | Aliases: MOE17.2	5.0	6.5	-1.5	-3.4	58.9%	-1.2
691	AT1G19380.1 | expressed protein | chr1:6698051-6698788 FORWARD | Aliases: F18O14.10, F18O14_10	7.1	5.9	1.2	3.4	59.1%	-1.2
692	AT2G45490.1 | Encodes a member of a family of Ser/Thr kinases whose activities peak during cell division. Transcripts are abundant in tissues rich in dividing cells like roots and flowers but are low or absent in fully expanded leaves and stems.	2.4	3.0	-0.6	-3.4	59.3%	-1.5
693	AT5G61230.1 | ankyrin repeat family protein, contains ankyrin repeats, Pfam:PF00023 | chr5:24644413-24646174 FORWARD | Aliases: MAF19.22, MAF19_22	8.5	7.6	0.9	3.3	59.3%	-1.2
694	AT5G65360.1 | histone H3, identical to histone H3 from Zea mays SP:P05203, Medicago sativa GI:166384, Encephalartos altensteinii SP:P08903, Pisum sativum SP:P02300; contains Pfam profile PF00125 Core histone H2A/H2B/H3/H4 | chr5:26137085-26137807 REVERSE | Aliases: MNA5.9	8.5	9.8	-1.3	-3.3	59.5%	-1.2
695	AT5G13310.1 | expressed protein | chr5:4263496-4265340 FORWARD | Aliases: T22N19.1	5.6	4.8	0.7	3.3	59.5%	-1.3
696	AT4G05070.1 | expressed protein | chr4:2593596-2594117 REVERSE | Aliases: C17L7.2	8.9	7.8	1.0	3.3	59.7%	-1.2
697	AT1G72280.1 | Symbol: AERO1 | endoplasmic reticulum oxidoreductin 1 (ERO1) family protein, contains Pfam domain, PF04137: Endoplasmic Reticulum Oxidoreductin 1 (ERO1) | chr1:27215513-27218168 REVERSE | Aliases: T9N14.18, T9N14_18	3.3	4.0	-0.8	-3.3	59.7%	-1.4
698	AT5G27980.1 | seed maturation family protein, similar to SP:P09444 Late embryogenesis abundant protein D-34 (LEA D-34) {Gossypium hirsutum}; contains Pfam profile PF04927: Seed maturation protein | chr5:10015803-10016704 REVERSE | Aliases: F15F15.50, F15F15_50	3.2	2.6	0.6	3.3	59.7%	-1.4
699	AT1G70700.2 | expressed protein, similar to expressed protein [Arabidopsis thaliana] (TAIR:At1g48500.1) | chr1:26658445-26660726 FORWARD | Aliases: F5A18.12, F5A18_12	2.7	2.3	0.4	3.3	59.7%	-1.9
700	AT3G02040.1 | Symbol: SRG3 | glycerophosphoryl diester phosphodiesterase family protein, contains Pfam profile PF03009: Glycerophosphoryl diester phosphodiesterase family | chr3:348174-350006 REVERSE | Aliases: F1C9.18, F1C9_18, SENESCENCE RELATED GENE 3	5.8	4.9	0.9	3.3	59.8%	-1.2
701	AT3G15590.1 | DNA-binding protein, putative, similar to DNA-binding protein (Triticum aestivum) GI:6958202; contains Pfam profile: PF01535 PPR repeat | chr3:5275408-5277994 REVERSE | Aliases: MQD17.5	10.1	10.7	-0.7	-3.3	59.8%	-1.5
702	AT2G38280.2 | Symbol: FAC1 | AMP deaminase, putative / myoadenylate deaminase, putative, similar to SP:P15274 AMP deaminase (EC 3.5.4.6) (Myoadenylate deaminase) {Saccharomyces cerevisiae}; contains Pfam profile PF00962: Adenosine/AMP deaminase | chr2:16040579-16045954 REVERSE | Aliases: EMBRYONIC FACTOR1, F16M14.21, F16M14_21	7.0	8.1	-1.1	-3.3	59.9%	-1.1
703	AT3G12280.1 | Symbol: RBR1 | retinoblastoma-related protein (RBR1), nearly identical to retinoblastoma-related protein (Arabidopsis thaliana) GI:8777927; contains Pfam profiles: PF01858 retinoblastoma-associated protein A domain, PF01857 retinoblastoma-associated protein B domain | chr3:3913419-3919087 REVERSE | Aliases: F28J15.11, RB, RBL1, RETINOBLASTOMA PROTEIN, RETINOBLASTOMA RELATED 1	5.2	6.4	-1.2	-3.3	59.9%	-1.2
704	AT1G10840.2 | Symbol: TIF3H1 |	8.1	9.4	-1.3	-3.3	59.9%	-1.2
705	AT4G35985.1 | senescence/dehydration-associated protein-related, similar to senescence-associated protein 12 (Hemerocallis hybrid cultivar) gi:3551958:gb:AAC34857; similar to early-responsive to dehydration stress ERD7 protein (Arabidopsis thaliana) gi:15320412:dbj:BAB63916 | chr4:17032271-17033865 REVERSE | Aliases: None	10.2	9.1	1.1	3.3	59.9%	-1.4
706	AT3G15353.1 | Symbol: MT3 | metallothionein protein, putative	13.0	11.1	1.9	3.3	60.0%	-1.2
707	AT1G78670.1 | gamma-glutamyl hydrolase, putative / gamma-Glu-X carboxypeptidase, putative / conjugase, putative, similar to gamma glutamyl hydrolase GI:1679658 SP:P93164 from (Glycine max) | chr1:29595848-29598392 FORWARD | Aliases: F9K20.29, F9K20_29	9.2	7.8	1.4	3.3	60.0%	-1.2
708	AT3G24360.1 | enoyl-CoA hydratase/isomerase family protein, similar to CHY1 (gi:8572760); contains Pfam profile PF00388 enoyl-CoA hydratase/isomerase family protein | chr3:8839735-8842838 REVERSE | Aliases: K7M2.21	4.2	5.2	-1.0	-3.3	60.0%	-1.2
709	AT1G70740.1 | protein kinase family protein, contains protein kinase domain, Pfam:PF00069 | chr1:26677294-26679543 REVERSE | Aliases: F5A18.8, F5A18_8	4.8	3.9	0.9	3.3	60.0%	-1.4
710	AT5G49910.1 | Symbol: cpHSC70 2 | heat shock protein 70 / HSP70 (HSC70-7), identical to heat shock protein 70 (Arabidopsis thaliana) GI:6746592 | chr5:20320640-20324039 FORWARD | Aliases: HEAT SHOCK PROTEIN 70, HEAT SHOCK PROTEIN 70 7, HSC70 7, K9P8.5, K9P8_5	8.7	9.8	-1.1	-3.3	60.0%	-1.2

Rank	Description	15.dpi	5.dpi	M	t	adj.q	B
711	AT2G19610.2 | zinc finger (C3HC4-type RING finger) family protein, contains a zinc finger, C3HC4 type (RING finger), signature, PROSITE:PS00518 | chr2:8491067-8493110 FORWARD | Aliases: F3P11.21, F3P11_21	4.7	3.8	0.9	3.3	60.0%	-1.3
712	AT4G31810.1 | enoyl-CoA hydratase/isomerase family protein, similar to CHY1 (gi:8572760); contains Pfam profile PF00388 enoyl-CoA hydratase/isomerase family protein | chr4:15387131-15390340 REVERSE | Aliases: F11C18.10, F11C18_10	7.1	8.8	-1.8	-3.3	60.3%	-1.2
713	AT4G28470.1 | similar to 26S proteasome regulatory subunit S2 (RPN1) [Arabidopsis thaliana] (TAIR:At2g20580.1); similar to Unknown (protein for MGC:83116) [Xenopus laevis] (GB:AAH68911.1); similar to MGC97650 protein [Xenopus tropicalis] (GB:NP_001015819.1); similar to putative 26S proteasome regulatory subunit S2 [Oryza sativa (japonica cultivar-group)] (GB:BAD54002.1); similar to putative proteasome 26S non-ATPase subunit 2 [Oryza sativa (japonica cultivar-group)] (GB:XP_450542.1); similar to MGC83233 protein [Xenopus laevis] (GB:AAH68957.1); contains InterPro domain Proteasome/cyclosome, regulatory subunit (InterPro:IPR002015) | chr4:14066882-14072549 REVERSE | Aliases: F20O9.150, F20O9_150	8.3	9.3	-1.0	-3.3	60.3%	-1.2
714	AT5G05440.1 | expressed protein, low similarity to cytokinin-specific binding protein (Vigna radiata) GI:4190976 | chr5:1609251-1610447 FORWARD | Aliases: K18I23.25, K18I23_25	7.8	6.4	1.4	3.3	60.3%	-1.1
715	AT5G64450.1 | expressed protein, contains Pfam profile PF04396: Protein of unknown function, DUF537 | chr5:25789175-25789987 FORWARD | Aliases: T12B11.4, T12B11_4	4.8	3.6	1.2	3.3	60.3%	-1.2
716	AT1G05950.1 | expressed protein | chr1:1804572-1807470 REVERSE | Aliases: T21E18.2, T21E18_2	3.7	4.9	-1.2	-3.3	60.3%	-1.2
717	AT4G30820.2 | cyclin-dependent kinase-activating kinase assembly factor-related / CDK-activating kinase assembly factor-related, contains similarity to CDK-activating kinase assembly factor MAT1 (RING finger protein MAT1) (Menage a trois) (CDK7/cyclin H assembly factor) (Swiss-Prot:P51950) (Marthasterias glacialis) | chr4:15006500-15008542 FORWARD | Aliases: F6I18.270, F6I18_270	5.5	4.6	0.9	3.3	60.3%	-1.3
718	AT2G21970.1 | Symbol: SEP2 | stress enhanced protein 2 (SEP2), nearly identical to stress enhanced protein 2; SEP2 (GI:7384980) (Arabidopsis thaliana) | chr2:9364063-9364996 REVERSE | Aliases: F7D8.29, F7D8_29, SEP2, STRESS ENHANCED PROTEIN 2	9.1	8.1	1.0	3.3	60.4%	-1.2
719	AT4G27970.1 | C4-dicarboxylate transporter/malic acid transport family protein, contains Pfam profile PF03595: C4-dicarboxylate transporter/malic acid transport protein | chr4:13918296-13920138 REVERSE | Aliases: T13J8.80, T13J8_80	7.6	8.6	-0.9	-3.3	60.5%	-1.3
720	AT4G35770.1 | Symbol: SEN1 | senescence-associated protein (SEN1), identical to senescence-associated protein GI:1046270 from (Arabidopsis thaliana) | chr4:16944946-16946096 FORWARD | Aliases: ATSEN1, DARK INDUCIBLE 1, DIN1, F4B14.3	3.1	2.5	0.6	3.3	60.5%	-1.5
721	AT1G49170.1 | expressed protein | chr1:18186934-18188102 REVERSE | Aliases: F27J15.6, F27J15_6	4.3	3.1	1.1	3.3	60.5%	-1.4
722	AT3G19130.1 | Symbol: ATRBP47B | RNA-binding protein, putative, similar to RNA Binding Protein 47 (Nicotiana plumbaginifolia) GI:9663769, DNA binding protein ACBF GB:AAC49850 from (Nicotiana tabacum); contains InterPro entry IPR000504: RNA-binding region RNP-1 (RNA recognition motif) (RRM) | chr3:6611219-6614050 REVERSE | Aliases: ATRBP47B, MVI11.3	9.5	8.6	0.9	3.3	60.5%	-1.4
723	AT1G66260.1 | RNA and export factor-binding protein, putative, similar to GI:7159943 from (Mus musculus) (RNA 6 (4), 638-650 (2000)) | chr1:24699303-24702093 REVERSE | Aliases: T6J19.1, T6J19_1	10.2	9.3	0.8	3.3	60.5%	-1.4
724	AT3G61980.1 | serine protease inhibitor, Kazal-type family protein, contains Pfam domain PF00050: Kazal-type serine protease inhibitor domain | chr3:22967114-22967858 REVERSE | Aliases: F21F14.150	7.8	6.7	1.1	3.3	60.5%	-1.3
725	AT2G42330.1 | D111/G-patch domain-containing protein, similar to SP:Q9ERA6 Tuftelin-interacting protein 11 {Mus musculus}; contains Pfam profile PF01585: G-patch domain | chr2:17638909-17641167 REVERSE | Aliases: MHK10.5, MHK10_5	5.1	4.3	0.8	3.3	60.5%	-1.3
726	AT1G74930.1 | encodes a member of the DREB subfamily A-5 of ERF/AP2 transcription factor family. The protein contains one AP2 domain. There are 15 members in this subfamily including RAP2.1, RAP2.9 and RAP2.10. | chr1:28147793-28148710 FORWARD | Aliases: F25A4.10, F25A4_10	4.0	3.3	0.7	3.3	60.5%	-1.4
727	AT5G24330.1 | PHD finger family protein / SET domain-containing protein, contains Pfam domain, PF00628: PHD-finger and PF00856: SET domain | chr5:8295248-8297000 REVERSE | Aliases: MOP9.7, MOP9_7	2.9	3.6	-0.7	-3.3	60.5%	-1.4
728	AT1G13220.2 | nuclear matrix constituent protein-related, similar to nuclear matrix constituent protein 1 (NMCP1) (Daucus carota) GI:2190187 | chr1:4515696-4520281 FORWARD | Aliases: F3F19.25, F3F19_25	5.2	6.4	-1.2	-3.3	60.5%	-1.2
729	AT1G10470.1 | Symbol: ARR4 | two-component responsive regulator / response regulator 4 (ARR4), identical to responce regulator1 GI:3273195 from (Arabidopsis thaliana); identical to cDNA ARR4 mRNA for response regulator 4 GI:3953596 | chr1:3442328-3443958 REVERSE | Aliases: ATRR1, IBC7, INDUCED BY CYTOKININ 7, RESPONSE REGULATOR 4, T10O24.8, T10O24_8	5.8	7.1	-1.3	-3.3	60.5%	-1.2

Rank	Description	15.dpi	5.dpi	M	t	adj.q	B
730	AT1G74690.1 | calmodulin-binding family protein, low similarity to SF16 protein (Helianthus annuus) GI:560150; contains Pfam profile PF00612: IQ calmodulin-binding motif | chr1:28064275-28068286 REVERSE | Aliases: F25A4.33, F25A4_33	5.9	7.3	-1.4	-3.3	60.6%	-1.2
731	AT5G46350.1 | Symbol: WRKY8 | WRKY family transcription factor, contains similarity to WRKY-type DNA-binding protein | chr5:18818445-18821267 REVERSE | Aliases: ATWRKY8, MPL12.15, MPL12_15, WRKY8	2.6	3.1	-0.4	-3.3	60.6%	-1.7
732	AT1G70550.2 | expressed protein, similar to hypothetical protein GB:AAD31338 (Arabidopsis thaliana) and to putative putative carboxyl-terminal peptidase GB:AAC16072 (Arabidopsis thaliana) contains Pfam profile PF03080: Arabidopsis proteins of unknown function | chr1:26600815-26603882 FORWARD | Aliases: F24J13.12, F24J13_12	4.0	5.1	-1.0	-3.3	60.6%	-1.2
733	AT4G23260.1 | protein kinase family protein, contains Pfam domain PF00069: Protein kinase domain | chr4:12167433-12169904 REVERSE | Aliases: F21P8.150, F21P8_150	2.6	3.5	-0.9	-3.3	60.6%	-1.5
734	AT5G67180.1 | AP2 domain-containing transcription factor, putative, similar to (SP:P47927) Floral homeotic protein APETALA2. (Mouse-ear cress) {Arabidopsis thaliana} | chr5:26819233-26821245 REVERSE | Aliases: K21H1.22, K21H1_22	4.2	3.4	0.8	3.3	60.6%	-1.4
735	AT1G11260.1 | glucose transporter (STP1), nearly identical to glucose transporter GB:P23586 SP:P23586 from (Arabidopsis thaliana) | chr1:3777329-3780337 FORWARD | Aliases: T28P6.9, T28P6_9	7.2	5.5	1.7	3.3	60.6%	-1.2
736	AT3G07540.1 | formin homology 2 domain-containing protein / FH2 domain-containing protein, contains formin homology 2 domain, Pfam:PF02181 | chr3:2404515-2407547 REVERSE | Aliases: F21O3.25	5.5	6.8	-1.3	-3.3	60.6%	-1.2
737	AT2G28790.1 | osmotin-like protein, putative, similar to SP:Q41350 Osmotin-like protein precursor {Lycopersicon esculentum}; contains Pfam profile PF00314: Thaumatin family | chr2:12361507-12362508 REVERSE | Aliases: F8N16.8, F8N16_8	3.9	5.2	-1.3	-3.3	60.6%	-1.2
738	AT2G41430.4 | Symbol: ERD15 | dehydration-induced protein (ERD15), identical to dehydration-induced protein ERD15 GI:710626 from (Arabidopsis thaliana) | chr2:17276388-17277620 FORWARD | Aliases: CID1, DEHYDRATION INDUCED PROTEIN, EARLY RESPONSIVE TO DEHYDRATION 15, LIGHT STRESS REGULATED 1, LSR1, T26J13.2	11.8	10.8	1.0	3.3	60.6%	-1.3
739	AT1G33030.1 | O-methyltransferase family 2 protein, similar to caffeic acid 3-O-methyltransferase (SP:Q00763) (Populus tremuloides), catechol O-methyltransferase (GI:4808524)(Thalictrum tuberosum) | chr1:11964756-11966256 REVERSE | Aliases: F9L11.18, F9L11_18	4.9	6.5	-1.7	-3.3	60.6%	-1.2
740	AT5G55300.1 | DNA topoisomerase I, identical to Swiss-Prot:P30181 DNA topoisomerase I (Arabidopsis thaliana) | chr5:22441812-22446664 REVERSE | Aliases: MTE17.1, MTE17_1	6.0	6.8	-0.8	-3.3	60.7%	-1.4
741	AT1G72030.1 | GCN5-related N-acetyltransferase (GNAT) family protein, contains Pfam profile PF00583: acetyltransferase, GNAT family | chr1:27114527-27115868 FORWARD | Aliases: F28P5.8, F28P5_8	8.8	7.0	1.8	3.3	60.9%	-1.4
742	AT3G04720.1 | Symbol: PR4 | hevein-like protein (HEL), identical to SP:P43082 Hevein-like protein precursor {Arabidopsis thaliana}; similar to SP:P09762 Wound-induced protein WIN2 precursor {Solanum tuberosum}; contains Pfam profile PF00187: Chitin recognition protein | chr3:1285573-1286568 REVERSE | Aliases: F7O18.21, F7O18_21, HEL, HEVEIN LIKE, PATHOGENESIS RELATED 4, PR 4	6.1	7.4	-1.4	-3.3	61.0%	-1.2
743	AT5G22650.2 | Symbol: HD2B | expressed protein, non-consensus AT donor splice site at exon 3, AC acceptor splice site at exon 4; | chr5:7534067-7536276 FORWARD | Aliases: ATHD2B, HD2, HDA4, HDT02, HDT2, HISTONE DEACETYLASE, HISTONE DEACETYLASE 2, HISTONE DEACETYLASE 2B, MDJ22.7, MDJ22_7	9.0	10.0	-1.0	-3.3	61.1%	-1.3
744	AT1G12320.1 | expressed protein | chr1:4189353-4190340 FORWARD | Aliases: F5O11.5, F5O11_5	7.3	6.1	1.1	3.3	61.1%	-1.3
745	AT5G35360.2 | Symbol: CAC2 | similar to methylcrotonyl-CoA carboxylase alpha chain, mitochondrial / 3-methylcrotonyl-CoA carboxylase 1 (MCCA) [Arabidopsis thaliana] (TAIR:At1g03090.2); similar to biotin carboxylase precursor [Glycine max] (GB:AAC23573.1); similar to biotin carboxylase precursor [Glycine max] (GB:AAC02267.1); similar to acetyl-CoA carboxylase [Glycine max] (GB:AAF80469.1); similar to biotin carboxylase subunit (GB:AAC41659.1); contains InterPro domain Carbamoyl-phosphate synthetase large chain, N-terminal (InterPro:IPR005481); contains InterPro domain Acetyl-CoA carboxylase, biotin carboxylase (InterPro:IPR004549); contains InterPro domain Biotin carboxylase, C-terminal (InterPro:IPR005482); contains InterPro domain Carbamoyl-phosphate synthase L chain, ATP-binding (InterPro:IPR005479) | chr5:13601388-13606337 FORWARD | Aliases: ACETYL COA CARBOXYLASE, T26D22.8, T26D22_8	10.3	11.2	-0.9	-3.3	61.1%	-1.7
746	AT5G04750.1 | F1F0-ATPase inhibitor protein, putative, similar to F1F0-ATPase inhibitor protein (Oryza sativa (japonica cultivar-group)) gi:5106371:dbj:BAA81661 | chr5:1372148-1374006 FORWARD | Aliases: MUK11.3	11.5	11.0	0.5	3.3	61.2%	-1.7
747	AT4G31210.1 | DNA topoisomerase family protein, similar to DNA Topoisomerase I (SP:Q9X3X7) {Zymomonas mobilis}	4.1	5.2	-1.1	-3.3	61.3%	-1.3

Rank	Description	15.dpi	5.dpi	M	t	adj.q	B
748	AT4G30470.1 | cinnamoyl-CoA reductase-related, similar to cinnamoyl-CoA reductase from Pinus taeda (GI:17978649), Saccharum officinarum (GI:3341511) | chr4:14894111-14896819 FORWARD | Aliases: F17I23.190, F17I23_190	8.1	7.2	0.9	3.3	61.3%	-1.4
749	AT1G02700.1 | expressed protein, similar to hypothetical protein GB:CAB80707 GI:7268598 from (Arabidopsis thaliana) | chr1:588329-589480 FORWARD | Aliases: T14P4.2, T14P4_2	5.0	3.9	1.1	3.3	61.3%	-1.2
750	AT4G01850.1 | S-adenosylmethionine synthetase 2 (SAM2), identical to S-adenosylmethionine synthetase 2 (Methionine adenosyltransferase 2, AdoMet synthetase 2) (Arabidopsis thaliana) SWISS-PROT:P17562 | chr4:796097-798285 REVERSE | Aliases: T7B11.11, T7B11_11	9.7	10.6	-0.9	-3.3	61.3%	-1.4
751	AT3G46940.1 | deoxyuridine 5'-triphosphate nucleotidohydrolase family, contains Pfam profile: PF00692 deoxyuridine 5'-triphosphate nucleotidohydrolase	5.9	7.3	-1.4	-3.3	61.3%	-1.2
752	AT1G67740.1 | Symbol: PSBY | photosystem II core complex proteins psbY, chloroplast (PSBY) / L-arginine metabolising enzyme, identical to SP:O49347 Photosystem II core complex proteins psbY, chloroplast precursor (L-arginine metabolising enzyme) (L-AME) (Contains: Photosystem II protein psbY-1 (psbY-A1); Photosystem II protein psbY-2 (psbY-A2)) (Arabidopsis thaliana) | chr1:25397731-25398817 REVERSE | Aliases: F12A21.13, F12A21_13, YCF32	7.6	5.9	1.7	3.3	61.5%	-1.3
753	AT5G19880.1 | peroxidase, putative, similar to peroxidase (Lycopersicon esculentum) gi:296910:emb:CAA50597 | chr5:6720386-6722477 REVERSE | Aliases: F28I16.30, F28I16_30	2.7	4.1	-1.4	-3.3	61.5%	-1.3
754	AT4G25810.1 | Symbol: XTR6 | xyloglucan:xyloglucosyl transferase, putative / xyloglucan endotransglycosylase, putative / endo-xyloglucan transferase, putative (XTR6), identical to xyloglucan endotransglycosylase-related protein GI:1244758 from (Arabidopsis thaliana) | chr4:13128655-13129893 FORWARD | Aliases: F14M19.90, F14M19_90, XYLOGLUCAN ENDOTRANSGLYCOSYLASE 6	10.6	8.6	2.0	3.3	61.5%	-1.3
755	AT4G30870.1 | similar to repair endonuclease family protein [Arabidopsis thaliana] (TAIR:At5g39770.1); similar to unnamed protein product [Debaryomyces hansenii CBS767] (GB:CAG90230.1); contains InterPro domain ERCC4 domain (InterPro:IPR006166) | chr4:15028592-15032467 FORWARD | Aliases: F6I18.220, F6I18_220	3.1	4.5	-1.4	-3.3	61.5%	-1.2
756	AT2G13360.2 | Symbol: AGT | serine-glyoxylate aminotransferase-related, similar to serine-glyoxylate aminotransferase	3.8	3.1	0.7	3.3	61.5%	-1.4
757	AT1G53400.1 | expressed protein | chr1:19928439-19930268 FORWARD | Aliases: F12M16.29, F12M16_29	9.7	8.9	0.9	3.3	61.5%	-1.3
758	AT4G16670.1 | expressed protein | chr4:9385241-9387776 FORWARD | Aliases: DL4360W, FCAALL.2	4.5	3.8	0.7	3.3	61.5%	-1.5
759	AT5G23460.1 | expressed protein | chr5:7912772-7913498 FORWARD | Aliases: K19M13.9, K19M13_9	9.5	8.5	1.0	3.3	61.6%	-1.2
760	AT3G59670.1 | expressed protein, similar to expressed protein [Arabidopsis thaliana] (TAIR:At4g37440.1); similar to unknown protein [Oryza sativa (japonica cultivar-group)] (GB:AAV32239.1) | chr3:22050454-22053637 FORWARD | Aliases: T16L24.220	4.8	5.9	-1.2	-3.3	61.7%	-1.2
761	AT5G61420.2 | Symbol: MYB28 | myb family transcription factor (MYB28), contains Pfam profile: PF00249 myb-like DNA-binding domain | chr5:24706377-24708021 REVERSE | Aliases: MFB13.22, MFB13_22	7.5	6.0	1.4	3.3	61.9%	-1.3
762	AT3G62110.1 | glycoside hydrolase family 28 protein / polygalacturonase (pectinase) family protein, weak similarity to polygalacturonase (Lycopersicon esculentum) GI:4325090; contains PF00295: Glycosyl hydrolases family 28	4.5	5.1	-0.7	-3.3	62.1%	-1.4
763	AT2G19350.1 | expressed protein | chr2:8383284-8384425 FORWARD | Aliases: F27F23.15, F27F23_15	7.2	6.2	1.1	3.3	62.1%	-1.4
764	AT5G24590.2 | Symbol: TIP | turnip crinkle virus-interacting protein / TCV-interacting protein (TIP), contains Pfam PF02365: No apical meristem (NAM) domain; similar to NAC2 (GI:6456751) {Arabidopsis thaliana}; identical to cDNA TIP mRNA, GI:9408600 | chr5:8416665-8418936 REVERSE | Aliases: ANAC091, K18P6.12, K18P6_12, TCV INTERACTING PROTEIN	5.3	4.7	0.6	3.3	62.1%	-1.5
765	AT3G07150.1 | hypothetical protein | chr3:2263812-2264582 REVERSE | Aliases: T1B9.19	3.9	3.1	0.8	3.3	62.1%	-1.4
766	AT3G25100.1 | Symbol: CDC45 | cell division control protein-related, contains weak similarity to cell division control protein 45 homolog (Suppressor of nda4 protein) (Swiss-Prot:O74113) (Schizosaccharomyces pombe) | chr3:9144006-9146216 FORWARD | Aliases: CELL DIVISION CYCLE 45, MJL12.4	5.0	6.8	-1.8	-3.3	62.2%	-1.3
767	AT1G21460.1 | nodulin MtN3 family protein, contains similarity to MTN3 (nodule development protein) GB:Y08726 GI:1619601 from (Medicago truncatula) | chr1:7511850-7513347 REVERSE | Aliases: F24J8.9, F24J8_9	5.0	3.8	1.1	3.3	62.3%	-1.3
768	AT4G34050.2 | caffeoyl-CoA 3-O-methyltransferase, putative, nearly identical to GI:2960356 (Populus balsamifera subsp. trichocarpa), GI:684942 (Medicago sativa subsp. sativa) isoform contains a GT-TG intron which removes an internal segment of the protein. | chr4:16310820-16311978 FORWARD | Aliases: F28A23.190, F28A23_190	9.2	11.0	-1.8	-3.2	62.3%	-1.3

Rank	Description	15.dpi	5.dpi	M	t	adj.q	B
769	AT2G37470.1 | histone H2B, putative, strong similarity to histone H2B from Lycopersicon esculentum GI:3021483, GI:3021485, Capsicum annuum SP:O49118; contains Pfam profile PF00125 Core histone H2A/H2B/H3/H4	8.0	9.8	-1.8	-3.2	62.3%	-1.3
770	AT3G07470.1 | expressed protein, contains Pfam profile PF04398: Protein of unknown function, DUF538 | chr3:2386903-2388477 REVERSE | Aliases: F21O3.18	6.2	5.5	0.8	3.2	62.3%	-1.4
771	AT1G08560.1 | Symbol: SYP111 |	7.8	6.7	1.1	3.2	62.3%	-1.4
772	AT4G28330.1 | expressed protein, similar to hypothetical protein [Arabidopsis thaliana] (TAIR:At4g28340.1) | chr4:14022393-14022993 REVERSE | Aliases: F20O9.10, F20O9_10	7.4	5.8	1.6	3.2	62.3%	-1.2
773	AT1G25550.1 | myb family transcription factor, contains Pfam domain, PF00249: Myb-like DNA-binding domain | chr1:8976525-8978080 FORWARD | Aliases: F2J7.21, F2J7_21	6.5	7.4	-0.9	-3.2	62.7%	-1.6
774	AT3G23330.1 | pentatricopeptide (PPR) repeat-containing protein, contains INTERPRO:IPR002885 PPR repeats | chr3:8347207-8349354 FORWARD | Aliases: MLM24.6	4.3	5.5	-1.2	-3.2	62.7%	-1.3
775	AT1G31750.1 | proline-rich family protein, contains proline rich extensin domains, INTERPRO:IPR002965 | chr1:11370640-11372058 REVERSE | Aliases: F5M6.24	5.2	3.7	1.6	3.2	62.7%	-1.3
776	AT2G37540.1 | short-chain dehydrogenase/reductase (SDR) family protein, contains INTERPRO family IPR002198 Short-chain dehydrogenase/reductase (SDR) superfamily | chr2:15758653-15761045 REVERSE | Aliases: F13M22.4, F13M22_4	3.7	2.8	0.9	3.2	62.7%	-1.6
777	AT1G31170.1 | parB-like nuclease domain-containing protein, contains Pfam profile PF02195: ParB-like nuclease domain | chr1:11133470-11134386 FORWARD | Aliases: F28K20.12, F28K20_12	8.4	7.4	1.1	3.2	62.7%	-1.3
778	AT5G67130.1 | expressed protein | chr5:26808592-26811253 FORWARD | Aliases: K21H1.9, K21H1_9	6.1	7.0	-0.9	-3.2	62.7%	-1.4
779	AT2G21100.1 | disease resistance-responsive protein-related / dirigent protein-related, similar to dirigent protein (Thuja plicata) gi:6694699:gb:AAF25360; similar to disease resistance response protein 206-d (Pisum sativum) gi:508844:gb:AAB18669	3.0	4.3	-1.4	-3.2	62.7%	-1.3
780	AT2G03280.2 | similar to expressed protein [Arabidopsis thaliana] (TAIR:At1g14020.1); similar to putative auxin-independent growth promoter [Oryza sativa (japonica cultivar-group)] (GB:BAD69015.1); contains InterPro domain Hypothetical plant protein (InterPro:IPR004348)	5.4	6.2	-0.8	-3.2	62.7%	-1.5
781	AT4G34590.1 | Symbol: GBF6 | bZIP transcription factor family protein, similar to common plant regulatory factor 7 GI:9650828 from (Petroselinum crispum) | chr4:16521866-16523235 FORWARD | Aliases: T4L20.170, T4L20_170	7.9	6.0	2.0	3.2	62.7%	-1.3
782	AT4G13195.1 | expressed protein | chr4:7662367-7663173 REVERSE | Aliases: None	2.7	3.4	-0.7	-3.2	62.7%	-1.6
783	AT3G58790.1 | glycosyl transferase family 8 protein, contains Pfam profile: PF01501 glycosyl transferase family 8; general stress protein gspA, Bacillus subtilis, PIR:S16423 | chr3:21753048-21757301 REVERSE | Aliases: T20N10.140	5.2	4.3	0.9	3.2	62.7%	-1.4
784	AT1G64690.1 | expressed protein | chr1:24041732-24042742 FORWARD | Aliases: F13O11.1, F13O11_1	5.5	4.5	1.1	3.2	62.7%	-1.3
785	AT2G48120.1 | Symbol: PAC | pale cress protein (PAC), identical to PAC (Arabidopsis thaliana) gi:1483213:emb:CAA65334	3.0	3.6	-0.6	-3.2	63.2%	-1.6
786	AT4G32800.1 | encodes a member of the DREB subfamily A-4 of ERF/AP2 transcription factor family. The protein contains one AP2 domain. There are 17 members in this subfamily including TINY. | chr4:15819528-15820875 FORWARD | Aliases: T16I18.10, T16I18_10	4.0	3.4	0.5	3.2	63.2%	-1.6
787	AT3G28340.1 | galactinol synthase, putative | chr3:10590184-10591776 REVERSE | Aliases: MZF16.17	5.7	4.8	1.0	3.2	63.2%	-1.3
788	AT1G26930.1 | kelch repeat-containing F-box family protein, contains Pfam:PF01344 Kelch motif, Pfam:PF00646 F-box domain | chr1:9336044-9337574 REVERSE | Aliases: T2P11.12, T2P11_12	7.1	8.3	-1.2	-3.2	63.2%	-1.5
789	AT1G12240.1 | Symbol: ATBETAFRUCT4 | beta-fructosidase (BFRUCT4) / beta-fructofuranosidase / invertase, vacuolar, identical to beta-fructosidase GI:1871503 from (Arabidopsis thaliana); contains Pfam profile PF00251:Glycosyl hydrolases family 32; identical to cDNA beta-fructosidase (vacuolar form) GI:1321683; similar to SP:Q43857 | chr1:4153650-4157642 FORWARD | Aliases: ATBETAFRUCT4, T28K15.3, T28K15_3	4.3	5.6	-1.3	-3.2	63.2%	-1.3
790	AT1G53520.1 | chalcone-flavanone isomerase-related, low similarity to GI:499036 (Vitis vinifera) | chr1:19980083-19981623 REVERSE | Aliases: F22G10.11	5.4	6.8	-1.4	-3.2	63.2%	-1.3

Rank	Description	15.dpi	5.dpi	M	t	adj.q	B
791	AT4G28310.1 | expressed protein | chr4:14017414-14018554 FORWARD | Aliases: F26K10.190, F26K10_190	4.3	6.1	-1.8	-3.2	63.2%	-1.3
792	AT2G44790.1 | Symbol: UCC2 | uclacyanin II, strong similarity to uclacyanin II GI:3399769 from (Arabidopsis thaliana); contains Pfam profile PF02298: Plastocyanin-like domain; identical to cDNA uclacyanin II GI:3399768 | chr2:18468981-18470384 REVERSE | Aliases: F16B22.32, UCLACYANIN 2	4.0	7.2	-3.2	-3.2	63.2%	-1.3
793	AT1G17460.1 | Symbol: TRFL3 | myb family transcription factor, contains Pfam PF00249: Myb-like DNA-binding domain | chr1:5998813-6002709 FORWARD | Aliases: F28G4.5, TRF LIKE 3	4.3	3.5	0.7	3.2	63.2%	-1.5
794	AT3G45730.1 | expressed protein | chr3:16800965-16801567 FORWARD | Aliases: T6D9.60	2.9	2.5	0.5	3.2	63.2%	-1.8
795	AT2G42760.1 | expressed protein | chr2:17803061-17804308 REVERSE | Aliases: F7D19.24, F7D19_24	8.8	7.5	1.3	3.2	63.2%	-1.4
796	AT1G28140.1 | expressed protein, contains similarity to cytochrome oxidase I GI:1289267 from (Xantholinus sp.)	6.9	5.8	1.1	3.2	63.2%	-1.3
797	AT2G35410.1 | 33 kDa ribonucleoprotein, chloroplast, putative / RNA-binding protein cp33, putative, similar to SP:P19684 33 kDa ribonucleoprotein, chloroplast precursor {Nicotiana sylvestris}; contains InterPro entry IPR000504: RNA-binding region RNP-1 (RNA recognition motif) (RRM) | chr2:14905407-14906829 FORWARD | Aliases: T32F12.21, T32F12_21	6.8	8.2	-1.4	-3.2	63.4%	-1.3
798	AT1G31710.1 | copper amine oxidase, putative, similar to copper amine oxidase (Lens culinaris) gi:15451834:gb:AAB34918	3.5	4.2	-0.7	-3.2	63.9%	-1.5
799	AT4G25920.1 | expressed protein, contains Pfam profile PF03478: Protein of unknown function (DUF295) | chr4:13165484-13166881 FORWARD | Aliases: F20B18.30, F20B18_30	2.6	2.1	0.4	3.2	63.9%	-1.8
800	AT5G07800.1 | flavin-containing monooxygenase family protein / FMO family protein, similar to flavin-containing monooxygenase 2 (FMO2) from Homo sapiens (GI:1834493); contains Pfam profile: PF00743 Flavin-binding monooxygenase-like | chr5:2486607-2489297 REVERSE | Aliases: MXM12.4, MXM12_4	9.1	10.4	-1.3	-3.2	63.9%	-1.3
801	AT5G39890.1 | expressed protein | chr5:15991621-15993498 FORWARD | Aliases: MYH19.8, MYH19_8	7.7	5.9	1.8	3.2	63.9%	-1.3
802	AT2G15780.1 | glycine-rich protein, similar to Blue copper protein precursor (SP:Q41001) {Pisum sativum}; contains a Pfam PF02298: Plastocyanin-like domain related to blue copper-binding protein; contains a domain related to blue copper-binding protein | chr2:6880658-6881823 REVERSE | Aliases: F19G14.22, F19G14_22	3.2	4.1	-0.9	-3.2	63.9%	-1.4
803	AT4G21680.1 | proton-dependent oligopeptide transport (POT) family protein, contains Pfam profile: PF00854 POT family | chr4:11517043-11519777 REVERSE | Aliases: F17L22.140, F17L22_140	7.4	5.8	1.6	3.2	63.9%	-1.3
804	AT1G66620.1 | seven in absentia (SINA) protein, putative, similar to SIAH1 protein (Brassica napus var. napus) GI:7657876; contains Pfam profile PF03145: Seven in absentia protein family | chr1:24856469-24857707 REVERSE | Aliases: T12I7.7, T12I7_7	5.8	7.8	-2.1	-3.2	63.9%	-1.3
805	AT3G52870.1 | calmodulin-binding family protein, contains Pfam profile PF00612: IQ calmodulin-binding motif | chr3:19604151-19606797 REVERSE | Aliases: F8J2.40	6.2	7.2	-1.0	-3.2	63.9%	-1.4
806	AT5G02260.1 | Symbol: ATEXPA9 | expansin, putative (EXP9), similar to expansin precursor GI:4138914 from (Lycopersicon esculentum); alpha-expansin gene family, PMID:11641069	3.0	2.4	0.6	3.2	63.9%	-1.7
807	AT1G19630.1 | Symbol: CYP722A1 | cytochrome P450 family protein, similar to Cytochrome P450 90A1 (SP:Q42569) (Arabidopsis thaliana) | chr1:6785638-6787958 REVERSE | Aliases: F14P1.4, F14P1_4	4.5	3.6	0.9	3.2	63.9%	-1.7
808	AT4G12840.1 | expressed protein, contains Pfam profile PF05212: Protein of unknown function (DUF707) | chr4:7533618-7535830 REVERSE | Aliases: T20K18.190, T20K18_190	4.5	5.3	-0.8	-3.2	63.9%	-1.5
809	AT2G17790.1 | similar to vacuolar protein sorting-associated protein 35 family protein / VPS35 family protein [Arabidopsis thaliana] (TAIR:At3g51310.1); similar to vacuolar protein sorting-associated protein 35 family protein / VPS35 family protein [Arabidopsis thaliana] (TAIR:At1g75850.1); similar to similar to vacuolar protein sorting 35; maternal-embryonic 3; vacuolar protein sorting 35 (yeast homolog) [Gallus gallus] (GB:NP_001005842.1); similar to unnamed protein product [Tetraodon nigroviridis] (GB:CAF93242.1); similar to novel protein simlar to human and mouse vacuolar protein sorting 35 (yeast) (VPS35) [Danio rerio] (GB:CAE49237.1); contains InterPro domain Vacuolar protein sorting-associated protein 35 (InterPro:IPR005378) | chr2:7740717-7746712 FORWARD | Aliases: T17A5.7, T17A5_7	6.1	7.3	-1.2	-3.2	63.9%	-1.4
810	AT5G53190.1 | nodulin MtN3 family protein, similar to MtN3 GI:1619602 (root nodule development) from (Medicago truncatula) | chr5:21589643-21591510 REVERSE | Aliases: MFH8.13, MFH8_13	6.6	5.1	1.5	3.2	63.9%	-1.3
811	AT3G09730.1 | expressed protein | chr3:2984023-2986265 FORWARD | Aliases: F11F8.32	2.7	3.4	-0.7	-3.2	64.1%	-1.6

Rank	Description	15.dpi	5.dpi	M	t	adj.q	B
812	AT3G43230.1 | zinc finger (FYVE type) family protein, contains Pfam domain PF01363: FYVE zinc finger | chr3:15218355-15220813 FORWARD | Aliases: F7K15.80	8.6	7.7	0.8	3.2	64.1%	-1.6
813	AT5G51260.1 | acid phosphatase, putative, similar to acid phosphatase-1(1); Apase-1(1) (Lycopersicon esculentum) GI:7705154, acid phosphatase (Glycine max) GI:3341443; contains Pfam profile PF03767: HAD superfamily (subfamily IIIB) phosphatase | chr5:20849409-20850918 REVERSE | Aliases: MWD22.21, MWD22_21	6.4	5.1	1.4	3.2	64.2%	-1.3
814	AT1G26240.1 | proline-rich extensin-like family protein, similar to hydroxyproline-rich glycoprotein precursor gi:727264:gb:AAA87902; contains proline-rich extensin domains, INTERPRO:IPR002965 | chr1:9078127-9079563 REVERSE | Aliases: F28B23.10, F28B23_10	2.0	2.4	-0.4	-3.2	64.2%	-1.9
815	AT5G11650.1 | hydrolase, alpha/beta fold family protein, contains Pfam profile PF00561: hydrolase, alpha/beta fold family; low similarity to monoglyceride lipase from (Homo sapiens) GI:14594904, (Mus musculus) GI:2632162 | chr5:3744981-3747126 FORWARD | Aliases: T22P22.40, T22P22_40	5.2	3.6	1.6	3.2	64.3%	-1.3
816	AT4G30800.1 | 40S ribosomal protein S11 (RPS11B), ribosomal protein S11, Arabidopsis thaliana,PIR2:C35542 | chr4:15001173-15002677 FORWARD | Aliases: T10C21.1	7.5	8.7	-1.2	-3.2	64.5%	-1.3
817	AT3G16140.1 | photosystem I reaction center subunit VI, chloroplast, putative / PSI-H, putative (PSAH1), identical to SP:Q9SUI7; similar to PSI-H precursor (Nicotiana sylvestris) GI:407353; contains Pfam profile PF03244: Photosystem I reaction centre subunit VI | chr3:5468515-5469482 REVERSE | Aliases: MSL1.18	9.6	8.3	1.3	3.2	64.5%	-1.3
818	AT1G13980.1 | Symbol: GN | pattern formation protein (EMB30) (GNOM), identical to SP:Q42510; contains Pfam profile PF01369: Sec7 domain | chr1:4788556-4794651 FORWARD | Aliases: EMB30, EMBRYO DEFECTIVE 30, F16A14.20, GNOM, VAN7	7.1	8.1	-1.0	-3.2	64.6%	-1.4
819	AT5G38510.1 | rhomboid family protein, contains Pfam profile PF01694: Rhomboid family | chr5:15435069-15437232 REVERSE | Aliases: MBB18.4, MBB18_4	5.8	4.8	1.0	3.2	64.6%	-1.3
820	AT5G07350.1 | tudor domain-containing protein / nuclease family protein, contains Pfam domains PF00567: Tudor domain and PF00565: Staphylococcal nuclease homologue | chr5:2320082-2325420 REVERSE | Aliases: T2I1.60, T2I1_60	8.4	9.3	-0.9	-3.2	64.7%	-1.5
821	AT3G28007.1 | nodulin MtN3 family protein, contains Pfam PF03083 MtN3/saliva family; similar to LIM7 GI:431154 (induced in meiotic prophase in lily microsporocytes) from (Lilium longiflorum)	3.1	2.5	0.6	3.2	64.7%	-1.7
822	AT3G05740.1 | DNA helicase (RECQI1), identical to DNA Helicase (Arabidopsis thaliana) GI:10944747 | chr3:1698118-1701405 FORWARD | Aliases: F18C1.21	3.5	5.0	-1.5	-3.2	64.7%	-1.3
823	AT3G50810.1 | integral membrane protein, putative, contains 4 transmembrane domains; contains Pfam PF04535 : Domain of unknown function (DUF588); similar to putative ethylene responsive element binding protein (GI:22135858) (Arabidopsis thaliana) | chr3:18898595-18900849 REVERSE | Aliases: F18B3.90	6.4	5.5	1.0	3.2	64.7%	-1.4
824	AT2G36220.1 | expressed protein | chr2:15199559-15200777 FORWARD | Aliases: F2H17.17, F2H17_17	3.8	2.6	1.2	3.2	64.8%	-1.3
825	AT2G43040.1 | Symbol: NPG1 | calmodulin-binding protein, similar to pollen-specific calmodulin-binding protein MPCBP GI:10086260 from (Zea mays); contains Pfam profile PF00515: TPR Domain | chr2:17902960-17906451 REVERSE | Aliases: MFL8.10, MFL8_10, NO POLLEN GERMINATION 1	4.7	5.5	-0.8	-3.2	64.8%	-1.6
826	AT1G49160.2 | Symbol: WNK7 | protein kinase family protein, contains protein kinase domain, Pfam:PF00069 | chr1:18182972-18185535 REVERSE | Aliases: F27J15.7, F27J15_7	4.2	5.8	-1.6	-3.2	64.8%	-1.4
827	AT1G32170.1 | Symbol: XTR4 | xyloglucan:xyloglucosyl transferase, putative / xyloglucan endotransglycosylase, putative / endo-xyloglucan transferase, putative (XTR4), identical to N-terminal partial sequence of xyloglucan endotransglycosylase-related protein XTR4 GI:1244754 from (Arabidopsis thaliana); similar to endoxyloglucan transferase (Arabidopsis thaliana) GI:5533311 | chr1:11575301-11577907 FORWARD | Aliases: F3C3.5, F3C3_5, XYLOGLUCAN ENDOTRANSGLYCOSYLASE 4, XYLOGLUCAN ENDOTRANSGLYCOSYLASE RELATED PROTEIN	7.0	5.3	1.7	3.2	64.8%	-1.4
828	AT3G58630.1 | expressed protein, similar to 6b-interacting protein 1 (NtSIP1) (Nicotiana tabacum) GI:18149189 | chr3:21694545-21696918 REVERSE | Aliases: F14P22.220	3.3	2.9	0.4	3.2	64.9%	-1.9
829	AT3G09690.2 | similar to hydrolase, alpha/beta fold family protein [Arabidopsis thaliana] (TAIR:At5g02970.1); similar to putative alpha/beta hydrolase [Oryza sativa (japonica cultivar-group)] (GB:AAP54670.1); similar to hypothetical protein [Oryza sativa (japonica cultivar-group)] (GB:AAM92284.1); contains InterPro domain Alpha/beta hydrolase fold (InterPro:IPR000073) | chr3:2972228-2974848 FORWARD | Aliases: F11F8.28	5.1	5.8	-0.7	-3.2	64.9%	-1.5
830	AT5G22550.2 | expressed protein, contains Pfam profile PF03140: Plant protein of unknown function | chr5:7483876-7485461 REVERSE | Aliases: MQJ16.9, MQJ16_9	2.6	2.2	0.4	3.2	65.3%	-2.0

Rank	Description	15.dpi	5.dpi	M	t	adj.q	B
831	AT5G26770.3 | expressed protein, similar to expressed protein [Arabidopsis thaliana] (TAIR:At3g05830.1); similar to hypothetical protein [Oryza sativa (japonica cultivar-group)] (GB:BAD86886.1) | chr5:9407855-9410008 REVERSE | Aliases: F2P16.30, F2P16_30	5.2	4.5	0.7	3.2	65.4%	-1.6
832	AT1G30440.1 | phototropic-responsive NPH3 family protein, contains NPH3 family domain, Pfam:PF03000 | chr1:10759457-10762376 FORWARD | Aliases: F26G16.2, F26G16_2	6.2	6.9	-0.7	-3.2	65.6%	-1.6
833	AT1G01510.1 | Symbol: AN | C-terminal binding protein (ANGUSTIFOLIA), nearly identical to C-terminal binding protein ANGUSTIFOLIA (Arabidopsis thaliana) GI:15408535; contains Pfam profile PF02826: D-isomer specific 2-hydroxyacid dehydrogenase, NAD binding domain | chr1:187211-190056 FORWARD | Aliases: ANGUSTIFOLIA, F22L4.6, F22L4_6	5.9	6.7	-0.8	-3.2	65.7%	-1.4
834	AT1G21000.1 | zinc-binding family protein, similar to zinc-binding protein (Pisum sativum) GI:16117799; contains Pfam profile PF04640 : Protein of unknown function, DUF597 | chr1:7337740-7339441 FORWARD | Aliases: F9H16.1, F9H16_1	6.8	5.9	0.9	3.2	65.7%	-1.5
835	AT1G64790.1 | translational activator family protein, similar to HsGCN1 (Homo sapiens) GI:2282576 | chr1:24068600-24085571 REVERSE | Aliases: F13O11.35, F13O11_35	5.3	6.2	-0.9	-3.2	65.7%	-1.5
836	AT1G64850.1 | calcium-binding EF hand family protein, contains INTERPRO:IPR002048 calcium-binding EF-hand domain | chr1:24100098-24101271 REVERSE | Aliases: F13O11.15, F13O11_15	10.2	9.6	0.5	3.2	65.7%	-1.9
837	AT3G47850.1 | expressed protein | chr3:17665064-17666984 REVERSE | Aliases: T23J7.180	6.2	5.2	1.0	3.2	65.7%	-1.4
838	AT5G64130.2 | expressed protein | chr5:25681625-25683425 REVERSE | Aliases: MHJ24.11, MHJ24_11	12.1	11.6	0.5	3.2	65.7%	-1.7
839	AT1G63800.1 | ubiquitin-conjugating enzyme 5 (UBC5), E2; identical to gi:431269, SP:P42749 | chr1:23671279-23672743 REVERSE | Aliases: T12P18.18, T12P18_18	10.0	8.6	1.4	3.2	65.7%	-1.5
840	AT4G14020.1 | rapid alkalinization factor (RALF) family protein | chr4:8095210-8095731 REVERSE | Aliases: DL3050C, FCAALL.138	4.7	3.4	1.3	3.2	65.7%	-1.4
841	AT3G60630.1 | scarecrow transcription factor family protein, scarecrow-like 6, Arabidopsis thaliana, EMBL:AF036303 | chr3:22421340-22423583 REVERSE | Aliases: T4C21.40	4.6	5.6	-0.9	-3.2	65.8%	-1.4
842	AT5G15120.1 | expressed protein | chr5:4898740-4900575 FORWARD | Aliases: F2G14.1	8.1	7.0	1.1	3.2	65.9%	-1.5
843	AT2G28900.1 | mitochondrial import inner membrane translocase subunit Tim17/Tim22/Tim23 family protein, contains Pfam PF02466: Mitochondrial import inner membrane translocase subunit Tim17 | chr2:12421191-12422655 REVERSE | Aliases: F8N16.19, F8N16_19	11.4	10.4	1.1	3.1	66.1%	-1.4
844	AT3G05060.1 | SAR DNA-binding protein, putative, strong similarity to SAR DNA-binding protein-1 (Pisum sativum) GI:3132696; contains Pfam profile PF01798: Putative snoRNA binding domain; encodes NOP58-like protein | chr3:1412873-1415958 REVERSE | Aliases: T12H1.2, T12H1_2	9.4	10.5	-1.1	-3.1	66.1%	-1.4
845	AT1G29395.1 | Symbol: COR414 TM1 | stress-responsive protein, putative, similar to cold acclimation WCOR413-like protein gamma form (Hordeum vulgare) gi:18449100:gb:AAL69988; similar to stress-regulated protein SAP1 (Xerophyta viscosa) gi:21360378:gb:AAM47505 | chr1:10288236-10289579 REVERSE | Aliases: None	4.7	3.6	1.1	3.1	66.2%	-1.4
846	AT3G15900.1 | expressed protein | chr3:5376257-5377089 REVERSE | Aliases: MVC8.2	7.6	6.6	1.1	3.1	66.2%	-1.4
847	AT2G39890.1 | Symbol: ProT1 | proline transporter 1 (ProT1), identical to proline transporter 1 GI:1769901 from (Arabidopsis thaliana) | chr2:16662757-16665409 FORWARD | Aliases: ATPROT1, PROLINE TRANSPORTER 1, T28M21.5, T28M21_5	8.0	6.7	1.3	3.1	66.2%	-1.5
848	AT5G40780.2 | Symbol: LHT1 | lysine and histidine specific transporter, putative, strong similarity to lysine and histidine specific transporter GI:2576361 from (Arabidopsis thaliana); contains Pfam profile PF01490: Transmembrane amino acid transporter protein | chr5:16340910-16344502 FORWARD | Aliases: K1B16.3, K1B16_3, LYSINE AND HISTIDINE SPECIFIC TRANSPORTER	8.7	10.0	-1.3	-3.1	66.2%	-1.5
849	AT5G52950.1 | expressed protein, ; expression supported by MPSS | chr5:21491113-21494165 REVERSE | Aliases: MXC20.3, MXC20_3	3.7	6.1	-2.4	-3.1	66.2%	-1.4
850	AT5G39570.1 | expressed protein | chr5:15861187-15863406 FORWARD | Aliases: MIJ24.6, MIJ24_6	13.1	13.6	-0.5	-3.1	66.2%	-1.8
851	AT3G48490.1 | expressed protein | chr3:17972274-17972890 FORWARD | Aliases: T29H11.260	3.8	4.8	-1.0	-3.1	66.5%	-1.4
852	AT1G32640.1 | Symbol: ATMYC2 |	6.7	5.6	1.2	3.1	66.6%	-1.4
853	AT5G54500.1 | Symbol: FQR1 | quinone reductase, putative, similar to 1,4-benzoquinone reductase (Phanerochaete chrysosporium)(GI:4454993); contains flavodoxin domain PF00258	8.5	9.4	-1.0	-3.1	66.8%	-1.6

Rank	Description	15.dpi	5.dpi	M	t	adj.q	B
854	AT3G24500.1 | ethylene-responsive transcriptional coactivator, putative, similar to ethylene-responsive transcriptional coactivator (Lycopersicon esculentum) gi:5669634:gb:AAD46402 | chr3:8918686-8919315 FORWARD | Aliases: MOB24.13	7.3	6.5	0.9	3.1	66.8%	-1.5
855	AT4G01120.1 | Symbol: GBF2 | G-box binding factor 2 (GBF2), identical to G-box binding factor 2 (GBF2) SP:P42775 from (Arabidopsis thaliana);contains Pfam profile: PF00170 bZIP transcription factor	8.0	6.5	1.5	3.1	66.8%	-1.5
856	AT3G27060.1 | ribonucleoside-diphosphate reductase small chain, putative / ribonucleotide reductase, putative, similar to ribonucleotide reductase R2 (Nicotiana tabacum) GI:1044912; contains Pfam profile PF00268: Ribonucleotide reductase, small chain | chr3:9981103-9982532 REVERSE | Aliases: MOJ10.15	6.5	8.3	-1.8	-3.1	66.8%	-1.4
857	AT5G43580.1 | protease inhibitor, putative, similar to SP:P19873 Inhibitor of trypsin and hageman factor (CMTI-V) {Cucurbita maxima}; contains Pfam profile PF00280: Potato inhibitor I family	2.9	4.1	-1.3	-3.1	66.9%	-1.4
858	AT4G00670.1 | similar to DNA-binding protein, putative [Arabidopsis thaliana] (TAIR:At2g45820.1); similar to OSJNBb0039L24.13 [Oryza sativa (japonica cultivar-group)] (GB:XP_473296.1); contains InterPro domain Remorin, C-terminal region (InterPro:IPR005516) | chr4:278335-279184 REVERSE | Aliases: F6N23.13, F6N23_13	5.0	3.4	1.6	3.1	66.9%	-1.4
859	AT1G26330.1 | expressed protein | chr1:9107612-9113507 FORWARD | Aliases: F28B23.2, F28B23_2	3.5	4.8	-1.3	-3.1	66.9%	-1.4
860	AT1G52710.1 | cytochrome c oxidase-related, similar to SP:P00428 Cytochrome c oxidase polypeptide Vb (EC 1.9.3.1) (VI) (Bovine) {Bos taurus} | chr1:19641999-19643891 REVERSE | Aliases: F6D8.4, F6D8_4	7.4	6.4	1.0	3.1	66.9%	-1.4
861	AT5G04320.1 | expressed protein | chr5:1209236-1212547 FORWARD | Aliases: T19N18.50, T19N18_50	4.5	5.5	-1.0	-3.1	67.3%	-1.5
862	AT3G11410.1 | protein phosphatase 2C, putative / PP2C, putative, identical to protein phosphatase 2C (PP2C) GB:P49598 (Arabidopsis thaliana); contains Pfam profile PF00481: Protein phosphatase 2C; identical to cDNA protein phosphatase 2C GI:633027 | chr3:3583889-3585796 REVERSE | Aliases: F24K9.8	6.4	5.4	1.0	3.1	67.4%	-1.5
863	AT3G50440.1 | similar to esterase, putative [Arabidopsis thaliana] (TAIR:At2g23620.1); similar to methyl jasmonate esterase [Solanum tuberosum] (GB:AAV87151.1); contains InterPro domain Esterase/lipase/thioesterase (InterPro:IPR000379); contains InterPro domain Alpha/beta hydrolase fold (InterPro:IPR000073) | chr3:18728292-18729475 REVERSE | Aliases: T20E23.40	8.4	6.5	1.8	3.1	67.5%	-1.5
864	AT5G16980.1 | NADP-dependent oxidoreductase, putative, strong similarity to probable NADP-dependent oxidoreductase (zeta-crystallin homolog) P1 (SP:Q39172)(gi:886428) and P2 (SP:Q39173)(gi:886430), Arabidopsis thaliana | chr5:5579146-5580783 REVERSE | Aliases: F2K13.130, F2K13_130	6.6	5.4	1.3	3.1	67.5%	-1.4
865	AT5G11980.1 | conserved oligomeric Golgi complex component-related / COG complex component-related, similar to SP:Q96MW5 Conserved oligomeric Golgi complex component 8 {Homo sapiens}; contains Pfam profile PF04124: Dor1-like family | chr5:3868653-3872270 REVERSE | Aliases: F14F18.150, F14F18_150	5.7	6.8	-1.1	-3.1	67.5%	-1.5
866	AT1G11380.1 | expressed protein, contains Pfam profile PF04749: Protein of unknown function, DUF614 | chr1:3832623-3834189 FORWARD | Aliases: T23J18.4, T23J18_4	5.9	4.4	1.5	3.1	67.5%	-1.5
867	AT5G04440.1 | expressed protein | chr5:1254949-1256954 FORWARD | Aliases: T19N18.1	7.2	6.2	1.0	3.1	67.6%	-1.5
868	AT5G08750.1 | zinc finger (C3HC4-type RING finger) family protein, contains Pfam profile: PF00097 zinc finger, C3HC4 type (RING finger) | chr5:2851980-2854496 FORWARD | Aliases: None	7.9	7.2	0.7	3.1	67.7%	-1.6
869	AT5G44520.1 | ribose 5-phosphate isomerase-related, low similarity to SP:P47968 Ribose 5-phosphate isomerase (EC 5.3.1.6) (Phosphoriboisomerase) {Mus musculus} | chr5:17951514-17953751 REVERSE | Aliases: MFC16.20, MFC16_20	6.3	5.4	0.9	3.1	67.7%	-1.5
870	AT1G24020.1 | Bet v I allergen family protein, similar to major pollen allergen Bet v 1 GB:CAA96544 GI:1321726 from (Betula pendula); contains Pfam profile PF00407: Pathogenesis-related protein Bet v I family | chr1:8500466-8501504 REVERSE | Aliases: T23E23.28, T23E23_28	3.6	4.3	-0.7	-3.1	67.7%	-1.6
871	AT5G20990.1 | Symbol: CHL6/CNX/CNX1/SIR4 | Involved in molybdenum cofactor (Moco) biosynthesis, inserting Mo into Molybdopterin. sir loss-of-function mutants are resistant to sirtinol, a modulator of auxin signaling. | chr5:7128477-7133459 REVERSE | Aliases: CHL6, CHLORATE RESISTANT 6, CNX, CNX1, COFACTOR OF NR/CHL6, MOLYBDENUM COFACTOR BIOSYNTHESIS ENZYME CNX1, SIR4, T10F18.20, T10F18_20	7.8	8.5	-0.7	-3.1	67.7%	-1.7
872	AT5G17980.1 | C2 domain-containing protein, contains INTERPRO:IPR000008 C2 domain | chr5:5953598-5956747 FORWARD | Aliases: MCM23.5, MCM23_5	4.4	3.7	0.7	3.1	67.7%	-1.6
873	AT5G43080.1 | Symbol: CYCA3;1 | cyclin, putative, similar to A-type cyclins from (Nicotiana tabacum) GI:1064931, (Catharanthus roseus) GI:2190259; contains Pfam profiles PF00134: Cyclin, N-terminal domain, PF02984: Cyclin, C-terminal domain | chr5:17310455-17312017 FORWARD | Aliases: CYCA3;1, Cyclin A3;1, MMG4.10, MMG4_10	3.8	4.7	-0.9	-3.1	68.2%	-1.4

Rank	Description	15.dpi	5.dpi	M	t	adj.q	B
874	AT4G38430.1 | member of KPP-like gene family, homolog of KPP (kinase partner protein) gene in tomato; expressed protein, contains Pfam profile PF03759: Domain of unknown function (DUF315) | chr4:17986461-17988801 FORWARD | Aliases: F22I13.200, F22I13_200, KINASE PARTNER PROTEIN LIKE, KPP LIKE	3.0	4.1	-1.1	-3.1	68.2%	-1.4
875	AT5G60690.1 | Symbol: REV | homeodomain-leucine zipper protein Revoluta (REV) / fascicular fiberless 1 (IFL1), identical to HD-zip transcription factor Revoluta (GI:9759333) {Arabidopsis thaliana}; contains Pfam profiles PF01852: START domain and PF00046: Homeobox domain | chr5:24414249-24419779 FORWARD | Aliases: HOMEODOMAIN LEUCINE ZIPPER PROTEIN, HOMEODOMAIN LEUCINE ZIPPER PROTEIN IFL1, IFL, IFL1, INTERFASCICULAR FIBERLESS 1, MUP24.16, MUP24_16, REVOLUTA	6.9	7.9	-1.0	-3.1	68.2%	-1.7
876	AT1G44900.1 | DNA replication licensing factor, putative, similar to DNA replication licensing factor MCM2 from {Xenopus laevis} SP:P55861, SP:P49736 {Homo sapiens}; contains Pfam profile PF00493: MCM2/3/5 family | chr1:16972701-16977030 FORWARD | Aliases: T12C22.19, T12C22_19	3.3	4.9	-1.6	-3.1	68.2%	-1.4
877	AT1G77640.1 | encodes a member of the DREB subfamily A-5 of ERF/AP2 transcription factor family. The protein contains one AP2 domain. There are 15 members in this subfamily including RAP2.1, RAP2.9 and RAP2.10. | chr1:29183631-29184563 FORWARD | Aliases: T5M16.23, T5M16_23	4.9	3.9	1.0	3.1	68.2%	-1.4
878	AT1G29980.2 | expressed protein, contains Pfam profile PF04862: Protein of unknown function, DUF642 | chr1:10503264-10504827 REVERSE | Aliases: T1P2.9, T1P2_9	5.0	6.9	-1.9	-3.1	68.2%	-1.4
879	AT5G59950.3 | RNA and export factor-binding protein, putative | chr5:24157421-24159084 FORWARD | Aliases: MMN10.26, MMN10_26	7.2	6.3	1.0	3.1	68.3%	-1.6
880	AT2G24590.1 | splicing factor, putative, similar to to RSZp22 protein (Arabidopsis thaliana) gi:2582645:emb:CAA05352 | chr2:10456710-10458240 FORWARD | Aliases: F25P17.11, F25P17_11	5.8	4.9	0.9	3.1	68.3%	-1.5
881	AT3G26830.1 | Symbol: PAD3 | cytochrome P450 71B15, putative (CYP71B15), Identical to Cytochrome P450 (SP:Q9LW27) (Arabidopsis thaliana); similar to cytochrome P450 71B2 GB:O65788 (Arabidopsis thaliana) | chr3:9889190-9890942 FORWARD | Aliases: CYP71B15, MDJ14.12, PHYTOALEXIN DEFICIENT 3	4.7	6.0	-1.3	-3.1	68.3%	-1.5
882	AT5G02520.1 | expressed protein | chr5:562392-564004 FORWARD | Aliases: T22P11.110, T22P11_110	3.9	5.2	-1.2	-3.1	68.3%	-1.6
883	AT5G54680.1 | basic helix-loop-helix (bHLH) family protein, similar to unknown protein (pir :B71406) | chr5:22234286-22236830 FORWARD | Aliases: K5F14.2, K5F14_2	9.7	9.0	0.8	3.1	68.3%	-1.7
884	AT1G16170.1 | expressed protein | chr1:5538731-5539790 REVERSE | Aliases: T24D18.25, T24D18_25	9.4	8.9	0.6	3.1	68.3%	-1.9
885	AT3G06030.1 | Symbol: ANP3 | NPK1-related protein kinase, putative (ANP3), similar to protein kinase (Nicotiana tabacum) gi:456309:dbj:BAA05648; identical to cDNA NPK1-related protein kinase 3 GI:2342426 | chr3:1818749-1822846 REVERSE | Aliases: F24F17.1, F24F17_1, MAPKKK12	4.5	5.5	-1.0	-3.1	68.3%	-1.5
886	AT5G40480.1 | Symbol: EMB3012 | expressed protein, ; expression supported by MPSS | chr5:16230589-16241210 FORWARD | Aliases: EMB3012, EMBRYO DEFECTIVE 3012, K21I16.30, K21I16_30	5.8	6.9	-1.1	-3.1	68.4%	-1.5
887	AT1G74410.1 | zinc finger (C3HC4-type RING finger) family protein, contains Pfam profile: PF00097 zinc finger, C3HC4 type (RING finger) | chr1:27969215-27971563 FORWARD | Aliases: F1M20.9, F1M20_9	6.3	5.5	0.8	3.1	68.6%	-1.5
888	AT1G70990.1 | proline-rich family protein | chr1:26770959-26771738 REVERSE | Aliases: F15H11.18, F15H11_18	4.4	3.7	0.7	3.1	68.6%	-1.7
889	AT1G58170.1 | disease resistance-responsive protein-related / dirigent protein-related, similar to dirigent protein (Thuja plicata) gi:6694699:gb:AAF25360; similar to pathogenesis-related protein (Pisum sativum) gi:4585273:gb:AAD25355 | chr1:21539793-21540501 FORWARD | Aliases: T15M6.17	4.8	5.4	-0.6	-3.1	68.6%	-1.7
890	AT5G05560.1 | Symbol: APC1 | E3 ubiquitin ligase, putative, E3, ubiquitin ligase; contains similarity to Apc1/Tsg24 protein, the largest subunit of human anaphase-promoting complex (APC/C) GI:11967711 from (Homo sapiens) | chr5:1641453-1654757 FORWARD | Aliases: MOP10.10, MOP10_10	3.8	4.8	-0.9	-3.1	68.6%	-1.5
891	AT3G06210.1 | similar to expressed protein [Arabidopsis thaliana] (TAIR:At5g18980.1); similar to BLE2 protein-like protein [Oryza sativa (japonica cultivar-group)] (GB:NP_910813.1); contains InterPro domain Armadillo repeat (InterPro:IPR000225) | chr3:1880753-1883497 FORWARD | Aliases: F28L1.15, F28L1_15	6.8	5.8	0.9	3.1	68.6%	-1.7
892	AT1G35230.1 | Symbol: AGP5 | arabinogalactan-protein (AGP5), identical to gi_3883128_gb_AAC77827 | chr1:12917127-12917741 FORWARD | Aliases: ARABINOGALACTAN PROTEIN 5, T9I1.2, T9I1_2	2.9	3.5	-0.6	-3.1	68.6%	-1.8
893	AT3G18500.1 | similar to endonuclease/exonuclease/phosphatase family protein [Arabidopsis thaliana] (TAIR:At1g73875.1); similar to endonuclease/exonuclease/phosphatase family protein-like [Oryza sativa (japonica cultivar-group)] (GB:BAD52532.1); contains InterPro domain Endonuclease/exonuclease/phosphatase family (InterPro:IPR005135) | chr3:6352544-6355446 FORWARD | Aliases: MYF24.23	5.1	4.1	1.0	3.1	68.6%	-1.5
894	AT2G37280.1 | ABC transporter family protein, similar to PDR5-like ABC transporter GI:1514643 from (Spirodela polyrhiza) | chr2:15657479-15663496 FORWARD | Aliases: F3G5.7, F3G5_7	6.1	7.1	-1.1	-3.1	68.6%	-1.6

Rank	Description	15.dpi	5.dpi	M	t	adj.q	B
895	AT3G54250.1 | mevalonate diphosphate decarboxylase, putative, similar to mevalonate diphosphate decarboxylase (Arabidopsis thaliana) gi:2288887:emb:CAA74700 | chr3:20093286-20095710 REVERSE | Aliases: F24B22.210	3.8	4.5	-0.7	-3.1	68.6%	-1.6
896	AT2G28200.1 | similar to zinc finger (C2H2 type) family protein [Arabidopsis thaliana] (TAIR:At5g04390.1); similar to putative zinc finger protein [Pisum sativum] (GB:CAA60828.1); contains InterPro domain Zn-finger, C2H2 type (InterPro:IPR007087)	6.2	5.2	1.0	3.1	68.6%	-1.6
897	AT3G05260.1 | short-chain dehydrogenase/reductase (SDR) family protein, contains INTERPRO family IPR002198 short-chain dehydrogenase/reductase (SDR) superfamily | chr3:1497495-1498979 REVERSE | Aliases: T12H1.23, T12H1_23	3.3	2.8	0.6	3.1	68.8%	-1.9
898	AT2G41180.1 | sigA-binding protein-related, low similarity to SigA binding protein (Arabidopsis thaliana) GI:6980074; contains Pfam domain PF05678: VQ motif | chr2:17172269-17173103 FORWARD | Aliases: T3K9.5, T3K9_5	4.3	3.5	0.8	3.1	68.9%	-1.6
899	AT5G54290.1 | cytochrome c biogenesis protein family, low similarity to cytochrome c biogenesis protein CcdA (Paracoccus pantotrophus) GI:11095328; contains Pfam profile PF02683: Cytochrome C biogenesis protein transmembrane region | chr5:22068263-22071604 FORWARD | Aliases: MDK4.11, MDK4_11	9.7	8.4	1.3	3.1	69.2%	-1.5
900	AT2G27970.1 | Symbol: CKS2 | cyclin-dependent kinase, putative / CDK, putative, similar to Cks1 protein (Arabidopsis thaliana) gi:2274859:emb:CAA03859 | chr2:11919295-11920348 REVERSE | Aliases: CDK subunit 2, CKS2, T1E2.11, T1E2_11	8.2	9.9	-1.7	-3.1	69.2%	-1.6
901	AT2G33710.1 | encodes a member of the ERF (ethylene response factor) subfamily B-4 of ERF/AP2 transcription factor family. The protein contains one AP2 domain. There are 7 members in this subfamily.	3.0	2.7	0.3	3.1	69.2%	-2.3
902	AT1G18540.1 | 60S ribosomal protein L6 (RPL6A), similar to 60S ribosomal protein L6 GI:7208784 from (Cicer arietinum) | chr1:6377314-6378571 REVERSE | Aliases: F25I16.12, F25I16_12	11.9	12.5	-0.6	-3.1	69.4%	-1.8
903	AT1G73600.2 | phosphoethanolamine N-methyltransferase 3, putative (NMT3), strong similarity to SP:Q9FR44 Phosphoethanolamine N-methyltransferase 1 (EC 2.1.1.103) (PEAMT 1) (AtNMT1) {Arabidopsis thaliana}; identical to SP:Q9C6B9 Putative phosphoethanolamine N-methyltransferase 3 {Arabidopsis thaliana} | chr1:27672813-27677230 FORWARD | Aliases: F6D5.1, F6D5_1	6.5	8.4	-1.9	-3.1	69.7%	-1.5
904	AT3G15620.2 | Symbol: UVR3 | similar to cryptochrome dash (CRYD) [Arabidopsis thaliana] (TAIR:At5g24850.1); similar to 6-4 photolyase [Dunaliella salina] (GB:AAX56342.1); contains InterPro domain FAD binding domain of DNA photolyase (InterPro:IPR005101); contains InterPro domain FAD binding domain of DNA photolyase, N-terminal (InterPro:IPR006051); contains InterPro domain DNA photolyase, N-terminal (InterPro:IPR006050) | chr3:5293313-5296599 REVERSE | Aliases: 6 4 PHOTOLYASE, MSJ11.2, UV REPAIR DEFECTIVE 4	3.1	3.7	-0.6	-3.1	69.7%	-1.8
905	AT5G06460.1 | Symbol: ATUBA2 |	7.7	8.6	-0.9	-3.1	69.7%	-1.6
906	AT5G18670.1 | Symbol: BMY3 | beta-amylase, putative (BMY3) / 1,4-alpha-D-glucan maltohydrolase, putative, almost identical to beta-amylase BMY3 GI:15149457 from (Arabidopsis thaliana); identical to cDNA putative beta-amylase BMY3 (BMY3) GI:15149456 | chr5:6225958-6228134 FORWARD | Aliases: T1A4.50, T1A4_50	7.2	6.6	0.7	3.1	69.7%	-1.7
907	AT3G15580.1 | Symbol: APG8H | autophagy 8i (APG8i), identical to autophagy 8i (Arabidopsis thaliana) GI:19912167; contains Pfam profile PF02991: Microtubule associated protein 1A/1B, light chain 3; supporting cDNA gi:21636957:gb:AF492760.1: | chr3:5273902-5275102 REVERSE | Aliases: MQD17.3	11.9	11.3	0.6	3.1	69.7%	-2.1
908	AT1G04250.1 | Symbol: AXR3 | auxin-responsive protein / indoleacetic acid-induced protein 17 (IAA17), Identical to SP:P93830 Auxin-responsive protein IAA17 (Indoleacetic acid-induced protein 17) {Arabidopsis thaliana}; ESTs gb:H36782 and gb:F14074 come from this gene | chr1:1136257-1138582 FORWARD | Aliases: AUXIN RESISTANT 3, F19P19.31, F19P19_31, IAA17	4.0	5.7	-1.7	-3.1	69.8%	-1.5
909	AT1G33055.1 | expressed protein | chr1:11972220-11972570 REVERSE | Aliases: None	6.9	3.9	3.0	3.1	69.8%	-1.5
910	AT2G15820.1 | pentatricopeptide (PPR) repeat-containing protein, contains Pfam profiles PF01535: PPR repeat, PF03161 LAGLIDADG DNA endonuclease family	3.7	4.3	-0.6	-3.1	69.8%	-1.9
911	AT3G13160.1 | pentatricopeptide (PPR) repeat-containing protein, contains Pfam profile PF01535: PPR repeat | chr3:4229978-4231293 REVERSE | Aliases: MJG19.11	5.6	7.1	-1.4	-3.1	69.8%	-1.5
912	AT3G50060.1 | myb family transcription factor, contains Pfam profile: PF00249 myb-like DNA-binding domain; identical to cDNA MYB-related protein (1107 bp) GI:1263096 | chr3:18568955-18570089 REVERSE | Aliases: F3A4.140	5.1	4.2	0.8	3.1	69.9%	-1.6
913	AT1G54990.1 | expressed protein | chr1:20515186-20517348 FORWARD | Aliases: F14C21.51, F14C21_51	7.1	7.9	-0.8	-3.1	70.0%	-1.6
914	AT5G12970.1 | C2 domain-containing protein, contains INTERPRO:IPR000008 C2 domain | chr5:4102138-4105330 FORWARD | Aliases: T24H18.140, T24H18_140	3.1	4.8	-1.7	-3.1	70.1%	-1.5

Rank	Description	15.dpi	5.dpi	M	t	adj.q	B
915	AT3G05190.1 | aminotransferase class IV family protein, contains Pfam profile: PF01063 aminotransferase class IV | chr3:1471403-1475790 FORWARD | Aliases: T12H1.16, T12H1_16	6.5	5.2	1.3	3.1	70.4%	-1.5
916	AT1G60710.1 | Symbol: ATB2 | aldo/keto reductase family protein, contains Pfam profile PF00248: oxidoreductase, aldo/keto reductase family | chr1:22358418-22360407 REVERSE | Aliases: F8A5.23, F8A5_23	10.4	11.7	-1.3	-3.1	70.4%	-1.9
917	AT1G15260.1 | expressed protein, EST gb:N65467 comes from this gene | chr1:5249755-5250568 FORWARD | Aliases: F9L1.20, F9L1_20	3.5	4.7	-1.2	-3.1	70.4%	-1.5
918	AT4G32340.1 | expressed protein | chr4:15612597-15614259 REVERSE | Aliases: F8B4.40	7.4	6.1	1.3	3.1	70.4%	-1.5
919	AT1G78780.2 | pathogenesis-related family protein, contains similarity to pathogen-related protein (GI:499073) (Swiss-Prot:P16273) (Hordeum vulgare) | chr1:29626237-29627338 REVERSE | Aliases: F9K20.18, F9K20_18	7.1	5.5	1.5	3.1	70.4%	-1.5
920	AT1G60970.1 | clathrin adaptor complex small chain family protein, contains Pfam profile: PF01217 clathrin adaptor complex small chain	7.8	6.2	1.7	3.1	70.4%	-1.5
921	AT1G53170.1 | Symbol: ATERF 8/ATERF8 |	3.7	4.7	-1.0	-3.1	70.4%	-1.6
922	AT4G26830.1 | glycosyl hydrolase family 17 protein, similar to elicitor inducible chitinase Nt-SubE76 GI:11071974 from (Nicotiana tabacum) | chr4:13495047-13496493 REVERSE | Aliases: F10M23.170, F10M23_170	4.2	3.4	0.8	3.0	70.4%	-1.7
923	AT1G66180.1 | aspartyl protease family protein, contains Pfam PF00026: Eukaryotic aspartyl protease profile; similar to CND41, chloroplast nucleoid DNA binding protein (GI:2541876) (Nicotiana tabacum) | chr1:24650851-24652476 FORWARD | Aliases: F15E12.7, F15E12_7	7.4	6.5	0.9	3.0	70.4%	-2.0
924	AT5G02550.1 | expressed protein | chr5:573052-573556 REVERSE | Aliases: T22P11.140, T22P11_140	3.5	4.7	-1.2	-3.0	70.4%	-1.7
925	AT1G33590.1 | disease resistance protein-related / LRR protein-related, contains leucine rich-repeat domains Pfam:PF00560, INTERPRO:IPR001611; similar to Hcr2-5D (Lycopersicon esculentum) gi:3894393:gb:AAC78596 | chr1:12177757-12179393 FORWARD | Aliases: T1E4.3, T1E4_3	5.7	6.5	-0.8	-3.0	70.6%	-1.6
926	AT5G15230.1 | Symbol: GASA4 | gibberellin-regulated protein 4 (GASA4) / gibberellin-responsive protein 4, identical to SP:P46690 Gibberellin-regulated protein 4 precursor {Arabidopsis thaliana} | chr5:4944906-4946219 FORWARD | Aliases: F8M21.120, F8M21_120	5.6	7.6	-2.0	-3.0	70.6%	-1.5
927	AT2G46920.2 | Symbol: POL | protein phosphatase 2C family protein / PP2C family protein, similar to protein phosphatase-2c (GI:3608412) (Mesembryanthemum crystallinum); contains Pfam PF00481 : Protein phosphatase 2C domain | chr2:19285056-19288283 REVERSE | Aliases: F14M4.25, POLTERGEIST	6.1	7.3	-1.2	-3.0	70.8%	-1.6
928	AT5G14340.1 | myb family transcription factor (MYB40), contains Pfam profile: PF00249 myb-like DNA-binding domain | chr5:4623370-4624850 FORWARD | Aliases: F18O22.130, F18O22_130	5.1	4.2	1.0	3.0	70.8%	-1.5
929	AT5G51200.1 | expressed protein | chr5:20822152-20836818 FORWARD | Aliases: MWD22.15, MWD22_15	5.7	6.9	-1.2	-3.0	70.8%	-1.6
930	AT2G01990.1 | expressed protein, similar to hypothetical protein [Arabidopsis thaliana] (TAIR:At1g14630.1); similar to hypothetical protein [Oryza sativa (japonica cultivar-group)] (GB:BAD38245.1) | chr2:465018-466723 REVERSE | Aliases: F14H20.6, F14H20_6	3.3	4.0	-0.7	-3.0	70.9%	-1.7
931	AT4G29140.1 | MATE efflux protein-related, several hypothetical proteins - Arabidopsis thaliana; contains Pfam profile PF01554: Uncharacterized membrane protein family | chr4:14368925-14370898 FORWARD | Aliases: F19B15.170, F19B15_170	9.2	7.9	1.3	3.0	71.0%	-1.5
932	AT3G07130.1 | serine/threonine protein phosphatase family protein, contains similarity to purple acid phosphatase (Arabidopsis thaliana) gi:20257489:gb:AAM15914 | chr3:2255716-2258011 REVERSE | Aliases: T1B9.21	6.8	7.7	-0.9	-3.0	71.1%	-1.6
933	AT1G31760.1 | SWIB complex BAF60b domain-containing protein, contains Pfam profile PF02201: BAF60b domain of the SWIB complex | chr1:11372407-11373463 REVERSE | Aliases: F5M6.23	6.3	5.4	1.0	3.0	71.1%	-1.6
934	AT1G13990.1 | expressed protein | chr1:4794761-4796594 FORWARD | Aliases: F7A19.8, F7A19_8	7.1	6.1	1.0	3.0	71.3%	-1.6
935	AT5G55820.1 | expressed protein | chr5:22603513-22611261 FORWARD | Aliases: MDF20.26, MDF20_26	2.8	3.3	-0.5	-3.0	71.4%	-1.9
936	AT1G70370.1 | BURP domain-containing protein / polygalacturonase, putative, similar to polygalacturonase isoenzyme 1 beta subunit (Lycopersicon esculentum) GI:170480; contains Pfam profile PF03181: BURP domain | chr1:26516385-26518851 REVERSE | Aliases: F17O7.9, F17O7_9	8.3	9.1	-0.8	-3.0	71.4%	-1.9
937	AT1G23850.1 | expressed protein | chr1:8425728-8427062 REVERSE | Aliases: T23E23.1, T23E23_1	6.2	5.2	1.0	3.0	71.8%	-1.6
938	AT2G32150.1 | haloacid dehalogenase-like hydrolase family protein, contains InterPro accession IPR005834: Haloacid dehalogenase-like hydrolase	5.8	4.7	1.1	3.0	71.9%	-1.5

Rank	Description	15.dpi	5.dpi	M	t	adj.q	B
939	AT2G06050.2 | Symbol: OPR3 | 12-oxophytodienoate reductase (OPR3) / delayed dehiscence1 (DDE1), nearly identical to DELAYED DEHISCENCE1 (GI:7688991) and to OPR3 (GI:10242314); contains Pfam profile PF00724:oxidoreductase, FAD/FMN-binding; identical to cDNA OPDA-reductase homolog GI:5059114 | chr2:2359112-2362124 REVERSE | Aliases: 12 OXO PHYTODIENOATE REDUCTASE, 12 OXOPHYTODIENOATE 10,11 REDUCTASE, F5K7.19, F5K7_19	11.0	9.3	1.7	3.0	72.1%	-1.6
940	AT1G74450.1 | expressed protein | chr1:27986170-27987941 FORWARD | Aliases: F1M20.13, F1M20_13	5.7	4.6	1.1	3.0	73.1%	-1.7
941	AT4G31790.2 | diphthine synthase, putative (DPH5), similar to	3.6	4.8	-1.2	-3.0	73.2%	-1.6
942	AT1G24010.1 | expressed protein | chr1:8498792-8499488 REVERSE | Aliases: T23E23.27, T23E23_27	2.7	3.1	-0.5	-3.0	73.3%	-2.0
943	AT2G46560.1 | transducin family protein / WD-40 repeat family protein, similar to CPY (GI:3096961) {Chironomus thummi}; contains Pfam PF00400: WD domain, G-beta repeat (8 copies, 3 weak):9780477:gb:BE522499.1:BE522499 | chr2:19122443-19132928 REVERSE | Aliases: F13A10.9	5.9	7.1	-1.2	-3.0	73.6%	-1.6
944	AT3G07560.1 | glycine-rich protein | chr3:2411327-2413420 REVERSE | Aliases: F21O3.27	8.8	7.7	1.1	3.0	73.6%	-1.8
945	AT1G28530.2 | expressed protein | chr1:10031983-10035485 REVERSE | Aliases: F3M18.3	3.9	4.6	-0.7	-3.0	73.6%	-1.7
946	AT2G46610.2 | arginine/serine-rich splicing factor, putative, similar to SP:P92964 Arginine/serine-rich splicing factor RSP31 {Arabidopsis thaliana} | chr2:19143672-19145751 REVERSE | Aliases: F13A10.14	7.2	5.7	1.5	3.0	73.6%	-1.6
947	AT2G44130.1 | kelch repeat-containing F-box family protein, very low similarity to SP:Q9ER30 Kelch-related protein 1 (Sarcosin) {Rattus norvegicus}; contains Pfam profiles PF01344: Kelch motif, PF00646: F-box domain | chr2:18260686-18262130 FORWARD | Aliases: F6E13.26	5.8	4.6	1.2	3.0	73.7%	-1.6
948	AT5G18040.1 | expressed protein | chr5:5972421-5974158 FORWARD | Aliases: MCM23.14, MCM23_14	10.1	9.3	0.8	3.0	73.7%	-1.7
949	AT5G14660.2 | Symbol: PDF1B | peptide deformylase, chloroplast / polypeptide deformylase 1B (PDF1B), identical to SP:Q9FUZ2 Peptide deformylase, chloroplast precursor (EC 3.5.1.88) (PDF) (Polypeptide deformylase) {Arabidopsis thaliana}; supporting cDNA gi:11320965:gb:AF269165.1:AF269165 | chr5:4726736-4728749 REVERSE | Aliases: DEF2, PEPTIDE DEFORMYLASE, PEPTIDE DEFORMYLASE 1B	4.1	5.1	-1.0	-3.0	73.8%	-1.7
950	AT1G14040.1 | similar to EXS family protein / ERD1/XPR1/SYG1 family protein [Arabidopsis thaliana] (TAIR:At2g03250.1); similar to EXS family protein / ERD1/XPR1/SYG1 family protein [Arabidopsis thaliana] (TAIR:At2g03260.1); similar to EXS family protein / ERD1/XPR1/SYG1 family protein [Arabidopsis thaliana] (TAIR:At2g03240.1); similar to EXS family protein / ERD1/XPR1/SYG1 family protein [Arabidopsis thaliana] (TAIR:At1g35350.1); similar to EXS family protein / ERD1/XPR1/SYG1 family protein [Arabidopsis thaliana] (TAIR:At1g26730.1); similar to putative PHO1-like protein [Oryza sativa (japonica cultivar-group)] (GB:XP_549873.1); contains InterPro domain SPX, N-terminal (InterPro:IPR004331); contains InterPro domain EXS, C-terminal (InterPro:IPR004342) | chr1:4810485-4814540 FORWARD | Aliases: F7A19.13, F7A19_13	5.9	6.8	-0.9	-3.0	74.0%	-1.7
951	AT1G14340.1 | RNA recognition motif (RRM)-containing protein | chr1:4897341-4898956 FORWARD | Aliases: F14L17.11, F14L17_11	7.8	6.6	1.2	3.0	74.0%	-1.7
952	AT3G29240.2 | expressed protein, similar to At1g33780 (Arabidopsis thaliana); contains Pfam profile PF02622: Uncharacterized ACR, COG1678 | chr3:11192843-11194223 FORWARD | Aliases: MXO21.9	7.7	6.9	0.7	3.0	74.0%	-1.7
953	AT3G45980.1 | histone H2B, identical to histone H2B Arabidopsis thaliana GI:2407802; contains Pfam profile PF00125 Core histone H2A/H2B/H3/H4 | chr3:16907801-16908998 REVERSE | Aliases: F16L2.190	10.3	11.4	-1.1	-3.0	74.0%	-1.8
954	AT1G06520.1 | Symbol: ATGPAT1/GPAT1 | Encodes a membrane associated mitochondrial localized protein with glycerol-3-phosphate acyltransferase activity.Expressed in flower buds and siliques. Homozygous mutant plants are male sterile and have abnormal glycerolipid levels. | chr1:1993977-1996111 REVERSE | Aliases: ATGPAT1, F12K11.15, F12K11_15, GPAT1	3.3	3.9	-0.5	-3.0	74.0%	-1.9
955	AT4G36600.1 | late embryogenesis abundant domain-containing protein / LEA domain-containing protein, low similarity to SP:P20075 Embryonic protein DC-8 {Daucus carota}; contains Pfam profile PF02987: Late embryogenesis abundant protein | chr4:17263656-17265155 FORWARD | Aliases: AP22.17, AP22_17	5.1	4.1	1.1	3.0	74.1%	-1.6
956	AT3G11710.1 | lysyl-tRNA synthetase, putative / lysine--tRNA ligase, putative, similar to SP:Q43776 Lysyl-tRNA synthetase (EC 6.1.1.6) (Lysine--tRNA ligase) {Lycopersicon esculentum}; contains Pfam profile PF00152: tRNA synthetases class II (D, K and N) | chr3:3702179-3705707 REVERSE | Aliases: T19F11.11, T19F11_11	9.6	10.5	-0.9	-3.0	74.1%	-1.7
957	AT5G42990.1 | ubiquitin-conjugating enzyme 18 (UBC18), E2; identical to gi:2801448 | chr5:17261219-17263182 REVERSE | Aliases: MBD2.19, MBD2_19	6.5	7.4	-1.0	-3.0	74.2%	-1.7

Rank	Description	15.dpi	5.dpi	M	t	adj.q	B
958	AT1G59760.1 | ATP-dependent RNA helicase, putative, similar to SP:P47047 ATP-dependent RNA helicase DOB1 {Saccharomyces cerevisiae}, HUA enhancer 2 (Arabidopsis thaliana) GI:16024936; contains Pfam profiles PF00270: DEAD/DEAH box helicase, PF00271: Helicase conserved C-terminal domain | chr1:21988136-21993838 REVERSE | Aliases: F23H11.8, F23H11_8	6.8	7.8	-0.9	-3.0	74.2%	-1.7
959	AT3G61090.1 | expressed protein, contains Pfam profile PF04396: Protein of unknown function, DUF537 | chr3:22620069-22620797 REVERSE | Aliases: T27I15.180	4.2	3.5	0.6	3.0	74.2%	-1.8
960	AT1G67120.1 | midasin-related, similar to Midasin (MIDAS-containing protein) (Swiss-Prot:Q12019) (Saccharomyces cerevisiae); similar to Midasin (MIDAS-containing protein) (Swiss-Prot:Q9NU22) (Homo sapiens); contains Prosite PS00017: ATP/GTP-binding site motif A (P-loop)	4.2	4.9	-0.7	-3.0	74.3%	-1.8
961	AT4G36540.2 | basic helix-loop-helix (bHLH) family protein, contains Pfam domain, PF00010: Helix-loop-helix DNA-binding domain	4.8	5.5	-0.7	-3.0	74.4%	-1.7
962	AT3G62300.1 | agenet domain-containing protein, contains Pfam PF05641: Agenet domain | chr3:23065079-23068163 FORWARD | Aliases: T17J13.260	6.1	7.1	-1.0	-3.0	74.4%	-1.6
963	AT2G17220.2 | protein kinase, putative, similar to protein kinase APK1A (Arabidopsis thaliana) Swiss-Prot:Q06548 | chr2:7494757-7497258 REVERSE | Aliases: T23A1.8, T23A1_8	7.9	6.4	1.5	3.0	74.4%	-1.6
964	AT3G12700.1 | aspartyl protease family protein, contains Pfam PF00026: Eukaryotic aspartyl protease; similar to CND41, chloroplast nucleoid DNA binding protein (GI:2541876) (Nicotiana tabacum) | chr3:4037077-4039141 FORWARD | Aliases: MBK21.7	4.8	5.8	-1.0	-3.0	74.4%	-1.6
965	AT5G42830.1 | transferase family protein, similar to anthranilate N-hydroxycinnamoyl/benzoyltransferase, Dianthus caryophyllus (gi:2239091); contains Pfam transferase family domain PF002458 | chr5:17193612-17195246 FORWARD | Aliases: MBD2.2, MBD2_2	4.9	5.8	-1.0	-3.0	74.4%	-1.6
966	AT4G25590.1 | similar to actin-depolymerizing factor, putative [Arabidopsis thaliana] (TAIR:At5g52360.1); similar to pollen specific actin-depolymerizing factor 1 [Nicotiana tabacum] (GB:AAL91666.1); contains InterPro domain Actin-binding, cofilin/tropomyosin type (InterPro:IPR002108) | chr4:13058945-13060116 REVERSE | Aliases: M7J2.40, M7J2_40	3.6	3.0	0.6	3.0	74.4%	-1.9
967	AT3G13560.3 | glycosyl hydrolase family 17 protein, similar to beta-1,3-glucanase GI:15150341 from (Camellia sinensis)	5.8	6.9	-1.1	-3.0	74.4%	-1.6
968	AT2G20680.1 | glycosyl hydrolase family 5 protein / cellulase family protein, similar to (1-4)-beta-mannan endohydrolase (Coffea arabica) GI:10178872; contains Pfam profile PF00150: Cellulase (glycosyl hydrolase family 5) | chr2:8927548-8930254 FORWARD | Aliases: F5H14.35, F5H14_35	5.3	5.9	-0.7	-3.0	74.4%	-1.9
969	AT3G03870.2 | expressed protein, predicted using genefinder | chr3:995224-997417 FORWARD | Aliases: F20H23.8, F20H23_8	5.3	4.5	0.8	3.0	74.4%	-1.7
970	AT1G30960.1 | GTP-binding protein (ERG), identical to GTP-binding protein ERG SP:O82653 from (Arabidopsis thaliana) | chr1:11037635-11039982 REVERSE | Aliases: F17F8.15	4.3	5.5	-1.2	-3.0	74.4%	-1.6
971	AT4G29170.1 | Mnd1 family protein, contains Pfam PF03962: Mnd1 family | chr4:14382845-14385381 FORWARD | Aliases: F19B15.200, F19B15_200	3.3	4.3	-1.1	-3.0	74.4%	-1.7
972	AT3G56870.1 | expressed protein | chr3:21063452-21066569 FORWARD | Aliases: T8M16.200	3.2	4.1	-0.9	-3.0	74.4%	-1.6
973	AT2G01760.1 | Symbol: ARR14 | two-component responsive regulator family protein / response regulator family protein, similar to response regulator 9 (GI:14189890) (Zea mays); similar to ARR1 protein (GI:4210449) (Arabidopsis thaliana); contains Pfam profile: PF00072 response regulator receiver domain	3.0	3.5	-0.6	-3.0	74.4%	-1.9
974	AT2G31650.1 | trithorax 1 (ATX-1) (TRX1), identical to trithorax-like protein 1 GI:12659210 from (Arabidopsis thaliana); characterized in Alvarez-Venegas R,et al, ATX-1, an Arabidopsis Homolog of Trithorax, Activates Flower Homeotic Genes.(Curr Biol. 2003 Apr 15;13(8):627-37 PMID: 12699618); contains Pfam profiles PF00856: SET domain, PF00855: PWWP domain, PF00628, PHD-finger; identical to cDNA	4.4	5.1	-0.7	-3.0	74.4%	-1.8
975	AT3G02840.1 | immediate-early fungal elicitor family protein, similar to immediate-early fungal elicitor protein CMPG1 (GI:14582200) (Petroselinum crispum) | chr3:618416-619693 FORWARD | Aliases: F13E7.22, F13E7_22	5.0	4.2	0.9	3.0	74.4%	-1.7
976	AT5G10750.1 | expressed protein | chr5:3398952-3400157 FORWARD | Aliases: MAJ23.2	6.2	5.2	1.0	3.0	74.4%	-1.7
977	AT3G61360.1 | pentatricopeptide (PPR) repeat-containing protein, contains Pfam profile PF01535: PPR repeat | chr3:22715279-22717113 REVERSE | Aliases: T20K12.260	3.5	4.3	-0.8	-3.0	74.4%	-1.7
978	AT1G78210.1 | hydrolase, alpha/beta fold family protein, low similarity to hydrolases from Rhodococcus sp. EtbD2 GI:3273241, EtbD1 GI:3273239; contains Pfam profile PF00561: hydrolase, alpha/beta fold family | chr1:29427611-29429268 REVERSE | Aliases: T11I11.15, T11I11_15	6.5	7.3	-0.8	-3.0	74.5%	-1.8
979	AT1G72790.1 | hydroxyproline-rich glycoprotein family protein, contains proline-rich extensin domains, INTERPRO:IPR002965 | chr1:27398409-27400461 FORWARD | Aliases: F28P22.2, F28P22_2	3.6	2.8	0.8	3.0	74.5%	-1.7

Rank	Description	15.dpi	5.dpi	M	t	adj.q	B
980	AT2G24170.1 | endomembrane protein 70, putative, similar to MURA transposase of maize Mutator transposon | chr2:10281224-10284066 REVERSE | Aliases: F27D4.8, F27D4_8	4.7	5.6	-0.9	-3.0	74.6%	-1.7
981	AT5G59780.3 | Symbol: MYB59 | myb family transcription factor (MYB59), contains PFAM profile: myb DNA binding domain PF00249 | chr5:24099423-24100612 REVERSE | Aliases: MTH12.19, MTH12_19	7.0	5.4	1.6	3.0	74.6%	-1.6
982	AT5G23090.4 | similar to TATA-binding protein-associated phosphoprotein Dr1 protein, putative [Arabidopsis thaliana] (TAIR:At5g08190.1); similar to repressor protein [Oryza sativa] (GB:AAL73485.1); contains InterPro domain Transcription factor CBF/NF-Y/archaeal histone (InterPro:IPR003958); contains InterPro domain Histone-fold/TFIID-TAF/NF-Y (InterPro:IPR007124) | chr5:7749073-7750541 FORWARD | Aliases: MYJ24.8, MYJ24_8	6.9	6.3	0.5	3.0	74.6%	-1.9
983	AT3G46010.1 | Symbol: ADF1 | actin-depolymerizing factor 1 (ADF1), identical to SP:Q39250 Actin-depolymerizing factor 1 (ADF-1) (AtADF1) {Arabidopsis thaliana} | chr3:16920391-16921805 REVERSE | Aliases: ACTIN DEPOLYMERIZING FACTOR 1, ATADF, ATADF1, F16L2.220	12.9	11.9	1.0	3.0	74.6%	-1.8
984	AT3G50240.1 | Symbol: KICP 02 | kinesin motor protein-related, KINESIN-LIKE PROTEIN KIF4, Homo sapiens, EMBL:AF179308	4.1	4.9	-0.9	-3.0	74.6%	-1.7
985	AT3G17900.1 | expressed protein | chr3:6128539-6133389 FORWARD | Aliases: MEB5.12	4.3	4.9	-0.6	-3.0	74.9%	-1.8
986	AT5G52220.1 | expressed protein, ; expression supported by MPSS | chr5:21224456-21224904 REVERSE | Aliases: F17P19.12, F17P19_12	5.3	6.9	-1.7	-3.0	74.9%	-1.6
987	AT4G33090.1 | Symbol: APM1 | aminopeptidase M, similar to SP:Q11011 Puromycin-sensitive aminopeptidase (EC 3.4.11.-) (PSA) {Mus musculus}; contains Pfam profile PF01433: Peptidase family M1	8.4	9.5	-1.2	-3.0	75.3%	-1.8
988	AT1G25275.3 | expressed protein | chr1:8860659-8861349 FORWARD | Aliases: None	11.5	11.0	0.6	3.0	75.3%	-2.3
989	AT4G25900.1 | aldose 1-epimerase family protein, similar to apospory-associated protein C; APOC (Chlamydomonas reinhardtii) GI:6970044 Pfam profile PF01263: Aldose 1-epimerase | chr4:13161388-13163607 FORWARD | Aliases: F14M19.180, F14M19_180	6.2	7.5	-1.3	-3.0	75.3%	-1.6
990	AT4G37560.1 | formamidase, putative / formamide amidohydrolase, putative, similar to SP:Q50228 Formamidase (EC 3.5.1.49) (Formamide amidohydrolase) {Methylophilus methylotrophus}; contains Pfam profile PF03069: Acetamidase/Formamidase family | chr4:17646402-17648616 FORWARD | Aliases: F19F18.50, F19F18_50	2.6	2.3	0.3	3.0	75.3%	-2.6
991	AT4G18130.1 | Symbol: PHYE | phytochrome E (PHYE), identical to SP:P42498 Phytochrome E {Arabidopsis thaliana} | chr4:10041878-10045960 REVERSE | Aliases: F15J5.100, F15J5_100, PHYTOCHROME DEFECTIVE E, PHYTOCHROME E	5.2	5.9	-0.7	-3.0	75.5%	-2.0
992	AT5G48910.1 | pentatricopeptide (PPR) repeat-containing protein, contains Pfam profile PF01535: PPR repeat | chr5:19850098-19852160 REVERSE | Aliases: K19E20.2, K19E20_2	5.2	5.9	-0.7	-3.0	75.5%	-1.7
993	AT2G43430.2 | Symbol: GLX2 1 |	8.1	7.0	1.1	3.0	75.5%	-1.9
994	AT2G16740.1 | ubiquitin-conjugating enzyme, putative, strong similarity to SP:P35133 Ubiquitin-conjugating enzyme E2-17 kDa 10 (EC 6.3.2.19) (Ubiquitin- protein ligase 10) (Ubiquitin carrier protein 10) {Arabidopsis thaliana}; contains Pfam profile PF00179: Ubiquitin-conjugating enzyme	6.3	5.0	1.2	3.0	75.5%	-1.6
995	AT1G03510.1 | pentatricopeptide (PPR) repeat-containing protein, contains Pfam profile PF01535: PPR repeat | chr1:876259-877548 REVERSE | Aliases: F21B7.13	3.3	3.7	-0.4	-3.0	75.9%	-2.4
996	AT4G28706.3 | pfkB-type carbohydrate kinase family protein, contains Pfam profile: PF00294 pfkB family carbohydrate kinase | chr4:14167761-14170748 FORWARD | Aliases: None	5.5	6.3	-0.8	-3.0	75.9%	-2.0
997	AT4G17840.1 | expressed protein | chr4:9918067-9920488 REVERSE | Aliases: T6K21.20, T6K21_20	7.2	6.2	1.1	2.9	76.1%	-1.7
998	AT4G31910.1 | transferase family protein, low similarity to anthranilate N-hydroxycinnamoyl/benzoyltransferase Dianthus caryophyllus GI:3288180, 10-deacetylbaccatin III-10-O-acetyl transferase Taxus cuspidata GI:6746554; contains Pfam profile PF02458 transferase family | chr4:15441088-15443906 FORWARD | Aliases: F11C18.110, F11C18_110	5.8	7.4	-1.6	-2.9	76.1%	-1.6
999	AT3G11660.1 | Symbol: NHL1 | harpin-induced family protein / HIN1 family protein / harpin-responsive family protein, similar to harpin-induced protein hin1 (GI:1619321) (Nicotiana tabacum) | chr3:3678841-3679949 REVERSE | Aliases: T19F11.6	5.7	4.4	1.3	2.9	76.1%	-1.7
1000	AT5G11320.2 | flavin-containing monooxygenase family protein / FMO family protein, similar to flavin-containing monooxygenases YUCCA (gi:16555352), YUCCA2 (gi:16555354), and YUCCA3 (gi:16555356) from Arabidopsis thaliana | chr5:3611991-3613362 REVERSE | Aliases: F2I11.210, F2I11_210	2.7	3.5	-0.7	-2.9	76.2%	-1.9

Rank	Description	15.dpi	5.dpi	M	t	adj.q	B
1001	AT2G18300.2 | basic helix-loop-helix (bHLH) family protein, contains Pfam profile: PF00010 helix-loop-helix DNA-binding domain ;supported by cDNA gi:20127067:gb:AF488597.1: | chr2:7959751-7961649 REVERSE | Aliases: T30D6.19, T30D6_19	2.6	3.0	-0.4	-2.9	76.3%	-2.3
1002	AT2G23810.1 | similar to senescence-associated family protein [Arabidopsis thaliana] (TAIR:At4g30430.1); similar to senescence-associated protein DH [Zea mays] (GB:AAV31120.1); contains InterPro domain CD9/CD37/CD63 antigen (InterPro:IPR000301) | chr2:10142685-10144531 REVERSE | Aliases: F27L4.1, F27L4_1	8.1	7.3	0.8	2.9	76.3%	-1.7
1003	AT3G47460.1 | Symbol: ATSMC2 |	3.4	4.2	-0.8	-2.9	76.6%	-2.0
1004	AT3G12320.1 | expressed protein | chr3:3923742-3925442 FORWARD | Aliases: T2E22.34	7.6	6.3	1.3	2.9	76.7%	-1.6
1005	AT2G34600.1 | expressed protein | chr2:14580251-14580899 FORWARD | Aliases: T31E10.6, T31E10_6	3.5	2.3	1.2	2.9	76.7%	-2.1
1006	AT3G49670.1 | leucine-rich repeat transmembrane protein kinase, putative, CLAVATA1 receptor kinase, Arabidopsis thaliana, EMBL:ATU96879 | chr3:18428553-18432091 FORWARD | Aliases: T16K5.20	5.1	6.5	-1.4	-2.9	76.8%	-1.7
1007	AT1G54540.1 | expressed protein | chr1:20371369-20372088 REVERSE | Aliases: F20D21.35, F20D21_35	2.8	4.1	-1.3	-2.9	77.1%	-1.7
1008	AT2G34357.1 | expressed protein | chr2:14506315-14512806 FORWARD | Aliases: None	5.7	7.0	-1.3	-2.9	77.2%	-1.7
1009	AT4G38400.1 | Symbol: ATEXLA2 | expansin family protein (EXPL2), contains Pfam profile: PF01357 pollen allergen; expansin-like gene, PMID:11641069, www.bio.psu.edu/expansins	7.1	5.7	1.4	2.9	77.2%	-1.7
1010	AT3G47250.3 | expressed protein, contains Pfam profile PF03140: Plant protein of unknown function | chr3:17410862-17412705 REVERSE | Aliases: F13I12.300	4.1	3.2	0.9	2.9	77.5%	-1.7
1011	AT5G66420.2 | similar to transcriptional regulator [Clostridium tetani E88] (GB:NP_782382.1) | chr5:26538900-26542307 REVERSE | Aliases: K1F13.7, K1F13_7	7.2	8.2	-1.0	-2.9	77.5%	-1.7
1012	AT4G05590.2 | similar to expressed protein [Arabidopsis thaliana] (TAIR:At4g22310.1); similar to light induced protein like protein [Oryza sativa (japonica cultivar-group)] (GB:XP_477903.1); contains InterPro domain Protein of unknown function UPF0041 (InterPro:IPR005336) | chr4:2907169-2908532 FORWARD | Aliases: F6H8.10, F6H8_10	5.2	4.1	1.1	2.9	77.5%	-1.7
1013	AT2G23030.1 | protein kinase, putative, similar to protein kinase 3 (Glycine max) GP:310582:gb:AAB68961 | chr2:9810582-9813759 REVERSE | Aliases: F21P24.9, F21P24_9	5.9	4.6	1.3	2.9	77.5%	-1.7
1014	AT2G28620.1 | kinesin motor protein-related | chr2:12272244-12277097 REVERSE | Aliases: T8O18.9, T8O18_9	3.7	4.5	-0.9	-2.9	77.5%	-1.8
1015	AT3G49530.1 | Symbol: ANAC062 | no apical meristem (NAM) family protein, contains Pfam PF02365: No apical meristem (NAM) domain; similar to NAC2 - Arabidopsis thaliana, EMBL:AF201456 | chr3:18373429-18375898 REVERSE | Aliases: ANAC062, T9C5.120	7.3	6.4	0.8	2.9	77.5%	-1.8
1016	AT5G61300.1 | expressed protein | chr5:24668131-24670002 REVERSE | Aliases: MFB13.7, MFB13_7	4.7	5.4	-0.8	-2.9	77.5%	-1.9
1017	AT1G48300.1 | expressed protein | chr1:17851024-17852466 FORWARD | Aliases: F11A17.15, F11A17_15	10.0	8.2	1.8	2.9	77.5%	-1.7
1018	AT1G80850.1 | methyladenine glycosylase family protein, similar to SP:P05100 DNA-3-methyladenine glycosylase I (EC 3.2.2.20) (3-methyladenine-DNA glycosylase I, constitutive) {Escherichia coli}; contains Pfam profile PF03352: Methyladenine glycosylase | chr1:30390499-30392164 REVERSE | Aliases: F23A5.20, F23A5_20	4.7	5.4	-0.7	-2.9	77.5%	-1.9
1019	AT1G72360.1 | encodes a member of the ERF (ethylene response factor) subfamily B-2 of ERF/AP2 transcription factor family. The protein contains one AP2 domain. There are 5 members in this subfamily including RAP2.2 AND RAP2.12. | chr1:27245474-27246489 FORWARD | Aliases: T10D10.17, T10D10_17	9.5	7.9	1.6	2.9	77.5%	-2.1
1020	AT3G06140.1 | zinc finger (C3HC4-type RING finger) family protein, contains Pfam profile: PF00097 Zinc finger, C3HC4 type (RING finger) | chr3:1856688-1858856 REVERSE | Aliases: F28L1.8, F28L1_8	3.7	4.6	-1.0	-2.9	77.5%	-1.7
1021	AT5G66250.3 | kinectin-related, contains weak similarity to kinectin (GI:3766232) (Vulpes vulpes) | chr5:26485761-26487170 FORWARD | Aliases: K1L20.3, K1L20_3	7.0	6.1	1.0	2.9	77.5%	-1.8
1022	AT5G50840.2 | expressed protein | chr5:20703046-20706144 FORWARD | Aliases: K7B16.6, K7B16_6	6.7	8.2	-1.5	-2.9	77.5%	-1.8

Rank	Description	15.dpi	5.dpi	M	t	adj.q	B
1023	AT4G14580.1 | Symbol: CIPK4 | CBL-interacting protein kinase 4 (CIPK4), identical to CBL-interacting protein kinase 4 (Arabidopsis thaliana) gi:13249503:gb:AAG01367; identical to cDNA calcineurin B-like (CBL) interacting protein kinase 4 (CIPK4) GI:13249502 | chr4:8367883-8369163 REVERSE | Aliases: CBL interacting protein kinase 4, DL3330C, FCAALL.259	8.8	7.9	0.9	2.9	77.5%	-1.9
1024	AT3G10340.1 | phenylalanine ammonia-lyase, putative, similar to phenylalanine ammonia-lyase GB:S48726 (Petroselinum crispum) | chr3:3204018-3208027 FORWARD | Aliases: F14P13.6	4.1	5.4	-1.3	-2.9	77.5%	-1.7
1025	AT5G67320.1 | WD-40 repeat family protein, strong similarity to unknown protein (ref:NP_005638.1) | chr5:26874380-26878337 FORWARD | Aliases: K8K14.4, K8K14_4	6.6	7.4	-0.8	-2.9	77.6%	-1.9
1026	AT1G26670.1 | Symbol: VTI12 | vesical transport v-SNARE 12 (VTI12) / vesicle soluble NSF attachment protein receptor VTI1b (VTI1B) receptor VTI1b, identical to SP:Q9SEL5 Vesicle transport v-SNARE 12 (AtVTI12) (Vesicle transport v-SNARE protein VTI1b) (Vesicle soluble NSF attachment protein receptor VTI1b) (AtVTI1b) {Arabidopsis thaliana} | chr1:9215951-9217990 FORWARD | Aliases: ATVTI12, T24P13.5, T24P13_5, VTI1B	10.1	9.5	0.6	2.9	77.6%	-2.2
1027	AT4G17240.1 | expressed protein | chr4:9666700-9668386 REVERSE | Aliases: DL4655C, FCAALL.387	2.7	3.0	-0.3	-2.9	77.7%	-2.6
1028	AT5G57960.1 | GTP-binding family protein, similar to SP:P25519 GTP-binding protein hflX {Escherichia coli} | chr5:23480024-23483726 REVERSE | Aliases: MTI20.22, MTI20_22	5.0	4.3	0.7	2.9	77.8%	-1.9
1029	AT2G43060.1 | expressed protein, similar to cDNA bHLH transcription factor (bHLH zeta gene) GI:32563005 | chr2:17916262-17917159 FORWARD | Aliases: MFL8.8	6.4	5.4	1.0	2.9	78.1%	-1.8
1030	AT1G11530.1 | Encodes a single cysteine active site thioredoxin-related protein, similar to thioredoxin H-type from Arabidopsis thaliana SP:P29448, Nicotiana tabacum SP:Q07090; contains Pfam profile: PF00085 Thioredoxin; | chr1:3874437-3875484 FORWARD | Aliases: T23J18.19, T23J18_19	6.6	5.9	0.7	2.9	78.1%	-2.0
1031	AT1G79380.1 | copine-related, low similarity to SP:Q99829 Copine I {Homo sapiens} | chr1:29865384-29868528 FORWARD | Aliases: T8K14.20, T8K14_20	7.4	5.6	1.7	2.9	78.1%	-1.7
1032	AT5G59550.1 | zinc finger (C3HC4-type RING finger) family protein, contains Pfam domain, PF00097: Zinc finger, C3HC4 type (RING finger) | chr5:24015485-24017019 REVERSE | Aliases: F2O15.22, F2O15_22	6.9	6.0	0.9	2.9	78.1%	-1.8
1033	AT3G16175.1 | thioesterase family protein, contains Pfam profile PF03061: thioesterase family protein | chr3:5480470-5482048 FORWARD | Aliases: None	3.7	3.0	0.7	2.9	78.1%	-2.0
1034	AT5G60860.1 | Ras-related GTP-binding protein, putative, similar to GTP-binding protein GI:303742 from (Pisum sativum)	5.6	4.4	1.2	2.9	78.1%	-1.8
1035	AT5G17160.1 | expressed protein | chr5:5639648-5642515 REVERSE | Aliases: F2K13.310, F2K13_310	3.9	4.7	-0.8	-2.9	78.1%	-1.8
1036	AT1G69070.1 | expressed protein | chr1:25970975-25975052 REVERSE | Aliases: F4N2.4, F4N2_4	3.9	4.5	-0.6	-2.9	78.1%	-2.0
1037	AT1G21890.1 | nodulin MtN21 family protein, similar to MtN21 GI:2598575 (root nodule development) from (Medicago truncatula) | chr1:7682573-7685651 REVERSE | Aliases: T26F17.11, T26F17_11	4.9	3.9	1.0	2.9	78.1%	-1.7
1038	ATCG00580.1 | Symbol: PSBE | PSII cytochrome b559. There have been many speculations about the function of Cyt b559, but the most favored at present is that it plays a protective role by acting as an electron acceptor or electron donor under conditions when electron flow through PSII is not optimized. | chrC:64071-64322 REVERSE | Aliases: PSBE	3.6	5.1	-1.5	-2.9	78.1%	-1.7
1039	AT3G19970.1 | expressed protein | chr3:6958654-6961466 FORWARD | Aliases: MZE19.2	4.8	3.8	0.9	2.9	78.1%	-1.8
1040	AT2G18700.1 | Symbol: ATTPS11 | glycosyl transferase family 20 protein / trehalose-phosphatase family protein, similar to trehalose-6-phosphate synthase SL-TPS/P (Selaginella lepidophylla) GI:4100325; contains Pfam profiles PF00982: Glycosyltransferase family 20, PF02358: Trehalose-phosphatase | chr2:8116125-8119092 FORWARD | Aliases: ATTPSB, MSF3.8, MSF3_8, TPS11	5.3	6.2	-0.9	-2.9	78.1%	-1.8
1041	AT1G20890.1 | expressed protein, Location of ESTs OAO242 5' end, gb:Z30466 and OAO242 3' end, gb:Z30467 | chr1:7265812-7267312 REVERSE | Aliases: F9H16.13, F9H16_13	6.1	5.3	0.7	2.9	78.1%	-1.8
1042	AT5G13960.1 | Symbol: SUVH4 | SET domain-containing protein (SUVH4), identical to SUVH4 (Arabidopsis thaliana) GI:13517749; contains Pfam profiles PF00856: SET domain, PF05033: Pre-SET motif, PF02182: YDG/SRA domain; identical to cDNA SUVH4 (SUVH4) GI:13517748 | chr5:4501449-4506190 FORWARD | Aliases: KRYPTONITE, KYP, MAC12.7, MAC12_7, SU(VAR)3 9 HOMOLOG 4	4.2	5.1	-0.9	-2.9	78.2%	-1.8

Rank	Description	15.dpi	5.dpi	M	t	adj.q	B
1043	AT4G11830.2 | Symbol: PLDGAMMA2 | phospholipase D gamma 2 / PLD gamma 2 (PLDGAMMA2), identical to SP:Q9T051 Phospholipase D gamma 2 (EC 3.1.4.4) (AtPLDgamma2) (PLD gamma 2) (Arabidopsis thaliana) | chr4:7115794-7121239 REVERSE | Aliases: AT4G11835, T26M18.40, T26M18_40	6.7	5.9	0.8	2.9	78.2%	-1.9
1044	AT5G37770.1 | Symbol: TCH2 | touch-responsive protein / calmodulin-related protein 2, touch-induced (TCH2), identical to calmodulin-related protein 2,touch-induced SP:P25070 from (Arabidopsis thaliana) | chr5:15016084-15016849 REVERSE | Aliases: K22F20.10, K22F20_10, TOUCH 2	6.1	4.5	1.6	2.9	78.2%	-1.7
1045	AT2G16440.1 | DNA replication licensing factor, putative, similar to SP:P49717 DNA replication licensing factor MCM4 (CDC21 homolog) {Mus musculus}, SP:P29458 Cdc21 protein {Schizosaccharomyces pombe}; contains Pfam profile PF00493: MCM2/3/5 family | chr2:7133446-7137750 REVERSE | Aliases: F16F14.6, F16F14_6	3.6	5.3	-1.7	-2.9	78.4%	-1.7
1046	AT3G57470.1 | peptidase M16 family protein / insulinase family protein, contains weak similarity to Pfam domain, PF05193: Peptidase M16 inactive domain | chr3:21280238-21286904 REVERSE | Aliases: T8H10.70	3.0	3.3	-0.4	-2.9	78.4%	-2.5
1047	AT1G23010.1 | multi-copper oxidase type I family protein, similar to SP:P07788 Spore coat protein A {Bacillus subtilis}; contains Pfam profile PF00394: Multicopper oxidase | chr1:8147253-8149801 FORWARD | Aliases: F19G10.5, F19G10_5	3.3	4.4	-1.1	-2.9	78.5%	-1.8
1048	AT4G30390.1 | expressed protein | chr4:14862015-14863191 REVERSE | Aliases: F17I23.270, F17I23_270	7.2	5.7	1.5	2.9	78.5%	-1.8
1049	AT3G48040.1 | Symbol: ROP10 | Rac-like GTP-binding protein (ARAC8), identical to rac GTP binding protein Arac8 GI:3702966 from (Arabidopsis thaliana)	4.2	4.9	-0.7	-2.9	78.6%	-1.9
1050	AT3G27170.1 | Symbol: CLC B | chloride channel protein (CLC-b), identical to CLC-b chloride channel protein GB:CAA96058 from (Arabidopsis thaliana) (J. Biol. Chem. 271 (52), 33632-33638 (1996)) | chr3:10025254-10028435 FORWARD | Aliases: ATCLC B, MYF5.4	7.3	6.2	1.1	2.9	78.6%	-1.8
1051	AT5G36210.1 | expressed protein | chr5:14265143-14270460 REVERSE | Aliases: MAB16.20	7.1	8.4	-1.3	-2.9	78.7%	-1.8
1052	AT5G56600.1 | Symbol: PRF3 | profilin 5 (PRO5) (PRF3), identical to SP:Q9FE63 Profilin 5 {Arabidopsis thaliana} | chr5:22926914-22928047 REVERSE | Aliases: MIK19.4, MIK19_4, PFN3, PROFILIN, PROFILIN 3	11.9	11.0	0.9	2.9	78.7%	-1.8
1053	AT1G08630.4 | similar to threonine aldolase family protein [Arabidopsis thaliana] (TAIR:At3g04520.1); similar to OSJNBa0073E02.3 [Oryza sativa (japonica cultivar-group)] (GB:CAE05443.2) | chr1:2743764-2747970 REVERSE | Aliases: F22O13.11, F22O13_11	4.1	3.4	0.8	2.9	78.7%	-1.8
1054	AT1G03820.1 | expressed protein, similar to arabinogalactan-protein; AGP (GI:1087015) {Pyrus communis}; similar to Protein E6. (SP:Q01197) {Gossypium hirsutum} | chr1:959927-960708 REVERSE | Aliases: F21M11.27, F21M11_27	5.0	6.9	-2.0	-2.9	78.7%	-1.7
1055	AT1G01230.1 | ORMDL family protein, contains Pfam domain PF04061: ORMDL family	9.2	8.0	1.2	2.9	78.7%	-2.0
1056	AT1G70520.1 | protein kinase family protein, contains Pfam domain, PF00069: Protein kinase domain | chr1:26588441-26591082 REVERSE | Aliases: F24J13.9, F24J13_9	4.9	5.6	-0.6	-2.9	78.7%	-2.0
1057	AT4G24160.2 | hydrolase, alpha/beta fold family protein, contains Pfam profile PF00561: hydrolase, alpha/beta fold family | chr4:12539787-12542499 FORWARD | Aliases: T19F6.150, T19F6_150	8.7	8.0	0.7	2.9	78.7%	-1.9
1058	AT2G35630.1 | Symbol: MOR1 | microtubule organization 1 protein (MOR1), identical to microtubule organization 1 protein GI:14317953 from (Arabidopsis thaliana) | chr2:14973757-14987661 FORWARD | Aliases: GEM1, MICROTUBULE ORGANIZATION 1, T20F21.17, T20F21_17	6.0	6.6	-0.6	-2.9	78.8%	-2.1
1059	AT1G68830.1 | STN7 protein kinase; required for state transitions, phosphorylation of the major antenna complex (LHCII) between PSII and PSI, and light adaptation | chr1:25876119-25879169 REVERSE | Aliases: STN7, T6L1.2, T6L1_2	5.8	5.1	0.7	2.9	79.0%	-1.9
1060	AT4G02440.1 | Symbol: EID1 | F-box family protein, to circadian clock coupling factor ZGT (GP:14210079) {Nicotiana tabacum}; contains Pfam PF00646: F-box domain | chr4:1072481-1073779 FORWARD | Aliases: EMPFINDLICHER IM DUNKELROTEN LICHT 1, T14P8.22, T14P8_22	5.9	5.2	0.7	2.9	79.0%	-2.0
1061	AT4G05160.1 | Encodes a peroxisomal protein involved in the activation of fatty acids through esterification with CoA. At4g05160 preferentially activates fatty acids with medium chain length (C6:0 and C7:0) as well as even-numbered long-chain fatty acids (C14:0,	10.2	10.7	-0.5	-2.9	79.0%	-2.3
1062	AT5G22340.2 | expressed protein | chr5:7394476-7396878 FORWARD | Aliases: MWD9.13, MWD9_13	5.2	6.0	-0.8	-2.9	79.0%	-1.9
1063	AT3G27280.2 | prohibitin, putative, strong similarity to prohibitin (Arabidopsis thaliana) GI:1946331; contains Pfam profile PF01145: SPFH domain / Band 7 family | chr3:10078077-10079658 FORWARD | Aliases: K17E12.10	6.1	6.9	-0.8	-2.9	79.0%	-1.9
1064	AT5G44190.1 | Symbol: GLK2 | myb family transcription factor (GLK2), contains Pfam profile: PF00249 myb-like DNA-binding domain | chr5:17815596-17818050 FORWARD | Aliases: ATGLK2, GOLDEN2 LIKE 2, GPRI2, MLN1.11, MLN1_11	3.8	3.2	0.6	2.9	79.0%	-2.0

Rank	Description	15.dpi	5.dpi	M	t	adj.q	B
1065	AT1G20230.1 | pentatricopeptide (PPR) repeat-containing protein, contains Pfam profile PF01535: PPR repeat | chr1:7009561-7011843 FORWARD | Aliases: T20H2.1, T20H2_1	3.5	4.3	-0.8	-2.9	79.0%	-1.8
1066	AT5G18290.1 | major intrinsic protein-related / MIP-related, contains weak similarity to Pfam profile: MIP PF00230; annotated based on segmental duplication | chr5:6055030-6056702 REVERSE | Aliases: F20L16.1	4.5	5.7	-1.2	-2.9	79.0%	-1.7
1067	AT1G76170.1 | expressed protein, contains Pfam profile PF01171: PP-loop family | chr1:28589073-28591119 REVERSE | Aliases: T23E18.11, T23E18_11	4.4	3.8	0.6	2.9	79.0%	-2.1
1068	AT3G55430.1 | glycosyl hydrolase family 17 protein / beta-1,3-glucanase, putative, similar to beta-1,3 glucanase GI:7414433 from (Pisum sativum); contains Pfam profile PF00332: Glycosyl hydrolases family 17	9.2	8.0	1.2	2.9	79.1%	-1.8
1069	AT5G18590.2 | kelch repeat-containing protein, identical to RanGAP1 interacting protein (GI:21950739) (Arabidopsis thaliana); similar to Tip elongation aberrant protein 1 (Cell polarity protein tea1) (SP:P87061) (Schizosaccharomyces pombe); contains Pfam PF01344: Kelch motif (5 repeats) | chr5:6177967-6182708 REVERSE | Aliases: T28N17.70, T28N17_70	6.7	7.5	-0.8	-2.9	79.3%	-2.1
1070	AT1G36160.1 | Symbol: ACC1 | similar to acetyl-CoA carboxylase 2 (ACC2) [Arabidopsis thaliana] (TAIR:At1g36180.1); similar to putative acetyl-CoA carboxylase [Oryza sativa (japonica cultivar-group)] (GB:AAP53321.1); similar to acetyl-CoA carboxylase [Medicago sativa] (GB:AAB42144.1); similar to acetyl-CoA carboxylase [Brassica napus] (GB:CAC19875.1); similar to acetyl-CoA carboxylase [Brassica napus] (GB:CAC19876.1); similar to acetyl CoA carboxylase (GB:AAA75528.1); contains InterPro domain Carboxyl transferase (InterPro:IPR000022); contains InterPro domain Carbamoyl-phosphate synthetase large chain, N-terminal (InterPro:IPR005481); contains InterPro domain Biotin carboxylase, C-terminal (InterPro:IPR005482); contains InterPro domain Biotin/lipoyl attachment (InterPro:IPR000089); contains InterPro domain Carbamoyl-phosphate synthase L chain, ATP-binding (InterPro:IPR005479) | chr1:13535377-13545220 FORWARD | Aliases: ACETYL COA CARBOXYLASE, ACETYL COENZYME A CARBOXYLASE 1, AT ACC1, EMB22, EMBRYO DEFECTIVE 22, F15C21.1, GK, GREEN BLIMP, GURKE, PAS3, PASTICCINO 3	6.2	7.4	-1.2	-2.9	79.4%	-1.8
1071	AT5G07340.1 | calnexin, putative, identical to calnexin homolog 2 from Arabidopsis thaliana (SP:Q38798), strong similarity to calnexin homolog 1, Arabidopsis thaliana, EMBL:AT08315 (SP:P29402); contains Pfam profile PF00262 calreticulin family | chr5:2317214-2319650 FORWARD | Aliases: T2I1.50, T2I1_50	5.8	6.8	-1.0	-2.9	79.4%	-1.8
1072	AT1G20350.1 | Symbol: ATTIM17 1 | mitochondrial import inner membrane translocase subunit Tim17, putative, similar to SP:Q9SP35 Mitochondrial import inner membrane translocase subunit TIM17 {Arabidopsis thaliana}; contains Pfam profile PF02466: Mitochondrial import inner membrane translocase subunit Tim17 | chr1:7043703-7044582 FORWARD | Aliases: F14O10.5, F14O10_5	3.5	3.1	0.5	2.9	79.4%	-2.3
1073	AT1G62710.1 | Symbol: BETA VPE | vacuolar processing enzyme beta / beta-VPE, identical to SP:Q39044 Vacuolar processing enzyme, beta-isozyme precursor (EC 3.4.22.-) (Beta-VPE) {Arabidopsis thaliana} | chr1:23227540-23230581 REVERSE | Aliases: BETAVPE, F23N19.7, F23N19_7	7.8	6.7	1.1	2.9	79.4%	-1.7
1074	AT3G63390.1 | expressed protein | chr3:23422141-23422943 REVERSE | Aliases: MAA21.20	9.3	8.5	0.9	2.9	79.4%	-1.9
1075	AT3G50340.1 | expressed protein, predicted protein, Arabidopsis thaliana | chr3:18676076-18677654 REVERSE | Aliases: F11C1.180	7.4	6.8	0.7	2.9	79.4%	-2.0
1076	AT3G15460.1 | brix domain-containing protein, contains Pfam domain, PF04427: Brix domain | chr3:5213343-5216268 REVERSE | Aliases: MJK13.12	6.2	7.3	-1.1	-2.9	79.4%	-1.8
1077	AT1G76930.2 | Symbol: ATEXT4 |	7.3	10.1	-2.8	-2.9	79.4%	-2.2
1078	AT1G17580.1 | Symbol: MYA1 | myosin, putative, similar to myosin GI:433663 from (Arabidopsis thaliana) | chr1:6039296-6049549 FORWARD | Aliases: ATMYA1, F1L3.28, F1L3_28	5.3	6.2	-0.8	-2.9	79.4%	-1.8
1079	AT5G35700.1 | fimbrin-like protein, putative, similar to fimbrin-like protein (ATFIM1) (Arabidopsis thaliana) GI:2905893, fimbrin (Schizosaccharomyces pombe) GI:3057144; contains Pfam profile PF00307: Calponin homology (CH) domain	8.4	9.7	-1.3	-2.9	79.4%	-1.9
1080	AT3G54260.1 | expressed protein, various predicted proteins, Arabidopsis thaliana | chr3:20095988-20097741 REVERSE | Aliases: F24B22.220	9.8	10.6	-0.9	-2.9	79.4%	-1.9
1081	AT3G56080.1 | dehydration-responsive protein-related, similar to early-responsive to dehydration stress ERD3 protein (Arabidopsis thaliana) GI:15320410; contains Pfam profile PF03141: Putative methyltransferase | chr3:20821288-20824015 REVERSE | Aliases: F18O21.40	4.1	3.4	0.7	2.9	79.4%	-1.9
1082	AT2G27760.1 | Symbol: ATIPT2 |	6.4	7.4	-1.0	-2.9	79.4%	-1.8
1083	AT4G30600.1 | signal recognition particle receptor alpha subunit family protein, similar to Signal recognition particle receptor alpha subunit (SR-alpha) (Docking protein alpha) (DP-alpha) (SP:P08240) (Homo sapiens}; similar to Signal recognition particle receptor alpha subunit	10.4	9.6	0.8	2.9	79.4%	-2.2
1084	AT2G05940.1 | protein kinase, putative, similar to auxin-regulated dual specificity cytosolic kinase (Lycopersicon esculentum) gi:14484938:gb:AAK62821	7.4	5.7	1.7	2.9	79.4%	-1.8

Rank	Description	15.dpi	5.dpi	M	t	adj.q	B
1085	AT3G06850.2 | Symbol: DIN3 | branched chain alpha-keto acid dehydrogenase E2 subunit (din3), identical to branched chain alpha-keto acid dehydrogenase E2 subunit (din3) (Arabidopsis thaliana) GI:7021284 | chr3:2157989-2160619 REVERSE | Aliases: DARK INDUCIBLE 3, F3E22.1, LTA1	7.0	7.6	-0.6	-2.9	79.5%	-2.1
1086	AT4G04955.1 | Symbol: ATALN | Encodes an allantoinase which is involved in allantoin degradation and assimilation. Gene expression was induced when allantoin was added to the medium. The insertion mutant, ataln m2-1, did not grow well on the MS medium where allantoin, instead of ammonium nitrate, was supplied. | chr4:2522032-2525278 REVERSE | Aliases: ATALN	6.5	5.7	0.9	2.9	79.5%	-1.9
1087	AT3G60510.2 | similar to enoyl-CoA hydratase/isomerase family protein [Arabidopsis thaliana] (TAIR:At4g31810.1); similar to enoyl-CoA-hydratase [Avicennia marina] (GB:AAF01467.1); contains InterPro domain Enoyl-CoA hydratase/isomerase (InterPro:IPR001753) | chr3:22367927-22371122 REVERSE | Aliases: T8B10.170	3.9	4.4	-0.6	-2.9	79.5%	-2.0
1088	AT1G49300.2 | similar to Ras-related GTP-binding protein, putative [Arabidopsis thaliana] (TAIR:At3g18820.1); similar to putative GTP-binding protein [Cucumis sativus] (GB:AAQ72787.1); contains InterPro domain Small GTP-binding protein domain (InterPro:IPR005225); contains InterPro domain Ras small GTPase, Rab type (InterPro:IPR003579); contains InterPro domain Ras small GTPase, Rho type (InterPro:IPR003578); contains InterPro domain Ras small GTPase, Ras type (InterPro:IPR003577); contains InterPro domain Ras GTPase superfamily (InterPro:IPR001806); contains InterPro domain GTP-binding nuclear protein Ran (InterPro:IPR002041) | chr1:18238417-18241195 FORWARD | Aliases: F13F21.26, F13F21_26	10.0	9.3	0.6	2.9	79.5%	-2.0
1089	AT1G17790.1 | DNA-binding bromodomain-containing protein, similar to SP:P13709 Female sterile homeotic protein (Fragile-chorion membrane protein) {Drosophila melanogaster}; contains Pfam profile PF00439: Bromodomain | chr1:6125356-6127397 REVERSE | Aliases: F2H15.2, F2H15_2	4.1	4.7	-0.5	-2.9	79.5%	-2.1
1090	AT3G57060.1 | non-SMC condensin subunit, XCAP-D2/Cnd1 family protein, similar to 13S condensin XCAP-D2 subunit (Xenopus laevis) GI:3764087; contains Pfam profile PF04594: Non-SMC condensin subunit, XCAP-D2/Cnd1 | chr3:21126057-21132030 REVERSE | Aliases: F24I3.140	3.3	3.9	-0.6	-2.9	79.5%	-2.0
1091	AT5G59870.1 | histone H2A, putative, similar to histone H2A Petroselinum crispum SP:P19177, Lycopersicon esculentum SP:P25469, Zea mays SP:P40280; contains Pfam profile PF00125 Core histone H2A/H2B/H3/H4 | chr5:24132608-24133444 REVERSE | Aliases: MMN10.22, MMN10_22	10.4	12.0	-1.7	-2.9	79.9%	-1.8
1092	AT5G03210.1 | expressed protein | chr5:762786-763320 REVERSE | Aliases: F15A17.240, F15A17_240	5.2	3.6	1.6	2.9	79.9%	-2.1
1093	AT5G46590.1 | Symbol: ANAC096 | no apical meristem (NAM) family protein, contains Pfam PF02365: No apical meristem (NAM) domain; | chr5:18922906-18924038 FORWARD | Aliases: ANAC096, F10E10.6, F10E10_6	4.4	3.0	1.3	2.9	79.9%	-1.8
1094	AT2G35820.1 | expressed protein | chr2:15057991-15059318 FORWARD | Aliases: F11F19.27, F11F19_27	5.4	3.7	1.6	2.9	79.9%	-1.7
1095	AT2G19900.1 | malate oxidoreductase, putative, similar to NADP-dependent malic enzyme (EC 1.1.1.40) (NADP-ME) (SP:P51615) {Vitis vinifera} | chr2:8598981-8602535 REVERSE | Aliases: F6F22.7, F6F22_7	6.7	5.3	1.3	2.9	80.0%	-1.7
1096	AT5G27450.2 | mevalonate kinase (MK), identical to mevalonate kinase (Arabidopsis thaliana) SWISS-PROT:P46086 | chr5:9690745-9693227 FORWARD | Aliases: F21A20.160, F21A20_160	6.3	7.4	-1.1	-2.9	80.0%	-1.8
1097	AT5G62520.2 | Symbol: SRO5 | expressed protein | chr5:25115253-25117133 FORWARD | Aliases: K19B1.13, K19B1_13, SIMILAR TO RCD ONE 5, SIMILAR TO RCD ONE 5, SRO5	5.4	4.1	1.3	2.9	80.0%	-1.8
1098	AT3G50950.2 | disease resistance protein (CC-NBS-LRR class), putative, domain signature CC-NBS-LRR exists, suggestive of a disease resistance protein.	5.2	4.1	1.1	2.9	80.0%	-1.8
1099	AT3G25900.2 | Symbol: ATHMT 1/HMT 1 | homocysteine S-methyltransferase 1 (HMT-1), identical to GB:AAF23821 from (Arabidopsis thaliana) | chr3:9482076-9484163 FORWARD | Aliases: ATHMT 1, HMT 1, MPE11.15	5.0	6.9	-1.8	-2.9	80.1%	-1.8
1100	AT3G11620.4 | expressed protein, similar to hypothetical protein DDB0187018 [Dictyostelium discoideum] (GB:EAL64046.1); contains InterPro domain Esterase/lipase/thioesterase (InterPro:IPR000379) | chr3:3669325-3672040 REVERSE | Aliases: T19F11.2	5.5	4.6	0.9	2.9	80.2%	-2.0
1101	AT3G03750.2 | SET domain-containing protein, low similarity to G9a (Homo sapiens) GI:287865; contains Pfam profiles PF00856: SET domain, PF05033: Pre-SET motif | chr3:939959-942101 FORWARD | Aliases: F20H23.22, F20H23_22	5.0	4.4	0.6	2.9	80.2%	-2.2
1102	AT5G60210.1 | cytoplasmic linker protein-related, contains weak similarity to cytoplasmic linker protein CLIP-170 (GI:2905649) (Gallus gallus)	3.7	4.9	-1.1	-2.8	80.4%	-1.8
1103	AT3G51800.2 | Symbol: ATG2 | metallopeptidase M24 family protein, similar to SP:P50580 Proliferation-associated protein 2G4 {Mus musculus}; contains Pfam profile PF00557: metallopeptidase family M24 | chr3:19222004-19224646 REVERSE | Aliases: ATEM1.5	10.6	11.5	-0.8	-2.8	80.4%	-2.3
1104	AT1G51500.1 | Symbol: CER5 | ABC transporter family protein, similar to GB:AAF61569 from (Bombyx mori) | chr1:19101465-19104735 REVERSE | Aliases: D3, ECERIFERUM 5, F5D21.6, F5D21_6	4.6	3.7	0.9	2.8	80.4%	-1.9

Rank	Description	15.dpi	5.dpi	M	t	adj.q	B
1105	AT1G04850.1 | ubiquitin-associated (UBA)/TS-N domain-containing protein, weak similarity to SP:P45974 Ubiquitin carboxyl-terminal hydrolase 5 (EC 3.1.2.15) {Homo sapiens}; contains Pfam profile PF00627: UBA/TS-N domain | chr1:1365118-1368792 REVERSE | Aliases: F13M7.16, F13M7_16	8.7	9.8	-1.1	-2.8	80.4%	-2.0
1106	AT2G39820.1 | eukaryotic translation initiation factor 6, putative / eIF-6, putative, similar to SP:O55135 Eukaryotic translation initiation factor 6 (eIF-6) (B4 integrin interactor) {Mus musculus}; contains Pfam profile PF01912: eIF-6 family | chr2:16625355-16626767 FORWARD | Aliases: T5I7.12, T5I7_12	5.9	4.8	1.1	2.8	80.4%	-1.8
1107	AT5G01200.1 | myb family transcription factor, contains PFAM profile: PF00249 myb-like DNA binding domain | chr5:77115-78542 FORWARD | Aliases: F7J8.180, F7J8_180	4.0	3.4	0.6	2.8	80.4%	-2.1
1108	AT4G19230.2 | Symbol: CYP707A1 | cytochrome P450 family protein, cytochrome P450, Arabidopsis thaliana; supported by cDNA: gi_15293092_gb_AY050980.1_	6.1	4.9	1.2	2.8	80.4%	-1.9
1109	AT2G38000.1 | chaperone protein dnaJ-related, weak similarity to Chaperone protein dnaJ (Swiss-Prot:Q9ZFC5) (Methylovorus sp.)	6.1	5.0	1.1	2.8	80.4%	-2.0
1110	AT1G69890.1 | expressed protein, contains Pfam profile: PF04601 protein of unknown function (DUF569 | chr1:26326774-26328267 REVERSE | Aliases: T17F3.8, T17F3_8	3.7	3.1	0.6	2.8	80.7%	-2.0
1111	AT4G01870.1 | tolB protein-related, contains weak similarity to TolB protein precursor (Swiss-Prot:P44677) (Haemophilus influenzae) | chr4:808376-810446 REVERSE | Aliases: T7B11.13, T7B11_13	6.7	8.1	-1.4	-2.8	80.7%	-1.8
1112	AT1G19180.2 | expressed protein | chr1:6622105-6623611 FORWARD | Aliases: T29M8.5, T29M8_5	6.5	5.0	1.5	2.8	80.8%	-1.8
1113	AT3G02380.1 | Symbol: COL2 | zinc finger protein CONSTANS-LIKE 2 (COL2), identical to putative flowering-time gene CONSTANS (COL2) GB:AAB67879 GI:1507699 SP:Q96502 (Arabidopsis thaliana) | chr3:487250-488700 REVERSE | Aliases: CONSTANS LIKE 2, F11A12.7, F11A12_7	3.6	3.2	0.5	2.8	80.8%	-2.2
1114	AT1G49630.3 | peptidase M16 family protein / insulinase family protein, contains Pfam domain, PF05193: Peptidase M16 inactive domain | chr1:18371261-18379049 REVERSE | Aliases: F14J22.13, F14J22_13	3.9	4.6	-0.6	-2.8	80.8%	-2.0
1115	AT5G65140.1 | trehalose-6-phosphate phosphatase, putative, similar to trehalose-6-phosphate phosphatase (AtTPPB) (Arabidopsis thaliana) GI:2944180; contains Pfam profile PF02358: Trehalose-phosphatase | chr5:26036814-26039430 REVERSE | Aliases: MQN23.7, MQN23_7	4.5	3.7	0.8	2.8	80.8%	-1.9
1116	AT1G14360.1 | UDP-galactose/UDP-glucose transporter, putative, very similar to UDP-galactose/UDP-glucose transporter (GI:22651763) {Arabidopsis thaliana} | chr1:4910624-4913147 REVERSE | Aliases: F14L17.13, F14L17_13	6.2	6.8	-0.6	-2.8	80.8%	-2.0
1117	AT4G08300.1 | nodulin MtN21 family protein, similar to MtN21 GI:2598575 (root nodule development) from (Medicago truncatula) | chr4:5244888-5248339 FORWARD | Aliases: T12G13.140, T12G13_140	4.3	3.0	1.2	2.8	80.8%	-1.8
1118	AT5G26330.1 | plastocyanin-like domain-containing protein / mavicyanin, putative, similar to mavicyanin SP:P80728 from (Cucurbita pepo) | chr5:9241549-9242704 REVERSE | Aliases: F9D12.16, F9D12_16	4.0	3.4	0.7	2.8	80.8%	-2.0
1119	AT5G46150.2 | LEM3 (ligand-effect modulator 3) family protein / CDC50 family protein, similar to GI:835763; GI:4966357; GI:4585976; GI:11994416 from (Arabidopsis thaliana) | chr5:18725043-18727820 REVERSE | Aliases: MCL19.21, MCL19_21	5.9	5.0	0.9	2.8	80.8%	-1.9
1120	AT2G35760.1 | integral membrane family protein, contains TIGRFAM TIGR01569 : plant integral membrane protein TIGR01569; contains Pfam PF04535 : Domain of unknown function (DUF588) | chr2:15039002-15040384 FORWARD | Aliases: T20F21.5, T20F21_5	7.6	6.5	1.1	2.8	80.8%	-1.8
1121	AT1G46264.1 | Symbol: AT HSFB4 | heat shock transcription factor family protein, contains Pfam profile: PF00447 HSF-type DNA-binding domain | chr1:17227282-17228720 FORWARD | Aliases: F2G19.8, F2G19_8, HSFB4	4.3	5.2	-0.9	-2.8	80.8%	-1.8
1122	AT1G35420.1 | dienelactone hydrolase family protein, low similarity to dienelactone hydrolase (Rhodococcus opacus) GI:23094407; contains Pfam profile PF01738: Dienelactone hydrolase family | chr1:13026292-13027645 FORWARD | Aliases: F12A4.4, F12A4_4	8.0	6.7	1.3	2.8	80.8%	-1.8
1123	AT3G61080.1 | fructosamine kinase family protein, contains Pfam PF03881: Fructosamine kinase | chr3:22618105-22620074 FORWARD | Aliases: T27I15.170	6.6	5.4	1.3	2.8	80.8%	-1.8
1124	AT4G11820.2 | Symbol: BAP1 | hydroxymethylglutaryl-CoA synthase / HMG-CoA synthase / 3-hydroxy-3-methylglutaryl coenzyme A synthase, identical to Hydroxymethylglutaryl-CoA synthase (EC 4.1.3.5) (HMG-CoA synthase) (3-hydroxy-3-methylglutaryl coenzyme A synthase) (Swiss-Prot:P54873) (Arabidopsis thaliana) | chr4:7108909-7112024 REVERSE | Aliases: MVA1, T26M18.30, T26M18_30	3.9	4.9	-1.0	-2.8	80.8%	-1.9
1125	AT2G28890.1 | protein phosphatase 2C family protein / PP2C family protein, similar to protein phosphatase-2c (GI:3608412) (Mesembryanthemum crystallinum); contains Pfam PF00481 : Protein phosphatase 2C domain | chr2:12412673-12415312 REVERSE | Aliases: F8N16.18, F8N16_18	8.1	8.7	-0.6	-2.8	80.8%	-2.0

Rank	Description	15.dpi	5.dpi	M	t	adj.q	B
1126	AT1G32100.1 | pinoresinol-lariciresinol reductase, putative, similar to pinoresinol-lariciresinol reductase TH1 (Tsuga heterophylla)(GI:7578915); contains isoflavone reductase domain PF02716	4.8	7.0	-2.2	-2.8	80.8%	-1.8
1127	AT1G34110.1 | leucine-rich repeat transmembrane protein kinase, putative, contains similarity to receptor protein kinase-like protein GI:10177178 from (Arabidopsis thaliana) | chr1:12417154-12421167 REVERSE | Aliases: F12G12.7, F12G12_7	5.0	4.2	0.8	2.8	80.8%	-1.9
1128	AT5G27390.1 | expressed protein, CG6949 - Drosophila melanogaster, EMBL:AE003739 | chr5:9671848-9676121 REVERSE | Aliases: F21A20.100, F21A20_100	6.1	7.0	-0.9	-2.8	80.8%	-1.8
1129	AT1G19310.1 | zinc finger (C3HC4-type RING finger) family protein, contains Pfam profile: PF00097 zinc finger, C3HC4 type (RING finger) | chr1:6676225-6677661 REVERSE | Aliases: F18O14.3, F18O14_3	8.0	6.9	1.1	2.8	80.8%	-1.8
1130	AT2G16990.2 | similar to expressed protein [Arabidopsis thaliana] (TAIR:At2g16980.2); similar to tetracycline transporter protein-like [Oryza sativa (japonica cultivar-group)] (GB:BAD28125.1) | chr2:7390161-7394936 FORWARD | Aliases: F6P23.2, F6P23_2	6.9	6.3	0.7	2.8	80.8%	-2.1
1131	AT3G03330.1 | short-chain dehydrogenase/reductase (SDR) family protein, contains INTERPRO family IPR002198 Short-chain dehydrogenase/reductase (SDR) superfamily | chr3:783339-786177 REVERSE | Aliases: T21P5.25, T21P5_25	9.4	8.5	0.9	2.8	80.8%	-2.0
1132	AT3G29270.2 | expressed protein | chr3:11235551-11237669 FORWARD | Aliases: MMF24.2	5.7	4.7	1.1	2.8	80.9%	-1.9
1133	AT1G52540.1 | protein kinase, putative, contains protein kinase domain, Pfam:PF00069 | chr1:19573727-19575873 REVERSE | Aliases: F6D8.24, F6D8_24	5.3	4.5	0.8	2.8	80.9%	-2.1
1134	AT1G06020.1 | pfkB-type carbohydrate kinase family protein, similar to fructokinase GI:2102693 from (Lycopersicon esculentum) | chr1:1824547-1826100 FORWARD | Aliases: T21E18.7, T21E18_7	3.5	4.0	-0.5	-2.8	80.9%	-2.3
1135	AT4G33920.1 | protein phosphatase 2C family protein / PP2C family protein, similar to Ser/Thr protein phosphatase 2C (PP2C6) (GI:15020818) (Arabidopsis thaliana); similar to protein phosphatase 2C (GI:3608412) (Mesembryanthemum crystallinum); contains Pfam PF00481 : Protein phosphatase 2C domain | chr4:16260689-16262984 FORWARD | Aliases: F17I5.110, F17I5_110	4.4	3.1	1.3	2.8	80.9%	-1.8
1136	AT1G17690.1 | expressed protein | chr1:6082748-6088333 REVERSE | Aliases: F11A6.3, F11A6_3	5.7	6.3	-0.6	-2.8	80.9%	-2.1
1137	AT5G64880.1 | expressed protein | chr5:25949516-25951373 FORWARD | Aliases: MXK3.11, MXK3_11	5.2	4.3	0.9	2.8	80.9%	-1.9
1138	AT1G35720.1 | Symbol: ANNAT1 |	9.8	12.5	-2.6	-2.8	80.9%	-1.8
1139	AT5G10320.3 | expressed protein | chr5:3244006-3246721 REVERSE | Aliases: F18D22.90, F18D22_90	4.5	5.1	-0.6	-2.8	80.9%	-2.1
1140	AT3G17390.1 | Symbol: MTO3 | S-adenosylmethionine synthetase, putative, similar to S-adenosylmethionine synthetase 2 (Methionine adenosyltransferase 2, AdoMet synthetase 2) (Catharanthus roseus) SWISS-PROT:Q96552 | chr3:5952193-5954088 REVERSE | Aliases: MGD8.26, S ADENOSYLMETHIONINE SYNTHETASE, SAMS3	11.1	11.8	-0.8	-2.8	80.9%	-2.2
1141	AT1G27880.1 | ATP-dependent DNA helicase, putative, similar to SP:O94761 ATP-dependent DNA helicase Q4 (RecQ4) {Homo sapiens}; contains Pfam profiles PF00270: DEAD/DEAH box helicase, PF00271: Helicase conserved C-terminal domain | chr1:9708923-9714065 FORWARD | Aliases: F28L5.4, F28L5_4	2.6	3.1	-0.5	-2.8	80.9%	-2.3
1142	AT5G25580.1 | expressed protein | chr5:8903508-8906314 FORWARD | Aliases: T14C9.120, T14C9_120	4.1	5.7	-1.6	-2.8	80.9%	-1.8
1143	AT3G10200.1 | dehydration-responsive protein-related, similar to early-responsive to dehydration stress ERD3 protein (Arabidopsis thaliana) GI:15320410; contains Pfam profile PF03141: Putative methyltransferase | chr3:3157613-3160186 FORWARD | Aliases: F14P13.20	5.6	6.7	-1.1	-2.8	80.9%	-1.8
1144	AT4G00030.1 | plastid-lipid associated protein PAP / fibrillin family protein, contains Pfam profile PF04755: PAP_fibrillin | chr4:13527-14413 FORWARD | Aliases: F6N15.13, F6N15_13	5.8	4.7	1.2	2.8	81.4%	-1.8
1145	AT5G67300.1 | myb family transcription factor, contains PFAM profile: myb DNA binding domain PF00249 | chr5:26871248-26872464 FORWARD | Aliases: K8K14.2, K8K14_2	8.7	6.5	2.2	2.8	81.4%	-1.9
1146	AT4G02500.1 | galactosyl transferase GMA12/MNN10 family protein, low similarity to alpha-1,2-galactosyltransferase, Schizosaccharomyces pombe (SP:Q09174) | chr4:1101507-1103610 FORWARD | Aliases: T10P11.20	5.5	6.6	-1.1	-2.8	81.4%	-1.9
1147	AT4G09890.1 | expressed protein | chr4:6218393-6218923 FORWARD | Aliases: T5L19.20, T5L19_20	7.1	6.0	1.1	2.8	81.4%	-1.9

Rank	Description	15.dpi	5.dpi	M	t	adj.q	B
1148	AT1G59890.1 | paired amphipathic helix repeat-containing protein, similar to transcription co-repressor Sin3 (Xenopus laevis) GI:4960210; contains Pfam profile PF02671: Paired amphipathic helix repeat | chr1:22047991-22054726 FORWARD | Aliases: F23H11.20, F23H11_20	4.7	5.7	-1.0	-2.8	81.5%	-1.8
1149	AT3G61260.1 | DNA-binding family protein / remorin family protein, similar to DNA-binding protein gi:601843 (Arabidopsis thaliana), remorin (Solanum tuberosum) GI:1881585; contains Pfam profiles PF03763: Remorin C-terminal region, PF03766: Remorin N-terminal region | chr3:22686226-22687763 REVERSE | Aliases: T20K12.160	8.4	7.0	1.4	2.8	81.5%	-1.9
1150	AT3G23890.1 | Symbol: TOPII | DNA topoisomerase, ATP-hydrolyzing / DNA topoisomerase II / DNA gyrase (TOP2), identical to SP:P30182 DNA topoisomerase II (EC 5.99.1.3) {Arabidopsis thaliana}	3.9	5.3	-1.3	-2.8	81.5%	-1.8
1151	AT5G26860.1 | Symbol: LON_ARA_ARA | similar to Lon protease, putative [Arabidopsis thaliana] (TAIR:At3g05790.1); similar to Lon protease, putative [Arabidopsis thaliana] (TAIR:At3g05780.1); similar to LON2 [Zea mays] (GB:AAC50021.1); similar to Lon protease, putative [Phytophthora infestans] (GB:CAI72282.1); similar to putative Lon2 protease [Oryza sativa (indica cultivar-group)] (GB:AAO34661.1); similar to putative ATP-dependent proteinase LON2 [Oryza sativa (japonica cultivar-group)] (GB:BAD30597.1); similar to Lon protease-like protein [Homo sapiens] (GB:CAA53625.1); contains InterPro domain AAA ATPase (InterPro:IPR003593); contains InterPro domain Peptidase family S16 (InterPro:IPR001984); contains InterPro domain Lon protease S16, C-terminal proteolytic (InterPro:IPR008269); contains InterPro domain ATP-dependent protease La, LON (InterPro:IPR003111); contains InterPro domain ATP-dependent protease La (InterPro:IPR004815); contains InterPro domain Peptidase family S16, active site (InterPro:IPR008268); contains InterPro domain AAA ATPase, central region (InterPro:IPR003959) | chr5:9451051-9457003 FORWARD | Aliases: F2P16.23, F2P16_23	5.0	5.8	-0.9	-2.8	81.5%	-1.8
1152	AT5G19690.1 | Symbol: STT3A | oligosaccharyl transferase STT3 subunit family protein, similar to SP:P39007 Oligosaccharyl transferase STT3 subunit {Saccharomyces cerevisiae}; contains Pfam profile PF02516: Oligosaccharyl transferase STT3 subunit	8.4	9.4	-1.0	-2.8	81.5%	-1.9
1153	AT4G22050.1 | aspartyl protease family protein, contains Pfam profile: PF00026 eukaryotic aspartyl protease | chr4:11683877-11685656 FORWARD | Aliases: F1N20.150, F1N20_150	2.6	3.6	-1.0	-2.8	81.5%	-1.9
1154	AT5G01940.1 | eukaryotic translation initiation factor 2B family protein / eIF-2B family protein, similar to SP:P41035 Eukaryotic translation initiation factor 2 subunit (eIF-2-beta) {Oryctolagus cuniculus}; contains Pfam profile PF01873: Domain found in IF2B/IF5 | chr5:363591-364766 REVERSE | Aliases: T20L15.210, T20L15_210	5.7	5.0	0.8	2.8	81.5%	-1.9
1155	AT3G49580.1 | expressed protein | chr3:18386254-18386762 REVERSE | Aliases: T9C5.170	7.0	10.2	-3.3	-2.8	81.5%	-1.8
1156	AT5G08100.2 | L-asparaginase / L-asparagine amidohydrolase, identical to Swiss-Prot:P50287 L-asparaginase (EC 3.5.1.1) (L-asparagine amidohydrolase) (Arabidopsis thaliana) | chr5:2593051-2594628 REVERSE | Aliases: T22D6.40, T22D6_40	10.1	9.2	0.9	2.8	81.5%	-2.0
1157	AT1G28380.1 | expressed protein | chr1:9963579-9966306 FORWARD | Aliases: F3M18.18, F3M18_18	5.7	4.9	0.9	2.8	81.5%	-2.0
1158	AT5G63400.2 | Symbol: ADK1 | similar to adenylate kinase, putative [Arabidopsis thaliana] (TAIR:At5g50370.1); similar to adenylate kinase-a [Oryza sativa] (GB:BAA01180.1); similar to adenylate kinase [Oryza sativa] (GB:BAA94761.1); contains InterPro domain Adenylate kinase, active site lid (InterPro:IPR007862); contains InterPro domain Adenylate kinase (InterPro:IPR000850) | chr5:25410227-25412090 REVERSE | Aliases: ADENYLATE KINASE 1, MLE2.3, MLE2_3	9.1	10.4	-1.3	-2.8	81.5%	-1.9
1159	AT5G62530.1 | Symbol: ALDH12A1 | delta-1-pyrroline-5-carboxylate dehydrogenase (P5CDH), identical to delta-1-pyrroline-5-carboxylate dehydrogenase precursor (Arabidopsis thaliana) gi:15383744:gb:AAK73756; identical to cDNA delta-1-pyrroline-5-carboxylate dehydrogenase precursor (P5CDH) nuclear gene for mitochondrial product GI:15383743; contains Pfam profile PF00171:aldehyde dehydrogenase (NAD) family protein | chr5:25116229-25120476 REVERSE | Aliases: ATP5CDH, K19B1.14, K19B1_14	6.9	8.5	-1.6	-2.8	81.5%	-1.8
1160	AT2G26690.1 | nitrate transporter (NTP2), identical to nitrate transporter (ntp2) (Arabidopsis thaliana) GI:4490321 | chr2:11354225-11358071 REVERSE | Aliases: F18A8.6, F18A8_6	3.0	3.6	-0.6	-2.8	81.7%	-2.2
1161	AT3G23400.1 | plastid-lipid associated protein PAP / fibrillin family protein, contains Pfam profile PF04755: PAP_fibrillin | chr3:8376454-8378307 REVERSE | Aliases: MLM24.13	9.8	8.1	1.7	2.8	81.7%	-1.9
1162	AT3G12170.1 | DNAJ heat shock N-terminal domain-containing protein, similar to SP:Q9QYI7 DnaJ homolog subfamily B member 8 (Mus musculus); contains Pfam profile: PF00226 DnaJ domain | chr3:3881028-3882662 FORWARD | Aliases: F28J15.2	3.8	5.5	-1.7	-2.8	81.7%	-1.8
1163	AT3G13430.2 | similar to zinc finger (C3HC4-type RING finger) family protein [Arabidopsis thaliana] (TAIR:At1g55530.1); similar to hypothetical protein [Oryza sativa (japonica cultivar-group)] (GB:AAT77283.1); contains InterPro domain Zn-finger, RING (InterPro:IPR001841) | chr3:4367514-4368886 FORWARD | Aliases: MRP15.6	3.6	2.9	0.7	2.8	81.7%	-2.0
1164	AT5G07060.1 | zinc finger (CCCH-type) family protein, contains Pfam domain, PF00642: Zinc finger C-x8-C-x5-C-x3-H type (and similar) | chr5:2194395-2195873 REVERSE | Aliases: MOJ9.23, MOJ9_23	3.4	2.8	0.7	2.8	81.7%	-2.4

Rank	Description	15.dpi	5.dpi	M	t	adj.q	B
1165	AT1G68670.1 | myb family transcription factor, contains Pfam domain, PF00249: Myb-like DNA-binding domain | chr1:25785745-25787737 FORWARD | Aliases: F24J5.9, F24J5_9	6.0	6.7	-0.7	-2.8	81.8%	-2.0
1166	AT4G02880.1 | expressed protein | chr4:1274512-1278165 FORWARD | Aliases: T5J8.20, T5J8_20	4.7	4.0	0.7	2.8	81.8%	-2.0
1167	AT5G25900.1 | Symbol: GA3 | ent-kaurene oxidase, putative (GA3) / cytochrome P450, identical to GA3 (Arabidopsis thaliana) GI:3342249; similar to ent-kaurene oxidase (Cucurbita maxima) GI:11934675; contains Pfam profile PF00067: Cytochrome P450 | chr5:9036046-9038399 FORWARD | Aliases: CYP701A3, ENT KAURENE OXIDASE, GA REQUIRING 3, T1N24.23, T1N24_23	6.9	7.6	-0.7	-2.8	81.8%	-2.0
1168	AT2G42950.1 | expressed protein | chr2:17870896-17873564 FORWARD | Aliases: F7D19.5, F7D19_5	6.6	7.1	-0.5	-2.8	81.8%	-2.3
1169	AT3G16330.1 | expressed protein | chr3:5535202-5536069 FORWARD | Aliases: MYA6.13	4.5	3.5	1.0	2.8	81.8%	-2.0
1170	AT5G39100.1 | Symbol: GLP6 | germin-like protein (GLP6), nearly identical to SP:P92997 Germin-like protein subfamily 1 member 13 precursor {Arabidopsis thaliana}; exon 2 interrupted by a stop codon, creating non-consensus donor and acceptor splice sites. | chr5:15670318-15671248 REVERSE | Aliases: GERMIN LIKE PROTEIN 6, MXF12.13, MXF12_13	3.6	4.4	-0.8	-2.8	82.0%	-1.9
1171	AT5G39960.1 | GTP-binding family protein, contains Pfam domain, PF01926: GTPase of unknown function | chr5:16011423-16014048 FORWARD | Aliases: MYH19.120, MYH19_120	4.8	5.5	-0.7	-2.8	82.1%	-2.0
1172	AT3G05200.1 | Symbol: ATL6 | zinc finger (C3HC4-type RING finger) family protein (ATL6), contains Pfam profile: PF00097: Zinc finger, C3HC4 type (RING finger) | chr3:1476988-1478736 FORWARD | Aliases: T12H1.17, T12H1_17	7.0	6.1	0.8	2.8	82.1%	-1.9
1173	AT2G40270.2 | protein kinase family protein, contains Pfam domain, PF00069: Protein kinase domain | chr2:16829012-16831539 REVERSE | Aliases: T7M7.19	5.9	5.1	0.8	2.8	82.1%	-2.2
1174	AT1G57770.1 | amine oxidase family, contains similarity to carotenoid isomerase (Lycopersicon esculentum) GI:19550437, phytoene dehydrogenase (PDH1) GI:433144 from (Cercospora nicotianae); contains Pfam profile PF01593 amine oxidase, flavin-containing | chr1:21398861-21401945 FORWARD | Aliases: F12K22.18	4.8	3.6	1.2	2.8	82.1%	-1.9
1175	AT1G17410.1 | nucleoside diphosphate kinase family protein, contains Pfam PF00334 : Nucleoside diphosphate kinase domain; similar to Nucleoside diphosphate kinase homolog 5 (NDK-H 5) (NDP kinase homolog 5) (nm23-H5) (Testis-specific nm23 homolog) (Inhibitor of p53-induced apoptosis-beta) (IPIA-beta) (SP:P56597) {Homo sapiens} | chr1:5968566-5969893 REVERSE | Aliases: F28G4.12, F28G4_12	6.4	5.6	0.7	2.8	82.1%	-2.1
1176	AT5G46740.1 | Symbol: UBP21 | ubiquitin-specific protease 21 (UBP21), identical to ubiquitin-specific protease 21 GI:11993482 (Arabidopsis thaliana) | chr5:18982498-18985969 REVERSE | Aliases: MZA15.16, MZA15_16, UBIQUITIN SPECIFIC PROTEASE 21	3.8	4.7	-0.9	-2.8	82.1%	-2.2
1177	AT2G18390.1 | Symbol: TTN5 | ADP-ribosylation factor-like protein 2 (ARL2), identical to ARL2 G-protein (Halimasch; HAL; TITAN5) GI:20514265 from (Arabidopsis thaliana); identical to cDNA ARL2 G-protein mRNA GI:20514264; contains Pfam profile PF00025: ADP-ribosylation factor family; contains TIGRfam profile TIGR00231: small GTP-binding protein domain | chr2:7995247-7996943 FORWARD | Aliases: ARG1 LIKE 2, ARL2, ATARLC1, HAL, HALLIMASCH, T30D6.10, T30D6_10, TITAN 5	9.7	10.3	-0.6	-2.8	82.1%	-2.3
1178	AT1G78280.1 | transcription factor jumonji (jmjC) domain-containing protein, contains Pfam PF00646: F-box domain; contains Pfam PF02373: jmjC domain; similar to apoptotic cell clearance receptor PtdSerR (GI:11037740) (Mus musculus) | chr1:29457448-29462548 FORWARD | Aliases: F3F9.18, F3F9_18	5.1	4.3	0.8	2.8	82.3%	-2.1
1179	AT2G22850.1 | bZIP transcription factor family protein, contains a bZIP transcription factor basic domain signature (PDOC00036) | chr2:9739555-9740422 REVERSE | Aliases: T20K9.6, T20K9_6	4.2	3.3	0.9	2.8	82.4%	-2.0
1180	AT1G76640.1 | calmodulin-related protein, putative, similar to regulator of gene silencing calmodulin-related protein GI:12963415 from (Nicotiana tabacum) | chr1:28770218-28770697 REVERSE | Aliases: F28O16.1, F28O16_1	4.3	3.5	0.9	2.8	82.4%	-2.0
1181	AT4G21070.1 | Symbol: ATBRCA1 | similar to zinc finger (C3HC4-type RING finger) family protein / BRCT domain-containing protein [Arabidopsis thaliana] (TAIR:At1g04020.2); similar to putative BRCT domain-containing protein [Oryza sativa (japonica cultivar-group)] (GB:AAV59289.1); contains InterPro domain BRCT (InterPro:IPR001357); contains InterPro domain Zn-finger, RING (InterPro:IPR001841) | chr4:11248089-11252735 FORWARD | Aliases: T13K14.230	2.6	3.2	-0.5	-2.8	82.4%	-2.2
1182	AT1G76020.1 | expressed protein | chr1:28535786-28537218 REVERSE | Aliases: T4O12.23, T4O12_23	4.7	3.7	1.0	2.8	82.4%	-2.1
1183	AT3G66658.2 | Symbol: ALDH22a1 | betaine-aldehyde dehydrogenase, putative, similar to betaine-aldehyde dehydrogenase, chloroplast precursor (BADH) (Spinacia oleracea) SWISS-PROT:P17202; contains non-consensus splice site (GC) at intron 13 | chr3:2095111-2099143 REVERSE | Aliases: T8E24.4, T8E24_4	5.1	6.1	-1.0	-2.8	82.5%	-1.9

Rank	Description	15.dpi	5.dpi	M	t	adj.q	B
1184	AT1G20120.1 | family II extracellular lipase, putative, similar to family II lipase EXL3 GI:15054386, SP:P40602 Anther-specific proline-rich protein APG precursor {Arabidopsis thaliana}; contains Pfam profile PF00657: GDSL-like Lipase/Acylhydrolase | chr1:6975379-6977235 FORWARD | Aliases: T20H2.29, T20H2_29	3.5	2.8	0.7	2.8	82.5%	-2.2
1185	AT1G61350.1 | armadillo/beta-catenin repeat family protein, armadillo/beta-catenin-like repeats, Pfam:PF00514 | chr1:22637547-22639559 FORWARD | Aliases: T1F9.16, T1F9_16	4.8	5.7	-0.9	-2.8	82.5%	-1.9
1186	AT2G32440.1 | Symbol: KAO2 | ent-kaurenoic acid hydroxylase, putative / cytochrome P450, putative, identical to ent-kaurenoic acid hydroxylase / cytochrome P450 CYP88A (GI:13021856) (Arabidopsis thaliana); similar to ent-kaurenoic acid hydroxylase (Arabidopsis thaliana) GI:13021853 | chr2:13782665-13785079 FORWARD | Aliases: CYP88A4, ENT KAURENOIC ACID HYDROXYLASE 2, T32F6.4, T32F6_4	5.0	4.2	0.8	2.8	82.5%	-2.0
1187	AT3G21650.1 | serine/threonine protein phosphatase 2A (PP2A) regulatory subunit B', putative, similar to SWISS-PROT:Q28653 serine/threonine protein phosphatase 2A, 56 kDa regulatory subunit, delta isoform (PP2A, B subunit, B' delta isoform, PP2A, B subunit, B56 delta isoform, PP2A, B subunit, PR61 delta isoform, PP2A, B subunit, R5 delta isoform, PP2A, B subunit, B'-gamma) (Oryctolagus cuniculus); contains Pfam domain, PF01603: Protein phosphatase 2A regulatory B subunit (B56 family) | chr3:7621578-7624083 REVERSE | Aliases: MIL23.8	4.8	5.4	-0.6	-2.8	82.5%	-2.1
1188	AT3G11700.1 | beta-Ig-H3 domain-containing protein / fasciclin domain-containing protein, contains Pfam profile PF02469: Fasciclin domain | chr3:3698845-3701499 FORWARD | Aliases: T19F11.10	7.1	6.4	0.6	2.8	82.7%	-2.2
1189	AT2G19970.1 | pathogenesis-related protein, putative, similar to pathogenesis-related protein 1 {Arabidopsis thaliana} GI:166805; contains Pfam profile PF00188: SCP-like extracellular protein | chr2:8630818-8631557 REVERSE | Aliases: T2G17.23, T2G17_23	3.4	4.1	-0.7	-2.8	82.7%	-2.1
1190	AT1G14620.1 | Symbol: DECOY | expressed protein | chr1:5014749-5016599 REVERSE | Aliases: DECOY, EXGT A2, T5E21.12, T5E21_12, XTR2	7.5	8.9	-1.4	-2.8	82.9%	-1.9
1191	AT3G54200.1 | expressed protein, hin1 protein, Nicotiana tabacum, PIR:T03265 | chr3:20076643-20077538 FORWARD | Aliases: F24B22.160	5.9	5.1	0.8	2.8	82.9%	-2.2
1192	AT4G27130.1 | eukaryotic translation initiation factor SUI1, putative, similar to SP:P32911 Protein translation factor SUI1 {Saccharomyces cerevisiae}; contains Pfam profile PF01253: Translation initiation factor SUI1 | chr4:13604601-13606454 REVERSE | Aliases: T24A18.80, T24A18_80	11.4	10.7	0.7	2.8	83.1%	-2.5
1193	AT1G72550.2 | tRNA synthetase beta subunit family protein, contains Pfam profiles: PF03484 phenylalanine-tRNA synthetase, B5 domain, PF03483 B3/4 domain; an isoform contains a non-consensus TG acceptor splice site at a terminal exon. | chr1:27323413-27327677 REVERSE | Aliases: F28P22.26, F28P22_26	8.2	9.5	-1.3	-2.8	83.1%	-2.0
1194	AT1G18710.1 | myb family transcription factor (MYB47), contains Pfam profile: PF00249 myb-like DNA-binding domain | chr1:6450586-6453106 FORWARD | Aliases: F6A14.18, F6A14_18	4.8	3.8	1.0	2.8	83.1%	-1.9
1195	AT4G26940.2 | galactosyltransferase family protein, contains Pfam profile: PF01762 galactosyltransferase | chr4:13529431-13532681 REVERSE | Aliases: F10M23.280, F10M23_280	6.1	5.2	0.9	2.8	83.1%	-2.0
1196	AT3G14940.1 | Symbol: ATPPC3 |	6.6	8.3	-1.7	-2.8	83.2%	-2.0
1197	AT5G20890.1 | chaperonin, putative, similar to SWISS-PROT:P78371- T-complex protein 1, beta subunit (TCP-1-beta) (Homo sapiens); contains Pfam:PF00118 domain, TCP-1/cpn60 chaperonin family | chr5:7086655-7090083 REVERSE | Aliases: F22D1.60, F22D1_60	9.2	10.2	-1.0	-2.8	83.2%	-2.3
1198	AT5G52850.1 | pentatricopeptide (PPR) repeat-containing protein, contains INTERPRO:IPR002885 PPR repeats | chr5:21432161-21434842 REVERSE | Aliases: MXC20.7, MXC20_7	3.4	4.0	-0.6	-2.8	83.2%	-2.5
1199	AT5G39040.1 | Symbol: ATTAP2 |	5.8	6.4	-0.5	-2.8	83.2%	-2.2
1200	AT1G16510.1 | auxin-responsive family protein, similar to indole-3-acetic acid induced protein (SP:D14414) (Vigna radiata.); ESTs gb:AA712892 and gb:Z17613 come from this gene | chr1:5644565-5645436 REVERSE | Aliases: F3O9.31, F3O9_31	5.9	5.3	0.6	2.8	83.2%	-2.2
1201	AT3G17970.1 | chloroplast outer membrane translocon subunit, putative, similar to Toc64 (Pisum sativum) GI:7453538; contains Pfam profile PF00515 TPR Domain | chr3:6147968-6151981 FORWARD | Aliases: AT3G17960, MEB5.19	4.8	5.3	-0.5	-2.8	83.2%	-2.2
1202	AT3G52390.2 | tatD-related deoxyribonuclease family protein, similar to SP:P27859 Deoxyribonuclease tatD (EC 3.1.21.-) (DNAse tatD) {Escherichia coli}; contains Pfam profile PF01026: TatD related DNase | chr3:19433692-19436161 REVERSE | Aliases: F22O6.230	6.4	7.6	-1.2	-2.8	83.2%	-1.9
1203	AT3G61640.1 | Symbol: AGP20 | arabinogalactan-protein (AGP20)	6.9	5.7	1.2	2.8	83.2%	-2.0
1204	AT5G40380.1 | protein kinase family protein, contains Pfam domain, PF00069: Protein kinase domain | chr5:16169375-16172405 FORWARD | Aliases: MPO12.90, MPO12_90	2.5	2.2	0.2	2.8	83.2%	-2.9

Rank	Description	15.dpi	5.dpi	M	t	adj.q	B
1205	AT2G24600.3 | ankyrin repeat family protein, contains ankyrin repeats, Pfam:PF00023 | chr2:10459238-10461603 REVERSE | Aliases: F25P17.10, F25P17_10	3.0	3.5	-0.6	-2.8	83.2%	-2.2
1206	AT3G48560.1 | Symbol: CSR1 | acetolactate synthase, chloroplast / acetohydroxy-acid synthase (ALS), nearly identical to SP:P17597 Acetolactate synthase, chloroplast precursor (EC 2.2.1.6, formerly EC 4.1.3.18) (Acetohydroxy-acid synthase) (ALS) {Arabidopsis thaliana} | chr3:18012297-18014566 REVERSE | Aliases: ACETOHYDROXY ACID SYNTHASE, ACETOLACTATE SYNTHASE, ACETOLACTATE SYNTHASE DEFICIENT, AHAS, ALS, CHLORSULFURON/IMIDAZOLINONE RESISTANT 1, IMIDAZOLE RESISTANT 1, IMR1, T8P19.70, TZP5	11.3	10.4	0.9	2.8	83.6%	-2.1
1207	AT1G24340.1 | Symbol: EMB2421 | similar to phenol 2-monooxygenase like FAD dependent oxidoreductase of plant origin in apicomplexans [Cryptosporidium parvum] (GB:EAK89698.1); contains InterPro domain Flavoprotein monooxygenase (InterPro:IPR000733); contains InterPro domain Monooxygenase, FAD-binding (InterPro:IPR002938); contains InterPro domain NAD-binding site (InterPro:IPR000205); contains InterPro domain Aromatic-ring hydroxylase (InterPro:IPR003042) | chr1:8635198-8639146 FORWARD | Aliases: EMB2421, EMBRYO DEFECTIVE 2421, F3I6.28, F3I6_28	6.9	7.9	-0.9	-2.8	83.6%	-1.9
1208	AT2G20725.1 | CAAX amino terminal protease family protein, contains Pfam profile PF02517 CAAX amino terminal protease family protein | chr2:8940657-8942568 REVERSE | Aliases: F5H14.3, F5H14_3	7.3	6.6	0.8	2.8	83.6%	-2.0
1209	AT2G34050.1 | expressed protein, contains Pfam PS00030: Eukaryotic putative RNA-binding region RNP-1 signature	5.5	7.0	-1.5	-2.8	83.7%	-1.9
1210	AT1G01380.1 | Symbol: ETC1 | myb family transcription factor, similar to myb homolog (CPC) GI:2346966 from (Arabidopsis thaliana) | chr1:147267-147935 FORWARD | Aliases: ENHANCER OF TRY AND CPC 1, F6F3.18, F6F3_18	6.3	5.3	1.0	2.8	83.7%	-2.0
1211	AT5G10560.1 | glycosyl hydrolase family 3 protein, beta-xylosidase, Aspergllus nidulans, EMBL:ANXLND | chr5:3336256-3339367 REVERSE | Aliases: F12B17.90, F12B17_90	5.7	6.8	-1.2	-2.8	83.7%	-2.0
1212	AT3G04950.1 | expressed protein, contains InterPro domain SEC-C motif (InterPro:IPR004027) | chr3:1371711-1373483 FORWARD | Aliases: T9J14.10, T9J14_10	3.0	3.8	-0.8	-2.8	83.7%	-2.0
1213	AT2G24520.1 | ATPase, plasma membrane-type, putative / proton pump, putative, strong similarity to P-type H(+)-transporting ATPase from (Phaseolus vulgaris) GI:758250, (Lycopersicon esculentum) GI:1621440, SP:Q03194 {Nicotiana plumbaginifolia}, (Solanum tuberosum) GI:435001; contains InterPro accession IPR001757: ATPase, E1-E2 type | chr2:10422512-10426863 FORWARD | Aliases: F25P17.18, F25P17_18	4.5	3.9	0.7	2.8	83.7%	-2.2
1214	AT4G18820.1 | expressed protein | chr4:10330382-10334279 FORWARD | Aliases: F28A21.230, F28A21_230	5.1	6.0	-0.9	-2.8	83.8%	-2.0
1215	AT1G73430.1 | sec34-like family protein, contains Pfam PF04136: Sec34-like family profile; similar to Conserved oligomeric Golgi complex component 3 (Vesicle docking protein SEC34 homolog) (p94) (Swiss-Prot:Q96JB2) (Homo sapiens) | chr1:27607316-27614729 FORWARD | Aliases: T9L24.37, T9L24_37	5.1	5.8	-0.7	-2.8	83.8%	-2.1
1216	AT1G08130.1 | DNA ligase / polydeoxyribonucleotide synthase (ATP), identical to SP:Q42572 DNA ligase (EC 6.5.1.1) (Polydeoxyribonucleotide synthase (ATP)) {Arabidopsis thaliana}; contains Pfam profiles: PF01068 ATP dependent DNA ligase domain, PF04679 ATP dependent DNA ligase C terminal region, PF04675 DNA ligase N terminus | chr1:2542699-2547940 REVERSE | Aliases: T6D22.23	5.5	7.2	-1.7	-2.8	83.8%	-2.0
1217	AT4G25620.1 | hydroxyproline-rich glycoprotein family protein, contains proline-rich extensin domains, INTERPRO:IPR002965; Common family member At5g52430 (Arabidopsis thaliana); | chr4:13066779-13069688 REVERSE | Aliases: M7J2.10, M7J2_10	5.3	4.5	0.8	2.8	83.8%	-2.0
1218	AT3G01400.1 | armadillo/beta-catenin repeat family protein, contains Pfam profile: PF00514 armadillo/beta-catenin-like repeats (4 copies) | chr3:151769-153224 FORWARD | Aliases: T13O15.4, T13O15_4	7.3	6.6	0.7	2.8	84.3%	-2.1
1219	AT4G30020.1 | subtilase family protein, contains similarity to meiotic serine proteinase TMP GI:6468325 from (Lycopersicon esculentum)	6.3	7.1	-0.8	-2.8	84.3%	-2.0
1220	AT5G64570.1 | Symbol: XYL4 | glycosyl hydrolase family 3 protein | chr5:25827341-25830561 REVERSE | Aliases: ATBXL4, MUB3.9, MUB3_9	5.4	6.4	-1.0	-2.8	84.3%	-2.0
1221	AT2G46600.1 | calcium-binding protein, putative, similar to EF-hand Ca2+-binding protein CCD1 (Triticum aestivum) GI:9255753 | chr2:19143090-19143643 FORWARD | Aliases: F13A10.13	7.9	6.8	1.1	2.8	84.3%	-2.1
1222	AT1G78770.1 | cell division cycle family protein, similar to cell division cycle protein GI:603230 from (Homo sapiens); contains Pfam profile PF00515 TPR Domain 148977 (apparently not full-length). | chr1:29621812-29626340 FORWARD | Aliases: F9K20.19, F9K20_19	2.5	3.3	-0.7	-2.8	84.3%	-2.3
1223	AT3G12930.1 | expressed protein, contains Pfam domain PF02410: Domain of unknown function DUF143 | chr3:4128414-4129963 FORWARD | Aliases: MGH6.4	4.0	4.8	-0.8	-2.8	84.3%	-1.9

Rank	Description	15.dpi	5.dpi	M	t	adj.q	B
1224	AT2G17230.1 | phosphate-responsive 1 family protein, similar to phi-1 (phosphate-induced gene) (Nicotiana tabacum) GI:3759184; contains Pfam profile PF04674: Phosphate-induced protein 1 conserved region | chr2:7501618-7503098 REVERSE | Aliases: T23A1.9, T23A1_9	6.6	4.9	1.7	2.7	84.4%	-1.9
1225	AT4G27270.1 | quinone reductase family protein, similar to 1,4-benzoquinone reductase (Phanerochaete chrysosporium)(GI:4454993); similar to Trp repressor binding protein (Escherichia coli)(SP:P30849); contains flavodoxin domain PF00258 | chr4:13661360-13663249 REVERSE | Aliases: M4I22.80, M4I22_80	5.1	5.9	-0.8	-2.7	84.4%	-2.2
1226	AT3G23740.1 | expressed protein | chr3:8551779-8554737 REVERSE | Aliases: MYM9.8	2.7	3.2	-0.4	-2.7	84.4%	-2.5
1227	AT2G38810.3 | histone H2A, putative, strong similarity to histone H2A.F/Z Arabidopsis thaliana GI:2407800; contains Pfam profile PF00125 Core histone H2A/H2B/H3/H4 | chr2:16226363-16227907 REVERSE | Aliases: F13I13.4, F13I13_4	7.2	8.8	-1.5	-2.7	84.4%	-1.9
1228	AT4G37730.1 | bZIP transcription factor family protein, contains Pfam profile: PF00170 bZIP transcription factor | chr4:17723660-17724909 FORWARD | Aliases: T28I19.10, T28I19_10	5.1	4.3	0.7	2.7	84.4%	-2.1
1229	AT4G29350.1 | Symbol: PRF2 | profilin 2 (PRO2) (PFN2) (PRF2), identical to profilin 2 SP:Q42418 GI:1353772 from (Arabidopsis thaliana); identical to cDNA profilin (PRF2) GI:9965570 | chr4:14450035-14451383 FORWARD | Aliases: F17A13.170, F17A13_170, PFN2, PROFILIN 2	14.2	13.6	0.6	2.7	84.4%	-2.5
1230	AT1G50580.1 | glycosyltransferase family protein, similar to UDP rhamnose: anthocyanidin-3-glucoside rhamnosyltransferase GB:CAA81057 GI:397567 from (Petunia x hybrida); contains Pfam profile: PF00201 UDP-glucoronosyl and UDP-glucosyl transferase	5.6	6.4	-0.8	-2.7	84.4%	-2.0
1231	AT4G11470.1 | protein kinase family protein, contains Pfam domain PF00069: Protein kinase domain | chr4:6967724-6970156 FORWARD | Aliases: F25E4.90, F25E4_90	2.7	3.0	-0.4	-2.7	84.4%	-2.6
1232	AT1G76990.4 | Symbol: ACR3 | similar to ACT domain containing protein (ACR4) [Arabidopsis thaliana] (TAIR:At1g69040.2); similar to ACT domain containing protein (ACR4) [Arabidopsis thaliana] (TAIR:At1g69040.1); similar to putative ACT domain containing protein, ACR4, with alternative splicing forms [Oryza sativa (japonica cultivar-group)] (GB:XP_468663.1); contains InterPro domain Amino acid-binding ACT (InterPro:IPR002912) | chr1:28937056-28940444 FORWARD | Aliases: F22K20.9, F22K20_9	5.4	4.3	1.1	2.7	84.6%	-2.0
1233	AT5G01300.1 | phosphatidylethanolamine-binding family protein, similar to cold-regulated protein (Hordeum vulgare subsp. vulgare) GI:10799810; contains Pfam profile PF01161: Phosphatidylethanolamine-binding protein	9.7	8.5	1.1	2.7	85.1%	-1.9
1234	AT1G44780.2 | expressed protein, similar to expressed protein [Arabidopsis thaliana] (TAIR:At4g08310.1); similar to conserved hypothetical protein [Plasmodium chabaudi] (GB:CAH74933.1) | chr1:16912148-16914710 FORWARD | Aliases: T12C22.5, T12C22_5	3.9	5.5	-1.6	-2.7	85.3%	-2.1
1235	AT1G78480.1 | prenyltransferase/squalene oxidase repeat-containing protein, similar to beta-Amyrin Synthase GI:3688600 from (Panax ginseng) | chr1:29530394-29531256 REVERSE | Aliases: F3F9.1	5.0	4.3	0.7	2.7	85.5%	-2.2
1236	AT5G19530.1 | Symbol: ACL5 | spermine/spermidine synthase family protein, similar to SP:P09158 Spermidine synthase (EC 2.5.1.16) (Putrescine aminopropyltransferase) {Escherichia coli}; contains Pfam profile PF01564: Spermine/spermidine synthase | chr5:6588960-6591183 REVERSE | Aliases: ACAULIS 5, SPERMINE SYNTHASE, T20D1.50, T20D1_50	2.6	3.6	-0.9	-2.7	85.6%	-1.9
1237	AT3G28180.1 | Symbol: ATCSLC04 | glycosyl transferase family 2 protein, similar to beta-(1-3)-glucosyl transferase GB:AAC62210 GI:3687658 from (Bradyrhizobium japonicum), cellulose synthase from Agrobacterium tumeficiens (gi:710492) and Agrobacterium radiobacter (gi:710493); contains Pfam glycosyl transferase, group 2 family protein domain PF00535	6.8	5.8	0.9	2.7	85.8%	-2.0
1238	AT5G12930.1 | expressed protein | chr5:4082893-4085300 REVERSE | Aliases: T24H18.90, T24H18_90	2.6	2.9	-0.3	-2.7	86.0%	-2.6
1239	AT2G44490.1 | glycosyl hydrolase family 1 protein, contains Pfam PF00232 : Glycosyl hydrolase family 1 domain; TIGRFAM TIGR01233: 6-phospho-beta-galactosidase; similar to anther-specific protein ATA27 (GI:2746341) (Arabidopsis thaliana) | chr2:18371859-18374801 FORWARD | Aliases: F4I1.30	4.6	5.9	-1.3	-2.7	86.0%	-1.9
1240	AT1G69935.1 | expressed protein | chr1:26345464-26346789 REVERSE | Aliases: None	6.6	5.7	1.0	2.7	86.0%	-1.9
1241	AT1G31320.1 | LOB domain protein 4 / lateral organ boundaries domain protein 4 (LBD4), identical to SP:Q9SHE9 LOB domain protein 4 {Arabidopsis thaliana} | chr1:11212998-11214363 FORWARD | Aliases: T19E23.11, T19E23_11	3.6	3.2	0.5	2.7	86.1%	-2.4
1242	AT5G10400.1 | histone H3, identical to several histone H3 proteins, including Zea mays SP:P05203, Medicago sativa GI:166384, Encephalartos altensteinii SP:P08903, Pisum sativum SP:P02300; contains Pfam profile PF00125 Core histone H2A/H2B/H3/H4 | chr5:3270290-3270953 REVERSE | Aliases: F12B17.250	6.5	8.8	-2.3	-2.7	86.1%	-2.0
1243	AT4G26320.1 | Symbol: AGP13 | arabinogalactan-protein (AGP13), identical to gi:10880503:gb:AAG24281 | chr4:13317109-13317479 REVERSE | Aliases: ARABINOGALACTAN PROTEIN 13, T25K17.130, T25K17_130	6.9	5.4	1.5	2.7	86.1%	-2.1

Rank	Description	15.dpi	5.dpi	M	t	adj.q	B
1244	AT5G04770.1 | Symbol: CAT6 | amino acid permease family protein, similar to cationic amino acid transporter-1 (Rattus norvegicus) GI:1589917; contains Pfam profile PF00324: Amino acid permease | chr5:1379081-1382420 FORWARD | Aliases: CATIONIC AMINO ACID TRANSPORTER 6, MUK11.10	5.6	6.3	-0.7	-2.7	86.1%	-2.1
1245	AT5G02780.1 | In2-1 protein, putative, similar to In2-1 (Zea mays) EMBL:X58573	4.8	6.5	-1.7	-2.7	86.1%	-2.0
1246	AT2G16400.1 | homeodomain-containing protein | chr2:7108412-7110973 REVERSE | Aliases: F16F14.10, F16F14_10	7.0	7.7	-0.7	-2.7	86.1%	-2.3
1247	AT4G19390.1 | expressed protein | chr4:10574779-10576439 REVERSE | Aliases: T5K18.170, T5K18_170	9.4	8.5	0.9	2.7	86.1%	-2.0
1248	AT1G21140.1 | nodulin, putative, similar to nodulin 21 (N-21) (Glycine max) SWISS-PROT:P16313 | chr1:7404372-7405270 FORWARD | Aliases: T22I11.3, T22I11_3	8.8	7.7	1.1	2.7	86.1%	-2.1
1249	AT4G16140.1 | proline-rich family protein, contains proline-rich extensin domains, INTERPRO:IPR002965 | chr4:9129058-9129853 FORWARD | Aliases: DL4110W, FCAALL.289	3.2	3.8	-0.7	-2.7	86.1%	-2.1
1250	AT1G63150.1 | pentatricopeptide (PPR) repeat-containing protein, contains Pfam profile PF01535: PPR repeat | chr1:23423061-23425244 FORWARD | Aliases: F16M19.13, F16M19_13	2.9	3.3	-0.4	-2.7	86.1%	-2.5
1251	AT1G61770.1 | DNAJ heat shock N-terminal domain-containing protein, similar to SP:Q9UBS4 DnaJ homolog subfamily B member 11 precursor Homo sapiens; contains Pfam profile PF00226 DnaJ domain | chr1:22813830-22816272 FORWARD | Aliases: T13M11.13, T13M11_13	7.7	8.4	-0.7	-2.7	86.1%	-2.1
1252	AT2G15960.1 | expressed protein | chr2:6954391-6954770 FORWARD | Aliases: F19G14.4, F19G14_4	4.6	3.2	1.4	2.7	86.1%	-2.0
1253	AT4G27657.1 | expressed protein | chr4:13813050-13813522 FORWARD | Aliases: None	4.7	3.2	1.5	2.7	86.2%	-2.4
1254	AT1G18470.2 | zinc finger (C3HC4-type RING finger) family protein, contains Pfam profile: PF00097 zinc finger, C3HC4 type | chr1:6356126-6360159 REVERSE | Aliases: F15H18.5, F15H18_5	6.8	5.7	1.1	2.7	86.2%	-2.0
1255	AT3G56170.1 | Symbol: CAN | Ca(2+)-dependent nuclease, identical to Ca(2+)-dependent nuclease (Arabidopsis thaliana) GI:7684292; supporting cDNA gi:7684291:dbj:D84226.1: | chr3:20853556-20855437 FORWARD | Aliases: CA 2+ DEPENDENT NUCLEASE, F18O21.130, F18O21_130	6.0	4.8	1.3	2.7	86.2%	-2.0
1256	AT3G15280.1 | expressed protein, very low similarity to cold regulated gene REP14 (Secale cereale) GI:20067233, late embryogenesis abundant protein (Picea glauca) GI:1161171 | chr3:5143975-5144700 REVERSE | Aliases: K7L4.8	4.4	3.2	1.3	2.7	86.2%	-1.9
1257	AT5G51545.1 | expressed protein | chr5:20953660-20954626 FORWARD | Aliases: None	6.6	5.5	1.0	2.7	86.2%	-1.9
1258	AT3G55840.1 | expressed protein | chr3:20727974-20729845 REVERSE | Aliases: F27K19.20	4.5	3.5	1.0	2.7	86.2%	-2.2
1259	AT3G27650.1 | LOB domain protein 25 / lateral organ boundaries domain protein 25 (LBD25), identical to LOB DOMAIN 25 (Arabidopsis thaliana) GI:17227166 | chr3:10239783-10241848 FORWARD | Aliases: MGF10.6	4.8	4.0	0.8	2.7	86.7%	-2.1
1260	AT2G34650.1 | Symbol: PID | protein kinase PINOID (PID), identical to protein kinase PINOID (Arabidopsis thaliana) gi:7208442:gb:AAF40202; contains protein kinase domain, Pfam:PF00069 | chr2:14596851-14598867 REVERSE | Aliases: ABR, ABRUPTUS, PINOID, PROTEIN KINASE PINOID, T31E10.1, T31E10_1	5.1	6.1	-1.0	-2.7	86.8%	-1.9
1261	AT5G41180.1 | leucine-rich repeat protein kinase, putative, contains leucine rich repeat (LRR) domains, Pfam:PF00560; contains protein kinase domain, Pfam:PF00069 | chr5:16501099-16504654 FORWARD | Aliases: MEE6.25, MEE6_25	4.2	5.0	-0.8	-2.7	86.8%	-2.1
1262	AT1G24510.2 | T-complex protein 1 epsilon subunit, putative / TCP-1-epsilon, putative / chaperonin, putative, identical to SWISS-PROT:O04450- T-complex protein 1, epsilon subunit (TCP-1-epsilon) (Arabidopsis thaliana); strong similarity to SP:P54411 T-complex protein 1, epsilon subunit (TCP-1-epsilon) (CCT-epsilon) (TCP-K36) {Avena sativa}; contains Pfam:PF00118 domain, TCP-1/cpn60 chaperonin family | chr1:8685298-8688231 REVERSE | Aliases: F21J9.17	9.1	9.9	-0.8	-2.7	86.8%	-2.6
1263	AT2G30360.1 | Symbol: CIPK11 |	7.8	6.5	1.4	2.7	86.8%	-2.1
1264	AT3G14890.2 | phosphoesterase, identical to phosphoesterase (Arabidopsis thaliana) GI:21630064; contains Pfam profile PF00645: Poly(ADP-ribose) polymerase and DNA-Ligase Zn-finger region | chr3:5008828-5013272 FORWARD | Aliases: K15M2.3	4.7	6.1	-1.4	-2.7	86.8%	-2.0
1265	AT3G46600.2 | scarecrow transcription factor family protein, scarecrow-like 11 - Arabidopsis thaliana, EMBL:AF036307 | chr3:17168929-17170950 FORWARD | Aliases: F12A12.120	5.3	4.8	0.5	2.7	86.8%	-2.3
1266	AT5G41870.1 | glycoside hydrolase family 28 protein / polygalacturonase (pectinase) family protein, weak similarity to polygalacturonase PG1 (Glycine max) GI:5669846; contains PF00295: Glycosyl hydrolases family 28	3.9	4.6	-0.6	-2.7	86.8%	-2.2

Rank	Description	15.dpi	5.dpi	M	t	adj.q	B
1267	AT5G60100.1 | Symbol: APRR3 | pseudo-response regulator 3 (APRR3), identical to pseudo-response regulator 3 GI:10281008 from (Arabidopsis thaliana) | chr5:24215441-24217728 REVERSE | Aliases: MGO3.8, MGO3_8, PRR3, PSEUDO RESPONSE REGULATOR 3	5.1	4.6	0.5	2.7	87.1%	-2.3
1268	AT2G28060.1 | protein kinase-related, similar to GAL83 protein (Solanum tuberosum) GI:5702015; contains Pfam profile PF04739: 5'-AMP-activated protein kinase, beta subunit, complex-interacting region; supporting cDNA gi:22652763:gb:AF491295.1: | chr2:11957063-11959279 REVERSE | Aliases: F24D13.15, F24D13_15	7.3	6.6	0.7	2.7	87.2%	-2.2
1269	AT5G46295.1 | expressed protein | chr5:18797023-18797429 REVERSE | Aliases: None	4.7	3.7	1.0	2.7	87.2%	-2.1
1270	AT4G26370.2 | antitermination NusB domain-containing protein, contains Pfam profile: PF01029 NusB family | chr4:13333733-13336184 REVERSE | Aliases: T25K17.4	4.2	3.4	0.7	2.7	87.2%	-2.2
1271	AT4G19120.2 | early-responsive to dehydration stress protein (ERD3), identical to ERD3 protein (Arabidopsis thaliana) GI:15320410; contains Pfam profile PF03141: Putative methyltransferase; identical to cDNA ERD3 GI:15320409 | chr4:10460306-10463113 REVERSE | Aliases: T18B16.90, T18B16_90	10.4	11.2	-0.8	-2.7	87.2%	-2.3
1272	AT5G03390.1 | expressed protein, contains Pfam profile PF03478: Protein of unknown function (DUF295) | chr5:834987-836750 FORWARD | Aliases: F12E4.130, F12E4_130	2.8	3.6	-0.8	-2.7	87.2%	-2.1
1273	AT3G60590.3 | expressed protein | chr3:22409203-22410728 FORWARD | Aliases: T4C21.1	4.7	4.0	0.8	2.7	87.2%	-2.3
1274	AT3G50350.1 | expressed protein | chr3:18683888-18684688 FORWARD | Aliases: F11C1.190	7.2	6.0	1.2	2.7	87.4%	-2.0
1275	AT2G42200.2 | squamosa promoter-binding protein-like 9 (SPL9), identical to squamosa promoter binding protein-like 9 (Arabidopsis thaliana) GI:5931673; contains Pfam profile PF03110: SBP domain | chr2:17594485-17596708 FORWARD | Aliases: T24P15.11, T24P15_11	3.3	4.1	-0.8	-2.7	87.4%	-2.2
1276	AT5G04420.3 | similar to acyl-CoA binding family protein [Arabidopsis thaliana] (TAIR:At3g05420.1); similar to acyl-CoA binding family protein [Arabidopsis thaliana] (TAIR:At3g05420.2); similar to putative transcription factor [Oryza sativa (japonica cultivar-group)] (GB:XP_470332.1); contains InterPro domain Kelch repeat (InterPro:IPR006652) | chr5:1246036-1249840 REVERSE | Aliases: T19N18.4	6.1	7.7	-1.6	-2.7	87.4%	-2.0
1277	AT5G66070.2 | similar to zinc finger (C3HC4-type RING finger) family protein [Arabidopsis thaliana] (TAIR:At2g17730.1); similar to putative zinc finger protein [Oryza sativa] (GB:AAL79729.1); contains InterPro domain Zn-finger, RING (InterPro:IPR001841) | chr5:26439081-26440458 FORWARD | Aliases: K2A18.15, K2A18_15	5.7	4.5	1.1	2.7	87.4%	-2.0
1278	AT3G62150.1 | multidrug resistant (MDR) ABC transporter, putative, similar to multidrug-resistant protein CjMDR1 GI:14715462 from (Coptis japonica); contains Pfam profiles PF00005: ABC transporter, PF00664: ABC transporter transmembrane region | chr3:23019730-23024554 REVERSE | Aliases: T17J13.110	7.4	6.8	0.7	2.7	87.4%	-2.1
1279	AT1G13030.1 | sphere organelles protein-related, contains weak similarity to Swiss-Prot:Q09003 sphere organelles protein SPH-1 (Sphere protein 1) (Xenopus laevis) | chr1:4444041-4447404 REVERSE | Aliases: F3F19.5, F3F19_5	5.3	5.9	-0.6	-2.7	87.4%	-2.3
1280	AT4G23180.1 | Symbol: CRK10 | receptor-like protein kinase 4, putative (RLK4), nearly identical to receptor-like protein kinase 4 (Arabidopsis thaliana) GI:13506745; contains Pfam domain, PF00069: Protein kinase domain; identical to cDNA receptor-like protein kinase 4 (RLK4) GI:13506744 | chr4:12138148-12140932 FORWARD | Aliases: CYSTEINE RICH RLK10, F21P8.70, F21P8_70, RECEPTOR LIKE PROTEIN KINASE 4, RLK4	4.8	5.5	-0.7	-2.7	87.4%	-2.2
1281	AT5G46580.1 | pentatricopeptide (PPR) repeat-containing protein, contains similarity to 67kD chloroplastic RNA-binding protein, P67.1 (Raphanus sativus) GI:9755886; contains Pfam profile PF01535: PPR repeat	3.9	4.6	-0.7	-2.7	87.6%	-2.1
1282	AT5G43340.1 | Symbol: PHT6 | inorganic phosphate transporter, identical to inorganic phosphate transporter (Arabidopsis thaliana) GI:3869190 | chr5:17410826-17412376 REVERSE | Aliases: MWF20.3, MWF20_3	3.7	3.1	0.6	2.7	87.9%	-2.3
1283	AT4G34750.1 | auxin-responsive protein, putative / small auxin up RNA (SAUR_E), contains similarity to indole-3-acetic acid induced protein ARG7 SP:P32295 from (Phaseolus aureus) | chr4:16577571-16578357 FORWARD | Aliases: F11I11.5	6.5	5.8	0.7	2.7	87.9%	-2.3
1284	AT5G64760.2 | similar to 26S proteasome regulatory subunit, putative (RPN5) [Arabidopsis thaliana] (TAIR:At5g09900.1); similar to 26S proteasome regulatory subunit, putative (RPN5) [Arabidopsis thaliana] (TAIR:At5g09900.2); similar to 26S proteasome regulatory particle non-ATPase subunit5 [Oryza sativa (japonica cultivar-group)] (GB:BAB78500.1); contains InterPro domain Proteasome component region PCI (InterPro:IPR000717) | chr5:25910702-25913781 REVERSE | Aliases: MVP7.9, MVP7_9	4.9	6.3	-1.4	-2.7	87.9%	-2.0
1285	AT4G28640.1 | Symbol: IAA11 | auxin-responsive protein / indoleacetic acid-induced protein 11 (IAA11), identical to SP:Q38829 Auxin-responsive protein IAA11 (Indoleacetic acid-induced protein 11) {Arabidopsis thaliana} | chr4:14142141-14143975 FORWARD | Aliases: T5F17.90, T5F17_90	6.6	5.9	0.7	2.7	87.9%	-2.2

Rank	Description	15.dpi	5.dpi	M	t	adj.q	B
1286	AT1G74850.1 | pentatricopeptide (PPR) repeat-containing protein, contains Pfam profile PF01535: PPR repeat | chr1:28122510-28125975 REVERSE | Aliases: F25A4.18, F25A4_18	4.1	4.5	-0.5	-2.7	88.0%	-2.3
1287	AT5G08535.2 | D111/G-patch domain-containing protein, contains Pfam profile PF01585: G-patch domain; contains a 2nt micro-exon, as identified using GeneSeqer.	9.2	8.5	0.7	2.7	88.1%	-2.6
1288	AT4G16340.1 | Symbol: SPK1 | adapter protein SPIKE1 (SPK1), One model reflects the alignment of a full-length cDNA sequence gi:18496702. There are multiple frame shifts in the gene model resulting in a truncated protein.	4.7	5.8	-1.0	-2.7	88.2%	-2.0
1289	AT3G57630.2 | exostosin family protein, contains Pfam profile: PF03016	4.7	5.6	-0.9	-2.7	88.5%	-2.1
1290	AT2G21650.1 | myb family transcription factor, contains PFAM profile: PF00249 myb-like DNA-binding domain | chr2:9266663-9267736 FORWARD | Aliases: F2G1.8, F2G1_8	2.7	3.1	-0.4	-2.7	88.5%	-2.4
1291	AT1G09575.1 | expressed protein, contains Pfam domain, PF04678: Protein of unknown function, DUF607 | chr1:3101673-3102927 REVERSE | Aliases: None	4.2	3.4	0.8	2.7	88.6%	-2.1
1292	AT1G28600.2 | similar to GDSL-motif lipase, putative [Arabidopsis thaliana] (TAIR:At1g28610.2); similar to lipase-like [Oryza sativa (japonica cultivar-group)] (GB:BAD68794.1); contains InterPro domain Lipolytic enzyme, G-D-S-L family (InterPro:IPR001087) | chr1:10050987-10053103 REVERSE | Aliases: F1K23.28, F1K23_28	4.2	3.7	0.5	2.7	88.9%	-2.4
1293	AT4G33040.1 | glutaredoxin family protein, contains INTERPRO Domain IPR002109, Glutaredoxin (thioltransferase)	6.0	5.3	0.7	2.7	88.9%	-2.4
1294	AT1G15790.2 | expressed protein | chr1:5438252-5440082 REVERSE | Aliases: F7H2.13, F7H2_13	4.4	3.9	0.5	2.7	88.9%	-2.4
1295	AT2G03220.1 | Symbol: FT1 | galactoside 2-alpha-L-fucosyltransferase / xyloglucan alpha-(1,2)-fucosyltransferase (FUT1) (FT1), identical to SP:Q9SWH5 Galactoside 2-alpha-L-fucosyltransferase (EC 2.4.1.69) (Xyloglucan alpha-(1,2)-fucosyltransferase) (AtFUT1) {Arabidopsis thaliana} | chr2:970256-972404 REVERSE | Aliases: A (1,2) FUCOSYLTRANSFERASE, ATFT1, ATFUT1, FUCOSYLTRANSFERASE 1, MUR2, MURUS 2, T18E12.11, T18E12_11, XYLOGLUCAN FUCOSYLTRANSFERASE	5.1	6.4	-1.3	-2.7	88.9%	-2.1
1296	AT4G37550.2 | similar to formamidase, putative / formamide amidohydrolase, putative [Arabidopsis thaliana] (TAIR:At4g37560.1); similar to formamidase [Methylophilus methylotrophus] (GB:CAA67953.1); contains InterPro domain Acetamidase/Formamidase (InterPro:IPR004304) | chr4:17643575-17645821 FORWARD | Aliases: F19F18.40, F19F18_40	6.4	7.2	-0.8	-2.7	88.9%	-2.0
1297	AT2G29650.3 | inorganic phosphate transporter, putative, similar to brain specific Na+-dependent inorganic phosphate cotransporter (Rattus norvegicus) GI:507415; contains Pfam profile PF00083: major facilitator superfamily protein	7.0	5.7	1.2	2.7	88.9%	-2.0
1298	AT3G22300.1 | Symbol: RPS10 | 40S ribosomal protein S10, mitochondrial (RPS10), identical to SP:P42797 40S ribosomal protein S10, mitochondrial precursor {Arabidopsis thaliana} | chr3:7885675-7886827 FORWARD | Aliases: MCB17.5, RIBOSOMAL PROTEIN S10	6.2	7.3	-1.1	-2.7	88.9%	-2.0
1299	AT1G61260.1 | expressed protein, contains similarity to cotton fiber expressed protein 1 (Gossypium hirsutum) gi:3264828:gb:AAC33276 | chr1:22597130-22598734 REVERSE | Aliases: F11P17.2, F11P17_2	5.1	3.3	1.8	2.7	89.0%	-2.0
1300	AT1G52800.1 | oxidoreductase, 2OG-Fe(II) oxygenase family protein, similar to GS-AOP loci (GI:16118889, GI:16118887, GI:16118891, GI:16118893); contains PF03171 2OG-Fe(II) oxygenase superfamily domain | chr1:19667712-19669030 FORWARD | Aliases: F14G24.7, F14G24_7	2.3	2.1	0.2	2.7	89.0%	-3.3
1301	AT4G35270.1 | RWP-RK domain-containing protein, similar to nodule inception protein GI:6448579 from (Lotus japonicus); contains Pfam profile: PF02042 RWP-RK domain | chr4:16777343-16780829 REVERSE | Aliases: F23E12.170, F23E12_170	6.0	4.9	1.0	2.7	89.0%	-2.1
1302	AT4G15850.1 | Symbol: ATRH1 | DEAD/DEAH box helicase, putative, similar to D-E-A-D box protein (Drosophila melanogaster) GI:499204; contains Pfam profiles PF00270: DEAD/DEAH box helicase, PF00271: Helicase conserved C-terminal domain | chr4:9001344-9004749 FORWARD | Aliases: DL3965W, FCAALL.401	3.5	4.2	-0.7	-2.7	89.0%	-2.1
1303	AT5G27330.1 | expressed protein | chr5:9632706-9634886 REVERSE | Aliases: F21A20.40, F21A20_40	5.5	7.7	-2.2	-2.7	89.0%	-2.0
1304	AT2G40480.1 | expressed protein, similar to expressed protein [Arabidopsis thaliana] (TAIR:At3g56270.1); similar to unknown protein [Oryza sativa (japonica cultivar-group)] (GB:AAU44189.1); contains InterPro domain Plant protein of unknown function DUF827 (InterPro:IPR008545) | chr2:16917442-16919670 FORWARD | Aliases: T2P4.17, T2P4_17	6.6	7.4	-0.8	-2.7	89.0%	-2.2
1305	AT5G27670.1 | histone H2A, putative, similar to histone H2A Lycopersicon esculentum SP:P25469, Pisum sativum SP:P25470, Petroselinum crispum SP:P19177; contains Pfam profile PF00125 Core histone H2A/H2B/H3/H4 | chr5:9792579-9793458 REVERSE | Aliases: T1G16.3	8.9	9.6	-0.8	-2.7	89.0%	-2.2

Rank	Description	15.dpi	5.dpi	M	t	adj.q	B
1306	AT4G02330.1 | pectinesterase family protein, contains Pfam profile: PF01095 pectinesterase | chr4:1032413-1035037 FORWARD | Aliases: ATPMEPCRB, T14P8.14, T14P8_14	4.2	5.5	-1.3	-2.7	89.0%	-2.1
1307	AT3G29810.1 | phytochelatin synthetase family protein / COBRA cell expansion protein COBL2, similar to phytochelatin synthetase (Hordeum vulgare subsp. vulgare) GI:29570314; identified in Roudier, et al, Plant Phys. (2002) 130:538-548 (PMID:12376623); contains Pfam profile PF04833: Phytochelatin synthetase-like conserved region | chr3:11730596-11732718 FORWARD | Aliases: K17E7.12	8.8	7.7	1.2	2.7	89.0%	-2.1
1308	AT2G44080.1 | expressed protein | chr2:18244261-18245249 FORWARD | Aliases: F6E13.21	9.5	8.7	0.8	2.7	89.0%	-2.5
1309	AT3G19515.1 | expressed protein | chr3:6766674-6769032 REVERSE | Aliases: MLD14.38	7.6	9.0	-1.4	-2.7	89.0%	-2.2
1310	AT4G25280.1 | adenylate kinase family protein, contains Pfam profile: PF00406 adenylate kinase	6.4	7.3	-1.0	-2.7	89.0%	-2.2
1311	AT5G37580.1 | expressed protein | chr5:14944543-14946800 REVERSE | Aliases: K12B20.4, K12B20_4	4.9	5.8	-0.8	-2.7	89.0%	-2.1
1312	AT5G14940.1 | proton-dependent oligopeptide transport (POT) family protein, contains Pfam profile: PF00854 POT family | chr5:4831751-4834315 REVERSE | Aliases: F2G14.60, F2G14_60	5.6	4.8	0.8	2.7	89.0%	-2.2
1313	AT4G28760.2 | expressed protein, predicted protein. Arabidopsis thaliana | chr4:14207467-14212050 FORWARD | Aliases: F16A16.130, F16A16_130	3.7	4.3	-0.6	-2.7	89.0%	-2.3
1314	AT1G11720.1 | starch synthase, putative, strong similarity to soluble-starch-synthase (Solanum tuberosum) GI:1911166 | chr1:3952511-3957044 FORWARD | Aliases: F25C20.13, F25C20_13	4.6	5.5	-0.9	-2.7	89.0%	-2.0
1315	AT1G05760.1 | Symbol: RTM1 | jacalin lectin family protein (RTM1), identical to gi:6503088 (GB:AAF14583) from (Arabidopsis thaliana) (Proc. Natl. Acad. Sci. U.S.A. 97 (1), 489-494 (2000)); contains Pfam profile PF01419 jacalin-like lectin domain	4.3	3.2	1.1	2.7	89.0%	-2.0
1316	AT3G15510.1 | Symbol: ATNAC2 |	4.3	3.5	0.8	2.7	89.0%	-2.1
1317	AT4G03320.1 | chloroplast protein import component-related, similar to P. sativum Tic20 chloroplast protein import component (GI:3769673) | chr4:1464467-1466164 FORWARD | Aliases: F4C21.25, F4C21_25	7.5	6.4	1.1	2.7	89.0%	-2.1
1318	AT1G60390.1 | BURP domain-containing protein / polygalacturonase, putative, similar to polygalacturonase isoenzyme 1 beta subunit GI:170480 from (Lycopersicon esculentum); contains Pfam profile PF03181: BURP domain	4.3	5.6	-1.2	-2.7	89.0%	-2.0
1319	AT1G62040.1 | autophagy 8c (APG8c), identical to autophagy 8c (Arabidopsis thaliana) GI:19912155; contains Pfam profile PF02991: Microtubule associated protein 1A/1B, light chain 3 | chr1:22936566-22938088 FORWARD | Aliases: F8K4.23, F8K4_23	9.4	8.6	0.8	2.7	89.0%	-2.3
1320	AT4G31530.1 | expressed protein | chr4:15282245-15284222 FORWARD | Aliases: F3L17.100, F3L17_100	5.8	5.2	0.6	2.7	89.0%	-2.3
1321	AT1G77420.1 | hydrolase, alpha/beta fold family protein, similar to monoglyceride lipase from (Homo sapiens) GI:14594904, (Mus musculus) GI:2632162; contains Pfam profile PF00561: hydrolase, alpha/beta fold family | chr1:29098393-29100610 FORWARD | Aliases: T5M16.2, T5M16_2	7.6	7.0	0.7	2.7	89.0%	-2.3
1322	AT1G09815.1 | DNA polymerase delta subunit 4 family, contains similarity to Swiss-Prot:Q9HCU8 DNA polymerase delta subunit 4 (DNA polymerase delta subunit p12) (Homo sapiens)	9.4	8.9	0.5	2.7	89.0%	-2.4
1323	AT3G01470.1 | Symbol: ATHB 1 |	9.2	8.0	1.1	2.7	89.0%	-2.1
1324	AT1G74000.1 | Symbol: SS3 | similar to strictosidine synthase family protein [Arabidopsis thaliana] (TAIR:At1g74020.1); similar to strictosidine synthase (GB:AAB40594.1); contains InterPro domain Strictosidine synthase (InterPro:IPR004141) | chr1:27832803-27835088 REVERSE | Aliases: ATSS 3 STRICTOSIDINE SYNTHASE, F2P9.13, F2P9_13, STRICTOSIDINE SYNTHASE 3	5.5	6.7	-1.2	-2.7	89.0%	-2.0
1325	AT5G20660.1 | 24 kDa vacuolar protein, putative, similar to 24 kDa vacuolar protein VP24 (Ipomoea batatas) gi:5821406:dbj:BAA83809 | chr5:6986237-6991045 FORWARD | Aliases: T1M15.60, T1M15_60	9.7	10.3	-0.6	-2.7	89.0%	-2.6
1326	AT5G23840.2 | MD-2-related lipid recognition domain-containing protein / ML domain-containing protein, contains Pfam profile PF02221: ML domain | chr5:8035770-8036985 FORWARD | Aliases: MRO11.12, MRO11_12	6.6	5.1	1.5	2.7	89.0%	-2.2
1327	AT4G16155.1 | dihydrolipoamide dehydrogenase 2, plastidic / lipoamide dehydrogenase 2 (PTLPD2), identical to plastidic lipoamide dehydrogenase from Arabidopsis thaliana (gi:7159284) | chr4:9153386-9157278 REVERSE | Aliases: None	8.2	9.1	-0.9	-2.7	89.0%	-2.0
1328	AT1G80180.1 | expressed protein | chr1:30161718-30162476 REVERSE | Aliases: F18B13.26, F18B13_26	7.2	6.2	1.0	2.7	89.2%	-2.0

Rank	Description	15.dpi	5.dpi	M	t	adj.q	B
1329	AT1G70810.1 | C2 domain-containing protein, similar to zinc finger and C2 domain protein GI:9957238 from (Arabidopsis thaliana) | chr1:26707792-26708898 FORWARD | Aliases: F15H11.6, F15H11_6	4.6	3.8	0.8	2.7	89.2%	-2.1
1330	AT2G25620.1 | protein phosphatase 2C, putative / PP2C, putative | chr2:10910021-10912323 REVERSE | Aliases: F3N11.7, F3N11_7	4.1	5.0	-0.9	-2.7	89.2%	-2.1
1331	AT1G64200.1 | Symbol: VHA E3 |	5.3	6.3	-1.0	-2.7	89.2%	-2.1
1332	AT1G23550.1 | Symbol: SRO2 | Encodes a protein with similarity to RCD1 but without the WWE domain. The protein does have a PARP signature upstream of the C-terminal protein interaction domain. The PARP signature may bind NAD+ and attach the ADP-ribose-moiety from NAD+ to the target molecule. Its presence suggests a role for the protein in ADP ribosylation. | chr1:8350901-8352221 FORWARD | Aliases: F28C11.18, SIMILAR TO RCD ONE 2, SIMILAR TO RCD ONE 2, SRO2	6.5	5.6	0.9	2.7	89.2%	-2.2
1333	AT5G10380.1 | zinc finger (C3HC4-type RING finger) family protein, contains Pfam profile: PF00097 zinc finger, C3HC4 type (RING finger) | chr5:3267719-3268814 FORWARD | Aliases: F12B17.270, F12B17_270	3.2	2.6	0.6	2.7	89.5%	-2.4
1334	AT5G52840.1 | NADH-ubiquinone oxidoreductase-related, contains weak similarity to NADH-ubiquinone oxidoreductase 13 kDa-B subunit (EC 1.6.5.3) (EC 1.6.99.3) (Complex I-13Kd-B) (CI-13Kd-B) (Complex I subunit B13) (Swiss-Prot:Q63362) (Rattus norvegicus) | chr5:21430880-21432232 FORWARD | Aliases: MXC20.6, MXC20_6	10.3	11.3	-1.0	-2.7	89.7%	-2.3
1335	AT5G51070.1 | Symbol: ERD1 | ATP-dependent Clp protease ATP-binding subunit (ClpD), (ERD1), SAG15/ERD1; identical to ERD1 protein GI:497629, SP:P42762 from (Arabidopsis thaliana); contains Pfam profile PF02861: Clp amino terminal domain | chr5:20781541-20785907 FORWARD | Aliases: CLPD, EARLY RESPONSIVE TO DEHYDRATION 1, K3K7.27, K3K7_27	7.4	6.3	1.1	2.7	89.7%	-2.1
1336	AT2G34510.1 | expressed protein, contains Pfam profile PF04862: Protein of unknown function, DUF642 | chr2:14551076-14553886 REVERSE | Aliases: T31E10.15, T31E10_15	5.6	4.5	1.1	2.7	89.7%	-2.0
1337	AT5G19330.2 | armadillo/beta-catenin repeat family protein / BTB/POZ domain-containing protein, contains armadillo/beta-catenin-like repeats, Pfam:PF00514 and a BTB/POZ domain, Pfam:PF00651 | chr5:6508302-6512746 REVERSE | Aliases: F7K24.80, F7K24_80	5.7	6.3	-0.7	-2.7	90.1%	-2.3
1338	AT3G09770.2 | zinc finger (C3HC4-type RING finger) family protein, contains Pfam domain, PF00097: Zinc finger, C3HC4 type (RING finger) | chr3:2996283-2998195 REVERSE | Aliases: F11F8.36	7.2	6.4	0.7	2.7	90.1%	-2.2
1339	AT4G24840.1 | expressed protein | chr4:12796257-12800948 REVERSE | Aliases: F6I7.50, F6I7_50	6.3	7.1	-0.8	-2.7	90.1%	-2.2
1340	AT1G58250.1 | Symbol: SAB | SABRE, putative, similar to SABRE (GI:719291) (Arabidopsis thaliana) | chr1:21590707-21605286 REVERSE | Aliases: F16M22.5, F16M22_5, SABRE	6.6	7.8	-1.2	-2.7	90.1%	-2.2
1341	AT5G19730.1 | pectinesterase family protein, contains Pfam profile: PF01095 pectinesterase | chr5:6670460-6673284 FORWARD | Aliases: T29J13.150, T29J13_150	3.3	4.9	-1.6	-2.7	90.1%	-2.0
1342	AT5G52390.1 | photoassimilate-responsive protein, putative, similar to PIR:S62698 photoassimilate-responsive protein precursor (clone PAR-1a) - common tobacco	5.3	7.8	-2.5	-2.6	90.2%	-2.1
1343	AT4G24380.2 | expressed protein, similar to expressed protein [Arabidopsis thaliana] (TAIR:At5g65400.1); similar to unknown protein [Oryza sativa (japonica cultivar-group)] (GB:XP_479601.1) | chr4:12612545-12614027 FORWARD | Aliases: T22A6.210, T22A6_210	9.4	8.3	1.2	2.6	90.3%	-2.2
1344	AT1G21010.1 | expressed protein | chr1:7346145-7346957 FORWARD | Aliases: F9H16.19, F9H16_19	4.2	3.8	0.4	2.6	90.7%	-2.5
1345	AT3G60660.1 | expressed protein | chr3:22432385-22434452 FORWARD | Aliases: T4C21.70	2.8	3.3	-0.6	-2.6	91.0%	-2.4
1346	AT1G65560.1 | allyl alcohol dehydrogenase, putative, similar to allyl alcohol dehydrogenase from Nicotiana tabacum (gi:6692816); similar to probable NADP-dependent oxidoreductase (zeta-crystallin homolog) P1 (SP:Q39172)(gi:886428) and P2 (SP:Q39173)(gi:886430), Arabidopsis thaliana | chr1:24375050-24377443 REVERSE | Aliases: F5I14.9, F5I14_9	4.4	5.3	-0.9	-2.6	91.0%	-2.2
1347	AT3G22260.2 | OTU-like cysteine protease family protein, contains Pfam profile PF02338: OTU-like cysteine protease | chr3:7871015-7873572 FORWARD | Aliases: MMP21.4	5.3	4.4	0.8	2.6	91.0%	-2.1
1348	AT1G68660.1 | expressed protein | chr1:25781514-25782824 REVERSE | Aliases: F24J5.10, F24J5_10	9.2	8.3	1.0	2.6	91.0%	-2.1
1349	AT1G61590.1 | protein kinase, putative, contains protein kinase domain, Pfam:PF00069; similar to serine/threonine protein kinase gi:1066501:gb:AAA81538 | chr1:22727166-22729739 REVERSE | Aliases: T25B24.6, T25B24_6	2.3	2.9	-0.5	-2.6	91.0%	-2.5

Rank	Description	15.dpi	5.dpi	M	t	adj.q	B
1350	AT1G74940.1 | senescence-associated protein-related, similar to senescence-associated protein SAG102 (GI:22331931) (Arabidopsis thaliana) | chr1:28149720-28150839 FORWARD | Aliases: F25A4.9, F25A4_9	8.4	7.5	0.9	2.6	91.0%	-2.2
1351	AT3G54140.1 | proton-dependent oligopeptide transport (POT) family protein, contains Pfam profile: PF00854 POT family | chr3:20056641-20059550 REVERSE | Aliases: F24B22.100	4.4	3.8	0.6	2.6	91.0%	-2.4
1352	AT4G27350.1 | expressed protein | chr4:13690827-13692800 REVERSE | Aliases: F27G19.7, M4I22.160, M4I22_160	7.4	6.7	0.7	2.6	91.0%	-2.4
1353	AT5G55790.1 | expressed protein, ; expression supported by MPSS | chr5:22597986-22598561 REVERSE | Aliases: MDF20.23, MDF20_23	3.5	2.8	0.7	2.6	91.0%	-2.5
1354	AT1G11130.1 | Symbol: SUB | leucine-rich repeat family protein / protein kinase family protein, contains leucine rich-repeat (LRR) domains Pfam:PF00560, INTERPRO:IPR001611; contains similarity to leucine-rich repeat transmembrane protein kinase 2 (Zea mays) gi:3360291:gb:AAC27895 | chr1:3722857-3727443 FORWARD | Aliases: AT1G11140, SCM, SCRAMBLED, STRUBBELIG, T19D16.8	3.4	3.8	-0.4	-2.6	91.1%	-2.5
1355	AT5G46770.1 | expressed protein | chr5:18994635-18995387 FORWARD | Aliases: MZA15.19, MZA15_19	2.9	3.2	-0.3	-2.6	91.1%	-3.1
1356	AT1G26560.1 | glycosyl hydrolase family 1 protein, contains Pfam PF00232 : Glycosyl hydrolase family 1 domain; TIGRFAM TIGR01233: 6-phospho-beta-galactosidase; similar to amygdalin hydrolase isoform AH I precursor (GI:16757966) (Prunus serotina)	5.1	4.4	0.7	2.6	91.2%	-2.3
1357	AT3G43800.1 | Symbol: ATGSTU27 | glutathione S-transferase, putative, glutathione transferase, papaya, PIR:T09781 | chr3:15671846-15672912 FORWARD | Aliases: T28A8.90	4.6	5.6	-1.0	-2.6	91.3%	-2.2
1358	AT1G09210.1 | calreticulin 2 (CRT2), identical to SP:Q38858 Calreticulin 2 precursor {Arabidopsis thaliana} | chr1:2972844-2976731 REVERSE | Aliases: T12M4.8, T12M4_8	8.0	9.1	-1.1	-2.6	91.3%	-2.3
1359	AT5G55400.1 | fimbrin-like protein, putative, similar to fimbrin-like protein (ATFIM1) (Arabidopsis thaliana) GI:2905893; contains Pfam profile PF00307: Calponin homology (CH) domain	7.7	9.9	-2.3	-2.6	91.3%	-2.2
1360	AT2G21380.1 | kinesin motor protein-related | chr2:9148711-9156389 FORWARD | Aliases: F3K23.14, F3K23_14	4.4	5.5	-1.2	-2.6	91.3%	-2.3
1361	AT3G52960.1 | peroxiredoxin type 2, putative, similar to type 2 peroxiredoxin (Brassica rapa subsp. pekinensis) GI:4928472; contains Pfam profile: PF00578 AhpC/TSA (alkyl hydroperoxide reductase and thiol-specific antioxidant) family | chr3:19650646-19651597 FORWARD | Aliases: F8J2.130	7.8	8.8	-1.0	-2.6	91.3%	-2.1
1362	AT3G07310.1 | expressed protein | chr3:2327824-2329787 REVERSE | Aliases: T1B9.2	5.1	4.2	0.9	2.6	91.4%	-2.1
1363	AT3G53410.1 | zinc finger (C3HC4-type RING finger) family protein, contains Pfam domain: PF00097: Zinc finger, C3HC4 type (RING finger) | chr3:19811930-19813298 REVERSE | Aliases: F4P12.110	6.1	5.5	0.6	2.6	91.4%	-2.3
1364	AT5G33290.1 | exostosin family protein, contains Pfam profile: PF03016 Exostosin family | chr5:12575486-12579250 FORWARD | Aliases: F19N2.10, F19N2_10	6.4	5.6	0.8	2.6	91.4%	-2.3
1365	AT4G15630.1 | integral membrane family protein, contains TIGRFAM TIGR01569 : plant integral membrane protein TIGR01569; contains Pfam PF04535 : Domain of unknown function (DUF588) | chr4:8917428-8918850 FORWARD | Aliases: DL3855W, FCAALL.230	3.4	4.0	-0.7	-2.6	91.4%	-2.3
1366	AT2G39720.1 | Symbol: RHC2A | zinc finger (C3HC4-type RING finger) family protein, contains Pfam domain, PF00097: Zinc finger, C3HC4 type (RING finger) | chr2:16574287-16575900 REVERSE | Aliases: RING H2 FINGER PROTEIN RHC2A, T5I7.2, T5I7_2	6.2	5.7	0.6	2.6	91.4%	-2.4
1367	AT3G14415.1 | (S)-2-hydroxy-acid oxidase, peroxisomal, putative / glycolate oxidase, putative / short chain alpha-hydroxy acid oxidase, putative, similar to (S)-2-hydroxy-acid oxidase, peroxisomal (Glycolate oxidase, GOX) (Short chain alpha-hydroxy acid oxidase) (Spinacia oleracea) SWISS-PROT:P05414 | chr3:4818674-4820755 FORWARD | Aliases: MOA2.13	4.4	3.3	1.0	2.6	91.5%	-2.1
1368	AT3G14420.4 | similar to (S)-2-hydroxy-acid oxidase, peroxisomal, putative / glycolate oxidase, putative / short chain alpha-hydroxy acid oxidase, putative [Arabidopsis thaliana] (TAIR:At3g14415.1); similar to glycolate oxidase [Zantedeschia aethiopica] (GB:AAO17067.1); contains InterPro domain FMN-dependent alpha-hydroxy acid dehydrogenase, active site (InterPro:IPR008259); contains InterPro domain FMN-dependent alpha-hydroxy acid dehydrogenase (InterPro:IPR000262)	4.4	3.3	1.0	2.6	91.5%	-2.1
1369	AT3G22900.1 | RNA polymerase Rpb7 N-terminal domain-containing protein, weak similarity to SP:P52433 DNA-directed RNA polymerase II 19 kDa polypeptide (EC 2.7.7.6) (RPB7) {Rattus norvegicus}; contains Pfam profile PF03876: RNA polymerase Rpb7, N-terminal domain | chr3:8115184-8116260 FORWARD | Aliases: F5N5.8	5.1	6.2	-1.0	-2.6	91.5%	-2.1

Rank	Description	15.dpi	5.dpi	M	t	adj.q	B
1370	AT5G67470.1 | formin homology 2 domain-containing protein / FH2 domain-containing protein, contains formin homology 2 domain, Pfam:PF02181 | chr5:26944061-26947686 FORWARD | Aliases: K9I9.3, K9I9_3	6.4	7.0	-0.6	-2.6	91.5%	-2.3
1371	AT2G27060.1 | leucine-rich repeat transmembrane protein kinase, putative | chr2:11558405-11561853 FORWARD | Aliases: T20P8.11, T20P8_11	5.1	5.7	-0.7	-2.6	91.5%	-2.3
1372	AT5G63920.1 | DNA topoisomerase III alpha, putative, similar to Swiss-Prot:Q9NG98 DNA topoisomerase III alpha (Drosophila melanogaster) | chr5:25591759-25598504 FORWARD | Aliases: MGI19.16	4.2	4.9	-0.7	-2.6	91.5%	-2.2
1373	AT1G01220.1 | GHMP kinase-related, contains similarity to L-fucose kinase (Homo sapiens) gi:21212956:emb:CAD29647 | chr1:91750-95552 FORWARD | Aliases: F6F3.3, F6F3_3	5.4	6.3	-0.9	-2.6	91.5%	-2.2
1374	AT1G72670.1 | calmodulin-binding family protein, low similarity to SF16 protein (Helianthus annuus) GI:560150; contains Pfam profile PF00612: IQ calmodulin-binding motif | chr1:27360357-27362443 REVERSE | Aliases: F28P22.14, F28P22_14	3.0	3.4	-0.3	-2.6	91.5%	-2.8
1375	AT2G01660.2 | 33 kDa secretory protein-related, contains Pfam PF01657: Domain of unknown function, duplicated in 33 KDa secretory proteins | chr2:290602-292569 REVERSE | Aliases: T8O11.17, T8O11_17	5.9	5.1	0.7	2.6	91.6%	-2.3
1376	AT4G37670.2 | GCN5-related N-acetyltransferase (GNAT) family protein / amino acid kinase family protein, similar to SP:P08205 from Escherichia coli ; contains Pfam profile PF00696: Amino acid kinase family | chr4:17696053-17698830 REVERSE | Aliases: F19F18.160, F19F18_160	4.7	5.9	-1.2	-2.6	91.6%	-2.1
1377	AT3G01620.1 | glycosyl transferase family 17 protein, low similarity to beta-1,4-mannosyl-glycoprotein beta-1,4-N-acetylglucosaminyltransferase (EC 2.4.1.144) from Mus musculus (SP:Q10470), Rattus norvegicus (SP:Q02527), Homo sapiens (SP:Q09327) ; contains Pfam profile PF04724 :Glycosyltransferase family 17 | chr3:235204-236932 REVERSE | Aliases: F4P13.16, F4P13_16	3.1	2.8	0.3	2.6	91.7%	-2.8
1378	AT1G17620.1 | expressed protein | chr1:6062227-6063284 FORWARD | Aliases: F11A6.25, F1L3.32, F1L3_32	6.6	5.4	1.2	2.6	91.9%	-2.1
1379	AT5G61660.1 | glycine-rich protein | chr5:24796441-24797156 REVERSE | Aliases: K11J9.19, K11J9_19	6.5	5.7	0.8	2.6	92.2%	-2.2
1380	AT4G34950.1 | nodulin family protein, similar to nodulin-like protein (Arabidopsis thaliana) GI:3329368, nodule-specific protein Nlj70 (Lotus japonicus) GI:3329366 | chr4:16642321-16644823 REVERSE | Aliases: F11I11.190, F11I11_190	7.8	6.3	1.6	2.6	92.3%	-2.2
1381	AT4G29690.1 | type I phosphodiesterase/nucleotide pyrophosphatase family protein, similar to SP:P22413 Ectonucleotide pyrophosphatase/phosphodiesterase 1 (Plasma-cell membrane glycoprotein PC-1) (Includes: Alkaline phosphodiesterase I (EC 3.1.4.1); Nucleotide pyrophosphatase (EC 3.6.1.9) {Homo sapiens}; contains Pfam profile PF01663: Type I phosphodiesterase / nucleotide pyrophosphatase | chr4:14540913-14542487 REVERSE | Aliases: T16L4.200, T16L4_200	2.8	3.8	-1.0	-2.6	92.3%	-2.2
1382	AT5G39840.1 | ATP-dependent RNA helicase, mitochondrial, putative, similar to mitochondrial RNA helicase (Arabidopsis thaliana) GI:5823579; contains Pfam profile PF00271: Helicase conserved C-terminal domain | chr5:15963997-15966414 FORWARD | Aliases: K13H13.20, K13H13_20	2.6	3.1	-0.5	-2.6	92.3%	-2.5
1383	AT1G75810.1 | expressed protein | chr1:28465326-28466007 FORWARD | Aliases: T4O12.4, T4O12_4	6.5	5.6	0.9	2.6	92.3%	-2.2
1384	AT5G41010.1 | DNA-directed RNA polymerases I, II, and III 7 kDa subunit, putative, similar to SP:P53803 DNA-directed RNA polymerases I, II, and III 7.0 kDa polypeptide (EC 2.7.7.6) (ABC10-alpha) (RPB7.0) (RPB10alpha) {Homo sapiens}; contains Pfam profile PF03604: DNA directed RNA polymerase, 7 kDa subunit | chr5:16441278-16442180 FORWARD | Aliases: MEE6.8, MEE6_8	10.8	10.4	0.4	2.6	92.3%	-2.6
1385	AT5G53880.1 | expressed protein | chr5:21890020-21890529 REVERSE | Aliases: K19P17.4, K19P17_4	10.2	8.8	1.4	2.6	92.3%	-2.3
1386	AT1G13340.1 | expressed protein, contains Pfam profile: PF03398 eukaryotic protein of unknown function, DUF292	4.9	3.9	1.0	2.6	92.3%	-2.2
1387	AT3G62190.1 | DNAJ heat shock N-terminal domain-containing protein, similar to SP:P39101 CAJ1 protein Saccharomyces cerevisiae; contains Pfam profile PF00226 DnaJ domain | chr3:23032096-23034307 FORWARD | Aliases: T17J13.150	9.4	8.8	0.6	2.6	92.3%	-2.6
1388	AT4G37750.1 | Symbol: ANT | ovule development protein aintegumenta (ANT), identical to ovule development protein aintegumenta (ANT) (GI:1244708)) (Arabidopsis thaliana) | chr4:17739508-17742395 FORWARD | Aliases: AINTEGUMENTA, CKC, CKC1, COMPLEMENTING A PROTEIN KINASE C MUTANT 1, DRAGON, DRG, T28I19.30, T28I19_30	3.9	5.1	-1.2	-2.6	92.3%	-2.1
1389	AT3G23200.1 | expressed protein | chr3:8281370-8282785 FORWARD | Aliases: K14B15.11	6.7	5.7	1.0	2.6	92.3%	-2.1
1390	AT5G10390.1 | histone H3, identical to histone H3 from Zea mays SP:P05203, Medicago sativa GI:166384, Encephalartos altensteinii SP:P08903, Pisum sativum SP:P02300; contains Pfam profile PF00125 Core histone H2A/H2B/H3/H4 | chr5:3268848-3269551 REVERSE | Aliases: F12B17.260	5.3	7.2	-2.0	-2.6	92.3%	-2.0

Rank	Description	15.dpi	5.dpi	M	t	adj.q	B
1391	AT5G42150.1 | expressed protein | chr5:16863378-16865507 FORWARD | Aliases: MJC20.26, MJC20_26	8.9	10.0	-1.1	-2.6	92.3%	-2.2
1392	AT5G02470.3 | Symbol: DPA | DP-2 transcription factor, putative (DPA), similar to Swiss-Prot:Q14188 transcription factor DP-2 (E2F dimerization partner 2) (Homo sapiens) | chr5:542356-544500 REVERSE | Aliases: DPA TRANSCRIPTION FACTOR, T22P11.60, T22P11_60	5.0	6.0	-1.0	-2.6	92.3%	-2.3
1393	AT5G64850.1 | expressed protein | chr5:25938670-25940580 FORWARD | Aliases: MXK3.7, MXK3_7	4.4	3.4	1.0	2.6	92.3%	-2.3
1394	AT2G15630.1 | pentatricopeptide (PPR) repeat-containing protein, contains Pfam profile PF01535: PPR repeat | chr2:6821603-6823486 FORWARD | Aliases: F9O13.18	3.8	4.7	-0.9	-2.6	92.3%	-2.1
1395	AT3G53310.1 | transcriptional factor B3 family protein, contains Pfam profile PF02362: B3 DNA binding domain | chr3:19777642-19779396 REVERSE | Aliases: F4P12.10	2.8	2.4	0.3	2.6	92.3%	-2.9
1396	AT1G05360.1 | expressed protein, Similar to Arabidopsis hypothetical protein PID:e326839 (gb:Z97337) contains transmembrane domains | chr1:1564621-1567464 FORWARD | Aliases: YUP8H12.2, YUP8H12_2	5.3	5.9	-0.6	-2.6	92.6%	-2.6
1397	AT1G66890.1 | expressed protein | chr1:24961221-24961777 FORWARD | Aliases: T4O24.4, T4O24_4	7.5	6.9	0.7	2.6	92.6%	-2.3
1398	AT3G09260.1 | Symbol: PYK10 | glycosyl hydrolase family 1 protein, contains Pfam PF00232 : Glycosyl hydrolase family 1 domain; TIGRFAM TIGR01233: 6-phospho-beta-galactosidase; almost identical to beta-glucosidase GI:1732570 from (Arabidopsis thaliana); similar to beta-glucosidase 1 (GI:12043529) (Arabidopsis thaliana) | chr3:2840486-2843784 REVERSE | Aliases: F3L24.13, PSR3.1	5.2	7.8	-2.6	-2.6	92.6%	-2.1
1399	AT4G24690.1 | ubiquitin-associated (UBA)/TS-N domain-containing protein / octicosapeptide/Phox/Bemp1 (PB1) domain-containing protein, contains Pfam profiles PF00627: Ubiquitin-associated (UBA)/TS-N domain, PF00569: Zinc finger ZZ type domain, PF00564: PB1 domain	8.5	7.5	1.0	2.6	92.6%	-2.3
1400	AT2G01850.1 | Symbol: EXGT A3 | xyloglucan:xyloglucosyl transferase / xyloglucan endotransglycosylase / endo-xyloglucan transferase (EXGT-A3), identical to endoxyloglucan transferase (Arabidopsis thaliana) GI:5533313 | chr2:385222-387343 FORWARD | Aliases: ATXTH27, T23K3.4, T23K3_4, XTH27	7.5	6.2	1.3	2.6	92.6%	-2.2
1401	AT3G15560.1 | expressed protein | chr3:5268599-5270125 FORWARD | Aliases: MQD17.1	3.2	2.9	0.3	2.6	92.6%	-3.0
1402	AT1G17230.1 | leucine-rich repeat family protein / protein kinase family protein, contains protein kinase domain, Pfam:PF00069; contains leucine-rich repeats, Pfam:PF00560 | chr1:5891317-5894848 FORWARD | Aliases: F20D23.7, F20D23_7	4.3	3.6	0.7	2.6	92.6%	-2.3
1403	AT1G74240.1 | mitochondrial substrate carrier family protein, contains Pfam profile: PF00153 mitochondrial carrier protein | chr1:27921057-27923800 FORWARD | Aliases: F1O17.9, F1O17_9	8.0	8.9	-0.9	-2.6	92.6%	-2.2
1404	AT1G69580.1 | myb family transcription factor, contains Pfam profile: PF00249 myb-like DNA-binding domain | chr1:26175802-26177275 FORWARD | Aliases: F24J1.30, F24J1_30	4.0	5.5	-1.6	-2.6	92.6%	-2.2
1405	AT1G79140.1 | expressed protein | chr1:29777505-29778801 REVERSE | Aliases: YUP8H12R.47, YUP8H12R_47	2.9	3.5	-0.6	-2.6	92.6%	-2.5
1406	AT4G14550.1 | Symbol: IAA14 | auxin-responsive AUX/IAA family protein, identical to IAA14 (GI:972931) (Arabidopsis thaliana); similar to SP:Q38825 Auxin-responsive protein IAA7 (Indoleacetic acid-induced protein 7) {Arabidopsis thaliana}	5.3	4.7	0.6	2.6	92.6%	-2.4
1407	AT3G44450.1 | expressed protein | chr3:16085084-16085607 REVERSE | Aliases: F14L2.1	6.1	5.0	1.1	2.6	92.6%	-2.1
1408	AT4G18810.1 | expressed protein, similar to UV-B and ozone similarly regulated protein 1 UOS1 (Pisum sativum) GI:20339364 | chr4:10322449-10325781 REVERSE | Aliases: F28A21.220, F28A21_220	6.8	5.5	1.2	2.6	92.6%	-2.1
1409	AT5G45930.1 | Symbol: CHLI2 | magnesium-chelatase subunit chlI, chloroplast, putative / Mg-protoporphyrin IX chelatase, putative, similar to SP:P161127 from Arabidopsis thaliana, SP:P93162 from Glycine max, SP:O22436 from Nicotiana tabacum; non-consensus AA donor splice site at exon 1, TG acceptor splice site at exon 2 | chr5:18645234-18646907 FORWARD | Aliases: CHL I2, K15I22.13, K15I22_13, MAGNESIUM CHELETASE I2	3.9	4.6	-0.7	-2.6	92.6%	-2.2
1410	AT1G75750.1 | Symbol: GASA1 | gibberellin-regulated protein 1 (GASA1) / gibberellin-responsive protein 1, identical to SP:P46689 Gibberellin-regulated protein 1 precursor {Arabidopsis thaliana}; supporting cDNA gi:887938:gb:U11766.1:ATU11766 | chr1:28445202-28446023 REVERSE | Aliases: F10A5.6, F10A5_6, GAST1 PROTEIN HOMOLOG	10.0	8.8	1.2	2.6	92.6%	-2.3
1411	AT2G35500.1 | shikimate kinase-related, low similarity to shikimate kinase precursor from Lycopersicon esculentum (SP:Q00497) | chr2:14921106-14923198 FORWARD | Aliases: T32F12.12, T32F12_12	6.6	5.5	1.1	2.6	92.6%	-2.1

Rank	Description	15.dpi	5.dpi	M	t	adj.q	B
1412	AT4G21100.1 | Symbol: DDB1B | UV-damaged DNA-binding protein, putative, similar to UV-damaged DNA binding protein (GI:12082087) (Oryza sativa) and damage-specific DNA binding protein 1, Homo sapiens, PIR2:I38908; contains Pfam PF03178 : CPSF A subunit region | chr4:11258804-11265320 REVERSE | Aliases: DAMAGED DNA BINDING PROTEIN 1 B, F7J7.40, F7J7_40	4.6	5.3	-0.7	-2.6	92.6%	-2.2
1413	AT5G48600.1 | Symbol: ATSMC3 |	3.6	4.8	-1.2	-2.6	92.6%	-2.2
1414	AT5G24170.1 | expressed protein | chr5:8189489-8191394 REVERSE | Aliases: K12G2.6, K12G2_6	4.6	4.0	0.6	2.6	92.6%	-2.3
1415	AT4G38890.1 | dihydrouridine synthase family protein, contains Pfam domain, PF01207: Dihydrouridine synthase (Dus) | chr4:18135903-18139094 REVERSE | Aliases: F19H22.4	8.6	9.4	-0.8	-2.6	92.6%	-2.2
1416	AT3G11770.1 | expressed protein | chr3:3721334-3722652 REVERSE | Aliases: F26K24.6	7.9	8.6	-0.7	-2.6	92.6%	-2.3
1417	AT3G06420.1 | autophagy 8h (APG8h), identical to autophagy 8h (Arabidopsis thaliana) GI:19912165; contains Pfam profile PF02991: Microtubule associated protein 1A/1B, light chain 3; supporting cDNA gi:19912164:dbj:AB073182.1: | chr3:1954996-1956399 REVERSE | Aliases: F24P17.11, F24P17_11	6.9	5.8	1.1	2.6	92.6%	-2.1
1418	AT5G23420.1 | Symbol: HMGB6 | high mobility group (HMG1/2) family protein, similar to high mobility group protein 2 HMG2 (Ipomoea nil) GI:1052956; contains Pfam profile PF00505: HMG (high mobility group) box | chr5:7888516-7890176 REVERSE | Aliases: K19M13.4, K19M13_4	2.8	3.7	-0.9	-2.6	92.6%	-2.3
1419	AT1G11480.1 | eukaryotic translation initiation factor-related, contains weak similarity to Swiss-Prot:P23588 eukaryotic translation initiation factor 4B (eIF-4B) (Homo sapiens) | chr1:3864153-3866969 REVERSE | Aliases: T23J18.15, T23J18_15	8.3	7.4	0.9	2.6	92.6%	-2.2
1420	AT1G08260.1 | Symbol: EMB2284 | DNA-directed DNA polymerase epsilon catalytic subunit, putative, similar to SP:Q07864 DNA polymerase epsilon, catalytic subunit A (EC 2.7.7.7) (DNA polymerase II subunit A) {Homo sapiens}; contains Pfam profiles: PF03175 DNA polymerase type B, organellar and viral, PF00136 DNA polymerase family B, PF03104 DNA polymerase family B, exonuclease domain | chr1:2590941-2606889 FORWARD | Aliases: EMB2284, EMBRYO DEFECTIVE 2284, T23G18.21, T23G18_21	4.6	5.1	-0.6	-2.6	92.6%	-2.3
1421	AT5G17610.1 | expressed protein | chr5:5803964-5805254 FORWARD | Aliases: K10A8.90, K10A8_90	7.9	7.4	0.5	2.6	92.6%	-2.4
1422	AT4G32590.4 | similar to ferredoxin-related [Arabidopsis thaliana] (TAIR:At3g16250.1); similar to 2Fe-2S iron-sulfur cluster protein-like [Oryza sativa (japonica cultivar-group)] (GB:XP_478158.1) | chr4:15721385-15722875 FORWARD | Aliases: F4D11.210, F4D11_210	5.8	5.2	0.6	2.6	92.6%	-2.3
1423	AT4G02195.1 | Symbol: SYP42 | syntaxin 42 (SYP42) / TLG2b, identical to SP:Q9SWH4 Syntaxin 42 (AtSYP42) (AtTLG2b) {Arabidopsis thaliana} | chr4:970009-972304 REVERSE | Aliases: ATSYP42, ATTLG2B, SYNTAXIN OF PLANTS 41, TLG2B	7.2	6.5	0.7	2.6	92.6%	-2.6
1424	AT4G23810.1 | Symbol: WRKY53 |	3.2	4.7	-1.5	-2.6	92.6%	-2.1
1425	AT3G23300.1 | dehydration-responsive protein-related, similar to early-responsive to dehydration stress ERD3 protein (Arabidopsis thaliana) GI:15320410; contains Pfam profile PF03141: Putative methyltransferase | chr3:8333155-8336153 FORWARD | Aliases: MLM24.3	7.9	8.6	-0.6	-2.6	92.6%	-2.7
1426	AT2G19810.1 | zinc finger (CCCH-type) family protein, contains Pfam domain, PF00642: Zinc finger C-x8-C-x5-C-x3-H type (and similar) | chr2:8557395-8558940 FORWARD | Aliases: F6F22.16, F6F22_16	6.6	6.0	0.6	2.6	92.6%	-2.3
1427	AT1G04040.1 | acid phosphatase class B family protein, similar to SP:P15490 STEM 28 kDa glycoprotein precursor (Vegetative storage protein A) {Glycine max}, acid phosphatase (Glycine max) GI:3341443; contains Pfam profile PF03767: HAD superfamily (subfamily IIIB) phosphatase; supporting cDNA gi:13926197:gb:AF370572.1:AF370572 | chr1:1042361-1043861 REVERSE | Aliases: F21M11.2, F21M11_2	7.9	8.6	-0.7	-2.6	92.7%	-2.3
1428	AT1G09160.2 | protein phosphatase 2C-related / PP2C-related, similar to GB:AAC16260 | chr1:2952773-2955606 REVERSE | Aliases: T12M4.15, T12M4_15	6.0	6.8	-0.8	-2.6	92.7%	-2.2
1429	AT4G20850.1 | Symbol: TPP2 | subtilase family protein, contains similarity to Tripeptidyl-peptidase II (EC 3.4.14.10) (TPP-II) (Tripeptidyl aminopeptidase) (Swiss-Prot:P29144) (Homo sapiens) | chr4:11160732-11169900 REVERSE | Aliases: T13K14.10, T13K14_10, TRIPEPTIDYL PEPTIDASE II	10.3	10.9	-0.6	-2.6	92.7%	-2.4
1430	AT2G35980.1 | Symbol: YLS9 | harpin-induced family protein (YLS9) / HIN1 family protein / harpin-responsive family protein, similar to harpin-induced protein hin1 (GI:1619321) (Nicotiana tabacum); identical to cDNA YLS9 mRNA for hin1 homolog GI:13122295 | chr2:15117667-15118550 FORWARD | Aliases: F11F19.11, F11F19_11, NHL10, YELLOW LEAF SPECIFIC GENE 9	2.0	2.3	-0.3	-2.6	92.7%	-3.1
1431	AT2G34690.1 | Symbol: ACD11 | expressed protein | chr2:14637430-14639029 FORWARD | Aliases: ACCELERATED CELL DEATH 11, T29F13.10, T29F13_10	10.3	9.8	0.6	2.6	92.8%	-2.4
1432	AT2G25250.1 | expressed protein | chr2:10760927-10761791 FORWARD | Aliases: T22F11.16, T22F11_16	5.5	4.2	1.4	2.6	93.3%	-2.2

Rank	Description	15.dpi	5.dpi	M	t	adj.q	B
1433	AT2G07680.1 | Symbol: ATMRP11 | ABC transporter family protein | chr2:3515959-3522488 FORWARD | Aliases: T5E7.1	4.2	5.5	-1.4	-2.6	93.4%	-2.2
1434	AT5G05790.1 | myb family transcription factor, contains Pfam profile: PF00249 myb-like DNA-binding domain | chr5:1740573-1742029 REVERSE | Aliases: MJJ3.20, MJJ3_20	9.1	8.2	0.9	2.6	93.4%	-2.4
1435	AT3G01420.1 | Symbol: ALPHA DOX1 | pathogen-responsive alpha-dioxygenase, putative, similar to pathogen-inducible alpha-dioxygenase (Nicotiana attenuata) GI:12539609; contains Pfam profile PF03098: Animal haem peroxidase | chr3:159473-162844 REVERSE | Aliases: ALPHA DIOXYGENASE 1, T13O15.6, T13O15_6	3.2	4.6	-1.4	-2.6	93.4%	-2.1
1436	AT2G40420.1 | amino acid transporter family protein, similar to neuronal glutamine transporter (Rattus norvegicus) GI:6978016; contains Pfam profile PF01490: Transmembrane amino acid transporter protein | chr2:16884318-16886292 FORWARD | Aliases: T3G21.19, T3G21_19	5.0	4.1	0.9	2.6	93.4%	-2.3
1437	AT4G32440.2 | agenet domain-containing protein, contains Pfam PF05641: Agenet domain | chr4:15656691-15659754 FORWARD | Aliases: F8B4.140, F8B4_140	5.3	4.6	0.7	2.6	93.5%	-2.3
1438	AT3G10180.1 | kinesin motor protein-related, similar to centromere protein E GB:4502781 (Homo sapiens)	2.9	3.2	-0.4	-2.6	93.5%	-2.8
1439	AT2G36230.1 | Symbol: BBM II II | N'-5'-phosphoribosyl-formimino-5-aminoimidazole-4-carboxamide ribonucleotide isomerase, identical to N'-5'-phosphoribosyl-formimino-5-aminoimidazole- 4-carboxamide ribonucleotide isomerase GI:3449284 from (Arabidopsis thaliana); contains Pfam profile: PF00977 histidine biosynthesis protein | chr2:15200779-15202617 REVERSE | Aliases: F2H17.16, F2H17_16, PHOSPHORIBOSYLFORMIMINO 5 AMINOIMIDAZOLE CARBOXAMIDE RIBOTIDE ISOMERASE	8.2	9.1	-0.9	-2.6	93.5%	-2.2
1440	AT5G58575.1 | expressed protein | chr5:23691244-23692462 FORWARD | Aliases: None	5.5	4.4	1.1	2.6	93.7%	-2.2
1441	AT2G42690.1 | lipase, putative, similar to lipase (Dianthus caryophyllus) GI:4103627; contains Pfam profile PF01764: Lipase | chr2:17783318-17784794 REVERSE | Aliases: F14N22.2	5.8	7.2	-1.4	-2.6	93.8%	-2.1
1442	AT1G54220.2 | similar to dihydrolipoamide S-acetyltransferase, putative [Arabidopsis thaliana] (TAIR:At3g13930.1); similar to dihydrolipoamide S-acetyltransferase [Zea mays] (GB:AAD46491.1); similar to unknown protein [Oryza sativa (japonica cultivar-group)] (GB:XP_477668.1); similar to putative dihydrolipoamide S-acetyltransferase [Oryza sativa (japonica cultivar-group)] (GB:XP_463813.1); contains InterPro domain Catalytic domain of components of various dehydrogenase complexes (InterPro:IPR001078); contains InterPro domain E3 binding domain (InterPro:IPR004167); contains InterPro domain Dihydrolipoamide acetyltransferase, long form (InterPro:IPR006257); contains InterPro domain 2-oxo acid dehydrogenase, lipoyl-binding site (InterPro:IPR003016); contains InterPro domain Biotin/lipoyl attachment (InterPro:IPR000089) | chr1:20249704-20253977 REVERSE | Aliases: F20D21.4, F20D21_4	5.6	6.5	-0.9	-2.6	93.8%	-2.2
1443	AT1G30260.1 | Symbol: AGL79 | expressed protein | chr1:10651035-10651712 FORWARD | Aliases: F12P21.9, F12P21_9	2.8	2.4	0.4	2.6	93.8%	-2.7
1444	AT2G43500.1 | RWP-RK domain-containing protein, low similarity to nodule inception protein (Lotus japonicus) GI:6448579; contains Pfam profile: PF02042 RWP-RK domain | chr2:18069792-18073527 FORWARD | Aliases: T1O24.24	6.1	5.3	0.9	2.6	93.9%	-2.2
1445	AT2G06005.2 | expressed protein, similar to expressed protein [Arabidopsis thaliana] (TAIR:At5g20580.1); similar to expressed protein [Arabidopsis thaliana] (TAIR:At5g20580.2); similar to unknown protein [Oryza sativa (japonica cultivar-group)] (GB:XP_468082.1) | chr2:2332575-2336108 FORWARD | Aliases: None	3.9	3.3	0.6	2.6	93.9%	-2.3
1446	AT3G10880.1 | hypothetical protein | chr3:3406300-3407136 FORWARD | Aliases: T7M13.4	3.1	3.6	-0.5	-2.6	94.3%	-2.5
1447	AT4G27470.1 | zinc finger (C3HC4-type RING finger) family protein, contains Pfam profile: PF00097 zinc finger, C3HC4 type (RING finger) | chr4:13735005-13736524 FORWARD | Aliases: F27G19.70, F27G19_70	4.4	3.5	1.0	2.6	94.4%	-2.1
1448	AT4G14965.1 | cytochrome b5 domain-containing protein, similar to SP:O15173 Membrane associated progesterone receptor component 2 (Steroid receptor protein DG6) {Homo sapiens}; contains Pfam profile PF00173: Heme/Steroid binding domain | chr4:8551240-8553549 FORWARD | Aliases: None	7.6	7.0	0.6	2.6	94.4%	-2.4
1449	AT3G22520.1 | expressed protein | chr3:7974769-7977597 FORWARD | Aliases: F16J14.8	4.7	5.6	-0.9	-2.6	94.5%	-2.3
1450	AT5G01890.1 | leucine-rich repeat transmembrane protein kinase, putative, leucine-rich receptor-like protein (LRPKm1) - Malus domestica, EMBL:AF053127	5.5	6.2	-0.7	-2.6	94.5%	-2.4
1451	AT4G02030.1 | expressed protein, similar to expressed protein [Arabidopsis thaliana] (TAIR:At1g21170.1); similar to unknown protein [Oryza sativa (japonica cultivar-group)] (GB:AAR07074.1) | chr4:892176-897318 FORWARD | Aliases: T10M13.4, T10M13_4	6.5	7.0	-0.6	-2.6	94.6%	-2.5
1452	AT1G17860.1 | trypsin and protease inhibitor family protein / Kunitz family protein, similar to LeMir (miraculin homolog) GI:2654440 from (Lycopersicon esculentum), tumor-related protein (Nicotiana tabacum) GI:1762933; contains Pfam profile PF00197: Trypsin and protease inhibitor | chr1:6149294-6150031 FORWARD | Aliases: F2H15.9, F2H15_9	5.9	7.8	-2.0	-2.6	94.6%	-2.1

Rank	Description	15.dpi	5.dpi	M	t	adj.q	B
1453	AT1G31050.1 | expressed protein | chr1:11075634-11076180 REVERSE | Aliases: F17F8.3	6.5	4.9	1.6	2.6	94.7%	-2.1
1454	AT5G06830.1 | expressed protein, contains Pfam profile: PF05600 protein of unknown function (DUF773) | chr5:2116784-2119630 REVERSE | Aliases: MPH15.20, MPH15_20	4.6	5.3	-0.7	-2.6	94.8%	-2.5
1455	AT3G63160.1 | expressed protein | chr3:23344488-23345009 REVERSE | Aliases: F16M2.10	5.3	4.6	0.7	2.6	94.8%	-2.4
1456	AT3G10450.2 | Symbol: SCPL7 | similar to serine carboxypeptidase S10 family protein [Arabidopsis thaliana] (TAIR:At1g73300.1); similar to serine carboxypeptidase S10 family protein [Arabidopsis thaliana] (TAIR:At1g73270.1); similar to serine carboxypeptidase S10 family protein [Arabidopsis thaliana] (TAIR:At1g73290.1); similar to serine carboxypeptidase S10 family protein [Arabidopsis thaliana] (TAIR:At1g73280.1); similar to serine carboxypeptidase S10 family protein [Arabidopsis thaliana] (TAIR:At5g36180.1); similar to OSJNBb0051N19.2 [Oryza sativa (japonica cultivar-group)] (GB:XP_474646.1); contains InterPro domain Serine carboxypeptidase (S10) (InterPro:IPR001563) | chr3:3249536-3252509 FORWARD | Aliases: F13M14.27, SCPL7	5.4	4.7	0.7	2.6	94.8%	-2.4
1457	AT5G47050.1 | expressed protein | chr5:19123567-19125575 FORWARD | Aliases: MQD22.19, MQD22_19	8.2	7.5	0.7	2.6	94.8%	-2.6
1458	AT3G55120.1 | Symbol: TT5 | chalcone-flavanone isomerase / chalcone isomerase (CHI), identical to SP:P41088 | chr3:20441091-20442447 REVERSE | Aliases: A11, CFI, CHALCONE FLAVANONE ISOMERASE, CHALCONE ISOMERASE, CHI, T15C9.120, TRANSPARENT TESTA 5	5.4	6.2	-0.7	-2.6	94.8%	-2.3
1459	AT1G67480.1 | kelch repeat-containing F-box family protein, similar to SKP1 interacting partner 6 (Arabidopsis thaliana) GI:10716957; contains Pfam profiles PF01344: Kelch motif, PF00646: F-box domain | chr1:25279958-25282381 FORWARD | Aliases: T1F15.5, T1F15_5	7.0	7.5	-0.5	-2.6	94.8%	-2.5
1460	AT1G35530.1 | DEAD/DEAH box helicase, putative, low similarity to RNA helicase/RNAseIII CAF protein (Arabidopsis thaliana) GI:6102610; contains Pfam profiles PF00270: DEAD/DEAH box helicase, PF00271: Helicase conserved C-terminal domain | chr1:13089972-13097056 FORWARD | Aliases: F15O4.40	2.3	2.8	-0.5	-2.6	94.9%	-2.7
1461	AT3G14570.1 | Symbol: ATGSL04 | glycosyl transferase family 48 protein, contains similarity to glucan synthases | chr3:4892650-4902635 FORWARD | Aliases: ATGSL4, GLUCAN SYNTHASE LIKE 4, GSL04, MIE1.7	8.0	6.9	1.1	2.6	94.9%	-2.4
1462	AT1G67470.1 | protein kinase family protein, contains protein kinase domain, Pfam:PF00069 | chr1:25275845-25277239 FORWARD | Aliases: T1F15.6	4.7	4.0	0.7	2.6	95.0%	-2.3
1463	AT4G21210.2 | expressed protein, similar to expressed protein [Arabidopsis thaliana] (TAIR:At3g01200.1); similar to hypothetical protein GSU0450 [Geobacter sulfurreducens PCA] (GB:NP_951509.1); contains InterPro domain Protein of unknown function DUF299 (InterPro:IPR005177) | chr4:11306955-11308752 FORWARD | Aliases: F7J7.150, F7J7_150	5.5	4.6	0.9	2.6	95.0%	-2.2
1464	AT5G11560.1 | PQQ enzyme repeat-containing protein, contains Pfam profile PF01011: PQQ enzyme repeat | chr5:3709482-3714042 REVERSE | Aliases: F15N18.150, F15N18_150	8.2	9.4	-1.2	-2.6	95.0%	-2.4
1465	AT2G01710.1 | DNAJ heat shock N-terminal domain-containing protein, simlar to AHM1 (Triticum aestivum) GI:6691467; contains Pfam profile PF00226: DnaJ domain | chr2:315725-316992 FORWARD | Aliases: T8O11.12, T8O11_12	5.3	4.7	0.6	2.6	95.0%	-2.5
1466	AT5G58420.1 | 40S ribosomal protein S4 (RPS4D), ribosomal protein S4, Arabidopsis thaliana, PIR:T48480	10.0	10.8	-0.8	-2.6	95.0%	-2.3
1467	AT1G07600.1 | Symbol: MT1A | metallothionein-like protein 1A (MT-1A) (MT-Q) (MT-2), identical to Metallothionein-like protein 1A (MT-1A) (MT-Q) (MT-2) SP:P43392 from (Arabidopsis thaliana) | chr1:2338901-2339318 REVERSE | Aliases: ATMT 2, ATMT Q, F22G5.2, LIGHT STRESS REGULATED 4, LSR4, METALLOTHIONEIN 1A, MT Q, MT1	12.9	12.2	0.7	2.6	95.0%	-3.2
1468	AT4G09830.1 | expressed protein | chr4:6188764-6190850 FORWARD | Aliases: F17A8.180, F17A8_180	9.4	8.5	0.9	2.6	95.0%	-2.5
1469	AT1G01360.1 | expressed protein, similar to hypothetical protein GB:CAB45785 GI:5262156 from (Arabidopsis thaliana) | chr1:142024-143177 FORWARD | Aliases: F6F3.16, F6F3_16	8.2	6.6	1.6	2.6	95.0%	-2.3
1470	AT4G12700.1 | expressed protein | chr4:7482463-7484402 REVERSE | Aliases: T20K18.50, T20K18_50	4.8	5.4	-0.6	-2.6	95.0%	-2.3
1471	AT5G36880.2 | similar to acyl-activating enzyme 17 (AAE17) [Arabidopsis thaliana] (TAIR:At5g23050.1); similar to Acetyl-CoA synthetase 2, isoform a [Homo sapiens] (GB:AAH12172.1); similar to OTTHUMP00000030713 [Homo sapiens] (GB:CAI19312.1); similar to OSJNBb0118P14.12 [Oryza sativa (japonica cultivar-group)] (GB:XP_472384.1); similar to OJ000315_02.12 [Oryza sativa (japonica cultivar-group)] (GB:CAE05367.3); similar to acetyl-CoA synthetase [Solanum tuberosum] (GB:CAA67130.1); contains InterPro domain AMP-dependent synthetase and ligase (InterPro:IPR000873) | chr5:14551843-14557516 REVERSE | Aliases: F5H8.15, F5H8_15	8.7	9.4	-0.7	-2.6	95.1%	-2.6
1472	AT1G66410.1 | Symbol: CAM4 | calmodulin-1/4 (CAM4), identical to calmodulin (Arabidopsis thaliana) GI:16223; nearly identical to SP:P25854 Calmodulin-1/4 {Arabidopsis thaliana} | chr1:24777880-24779516 REVERSE | Aliases: ACAM 4, CALMODULIN 4, T27F4.1, T27F4_1	13.7	13.1	0.6	2.6	95.1%	-2.6

Rank	Description	15.dpi	5.dpi	M	t	adj.q	B
1473	AT4G29160.2 | SNF7 family protein, contains Pfam domain, PF03357: SNF7 family | chr4:14380810-14382672 FORWARD | Aliases: F19B15.190, F19B15_190	9.3	8.6	0.7	2.6	95.2%	-2.5
1474	AT1G80190.1 | expressed protein | chr1:30164368-30166007 FORWARD | Aliases: F18B13.27, F18B13_27	4.5	5.6	-1.1	-2.6	95.4%	-2.3
1475	AT5G38690.1 | expressed protein | chr5:15496407-15500291 REVERSE | Aliases: MBB18.24, MBB18_24	4.0	4.6	-0.7	-2.6	95.4%	-2.5
1476	AT2G21660.2 | Symbol: ATGRP7 |	12.4	10.2	2.1	2.6	95.5%	-2.2
1477	AT1G69250.2 | nuclear transport factor 2 (NTF2) family protein / RNA recognition motif (RRM)-containing protein, contains Pfam profiles PF02136: Nuclear transport factor 2 (NTF2) domain, PF00076: RNA recognition motif (a.k.a. RRM, RBD, or RNP domain) | chr1:26036558-26039167 FORWARD | Aliases: F4N2.20	4.6	5.8	-1.2	-2.6	95.7%	-2.4
1478	AT3G20370.1 | meprin and TRAF homology domain-containing protein / MATH domain-containing protein, similar to ubiquitin-specific protease 12 (Arabidopsis thaliana) GI:11993471; contains Pfam profile PF00917: MATH domain | chr3:7105377-7107231 FORWARD | Aliases: MQC12.15	3.8	5.0	-1.2	-2.5	95.7%	-2.1
1479	AT1G28540.1 | expressed protein | chr1:10035650-10036927 FORWARD | Aliases: F3M18.29	8.1	7.2	0.9	2.5	95.7%	-2.5
1480	AT4G38225.3 | expressed protein | chr4:17927216-17928888 FORWARD | Aliases: None	7.1	6.1	1.0	2.5	95.7%	-2.1
1481	AT4G35140.1 | transducin family protein / WD-40 repeat family protein, contains 6 (3 significant) WD-40 repeats; similar to PC326 protein (GI:200241) (PIR2:S37694) (Mus musculus); Human (H326) mRNA, Homo sapiens, gb:U06631 | chr4:16723726-16726597 REVERSE | Aliases: T12J5.10, T12J5_10	6.2	5.4	0.8	2.5	95.7%	-2.3
1482	AT5G23330.1 | riboflavin biosynthesis protein-related, contains weak similarity to Riboflavin biosynthesis protein ribF. (Swiss-Prot:P08391) (Escherichia coli O157:H7) | chr5:7854132-7855816 FORWARD | Aliases: MKD15.19, MKD15_19	8.4	7.7	0.7	2.5	95.8%	-2.3
1483	AT3G11020.1 | Symbol: DREB2B |	5.0	4.4	0.5	2.5	95.8%	-2.7
1484	AT1G68500.1 | expressed protein | chr1:25705666-25706174 FORWARD | Aliases: T26J14.7, T26J14_7	9.3	8.0	1.3	2.5	95.8%	-2.4
1485	AT1G77550.1 | expressed protein, similar to hypothetical protein LOC223723 [Mus musculus] (GB:NP_898838.1); contains InterPro domain Tubulin-tyrosine ligase (InterPro:IPR004344) | chr1:29143197-29147859 REVERSE | Aliases: T5M16.14, T5M16_14	6.2	6.9	-0.7	-2.5	96.1%	-2.5
1486	AT3G04470.1 | similar to ankyrin repeat family protein [Arabidopsis thaliana] (TAIR:At1g04780.1); similar to hypothetical protein [Oryza sativa (japonica cultivar-group)] (GB:XP_477804.1); contains InterPro domain Ankyrin (InterPro:IPR002110) | chr3:1189646-1192644 REVERSE | Aliases: T27C4.12, T27C4_12	6.5	7.3	-0.7	-2.5	96.4%	-2.5
1487	AT4G24180.1 | similar to thaumatin, putative [Arabidopsis thaliana] (TAIR:At4g38660.1); similar to putative thaumatin-like protein [Oryza sativa (japonica cultivar-group)] (GB:AAP52107.1); contains InterPro domain Thaumatin, pathogenesis-related (InterPro:IPR001938) | chr4:12550366-12551309 REVERSE | Aliases: T22A6.10, T22A6_10	4.5	3.5	1.0	2.5	96.4%	-2.2
1488	AT5G25610.1 | Symbol: RD22 | dehydration-responsive protein (RD22), identical to SP:Q08298 Dehydration-responsive protein RD22 precursor {Arabidopsis thaliana} | chr5:8914236-8916724 REVERSE | Aliases: T14C9.150, T14C9_150	3.3	2.8	0.5	2.5	96.4%	-2.5
1489	AT4G30700.1 | pentatricopeptide (PPR) repeat-containing protein, contains INTERPRO:IPR002885 PPR repeats | chr4:14962499-14965020 REVERSE | Aliases: T10C21.50, T10C21_50	3.5	4.2	-0.7	-2.5	96.4%	-2.3
1490	AT4G31840.1 | plastocyanin-like domain-containing protein | chr4:15401646-15402623 FORWARD | Aliases: F11C18.40, F11C18_40	3.8	4.7	-0.9	-2.5	96.4%	-2.2
1491	AT2G19310.1 | expressed protein | chr2:8376876-8377488 REVERSE | Aliases: F27F23.11, F27F23_11	6.5	5.5	1.1	2.5	96.4%	-2.3
1492	AT3G50380.1 | expressed protein | chr3:18697424-18711514 REVERSE | Aliases: F11C1.220	4.7	5.8	-1.1	-2.5	96.4%	-2.3
1493	AT5G63640.1 | VHS domain-containing protein / GAT domain-containing protein, weak similarity to hepatocyte growth factor-regulated tyrosine kinase substrate HRS isoform 2 (Homo sapiens) GI:9022389; contains Pfam profiles PF00790: VHS domain, PF03127: GAT domain | chr5:25494982-25498715 FORWARD | Aliases: MBK5.12, MBK5_12	4.9	4.2	0.8	2.5	96.4%	-2.3
1494	AT5G52650.1 | 40S ribosomal protein S10 (RPS10C), contains similarity to 40S ribosomal protein S10 | chr5:21372841-21374301 REVERSE | Aliases: F6N7.14, F6N7_14	10.9	11.5	-0.6	-2.5	96.5%	-2.5
1495	AT4G37590.1 | phototropic-responsive NPH3 family protein, contains NPH3 family domain, Pfam:PF03000 | chr4:17662836-17666056 REVERSE | Aliases: F19F18.80, F19F18_80	5.2	5.9	-0.8	-2.5	96.5%	-2.5

Rank	Description	15.dpi	5.dpi	M	t	adj.q	B
1496	AT2G01300.1 | expressed protein | chr2:151062-151728 REVERSE | Aliases: F10A8.18, F10A8_18	2.9	2.5	0.5	2.5	96.5%	-2.8
1497	AT1G65680.1 | Symbol: ATEXPB2 | similar to beta-expansin, putative (EXPB4) [Arabidopsis thaliana] (TAIR:At2g45110.1); similar to cim1 protein - soybean (GB:S48032); contains InterPro domain Expansin 45, endoglucanase-like domain (InterPro:IPR007112); contains InterPro domain Major pollen allergen Lol pI (InterPro:IPR005795); contains InterPro domain Expansin/Lol pI (InterPro:IPR007118); contains InterPro domain Pollen allergen/expansin, C-terminal (InterPro:IPR007117) | chr1:24430929-24432062 FORWARD | Aliases: ARABIDOPSIS THALIANA EXPANSIN B2, ATEXPB2, ATHEXP BETA 1.4, EXPB2, F1E22.6	3.1	3.7	-0.6	-2.5	96.5%	-2.5
1498	AT1G69310.2 | Symbol: WRKY57 |	3.3	2.9	0.4	2.5	96.5%	-2.8
1499	AT4G21800.2 | ATP-binding family protein, contains Pfam domain, PF03029: Conserved hypothetical ATP binding protein | chr4:11572877-11575504 FORWARD | Aliases: F17L22.260, F17L22_260	6.2	6.9	-0.7	-2.5	96.5%	-2.4
1500	AT4G22740.2 | glycine-rich protein | chr4:11942812-11945967 REVERSE | Aliases: T12H17.1	10.9	9.7	1.1	2.5	96.6%	-2.4
1501	AT3G05630.1 | Symbol: PLDP2 | phospholipase D, putative (PLDP2), identical to SP:Q9M9W8 Phospholipase D p2 (EC 3.1.4.4) (AtPLDp2) (Phospholipase D2 PHOX and PX containing domain) (Phospholipase D zeta 2) (PLDzeta2) {Arabidopsis thaliana}; similar to phospholipase D GB:BAA24577 from (Rattus norvegicus); contains Pfam profile: PF00614 phospholipase D, PF00169 PH domain, PF00787 PX domain | chr3:1635265-1640349 FORWARD | Aliases: F18C1.10, F18C1_10, PLDZETA2	6.6	5.4	1.2	2.5	96.7%	-2.2
1502	AT4G35880.1 | aspartyl protease family protein, contains Eukaryotic and viral aspartyl proteases active site, PROSITE:PS00141 | chr4:16993342-16995821 FORWARD | Aliases: F4B14.150, F4B14_150	4.2	4.9	-0.7	-2.5	96.8%	-2.5
1503	AT5G63570.1 | Symbol: GSA1 | glutamate-1-semialdehyde 2,1-aminomutase 1 (GSA 1) / glutamate-1-semialdehyde aminotransferase 1 (GSA-AT 1), identical to GSA 1 (SP:P42799) | chr5:25469103-25471067 FORWARD | Aliases: GLUTAMATE 1 SEMIALDEHYDE 2,1 AMINOMUTASE, MBK5.3, MBK5_3	7.7	8.8	-1.1	-2.5	96.9%	-2.3
1504	AT1G73220.1 | sugar transporter family protein, contains Pfam profile: PF00083 sugar (and other) transporter | chr1:27541937-27544352 FORWARD | Aliases: T18K17.11, T18K17_11	3.5	2.9	0.6	2.5	96.9%	-2.6
1505	AT1G61610.1 | S-locus lectin protein kinase family protein, similar to KI domain interacting kinase 1 (Zea mays) gi:2735017:gb:AAB93834; contains S-locus glycoprotein family domain, Pfam:PF00954 | chr1:22737137-22740174 FORWARD | Aliases: T25B24.4, T25B24_4	2.6	2.9	-0.3	-2.5	96.9%	-3.0
1506	AT5G22860.2 | serine carboxypeptidase S28 family protein, contains Pfam profile: PF05577 serine carboxypeptidase S28 | chr5:7639855-7642987 REVERSE | Aliases: MRN17.9, MRN17_9	4.2	3.5	0.7	2.5	96.9%	-2.5
1507	AT5G05180.2 | expressed protein | chr5:1536227-1538493 FORWARD | Aliases: K2A11.5, K2A11_5	5.9	6.9	-1.0	-2.5	96.9%	-2.2
1508	AT1G05140.1 | membrane-associated zinc metalloprotease, putative, similar to Hypothetical zinc metalloprotease All3971 (SP:Q8YQ64) (strain PCC 7120) {Anabaena sp.}; Similar to Synechocystis hypothetical protein (gb:D90908);contains Pfam PF00595: PDZ domain (Also known as DHR or GLGF); contains TIGRFAM TIGR00054: membrane-associated zinc metalloprotease, putative | chr1:1482547-1484049 FORWARD | Aliases: YUP8H12.25, YUP8H12_25	8.5	7.8	0.7	2.5	96.9%	-2.6
1509	AT5G57620.1 | Symbol: MYB36 | myb family transcription factor (MYB36), contains PFAM profile: myb DNA binding domain PF00249 | chr5:23352051-23353792 FORWARD | Aliases: MUA2.20, MUA2_20	3.7	4.3	-0.6	-2.5	97.0%	-2.5
1510	AT3G14740.2 | PHD finger family protein, similar to zinc-finger protein BR140 (PIR:JC2069)(Homo sapiens); contains PHD-finger domain PF00628 | chr3:4952068-4953430 REVERSE | Aliases: MIE1.25	5.0	5.8	-0.9	-2.5	97.0%	-2.3
1511	AT3G01350.1 | proton-dependent oligopeptide transport (POT) family protein, contains Pfam profile: PF00854 POT family | chr3:135031-137467 FORWARD | Aliases: T13O15.11	4.5	3.7	0.8	2.5	97.0%	-2.3
1512	AT2G46400.1 | Symbol: WRKY46 |	4.5	3.8	0.7	2.5	97.0%	-2.4
1513	AT5G27930.2 | protein phosphatase 2C, putative / PP2C, putative, protein phosphatase-2C, Mesembryanthemum crystallinum, EMBL:AF075581 | chr5:9958102-9961170 REVERSE | Aliases: F15F15.3	5.0	4.2	0.8	2.5	97.0%	-2.3
1514	AT5G03840.1 | Symbol: TFL1 | terminal flower 1 protein (TFL1), identical go SP:P93003 TERMINAL FLOWER 1 protein {Arabidopsis thaliana}; contains Pfam profile PF01161: Phosphatidylethanolamine-binding protein | chr5:1024640-1025811 REVERSE | Aliases: MED24.6, TERMINAL FLOWER 1, TFL 1	3.8	4.2	-0.4	-2.5	97.0%	-2.8
1515	AT3G18610.1 | nucleolin, putative, contains Pfam profile: PF00076 RNA recognition motif | chr3:6404276-6407828 REVERSE | Aliases: K24M9.10	7.4	6.3	1.0	2.5	97.1%	-2.2
1516	AT5G13190.1 | expressed protein | chr5:4204820-4206408 REVERSE | Aliases: T19L5.5	9.5	8.7	0.8	2.5	97.2%	-2.4
1517	AT3G14700.1 | expressed protein | chr3:4940325-4941405 FORWARD | Aliases: MIE1.20	6.2	5.1	1.1	2.5	97.3%	-2.2

Rank	Description	15.dpi	5.dpi	M	t	adj.q	B
1518	AT2G32830.1 | Symbol: PHT5 | inorganic phosphate transporter (PHT5), identical to inorganic phosphate transporter (PHT5) (Arabidopsis thaliana) GI:2780345 | chr2:13934776-13936790 REVERSE | Aliases: F24L7.2	6.3	5.0	1.3	2.5	97.5%	-2.2
1519	AT1G06160.1 | encodes a member of the ERF (ethylene response factor) subfamily B-3 of ERF/AP2 transcription factor family. The protein contains one AP2 domain. There are 18 members in this subfamily including ATERF-1, ATERF-2, AND ATERF-5. | chr1:1883003-1883933 FORWARD | Aliases: F9P14.2, F9P14_2	4.1	3.0	1.1	2.5	97.5%	-2.2
1520	AT5G02230.2 | haloacid dehalogenase-like hydrolase family protein, contains InterPro accession IPR005834: Haloacid dehalogenase-like hydrolase	4.4	3.2	1.1	2.5	97.5%	-2.4
1521	AT3G06760.1 | drought-responsive family protein, similar to drought-induced mRNA, Di19 (Arabidopsis thaliana) gi:469110:emb:CAA55321 | chr3:2132977-2134562 FORWARD | Aliases: F3E22.10	6.8	5.9	0.8	2.5	97.5%	-2.4
1522	AT4G24450.1 | similar to starch excess protein (SEX1) [Arabidopsis thaliana] (TAIR:At1g10760.1); similar to starch associated protein R1 [Solanum tuberosum] (GB:AAK11735.1); similar to R1 [Citrus reticulata] (GB:AAM18228.1); similar to SPR1b [Ostreococcus tauri] (GB:AAS88899.1)	4.3	3.3	1.0	2.5	97.6%	-2.3
1523	AT1G37130.1 | Symbol: NIA2 | nitrate reductase 2 (NR2), identical to SP:P11035 Nitrate reductase 2 (formerly EC 1.6.6.1) (NR2) {Arabidopsis thaliana}	11.1	9.7	1.4	2.5	97.6%	-2.5
1524	AT1G33170.1 | dehydration-responsive family protein, similar to early-responsive to dehydration stress ERD3 protein (Arabidopsis thaliana) GI:15320410; contains Pfam profile PF03141: Putative methyltransferase | chr1:12027064-12030519 FORWARD | Aliases: T9L6.6, T9L6_6	5.7	6.2	-0.5	-2.5	97.6%	-2.7
1525	AT5G03455.1 | Symbol: CDC25 | rhodanese-like domain-containing protein, contains Rhodanese-like domain PF:00581 | chr5:862558-864107 FORWARD | Aliases: ARATH;CDC25	10.6	9.9	0.7	2.5	97.6%	-2.9
1526	AT4G12410.1 | auxin-responsive family protein, similar to GP:546362 small auxin up RNA {Arabidopsis thaliana}; auxin-induced protein 10A -Glycine max,PID:g255579 | chr4:7342953-7343587 REVERSE | Aliases: T1P17.3	8.5	7.3	1.2	2.5	97.6%	-2.2
1527	AT1G50490.1 | Symbol: UBC20 | ubiquitin-conjugating enzyme 20 (UBC20), nearly identical to ubiquitin-conjugating enzyme UBC20 (Arabidopsis thaliana) GI:22530867; contains Pfam profile PF00179: Ubiquitin-conjugating enzyme	7.3	8.4	-1.1	-2.5	97.6%	-2.3
1528	AT5G49760.1 | leucine-rich repeat family protein / protein kinase family protein, contains Pfam domains PF00560: Leucine Rich Repeat and PF00069: Protein kinase domain | chr5:20233598-20238525 FORWARD | Aliases: K2I5.13, K2I5_13	5.1	6.1	-1.0	-2.5	97.6%	-2.3
1529	AT4G15890.1 | expressed protein | chr4:9017468-9022997 FORWARD | Aliases: DL3985W, FCAALL.407	4.9	5.9	-1.0	-2.5	97.7%	-2.3
1530	AT5G18410.2 | Symbol: KLK/PIR/PIR121/PIRP/SRA1 | expressed protein, similar to p53 inducible protein (Homo sapiens) GI:5616320 | chr5:6098571-6104982 REVERSE | Aliases: F20L16.130, F20L16_130, KLK, PIR, PIR121, PIROGI, PIRP, SRA1	5.7	6.5	-0.8	-2.5	97.7%	-2.6
1531	AT2G04850.1 | auxin-responsive protein-related, related to auxin-induced protein AIR12 GI:11357190 (Arabidopsis thaliana) | chr2:1704280-1705775 FORWARD | Aliases: F28I8.9, F28I8_9	4.5	4.0	0.5	2.5	97.7%	-2.7
1532	AT3G18110.1 | Symbol: EMB1270 | pentatricopeptide (PPR) repeat-containing protein, contains Pfam profile: PF01535 PPR repeat | chr3:6204810-6209697 REVERSE | Aliases: EMB1270, EMBRYO DEFECTIVE 1270, MRC8.9	5.7	6.8	-1.1	-2.5	97.7%	-2.2
1533	AT5G60250.1 | zinc finger (C3HC4-type RING finger) family protein, contains a Prosite:PS00518 Zinc finger, C3HC4 type (RING finger), signature and Pfam domain PF01485: IBR domain | chr5:24269384-24272066 FORWARD | Aliases: F15L12.13, F15L12_13	4.3	3.7	0.6	2.5	97.7%	-2.6
1534	AT2G31230.1 | Symbol: ATERF15 | encodes a member of the ERF (ethylene response factor) subfamily B-3 of ERF/AP2 transcription factor family. The protein contains one AP2 domain. There are 18 members in this subfamily including ATERF-1, ATERF-2, AND ATERF-5. | chr2:13313670-13314552 REVERSE | Aliases: ATERF15, F16D14.7, F16D14_7	4.0	3.4	0.6	2.5	97.7%	-2.5
1535	AT3G09030.1 | potassium channel tetramerisation domain-containing protein, contains Pfam profile PF02214: K+ channel tetramerisation domain | chr3:2754787-2756511 FORWARD | Aliases: T16O11.1	5.2	4.7	0.5	2.5	97.7%	-2.8
1536	AT3G15770.2 | expressed protein, similar to expressed protein [Arabidopsis thaliana] (TAIR:At5g25360.1); similar to hypothetical protein [Picea mariana] (GB:AAC32109.1) | chr3:5340081-5341346 FORWARD | Aliases: MSJ11.17	6.4	5.5	0.9	2.5	97.8%	-2.5
1537	AT3G27880.1 | expressed protein | chr3:10339449-10340673 FORWARD | Aliases: K16N12.10	5.7	4.7	1.0	2.5	98.0%	-2.4
1538	AT1G54130.1 | Symbol: RSH3 | RelA/SpoT protein, putative (RSH3), identical to RSH3 (RelA/SpoT homolog) GI:7141308 from (Arabidopsis thaliana); contains Pfam profiles PF01966: HD domain, PF04607: Region found in RelA / SpoT proteins | chr1:20214540-20217720 FORWARD | Aliases: AT RSH3, F15I1.23, F15I1_23, RELA/SPOT HOMOLOG 3, RSH3	8.4	7.4	1.0	2.5	98.0%	-2.4

Rank	Description	15.dpi	5.dpi	M	t	adj.q	B
1539	AT5G26280.1 | meprin and TRAF homology domain-containing protein / MATH domain-containing protein, low similarity to ubiquitin-specific protease 12 (Arabidopsis thaliana) GI:11993471; contains Pfam profile PF00917: MATH domain | chr5:9208715-9210575 FORWARD | Aliases: F9D12.7	4.1	5.0	-0.9	-2.5	98.0%	-2.4
1540	AT3G10985.1 | wound-responsive protein-related, similar to SP:P20144 Wound-induced protein 1 {Solanum tuberosum} | chr3:3442534-3443451 FORWARD | Aliases: None	4.8	3.6	1.2	2.5	98.0%	-2.2
1541	AT1G09200.1 | histone H3, identical to histone H3 from Zea mays SP:P05203, Medicago sativa GI:166384, Encephalartos altensteinii SP:P08903, Pisum sativum SP:P02300; contains Pfam profile PF00125 Core histone H2A/H2B/H3/H4 | chr1:2971595-2972201 REVERSE | Aliases: T12M4.9	9.2	11.3	-2.1	-2.5	98.0%	-2.3
1542	AT1G80310.1 | expressed protein | chr1:30199510-30201446 FORWARD | Aliases: F5I6.6, F5I6_6	7.3	6.5	0.8	2.5	98.0%	-2.4
1543	AT5G50900.1 | armadillo/beta-catenin repeat family protein, armadillo/beta-catenin-like repeats, Pfam:PF00514 | chr5:20722030-20724004 REVERSE | Aliases: K3K7.4, K3K7_4	8.4	7.7	0.7	2.5	98.0%	-2.6
1544	AT5G03300.1 | Symbol: ADK2 | adenosine kinase 2 (ADK2), contains Pfam profile: PF00294 pfkB family carbohydrate kinase; identical to cDNA adenosine kinase 2 (ADK2) GI:12017763 | chr5:796441-799153 FORWARD | Aliases: ADENOSINE KINASE 2, F12E4.30, F12E4_30	10.5	11.4	-1.0	-2.5	98.0%	-2.5
1545	AT4G03020.1 | transducin family protein / WD-40 repeat family protein, contains 6 WD-40 repeats (PF00400); similar to L. erythrorhizon LEC14B, GenBank accession number Q40153	7.1	8.2	-1.2	-2.5	98.1%	-2.3
1546	AT4G22470.1 | protease inhibitor/seed storage/lipid transfer protein (LTP) family protein, similar to hydroxyproline-rich glycoprotein DZ-HRGP from Volvox carteri f. nagariensis GP:6523547; contains Pfam profile PF00234 Protease inhibitor/seed storage/LTP family	2.5	3.2	-0.7	-2.5	98.1%	-2.7
1547	AT5G51660.1 | cleavage and polyadenylation specificity factor (CPSF) A subunit C-terminal domain-containing protein, similar to SP:Q9EPU4 Cleavage and polyadenylation specificity factor, 160 kDa subunit (CPSF 160 kDa subunit) {Mus musculus}; contains Pfam profile PF03178: CPSF A subunit region	5.2	5.8	-0.6	-2.5	98.1%	-2.6
1548	AT1G23070.1 | expressed protein, contains Pfam profile PF03619: Domain of unknown function | chr1:8174000-8175747 REVERSE | Aliases: T26J12.15, T26J12_15	4.1	3.5	0.6	2.5	98.1%	-2.6
1549	AT2G34840.1 | coatomer protein epsilon subunit family protein / COPE family protein, similar to SP:O14579 Coatomer epsilon subunit (Epsilon-coat protein) (Epsilon-COP) from Homo sapiens, SP:Q60445 from Cricetulus griseus | chr2:14709591-14711432 FORWARD | Aliases: F19I3.7, F19I3_7	6.4	7.1	-0.7	-2.5	98.2%	-2.5
1550	AT5G41400.1 | zinc finger (C3HC4-type RING finger) family protein, similar to RING-H2 finger protein RHA1a (Arabidopsis thaliana) GI:3790554; contains Pfam profile PF00097: Zinc finger, C3HC4 type (RING finger) | chr5:16586605-16587405 REVERSE | Aliases: MYC6.11, MYC6_11	3.7	3.0	0.7	2.5	98.2%	-2.5
1551	AT1G74030.1 | enolase, putative, similar to Swiss-Prot:P15007 enolase (EC 4.2.1.11) (2-phosphoglycerate dehydratase)(2-phospho-D- glycerate hydro-lyase) (Drosophila melanogaster) | chr1:27842845-27845592 REVERSE | Aliases: F2P9.10, F2P9_10	10.0	11.4	-1.4	-2.5	98.2%	-2.4
1552	AT4G30400.1 | zinc finger (C3HC4-type RING finger) family protein, similar to RING-H2 finger protein RHX1a (Arabidopsis thaliana) GI:3790591; contains Pfam profile PF00097: Zinc finger, C3HC4 type (RING finger) | chr4:14866952-14868634 FORWARD | Aliases: F17I23.260, F17I23_260	4.1	4.6	-0.5	-2.5	98.2%	-2.7
1553	AT3G17350.1 | expressed protein | chr3:5934100-5935042 FORWARD | Aliases: MGD8.19	4.6	5.5	-1.0	-2.5	98.2%	-2.2
1554	AT1G50140.1 | AAA-type ATPase family protein, contains Pfam domain, PF00004: ATPase, AAA family | chr1:18573449-18579492 REVERSE | Aliases: F2J10.1, F2J10_1	5.8	6.7	-0.9	-2.5	98.2%	-2.5
1555	AT5G55230.1 | microtubule associated protein (MAP65/ASE1) family protein, low similarity to protein regulating cytokinesis 1 (PRC1) (Homo sapiens) GI:2865521; contains Pfam profile PF03999: Microtubule associated protein (MAP65/ASE1 family) | chr5:22419276-22422718 FORWARD | Aliases: MCO15.18, MCO15_18	7.1	7.9	-0.8	-2.5	98.2%	-2.5
1556	AT4G23240.1 | protein kinase family protein, contains Pfam PF00069: Protein kinase domain | chr4:12160512-12161964 REVERSE | Aliases: F21P8.130, F21P8_130	3.6	3.0	0.6	2.5	98.2%	-2.7
1557	AT5G59720.1 | Symbol: HSP18.2 | 18.1 kDa class I heat shock protein (HSP18.1-CI), identical to 18.2 kDa class I heat shock protein (HSP 18.2) (SP:P19037)(Arabidopsis thaliana); contains Pfam profile: PF00011 Hsp20/alpha crystallin family	4.1	4.8	-0.7	-2.5	98.2%	-2.3
1558	AT2G20390.1 | expressed protein | chr2:8803364-8805299 REVERSE | Aliases: F11A3.6, F11A3_6	7.5	8.3	-0.8	-2.5	98.2%	-2.3
1559	AT5G50720.1 | Symbol: ATHVA22E | ABA-responsive protein (HVA22e), identical to AtHVA22e (Arabidopsis thaliana) GI:11225589 | chr5:20650422-20651778 REVERSE | Aliases: ATHVA22E, MFB16.12, MFB16_12	8.2	7.0	1.2	2.5	98.4%	-2.2

Rank	Description	15.dpi	5.dpi	M	t	adj.q	B
1560	AT5G10740.1 | protein phosphatase 2C-related / PP2C-related, protein phosphatase 2C, alfalfa, PIR:T09640 | chr5:3393570-3396177 REVERSE | Aliases: MAJ23.3	7.2	6.6	0.6	2.5	98.4%	-2.7
1561	AT1G76060.1 | Symbol: EMB1793 | complex 1 family protein / LVR family protein, contains Pfam PF05347: Complex 1 protein (LYR family) | chr1:28547998-28548744 REVERSE | Aliases: EMBRYO DEFECTIVE 1793, T4O12.26, T4O12_26	7.1	6.0	1.1	2.5	98.4%	-2.4
1562	AT4G40000.1 | NOL1/NOP2/sun family protein, contains Pfam profile PF01189: NOL1/NOP2/sun family | chr4:18543348-18547517 REVERSE | Aliases: T5J17.170, T5J17_170	3.4	4.2	-0.8	-2.5	98.4%	-2.5
1563	AT3G24320.1 | Symbol: CHM | DNA mismatch repair MutS family (MSH1), low similarity to SP:Q56239 DNA mismatch repair protein mutS {Thermus aquaticus}; contains Pfam profiles PF05190: MutS family domain IV, PF01624: MutS domain I, PF01541: Endo/excinuclease amino terminal domain	3.4	4.1	-0.8	-2.5	98.4%	-2.3
1564	AT4G23540.1 | expressed protein, probable membrane protein YPL012w, Saccharomyces cerevisiae, PIR2:S59681 | chr4:12279918-12284693 REVERSE | Aliases: F16G20.240, F16G20_240	4.2	5.2	-0.9	-2.5	98.6%	-2.5
1565	AT5G64470.2 | expressed protein, similar to unknown protein (gb:AAD15463.1) | chr5:25793251-25795189 FORWARD | Aliases: T12B11.6, T12B11_6	4.3	4.8	-0.5	-2.5	98.6%	-2.6
1566	AT1G07870.1 | protein kinase family protein, contains protein kinase domain, Pfam:PF00069 | chr1:2429696-2432018 REVERSE | Aliases: F24B9.4, F24B9_4	7.3	6.3	1.0	2.5	98.6%	-2.3
1567	AT5G49880.1 | mitotic checkpoint family protein, similar to mitotic checkpoint protein isoform MAD1a (Homo sapiens) GI:4580767; contains Pfam profile PF05557: Mitotic checkpoint protein | chr5:20299391-20305310 FORWARD | Aliases: K9P8.2, K9P8_2	5.8	6.7	-0.9	-2.5	98.6%	-2.3
1568	AT5G55670.1 | RNA recognition motif (RRM)-containing protein | chr5:22561166-22564027 REVERSE | Aliases: MDF20.11, MDF20_11	5.9	5.3	0.6	2.5	98.6%	-2.4
1569	AT2G28420.1 | lactoylglutathione lyase family protein / glyoxalase I family protein, contains glyoxalase family protein domain, Pfam:PF00903 | chr2:12165000-12165868 FORWARD | Aliases: T1B3.6, T1B3_6	2.5	2.1	0.3	2.5	98.6%	-3.2
1570	AT3G23160.1 | expressed protein, contains Pfam domain PF05003: protein of unknown function (DUF668); expression supported by MPSS | chr3:8260066-8261634 REVERSE | Aliases: K14B15.5	4.1	4.9	-0.8	-2.5	98.6%	-2.3
1571	AT3G53920.1 | Symbol: SIGC | RNA polymerase sigma subunit SigC (sigC) / sigma factor 3 (SIG3), identical to sigma factor SigC (Arabidopsis thaliana) GI:5478585, sigma factor 3 (SIG3) (Arabidopsis thaliana) GI:2353175, plastid RNA polymerase sigma-subunit (Arabidopsis thaliana) GI:2398853; contains Pfam profiles PF04545: Sigma-70, region 4, PF04539: Sigma-70 region 3, PF04542: Sigma-70 region 2 | chr3:19971847-19974927 REVERSE | Aliases: F5K20.220, RNA POLYMERASE SIGMA SUBUNIT, SIG3	7.2	6.3	0.9	2.5	98.6%	-2.4
1572	AT5G59910.1 | histone H2B, nearly identical to histone H2B Arabidopsis thaliana GI:2407802; contains Pfam profile PF00125 Core histone H2A/H2B/H3/H4 | chr5:24144354-24145161 FORWARD | Aliases: MMN10.15, MMN10_15	10.3	10.9	-0.6	-2.5	98.6%	-2.6
1573	AT1G49580.1 | calcium-dependent protein kinase, putative / CDPK, putative, similar to calcium/calmodulin-dependent protein kinase CaMK3 (Nicotiana tabacum) gi:16904226:gb:AAL30820; contains protein kinase domain, Pfam:PF00069; contains serine/threonine protein kinase domain, INTERPRO:IPR002290	4.0	4.9	-0.9	-2.5	98.6%	-2.3
1574	AT4G10280.1 | expressed protein | chr4:6374742-6376107 REVERSE | Aliases: T9A4.15	4.4	5.6	-1.2	-2.5	98.6%	-2.4
1575	AT4G28025.1 | expressed protein | chr4:13935641-13937388 REVERSE | Aliases: None	8.8	8.2	0.6	2.5	98.6%	-2.4
1576	AT2G39650.1 | expressed protein, contains Pfam profile PF04720: Protein of unknown function (DUF506) | chr2:16534922-16536352 REVERSE | Aliases: F12L6.31, F12L6_31	4.4	3.9	0.6	2.5	98.6%	-2.6
1577	AT4G37910.1 | Symbol: MTHSC70 1 | heat shock protein 70, mitochondrial, putative / HSP70, mitochondrial, putative, strong similarity to SP:Q01899 Heat shock 70 kDa protein, mitochondrial precursor {Phaseolus vulgaris} | chr4:17825074-17828171 REVERSE | Aliases: F20D10.30, F20D10_30, MTHSC70 1	9.6	10.7	-1.1	-2.5	98.6%	-2.4
1578	AT4G26460.1 | similar to S-adenosyl-L-methionine:carboxyl methyltransferase family protein [Arabidopsis thaliana] (TAIR:At4g26420.1) | chr4:13369541-13370019 FORWARD | Aliases: M3E9.110, M3E9_110	3.1	2.8	0.4	2.5	98.8%	-2.9
1579	AT1G50030.1 | Symbol: TOR | target of rapamycin protein (TOR), identical to pTOR (Arabidopsis thaliana) GI:12002902; contains Pfam profiles PF00454 Phosphatidylinositol 3- and 4-kinase, PF02259 FAT domain, PF02260 FATC domain | chr1:18526109-18543663 REVERSE | Aliases: F2J10.19, F2J10_19, TARGET OF RAPAMYCIN, TOR	5.5	6.3	-0.8	-2.5	98.9%	-2.4
1580	AT4G23680.1 | major latex protein-related / MLP-related, low similarity to major latex protein {Papaver somniferum}(GI:294060) ; contains Pfam profile PF00407: Pathogenesis-related protein Bet v I family | chr4:12336196-12337486 REVERSE | Aliases: F9D16.150, F9D16_150	3.1	3.5	-0.4	-2.5	99.0%	-2.8

Rank	Description	15.dpi	5.dpi	M	t	adj.q	B
1581	ATCG00800.1 | Symbol: RPS3 | encodes a chloroplast ribosomal protein S3, a constituent of the small subunit of the ribosomal complex | chrC:82826-83482 REVERSE | Aliases: RPS3	11.0	11.6	-0.6	-2.5	99.0%	-2.6
1582	AT5G09650.1 | inorganic pyrophosphatase family protein, similar to SP:Q15181 Inorganic pyrophosphatase (EC 3.6.1.1) (Pyrophosphate {Homo sapiens}; contains Pfam profile PF00719: inorganic pyrophosphatase | chr5:2991156-2993214 REVERSE | Aliases: F17I14.160, F17I14_160	9.3	10.4	-1.2	-2.5	99.0%	-2.5
1583	AT5G56520.1 | expressed protein | chr5:22902397-22903141 FORWARD | Aliases: MKN22.3, MKN22_3	5.1	4.4	0.7	2.5	99.0%	-2.4
1584	AT1G77600.1 | expressed protein, weak similarity to Pds5 (GI:16751524) (Schizosaccharomyces pombe); weak similarity to androgen-induced prostate proliferative shutoff associated protein (GI:4559410) (Homo sapiens) | chr1:29157642-29167856 REVERSE | Aliases: T5M16.19, T5M16_19	4.3	4.9	-0.7	-2.5	99.1%	-2.6
1585	AT2G26560.1 | patatin, putative, similar to patatin-like latex allergen (Hevea brasiliensis)(PMID:10589016); contains patatin domain PF01734 | chr2:11300847-11302881 REVERSE | Aliases: T9J22.23, T9J22_23	3.3	3.7	-0.4	-2.5	99.1%	-2.8
1586	AT2G24260.1 | basic helix-loop-helix (bHLH) family protein, contains Pfam profile: PF00010 helix-loop-helix DNA-binding domain	6.9	6.2	0.6	2.5	99.2%	-2.6
1587	AT1G30135.1 | expressed protein | chr1:10596476-10597222 FORWARD | Aliases: None	2.7	2.4	0.3	2.5	99.3%	-3.2
1588	AT3G02110.1 | Symbol: SCPL25 |	3.3	3.9	-0.6	-2.5	99.4%	-2.4
1589	AT3G19000.2 | oxidoreductase, 2OG-Fe(II) oxygenase family protein, similar to SP:P24397 Hyoscyamine 6-dioxygenase (EC 1.14.11.11) (Hyoscyamine 6-beta-hydroxylase) {Hyoscyamus niger}, SP:Q05965 Naringenin,2-oxoglutarate 3-dioxygenase (EC 1.14.11.9) (Flavonone- 3-hydroxylase) (F3H) (FHT) {Matthiola incana}; contains Pfam profile PF03171: oxidoreductase, 2OG-Fe(II) oxygenase family	6.6	7.5	-0.9	-2.5	99.5%	-2.5
1590	AT2G41500.1 | WD-40 repeat family protein / small nuclear ribonucleoprotein Prp4p-related, similar to U4/U6 small nuclear ribonucleoprotein hPrp4 (GP:2708305) {Homo sapiens}; contains Pfam PF00400: WD domain, G-beta repeat (7 copies):19877698:gb:AU238529.1:AU238529 | chr2:17311209-17314504 REVERSE | Aliases: T26J13.9, T26J13_9	6.5	7.1	-0.6	-2.5	99.5%	-2.5
1591	AT1G10350.1 | DNAJ heat shock protein, putative, similar to SP:Q9QYJ3 DnaJ homolog subfamily B member 1 (Heat shock 40 kDa protein 1) {Mus musculus}; contains Pfam profile PF00226: DnaJ domain | chr1:3393409-3395057 REVERSE | Aliases: F14N23.23, F14N23_23	5.8	6.5	-0.7	-2.5	99.5%	-2.4
1592	AT2G35190.1 | Symbol: NPSN11 |	6.2	7.3	-1.1	-2.5	99.6%	-2.4
1593	AT4G34190.1 | Symbol: SEP1 | stress enhanced protein 1 (SEP1), identical to stress enhanced protein 1 (SEP1) GI:7384978 from (Arabidopsis thaliana) | chr4:16372394-16373630 REVERSE | Aliases: F10M10.5, SEP1, STRESS ENHANCED PROTEIN 1	7.4	6.6	0.8	2.5	99.6%	-2.5
1594	AT1G76350.1 | RWP-RK domain-containing protein, similar to nodule inception protein (Lotus japonicus) GI:6448579; contains Pfam profile: PF02042 RWP-RK domain	5.1	4.4	0.7	2.5	99.6%	-2.6
1595	AT4G21180.1 | DNAJ heat shock N-terminal domain-containing protein / sec63 domain-containing protein, similar to SP:Q9UGP8 Translocation protein SEC63 homolog {Homo sapiens}; contains Pfam profiles PF00226 DnaJ domain, PF02889 Sec63 domain | chr4:11288939-11292776 FORWARD | Aliases: F7J7.120, F7J7_120	4.3	4.8	-0.5	-2.5	99.8%	-2.6
1596	AT5G54180.1 | mitochondrial transcription termination factor-related / mTERF-related, contains Pfam profile PF02536: mTERF | chr5:22005732-22007454 FORWARD | Aliases: K18G13.5, K18G13_5	5.1	5.7	-0.7	-2.5	99.8%	-2.4
1597	AT3G55460.1 | Symbol: SCL30 | SC35-like splicing factor, 30 kD (SCL30), nearly identical to SC35-like splicing factor SCL30, 30 kD (Arabidopsis thaliana) GI:9843657; Serine/arginine-rich protein/putative splicing factor, Arabidopdis thaliana, EMBL:AF099940; contains Pfam profile PF00076: RNA recognition motif. (a.k.a. RRM, RBD, or RNP domain) | chr3:20571801-20574709 FORWARD | Aliases: T22E16.120	7.4	6.6	0.7	2.5	99.9%	-2.5
1598	AT5G11600.1 | expressed protein | chr5:3733685-3735016 REVERSE | Aliases: T22P22.2	6.5	5.8	0.7	2.5	100.0%	-2.6
1599	AT2G36910.1 | Symbol: ATPGP1 |	8.2	9.0	-0.7	-2.5	100.0%	-2.5
1600	AT3G43610.1 | tubulin family protein | chr3:15528760-15534915 REVERSE | Aliases: F22J12.50	3.2	4.1	-0.8	-2.5	100.0%	-2.6
1601	AT5G14970.1 | expressed protein | chr5:4847281-4848868 FORWARD | Aliases: F2G14.90, F2G14_90	8.6	7.9	0.7	2.5	100.0%	-2.5
1602	AT5G12390.1 | expressed protein | chr5:4010444-4012040 REVERSE | Aliases: None	9.6	8.7	0.9	2.5	100.0%	-2.6

Rank	Description	15.dpi	5.dpi	M	t	adj.q	B
1603	AT5G23940.1 | Symbol: EMB3009 | transferase family protein, similar to anthranilate N-hydroxycinnamoyl/benzoyltransferase, Dianthus caryophyllus (gi:2239091); contains Pfam transferase family domain PF002458 | chr5:8076332-8079796 REVERSE | Aliases: EMB3009, EMBRYO DEFECTIVE 3009, MRO11.2, MRO11_2	2.8	2.5	0.4	2.5	100.0%	-3.1
1604	AT5G65660.1 | hydroxyproline-rich glycoprotein family protein | chr5:26262475-26263605 REVERSE | Aliases: K21L13.18, K21L13_18	9.2	10.0	-0.7	-2.5	100.0%	-2.5
1605	AT3G54000.3 | expressed protein | chr3:20007585-20009601 FORWARD | Aliases: F5K20.300	3.7	3.1	0.6	2.5	100.0%	-2.6
1606	AT5G20980.1 | 5-methyltetrahydropteroyltriglutamate--homocysteine methyltransferase, putative / vitamin-B12-independent methionine synthase, putative / cobalamin-independent methionine synthase, putative, strong similarity to SP:O50008 5-methyltetrahydropteroyltriglutamate--homocysteine methyltransferase (EC 2.1.1.14) (Vitamin-B12-independent methionine synthase isozyme) (Cobalamin-independent methionine synthase isozyme) {Arabidopsis thaliana}; contains Pfam profile PF01717: Methionine synthase, vitamin-B12 independent | chr5:7124116-7128361 REVERSE | Aliases: F22D1.150, F22D1_150	4.3	5.0	-0.8	-2.5	100.0%	-2.6
1607	AT5G55540.1 | expressed protein | chr5:22513422-22517769 REVERSE | Aliases: MTE17.26, MTE17_26	5.9	6.6	-0.7	-2.5	100.0%	-2.4
1608	AT4G38480.1 | transducin family protein / WD-40 repeat family protein, contains contains Pfam PF00400: WD domain, G-beta repeat (7 copies, 3 weak);similar to gene PC326 protein - mouse, PIR2:S37694 | chr4:18003910-18006134 FORWARD | Aliases: F20M13.40, F20M13_40	3.3	3.6	-0.4	-2.5	100.0%	-2.9
1609	AT5G53850.3 | haloacid dehalogenase-like hydrolase family protein, low similarity to enolase-phosphatase E-1 enzyme (Klebsiella oxytoca) GI:401712; contains InterPro accession IPR005834: Haloacid dehalogenase-like hydrolase | chr5:21878223-21882133 REVERSE | Aliases: K19P17.1, K19P17_1	8.9	9.5	-0.6	-2.5	100.0%	-2.7
1610	AT5G25240.1 | expressed protein | chr5:8746608-8747200 REVERSE | Aliases: F21J6.107, F21J6_107	5.5	4.4	1.1	2.5	100.0%	-2.5
1611	AT4G08330.1 | expressed protein | chr4:5254654-5256701 REVERSE | Aliases: T28D5.20, T28D5_20	4.7	4.1	0.6	2.5	100.0%	-2.5
1612	AT1G69600.1 | zinc finger homeobox family protein / ZF-HD homeobox family protein | chr1:26186133-26186861 FORWARD | Aliases: F24J1.29, F24J1_29	4.0	4.8	-0.9	-2.5	100.0%	-2.3
1613	AT1G09620.1 | similar to leucyl-tRNA synthetase, putative / leucine--tRNA ligase, putative [Arabidopsis thaliana] (TAIR:At4g04350.1); similar to leucyl tRNA synthetase [Homo sapiens] (GB:BAA95667.1); similar to SYLC_HUMAN Leucyl-tRNA synthetase, cytoplasmic (Leucine--tRNA ligase) (LeuRS) (GB:Q9P2J5); similar to KIAA1352 protein [Homo sapiens] (GB:BAA92590.1); similar to MGC82093 protein [Xenopus laevis] (GB:AAH79713.1); similar to PREDICTED: similar to Leucyl-tRNA synthetase, cytoplasmic (Leucine--tRNA ligase) (LeuRS) [Canis familiaris] (GB:XP_535229.1); contains InterPro domain Aminoacyl-tRNA synthetase, class I (InterPro:IPR001412); contains InterPro domain Leucyl-tRNA synthetase archae/euk cytosolic, class Ia (InterPro:IPR004493); contains InterPro domain Aminoacyl-tRNA synthetase, class Ia (InterPro:IPR002300) | chr1:3112230-3116700 REVERSE | Aliases: F21M12.1, F21M12_1	5.1	6.1	-0.9	-2.5	100.0%	-2.5
1614	AT4G39840.1 | expressed protein | chr4:18484895-18486808 FORWARD | Aliases: T5J17.10, T5J17_10	4.4	4.9	-0.4	-2.5	100.0%	-2.9
1615	AT5G40460.1 | expressed protein | chr5:16219475-16220160 FORWARD | Aliases: K21I16.3, K21I16_3	3.9	3.0	0.9	2.5	100.0%	-2.5
1616	AT1G56720.2 | protein kinase family protein, contains protein kinase domain, Pfam:PF00069 | chr1:21267093-21270033 REVERSE | Aliases: F25P12.84, F25P12_84	3.2	4.1	-0.9	-2.5	100.0%	-2.5
1617	AT4G10330.1 | glycine-rich protein | chr4:6406537-6407773 FORWARD | Aliases: F24G24.130, F24G24_130	8.3	7.6	0.7	2.5	100.0%	-2.7
1618	AT4G30840.1 | WD-40 repeat protein family | chr4:15017625-15019741 REVERSE | Aliases: F6I18.250, F6I18_250	4.7	5.7	-1.1	-2.5	100.0%	-2.2
1619	AT5G15490.1 | UDP-glucose 6-dehydrogenase, putative, very strong similarity to SP:Q96558 UDP-glucose 6-dehydrogenase (EC 1.1.1.22) (UDP-Glc dehydrogenase) (UDP-GlcDH) (UDPGDH) {Glycine max}; contains Pfam profiles PF03721: UDP-glucose/GDP-mannose dehydrogenase family NAD binding domain, PF00984: UDP-glucose/GDP-mannose dehydrogenase family central domain, PF03720: UDP-glucose/GDP-mannose dehydrogenase family UDP binding domain	5.1	7.5	-2.4	-2.5	100.0%	-2.3
1620	AT3G07990.1 | Symbol: SCPL27 |	2.6	3.1	-0.5	-2.5	100.0%	-2.6
1621	AT4G30110.1 | Symbol: HMA2 | ATPase E1-E2 type family protein / haloacid dehalogenase-like hydrolase family protein / heavy-metal-associated domain-containing protein, similar to cadmium efflux pump protein from Geobacillus stearothermophilus (GI:16753175), cadmium resistance protein B from Staphylococcus aureus (GI:14020985) | chr4:14720259-14724583 REVERSE | Aliases: F6G3.140, F6G3_140	5.3	4.2	1.1	2.5	100.0%	-2.3
1622	AT2G25530.1 | AFG1-like ATPase family protein, contains Pfam profile: PF03969 AFG1-like ATPase	6.7	7.8	-1.1	-2.5	100.0%	-2.4
1623	AT2G42530.1 | cold-responsive protein / cold-regulated protein (cor15b), nearly identical to cold-regulated gene cor15b (Arabidopsis thaliana) GI:456016; contains Pfam profile PF02987: Late embryogenesis abundant protein | chr2:17716114-17717023 REVERSE | Aliases: F14N22.20, F14N22_20	6.5	5.1	1.4	2.5	100.0%	-2.3
1624	AT5G11970.1 | expressed protein | chr5:3863072-3863756 REVERSE | Aliases: F14F18.140, F14F18_140	7.2	5.8	1.5	2.5	100.0%	-2.3

Rank	Description	15.dpi	5.dpi	M	t	adj.q	B
1625	AT1G18850.1 | expressed protein | chr1:6504797-6506412 REVERSE | Aliases: F6A14.6, F6A14_6	5.0	6.0	-1.0	-2.5	100.0%	-2.5
1626	AT3G19200.1 | expressed protein | chr3:6649484-6650012 REVERSE | Aliases: MVI11.12	2.9	3.3	-0.4	-2.5	100.0%	-2.8
1627	AT5G36940.1 | Symbol: CAT3 | amino acid permease family protein, similar to SP:Q09143 High-affinity cationic amino acid transporter-1 (CAT-1) {Mus musculus}; contains Pfam profile PF00324: Amino acid permease | chr5:14607424-14611614 FORWARD | Aliases: CATIONIC AMINO ACID TRANSPORTER 3, MLF18.60, MLF18_60	6.4	5.6	0.8	2.5	100.0%	-2.4
1628	AT3G51500.1 | expressed protein, predicted protein, Oryza sativa, EMBL:AP000367 | chr3:19119113-19119866 FORWARD | Aliases: F26O13.140	4.7	3.9	0.8	2.5	100.0%	-2.5
1629	AT1G12050.1 | fumarylacetoacetase, putative, similar to fumarylacetoacetase (Fumarylacetoacetate hydrolase, Beta-diketonase, FAA)(Rattus norvegicus) SWISS-PROT:P25093	6.9	7.9	-1.0	-2.5	100.0%	-2.5
1630	AT5G38030.1 | MATE efflux family protein, similar to ripening regulated protein DDTFR18 (Lycopersicon esculentum) GI:12231296; contains Pfam profile PF01554: Uncharacterized membrane protein family; putative multidrug efflux protein NorM - Vibrio parahaemolyticus, EMBL:AB010463	2.9	3.6	-0.7	-2.5	100.0%	-2.4
1631	AT1G05320.3 | similar to expressed protein [Arabidopsis thaliana] (TAIR:At2g32240.1); similar to unnamed protein product [Candida glabrata CBS138] (GB:CAG58365.1) | chr1:1553607-1557769 FORWARD | Aliases: YUP8H12.6, YUP8H12_6	5.0	5.9	-1.0	-2.5	100.0%	-2.3
1632	AT4G36515.1 | expressed protein | chr4:17229557-17230299 REVERSE | Aliases: None	9.9	8.7	1.2	2.5	100.0%	-2.3
1633	AT1G31010.1 | expressed protein, contains Pfam PF05329: Protein of unknown function (DUF731) | chr1:11054733-11056859 REVERSE | Aliases: F17F8.7	4.6	5.1	-0.5	-2.5	100.0%	-2.9
1634	AT2G27090.1 | expressed protein, contains Pfam domains, PF04782: Protein of unknown function (DUF632) and PF04783: Protein of unknown function (DUF630) | chr2:11573366-11578890 REVERSE | Aliases: T20P8.14, T20P8_14	5.5	4.6	0.9	2.5	100.0%	-2.5
1635	AT4G18170.1 | Symbol: WRKY28 |	5.5	6.4	-0.9	-2.5	100.0%	-2.3
1636	AT3G44340.2 | Symbol: CEF | similar to sec23/sec24 transport protein-related [Arabidopsis thaliana] (TAIR:At4g32640.1); similar to PREDICTED: similar to SEC24-related protein C; protein transport protein SEC24C; SEC24 (S. cerevisiae) related gene family, member C [Gallus gallus] (GB:XP_421617.1); contains InterPro domain Sec23/Sec24 trunk domain (InterPro:IPR006896); contains InterPro domain Sec23/Sec24 zinc finger (InterPro:IPR006895); contains InterPro domain Sec23/Sec24 helical domain (InterPro:IPR006900); contains InterPro domain Proline-rich region (InterPro:IPR000694); contains InterPro domain Gelsolin region (InterPro:IPR007123) | chr3:16022608-16031241 REVERSE | Aliases: T22K7.20	6.1	6.9	-0.8	-2.4	100.0%	-2.6
1637	AT1G71230.1 | Symbol: AJH2 | COP9 signalosome subunit 5A / CSN subunit 5A (CSN5A) / c-JUN coactivator protein AJH2, putative (AJH2), COP9 complex subunit CSN5-2; identical to c-Jun coactivator protein AJH2 GI:3641312 from (Arabidopsis thaliana); identical to cDNA CSN complex subunit 5A (CSN5A) GI:18056660; contains Pfam profile PF01398: Mov34/MPN/PAD-1 family | chr1:26856123-26858058 FORWARD | Aliases: AJH2, CSN5, CSN5A, F3I17.12, F3I17_12	4.3	5.1	-0.8	-2.4	100.0%	-2.6
1638	AT2G17820.1 | Symbol: ATHK1 | histidine kinase 1, 99% identical to GP:4586626	3.8	4.9	-1.1	-2.4	100.0%	-2.4
1639	AT2G39510.1 | nodulin MtN21 family protein, similar to MtN21 GI:2598575 (root nodule development) from (Medicago truncatula) | chr2:16498221-16500210 REVERSE | Aliases: F12L6.17, F12L6_17	6.5	5.4	1.2	2.4	100.0%	-2.2
1640	AT5G57700.2 | BNR/Asp-box repeat family protein, contains Pfam PF02012: BNR/Asp-box repeat | chr5:23392100-23394348 FORWARD | Aliases: MRI1.6, MRI1_6	6.9	7.8	-0.9	-2.4	100.0%	-2.6
1641	AT5G10910.1 | mraW methylase family protein, contains Pfam profile PF01795: MraW methylase family | chr5:3439554-3441583 FORWARD | Aliases: T30N20.180, T30N20_180	5.1	6.2	-1.1	-2.4	100.0%	-2.5
1642	AT1G03730.1 | expressed protein, similar to ESTs gb:AA605440 and gb:H37232 | chr1:930828-931895 REVERSE | Aliases: F21B7.33	6.3	5.7	0.6	2.4	100.0%	-2.6
1643	AT3G61820.1 | aspartyl protease family protein, contains Pfam domain, PF00026: eukaryotic aspartyl protease | chr3:22890779-22892605 REVERSE | Aliases: F21F14.7	4.5	5.6	-1.1	-2.4	100.0%	-2.4
1644	AT4G18240.1 | starch synthase-related protein, contains similarity to starch synthase GI:4582783 from (Vigna unguiculata) | chr4:10082163-10087214 FORWARD | Aliases: T9A21.90, T9A21_90	4.4	4.9	-0.5	-2.4	100.0%	-2.6
1645	AT3G16000.1 | Symbol: MFP1 | matrix-localized MAR DNA-binding protein-related, similar to matrix-localized MAR DNA binding protein MFP1 GI:1771158 from (Lycopersicon esculentum) | chr3:5430895-5433823 REVERSE | Aliases: MAR BINDING FILAMENT LIKE PROTEIN 1, MSL1.8	6.2	7.0	-0.8	-2.4	100.0%	-2.4
1646	AT5G53560.1 | Symbol: ATB5 A |	12.3	12.8	-0.5	-2.4	100.0%	-3.3

Rank	Description	15.dpi	5.dpi	M	t	adj.q	B
1647	AT5G48650.1 | nuclear transport factor 2 (NTF2) family protein / RNA recognition motif (RRM)-containing protein | chr5:19743960-19746880 FORWARD | Aliases: K15N18.17, K15N18_17	3.8	4.8	-0.9	-2.4	100.0%	-2.5
1648	AT2G44670.1 | senescence-associated protein-related, similar to senescence-associated protein SAG102 (GI:22331931) (Arabidopsis thaliana); | chr2:18432255-18433135 FORWARD | Aliases: F16B22.16	10.0	8.7	1.3	2.4	100.0%	-2.4
1649	AT3G60240.1 | Symbol: EIF4G | MIF4G domain-containing protein / MA3 domain-containing protein, similar to eukaryotic protein synthesis initiation factor (Homo sapiens) GI:3941724; contains Pfam profiles PF02854: MIF4G domain, PF02847: MA3 domain	8.5	9.2	-0.8	-2.4	100.0%	-2.6
1650	AT4G33100.1 | expressed protein, contains Pfam PF05254: Uncharacterised protein family (UPF0203) | chr4:15971177-15972492 FORWARD | Aliases: F4I10.30, F4I10_30	7.5	6.8	0.7	2.4	100.0%	-2.4
1651	AT5G63810.1 | Symbol: BGAL10 |	4.3	4.9	-0.6	-2.4	100.0%	-2.5
1652	AT5G09840.1 | expressed protein, contains Pfam profile PF04396: Protein of unknown function, DUF537 | chr5:3058998-3061971 FORWARD | Aliases: MYH9.5, MYH9_5	2.3	2.6	-0.2	-2.4	100.0%	-3.4
1653	AT1G48440.1 | expressed protein | chr1:17910669-17912191 FORWARD | Aliases: T1N15.5, T1N15_5	11.3	10.6	0.7	2.4	100.0%	-3.0
1654	AT3G51670.1 | SEC14 cytosolic factor family protein / phosphoglyceride transfer family protein, similar to polyphosphoinositide binding protein Ssh2p (GI:2739046) {Glycine max};; contains Pfam PF00650 : CRAL/TRIO domain; contains Pfam PF03765 : CRAL/TRIO, N-terminus | chr3:19179829-19182129 FORWARD | Aliases: T18N14.50	6.2	7.8	-1.6	-2.4	100.0%	-2.3
1655	AT5G06950.3 | Symbol: AHBP 1B | similar to bZIP family transcription factor [Arabidopsis thaliana] (TAIR:At3g12250.1); similar to bZIP family transcription factor [Arabidopsis thaliana] (TAIR:At3g12250.2); similar to TGA-type basic leucine zipper protein TGA2.1 [Phaseolus vulgaris] (GB:AAK84889.1); contains InterPro domain Basic-leucine zipper (bZIP) transcription factor (InterPro:IPR004827) | chr5:2151038-2154554 FORWARD | Aliases: MOJ9.12, MOJ9_12, TGA2	4.6	4.0	0.6	2.4	100.0%	-2.6
1656	AT1G79975.2 | expressed protein | chr1:30089065-30090457 FORWARD | Aliases: None	5.6	4.8	0.7	2.4	100.0%	-2.5
1657	AT3G08650.2 | metal transporter family protein, contains ZIP Zinc transporter domain, Pfam:PF02535 | chr3:2624467-2627376 REVERSE | Aliases: F17O14.12	8.3	7.6	0.7	2.4	100.0%	-2.7
1658	AT2G26040.1 | Bet v I allergen family protein, similar to ribonucleases from {Panax ginseng} SP:P80890, SP:P80889, SP:Q05736 Pathogenesis-related protein 1 (AOPR1) {Asparagus officinalis}; contains Pfam profile PF00407: Pathogenesis-related protein Bet v I family | chr2:11101910-11102482 REVERSE | Aliases: T19L18.15, T19L18_15	4.9	4.1	0.8	2.4	100.0%	-2.5
1659	AT2G26440.1 | pectinesterase family protein, contains Pfam profile: PF01095 pectinesterase | chr2:11254461-11256562 FORWARD | Aliases: T9J22.11, T9J22_11	5.2	6.1	-0.9	-2.4	100.0%	-2.3
1660	AT5G02150.2 | similar to armadillo/beta-catenin repeat family protein [Arabidopsis thaliana] (TAIR:At3g09350.1); similar to Zgc:55259 protein [Danio rerio] (GB:AAH49402.1) | chr5:424290-426045 REVERSE | Aliases: T7H20.200, T7H20_200	5.7	5.0	0.8	2.4	100.0%	-2.5
1661	AT1G25400.1 | expressed protein, similar to unknown protein GI:6714347 from (Arabidopsis thaliana) | chr1:8911117-8912385 REVERSE | Aliases: F2J7.13, F2J7_13	4.9	4.0	0.8	2.4	100.0%	-2.4
1662	AT1G09450.1 | haspin-related, similar to haspin (GI:9229937) {Mus musculus} and haploid germ cell-specific nuclear protein kinase (GI:13561418) {Mus musculus}	2.6	3.1	-0.4	-2.4	100.0%	-2.9
1663	AT1G80460.2 | Symbol: NHO1 | similar to glycerol kinase [Pandanus amaryllifolius] (GB:AAR88660.1); contains InterPro domain Carbohydrate kinase, FGGY (InterPro:IPR000577); contains InterPro domain Glycerol kinase (InterPro:IPR005999) | chr1:30251637-30254197 REVERSE | Aliases: GLI1, T21F11.21, T21F11_21	9.7	10.5	-0.7	-2.4	100.0%	-2.8
1664	AT1G09950.1 | transcription factor-related, low similarity to tumor-related protein (Nicotiana glauca x Nicotiana langsdorffii) GI:688423, SP:P23923 Transcription factor HBP-1b {Triticum aestivum} | chr1:3240608-3241576 REVERSE | Aliases: F21M12.34, F21M12_34	4.9	3.9	1.0	2.4	100.0%	-2.4
1665	AT5G56795.1 | Symbol: MT1B | one of the five metallothioneins (MTs) genes identified in Arabidopsis. MTs are cysteine-rich proteins required for heavy metal tolerance. The MT1b gene, however, is indicated to be inactive. | chr5:22988283-22990379 REVERSE | Aliases: None	12.8	12.0	0.7	2.4	100.0%	-3.6
1666	AT5G16130.1 | 40S ribosomal protein S7 (RPS7C), 40S ribosomal protein S7 homolog - Brassica oleracea, EMBL:AF144752 | chr5:5268424-5270091 FORWARD | Aliases: T21H19.50, T21H19_50	10.9	11.6	-0.7	-2.4	100.0%	-2.5

Rank	Description	15.dpi	5.dpi	M	t	adj.q	B
1667	AT5G52570.1 | Symbol: BETA OHASE 2 | beta-carotene hydroxylase, putative, similar to GI:1575296, beta-carotene hydroxylase | chr5:21351630-21354206 REVERSE | Aliases: BETA CAROTENE HYDROXYLASE 2, F6N7.5, F6N7_5	7.1	6.2	0.9	2.4	100.0%	-2.3
1668	AT3G54960.1 | Symbol: ATPDIL1 3 | Encodes a protein disulfide isomerase-like (PDIL) protein, a member of a multigene family within the thioredoxin (TRX) superfamily.	7.8	8.9	-1.1	-2.4	100.0%	-2.3
1669	AT3G18690.1 | Symbol: MKS1 | VQ motif-containing protein, contains PF05678: VQ motif | chr3:6429540-6430508 REVERSE | Aliases: MVE11.5	5.9	5.3	0.7	2.4	100.0%	-2.6
1670	AT2G16450.1 | F-box family protein, contains Pfam PF00646: F-box domain; contains TIGRFAM TIGR01640 : F-box protein interaction domain	3.3	3.0	0.4	2.4	100.0%	-3.1
1671	AT5G07880.1 | Symbol: SNAP29 |	3.7	3.1	0.6	2.4	100.0%	-2.5
1672	AT2G17870.1 | cold-shock DNA-binding family protein, contains Pfam domains, PF00313: 'Cold-shock' DNA-binding domain and PF00098: Zinc knuckle	10.2	9.3	0.8	2.4	100.0%	-2.8
1673	AT1G76410.1 | zinc finger (C3HC4-type RING finger) family protein, contains Pfam domain, PF00097: Zinc finger, C3HC4 type (RING finger) | chr1:28673742-28674540 FORWARD | Aliases: F15M4.9	5.3	4.5	0.8	2.4	100.0%	-2.4
1674	AT1G22190.1 | AP2 domain-containing transcription factor, putative, similar to AP2 domain containing protein RAP2.4 GI:2281633 from (Arabidopsis thaliana) | chr1:7835771-7837277 FORWARD | Aliases: F16L1.8, F16L1_8	5.2	4.0	1.1	2.4	100.0%	-2.3
1675	AT5G64310.1 | Symbol: AGP1 | arabinogalactan-protein (AGP1), identical to gi:3883120 gb:AAC77823 | chr5:25739159-25739872 FORWARD | Aliases: ARABINOGALACTAN PROTEIN 1, MSJ1.15, MSJ1_15	10.4	9.2	1.2	2.4	100.0%	-2.4
1676	AT4G20350.1 | similar to LOC495973 protein [Xenopus laevis] (GB:AAH87349.1) | chr4:10988405-10989851 FORWARD | Aliases: F9F13.6, F9F13_6	3.4	3.9	-0.5	-2.4	100.0%	-2.9
1677	AT5G42860.1 | expressed protein | chr5:17200294-17202144 REVERSE | Aliases: MBD2.5, MBD2_5	5.7	6.4	-0.7	-2.4	100.0%	-2.6
1678	AT4G12040.2 | zinc finger (AN1-like) family protein, contains Pfam domains, PF01428: AN1-like Zinc finger and PF01754: A20-like zinc finger | chr4:7214704-7215979 FORWARD | Aliases: F16J13.110, F16J13_110	9.2	8.2	1.0	2.4	100.0%	-2.8
1679	AT2G38330.1 | MATE efflux family protein, low similarity to enhanced disease susceptibility 5 (Arabidopsis thaliana) GI:16589070; contains Pfam profile PF01554: Uncharacterized membrane protein family | chr2:16071602-16075369 FORWARD | Aliases: T19C21.18, T19C21_18	7.2	6.4	0.8	2.4	100.0%	-2.4
1680	AT4G26200.1 | Symbol: ACS7 | 1-aminocyclopropane-1-carboxylate synthase, putative / ACC synthase, putative, similar to ACC synthase from Malus x domestica, GI:1658062 (U73816), Pyrus pyrifolia GI:4586411 | chr4:13275316-13277197 FORWARD | Aliases: T25K17.10, T25K17_10	5.0	6.0	-1.1	-2.4	100.0%	-2.3
1681	AT3G57160.1 | expressed protein | chr3:21168589-21169795 REVERSE | Aliases: None	7.7	6.8	0.9	2.4	100.0%	-2.4
1682	AT1G77310.1 | wound-responsive protein, putative, similar to wound-responsive protein 14.05 (GI:16506638) (Castanea sativa) | chr1:29056416-29061233 FORWARD | Aliases: F2P24.2, F2P24_2	2.6	2.9	-0.3	-2.4	100.0%	-3.2
1683	AT4G23720.1 | expressed protein, similar to expressed protein [Arabidopsis thaliana] (TAIR:At4g01140.1); similar to hypothetical protein-like protein [Sorghum bicolor] (GB:AAO16691.1) | chr4:12358685-12359855 FORWARD | Aliases: F9D16.190, F9D16_190	4.0	4.5	-0.5	-2.4	100.0%	-2.8
1684	AT1G48320.1 | thioesterase family protein, similar to ComAB (Bacillus licheniformis) GI:1834379; contains Pfam profile PF03061: thioesterase family protein | chr1:17858481-17859316 REVERSE | Aliases: F11A17.13, F11A17_13	6.0	6.7	-0.7	-2.4	100.0%	-2.6
1685	AT4G02840.1 | small nuclear ribonucleoprotein D1, putative / snRNP core protein D1, putative / Sm protein D1, putative, similar to small nuclear ribonucleoprotein Sm D1 (snRNP core protein D1, Sm-D1, Sm-D autoantigen) (Mouse) SWISS-PROT:P13641 | chr4:1264627-1266486 FORWARD | Aliases: T5J8.16, T5J8_16	8.7	9.3	-0.6	-2.4	100.0%	-2.6
1686	AT5G57230.1 | expressed protein | chr5:23207798-23208711 FORWARD | Aliases: MJB24.4, MJB24_4	6.2	7.0	-0.8	-2.4	100.0%	-2.5
1687	AT3G10610.1 | 40S ribosomal protein S17 (RPS17C), similar to 40S ribosomal protein S17 GB:AAD50774 (Lycopersicon esculentum) | chr3:3319041-3320154 FORWARD | Aliases: F13M14.10	8.1	8.9	-0.8	-2.4	100.0%	-2.5
1688	AT3G02360.2 | 6-phosphogluconate dehydrogenase family protein, contains Pfam profiles: PF00393 6-phosphogluconate dehydrogenase C-terminal domain, PF03446 NAD binding domain of 6-phosphogluconate ;similar to 6-phosphogluconate dehydrogenase GB:BAA22812 GI:2529229 (Glycine max) | chr3:482035-484154 FORWARD | Aliases: F11A12.5, F11A12_5	12.0	12.7	-0.7	-2.4	100.0%	-3.2
1689	AT5G20550.1 | oxidoreductase, 2OG-Fe(II) oxygenase family protein, similar to leucoanthocyanidin dioxygenase (Malus domestica)(SP:P51091), flavonol synthase (Petunia x hybrida)(GI:311658); contains PF03171 2OG-Fe(II) oxygenase superfamily domain | chr5:6952419-6953883 REVERSE | Aliases: F7C8.140, F7C8_140	6.9	8.2	-1.3	-2.4	100.0%	-2.3

Rank	Description	15.dpi	5.dpi	M	t	adj.q	B
1690	AT5G46640.1 | DNA-binding family protein, contains a AT hook motif (DNA binding motifs with a preference for A/T rich regions), Pfam:PF02178 | chr5:18941712-18943802 FORWARD | Aliases: MZA15.3, MZA15_3	5.4	4.7	0.8	2.4	100.0%	-2.4
1691	AT5G53570.1 | RabGAP/TBC domain-containing protein, similar to GTPase activating protein (Yarrowia lipolytica) GI:2370595; contains Pfam profile PF00566: TBC domain | chr5:21777816-21780688 REVERSE | Aliases: MNC6.11, MNC6_11	6.5	5.5	1.0	2.4	100.0%	-2.5
1692	AT1G70330.1 | Symbol: ENT1,AT | equilibrative nucleoside transporter family protein, contains similarity to SWISS-PROT:Q14542 equilibrative nucleoside transporter 2 (Equilibrative nitrobenzylmercaptopurine riboside-insensitive nucleoside transporter, Equilibrative NBMPR-insensitive nucleoside transporter, Nucleoside transporter, ei-type, 36 kDa nucleolar protein HNP36, Hydrophobic nucleolar protein, 36 kDa, Delayed-early response protein 12) (Homo sapiens) | chr1:26506572-26508282 FORWARD | Aliases: EQUILIBRATIVE NUCLEOTIDE TRANSPORTER 1	7.8	6.3	1.6	2.4	100.0%	-2.4
1693	AT3G04070.1 | Symbol: ANAC047 | no apical meristem (NAM) family protein, contains Pfam PF02365: No apical meristem (NAM) domain; similar to NAM GB:CAA63101 (Petunia x hybrida) | chr3:1061533-1063101 REVERSE | Aliases: ANAC047, T11I18.18, T11I18_18	5.2	4.3	0.9	2.4	100.0%	-2.4
1694	AT5G11430.1 | transcription elongation factor-related, contains weak similarity to transcription elongation factors | chr5:3648476-3652405 FORWARD | Aliases: F15N18.20, F15N18_20	4.6	5.3	-0.7	-2.4	100.0%	-2.5
1695	AT2G27260.1 | expressed protein | chr2:11676733-11677722 FORWARD | Aliases: F12K2.16, F12K2_16	6.8	6.2	0.6	2.4	100.0%	-2.5
1696	AT1G09850.1 | Symbol: XBCP3 | cysteine protease, papain-like (XBCP3), identical to papain-like cysteine peptidase XBCP3 GI:14600257 from (Arabidopsis thaliana); contains Pfam profiles PF00112: Papain family cysteine protease and PF00396: Granulin | chr1:3201801-3204152 FORWARD | Aliases: F21M12.24, F21M12_24	7.2	8.2	-1.0	-2.4	100.0%	-2.5
1697	AT1G02160.1 | expressed protein | chr1:410785-411590 FORWARD | Aliases: T6A9.18	10.8	10.1	0.7	2.4	100.0%	-2.9
1698	AT2G46735.1 | expressed protein | chr2:19211255-19212182 FORWARD | Aliases: None	4.5	3.9	0.6	2.4	100.0%	-2.6
1699	AT3G24190.1 | ABC1 family protein, contains Pfam domain, PF03109: ABC1 family | chr3:8743297-8747861 FORWARD | Aliases: MUJ8.9	7.2	6.4	0.8	2.4	100.0%	-2.6
1700	AT4G13720.1 | inosine triphosphate pyrophosphatase, putative / HAM1 family protein, contains Pfam profile PF01725: Ham1 family; similar to inosine triphosphate pyrophosphatase (GI:13398328) (Homo sapiens) | chr4:7966976-7968900 REVERSE | Aliases: F18A5.110, F18A5_110	7.8	8.6	-0.8	-2.4	100.0%	-2.4
1701	AT5G60840.1 | expressed protein, predicted protein, Drosophila melanogaster | chr5:24490560-24491717 REVERSE | Aliases: MAE1.8, MAE1_8	4.3	5.3	-1.0	-2.4	100.0%	-2.4
1702	AT4G31830.1 | expressed protein | chr4:15400610-15401138 REVERSE | Aliases: F11C18.30, F11C18_30	3.1	2.8	0.3	2.4	100.0%	-3.1
1703	AT3G10040.1 | expressed protein, est match | chr3:3096420-3098076 REVERSE | Aliases: T22K18.13	7.2	5.8	1.3	2.4	100.0%	-2.4
1704	AT4G23740.1 | leucine-rich repeat transmembrane protein kinase, putative, receptor-like protein kinase - Arabidopsis thaliana RKL1, PID:g4008006 | chr4:12366472-12369348 FORWARD | Aliases: F9D16.210, F9D16_210	4.6	5.2	-0.6	-2.4	100.0%	-2.8
1705	ATCG00570.1 | Symbol: PSBF | PSII cytochrome b559 | chrC:63942-64061 REVERSE | Aliases: PSBF	8.2	10.0	-1.7	-2.4	100.0%	-2.4
1706	AT1G80270.2 | DNA-binding protein, putative, similar to DNA-binding protein (Triticum aestivum) GI:6958202; contains Pfam profile: PF01535 PPR repeat | chr1:30185926-30188399 FORWARD | Aliases: F5I6.2, F5I6_2	7.4	8.1	-0.7	-2.4	100.0%	-2.5
1707	AT3G47930.1 | Symbol: ATGLDH |	7.9	9.0	-1.1	-2.4	100.0%	-2.4
1708	AT3G13235.1 | ubiquitin family protein, contains INTERPRO:IPR000626 ubiquitin domain | chr3:4271343-4274436 REVERSE | Aliases: None	4.4	5.0	-0.6	-2.4	100.0%	-2.7
1709	AT5G50240.1 | Symbol: PIMT2 | protein-L-isoaspartate O-methyltransferase, putative / PIMT, putative, similar to SP:Q42539 Protein-L-isoaspartate O-methyltransferase (EC 2.1.1.77) (Protein- beta-aspartate methyltransferase) (PIMT) (Protein L-isoaspartyl methyltransferase) (L-isoaspartyl protein carboxyl methyltransferase) {Arabidopsis thaliana}; contains Pfam profile PF01135: Protein-L-isoaspartate(D-aspartate) O-methyltransferase (PCMT) | chr5:20468477-20469687 FORWARD | Aliases: K6A12.10, K6A12_10, PROTEIN L ISOASPARTATE METHYLTRANSFERASE 2	6.2	5.5	0.7	2.4	100.0%	-2.5
1710	AT1G36980.1 | expressed protein | chr1:14027484-14029382 FORWARD | Aliases: T32E20.29, T32E20_29	10.2	9.7	0.5	2.4	100.0%	-3.1
1711	AT2G37660.1 | expressed protein | chr2:15802392-15804137 REVERSE | Aliases: F13M22.16, F13M22_16	9.1	10.0	-1.0	-2.4	100.0%	-2.4
1712	AT2G37520.1 | PHD finger family protein, contains Pfam domain, PF00628: PHD-finger | chr2:15751847-15756866 REVERSE | Aliases: F13M22.2, F13M22_2	3.6	3.2	0.3	2.4	100.0%	-3.0

Rank	Description	15.dpi	5.dpi	M	t	adj.q	B
1713	AT3G55800.1 | Symbol: SBPASE |	8.6	7.5	1.1	2.4	100.0%	-2.3
1714	AT4G16500.1 | cysteine protease inhibitor family protein / cystatin family protein, similar to SP:Q06445 Cysteine proteinase inhibitor (Cystatin) {Vigna unguiculata}; contains Pfam profile PF00031: Cystatin domain | chr4:9301413-9302045 REVERSE | Aliases: DL4275C, FCAALL.171	10.9	11.5	-0.6	-2.4	100.0%	-3.0
1715	AT2G04039.2 | expressed protein | chr2:1333268-1334563 FORWARD | Aliases: F3L12.11, F3L12_11	3.2	2.8	0.4	2.4	100.0%	-3.0
1716	AT2G38905.1 | hydrophobic protein, putative / low temperature and salt responsive protein, putative, strong similarity to SP:Q9ZNQ7 Hydrophobic protein RCI2A (Low temperature and salt responsive protein LTI6A) {Arabidopsis thaliana}; contains Pfam profile PF01679: Uncharacterized protein family | chr2:16251177-16251562 REVERSE | Aliases: None	7.4	5.8	1.6	2.4	100.0%	-2.3
1717	AT5G55490.1 | Symbol: GEX1 | expressed protein, similar to expressed protein [Arabidopsis thaliana] (TAIR:At1g68790.1); similar to hypothetical protein S250_18C08.13 [Sorghum bicolor] (GB:AAL73526.1) | chr5:22495938-22498374 REVERSE | Aliases: GAMETE EXPRESSED PROTEIN1, MTE17.20, MTE17_20	3.1	2.7	0.4	2.4	100.0%	-3.2
1718	AT4G34260.1 | expressed protein | chr4:16398119-16401814 FORWARD | Aliases: F10M10.30, F10M10_30	5.7	6.4	-0.8	-2.4	100.0%	-2.5
1719	AT1G69850.1 | Symbol: NTL1 | nitrate transporter (NTL1), identical to nitrate transporter (NTL1) GI:3377517 (Arabidopsis thaliana) | chr1:26300339-26304109 REVERSE | Aliases: ATNRT1:2, NITRATE TRANSPORTER, T17F3.12, T17F3_12	8.3	7.2	1.1	2.4	100.0%	-2.5
1720	AT2G43690.1 | lectin protein kinase, putative, similar to receptor-like kinase LECRK1 (Arabidopsis thaliana) gi:2150023:gb:AAB58725 | chr2:18119666-18121660 FORWARD | Aliases: F18O19.20	3.0	3.4	-0.4	-2.4	100.0%	-2.9
1721	AT5G50390.1 | pentatricopeptide (PPR) repeat-containing protein, contains INTERPRO:IPR002885 PPR repeats | chr5:20538015-20540206 REVERSE | Aliases: MXI22.11, MXI22_11	4.2	4.7	-0.5	-2.4	100.0%	-2.8
1722	AT1G28520.1 | expressed protein | chr1:10029179-10031969 FORWARD | Aliases: F3M18.4, F3M18_4	4.7	4.2	0.4	2.4	100.0%	-2.9
1723	AT4G01710.1 | Symbol: CRK | actin polymerization factor protein-related, similar to human ARP2/3 complex 16 kd subunit, GenBank accession number O15511 likely functions to control the polymerization of actin | chr4:735753-736604 FORWARD | Aliases: ARPC5, CROOKED, T15B16.22, T15B16_22	7.5	6.4	1.1	2.4	100.0%	-2.5
1724	AT5G42030.1 | expressed protein, similar to unknown protein (emb:CAB66408.1) | chr5:16828623-16830333 REVERSE | Aliases: MJC20.13, MJC20_13	4.4	5.4	-0.9	-2.4	100.0%	-2.5
1725	AT3G10970.2 | haloacid dehalogenase-like hydrolase family protein, low similarity to genetic modifier (Zea mays) GI:10444400; contains InterPro accession IPR005834: Haloacid dehalogenase-like hydrolase | chr3:3433078-3436284 REVERSE | Aliases: F9F8.21	7.9	7.2	0.6	2.4	100.0%	-2.6
1726	AT3G29970.1 | germination protein-related, similar to HvB12D (Hordeum vulgare subsp. vulgare) gi:471319:emb:CAA54065 | chr3:11747709-11749049 FORWARD | Aliases: K17E7.15	5.9	3.2	2.6	2.4	100.0%	-2.8
1727	AT5G54910.1 | DEAD/DEAH box helicase, putative	4.8	6.2	-1.4	-2.4	100.0%	-2.4
1728	AT4G15790.1 | expressed protein | chr4:8982253-8984216 FORWARD | Aliases: DL3935W, FCAALL.69	5.3	6.1	-0.9	-2.4	100.0%	-2.6
1729	AT2G01080.1 | expressed protein | chr2:77888-79333 FORWARD | Aliases: F23H14.5, F23H14_5	5.8	5.4	0.5	2.4	100.0%	-2.8
1730	AT2G20740.3 | expressed protein | chr2:8943759-8944591 FORWARD | Aliases: AT2G20730, F5H14.29, F5H14_29	6.1	5.4	0.7	2.4	100.0%	-2.7
1731	AT1G12250.1 | thylakoid lumenal protein-related, weak similarity to SP:O22160 Thylakoid lumenal 15 kDa protein, chloroplast precursor (p15) {Arabidopsis thaliana}; contains Pfam profile PF00805: Pentapeptide repeats (8 copies) | chr1:4159268-4161453 FORWARD | Aliases: T28K15.2, T28K15_2	5.8	5.4	0.4	2.4	100.0%	-2.8
1732	AT5G50810.1 | Symbol: TIM8 | mitochondrial import inner membrane translocase (TIM8), identical to mitochondrial import inner membrane translocase subunit Tim8 (Arabidopsis thaliana) Swiss-Prot:Q9XGY4; contains Pfam domain, PF02953: Tim10/DDP family zinc finger | chr5:20692860-20693816 REVERSE | Aliases: K7B16.3, K7B16_3	7.6	8.6	-1.0	-2.4	100.0%	-2.4
1733	AT5G05450.1 | DEAD/DEAH box helicase, putative (RH18) | chr5:1612050-1615337 FORWARD | Aliases: K18I23.26, K18I23_26	5.9	7.7	-1.8	-2.4	100.0%	-2.4
1734	AT4G31700.1 | Symbol: RPS6 | 40S ribosomal protein S6 (RPS6A), ribosomal protein S6, Arabidopsis thaliana, PID:g2662469 | chr4:15346083-15347731 REVERSE | Aliases: F28M20.110, F28M20_110, RIBOSOMAL PROTEIN S6	11.0	11.9	-0.9	-2.4	100.0%	-2.7
1735	AT4G27090.1 | 60S ribosomal protein L14 (RPL14B), ribosomal protein L14 - Human,PIR3:JC5954 | chr4:13593911-13595257 REVERSE | Aliases: T24A18.40, T24A18_40	12.6	13.1	-0.5	-2.4	100.0%	-3.0

Rank	Description	15.dpi	5.dpi	M	t	adj.q	B
1736	AT4G19130.1 | replication protein-related, similar to replication protein A 70kDa (Oryza sativa) GI:13536993; contains Pfam profile PF00098: Zinc knuckle | chr4:10466488-10468071 REVERSE | Aliases: T18B16.100, T18B16_100	4.4	5.1	-0.7	-2.4	100.0%	-2.5
1737	AT3G16800.3 | similar to protein phosphatase 2C, putative / PP2C, putative [Arabidopsis thaliana] (TAIR:At3g05640.1); similar to protein phosphatase 2C, putative / PP2C, putative [Arabidopsis thaliana] (TAIR:At3g05640.2); similar to putative protein phosphatase 2C [Oryza sativa (japonica cultivar-group)] (GB:XP_483187.1); contains InterPro domain Protein phosphatase 2C-like (InterPro:IPR001932) | chr3:5721026-5723187 FORWARD | Aliases: K20I9.2	5.4	4.6	0.8	2.4	100.0%	-2.4
1738	AT5G58730.1 | pfkB-type carbohydrate kinase family protein, contains Pfam profile: PF00294 pfkB family carbohydrate kinase | chr5:23740634-23742691 REVERSE | Aliases: MZN1.17, MZN1_17	7.4	6.6	0.8	2.4	100.0%	-2.6
1739	AT3G44735.1 | phytosulfokines-related, contains similarities to phytosulfokines from (Arabidopsis thaliana) | chr3:16302187-16303137 REVERSE | Aliases: None	6.3	5.4	0.9	2.4	100.0%	-2.4
1740	AT4G03560.1 | Symbol: ATTPC1 |	7.4	8.2	-0.8	-2.4	100.0%	-2.6
1741	AT2G18330.1 | AAA-type ATPase family protein, contains Pfam profile: PF00004 ATPase family associated with various cellular activities (AAA) | chr2:7972739-7976187 FORWARD | Aliases: T30D6.16, T30D6_16	6.3	7.3	-1.0	-2.4	100.0%	-2.6
1742	AT1G80820.1 | Symbol: CCR2 | cinnamoyl-CoA reductase, putative, identical to CCR2 (GI:12407990), similar to cinnamoyl CoA reductase from Eucalyptus gunnii (GI:2058311) | chr1:30375465-30377562 FORWARD | Aliases: CINNAMOYL COA REDUCTASE, F23A5.17, F23A5_17	8.3	9.8	-1.5	-2.4	100.0%	-2.5
1743	AT3G58970.1 | magnesium transporter CorA-like family protein, contains Pfam profile PF01544: CorA-like Mg2+ transporter protein | chr3:21800491-21802336 REVERSE | Aliases: F17J16.20	7.5	7.0	0.5	2.4	100.0%	-2.9
1744	AT1G68060.1 | Symbol: ATMAP70 1 | Encodes a microtubule associated protein (MAP70-1). Expressed in all tissues. | chr1:25514659-25518163 REVERSE | Aliases: ATMAP70 1, T23K23.9, T23K23_9	4.2	4.7	-0.5	-2.4	100.0%	-2.8
1745	AT5G40200.1 | DegP protease, putative, contains similarity to DegP2 protease GI:13172275 from (Arabidopsis thaliana) | chr5:16087558-16090542 FORWARD | Aliases: MSN9.100, MSN9_100	5.6	6.6	-1.0	-2.4	100.0%	-2.6
1746	AT1G79310.1 | latex-abundant protein, putative (AMC4) / caspase family protein, similar to latex-abundant protein (Hevea brasiliensis) gb:AAD13216; contains Pfam profile PF00656: ICE-like protease (caspase) p20 domain | chr1:29838879-29840438 FORWARD | Aliases: YUP8H12R.7, YUP8H12R_7	3.2	2.9	0.3	2.4	100.0%	-3.0
1747	AT3G14220.1 | GDSL-motif lipase/hydrolase family protein, similar to myrosinase-associated proteins GI:1769968, GI:1769970 from (Brassica napus); contains InterPro Entry IPR001087 Lipolytic enzyme, G-D-S-L family; contains 1 predicted transmembrane domain; | chr3:4732900-4734616 FORWARD | Aliases: MLE3.1	8.0	8.7	-0.7	-2.4	100.0%	-2.5
1748	AT3G27110.2 | peptidase M48 family protein, contains Pfam domain, PF01435: Peptidase family M48 | chr3:9999133-10001412 FORWARD | Aliases: MOJ10.19	4.7	3.6	1.1	2.4	100.0%	-2.3
1749	AT3G19720.2 | Symbol: ARC5 | dynamin family protein, identical to cDNA dynamin-like protein (ARC5) GI: 30349145; contains Pfam profile PF00350: Dynamin family | chr3:6850377-6855519 REVERSE | Aliases: ACCUMULATION AND REPLICATION OF CHLOROPLAST 5, MMB12.21	4.2	4.6	-0.4	-2.4	100.0%	-2.9
1750	AT3G02820.1 | zinc knuckle (CCHC-type) family protein, contains Pfam domain, PF00098: Zinc knuckle | chr3:611543-613390 FORWARD | Aliases: F13E7.24, F13E7_24	3.8	4.6	-0.8	-2.4	100.0%	-2.7
1751	AT1G59640.2 | Symbol: ZCW32 | basic helix-loop-helix (bHLH) family protein | chr1:21913300-21914837 REVERSE | Aliases: T30E16.21, T30E16_21, ZCW32	7.7	6.7	1.0	2.4	100.0%	-2.4
1752	AT2G04660.1 | Symbol: APC2 | E3 ubiquitin ligase, putative, E3, ubiquitin ligase; contains similarity to anaphase-promoting complex subunit 2 GI:6180009 from (Homo sapiens) | chr2:1624738-1629159 FORWARD | Aliases: F28I8.30, F28I8_30	4.2	4.8	-0.6	-2.4	100.0%	-2.8
1753	AT1G70300.1 | Symbol: KUP6 | potassium transporter, putative, similar to potassium transporter HAK2p (Mesembryanthemum crystallinum) gi:14091471:gb:AAK53759; KUP/HAK/KT Transporter family member, PMID:11500563; contains Pfam profile PF02705: K+ potassium transporter | chr1:26481543-26485293 REVERSE | Aliases: F17O7.17, F17O7_17	5.3	6.0	-0.7	-2.4	100.0%	-2.5
1754	AT1G22060.1 | expressed protein | chr1:7773052-7780575 REVERSE | Aliases: F2E2.13, F2E2_13	3.9	4.6	-0.7	-2.4	100.0%	-2.7
1755	AT1G77010.1 | pentatricopeptide (PPR) repeat-containing protein, contains INTERPRO:IPR002885 PPR repeats | chr1:28947604-28949691 FORWARD | Aliases: F22K20.11, F22K20_11	4.0	4.7	-0.6	-2.4	100.0%	-2.8
1756	AT1G63420.1 | expressed protein | chr1:23519539-23522575 FORWARD | Aliases: F2K11.20, F2K11_20	8.3	7.7	0.6	2.4	100.0%	-2.6

Rank	Description	15.dpi	5.dpi	M	t	adj.q	B
1757	AT1G51170.1 | protein kinase family protein | chr1:18957126-18958560 REVERSE | Aliases: F23H24.1	3.8	4.9	-1.0	-2.4	100.0%	-2.4
1758	AT3G46030.1 | histone H2B, putative, strong similarity to histone H2B Arabidopsis thaliana GI:2407802, Gossypium hirsutum SP:O22582, Lycopersicon esculentum GI:3021489; contains Pfam profile PF00125 Core histone H2A/H2B/H3/H4 | chr3:16924433-16925116 REVERSE | Aliases: F16L2.240	9.3	10.2	-0.9	-2.4	100.0%	-2.6
1759	AT5G48000.4 | Symbol: CYP708A2 | similar to cytochrome P450 family protein [Arabidopsis thaliana] (TAIR:At1g78490.1); similar to putative cytochrome P450 [Oryza sativa (japonica cultivar-group)] (GB:CAD30852.1); contains InterPro domain Cytochrome P450 (InterPro:IPR001128); contains InterPro domain E-class P450, group IV (InterPro:IPR002403) | chr5:19461364-19464500 REVERSE | Aliases: MDN11.7, MDN11_7	2.6	3.1	-0.5	-2.4	100.0%	-2.7
1760	AT1G01725.1 | expressed protein | chr1:269794-270775 REVERSE | Aliases: None	8.6	7.5	1.1	2.4	100.0%	-2.5
1761	AT3G49860.1 | Symbol: ATARLA1B | ADP-ribosylation factor, putative, similar to GTP-binding ADP-ribosylation factor homolog 1 protein (SP:P25160) (Drosophila melanogaster) and various ADP-RIBOSYLATION FACTOR (ARF) - like proteins; contains PF00025: ADP-ribosylation factor family domain	8.9	9.4	-0.5	-2.4	100.0%	-3.0
1762	AT3G02260.1 | Symbol: BIG | auxin transport protein (BIG), nearly identical to auxin transport protein; BIG (Arabidopsis thaliana) GI:21779966; contains Pfam profiles PF02207: Putative zinc finger in N-recognin, PF00569: Zinc finger ZZ type | chr3:430991-448496 REVERSE | Aliases: ASA1, ATTENUATED SHADE AVOIDANCE 1, DARK OVER EXPRESSION OF CAB 1, DOC1, F14P3.3, F14P3_3, LOW PHOSPHATE RESISTANT ROOT 1, LPR1, TIR3, TRANSPORT INHIBITOR RESPONSE 3, UMB1	6.1	6.8	-0.7	-2.4	100.0%	-2.8
1763	AT1G56280.2 | Symbol: ATDI19 |	10.0	9.0	1.0	2.4	100.0%	-2.9
1764	AT5G61440.1 | thioredoxin family protein, low similarity to thioredoxin (Callithrix jacchus) GI:13560979; contains Pfam profile: PF00085 Thioredoxin | chr5:24724726-24726119 FORWARD | Aliases: MFB13.8, MFB13_8	6.1	5.2	0.9	2.4	100.0%	-2.5
1765	AT1G10370.1 | Symbol: ATGSTU17 | glutathione S-transferase, putative (ERD9), similar to glutathione S-transferase TSI-1 (Aegilops tauschii) gi:2190992 gb:AAD10129; similar to ESTs gb:R29860, emb:Z29757, and emb:Z29758; identical to cDNA ERD9 mRNA for glutathione S-transferase,	3.6	4.5	-0.9	-2.4	100.0%	-2.6
1766	AT5G40890.2 | Symbol: ATCLC A | similar to chloride channel protein (CLC-c) [Arabidopsis thaliana] (TAIR:At5g49890.1); similar to chloride channel-like (CLC) protein, putative [Arabidopsis thaliana] (TAIR:At5g33280.1); similar to chloride channel protein (CLC-b) [Arabidopsis thaliana] (TAIR:At3g27170.1); similar to chloride channel [Oryza sativa (japonica cultivar-group)] (GB:XP_466225.1); similar to chloride channel [Oryza sativa (japonica cultivar-group)] (GB:BAB97268.1); similar to chloride channel [Oryza sativa (japonica cultivar-group)] (GB:BAB97267.1); similar to chloride channel Stclc1 [Solanum tuberosum] (GB:CAA71369.1); similar to C1C-Nt1 [Nicotiana tabacum] (GB:CAA64829.1); contains InterPro domain Cl- channel, voltage gated (InterPro:IPR001807); contains InterPro domain Chloride channel plant CLC (InterPro:IPR002251); contains InterPro domain CBS domain (InterPro:IPR000644) | chr5:16398542-16401547 REVERSE | Aliases: CHLORIDE CHANNEL A, CLC A, CLCA, MHK7.12, MHK7_12	5.9	6.7	-0.8	-2.4	100.0%	-2.5
1767	AT3G21520.1 | expressed protein, contains Pfam profile PF05078: Protein of unknown function (DUF679) | chr3:7581965-7582799 FORWARD | Aliases: MIL23.9	3.8	4.4	-0.6	-2.4	100.0%	-2.6
1768	ATCG00720.1 | Symbol: PETB | Encodes the cytochrome b(6) subunit of the cytochrome b6f complex. | chrC:74841-76292 FORWARD | Aliases: PETB	9.2	7.8	1.4	2.4	100.0%	-2.4
1769	AT2G16940.1 | RNA recognition motif (RRM)-containing protein | chr2:7349723-7354490 REVERSE | Aliases: F12A24.12, F12A24_12	5.8	6.6	-0.8	-2.4	100.0%	-2.7
1770	AT1G44414.1 | expressed protein | chr1:16850221-16850526 FORWARD | Aliases: T18F15.8, T18F15_8	3.7	3.1	0.6	2.4	100.0%	-2.7
1771	AT2G22910.1 | GCN5-related N-acetyltransferase (GNAT) family protein / amino acid kinase family protein, similar to SP:P08205 Amino-acid acetyltransferase (EC 2.3.1.1) (N-acetylglutamate synthase) {Escherichia coli}; contains Pfam profiles PF00696: Amino acid kinase family, PF00583: acetyltransferase, GNAT family | chr2:9756949-9759928 FORWARD | Aliases: T20K9.12, T20K9_12	5.6	6.6	-1.0	-2.4	100.0%	-2.7
1772	AT1G75310.1 | Symbol: AUL1 | DNAJ heat shock N-terminal domain-containing protein, low similarity to SP:Q27974 Auxilin {Bos taurus}; contains Pfam profile PF00226: DnaJ domain | chr1:28264665-28269785 FORWARD | Aliases: F22H5.15	6.0	5.3	0.6	2.4	100.0%	-2.6
1773	AT2G41950.1 | expressed protein | chr2:17520157-17521921 FORWARD | Aliases: T6D20.26, T6D20_26	6.3	7.2	-0.9	-2.4	100.0%	-2.5
1774	AT4G29820.1 | expressed protein | chr4:14595686-14597487 REVERSE | Aliases: F27B13.60, F27B13_60	4.6	3.9	0.6	2.4	100.0%	-2.8
1775	AT1G77090.1 | thylakoid lumenal 29.8 kDa protein, identical to SP:O49292:TL30_ARATH (Arabidopsis thaliana);contains a PsbP domain AF370571; SIMILAR TO GI:13926195--F22K20.16 | chr1:28965291-28966816 REVERSE | Aliases: F22K20.16, F22K20_16	6.3	5.2	1.1	2.4	100.0%	-2.4
1776	AT5G23000.1 | myb family transcription factor (MYB37), contains PFAM profile: myb DNA binding domain PF00249; | chr5:7696237-7697930 FORWARD | Aliases: T20O7.2, T20O7_2	2.6	3.0	-0.4	-2.4	100.0%	-2.9

Rank	Description	15.dpi	5.dpi	M	t	adj.q	B
1777	AT2G21090.1 | pentatricopeptide (PPR) repeat-containing protein, contains INTERPRO:IPR002885 PPR repeats | chr2:9052597-9054657 REVERSE | Aliases: F26H11.15, F26H11_15	4.2	4.7	-0.5	-2.4	100.0%	-2.7
1778	AT1G27360.2 | squamosa promoter-binding protein-like 11 (SPL11), identical to squamosa promoter binding protein-like 11 (Arabidopsis thaliana) GI:5931665; contains Pfam profile PF03110: SBP domain | chr1:9501064-9503856 FORWARD | Aliases: F17L21.14, F17L21_14	4.3	5.0	-0.7	-2.4	100.0%	-2.6
1779	AT5G27850.1 | 60S ribosomal protein L18 (RPL18C), 60S ribosomal protein L18, Arabidopsis thaliana, SWISSPROT:RL18_ARATH | chr5:9873160-9874602 FORWARD | Aliases: F14I23.10, F14I23_10	10.6	11.3	-0.7	-2.4	100.0%	-2.7
1780	AT5G03460.1 | expressed protein | chr5:864280-865672 FORWARD | Aliases: F12E4.230, F12E4_230	8.2	7.4	0.9	2.4	100.0%	-2.6
1781	AT2G36950.1 | heavy-metal-associated domain-containing protein, nearly identical to farnesylated protein ATFP2 (GI:4097545) Pfam profile PF00403: Heavy-metal-associated domain | chr2:15522212-15524010 FORWARD | Aliases: T1J8.13, T1J8_13	10.2	9.1	1.1	2.4	100.0%	-2.7
1782	AT3G50940.1 | AAA-type ATPase family protein, contains Pfam profile: ATPase family PF00004 | chr3:18944744-18946592 FORWARD | Aliases: F18B3.220	3.2	2.7	0.4	2.4	100.0%	-3.1
1783	AT1G33420.1 | PHD finger family protein, contains Pfam profile: PF00628: PHD-finger | chr1:12120866-12123556 REVERSE | Aliases: F10C21.9, F10C21_9	5.5	6.1	-0.5	-2.4	100.0%	-2.8
1784	AT3G33520.1 | Symbol: ATARP6 |	5.7	7.0	-1.2	-2.4	100.0%	-2.6
1785	AT1G63080.1 | pentatricopeptide (PPR) repeat-containing protein, contains Pfam profile PF01535: PPR repeat | chr1:23392549-23394393 REVERSE | Aliases: F16M19.17, F16M19_17	5.3	5.8	-0.5	-2.4	100.0%	-3.1
1786	AT2G36160.1 | 40S ribosomal protein S14 (RPS14A) | chr2:15176897-15178428 FORWARD | Aliases: F9C22.9, F9C22_9	10.8	11.3	-0.5	-2.4	100.0%	-3.3
1787	AT5G60930.1 | chromosome-associated kinesin, putative, microtubule-associated motor KIF4 , Mus musculus, PIR:A54803 | chr5:24532624-24539737 REVERSE | Aliases: MSL3.50, MSL3_50	3.6	4.3	-0.7	-2.4	100.0%	-2.8
1788	AT2G39370.1 | expressed protein | chr2:16451208-16452344 REVERSE | Aliases: F12L6.3, F12L6_3	4.7	5.2	-0.5	-2.3	100.0%	-2.9
1789	AT2G24230.1 | leucine-rich repeat transmembrane protein kinase, putative | chr2:10308897-10311892 REVERSE | Aliases: F27D4.14, F27D4_14	3.5	4.0	-0.4	-2.3	100.0%	-2.8
1790	AT5G16380.1 | expressed protein, contains Pfam profile PF04398: Protein of unknown function, DUF538 | chr5:5359413-5360713 REVERSE | Aliases: MQK4.11, MQK4_11	3.4	2.7	0.7	2.3	100.0%	-2.8
1791	AT5G43590.1 | patatin, putative, similar to patatin-like latex allergen (Hevea brasiliensis)(PMID:10589016); contains patatin domain PF01734 | chr5:17526595-17528704 REVERSE | Aliases: K9D7.9, K9D7_9	2.5	2.9	-0.4	-2.3	100.0%	-3.1
1792	AT5G50350.1 | expressed protein | chr5:20515702-20518906 REVERSE | Aliases: MXI22.6, MXI22_6	4.8	5.5	-0.6	-2.3	100.0%	-2.7
1793	AT2G19170.1 | Symbol: SLP3 | subtilase family protein, contains similarity to meiotic serine proteinase TMP GI:6468325 from (Lycopersicon esculentum)	3.4	3.8	-0.4	-2.3	100.0%	-3.0
1794	AT4G30310.3 | ribitol kinase, putative, similar to ribitol kinase (Klebsiella pneumoniae) gi:2905643:gb:AAC26495 | chr4:14832322-14835098 FORWARD | Aliases: F17I23.350, F17I23_350	6.4	7.0	-0.6	-2.3	100.0%	-2.8
1795	AT5G67260.1 | Symbol: CYCD3;2 | cyclin family protein, similar to cyclin D3.1 protein (Nicotiana tabacum) GI:4160300; contains Pfam profiles PF00134: Cyclin, N-terminal domain, PF02984: Cyclin, C-terminal domain | chr5:26853374-26855037 FORWARD | Aliases: CYCD3;2, Cyclin D3;2, K3G17.2, K3G17_2	5.0	5.9	-0.9	-2.3	100.0%	-2.7
1796	AT1G03780.2 | targeting protein-related, similar to microtubule-associated protein / targeting protein for Xklp2 ((TPX2) GI:8926138) {Homo sapiens}; similar to Restricted expression proliferation associated protein 100 (p100) (Differentially expressed in lung cells 2) (DIL-2) (Targeting protein for Xklp2) (C20orf1 protein) (C20orf2 protein) (Protein FLS353)(SP:Q9ULW0) {Homo sapiens} | chr1:947643-951729 REVERSE | Aliases: F21M11.31, F21M11_31	4.4	3.7	0.6	2.3	100.0%	-2.7
1797	AT4G04940.1 | transducin family protein / WD-40 repeat family protein, contains seven G-protein beta WD-40 repeats | chr4:2511026-2517191 REVERSE | Aliases: T1J1.5, T1J1_5	4.1	4.8	-0.7	-2.3	100.0%	-2.6
1798	ATMG00150.1 | Symbol: ORF116 |	2.6	2.9	-0.3	-2.3	100.0%	-3.4
1799	AT5G37360.1 | expressed protein | chr5:14822459-14825454 REVERSE | Aliases: MNJ8.18, MNJ8_18	6.8	6.2	0.7	2.3	100.0%	-2.5

Rank	Description	15.dpi	5.dpi	M	t	adj.q	B
1800	AT4G24700.1 | expressed protein | chr4:12744632-12745323 REVERSE | Aliases: F22K18.100, F22K18_100	3.6	3.1	0.5	2.3	100.0%	-2.8
1801	AT5G16910.1 | Symbol: ATCSLD2 | cellulose synthase family protein, similar to gi:2827143 cellulose synthase catalytic subunit, Arabidopsis thaliana, gi:9622886 cellulose synthase-7 from Zea mays | chr5:5561682-5565583 FORWARD | Aliases: CSLD2, F2K13.60, F2K13_60	6.7	6.1	0.6	2.3	100.0%	-2.7
1802	AT1G36950.1 | zinc finger protein-related, contains similarity to zinc finger proteins (C3HC4-type RING finger) | chr1:14012114-14013263 REVERSE | Aliases: T32E20.33, T32E20_33	2.1	1.9	0.1	2.3	100.0%	-4.0
1803	AT4G00070.1 | zinc finger protein-related, contains similarity to zinc finger proteins (C3HC4-type RING finger) | chr4:29672-31426 REVERSE | Aliases: F6N15.15, F6N15_15	2.1	1.9	0.1	2.3	100.0%	-4.0
1804	AT1G19270.1 | ubiquitin interaction motif-containing protein / LIM domain-containing protein, weak similarity to LIM-homeobox protein (Mus musculus) GI:2149584, Hic-5 (Mus musculus) GI:664955; contains Pfam profiles PF02809: Ubiquitin interaction motif, PF00412: LIM domain | chr1:6662492-6666019 FORWARD | Aliases: T29M8.14, T29M8_14	5.0	4.5	0.5	2.3	100.0%	-2.7
1805	AT5G06590.1 | expressed protein | chr5:2016646-2018836 FORWARD | Aliases: F15M7.12, F15M7_12	8.5	9.6	-1.1	-2.3	100.0%	-2.4
1806	AT3G53340.1 | CCAAT-box binding transcription factor, putative, similar to CAAT-box DNA binding protein subunit B (NF-YB) (SP:P25209) (GI:22380) (Zea mays); contains Pfam PF00808 : Histone-like transcription factor (CBF/NF-Y) and archaeal histone	5.6	6.5	-0.9	-2.3	100.0%	-2.5
1807	AT1G22780.1 | Symbol: PFL | 40S ribosomal protein S18 (RPS18A), Match to ribosomal S18 gene mRNA gb:Z28701, DNA gb:Z23165 from A. thaliana. ESTs gb:T21121, gb:Z17755, gb:R64776 and gb:R30430 come from this gene | chr1:8067853-8069319 FORWARD | Aliases: 40S RIBOSOMAL PROTEIN S18, PFL1, POINTED FIRST LEAVES, RPS18A, T22J18.5, T22J18_5	10.8	11.4	-0.6	-2.3	100.0%	-2.8
1808	AT1G05570.1 | Symbol: CALS1 | similar to glycosyl transferase family 48 protein [Arabidopsis thaliana] (TAIR:At5g13000.1); similar to glycosyl transferase family 48 protein [Arabidopsis thaliana] (TAIR:At2g31960.1); similar to glycosyl transferase family 48 protein [Arabidopsis thaliana] (TAIR:At1g06490.1); similar to glycosyl transferase family 48 protein [Arabidopsis thaliana] (TAIR:At2g13680.1); similar to glycosyl transferase family 48 protein [Arabidopsis thaliana] (TAIR:At3g59100.1); similar to putative callose synthase 1 catalytic subunit [Oryza sativa (japonica cultivar-group)] (GB:BAD72533.1); similar to Putative glucan synthase [Oryza sativa (japonica cultivar-group)] (GB:NP_912480.1); similar to OJ1029_F04.4 [Oryza sativa (japonica cultivar-group)] (GB:NP_918100.1); similar to putative beta-1,3-glucan synthase [Nicotiana alata] (GB:AAK49452.2); similar to putative callose synthase 1 catalytic subunit [Oryza sativa (japonica cultivar-group)] (GB:BAD62105.1); contains InterPro domain Glycosyl transferase, family 48 (InterPro:IPR003440) | chr1:1647719-1658685 REVERSE | Aliases: ATGSL06, ATGSL6, CALLOSE SYNTHASE 1, F3F20.1, F3F20_1, GLUCAN SYNTHASE LIKE 6, GSL06, GSL6	6.6	5.8	0.8	2.3	100.0%	-2.4
1809	AT1G67720.1 | leucine-rich repeat family protein / protein kinase family protein, contains similarity to light repressible receptor protein kinase (Arabidopsis thaliana) gi:1321686:emb:CAA66376; contains Pfam doamins PF00069: Protein kinase domain and PF00560: Leucine Rich Repeat | chr1:25390004-25394736 FORWARD | Aliases: F12A21.30	6.9	6.1	0.7	2.3	100.0%	-2.6
1810	AT5G01770.1 | Encodes one of two Arabidopsis RAPTOR/KOG1 homologs. RAPTOR proteins are binding partners of the target of rapamycin kinase that is present in all eukaryotes and play a central role in the stimulation of cell growth and metabolism in response to nutrients. Mutations in this gene have no visible effects on embryo or plant development. | chr5:294536-301770 REVERSE | Aliases: ATRAPTOR2, RAPTOR2, T20L15.40, T20L15_40	3.8	4.4	-0.6	-2.3	100.0%	-2.8
1811	AT3G56400.1 | Symbol: WRKY70 |	4.8	4.1	0.7	2.3	100.0%	-2.6
1812	AT5G23880.1 | Symbol: CPSF100 | cleavage and polyadenylation specificity factor, identical to cleavage and polyadenylation specificity factor (Arabidopsis thaliana) SWISS-PROT:Q9LKF9 | chr5:8052507-8058347 FORWARD | Aliases: CLEAVAGE AND POLYADENYLATION SPECIFICITY FACTOR, EMB1265, EMBRYO DEFECTIVE 1265	5.4	6.0	-0.6	-2.3	100.0%	-2.8
1813	AT1G22510.1 | zinc finger (C3HC4-type RING finger) family protein, contains Pfam profile: PF00097 zinc finger, C3HC4 type (RING finger) | chr1:7950808-7952659 REVERSE | Aliases: F12K8.14, F12K8_14	6.0	4.7	1.3	2.3	100.0%	-2.5
1814	AT5G60040.1 | Symbol: NRPC1 | DNA-directed RNA polymerase, putative, similar to SP:P04051 DNA-directed RNA polymerase III largest subunit (EC 2.7.7.6) {Saccharomyces cerevisiae}; contains InterPro accession IPR000722: RNA polymerase, alpha subunit | chr5:24190816-24200495 FORWARD | Aliases: MGO3.13, MGO3_13	4.1	4.9	-0.8	-2.3	100.0%	-2.5
1815	AT3G60830.1 | Symbol: ATARP7 |	6.9	7.6	-0.8	-2.3	100.0%	-2.6
1816	AT1G70000.1 | DNA-binding family protein, contains Pfam domains, PF00249: Myb-like DNA-binding domain and PF00098: Zinc knuckle | chr1:26366941-26368362 REVERSE | Aliases: F20P5.26, F20P5_26	4.9	4.1	0.8	2.3	100.0%	-2.6
1817	AT3G56620.1 | integral membrane family protein / nodulin MtN21-related, similar to MtN21 GI:2598575 (root nodule development) from (Medicago truncatula) | chr3:20983482-20985585 REVERSE | Aliases: T5P19.270	7.1	6.2	0.9	2.3	100.0%	-2.5

Rank	Description	15.dpi	5.dpi	M	t	adj.q	B
1818	AT1G03970.1 | Symbol: GBF4 | G-box binding factor 4 (GBF4), identical to G-box binding factor 4 (GBF4) SP:P42777 from (Arabidopsis thaliana) | chr1:1018099-1019207 FORWARD | Aliases: F21M11.10, F21M11_10	7.7	6.8	0.9	2.3	100.0%	-2.7
1819	AT3G20820.1 | leucine-rich repeat family protein, contains similarity to Cf-2.1 (Lycopersicon pimpinellifolium) gi:1184075:gb:AAC15779; contains leucine rich-repeat domains Pfam:PF00560, INTERPRO:IPR001611 | chr3:7280884-7282183 FORWARD | Aliases: MOE17.11	3.3	4.1	-0.8	-2.3	100.0%	-2.5
1820	AT1G24280.1 | Symbol: G6PD3 | Encodes a plastidic glucose-6-phosphate dehydrogenase that is sensitive to reduction by DTT and whose mRNA is most highly expressed in root. | chr1:8609434-8612569 FORWARD | Aliases: F3I6.22, F3I6_22, G6PD3, GLUCOSE 6 PHOSPHATE DEHYDROGENASE 3	8.4	9.0	-0.6	-2.3	100.0%	-2.8
1821	AT4G38180.1 | far-red impaired responsive protein, putative, similar to far-red impaired response protein FAR1 (Arabidopsis thaliana) gi:5764395:gb:AAD51282; contains Pfam:PF03101 domain: FAR1 family and PF04434: SWIM zinc finger | chr4:17906508-17909671 REVERSE | Aliases: F20D10.300, F20D10_300	3.8	4.3	-0.5	-2.3	100.0%	-2.9
1822	AT3G57120.1 | protein kinase family protein, contains eukaryotic protein kinase domain, INTERPRO:IPR000719 | chr3:21149884-21151589 FORWARD | Aliases: F24I3.200	5.0	4.6	0.4	2.3	100.0%	-3.1
1823	AT1G20030.2 | pathogenesis-related thaumatin family protein, similar to receptor serine/threonine kinase PR5K (Arabidopsis thaliana) GI:1235680; contains Pfam profile PF00314: Thaumatin family | chr1:6945416-6947335 FORWARD | Aliases: T20H2.19, T20H2_19	3.8	3.0	0.8	2.3	100.0%	-2.6
1824	AT3G54500.2 | expressed protein | chr3:20186382-20189958 REVERSE | Aliases: T14E10.70	5.0	4.1	0.9	2.3	100.0%	-2.5
1825	AT5G09220.1 | Symbol: AAP2 | amino acid permease 2 (AAP2), identical to amine acid permease AAP2 (Arabidopsis thaliana) GI:510236 | chr5:2866253-2869055 FORWARD | Aliases: AMINO ACID PERMEASE 2, T2K12.6	12.2	9.9	2.3	2.3	100.0%	-2.5
1826	AT2G22190.1 | similar to trehalose-6-phosphate phosphatase, putative [Arabidopsis thaliana] (TAIR:At4g39770.1); similar to putative trehalose-phosphatase [Oryza sativa (japonica cultivar-group)] (GB:XP_482349.1); contains InterPro domain Trehalose-phosphatase (InterPro:IPR003337); contains InterPro domain HAD-superfamily hydrolase, subfamily IIB (InterPro:IPR006379) | chr2:9440845-9443624 REVERSE | Aliases: T26C19.15, T26C19_15	3.4	2.9	0.5	2.3	100.0%	-2.9
1827	AT2G01750.1 | Symbol: ATMAP70 3 | Encodes a microtubule associated protein (MAP70-3). Expressed in all tissues. | chr2:328350-332112 FORWARD | Aliases: ATMAP70 3, T8O11.8, T8O11_8	3.9	4.5	-0.6	-2.3	100.0%	-2.8
1828	AT1G09780.1 | 2,3-biphosphoglycerate-independent phosphoglycerate mutase, putative / phosphoglyceromutase, putative, strong similarity to SP:Q42908 2,3-bisphosphoglycerate-independent phosphoglycerate mutase (EC 5.4.2.1) (Phosphoglyceromutase) {Mesembryanthemum crystallinum}; contains Pfam profile PF01676: Metalloenzyme superfamily | chr1:3165372-3167871 REVERSE | Aliases: F21M12.16, F21M12_16	10.7	11.9	-1.2	-2.3	100.0%	-3.1
1829	AT3G19440.1 | pseudouridine synthase family protein, low similarity to SP:P23851 Ribosomal large subunit pseudouridine synthase C (EC 4.2.1.70) (Pseudouridylate synthase) (Uracil hydrolyase) {Escherichia coli}; contains Pfam profile PF00849: RNA pseudouridylate synthase	3.7	4.6	-0.9	-2.3	100.0%	-2.6
1830	AT1G01120.1 | Symbol: KCS1 | fatty acid elongase 3-ketoacyl-CoA synthase 1 (KCS1), nearly identical to GB:AAC99312 GI:4091810 from (Arabidopsis thaliana) | chr1:57269-59167 REVERSE | Aliases: T25K16.11, T25K16_11	3.7	4.7	-1.0	-2.3	100.0%	-2.4
1831	AT2G40550.1 | expressed protein | chr2:16941914-16945213 FORWARD | Aliases: T2P4.10, T2P4_10	5.4	7.1	-1.7	-2.3	100.0%	-2.4
1832	AT1G69870.1 | proton-dependent oligopeptide transport (POT) family protein, contains Pfam profile: PF00854 POT family | chr1:26319690-26323883 FORWARD | Aliases: T17F3.10, T17F3_10	2.9	2.5	0.3	2.3	100.0%	-3.1
1833	AT4G37180.2 | myb family transcription factor, contains Pfam domain, PF00249: Myb-like DNA-binding domain | chr4:17504558-17506361 FORWARD | Aliases: AP22.100, AP22_100	3.0	3.5	-0.6	-2.3	100.0%	-2.8
1834	AT4G17030.1 | Symbol: ATEXLB1 | expansin-related, identical to SWISS-PROT:O23547 expansin-related protein 1 precursor (At-EXPR1)(Arabidopsis thaliana); related to expansins, http://www.bio.psu.edu/expansins/ | chr4:9581605-9583309 REVERSE | Aliases: ARABIDOPSIS THALIANA EXPANSIN LIKE B1, AT EXPR, ATEXPR1, ATHEXP BETA 3.1, DL4545C, EXPR, FCAALL.341	3.6	2.7	1.0	2.3	100.0%	-2.4
1835	AT1G03110.1 | transducin family protein / WD-40 repeat family protein, contains 4 WD-40 repeats (PF00400); similar to WD-repeat domain 4 protein (GI:9955698) (Mus musculus) | chr1:749246-751963 FORWARD | Aliases: F10O3.6, F10O3_6	4.5	5.4	-0.8	-2.3	100.0%	-2.7
1836	AT1G55740.1 | alkaline alpha galactosidase, putative, similar to alkaline alpha galactosidase I (Cucumis melo) GI:29838629; contains Pfam profile PF05691: Raffinose synthase or seed imbibition protein Sip1 | chr1:20839033-20842442 REVERSE | Aliases: F20N2.14	6.5	6.0	0.5	2.3	100.0%	-3.0
1837	AT1G10650.1 | expressed protein | chr1:3524019-3526336 REVERSE | Aliases: F20B24.9, F20B24_9	8.7	8.2	0.5	2.3	100.0%	-3.0

Rank	Description	15.dpi	5.dpi	M	t	adj.q	B
1838	AT1G14020.1 | expressed protein, contains Pfam PF03138: Plant protein family. The function of this family of plant proteins is unknown; previously annotated as 'growth regulator protein -related' based on similarity to axi 1 protein (GB:X80301) (GI:559920) from (Nicotiana tabacum), which, due to scienitific fraud was retracted. Retraction in: Schell J. EMBO J 1999 May 17;18(10):2908. PMID:10400497. | chr1:4802728-4805323 FORWARD | Aliases: F7A19.11, F7A19_11	4.7	5.5	-0.9	-2.3	100.0%	-2.6
1839	AT1G11870.3 | seryl-tRNA synthetase, putative / serine--tRNA ligase, putative, similar to PIR:T03949 serine--tRNA ligase (EC 6.1.1.11) serS {Zea mays}; contains Pfam profiles PF00587: tRNA synthetase class II core domain (G, H, P, S and T), PF02403: Seryl-tRNA synthetase N-terminal domain | chr1:4003845-4006737 FORWARD | Aliases: F12F1.29, F12F1_29	4.5	5.3	-0.8	-2.3	100.0%	-2.6
1840	AT1G28330.3 | Symbol: DRM1 | dormancy-associated protein, putative (DRM1), identical to dormancy-associated protein (Arabidopsis thaliana) GI:2995990; similar to dormancy-associated protein GI:2605887 from (Pisum sativum); contains Pfam profile PF05564: Dormancy/auxin associated protein | chr1:9933896-9935251 REVERSE | Aliases: DORMANCY ASSOCIATED PROTEIN 1, DRM1, F3H9.1, F3H9_1	6.3	4.8	1.5	2.3	100.0%	-2.6
1841	AT3G06200.1 | guanylate kinase, putative, similar to guanylate kinase (GMP kinase) (Bacillus halodurans) Swiss-Prot:Q9K9Y2; contains Pfam profile: PF00625 guanylate kinase | chr3:1877600-1879244 FORWARD | Aliases: F28L1.14, F28L1_14	6.3	5.5	0.8	2.3	100.0%	-2.6
1842	AT2G21740.1 | expressed protein | chr2:9289066-9289443 REVERSE | Aliases: F7D8.6, F7D8_6	2.2	2.5	-0.3	-2.3	100.0%	-3.2
1843	AT5G27540.2 | Symbol: EMB2473 | similar to GTP-binding protein-related [Arabidopsis thaliana] (TAIR:At3g63150.1); similar to GTP-binding protein-related [Arabidopsis thaliana] (TAIR:At3g05310.1); similar to rac-GTP binding protein -like [Oryza sativa (japonica cultivar-group)] (GB:NP_915455.1); similar to putative mitochondrial Rho 1 [Oryza sativa (japonica cultivar-group)] (GB:BAD81741.1); contains InterPro domain Small GTP-binding protein domain (InterPro:IPR005225); contains InterPro domain Ras small GTPase, Rab type (InterPro:IPR003579); contains InterPro domain Ras small GTPase, Rho type (InterPro:IPR003578); contains InterPro domain Ras GTPase superfamily (InterPro:IPR001806); contains InterPro domain Calcium-binding EF-hand (InterPro:IPR002048); contains InterPro domain ATP/GTP-binding site motif A (P-loop) (InterPro:IPR001687) | chr5:9722577-9727457 FORWARD | Aliases: EMB2473, EMBRYO DEFECTIVE 2473, F21A20.250, F21A20_250	5.8	7.0	-1.2	-2.3	100.0%	-2.6
1844	AT4G20270.1 | leucine-rich repeat transmembrane protein kinase, putative, CLAVATA1 receptor kinase, Arabidopsis th., PATX:G2160756 | chr4:10949671-10953089 FORWARD | Aliases: F1C12.190, F1C12_190	6.7	7.3	-0.6	-2.3	100.0%	-3.0
1845	AT4G13030.2 | expressed protein | chr4:7607264-7609840 FORWARD | Aliases: F25G13.120, F25G13_120	6.2	5.4	0.7	2.3	100.0%	-2.5
1846	AT3G24760.1 | F-box family protein, ; similar to SKP1 interacting partner 2 (SKIP2) TIGR_Ath1:At5g67250 | chr3:9040095-9041841 FORWARD | Aliases: K7P8.5	7.2	6.6	0.6	2.3	100.0%	-2.8
1847	AT1G08070.1 | pentatricopeptide (PPR) repeat-containing protein, contains Pfam profile PF01535: PPR repeat | chr1:2514189-2516602 REVERSE | Aliases: T6D22.15, T6D22_15	3.8	4.4	-0.6	-2.3	100.0%	-2.9
1848	AT2G42450.1 | lipase class 3 family protein, similar to calmodulin-binding heat-shock protein CaMBP (Nicotiana tabacum) GI:1087073; contains Pfam profile PF01764: Lipase, PF03893: Lipase 3 N-terminal region	4.5	5.3	-0.8	-2.3	100.0%	-2.5
1849	AT3G16460.2 | similar to jacalin lectin family protein [Arabidopsis thaliana] (TAIR:At1g52040.1); similar to myrosinase-binding protein [Brassica napus] (GB:AAC08048.1); contains InterPro domain Jacalin-related lectin (InterPro:IPR001229)	6.5	7.3	-0.9	-2.3	100.0%	-2.6
1850	AT1G77120.1 | Symbol: ADH1 | alcohol dehydrogenase (ADH), identical to alcohol dehydrogenase GI:469467 from (Arabidopsis thaliana) | chr1:28980345-28982311 FORWARD | Aliases: ADH, ALCOHOL DEHYDROGENASE, ALCOHOL DEHYDROGENASE 1, F22K20.19, F22K20_19	13.5	12.4	1.1	2.3	100.0%	-2.8
1851	AT1G60220.1 | similar to Ulp1 protease family protein [Arabidopsis thaliana] (TAIR:At1g10570.2); similar to Ulp1 protease family protein [Arabidopsis thaliana] (TAIR:At1g10570.1); similar to OSJNBb0021A09.14 [Oryza sativa (japonica cultivar-group)] (GB:NP_916015.1); contains InterPro domain SUMO/Sentrin/Ubl1 specific protease (InterPro:IPR003653) | chr1:22211890-22215816 FORWARD | Aliases: T13D8.11, T13D8_11	8.1	8.6	-0.5	-2.3	100.0%	-3.0
1852	AT1G10290.1 | Symbol: ADL6 | dynamin-like protein 6 (ADL6), identical to dynamin-like protein 6 (ADL6) (Arabidopsis thaliana) GI:6651399; contains Pfam profiles PF01031: Dynamin central region, PF00350: Dynamin family, PF02212: Dynamin GTPase effector domain, PF00169: PH domain | chr1:3370563-3377465 FORWARD | Aliases: ADL6, DRP2A, DYNAMIN LIKE PROTEIN 6, F14N23.17, F14N23_17	6.3	6.9	-0.6	-2.3	100.0%	-2.7
1853	AT2G29560.1 | enolase, putative, similar to enolase (Spinacia oleracea) gi:8919731:emb:CAB96173 | chr2:12653666-12656983 FORWARD | Aliases: F16P2.6, F16P2_6	7.4	8.2	-0.8	-2.3	100.0%	-2.8
1854	AT5G19500.1 | tryptophan/tyrosine permease family protein, contains Pfam profile PF03222: Tryptophan/tyrosine permease family | chr5:6578954-6581947 FORWARD | Aliases: T20D1.20, T20D1_20	4.1	3.6	0.5	2.3	100.0%	-2.8
1855	AT1G25380.1 | mitochondrial substrate carrier family protein, contains Pfam profile: PF00153 mitochondrial carrier protein | chr1:8903522-8905989 FORWARD | Aliases: F4F7.45	7.5	8.3	-0.8	-2.3	100.0%	-2.9

Rank	Description	15.dpi	5.dpi	M	t	adj.q	B
1856	AT2G27580.1 | zinc finger (AN1-like) family protein, contains Pfam domains, PF01428: AN1-like Zinc finger and PF01754: A20-like zinc finger | chr2:11783488-11784539 REVERSE | Aliases: F10A12.25, F10A12_25	6.8	5.9	0.8	2.3	100.0%	-2.7
1857	AT5G15980.1 | pentatricopeptide (PPR) repeat-containing protein, contains Pfam profile PF01535: PPR repeat | chr5:5213084-5215623 FORWARD | Aliases: F1N13.120, F1N13_120	5.7	6.7	-1.0	-2.3	100.0%	-2.7
1858	AT1G60690.1 | aldo/keto reductase family protein, contains Pfam profile PF00248: oxidoreductase, aldo/keto reductase family | chr1:22353557-22355333 REVERSE | Aliases: F8A5.21, F8A5_21	6.5	7.1	-0.6	-2.3	100.0%	-2.7
1859	AT2G33210.1 | chaperonin, putative, similar to SWISS-PROT:Q05046- chaperonin CPN60-2, mitochondrial precursor (HSP60-2) (Cucurbita maxima); contains Pfam:PF00118 domain, TCP-1/cpn60 chaperonin family | chr2:14081986-14085797 REVERSE | Aliases: F25I18.5, F25I18_5	8.7	9.7	-1.0	-2.3	100.0%	-2.6
1860	AT1G80910.1 | expressed protein | chr1:30405628-30408934 FORWARD | Aliases: F23A5.27, F23A5_27	5.6	6.3	-0.7	-2.3	100.0%	-2.6
1861	AT3G18215.1 | expressed protein, contains Pfam profile PF04654: Protein of unknown function, DUF599 | chr3:6240890-6242577 FORWARD | Aliases: None	8.6	6.9	1.7	2.3	100.0%	-2.7
1862	AT5G14150.1 | expressed protein, contains Pfam profile PF04862: Protein of unknown function, DUF642 | chr5:4565174-4566907 REVERSE | Aliases: MUA22.15, MUA22_15	3.0	3.3	-0.3	-2.3	100.0%	-3.2
1863	AT1G74600.1 | pentatricopeptide (PPR) repeat-containing protein, contains INTERPRO:IPR002885 PPR repeats | chr1:28028814-28031501 REVERSE | Aliases: F1M20.28, F1M20_28	4.2	4.7	-0.5	-2.3	100.0%	-3.0
1864	AT2G35736.1 | expressed protein, ; expression supported by MPSS | chr2:15030163-15030333 REVERSE | Aliases: None	5.8	5.0	0.8	2.3	100.0%	-2.5
1865	AT4G37260.1 | Symbol: MYB73 | myb family transcription factor (MYB73), contains Pfam profile: PF00249 myb-like DNA-binding domain | chr4:17540484-17541793 FORWARD | Aliases: AP22.97, AP22_97, MYB73	8.9	8.0	1.0	2.3	100.0%	-2.7
1866	AT2G45820.1 | DNA-binding protein, putative, identical to DNA-binding protein gi:601843:gb:AAA57124 (Arabidopsis thaliana); contains Pfam domain, PF03766: Remorin, N-terminal region; contains Pfam domain, PF03763: Remorin, C-terminal region | chr2:18870032-18871735 REVERSE | Aliases: F4I18.20	9.8	9.1	0.7	2.3	100.0%	-2.8
1867	AT5G52020.1 | encodes a member of the DREB subfamily A-4 of ERF/AP2 transcription factor family. The protein contains one AP2 domain. There are 17 members in this subfamily including TINY. | chr5:21141184-21142174 REVERSE | Aliases: MSG15.10, MSG15_10	4.3	3.4	0.9	2.3	100.0%	-2.5
1868	AT1G13260.1 | Symbol: RAV1 | DNA-binding protein RAV1 (RAV1), identical to SP:Q9ZWM9 DNA-binding protein RAV1 {Arabidopsis thaliana}, RAV1 GI:3868857 from (Arabidopsis thaliana) | chr1:4542165-4543739 FORWARD | Aliases: RAV1, T6J4.2, T6J4_2	7.6	6.8	0.7	2.3	100.0%	-3.0
1869	AT5G50130.2 | short-chain dehydrogenase/reductase (SDR) family protein, contains INTERPRO family IPR002198 Short-chain dehydrogenase/reductase (SDR) superfamily | chr5:20407223-20410410 FORWARD | Aliases: MPF21.15, MPF21_15	3.5	4.2	-0.7	-2.3	100.0%	-2.7
1870	AT3G27400.1 | pectate lyase family protein, similar to pectate lyase GP:7547009 from (Vitis vinifera); contains Pfam profile: PF00544 pectate lyase | chr3:10141560-10144462 FORWARD | Aliases: K1G2.22	8.8	10.3	-1.4	-2.3	100.0%	-2.4
1871	AT5G44310.2 | late embryogenesis abundant domain-containing protein / LEA domain-containing protein, low similarity to 51 kDa seed maturation protein (Glycine max) GI:414977; contains Pfam profile PF02987: Late embryogenesis abundant protein | chr5:17865100-17866739 REVERSE | Aliases: K9L2.7, K9L2_7	5.3	4.5	0.8	2.3	100.0%	-2.8
1872	AT3G02140.1 | expressed protein | chr3:385324-386545 REVERSE | Aliases: F1C9.7	7.9	6.9	1.0	2.3	100.0%	-2.7
1873	AT1G30610.1 | Symbol: EMB2279 | pentatricopeptide (PPR) repeat-containing protein, contains Pfam profile PF01535: PPR repeat | chr1:10846658-10850499 FORWARD | Aliases: EMB2279, EMBRYO DEFECTIVE 2279, T5I8.6, T5I8_6	6.3	7.0	-0.7	-2.3	100.0%	-2.9
1874	AT4G36740.1 | homeobox-leucine zipper family protein, similar to CRHB7 (GP:3868841) {Ceratopteris richardii} and to homeotic protein VAHOX1 (PIR:T07734) (Lycopersicon esculentum) | chr4:17314653-17316318 REVERSE | Aliases: AP22.8, AP22_8, HB 5	3.2	2.8	0.4	2.3	100.0%	-2.9
1875	AT5G58070.1 | lipocalin, putative, similar to temperature stress-induced lipocalin (Triticum aestivum) GI:18650668 | chr5:23517287-23518435 REVERSE | Aliases: K21L19.9, K21L19_9	11.7	9.8	1.9	2.3	100.0%	-2.5
1876	AT2G28560.2 | Symbol: ATRAD51B/RAD51B | similar to DNA repair family protein [Arabidopsis thaliana] (TAIR:At2g45280.1); similar to putative DNA repair protein RAD51 [Oryza sativa (japonica cultivar-group)] (GB:XP_475513.1); contains InterPro domain AAA ATPase (InterPro:IPR003593); contains InterPro domain RecA bacterial DNA recombination protein (InterPro:IPR001553) | chr2:12243692-12246201 REVERSE | Aliases: ATRAD51B, RAD51B, T17D12.12, T17D12_12	3.3	3.0	0.3	2.3	100.0%	-3.2

Rank	Description	15.dpi	5.dpi	M	t	adj.q	B
1877	AT5G48480.1 | expressed protein | chr5:19661901-19663103 FORWARD | Aliases: MJE7.12, MJE7_12	7.4	8.5	-1.2	-2.3	100.0%	-2.5
1878	AT2G38010.2 | ceramidase family protein, contains Pfam domain, PF04734: Neutral/alkaline nonlysosomal ceramidase | chr2:15913940-15917009 FORWARD | Aliases: T8P21.8, T8P21_8	6.6	7.1	-0.6	-2.3	100.0%	-2.9
1879	AT3G20420.1 | ribonuclease III family protein, similar to CAF protein (RNA helicase/RNAseIII) (Arabidopsis thaliana) GI:6102610; contains Pfam profiles: PF00636 RNase3 domain, PF00035 Double-stranded RNA binding motif | chr3:7119288-7120925 REVERSE | Aliases: MQC12.21	3.8	4.5	-0.6	-2.3	100.0%	-2.6
1880	AT5G06190.1 | expressed protein | chr5:1876035-1876638 REVERSE | Aliases: MBL20.7, MBL20_7	6.7	5.1	1.6	2.3	100.0%	-2.6
1881	AT3G60260.3 | similar to phagocytosis and cell motility protein ELMO1-related [Arabidopsis thaliana] (TAIR:At2g44770.1); similar to OSJNBb0038F03.14 [Oryza sativa (japonica cultivar-group)] (GB:XP_473390.1); contains InterPro domain Protein of unknown function DUF609 (InterPro:IPR006816) | chr3:22285480-22288123 FORWARD | Aliases: F27H5.50	6.8	6.0	0.8	2.3	100.0%	-2.8
1882	AT5G58220.3 | similar to putative transthyretin, having alternative splicing products [Oryza sativa (japonica cultivar-group)] (GB:AAR06357.1); contains InterPro domain Transthyretin (InterPro:IPR000895) | chr5:23571153-23573092 REVERSE | Aliases: MCK7.9, MCK7_9	8.0	7.3	0.7	2.3	100.0%	-2.6
1883	AT5G06870.1 | Symbol: PGIP2 | polygalacturonase inhibiting protein 2 (PGIP2), identical to polygalacturonase inhibiting protein 2 (PGIP2) (Arabidopsis thaliana) gi:7800201:gb:AAF69828; contains leucine rich-repeat (LRR) domains Pfam:PF00560, INTERPRO:IPR001611 | chr5:2133919-2135162 FORWARD | Aliases: MOJ9.4, MOJ9_4, POLYGALACTURONASE INHIBITING PROTEIN 2	3.5	2.9	0.5	2.3	100.0%	-2.8
1884	AT3G08700.1 | ubiquitin-conjugating enzyme, putative, strong similar to ubiquitin-conjugating enzymes E2-17 from (Arabidopsis thaliana) SP:P35134, SP:P35132, SP:P35133; contains Pfam profile PF00179: Ubiquitin-conjugating enzyme | chr3:2643488-2644551 FORWARD | Aliases: F17O14.17	4.0	3.7	0.4	2.3	100.0%	-3.1
1885	AT2G41100.3 | Symbol: TCH3 | similar to calmodulin-2/3/5 (CAM2) (CAL1) [Arabidopsis thaliana] (TAIR:At2g41110.1); similar to calmodulin-2/3/5 (CAM3) [Arabidopsis thaliana] (TAIR:At3g56800.1); similar to calmodulin-2/3/5 (CAM5) (TCH1) [Arabidopsis thaliana] (TAIR:At2g27030.1); similar to calmodulin-2/3/5 (CAM5) (TCH1) [Arabidopsis thaliana] (TAIR:At2g27030.3); similar to calmodulin-7 (CAM7) [Arabidopsis thaliana] (TAIR:At3g43810.1); similar to CALM_PATSP Calmodulin (CaM) (GB:P02595); contains InterPro domain Calcium-binding EF-hand (InterPro:IPR002048); contains InterPro domain Flagellar calcium-binding protein (calflagin) (InterPro:IPR003299) | chr2:17145157-17146690 FORWARD | Aliases: ATCAL5, CALMODULIN LIKE 5, T3K9.13, T3K9_13, TOUCH 3	6.0	5.0	1.0	2.3	100.0%	-2.6
1886	AT1G52160.1 | metallo-beta-lactamase family protein | chr1:19424442-19428108 REVERSE | Aliases: F9I5.1, F9I5_1	6.2	6.7	-0.5	-2.3	100.0%	-3.1
1887	AT1G57590.1 | similar to pectinacetylesterase, putative [Arabidopsis thaliana] (TAIR:At5g26670.1); similar to putative pectinacetylesterase precursor [Oryza sativa (japonica cultivar-group)] (GB:NP_918013.1); contains InterPro domain Pectinacetylesterase (InterPro:IPR004963) | chr1:21331025-21333427 REVERSE | Aliases: T8L23.6, T8L23_6	4.2	3.6	0.6	2.3	100.0%	-2.8
1888	ATCG00750.1 | Symbol: RPS11 | 30S chloroplast ribosomal protein S11 | chrC:78960-79376 REVERSE | Aliases: RPS11	7.4	9.0	-1.6	-2.3	100.0%	-2.6
1889	AT4G24130.1 | expressed protein, contains Pfam profile PF04398: Protein of unknown function, DUF538 | chr4:12527785-12528602 FORWARD | Aliases: T19F6.120, T19F6_120	3.0	4.0	-1.0	-2.3	100.0%	-2.6
1890	AT5G22330.1 | TATA box-binding protein-interacting protein-related, similar to TATA box-binding protein-interacting protein SP:O35753 from (Mus musculus) | chr5:7390799-7394197 REVERSE | Aliases: MWD9.12, MWD9_12	5.6	6.4	-0.8	-2.3	100.0%	-2.7
1891	AT1G10120.1 | similar to basic helix-loop-helix (bHLH) family protein [Arabidopsis thaliana] (TAIR:At1g68920.1); similar to TA1 protein-like [Oryza sativa (japonica cultivar-group)] (GB:BAD34345.1); contains InterPro domain Basic helix-loop-helix dimerization domain bHLH (InterPro:IPR001092) | chr1:3304044-3306190 REVERSE | Aliases: T27I1.15, T27I1_15	6.2	7.0	-0.8	-2.3	100.0%	-2.6
1892	AT4G02400.1 | U3 ribonucleoprotein (Utp) family protein, contains Pfam profile: PF04615 Utp14 protein | chr4:1054243-1058236 FORWARD | Aliases: T14P8.20, T14P8_20	5.9	7.3	-1.4	-2.3	100.0%	-2.5
1893	AT4G01575.1 | serine protease inhibitor, Kazal-type family protein, contains Pfam domain PF00050: Kazal-type serine protease inhibitor domain | chr4:681868-682583 REVERSE | Aliases: T15B16.19, T15B16_19	5.7	5.0	0.7	2.3	100.0%	-2.6
1894	AT1G49500.1 | expressed protein | chr1:18325476-18325952 REVERSE | Aliases: F13F21.6, F13F21_6	4.0	4.8	-0.8	-2.3	100.0%	-2.6
1895	AT4G27820.1 | glycosyl hydrolase family 1 protein, contains Pfam PF00232 : Glycosyl hydrolase family 1 domain; TIGRFAM TIGR01233: 6-phospho-beta-galactosidase; similar to hydroxyisourate hydrolase (GI:19569603) (Glycine max) | chr4:13857764-13860577 REVERSE | Aliases: T27E11.60, T27E11_60	4.1	3.6	0.5	2.3	100.0%	-2.8

Rank	Description	15.dpi	5.dpi	M	t	adj.q	B
1896	AT5G48870.1 | Symbol: SAD1 | small nuclear ribonucleoprotein, putative / snRNP, putative / Sm protein, putative, similar to U6 snRNA-associated Sm-like protein LSm5 (Homo sapiens) SWISS-PROT:Q9Y4Y9	9.9	10.3	-0.5	-2.3	100.0%	-2.9
1897	AT1G65010.1 | expressed protein, similar to endosome-associated protein (GI:1016368) (Homo sapiens); similar to Centromeric protein E (CENP-E protein) (Swiss-Prot:Q02224) (Homo sapiens) | chr1:24153577-24157937 FORWARD | Aliases: F13O11.30, F13O11_30	4.2	5.3	-1.1	-2.3	100.0%	-2.6
1898	AT4G39380.1 | expressed protein | chr4:18309148-18312718 REVERSE | Aliases: F23K16.10, F23K16_10	3.0	3.3	-0.3	-2.3	100.0%	-3.2
1899	AT1G24360.1 | 3-oxoacyl-(acyl-carrier protein) reductase, chloroplast / 3-ketoacyl-acyl carrier protein reductase, identical to 3-oxoacyl-(acyl-carrier protein) reductase SP:P33207 from (Arabidopsis thaliana) | chr1:8640725-8643467 FORWARD | Aliases: F21J9.2, F21J9.34, F21J9_34	10.7	11.3	-0.7	-2.3	100.0%	-2.9
1900	AT3G01660.1 | expressed protein, similar to putative protein GB:CAB45319 (Arabidopsis thaliana) | chr3:245485-246439 FORWARD | Aliases: F4P13.20, F4P13_20	4.9	4.1	0.9	2.3	100.0%	-2.5
1901	AT5G24970.1 | ABC1 family protein, contains Pfam domain, PF03109: ABC1 family | chr5:8604339-8608820 REVERSE | Aliases: F6A4.180, F6A4_180	6.4	7.3	-0.9	-2.3	100.0%	-2.6
1902	AT1G27290.1 | expressed protein | chr1:9481546-9482899 FORWARD | Aliases: F17L21.8, F17L21_8	7.2	6.6	0.7	2.3	100.0%	-2.8
1903	AT4G16260.1 | glycosyl hydrolase family 17 protein, similar to glucan endo-1,3-beta-glucosidase, basic vacuolar isoform precursor SP:P52407 from (Hevea brasiliensis) | chr4:9200025-9201544 REVERSE | Aliases: DL4170C, FCAALL.386	3.3	4.2	-0.8	-2.3	100.0%	-2.6
1904	AT1G56580.1 | expressed protein, contains Pfam profile PF04398: Protein of unknown function, DUF538 | chr1:21201924-21202621 REVERSE | Aliases: F25P12.97, F25P12_97	10.0	10.8	-0.8	-2.3	100.0%	-3.1
1905	AT4G21120.1 | Symbol: AAT1 | amino acid permease family protein, similar to cationic amino acid transporter-1 (Rattus norvegicus) GI:1589917; contains Pfam profile PF00324: Amino acid permease | chr4:11270056-11273982 FORWARD | Aliases: CAT1, CATIONIC AMINO ACID TRANSPORTER 1, F7J7.60, F7J7_60	4.8	4.3	0.4	2.3	100.0%	-2.9
1906	AT5G08260.1 | Symbol: SCPL35 |	4.2	4.9	-0.6	-2.3	100.0%	-3.0
1907	AT5G10900.1 | calcineurin-like phosphoesterase family protein, contains Pfam profile: PF00149 calcineurin-like phosphoesterase	2.4	2.6	-0.3	-2.3	100.0%	-3.4
1908	AT5G41000.1 | oligopeptide transporter OPT family protein, contains Pfam profile PF03169: OPT oligopeptide transporter protein	3.6	4.4	-0.8	-2.3	100.0%	-2.7
1909	AT1G51060.1 | histone H2A, putative, similar to histone H2A GI:7595337 from Arabidopsis thaliana, Triticum aestivum GI:536892, Picea abies SP:P35063; contains Pfam profile PF00125 Core histone H2A/H2B/H3/H4	8.9	9.9	-1.0	-2.3	100.0%	-2.7
1910	AT5G67160.1 | transferase family protein, similar to anthranilate N-hydroxycinnamoyl/benzoyltransferase, Dianthus caryophyllus (gi:2239091); contains Pfam transferase family domain PF002458 | chr5:26814839-26816267 REVERSE | Aliases: K21H1.12, K21H1_12	8.1	6.9	1.2	2.3	100.0%	-2.5
1911	AT1G61550.1 | S-locus protein kinase, putative, similar to receptor protein kinase (Ipomoea trifida) gi:836954:gb:AAC23542; contains S-locus glycoprotein family domain, Pfam:PF00954 | chr1:22708531-22711491 REVERSE | Aliases: T25B24.10, T25B24_10	4.3	5.1	-0.8	-2.3	100.0%	-2.5
1912	AT1G31910.1 | GHMP kinase family protein, contains TIGRFAM profile TIGR01219: phosphomevalonate kinase; contains Pfam PF00288: GHMP kinases putative ATP-binding protein domain; similar to Phosphomevalonate kinase (EC 2.7.4.2) (Swiss-Prot:P24521) (Saccharomyces cerevisiae) | chr1:11459030-11461805 FORWARD | Aliases: F5M6.9, F5M6_9	12.0	12.3	-0.3	-2.3	100.0%	-3.5
1913	AT5G58250.1 | expressed protein | chr5:23576703-23577739 FORWARD | Aliases: MCK7.12, MCK7_12	6.1	7.1	-1.1	-2.3	100.0%	-2.5
1914	AT4G14010.1 | Symbol: RALFL32 | rapid alkalinization factor (RALF) family protein, similar to RALF precursor (Nicotiana tabacum) GI:16566316 | chr4:8092832-8093377 REVERSE | Aliases: DL3045C, FCAALL.140, RALF LIKE 32	9.0	8.3	0.8	2.3	100.0%	-3.0
1915	AT3G59820.2 | similar to calcium-binding EF hand family protein [Arabidopsis thaliana] (TAIR:At1g65540.1); similar to OSJNBa0067K08.12 [Oryza sativa (japonica cultivar-group)] (GB:XP_473035.1) | chr3:22109005-22112736 REVERSE | Aliases: F24G16.90	6.9	8.1	-1.2	-2.3	100.0%	-2.5
1916	AT2G32240.1 | expressed protein, contains Pfam profile: PF04508 viral A-type inclusion protein repeat | chr2:13691376-13695401 REVERSE | Aliases: F22D22.1, F22D22_1	9.7	8.8	0.9	2.3	100.0%	-2.9
1917	AT5G16610.2 | expressed protein | chr5:5444967-5448316 REVERSE | Aliases: MTG13.5, MTG13_5	5.2	4.9	0.4	2.3	100.0%	-3.1
1918	AT2G40030.1 | Symbol: NRPD1b |	3.6	4.0	-0.4	-2.3	100.0%	-3.0

Rank	Description	15.dpi	5.dpi	M	t	adj.q	B
1919	AT5G65685.1 | soluble glycogen synthase-related, contains weak similarity to Soluble glycogen synthase, chloroplast precursor (EC 2.4.1.11) (SS III) (Swiss-Prot:Q43846) (Solanum tuberosum) | chr5:26290361-26293145 REVERSE | Aliases: None	6.8	6.2	0.6	2.3	100.0%	-2.7
1920	AT2G46410.1 | Symbol: CPC | myb-related protein CAPRICE (CPC), identical to myb-related protein CAPRICE (CPC) GI:2346965 from (Arabidopsis thaliana) | chr2:19056245-19057414 REVERSE | Aliases: CAPRICE, F11C10.10, MYB FAMILY TRANSCRIPTION FACTOR CPC	5.2	4.5	0.7	2.3	100.0%	-2.8
1921	AT5G22270.1 | expressed protein, similar to unknown protein (gb AAF02129.1) | chr5:7372236-7373105 REVERSE | Aliases: T6G21.5	3.2	5.2	-2.0	-2.3	100.0%	-2.5
1922	AT3G10020.1 | expressed protein | chr3:3091048-3091771 REVERSE | Aliases: T22K18.16	8.8	8.1	0.8	2.3	100.0%	-2.9
1923	AT1G74370.1 | zinc finger (C3HC4-type RING finger) family protein, contains Pfam profile: PF00097 zinc finger, C3HC4 type (RING finger) | chr1:27961323-27963018 REVERSE | Aliases: F1M20.5, F1M20_5	6.6	6.0	0.6	2.3	100.0%	-2.7
1924	AT3G16450.3 | similar to jacalin lectin family protein [Arabidopsis thaliana] (TAIR:At3g16440.1); similar to myrosinase binding protein [Brassica napus] (GB:CAA70587.1); contains InterPro domain Jacalin-related lectin (InterPro:IPR001229)	4.5	5.2	-0.8	-2.3	100.0%	-2.6
1925	AT5G11090.1 | serine-rich protein-related, contains some similarity to serine-rich proteins | chr5:3524681-3525847 FORWARD | Aliases: T5K6.80, T5K6_80	5.5	4.4	1.1	2.3	100.0%	-2.6
1926	AT3G03110.1 | Symbol: XPO1B | exportin 1, putative, strong similarity to Exportin1 (XPO1) protein (Arabidopsis thaliana) GI:7671510; contains Pfam profile PF03810: Importin-beta N-terminal domain | chr3:708943-717100 FORWARD | Aliases: CRM1B, T17B22.20, T17B22_20	3.9	4.7	-0.8	-2.3	100.0%	-2.8
1927	AT3G62390.1 | expressed protein, several hypothetical proteins - Arabidopsis thaliana | chr3:23097996-23100166 REVERSE | Aliases: T12C14.90	4.0	4.7	-0.7	-2.3	100.0%	-2.7
1928	AT5G62860.1 | F-box family protein-related, contains TIGRFAM TIGR01640: F-box protein interaction domain | chr5:25249858-25251793 FORWARD | Aliases: MQB2.19, MQB2_19	3.5	3.1	0.4	2.3	100.0%	-3.0
1929	AT5G41700.4 | Symbol: UBC8 | ubiquitin-conjugating enzyme 8 (UBC8), E2; identical to gi:297882, SP:P35131 | chr5:16693070-16694737 FORWARD | Aliases: ATUBC8, MBK23.24, MBK23_24, UBC8, UBIQUITIN CONJUGATING ENZYME, UBIQUITIN CONJUGATING ENZYME 8	12.8	12.2	0.6	2.3	100.0%	-3.2
1930	AT5G41190.1 | expressed protein, ; expression supported by MPSS | chr5:16504856-16507240 REVERSE | Aliases: MEE6.26, MEE6_26	6.2	6.9	-0.7	-2.3	100.0%	-2.8
1931	AT5G23020.1 | Symbol: MAM L | 2-isopropylmalate synthase 2 (IMS2), identical to 2-isopropylmalate synthase (IMS2) (Arabidopsis thaliana) GI:12330689 | chr5:7718206-7721842 FORWARD | Aliases: 2 ISOPROPYLMALATE SYNTHASE 2, IMS2, METHYLTHIOALKYMALATE SYNTHASE LIKE, MYJ24.1, MYJ24_1	10.0	9.7	0.3	2.3	100.0%	-3.3
1932	AT3G26770.1 | short-chain dehydrogenase/reductase (SDR) family protein, similar to sex determination protein tasselseed 2 SP:P50160 from (Zea mays) | chr3:9846721-9848385 FORWARD | Aliases: MDJ14.1	10.1	9.3	0.8	2.3	100.0%	-2.6
1933	AT5G53870.1 | plastocyanin-like domain-containing protein, contains similarity to SP:Q02917 Early nodulin 55-2 precursor {Glycine max}; PF02298: Plastocyanin-like domain | chr5:21887259-21888454 REVERSE | Aliases: K19P17.3, K19P17_3	2.3	2.1	0.2	2.3	100.0%	-3.9
1934	AT5G40390.1 | raffinose synthase family protein, similar to galactinol-raffinose galactosyltransferase (Vigna angularis) GI:6634701, seed imbibition protein GB:AAA32975 GI:167100 from (Hordeum vulgare); contains Pfam profile PF05691: Raffinose synthase or seed imbibition protein Sip1 | chr5:16178743-16182585 FORWARD | Aliases: MPO12.100, MPO12_100	5.5	6.2	-0.7	-2.3	100.0%	-2.6
1935	AT5G04150.1 | basic helix-loop-helix (bHLH) family protein, contains Pfam profile: PF00010 helix-loop-helix DNA-binding domain; PMID: 12679534	3.3	3.8	-0.5	-2.3	100.0%	-2.8
1936	AT5G02870.1 | 60S ribosomal protein L4/L1 (RPL4D), 60S roibosomal protein L4, Arabidopsis thaliana, EMBL:CAA79104 | chr5:657784-659716 FORWARD | Aliases: F9G14.180, F9G14_180	11.3	11.8	-0.5	-2.3	100.0%	-3.0
1937	AT5G19250.1 | expressed protein | chr5:6471951-6472947 FORWARD | Aliases: T24G5.150, T24G5_150	4.7	5.4	-0.6	-2.3	100.0%	-2.9
1938	AT1G27370.3 | similar to squamosa promoter-binding protein-like 11 (SPL11) [Arabidopsis thaliana] (TAIR:At1g27360.2); similar to squamosa promoter-binding protein-like 11 (SPL11) [Arabidopsis thaliana] (TAIR:At1g27360.1); similar to squamosa promoter binding protein 2-like [Oryza sativa (japonica cultivar-group)] (GB:BAD54038.1); contains InterPro domain SBP (InterPro:IPR004333) | chr1:9505126-9508267 REVERSE | Aliases: F17L21.15, F17L21_15	3.5	4.0	-0.6	-2.3	100.0%	-2.8
1939	AT5G05000.3 | Symbol: OEP34 | translocate of chloroplast 34 (TOC34) / GTP-binding protein (OEP34), contains Pfam PF04548: AIG1 family;contains TIGRFAM TIGR00991: GTP-binding protein and TIGR00231: small GTP-binding protein domain; 99.7% identical to atToc34 protein (GI:11557975) (Arabidopsis thaliana); similar to Chain A,	8.6	9.4	-0.8	-2.3	100.0%	-2.6
1940	AT1G22600.1 | expressed protein | chr1:7987297-7989176 REVERSE | Aliases: F12K8.5, F12K8_5	3.7	2.7	0.9	2.3	100.0%	-2.8

Rank	Description	15.dpi	5.dpi	M	t	adj.q	B
1941	AT4G26300.1 | Symbol: EMB1027 | arginyl-tRNA synthetase, putative / arginine--tRNA ligase, putative, similar to SP:P37880 Arginyl-tRNA synthetase (EC 6.1.1.19) (Arginine--tRNA ligase) (ArgRS) {Cricetulus longicaudatus}; contains Pfam profiles PF00750: arginyl-tRNA synthetase, PF03485: arginyl-tRNA synthetase N-terminal domain | chr4:13307986-13313118 REVERSE | Aliases: EMB1027, EMBRYO DEFECTIVE 1027, T25K17.110, T25K17_110	8.4	9.4	-1.0	-2.3	100.0%	-2.7
1942	AT5G07590.1 | WD-40 repeat protein family, contains 3 WD-40 repeats (PF00400); similarity to WD-repeat protein 8 (WDR8)(SP:Q9P2S5) (HOMO SAPIENS) | chr5:2401621-2403855 REVERSE | Aliases: MBK20.2, MBK20_2	4.5	5.0	-0.5	-2.3	100.0%	-2.9
1943	AT5G66410.1 | expressed protein | chr5:26537032-26538922 FORWARD | Aliases: K1F13.6, K1F13_6	8.7	9.4	-0.7	-2.3	100.0%	-2.7
1944	AT1G18680.1 | HNH endonuclease domain-containing protein, contains Pfam domain PF01844: HNH endonuclease | chr1:6431930-6433437 REVERSE | Aliases: F6A14.21, F6A14_21	6.3	5.7	0.6	2.3	100.0%	-2.7
1945	AT3G47210.1 | expressed protein, contains Pfam profile PF03140: Plant protein of unknown function | chr3:17396298-17398229 FORWARD | Aliases: F13I12.260	5.5	4.7	0.8	2.3	100.0%	-2.6
1946	AT3G02920.1 | replication protein-related, similar to replication protein A 30kDa (Oryza sativa (japonica cultivar-group)) GI:13516746; contains InterPro entry IPR004365: OB-fold nucleic acid binding domain	5.9	6.8	-1.0	-2.3	100.0%	-2.7
1947	AT3G01640.1 | GHMP kinase family protein, contains GHMP kinases putative ATP-binding protein domain, Pfam:PF00288 | chr3:239388-241450 FORWARD | Aliases: F4P13.18, F4P13_18	6.1	6.8	-0.7	-2.3	100.0%	-2.6
1948	AT5G47550.1 | cysteine protease inhibitor, putative / cystatin, putative, similar to SP:P09229 Cysteine proteinase inhibitor-I (Oryzacystatin-I) {Oryza sativa}; contains Pfam profile PF00031: Cystatin domain | chr5:19303676-19304221 REVERSE | Aliases: MNJ7.14, MNJ7_14	6.2	7.2	-1.0	-2.3	100.0%	-2.5
1949	AT4G36810.1 | Symbol: GGPS1 | geranylgeranyl pyrophosphate synthase (GGPS1) / GGPP synthetase / farnesyltranstransferase, identical to GI:413730 to geranylgeranyl pyrophosphate synthase, chloroplast precursor GB:P34802 from (Arabidopsis thaliana) synonymous with farnesyltranstransferase | chr4:17343378-17344869 FORWARD | Aliases: GERANYLGERANYL PYROPHOSPHATE SYNTHASE 1	6.1	5.5	0.6	2.3	100.0%	-2.9
1950	AT3G19590.1 | WD-40 repeat family protein / mitotic checkpoint protein, putative, contains 5 WD-40 repeats (PF00400) (1 weak); similar to testis mitotic checkpoint protein BUB3 (GB:AAC28439,SP:O43684)(Homo sapiens)	5.8	6.6	-0.8	-2.3	100.0%	-2.7
1951	AT5G44110.2 | Symbol: POP1 | similar to ABC transporter family protein [Arabidopsis thaliana] (TAIR:At1g03905.1); similar to hypothetical protein UM03153.1 [Ustilago maydis 521] (GB:EAK84383.1); contains InterPro domain ABC transporter (InterPro:IPR003439); contains InterPro domain ATP/GTP-binding site motif A (P-loop) (InterPro:IPR001687)	8.6	9.4	-0.8	-2.3	100.0%	-2.9
1952	AT3G02770.1 | dimethylmenaquinone methyltransferase family protein, similar to bacterial S-adenosylmethionine:2-demethylmenaquinone methyltransferases; contains Pfam profile PF03737: Dimethylmenaquinone methyltransferase | chr3:600900-602218 REVERSE | Aliases: F13E7.29, F13E7_29	10.0	9.1	0.9	2.3	100.0%	-2.6
1953	AT2G21045.1 | similar to senescence-associated family protein [Arabidopsis thaliana] (TAIR:At5g66170.1); similar to Ntdin [Nicotiana tabacum] (GB:BAA88985.2); contains InterPro domain Rhodanese-like (InterPro:IPR001763) | chr2:9034933-9036101 FORWARD | Aliases: None	4.0	5.0	-1.0	-2.3	100.0%	-2.5
1954	AT3G20440.1 | Symbol: EMB2729 | similar to 1,4-alpha-glucan branching enzyme / starch branching enzyme class II (SBE2-1) [Arabidopsis thaliana] (TAIR:At2g36390.1); similar to glucan (1,4-alpha-), branching enzyme 1 [Mus musculus] (GB:NP_083079.1) | chr3:7123203-7130442 REVERSE | Aliases: EMB2729, EMBRYO DEFECTIVE 2729, MQC12.20	3.1	3.8	-0.7	-2.3	100.0%	-2.7
1955	AT1G32190.1 | expressed protein | chr1:11592935-11595962 REVERSE | Aliases: F3C3.3, F3C3_3	4.2	4.7	-0.5	-2.3	100.0%	-2.9
1956	AT5G52550.1 | expressed protein | chr5:21344938-21347362 REVERSE | Aliases: F6N7.3, F6N7_3	6.1	5.5	0.6	2.3	100.0%	-2.8
1957	AT5G23110.1 | zinc finger (C3HC4-type RING finger) family protein, contains Pfam profile: PF00097 zinc finger, C3HC4 type (RING finger) | chr5:7758310-7775512 FORWARD | Aliases: MYJ24.15, MYJ24_15	4.7	5.6	-0.9	-2.3	100.0%	-2.8
1958	AT3G49180.1 | transducin family protein / WD-40 repeat family protein, contains 5 WD-40 repeats (PF00400); GTP-binding protein beta chain homolog, Nicotiana tabacum, PIR:T16970 | chr3:18240751-18243209 FORWARD | Aliases: F2K15.40	4.4	4.9	-0.5	-2.3	100.0%	-2.9
1959	AT5G17930.1 | similar to MIF4G domain-containing protein / MA3 domain-containing protein [Arabidopsis thaliana] (TAIR:At1g80930.1); similar to nucleolar protein with MIF4G domain 1 [Homo sapiens] (GB:NP_612409.1); contains InterPro domain Initiation factor eIF-4 gamma, MA3 (InterPro:IPR003891); contains InterPro domain Initiation factor eIF-4 gamma, middle (InterPro:IPR003890) | chr5:5940000-5944428 REVERSE | Aliases: MCM23.1, MCM23_1	6.1	6.9	-0.9	-2.3	100.0%	-2.7

Rank	Description	15.dpi	5.dpi	M	t	adj.q	B
1960	AT2G02170.1 | remorin family protein, contains Pfam domain, PF03763: Remorin, C-terminal region | chr2:556492-558797 REVERSE | Aliases: F5O4.6, F5O4_6	5.1	5.8	-0.7	-2.3	100.0%	-2.7
1961	AT1G11960.1 | early-responsive to dehydration protein-related / ERD protein-related, low similarity to ERD4 protein (Arabidopsis thaliana) GI:15375406	5.1	4.4	0.7	2.3	100.0%	-2.9
1962	AT3G60440.1 | similar to expressed protein [Arabidopsis thaliana] (TAIR:At3g60450.1); similar to COG0406: Fructose-2,6-bisphosphatase [Trichodesmium erythraeum IMS101] (GB:ZP_00325866.1) | chr3:22348768-22350421 FORWARD | Aliases: T8B10.100	4.3	4.9	-0.7	-2.3	100.0%	-2.6
1963	AT1G51680.2 | Symbol: 4CL1 | 4-coumarate--CoA ligase 1 / 4-coumaroyl-CoA synthase 1 (4CL1), identical to SP:Q42524 4-coumarate--CoA ligase 1 (EC 6.2.1.12) (4CL 1) (4-coumaroyl-CoA synthase 1) {Arabidopsis thaliana} | chr1:19163097-19165220 REVERSE | Aliases: 4 COUMARATE:COA LIGASE, 4 COUMARATE:COA LIGASE 1, 4CL.1, AT4CL1, F19C24.11, F19C24_11	8.8	11.0	-2.2	-2.3	100.0%	-2.8
1964	AT5G10440.1 | Symbol: CYCD4;2 | cyclin family protein, similar to cyclin D2.1 protein (Nicotiana tabacum) GI:4160298; contains Pfam profile PF00134: Cyclin, N-terminal domain | chr5:3280517-3282393 REVERSE | Aliases: CYCLIN D4;2, F12B17.210, F12B17_210	3.3	3.8	-0.5	-2.3	100.0%	-2.8
1965	AT2G23680.1 | stress-responsive protein, putative, similar to cold acclimation WCOR413-like protein gamma form (Hordeum vulgare) gi:18449100:gb:AAL69988; similar to stress-regulated protein SAP1 (Xerophyta viscosa) gi:21360378:gb:AAM47505	3.6	3.2	0.3	2.3	100.0%	-3.4
1966	AT5G09830.1 | BolA-like family protein, contains Pfam profile: PF01722 BolA-like protein | chr5:3057658-3058822 REVERSE | Aliases: MYH9.4, MYH9_4	9.8	9.2	0.6	2.3	100.0%	-2.8
1967	AT3G52155.1 | expressed protein | chr3:19354673-19355827 FORWARD | Aliases: None	3.4	3.0	0.4	2.3	100.0%	-3.0
1968	AT1G02450.1 | NPR1/NIM1-interacting protein 1 (NIMIN-1), identical to NIMIN-1 protein (Arabidopsis thaliana) gi:12057154:emb:CAC19844; identical to cDNA NIMIN-1 protein (nimin-1 gene)GI:12057153 | chr1:497976-498516 REVERSE | Aliases: T6A9.23	3.8	3.2	0.5	2.3	100.0%	-3.1
1969	AT5G44130.1 | fasciclin-like arabinogalactan-protein, putative, similar to gi_13377784_gb_AAK20861 | chr5:17778317-17779230 FORWARD | Aliases: MLN1.5, MLN1_5	5.2	4.3	0.9	2.3	100.0%	-2.6
1970	AT5G16310.1 | ubiquitin carboxyl-terminal hydrolase family 1 protein, similar to 26S proteasome regulatory complex subunit p37A (Drosophila melanogaster) GI:6434962; contains Pfam profile PF01088: Ubiquitin carboxyl-terminal hydrolase, family 1 | chr5:5342062-5344286 REVERSE | Aliases: MQK4.3, MQK4_3	4.8	5.4	-0.6	-2.3	100.0%	-2.7
1971	AT5G09420.1 | chloroplast outer membrane translocon subunit, putative, similar to component of chloroplast outer membrane translocon Toc64 (Pisum sativum) GI:7453538; contains Pfam profiles PF01425: Amidase, PF00515: TPR Domain | chr5:2928317-2931950 FORWARD | Aliases: T5E8.220, T5E8_220	3.3	3.8	-0.5	-2.3	100.0%	-3.0
1972	AT3G02540.2 | ubiquitin family protein, contains Pfam profiles PF00240: Ubiquitin family, PF00627: UBA/TS-N domain; | chr3:533461-536302 REVERSE | Aliases: F16B3.17, F16B3_17	8.2	7.1	1.1	2.3	100.0%	-2.6
1973	AT2G32120.2 | Symbol: HSP70T 2 | heat shock protein 70 family protein / HSP70 family protein, similar to SP:P22953 Heat shock cognate 70 kDa protein 1 (Hsc70.1) {Arabidopsis thaliana}; contains InterPro accession IPR001023: Heat shock protein Hsp70 | chr2:13658587-13660998 REVERSE | Aliases: F22D22.13, F22D22_13, HSP70T 2	5.4	5.0	0.5	2.3	100.0%	-2.8
1974	AT5G54650.2 | formin homology 2 domain-containing protein / FH2 domain-containing protein, contains formin homology 2 domain, Pfam:PF02181 | chr5:22214855-22219249 REVERSE | Aliases: MRB17.15, MRB17_15	4.8	5.5	-0.7	-2.3	100.0%	-2.8
1975	AT5G37930.1 | seven in absentia (SINA) family protein, similar to SIAH1 protein (Brassica napus var. napus) GI:7657876; contains Pfam profile PF03145: Seven in absentia protein family | chr5:15119430-15120899 REVERSE | Aliases: K18L3.13, K18L3_13	6.4	5.7	0.6	2.3	100.0%	-2.7
1976	AT1G14400.2 | Symbol: UBC1 | ubiquitin-conjugating enzyme 1 (UBC1), E2; identical to gi:431259, SP:P25865 | chr1:4927021-4928528 REVERSE | Aliases: ATUBC1, F14L17.17, F14L17_17, UBIQUITIN CARRIER PROTEIN 1	10.8	10.1	0.7	2.3	100.0%	-3.1
1977	AT1G03400.1 | 2-oxoglutarate-dependent dioxygenase, putative, similar to 2A6 (GI:599622) and tomato ethylene synthesis regulatory protein E8 (SP:P10967); similar to ESTs emb:Z34690, gb:T04168, gb:H37738, gb:T76913, gb:T43801, amd gb:T21964 | chr1:842746-844189 REVERSE | Aliases: F21B7.39, F21B7_39	4.6	3.7	1.0	2.3	100.0%	-2.7
1978	AT3G21400.1 | expressed protein | chr3:7534639-7536515 REVERSE | Aliases: MHC9.8	5.9	6.6	-0.7	-2.3	100.0%	-2.8
1979	AT2G28350.1 | Symbol: ARF10 | auxin-responsive factor (ARF10), similar to auxin response factor 10 GI:6165644 from (Arabidopsis thaliana); identical to cDNA auxin response factor 10 (ARF10) mRNA, partial cds GI:6165643 | chr2:12121408-12123914 FORWARD | Aliases: ARF10, AUXIN RESPONSE FACTOR 10, T1B3.13, T1B3_13	6.5	5.9	0.6	2.3	100.0%	-3.0
1980	AT5G13880.1 | expressed protein | chr5:4477356-4478111 REVERSE | Aliases: MAC12.16, MAC12_16	4.1	3.7	0.4	2.3	100.0%	-3.1

Rank	Description	15.dpi	5.dpi	M	t	adj.q	B
1981	AT3G53260.1 | Symbol: PAL2 | phenylalanine ammonia-lyase 2 (PAL2), nearly identical to SP:P45724 | chr3:19755029-19757758 REVERSE | Aliases: T4D2.190	7.3	8.8	-1.5	-2.3	100.0%	-2.9
1982	AT2G39400.1 | hydrolase, alpha/beta fold family protein, similar to monoglyceride lipase from (Homo sapiens) GI:14594904, (Mus musculus) GI:2632162; contains Pfam profile PF00561: hydrolase, alpha/beta fold family | chr2:16459721-16461879 FORWARD | Aliases: F12L6.6, F12L6_6	2.6	2.8	-0.3	-2.3	100.0%	-3.5
1983	AT5G61400.1 | pentatricopeptide (PPR) repeat-containing protein, contains Pfam profile PF01535: PPR repeat | chr5:24698776-24700740 FORWARD | Aliases: MFB13.18, MFB13_18	3.3	3.6	-0.3	-2.3	100.0%	-3.2
1984	AT2G31725.1 | expressed protein | chr2:13493394-13494522 REVERSE | Aliases: T9H9.25, T9H9_25	5.0	5.5	-0.5	-2.3	100.0%	-2.9
1985	AT5G04850.1 | SNF7 family protein, contains Pfam domain, PF03357: SNF7 family | chr5:1408063-1409893 REVERSE | Aliases: MUK11.17, MUK11_17	11.3	10.5	0.8	2.2	100.0%	-2.8
1986	AT1G67710.1 | Symbol: ARR11 | two-component responsive regulator family protein / response regulator family protein, contains Pfam profile: PF00072 response regulator receiver domain; contains similarity to response regulator proteins from (Arabidopsis thaliana) | chr1:25380414-25382807 REVERSE | Aliases: F12A21.15, F12A21_15, RESPONSE REGULATOR 11	4.7	4.1	0.6	2.2	100.0%	-2.9
1987	AT2G25420.1 | transducin family protein / WD-40 repeat family protein, contains Pfam PF00400: WD domain, G-beta repeat (3 repeats) | chr2:10824096-10828129 FORWARD | Aliases: F13B15.8, F13B15_8	4.0	4.6	-0.5	-2.2	100.0%	-3.0
1988	AT5G61760.1 | Symbol: ATIPK2BETA | inositol polyphosphate 6-/3-/5-kinase 2b (IPK2b), contains Pfam domain, PF03770: inositol polyphosphate kinase | chr5:24830955-24832657 REVERSE | Aliases: IPK2B, MAC9.7, MAC9_7	6.8	7.3	-0.5	-2.2	100.0%	-3.0
1989	AT2G36660.1 | Symbol: PAB7 | polyadenylate-binding protein, putative / PABP, putative. Member of the class III family of PABP proteins. | chr2:15368400-15371477 REVERSE | Aliases: F13K3.6, F13K3_6, PAB7, POLY(A) BINDING PROTEIN 7	4.2	4.8	-0.6	-2.2	100.0%	-2.7
1990	AT1G06420.1 | expressed protein, ; expression supported by MPSS | chr1:1958986-1959651 FORWARD | Aliases: T2D23.12, T2D23_12	3.8	5.2	-1.4	-2.2	100.0%	-2.6
1991	AT2G01170.2 | amino acid permease family protein, weak similarity to GABA permease (Emericella nidulans) GI:4972245; contains Pfam profile PF00324: Amino acid permease | chr2:102210-104514 REVERSE | Aliases: F10A8.5, F10A8_5	6.1	5.0	1.0	2.2	100.0%	-2.5
1992	AT2G23420.1 | nicotinate phosphoribosyltransferase family protein / NAPRTase family protein, contains Pfam domain PF04095: Nicotinate phosphoribosyltransferase (NAPRTase) | chr2:9978845-9982272 FORWARD | Aliases: F26B6.7, F26B6_7	4.1	4.7	-0.6	-2.2	100.0%	-3.0
1993	AT4G16570.1 | protein arginine N-methyltransferase-related, contains weak similarity to protein arginine N-methyltransferase 2 (EC 2.1.1.-) (Swiss-Prot:P55345) (Homo sapiens)	3.5	4.1	-0.6	-2.2	100.0%	-3.0
1994	AT1G67600.1 | expressed protein, contains Pfam domain, PF02681: Uncharacterized BCR, COG1963 | chr1:25340090-25341325 REVERSE | Aliases: F12A21.27, F12A21_27	4.1	3.7	0.4	2.2	100.0%	-3.2
1995	AT5G50420.1 | expressed protein, strong similarity to unknown protein (gb:AAD50008.1) | chr5:20547433-20550082 REVERSE | Aliases: MXI22.14, MXI22_14	4.5	5.0	-0.6	-2.2	100.0%	-2.9
1996	AT1G22540.1 | proton-dependent oligopeptide transport (POT) family protein, contains Pfam profile: PF00854 POT family | chr1:7964031-7966425 FORWARD | Aliases: F12K8.12, F12K8_12	6.0	5.4	0.6	2.2	100.0%	-2.8
1997	AT1G50380.1 | prolyl oligopeptidase family protein, similar to oligopeptidase B (Leishmania major) GI:4581757; contains Pfam profiles PF00326: prolyl oligopeptidase family, PF02897: Prolyl oligopeptidase, N-terminal beta-propeller domain | chr1:18665977-18670146 FORWARD | Aliases: F14I3.4, F14I3_4	8.3	9.4	-1.1	-2.2	100.0%	-2.8
1998	AT1G79070.1 | SNARE-associated protein-related, contains weak similarity to SNARE-associated protein snapin (Mus musculus) gi:4206090:gb:AAD11418 | chr1:29749584-29750708 REVERSE | Aliases: YUP8H12R.31, YUP8H12R_31	3.7	3.3	0.4	2.2	100.0%	-3.1
1999	AT1G05630.2 | similar to endonuclease/exonuclease/phosphatase family protein [Arabidopsis thaliana] (TAIR:At1g65580.1); similar to endonuclease/exonuclease/phosphatase family protein [Arabidopsis thaliana] (TAIR:At2g43900.1); similar to PREDICTED: similar to Type II inositol-1,4,5-trisphosphate 5-phosphatase precursor (Phosphoinositide 5-phosphatase) (5PTase) (75 kDa inositol polyphosphate-5-phosphatase) [Rattus norvegicus] (GB:XP_342910.2); contains InterPro domain Endonuclease/exonuclease/phosphatase family (InterPro:IPR005135); contains InterPro domain Inositol polyphosphate related phosphatase family (InterPro:IPR000300); contains InterPro domain G-protein beta WD-40 repeat (InterPro:IPR001680) | chr1:1682446-1687353 FORWARD | Aliases: F3F20.8, F3F20_8	3.2	3.6	-0.4	-2.2	100.0%	-3.0
2000	AT2G37330.1 | Symbol: ALS3 | expressed protein, and genefinder | chr2:15676217-15677744 FORWARD | Aliases: ALUMINUM SENSITIVE 3, F3G5.12, F3G5_12	7.2	6.2	1.0	2.2	100.0%	-2.6

Rank	Description	15.dpi	5.dpi	M	t	adj.q	B
2001	AT1G77940.1 | 60S ribosomal protein L30 (RPL30B), similar to ribosomal protein L30 GI:388034 from (Homo sapiens) | chr1:29308791-29310259 REVERSE | Aliases: F28K19.15, F28K19_15	11.8	12.3	-0.5	-2.2	100.0%	-3.2
2002	AT2G37760.3 | aldo/keto reductase family protein, similar to chalcone reductase (Sesbania rostrata)(GI:2792155), and aldose reductase ALDRXV4 (Xerophyta viscosa)(GI:4539944), (Hordeum vulgare)(GI:728592) | chr2:15838948-15840909 FORWARD | Aliases: T8P21.6	9.4	10.3	-1.0	-2.2	100.0%	-2.8
2003	AT3G44020.1 | thylakoid lumenal P17.1 protein, contains Pfam PF00684 : DnaJ central domain (4 repeats); SP:Q9LXV9; PMID:11719511; supported by EST AI997589. | chr3:15812359-15813678 FORWARD | Aliases: T15B3.160	3.6	3.2	0.4	2.2	100.0%	-3.0
2004	AT4G24990.1 | Symbol: ATGP4 | ubiquitin family protein, contains INTERPRO:IPR000626 ubiquitin domain | chr4:12849719-12851643 REVERSE | Aliases: F13M23.130, F13M23_130	9.5	8.7	0.8	2.2	100.0%	-2.9
2005	AT1G21065.1 | expressed protein | chr1:7374199-7375778 FORWARD | Aliases: None	9.2	8.5	0.8	2.2	100.0%	-3.1
2006	AT1G15210.1 | ABC transporter family protein, Similar to gb:Z70524 GI:1514643 PDR5-like ABC transporter from Spirodela polyrrhiza and is a member of the PF:00005 ABC transporter family. ESTs gb:N97039 and gb:T43169 come from this gene | chr1:5231343-5236568 REVERSE | Aliases: F9L1.15, F9L1_15	6.1	7.0	-0.9	-2.2	100.0%	-2.7
2007	AT5G37055.1 | zinc finger (HIT type) family protein, contains Pfam profile: PF04438 HIT zinc finger | chr5:14658781-14659670 REVERSE | Aliases: None	5.4	4.9	0.5	2.2	100.0%	-3.0
2008	AT3G57530.1 | Symbol: CPK32 | calcium-dependent protein kinase, putative / CDPK, putative, similar to calmodulin-domain protein kinase CDPK isoform 7 (Arabidopsis thaliana) gi:1399277:gb:AAB03247 | chr3:21307531-21310568 REVERSE | Aliases: T8H10.130	6.7	5.7	1.0	2.2	100.0%	-2.6
2009	AT5G56740.1 | histone acetyltransferase family protein, similar to histone acetyltransferase HAT B (Zea mays) GI:5579441; identical to cDNA histone acetyltransferase partial cds GI:21637256	3.7	4.8	-1.1	-2.2	100.0%	-2.8
2010	AT3G03020.1 | expressed protein | chr3:680951-682006 FORWARD | Aliases: F13E7.3, F13E7_3	4.2	3.6	0.6	2.2	100.0%	-2.9
2011	AT5G62290.1 | nucleotide-sensitive chloride conductance regulator (ICln) family protein, contains PF03517: Nucleotide-sensitive chloride conductance regulator (ICln) | chr5:25036713-25038357 FORWARD | Aliases: MMI9.12, MMI9_12	5.6	6.1	-0.5	-2.2	100.0%	-2.9
2012	AT1G75370.1 | SEC14 cytosolic factor, putative / phosphatidylinositol transfer-like protein, putative, similar to phosphatidylinositol transfer-like protein III (GI:14486705) (Lotus japonicus); contains Pfam PF00650 : CRAL/TRIO domain; contains Pfam PF03765 : CRAL/TRIO, N-terminu | chr1:28280105-28283559 REVERSE | Aliases: F1B16.10, F1B16_10	5.3	5.8	-0.5	-2.2	100.0%	-2.9
2013	AT4G26900.1 | Symbol: AT HF | imidazole glycerol phosphate synthase hisHF, chloroplast / IGP synthase / ImGPP synthase / IGPS, identical to SP:Q9SZ30 Imidazole glycerol phosphate synthase hisHF, chloroplast precursor (IGP synthase) {Arabidopsis thaliana} | chr4:13515488-13520317 FORWARD | Aliases: HISHF	8.5	9.4	-0.9	-2.2	100.0%	-2.6
2014	AT3G52400.1 | Symbol: SYP122 |	5.7	4.7	1.0	2.2	100.0%	-2.6
2015	AT5G04410.1 | Symbol: NAC2 | no apical meristem (NAM) family protein, contains Pfam PF02365: No apical meristem (NAM) protein; supporting cDNA gi:6456750:gb:AF201456.1:AF201456 | chr5:1243759-1247015 FORWARD | Aliases: ANAC078, T19N18.11	8.6	8.0	0.6	2.2	100.0%	-3.0
2016	AT1G65000.1 | expressed protein | chr1:24150707-24152352 FORWARD | Aliases: F13O11.34, F13O11_34	6.4	5.8	0.6	2.2	100.0%	-2.8
2017	AT1G51200.1 | zinc finger (AN1-like) family protein, contains Pfam domains, PF01428: AN1-like Zinc finger and PF01754: A20-like zinc finger | chr1:18988102-18990236 FORWARD | Aliases: F11M15.7, F11M15_7	11.0	9.9	1.0	2.2	100.0%	-2.9
2018	AT3G62360.1 | expressed protein | chr3:23083761-23091518 REVERSE | Aliases: T12C14.60	7.5	8.5	-1.0	-2.2	100.0%	-2.8
2019	AT2G45320.1 | expressed protein | chr2:18691190-18693219 REVERSE | Aliases: F4L23.17	7.0	6.5	0.5	2.2	100.0%	-3.0
2020	AT5G05940.1 | member of KPP-like gene family, homolog of KPP (kinase partner protein) gene in tomato; expressed protein, contains Pfam profile PF03759: Domain of unknown function (DUF315) | chr5:1785928-1788437 FORWARD | Aliases: K18J17.10, K18J17_10, KINASE PARTNER PROTEIN LIKE, KPP LIKE	2.8	3.4	-0.5	-2.2	100.0%	-3.0
2021	AT3G53420.2 | Symbol: PIP2A | similar to plasma membrane intrinsic protein 2B (PIP2B) / aquaporin PIP2.2 (PIP2.2) [Arabidopsis thaliana] (TAIR:At2g37170.1); similar to Plasma membrane aquaporin (PAQ2) [Raphanus sativus] (GB:BAA32778.1); contains InterPro domain MIP family (InterPro:IPR000425)	4.7	3.9	0.8	2.2	100.0%	-2.7
2022	AT5G63060.1 | similar to SEC14 cytosolic factor family protein / phosphoglyceride transfer family protein [Arabidopsis thaliana] (TAIR:At4g08690.1); similar to sec14 like protein [Oryza sativa (japonica cultivar-group)] (GB:XP_463685.1); contains InterPro domain Cellular retinaldehyde-binding)/triple function, C-terminal (InterPro:IPR001251) | chr5:25311788-25314075 REVERSE | Aliases: MDC12.2, MDC12_2	4.3	3.8	0.5	2.2	100.0%	-3.0

Rank	Description	15.dpi	5.dpi	M	t	adj.q	B
2023	AT1G60160.1 | potassium transporter family protein, similar to potassium transporter HAK2p (Mesembryanthemum crystallinum) gi:14091471:gb:AAK53759; KUP/HAK/KT Transporter family member, PMID:11500563; contains Pfam profile PF02705: K+ potassium transporter | chr1:22191995-22195060 REVERSE | Aliases: T13D8.5, T13D8_5	4.0	3.6	0.4	2.2	100.0%	-3.1
2024	AT4G36000.1 | pathogenesis-related thaumatin family protein, similar to thaumatin-like protein precursor (Pyrus pyrifolia) GI:3241854; contains Pfam profile PF00314: Thaumatin family	3.5	3.2	0.3	2.2	100.0%	-3.4
2025	AT2G36310.1 | inosine-uridine preferring nucleoside hydrolase family protein, similar to Chain A, Crystal Structure Of Nucleoside Hydrolase From Leishmania MajorGI:8569431; contains Pfam profile PF01156: Inosine-uridine preferring nucleoside hydrolase | chr2:15231576-15233951 REVERSE | Aliases: F2H17.8, F2H17_8	7.0	7.7	-0.7	-2.2	100.0%	-2.9
2026	AT2G03310.1 | expressed protein | chr2:1006253-1007180 REVERSE | Aliases: T4M8.26, T4M8_26	5.1	4.6	0.6	2.2	100.0%	-2.9
2027	AT3G03960.1 | chaperonin, putative, similar to SWISS-PROT:P42932- T-complex protein 1, theta subunit (TCP-1-theta) (Mus musculus); contains Pfam:PF00118 domain, TCP-1/cpn60 chaperonin family	6.1	7.2	-1.1	-2.2	100.0%	-2.7
2028	AT5G41350.1 | zinc finger (C3HC4-type RING finger) family protein, contains Pfam profile: PF00097 zinc finger, C3HC4 type (RING finger) | chr5:16558751-16561141 REVERSE | Aliases: MYC6.6, MYC6_6	6.3	5.8	0.5	2.2	100.0%	-3.0
2029	AT1G17710.1 | similar to expressed protein [Arabidopsis thaliana] (TAIR:At1g73010.1); similar to putative phosphatase [Lycopersicon esculentum] (GB:CAD30863.1); contains InterPro domain HAD-superfamily subfamily IB hydrolase, hypothetical 1 (InterPro:IPR006384); contains InterPro domain HAD-superfamily hydrolase, subfamily IB (PSPase-like) (InterPro:IPR006383) | chr1:6090553-6092011 REVERSE | Aliases: F11A6.5, F11A6_5	4.0	3.4	0.6	2.2	100.0%	-2.9
2030	AT3G59010.1 | pectinesterase family protein, contains Pfam profile: PF01095 pectinesterase | chr3:21813782-21816191 REVERSE | Aliases: F17J16.60	4.6	3.9	0.7	2.2	100.0%	-2.9
2031	AT1G58270.1 | Symbol: ZW9 | meprin and TRAF homology domain-containing protein / MATH domain-containing protein, similar to ubiquitin-specific protease 12 (Arabidopsis thaliana) GI:11993471; contains Pfam profile PF00917: MATH domain | chr1:21615781-21617782 REVERSE | Aliases: F19C14.11, F19C14_11	7.9	9.0	-1.1	-2.2	100.0%	-2.9
2032	AT5G06320.1 | Symbol: NHL3 | harpin-induced family protein / HIN1 family protein / harpin-responsive family protein / NDR1/HIN1-like protein 3, similar to harpin-induced protein hin1 (GI:1619321)(Nicotiana tabacum) | chr5:1930625-1931769 REVERSE | Aliases: MHF15.16, MHF15_16	6.8	5.9	0.9	2.2	100.0%	-2.9
2033	AT1G69523.1 | UbiE/COQ5 methyltransferase family protein, low similarity to SP:Q05197 Phosphatidylethanolamine N-methyltransferase (EC 2.1.1.17) (Rhodopseudomonas sphaeroides) {Rhodobacter sphaeroides}; contains Pfam profile PF01209: methlytransferase, UbiE/COQ5 family | chr1:26133214-26134912 FORWARD | Aliases: None	3.6	3.0	0.5	2.2	100.0%	-3.1
2034	AT5G57130.1 | similar to heat shock protein-related [Arabidopsis thaliana] (TAIR:At4g29920.1); similar to heat shock protein-related-like [Oryza sativa (japonica cultivar-group)] (GB:XP_465522.1) | chr5:23162206-23166794 FORWARD | Aliases: MUL3.8, MUL3_8	4.8	5.1	-0.3	-2.2	100.0%	-3.5
2035	AT3G51010.1 | expressed protein | chr3:18957681-18959137 REVERSE | Aliases: F24M12.50	6.5	7.7	-1.2	-2.2	100.0%	-2.7
2036	AT1G23040.1 | hydroxyproline-rich glycoprotein family protein, contains proline-rich domains, INTERPRO:IPR000694 | chr1:8164948-8165851 FORWARD | Aliases: F19G10.19	6.6	5.9	0.7	2.2	100.0%	-2.7
2037	AT5G23580.1 | Symbol: CDPK9 | calcium-dependent protein kinase 9 (CDPK9), identical to calcium-dependent protein kinase (Arabidopsis thaliana) gi:836938:gb:AAA67653; contains protein kinase domain, Pfam:PF00069; contains EF hand domain (calcium-binding EF-hand), Pfam:PF00036, INTERPRO:IPR002048 | chr5:7949989-7952535 REVERSE | Aliases: ATCDPK9, CALCIUM DEPENDENT PROTEIN KINASE, CALCIUM DEPENDENT PROTEIN KINASE 9, CPK12, MQM1.15, MQM1_15	6.4	7.3	-1.0	-2.2	100.0%	-2.8
2038	AT1G32360.1 | zinc finger (CCCH-type) family protein, contains Pfam domain, PF00642: Zinc finger C-x8-C-x5-C-x3-H type (and similar) | chr1:11672911-11675319 FORWARD | Aliases: F27G20.10	7.4	6.8	0.5	2.2	100.0%	-2.9
2039	AT3G02690.1 | integral membrane family protein, similar to PecM protein (GI:5852331) {Vogesella indigofera} and PecM protein (SP:P42194) (Erwinia chrysanthemi) | chr3:579593-582136 FORWARD | Aliases: F16B3.32, F16B3_32	7.4	6.5	0.9	2.2	100.0%	-2.6
2040	AT5G35735.1 | auxin-responsive family protein, similar to auxin-induced protein AIR12 GI:11357190 (Arabidopsis thaliana) | chr5:13917982-13920227 REVERSE | Aliases: None	7.0	5.8	1.1	2.2	100.0%	-2.8
2041	AT2G41370.1 | Symbol: BOP2 | ankyrin repeat family protein / BTB/POZ domain-containing protein, contains Pfam domain, PF00023: Ankyrin repeat and Pfam domain, PF00651: BTB/POZ domain | chr2:17244823-17247518 REVERSE | Aliases: BLADE ON PETIOLE2, F13H10.8, F13H10_8	4.4	4.9	-0.5	-2.2	100.0%	-2.9

Rank	Description	15.dpi	5.dpi	M	t	adj.q	B
2042	AT2G42880.1 | Symbol: ATMPK20 | mitogen-activated protein kinase, putative / MAPK, putative (MPK20), mitogen-activated protein kinase (MAPK)(AtMPK20), PMID:12119167 | chr2:17847465-17851439 REVERSE | Aliases: F7D19.12, F7D19_12	7.5	8.5	-0.9	-2.2	100.0%	-2.8
2043	AT2G36620.1 | 60S ribosomal protein L24 (RPL24A) | chr2:15357486-15358944 REVERSE | Aliases: F13K3.2, F13K3_2	10.4	10.9	-0.6	-2.2	100.0%	-2.9
2044	AT5G17490.1 | Symbol: RGL3 | gibberellin response modulator, putative / gibberellin-responsive modulator, putative, putative member of the VHIID domain transcription factor family RGAL - Arabidopsis thaliana, EMBL:AJ224957 | chr5:5764065-5766079 REVERSE | Aliases: K3M16.60, K3M16_60, RGA LIKE PROTEIN	3.0	3.7	-0.6	-2.2	100.0%	-2.9
2045	AT1G20490.1 | AMP-dependent synthetase and ligase family protein, similar to SP:Q42524 and SP:Q9S725; contains Pfam AMP-binding enzyme domain PF00501 | chr1:7097283-7099685 REVERSE | Aliases: F5M15.28, F5M15_28	4.3	5.0	-0.7	-2.2	100.0%	-3.0
2046	AT4G17550.1 | transporter-related, similar to cAMP inducible 2 protein (Mus musculus) GI:4580997, glycerol 3-phosphate permease (Homo sapiens) GI:7543982; contains Pfam profile PF00083: major facilitator superfamily protein | chr4:9777661-9779751 REVERSE | Aliases: DL4810C, FCAALL.41	5.0	4.2	0.8	2.2	100.0%	-2.9
2047	AT1G25560.1 | AP2 domain-containing transcription factor, putative, similar to DNA-binding protein RAV2 GI:3868859 from (Arabidopsis thaliana)	5.2	4.7	0.5	2.2	100.0%	-2.9
2048	AT2G02760.1 | Symbol: ATUBC2 |	11.6	11.1	0.6	2.2	100.0%	-3.3
2049	AT4G08770.1 | peroxidase, putative, identical to class III peroxidase ATP38 (Arabidopsis thaliana) gi:17530568:gb:AAL40851; similar to peroxidase C2 precursor (Armoracia rusticana) SWISS-PROT: P17179; identical to cDNA class III peroxidase ATP38 GI:17530567 | chr4:5598112-5600309 REVERSE | Aliases: T32A17.80, T32A17_80	5.2	6.6	-1.4	-2.2	100.0%	-2.7
2050	AT4G38780.1 | splicing factor, putative, strong similarity to splicing factor Prp8 (Homo sapiens) GI:3661610; contains Pfam profile PF01398: Mov34/MPN/PAD-1 family | chr4:18101371-18111023 REVERSE | Aliases: T9A14.60, T9A14_60	3.0	2.6	0.4	2.2	100.0%	-3.2
2051	AT3G53980.2 | protease inhibitor/seed storage/lipid transfer protein (LTP) family protein, contains Pfam protease inhibitor/seed storage/LTP family domain PF00234 | chr3:19998559-19999237 REVERSE | Aliases: F5K20.280	5.8	6.9	-1.1	-2.2	100.0%	-2.7
2052	AT1G31930.2 | extra-large guanine nucleotide binding protein, putative / G-protein, putative, similar to extra-large G-protein (XLG) (Arabidopsis thaliana) GI:3201680; contains Pfam profile PF00503: G-protein alpha subunit	3.9	4.6	-0.7	-2.2	100.0%	-3.0
2053	AT1G69020.1 | prolyl oligopeptidase family protein, similar to SP:Q59536 Protease II (EC 3.4.21.83) (Oligopeptidase B) {Moraxella lacunata}; contains Pfam profiles PF00326: prolyl oligopeptidase family, PF02897: Prolyl oligopeptidase, N-terminal beta-propeller domain; contains non-consensus GA donor splice site at intron 5 | chr1:25947549-25950860 REVERSE | Aliases: T6L1.20, T6L1_20	4.6	4.1	0.5	2.2	100.0%	-2.9
2054	AT3G09620.1 | DEAD/DEAH box helicase, putative, similar to RNA helicase GB:A57514 GI:897915 from (Rattus norvegicus); contains Pfam profiles PF00270: DEAD/DEAH box helicase, PF00271: Helicase conserved C-terminal domain | chr3:2949157-2952210 REVERSE | Aliases: F11F8.21	3.9	3.4	0.6	2.2	100.0%	-2.8
2055	AT1G54960.1 | Symbol: ANP2 | similar to NPK1-related protein kinase, putative (ANP1) [Arabidopsis thaliana] (TAIR:At1g09000.1); similar to protein kinase [Nicotiana tabacum] (GB:BAA05648.1); contains InterPro domain Serine/threonine protein kinase, active site (InterPro:IPR008271); contains InterPro domain Tyrosine protein kinase (InterPro:IPR001245); contains InterPro domain Serine/threonine protein kinase (InterPro:IPR002290); contains InterPro domain Protein kinase (InterPro:IPR000719)	4.4	5.0	-0.6	-2.2	100.0%	-2.8
2056	AT5G45430.1 | protein kinase, putative, contains similarity to male germ cell-associated kinase (Homo sapiens) gi:23268497:gb:AAN16405	6.1	5.1	1.0	2.2	100.0%	-2.7
2057	AT2G01210.1 | leucine-rich repeat transmembrane protein kinase, putative | chr2:119440-121843 REVERSE | Aliases: F10A8.9, F10A8_9	3.5	4.2	-0.6	-2.2	100.0%	-2.8
2058	AT4G39260.4 | Symbol: ATGRP8 |	11.7	9.9	1.8	2.2	100.0%	-2.6
2059	AT1G53050.1 | protein kinase family protein, contains Pfam domain, PF00069: Protein kinase domain | chr1:19775713-19779415 FORWARD | Aliases: F8L10.9, F8L10_9	5.8	6.8	-0.9	-2.2	100.0%	-2.8
2060	AT5G07030.1 | similar to aspartyl protease family protein [Arabidopsis thaliana] (TAIR:At3g54400.1); similar to putative nucleoid DNA-binding-like protein [Oryza sativa (japonica cultivar-group)] (GB:XP_463752.1); contains InterPro domain Aspartic protease A1, pepsin (InterPro:IPR001461) | chr5:2183361-2185973 REVERSE | Aliases: MOJ9.20, MOJ9_20	3.1	4.9	-1.7	-2.2	100.0%	-2.6
2061	AT3G26000.1 | F-box family protein, contains Pfam PF00646: F-box domain; similar to SKP1 interacting partner 2 (SKIP2) TIGR_Ath1:At5g67250 | chr3:9508015-9510495 REVERSE | Aliases: MPE11.17	6.7	6.2	0.5	2.2	100.0%	-3.2
2062	AT3G16720.1 | Symbol: ATL2 | zinc finger (C3HC4-type RING finger) family protein, contains Pfam domain, PF00097: Zinc finger, C3HC4 type (RING finger) | chr3:5692744-5694133 FORWARD | Aliases: MGL6.26	5.3	4.7	0.6	2.2	100.0%	-3.0

Rank	Description	15.dpi	5.dpi	M	t	adj.q	B
2063	AT4G40045.1 | expressed protein | chr4:18559778-18560651 REVERSE | Aliases: None	7.1	6.4	0.7	2.2	100.0%	-2.8
2064	AT1G49560.1 | myb family transcription factor, contains Pfam profile: PF00249 myb-like DNA-binding domain | chr1:18346252-18348005 FORWARD | Aliases: F14J22.20, F14J22_20	3.5	4.0	-0.5	-2.2	100.0%	-2.9
2065	AT3G54930.1 | serine/threonine protein phosphatase 2A (PP2A) regulatory subunit B', putative, similar to SWISS-PROT:Q28653 serine/threonine protein phosphatase 2A, 56 kDa regulatory subunit, delta isoform (PP2A, B subunit, B' delta isoform, PP2A, B subunit, B56 delta isoform, PP2A, B subunit, PR61 delta isoform, PP2A, B subunit, R5 delta isoform, PP2A, B subunit, B'-gamma) (Oryctolagus cuniculus); contains Pfam domain, PF01603: Protein phosphatase 2A regulatory B subunit (B56 family) | chr3:20362062-20363637 REVERSE | Aliases: T15C9.2	3.6	4.0	-0.4	-2.2	100.0%	-3.2
2066	AT3G56120.1 | Met-10+ like family protein, non-consensus TT donor splice site at exon 4 ; contains Pfam profile PF02475: Met-10+ like-protein | chr3:20834129-20837522 FORWARD | Aliases: F18O21.80	6.2	7.3	-1.1	-2.2	100.0%	-2.7
2067	AT2G10950.1 | BSD domain-containing protein, contains Pfam profile PF03909: BSD domain	7.4	6.8	0.6	2.2	100.0%	-3.1
2068	AT1G30090.1 | kelch repeat-containing F-box family protein, similar to SP:O95198 Kelch-like protein 2 (Actin-binding protein Mayven) {Homo sapiens}; contains Pfam profiles PF01344: Kelch motif, PF00646: F-box domain | chr1:10559545-10561407 REVERSE | Aliases: T2H7.11, T2H7_11	7.7	6.7	1.0	2.2	100.0%	-2.8
2069	AT1G02910.1 | tetratricopeptide repeat (TPR)-containing protein, contains Pfam profile PF00515: TPR Domain | chr1:655577-658148 REVERSE | Aliases: F22D16.9, F22D16_9	5.6	5.0	0.6	2.2	100.0%	-2.9
2070	AT3G63420.2 | Symbol: AGG1 | heterotrimeric G protein gamma-subunit (AGG1) | chr3:23428292-23429673 FORWARD | Aliases: HETEROTRIMERIC G PROTEIN GAMMA SUBUNIT, MAA21.50	8.6	7.9	0.7	2.2	100.0%	-2.9
2071	AT5G04470.1 | expressed protein | chr5:1266692-1267417 REVERSE | Aliases: T19N18.8	7.1	6.4	0.7	2.2	100.0%	-2.9
2072	AT1G59740.1 | proton-dependent oligopeptide transport (POT) family protein, contains Pfam profile: PF00854 POT family | chr1:21971736-21976076 FORWARD | Aliases: F23H11.6, F23H11_6	7.4	6.6	0.8	2.2	100.0%	-3.0
2073	AT5G05860.1 | Symbol: UGT76C2 | UDP-glucoronosyl/UDP-glucosyl transferase family protein, contains Pfam profile: PF00201 UDP-glucoronosyl and UDP-glucosyl transferase | chr5:1765508-1767456 FORWARD | Aliases: MJJ3.28, MJJ3_28	5.0	5.8	-0.9	-2.2	100.0%	-2.8
2074	AT4G23490.1 | fringe-related protein, + weak similarity to Fringe (Schistocerca gregaria)(GI:6573138);Fringe encodes an extracellular protein that regulates Notch signalling. | chr4:12251442-12254252 REVERSE | Aliases: F16G20.2	3.5	4.0	-0.5	-2.2	100.0%	-3.0
2075	AT5G62850.1 | nodulin MtN3 family protein, contains Pfam PF03083 MtN3/saliva family; similar to LIM7 (cDNAs induced in meiotic prophase in lily microsporocytes) GI:431154 from (Lilium longiflorum) | chr5:25247904-25248503 REVERSE | Aliases: MQB2.17, MQB2_17	4.2	3.7	0.5	2.2	100.0%	-3.2
2076	AT4G33070.1 | pyruvate decarboxylase, putative, strong similarity to SP:P51846 Pyruvate decarboxylase isozyme 2 (EC 4.1.1.1) (PDC) {Nicotiana tabacum}; contains InterPro entry IPR000399: Pyruvate decarboxylase | chr4:15952292-15954774 REVERSE | Aliases: F4I10.4	12.2	10.7	1.5	2.2	100.0%	-2.8
2077	AT5G10920.1 | argininosuccinate lyase, putative / arginosuccinase, putative, similar to argininosuccinate lyase (Nostoc punctiforme) GI:7672743; contains Pfam profile PF00206: Lyase | chr5:3441753-3444143 FORWARD | Aliases: T30N20.190, T30N20_190	7.2	8.6	-1.3	-2.2	100.0%	-2.6
2078	AT1G63480.1 | DNA-binding family protein, contains a AT hook motif (DNA binding motifs with a preference for A/T rich regions), Pfam:PF02178 | chr1:23543222-23545802 REVERSE | Aliases: F2K11.15, F2K11_15	4.3	4.7	-0.4	-2.2	100.0%	-3.2
2079	AT1G06720.1 | expressed protein, contains Pfam domain, PF04950: Protein of unknown function (DUF663) | chr1:2060443-2067322 REVERSE | Aliases: F4H5.19, F4H5_19	6.2	7.7	-1.5	-2.2	100.0%	-2.8
2080	AT1G33990.1 | hydrolase, alpha/beta fold family protein, similar to polyneuridine aldehyde esterase GI:6651393 from (Rauvolfia serpentina), SP:Q40708 PIR7A protein {Oryza sativa}, ethylene-induced esterase (Citrus sinensis) GI:14279437; contains Pfam profile PF00561: hydrolase, alpha/beta fold family | chr1:12355548-12358237 FORWARD | Aliases: F12G12.19, F12G12_19	7.6	8.3	-0.7	-2.2	100.0%	-2.8
2081	AT1G79900.1 | mitochondrial substrate carrier family protein, contains Pfam profile: PF00153 mitochondrial carrier protein | chr1:30056972-30058624 REVERSE | Aliases: F19K16.14, F19K16_14	4.6	3.9	0.7	2.2	100.0%	-2.8
2082	AT1G15920.2 | CCR4-NOT transcription complex protein, putative, similar to SWISS-PROT:Q60809 CCR4-NOT transcription complex, subunit 7 (CCR4-associated factor 1, (CAF1) (Mus musculus) | chr1:5469397-5471143 FORWARD | Aliases: T24D18.2, T24D18_2	9.2	8.6	0.6	2.2	100.0%	-2.9

Rank	Description	15.dpi	5.dpi	M	t	adj.q	B
2083	AT4G30450.1 | glycine-rich protein | chr4:14886033-14886681 REVERSE | Aliases: F17I23.210, F17I23_210	8.7	8.1	0.5	2.2	100.0%	-3.0
2084	AT4G00570.1 | malate oxidoreductase, putative, similar to NAD-dependent malic enzyme 59 kDa isoform, mitochondrial precursor (EC 1.1.1.39) (NAD-ME) (SP:P37225) {Solanum tuberosum} | chr4:242516-246736 REVERSE | Aliases: F6N23.16, F6N23_16	8.2	9.1	-1.0	-2.2	100.0%	-2.8
2085	AT2G41200.1 | expressed protein | chr2:17178704-17180208 FORWARD | Aliases: T3K9.3, T3K9_3	4.0	3.5	0.5	2.2	100.0%	-2.9
2086	AT5G60640.2 | Symbol: ATPDIL1 4 | thioredoxin family protein, similar to protein disulfide isomerase GI:5902592 from (Volvox carteri f. nagariensis), GI:2708314 from Chlamydomonas reinhardtii; contains Pfam profile: PF00085 Thioredoxin | chr5:24388186-24391264 REVERSE | Aliases: ATPDIL1 4, MUP24.6, MUP24_6, PDI LIKE 1 4	11.4	11.8	-0.4	-2.2	100.0%	-3.6
2087	AT3G12520.1 | Symbol: SULTR4;2 | sulfate transporter family protein, similar to sulfate transporter (Arabidopsis thaliana) GI:3777483; contains Pfam profiles PF00916: Sulfate transporter family, PF01740: STAS domain | chr3:3967823-3971925 REVERSE | Aliases: SULFATE TRANSPORTER, T2E22.36	5.7	6.4	-0.7	-2.2	100.0%	-3.1
2088	AT5G59020.1 | expressed protein | chr5:23844262-23846829 REVERSE | Aliases: K19M22.16, K19M22_16	3.9	4.5	-0.7	-2.2	100.0%	-2.8
2089	AT3G62870.1 | 60S ribosomal protein L7A (RPL7aB), 60S RIBOSOMAL PROTEIN L7A - Oryza sativa, SWISSPROT:RL7A_ORYSA | chr3:23253640-23255328 REVERSE | Aliases: F26K9.300	11.7	12.1	-0.4	-2.2	100.0%	-3.1
2090	AT4G05040.3 | similar to ankyrin repeat family protein [Arabidopsis thaliana] (TAIR:At4g14400.1); similar to ankyrin repeat family protein [Arabidopsis thaliana] (TAIR:At4g14390.1); similar to hypothetical protein [Oryza sativa (japonica cultivar-group)] (GB:XP_475255.1); contains InterPro domain Ankyrin (InterPro:IPR002110)	4.0	3.5	0.5	2.2	100.0%	-3.1
2091	AT3G55920.1 | peptidyl-prolyl cis-trans isomerase, putative / cyclophilin, putative / rotamase, putative, similar to cyclophilin (Arabidopsis thaliana) gi:2443755:gb:AAB71401	6.9	7.5	-0.6	-2.2	100.0%	-3.0
2092	AT1G75680.1 | glycosyl hydrolase family 9 protein, similar to endo-beta-1,4-glucanase GB:AAC12685 GI:3025470 from (Pinus radiata) | chr1:28420713-28423190 REVERSE | Aliases: F10A5.13, F10A5_13	8.8	9.3	-0.5	-2.2	100.0%	-3.2
2093	AT3G46110.2 | expressed protein | chr3:16943097-16944627 FORWARD | Aliases: F12M12.80	4.4	5.1	-0.7	-2.2	100.0%	-2.7
2094	AT1G15480.1 | DNA-binding protein, putative, similar to DNA-binding protein (Triticum aestivum) GI:6958202; contains Pfam profile PF01535: PPR repeat | chr1:5318208-5320589 FORWARD | Aliases: F9L1.43, F9L1_43	4.4	5.1	-0.7	-2.2	100.0%	-2.8
2095	AT1G78180.1 | mitochondrial substrate carrier family protein, contains Pfam profile: PF00153 mitochondrial carrier protein | chr1:29421821-29423868 FORWARD | Aliases: T11I11.12, T11I11_12	3.9	4.4	-0.5	-2.2	100.0%	-3.0
2096	AT5G44080.1 | bZIP transcription factor family protein, contains Pfam profile: PF00170 bZIP transcription factor | chr5:17755879-17757098 REVERSE | Aliases: MRH10.19, MRH10_19	6.4	5.5	0.9	2.2	100.0%	-3.0
2097	AT1G01710.1 | acyl-CoA thioesterase family protein, contains Pfam profiles: PF02551 acyl-CoA thioesterase, PF00027 cyclic nucleotide-binding domain | chr1:262828-267771 FORWARD | Aliases: T1N6.10, T1N6.9, T1N6_10, T1N6_9	5.7	6.3	-0.5	-2.2	100.0%	-3.1
2098	AT5G13680.1 | IKI3 family protein, weak similarity to SP:O95163 IkappaB kinase complex-associated protein (IKK complex-associated protein) (p150) {Homo sapiens}; contains Pfam profile PF04762: IKI3 family | chr5:4410364-4415474 REVERSE | Aliases: MSH12.15, MSH12_15	5.9	6.4	-0.6	-2.2	100.0%	-2.9
2099	AT2G21120.1 | expressed protein | chr2:9058829-9061694 REVERSE | Aliases: F26H11.12, F26H11_12	6.0	5.1	0.9	2.2	100.0%	-2.7
2100	AT4G30150.1 | expressed protein | chr4:14742458-14749993 FORWARD | Aliases: F6G3.180, F6G3_180	5.4	5.9	-0.5	-2.2	100.0%	-3.0
2101	AT3G48510.1 | expressed protein | chr3:17978341-17979523 REVERSE | Aliases: T8P19.20	7.3	6.2	1.1	2.2	100.0%	-2.6
2102	AT3G63480.2 | kinesin heavy chain, putative, kinesin heavy chain, Syncephalastrum racemosum, SWISSPROT:KINH_SYNRA | chr3:23450847-23454981 REVERSE | Aliases: MAA21.110	3.5	4.1	-0.5	-2.2	100.0%	-2.9
2103	AT3G54670.2 | Symbol: TTN8 | similar to structural maintenance of chromosomes (SMC) family protein [Arabidopsis thaliana] (TAIR:At5g48600.1); similar to hypothetical protein UM03243.1 [Ustilago maydis 521] (GB:EAK84348.1); similar to unnamed protein product [Debaryomyces hansenii CBS767] (GB:CAG89604.1); contains InterPro domain Structural maintenance of chromosome protein SMC, C-terminal (InterPro:IPR003405) | chr3:20246656-20254938 FORWARD | Aliases: ATSMC1, SMC1, STRUCTURAL MAINTENANCE OF CHROMOSOMES 1, T5N23.30, TITAN8	4.7	5.4	-0.7	-2.2	100.0%	-3.1
2104	AT5G08110.1 | DEAD/DEAH box helicase, putative, several putative ATP-dependent helicases | chr5:2594971-2600115 REVERSE | Aliases: T22D6.50, T22D6_50	4.2	4.7	-0.4	-2.2	100.0%	-3.1

Rank	Description	15.dpi	5.dpi	M	t	adj.q	B
2105	AT3G26720.1 | glycosyl hydrolase family 38 protein, similar to lysosomal alpha-mannosidase GI:3522867 from (Homo sapiens) | chr3:9817891-9824423 FORWARD | Aliases: MLJ15.19	7.3	8.3	-0.9	-2.2	100.0%	-2.8
2106	AT2G30160.1 | mitochondrial substrate carrier family protein, contains Pfam profile: PF00153 mitochondrial carrier protein | chr2:12884915-12886726 FORWARD | Aliases: T27E13.10, T27E13_10	5.0	5.9	-0.9	-2.2	100.0%	-2.7
2107	AT1G61360.1 | S-locus lectin protein kinase family protein, contains protein kinase domain, Pfam:PF00069; contains S-locus glycoprotein family domain, Pfam:PF00954 | chr1:22641393-22644681 REVERSE | Aliases: T1F9.15, T1F9_15	5.9	6.8	-1.0	-2.2	100.0%	-2.9
2108	AT3G29230.1 | pentatricopeptide (PPR) repeat-containing protein, contains INTERPRO:IPR002885 PPR repeats | chr3:11190040-11191842 FORWARD | Aliases: MXO21.8	6.4	7.3	-0.9	-2.2	100.0%	-2.6
2109	AT1G12010.1 | 1-aminocyclopropane-1-carboxylate oxidase, putative / ACC oxidase, putative, Strong similarity to amino-cyclopropane-carboxylic acid oxidase (ACC ox2) (GI:559407) from Brassica napus. ESTs gb:Z48548 and gb:Z48549 come from this gene	8.1	7.0	1.1	2.2	100.0%	-3.0
2110	AT1G68470.1 | exostosin family protein, contains Pfam profile: PF03016	9.2	10.0	-0.8	-2.2	100.0%	-2.9
2111	AT5G52200.1 | expressed protein | chr5:21219865-21221976 REVERSE | Aliases: F17P19.10, F17P19_10	8.4	7.9	0.6	2.2	100.0%	-3.0
2112	AT2G38700.1 | Symbol: MVD1 | mevalonate diphosphate decarboxylase (MVD1), identical to mevalonate diphosphate decarboxylase (Arabidopsis thaliana) gi:2288887:emb:CAA74700 | chr2:16187774-16191023 REVERSE | Aliases: ATMVD1, MEVALONATE DIPHOSPHATE DECARBOXYLASE, T6A23.10, T6A23_10	6.0	7.5	-1.5	-2.2	100.0%	-2.7
2113	AT3G61060.2 | Symbol: ATPP2 A13 | F-box family protein / lectin-related, low similarity to PP2 lectin polypeptide (Cucurbita maxima) GI:410437; contains Pfam profile PF00646: F-box domain | chr3:22613885-22615553 FORWARD | Aliases: ATPP2 A13, T27I15.150	5.4	4.2	1.2	2.2	100.0%	-2.7
2114	AT1G53670.1 | transcription factor-related, similar to pilin-like transcription factor (Homo sapiens) GI:5059062; contains Pfam profile PF01641: SelR domain | chr1:20040344-20041871 FORWARD | Aliases: F22G10.17, F22G10_17	8.1	7.4	0.7	2.2	100.0%	-2.9
2115	AT1G08710.1 | F-box family protein, similar to ESTs gb:T22270 and gb:T76886 ; similar to SKP1 interacting partner 2 (SKIP2) TIGR_Ath1:At5g67250 | chr1:2771636-2773463 FORWARD | Aliases: F22O13.19, F22O13_19	5.9	4.8	1.1	2.2	100.0%	-2.7
2116	AT1G54500.1 | rubredoxin family protein, similar to SP:P00270 Rubredoxin (Rd) {Desulfovibrio gigas}; contains Pfam profile PF00301: Rubredoxin	7.7	7.0	0.7	2.2	100.0%	-2.8
2117	AT5G45380.1 | sodium:solute symporter family protein, contains Pfam profile: PF00474 sodium:solute symporter family | chr5:18408351-18413053 FORWARD | Aliases: MFC19.5, MFC19_5	5.2	4.3	0.9	2.2	100.0%	-2.7
2118	AT4G22770.1 | DNA-binding family protein, contains a AT hook motif (DNA binding motifs with a preference for A/T rich regions), Pfam:PF02178 | chr4:11963639-11965695 REVERSE | Aliases: T12H17.160, T12H17_160	5.2	4.5	0.8	2.2	100.0%	-2.7
2119	AT2G44090.2 | expressed protein, similar to expressed protein [Arabidopsis thaliana] (TAIR:At3g59910.1); similar to expressed protein [Oryza sativa (japonica cultivar-group)] (GB:AAX96769.1)	5.6	6.2	-0.6	-2.2	100.0%	-2.9
2120	AT1G57540.2 | expressed protein, contains similarity to T-box domain GI:5931592 from (Cynops pyrrhogaster) | chr1:21313995-21315077 REVERSE | Aliases: F25P12.4	10.1	9.3	0.8	2.2	100.0%	-2.8
2121	AT5G06690.2 | similar to thioredoxin family protein [Arabidopsis thaliana] (TAIR:At5g04260.1); similar to putative hioredoxin 5 [Oryza sativa (japonica cultivar-group)] (GB:XP_468041.1); contains InterPro domain Thioredoxin domain 2 (InterPro:IPR006663) | chr5:2060504-2061957 REVERSE | Aliases: MPH15.4, MPH15_4, THIOREDOXIN LIKE 5	4.6	4.2	0.3	2.2	100.0%	-3.3
2122	AT1G60490.1 | Symbol: ATVPS34 | phosphatidylinositol 3-kinase (PI3K), identical to SP:P42339 Phosphatidylinositol 3-kinase (EC 2.7.1.137) (PI3-kinase) (PtdIns-3-kinase) (PI3K) {Arabidopsis thaliana} | chr1:22289249-22293966 REVERSE | Aliases: F8A5.4, F8A5_4, PHOSPHATIDYLINOSITOL 3 KINASE	5.9	6.5	-0.6	-2.2	100.0%	-3.0
2123	AT5G23400.1 | disease resistance family protein / LRR family protein, similar to disease resistance protein (Lycopersicon esculentum) gi:3894383:gb:AAC78591; contains leucine rich-repeat domain Pfam:PF00560, INTERPRO:IPR001611 | chr5:7880338-7882637 FORWARD | Aliases: K19M13.1, K19M13_1	4.2	4.9	-0.7	-2.2	100.0%	-2.8
2124	AT5G45600.1 | Symbol: GAS41 | YEATS family protein, contains Pfam domain PF03366: YEATS family | chr5:18505129-18507096 FORWARD | Aliases: K2N11.8, K2N11_8	7.4	8.4	-1.0	-2.2	100.0%	-2.9

Rank	Description	15.dpi	5.dpi	M	t	adj.q	B
2125	AT3G26370.1 | expressed protein, contains Pfam PF03138: Plant protein family. The function of this family of plant proteins is unknown; | chr3:9657983-9661164 FORWARD | Aliases: F20C19.9	6.1	6.9	-0.8	-2.2	100.0%	-2.7
2126	AT3G55890.1 | yippee family protein, similar to mdgl-1 (Mus musculus) GI:10441648, Yippee protein (Drosophila melanogaster) GI:5713279; contains Pfam profile PF03226: Yippee putative zinc-binding protein | chr3:20751796-20752981 FORWARD | Aliases: F27K19.70	4.3	3.7	0.6	2.2	100.0%	-2.9
2127	AT3G05050.1 | protein kinase family protein, contains Pfam domain, PF00069: Protein kinase domain | chr3:1408619-1411807 REVERSE | Aliases: T12H1.1, T12H1_1	4.8	5.4	-0.6	-2.2	100.0%	-2.8
2128	AT1G60680.1 | aldo/keto reductase family protein, contains Pfam profile PF00248: oxidoreductase, aldo/keto reductase family | chr1:22350775-22352913 REVERSE | Aliases: F8A5.20, F8A5_20	11.2	11.7	-0.4	-2.2	100.0%	-3.5
2129	AT2G40765.1 | expressed protein | chr2:17019276-17020403 FORWARD | Aliases: None	10.5	11.1	-0.5	-2.2	100.0%	-3.0
2130	AT1G78140.1 | methyltransferase-related, similar to Probable delta(24)-sterol C-methyltransferase (Swiss-Prot:O14321) (Schizosaccharomyces pombe); similar to C5-O-methyltransferase (GI:5921167) (Streptomyces avermitilis); similar to S-adenosyl-methionine-sterol-C- methyltransferase (GI:3560474) (Nicotiana tabacum) | chr1:29406378-29408839 REVERSE | Aliases: T11I11.8, T11I11_8	8.8	7.6	1.3	2.2	100.0%	-3.0
2131	AT3G09660.1 | minichromosome maintenance family protein / MCM family protein, similar to SP:P49717 DNA replication licensing factor MCM4 (CDC21 homolog) {Mus musculus}; contains Pfam profile PF00493: MCM2/3/5 family | chr3:2961319-2966171 REVERSE | Aliases: F11F8.25	4.2	5.1	-0.9	-2.2	100.0%	-2.8
2132	AT3G24240.1 | leucine-rich repeat transmembrane protein kinase, putative, similar to CLV1 receptor kinase GB:AAB58929 from (Arabidopsis thaliana) | chr3:8780558-8784157 FORWARD | Aliases: K13K6.1	4.5	5.1	-0.6	-2.2	100.0%	-2.9
2133	AT1G68740.1 | EXS family protein / ERD1/XPR1/SYG1 family protein, similar to PHO1 protein (Arabidopsis thaliana) GI:20069032; contains Pfam profiles PF03105: SPX domain, PF03124: EXS family | chr1:25816112-25820320 REVERSE | Aliases: F24J5.2, F24J5_2	5.0	5.6	-0.6	-2.2	100.0%	-2.9
2134	AT2G21780.1 | expressed protein | chr2:9298116-9298749 FORWARD | Aliases: F7D8.10, F7D8_10	3.2	2.8	0.4	2.2	100.0%	-3.3
2135	AT5G25640.1 | expressed protein, ; expression supported by MPSS | chr5:8949852-8950334 REVERSE | Aliases: T14C9.180, T14C9_180	4.9	5.5	-0.6	-2.2	100.0%	-2.9
2136	AT1G15100.1 | Symbol: RHA2A | zinc finger (C3HC4-type RING finger) family protein, contains Pfam profile: PF00097 zinc finger, C3HC4 type (RING finger) | chr1:5193421-5194198 REVERSE | Aliases: RING H2 FINGER PROTEIN RHA2A	6.8	5.8	1.0	2.2	100.0%	-2.8
2137	AT4G10030.1 | hydrolase, alpha/beta fold family protein, contains Pfam profile PF00561: hydrolase, alpha/beta fold family | chr4:6269787-6273237 REVERSE | Aliases: T5L19.160, T5L19_160	6.9	6.1	0.8	2.2	100.0%	-2.9
2138	AT3G52610.1 | expressed protein | chr3:19521459-19523636 FORWARD | Aliases: F3C22.10	5.9	6.4	-0.5	-2.2	100.0%	-3.0
2139	AT3G43920.1 | Symbol: DCL3 | ribonuclease III family protein, similar to RNA helicase/RNAseIII CAF protein (Arabidopsis thaliana) GI:6102610; contains Pfam profiles PF02170: PAZ domain, PF00636: RNase3 domain | chr3:15764535-15771817 FORWARD | Aliases: DICER LIKE 3, T15B3.60	5.0	5.6	-0.6	-2.2	100.0%	-2.8
2140	AT1G75150.1 | similar to expressed protein [Arabidopsis thaliana] (TAIR:At3g28770.1); similar to retinitis pigmentosa GTPase regulator-like protein [Takifugu rubripes] (GB:AAG00554.1) | chr1:28208048-28212234 REVERSE | Aliases: F22H5.12, F22H5_12	2.8	3.6	-0.8	-2.2	100.0%	-3.1
2141	AT2G33420.1 | expressed protein | chr2:14165781-14169603 FORWARD | Aliases: F4P9.19, F4P9_19	3.3	2.9	0.3	2.2	100.0%	-3.4
2142	AT3G50650.1 | scarecrow-like transcription factor 7 (SCL7) | chr3:18817239-18819191 REVERSE | Aliases: T3A5.30	4.6	4.2	0.4	2.2	100.0%	-3.1
2143	AT1G65960.1 | Symbol: GAD2 | similar to glutamate decarboxylase 1 (GAD 1) [Arabidopsis thaliana] (TAIR:At5g17330.1); similar to glutamate decarboxylase [Nicotiana tabacum] (GB:AAM48129.1); similar to DCE_PETHY Glutamate decarboxylase (GAD) (GB:Q07346); contains InterPro domain Pyridoxal-dependent decarboxylase (InterPro:IPR002129) | chr1:24558084-24561314 FORWARD | Aliases: F12P19.12, F12P19_12, GLUTAMATE DECARBOXYLASE 2	7.0	8.0	-1.0	-2.2	100.0%	-3.0
2144	AT3G20490.1 | expressed protein | chr3:7153600-7156542 REVERSE | Aliases: K10D20.3	3.8	4.2	-0.4	-2.2	100.0%	-3.3
2145	AT4G22930.1 | Symbol: PYR4 | dihydroorotase, mitochondrial / DHOase (PYR4), identical to SP:O04904 Dihydroorotase, mitochondrial precursor (EC 3.5.2.3) (DHOase) {Arabidopsis thaliana} | chr4:12018906-12021602 FORWARD | Aliases: DIHYDROOROTASE, F7H19.110, F7H19_110	7.0	7.9	-0.9	-2.2	100.0%	-2.8
2146	AT2G27490.2 | dephospho-CoA kinase family, similar to Dephospho-CoA kinase (EC 2.7.1.24) (Dephosphocoenzyme A kinase). (Swiss-Prot:Q92BF2) (Listeria innocua); contains Pfam profile PF01121: Dephospho-CoA kinase | chr2:11755165-11757259 REVERSE | Aliases: F10A12.17, F10A12_17	8.0	7.5	0.5	2.2	100.0%	-3.1

Rank	Description	15.dpi	5.dpi	M	t	adj.q	B
2147	AT5G16300.3 | similar to putative low density lipoprotein [Oryza sativa (japonica cultivar-group)] (GB:NP_916361.1) | chr5:5338122-5342781 FORWARD | Aliases: MQK4.2, MQK4_2	5.7	6.7	-1.0	-2.2	100.0%	-3.0
2148	AT4G07820.1 | pathogenesis-related protein, putative, similar to SP:P33154 Pathogenesis-related protein 1 precursor (PR-1) {Arabidopsis thaliana}; contains Pfam profile PF00188: SCP-like extracellular protein | chr4:4636861-4637348 REVERSE | Aliases: F5K24.6, F5K24_6	3.7	4.4	-0.8	-2.2	100.0%	-2.7
2149	AT1G64890.1 | integral membrane transporter family protein, contains 11 transmembrane domains; contains Pfam PF03092: BT1 family; contains TIGRFAMS TIGR00788: folate/biopterin transporter | chr1:24113205-24115063 FORWARD | Aliases: F13O11.19, F13O11_19	6.6	5.4	1.2	2.2	100.0%	-2.9
2150	AT3G50530.1 | Symbol: CRK | calcium-dependent protein kinase, putative / CDPK, putative, similar to calcium/calmodulin-dependent protein kinase CaMK3 (Nicotiana tabacum) gi:16904226:gb:AAL30820 | chr3:18764522-18767754 FORWARD | Aliases: T20E23.130	6.1	6.7	-0.6	-2.2	100.0%	-3.1
2151	AT3G15480.1 | expressed protein | chr3:5226255-5227794 REVERSE | Aliases: MJK13.14	8.4	7.4	1.0	2.2	100.0%	-2.9
2152	AT5G27430.1 | signal peptidase subunit family protein, contains Pfam profile: PF04573 signal peptidase subunit | chr5:9687390-9689366 FORWARD | Aliases: F21A20.140, F21A20_140	5.1	6.1	-1.0	-2.2	100.0%	-2.9
2153	AT4G28980.2 | Symbol: CAK1AT |	5.1	4.8	0.3	2.2	100.0%	-3.4
2154	AT4G26060.1 | expressed protein | chr4:13214755-13216470 REVERSE | Aliases: F20B18.170, F20B18_170	8.3	7.6	0.7	2.2	100.0%	-3.0
2155	AT1G55590.1 | F-box family protein, ; similar to SKP1 interacting partner 2 (SKIP2) TIGR_Ath1:At5g67250 | chr1:20772986-20775537 REVERSE | Aliases: F20N2.2	5.7	5.2	0.6	2.2	100.0%	-2.9
2156	AT3G17920.1 | leucine-rich repeat family protein, contains leucine rich repeat (LRR) domains, Pfam:PF00560 | chr3:6137261-6142029 FORWARD | Aliases: MEB5.14	5.7	5.1	0.6	2.2	100.0%	-3.0
2157	AT4G39770.1 | trehalose-6-phosphate phosphatase, putative, similar to trehalose-6-phosphate phosphatase (AtTPPB) (Arabidopsis thaliana) GI:2944180; contains Pfam profile PF02358: Trehalose-phosphatase | chr4:18448974-18451287 REVERSE | Aliases: T19P19.160, T19P19_160	2.8	2.4	0.4	2.2	100.0%	-3.4
2158	AT4G18140.2 | similar to NLI interacting factor (NIF) family protein [Arabidopsis thaliana] (TAIR:At5g46410.1)	4.4	3.9	0.6	2.2	100.0%	-2.9
2159	AT2G15890.1 | expressed protein | chr2:6927451-6929084 REVERSE | Aliases: F19G14.11, F19G14_11	6.1	5.5	0.5	2.2	100.0%	-2.9
2160	AT5G16200.1 | 50S ribosomal protein-related, contains weak similarity to Cyanelle 50S ribosomal protein L35. (Swiss-Prot:P14810) (Cyanophora paradoxa)	5.8	5.1	0.7	2.2	100.0%	-2.8
2161	AT5G41670.2 | 6-phosphogluconate dehydrogenase family protein, contains Pfam profiles: PF00393 6-phosphogluconate dehydrogenase C-terminal domain, PF03446 NAD binding domain of 6-phosphogluconate | chr5:16682558-16684399 REVERSE | Aliases: MBK23.20, MBK23_20	10.1	10.7	-0.5	-2.2	100.0%	-3.3
2162	AT5G22440.1 | 60S ribosomal protein L10A (RPL10aC) | chr5:7435128-7436642 REVERSE | Aliases: MWD9.24, MWD9_24	7.1	8.3	-1.2	-2.2	100.0%	-2.8
2163	AT1G14610.1 | Symbol: TWN2 | valyl-tRNA synthetase / valine--tRNA ligase (VALRS), nearly identical to SP:P93736 Valyl-tRNA synthetase (EC 6.1.1.9) (Valine--tRNA ligase) (ValRS) {Arabidopsis thaliana} | chr1:5008239-5014512 REVERSE | Aliases: T5E21.11, T5E21_11, TWIN 2, VALRS, VALYL TRNA SYNTHETASE	9.7	10.5	-0.8	-2.2	100.0%	-3.4
2164	AT4G38260.1 | expressed protein, contains Pfam PF05742: Protein of unknown function (DUF833) | chr4:17936342-17938266 REVERSE | Aliases: F22I13.30, F22I13_30	6.3	4.9	1.4	2.2	100.0%	-2.8
2165	AT2G24190.1 | short-chain dehydrogenase/reductase (SDR) family protein, contains INTERPRO family IPR002198 short-chain dehydrogenase/reductase (SDR) superfamily | chr2:10290820-10292014 REVERSE | Aliases: F27D4.10, F27D4_10	9.2	10.0	-0.8	-2.2	100.0%	-2.8
2166	AT2G27050.1 | Symbol: EIL1 | ethylene-insensitive3-like1 (EIL1), identical to ethylene-insensitive3-like1 GI:2224927 from (Arabidopsis thaliana) | chr2:11552873-11555371 FORWARD | Aliases: ETHYLENE INSENSITIVE3 LIKE 1, T20P8.10, T20P8_10	6.4	5.7	0.7	2.2	100.0%	-2.9
2167	AT5G01380.1 | expressed protein | chr5:155638-157521 REVERSE | Aliases: T10O8.90, T10O8_90	5.4	4.2	1.1	2.2	100.0%	-2.7
2168	AT5G44560.1 | SNF7 family protein, contains Pfam domain, PF03357: SNF7 family | chr5:17963206-17965627 FORWARD | Aliases: MFC16.25, MFC16_25	7.7	8.7	-1.0	-2.2	100.0%	-2.6
2169	AT2G16430.2 | purple acid phosphatase (PAP10), identical to purple acid phosphatase (PAP10) GI:20257482 from (Arabidopsis thaliana) | chr2:7127501-7129901 REVERSE | Aliases: F16F14.7, F16F14_7	5.4	6.7	-1.4	-2.2	100.0%	-2.8

Rank	Description	15.dpi	5.dpi	M	t	adj.q	B
2170	AT3G50110.1 | phosphatase-related, similar to PTEN1 GI:5566292 from (Drosophila melanogaster); contains prosite evidence: PS00383: Tyrosine specific protein phosphatases active site | chr3:18591592-18595518 REVERSE | Aliases: F3A4.190	5.2	6.0	-0.7	-2.2	100.0%	-2.9
2171	AT1G01160.1 | Symbol: GIF2 | SSXT protein-related / transcription co-activator-related, similar to SYT/SSX4 fusion protein (GI:11127695) (Homo sapiens); supporting cDNA gi:21539891:gb:AY102640.1:; contains Pfam profile PF05030: SSXT protein (N-terminal region) | chr1:72357-74096 FORWARD | Aliases: F6F3.29, GRF1 INTERACTING FACTOR 2	4.9	3.8	1.2	2.2	100.0%	-2.9
2172	AT4G31800.2 | Symbol: WRKY18 |	5.6	4.7	0.9	2.1	100.0%	-2.9
2173	AT4G31560.1 | expressed protein | chr4:15295207-15296233 FORWARD | Aliases: F3L17.130, F3L17_130	6.0	5.2	0.8	2.1	100.0%	-2.7
2174	AT1G28280.1 | VQ motif-containing protein, contains PF05678: VQ motif | chr1:9886064-9887465 REVERSE | Aliases: F3H9.7, F3H9_7	4.5	5.0	-0.6	-2.1	100.0%	-3.0
2175	AT3G03180.1 | Got1-like family protein, contains Pfam profile: PF04178 Got1-like family | chr3:733394-734434 FORWARD | Aliases: T17B22.13, T17B22_13	6.0	5.6	0.4	2.1	100.0%	-3.1
2176	AT2G34200.1 | zinc finger (C3HC4-type RING finger) family protein | chr2:14447727-14449084 FORWARD | Aliases: F13P17.4, F13P17_4	6.3	5.5	0.8	2.1	100.0%	-2.7
2177	AT5G57060.2 | expressed protein | chr5:23107424-23109196 FORWARD | Aliases: MHM17.20, MHM17_20	6.2	5.4	0.8	2.1	100.0%	-2.9
2178	AT4G37190.2 | similar to misato [Mus musculus] (GB:NP_659147.1) | chr4:17507253-17510102 FORWARD | Aliases: AP22.7, AP22_7	2.8	3.1	-0.3	-2.1	100.0%	-3.3
2179	AT3G04630.3 | Symbol: WDL1 | similar to expressed protein [Arabidopsis thaliana] (TAIR:At1g54460.1); similar to BRI1-KD interacting protein 116 [Oryza sativa (japonica cultivar-group)] (GB:BAD11344.1) | chr3:1258804-1260824 FORWARD | Aliases: F7O18.11, F7O18_11	3.7	4.9	-1.2	-2.1	100.0%	-2.9
2180	AT2G21430.1 | cysteine proteinase A494, putative / thiol protease, putative, identical to SP:P43295 Probable cysteine proteinase A494 precursor (Arabidopsis thaliana); strong similarity to cysteine proteinase RD19A (thiol protease) GI:435618, SP:P43296 from (Arabidopsis thaliana) | chr2:9178971-9180399 REVERSE | Aliases: F3K23.19, F3K23_19	5.4	4.4	1.0	2.1	100.0%	-2.7
2181	AT3G14595.1 | expressed protein | chr3:4908957-4910483 FORWARD | Aliases: None	10.6	10.2	0.5	2.1	100.0%	-3.3
2182	AT3G16570.1 | Symbol: RALFL23 | rapid alkalinization factor (RALF) family protein, similar to RALF precursor (Nicotiana tabacum) GI:16566316 | chr3:5644704-5645420 FORWARD | Aliases: MGL6.3, RALF LIKE 23	7.0	6.2	0.8	2.1	100.0%	-2.9
2183	AT1G32520.1 | expressed protein, similar to hypothetical protein [Oryza sativa (japonica cultivar-group)] (GB:XP_475242.1) | chr1:11758198-11759260 REVERSE | Aliases: F5D14.31, F5D14_31	4.5	3.9	0.6	2.1	100.0%	-3.3
2184	AT5G16240.1 | acyl-(acyl-carrier-protein) desaturase, putative / stearoyl-ACP desaturase, putative, similar to Acyl-(acyl-carrier protein) desaturase from Sesamum indicum GI:575942, Cucumis sativus SP:P32061, Ricinus communis SP:P22337; contains Pfam profile PF03405 Fatty acid desaturase | chr5:5306810-5309935 FORWARD | Aliases: T21H19.160, T21H19_160	5.2	4.6	0.6	2.1	100.0%	-2.8
2185	AT3G05250.1 | zinc finger (C3HC4-type RING finger) family protein, contains Pfam profile: PF00097 zinc finger, C3HC4 type (RING finger) | chr3:1495603-1497475 FORWARD | Aliases: T12H1.22, T12H1_22	6.5	5.8	0.6	2.1	100.0%	-3.0
2186	AT4G33170.1 | pentatricopeptide (PPR) repeat-containing protein, contains Pfam profile PF01535: PPR repeat | chr4:15995704-15998676 REVERSE | Aliases: F4I10.100, F4I10_100	3.0	3.3	-0.3	-2.1	100.0%	-3.5
2187	AT4G30860.1 | SET domain-containing protein, low similarity to IL-5 promoter REII-region-binding protein (Homo sapiens) GI:12642795; contains Pfam profile PF00856: SET domain	3.9	4.8	-0.8	-2.1	100.0%	-2.7
2188	AT1G55620.2 | Symbol: CLC F | voltage-gated chloride channel family protein, contains Pfam profiles PF00654: Voltage gated chloride channel, PF00571: CBS domain | chr1:20790872-20794855 REVERSE | Aliases: ATCLC F, CHLORIDE CHANNEL F, F20N2.5	3.6	4.4	-0.8	-2.1	100.0%	-2.9
2189	AT1G04810.1 | 26S proteasome regulatory subunit, putative, contains similarity to 26S proteasome regulatory subunit S1 SP:O88761, GI:3288594 from (Rattus norvegicus) | chr1:1350179-1355441 FORWARD | Aliases: F13M7.20, F13M7_20	6.4	7.3	-0.9	-2.1	100.0%	-3.0
2190	AT2G35110.2 | Symbol: GRL/NAP1/NAPP | similar to similar to Mus musculus (Mouse). Similar to NCK-associated protein 1 [Dictyostelium discoideum] (GB:AAO52562.1) | chr2:14802794-14811009 REVERSE | Aliases: GRL, NAP1, NAPP, T4C15.22, T4C15_22	4.5	5.1	-0.7	-2.1	100.0%	-3.1
2191	AT1G21310.1 | Symbol: ATEXT3 |	3.3	5.4	-2.1	-2.1	100.0%	-2.6

Rank	Description	15.dpi	5.dpi	M	t	adj.q	B
2192	AT3G49490.1 | expressed protein | chr3:18355729-18359665 REVERSE | Aliases: T9C5.90	8.3	8.7	-0.4	-2.1	100.0%	-3.2
2193	AT5G42400.1 | SET domain-containing protein (TXR7), contains Pfam profile PF00856: SET domain | chr5:16971697-16977899 REVERSE | Aliases: MDH9.9, MDH9_9	4.7	5.3	-0.6	-2.1	100.0%	-3.0
2194	AT3G20410.1 | Symbol: CPK9 | calmodulin-domain protein kinase isoform 9 (CPK9), identical to calmodulin-domain protein kinase CDPK isoform 9 (Arabidopsis thaliana) gi:1399265:gb:AAB03242 | chr3:7116207-7119127 FORWARD | Aliases: CALMODULIN DOMAIN PROTEIN KINASE 9, MQC12.23	5.2	5.9	-0.7	-2.1	100.0%	-2.9
2195	AT3G23560.1 | Symbol: ALF5 | MATE efflux family protein, similar to ripening regulated protein DDTFR18 (Lycopersicon esculentum) GI:12231296; contains Pfam profile: PF01554 uncharacterized membrane protein family | chr3:8454134-8456688 REVERSE | Aliases: ABERRANT LATERAL ROOT FORMATION 5, MDB19.4	5.1	4.4	0.7	2.1	100.0%	-3.0
2196	AT5G64905.1 | expressed protein | chr5:25956800-25957285 FORWARD | Aliases: None	2.7	3.3	-0.6	-2.1	100.0%	-3.0
2197	AT1G46768.1 | Symbol: RAP2.1 |	4.1	3.5	0.5	2.1	100.0%	-2.9
2198	AT3G25490.1 | wall-associated kinase, putative, similar to wall-associated kinase 4 GB:CAA08793 from (Arabidopsis thaliana) | chr3:9242962-9244722 FORWARD | Aliases: MWL2.11	2.6	2.4	0.2	2.1	100.0%	-3.9
2199	AT3G10640.2 | SNF7 family protein, contains Pfam domain, PF03357: SNF7 family | chr3:3323315-3325051 REVERSE | Aliases: F13M14.7	8.6	7.8	0.8	2.1	100.0%	-3.0
2200	AT3G56880.1 | VQ motif-containing protein, contains PF05678: VQ motif | chr3:21070894-21071944 FORWARD | Aliases: T8M16.210	9.3	8.5	0.8	2.1	100.0%	-3.1
2201	AT5G25500.1 | expressed protein, ; expression supported by MPSS | chr5:8881121-8882383 FORWARD | Aliases: T14C9.40, T14C9_40	4.9	5.6	-0.7	-2.1	100.0%	-2.8
2202	AT3G21670.1 | nitrate transporter (NTP3), nearly identical to nitrate transporter (Arabidopsis thaliana) GI:4490323; contains Pfam profile: PF00854 POT family	5.0	4.2	0.8	2.1	100.0%	-2.9
2203	AT2G34680.1 | Symbol: AIR9 | similar to leucine-rich repeat family protein [Arabidopsis thaliana] (TAIR:At1g78230.1); similar to putative leucine rich repeat protein [Oryza sativa (japonica cultivar-group)] (GB:XP_476635.1); contains InterPro domain Leucine-rich repeat (InterPro:IPR001611) | chr2:14623701-14636208 REVERSE | Aliases: T29F13.11, T29F13_11	7.4	8.0	-0.7	-2.1	100.0%	-2.9
2204	AT1G06760.1 | histone H1, putative, similar to histone H1-1 GB:CAA44312 GI:16314 from (Arabidopsis thaliana); identical to cDNA H1-1C mRNA for histone H1-1 (partial) GI:732560 | chr1:2076503-2077697 REVERSE | Aliases: F4H5.15, F4H5_15	9.6	10.3	-0.7	-2.1	100.0%	-3.5
2205	AT5G27920.1 | F-box family protein, contains similarity to leucine-rich repeats containing F-box protein FBL3 GI:5919219 from (Homo sapiens) | chr5:9941909-9944782 REVERSE | Aliases: F15F15.2	6.4	5.9	0.5	2.1	100.0%	-3.1
2206	AT3G13880.1 | pentatricopeptide (PPR) repeat-containing protein, contains INTERPRO:IPR002885 PPR repeats | chr3:4572187-4574433 FORWARD | Aliases: MCP4.10	2.8	3.0	-0.3	-2.1	100.0%	-3.5
2207	AT2G02500.1 | Symbol: ATMEPCT/ISPD | expressed protein, contains Pfam profile: PF01128 uncharacterized protein family UPF0007; identical to GP:12697583 2-C-methyl-D-erythritol 4-phosphate cytidyltransferase {Arabidopsis thaliana}; identical to cDNA 4-Diphosphocytidyl-2C-methyl-D-erythritol synthase (ISPD) GI:7385140 | chr2:670877-673154 REVERSE | Aliases: 2 C METHYL D ERYTHRITOL 4 PHOSPHATE CYTIDYLTRANSFERASE, 4 DIPHOSPHOCYTIDYL 2C METHYL D ERYTHRITOL SYNTHASE, T8K22.20, T8K22_20	5.1	5.8	-0.7	-2.1	100.0%	-2.8
2208	AT4G32950.1 | protein phosphatase 2C, putative / PP2C, putative, phosphoprotein phosphatase, Arabidopsis thaliana, PIR2:S55457	6.4	5.4	1.0	2.1	100.0%	-2.7
2209	AT2G13790.1 | Symbol: ATSERK4 | leucine-rich repeat family protein / protein kinase family protein | chr2:5748917-5753875 FORWARD | Aliases: AT2G13780, ATSERK4, F13J11.14, F13J11_14, SOMATIC EMBRYOGENESIS RECEPTOR LIKE KINASE 4	2.9	2.6	0.3	2.1	100.0%	-3.5
2210	AT4G32150.1 | Symbol: VAMP7C |	7.4	6.8	0.5	2.1	100.0%	-3.0
2211	AT3G16850.1 | glycoside hydrolase family 28 protein / polygalacturonase (pectinase) family protein, weak similarity to SP:P05117 Polygalacturonase 2A precursor (EC 3.2.1.15) (Pectinase) {Lycopersicon esculentum}; contains PF00295: Glycosyl hydrolases family 28 | chr3:5748580-5751303 FORWARD | Aliases: K20I9.8	5.5	4.8	0.7	2.1	100.0%	-2.8
2212	AT2G38460.1 | iron transporter-related, contains transmembrane domains; similar to iron-regulated transporter IREG1 (GI:7264727) (Mus musculus); similar to ferroportin1 (GI:7109247) (Mus musculus) | chr2:16110681-16113255 FORWARD | Aliases: T19C21.5, T19C21_5	7.4	6.7	0.6	2.1	100.0%	-2.9

Rank	Description	15.dpi	5.dpi	M	t	adj.q	B
2213	AT4G08690.2 | similar to SEC14 cytosolic factor family protein / phosphoglyceride transfer family protein [Arabidopsis thaliana] (TAIR:At1g22180.2); similar to putative sec14 like protein [Oryza sativa (japonica cultivar-group)] (GB:BAD81256.1); similar to unnamed protein product [Oryza sativa (japonica cultivar-group)] (GB:NP_912885.1); contains InterPro domain Cellular retinaldehyde-binding)/triple function, C-terminal (InterPro:IPR001251); contains InterPro domain Cellular retinaldehyde-binding/triple function, N-terminal (InterPro:IPR008273) | chr4:5550937-5552953 REVERSE | Aliases: T32A17.1	5.0	5.4	-0.5	-2.1	100.0%	-3.2
2214	AT5G58260.1 | Encodes subunit NDH-N of NAD(P)H:plastoquinone dehydrogenase complex (Ndh complex) present in the thylakoid membrane of chloroplasts. This subunit is thought to be required for Ndh complex assembly. | chr5:23578108-23579167 REVERSE | Aliases: MCK7.13, MCK7_13, NDH N	4.2	3.8	0.4	2.1	100.0%	-3.2
2215	AT2G41410.1 | calmodulin, putative, identical to SP:P30188 Calmodulin-like protein {Arabidopsis thaliana} | chr2:17268806-17269962 REVERSE | Aliases: F13H10.4, F13H10_4	7.8	7.1	0.7	2.1	100.0%	-3.0
2216	AT3G45040.1 | phosphatidate cytidylyltransferase family protein, weak similarity to SP:P20048 Dolichol kinase (EC 2.7.1.108) {Saccharomyces cerevisiae}; contains Pfam profile: PF01148 phosphatidate cytidylyltransferase | chr3:16483647-16486738 REVERSE | Aliases: F14D17.110	7.3	6.7	0.5	2.1	100.0%	-2.9
2217	AT2G42040.1 | expressed protein | chr2:17550112-17552308 FORWARD | Aliases: T6D20.7, T6D20_7	7.1	5.7	1.3	2.1	100.0%	-2.7
2218	AT5G40040.1 | 60S acidic ribosomal protein P2 (RPP2E), acidic ribosomal protein P2, Parthenium argentatum,SWISSPROT:RLA2_PARAR	5.0	6.0	-1.0	-2.1	100.0%	-2.7
2219	AT1G27460.1 | Symbol: NPGR1 | calmodulin-binding protein, similar to calmodulin-binding protein MPCBP (Zea mays) GI:10086260; contains Pfam profile PF00515: TPR Domain | chr1:9534519-9537548 FORWARD | Aliases: F17L21.25, F17L21_25, NO POLLEN GERMINATION RELATED 1	3.9	4.8	-0.9	-2.1	100.0%	-2.7
2220	AT5G20600.1 | expressed protein | chr5:6966206-6968035 REVERSE | Aliases: F7C8.190, F7C8_190	5.8	6.6	-0.8	-2.1	100.0%	-2.8
2221	AT2G01270.1 | Symbol: ATQSOX2 | Encodes a protein disulfide isomerase-like (PDIL) protein, a member of a multigene family within the thioredoxin (TRX) superfamily. This protein also belongs to the quiescin-sulfhydryl oxidase (QSOX) family, which possess an Erv1-like domain at the COOH terminus in addition to a TRX domain. | chr2:139334-142559 FORWARD | Aliases: ATQSOX2, F10A8.15, F10A8_15	8.0	6.8	1.2	2.1	100.0%	-2.7
2222	AT1G60660.1 | Symbol: B5 #5 | cytochrome b5 domain-containing protein, contains InterPro accession IPR001199: Cytochrome b5 | chr1:22346079-22346679 REVERSE | Aliases: F8A5.18, F8A5_18	7.6	8.4	-0.8	-2.1	100.0%	-2.8
2223	AT5G61790.1 | calnexin 1 (CNX1), identical to calnexin homolog 1, Arabidopsis thaliana, EMBL:AT08315 (SP:P29402) | chr5:24844328-24846981 REVERSE | Aliases: MAC9.15, MAC9_15	7.1	8.1	-1.1	-2.1	100.0%	-2.9
2224	AT5G43710.1 | glycoside hydrolase family 47 protein, similar to mannosyl-oligosaccharide 1,2-alpha-mannosidase IB (Mus musculus)(SP:P39098) | chr5:17569357-17573856 REVERSE | Aliases: MQD19.4, MQD19_4	4.8	5.6	-0.8	-2.1	100.0%	-2.9
2225	AT4G16410.1 | expressed protein, contains Pfam PF05421: Protein of unknown function (DUF751) | chr4:9261708-9262614 REVERSE | Aliases: DL4233C, FCAALL.144	7.0	6.0	0.9	2.1	100.0%	-2.7
2226	AT1G31660.1 | bystin family, contains Pfam profile: PF05291 Bystin | chr1:11331157-11333519 REVERSE | Aliases: F27M3.14, F27M3_14	4.3	4.9	-0.6	-2.1	100.0%	-3.1
2227	AT1G55325.1 | expressed protein | chr1:20641103-20650885 FORWARD | Aliases: None	2.7	3.0	-0.3	-2.1	100.0%	-3.4
2228	AT1G03190.2 | Symbol: UVH6 | DNA repair protein / transcription factor protein (UVH6), identical to DNA repair/transcription factor protein (UVH6) gi:22651569 gb:AY090788 | chr1:775622-780062 FORWARD | Aliases: ATXPD, ULTRAVIOLET HYPERSENSITIVE 6	4.5	5.2	-0.7	-2.1	100.0%	-3.0
2229	AT1G23860.2 | Symbol: SRZ 21 |	6.5	5.5	1.1	2.1	100.0%	-2.8
2230	AT4G13830.2 | Symbol: J20 | DNAJ heat shock N-terminal domain-containing protein (J20), identical to DnaJ-like protein (Arabidopsis thaliana) GI:6691127; similar to SP:Q05646 Chaperone protein dnaJ Erysipelothrix rhusiopathiae, J11 protein (Arabidopsis thaliana) GI:9843641; contains Pfam profile PF00226 DnaJ domain | chr4:8011420-8012749 FORWARD | Aliases: DNAJ LIKE 20, F18A5.220, F18A5_220	4.6	4.2	0.5	2.1	100.0%	-3.2
2231	AT1G32690.1 | expressed protein, similar to hypothetical protein GB:AAC61817 GI:3668085 from (Arabidopsis thaliana) | chr1:11820888-11821690 REVERSE | Aliases: F6N18.23, F6N18_23	6.1	5.4	0.7	2.1	100.0%	-2.9
2232	AT4G08920.1 | Symbol: CRY1 | cryptochrome 1 apoprotein (CRY1) / flavin-type blue-light photoreceptor (HY4), contains Pfam PF03441: FAD binding domain of DNA photolyase; member of Pfam PF00875: deoxyribodipyrimidine photolyase superfamily; 99% identical to Cryptochrome 1 apoprotein (Blue light photoreceptor) (flavin-type blue-light photoreceptor) (SP:Q43125) (Arabidopsis thaliana) | chr4:5724131-5727250 FORWARD | Aliases: BLU1, BLUE LIGHT UNINHIBITED 1, CRYPTOCHROME 1, CRYPTOCHROME 1 APOPROTEIN (BLUE LIGHT PHOTORECEPTOR, ELONGATED HYPOCOTYL 4, HY4, T3H13.14, T3H13_14	5.0	4.6	0.4	2.1	100.0%	-3.2

Rank	Description	15.dpi	5.dpi	M	t	adj.q	B
2233	AT1G10750.1 | expressed protein, similar to gi 3128199 F4I1.5 putative proteinase from Arabidopsis thaliana BAC gb AC004521 | chr1:3574808-3578475 REVERSE | Aliases: T16B5.11, T16B5_11	5.9	6.9	-1.1	-2.1	100.0%	-2.6
2234	AT5G48485.1 | Symbol: DIR1 | protease inhibitor/seed storage/lipid transfer protein (LTP) family protein, contains Pfam protease inhibitor/seed storage/LTP family domain PF00234 | chr5:19663458-19664028 REVERSE | Aliases: DEFECTIVE IN INDUCED RESISTANCE 1	3.7	3.3	0.4	2.1	100.0%	-3.5
2235	AT1G79760.1 | Symbol: DTA4 | expressed protein | chr1:30016947-30018045 FORWARD | Aliases: DOWNSTREAM TARGET OF AGL15 4, F19K16.26, F19K16_26	3.3	4.4	-1.1	-2.1	100.0%	-3.1
2236	AT5G01600.1 | Symbol: ATFER1 |	10.7	11.6	-0.9	-2.1	100.0%	-2.9
2237	AT5G57500.1 | expressed protein | chr5:23303463-23304797 REVERSE | Aliases: MUA2.7, MUA2_7	3.0	2.7	0.3	2.1	100.0%	-3.4
2238	AT1G55150.1 | DEAD box RNA helicase, putative (RH20), similar to ethylene-responsive RNA helicase GI:5669638 from (Lycopersicon esculentum); contains Pfam profiles PF00270: DEAD/DEAH box helicase, PF00271: Helicase conserved C-terminal domain | chr1:20578151-20580977 FORWARD | Aliases: T7N22.9, T7N22_9	5.6	6.2	-0.6	-2.1	100.0%	-3.1
2239	AT5G24270.1 | Symbol: SOS3 | calcineurin B-like protein, putative / calcium sensor homolog (SOS3), identical to calcium sensor homolog (Arabidopsis thaliana) GI:3309575; similar to calcineurin B-like protein 8 (GI:15866276) (Arabidopsis thaliana) | chr5:8238784-8240182 REVERSE | Aliases: CALCINEURIN B LIKE PROTEIN 4, CBL4, MOP9.19, MOP9_19, SALT OVERLY SENSITIVE 3	3.4	4.3	-1.0	-2.1	100.0%	-2.6
2240	AT1G56050.1 | GTP-binding protein-related, similar to GTP-binding protein GI:10176676 from (Bacillus halodurans) | chr1:20967384-20970006 FORWARD | Aliases: T6H22.14, T6H22_14	4.3	5.2	-0.9	-2.1	100.0%	-2.7
2241	AT5G61210.1 | Symbol: SNAP33 |	11.1	9.8	1.2	2.1	100.0%	-2.9
2242	AT2G23540.1 | GDSL-motif lipase/hydrolase family protein, similar to family II lipase EXL1 GI:15054382 from (Arabidopsis thaliana); contains Pfam profile PF00657: GDSL-like Lipase/Acylhydrolase | chr2:10031433-10033265 FORWARD | Aliases: F26B6.19, F26B6_19	2.9	4.0	-1.1	-2.1	100.0%	-2.6
2243	AT4G36920.1 | Symbol: AP2 | floral homeotic protein APETALA2 (AP2), Identical to (SP:P47927) Floral homeotic protein APETALA2. (Mouse-ear cress) {Arabidopsis thaliana} | chr4:17400844-17403329 FORWARD | Aliases: AP22.49, AP22_49, APETALA 2, APETALA2, FL1, FLO2, FLORAL HOMEOTIC PROTEIN APETALA 2, FLOWER1	5.4	4.7	0.7	2.1	100.0%	-2.8
2244	AT2G22610.1 | kinesin motor protein-related | chr2:9606630-9611827 FORWARD | Aliases: T9I22.5, T9I22_5	4.2	5.0	-0.8	-2.1	100.0%	-2.9
2245	AT3G06530.1 | BAP28-related, similar to Protein BAP28 (Swiss-Prot:Q9H583) (Homo sapiens) | chr3:2022602-2033643 FORWARD | Aliases: F5E6.14, F5E6_14	4.2	4.6	-0.4	-2.1	100.0%	-3.2
2246	AT3G58520.1 | expressed protein | chr3:21654333-21656463 REVERSE | Aliases: F14P22.110	3.1	3.4	-0.2	-2.1	100.0%	-3.8
2247	AT3G63050.1 | expressed protein | chr3:23308866-23309364 REVERSE | Aliases: T20O10.150	4.2	3.7	0.5	2.1	100.0%	-3.2
2248	AT3G24890.1 | Symbol: ATVAMP728 | synaptobrevin-related, similar to SYNAPTOBREVIN-RELATED PROTEIN GB:P47192 from (Arabidopsis thaliana) (Proc. Natl. Acad. Sci. U.S.A. (1992) 89(9), 3894-3898); contains Pfam profile PF00957: synaptobrevin	4.7	3.8	0.8	2.1	100.0%	-2.9
2249	AT5G43690.1 | sulfotransferase family protein, similar to steroid sulfotransferase 3 (Brassica napus) GI:3420008, steroid sulfotransferase 1 (Brassica napus) GI:3420004; contains Pfam profile PF00685: Sulfotransferase domain | chr5:17563500-17564561 FORWARD | Aliases: MQD19.2, MQD19_2	4.6	5.4	-0.8	-2.1	100.0%	-2.8
2250	AT4G37310.1 | Symbol: CYP81H1 | cytochrome P450, putative | chr4:17555921-17558887 REVERSE | Aliases: F6G17.6	4.6	5.8	-1.2	-2.1	100.0%	-2.7
2251	AT2G30830.1 | 2-oxoglutarate-dependent dioxygenase, putative, similar to 2A6 (GI:599622) and tomato ethylene synthesis regulatory protein E8 (SP:P10967) | chr2:13139784-13141361 REVERSE | Aliases: F7F1.4, F7F1_4	3.7	3.2	0.6	2.1	100.0%	-3.5
2252	AT3G55770.5 | similar to LIM domain-containing protein [Arabidopsis thaliana] (TAIR:At2g39900.1); similar to LIM domain protein [Gossypium hirsutum] (GB:AAL38006.1); contains InterPro domain Zn-binding protein, LIM (InterPro:IPR001781) | chr3:20714379-20716299 FORWARD | Aliases: F1I16.180	10.6	9.8	0.8	2.1	100.0%	-3.5
2253	AT1G16850.1 | expressed protein | chr1:5764800-5765644 REVERSE | Aliases: F17F16.17, F6I1.15, F6I1_15	5.0	4.4	0.6	2.1	100.0%	-3.0
2254	AT4G38010.1 | pentatricopeptide (PPR) repeat-containing protein, contains Pfam profile PF01535: PPR repeat | chr4:17859576-17861255 REVERSE | Aliases: F20D10.130, F20D10_130	3.8	4.1	-0.3	-2.1	100.0%	-3.3
2255	AT5G01180.1 | proton-dependent oligopeptide transport (POT) family protein, contains Pfam profile: PF00854 POT family | chr5:61016-63847 REVERSE | Aliases: F7J8.160, F7J8_160	2.7	3.0	-0.3	-2.1	100.0%	-3.4

Rank	Description	15.dpi	5.dpi	M	t	adj.q	B
2256	AT3G14180.1 | expressed protein, similar to 6b-interacting protein 1 (NtSIP1) (Nicotiana tabacum) GI:18149189 | chr3:4707120-4708855 REVERSE | Aliases: MAG2.16	5.0	4.2	0.8	2.1	100.0%	-3.0
2257	AT5G58787.2 | similar to expressed protein [Arabidopsis thaliana] (TAIR:At3g47160.1); similar to zinc finger protein family-like [Oryza sativa (japonica cultivar-group)] (GB:XP_479418.1); contains InterPro domain Zn-finger, RING (InterPro:IPR001841) | chr5:23759676-23761845 FORWARD | Aliases: None	7.1	6.5	0.6	2.1	100.0%	-3.0
2258	AT3G48550.1 | expressed protein, putative zinc-finger protein - Arabidopsis thaliana,PID:g4406777 | chr3:18004984-18005677 FORWARD | Aliases: T8P19.60	3.6	3.1	0.5	2.1	100.0%	-3.1
2259	AT5G05750.1 | DNAJ heat shock N-terminal domain-containing protein, similar to SP:Q9QYI4 DnaJ homolog subfamily B member 12 Mus musculus; contains Pfam profile PF00226 DnaJ domain | chr5:1727430-1728934 FORWARD | Aliases: MJJ3.16, MJJ3_16	5.4	4.3	1.1	2.1	100.0%	-2.9
2260	AT2G14890.2 | Symbol: AGP9 | arabinogalactan-protein (AGP9), identical to gi:10880495:gb:AAG24277 | chr2:6406703-6408064 FORWARD | Aliases: ARABINOGALACTAN PROTEIN 9, T26I20.5, T26I20_5	8.9	9.7	-0.8	-2.1	100.0%	-2.9
2261	AT4G17500.1 | Symbol: ATERF 1 | encodes a member of the ERF (ethylene response factor) subfamily B-3 of ERF/AP2 transcription factor family (ATERF-1). The protein contains one AP2 domain. There are 18 members in this subfamily including ATERF-1, ATERF-2, AND ATERF-5. | chr4:9759337-9760353 FORWARD | Aliases: DL4785W, ETHYLENE RESPONSIVE ELEMENT BINDING FACTOR 1, FCAALL.123	6.1	5.5	0.6	2.1	100.0%	-3.0
2262	AT4G29900.1 | Symbol: ACA10 | calcium-transporting ATPase, plasma membrane-type, putative / Ca2+-ATPase, putative (ACA10), identical to SP:Q9SZR1 Potential calcium-transporting ATPase 10, plasma membrane-type (EC 3.6.3.8) (Ca(2+)-ATPase isoform 10) {Arabidopsis thaliana}; similar to SP:Q9LF79 Calcium-transporting ATPase 8, plasma membrane-type (EC 3.6.3.8) (Ca(2+)-ATPase isoform 8) {Arabidopsis thaliana} | chr4:14610889-14618781 REVERSE | Aliases: ATACA10, AUTOINHIBITED CA(2+) ATPASE, F27B13.140, F27B13_140	7.0	6.3	0.7	2.1	100.0%	-3.0
2263	AT1G07640.3 | Symbol: OBP2 | similar to Dof-type zinc finger domain-containing protein [Arabidopsis thaliana] (TAIR:At2g28810.1); similar to putative DNA-binding protein [Oryza sativa (japonica cultivar-group)] (GB:XP_470142.1); contains InterPro domain Zn-finger, Dof type (InterPro:IPR003851) | chr1:2354351-2356224 REVERSE | Aliases: F24B9.30, F24B9_30, ZINC FINGER PROTEIN OBP2	6.7	6.0	0.6	2.1	100.0%	-2.8
2264	AT1G50430.1 | Symbol: DWF5 | 7-dehydrocholesterol reductase / 7-DHC reductase / sterol delta-7-reductase (ST7R) / dwarf5 protein (DWF5), identical to SP:Q9LDU6 7-dehydrocholesterol reductase (EC 1.3.1.21) (7-DHC reductase) (Sterol delta-7-reductase) (Dwarf5 protein) {Arabidopsis thaliana} | chr1:18685614-18689307 REVERSE | Aliases: 7RED, DWARF 5, F11F12.21, F11F12_21, LE, LEPIDA, PA, PARVA, ST7R, STEROL DELTA 7 REDUCTASE, STEROL DELTA7 REDUCTASE	8.7	9.3	-0.5	-2.1	100.0%	-3.1
2265	AT5G18070.1 | Symbol: DRT101 |	6.0	6.6	-0.6	-2.1	100.0%	-3.0
2266	AT1G15020.2 | Symbol: ATQSOX1 | thioredoxin family protein, low similarity to FAD-dependent sulfhydryl oxidase-2 (Rattus norvegicus) GI:12483919; contains Pfam profiles PF00085: Thioredoxin, PF04777: Erv1 / Alr family | chr1:5173120-5176215 REVERSE | Aliases: ATQSOX1, QUIESCIN SULFHYDRYL OXIDASE 1, T15D22.7, T15D22_7	5.5	4.9	0.5	2.1	100.0%	-3.2
2267	AT2G24180.1 | Symbol: CYP71B6 | cytochrome P450 family protein | chr2:10288927-10290815 FORWARD | Aliases: CYTOCHROME P450 71B6, CYTOCHROME P450 MONOOXYGENASE 71B6, F27D4.9, F27D4_9	7.3	8.0	-0.7	-2.1	100.0%	-3.0
2268	AT3G51890.1 | expressed protein, protein At2g40060 - Arabidopsis thaliana, EMBL:AF002109 | chr3:19260457-19261942 REVERSE | Aliases: ATEM1.14	7.1	6.2	0.9	2.1	100.0%	-2.8
2269	AT3G05360.1 | disease resistance family protein / LRR family protein, contains leucine rich-repeat domains Pfam:PF00560, INTERPRO:IPR001611; similar to elicitor-inducible LRR receptor-like protein EILP (Nicotiana tabacum) gi:6635236:dbj:BAA88636; similar to Cf-2.2 (Lycopersicon pimpinellifolium) gi:1184077:gb:AAC15780 | chr3:1530741-1533294 REVERSE | Aliases: F22F7.23	3.0	3.5	-0.4	-2.1	100.0%	-3.2
2270	AT5G57950.1 | 26S proteasome regulatory subunit, putative, contains similarity to 26S proteasome non-ATPase subunit 9 SP:O00233 from (Homo sapiens) | chr5:23477935-23479614 FORWARD | Aliases: MTI20.21, MTI20_21	4.9	4.4	0.6	2.1	100.0%	-3.1
2271	AT5G18840.1 | sugar transporter, putative, similar to ERD6 protein {Arabidopsis thaliana} GI:3123712, sugar-porter family protein 1 (Arabidopsis thaliana) GI:14585699; contains Pfam profile PF00083: major facilitator superfamily protein | chr5:6282883-6286709 FORWARD | Aliases: F17K4.90, F17K4_90	3.7	3.4	0.3	2.1	100.0%	-3.6
2272	AT1G12130.1 | flavin-containing monooxygenase family protein / FMO family protein, contains similarity to flavin-containing monooxygenase 2 (FMO2) from Homo sapiens (GI:1834493); contains Pfam profile PF00743 Flavin-binding monooxygenase-like | chr1:4118592-4120869 FORWARD | Aliases: T28K15.13, T28K15_13	2.8	3.0	-0.2	-2.1	100.0%	-3.9
2273	AT5G06140.1 | phox (PX) domain-containing protein, similar to SP:O60749 Sorting nexin 2 {Homo sapiens}; contains Pfam profile PF00787: PX domain | chr5:1856009-1858767 REVERSE | Aliases: K16F4.11, K16F4_11	6.5	7.4	-0.8	-2.1	100.0%	-2.9

Rank	Description	15.dpi	5.dpi	M	t	adj.q	B
2274	AT3G13340.1 | WD-40 repeat family protein, contains Pfam profile: PF00400 WD domain, G-beta repeat (3 copies, 1 significant); similar to Trp-Asp repeat protein (PIR:T40094) (Schizosaccharomyces) | chr3:4331766-4335080 FORWARD | Aliases: MDC11.14	3.7	3.2	0.5	2.1	100.0%	-3.2
2275	AT2G28800.2 | Symbol: ALB3 | chloroplast membrane protein (ALBINO3), Oxa1p homolog {PMID:11148275}; identical to chloroplast membrane protein ALBINO3 (Arabidopsis thaliana) GI:2209332 | chr2:12364233-12366329 REVERSE | Aliases: ABL3, ALBINA 3, ALBINO 3, CHLOROPLAST MEMBRANE PROTEIN ALB3, F8N16.9, F8N16_9	8.7	7.6	1.1	2.1	100.0%	-2.8
2276	AT5G07330.1 | expressed protein | chr5:2315981-2316875 FORWARD | Aliases: T2I1.40, T2I1_40	4.7	3.7	1.0	2.1	100.0%	-2.8
2277	AT1G72520.1 | lipoxygenase, putative, similar to lipoxygenase gi:1495804 (Solanum tuberosum), gi:1654140 (Lycopersicon esculentum), GB:CAB56692 (Arabidopsis thaliana)	5.3	4.6	0.8	2.1	100.0%	-2.8
2278	AT2G32060.3 | 40S ribosomal protein S12 (RPS12C) | chr2:13646122-13647257 REVERSE | Aliases: F22D22.19, F22D22_19	10.8	11.5	-0.7	-2.1	100.0%	-3.1
2279	AT1G76140.1 | similar to prolyl oligopeptidase, putative / prolyl endopeptidase, putative / post-proline cleaving enzyme, putative [Arabidopsis thaliana] (TAIR:At1g20380.1); similar to putative prolyl endopeptidase [Oryza sativa (japonica cultivar-group)] (GB:NP_908392.1); similar to OTTHUMP00000040498 [Homo sapiens] (GB:CAH72545.1); similar to OSJNBa0084K11.14 [Oryza sativa (japonica cultivar-group)] (GB:XP_473492.1); similar to similar to Prolyl endopeptidase (Post-proline cleaving enzyme) (PE) [Gallus gallus] (GB:NP_001006410.1); similar to prolyl endopeptidase [Mus musculus] (GB:NP_035286.1); contains InterPro domain Prolyl oligopeptidase serine protease (S9A) (InterPro:IPR002470); contains InterPro domain Prolyl oligopeptidase (InterPro:IPR001375); contains InterPro domain Prolyl oligopeptidase, N-terminal beta-propeller domain (InterPro:IPR004106) | chr1:28576024-28579930 FORWARD | Aliases: T23E18.8, T23E18_8	8.9	9.7	-0.8	-2.1	100.0%	-3.1
2280	AT5G52300.2 | Symbol: RD29B | similar to stress-responsive protein-related [Arabidopsis thaliana] (TAIR:At4g25580.1); similar to cor78 (GB:AAA32776.1) | chr5:21254282-21256755 FORWARD | Aliases: K24M7.3, K24M7_3, LTI65, RESPONSIVE TO DESSICATION 29B	7.2	6.3	0.9	2.1	100.0%	-2.9
2281	AT1G15170.1 | MATE efflux family protein, similar to ripening regulated protein DDTFR18 (Lycopersicon esculentum) GI:12231296; contains Pfam profile PF01554: Uncharacterized membrane protein family | chr1:5220636-5222968 FORWARD | Aliases: F9L1.11, F9L1_11	5.4	4.8	0.6	2.1	100.0%	-2.9
2282	AT5G61990.1 | pentatricopeptide (PPR) repeat-containing protein, contains Pfam profile PF01535: PPR repeat | chr5:24916832-24920343 REVERSE | Aliases: MTG10.2, MTG10_2	3.4	3.7	-0.3	-2.1	100.0%	-3.5
2283	AT5G40870.1 | uracil phosphoribosyltransferase, putative / UMP pyrophosphorylase, putative / UPRTase, putative, similar to SP:O65583 Uracil phosphoribosyltransferase (EC 2.4.2.9) (UMP pyrophosphorylase) (UPRTase) {Arabidopsis thaliana}; contains Pfam profile PF00485: Phosphoribulokinase / Uridine kinase family | chr5:16392155-16395876 FORWARD | Aliases: MHK7.10, MHK7_10	6.5	7.5	-1.0	-2.1	100.0%	-3.0
2284	AT1G15430.2 | expressed protein | chr1:5304454-5306007 FORWARD | Aliases: F9L1.39, F9L1_39	8.1	6.9	1.2	2.1	100.0%	-2.9
2285	AT3G19520.1 | expressed protein, contains Pfam profile PF04776: Protein of unknown function (DUF626) | chr3:6772696-6774449 FORWARD | Aliases: T31J18.2	7.1	6.6	0.5	2.1	100.0%	-3.1
2286	AT4G35830.1 | aconitate hydratase, cytoplasmic / citrate hydro-lyase / aconitase (ACO), identical to SP:Q42560 Aconitate hydratase, cytoplasmic (EC 4.2.1.3) (Citrate hydro-lyase) (Aconitase) {Arabidopsis thaliana}; contains Pfam profiles PF00330: Aconitase family (aconitate hydratase), PF00694: Aconitase C-terminal domain | chr4:16972602-16978061 REVERSE | Aliases: F4B14.100, F4B14_100	9.5	10.0	-0.5	-2.1	100.0%	-3.4
2287	AT2G06000.2 | pentatricopeptide (PPR) repeat-containing protein, contains Pfam profile PF01535: PPR repeat | chr2:2327391-2329662 REVERSE | Aliases: T6P5.20, T6P5_20	4.6	5.0	-0.5	-2.1	100.0%	-3.2
2288	AT5G66750.1 | Symbol: DDM1 | SNF2 domain-containing protein / helicase domain-containing protein, similar to proliferation-associated SNF2-like protein (Homo sapiens) GI:8980660; contains Pfam profiles PF00271: Helicase conserved C-terminal domain, PF00176: SNF2 family N-terminal domain | chr5:26666177-26670299 FORWARD | Aliases: DECREASED DNA METHYLATION 1, MSN2.14, MSN2_14, SOM1, SOM4, SOMNIFEROUS 1	2.9	3.6	-0.7	-2.1	100.0%	-3.2
2289	AT1G62045.1 | expressed protein | chr1:22938353-22939008 REVERSE | Aliases: None	6.3	5.4	0.9	2.1	100.0%	-2.9
2290	AT3G15080.1 | exonuclease family protein, contains exonuclease domain, Pfam:PF00929 | chr3:5074272-5076528 FORWARD | Aliases: K15M2.23	6.2	7.0	-0.8	-2.1	100.0%	-3.0
2291	AT5G64990.1 | Ras-related GTP-binding protein, putative, similar to GTP-binding protein GI:550072 from (Homo sapiens) | chr5:25980788-25982018 REVERSE | Aliases: MXK3.22, MXK3_22	6.0	5.0	1.0	2.1	100.0%	-2.7
2292	AT3G60840.1 | microtubule associated protein (MAP65/ASE1) family protein, low similarity to protein regulating cytokinesis 1 (PRC1) (Homo sapiens) GI:2865521; contains Pfam profile PF03999: Microtubule associated protein (MAP65/ASE1 family) | chr3:22488548-22491438 REVERSE | Aliases: T4C21.250	3.6	4.1	-0.5	-2.1	100.0%	-3.1

Rank	Description	15.dpi	5.dpi	M	t	adj.q	B
2293	AT5G22920.1 | zinc finger (C3HC4-type RING finger) family protein, contains Pfam profiles:PF05495 CHY zinc finger, PF00097 zinc finger, C3HC4 type (RING finger) | chr5:7664994-7667268 FORWARD | Aliases: MRN17.15, MRN17_15	8.2	7.4	0.8	2.1	100.0%	-3.2
2294	AT1G58100.1 | TCP family transcription factor, putative, similar to auxin-induced basic helix-loop-helix transcription factor GI:5731257 from (Gossypium hirsutum) | chr1:21516112-21517592 REVERSE | Aliases: T15M6.11	6.4	5.8	0.6	2.1	100.0%	-3.2
2295	AT3G23150.1 | Symbol: ETR2 | ethylene receptor, putative (ETR2), similar to putative ethylene receptor; ETR2 (Arabidopsis thaliana) gi:3687654:gb:AAC62208. | chr3:8254670-8257742 FORWARD | Aliases: ETHYLENE RESPONSE 2, ETR2, K14B15.9	5.9	5.0	0.9	2.1	100.0%	-2.9
2296	AT4G17360.1 | formyltetrahydrofolate deformylase, putative, similar to formyltetrahydrofolate deformylase (strain PCC 6803- Synechocystis sp.) SWISS-PROT:Q55135 | chr4:9703187-9705480 REVERSE | Aliases: DL4715C, FCAALL.417	4.4	3.9	0.5	2.1	100.0%	-3.0
2297	AT3G07790.1 | DGCR14-related, similar to DGCR14 protein (DiGeorge syndrome critical region 14) (ES2 protein) (Swiss-Prot:Q96DF8) (Homo sapiens) | chr3:2487375-2489226 FORWARD | Aliases: MLP3.24	8.4	7.5	0.9	2.1	100.0%	-2.9
2298	AT2G03670.1 | Symbol: CDC48B |	3.9	4.7	-0.8	-2.1	100.0%	-3.0
2299	AT1G65295.1 | expressed protein | chr1:24255174-24255966 FORWARD | Aliases: None	6.7	5.8	0.8	2.1	100.0%	-2.9
2300	AT1G73930.2 | similar to FLJ00229 protein [Homo sapiens] (GB:BAB84982.1) | chr1:27798781-27801642 REVERSE | Aliases: F2P9.20, F2P9_20	5.8	6.3	-0.5	-2.1	100.0%	-3.1
2301	AT2G17660.1 | nitrate-responsive NOI protein, putative, similar to nitrate-induced NOI protein (Zea mays) GI:2642213 | chr2:7680364-7680794 FORWARD | Aliases: T17A5.20, T17A5_20	4.4	3.3	1.1	2.1	100.0%	-2.9
2302	AT1G79210.1 | 20S proteasome alpha subunit B, putative, nearly identical to SP:O23708 Proteasome subunit alpha type 2 (EC 3.4.25.1) (20S proteasome alpha subunit B) {Arabidopsis thaliana} and to At1g16470 | chr1:29800987-29803624 REVERSE | Aliases: YUP8H12R.19, YUP8H12R_19	7.2	8.3	-1.1	-2.1	100.0%	-2.9
2303	AT5G57930.2 | Symbol: APO2 | expressed protein | chr5:23471843-23473719 FORWARD | Aliases: ACCUMULATION OF PHOTOSYSTEM ONE 2, EMB1629, EMBRYO DEFECTIVE 1629, MTI20.19, MTI20_19	7.2	6.7	0.5	2.1	100.0%	-3.0
2304	AT2G32020.1 | GCN5-related N-acetyltransferase (GNAT) family protein, contains Pfam profile PF00583: acetyltransferase, GNAT family | chr2:13638139-13638845 REVERSE | Aliases: F22D22.23, F22D22_23	3.6	3.2	0.4	2.1	100.0%	-3.6
2305	AT2G40820.1 | proline-rich family protein, contains proline rich extensin domains, INTERPRO:IPR002965 | chr2:17041814-17046552 REVERSE | Aliases: T20B5.2, T20B5_2	4.5	5.0	-0.5	-2.1	100.0%	-3.0
2306	AT4G36010.2 | similar to pathogenesis-related thaumatin family protein [Arabidopsis thaliana] (TAIR:At2g17860.1); similar to putative thaumatin-like protein [Oryza sativa (japonica cultivar-group)] (GB:AAP52110.1); contains InterPro domain Thaumatin, pathogenesis-related (InterPro:IPR001938) | chr4:17039066-17041134 REVERSE | Aliases: T19K4.140, T19K4_140	4.7	3.9	0.8	2.1	100.0%	-2.8
2307	AT1G22740.1 | Symbol: RAB7 | Ras-related protein (RAB7) / AtRab75 / small GTP-binding protein, putative, identical to SP:O04157 Ras-related protein Rab7 (AtRab75) (Arabidopsis thaliana) | chr1:8049089-8050697 FORWARD | Aliases: T22J18.9, T22J18_9	4.6	4.0	0.6	2.1	100.0%	-3.0
2308	AT3G20790.1 | oxidoreductase family protein, weak similarity to SP:Q07982 Glucose--fructose oxidoreductase precursor (EC 1.1.99.28) {Zymomonas mobilis}; contains Pfam profiles PF01408: Oxidoreductase family NAD-binding Rossmann fold, PF02894: Oxidoreductase family C-terminal alpha/beta domain | chr3:7268768-7271517 FORWARD | Aliases: MOE17.10	8.8	9.5	-0.8	-2.1	100.0%	-2.9
2309	AT1G12000.1 | pyrophosphate--fructose-6-phosphate 1-phosphotransferase beta subunit, putative / pyrophosphate-dependent 6-phosphofructose-1-kinase, putative, strong similarity to SP:Q41141 Pyrophosphate--fructose 6-phosphate 1-phosphotransferase beta subunit (EC 2.7.1.90) (PFP) ((PPI-PFK) {Ricinus communis}; contains Pfam profile PF00365: Phosphofructokinase | chr1:4049894-4053833 REVERSE | Aliases: F12F1.13, F12F1_13	7.5	9.2	-1.7	-2.1	100.0%	-2.9
2310	AT1G68560.1 | Symbol: XYL1 | alpha-xylosidase (XYL1), identical to alpha-xylosidase precursor GB:AAD05539 GI:4163997 from (Arabidopsis thaliana); contains Pfam profile PF01055: Glycosyl hydrolases family 31; identical to cDNA alpha-xylosidase precursor (XYL1) partial cds GI:4163996 | chr1:25737845-25741658 REVERSE | Aliases: F24J5.20, F24J5_20	3.5	4.4	-0.9	-2.1	100.0%	-2.9
2311	AT3G28350.1 | hypothetical protein | chr3:10609596-10611901 FORWARD | Aliases: MFJ20.3	2.7	2.5	0.2	2.1	100.0%	-3.8
2312	AT2G47630.1 | esterase/lipase/thioesterase family protein, similar to monoglyceride lipase from (Homo sapiens) GI:14594904, (Mus musculus) GI:2632162; contains Interpro entry IPR000379 | chr2:19541573-19544073 FORWARD | Aliases: F17A22.2	6.4	7.4	-1.1	-2.1	100.0%	-3.1

Rank	Description	15.dpi	5.dpi	M	t	adj.q	B
2313	AT4G19880.3 | similar to expressed protein [Arabidopsis thaliana] (TAIR:At5g45020.1); similar to putative transferase [Escherichia coli O157:H7 EDL933] (GB:AAG58235.1) | chr4:10784401-10786433 REVERSE | Aliases: T16H5.240, T16H5_240	8.9	9.4	-0.4	-2.1	100.0%	-3.6
2314	AT1G04190.1 | tetratricopeptide repeat (TPR)-containing protein, low similarity to protein antigen LmSTI1 (Leishmania major) GI:1698880; contains Pfam profile PF00515 TPR Domain; EST gb:Z47802 and gb:Z48402 come from this gene | chr1:1106427-1108707 REVERSE | Aliases: F20D22.4, F20D22_4	7.5	8.5	-1.0	-2.1	100.0%	-2.9
2315	AT2G46520.1 | cellular apoptosis susceptibility protein, putative / importin-alpha re-exporter, putative, similar to cellular apoptosis susceptibility protein (Homo sapiens) GI:3598795, SP:Q9ERK4 Importin-alpha re-exporter (Chromosome segregation 1-like protein) (Cellular apoptosis susceptibility protein) {Mus musculus}; contains Pfam profiles PF03810: Importin-beta N-terminal domain, PF03378: CAS/CSE protein C-terminus | chr2:19103840-19107344 FORWARD | Aliases: F11C10.33	4.5	5.0	-0.6	-2.1	100.0%	-3.1
2316	AT5G02880.1 | Symbol: UPL4 | HECT-domain-containing protein / ubiquitin-transferase family protein / armadillo/beta-catenin-like repeat-containing protein, similar to SP:Q14669 Thyroid receptor interacting protein 12 (TRIP12) {Homo sapiens}; contains Pfam profiles PF00632: HECT-domain (ubiquitin-transferase), PF00514: Armadillo/beta-catenin-like repeat | chr5:662641-669105 FORWARD | Aliases: F9G14.190, F9G14_190	5.7	6.4	-0.6	-2.1	100.0%	-2.9
2317	AT4G34880.1 | amidase family protein, similar to peptide amidase (Stenotrophomonas maltophilia) GI:19744118; contains Pfam profile PF01425: Amidase | chr4:16615554-16617429 FORWARD | Aliases: F11I11.120, F11I11_120	3.3	4.1	-0.8	-2.1	100.0%	-3.1
2318	AT4G37170.1 | pentatricopeptide (PPR) repeat-containing protein, contains Pfam profile PF01535: PPR repeat | chr4:17498574-17500649 REVERSE | Aliases: AP22.43, AP22_43	4.3	5.0	-0.7	-2.1	100.0%	-3.2
2319	AT1G16710.1 | TAZ zinc finger family protein / zinc finger (ZZ type) family protein, contains Pfam profiles PF02135: TAZ zinc finger, PF00569: Zinc finger, ZZ type | chr1:5714685-5722359 FORWARD | Aliases: F19K19.17	4.2	3.6	0.6	2.1	100.0%	-3.1
2320	AT5G27470.1 | seryl-tRNA synthetase / serine--tRNA ligase, identical to SP:Q39230 Seryl-tRNA synthetase (EC 6.1.1.11) (Serine--tRNA ligase) (SerRS) {Arabidopsis thaliana} | chr5:9695011-9697392 FORWARD | Aliases: F21A20.180, F21A20_180	7.8	8.4	-0.7	-2.1	100.0%	-3.2
2321	AT3G01430.1 | expressed protein | chr3:165415-166254 REVERSE | Aliases: T13O15.7, T13O15_7	5.7	5.1	0.6	2.1	100.0%	-3.0
2322	AT3G54010.2 | Symbol: PAS1 | peptidyl-prolyl cis-trans isomerase, putative / FK506-binding protein, putative / pasticcino 1-D (PAS1-D), nearly identical to pasticcino 1-D (Arabidopsis thaliana) GI:3080740 | chr3:20011867-20016161 FORWARD | Aliases: F5K20.310, PASTICCINO 1	8.7	9.4	-0.6	-2.1	100.0%	-3.3
2323	AT5G65480.1 | expressed protein | chr5:26193247-26195140 FORWARD | Aliases: MNA5.18	9.8	9.1	0.7	2.1	100.0%	-3.2
2324	AT2G36880.1 | S-adenosylmethionine synthetase, putative, similar to S-adenosylmethionine synthetase 3 (Methionine adenosyltransferase 3, AdoMet synthetase 3) (Lycopersicon esculentum) SWISS-PROT:P43282 | chr2:15486445-15488486 REVERSE | Aliases: T1J8.6, T1J8_6	7.0	8.7	-1.7	-2.1	100.0%	-2.9
2325	AT2G20260.1 | photosystem I reaction center subunit IV, chloroplast, putative / PSI-E, putative (PSAE2), identical to SP:Q9S714; similar to SP:P12354 Photosystem I reaction center subunit IV, chloroplast precursor (PSI-E) {Spinacia oleracea}; contains Pfam profile PF02427: Photosystem I reaction centre subunit IV / PsaE | chr2:8743818-8744843 FORWARD | Aliases: F11A3.19, F11A3_19	7.3	6.6	0.8	2.1	100.0%	-3.0
2326	AT2G46230.1 | expressed protein, contains Pfam profile: PF04900 protein of unknown function, DUF652 | chr2:18991045-18993032 REVERSE | Aliases: T3F17.12	7.7	8.6	-0.9	-2.1	100.0%	-3.0
2327	AT1G32410.2 | vacuolar protein sorting 55 family protein / VPS55 family protein, contains Pfam domain PF04133: Vacuolar protein sorting 55 | chr1:11693837-11695534 FORWARD | Aliases: F5D14.32	7.9	7.2	0.7	2.1	100.0%	-3.2
2328	AT2G16710.1 | hesB-like domain-containing protein, similar to IscA (putative iron-sulfur cluster assembly protein) (Azotobacter vinelandii) GI:2271523; contains Pfam profile PF01521: HesB-like domain | chr2:7255255-7257367 FORWARD | Aliases: T24I21.12, T24I21_12	10.3	9.5	0.8	2.1	100.0%	-3.2
2329	AT4G33280.1 | similar to transcriptional factor B3 family protein [Arabidopsis thaliana] (TAIR:At3g18990.1); similar to putative auxin response factor [Oryza sativa (japonica cultivar-group)] (GB:XP_463202.1); contains InterPro domain Transcriptional factor B3 (InterPro:IPR003340) | chr4:16047361-16049359 REVERSE | Aliases: F17M5.40, F17M5_40	4.0	3.5	0.6	2.1	100.0%	-3.0
2330	AT2G40400.2 | expressed protein, similar to GI:7572912 (At3g56140)(Arabidopsis thaliana) | chr2:16876412-16880336 FORWARD | Aliases: T3G21.17, T3G21_17	4.8	5.7	-0.9	-2.1	100.0%	-3.0
2331	AT2G31690.1 | lipase class 3 family protein, similar to DEFECTIVE IN ANTHER DEHISCENCE1 (Arabidopsis thaliana) GI:16215706; contains Pfam profile PF01764: Lipase | chr2:13483170-13484624 REVERSE | Aliases: T9H9.21, T9H9_21	2.0	2.2	-0.2	-2.1	100.0%	-4.4

Rank	Description	15.dpi	5.dpi	M	t	adj.q	B
2332	AT4G18070.4 | expressed protein, similar to expressed protein [Arabidopsis thaliana] (TAIR:At1g29530.1); similar to hypothetical protein DDB0183896 [Dictyostelium discoideum] (GB:EAL61706.1); contains domain SER_RICH (PS50324) | chr4:10030709-10032947 FORWARD | Aliases: F15J5.40, F15J5_40	6.7	5.8	0.9	2.1	100.0%	-3.2
2333	AT5G39320.1 | UDP-glucose 6-dehydrogenase, putative, very strong similarity to SP:Q96558 UDP-glucose 6-dehydrogenase (EC 1.1.1.22) (UDP-Glc dehydrogenase) (UDP-GlcDH) (UDPGDH) {Glycine max}; contains Pfam profiles PF03721: UDP-glucose/GDP-mannose dehydrogenase family NAD binding domain, PF00984: UDP-glucose/GDP-mannose dehydrogenase family central domain, PF03720: UDP-glucose/GDP-mannose dehydrogenase family UDP binding domain	3.8	4.8	-1.0	-2.1	100.0%	-2.9
2334	AT3G23805.1 | Symbol: RALFL24 | rapid alkalinization factor (RALF) family protein, similar to RALF precursor (Nicotiana tabacum) GI:16566316 | chr3:8586389-8587063 FORWARD | Aliases: RALF LIKE 24	5.7	6.5	-0.7	-2.1	100.0%	-3.0
2335	AT2G47250.1 | RNA helicase, putative, similar to SP:P53131 Pre-mRNA splicing factor RNA helicase PRP43 (Helicase JA1) {Saccharomyces cerevisiae}; contains Pfam profiles PF04408: Helicase associated domain (HA2), PF00271: Helicase conserved C-terminal domain | chr2:19406765-19410107 REVERSE | Aliases: T8I13.9	7.4	8.1	-0.6	-2.1	100.0%	-3.0
2336	AT5G19510.1 | elongation factor 1B alpha-subunit 2 (eEF1Balpha2), identical to elongation factor 1B alpha-subunit (Arabidopsis thaliana) GI:6686821 | chr5:6581660-6583213 REVERSE | Aliases: T20D1.30, T20D1_30	11.3	11.7	-0.4	-2.1	100.0%	-3.3
2337	AT2G38140.1 | Symbol: PSRP4 | chloroplast 30S ribosomal protein S31 (PSRP4) | chr2:15987961-15988676 FORWARD | Aliases: 30S RIBOSOMAL PROTEIN S31, F16M14.7, F16M14_7, PLASTID SPECIFIC RIBOSOMAL PROTEIN 4	8.8	9.7	-0.8	-2.1	100.0%	-2.9
2338	AT1G03520.1 | glycosyltransferase family 14 protein / core-2/I-branching enzyme family protein, contains Pfam profile PF02485: Core-2/I-Branching enzyme | chr1:877863-880159 REVERSE | Aliases: F21B7.14	5.7	5.2	0.5	2.1	100.0%	-3.2
2339	AT4G25240.1 | Symbol: SKS1 | multi-copper oxidase type I family protein, pollen-specific protein precursor -Nicotiana tabacum, PID:g19902; contains Pfam profile: PF00394 Multicopper oxidase	3.0	3.4	-0.3	-2.1	100.0%	-3.4
2340	AT3G29330.1 | expressed protein | chr3:11258939-11260290 REVERSE | Aliases: MUO10.3	4.6	4.1	0.4	2.1	100.0%	-3.2
2341	AT2G28600.1 | expressed protein | chr2:12258862-12261900 FORWARD | Aliases: T8O18.11, T8O18_11	5.7	6.5	-0.8	-2.1	100.0%	-2.8
2342	AT1G29530.1 | expressed protein | chr1:10324731-10326721 REVERSE | Aliases: F15D2.11, F15D2_11	4.7	5.5	-0.7	-2.1	100.0%	-2.8
2343	AT4G23860.2 | PHD finger protein-related, similar to Pfam profiles PF02207: Putative zinc finger in N-recognin, weak hit to PF00628: PHD-finger | chr4:12409033-12411575 FORWARD | Aliases: T32A16.30, T32A16_30	3.6	4.0	-0.5	-2.1	100.0%	-3.1
2344	AT1G17100.1 | SOUL heme-binding family protein, similar to SOUL protein (Mus musculus) GI:4886906; contains Pfam profile PF04832: SOUL heme-binding protein | chr1:5844729-5846215 FORWARD | Aliases: F20D23.21, F20D23_21	9.0	8.4	0.6	2.1	100.0%	-2.9
2345	AT5G46680.1 | pentatricopeptide (PPR) repeat-containing protein, contains Pfam profile PF01535: PPR repeat | chr5:18958196-18959789 FORWARD | Aliases: MZA15.9, MZA15_9	5.1	6.3	-1.2	-2.1	100.0%	-2.9
2346	AT2G30200.2 | similar to malonyl-CoA:ACP transacylase [Perilla frutescens] (GB:AAG43518.1); contains InterPro domain Acyl transferase domain (InterPro:IPR001227) | chr2:12890074-12892845 REVERSE | Aliases: T27E13.6, T27E13_6	9.7	10.7	-1.0	-2.1	100.0%	-2.9
2347	AT1G26960.1 | homeobox-leucine zipper protein, putative / HD-ZIP transcription factor, putative, similar to homeobox-leucine zipper protein HAT7 (HD-ZIP protein 7) (HD-ZIP protein ATHB-3 (SP:Q00466: (Arabidopsis thaliana); similar to Helianthus annuus gi:349379, and carrot, gi:1435022. Contains Homeobox domain motif | chr1:9355907-9357437 FORWARD | Aliases: T2P11.15, T2P11_15	3.1	2.8	0.3	2.1	100.0%	-3.7
2348	AT2G23560.1 | hydrolase, alpha/beta fold family protein, similar to ethylene-induced esterase (Citrus sinensis) GI:14279437, polyneuridine aldehyde esterase (Rauvolfia serpentina) GI:6651393; contains Pfam profile PF00561: hydrolase, alpha/beta fold family | chr2:10036416-10037362 REVERSE | Aliases: F26B6.21, F26B6_21	7.7	8.5	-0.8	-2.1	100.0%	-3.0
2349	AT5G24490.1 | 30S ribosomal protein, putative, similar to SP:P19954 Plastid-specific 30S ribosomal protein 1, chloroplast precursor (CS-S5) (CS5) (S22) (Ribosomal protein 1) (PSRP-1) {Spinacia oleracea}; contains Pfam profile PF02482: Sigma 54 modulation protein / S30EA ribosomal protein | chr5:8365624-8367429 FORWARD | Aliases: T31K7.7, T31K7_7	8.3	9.0	-0.7	-2.1	100.0%	-2.9
2350	AT5G56250.2 | expressed protein | chr5:22785803-22790118 REVERSE | Aliases: K24C1.6, K24C1_6	3.6	3.3	0.3	2.1	100.0%	-3.7
2351	AT1G76080.1 | thioredoxin family protein, low similarity to thioredoxin (TRX) (Fasciola hepatica) GI:6687568; contains Pfam profile PF00085: Thioredoxin | chr1:28552680-28554313 REVERSE | Aliases: T23E18.2, T23E18_2	8.3	7.6	0.7	2.1	100.0%	-2.8

Rank	Description	15.dpi	5.dpi	M	t	adj.q	B
2352	AT1G05850.1 | Symbol: ELP | chitinase-like protein 1 (CTL1), similar to class I chitinase GI:7798656 from (Halimolobos perplexa var. perplexa); contains Pfam profile PF00182: Chitinase class I; identical to cDNA chitinase-like protein 1 (CTL1) CTL1-ELP1 allele GI:17226328 | chr1:1766502-1768662 REVERSE | Aliases: CHITINASE LIKE PROTEIN 1, CTL1, ECTOPIC DEPOSITION OF LIGNIN IN PITH, ELP1, T20M3.12, T20M3_12	10.1	8.7	1.3	2.1	100.0%	-3.3
2353	AT5G37540.1 | aspartyl protease family protein, weak similarity to CND41, chloroplast nucleoid DNA binding protein (Nicotiana tabacum) GI:2541876; contains Prosite PS00141: Eukaryotic and viral aspartyl proteases active site; contains 1 predicted transmembrane domain | chr5:14930065-14931661 FORWARD | Aliases: MPA22.8, MPA22_8	4.9	4.2	0.8	2.1	100.0%	-3.0
2354	AT1G17070.1 | D111/G-patch domain-containing protein, Similar to SP:Q9ERA6 Tuftelin-interacting protein 11 {Mus musculus}; contains Pfam profile PF01585: G-patch domain | chr1:5837583-5840261 FORWARD | Aliases: F20D23.23, F20D23_23	5.3	6.1	-0.8	-2.1	100.0%	-2.9
2355	AT1G29570.1 | zinc finger protein-related, contains similarity to zinc finger proteins (CCCH type) | chr1:10335896-10337840 FORWARD | Aliases: F15D2.36, F15D2_36	2.9	2.7	0.2	2.1	100.0%	-3.8
2356	AT2G47800.1 | Symbol: ATMRP4 |	4.1	4.7	-0.6	-2.1	100.0%	-3.4
2357	AT1G09010.1 | glycoside hydrolase family 2 protein, low similarity to mannosidase (gi:5359712) from Cellulomonas fimi | chr1:2895169-2899385 REVERSE | Aliases: F7G19.12, F7G19_12	6.1	6.8	-0.7	-2.1	100.0%	-3.3
2358	AT2G14170.1 | Symbol: ALDH6B2 | methylmalonate-semialdehyde dehydrogenase, putative, similar to methylmalonate-semialdehyde dehydrogenase (acylating), mitochondrial precursor (MMSDH) (Rattus norvegicus) SWISS-PROT:Q02253 | chr2:5984438-5988981 REVERSE | Aliases: T22C12.10, T22C12_10	11.0	10.7	0.3	2.1	100.0%	-3.7
2359	AT4G26860.1 | alanine racemase family protein, contains Pfam domain, PF01168: Alanine racemase, N-terminal domain | chr4:13503092-13504756 REVERSE | Aliases: F10M23.200, F10M23_200	6.7	6.2	0.5	2.1	100.0%	-3.1
2360	AT1G19525.1 | pentatricopeptide (PPR) repeat-containing protein, contains Pfam profile PF01535: PPR repeat | chr1:6761511-6762769 FORWARD | Aliases: None	5.9	6.7	-0.8	-2.1	100.0%	-3.0
2361	AT5G05980.2 | Symbol: ATDFB | similar to dihydrofolate synthetase/folylpolyglutamate synthetase (DHFS/FPGS4) [Arabidopsis thaliana] (TAIR:At3g55630.3); similar to folylpoly-gammaglutamate synthetase precursor [Cricetulus griseus] (GB:AAK69546.1); contains InterPro domain Folylpolyglutamate synthetase (InterPro:IPR001645) | chr5:1799491-1804296 REVERSE | Aliases: A. THALIANA DHFS FPGS HOMOLOG B, K18J17.17, K18J17_17	8.3	8.8	-0.5	-2.1	100.0%	-3.1
2362	AT3G45960.2 | Symbol: ATEXLA3 | similar to expansin family protein (EXPL2) [Arabidopsis thaliana] (TAIR:At4g38400.1); similar to putative pollen allergen [Oryza sativa (japonica cultivar-group)] (GB:AAP54861.1); contains InterPro domain Expansin 45, endoglucanase-like domain (InterPro:IPR007112); contains InterPro domain Major pollen allergen Lol pI (InterPro:IPR005795); contains InterPro domain Expansin/Lol pI (InterPro:IPR007118); contains InterPro domain Pollen allergen/expansin, C-terminal (InterPro:IPR007117) | chr3:16903741-16904884 FORWARD | Aliases: ARABIDOPSIS THALIANA EXPANSIN LIKE A3, ATEXPL3, ATHEXP BETA 2.3, EXPL3, F16L2.170, F16L2_170	7.6	6.6	1.0	2.1	100.0%	-3.2
2363	AT4G05180.1 | oxygen-evolving enhancer protein 3, chloroplast, putative (PSBQ2), identical to SP:Q41932 Oxygen-evolving enhancer protein 3-2, chloroplast precursor (OEE3) (16 kDa subunit of oxygen evolving system of photosystem II) (OEC 16 kDa subunit) {Arabidopsis thaliana}; similar to SP:P12301 Oxygen-evolving enhancer protein 3, chloroplast precursor (OEE3) (16 kDa subunit of oxygen evolving system of photosystem II) (OEC 16 kDa subunit) {Spinacia oleracea}; contains Pfam profile PF05757: Oxygen evolving enhancer protein 3 (PsbQ) | chr4:2671820-2673241 REVERSE | Aliases: C17L7.100, C17L7_100	9.7	8.4	1.3	2.1	100.0%	-2.8
2364	AT1G12775.1 | similar to helicase domain-containing protein / pentatricopeptide (PPR) repeat-containing protein [Arabidopsis thaliana] (TAIR:At1g12700.1); similar to pentatricopeptide (PPR) repeat-containing protein [Arabidopsis thaliana] (TAIR:At1g12300.1); similar to pentatricopeptide (PPR) repeat-containing protein [Arabidopsis thaliana] (TAIR:At1g12620.1); similar to pentatricopeptide (PPR) repeat-containing protein [Arabidopsis thaliana] (TAIR:At1g62590.1); similar to pentatricopeptide (PPR) repeat-containing protein [Arabidopsis thaliana] (TAIR:At3g22470.1); similar to fertility restorer homologue A [Raphanus sativus] (GB:CAD80166.1); contains InterPro domain PPR repeat (InterPro:IPR002885) | chr1:4353904-4355927 FORWARD | Aliases: None	4.3	4.8	-0.5	-2.1	100.0%	-3.2
2365	AT5G37790.1 | protein kinase family protein, contains protein kinase domain, Pfam:PF00069 | chr5:15025402-15028382 REVERSE | Aliases: K22F20.5, K22F20_5	5.7	6.3	-0.6	-2.1	100.0%	-3.3
2366	AT4G29510.1 | protein arginine N-methyltransferase, putative, similar to protein arginine N-methyltransferase 1-variant 2 (Homo sapiens) GI:7453575 | chr4:14491631-14494007 FORWARD | Aliases: T16L4.20, T16L4_20	7.6	8.8	-1.2	-2.1	100.0%	-3.0
2367	AT1G54290.1 | eukaryotic translation initiation factor SUI1, putative, similar to P:P32911 Protein translation factor SUI1 {Saccharomyces cerevisiae}; contains Pfam profile PF01253: Translation initiation factor SUI1 | chr1:20272152-20273774 REVERSE | Aliases: F20D21.11, F20D21_11	8.4	7.4	0.9	2.1	100.0%	-2.8
2368	AT5G42000.1 | ORMDL family protein, contains Pfam domain PF04061: ORMDL family	6.1	5.1	1.0	2.1	100.0%	-3.1

Rank	Description	15.dpi	5.dpi	M	t	adj.q	B
2369	AT3G55510.1 | expressed protein | chr3:20589963-20593382 FORWARD | Aliases: T22E16.170	4.2	5.2	-0.9	-2.1	100.0%	-2.9
2370	AT4G39190.1 | expressed protein, ; expression supported by MPSS | chr4:18252026-18252859 REVERSE | Aliases: T22F8.90, T22F8_90	5.3	4.5	0.8	2.1	100.0%	-3.2
2371	AT3G43960.1 | cysteine proteinase, putative, contains similarity to cysteine proteinase RD21A (thiol protease) GI:435619, SP:P43297 from (Arabidopsis thaliana) | chr3:15785042-15786644 REVERSE | Aliases: T15B3.100	3.6	5.0	-1.3	-2.1	100.0%	-2.9
2372	AT4G22530.1 | embryo-abundant protein-related, similar to embryo-abundant protein (Picea glauca) GI:1350531 | chr4:11859015-11860159 REVERSE | Aliases: F7K2.110, F7K2_110	6.4	7.2	-0.8	-2.1	100.0%	-2.9
2373	AT2G29680.2 | Symbol: CDC6 | cell division control protein CDC6, putative, almost identical to DNA replication protein CDC6 GI:18056480 from (Arabidopsis thaliana); identical to cDNA CDC6 protein (2g29680 gene) GI:18056479 | chr2:12696626-12700101 FORWARD | Aliases: T27A16.22, T27A16_22, cell division control protein 6	2.9	3.2	-0.3	-2.1	100.0%	-3.5
2374	AT5G65080.1 | Symbol: MAF5 | MADS-box family protein | chr5:26014730-26019691 FORWARD | Aliases: F15O5.4, F15O5_4, MADS AFFECTING FLOWERING 5 VARIANT I, MADS AFFECTING FLOWERING 5 VARIANT II, MAF5.1	3.0	2.4	0.6	2.1	100.0%	-3.1
2375	AT5G64340.1 | expressed protein | chr5:25747561-25750539 REVERSE | Aliases: MSJ1.18, MSJ1_18	3.7	4.3	-0.6	-2.1	100.0%	-3.1
2376	AT1G79660.1 | expressed protein | chr1:29980756-29981666 FORWARD | Aliases: F20B17.9, F20B17_9	10.9	10.1	0.9	2.1	100.0%	-3.6
2377	AT5G62910.1 | expressed protein, predicted proteins, Arabidopsis thaliana | chr5:25267960-25269430 FORWARD | Aliases: MQB2.23, MQB2_23	5.9	5.2	0.7	2.1	100.0%	-3.2
2378	AT1G52730.2 | transducin family protein / WD-40 repeat family protein, contains 7 WD-40 repeats (PF00400); similar to UNR-interacting protein (WD-40 repeat protein PT-WD) (SP:Q9Y3F4) (Homo sapiens) | chr1:19646158-19648802 FORWARD | Aliases: F6D8.2, F6D8_2	8.4	8.9	-0.5	-2.1	100.0%	-3.3
2379	AT4G25380.1 | zinc finger (AN1-like) family protein, contains Pfam domains, PF01428: AN1-like Zinc finger and PF01754: A20-like zinc finger | chr4:12975946-12976338 REVERSE | Aliases: T30C3.50, T30C3_50	3.7	3.3	0.4	2.1	100.0%	-3.3
2380	AT4G19610.1 | RNA recognition motif (RRM)-containing protein, contains InterPro entry IPR000504: RNA-binding region RNP-1 (RNA recognition motif) (RRM) | chr4:10677675-10681633 FORWARD | Aliases: F24J7.162, F24J7_162	6.6	7.3	-0.7	-2.1	100.0%	-3.2
2381	AT5G04340.1 | Symbol: C2H2 | zinc finger (C2H2 type) family protein, contains Pfam domain, PF00096: Zinc finger, C2H2 type | chr5:1216132-1217107 REVERSE | Aliases: C2H2 ZINC FINGER TRANSCRIPTION FACTOR, T19N18.70, T19N18_70	4.5	3.4	1.1	2.1	100.0%	-2.8
2382	AT5G60730.1 | anion-transporting ATPase family protein, low similarity to SP:O43681 Arsenical pump-driving ATPase (EC 3.6.3.16) (Arsenite-translocating ATPase) (Arsenical resistance ATPase) (Arsenite-transporting ATPase) (ARSA) (ASNA-I) {Homo sapiens}; contains Pfam profile PF02374: Anion-transporting ATPase | chr5:24439961-24442718 FORWARD | Aliases: MUP24.8, MUP24_8	3.5	3.9	-0.4	-2.1	100.0%	-3.3
2383	AT4G25650.2 | Rieske (2Fe-2S) domain-containing protein, similar to cell death suppressor protein lls1 from Zea mays (gi:1935909), Rieske iron-sulfur protein Tic55 from Pisum sativum (gi:2764524); contains Pfam PF00355 Rieske (2Fe-2S) domain | chr4:13080914-13083206 REVERSE | Aliases: L73G19.30, L73G19_30	9.5	8.8	0.7	2.0	100.0%	-3.2
2384	AT5G13410.1 | immunophilin / FKBP-type peptidyl-prolyl cis-trans isomerase family protein, SP:Q9LYR5; similar to FK506-binding protein (Peptidyl-prolyl cis-trans isomerase) (PPiase) (Rotamase) (SP:P25138) ({Neisseria meningitidis); peptidyl-prolyl cis-trans isomerase, Spodoptera frugiperda, EMBL:SF15038; contains Pfam PF00254: peptidyl-prolyl cis-trans isomerase, FKBP-type | chr5:4299744-4301770 REVERSE | Aliases: T22N19.60, T22N19_60	7.3	6.3	1.0	2.0	100.0%	-2.9
2385	AT5G28770.3 | Symbol: BZO2H3 |	4.5	3.7	0.9	2.0	100.0%	-3.0
2386	AT2G02370.1 | expressed protein | chr2:621154-623343 FORWARD | Aliases: T16F16.16, T16F16_16	6.4	5.6	0.9	2.0	100.0%	-2.9
2387	AT1G18280.1 | protease inhibitor/seed storage/lipid transfer protein (LTP) family protein, similar to lipid transfer protein GI:2627141 from (Picea abies); contains Pfam profile PF00234 Protease inhibitor/seed storage/LTP family | chr1:6294333-6295356 FORWARD | Aliases: T10O22.25, T10O22_25	3.3	2.9	0.3	2.0	100.0%	-3.5
2388	AT5G55730.1 | Symbol: FLA1 | fasciclin-like arabinogalactan-protein (FLA1), identical to gi:13377776::AAK20857:13377775:gb:AF333970 | chr5:22575374-22577687 REVERSE | Aliases: MDF20.17, MDF20_17, fasciclin like arabinogalactan protein 1	3.8	4.5	-0.7	-2.0	100.0%	-2.9
2389	AT5G48070.1 | Symbol: ATXTH20 | putative xyloglucan endotransglycosylase/hydrolase, expressed primarily in the stele of mature non-elongating regions of both the main and the lateral root. Is expressed in lateral root primordia but expression ceases after lateral root begins to grow.	2.9	3.5	-0.6	-2.0	100.0%	-3.0
2390	AT1G11310.1 | Symbol: MLO2 | seven transmembrane MLO family protein / MLO-like protein 2 (MLO2), idenctical to membrane protein Mlo2 (Arabidopsis thaliana) gi:14091574:gb:AAK53795; similar to Mlo (Hordeum vulgare subsp. vulgare) gi:1877221:emb:CAB06083 SWISS-PROT:P93766 | chr1:3800747-3804144 REVERSE | Aliases: T28P6.4, T28P6_4	5.5	6.4	-0.9	-2.0	100.0%	-3.0

Rank	Description	15.dpi	5.dpi	M	t	adj.q	B
2391	AT1G71000.1 | DNAJ heat shock N-terminal domain-containing protein, similar to SP:O35723 DnaJ homolog subfamily B member 3 Mus musculus, SP:Q9QYI7 DnaJ homolog subfamily B member 8 Mus musculus; contains Pfam profile PF00226 DnaJ domain | chr1:26772998-26773773 REVERSE | Aliases: F15H11.19, F15H11_19	3.0	2.7	0.3	2.0	100.0%	-3.8
2392	AT1G51090.1 | heavy-metal-associated domain-containing protein, contains Pfam profile PF00403: Heavy-metal-associated domain | chr1:18936605-18937618 FORWARD | Aliases: F23H24.15, F23H24_15	2.9	2.6	0.3	2.0	100.0%	-3.5
2393	AT4G28220.1 | NADH dehydrogenase-related, similar to 64 kDa mitochondrial NADH dehydrogenase (Neurospora crassa) GI:4753821, alternative NADH-dehydrogenase (Yarrowia lipolytica) GI:3718005; contains Pfam profile PF00070: Pyridine nucleotide-disulphide oxidoreductase | chr4:13993003-13998353 FORWARD | Aliases: F26K10.100, F26K10_100	5.3	5.9	-0.6	-2.0	100.0%	-3.0
2394	AT1G31150.1 | expressed protein, EST gb:Z33866 comes from this gene | chr1:11120078-11122568 FORWARD | Aliases: F28K20.8, F28K20_8	4.4	5.0	-0.6	-2.0	100.0%	-3.0
2395	AT1G18630.1 | glycine-rich RNA-binding protein, putative, similar to glycine-rich RNA-binding protein from {Sorghum bicolor} SP:Q99070, GI:1778373 from (Pisum sativum); contains InterPro entry IPR000504: RNA-binding region RNP-1 (RNA recognition motif) (RRM) | chr1:6414717-6416445 FORWARD | Aliases: F25I16.4, F25I16_4	6.1	5.6	0.5	2.0	100.0%	-3.2
2396	AT4G39670.1 | expressed protein | chr4:18410166-18410994 FORWARD | Aliases: T19P19.60, T19P19_60	4.4	3.3	1.0	2.0	100.0%	-2.8
2397	AT5G50740.1 | copper chaperone (CCH)-related, low similarity to copper homeostasis factor (GI:3168840)(PMID: 9701579); similar to farnesylated protein ATFP3 (GI:4097547); contains Pfam profile PF00403: Heavy-metal-associated domain | chr5:20654374-20655086 REVERSE | Aliases: MFB16.14, MFB16_14	3.9	4.5	-0.6	-2.0	100.0%	-3.1
2398	AT3G25640.1 | expressed protein, contains Pfam profile PF04759: Protein of unknown function, DUF617 | chr3:9334956-9336041 FORWARD | Aliases: T5M7.9	3.3	4.2	-0.9	-2.0	100.0%	-2.9
2399	AT2G40960.1 | expressed protein | chr2:17102600-17104527 FORWARD | Aliases: T20B5.16, T20B5_16	3.5	4.0	-0.5	-2.0	100.0%	-3.1
2400	AT2G37570.2 | Symbol: SLT1 | expressed protein | chr2:15767955-15770461 REVERSE | Aliases: F13M22.7, F13M22_7, SLT1 PROTEIN, SODIUM AND LITHIUM TOLERANT 1	5.5	6.5	-0.9	-2.0	100.0%	-3.0
2401	AT5G44010.1 | expressed protein | chr5:17724192-17726426 REVERSE | Aliases: MRH10.12, MRH10_12	2.3	2.6	-0.2	-2.0	100.0%	-3.9
2402	AT1G31920.1 | pentatricopeptide (PPR) repeat-containing protein, contains Pfam profile PF01535: PPR repeat | chr1:11461835-11463782 REVERSE | Aliases: F5M6.8, F5M6_8	3.4	3.9	-0.6	-2.0	100.0%	-3.3
2403	AT1G33330.1 | peptide chain release factor, putative, similar to peptide chain release factor 2 (Campylobacter jejuni) GI:6469929; contains Pfam profile PF00472: Peptidyl-tRNA hydrolase domain | chr1:12084931-12086508 FORWARD | Aliases: F10C21.2	6.2	5.6	0.6	2.0	100.0%	-2.9
2404	AT1G26450.1 | beta-1,3-glucanase-related, similar to beta-1,3-glucanase GI:15150341 from (Camellia sinensis); C-terminal homology only	3.2	2.6	0.6	2.0	100.0%	-3.0
2405	AT4G24440.2 | transcription initiation factor IIA gamma chain / TFIIA-gamma (TFIIA-S), identical to transcription initiation factor IIA gamma chain SP:Q39236 from (Arabidopsis thaliana); | chr4:12633190-12634767 FORWARD | Aliases: T22A6.270, T22A6_270	9.0	8.0	0.9	2.0	100.0%	-3.0
2406	AT5G58300.1 | leucine-rich repeat transmembrane protein kinase, putative | chr5:23589105-23592587 FORWARD | Aliases: MCK7.17, MCK7_17	4.0	4.9	-0.9	-2.0	100.0%	-2.9
2407	AT3G18190.1 | chaperonin, putative, similar to SWISS-PROT:P50991- T-complex protein 1, delta subunit (TCP-1-delta) (Homo sapiens); contains Pfam:PF00118 domain, TCP-1/cpn60 chaperonin family	6.1	6.8	-0.7	-2.0	100.0%	-3.1
2408	AT4G33470.1 | histone deacetylase family protein, similar to histone deacetylase 10 isoform alpha (Homo sapiens) GI:15213865; contains Pfam profile PF00850: Histone deacetylase family | chr4:16102598-16105484 REVERSE | Aliases: F17M5.230, F17M5_230	3.5	3.2	0.4	2.0	100.0%	-3.4
2409	ATCG00810.1 | Symbol: RPL22 | encodes a chloroplast ribosomal protein L22, a constituent of the large subunit of the ribosomal complex | chrC:83467-83949 REVERSE | Aliases: RPL22	8.4	10.4	-1.9	-2.0	100.0%	-3.2
2410	AT5G22880.1 | histone H2B, putative, strong similarity to histone H2B-3 Lycopersicon esculentum GI:3021485, H2B Gossypium hirsutum SP:O22582; contains Pfam profile PF00125 Core histone H2A/H2B/H3/H4	7.9	9.2	-1.4	-2.0	100.0%	-2.8
2411	AT4G10320.1 | isoleucyl-tRNA synthetase, putative / isoleucine--tRNA ligase, putative, similar to SP:P41252 Isoleucyl-tRNA synthetase, cytoplasmic (EC 6.1.1.5) (Isoleucine--tRNA ligase) (IleRS) (IRS) {Homo sapiens}; contains Pfam profile PF00133: tRNA synthetases class I (I, L, M and V) | chr4:6397326-6404505 REVERSE | Aliases: F24G24.120, F24G24_120	10.2	10.9	-0.7	-2.0	100.0%	-3.2

Rank	Description	15.dpi	5.dpi	M	t	adj.q	B
2412	AT5G66240.2 | transducin family protein / WD-40 repeat family protein, contains Pfam PF00400: WD domain, G-beta repeat (4 copies, 1 weak); similar to Will die slowly protein. {Drosophila melanogaster} (SP:Q9V3J8) {Drosophila melanogaster}	8.6	8.0	0.5	2.0	100.0%	-3.4
2413	AT5G03560.1 | expressed protein | chr5:900462-902740 REVERSE | Aliases: F12E4.360, F12E4_360	6.1	5.4	0.7	2.0	100.0%	-3.0
2414	AT1G31940.1 | expressed protein | chr1:11470039-11471320 REVERSE | Aliases: F5M6.6	6.7	5.8	0.9	2.0	100.0%	-3.2
2415	AT5G08280.1 | hydroxymethylbilane synthase / porphobilinogen deaminase, chloroplast / pre-uroporphyrinogen synthase, identical to SP:Q43316 | chr5:2663191-2665793 REVERSE | Aliases: F8L15.10, F8L15_10	5.2	6.4	-1.2	-2.0	100.0%	-2.9
2416	AT3G10620.1 | diadenosine 5',5'''-P1,P4-tetraphosphate hydrolase, putative, similar to diadenosine 5',5'''-P1,P4-tetraphosphate hydrolase from (Lupinus angustifolius) GI:1888557, (Hordeum vulgare subsp. vulgare) GI:2564253; contains Pfam profile PF00293: NUDIX domain | chr3:3320268-3322022 FORWARD | Aliases: F13M14.9	7.1	8.1	-0.9	-2.0	100.0%	-2.8
2417	AT3G51720.1 | expressed protein, contains Pfam PF05701: Plant protein of unknown function (DUF827) | chr3:19196030-19197613 FORWARD | Aliases: T18N14.100	6.8	8.0	-1.2	-2.0	100.0%	-2.9
2418	AT5G51910.2 | TCP family transcription factor, putative, similar to PCF2 ((GI:2580440) Oryza sativa) | chr5:21111811-21113058 REVERSE | Aliases: MJM18.6, MJM18_6	3.7	4.2	-0.5	-2.0	100.0%	-3.3
2419	AT2G25100.1 | ribonuclease HII family protein, contains Pfam profile: PF01351 ribonuclease HII	5.0	5.6	-0.6	-2.0	100.0%	-3.1
2420	AT2G32410.2 | similar to auxin-resistance protein AXR1 (AXR1) [Arabidopsis thaliana] (TAIR:At1g05180.1); similar to putative ubiquitin-activating enzyme (alternative splicing product) [Oryza sativa (japonica cultivar-group)] (GB:XP_470134.1); similar to putative ubiquitin-activating enzyme (alternative splicing product) [Oryza sativa (japonica cultivar-group)] (GB:XP_470133.1); contains InterPro domain UBA/THIF-type NAD/FAD binding fold (InterPro:IPR000594) | chr2:13764670-13768154 FORWARD | Aliases: T32F6.7, T32F6_7	3.9	4.2	-0.3	-2.0	100.0%	-3.6
2421	AT4G00990.1 | transcription factor jumonji (jmjC) domain-containing protein, contains Pfam domain, PF02373: jmjC domain | chr4:426944-431821 FORWARD | Aliases: F3I3.10	5.5	5.9	-0.4	-2.0	100.0%	-3.3
2422	AT4G15930.1 | dynein light chain, putative, similar to dynein light chain 2 (Mus musculus) GI:15545995; contains Pfam profile PF01221: Dynein light chain type 1 | chr4:9036236-9038077 FORWARD | Aliases: DL4005W, FCAALL.238	10.5	10.0	0.5	2.0	100.0%	-3.4
2423	AT3G47590.1 | esterase/lipase/thioesterase family protein, low similarity to cinnamoyl ester hydrolase CinI (Butyrivibrio fibrisolvens) GI:1622732; contains Interpro entry IPR000379 | chr3:17546605-17548508 REVERSE | Aliases: F1P2.140	7.8	8.5	-0.6	-2.0	100.0%	-3.0
2424	AT4G36180.1 | leucine-rich repeat family protein, contains protein kinase domain, Pfam:PF00069; contains leucine-rich repeats, Pfam:PF00560 | chr4:17120088-17123844 REVERSE | Aliases: F23E13.70, F23E13_70	4.8	5.8	-1.0	-2.0	100.0%	-3.1
2425	AT3G05400.1 | sugar transporter, putative, similar to sugar-porter family proteins 1 and 2 (Arabidopsis thaliana) GI:14585699, GI:14585701, integral membrane protein GB:U43629 from (Beta vulgaris); contains Pfam profile PF00083: major facilitator superfamily protein | chr3:1549592-1554154 FORWARD | Aliases: F22F7.16, F22F7_16	4.5	5.5	-1.0	-2.0	100.0%	-2.8
2426	AT5G43360.1 | Symbol: PHT3 | inorganic phosphate transporter (PHT3), identical to inorganic phosphate transporter (Arabidopsis thaliana) GI:2780347 | chr5:17426752-17428442 FORWARD | Aliases: ATPT4, MWF20.5, MWF20_5	6.8	5.0	1.9	2.0	100.0%	-3.0
2427	AT1G14000.1 | protein kinase family protein / ankyrin repeat family protein, contains Pfam profiles: PF00069 protein kinase domain, PF00023 ankyrin repeat | chr1:4797355-4800278 FORWARD | Aliases: F7A19.9, F7A19_9	7.9	8.6	-0.8	-2.0	100.0%	-3.1
2428	AT1G51440.1 | lipase class 3 family protein, similar to DEFECTIVE IN ANTHER DEHISCENCE1 (Arabidopsis thaliana) GI:16215706, lipase (Dianthus caryophyllus) GI:4103627; contains Pfam profile PF01764: Lipase | chr1:19074585-19076362 FORWARD | Aliases: F5D21.19, F5D21_19	3.3	3.8	-0.5	-2.0	100.0%	-3.1
2429	AT4G02800.1 | expressed protein, similar to A. thaliana hypothetical protein T6B20.12 (1946366) | chr4:1250077-1251681 FORWARD | Aliases: T5J8.12, T5J8_12	3.2	3.5	-0.4	-2.0	100.0%	-3.4
2430	AT5G09620.1 | octicosapeptide/Phox/Bem1p (PB1) domain-containing protein, predicted proteins, Arabidopsis thaliana and Drosophila melanogaster contains Pfam profile PF00564: PB1 domain | chr5:2983450-2985436 REVERSE | Aliases: F17I14.190, F17I14_190	7.6	7.1	0.5	2.0	100.0%	-3.4
2431	AT3G57070.1 | glutaredoxin family protein, contains Pfam profile PF00462: Glutaredoxin	4.3	4.0	0.3	2.0	100.0%	-3.5

Rank	Description	15.dpi	5.dpi	M	t	adj.q	B
2432	AT3G46790.1 | Symbol: CRR2 | pentatricopeptide (PPR) repeat-containing protein, contains INTERPRO:IPR002885 PPR repeats | chr3:17242758-17244933 REVERSE | Aliases: CHLORORESPIRATORY REDUCTION 2, T6H20.180	6.3	7.0	-0.7	-2.0	100.0%	-3.5
2433	AT5G53060.1 | KH domain-containing protein | chr5:21532252-21535663 FORWARD | Aliases: MNB8.12, MNB8_12	4.9	5.6	-0.6	-2.0	100.0%	-3.1
2434	AT5G55830.1 | lectin protein kinase, putative, similar to receptor lectin kinase 3 (Arabidopsis thaliana) gi:4100060:gb:AAD00733; contains protein kinase domain, Pfam:PF00069; contains legume lectins alpha and beta domains, Pfam:PF00138 and Pfam:PF00139 | chr5:22611881-22614069 FORWARD | Aliases: MDF20.27, MDF20_27	2.8	3.3	-0.4	-2.0	100.0%	-3.4
2435	AT3G53560.1 | chloroplast lumen common family protein | chr3:19870605-19872009 REVERSE | Aliases: F4P12.260	6.3	7.1	-0.8	-2.0	100.0%	-3.0
2436	AT5G33300.1 | chromosome-associated kinesin-related, contains weak similarity to chromosome-associated kinesin KIF4A (Chromokinesin) (Swiss-Prot:P33174) (Mus musculus) | chr5:12579660-12582970 REVERSE | Aliases: F19N2.20, F19N2_20	4.1	4.6	-0.5	-2.0	100.0%	-3.2
2437	AT5G54660.1 | heat shock protein-related, contains weak similarity to 17.6 kDa class I heat shock protein (HSP 17.6) (Swiss-Prot:P13853) (Arabidopsis thaliana) | chr5:22221131-22222378 FORWARD | Aliases: MRB17.16, MRB17_16	7.0	6.3	0.7	2.0	100.0%	-3.2
2438	AT3G44200.1 | protein kinase family protein, contains protein kinase domain, Pfam:PF00069; contains serine/threonine protein kinase domain, INTERPRO:IPR002290	4.7	5.3	-0.6	-2.0	100.0%	-3.0
2439	AT4G12080.1 | DNA-binding family protein, contains a AT hook motif (DNA binding motifs with a preference for A/T rich regions), Pfam:PF02178 | chr4:7239213-7241488 FORWARD | Aliases: F16J13.150, F16J13_150	7.9	7.0	0.8	2.0	100.0%	-3.1
2440	AT3G24820.1 | BSD domain-containing protein, contains Pfam profile PF03909: BSD domain	5.9	5.4	0.5	2.0	100.0%	-3.2
2441	AT5G14750.1 | Symbol: WER | myb family transcription factor (MYB66) / werewolf (WER), contains PFAM profile: Myb DNA binding domain PF00249; identical to cDNA putative transcription factor (MYB66) mRNA, partial cds GI:3941491; identical to GP:9755743 myb transcription factor werewolf (WER)/ MYB66 {Arabidopsis thaliana} | chr5:4763455-4764741 REVERSE | Aliases: T9L3.50, T9L3_50, WER1, WEREWOLF 1	2.5	2.8	-0.3	-2.0	100.0%	-3.8
2442	AT2G26700.1 | protein kinase family protein, contains Pfam PF00069: Protein kinase domain | chr2:11375691-11378092 FORWARD | Aliases: F18A8.7, F18A8_7	2.4	2.8	-0.4	-2.0	100.0%	-3.4
2443	AT1G51940.1 | protein kinase family protein / peptidoglycan-binding LysM domain-containing protein, contains protein kinases ATP-binding region signature, PROSITE:PS00107 | chr1:19299598-19302787 REVERSE | Aliases: T14L22.13, T14L22_13	6.2	6.8	-0.6	-2.0	100.0%	-3.0
2444	AT5G05270.2 | chalcone-flavanone isomerase family protein, contains very low similarity to chalcone-flavonone isomerase (chalcone isomerase), GI:1705761 from Vitis vinifera; contains Pfam profile PF02431: Chalcone-flavanone isomerase | chr5:1563431-1565007 FORWARD | Aliases: K18I23.7, K18I23_7	4.1	4.7	-0.5	-2.0	100.0%	-3.1
2445	AT2G21510.1 | DNAJ heat shock N-terminal domain-containing protein, similar to SP:P39101 CAJ1 protein, Saccharomyces cerevisiae; contains Pfam profile PF00226 DnaJ domain | chr2:9217735-9220138 REVERSE | Aliases: F3K23.27, F3K23_27	3.9	3.3	0.6	2.0	100.0%	-3.1
2446	AT2G32770.3 | purple acid phosphatase (PAP13), identical to purple acid phosphatase (PAP13) (Arabidopsis thaliana) GI:20257489; contains Pfam profile PF00149: Ser/Thr protein phosphatase | chr2:13902915-13905354 FORWARD | Aliases: F24L7.9, F24L7_9	2.9	3.3	-0.3	-2.0	100.0%	-3.4
2447	AT1G15230.1 | expressed protein, ESTs gb:R30529, gb:Z48463, gb:Z48467, gb:AA597369 and gb:AA394772 come from this gene	6.6	5.9	0.7	2.0	100.0%	-3.2
2448	AT4G02170.1 | similar to expressed protein [Arabidopsis thaliana] (TAIR:At5g38700.1); similar to disease resistance gene [Pinus sylvestris] (GB:AAR36911.1) | chr4:958049-958641 FORWARD | Aliases: T10M13.17, T10M13_17	2.5	2.3	0.2	2.0	100.0%	-4.0
2449	AT3G05580.1 | serine/threonine protein phosphatase, putative, similar to serine/threonine protein phosphatase PP1 isozyme 8 SP:O82734 from (Arabidopsis thaliana) | chr3:1617853-1619995 REVERSE | Aliases: F18C1.15, F18C1_15	5.8	5.0	0.8	2.0	100.0%	-3.0
2450	AT5G42480.1 | Symbol: ARC6 | DNAJ plastid division protein (ARC6), almost identical to dnaJ plastid division protein ARC6 (GI:33436339) (Arabidopsis thaliana);low similarity to cell division protein Ftn2 (Synechococcus sp. PCC 7942) GI:16226084; contains Pfam profile PF00226: DnaJ domain | chr5:17002410-17005668 FORWARD | Aliases: ACCUMULATION AND REPLICATION OF CHLOROPLASTS 6, MDH9.18, MDH9_18	8.4	8.8	-0.5	-2.0	100.0%	-3.2
2451	AT5G56090.1 | cytochrome oxidase assembly family protein, contains PF02628: Cytochrome oxidase assembly protein | chr5:22731744-22734061 FORWARD | Aliases: MDA7.15, MDA7_15	7.3	8.6	-1.3	-2.0	100.0%	-2.9
2452	AT2G26340.1 | expressed protein | chr2:11222353-11223701 FORWARD | Aliases: T1D16.2, T1D16_2	7.5	6.9	0.6	2.0	100.0%	-3.0

Rank	Description	15.dpi	5.dpi	M	t	adj.q	B
2453	AT1G24625.1 | zinc finger (C2H2 type) family protein (ZFP7), identical to zinc finger protein, ZFP7 gi:790685:gb:AAA87303; contains Pfam domain, PF00096: Zinc finger, C2H2 type | chr1:8725869-8727198 REVERSE | Aliases: F21J9.29	6.6	6.2	0.5	2.0	100.0%	-3.5
2454	AT5G23730.1 | similar to transducin family protein / WD-40 repeat family protein [Arabidopsis thaliana] (TAIR:At5g52250.1); similar to COP1 homolog [Lycopersicon esculentum] (GB:AAC98912.1); contains InterPro domain G-protein beta WD-40 repeat (InterPro:IPR001680) | chr5:8005237-8006633 FORWARD | Aliases: MRO11.23, MRO11_23	6.9	6.2	0.7	2.0	100.0%	-3.2
2455	AT3G46230.1 | Symbol: ATHSP17.4 | 17.4 kDa class I heat shock protein (HSP17.4-CI), identical to 17.4 kDa class I heat shock protein SP:P19036 from (Arabidopsis thaliana) | chr3:16994886-16995826 REVERSE | Aliases: F12M12.200, SMALL HEAT SHOCK PROTEIN 17.4	6.2	5.5	0.8	2.0	100.0%	-3.0
2456	AT2G31180.1 | myb family transcription factor (MYB14), similar to myb-related transcription factor GI:1370140 from (Lycopersicon esculentum) | chr2:13293798-13295252 REVERSE | Aliases: F16D14.2	3.8	4.3	-0.5	-2.0	100.0%	-3.3
2457	AT3G57040.1 | Symbol: ARR9 | two-component responsive regulator / response reactor 4 (RR4), identical to responce reactor4 GI:3273202 from (Arabidopsis thaliana); contains Pfam profile: PF00072 response regulator receiver domain | chr3:21120593-21122411 FORWARD | Aliases: ATRR4, F24I3.120, F24I3_120, RESPONSE REACTOR 4	3.6	4.5	-0.9	-2.0	100.0%	-2.9
2458	AT1G69920.1 | Symbol: ATGSTU12 | glutathione S-transferase, putative, similar to glutathione transferase GB:CAA09188 (Alopecurus myosuroides); supported by cDNA gi:15451157 gb:AY050343. | chr1:26337913-26339206 REVERSE | Aliases: T17F3.5, T17F3_5	4.9	6.1	-1.1	-2.0	100.0%	-2.7
2459	AT4G34350.1 | Symbol: CLB6 | Arabidopsis ISPH is involved in the plastid nonmevalonate pathway of isoprenoid biosynthesis. It was shown to complement the lethal phenotype of E.coli ispH mutant and is therefore most likely encodes a protein with 4-hydroxy-3-methylbut-2-en-1-yl diphosphate reductase activity involved in the last step of mevalonate-independent isopentenyl biosynthesis. | chr4:16428484-16431117 REVERSE | Aliases: CHLOROPLAST BIOGENESIS, F10M10.120, F10M10_120, ISPH	10.1	9.4	0.8	2.0	100.0%	-3.1
2460	AT2G46420.1 | expressed protein | chr2:19060759-19064163 FORWARD | Aliases: F11C10.11	6.6	5.1	1.5	2.0	100.0%	-2.8
2461	NA	4.7	4.2	0.5	2.0	100.0%	-3.6
2462	AT1G20110.1 | zinc finger (FYVE type) family protein, contains Pfam profile: PF01363 FYVE zinc finger | chr1:6971476-6974812 FORWARD | Aliases: T20H2.10, T20H2_10	9.1	8.5	0.5	2.0	100.0%	-3.3
2463	AT1G53540.1 | 17.6 kDa class I small heat shock protein (HSP17.6C-CI) (AA 1-156), identical to (17.6 kDa class I heat shock protein (HSP 17.6) (AA 1-156)(SP:P13853) (GI:4376161) (Arabidopsis thaliana) (Nucleic Acids Res. 17 (19), 7995 (1989)) | chr1:19984130-19984775 FORWARD | Aliases: F22G10.20	5.7	5.0	0.7	2.0	100.0%	-3.1
2464	AT3G06580.1 | Symbol: GAL1 | galactokinase (GAL1), identical to galactokinase (Galactose kinase) (Arabidopsis thaliana) SWISS-PROT:Q9SEE5 | chr3:2048911-2052031 REVERSE | Aliases: F5E6.9, F5E6_9, GALACTOSE KINASE 1	9.1	10.4	-1.3	-2.0	100.0%	-3.0
2465	AT5G63850.1 | Symbol: AAP4 | amino acid transporter 4, putative (AAP4), identical to amino acid transporter GI:608671 from (Arabidopsis thaliana); | chr5:25568281-25570746 FORWARD | Aliases: MGI19.6, MGI19_6	11.2	8.5	2.7	2.0	100.0%	-2.8
2466	AT1G70510.1 | Symbol: KNAT2 | homeobox protein knotted-1 like 2 (KNAT2) (K1), identical to homeobox protein knotted-1 like 2 (KNAT2/ ATK1) SP: from (Arabidopsis thaliana) | chr1:26580168-26586094 FORWARD | Aliases: F24J13.8, F24J13_8, KNOTTED LIKE FROM ARABIDOPSIS THALIANA 2	3.1	2.7	0.3	2.0	100.0%	-3.6
2467	AT5G09530.1 | hydroxyproline-rich glycoprotein family protein, contains proline-rich extensin domains, INTERPRO:IPR002965 | chr5:2959735-2961272 REVERSE | Aliases: T5E8.2	2.8	3.5	-0.7	-2.0	100.0%	-3.3
2468	AT5G56270.1 | Symbol: WRKY2 | WRKY family transcription factor | chr5:22796919-22800494 FORWARD | Aliases: ATWRKY2, MXK23.1, MXK23_1	4.9	4.4	0.5	2.0	100.0%	-3.1
2469	AT5G16440.1 | Symbol: IPP1 | similar to MutT/nudix family protein [Arabidopsis thaliana] (TAIR:At1g79690.1); similar to isopentenyl pyrophosphate:dimethyllallyl pyrophosphate isomerase [Adonis palaestina] (GB:AAF29973.1); similar to isopentenyl pyrophosphate isomerase [Pueraria montana var. lobata] (GB:AAQ84167.1); contains InterPro domain Isopentenyl-diphosphate delta-isomerase (InterPro:IPR002667); contains InterPro domain NUDIX hydrolase (InterPro:IPR000086) | chr5:5371743-5373738 FORWARD | Aliases: IDI1, ISOPENTENYL DIPHOSPHATE ISOMERASE, MQK4.17, MQK4_17	6.2	7.9	-1.7	-2.0	100.0%	-3.0
2470	AT1G55900.1 | Symbol: TIM50 | NLI interacting factor (NIF) family protein, contains Pfam profile PF03031: NLI interacting factor | chr1:20906733-20909393 FORWARD | Aliases: EMB1860, EMBRYO DEFECTIVE 1860, F14J16.15, F14J16_15	6.4	7.4	-1.0	-2.0	100.0%	-2.9
2471	AT3G47160.1 | expressed protein, similar to MTD2 (Medicago truncatula) GI:9294812; supporting cDNA gi:25083983:gb:BT002136.1: | chr3:17370976-17372990 REVERSE | Aliases: F13I12.210	6.8	6.0	0.8	2.0	100.0%	-2.9

Rank	Description	15.dpi	5.dpi	M	t	adj.q	B
2472	AT1G54690.1 | histone H2A, putative, strong similarity to histone H2A GI:3204129 SP:O65759 from Cicer arietinum, Picea abies SP:P35063; contains Pfam profile PF00125 Core histone H2A/H2B/H3/H4 | chr1:20417983-20418954 REVERSE | Aliases: T22H22.12, T22H22_12	9.4	10.7	-1.3	-2.0	100.0%	-2.9
2473	AT4G02200.2 | drought-responsive family protein, similar to drought-induced mRNA, Di19 (Arabidopsis thaliana) gi:469110:emb:CAA55321 | chr4:972707-974692 FORWARD | Aliases: T2H3.13	8.3	7.5	0.8	2.0	100.0%	-3.1
2474	AT4G11270.1 | transducin family protein / WD-40 repeat family protein, contains 5 WD-40 repeats (PF00400); related to TGF-beta resistance-associated protein TRAG (GI:15624071) {Mus musculus}; similar to beta-transducin repeats containing protein - Homo sapiens,PID:e1284220; 3' EST no_NP:TC8031 | chr4:6854457-6859660 FORWARD | Aliases: F8L21.60, F8L21_60	6.9	7.5	-0.7	-2.0	100.0%	-3.2
2475	AT1G68880.1 | bZIP transcription factor family protein, similar to common plant regulatory factor 6 GI:9650826 from (Petroselinum crispum); contains Pfam profile: PF00170 bZIP transcription factor | chr1:25898162-25898578 REVERSE | Aliases: T6L1.5	4.6	5.6	-1.0	-2.0	100.0%	-3.0
2476	AT1G31360.1 | DNA helicase, putative (RECQl2), nearly identical to DNA Helicase (Arabidopsis thaliana) GI:11121445 | chr1:11232314-11237511 FORWARD | Aliases: T19E23.16, T19E23_16	3.2	3.9	-0.7	-2.0	100.0%	-3.5
2477	AT2G42430.1 | Symbol: LBD16 | LOB domain protein 16 / lateral organ boundaries domain protein 16 (LBD16), identical to LOB DOMAIN 16 (Arabidopsis thaliana) GI:17227162 | chr2:17670522-17672370 FORWARD | Aliases: MHK10.15, MHK10_15	4.2	4.8	-0.6	-2.0	100.0%	-3.1
2478	AT1G22920.2 | Symbol: AJH1 | COP9 signalosome subunit 5B / CSN subunit 5B (CSN5B) / c-JUN coactivator protein AJH1, putative (AJH1), COP9 complex subunit CSN5-1; identical to Arabidopsis homologs of a c-Jun coactivator AJH1 GI:3641314 from (Arabidopsis thaliana); identical to cDNA CSN complex subunit 5B (CSN5B) GI:18056662; contains Pfam profile PF01398: Mov34/MPN/PAD-1 family | chr1:8109703-8112040 REVERSE | Aliases: CSN5, CSN5B, F19G10.12, F19G10_12, JAB1, PUTATIVE JUN KINASE ACTIVATOR	6.8	7.9	-1.1	-2.0	100.0%	-3.0
2479	AT1G26380.1 | FAD-binding domain-containing protein, similar to SP:P30986 reticuline oxidase precursor (Berberine-bridge-forming enzyme) (BBE) (Tetrahydroprotoberberine synthase) (Eschscholzia californica); contains PF01565 FAD binding domain | chr1:9126724-9128515 REVERSE | Aliases: T1K7.24, T1K7_24	2.3	2.5	-0.2	-2.0	100.0%	-4.0
2480	AT5G39970.1 | expressed protein, low similarity to up-regulated by thyroid hormone in tadpoles; expressed specifically in the tail and only at metamorphosis; membrane bound or extracellular protein; C-terminal basic region (Xenopus laevis) GI:1234787 | chr5:16015253-16018850 FORWARD | Aliases: MYH19.17, MYH19_17	4.7	3.9	0.8	2.0	100.0%	-3.0
2481	AT1G62300.1 | Symbol: WRKY6 | WRKY family transcription factor, similar to putative DNA-binding protein GI:7268215 from (Arabidopsis thaliana) | chr1:23020348-23022944 REVERSE | Aliases: F19K23.22, F19K23_22	4.6	5.6	-1.0	-2.0	100.0%	-2.9
2482	AT2G47450.1 | Symbol: CAO | chloroplast signal recognition particle component (CAO), nearly identical to CAO (Arabidopsis thaliana) GI:4102582 | chr2:19479751-19481384 FORWARD | Aliases: CHAOS, CHLOROPHYLL A/B BINDING PROTEIN HARVESTING ORGANELLE SPECIFIC, CPSRP43, T30B22.25	9.6	8.7	0.9	2.0	100.0%	-3.1
2483	AT2G29630.2 | thiamine biosynthesis family protein / thiC family protein, contains Pfam profile: PF01964 ThiC family | chr2:12674111-12677388 FORWARD | Aliases: T27A16.27, T27A16_27	7.5	6.5	1.0	2.0	100.0%	-2.8
2484	AT1G15370.1 | expressed protein | chr1:5287057-5288506 FORWARD | Aliases: F9L1.32, F9L1_32	9.1	8.4	0.7	2.0	100.0%	-3.2
2485	AT5G13760.1 | expressed protein, similar to unknown protein (gb AAF63775.1) | chr5:4442017-4444938 FORWARD | Aliases: MXE10.1, MXE10_1	4.7	3.9	0.8	2.0	100.0%	-3.1
2486	AT2G20810.1 | glycosyl transferase family 8 protein, contains Pfam profile: PF01501 glycosyl transferase family 8 | chr2:8964874-8966861 FORWARD | Aliases: F5H14.44	6.3	6.8	-0.5	-2.0	100.0%	-3.1
2487	AT2G41350.2 | similar to hypothetical protein [Arabidopsis thaliana] (TAIR:At2g21980.1); similar to unnamed protein product [Mus musculus] (GB:BAC36526.1) | chr2:17238428-17240536 FORWARD | Aliases: F13H10.10, F13H10_10	7.4	8.1	-0.7	-2.0	100.0%	-3.1
2488	AT5G64980.1 | expressed protein | chr5:25978001-25980461 REVERSE | Aliases: MXK3.21, MXK3_21	3.6	4.1	-0.6	-2.0	100.0%	-3.0
2489	AT4G16450.1 | expressed protein | chr4:9280104-9280740 FORWARD | Aliases: DL4250W, FCAALL.388	10.3	10.8	-0.5	-2.0	100.0%	-3.5
2490	AT1G29680.1 | expressed protein | chr1:10377503-10378947 REVERSE | Aliases: F15D2.23, F15D2_23	3.3	2.9	0.4	2.0	100.0%	-3.6
2491	AT1G29250.1 | expressed protein, contains TIGRFAM TIGR00285: conserved hypothetical protein TIGR00285 | chr1:10223252-10224713 REVERSE | Aliases: F28N24.7, F28N24_7	9.3	10.0	-0.7	-2.0	100.0%	-3.0
2492	AT1G64680.1 | expressed protein | chr1:24039684-24041064 FORWARD | Aliases: F1N19.25, F1N19_25	5.5	4.9	0.7	2.0	100.0%	-3.0

Rank	Description	15.dpi	5.dpi	M	t	adj.q	B
2493	AT5G62440.1 | expressed protein | chr5:25089709-25091215 REVERSE | Aliases: AT5G62450, K19B1.5, K19B1_5	7.2	7.6	-0.5	-2.0	100.0%	-3.4
2494	AT4G37970.1 | mannitol dehydrogenase, putative, similar to ELI3-2 (SP:Q02972), sinapyl alcohol dehydrogenase (Populus tremuloides) (gi:14279694); contains Pfam zinc-binding dehydrogenase domain PF00107 | chr4:17849666-17852233 FORWARD | Aliases: F20D10.90, F20D10_90	5.8	6.6	-0.9	-2.0	100.0%	-2.9
2495	AT5G06700.1 | expressed protein, strong similarity to unknown protein (emb:CAB82953.1)	7.9	8.8	-0.8	-2.0	100.0%	-3.1
2496	AT1G74430.1 | Symbol: MYB95 | myb family transcription factor (MYB95), contains Pfam profile: PF00249 myb-like DNA-binding domain | chr1:27978842-27981095 FORWARD | Aliases: F1M20.11, F1M20_11, MYB95	3.7	3.1	0.6	2.0	100.0%	-3.3
2497	AT3G22150.1 | pentatricopeptide (PPR) repeat-containing protein, contains INTERPRO:IPR002885 PPR repeats | chr3:7812975-7815497 FORWARD | Aliases: MKA23.11	3.1	3.5	-0.5	-2.0	100.0%	-3.3
2498	AT5G27740.1 | expressed protein | chr5:9823744-9827068 FORWARD | Aliases: T1G16.70, T1G16_70	4.8	5.5	-0.7	-2.0	100.0%	-3.0
2499	AT3G20560.1 | Symbol: ATPDIL5 3 | Encodes a protein disulfide isomerase-like (PDIL) protein, a member of a multigene family within the thioredoxin (TRX) superfamily.	6.4	7.1	-0.8	-2.0	100.0%	-3.0
2500	AT5G15810.1 | N2,N2-dimethylguanosine tRNA methyltransferase family protein, similar to SP:Q9P804 N(2),N(2)-dimethylguanosine tRNA methyltransferase (EC 2.1.1.32) (tRNA(guanine-26,N(2)-N(2)) methyltransferase) {Schizosaccharomyces pombe}; contains Pfam profile PF02005: N2,N2-dimethylguanosine tRNA methyltransferase | chr5:5157801-5161361 FORWARD | Aliases: F14F8.190, F14F8_190	6.7	7.4	-0.6	-2.0	100.0%	-3.1
2501	AT5G38980.1 | expressed protein | chr5:15624001-15624579 FORWARD | Aliases: K15E6.160, K15E6_160	3.1	2.8	0.3	2.0	100.0%	-3.6
2502	AT3G56900.1 | aladin-related / adracalin-related, weak similarity to SP:Q9NRG9 Aladin (Adracalin) (GL003) {Homo sapiens}; non-consensus AT-AC splice sites at intron 6	5.5	6.4	-0.9	-2.0	100.0%	-2.9
2503	AT3G05230.2 | similar to signal peptidase subunit family protein [Arabidopsis thaliana] (TAIR:At5g27430.1); similar to signal peptidase protein-like [Cucumis melo] (GB:AAT52230.1); contains InterPro domain Signal peptidase subunit (InterPro:IPR007653) | chr3:1492161-1493395 FORWARD | Aliases: T12H1.20, T12H1_20	6.4	7.1	-0.7	-2.0	100.0%	-3.0
2504	AT1G25250.1 | zinc finger (C2H2 type) family protein, contains zinc finger, C2H2 type, domain, PROSITE:PS00028	2.6	2.4	0.2	2.0	100.0%	-4.0
2505	AT1G10270.1 | pentatricopeptide (PPR) repeat-containing protein, contains Pfam profile: PF01535 PPR repeat; similar to ESTs gb:R30192 and gb:AA651017 | chr1:3363506-3366490 FORWARD | Aliases: F14N23.15, F14N23_15	7.4	7.9	-0.5	-2.0	100.0%	-3.1
2506	AT1G53460.1 | expressed protein | chr1:19958158-19960300 REVERSE | Aliases: T3F20.22, T3F20_22	9.8	10.4	-0.6	-2.0	100.0%	-3.1
2507	AT1G35550.1 | elongation factor Tu C-terminal domain-containing protein, similar to SP:P13905 Elongation factor 1-alpha (EF-1-alpha) {Arabidopsis thaliana}; contains Pfam profile PF03143: Elongation factor Tu C-terminal domain | chr1:13112484-13113014 FORWARD | Aliases: F15O4.37	5.7	6.4	-0.7	-2.0	100.0%	-2.9
2508	AT5G49280.1 | hydroxyproline-rich glycoprotein family protein, contains proline-rich extensin domains, INTERPRO:IPR002965 | chr5:19993820-19994675 REVERSE | Aliases: K21P3.16, K21P3_16	5.8	5.1	0.7	2.0	100.0%	-3.0
2509	AT1G62830.1 | amine oxidase family protein / SWIRM domain-containing protein, contains Pfam profile: PF01593 Flavin containing amine oxidase | chr1:23268155-23270867 REVERSE | Aliases: F23N19.21, F23N19_21	4.8	5.2	-0.4	-2.0	100.0%	-3.4
2510	AT2G27810.2 | similar to xanthine/uracil permease family protein [Arabidopsis thaliana] (TAIR:At4g38050.1); similar to putative permease [Gossypium hirsutum] (GB:AAT64019.1); contains InterPro domain Xanthine/uracil/vitamin C permease family (InterPro:IPR006043)	4.3	5.0	-0.6	-2.0	100.0%	-3.1
2511	AT3G17770.1 | dihydroxyacetone kinase family protein, contains Pfam domains, PF02733: DAK1 domain and PF02734: DAK2 domain | chr3:6081677-6085963 REVERSE | Aliases: MIG5.7	6.0	6.7	-0.7	-2.0	100.0%	-3.1
2512	AT5G18150.1 | expressed protein, similar to unknown protein (emb:CAB87627.1) | chr5:6000016-6000333 REVERSE | Aliases: MRG7.11, MRG7_11	8.0	7.5	0.5	2.0	100.0%	-3.2
2513	AT1G64960.1 | expressed protein | chr1:24133581-24137712 FORWARD | Aliases: F13O11.26, F13O11_26	5.2	5.6	-0.4	-2.0	100.0%	-3.6
2514	AT5G43180.1 | expressed protein, contains Pfam profile PF04654: Protein of unknown function, DUF599 | chr5:17353566-17355080 REVERSE | Aliases: K24F5.2, K24F5_2	5.1	4.0	1.1	2.0	100.0%	-2.9
2515	AT5G06730.1 | peroxidase, putative, similar to peroxidase (Arabidopsis thaliana) gi:1491617:emb:CAA68212 | chr5:2079956-2081685 REVERSE | Aliases: MPH15.9, MPH15_9	6.2	7.8	-1.6	-2.0	100.0%	-2.8

Rank	Description	15.dpi	5.dpi	M	t	adj.q	B
2516	AT2G45790.1 | eukaryotic phosphomannomutase family protein, contains Pfam profile: PF03332 eukaryotic phosphomannomutase | chr2:18862853-18865442 FORWARD | Aliases: F4I18.23	9.9	10.7	-0.8	-2.0	100.0%	-3.3
2517	AT1G28395.3 | expressed protein | chr1:9969428-9971504 REVERSE | Aliases: None	7.6	8.5	-0.9	-2.0	100.0%	-3.0
2518	AT3G45850.1 | kinesin motor protein-related, kinesin-related protein TKRP125, Nicotiana tabacum, PIR:T02017 | chr3:16866597-16871935 REVERSE | Aliases: F16L2.60	4.1	4.6	-0.5	-2.0	100.0%	-3.5
2519	AT3G60860.1 | guanine nucleotide exchange family protein, similar to guanine nucleotide exchange factor, Homo sapiens, GI:5456754; contains Pfam profile PF01369: Sec7 domain | chr3:22495779-22502555 FORWARD | Aliases: T4C21.270	7.4	8.2	-0.8	-2.0	100.0%	-3.2
2520	AT5G55140.1 | ribosomal protein L30 family protein, contains similarity to 50S ribosomal protein L30 | chr5:22398262-22399254 FORWARD | Aliases: MCO15.9, MCO15_9	6.7	5.6	1.0	2.0	100.0%	-2.9
2521	AT1G08280.1 | glycosyl transferase family 29 protein / sialyltransferase family protein, contains Pfam profile: PF00777 sialyltransferase (Glycosyltransferase family 29) | chr1:2608405-2609850 FORWARD | Aliases: T23G18.14, T23G18_14	5.5	6.4	-0.9	-2.0	100.0%	-2.9
2522	AT3G20300.1 | expressed protein | chr3:7079623-7081925 REVERSE | Aliases: MQC12.4	4.2	3.8	0.4	2.0	100.0%	-3.5
2523	AT5G64260.1 | Symbol: MSJ1.10 | phosphate-responsive protein, putative, similar to phi-1 (phosphate-induced gene) (Nicotiana tabacum) GI:3759184; contains Pfam profile PF04674: Phosphate-induced protein 1 conserved region	7.3	6.2	1.2	2.0	100.0%	-2.8
2524	AT5G13830.1 | FtsJ-like methyltransferase family protein, contains Pfam profile: PF01728 FtsJ-like methyltransferase | chr5:4467366-4468490 FORWARD | Aliases: MAC12.22, MAC12_22	4.0	4.4	-0.4	-2.0	100.0%	-3.4
2525	AT4G31900.1 | chromatin remodeling factor, putative, strong similarity to chromatin remodeling factor CHD3 (PICKLE) (Arabidopsis thaliana) GI:6478518; contains Pfam profiles PF00271: Helicase conserved C-terminal domain, PF00176: SNF2 family N-terminal domain | chr4:15431534-15438449 FORWARD | Aliases: F11C18.100, F11C18_100	2.3	2.6	-0.3	-2.0	100.0%	-3.8
2526	AT1G48850.3 | Symbol: EMB1144 | similar to 3-phosphoshikimate 1-carboxyvinyltransferase, putative / 5-enolpyruvylshikimate-3-phosphate, putative / EPSP synthase, putative [Arabidopsis thaliana] (TAIR:At1g48860.1); similar to chorismate synthase 1 [Lycopersicon esculentum] (GB:CAA79859.1); contains InterPro domain Chorismate synthase (InterPro:IPR000453) | chr1:18068245-18071758 REVERSE | Aliases: EMB1144, EMBRYO DEFECTIVE 1144, T24P22.3, T24P22_3	10.9	11.6	-0.7	-2.0	100.0%	-3.5
2527	AT5G09900.2 | Symbol: EMB2107 | 26S proteasome regulatory subunit, putative (RPN5), p55 protein-like | chr5:3089279-3092537 REVERSE | Aliases: EMB2107, EMBRYO DEFECTIVE 2107, MYH9.11, MYH9_11	7.7	8.5	-0.7	-2.0	100.0%	-3.3
2528	AT1G02280.1 | Symbol: TOC33 | GTP-binding protein (TOC33), identical to atToc33 protein (GI:11557973) (Arabidopsis thaliana); Carboxyl-terminal end highly similar to GTP-binding protein SP:U43377, location of EST gb:AA394770 and gb:R30089; identical to cDNA for chloroplast atToc33 protein GI:11557972	7.0	8.0	-1.0	-2.0	100.0%	-2.9
2529	AT2G15760.1 | calmodulin-binding protein, similar to AR781 GI:1669593 from (Arabidopsis thaliana); AR781 complements pheromone receptor deficient mutant of Shizosaccharomyces pombe | chr2:6872692-6873959 REVERSE | Aliases: F9O13.1	5.9	6.3	-0.4	-2.0	100.0%	-3.4
2530	AT5G52470.1 | Symbol: FIB1 | fibrillarin 1 (FBR1) (FIB1) (SKIP7), identical to fibrillarin 1 GI:9965653 from (Arabidopsis thaliana); C-terminus identical to SKP1 interacting partner 7 GI:10716959 from (Arabidopsis thaliana); contains Pfam domain PF01269: Fibrillarin | chr5:21311454-21313902 FORWARD | Aliases: ATFBR1, ATFIB1, FBR1, FIBRILLARIN 1, K24M7.22, K24M7_22, SKIP7, SKP1 INTERACTING PARTNER 7	11.0	11.4	-0.4	-2.0	100.0%	-3.7
2531	AT3G63180.1 | expressed protein | chr3:23347293-23351211 REVERSE | Aliases: F16M2.30	4.4	4.9	-0.5	-2.0	100.0%	-3.5
2532	AT3G18760.1 | ribosomal protein S6 family protein, contains TIGRFAM profile TIGR00166: ribosomal protein S6 | chr3:6457696-6459633 REVERSE | Aliases: MVE11.12	7.3	8.1	-0.8	-2.0	100.0%	-3.0
2533	AT5G57160.1 | Symbol: ATLIG4 |	5.8	6.5	-0.7	-2.0	100.0%	-3.2
2534	AT5G53120.3 | Symbol: SPDS3 | spermidine synthase, putative / putrescine aminopropyltransferase, putative, similar to SP:O82147 Spermidine synthase (EC 2.5.1.16) (Putrescine aminopropyltransferase) (SPDSY) {Coffea arabica}; contains Pfam profile PF01564: Spermine/spermidine synthase | chr5:21552058-21555474 FORWARD | Aliases: MFH8.5, MFH8_5, SPERMIDINE SYNTHASE 3, SPMS	10.4	11.1	-0.7	-2.0	100.0%	-3.5
2535	AT5G64410.1 | Symbol: ATOPT4 |	3.8	4.7	-1.0	-2.0	100.0%	-2.8

Rank	Description	15.dpi	5.dpi	M	t	adj.q	B
2536	AT3G20520.1 | glycerophosphoryl diester phosphodiesterase family protein, contains Pfam PF03009 : Glycerophosphoryl diester phosphodiesterase family; similar to glycerophosphodiester phosphodiesterase (GI:1399038) (Borrelia hermsii) | chr3:7162834-7165865 FORWARD | Aliases: K10D20.6	3.7	3.3	0.4	2.0	100.0%	-3.4
2537	AT5G17620.1 | expressed protein | chr5:5805762-5808563 REVERSE | Aliases: K10A8.100, K10A8_100	5.4	6.3	-0.9	-2.0	100.0%	-3.2
2538	AT1G09220.1 | pentatricopeptide (PPR) repeat-containing protein, contains Pfam profile PF01535: PPR repeat | chr1:2977794-2978970 REVERSE | Aliases: T12M4.7, T12M4_7	3.0	3.3	-0.3	-2.0	100.0%	-3.8
2539	AT3G15120.1 | AAA-type ATPase family protein, contains PROSITE domains, PS00674: AAA-protein family signature and PS00017: ATP/GTP-binding site motif A (P-loop) | chr3:5088494-5095489 REVERSE | Aliases: F4B12.4	6.2	6.7	-0.5	-2.0	100.0%	-3.3
2540	AT5G01740.1 | expressed protein, wound-inducible protein wun1 protein - Solanum tuberosum, PIR:JQ0398 | chr5:280719-281442 FORWARD | Aliases: T20L15.10, T20L15_10	4.6	3.8	0.8	2.0	100.0%	-2.9
2541	AT3G24120.2 | myb family transcription factor, contains Pfam profile: PF00249 myb-like DNA-binding domain | chr3:8705643-8708322 REVERSE | Aliases: MUJ8.2	6.7	5.9	0.8	2.0	100.0%	-3.3
2542	AT1G29160.1 | Dof-type zinc finger domain-containing protein, similar to ascorbate oxidase promoter-binding protein GB:D45066 GI:853689 from (Cucurbita maxima) | chr1:10183783-10184310 REVERSE | Aliases: F28N24.35	6.5	6.2	0.3	2.0	100.0%	-3.5
2543	AT1G19835.1 | expressed protein, contains Pfam PF05911: Plant protein of unknown function (DUF869) | chr1:6855943-6860323 REVERSE | Aliases: F14P1.24, F14P1_24	8.7	9.2	-0.6	-2.0	100.0%	-3.4
2544	AT3G22750.1 | protein kinase, putative, similar to protein kinase ATMRK1 (Arabidopsis thaliana) gi:2351097:dbj:BAA22079 | chr3:8037223-8039910 REVERSE | Aliases: MWI23.12	4.7	5.3	-0.6	-2.0	100.0%	-3.0
2545	AT1G14150.2 | oxygen evolving enhancer 3 (PsbQ) family protein, extrinsic pII protein; contains Pfam profile PF05757: Oxygen evolving enhancer protein 3 (PsbQ) | chr1:4839850-4840449 FORWARD | Aliases: F7A19.23, F7A19_23	3.1	2.6	0.5	2.0	100.0%	-3.4
2546	AT1G70130.1 | lectin protein kinase, putative, similar to receptor lectin kinase 3 (Arabidopsis thaliana) gi:4100060:gb:AAD00733 | chr1:26413406-26415464 REVERSE | Aliases: F20P5.15, F20P5_15	2.8	3.2	-0.3	-2.0	100.0%	-3.5
2547	AT5G39250.1 | F-box family protein, ; similar to SKP1 interacting partner 2 (SKIP2) TIGR_Ath1:At5g67250 | chr5:15741758-15742984 FORWARD | Aliases: K3K3.16, K3K3_16	6.0	5.6	0.4	2.0	100.0%	-3.3
2548	AT3G54720.1 | Symbol: AMP1 | glutamate carboxypeptidase, putative (AMP1), identical to GI:15624092 glutamate carboxypeptidase {Arabidopsis thaliana}; ileal peptidase, Rattus norvegicus, EMBL:AF009921; identical to cDNA glutamate carboxypeptidase (AMP1) GI:15624091; contains Pfam profiles PF04389: Peptidase family M28, PF04253: Transferrin receptor-like dimerisation domain and PF02225 PA domain	4.8	5.3	-0.5	-2.0	100.0%	-3.3
2549	AT1G48240.1 | Symbol: ATNPSN12 | novel plant SNARE 12 (NPSN12), identical to Novel plant SNARE 12 (AtNPSN12) (Swiss-Prot:Q9LNH6) (Arabidopsis thaliana) | chr1:17812676-17815563 REVERSE | Aliases: F21D18.4, NPSN12	7.3	6.1	1.1	2.0	100.0%	-3.1
2550	AT3G47560.2 | esterase/lipase/thioesterase family protein, low similarity to cinnamoyl ester hydrolase CinI (Butyrivibrio fibrisolvens) GI:1622732; contains Interpro entry IPR000379 | chr3:17536162-17537963 REVERSE | Aliases: F1P2.110	6.5	7.6	-1.1	-2.0	100.0%	-2.9
2551	AT4G24190.2 | Symbol: SHD | shepherd protein (SHD) / clavata formation protein, putative, nearly identical to SHEPHERD (Arabidopsis thaliana) GI:19570872; contains Pfam profiles PF02518: ATPase, histidine kinase-, DNA gyrase B-, and HSP90-like domain protein, PF00183: Hsp90 protein | chr4:12551731-12555915 REVERSE | Aliases: SHEPHERD, T22A6.20, T22A6_20	11.2	12.0	-0.8	-2.0	100.0%	-3.4
2552	AT3G21610.2 | expressed protein, similar to expressed protein [Arabidopsis thaliana] (TAIR:At1g67600.1); similar to unknown protein [Oryza sativa (japonica cultivar-group)] (GB:BAD82112.1); contains InterPro domain Protein of unknown function DUF212 (InterPro:IPR003832)	7.2	6.5	0.7	2.0	100.0%	-3.3
2553	AT1G19110.1 | inter-alpha-trypsin inhibitor heavy chain-related, similar to SP:Q61704 Inter-alpha-trypsin inhibitor heavy chain H3 precursor {Mus musculus}; contains Pfam profile PF00092: von Willebrand factor type A domain | chr1:6602117-6605899 FORWARD | Aliases: F14D16.26, F14D16_26	8.8	8.4	0.4	2.0	100.0%	-3.3
2554	AT1G01480.2 | Symbol: ACS2 | 1-aminocyclopropane-1-carboxylate synthase 2 / ACC synthase 2 (ACS2) (ACC1), identical to 1-aminocyclopropane-1-carboxylate synthase SP:Q06402 (GI:166578) from (Arabidopsis thaliana)	3.7	4.2	-0.6	-2.0	100.0%	-3.2
2555	AT1G53390.1 | ABC transporter family protein, similar to ATP-binding cassette, sub-family G, member 2 (Placenta-specific ATP- binding cassette transporter) (Breast cancer resistance protein) SP:Q9UNQ0 from (Homo sapiens) | chr1:19921715-19927475 FORWARD | Aliases: F12M16.28, F12M16_28	4.9	5.4	-0.5	-2.0	100.0%	-3.2

Rank	Description	15.dpi	5.dpi	M	t	adj.q	B
2556	AT1G26100.1 | cytochrome B561 family protein, contains Pfam domain, PF03188: Cytochrome b561 | chr1:9022588-9024068 REVERSE | Aliases: F14G11.7, F14G11_7	5.5	5.9	-0.4	-2.0	100.0%	-3.4
2557	AT1G12450.1 | expressed protein | chr1:4244308-4245990 FORWARD | Aliases: F5O11.19, F5O11_19	4.3	3.8	0.5	2.0	100.0%	-3.4
2558	AT1G68810.1 | basic helix-loop-helix (bHLH) family protein, contains Pfam profile: PF00010 helix-loop-helix DNA-binding domain	5.5	4.8	0.7	2.0	100.0%	-3.0
2559	AT1G19860.1 | zinc finger (CCCH-type) family protein, contains Pfam domain, PF00642: Zinc finger C-x8-C-x5-C-x3-H type (and similar) | chr1:6891420-6894708 REVERSE | Aliases: F6F9.9, F6F9_9	4.2	4.7	-0.5	-2.0	100.0%	-3.4
2560	AT3G53950.1 | glyoxal oxidase-related, contains similarity to glyoxal oxidase precursor (Phanerochaete chrysosporium) gi:1050302:gb:AAA87594 | chr3:19986597-19988417 FORWARD | Aliases: F5K20.250	6.2	5.5	0.6	2.0	100.0%	-3.3
2561	AT5G63950.1 | SNF2 domain-containing protein / helicase domain-containing protein, low similarity to SP:Q03468 Excision repair protein ERCC-6 (Cockayne syndrome protein CSB) {Homo sapiens}; contains PFam profiles PF00271: Helicase conserved C-terminal domain, PF00176: SNF2 family N-terminal domain | chr5:25609182-25615731 REVERSE | Aliases: MBM17.5, MBM17_5	3.5	3.9	-0.3	-2.0	100.0%	-3.5
2562	AT2G42540.2 | Symbol: COR15A |	6.5	5.0	1.5	2.0	100.0%	-2.9
2563	AT2G23950.1 | leucine-rich repeat family protein / protein kinase family protein, contains Pfam domains PF00560: Leucine Rich Repeat and PF00069: Protein kinase domain | chr2:10194171-10197365 REVERSE | Aliases: T29E15.15, T29E15_15	5.0	4.2	0.8	2.0	100.0%	-3.0
2564	AT3G53540.1 | expressed protein | chr3:19857478-19861946 REVERSE | Aliases: F4P12.240	5.3	5.8	-0.5	-2.0	100.0%	-3.3
2565	AT1G02750.1 | drought-responsive family protein, contains similarity to drought-induced mRNA, Di19 (Arabidopsis thaliana) gi:469110:emb:CAA55321 | chr1:602673-604286 FORWARD | Aliases: T14P4.27	5.0	4.4	0.6	2.0	100.0%	-3.1
2566	AT3G25980.1 | mitotic spindle checkpoint protein, putative (MAD2), identical to Swiss-Prot:Q9LU93 mitotic spindle checkpoint protein MAD2 (Arabidopsis thaliana) | chr3:9504469-9505639 FORWARD | Aliases: MPE11.16	5.0	4.5	0.4	2.0	100.0%	-3.5
2567	AT5G59480.2 | haloacid dehalogenase-like hydrolase family protein, low similarity to SP:P53078 SSM1 protein {Saccharomyces cerevisiae}; contains InterPro accession IPR005834: Haloacid dehalogenase-like hydrolase | chr5:23996649-23998793 REVERSE | Aliases: F2O15.10, F2O15_10	8.1	9.6	-1.5	-2.0	100.0%	-2.9
2568	AT3G56130.3 | similar to biotin/lipoyl attachment domain-containing protein [Arabidopsis thaliana] (TAIR:At1g52670.1); similar to acetyl-CoA carboxylasesubunit (biotin carboxyl carrier subunit) [Geobacillus kaustophilus HTA426] (GB:YP_148253.1); contains InterPro domain Biotin/lipoyl attachment (InterPro:IPR000089) | chr3:20837637-20840249 FORWARD | Aliases: F18O21.90	4.4	5.4	-1.0	-2.0	100.0%	-3.0
2569	AT3G53320.1 | expressed protein | chr3:19780154-19783347 REVERSE | Aliases: F4P12.20	4.8	5.3	-0.5	-2.0	100.0%	-3.5
2570	AT5G66010.1 | heterogeneous nuclear ribonucleoprotein, putative / hnRNP, putative, similar to Heterogeneous nuclear ribonucleoprotein SP:P55795, SP:P31943, SP:P52597 {Homo sapiens}; contains InterPro entry IPR000504: RNA-binding region RNP-1 (RNA recognition motif) (RRM) domain	5.7	5.2	0.5	2.0	100.0%	-3.3
2571	AT2G37310.1 | pentatricopeptide (PPR) repeat-containing protein, contains Pfam profile PF01535: PPR repeat | chr2:15672181-15674154 REVERSE | Aliases: F3G5.10, F3G5_10	3.1	3.4	-0.3	-2.0	100.0%	-3.5
2572	AT5G03340.1 | cell division cycle protein 48, putative / CDC48, putative, very strong similarity to SP:P54609 Cell division cycle protein 48 homolog {Arabidopsis thaliana}; contains Pfam profiles PF00004: ATPase AAA family, PF02359: Cell division protein 48 (CDC48) N-terminal domain; supporting cDNA gi:26449351:dbj:AK117125.1: | chr5:809947-813227 REVERSE | Aliases: F12E4.70, F12E4_70	6.4	7.0	-0.7	-2.0	100.0%	-3.0
2573	AT1G05940.1 | Symbol: CAT9 | amino acid permease family protein, low similarity to SP:P30823 High-affinity cationic amino acid transporter-1 (CAT-1) {Rattus norvegicus}; contains Pfam profile PF00324: Amino acid permease | chr1:1801177-1803975 REVERSE | Aliases: CATIONIC AMINO ACID TRANSPORTER 9, T21E18.1, T21E18_1	7.2	6.7	0.5	2.0	100.0%	-3.3
2574	AT1G74310.1 | Symbol: ATHSP101 | heat shock protein 101 (HSP101), identical to heat shock protein 101 GI:6715468 GB:AAF26423 from (Arabidopsis thaliana) | chr1:27939597-27943851 REVERSE | Aliases: F1O17.2, F1O17_2, HEAT SHOCK PROTEIN 101, HEAT SHOCK PROTEIN ATHSP101, HOT1, HSP101	5.7	5.2	0.5	2.0	100.0%	-3.2
2575	AT1G67360.2 | rubber elongation factor (REF) family protein, contains Pfam profile: PF05755 rubber elongation factor protein (REF) | chr1:25240474-25241757 REVERSE | Aliases: F1N21.18	7.8	7.1	0.7	2.0	100.0%	-3.3
2576	AT3G04490.1 | exportin-related, weak similarity to Exportin 4 (Exp4) (Swiss-Prot:Q9ESJ0) (Mus musculus) and (Swiss-Prot:Q9C0E2) (Homo sapiens)	5.0	5.9	-1.0	-2.0	100.0%	-2.9

Rank	Description	15.dpi	5.dpi	M	t	adj.q	B
2577	AT3G26580.1 | expressed protein | chr3:9760124-9761781 FORWARD | Aliases: MFE16.11	9.2	8.4	0.9	2.0	100.0%	-3.1
2578	AT3G05750.2 | similar to expressed protein [Arabidopsis thaliana] (TAIR:At5g26910.1); similar to expressed protein [Arabidopsis thaliana] (TAIR:At3g58650.1); similar to serine-threonine rich antigen [Staphylococcus aureus] (GB:AAL58470.1)	2.7	3.0	-0.3	-2.0	100.0%	-3.7
2579	AT1G36380.1 | expressed protein, similar to vanilloid receptor-like protein GB:AAD26363 from (Homo sapiens) | chr1:13702026-13702844 FORWARD | Aliases: F7F23.10, F7F23_10	7.3	6.5	0.8	2.0	100.0%	-3.3
2580	AT4G26555.1 | immunophilin / FKBP-type peptidyl-prolyl cis-trans isomerase family protein, similar to FK506-binding protein (Peptidyl-prolyl cis-trans isomerase) (PPiase) (Rotamase) (SP:P25138) ({Neisseria meningitidis); contains Pfam profile: PF00254 FKBP-type peptidyl-prolyl cis-trans isomerases; similar to FK506-binding protein 39 kDa (Peptidyl-prolyl cis-trans isomerase) (PPiase) (EC 5.2.1.8) (SP:O74191) {Schizosaccharomyces pombe} | chr4:13404485-13406210 REVERSE | Aliases: None	6.2	5.6	0.6	2.0	100.0%	-3.3
2581	AT4G23960.1 | F-box family protein, similar to SKP1 interacting partner 2 (SKIP2) TIGR_Ath1:At5g67250 | chr4:12444775-12445333 FORWARD | Aliases: T32A16.130, T32A16_130	3.4	3.0	0.4	2.0	100.0%	-3.5
2582	AT5G20230.1 | Symbol: ATBCB | plastocyanin-like domain-containing protein | chr5:6826508-6827601 FORWARD | Aliases: ARABIDOPSIS BLUE COPPER BINDING PROTEIN, BCB, F5O24.120, F5O24_120	5.9	4.6	1.3	2.0	100.0%	-3.0
2583	AT1G63880.1 | disease resistance protein (TIR-NBS-LRR class), putative, domain signature TIR-NBS-LRR exists, suggestive of a disease resistance protein. | chr1:23715939-23719787 REVERSE | Aliases: T12P18.10, T12P18_10	2.9	2.6	0.3	2.0	100.0%	-3.6
2584	AT1G72650.2 | Symbol: TRFL6 | myb family transcription factor, contains Pfam PF00249: Myb-like DNA-binding domain | chr1:27353129-27357402 FORWARD | Aliases: F28P22.16, F28P22_16, TRF LIKE 6	6.4	5.7	0.6	2.0	100.0%	-3.1
2585	AT2G04530.1 | Symbol: CPZ | RNase Z, 97% identical to RNase Z (GI:20975607) (Arabidopsis thaliana); similar to RNase Z (GI:20975609) (Arabidopsis thaliana); identical to cDNA RNase Z (At2g04530) GI:20975606 | chr2:1576768-1578738 FORWARD | Aliases: T1O3.6, T1O3_6	5.2	6.1	-0.9	-2.0	100.0%	-2.8
2586	AT4G00080.1 | invertase/pectin methylesterase inhibitor family protein, low similarity to pectinesterase from Lycopersicon esculentum SP:Q43143, Arabidopsis thaliana SP:Q42534; contains Pfam profile PF04043: Plant invertase/pectin methylesterase inhibitor | chr4:32893-33705 FORWARD | Aliases: F6N15.9, F6N15_9	2.3	2.1	0.2	2.0	100.0%	-4.1
2587	AT5G60850.1 | Symbol: OBP4 | Dof-type zinc finger domain-containing protein, similar to zinc finger protein OBP4 gi:5059396 from (Arabidopsis thaliana); EMBL:AF155817 | chr5:24497675-24499094 FORWARD | Aliases: MAE1.2, MAE1_2, ZINC FINGER PROTEIN OBP4	4.8	4.3	0.5	2.0	100.0%	-3.2
2588	AT1G29970.1 | expressed protein, similar to GI:3128228 from (Arabidopsis thaliana) (Nature 402 (6763), 761-768 (1999))	6.9	6.5	0.4	2.0	100.0%	-3.4
2589	AT3G25480.1 | rhodanese-like domain-containing protein, contains Rhodanese-like domain PF:00581 | chr3:9236435-9237844 REVERSE | Aliases: MWL2.9	3.7	4.1	-0.4	-2.0	100.0%	-3.4
2590	AT3G23550.1 | MATE efflux family protein, similar to ripening regulated protein DDTFR18 (Lycopersicon esculentum) GI:12231296; contains Pfam profile: PF01554 uncharacterized membrane protein family | chr3:8448321-8450704 REVERSE | Aliases: MDB19.3	4.8	4.1	0.7	2.0	100.0%	-3.2
2591	AT4G34480.1 | glycosyl hydrolase family 17 protein, similar to glucan endo-1,3-beta-glucosidase precursor SP:P52409 from (Triticum aestivum) | chr4:16481884-16483992 REVERSE | Aliases: T4L20.60, T4L20_60	4.2	3.7	0.5	2.0	100.0%	-3.3
2592	AT1G07130.1 | OB-fold nucleic acid binding domain-containing protein, contains InterPro entry IPR004365: OB-fold nucleic acid binding domain | chr1:2187775-2188732 FORWARD | Aliases: F10K1.17, F10K1_17	3.5	3.3	0.3	2.0	100.0%	-3.8
2593	AT3G03530.1 | Symbol: NPC4 | phosphoesterase family protein, low similarity to SP:P95246 Phospholipase C 2 precursor (EC 3.1.4.3) {Mycobacterium tuberculosis}; contains Pfam profile PF04185: Phosphoesterase family	2.6	2.9	-0.3	-2.0	100.0%	-3.9
2594	AT3G03540.1 | phosphoesterase family protein, similar to SP:P95246 Phospholipase C 2 precursor (EC 3.1.4.3) {Mycobacterium tuberculosis}; contains Pfam profile PF04185: Phosphoesterase family | chr3:846357-848304 REVERSE | Aliases: T21P5.4	2.6	2.9	-0.3	-2.0	100.0%	-3.9
2595	AT5G60670.1 | 60S ribosomal protein L12 (RPL12C), 60S RIBOSOMAL PROTEIN L12 (like), Arabidopsis thaliana, PIR:T45883 | chr5:24398136-24398819 REVERSE | Aliases: MUP24.13, MUP24_13	9.1	9.7	-0.6	-2.0	100.0%	-3.2
2596	AT4G21820.1 | calmodulin-binding family protein, contains IQ calmodulin-binding motif, Pfam:PF00612 | chr4:11577536-11584065 FORWARD | Aliases: T8O5.30, T8O5_30	2.6	2.8	-0.2	-2.0	100.0%	-3.9

Rank	Description	15.dpi	5.dpi	M	t	adj.q	B
2597	AT4G23800.1 | high mobility group (HMG1/2) family protein, similar to HMG2B (Homo sapiens) GI:32335; contains Pfam profile PF00505: HMG (high mobility group) box | chr4:12390177-12393008 FORWARD | Aliases: None	4.0	5.3	-1.3	-2.0	100.0%	-2.9
2598	AT5G23050.1 | acyl-activating enzyme 17 (AAE17), nearly identical to acyl-activating enzyme 17 (Arabidopsis thaliana) GI:29893266; similar to acetyl-CoA synthetase (SP:P27095) from Methanothrix soehngenii; contains Pfam AMP-binding enzyme domain PF00501; identical to cDNA acyl-activating enzyme 17 (At5g23050) GI:29893265 | chr5:7731272-7735442 REVERSE | Aliases: MYJ24.4, MYJ24_4	7.0	7.6	-0.6	-2.0	100.0%	-3.4
2599	AT3G58780.1 | Symbol: SHP1 | agamous-like MADS box protein AGL1 / shatterproof 1 (AGL1) (SHP1), identical to SP:P29381 Agamous-like MADS box protein AGL1 (Protein Shatterproof 1) {Arabidopsis thaliana} | chr3:21749437-21752884 FORWARD | Aliases: AGL1, SHATTERPROOF 1, T20N10.130	4.1	3.6	0.5	2.0	100.0%	-3.2
2600	AT5G01880.1 | zinc finger (C3HC4-type RING finger) family protein, contains Pfam domain, PF00097: Zinc finger, C3HC4 type (RING finger) | chr5:338896-340250 FORWARD | Aliases: T20L15.150, T20L15_150	5.3	4.5	0.8	2.0	100.0%	-3.0
2601	AT1G18040.1 | Symbol: CDKD1;3 | cell division protein kinase, putative, similar to cell division protein kinase 7 (Homo sapiens) SWISS-PROT:P50613 | chr1:6206864-6209493 REVERSE | Aliases: CDKD1;3, Cyclin dependent kinase D1;3, T10F20.5, T10F20_5	5.6	6.4	-0.9	-2.0	100.0%	-3.0
2602	AT3G23750.1 | leucine-rich repeat family protein / protein kinase family protein, contains Pfam domains PF00560: Leucine Rich Repeat and PF00069: Protein kinase domain | chr3:8558339-8561435 FORWARD | Aliases: MYM9.9	4.9	5.6	-0.7	-2.0	100.0%	-3.2
2603	AT2G05630.1 | Symbol: atg8d | autophagy 8d (APG8d), identical to autophagy 8d (Arabidopsis thaliana) GI:19912157; contains Pfam profile PF02991: Microtubule associated protein 1A/1B, light chain 3 | chr2:2082983-2084879 REVERSE | Aliases: T20G20.2, T20G20_2	9.2	8.5	0.7	2.0	100.0%	-3.4
2604	AT5G25590.1 | expressed protein, contains Pfam profile PF04783: Protein of unknown function (DUF630) | chr5:8906601-8909923 REVERSE | Aliases: T14C9.130, T14C9_130	3.5	3.8	-0.3	-2.0	100.0%	-3.5
2605	AT1G04200.1 | expressed protein, Contains similarity to gb:Z69902 from C. elegans | chr1:1109489-1114049 FORWARD | Aliases: F20D22.3, F20D22_3	4.8	5.5	-0.7	-2.0	100.0%	-3.2
2606	NA	2.8	2.6	0.2	2.0	100.0%	-4.1
2607	AT3G05880.1 | Symbol: RCI2A | hydrophobic protein (RCI2A) / low temperature and salt responsive protein (LTI6A), identical to SP:Q9ZNQ7 Hydrophobic protein RCI2A (Low temperature and salt responsive protein LTI6A) {Arabidopsis thaliana} | chr3:1755503-1756546 REVERSE | Aliases: F10A16.18, F10A16_18, RARE COLD INDUCIBLE 2A	11.4	10.8	0.6	2.0	100.0%	-3.6
2608	AT4G18880.1 | Symbol: AT HSFA4A | heat shock transcription factor 21 (HSF21), identical to heat shock transcription factor 21 (Arabidopsis thaliana) GI:3399765; contains Pfam profile: PF00447 HSF-type DNA-binding domain | chr4:10347593-10349573 REVERSE | Aliases: F13C5.50, F13C5_50, HSFA4A	4.4	3.8	0.6	2.0	100.0%	-3.2
2609	AT1G66430.1 | pfkB-type carbohydrate kinase family protein, contains Pfam profile: PF00294 pfkB family carbohydrate kinase | chr1:24781959-24784278 FORWARD | Aliases: F28G11.11, F28G11_11	4.8	5.6	-0.8	-2.0	100.0%	-3.1
2610	AT5G59880.2 | Symbol: ADF3 | actin-depolymerizing factor 3 (ADF3), identical to SP:Q9ZSK4 Actin-depolymerizing factor 3 (ADF 3) (AtADF3) {Arabidopsis thaliana} | chr5:24137457-24139105 FORWARD | Aliases: ACTIN DEPOLYMERIZING FACTOR 3, MMN10.4, MMN10_4	13.2	12.6	0.6	2.0	100.0%	-3.3
2611	AT5G49630.1 | Symbol: AAP6 | amino acid permease 6 (AAP6), identical to amino acid permease 6 (AAP6) (Arabidopsis thaliana) GI:1769887 | chr5:20159696-20163712 REVERSE | Aliases: AMINO ACID PERMEASE 6, MNI5.1, MNI5_1	12.1	10.9	1.2	2.0	100.0%	-3.0
2612	AT1G57630.1 | disease resistance protein (TIR class), putative, domain signature TIR exists, suggestive of a disease resistance protein. | chr1:21349304-21349822 FORWARD | Aliases: T8L23.10, T8L23_10	2.9	3.3	-0.4	-2.0	100.0%	-3.6
2613	AT1G14730.1 | similar to cytochrome B561 family protein [Arabidopsis thaliana] (TAIR:At4g25570.1); similar to putative cytochrome protein [Oryza sativa (japonica cultivar-group)] (GB:XP_469562.1); contains InterPro domain Cytochrome b561 / ferric reductase transmembrane (InterPro:IPR006593); contains InterPro domain Cytochrome b561 (InterPro:IPR004877) | chr1:5073100-5074774 FORWARD | Aliases: F10B6.13, F10B6_13	8.7	8.3	0.4	2.0	100.0%	-3.4
2614	AT1G23710.1 | expressed protein | chr1:8385240-8386415 FORWARD | Aliases: F5O8.26, F5O8_26	5.5	4.1	1.3	2.0	100.0%	-3.0
2615	AT4G27900.2 | expressed protein | chr4:13890670-13893079 FORWARD | Aliases: T13J8.10, T13J8_10	5.9	5.3	0.6	2.0	100.0%	-3.0
2616	AT3G18080.1 | glycosyl hydrolase family 1 protein, contains Pfam PF00232 : Glycosyl hydrolase family 1 domain; TIGRFAM TIGR01233: 6-phospho-beta-galactosidase; similar to beta-glucosidase BGQ60 precursor GB:A57512 (Hordeum vulgare); similar to beta-mannosidase enzyme (GI:17226270) (Lycopersicon esculentum) | chr3:6191565-6194458 FORWARD | Aliases: MRC8.20	6.2	7.3	-1.1	-2.0	100.0%	-3.0

Rank	Description	15.dpi	5.dpi	M	t	adj.q	B
2617	AT5G67510.1 | 60S ribosomal protein L26 (RPL26B) | chr5:26955039-26955677 REVERSE | Aliases: K9I9.7, K9I9_7	8.1	8.8	-0.7	-2.0	100.0%	-3.1
2618	AT3G42600.1 | hypothetical protein | chr3:14708749-14709408 FORWARD | Aliases: T12K4.50	2.6	2.8	-0.2	-2.0	100.0%	-4.3
2619	AT2G20840.1 | secretory carrier membrane protein (SCAMP) family protein, contains Pfam domain, PF04144: SCAMP family | chr2:8978843-8981551 REVERSE | Aliases: F5H14.19, F5H14_19	5.2	4.8	0.4	2.0	100.0%	-3.3
2620	AT4G37280.1 | MRG family protein, contains Pfam domain PF05712: MRG | chr4:17546578-17549409 REVERSE | Aliases: AP22.79, AP22_79	7.2	7.9	-0.7	-2.0	100.0%	-3.1
2621	AT5G55760.1 | transcriptional regulator Sir2 family protein, contains Pfam domain PF02146: transcriptional regulator, Sir2 family | chr5:22584448-22588189 FORWARD | Aliases: MDF20.20, MDF20_20	5.8	6.3	-0.6	-2.0	100.0%	-3.3
2622	AT5G44380.1 | FAD-binding domain-containing protein, similar to SP:P30986 reticuline oxidase precursor (Berberine-bridge-forming enzyme) (BBE) (Tetrahydroprotoberberine synthase) (Eschscholzia californica); contains PF01565 FAD binding domain | chr5:17895949-17898666 REVERSE | Aliases: K9L2.18, K9L2_18	3.3	4.0	-0.7	-2.0	100.0%	-3.2
2623	AT3G23050.2 | Symbol: IAA7 | auxin-responsive protein / indoleacetic acid-induced protein 7 (IAA7), identical to SP:Q38825:AXI7_ARATH Auxin-responsive protein IAA7 (Indoleacetic acid-induced protein 7) | chr3:8194718-8196521 FORWARD | Aliases: AUXIN RESISTANT 2, AXR2, MXC7.8	5.3	6.2	-0.9	-2.0	100.0%	-2.9
2624	AT1G76100.1 | plastocyanin, identical to plastocyanin GI:1865683 from (Arabidopsis thaliana) | chr1:28558881-28559651 REVERSE | Aliases: T23E18.3, T23E18_3	5.5	4.6	0.9	2.0	100.0%	-2.8
2625	AT3G55640.1 | mitochondrial substrate carrier family protein, contains Pfam profile: PF00153 mitochondrial carrier protein | chr3:20650818-20653638 FORWARD | Aliases: F1I16.50	3.8	3.5	0.3	2.0	100.0%	-3.7
2626	AT1G28680.1 | transferase family protein, similar to elicitor inducible gene product EIG-I24 (Nicotiana tabacum) (gi:10798748); contains Pfam transferase family domain PF00248 | chr1:10078175-10080015 FORWARD | Aliases: F1K23.12, F1K23_12	8.7	9.4	-0.8	-2.0	100.0%	-3.5
2627	AT1G26290.1 | expressed protein | chr1:9098038-9098542 FORWARD | Aliases: F28B23.5, F28B23_5	3.6	3.1	0.5	2.0	100.0%	-3.5
2628	AT5G67420.1 | LOB domain protein 37 / lateral organ boundaries domain protein 37 (LBD37), identical to LOB DOMAIN 37 (Arabidopsis thaliana) GI:17227170 | chr5:26921515-26923252 REVERSE | Aliases: K8K14.16, K8K14_16	3.9	5.0	-1.1	-2.0	100.0%	-2.9
2629	AT4G37200.1 | Symbol: HCF164 |	7.5	6.9	0.5	2.0	100.0%	-3.2
2630	AT1G59750.3 | Symbol: ARF1 | similar to transcriptional factor B3 family protein / auxin-responsive factor AUX/IAA-related [Arabidopsis thaliana] (TAIR:At2g46530.1); similar to OSJNBa0064D20.11 [Oryza sativa (japonica cultivar-group)] (GB:CAE04227.2); contains InterPro domain Transcriptional factor B3 (InterPro:IPR003340); contains InterPro domain AUX/IAA protein (InterPro:IPR003311) | chr1:21983067-21988098 FORWARD | Aliases: ARF1, AUXIN RESPONSE FACTOR 1, F23H11.7, F23H11_7	5.8	6.2	-0.4	-2.0	100.0%	-3.3
2631	AT1G76940.1 | RNA recognition motif (RRM)-containing protein, contains Pfam PF00076: RNA recognition motif. (a.k.a. RRM, RBD, or RNP domain); similar to RNA-binding protein with multiple splicing homolog (RBP-MS) (HEart, RRM Expressed Sequence) (Hermes) (Swiss-Prot:Q9YGI5) (Xenopus laevis); similar to RNA-binding protein with multiple splicing (RBP-MS) (Swiss-Prot:Q93062) (Homo sapiens) | chr1:28907322-28909149 REVERSE | Aliases: F22K20.4, F22K20_4	5.3	4.8	0.5	2.0	100.0%	-3.3
2632	AT3G27870.1 | haloacid dehalogenase-like hydrolase family protein, similar to Potential phospholipid-transporting ATPase (EC 3.6.3.1) from {Mus musculus} SP:P98200, Homo sapiens SP:O43520, {Arabidopsis thaliana} SP:P98204; contains InterPro accession IPR005834: Haloacid dehalogenase-like hydrolase | chr3:10332187-10336542 FORWARD | Aliases: K16N12.23	3.5	3.2	0.3	2.0	100.0%	-3.7
2633	AT1G16650.1 | expressed protein | chr1:5687936-5691191 FORWARD | Aliases: F19K19.6, F19K19_6	4.4	3.9	0.5	2.0	100.0%	-3.3
2634	AT1G04490.1 | expressed protein | chr1:1218004-1219913 REVERSE | Aliases: F19P19.4, F19P19_4	3.9	3.5	0.4	2.0	100.0%	-3.5
2635	AT3G27340.2 | similar to B1065G12.33 [Oryza sativa (japonica cultivar-group)] (GB:NP_915228.1)	8.2	7.6	0.6	2.0	100.0%	-3.1
2636	AT3G18860.2 | transducin family protein / WD-40 repeat family protein, contains seven G-protein beta WD-40 repeats; similar to phospholipase a-2-activating protein SP:P27612 from (Mus musculus) | chr3:6501688-6508542 FORWARD | Aliases: MCB22.3	7.8	8.6	-0.8	-2.0	100.0%	-3.3
2637	AT1G77760.1 | Symbol: NIA1 | nitrate reductase 1 (NR1), identical to SP:P11832 Nitrate reductase 1 (formerly EC 1.6.6.1) (NR1){Arabidopsis thaliana} | chr1:29240697-29244339 REVERSE | Aliases: GNR1, NITRATE REDUCTASE, NITRATE REDUCTASE 1, NITRATE REDUCTASE DEFECTIVE 1, NR1, T32E8.9, T32E8_9	9.3	7.5	1.8	2.0	100.0%	-3.1
2638	AT4G04790.1 | similar to pentatricopeptide (PPR) repeat-containing protein [Arabidopsis thaliana] (TAIR:At4g21880.1); similar to B1358B12.18 [Oryza sativa (japonica cultivar-group)] (GB:XP_472769.1); contains InterPro domain PPR repeat (InterPro:IPR002885) | chr4:2434905-2439401 REVERSE | Aliases: T4B21.17, T4B21_17	4.0	4.6	-0.6	-2.0	100.0%	-3.4

Rank	Description	15.dpi	5.dpi	M	t	adj.q	B
2639	AT1G18840.2 | similar to calmodulin-binding family protein [Arabidopsis thaliana] (TAIR:At1g74690.1); similar to calmodulin-binding family protein-like [Oryza sativa (japonica cultivar-group)] (GB:BAD87380.1); contains InterPro domain IQ calmodulin-binding region (InterPro:IPR000048) | chr1:6500839-6504388 REVERSE | Aliases: F6A14.7, F6A14_7	3.9	4.4	-0.5	-2.0	100.0%	-3.2
2640	AT2G01620.1 | expressed protein | chr2:278126-279295 FORWARD | Aliases: T8O11.21, T8O11_21	7.9	7.3	0.6	2.0	100.0%	-3.2
2641	AT1G04900.1 | expressed protein, contains Pfam PF02636: Uncharacterized ACR, COG1565 | chr1:1383840-1388520 FORWARD | Aliases: F13M7.11, F13M7_11	4.1	4.7	-0.6	-2.0	100.0%	-3.2
2642	AT2G38170.3 | Symbol: CAX1 | calcium exchanger (CAX1), identical to high affinity calcium antiporter CAX1 (Arabidopsis thaliana) gi:9256741:gb:AAB05913, except a possible frameshift at base 58008. Sequence has been confirmed with 5 sequencing reads.; Ca2+:Cation Antiporter (CaCA) Family member PMID:11500563 | chr2:15997127-16000354 REVERSE | Aliases: ATCAX1, F16M14.10, F16M14_10, HIGH AFFINITY CALCIUM ANTIPORTER CAX1, RARE COLD INDUCIBLE4, RCI4	7.3	8.1	-0.8	-2.0	100.0%	-3.0
2643	AT2G02570.3 | expressed protein, similar to hypothetical protein DDB0192063 [Dictyostelium discoideum] (GB:EAL60630.1); contains InterPro domain Tudor domain (InterPro:IPR002999) | chr2:697950-700908 REVERSE | Aliases: T8K22.13, T8K22_13	6.9	6.1	0.8	2.0	100.0%	-3.2
2644	AT1G60080.1 | 3' exoribonuclease family domain 1-containing protein, similar to SP:Q96B26 Exosome complex exonuclease RRP43 (EC 3.1.13.-) (Ribosomal RNA processing protein 43) {Homo sapiens}; contains Pfam profile PF01138: 3' exoribonuclease family, domain 1 | chr1:22155895-22158246 REVERSE | Aliases: T2K10.14, T2K10_14	5.8	6.4	-0.6	-2.0	100.0%	-3.0
2645	AT4G34490.1 | Symbol: ATCAP1 |	8.2	8.8	-0.6	-2.0	100.0%	-3.3
2646	AT3G52730.1 | ubiquinol-cytochrome C reductase UQCRX/QCR9-like family protein, contains Pfam profile: PF05365 ubiquinol-cytochrome C reductase, UQCRX/QCR9 like | chr3:19553909-19555242 REVERSE | Aliases: F3C22.130	11.6	12.1	-0.4	-2.0	100.0%	-3.6
2647	AT1G49670.1 | ARP protein (REF), identical to ARP protein GB:CAA89858 GI:886434 from (Arabidopsis thaliana); contains Pfam profile PF00107: oxidoreductase, zinc-binding dehydrogenase family | chr1:18384930-18389775 REVERSE | Aliases: F14J22.10, F14J22_10	10.0	10.8	-0.8	-2.0	100.0%	-3.3
2648	AT1G10900.1 | phosphatidylinositol-4-phosphate 5-kinase family protein, similar to phosphatidylinositol-4-phosphate 5-kinase AtPIP5K1 (Arabidopsis thaliana) GI:3702691; contains Pfam profiles PF01504: Phosphatidylinositol-4-phosphate 5-Kinase, PF02493: MORN repeat | chr1:3632274-3637141 REVERSE | Aliases: T19D16.18, T19D16_18	4.9	5.5	-0.6	-2.0	100.0%	-3.2
2649	AT1G30330.2 | Symbol: ARF6 | similar to auxin-responsive factor (ARF8) [Arabidopsis thaliana] (TAIR:At5g37020.1); similar to auxin response factor 6b [Oryza sativa] (GB:BAB85915.1); similar to OSJNBb0004A17.5 [Oryza sativa (japonica cultivar-group)] (GB:XP_474307.1); similar to auxin response factor 4 [Cucumis sativus] (GB:BAD19064.1); similar to putative auxin response factor [Oryza sativa (japonica cultivar-group)] (GB:BAD45924.1); similar to putative auxin response transcription factor(ARF6) [Oryza sativa (japonica cultivar-group)] (GB:XP_464221.1); contains InterPro domain Transcriptional factor B3 (InterPro:IPR003340) | chr1:10685804-10690018 REVERSE | Aliases: ARF6, AUXIN RESPONSE FACTOR 6, T4K22.6, T4K22_6	5.2	5.8	-0.6	-2.0	100.0%	-3.1
2650	AT4G00585.1 | expressed protein | chr4:250955-252532 REVERSE | Aliases: None	11.0	11.6	-0.6	-2.0	100.0%	-3.5
2651	AT5G54770.1 | Symbol: THI1 | thiazole biosynthetic enzyme, chloroplast (ARA6) (THI1) (THI4), identical to SP:Q38814 Thiazole biosynthetic enzyme, chloroplast precursor (ARA6) {Arabidopsis thaliana}	12.8	11.4	1.4	2.0	100.0%	-2.9
2652	AT1G52870.2 | peroxisomal membrane protein-related, contains weak similarity to Swiss-Prot:Q07066 22 kDa peroxisomal membrane protein (Rattus norvegicus) | chr1:19689415-19691531 FORWARD | Aliases: F14G24.14, F14G24_14	7.1	6.0	1.1	2.0	100.0%	-3.0
2653	AT3G53880.1 | aldo/keto reductase family protein, similar to chalcone reductase (Sesbania rostrata)(GI:2792155), and aldose reductase ALDRXV4 (Xerophyta viscosa)(GI:4539944), (Hordeum vulgare)(GI:728592) | chr3:19964151-19966268 FORWARD | Aliases: F5K20.180	6.4	6.9	-0.5	-2.0	100.0%	-3.4
2654	AT5G09460.1 | expressed protein | chr5:2944438-2945418 REVERSE | Aliases: T5E8.260, T5E8_260	5.0	5.9	-0.8	-2.0	100.0%	-3.0
2655	AT4G31140.1 | glycosyl hydrolase family 17 protein, similar to elicitor inducible chitinase Nt-SubE76 GI:11071974 from (Nicotiana tabacum) | chr4:15141294-15143397 FORWARD | Aliases: F6E21.60, F6E21_60	6.0	5.3	0.7	2.0	100.0%	-3.2
2656	AT2G18450.1 | Symbol: SDH1 2 |	5.6	4.4	1.3	2.0	100.0%	-3.0
2657	AT1G02970.1 | Symbol: WEE1 | protein kinase, putative, similar to Wee1-like protein GI:5821717 from (Zea mays) | chr1:673306-676378 FORWARD | Aliases: F22D16.3, F22D16_3, WEE1, WEE1 kinase	2.8	3.2	-0.4	-2.0	100.0%	-3.6
2658	AT1G64500.1 | glutaredoxin family protein | chr1:23956896-23958155 FORWARD | Aliases: F1N19.7, F1N19_7	5.1	4.6	0.5	2.0	100.0%	-3.4

Rank	Description	15.dpi	5.dpi	M	t	adj.q	B
2659	AT1G44890.1 | expressed protein | chr1:16965584-16967406 FORWARD | Aliases: T12C22.18, T12C22_18	7.4	6.6	0.8	2.0	100.0%	-3.1
2660	AT3G27770.1 | expressed protein | chr3:10286072-10288979 REVERSE | Aliases: MGF10.17	10.3	9.1	1.2	2.0	100.0%	-3.3
2661	AT2G37860.3 | Symbol: LCD1 | similar to expressed protein [Arabidopsis thaliana] (TAIR:At5g22790.1); similar to chloroplast lumen common protein family-like protein [Oryza sativa (japonica cultivar-group)] (GB:XP_477219.1); contains domain GLY_RICH (PS50315) | chr2:15863710-15865998 FORWARD | Aliases: LOWER CELL DENSITY 1, T8P21.23, T8P21_23	7.5	8.9	-1.4	-2.0	100.0%	-3.0
2662	AT5G44350.1 | ethylene-responsive nuclear protein -related, contains weak similarity to ethylene-regulated nuclear protein ERT2 (Arabidopsis thaliana) gi:2765442:emb:CAA75349	5.4	4.8	0.6	2.0	100.0%	-3.3
2663	AT2G45280.1 | Symbol: ATRAD51C | DNA repair family protein, contains similarity to Swiss-Prot:O43502 DNA repair protein RAD51 homolog 3 (Homo sapiens) | chr2:18677083-18679351 FORWARD | Aliases: F4L23.21, RAD51C	5.2	4.6	0.6	2.0	100.0%	-3.2
2664	AT1G65270.3 | expressed protein | chr1:24245411-24248615 FORWARD | Aliases: T8F5.4, T8F5_4	8.4	7.5	0.8	2.0	100.0%	-3.2
2665	AT2G33530.1 | Symbol: SCPL46 |	6.8	7.4	-0.6	-2.0	100.0%	-3.0
2666	AT4G34220.1 | leucine-rich repeat transmembrane protein kinase, putative, protein kinase TMKL1, Arabidopsis thaliana, PID:E353150 | chr4:16381510-16384198 REVERSE | Aliases: F10M10.12	3.1	3.4	-0.4	-2.0	100.0%	-3.5
2667	AT2G34585.1 | expressed protein | chr2:14575126-14575716 REVERSE | Aliases: None	9.3	8.8	0.6	2.0	100.0%	-3.9
2668	AT5G08420.1 | expressed protein | chr5:2713414-2716180 FORWARD | Aliases: F8L15.150, F8L15_150	6.5	7.7	-1.2	-1.9	100.0%	-3.0
2669	AT1G22150.1 | Symbol: SULTR1;3 | sulfate transporter (Sultr1;3), identical to sulfate tansporter Sultr1;3 (Arabidopsis thaliana) GI:10716805; contains Pfam profile PF00916: Sulfate transporter family; contains Pfam profile PF01740: STAS domain; contains TIGRfam profile TIGR00815: sulfate permease | chr1:7818350-7821333 FORWARD | Aliases: F2E2.22, F2E2_22, SULFATE TANSPORTER SULTR1;3	4.2	3.7	0.5	1.9	100.0%	-3.2
2670	AT3G17000.1 | ubiquitin-conjugating enzyme, putative, similar to Non-Canonical UBiquitin Conjugating Enzyme 1 (NCUBE1) from (Gallus gallus) GI:7362937, (Mus musculus) GI:7363050, (Homo sapiens) GI:7362973; contains Pfam profile PF00179: Ubiquitin-conjugating enzyme | chr3:5797185-5799689 FORWARD | Aliases: K14A17.7	7.6	6.3	1.3	1.9	100.0%	-3.0
2671	AT1G79280.1 | expressed protein, weak similarity to Nucleoprotein TPR (Swiss-Prot:P12270) (Homo sapiens) | chr1:29824069-29837702 REVERSE | Aliases: YUP8H12R.12, YUP8H12R_12	7.0	7.9	-0.9	-1.9	100.0%	-3.3
2672	AT1G78450.1 | SOUL heme-binding family protein, weak similarity to SOUL protein (Mus musculus) GI:4886906; contains Pfam profile PF04832: SOUL heme-binding protein | chr1:29521882-29522826 REVERSE | Aliases: F3F9.4	4.5	3.6	0.9	1.9	100.0%	-2.9
2673	AT3G53730.1 | histone H4, identical to histone H4 from Lycopersicon esculentum GI:297150, Lolium temulentum SP:P02308, Acropora formosa GI:455652, Citrus jambhiri GI:16797797	11.8	12.6	-0.8	-1.9	100.0%	-3.5
2674	ATMG00720.1 | Symbol: ORF107D | hypothetical protein | chrM:209499-209822 FORWARD | Aliases: ORF107D	2.2	2.5	-0.3	-1.9	100.0%	-3.8
2675	AT1G23340.2 | expressed protein, similar to At1g70550, At1g10750 contains Pfam profile PF03080: Arabidopsis proteins of unknown function | chr1:8283633-8286517 REVERSE | Aliases: F26F24.22, F26F24_22	3.6	4.2	-0.6	-1.9	100.0%	-3.2
2676	AT1G51650.1 | ATP synthase epsilon chain, mitochondrial, identical to ATP synthase epsilon chain, mitochondrial SP:Q96253 from (Arabidopsis thaliana) | chr1:19156283-19157597 FORWARD | Aliases: F19C24.25, F19C24_25	12.0	12.3	-0.3	-1.9	100.0%	-3.9
2677	AT1G23560.1 | expressed protein, contains Pfam profile PF02713: Domain of unknown function DUF220 | chr1:8352604-8354251 REVERSE | Aliases: F28C11.32	4.4	5.2	-0.8	-1.9	100.0%	-3.1
2678	AT2G20340.1 | tyrosine decarboxylase, putative, similar to tyrosine/dopa decarboxylase (Papaver somniferum) GI:3282527, SP:Q06087 Tyrosine decarboxylase 3 (EC 4.1.1.25) {Petroselinum crispum}; contains Pfam profile PF00282: Pyridoxal-dependent decarboxylase conserved domain | chr2:8786816-8789853 FORWARD | Aliases: F11A3.11, F11A3_11	3.2	3.9	-0.7	-1.9	100.0%	-3.2
2679	AT3G09830.2 | protein kinase, putative, similar to protein kinase (Lophopyrum elongatum) gi:13022177:gb:AAK11674 | chr3:3016545-3018988 FORWARD | Aliases: F8A24.12	5.2	4.5	0.7	1.9	100.0%	-3.1

Rank	Description	15.dpi	5.dpi	M	t	adj.q	B
2680	AT1G14690.1 | similar to microtubule associated protein (MAP65/ASE1) family protein [Arabidopsis thaliana] (TAIR:At5g55230.1); similar to Unknown protein [Oryza sativa (japonica cultivar-group)] (GB:XP_470643.1); contains InterPro domain MAP65/ASE1 (InterPro:IPR007145) | chr1:5051758-5055247 REVERSE | Aliases: F10B6.8, F10B6_8	8.0	8.7	-0.7	-1.9	100.0%	-3.2
2681	AT5G34930.1 | arogenate dehydrogenase, identical to arogenate dehydrogenase GI:16903098 from (Arabidopsis thaliana); contains Pfam profile: PF02153: prephenate dehydrogenase | chr5:13250604-13252797 FORWARD | Aliases: T2L5.1	5.0	5.5	-0.5	-1.9	100.0%	-3.4
2682	AT3G02250.1 | expressed protein, contains Pfam PF03138: Plant protein family. The function of this family of plant proteins is unknown; previously annotated as 'auxin-independent growth promoter -related' based on similarity to axi 1 protein (GB:X80301) (GI:559920) from (Nicotiana tabacum), which, due to scienitific fraud was retracted. Retraction in: Schell J. EMBO J 1999 May 17;18(10):2908. PMID:10400497. | chr3:423808-427068 REVERSE | Aliases: F14P3.10, F14P3_10	3.3	3.6	-0.4	-1.9	100.0%	-3.7
2683	AT5G44730.2 | haloacid dehalogenase-like hydrolase family protein, low similarity to SP:Q94915 Rhythmically expressed gene 2 protein (DREG-2) {Drosophila melanogaster}; contains InterPro accession IPR005834: Haloacid dehalogenase-like hydrolase | chr5:18062758-18064135 REVERSE | Aliases: K23L20.7, K23L20_7	6.9	6.1	0.8	1.9	100.0%	-3.1
2684	AT4G30935.1 | WRKY family transcription factor, contains Pfam profile: PF03106	6.8	6.2	0.5	1.9	100.0%	-3.2
2685	ATCG00440.1 | Symbol: NDHC | Encodes NADH dehydrogenase D3 subunit of the chloroplast NAD(P)H dehydrogenase complex | chrC:50001-50363 REVERSE | Aliases: NDHC	11.3	10.4	0.9	1.9	100.0%	-3.5
2686	AT5G51140.1 | pseudouridine synthase family protein, low similarity to SP:P23851 Ribosomal large subunit pseudouridine synthase C (EC 4.2.1.70) (Pseudouridylate synthase) (Uracil hydrolyase) {Escherichia coli}; contains Pfam profile PF00849: RNA pseudouridylate synthase	8.9	8.3	0.5	1.9	100.0%	-3.2
2687	AT4G31970.1 | Symbol: CYP82C2 | cytochrome P450 family protein, similar to cytochrome P450 82C1 (CYP82C1p)(GI:2739004) (Glycine max); flavonoid 3 ,5'-hydroxylase, Campanula medium, PATCHX:D1003951 | chr4:15462414-15464364 FORWARD | Aliases: F11C18.12	2.7	3.0	-0.3	-1.9	100.0%	-3.9
2688	AT3G12510.1 | expressed protein | chr3:3967134-3967804 FORWARD | Aliases: T2E22.17	6.9	6.2	0.7	1.9	100.0%	-3.0
2689	AT1G06570.1 | Symbol: PDS1 | 4-hydroxyphenylpyruvate dioxygenase (HPD), identical to 4-hydroxyphenylpyruvate dioxygenase (HPD) SP:P93836 (Arabidopsis thaliana (Mouse-ear cress)) | chr1:2011885-2013544 REVERSE | Aliases: 4 HYDROXYPHENYLPYRUVATE DIOXYGENASE, F12K11.9, F12K11_9, HPD, P HYDROXYPHENYLPYRUVATE DIOXYGENASE, PHYTOENE DESATURATION 1	10.1	8.9	1.2	1.9	100.0%	-3.2
2690	AT4G16630.1 | DEAD/DEAH box helicase, putative (RH28), identical to cDNA DEAD box RNA helicase, RH28 GI:3776026 | chr4:9362011-9366770 REVERSE | Aliases: DL4340C, FCAALL.424	4.0	5.4	-1.4	-1.9	100.0%	-2.9
2691	AT4G02990.1 | mitochondrial transcription termination factor family protein / mTERF family protein, weak similarity to mtDBP protein (Paracentrotus lividus) GI:4584695; contains Pfam profile PF02536: mTERF | chr4:1321859-1324004 FORWARD | Aliases: T4I9.13, T4I9_13	3.8	4.3	-0.5	-1.9	100.0%	-3.3
2692	AT5G59010.1 | protein kinase-related, low similarity to serine/threonine/tyrosine-specific protein kinase APK1, Arabidopsis thaliana, SP:Q06548 PIR:S28615; contains Pfam profile: PF00069 Eukaryotic protein kinase domain | chr5:23837594-23840476 REVERSE | Aliases: K19M22.7, K19M22_7	3.6	4.1	-0.4	-1.9	100.0%	-3.4
2693	AT1G79350.1 | Symbol: EMB1135 | DNA-binding protein, putative, contains Pfam PF00628: PHD-finger domain; contains TIGRFAMS TIGR01053: zinc finger domain, LSD1 subclass; contains Pfam PF00271: Helicase conserved C-terminal domain; similar to WSSV086 (GI:19481678)(shrimp white spot syndrome virus); similar to nuclear protein Np95 (GI:17939938) (Mus musculus) | chr1:29849548-29858307 REVERSE | Aliases: EMB1135, EMBRYO DEFECTIVE 1135, YUP8H12R.3, YUP8H12R_3	7.4	8.2	-0.9	-1.9	100.0%	-3.3
2694	AT1G07450.1 | tropinone reductase, putative / tropine dehydrogenase, putative, similar to tropinone reductase SP:P50165 from (Datura stramonium) | chr1:2288004-2289253 REVERSE | Aliases: F22G5.20, F22G5_20	3.1	2.7	0.3	1.9	100.0%	-3.7
2695	AT5G61130.1 | glycosyl hydrolase family protein 17, similar to beta-1,3-glucanase GI:15150341 from (Camellia sinensis); C-terminal homology only | chr5:24604456-24606806 REVERSE | Aliases: MAF19.13, MAF19_13	3.8	4.4	-0.6	-1.9	100.0%	-3.2
2696	AT5G64930.1 | Symbol: CPR5 | CPR5 protein, putative, Constitutive expressor of Pathogensis Related genes 5 (cpr5); regulator of disease resistance and senescence (Plant J. (2001)26(4)409-420. | chr5:25962938-25965565 REVERSE | Aliases: CONSTITUTIVE EXPRESSION OF PR GENES 5, HYS1, MXK3.16, MXK3_16	6.8	6.4	0.5	1.9	100.0%	-3.3
2697	AT2G38310.1 | expressed protein, low similarity to early flowering protein 1 (Asparagus officinalis) GI:1572683, SP:P80889 Ribonuclease 1 (EC 3.1.-.-) {Panax ginseng}	8.6	7.8	0.9	1.9	100.0%	-3.2
2698	AT2G38740.1 | haloacid dehalogenase-like hydrolase family protein, similar to SP:P71447 Beta-phosphoglucomutase (EC 5.4.2.6) {Lactococcus lactis}; contains InterPro accession IPR005834: Haloacid dehalogenase-like hydrolase	7.9	8.8	-0.9	-1.9	100.0%	-3.3

Rank	Description	15.dpi	5.dpi	M	t	adj.q	B
2699	AT3G15430.2 | regulator of chromosome condensation (RCC1) family protein, low similarity to UVB-resistance protein UVR8 (Arabidopsis thaliana) GI:5478530; contains Pfam profile PF00415: Regulator of chromosome condensation (RCC1) | chr3:5208766-5211752 FORWARD | Aliases: MJK13.9	7.0	6.5	0.5	1.9	100.0%	-3.2
2700	AT1G06560.1 | NOL1/NOP2/sun family protein, contains Pfam profile PF01189: NOL1/NOP2/sun family | chr1:2007629-2012200 FORWARD | Aliases: F12K11.10, F12K11_10	4.7	5.3	-0.7	-1.9	100.0%	-3.2
2701	AT2G42890.2 | Symbol: MEI2 | RNA recognition motif (RRM)-containing protein | chr2:17857639-17861607 FORWARD | Aliases: F7D19.11, F7D19_11	6.2	5.3	0.9	1.9	100.0%	-2.9
2702	AT4G23850.1 | long-chain-fatty-acid--CoA ligase / long-chain acyl-CoA synthetase, nearly identical to acyl-CoA synthetase (MF7P) from Brassica napus (gi:1617270) | chr4:12403477-12408343 REVERSE | Aliases: T32A16.20, T32A16_20	8.0	8.7	-0.7	-1.9	100.0%	-3.3
2703	AT3G07610.1 | transcription factor jumonji (jmjC) domain-containing protein, contains Pfam domain, PF02373: jmjC domain | chr3:2426154-2432882 FORWARD | Aliases: MLP3.6	3.7	4.1	-0.4	-1.9	100.0%	-3.4
2704	AT1G78995.1 | expressed protein | chr1:29719972-29721004 REVERSE | Aliases: None	4.1	3.7	0.5	1.9	100.0%	-3.4
2705	AT3G16220.1 | expressed protein, similar to CGI-18 protein GB:AAD27727 (Homo sapiens) | chr3:5497620-5499241 FORWARD | Aliases: MYA6.3	5.5	4.8	0.8	1.9	100.0%	-2.9
2706	AT5G28290.1 | protein kinase, putative, similar to LSTK-1-like kinase (Lycopersicon esculentum) GI:15637110; contains Pfam profile: PF00069 Eukaryotic protein kinase domain | chr5:10278695-10282618 REVERSE | Aliases: T8M17.60, T8M17_60	3.3	3.7	-0.4	-1.9	100.0%	-3.6
2707	AT3G28860.1 | Symbol: ATMDR1/ATMDR11/PGP19 | Belongs to the family of ATP-binding cassette (ABC) transporters. Also known as AtMDR11 and PGP19. Possibly regulates auxin-dependent responses by influencing basipetal auxin transport in the root. Acts upstream of phyA in regulating hypocotyl elongation and gravitropic response. Exerts nonredundant, partially overlapping functions with the ABC transporter encoded by AtPGP1. | chr3:10871275-10878743 REVERSE | Aliases: ATMDR1, ATMDR11, MLD15.2, PGP19	5.6	6.2	-0.6	-1.9	100.0%	-3.3
2708	AT5G14520.1 | pescadillo-related, similar to pescadillo (Zebrafish, Danio rerio) SWISS-PROT:P79741 | chr5:4680792-4684333 REVERSE | Aliases: T15N1.10, T15N1_10	7.7	8.2	-0.5	-1.9	100.0%	-3.4
2709	AT5G54760.1 | eukaryotic translation initiation factor SUI1, putative, similar to SP:P32911 Protein translation factor SUI1 {Saccharomyces cerevisiae}; contains Pfam profile PF01253: Translation initiation factor SUI1 | chr5:22261112-22262961 FORWARD | Aliases: MBG8.2, MBG8_2	11.3	10.7	0.7	1.9	100.0%	-3.5
2710	AT3G59430.3 | expressed protein | chr3:21975042-21977324 REVERSE | Aliases: F25L23.290	2.6	2.3	0.3	1.9	100.0%	-3.9
2711	AT3G17940.1 | aldose 1-epimerase family protein, similar to ALDOSE 1-EPIMERASE PRECURSOR GB:P05149 (SP:P05149) from (Acinetobacter calcoaceticus); contains Pfam profile PF01263 Aldose 1-epimerase | chr3:6143525-6145324 REVERSE | Aliases: MEB5.16	9.4	9.9	-0.5	-1.9	100.0%	-3.6
2712	AT4G35850.1 | pentatricopeptide (PPR) repeat-containing protein, low similarity to CRP1 (Zea mays) GI:3289002; contains Pfam profile PF01535: PPR repeat | chr4:16983528-16987203 FORWARD | Aliases: F4B14.120, F4B14_120	8.2	9.2	-1.0	-1.9	100.0%	-3.1
2713	AT1G80660.1 | Symbol: AHA9 | ATPase 9, plasma membrane-type, putative / proton pump 9, putative / proton-exporting ATPase, putative, strong similarity to SP:Q42556 ATPase 9, plasma membrane-type (EC 3.6.3.6) (Proton pump 9) {Arabidopsis thaliana}; contains InterPro accession IPR001757: ATPase, E1-E2 type | chr1:30321119-30324840 REVERSE | Aliases: F23A5.1, F23A5_1	2.5	2.7	-0.2	-1.9	100.0%	-3.8
2714	AT3G06910.1 | Ulp1 protease family protein, similar to sentrin/SUMO-specific protease (Homo sapiens) GI:6906859; contains Pfam profile PF02902: Ulp1 protease family, C-terminal catalytic domain | chr3:2178636-2181203 REVERSE | Aliases: F17A9.6	3.4	3.8	-0.4	-1.9	100.0%	-3.7
2715	AT2G41420.1 | proline-rich family protein, contains proline rich extensin domains, INTERPRO:IPR002965 | chr2:17273555-17275102 REVERSE | Aliases: F13H10.3, F13H10_3	7.2	5.7	1.4	1.9	100.0%	-3.1
2716	AT4G32060.1 | calcium-binding EF hand family protein, contains INTERPRO:IPR002048 calcium-binding EF-hand domain | chr4:15501084-15503599 FORWARD | Aliases: F10N7.140, F10N7_140	9.4	8.9	0.5	1.9	100.0%	-3.4
2717	AT1G11545.1 | xyloglucan:xyloglucosyl transferase, putative / xyloglucan endotransglycosylase, putative / endo-xyloglucan transferase, putative, similar to endo-xyloglucan transferase GI:2244732 from (Gossypium hirsutum) | chr1:3878550-3880360 REVERSE | Aliases: T23J18.21, T23J18_21	3.4	4.1	-0.7	-1.9	100.0%	-3.4
2718	AT1G19150.1 | Symbol: LHCA2*1 | chlorophyll A-B binding protein, putative / LHCI type II, putative, very strong similarity to PSI type II chlorophyll a/b-binding protein Lhca2*1 GI:541565 from (Arabidopsis thaliana); contains Pfam profile: PF00504 chlorophyll A-B binding protein | chr1:6612740-6613963 FORWARD | Aliases: PSI TYPE II CHLOROPHYLL A/B BINDING PROTEIN, T29M8.2, T29M8_2	3.9	3.2	0.7	1.9	100.0%	-3.1

Rank	Description	15.dpi	5.dpi	M	t	adj.q	B
2719	AT1G09330.1 | expressed protein, contains 3 transmembrane domains; contains Pfam profile PF05832: Eukaryotic protein of unknown function | chr1:3012764-3015093 REVERSE | Aliases: T31J12.5, T31J12_5	9.9	9.3	0.6	1.9	100.0%	-3.5
2720	AT2G30490.1 | Symbol: ATC4H | trans-cinnamate 4-monooxygenase / cinnamic acid 4-hydroxylase (C4H) (CA4H) / cytochrome P450 73 (CYP73) (CYP73A5), identical to SP:P92994: Trans-cinnamate 4-monooxygenase (EC 1.14.13.11) (Cinnamic acid 4-hydroxylase) (CA4H) (C4H) (P450C4H) (Cytochrome P450 73). {Arabidopsis thaliana}; molecular marker C4H (GB:U71080) | chr2:13000740-13002847 REVERSE | Aliases: C4H, CINNAMATE 4 HYDROXYLASE, CINNAMATE 4 HYDROXYLASE, CYP73A5, T6B20.16, T6B20_16	10.2	11.0	-0.8	-1.9	100.0%	-3.5
2721	AT1G77890.1 | expressed protein | chr1:29297017-29301054 FORWARD | Aliases: AT1G77900, F28K19.10, F28K19_10	4.5	3.9	0.6	1.9	100.0%	-3.4
2722	AT1G50010.1 | Symbol: TUA2 | tubulin alpha-2/alpha-4 chain (TUA2), identical to tubulin alpha-2/alpha-4 chain SP:P29510 GB:P29510 from (Arabidopsis thaliana)	9.1	6.9	2.2	1.9	100.0%	-3.0
2723	AT2G47190.1 | Symbol: MYB2 | myb family transcription factor (MYB2), contains Pfam profile: PF00249 myb-like DNA-binding domain | chr2:19383272-19384601 FORWARD | Aliases: ATMYB2, T8I13.3	3.4	3.0	0.4	1.9	100.0%	-3.4
2724	AT2G19760.1 | Symbol: PRF1 | profilin 1 (PRO1) (PFN1) (PRF1) / allergen Ara t 8, identical to profilin 1 (Allergen Ara t 8) SP:Q42449 GI:1353770 from (Arabidopsis thaliana) | chr2:8523869-8525249 REVERSE | Aliases: F6F22.21, F6F22_21, PFN1, PROFILIN 1	12.9	12.4	0.5	1.9	100.0%	-3.7
2725	AT5G11410.1 | protein kinase family protein, contains eukaryotic protein kinase domain, INTERPRO:IPR000719 | chr5:3638432-3639884 REVERSE | Aliases: F15N18.1	2.2	2.3	-0.2	-1.9	100.0%	-4.2
2726	AT5G12840.4 | Symbol: HAP2A | CCAAT-binding transcription factor (CBF-B/NF-YA) family protein, contains Pfam profile: PF02045 CCAAT-binding transcription factor (CBF-B/NF-YA) subunit B | chr5:4050808-4053591 REVERSE | Aliases: ATHAP2A, EMB2220, EMBRYO DEFECTIVE 2220, HAP2, T24H18.10, T24H18_10	4.4	4.1	0.4	1.9	100.0%	-3.5
2727	AT5G10480.1 | Symbol: PAS2 | protein tyrosine phosphatase-like protein, putative (PAS2), identical to PEPINO/PASTICCINO2 protein GI:24411193, GI:24575153 from (Arabidopsis thaliana); contains Pfam:04387: protein tyrosine phosphatase-like protein	4.9	5.8	-0.9	-1.9	100.0%	-3.2
2728	AT2G27000.1 | Symbol: CYP705A8 | cytochrome P450 family protein | chr2:11530382-11532173 REVERSE | Aliases: T20P8.5, T20P8_5	3.9	4.7	-0.8	-1.9	100.0%	-3.1
2729	AT2G04280.1 | expressed protein | chr2:1479815-1482086 REVERSE | Aliases: T23O15.9, T23O15_9	7.5	8.2	-0.7	-1.9	100.0%	-3.4
2730	AT5G10190.1 | transporter-related, low similarity to spinster membrane proteins from (Drosophila melanogaster) GI:12003974, GI:12003976, GI:12003972, GI:12003970; contains Pfam profile PF00083: major facilitator superfamily protein | chr5:3199134-3201364 FORWARD | Aliases: F18D22.5	6.2	5.2	1.0	1.9	100.0%	-3.2
2731	AT1G02680.1 | transcription initiation factor IID (TFIID) 18 kDa subunit (TAFII-18) family protein, similar to transcription factor TFIID (SP:Q15543) (Homo sapiens); contains Pfam PF02269: Transcription initiation factor IID, 18kD subunit	5.9	5.2	0.7	1.9	100.0%	-3.2
2732	AT2G45740.3 | similar to peroxisomal biogenesis factor 11 family protein / PEX11 family protein [Arabidopsis thaliana] (TAIR:At3g61070.1); similar to peroxisomal biogenesis factor 11 protein-like [Oryza sativa (japonica cultivar-group)] (GB:XP_550574.1); contains InterPro domain Peroxisomal biogenesis factor 11 (InterPro:IPR008733) | chr2:18846662-18848414 FORWARD | Aliases: F4I18.28	7.3	6.4	1.0	1.9	100.0%	-3.0
2733	AT3G50910.1 | expressed protein | chr3:18930494-18933219 FORWARD | Aliases: F18B3.190	9.9	9.3	0.6	1.9	100.0%	-3.2
2734	AT1G09320.1 | agenet domain-containing protein, contains Pfam PF05641: Agenet domain | chr1:3009943-3012354 REVERSE | Aliases: T31J12.4, T31J12_4	4.3	5.2	-0.8	-1.9	100.0%	-3.1
2735	AT2G21185.1 | expressed protein | chr2:9083518-9084129 REVERSE | Aliases: None	7.6	6.9	0.7	1.9	100.0%	-3.0
2736	AT5G47670.1 | CCAAT-box binding transcription factor family protein / leafy cotyledon 1-related (L1L), supporting cDNA gi:27372446:gb:AY138461.1:; contains Pfam PF00808 : Histone-like transcription factor (CBF/NF-Y) and archaeal histone; contains similarity to CCAAT-binding transcription factor subunit A (CBF-A) (NF-Y protein chain B) (NF-YB) (CAAT-box DNA binding protein subunit B) (Swiss-Prot:P25209) (Zea mays) | chr5:19332287-19333616 FORWARD | Aliases: MNJ7.26, MNJ7_26	3.0	2.8	0.3	1.9	100.0%	-3.8
2737	AT3G21690.1 | MATE efflux family protein, similar to ripening regulated protein DDTFR18 (Lycopersicon esculentum) GI:12231296; contains Pfam profile: PF01554 MatE uncharacterized membrane protein family | chr3:7638672-7642212 FORWARD | Aliases: MIL23.25	5.0	4.5	0.5	1.9	100.0%	-3.5
2738	AT2G46100.1 | expressed protein | chr2:18960353-18962069 FORWARD | Aliases: T3F17.25	9.6	8.8	0.8	1.9	100.0%	-2.9
2739	AT3G11830.1 | chaperonin, putative, similar to SWISS-PROT:P80313 T-complex protein 1, eta subunit (TCP-1-eta) (Mus musculus); contains Pfam:PF00118 domain, TCP-1/cpn60 chaperonin family | chr3:3732670-3736377 FORWARD | Aliases: F26K24.12	7.4	8.4	-1.0	-1.9	100.0%	-3.1

Rank	Description	15.dpi	5.dpi	M	t	adj.q	B
2740	AT5G56190.2 | WD-40 repeat family protein, contains 3 (2 significant) WD-40 repeats (PF0400); similar to beta transducin-like protein HET-E2C*40 (GI:17225208) (Podospora anserina) | chr5:22759258-22762403 FORWARD | Aliases: MDA7.25, MDA7_25	8.1	7.7	0.4	1.9	100.0%	-3.6
2741	AT2G45170.2 | autophagy 8e (APG8e), identical to autophagy 8e (Arabidopsis thaliana) GI:19912159; contains Pfam profile PF02991: Microtubule associated protein 1A/1B, light chain 3 | chr2:18631456-18632773 FORWARD | Aliases: T14P1.2	7.4	6.5	0.8	1.9	100.0%	-3.2
2742	AT1G12640.1 | membrane bound O-acyl transferase (MBOAT) family protein, low similarity to porcupine from (Xenopus laevis) GI:6714514, GI:6714520, GI:6714518, GI:6714516; contains Pfam profile PF03062: MBOAT family | chr1:4303315-4305786 REVERSE | Aliases: T12C24.17, T12C24_17	7.7	6.5	1.3	1.9	100.0%	-3.2
2743	AT4G19100.1 | expressed protein | chr4:10453464-10454370 FORWARD | Aliases: None	4.2	3.8	0.4	1.9	100.0%	-3.5
2744	AT3G09820.2 | Symbol: ADK1 | adenosine kinase 1 (ADK1) / adenosine 5'-phosphotransferase 1, identical to adenosine kinase 1 /adenosine 5'-phosphotransferase 1 SP:Q9SF85 from (Arabidopsis thaliana) | chr3:3012099-3014949 FORWARD | Aliases: ADENOSINE KINASE 1, F8A24.13	10.7	11.9	-1.3	-1.9	100.0%	-3.1
2745	AT5G22690.1 | disease resistance protein (TIR-NBS-LRR class), putative, domain signature TIR-NBS-LRR exists, suggestive of a disease resistance protein. | chr5:7541274-7545185 FORWARD | Aliases: MDJ22.11, MDJ22_11	3.5	3.2	0.3	1.9	100.0%	-3.6
2746	AT2G36010.3 | Symbol: E2F3 | E2F transcription factor-3 (E2F3), identical to E2F transcription factor-3 E2F3 (Arabidopsis thaliana) gi:10443853:gb:AAG17610 | chr2:15126621-15130683 FORWARD | Aliases: ATE2FA, E2F TRANSCRIPTION FACTOR 3, E2F TRANSCRIPTION FACTOR 3 E2F3, F11F19.8, F11F19_8	2.5	2.7	-0.2	-1.9	100.0%	-4.1
2747	AT1G50710.1 | expressed protein | chr1:18788177-18791470 REVERSE | Aliases: F17J6.23, F17J6_23	5.6	6.3	-0.6	-1.9	100.0%	-3.2
2748	AT3G21060.1 | transducin family protein / WD-40 repeat family protein, contains 4 WD-40 repeats (PF00400); similar to Retinoblastoma-binding protein 5 (RBBP-5) (Homo sapiens)(RBQ-3) | chr3:7377440-7380717 FORWARD | Aliases: MSA6.10	4.1	4.7	-0.6	-1.9	100.0%	-3.4
2749	AT5G54520.1 | WD-40 repeat family protein, contains 5 WD-40 repeats (PF00400); similar to pre-mRNA splicing factor PRP17 (SP:O60508) (Homo sapiens) | chr5:22163740-22166346 REVERSE | Aliases: MRB17.2, MRB17_2	5.3	4.8	0.5	1.9	100.0%	-3.2
2750	AT5G62570.1 | calmodulin-binding protein, similar to calmodulin-binding protein TCB60 GI:1698548 from (Nicotiana tabacum) | chr5:25131617-25134193 FORWARD | Aliases: K19B1.18, K19B1_18	6.4	5.9	0.6	1.9	100.0%	-3.2
2751	AT1G61100.1 | disease resistance protein (TIR class), putative, domain signature TIR exists, suggestive of a disease resistance protein. | chr1:22512203-22515936 REVERSE | Aliases: F11P17.17, F11P17_17	6.8	6.1	0.7	1.9	100.0%	-3.1
2752	AT1G10140.1 | expressed protein, similar to EST gb:AA598098 | chr1:3322804-3323550 REVERSE | Aliases: F14N23.2, F14N23_2	6.2	5.9	0.3	1.9	100.0%	-3.6
2753	AT1G72070.1 | DNAJ heat shock N-terminal domain-containing protein, similar to TCJ2 (Trypanosoma cruzi) GI:886414; contains Pfam profile: PF00226: DnaJ domain	2.7	3.0	-0.3	-1.9	100.0%	-3.7
2754	AT1G49470.1 | expressed protein, contains Pfam profile PF04819: Family of unknown function (DUF716) (Plant viral-response family) | chr1:18314162-18315302 FORWARD | Aliases: F13F21.10, F13F21_10	6.6	6.2	0.5	1.9	100.0%	-3.4
2755	AT1G16290.1 | expressed protein | chr1:5570747-5573532 REVERSE | Aliases: F3O9.9, F3O9_9	4.1	3.7	0.5	1.9	100.0%	-3.5
2756	AT2G38180.1 | GDSL-motif lipase/hydrolase family protein, similar to SP:P41734 Isoamyl acetate-hydrolyzing esterase (EC 3.1.-.-) {Saccharomyces cerevisiae}; contains Pfam profile PF00657: Lipase/Acylhydrolase with GDSL-like motif | chr2:16004221-16005984 FORWARD | Aliases: F16M14.11, F16M14_11	6.8	7.3	-0.5	-1.9	100.0%	-3.5
2757	AT2G36530.1 | Symbol: LOS2 | enolase, identical to SWISS-PROT:P25696 enolase (EC 4.2.1.11) (2-phosphoglycerate dehydratase)(2-phospho-D- glycerate hydro-lyase) (Arabidopsis thaliana) | chr2:15327835-15330945 REVERSE | Aliases: F1O11.16, F1O11_16	11.7	12.7	-1.0	-1.9	100.0%	-3.6
2758	AT1G55960.1 | expressed protein, weak similarity to SP:P53808 Phosphatidylcholine transfer protein (PC-TP) {Mus musculus} | chr1:20931308-20933682 REVERSE | Aliases: F14J16.24, F14J16_24	8.6	8.0	0.6	1.9	100.0%	-3.3
2759	AT5G62980.1 | dihydroneopterin aldolase, putative, similar to SP:O33725 Dihydroneopterin aldolase (EC 4.1.2.25) (DHNA) {Streptococcus pyogenes}; contains Pfam profile PF02152: dihydroneopterin aldolase | chr5:25293260-25294418 FORWARD | Aliases: MJH22.3, MJH22_3	7.4	8.1	-0.8	-1.9	100.0%	-3.0
2760	AT1G15400.2 | expressed protein, ESTs gb:H37295 and gb:R64895 come from this gene | chr1:5296220-5296897 REVERSE | Aliases: F9L1.35, F9L1_35	8.6	8.1	0.6	1.9	100.0%	-3.1
2761	AT1G19025.1 | DNA cross-link repair protein-related, contains weak similarity to Swiss-Prot:P30620 DNA cross-LINK repair protein PSO2/SNM1 (Saccharomyces cerevisiae) | chr1:6568844-6570930 FORWARD | Aliases: None	2.9	3.2	-0.3	-1.9	100.0%	-3.7

Rank	Description	15.dpi	5.dpi	M	t	adj.q	B
2762	AT3G61190.1 | Symbol: BAP1 | BON1-associated protein 1 (BAP1), identical to BON1-associated protein 1 (Arabidopsis thaliana) GI:15487384; contains Pfam profile PF00168: C2 domain; supporting cDNA gi:15487383:gb:AY045765.1: | chr3:22661502-22662424 REVERSE | Aliases: BON ASSOCIATION PROTEIN 1, T20K12.90	5.2	4.3	0.9	1.9	100.0%	-3.1
2763	AT3G60380.1 | expressed protein | chr3:22327888-22330241 REVERSE | Aliases: T8B10.40	6.7	7.5	-0.8	-1.9	100.0%	-3.3
2764	AT1G23230.1 | expressed protein | chr1:8244470-8251730 FORWARD | Aliases: F26F24.8, F26F24_8	5.3	6.0	-0.7	-1.9	100.0%	-3.2
2765	AT3G02210.1 | phytochelatin synthetase family protein / COBRA cell expansion protein COBL3, similar to phytochelatin synthetase (Hordeum vulgare subsp. vulgare) GI:29570314; identified in Roudier, et al, Plant Phys. (2002) 130:538-548 (PMID:12376623); supported by cDNA: gi:26452134:dbj:AK118555.1; contains Pfam profile PF04833: Phytochelatin synthetase-like conserved region | chr3:409208-411659 REVERSE | Aliases: F14P3.14, F14P3_14	4.5	4.9	-0.4	-1.9	100.0%	-3.4
2766	AT1G53590.1 | C2 domain-containing protein | chr1:20000118-20003933 FORWARD | Aliases: F22G10.28	4.8	5.4	-0.6	-1.9	100.0%	-3.2
2767	AT2G41690.1 | Symbol: AT HSFB3 | heat shock transcription factor family protein, contains Pfam profile: PF00447 HSF-type DNA-binding domain | chr2:17388801-17389655 FORWARD | Aliases: HSFB3, T32G6.21, T32G6_21	3.3	3.7	-0.4	-1.9	100.0%	-3.6
2768	AT4G00830.2 | similar to RNA recognition motif (RRM)-containing protein [Arabidopsis thaliana] (TAIR:At3g52660.1); similar to putative RNA-binding protein [Oryza sativa (japonica cultivar-group)] (GB:AAP52145.1); contains InterPro domain RNA-binding region RNP-1	7.3	6.8	0.5	1.9	100.0%	-3.4
2769	AT5G64860.1 | Symbol: DPE1 | 4-alpha-glucanotransferase, putative / disproportionating enzyme, putative, similar to 4-alpha-glucanotransferase SP:Q06801 from (Solanum tuberosum) | chr5:25942400-25946034 REVERSE | Aliases: DISPROPORTIONATING ENZYME, MXK3.9, MXK3_9	6.9	7.7	-0.8	-1.9	100.0%	-3.0
2770	AT3G45000.1 | SNF7 family protein, contains Pfam profile PF03357: SNF7 family | chr3:16471333-16472392 FORWARD | Aliases: F14D17.70	3.0	2.7	0.3	1.9	100.0%	-4.0
2771	AT4G17520.1 | nuclear RNA-binding protein, putative, similar to nuclear RNA binding protein GI:6492264 from (Arabidopsis thaliana) | chr4:9771343-9773605 FORWARD | Aliases: DL4795W, FCAALL.142	9.8	10.5	-0.7	-1.9	100.0%	-3.4
2772	AT3G19240.1 | expressed protein | chr3:6664256-6666564 FORWARD | Aliases: MVI11.21	10.0	9.2	0.8	1.9	100.0%	-3.1
2773	AT2G01420.2 | Symbol: PIN4 | auxin transport protein, putative, similar to auxin transport protein PIN7(Arabidopsis thaliana) gi:5817305:gb:AAD52697 | chr2:180104-183327 REVERSE | Aliases: AUXIN TRANSPORTER SPLICE VARIANT B, F10A8.27, PIN FORMED 4	3.4	3.8	-0.4	-1.9	100.0%	-3.5
2774	AT3G03580.1 | pentatricopeptide (PPR) repeat-containing protein, contains INTERPRO:IPR002885 PPR repeats | chr3:860519-863359 REVERSE | Aliases: T12J13.14, T12J13_14	3.7	4.2	-0.5	-1.9	100.0%	-3.4
2775	AT3G62290.1 | Gene encoding ADP-ribosylation factor and similar to other ARFs and ARF-like proteins. Members of this family are known to be essential for vesicle coating and uncoating and functions in GTP-binding. The gene is shown to play a role in cell division, cell expansion and cellulose production using antisense construct. | chr3:23062627-23064719 FORWARD | Aliases: T17J13.250	12.4	11.8	0.6	1.9	100.0%	-3.7
2776	AT5G58790.1 | expressed protein, ; expression supported by MPSS | chr5:23762232-23763018 FORWARD | Aliases: MZN1.28	5.2	4.6	0.6	1.9	100.0%	-3.5
2777	AT5G61960.2 | Symbol: AML1 | similar to RNA recognition motif (RRM)-containing protein [Arabidopsis thaliana] (TAIR:At5g07290.1); similar to AML1 [Lycopersicon esculentum] (GB:AAT39005.1); similar to Mei2-like protein [Hordeum vulgare subsp. vulgare] (GB:AAL85701.1); contains InterPro domain RNA recognition motif 2 (InterPro:IPR007201); contains InterPro domain RNA-binding region RNP-1 (RNA recognition motif) (InterPro:IPR000504) | chr5:24895746-24901376 REVERSE | Aliases: ARABIDOPSIS MEI2 LIKE PROTEIN 1, K22G18.10, K22G18_10	6.0	7.2	-1.2	-1.9	100.0%	-3.1
2778	AT2G18790.1 | Symbol: PHYB | phytochrome B (PHYB), Identical to SP:P14713 Phytochrome B {Arabidopsis thaliana} | chr2:8146963-8151512 FORWARD | Aliases: HY3, MSF3.17, MSF3_17, OOP1, OUT OF PHASE 1, PHYTOCHROME B	5.7	6.6	-0.9	-1.9	100.0%	-3.3
2779	AT4G38210.1 | Symbol: ATEXPA20 | expansin, putative (EXP20), similar to alpha-expansin 3 GI:6942322 from (Triphysaria versicolor); alpha-expansin gene family, PMID:11641069 | chr4:17922750-17923967 REVERSE | Aliases: ARABIDOPSIS THALIANA EXPANSIN A20, ATEXP20, ATHEXP ALPHA 1.23, EXP20, F20D10.330, F20D10_330	5.0	5.8	-0.9	-1.9	100.0%	-3.0
2780	AT4G20170.1 | expressed protein | chr4:10896689-10899151 FORWARD | Aliases: F1C12.90, F1C12_90	7.7	7.1	0.5	1.9	100.0%	-3.3
2781	AT5G03440.2 | expressed protein | chr5:856871-858404 REVERSE | Aliases: F12E4.200, F12E4_200	6.2	5.5	0.7	1.9	100.0%	-3.3
2782	AT5G07010.1 | sulfotransferase family protein, similar to steroid sulfotransferase 3 (Brassica napus) GI:3420008, steroid sulfotransferase 1 (Brassica napus) GI:3420004; contains Pfam profile PF00685: Sulfotransferase domain | chr5:2174679-2176107 REVERSE | Aliases: MOJ9.18, MOJ9_18	3.3	3.0	0.3	1.9	100.0%	-3.6
2783	AT3G01015.1 | expressed protein, ; expression supported by MPSS | chr3:1805-4024 REVERSE | Aliases: TEL3N.1, TEL3N_1	2.8	2.6	0.2	1.9	100.0%	-4.0

Rank	Description	15.dpi	5.dpi	M	t	adj.q	B
2784	AT2G21710.1 | Symbol: EMB2219 | mitochondrial transcription termination factor-related / mTERF-related, contains Pfam profile PF02536: mTERF | chr2:9277966-9280498 FORWARD | Aliases: EMB2219, EMBRYO DEFECTIVE 2219, F7D8.3, F7D8_3	3.3	3.8	-0.4	-1.9	100.0%	-3.4
2785	AT5G02290.2 | Symbol: NAK | protein kinase, putative, similar to protein kinase APK1A (Arabidopsis thaliana) SWISS-PROT:Q06548 | chr5:470194-472782 REVERSE | Aliases: PROTEIN KINASE NAK, T1E22.50, T1E22_50	7.7	7.0	0.7	1.9	100.0%	-3.3
2786	AT2G05170.1 | Symbol: ATVPS11 | vacuolar protein sorting 11 family protein / VPS11 family protein, similar to Vacuolar protein sorting 11 (hVPS11) (PP3476) (Swiss-Prot:Q9H270) (Homo sapiens); similar to Vacuolar biogenesis protein END1 (PEP5 protein) (Vacuolar protein sorting 11) (Swiss-Prot:P12868) (Saccharomyces cerevisiae) | chr2:1869882-1873809 FORWARD | Aliases: F5G3.7, F5G3_7	3.7	4.7	-1.0	-1.9	100.0%	-3.2
2787	AT1G21650.1 | preprotein translocase secA family protein, contains Pfam profiles: PF01043 SecA protein, amino terminal region, PF00400 WD domain, G-beta repeat, PF00097 zinc finger, C3HC4 type (RING finger)	2.8	3.1	-0.3	-1.9	100.0%	-3.8
2788	AT3G28970.1 | expressed protein, contains Pfam domain PF03556: Domain of unknown function (DUF298) | chr3:10989024-10991122 REVERSE | Aliases: K5K13.10	8.1	8.5	-0.5	-1.9	100.0%	-3.7
2789	AT3G11590.1 | expressed protein | chr3:3660383-3663740 FORWARD | Aliases: F24K9.26	6.7	7.3	-0.6	-1.9	100.0%	-3.4
2790	AT3G12360.1 | ankyrin repeat family protein, contains ankyrin repeat domains, Pfam:PF00023 | chr3:3934085-3936701 FORWARD | Aliases: T2E22.31	8.7	8.3	0.4	1.9	100.0%	-3.7
2791	AT5G03960.1 | calmodulin-binding family protein | chr5:1068275-1069977 REVERSE | Aliases: F8F6.170, F8F6_170	4.9	4.1	0.8	1.9	100.0%	-3.1
2792	AT5G25540.1 | Symbol: CID6 | Expressed protein contains PAM2 PABC interacting domain.	6.9	6.5	0.4	1.9	100.0%	-3.5
2793	AT5G39130.1 | germin-like protein, putative, identical to germin-like protein subfamily 1 member 16 (SP:Q9FIC8) | chr5:15682730-15683667 REVERSE | Aliases: MXF12.140, MXF12_140	2.6	2.9	-0.2	-1.9	100.0%	-4.3
2794	AT4G05110.1 | equilibrative nucleoside transporter, putative (ENT6), identical to putative equilibrative nucleoside transporter ENT6 (Arabidopsis thaliana) GI:16518997; contains similarity to SWISS-PROT:O54699 equilibrative nucleoside transporter 2 (Equilibrative nitrobenzylmercaptopurine riboside-insensitive nucleoside transporter, Equilibrative NBMPR-insensitive nucleoside transporter, Nucleoside transporter, ei-type) (Rattus norvegicus); contains Pfam profile PF01733: Nucleoside transporter | chr4:2626403-2628828 REVERSE | Aliases: C17L7.30, C17L7_30	4.2	3.5	0.7	1.9	100.0%	-3.0
2795	AT4G05120.1 | equilibrative nucleoside transporter, putative (ENT3), identical to putative equilibrative nucleoside transporter ENT3 (Arabidopsis thaliana) GI:16518993; contains similarity to SWISS-PROT:O54699 equilibrative nucleoside transporter 2 (Equilibrative nitrobenzylmercaptopurine riboside-insensitive nucleoside transporter, Equilibrative NBMPR-insensitive nucleoside transporter, Nucleoside transporter, ei-type) (Rattus norvegicus); contains Pfam profile PF01733: Nucleoside transporter | chr4:2632884-2634822 REVERSE | Aliases: C17L7.40, C17L7_40	4.2	3.5	0.7	1.9	100.0%	-3.0
2796	AT2G19830.1 | SNF7 family protein, contains Pfam domain, PF03357: SNF7 family | chr2:8564931-8566800 REVERSE | Aliases: F6F22.14, F6F22_14	7.8	7.2	0.5	1.9	100.0%	-3.4
2797	AT5G47580.1 | expressed protein, strong similarity to unknown protein (pir::E71441) | chr5:19312072-19314644 REVERSE | Aliases: MNJ7.17, MNJ7_17	5.3	5.8	-0.5	-1.9	100.0%	-3.4
2798	AT2G47770.1 | benzodiazepine receptor-related, contains weak similarity to Peripheral-type benzodiazepine receptor (PBR) (PKBS) (Mitochondrial benzodiazepine receptor) (Swiss-Prot:P30536) (Homo sapiens) | chr2:19575534-19576577 FORWARD | Aliases: F17A22.16	5.6	4.9	0.6	1.9	100.0%	-3.2
2799	AT2G04940.1 | scramblase-related, weak similarity to Phospholipid scramblase 1 (PL scramblase 1) (Ca(2)-dependent phospholipid scramblase 1) (Transplantability associated protein 1) (TRA1) (NOR1) (Swiss-Prot:Q9JJ00) (Mus musculus); weak similarity to Phospholipid scramblase 4 (PL scramblase 4) (Ca(2)-dependent phospholipid scramblase 4) (Swiss-Prot:Q9NRQ2) (Homo sapiens) | chr2:1736482-1740385 REVERSE | Aliases: F1O13.7, F1O13_7	6.4	5.8	0.5	1.9	100.0%	-3.4
2800	AT5G01210.1 | transferase family protein, contains Pfam profile PF02458 transferase family | chr5:84473-86274 FORWARD | Aliases: F7J8.190, F7J8_190	11.5	10.7	0.7	1.9	100.0%	-3.5
2801	AT3G07580.1 | expressed protein | chr3:2420975-2422667 REVERSE | Aliases: MLP3.3	4.6	4.1	0.4	1.9	100.0%	-3.3
2802	AT5G58120.1 | disease resistance protein (TIR-NBS-LRR class), putative, domain signature TIR-NBS-LRR exists, suggestive of a disease resistance protein. | chr5:23534718-23538293 FORWARD | Aliases: K21L19.1, K21L19_1	4.1	3.8	0.3	1.9	100.0%	-3.7
2803	AT2G31840.1 | expressed protein | chr2:13544989-13546835 REVERSE | Aliases: F20M17.12, F20M17_12	5.6	6.2	-0.6	-1.9	100.0%	-3.3
2804	AT3G49830.1 | DNA helicase-related, similar to DNA helicase GI:4521249 from (Mus musculus) | chr3:18493368-18494925 FORWARD | Aliases: T16K5.180	2.7	2.4	0.2	1.9	100.0%	-4.0

Rank	Description	15.dpi	5.dpi	M	t	adj.q	B
2805	AT1G13160.1 | SDA1 family protein, contains Pfam PF05285: SDA1 domain; similar to mystery 45A (GI:16797816){Drosophila melanogaster} | chr1:4484597-4488525 FORWARD | Aliases: F3F19.18, F3F19_18	6.6	7.2	-0.6	-1.9	100.0%	-3.4
2806	AT1G32260.1 | expressed protein, identical to hypothetical protein GB:AAF81322 GI:8920600 from (Arabidopsis thaliana) | chr1:11640562-11641764 REVERSE | Aliases: F27G20.13	4.8	5.2	-0.4	-1.9	100.0%	-3.4
2807	AT2G01610.1 | invertase/pectin methylesterase inhibitor family protein, low similarity to SP:Q42534 Pectinesterase 2 precursor (EC 3.1.1.11) (Pectin methylesterase 2) (PE 2) {Arabidopsis thaliana}; contains Pfam profile PF04043: Plant invertase/pectin methylesterase inhibitor | chr2:274123-274819 REVERSE | Aliases: T8O11.22, T8O11_22	2.9	3.4	-0.4	-1.9	100.0%	-3.4
2808	AT5G49660.1 | leucine-rich repeat transmembrane protein kinase, putative, contains leucine rich repeat (LRR) domains, Pfam:PF00560; contains protein kinase domain, Pfam:PF00069 | chr5:20178432-20181955 REVERSE | Aliases: MNI5.4, MNI5_4	6.1	6.6	-0.5	-1.9	100.0%	-3.3
2809	AT5G58660.1 | oxidoreductase, 2OG-Fe(II) oxygenase family protein, similar to ACC oxidase, Lycopersicon esculentum (SP:P05116), gibberellin 3B-hydroxylase, Latuca sativa (gi:4164145); contains Pfam domain PF03171, 2OG-Fe(II) oxygenase superfamily	4.7	4.1	0.6	1.9	100.0%	-3.2
2810	AT2G42240.1 | RNA recognition motif (RRM)-containing protein, similar to RNA-binding protein (Hermes) from {Gallus gallus} SP:Q9W6I1, {Xenopus laevis} SP:Q9YGP5, {Mus musculus} SP:Q9WVB0; contains InterPro entry IPR000504: RNA-binding region RNP-1 (RNA recognition motif) (RRM) | chr2:17603967-17604642 FORWARD | Aliases: T24P15.15, T24P15_15	3.3	3.0	0.3	1.9	100.0%	-3.7
2811	AT5G46420.1 | 16S rRNA processing protein RimM family, contains weak similarity to Swiss-Prot:O74933 UDP-N-acetylglucosamine pyrophosphorylase (Candida albicans); contains Pfam profiles PF01782: 16S rRNA processing protein RimM, PF05239: PRC-barrel domain | chr5:18847182-18850180 FORWARD | Aliases: K11I1.1, K11I1_1	6.0	6.5	-0.6	-1.9	100.0%	-3.3
2812	AT2G39090.2 | similar to cell division cycle family protein / CDC family protein [Arabidopsis thaliana] (TAIR:At3g48150.1); similar to IVR-like protein [Nicotiana tabacum] (GB:CAA08776.1); contains InterPro domain TPR repeat (InterPro:IPR001440) | chr2:16319760-16324378 REVERSE | Aliases: T7F6.26, T7F6_26	3.3	3.9	-0.6	-1.9	100.0%	-3.5
2813	AT3G04340.1 | Symbol: EMB2458 | FtsH protease family protein, similar to chloroplast FtsH protease (Arabidopsis thaliana) GI:1483215; contains Pfam profiles PF01434: Peptidase family M41, PF00004: ATPase AAA family | chr3:1146807-1152455 REVERSE | Aliases: EMB2458, EMBRYO DEFECTIVE 2458, T6K12.4, T6K12_4	4.3	4.7	-0.5	-1.9	100.0%	-3.3
2814	AT4G36680.1 | pentatricopeptide (PPR) repeat-containing protein, low similarity to fertility restorer (Petunia x hybrida) GI:22128587; contains Pfam profile PF01535: PPR repeat; identical to cDNA membrane-associated salt-inducible protein like GI:2632060	6.8	7.5	-0.7	-1.9	100.0%	-3.1
2815	AT3G15810.1 | expressed protein, contains Pfam profile PF04525: Protein of unknown function (DUF567) | chr3:5347833-5349295 REVERSE | Aliases: MSJ11.21	7.0	6.6	0.5	1.9	100.0%	-3.5
2816	AT3G11745.1 | expressed protein | chr3:3713769-3714585 REVERSE | Aliases: None	4.4	3.9	0.5	1.9	100.0%	-3.4
2817	AT3G21240.1 | Symbol: 4CL2 | 4-coumarate--CoA ligase 2 / 4-coumaroyl-CoA synthase 2 (4CL2), identical to SP:Q9S725 4-coumarate--CoA ligase 2 (EC 6.2.1.12) (4CL 2) (4-coumaroyl-CoA synthase 2) {Arabidopsis thaliana} | chr3:7454282-7457385 REVERSE | Aliases: COUMARATE:COA LIGASE, AT4CL2, MXL8.10	10.7	11.7	-1.0	-1.9	100.0%	-3.7
2818	AT3G16290.1 | Symbol: EMB2083 | FtsH protease, putative, contains similarity to cell division protein FtsH GI:1652085 from (Synechocystis sp. PCC 6803) | chr3:5521193-5525001 REVERSE | Aliases: EMB2083, EMBRYO DEFECTIVE 2083, MYA6.12	5.5	6.0	-0.5	-1.9	100.0%	-3.5
2819	AT4G15260.1 | UDP-glucoronosyl/UDP-glucosyl transferase family protein, contains Pfam profile: PF00201 UDP-glucoronosyl and UDP-glucosyl transferase | chr4:8713689-8715339 FORWARD | Aliases: DL3675W, FCAALL.250	5.3	4.3	1.0	1.9	100.0%	-3.2
2820	AT2G31390.1 | pfkB-type carbohydrate kinase family protein, contains Pfam profile: PF00294 pfkB family carbohydrate kinase | chr2:13390140-13393286 REVERSE | Aliases: T28P16.12, T28P16_12	11.2	11.7	-0.5	-1.9	100.0%	-3.7
2821	AT1G08840.1 | Symbol: EMB2411 | DNA replication helicase, putative, similar to helicase (Xenopus laevis) gi:18845092:gb:AAL79550 | chr1:2829582-2838372 REVERSE | Aliases: EMB2411, EMBRYO DEFECTIVE 2411, F7G19.26	2.8	3.1	-0.2	-1.9	100.0%	-4.0
2822	AT5G13220.3 | expressed protein | chr5:4218920-4220770 FORWARD | Aliases: T31B5.40, T31B5_40	4.1	2.5	1.6	1.9	100.0%	-3.6
2823	AT1G77660.1 | MORN (Membrane Occupation and Recognition Nexus) repeat-containing protein /phosphatidylinositol-4-phosphate 5-kinase-related, low similarity to phosphatidylinositol-4-phosphate 5-kinase AtPIP5K1 (Arabidopsis thaliana) GI:3702691; contains Pfam profile PF02493: MORN repeat | chr1:29190967-29192394 REVERSE | Aliases: T5M16.25, T5M16_25	3.7	3.2	0.5	1.9	100.0%	-3.4

Rank	Description	15.dpi	5.dpi	M	t	adj.q	B
2824	AT1G43000.1 | zinc-binding family protein, similar to zinc-binding protein (Pisum sativum) GI:16117799; contains Pfam profile PF04640 : Protein of unknown function, DUF597 | chr1:16143256-16144095 FORWARD | Aliases: F13A11.6, F13A11_6	3.0	3.3	-0.3	-1.9	100.0%	-3.7
2825	AT3G08880.1 | expressed protein | chr3:2703175-2703598 FORWARD | Aliases: T16O11.19	3.1	3.4	-0.3	-1.9	100.0%	-3.9
2826	AT1G13890.1 | Symbol: SNAP30 |	2.9	2.6	0.3	1.9	100.0%	-4.0
2827	AT3G03570.1 | expressed protein, similar to hypothetical protein GB:CAB38918 (Arabidopsis thaliana) | chr3:856903-860476 FORWARD | Aliases: T12J13.15, T12J13_15	3.5	3.9	-0.4	-1.9	100.0%	-3.4
2828	AT1G30040.2 | similar to gibberellin 2-oxidase / GA2-oxidase (GA2OX3) [Arabidopsis thaliana] (TAIR:At2g34555.1); similar to GA 2-oxidase 2 [Nerium oleander] (GB:AAT92094.1); contains InterPro domain 2OG-Fe(II) oxygenase superfamily (InterPro:IPR005123) | chr1:10537632-10539172 FORWARD | Aliases: T1P2.6, T1P2_6	5.6	6.4	-0.8	-1.9	100.0%	-3.0
2829	AT3G11530.2 | vacuolar protein sorting 55 family protein / VPS55 family protein, contains Pfam domain PF04133: Vacuolar protein sorting 55 | chr3:3628599-3630416 REVERSE | Aliases: F24K9.21	7.7	7.1	0.6	1.9	100.0%	-3.3
2830	AT1G70090.1 | glycosyl transferase family 8 protein, contains Pfam profile: PF01501 glycosyl transferase family 8 | chr1:26404453-26406060 FORWARD | Aliases: F20P5.18, F20P5_18	5.2	4.7	0.5	1.9	100.0%	-3.3
2831	AT1G22800.1 | expressed protein, similar to Biotin synthesis protein bioC. {Serratia marcescens} (SP:P36571); ESTs gb:Z34075, gb:Z34835 and gb:AA404888 come from this gene | chr1:8071924-8074346 FORWARD | Aliases: T22J18.3, T22J18_3	6.7	7.5	-0.7	-1.9	100.0%	-3.0
2832	AT4G24270.2 | RNA recognition motif (RRM)-containing protein, low similarity to tumor-rejection antigen SART3 (Mus musculus) GI:7637845; contains INTERPRO:IPR000504 RNA-binding region RNP-1 (RNA recognition motif) domain | chr4:12581611-12587394 REVERSE | Aliases: T22A6.100, T22A6_100	4.7	5.1	-0.4	-1.9	100.0%	-3.4
2833	AT5G45610.1 | expressed protein | chr5:18513538-18516900 REVERSE | Aliases: MRA19.1, MRA19_1	4.2	4.6	-0.4	-1.9	100.0%	-3.4
2834	AT1G42540.1 | Symbol: ATGLR3.3 | glutamate receptor family protein (GLR3.3), plant glutamate receptor family, PMID:11379626 | chr1:15975929-15979143 FORWARD | Aliases: GLR3.3, T8D8.1, T8D8_1	5.0	5.5	-0.6	-1.9	100.0%	-3.3
2835	AT5G65750.1 | 2-oxoglutarate dehydrogenase E1 component, putative / oxoglutarate decarboxylase, putative / alpha-ketoglutaric dehydrogenase, putative, similar to SP:P20967 2-oxoglutarate dehydrogenase E1 component, mitochondrial precursor (EC 1.2.4.2) (Alpha-ketoglutarate dehydrogenase) {Saccharomyces cerevisiae}; contains Pfam profiles PF02779: Transketolase, pyridine binding domain, PF00676: Dehydrogenase E1 component | chr5:26320932-26325415 FORWARD | Aliases: MPA24.10, MPA24_10	8.3	8.9	-0.7	-1.9	100.0%	-3.0
2836	AT5G11710.1 | epsin N-terminal homology (ENTH) domain-containing protein / clathrin assembly protein-related, contains Pfam PF01417: ENTH domain. ENTH (Epsin N-terminal homology) domain; similar to Af10-protein (GI:1724114) (Avena fatua); similar to clathrin assembly protein AP180 (GI:6492344) (Xenopus laevis); Af10-protein, Avena fatua, EMBL:U80041 | chr5:3772865-3776663 FORWARD | Aliases: T22P22.100, T22P22_100	6.1	5.7	0.4	1.9	100.0%	-3.4
2837	AT1G66980.1 | protein kinase family protein / glycerophosphoryl diester phosphodiesterase family protein, similar to leaf rust resistance kinase Lr10 GI:1680685 from (Triticum aestivum); contains Pfam profiles PF03009: Glycerophosphoryl diester phosphodiesterase family, PF00069: Protein kinase domain | chr1:25000972-25005624 REVERSE | Aliases: F1O19.6, F1O19_6	3.5	3.2	0.4	1.9	100.0%	-3.5
2838	AT1G08700.1 | presenilin family protein, similar to SP:P52166 Presenilin sel-12 {Caenorhabditis elegans}; contains Pfam profile PF01080: Presenilin | chr1:2769818-2771480 REVERSE | Aliases: F22O13.18, F22O13_18	8.5	8.9	-0.4	-1.9	100.0%	-3.6
2839	AT5G14030.1 | translocon-associated protein beta (TRAPB) family protein, low similarity to SP:P23438 Translocon-associated protein, beta subunit precursor (TRAP-beta) (Signal sequence receptor beta subunit) {Canis familiaris}; contains Pfam profile PF05753: Translocon-associated protein beta (TRAPB) | chr5:4526813-4528411 FORWARD | Aliases: MUA22.2, MUA22_2	9.9	10.5	-0.6	-1.9	100.0%	-3.4
2840	AT4G09490.1 | RNase H domain-containing protein, contains Pfam profile PF00075: RNase H | chr4:6015559-6016572 REVERSE | Aliases: T15G18.90, T15G18_90	4.1	3.4	0.7	1.9	100.0%	-3.1

Rank	Description	15.dpi	5.dpi	M	t	adj.q	B
2841	AT1G11280.4 | similar to S-locus protein kinase, putative [Arabidopsis thaliana] (TAIR:At1g61390.1); similar to S-locus protein kinase, putative [Arabidopsis thaliana] (TAIR:At1g61480.1); similar to S-locus protein kinase, putative [Arabidopsis thaliana] (TAIR:At1g61490.1); similar to S-locus lectin protein kinase family protein [Arabidopsis thaliana] (TAIR:At1g61370.1); similar to S-locus protein kinase, putative [Arabidopsis thaliana] (TAIR:At1g61380.1); similar to receptor kinase 5 [Brassica rapa] (GB:BAB69683.1); similar to KI domain interacting kinase 1 [Zea mays] (GB:AAB93834.1); contains InterPro domain Serine/threonine protein kinase, active site (InterPro:IPR008271); contains InterPro domain Apple-like (InterPro:IPR003609); contains InterPro domain Tyrosine protein kinase (InterPro:IPR001245); contains InterPro domain Serine/threonine protein kinase (InterPro:IPR002290); contains InterPro domain Curculin-like (mannose-binding) lectin (InterPro:IPR001480); contains InterPro domain Protein kinase (InterPro:IPR000719); contains InterPro domain S-locus glycoprotein (InterPro:IPR000858) | chr1:3787334-3790812 REVERSE | Aliases: T28P6.7, T28P6_7	6.4	7.1	-0.7	-1.9	100.0%	-3.3
2842	AT4G26110.1 | nucleosome assembly protein (NAP), putative, similar to nucleosome assembly protein 1 (Glycine max) GI:1161252; contains Pfam profile PF00956: Nucleosome assembly protein (NAP)	6.8	7.4	-0.6	-1.9	100.0%	-3.4
2843	AT5G64330.1 | Symbol: NPH3 | non-phototropic hypocotyl 3 (NPH3), identical to non-phototropic hypocotyl 3 (Arabidopsis thaliana) gi:6224712:gb:AAF05914, PMID:10542152 | chr5:25744563-25748328 FORWARD | Aliases: JK218, MSJ1.17, MSJ1_17, NON PHOTOTROPIC HYPOCOTYL 3, ROOT PHOTOTROPISM 3, RPT3	4.9	5.4	-0.5	-1.9	100.0%	-3.3
2844	AT5G36160.1 | aminotransferase-related, similar to nicotianamine aminotransferase B GI:6469087 from (Hordeum vulgare subsp. vulgare) | chr5:14250343-14252364 REVERSE | Aliases: MAB16.11, MAB16_11	6.8	8.0	-1.2	-1.9	100.0%	-3.3
2845	AT5G02740.2 | expressed protein | chr5:616492-618595 FORWARD | Aliases: F9G14.50, F9G14_50	5.7	6.6	-0.9	-1.9	100.0%	-3.3
2846	AT3G62560.1 | GTP-binding protein, putative, similar to GTP-binding protein SAR1A (SP:O04834) (Arabidopsis thaliana); small GTP-binding protein Bsar1a - Brassica campestris, EMBL:U55035 | chr3:23148459-23150021 FORWARD | Aliases: T12C14.260	9.3	8.4	0.9	1.9	100.0%	-3.3
2847	AT2G24970.1 | expressed protein | chr2:10627198-10628386 REVERSE | Aliases: F27C12.11, F27C12_11	4.8	6.1	-1.2	-1.9	100.0%	-3.0
2848	AT1G21680.1 | expressed protein, similar to TolB protein precursor (SP:Q9ZDM5) {Rickettsia prowazekii}; ESTs gb:N96028, gb:F14286, gb:T20680, gb:F14443, gb:AA657300 and gb:N65244 come from this gene	9.8	8.5	1.2	1.9	100.0%	-3.3
2849	AT1G09560.1 | Symbol: GLP5 | germin-like protein (GLP4) (GLP5), identical to Arabidopsis germin-like protein subfamily 2 member 1 (SP:P94014); Location of EST 180L10T7, gi:906417 | chr1:3093841-3094864 FORWARD | Aliases: F14J9.22, F14J9_22, GERMIN LIKE PROTEIN 5, GLP5	8.0	8.9	-0.9	-1.9	100.0%	-3.0
2850	AT3G20100.1 | Symbol: CYP705A19 | cytochrome P450 family protein, similar to Cytochrome P450 93A3 (P450 CP5) (SP:O81973) (Glycine max); contains Pfam profile: PF00067 cytochrome P450; supported by full-length cDNA: Ceres:149380. | chr3:7019001-7020907 FORWARD | Aliases: MAL21.14	7.8	7.4	0.4	1.9	100.0%	-3.4
2851	AT4G13280.1 | terpene synthase/cyclase family protein, predicted protein, Arabidopsis thaliana	2.9	3.2	-0.4	-1.9	100.0%	-3.6
2852	AT1G80160.1 | lactoylglutathione lyase family protein / glyoxalase I family protein, contains glyoxalase family protein domain, Pfam:PF00903 | chr1:30155895-30157082 FORWARD | Aliases: F18B13.24, F18B13_24	2.9	2.7	0.2	1.9	100.0%	-4.0
2853	AT3G55605.1 | mitochondrial glycoprotein family protein / MAM33 family protein, low similarity to SUAPRGA1 (Emericella nidulans) GI:6562379; contains Pfam profile PF02330: Mitochondrial glycoprotein	6.7	7.4	-0.6	-1.9	100.0%	-3.1
2854	AT2G38060.1 | transporter-related, low similarity to vesicular glutamate transporter 3 (Homo sapiens) GI:21213895, brain specific Na+-dependent inorganic phosphate cotransporter (Rattus norvegicus) GI:507415; contains Pfam profile PF00083: major facilitator superfamily protein | chr2:15929805-15932701 REVERSE | Aliases: T8P21.3, T8P21_3	4.4	4.9	-0.5	-1.9	100.0%	-3.3
2855	AT1G76185.1 | expressed protein | chr1:28595219-28596412 FORWARD | Aliases: None	5.3	4.5	0.8	1.9	100.0%	-3.1
2856	AT1G55210.1 | disease resistance response protein-related/ dirigent protein-related, smimilar to dirigent protein (Thuja plicata) gi:6694699:gb:AAF25360; similar to pathogenesis-related protein (Pisum sativum) gi:4585273:gb:AAD25355 | chr1:20601536-20602375 REVERSE | Aliases: F7A10.7, F7A10_7	6.3	7.2	-0.8	-1.9	100.0%	-3.4
2857	AT1G09340.1 | expressed protein | chr1:3015388-3018236 FORWARD | Aliases: None	8.1	7.2	0.9	1.9	100.0%	-2.9
2858	AT4G01026.1 | expressed protein | chr4:447090-448510 FORWARD | Aliases: None	5.6	4.9	0.6	1.9	100.0%	-3.3
2859	AT1G77250.1 | PHD finger family protein, contains Pfam domain, PF00628: PHD-finger | chr1:29025201-29028349 REVERSE | Aliases: T14N5.11, T14N5_11	5.3	6.1	-0.7	-1.9	100.0%	-3.3
2860	AT2G29260.1 | tropinone reductase, putative / tropine dehydrogenase, putative, similar to tropinone reductase SP:P50165 from (Datura stramonium) | chr2:12589600-12591178 FORWARD | Aliases: F16P2.36, F16P2_36	4.2	4.7	-0.5	-1.9	100.0%	-3.4

Rank	Description	15.dpi	5.dpi	M	t	adj.q	B
2861	AT2G26600.2 | glycosyl hydrolase family 17 protein | chr2:11323530-11325561 FORWARD | Aliases: T9J22.27, T9J22_27	7.5	7.1	0.4	1.9	100.0%	-3.5
2862	AT5G47210.1 | nuclear RNA-binding protein, putative, similar to nuclear RNA binding protein GI:6492264 from (Arabidopsis thaliana) | chr5:19186249-19188446 REVERSE | Aliases: MQL5.6, MQL5_6	11.5	12.0	-0.6	-1.9	100.0%	-3.9
2863	AT5G42980.1 | Symbol: ATTRX3 |	13.4	13.1	0.3	1.9	100.0%	-4.0
2864	AT1G75690.1 | chaperone protein dnaJ-related, contains Pfam PF00684 : DnaJ central domain (4 repeats); similar to bundle sheath defective protein 2 (GI:4732091) (Zea mays) | chr1:28425771-28426880 REVERSE | Aliases: F10A5.12, F10A5_12	6.7	5.7	1.0	1.9	100.0%	-3.0
2865	AT5G49840.1 | ATP-dependent Clp protease ATP-binding subunit ClpX, putative, similar to CLP protease regulatory subunit CLPX GI:2674203 from (Arabidopsis thaliana); non-consensus splice donor GC at exon 4; non-consensus splice donor AA at exon 7 | chr5:20272469-20276261 FORWARD | Aliases: K21G20.5, K21G20_5	5.9	5.4	0.5	1.9	100.0%	-3.2
2866	AT2G37420.1 | kinesin motor protein-related | chr2:15707410-15712422 FORWARD | Aliases: F3G5.21, F3G5_21	4.4	5.4	-1.0	-1.9	100.0%	-3.2
2867	AT3G11840.1 | U-box domain-containing protein, low similarity to immediate-early fungal elicitor protein CMPG1 (Petroselinum crispum) GI:14582200; contains Pfam profile PF04564: U-box domain	4.6	4.0	0.6	1.9	100.0%	-3.2
2868	AT2G45960.1 | Symbol: PIP1B | plasma membrane intrinsic protein 1B (PIP1B) / aquaporin PIP1.2 (PIP1.2) / transmembrane protein A (TMPA), identical to plasma membrane intrinsic protein 1B SP:Q06611 from (Arabidopsis thaliana) | chr2:18917384-18919035 FORWARD | Aliases: ATHH2, F4I18.6, PIP1;2, PIP1B, TMP A	6.4	5.5	0.9	1.9	100.0%	-3.0
2869	AT1G21560.1 | expressed protein, similar to hypothetical protein [Arabidopsis thaliana] (TAIR:At4g01170.1); similar to unknown protein [Oryza sativa (japonica cultivar-group)] (GB:AAV24769.1) | chr1:7554829-7557014 FORWARD | Aliases: F24J8.19, F24J8_19	6.0	6.6	-0.6	-1.9	100.0%	-3.2
2870	AT5G55660.1 | expressed protein, similar to unknown protein (pir::T08929) | chr5:22556601-22560700 FORWARD | Aliases: MDF20.10, MDF20_10	5.3	6.5	-1.2	-1.9	100.0%	-3.2
2871	AT5G51440.1 | 23.5 kDa mitochondrial small heat shock protein (HSP23.5-M), similar to heat shock 22 kDa protein, mitochondrial precursor SP:Q96331 from (Arabidopsis thaliana); identified in Scharf, K-D., et al,Cell Stress & Chaperones (2001) 6: 225-237. | chr5:20908389-20909437 FORWARD | Aliases: MFG13.15, MFG13_15	3.5	3.2	0.3	1.9	100.0%	-3.7
2872	AT2G26730.1 | leucine-rich repeat transmembrane protein kinase, putative | chr2:11395485-11398719 FORWARD | Aliases: F18A8.10, F18A8_10	4.0	4.6	-0.6	-1.9	100.0%	-3.2
2873	AT5G13770.1 | pentatricopeptide (PPR) repeat-containing protein, contains Pfam profile PF01535: PPR repeat | chr5:4445426-4447839 FORWARD | Aliases: MXE10.6, MXE10_6	8.6	7.2	1.4	1.9	100.0%	-3.2
2874	AT4G05020.1 | NADH dehydrogenase-related, similar to alternative NADH-dehydrogenase (Yarrowia lipolytica) GI:3718005, 64 kDa mitochondrial NADH dehydrogenase (Neurospora crassa) GI:4753821; contains Pfam profile PF00070: Pyridine nucleotide-disulphide oxidoreductase | chr4:2572693-2576593 FORWARD | Aliases: C17L7.11	5.2	6.8	-1.5	-1.9	100.0%	-3.2
2875	AT3G18295.1 | expressed protein | chr3:6281210-6282087 FORWARD | Aliases: MIE15.10	3.5	3.3	0.3	1.9	100.0%	-3.8
2876	AT1G74420.2 | Symbol: FUT3 | xyloglucan fucosyltransferase, putative (FUT3), identical to SP:Q9CA71 Probable fucosyltransferase 3 (EC 2.4.1.-) (AtFUT3) {Arabidopsis thaliana}; similar to SP:Q9SWH5 Galactoside 2-alpha-L-fucosyltransferase (EC 2.4.1.69) (Xyloglucan alpha-(1,2)-fucosyltransferase) (AtFUT1) {Arabidopsis thaliana} | chr1:27971555-27973464 REVERSE | Aliases: ATFUT3, F1M20.10, F1M20_10	3.1	3.7	-0.5	-1.9	100.0%	-3.5
2877	AT3G11440.1 | Symbol: ATMYB65 | myb family transcription factor (MYB65), contains Pfam profile: PF00249 myb-like DNA-binding domain | chr3:3602100-3605110 FORWARD | Aliases: F24K9.11, MYB65	5.3	4.8	0.5	1.9	100.0%	-3.5
2878	AT2G24940.1 | cytochrome b5 domain-containing protein, similar to SP:P70580 Membrane associated progesterone receptor component 1 {Rattus norvegicus}; contains Pfam profile PF00173: Heme/Steroid binding domain | chr2:10616473-10619311 FORWARD | Aliases: F27C12.14, F27C12_14	12.8	12.0	0.8	1.9	100.0%	-3.4
2879	AT2G24945.1 | expressed protein | chr2:10616492-10619311 FORWARD | Aliases: None	12.8	12.0	0.8	1.9	100.0%	-3.4
2880	AT2G45140.1 | vesicle-associated membrane protein, putative / VAMP, putative, similar to VAP27 GI:6688926 (Nicotiana plumbaginifolia) | chr2:18617881-18620365 FORWARD | Aliases: T14P1.5	7.9	7.4	0.5	1.9	100.0%	-3.4
2881	AT1G10020.1 | expressed protein | chr1:3269606-3271989 REVERSE | Aliases: T27I1.4, T27I1_4	4.5	3.9	0.5	1.9	100.0%	-3.4
2882	AT1G20480.1 | 4-coumarate--CoA ligase family protein / 4-coumaroyl-CoA synthase family protein, similar to SP:Q9S725 from Arabidopsis thaliana and SP:P17814 from Oryza sativa; contains Pfam AMP-binding enzyme domain PF00501 | chr1:7094250-7097104 REVERSE | Aliases: F5M15.29, F5M15_29	4.8	4.3	0.4	1.9	100.0%	-3.3

Rank	Description	15.dpi	5.dpi	M	t	adj.q	B
2883	AT4G24290.2 | expressed protein | chr4:12594349-12598038 FORWARD | Aliases: T22A6.120, T22A6_120	3.3	3.0	0.3	1.9	100.0%	-3.8
2884	AT3G25160.1 | ER lumen protein retaining receptor family protein, similar to SP:P24390 ER lumen protein retaining receptor 1 (KDEL receptor 1) {Homo sapiens}; contains Pfam profile PF00810: ER lumen protein retaining receptor | chr3:9160175-9162074 REVERSE | Aliases: MJL12.10	3.2	2.7	0.5	1.9	100.0%	-3.4
2885	AT4G40070.1 | similar to zinc finger (C3HC4-type RING finger) family protein (ATL6) [Arabidopsis thaliana] (TAIR:At3g05200.1); similar to RING/C3HC4/PHD zinc finger-like protein [Cucumis melo] (GB:AAO45753.1); contains InterPro domain Zn-finger, RING (InterPro:IPR001841) | chr4:18576412-18577768 FORWARD | Aliases: T5J17.240, T5J17_240	6.3	5.7	0.6	1.9	100.0%	-3.4
2886	AT2G01640.1 | expressed protein | chr2:283003-284124 FORWARD | Aliases: T8O11.19, T8O11_19	5.0	5.6	-0.6	-1.9	100.0%	-3.4
2887	AT1G45976.1 | Symbol: SBP1 | expressed protein | chr1:17201791-17203173 FORWARD | Aliases: F2G19.22, F2G19_22, S RIBONUCLEASE BINDING PROTEIN 1	7.7	7.1	0.5	1.9	100.0%	-3.5
2888	AT5G65520.1 | expressed protein | chr5:26206951-26207817 REVERSE | Aliases: K21L13.2, K21L13_2	4.1	3.7	0.4	1.9	100.0%	-3.8
2889	AT2G31740.1 | expressed protein | chr2:13497985-13502187 REVERSE | Aliases: F20M17.22, F20M17_22	6.8	7.3	-0.5	-1.9	100.0%	-3.4
2890	AT3G53650.1 | histone H2B, putative, similar to histone H2B from Lycopersicon esculentum, PIR:T06389 GI:3021483, Gossypium hirsutum SP:O22582, Capsicum annuum SP:O49118; contains Pfam profile PF00125 Core histone H2A/H2B/H3/H4 | chr3:19900336-19900752 FORWARD | Aliases: F4P12.350	6.5	7.7	-1.2	-1.9	100.0%	-3.0
2891	AT5G25210.1 | expressed protein | chr5:8723961-8725774 FORWARD | Aliases: F21J6.105, F21J6_105	3.3	3.0	0.3	1.9	100.0%	-3.7
2892	AT4G24060.1 | Dof-type zinc finger domain-containing protein, Dof zinc finger protein - Oryza sativa,PID:d1042342 | chr4:12503821-12505656 FORWARD | Aliases: T19F6.50, T19F6_50	5.2	4.6	0.6	1.9	100.0%	-3.5
2893	AT3G02650.1 | pentatricopeptide (PPR) repeat-containing protein, contains Pfam profile PF01535: PPR repeat | chr3:566278-570110 FORWARD | Aliases: F16B3.28, F16B3_28	6.7	7.6	-0.9	-1.9	100.0%	-3.2
2894	AT3G17860.2 | expressed protein | chr3:6121059-6123017 FORWARD | Aliases: MEB5.8	4.6	4.2	0.4	1.9	100.0%	-3.4
2895	AT2G29050.1 | rhomboid family protein, contains PFAM domain PF01694, Rhomboid family | chr2:12485223-12487449 FORWARD | Aliases: T9I4.13, T9I4_13	3.8	4.4	-0.6	-1.9	100.0%	-3.2
2896	AT1G30570.1 | protein kinase family protein, contains protein kinase domain, Pfam:PF00069 | chr1:10828915-10831464 FORWARD | Aliases: T5I8.2, T5I8_2	3.6	4.1	-0.5	-1.9	100.0%	-3.6
2897	AT2G13540.1 | Symbol: ABH1 | mRNA cap-binding protein (ABH1), identical to mRNA cap binding protein (Arabidopsis thaliana) GI:15192738; contains Pfam profile PF02854: MIF4G domain; identical to cDNA nuclear cap-binding protein CBP80 GI:8515770 | chr2:5643966-5650039 FORWARD | Aliases: ABA HYPERSENSITIVE 1, CBP80, NUCLEAR CAP BINDING PROTEIN CBP80, T10F5.8, T10F5_8	6.2	6.7	-0.5	-1.9	100.0%	-3.4
2898	AT5G39350.1 | pentatricopeptide (PPR) repeat-containing protein, contains Pfam profile PF01535: PPR repeat | chr5:15768157-15770190 FORWARD | Aliases: MUL8.30, MUL8_30	4.5	5.0	-0.5	-1.9	100.0%	-3.3
2899	AT1G57680.2 | expressed protein | chr1:21366153-21368351 REVERSE | Aliases: T8L23.15, T8L23_15	6.5	5.3	1.2	1.9	100.0%	-3.2
2900	AT1G54720.1 | early-responsive to dehydration protein-related / ERD protein-related, similar to ERD6 protein (Arabidopsis thaliana) GI:3123712	2.2	2.0	0.2	1.9	100.0%	-4.3
2901	AT1G11940.1 | expressed protein, contains Pfam profile PF03267: Arabidopsis protein of unknown function, DUF266 | chr1:4031578-4034076 REVERSE | Aliases: F12F1.19, F12F1_19	4.6	5.2	-0.6	-1.9	100.0%	-3.3
2902	ATCG00160.1 | Symbol: RPS2 | Chloroplast ribosomal protein S2	6.6	7.5	-0.9	-1.9	100.0%	-3.4
2903	AT5G54580.1 | RNA recognition motif (RRM)-containing protein, low similarity to RNA-binding protein RGP-3 (Nicotiana sylvestris) GI:1009363; contains InterPro entry IPR000504: RNA-binding region RNP-1 (RNA recognition motif) (RRM)	8.7	8.1	0.6	1.9	100.0%	-3.3
2904	AT3G11880.1 | expressed protein | chr3:3751666-3754066 FORWARD | Aliases: F26K24.17	6.7	6.3	0.4	1.9	100.0%	-3.5
2905	AT1G14260.2 | zinc finger (C3HC4-type RING finger) family protein, contains Pfam domain, PF00097: Zinc finger, C3HC4 type (RING finger) | chr1:4872900-4874821 FORWARD | Aliases: F14L17.2, F14L17_2	3.3	2.9	0.4	1.9	100.0%	-3.6

Rank	Description	15.dpi	5.dpi	M	t	adj.q	B
2906	AT5G65950.2 | expressed protein, similar to unknown protein [Oryza sativa (japonica cultivar-group)] (GB:BAD33026.1) | chr5:26398945-26402447 FORWARD | Aliases: K14B20.12, K14B20_12	8.1	8.7	-0.6	-1.9	100.0%	-3.3
2907	AT5G54780.1 | similar to RabGAP/TBC domain-containing protein [Arabidopsis thaliana] (TAIR:At4g27100.1); similar to putative GTPase activating protein [Oryza sativa (japonica cultivar-group)] (GB:XP_468334.1); contains InterPro domain RabGAP/TBC domain (InterPro:IPR000195) | chr5:22265673-22269089 REVERSE | Aliases: MBG8.4, MBG8_4	3.5	3.9	-0.4	-1.9	100.0%	-3.4
2908	AT4G08150.1 | Symbol: KNAT1 | homeobox protein knotted-1 like 1 (KNAT1), identical to homeobox protein knotted-1 like 1 (KNAT1) SP:P46639 from (Arabidopsis thaliana) | chr4:5147697-5150963 REVERSE | Aliases: BP, BP1, BREVIPEDICELLUS, BREVIPEDICELLUS 1, F9M13.2, F9M13_2, KNOTTED LIKE HOMEOBOX PROTEIN	3.3	2.9	0.4	1.9	100.0%	-3.6
2909	AT4G37410.1 | Symbol: CYP81F4 | cytochrome P450, putative, similar to cytochrome p450 SP:O65790 from (Arabidopsis thaliana) | chr4:17590766-17592914 FORWARD | Aliases: F6G17.60, F6G17_60	6.2	7.2	-1.0	-1.9	100.0%	-3.4
2910	AT1G19850.1 | Symbol: MP | transcription factor MONOPTEROS (MP) / auxin-responsive protein (IAA24) / auxin response factor 5 (ARF5), identical to transcription factor MONOPTEROS (MP/IAA24/ARF5) SP:P93024 from (Arabidopsis thaliana) | chr1:6886870-6891365 FORWARD | Aliases: ARF5, F6F9.10, F6F9_10, IAA24, MONOPTEROS, MP, TRANSCRIPTION FACTOR IAA24	3.9	4.3	-0.3	-1.9	100.0%	-3.6
2911	AT2G35900.1 | expressed protein | chr2:15077076-15078732 REVERSE | Aliases: F11F19.19, F11F19_19	7.8	6.8	1.0	1.9	100.0%	-3.0
2912	AT5G45680.1 | FK506-binding protein 1 (FKBP13), identical to Probable FKBP-type peptidyl-prolyl cis-trans isomerase 3, chloroplast precursor (Ppiase) (Rotamase) (SP:Q9SCY2) / FK506 binding protein 1 (GI:21535744) (Arabidopsis thaliana); contains Pfam PF00254: peptidyl-prolyl cis-trans isomerase, FKBP-type | chr5:18548107-18549508 FORWARD | Aliases: MRA19.7, MRA19_7	4.8	4.2	0.5	1.9	100.0%	-3.3
2913	AT4G25390.2 | protein kinase family protein, contains protein kinase domain, Pfam:PF00069 | chr4:12977351-12979578 FORWARD | Aliases: T30C3.60, T30C3_60	5.7	4.8	0.9	1.9	100.0%	-3.2
2914	AT1G23280.1 | MAK16 protein-related, contains similarity to MAK16 protein SP:P10962 from (Saccharomyces cerevisiae) | chr1:8260854-8262639 REVERSE | Aliases: F26F24.12, F26F24_12	5.1	6.3	-1.2	-1.9	100.0%	-3.2
2915	AT1G20900.1 | Symbol: ESC | DNA-binding protein-related, contains Pfam domain PF03479: Domain of unknown function (DUF296), found in AT-hook motifs Pfam:PF02178 | chr1:7272545-7274061 FORWARD | Aliases: ESC, ESCAROLA, F9H16.12, F9H16_12	5.6	5.0	0.6	1.9	100.0%	-3.1
2916	AT3G23250.2 | similar to myb family transcription factor [Arabidopsis thaliana] (TAIR:At1g06180.1); similar to myb-related transcription factor LBM4 [Nicotiana tabacum] (GB:BAA88224.1); contains InterPro domain Myb DNA-binding domain (InterPro:IPR001005)	5.9	5.1	0.8	1.9	100.0%	-3.1
2917	AT3G21390.1 | mitochondrial substrate carrier family protein | chr3:7531408-7534544 FORWARD | Aliases: MHC9.7	8.3	7.6	0.7	1.9	100.0%	-3.2
2918	AT5G66470.1 | expressed protein | chr5:26558965-26561585 REVERSE | Aliases: K1F13.13, K1F13_13	4.8	5.5	-0.7	-1.9	100.0%	-3.1
2919	AT4G31580.1 | Symbol: SRZ 22 |	7.8	7.1	0.8	1.9	100.0%	-3.1
2920	AT4G17830.1 | peptidase M20/M25/M40 family protein, similar to acetylornithine deacetylase (Acetylornithinase, AO; N-acetylornithinase, NAO) (Dictyostelium discoideum) SWISS-PROT:P54638 | chr4:9915893-9918256 FORWARD | Aliases: T6K21.10	5.7	6.4	-0.7	-1.9	100.0%	-3.3
2921	AT2G13820.2 | protease inhibitor/seed storage/lipid transfer protein (LTP) family protein, contains Pfam protease inhibitor/seed storage/LTP family domain PF00234 | chr2:5782333-5783371 REVERSE | Aliases: F17L24.13, F17L24_13	6.0	6.6	-0.6	-1.9	100.0%	-3.2
2922	AT5G43870.1 | expressed protein | chr5:17649573-17653125 REVERSE | Aliases: F6B6.1, F6B6_1	3.6	4.1	-0.6	-1.9	100.0%	-3.2
2923	AT3G51550.1 | protein kinase family protein, contains protein kinase domain, Pfam:PF00069 | chr3:19128563-19131840 REVERSE | Aliases: F26O13.190	5.6	4.8	0.8	1.9	100.0%	-3.3
2924	AT1G52630.2 | expressed protein, contains Pfam PF03138: Plant protein family. The function of this family of plant proteins is unknown; | chr1:19610003-19612215 REVERSE | Aliases: F6D8.15, F6D8_15	3.6	3.3	0.3	1.9	100.0%	-3.7
2925	AT3G04610.1 | Symbol: FLK | KH domain-containing protein, similar putative nucleic acid binding protein GB:CAB39665 (Arabidopsis thaliana); Pfam HMM hit: KH domain family of RNA binding proteins | chr3:1250568-1254878 REVERSE | Aliases: F7O18.9, F7O18_9, FLK, FLOWERING LOCUS KH DOMAIN	7.1	7.6	-0.5	-1.9	100.0%	-3.4
2926	AT2G01190.1 | octicosapeptide/Phox/Bem1p (PB1) domain-containing protein, Pfam profile PF00564: PB1 domain | chr2:114974-117639 FORWARD | Aliases: F10A8.7, F10A8_7	5.2	4.7	0.4	1.9	100.0%	-3.5
2927	AT4G23885.1 | expressed protein | chr4:12419404-12420477 FORWARD | Aliases: None	9.1	9.7	-0.5	-1.9	100.0%	-3.5

Rank	Description	15.dpi	5.dpi	M	t	adj.q	B
2928	AT2G23470.1 | expressed protein, similar to expressed protein [Arabidopsis thaliana] (TAIR:At5g49820.1); similar to unknown protein [Oryza sativa (japonica cultivar-group)] (GB:BAD82127.1); contains InterPro domain Protein of unknown function DUF647 (InterPro:IPR006968)	3.3	3.9	-0.6	-1.9	100.0%	-3.5
2929	AT5G46070.1 | guanylate-binding family protein, contains Pfam domains PF02263: Guanylate-binding protein, N-terminal domain and PF02841: Guanylate-binding protein, C-terminal domain | chr5:18700695-18705934 FORWARD | Aliases: MCL19.12, MCL19_12	4.6	5.0	-0.5	-1.9	100.0%	-3.5
2930	AT3G56040.1 | expressed protein | chr3:20803052-20808551 REVERSE | Aliases: F27K19.220	4.2	4.7	-0.5	-1.9	100.0%	-3.3
2931	AT1G67910.1 | expressed protein | chr1:25474972-25476008 REVERSE | Aliases: T23K23.24, T23K23_24	4.2	4.9	-0.7	-1.9	100.0%	-3.3
2932	AT3G07760.2 | expressed protein | chr3:2476450-2478204 FORWARD | Aliases: MLP3.21	7.4	6.5	0.9	1.9	100.0%	-3.4
2933	AT3G52430.1 | Symbol: PAD4 | phytoalexin-deficient 4 protein (PAD4), identical to phytoalexin-deficient 4 protein (Arabidopsis thaliana) GI:6457331; contains Pfam profile PF01764: Lipase | chr3:19442349-19445381 FORWARD | Aliases: F22O6.190, PAD4, PHYTOALEXIN DEFICIENT 4, PHYTOALEXIN DEFICIENT 4	4.3	5.0	-0.8	-1.9	100.0%	-3.3
2934	AT1G22530.1 | SEC14 cytosolic factor family protein / phosphoglyceride transfer family protein, contains Pfam PF00650 : CRAL/TRIO domain; contains Pfam PF03765 : CRAL/TRIO, N-terminus; similar to SEC14-like protein 2 (Alpha-tocopherol associated protein) (TAP) (bTAP) (Fragment) (SP:P58875) {Bos taurus} | chr1:7955482-7958431 REVERSE | Aliases: F12K8.13, F12K8_13	8.6	7.2	1.5	1.9	100.0%	-3.1
2935	AT3G56730.1 | expressed protein, contains Pfam profile PF04396: Protein of unknown function, DUF537 | chr3:21025166-21026053 FORWARD | Aliases: T8M16.60	8.3	7.6	0.7	1.9	100.0%	-3.5
2936	AT5G11670.1 | malate oxidoreductase, putative, similar to NADP-dependent malic enzyme (EC 1.1.1.40) (NADP-ME) (SP:P12628) {Phaseolus vulgaris}	10.4	11.0	-0.6	-1.9	100.0%	-3.8
2937	AT5G09410.1 | Symbol: EICBP.B | calmodulin-binding protein, similar to another ethylene-upregulated calmodulin-binding protein ER1 GI:11612392 from (Nicotiana tabacum) | chr5:2921458-2927417 FORWARD | Aliases: EICBP.B, ETHYLENE INDUCED CALMODULIN BINDING PROTEIN, T5E8.210, T5E8_210	8.2	8.9	-0.7	-1.9	100.0%	-3.8
2938	AT3G13140.1 | hydroxyproline-rich glycoprotein family protein | chr3:4226661-4227384 REVERSE | Aliases: MJG19.9	2.5	2.8	-0.3	-1.9	100.0%	-4.0
2939	AT1G62640.2 | Symbol: KAS III | similar to beta-ketoacyl-acyl carrier protein synthase III [Glycine max] (GB:AAF70509.1); contains InterPro domain Beta-ketoacyl-acyl carrier protein synthase III (FabH) (InterPro:IPR004655) | chr1:23195909-23198740 FORWARD | Aliases: 3 KETOACYL ACYL CARRIER PROTEIN SYNTHASE III, T3P18.20, T3P18_20	8.2	8.6	-0.5	-1.9	100.0%	-3.6
2940	AT5G07870.1 | transferase family protein, similar to anthranilate N-hydroxycinnamoyl/benzoyltransferase, Dianthus caryophyllus (gi:2239091); contains Pfam transferase family domain PF002458 | chr5:2513795-2515419 FORWARD | Aliases: F13G24.70, F13G24_70	3.5	4.0	-0.4	-1.9	100.0%	-3.4
2941	AT5G66980.1 | transcriptional factor B3 family protein, contains Pfam profile PF02362: B3 DNA binding domain | chr5:26758789-26760052 FORWARD | Aliases: K8A10.5, K8A10_5	3.4	3.0	0.4	1.9	100.0%	-3.7
2942	AT2G31190.1 | expressed protein, contains Pfam domain, PF04884: Protein of unknown function, DUF647 | chr2:13298303-13300839 REVERSE | Aliases: F16D14.3, F16D14_3	5.4	5.9	-0.6	-1.9	100.0%	-3.3
2943	AT5G23850.1 | expressed protein | chr5:8038129-8040934 FORWARD | Aliases: MRO11.11, MRO11_11	6.6	7.3	-0.7	-1.9	100.0%	-3.1
2944	AT5G10170.1 | inositol-3-phosphate synthase, putative / myo-inositol-1-phosphate synthase, putative / MI-1-P synthase, putative, very strong similarity to SP:Q38862 Myo-inositol-1-phosphate synthase isozyme 2 (EC 5.5.1.4) (MI-1-P synthase 2) (IPS 2) {Arabidopsis thaliana}; identical to SP:Q9LX12: Probable inositol-3-phosphate synthase isozyme 3 (EC 5.5.1.4) (Myo- inositol-1-phosphate synthase 3) (MI-1-P synthase 3) (IPS 3) {Arabidopsis thaliana}; contains Pfam profile PF01658: Myo-inositol-1-phosphate synthase | chr5:3187278-3190252 REVERSE | Aliases: T31P16.160, T31P16_160	4.0	4.5	-0.5	-1.9	100.0%	-3.4
2945	AT1G66480.1 | expressed protein | chr1:24809433-24811129 REVERSE | Aliases: F28G11.8, F28G11_8	4.3	3.9	0.4	1.9	100.0%	-3.5
2946	AT5G62670.1 | ATPase, plasma membrane-type, putative / proton pump, putative, strong similarity to P-type H(+)-transporting ATPase from Nicotiana plumbaginifolia (SP:Q08435, SP:Q08436), Lycopersicon esculentum (GI:5901757, SP:P22180), Solanum tuberosum (GI:435003); contains InterPro accession IPR001757: ATPase, E1-E2 type | chr5:25176456-25183574 FORWARD | Aliases: MRG21.9, MRG21_9	8.2	9.2	-1.0	-1.9	100.0%	-3.0
2947	AT5G36890.2 | similar to glycosyl hydrolase family 1 protein [Arabidopsis thaliana] (TAIR:At1g26560.1); similar to putative latex cyanogenic beta glucosidase [Oryza sativa (japonica cultivar-group)] (GB:BAD82183.1); contains InterPro domain Glycoside hydrolase, family 1 (InterPro:IPR001360) | chr5:14558580-14563459 REVERSE | Aliases: MLF18.1, MLF18_1	7.1	8.2	-1.1	-1.9	100.0%	-3.4
2948	AT3G09000.1 | proline-rich family protein | chr3:2745649-2748464 FORWARD | Aliases: T16O11.4	5.1	5.5	-0.4	-1.9	100.0%	-3.7

Rank	Description	15.dpi	5.dpi	M	t	adj.q	B
2949	AT2G17720.1 | oxidoreductase, 2OG-Fe(II) oxygenase family protein, similar to prolyl 4-hydroxylase, alpha subunit, from Gallus gallus (GI:212530), Rattus norvegicus (GI:474940), Mus musculus (SP:Q60715); contains PF03171 2OG-Fe(II) oxygenase superfamily domain | chr2:7704418-7706636 FORWARD | Aliases: T17A5.10, T17A5_10	7.4	8.0	-0.6	-1.9	100.0%	-3.5
2950	AT3G29030.1 | Symbol: ATEXPA5 | expansin, putative (EXP5), identical to expansin At-EXP5 GB:AAB38071 from (Arabidopsis thaliana); alpha-expansin gene family, PMID:11641069 | chr3:11012545-11014595 REVERSE | Aliases: ARABIDOPSIS THALIANA EXPANSIN A5, ATEXP5, ATHEXP ALPHA 1.4, EXPANSIN 5, K5K13.14	4.2	3.6	0.5	1.9	100.0%	-3.4
2951	AT2G48020.2 | sugar transporter, putative, similar to ERD6 protein {Arabidopsis thaliana} GI:3123712, sugar-porter family proteins 1 and 2 (Arabidopsis thaliana) GI:14585699, GI:14585701; contains Pfam profile PF00083: major facilitator superfamily protein | chr2:19651325-19654250 FORWARD | Aliases: T9J23.17	8.0	7.4	0.6	1.9	100.0%	-3.3
2952	AT4G16150.1 | calmodulin-binding protein, similar to anther ethylene-upregulated calmodulin-binding protein ER1 GI:11612392 from (Nicotiana tabacum) | chr4:9148239-9153064 FORWARD | Aliases: DL4115W, FCAALL.291	6.5	7.4	-0.9	-1.9	100.0%	-3.5
2953	AT5G62710.1 | leucine-rich repeat family protein / protein kinase family protein, contains protein kinase domain, Pfam:PF00069; contains leucine-rich repeats, Pfam:PF00560 | chr5:25204529-25207826 FORWARD | Aliases: MQB2.1, MQB2_1	3.5	3.8	-0.3	-1.9	100.0%	-3.6
2954	AT1G05060.1 | expressed protein | chr1:1451057-1452871 REVERSE | Aliases: T7A14.7, T7A14_7	6.3	5.3	1.0	1.8	100.0%	-3.1
2955	AT1G49860.1 | Symbol: ATGSTF14 | glutathione S-transferase, putative, similar to GI:860955 from (Hyoscyamus muticus) (Plant Physiol. 109 (1), 253-260 (1995))	2.5	3.5	-1.0	-1.8	100.0%	-2.9
2956	AT4G19810.1 | glycosyl hydrolase family 18 protein, similar to chitinase/lysozyme GI:467689 from (Nicotiana tabacum) | chr4:10764046-10765763 REVERSE | Aliases: T16H5.170, T16H5_170	3.5	4.4	-0.9	-1.8	100.0%	-3.2
2957	AT1G16610.2 | Symbol: SR45 | arginine/serine-rich protein, putative (SR45), similar to arginine/serine-rich protein GI:6601502 from (Arabidopsis thaliana) | chr1:5675723-5678734 REVERSE | Aliases: ARGININE/SERINE RICH PROTEIN SR45, F19K19.9, F19K19_9	7.5	8.3	-0.8	-1.8	100.0%	-3.1
2958	AT5G51100.1 | Symbol: FSD2 | superoxide dismutase (Fe), putative / iron superoxide dismutase, putative, similar to Fe-superoxide dismutase precursor (Medicago sativa) gi:16974682:gb:AAL32441 | chr5:20790522-20792898 REVERSE | Aliases: FE SUPEROXIDE DISMUTASE 2, MWD22.4, MWD22_4	4.1	4.8	-0.8	-1.8	100.0%	-3.2
2959	AT4G35620.1 | Symbol: CYCB2;2 | cyclin 2b (CYC2b), identical to cyclin 2b protein (Arabidopsis thaliana) GI:509423 | chr4:16901647-16903916 FORWARD | Aliases: CYCB2;2, Cyclin B2;2, F8D20.130, F8D20_130	4.1	4.6	-0.5	-1.8	100.0%	-3.4
2960	AT2G22230.1 | beta-hydroxyacyl-ACP dehydratase, putative, similar to beta-hydroxyacyl-ACP dehydratase from Toxoplasma gondii (GI:3850997); contains Pfam profile PF01377 Thioester dehydratase	5.3	6.1	-0.8	-1.8	100.0%	-3.2
2961	AT4G15430.1 | early-responsive to dehydration protein-related / ERD protein-related, similar to ERD4 protein (early-responsive to dehydration stress) (Arabidopsis thaliana) GI:15375406; contains Pfam profile PF02714: Domain of unknown function DUF221 | chr4:8827876-8831503 FORWARD | Aliases: DL3760W, FCAALL.292	4.5	4.1	0.4	1.8	100.0%	-3.5
2962	AT4G37780.1 | myb family transcription factor (MYB87), identical to AtMYB87 R2R3-MYB transcription factor GI:2832559 from (Arabidopsis thaliana); contains Pfam profile: PF00249 myb-like DNA-binding domain | chr4:17758237-17759493 REVERSE | Aliases: T28I19.60, T28I19_60	3.7	4.5	-0.8	-1.8	100.0%	-3.1
2963	AT4G20230.1 | terpene synthase/cyclase family protein, vetispiradiene synthase, Hyoscyamus muticus, PATX:G763421 | chr4:10924221-10926761 REVERSE | Aliases: F1C12.150, F1C12_150	2.4	2.8	-0.4	-1.8	100.0%	-3.7
2964	AT2G27710.3 | 60S acidic ribosomal protein P2 (RPP2B) | chr2:11823868-11824944 FORWARD | Aliases: F15K20.19, F15K20_19	9.6	10.6	-0.9	-1.8	100.0%	-3.2
2965	AT3G48150.1 | Symbol: APC8 | cell division cycle family protein / CDC family protein, similar to cell division cycle protein 23 (Homo sapiens) GI:3283051, anaphase-promoting complex subunit 8 (Homo sapiens) GI:6180017; contains Pfam profile PF00515: TPR Domain | chr3:17790641-17793630 REVERSE | Aliases: CDC23, T24C20.30	6.2	6.8	-0.6	-1.8	100.0%	-3.4
2966	AT5G54130.1 | calcium-binding EF hand family protein, contains INTERPRO:IPR002048 calcium-binding EF-hand domain | chr5:21981074-21982505 FORWARD | Aliases: MJP23.11, MJP23_11	4.3	3.6	0.7	1.8	100.0%	-3.1
2967	AT4G28050.1 | senescence-associated protein, putative, similar to senescence-associated protein 5 (Hemerocallis hybrid cultivar) gi:3551954:gb:AAC34855 | chr4:13942013-13943738 REVERSE | Aliases: T13J8.160, T13J8_160	3.9	4.3	-0.4	-1.8	100.0%	-3.6
2968	AT1G61667.1 | expressed protein, contains Pfam profile PF04398: Protein of unknown function, DUF538 | chr1:22771328-22772160 FORWARD | Aliases: None	6.0	5.1	1.0	1.8	100.0%	-3.0

Rank	Description	15.dpi	5.dpi	M	t	adj.q	B
2969	AT3G54050.1 | fructose-1,6-bisphosphatase, putative / D-fructose-1,6-bisphosphate 1-phosphohydrolase, putative / FBPase, putative, strong similarity to fructose-1,6-bisphosphatase (Brassica napus) GI:289367; identical to SP:P25851 Fructose-1,6-bisphosphatase, chloroplast precursor (EC 3.1.3.11) (D-fructose-1,6-bisphosphate 1-phosphohydrolase) (FBPase) {Arabidopsis thaliana}; contains Pfam profile PF00316: fructose-1,6-bisphosphatase | chr3:20027891-20029726 FORWARD | Aliases: F24B22.10	3.8	3.4	0.4	1.8	100.0%	-3.4
2970	AT5G47870.1 | expressed protein | chr5:19401600-19403095 REVERSE | Aliases: MCA23.21, MCA23_21	7.7	8.1	-0.4	-1.8	100.0%	-3.5
2971	AT3G53020.1 | 60S ribosomal protein L24 (RPL24B), 60S ribosomal protein L24, Arabidopsis thaliana, EMBL:AC006282 | chr3:19671545-19673003 REVERSE | Aliases: F8J2.190	11.8	12.1	-0.3	-1.8	100.0%	-3.9
2972	AT2G42210.4 | expressed protein, similar to hypothetical protein [Oryza sativa (japonica cultivar-group)] (GB:XP_450480.1); contains InterPro domain Mitochondrial import inner membrane translocase, subunit Tim17/22 (InterPro:IPR003397) | chr2:17597291-17598930 FORWARD | Aliases: T24P15.12, T24P15_12	10.9	11.5	-0.6	-1.8	100.0%	-3.6
2973	AT1G71010.1 | phosphatidylinositol-4-phosphate 5-kinase family protein, low similarity to phosphatidylinositol 3,5-kinase (Candida albicans) GI:14571648; contains Pfam profile PF01504: Phosphatidylinositol-4-phosphate 5-Kinase | chr1:26786501-26792630 FORWARD | Aliases: F23N20.21	8.3	7.9	0.4	1.8	100.0%	-3.5
2974	AT2G34040.2 | apoptosis inhibitory 5 (API5) family protein, contains Pfam domain PF05918 Apoptosis inhibitory protein 5 (API5)	6.4	7.1	-0.7	-1.8	100.0%	-3.4
2975	AT3G11420.1 | fringe-related protein, similar to hypothetical protein GB:AAC23643 (Arabidopsis thaliana) + weak similarity to Fringe (Schistocerca gregaria)(GI:6573138);Fringe encodes an extracellular protein that regulates Notch signalling. | chr3:3591481-3594539 FORWARD | Aliases: F24K9.9	5.0	4.5	0.4	1.8	100.0%	-3.4
2976	AT1G14460.1 | DNA polymerase-related, weak similarity to DNA polymerase III holoenzyme tau subunit (Thermus thermophilus) GI:2583049 | chr1:4948743-4952922 REVERSE | Aliases: F14L17.24, F14L17_24	5.0	5.4	-0.4	-1.8	100.0%	-3.5
2977	AT1G74950.1 | expressed protein | chr1:28152236-28154055 REVERSE | Aliases: F25A4.8, F25A4_8	6.8	5.5	1.3	1.8	100.0%	-3.1
2978	AT5G17280.1 | expressed protein, similar to unknown protein (pir::T41692) | chr5:5686301-5686889 FORWARD | Aliases: MKP11.13, MKP11_13	6.8	6.0	0.8	1.8	100.0%	-3.3
2979	AT1G53140.1 | dynamin family protein, low similarity to dynamin-like protein E (Arabidopsis thaliana) GI:19423872; contains Pfam profile PF00350: Dynamin family | chr1:19802906-19806262 FORWARD | Aliases: F8L10.1, F8L10_1	5.2	5.5	-0.3	-1.8	100.0%	-3.8
2980	AT3G01210.1 | similar to RNA recognition motif (RRM)-containing protein [Arabidopsis thaliana] (TAIR:At1g14340.1); similar to hypothetical protein [Solanum demissum] (GB:AAT40482.1); contains InterPro domain RNA-binding region RNP-1 (RNA recognition motif) (InterPro:IPR000504)	6.2	5.3	0.9	1.8	100.0%	-3.4
2981	AT3G07370.1 | tetratricopeptide repeat (TPR)-containing protein / U-box domain-containing protein, similar to serologically defined colon cancer antigen 7 GB:5031963 GI:3170178 (Homo sapiens); | chr3:2358091-2360555 REVERSE | Aliases: F21O3.8	4.9	4.5	0.4	1.8	100.0%	-3.6
2982	AT2G18660.1 | expansin family protein (EXPR3), identical to Expansin-related protein 3 precursor (Ath-ExpGamma-1.2) (Swiss-Prot:Q9ZV52) (Arabidopsis thaliana); contains Prosite PS00092: N-6 Adenine-specific DNA methylases signature; | chr2:8097740-8098738 REVERSE | Aliases: MSF3.4, MSF3_4	3.1	2.6	0.5	1.8	100.0%	-3.7
2983	AT3G62680.1 | Symbol: PRP3 | proline-rich family protein, contains proline-rich region, INTERPRO:IPR000694 | chr3:23193698-23194970 FORWARD | Aliases: ATPRP3, F26K9.110, PROLINE RICH PROTEIN 3	4.6	5.3	-0.7	-1.8	100.0%	-3.2
2984	AT1G13120.1 | Symbol: EMB1745 | expressed protein, contains Prosite PS00012: Phosphopantetheine attachment site; similar to GLE1 (GI:3288817) {Homo sapiens}; EST gb:N37870 comes from this gene | chr1:4469181-4473211 REVERSE | Aliases: EMB1745, EMBRYO DEFECTIVE 1745, F3F19.14, F3F19_14	6.1	6.7	-0.5	-1.8	100.0%	-3.4
2985	AT5G22710.1 | expressed protein | chr5:7547800-7549372 REVERSE | Aliases: MDJ22.13, MDJ22_13	4.0	3.5	0.5	1.8	100.0%	-3.4
2986	AT5G04020.1 | calmodulin-binding protein-related (PICBP), contains similarity to potato calmodulin-binding protein PCBP GI:17933110 from (Solanum tuberosum) | chr5:1081610-1086545 REVERSE | Aliases: F8F6.230, F8F6_230	5.7	6.2	-0.5	-1.8	100.0%	-3.4
2987	AT5G09990.1 | expressed protein | chr5:3124569-3125073 FORWARD | Aliases: MYH9.1	3.6	3.1	0.5	1.8	100.0%	-3.8
2988	AT5G08470.1 | Symbol: PEX1 | peroxisome biogenesis protein (PEX1), identical to peroxisome biogenesis protein PEX1 (Arabidopsis thaliana) gi:12006272:gb:AAG44817; contains Pfam profile PF00004: ATPase, AAA family; identical to cDNA peroxisome biogenesis protein PEX1 (PEX1) mRNA, partial cds GI:12006271 | chr5:2735926-2743057 FORWARD | Aliases: F8L15.15, PEROXISOME 1	3.5	3.9	-0.4	-1.8	100.0%	-3.6
2989	AT2G29990.1 | pyridine nucleotide-disulphide oxidoreductase family protein, similar to SP:P32340 Rotenone-insensitive NADH-ubiquinone oxidoreductase, mitochondrial precursor (EC 1.6.5.3) (Internal NADH dehydrogenase) {Saccharomyces cerevisiae} ; contains Pfam profile PF00070: Pyridine nucleotide-disulphide oxidoreductase | chr2:12800639-12802990 REVERSE | Aliases: F23F1.9, F23F1_9	6.8	6.1	0.7	1.8	100.0%	-3.2

Rank	Description	15.dpi	5.dpi	M	t	adj.q	B
2990	AT2G06530.1 | SNF7 family protein, contains Pfam domain, PF03357: SNF7 family | chr2:2588542-2590442 REVERSE | Aliases: T12H3.8, T12H3_8	9.7	9.0	0.7	1.8	100.0%	-3.4
2991	AT2G19990.1 | Symbol: PR 1 LIKE | pathogenesis-related protein 1 (PR-1), identical to pathogenesis-related protein 1 {Arabidopsis thaliana} GI:166805; contains an extracellular proteins SCP/Tpx-1/Ag5/PR-1/Sc7 signature (PDOC00772) | chr2:8637146-8637676 REVERSE | Aliases: PATHOGENESIS RELATED PROTEIN 1 LIKE, PR 1 LIKE, T2G17.21, T2G17_21	2.6	2.9	-0.3	-1.8	100.0%	-3.7
2992	AT1G10490.1 | expressed protein, contains Pfam profile PF05127: Putative ATPase (DUF699) | chr1:3453230-3460621 FORWARD | Aliases: T10O24.10, T10O24_10	3.5	3.9	-0.4	-1.8	100.0%	-3.6
2993	AT2G45160.1 | scarecrow transcription factor family protein | chr2:18624937-18627225 REVERSE | Aliases: T14P1.3	4.9	5.3	-0.4	-1.8	100.0%	-3.7
2994	AT4G24740.2 | Symbol: AFC2 | protein kinase (AFC2), identical to protein kinase AFC2 (Arabidopsis thaliana) SWISS-PROT:P51567	7.1	6.5	0.6	1.8	100.0%	-3.3
2995	AT2G30460.1 | expressed protein, contains 4 predicted transmembrane domains; similar to c_pp004044298r (GI:14597790) (Physcomitrella patens) | chr2:12983526-12985566 REVERSE | Aliases: T6B20.18, T6B20_18	3.8	4.5	-0.7	-1.8	100.0%	-3.2
2996	AT5G56150.2 | ubiquitin-conjugating enzyme, putative, strong similarity to ubiquitin-conjugating enzyme UBC2 (Mesembryanthemum crystallinum) GI:5762457, UBC4 (Pisum sativum) GI:456568; contains Pfam profile PF00179: Ubiquitin-conjugating enzyme | chr5:22746408-22748409 FORWARD | Aliases: MDA7.21, MDA7_21	11.1	11.5	-0.4	-1.8	100.0%	-3.9
2997	AT5G13560.1 | expressed protein, weak similarity to SP:O42184 Restin (Cytoplasmic linker protein-170) (CLIP-170) {Gallus gallus} | chr5:4360634-4366413 REVERSE | Aliases: T6I14.4	7.6	6.8	0.8	1.8	100.0%	-3.3
2998	AT5G19200.1 | short-chain dehydrogenase/reductase (SDR) family protein, similar to follicular variant translocation protein 1 precursor (FVT-1) SP:Q06136 from (Homo sapiens) | chr5:6458997-6460858 FORWARD | Aliases: T24G5.100, T24G5_100	4.3	4.0	0.3	1.8	100.0%	-3.7
2999	AT4G28940.1 | nucleosidase-related, contains weak similarity to MTA/SAH nucleosidase (P46). (Swiss-Prot:P24247) (Shigella flexneri) | chr4:14275811-14277101 FORWARD | Aliases: F25O24.60, F25O24_60	3.0	3.3	-0.3	-1.8	100.0%	-3.8
3000	AT4G02430.2 | pre-mRNA splicing factor, putative / SR1 protein, putative, strong similarity to SP:O22315 Pre-mRNA splicing factor SF2 (SR1 protein) {Arabidopsis thaliana}; cDNA NCBI_gi:15810292 supports a truncated version while protein evidence supports a longer model.	5.4	4.6	0.8	1.8	100.0%	-3.4
3001	AT2G28130.1 | expressed protein | chr2:11995753-11998694 FORWARD | Aliases: F24D13.8, F24D13_8	4.6	5.0	-0.4	-1.8	100.0%	-3.7
3002	AT3G18524.1 | Symbol: MSH2 | DNA mismatch repair protein MSH2 (MSH2), identical to SP:O24617 DNA mismatch repair protein MSH2 (AtMsh2) {Arabidopsis thaliana}	3.8	4.3	-0.5	-1.8	100.0%	-3.4
3003	AT1G62870.1 | similar to expressed protein [Arabidopsis thaliana] (TAIR:At1g12380.1); similar to OSJNBa0013A04.12 [Oryza sativa (japonica cultivar-group)] (GB:CAE05175.2); contains domain HIS_RICH (PS50316)	3.3	4.1	-0.7	-1.8	100.0%	-3.3
3004	AT3G02070.1 | OTU-like cysteine protease family protein, contains Pfam profile PF02338: OTU-like cysteine protease | chr3:360928-363336 FORWARD | Aliases: F1C9.14, F1C9_14	5.1	4.5	0.6	1.8	100.0%	-3.1
3005	AT2G45980.1 | expressed protein | chr2:18924425-18926182 REVERSE | Aliases: F4I18.37, F4I18_37	8.2	7.3	0.9	1.8	100.0%	-3.2
3006	AT3G49010.3 | Symbol: ATBBC1 |	12.6	13.2	-0.5	-1.8	100.0%	-3.9
3007	AT3G44590.2 | 60S acidic ribosomal protein P2 (RPP2D), acidic ribosomal protein P2, maize, PIR:S54179	9.9	10.4	-0.5	-1.8	100.0%	-3.4
3008	AT5G45280.2 | pectinacetylesterase, putative, similar to pectinacetylesterase precursor GI:1431629 from (Vigna radiata) | chr5:18362822-18366919 FORWARD | Aliases: K9E15.6, K9E15_6	4.9	5.3	-0.4	-1.8	100.0%	-3.4
3009	AT1G68550.2 | encodes a member of the ERF (ethylene response factor) subfamily B-6 of ERF/AP2 transcription factor family. The protein contains one AP2 domain. There are 12 members in this subfamily including RAP2.11. | chr1:25729118-25731049 REVERSE | Aliases: T26J14.12, T26J14_12	4.6	5.0	-0.4	-1.8	100.0%	-3.5
3010	AT3G12750.1 | Symbol: ZIP1 | zinc transporter (ZIP1), identical to putative zinc transporter GB:AAC24197 from (Arabidopsis thaliana), (Proc. Natl. Acad. Sci. U.S.A. 95 (12), 7220-7224 (1998)); member of the Zinc (Zn2+)-Iron (Fe2+) permease (ZIP) family, PMID:11500563 | chr3:4051658-4053187 REVERSE | Aliases: MBK21.8, ZRT, IRT LIKE PROTEIN	4.7	4.2	0.6	1.8	100.0%	-3.4
3011	AT5G09980.1 | expressed protein | chr5:3122757-3123909 FORWARD | Aliases: MYH9.20, MYH9_20	4.3	3.6	0.7	1.8	100.0%	-3.7

Rank	Description	15.dpi	5.dpi	M	t	adj.q	B
3012	AT5G18470.1 | curculin-like (mannose-binding) lectin family protein, contains Pfam profile: PF01453 lectin (probable mannose binding) | chr5:6127799-6129679 FORWARD | Aliases: T28N17.6	3.9	4.4	-0.5	-1.8	100.0%	-3.5
3013	AT2G29080.1 | Symbol: FTSH3 | encodes an FtsH protease that is localized to the mitochondrion	7.2	8.2	-1.0	-1.8	100.0%	-3.4
3014	AT4G27750.1 | expressed protein | chr4:13841694-13843639 FORWARD | Aliases: T29A15.240, T29A15_240	10.3	9.5	0.8	1.8	100.0%	-3.3
3015	AT1G16060.2 | ovule development protein, putative, similar to ovule development protein AINTEGUMENTA (GI:1209099)(Arabidopsis thaliana) | chr1:5508537-5511729 FORWARD | Aliases: T24D18.16, T24D18_16	3.0	3.7	-0.6	-1.8	100.0%	-3.3
3016	AT2G37110.1 | expressed protein, contains Pfam profile PF04749: Protein of unknown function, DUF614 | chr2:15599636-15601072 FORWARD | Aliases: T2N18.13, T2N18_13	9.2	8.5	0.7	1.8	100.0%	-3.6
3017	AT1G09430.1 | Symbol: ACLA 3 |	8.4	9.0	-0.6	-1.8	100.0%	-3.6
3018	AT1G50575.1 | lysine decarboxylase family protein, contains Pfam profile PF03641: lysine decarboxylase family | chr1:18732387-18734055 REVERSE | Aliases: None	6.8	7.5	-0.7	-1.8	100.0%	-3.1
3019	AT3G06450.1 | anion exchange family protein, similar to putative Anion exchanger family members: GB:AAD39673, GB:AAD55295 (Arabidopsis thaliana) | chr3:1975965-1980042 REVERSE | Aliases: F24P17.6, F24P17_6	7.1	7.7	-0.6	-1.8	100.0%	-3.2
3020	AT1G23890.2 | NHL repeat-containing protein, contains Pfam profile PF01436: NHL repeat	6.3	5.3	1.0	1.8	100.0%	-3.3
3021	AT1G16870.1 | mitochondrial 28S ribosomal protein S29-related, contains weak similarity to Swiss-Prot:P51398 mitochondrial 28S ribosomal protein S29 (MRP-S29, Death-associated protein 3, DAP-3) (Homo sapiens) | chr1:5770728-5773241 REVERSE | Aliases: F17F16.14	7.5	8.5	-0.9	-1.8	100.0%	-3.2
3022	AT4G32970.1 | expressed protein, low similarity to SP:Q13061 Triadin {Homo sapiens} | chr4:15910348-15914328 REVERSE | Aliases: F26P21.90, F26P21_90	4.9	5.9	-0.9	-1.8	100.0%	-3.1
3023	AT4G38920.1 | vacuolar ATP synthase 16 kDa proteolipid subunit 3 / V-ATPase 16 kDa proteolipid subunit 3 (AVAP3) (AVA-P3), identical to SP:P59227 Vacuolar ATP synthase 16 kDa proteolipid subunit 1/3/5 (EC 3.6.3.14) (V-ATPase 16 kDa proteolipid subunit 1/3/5) {Arabidopsis thaliana}; contains Pfam profile PF00137: ATP synthase subunit C | chr4:18147205-18149261 FORWARD | Aliases: F19H22.20	11.0	10.3	0.8	1.8	100.0%	-3.7
3024	AT4G30440.1 | Symbol: GAE1 | NAD-dependent epimerase/dehydratase family protein, similar to nucleotide sugar epimerase from Vibrio vulnificus GI:3093975 (PID:g3093975), WbnF (Escherichia coli) GI:5739472, CAPI protein {Staphylococcus aureus} SP:P39858; contains Pfam profile PF01370 NAD dependent epimerase/dehydratase family	8.1	7.6	0.6	1.8	100.0%	-3.5
3025	AT5G13120.1 | peptidyl-prolyl cis-trans isomerase cyclophilin-type family protein, contains Pfam domain, PF00160: peptidyl-prolyl cis-trans isomerase, cyclophilin-type | chr5:4162506-4164787 REVERSE | Aliases: T19L5.80, T19L5_80	9.0	9.6	-0.5	-1.8	100.0%	-3.8
3026	AT4G34555.1 | 40S ribosomal protein S25, putative | chr4:16504235-16505477 REVERSE | Aliases: None	7.7	8.6	-0.9	-1.8	100.0%	-3.1
3027	AT3G25660.1 | glutamyl-tRNA(Gln) amidotransferase, putative, similar to SP:O06491 Glutamyl-tRNA(Gln) amidotransferase subunit A (EC 6.3.5.-) {Bacillus subtilis}; contains Pfam profile PF01425: Amidase | chr3:9340678-9343441 REVERSE | Aliases: T5M7.15, T5M7_15	6.9	7.3	-0.4	-1.8	100.0%	-3.5
3028	AT3G05040.1 | Symbol: HST | similar to P0503E05.11 [Oryza sativa (japonica cultivar-group)] (GB:NP_918909.1) | chr3:1401322-1408101 REVERSE | Aliases: HASTY, T9J14.1, T9J14_1	4.0	4.6	-0.5	-1.8	100.0%	-3.3
3029	AT4G15530.3 | similar to pyruvate,orthophosphate dikinase [Flaveria brownii] (GB:CAA55784.1); similar to pyruvate,orthophosphate dikinase [Mesembryanthemum crystallinum] (GB:CAA57872.1); similar to pyruvate,orthophosphate dikinase [Mesembryanthemum crystallinum] (GB:CAA55143.1); similar to cold stable pyruvate, orthophosphate dikinase (GB:AAA86940.1); similar to pyruvate, phosphate dikinase (EC 2.7.9.1) precursor, chloroplast - Flaveria brownii (GB:S56649); contains InterPro domain Pyruvate phosphate dikinase, PEP/pyruvate-binding (InterPro:IPR002192); contains InterPro domain PEP-utilizing enzyme (InterPro:IPR000121); contains InterPro domain PEP-utilising enzyme, mobile region (InterPro:IPR008279) | chr4:8864826-8870905 REVERSE | Aliases: DL3805C, FCAALL.325	5.7	5.0	0.7	1.8	100.0%	-3.1
3030	AT3G06350.1 | Symbol: EMB3004 | dehydroquinate dehydratase, putative / shikimate dehydrogenase, putative, similar to dehydroquinate dehydratase/shikimate dehydrogenase (Nicotiana tabacum)(GI:535771), dehydroquinate dehydratase/shikimate:NADP oxidoreductase (Lycopersicon esculentum)(GI:3169883) | chr3:1924368-1927759 REVERSE | Aliases: EMB3004, EMBRYO DEFECTIVE 3004, F24P17.18, F24P17_18	9.5	10.1	-0.7	-1.8	100.0%	-3.3

Rank	Description	15.dpi	5.dpi	M	t	adj.q	B
3031	AT2G19730.2 | similar to 60S ribosomal protein L28 (RPL28C) [Arabidopsis thaliana] (TAIR:At4g29410.1); similar to putative 60S ribosomal L28 protein [Oryza sativa (japonica cultivar-group)] (GB:AAV67824.1); contains InterPro domain Ribosomal L28e protein (InterPro:IPR002672) | chr2:8518589-8520303 FORWARD | Aliases: F6F22.24, F6F22_24	11.2	11.7	-0.5	-1.8	100.0%	-3.5
3032	AT5G03490.1 | UDP-glucoronosyl/UDP-glucosyl transferase family protein, contains Pfam profile: PF00201 UDP-glucoronosyl and UDP-glucosyl transferase | chr5:871459-873046 FORWARD | Aliases: F12E4.260, F12E4_260	6.8	6.2	0.6	1.8	100.0%	-3.5
3033	AT4G15120.1 | VQ motif-containing protein, contains PF05678: VQ motif | chr4:8634722-8635558 FORWARD | Aliases: DL3605W, FCAALL.67	3.0	3.4	-0.4	-1.8	100.0%	-3.5
3034	AT5G55920.1 | nucleolar protein, putative, similar to SP:P46087 Proliferating-cell nucleolar antigen p120 (Proliferation-associated nucleolar protein p120) {Homo sapiens}, SP:P40991 Nucleolar protein NOP2 {Saccharomyces cerevisiae}; contains Pfam profile PF01189: NOL1/NOP2/sun family | chr5:22662709-22666673 REVERSE | Aliases: None	9.2	9.9	-0.6	-1.8	100.0%	-3.4
3035	AT1G51140.1 | basic helix-loop-helix (bHLH) family protein, contains Pfam profile: PF00010 helix-loop-helix DNA-binding domain	5.5	4.9	0.7	1.8	100.0%	-3.3
3036	AT5G53920.1 | ribosomal protein L11 methyltransferase-related, similar to ribosomal protein L11 methyltransferase; PrmA (Escherichia coli) GI:455655 | chr5:21909350-21911493 FORWARD | Aliases: K19P17.9, K19P17_9	2.7	3.0	-0.3	-1.8	100.0%	-3.9
3037	AT3G55370.2 | Symbol: OBP3 | Dof-type zinc finger domain-containing protein | chr3:20538053-20540268 FORWARD | Aliases: OBF BINDING PROTEIN 3, T22E16.30, ZINC FINGER PROTEIN OBP3	3.3	3.0	0.4	1.8	100.0%	-3.7
3038	AT4G37610.1 | TAZ zinc finger family protein / BTB/POZ domain-containing protein, contains Pfam PF00651 : BTB/POZ domain; contains Pfam PF02135 : TAZ zinc finger; similar to Speckle-type POZ protein (SP:O43791) (Homo sapiens) | chr4:17670475-17672334 REVERSE | Aliases: F19F18.100, F19F18_100	7.6	6.9	0.7	1.8	100.0%	-3.4
3039	AT4G14200.1 | expressed protein | chr4:8185365-8188719 REVERSE | Aliases: DL3140C, FCAALL.116	2.9	3.2	-0.3	-1.8	100.0%	-3.6
3040	AT4G28740.1 | expressed protein | chr4:14200995-14202651 FORWARD | Aliases: F16A16.150, F16A16_150	8.1	7.5	0.6	1.8	100.0%	-3.4
3041	AT1G06180.1 | Symbol: ATMYB13 | myb family transcription factor, identical to	2.5	2.9	-0.4	-1.8	100.0%	-3.7
3042	AT1G74020.1 | Symbol: SS2 | strictosidine synthase family protein, similar to strictosidine synthase (Rauvolfia serpentina)(SP:P15324); contains strictosidine synthase domain PF03088 | chr1:27838792-27840970 REVERSE | Aliases: ATSS 2 STRICTOSIDINE SYNTHASE, F2P9.11, F2P9_11, STRICTOSIDINE SYNTHASE 2	11.0	11.4	-0.4	-1.8	100.0%	-3.7
3043	AT4G03070.1 | Symbol: AOP1 | 2-oxoglutarate-dependent dioxygenase (AOP1.2), identical to GI:16118887; contains PF03171: 2OG-Fe(II) oxygenase superfamily domain | chr4:1358432-1359644 FORWARD | Aliases: AOP, AOP1.1, T4I9.5, T4I9_5	3.3	2.9	0.4	1.8	100.0%	-3.6
3044	AT3G21220.1 | Symbol: ATMKK5 |	4.5	4.1	0.4	1.8	100.0%	-3.7
3045	AT4G26780.1 | Symbol: AR192 | co-chaperone grpE family protein, similar to chaperone GrpE type 2 (Nicotiana tabacum) GI:3851640; contains Pfam profile PF01025: co-chaperone GrpE | chr4:13484845-13486777 REVERSE | Aliases: F10M23.120, F10M23_120	7.5	8.3	-0.7	-1.8	100.0%	-3.3
3046	AT2G29760.1 | pentatricopeptide (PPR) repeat-containing protein, contains Pfam profile PF01535: PPR repeat | chr2:12719961-12722177 FORWARD | Aliases: T27A16.14, T27A16_14	4.5	4.9	-0.4	-1.8	100.0%	-3.6
3047	AT1G47900.1 | expressed protein | chr1:17650813-17654704 REVERSE | Aliases: T6B12.1, T6B12_1	6.0	6.7	-0.7	-1.8	100.0%	-3.4
3048	AT1G07030.1 | mitochondrial substrate carrier family protein, contains Pfam profile: PF00153 mitochondrial carrier protein | chr1:2158419-2160808 REVERSE | Aliases: F10K1.26, F10K1_26	5.1	5.5	-0.4	-1.8	100.0%	-3.6
3049	AT2G32380.1 | expressed protein | chr2:13753688-13754868 REVERSE | Aliases: T32F6.10, T32F6_10	3.6	4.2	-0.5	-1.8	100.0%	-3.5
3050	AT3G15530.2 | expressed protein | chr3:5252882-5254465 REVERSE | Aliases: MJK13.19	7.5	6.9	0.6	1.8	100.0%	-3.5
3051	AT1G29710.1 | pentatricopeptide (PPR) repeat-containing protein, contains Pfam profile PF01535: PPR repeat | chr1:10387659-10389086 FORWARD | Aliases: T3M22.4, T3M22_4	3.2	3.4	-0.2	-1.8	100.0%	-4.0
3052	AT2G40330.1 | Bet v I allergen family protein, contains Pfam profile PF00407: Pathogenesis-related protein Bet v I family | chr2:16851942-16853012 REVERSE | Aliases: T7M7.15	4.6	3.9	0.7	1.8	100.0%	-3.2

Rank	Description	15.dpi	5.dpi	M	t	adj.q	B
3053	AT3G62030.1 | Symbol: ROC4 | peptidyl-prolyl cis-trans isomerase, chloroplast / cyclophilin / rotamase / cyclosporin A-binding protein (ROC4), identical to peptidyl-prolyl cis-trans isomerase, chloroplast precursor, PPIase (cyclophilin, cyclosporin A-binding protein) (Arabidopsis thaliana) SWISS-PROT:P34791; identical to cDNA nuclear-encoded chloroplast stromal cyclophilin (ROC4) GI:405130 | chr3:22984585-22986345 FORWARD | Aliases: PEPTIDYLPROLYL ISOMERASE ROC4, ROTAMASE CYCLOPHILIN 4, ROTAMASE CYP 4, T17J13.1	9.0	9.9	-0.9	-1.8	100.0%	-3.1
3054	AT2G19430.1 | transducin family protein / WD-40 repeat family protein, similar to WD-repeat protein 5 (SP:Q9UGP9) (Homo sapiens); contains 7 Trp-Asp WD-40 repeats | chr2:8422241-8424998 FORWARD | Aliases: F27F23.25	5.1	4.6	0.6	1.8	100.0%	-3.4
3055	AT3G07210.1 | expressed protein, predicted using genefinder | chr3:2292804-2295458 REVERSE | Aliases: T1B9.12	3.4	3.9	-0.5	-1.8	100.0%	-3.4
3056	AT5G13100.1 | expressed protein | chr5:4156663-4158539 FORWARD | Aliases: T19L5.60, T19L5_60	6.1	5.5	0.6	1.8	100.0%	-3.2
3057	AT1G51660.1 | Symbol: ATMKK4 |	4.3	3.9	0.4	1.8	100.0%	-3.7
3058	AT2G32030.1 | GCN5-related N-acetyltransferase (GNAT) family protein, contains Pfam profile PF00583: acetyltransferase, GNAT family | chr2:13639545-13640412 REVERSE | Aliases: F22D22.22, F22D22_22	4.1	3.7	0.4	1.8	100.0%	-3.6
3059	AT5G47800.1 | phototropic-responsive NPH3 family protein, contains NPH3 family domain, Pfam:PF03000 | chr5:19371053-19373526 FORWARD | Aliases: MCA23.12, MCA23_12	3.2	3.5	-0.3	-1.8	100.0%	-3.8
3060	AT1G11420.1 | agenet domain-containing protein, contains Pfam PF05641: Agenet domain | chr1:3844581-3846872 REVERSE | Aliases: T23J18.9, T23J18_9	5.0	6.0	-1.0	-1.8	100.0%	-3.3
3061	AT4G38470.1 | protein kinase family protein, similar to protein kinase (gi:170047) from Glycine max; contains Pfam protein kinase domain PF00069 | chr4:17999426-18003675 FORWARD | Aliases: F20M13.30, F20M13_30	6.1	6.8	-0.7	-1.8	100.0%	-3.5
3062	AT1G12030.1 | expressed protein, contains Pfam profile PF04720: Protein of unknown function (DUF506) | chr1:4064749-4066317 REVERSE | Aliases: F12F1.10, F12F1_10	3.8	4.5	-0.7	-1.8	100.0%	-3.3
3063	AT4G38670.1 | pathogenesis-related thaumatin family protein, similar to receptor serine/threonine kinase PR5K (Arabidopsis thaliana) GI:1235680; contains Pfam profile PF00314: Thaumatin family | chr4:18069610-18071346 REVERSE | Aliases: T9A14.1	3.9	3.1	0.8	1.8	100.0%	-3.5
3064	AT2G45130.1 | SPX (SYG1/Pho81/XPR1) domain-containing protein, weak similarity to NUC-2 (Neurospora crassa) GI:1399532, xenotropic and polytropic murine leukemia virus receptor (Mus musculus castaneus) GI:6093320; contains Pfam profile PF03105: SPX domain | chr2:18613485-18614928 FORWARD | Aliases: T14P1.6	4.1	3.4	0.7	1.8	100.0%	-3.5
3065	AT5G13840.1 | WD-40 repeat family protein, contains 6 WD-40 repeats (PF00400); similar to Fzr1 (GI:6463679){Homo sapiens} | chr5:4468519-4470994 REVERSE | Aliases: MAC12.21, MAC12_21	7.0	7.7	-0.7	-1.8	100.0%	-3.5
3066	AT4G10790.1 | UBX domain-containing protein, low similarity to SP:Q9UNN5 FAS-associated factor 1 (FAF1 protein) {Homo sapiens}; contains Pfam profile PF00789: UBX domain | chr4:6640609-6643058 REVERSE | Aliases: T12H20.9, T12H20_9	7.1	6.4	0.6	1.8	100.0%	-3.3
3067	AT1G71400.1 | disease resistance family protein / LRR family protein, contains leucine rich-repeat domains Pfam:PF00560, INTERPRO:IPR001611; similar to Hcr2-5D (Lycopersicon esculentum) gi:3894393:gb:AAC78596 | chr1:26913497-26916259 FORWARD | Aliases: F3I17.30, F3I17_30	5.0	5.5	-0.5	-1.8	100.0%	-3.3
3068	AT5G04790.1 | expressed protein | chr5:1388156-1388320 REVERSE | Aliases: MUK11.11	7.5	6.5	1.0	1.8	100.0%	-3.0
3069	AT5G15750.1 | RNA-binding S4 domain-containing protein, 40S RIBOSOMAL PROTEINs - different species | chr5:5141194-5142861 FORWARD | Aliases: F14F8.130, F14F8_130	8.3	8.7	-0.4	-1.8	100.0%	-3.4
3070	AT5G65020.1 | Symbol: ANNAT2 |	12.1	12.9	-0.8	-1.8	100.0%	-3.5
3071	AT3G09210.1 | KOW domain-containing transcription factor family protein, ; est match | chr3:2825290-2826886 REVERSE | Aliases: F3L24.8	8.0	8.7	-0.7	-1.8	100.0%	-3.1
3072	AT3G08940.2 | Symbol: LHCB4.2 | chlorophyll A-B binding protein (LHCB4.2), contains Pfam profile: PF00504 chlorophyll A-B binding protein; identical to Lhcb4.2 protein GB:AAD28774 (Arabidopsis thaliana) | chr3:2717681-2718874 FORWARD | Aliases: T16O11.12	8.2	7.2	1.0	1.8	100.0%	-3.2
3073	AT1G64405.1 | expressed protein | chr1:23927395-23927999 FORWARD | Aliases: None	2.9	3.5	-0.7	-1.8	100.0%	-3.3

Rank	Description	15.dpi	5.dpi	M	t	adj.q	B
3074	AT5G44800.1 | chromodomain-helicase-DNA-binding family protein / CHD family protein, similar to chromatin remodeling factor CHD3 (PICKLE) (Arabidopsis thaliana) GI:6478518; contains Pfam profiles PF00271: Helicase conserved C-terminal domain, PF00176: SNF2 family N-terminal domain, PF00628: PHD-finger, PF00385: 'chromo' (CHRromatin Organization MOdifier) | chr5:18100886-18109347 REVERSE | Aliases: K23L20.15, K23L20_15	3.5	3.8	-0.3	-1.8	100.0%	-3.9
3075	AT1G03350.1 | BSD domain-containing protein, contains Pfam profile PF03909: BSD domain	7.4	7.0	0.4	1.8	100.0%	-3.7
3076	AT3G59660.1 | C2 domain-containing protein / GRAM domain-containing protein, low similarity to GLUT4 vesicle protein (Rattus norvegicus) GI:4193489; contains Pfam profiles PF00168: C2 domain, PF02893: GRAM domain | chr3:22045155-22049634 REVERSE | Aliases: T16L24.210	5.3	5.9	-0.6	-1.8	100.0%	-3.2
3077	AT1G22550.1 | proton-dependent oligopeptide transport (POT) family protein, contains Pfam profile: PF00854 POT family | chr1:7966522-7968630 REVERSE | Aliases: F12K8.11, F12K8_11	6.9	6.3	0.6	1.8	100.0%	-3.2
3078	AT2G30390.1 | ferrochelatase II, identical to Swiss-Prot:O04921 ferrochelatase II, chloroplast precursor (EC 4.99.1.1) (Protoheme ferro-lyase) (Heme synthetase) (Arabidopsis thaliana) | chr2:12958104-12961167 REVERSE | Aliases: T9D9.1	7.7	8.4	-0.7	-1.8	100.0%	-3.2
3079	AT4G32730.2 | Symbol: PC MYB1 | myb family transcription factor, identical to PC-MYB1 GI:5678826 from (Arabidopsis thaliana);	3.5	3.9	-0.4	-1.8	100.0%	-3.9
3080	AT4G11550.1 | DC1 domain-containing protein, contains Pfam profile PF03107: DC1 domain	3.1	3.3	-0.3	-1.8	100.0%	-3.9
3081	AT5G28650.1 | Symbol: WRKY74 |	5.1	5.6	-0.5	-1.8	100.0%	-3.4
3082	AT4G37640.1 | Symbol: ACA2 | calcium-transporting ATPase 2, plasma membrane-type / Ca(2+)-ATPase isoform 2 (ACA2), identical to SP:O81108 Calcium-transporting ATPase 2, plasma membrane-type (EC 3.6.3.8) (Ca(2+)-ATPase isoform 2) {Arabidopsis thaliana} | chr4:17682971-17686935 REVERSE | Aliases: CALCIUM ATPASE 2, F19F18.130, F19F18_130	6.2	6.9	-0.8	-1.8	100.0%	-3.6
3083	AT3G13000.2 | expressed protein, contains Pfam profile PF04784: Protein of unknown function, DUF547 | chr3:4157975-4161948 REVERSE | Aliases: MGH6.15, MGH6_15	3.2	2.8	0.3	1.8	100.0%	-3.8
3084	AT1G70560.1 | alliinase C-terminal domain-containing protein, contains Pfam profiles: PF04864 allinase C-terminal domain, PF04863 alliinase EGF-like domain | chr1:26608518-26611166 FORWARD | Aliases: F24J13.13, F24J13_13	6.0	7.0	-0.9	-1.8	100.0%	-3.0
3085	AT5G25820.1 | exostosin family protein, contains Pfam profile: PF03016 exostosin family | chr5:8997186-9000318 REVERSE | Aliases: F18A17.70, F18A17_70	4.4	3.5	0.8	1.8	100.0%	-3.0
3086	AT2G02850.1 | Symbol: ARPN | plastocyanin-like domain-containing protein / plantacyanin, putative, similar to plantacyanin GI:3395754 from (Spinacia oleracea) | chr2:826542-827785 REVERSE | Aliases: PLANTACYANIN, T17M13.2, T17M13_2	3.6	4.6	-0.9	-1.8	100.0%	-3.2
3087	AT4G31600.2 | similar to integral membrane family protein [Arabidopsis thaliana] (TAIR:At5g19980.1); similar to hypothetical protein DDB0202972 [Dictyostelium discoideum] (GB:EAL69125.1) | chr4:15314891-15317141 REVERSE | Aliases: F28M20.210, F28M20_210	4.1	4.5	-0.4	-1.8	100.0%	-3.7
3088	AT1G70890.1 | major latex protein-related / MLP-related, low similarity to major latex protein {Papaver somniferum}(GI:294062) ; contains Pfam profile PF00407: Pathogenesis-related protein Bet v I family | chr1:26729288-26730206 REVERSE | Aliases: F15H11.12, F15H11_12	4.6	5.2	-0.7	-1.8	100.0%	-3.4
3089	AT1G04530.1 | expressed protein | chr1:1234198-1236033 REVERSE | Aliases: T1G11.22, T1G11_22	5.4	6.1	-0.7	-1.8	100.0%	-3.3
3090	AT1G08830.1 | Symbol: CSD1 | superoxide dismutase (Cu-Zn) (SODCC) / copper/zinc superoxide dismutase (CSD1), identical to SWISS-PROT: P24704 | chr1:2827089-2829260 FORWARD | Aliases: F22O13.32, F22O13_32	12.0	12.8	-0.8	-1.8	100.0%	-4.3
3091	AT5G47990.1 | Symbol: CYP705A5 | cytochrome P450 family protein, similar to Cytochrome P450 93A3 (P450 CP5) (SP:O81973) (Glycine max); | chr5:19452053-19453915 FORWARD | Aliases: MDN11.4, MDN11_4	2.5	2.8	-0.3	-1.8	100.0%	-3.7
3092	AT5G66090.1 | expressed protein | chr5:26442962-26444394 FORWARD | Aliases: K2A18.17, K2A18_17	4.4	3.9	0.5	1.8	100.0%	-3.5
3093	AT5G42880.1 | expressed protein, contains Pfam profile PF05701: Plant protein of unknown function (DUF827) | chr5:17208986-17211319 FORWARD | Aliases: MBD2.7, MBD2_7	4.7	5.6	-0.9	-1.8	100.0%	-3.5
3094	AT1G54680.1 | expressed protein | chr1:20416721-20418149 FORWARD | Aliases: T22H22.11, T22H22_11	9.3	8.5	0.8	1.8	100.0%	-3.4
3095	AT3G04260.1 | SAP domain-containing protein, contains Pfam domain PF02037: SAP domain | chr3:1123030-1127522 REVERSE | Aliases: T6K12.12, T6K12_12	4.8	5.5	-0.6	-1.8	100.0%	-3.2

Rank	Description	15.dpi	5.dpi	M	t	adj.q	B
3096	AT5G47180.2 | vesicle-associated membrane family protein / VAMP family protein, similar to VAP27 GI:6688926 (Nicotiana plumbaginifolia), to VAMP-associated protein B GI:4240464 (Rattus norvegicus) and to Vesicle-associated membrane protein/synaptobrevin binding protein (VAP-33) (SP:Q16943)(Aplysia californica) | chr5:19178372-19181010 REVERSE | Aliases: MQL5.3, MQL5_3	5.7	5.0	0.7	1.8	100.0%	-3.5
3097	AT3G02760.1 | histidyl-tRNA synthetase, putative / histidine--tRNA ligase, putative, similar to SP:P12081 Histidyl-tRNA synthetase (EC 6.1.1.21) (Histidine--tRNA ligase) (HisRS) {Homo sapiens}; contains Pfam profiles PF00587: tRNA synthetase class II core domain (G, H, P, S and T), PF03129: Anticodon binding domain | chr3:597366-599445 REVERSE | Aliases: F13E7.30, F13E7_30	8.1	9.1	-1.0	-1.8	100.0%	-3.7
3098	AT5G65640.1 | basic helix-loop-helix (bHLH) family protein | chr5:26254285-26256310 FORWARD | Aliases: K21L13.16, K21L13_16	3.5	4.2	-0.7	-1.8	100.0%	-3.3
3099	AT5G50010.1 | expressed protein | chr5:20365841-20367936 FORWARD | Aliases: K9P8.17	3.6	4.2	-0.6	-1.8	100.0%	-3.4
3100	AT1G71020.1 | armadillo/beta-catenin repeat family protein / U-box domain-containing protein, contains Pfam domain, PF00514: Armadillo/beta-catenin-like repeats and Pfam, PF04564: U-box domain | chr1:26794259-26796837 REVERSE | Aliases: F23N20.1, F23N20_1	3.5	3.9	-0.3	-1.8	100.0%	-3.7
3101	AT4G17190.2 | Symbol: FPS2 | farnesyl pyrophosphate synthetase 2 (FPS2) / FPP synthetase 2 / farnesyl diphosphate synthase 2, identical to SP:Q43315 Farnesyl pyrophosphate synthetase 2 (FPP synthetase 2) (FPS 2) (Farnesyl diphosphate synthetase 2) (Includes: Dimethylallyltransferase (EC 2.5.1.1); Geranyltranstransferase (EC 2.5.1.10)) {Arabidopsis thaliana} | chr4:9648552-9650857 REVERSE | Aliases: DL4630C, FARNESYL DIPHOSPHATE SYNTHASE 2, FCAALL.91	6.3	7.2	-1.0	-1.8	100.0%	-3.0
3102	AT4G07400.1 | Symbol: VFB3 | F-box family protein (FBL8) (FBL24), contains similarity to SKP1 interacting partner 2 GI:10716949 from (Arabidopsis thaliana); contains Pfam PF00646: F-box domain | chr4:4197844-4199508 REVERSE | Aliases: F28D6.13, F28D6_13, VFB3, VIER F BOX PROTEINE 3	4.6	5.4	-0.7	-1.8	100.0%	-3.4
3103	AT5G46210.1 | Symbol: CUL4 | cullin, putative, similar to SP:Q13619 Cullin homolog 4A (CUL-4A) {Homo sapiens}; contains Pfam profile PF00888: Cullin family | chr5:18748645-18754037 REVERSE | Aliases: CUL4, MDE13.3, MDE13_3	9.4	10.0	-0.5	-1.8	100.0%	-3.6
3104	AT2G05620.1 | Symbol: PGR5 | expressed protein | chr2:2081050-2081837 REVERSE | Aliases: T20G20.3, T20G20_3	9.2	8.3	0.9	1.8	100.0%	-3.3
3105	AT4G26840.1 | Symbol: SUM1 | ubiquitin-like protein (SMT3), identical to Ubiquitin-like protein SMT3 SP:P55852 from(Arabidopsis thaliana); identical to cDNA SMT3 protein GI:1707371 | chr4:13497334-13498733 FORWARD | Aliases: F10M23.180, F10M23_180, SMALL UBIQUITIN LIKE MODIFIER 1, SUMO 1, SUMO1	9.6	8.9	0.7	1.8	100.0%	-3.8
3106	AT5G38700.1 | similar to expressed protein [Arabidopsis thaliana] (TAIR:At4g02170.1); similar to disease resistance gene [Pinus sylvestris] (GB:AAR36911.1) | chr5:15506825-15507468 FORWARD | Aliases: MBB18.26, MBB18_26	3.1	2.7	0.4	1.8	100.0%	-3.5
3107	AT2G45530.1 | zinc finger (C3HC4-type RING finger) family protein, contains Pfam profile: PF00097 zinc finger, C3HC4 type (RING finger) | chr2:18763552-18765002 FORWARD | Aliases: F17K2.6	8.0	7.4	0.5	1.8	100.0%	-3.4
3108	AT5G11510.2 | Symbol: MYB3R 4 | similar to myb family transcription factor [Arabidopsis thaliana] (TAIR:At4g32730.1); similar to myb family transcription factor [Arabidopsis thaliana] (TAIR:At4g32730.2); similar to Myb [Nicotiana tabacum] (GB:BAB70510.1); contains InterPro domain Myb DNA-binding domain (InterPro:IPR001005) | chr5:3680453-3683634 FORWARD | Aliases: F15N18.100, F15N18_100	3.5	3.9	-0.5	-1.8	100.0%	-3.6
3109	AT1G55090.1 | carbon-nitrogen hydrolase family protein, low similarity to SP:P71911 Glutamine-dependent NAD(+) synthetase (EC 6.3.5.1) {Mycobacterium tuberculosis}; contains Pfam profile PF00795: hydrolase, carbon-nitrogen family | chr1:20558104-20561855 FORWARD | Aliases: T7N22.4, T7N22_4	4.5	5.0	-0.5	-1.8	100.0%	-3.5
3110	AT4G26850.1 | Symbol: VTC2 | expressed protein | chr4:13499016-13501654 REVERSE | Aliases: F10M23.190, F10M23_190, VITAMIN C DEFECTIVE 2	8.7	7.8	0.9	1.8	100.0%	-3.3
3111	AT5G53750.1 | similar to CBS domain-containing protein [Arabidopsis thaliana] (TAIR:At4g27460.1); similar to OSJNBb0017I01.11 [Oryza sativa (japonica cultivar-group)] (GB:XP_474370.1); contains InterPro domain CBS domain (InterPro:IPR000644) | chr5:21834593-21836064 FORWARD | Aliases: MGN6.11, MGN6_11	6.0	5.5	0.5	1.8	100.0%	-3.4
3112	AT1G73180.1 | eukaryotic translation initiation factor-related, similar to eukaryotic translation initiation factor 2A (GI:21956484) (Homo sapiens); similar to Eukaryotic translation initiation factor 3 subunit 9 (eIF-3 eta) (eIF3 p116) (eIF3 p110) (eIF3b) (Swiss-Prot:P55884) (Homo sapiens) | chr1:27521577-27525290 FORWARD | Aliases: T18K17.15, T18K17_15	7.6	8.1	-0.5	-1.8	100.0%	-3.3
3113	AT2G22620.1 | expressed protein | chr2:9611881-9617675 REVERSE | Aliases: T9I22.6, T9I22_6	2.9	3.6	-0.7	-1.8	100.0%	-3.3
3114	AT5G17170.1 | rubredoxin family protein, contains Pfam profile PF00301: Rubredoxin | chr5:5649205-5651160 FORWARD | Aliases: MKP11.2, MKP11_2	8.8	8.0	0.7	1.8	100.0%	-3.1
3115	AT5G06370.1 | NC domain-containing protein, contains Pfam domain, PF04970: NC domain | chr5:1947140-1948574 REVERSE | Aliases: MHF15.11, MHF15_11	7.9	7.3	0.6	1.8	100.0%	-3.4

Rank	Description	15.dpi	5.dpi	M	t	adj.q	B
3116	AT5G60340.1 | maoC-like dehydratase domain-containing protein, contains similarity to (R)-specific enoyl-CoA hydratase PhaJ1 (Pseudomonas oleovorans) gi:22506675:gb:AAM97601; contains Pfam domain PF01575: MaoC like domain | chr5:24291105-24293117 REVERSE | Aliases: K9B18.5, K9B18_5	2.7	2.9	-0.3	-1.8	100.0%	-4.0
3117	AT4G21110.1 | G10 family protein, contains Pfam profile: PF01125 G10 protein | chr4:11267196-11268968 FORWARD | Aliases: F7J7.50, F7J7_50	10.7	10.2	0.5	1.8	100.0%	-3.6
3118	AT2G31350.2 | hydroxyacylglutathione hydrolase, putative / glyoxalase II, putative, similar to glyoxalase II isozyme (Arabidopsis thaliana) gi:2570338:gb:AAC49865 | chr2:13375370-13378070 FORWARD | Aliases: T28P16.16, T28P16_16	9.4	8.8	0.6	1.8	100.0%	-3.6
3119	AT1G67630.1 | DNA polymerase alpha subunit B family, contains Pfam profile: PF04058 DNA polymerase alpha subunit B | chr1:25348103-25352069 REVERSE | Aliases: F12B7.19, F12B7_19	4.3	4.9	-0.6	-1.8	100.0%	-3.5
3120	AT4G26720.1 | Symbol: PPX1 | serine/threonine protein phosphatase PP-X isozyme 1 (PPX1), identical to SP:P48529 Serine/threonine protein phosphatase PP-X isozyme 1 (EC 3.1.3.16) {Arabidopsis thaliana}; contains Pfam profile PF00149: Ser/Thr protein phosphatase | chr4:13470040-13472326 REVERSE | Aliases: EP124, EP129, F10M23.60, F10M23_60, PPX 1	6.0	5.7	0.3	1.8	100.0%	-3.8
3121	AT3G17010.1 | transcriptional factor B3 family protein, contains Pfam profile PF02362: B3 DNA binding domain. Activated by AGAMOUS ina a cal-1, ap1-1 background. Expressed in stamen primordia, the placental region of developing carpels and the ovary. | chr3:5800466-5802474 FORWARD | Aliases: K14A17.8	4.4	3.9	0.5	1.8	100.0%	-3.9
3122	AT1G13700.1 | glucosamine/galactosamine-6-phosphate isomerase family protein, similar to SP:O95336 6-phosphogluconolactonase (EC 3.1.1.31) (6PGL) {Homo sapiens}; contains Pfam profile PF01182: Glucosamine-6-phosphate isomerase/6-phosphogluconolactonase | chr1:4693959-4695979 REVERSE | Aliases: F21F23.14, F21F23_14	3.6	4.1	-0.6	-1.8	100.0%	-3.6
3123	AT5G14270.1 | DNA-binding bromodomain-containing protein, contains bromodomain, INTERPRO:IPR001487 | chr5:4604735-4608908 FORWARD | Aliases: F18O22.60, F18O22_60	7.5	6.9	0.7	1.8	100.0%	-3.3
3124	AT3G56690.1 | Symbol: CIP111 |	4.8	5.4	-0.6	-1.8	100.0%	-3.4
3125	AT1G72480.1 | expressed protein | chr1:27289727-27292001 REVERSE | Aliases: T10D10.5, T10D10_5	4.7	5.2	-0.6	-1.8	100.0%	-3.4
3126	AT2G21950.1 | SKP1 interacting partner 6 (SKIP6), identical to SKP1 interacting partner 6 GI:10716957 from (Arabidopsis thaliana) | chr2:9360254-9361725 REVERSE | Aliases: F7D8.27, F7D8_27	7.1	6.6	0.5	1.8	100.0%	-3.6
3127	AT2G39880.1 | Symbol: MYB25 | myb family transcription factor (MYB25), contains Pfam profile: PF00249 myb-like DNA-binding domain | chr2:16655023-16656557 REVERSE | Aliases: T28M21.4, T28M21_4	5.1	5.5	-0.4	-1.8	100.0%	-3.4
3128	AT1G03830.1 | guanylate-binding family protein, contains Pfam domains PF02263: Guanylate-binding protein, N-terminal domain and PF02841: Guanylate-binding protein, C-terminal domain | chr1:962127-966621 REVERSE | Aliases: F21M11.26, F21M11_26	4.9	5.7	-0.8	-1.8	100.0%	-3.7
3129	AT3G26560.1 | ATP-dependent RNA helicase, putative, similar to SP:Q14562 ATP-dependent helicase DDX8 (RNA helicase HRH1) (DEAH-box protein 8) {Homo sapiens}; contains Pfam profiles PF04408: Helicase associated domain (HA2), PF00271: Helicase conserved C-terminal domain, PF00575: S1 RNA binding domain | chr3:9751166-9755009 REVERSE | Aliases: MFE16.8	6.5	7.4	-0.9	-1.8	100.0%	-3.2
3130	AT2G26380.1 | disease resistance protein-related / LRR protein-related, contains leucine rich-repeat domains Pfam:PF00560, INTERPRO:IPR001611; similar to Hcr2-2A (Lycopersicon pimpinellifolium) gi:3894389:gb:AAC78594 | chr2:11233693-11235135 REVERSE | Aliases: T9J22.5, T9J22_5	3.5	3.1	0.4	1.8	100.0%	-3.6
3131	AT1G76130.1 | Symbol: ATAMY2 |	4.8	4.2	0.6	1.8	100.0%	-3.2
3132	AT3G62700.1 | Symbol: ATMRP10 | glutathione-conjugate transporter, putative, similar to glutathione-conjugate transporter AtMRP4 GI:2959767 from (Arabidopsis thaliana) | chr3:23201243-23206738 REVERSE | Aliases: F26K9.130	7.0	7.8	-0.8	-1.8	100.0%	-3.5
3133	AT5G17000.1 | NADP-dependent oxidoreductase, putative, strong similarity to probable NADP-dependent oxidoreductase (zeta-crystallin homolog) P1 (SP:Q39172)(gi:886428) and P2 (SP:Q39173)(gi:886430), Arabidopsis thaliana | chr5:5584804-5587066 REVERSE | Aliases: F2K13.150, F2K13_150	9.0	8.2	0.8	1.8	100.0%	-3.3
3134	AT5G22300.1 | Symbol: NIT4 | nitrilase 4 (NIT4), identical to SP:P46011 Nitrilase 4 (EC 3.5.5.1) {Arabidopsis thaliana} | chr5:7379221-7381928 FORWARD | Aliases: NITRILASE 4, T6G21.6	5.6	4.9	0.7	1.8	100.0%	-3.1
3135	AT3G56760.1 | calcium-dependent protein kinase, putative / CDPK, putative, similar to calcium/calmodulin-dependent protein kinase CaMK3 (Nicotiana tabacum) gi:16904226:gb:AAL30820 | chr3:21031537-21034735 REVERSE | Aliases: T8M16.90	6.3	6.8	-0.5	-1.8	100.0%	-3.3

Rank	Description	15.dpi	5.dpi	M	t	adj.q	B
3136	AT5G07220.1 | BAG domain-containing protein, contains Pfam:PF02179 BAG domain | chr5:2265257-2266968 REVERSE | Aliases: T28J14.160, T28J14_160	5.8	5.1	0.7	1.8	100.0%	-3.2
3137	AT4G35020.1 | Symbol: ROP6 | Rac-like GTP-binding protein (ARAC3) / Rho-like GTP-binding protein (ROP6), identical to SP:Q38912 RAC-like GTP binding protein ARAC3 (GTP-binding protein ROP6) {Arabidopsis thaliana}; identical to cDNA Rho-like GTP binding protein (Rop6) GI:2645642 | chr4:16672945-16674776 FORWARD | Aliases: ATROP6, M4E13.80, M4E13_80, RHO1PS	6.7	7.2	-0.6	-1.8	100.0%	-3.3
3138	AT2G15690.1 | pentatricopeptide (PPR) repeat-containing protein, contains Pfam profile PF01535: PPR repeat | chr2:6838077-6840859 REVERSE | Aliases: F9O13.34	5.8	6.6	-0.8	-1.8	100.0%	-3.1
3139	AT5G10770.1 | chloroplast nucleoid DNA-binding protein, putative, similar to CND41, chloroplast nucleoid DNA binding protein (Nicotiana tabacum) GI:2541876; contains Pfam profile PF00026: Eukaryotic aspartyl protease | chr5:3403120-3405449 REVERSE | Aliases: T30N20.40, T30N20_40	4.1	3.4	0.8	1.8	100.0%	-3.6
3140	AT3G10190.1 | calmodulin, putative, similar to calmodulin NtCaM13 (Nicotiana tabacum) GI:14625425, calmodulin GB:AAA34015 (Glycine max); contains INTERPRO:IPR002048 calcium-binding EF-hand domain | chr3:3155303-3156145 FORWARD | Aliases: F14P13.21	4.2	3.6	0.6	1.8	100.0%	-3.5
3141	AT1G16750.1 | expressed protein, contains Pfam profile PF04784: Protein of unknown function, DUF547 | chr1:5729090-5731863 REVERSE | Aliases: F19K19.16	7.5	6.5	1.0	1.8	100.0%	-3.0
3142	AT2G28650.1 | exocyst subunit EXO70 family protein, contains Pfam domain PF03081: Exo70 exocyst complex subunit | chr2:12296229-12298122 REVERSE | Aliases: T8O18.6, T8O18_6	5.8	5.2	0.6	1.8	100.0%	-3.6
3143	AT5G12340.1 | expressed protein, ; expression supported by MPSS | chr5:3992949-3993611 REVERSE | Aliases: None	3.4	4.0	-0.6	-1.8	100.0%	-3.4
3144	AT1G62250.2 | expressed protein | chr1:22999169-23001221 REVERSE | Aliases: F19K23.17, F19K23_17	6.7	5.9	0.8	1.8	100.0%	-3.4
3145	AT3G55280.1 | 60S ribosomal protein L23A (RPL23aB), various ribosomal L23a proteins | chr3:20511312-20512686 FORWARD | Aliases: T26I12.160	9.0	9.9	-0.9	-1.8	100.0%	-3.2
3146	AT3G51270.1 | RIO1 family protein, contains Pfam profile PF01163: RIO1 family | chr3:19044830-19047751 FORWARD | Aliases: F24M12.310	7.0	7.8	-0.8	-1.8	100.0%	-3.3
3147	AT4G14680.1 | Symbol: APS3 | sulfate adenylyltransferase 3 / ATP-sulfurylase 3 (APS3), identical to ATP sulfurylase (APS3) (Arabidopsis thaliana) GI:1575327 | chr4:8413286-8415349 REVERSE | Aliases: DL3380C, FCAALL.128	8.4	9.6	-1.2	-1.8	100.0%	-3.3
3148	AT1G16920.1 | Symbol: RAB11 | Ras-related GTP-binding protein, putative, similar to GTP binding protein GI:218228 from (Vicia faba); identical to cDNA small GTP-binding protein (Rab11) GI:451859 | chr1:5787323-5789242 REVERSE | Aliases: ATRABA4B, F17F16.26, RAB11	7.8	8.9	-1.0	-1.8	100.0%	-3.2
3149	AT3G51880.2 | Symbol: HMGB1 | high mobility group protein alpha (HMGalpha) / HMG protein alpha, nearly identical to HMG protein (HMGalpha) (Arabidopsis thaliana) GI:2832357; contains Pfam profile PF00505: HMG (high mobility group) box | chr3:19257980-19260072 REVERSE | Aliases: ATEM1.13, HIGH MOBILITY GROUP B 1, HMG ALPHA, NFD1	9.1	8.5	0.6	1.8	100.0%	-3.5
3150	AT5G08610.1 | DEAD box RNA helicase (RH26), strong similarity to RNA helicase RH26 (Arabidopsis thaliana) GI:3776025; contains Pfam profiles PF00270: DEAD/DEAH box helicase, PF00271: Helicase conserved C-terminal domain; identical to cDNA DEAD box RNA helicase, RH26 GI:3776024 | chr5:2790296-2794216 FORWARD | Aliases: MAH20.17, MAH20_17	6.1	6.9	-0.8	-1.8	100.0%	-3.1
3151	AT2G22250.3 | similar to aminotransferase class I and II family protein [Arabidopsis thaliana] (TAIR:At1g77670.1); similar to aspartate aminotransferase [Pinus pinaster] (GB:CAF31327.1); contains InterPro domain Aminotransferase, class I and II (InterPro:IPR004839); contains InterPro domain Aminotransferases class-I pyridoxal-phosphate-binding site (InterPro:IPR004838) | chr2:9464890-9467999 REVERSE | Aliases: T26C19.9, T26C19_9	8.1	9.1	-1.0	-1.8	100.0%	-3.4
3152	AT1G55680.1 | WD-40 repeat family protein, contains 2 (1 significant) WD-40 repeats (PF0400); similar to Trp-Asp repeat protein (PIR:T40094) (Schizosaccharomyces) | chr1:20808258-20811544 REVERSE | Aliases: F20N2.10	5.3	4.6	0.6	1.8	100.0%	-3.4
3153	AT1G28200.1 | Symbol: FIP1 | GRAM domain-containing protein / ABA-responsive protein-related, similar to ABA-responsive protein (Hordeum vulgare) GI:4103635; contains Pfam profile PF02893: GRAM domain | chr1:9850134-9852353 REVERSE | Aliases: F3H9.14, F3H9_14, FH INTERACTING PROTEIN 1, VIRF INTERACTING PROTEIN 1	8.5	7.7	0.8	1.8	100.0%	-3.4
3154	AT5G48800.1 | phototropic-responsive NPH3 family protein, contains NPH3 family domain, Pfam:PF03000 | chr5:19803990-19806733 FORWARD | Aliases: K24G6.13, K24G6_13	3.7	4.3	-0.5	-1.8	100.0%	-3.5
3155	AT2G32090.2 | lactoylglutathione lyase family protein / glyoxalase I family protein, contains glyoxalase family protein domain, Pfam:PF00903 | chr2:13651704-13652906 FORWARD | Aliases: F22D22.16, F22D22_16	10.6	9.8	0.8	1.8	100.0%	-3.7

Rank	Description	15.dpi	5.dpi	M	t	adj.q	B
3156	AT5G65450.1 | ubiquitin carboxyl-terminal hydrolase family protein / zinc finger (MYND type) family protein, similar to ubiquitin-specific protease 16 (UBP16) (Arabidopsis thaliana) GI:11993477; contains Pfam profiles PF00443: Ubiquitin carboxyl-terminal hydrolase, PF01753: MYND finger	6.5	7.2	-0.6	-1.8	100.0%	-3.3
3157	AT5G40610.1 | glycerol-3-phosphate dehydrogenase (NAD+) / GPDH, strong similarity to SP:P52425 Glycerol-3-phosphate dehydrogenase (NAD+) (EC 1.1.1.8) {Cuphea lanceolata}; contains Pfam profile PF01210: NAD-dependent glycerol-3-phosphate dehydrogenase | chr5:16282090-16284520 REVERSE | Aliases: MNF13.130, MNF13_130	7.1	7.8	-0.7	-1.8	100.0%	-3.2
3158	AT2G41120.1 | expressed protein | chr2:17147973-17149497 REVERSE | Aliases: T3K9.11, T3K9_11	4.0	3.5	0.5	1.8	100.0%	-3.4
3159	AT5G53180.1 | polypyrimidine tract-binding protein, putative / heterogeneous nuclear ribonucleoprotein, putative, similar to Polypyrimidine tract-binding protein 1 (PTB) (Heterogeneous nuclear ribonucleoprotein I) (hnRNP I) from {Rattus norvegicus} SP:Q00438, {Homo sapiens} SP:P26599; contains InterPro entry IPR000504: RNA-binding region RNP-1 (RNA recognition motif) (RRM) | chr5:21585426-21589115 REVERSE | Aliases: MFH8.12, MFH8_12	8.1	7.6	0.5	1.8	100.0%	-3.4
3160	AT2G25140.1 | heat shock protein 100, putative / HSP100, putative / heat shock protein clpB, putative / HSP100/ClpB, putative, similar to HSP100/ClpB GI:9651530 (Phaseolus lunatus) | chr2:10704782-10709231 REVERSE | Aliases: F13D4.100, F13D4_100	6.0	6.5	-0.4	-1.8	100.0%	-3.5
3161	AT1G18290.1 | expressed protein | chr1:6297429-6297959 FORWARD | Aliases: F15H18.19	3.2	2.8	0.3	1.8	100.0%	-3.7
3162	AT5G55180.1 | glycosyl hydrolase family 17 protein, similar to elicitor inducible chitinase Nt-SubE76 GI:11071974 from (Nicotiana tabacum) | chr5:22406012-22407938 FORWARD | Aliases: MCO15.13, MCO15_13	8.5	8.1	0.5	1.8	100.0%	-3.5
3163	AT1G76120.2 | tRNA pseudouridine synthase family protein, similar to SP:Q9Y606 tRNA pseudouridine synthase A (EC 4.2.1.70) (Uracil hydrolyase) {Homo sapiens}; contains Pfam profile PF01416: tRNA pseudouridine synthase | chr1:28563568-28565330 REVERSE | Aliases: T23E18.5, T23E18_5	4.3	4.7	-0.4	-1.8	100.0%	-3.4
3164	AT2G26390.1 | serpin, putative / serine protease inhibitor, putative, similar to phloem serpin-1 (Cucurbita maxima) GI:9937311; contains Pfam profile PF00079: Serpin (serine protease inhibitor) | chr2:11236165-11237569 REVERSE | Aliases: T9J22.6, T9J22_6	3.3	3.6	-0.3	-1.8	100.0%	-4.0
3165	AT3G10940.1 | protein phosphatase-related, similar to protein phosphatase PTPKIS1 protein (GI:11595504) (Arabidopsis thaliana)	6.3	6.8	-0.5	-1.8	100.0%	-3.5
3166	AT1G16520.1 | expressed protein | chr1:5648763-5651160 FORWARD | Aliases: F3O9.32	6.5	7.3	-0.8	-1.8	100.0%	-3.3
3167	AT1G67330.1 | expressed protein, contains Pfam profile PF04669: Protein of unknown function (DUF579) | chr1:25217515-25218834 FORWARD | Aliases: F1N21.15	3.3	4.5	-1.2	-1.8	100.0%	-3.2
3168	AT2G37500.1 | arginine biosynthesis protein ArgJ family, contains Pfam profile: PF01960 ArgJ family | chr2:15746418-15749836 REVERSE | Aliases: F3G5.29, F3G5_29	7.7	8.5	-0.8	-1.8	100.0%	-3.3
3169	AT5G16920.1 | expressed protein | chr5:5567014-5568131 FORWARD | Aliases: F2K13.70, F2K13_70	1.9	2.0	-0.1	-1.8	100.0%	-4.7
3170	AT1G69210.1 | expressed protein | chr1:26021765-26023721 REVERSE | Aliases: F4N2.17, F4N2_17	3.6	4.0	-0.4	-1.8	100.0%	-3.7
3171	AT2G20080.2 | expressed protein | chr2:8670011-8672107 REVERSE | Aliases: T2G17.12, T2G17_12	2.3	2.6	-0.2	-1.8	100.0%	-4.3
3172	AT1G67560.1 | lipoxygenase family protein, similar to 13-lipoxygenase GB:CAA65269 (Solanum tuberosum), gi:1654140 (Lycopersicon esculentum) | chr1:25323562-25327927 FORWARD | Aliases: F12B7.11, F12B7_11	7.9	7.3	0.6	1.8	100.0%	-3.4
3173	AT1G28050.1 | zinc finger (B-box type) family protein | chr1:9775515-9777797 REVERSE | Aliases: F13K9.15, F13K9_15	5.2	4.7	0.5	1.8	100.0%	-3.6
3174	AT5G61620.1 | myb family transcription factor, contains Pfam profile: PF00249 myb-like DNA-binding domain | chr5:24789609-24790733 FORWARD | Aliases: K11J9.15, K11J9_15	3.2	2.9	0.3	1.8	100.0%	-3.8
3175	AT5G65460.1 | kinesin motor protein-related, contains similarity to kinesin heavy chain | chr5:26179057-26186227 REVERSE | Aliases: MNA5.20, MNA5_20	3.2	3.7	-0.5	-1.8	100.0%	-3.7
3176	AT4G27440.2 | Symbol: PORB | similar to protochlorophyllide reductase A, chloroplast / PCR A / NADPH-protochlorophyllide oxidoreductase A (PORA) [Arabidopsis thaliana] (TAIR:At5g54190.1); similar to NADPH-protochlorophyllide oxidoreductase [Cucumis sativus] (GB:BAA21089.1); similar to protochlorophyllide reductase (EC 1.3.1.33) precursor - garden pea (GB:S20941); similar to protochlorophyllide reductase [Pisum sativum] (GB:CAA44786.1); similar to NADPH:protochlorophyllide oxidoreductase [Nicotiana tabacum] (GB:BAB93003.1); similar to NADPH:protochlorophyllide oxidoreductase [Daucus carota] (GB:AAF20949.1); contains InterPro domain Glucose/ribitol dehydrogenase (InterPro:IPR002347); contains InterPro domain Light-dependent protochlorophyllide reductase (InterPro:IPR005979); contains InterPro domain Short-chain dehydrogenase/reductase SDR (InterPro:IPR002198)	6.4	7.3	-0.9	-1.8	100.0%	-3.3

Rank	Description	15.dpi	5.dpi	M	t	adj.q	B
3177	AT3G49690.1 | myb family transcription factor, contains PFAM profile: myb DNA binding domain PF00249 | chr3:18438821-18440186 FORWARD | Aliases: T16K5.40	5.0	5.9	-0.9	-1.8	100.0%	-3.3
3178	AT1G08770.1 | prenylated rab acceptor (PRA1) family protein, weak similarity to prenylated Rab acceptor 1 (PRA1) (Homo sapiens) GI:4877285; contains Pfam profile PF03208: Prenylated rab acceptor (PRA1) | chr1:2808886-2809747 FORWARD | Aliases: F22O13.26, F22O13_26	4.8	4.5	0.4	1.8	100.0%	-3.8
3179	AT5G63905.1 | expressed protein | chr5:25588084-25588812 REVERSE | Aliases: None	5.6	4.7	0.8	1.8	100.0%	-3.3
3180	AT2G36270.1 | Symbol: ABI5 | bZIP transcription factor family protein / ABA-responsive element-binding protein, putative, similar to ABA-responsive element binding protein 1 (AREB1) GI:9967417 from (Arabidopsis thaliana); contains a bZIP transcription factor basic domain signature (PDOC00036)	5.5	4.8	0.7	1.8	100.0%	-3.4
3181	AT3G56800.1 | Symbol: CAM3 | calmodulin-2/3/5 (CAM3), identical to calmodulin GI:474183 from (Arabidopsis thaliana); almost identical to calmodulin-2/3/5 SP:P25069 (Arabidopsis thaliana) | chr3:21045656-21047053 REVERSE | Aliases: ACAM 3, CALMODULIN 3, T8M16.130	11.4	10.9	0.5	1.8	100.0%	-3.6
3182	AT5G40520.1 | similar to hypothetical protein [Arabidopsis thaliana] (TAIR:At5g07170.1); similar to B1158C05.16 [Oryza sativa (japonica cultivar-group)] (GB:NP_917835.1) | chr5:16248482-16251536 REVERSE | Aliases: MNF13.3, MNF13_3	3.8	4.2	-0.4	-1.8	100.0%	-3.7
3183	AT4G26870.1 | aspartyl-tRNA synthetase, putative / aspartate--tRNA ligase, putative, simialr to aspartate-tRNA ligase (EC 6.1.1.12) from Drosophila melanogaster GI:4512034, Homo sapiens SP:P14868, Rattus norvegicus SP:P15178; contains Pfam profile PF00152 tRNA synthetases class II (D, K and N)	6.4	7.0	-0.6	-1.8	100.0%	-3.5
3184	AT1G80070.1 | Symbol: SUS2 | splicing factor, putative, strong similarity to splicing factor Prp8 (Homo sapiens) GI:3661610; contains Pfam profile PF01398: Mov34/MPN/PAD-1 family | chr1:30122945-30132704 FORWARD | Aliases: ABNORMAL SUSPENSOR 2, EMB14, EMB158, EMB177, EMB33, EMBRYO DEFECTIVE 14, EMBRYO DEFECTIVE 177, EMBRYO DEFECTIVE 33, F18B13.15, F18B13_15	8.3	9.1	-0.8	-1.8	100.0%	-3.3
3185	AT5G35180.1 | expressed protein | chr5:13441648-13450248 FORWARD | Aliases: T25C13.60, T25C13_60	6.3	7.2	-0.9	-1.8	100.0%	-3.3
3186	AT1G80750.1 | 60S ribosomal protein L7 (RPL7A), similar to ribosomal protein L7 GB:AAA03081 GI:307388 from (Homo sapiens) | chr1:30353715-30355456 FORWARD | Aliases: F23A5.10, F23A5_10	7.9	8.8	-0.9	-1.8	100.0%	-3.2
3187	AT5G19180.1 | Symbol: ECR1 | ubiquitin activating enzyme, putative (ECR1), identical to putative ubiquitin activating enzyme E1 (Arabidopsis thaliana) GI:2952433; similar to NEDD8 activating enzyme (Mus musculus) GI:17061821 | chr5:6453279-6456016 FORWARD | Aliases: E1 C TERMINAL RELATED 1, T24G5.80, T24G5_80	5.9	6.8	-0.9	-1.8	100.0%	-3.4
3188	AT3G30220.1 | expressed protein | chr3:11880345-11880937 REVERSE | Aliases: MIL15.3	2.0	2.1	-0.1	-1.8	100.0%	-4.6
3189	AT2G44850.1 | expressed protein | chr2:18505747-18507898 FORWARD | Aliases: T13E15.14	4.0	3.6	0.4	1.8	100.0%	-3.7
3190	AT4G26710.2 | ATP synthase subunit H family protein, contains similarity to Swiss-Prot:O15342 Vacuolar ATP synthase subunit H (V-ATPase H subunit) (Vacuolar proton pump H subunit) (V-ATPase M9.2 subunit) (V-ATPase 9.2 kDa membrane accessory protein) (Homo sapiens) | chr4:13468603-13469941 FORWARD | Aliases: F10M23.50, F10M23_50	10.6	9.9	0.7	1.8	100.0%	-4.0
3191	AT2G46690.1 | auxin-responsive family protein, similar to indole-3-acetic acid induced protein ARG7 (SP:P32295) (Phaseolus aureus) | chr2:19187800-19188537 FORWARD | Aliases: T3A4.7	8.9	8.4	0.5	1.8	100.0%	-3.5
3192	AT5G60940.2 | transducin family protein / WD-40 repeat family protein, contains 6 WD-40 repeats (PF00400); similar to cleavage stimulation factor 50K chain Homo sapiens, PIR:A45142 | chr5:24540247-24543038 FORWARD | Aliases: MSL3.60, MSL3_60	3.9	4.3	-0.4	-1.8	100.0%	-3.7
3193	AT5G19430.1 | zinc finger (C3HC4-type RING finger) family protein, contains Pfam profile: PF00097 zinc finger, C3HC4 type (RING finger) | chr5:6553827-6555833 FORWARD | Aliases: F7K24.180, F7K24_180	6.3	5.7	0.7	1.8	100.0%	-3.6
3194	AT2G03760.1 | Symbol: ST | steroid sulfotransferase, putative, strong similarity to steroid sulfotransferases from (Brassica napus) GI:3420008, GI:3420004, GI:3420006; contains Pfam profile PF00685: Sulfotransferase domain | chr2:1149334-1150660 REVERSE | Aliases: F19B11.21, F19B11_21, RAR047, SULFOTRANSFERASE, SULFURTRANSFERASE	3.5	4.0	-0.5	-1.8	100.0%	-3.4
3195	AT5G63200.1 | tetratricopeptide repeat (TPR)-containing protein, low similarity to SP:P28290 Sperm-specific antigen 2 (Cleavage signal-1 protein) (CS-1) Homo sapiens; contains Pfam profile PF00515: TPR Domain | chr5:25366237-25370228 FORWARD | Aliases: AT5G63210, MDC12.17, MDC12_17	5.7	5.4	0.3	1.8	100.0%	-3.9
3196	AT1G77590.1 | Symbol: LACS9 | long-chain-fatty-acid--CoA ligase family protein / long-chain acyl-CoA synthetase family protein (LACS9), similar to LACS 3 (SP:O95573) from Homo Sapiens, LACS 3 (SP:Q63151) from Rattus norvegicus; contains Pfam HMM hit: AMP-binding enzymes PF00501 | chr1:29153150-29156670 REVERSE | Aliases: LONG CHAIN ACYL COA SYNTHETASE 9, T5M16.18, T5M16_18	4.9	5.5	-0.6	-1.8	100.0%	-3.3

Rank	Description	15.dpi	5.dpi	M	t	adj.q	B
3197	AT4G12120.1 | Symbol: SEC1B | cytokinesis-related Sec1 protein, putative, similar to cytokinesis-related Sec1 protein KEULE (Arabidopsis thaliana) gi:12659318:gb:AAK01291; contains Pfam domain, PF00995: Sec1 family | chr4:7256532-7261174 REVERSE | Aliases: ATSEC1B, F16J13.190, F16J13_190, SEC1B	5.4	5.8	-0.4	-1.8	100.0%	-3.6
3198	AT1G55265.1 | expressed protein, contains Pfam profile PF04398: Protein of unknown function, DUF538 | chr1:20620980-20621668 FORWARD | Aliases: None	4.6	4.1	0.5	1.8	100.0%	-3.3
3199	AT5G27110.1 | pentatricopeptide (PPR) repeat-containing protein, contains INTERPRO:IPR002885 PPR repeats | chr5:9538575-9540650 REVERSE | Aliases: T21B4.20, T21B4_20	5.2	5.7	-0.5	-1.8	100.0%	-3.4
3200	AT1G35780.1 | expressed protein | chr1:13278486-13281472 REVERSE | Aliases: F14D7.8, F14D7_8	5.2	5.7	-0.5	-1.8	100.0%	-3.6
3201	AT1G30890.1 | integral membrane HRF1 family protein, contains Pfam domain PF03878: Hrf1 family	7.2	6.6	0.6	1.8	100.0%	-3.5
3202	AT5G44240.1 | haloacid dehalogenase-like hydrolase family protein, similar to Potential phospholipid-transporting ATPase (EC 3.6.3.1) from {Mus musculus} SP:P70704, {Bos taurus} SP:Q29449; contains InterPro accession IPR005834: Haloacid dehalogenase-like hydrolase | chr5:17834846-17840933 FORWARD | Aliases: MLN1.17, MLN1_17	5.6	6.3	-0.6	-1.8	100.0%	-3.3
3203	AT3G28900.1 | 60S ribosomal protein L34 (RPL34C), similar to 60S ribosomal protein L34 GB:P41098 (Nicotiana tabacum) | chr3:10903924-10905686 REVERSE | Aliases: MLD15.7	7.7	8.8	-1.1	-1.8	100.0%	-3.2
3204	AT5G12150.1 | pleckstrin homology (PH) domain-containing protein / RhoGAP domain-containing protein, weak similarity to glucocorticoid receptor DNA binding factor 1 (Canis familiaris) GI:23266717; contains Pfam profiles PF00169: PH domain, PF00620: RhoGAP domain	6.7	7.3	-0.6	-1.8	100.0%	-3.4
3205	AT1G53280.1 | DJ-1 family protein, similar to DJ-1 protein (Homo sapiens) GI:1780755; similar to DJ-1 beta (GI:18642508) (Drosophila melanogaster); contains Pfam profile: PF01965 ThiJ/PfpI family; TIGRFAM TIGR01383: DJ-1 family protein | chr1:19867968-19871057 REVERSE | Aliases: F12M16.18, F12M16_18	10.1	10.5	-0.4	-1.8	100.0%	-3.8
3206	AT2G33450.1 | 50S ribosomal protein L28, chloroplast (CL28) | chr2:14180655-14181609 FORWARD | Aliases: F4P9.22, F4P9_22	5.6	6.5	-0.9	-1.8	100.0%	-3.1
3207	AT3G13222.1 | Symbol: GIP1 | expressed protein | chr3:4251021-4254244 REVERSE | Aliases: GBF INTERACTING PROTEIN 1, MJG19.5	3.9	4.4	-0.5	-1.8	100.0%	-3.5
3208	AT3G53240.1 | leucine-rich repeat family protein, contains leucine rich-repeat (LRR) domains Pfam:PF00560, INTERPRO:IPR001611; contains similarity to Cf-2.1 (Lycopersicon pimpinellifolium) gi:1184075:gb:AAC15779 | chr3:19744791-19750159 FORWARD | Aliases: T4D2.170	4.5	4.0	0.4	1.8	100.0%	-3.5
3209	AT2G30600.4 | similar to BTB/POZ domain-containing protein [Arabidopsis thaliana] (TAIR:At2g46260.1); similar to similar to Dictyostelium discoideum (Slime mold). MigA (GB:AAS45368.1); contains InterPro domain BTB/POZ domain (InterPro:IPR000210) | chr2:13043953-13048761 FORWARD | Aliases: AT2G30610, T6B20.5, T6B20_5	6.5	5.8	0.7	1.8	100.0%	-3.9
3210	AT3G61610.1 | aldose 1-epimerase family protein, similar to apospory-associated protein C; APOC (Chlamydomonas reinhardtii) GI:6970044; contains Pfam profile PF01263: Aldose 1-epimerase | chr3:22810002-22812180 FORWARD | Aliases: F15G16.1	3.1	3.4	-0.3	-1.8	100.0%	-3.8
3211	AT5G52920.1 | pyruvate kinase, putative, similar to pyruvate kinase isozyme G, chloroplast precursor (Nicotiana tabacum) SWISS-PROT:Q40546 | chr5:21480769-21484043 FORWARD | Aliases: MXC20.15, MXC20_15	9.8	10.5	-0.7	-1.8	100.0%	-3.8
3212	AT1G02020.2 | nitroreductase family protein, contains Pfam PF00881: nitroreductase family protein profile; contains Prosite PS00343: Gram-positive cocci surface proteins 'anchoring' hexapeptide | chr1:352637-354969 REVERSE | Aliases: T7I23.2, T7I23_2	6.2	5.6	0.6	1.8	100.0%	-3.3
3213	AT1G75040.1 | Symbol: PR5 | pathogenesis-related protein 5 (PR-5), identical to SP:P28493 Pathogenesis-related protein 5 precursor (PR-5) {Arabidopsis thaliana}; contains Pfam profile: PF00314 Thaumatin family | chr1:28181364-28182601 FORWARD | Aliases: PATHOGENESIS RELATED GENE 5, PATHOGENESIS RELATED PROTEIN 5	3.9	4.4	-0.6	-1.8	100.0%	-3.4
3214	AT5G46570.1 | protein kinase family protein, contains protein kinase domain, Pfam:PF00069 | chr5:18911629-18914633 FORWARD | Aliases: F10E10.4, F10E10_4	5.2	6.0	-0.7	-1.8	100.0%	-3.2
3215	AT3G12640.1 | RNA recognition motif (RRM)-containing protein, contains InterPro entry IPR000504: RNA-binding region RNP-1 (RNA recognition motif) (RRM) | chr3:4014462-4017851 FORWARD | Aliases: T2E22.5	5.2	5.8	-0.6	-1.8	100.0%	-3.5
3216	AT1G53560.1 | expressed protein | chr1:19988423-19989649 REVERSE | Aliases: F22G10.10	5.8	5.2	0.6	1.8	100.0%	-3.5
3217	AT2G24762.1 | expressed protein | chr2:10566463-10567213 FORWARD | Aliases: None	4.3	4.9	-0.6	-1.8	100.0%	-3.5

Rank	Description	15.dpi	5.dpi	M	t	adj.q	B
3218	AT4G17760.2 | similar to Rad1-like protein [Oryza sativa (japonica cultivar-group)] (GB:XP_550603.1) | chr4:9871803-9873411 FORWARD | Aliases: DL4915W, FCAALL.108	3.2	3.5	-0.4	-1.8	100.0%	-3.8
3219	AT1G36310.2 | similar to hypothetical protein LOC67667 [Mus musculus] (GB:NP_080579.1); similar to unnamed protein product [Mus musculus] (GB:BAC38223.1); contains InterPro domain Generic methyltransferase (InterPro:IPR001601); contains InterPro domain SAM (and some other nucleotide) binding motif (InterPro:IPR000051) | chr1:13670711-13672337 FORWARD | Aliases: F7F23.3, F7F23_3	6.6	7.4	-0.7	-1.8	100.0%	-3.2
3220	AT3G59360.2 | nucleotide-sugar transporter family protein, low similarity to SP:P78382 CMP-sialic acid transporter {Homo sapiens}; contains Pfam profile PF04142: Nucleotide-sugar transporter	4.5	3.9	0.6	1.8	100.0%	-3.4
3221	AT4G34680.2 | similar to zinc finger (GATA type) family protein [Arabidopsis thaliana] (TAIR:At4g36240.1); similar to zinc finger (GATA type) family protein [Arabidopsis thaliana] (TAIR:At5g66320.1); similar to zinc finger (GATA type) family protein [Arabidopsis thaliana] (TAIR:At5g66320.2); similar to AG-motif binding protein-3 [Nicotiana tabacum] (GB:BAC98493.1); contains InterPro domain Zn-finger, GATA type (InterPro:IPR000679) | chr4:16553395-16554735 FORWARD | Aliases: T4L20.260, T4L20_260	5.9	5.6	0.3	1.8	100.0%	-3.7
3222	AT4G13270.1 | expressed protein | chr4:7731276-7733162 FORWARD | Aliases: T9E8.10, T9E8_10	5.1	4.6	0.6	1.8	100.0%	-3.6
3223	AT4G12800.1 | photosystem I reaction center subunit XI, chloroplast (PSI-L) / PSI subunit V, identical to Photosystem I reaction center subunit XI, chloroplast precursor (PSI-L) (PSI subunit V) (Swiss-Prot:Q9SUI4) (Arabidopsis thaliana); contains Pfam profile PF02605: photosystem I reaction center subunit XI; contains 2 transmembrane domains | chr4:7521319-7522667 FORWARD | Aliases: T20K18.150, T20K18_150	8.8	8.0	0.9	1.8	100.0%	-3.1
3224	AT5G48810.1 | Symbol: ATB5 B |	11.7	11.3	0.5	1.8	100.0%	-3.7
3225	AT1G62085.1 | mitochondrial transcription termination factor family protein / mTERF family protein, contains Pfam profile PF02536: mTERF | chr1:22952422-22953807 REVERSE | Aliases: None	2.7	3.0	-0.3	-1.8	100.0%	-4.1
3226	AT1G60190.1 | armadillo/beta-catenin repeat family protein / U-box domain-containing protein, contains Pfam domain, PF00514: Armadillo/beta-catenin-like repeats and Pfam, PF04564: U-box domain | chr1:22202068-22204343 FORWARD | Aliases: T13D8.8, T13D8_8	6.8	6.3	0.5	1.8	100.0%	-3.5
3227	AT1G19690.1 | expressed protein, similar to (SP:P76370) Protein yeeZ precursor. {Escherichia coli O157:H7} | chr1:6807706-6809560 REVERSE | Aliases: F14P1.35, F14P1_35	6.8	6.1	0.7	1.8	100.0%	-3.2
3228	AT1G60380.1 | apical meristem formation protein-related, contains similarity to CUC1 (Arabidopsis thaliana) gi:12060422:dbj:BAB20598 and NAM (Petunia x hybrida) gi:1279640:emb:CAA63101 | chr1:22250120-22251076 REVERSE | Aliases: T13D8.25, T13D8_25	2.6	2.8	-0.2	-1.8	100.0%	-4.2
3229	AT5G05840.1 | expressed protein, contains Pfam profile PF04788: Protein of unknown function (DUF620); expression supported by MPSS | chr5:1757269-1760459 REVERSE | Aliases: MJJ3.26, MJJ3_26	3.5	3.9	-0.4	-1.8	100.0%	-3.8
3230	AT5G18170.1 | Symbol: GDH1 | glutamate dehydrogenase 1 (GDH1), identical to glutamate dehydrogenase 1 (GDH 1) (Arabidopsis thaliana) SWISS-PROT:Q43314 | chr5:6006046-6008451 FORWARD | Aliases: GLUTAMATE DECARBOXYLASE 1, GLUTAMATE DEHYDROGENASE 1, GLUTAMATE DEHYDROGENASE DEFICIENT 1, MRG7.13, MRG7_13	7.6	6.8	0.8	1.8	100.0%	-3.4
3231	AT1G44100.1 | Symbol: AAP5 | amino acid permease 5, putative (AAP5), nearly identical to amino acid permease (AAP5) GI:608673 from (Arabidopsis thaliana) | chr1:16766845-16769973 REVERSE | Aliases: T7O23.19, T7O23_19	5.5	4.8	0.7	1.8	100.0%	-3.3
3232	AT3G61650.1 | tubulin gamma-1 chain / gamma-1 tubulin (TUBG1), identical to SP:P38557 Tubulin gamma-1 chain (Gamma-1 tubulin) {Arabidopsis thaliana} | chr3:22823576-22825986 REVERSE | Aliases: F15G16.40	4.2	4.9	-0.7	-1.8	100.0%	-3.2
3233	AT5G05620.1 | tubulin gamma-2 chain / gamma-2 tubulin (TUBG2), identical to SP:P38558 Tubulin gamma-2 chain (Gamma-2 tubulin) {Arabidopsis thaliana} | chr5:1679341-1681720 FORWARD | Aliases: MJJ3.10, MJJ3_10	4.2	4.9	-0.7	-1.8	100.0%	-3.2
3234	AT5G05160.1 | leucine-rich repeat transmembrane protein kinase, putative | chr5:1528001-1530063 FORWARD | Aliases: K2A11.3, K2A11_3	9.1	9.7	-0.5	-1.8	100.0%	-3.6
3235	AT4G24260.1 | endo-1,4-beta-glucanase, putative / cellulase, putative, similar to endo-1,4-beta-D-glucanase; cellulase GI:5689613 from (Brassica napus) | chr4:12577881-12580143 REVERSE | Aliases: T22A6.90, T22A6_90	6.7	7.7	-1.0	-1.8	100.0%	-3.1
3236	AT1G03040.1 | basic helix-loop-helix (bHLH) family protein, component of the pyruvate dehydrogenase complex E3, contains PF:00010 helix-loop-helix DNA-binding domain. ESTs gb:T45640 and gb:T22783 come from this gene | chr1:703906-706631 REVERSE | Aliases: F10O3.14, F10O3_14	8.2	6.9	1.3	1.8	100.0%	-3.3

Rank	Description	15.dpi	5.dpi	M	t	adj.q	B
3237	AT5G25510.1 | serine/threonine protein phosphatase 2A (PP2A) regulatory subunit B', putative, similar to SWISS-PROT:Q28653 serine/threonine protein phosphatase 2A, 56 kDa regulatory subunit, delta isoform (PP2A, B subunit, B' delta isoform, PP2A, B subunit, B56 delta isoform, PP2A, B subunit, PR61 delta isoform, PP2A, B subunit, R5 delta isoform, PP2A, B subunit, B'-gamma) (Oryctolagus cuniculus); contains Pfam domain, PF01603: Protein phosphatase 2A regulatory B subunit (B56 family) | chr5:8882471-8884837 REVERSE | Aliases: T14C9.50, T14C9_50	4.9	5.4	-0.5	-1.8	100.0%	-3.4
3238	AT4G21940.1 | Symbol: CPK15 | calcium-dependent protein kinase, putative / CDPK, putative, similar to calcium-dependent protein kinase (Nicotiana tabacum) gi:3283996:gb:AAC25423 | chr4:11640819-11643653 FORWARD | Aliases: F1N20.5	3.4	3.8	-0.4	-1.8	100.0%	-3.8
3239	AT4G26230.1 | 60S ribosomal protein L31 (RPL31B), ribosomal protein L31, Nicotiana glutinosa, U23784 | chr4:13285837-13286800 FORWARD | Aliases: T25K17.40, T25K17_40	11.6	12.0	-0.4	-1.8	100.0%	-3.8
3240	AT1G80360.1 | aminotransferase class I and II family protein, low similarity to GI:14278621 Aromatic Aminotransferase from Pyrococcus horikoshii | chr1:30213391-30215579 REVERSE | Aliases: F5I6.11, F5I6_11	7.7	8.4	-0.6	-1.8	100.0%	-3.4
3241	AT1G79590.2 | Symbol: SYP52 | similar to syntaxin 51 (SYP51) [Arabidopsis thaliana] (TAIR:At1g16240.1); similar to putative syntaxin of plants 52 [Oryza sativa (japonica cultivar-group)] (GB:XP_463898.1); contains InterPro domain Target SNARE coiled-coil domain (InterPro:IPR000727) | chr1:29951130-29953667 FORWARD | Aliases: ATSYP52, F20B17.2, F20B17_2, SYNTAXIN OF PLANTS 52	7.5	6.7	0.8	1.8	100.0%	-3.3
3242	AT2G16880.1 | pentatricopeptide (PPR) repeat-containing protein, contains Pfam profile PF01535: PPR repeat | chr2:7319228-7321615 REVERSE | Aliases: F12A24.6, F12A24_6	3.6	3.9	-0.3	-1.8	100.0%	-4.0
3243	AT5G37830.1 | hydantoinase/oxoprolinase family protein, contains Pfam profiles: PF02538 hydantoinase B/oxoprolinase, PF01968 hydantoinase/oxoprolinase, PF05378 hydantoinase/oxoprolinase N-terminal region | chr5:15073551-15078007 REVERSE | Aliases: K22F20.70, K22F20_70	5.8	6.2	-0.4	-1.8	100.0%	-3.7
3244	AT2G43030.1 | ribosomal protein L3 family protein, contains Pfam profile PF00297: ribosomal protein L3	7.9	8.8	-1.0	-1.8	100.0%	-3.2
3245	AT3G61250.1 | myb family transcription factor (MYB17), contains PFAM profile: Myb-like DNA-binding domain PF00249 | chr3:22681977-22683713 FORWARD | Aliases: T20K12.150	2.9	3.1	-0.3	-1.8	100.0%	-3.9
3246	AT4G21430.1 | similar to transcription factor jumonji (jmjC) domain-containing protein [Arabidopsis thaliana] (TAIR:At1g62310.1); similar to putative DNA-binding protein PD3, chloroplast [Oryza sativa (japonica cultivar-group)] (GB:XP_468520.1); contains InterPro domain Transcription factor jumonji, jmjC (InterPro:IPR003347)	5.4	5.9	-0.5	-1.8	100.0%	-3.7
3247	AT5G06960.2 | Symbol: OBF5 | bZIP family transcription factor (OBF5), identical to bZIP family transcription factor (OBF5) GI:414615 from (Arabidopsis thaliana) | chr5:2154823-2157647 FORWARD | Aliases: MOJ9.13, MOJ9_13, OBF5, OCS ELEMENT BINDING FACTOR 5, TGA5	6.1	5.6	0.5	1.8	100.0%	-3.3
3248	AT3G02860.2 | expressed protein | chr3:625187-627374 REVERSE | Aliases: F13E7.20, F13E7_20	6.0	5.6	0.4	1.8	100.0%	-3.6
3249	AT1G73970.1 | expressed protein | chr1:27820475-27823735 FORWARD | Aliases: F2P9.16, F2P9_16	5.9	6.3	-0.5	-1.8	100.0%	-3.4
3250	AT1G12800.1 | S1 RNA-binding domain-containing protein, contains Pfam domain, PF00575: S1 RNA binding domain | chr1:4361549-4365314 REVERSE | Aliases: F13K23.5, F13K23_5	6.5	7.4	-0.9	-1.8	100.0%	-3.2
3251	AT2G30550.2 | lipase class 3 family protein, similar to DEFECTIVE IN ANTHER DEHISCENCE1 (Arabidopsis thaliana) GI:16215706; contains Pfam profile PF01764: Lipase | chr2:13021925-13024230 FORWARD | Aliases: T6B20.10, T6B20_10	7.0	6.1	0.9	1.8	100.0%	-3.4
3252	AT3G58700.1 | 60S ribosomal protein L11 (RPL11B), ribosomal protein L11, cytosolic, Arabidopsis thaliana, PIR:S49033 | chr3:21722540-21723930 FORWARD | Aliases: T20N10.50	9.9	10.3	-0.4	-1.7	100.0%	-3.5
3253	AT5G07240.1 | calmodulin-binding family protein, contains Pfam profile PF00612: IQ calmodulin-binding motif | chr5:2271920-2274288 FORWARD | Aliases: T28J14.180, T28J14_180	3.9	3.4	0.5	1.7	100.0%	-3.4
3254	AT1G01820.1 | peroxisomal biogenesis factor 11 family protein / PEX11 family protein, contains Pfam PF05648: Peroxisomal biogenesis factor 11 (PEX11) | chr1:296001-298109 REVERSE | Aliases: T1N6.24, T1N6_24	7.8	7.4	0.4	1.7	100.0%	-3.7
3255	AT3G26085.1 | CAAX amino terminal protease family protein, contains Pfam profile PF02517 CAAX amino terminal protease family protein | chr3:9532006-9533850 FORWARD | Aliases: None	5.8	5.4	0.4	1.7	100.0%	-3.5
3256	AT2G30400.1 | ovate family protein, 57% similar to ovate protein (GI:23429649) (Lycopersicon esculentum); contains TIGRFAM TIGR01568 : uncharacterized plant-specific domain TIGR01568	4.2	3.5	0.7	1.7	100.0%	-3.3

Rank	Description	15.dpi	5.dpi	M	t	adj.q	B
3257	AT1G31020.1 | thioredoxin o (TRXO2), similar to thioredoxin 2 from Saccharomyces cerevisiae GI:173050, 3'-end of protein contains similarity to thioredoxins; contains Pfam profile: PF00085 Thioredoxin; identical to cDNA thioredoxin o (TRXO2) GI:15081458	6.7	6.2	0.5	1.7	100.0%	-3.6
3258	AT3G63440.1 | similar to FAD-binding domain-containing protein / cytokinin oxidase family protein [Arabidopsis thaliana] (TAIR:At2g41510.1); similar to putative cytokinin oxidase [Oryza sativa (japonica cultivar-group)] (GB:XP_463696.1); similar to putative cytokinin dehydrogenase [Oryza sativa (japonica cultivar-group)] (GB:BAD87612.1); similar to cytokinin oxidase 3 [Zea mays] (GB:CAE55201.1); contains InterPro domain FAD linked oxidase, N-terminal (InterPro:IPR006094) | chr3:23435132-23437240 FORWARD | Aliases: MAA21.70	6.7	5.8	0.9	1.7	100.0%	-3.1
3259	AT5G59440.2 | thymidylate kinase family protein, similar to thymidylate kinase GI:291900 | chr5:23988461-23990097 FORWARD | Aliases: F2O15.12, F2O15_12	6.5	5.7	0.8	1.7	100.0%	-3.2
3260	AT2G24100.1 | expressed protein, similar to expressed protein [Arabidopsis thaliana] (TAIR:At4g30780.1); similar to hypothetical protein [Pinus pinaster] (GB:CAC84497.1) | chr2:10251966-10253999 FORWARD | Aliases: F27D4.1, F27D4_1	6.1	5.0	1.1	1.7	100.0%	-3.3
3261	AT3G03970.3 | expressed protein | chr3:1028004-1030612 REVERSE | Aliases: T11I18.8, T11I18_8	4.2	4.6	-0.5	-1.7	100.0%	-3.6
3262	AT2G20240.1 | expressed protein | chr2:8734713-8737581 REVERSE | Aliases: F11A3.21, F11A3_21	2.7	2.9	-0.2	-1.7	100.0%	-4.0
3263	AT3G06040.2 | ribosomal protein L12 family protein, contains similarity to 50S ribosomal protein L12-C, chloroplast precursor GB:P36212 from (Arabidopsis thaliana) | chr3:1824382-1825634 REVERSE | Aliases: F24F17.2, F24F17_2	5.1	6.0	-0.9	-1.7	100.0%	-3.3
3264	AT3G03060.1 | AAA-type ATPase family protein, contains a ATP/GTP-binding site motif A (P-loop), PROSITE:PS00017 | chr3:692195-695431 FORWARD | Aliases: T17B22.25, T17B22_25	4.6	5.2	-0.6	-1.7	100.0%	-3.4
3265	AT3G05430.1 | PWWP domain-containing protein, contains Pfam profile:PF00855 PWWP domain | chr3:1567631-1571078 FORWARD | Aliases: F22F7.12, F22F7_12	3.8	4.1	-0.3	-1.7	100.0%	-4.0
3266	AT3G57540.1 | remorin family protein, contains Pfam domain, PF03763: Remorin, C-terminal region | chr3:21312374-21314038 REVERSE | Aliases: T8H10.140	8.7	7.6	1.1	1.7	100.0%	-3.6
3267	AT1G27200.1 | expressed protein, contains Pfam PF01697: Domain of unknown function | chr1:9449536-9451737 REVERSE | Aliases: T7N9.26, T7N9_26	3.5	3.3	0.2	1.7	100.0%	-4.1
3268	AT1G61920.1 | expressed protein | chr1:22892122-22892433 FORWARD | Aliases: F8K4.11, F8K4_11	7.6	6.7	0.8	1.7	100.0%	-3.5
3269	AT5G64550.1 | loricrin-related, contains weak similarity to Loricrin (Swiss-Prot:P23490) (Homo sapiens) | chr5:25818754-25822206 REVERSE | Aliases: MUB3.7, MUB3_7	5.5	5.1	0.4	1.7	100.0%	-3.6
3270	AT1G06630.1 | F-box family protein, contains F-box domain Pfam:PF00646	4.6	4.2	0.4	1.7	100.0%	-3.6
3271	AT3G10860.1 | ubiquinol-cytochrome C reductase complex ubiquinone-binding protein, putative / ubiquinol-cytochrome C reductase complex 8.2 kDa protein, putative, similar to ubiquinol--cytochrome c reductase GI:633687 from (Solanum tuberosum) | chr3:3399741-3400732 FORWARD | Aliases: T7M13.6	13.2	13.4	-0.2	-1.7	100.0%	-4.7
3272	AT5G40450.1 | expressed protein | chr5:16202027-16212756 REVERSE | Aliases: MPO12.160, MPO12_160	9.4	8.6	0.7	1.7	100.0%	-3.7
3273	AT2G20550.2 | similar to DNAJ heat shock family protein [Arabidopsis thaliana] (TAIR:At2g20560.1); similar to DnaJ like protein [Lycopersicon esculentum] (GB:CAC16088.2); contains InterPro domain Heat shock protein DnaJ (InterPro:IPR003095); contains InterPro domain Chaperone DnaJ, C-terminal (InterPro:IPR002939) | chr2:8852883-8854392 REVERSE | Aliases: T13C7.14, T13C7_14	4.7	4.3	0.4	1.7	100.0%	-3.5
3274	AT1G56690.1 | pentatricopeptide (PPR) repeat-containing protein, contains INTERPRO:IPR002885 PPR repeats | chr1:21257482-21259596 FORWARD | Aliases: F25P12.87, F25P12_87	2.6	2.9	-0.2	-1.7	100.0%	-4.1
3275	AT1G80440.1 | kelch repeat-containing F-box family protein, similar to SP:Q9ER30 Kelch-related protein 1 (Sarcosin) {Rattus norvegicus}; contains Pfam profiles PF01344: Kelch motif, PF00646: F-box domain | chr1:30246499-30247956 FORWARD | Aliases: T21F11.23, T21F11_23	5.4	4.5	0.9	1.7	100.0%	-3.3
3276	AT3G44750.1 | Symbol: HD2A | histone deacetylase, putative (HD2A), contains Pfam domain, PF00096: Zinc finger, C2H2 type; identical to cDNA putative histone deacetylase (HD2A) GI:11066134 | chr3:16308970-16310733 FORWARD | Aliases: ATHD2A, HDA3, HDT1, HISTONE DEACETYLASE 2A, T32N15.8	7.7	8.5	-0.8	-1.7	100.0%	-3.2
3277	AT4G12550.1 | Symbol: AIR1 | protease inhibitor/seed storage/lipid transfer protein (LTP) family protein, similar to pEARLI 1 (Accession No. L43080): an Arabidopsis member of a conserved gene family (PGF95-099), Plant Physiol. 109 (4), 1497 (1995); contains Pfam protease inhibitor/seed storage/LTP family domain PF00234; identical to cDNA AIR1 mRNA, partial cds GI:3695016	2.2	3.4	-1.2	-1.7	100.0%	-3.0

Rank	Description	15.dpi	5.dpi	M	t	adj.q	B
3278	AT4G19500.1 | disease resistance protein (TIR-NBS-LRR class), putative, domain signature TIR-NBS-LRR exists, suggestive of a disease resistance protein. A false intron was added between exons 2 and 3 to circumvent a frameshift caused by a sequencing error, as per Blake Meyers (bcmeyers@vegmail.ucdavis.edu) | chr4:10625798-10630150 FORWARD | Aliases: F24J7.60, F24J7_60	5.7	6.3	-0.6	-1.7	100.0%	-3.5
3279	AT2G25270.1 | expressed protein | chr2:10766828-10769797 FORWARD | Aliases: T22F11.14, T22F11_14	6.7	7.5	-0.8	-1.7	100.0%	-3.1
3280	AT4G20980.3 | similar to eukaryotic translation initiation factor 3 subunit 7, putative / eIF-3 zeta, putative / eIF3d, putative [Arabidopsis thaliana] (TAIR:At5g44320.1); similar to ENSANGP00000015368 [Anopheles gambiae str. PEST] (GB:XP_312631.2); contains InterPro domain Eukaryotic translation initiation factor 3, subunit 7 (InterPro:IPR007783) | chr4:11216955-11219330 FORWARD | Aliases: T13K14.140, T13K14_140	7.3	8.2	-0.9	-1.7	100.0%	-3.4
3281	AT5G43990.2 | SET domain-containing protein, identical to SET domain protein SUVR2 (Arabidopsis thaliana) GI:15290521; contains Pfam profiles PF00856: SET domain, PF05033: Pre-SET motif; identical to cDNA SET domain protein SUVR2 GI:15290520 | chr5:17715116-17719190 FORWARD | Aliases: MRH10.10, MRH10_10	4.5	5.1	-0.6	-1.7	100.0%	-3.4
3282	AT3G20330.1 | aspartate carabmoyltransferase, chloroplast / aspartate transcarbamylase / ATCase (PYRB), identical to SP:P49077 Aspartate carbamoyltransferase, chloroplast precursor (EC 2.1.3.2) (Aspartate transcarbamylase) (ATCase) {Arabidopsis thaliana}	7.9	9.0	-1.1	-1.7	100.0%	-3.3
3283	AT4G23750.2 | encodes a member of the ERF (ethylene response factor) subfamily B-5 of ERF/AP2 transcription factor family. The protein contains one AP2 domain. There are 7 members in this subfamily.	3.4	3.9	-0.5	-1.7	100.0%	-3.6
3284	AT3G11964.1 | S1 RNA-binding domain-containing protein, similar to SP:Q05022 rRNA biogenesis protein RRP5 {Saccharomyces cerevisiae}; contains Pfam profile PF00575: S1 RNA binding domain | chr3:3793963-3806632 REVERSE | Aliases: MEC18.13	5.7	6.6	-0.9	-1.7	100.0%	-3.2
3285	AT5G24930.1 | zinc finger (B-box type) family protein, similar to CONSTANS-like protein 1 GI:4091804 from (Malus x domestica) | chr5:8589237-8591239 FORWARD | Aliases: F6A4.140, F6A4_140	8.5	8.0	0.5	1.7	100.0%	-3.7
3286	AT4G27180.1 | Symbol: ATK2 | kinesin-like protein B (KATB) | chr4:13614866-13619162 REVERSE | Aliases: ARABIDOPSIS THALIANA KINESIN 2, KATB, KINESIN LIKE PROTEIN B, T24A18.130, T24A18_130	5.5	6.3	-0.8	-1.7	100.0%	-3.5
3287	AT5G25890.1 | Symbol: IAA28 | auxin-responsive protein / indoleacetic acid-induced protein 28 (IAA28), identical to SP:Q9XFM0:AXIS_ARATH Auxin-responsive protein IAA28 (Indoleacetic acid-induced protein 28) {Arabidopsis thaliana}	6.0	5.4	0.6	1.7	100.0%	-3.4
3288	AT1G53160.2 | Symbol: SPL4 | squamosa promoter-binding protein-like 4 (SPL4), nearly identical to squamosa promoter binding protein-like 4 (Arabidopsis thaliana) GI:5931657; contains Pfam profile PF03110: SBP domain | chr1:19810087-19811276 FORWARD | Aliases: F8L10.12, F8L10_12, SQUAMOSA PROMOTER BINDING PROTEIN LIKE 4	3.5	3.2	0.2	1.7	100.0%	-4.1
3289	AT4G28390.1 | Symbol: AAC3 | ADP, ATP carrier protein, mitochondrial, putative / ADP/ATP translocase, putative / adenine nucleotide translocator, putative, similar to mitochondrial ADP,ATP carrier protein SP:P12857 from (Zea mays) | chr4:14040745-14043251 REVERSE | Aliases: ADP/ATP CARRIER 3, ATAAC3, F20O9.60, F20O9_60	7.4	6.6	0.8	1.7	100.0%	-3.2
3290	AT5G06820.1 | leucine-rich repeat transmembrane protein kinase, putative | chr5:2112995-2116664 FORWARD | Aliases: MPH15.19, MPH15_19	3.0	2.8	0.3	1.7	100.0%	-4.0
3291	AT1G75180.3 | expressed protein | chr1:28219134-28222046 REVERSE | Aliases: F22H5.9, F22H5_9	5.0	4.6	0.4	1.7	100.0%	-3.9
3292	AT5G61590.1 | encodes a member of the ERF (ethylene response factor) subfamily B-3 of ERF/AP2 transcription factor family. The protein contains one AP2 domain. There are 18 members in this subfamily including ATERF-1, ATERF-2, AND ATERF-5. | chr5:24781664-24782550 REVERSE | Aliases: K11J9.4, K11J9_4	6.6	5.7	0.9	1.7	100.0%	-3.5
3293	AT3G13030.2 | similar to hAT dimerisation domain-containing protein [Arabidopsis thaliana] (TAIR:At3g13020.1); similar to hypothetical protein [Oryza sativa (japonica cultivar-group)] (GB:NP_910050.1); contains InterPro domain HAT dimerisation (InterPro:IPR008906); contains InterPro domain Protein of unknown function DUF659 (InterPro:IPR007021) | chr3:4169548-4171785 REVERSE | Aliases: MGH6.16	4.1	4.5	-0.4	-1.7	100.0%	-3.7
3294	AT5G27380.1 | Symbol: GSH2 | glutathione synthetase (GSH2), non-consensus AT donor splice site at exon 6, AC acceptor splice site at exon 7; identical to Swiss-Prot:P46416 glutathione synthetase, chloroplast precursor (Glutathione synthase) (GSH synthetase) (GSH-S) (Arabidopsis thaliana) | chr5:9668039-9670858 REVERSE | Aliases: F21A20.90, F21A20_90, GLUTATHIONE SYNTHETASE, GSHB	10.1	10.4	-0.3	-1.7	100.0%	-4.1
3295	AT2G40010.1 | 60S acidic ribosomal protein P0 (RPP0A) | chr2:16715457-16717526 REVERSE | Aliases: T28M21.17, T28M21_17	7.3	5.8	1.5	1.7	100.0%	-3.2
3296	AT2G43535.1 | Encodes a defensin-like (DEFL) family protein. | chr2:18078050-18078739 FORWARD | Aliases: None	3.7	4.7	-1.1	-1.7	100.0%	-3.3
3297	AT1G24260.2 | Symbol: SEP3 | MADS-box protein (AGL9), strongly similar to GB:O22456, MADS-box protein, Location of EST gb:H37053 | chr1:8593631-8595857 REVERSE | Aliases: AGAMOUS LIKE 9, AGL9, F3I6.19, F3I6_19, SEPALLATA3, TRANSCRIPTION FACTOR AGL9	2.3	2.2	0.1	1.7	100.0%	-4.6

Rank	Description	15.dpi	5.dpi	M	t	adj.q	B
3298	AT5G20850.1 | Symbol: ATRAD51 | DNA repair protein RAD51, putative, identical to Rad51-like protein (Arabidopsis thaliana) GI:2388778; strong similarity to SP:Q06609 DNA repair protein RAD51 homolog 1 {Homo sapiens}; contains Pfam profile PF00633: Helix-hairpin-helix motif | chr5:7070588-7072953 REVERSE | Aliases: F22D1.20, F22D1_20, RAD51	3.6	4.0	-0.4	-1.7	100.0%	-3.9
3299	AT1G03620.1 | phagocytosis and cell motility protein ELMO1-related, contains weak similarity to ELMO1 (Mus musculus) gi:16118551:gb:AAL14464 | chr1:904318-906012 REVERSE | Aliases: F21B7.23	3.7	3.1	0.5	1.7	100.0%	-3.5
3300	AT3G62380.1 | expressed protein, weak similarity to S locus F-box (SLF)-S2 protein (Antirrhinum hispanicum) GI:13161526 | chr3:23094584-23095827 REVERSE | Aliases: T12C14.80	2.8	2.6	0.2	1.7	100.0%	-4.1
3301	AT5G20430.1 | mob1/phocein family protein, contains Pfam profile: PF03637 mob1/phocein family | chr5:6904707-6905393 FORWARD | Aliases: F7C8.20, F7C8_20	3.0	2.6	0.4	1.7	100.0%	-3.8
3302	AT5G20440.1 | similar to mob1/phocein family protein [Arabidopsis thaliana] (TAIR:At5g45550.1); similar to putative Mob1-like protein [Oryza sativa (japonica cultivar-group)] (GB:XP_463026.1); similar to putative Mob1/phocein family protein [Oryza sativa (japonica cultivar-group)] (GB:AAT76373.1); contains InterPro domain Mob1/phocein family (InterPro:IPR005301) | chr5:6908668-6909998 FORWARD | Aliases: F7C8.30, F7C8_30	3.0	2.6	0.4	1.7	100.0%	-3.8
3303	AT1G45000.1 | 26S proteasome regulatory complex subunit p42D, putative, similar to 26S proteasome regulatory complex subunit p42D (Drosophila melanogaster) gi:6434958:gb:AAF08391 | chr1:17011584-17014326 FORWARD | Aliases: F27F5.8, F27F5_8	10.1	10.6	-0.5	-1.7	100.0%	-3.7
3304	AT1G78370.1 | Symbol: ATGSTU20 | glutathione S-transferase, putative, similar to 2,4-D inducible glutathione S-transferase GI:2920666 from (Glycine max) | chr1:29489165-29490183 REVERSE | Aliases: F3F9.23, F3F9_23	3.3	3.8	-0.5	-1.7	100.0%	-3.5
3305	AT1G75100.1 | expressed protein, low similarity to SP:O14976 Cyclin G-associated kinase (EC 2.7.1.-) {Homo sapiens} | chr1:28194604-28197461 REVERSE | Aliases: F9E10.5, F9E10_5	4.4	3.9	0.5	1.7	100.0%	-3.5
3306	AT3G11150.1 | expressed protein | chr3:3492873-3494655 REVERSE | Aliases: F11B9.8	3.7	3.3	0.4	1.7	100.0%	-3.7
3307	AT3G60900.1 | Symbol: FLA10 | fasciclin-like arabinogalactan-protein (FLA10) | chr3:22510335-22512214 REVERSE | Aliases: T4C21.310	2.1	2.2	-0.2	-1.7	100.0%	-4.4
3308	AT3G62010.1 | expressed protein | chr3:22975652-22982754 FORWARD | Aliases: F21F14.180	6.2	5.6	0.6	1.7	100.0%	-3.6
3309	AT2G18730.1 | diacylglycerol kinase, putative, contains INTERPRO domain, IPR001206, DAG-kinase catalytic domain | chr2:8125912-8129003 FORWARD | Aliases: MSF3.11, MSF3_11	4.8	4.4	0.4	1.7	100.0%	-3.7
3310	AT5G64580.1 | AAA-type ATPase family protein, similar to zinc dependent protease (Arabidopsis thaliana) GI:7650138; contains Pfam profile PF00004: ATPase AAA family | chr5:25834318-25838691 REVERSE | Aliases: MUB3.10, MUB3_10	4.6	5.3	-0.7	-1.7	100.0%	-3.3
3311	AT3G48750.1 | Symbol: CDC2A | A-type cyclin-dependent kinase. Together with its specific inhibitor, the Kip-related protein, KRP2 they regulate the mitosis-to-endocycle transition during leaf development. | chr3:18082533-18085626 FORWARD | Aliases: CDC2AAT, CDK2, CDKA;1, CELL DIVISION CONTROL 2, CYCLIN DEPENDENT KINASE A;1, P34CDC2, T21J18.20	6.7	7.7	-1.1	-1.7	100.0%	-3.2
3312	AT5G49460.1 | Symbol: ACLB 2 |	5.4	6.3	-1.0	-1.7	100.0%	-3.5
3313	AT1G74160.1 | expressed protein | chr1:27890797-27895084 FORWARD | Aliases: F9E11.1, F9E11_1	3.8	4.3	-0.5	-1.7	100.0%	-3.5
3314	AT5G54260.1 | Symbol: MRE11 | DNA repair and meiosis protein (Mre11), identical to DNA repair and meiosis protein (Mre11) GI:5524769 from (Arabidopsis thaliana) | chr5:22049295-22055217 FORWARD | Aliases: MDK4.8, MDK4_8	2.8	3.1	-0.3	-1.7	100.0%	-4.0
3315	AT2G45540.1 | WD-40 repeat family protein / beige-related, contains Pfam PF02138: Beige/BEACH domain; contains Pfam PF00400: WD domain, G-beta repeat (3 copies) | chr2:18763579-18779303 REVERSE | Aliases: F17K2.7	4.2	4.9	-0.7	-1.7	100.0%	-3.4
3316	AT1G32760.1 | glutaredoxin family protein, contains Pfam profile PF00462: Glutaredoxin	5.3	4.9	0.4	1.7	100.0%	-3.8
3317	AT3G13760.1 | DC1 domain-containing protein, contains Pfam profile PF03107: DC1 domain	5.2	4.6	0.6	1.7	100.0%	-3.2
3318	AT3G13845.1 | expressed protein | chr3:4557333-4558586 REVERSE | Aliases: None	9.6	10.2	-0.5	-1.7	100.0%	-3.8
3319	AT4G28290.1 | expressed protein | chr4:14011875-14012589 REVERSE | Aliases: F26K10.170, F26K10_170	5.4	4.5	0.9	1.7	100.0%	-3.4

Rank	Description	15.dpi	5.dpi	M	t	adj.q	B
3320	AT1G26230.1 | chaperonin, putative, similar to SWISS-PROT:P08927- RuBisCO subunit binding-protein beta subunit, chloroplast precursor (60 kDa chaperonin beta subunit, CPN-60 beta) (Pisum sativum); contains Pfam:PF00118 domain, TCP-1/cpn60 chaperonin family | chr1:9072375-9075266 REVERSE | Aliases: F28B23.11, F28B23_11	4.8	4.2	0.6	1.7	100.0%	-3.2
3321	AT5G62580.1 | Symbol: VEX1 | Encodes a protein that is expressed in vegetative cells of pollen. | chr5:25137089-25139752 FORWARD | Aliases: K19B1.19, K19B1_19, VEGETATIVE CELL EXPRESSED1, VEX1	5.0	5.4	-0.5	-1.7	100.0%	-3.5
3322	AT4G24630.1 | zinc finger (DHHC type) family protein, contains Pfam profile PF01529: DHHC zinc finger domain | chr4:12714929-12717121 FORWARD | Aliases: F22K18.170, F22K18_170	3.3	3.0	0.3	1.7	100.0%	-4.0
3323	AT5G50335.1 | expressed protein | chr5:20506320-20506953 REVERSE | Aliases: None	2.7	2.6	0.2	1.7	100.0%	-4.5
3324	AT1G55050.1 | expressed protein, similar to expressed protein [Arabidopsis thaliana] (TAIR:At1g09040.1); similar to expressed protein [Arabidopsis thaliana] (TAIR:At1g09050.1); similar to hypothetical protein DDB0188541 [Dictyostelium discoideum] (GB:EAL62571.1)	2.4	2.6	-0.2	-1.7	100.0%	-4.3
3325	AT2G37510.1 | RNA-binding protein, putative, similar to SP:P10979 Glycine-rich RNA-binding, abscisic acid-inducible protein {Zea mays}; contains InterPro entry IPR000504: RNA-binding region RNP-1 (RNA recognition motif) (RRM) | chr2:15750070-15751619 REVERSE | Aliases: F3G5.30, F3G5_30	4.9	5.6	-0.6	-1.7	100.0%	-3.3
3326	AT1G09740.1 | ethylene-responsive protein, putative, similar to ER6 protein (Lycopersicon esculentum) GI:5669654; contains Pfam profile PF00582: universal stress protein family | chr1:3154534-3156252 FORWARD | Aliases: F21M12.12, F21M12_12	6.9	5.6	1.3	1.7	100.0%	-3.3
3327	AT1G65220.1 | eIF4-gamma/eIF5/eIF2-epsilon domain-containing protein, low similarity to SP:P47823 Translation initiation factor eIF-2B epsilon subunit (eIF-2B GDP-GTP exchange factor) {Oryctolagus cuniculus}; contains Pfam profile PF02020: eIF4-gamma/eIF5/eIF2-epsilon | chr1:24229774-24232633 FORWARD | Aliases: T23K8.13, T23K8_13	8.6	8.2	0.4	1.7	100.0%	-3.9
3328	AT3G16730.1 | expressed protein, ; expression supported by MPSS | chr3:5695639-5698869 REVERSE | Aliases: MGL6.20	3.5	3.9	-0.3	-1.7	100.0%	-3.8
3329	AT1G35310.1 | Bet v I allergen family protein, similar to Csf-2 (Cucumis sativus)(GI:5762258)(J Am Soc Hortic Sci 124, 136-139 (1999)) ; contains Pfam profile PF00407: Pathogenesis-related protein Bet v I family | chr1:12956137-12957048 REVERSE | Aliases: T9I1.8, T9I1_8	3.0	2.9	0.1	1.7	100.0%	-4.5
3330	AT4G01560.1 | brix domain-containing protein, contains Pfam domain, PF04427: Brix domain | chr4:677054-679229 REVERSE | Aliases: F11O4.6, F11O4_6	4.6	5.3	-0.6	-1.7	100.0%	-3.7
3331	AT3G12160.1 | Ras-related GTP-binding family protein, similar to ras-related GTP-binding protein RGP1 SP:P25766 from (Oryza sativa);contains Pfam profile: PF00071 Ras family | chr3:3879502-3880444 REVERSE | Aliases: T21B14.2	3.0	2.7	0.3	1.7	100.0%	-4.1
3332	AT1G74470.1 | geranylgeranyl reductase, identical to geranylgeranyl reductase GB:Y14044 (Arabidopsis thaliana) (involvement: chlorophyll, the tocopherol and the phylloquinone pathways Eur J Biochem 1998 Jan 15;251(1-2):413-7) | chr1:27994826-27996667 FORWARD | Aliases: F1M20.15, F1M20_15	11.2	9.9	1.3	1.7	100.0%	-3.3
3333	AT5G01790.1 | expressed protein, similar to expressed protein [Arabidopsis thaliana] (TAIR:At2g40475.1); similar to unknown protein [Oryza sativa (japonica cultivar-group)] (GB:XP_475660.1) | chr5:304894-305693 REVERSE | Aliases: T20L15.60, T20L15_60	3.7	3.3	0.4	1.7	100.0%	-3.5
3334	AT5G65670.2 | Symbol: IAA9 | auxin-responsive protein / indoleacetic acid-induced protein 9 (IAA9), identical to SP:Q38827 Auxin-responsive protein IAA9 (Indoleacetic acid-induced protein 9) {Arabidopsis thaliana}	6.0	6.9	-0.9	-1.7	100.0%	-3.5
3335	AT4G37740.1 | Symbol: AtGRF2 |	3.7	4.5	-0.8	-1.7	100.0%	-3.4
3336	AT4G09140.1 | Symbol: ATMLH1 |	3.9	4.2	-0.4	-1.7	100.0%	-3.9
3337	AT3G13640.1 | Symbol: ATRLI1 |	4.3	3.9	0.5	1.7	100.0%	-3.7
3338	AT3G55060.1 | expressed protein, contains weak similarity to intracellular protein transport protein USO1 (Swiss-Prot:P25386) (Saccharomyces cerevisiae); expression supported by MPSS	3.5	4.0	-0.5	-1.7	100.0%	-3.8
3339	AT3G10350.1 | anion-transporting ATPase family protein, similar to SP:O43681 Arsenical pump-driving ATPase (EC 3.6.3.16) (Arsenite-translocating ATPase) (Arsenical resistance ATPase) (Arsenite-transporting ATPase) (ARSA) (ASNA-I) {Homo sapiens}; contains Pfam profile PF02374: Anion-transporting ATPase; contains non-consensus GA donor splice site at intron 5 | chr3:3208244-3211299 FORWARD | Aliases: F14P13.5	7.8	7.3	0.5	1.7	100.0%	-3.4
3340	AT4G31070.1 | pentatricopeptide (PPR) repeat-containing protein, contains INTERPRO:IPR002885 PPR repeats | chr4:15118702-15120543 REVERSE | Aliases: F6I18.20, F6I18_20	2.9	3.0	-0.1	-1.7	100.0%	-4.6

Rank	Description	15.dpi	5.dpi	M	t	adj.q	B
3341	AT3G23410.1 | alcohol oxidase-related, similar to long chain fatty alcohol oxidase from Candida cloacae (GI:6983581), Candida tropicalis (GI:6983594) | chr3:8382818-8386155 FORWARD | Aliases: MLM24.14	6.7	5.9	0.8	1.7	100.0%	-3.4
3342	AT5G46250.2 | RNA recognition motif (RRM)-containing protein, contains similarity to RNA-binding protein; contains InterPro entry IPR000504: RNA-binding region RNP-1 (RNA recognition motif) (RRM) | chr5:18772535-18775505 FORWARD | Aliases: MPL12.3, MPL12_3	10.4	10.0	0.4	1.7	100.0%	-3.9
3343	AT3G60470.1 | expressed protein, contains Pfam profile PF03140: Plant protein of unknown function | chr3:22355788-22357859 FORWARD | Aliases: T8B10.130	3.0	2.7	0.3	1.7	100.0%	-3.8
3344	AT2G31090.1 | expressed protein | chr2:13263215-13263788 FORWARD | Aliases: T16B12.10, T16B12_10	9.9	9.4	0.5	1.7	100.0%	-3.8
3345	AT5G01450.1 | expressed protein | chr5:183329-186444 REVERSE | Aliases: T10O8.160, T10O8_160	5.8	4.9	0.9	1.7	100.0%	-3.5
3346	AT2G27980.1 | expressed protein | chr2:11920706-11926818 REVERSE | Aliases: T1E2.10, T1E2_10	3.4	3.1	0.3	1.7	100.0%	-3.8
3347	AT3G57010.1 | strictosidine synthase family protein, similar to strictosidine synthase (Rauvolfia serpentina)(SP:P15324) | chr3:21106633-21108430 REVERSE | Aliases: F24I3.90	9.0	9.9	-0.9	-1.7	100.0%	-3.3
3348	AT1G75800.1 | pathogenesis-related thaumatin family protein, similar to receptor serine/threonine kinase PR5K (Arabidopsis thaliana) GI:1235680; contains Pfam profile: PF00314 Thaumatin family | chr1:28462443-28464515 FORWARD | Aliases: T4O12.3, T4O12_3	6.2	5.5	0.7	1.7	100.0%	-3.8
3349	AT5G12160.1 | expressed protein, similar to unknown protein (pir::T09909) | chr5:3931650-3932090 FORWARD | Aliases: MXC9.12, MXC9_12	3.7	3.3	0.4	1.7	100.0%	-3.9
3350	AT2G24050.1 | MIF4G domain-containing protein / MA3 domain-containing protein, similar to SP:Q03387 Eukaryotic initiation factor (iso)4F subunit P82-34 (eIF-(iso)4F P82-34) {Triticum aestivum}; contains Pfam profiles PF02854: MIF4G domain, PF02847: MA3 domain | chr2:10232305-10235718 REVERSE | Aliases: T29E15.25, T29E15_25	6.5	7.0	-0.5	-1.7	100.0%	-3.7
3351	AT3G54130.1 | josephin family protein, contains Pfam domain PF02099: Josephin | chr3:20053683-20055504 REVERSE | Aliases: F24B22.90	4.4	4.7	-0.3	-1.7	100.0%	-3.8
3352	AT5G16760.1 | Encodes a inositol 1,3,4-trisphosphate 5/6-kinase. | chr5:5509677-5511235 FORWARD | Aliases: F5E19.100, F5E19_100, INOSITOL 1,3,4 TRISPHOSPHATE 5/6 KINASE	7.7	8.2	-0.5	-1.7	100.0%	-3.6
3353	AT3G09085.1 | expressed protein | chr3:2782156-2782688 FORWARD | Aliases: None	7.8	6.9	0.9	1.7	100.0%	-3.4
3354	AT3G14200.1 | DNAJ heat shock N-terminal domain-containing protein, low similarity to SP:O75190:DJB6_HUMAN DnaJ homolog subfamily B member 6 (Heat shock protein J2) {Homo sapiens}; contains Pfam profile PF00226 DnaJ domain	5.6	5.1	0.4	1.7	100.0%	-3.7
3355	AT1G70660.1 | ubiquitin-conjugating enzyme family protein, similar to TRAF6-regulated IKK activator 1 beta Uev1A (Homo sapiens) GI:10880969; contains Pfam profile PF00179: Ubiquitin-conjugating enzyme | chr1:26644258-26645810 FORWARD | Aliases: F5A18.16, F5A18_16	7.8	8.4	-0.6	-1.7	100.0%	-3.3
3356	AT5G39900.1 | similar to GTP-binding protein LepA, putative [Arabidopsis thaliana] (TAIR:At5g08650.1); similar to hypothetical protein LOC231279 isoform a [Mus musculus] (GB:NP_766299.1); similar to PREDICTED: similar to Hypothetical protein FLJ13220 [Canis familiaris] (GB:XP_539246.1); similar to PREDICTED: similar to expressed sequence AA407526 isoform a [Rattus norvegicus] (GB:XP_223381.3); similar to unnamed protein product [Homo sapiens] (GB:BAB14507.1); similar to Hypothetical protein FLJ13220 [Homo sapiens] (GB:AAH36768.1); contains InterPro domain GTP-binding protein LepA (InterPro:IPR006297); contains InterPro domain Small GTP-binding protein domain (InterPro:IPR005225); contains InterPro domain Elongation factor G, C-terminal (InterPro:IPR000640); contains InterPro domain Elongation factor, GTP-binding (InterPro:IPR000795) | chr5:15993775-15996229 REVERSE | Aliases: MYH19.60, MYH19_60	3.4	3.7	-0.3	-1.7	100.0%	-4.2
3357	AT4G22010.1 | Symbol: SKS4 | multi-copper oxidase type I family protein, similar to pollen-specific BP10 protein (SP:Q00624)(Brassica napus); contains Pfam profile: PF00394 Multicopper oxidase | chr4:11663336-11666727 FORWARD | Aliases: F1N20.110, F1N20_110, SKS4	2.7	3.3	-0.5	-1.7	100.0%	-3.5
3358	AT2G23120.1 | expressed protein | chr2:9849123-9849714 FORWARD | Aliases: F21P24.18, F21P24_18	11.6	12.1	-0.5	-1.7	100.0%	-4.4
3359	AT3G46210.4 | similar to hypothetical protein [Arabidopsis thaliana] (TAIR:At2g07110.1); similar to putative exosome component [Oryza sativa (japonica cultivar-group)] (GB:XP_470449.1); contains InterPro domain 3' exoribonuclease (InterPro:IPR001247) | chr3:16987739-16989877 REVERSE | Aliases: F12M12.180	5.3	5.8	-0.6	-1.7	100.0%	-3.5
3360	AT3G47380.1 | invertase/pectin methylesterase inhibitor family protein, low similarity to SP:Q42534 Pectinesterase 2 precursor (EC 3.1.1.11) (Pectin methylesterase 2) (PE 2) {Arabidopsis thaliana}; contains Pfam profile PF04043: Plant invertase/pectin methylesterase inhibitor | chr3:17468780-17469555 FORWARD | Aliases: T21L8.130	3.1	3.5	-0.4	-1.7	100.0%	-3.7

Rank	Description	15.dpi	5.dpi	M	t	adj.q	B
3361	AT3G20475.1 | similar to DNA mismatch repair protein MSH3 (MSH3) [Arabidopsis thaliana] (TAIR:At4g25540.1); similar to hypothetical protein [Neurospora crassa] (GB:XP_332076.1); contains InterPro domain DNA mismatch repair protein MutS, C-terminal (InterPro:IPR000432) | chr3:7148223-7150943 FORWARD | Aliases: MQC12.27	2.4	2.6	-0.2	-1.7	100.0%	-4.1
3362	AT1G76660.1 | expressed protein | chr1:28773774-28776260 REVERSE | Aliases: F28O16.3, F28O16_3	4.0	4.4	-0.4	-1.7	100.0%	-3.8
3363	AT3G56370.1 | leucine-rich repeat transmembrane protein kinase, putative, leucine-rich receptor-like protein kinase - Malus domestica, EMBL:AF053127 | chr3:20910078-20913960 REVERSE | Aliases: T5P19.20	3.1	3.4	-0.4	-1.7	100.0%	-3.7
3364	AT2G31970.1 | Symbol: RAD50 | DNA repair-recombination protein (RAD50), identical to DNA repair-recombination protein GI:7110148 from (Arabidopsis thaliana) | chr2:13607589-13616146 FORWARD | Aliases: ATRAD50, DNA REPAIR RECOMBINATION PROTEIN, F22D22.28, F22D22_28	3.4	3.8	-0.4	-1.7	100.0%	-3.8
3365	AT3G11650.1 | Symbol: NHL2 | harpin-induced family protein / HIN1 family protein / harpin-responsive family protein / NDR1/HIN1-like protein 2, identical to NDR1/HIN1-Like protein 2 (GP:9502174) (Arabidopsis thaliana); similar to hin1 GB:CAA68848 (Nicotiana tabacum) | chr3:3676152-3677043 REVERSE | Aliases: T19F11.5	3.9	3.5	0.4	1.7	100.0%	-3.9
3366	AT1G07240.1 | UDP-glucoronosyl/UDP-glucosyl transferase family protein, contains Pfam profile: PF00201 UDP-glucoronosyl and UDP-glucosyl transferase | chr1:2223690-2225447 FORWARD | Aliases: F10K1.5, F10K1_5	5.6	5.1	0.5	1.7	100.0%	-3.4
3367	AT1G69930.1 | Symbol: ATGSTU11 | glutathione S-transferase, putative, similar to glutathione transferase GB:CAA09188 (Alopecurus myosuroides)	5.9	6.9	-1.0	-1.7	100.0%	-3.0
3368	AT5G47830.2 | expressed protein | chr5:19390124-19391557 REVERSE | Aliases: MCA23.17, MCA23_17	6.0	5.6	0.5	1.7	100.0%	-3.5
3369	AT3G18170.1 | expressed protein, contains Pfam domain, PF04577: Protein of unknown function (DUF563) | chr3:6228348-6229668 FORWARD | Aliases: MRC8.17	5.2	4.0	1.1	1.7	100.0%	-3.1
3370	AT5G49900.1 | expressed protein, contains Pfam domain PF04685: Protein of unknown function, DUF608 | chr5:20314349-20319572 REVERSE | Aliases: K9P8.4, K9P8_4	7.8	8.2	-0.4	-1.7	100.0%	-3.6
3371	AT1G06490.1 | Symbol: ATGSL07 | glycosyl transferase family 48 protein, contains Pfam profile: PF02364 1,3-beta-glucan synthase | chr1:1978761-1989045 FORWARD | Aliases: ATGSL7, F12K11.17, F12K11_17, GLUCAN SYNTHASE LIKE 7, GSL07	4.7	4.3	0.4	1.7	100.0%	-3.7
3372	AT5G26850.3 | similar to cyclin-related [Arabidopsis thaliana] (TAIR:At2g41830.1); similar to cyclin-like protein [Oryza sativa (japonica cultivar-group)] (GB:XP_477071.1); similar to cyclin-like protein [Oryza sativa (japonica cultivar-group)] (GB:XP_464126.1) | chr5:9445443-9450822 FORWARD | Aliases: F2P16.24, F2P16_24	4.4	4.9	-0.5	-1.7	100.0%	-3.6
3373	AT1G26750.1 | expressed protein | chr1:9246774-9248261 FORWARD | Aliases: T24P13.13, T24P13_13	7.1	7.9	-0.8	-1.7	100.0%	-3.2
3374	AT1G26790.1 | Dof-type zinc finger domain-containing protein, similar to H-protein promoter binding factor-2b GI:3386548 from (Arabidopsis thaliana) | chr1:9273844-9275299 REVERSE | Aliases: T24P13.17, T24P13_17	2.9	2.6	0.3	1.7	100.0%	-3.9
3375	AT3G58120.1 | bZIP transcription factor family protein, contains Pfam profile: PF00170 bZIP transcription factor ;supported by cDNA gi:15100054:gb:AF401300.1:AF401300	3.1	2.8	0.3	1.7	100.0%	-3.9
3376	AT5G17410.2 | tubulin family protein, similar to spindle pole body protein (Homo sapiens)(GI:2801701)(PMID:9566967), gamma-tubulin ring protein Dgrip84 (Drosophila melanogaster)(GI:4689225)(PMID: 10037793) | chr5:5730612-5737366 FORWARD | Aliases: T10B6.70, T10B6_70	3.2	3.6	-0.4	-1.7	100.0%	-3.8
3377	AT5G40500.1 | expressed protein | chr5:16245062-16245839 FORWARD | Aliases: MNF13.2, MNF13_2	4.7	4.2	0.5	1.7	100.0%	-3.4
3378	AT3G18750.2 | Symbol: WNK6 | similar to protein kinase family protein [Arabidopsis thaliana] (TAIR:At1g49160.2); similar to protein kinase family protein [Arabidopsis thaliana] (TAIR:At1g49160.1); similar to putative MAP kinase [Oryza sativa (japonica cultivar-group)] (GB:BAD27820.1); contains InterPro domain Serine/threonine protein kinase, active site (InterPro:IPR008271); contains InterPro domain Tyrosine protein kinase (InterPro:IPR001245); contains InterPro domain Serine/threonine protein kinase (InterPro:IPR002290); contains InterPro domain Protein kinase (InterPro:IPR000719) | chr3:6454116-6457503 REVERSE | Aliases: MVE11.20, ZIK5	3.6	4.4	-0.7	-1.7	100.0%	-3.4
3379	AT5G61560.1 | protein kinase family protein, contains protein kinase domain, Pfam:PF00069 | chr5:24770094-24773944 FORWARD | Aliases: K11J9.9, K11J9_9	4.6	5.1	-0.5	-1.7	100.0%	-3.5
3380	AT3G27080.1 | Symbol: TOM20 3 | mitochondrial import receptor subunit TOM20-3 / translocase of outer membrane 20 kDa subunit 3 (TOM20-3), identical to mitochondrial import receptor subunit TOM20-3 SP:P82874 from (Arabidopsis thaliana) | chr3:9986319-9987866 REVERSE | Aliases: MOJ10.22, TRANSLOCASE OUTER MEMBRANE 20 3	9.0	9.6	-0.6	-1.7	100.0%	-3.3

Rank	Description	15.dpi	5.dpi	M	t	adj.q	B
3381	AT5G01220.1 | Symbol: SQD2 | UDP-sulfoquinovose:DAG sulfoquinovosyltransferase / sulfolipid synthase (SQD2), identical to GI:20302857	3.9	3.6	0.3	1.7	100.0%	-3.9
3382	AT3G04820.1 | expressed protein, contains PFam profile PF01142: Uncharacterized protein family UPF0024; expression supported by MPSS | chr3:1321537-1325955 REVERSE | Aliases: T9J14.23, T9J14_23	3.4	3.8	-0.4	-1.7	100.0%	-3.8
3383	AT4G36830.1 | GNS1/SUR4 membrane family protein, weak similarity to long chain polyunsaturated fatty acid elongation enzyme (Isochrysis galbana) GI:17226123; contains Pfam profile PF01151: GNS1/SUR4 family | chr4:17349488-17350384 FORWARD | Aliases: AP22.81, AP22_81	4.5	3.7	0.8	1.7	100.0%	-3.3
3384	AT5G62490.1 | Symbol: ATHVA22B | ABA-responsive protein (HVA22b), identical to AtHVA22b (Arabidopsis thaliana) GI:4884934 | chr5:25107356-25108781 FORWARD | Aliases: ATHVA22B, K19B1.10, K19B1_10	4.1	3.7	0.4	1.7	100.0%	-3.7
3385	AT2G43660.2 | glycosyl hydrolase family protein 17, similar to glucan endo-1,3-beta-glucosidase precursor SP:P52409 from (Triticum aestivum); C terminal homology only	6.5	7.4	-0.9	-1.7	100.0%	-3.2
3386	AT1G59700.1 | Symbol: ATGSTU16 | glutathione S-transferase, putative, similar to glutathione S-transferase GB:AAF29773 GI:6856103 from (Gossypium hirsutum) | chr1:21940094-21941624 FORWARD | Aliases: F23H11.1, F23H11_1	4.0	3.5	0.5	1.7	100.0%	-3.7
3387	AT5G47790.1 | forkhead-associated domain-containing protein / FHA domain-containing protein | chr5:19368531-19370670 FORWARD | Aliases: MCA23.11, MCA23_11	5.1	4.8	0.4	1.7	100.0%	-3.8
3388	AT1G71990.1 | Symbol: FUT13 | alpha-(1,4)-fucosyltransferase / galactoside 3(4)-L-fucosyltransferase (FUT13) (FucTC), identical to SP:Q9C8W3 Alpha-(1,4)-fucosyltransferase (EC 2.4.1.-) (FT4-M) (Galactoside 3(4)- L-fucosyltransferase) (FucTC) (AtFUT13) {Arabidopsis thaliana}; identical to cDNA alpha1,3-fucosyltransferase homologue (FucTC) GI:13992485 | chr1:27103946-27105924 FORWARD | Aliases: ATFUT13, F17M19.14, F17M19_14, FT4 M, FUCTC	5.7	5.0	0.6	1.7	100.0%	-3.4
3389	AT5G63980.1 | Symbol: SAL1 | 3'(2'),5'-bisphosphate nucleotidase / inositol polyphosphate 1-phosphatase / FIERY1 protein (FRY1) (SAL1), identical to SP:Q42546 3'(2'),5'-bisphosphate nucleotidase (EC 3.1.3.7) (3'(2'),5- bisphosphonucleoside 3'(2')-phosphohydrolase) (DPNPase) {Arabidopsis thaliana}; identical to cDNA inositol polyphosphate 1-phosphatase FIERY1 (FRY1) GI:15281147 | chr5:25627089-25629157 FORWARD | Aliases: ATSAL1, FIERY 1, FRY1, HIGH EXPRESSION OF OSMOTICALLY RESPONSIVE GENES, HOS2, MBM17.8, MBM17_8	6.3	7.2	-0.9	-1.7	100.0%	-3.6
3390	AT1G69530.3 | Symbol: ATEXPA1 | expansin, putative (EXP1), identical to expansin (At-EXP1) (Arabidopsis thaliana) GI:1041702; alpha-expansin gene family, PMID:11641069 | chr1:26145499-26147159 FORWARD | Aliases: ARABIDOPSIS THALIANA EXPANSIN A1, AT EXP1, ATEXP1, ATHEXP ALPHA 1.2, EXP1, F10D13.18, F10D13_18	8.9	6.9	2.0	1.7	100.0%	-3.1
3391	AT4G39170.1 | SEC14 cytosolic factor, putative / phosphoglyceride transfer protein, putative, similar to phosphatidylinositol transfer-like protein IV (GI:14486707) (Lotus japonicus) and phosphatidylinositol-phosphatidylcholine transfer protein SEC14, Yarrowia lipolytica, PIR2:S43745;contains Pfam PF00650 : CRAL/TRIO domain; contains Pfam PF03765 : CRAL/TRIO, N-terminus | chr4:18240667-18243772 FORWARD | Aliases: T22F8.70, T22F8_70	6.6	7.5	-0.9	-1.7	100.0%	-3.5
3392	AT3G04600.2 | tRNA synthetase class I (W and Y) family protein, contains Pfam profile: PF00579 tRNA synthetases class I (W and Y) | chr3:1242993-1246184 FORWARD | Aliases: F7O18.7, F7O18_7	5.6	6.1	-0.5	-1.7	100.0%	-3.5
3393	AT1G17280.1 | ubiquitin-conjugating enzyme, putative, similar to ubiquitin conjugating enzyme 6 from (Homo sapiens) GI:14029267, (Mus musculus) GI:14029263; contains Pfam profile PF00179: Ubiquitin-conjugating enzyme | chr1:5916864-5920051 REVERSE | Aliases: F20D23.1, F20D23_1	5.8	4.5	1.3	1.7	100.0%	-3.3
3394	AT5G50430.3 | similar to ubiquitin-conjugating enzyme, putative [Arabidopsis thaliana] (TAIR:At1g17280.1); similar to probable ubiquitin-conjugating enzyme E2 [Picea mariana] (GB:AAC32141.1); contains InterPro domain Ubiquitin-conjugating enzymes (InterPro:IPR000608) | chr5:20551399-20554343 REVERSE | Aliases: MXI22.15, MXI22_15	5.8	4.5	1.3	1.7	100.0%	-3.3
3395	AT1G29340.1 | armadillo/beta-catenin repeat family protein / U-box domain-containing protein, contains Pfam domain, PF00514: Armadillo/beta-catenin-like repeats and Pfam, PF04564: U-box domain | chr1:10264381-10266804 FORWARD | Aliases: F15D2.34, F15D2_34	7.1	6.6	0.5	1.7	100.0%	-3.6
3396	AT3G21540.1 | transducin family protein / WD-40 repeat family protein, contains Pfam profile: PF00400 WD domain, G-beta repeat (10 copies); similar to WD-repeat protein 3 (SP:Q9UNX4) (Homo sapiens) | chr3:7585952-7590862 REVERSE | Aliases: MIL23.11	5.0	5.7	-0.7	-1.7	100.0%	-3.4
3397	AT4G26530.2 | similar to fructose-bisphosphate aldolase, cytoplasmic [Arabidopsis thaliana] (TAIR:At4g26520.1); similar to fructose-bisphosphate aldolase [Glycine max] (GB:AAR86689.1); similar to fructose 1,6, bisphosphate aldolase [Salicornia herbacea] (GB:AAR84667.1); contains InterPro domain Fructose-bisphosphate aldolase, class-I (InterPro:IPR000741) | chr4:13391351-13393126 FORWARD | Aliases: M3E9.40, M3E9_40	2.3	2.1	0.1	1.7	100.0%	-4.5
3398	AT3G08710.1 | thioredoxin family protein, similar to thioredoxin H-type GB:P29448 SP:P29448 (Arabidopsis thaliana), Thioredoxin H-type 2 (TRX-H2) SP:Q07090 {Nicotiana tabacum}; contains Pfam profile: PF00085 Thioredoxin | chr3:2645223-2646496 FORWARD | Aliases: F17O14.18	10.4	9.7	0.7	1.7	100.0%	-3.8

Rank	Description	15.dpi	5.dpi	M	t	adj.q	B
3399	AT1G14345.1 | expressed protein, contains one transmembrane domain | chr1:4899141-4899974 FORWARD | Aliases: None	4.2	3.9	0.4	1.7	100.0%	-3.8
3400	AT1G30400.2 | Symbol: ATMRP1 |	5.6	6.3	-0.7	-1.7	100.0%	-3.4
3401	AT2G35710.3 | glycogenin glucosyltransferase (glycogenin)-related, low similarity to glycogenin-2 from Homo sapiens (SP:O15488) | chr2:15020656-15021226 REVERSE | Aliases: T20F21.27	4.1	3.8	0.3	1.7	100.0%	-4.1
3402	AT2G47200.1 | expressed protein | chr2:19384799-19385401 REVERSE | Aliases: T8I13.4	2.8	3.6	-0.8	-1.7	100.0%	-3.2
3403	AT2G29700.1 | Symbol: ATPH1 | pleckstrin homology (PH) domain-containing protein (PH1), identical to AtPH1 (Arabidopsis thaliana) GI:5926716; contains Pfam profile PF00169: PH domain | chr2:12704497-12705244 FORWARD | Aliases: ARABIDOPSIS THALIANA PLECKSTRIN HOMOLOGUE 1, T27A16.20, T27A16_20	10.6	9.7	1.0	1.7	100.0%	-3.9
3404	AT2G23380.1 | Symbol: CLF | curly leaf protein (CURLY LEAF) / polycomb-group protein, identical to polycomb group (Arabidopsis thaliana) GI:1903019 (curly leaf); contains Pfam profile PF00856: SET domain | chr2:9962650-9967197 FORWARD | Aliases: CLF, CURLY LEAF, F26B6.3, F26B6_3, ICU1, INCURVATA 1	4.7	5.3	-0.5	-1.7	100.0%	-3.6
3405	AT1G30820.1 | CTP synthase, putative / UTP--ammonia ligase, putative, similar to SP:P17812 CTP synthase (EC 6.3.4.2) (UTP--ammonia ligase) {Homo sapiens}; contains Pfam profile PF00117: glutamine amidotransferase class-I; similar to ESTs gb:AA660762, gb:AA220982, dbj:AU008137, gb:AI054783, and gb:AA100804 | chr1:10944894-10949431 REVERSE | Aliases: T17H7.12, T17H7_12	6.5	5.6	0.9	1.7	100.0%	-3.4
3406	AT4G09340.1 | SPla/RYanodine receptor (SPRY) domain-containing protein, low similarity to RanBPM (Homo sapiens) GI:15080674; contains Pfam profile PF00622: SPRY domain | chr4:5924599-5927632 REVERSE | Aliases: AT4G09330, T30A10.100, T30A10_100	6.0	7.0	-0.9	-1.7	100.0%	-3.5
3407	AT3G55830.1 | Symbol: EPC1 | glycosyltransferase family protein 47, similar to exostose-related protein 2, Homo sapiens, PIR:JC5935 (SP:Q93063), EXTL2, Mus musculus (GI:10443633) | chr3:20726080-20729017 FORWARD | Aliases: ECTOPICALLY PARTING CELLS, F27K19.10	7.6	7.0	0.6	1.7	100.0%	-3.3
3408	AT5G07390.1 | Symbol: ATRBOHA | respiratory burst oxidase protein A (RbohA) / NADPH oxidase, identical to respiratory burst oxidase protein A from Arabidopsis thaliana (gi:3242781) | chr5:2335896-2339914 REVERSE | Aliases: ATRBOHA, RESPIRATORY BURST OXIDASE HOMOLOG A, T2I1.100, T2I1_100	3.4	4.4	-1.0	-1.7	100.0%	-3.2
3409	AT3G16770.1 | Symbol: ATEBP/RAP2.3 | Encodes a member of the ERF (ethylene response factor) subfamily B-2 of ERF/AP2 transcription factor family (RAP2.3). The protein contains one AP2 domain. There are 5 members in this subfamily including RAP2.2 AND RAP2.12. | chr3:5705721-5707029 FORWARD | Aliases: ATEBP, MGL6.1, RAP2.3, RELATED TO AP2 3	11.4	10.5	0.8	1.7	100.0%	-3.8
3410	AT2G47010.2 | expressed protein | chr2:19324195-19326467 FORWARD | Aliases: F14M4.16	3.1	3.3	-0.2	-1.7	100.0%	-4.3
3411	AT5G47220.1 | Symbol: ATERF 2/ATERF2/ERF2 | encodes a member of the ERF (ethylene response factor) subfamily B-3 of ERF/AP2 transcription factor family (ATERF-2). The protein contains one AP2 domain. There are 18 members in this subfamily including ATERF-1, ATERF-2, AND ATERF-5. Functions as activator of GCC box??dependent transcription. | chr5:19189089-19190050 REVERSE | Aliases: ATERF 2, ATERF2, ERF2, ETHYLENE RESPONSE FACTOR 2, ETHYLENE RESPONSIVE ELEMENT BINDING FACTOR 2, MQL5.7, MQL5_7	4.2	3.8	0.4	1.7	100.0%	-3.6
3412	AT1G21590.1 | protein kinase family protein, contains Pfam domain, PF00069: Protein kinase domain | chr1:7566221-7569890 REVERSE | Aliases: F24J8.18, F24J8_18	3.5	4.0	-0.5	-1.7	100.0%	-3.7
3413	AT2G40290.3 | similar to eukaryotic translation initiation factor 2 subunit 1, putative / eIF-2A, putative / eIF-2-alpha, putative [Arabidopsis thaliana] (TAIR:At5g05470.1); similar to hypothetical protein [Neurospora crassa] (GB:XP_328983.1); contains InterPro domain RNA binding S1 (InterPro:IPR003029) | chr2:16835844-16838132 REVERSE | Aliases: T7M7.14	7.1	8.1	-1.1	-1.7	100.0%	-3.3
3414	AT5G18830.2 | Symbol: SPL7 | squamosa promoter-binding protein-like 7 (SPL7), identical to squamosa promoter binding protein-like 7 (Arabidopsis thaliana) GI:5931635; contains Pfam profile PF03110: SBP domain | chr5:6276024-6280683 FORWARD | Aliases: F17K4.80, F17K4_80, SQUAMOSA PROMOTER BINDING PROTEIN LIKE 7	3.9	4.5	-0.6	-1.7	100.0%	-3.5
3415	AT5G20740.1 | invertase/pectin methylesterase inhibitor family protein, low similarity to pectinesterase from Arabidopsis thaliana SP:Q42534, Lycopersicon esculentum SP:Q43143; contains Pfam profile PF04043: Plant invertase/pectin methylesterase inhibitor | chr5:7025688-7026534 REVERSE | Aliases: T1M15.140, T1M15_140	5.2	6.4	-1.2	-1.7	100.0%	-3.0
3416	AT1G23480.3 | Symbol: ATCSLA03 | similar to glycosyl transferase family 2 protein [Arabidopsis thaliana] (TAIR:At5g03760.1); similar to glycosyl transferase family 2 protein [Arabidopsis thaliana] (TAIR:At1g24070.1); similar to glycosyl transferase family 2 protein [Arabidopsis thaliana] (TAIR:At3g56000.1); similar to glycosyl transferase family 2 protein [Arabidopsis thaliana] (TAIR:At4g13410.1); similar to glycosyl transferase family 2 protein [Arabidopsis thaliana] (TAIR:At5g16190.1); similar to glycosyltransferase 10 [Ipomoea trifida] (GB:AAQ62572.1); similar to glycosyltransferase 5 [Ipomoea trifida] (GB:AAQ62571.1); similar to glycosyltransferase 1 [Ipomoea trifida] (GB:AAQ62570.1); similar to TPA: cellulose synthase-like A1 [Oryza sativa (japonica cultivar-group)] (GB:DAA01743.1); contains InterPro domain Glycosyl transferase, family 2 (InterPro:IPR001173) | chr1:8333685-8336374 FORWARD | Aliases: ATCSLA3, CSLA03, F28C11.11	3.0	3.6	-0.6	-1.7	100.0%	-3.4

Rank	Description	15.dpi	5.dpi	M	t	adj.q	B
3417	AT1G12990.1 | glycosyl transferase family 17 protein, low similarity to beta-1,4-mannosyl-glycoprotein beta-1,4-N-acetylglucosaminyltransferase (EC 2.4.1.144) from Mus musculus (SP:Q10470), Rattus norvegicus (SP:Q02527), Homo sapiens (SP:Q09327); contains Pfam profile PF04724 :Glycosyltransferase family 17 | chr1:4433638-4436068 FORWARD | Aliases: F3F19.2, F3F19_2	3.2	2.9	0.3	1.7	100.0%	-3.9
3418	AT3G09850.1 | D111/G-patch domain-containing protein, contains Pfam profile PF01585: G-patch domain | chr3:3023216-3025856 REVERSE | Aliases: F8A24.10	8.1	8.4	-0.4	-1.7	100.0%	-3.6
3419	AT1G22290.1 | 14-3-3 protein GF14, putative (GRF10), similar to 14-3-3 protein GF14 epsilon GI:5802798 from (Arabidopsis thaliana) | chr1:7876955-7877904 REVERSE | Aliases: T16E15.9, T16E15_9	2.2	2.4	-0.2	-1.7	100.0%	-4.4
3420	AT3G50080.1 | Symbol: VFB2 | F-box family protein (FBL16), contains similarity to SKP1 interacting partner 2 GI:10716949 from (Arabidopsis thaliana); contains Pfam profile: PF00646 F-box domain | chr3:18583719-18585497 FORWARD | Aliases: F3A4.160, VFB2, VIER F BOX PROTEINE 2	6.7	6.1	0.6	1.7	100.0%	-3.4
3421	AT4G12890.1 | gamma interferon responsive lysosomal thiol reductase family protein / GILT family protein, similar to SP:P13284 Gamma-interferon inducible lysosomal thiol reductase precursor {Homo sapiens}; contains Pfam profile PF03227: Gamma interferon inducible lysosomal thiol reductase (GILT) | chr4:7546375-7547569 FORWARD | Aliases: T20K18.240, T20K18_240	3.1	2.8	0.3	1.7	100.0%	-4.0
3422	AT2G16600.1 | Symbol: ROC3 | peptidyl-prolyl cis-trans isomerase, cytosolic / cyclophilin / rotamase (ROC3), identical to cytosolic cyclophilin (Arabidopsis thaliana) GI:1305455 | chr2:7207889-7208650 FORWARD | Aliases: CYCLOPHILIN, T24I21.1, T24I21_1	8.3	9.9	-1.6	-1.7	100.0%	-3.2
3423	AT2G29360.1 | tropinone reductase, putative / tropine dehydrogenase, putative, similar to tropinone reductase SP:P50165 from (Datura stramonium) | chr2:12610902-12612312 FORWARD | Aliases: F16P2.26, F16P2_26	3.6	3.2	0.4	1.7	100.0%	-3.8
3424	AT3G05560.2 | 60S ribosomal protein L22-2 (RPL22B), identical to 60S ribosomal protein L22-2 SP:Q9M9W1 from (Arabidopsis thaliana) | chr3:1614221-1615449 FORWARD | Aliases: F18C1.17, F18C1_17	12.2	12.6	-0.4	-1.7	100.0%	-4.2
3425	AT2G27510.1 | ferredoxin, putative, similar to non-photosynthetic ferredoxin from Citrus sinensis (GI:1360725), Ferredoxin, root R-B2 from Raphanus sativus (SP:P14937); contains Pfam profile PF00111 2Fe-2S iron-sulfur cluster binding domain | chr2:11765157-11766554 REVERSE | Aliases: F10A12.19, F10A12_19	11.8	11.4	0.5	1.7	100.0%	-3.8
3426	AT4G28950.1 | Symbol: ARAC7 | Rac-like GTP-binding protein (ARAC7), identical to rac GTP binding protein Arac7 GI:3702962 from (Arabidopsis thaliana)	3.4	3.8	-0.3	-1.7	100.0%	-4.0
3427	AT5G37730.1 | expressed protein | chr5:15003263-15004147 REVERSE | Aliases: K12B20.20, K12B20_20	2.3	2.1	0.2	1.7	100.0%	-4.7
3428	AT5G55190.1 | Symbol: RAN3 | Ras-related GTP-binding protein (RAN3), identical to atran3 (Arabidopsis thaliana) GI:2058280 | chr5:22409402-22411392 FORWARD | Aliases: MCO15.14, MCO15_14	10.9	11.3	-0.4	-1.7	100.0%	-3.8
3429	AT2G02800.2 | Symbol: APK2B | protein kinase (APK2b), identical to protein kinase APK2b (Arabidopsis thaliana) gi:2852449:dbj:BAA24695 | chr2:795514-799441 REVERSE | Aliases: PROTEIN KINASE 2B, T20F6.6, T20F6_6	9.0	8.2	0.9	1.7	100.0%	-3.5
3430	AT5G63290.1 | coproporphyrinogen oxidase-related, low similarity to coproporphyrinogen III oxidase from Geobacillus stearothermophilus (GI:2104798); contains Pfam profile PF04055: radical SAM domain protein	4.2	4.7	-0.5	-1.7	100.0%	-3.4
3431	AT1G61370.1 | S-locus lectin protein kinase family protein, contains Pfam domains, PF00954: S-locus glycoprotein family, PF00069: Protein kinase domain, and PF01453: Lectin (probable mannose binding) | chr1:22645761-22648812 REVERSE | Aliases: T1F9.14, T1F9_14	5.9	5.3	0.5	1.7	100.0%	-3.5
3432	AT5G38140.1 | histone-like transcription factor (CBF/NF-Y) family protein, similar to CCAAT-binding transcription factor subunit AAB-1 (GI:2583171) (Neurospora crassa); contains a CBF/NF-Y subunit signature (PDOC00578) presernt in members of histone-like transcription factor family; contains Pfam PF00808 : Histone-like transcription factor (CBF/NF-Y) and archaeal histone	3.6	4.1	-0.5	-1.7	100.0%	-3.5
3433	AT5G57360.1 | Symbol: ZTL | F-box family protein / LOV kelch protein 1 (LKP1), E3 ubiquitin ligase SCF complex F-box subunit; identical to clock-associated PAS protein ZTL; ZEITLUPE GI:7839456, LOV kelch protein 1 GI:11610573, Adagio 1 GI:13487068 from (Arabidopsis thaliana); contains Pfam profile PF01344: Kelch motif; identical to cDNA Adagio 1 (ADO1) GI:13487067; identical to cDNA LKP1 mRNA for LOV kelch protein 1, GI:11610572 | chr5:23258653-23261777 FORWARD | Aliases: ADO1, FKF1 LIKE PROTEIN 2, FKL2, LKP1, LOV KELCH PROTEIN 1, MSF19.2, MSF19_2, ZEITLUPE, ZTL	4.1	3.7	0.4	1.7	100.0%	-3.9
3434	AT1G70950.1 | expressed protein | chr1:26756351-26759011 FORWARD | Aliases: F15H11.15, F15H11_15	3.8	4.1	-0.3	-1.7	100.0%	-4.0
3435	AT2G24390.1 | similar to avirulence-responsive protein-related / avirulence induced gene (AIG) protein-related [Arabidopsis thaliana] (TAIR:At4g31310.1); similar to avrRpt2-induced protein 2-like [Oryza sativa (japonica cultivar-group)] (GB:XP_464100.1) | chr2:10380811-10382032 FORWARD | Aliases: T28I24.12, T28I24_12	3.7	3.4	0.3	1.7	100.0%	-3.8

Rank	Description	15.dpi	5.dpi	M	t	adj.q	B
3436	AT5G16715.1 | Symbol: EMB2247 | tRNA synthetase class I (I, L, M and V) family protein, similar to SP:P11931 Valyl-tRNA synthetase (EC 6.1.1.9) (Valine--tRNA ligase) (VALRS) {Bacillus stearothermophilus}; contains Pfam profile PF00133: tRNA synthetases class I (I, L, M and V) | chr5:5485356-5493407 FORWARD | Aliases: EMB2247, EMBRYO DEFECTIVE 2247	4.7	5.3	-0.6	-1.7	100.0%	-3.4
3437	AT5G20790.1 | expressed protein, predicted protein, Arabidopsis thaliana | chr5:7039913-7040656 REVERSE | Aliases: T1M15.190, T1M15_190	4.6	3.9	0.7	1.7	100.0%	-3.3
3438	AT1G09510.1 | similar to Eucalyptus gunnii alcohol dehydrogenase of unknown physiological function (GI:1143445), Vigna unguiculata (gi:1854445), NOT a cinnamyl-alcohol dehydrogenase | chr1:3069387-3072052 FORWARD | Aliases: F14J9.17, F14J9_17	2.5	2.7	-0.2	-1.7	100.0%	-4.2
3439	AT4G30830.1 | expressed protein, weak similarity to M protein type 1 (Streptococcus pyogenes) GI:311758; contains Pfam profile PF04576: Protein of unknown function, DUF593 | chr4:15015276-15016983 REVERSE | Aliases: F6I18.260, F6I18_260	4.4	3.8	0.6	1.7	100.0%	-3.7
3440	AT1G76790.1 | O-methyltransferase family 2 protein, similar to caffeic acid O-methyltransferase (Catharanthus roseus)(GI:18025321), catechol O-methyltransferase GB:CAA55358 (Vanilla planifolia) | chr1:28827080-28828567 REVERSE | Aliases: F28O16.16, F28O16_16	9.8	9.0	0.8	1.7	100.0%	-3.7
3441	AT1G55450.1 | embryo-abundant protein-related, similar to embryo-abundant protein GI:1350531 from (Picea glauca) | chr1:20708593-20710556 REVERSE | Aliases: T5A14.14, T5A14_14	5.2	5.8	-0.6	-1.7	100.0%	-3.6
3442	AT3G51240.1 | Symbol: F3H | naringenin 3-dioxygenase / flavanone 3-hydroxylase (F3H), identical to GI:3790548	7.6	8.4	-0.8	-1.7	100.0%	-3.7
3443	AT1G59650.1 | Symbol: CW14 | expressed protein, ; supporting cDNA gi:6520232:dbj:AB028233.1: | chr1:21923410-21926588 FORWARD | Aliases: CW14, T30E16.22, T30E16_22	7.2	7.6	-0.4	-1.7	100.0%	-3.7
3444	AT2G36480.1 | zinc finger (C2H2-type) family protein, weak similarity to S-locus protein 4 (GI:6069478) (Brassica rapa); weak similarity to Pre-mRNA cleavage complex II protein Pcf11 (Fragment) (Swiss-Prot:O94913) (Homo sapiens); contains Prosite PS00028: Zinc finger, C2H2 type, domain	5.6	5.9	-0.4	-1.7	100.0%	-3.8
3445	AT3G05320.1 | expressed protein | chr3:1513456-1514688 REVERSE | Aliases: T12H1.29, T12H1_29	5.5	4.6	0.9	1.7	100.0%	-3.5
3446	AT4G34120.1 | CBS domain-containing protein, contains Pfam profile PF00571: CBS domain	8.0	7.3	0.7	1.7	100.0%	-3.4
3447	AT3G12530.2 | expressed protein, similar to unknown protein [Oryza sativa (japonica cultivar-group)] (GB:AAD38294.1); contains InterPro domain Protein of unknown function DUF392 (InterPro:IPR007257)	3.3	3.7	-0.4	-1.7	100.0%	-3.7
3448	AT2G36330.1 | integral membrane protein, putative, contains 4 transmembrane domains; contains Pfam PF04535 : Domain of unknown function (DUF588); similar to putative ethylene responsive element binding protein (GI:22135858) (Arabidopsis thaliana) | chr2:15239349-15242377 FORWARD | Aliases: F2H17.6, F2H17_6	6.1	5.5	0.7	1.7	100.0%	-3.6
3449	AT1G11100.1 | SNF2 domain-containing protein / helicase domain-containing protein / zinc finger protein-related, similar to RUSH-1alpha (Oryctolagus cuniculus) GI:1655930; contains Pfam profiles PF00271: Helicase conserved C-terminal domain, PF00176: SNF2 family N-terminal domain | chr1:3703358-3709302 REVERSE | Aliases: T19D16.2, T19D16_2	4.7	5.2	-0.5	-1.7	100.0%	-3.6
3450	AT1G07570.2 | Symbol: APK1A | protein kinase (APK1a), identical to Protein kinase APK1A from (Arabidopsis thaliana) SWISS-PROT:Q06548	4.9	4.5	0.5	1.7	100.0%	-3.5
3451	AT4G38270.1 | glycosyl transferase family 8 protein, contains Pfam profile: PF01501 glycosyl transferase family 8 | chr4:17938366-17941547 FORWARD | Aliases: F22I13.40, F22I13_40	6.4	6.9	-0.5	-1.7	100.0%	-3.6
3452	AT2G01250.1 | 60S ribosomal protein L7 (RPL7B)	11.5	11.9	-0.5	-1.7	100.0%	-4.1
3453	AT1G67110.1 | Symbol: CYP735A2 | cytochrome P450, putative, similar to Cytochrome P450 72A1 (SP:Q05047) (Catharanthus roseus); | chr1:25065394-25069080 REVERSE | Aliases: F5A8.3, F5A8_3	6.5	7.2	-0.7	-1.7	100.0%	-3.4
3454	AT2G37920.1 | Symbol: EMB1513 | expressed protein | chr2:15876187-15877149 REVERSE | Aliases: EMB1513, EMBRYO DEFECTIVE 1513, T8P21.17, T8P21_17	5.1	4.6	0.4	1.7	100.0%	-3.8
3455	AT1G05910.1 | cell division cycle protein 48-related / CDC48-related, similar to SP:P54609 Cell division cycle protein 48 homolog {Arabidopsis thaliana}; contains Pfam profiles PF00004: ATPase AAA family, PF00439: Bromodomain | chr1:1790223-1796646 FORWARD | Aliases: T20M3.19, T20M3_19	5.4	5.9	-0.5	-1.7	100.0%	-3.6
3456	AT3G14075.1 | lipase class 3 family protein, low similarity to calmodulin-binding heat-shock protein CaMBP (Nicotiana tabacum) GI:1087073; contains Pfam profile PF01764: Lipase, PF03893: Lipase 3 N-terminal region | chr3:4663609-4666975 REVERSE | Aliases: None	6.8	6.3	0.5	1.7	100.0%	-3.7
3457	AT1G34380.1 | 5'-3' exonuclease family protein, similar to DNA polymerase type I (Rhodothermus sp. 'ITI 518') gi:4090935:gb:AAC98908 ; contains Pfam profile PF02739: 5'-3' exonuclease, N-terminal resolvase-like domain | chr1:12552633-12554298 REVERSE | Aliases: F7P12.3, F7P12_3	4.0	4.6	-0.7	-1.7	100.0%	-3.5

Rank	Description	15.dpi	5.dpi	M	t	adj.q	B
3458	AT5G02570.1 | histone H2B, putative, similar to histone H2B-2 Lycopersicon esculentum GI:3021483, Gossypium hirsutum SP:O22582, Asparagus officinalis GI:563329; contains Pfam profile PF00125 Core histone H2A/H2B/H3/H4 | chr5:576740-577138 REVERSE | Aliases: T22P11.160, T22P11_160	6.9	8.1	-1.2	-1.7	100.0%	-3.2
3459	AT4G01970.1 | similar to raffinose synthase family protein [Arabidopsis thaliana] (TAIR:At5g40390.1); similar to raffinose synthase [Cucumis sativus] (GB:AAD02832.1); similar to galactinol-raffinose galactosyltransferase [Vigna angularis] (GB:CAB64363.1); similar to putative alkaline alpha galactosidase I [Oryza sativa (japonica cultivar-group)] (GB:XP_550270.1); similar to putative raffinose synthase [Oryza sativa (japonica cultivar-group)] (GB:NP_909442.1); similar to stachyose synthase [Pisum sativum] (GB:CAD55555.1); contains InterPro domain Raffinose synthase (InterPro:IPR008811) | chr4:853927-857358 REVERSE | Aliases: T7B11.23, T7B11_23	2.9	3.3	-0.4	-1.7	100.0%	-3.8
3460	AT4G10710.1 | transcriptional regulator-related, similar to chromatin-specific transcription elongation factor FACT 140 kDa subunit (GI:5499741) (Homo sapiens) | chr4:6601985-6606350 REVERSE | Aliases: T12H20.3, T12H20_3	7.1	7.6	-0.5	-1.7	100.0%	-3.8
3461	AT1G66830.1 | leucine-rich repeat transmembrane protein kinase, putative, contains Pfam profiles: PF00069: Eukaryotic protein kinase domain, multiple PF00560: Leucine Rich Repeat | chr1:24934328-24936581 REVERSE | Aliases: F4N21.23, F4N21_23	3.4	3.8	-0.4	-1.7	100.0%	-3.6
3462	AT2G27830.1 | expressed protein | chr2:11867295-11868552 FORWARD | Aliases: F15K20.7, F15K20_7	4.2	3.3	0.9	1.7	100.0%	-3.1
3463	AT5G28840.1 | NAD-dependent epimerase/dehydratase family protein, similar to sugar epimerase BlmG from Streptomyces verticillus GI:9937230; contains Pfam profile PF01370 NAD dependent epimerase/dehydratase family | chr5:10862177-10864906 REVERSE | Aliases: F7P1.20, F7P1_20	8.0	9.0	-1.0	-1.7	100.0%	-3.3
3464	AT2G39750.1 | dehydration-responsive family protein, similar to early-responsive to dehydration stress ERD3 protein (Arabidopsis thaliana) GI:15320410; contains Pfam profile PF03141: Putative methyltransferase | chr2:16585902-16589482 REVERSE | Aliases: T5I7.5, T5I7_5	7.8	8.1	-0.4	-1.7	100.0%	-3.6
3465	AT2G23050.1 | phototropic-responsive NPH3 family protein, contains NPH3 family domain, Pfam:PF03000 | chr2:9817737-9819740 FORWARD | Aliases: F21P24.11, F21P24_11	2.7	3.1	-0.4	-1.7	100.0%	-3.8
3466	AT1G04560.1 | AWPM-19-like membrane family protein, contains Pfam PF05512: AWPM-19-like family; similar to late embryogenesis abundant protein, (Lea) with hydrophobic domain, high pI value (11.6); 15kD protein; putative (GI:310570) {Glycine max} | chr1:1244986-1246057 FORWARD | Aliases: T1G11.19, T1G11_19	3.4	3.1	0.4	1.7	100.0%	-3.9
3467	AT5G13330.1 | encodes a member of the ERF (ethylene response factor) subfamily B-4 of ERF/AP2 transcription factor family. The protein contains one AP2 domain. There are 7 members in this subfamily.	6.5	5.9	0.6	1.7	100.0%	-3.5
3468	AT5G43700.1 | Symbol: ATAUX2 11 | auxin-responsive protein / indoleacetic acid-induced protein 4 (IAA4) / auxin-induced protein (AUX2-11), identical to SP:P33077 Auxin-responsive protein IAA4 (Indoleacetic acid-induced protein 4) (Auxin-induced protein AUX2-11) {Arabidopsis thaliana} | chr5:17567460-17568808 FORWARD | Aliases: IAA4, MQD19.3, MQD19_3	5.1	5.3	-0.2	-1.7	100.0%	-4.2
3469	AT5G65840.1 | expressed protein | chr5:26360956-26363289 REVERSE | Aliases: K14B20.1, K14B20_1	4.2	3.8	0.4	1.7	100.0%	-3.7
3470	AT1G68460.1 | Symbol: ATIPT1 |	3.1	3.6	-0.5	-1.7	100.0%	-3.5
3471	AT2G47780.1 | rubber elongation factor (REF) protein-related, similar to Small rubber particle protein (SRPP) (22 kDa rubber particle protein) (22 kDa RPP) (Latex allergen Hev b 3) (27 kDa natural rubber allergen) (Swiss-Prot:O82803) (Hevea brasiliensis); similar to Stress-related protein (Swiss-Prot:Q9SW70) (Vitis riparia) | chr2:19577132-19578494 FORWARD | Aliases: F17A22.17	7.2	6.3	0.9	1.7	100.0%	-3.1
3472	AT5G35750.1 | Symbol: AHK2 | histidine kinase (AHK2), identical to histidine kinase AHK2 (Arabidopsis thaliana) gi:13537196:dbj:BAB40774 | chr5:13928601-13933567 REVERSE | Aliases: ARABIDOPSIS HISTIDINE KINASE 2, MXH1.16, MXH1_16	4.4	3.7	0.7	1.7	100.0%	-3.3
3473	AT4G13020.3 | Symbol: MHK | similar to protein kinase, putative [Arabidopsis thaliana] (TAIR:At4g19110.1); similar to protein kinase, putative [Arabidopsis thaliana] (TAIR:At4g19110.2); similar to putative Cdc2-related protein kinase CRK2 [Beta vulgaris] (GB:CAB90209.1); contains InterPro domain Serine/threonine protein kinase, active site (InterPro:IPR008271); contains InterPro domain Tyrosine protein kinase (InterPro:IPR001245); contains InterPro domain Serine/threonine protein kinase (InterPro:IPR002290); contains InterPro domain Protein kinase (InterPro:IPR000719) | chr4:7603823-7607152 FORWARD | Aliases: F25G13.110, F25G13_110, PROTEIN KINASE	5.2	5.8	-0.6	-1.7	100.0%	-3.4
3474	AT2G32910.1 | expressed protein | chr2:13966127-13969195 FORWARD | Aliases: T21L14.15, T21L14_15	6.4	7.1	-0.7	-1.7	100.0%	-3.7
3475	AT2G37080.1 | myosin heavy chain-related, low similarity to myosin heavy chain (Rana catesbeiana) GI:4249701 | chr2:15588486-15592265 REVERSE | Aliases: T2N18.16, T2N18_16	6.3	7.2	-0.8	-1.7	100.0%	-3.6
3476	AT5G27240.1 | DNAJ heat shock N-terminal domain-containing protein | chr5:9597555-9600869 FORWARD | Aliases: T21B4.150, T21B4_150	3.1	3.3	-0.2	-1.7	100.0%	-4.2

Rank	Description	15.dpi	5.dpi	M	t	adj.q	B
3477	AT1G61680.1 | terpene synthase/cyclase family protein, similar to 1,8-cineole synthase (GI:3309117)(Salvia officinalis); contains Pfam profile: PF01397 terpene synthase family | chr1:22776120-22778352 REVERSE | Aliases: T13M11.3, T13M11_3	2.2	2.4	-0.2	-1.7	100.0%	-4.6
3478	AT3G22840.1 | Symbol: ELIP1 | chlorophyll A-B binding family protein / early light-induced protein (ELIP), identical to early light-induced protein; ELIP (Arabidopsis thaliana) GI:1872544; contains Pfam profile: PF00504 chlorophyll A-B binding protein; identical to cDNA early light-induced protein GI:1872543	10.1	9.0	1.1	1.7	100.0%	-3.1
3479	AT5G56710.1 | 60S ribosomal protein L31 (RPL31C) | chr5:22961062-22962196 REVERSE | Aliases: MIK19.16, MIK19_16	11.7	12.3	-0.6	-1.7	100.0%	-3.7
3480	AT3G22780.1 | Symbol: TSO1 | CXC domain protein (TSO1), identical to CXC domain protein TSO1 (Arabidopsis thaliana) GI:7767425 | chr3:8048809-8052819 FORWARD | Aliases: MWI23.15	6.6	7.4	-0.8	-1.7	100.0%	-3.6
3481	AT4G11010.1 | Symbol: NDPK3 | nucleoside diphosphate kinase 3, mitochondrial (NDK3), identical to Nucleoside diphosphate kinase III, mitochondrial precursor (NDK III) (NDP kinase III) (NDPK III) (SP:O49203) (Arabidopsis thaliana); contains Pfam PF00334 : Nucleoside diphosphate kinase domain;	10.3	11.0	-0.7	-1.7	100.0%	-3.6
3482	AT5G09670.2 | loricrin-related, contains weak similarity to Loricrin (Swiss-Prot:P23490) (Homo sapiens) | chr5:2995913-2998917 REVERSE | Aliases: F17I14.140, F17I14_140	7.9	7.5	0.5	1.7	100.0%	-3.6
3483	AT4G26260.1 | Symbol: MIOX4 | expressed protein, similar to myo-inositol oxygenase (Sus scrofa) gi:17432544:gb:AAL39076 | chr4:13297948-13300186 FORWARD | Aliases: MYO INOSITOL OXYGENASE 4, T25K17.70, T25K17_70	8.3	7.1	1.2	1.7	100.0%	-3.0
3484	AT5G01710.1 | expressed protein | chr5:263516-265613 REVERSE | Aliases: F7A7.230, F7A7_230	9.0	8.6	0.4	1.7	100.0%	-3.9
3485	AT3G62890.1 | similar to pentatricopeptide (PPR) repeat-containing protein [Arabidopsis thaliana] (TAIR:At5g48910.1); similar to pentatricopeptide (PPR) repeat-containing protein-like [Oryza sativa (japonica cultivar-group)] (GB:NP_919101.1); contains InterPro domain PPR repeat (InterPro:IPR002885) | chr3:23257132-23259036 FORWARD | Aliases: F26K9.320	3.5	3.9	-0.4	-1.7	100.0%	-3.7
3486	AT5G67100.1 | DNA-directed DNA polymerase alpha catalytic subunit, putative, similar to SP:O48653 DNA polymerase alpha catalytic subunit (EC 2.7.7.7) {Oryza sativa}; contains Pfam profiles: PF03175 DNA polymerase type B, organellar and viral, PF00136 DNA polymerase family B, PF03104 DNA polymerase family B, exonuclease domain | chr5:26794220-26802330 FORWARD | Aliases: K21H1.14, K21H1_14	3.1	3.4	-0.3	-1.7	100.0%	-4.1
3487	AT5G48360.1 | formin homology 2 domain-containing protein / FH2 domain-containing protein, contains formin homology 2 domain, Pfam:PF02181 | chr5:19612883-19615718 FORWARD | Aliases: K23F3.8, K23F3_8	3.1	3.7	-0.6	-1.7	100.0%	-3.7
3488	AT4G13780.1 | methionine--tRNA ligase, putative / methionyl-tRNA synthetase, putative / MetRS, putative, similar to methionyl-tRNA synthetase (Oryza sativa) GI:4091008; contains Pfam profiles PF00133: tRNA synthetases class I (I, L, M and V), PF01588: Putative tRNA binding domain | chr4:7993198-7998511 REVERSE | Aliases: F18A5.170, F18A5_170	9.3	9.9	-0.5	-1.7	100.0%	-3.8
3489	AT5G61140.1 | DEAD box RNA helicase, putative, similar to ASC-1 complex subunit P200 (Homo sapiens) GI:12061185; contains Pfam profiles PF00270: DEAD/DEAH box helicase, PF00271: Helicase conserved C-terminal domain, PF02889: Sec63 domain | chr5:24607225-24620740 FORWARD | Aliases: MAF19.14, MAF19_14	5.6	6.1	-0.5	-1.7	100.0%	-3.7
3490	AT3G14980.1 | PHD finger transcription factor, putative, contains Pfam profile: PF00628 PHD-finger | chr3:5039938-5044818 REVERSE | Aliases: K15M2.12	5.1	5.6	-0.5	-1.7	100.0%	-3.5
3491	AT5G42440.1 | protein kinase family protein, contains protein kinase domain, Pfam:PF00069 | chr5:16990532-16991802 REVERSE | Aliases: MDH9.13, MDH9_13	3.7	3.4	0.3	1.7	100.0%	-4.0
3492	AT4G27010.1 | expressed protein, ; expression supported by MPSS | chr4:13558472-13568302 FORWARD | Aliases: F10M23.350, F10M23_350	3.9	4.5	-0.6	-1.7	100.0%	-3.6
3493	AT1G03687.2 | DTW domain-containing protein, contains Pfam PF03942: DTW domain	4.8	5.3	-0.5	-1.7	100.0%	-3.4
3494	AT5G19110.1 | extracellular dermal glycoprotein-related / EDGP-related, similar to extracellular dermal glycoprotein EDGP precursor (Daucus carota) GI:285741 | chr5:6411563-6413172 REVERSE | Aliases: T24G5.10, T24G5_10	6.3	5.3	1.0	1.7	100.0%	-3.2
3495	AT5G61900.3 | Symbol: BON1 | copine BONZAI1 (BON1), nearly identical to BONZAI1 (Arabidopsis thaliana) GI:15487382; contains Pfam profile PF00168: C2 domain | chr5:24872887-24876453 REVERSE | Aliases: BON, BONSAI, BONZAI1, COPINE 1, CPN1, K22G18.2, K22G18_2	6.5	6.1	0.5	1.7	100.0%	-3.7
3496	AT1G06110.1 | F-box family protein, contains similarity to F-box protein FBX3 GI:6103643 from (Homo sapiens) ; similar to SKP1 interacting partner 2 (SKIP2) TIGR_Ath1:At5g67250 | chr1:1853076-1855021 REVERSE | Aliases: T21E18.16, T21E18_16	6.4	6.0	0.4	1.7	100.0%	-3.5

Rank	Description	15.dpi	5.dpi	M	t	adj.q	B
3497	AT1G71980.1 | protease-associated zinc finger (C3HC4-type RING finger) family protein, identical to ReMembR-H2 protein JR702 (Arabidopsis thaliana) gi:6942149:gb:AAF32326; contains Pfam domain, PF02225: protease-associated (PA) domain and Pfam domain, PF00097: Zinc finger, C3HC4 type (RING finger); identical to cDNA ReMembR-H2 protein JR702 mRNA, partial cds GI:6942148 | chr1:27101628-27103801 FORWARD | Aliases: F17M19.13, F17M19_13	5.7	4.9	0.7	1.7	100.0%	-3.4
3498	AT1G50630.2 | similar to expressed protein [Arabidopsis thaliana] (TAIR:At3g20300.1); similar to OSJNBa0064H22.7 [Oryza sativa (japonica cultivar-group)] (GB:XP_462655.1) | chr1:18755124-18757337 REVERSE | Aliases: F11F12.5, F11F12_5	4.5	3.8	0.7	1.7	100.0%	-3.4
3499	AT5G24740.1 | expressed protein | chr5:8469954-8489706 REVERSE | Aliases: T4C12.10	5.2	5.8	-0.7	-1.7	100.0%	-3.6
3500	AT5G15170.1 | tyrosyl-DNA phosphodiesterase-related, similar to Chain A, Crystal Structure Of Human Tyrosyl-Dna Phosphodiesterase (Tdp1) (Homo sapiens) GI:20150581 | chr5:4926097-4929505 REVERSE | Aliases: F8M21.60, F8M21_60	4.3	4.8	-0.6	-1.7	100.0%	-3.5
3501	AT1G67920.1 | expressed protein | chr1:25477207-25477803 FORWARD | Aliases: T23K23.23, T23K23_23	3.9	4.9	-0.9	-1.7	100.0%	-3.4
3502	AT3G48610.1 | phosphoesterase family protein, low similarity to SP:P95246 Phospholipase C 2 precursor (EC 3.1.4.3) {Mycobacterium tuberculosis}; contains Pfam profile PF04185: Phosphoesterase family	7.7	8.5	-0.8	-1.7	100.0%	-3.3
3503	AT5G42520.1 | expressed protein | chr5:17017511-17019835 FORWARD | Aliases: MDH9.22, MDH9_22	8.8	9.2	-0.4	-1.7	100.0%	-3.8
3504	AT3G44600.1 | peptidyl-prolyl cis-trans isomerase cyclophilin-type family protein, similar to SP:P87051 Peptidyl-prolyl cis-trans isomerase cyp2 (EC 5.2.1.8) (Cyclophilin cyp2) {Schizosaccharomyces pombe}; contains Pfam profiles PF00160: peptidyl-prolyl cis-trans isomerase cyclophilin-type, PF00400: WD domain G-beta repeat | chr3:16175922-16180249 REVERSE | Aliases: F14L2.150	5.1	5.6	-0.5	-1.7	100.0%	-3.4
3505	AT2G21170.1 | Symbol: TIM | triosephosphate isomerase, chloroplast, putative, similar to Triosephosphate isomerase, chloroplast precursor: SP:P48496 from Spinacia oleracea, SP:P46225 from Secale cereale | chr2:9077835-9080304 REVERSE | Aliases: F26H11.7, F26H11_7, TRIOSEPHOSPHATE ISOMERASE	11.0	11.7	-0.6	-1.7	100.0%	-3.9
3506	AT5G63700.1 | zinc finger (C3HC4 type RING finger) family protein, contains Pfam PF03126: Plus-3 domain; contains Pfam PF02201: BAF60b domain of the SWIB complex; contains Pfam PF00628: PHD-finger domain; contains Prosite Zinc finger, C3HC4 type (RING finger), signature; similar to CPRF interacting protein (GI:9588690) (Petroselinum crispum) | chr5:25511160-25514858 REVERSE | Aliases: MBK5.18, MBK5_18	5.4	6.1	-0.7	-1.7	100.0%	-3.5
3507	AT5G35910.1 | 3'-5' exonuclease domain-containing protein / helicase and RNase D C-terminal domain-containing protein / HRDC domain-containing protein, low similarity to SP:Q01780 Polymyositis/scleroderma autoantigen 2 {Homo sapiens}; contains Pfam profiles PF00570: HRDC domain, PF01612: 3'-5' exonuclease | chr5:14046916-14052635 REVERSE | Aliases: MIK22.22, MIK22_22	4.2	4.7	-0.5	-1.7	100.0%	-4.0
3508	AT1G22750.3 | expressed protein, similar to unknown protein [Oryza sativa (japonica cultivar-group)] (GB:BAD68607.1) | chr1:8050851-8052893 FORWARD | Aliases: T22J18.8, T22J18_8	5.6	4.9	0.7	1.7	100.0%	-3.4
3509	AT1G07150.1 | Symbol: MAPKKK13 | protein kinase family protein, contains eukaryotic protein kinase domain, INTERPRO:IPR000719	4.7	4.2	0.5	1.7	100.0%	-3.6
3510	AT3G49810.1 | U-box domain-containing protein, contains Pfam profile PF04564: U-box domain | chr3:18485522-18487819 REVERSE | Aliases: T16K5.160	3.2	3.5	-0.2	-1.7	100.0%	-4.2
3511	AT5G54900.1 | Symbol: ATRBP45A | RNA-binding protein 45 (RBP45), putative, contains similarity to polyadenylate-binding protein 5 | chr5:22312609-22315572 FORWARD | Aliases: ATRBP45A, MBG8.17, MBG8_17	10.2	10.9	-0.7	-1.7	100.0%	-3.7
3512	AT1G01100.2 | 60S acidic ribosomal protein P1 (RPP1A), similar to 60S ACIDIC RIBOSOMAL PROTEIN P1 GB:O23095 from (Arabidopsis thaliana) | chr1:50091-51182 REVERSE | Aliases: T25K16.9, T25K16_9	11.6	12.1	-0.5	-1.7	100.0%	-4.0
3513	AT2G28740.1 | Symbol: HIS4 | histone H4, identical to histone H4 from Lycopersicon esculentum GI:297150, Lolium temulentum SP:P02308, Acropora formosa GI:455652, Citrus jambhiri GI:16797797	8.2	9.0	-0.8	-1.7	100.0%	-3.2
3514	AT3G03210.1 | expressed protein | chr3:740777-742316 FORWARD | Aliases: T17B22.10, T17B22_10	6.2	5.7	0.6	1.7	100.0%	-3.6
3515	AT4G17610.1 | tRNA/rRNA methyltransferase (SpoU) family protein, similar to TAR RNA loop binding protein (Homo sapiens) GI:1184692; contains Pfam profile PF00588: SpoU rRNA Methylase (RNA methyltransferase, TrmH) family | chr4:9805665-9814923 REVERSE | Aliases: DL4840C, FCAALL.71	7.5	6.9	0.6	1.7	100.0%	-3.5
3516	AT3G14120.2 | expressed protein, similar to Nuclear pore complex protein Nup107 (Nucleoporin Nup107) (107 kDa nucleoporin) (p105) (Swiss-Prot:P52590) (Rattus norvegicus) | chr3:4677821-4685645 FORWARD | Aliases: MAG2.8	5.1	5.5	-0.5	-1.7	100.0%	-3.6

Rank	Description	15.dpi	5.dpi	M	t	adj.q	B
3517	AT5G51590.1 | DNA-binding protein-related, contains Pfam domain PF03479: Domain of unknown function (DUF296), found in AT-hook motifs Pfam:PF02178 | chr5:20973793-20976339 REVERSE | Aliases: K17N15.14, K17N15_14	6.1	6.9	-0.8	-1.7	100.0%	-3.3
3518	AT3G10480.2 | Symbol: ANAC050 | no apical meristem (NAM) family protein, similar to to NAC2 (GI:645671) (Arabidopsis thaliana); contains Pfam PF02365: No apical meristem (NAM) protein; N-terminus similar to unknown protein GB:AAD25613 (Arabidopsis thaliana) | chr3:3264362-3267068 FORWARD | Aliases: ANAC050, F13M14.24	3.3	2.9	0.4	1.7	100.0%	-3.7
3519	AT3G10490.2 | Symbol: ANAC051/ANAC052 | no apical meristem (NAM) family protein, similar to to NAC2 (GI:645671) (Arabidopsis thaliana); contains Pfam PF02365: No apical meristem (NAM) protein | chr3:3267877-3270888 FORWARD | Aliases: ANAC051, ANAC052, F13M14.23	3.3	2.9	0.4	1.7	100.0%	-3.7
3520	AT4G38060.2 | expressed protein | chr4:17873003-17874801 REVERSE | Aliases: F20D10.180, F20D10_180	7.6	6.6	1.0	1.7	100.0%	-3.4
3521	AT5G41970.1 | similar to expressed protein [Arabidopsis thaliana] (TAIR:At3g49320.1); similar to LOC496075 protein [Xenopus laevis] (GB:AAH87491.1); contains InterPro domain Metal-dependent protein hydrolase (InterPro:IPR003226) | chr5:16808411-16810485 FORWARD | Aliases: MJC20.7, MJC20_7	7.8	8.3	-0.6	-1.7	100.0%	-3.6
3522	AT5G35980.2 | similar to protein kinase family protein [Arabidopsis thaliana] (TAIR:At2g40120.1); similar to putative protein kinase YakA [Oryza sativa (japonica cultivar-group)] (GB:XP_467340.1); contains InterPro domain Serine/threonine protein kinase, active site (InterPro:IPR008271); contains InterPro domain Tyrosine protein kinase (InterPro:IPR001245); contains InterPro domain Serine/threonine protein kinase (InterPro:IPR002290); contains InterPro domain Protein kinase (InterPro:IPR000719) | chr5:14145214-14151682 FORWARD | Aliases: MEE13.9, MEE13_9	4.9	5.4	-0.5	-1.7	100.0%	-3.8
3523	AT5G12980.1 | rcd1-like cell differentiation protein, putative, similar to protein involved in sexual development (Schizosaccharomyces pombe) GI:1620896; contains Pfam profile PF04078: Cell differentiation family, Rcd1-like | chr5:4105386-4108179 REVERSE | Aliases: T24H18.150, T24H18_150	6.2	7.1	-0.8	-1.7	100.0%	-3.3
3524	AT1G76405.2 | expressed protein | chr1:28666817-28668683 FORWARD | Aliases: None	5.9	6.8	-0.8	-1.7	100.0%	-3.3
3525	AT4G16720.1 | 60S ribosomal protein L15 (RPL15A) | chr4:9399987-9401404 REVERSE | Aliases: DL4385C, FCAALL.416	11.7	12.0	-0.3	-1.7	100.0%	-4.1
3526	AT2G01200.2 | Symbol: IAA32 | auxin-responsive AUX/IAA family protein, contains Pfam profile: PF02309 AUX/IAA family | chr2:118017-119341 FORWARD | Aliases: F10A8.8, F10A8_8	4.9	3.6	1.3	1.7	100.0%	-3.1
3527	AT1G75780.1 | Symbol: TUB1 | tubulin beta-1 chain (TUB1), nearly identical to SP:P12411 Tubulin beta-1 chain {Arabidopsis thaliana} | chr1:28454802-28457301 REVERSE | Aliases: F10A5.3, F10A5_3	4.3	4.8	-0.5	-1.7	100.0%	-3.6
3528	AT2G29350.3 | Symbol: SAG13 | similar to tropinone reductase, putative / tropine dehydrogenase, putative [Arabidopsis thaliana] (TAIR:At2g29290.1); similar to putative pfam00106, adh_short, short chain dehydrogenase [Oryza sativa (japonica cultivar-group)] (GB:NP_912375.1); contains InterPro domain Glucose/ribitol dehydrogenase (InterPro:IPR002347); contains InterPro domain Short-chain dehydrogenase/reductase SDR (InterPro:IPR002198)	2.9	3.3	-0.4	-1.7	100.0%	-3.7
3529	AT5G40060.1 | disease resistance protein (TIR-NBS-LRR class), putative, domain signature TIR-NBS-LRR exists, suggestive of a disease resistance protein. False intron created at intron 2 to escape a frameshift in the BAC sequence. | chr5:16051816-16055861 FORWARD | Aliases: MUD12.40, MUD12_40	2.9	2.6	0.2	1.7	100.0%	-4.2
3530	AT1G66070.1 | translation initiation factor-related, similar to Eukaryotic translation initiation factor 3 subunit 1 (eIF-3 alpha) (eIF3 p35) (eIF3j) (Swiss-Prot:O75822) (Homo sapiens) | chr1:24599549-24602181 REVERSE | Aliases: F15E12.10, F15E12_10	5.1	5.5	-0.4	-1.7	100.0%	-3.8
3531	AT4G03100.1 | rac GTPase activating protein, putative, similar to rac GTPase activating protein 3 (Lotus japonicus) GI:3695063; contains Pfam profile PF00620: RhoGAP domain | chr4:1374281-1376231 FORWARD | Aliases: F4C21.2	3.0	3.3	-0.3	-1.7	100.0%	-4.1
3532	AT3G50830.1 | Symbol: COR413 PM2 | stress-responsive protein, putative, similar to cold acclimation WCOR413-like protein gamma form (Hordeum vulgare) gi:18449100:gb:AAL69988; similar to stress-regulated protein SAP1 (Xerophyta viscosa) gi:21360378:gb:AAM47505; identical to cDNA cold acclimation protein WCOR413-like protein beta form GI:10121842 | chr3:18904826-18906476 REVERSE | Aliases: ATCOR413 PM2, F18B3.110, F18B3_110	7.5	6.0	1.5	1.7	100.0%	-3.5
3533	AT5G08560.1 | transducin family protein / WD-40 repeat family protein, contains 7 WD-40 repeats (PF00400); similar to will die slowly protein (WDS) (SP:Q9V3J8) (Drosophila melanogaster) | chr5:2770875-2774673 REVERSE | Aliases: MAH20.12, MAH20_12	8.9	9.2	-0.4	-1.7	100.0%	-3.8
3534	AT3G60245.1 | 60S ribosomal protein L37a (RPL37aC) | chr3:22279721-22280981 FORWARD | Aliases: None	13.6	13.9	-0.3	-1.7	100.0%	-4.1
3535	AT1G23440.2 | pyrrolidone-carboxylate peptidase family protein, similar to Pyrrolidone-carboxylate peptidase (Swiss-Prot:O58321) (Pyrococcus horikoshii); contains Prosite PS00141: Eukaryotic and viral aspartyl proteases active site | chr1:8321694-8323359 FORWARD | Aliases: F28C11.26, F28C11_26	7.4	6.7	0.7	1.7	100.0%	-3.7
3536	AT3G12400.1 | tumour susceptibility gene 101 (TSG101) family protein, contains Pfam profile PF05743: Tumour susceptibility gene 101 protein (TSG101); similar to Tumor susceptibility gene 101 protein (Swiss-Prot:Q99816) (Homo sapiens) | chr3:3944303-3945858 REVERSE | Aliases: T2E22.28	7.1	6.7	0.4	1.7	100.0%	-3.7

Rank	Description	15.dpi	5.dpi	M	t	adj.q	B
3537	AT2G48040.1 | expressed protein | chr2:19658266-19659835 REVERSE | Aliases: T9J23.19	5.5	6.1	-0.6	-1.7	100.0%	-3.5
3538	AT5G41620.1 | expressed protein, weak similarity to microtubule binding protein D-CLIP-190 (GI:2773363) (Drosophila melanogaster); weak similarity to Synaptonemal complex protein 1 (SCP-1 protein) (Swiss-Prot:Q15431) (Homo sapiens); weak similarity to DNA double-strand break repair rad50 ATPase. (Swiss-Prot:P58301) (Pyrococcus furiosus) | chr5:16663666-16666196 FORWARD | Aliases: MBK23.17, MBK23_17	6.1	6.8	-0.7	-1.7	100.0%	-3.6
3539	AT4G01730.1 | zinc finger (DHHC type) family protein, contains Pfam profile PF01529: DHHC zinc finger domain | chr4:749922-752034 FORWARD | Aliases: T15B16.11, T15B16_11	4.8	4.4	0.4	1.7	100.0%	-3.7
3540	AT2G47000.1 | multidrug resistant (MDR) ABC transporter, putative, similar to multidrug-resistant protein CjMDR1 (Coptis japonica) GI:14715462, MDR-like p-glycoprotein (Arabidopsis thaliana) GI:24324262; contains Pfam profiles PF00005: ABC transporter, PF00664: ABC transporter transmembrane region | chr2:19316937-19321819 REVERSE | Aliases: F14M4.17	4.4	5.1	-0.7	-1.7	100.0%	-3.4
3541	AT5G04510.2 | Symbol: PDK1 | 3-phosphoinositide-dependent protein kinase, putative, similar to 3-phosphoinositide-dependent protein kinase-1 (Oryza sativa) gi:5001830:gb:AAD37166 | chr5:1286938-1289903 FORWARD | Aliases: 3 PHOSPHOINOSITIDE DEPENDENT PROTEIN KINASE 1, ATPDK1, T32M21.110, T32M21_110	4.3	4.9	-0.6	-1.7	100.0%	-3.5
3542	AT4G13640.2 | similar to myb family transcription factor [Arabidopsis thaliana] (TAIR:At3g24120.2); similar to transfactor-like [Oryza sativa (japonica cultivar-group)] (GB:XP_481813.1); contains InterPro domain Myb DNA-binding domain (InterPro:IPR001005); contains InterPro domain Myb-like DNA-binding domain, SHAQKYF class (InterPro:IPR006447) | chr4:7936602-7938626 REVERSE | Aliases: F18A5.30, F18A5_30	6.3	5.9	0.4	1.7	100.0%	-3.7
3543	AT4G30380.1 | expansin-related, similar to blight-associated protein p12 precursor (Citrus jambhiri) gi:4102727:gb:AAD03398; similar to beta-expansin (Oryza sativa) gi:8118428:gb:AAF72986; expansin-related gene, PMID:11641069, www.bio.psu.edu/expansins | chr4:14860492-14861000 FORWARD | Aliases: F17I23.280, F17I23_280	2.3	2.1	0.2	1.7	100.0%	-4.6
3544	AT5G61600.1 | encodes a member of the ERF (ethylene response factor) subfamily B-3 of ERF/AP2 transcription factor family. The protein contains one AP2 domain. There are 18 members in this subfamily including ATERF-1, ATERF-2, AND ATERF-5. | chr5:24783612-24784656 REVERSE | Aliases: K11J9.13, K11J9_13	6.3	5.5	0.8	1.7	100.0%	-3.5
3545	AT5G06770.1 | KH domain-containing protein / zinc finger (CCCH type) family protein, contains Pfam domains PF00013: KH domain and PF00642: Zinc finger C-x8-C-x5-C-x3-H type (and similar) | chr5:2090704-2092951 REVERSE | Aliases: MPH15.13, MPH15_13	4.6	4.3	0.3	1.7	100.0%	-4.0
3546	AT1G68410.2 | similar to protein phosphatase 2C-related / PP2C-related [Arabidopsis thaliana] (TAIR:At1g09160.2); similar to protein phosphatase 2C-related / PP2C-related [Arabidopsis thaliana] (TAIR:At1g09160.1); similar to protein phosphatase 2C-like [Oryza sativa (japonica cultivar-group)] (GB:XP_479610.1); contains InterPro domain Protein phosphatase 2C-like (InterPro:IPR001932) | chr1:25653662-25656682 REVERSE | Aliases: T2E12.9, T2E12_9	7.1	6.6	0.5	1.7	100.0%	-3.5
3547	AT1G50200.1 | Symbol: ALATS | aminoacyl-tRNA synthetase family protein, contains Pfam profiles: PF01411 tRNA synthetases class II (A), PF02272 DHHA1 domain | chr1:18594882-18602149 REVERSE | Aliases: ACD, ALANYL TRNA SYNTHETASE, ALARS, F14I3.17, F14I3_17	9.8	10.1	-0.4	-1.7	100.0%	-3.9
3548	AT3G17250.1 | protein phosphatase 2C-related / PP2C-related, similar to protein phosphatase-2C GB:AAC36698 from (Mesembryanthemum crystallinum) | chr3:5892567-5894452 REVERSE | Aliases: MGD8.13	5.2	4.7	0.5	1.7	100.0%	-3.5
3549	AT2G20710.2 | pentatricopeptide (PPR) repeat-containing protein, low similarity to DNA-binding protein (Triticum aestivum) GI:6958202; contains Pfam profile PF01535: PPR repeat | chr2:8933087-8935297 FORWARD | Aliases: F5H14.32, F5H14_32	4.5	5.2	-0.7	-1.7	100.0%	-3.4
3550	AT5G48350.1 | expressed protein | chr5:19610792-19611391 FORWARD | Aliases: K23F3.7, K23F3_7	2.7	2.3	0.3	1.7	100.0%	-4.4
3551	AT2G21900.1 | Symbol: WRKY59 |	2.3	2.1	0.2	1.7	100.0%	-4.1
3552	AT3G47520.1 | Symbol: MDH | malate dehydrogenase (NAD), chloroplast (MDH), identical to chloroplast NAD-malate dehydrogenase (Arabidopsis thaliana) GI:3256066; contains InterPro entry IPR001236: Lactate/malate dehydrogenase; contains Pfam profiles PF00056: lactate/malate dehydrogenase, NAD binding domain and PF02866: lactate/malate dehydrogenase, alpha/beta C-terminal domain | chr3:17524259-17526026 FORWARD | Aliases: F1P2.70, MALATE DEHYDROGENASE	9.8	10.5	-0.7	-1.7	100.0%	-3.6
3553	AT3G48210.1 | expressed protein | chr3:17860344-17862663 FORWARD | Aliases: T24C20.90	3.0	3.3	-0.3	-1.7	100.0%	-4.1
3554	AT3G44140.1 | expressed protein | chr3:15896064-15896367 REVERSE | Aliases: F26G5.90	9.1	8.4	0.6	1.7	100.0%	-3.6
3555	AT3G06130.1 | heavy-metal-associated domain-containing protein, contains Pfam heavy metal associated domain PF00403 | chr3:1852899-1855035 REVERSE | Aliases: F28L1.7, F28L1_7	5.4	6.0	-0.6	-1.7	100.0%	-3.5
3556	AT3G21430.1 | expressed protein | chr3:7544634-7550830 REVERSE | Aliases: MHC9.11	5.2	5.7	-0.5	-1.7	100.0%	-3.7

Rank	Description	15.dpi	5.dpi	M	t	adj.q	B
3557	AT4G26240.1 | expressed protein | chr4:13286786-13288980 REVERSE | Aliases: T25K17.50, T25K17_50	6.5	6.1	0.5	1.7	100.0%	-3.7
3558	AT1G26180.1 | expressed protein | chr1:9054738-9056837 FORWARD | Aliases: F28B23.14, F28B23_14	6.0	5.3	0.7	1.7	100.0%	-3.3
3559	AT2G27180.1 | expressed protein | chr2:11627311-11628252 FORWARD | Aliases: T22O13.5, T22O13_5	4.0	3.5	0.5	1.7	100.0%	-3.8
3560	AT3G52640.3 | expressed protein, similar to hypothetical protein DDB0187645 [Dictyostelium discoideum] (GB:EAL63547.1)	5.5	5.9	-0.4	-1.7	100.0%	-3.8
3561	AT5G49580.1 | DNAJ heat shock N-terminal domain-containing protein, contains similarity to S-locus protein 5 GI:6069485 from (Brassica rapa); contains Pfam profile PF00226 DnaJ domain | chr5:20141049-20144039 REVERSE | Aliases: K6M13.13, K6M13_13	7.8	8.4	-0.5	-1.7	100.0%	-3.7
3562	AT3G01780.1 | expressed protein, est hit, | chr3:279067-283554 FORWARD | Aliases: F28J7.11, F28J7_11	8.2	9.0	-0.8	-1.7	100.0%	-3.6
3563	AT4G32760.1 | similar to VHS domain-containing protein / GAT domain-containing protein [Arabidopsis thaliana] (TAIR:At3g08790.1); similar to putative VHS domain-containing protein [Oryza sativa (japonica cultivar-group)] (GB:XP_464916.1); contains InterPro domain GAT domain (InterPro:IPR004152); contains InterPro domain VHS (InterPro:IPR002014) | chr4:15799144-15804180 FORWARD | Aliases: F4D11.40, F4D11_40	6.3	5.7	0.6	1.7	100.0%	-3.6
3564	AT5G58670.1 | Symbol: ATPLC1 |	5.5	5.9	-0.4	-1.7	100.0%	-3.7
3565	AT1G06950.1 | Symbol: ATTIC110/TIC110 | Encodes a protein thought to be a part of the translocon at the chloroplast inner envelope. Involved in protein import into the chloroplast and chloroplast biogenesis. | chr1:2130154-2135664 REVERSE | Aliases: ATTIC110, F10K1.33, TIC110	8.0	8.9	-1.0	-1.7	100.0%	-3.5
3566	AT3G22980.1 | elongation factor Tu family protein, similar to eukaryotic translation elongation factor 2 GB:NP_001952 (Homo sapiens) | chr3:8160276-8163323 REVERSE | Aliases: MXC7.1	4.1	4.4	-0.3	-1.7	100.0%	-4.0
3567	AT1G70985.1 | hydroxyproline-rich glycoprotein family protein, contains proline-rich extensin domains, INTERPRO:IPR002965 | chr1:26768334-26768947 REVERSE | Aliases: None	3.5	3.2	0.3	1.7	100.0%	-3.9
3568	AT1G76810.1 | eukaryotic translation initiation factor 2 family protein / eIF-2 family protein, similar to IF2 protein (Drosophila melanogaster) GI:7108770; contains Pfam profile PF03144: Elongation factor Tu domain 2 | chr1:28835900-28841480 REVERSE | Aliases: F28O16.18, F28O16_18	6.0	7.0	-1.0	-1.7	100.0%	-3.3
3569	AT1G58440.1 | Symbol: XF1 | squalene monooxygenase, putative / squalene epoxidase, putative, similar to SP:O65404 (SE 1,1), SP:O65402 (SE 1,2) 6566341 dbj AB008021.1 AB008021 | chr1:21717507-21720955 REVERSE | Aliases: F9K23.3, F9K23_3, XF1	3.8	4.4	-0.5	-1.7	100.0%	-3.4
3570	AT5G25800.1 | exonuclease family protein, contains exonuclease domain, Pfam:PF00929 | chr5:8979625-8982876 REVERSE | Aliases: F18A17.50, F18A17_50	4.5	4.9	-0.4	-1.7	100.0%	-3.6
3571	AT5G65040.1 | senescence-associated protein-related, similar to senescence-associated protein SAG102 (GI:22331931) (Arabidopsis thaliana) | chr5:25994847-25995643 REVERSE | Aliases: MXK3.29, MXK3_29	7.4	6.7	0.7	1.7	100.0%	-3.4
3572	AT2G40660.1 | tRNA-binding region domain-containing protein, similar to SP:Q12904 Multisynthetase complex auxiliary component p43 (Contains: Endothelial-monocyte activating polypeptide II (EMAP-II) (Small inducible cytokine subfamily E member 1)) {Homo sapiens}; contains Pfam profile PF01588: Putative tRNA binding domain | chr2:16973011-16976075 FORWARD | Aliases: T7D17.16, T7D17_16	8.1	8.7	-0.7	-1.7	100.0%	-3.6
3573	AT5G59845.1 | gibberellin-regulated family protein, similar to SP:P27057 GAST1 protein precursor {Lycopersicon esculentum}; contains Pfam profile PF02704: Gibberellin regulated protein | chr5:24128550-24129238 FORWARD | Aliases: None	4.0	4.7	-0.7	-1.7	100.0%	-3.6
3574	AT3G51600.1 | Symbol: LTP5 | nonspecific lipid transfer protein 5 (LTP5), identical to SP:Q9XFS7 Nonspecific lipid-transfer protein 5 (LTP 5) {Arabidopsis thaliana} | chr3:19149373-19150231 REVERSE | Aliases: LIPID TRANSFER PROTEIN 5, T18N14.5	3.2	3.5	-0.3	-1.7	100.0%	-4.0
3575	AT5G35170.2 | similar to adenylate kinase, chloroplast, putative / ATP-AMP transphosphorylase, putative [Arabidopsis thaliana] (TAIR:At5g47840.1); similar to putative adenylate kinase, chloroplast (ATP-AMP transphosphorylase) [Oryza sativa (japonica cultivar-group)] (GB:XP_479721.1); contains InterPro domain Adenylate kinase, subfamily (InterPro:IPR006259); contains InterPro domain Adenylate kinase (InterPro:IPR000850)	4.2	4.8	-0.5	-1.7	100.0%	-3.5
3576	AT3G12110.1 | Symbol: ACT11 | actin 11 (ACT11), identical to SP:P53496 Actin 11 {Arabidopsis thaliana} | chr3:3857860-3859804 FORWARD | Aliases: ACTIN 11, T21B14.7	8.2	7.4	0.8	1.7	100.0%	-3.3
3577	AT5G47530.1 | auxin-responsive protein, putative, similar to auxin-induced protein AIR12 (GI:11357190) (Arabidopsis thaliana); similar to stromal cell derived factor receptor 2 (GI:20381292) (Mus musculus) | chr5:19298601-19300288 FORWARD | Aliases: MNJ7.12, MNJ7_12	2.7	3.2	-0.5	-1.7	100.0%	-3.4

Rank	Description	15.dpi	5.dpi	M	t	adj.q	B
3578	AT5G18660.1 | Symbol: PCB2 | Encodes a protein with 3,8-divinyl protochlorophyllide a 8-vinyl reductase activity. Mutants accumulate divinyl chlorophyll rather than monovinyl chlorophyll. | chr5:6220783-6222230 REVERSE | Aliases: DVR, PALE GREEN AND CHLOROPHYLL B REDUCED 2, T1A4.40, T1A4_40	5.9	6.7	-0.8	-1.7	100.0%	-3.1
3579	AT4G21720.1 | expressed protein | chr4:11542573-11544286 FORWARD | Aliases: F17L22.180, F17L22_180	4.5	4.1	0.4	1.7	100.0%	-3.8
3580	AT3G27660.1 | glycine-rich protein / oleosin, identical to oleosin isoform GB:S71286 from (Arabidopsis thaliana); identical to cDNA oleosin (isoform Atol2) GI:987013 | chr3:10244995-10246260 FORWARD | Aliases: MGF10.3	5.2	4.7	0.5	1.7	100.0%	-3.8
3581	AT3G17300.1 | expressed protein | chr3:5907240-5908898 FORWARD | Aliases: MGD8.14	9.3	9.8	-0.6	-1.7	100.0%	-3.5
3582	AT3G12120.1 | Symbol: FAD2 | omega-6 fatty acid desaturase, endoplasmic reticulum (FAD2) / delta-12 desaturase, identical to omega-6 fatty acid desaturase, endoplasmic reticulum (FAD2) SP:P46313 (Arabidopsis thaliana (Mouse-ear cress)) (Plant Cell 6:147-158(1994)) | chr3:3860291-3863036 REVERSE | Aliases: DELTA 12 DESATURASE, FATTY ACID DESATURASE 2, T21B14.6	12.2	11.2	1.0	1.7	100.0%	-4.0
3583	AT5G02580.2 | expressed protein | chr5:579850-580785 FORWARD | Aliases: T22P11.170, T22P11_170	3.7	3.4	0.3	1.7	100.0%	-4.0
3584	AT5G26110.1 | expressed protein | chr5:9117910-9119809 REVERSE | Aliases: T1N24.12, T1N24_12	5.0	5.8	-0.8	-1.7	100.0%	-3.4
3585	AT1G74840.1 | myb family transcription factor, similar to myb-related transcription activator GI:9279717 from (Arabidopsis thaliana) | chr1:28119558-28121066 REVERSE | Aliases: F25A4.19, F25A4_19	9.2	8.5	0.7	1.7	100.0%	-3.8
3586	AT5G09270.2 | expressed protein | chr5:2878885-2880817 FORWARD | Aliases: T2K12.7	6.5	7.0	-0.5	-1.7	100.0%	-3.7
3587	AT5G46860.1 | Symbol: VAM3 | syntaxin 22 (SYP22) (VAM3), identical to GP:8809669: syntaxin related protein AtVam3p (Arabidopsis thaliana) | chr5:19029248-19031194 REVERSE | Aliases: ATSYP22, ATVAM3, SYP22	6.5	6.0	0.4	1.7	100.0%	-3.8
3588	AT2G43810.1 | small nuclear ribonucleoprotein F, putative / U6 snRNA-associated Sm-like protein, putative / Sm protein F, putative, similar to SWISS-PROT:Q9Y4Y8 U6 snRNA-associated Sm-like protein LSm6 (Mus musculus) | chr2:18156388-18158371 FORWARD | Aliases: F18O19.8	8.3	7.5	0.7	1.7	100.0%	-3.4
3589	AT1G60650.2 | glycine-rich RNA-binding protein, putative, similar to RNA binding protein(RZ-1) GI:1435061 from (Nicotiana sylvestris); contains InterPro entry IPR000504: RNA-binding region RNP-1 (RNA recognition motif) (RRM) | chr1:22343552-22346002 FORWARD | Aliases: F8A5.17, F8A5_17	6.6	6.0	0.6	1.7	100.0%	-3.5
3590	AT2G35750.1 | expressed protein | chr2:15036908-15037369 REVERSE | Aliases: T20F21.6, T20F21_6	4.6	3.7	0.9	1.7	100.0%	-3.5
3591	AT4G29660.1 | Symbol: EMB2752 | expressed protein, predicted proteins, Arabidopsis thaliana | chr4:14534269-14535298 REVERSE | Aliases: EMB2752, EMBRYO DEFECTIVE 2752, T16L4.170, T16L4_170	8.6	8.1	0.5	1.7	100.0%	-3.6
3592	AT4G18990.1 | xyloglucan:xyloglucosyl transferase, putative / xyloglucan endotransglycosylase, putative / endo-xyloglucan transferase, putative, similar to xyloglucan endotransglycosylase-related protein XTR4 GI:1244754 from (Arabidopsis thaliana) | chr4:10401921-10404259 REVERSE | Aliases: F13C5.160, F13C5_160	2.3	2.1	0.2	1.7	100.0%	-4.4
3593	AT5G11580.1 | UVB-resistance protein-related / regulator of chromosome condensation (RCC1) family protein, contains Pfam PF00415 : Regulator of chromosome condensation (RCC1); similar to rjs protein (GI:3414809) (Mus musculus); similar to HERC2 (GI:4079809) (Homo sapiens);similar to UVB-resistance protein UVR8 (GI:10177674) {Arabidopsis thaliana} | chr5:3718811-3721335 FORWARD | Aliases: F15N18.170, F15N18_170	5.9	5.4	0.5	1.7	100.0%	-3.7
3594	AT2G28670.1 | disease resistance-responsive family protein / fibroin-related, contains similarity to silk fibroin heavy chain (Bombyx mori) gi:765323:gb:AAB31861; contains disease resistance response protien domain Pfam:FP03018 | chr2:12307336-12308859 REVERSE | Aliases: T8O18.4, T8O18_4	5.8	6.4	-0.7	-1.7	100.0%	-3.3
3595	AT3G10530.1 | transducin family protein / WD-40 repeat family protein, contains 2 WD-40 repeats (PF00400); BING4 (gi:3811380) {Mus musculus}; similar to hypothetical protein GB:P40055 (Saccharomyces cerevisiae) | chr3:3286018-3288885 FORWARD | Aliases: F13M14.19	6.4	7.1	-0.7	-1.7	100.0%	-3.4
3596	AT4G25470.1 | Symbol: CBF2 | encodes a member of the DREB subfamily A-1 of ERF/AP2 transcription factor family (CBF2). The protein contains one AP2 domain. There are six members in this subfamily, including CBF1, CBF2, and CBF3. This gene is involved in response to low temperature and abscisic acid. | chr4:13015287-13016230 REVERSE | Aliases: DRE/CRT BINDING PROTEIN, DREB1C, T30C3.12	4.4	3.9	0.5	1.7	100.0%	-3.5
3597	AT5G58570.1 | expressed protein | chr5:23689929-23690513 REVERSE | Aliases: MZN1.2, MZN1_2	2.6	2.3	0.2	1.7	100.0%	-4.3
3598	AT3G16890.1 | pentatricopeptide (PPR) repeat-containing protein, contains Pfam profile PF01535: PPR repeat | chr3:5768407-5770386 REVERSE | Aliases: K14A17.14	2.6	2.8	-0.2	-1.7	100.0%	-4.5

Rank	Description	15.dpi	5.dpi	M	t	adj.q	B
3599	AT3G28780.1 | similar to expressed protein [Arabidopsis thaliana] (TAIR:At3g28840.1); similar to unnamed protein product [Yarrowia lipolytica CLIB99] (GB:CAG83749.1); similar to unnamed protein product [Candida glabrata CBS138] (GB:CAG60731.1); contains domain GLY_RICH (PS50315); contains domain SER_RICH (PS50324) | chr3:10807155-10809695 REVERSE | Aliases: T19N8.7	3.6	3.4	0.2	1.7	100.0%	-4.3
3600	AT3G63300.2 | expressed protein | chr3:23395963-23398817 FORWARD | Aliases: MAA21.9	3.7	4.3	-0.7	-1.7	100.0%	-3.5
3601	AT3G04580.2 | Symbol: EIN4 | ethylene receptor, putative (EIN4), similar to ethylene receptor GB:AAC31123 (Malus domestica), identical to putative ethylene receptor GB:AAD02485 (Arabidopsis thaliana); Pfam HMM hit: response regulator receiver domain, signal C terminal domain | chr3:1235289-1238499 REVERSE | Aliases: ETHYLENE INSENSITIVE 4, F7O18.5, F7O18_5	4.6	5.0	-0.4	-1.7	100.0%	-3.7
3602	AT1G08230.1 | amino acid transporter family protein, low similarity to amino acid permease (Oryza sativa) GI:7415521; contains Pfam profile PF01490: Transmembrane amino acid transporter protein | chr1:2583712-2585216 REVERSE | Aliases: T23G18.9, T23G18_9	3.5	3.2	0.4	1.7	100.0%	-3.9
3603	AT2G28755.1 | UDP-D-glucuronate carboxy-lyase-related, contains similarity to UDP-D-glucuronate carboxy-lyase GI:13591616 from (Pisum sativum)	2.7	2.9	-0.2	-1.7	100.0%	-4.2
3604	AT3G63170.1 | expressed protein | chr3:23345327-23347732 FORWARD | Aliases: F16M2.20	6.7	7.3	-0.6	-1.7	100.0%	-3.6
3605	AT2G19750.1 | 40S ribosomal protein S30 (RPS30A) | chr2:8522119-8522888 FORWARD | Aliases: F6F22.22, F6F22_22	7.5	8.2	-0.7	-1.7	100.0%	-3.4
3606	AT3G15980.3 | coatomer protein complex, subunit beta 2 (beta prime), putative, contains 7 WD-40 repeats (PF00400) (1 weak); identical to coatomer protein complex, beta prime (beta'-COP) protein {Arabidopsis thaliana} (GI:9294445); similar to Coatomer beta' subunit (Beta'-coat protein) (Beta'-COP) (p102) (SP:P35606) (Homo sapiens) | chr3:5411678-5418451 REVERSE | Aliases: MSL1.4	5.8	6.7	-0.9	-1.7	100.0%	-3.5
3607	AT3G19270.1 | Symbol: CYP707A4 | cytochrome P450 family protein, similar to Cytochrome P450 85 (SP:Q43147) {Lycopersicon esculentum};	7.4	6.4	1.1	1.7	100.0%	-3.3
3608	AT5G05320.1 | monooxygenase, putative (MO3), similar to GI:3426064; identical to cDNA monooxygenase 3, partial GI:3426065 | chr5:1575007-1576732 REVERSE | Aliases: K18I23.12, K18I23_12	6.7	6.2	0.5	1.7	100.0%	-3.7
3609	AT3G03250.1 | Symbol: UGP | Is thought to encodes a cytosolic UDP-glucose pyrophosphorylase with strong similarity to UTP--glucose-1-phosphate uridylyltransferase (SwissProt P19595, EC 2.7.7.9, UDP-glucose pyrophosphorylase <i>Solanum tuberosum</i>). | chr3:749486-754166 REVERSE | Aliases: T17B22.6, T17B22_6, UGP, UGPASE	9.1	10.3	-1.2	-1.7	100.0%	-3.6
3610	AT1G61400.1 | S-locus protein kinase, putative, similar to KI domain interacting kinase 1 (Zea mays) gi:2735017:gb:AAB93834; contains S-locus glycoprotein family domain, Pfam:PF00954	3.5	3.8	-0.2	-1.7	100.0%	-4.2
3611	AT3G57480.1 | zinc finger (C2H2 type, AN1-like) family protein, contains Pfam domain, PF00096: Zinc finger, C2H2 type; contains Pfam domain, PF01428: AN1-like Zinc finger | chr3:21288917-21290331 REVERSE | Aliases: T8H10.80	5.8	5.4	0.4	1.7	100.0%	-3.8
3612	AT3G49250.1 | expressed protein | chr3:18269246-18271983 REVERSE | Aliases: F2K15.110	4.4	4.8	-0.4	-1.7	100.0%	-3.6
3613	AT5G01750.2 | expressed protein, contains Pfam profile PF04525: Protein of unknown function (DUF567) | chr5:289762-291323 FORWARD | Aliases: T20L15.20, T20L15_20	9.1	8.1	1.0	1.7	100.0%	-3.6
3614	AT4G22840.1 | bile acid:sodium symporter family protein, low similarity to SP:Q12908 Ileal sodium/bile acid cotransporter {Homo sapiens}; contains Pfam profile PF01758: Sodium Bile acid symporter family | chr4:11991508-11993781 REVERSE | Aliases: F7H19.20	4.7	4.3	0.4	1.6	100.0%	-3.8
3615	AT3G57300.1 | transcriptional activator, putative, similar to transcriptional activator SRCAP (Homo sapiens) GI:5106572; contains Pfam profiles PF00271: Helicase conserved C-terminal domain, PF00176: SNF2 family N-terminal domain | chr3:21210591-21218864 FORWARD | Aliases: F28O9.150	7.3	8.1	-0.7	-1.6	100.0%	-3.5
3616	AT5G23070.1 | thymidine kinase, putative, similar to thymidine kinase (Oryza sativa) SWISS-PROT:O81263	6.3	6.8	-0.4	-1.6	100.0%	-3.5
3617	AT4G38810.2 | calcium-binding EF hand family protein, contains INTERPRO:IPR002048 calcium-binding EF-hand domain | chr4:18115277-18119071 REVERSE | Aliases: T9A14.90, T9A14_90	5.9	5.4	0.5	1.6	100.0%	-3.5
3618	AT3G53200.1 | myb family transcription factor (MYB27), similar to myb-related DNA-binding protein GI:6467223 from (Arabidopsis thaliana); contains PFAM profile: myb DNA binding domain PF00249 | chr3:19729260-19730440 REVERSE | Aliases: T4D2.130	3.5	3.9	-0.4	-1.6	100.0%	-4.0
3619	AT4G17410.1 | expressed protein | chr4:9717226-9721937 FORWARD | Aliases: DL4740W, FCAALL.426	3.0	2.7	0.2	1.6	100.0%	-4.0
3620	AT5G48470.1 | expressed protein | chr5:19658987-19661588 FORWARD | Aliases: MJE7.11, MJE7_11	4.9	5.3	-0.4	-1.6	100.0%	-3.7

Rank	Description	15.dpi	5.dpi	M	t	adj.q	B
3621	AT3G50900.1 | expressed protein | chr3:18929000-18929599 FORWARD | Aliases: F18B3.180	4.0	4.8	-0.8	-1.6	100.0%	-3.4
3622	AT1G03850.2 | glutaredoxin family protein, contains INTERPRO Domain IPR002109, Glutaredoxin (thioltransferase)	4.1	4.9	-0.8	-1.6	100.0%	-3.2
3623	AT5G45130.1 | Symbol: RHA1 | Ras-related protein (RHA1) / small GTP-binding protein, identical to Ras-related protein RHA1 SP:P31582 from (Arabidopsis thaliana) | chr5:18261493-18263670 FORWARD | Aliases: K17O22.15, K17O22_15	11.3	10.8	0.5	1.6	100.0%	-4.0
3624	AT5G67200.1 | leucine-rich repeat transmembrane protein kinase, putative | chr5:26830951-26833792 REVERSE | Aliases: K21H1.16, K21H1_16	3.6	4.0	-0.4	-1.6	100.0%	-3.7
3625	AT5G20320.1 | DEAD/DEAH box helicase, putative, similar to CAF protein (Arabidopsis thaliana) GI:6102610; contains Pfam profiles PF00270: DEAD/DEAH box helicase, PF00271: Helicase conserved C-terminal domain, PF03368: Domain of unknown function, PF00636: RNase3 domain, PF00035: Double-stranded RNA binding motif | chr5:6859342-6869070 REVERSE | Aliases: F5O24.210, F5O24_210	6.5	7.2	-0.7	-1.6	100.0%	-3.5
3626	AT1G06190.1 | expressed protein | chr1:1892272-1894137 REVERSE | Aliases: F9P14.5, F9P14_5	5.2	4.8	0.4	1.6	100.0%	-3.6
3627	AT5G15920.1 | structural maintenance of chromosomes (SMC) family protein (MSS2), similar to SMC-related protein MSS2 (Arabidopsis thaliana) GI:9965743; contains Pfam profiles PF02483: SMC family C-terminal domain, PF02463: RecF/RecN/SMC N terminal domain	3.8	4.2	-0.5	-1.6	100.0%	-3.7
3628	AT5G60990.1 | DEAD/DEAH box helicase, putative (RH10), probable replication protein A1, Oryza sativa, EMBL:AF009179 | chr5:24563658-24566565 REVERSE | Aliases: MSL3.110, MSL3_110	6.3	7.1	-0.8	-1.6	100.0%	-3.4
3629	AT3G45140.1 | Symbol: LOX2 | lipoxygenase (LOX2), identical to SP:P38418 | chr3:16536395-16540337 FORWARD | Aliases: ATLOX2, LIPOXYGENASE 2, T14D3.80	2.8	3.1	-0.3	-1.6	100.0%	-4.1
3630	AT5G39720.1 | avirulence-responsive protein-related / avirulence induced gene (AIG) protein-related, similar to SP:P54121 AIG2 protein {Arabidopsis thaliana} | chr5:15916874-15917881 FORWARD | Aliases: MKM21.1	7.1	6.2	0.9	1.6	100.0%	-3.2
3631	AT1G52720.1 | expressed protein | chr1:19644567-19645333 FORWARD | Aliases: F6D8.3, F6D8_3	7.0	6.3	0.7	1.6	100.0%	-3.6
3632	AT3G48710.1 | expressed protein, putative protein - Arabidopsis thaliana, EMBL:AL078465.1 | chr3:18051962-18055173 FORWARD | Aliases: T8P19.220	5.4	6.6	-1.2	-1.6	100.0%	-3.5
3633	AT2G43140.1 | basic helix-loop-helix (bHLH) family protein, contains Pfam profile PF00010: Helix-loop-helix DNA-binding domain	3.5	3.1	0.4	1.6	100.0%	-3.8
3634	AT3G29610.1 | hypothetical protein | chr3:11412081-11412569 FORWARD | Aliases: MTO24.8	2.5	2.7	-0.2	-1.6	100.0%	-4.3
3635	AT2G23620.1 | esterase, putative, similar to ethylene-induced esterase (Citrus sinensis) GI:14279437, polyneuridine aldehyde esterase (Rauvolfia serpentina) GI:6651393; contains Pfam profile PF00561: hydrolase, alpha/beta fold family | chr2:10054459-10056371 REVERSE | Aliases: F26B6.27, F26B6_27	7.9	6.7	1.2	1.6	100.0%	-3.5
3636	AT2G04240.2 | zinc finger (C3HC4-type RING finger) family protein, contains Pfam profile: PF00097 zinc finger, C3HC4 type (RING finger) | chr2:1461627-1462934 REVERSE | Aliases: T23O15.13, T23O15_13	6.2	5.6	0.6	1.6	100.0%	-3.6
3637	AT3G58570.1 | DEAD box RNA helicase, putative, similar to SP:O00571 DEAD-box protein 3 (Helicase-like protein 2) {Homo sapiens}, DEAD box RNA helicase DDX3 (Homo sapiens) GI:3523150; contains Pfam profiles PF00270: DEAD/DEAH box helicase, PF00271: Helicase conserved C-terminal domain | chr3:21667481-21671509 FORWARD | Aliases: F14P22.160	6.5	6.1	0.4	1.6	100.0%	-4.1
3638	AT2G32790.1 | ubiquitin-conjugating enzyme, putative, similar to ubiquitin conjugating enzyme from (Oryza sativa) GI:1373001, {Arabidopsis thaliana} SP:P35134, SP:P35131; contains Pfam profile PF00179: Ubiquitin-conjugating enzyme	4.1	3.7	0.4	1.6	100.0%	-3.7
3639	AT1G22985.1 | encodes a member of the ERF (ethylene response factor) subfamily B-5 of ERF/AP2 transcription factor family. The protein contains one AP2 domain. There are 7 members in this subfamily.	6.6	6.0	0.6	1.6	100.0%	-3.7
3640	AT3G10780.1 | emp24/gp25L/p24 family protein, similar to SP:O35587 Transmembrane protein Tmp21 precursor (21 kDa Transmembrane trafficking protein) {Mesocricetus auratus}; contains Pfam profile: PF01105 emp24/gp25L/p24 family; contains non-consensus TG acceptor splice site at exon 3 | chr3:3375166-3376339 FORWARD | Aliases: T7M13.14	3.9	3.5	0.5	1.6	100.0%	-3.7
3641	AT5G39590.1 | expressed protein | chr5:15868639-15871194 FORWARD | Aliases: MIJ24.8, MIJ24_8	6.7	7.7	-0.9	-1.6	100.0%	-3.5
3642	AT2G16640.1 | Symbol: ATTOC132/TOC132 | chloroplast outer membrane protein, putative, similar to chloroplast protein import component Toc159 (Pisum sativum) GI:8489806, chloroplast outer envelope protein 86 (Pisum sativum) GI:599958, GTP-binding protein (Pisum sativum) GI:576509 | chr2:7218194-7222612 REVERSE | Aliases: ATTOC132, T24I21.5, T24I21_5, TOC132	6.7	7.3	-0.6	-1.6	100.0%	-3.8

Rank	Description	15.dpi	5.dpi	M	t	adj.q	B
3643	AT2G13440.1 | glucose-inhibited division family A protein, similar to GidA from Pseudomonas syringae (GI:10764670); contains Pfam profile PF01134 Glucose inhibited division protein A	4.5	4.9	-0.3	-1.6	100.0%	-3.8
3644	AT1G04780.1 | ankyrin repeat family protein, contains Pfam PF00023: Ankyrin repeat | chr1:1340668-1343583 REVERSE | Aliases: F13M7.23	7.1	8.0	-0.9	-1.6	100.0%	-3.4
3645	AT2G17420.1 | Symbol: NTRA | similar to thioredoxin reductase 1 / NADPH-dependent thioredoxin reductase 1 (NTR1) [Arabidopsis thaliana] (TAIR:At4g35460.1); similar to NADPH-thioredoxin reductase [Triticum aestivum] (GB:CAD19162.1); contains InterPro domain Thioredoxin reductase (InterPro:IPR005982); contains InterPro domain Pyridine nucleotide-disulphide oxidoreductase, class I (InterPro:IPR001100); contains InterPro domain FAD-dependent pyridine nucleotide-disulphide oxidoreductase (InterPro:IPR001327); contains InterPro domain Pyridine nucleotide-disulphide oxidoreductase, class-II, active site (InterPro:IPR008255); contains InterPro domain Adrenodoxin reductase (InterPro:IPR000759); contains InterPro domain Pyridine nucleotide-disulphide oxidoreductase, class-II (InterPro:IPR000103) | chr2:7571427-7573621 FORWARD | Aliases: ATNTRA, F5J6.18, F5J6_18	12.2	12.5	-0.3	-1.6	100.0%	-4.6
3646	AT4G35460.1 | Symbol: NTR1 | thioredoxin reductase 1 / NADPH-dependent thioredoxin reductase 1 (NTR1), identical to SP:Q39243	12.2	12.5	-0.3	-1.6	100.0%	-4.6
3647	AT1G13410.1 | pentatricopeptide (PPR) repeat-containing protein, contains INTERPRO:IPR002885 PPR repeats | chr1:4601636-4603171 FORWARD | Aliases: T6J4.15, T6J4_15	3.0	3.3	-0.3	-1.6	100.0%	-4.1
3648	AT5G18090.1 | transcriptional factor B3 family protein, contains Pfam profile PF02362: B3 DNA binding domain | chr5:5985390-5987163 FORWARD | Aliases: MRG7.5, MRG7_5	3.5	3.2	0.3	1.6	100.0%	-4.0
3649	AT1G52700.1 | phospholipase/carboxylesterase family protein, similar to lysophospholipase I (Mus musculus) GI:1864159; contains Pfam profile PF02230: Phospholipase/Carboxylesterase family | chr1:19634794-19637108 REVERSE | Aliases: F6D8.5, F6D8_5	3.8	3.4	0.3	1.6	100.0%	-3.8
3650	AT5G43790.1 | pentatricopeptide (PPR) repeat-containing protein, contains Pfam profile PF01535: PPR repeat | chr5:17609234-17610708 REVERSE | Aliases: MQD19.14, MQD19_14	4.5	4.9	-0.4	-1.6	100.0%	-3.8
3651	AT3G11820.2 | Symbol: SYP121 |	6.4	5.7	0.7	1.6	100.0%	-3.4
3652	AT2G29590.1 | thioesterase family protein, contains Pfam profile PF03061: thioesterase family protein | chr2:12661533-12662525 REVERSE | Aliases: F16P2.3, F16P2_3	7.6	7.2	0.4	1.6	100.0%	-3.8
3653	AT3G04680.2 | pre-mRNA cleavage complex family protein, similar to Pre-mRNA cleavage complex II protein Clp1 (Swiss-Prot:Q92989) (Homo sapiens) | chr3:1269859-1272922 REVERSE | Aliases: F7O18.15, F7O18_15	6.4	6.8	-0.5	-1.6	100.0%	-3.6
3654	AT2G38340.1 | encodes a member of the DREB subfamily A-2 of ERF/AP2 transcription factor family. The protein contains one AP2 domain. There are eight members in this subfamily including DREB2A AND DREB2B that are involved in response to drought. | chr2:16074474-16075369 REVERSE | Aliases: T19C21.17, T19C21_17	2.9	2.7	0.2	1.6	100.0%	-4.4
3655	AT5G20570.1 | Symbol: RBX1 | ring-box protein-related, similar to ring-box protein 1 GI:4769004 from (Homo sapiens) | chr5:6956662-6958313 REVERSE | Aliases: F7C8.160, F7C8_160, HRT1, ROC1	10.5	10.0	0.6	1.6	100.0%	-3.7
3656	AT2G27360.1 | lipase, putative, similar to lipase (Arabidopsis thaliana) GI:1145627; contains Pfam profile PF00657: Lipase/Acylhydrolase with GDSL-like motif | chr2:11713276-11715120 FORWARD | Aliases: F12K2.6, F12K2_6	4.6	3.9	0.7	1.6	100.0%	-3.4
3657	AT3G29140.1 | hypothetical protein | chr3:11107042-11107381 FORWARD | Aliases: MXE2.16	6.1	5.0	1.1	1.6	100.0%	-3.4
3658	AT4G39090.1 | Symbol: RD19 | cysteine proteinase RD19a (RD19A) / thiol protease, identical to cysteine proteinase RD19a, thiol protease SP:P43296, GI:435618 from (Arabidopsis thaliana) | chr4:18214569-18217476 REVERSE | Aliases: EMB3005, EMBRYO DEFECTIVE 3005, F19H22.190, F19H22_190, RD19A, RESPONSIVE TO DEHYDRATION 19	11.5	10.5	1.0	1.6	100.0%	-3.8
3659	AT5G61290.1 | flavin-containing monooxygenase family protein / FMO family protein, low similarity to FMO3 from Homo sapiens (SP:P31513); contains Pfam profile: PF00743 Flavin-binding monooxygenase-like; supported by full-length cDNA Ceres:14492 | chr5:24665784-24668492 FORWARD | Aliases: MFB13.9, MFB13_9	3.0	3.3	-0.3	-1.6	100.0%	-4.0
3660	AT1G74460.1 | GDSL-motif lipase/hydrolase family protein, similar to family II lipases EXL3 GI:15054386, EXL1 GI:15054382, EXL2 GI:15054384 from (Arabidopsis thaliana); contains Pfam profile: PF00657 lipase/acylhydrolase with GDSL-like motif | chr1:27991622-27993462 REVERSE | Aliases: F1M20.14, F1M20_14	3.7	4.4	-0.6	-1.6	100.0%	-3.3
3661	AT4G29010.1 | Symbol: AIM1 | abnormal inflorescence meristem 1 / fatty acid multifunctional protein (AIM1), identical to gi:4337025; contains Pfam profiles PF02737 (3-hydroxyacyl-CoA dehydrogenase, NAD binding domain), PF00378 (enoyl-CoA hydratase/isomerase family protein), PF00725 (3-hydroxyacyl-CoA dehydrogenase) | chr4:14296679-14302066 REVERSE | Aliases: ABNORMAL INFLORESCENCE MERISTEM, F19B15.40, F19B15_40	8.9	9.7	-0.8	-1.6	100.0%	-3.8

Rank	Description	15.dpi	5.dpi	M	t	adj.q	B
3662	AT2G04790.3 | expressed protein | chr2:1679525-1680540 FORWARD | Aliases: F28I8.17, F28I8_17	3.1	2.8	0.3	1.6	100.0%	-4.0
3663	AT4G36730.2 | Symbol: GBF1 | G-box binding factor 1 (GBF1), identical to G-box binding factor 1 SP:P42774 from (Arabidopsis thaliana); contains Pfam profile: PF00170 bZIP transcription factor | chr4:17309623-17312480 REVERSE | Aliases: AP22.21, AP22_21	8.1	7.4	0.8	1.6	100.0%	-3.7
3664	AT3G50790.1 | late embryogenesis abundant protein, putative / LEA protein, putative, similar to Picea glauca late embryogenesis abundant protein (EMB8), PID:g1350545 SP:Q40863; contains Pfam profile PF00561: hydrolase, alpha/beta fold family | chr3:18890904-18892963 REVERSE | Aliases: F18B3.70	5.8	6.4	-0.6	-1.6	100.0%	-3.5
3665	AT1G74390.1 | exonuclease family protein, contains exonuclease domain, Pfam:PF00929 | chr1:27965198-27967508 REVERSE | Aliases: F1M20.7, F1M20_7	5.4	4.9	0.5	1.6	100.0%	-3.5
3666	AT5G62390.1 | calmodulin-binding family protein, contains IQ calmodulin-binding motif, Pfam:PF00612 | chr5:25069275-25071515 REVERSE | Aliases: MMI9.22, MMI9_22	6.6	7.3	-0.8	-1.6	100.0%	-3.5
3667	AT3G18870.1 | mitochondrial transcription termination factor-related / mTERF-related, contains Pfam profile PF02536: mTERF | chr3:6508226-6509349 REVERSE | Aliases: MCB22.4	3.1	2.8	0.3	1.6	100.0%	-4.0
3668	AT1G25375.1 | metallo-beta-lactamase family protein | chr1:8900189-8903244 REVERSE | Aliases: None	6.3	6.7	-0.4	-1.6	100.0%	-4.0
3669	AT3G52105.1 | expressed protein | chr3:19334310-19334805 FORWARD | Aliases: None	5.7	4.5	1.1	1.6	100.0%	-3.2
3670	AT2G23700.1 | expressed protein, contains Pfam profile PF04784: Protein of unknown function, DUF547 | chr2:10083449-10087312 REVERSE | Aliases: F27L4.12, F27L4_12	3.4	4.1	-0.7	-1.6	100.0%	-3.8
3671	AT4G18480.1 | Symbol: CHLI1 | magnesium-chelatase subunit chlI, chloroplast / Mg-protoporphyrin IX chelatase (CHLI) (CS) (CH42), identical to SP:P161127 Magnesium-chelatase subunit chlI, chloroplast precursor (Mg-protoporphyrin IX chelatase) (Protein CS/CH-42) {Arabidopsis thaliana} | chr4:10201683-10203454 REVERSE | Aliases: CH 42, CH42, CHLORATA, CHLORINA 42, F28J12.140, F28J12_140, PROTOPORPHYRIN IX MG CHELATASE	8.2	9.0	-0.8	-1.6	100.0%	-3.4
3672	AT5G26980.2 | Symbol: SYP41 | similar to syntaxin, putative [Arabidopsis thaliana] (TAIR:At3g05710.2); similar to putative syntaxin protein [Oryza sativa (japonica cultivar-group)] (GB:NP_910328.1); contains InterPro domain Target SNARE coiled-coil domain (InterPro:IPR000727); contains InterPro domain Syntaxin, N-terminal (InterPro:IPR006011) | chr5:9488153-9490819 REVERSE | Aliases: ATSYP41, ATTLG2A, F2P16.16, F2P16_16, SYNTAXIN OF PLANTS 41	5.2	4.8	0.4	1.6	100.0%	-3.7
3673	AT4G16566.1 | histidine triad family protein / HIT family protein, contains Pfam domain, PF01230: HIT family (histidine triad protein family) | chr4:9334648-9335980 REVERSE | Aliases: None	7.2	6.5	0.7	1.6	100.0%	-3.5
3674	AT3G16620.1 | Symbol: ATTOC120 | chloroplast outer membrane protein, putative, similar to chloroplast protein import component Toc159 (Pisum sativum) GI:8489806, chloroplast outer envelope protein 86 (Pisum sativum) GI:599958, GTP-binding protein (Pisum sativum) GI:576509 | chr3:5658475-5661969 REVERSE | Aliases: MGL6.15	4.7	5.2	-0.5	-1.6	100.0%	-3.5
3675	AT3G06740.1 | zinc finger (GATA type) family protein | chr3:2126425-2127473 FORWARD | Aliases: F3E22.12	6.1	6.9	-0.8	-1.6	100.0%	-3.5
3676	AT4G24710.1 | similar to AAA-type ATPase family protein [Arabidopsis thaliana] (TAIR:At2g03670.1); similar to Hypothetical protein MGC76197 [Xenopus tropicalis] (GB:AAH63217.1); contains InterPro domain AAA ATPase (InterPro:IPR003593); contains InterPro domain Chaperonin clpA/B (InterPro:IPR001270); contains InterPro domain AAA ATPase, central region (InterPro:IPR003959) | chr4:12745557-12749005 REVERSE | Aliases: F22K18.90, F22K18_90	3.6	3.9	-0.3	-1.6	100.0%	-3.8
3677	AT1G68590.1 | plastid-specific 30S ribosomal protein 3, putative / PSRP-3, putative, similar to SP:P82412 Plastid-specific 30S ribosomal protein 3, chloroplast precursor (PSRP-3) {Spinacia oleracea}; contains Pfam profile PF04839: Plastid and cyanobacterial ribosomal protein (PSRP-3 / Ycf65) | chr1:25761072-25761890 REVERSE | Aliases: F24J5.17, F24J5_17	5.4	6.2	-0.8	-1.6	100.0%	-3.2
3678	AT1G47240.1 | Symbol: NRAMP2 |	6.9	6.3	0.6	1.6	100.0%	-3.6
3679	AT2G28910.1 | Symbol: CXIP4 | CAX-interacting protein 4 (CAXIP4), contains Pfam domain PF00098: Zinc knuckle; identical to cDNA CAX-interacting protein 4 GI:27651998	12.2	11.7	0.5	1.6	100.0%	-4.3
3680	AT5G14610.1 | similar to DEAD box RNA helicase (DRH1) [Arabidopsis thaliana] (TAIR:At3g01540.2); similar to DEAD box RNA helicase (DRH1) [Arabidopsis thaliana] (TAIR:At3g01540.1); similar to DEAD box RNA helicase (DRH1) [Arabidopsis thaliana] (TAIR:At3g01540.3); similar to putative RNA helicase, DRH1 [Oryza sativa (japonica cultivar-group)] (GB:XP_462826.1); similar to ATP-dependent RNA helicase DB10 - wood tobacco (GB:S42639); similar to putative p68 RNA helicase [Oryza sativa (japonica cultivar-group)] (GB:XP_550287.1); contains InterPro domain WW/Rsp5/WWP domain (InterPro:IPR001202); contains InterPro domain ATP-dependent helicase, DEAD-box (InterPro:IPR000629); contains InterPro domain Helicase, C-terminal (InterPro:IPR001650); contains InterPro domain DEAD/DEAH box helicase (InterPro:IPR001410) | chr5:4710575-4715072 FORWARD | Aliases: T15N1.100, T15N1_100	6.5	6.9	-0.4	-1.6	100.0%	-3.7

Rank	Description	15.dpi	5.dpi	M	t	adj.q	B
3681	AT2G25570.1 | expressed protein | chr2:10892776-10894999 REVERSE | Aliases: F3N11.2, F3N11_2	7.2	7.9	-0.7	-1.6	100.0%	-3.5
3682	AT5G10160.1 | beta-hydroxyacyl-ACP dehydratase, putative, similar to beta-hydroxyacyl-ACP dehydratase from Toxoplasma gondii (GI:3850997); contains Pfam profile PF01377 Thioester dehydratase	7.0	8.1	-1.1	-1.6	100.0%	-3.3
3683	AT2G39460.1 | Symbol: ATRPL23A | 60S ribosomal protein L23A (RPL23aA), identical to GB:AF034694 | chr2:16482001-16483163 FORWARD | Aliases: F12L6.12, F12L6_12, RIBOSOMAL PROTEIN L23A, RPL23A	10.5	11.2	-0.8	-1.6	100.0%	-3.4
3684	AT5G53650.1 | expressed protein | chr5:21808403-21809666 FORWARD | Aliases: MNC6.19, MNC6_19	8.7	8.1	0.6	1.6	100.0%	-3.9
3685	AT3G49500.1 | Symbol: RDR6 | RNA-dependent RNA polymerase (SDE1), identical to RNA-dependent RNA polymerase (Arabidopsis thaliana) gi:8248473:gb:AAF74208 | chr3:18360178-18364190 REVERSE | Aliases: RNA DEPENDENT RNA POLYMERASE 6, SDE1, SGS2, SILENCING DEFECTIVE 1, SUPPRESSOR OF GENE SILENCING 2, T9C5.95	5.6	6.1	-0.6	-1.6	100.0%	-3.7
3686	AT5G23920.1 | expressed protein | chr5:8073227-8074173 REVERSE | Aliases: MRO11.4, MRO11_4	6.2	6.9	-0.6	-1.6	100.0%	-3.5
3687	AT5G56640.1 | Symbol: MIOX5 | expressed protein, similar to myo-inositol oxygenase (Sus scrofa) gi:17432544:gb:AAL39076 | chr5:22944555-22946869 REVERSE | Aliases: MIK19.9, MIK19_9	11.3	10.6	0.7	1.6	100.0%	-3.3
3688	AT2G17300.1 | expressed protein | chr2:7529547-7530018 REVERSE | Aliases: F5J6.6, F5J6_6	4.1	3.8	0.3	1.6	100.0%	-3.8
3689	AT2G18120.1 | lateral root primordium (LRP) protein-related, similar to lateral root primordium 1 (LRP1) (Arabidopsis thaliana) GI:882341; contains Pfam profile PF05142: Domain of unknown function (DUF702) | chr2:7883697-7884763 REVERSE | Aliases: F8D23.10, F8D23_10	2.8	2.4	0.4	1.6	100.0%	-4.1
3690	AT5G55310.1 | Symbol: TOP1 | DNA topoisomerase I, putative, similar to Swiss-Prot:P30181 DNA topoisomerase I (Arabidopsis thaliana) | chr5:22447615-22452285 REVERSE | Aliases: MTE17.2, MTE17_2, TOPOISOMERASE I	7.8	8.5	-0.7	-1.6	100.0%	-3.9
3691	AT2G42980.1 | aspartyl protease family protein, contains pfam profile: PF00026 eukaryotic aspartyl protease | chr2:17882082-17883665 REVERSE | Aliases: F23E6.3, F23E6_3	2.5	2.7	-0.3	-1.6	100.0%	-4.3
3692	AT3G54040.1 | photoassimilate-responsive protein-related, contains weak similarity to mRNA inducible by sucrose and salicylic acid expressed in sugar-accumulating tobacco plants (GI:871487) (Nicotiana tabacum) | chr3:20024739-20026415 REVERSE | Aliases: F5K20.340	3.4	4.5	-1.0	-1.6	100.0%	-3.3
3693	AT1G26920.1 | expressed protein, Location of EST 228A16T7A, gb:N65686 | chr1:9329499-9330385 FORWARD | Aliases: T2P11.11, T2P11_11	5.0	4.6	0.4	1.6	100.0%	-3.8
3694	AT1G16070.2 | similar to F-box family protein / tubby family protein [Arabidopsis thaliana] (TAIR:At1g47270.1); similar to tubby like protein 3 [Homo sapiens] (GB:AAC95431.1); contains InterPro domain Tubby (InterPro:IPR000007) | chr1:5511868-5513890 REVERSE | Aliases: T24D18.17, T24D18_17	2.5	2.7	-0.2	-1.6	100.0%	-4.3
3695	AT5G16000.1 | leucine-rich repeat family protein / protein kinase family protein, contains Pfam domains PF00560: Leucine Rich Repeat and PF00069: Protein kinase domain | chr5:5224146-5227291 FORWARD | Aliases: F1N13.140, F1N13_140	5.3	5.8	-0.5	-1.6	100.0%	-3.6
3696	AT3G15030.2 | Symbol: TCP4 | TCP family transcription factor, putative, similar to TCP3 GB:AAC24010 (Arabidopsis thaliana) | chr3:5062026-5064045 FORWARD | Aliases: K15M2.17	3.9	4.2	-0.4	-1.6	100.0%	-3.7
3697	AT4G09900.1 | hydrolase, alpha/beta fold family protein, similar to ethylene-induced esterase (Citrus sinensis) GI:14279437, polyneuridine aldehyde esterase (Rauvolfia serpentina) GI:6651393, SP:Q40708 PIR7A protein {Oryza sativa}; contains Pfam profile PF00561: hydrolase, alpha/beta fold family | chr4:6221661-6224130 REVERSE | Aliases: AT4G09910, T5L19.30, T5L19_30	3.7	3.3	0.4	1.6	100.0%	-3.9
3698	AT5G66200.1 | armadillo/beta-catenin repeat family protein, contains Pfam profile: PF00514 armadillo/beta-catenin-like repeat | chr5:26470686-26473048 REVERSE | Aliases: K2A18.28, K2A18_28	6.3	5.8	0.5	1.6	100.0%	-3.8
3699	AT4G22920.1 | expressed protein | chr4:12016536-12018503 REVERSE | Aliases: F7H19.100, F7H19_100	6.2	5.8	0.4	1.6	100.0%	-3.9
3700	AT1G16530.1 | LOB domain protein 3 / lateral organ boundaries domain protein 3 (LBD3), identical to SP:Q9SA51 LOB domain protein 3 {Arabidopsis thaliana}; identical to ASYMMETRIC LEAVES2-like protein 9 (Arabidopsis thaliana) GI:19918989 | chr1:5651463-5652832 REVERSE | Aliases: F3O9.33, F3O9_33	4.1	4.7	-0.6	-1.6	100.0%	-3.5
3701	AT3G03120.1 | Symbol: ATARFB1C | Gene encoding ADP-ribosylation factor and similar to ADP-ribosylation factor 1; ARF 1 (GP:385340) {Drosophila melanogaster}, other ARFs and ARF-like proteins. Members of this family are known to be essential for vesicle coating and uncoating and functions in GTP-binding. | chr3:717186-719322 FORWARD | Aliases: ATARFB1C, T17B22.19, T17B22_19	6.1	5.8	0.3	1.6	100.0%	-3.9

Rank	Description	15.dpi	5.dpi	M	t	adj.q	B
3702	AT1G62670.1 | pentatricopeptide (PPR) repeat-containing protein, contains multiple PPR repeats Pfam Profile: PF01535 | chr1:23208438-23210330 REVERSE | Aliases: F23N19.4, F23N19_4	3.1	3.6	-0.5	-1.6	100.0%	-4.0
3703	AT4G38090.2 | expressed protein, contains Pfam PF01205: Uncharacterized protein family UPF0029	6.6	6.0	0.6	1.6	100.0%	-3.6
3704	AT4G18465.1 | RNA helicase, putative, similar to SP:Q14562 ATP-dependent helicase DDX8 (RNA helicase HRH1) (DEAH-box protein 8) {Homo sapiens}; contains Pfam profiles PF04408: Helicase associated domain (HA2), PF00271: Helicase conserved C-terminal domain | chr4:10197067-10201622 FORWARD | Aliases: None	3.6	4.1	-0.5	-1.6	100.0%	-3.6
3705	AT3G17120.2 | expressed protein | chr3:5841323-5843491 FORWARD | Aliases: K14A17.19	5.7	5.0	0.7	1.6	100.0%	-3.3
3706	AT5G28740.1 | transcription-coupled DNA repair protein-related, similar to XAB2 (XPA-binding protein 2) (Homo sapiens) GI:10566459 | chr5:10780778-10783915 FORWARD | Aliases: T32B20.1, T32B20_1	6.2	7.0	-0.8	-1.6	100.0%	-3.6
3707	AT5G10970.1 | zinc finger (C2H2 type) family protein, contains zinc finger, C2H2 type domain, Prosite:PS00028 | chr5:3469269-3470087 FORWARD | Aliases: T30N20.240, T30N20_240	2.9	2.7	0.3	1.6	100.0%	-4.0
3708	AT1G31335.1 | expressed protein | chr1:11216895-11217374 REVERSE | Aliases: None	5.4	4.7	0.7	1.6	100.0%	-3.5
3709	AT3G14050.1 | Symbol: RSH2 | RelA/SpoT protein, putative (RSH2), nearly identical to RelA/SpoT homolog RSH2 (Arabidopsis thaliana) GI:7141306; contains Pfam profiles PF01966: HD domain, PF04607: Region found in RelA / SpoT proteins | chr3:4650768-4653921 REVERSE | Aliases: AT RSH2, MDC16.19, RELA SPOT HOMOLOG	7.1	6.6	0.5	1.6	100.0%	-3.5
3710	AT3G19553.1 | amino acid permease family protein, weak similarity to aspartate/glutamate transporter 1 (Mus musculus) GI:21322754; contains Pfam profile PF00324: Amino acid permease | chr3:6790748-6792827 REVERSE | Aliases: T31J18.6	5.6	4.8	0.8	1.6	100.0%	-3.6
3711	AT3G63060.1 | circadian clock coupling factor, putative, similar to gb:AAK56924 circadian clock coupling factor ZGT {Nicotiana tabacum} | chr3:23311265-23312448 REVERSE | Aliases: T20O10.160	3.3	3.1	0.2	1.6	100.0%	-4.3
3712	AT2G22500.1 | mitochondrial substrate carrier family protein, contains Pfam profile: PF00153 mitochondrial carrier protein | chr2:9570304-9571831 REVERSE | Aliases: F14M13.10, F14M13_10	7.1	6.3	0.8	1.6	100.0%	-3.9
3713	AT5G05950.1 | expressed protein | chr5:1788768-1789439 FORWARD | Aliases: K18J17.12, K18J17_12	4.9	4.4	0.5	1.6	100.0%	-3.7
3714	AT5G26040.2 | Symbol: HDA2 | histone deacetylase family protein (HDA2), identical to HDA2 (Arabidopsis thaliana) GI:21105771; similar to SP:Q96DB2 Histone deacetylase 11 (HD11) {Homo sapiens}; contains Pfam profile PF00850: Histone deacetylase family | chr5:9098557-9101639 REVERSE | Aliases: HISTONE DEACETYLASE, T1N24.9, T1N24_9	3.9	3.5	0.4	1.6	100.0%	-3.8
3715	AT5G64180.1 | expressed protein | chr5:25694306-25695518 FORWARD | Aliases: MSJ1.2, MSJ1_2	5.6	5.0	0.5	1.6	100.0%	-3.7
3716	AT2G18220.1 | expressed protein, contains Pfam domain PF03715: Uncharacterised protein family (UPF0120) | chr2:7935336-7938933 FORWARD | Aliases: T30D6.27, T30D6_27	4.9	6.1	-1.2	-1.6	100.0%	-3.5
3717	AT1G63290.1 | ribulose-phosphate 3-epimerase, cytosolic, putative / pentose-5-phosphate 3-epimerase, putative, strong similarity to D-ribulose-5-phosphate 3-epimerase (Oryza sativa) GI:6007803; contains Pfam profile PF00834: Ribulose-phosphate 3 epimerase family	8.6	9.2	-0.7	-1.6	100.0%	-3.8
3718	AT1G50910.1 | expressed protein | chr1:18870751-18873507 REVERSE | Aliases: F8A12.13, F8A12_13	2.5	2.7	-0.2	-1.6	100.0%	-4.3
3719	AT5G65930.2 | Symbol: ZWI | kinesin-like calmodulin-binding protein (ZWICHEL), identical to kinesin-like protein GI:2224925 from (Arabidopsis thaliana) | chr5:26387427-26393746 REVERSE | Aliases: K14B20.10, K14B20_10, KCBP, KINESIN LIKE CALMODULIN BINDING PROTEIN, PKCBP, ZWICHEL	4.3	5.0	-0.6	-1.6	100.0%	-3.6
3720	AT2G35010.1 | thioredoxin family protein, similar to SP:Q42443 Thioredoxin H-type (TRX-H) {Oryza sativa}; contains Pfam profile: PF00085 Thioredoxin | chr2:14761415-14763122 FORWARD | Aliases: F19I3.24, F19I3_24	6.4	7.2	-0.8	-1.6	100.0%	-3.7
3721	AT1G01570.1 | fringe-related protein, + similar to hypothetical protein GB:AAC23643 (Arabidopsis thaliana) + weak similarity to Fringe (Schistocerca gregaria)(GI:6573138);Fringe encodes an extracellular protein that regulates Notch signalling. | chr1:205176-207435 FORWARD | Aliases: F22L4.11, F22L4_11	4.2	4.7	-0.5	-1.6	100.0%	-3.8
3722	AT4G17970.1 | expressed protein, contains Pfam profile PF01027: Uncharacterized protein family UPF0005	5.2	4.5	0.7	1.6	100.0%	-3.2
3723	AT5G13470.1 | expressed protein | chr5:4318565-4319834 FORWARD | Aliases: T6I14.1	5.3	4.8	0.4	1.6	100.0%	-3.6
3724	AT1G80170.1 | polygalacturonase, putative / pectinase, putative, similar to polygalacturonase GI:7381227 from (Lycopersicon esculentum); contains Pfam profile PF00295: Glycosyl hydrolases family 28 (polygalacturonases) | chr1:30158300-30160584 REVERSE | Aliases: F18B13.25, F18B13_25	5.2	5.8	-0.6	-1.6	100.0%	-3.6

Rank	Description	15.dpi	5.dpi	M	t	adj.q	B
3725	AT3G05390.1 | expressed protein, ; expression supported by MPSS | chr3:1546591-1547982 REVERSE | Aliases: F22F7.17, F22F7_17	4.5	5.1	-0.6	-1.6	100.0%	-3.6
3726	AT1G30220.1 | sugar transporter family protein, similar to SP:Q96QE2 Proton myo-inositol co-transporter (Hmit) (Homo sapiens); contains Pfam profile PF00083: major facilitator superfamily protein | chr1:10632805-10635455 REVERSE | Aliases: F12P21.2, F12P21_2	5.0	4.2	0.8	1.6	100.0%	-3.5
3727	AT2G46800.2 | Symbol: ZAT1 | zinc transporter (ZAT), identical to zinc transporter ZAT (Arabidopsis thaliana) gi:4206640:gb:AAD11757; member of the cation diffusion facilitator (CDF) family, or cation efflux (CE) family, PMID:11500563 | chr2:19244503-19246474 FORWARD | Aliases: ATMTP1, F19D11.8, MTP1, ZINC TRANSPORTER OF ARABIDOPSIS THALIANA, ZINC TRANSPORTER ZAT	10.6	10.0	0.5	1.6	100.0%	-4.0
3728	AT4G39720.1 | VQ motif-containing protein, contains PF05678: VQ motif | chr4:18429886-18430858 REVERSE | Aliases: T19P19.110, T19P19_110	4.2	3.9	0.3	1.6	100.0%	-4.0
3729	AT3G24340.1 | SNF2 domain-containing protein / helicase domain-containing protein, similar to SP:P41410 DNA repair protein rhp54 (RAD54 homolog) {Schizosaccharomyces pombe}; contains PFam profiles PF00271: Helicase conserved C-terminal domain, PF00176: SNF2 family N-terminal domain | chr3:8832092-8835729 REVERSE | Aliases: K7M2.11	2.5	2.8	-0.3	-1.6	100.0%	-4.3
3730	AT1G29880.1 | glycyl-tRNA synthetase / glycine--tRNA ligase, identical to SP:O23627 Glycyl-tRNA synthetase (EC 6.1.1.14) (Glycine--tRNA ligase) (GlyRS) {Arabidopsis thaliana}	8.1	8.8	-0.6	-1.6	100.0%	-3.4
3731	AT2G39000.3 | GCN5-related N-acetyltransferase (GNAT) family protein, contains Pfam profile PF00583: acetyltransferase, GNAT family | chr2:16293414-16295045 REVERSE | Aliases: T7F6.17, T7F6_17	7.6	6.9	0.7	1.6	100.0%	-3.6
3732	AT4G14630.1 | Symbol: GLP9 | germin-like protein (GLP9), identical to germin-like protein subfamily 1 member 8 (SP:Q9LEA7) | chr4:8392915-8393802 FORWARD | Aliases: DL3355W, FCAALL.278, GERMIN LIKE PROTEIN 9	2.4	2.9	-0.6	-1.6	100.0%	-3.7
3733	AT1G05290.1 | expressed protein | chr1:1539151-1540545 REVERSE | Aliases: YUP8H12.10, YUP8H12_10	2.7	2.9	-0.2	-1.6	100.0%	-4.4
3734	AT5G66570.1 | Symbol: PSBO 1 |	8.4	7.0	1.4	1.6	100.0%	-3.3
3735	AT2G41880.1 | Symbol: GK 1 | guanylate kinase 1 (GK-1), identical to guanylate kinase (GK-1) (Arabidopsis thaliana) gi:7861795:gb:AAF70408 | chr2:17481713-17484301 FORWARD | Aliases: AGK1, GUANYLATE KINASE 1, T11A7.23	6.6	5.8	0.7	1.6	100.0%	-3.5
3736	AT5G09770.1 | ribosomal protein L17 family protein, contains Pfam profile: PF01196 ribosomal protein L17 | chr5:3035047-3036543 REVERSE | Aliases: F17I14.40, F17I14_40	8.6	9.2	-0.6	-1.6	100.0%	-3.5
3737	AT4G16390.1 | chloroplastic RNA-binding protein P67, putative, nearly identical to 67kD chloroplastic RNA-binding protein, P67 (Arabidopsis thaliana) GI:9755842 | chr4:9257980-9260300 FORWARD | Aliases: DL4225W, FCAALL.354	6.6	7.2	-0.6	-1.6	100.0%	-3.6
3738	AT1G55500.1 | expressed protein, contains Pfam profile PF04146: YT521-B-like family | chr1:20723241-20726399 FORWARD | Aliases: T5A14.10, T5A14_10	5.3	5.7	-0.4	-1.6	100.0%	-3.7
3739	AT2G16250.1 | leucine-rich repeat transmembrane protein kinase, putative | chr2:7046764-7050015 REVERSE | Aliases: F16F14.25, F16F14_25	3.9	3.5	0.3	1.6	100.0%	-3.9
3740	AT3G56640.1 | exocyst complex subunit Sec15-like family protein, contains Pfam profile PF04091: Exocyst complex subunit Sec15-like | chr3:20992946-20995315 FORWARD | Aliases: T5P19.290	3.6	3.8	-0.2	-1.6	100.0%	-4.1
3741	AT2G39730.3 | Symbol: RCA | ribulose bisphosphate carboxylase/oxygenase activase / RuBisCO activase, identical to SWISS-PROT:P10896 ribulose bisphosphate carboxylase/oxygenase activase, chloroplast precursor (RuBisCO activase, RA)(Arabidopsis thaliana) | chr2:16577824-16580597 REVERSE | Aliases: RUBISCO ACTIVASE, T5I7.18	9.5	8.1	1.5	1.6	100.0%	-3.4
3742	AT4G04470.1 | Symbol: PMP22 | peroxisomal membrane protein 22 kDa (PMP22), identical to peroxisomal membrane protein (Arabidopsis thaliana) gi:3980254:emb:CAA06834 | chr4:2227659-2229256 REVERSE | Aliases: 22 KD PEROXISOMAL MEMBRANE PROTEIN, T26N6.9, T26N6_9	7.7	8.2	-0.5	-1.6	100.0%	-3.8
3743	AT3G07060.1 | Symbol: EMB1974 | expressed protein, ; expression supported by MPSS | chr3:2233000-2236919 FORWARD | Aliases: EMB1974, EMBRYO DEFECTIVE 1974, F17A9.23	3.5	3.8	-0.3	-1.6	100.0%	-4.0
3744	AT2G46110.1 | ketopantoate hydroxymethyltransferase family protein, similar to SP:Q9Y7B6 3-methyl-2-oxobutanoate hydroxymethyltransferase (EC 2.1.2.11) (Ketopantoate hydroxymethyltransferase) {Emericella nidulans}; contains Pfam profile PF02548: Ketopantoate hydroxymethyltransferase | chr2:18961532-18963603 REVERSE | Aliases: T3F17.24	8.2	8.9	-0.7	-1.6	100.0%	-3.3

Rank	Description	15.dpi	5.dpi	M	t	adj.q	B
3745	AT5G02010.1 | member of KPP-like gene family, homolog of KPP (kinase partner protein) gene in tomato; expressed protein, contains Pfam profile PF03759: Domain of unknown function (DUF315) | chr5:383150-385847 FORWARD | Aliases: KINASE PARTNER PROTEIN LIKE, KPP LIKE, T7H20.60, T7H20_60	2.9	3.3	-0.4	-1.6	100.0%	-3.8
3746	AT5G10100.1 | trehalose-6-phosphate phosphatase, putative, similar to trehalose-6-phosphate phosphatase (AtTPPB) (Arabidopsis thaliana) GI:2944180; contains Pfam profile PF02358: Trehalose-phosphatase | chr5:3157981-3160446 FORWARD | Aliases: T31P16.90, T31P16_90	3.1	2.7	0.4	1.6	100.0%	-3.8
3747	AT5G63540.1 | expressed protein, similar to expressed protein [Arabidopsis thaliana] (TAIR:At5g19950.2); similar to expressed protein [Arabidopsis thaliana] (TAIR:At5g19950.3); similar to expressed protein [Arabidopsis thaliana] (TAIR:At5g19950.1); similar to hypothetical protein LOC549050 [Xenopus tropicalis] (GB:NP_001016296.1) | chr5:25458009-25461649 REVERSE | Aliases: MLE2.17, MLE2_17	3.0	3.3	-0.2	-1.6	100.0%	-4.2
3748	AT3G25610.1 | haloacid dehalogenase-like hydrolase family protein, similar to Potential phospholipid-transporting ATPase (EC 3.6.3.1) from Mus musculus (SP:P98200, SP:P70704), {Bos taurus} SP:Q29449; contains InterPro accession IPR005834: Haloacid dehalogenase-like hydrolase | chr3:9310179-9314590 REVERSE | Aliases: T5M7.10	4.4	4.9	-0.6	-1.6	100.0%	-3.4
3749	AT4G26020.1 | expressed protein, weak similarity to cardiac muscle factor 1 (Gallus gallus) GI:14422164 | chr4:13202022-13204037 REVERSE | Aliases: F20B18.130, F20B18_130	2.5	2.7	-0.2	-1.6	100.0%	-4.6
3750	AT5G64230.1 | expressed protein | chr5:25709679-25711279 FORWARD | Aliases: MSJ1.7, MSJ1_7	4.3	3.8	0.5	1.6	100.0%	-3.8
3751	AT5G45750.1 | Ras-related GTP-binding protein, putative, similar to GTP-binding protein GI:303744 from (Pisum sativum)	8.7	9.4	-0.7	-1.6	100.0%	-3.7
3752	AT2G20540.1 | pentatricopeptide (PPR) repeat-containing protein, contains INTERPRO:IPR002885 PPR repeats | chr2:8851241-8852845 FORWARD | Aliases: T13C7.13, T13C7_13	3.5	4.1	-0.6	-1.6	100.0%	-3.6
3753	AT2G38370.1 | expressed protein | chr2:16079148-16081269 REVERSE | Aliases: T19C21.14, T19C21_14	6.7	7.8	-1.1	-1.6	100.0%	-3.8
3754	AT3G57780.1 | expressed protein | chr3:21410743-21413303 REVERSE | Aliases: F15B8.30	3.0	3.2	-0.3	-1.6	100.0%	-4.0
3755	AT5G02270.1 | Symbol: ATNAP9 |	10.2	10.6	-0.4	-1.6	100.0%	-4.3
3756	AT1G18070.2 | similar to elongation factor 1-alpha / EF-1-alpha [Arabidopsis thaliana] (TAIR:At1g07930.1); similar to elongation factor 1-alpha / EF-1-alpha [Arabidopsis thaliana] (TAIR:At5g60390.1); similar to elongation factor 1-alpha / EF-1-alpha [Arabidopsis thaliana] (TAIR:At1g07920.1); similar to elongation factor 1-alpha / EF-1-alpha [Arabidopsis thaliana] (TAIR:At1g07940.1); similar to OSJNBb0067G11.10 [Oryza sativa (japonica cultivar-group)] (GB:XP_471489.1); similar to SUP2 gene product (GB:AAA79033.1); contains InterPro domain Elongation factor, GTP-binding (InterPro:IPR000795); contains InterPro domain Yeast eukaryotic release factor (InterPro:IPR003285); contains InterPro domain Elongation factor Tu, domain 2 (InterPro:IPR004161); contains InterPro domain Elongation factor Tu, C-terminal (InterPro:IPR004160) | chr1:6213736-6218328 REVERSE | Aliases: T10F20.8	6.8	7.9	-1.1	-1.6	100.0%	-3.7
3757	AT4G31050.1 | lipoyltransferase (LIP2p), identical to lipoyltransferase (LIP2p) (Arabidopsis thaliana) GI:15887052; supporting cDNA gi:15887051:dbj:AB072390.1: | chr4:15114340-15115639 FORWARD | Aliases: F6I18.40, F6I18_40	3.7	3.4	0.3	1.6	100.0%	-3.8
3758	AT3G61870.2 | expressed protein, hypothetical protein - Synechocystis sp. (strain PCC 6803), PIR:S75899 | chr3:22913627-22915034 FORWARD | Aliases: F21F14.40	6.9	6.2	0.7	1.6	100.0%	-3.3
3759	AT3G12040.1 | DNA-3-methyladenine glycosylase (MAG), identical to DNA-3-methlyadenine glycosylase (MAG) SP:Q39147 from (Arabidopsis thaliana); contains Pfam profile: PF02245 methylpurine-DNA glycosylase (MPG) | chr3:3835135-3836802 REVERSE | Aliases: MEC18.4	3.8	3.3	0.4	1.6	100.0%	-3.8
3760	AT3G06780.1 | glycine-rich protein | chr3:2143494-2144527 FORWARD | Aliases: F3E22.8	4.8	4.5	0.3	1.6	100.0%	-4.0
3761	AT4G32680.1 | expressed protein | chr4:15763242-15765119 REVERSE | Aliases: F4D11.120, F4D11_120	4.8	4.2	0.6	1.6	100.0%	-3.9
3762	AT3G07390.1 | Symbol: AIR12 | auxin-responsive protein / auxin-induced protein (AIR12), identical (with 7 residue gap) to auxin-induced protein AIR12 GI:11357190 (Arabidopsis thaliana) | chr3:2365307-2366502 FORWARD | Aliases: F21O3.10	4.7	3.7	1.0	1.6	100.0%	-3.3
3763	AT3G25530.2 | similar to 6-phosphogluconate dehydrogenase NAD-binding domain-containing protein [Arabidopsis thaliana] (TAIR:At1g17650.1); similar to oxidoreductase-like [Oryza sativa (japonica cultivar-group)] (GB:BAD45192.1); contains InterPro domain 3-hydroxyisobutyrate dehydrogenase (InterPro:IPR002204); contains InterPro domain 6-phosphogluconate dehydrogenase, NAD binding domain (InterPro:IPR006115) | chr3:9273037-9274830 REVERSE | Aliases: MWL2.23	11.4	12.0	-0.5	-1.6	100.0%	-4.2
3764	AT1G22970.1 | expressed protein | chr1:8130705-8132197 REVERSE | Aliases: F19G10.8, F19G10_8	5.0	5.5	-0.5	-1.6	100.0%	-3.7

Rank	Description	15.dpi	5.dpi	M	t	adj.q	B
3765	AT5G62070.1 | calmodulin-binding family protein, contains Pfam profile PF00612: IQ calmodulin-binding motif | chr5:24947145-24949802 REVERSE | Aliases: MTG10.10, MTG10_10	4.0	4.3	-0.3	-1.6	100.0%	-4.1
3766	AT1G16280.1 | DEAD/DEAH box helicase, putative, similar to gb:L13612 DEAD-box protein (dbp45A) from Drosophila melanogaster and is a member of PF:00270 DEAD/DEAH box helicase family | chr1:5568476-5570481 REVERSE | Aliases: F3O9.8, F3O9_8	4.3	4.9	-0.6	-1.6	100.0%	-3.7
3767	AT4G39955.1 | hydrolase, alpha/beta fold family protein, low similarity to SP:P24640:LIP3_MORSP Lipase 3 precursor (EC 3.1.1.3) (Triacylglycerol lipase) {Moraxella sp}; contains Pfam profile PF00561: hydrolase, alpha/beta fold family | chr4:18530537-18532253 FORWARD | Aliases: None	7.2	6.7	0.5	1.6	100.0%	-3.6
3768	AT1G76710.2 | SET domain-containing protein (ASHH1), low similarity to huntingtin interacting protein 1 (Homo sapiens) GI:12697196; contains Pfam profile PF00856: SET domain; identical to cDNA ASH1-like protein 1 (ASHH1) partial cds GI:15488417 | chr1:28794623-28797475 REVERSE | Aliases: F28O16.8, F28O16_8	5.7	6.2	-0.5	-1.6	100.0%	-3.6
3769	AT1G72190.1 | oxidoreductase family protein, similar to D-3-phosphoglycerate dehydrogenase from Arabidopsis thaliana (SP:O04130), glyoxylate reductase from Homo sapiens (gi:6002730); contains Pfam D-isomer specific 2-hydroxyacid dehydrogenase, NAD binding domain PF02826	6.7	6.3	0.3	1.6	100.0%	-3.8
3770	AT5G02100.1 | oxysterol-binding family protein, similar to SWH1 (Saccharomyces cerevisiae) GI:402658; contains Pfam profile PF01237: Oxysterol-binding protein	8.0	8.5	-0.5	-1.6	100.0%	-3.8
3771	AT1G47670.1 | amino acid transporter family protein, similar to lysine and histidine specific transporter GI:2576361 from (Arabidopsis thaliana); contains Pfam profile PF01490: Transmembrane amino acid transporter protein | chr1:17539107-17541973 REVERSE | Aliases: F16N3.4, F16N3_4	4.8	5.1	-0.4	-1.6	100.0%	-3.9
3772	AT1G44770.1 | expressed protein | chr1:16909979-16911903 REVERSE | Aliases: T12C22.4, T12C22_4	8.5	8.2	0.4	1.6	100.0%	-3.9
3773	AT4G01660.1 | Symbol: ABC1At |	7.1	7.6	-0.5	-1.6	100.0%	-3.8
3774	AT4G02070.1 | Symbol: MSH6 | DNA mismatch repair protein MSH6-1 (MSH6-1) (AGAA.3), identical to SP:O04716 DNA mismatch repair protein MSH6-1 (AtMsh6-1) cress) {Arabidopsis thaliana}	3.8	4.2	-0.3	-1.6	100.0%	-3.9
3775	AT5G13550.1 | Symbol: SULTR4;1 | sulfate transporter family protein, similar to sulfate transporter (Arabidopsis thaliana) GI:3777483; contains Pfam profiles PF00916: Sulfate transporter family, PF01740: STAS domain | chr5:4355279-4359555 REVERSE | Aliases: None	4.7	5.1	-0.4	-1.6	100.0%	-3.7
3776	AT1G80790.1 | XH/XS domain-containing protein / XS zinc finger domain-containing protein, contains Pfam domains PF03469: XH domain, PF03468: XS domain and PF03470: XS zinc finger domain | chr1:30364615-30367748 FORWARD | Aliases: F23A5.14, F23A5_14	5.5	7.2	-1.7	-1.6	100.0%	-3.3
3777	AT4G34640.1 | Symbol: SQS1 | farnesyl-diphosphate farnesyltransferase 1 / squalene synthase 1 (SQS1), identical to SP:P53799 Farnesyl-diphosphate farnesyltransferase (EC 2.5.1.21) (Squalene synthetase) (SQS) (SS) (FPP:FPP farnesyltransferase) {Arabidopsis thaliana}; non-consensus GC donor splice site at exon 3, squalene synthase 2 (SQS2), Arabidopsis thaliana | chr4:16538287-16541913 FORWARD | Aliases: ERG9, SQUALENE SYNTHASE, SQUALENE SYNTHASE 1, T4L20.220, T4L20_220	7.7	8.3	-0.6	-1.6	100.0%	-3.7
3778	AT2G29995.1 | expressed protein | chr2:12803987-12806496 REVERSE | Aliases: None	5.1	4.3	0.8	1.6	100.0%	-3.4
3779	AT5G54850.1 | expressed protein | chr5:22297809-22298589 REVERSE | Aliases: MBG8.11, MBG8_11	4.3	4.6	-0.3	-1.6	100.0%	-4.0
3780	AT5G12260.1 | expressed protein | chr5:3966366-3968879 FORWARD | Aliases: MXC9.22, MXC9_22	6.1	6.4	-0.2	-1.6	100.0%	-4.0
3781	AT1G72320.3 | pumilio/Puf RNA-binding domain-containing protein, contains Pfam profile: PF00806 Pumilio-family RNA binding domains (aka PUM-HD, Pumilio homology domain) (5 copies) | chr1:27231822-27236658 REVERSE | Aliases: T9N14.7	7.4	8.4	-1.0	-1.6	100.0%	-3.5
3782	AT1G20870.1 | expressed protein | chr1:7259091-7260764 REVERSE | Aliases: F9H16.15, F9H16_15	4.5	5.0	-0.5	-1.6	100.0%	-3.5
3783	AT3G15095.1 | expressed protein | chr3:5081961-5084057 FORWARD | Aliases: K15M2.25	4.6	5.2	-0.6	-1.6	100.0%	-3.5
3784	AT5G53330.1 | expressed protein | chr5:21656443-21658189 FORWARD | Aliases: K19E1.13, K19E1_13	7.3	6.8	0.5	1.6	100.0%	-3.8
3785	AT4G00355.4 | similar to expressed protein [Arabidopsis thaliana] (TAIR:At2g45980.1); similar to OSJNBa0019G23.5 [Oryza sativa (japonica cultivar-group)] (GB:XP_474580.1) | chr4:154721-156376 FORWARD | Aliases: None	7.1	6.3	0.8	1.6	100.0%	-3.5
3786	AT2G32220.1 | 60S ribosomal protein L27 (RPL27A) | chr2:13685996-13686600 FORWARD | Aliases: F22D22.3, F22D22_3	7.5	6.8	0.7	1.6	100.0%	-3.4
3787	AT1G05100.1 | Symbol: MAPKKK18 | protein kinase family protein, contains protein kinase domain, Pfam:PF00069 | chr1:1469609-1470880 FORWARD | Aliases: T7A14.2, T7A14_2	4.6	4.2	0.4	1.6	100.0%	-3.9

Rank	Description	15.dpi	5.dpi	M	t	adj.q	B
3788	AT1G66580.1 | 60S ribosomal protein L10 (RPL10C), contains Pfam profile: PF00826: Ribosomal L10 | chr1:24842828-24844275 FORWARD | Aliases: T12I7.3, T12I7_3	11.7	12.1	-0.4	-1.6	100.0%	-3.8
3789	AT3G61690.1 | expressed protein | chr3:22839324-22845011 FORWARD | Aliases: F15G16.80	5.1	5.6	-0.5	-1.6	100.0%	-3.7
3790	AT3G54090.1 | pfkB-type carbohydrate kinase family protein, contains Pfam profile: PF00294 pfkB family carbohydrate kinase | chr3:20039102-20040964 FORWARD | Aliases: F24B22.50	5.4	6.2	-0.8	-1.6	100.0%	-3.4
3791	AT2G28250.2 | similar to protein kinase family protein [Arabidopsis thaliana] (TAIR:At1g10620.1); similar to putative receptor protein kinase PERK1 [Oryza sativa (japonica cultivar-group)] (GB:XP_463824.1); contains InterPro domain Serine/threonine protein kinase, active site (InterPro:IPR008271); contains InterPro domain Tyrosine protein kinase (InterPro:IPR001245); contains InterPro domain Serine/threonine protein kinase (InterPro:IPR002290); contains InterPro domain Protein kinase (InterPro:IPR000719) | chr2:12050559-12053614 FORWARD | Aliases: T3B23.8, T3B23_8	3.6	4.4	-0.8	-1.6	100.0%	-3.3
3792	AT4G36970.1 | remorin family protein, contains Pfam domain, PF03763: Remorin, C-terminal region | chr4:17429739-17431680 REVERSE | Aliases: AP22.30, AP22_30	5.2	5.8	-0.6	-1.6	100.0%	-3.6
3793	AT5G64220.2 | similar to ethylene-responsive calmodulin-binding protein, putative (SR1) [Arabidopsis thaliana] (TAIR:At2g22300.1); similar to calmodulin-binding protein [Arabidopsis thaliana] (TAIR:At5g09410.1); similar to putative calmodulin-binding transcription factor [Oryza sativa (japonica cultivar-group)] (GB:AAR06369.1); similar to anther ethylene-upregulated protein ER1 [Nicotiana tabacum] (GB:AAG39222.1); similar to putative calmodulin binding protein similar to ER66 [Oryza sativa (japonica cultivar-group)] (GB:AAP53419.1); contains InterPro domain IQ calmodulin-binding region (InterPro:IPR000048); contains InterPro domain CG-1 domain (InterPro:IPR005559); contains InterPro domain Ankyrin (InterPro:IPR002110) | chr5:25703434-25709476 FORWARD | Aliases: MSJ1.6, MSJ1_6	6.4	7.3	-0.9	-1.6	100.0%	-3.6
3794	AT3G24200.2 | similar to putative oxygenase [Oryza sativa (japonica cultivar-group)] (GB:XP_470364.1); contains InterPro domain Flavoprotein monooxygenase (InterPro:IPR000733); contains InterPro domain NAD-binding site (InterPro:IPR000205); contains InterPro domain Aromatic-ring hydroxylase (InterPro:IPR003042); contains InterPro domain UbiH/COQ6 monooxygenase (InterPro:IPR000689) | chr3:8747838-8751376 REVERSE | Aliases: MUJ8.10	10.2	9.5	0.7	1.6	100.0%	-3.8
3795	AT2G36870.1 | xyloglucan:xyloglucosyl transferase, putative / xyloglucan endotransglycosylase, putative / endo-xyloglucan transferase, putative, similar to cellulase (xyloglucan endo-transglycosylase) GI:311835 from (Tropaeolum majus) | chr2:15479724-15481760 REVERSE | Aliases: T1J8.5, T1J8_5	3.0	3.5	-0.5	-1.6	100.0%	-3.8
3796	AT1G16350.1 | inosine-5'-monophosphate dehydrogenase, putative, strong similarity to SP:P47996 gb:L34684 inosine monophosphate dehydrogenase (IMPDH) from Arabidopsis thaliana; member of the PF:00478 IMP dehydrogenase family | chr1:5590945-5592866 FORWARD | Aliases: F3O9.15, F3O9_15	8.1	8.9	-0.8	-1.6	100.0%	-3.7
3797	AT2G25830.1 | YebC-related, contains Pfam profile PF01709: Domain of unknown function; contains TIGRFAM TIGR01033: conserved hypothetical protein; similar to YebC (GI:5771432) (Legionella pneumophila) | chr2:11025846-11028768 REVERSE | Aliases: F17H15.14, F17H15_14	6.0	7.0	-1.0	-1.6	100.0%	-3.4
3798	AT1G63460.1 | glutathione peroxidase, putative, contains Pfam profile: PF00255 glutathione peroxidases	9.8	10.2	-0.4	-1.6	100.0%	-3.9
3799	AT5G05710.1 | pleckstrin homology (PH) domain-containing protein, similar to AtPH1 (Arabidopsis thaliana) GI:5926716; contains Pfam profile PF00169: PH domain	10.5	9.9	0.6	1.6	100.0%	-3.5
3800	AT2G30590.1 | Symbol: WRKY21 |	4.2	3.7	0.5	1.6	100.0%	-3.7
3801	AT4G22200.1 | Symbol: AKT2 | potassium channel protein 2 (AKT2) (AKT3), identical to potassium channel (Arabidopsis thaliana) gi:1100898:gb:AAA97865; Note: also identical to AKT3 (Arabidopsis thaliana) gi:1172218:gb:AAA96153, which is a truncated version of AKT2, PMID:10852932; member of the 1 pore, 6 transmembrane (1P/6TM- Shaker-type) K+ channel family, PMID:11500563; identical to cDNA inward-rectifying K+ channel (AKT3) GI:1172219	2.5	2.7	-0.2	-1.6	100.0%	-4.4
3802	AT3G44850.1 | protein kinase-related, contains eukaryotic protein kinase domain, INTERPRO:IPR000719 | chr3:16385602-16387916 REVERSE | Aliases: F28D10.40	4.6	5.5	-0.9	-1.6	100.0%	-3.6
3803	AT4G17890.2 | Symbol: UBP20 | similar to human Rev interacting-like family protein / hRIP family protein [Arabidopsis thaliana] (TAIR:At5g46750.1); similar to putative zinc finger protein [Oryza sativa (japonica cultivar-group)] (GB:AAO66537.1); contains InterPro domain Human Rev interacting-like protein (hRIP) (InterPro:IPR001164)	7.7	8.5	-0.8	-1.6	100.0%	-3.5
3804	AT1G03980.1 | phytochelatin synthase 2 (PCS2), identical to phytochelatin synthase (Arabidopsis thaliana) gi:16519291:gb:AAK94671 | chr1:1018106-1022107 REVERSE | Aliases: F21M11.9, F21M11_9	3.2	3.4	-0.3	-1.6	100.0%	-4.1
3805	AT1G10210.2 | Symbol: ATMPK1 |	3.0	2.7	0.3	1.6	100.0%	-4.1
3806	AT5G46780.2 | VQ motif-containing protein, contains PF05678: VQ motif | chr5:18996204-18997283 FORWARD | Aliases: MZA15.20, MZA15_20	6.1	5.1	1.0	1.6	100.0%	-3.5

Rank	Description	15.dpi	5.dpi	M	t	adj.q	B
3807	AT5G44610.1 | DREPP plasma membrane polypeptide-related, contains Pfam profile: PF05558 DREPP plasma membrane polypeptide | chr5:18010792-18011895 REVERSE | Aliases: K15C23.5, K15C23_5	6.3	5.4	0.9	1.6	100.0%	-3.3
3808	AT1G24310.1 | expressed protein | chr1:8624050-8626394 FORWARD | Aliases: F3I6.25, F3I6_25	4.1	4.4	-0.3	-1.6	100.0%	-3.9
3809	AT5G41460.1 | fringe-related protein, strong similarity to unknown protein (pir::T13026) similarity to predicted proteins + similar to hypothetical protein GB:AAC23643 (Arabidopsis thaliana) + weak similarity to Fringe (Schistocerca gregaria)(GI:6573138);Fringe encodes an extracellular protein that regulates Notch signalling. | chr5:16606826-16609469 REVERSE | Aliases: MYC6.16, MYC6_16	3.0	3.2	-0.3	-1.6	100.0%	-4.0
3810	AT1G65590.1 | glycosyl hydrolase family 20 protein, contains Pfam PF00728: Glycosyl hydrolase family 20, catalytic domain; contains Pfam PF02838: Glycosyl hydrolase family 20, domain 2; similar to Beta-hexosaminidase beta chain precursor (EC 3.2.1.52) (N-acetyl-beta- glucosaminidase) (Beta-N-acetylhexosaminidase) (Hexosaminidase B) (Swiss-Prot:P07686) (Homo sapiens) | chr1:24389594-24394870 FORWARD | Aliases: F5I14.12, F5I14_12	2.2	1.9	0.3	1.6	100.0%	-4.3
3811	AT3G26410.1 | expressed protein | chr3:9670653-9672565 REVERSE | Aliases: F20C19.14	4.0	4.4	-0.4	-1.6	100.0%	-3.9
3812	AT1G24620.1 | polcalcin, putative / calcium-binding pollen allergen, putative, similar to polcalcin Jun o 2 (calcium-binding pollen allergen Jun o 2) SP:O64943 from (Juniperus oxycedrus) | chr1:8723698-8724445 REVERSE | Aliases: F21J9.28	3.5	3.2	0.3	1.6	100.0%	-4.1
3813	AT1G52420.1 | glycosyl transferase family 1 protein, contains Pfam profile: PF00534 Glycosyl transferases group 1 | chr1:19531518-19534956 FORWARD | Aliases: F19K6.13	9.6	9.2	0.4	1.6	100.0%	-3.8
3814	AT2G14860.1 | peroxisomal membrane protein 22 kDa, putative, similar to 22 kDa peroxisomal membrane protein {Mus musculus} SWISS-PROT:P42925, NCBI_gi:454833 | chr2:6394902-6396645 REVERSE | Aliases: T26I20.2, T26I20_2	3.5	4.1	-0.5	-1.6	100.0%	-3.5
3815	AT4G18300.1 | eIF4-gamma/eIF5/eIF2-epsilon domain-containing protein, similar to SP:Q64350 Translation initiation factor eIF-2B epsilon subunit (eIF-2B GDP-GTP exchange factor) {Rattus norvegicus}; contains Pfam profile PF02020: eIF4-gamma/eIF5/eIF2-epsilon | chr4:10118863-10120992 FORWARD | Aliases: T9A21.150, T9A21_150	4.0	4.5	-0.5	-1.6	100.0%	-3.9
3816	AT1G68290.1 | bifunctional nuclease, putative, similar to bifunctional nuclease (Zinnia elegans) gi:4099833:gb:AAD00694 | chr1:25600334-25602101 FORWARD | Aliases: T22E19.8, T22E19_8	2.4	2.8	-0.4	-1.6	100.0%	-4.0
3817	AT4G33060.1 | peptidyl-prolyl cis-trans isomerase cyclophilin-type family protein, contains Pfam domain, PF00160: peptidyl-prolyl cis-trans isomerase, cyclophilin-type | chr4:15948507-15952172 FORWARD | Aliases: F4I10.3	6.2	7.0	-0.8	-1.6	100.0%	-3.6
3818	AT5G62575.2 | expressed protein | chr5:25134749-25136118 FORWARD | Aliases: None	7.2	7.7	-0.5	-1.6	100.0%	-3.5
3819	AT4G18750.1 | pentatricopeptide (PPR) repeat-containing protein, contains INTERPRO:IPR002885 PPR repeats | chr4:10304861-10307476 FORWARD | Aliases: F28A21.160, F28A21_160	3.0	3.2	-0.2	-1.6	100.0%	-4.3
3820	AT3G23870.1 | permease-related, low similarity to purine permease (Arabidopsis thaliana) GI:7620007; contains 9 predicted transmembrane domains; contains Pfam PF05653: Protein of unknown function (DUF803); identified as COG0697, Permeases of the drug/metabolite transporter (DMT) superfamily | chr3:8619199-8621914 FORWARD | Aliases: F14O13.5	3.7	3.3	0.5	1.6	100.0%	-3.6
3821	AT3G07690.1 | similar to NAD-dependent glycerol-3-phosphate dehydrogenase family protein [Arabidopsis thaliana] (TAIR:At2g41540.1); similar to NAD-dependent glycerol-3-phosphate dehydrogenase family protein [Arabidopsis thaliana] (TAIR:At2g41540.2); similar to putative glycerol-3-phosphate dehydrogenase [Oryza sativa (japonica cultivar-group)] (GB:NP_915827.1); similar to putative glycerol-3-phosphate dehydrogenase [Oryza sativa (japonica cultivar-group)] (GB:AAU44049.1); contains InterPro domain NAD-dependent glycerol-3-phosphate dehydrogenase (InterPro:IPR006168) | chr3:2457114-2459700 FORWARD | Aliases: MLP3.14	5.5	6.0	-0.5	-1.6	100.0%	-3.6
3822	AT3G53400.1 | expressed protein | chr3:19809129-19811114 FORWARD | Aliases: F4P12.100	6.9	7.5	-0.6	-1.6	100.0%	-3.4
3823	AT1G09300.1 | metallopeptidase M24 family protein, similar to SP:P15034 Xaa-Pro aminopeptidase (EC 3.4.11.9) (X-Pro aminopeptidase) (Aminopeptidase P II) (Aminoacylproline aminopeptidase) {Escherichia coli}; contains Pfam profiles PF00557: metallopeptidase family M24, PF05195: Aminopeptidase P, N-terminal domain | chr1:3003928-3008215 FORWARD | Aliases: T31J12.2, T31J12_2	5.8	6.2	-0.4	-1.6	100.0%	-3.8
3824	AT4G27490.1 | 3' exoribonuclease family domain 1-containing protein, contains Pfam PF01138: 3' exoribonuclease family, domain 1 | chr4:13739812-13741809 FORWARD | Aliases: F27G19.90, F27G19_90	5.8	6.7	-0.9	-1.6	100.0%	-3.3
3825	AT4G36420.1 | ribosomal protein L12 family protein | chr4:17203034-17204315 REVERSE | Aliases: AP22.77, AP22_77	3.1	3.4	-0.3	-1.6	100.0%	-4.0

Rank	Description	15.dpi	5.dpi	M	t	adj.q	B
3826	AT5G53770.1 | nucleotidyltransferase family protein, contains Pfam domains PF03828: PAP/25A associated domain, PF01909: Nucleotidyltransferase domain | chr5:21843885-21847209 FORWARD | Aliases: MGN6.13, MGN6_13	4.0	5.0	-1.0	-1.6	100.0%	-3.8
3827	AT5G42850.2 | expressed protein | chr5:17199108-17199807 REVERSE | Aliases: MBD2.4, MBD2_4	7.4	8.0	-0.6	-1.6	100.0%	-3.9
3828	AT1G07745.1 | Symbol: ATRAD51D/RAD51D | DNA repair family protein, contains similarity to Swiss-Prot:O75771 DNA repair protein RAD51 homolog 4 (R51H3) (TRAD) (Homo sapiens) | chr1:2400794-2402477 REVERSE | Aliases: ATRAD51D, F24B9.14, F24B9_14, RAD51D	2.7	2.5	0.2	1.6	100.0%	-4.3
3829	AT1G01730.1 | expressed protein | chr1:270967-272021 FORWARD | Aliases: T1N6.14, T1N6_14	7.7	7.3	0.3	1.6	100.0%	-3.9
3830	AT1G70360.1 | F-box protein-related | chr1:26512441-26512988 FORWARD | Aliases: F17O7.10, F17O7_10	5.7	5.2	0.4	1.6	100.0%	-4.1
3831	AT1G52140.1 | expressed protein | chr1:19411033-19411923 REVERSE | Aliases: F5F19.20, F5F19_20	3.8	3.4	0.4	1.6	100.0%	-4.0
3832	AT1G21740.1 | expressed protein, contains Pfam domains, PF04782: Protein of unknown function (DUF632) and PF04783: Protein of unknown function (DUF630) | chr1:7641569-7645067 FORWARD | Aliases: F8K7.18, F8K7_18	4.3	4.7	-0.4	-1.6	100.0%	-3.8
3833	AT5G01240.2 | amino acid permease, putative, strong similarity to AUX1 GI:1531758 from (Arabidopsis thaliana); contains Pfam profile PF01490: Transmembrane amino acid transporter protein | chr5:98532-101830 FORWARD | Aliases: F7J8.220, F7J8_220	4.9	4.6	0.3	1.6	100.0%	-3.9
3834	AT1G14180.2 | expressed protein | chr1:4847159-4849200 FORWARD | Aliases: F7A19.26	3.3	3.7	-0.4	-1.6	100.0%	-3.8
3835	AT1G53200.2 | expressed protein | chr1:19844044-19847071 REVERSE | Aliases: F12M16.11, F12M16_11	3.9	4.3	-0.4	-1.6	100.0%	-3.8
3836	AT5G64140.1 | Symbol: RPS28 | 40S ribosomal protein S28 (RPS28C) | chr5:25684550-25685518 REVERSE | Aliases: MHJ24.12, MHJ24_12, RIBOSOMAL PROTEIN S28	10.8	11.3	-0.5	-1.6	100.0%	-4.0
3837	AT1G69330.1 | zinc finger (C3HC4-type RING finger) family protein, contains Pfam profile: PF00097 zinc finger, C3HC4 type (RING finger) | chr1:26067479-26069168 REVERSE | Aliases: F23O10.9, F23O10_9	4.4	3.8	0.6	1.6	100.0%	-3.7
3838	AT2G44360.1 | expressed protein | chr2:18327499-18328766 REVERSE | Aliases: F4I1.17	6.9	7.5	-0.5	-1.6	100.0%	-3.7
3839	AT2G40490.1 | uroporphyrinogen decarboxylase, putative / UPD, putative, similar to uroporphyrinogen decarboxylase (chloroplast) from Nicotiana tabacum (SP:Q42967), Hordeum vulgare (SP:Q42855), Zea mays (SP:O81220); contains Pfam domain uroporphyrinogen decarboxylase (URO-D) PF01208 | chr2:16919940-16922255 FORWARD | Aliases: T2P4.16, T2P4_16	5.1	5.5	-0.5	-1.6	100.0%	-3.8
3840	AT1G12110.1 | Symbol: NRT1.1 |	8.1	7.6	0.5	1.6	100.0%	-3.6
3841	AT1G70160.1 | expressed protein, similar to hypothetical protein GI:4455225 from (Arabidopsis thaliana) | chr1:26423612-26426188 FORWARD | Aliases: F20P5.12, F20P5_12	8.1	9.0	-0.9	-1.6	100.0%	-3.6
3842	AT1G61990.1 | mitochondrial transcription termination factor-related / mTERF-related, contains Pfam profile PF02536: mTERF | chr1:22914870-22916600 FORWARD | Aliases: F8K4.18, F8K4_18	4.8	5.5	-0.8	-1.6	100.0%	-3.5
3843	AT1G32210.1 | Symbol: ATDAD1 |	10.1	9.5	0.6	1.6	100.0%	-3.8
3844	AT1G78340.1 | Symbol: ATGSTU22 | glutathione S-transferase, putative, similar to glutathione transferase GI:2853219 from (Carica papaya) | chr1:29477785-29478756 REVERSE | Aliases: F3F9.13, F3F9_13	5.7	6.5	-0.8	-1.6	100.0%	-3.5
3845	AT5G54640.1 | Symbol: RAT5 | histone H2A, identical to histone H2A Arabidopsis thaliana GI:7595337 | chr5:22213703-22214713 FORWARD | Aliases: MRB17.14, MRB17_14, RESISTANT TO AGROBACTERIUM TRANSFORMATION 5	8.8	9.7	-0.8	-1.6	100.0%	-3.8
3846	AT1G60180.1 | expressed protein, similar to hypothetical protein GI:6017113 from (Arabidopsis thaliana) | chr1:22199190-22200350 REVERSE | Aliases: T13D8.7, T13D8_7	2.9	2.7	0.2	1.6	100.0%	-4.3
3847	AT5G56170.1 | expressed protein, contains similarity to GPI-anchored protein | chr5:22753195-22754512 FORWARD | Aliases: MDA7.23, MDA7_23	9.1	8.6	0.5	1.6	100.0%	-4.3
3848	AT5G02460.1 | Dof-type zinc finger domain-containing protein, zinc finger protein OBP3, Arabidopsis thaliana, EMBL:AF155818 | chr5:539247-541056 REVERSE | Aliases: T22P11.50, T22P11_50	2.4	2.2	0.2	1.6	100.0%	-4.6
3849	AT1G53070.1 | legume lectin family protein, contains Pfam domain, PF00139: Legume lectins beta domain | chr1:19782039-19783041 FORWARD | Aliases: F8L10.7, F8L10_7	3.0	3.2	-0.2	-1.6	100.0%	-4.1

Rank	Description	15.dpi	5.dpi	M	t	adj.q	B
3850	AT3G21790.1 | UDP-glucoronosyl/UDP-glucosyl transferase family protein, contains Pfam profile: PF00201 UDP-glucoronosyl and UDP-glucosyl transferase | chr3:7676934-7678421 REVERSE | Aliases: MSD21.15	6.3	5.4	0.9	1.6	100.0%	-3.2
3851	AT1G48090.2 | C2 domain-containing protein, contains Pfam profile: PF00168 C2 domain | chr1:17739966-17761639 REVERSE | Aliases: F21D18.22	4.6	5.5	-0.9	-1.6	100.0%	-3.6
3852	AT4G15390.1 | transferase family protein, similar to alcohol acyltransferase (Fragaria x ananassa)(GI:10121328)(PMID:10810141), deacetylvindoline 4-O-acetyltransferase (Catharanthus roseus)(GI:4091808)(PMID:9681034) | chr4:8792812-8794293 REVERSE | Aliases: DL3740C, FCAALL.282	3.4	4.1	-0.7	-1.6	100.0%	-3.3
3853	AT4G04800.1 | methionine sulfoxide reductase domain-containing protein / SelR domain-containing protein, low similarity to pilin-like transcription factor (Homo sapiens) GI:5059062, SP:P14930 Peptide methionine sulfoxide reductase msrA/msrB (EC 1.8.4.6) {Neisseria gonorrhoeae}; contains Pfam profile PF01641: SelR domain | chr4:2439548-2441203 FORWARD | Aliases: T4B21.6, T4B21_6	6.0	5.6	0.4	1.6	100.0%	-3.9
3854	AT1G54340.1 | Symbol: ICDH | isocitrate dehydrogenase, putative / NADP+ isocitrate dehydrogenase, putative, strong similarity to NADP-isocitrate dehydrogenase GI:5764653 from (Citrus limon); Nicotiana tabacum SP:P50218 | chr1:20287147-20290453 FORWARD | Aliases: F20D21.16, F20D21_16, isocitrate dehydrogenase	5.9	5.3	0.7	1.6	100.0%	-3.6
3855	AT2G31410.1 | expressed protein | chr2:13399099-13399928 REVERSE | Aliases: T28P16.10, T28P16_10	4.8	5.4	-0.6	-1.6	100.0%	-3.5
3856	AT1G80400.1 | zinc finger (C3HC4-type RING finger) family protein, low similarity to SP:Q90972 RING finger protein 13 {Gallus gallus}; contains Pfam profile: PF00097 zinc finger, C3HC4 type (RING finger) | chr1:30230068-30232931 FORWARD | Aliases: F5I6.15, F5I6_15	6.7	6.2	0.5	1.6	100.0%	-3.6
3857	AT1G35190.1 | oxidoreductase, 2OG-Fe(II) oxygenase family protein, low similarity to hyoscyamine 6-dioxygenase hydroxylase from Hyoscyamus niger (GB:P24397)(SP:P24397), Atropa belladona (gi:4996123); contains Pfam domain PF03171, 2OG-Fe(II) oxygenase superfamily	8.2	7.5	0.7	1.6	100.0%	-3.7
3858	AT3G13860.1 | chaperonin, putative, similar to SWISS-PROT:P29197- chaperonin CPN60, mitochondrial precursor (HSP60) (Arabidopsis thaliana) ; contains Pfam:PF00118 domain, TCP-1/cpn60 chaperonin family | chr3:4561524-4565256 REVERSE | Aliases: MCP4.9	9.8	10.3	-0.6	-1.6	100.0%	-3.6
3859	AT1G76550.1 | pyrophosphate--fructose-6-phosphate 1-phosphotransferase alpha subunit, putative / pyrophosphate-dependent 6-phosphofructose-1-kinase, putative, strong similarity to SP:Q41140 Pyrophosphate--fructose 6-phosphate 1-phosphotransferase alpha subunit (EC 2.7.1.90) (PFP) (PPI-PFK) {Ricinus communis}; contains Pfam profile PF00365: Phosphofructokinase | chr1:28727596-28731900 REVERSE | Aliases: F14G6.15, F14G6_15	7.8	9.2	-1.4	-1.6	100.0%	-3.3
3860	AT3G14920.1 | expressed protein | chr3:5018282-5020280 FORWARD | Aliases: K15M2.6	7.7	8.6	-0.9	-1.6	100.0%	-3.6
3861	AT2G02480.1 | Symbol: STI | DNA polymerase-related, weak similarity to DNA polymerase III holoenzyme tau subunit (Thermus thermophilus) GI:2583049 | chr2:660742-665634 FORWARD | Aliases: DNA POLYMERASE III LIKE PROTEIN, GAMMA SUBUNIT, STICHEL, T8K22.22, T8K22_22	3.9	4.2	-0.3	-1.6	100.0%	-4.0
3862	AT4G02560.1 | Symbol: LD | homeobox protein LUMINIDEPENDENS (LD), identical to Homeobox protein LUMINIDEPENDENS (Swiss-Prot:Q38796) (Arabidopsis thaliana) | chr4:1123492-1128397 REVERSE | Aliases: LUMINIDEPENDENS, T10P11.15, T10P11_15	5.7	6.4	-0.6	-1.6	100.0%	-3.7
3863	AT1G43710.1 | Symbol: EMB1075 | serine decarboxylase, identical to serine decarboxylase (Arabidopsis thaliana) GI:15011302; contains Pfam profile PF00282: Pyridoxal-dependent decarboxylase conserved domain	9.2	9.6	-0.4	-1.6	100.0%	-4.0
3864	AT5G52810.1 | ornithine cyclodeaminase/mu-crystallin family protein, contains Pfam PF02423: Ornithine cyclodeaminase/mu-crystallin family | chr5:21416557-21417829 FORWARD | Aliases: MXC20.2, MXC20_2	4.4	4.7	-0.3	-1.6	100.0%	-4.0
3865	AT1G50700.1 | Symbol: CPK33 | calcium-dependent protein kinase, putative / CDPK, putative, similar to calmodulin-domain protein kinase CDPK isoform 9 (Arabidopsis thaliana) gi:1399265:gb:AAB03242 | chr1:18785882-18788053 FORWARD | Aliases: F17J6.22, F17J6_22	4.7	5.2	-0.5	-1.6	100.0%	-3.6
3866	AT5G20300.3 | similar to chloroplast outer membrane protein, putative [Arabidopsis thaliana] (TAIR:At3g16620.1); similar to chloroplast Toc125 [Physcomitrella patens] (GB:AAS47583.1)	6.2	6.9	-0.7	-1.6	100.0%	-3.9
3867	AT5G18750.1 | DNAJ heat shock N-terminal domain-containing protein, contains Pfam profile PF00226 DnaJ domain | chr5:6255179-6257934 FORWARD | Aliases: F17K4.2	4.9	5.4	-0.4	-1.6	100.0%	-3.6
3868	AT1G07230.1 | phosphoesterase family protein, low similarity to SP:P95246 Phospholipase C 2 precursor (EC 3.1.4.3) {Mycobacterium tuberculosis}; contains Pfam profile PF04185: Phosphoesterase family	6.8	5.7	1.1	1.6	100.0%	-3.5
3869	AT5G50730.1 | expressed protein, ; expression supported by MPSS | chr5:20653209-20653628 REVERSE | Aliases: MFB16.13, MFB16_13	6.2	7.3	-1.1	-1.6	100.0%	-3.5
3870	AT1G76510.2 | ARID/BRIGHT DNA-binding domain-containing protein, contains Pfam profile PF01388: ARID/BRIGHT DNA binding domain	6.1	6.7	-0.6	-1.6	100.0%	-3.7

Rank	Description	15.dpi	5.dpi	M	t	adj.q	B
3871	AT3G46480.1 | oxidoreductase, 2OG-Fe(II) oxygenase family protein, low similarity to gibberellin 20-oxidase (gi:4678370); contains Pfam domain PF03171, 2OG-Fe(II) oxygenase superfamily | chr3:17114158-17116579 FORWARD | Aliases: F12A12.1	3.5	3.0	0.5	1.6	100.0%	-3.7
3872	AT3G22830.1 | Symbol: AT HSFA6B | heat shock transcription factor family protein, contains Pfam profile: PF00447 HSF-type DNA-binding domain | chr3:8078827-8081058 FORWARD | Aliases: HSFA6B, MWI23.20	5.4	4.6	0.8	1.6	100.0%	-3.4
3873	AT4G25730.1 | FtsJ-like methyltransferase family protein, contains Pfam profile: PF01728 FtsJ-like methyltransferase | chr4:13102688-13106789 REVERSE | Aliases: F14M19.10, F14M19_10	5.2	5.7	-0.4	-1.6	100.0%	-3.7
3874	AT1G29470.2 | similar to dehydration-responsive protein-related [Arabidopsis thaliana] (TAIR:At5g64030.1); similar to dehydration-responsive protein-related [Arabidopsis thaliana] (TAIR:At3g51070.1); similar to dehydration-responsive protein-related [Arabidopsis thaliana] (TAIR:At2g34300.1); similar to OSJNBb0020J19.14 [Oryza sativa (japonica cultivar-group)] (GB:XP_474482.1); similar to ankyrin-like protein [Oryza sativa (japonica cultivar-group)] (GB:BAD82580.1); contains InterPro domain Putative methyltransferase DUF248 (InterPro:IPR004159) | chr1:10310231-10313741 REVERSE | Aliases: F15D2.5, F15D2_5	9.9	10.3	-0.4	-1.6	100.0%	-4.0
3875	AT4G18740.2 | expressed protein | chr4:10303425-10304583 REVERSE | Aliases: F28A21.150, F28A21_150	3.4	3.0	0.4	1.6	100.0%	-4.0
3876	AT5G52430.1 | hydroxyproline-rich glycoprotein family protein, Common family member At4g25620 (Arabidopsis thaliana) | chr5:21298999-21302431 REVERSE | Aliases: K24M7.18, K24M7_18	4.4	4.0	0.3	1.6	100.0%	-3.9
3877	AT3G17360.1 | kinesin motor protein-related, similar to KLP2 protein GB:CAA63826 from (Xenopus laevis)	2.2	2.3	-0.1	-1.6	100.0%	-4.9
3878	AT3G46780.1 | expressed protein | chr3:17239658-17242186 FORWARD | Aliases: T6H20.190	2.4	2.2	0.2	1.6	100.0%	-4.6
3879	AT3G47530.1 | pentatricopeptide (PPR) repeat-containing protein, contains Pfam profile PF01535: PPR repeat | chr3:17528367-17530142 REVERSE | Aliases: F1P2.80	4.8	5.1	-0.3	-1.6	100.0%	-3.8
3880	AT3G24560.3 | Symbol: RSY3 | expressed protein, contains Pfam profile PF01171: PP-loop family | chr3:8963767-8966968 FORWARD | Aliases: MOB24.14, RASPBERRY 3	3.2	2.8	0.4	1.6	100.0%	-3.9
3881	AT5G06240.1 | Symbol: EMB2735 | expressed protein | chr5:1888314-1889697 FORWARD | Aliases: EMB2735, EMBRYO DEFECTIVE 2735, MBL20.12, MBL20_12	5.9	6.3	-0.4	-1.6	100.0%	-3.8
3882	AT2G22310.1 | Symbol: ATUBP4 |	6.0	6.4	-0.4	-1.6	100.0%	-3.7
3883	AT3G56270.1 | expressed protein, contains Pfam PF05701: Plant protein of unknown function (DUF827) | chr3:20880979-20883028 FORWARD | Aliases: F18O21.230	3.8	4.2	-0.4	-1.6	100.0%	-3.9
3884	AT5G18770.1 | F-box family protein, contains F-box domain Pfam:PF00646	3.3	3.0	0.3	1.6	100.0%	-4.2
3885	AT2G47520.1 | encodes a member of the ERF (ethylene response factor) subfamily B-2 of ERF/AP2 transcription factor family. The protein contains one AP2 domain. There are 5 members in this subfamily including RAP2.2 AND RAP2.12. | chr2:19509917-19510602 REVERSE | Aliases: T30B22.18	4.6	4.0	0.7	1.6	100.0%	-3.5
3886	AT1G56200.1 | Symbol: EMB1303 | expressed protein | chr1:21034480-21035721 FORWARD | Aliases: EMB1303, EMBRYO DEFECTIVE 1303, F14G9.26, F14G9_26	9.5	9.0	0.5	1.6	100.0%	-3.9
3887	AT5G10550.1 | DNA-binding bromodomain-containing protein, low similarity to kinase (Gallus gallus) GI:1370092; contains Pfam profile PF00439: Bromodomain | chr5:3332856-3335233 REVERSE | Aliases: F12B17.100, F12B17_100	5.6	6.1	-0.6	-1.6	100.0%	-3.7
3888	AT3G15010.2 | RNA recognition motif (RRM)-containing protein, similar to UBP1 interacting protein 1a (Arabidopsis thaliana) GI:19574236; contains Pfam profile: PF00076 RNA recognition motif. (a.k.a. RRM, RBD, or RNP domain) | chr3:5052738-5054949 FORWARD | Aliases: K15M2.15	6.5	6.0	0.6	1.6	100.0%	-4.1
3889	AT3G53440.2 | expressed protein | chr3:19821766-19823648 FORWARD | Aliases: F4P12.140	3.4	3.6	-0.2	-1.6	100.0%	-4.2
3890	AT5G03680.1 | trihelix DNA-binding protein, putative, similar to DNA-binding protein DF1 (Pisum sativum) GI:13646986 | chr5:957857-960759 FORWARD | Aliases: F17C15.100, F17C15_100	4.3	3.9	0.4	1.6	100.0%	-3.7
3891	AT3G09710.1 | Symbol: IQD1 | calmodulin-binding family protein, low similarity to SF16 protein (Helianthus annuus) GI:560150; contains Pfam profile PF00612: IQ calmodulin-binding motif | chr3:2976669-2979274 REVERSE | Aliases: F11F8.30, IQ DOMAIN 1	3.2	3.0	0.3	1.6	100.0%	-4.1
3892	AT1G74150.1 | kelch repeat-containing protein, low similarity to rngB protein, Dictyostelium discoideum, PIR:S68824; contains Pfam profile PF01344: Kelch motif | chr1:27884189-27887157 FORWARD | Aliases: F9E11.8, F9E11_8	2.6	2.9	-0.2	-1.6	100.0%	-4.5
3893	AT2G35733.1 | expressed protein | chr2:15029755-15029946 REVERSE | Aliases: None	5.7	5.2	0.5	1.6	100.0%	-3.8

Rank	Description	15.dpi	5.dpi	M	t	adj.q	B
3894	AT2G22830.1 | squalene monooxygenase, putative / squalene epoxidase, putative, similar to SP:O65404 (SE 1,1), SP:O65402 (SE 1,2), SP:O65403 (SE 2) | chr2:9730768-9733350 REVERSE | Aliases: T20K9.4, T20K9_4	2.9	3.1	-0.3	-1.6	100.0%	-4.1
3895	AT4G23450.1 | zinc finger (C3HC4-type RING finger) family protein, contains Pfam profile: PF00097 zinc finger, C3HC4 type | chr4:12240837-12242716 REVERSE | Aliases: F16G20.150, F16G20_150	3.9	3.5	0.4	1.6	100.0%	-3.7
3896	AT4G34138.1 | UDP-glucoronosyl/UDP-glucosyl transferase family protein, contains Pfam profile: PF00201 UDP-glucoronosyl and UDP-glucosyl transferase | chr4:16348110-16349986 REVERSE | Aliases: None	5.4	6.2	-0.9	-1.6	100.0%	-3.6
3897	AT4G11380.1 | beta-adaptin, putative, strong similarity to SP:Q10567 Adapter-related protein complex 1 beta 1 subunit (Beta-adaptin 1) (Homo sapiens), beta-adaptin (Drosophila melanogaster) GI:434902; contains Pfam profile: PF01602 Adaptin N terminal region | chr4:6920503-6925964 FORWARD | Aliases: F8L21.170, F8L21_170	7.5	8.5	-1.0	-1.6	100.0%	-3.6
3898	AT3G53110.1 | Symbol: LOS4 | DEAD/DEAH box helicase, putative, RNA helicase, Mus musculus, PIR:I49731	9.0	9.4	-0.4	-1.6	100.0%	-4.1
3899	AT3G56710.1 | Symbol: SIB1 | sigA-binding protein, identical to SigA binding protein (Arabidopsis thaliana) gi:6980074:gb:AAF34713; contains Pfam PF05678: VQ motif | chr3:21017729-21018544 REVERSE | Aliases: SIGMA FACTOR BINDING PROTEIN 1, T8M16.40	4.3	3.9	0.4	1.6	100.0%	-3.6
3900	AT4G21900.1 | MATE efflux family protein, similar to ripening regulated protein DDTFR18 (Lycopersicon esculentum) GI:12231296; contains Pfam profile PF01554: MatE | chr4:11613291-11623531 REVERSE | Aliases: T8O5.110, T8O5_110	6.5	5.9	0.6	1.6	100.0%	-3.5
3901	AT4G21910.3 | MATE efflux family protein, similar to ripening regulated protein DDTFR18 (Lycopersicon esculentum) GI:12231296; contains Pfam profile PF01554: Uncharacterized membrane protein family | chr4:11625579-11631029 REVERSE | Aliases: T8O5.120, T8O5_120	6.5	5.9	0.6	1.6	100.0%	-3.5
3902	ATCG00480.1 | Symbol: ATPB | chloroplast-encoded gene for beta subunit of ATP synthase | chrC:52660-54156 REVERSE | Aliases: ATPB	8.9	9.9	-1.0	-1.6	100.0%	-3.6
3903	AT5G66055.2 | Symbol: EMB2036 | ankyrin repeat protein / AKRP (AKR), identical to ankyrin repeat protein (AKRP) (Arabidopsis thaliana) SWISS-PROT:Q05753 | chr5:26434382-26436490 REVERSE | Aliases: EMB2036, EMBRYO DEFECTIVE 2036	3.9	3.5	0.4	1.6	100.0%	-4.0
3904	AT1G31810.1 | similar to formin homology 2 domain-containing protein / FH2 domain-containing protein [Arabidopsis thaliana] (TAIR:At5g58160.1); similar to formin homology 2 domain-containing protein / FH2 domain-containing protein [Arabidopsis thaliana] (TAIR:At2g25050.1); similar to diaphanous homologue-like [Oryza sativa (japonica cultivar-group)] (GB:XP_468248.1); similar to putative diaphanous homologue [Oryza sativa (japonica cultivar-group)] (GB:XP_478998.1); similar to putative diaphanous 1 [Oryza sativa (japonica cultivar-group)] (GB:XP_481245.1); contains InterPro domain Actin-binding FH2 (InterPro:IPR003104); contains InterPro domain RNA polymerase Rpb1, domain 5 (InterPro:IPR007081)	5.4	6.1	-0.7	-1.6	100.0%	-3.5
3905	AT1G68400.1 | leucine-rich repeat transmembrane protein kinase, putative, similar to receptor kinase GB:AAA33715 from (Petunia integrifolia) | chr1:25649702-25652609 REVERSE | Aliases: T2E12.5, T2E12_5	3.3	3.6	-0.3	-1.6	100.0%	-3.9
3906	AT1G52920.1 | lanthionine synthetase C-like family protein, contains Pfam domain, PF05147: Lanthionine synthetase C-like protein | chr1:19713028-19714689 REVERSE | Aliases: F14G24.19, F14G24_19	6.1	5.2	0.9	1.6	100.0%	-3.4
3907	AT3G07970.1 | polygalacturonase, putative / pectinase, putative, similar to polygalacturonase precursor (Cucumis melo) GI:3320462; contains PF00295: Glycosyl hydrolases family 28 (polygalacturonases); contains non-consensus AA donor splice site at exon 2	2.6	2.9	-0.3	-1.6	100.0%	-4.2
3908	AT3G56310.2 | alpha-galactosidase, putative / melibiase, putative / alpha-D-galactoside galactohydrolase, putative, similar to alpha-galactosidase SP:Q42656 from (Coffea arabica) | chr3:20893806-20897302 FORWARD | Aliases: F18O21.270	11.1	10.7	0.4	1.6	100.0%	-4.0
3909	AT2G03460.1 | similar to kelch repeat-containing F-box family protein [Arabidopsis thaliana] (TAIR:At5g51250.1) | chr2:1043229-1044764 REVERSE | Aliases: T4M8.11, T4M8_11	4.1	3.6	0.5	1.6	100.0%	-3.9
3910	AT3G05675.2 | expressed protein | chr3:1658277-1660312 REVERSE | Aliases: None	3.9	4.3	-0.3	-1.6	100.0%	-4.0
3911	AT1G70940.1 | Symbol: PIN3 | auxin transport protein, putative (PIN3), similar to auxin transport protein (Arabidopsis thaliana) gi:5817301:gb:AAD52695 | chr1:26746716-26750057 FORWARD | Aliases: F15H11.14, F15H11_14, PIN FORMED 3, PIN3	4.8	5.5	-0.7	-1.6	100.0%	-3.6
3912	AT4G31500.1 | Symbol: CYP83B1 | cytochrome P450 83B1 (CYP83B1), Identical to Cytochrome P450 (SP:O65782)(Arabidopsis thaliana) | chr4:15273477-15275316 REVERSE | Aliases: ALTERED TRYPTOPHAN REGULATION 4, ATR4, CYTOCHROME P450 MONOOXYGENASE 83B1, F3L17.70, F3L17_70, RED LIGHT ELONGATED 1, RED1, RNT1, SUPERROOT 2, SUR2	5.6	6.4	-0.8	-1.6	100.0%	-3.5

Rank	Description	15.dpi	5.dpi	M	t	adj.q	B
3913	AT2G02090.1 | SNF2 domain-containing protein / helicase domain-containing protein, similar to transcriptional activator SRCAP (Homo sapiens) GI:5106572; contains Pfam profiles PF00271: Helicase conserved C-terminal domain, PF00176: SNF2 family N-terminal domain | chr2:523283-527011 FORWARD | Aliases: F5O4.14, F5O4_14	6.7	7.2	-0.4	-1.6	100.0%	-3.9
3914	AT1G03420.1 | expressed protein, similar to gb:T45484, emb:Z30724, and emb:Z30531 | chr1:846790-847682 FORWARD | Aliases: F21B7.4, F21B7_4	5.4	5.1	0.4	1.6	100.0%	-3.9
3915	AT2G41620.1 | nucleoporin interacting component family protein, contains Pfam profile PF04097: Nucleoporin interacting component | chr2:17357223-17362126 REVERSE | Aliases: T32G6.14, T32G6_14	5.4	6.2	-0.8	-1.6	100.0%	-3.7
3916	AT3G12650.1 | expressed protein | chr3:4017837-4018638 REVERSE | Aliases: T2E22.4	9.1	8.7	0.4	1.6	100.0%	-3.9
3917	AT5G67600.1 | expressed protein | chr5:26976797-26977535 REVERSE | Aliases: K9I9.17, K9I9_17	10.3	9.6	0.8	1.6	100.0%	-3.8
3918	AT3G20390.1 | endoribonuclease L-PSP family protein, contains Pfam domain PF01042: Endoribonuclease L-PSP | chr3:7109955-7111830 REVERSE | Aliases: MQC12.17	11.3	11.9	-0.6	-1.6	100.0%	-3.9
3919	AT5G05610.2 | PHD finger family protein, contains Pfam domain, PF00628: PHD-finger | chr5:1676941-1679105 REVERSE | Aliases: MOP10.15, MOP10_15	6.3	5.7	0.6	1.6	100.0%	-3.6
3920	AT4G15660.1 | glutaredoxin family protein, contains INTERPRO Domain IPR002109, Glutaredoxin (thioltransferase)	3.0	3.2	-0.2	-1.6	100.0%	-4.4
3921	AT4G32850.8 | Symbol: nPAP | similar to nucleotidyltransferase family protein [Arabidopsis thaliana] (TAIR:At1g17980.1); similar to nucleotidyltransferase family protein [Arabidopsis thaliana] (TAIR:At2g25850.1); similar to nucleotidyltransferase family protein [Arabidopsis thaliana] (TAIR:At2g25850.2); similar to putative polynucleotide adenylyltransferase [Oryza sativa (indica cultivar-group)] (GB:AAW68018.1); similar to putative polynucleotide adenylyltransferase [Oryza sativa (indica cultivar-group)] (GB:AAW68015.1); similar to putative poly(A) polymerase [Oryza sativa (japonica cultivar-group)] (GB:XP_464757.1); contains InterPro domain Poly(A) polymerase, predicted RNA-binding domain (InterPro:IPR007010); contains InterPro domain Poly(A) polymerase, central region (InterPro:IPR007012); contains InterPro domain PAP/25A core domain (InterPro:IPR001201); contains InterPro domain DNA polymerase, beta-like region (InterPro:IPR002934) | chr4:15849776-15854464 FORWARD | Aliases: NUCLEAR POLY(A) POLYMERASE, T16I18.60, T16I18_60	6.6	6.2	0.4	1.6	100.0%	-3.8
3922	AT4G26600.1 | nucleolar protein, putative, similar to SP:P46087 Proliferating-cell nucleolar antigen p120 (Proliferation-associated nucleolar protein p120) {Homo sapiens}; contains Pfam profile PF01189: NOL1/NOP2/sun family | chr4:13419573-13423775 FORWARD | Aliases: T15N24.50, T15N24_50	3.2	3.8	-0.6	-1.6	100.0%	-4.2
3923	AT5G17310.2 | UTP--glucose-1-phosphate uridylyltransferase, putative / UDP-glucose pyrophosphorylase, putative / UGPase, putative, strong similarity to SP:P19595 UTP--glucose-1-phosphate uridylyltransferase (EC 2.7.7.9) (UDP-glucose pyrophosphorylase) (UDPGP) (UGPase) {Solanum tuberosum}; contains Pfam profile PF01704: UTP--glucose-1-phosphate uridylyltransferase | chr5:5696649-5700924 REVERSE | Aliases: MKP11.26, MKP11_26	8.6	9.6	-1.0	-1.6	100.0%	-3.4
3924	AT5G45140.1 | Symbol: NRPC2 | similar to DNA-directed RNA polymerase II 135 kDa polypeptide / RNA polymerase II subunit 2 (RPB135) (RPB2) (RP140) [Arabidopsis thaliana] (TAIR:At4g21710.1); similar to hypothetical protein [Homo sapiens] (GB:CAD97689.1); similar to SPAC4G9.08c [Schizosaccharomyces pombe] (GB:CAA93558.1); similar to RNA polymerase III subunit RPC2 [Homo sapiens] (GB:AAM18214.1); similar to unnamed protein product [Tetraodon nigroviridis] (GB:CAG03415.1); similar to Polymerase (RNA) III (DNA directed) polypeptide B [Homo sapiens] (GB:AAH46238.1); contains InterPro domain RNA polymerase Rpb2, domain 3 (InterPro:IPR007645); contains InterPro domain RNA polymerase Rpb2, domain 7 (InterPro:IPR007641); contains InterPro domain RNA polymerase Rpb2, domain 4 (InterPro:IPR007646); contains InterPro domain RNA polymerase beta subunit (InterPro:IPR007644); contains InterPro domain RNA polymerase Rpb2, domain 2 (InterPro:IPR007642); contains InterPro domain RNA polymerase Rpb2, domain 6 (InterPro:IPR007120); contains InterPro domain RNA polymerase Rpb2, domain 5 (InterPro:IPR007647); contains InterPro domain RNA polymerase, beta subunit (InterPro:IPR007121) | chr5:18264455-18275365 REVERSE | Aliases: K18C1.1, K18C1_1	5.8	6.4	-0.6	-1.6	100.0%	-3.5
3925	AT2G21690.1 | RNA-binding protein, putative, similar to Glycine-rich RNA-binding protein from {Sinapis alba} SP:P49311, {Brassica napus} SP:Q05966, {Arabidopsis thaliana} SP:Q03251; contains InterPro entry IPR000504: RNA-binding region RNP-1 (RNA recognition motif) (RRM) | chr2:9277194-9277677 REVERSE | Aliases: F7D8.1, F7D8_1	3.0	2.8	0.2	1.6	100.0%	-4.5
3926	AT2G38600.1 | acid phosphatase class B family protein, similar to acid phosphatase (Glycine max) GI:3341443; contains Pfam profile PF03767: HAD superfamily (subfamily IIIB) phosphatase | chr2:16152985-16153935 FORWARD | Aliases: T6A23.20, T6A23_20	4.7	4.0	0.6	1.6	100.0%	-3.5
3927	AT4G24670.2 | alliinase family protein, contains Pfam profiles: PF04864 allinase C-terminal domain, PF04863 alliinase EGF-like domain | chr4:12727844-12731284 REVERSE | Aliases: F22K18.130, F22K18_130	4.2	3.6	0.6	1.6	100.0%	-3.8
3928	AT3G17520.1 | late embryogenesis abundant domain-containing protein / LEA domain-containing protein, low similarity to PIR:S04045:S04045 embryonic abundant protein D-29 (Gossypium hirsutum); contains Pfam profile PF02987: Late embryogenesis abundant protein | chr3:5999295-6000376 REVERSE | Aliases: MKP6.25	4.1	4.7	-0.6	-1.6	100.0%	-3.6

Rank	Description	15.dpi	5.dpi	M	t	adj.q	B
3929	AT1G15440.2 | transducin family protein / WD-40 repeat family protein, Strong similarity to gb X95263 Periodic tryptophan protein 2 gene (PWP2) from Homo sapiens and contains 6 WD40, G-beta repeat domains | chr1:5306056-5309509 REVERSE | Aliases: F9L1.40, F9L1_40	6.7	7.1	-0.4	-1.6	100.0%	-3.9
3930	AT3G46220.1 | expressed protein | chr3:16990060-16995776 FORWARD | Aliases: F12M12.190	6.2	6.7	-0.5	-1.6	100.0%	-3.6
3931	AT5G15730.1 | serine/threonine protein kinase, putative, similar to protein-serine/threonine kinase (Nicotiana tabacum) gi:505146:dbj:BAA06538	5.3	4.9	0.4	1.6	100.0%	-3.9
3932	AT2G31370.5 | similar to bZIP transcription factor, putative (bZIP69) [Arabidopsis thaliana] (TAIR:At1g06070.1); similar to RF2a [Oryza sativa] (GB:AAC49832.1); contains InterPro domain Basic-leucine zipper (bZIP) transcription factor (InterPro:IPR004827) | chr2:13386110-13388600 FORWARD | Aliases: T28P16.14, T28P16_14	7.0	6.2	0.8	1.6	100.0%	-3.6
3933	AT5G12200.1 | dihydropyrimidinase / DHPase / dihydropyrimidine amidohydrolase / hydantoinase (PYD2), identical to dihydropyrimidine amidohydrolase (PYD2) (Arabidopsis thaliana) GI:28194047; similar to SP:Q9EQF5 Dihydropyrimidinase (EC 3.5.2.2) (DHPase) (Hydantoinase) (DHP) {Mus musculus}; contains Pfam profile PF01979: Amidohydrolase family	9.3	8.7	0.6	1.6	100.0%	-3.6
3934	AT5G14960.1 | Symbol: DEL2/E2FD/E2L1 |	3.1	3.3	-0.2	-1.6	100.0%	-4.2
3935	AT5G18490.1 | expressed protein | chr5:6134154-6136767 REVERSE | Aliases: T28N17.2	4.8	4.2	0.5	1.6	100.0%	-3.5
3936	AT5G56020.1 | expressed protein | chr5:22701715-22703595 FORWARD | Aliases: MDA7.6, MDA7_6	7.3	6.7	0.6	1.6	100.0%	-3.6
3937	AT3G48930.1 | Symbol: EMB1080 | 40S ribosomal protein S11 (RPS11A) | chr3:18151756-18153251 REVERSE | Aliases: EMB1080, EMBRYO DEFECTIVE 1080, T2I13.230	11.4	11.8	-0.3	-1.6	100.0%	-4.1
3938	AT1G73800.1 | calmodulin-binding protein, similar to calmodulin-binding protein TCB60 GI:1698548 from (Nicotiana tabacum) | chr1:27749109-27750369 REVERSE | Aliases: F25P22.22, F25P22_22	4.1	3.7	0.4	1.6	100.0%	-3.7
3939	AT5G57940.3 | Symbol: ATCNGC5 | cyclic nucleotide-regulated ion channel / cyclic nucleotide-gated channel (CNGC5), identical to cyclic nucleotide and calmodulin-regulated ion channel (cngc5) GI:4581205 from (Arabidopsis thaliana)	6.9	6.1	0.8	1.6	100.0%	-3.5
3940	AT1G13320.2 | Symbol: PDF2 | similar to serine/threonine protein phosphatase 2A (PP2A) regulatory subunit A (RCN1) [Arabidopsis thaliana] (TAIR:At1g25490.1); similar to serine/threonine protein phosphatase 2A (PP2A) 65 KDa regulatory subunit A [Arabidopsis thaliana] (TAIR:At3g25800.1); similar to protein phosphatase [Cicer arietinum] (GB:CAA10285.1); similar to serine/threonine protein phosphatase type 2A regulatory subunit A (GB:AAB60713.1); similar to Ser/Thr specific protein phosphatase 2A A regulatory subunit beta isoform [Medicago sativa subsp. x varia] (GB:AAG29594.1); similar to protein phosphatase 2A [Nicotiana tabacum] (GB:CAA66487.1); similar to phosphatase 2A regulatory A subunit [Oryza sativa (japonica cultivar-group)] (GB:XP_450276.1); contains InterPro domain HEAT repeat (InterPro:IPR000357) | chr1:4563472-4567747 REVERSE | Aliases: PR65, T6J4.8, T6J4_8	6.9	8.3	-1.4	-1.6	100.0%	-3.4
3941	AT1G73177.1 | expressed protein | chr1:27520248-27521515 REVERSE | Aliases: None	8.2	7.8	0.4	1.6	100.0%	-3.8
3942	AT5G22590.1 | hypothetical protein | chr5:7504539-7505841 FORWARD | Aliases: MDJ22.1, MDJ22_1	2.9	2.7	0.2	1.6	100.0%	-4.7
3943	AT5G15510.1 | expressed protein | chr5:5034123-5036798 FORWARD | Aliases: T20K14.120, T20K14_120	5.3	5.8	-0.5	-1.6	100.0%	-3.5
3944	AT1G57560.1 | myb family transcription factor (MYB50), similar to DNA-binding protein GI:19058 from (Hordeum vulgare) | chr1:21320493-21321729 FORWARD | Aliases: T8L23.3, T8L23_3	3.0	3.7	-0.7	-1.6	100.0%	-3.5
3945	AT3G52140.1 | tetratricopeptide repeat (TPR)-containing protein, contains Pfam profile PF00515: TPR Domain | chr3:19343907-19352635 FORWARD | Aliases: F4F15.250	5.7	6.3	-0.6	-1.6	100.0%	-3.8
3946	AT5G22640.1 | Symbol: EMB1211 | MORN (Membrane Occupation and Recognition Nexus) repeat-containing protein, contains Pfam profile PF02493: MORN repeat | chr5:7529371-7533787 FORWARD | Aliases: EMB1211, EMBRYO DEFECTIVE 1211, MDJ22.6, MDJ22_6	5.6	6.2	-0.6	-1.6	100.0%	-3.9
3947	AT4G14100.1 | expressed protein | chr4:8120715-8122406 FORWARD | Aliases: DL3085W, FCAALL.191	5.2	4.6	0.5	1.6	100.0%	-3.8
3948	AT5G42240.1 | Symbol: SCPL42 |	4.3	4.7	-0.4	-1.6	100.0%	-3.6
3949	AT4G18570.1 | proline-rich family protein, common family members: At3g25690, At4g04980, At5g61090 (Arabidopsis thaliana) | chr4:10231229-10234704 FORWARD | Aliases: F28J12.8	3.6	3.9	-0.3	-1.6	100.0%	-4.0
3950	AT1G31440.1 | SH3 domain-containing protein 1 (SH3P1), nearly identical to SH3 domain-containing protein 1 (Arabidopsis thaliana) GI:16974676; contains Pfam profile PF00018: SH3 domain | chr1:11255817-11258685 REVERSE | Aliases: T8E3.10, T8E3_10	5.8	5.4	0.4	1.6	100.0%	-3.7

Rank	Description	15.dpi	5.dpi	M	t	adj.q	B
3951	AT5G15400.1 | U-box domain-containing protein, contains Pfam profile PF04564: U-box domain | chr5:4997716-5003143 REVERSE | Aliases: T20K14.10, T20K14_10	6.5	7.0	-0.6	-1.6	100.0%	-3.8
3952	AT1G23140.1 | C2 domain-containing protein, similar to zinc finger and C2 domain protein GI:9957238 from (Arabidopsis thaliana) | chr1:8202351-8203201 REVERSE | Aliases: T26J12.9, T26J12_9	6.0	5.5	0.6	1.6	100.0%	-3.5
3953	AT1G51620.1 | protein kinase family protein, contains protein kinase domain, Pfam:PF00069 | chr1:19143798-19145455 FORWARD | Aliases: F19C24.15, F19C24_15	2.4	2.3	0.2	1.6	100.0%	-4.4
3954	AT1G18200.1 | Ras-related GTP-binding family protein, contains Pfam profile: PF00071 Ras family | chr1:6264240-6266652 REVERSE | Aliases: T10F20.21	3.6	3.3	0.3	1.6	100.0%	-3.9
3955	AT1G75200.1 | flavodoxin family protein / radical SAM domain-containing protein, contains Pfam profiles PF00258: Flavodoxin, PF04055: radical SAM domain protein | chr1:28224287-28227368 REVERSE | Aliases: F22H5.13, F22H5_13	5.2	5.8	-0.6	-1.6	100.0%	-3.5
3956	AT4G10450.1 | 60S ribosomal protein L9 (RPL90D), ribosomal protein L9, cytosolic - garden pea, PIR2:S19978 | chr4:6462949-6464521 REVERSE | Aliases: F7L13.30, F7L13_30	5.5	6.1	-0.6	-1.6	100.0%	-3.5
3957	AT5G19090.2 | heavy-metal-associated domain-containing protein, contains Pfam heavy-metal-associated domain PF00403; glycine-rich protein GRP22, rape, PIR:S31415; isoform contains a non-consensus TG-acceptor splice site at intron 3 | chr5:6387491-6390026 FORWARD | Aliases: T16G12.130, T16G12_130	6.4	5.9	0.5	1.6	100.0%	-4.0
3958	AT2G19385.1 | expressed protein, weak similarity to Cell growth regulating nucleolar protein (Swiss-Prot:Q08288) (Mus musculus) | chr2:8394117-8395794 FORWARD | Aliases: None	5.4	6.0	-0.6	-1.6	100.0%	-3.5
3959	AT1G72300.1 | leucine-rich repeat transmembrane protein kinase, putative, similar to GI:3641252 from (Malus x domestica) (Plant Mol. Biol. 40 (6), 945-957 (1999)) | chr1:27221162-27224738 REVERSE | Aliases: T9N14.20, T9N14_20	5.9	5.3	0.6	1.6	100.0%	-3.7
3960	AT3G13110.1 | Symbol: AtSerat2;2 | serine O-acetyltransferase (SAT-1), identical to serine acetyltransferase (Sat-1) GI:1184048 (Arabidopsis thaliana) | chr3:4214624-4216309 REVERSE | Aliases: MJG19.1, SAT 1, SAT A, SERINE ACETYLTRANSFERASE 1	8.6	7.7	0.9	1.6	100.0%	-3.6
3961	AT4G23920.1 | UDP-glucose 4-epimerase, putative / UDP-galactose 4-epimerase, putative / Galactowaldenase, putative, similar to UDP-galactose 4-epimerase from Arabidopsis thaliana SP:Q42605, Cyamopsis tetragonoloba GI:3021357 (AJ005082) | chr4:12431287-12433874 FORWARD | Aliases: T32A16.90, T32A16_90	4.4	4.7	-0.3	-1.6	100.0%	-4.1
3962	AT1G15830.1 | expressed protein | chr1:5448721-5450172 FORWARD | Aliases: F7H2.23, F7H2_23	6.1	5.4	0.6	1.6	100.0%	-3.7
3963	AT1G26300.2 | BSD domain-containing protein, contains Pfam profile PF03909: BSD domain	5.0	4.7	0.2	1.6	100.0%	-4.4
3964	AT1G74230.1 | glycine-rich RNA-binding protein, similar to RNA-binding protein GB:S46286 from (Nicotiana sylvestris) | chr1:27918367-27920744 FORWARD | Aliases: F1O17.10, F1O17_10	9.1	8.7	0.4	1.6	100.0%	-3.9
3965	AT1G69200.1 | pfkB-type carbohydrate kinase family protein, contains Pfam profile: PF00294 pfkB family carbohydrate kinase | chr1:26019687-26022528 FORWARD | Aliases: F4N2.16, F4N2_16	3.7	4.3	-0.5	-1.6	100.0%	-3.5
3966	AT5G17150.1 | expressed protein | chr5:5638872-5639594 REVERSE | Aliases: F2K13.300, F2K13_300	2.8	2.6	0.2	1.6	100.0%	-4.6
3967	AT5G05460.1 | similar to glycosyl hydrolase family 85 protein [Arabidopsis thaliana] (TAIR:At3g11040.1); similar to PREDICTED: similar to RIKEN cDNA D230014K01 [Rattus norvegicus] (GB:XP_221141.2); contains InterPro domain Glycoside hydrolase, family 85 (InterPro:IPR005201) | chr5:1615616-1618903 FORWARD | Aliases: K18I23.27, K18I23_27	5.0	5.3	-0.3	-1.6	100.0%	-4.0
3968	AT1G08810.2 | Symbol: MYB60 | myb family transcription factor (MYB60)	2.1	2.0	0.1	1.6	100.0%	-4.9
3969	AT1G22850.1 | expressed protein | chr1:8080191-8082864 REVERSE | Aliases: F19G10.23	6.8	6.0	0.8	1.6	100.0%	-3.6
3970	AT3G18380.2 | expressed protein | chr3:6310742-6313305 REVERSE | Aliases: MYF24.10	6.0	5.3	0.7	1.6	100.0%	-3.7
3971	AT5G58310.1 | hydrolase, alpha/beta fold family protein, low similarity to SP:Q40708 PIR7A protein {Oryza sativa}, polyneuridine aldehyde esterase (Rauvolfia serpentina) GI:6651393, ethylene-induced esterase (Citrus sinensis) GI:14279437; contains Pfam profile PF00561: hydrolase, alpha/beta fold family | chr5:23591706-23593310 REVERSE | Aliases: MCK7.18, MCK7_18	2.4	2.3	0.2	1.6	100.0%	-4.5

Rank	Description	15.dpi	5.dpi	M	t	adj.q	B
3972	AT1G54140.1 | Symbol: TAFII21 | transcription initiation factor IID (TFIID) 31 kDa subunit (TAFII-31) family protein, contains Pfam profile: PF02291 transcription initiation factor IID, 31kD subunit | chr1:20217637-20218887 REVERSE | Aliases: F15I1.24, F15I1_24, TATA BINDING PROTEIN ASSOCIATED FACTOR 21KDA SUBUNI, TATA BINDING PROTEIN ASSOCIATED FACTOR 21KDA SUBUNIT	7.2	7.8	-0.6	-1.6	100.0%	-3.5
3973	AT1G58180.3 | similar to carbonic anhydrase family protein / carbonate dehydratase family protein [Arabidopsis thaliana] (TAIR:At4g33580.1); similar to carbonic anhydrase 2 [Flaveria bidentis] (GB:AAO17573.1); contains InterPro domain Carbonic anhydrase, prokaryotic (InterPro:IPR001765) | chr1:21541600-21543579 REVERSE | Aliases: T15M6.18	5.4	4.9	0.5	1.6	100.0%	-3.7
3974	AT5G14470.1 | GHMP kinase-related, contains similarity to D-glycero-D-manno-heptose 7-phosphate kinase (Aneurinibacillus thermoaerophilus) gi:13491143:gb:AAK27850 | chr5:4662807-4664650 REVERSE | Aliases: F18O22.260, F18O22_260	3.2	3.0	0.2	1.6	100.0%	-4.5
3975	AT4G33130.2 | expressed protein, weak similarity to Max-interacting transcriptional repressor (Mus musculus) GI:1184157 | chr4:15979053-15980658 REVERSE | Aliases: F4I10.60, F4I10_60	4.1	4.3	-0.2	-1.6	100.0%	-4.3
3976	AT5G17340.1 | expressed protein, weak similarity to M3.4 protein (Brassica napus) GI:4574746 | chr5:5715626-5716270 REVERSE | Aliases: MKP11.1, MKP11_1	2.6	2.4	0.2	1.6	100.0%	-4.7
3977	AT1G74400.1 | pentatricopeptide (PPR) repeat-containing protein, contains Pfam profile: PF01535 domain of unknown function | chr1:27967614-27969002 FORWARD | Aliases: F1M20.8, F1M20_8	3.3	3.6	-0.3	-1.6	100.0%	-4.2
3978	AT5G63750.1 | IBR domain-containing protein, contains a Prosite:PS00518 Zinc finger, C3HC4 type (RING finger), signature and Pfam:PF01485 IBR domain | chr5:25530220-25532164 REVERSE | Aliases: MBK5.23, MBK5_23	5.1	4.7	0.5	1.6	100.0%	-3.7
3979	AT5G10360.1 | Symbol: EMB3010 | 40S ribosomal protein S6 (RPS6B) | chr5:3258475-3260228 REVERSE | Aliases: EMB3010, EMBRYO DEFECTIVE 3010, F12B17.290, F12B17_290	10.5	10.9	-0.4	-1.6	100.0%	-4.2
3980	AT2G39795.1 | mitochondrial glycoprotein family protein / MAM33 family protein, low similarity to SUAPRGA1 (Emericella nidulans) GI:6562379; contains Pfam profile PF02330: Mitochondrial glycoprotein	6.4	6.9	-0.5	-1.6	100.0%	-3.7
3981	AT2G13500.1 | hypothetical protein, similar to zinc finger protein (Arabidopsis thaliana) GI:976277 | chr2:5635018-5636181 FORWARD | Aliases: T10F5.4, T10F5_4	2.2	2.3	-0.1	-1.6	100.0%	-4.9
3982	AT1G34020.1 | transporter-related, low similarity to UDP-sugar transporter (Drosophila melanogaster) GI:14971008, UDP-glucuronic acid transporter (Homo sapiens) GI:11463949 | chr1:12366867-12369158 FORWARD | Aliases: F12G12.16, F12G12_16	5.2	5.9	-0.7	-1.6	100.0%	-3.7
3983	AT3G62910.1 | Symbol: APG3 | peptide chain release factor, putative, similar to peptide chain release factor 1 (Escherichia coli) GI:147567; contains Pfam profiles PF00472: Peptidyl-tRNA hydrolase domain, PF03462: PCRF domain | chr3:23268530-23271402 REVERSE | Aliases: ALBINO AND PALE GREEN, T20O10.10	4.8	5.3	-0.5	-1.6	100.0%	-3.6
3984	AT1G75500.1 | nodulin MtN21 family protein, similar to MtN21 GB:CAA75575 GI:2598575 from (Medicago truncatula) (Mol. Plant Microbe Interact. 9 (4), 233-242 (1996)); contains Pfam profile PF00892: Integral membrane protein | chr1:28341453-28343821 REVERSE | Aliases: F1B16.19	3.3	4.4	-1.1	-1.6	100.0%	-3.3
3985	AT5G48960.1 | 5' nucleotidase family protein, low similarity to SP:P49902 Cytosolic purine 5'-nucleotidase (EC 3.1.3.5) (5'-nucleotidase cytosolic II) {Homo sapiens}; contains Pfam profile PF05761: 5' nucleotidase family | chr5:19866769-19870790 FORWARD | Aliases: K19E20.8, K19E20_8	4.7	4.2	0.4	1.6	100.0%	-3.6
3986	AT5G36950.1 | DegP protease, putative, contains similarity to DegP2 protease GI:13172275 from (Arabidopsis thaliana) | chr5:14612211-14615570 FORWARD | Aliases: MLF18.70, MLF18_70	5.6	6.2	-0.6	-1.6	100.0%	-3.4
3987	AT2G46910.1 | plastid-lipid associated protein PAP / fibrillin family protein, contains Pfam profile PF04755: PAP_fibrillin | chr2:19279350-19281071 FORWARD | Aliases: F14M4.46	4.4	4.8	-0.4	-1.6	100.0%	-3.9
3988	AT1G62930.1 | similar to pentatricopeptide (PPR) repeat-containing protein [Arabidopsis thaliana] (TAIR:At1g63130.1); similar to pentatricopeptide (PPR) repeat-containing protein [Arabidopsis thaliana] (TAIR:At1g63080.1); similar to pentatricopeptide (PPR) repeat-containing protein [Arabidopsis thaliana] (TAIR:At1g62670.1); similar to pentatricopeptide (PPR) repeat-containing protein [Arabidopsis thaliana] (TAIR:At1g62910.1); similar to pentatricopeptide (PPR) repeat-containing protein [Arabidopsis thaliana] (TAIR:At1g62590.1); similar to fertility restorer homologue [Raphanus sativus] (GB:CAD61286.1); contains InterPro domain PPR repeat (InterPro:IPR002885) | chr1:23310199-23312348 FORWARD | Aliases: F16P17.7, F16P17_7	6.2	7.1	-0.8	-1.6	100.0%	-3.8
3989	AT2G16260.1 | glycine-rich RNA-binding protein, putative, similar to Glycine-rich RNA-binding protein from {Daucus carota} SP:Q03878, {Sinapis alba} SP:P49311, {Brassica napus} SP:Q05966, {Arabidopsis thaliana} SP:Q03251; contains InterPro entry IPR000504: RNA-binding region RNP-1 (RNA recognition motif) (RRM) | chr2:7051897-7052584 FORWARD | Aliases: F16F14.24, F16F14_24	4.8	4.3	0.4	1.6	100.0%	-3.9

Rank	Description	15.dpi	5.dpi	M	t	adj.q	B
3990	AT1G20370.1 | tRNA pseudouridine synthase family protein, similar to SP:Q9WU56 tRNA pseudouridine synthase A (EC 4.2.1.70) (Uracil hydrolyase) {Mus musculus}; contains Pfam profile PF01416: tRNA pseudouridine synthase | chr1:7051567-7053631 REVERSE | Aliases: F14O10.3, F14O10_3	3.9	4.2	-0.4	-1.6	100.0%	-4.0
3991	AT4G11570.2 | haloacid dehalogenase-like hydrolase family protein, similar to genetic modifier (Zea mays) GI:10444400; contains InterPro accession IPR005834: Haloacid dehalogenase-like hydrolase | chr4:7004329-7006075 FORWARD | Aliases: F25E4.190, F25E4_190	7.6	6.9	0.7	1.6	100.0%	-3.8
3992	AT1G32050.1 | secretory carrier membrane protein (SCAMP) family protein, contains Pfam domain, PF04144: SCAMP family | chr1:11528461-11531261 FORWARD | Aliases: T12O21.5, T12O21_5	7.9	6.9	1.0	1.6	100.0%	-3.7
3993	AT2G26430.1 | Symbol: RCY1 | ania-6a type cyclin (RCY1), nearly identical to ania-6a type cyclin (Arabidopsis thaliana) GI:13924511 | chr2:11250297-11253107 REVERSE | Aliases: ARGININE RICH CYCLIN 1, ATRCY1, T9J22.10, T9J22_10	6.7	6.4	0.4	1.6	100.0%	-3.9
3994	AT4G02020.1 | Symbol: EZA1 | zeste-like protein 1 (EZA1), identical to enhancer of zeste-like protein 1(EZA1) (GI:4185507) (Arabidopsis thaliana); similar to polycomb group (Arabidopsis thaliana) GI:1903019 (curly leaf); contains Pfam profile PF00856: SET domain | chr4:886600-891955 FORWARD | Aliases: T10M13.3, T10M13_3	8.1	7.6	0.5	1.6	100.0%	-3.9
3995	AT4G38750.1 | expressed protein | chr4:18084902-18090840 REVERSE | Aliases: T9A14.30, T9A14_30	6.7	7.0	-0.3	-1.6	100.0%	-4.2
3996	AT1G75090.1 | methyladenine glycosylase family protein, similar to SP:P05100 DNA-3-methyladenine glycosylase I (EC 3.2.2.20) (3-methyladenine-DNA glycosylase I, constitutive) {Escherichia coli}; contains Pfam profile PF03352: Methyladenine glycosylase | chr1:28191112-28193386 REVERSE | Aliases: F9E10.6, F9E10_6	3.4	3.7	-0.3	-1.6	100.0%	-4.2
3997	AT2G45650.1 | Symbol: AGL6 | MADS-box protein (AGL6) | chr2:18811424-18813596 FORWARD | Aliases: F17K2.18	2.5	2.3	0.2	1.6	100.0%	-4.7
3998	AT5G04280.1 | glycine-rich RNA-binding protein	5.7	5.0	0.8	1.6	100.0%	-3.6
3999	AT5G08220.1 | expressed protein | chr5:2642281-2642992 REVERSE | Aliases: F8L15.7	2.9	2.7	0.2	1.6	100.0%	-4.4
4000	AT1G57943.1 | Symbol: ATPUP17 | purine permease-related, low similarity to purine permease (Arabidopsis thaliana) GI:7620007; contains Pfam profile PF03151: Domain of unknown function, DUF250 | chr1:21441267-21442554 REVERSE | Aliases: F13D13.4, F13D13_4	4.8	4.3	0.5	1.6	100.0%	-3.8
4001	AT1G49490.1 | leucine-rich repeat family protein / extensin family protein, contains similarity to disease resistance protein GI:3894383 from (Lycopersicon esculentum); contains leucine-rich repeats, Pfam:PF00560; contains proline rich extensin domains, INTERPRO:IPR002965 | chr1:18321231-18323774 REVERSE | Aliases: F13F21.7, F13F21_7	2.6	2.8	-0.2	-1.6	100.0%	-4.7
4002	AT1G72800.1 | nuM1-related, contains similarity with nuM1 GI:1279563 from (Medicago sativa) | chr1:27400878-27402162 FORWARD | Aliases: F28P22.1, F28P22_1	5.8	4.9	0.9	1.6	100.0%	-3.5
4003	AT3G25140.1 | Symbol: QUA1 | glycosyl transferase family 8 protein, contains Pfam profile: PF01501 glycosyl transferase family 8 | chr3:9154718-9156851 FORWARD | Aliases: MJL12.8, QUASIMODO1	11.1	11.4	-0.3	-1.6	100.0%	-4.2
4004	AT3G05530.1 | Symbol: RPT5A | 26S proteasome AAA-ATPase subunit (RPT5a), identical to GB:AAF22525 GI:6652886 from (Arabidopsis thaliana) | chr3:1603438-1606237 FORWARD | Aliases: 19S PROTEASOME REGULATORY COMPLEX SUBUNIT S6A, 26S PROTEASOME AAA ATPASE SUBUNIT, ATS6A.2, F22F7.1, F22F7_1	8.7	9.6	-0.9	-1.6	100.0%	-3.6
4005	AT2G34300.2 | similar to dehydration-responsive protein-related [Arabidopsis thaliana] (TAIR:At1g29470.1); similar to dehydration-responsive family protein [Arabidopsis thaliana] (TAIR:At2g40280.1); similar to dehydration-responsive protein-related [Arabidopsis thaliana] (TAIR:At5g64030.1); similar to dehydration-responsive protein-related [Arabidopsis thaliana] (TAIR:At3g51070.1); similar to OSJNBb0020J19.14 [Oryza sativa (japonica cultivar-group)] (GB:XP_474482.1); similar to ankyrin-like protein [Oryza sativa (japonica cultivar-group)] (GB:BAD82580.1); contains InterPro domain Putative methyltransferase DUF248 (InterPro:IPR004159) | chr2:14480744-14484343 REVERSE | Aliases: F13P17.14, F13P17_14	7.7	8.3	-0.6	-1.6	100.0%	-3.8
4006	AT1G78600.1 | zinc finger (B-box type) family protein, similar to zinc finger protein GI:3618316 from (Oryza sativa) | chr1:29572104-29573752 FORWARD | Aliases: T30F21.7, T30F21_7	5.9	5.6	0.3	1.6	100.0%	-4.1
4007	AT4G16250.1 | Symbol: PHYD | phytochrome D (PHYD), nearly identical to SP:P42497 Phytochrome D {Arabidopsis thaliana}	3.9	4.1	-0.3	-1.6	100.0%	-4.0
4008	AT4G22950.1 | Symbol: AGL19 | MADS-box protein (AGL19), MADS-box protein AGL14, Arabidopsis thaliana, gb:U20184 | chr4:12023926-12027432 REVERSE | Aliases: AGAMOUS LIKE 19, F7H19.130, F7H19_130, GL19	4.4	4.7	-0.3	-1.6	100.0%	-3.9
4009	AT5G39340.1 | Symbol: AHP3 | two-component phosphorelay mediator 2 (HP2), nearly identical to ATHP2 (Arabidopsis thaliana) GI:4156243	9.1	8.8	0.3	1.6	100.0%	-4.0
4010	AT2G23670.1 | expressed protein | chr2:10070235-10070943 REVERSE | Aliases: F26B6.28	5.9	5.4	0.5	1.6	100.0%	-3.4

Rank	Description	15.dpi	5.dpi	M	t	adj.q	B
4011	AT1G29690.1 | Symbol: CAD1 | Encodes a protein containing a domain with significant homology to the MACPF (membrane attack complex and perforin) domain of complements and perforin proteins that are involved in innate immunity in animals. Transgenic <i>cad1-1</i> mutant plants show lesions seen in the hypersensitive response, as well as a spontaneous activation of expression of pathogenesis-related genes and leading to a 32-fold increase in salicylic acid (SA). CAD1 is postulated to act as a negative regulator controlling SA-mediated pathway of programmed cell death in plant immunity. | chr1:10379012-10382109 REVERSE | Aliases: CAD1, F15D2.24, F15D2_24	3.9	3.5	0.4	1.6	100.0%	-3.8
4012	AT3G10360.1 | pumilio/Puf RNA-binding domain-containing protein, similar to RNA binding protein PufA GB:AAD39751 (Dictyostelium discoideum) and similar to Pumilio protein GB:A46221 (Drosophila sp.) | chr3:3211281-3215149 REVERSE | Aliases: F14P13.4	3.6	4.0	-0.4	-1.6	100.0%	-3.9
4013	AT1G11660.1 | heat shock protein, putative, strong similarity to gb:Z70314 heat-shock protein from Arabidopsis thaliana and is a member of the PF:00012 Hsp70 protein family | chr1:3921056-3924507 FORWARD | Aliases: F25C20.19, F25C20_19	7.8	8.2	-0.5	-1.6	100.0%	-3.9
4014	AT2G34260.2 | transducin family protein / WD-40 repeat family protein, similar to Vegetatible incompatibility protein HET-E-1 (SP:Q00808) {Podospora anserina}	7.0	7.6	-0.6	-1.6	100.0%	-3.6
4015	AT3G11900.1 | Symbol: ANT1 | amino acid transporter family protein, low similarity to proton/amino acid transporter 1 (Mus musculus) GI:21908024; belongs to INTERPRO:IPR002422 amino acid/polyamine transporter, family II | chr3:3758458-3760384 FORWARD | Aliases: AROMATIC AND NEUTRAL TRANSPORTER 1, F26K24.19, F26K24_19	6.2	5.8	0.5	1.6	100.0%	-3.8
4016	AT1G48370.1 | oligopeptide transporter OPT family protein, similar to iron-phytosiderophore transporter protein yellow stripe 1 (Zea mays) GI:10770865; contains Pfam profile PF03169: OPT oligopeptide transporter protein | chr1:17878183-17881065 FORWARD | Aliases: F11A17.8, F11A17_8	5.2	5.7	-0.5	-1.6	100.0%	-3.8
4017	AT2G15695.1 | expressed protein, contains Pfam PF05705: Eukaryotic protein of unknown function (DUF829) | chr2:6840962-6843506 REVERSE | Aliases: None	6.0	5.4	0.7	1.6	100.0%	-3.8
4018	AT1G72660.3 | similar to developmentally regulated GTP-binding protein (DRG1) [Arabidopsis thaliana] (TAIR:At1g17470.1); similar to developmentally regulated GTP binding protein [Pisum sativum] (GB:AAB67829.1); contains InterPro domain Small GTP-binding protein domain (InterPro:IPR005225); contains InterPro domain TGS domain (InterPro:IPR004095); contains InterPro domain GTP1/OBG domain (InterPro:IPR006074); contains InterPro domain GTP1/OBG (InterPro:IPR006073); contains InterPro domain GTP1/OBG sub-domain (InterPro:IPR006169) | chr1:27357542-27360332 FORWARD | Aliases: F28P22.15, F28P22_15	5.5	4.9	0.6	1.6	100.0%	-3.6
4019	AT1G75280.1 | isoflavone reductase, putative, identical to SP:P52577 Isoflavone reductase homolog P3 (EC 1.3.1.-) {Arabidopsis thaliana}; contains Pfam profile PF02716: isoflavone reductase. Involved in response to oxidative stress. | chr1:28255622-28257280 FORWARD | Aliases: F22H5.17, F22H5_17, P3	11.1	12.4	-1.4	-1.6	100.0%	-3.9
4020	AT5G14780.1 | Symbol: FDH | formate dehydrogenase (FDH), identical to GI:7677266 | chr5:4776987-4779497 FORWARD | Aliases: FORMATE DEHYDROGENASE, T9L3.80, T9L3_80	9.9	9.0	0.9	1.6	100.0%	-3.5
4021	AT1G71130.1 | encodes a member of the ERF (ethylene response factor) subfamily B-5 of ERF/AP2 transcription factor family. The protein contains one AP2 domain. There are 7 members in this subfamily.	6.8	6.4	0.4	1.6	100.0%	-3.9
4022	AT3G07320.1 | glycosyl hydrolase family 17 protein, similar to beta-1,3-glucanase from GI:6714534 (Salix gilgiana) | chr3:2332077-2334057 REVERSE | Aliases: T1B9.1	4.8	5.6	-0.8	-1.6	100.0%	-3.8
4023	AT5G64510.1 | expressed protein | chr5:25801630-25803533 FORWARD | Aliases: MUB3.3, MUB3_3	5.6	4.9	0.7	1.6	100.0%	-3.6
4024	AT5G02480.1 | expressed protein, p | chr5:547561-549910 FORWARD | Aliases: T22P11.70, T22P11_70	7.8	7.4	0.3	1.6	100.0%	-3.9
4025	AT2G40080.1 | Symbol: ELF4 | expressed protein | chr2:16741372-16741990 REVERSE | Aliases: T28M21.24, T28M21_24	7.7	7.1	0.6	1.6	100.0%	-3.6
4026	AT3G13674.1 | expressed protein | chr3:4474784-4475888 REVERSE | Aliases: MMM17.23	3.1	3.5	-0.4	-1.6	100.0%	-3.8
4027	AT1G21770.1 | expressed protein | chr1:7651635-7652275 REVERSE | Aliases: F8K7.21, F8K7_21	10.2	9.5	0.6	1.6	100.0%	-4.0
4028	AT3G02780.1 | Symbol: IPP2 | isopentenyl-diphosphate delta-isomerase II / isopentenyl diphosphate:dimethylallyl diphosphate isomerase II (IPP2), identical to isopentenyl diphosphate:dimethylallyl diphosphate isomerase (IPP2) GB:U49259 (Arabidopsis thaliana) | chr3:602440-604682 REVERSE | Aliases: F13E7.28, F13E7_28, IDI2, IPIAT1, ISOPENTENYL DIPHOSPHATE ISOMERASE 2, ISOPENTENYL PYROPHOSPHATE:DIMETHYLLALLYL PYROPHOSPHATE ISOMERASE, ISOPENTENYL PYROPHOSPHATE:DIMETHYLLALLYL PYROPHOSPHATE ISOMERASE 2	11.2	11.6	-0.4	-1.6	100.0%	-4.0
4029	AT5G58650.1 | expressed protein | chr5:23716934-23717452 REVERSE | Aliases: MZN1.10, MZN1_10	7.7	7.2	0.5	1.5	100.0%	-3.8

Rank	Description	15.dpi	5.dpi	M	t	adj.q	B
4030	AT1G55000.3 | expressed protein, similar to hypothetical protein [Oryza sativa (japonica cultivar-group)] (GB:AAX95630.1); contains InterPro domain Peptidoglycan-binding LysM (InterPro:IPR002482) | chr1:20518304-20520116 FORWARD | Aliases: F14C21.53, F14C21_53	5.7	5.0	0.7	1.5	100.0%	-3.8
4031	AT4G21060.1 | galactosyltransferase family protein, contains Pfam profile: PF01762 galactosyltransferase | chr4:11240741-11244871 FORWARD | Aliases: T13K14.220, T13K14_220	4.3	5.1	-0.8	-1.5	100.0%	-3.5
4032	AT4G17330.1 | Symbol: ATG2484 1 | agenet domain-containing protein, contains Pfam PF05641: Agenet domain | chr4:9688900-9693875 REVERSE | Aliases: DL4700C, FCAALL.411	7.0	7.6	-0.6	-1.5	100.0%	-4.0
4033	AT5G17990.1 | Symbol: TRP1 | anthranilate phosphoribosyltransferase, identical to anthranilate phosphoribosyltransferase, chloroplast precursor (EC 2.4.2.18) SP:Q02166 from (Arabidopsis thaliana) | chr5:5957215-5959906 FORWARD | Aliases: MCM23.6, MCM23_6, PAT1, PHOSPHORIBOSYLANTHRANILATE TRANSFERASE, PHOSPHORIBOSYLANTHRANILATE TRANSFERASE 1, TRYPTOPHAN BIOSYNTHESIS 1	9.1	10.1	-0.9	-1.5	100.0%	-3.5
4034	AT2G43550.1 | Encodes a defensin-like (DEFL) family protein. | chr2:18080311-18080982 FORWARD | Aliases: T1O24.29	3.9	4.4	-0.4	-1.5	100.0%	-3.7
4035	AT1G50640.1 | Symbol: ATERF3/ERF3 | encodes a member of the ERF (ethylene response factor) subfamily B-1 of ERF/AP2 transcription factor family (ATERF-3). The protein contains one AP2 domain. There are 15 members in this subfamily including ATERF-3, ATERF-4, ATERF-7, and leafy petiole. | chr1:18760816-18762101 REVERSE | Aliases: ATERF 3, ATERF3, ERF3, ETHYLENE RESPONSIVE ELEMENT BINDING FACTOR 3, F11F12.4, F11F12_4	3.1	2.8	0.2	1.5	100.0%	-4.3
4036	AT5G02490.1 | heat shock cognate 70 kDa protein 2 (HSC70-2) (HSP70-2), identical to SP:P22954 Heat shock cognate 70 kDa protein 2 (Hsc70.2) {Arabidopsis thaliana} | chr5:550033-552643 REVERSE | Aliases: T22P11.80, T22P11_80	7.8	8.4	-0.7	-1.5	100.0%	-3.4
4037	AT2G21960.1 | expressed protein | chr2:9361999-9364499 FORWARD | Aliases: F7D8.28, F7D8_28	8.2	7.5	0.6	1.5	100.0%	-3.5
4038	AT1G28400.1 | expressed protein, similar to E6 (GI:1000090) (Gossypium barbadense) | chr1:9972370-9973779 REVERSE | Aliases: F3M18.16, F3M18_16	12.8	13.1	-0.3	-1.5	100.0%	-4.1
4039	AT5G01370.1 | expressed protein | chr5:152529-154350 FORWARD | Aliases: T10O8.80, T10O8_80	5.8	6.5	-0.7	-1.5	100.0%	-3.6
4040	AT5G54170.1 | expressed protein, weak similarity to SP:Q9UKL6 Phosphatidylcholine transfer protein (PC-TP) {Homo sapiens} | chr5:22002104-22004280 REVERSE | Aliases: K18G13.4, K18G13_4	4.5	4.2	0.4	1.5	100.0%	-3.9
4041	AT2G38410.1 | VHS domain-containing protein / GAT domain-containing protein, weak similarity to hepatocyte growth factor-regulated tyrosine kinase substrate HRS isoform 2 (Homo sapiens) GI:9022389; contains Pfam profiles PF00790: VHS domain, PF03127: GAT domain | chr2:16093708-16097286 REVERSE | Aliases: T19C21.10, T19C21_10	3.9	3.4	0.5	1.5	100.0%	-3.9
4042	AT4G39780.1 | encodes a member of the DREB subfamily A-6 of ERF/AP2 transcription factor family. The protein contains one AP2 domain. There are 8 members in this subfamily including RAP2.4. | chr4:18457951-18459174 REVERSE | Aliases: T19P19.170, T19P19_170	4.3	3.7	0.6	1.5	100.0%	-3.4
4043	AT2G45290.1 | transketolase, putative, strong similarity to transketolase 1 (Capsicum annuum) GI:3559814; contains Pfam profiles PF02779: Transketolase, pyridine binding domain, PF02780: Transketolase, C-terminal domain, PF00456: Transketolase, thiamine diphosphate binding domain | chr2:18679756-18682980 FORWARD | Aliases: F4L23.20	6.2	7.4	-1.2	-1.5	100.0%	-3.5
4044	AT2G25150.1 | transferase family protein, similar to 10-deacetylbaccatin III-10-O-acetyl transferase (gi:6746554), 2-debenzoyl-7,13-diacetylbaccatin III-2-O-benzoyl transferase (gi:11559716) from Taxus cuspidata; contains Pfam transferase family domain PF00248; contains EST gb:R65039 | chr2:10709442-10711373 REVERSE | Aliases: F13D4.110, F13D4_110	2.5	2.3	0.2	1.5	100.0%	-4.6
4045	AT1G78660.3 | gamma-glutamyl hydrolase, putative / gamma-Glu-X carboxypeptidase, putative / conjugase, putative, similar to SP:O65355 Gamma-glutamyl hydrolase precursor (EC 3.4.19.9) (Gamma-Glu-X carboxypeptidase) (Conjugase) (GH) {Arabidopsis thaliana} | chr1:29590570-29593185 FORWARD | Aliases: T30F21.1, T30F21_1	7.1	8.0	-0.9	-1.5	100.0%	-3.7
4046	AT5G67240.1 | similar to exonuclease family protein [Arabidopsis thaliana] (TAIR:At3g50100.1); similar to hypothetical protein AN7566.2 [Aspergillus nidulans FGSC A4] (GB:EAA62146.1); contains InterPro domain Exonuclease (InterPro:IPR006055) | chr5:26841462-26845395 REVERSE | Aliases: K21H1.20, K21H1_20	6.5	7.8	-1.3	-1.5	100.0%	-3.5
4047	AT4G17770.1 | Symbol: ATTPS5 |	7.9	8.4	-0.5	-1.5	100.0%	-4.0
4048	AT5G23230.1 | isochorismatase hydrolase family protein, low similarity to SP:P45743 Isochorismatase (EC 3.3.2.1) (2,3 dihydro-2,3 dihydroxybenzoate synthase) (Superoxide-inducible protein 1) (SOI1) {Bacillus subtilis}; contains Pfam profile PF00857: isochorismatase family protein | chr5:7825885-7826604 REVERSE | Aliases: MKD15.9, MKD15_9	3.9	3.6	0.3	1.5	100.0%	-4.1

Rank	Description	15.dpi	5.dpi	M	t	adj.q	B
4049	AT5G47620.3 | heterogeneous nuclear ribonucleoprotein, putative / hnRNP, putative | chr5:19319496-19321316 REVERSE | Aliases: MNJ7.21, MNJ7_21	10.1	9.8	0.3	1.5	100.0%	-4.1
4050	AT2G29980.2 | Symbol: FAD3 | omega-3 fatty acid desaturase, endoplasmic reticulum (FAD3), identical to SP:48623 | chr2:12788668-12792114 REVERSE | Aliases: F23F1.10, F23F1_10, FATTY ACID DESATURASE 3, OMEGA 3 FATTY ACID DESATURASE, ENDOPLASMIC RETICULUM18:2 DESATURASE (OMEGA 3 FATTY ACID DESATURASE)	7.4	8.7	-1.3	-1.5	100.0%	-3.4
4051	AT2G33770.1 | ubiquitin-conjugating enzyme family protein, low similarity to ubiquitin-conjugating BIR-domain enzyme APOLLON (Homo sapiens) GI:8489831, ubiquitin-conjugating enzyme (Mus musculus) GI:3319990; contains Pfam profile PF00179: Ubiquitin-conjugating enzyme | chr2:14284635-14290116 REVERSE | Aliases: T1B8.8, T1B8_8	5.7	6.8	-1.1	-1.5	100.0%	-3.8
4052	AT2G10920.1 | expressed protein | chr2:4308965-4309216 REVERSE | Aliases: F16G22.2, F16G22_2	7.2	6.1	1.0	1.5	100.0%	-3.5
4053	AT4G07410.1 | transducin family protein / WD-40 repeat family protein, contains 7 WD-40 repeats (PF00400) (2 weak); similar to Vegetatible incompatibility protein HET-E-1 (SP:Q00808) {Podospora anserina} | chr4:4201246-4205221 REVERSE | Aliases: F28D6.14, F28D6_14	3.7	4.4	-0.6	-1.5	100.0%	-3.8
4054	AT1G69010.1 | basic helix-loop-helix (bHLH) family protein, contains Pfam profile: PF00010 helix-loop-helix DNA-binding domain	6.1	5.1	1.0	1.5	100.0%	-3.7
4055	AT4G12830.1 | hydrolase, alpha/beta fold family protein, low similarity to haloalkane dehalogenase from (Mycobacterium avium subsp. avium) GI:14422311, (Pseudomonas pavonaceae) GI:6689030; contains Pfam profile PF00561: hydrolase, alpha/beta fold family | chr4:7531138-7533584 FORWARD | Aliases: T20K18.180, T20K18_180	3.6	3.3	0.3	1.5	100.0%	-4.0
4056	AT3G63520.1 | Symbol: CCD1 | 9-cis-epoxycarotenoid dioxygenase / neoxanthin cleavage enzyme / NCED1 / carotenoid cleavage dioxygenase 1 (CCD1), identical to putative 9-cis-epoxy-carotenoid dioxygenase (GI:3096910); contains Pfam profile PF03055: Retinal pigment epithelial membrane protein | chr3:23463915-23466871 FORWARD | Aliases: ATCCD1, ATNCED1, CAROTENOID CLEAVAGE DIOXYGENASE 1, MAA21.150	9.1	9.6	-0.5	-1.5	100.0%	-3.8
4057	AT1G09390.1 | GDSL-motif lipase/hydrolase family protein, Similar to early nodulin ENOD8 (Medicago sativa) GI:304037, lanatoside 15'-O-acetylesterase (Digitalis lanata) GI:3688284, elicitor-induced glycoprotein iEP4 (Daucus carota) GI:1911765; contains InterPro Entry IPR001087 Lipolytic enzyme, G-D-S-L family	5.3	5.7	-0.4	-1.5	100.0%	-3.8
4058	AT5G50280.1 | Symbol: EMB1006 | pentatricopeptide (PPR) repeat-containing protein, contains Pfam profile PF01535: PPR repeat | chr5:20476464-20478730 FORWARD | Aliases: EMB1006, EMBRYO DEFECTIVE 1006, K6A12.14, K6A12_14	3.9	4.4	-0.5	-1.5	100.0%	-3.7
4059	AT1G04680.1 | pectate lyase family protein, similar to pectate lyase GP:14531296 from (Fragaria x ananassa) | chr1:1303528-1307881 REVERSE | Aliases: T1G11.7, T1G11_7	3.7	4.5	-0.8	-1.5	100.0%	-3.8
4060	AT1G13300.1 | myb family transcription factor, contains Pfam domain, PF00249: Myb-like DNA-binding domain | chr1:4556889-4558705 FORWARD | Aliases: T6J4.6, T6J4_6	5.2	5.7	-0.5	-1.5	100.0%	-3.6
4061	AT3G60940.1 | expressed protein, contains Pfam profile PF04396: Protein of unknown function, DUF537; expression supported by MPSS | chr3:22532590-22534004 REVERSE | Aliases: T27I15.30	2.6	2.4	0.2	1.5	100.0%	-4.3
4062	AT3G18050.1 | expressed protein | chr3:6180865-6182860 FORWARD | Aliases: MRC8.3	2.6	2.8	-0.3	-1.5	100.0%	-4.3
4063	AT1G69230.2 | expressed protein | chr1:26029981-26031371 REVERSE | Aliases: F4N2.18	4.9	4.2	0.6	1.5	100.0%	-3.7
4064	AT1G62200.1 | proton-dependent oligopeptide transport (POT) family protein, contains Pfam profile: PF00854 POT family ; contains non-consensus GA donor site at intron 4 | chr1:22985701-22988024 REVERSE | Aliases: F19K23.13, F19K23_13	7.2	6.8	0.4	1.5	100.0%	-3.8
4065	AT3G06730.1 | thioredoxin family protein, contains Pfam profile: PF00085 Thioredoxin | chr3:2124240-2126148 FORWARD | Aliases: F3E22.13	4.7	5.3	-0.6	-1.5	100.0%	-3.4
4066	AT4G14960.2 | Symbol: TUA6 | tubulin alpha-6 chain (TUA6), nearly identical to SP:P29511 Tubulin alpha-6 chain {Arabidopsis thaliana}	7.6	4.6	3.0	1.5	100.0%	-3.6
4067	AT1G75560.1 | zinc knuckle (CCHC-type) family protein, contains Pfam domain, PF00098: Zinc knuckle | chr1:28374802-28376902 REVERSE | Aliases: F10A5.22, F10A5_22	4.7	3.1	1.6	1.5	100.0%	-3.6
4068	AT3G06650.1 | Symbol: ACLB 1 |	5.6	6.2	-0.6	-1.5	100.0%	-3.6
4069	AT1G56340.2 | Symbol: CRT1 | similar to calreticulin 2 (CRT2) [Arabidopsis thaliana] (TAIR:At1g09210.1); similar to calreticulin [Beta vulgaris subsp. vulgaris] (GB:CAA05161.1); similar to calreticulin [Nicotiana plumbaginifolia] (GB:CAA95999.1); similar to calreticulin (GB:AAA80652.1); similar to calcium-binding protein calreticulin [Prunus armeniaca] (GB:AAD32207.1); similar to CRTC_RICCO Calreticulin precursor (GB:P93508); contains InterPro domain Calreticulin (InterPro:IPR001580) | chr1:21093545-21096322 REVERSE | Aliases: CALRETICULIN 1, F14G9.5, F14G9_5	10.0	10.6	-0.6	-1.5	100.0%	-3.6

Rank	Description	15.dpi	5.dpi	M	t	adj.q	B
4070	AT2G47730.1 | Symbol: ATGSTF8 | glutathione S-transferase 6 (GST6), identical to GB:X95295. Based on identical cDNA hits, the translation is now 40 AAs longer at the N-terminal, and start of exon2 is also corrected. | chr2:19565270-19566533 FORWARD | Aliases: ATGSTF5, F17A22.12, GLUTATHIONE S TRANSFERASE 16, GLUTATHIONE S TRANSFERASE 8, GST6, GSTF8	7.3	8.4	-1.1	-1.5	100.0%	-3.7
4071	AT3G03920.1 | Gar1 RNA-binding region family protein, contains Pfam profile PF04410: Gar1 protein RNA binding region | chr3:1009005-1010477 REVERSE | Aliases: F20H23.3, F20H23_3	9.5	8.8	0.7	1.5	100.0%	-3.5
4072	AT3G24315.1 | sec20 family protein, contains Pfam PF03908: Sec20 | chr3:8820464-8822810 REVERSE | Aliases: None	9.6	9.1	0.5	1.5	100.0%	-3.9
4073	AT3G20780.1 | Symbol: ATTOP6B | topoisomerase 6 subunit B (TOP6B), nearly identical to topoisomerase 6 subunit B (Arabidopsis thaliana) GI:12331188 | chr3:7263906-7268578 REVERSE | Aliases: BIN3, BRASSINOSTEROID INSENSITIVE 3, ELONGATED HYPOCOTYL 6, HYP6, MOE17.8	3.6	4.2	-0.6	-1.5	100.0%	-3.7
4074	AT2G17630.1 | phosphoserine aminotransferase, putative, similar to Phosphoserine aminotransferase, chloroplast precursor (PSAT) (SP:Q96255) (Arabidopsis thaliana); contains TIGRFAM TIGR01364: phosphoserine aminotransferase; contains Pfam PF00266: aminotransferase, class V | chr2:7673667-7675138 FORWARD | Aliases: T19E12.3, T19E12_3	7.4	8.3	-0.9	-1.5	100.0%	-3.6
4075	AT5G01810.2 | Symbol: CIPK15 |	7.9	7.0	0.9	1.5	100.0%	-3.9
4076	AT1G27720.1 | transcription initiation factor IID (TFIID) component TAF4 family protein, contains Pfam profile PF05236: Transcription initiation factor TFIID component TAF4 family | chr1:9643338-9647363 REVERSE | Aliases: T22C5.17, T22C5_17	3.8	4.1	-0.4	-1.5	100.0%	-4.1
4077	AT3G22060.1 | contains Pfam profile: PF01657 Domain of unknown function that is usually associated with protein kinase domain Pfam:PF00069, however this protein does not have the protein kinase domain | chr3:7771023-7772347 FORWARD | Aliases: MZN24.26	2.8	3.1	-0.3	-1.5	100.0%	-4.0
4078	AT5G04360.1 | pullulanase, putative / starch debranching enzyme, putative, similar to pullulanase (Spinacia oleracea) GI:634093 (EC 3.2.1.41); contains Pfam profiles PF00128: Alpha amylase catalytic domain, PF02922: Isoamylase N-terminal domain | chr5:1221531-1228612 FORWARD | Aliases: T19N18.90, T19N18_90	4.8	4.1	0.7	1.5	100.0%	-3.7
4079	AT4G36940.1 | similar to putative nicotinate phosphoribosyltransferase [Oryza sativa (japonica cultivar-group)] (GB:XP_470349.1); similar to nicotinate phosphoribosyltransferase-like protein [Oryza sativa] (GB:AAP69615.1); similar to OSJNBa0042L16.13 [Oryza sativa (japonica cultivar-group)] (GB:NP_910116.2); contains InterPro domain Nicotinate phosphoribosyltransferase related (InterPro:IPR006405); contains InterPro domain Nicotinate phosphoribosyltransferase and related (InterPro:IPR007229) | chr4:17416852-17420248 FORWARD | Aliases: AP22.31, AP22_31	4.7	5.3	-0.6	-1.5	100.0%	-3.9
4080	AT5G37020.1 | Symbol: ARF8 | auxin-responsive factor (ARF8), identical to auxin response factor 8 GI:4104931 from (Arabidopsis thaliana) | chr5:14647258-14651617 FORWARD | Aliases: AUXIN RESPONSE FACTOR 8, K15O15.1, K15O15_1	4.4	4.7	-0.3	-1.5	100.0%	-4.1
4081	AT4G15910.1 | Symbol: ATDI21 |	11.2	11.7	-0.4	-1.5	100.0%	-3.7
4082	AT1G05205.1 | expressed protein | chr1:1509212-1510280 REVERSE | Aliases: None	10.1	10.5	-0.4	-1.5	100.0%	-4.0
4083	AT3G19800.1 | expressed protein | chr3:6876120-6878156 FORWARD | Aliases: MPN9.4	6.1	5.4	0.6	1.5	100.0%	-3.4
4084	AT5G01540.1 | lectin protein kinase, putative, similar to receptor lectin kinase 3 (Arabidopsis thaliana) gi:4100060:gb:AAD00733; contains protein kinase domain, Pfam:PF00069; contains legume lectins alpha and beta domains, Pfam:PF00138 and Pfam:PF00139 | chr5:210978-213471 REVERSE | Aliases: F7A7.60, F7A7_60	2.6	2.9	-0.3	-1.5	100.0%	-4.3
4085	AT4G14615.1 | expressed protein | chr4:8383765-8385074 FORWARD | Aliases: None	10.4	10.8	-0.4	-1.5	100.0%	-4.2
4086	AT1G67430.1 | 60S ribosomal protein L17 (RPL17B), similar to ribosomal protein GI:19101 from (Hordeum vulgare)	11.9	12.4	-0.5	-1.5	100.0%	-4.1
4087	AT2G41680.1 | thioredoxin reductase, putative / NADPH-dependent thioredoxin reductase, putative, The last 2 exons encode thioredoxin. There is an EST match to exons 5-7, and the distance between exon 7 and exon 8 is only 90bp. It is unlikely this is two separate genes, but more likely a hybrid protein. | chr2:17383233-17386122 REVERSE | Aliases: T32G6.20, T32G6_20	7.2	7.7	-0.5	-1.5	100.0%	-3.8
4088	AT5G05420.1 | immunophilin, putative / FKBP-type peptidyl-prolyl cis-trans isomerase, putative, contains similarity to peptidyl-prolyl cis-trans isomerase | chr5:1604041-1604824 REVERSE | Aliases: K18I23.23, K18I23_23	4.8	4.4	0.4	1.5	100.0%	-4.0
4089	AT2G01830.3 | Symbol: WOL | histidine kinase (AHK4) (WOL), identical to histidine kinase AHK4 (Arabidopsis thaliana) gi:13537200:dbj:BAB40776; contains Pfam profiles PF03924: CHASE domain, PF02518: ATPase, histidine kinase-, DNA gyrase B-, and HSP90-like domain protein, PF00512: His Kinase A (phosphoacceptor) domain, PF00072: Response regulator receiver domain | chr2:362982-369706 REVERSE | Aliases: AHK4, CRE1, CYTOKININ RESPONSE 1, T23K3.2, T23K3_2, WOL, WOL1	4.6	5.2	-0.5	-1.5	100.0%	-3.9

Rank	Description	15.dpi	5.dpi	M	t	adj.q	B
4090	AT1G48950.1 | expressed protein, similar to expressed protein [Arabidopsis thaliana] (TAIR:At1g17210.1); similar to hypothetical protein [Oryza sativa (japonica cultivar-group)] (GB:BAD52672.1) | chr1:18110565-18113905 FORWARD | Aliases: F27K7.14	5.9	6.4	-0.5	-1.5	100.0%	-3.6
4091	AT1G21690.2 | Symbol: EMB1968 | replication factor C 37 kDa, putative, Similar to SWISS-PROT:P35249 activator 1 37 kDa subunit (Replication factor C 37 kDa subunit, A1 37 kDa subunit, RF-C 37 kDa subunit, RFC37) (Homo sapiens); contains Pfam domain, PF00004: ATPase, AAA family	5.3	5.7	-0.4	-1.5	100.0%	-4.1
4092	AT3G22420.2 | Symbol: WNK2 | protein kinase family protein, contains protein kinase domain, Pfam:PF00069 | chr3:7946533-7949081 FORWARD | Aliases: MCB17.15, PROTEIN KINASE, WITH NO K 2, ZIK3	6.3	7.1	-0.7	-1.5	100.0%	-3.3
4093	AT3G12690.3 | protein kinase, putative, similar to viroid symptom modulation protein (Lycopersicon esculentum) gi:7672777:gb:AAF66637 | chr3:4030462-4032820 REVERSE | Aliases: MBK21.5	4.1	3.9	0.3	1.5	100.0%	-4.1
4094	AT1G49970.1 | Symbol: CLPR1 | ATP-dependent Clp protease proteolytic subunit (ClpR1) (nClpP5), identical to nClpP5 GB:BAA82069 GI:5360595 from (Arabidopsis thaliana); identical to cDNA nClpP5 (nuclear encoded ClpP5) GI:5360594 | chr1:18505201-18508277 REVERSE | Aliases: F2J10.14, F2J10_14, NCLPP5	10.5	10.0	0.5	1.5	100.0%	-4.0
4095	AT3G23590.1 | expressed protein | chr3:8467456-8473626 FORWARD | Aliases: MDB19.7	6.0	5.6	0.4	1.5	100.0%	-3.9
4096	AT4G04180.1 | AAA-type ATPase family protein, contains Pfam domain, PF00004: ATPase, AAA family | chr4:2020434-2023990 FORWARD | Aliases: T27D20.13, T27D20_13	4.5	4.9	-0.4	-1.5	100.0%	-3.7
4097	AT1G07970.1 | expressed protein | chr1:2469288-2472620 REVERSE | Aliases: T6D22.6, T6D22_6	5.0	5.4	-0.4	-1.5	100.0%	-3.8
4098	AT1G50170.1 | expressed protein, similar to Cbi protein (GI:3724039) (Bacillus megaterium); similar to YlnE protein (GI:2462961) (Bacillus subtilis); similar to NirR (GI:4433637) (Staphylococcus carnosus) | chr1:18585692-18587392 FORWARD | Aliases: F14I3.20, F14I3_20	6.0	5.4	0.6	1.5	100.0%	-3.8
4099	AT5G41380.1 | expressed protein, similar to hypothetical protein [Arabidopsis thaliana] (TAIR:At1g63820.1); similar to 'unknown protein, contains CCT motif, PF06203' [Oryza sativa (japonica cultivar-group)] (GB:XP_475389.1) | chr5:16578506-16580960 REVERSE | Aliases: MYC6.9, MYC6_9	3.1	2.8	0.2	1.5	100.0%	-4.2
4100	AT5G23450.3 | Symbol: ATLCBK1 | similar to diacylglycerol kinase family protein [Arabidopsis thaliana] (TAIR:At4g21540.1); similar to putative sphingosine kinase [Oryza sativa (japonica cultivar-group)] (GB:AAP54628.1); similar to OSJNBb0103I08.5 [Oryza sativa (japonica cultivar-group)] (GB:XP_473364.1); contains InterPro domain Diacylglycerol kinase, catalytic domain (InterPro:IPR001206) | chr5:7904711-7909588 REVERSE | Aliases: A. THALIANA LONG CHAIN BASE (LCB) KINASE 1, K19M13.8, K19M13_8, SPHINGOSINE KINASE	4.9	4.4	0.5	1.5	100.0%	-3.7
4101	AT5G63880.1 | SNF7 family protein, contains Pfam domain, PF03357: SNF7 family | chr5:25580964-25583175 FORWARD | Aliases: MGI19.8, MGI19_8	8.5	8.2	0.3	1.5	100.0%	-4.0
4102	AT4G36450.1 | Symbol: ATMPK14 | mitogen-activated protein kinase, putative / MAPK, putative (MPK14), mitogen-activated protein kinase (MAPK)(AtMPK14), PMID:12119167 | chr4:17210248-17211416 REVERSE | Aliases: AP22.98, AP22_98	3.5	3.3	0.3	1.5	100.0%	-4.2
4103	AT4G12600.1 | ribosomal protein L7Ae/L30e/S12e/Gadd45 family protein, Similar to NHP2/L7Ae family proteins, see SWISSPROT:P32495 and PMID:2063628. | chr4:7453199-7454478 FORWARD | Aliases: T1P17.190, T1P17_190	9.9	10.5	-0.6	-1.5	100.0%	-3.7
4104	AT3G28730.1 | Symbol: ATHMG | structure-specific recognition protein 1 / high mobility group protein / HMG protein, nearly identical to SP:Q05153 Structure-specific recognition protein 1 homolog (HMG protein) {Arabidopsis thaliana}; contains Pfam profile PF00505: HMG (high mobility group) box; contains Pfam profile PF03531: Structure-specific recognition protein | chr3:10786126-10790386 FORWARD | Aliases: HIGH MOBILITY GROUP, T19N8.3	8.8	9.0	-0.3	-1.5	100.0%	-4.0
4105	AT4G02150.1 | Symbol: ATIMPALPHA3/MOS6 | Encodes IMPORTIN ALPHA 3. Mutant plants act as suppressors of snc1 response and salicylic acid accumulation. Located in the nucleus. Involved in protein import. | chr4:950649-953689 REVERSE | Aliases: ATIMPALPHA3, MOS6, T10M13.16, T10M13_16	8.0	9.0	-0.9	-1.5	100.0%	-3.8
4106	AT3G27210.1 | expressed protein | chr3:10046423-10048580 REVERSE | Aliases: K17E12.3	4.4	3.9	0.5	1.5	100.0%	-3.8
4107	AT2G45880.1 | glycosyl hydrolase family 14 protein, similar to beta-amylase GI:13560977 from (Castanea crenata) | chr2:18885593-18889649 REVERSE | Aliases: F4I18.14	4.1	3.7	0.4	1.5	100.0%	-4.2
4108	AT4G34240.2 | Symbol: ALDH3I1 | aldehyde dehydrogenase (ALDH3), similar to aldehyde dehydrogenase (Arabidopsis thaliana) gi:17065876:emb:CAC84903; contains Pfam profile PF00171: aldehyde dehydrogenase (NAD) family protein; identical to cDNA aldehyde dehydrogenase (ALDH3 gene) GI:17065875, aldehyde dehydrogenase (Arabidopsis thaliana) GI:17065876 | chr4:16389782-16392086 FORWARD | Aliases: ALDH3, F10M10.10, F10M10_10	6.8	7.4	-0.6	-1.5	100.0%	-3.5
4109	AT1G52640.1 | pentatricopeptide (PPR) repeat-containing protein, contains Pfam profile PF01535: PPR repeat | chr1:19612525-19614096 REVERSE | Aliases: F6D8.14, F6D8_14	2.7	2.9	-0.3	-1.5	100.0%	-4.2

Rank	Description	15.dpi	5.dpi	M	t	adj.q	B
4110	AT5G16390.2 | Symbol: BCCP1 | biotin carboxyl carrier protein 1 (BCCP1), identical to biotin carboxyl carrier protein of acetyl-CoA carboxylase precursor (Arabidopsis thaliana) gi:9759121:dbj:BAB09606 | chr5:5360844-5363126 REVERSE | Aliases: BCCP, BIOTIN CARBOXYL CARRIER, BIOTIN CARBOXYL CARRIER PROTEIN 1, CAC1A, MQK4.12, MQK4_12	6.3	6.8	-0.5	-1.5	100.0%	-3.7
4111	AT1G15220.2 | cytochrome c biogenesis protein family, contains Pfam PF03918: Cytochrome C biogenesis protein; similar to Cytochrome c-type biogenesis protein cycL precursor.(SP:P45405) {Bradyrhizobium japonicum} | chr1:5240024-5241516 REVERSE | Aliases: F9L1.17, F9L1_17	3.6	3.3	0.3	1.5	100.0%	-3.9
4112	AT3G51420.1 | strictosidine synthase family protein, similar to hemomucin (Drosophila melanogaster)(GI:1280434), strictosidine synthase (Rauvolfia serpentina)(SP:P15324); contains strictosidine synthase domain PF03088 | chr3:19095014-19096837 FORWARD | Aliases: F26O13.60	2.7	2.4	0.3	1.5	100.0%	-4.3
4113	AT3G23920.1 | beta-amylase, putative / 1,4-alpha-D-glucan maltohydrolase, putative, similar to beta-amylase enzyme (Arabidopsis thaliana) GI:6065749, beta-amylase PCT-BMYI from (Solanum tuberosum); contains Pfam profile PF01373: Glycosyl hydrolase family 14 | chr3:8641576-8644456 FORWARD | Aliases: F14O13.12	6.0	5.2	0.8	1.5	100.0%	-3.8
4114	AT5G45760.2 | transducin family protein / WD-40 repeat family protein | chr5:18578338-18580422 FORWARD | Aliases: MRA19.19, MRA19_19	5.9	6.4	-0.5	-1.5	100.0%	-3.7
4115	AT4G03180.1 | expressed protein | chr4:1403201-1405570 FORWARD | Aliases: F4C21.10, F4C21_10	6.5	6.9	-0.4	-1.5	100.0%	-3.9
4116	AT3G02180.2 | Symbol: SP1L | expressed protein | chr3:404891-405656 FORWARD | Aliases: F1C9.3, SPIRAL 1 LIKE	9.9	10.3	-0.4	-1.5	100.0%	-3.9
4117	AT3G18270.1 | Symbol: CYP77A5P | mandelate racemase/muconate lactonizing enzyme family protein, low similarity to cis,cis-muconate lactonizing enzyme (Burkholderia sp. TH2) GI:23491535; contains Pfam profile: PF01188 Mandelate racemase/muconate lactonizing enzyme, C-terminal domain, PF02746: Mandelate racemase/muconate lactonizing enzyme, N-terminal domain | chr3:6261917-6264315 FORWARD | Aliases: MIE15.6	4.4	4.0	0.3	1.5	100.0%	-3.9
4118	AT3G10210.1 | expressed protein, similar to putative protein GB:CAA20045 (Arabidopsis thaliana) | chr3:3160244-3161599 REVERSE | Aliases: F14P13.19	5.0	4.6	0.5	1.5	100.0%	-3.8
4119	AT1G63670.1 | expressed protein | chr1:23611085-23613666 REVERSE | Aliases: F24D7.14, F24D7_14	3.0	3.4	-0.3	-1.5	100.0%	-4.0
4120	AT3G51895.1 | Symbol: SULTR3;1 | sulfate transporter (ST1), identical to sulfate transporter (Arabidopsis thaliana) GI:2285885 | chr3:19262274-19266738 REVERSE | Aliases: AST12, ATST1, SULFATE TRANSPORTER 1	4.6	4.1	0.5	1.5	100.0%	-3.6
4121	AT2G18250.1 | cytidylyltransferase domain-containing protein, similar to CoA synthase (Mus musculus) GI:21780289, bifunctional phosphopantetheine adenylyl transferase / dephospho CoA kinase (Sus scrofa) GI:20465246; contains Pfam profile PF01467: Cytidylyltransferase | chr2:7947107-7948581 FORWARD | Aliases: T30D6.24, T30D6_24	8.8	7.8	1.1	1.5	100.0%	-3.6
4122	AT5G67070.1 | Symbol: RALFL34 | rapid alkalinization factor (RALF) family protein, similar to RALF precursor (Nicotiana tabacum) GI:16566316 | chr5:26785321-26786072 FORWARD | Aliases: K21H1.3, K21H1_3, RALF LIKE 34	4.5	5.1	-0.5	-1.5	100.0%	-3.6
4123	AT5G08430.1 | SWIB complex BAF60b domain-containing protein / plus-3 domain-containing protein / GYF domain-containing protein, similar to CPRF interacting protein (Petroselinum crispum) GI:9588690; contains Pfam profiles PF02201: BAF60b domain of the SWIB complex, PF03126: Plus-3 domain, PF02213: GYF domain; contains non-conensus AT-AC splice sites at intron 5 | chr5:2716701-2720213 FORWARD | Aliases: F8L15.160, F8L15_160	7.1	7.6	-0.5	-1.5	100.0%	-4.1
4124	AT3G53150.1 | UDP-glucoronosyl/UDP-glucosyl transferase family protein, contains Pfam profile: PF00201 UDP-glucoronosyl and UDP-glucosyl transferase | chr3:19708714-19710237 REVERSE | Aliases: T4D2.80	3.0	3.8	-0.8	-1.5	100.0%	-3.4
4125	AT5G62990.1 | Symbol: EMB1692 | expressed protein | chr5:25295054-25296538 FORWARD | Aliases: EMB1692, EMBRYO DEFECTIVE 1692, MJH22.4, MJH22_4	3.2	3.5	-0.3	-1.5	100.0%	-4.4
4126	AT2G23960.1 | defense-related protein, putative, similar to defense-related protein (Brassica carinata) GI:14009290 | chr2:10203139-10204690 FORWARD | Aliases: T29E15.16, T29E15_16	2.8	3.2	-0.4	-1.5	100.0%	-3.8
4127	AT1G66660.1 | seven in absentia (SINA) protein, putative, similar to SIAH2 protein (Brassica napus var. napus) GI:7657878; contains Pfam profile PF03145: Seven in absentia protein family | chr1:24865539-24867031 REVERSE | Aliases: F4N21.20, F4N21_20	4.9	4.5	0.4	1.5	100.0%	-3.8
4128	AT1G43900.1 | protein phosphatase 2C, putative / PP2C, putative, similar to protein phosphatase type 2C GI:4336436 from (Lotus japonicus) | chr1:16656485-16658885 FORWARD | Aliases: F9C16.6, F9C16_6	9.2	9.6	-0.4	-1.5	100.0%	-4.1
4129	AT2G35340.1 | RNA helicase, putative, similar to ATP-dependent RNA helicase #3 (Homo sapiens) GI:3107913; contains Pfam profiles PF04408: Helicase associated domain (HA2), PF00271: Helicase conserved C-terminal domain | chr2:14879807-14886694 FORWARD | Aliases: T32F12.28, T32F12_28	3.2	3.4	-0.2	-1.5	100.0%	-4.4

Rank	Description	15.dpi	5.dpi	M	t	adj.q	B
4130	AT4G35950.1 | Symbol: ARAC6 | Rac-like GTP-binding protein (ARAC6), identical to rac-like GTP binding protein Arac6 GI:3406757 from (Arabidopsis thaliana) | chr4:17023840-17025866 REVERSE | Aliases: RAC2, T19K4.80	7.9	8.4	-0.4	-1.5	100.0%	-3.8
4131	AT4G02590.2 | similar to basic helix-loop-helix (bHLH) family protein [Arabidopsis thaliana] (TAIR:At1g03040.1); similar to bHLH transcription factor PTF1-like protein [Oryza sativa (japonica cultivar-group)] (GB:BAD29274.1); contains InterPro domain Basic helix-loop-helix dimerization domain bHLH (InterPro:IPR001092) | chr4:1137508-1140490 REVERSE | Aliases: T10P11.13, T10P11_13	5.1	4.4	0.7	1.5	100.0%	-4.0
4132	AT1G64970.1 | Symbol: G TMT | expressed protein | chr1:24137050-24139723 REVERSE | Aliases: F13O11.27, F13O11_27, GAMMA TOCOPHEROL METHYLTRANSFERASE, TMT1, VITAMIN E DEFICIENT 4, VTE4	4.3	3.9	0.4	1.5	100.0%	-3.7
4133	AT2G20930.1 | expressed protein | chr2:9007700-9008818 REVERSE | Aliases: F5H14.10, F5H14_10	6.6	7.0	-0.5	-1.5	100.0%	-3.9
4134	AT1G22110.1 | expressed protein | chr1:7801405-7802462 REVERSE | Aliases: F2E2.18, F2E2_18	3.8	3.5	0.3	1.5	100.0%	-4.0
4135	AT5G13090.1 | expressed protein, predicted proteins - Arabidopsis thaliana | chr5:4153589-4154675 REVERSE | Aliases: T19L5.50, T19L5_50	5.7	5.3	0.4	1.5	100.0%	-3.8
4136	AT2G42770.1 | peroxisomal membrane 22 kDa family protein, contains Mpv17 / PMP22 family domain, Pfam:PF04117 | chr2:17805363-17807083 REVERSE | Aliases: F7D19.23, F7D19_23	7.7	7.0	0.7	1.5	100.0%	-3.3
4137	AT4G32900.2 | expressed protein | chr4:15878751-15881044 REVERSE | Aliases: F26P21.20, F26P21_20	3.6	3.1	0.4	1.5	100.0%	-3.8
4138	AT1G33290.2 | sporulation protein-related, isoform contains non-consensus AT-donor acceptor site at intron 6; similar to Stage III sporulation protein AA. (Swiss-Prot:Q01367) (Bacillus subtilis); similar to SpoIIIAA (GI:1303904) (Bacillus subtilis); similar to stage III sporulation protein AA (GI:18145497) (Clostridium perfringens str. 13) | chr1:12074090-12076081 FORWARD | Aliases: T16O9.17, T16O9_17	7.5	6.8	0.7	1.5	100.0%	-3.8
4139	AT2G25070.1 | protein phosphatase 2C, putative / PP2C, putative | chr2:10670319-10673043 REVERSE | Aliases: F13D4.1	9.8	9.5	0.4	1.5	100.0%	-4.3
4140	AT1G19660.2 | similar to wound-responsive protein-related [Arabidopsis thaliana] (TAIR:At1g75380.2); similar to wound-responsive protein-related [Arabidopsis thaliana] (TAIR:At1g75380.1); similar to wound-responsive protein-related [Arabidopsis thaliana] (TAIR:At1g75380.3); similar to putative wound inductive gene [Oryza sativa (japonica cultivar-group)] (GB:XP_481914.1) | chr1:6799972-6802643 REVERSE | Aliases: F14P1.1, F14P1_1	10.0	8.7	1.3	1.5	100.0%	-3.5
4141	AT1G75380.3 | wound-responsive protein-related, similar to wound inducive gene GI:8096273 from (Nicotiana tabacum) | chr1:28285050-28287694 REVERSE | Aliases: F1B16.9, F1B16_9	10.0	8.7	1.3	1.5	100.0%	-3.5
4142	AT1G22240.1 | pumilio/Puf RNA-binding domain-containing protein, contains Pfam profile: PF00806 pumilio-family RNA binding domain | chr1:7853073-7854952 FORWARD | Aliases: F16L1.3, F16L1_3	3.0	2.8	0.2	1.5	100.0%	-4.3
4143	AT4G33970.1 | leucine-rich repeat family protein / extensin family protein, similar to extensin-like protein (Lycopersicon esculentum) gi:5917664:gb:AAD55979; contains leucine-rich repeats, Pfam:PF00560; contains proline rich extensin domains, INTERPRO:IPR002965	2.4	2.3	0.2	1.5	100.0%	-4.5
4144	AT5G65630.1 | DNA-binding bromodomain-containing protein, similar to 5.9 kb fsh membrane protein (Drosophila melanogaster) GI:157455; contains Pfam profile PF00439: Bromodomain | chr5:26243058-26245675 REVERSE | Aliases: K21L13.15, K21L13_15	4.6	5.2	-0.6	-1.5	100.0%	-3.9
4145	AT5G17060.1 | Gene encoding ADP-ribosylation factor and similar to ADP-ribosylation factor 1; ARF 1 (GP:385340) {Drosophila melanogaster), other ARFs and ARF-like proteins. Members of this family are known to be essential for vesicle coating and uncoating and functions in GTP-binding. | chr5:5610809-5613063 FORWARD | Aliases: F2K13.210, F2K13_210	6.9	6.3	0.6	1.5	100.0%	-4.0
4146	AT2G27010.1 | Symbol: CYP705A9 | cytochrome P450 family protein, similar to Cytochrome P450 93A1 (SP:Q42798) {Glycine max}; | chr2:11533246-11534932 REVERSE | Aliases: T20P8.6, T20P8_6	4.4	3.4	1.0	1.5	100.0%	-3.6
4147	AT5G09310.1 | expressed protein | chr5:2887194-2888528 REVERSE | Aliases: T5E8.110, T5E8_110	7.9	7.1	0.8	1.5	100.0%	-3.7
4148	AT5G55220.1 | trigger factor type chaperone family protein, contains Pfam profiles PF05697: Bacterial trigger factor protein (TF), PF05698: Bacterial trigger factor protein (TF) C-terminus, PF00254: peptidyl-prolyl cis-trans isomerase, FKBP-type | chr5:22414842-22418062 FORWARD | Aliases: MCO15.17, MCO15_17	3.3	3.7	-0.4	-1.5	100.0%	-3.9
4149	AT5G60360.2 | Symbol: AALP | similar to cysteine proteinase, putative [Arabidopsis thaliana] (TAIR:At3g45310.1); similar to cysteine protease [Nicotiana tabacum] (GB:BAA96501.1); contains InterPro domain Papain cysteine protease (C1) (InterPro:IPR000668); contains InterPro domain Eukaryotic thiol (cysteine) protease (InterPro:IPR000169) | chr5:24297123-24299623 FORWARD | Aliases: MUF9.4, MUF9_4	11.4	10.4	1.0	1.5	100.0%	-3.9

Rank	Description	15.dpi	5.dpi	M	t	adj.q	B
4150	AT5G38830.1 | tRNA synthetase class I (C) family protein, similar to SP:Q06752 Cysteinyl-tRNA synthetase (EC 6.1.1.16) (Cysteine--tRNA ligase) (CysRS) {Bacillus subtilis}; contains Pfam profile PF01406: tRNA synthetases class I (C) | chr5:15562994-15565382 REVERSE | Aliases: K15E6.3, K15E6_3	8.3	9.0	-0.7	-1.5	100.0%	-3.8
4151	AT4G11130.1 | Symbol: RDR2 | RNA-dependent RNA polymerase, putative, similar to RNA-directed RNA polymerase (Lycopersicon esculentum) gi:4038592:emb:CAA71421	4.4	4.7	-0.3	-1.5	100.0%	-4.2
4152	AT4G01940.1 | Symbol: NFU1 | nitrogen fixation NifU-like family protein, similar to apricot NifU homolog partial CDS, GenBank accession number U95179; contains Pfam profile: PF01106 NifU-like domain | chr4:841989-843459 REVERSE | Aliases: ATCNFU1, T7B11.20, T7B11_20	5.2	4.7	0.5	1.5	100.0%	-3.6
4153	AT4G00780.1 | meprin and TRAF homology domain-containing protein / MATH domain-containing protein, contains Pfam profile PF00917: MATH domain | chr4:334733-336310 FORWARD | Aliases: A_TM018A10.12, A_TM018A10_12, T18A10.3, T18A10_3	2.5	2.3	0.2	1.5	100.0%	-4.5
4154	AT2G25300.1 | similar to galactosyltransferase family protein [Arabidopsis thaliana] (TAIR:At4g32120.1); similar to beta 1,3-glycosyltransferase-like protein I [Lycopersicon esculentum] (GB:CAD30015.1); contains InterPro domain Glycosyl transferase, family 31 (InterPro:IPR002659) | chr2:10778773-10781373 REVERSE | Aliases: T22F11.11, T22F11_11	7.3	6.7	0.6	1.5	100.0%	-3.7
4155	AT1G68130.1 | zinc finger (C2H2 type) family protein, contains Pfam domain, PF00096: Zinc finger, C2H2 type | chr1:25535907-25538249 FORWARD | Aliases: T23K23.2, T23K23_2	4.0	3.4	0.6	1.5	100.0%	-3.8
4156	AT3G14900.1 | expressed protein | chr3:5013299-5015284 REVERSE | Aliases: K15M2.4	4.5	5.5	-1.0	-1.5	100.0%	-3.6
4157	AT3G07000.1 | DC1 domain-containing protein, contains Pfam profile PF03107: DC1 domain	4.6	4.2	0.4	1.5	100.0%	-3.8
4158	AT3G15610.1 | transducin family protein / WD-40 repeat family protein, contains 7 WD-40 repeats (PF00400); similar to serine/threonine kinase receptor associated protein GB:NP_035629 (SP:Q9Z1Z2) (Mus musculus); UNR-interacting protein GB:NP_009109 (Homo sapiens)	9.9	10.2	-0.3	-1.5	100.0%	-4.3
4159	AT5G49110.1 | expressed protein, ; expression supported by MPSS | chr5:19917429-19923151 FORWARD | Aliases: K20J1.8, K20J1_8	3.5	3.8	-0.2	-1.5	100.0%	-4.4
4160	AT4G21960.1 | Symbol: PRXR1 | peroxidase 42 (PER42) (P42) (PRXR1), identical to SP:Q9SB81 Peroxidase 42 precursor (EC 1.11.1.7) (Atperox P42) (PRXR1) (ATP1a/ATP1b) {Arabidopsis thaliana} | chr4:11646186-11648373 REVERSE | Aliases: F1N20.3, PEROXIDASE PRXR1	13.0	12.3	0.7	1.5	100.0%	-4.6
4161	AT3G55140.2 | pectate lyase family protein, similar to pollen allergen Amb a 1.3 SP:P27761 from (Ambrosia artemisiifolia) | chr3:20449760-20451404 FORWARD | Aliases: T26I12.20	7.2	7.7	-0.6	-1.5	100.0%	-3.6
4162	AT2G35650.1 | Symbol: ATCSLA07 | glycosyl transferase family 2 protein, similar to beta-(1-3)-glucosyl transferase GB:AAC62210 GI:3687658 from (Bradyrhizobium japonicum), cellulose synthase from Agrobacterium tumeficiens (gi:710492) and Agrobacterium radiobacter (gi:710493); contains Pfam glycosyl transferase, group 2 family protein domain PF00535; identical to cDNA for partial mRNA for glycosyltransferase (cslA07 gene) GI:28551963 | chr2:14992567-14995436 FORWARD | Aliases: ATCSLA7, CELLULOSE SYNTHASE LIKE, CSLA07, CSLA7, T20F21.16, T20F21_16	5.6	5.9	-0.3	-1.5	100.0%	-4.1
4163	AT1G09140.2 | Symbol: ATSRP30.1 | similar to pre-mRNA splicing factor SF2 (SF2) / SR1 protein [Arabidopsis thaliana] (TAIR:At1g02840.1); similar to pre-mRNA splicing factor SF2 (SF2) / SR1 protein [Arabidopsis thaliana] (TAIR:At1g02840.3); similar to ASF/SF2-like pre-mRNA splicing factor SRP32 [Zea mays] (GB:AAU29328.1); contains InterPro domain RNA-binding region RNP-1 (RNA recognition motif) (InterPro:IPR000504) | chr1:2942566-2945961 REVERSE | Aliases: ATSRP30, T12M4.19, T12M4_19	6.3	5.7	0.6	1.5	100.0%	-3.8
4164	AT1G33820.1 | hypothetical protein | chr1:12278418-12279431 REVERSE | Aliases: F14M2.4, F14M2_4	2.4	2.6	-0.2	-1.5	100.0%	-4.9
4165	AT5G56100.1 | glycine-rich protein / oleosin, contains Pfam PF01277: Oleosin domain | chr5:22734341-22734977 FORWARD | Aliases: MDA7.16, MDA7_16	7.7	6.6	1.1	1.5	100.0%	-3.7
4166	AT5G54110.1 | Symbol: ATMAMI |	6.9	6.6	0.3	1.5	100.0%	-4.1
4167	AT2G35160.1 | Symbol: SUVH5 | SET domain-containing protein (SUVH5), identical to SUVH5 (Arabidopsis thaliana) GI:13517751; contains Pfam profiles PF00856: SET domain, PF05033: Pre-SET motif, PF02182: YDG/SRA domain; identical to cDNA SUVH5 (SUVH5) GI:13517750	3.8	4.1	-0.3	-1.5	100.0%	-4.2
4168	AT1G49600.1 | Symbol: ATRBP47A | RNA-binding protein 47 (RBP47), putative, similar to DNA binding protein ACBF GB:U90212 GI:1899187 from (Nicotiana tabacum) | chr1:18360554-18363818 REVERSE | Aliases: ATRBP47A, F14J22.16, F14J22_16	5.0	5.5	-0.5	-1.5	100.0%	-3.8
4169	AT2G07340.2 | prefoldin-related KE2 family protein, contains similarity to Swiss-Prot:O60925 prefoldin subunit 1 (Homo sapiens); contains Pfam domain, PF01920: KE2 family protein | chr2:3045556-3046716 FORWARD | Aliases: T13E11.11, T13E11_11	9.6	10.0	-0.4	-1.5	100.0%	-3.9

Rank	Description	15.dpi	5.dpi	M	t	adj.q	B
4170	AT5G08190.1 | TATA-binding protein-associated phosphoprotein Dr1 protein, putative, similar to Dr1 protein homolog (SP:P49592) (Arabidopsis thaliana); similar to TATA-binding protein-associated phosphoprotein (Down-regulator of transcription 1) (DR1 protein) (SP:Q01658) (Homo sapiens); contains Pfam PF00808 : Histone-like transcription factor (CBF/NF-Y) and archaeal histone | chr5:2635907-2637431 FORWARD | Aliases: T22D6.130, T22D6_130	5.5	5.0	0.5	1.5	100.0%	-3.8
4171	AT1G63300.1 | expressed protein, similar to Intracellular protein transport protein USO1 (Swiss-Prot:P25386) (Saccharomyces cerevisiae); similar to Myosin II heavy chain, non muscle (Swiss-Prot:P08799) (Dictyostelium discoideum) | chr1:23485858-23489885 FORWARD | Aliases: F9N12.8, F9N12_8	3.9	4.4	-0.5	-1.5	100.0%	-3.9
4172	AT3G58650.1 | expressed protein | chr3:21707223-21709828 REVERSE | Aliases: F14P22.240	2.8	3.0	-0.2	-1.5	100.0%	-4.6
4173	AT4G18010.2 | Symbol: IP5PII |	5.6	4.9	0.6	1.5	100.0%	-3.8
4174	AT4G34340.1 | expressed protein | chr4:16426643-16428014 REVERSE | Aliases: F10M10.110, F10M10_110	4.3	4.9	-0.7	-1.5	100.0%	-3.7
4175	AT1G63810.1 | nucleolar RNA-associated family protein / Nrap family protein, contains Pfam profile PF03813: Nrap protein; similar to nucleolar RNA-associated protein alpha (GI:18539461) (Mus musculus) | chr1:23673215-23679223 REVERSE | Aliases: T12P18.17, T12P18_17	4.3	4.9	-0.6	-1.5	100.0%	-3.7
4176	AT5G27560.1 | expressed protein, hypothetical protein slr1702 - Synechocystis sp., PIR:S75312 | chr5:9731731-9733921 FORWARD | Aliases: F15A18.20, F15A18_20	8.4	7.9	0.5	1.5	100.0%	-3.6
4177	AT5G10260.1 | Ras-related GTP-binding protein, putative, similar to Ras-related protein Rab-6A SP:P20340 from (Homo sapiens) | chr5:3220064-3221516 FORWARD | Aliases: F18D22.30, F18D22_30	3.6	4.0	-0.5	-1.5	100.0%	-3.8
4178	AT3G62790.1 | NADH-ubiquinone oxidoreductase-related, contains weak similarity to NADH-ubiquinone oxidoreductase 15 kDa subunit (EC 1.6.5.3) (EC 1.6.99.3) (Complex I-15 kDa) (CI-15 kDa) (Swiss-Prot:O43920) (Homo sapiens) | chr3:23234059-23235283 REVERSE | Aliases: F26K9.220	10.7	11.0	-0.3	-1.5	100.0%	-4.3
4179	AT5G22570.1 | Symbol: WRKY38 |	2.6	2.3	0.2	1.5	100.0%	-4.6
4180	AT3G03950.3 | similar to YT521-B-like family protein [Arabidopsis thaliana] (TAIR:At5g61020.2); similar to YT521-B-like family protein [Arabidopsis thaliana] (TAIR:At5g61020.1); similar to high-glucose-regulated protein 8-like [Oryza sativa (japonica cultivar-group)] (GB:XP_476753.1); contains InterPro domain YT521-B-like protein (InterPro:IPR007275) | chr3:1021267-1024016 FORWARD | Aliases: T11I18.6, T11I18_6	7.3	8.2	-0.9	-1.5	100.0%	-3.4
4181	AT2G21290.1 | expressed protein | chr2:9119937-9120411 FORWARD | Aliases: F3K23.5, F3K23_5	11.0	10.6	0.4	1.5	100.0%	-4.4
4182	AT5G66960.1 | prolyl oligopeptidase family protein, similar to OpdB (Treponema denticola) GI:13786054; contains Pfam profiles PF00326: prolyl oligopeptidase family, PF02897: Prolyl oligopeptidase, N-terminal beta-propeller domain | chr5:26753435-26756719 REVERSE | Aliases: K8A10.3, K8A10_3	3.3	3.5	-0.2	-1.5	100.0%	-4.6
4183	AT2G02180.1 | Symbol: TOM3 | tobamovirus multiplication protein 3 (TOM3), identical to tobamovirus multiplication protein (TOM3) GI:15425641 from (Arabidopsis thaliana) | chr2:560863-563232 FORWARD | Aliases: F5O4.5, F5O4_5	5.7	5.3	0.4	1.5	100.0%	-3.8
4184	AT4G10930.1 | expressed protein | chr4:6705143-6708842 REVERSE | Aliases: F25I24.140, F25I24_140	4.8	5.3	-0.5	-1.5	100.0%	-4.1
4185	AT1G09420.1 | Symbol: G6PD4 | Encodes a protein similar to glucose-6-phosphate dehydrogenase but, based on amino acid differences in the active site and lack of activity, does not encode a functional G6PDH. The amino acid sequence for the consensus sequence of the G6PDH active site (DHYLGKE) differs in three places in this protein. gc exon splice site at 20574 is based on protein alignment, and is not confirmed experimentally. | chr1:3038464-3041806 REVERSE | Aliases: F14J9.8, F14J9_8, G6PD4, GLUCOSE 6 PHOSPHATE DEHYDROGENASE 4	5.2	4.9	0.4	1.5	100.0%	-3.8
4186	AT5G23130.1 | peptidoglycan-binding LysM domain-containing protein, contains Pfam profile PF01476: LysM domain	3.3	3.0	0.3	1.5	100.0%	-4.2
4187	AT5G62110.1 | expressed protein | chr5:24960130-24963307 REVERSE | Aliases: MTG10.14, MTG10_14	2.3	2.4	-0.1	-1.5	100.0%	-5.1
4188	AT1G79780.1 | integral membrane protein, putative, contains 1 transmembrane domain; contains plant integral membrane protein domain, TIGR01569; contains Pfam PF04535 : Domain of unknown function (DUF588); similar to putative ethylene responsive element binding protein (GI:22135858) (Arabidopsis thaliana) | chr1:30020823-30021749 FORWARD | Aliases: F20B17.26, F20B17_26	4.1	3.8	0.3	1.5	100.0%	-4.1
4189	AT5G15550.2 | transducin family protein / WD-40 repeat family protein, similar to YTM1 - Homo sapiens, EMBL:AF242546; contains Pfam PF00400: WD domain, G-beta repeat (7 copies,1 weak); | chr5:5058839-5062090 REVERSE | Aliases: T20K14.160, T20K14_160	6.6	7.2	-0.6	-1.5	100.0%	-3.6
4190	AT1G14820.3 | SEC14 cytosolic factor family protein / phosphoglyceride transfer family protein, contains Pfam PF00650 : CRAL/TRIO domain; similar to phosphatidylinositol/phosphatidylcholine transfer protein SP:P24280 (Saccharomyces cerevisiae (Baker's yeast)) | chr1:5105109-5106790 REVERSE | Aliases: F10B6.40, F10B6_40	7.2	7.7	-0.5	-1.5	100.0%	-3.5

Rank	Description	15.dpi	5.dpi	M	t	adj.q	B
4191	AT4G14540.1 | CCAAT-box binding transcription factor subunit B (NF-YB) (HAP3) (AHAP3) family, contains Pfam PF00808 : Histone-like transcription factor (CBF/NF-Y) and archaeal histone; similar to LEC1-like protein (GI:22536010) (Phaseolus coccineus) | chr4:8344612-8345214 FORWARD | Aliases: DL3310W, FCAALL.252	7.0	6.3	0.7	1.5	100.0%	-3.5
4192	AT2G16700.1 | Symbol: ADF5 | actin-depolymerizing factor 5 (ADF5), identical to SP:Q9ZNT3 Actin-depolymerizing factor 5 (ADF-5) (AtADF5) {Arabidopsis thaliana} | chr2:7251704-7252823 FORWARD | Aliases: ACTIN DEPOLYMERIZING FACTOR 5, T24I21.11, T24I21_11	7.5	7.1	0.4	1.5	100.0%	-3.8
4193	NA	12.2	12.4	-0.2	-1.5	100.0%	-4.7
4194	AT1G53430.1 | leucine-rich repeat family protein / protein kinase family protein, contains Pfam domains PF00560: Leucine Rich Repeat and PF00069: Protein kinase domain | chr1:19938835-19944756 FORWARD | Aliases: T3F20.25, T3F20_25	5.3	4.4	0.8	1.5	100.0%	-3.6
4195	AT3G53960.1 | proton-dependent oligopeptide transport (POT) family protein, contains Pfam profile: PF00854 POT family | chr3:19989100-19991912 REVERSE | Aliases: F5K20.260	5.7	5.2	0.6	1.5	100.0%	-3.5
4196	AT2G42740.1 | Symbol: RPL16A |	8.3	8.7	-0.5	-1.5	100.0%	-3.7
4197	AT2G16750.1 | protein kinase family protein, contains Pfam domain, PF00069: Protein kinase domain | chr2:7278094-7281775 FORWARD | Aliases: T24I21.16, T24I21_16	2.3	2.7	-0.4	-1.5	100.0%	-4.3
4198	AT1G48550.1 | vacuolar protein sorting-associated protein 26 family protein / VPS26 family protein, contains Pfam profile PF03643: Vacuolar protein sorting-associated protein 26 | chr1:17954247-17956361 REVERSE | Aliases: T1N15.17, T1N15_17	4.8	4.4	0.3	1.5	100.0%	-4.0
4199	AT5G58230.1 | Symbol: MSI1 | WD-40 repeat protein (MSI1), contains 6 WD-40 repeats (PF0400); identical to WD-40 repeat protein (SP:O22467) (Arabidopsis thaliana) | chr5:23573258-23575471 FORWARD | Aliases: MCK7.10, MCK7_10, MEDICIS, MULTICOPY SUPRESSOR OF IRA1	6.7	7.4	-0.7	-1.5	100.0%	-3.8
4200	AT1G68820.1 | membrane protein, putative, contains 7 transmembrane domains; similar to inhibitor of apoptosis-2 IAP-2 (GI:20043383) (Mamestra configurata nucleopolyhedrovirus) | chr1:25869337-25872648 FORWARD | Aliases: F14K14.7, F14K14_7	5.9	5.4	0.5	1.5	100.0%	-3.5
4201	AT5G58950.1 | protein kinase family protein, concontains protein kinase domain, Pfam:PF00069 | chr5:23818154-23820868 REVERSE | Aliases: K19M22.20, K19M22_20	8.5	8.1	0.5	1.5	100.0%	-3.9
4202	AT3G23210.1 | basic helix-loop-helix (bHLH) family protein, similar to hypothetical protein GB:CAB10220 from (Arabidopsis thaliana) | chr3:8283097-8285087 REVERSE | Aliases: K14B15.12	6.1	5.8	0.3	1.5	100.0%	-4.0
4203	AT1G72390.1 | expressed protein | chr1:27250615-27255238 FORWARD | Aliases: T10D10.14, T10D10_14	4.2	4.5	-0.3	-1.5	100.0%	-3.9
4204	AT3G54990.1 | Symbol: SMZ | similar to AP2 domain-containing transcription factor, putative [Arabidopsis thaliana] (TAIR:At2g39250.1); similar to PHAP2B protein [Petunia x hybrida] (GB:AAD39440.1); contains InterPro domain Pathogenesis-related transcriptional factor and ERF (InterPro:IPR001471) | chr3:20384695-20387499 REVERSE | Aliases: SCHLAFMUTZE, T15C9.6	4.9	4.2	0.7	1.5	100.0%	-3.6
4205	AT2G07240.1 | Ulp1 protease family protein, contains Pfam profile PF02902: Ulp1 protease family, C-terminal catalytic domain | chr2:3007099-3012136 REVERSE | Aliases: T13E11.1, T13E11_1	3.4	3.6	-0.2	-1.5	100.0%	-4.3
4206	AT2G07360.1 | SH3 domain-containing protein, contains Pfam profile PF00018: SH3 domain	4.3	4.5	-0.3	-1.5	100.0%	-4.3
4207	AT4G24800.2 | similar to MA3 domain-containing protein [Arabidopsis thaliana] (TAIR:At5g63190.2); similar to MA3 domain-containing protein [Arabidopsis thaliana] (TAIR:At3g48390.1); similar to MA3 domain-containing protein [Arabidopsis thaliana] (TAIR:At5g63190.1); similar to OSJNBb0011N17.20 [Oryza sativa (japonica cultivar-group)] (GB:XP_472924.1); contains InterPro domain Initiation factor eIF-4 gamma, MA3 (InterPro:IPR003891) | chr4:12781772-12785207 FORWARD | Aliases: F6I7.10, F6I7_10	5.4	5.8	-0.4	-1.5	100.0%	-3.9
4208	AT5G05520.1 | outer membrane OMP85 family protein, contains Pfam profile PF01103: outer membrane protein, OMP85 family	6.5	7.0	-0.5	-1.5	100.0%	-3.9
4209	AT1G02710.1 | glycine-rich protein | chr1:589706-589996 REVERSE | Aliases: T14P4.26, T14P4_26	6.5	5.9	0.7	1.5	100.0%	-3.7
4210	AT1G06830.1 | glutaredoxin family protein, contains INTERPRO Domain IPR002109, Glutaredoxin (thioltransferase)	3.8	3.3	0.5	1.5	100.0%	-4.0
4211	AT2G13290.2 | glycosyl transferase family 17 protein, low similarity to beta-1,4-mannosyl-glycoprotein beta-1,4-N-acetylglucosaminyltransferase (EC 2.4.1.144) from Mus musculus (SP:Q10470), Rattus norvegicus (SP:Q02527), Homo sapiens (SP:Q09327) ; contains Pfam profile PF04724 :Glycosyltransferase family 17 | chr2:5518678-5520347 FORWARD | Aliases: F15O11.7, F15O11_7	3.9	3.5	0.3	1.5	100.0%	-4.1

Rank	Description	15.dpi	5.dpi	M	t	adj.q	B
4212	AT3G43520.1 | expressed protein, contains Pfam profile PF03647: Uncharacterised protein family (UPF0136) | chr3:15417012-15418896 FORWARD | Aliases: T18D12.90	10.3	9.6	0.7	1.5	100.0%	-4.0
4213	AT1G47870.1 | Symbol: E2FC | E2F transcription factor-2 (E2F2) / transcription factor E2Fc (E2Fc), identical to transcription factor E2Fc (Arabidopsis thaliana) GI:19578311; contains Pfam profile PF02319: Transcription factor E2F/dimerisation partner; identical to cDNA E2F transcription factor-2 E2F2 GI:10443850 | chr1:17637228-17640210 FORWARD | Aliases: ATE2F2, E2FC, E2FC TRANSCRIPTION FACTOR, T2E6.2, T2E6_2, TRANSCRIPTION FACTOR 2 E2F2	6.2	6.6	-0.4	-1.5	100.0%	-3.8
4214	ATMG00640.1 | Symbol: ORF25 | encodes a plant b subunit of mitochondrial ATP synthase based on structural similarity and the presence in the F(0) complex. | chrM:188084-188662 REVERSE | Aliases: ORF25	4.4	3.5	0.9	1.5	100.0%	-3.7
4215	AT2G21340.2 | enhanced disease susceptibility protein, putative / salicylic acid induction deficient protein, putative, similar to enhanced disease susceptibility 5 (Arabidopsis thaliana) GI:16589070 | chr2:9139638-9143524 FORWARD | Aliases: F3K23.10, F3K23_10	6.3	6.6	-0.3	-1.5	100.0%	-4.0
4216	AT3G59530.2 | strictosidine synthase family protein, similar to strictosidine synthase (Rauvolfia serpentina)(SP:P15324) | chr3:22004259-22006238 FORWARD | Aliases: T16L24.80	4.8	4.4	0.4	1.5	100.0%	-4.2
4217	AT1G06450.1 | CCR4-NOT transcription complex protein, putative, similar to SWISS-PROT:Q9UFF9 CCR4-NOT transcription complex, subunit 8 (CAF1-like protein, CALIFp) (Homo sapiens) | chr1:1965839-1967239 FORWARD | Aliases: F12K11.20, F12K11_20	3.7	4.3	-0.6	-1.5	100.0%	-3.8
4218	AT3G05500.1 | rubber elongation factor (REF) family protein, contains Pfam profile: PF05755 rubber elongation factor protein (REF) | chr3:1593440-1595021 FORWARD | Aliases: F22F7.5, F22F7_5	8.7	7.8	0.9	1.5	100.0%	-3.8
4219	AT3G55970.1 | oxidoreductase, 2OG-Fe(II) oxygenase family protein, similar to leucoanthocyanidin dioxygenase, Malus domestica, SP:P51091; contains Pfam profile PF03171: oxidoreductase, 2OG-Fe(II) oxygenase family | chr3:20777718-20780303 REVERSE | Aliases: F27K19.150	3.4	3.8	-0.4	-1.5	100.0%	-3.7
4220	AT4G21300.1 | pentatricopeptide (PPR) repeat-containing protein, contains INTERPRO:IPR002885 PPR repeats | chr4:11336489-11339062 FORWARD | Aliases: T6K22.30, T6K22_30	3.5	3.3	0.2	1.5	100.0%	-4.3
4221	AT4G13690.1 | expressed protein | chr4:7953658-7955090 REVERSE | Aliases: F18A5.80, F18A5_80	3.7	4.3	-0.6	-1.5	100.0%	-3.7
4222	AT2G18440.1 | Symbol: GUT15 | expressed protein | chr2:8002811-8004000 REVERSE | Aliases: GENE WITH UNSTABLE TRANSCRIPT 15, GUT15, T30D6.5, T30D6_5	8.5	7.7	0.8	1.5	100.0%	-3.9
4223	AT3G07040.1 | Symbol: RPM1 | disease resistance protein RPM1 (CC-NBS-LRR class), putative, domain signature CC-NBS-LRR exists, suggestive of a disease resistance protein. Identical to RPM1 (gi:1361985) | chr3:2225958-2229030 REVERSE | Aliases: DISEASE RESISTANCE PROTEIN RPM1, F17A9.20, RESISTANCE TO P. SYRINGAE PV MACULICOLA 1, RESISTANCE TO PSEUDOMONAS SYRINGAE 3, RPS3	3.4	3.1	0.3	1.5	100.0%	-4.2
4224	AT1G63770.2 | peptidase M1 family protein, similar to SP:P04825 Aminopeptidase N (EC 3.4.11.2) (Alpha-aminoacylpeptide hydrolase) {Escherichia coli}; contains Pfam profile PF01433: Peptidase family M1 | chr1:23661169-23667906 REVERSE | Aliases: F24D7.4, F24D7_4	10.4	11.1	-0.7	-1.5	100.0%	-4.0
4225	AT5G53450.3 | Symbol: ORG1 | expressed protein, contains InterPro domain Serine/threonine protein kinase (InterPro:IPR002290); contains InterPro domain Protein kinase (InterPro:IPR000719) | chr5:21705999-21709677 FORWARD | Aliases: MYN8.6, MYN8_6	7.8	7.4	0.4	1.5	100.0%	-4.0
4226	AT1G51420.1 | sucrose-phosphatase, putative, similar to sucrose-phosphatase (SPP1) (Arabidopsis thaliana) GI:11127757; contains Pfam profile PF05116: Sucrose-6F-phosphate phosphohydrolase | chr1:19068520-19070372 REVERSE | Aliases: F5D21.9, F5D21_9	4.0	4.8	-0.8	-1.5	100.0%	-3.7
4227	AT1G16020.2 | expressed protein | chr1:5498159-5501352 FORWARD | Aliases: T24D18.12, T24D18_12	4.9	5.3	-0.4	-1.5	100.0%	-3.9
4228	AT2G44120.2 | 60S ribosomal protein L7 (RPL7C)	8.3	8.8	-0.6	-1.5	100.0%	-3.9
4229	AT4G17430.1 | expressed protein, weak similarity to CigA protein (Mucor circinelloides) GI:9717020 | chr4:9724852-9726840 REVERSE | Aliases: DL4750C, FCAALL.1	4.7	5.1	-0.4	-1.5	100.0%	-3.9
4230	AT3G51250.1 | senescence/dehydration-associated protein-related, similar to senescence-associated protein 12 (Hemerocallis hybrid cultivar) gi:3551958:gb:AAC34857; similar to early-responsive to dehydration stress ERD7 protein (Arabidopsis thaliana) gi:15320412:dbj:BAB63916 | chr3:19039182-19041655 FORWARD | Aliases: F24M12.290	7.1	6.5	0.6	1.5	100.0%	-3.6
4231	AT2G16060.1 | Symbol: AHB1 | non-symbiotic hemoglobin 1 (HB1) (GLB1), identical to SP:O24520 Non-symbiotic hemoglobin 1 (Hb1) (ARAth GLB1) {Arabidopsis thaliana} | chr2:6989703-6990713 REVERSE | Aliases: ARATH GLB1, F7H1.8, F7H1_8, GLB1, HEMOGLOBIN, NSHB1	10.6	10.1	0.5	1.5	100.0%	-3.7

Rank	Description	15.dpi	5.dpi	M	t	adj.q	B
4232	AT3G15180.1 | proteasome-related, similar to 26S proteasome non-ATPase regulatory subunit 5 (26S proteasome subunit S5B) (26S protease subunit S5 basic) (Swiss-Prot:Q16401) (Homo sapiens) | chr3:5111900-5115993 FORWARD | Aliases: F4B12.9	6.0	6.3	-0.2	-1.5	100.0%	-4.2
4233	AT3G60200.1 | expressed protein, hypothetical proteins At2g44600 - Arabidopsis thaliana, EMBL:AAC27462	4.3	4.0	0.3	1.5	100.0%	-4.1
4234	AT5G02560.1 | histone H2A, putative, similar to histone H2A from Pisum sativum SP:P25470, Zea mays SP:P40280, Petroselinum crispum SP:P19177; contains Pfam profile PF00125 Core histone H2A/H2B/H3/H4	7.8	7.5	0.3	1.5	100.0%	-4.0
4235	AT1G09280.2 | expressed protein, contains Pfam profile: PF03959 domain of unknown function (DUF341) | chr1:2997552-3001296 REVERSE | Aliases: T12M4.1, T12M4_1	7.1	7.4	-0.4	-1.5	100.0%	-4.3
4236	AT1G26170.1 | importin beta-2 subunit family protein, similar to Importin9 isoform 1 (Mus musculus) GI:15186756; contains Pfam profile PF03810: Importin-beta N-terminal domain | chr1:9047526-9054425 REVERSE | Aliases: F28B23.15, F28B23_15	3.5	3.8	-0.3	-1.5	100.0%	-4.2
4237	AT2G35210.2 | human Rev interacting-like family protein / hRIP family protein, similar to ARFGAP1 protein GI:7211442 from (Homo sapiens); contains InterPro accession IPR001164: Human Rev interacting-like protein (hRIP) | chr2:14843193-14845228 FORWARD | Aliases: T4C15.12, T4C15_12	3.2	2.9	0.3	1.5	100.0%	-4.4
4238	AT5G07020.1 | proline-rich family protein | chr5:2180503-2182387 REVERSE | Aliases: MOJ9.19, MOJ9_19	7.2	6.5	0.7	1.5	100.0%	-3.5
4239	AT3G51510.1 | expressed protein | chr3:19120041-19121030 FORWARD | Aliases: F26O13.150	7.2	6.4	0.8	1.5	100.0%	-3.3
4240	AT2G18950.1 | Symbol: HPT1 | homogentisate phytylprenyltransferase family protein (HPT1) / tocopherol phytyltransferase family protein (TPT1), identical to gi:17104828; contains Pfam profile PF01040: UbiA prenyltransferase family; identical to cDNA tocopherol polyprenyltransferase (TPT1) GI:17104827 | chr2:8214424-8217456 FORWARD | Aliases: ATHPT, F19F24.15, F19F24_15, HOMOGENTISATE PHYTYLTRANSFERASE, HOMOGENTISATE PHYTYLTRANSFERASE 1, HPT, TOCOPHEROL PHYTYLTRANSFERASE, TOCOPHEROL POLYPRENYLTRANSFERASE, TPT1, VITAMIN E 2, VTE2	5.2	4.6	0.6	1.5	100.0%	-3.6
4241	AT2G37060.3 | similar to CCAAT-box binding transcription factor, putative [Arabidopsis thaliana] (TAIR:At3g53340.1); similar to CAAT-box DNA binding protein subunit B (NF-YB) [Zea mays] (GB:CAA42234.1); contains InterPro domain Transcription factor CBF/NF-Y/archaeal histone (InterPro:IPR003958); contains InterPro domain Histone-like transcription factor/archaeal histone/topoisomerase (InterPro:IPR003957); contains InterPro domain Histone-fold/TFIID-TAF/NF-Y (InterPro:IPR007124); contains InterPro domain Histone-like transcription factor CBF/NF-Y/archaeal histone, subunit A (InterPro:IPR003956) | chr2:15583103-15584956 FORWARD | Aliases: T2N18.18, T2N18_18	3.5	4.1	-0.6	-1.5	100.0%	-3.6
4242	AT5G37780.1 | Symbol: CAM1 | calmodulin-1/4 (CAM1), identical to calmodulin 4 (Arabidopsis thaliana) GI:16223, SP:P25854 Calmodulin-1/4 {Arabidopsis thaliana} | chr5:15021763-15023435 REVERSE | Aliases: ACAM 1, CALMODULIN 1, CAM1, K22F20.20, K22F20_20, TCH1	12.2	11.5	0.7	1.5	100.0%	-4.3
4243	AT3G08990.1 | yippee family protein, similar to qdgl-1 (Coturnix coturnix) GI:10441650, Yippee protein (Drosophila melanogaster) GI:5713279; contains Pfam profile PF03226: Yippee putative zinc-binding protein | chr3:2743081-2744937 FORWARD | Aliases: T16O11.5	6.1	5.6	0.5	1.5	100.0%	-3.7
4244	AT1G30550.1 | expressed protein, similar to PIMT (GI:15127914) (Mus musculus); similar to hypothetical protein GB:AAF19758 GI:6634778 from (Arabidopsis thaliana) | chr1:10820682-10823623 REVERSE | Aliases: F26G16.17, F26G16_17	4.4	3.9	0.5	1.5	100.0%	-4.0
4245	AT1G22380.1 | similar to UDP-glucose glucosyltransferase, putative [Arabidopsis thaliana] (TAIR:At1g78270.1); similar to UDP-glucoronosyl/UDP-glucosyl transferase family protein [Arabidopsis thaliana] (TAIR:At1g22340.1); similar to UDP-glucoronosyl/UDP-glucosyl transferase family protein [Arabidopsis thaliana] (TAIR:At1g22360.1); similar to UDP-glucoronosyl/UDP-glucosyl transferase family protein [Arabidopsis thaliana] (TAIR:At1g22400.1); similar to UDP-glucoronosyl/UDP-glucosyl transferase family protein [Arabidopsis thaliana] (TAIR:At1g22370.2); similar to glycosyltransferase NTGT5a [Nicotiana tabacum] (GB:BAD93689.1); contains InterPro domain UDP-glucoronosyl/UDP-glucosyl transferase (InterPro:IPR002213) | chr1:7900376-7902321 REVERSE | Aliases: F12K8.28	3.1	2.8	0.3	1.5	100.0%	-4.2
4246	AT1G26190.1 | phosphoribulokinase/uridine kinase family protein, weak similarity to SP:Q59190 Uridine kinase (EC 2.7.1.48) (Uridine monophosphokinase) (Cytidine monophosphokinase) {Borrelia burgdorferi}; contains Pfam profile PF00485: Phosphoribulokinase / Uridine kinase family	3.2	3.5	-0.3	-1.5	100.0%	-4.1
4247	AT1G23760.1 | Symbol: JP630 | BURP domain-containing protein / polygalacturonase, putative, similar to polygalacturonase isoenzyme 1 beta subunit (Lycopersicon esculentum) GI:170480; contains Pfam profile PF03181: BURP domain | chr1:8402035-8404280 FORWARD | Aliases: F5O8.31, F5O8_31, JP630	3.7	4.6	-0.9	-1.5	100.0%	-3.5
4248	AT4G35840.1 | zinc finger (C3HC4-type RING finger) family protein, contains a TG non-consensus donor splice site at exon 2; contains Pfam profile PF00097: Zinc finger, C3HC4 type (RING finger) | chr4:16980965-16982368 FORWARD | Aliases: F4B14.110, F4B14_110	9.0	8.2	0.8	1.5	100.0%	-3.8
4249	AT5G25470.2 | expressed protein | chr5:8865732-8867688 REVERSE | Aliases: T14C9.20, T14C9_20	5.3	4.7	0.7	1.5	100.0%	-3.4
4250	AT4G27850.1 | proline-rich family protein, contains proline-rich extensin domains, INTERPRO:IPR002965 | chr4:13870388-13873515 FORWARD | Aliases: T27E11.90, T27E11_90	3.3	3.1	0.2	1.5	100.0%	-4.3

Rank	Description	15.dpi	5.dpi	M	t	adj.q	B
4251	AT1G78895.1 | expressed protein | chr1:29663009-29664869 REVERSE | Aliases: None	6.2	5.2	1.0	1.5	100.0%	-3.6
4252	AT3G06170.1 | TMS membrane family protein / tumour differentially expressed (TDE) family protein, contains Pfam domain, PF03348: TMS membrane protein/tumour differentially expressed protein (TDE) | chr3:1867518-1869944 FORWARD | Aliases: F28L1.11, F28L1_11	5.7	4.7	1.1	1.5	100.0%	-3.7
4253	AT3G05940.1 | expressed protein, contains Pfam profile PF03619: Domain of unknown function | chr3:1777370-1779654 REVERSE | Aliases: F2O10.10	5.3	4.8	0.5	1.5	100.0%	-3.7
4254	AT3G23900.2 | similar to expressed protein [Arabidopsis thaliana] (TAIR:At3g28770.1); similar to B52-prov protein [Xenopus laevis] (GB:AAH44265.1); contains InterPro domain Filamin/ABP280 repeat (InterPro:IPR001298); contains InterPro domain RNA-binding region RNP-1 (RNA recognition motif) (InterPro:IPR000504) | chr3:8632698-8636052 REVERSE | Aliases: F14O13.8	4.3	5.3	-1.0	-1.5	100.0%	-3.4
4255	AT3G60680.1 | expressed protein, contains Pfam profile PF04859: Plant protein of unknown function (DUF641 | chr3:22440935-22443017 FORWARD | Aliases: T4C21.90	4.3	3.9	0.4	1.5	100.0%	-3.9
4256	AT5G08310.1 | pentatricopeptide (PPR) repeat-containing protein, contains Pfam profile PF01535: PPR repeat | chr5:2670135-2675255 REVERSE | Aliases: F8L15.40, F8L15_40	3.6	4.2	-0.6	-1.5	100.0%	-3.9
4257	AT5G52760.1 | heavy-metal-associated domain-containing protein, contains Pfam profile PF00403: Heavy-metal-associated domain | chr5:21404052-21404922 FORWARD | Aliases: F6N7.25, F6N7_25	2.4	2.6	-0.2	-1.5	100.0%	-4.6
4258	AT1G05040.1 | expressed protein | chr1:1447757-1448447 REVERSE | Aliases: T7A14.9, T7A14_9	2.4	2.2	0.1	1.5	100.0%	-4.7
4259	AT3G16260.1 | metallo-beta-lactamase family protein | chr3:5509418-5513210 FORWARD | Aliases: MYA6.7	5.8	6.5	-0.7	-1.5	100.0%	-3.7
4260	AT1G01240.3 | expressed protein | chr1:99894-101834 FORWARD | Aliases: F633.5, F633_5, F6F3.27	7.0	6.5	0.5	1.5	100.0%	-4.0
4261	AT5G63110.1 | Symbol: HDA6 | histone deacetylase, putative, similar to SP:O22446 Histone deacetylase (HD) {Arabidopsis thaliana}; contains Pfam profile PF00850: Histone deacetylase family | chr5:25332863-25335573 REVERSE | Aliases: ATHDA6, AXE1, CAT1, CONSTITUTIVE EXPRESSION OF AUXIN RESPONSE TRANSGENES 1, HISTONE DEACETYLASE 6, MDC12.7, MDC12_7, RNA MEDIATED TRANSCRIPTIONAL SILENCING 1, RPD3B, RTS1	6.3	6.7	-0.4	-1.5	100.0%	-3.7
4262	AT1G77920.1 | bZIP family transcription factor, contains Pfam profile: PF00170 bZIP transcription factor | chr1:29303299-29305757 FORWARD | Aliases: F28K19.13, F28K19_13	7.9	7.5	0.4	1.5	100.0%	-3.8
4263	AT5G08360.1 | expressed protein | chr5:2689714-2690973 FORWARD | Aliases: F8L15.90, F8L15_90	4.3	3.8	0.5	1.5	100.0%	-3.6
4264	AT4G25710.1 | kelch repeat-containing F-box family protein, similar to SKP1 interacting partner 6 (Arabidopsis thaliana) GI:10716957; contains Pfam profiles PF01344: Kelch motif, PF00646: F-box domain | chr4:13098150-13099540 REVERSE | Aliases: L73G19.90, L73G19_90	6.3	5.8	0.4	1.5	100.0%	-3.8
4265	AT3G47990.1 | zinc finger (C3HC4-type RING finger) family protein, contains Pfam profile: PF00097 zinc finger, C3HC4 type (RING finger) | chr3:17724129-17727293 REVERSE | Aliases: T17F15.140	6.8	5.9	0.9	1.5	100.0%	-3.8
4266	AT1G20910.1 | ARID/BRIGHT DNA-binding domain-containing protein, low similarity to Chain A, Human Mrf-2 Domain (Homo sapiens) GI:14278238; contains Pfam profile PF01388: ARID/BRIGHT DNA binding domain | chr1:7276932-7281127 REVERSE | Aliases: F9H16.11, F9H16_11	5.3	5.8	-0.5	-1.5	100.0%	-3.8
4267	AT3G26640.1 | transducin family protein / WD-40 repeat family protein, contains 3 WD-40 repeats (PF00400); similar to ATAN11 (GI:2290528) (Arabidopsis thaliana) (Genes Dev. 11 (11), 1422-1434 (1997)); contains Pfam profile: PF00400 WD domain, G-beta repeat (3 copies) | chr3:9794459-9795694 FORWARD | Aliases: MLJ15.4	6.0	5.5	0.5	1.5	100.0%	-3.7
4268	AT2G35270.1 | DNA-binding protein-related, contains Pfam domain PF03479: Domain of unknown function (DUF296), found in AT-hook motifs Pfam:PF02178 | chr2:14864100-14864957 FORWARD | Aliases: T4C15.6, T4C15_6	4.8	3.9	0.9	1.5	100.0%	-3.6
4269	AT1G11780.1 | oxidoreductase, 2OG-Fe(II) oxygenase family protein, low similarity to alkB proteins from Homo sapiens (SP:Q13686), Caulobacter crescentus (GI:2055386); contains Pfam profile PF03171: oxidoreductase, 2OG-Fe(II) oxygenase family | chr1:3976838-3979194 REVERSE | Aliases: F25C20.6, F25C20_6	4.5	5.0	-0.5	-1.5	100.0%	-3.7
4270	AT1G43640.1 | F-box family protein / tubby family protein, contains Pfam profile: PF01167: Tub family; contains Pfam PF00646: F-box domain; similar to Chain A, C-Terminal Domain Of Mouse Brain Tubby Protein Length(GI:6730158) (Mus musculus); similar to Tubby related protein 1 (Tubby-like protein 1) (Swiss-Prot:O00294) (Homo sapiens); similar to phosphodiesterase (GI:467578) (Mus musculus) | chr1:16441875-16444284 REVERSE | Aliases: T10P12.9, T10P12_9	3.0	2.8	0.3	1.5	100.0%	-4.3
4271	AT3G07190.1 | expressed protein | chr3:2285811-2287161 REVERSE | Aliases: T1B9.14	6.1	5.6	0.5	1.5	100.0%	-3.6
4272	AT2G35610.1 | expressed protein | chr2:14954444-14958326 REVERSE | Aliases: T20F21.19, T20F21_19	4.9	5.5	-0.6	-1.5	100.0%	-3.6

Rank	Description	15.dpi	5.dpi	M	t	adj.q	B
4273	AT4G01210.1 | glycosyltransferase family protein 1, contains Pfam profile: PF00534 Glycosyl transferases group 1 | chr4:507738-512782 REVERSE | Aliases: F2N1.24, F2N1_24	7.5	8.3	-0.8	-1.5	100.0%	-4.0
4274	AT4G04330.1 | expressed protein | chr4:2116551-2118592 REVERSE | Aliases: T19B17.5, T19B17_5	3.0	2.7	0.3	1.5	100.0%	-4.3
4275	AT1G32060.1 | phosphoribulokinase (PRK) / phosphopentokinase, nearly identical to SP:P25697 Phosphoribulokinase, chloroplast precursor (EC 2.7.1.19) (Phosphopentokinase) (PRKASE) (PRK) {Arabidopsis thaliana} | chr1:11532530-11534619 FORWARD | Aliases: T12O21.4, T12O21_4	4.2	3.8	0.4	1.5	100.0%	-3.8
4276	AT3G07010.1 | pectate lyase family protein, similar to pectate lyase GP:14531296 from (Fragaria x ananassa) | chr3:2212734-2216489 REVERSE | Aliases: F17A9.16	3.8	4.3	-0.5	-1.5	100.0%	-3.9
4277	AT4G30690.1 | translation initiation factor 3 (IF-3) family protein, similar to SP:P33319 Translation initiation factor IF-3 {Proteus vulgaris}; contains Pfam profiles PF00707: Translation initiation factor IF-3, C-terminal domain, PF05198: Translation initiation factor IF-3, N-terminal domain	7.3	6.1	1.2	1.5	100.0%	-3.7
4278	AT1G04660.1 | glycine-rich protein | chr1:1300485-1301772 REVERSE | Aliases: T1G11.8, T1G11_8	6.7	6.2	0.5	1.5	100.0%	-4.1
4279	AT4G16100.1 | expressed protein | chr4:9105731-9108169 FORWARD | Aliases: DL4090W, FCAALL.70	5.6	5.1	0.4	1.5	100.0%	-4.0
4280	AT1G27150.1 | expressed protein | chr1:9429044-9432235 FORWARD | Aliases: T7N9.21, T7N9_21	8.9	9.2	-0.3	-1.5	100.0%	-4.2
4281	AT5G35840.1 | Symbol: PHYC | phytochrome C (PHYC), identical to SP:P14714 Phytochrome C {Arabidopsis thaliana} | chr5:14025056-14028994 FORWARD | Aliases: MIK22.15, MIK22_15, PHYTOCHROME C, PHYTOCHROME DEFECTIVE C	4.5	5.0	-0.5	-1.5	100.0%	-3.9
4282	AT1G60070.1 | gamma-adaptin, putative, similar to gamma-adaptin GI:2765190 from (Homo sapiens); contains Pfam profiles PF01602: Adaptin N terminal region, PF02883: Adaptin C-terminal domain | chr1:22146347-22153116 REVERSE | Aliases: T2K10.12, T2K10_12	5.8	6.6	-0.8	-1.5	100.0%	-4.0
4283	AT5G24760.2 | alcohol dehydrogenase, putative, similar to alcohol dehydrogenase from Solanum tuberosum (SP:p14673); contains Pfam zinc-binding dehydrogenase domain PF00107 | chr5:8494795-8497303 REVERSE | Aliases: T4C12.30	8.6	9.2	-0.6	-1.5	100.0%	-3.8
4284	AT1G70650.1 | zinc finger (Ran-binding) family protein, contains Pfam domain, PF00641: Zn-finger in Ran binding protein and others | chr1:26641740-26643867 FORWARD | Aliases: F5A18.17, F5A18_17	3.5	3.9	-0.4	-1.5	100.0%	-4.2
4285	AT3G01720.1 | expressed protein | chr3:262258-265912 REVERSE | Aliases: F4P13.27	7.3	7.9	-0.7	-1.5	100.0%	-3.7
4286	AT5G39740.1 | 60S ribosomal protein L5 (RPL5B), ribosomal protein L5, rice | chr5:15920551-15922688 FORWARD | Aliases: MKM21.30, MKM21_30	8.1	9.4	-1.2	-1.5	100.0%	-3.7
4287	AT5G52400.1 | Symbol: CYP715A1 | cytochrome P450 family protein, similar to Cytochrome P450 72A1 (SP:Q05047) (cytochrome P450 lxxii hydroxylase) (ge10h) (Catharanthus roseus) | chr5:21290175-21292735 FORWARD | Aliases: K24M7.14, K24M7_14	3.5	3.1	0.3	1.5	100.0%	-4.2
4288	AT3G54940.3 | cysteine proteinase, putative, contains similarity to cysteine proteinase GI:479060 from (Glycine max) | chr3:20365330-20367245 FORWARD | Aliases: F28P10.80	3.9	3.0	1.0	1.5	100.0%	-3.6
4289	AT1G14270.2 | CAAX amino terminal protease family protein, contains Pfam profile PF02517: CAAX amino terminal protease family | chr1:4874870-4877221 REVERSE | Aliases: F14L17.3, F14L17_3	5.7	5.3	0.4	1.5	100.0%	-3.9
4290	AT3G15060.1 | Ras-related GTP-binding family protein, similar to GTP-binding protein GI:303742 from (Pisum sativum); contains Pfam profile: PF00071 ras family	4.8	4.3	0.5	1.5	100.0%	-3.8
4291	AT4G27260.1 | Symbol: GH3.5 | encodes an IAA-amido synthase that conjugates Asp and other amino acids to auxin in vitro. Lines carrying insertions in this gene are hypersensitive to auxin. | chr4:13653624-13656210 FORWARD | Aliases: GH3.5, M4I22.70, M4I22_70	4.5	4.2	0.3	1.5	100.0%	-4.0
4292	AT5G64650.1 | ribosomal protein L17 family protein, contains Pfam profile: PF01196 ribosomal protein L17 | chr5:25856586-25858035 REVERSE | Aliases: MUB3.17, MUB3_17	7.2	7.8	-0.6	-1.5	100.0%	-3.6
4293	AT3G19470.2 | similar to F-box family protein-related [Arabidopsis thaliana] (TAIR:At3g22770.1); similar to S2 self-incompatibility locus-linked putative F-box protein S2-A113 [Petunia integrifolia subsp. inflata] (GB:AAR15912.2); contains InterPro domain Cyclin-like F-box (InterPro:IPR001810); contains InterPro domain F-box protein interaction domain (InterPro:IPR006527)	3.8	4.3	-0.5	-1.5	100.0%	-3.7
4294	AT1G66530.1 | arginyl-tRNA synthetase, putative / arginine--tRNA ligase, putative, similar to SP:P37880 Arginyl-tRNA synthetase (EC 6.1.1.19) (Arginine--tRNA ligase) (ArgRS) {Cricetulus longicaudatus}; contains Pfam profiles PF00750: arginyl-tRNA synthetase, PF03485: arginyl-tRNA synthetase N-terminal domain | chr1:24822574-24826005 REVERSE | Aliases: F28G11.14, F28G11_14	8.8	9.7	-0.8	-1.5	100.0%	-3.6

Rank	Description	15.dpi	5.dpi	M	t	adj.q	B
4295	AT4G16745.1 | exostosin family protein, contains Pfam PF03016: Exostosin family | chr4:9411914-9414133 FORWARD | Aliases: None	3.6	3.1	0.5	1.5	100.0%	-3.8
4296	AT3G12860.1 | nucleolar protein Nop56, putative, similar to XNop56 protein (Xenopus laevis) GI:14799394; contains Pfam profile PF01798: Putative snoRNA binding domain	6.1	6.7	-0.6	-1.5	100.0%	-3.8
4297	AT5G61020.2 | YT521-B-like family protein, contains Pfam profile PF04146: YT521-B-like family | chr5:24574427-24577105 REVERSE | Aliases: MAF19.3, MAF19_3	6.9	7.8	-0.9	-1.5	100.0%	-3.4
4298	AT4G25480.1 | Symbol: DREB1A |	3.4	2.9	0.5	1.5	100.0%	-3.9
4299	AT5G14710.1 | expressed protein | chr5:4746365-4747936 FORWARD | Aliases: T9L3.10, T9L3_10	7.2	6.5	0.7	1.5	100.0%	-3.7
4300	AT1G11170.2 | expressed protein, contains Pfam profile PF05212: Protein of unknown function (DUF707) | chr1:3741129-3744675 FORWARD | Aliases: T28P6.16, T28P6_16	3.8	3.5	0.3	1.5	100.0%	-4.0
4301	AT1G73230.1 | nascent polypeptide-associated complex (NAC) domain-containing protein, similar to SP:P20290 Transcription factor BTF3 (RNA polymerase B transcription factor 3) {Homo sapiens}; contains Pfam profile PF01849: NAC domain | chr1:27543969-27545603 REVERSE | Aliases: T18K17.10, T18K17_10	10.1	10.8	-0.7	-1.5	100.0%	-3.7
4302	AT4G35510.1 | expressed protein | chr4:16860457-16862377 REVERSE | Aliases: F8D20.20, F8D20_20	5.9	6.5	-0.6	-1.5	100.0%	-3.6
4303	AT4G27510.1 | expressed protein | chr4:13745615-13749037 REVERSE | Aliases: F27G19.110, F27G19_110	3.9	4.3	-0.4	-1.5	100.0%	-3.9
4304	AT1G03320.1 | expressed protein | chr1:817027-817767 FORWARD | Aliases: F15K9.8, F15K9_8	2.7	2.5	0.2	1.5	100.0%	-4.4
4305	AT5G27050.1 | MADS-box family protein, contains similarity to hypothetical proteins of (Arabidopsis thaliana) | chr5:9520279-9520641 FORWARD | Aliases: F15P11.2, F15P11_2	3.9	3.7	0.3	1.5	100.0%	-4.3
4306	AT1G60590.1 | polygalacturonase, putative / pectinase, putative, similar to polygalacturonase PG1 (GI:5669846), PG2 (GI:5669848) from (Glycine max); contains PF00295: Glycosyl hydrolases family 28 (polygalacturonases) | chr1:22317835-22320635 REVERSE | Aliases: F8A5.12, F8A5_12	2.5	2.7	-0.2	-1.5	100.0%	-4.6
4307	AT4G22820.2 | zinc finger (AN1-like) family protein, contains Pfam domains, PF01428: AN1-like Zinc finger and PF01754: A20-like zinc finger | chr4:11987720-11988960 REVERSE | Aliases: F7H19.10	7.3	6.4	0.9	1.5	100.0%	-3.7
4308	AT1G10870.1 | ARF GTPase-activating domain-containing protein | chr1:3616610-3623757 REVERSE | Aliases: T19D16.20, T19D16_20	5.7	6.3	-0.5	-1.5	100.0%	-3.6
4309	AT4G23640.1 | Symbol: TRH1 | potassium transporter / tiny root hair 1 protein (TRH1), identical to tiny root hair 1 protein (Arabidopsis thaliana) gi:11181958:emb:CAC16137; KUP/HAK/KT Transporter family member, PMID:11500563; identical to cDNA mRNA for tiny root hair 1 protein (trh1) GI:11181957 | chr4:12320162-12324479 REVERSE | Aliases: ATKT3, F9D16.110, F9D16_110, KUP4, TINY ROOT HAIR 1	5.5	5.0	0.5	1.5	100.0%	-3.7
4310	AT4G15210.2 | Symbol: ATBETA AMY | beta-amylase (BMY1) / 1,4-alpha-D-glucan maltohydrolase, identical to Beta-amylase (EC 3.2.1.2) (1,4-alpha-D-glucan maltohydrolase) SP:P25853 (Arabidopsis thaliana) | chr4:8666338-8669470 REVERSE | Aliases: AT BETA AMY, BETA AMYLASE, DL3650C, FCAALL.97, RAM1, REDUCED BETA AMYLASE	2.2	2.4	-0.2	-1.5	100.0%	-5.0
4311	AT5G18440.1 | expressed protein | chr5:6113250-6115750 FORWARD | Aliases: F20L16.160, F20L16_160	5.3	5.6	-0.3	-1.5	100.0%	-4.1
4312	AT5G50375.1 | Symbol: CPI1 | cyclopropyl isomerase (CPI1) | chr5:20528893-20531468 FORWARD | Aliases: CYCLOEUCALENOL CYCLOISOMERASE, CYCLOPROPYL ISOMERASE	5.2	5.7	-0.5	-1.5	100.0%	-4.0
4313	AT1G07350.2 | transformer serine/arginine-rich ribonucleoprotein, putative, similar to GB:Y09506 from (Nicotiana tabacum) (Plant Mol. Biol. 35 (3), 261-269 (1997)) | chr1:2257499-2260087 REVERSE | Aliases: F22G5.31, F22G5_31	6.0	5.2	0.9	1.5	100.0%	-3.7
4314	AT5G59380.1 | Symbol: MBD6 | methyl-CpG-binding domain-containing protein, similar to testis specific methyl-CpG binding protein MBD2 (Homo sapiens) GI:3800801; contains Pfam profile PF01429: Methyl-CpG binding domain | chr5:23969487-23970933 FORWARD | Aliases: F2O15.4, F2O15_4	6.5	6.0	0.5	1.5	100.0%	-3.8
4315	AT3G01340.2 | similar to transducin family protein / WD-40 repeat family protein [Arabidopsis thaliana] (TAIR:At2g30050.1); similar to putative Sec13p [Oryza sativa (japonica cultivar-group)] (GB:XP_477253.1); contains InterPro domain G-protein beta WD-40 repeat (InterPro:IPR001680) | chr3:127355-128973 REVERSE | Aliases: T22N4.3, T22N4_3	7.2	5.9	1.3	1.5	100.0%	-3.7
4316	AT1G16370.1 | transporter-related, low similarity to organic cation transporter OCTN1 from (Homo sapiens) GI:2605501, (Mus musculus) GI:4126605, (Rattus norvegicus) GI:5679326; contains Pfam profile PF00083: major facilitator superfamily protein | chr1:5596688-5598441 FORWARD | Aliases: F3O9.17, F3O9_17	5.0	4.7	0.4	1.5	100.0%	-3.9

Rank	Description	15.dpi	5.dpi	M	t	adj.q	B
4317	AT1G73630.1 | calcium-binding protein, putative, similar to calcium binding protein GI:14589311 from (Sesbania rostrata); contains Pfam profile: PF00036 EF hand (4 copies) | chr1:27688397-27689114 FORWARD | Aliases: F25P22.4, F25P22_4	2.8	2.5	0.3	1.5	100.0%	-3.9
4318	AT1G03380.1 | expressed protein | chr1:835825-840891 FORWARD | Aliases: F21B7.1	5.2	4.7	0.5	1.5	100.0%	-3.8
4319	AT1G72890.1 | disease resistance protein (TIR-NBS class), putative, domain signature TIR-NBS exists, suggestive of a disease resistance protein. | chr1:27433608-27435378 FORWARD | Aliases: F3N23.9, F3N23_9	5.1	4.6	0.5	1.5	100.0%	-3.7
4320	AT4G22270.1 | expressed protein | chr4:11773340-11775984 FORWARD | Aliases: T10I14.100, T10I14_100	4.3	3.9	0.4	1.5	100.0%	-3.7
4321	AT5G11760.1 | expressed protein | chr5:3789293-3790453 FORWARD | Aliases: T22P22.150, T22P22_150	7.9	7.1	0.8	1.5	100.0%	-3.7
4322	AT2G19790.1 | clathrin adaptor complex small chain family protein, contains Pfam profile: PF01217 clathrin adaptor complex small chain	9.9	8.6	1.2	1.5	100.0%	-3.5
4323	NA	6.5	5.8	0.8	1.5	100.0%	-3.9
4324	AT1G26665.2 | expressed protein | chr1:9214164-9215538 FORWARD | Aliases: None	8.8	8.3	0.5	1.5	100.0%	-3.7
4325	AT4G28085.2 | expressed protein | chr4:13959976-13960578 FORWARD | Aliases: None	3.3	3.0	0.3	1.5	100.0%	-4.2
4326	AT3G53270.4 | expressed protein, endopeptidase Clp ATP-binding chain C, Chlamydia pneumoniae, PIR:G72079 | chr3:19761025-19764083 REVERSE | Aliases: T4D2.1	4.8	5.1	-0.3	-1.5	100.0%	-4.2
4327	AT5G18210.1 | short-chain dehydrogenase/reductase (SDR) family protein, similar to short-chain type dehydrogenase/reductase SP:Q08632 (Picea abies) | chr5:6017867-6019995 FORWARD | Aliases: MRG7.17, MRG7_17	5.0	4.7	0.2	1.5	100.0%	-4.3
4328	AT1G56180.1 | expressed protein | chr1:21029569-21031729 REVERSE | Aliases: F14G9.20	4.6	4.0	0.6	1.5	100.0%	-3.8
4329	AT1G18800.1 | nucleosome assembly protein (NAP) family protein, similar to SP:Q01105:SET protein (HLA-DR associated protein II) (PHAPII) (Phosphatase 2A inhibitor I2PP2A) {Homo sapiens}; contains Pfam profile PF00956: Nucleosome assembly protein (NAP) | chr1:6481104-6483587 REVERSE | Aliases: F6A14.10, F6A14_10	3.4	3.9	-0.5	-1.5	100.0%	-4.0
4330	AT2G17130.2 | Symbol: IDH2 | isocitrate dehydrogenase subunit 2 / NAD+ isocitrate dehydrogenase subunit 2, nearly identical to NAD+ dependent isocitrate dehydrogenase subunit 2 (Arabidopsis thaliana) GI:1766048 | chr2:7467896-7469636 REVERSE | Aliases: F6P23.14, F6P23_14, ISOCITRATE DEHYDROGENASE SUBUNIT 2, NAD+ DEPENDENT ISOCITRATE DEHYDROGENASE SUBUNIT 2	8.9	9.5	-0.6	-1.5	100.0%	-3.9
4331	AT5G43560.2 | meprin and TRAF homology domain-containing protein / MATH domain-containing protein, weak similarity to ubiquitin-specific protease 12 (Arabidopsis thaliana) GI:11993471; contains Pfam profile PF00917: MATH domain | chr5:17517656-17522873 FORWARD | Aliases: K9D7.6, K9D7_6	8.1	8.5	-0.4	-1.5	100.0%	-3.9
4332	AT1G12080.2 | expressed protein | chr1:4084203-4085041 FORWARD | Aliases: F12F1.4, F12F1_4	4.6	3.4	1.2	1.5	100.0%	-3.4
4333	AT4G20280.1 | transcription initiation factor IID (TFIID) 28 kDa subunit (TAFII-28) family protein, similar to SP:Q15544 Transcription initiation factor TFIID 28 kDa subunit (TAFII-28) (TAFII28) (TFIID subunit p30-beta) {Homo sapiens}; contains Pfam profile PF04719: hTAFII28-like protein conserved region | chr4:10953557-10954983 FORWARD | Aliases: F1C12.195, F1C12_195	7.6	7.2	0.4	1.5	100.0%	-3.8
4334	AT4G19650.1 | similar to mitochondrial transcription termination factor-related / mTERF-related [Arabidopsis thaliana] (TAIR:At5g45113.1); similar to unknown protein [Oryza sativa (japonica cultivar-group)] (GB:NP_912680.1); contains InterPro domain Mitochodrial transcription termination factor-related (InterPro:IPR003690) | chr4:10691684-10695176 FORWARD | Aliases: T16H5.10, T16H5_10	2.4	2.2	0.2	1.5	100.0%	-4.7
4335	AT1G33270.2 | patatin-related, contains Patatin domain PF01734	7.3	6.5	0.8	1.5	100.0%	-3.6
4336	AT3G28620.1 | zinc finger (C3HC4-type RING finger) family protein, low similarity to RING-H2 finger protein RHA2b (Arabidopsis thaliana) GI:3790571; contains Pfam profile PF00097: Zinc finger, C3HC4 type (RING finger) | chr3:10729063-10729698 FORWARD | Aliases: MZN14.9	2.9	2.7	0.3	1.5	100.0%	-4.5
4337	AT5G63650.1 | serine/threonine protein kinase, putative, similar to serine/threonine-protein kinase ASK2(Arabidopsis thaliana), SWISS-PROT:P43292; contains protein kinase domain, Pfam:PF00069 | chr5:25498743-25500945 REVERSE | Aliases: MBK5.13, MBK5_13	5.7	5.1	0.6	1.5	100.0%	-3.8
4338	AT3G52760.1 | integral membrane Yip1 family protein, contains Pfam domain, PF04893: Yip1 domain | chr3:19564657-19565790 REVERSE | Aliases: F3C22.160	6.0	5.7	0.3	1.5	100.0%	-4.1

Rank	Description	15.dpi	5.dpi	M	t	adj.q	B
4339	AT3G08810.1 | kelch repeat-containing F-box family protein, contains F-box domain Pfam:PF00646 and Kelch motif Pfam:PF01344 | chr3:2675637-2676699 REVERSE | Aliases: F17O14.28	4.2	3.7	0.4	1.5	100.0%	-4.0
4340	AT5G55070.1 | 2-oxoacid dehydrogenase family protein, similar to SP:Q01205 Dihydrolipoamide succinyltransferase component of 2-oxoglutarate dehydrogenase complex, mitochondrial precursor (EC 2.3.1.61) {Rattus norvegicus}; contains Pfam profiles PF00198: 2-oxo acid dehydrogenases acyltransferase (catalytic domain), PF00364: Biotin-requiring enzyme | chr5:22364490-22368043 FORWARD | Aliases: MCO15.2, MCO15_2	9.1	10.1	-1.0	-1.5	100.0%	-3.6
4341	AT4G10800.1 | expressed protein, predicted proteins, Arabidopsis thaliana | chr4:6643659-6644841 FORWARD | Aliases: F25I24.10, F25I24_10	3.7	4.0	-0.3	-1.5	100.0%	-4.4
4342	AT2G33810.1 | Symbol: SPL3 | squamosa promoter-binding protein-like 3 (SPL3), identical to squamosa-promoter binding protein like 3 (Arabidopsis thaliana) GI:2462081; contains Pfam profile PF03110: SBP domain | chr2:14312077-14313148 FORWARD | Aliases: SQUAMOSA PROMOTER BINDING PROTEIN LIKE 3, T1B8.11, T1B8_11	2.9	3.1	-0.2	-1.5	100.0%	-4.6
4343	AT1G53320.1 | F-box family protein / tubby family protein (TULP7), similar to Tubby related protein 2 (Tubby-like protein 2) (P4-6 protein) (Fragment) (SP:P46686) (Mus musculus); similar to phosphodiesterase (GI:467578) (Mus musculus); similar to Tubby protein homolog 1. (Swiss-Prot:Q09306) (Caenorhabditis elegans) contains Pfam profile: PF01167: Tub family; contains Pfam PF00646: F-box domain | chr1:19894682-19897159 REVERSE | Aliases: F12M16.22, F12M16_22	11.1	10.8	0.3	1.5	100.0%	-4.5
4344	AT1G19330.1 | expressed protein | chr1:6680558-6683498 REVERSE | Aliases: F18O14.5, F18O14_5	6.4	7.0	-0.6	-1.5	100.0%	-3.6
4345	AT5G46390.2 | peptidase S41 family protein, similar to C-terminal peptidase of the D1 protein (Hordeum vulgare subsp vulgare) GI:1296805; contains Pfam profiles PF03572: Peptidase family S41B, PF00595: PDZ domain (Also known as DHR or GLGF) | chr5:18833831-18836574 FORWARD | Aliases: MPL12.19, MPL12_19	5.1	4.7	0.5	1.5	100.0%	-3.7
4346	AT2G41450.1 | GCN5-related N-acetyltransferase (GNAT) family protein, low similarity to Swift (Xenopus laevis) GI:14164561; contains Pfam profiles PF00583: acetyltransferase, GNAT family, PF00533: BRCA1 C Terminus (BRCT) domain | chr2:17285268-17292165 FORWARD | Aliases: T26J13.4, T26J13_4	2.3	2.2	0.1	1.5	100.0%	-5.0
4347	AT2G19450.1 | Symbol: TAG1 | diacylglycerol O-acyltransferase / acyl CoA:diacylglycerol acyltransferase (DGAT), identical to gi:5050913, gi:6625553 | chr2:8433288-8436818 FORWARD | Aliases: ABX45, AS11, ATDGAT, DGAT1, DIACYLGLYCEROL ACYLTRANSFERASE, F27F23.24, RDS1, REGULATION OF DESATURASES IN SEEDS 1, TRIACYLGLYCEROL BIOSYNTHESIS DEFECT 1	6.6	7.0	-0.5	-1.5	100.0%	-3.8
4348	AT4G36290.1 | ATP-binding region, ATPase-like domain-containing protein, low similarity to microrchidia (Mus musculus) GI:5410255; contains Pfam profile PF02518: ATPase, histidine kinase-, DNA gyrase B-, and HSP90-like domain protein | chr4:17169467-17173208 REVERSE | Aliases: F23E13.180, F23E13_180	4.2	4.5	-0.3	-1.5	100.0%	-4.2
4349	AT5G22370.1 | Symbol: EMB1705 | ATP-binding family protein, contains Pfam domain, PF03029: Conserved hypothetical ATP binding protein	3.1	3.5	-0.4	-1.5	100.0%	-4.2
4350	AT1G20760.1 | calcium-binding EF hand family protein, contains INTERPRO:IPR002048 calcium-binding EF-hand domain | chr1:7209382-7214982 FORWARD | Aliases: F2D10.25, F2D10_25	5.7	4.9	0.8	1.5	100.0%	-3.7
4351	AT1G33970.3 | similar to avirulence-responsive protein / avirulence induced gene (AIG1) [Arabidopsis thaliana] (TAIR:At1g33960.1); similar to putative NTGP4 [Oryza sativa (japonica cultivar-group)] (GB:XP_466219.1); contains InterPro domain AIG1 family (InterPro:IPR006703) | chr1:12349440-12351224 REVERSE | Aliases: F12G12.21, F12G12_21	7.0	6.5	0.5	1.5	100.0%	-3.9
4352	AT2G38650.1 | glycosyl transferase family 8 protein, contains Pfam profile: PF01501 glycosyl transferase family 8 | chr2:16168886-16172804 REVERSE | Aliases: T6A23.15, T6A23_15	5.5	5.9	-0.3	-1.5	100.0%	-4.0
4353	AT5G65970.1 | Symbol: MLO10 | seven transmembrane MLO family protein / MLO-like protein 10 (MLO10), identical to membrane protein Mlo10 (Arabidopsis thaliana) gi:14091590:gb:AAK53803; similar to MLO protein SWISS-PROT:P93766, NCBI_gi:1877221 (Hordeum vulgare)(Barley) | chr5:26404654-26407678 REVERSE | Aliases: K2A18.3, K2A18_3	5.7	6.2	-0.5	-1.5	100.0%	-3.7
4354	AT3G59420.1 | Symbol: ACR4 | receptor protein kinase, putative (ACR4), identical to putative receptor protein kinase ACR4 (Arabidopsis thaliana) GI:20302590; contains protein kinase domain, Pfam:PF00069 | chr3:21970624-21974018 REVERSE | Aliases: F25L23.280	3.3	3.7	-0.4	-1.5	100.0%	-4.1
4355	AT2G32300.1 | Symbol: UCC1 | uclacyanin I, identical to uclacyanin I GI:3399767 from (Arabidopsis thaliana); contains Pfam profile PF02298: Plastocyanin-like domain; identical to cDNA uclacyanin I GI:3399766 | chr2:13729504-13730667 FORWARD | Aliases: UCLACYANIN 1	3.1	3.7	-0.6	-1.5	100.0%	-3.4
4356	AT3G03160.1 | expressed protein | chr3:729775-730474 FORWARD | Aliases: T17B22.15, T17B22_15	10.0	9.7	0.3	1.5	100.0%	-4.4
4357	AT5G61540.3 | similar to L-asparaginase, putative / L-asparagine amidohydrolase, putative [Arabidopsis thaliana] (TAIR:At3g16150.1); similar to hypothetical protein FG02189.1 [Gibberella zeae PH-1] (GB:EAA69124.1); contains InterPro domain Asparaginase 2 family (InterPro:IPR000246) | chr5:24762205-24764725 FORWARD | Aliases: K11J9.7, K11J9_7	4.0	3.7	0.3	1.5	100.0%	-4.2

Rank	Description	15.dpi	5.dpi	M	t	adj.q	B
4358	AT1G75270.1 | dehydroascorbate reductase, putative, similar to GI:6939839 from (Oryza sativa) | chr1:28253736-28255010 REVERSE | Aliases: F22H5.1, F22H5_1	9.5	10.2	-0.6	-1.5	100.0%	-3.8
4359	AT2G34720.1 | CCAAT-binding transcription factor (CBF-B/NF-YA) family protein, contains Pfam profile: PF02045 CCAAT-binding transcription factor (CBF-B/NF-YA) subunit B | chr2:14656846-14658692 REVERSE | Aliases: T29F13.7, T29F13_7	4.9	4.5	0.4	1.5	100.0%	-3.8
4360	AT4G19210.1 | Symbol: ATRLI2 |	7.6	8.9	-1.3	-1.5	100.0%	-3.8
4361	AT1G27030.1 | expressed protein | chr1:9381790-9383970 FORWARD | Aliases: T7N9.9, T7N9_9	4.9	5.5	-0.5	-1.5	100.0%	-3.7
4362	AT1G34370.3 | similar to zinc finger (C2H2 type) family protein [Arabidopsis thaliana] (TAIR:At5g22890.1); similar to putative zinc finger protein [Oryza sativa (japonica cultivar-group)] (GB:XP_470361.1); contains InterPro domain Zn-finger, C2H2 type (InterPro:IPR007087) | chr1:12550455-12552677 FORWARD | Aliases: F7P12.7, F7P12_7	8.9	8.4	0.5	1.5	100.0%	-3.9
4363	AT5G40750.1 | expressed protein | chr5:16325584-16327047 FORWARD | Aliases: MNF13.30, MNF13_30	2.1	2.2	-0.1	-1.5	100.0%	-4.9
4364	AT4G36470.1 | S-adenosyl-L-methionine:carboxyl methyltransferase family protein, similar to SAM:jasmonic acid carboxyl methyltransferase (JMT)(GI:13676829) and to SAM:salicylic acid carboxyl methyltransferase (SAMT)(GI:6002712)(Clarkia breweri) | chr4:17215131-17216478 REVERSE | Aliases: AP22.82, AP22_82	3.4	3.6	-0.2	-1.5	100.0%	-4.3
4365	AT2G33255.1 | haloacid dehalogenase-like hydrolase family protein, contains InterPro accession IPR005834: Haloacid dehalogenase-like hydrolase	6.1	6.9	-0.8	-1.5	100.0%	-3.8
4366	AT3G02530.1 | chaperonin, putative, similar to SWISS-PROT:P80317- T-complex protein 1, zeta subunit (TCP-1-zeta) (Mus musculus); contains Pfam:PF00118 domain, TCP-1/cpn60 chaperonin family | chr3:528581-532589 REVERSE | Aliases: F16B3.16, F16B3_16	7.9	8.9	-1.0	-1.5	100.0%	-3.8
4367	AT2G03390.1 | uvrB/uvrC motif-containing protein, contains Pfam profile PF02151: UvrB/uvrC motif | chr2:1030399-1032619 REVERSE | Aliases: T4M8.18, T4M8_18	5.6	5.3	0.2	1.5	100.0%	-4.3
4368	AT1G15670.1 | kelch repeat-containing F-box family protein, similar to SP:Q9ER30 Kelch-related protein 1 (Sarcosin) {Rattus norvegicus}; contains Pfam profiles PF01344: Kelch motif, PF00646: F-box domain | chr1:5389947-5391485 FORWARD | Aliases: F7H2.1, F7H2_1	4.0	3.3	0.7	1.5	100.0%	-3.5
4369	AT4G21380.1 | Symbol: ARK3 | S-locus protein kinase, putative (ARK3), identical to PIR:T05180:T05180 S-receptor kinase ARK3 precursor - (Arabidopsis thaliana) | chr4:11388936-11393237 REVERSE | Aliases: T6K22.110, T6K22_110	3.0	3.2	-0.2	-1.5	100.0%	-4.4
4370	AT1G26390.1 | FAD-binding domain-containing protein, similar to SP:P30986 reticuline oxidase precursor (Berberine-bridge-forming enzyme) (BBE) (Tetrahydroprotoberberine synthase) (Eschscholzia californica); contains PF01565 FAD binding domain | chr1:9130004-9131766 REVERSE | Aliases: T1K7.23, T1K7_23	2.7	2.9	-0.2	-1.5	100.0%	-4.3
4371	AT4G30570.1 | GDP-mannose pyrophosphorylase, putative, similar to GDP-mannose pyrophosphorylase (Arabidopsis thaliana) GI:3598958; contains Pfam profile PF00483: Nucleotidyl transferase | chr4:14930683-14931957 REVERSE | Aliases: F17I23.90, F17I23_90	4.4	4.0	0.4	1.5	100.0%	-4.2
4372	AT4G04610.1 | Symbol: APR1 | 5'-adenylylsulfate reductase (APR1) / PAPS reductase homolog (PRH19), identical to 5'-adenylylsulfate reductase (Arabidopsis thaliana) GI:2738756; identical to cDNA PAPS reductase homolog (PRH19) GI:1710111 | chr4:2325001-2327011 FORWARD | Aliases: 3' PHOSPHOADENOSINE 5' PHOSPHOSULFATE REDUCTASE, APR, APS REDUCTASE, ATAPR1, F4H6.13, F4H6_13, PAPS REDUCTASE HOMOLOG 19, PRH19	8.3	9.1	-0.8	-1.5	100.0%	-3.8
4373	AT1G65290.1 | acyl carrier family protein / ACP family protein, similar to SP:P53665 Acyl carrier protein, mitochondrial precursor (ACP) (NADH-ubiquinone oxidoreductase 9.6 kDa subunit) (MtACP-1) {Arabidopsis thaliana}; contains InterPro accession IPR003881: Isochorismatase | chr1:24252515-24254065 REVERSE | Aliases: T8F5.6, T8F5_6	10.3	10.9	-0.6	-1.5	100.0%	-4.0
4374	AT4G26650.2 | similar to RNA recognition motif (RRM)-containing protein [Arabidopsis thaliana] (TAIR:At5g55550.3); similar to RNA recognition motif (RRM)-containing protein [Arabidopsis thaliana] (TAIR:At5g55550.2); similar to RNA recognition motif (RRM)-containing protein [Arabidopsis thaliana] (TAIR:At5g55550.1); similar to putative RNA-binding protein [Oryza sativa (japonica cultivar-group)] (GB:AAP54226.1); contains InterPro domain RNA-binding region RNP-1 (RNA recognition motif) (InterPro:IPR000504)	6.3	6.7	-0.4	-1.5	100.0%	-3.9
4375	AT3G08740.1 | elongation factor P (EF-P) family protein, similar to SP:P33398 Elongation factor P (EF-P) {Escherichia coli O157:H7}; contains Pfam profile PF01132: Elongation factor P (EF-P)	7.9	8.7	-0.7	-1.5	100.0%	-3.4
4376	AT5G13460.1 | calmodulin-binding family protein, low similarity to SF16 protein (Helianthus annuus) GI:560150; contains Pfam profile PF00612: IQ calmodulin-binding motif | chr5:4315870-4318363 FORWARD | Aliases: T22N19.110, T22N19_110	5.1	5.5	-0.4	-1.5	100.0%	-3.9

Rank	Description	15.dpi	5.dpi	M	t	adj.q	B
4377	AT1G19980.1 | cytomatrix protein-related, contains weak similarity to CAST1 (Rattus norvegicus) gi:22138113:gb:AAL07517 | chr1:6933022-6934993 FORWARD | Aliases: T20H2.28, T20H2_28	4.7	5.1	-0.3	-1.5	100.0%	-3.8
4378	AT1G01920.2 | similar to ribulose-1,5 bisphosphate carboxylase oxygenase large subunit N-methyltransferase, putative [Arabidopsis thaliana] (TAIR:At1g14030.1); similar to hypothetical protein [Oryza sativa (japonica cultivar-group)] (GB:NP_910361.1); contains InterPro domain Nuclear protein SET (InterPro:IPR001214) | chr1:316128-319637 FORWARD | Aliases: F22M8.5, F22M8_5	5.0	5.5	-0.5	-1.5	100.0%	-3.8
4379	AT1G35430.1 | expressed protein | chr1:13030531-13031130 REVERSE | Aliases: F12A4.3, F12A4_3	6.0	5.4	0.6	1.5	100.0%	-3.9
4380	AT4G32870.1 | expressed protein, hypothetical protein F17H15.20 Arabidopsis thaliana chromosome II BAC F17H15, PID:g3643606 | chr4:15862147-15862745 FORWARD | Aliases: T16I18.80, T16I18_80	10.0	10.4	-0.4	-1.5	100.0%	-4.4
4381	AT3G53220.1 | thioredoxin family protein, low similarity to SP:P29451 Thioredoxin (Rhesus macaque) {Macaca mulatta}; contains Pfam profile: PF00085 Thioredoxin | chr3:19732972-19733680 FORWARD | Aliases: T4D2.150	3.2	3.4	-0.2	-1.5	100.0%	-4.3
4382	AT2G17730.1 | zinc finger (C3HC4-type RING finger) family protein, contains Pfam PF00097: Zinc finger, C3HC4 type (RING finger) domain; similar to RING-H2 finger protein RHA3a (GI:3790573) (Arabidopsis thaliana); similar to ReMembR-H2 protein JR700 (GI:6942147) (Arabidopsis thaliana) | chr2:7710938-7712832 FORWARD | Aliases: T17A5.9, T17A5_9	6.2	5.6	0.5	1.5	100.0%	-3.7
4383	AT2G29690.1 | Symbol: ASA2 | anthranilate synthase, alpha subunit, component I-2 (ASA2), identical to SP:P32069 | chr2:12700948-12704042 FORWARD | Aliases: ATHANSYNAB, T27A16.21, T27A16_21	7.7	8.2	-0.6	-1.5	100.0%	-3.6
4384	AT4G12430.1 | trehalose-6-phosphate phosphatase, putative, similar to trehalose-6-phosphate phosphatase (AtTPPB) (Arabidopsis thaliana) GI:2944180; contains Pfam profile PF02358: Trehalose-phosphatase | chr4:7365330-7368727 REVERSE | Aliases: T1P17.20, T1P17_20	3.7	4.1	-0.4	-1.5	100.0%	-3.9
4385	AT1G79490.1 | Symbol: EMB2217 | pentatricopeptide (PPR) repeat-containing protein, low similarity to fertility restorer (Petunia x hybrida) GI:22128587; contains Pfam profile PF01535: PPR repeat | chr1:29905352-29908082 FORWARD | Aliases: EMB2217, EMBRYO DEFECTIVE 2217, T8K14.9, T8K14_9	4.9	5.3	-0.5	-1.5	100.0%	-3.8
4386	AT3G63200.1 | patatin-related, low similarity to patatin-like latex protein allergen Hev b 7 - Hevea brasiliensis, EMBL:AF113546; contains patatin domain PF01734 | chr3:23356877-23358539 FORWARD | Aliases: F16M2.50	5.1	5.8	-0.7	-1.5	100.0%	-3.6
4387	AT5G17400.1 | ADP, ATP carrier protein, mitochondrial, putative / ADP/ATP translocase, putative / adenine nucleotide translocator, putative, similar to SWISS-PROT:Q09188 ADP,ATP carrier protein (ADP/ATP translocase) (Schizosaccharomyces pombe); contains Pfam profile: PF00153 mitochondrial carrier protein | chr5:5728793-5730378 REVERSE | Aliases: T10B6.60, T10B6_60	5.8	5.4	0.3	1.5	100.0%	-4.2
4388	AT1G03055.2 | expressed protein | chr1:710063-711803 REVERSE | Aliases: None	2.9	2.7	0.2	1.5	100.0%	-4.4
4389	AT5G59980.1 | RNase P subunit p30 family protein, contains Pfam PF01876: RNase P subunit p30 | chr5:24164433-24166953 FORWARD | Aliases: MMN10.23, MMN10_23	3.2	3.5	-0.3	-1.5	100.0%	-3.9
4390	AT1G02720.2 | glycosyl transferase family 8 protein, low similarity to putative glycosyl transferase from Neisseria gonorrhoeae (GI:595812); contains Pfam glycosyl transferase family 8 domain PF01501 | chr1:591826-593289 FORWARD | Aliases: T14P4.1, T14P4_1	4.0	4.3	-0.3	-1.5	100.0%	-4.3
4391	AT3G03980.1 | short-chain dehydrogenase/reductase (SDR) family protein, similar to short-chain type dehydrogenase/reductase SP:Q08632 (Picea abies) | chr3:1031749-1033214 FORWARD | Aliases: T11I18.9, T11I18_9	8.8	9.8	-0.9	-1.5	100.0%	-3.4
4392	AT3G17710.1 | F-box family protein, contains F-box domain Pfam:PF00646	3.9	3.5	0.3	1.5	100.0%	-4.1
4393	AT5G46110.3 | Symbol: APE2 | similar to glucose-6-phosphate/phosphate translocator, putative [Arabidopsis thaliana] (TAIR:At5g54800.1); similar to triose phosphate/phosphate translocator precursor [Mesembryanthemum crystallinum] (GB:AAF86906.1); contains InterPro domain Tpt phosphate/phosphoenolpyruvate translocator (InterPro:IPR004696) | chr5:18714477-18717883 FORWARD | Aliases: ACCLIMATION OF PHOTOSYNTHESIS TO ENVIRONMENT, MCL19.16, MCL19_16, TPT	6.5	5.5	1.0	1.5	100.0%	-3.6
4394	AT1G18170.1 | immunophilin / FKBP-type peptidyl-prolyl cis-trans isomerase family protein, similar to (Peptidyl-prolyl cis-trans isomerase) (PPiase) (Rotamase) (SP:Q26486) (Spodoptera frugiperda); contains Pfam profile: PF00254 FKBP-type peptidyl-prolyl cis-trans isomerases | chr1:6254222-6255683 FORWARD | Aliases: T10F20.17	4.7	4.1	0.6	1.5	100.0%	-3.8
4395	AT4G05450.1 | adrenodoxin-like ferredoxin 2, similar to SP:P46656 Adrenodoxin, mitochondrial precursor (Adrenal ferredoxin) from Mus musculus, SP:P10109 Adrenodoxin, mitochondrial precursor (Hepatoredoxin) from Homo sapiens, SP:P29330 Adrenodoxin from Ovis aries; contains Pfam profile: PF00111 2Fe-2S iron-sulfur cluster binding domains; identical to cDNA GI: 28192430 | chr4:2758653-2760895 FORWARD | Aliases: C6L9.130, C6L9_130	5.2	5.6	-0.4	-1.5	100.0%	-3.9

Rank	Description	15.dpi	5.dpi	M	t	adj.q	B
4396	AT1G17720.2 | Symbol: ATB BETA | serine/threonine protein phosphatase 2A (PP2A) 55 kDa regulatory subunit B, identical to type 2A protein serine/threonine phosphatase 55 kDa B regulatory subunit (GI:1408460) (Arabidopsis thaliana); similar to 55 kDa B regulatory subunit of phosphatase 2A GI:710330; contains Pfam PF00400: WD domain, G-beta repeat (5 copies, 3 weak) | chr1:6093201-6098326 REVERSE | Aliases: F11A6.6, F11A6_6	3.0	2.6	0.4	1.5	100.0%	-4.3
4397	AT3G44100.1 | MD-2-related lipid recognition domain-containing protein / ML domain-containing protein, contains Pfam profile PF02221: ML domain | chr3:15877028-15878291 REVERSE | Aliases: F26G5.50	8.8	9.2	-0.4	-1.5	100.0%	-4.3
4398	AT3G16340.1 | ABC transporter family protein, similar to PDR5-like ABC transporter GI:1514643 from (Spirodela polyrhiza); contains Pfam profile: PF00005 ABC transporter | chr3:5539903-5546410 FORWARD | Aliases: MYA6.19	5.1	5.7	-0.6	-1.5	100.0%	-3.8
4399	AT5G14170.1 | SWIB complex BAF60b domain-containing protein, similar to brahma associated protein 60 kDa (Drosophila melanogaster) GI:3378134, SWI/SNF-related, matrix-associated, actin-dependent regulator of chromatin D1 (Homo sapiens) GI:4566530; contains Pfam profile PF02201: BAF60b domain of the SWIB complex | chr5:4568549-4570943 REVERSE | Aliases: MUA22.17, MUA22_17	6.4	6.7	-0.3	-1.5	100.0%	-4.0
4400	AT3G43430.1 | zinc finger (C3HC4-type RING finger) family protein, contains Pfam profile: PF00097 zinc finger, C3HC4 type (RING finger) | chr3:15365547-15366275 REVERSE | Aliases: T5C2.130	4.7	4.1	0.6	1.5	100.0%	-3.6
4401	AT1G31120.1 | Symbol: KUP10 | potassium transporter family protein, similar to HAK2 (Hordeum vulgare) GI:7108599, potassium transporter (Arabidopsis thaliana) gi:2654088:gb:AAB87687; KUP/HAK/KT Transporter family member, PMID:11500563; contains Pfam profile PF02705: K+ potassium transporter | chr1:11104282-11108012 REVERSE | Aliases: F28K20.5, F28K20_5	4.4	4.9	-0.5	-1.5	100.0%	-3.9
4402	AT1G03000.1 | Symbol: PEX6 | AAA-type ATPase family protein, contains Pfam domain, PF00004: ATPase, AAA family ('A'TPases 'A'ssociated with diverse cellular 'A'ctivities) | chr1:687908-692476 REVERSE | Aliases: F10O3.18, F10O3_18, PEROXIN6	5.4	5.8	-0.4	-1.5	100.0%	-4.0
4403	AT1G09910.1 | expressed protein | chr1:3220037-3224543 REVERSE | Aliases: F21M12.30, F21M12_30	6.9	7.4	-0.4	-1.5	100.0%	-3.8
4404	AT5G16970.1 | NADP-dependent oxidoreductase, putative (P1), identical to probable NADP-dependent oxidoreductase P1, zeta-crystallin homolog (SP:Q39172)(gi:886428), Arabidopsis thaliana; similar to allyl alcohol dehydrogenase (Nicotiana tabacum) GI:6692816; contains Pfam profile PF00107: oxidoreductase, zinc-binding dehydrogenase family | chr5:5576171-5578049 REVERSE | Aliases: F2K13.120, F2K13_120	9.4	9.0	0.4	1.5	100.0%	-4.1
4405	AT3G13280.1 | expressed protein, contains Pfam profile PF04396: Protein of unknown function, DUF537; expression supported by MPSS | chr3:4295334-4297007 REVERSE | Aliases: MDC11.11	3.6	3.4	0.2	1.5	100.0%	-4.5
4406	AT1G71760.1 | expressed protein | chr1:26993076-26994161 FORWARD | Aliases: F14O23.14, F14O23_14	3.1	3.5	-0.4	-1.5	100.0%	-3.9
4407	AT1G62530.1 | expressed protein | chr1:23149997-23150961 REVERSE | Aliases: T3P18.9, T3P18_9	2.6	2.5	0.2	1.5	100.0%	-4.6
4408	AT5G24380.1 | Symbol: YSL2 | similar to transporter, putative [Arabidopsis thaliana] (TAIR:At5g53550.1); similar to oligopeptide transporter OPT family protein [Arabidopsis thaliana] (TAIR:At5g41000.1); similar to oligopeptide transporter OPT family protein [Arabidopsis thaliana] (TAIR:At1g65730.1); similar to oligopeptide transporter OPT family protein [Arabidopsis thaliana] (TAIR:At3g17650.1); similar to transporter, putative [Arabidopsis thaliana] (TAIR:At4g24120.1); similar to OSJNBb0103I08.15 [Oryza sativa (japonica cultivar-group)] (GB:XP_473374.1); similar to putative iron-phytosiderophore transporter protein yellow stripe 1 [Oryza sativa (japonica cultivar-group)] (GB:XP_467066.1); similar to iron transport protein 1 [Oryza sativa] (GB:AAS49493.1); similar to hypothetical protein [Oryza sativa (japonica cultivar-group)] (GB:BAD90812.1); similar to iron-phytosiderophore transporter protein yellow stripe 1 [Zea mays] (GB:AAG17016.2); contains InterPro domain Oligopeptide transporter OPT superfamily (InterPro:IPR004813) | chr5:8323898-8326708 FORWARD | Aliases: ATYSL2, K16H17.9, K16H17_9, YELLOW STRIPE LIKE 2	7.1	6.5	0.6	1.5	100.0%	-3.8
4409	AT3G28740.1 | cytochrome P450 family protein, contains Pfam profile: PF00067 cytochrome P450 | chr3:10789935-10791790 REVERSE | Aliases: T19N8.17	6.5	6.0	0.5	1.5	100.0%	-4.0
4410	AT3G07700.2 | ABC1 family protein, contains Pfam domain, PF03109: ABC1 family | chr3:2459497-2463648 REVERSE | Aliases: MLP3.15	4.3	3.9	0.4	1.5	100.0%	-4.0
4411	AT4G38380.1 | MATE efflux protein-related, T19C21.18 Arabidopsis thaliana chromosome II BAC T19C21 genomic sequence, PID:g3395439 | chr4:17972122-17974781 REVERSE | Aliases: F22I13.150, F22I13_150	4.5	4.2	0.3	1.5	100.0%	-4.0
4412	AT5G09960.1 | expressed protein, similar to unknown protein (emb:CAB61744.1) | chr5:3106321-3107830 FORWARD | Aliases: MYH9.17, MYH9_17	8.7	7.8	0.9	1.5	100.0%	-3.6
4413	AT3G25920.1 | Symbol: RPL15 | 50S ribosomal protein L15, chloroplast (CL15), identical to GB:P25873 from (Arabidopsis thaliana) | chr3:9491321-9493855 REVERSE | Aliases: MPE11.32, RIBOSOMAL PROTEIN CL15	8.8	9.4	-0.5	-1.5	100.0%	-3.6
4414	AT5G44390.1 | FAD-binding domain-containing protein, similar to SP:P30986 reticuline oxidase precursor (Berberine-bridge-forming enzyme) (BBE) (Tetrahydroprotoberberine synthase) (Eschscholzia californica); contains PF01565 FAD binding domain | chr5:17899423-17902231 REVERSE | Aliases: K9L2.19, K9L2_19	2.5	2.7	-0.3	-1.5	100.0%	-4.2

Rank	Description	15.dpi	5.dpi	M	t	adj.q	B
4415	AT2G39700.1 | Symbol: ATEXPA4 | expansin, putative (EXP4), similar to alpha-expansin 6 precursor GI:16923359 from (Cucumis sativus); alpha-expansin gene family, PMID:11641069 | chr2:16550910-16552662 REVERSE | Aliases: ARABIDOPSIS THALIANA EXPANSIN A4, ATEXP4, ATHEXP ALPHA 1.6, F17A14.7, F17A14_7	7.6	5.5	2.1	1.5	100.0%	-3.4
4416	AT1G77360.1 | pentatricopeptide (PPR) repeat-containing protein, contains Pfam profile PF01535: PPR repeat | chr1:29076877-29078322 REVERSE | Aliases: F2P24.7, F2P24_7	4.1	4.5	-0.4	-1.5	100.0%	-4.0
4417	AT5G12330.3 | Symbol: LRP1 | lateral root primordium 1 (LRP1), identical to lateral root primordium 1 (LRP1) (Arabidopsis thaliana) GI:882341; contains Pfam profile PF05142: Domain of unknown function (DUF702) | chr5:3988194-3989648 REVERSE | Aliases: LATERAL ROOT PRIMORDIUM 1	5.6	6.0	-0.5	-1.5	100.0%	-3.9
4418	AT5G26720.1 | expressed protein | chr5:9304028-9304746 FORWARD | Aliases: None	6.1	5.6	0.5	1.5	100.0%	-4.1
4419	AT2G20280.1 | zinc finger (CCCH-type) family protein, contains Pfam domain, PF00642: Zinc finger C-x8-C-x5-C-x3-H type (and similar) | chr2:8746992-8749430 REVERSE | Aliases: F11A3.17, F11A3_17	6.8	7.5	-0.7	-1.5	100.0%	-4.1
4420	AT1G63050.1 | membrane bound O-acyl transferase (MBOAT) family protein, low similarity to porcupine from (Xenopus laevis) GI:6714514, GI:6714520, GI:6714518, GI:6714516; contains Pfam profile PF03062: MBOAT family | chr1:23379549-23381927 REVERSE | Aliases: F16M19.14, F16M19_14	7.0	7.7	-0.6	-1.5	100.0%	-3.9
4421	AT5G47750.1 | protein kinase, putative, similar to protein kinase G11A (Oryza sativa) SWISS-PROT:P47997 | chr5:19356929-19359582 REVERSE | Aliases: MCA23.7, MCA23_7	5.5	5.2	0.3	1.5	100.0%	-4.2
4422	AT1G16970.1 | Symbol: KU70 | Ku70-like protein, identical to Ku70-like protein GI:12006424 from (Arabidopsis thaliana); contains Pfam profiles PF03731: Ku70/Ku80 N-terminal alpha/beta domain, PF02735: Ku70/Ku80 beta-barrel domain, PF03730: Ku70/Ku80 C-terminal arm, and PF02037: SAP domain; contains TIGRfam profile TIGR00578: ATP-dependent DNA helicase ii, 70 kDa subunit | chr1:5800904-5805767 REVERSE | Aliases: F6I1.2, F6I1_2, KU70 LIKE PROTEIN	4.4	4.7	-0.4	-1.5	100.0%	-3.9
4423	AT5G49930.1 | Symbol: EMB1441 | zinc knuckle (CCHC-type) family protein, contains Pfam domain, PF00098: Zinc knuckle | chr5:20325261-20330034 REVERSE | Aliases: EMB1441, EMBRYO DEFECTIVE 1441, K9P8.7, K9P8_7	8.0	8.4	-0.4	-1.5	100.0%	-4.1
4424	AT5G45590.1 | expressed protein | chr5:18501734-18503505 REVERSE | Aliases: K2N11.6, K2N11_6	4.5	5.2	-0.8	-1.5	100.0%	-3.5
4425	AT3G15360.1 | Symbol: ATHM4 | thioredoxin M-type 4, chloroplast (TRX-M4), nearly identical to SP:Q9SEU6 Thioredoxin M-type 4, chloroplast precursor (TRX-M4) {Arabidopsis thaliana} | chr3:5188395-5189704 FORWARD | Aliases: MJK13.20	8.6	8.0	0.7	1.5	100.0%	-3.9
4426	AT2G22240.2 | inositol-3-phosphate synthase isozyme 2 / myo-inositol-1-phosphate synthase 2 / MI-1-P synthase 2 / IPS 2, identical to SP:Q38862 Myo-inositol-1-phosphate synthase isozyme 2 (EC 5.5.1.4) (MI-1-P synthase 2) (IPS 2) {Arabidopsis thaliana} | chr2:9458255-9461159 REVERSE | Aliases: T26C19.10, T26C19_10	5.0	5.7	-0.6	-1.5	100.0%	-3.6
4427	AT5G55120.1 | expressed protein, strong similarity to unknown protein (pir::T04808) | chr5:22386321-22389093 FORWARD | Aliases: MCO15.7, MCO15_7	4.8	5.2	-0.3	-1.5	100.0%	-4.1
4428	AT1G05560.1 | Symbol: UGT1 | UDP-glucose transferase (UGT75B2), similar to UDP-glucose:indole-3-acetate beta-D-glucosyltransferase GI:2149127 from (Arabidopsis thaliana); identical to cDNA UDP-glucosyltransferase (UGT75B2) GI:13661274 | chr1:1645497-1647146 REVERSE | Aliases: T25N20.21, UDP GLUCOSYLTRANSFERASE, UGT75B2	4.8	4.2	0.7	1.5	100.0%	-3.8
4429	AT1G54450.1 | calcium-binding EF-hand family protein, contains Pfam profile: PF00036 EF hand | chr1:20340222-20343202 REVERSE | Aliases: F20D21.27, F20D21_27	4.1	4.4	-0.3	-1.5	100.0%	-4.3
4430	AT3G09890.1 | ankyrin repeat family protein, contains ankyrin repeats, Pfam:PF00023 | chr3:3032564-3034448 FORWARD | Aliases: F8A24.6	5.0	4.5	0.5	1.5	100.0%	-3.7
4431	AT3G01370.1 | expressed protein, contains Pfam domain, PF04581: Protein of unknown function (DUF578) | chr3:139018-143699 FORWARD | Aliases: T13O15.1, T13O15_1	5.3	5.8	-0.5	-1.5	100.0%	-3.8
4432	AT1G64580.1 | pentatricopeptide (PPR) repeat-containing protein, low similarity to fertility restorer (Petunia x hybrida) GI:22128587; contains Pfam profile PF01535: PPR repeat | chr1:23988741-23992412 REVERSE | Aliases: F1N19.15, F1N19_15	3.9	4.1	-0.2	-1.5	100.0%	-4.3
4433	AT1G22710.1 | Symbol: SUC2 | sucrose transporter / sucrose-proton symporter (SUC2), nearly identical to sucrose-proton symporter SUC2 (Arabidopsis thaliana) GI:407092 | chr1:8030630-8033106 REVERSE | Aliases: ATSUC2, SUCROSE H+ SYMPORTER, SUT1, T22J18.12, T22J18_12	4.7	4.1	0.6	1.5	100.0%	-4.0
4434	AT4G29270.1 | acid phosphatase class B family protein, similar to acid phosphatase (Glycine max) GI:3341443; contains Pfam profile PF03767: HAD superfamily (subfamily IIIB) phosphatase | chr4:14423671-14424888 REVERSE | Aliases: F17A13.90, F17A13_90	3.4	3.8	-0.5	-1.5	100.0%	-3.7

Rank	Description	15.dpi	5.dpi	M	t	adj.q	B
4435	AT1G70310.1 | Symbol: SPDS2 | spermidine synthase 2 (SPDSYN2) / putrescine aminopropyltransferase 2, identical to SP:O48661 Spermidine synthase 2 (EC 2.5.1.16) (Putrescine aminopropyltransferase 2) (SPDSY 2) {Arabidopsis thaliana} | chr1:26488969-26491076 REVERSE | Aliases: F17O7.16, F17O7_16, SPERMIDINE SYNTHASE 2	11.5	12.0	-0.5	-1.5	100.0%	-4.2
4436	AT2G25210.1 | 60S ribosomal protein L39 (RPL39A) | chr2:10746934-10747631 FORWARD | Aliases: T22F11.20, T22F11_20	10.3	11.0	-0.6	-1.5	100.0%	-3.6
4437	AT5G28060.1 | 40S ribosomal protein S24 (RPS24B), 40S ribosomal protein S19, Cyanophora paradoxa, EMBL:CPA245654 | chr5:10069553-10070897 REVERSE | Aliases: F15F15.130, F15F15_130	8.8	9.6	-0.8	-1.5	100.0%	-3.6
4438	AT1G08190.1 | vacuolar assembly protein, putative (VPS41), 99.8% identical to Vacuolar assembly protein VPS41 homolog (SP:P93043) (Arabidopsis thaliana); similar to vacuolar assembly protein vps41 GI:1835787 from (Lycopersicon esculentum)	5.2	5.7	-0.5	-1.5	100.0%	-3.7
4439	AT4G20030.1 | RNA recognition motif (RRM)-containing protein, low similarity to heterogeneous nuclear ribonucleoprotein G (Mus musculus) GI:5579009; contains InterPro entry IPR000504: RNA-binding region RNP-1 (RNA recognition motif) (RRM)	4.9	4.5	0.5	1.5	100.0%	-3.8
4440	AT2G03240.1 | EXS family protein / ERD1/XPR1/SYG1 family protein, similar to PHO1 protein (Arabidopsis thaliana) GI:20069032; contains Pfam profiles PF03105: SPX domain, PF03124: EXS family	4.8	4.1	0.7	1.5	100.0%	-3.8
4441	AT3G07360.3 | similar to armadillo/beta-catenin repeat family protein / U-box domain-containing protein [Arabidopsis thaliana] (TAIR:At5g18320.1); similar to OSJNBa0020I02.15 [Oryza sativa (japonica cultivar-group)] (GB:XP_472015.1); contains InterPro domain Armadillo repeat (InterPro:IPR000225) | chr3:2354725-2356845 FORWARD | Aliases: F21O3.7	5.2	4.8	0.4	1.5	100.0%	-4.2
4442	AT2G41870.1 | remorin family protein, contains Pfam domain, PF03763: Remorin, C-terminal region | chr2:17477944-17480014 REVERSE | Aliases: T11A7.3, T11A7_3	5.7	5.2	0.4	1.5	100.0%	-4.2
4443	AT3G14230.3 | Symbol: RAP2.2 |	5.6	4.7	0.9	1.5	100.0%	-3.8
4444	AT3G57090.1 | expressed protein | chr3:21139509-21141167 FORWARD | Aliases: F24I3.170	9.3	8.6	0.7	1.5	100.0%	-3.9
4445	AT1G78120.1 | tetratricopeptide repeat (TPR)-containing protein, low similarity to SP:Q99615 DnaJ homolog subfamily C member 7 (Tetratricopeptide repeat protein 2) {Homo sapiens}; contains Pfam profile PF00515: TPR Domain | chr1:29398914-29400896 REVERSE | Aliases: T11I11.6, T11I11_6	5.6	5.2	0.4	1.5	100.0%	-3.8
4446	AT2G43780.1 | expressed protein | chr2:18143521-18143724 REVERSE | Aliases: F18O19.11	10.7	11.0	-0.3	-1.5	100.0%	-4.3
4447	AT5G57590.1 | Symbol: BIO1 | aminotransferase class III family protein, low similarity to 7,8-diaminopelargonic acid aminotransferase BioA (Mesorhizobium loti) GI:12044306; contains Pfam profile PF00202: aminotransferase, class III | chr5:23335734-23338765 REVERSE | Aliases: BIOTIN AUXOTROPH 1, MUA2.17, MUA2_17	5.8	6.5	-0.6	-1.5	100.0%	-3.8
4448	AT3G16990.1 | TENA/THI-4 family protein, contains Pfam profile: PF03070 TENA/THI-4 family | chr3:5795845-5796811 REVERSE | Aliases: K14A17.22	7.8	7.5	0.3	1.5	100.0%	-4.4
4449	AT2G06990.1 | Symbol: HEN2 | HUA enhancer 2 (HEN2) / DExH-box RNA helicase, putative, nearly identical to HUA enhancer 2 (Arabidopsis thaliana) GI:16024936 | chr2:2894937-2901075 FORWARD | Aliases: HUA ENHANCER 2, T4E14.10, T4E14_10	5.0	5.4	-0.3	-1.5	100.0%	-4.2
4450	AT5G42450.1 | pentatricopeptide (PPR) repeat-containing protein, contains Pfam profile PF01535: PPR repeat | chr5:16994871-16996244 FORWARD | Aliases: MDH9.15, MDH9_15	3.3	3.7	-0.4	-1.5	100.0%	-4.1
4451	AT2G24450.1 | fasciclin-like arabinogalactan family protein, similar to fasciclin-like arabinogalactan-protein 1 (Arabidopsis thaliana) gi:13377776:gb:AAK20857 | chr2:10399871-10401003 REVERSE | Aliases: T28I24.18, T28I24_18	2.6	2.8	-0.2	-1.5	100.0%	-4.6
4452	AT5G59770.1 | similar to protein tyrosine phosphatase-like protein, putative (PAS2) [Arabidopsis thaliana] (TAIR:At5g10480.1); similar to Protein tyrosine phosphatase-like (proline instead of catalytic arginine), member b [Homo sapiens] (GB:AAH49369.1); similar to hypothetical protein [Pongo pygmaeus] (GB:CAH90857.1); similar to Similar to RIKEN cDNA 6330408J20 gene [Danio rerio] (GB:AAH44396.1); contains InterPro domain Protein tyrosine phosphatase-like protein, PTPLA (InterPro:IPR007482)	5.4	4.9	0.5	1.5	100.0%	-3.7
4453	AT4G21980.1 | Symbol: APG8A | autophagy 8a (APG8a), identical to autophagy 8a (Arabidopsis thaliana) GI:19912151; contains Pfam profile PF02991: Microtubule associated protein 1A/1B, light chain 3 | chr4:11655742-11656980 FORWARD | Aliases: ATG8A, F1N20.80, F1N20_80	7.4	6.8	0.6	1.5	100.0%	-3.7
4454	AT3G51960.2 | bZIP family transcription factor, contains Pfam profile: PF00170 bZIP transcription factor | chr3:19293579-19295124 REVERSE | Aliases: F4F15.70	3.4	3.2	0.2	1.5	100.0%	-4.4
4455	AT4G22160.2 | expressed protein | chr4:11733135-11734039 FORWARD | Aliases: T10I14.8	6.2	5.7	0.5	1.5	100.0%	-3.7
4456	AT2G42700.1 | expressed protein | chr2:17785429-17789167 FORWARD | Aliases: F14N22.4	6.0	6.5	-0.5	-1.5	100.0%	-3.8

Rank	Description	15.dpi	5.dpi	M	t	adj.q	B
4457	AT4G02550.3 | similar to expressed protein [Arabidopsis thaliana] (TAIR:At4g02210.1); similar to putative transposase [Oryza sativa (japonica cultivar-group)] (GB:AAP51979.1); similar to putative polyprotein [Oryza sativa (japonica cultivar-group)] (GB:AAV43964.1) | chr4:1120421-1121725 REVERSE | Aliases: T10P11.16, T10P11_16	3.9	3.5	0.4	1.5	100.0%	-4.1
4458	AT5G27620.1 | Symbol: CYCH;1 |	4.3	4.9	-0.5	-1.5	100.0%	-3.9
4459	AT3G08590.2 | 2,3-biphosphoglycerate-independent phosphoglycerate mutase, putative / phosphoglyceromutase, putative, strong similarity to SP:Q42908 2,3-bisphosphoglycerate-independent phosphoglycerate mutase (EC 5.4.2.1) (Phosphoglyceromutase) {Mesembryanthemum crystallinum}; contains Pfam profile PF01676: Metalloenzyme superfamily | chr3:2608483-2611333 REVERSE | Aliases: F17O14.6	10.9	11.6	-0.7	-1.5	100.0%	-4.2
4460	AT5G48640.1 | cyclin family protein, similar to SP:P55168 Cyclin C {Gallus gallus}; contains Pfam profile PF00134: Cyclin, N-terminal domain | chr5:19740759-19742915 REVERSE | Aliases: K15N18.11, K15N18_11	6.2	6.7	-0.5	-1.5	100.0%	-3.9
4461	AT1G04160.1 | Symbol: XIB | myosin family protein, contains Pfam profiles: PF02736 myosin N-terminal SH3-like domain, PF00063 myosin head (motor domain), PF00612 IQ calmodulin-binding motif, PF01843: DIL domain | chr1:1086494-1096145 FORWARD | Aliases: ATXIB, F20D22.7, F20D22_7	3.9	4.2	-0.4	-1.5	100.0%	-4.0
4462	AT4G19110.2 | protein kinase, putative, contains protein kinase domain, Pfam:PF00069 | chr4:10454540-10459309 REVERSE | Aliases: T18B16.80, T18B16_80	6.5	5.5	1.0	1.4	100.0%	-3.6
4463	AT2G16780.1 | Symbol: MSI2 | WD-40 repeat protein (MSI2), contains 5 WD-40 repeats (PF0400); identical to WD-40 repeat protein MSI2 (SP:O22468) (Arabidopsis thaliana) WD-40 repeats (PF0400); | chr2:7288544-7290715 REVERSE | Aliases: MSI02, MSI2, NFC02, NFC2, T24I21.19, T24I21_19	6.9	7.6	-0.7	-1.4	100.0%	-3.5
4464	AT2G36320.1 | zinc finger (AN1-like) family protein, contains Pfam domain, PF01428: AN1-like Zinc finger | chr2:15235867-15237575 FORWARD | Aliases: F2H17.7, F2H17_7	7.3	6.6	0.8	1.4	100.0%	-3.6
4465	AT5G24580.2 | copper-binding family protein, similar to copper homeostasis factor gi:3168840 from Arabidopsis thaliana; contains Pfam profile PF00403: Heavy-metal-associated domain | chr5:8410261-8412268 REVERSE | Aliases: K18P6.11, K18P6_11	6.3	5.7	0.7	1.4	100.0%	-3.4
4466	AT4G21170.1 | pentatricopeptide (PPR) repeat-containing protein, contains Pfam profile PF01535: PPR repeat | chr4:11286766-11288641 REVERSE | Aliases: F7J7.110, F7J7_110	4.8	5.3	-0.5	-1.4	100.0%	-4.0
4467	AT5G42050.1 | expressed protein, similar to gda-1 (Pisum sativum) GI:2765418 | chr5:16832717-16834521 FORWARD | Aliases: MJC20.15, MJC20_15	7.9	6.9	0.9	1.4	100.0%	-3.9
4468	AT3G06840.1 | expressed protein | chr3:2156868-2157856 FORWARD | Aliases: F3E22.2	2.4	2.6	-0.2	-1.4	100.0%	-4.5
4469	AT2G14835.2 | zinc finger (C3HC4-type RING finger) family protein, contains Pfam profile: PF00097 zinc finger, C3HC4 type (RING finger) | chr2:6373993-6378116 FORWARD | Aliases: None	4.7	4.4	0.4	1.4	100.0%	-4.1
4470	AT2G34070.1 | expressed protein | chr2:14394505-14397309 REVERSE | Aliases: T14G11.19, T14G11_19	4.9	5.3	-0.4	-1.4	100.0%	-3.8
4471	AT4G16160.2 | mitochondrial import inner membrane translocase subunit Tim17/Tim22/Tim23 family protein, contains Pfam PF02466: Mitochondrial import inner membrane translocase subunit Tim17 | chr4:9157530-9158948 FORWARD | Aliases: DL4120W, FCAALL.207	2.7	2.6	0.2	1.4	100.0%	-4.6
4472	AT1G32790.2 | Symbol: CID11 | similar to RNA-binding protein, putative [Arabidopsis thaliana] (TAIR:At4g10610.1); similar to putative RNA-binding protein RBP37 [Oryza sativa (japonica cultivar-group)] (GB:XP_479783.1); contains InterPro domain RNA-binding region RNP-1 (RNA recognition motif) (InterPro:IPR000504) | chr1:11874684-11877367 REVERSE | Aliases: CID11, F6N18.17, F6N18_17	9.6	8.6	0.9	1.4	100.0%	-3.8
4473	AT1G53980.1 | polyubiquitin-related, contains similarity to polyubiquitin GI:166336 from (Aglaothamnion neglectum) | chr1:20153002-20153277 REVERSE | Aliases: T18A20.18	3.5	3.2	0.3	1.4	100.0%	-4.4
4474	AT1G12160.1 | flavin-containing monooxygenase family protein / FMO family protein, similar to flavin-containing monooxygenase FMO2 from Homo sapiens (SP:Q99518); contains Pfam profile PF00743 Flavin-binding monooxygenase-like | chr1:4126064-4128308 FORWARD | Aliases: T28K15.10, T28K15_10	8.1	8.6	-0.5	-1.4	100.0%	-3.7
4475	AT3G17820.1 | Symbol: ATGSKB6 | glutamine synthetase (GS1), identical to glutamine synthetase, cytosolic isozyme (glutamate-- ammonia ligase, GS1) (Arabidopsis thaliana) SWISS-PROT:Q9LVI8 | chr3:6097420-6099601 FORWARD | Aliases: GLN1;3, MEB5.4	11.5	12.0	-0.6	-1.4	100.0%	-3.6
4476	AT2G05210.1 | expressed protein, similar to expressed protein [Arabidopsis thaliana] (TAIR:At5g06310.1) | chr2:1891769-1894359 FORWARD | Aliases: F5G3.11, F5G3_11	3.3	3.8	-0.4	-1.4	100.0%	-3.9

Rank	Description	15.dpi	5.dpi	M	t	adj.q	B
4477	AT4G23890.1 | expressed protein, hypothetical protein, Synechocystis sp., PIR:S76577 | chr4:12420424-12421474 REVERSE | Aliases: T32A16.60, T32A16_60	5.5	4.9	0.6	1.4	100.0%	-3.7
4478	AT2G32100.1 | ovate protein-related, contains TIGRFAM TIGR01568 : uncharacterized plant-specific domain TIGR01568 | chr2:13654929-13655862 FORWARD | Aliases: F22D22.15, F22D22_15	2.3	2.5	-0.2	-1.4	100.0%	-4.4
4479	AT1G66220.1 | subtilase family protein, contains similarity to subtilase; SP1 GI:9957714 from (Oryza sativa); contains Pfam profiles: PF00082 Subtilase family (3 copies) | chr1:24674199-24677324 FORWARD | Aliases: T6J19.4, T6J19_4	2.7	2.5	0.2	1.4	100.0%	-4.3
4480	AT5G58090.1 | glycosyl hydrolase family 17 protein, similar to 3-glucanase GI:18483232 from (Sorghum bicolor) | chr5:23522592-23524514 REVERSE | Aliases: K21L19.12, K21L19_12	8.0	7.1	0.9	1.4	100.0%	-3.8
4481	AT4G18593.1 | dual specificity protein phosphatase-related, contains weak similarity to Dual specificity protein phosphatase 12 (EC 3.1.3.48) (EC 3.1.3.16) (Dual-specificity tyrosine phosphatase YVH1) (Swiss-Prot:Q9UNI6) (Homo sapiens) | chr4:10237583-10238847 FORWARD | Aliases: None	8.8	8.4	0.4	1.4	100.0%	-3.8
4482	AT5G47890.1 | NADH-ubiquinone oxidoreductase B8 subunit, putative, similar to SP:O43678 NADH-ubiquinone oxidoreductase B8 subunit (EC 1.6.5.3) (EC 1.6.99.3) (Complex I-B8) (CI-B8) {Homo sapiens}; contains Pfam profile PF05047: Mitochondrial ribosomal protein L51 / S25 / CI-B8 domain | chr5:19405963-19407852 FORWARD | Aliases: MCA23.23, MCA23_23	10.5	10.9	-0.4	-1.4	100.0%	-4.3
4483	AT4G11370.1 | Symbol: RHA1A | zinc finger (C3HC4-type RING finger) family protein, strong similarity to RING-H2 finger protein RHA1a (Arabidopsis thaliana) GI:3790554; contains Pfam profile PF00097: Zinc finger, C3HC4 type (RING finger) | chr4:6907669-6908378 FORWARD | Aliases: F8L21.160, F8L21_160, RING H2 FINGER PROTEIN RHA1A	3.0	3.2	-0.2	-1.4	100.0%	-4.5
4484	AT5G09860.1 | nuclear matrix protein-related, low similarity to nuclear matrix protein p84 (Homo sapiens) GI:550058 | chr5:3066641-3070934 FORWARD | Aliases: MYH9.7, MYH9_7	5.9	6.3	-0.4	-1.4	100.0%	-3.8
4485	AT5G10430.1 | Symbol: AGP4 | arabinogalactan-protein (AGP4), identical to gi_3883126_gb_AAC77826 | chr5:3277533-3278314 REVERSE | Aliases: ARABINOGALACTAN PROTEIN 4, ATAGP4, F12B17.220, F12B17_220	4.4	3.7	0.6	1.4	100.0%	-3.6
4486	AT4G32140.1 | expressed protein | chr4:15522905-15525433 REVERSE | Aliases: F10N7.50, F10N7_50	5.8	5.3	0.5	1.4	100.0%	-3.8
4487	AT5G16540.3 | Symbol: ZFN3 | zinc finger (CCCH-type) family protein, identical to zinc finger protein 3 (Arabidopsis thaliana) gi:4689376:gb:AAD27875; contains Pfam domain, PF00642: Zinc finger C-x8-C-x5-C-x3-H type (and similar)	5.4	4.9	0.5	1.4	100.0%	-3.9
4488	AT5G12310.1 | zinc finger (C3HC4-type RING finger) family protein, contains Pfam profile: PF00097 zinc finger, C3HC4 type (RING finger) | chr5:3980227-3982443 REVERSE | Aliases: None	8.4	7.9	0.4	1.4	100.0%	-4.0
4489	AT4G30760.2 | expressed protein, contains Pfam profile PF04396: Protein of unknown function, DUF537 | chr4:14982528-14983425 FORWARD | Aliases: T10C21.110, T10C21_110	8.9	9.1	-0.1	-1.4	100.0%	-4.8
4490	AT1G48750.1 | protease inhibitor/seed storage/lipid transfer protein (LTP) family protein, similar to TED4 (Zinnia elegans) GI:493721; contains Pfam protease inhibitor/seed storage/LTP family domain PF00234	12.3	11.8	0.5	1.4	100.0%	-4.5
4491	AT3G61310.1 | DNA-binding family protein, contains a AT hook motif (DNA binding motifs with a preference for A/T rich regions), Pfam:PF02178 | chr3:22701167-22703797 REVERSE | Aliases: T20K12.210	5.3	4.8	0.5	1.4	100.0%	-3.6
4492	AT5G02030.1 | Symbol: LSN | homeodomain protein (BELLRINGER), several homeodomain proteins; | chr5:395631-399038 FORWARD | Aliases: BELLRINGER, BLR, HB 6, LARSON, PENNYWISE, PNY, REPLUMLESS, RPL, T7H20.80, T7H20_80	6.4	5.7	0.7	1.4	100.0%	-3.5
4493	AT3G42130.1 | glycine-rich protein | chr3:14309227-14309869 REVERSE | Aliases: F4M19.90	7.0	6.3	0.8	1.4	100.0%	-3.6
4494	AT3G49990.1 | expressed protein | chr3:18543308-18545257 REVERSE | Aliases: F3A4.70	5.9	6.6	-0.7	-1.4	100.0%	-3.9
4495	AT4G16440.1 | iron hydrogenase family protein, contains Pfam profiles: PF02906 Iron only hydrogenase large subunit, C-terminal domain, PF02256 Iron hydrogenase small subunit | chr4:9269286-9271532 REVERSE | Aliases: DL4245C, FCAALL.367	6.7	7.3	-0.6	-1.4	100.0%	-3.9
4496	AT5G23430.2 | transducin family protein / WD-40 repeat family protein, contains 6 WD-40 repeats (PF00400); katanin p80 subunit (GI:3005601) (Strongylocentrotus purpuratus) | chr5:7893674-7900390 REVERSE | Aliases: K19M13.6, K19M13_6	4.2	4.6	-0.4	-1.4	100.0%	-3.9

Rank	Description	15.dpi	5.dpi	M	t	adj.q	B
4497	AT5G03030.1 | DNAJ heat shock N-terminal domain-containing protein, contains Pfam profile PF00226 DnaJ domain; DNAJ domain-containing protein, Homo sapiens, EMBL:AF126743 | chr5:708341-709701 REVERSE | Aliases: F15A17.60, F15A17_60	8.2	7.8	0.4	1.4	100.0%	-4.0
4498	AT1G31280.1 | Symbol: AGO2 | PAZ domain-containing protein / piwi domain-containing protein, similar to SP:O04379 Argonaute protein (AGO1) {Arabidopsis thaliana}, SP:Q9XGW1 PINHEAD protein (ZWILLE protein) {Arabidopsis thaliana}; contains Pfam profiles PF02171: Piwi domain, PF02170: PAZ domain | chr1:11181758-11185093 FORWARD | Aliases: T19E23.7, T19E23_7	3.0	3.4	-0.4	-1.4	100.0%	-4.0
4499	AT1G28090.2 | polynucleotide adenylyltransferase family protein, low similarity to SP:P13685 Poly(A) polymerase (EC 2.7.7.19) {Escherichia coli O157:H7}; contains Pfam profile PF01743: polyA polymerase family protein | chr1:9795774-9799008 FORWARD | Aliases: F13K9.19, F13K9_19	2.5	2.6	-0.2	-1.4	100.0%	-4.6
4500	AT5G44210.1 | Symbol: ATERF 9/ATERF9/ERF9 | encodes a member of the ERF (ethylene response factor) subfamily B-1 of ERF/AP2 transcription factor family (ATERF-9). The protein contains one AP2 domain. There are 15 members in this subfamily including ATERF-3, ATERF-4, ATERF-7, and leafy petiole. | chr5:17823699-17824760 FORWARD | Aliases: ATERF 9, ATERF9, ERF9, MLN1.14, MLN1_14	3.0	3.7	-0.7	-1.4	100.0%	-3.6
4501	AT1G33560.1 | Symbol: ADR1 | disease resistance protein (CC-NBS-LRR class), putative, domain signature CC-NBS-LRR exists, suggestive of a disease resistance protein.	3.4	3.8	-0.4	-1.4	100.0%	-3.7
4502	AT4G13200.1 | expressed protein, hypothetical protein sll1769 - Synechocystis sp,PID:d1018406 | chr4:7668146-7669197 REVERSE | Aliases: F17N18.90, F17N18_90	6.5	6.2	0.3	1.4	100.0%	-4.1
4503	AT2G20780.1 | mannitol transporter, putative, similar to mannitol transporter (Apium graveolens var. dulce) GI:12004316; contains Pfam profile PF00083: major facilitator superfamily protein	5.2	4.9	0.3	1.4	100.0%	-4.0
4504	AT5G15740.1 | expressed protein, contains Pfam PF03138: Plant protein family. The function of this family of plant proteins is unknown; | chr5:5134530-5137629 REVERSE | Aliases: F14F8.120, F14F8_120	4.1	4.5	-0.5	-1.4	100.0%	-4.0
4505	AT2G19080.1 | metaxin-related, contains 1 transmembrane domain; similar to Metaxin 1 (component of a preprotein import complex) (Swiss-Prot:P47802) (Mus musculus); | chr2:8269839-8272046 FORWARD | Aliases: METAXIN, T20K24.9, T20K24_9	5.5	6.0	-0.5	-1.4	100.0%	-3.9
4506	AT4G11210.1 | disease resistance-responsive family protein / dirigent family protein, similar to dirigent protein (Thuja plicata) gi:6694699:gb:AAF25360; similar to disease resistance response protein 206-d (Pisum sativum) gi:508844:gb:AAB18669 | chr4:6832687-6833241 FORWARD | Aliases: F8L21.1	3.9	3.4	0.5	1.4	100.0%	-3.7
4507	AT1G30540.1 | ATPase, BadF/BadG/BcrA/BcrD-type family, contains Pfam PF01869: BadF/BadG/BcrA/BcrD ATPase family; similar to Transcriptional regulators of NagC/XylR (ROK) family, sugar kinase (GI:15023013) (Clostridium acetobutylicum); similar to N-acetylglucosamine kinase (EC 2.7.1.59) (GlcNAc kinase) (Swiss-Prot:Q9UJ70) (Homo sapiens) | chr1:10816756-10819309 REVERSE | Aliases: F26G16.16, F26G16_16	5.2	4.7	0.5	1.4	100.0%	-3.8
4508	AT3G32940.1 | expressed protein | chr3:13494664-13497497 REVERSE | Aliases: T13O13.2	4.9	5.8	-0.8	-1.4	100.0%	-3.8
4509	AT3G54290.1 | expressed protein, contains Pfam PF03794: Domain of Unknown function | chr3:20115377-20117022 REVERSE | Aliases: F24B22.250	7.7	7.4	0.4	1.4	100.0%	-4.2
4510	AT3G13980.1 | expressed protein | chr3:4619045-4620269 REVERSE | Aliases: MDC16.10	4.0	4.4	-0.3	-1.4	100.0%	-4.0
4511	AT3G50930.1 | AAA-type ATPase family protein, contains Pfam profile: ATPase family PF00004 | chr3:18940507-18942820 FORWARD | Aliases: F18B3.210	3.2	3.4	-0.2	-1.4	100.0%	-4.7
4512	AT5G06210.1 | RNA-binding protein, putative, contains similarity to RNA-binding protein from (Nicotiana tabacum) GI:15822703, (Nicotiana sylvestris) GI:624925, (Solanum tuberosum) GI:15822705; contains InterPro entry IPR000504: RNA-binding region RNP-1 (RNA recognition motif) (RRM) | chr5:1878472-1879741 FORWARD | Aliases: MBL20.9, MBL20_9	9.9	9.5	0.4	1.4	100.0%	-3.8
4513	AT3G60810.2 | expressed protein | chr3:22481494-22483079 FORWARD | Aliases: T4C21.220	6.8	7.1	-0.4	-1.4	100.0%	-3.8
4514	AT2G24140.1 | expressed protein, contains Pfam PF04576: Protein of unknown function, DUF593 | chr2:10269634-10271113 REVERSE | Aliases: F27D4.5, F27D4_5	3.7	4.0	-0.3	-1.4	100.0%	-4.6
4515	AT2G42120.2 | DNA polymerase delta small subunit-related, similar to DNA polymerase delta small subunit SP:Q9LRE5 from (Oryza sativa) | chr2:17570254-17573110 REVERSE | Aliases: T24P15.3, T24P15_3	4.7	5.2	-0.5	-1.4	100.0%	-3.8
4516	AT5G10980.1 | histone H3, identical to HISTONE H3.2, MINOR, Medicago sativa, SWISSPROT:P11105, histone H3 variant H3.3 Lycopersicon esculentum GI:1435157; contains Pfam profile PF00125 Core histone H2A/H2B/H3/H4 | chr5:3472429-3473442 REVERSE | Aliases: T30N20.250, T30N20_250	11.6	12.0	-0.3	-1.4	100.0%	-4.4

Rank	Description	15.dpi	5.dpi	M	t	adj.q	B
4517	AT5G65430.2 | Symbol: GRF8 | 14-3-3 protein GF14 kappa (GRF8), identical to 14-3-3 protein GF14 kappa GI:5802794, SP:P48348 from (Arabidopsis thaliana) | chr5:26165427-26167568 REVERSE | Aliases: GF14 KAPPA, GF14 KAPPA ISOFORM, MNA5.16, MNA5_16	10.3	9.4	0.9	1.4	100.0%	-3.9
4518	AT1G09810.1 | expressed protein, contains Pfam profile PF04146: YT521-B-like family | chr1:3180964-3182832 REVERSE | Aliases: F21M12.20, F21M12_20	4.4	4.9	-0.5	-1.4	100.0%	-3.8
4519	AT4G39830.1 | L-ascorbate oxidase, putative, similar to SP:P14133 L-ascorbate oxidase precursor (EC 1.10.3.3) (Ascorbase) {Cucumis sativus}; contains Pfam profile PF00394: Multicopper oxidase | chr4:18478923-18481337 FORWARD | Aliases: T5J17.9	4.0	4.3	-0.3	-1.4	100.0%	-4.0
4520	AT1G74050.1 | 60S ribosomal protein L6 (RPL6C), similar to 60S ribosomal protein L6 (YL 16 like) GB:CAB57309 from (Cyanophora paradoxa) | chr1:27850710-27852467 REVERSE | Aliases: F2P9.8, F2P9_8	11.9	12.1	-0.2	-1.4	100.0%	-4.7
4521	AT1G74060.1 | 60S ribosomal protein L6 (RPL6B), similar to 60S ribosomal protein L6 (YL 16 like) GB:CAB57309 from (Cyanophora paradoxa) | chr1:27853497-27855028 REVERSE | Aliases: F2P9.7, F2P9_7	11.9	12.1	-0.2	-1.4	100.0%	-4.7
4522	AT1G53440.1 | leucine-rich repeat family protein / protein kinase family protein, contains Pfam domains PF00560: Leucine Rich Repeat and PF00069: Protein kinase domain | chr1:19949627-19955230 FORWARD | Aliases: T3F20.24, T3F20_24	5.1	5.5	-0.3	-1.4	100.0%	-4.0
4523	AT1G63020.1 | Symbol: NRPD1a |	5.6	6.3	-0.7	-1.4	100.0%	-3.5
4524	AT1G12620.1 | pentatricopeptide (PPR) repeat-containing protein, contains Pfam profile: PF01535 PPR repeat | chr1:4294592-4297082 REVERSE | Aliases: T12C24.15, T12C24_15	4.7	5.3	-0.6	-1.4	100.0%	-3.8
4525	AT1G29820.1 | expressed protein | chr1:10437646-10440770 FORWARD | Aliases: F1N18.14, F1N18_14	6.9	7.3	-0.4	-1.4	100.0%	-4.0
4526	AT1G13170.1 | oxysterol-binding family protein, similar to SP:P16258 Oxysterol-binding protein 1 {Oryctolagus cuniculus}; contains Pfam profiles PF00169: PH domain, PF01237: Oxysterol-binding protein | chr1:4488622-4492578 REVERSE | Aliases: F3F19.19, F3F19_19	4.2	4.8	-0.6	-1.4	100.0%	-4.0
4527	AT1G01070.2 | nodulin MtN21 family protein, similar to MtN21 GI:2598575 (root nodule development) from (Medicago truncatula) | chr1:38753-40927 REVERSE | Aliases: T25K16.7, T25K16_7	9.6	8.9	0.7	1.4	100.0%	-3.6
4528	AT2G38820.2 | expressed protein, contains Pfam profile PF04720: Protein of unknown function (DUF506) | chr2:16228914-16230740 FORWARD | Aliases: F13I13.5, F13I13_5	5.2	5.5	-0.3	-1.4	100.0%	-4.2
4529	AT4G17660.1 | protein kinase, putative, similar to protein kinase (Lophopyrum elongatum) gi:13022177:gb:AAK11674 | chr4:9831372-9833019 FORWARD | Aliases: DL4865W, FCAALL.77	2.7	2.9	-0.2	-1.4	100.0%	-4.4
4530	AT1G27480.1 | lecithin:cholesterol acyltransferase family protein / LACT family protein, similar to LCAT-like lysophospholipase (LLPL) (Homo sapiens) GI:4589720; contains Pfam profile PF02450: Lecithin:cholesterol acyltransferase (phosphatidylcholine-sterol acyltransferase) | chr1:9544501-9546272 FORWARD | Aliases: F17L21.27, F17L21_27	4.9	4.4	0.5	1.4	100.0%	-3.5
4531	AT4G13850.2 | Symbol: ATGRP2 |	8.1	7.1	1.0	1.4	100.0%	-3.6
4532	AT4G39100.1 | Symbol: SHL1 | PHD finger family protein / bromo-adjacent homology (BAH) domain-containing protein, contains Pfam domain, PF00628: PHD-finger and PF01426: BAH domain | chr4:18217933-18220276 REVERSE | Aliases: SHORT LIFE, T22F8.2	6.6	6.1	0.4	1.4	100.0%	-4.2
4533	AT5G56590.1 | glycosyl hydrolase family 17 protein, similar to elicitor inducible chitinase Nt-SubE76 GI:11071974 from (Nicotiana tabacum) | chr5:22924549-22926715 FORWARD | Aliases: MIK19.3, MIK19_3	6.9	7.3	-0.5	-1.4	100.0%	-3.9
4534	AT3G28850.1 | glutaredoxin family protein | chr3:10849906-10851359 FORWARD | Aliases: T19N8.15	6.6	6.2	0.5	1.4	100.0%	-4.0
4535	AT4G14660.1 | RNA polymerase Rpb7 N-terminal domain-containing protein, similar to SP:P52433 DNA-directed RNA polymerase II 19 kDa polypeptide (EC 2.7.7.6) (RPB7) {Rattus norvegicus}; contains Pfam profile PF03876: RNA polymerase Rpb7, N-terminal domain | chr4:8406330-8407042 REVERSE | Aliases: DL3370C, FCAALL.283	4.9	5.2	-0.3	-1.4	100.0%	-4.2
4536	AT1G21410.1 | F-box family protein, similar to SKP1 interacting partner 2 (SKIP2) TIGR_Ath1:At5g67250	7.7	7.2	0.5	1.4	100.0%	-3.9
4537	AT4G29410.1 | 60S ribosomal protein L28 (RPL28C), unknown protein chromosome II BAC F6F22 - Arabidopsis thaliana,PID:g3687251 | chr4:14468250-14470173 REVERSE | Aliases: F17A13.230, F17A13_230	11.0	11.4	-0.5	-1.4	100.0%	-4.1

Rank	Description	15.dpi	5.dpi	M	t	adj.q	B
4538	AT2G40910.2 | F-box protein-related, similar to F-box protein family, AtFBX9 (GI:20197985) (Arabidopsis thaliana); similar to F-box protein family, AtFBX8 (GI:20197464) (Arabidopsis thaliana) | chr2:17076946-17078735 REVERSE | Aliases: T20B5.11, T20B5_11	3.8	3.4	0.4	1.4	100.0%	-4.1
4539	AT5G48170.1 | F-box family protein, contains Pfam PF00646: F-box domain; similar to unknown protein (pir::T09884); | chr5:19549763-19550428 REVERSE | Aliases: MIF21.6, MIF21_6	2.7	2.6	0.2	1.4	100.0%	-4.7
4540	AT3G01650.1 | copine-related, low similarity to SP:Q99829 Copine I {Homo sapiens} | chr3:242018-245275 FORWARD | Aliases: F4P13.19, F4P13_19	5.6	5.2	0.3	1.4	100.0%	-3.9
4541	AT5G39940.1 | expressed protein | chr5:16004781-16007929 FORWARD | Aliases: MYH19.13, MYH19_13	4.0	4.2	-0.3	-1.4	100.0%	-4.2
4542	AT3G49880.1 | glycosyl hydrolase family protein 43, contains similarity to xylanase GI:2645416 from (Caldicellulosiruptor saccharolyticus) | chr3:18507234-18509698 FORWARD | Aliases: T16K5.230	4.5	4.2	0.4	1.4	100.0%	-3.9
4543	AT1G22840.1 | cytochrome c, putative, similar to cytochrome c (Pumpkin, Winter squash) SWISS-PROT:P00051 | chr1:8079279-8080563 FORWARD | Aliases: F19G10.20, F19G10_20	11.9	12.3	-0.3	-1.4	100.0%	-4.3
4544	AT3G19660.1 | expressed protein | chr3:6825527-6826012 REVERSE | Aliases: MMB12.13	5.9	5.4	0.5	1.4	100.0%	-3.7
4545	AT1G62150.1 | mitochondrial transcription termination factor-related / mTERF-related, contains Pfam profile PF02536: mTERF | chr1:22973694-22975429 REVERSE | Aliases: F19K23.9, F19K23_9	5.4	5.8	-0.5	-1.4	100.0%	-3.7
4546	AT1G49740.1 | expressed protein, similar to MAP3K-like protein kinase GB:CAB16796 GI:4006878 from (Arabidopsis thaliana) | chr1:18411247-18413690 FORWARD | Aliases: F14J22.5, F14J22_5	6.8	7.3	-0.5	-1.4	100.0%	-3.8
4547	AT5G24120.1 | Symbol: SIGE | RNA polymerase sigma subunit SigE (sigE) / sigma-like factor (SIG5), identical to RNA polymerase sigma subunit SigE (Arabidopsis thaliana) GI:4972299, sigma-like factor (Arabidopsis thaliana) GI:4033838; contains Pfam profiles PF04545: Sigma-70, region 4, PF04539: Sigma-70 region 3, PF04542: Sigma-70 region 2 | chr5:8157554-8160231 REVERSE | Aliases: MLE8.4, MLE8_4, SIG5, SIGMA FACTOR	6.2	5.7	0.5	1.4	100.0%	-3.9
4548	AT5G55060.1 | similar to expressed protein [Arabidopsis thaliana] (TAIR:At5g58510.1); similar to unnamed protein product [Tetraodon nigroviridis] (GB:CAF94658.1) | chr5:22359183-22363803 FORWARD | Aliases: MCO15.1, MCO15_1	5.2	5.9	-0.7	-1.4	100.0%	-4.0
4549	AT1G15880.1 | Symbol: GOS11 | Golgi SNARE 11 protein, identical to Golgi SNARE 11 protein (GI:13898893) {Arabidopsis thaliana}; similar to putative cis-Golgi SNARE protein GI:2583133 from (Arabidopsis thaliana);similar toSP:O95249: 28 kDa Golgi SNARE protein (28 kDa cis-Golgi SNARE p28) (GOS-28). (Homo sapiens); similar to Golgi SNARE protein (Golgi SNAP receptor complex member 1) (28 kDa cis-Golgi SNARE p28) (GOS-28) (SP:Q62931) {Rattus norvegicus}	8.5	7.9	0.5	1.4	100.0%	-4.0
4550	AT1G47250.1 | Symbol: PAF2 | 20S proteasome alpha subunit F2 (PAF2) (PRC2B) (PRS1), identical to GB:AAC32063 from (Arabidopsis thaliana) (Genetics 149 (2), 677-692 (1998)); identical to cDNA proteasome subunit prc2b GI:2511585 | chr1:17321617-17324100 FORWARD | Aliases: 20S PROTEASOME SUBUNIT PAF2, F8G22.3, F8G22_3	9.1	10.0	-0.9	-1.4	100.0%	-3.6
4551	AT3G27960.1 | kinesin light chain-related, low similarity to kinesin light chain (Plectonema boryanum) GI:2645229; contains Pfam profile PF00515: TPR Domain | chr3:10381617-10383830 REVERSE | Aliases: K24A2.5	6.1	5.1	0.9	1.4	100.0%	-3.7
4552	AT1G17470.1 | Symbol: ATDRG1 |	9.4	9.9	-0.5	-1.4	100.0%	-3.9
4553	AT3G49920.1 | porin, putative, similar to SP:P42055 34 kDa outer mitochondrial membrane protein porin (Voltage-dependent anion-selective channel protein) (VDAC) {Solanum tuberosum}; contains Pfam profile PF01459: Eukaryotic porin | chr3:18516821-18518684 REVERSE | Aliases: F3A4.1	4.9	4.5	0.5	1.4	100.0%	-4.0
4554	AT4G26520.1 | fructose-bisphosphate aldolase, cytoplasmic, identical to SP:P22197 Fructose-bisphosphate aldolase, cytoplasmic isozyme (EC 4.1.2.13) {Arabidopsis thaliana} | chr4:13388683-13390381 FORWARD | Aliases: M3E9.50, M3E9_50	2.7	2.9	-0.2	-1.4	100.0%	-4.3
4555	AT2G24350.1 | RNA recognition motif (RRM)-containing protein, low similarity to poly(A) binding protein II from (Xenopus laevis) GI:11527140, (Mus musculus) GI:2351846, (Bos taurus) GI:1051125; contains INTERPRO:IPR000504 RNA-binding region RNP-1 (RNA recognition motif) domain | chr2:10368206-10371434 FORWARD | Aliases: T28I24.8, T28I24_8	2.6	2.9	-0.3	-1.4	100.0%	-4.3
4556	AT3G44280.1 | expressed protein | chr3:15979580-15982083 REVERSE | Aliases: T10D17.70	6.5	7.2	-0.7	-1.4	100.0%	-3.8
4557	AT3G13070.1 | CBS domain-containing protein / transporter associated domain-containing protein, similar to SP:Q54318 Hemolysin C (Serpulina hyodysenteriae) {Treponema hyodysenteriae}; contains Pfam profiles PF00571: CBS domain, PF03471: Transporter associated domain, PF01595: Domain of unknown function | chr3:4191518-4195119 REVERSE | Aliases: MJG19.2	9.8	10.3	-0.5	-1.4	100.0%	-4.4

Rank	Description	15.dpi	5.dpi	M	t	adj.q	B
4558	AT1G28110.2 | Symbol: SCPL45 |	9.4	10.0	-0.5	-1.4	100.0%	-3.8
4559	AT3G17880.1 | Symbol: ATTDX | tetratricoredoxin (TDX), identical to tetratricoredoxin (Arabidopsis thaliana) GI:18041544; similar to SP:Q42443 Thioredoxin H-type (TRX-H) (Phloem sap 13 kDa protein-1) {Oryza sativa}; contains Pfam profile: PF00085 Thioredoxin | chr3:6123452-6126276 FORWARD | Aliases: AT3G17870, MEB5.24, TETRATICOPEPTIDE DOMAIN CONTAINING THIOREDOXIN, TETRATRICOREDOXIN	7.1	6.6	0.5	1.4	100.0%	-4.2
4560	AT5G04540.1 | expressed protein | chr5:1296443-1302429 REVERSE | Aliases: T32M21.150, T32M21_150	7.1	6.5	0.5	1.4	100.0%	-3.8
4561	AT3G12390.1 | nascent polypeptide associated complex alpha chain protein, putative / alpha-NAC, putative, similar to alpha-NAC, non-muscle form (Mus musculus) GI:1666690; contains Pfam profiles PF01849: NAC domain, PF00627: UBA/TS-N domain | chr3:3942266-3943789 FORWARD | Aliases: T2E22.29	10.7	11.0	-0.3	-1.4	100.0%	-4.1
4562	AT3G23430.1 | Symbol: PHO1 | phosphate transporter, putative (PHO1), identical to PHO1 protein (Arabidopsis thaliana) GI:20069032; supporting cDNA gi:20069031:gb:AF474076.1:; contains Pfam profiles PF03124: EXS family and PF03105: SPX domain | chr3:8387521-8393303 REVERSE | Aliases: MLM24.26, PHOSPHATE 1	7.3	6.6	0.7	1.4	100.0%	-3.9
4563	AT3G51750.2 | expressed protein | chr3:19207482-19208313 REVERSE | Aliases: T18N14.130	4.7	4.0	0.6	1.4	100.0%	-3.9
4564	AT1G23020.1 | ferric-chelate reductase, putative, similar to ferric-chelate reductase (FRO1) (Pisum sativum) GI:15341529; contains Pfam profile PF01794: Ferric reductase like transmembrane component	6.0	6.6	-0.6	-1.4	100.0%	-3.6
4565	AT4G01790.1 | ribosomal protein L7Ae/L30e/S12e/Gadd45 family protein / ribonuclease P-related, similar to human RNaseP-associate protein P38, GenBank accession number U77664 | chr4:769868-770728 FORWARD | Aliases: T7B11.5	3.2	3.0	0.2	1.4	100.0%	-4.4
4566	AT3G60850.1 | expressed protein | chr3:22492869-22495077 FORWARD | Aliases: T4C21.260	5.0	4.6	0.4	1.4	100.0%	-3.9
4567	AT3G19080.1 | SWIB complex BAF60b domain-containing protein, contains Pfam profile PF02201: BAF60b domain of the SWIB complex | chr3:6596088-6598673 FORWARD | Aliases: K13E13.21	3.0	3.4	-0.4	-1.4	100.0%	-4.2
4568	AT5G42220.1 | ubiquitin family protein, contains INTERPRO:IPR000626 ubiquitin domain | chr5:16889582-16894894 FORWARD | Aliases: K5J14.2, K5J14_2	7.0	7.5	-0.4	-1.4	100.0%	-4.0
4569	AT2G31870.1 | Symbol: TEJ | poly (ADP-ribose) glycohydrolase (PARG) family protein, similar to poly(ADP-ribose) glycohydrolase (Bos taurus) GI:2062407; contains Pfam domain, PF05028: poly (ADP-ribose) glycohydrolase (PARG) | chr2:13557076-13560833 REVERSE | Aliases: F20M17.9, F20M17_9	4.6	4.2	0.3	1.4	100.0%	-4.0
4570	AT5G48220.1 | indole-3-glycerol phosphate synthase, putative, similar to SP:P49572 | chr5:19567071-19569460 FORWARD | Aliases: MIF21.11, MIF21_11	8.3	7.8	0.4	1.4	100.0%	-3.8
4571	AT3G47960.1 | proton-dependent oligopeptide transport (POT) family protein, contains Pfam profile: PF00854 POT family | chr3:17708927-17711754 REVERSE | Aliases: T17F15.170	4.0	3.6	0.4	1.4	100.0%	-3.9
4572	AT1G34410.1 | Symbol: ARF21 | transcriptional factor B3 family protein / auxin-responsive factor AUX/IAA-related, contains Pfam profile: PF02309 AUX/IAA family | chr1:12577700-12580802 FORWARD | Aliases: ARF21, AUXIN RESPONSE FACTOR 21, F12K21.26, F12K21_26	2.3	2.1	0.2	1.4	100.0%	-4.4
4573	AT3G55600.1 | expressed protein, predicted proteins, Arabidopsis thaliana | chr3:20631661-20633449 FORWARD | Aliases: F1I16.10	5.6	5.3	0.4	1.4	100.0%	-4.0
4574	AT3G29110.1 | terpene synthase/cyclase family protein, contains Pfam profile: PF01397 terpene synthase family; similar to epidermal germacrene C synthase GB:AAC39431 (Lycopersicon esculentum), (+)-delta-cadinene synthase GB:P93665 (Gossypium hirsutum) | chr3:11083389-11087128 REVERSE | Aliases: MXE2.1	3.7	3.4	0.3	1.4	100.0%	-4.4
4575	AT2G42070.1 | MutT/nudix family protein, similar to SP:Q58549 ADP-ribose pyrophosphatase (EC 3.6.1.13) {Methanococcus jannaschii}; contains Pfam profile PF00293: NUDIX domain	4.7	4.4	0.3	1.4	100.0%	-4.1
4576	AT1G59990.1 | DEAD/DEAH box helicase, putative (RH22), similar to RNA helicase GI:3776015 from (Arabidopsis thaliana); contains Pfam profiles PF00271: Helicase conserved C-terminal domain, PF00270: DEAD/DEAH box helicase; matches EST OAO811-2 | chr1:22093904-22096667 REVERSE | Aliases: T2K10.4, T2K10_4	7.5	8.2	-0.7	-1.4	100.0%	-3.7
4577	AT4G36690.3 | Symbol: ATU2AF65A | U2 snRNP auxiliary factor large subunit, putative, similar to U2 snRNP auxiliary factor, large subunit (Nicotiana plumbaginifolia) GI:3850823 | chr4:17294019-17297725 REVERSE | Aliases: AP22.29, AP22_29, ATU2AF65A	7.9	7.4	0.5	1.4	100.0%	-3.9

Rank	Description	15.dpi	5.dpi	M	t	adj.q	B
4578	AT3G25990.1 | DNA-binding protein GT-1-related, similar to GT-1 GI:598073 from (Arabidopsis thaliana) | chr3:9505922-9507940 REVERSE | Aliases: MPE11.20	3.4	3.7	-0.3	-1.4	100.0%	-4.0
4579	AT4G30560.1 | Symbol: ATCNGC9 | cyclic nucleotide-regulated ion channel, putative, similar to cyclic nucleotide and calmodulin-regulated ion channel cngc6 GI:4581207 from (Arabidopsis thaliana) | chr4:14926980-14929687 REVERSE | Aliases: CNGC9, CYCLIC NUCLEOTIDE GATED CHANNEL 9, F17I23.100, F17I23_100	4.5	4.9	-0.4	-1.4	100.0%	-4.0
4580	AT5G54200.1 | WD-40 repeat family protein, contains Pfam PF00400: WD domain, G-beta repeat; similar to WD-repeat protein 5 (WD repeat protein BIG-3) (SP: Q9UGP9) (Homo sapiens) | chr5:22010575-22014302 REVERSE | Aliases: K18G13.8, K18G13_8	3.6	3.9	-0.3	-1.4	100.0%	-4.3
4581	AT4G03190.1 | Symbol: GRH1 | F-box family protein (FBL18), almost identical to GRR1-like protein 1 GI:12658970 from (Arabidopsis thaliana); similar to leucine-rich repeats containing F-box protein FBL3 (GI:5919219) (Homo sapiens); similar to F-box protein FBL2 (GI:6063090) (Homo sapiens) | chr4:1404445-1407139 REVERSE | Aliases: ATFBL18, ATGER1, ATGRH1, F4C21.11, F4C21_11, GRR1 LIKE PROTEIN 1	3.5	3.8	-0.3	-1.4	100.0%	-4.4
4582	AT3G58710.2 | Symbol: WRKY69 |	6.1	5.5	0.7	1.4	100.0%	-3.8
4583	AT4G37430.1 | Symbol: CYP91A2 | cytochrome P450 81F1 (CYP81F1) (CYP91A2), identical to cytochrome P450 81F1 (91A2) (SP:O65790) (Arabidopsis thaliana)	5.1	5.4	-0.3	-1.4	100.0%	-4.1
4584	AT3G61890.1 | Symbol: ATHB 12 | homeobox-leucine zipper protein 12 (HB-12) / HD-ZIP transcription factor 12, identical to homeobox-leucine zipper protein ATHB-12 (GI:6899887) (Arabidopsis thaliana)	4.3	3.7	0.6	1.4	100.0%	-4.0
4585	AT1G64400.1 | long-chain-fatty-acid--CoA ligase, putative / long-chain acyl-CoA synthetase, putative, similar to GI:1617270 (MF7P) from (Brassica napus) | chr1:23919261-23923446 REVERSE | Aliases: F15H21.7, F15H21_7	2.7	3.3	-0.6	-1.4	100.0%	-3.5
4586	AT5G06180.2 | expressed protein, similar to unknown protein (sp:Q9ZE28) | chr5:1873166-1876045 FORWARD | Aliases: MBL20.6, MBL20_6	4.6	4.9	-0.3	-1.4	100.0%	-4.1
4587	AT1G63990.1 | DNA topoisomerase VIA, putative (SPO11-2), similar to topoisomerase 6 subunit A (spo11) (Arabidopsis thaliana) GI:12331186; contains Pfam profile PF04406: Type IIB DNA topoisomerase; identical to cDNA putative topoisomerase VIA (SPO11 gene 2) GI:7270976 | chr1:23746505-23749037 REVERSE | Aliases: F22C12.24, F22C12_24	2.2	2.3	-0.1	-1.4	100.0%	-4.9
4588	AT3G53570.4 | Symbol: AFC1 | similar to protein kinase (AFC2) [Arabidopsis thaliana] (TAIR:At4g24740.1); similar to putative protein kinase (AME2/AFC1) [Oryza sativa (japonica cultivar-group)] (GB:BAD81689.1); similar to putative protein kinase AFC1 [Oryza sativa (japonica cultivar-group)] (GB:NP_915397.1); contains InterPro domain Serine/threonine protein kinase, active site (InterPro:IPR008271); contains InterPro domain Tyrosine protein kinase (InterPro:IPR001245); contains InterPro domain Serine/threonine protein kinase (InterPro:IPR002290); contains InterPro domain Protein kinase (InterPro:IPR000719); contains InterPro domain Proline-rich region (InterPro:IPR000694) | chr3:19872157-19875566 REVERSE | Aliases: AME2, ARABIDOPSIS FUS3 COMPLEMENTING GENE 1, CLK/STY (LAMMER TYPE) PROTEIN KINASE, F4P12.270	7.0	6.3	0.7	1.4	100.0%	-3.9
4589	AT3G50590.1 | transducin family protein / WD-40 repeat family protein, contains 3 WD-40 repeats (PF00400); some similarity to s-tomosyn isoform (GI:4689231)(Rattus norvegicus); contains non-consensus AT-AC splice sites at intron 18 | chr3:18782000-18790483 FORWARD | Aliases: AT3G50600, T20E23.190	4.3	4.6	-0.3	-1.4	100.0%	-4.3
4590	AT4G37890.2 | zinc finger (C3HC4-type RING finger) family protein, contains Pfam profiles PF00097: Zinc finger, C3HC4 type (RING finger), PF00092: von Willebrand factor type A domain	4.9	5.3	-0.5	-1.4	100.0%	-3.9
4591	AT2G01540.1 | C2 domain-containing protein, similar to zinc finger and C2 domain protein GI:9957238 from (Arabidopsis thaliana) | chr2:242122-243408 REVERSE | Aliases: F2I9.16, F2I9_16	6.2	5.8	0.4	1.4	100.0%	-4.2
4592	AT5G03650.1 | Symbol: SBE2.2 |	9.0	8.2	0.9	1.4	100.0%	-3.7
4593	AT2G23040.1 | expressed protein, ; expression supported by MPSS | chr2:9814973-9816289 REVERSE | Aliases: F21P24.10, F21P24_10	2.8	3.1	-0.3	-1.4	100.0%	-4.3
4594	AT5G05220.1 | expressed protein | chr5:1550268-1550929 FORWARD | Aliases: K18I23.2, K18I23_2	3.7	3.5	0.2	1.4	100.0%	-4.7
4595	AT4G38950.1 | kinesin motor family protein, similar to AtNACK1 kinesin-like protein (GI:19979627) (Arabidopsis thaliana); similar to kinesin-like protein NACK1 (GI:19570247) (Nicotiana tabacum) | chr4:18154466-18158455 REVERSE | Aliases: F19H22.50, F19H22_50	3.6	4.2	-0.6	-1.4	100.0%	-3.9
4596	AT1G09530.2 | Symbol: PIF3 | phytochrome interacting factor 3 (PIF3), identical to phytochrome interacting factor 3 (PIF3) GI:3929585 from (Arabidopsis thaliana) | chr1:3076604-3079541 FORWARD | Aliases: F14J9.19, F14J9_19, PAP3, PHOTOCURRENT 1, PHYTOCHROME INTERACTING FACTOR 3, PHYTOCHROME ASSOCIATED PROTEIN 3, POC1	4.1	4.5	-0.4	-1.4	100.0%	-4.0
4597	AT2G02360.1 | Symbol: ATPP2 B10 | F-box family protein / SKP1 interacting partner 3-related, contains similarity to SKP1 interacting partner 3 GI:10716951 from (Arabidopsis thaliana) | chr2:619407-620743 REVERSE | Aliases: ATPP2 B10, T16F16.15, T16F16_15	4.1	3.7	0.5	1.4	100.0%	-3.8

Rank	Description	15.dpi	5.dpi	M	t	adj.q	B
4598	AT1G16720.1 | expressed protein | chr1:5723081-5726471 FORWARD | Aliases: F19K19.14	8.4	7.9	0.4	1.4	100.0%	-4.0
4599	AT1G72180.1 | leucine-rich repeat transmembrane protein kinase, putative, similar to GI:3641252 from (Malus x domestica) (Plant Mol. Biol. 40 (6), 945-957 (1999)) | chr1:27166482-27171092 FORWARD | Aliases: T9N14.3, T9N14_3	4.6	4.2	0.4	1.4	100.0%	-4.0
4600	AT5G49950.1 | embryogenesis-associated protein-related, contains weak similarity to Embryogenesis-associated protein EMB8 (Swiss-Prot:Q40863) (Picea glauca) | chr5:20336442-20340842 REVERSE | Aliases: K9P8.9, K9P8_9	7.1	7.4	-0.3	-1.4	100.0%	-4.1
4601	AT1G55020.1 | Symbol: LOX1 | lipoxygenase (LOX1), identical to SP:Q06327 | chr1:20529375-20533940 FORWARD | Aliases: LIPOXYGENASE	4.7	5.4	-0.7	-1.4	100.0%	-3.7
4602	AT3G62600.1 | DNAJ heat shock family protein, similar to DnaJ homolog subfamily B member 11 precursor (SP:Q99KV1){Mus musculus}; contains Pfam PF00226: DnaJ domain; contains PfaPF01556: DnaJ C terminal regionm | chr3:23161766-23164486 REVERSE | Aliases: F26K9.30	9.5	10.2	-0.6	-1.4	100.0%	-4.1
4603	AT5G21160.1 | La domain-containing protein / proline-rich family protein, contains proline-rich extensin domains, INTERPRO:IPR002965, PF05383: La domain | chr5:7198744-7204093 REVERSE | Aliases: T10F18.190, T10F18_190	4.7	5.1	-0.4	-1.4	100.0%	-4.4
4604	AT2G40760.1 | rhodanese-like domain-containing protein, contains rhodanese-like domain PF00581	7.6	8.0	-0.4	-1.4	100.0%	-3.9
4605	AT5G13930.1 | Symbol: CHS | chalcone synthase / naringenin-chalcone synthase, identical to SP:P13114 | chr5:4488692-4490266 FORWARD | Aliases: ATCHS, CHALCONE SYNTHASE, CHALCONE/STILBENE SYNTHASE, MAC12.28, MAC12_28, NARINGENIN CHALCONE SYNTHASE, TESTA 4 PROTEIN, TRANSPARENT TESTA 4, TT4	5.7	6.4	-0.7	-1.4	100.0%	-3.7
4606	AT4G36960.1 | RNA recognition motif (RRM)-containing protein, similar to SP:P48809 Heterogeneous nuclear ribonucleoprotein 27C (hnRNP 48) {Drosophila melanogaster}; contains InterPro entry IPR000504: RNA-binding region RNP-1 (RNA recognition motif) (RRM); non-consensus TA donor splice site at exon 6 | chr4:17426927-17429757 FORWARD | Aliases: AP22.66, AP22_66	6.2	5.8	0.4	1.4	100.0%	-3.9
4607	AT2G33860.1 | Symbol: ETT | auxin-responsive factor (ARF3) / ETTIN protein (ETT), identical to ETTIN GB:AF007788 from (Arabidopsis thaliana) | chr2:14332345-14335847 REVERSE | Aliases: ARF3, AUXIN RESPONSE TRANSCRIPTION FACTOR 3, ETTIN, T1B8.30, T1B8_30	6.3	6.8	-0.4	-1.4	100.0%	-3.8
4608	AT1G01500.1 | expressed protein, identical to cDNA unknown protein GI:1922247 embY10087.1 | chr1:185133-186923 FORWARD | Aliases: F22L4.17, F22L4_17	6.7	6.5	0.3	1.4	100.0%	-4.2
4609	AT3G55410.1 | 2-oxoglutarate dehydrogenase E1 component, putative / oxoglutarate decarboxylase, putative / alpha-ketoglutaric dehydrogenase, putative, similar to SP:P20967 2-oxoglutarate dehydrogenase E1 component, mitochondrial precursor (EC 1.2.4.2) (Alpha-ketoglutarate dehydrogenase) {Saccharomyces cerevisiae}; contains Pfam profiles PF02779: Transketolase, pyridine binding domain, PF00676: Dehydrogenase E1 component | chr3:20552412-20557011 FORWARD | Aliases: T22E16.70	6.8	7.5	-0.7	-1.4	100.0%	-4.1
4610	AT5G55280.1 | Symbol: FTSZ1 1 | cell division protein FtsZ, chloroplast, putative (FTSZ), identical to SP:Q42545 Cell division protein ftsZ homolog, chloroplast precursor {Arabidopsis thaliana}; similar to FtsZ1 (Tagetes erecta) GI:8896066; contains Pfam profiles PF00091: Tubulin/FtsZ family, GTPase domain, PF03953: Tubulin/FtsZ family, C-terminal domain | chr5:22437882-22439838 REVERSE | Aliases: ATFTSZ1 1, CPFTSZ, MCO15.23, MCO15_23	8.5	9.1	-0.6	-1.4	100.0%	-3.6
4611	AT1G03610.1 | expressed protein | chr1:900886-903002 FORWARD | Aliases: F21B7.22	5.2	4.7	0.4	1.4	100.0%	-3.9
4612	AT3G15350.2 | glycosyltransferase family 14 protein / core-2/I-branching enzyme family protein, contains Pfam profile: PF02485 Core-2/I-Branching enzyme | chr3:5166249-5169527 FORWARD | Aliases: K7L4.15	4.0	4.2	-0.3	-1.4	100.0%	-4.1
4613	AT1G71950.1 | expressed protein, similar to Pi starvation-induced protein GB:BAA06151 from (Nicotiana tabacum)	8.7	8.2	0.5	1.4	100.0%	-4.0
4614	AT5G63960.1 | DNA-directed DNA polymerase delta catalytic subunit, putative (POLD1), similar to DNA polymerase delta (Glycine max) GI:2895198, OsPol delta large subunit (Oryza sativa (japonica cultivar-group) GI:9188570; contains Pfam profiles: PF03175 DNA polymerase type B, organellar and viral, PF00136 DNA polymerase family B, PF03104 DNA polymerase family B, exonuclease domain	4.2	4.6	-0.4	-1.4	100.0%	-3.8
4615	AT3G27690.1 | Symbol: LHCB2:4 | chlorophyll A-B binding protein (LHCB2:4), nearly identical to Lhcb2 protein (Arabidopsis thaliana) GI:4741950; similar to chlorophyll A-B binding protein 151 precursor (LHCP) GB:P27518 from (Gossypium hirsutum); contains Pfam PF00504: Chlorophyll A-B binding protein | chr3:10257184-10258248 FORWARD | Aliases: LHCB2.3, MGF10.10	7.4	6.6	0.7	1.4	100.0%	-3.5
4616	AT1G74590.1 | Symbol: ATGSTU10 | glutathione S-transferase, putative, similar to putative glutathione S-transferase GB:CAA10060 (Arabidopsis thaliana); contains Pfam profile: PF00043 Glutathione S-transferases | chr1:28027288-28028387 REVERSE | Aliases: F1M20.27, F1M20_27	3.1	3.9	-0.8	-1.4	100.0%	-3.6

Rank	Description	15.dpi	5.dpi	M	t	adj.q	B
4617	AT4G09570.1 | Symbol: CPK4 | calcium-dependent protein kinase, putative / CDPK, putative, similar to calcium-dependent protein kinase (Arabidopsis thaliana) gi:604881:dbj:BAA04830; contains protein kinase domain, Pfam:PF00069; contains EF hand domain (calcium-binding EF-hand), Pfam:PF00036, INTERPRO:IPR002048 | chr4:6049518-6052332 FORWARD | Aliases: T25P22.10, T25P22_10	9.0	9.4	-0.5	-1.4	100.0%	-4.2
4618	AT3G46960.1 | similar to HUA enhancer 2 (HEN2) / DExH-box RNA helicase, putative [Arabidopsis thaliana] (TAIR:At2g06990.1); similar to superkiller viralicidic activity 2-like (S. cerevisiae) [Homo sapiens] (GB:CAI41730.1); similar to superkiller viralicidic activity 2-like (S. cerevisiae) [Homo sapiens] (GB:CAI41863.1); similar to similar to putative DEAD/DEAH box helicase [Schizosaccharomyces pombe] [Dictyostelium discoideum] (GB:AAO51181.1); similar to superkiller viralicidic activity 2-like [Rattus norvegicus] (GB:NP_998724.1); similar to superkiller viralicidic activity 2-like [Mus musculus] (GB:NP_067312.1); contains InterPro domain Helicase, C-terminal (InterPro:IPR001650); contains InterPro domain DEAD/DEAH box helicase (InterPro:IPR001410) | chr3:17301878-17309052 REVERSE | Aliases: F13I12.10	7.1	7.3	-0.2	-1.4	100.0%	-4.3
4619	AT1G24090.1 | RNase H domain-containing protein, very low similarity to GAG-POL precursor (Oryza sativa (japonica cultivar-group)) GI:5902445; contains Pfam profiles PF00075: RNase H, PF04134: Protein of unknown function, DUF393 | chr1:8520823-8524679 FORWARD | Aliases: T23E23.25	3.0	3.2	-0.2	-1.4	100.0%	-4.7
4620	AT4G22330.1 | Symbol: ATCES1 |	5.7	5.0	0.7	1.4	100.0%	-4.0
4621	AT1G10670.2 | Symbol: ACLA 1 |	11.0	11.5	-0.5	-1.4	100.0%	-4.3
4622	AT4G23050.2 | protein kinase, putative, similar to MAP3K delta-1 protein kinase (Arabidopsis thaliana) gi:2253010:emb:CAA74591; contains Pfam PF00069 Protein kinase domain and PF00989 PAS domain | chr4:12080071-12084267 FORWARD | Aliases: F7H19.240, F7H19_240	7.3	6.9	0.4	1.4	100.0%	-3.9
4623	AT4G25700.2 | Symbol: BETA OHASE 1 | similar to beta-carotene hydroxylase, putative [Arabidopsis thaliana] (TAIR:At5g52570.1); similar to beta-carotene hydroxylase [Citrus unshiu] (GB:AAG10793.1); contains InterPro domain Carotene hydroxylase (InterPro:IPR005596); contains InterPro domain SUR2-type hydroxylase/desaturase, catalytic domain (InterPro:IPR006087) | chr4:13094782-13095944 REVERSE | Aliases: BETA HYDROXYLASE 1, L73G19.80, L73G19_80	8.2	7.7	0.5	1.4	100.0%	-3.8
4624	AT5G23740.1 | Symbol: RPS11 BETA | 40S ribosomal protein S11 (RPS11C)	11.3	11.7	-0.4	-1.4	100.0%	-4.0
4625	AT5G13800.2 | hydrolase, alpha/beta fold family protein, low similarity to hydrolase (Terrabacter sp. DBF63) GI:14196240; contains Pfam profile PF00561: hydrolase, alpha/beta fold family | chr5:4451840-4454559 REVERSE | Aliases: MAC12.25, MAC12_25	6.5	6.1	0.4	1.4	100.0%	-4.0
4626	AT5G62210.1 | embryo-specific protein-related, contains weak similarity to embryo-specific protein 3 (GI:3335171) (Arabidopsis thaliana) | chr5:25003553-25004715 REVERSE | Aliases: MMI9.4, MMI9_4	4.1	4.5	-0.4	-1.4	100.0%	-4.0
4627	AT2G40430.1 | expressed protein, identical to Protein At2g40430 (Swiss-Prot:O22892) (Arabidopsis thaliana); similar to Glioma tumor suppressor candidate region gene 2 protein (p60) (Swiss-Prot:Q9NZM5) (Homo sapiens) | chr2:16886297-16889505 REVERSE | Aliases: T2P4.22	9.4	9.8	-0.4	-1.4	100.0%	-3.9
4628	AT1G51390.2 | Symbol: NFU5 | similar to nitrogen fixation NifU-like family protein [Arabidopsis thaliana] (TAIR:At3g20970.1); similar to hypothetical protein UM04972.1 [Ustilago maydis 521] (GB:EAK85802.1); contains InterPro domain Nitrogen-fixing NifU, C-terminal (InterPro:IPR001075)	4.4	4.7	-0.3	-1.4	100.0%	-4.2
4629	AT5G25110.1 | Symbol: CIPK25 |	6.0	5.0	1.0	1.4	100.0%	-3.6
4630	AT5G61640.1 | Symbol: PMSR1 | peptide methionine sulfoxide reductase, putative, similar to peptide methionine sulfoxide reductase (msr) (Arabidopsis thaliana) GI:4884033; contains Pfam profile PF01625: Peptide methionine sulfoxide reductase | chr5:24792261-24793616 FORWARD | Aliases: K11J9.18, K11J9_18, PEPTIDEMETHIONINE SULFOXIDE REDUCTASE 1	5.3	4.6	0.7	1.4	100.0%	-3.8
4631	AT2G27150.1 | Symbol: AAO3 | aldehyde oxidase 3 (AAO3), identical to GP:3172044:gnl:PID:d1029570:AB010080 | chr2:11608832-11614239 FORWARD | Aliases: ALDEHYDE OXIDASE 3, F20F1.2, F20F1_2	7.0	7.4	-0.4	-1.4	100.0%	-4.0
4632	AT5G50120.1 | transducin family protein / WD-40 repeat family protein, Similar to En/Spm-like transposon protein (gi:2739374)(Arabidopsis thaliana); similar to GTP-binding regulatory protein and WD-repeat protein; contains 7 WD-40 repeats	5.1	4.6	0.6	1.4	100.0%	-3.8
4633	AT2G34820.1 | basic helix-loop-helix (bHLH) family protein, contains Pfam profile: PF00010 helix-loop-helix DNA-binding domain	2.2	2.3	-0.1	-1.4	100.0%	-4.9
4634	AT2G27790.1 | expressed protein | chr2:11854412-11856362 REVERSE | Aliases: F15K20.11, F15K20_11	3.5	3.3	0.2	1.4	100.0%	-4.3
4635	AT1G31770.1 | ABC transporter family protein, contains Pfam profile: PF00005: ABC transporter | chr1:11374874-11377775 REVERSE | Aliases: F5M6.22	6.1	6.8	-0.8	-1.4	100.0%	-3.8
4636	AT2G41000.1 | Symbol: LCR51 | DNAJ heat shock N-terminal domain-containing protein, similar to SP:O75190 DnaJ homolog subfamily B member 6 (Heat shock protein J2) Homo sapiens; contains Pfam profile PF00226 DnaJ domain | chr2:17117906-17120673 FORWARD | Aliases: LCR51, Low molecular weight cysteine rich 51, T3K9.23, T3K9_23	4.9	4.5	0.4	1.4	100.0%	-4.2

Rank	Description	15.dpi	5.dpi	M	t	adj.q	B
4637	AT4G27370.1 | Symbol: VIIIB | myosin family protein, contains Pfam profiles: PF00063 myosin head (motor domain), PF00612 IQ calmodulin-binding motif | chr4:13694887-13700786 REVERSE | Aliases: F27G19.14, TVIIIB	4.0	4.3	-0.3	-1.4	100.0%	-4.2
4638	AT4G36880.1 | cysteine proteinase, putative, strong similarity to cysteine proteinase COT44 precursor SP:P25251 from (Brassica napus) (Rape) | chr4:17374459-17376220 REVERSE | Aliases: AP22.67, AP22_67	5.0	4.5	0.5	1.4	100.0%	-3.8
4639	AT5G19440.1 | similar to Eucalyptus gunnii alcohol dehydrogenase of unknown physiological function (GI:1143445), apple tree, PIR:T16995; NOT a cinnamyl-alcohol dehydrogenase | chr5:6556422-6558344 FORWARD | Aliases: F7K24.190, F7K24_190	10.3	11.2	-0.9	-1.4	100.0%	-3.9
4640	AT1G73510.1 | expressed protein | chr1:27646312-27646779 REVERSE | Aliases: T9L24.31, T9L24_31	2.5	2.4	0.2	1.4	100.0%	-4.7
4641	AT5G13530.1 | protein kinase family protein / ankyrin repeat family protein, contains similarity to ankyrin-related gene UNC-44 gi:790608:gb:AAA85854; contains Pfam domains PF00023: Ankyrin repeat and PF00069: Protein kinase domain | chr5:4345621-4351150 FORWARD | Aliases: T6I14.60, T6I14_60	2.6	2.4	0.2	1.4	100.0%	-4.9
4642	AT5G18690.1 | hydroxyproline-rich glycoprotein family protein, contains proline-rich extensin domains, INTERPRO:IPR002965; Common family member: At3g06360 (Arabidopsis thaliana) | chr5:6233521-6234128 REVERSE | Aliases: T1A4.70, T1A4_70	2.8	3.6	-0.7	-1.4	100.0%	-4.0
4643	AT2G17800.1 | Symbol: ARAC1 | Rac-like GTP-binding protein (ARAC1) (ATGP2), identical to Rac-like GTP-binding protein (ARAC1) SP:Q38902 from (Arabidopsis thaliana) | chr2:7746954-7749237 FORWARD | Aliases: ATGP2, T17A5.14, T17A5_14	7.1	7.6	-0.5	-1.4	100.0%	-3.9
4644	AT5G09510.1 | 40S ribosomal protein S15 (RPS15D), ribosomal protein S15 - Arabidopsis thaliana, EMBL:Z23161 | chr5:2955114-2956674 REVERSE | Aliases: T5E8.310, T5E8_310	7.9	8.6	-0.6	-1.4	100.0%	-3.9
4645	AT1G47230.2 | Symbol: CYCA3;4 | cyclin, putative, similar to cyclin A-like protein (Nicotiana tabacum) GI:1064931, A-type cyclin (Catharanthus roseus) GI:2190259; contains Pfam profiles PF00134: Cyclin, N-terminal domain, PF02984: Cyclin, C-terminal domain | chr1:17309017-17311674 FORWARD | Aliases: CYCA3;4, Cyclin A3;4, F8G22.5, F8G22_5	4.5	4.9	-0.4	-1.4	100.0%	-4.0
4646	AT5G24400.1 | Symbol: EMB2024 | glucosamine/galactosamine-6-phosphate isomerase family protein, low similarity to SP:O95336 6-phosphogluconolactonase (EC 3.1.1.31) (6PGL) {Homo sapiens}; contains Pfam profile PF01182: Glucosamine-6-phosphate isomerase/6-phosphogluconolactonase	8.0	8.6	-0.7	-1.4	100.0%	-3.9
4647	AT5G45160.1 | root hair defective 3 GTP-binding (RHD3) family protein, contains Pfam profile: PF05879 root hair defective 3 GTP-binding protein (RHD3) family | chr5:18282218-18287510 FORWARD | Aliases: K18C1.4, K18C1_4	6.6	5.9	0.6	1.4	100.0%	-4.0
4648	AT5G37670.1 | 15.7 kDa class I-related small heat shock protein-like (HSP15.7-CI), contains Pfam profile: PF00011 Hsp20/alpha crystallin family; identified in Scharf, K-D., et al,Cell Stress & Chaperones (2001) 6: 225-237.	7.3	6.7	0.6	1.4	100.0%	-3.9
4649	AT5G02540.1 | short-chain dehydrogenase/reductase (SDR) family protein, contains INTERPRO family IPR002198 Short-chain dehydrogenase/reductase (SDR) superfamily | chr5:568524-570918 FORWARD | Aliases: T22P11.130, T22P11_130	2.2	2.4	-0.2	-1.4	100.0%	-4.7
4650	AT1G05805.1 | basic helix-loop-helix (bHLH) family protein | chr1:1744773-1747692 FORWARD | Aliases: None	4.4	4.0	0.4	1.4	100.0%	-3.9
4651	AT2G37430.1 | zinc finger (C2H2 type) family protein (ZAT11), contains Pfam domain, PF00096: Zinc finger, C2H2 type | chr2:15713440-15714303 FORWARD | Aliases: F3G5.22, F3G5_22	2.4	2.6	-0.2	-1.4	100.0%	-4.5
4652	AT1G69840.4 | band 7 family protein, strong similarity to hypersensitive-induced response protein (Zea mays) GI:7716466; contains Pfam profile PF01145: SPFH domain / Band 7 family | chr1:26297331-26299298 REVERSE | Aliases: T17F3.13, T17F3_13	8.1	6.9	1.1	1.4	100.0%	-3.6
4653	AT1G18060.1 | expressed protein | chr1:6211867-6213358 REVERSE | Aliases: T10F20.7	7.1	6.7	0.4	1.4	100.0%	-3.9
4654	AT3G56820.1 | expressed protein | chr3:21050790-21052724 REVERSE | Aliases: T8M16.150	7.1	6.8	0.3	1.4	100.0%	-4.1
4655	AT2G34620.1 | mitochondrial transcription termination factor-related / mTERF-related, contains Pfam profile PF02536: mTERF | chr2:14584275-14585367 FORWARD | Aliases: T31E10.4, T31E10_4	2.7	2.9	-0.2	-1.4	100.0%	-4.5
4656	AT3G59780.1 | expressed protein | chr3:22097904-22101421 FORWARD | Aliases: F24G16.50	7.7	8.4	-0.6	-1.4	100.0%	-3.5
4657	AT3G10690.1 | DNA gyrase subunit A family protein, similar to SP:P94605 DNA gyrase subunit A (EC 5.99.1.3). {Clostridium acetobutylicum}; contains Pfam profiles PF00521: DNA gyrase/topoisomerase IV A subunit, PF03989: DNA gyrase C-terminal domain beta-propeller | chr3:3339397-3346345 REVERSE | Aliases: T7M13.23	4.4	5.3	-0.9	-1.4	100.0%	-3.8
4658	AT5G41960.1 | expressed protein | chr5:16806942-16808270 FORWARD | Aliases: MJC20.6, MJC20_6	7.8	7.4	0.4	1.4	100.0%	-4.0

Rank	Description	15.dpi	5.dpi	M	t	adj.q	B
4659	AT1G52260.1 | Symbol: ATPDIL1 5 | Encodes a protein disulfide isomerase-like (PDIL) protein, a member of a multigene family within the thioredoxin (TRX) superfamily.	5.8	6.3	-0.5	-1.4	100.0%	-3.9
4660	AT1G47128.1 | cysteine proteinase (RD21A) / thiol protease, identical to SP:P43297 Cysteine proteinase RD21A precursor (EC 3.4.22.-) {Arabidopsis thaliana}, thiol protease RD21A SP:P43297 from (Arabidopsis thaliana) | chr1:17285265-17288110 REVERSE | Aliases: F2G19.31, F2G19_31	9.5	8.8	0.7	1.4	100.0%	-4.2
4661	AT3G27820.1 | monodehydroascorbate reductase, putative, similar to cytosolic monodehydroascorbate reductase GB:BAA77214 (Oryza sativa)	7.6	8.0	-0.4	-1.4	100.0%	-4.1
4662	AT4G11670.1 | expressed protein, contains Pfam PF05664: Protein of unknown function (DUF810) | chr4:7044395-7053085 REVERSE | Aliases: T5C23.100, T5C23_100	4.9	5.4	-0.5	-1.4	100.0%	-3.8
4663	AT5G49800.1 | expressed protein | chr5:20251868-20253410 REVERSE | Aliases: K21G20.1, K21G20_1	3.2	3.5	-0.3	-1.4	100.0%	-4.3
4664	AT5G49670.1 | expressed protein | chr5:20193611-20198897 FORWARD | Aliases: K2I5.3, K2I5_3	3.2	2.9	0.3	1.4	100.0%	-4.4
4665	AT5G58520.1 | protein kinase family protein, contains protein kinase domain, Pfam:PF00069 | chr5:23672476-23675388 FORWARD | Aliases: MQJ2.14, MQJ2_14	5.7	6.2	-0.4	-1.4	100.0%	-3.8
4666	AT1G65650.1 | ubiquitin carboxyl-terminal hydrolase family 1 protein, similar to 26S proteasome regulatory complex subunit p37A (Drosophila melanogaster) GI:6434962; contains Pfam profile PF01088: Ubiquitin carboxyl-terminal hydrolase, family 1 | chr1:24418678-24421372 REVERSE | Aliases: F1E22.3	7.8	8.6	-0.8	-1.4	100.0%	-3.9
4667	AT5G02840.3 | similar to myb family transcription factor [Arabidopsis thaliana] (TAIR:At3g09600.1); similar to myb family transcription factor-like [Oryza sativa (japonica cultivar-group)] (GB:BAD29385.1); contains InterPro domain Myb DNA-binding domain (InterPro:IPR001005); contains InterPro domain Myb-like DNA-binding domain, SHAQKYF class (InterPro:IPR006447) | chr5:648702-651956 FORWARD | Aliases: F9G14.150, F9G14_150	6.2	5.2	0.9	1.4	100.0%	-3.8
4668	AT3G30260.1 | MADS-box protein (AGL79), similar to GB:Q38876 from (Arabidopsis thaliana) (Plant Cell 7 (11), 1763-1771 (1995)); contains Pfam profile PF00319: SRF-type transcription factor (DNA-binding and dimerisation domain) | chr3:11911598-11915359 FORWARD | Aliases: T6J22.1	3.2	3.0	0.2	1.4	100.0%	-4.9
4669	AT3G47640.1 | basic helix-loop-helix (bHLH) family protein, contains Pfam profile: PF00010 helix-loop-helix DNA-binding domain	4.0	3.7	0.3	1.4	100.0%	-4.0
4670	AT1G71260.1 | expressed protein | chr1:26865327-26867080 REVERSE | Aliases: F3I17.9, F3I17_9	7.4	8.2	-0.8	-1.4	100.0%	-3.7
4671	AT5G24870.2 | zinc finger (C3HC4-type RING finger) family protein, similar to Pfam domain, PF00097: Zinc finger, C3HC4 type (RING finger) | chr5:8544788-8547116 REVERSE | Aliases: F6A4.80, F6A4_80	4.9	4.3	0.6	1.4	100.0%	-3.6
4672	AT3G12830.1 | auxin-responsive family protein, similar to auxin-induced protein (SP:P33082) (Glycine max) | chr3:4078658-4079615 REVERSE | Aliases: MBK21.19	4.5	3.9	0.6	1.4	100.0%	-4.0
4673	AT2G45600.1 | expressed protein, low similarity to PrMC3 (Pinus radiata) GI:5487873 | chr2:18796662-18798026 FORWARD | Aliases: F17K2.13	3.8	4.3	-0.5	-1.4	100.0%	-3.8
4674	AT1G17550.1 | Symbol: HAB2 | protein phosphatase 2C-related / PP2C-related, similar to protein phosphatase 2C GI:3242077 from (Arabidopsis thaliana) | chr1:6034355-6037282 FORWARD | Aliases: F1L3.38	6.4	6.1	0.3	1.4	100.0%	-4.3
4675	AT4G25820.1 | Symbol: XTR9 | xyloglucan:xyloglucosyl transferase / xyloglucan endotransglycosylase / endo-xyloglucan transferase (XTR9), identical to xyloglucan endotransglycosylase GI:4218963 from (Arabidopsis thaliana) | chr4:13130306-13131797 FORWARD | Aliases: F14M19.100, F14M19_100, XYLOGLUCAN ENDOTRANSGLYCOSYLASE 9	4.4	3.9	0.5	1.4	100.0%	-3.8
4676	AT2G36720.1 | PHD finger transcription factor, putative | chr2:15400150-15406497 FORWARD | Aliases: F13K3.12, F13K3_12	5.2	5.7	-0.5	-1.4	100.0%	-4.0
4677	AT5G63870.3 | Symbol: PP7 | serine/threonine protein phosphatase (PP7), identical to PP7 (Arabidopsis thaliana) GI:2791900 | chr5:25578398-25580500 REVERSE | Aliases: MGI19.12, MGI19_12	4.2	4.6	-0.4	-1.4	100.0%	-3.9
4678	AT5G63040.2 | expressed protein, similar to unknown protein (emb:CAB81845.1) | chr5:25305372-25307638 FORWARD | Aliases: MJH22.10, MJH22_10	3.8	4.1	-0.3	-1.4	100.0%	-4.1
4679	AT1G16840.4 | expressed protein | chr1:5762706-5764027 REVERSE | Aliases: F17F16.27	7.7	7.0	0.8	1.4	100.0%	-3.8
4680	AT2G26120.1 | glycine-rich protein | chr2:11130508-11130895 FORWARD | Aliases: T19L18.7, T19L18_7	5.1	4.5	0.6	1.4	100.0%	-3.8
4681	AT5G04870.1 | Symbol: CPK1 | calcium-dependent protein kinase isoform AK1 (AK1), identical to calcium-dependent protein kinase, isoform AK1 (CDPK) (Arabidopsis thaliana) SWISS-PROT:Q06850; contains protein kinase domain, Pfam:PF00069; contains EF hand domain (calcium-binding EF-hand), Pfam:PF00036, INTERPRO:IPR002048 | chr5:1416784-1420339 REVERSE | Aliases: AK1, ATCPK1, CALCIUM DEPENDENT PROTEIN KINASE, MUK11.19, MUK11_19	8.5	8.9	-0.4	-1.4	100.0%	-4.5

Rank	Description	15.dpi	5.dpi	M	t	adj.q	B
4682	AT5G66280.1 | Symbol: GMD1 | GDP-D-mannose 4,6-dehydratase, putative, strong similarity to GDP-D-mannose-4,6-dehydratase (Arabidopsis thaliana) GI:1764100 | chr5:26493392-26495344 FORWARD | Aliases: GDP D MANNOSE 4,6 DEHYDRATASE 1, K1L20.6, K1L20_6	3.9	4.3	-0.4	-1.4	100.0%	-4.0
4683	AT1G26260.3 | similar to basic helix-loop-helix (bHLH) family protein [Arabidopsis thaliana] (TAIR:At1g68920.2); similar to hypothetical protein [Oryza sativa (japonica cultivar-group)] (GB:NP_910691.1); similar to putative TA1 protein [Oryza sativa (japonica cultivar-group)] (GB:BAD68029.1); contains InterPro domain Basic helix-loop-helix dimerization domain bHLH (InterPro:IPR001092) | chr1:9087201-9089365 FORWARD | Aliases: F28B23.8, F28B23_8	2.7	3.0	-0.2	-1.4	100.0%	-4.4
4684	AT1G73690.1 | Symbol: CDKD1;1 | cell division protein kinase, putative, similar to cell division protein kinase 7 (Homo sapiens) SWISS-PROT:P50613 | chr1:27718609-27720942 FORWARD | Aliases: CDKD1;1, Cyclin dependent kinase D1;1, F25P22.11, F25P22_11	3.3	3.9	-0.6	-1.4	100.0%	-4.2
4685	AT3G04590.2 | DNA-binding family protein, contains a AT hook motif (DNA binding motifs with a preference for A/T rich regions), Pfam:PF02178 | chr3:1238859-1241646 REVERSE | Aliases: F7O18.6, F7O18_6	5.4	5.0	0.4	1.4	100.0%	-3.8
4686	AT1G05350.1 | thiF family protein, low similarity to SP:P30138 Adenylyltransferase thiF (EC 2.7.7.-) {Escherichia coli}; contains Pfam profile PF00899: ThiF family | chr1:1560890-1564004 REVERSE | Aliases: YUP8H12.3, YUP8H12_3	7.8	8.4	-0.6	-1.4	100.0%	-3.7
4687	AT2G46060.2 | transmembrane protein-related, contains weak similarity to Swiss-Prot:Q9HCN3 transmembrane protein 8 precursor (M83 protein) (Homo sapiens) | chr2:18948172-18952129 REVERSE | Aliases: T3F17.29	5.7	5.3	0.5	1.4	100.0%	-4.0
4688	AT5G09390.2 | similar to SMC2orf [Podocoryne carnea] (GB:CAA08790.1) | chr5:2913535-2915924 FORWARD | Aliases: T5E8.190, T5E8_190	7.8	7.5	0.3	1.4	100.0%	-4.1
4689	AT3G55000.1 | Symbol: TON1A | tonneau family protein, similar to tonneau 1b (GI:11494367) (Arabidopsis thaliana) | chr3:20392485-20394764 FORWARD | Aliases: T15C9.7, TON1, TONNEAU 1	4.8	5.3	-0.5	-1.4	100.0%	-4.1
4690	AT5G27720.1 | Symbol: EMB1644 | small nuclear ribonucleoprotein, putative / snRNP, putative / Sm protein, putative, similar to SWISS-PROT:Q9QXA5 U6 snRNA-associated Sm-like protein LSm4 (Mus musculus) | chr5:9815579-9817576 FORWARD | Aliases: EMB1644, EMBRYO DEFECTIVE 1644, T1G16.50, T1G16_50	8.2	7.7	0.5	1.4	100.0%	-3.9
4691	AT1G22020.1 | glycine hydroxymethyltransferase, putative / serine hydroxymethyltransferase, putative / serine/threonine aldolase, putative, similar to serine hydroxymethyltransferase (Chlamydomonas reinhardtii) GI:17066746; contains Pfam profile PF00464: serine hydroxymethyltransferase	4.6	4.2	0.4	1.4	100.0%	-4.0
4692	AT3G16190.1 | isochorismatase hydrolase family protein, low similarity to SP:P32400 N-carbamoylsarcosine amidase (EC 3.5.1.59) (N-carbamoylsarcosine amidohydrolase) {Arthrobacter sp}; contains Pfam profile PF00857: isochorismatase family protein | chr3:5489707-5491254 REVERSE | Aliases: MYA6.5	8.0	7.6	0.5	1.4	100.0%	-3.8
4693	AT5G24150.2 | Symbol: SQP1 | similar to squalene monooxygenase 1,2 / squalene epoxidase 1,2 (SQP1,2) [Arabidopsis thaliana] (TAIR:At5g24160.1); similar to squalene monooxygenase 2 / squalene epoxidase 2 (SQP2) [Arabidopsis thaliana] (TAIR:At5g24140.1); similar to squalene epoxidase homologue [Brassica napus] (GB:CAA06770.1); contains InterPro domain Flavoprotein monooxygenase (InterPro:IPR000733); contains InterPro domain NAD-binding site (InterPro:IPR000205); contains InterPro domain Aromatic-ring hydroxylase (InterPro:IPR003042) | chr5:8172319-8175434 REVERSE | Aliases: K12G2.2, K12G2_2	3.7	3.5	0.3	1.4	100.0%	-4.3
4694	AT4G15980.1 | pectinesterase family protein, contains Pfam profile: PF01095 pectinesterase | chr4:9057478-9059995 REVERSE | Aliases: DL4030C, FCAALL.248	2.0	2.1	-0.2	-1.4	100.0%	-4.8
4695	AT1G49540.1 | transducin family protein / WD-40 repeat family protein, similar to signal transducer and activator of transcription interacting protein 1 (GI:15929722) {Mus musculus}; similar to hypothetical protein GB:AAD43147 GI:5430747 from (Arabidopsis thaliana); contains Pfam PF00400: WD domain, G-beta repeat (11 copies, 2 weak)	4.5	4.8	-0.3	-1.4	100.0%	-4.2
4696	AT1G51915.1 | cryptdin protein-related, contains weak similarity to Swiss-Prot:P17533 cryptdin-related protein 1C precursor (CRS1C) (Mus musculus) | chr1:19297147-19297638 FORWARD | Aliases: None	2.0	2.0	0.1	1.4	100.0%	-5.4
4697	AT1G14370.1 | Symbol: APK2A | protein kinase (APK2a), identical to protein kinase APK2a GI:2852447 from (Arabidopsis thaliana) | chr1:4915662-4918303 FORWARD | Aliases: F14L17.14, F14L17_14, PROTEIN KINASE 2A	5.2	4.8	0.5	1.4	100.0%	-3.8
4698	AT2G31320.1 | poly (ADP-ribose) polymerase, putative / NAD(+) ADP-ribosyltransferase, putative / poly(ADP-ribose) synthetase, putative, similar to poly(ADP)-ribose polymerase (Zea mays) GI:3928871 ; contains Pfam profiles PF00644: Poly(ADP-ribose) polymerase catalytic domain, PF00645: Poly(ADP-ribose) polymerase and DNA-Ligase Zn-finger region, PF02877: Poly(ADP-ribose) polymerase, regulatory domain, PF00533: BRCA1 C Terminus (BRCT) domain | chr2:13360898-13366805 REVERSE | Aliases: F16D14.16, F16D14_16	5.1	5.6	-0.4	-1.4	100.0%	-3.8
4699	AT4G34110.1 | Symbol: PAB2 | polyadenylate-binding protein 2 (PABP2), non-consensus TA donor splice site at exon 2, polyadenylate-binding protein - Triticum aestivum (common wheat),PIR:T06979 | chr4:16336392-16340102 FORWARD | Aliases: F28A23.130, F28A23_130, POLY(A) BINDING PROTEIN 2	10.9	10.2	0.8	1.4	100.0%	-3.9
4700	AT2G22600.1 | KH domain-containing protein | chr2:9603697-9606130 FORWARD | Aliases: T9I22.4, T9I22_4	4.8	5.2	-0.4	-1.4	100.0%	-3.8

Rank	Description	15.dpi	5.dpi	M	t	adj.q	B
4701	AT1G08250.1 | prephenate dehydratase family protein, contains similarity to prephenate dehydratase GI:1008717 from (Amycolatopsis methanolica)	6.1	6.4	-0.4	-1.4	100.0%	-4.0
4702	AT3G25220.1 | Symbol: FKBP15 1 | FK506-binding protein 2-1 (FKBP15-1) / immunophilin / peptidyl-prolyl cis-trans isomerase / rotamase, identical to SP:Q38935 FK506-binding protein 2-1 precursor (EC 5.2.1.8) (Peptidyl-prolyl cis- trans isomerase) (PPiase) (Rotamase) (15 kDa FKBP) (FKBP-15-1) {Arabidopsis thaliana}, immunophilin (FKBP15-1) GB:U52046 (Arabidopsis thaliana) (Proc. Natl. Acad. Sci. U.S.A. 93 (14), 6964-6969 (1996)) | chr3:9183855-9185849 FORWARD | Aliases: MJL12.19	9.8	10.1	-0.3	-1.4	100.0%	-4.2
4703	AT2G10870.1 | expressed protein | chr2:4290762-4291013 FORWARD | Aliases: F16G22.7, F16G22_7	4.0	3.7	0.3	1.4	100.0%	-4.3
4704	AT2G30920.1 | Symbol: ATCOQ3 |	6.7	6.2	0.5	1.4	100.0%	-3.8
4705	AT5G16010.1 | 3-oxo-5-alpha-steroid 4-dehydrogenase family protein / steroid 5-alpha-reductase family protein, similar to steroid 5alpha-reductase - Rattus norvegicus, PIR:A34239 (SP:24008); contains Pfam 3-oxo-5-alpha-steroid 4-dehydrogenase domain PF02544 | chr5:5227927-5229119 FORWARD | Aliases: F1N13.150, F1N13_150	9.1	8.2	0.9	1.4	100.0%	-3.7
4706	AT1G70850.3 | similar to Bet v I allergen family protein [Arabidopsis thaliana] (TAIR:At1g70830.1); similar to Bet v I allergen family protein [Arabidopsis thaliana] (TAIR:At1g70830.2); similar to Csf-2 [Cucumis sativus] (GB:BAA83470.1); contains InterPro domain Bet v I allergen (InterPro:IPR000916) | chr1:26718528-26720445 REVERSE | Aliases: F15H11.10, F15H11_10	4.5	5.0	-0.5	-1.4	100.0%	-3.9
4707	AT4G29750.1 | expressed protein, contains Pfam domain, PF04581: Protein of unknown function (DUF578) | chr4:14569734-14572810 FORWARD | Aliases: T16L4.260, T16L4_260	5.0	5.5	-0.5	-1.4	100.0%	-3.9
4708	AT5G65390.1 | Symbol: AGP7 | arabinogalactan-protein (AGP7) | chr5:26145810-26146564 REVERSE | Aliases: MNA5.12, MNA5_12	7.1	6.6	0.5	1.4	100.0%	-3.9
4709	AT1G03905.1 | ABC transporter family protein, similar to NBD-like protein GB:AAD20643 | chr1:993477-995593 FORWARD | Aliases: None	6.5	7.1	-0.6	-1.4	100.0%	-3.8
4710	AT5G14120.1 | nodulin family protein, similar to nodulin-like protein (Arabidopsis thaliana) GI:3329368, nodule-specific protein Nlj70 (Lotus japonicus) GI:3329366 | chr5:4555984-4559116 FORWARD | Aliases: MUA22.12, MUA22_12	7.1	6.6	0.5	1.4	100.0%	-3.8
4711	AT1G48450.2 | similar to expressed protein [Arabidopsis thaliana] (TAIR:At3g17800.1); similar to unknown [Brassica napus] (GB:AAK30572.1); contains InterPro domain Protein of unknown function DUF760 (InterPro:IPR008479) | chr1:17912171-17914113 REVERSE | Aliases: T1N15.6, T1N15_6	9.5	9.1	0.4	1.4	100.0%	-4.1
4712	AT3G49710.1 | pentatricopeptide (PPR) repeat-containing protein, contains Pfam profile PF01535: PPR repeat | chr3:18448830-18450995 FORWARD | Aliases: T16K5.60	5.1	5.9	-0.8	-1.4	100.0%	-4.0
4713	AT2G01980.1 | Symbol: SOS1 | sodium proton exchanger, putative (NHX7) (SOS1), identical to putative Na+/H+ antiporter SOS1 (Arabidopsis thaliana) gi:8515714:gb:AAF76139; Member of The Monovalent Cation:Proton Antiporter (CPA1) Family, PMID:11500563 | chr2:457009-463325 FORWARD | Aliases: ATSOS1, F14H20.5, F14H20_5, SALT OVERLY SENSITIVE 1	5.4	5.8	-0.4	-1.4	100.0%	-3.9
4714	AT1G76420.1 | Symbol: CUC3 | no apical meristem (NAM) family protein, N-term similar to N-term of NAM GB:CAA63101 (Petunia x hybrida) (apical meristem formation), CUC2 GB:BAA19529 (Arabidopsis thaliana), GRAB2 protein GB:CAA09372 (Triticum sp.)	2.1	2.0	0.1	1.4	100.0%	-5.1
4715	AT3G03840.1 | auxin-responsive protein, putative, similar to auxin-inducible SAUR (Small Auxin Up RNAs) (GI:3043536) (Raphanus sativus) | chr3:981079-981805 FORWARD | Aliases: F20H23.14, F20H23_14	2.1	2.0	0.1	1.4	100.0%	-5.3
4716	AT1G73400.1 | pentatricopeptide (PPR) repeat-containing protein, contains Pfam profile PF01535: PPR repeat | chr1:27602073-27603473 FORWARD | Aliases: T9L24.39, T9L24_39	3.3	3.7	-0.3	-1.4	100.0%	-4.2
4717	AT2G20690.1 | lumazine-binding family protein, SP:P50854 Riboflavin synthase alpha chain (EC 2.5.1.9) {Actinobacillus pleuropneumoniae}; contains Pfam profile PF00677: Lumazine binding domain | chr2:8930386-8932052 FORWARD | Aliases: F5H14.34, F5H14_34	5.3	4.9	0.4	1.4	100.0%	-4.2
4718	AT1G76280.2 | similar to pentatricopeptide (PPR) repeat-containing protein [Arabidopsis thaliana] (TAIR:At2g18940.1); similar to P0529E05.10 [Oryza sativa (japonica cultivar-group)] (GB:NP_915531.1); contains InterPro domain PPR repeat (InterPro:IPR002885) | chr1:28623258-28627625 REVERSE | Aliases: F15M4.22	3.4	3.7	-0.3	-1.4	100.0%	-4.4
4719	AT5G17270.1 | tetratricopeptide repeat (TPR)-containing protein, contains Pfam profile PF00515 TPR Domain | chr5:5679997-5685599 FORWARD | Aliases: MKP11.12, MKP11_12	4.4	4.9	-0.5	-1.4	100.0%	-3.8
4720	AT5G27660.1 | DegP protease, putative, similar to Serine protease HTRA2, mitochondrial precursor (High temperature requirement protein A2) (HtrA2) (Omi stress-regulated endoprotease) (Serine proteinase OMI) (SP:O43464) {Homo sapiens} | chr5:9789902-9792427 REVERSE | Aliases: T1G16.1	4.4	4.8	-0.4	-1.4	100.0%	-4.0

Rank	Description	15.dpi	5.dpi	M	t	adj.q	B
4721	AT3G17170.1 | Symbol: RFC3 | ribosomal protein S6 family protein (RFC3), annotation temporarily based on supporting cDNA gi:15620809:dbj:AB057424.1:; contains TIGRfam TIGR00166 and Pfam PF01250 profiles ribosomal protein S6. | chr3:5852646-5854912 REVERSE | Aliases: K14A17.25, REGULATOR OF FATTY ACID COMPOSITION 3	5.5	6.6	-1.1	-1.4	100.0%	-3.7
4722	AT3G49870.1 | Symbol: ATARLA1C | ADP-ribosylation factor, putative, similar to ADP-ribosylation factor-like protein 1 (SP:P40616) (Homo sapiens); ARF3 ADP-RIBOSYLATION FACTOR,GP:453191 Arabidopsis thaliana; contains domain PF00025: ADP-ribosylation factor family | chr3:18503435-18505124 REVERSE | Aliases: ATARLA1C, T16K5.220	9.2	8.8	0.4	1.4	100.0%	-3.9
4723	AT1G21840.1 | expressed protein, weak similarity to SP:Q07402 Urease accessory protein ureF (strain TB-90) {Bacillus sp.} | chr1:7666489-7667657 FORWARD | Aliases: T26F17.5	5.5	5.1	0.5	1.4	100.0%	-4.0
4724	AT2G29410.1 | Symbol: MTPB1 | zinc transporter, putative, similar to zinc transporter ZAT (Arabidopsis thaliana) gi:4206640:gb:AAD11757; similar to zinc transporter ZnT-2 (Rattus norvegicus) gi:1256378:gb:AAB02775; member of the cation diffusion facilitator (CDF) family, or cation efflux (CE) family, PMID:11500563 | chr2:12623652-12625064 FORWARD | Aliases: ATMTPB1, F16P2.21, F16P2_21	2.7	3.1	-0.4	-1.4	100.0%	-4.0
4725	AT5G04000.1 | expressed protein | chr5:1079102-1079839 REVERSE | Aliases: F8F6.210, F8F6_210	5.6	4.8	0.8	1.4	100.0%	-3.6
4726	AT5G22830.1 | Symbol: GMN10 | magnesium transporter CorA-like family protein, weak similarity to SP:Q01926 RNA splicing protein MRS2, mitochondrial precursor {Saccharomyces cerevisiae}; contains Pfam profile PF01544: CorA-like Mg2+ transporter protein; supporting cDNA gi:12007446:gb:AF322255.1:AF322255 | chr5:7627653-7631371 FORWARD | Aliases: GMN10, MRN17.6, MRN17_6	6.9	6.6	0.3	1.4	100.0%	-4.1
4727	AT1G13020.1 | eukaryotic translation initiation factor, putative (EIF4B5), eukaryotic initiation factor 4B (GI:6739522) {Arabidopsis thaliana}; EST gb:T22808 comes from this gene | chr1:4440542-4443682 REVERSE | Aliases: F3F19.4, F3F19_4	5.5	6.0	-0.5	-1.4	100.0%	-3.9
4728	AT2G31730.1 | ethylene-responsive protein, putative, similar to ethylene-inducible ER33 protein (Lycopersicon esculentum) gi:5669656:gb:AAD46413 | chr2:13494769-13495670 REVERSE | Aliases: T9H9.27	3.8	4.3	-0.5	-1.4	100.0%	-3.8
4729	AT3G62620.1 | sucrose-phosphatase-related, contains weak similarity to sucrose-phosphatase (Arabidopsis thaliana) gi:11127757:gb:AAG31075 | chr3:23170779-23172830 FORWARD | Aliases: F26K9.50	3.8	4.1	-0.3	-1.4	100.0%	-4.1
4730	AT5G44785.2 | expressed protein, contains Pfam PF05329: Protein of unknown function (DUF731) | chr5:18087918-18090356 REVERSE | Aliases: None	6.2	6.7	-0.5	-1.4	100.0%	-3.9
4731	AT5G57440.1 | Symbol: GS1 | haloacid dehalogenase-like hydrolase family protein, similar to SP:Q08623 GS1 protein {Homo sapiens}; contains InterPro accession IPR005834: Haloacid dehalogenase-like hydrolase	4.7	5.5	-0.8	-1.4	100.0%	-3.6
4732	AT2G16570.1 | Symbol: ATASE | amidophosphoribosyltransferase / glutamine phosphoribosylpyrophosphate amidotransferase / phosphoribosyldiphosphate 5-amidotransferase, identical to amidophosphoribosyltransferase (Arabidopsis thaliana) GI:469193; identical to cDNA amidophosphoribosyltransferase GI:469192 | chr2:7187227-7189275 REVERSE | Aliases: ATASE1, F1P15.5, F1P15_5, GLN PHOSPHORIBOSYL PYROPHOSPHATE AMIDOTRANSFERASE 1	7.7	8.3	-0.6	-1.4	100.0%	-3.8
4733	AT1G26570.1 | UDP-glucose 6-dehydrogenase, putative, strong similarity to SP:Q96558 UDP-glucose 6-dehydrogenase (EC 1.1.1.22) (UDP-Glc dehydrogenase) (UDP-GlcDH) (UDPGDH) {Glycine max}; contains Pfam profiles PF03721: UDP-glucose/GDP-mannose dehydrogenase family NAD binding domain, PF00984: UDP-glucose/GDP-mannose dehydrogenase family central domain, PF03720: UDP-glucose/GDP-mannose dehydrogenase family UDP binding domain | chr1:9182191-9184380 FORWARD | Aliases: T1K7.6, T1K7_6	4.8	5.2	-0.4	-1.4	100.0%	-3.9
4734	AT4G13360.1 | similar to enoyl-CoA hydratase/isomerase family protein [Arabidopsis thaliana] (TAIR:At3g24360.1); similar to ENSANGP00000024573 [Anopheles gambiae str. PEST] (GB:XP_312972.2); contains InterPro domain Enoyl-CoA hydratase/isomerase (InterPro:IPR001753) | chr4:7775124-7778223 FORWARD | Aliases: T9E8.100, T9E8_100	7.2	8.7	-1.4	-1.4	100.0%	-3.9
4735	AT2G17845.1 | short-chain dehydrogenase/reductase (SDR) family protein, contains similarity to 3-oxoacyl-(acyl-carrier protein) reductase SP:P51831 from (Bacillus subtilis) | chr2:7765928-7766963 FORWARD | Aliases: None	3.3	3.0	0.2	1.4	100.0%	-4.5
4736	AT5G07440.1 | Symbol: GDH2 | glutamate dehydrogenase 2 (GDH2), identical to glutamate dehydrogenase 2 (GDH 2) (Arabidopsis thaliana) SWISS-PROT:Q38946 | chr5:2355938-2358195 FORWARD | Aliases: GLUTAMATE DEHYDROGENASE 2, T2I1.150, T2I1_150	8.7	9.2	-0.5	-1.4	100.0%	-4.2
4737	AT4G20070.1 | peptidase M20/M25/M40 family protein, contains similarity to hydantoin utilization protein C (Pseudomonas sp.) SWISS-PROT:Q01264; contains Pfam profile PF01546: Peptidase family M20/M25/M40 | chr4:10861465-10864705 FORWARD | Aliases: F18F4.170, F18F4_170	5.5	5.2	0.3	1.4	100.0%	-4.3
4738	AT2G30730.1 | serine/threonine protein kinase, putative, similar to Pto kinase interactor 1 (serine/threonine protein kinase) (Lycopersicon esculentum) gi:3668069:gb:AAC61805; contains protein kinase domain, Pfam:PF00069 | chr2:13100222-13101754 FORWARD | Aliases: T11J7.12, T11J7_12	2.3	2.1	0.1	1.4	100.0%	-5.1

Rank	Description	15.dpi	5.dpi	M	t	adj.q	B
4739	AT3G59980.1 | tRNA-binding region domain-containing protein, similar to SP:O54873 Multisynthetase complex auxiliary component p43 (Contains: Endothelial-monocyte activating polypeptide II (EMAP-II) (Small inducible cytokine subfamily E member 1)) {Cricetulus griseus}; contains Pfam profile PF01588: Putative tRNA binding domain | chr3:22165221-22166618 REVERSE | Aliases: F24G16.250	7.6	8.1	-0.4	-1.4	100.0%	-3.8
4740	AT3G47120.1 | RNA recognition motif (RRM)-containing protein, contains InterPro entry IPR000504: RNA-binding region RNP-1 (RNA recognition motif) (RRM) | chr3:17362126-17363807 REVERSE | Aliases: F13I12.170	6.9	6.3	0.6	1.4	100.0%	-3.9
4741	AT1G22440.1 | alcohol dehydrogenase, putative, similar to alcohol dehydrogenase ADH GI:7705214 from (Lycopersicon esculentum); contains Pfam zinc-binding dehydrogenase domain PF00107	6.5	7.1	-0.6	-1.4	100.0%	-4.0
4742	AT1G04080.1 | hydroxyproline-rich glycoprotein family protein, Contains similarity to pre-mRNA processing protein PRP39 gb L29224 from S. cerevisiae. ESTs gb:R64908 and gb:T88158, gb:N38703 and gb:AA651043 come from this gene | chr1:1051375-1056750 FORWARD | Aliases: F20D22.14	8.1	8.6	-0.5	-1.4	100.0%	-4.3
4743	AT5G29000.2 | myb family transcription factor, contains Pfam profile: PF00249 myb-like DNA-binding domain | chr5:11022087-11024279 REVERSE | Aliases: F3F24.100, F3F24_100	5.3	4.8	0.4	1.4	100.0%	-3.9
4744	AT3G02560.2 | 40S ribosomal protein S7 (RPS7B), similar to ribosomal protein S7 GB:AAD26256 from (Secale cereale) | chr3:541807-543341 FORWARD | Aliases: F16B3.19, F16B3_19	10.1	10.7	-0.6	-1.4	100.0%	-4.2
4745	AT3G04810.2 | similar to protein kinase family protein [Arabidopsis thaliana] (TAIR:At1g54510.1); similar to protein kinase, putative [Arabidopsis thaliana] (TAIR:At5g28290.1); similar to putative LSTK-1-like kinase [Oryza sativa (japonica cultivar-group)] (GB:AAR01739.1); similar to LSTK-1-like kinase [Lycopersicon esculentum] (GB:AAL04423.1); contains InterPro domain Serine/threonine protein kinase, active site (InterPro:IPR008271); contains InterPro domain Tyrosine protein kinase (InterPro:IPR001245); contains InterPro domain Serine/threonine protein kinase (InterPro:IPR002290); contains InterPro domain Protein kinase (InterPro:IPR000719) | chr3:1317266-1321300 FORWARD | Aliases: T9J14.24, T9J14_24	3.9	4.7	-0.8	-1.4	100.0%	-3.6
4746	AT1G70480.2 | expressed protein, similar to expressed protein [Arabidopsis thaliana] (TAIR:At1g23560.1); contains InterPro domain Protein of unknown function DUF220 (InterPro:IPR003863) | chr1:26565722-26567472 FORWARD | Aliases: F24J13.5, F24J13_5	9.6	8.8	0.8	1.4	100.0%	-3.9
4747	AT4G35180.1 | amino acid transporter family protein, similar to amino acid permease 1 GI:976402 from (Nicotiana sylvestris); contains Pfam profile PF01490: Transmembrane amino acid transporter protein | chr4:16738344-16740371 REVERSE | Aliases: T12J5.50, T12J5_50	3.7	3.4	0.2	1.4	100.0%	-4.6
4748	AT3G17020.1 | universal stress protein (USP) family protein, similar to early nodulin ENOD18 (Vicia faba) GI:11602747; contains Pfam profile PF00582: universal stress protein family | chr3:5802555-5804130 REVERSE | Aliases: K14A17.9	9.9	8.8	1.1	1.4	100.0%	-4.2
4749	AT5G65470.1 | expressed protein, contains Pfam PF03138: Plant protein family. The function of this family of plant proteins is unknown; | chr5:26189358-26192259 FORWARD | Aliases: MNA5.21, MNA5_21	8.4	7.9	0.4	1.4	100.0%	-4.1
4750	AT5G10690.1 | pentatricopeptide (PPR) repeat-containing protein / CBS domain-containing protein, contains CBS and PPR domain repeats | chr5:3374394-3377431 REVERSE | Aliases: MAJ23.50, MAJ23_50	6.1	6.5	-0.5	-1.4	100.0%	-3.7
4751	AT5G23250.1 | succinyl-CoA ligase (GDP-forming) alpha-chain, mitochondrial, putative / succinyl-CoA synthetase, alpha chain, putative / SCS-alpha, putative, similar to SP:P36967 Succinyl-CoA ligase (GDP-forming) alpha-chain, mitochondrial precursor (EC 6.2.1.4) (Succinyl-CoA synthetase, alpha chain) (SCS-alpha) {Dictyostelium discoideum}; contains Pfam profiles PF00549: CoA-ligase, PF02629: CoA binding domain | chr5:7830392-7832846 FORWARD | Aliases: MKD15.11, MKD15_11	10.5	11.1	-0.6	-1.4	100.0%	-3.9
4752	AT2G27230.2 | similar to expressed protein [Arabidopsis thaliana] (TAIR:At1g64625.1); similar to transcription factor-like [Oryza sativa (japonica cultivar-group)] (GB:BAD29456.1) | chr2:11657437-11661221 FORWARD | Aliases: F12K2.19, F12K2_19	7.2	7.7	-0.5	-1.4	100.0%	-3.9
4753	AT1G13610.2 | similar to expressed protein [Arabidopsis thaliana] (TAIR:At3g30380.1); similar to putative Cgi67 serine protease [Oryza sativa (japonica cultivar-group)] (GB:XP_464411.1); contains InterPro domain Esterase/lipase/thioesterase (InterPro:IPR000379) | chr1:4663832-4666028 REVERSE | Aliases: F21F23.4	5.8	5.2	0.6	1.4	100.0%	-3.8
4754	AT4G32830.1 | Encodes a member of a family of Ser/Thr kinases whose activities peak during cell division. Transcripts are abundant in tissues rich in dividing cells like roots and flowers but are low or absent in fully expanded leaves and stems.	5.8	5.3	0.5	1.4	100.0%	-4.0
4755	AT1G72240.1 | expressed protein, similar to expressed protein [Arabidopsis thaliana] (TAIR:At1g22470.1); contains domain SER_RICH (PS50324) | chr1:27193841-27194411 FORWARD | Aliases: T9N14.5, T9N14_5	4.8	3.9	0.9	1.4	100.0%	-3.7
4756	AT4G03140.1 | short-chain dehydrogenase/reductase (SDR) family protein, similar to stem secoisolariciresinol dehydrogenase GI:13752458 from {Forsythia x intermedia}; similar to sex determination protein tasselseed 2 SP:P50160 from (Zea mays) | chr4:1392601-1393662 FORWARD | Aliases: F4C21.6, F4C21_6	2.8	3.3	-0.5	-1.4	100.0%	-3.9

Rank	Description	15.dpi	5.dpi	M	t	adj.q	B
4757	AT2G21130.1 | peptidyl-prolyl cis-trans isomerase / cyclophilin (CYP2) / rotamase, identical to cyclophilin (Arabidopsis thaliana) gi:2443757:gb:AAB71402; similar to peptidyl-prolyl cis-trans isomerase, PPIase (cyclophilin, cyclosporin A-binding protein) (Arabidopsis thaliana) SWISS-PROT:P34790 | chr2:9062479-9063313 REVERSE | Aliases: F26H11.11, F26H11_11	11.4	10.5	0.9	1.4	100.0%	-3.5
4758	AT1G21880.2 | peptidoglycan-binding LysM domain-containing protein, contains Pfam profile PF01476: LysM domain	5.5	6.4	-0.9	-1.4	100.0%	-3.9
4759	AT2G02950.1 | Symbol: PKS1 | phytochrome kinase substrate 1 (PKS1), identical to Swiss-Prot:Q9SWI1 phytochrome kinase substrate 1 (Arabidopsis thaliana) | chr2:854946-856537 REVERSE | Aliases: PHYTOCHROME KINASE SUBSTRATE 1, T17M13.12, T17M13_12	2.5	2.3	0.2	1.4	100.0%	-4.9
4760	AT1G52560.1 | 26.5 kDa class I small heat shock protein-like (HSP26.5-P), contains Pfam profile: PF00011 Hsp20/alpha crystallin family; identified in Scharf, K-D., et al,Cell Stress & Chaperones (2001) 6: 225-237. | chr1:19578429-19579435 REVERSE | Aliases: F6D8.22, F6D8_22	3.2	3.5	-0.3	-1.4	100.0%	-4.2
4761	AT3G04940.1 | Symbol: ATCYSD1 | cysteine synthase, putative / O-acetylserine (thiol)-lyase, putative / O-acetylserine sulfhydrylase, putative, similar to O-acetylserine(thiol) lyase (EC 4.2.99.8) (Brassica juncea) GI:2245144; contains Pfam profile PF00291: Pyridoxal-phosphate dependent enzyme	6.5	6.9	-0.4	-1.4	100.0%	-3.9
4762	AT3G27260.1 | Symbol: GTE8 | DNA-binding bromodomain-containing protein, contains bromodomain, INTERPRO:IPR001487 | chr3:10068875-10073898 FORWARD | Aliases: GLOBAL TRANSCRIPTION FACTOR GROUP E, K17E12.8	6.4	5.7	0.6	1.4	100.0%	-3.7
4763	AT5G13450.2 | similar to F1-ATP synthase delta subunit [Ipomoea batatas] (GB:BAA77508.1); similar to oligomycin sensitivity conferring protein [Silene latifolia] (GB:AAN38066.1); contains InterPro domain H+-transporting two-sector ATPase, delta (OSCP) subunit (InterPro:IPR000711) | chr5:4310317-4312015 REVERSE | Aliases: T22N19.100, T22N19_100	10.6	11.4	-0.8	-1.4	100.0%	-3.9
4764	AT1G48880.1 | similar to expressed protein [Arabidopsis thaliana] (TAIR:At3g06080.2); similar to leaf senescence related protein-like [Oryza sativa (japonica cultivar-group)] (GB:BAD81676.1); contains InterPro domain Protein of unknown function DUF231 (InterPro:IPR004253) | chr1:18084701-18086593 FORWARD | Aliases: F27K7.9, F27K7_9	4.5	4.8	-0.3	-1.4	100.0%	-4.2
4765	AT1G63710.1 | Symbol: CYP86A7 | cytochrome P450, putative, similar to cytochrome P450 GB:O23066 (Arabidopsis thaliana) | chr1:23635841-23637605 REVERSE | Aliases: F24D7.10, F24D7_10	2.6	2.5	0.2	1.4	100.0%	-4.7
4766	AT5G06670.1 | kinesin motor protein-related | chr5:2048244-2055020 REVERSE | Aliases: F15M7.20, F15M7_20	6.3	5.9	0.3	1.4	100.0%	-4.0
4767	AT5G26210.1 | PHD finger family protein, contains Pfam domain, PF00628: PHD-finger | chr5:9157982-9160357 REVERSE | Aliases: T19G15.60, T19G15_60	5.6	5.3	0.4	1.4	100.0%	-4.0
4768	AT1G15840.1 | expressed protein | chr1:5451427-5452369 FORWARD | Aliases: F7H2.17, F7H2_17	7.3	6.6	0.7	1.4	100.0%	-3.9
4769	AT5G47400.1 | expressed protein | chr5:19246389-19248749 FORWARD | Aliases: MQL5.26, MQL5_26	3.0	2.7	0.3	1.4	100.0%	-4.3
4770	AT5G11230.1 | phosphate translocator-related, low similarity to phosphoenolpyruvate/phosphate translocator precursor (Mesembryanthemum crystallinum) GI:9295275, SP:P52178 Triose phosphate/phosphate translocator, non-green plastid, chloroplast precursor (CTPT) {Brassica oleracea} | chr5:3580563-3581618 FORWARD | Aliases: F2I11.120, F2I11_120	4.5	4.8	-0.3	-1.4	100.0%	-4.2
4771	AT4G27460.1 | CBS domain-containing protein | chr4:13732916-13734310 FORWARD | Aliases: F27G19.60, F27G19_60	3.7	3.3	0.4	1.4	100.0%	-4.0
4772	AT3G48070.2 | similar to expressed protein [Arabidopsis thaliana] (TAIR:At5g62910.1); similar to OSJNBa0060P14.3 [Oryza sativa (japonica cultivar-group)] (GB:CAD41040.2); contains domain SER_RICH (PS50324) | chr3:17761697-17763746 FORWARD | Aliases: T17F15.60	5.8	5.1	0.7	1.4	100.0%	-3.7
4773	AT5G15830.1 | bZIP transcription factor family protein, similar to common plant regulatory factor 7 GI:9650828 from (Petroselinum crispum); contains Pfam profile: PF00170 bZIP transcription factor | chr5:5168546-5169364 FORWARD | Aliases: F14F8.210, F14F8_210	4.1	3.5	0.6	1.4	100.0%	-3.7
4774	AT2G41550.1 | expressed protein | chr2:17335617-17338211 REVERSE | Aliases: T32G6.7, T32G6_7	5.0	5.5	-0.4	-1.4	100.0%	-3.9
4775	AT5G19970.1 | expressed protein, ; expression supported by MPSS | chr5:6747552-6748643 FORWARD | Aliases: F28I16.120, F28I16_120	3.5	3.0	0.5	1.4	100.0%	-3.7
4776	AT5G64700.1 | nodulin MtN21 family protein, similar to MtN21 GI:2598575 (root nodule development) from (Medicago truncatula); contains Pfam profile PF00892: Integral membrane protein	3.5	3.2	0.3	1.4	100.0%	-4.2
4777	AT1G79190.1 | expressed protein | chr1:29793532-29799063 FORWARD | Aliases: YUP8H12R.20, YUP8H12R_20	4.8	5.2	-0.4	-1.4	100.0%	-4.3
4778	AT3G49050.1 | lipase class 3 family protein / calmodulin-binding heat-shock protein, putative, calmodulin-binding heat-shock protein, Nicotiana tabacum, PIR:T04107 | chr3:18191953-18194737 FORWARD | Aliases: T2J13.110	4.5	5.1	-0.6	-1.4	100.0%	-4.0

Rank	Description	15.dpi	5.dpi	M	t	adj.q	B
4779	AT4G16840.1 | expressed protein | chr4:9474904-9475542 FORWARD | Aliases: DL4445W, FCAALL.92	4.2	3.8	0.3	1.4	100.0%	-4.0
4780	AT1G32200.2 | Symbol: ATS1 | glycerol-3-phosphate acyltransferase, chloroplast (ATS1), identical to SP:Q43307:PLSB_ARATH Glycerol-3-phosphate acyltransferase, chloroplast precursor (EC 2.3.1.15) (GPAT) (ATS1) {Arabidopsis thaliana}; contains Pfam profile PF01553: Acyltransferase | chr1:11602064-11605040 REVERSE | Aliases: ACT1, ACYLTRANSFERASE 1, F3C3.13, F3C3_13, GLYCEROL 3 PHOSPHATE ACYLTRANSFERASE	6.5	7.0	-0.5	-1.4	100.0%	-3.8
4781	AT3G58770.1 | expressed protein, ; expression supported by MPSS | chr3:21743497-21746160 FORWARD | Aliases: T20N10.120	2.7	2.9	-0.2	-1.4	100.0%	-4.6
4782	AT2G45710.1 | 40S ribosomal protein S27 (RPS27A) | chr2:18838235-18839281 FORWARD | Aliases: F4I18.31	8.9	9.4	-0.6	-1.4	100.0%	-3.8
4783	AT2G32730.1 | 26S proteasome regulatory subunit, putative, contains similarity to 26S proteasome regulatory subunit S1 SP:O88761, GI:3288594 from (Rattus norvegicus) | chr2:13887137-13892813 FORWARD | Aliases: F24L7.13, F24L7_13	5.5	6.4	-0.9	-1.4	100.0%	-3.8
4784	AT5G38220.3 | similar to expressed protein [Arabidopsis thaliana] (TAIR:At1g66900.1); similar to Cgi67 serine protease-like [Oryza sativa (japonica cultivar-group)] (GB:BAD82560.1); contains InterPro domain Esterase/lipase/thioesterase (InterPro:IPR000379) | chr5:15286057-15288616 FORWARD | Aliases: MXA21.9, MXA21_9	5.2	4.7	0.5	1.4	100.0%	-3.8
4785	AT4G14030.1 | selenium-binding protein, putative, contains Pfam profile PF05694: 56kDa selenium binding protein (SBP56); identical to Putative selenium-binding protein (Swiss-Prot:O23264) (Arabidopsis thaliana); similar to selenium binding protein (GI:15485232) (Arabidopsis thaliana); identical to cDNA from partial mRNA for selenium binding protein (sbp gene) GI:15485231	6.4	7.5	-1.1	-1.4	100.0%	-4.1
4786	AT4G14040.1 | selenium-binding protein, putative, contains Pfam profile PF05694: 56kDa selenium binding protein (SBP56); similar to Putative selenium-binding protein (Swiss-Prot:O23264) (Arabidopsis thaliana); similar to selenium binding protein (GI:15485232) (Arabidopsis thaliana) | chr4:8100344-8102870 REVERSE | Aliases: DL3061C, FCAALL.29	6.4	7.5	-1.1	-1.4	100.0%	-4.1
4787	AT4G11870.1 | expressed protein | chr4:7140489-7140713 REVERSE | Aliases: T26M18.80, T26M18_80	3.7	4.4	-0.7	-1.4	100.0%	-4.1
4788	AT4G03330.1 | Symbol: SYP123 |	3.4	4.0	-0.6	-1.4	100.0%	-3.6
4789	AT1G01370.2 | Symbol: HTR12 | similar to histone H3.2 [Arabidopsis thaliana] (TAIR:At4g40030.1); similar to histone H3, putative [Arabidopsis thaliana] (TAIR:At1g19890.1); similar to histone H3.2 [Arabidopsis thaliana] (TAIR:At4g40040.1); similar to histone H3 [Arabidopsis thaliana] (TAIR:At5g10980.1); similar to histone H3 like protein [Arabis gemmifera] (GB:BAC79431.1); contains InterPro domain Histone-fold/TFIID-TAF/NF-Y (InterPro:IPR007124); contains InterPro domain Histone H3 (InterPro:IPR000164); contains InterPro domain Histone core (InterPro:IPR007125) | chr1:143717-145684 FORWARD | Aliases: F6F3.17, F6F3_17	2.7	3.0	-0.3	-1.4	100.0%	-4.3
4790	AT3G51730.1 | saposin B domain-containing protein, contains Pfam profiles: PF00026 eukaryotic aspartyl protease, PF03489 surfactant protein B, PF05184 saposin-like type B, region 1 | chr3:19197645-19199670 FORWARD | Aliases: T18N14.110	8.3	7.1	1.2	1.4	100.0%	-3.8
4791	AT5G40550.1 | expressed protein | chr5:16258333-16260609 FORWARD | Aliases: MNF13.7, MNF13_7	3.6	4.0	-0.4	-1.4	100.0%	-4.2
4792	AT3G12130.1 | KH domain-containing protein / zinc finger (CCCH type) family protein | chr3:3864158-3866596 REVERSE | Aliases: T21B14.5	5.6	5.3	0.3	1.4	100.0%	-4.0
4793	AT2G44510.1 | p21Cip1-binding protein-related, similar to p21Cip1-binding protein TOK-1beta (GI:7339688) (Homo sapiens); similar to BRCA2 and Cip1/p21 interacting protein (GI:25989375) (Homo sapiens) | chr2:18384147-18386255 FORWARD | Aliases: F4I1.32	4.2	4.5	-0.3	-1.4	100.0%	-4.1
4794	AT5G02680.1 | expressed protein | chr5:605710-606763 FORWARD | Aliases: F9G14.1	4.1	3.5	0.5	1.4	100.0%	-3.8
4795	AT1G34470.1 | permease-related, low similarity to purine permease (Arabidopsis thaliana) GI:7620007 | chr1:12604278-12607011 FORWARD | Aliases: F12K21.21, F12K21_21	6.0	5.7	0.3	1.4	100.0%	-4.1
4796	AT3G09010.1 | protein kinase family protein, contains protein kinase domain, Pfam:PF00069 | chr3:2749958-2752281 FORWARD | Aliases: T16O11.3	3.9	3.6	0.3	1.4	100.0%	-4.0
4797	AT1G32370.4 | Symbol: TOM2B | expressed protein | chr1:11676941-11678427 FORWARD | Aliases: F5D14.14, F5D14_14, TTM1	5.0	4.6	0.4	1.4	100.0%	-4.0
4798	AT5G57815.1 | cytochrome c oxidase subunit 6b, putative, similar to subunit 6b of cytochrome c oxidase (Arabidopsis thaliana) gi:6518353:dbj:BAA87883 | chr5:23443903-23445077 FORWARD | Aliases: None	7.4	6.5	0.9	1.4	100.0%	-3.7
4799	AT2G21860.1 | violaxanthin de-epoxidase-related, contains weak similarity to violaxanthin de-epoxidase precursor gi:1438875:gb:AAC49373 | chr2:9325338-9327082 REVERSE | Aliases: F7D8.18, F7D8_18	7.0	6.4	0.6	1.4	100.0%	-3.6
4800	AT1G19970.1 | ER lumen protein retaining receptor family protein, similar to SP:P33946 ER lumen protein retaining receptor 1 (KDEL receptor 1) {Bos taurus}; contains Pfam profile PF00810: ER lumen protein retaining receptor | chr1:6931009-6932680 REVERSE | Aliases: T20H2.24, T20H2_24	4.4	4.7	-0.3	-1.4	100.0%	-4.1

Rank	Description	15.dpi	5.dpi	M	t	adj.q	B
4801	AT1G60780.1 | clathrin adaptor complexes medium subunit family protein, contains Pfam profile: PF00928 adaptor complexes medium subunit family	7.5	8.3	-0.9	-1.4	100.0%	-4.0
4802	AT1G22080.1 | expressed protein | chr1:7792473-7794580 REVERSE | Aliases: F2E2.15, F2E2_15	3.3	3.1	0.2	1.4	100.0%	-4.5
4803	AT2G32400.1 | Symbol: GLR5 | glutamate receptor family protein (GLR3.7) (GLR5), identical to Glr5 (Arabidopsis thaliana) gi:6644388:gb:AAF21042; plant glutamate receptor family, PMID:11379626 | chr2:13759742-13763930 REVERSE | Aliases: ATGLR3.7, GLR3.7, GLR5, T32F6.8, T32F6_8	4.0	4.2	-0.2	-1.4	100.0%	-4.4
4804	AT5G47920.1 | expressed protein, similar to unknown protein (emb:CAB67623.1) | chr5:19420399-19421314 REVERSE | Aliases: MCA23.26, MCA23_26	3.3	3.1	0.2	1.4	100.0%	-4.3
4805	AT3G49240.1 | Symbol: EMB1796 | pentatricopeptide (PPR) repeat-containing protein, contains Pfam profile PF01535: PPR repeat | chr3:18266964-18269085 FORWARD | Aliases: EMB1796, EMBRYO DEFECTIVE 1796, F2K15.100	5.3	6.0	-0.7	-1.4	100.0%	-3.9
4806	AT3G04780.1 | Encodes a protein with little sequence identity with any other protein of known structure or function. Part of this protein shows a 42% sequence identity with the C-terminal domain of the 32-kD human thioredoxin-like protein. | chr3:1311253-1313096 REVERSE | Aliases: F7O18.27, F7O18_27	6.5	7.2	-0.7	-1.4	100.0%	-3.8
4807	AT1G79200.1 | expressed protein | chr1:29799281-29800933 FORWARD | Aliases: YUP8H12R.41, YUP8H12R_41	4.7	5.1	-0.4	-1.4	100.0%	-3.9
4808	AT1G07720.1 | beta-ketoacyl-CoA synthase family protein, similar to GB:AAC99312 from (Arabidopsis thaliana) (Plant J. (1999) In press)	2.2	2.1	0.1	1.4	100.0%	-4.9
4809	AT5G43600.1 | N-carbamyl-L-amino acid hydrolase, putative, similar to N-carbamoyl-L-amino acid hydrolase (Bacillus stearothermophilus) SWISS-PROT:Q53389 | chr5:17529703-17532722 FORWARD | Aliases: K9D7.10, K9D7_10	7.3	6.7	0.7	1.4	100.0%	-3.9
4810	AT2G19720.1 | Symbol: RPS15AB | 40S ribosomal protein S15A (RPS15aB) | chr2:8516562-8518399 REVERSE | Aliases: F6F22.25, F6F22_25, RPS15AB	6.4	7.2	-0.7	-1.4	100.0%	-3.6
4811	ATCG00560.1 | Symbol: PSBL | PSII L protein | chrC:63804-63920 REVERSE | Aliases: PSBL	6.9	8.2	-1.2	-1.4	100.0%	-3.8
4812	AT4G12880.1 | plastocyanin-like domain-containing protein | chr4:7544379-7545301 REVERSE | Aliases: T20K18.230, T20K18_230	6.2	7.3	-1.1	-1.4	100.0%	-4.0
4813	AT2G35155.1 | expressed protein | chr2:14826073-14829044 REVERSE | Aliases: None	5.3	5.9	-0.6	-1.4	100.0%	-3.7
4814	AT5G52050.1 | MATE efflux protein-related, contains Pfam profile PF01554: Uncharacterized membrane protein family | chr5:21156088-21157869 FORWARD | Aliases: MSG15.13, MSG15_13	7.1	6.2	0.9	1.4	100.0%	-3.4
4815	AT2G34060.1 | peroxidase, putative, similar to peroxidase ATP20a {Arabidopsis thaliana} GP:9757794:dbj:BAB08292 | chr2:14391993-14393748 FORWARD | Aliases: T14G11.18, T14G11_18	3.1	3.4	-0.3	-1.4	100.0%	-4.3
4816	AT5G16070.1 | chaperonin, putative, similar to SWISS-PROT:P80317 T-complex protein 1, zeta subunit (TCP-1-zeta) (Mus musculus); contains Pfam:PF00118 domain, TCP-1/cpn60 chaperonin family | chr5:5247357-5251182 REVERSE | Aliases: F1N13.210, F1N13_210	8.7	9.6	-0.9	-1.4	100.0%	-3.7
4817	AT3G09920.2 | similar to phosphatidylinositol-4-phosphate 5-kinase family protein [Arabidopsis thaliana] (TAIR:At1g60890.1); similar to phosphatidylinositol-4-phosphate 5-kinase family protein [Arabidopsis thaliana] (TAIR:At1g10900.1); similar to phosphatidylinositol-4-phosphate 5-kinase [Oryza sativa (japonica cultivar-group)] (GB:CAD27794.1); similar to Putative Phosphatidylinositol-4-phosphate 5-Kinase [Oryza sativa (japonica cultivar-group)] (GB:AAX95508.1); contains InterPro domain Phosphatidylinositol-4-phosphate 5-kinase (InterPro:IPR002498); contains InterPro domain MORN motif (InterPro:IPR003409) | chr3:3040308-3044772 REVERSE | Aliases: F8A24.2	4.8	5.4	-0.5	-1.4	100.0%	-3.9
4818	AT5G17510.1 | expressed protein | chr5:5769975-5772397 REVERSE | Aliases: K3M16.80, K3M16_80	3.1	2.8	0.3	1.4	100.0%	-4.2
4819	AT1G15980.1 | expressed protein | chr1:5489292-5491303 FORWARD | Aliases: T24D18.8, T24D18_8	7.4	6.7	0.7	1.4	100.0%	-3.5
4820	AT5G23690.1 | polynucleotide adenylyltransferase family protein, low similarity to SP:P13685 Poly(A) polymerase (EC 2.7.7.19) {Escherichia coli O157:H7}; contains Pfam profile PF01743: polyA polymerase family protein | chr5:7987543-7990550 FORWARD | Aliases: MQM1.4, MQM1_4	4.3	4.6	-0.3	-1.4	100.0%	-4.3
4821	AT5G13690.1 | alpha-N-acetylglucosaminidase family / NAGLU family, contains Pfam profile: PF05089 alpha-N-acetylglucosaminidase (NAGLU) | chr5:4415757-4420374 FORWARD | Aliases: MSH12.16, MSH12_16	4.3	4.7	-0.5	-1.4	100.0%	-4.0
4822	AT3G20280.2 | PHD finger family protein, contains Pfam profile: PF00628 PHD-finger | chr3:7071199-7074647 FORWARD | Aliases: MQC12.7	2.3	2.5	-0.1	-1.4	100.0%	-4.9
4823	AT1G33500.1 | hypothetical protein | chr1:12152754-12154665 FORWARD | Aliases: F10C21.15, F10C21_15	2.7	3.0	-0.2	-1.4	100.0%	-4.8
4824	AT5G14100.1 | Symbol: ATNAP14 | ABC transporter family protein, contains similarity to ABC transporter, ATP-binding protein | chr5:4549440-4551845 REVERSE | Aliases: MUA22.10, MUA22_10	3.6	3.3	0.3	1.4	100.0%	-4.0

Rank	Description	15.dpi	5.dpi	M	t	adj.q	B
4825	AT3G62260.2 | protein phosphatase 2C, putative / PP2C, putative, phosphoprotein phosphatase (EC 3.1.3.16) 1A-alpha - Homo sapiens, PIR:S22423 | chr3:23049265-23051545 REVERSE | Aliases: T17J13.220	6.4	5.9	0.5	1.4	100.0%	-4.2
4826	AT3G26900.1 | shikimate kinase family protein, similar to shikimate kinase precursor GB:CAA45121 (Lycopersicon esculentum); contains Pfam shikimate kinase domain PF01202 | chr3:9913449-9915715 REVERSE | Aliases: MDJ14.24	7.2	6.7	0.5	1.4	100.0%	-3.8
4827	AT2G31800.1 | ankyrin protein kinase, putative, similar to ankyrin-kinase (Medicago truncatula) gi:18700701:gb:AAL78674; contains Pfam profile PF00023: Ankyrin repeat; identical to cDNA calcineurin B-like protein 10 (CBL10) GI:29150247; blastp match of 67% identity and 1.9e-200 P-value to GP:18700701:gb:AAL78674.1:AF458699_1:AF458699 ankyrin-kinase {Medicago truncatula} | chr2:13526787-13530775 REVERSE | Aliases: F20M17.16, F20M17_16	5.9	5.5	0.5	1.4	100.0%	-3.8
4828	AT3G14950.1 | tetratricopeptide repeat (TPR)-containing protein, low similarity to SP:Q99615 DnaJ homolog subfamily C member 7 (Tetratricopeptide repeat protein 2) {Homo sapiens}; contains Pfam profile PF00515: TPR Domain | chr3:5030223-5032899 REVERSE | Aliases: K15M2.9	2.5	2.7	-0.2	-1.4	100.0%	-4.6
4829	AT5G61460.1 | Symbol: MIM | structural maintenance of chromosomes (SMC) family protein, very strong similarity to SMC-like protein (MIM) (Arabidopsis thaliana) GI:5880614; contains Pfam profile PF02463: RecF/RecN/SMC N terminal domain | chr5:24731302-24739166 REVERSE | Aliases: MCI2.2, MCI2_2	3.5	3.8	-0.3	-1.4	100.0%	-4.1
4830	AT2G14120.2 | dynamin-like protein 2b (ADL2b), identical to dynamin like protein 2b (ADL2b) (Arabidopsis thaliana) GI:19032339	7.5	8.1	-0.6	-1.4	100.0%	-4.0
4831	AT5G35200.1 | epsin N-terminal homology (ENTH) domain-containing protein, contains Pfam PF01417: ENTH domain. ENTH (Epsin N-terminal homology) domain; similar to clathrin assembly protein short form, Rattus norvegicus, EMBL:AF041373;similar to Chain A, Calm-N N-Terminal Domain Of Clathrin Assembly Lymphoid Myeloid Leukaemia Protein, Pi(4,5)p2 Complex (GP:13399999) {Homo sapiens} | chr5:13479392-13483281 REVERSE | Aliases: T25C13.80, T25C13_80	6.5	5.9	0.6	1.4	100.0%	-4.1
4832	AT5G47110.1 | lil3 protein, putative, similar to Lil3 protein (Arabidopsis thaliana) gi:4741966:gb:AAD28780 | chr5:19151215-19152544 REVERSE | Aliases: K14A3.6, K14A3_6	9.0	8.4	0.6	1.4	100.0%	-3.8
4833	AT5G25190.1 | encodes a member of the ERF (ethylene response factor) subfamily B-6 of ERF/AP2 transcription factor family. The protein contains one AP2 domain. There are 12 members in this subfamily including RAP2.11. | chr5:8706793-8707739 REVERSE | Aliases: F21J6.103, F21J6_103	2.9	3.1	-0.2	-1.4	100.0%	-4.4
4834	AT5G11480.1 | expressed protein | chr5:3669198-3671575 REVERSE | Aliases: F15N18.70, F15N18_70	8.1	8.6	-0.5	-1.4	100.0%	-3.8
4835	AT4G24370.1 | expressed protein | chr4:12611038-12612141 REVERSE | Aliases: T22A6.200, T22A6_200	4.6	4.2	0.5	1.4	100.0%	-3.9
4836	AT4G34265.2 | expressed protein | chr4:16402284-16403827 FORWARD | Aliases: None	8.0	7.3	0.8	1.4	100.0%	-3.7
4837	AT2G25170.1 | Symbol: PKL | chromatin remodeling factor CHD3 (PICKLE), identical to chromatin remodeling factor CHD3 (Arabidopsis thaliana) GI:6478518 | chr2:10721485-10731065 FORWARD | Aliases: CHD3, CHD3 PROTEIN, F13D4.130, F13D4_130, GYM, GYMNOS, PICKLE	7.2	7.8	-0.6	-1.4	100.0%	-4.0
4838	AT5G08580.1 | calcium-binding EF hand family protein, contains INTERPRO:IPR002048 calcium-binding EF-hand domain | chr5:2780513-2783067 REVERSE | Aliases: MAH20.14, MAH20_14	6.5	7.1	-0.6	-1.4	100.0%	-4.0
4839	AT1G17870.1 | S2P-like putative metalloprotease, also contain transmembrane helices near their C-termini and many of them, five of seven, contain a conserved zinc-binding motif HEXXH. Homolog of EGY1. Each of the EGY1 and EGY-like proteins share two additional highly conserved motifs, the previously reported NPDG motif (aa 442??454 in EGY1, Rudner et al., 1999) and a newly defined GNLR motif (aa 171??179 in EGY1). The GNLR motif is a novel signature motif unique to EGY1 and EGY-like proteins as well as other EGY1 orthologs found in cyanobacteria. | chr1:6150029-6152215 REVERSE | Aliases: ATEGY3, F2H15.10, F2H15_10	4.8	4.5	0.3	1.4	100.0%	-4.2
4840	AT2G24060.1 | translation initiation factor 3 (IF-3) family protein, similar to SP:P33319 Translation initiation factor IF-3 {Proteus vulgaris}; contains Pfam profiles PF00707: Translation initiation factor IF-3, C-terminal domain, PF05198: Translation initiation factor IF-3, N-terminal domain	7.9	8.6	-0.7	-1.4	100.0%	-3.7
4841	AT5G57280.1 | expressed protein | chr5:23221696-23223877 FORWARD | Aliases: MJB24.9, MJB24_9	8.2	8.8	-0.5	-1.4	100.0%	-3.8
4842	AT4G15955.1 | epoxide hydrolase-related, similar to epoxide hydrolase GI:1109600 from (Arabidopsis thaliana) | chr4:9043727-9045613 REVERSE | Aliases: None	2.5	2.3	0.2	1.4	100.0%	-4.5
4843	AT3G48500.1 | Symbol: PDE312 |	5.1	5.6	-0.5	-1.4	100.0%	-4.0
4844	AT4G30660.1 | hydrophobic protein, putative / low temperature and salt responsive protein, putative, similar to SP:Q9ZNQ7 Hydrophobic protein RCI2A (Low temperature and salt responsive protein LTI6A) {Arabidopsis thaliana}; contains Pfam profile PF01679: Uncharacterized protein family | chr4:14955453-14956069 FORWARD | Aliases: T10C21.10, T10C21_10	9.3	8.9	0.4	1.4	100.0%	-4.0

Rank	Description	15.dpi	5.dpi	M	t	adj.q	B
4845	AT1G61490.1 | S-locus protein kinase, putative, similar to KI domain interacting kinase 1 (Zea mays) gi:2735017:gb:AAB93834; contains S-locus glycoprotein family domain, Pfam:PF00954	3.1	3.4	-0.3	-1.4	100.0%	-4.6
4846	AT2G20950.4 | expressed protein | chr2:9010220-9013019 FORWARD | Aliases: F26H11.29	6.2	5.3	1.0	1.4	100.0%	-3.6
4847	AT2G22200.1 | encodes a member of the DREB subfamily A-6 of ERF/AP2 transcription factor family. The protein contains one AP2 domain. There are 8 members in this subfamily including RAP2.4. | chr2:9450208-9451503 REVERSE | Aliases: T26C19.14, T26C19_14	2.8	2.6	0.2	1.4	100.0%	-4.7
4848	AT1G34200.1 | oxidoreductase family protein, similar to AX110P (Daucus carota) GI:285739; contains Pfam profiles PF01408: Oxidoreductase family NAD-binding Rossmann fold, PF02894: Oxidoreductase family C-terminal alpha/beta domain	4.6	4.3	0.4	1.4	100.0%	-3.9
4849	AT1G30510.3 | ferredoxin--NADP(+) reductase, putative / adrenodoxin reductase, putative, strong similarity to SP:P41345 Ferredoxin--NADP reductase, root isozyme, chloroplast precursor (EC 1.18.1.2) (FNR) {Oryza sativa}, ferredoxin-NADP reductase precursor (Zea mays) GI:500751 | chr1:10806968-10809144 REVERSE | Aliases: F26G16.13, F26G16_13	9.8	9.3	0.5	1.4	100.0%	-3.9
4850	AT5G40540.1 | protein kinase, putative, similar to protein kinase ATN1 (Arabidopsis thaliana) gi:1054633:emb:CAA63387 | chr5:16254609-16256818 FORWARD | Aliases: MNF13.60, MNF13_60	7.0	6.6	0.4	1.4	100.0%	-3.9
4851	AT4G10050.1 | hydrolase, alpha/beta fold family protein, similar to protein phosphatase methylesterase-1 (Homo sapiens) GI:5533003; contains Pfam profile PF00561: hydrolase, alpha/beta fold family | chr4:6284447-6287287 REVERSE | Aliases: F28M11.4	9.1	9.7	-0.6	-1.4	100.0%	-3.9
4852	AT2G28510.1 | Dof-type zinc finger domain-containing protein, similar to elicitor-responsive Dof protein ERDP GI:6092016 from (Pisum sativum) | chr2:12206175-12207842 REVERSE | Aliases: T17D12.7, T17D12_7	5.2	5.5	-0.3	-1.4	100.0%	-4.4
4853	AT3G16857.2 | Symbol: ARR1 | two-component responsive regulator family protein / response regulator family protein, contains Pfam profile: PF00072 response regulator receiver domain; similar to ARR1 protein GB:BAA74528 from (Arabidopsis thaliana) (Plant Cell Physiol. (1998) 39 (11), 1232-1239) | chr3:5755930-5759376 FORWARD | Aliases: ARABIDOPSIS RESPONSE REGULATOR 1, MUH15.5	4.4	4.1	0.4	1.4	100.0%	-4.0
4854	AT4G17215.1 | expressed protein | chr4:9655848-9656588 FORWARD | Aliases: None	3.5	4.1	-0.5	-1.4	100.0%	-3.8
4855	AT1G21190.1 | small nuclear ribonucleoprotein, putative / snRNP, putative / Sm protein, putative, similar to SWISS-PROT:Q9Y4Z1 U6 snRNA-associated Sm-like protein LSm3 (MDS017) (Mouse) | chr1:7419667-7420892 REVERSE | Aliases: F16F4.12, F16F4_12	6.5	6.1	0.5	1.4	100.0%	-3.8
4856	AT3G66652.1 | fip1 motif-containing protein, contains Pfam profile PF05182: Fip1 motif	4.6	5.3	-0.7	-1.4	100.0%	-3.9
4857	AT5G24010.1 | protein kinase family protein, contains protein kinase domain, Pfam:PF00069 | chr5:8113798-8116618 FORWARD | Aliases: MZF18.11, MZF18_11	6.5	6.0	0.5	1.4	100.0%	-3.9
4858	AT2G02960.5 | zinc finger (C3HC4-type RING finger) family protein, contains Pfam profile PF00097: Zinc finger, C3HC4 type (RING finger); contains PROSITE PS00190: Cytochrome c family heme-binding site signature | chr2:862086-864358 REVERSE | Aliases: T17M13.13, T17M13_13	6.5	6.0	0.5	1.4	100.0%	-3.9
4859	AT5G19310.1 | homeotic gene regulator, putative, similar to SP:P25439 Homeotic gene regulator (Brahma protein) {Drosophila melanogaster}; contains Pfam profiles PF00271: Helicase conserved C-terminal domain, PF00176: SNF2 family N-terminal domain	4.1	5.6	-1.5	-1.4	100.0%	-3.8
4860	AT1G78580.1 | Symbol: ATTPS1 |	7.2	6.7	0.5	1.4	100.0%	-4.0
4861	AT1G08350.1 | endomembrane protein 70 family protein, KNOLLE; similar to putative endosomal protein GB:AAD20090 GI:4406780 from (Arabidopsis thaliana)	4.6	4.8	-0.3	-1.4	100.0%	-4.3
4862	AT4G02940.1 | oxidoreductase, 2OG-Fe(II) oxygenase family protein, similar to A. thaliana hypothetical protein T13L16.2, GenBank accession number 2708738; contains Pfam domain PF03171 2OG-Fe(II) oxygenase superfamily | chr4:1306503-1310938 FORWARD | Aliases: T4I9.18, T4I9_18	5.6	5.1	0.5	1.4	100.0%	-3.8
4863	AT4G14150.1 | Symbol: PAKRP1 |	6.1	7.0	-0.9	-1.4	100.0%	-3.8
4864	AT1G17080.1 | expressed protein | chr1:5839679-5841559 REVERSE | Aliases: F20D23.22, F20D23_22	9.4	8.8	0.6	1.4	100.0%	-4.0
4865	AT4G15470.1 | expressed protein, low similarity to N-methyl-D-aspartate receptor-associated protein (Drosophila melanogaster) GI:567104; contains Pfam profile PF01027: Uncharacterized protein family UPF0005	11.4	10.6	0.8	1.4	100.0%	-4.1
4866	AT3G59650.1 | mitochondrial ribosomal protein L51/S25/CI-B8 family protein, low similarity to mitochondrial ribosomal protein bMRP36a (Mus musculus) GI:13559406; contains Pfam profile PF05047: Mitochondrial ribosomal protein L51 / S25 / CI-B8 domain | chr3:22044152-22045134 FORWARD | Aliases: T16L24.200	7.2	7.7	-0.6	-1.4	100.0%	-3.6

Rank	Description	15.dpi	5.dpi	M	t	adj.q	B
4867	AT3G44940.1 | expressed protein | chr3:16424319-16426226 REVERSE | Aliases: F14D17.10	3.0	2.8	0.2	1.4	100.0%	-4.6
4868	AT5G51820.1 | Symbol: PGM | phosphoglucomutase, chloroplast (PGM) (PGMP) / glucose phosphomutase, identical to SP:Q9SCY0 Phosphoglucomutase, chloroplast precursor (EC 5.4.2.2) (Glucose phosphomutase) (PGM) {Arabidopsis thaliana} | chr5:21080594-21085283 REVERSE | Aliases: ATPGMP, MIO24.4, MIO24_4, PGM1, PHOSPHOGLUCOMUTASE, STARCH FREE 1, STF1	8.1	8.7	-0.6	-1.4	100.0%	-3.7
4869	AT4G12990.1 | expressed protein | chr4:7595041-7595445 REVERSE | Aliases: F25G13.80, F25G13_80	6.5	5.5	1.0	1.4	100.0%	-3.8
4870	AT5G51990.1 | Symbol: CBF4 | encodes a member of the DREB subfamily A-1 of ERF/AP2 transcription factor family (CBF4). The protein contains one AP2 domain. There are six members in this subfamily, including CBF1, CBF2, and CBF3. This gene is involved in response to drought stress and abscisic acid treatment, but not to low temperature. | chr5:21134339-21135013 REVERSE | Aliases: CBF4, MSG15.8, MSG15_8	3.4	3.0	0.4	1.4	100.0%	-4.0
4871	AT4G17870.1 | expressed protein | chr4:9928691-9929531 FORWARD | Aliases: T6K21.50, T6K21_50	7.6	7.0	0.7	1.4	100.0%	-3.9
4872	AT4G31520.1 | SDA1 family protein, contains Pfam profile PF05285: SDA1	3.2	2.9	0.2	1.4	100.0%	-4.6
4873	AT1G65070.1 | DNA mismatch repair MutS family protein, contains Pfam profile PF00488: MutS domain V | chr1:24176795-24179907 REVERSE | Aliases: F16G16.7, F16G16_7	4.0	4.5	-0.5	-1.4	100.0%	-3.9
4874	AT4G31400.1 | expressed protein | chr4:15237417-15239062 FORWARD | Aliases: F3L17.7	3.3	3.7	-0.4	-1.4	100.0%	-4.1
4875	AT4G19660.1 | Symbol: NPR4 | ankyrin repeat family protein / BTB/POZ domain-containing protein, contains Pfam domain, PF00023: Ankyrin repeat and Pfam domain, PF00651: BTB/POZ domain | chr4:10696276-10698253 REVERSE | Aliases: NPR1 LIKE PROTEIN 4, T16H5.20, T16H5_20	4.0	4.6	-0.5	-1.4	100.0%	-3.8
4876	AT3G44890.1 | Symbol: RPL9 | 50S ribosomal protein L9, chloroplast (CL9), contains Pfam profile PF03948: Ribosomal protein L9, C-terminal domain; contains Pfam profile PF01281: ribosomal protein L9, N-terminal domain; contains TIGRfam profile TIGR00158: ribosomal protein L9 | chr3:16397420-16399451 FORWARD | Aliases: F28D10.80, RP19	6.9	7.6	-0.7	-1.4	100.0%	-3.6
4877	AT3G06750.1 | hydroxyproline-rich glycoprotein family protein, contains proline-rich extensin domains, INTERPRO:IPR002965 | chr3:2129629-2130321 REVERSE | Aliases: F3E22.11	5.8	5.4	0.5	1.4	100.0%	-4.0
4878	AT3G21230.1 | 4-coumarate--CoA ligase, putative / 4-coumaroyl-CoA synthase, putative (4CL), similar to 4CL2 (gi:12229665) and 4CL1 (gi:12229649) from (Arabidopsis thaliana), 4CL1 (gi:12229631) from Nicotiana tabacum | chr3:7448046-7452006 REVERSE | Aliases: MXL8.9	8.1	8.7	-0.6	-1.4	100.0%	-4.0
4879	AT3G27010.1 | Symbol: AT TCP20 | TCP family transcription factor, putative, similar to PCF2 ((GI:2580440) Oryza sativa) | chr3:9958619-9960135 REVERSE | Aliases: MOJ10.8, PCF1	5.4	5.1	0.3	1.4	100.0%	-4.1
4880	AT5G08540.1 | expressed protein, similar to unknown protein (pir::T27191) | chr5:2763824-2766156 FORWARD | Aliases: MAH20.10, MAH20_10	6.9	7.6	-0.7	-1.4	100.0%	-3.8
4881	AT3G58010.1 | expressed protein | chr3:21486790-21488475 REVERSE | Aliases: T10K17.220	6.4	6.9	-0.4	-1.4	100.0%	-3.9
4882	AT3G12550.1 | XH/XS domain-containing protein / XS zinc finger domain-containing protein, contains Pfam domains PF03469: XH domain, PF03468: XS domain and PF03470: XS zinc finger domain | chr3:3978676-3981379 FORWARD | Aliases: T2E22.14	3.1	3.4	-0.3	-1.4	100.0%	-4.3
4883	AT4G25490.1 | Symbol: CBF1 | Transcriptional activator that binds to the DRE/CRT regulatory element and induces COR (cold-regulated) gene expression increasing plant freezing tolerance. It encodes a member of the DREB subfamily A-1 of ERF/AP2 transcription factor family (CBF1). The protein contains one AP2 domain. There are six members in this subfamily, including CBF1, CBF2, and CBF3. This gene is involved in response to low temperature and abscisic acid. | chr4:13021790-13022735 REVERSE | Aliases: C REPEAT/DRE BINDING FACTOR 1, DREB1B, T30C3.11, TRANSCRIPTIONAL ACTIVATOR CBF1	3.4	3.2	0.3	1.4	100.0%	-4.2
4884	AT3G16240.1 | Symbol: DELTA TIP | delta tonoplast integral protein (delta-TIP), identical to delta tonoplast integral protein (delta-TIP) (GI:9279707)(GB:U39485) (Arabidopsis thaliana) (Plant Cell 8 (4), 587-599 (1996)) | chr3:5505430-5507056 FORWARD | Aliases: AQP1, ATTIP2;1, DELTA TIP1, MYA6.10, MYA6_10, TIP2;1	5.9	6.4	-0.4	-1.4	100.0%	-4.0
4885	AT1G26130.1 | haloacid dehalogenase-like hydrolase family protein, similar to Potential phospholipid-transporting ATPase (EC 3.6.3.1) from Homo sapiens (SP:Q9Y2Q0, SP:O43520), Mus musculus (SP:P98200, SP:P70704), {Bos taurus} SP:Q29449; contains InterPro accession IPR005834: Haloacid dehalogenase-like hydrolase | chr1:9033587-9038233 FORWARD | Aliases: F28B23.19, F28B23_19	3.2	3.5	-0.2	-1.4	100.0%	-4.4
4886	AT1G20330.1 | Symbol: SMT2 | S-adenosyl-methionine-sterol-C-methyltransferase, identical to sterol-C-methyltransferase GI:1061040 from (Arabidopsis thaliana)	7.4	6.6	0.8	1.4	100.0%	-3.8
4887	AT1G01600.1 | Symbol: CYP86A4 | cytochrome P450, putative, similar to cytochrome P450 GI:10442763 from (Triticum aestivum) | chr1:219131-221286 FORWARD | Aliases: F22L4.14, F22L4_14	5.0	4.6	0.4	1.4	100.0%	-4.2

Rank	Description	15.dpi	5.dpi	M	t	adj.q	B
4888	AT5G24600.1 | expressed protein, contains Pfam profile PF04654: Protein of unknown function, DUF599 | chr5:8421632-8423483 REVERSE | Aliases: K18P6.13, K18P6_13	5.0	4.5	0.5	1.4	100.0%	-3.8
4889	AT4G19400.1 | expressed protein | chr4:10580838-10582000 REVERSE | Aliases: T5K18.180, T5K18_180	7.5	7.9	-0.4	-1.4	100.0%	-4.1
4890	AT1G61430.1 | S-locus protein kinase, putative, similar to receptor protein kinase (Ipomoea trifida) gi:836954:gb:AAC23542; contains S-locus glycoprotein family domain, Pfam:PF00954 | chr1:22668334-22672025 REVERSE | Aliases: T1F9.8, T1F9_8	2.8	2.9	-0.2	-1.4	100.0%	-5.0
4891	AT1G77630.1 | peptidoglycan-binding LysM domain-containing protein, contains Pfam profile PF01476: LysM domain	3.1	3.5	-0.4	-1.4	100.0%	-4.1
4892	AT1G29220.1 | transcriptional regulator family protein, similar to Transcriptional regulator protein HCNGP (Swiss-Prot:Q02614) (Mus musculus) | chr1:10210506-10212679 REVERSE | Aliases: F28N24.10, F28N24_10	6.6	6.2	0.4	1.4	100.0%	-3.9
4893	AT5G41770.1 | crooked neck protein, putative / cell cycle protein, putative, similar to Swiss-Prot:P17886 crooked neck protein (Drosophila melanogaster) | chr5:16735224-16738450 FORWARD | Aliases: K16L22.5, K16L22_5	5.9	6.6	-0.7	-1.4	100.0%	-3.9
4894	AT2G17880.1 | DNAJ heat shock protein, putative, similar to J11 protein (Arabidopsis thaliana) GI:9843641; contains Pfam profile PF00226 DnaJ domain | chr2:7774047-7775013 REVERSE | Aliases: T13L16.10, T13L16_10	2.6	2.4	0.2	1.4	100.0%	-4.5
4895	AT1G27300.1 | expressed protein | chr1:9483250-9484576 FORWARD | Aliases: F17L21.9, F17L21_9	7.1	6.7	0.4	1.4	100.0%	-3.8
4896	AT5G66060.1 | oxidoreductase, 2OG-Fe(II) oxygenase family protein, similar to prolyl 4-hydroxylase, alpha subunit, from Rattus norvegicus (GI:474940), Mus musculus (SP:Q60715), Homo sapiens (GI:18073925); contains PF03171 2OG-Fe(II) oxygenase superfamily domain | chr5:26436707-26438156 FORWARD | Aliases: K2A18.14, K2A18_14	3.7	3.3	0.4	1.4	100.0%	-4.1
4897	AT2G39980.1 | transferase family protein, contains Pfam profile PF02458 transferase family | chr2:16695379-16697034 REVERSE | Aliases: T28M21.14, T28M21_14	6.2	5.4	0.9	1.4	100.0%	-3.9
4898	AT1G67490.2 | Symbol: GCS1 | similar to alpha-glucosidase, putative [Arabidopsis thaliana] (TAIR:At1g24320.1); similar to unnamed protein product [Tetraodon nigroviridis] (GB:CAG11506.1); contains InterPro domain Glycoside hydrolase, family 63 (InterPro:IPR004888) | chr1:25284114-25290274 REVERSE | Aliases: GLUCOSIDASE 1, KNF, KNOPF, T1F15.4, T1F15_4	5.4	5.8	-0.4	-1.4	100.0%	-4.2
4899	AT3G25620.1 | ABC transporter family protein, similar to GB:AAC61893 from (Bactrocera tryoni) (Insect Mol. Biol. 6 (4), 343-356 (1997)) | chr3:9317311-9320805 REVERSE | Aliases: T5M7.11	4.5	4.9	-0.4	-1.4	100.0%	-3.8
4900	AT5G02450.1 | 60S ribosomal protein L36 (RPL36C), 60S ribosomal protein L36, Arabidopsis thaliana, EMBL:AC004684 | chr5:533119-534635 FORWARD | Aliases: T22P11.40, T22P11_40	11.0	11.3	-0.4	-1.4	100.0%	-4.4
4901	AT1G27090.1 | glycine-rich protein | chr1:9403898-9406243 REVERSE | Aliases: T7N9.15, T7N9_15	5.7	5.1	0.6	1.4	100.0%	-3.8
4902	AT5G24300.1 | starch synthase, putative, similar to starch synthase I-1 GI:9369334 from (Triticum aestivum) | chr5:8266802-8271199 FORWARD | Aliases: MOP9.12, MOP9_12	9.2	8.5	0.7	1.4	100.0%	-3.7
4903	AT5G60220.1 | senescence-associated family protein, similar to senescence-associated protein 5 (Hemerocallis hybrid cultivar) gi:3551954:gb:AAC34855 | chr5:24266183-24267291 FORWARD | Aliases: F15L12.11, F15L12_11	3.2	3.0	0.3	1.4	100.0%	-4.3
4904	AT3G05327.1 | similar to cyclin family protein [Arabidopsis thaliana] (TAIR:At3g21870.1); similar to PREG-like protein [Picea mariana] (GB:AAC32127.1); contains InterPro domain Cyclin, N-terminal domain (InterPro:IPR006671) | chr3:1517587-1518405 REVERSE | Aliases: None	2.6	2.4	0.2	1.4	100.0%	-4.4
4905	AT2G04370.1 | hypothetical protein | chr2:1523760-1524118 REVERSE | Aliases: T23O15.2	2.4	2.6	-0.2	-1.4	100.0%	-5.1
4906	AT3G43150.1 | hypothetical protein | chr3:15154683-15155333 FORWARD | Aliases: T6L19.10	2.4	2.2	0.2	1.4	100.0%	-4.6
4907	AT1G29080.1 | peptidase C1A papain family protein, contains similarity to cysteine protease SPCP1 GI:13491750 from (Ipomoea batatas); contains Pfam profile PF00112: Papain family cysteine protease | chr1:10157480-10158660 REVERSE | Aliases: F28N24.27, F28N24_27	2.3	2.1	0.2	1.4	100.0%	-4.7
4908	AT2G46650.1 | Symbol: B5 #1 | cytochrome b5, putative, similar to cytochome b5 GI:2695711 from (Olea europaea) | chr2:19158723-19159663 FORWARD | Aliases: T3A4.3, T3A4_3	5.0	4.0	1.0	1.4	100.0%	-3.6
4909	AT1G63130.1 | pentatricopeptide (PPR) repeat-containing protein, contains Pfam profile PF01535: PPR repeat | chr1:23416395-23418814 FORWARD | Aliases: F16M19.5, F16M19_5	6.5	7.0	-0.6	-1.4	100.0%	-3.6

Rank	Description	15.dpi	5.dpi	M	t	adj.q	B
4910	AT2G19180.1 | expressed protein | chr2:8331798-8333040 FORWARD | Aliases: T20K24.20, T20K24_20	2.7	2.5	0.2	1.4	100.0%	-4.5
4911	AT5G16880.3 | VHS domain-containing protein / GAT domain-containing protein, weak similarity to hepatocyte growth factor-regulated tyrosine kinase substrate (Homo sapiens) GI:2731383; contains Pfam profiles PF00790: VHS domain, PF03127: GAT domain | chr5:5548993-5551466 FORWARD | Aliases: F2K13.30, F2K13_30	8.0	7.3	0.7	1.4	100.0%	-3.9
4912	AT5G22540.1 | expressed protein, contains Pfam profile PF03140: Plant protein of unknown function | chr5:7481497-7482902 REVERSE | Aliases: MQJ16.8, MQJ16_8	2.9	3.1	-0.3	-1.4	100.0%	-4.3
4913	AT3G15040.1 | expressed protein, contains Pfam profile PF04520: Protein of unknown function, DUF584 | chr3:5064711-5066697 REVERSE | Aliases: K15M2.18	6.1	5.8	0.3	1.4	100.0%	-4.0
4914	AT4G02640.2 | Symbol: BZO2H1 |	7.0	6.3	0.7	1.4	100.0%	-3.8
4915	AT5G66040.2 | senescence-associated family protein, almost identical to ketoconazole resistant protein GI:928938 from (Arabidopsis thaliana) full-length cDNA: Ceres:101608. | chr5:26427756-26428553 FORWARD | Aliases: K2A18.11, K2A18_11	6.8	6.2	0.6	1.4	100.0%	-3.7
4916	AT4G18050.1 | ABC transporter family protein, contains Pfam profile: PF00005 ABC transporter; similar to multidrug-resistant protein CjMDR1 GI:14715462 from (Coptis japonica)	2.8	2.6	0.3	1.4	100.0%	-4.3
4917	AT4G33310.1 | expressed protein | chr4:16056160-16056420 FORWARD | Aliases: F17M5.70, F17M5_70	4.7	4.3	0.5	1.4	100.0%	-3.9
4918	AT3G46430.1 | expressed protein | chr3:17098672-17099482 FORWARD | Aliases: F18L15.150, F18L15_150	10.1	10.6	-0.5	-1.4	100.0%	-4.2
4919	AT5G25450.1 | ubiquinol-cytochrome C reductase complex 14 kDa protein, putative, similar to SP:P48502 Ubiquinol-cytochrome C reductase complex 14 kDa protein (EC 1.10.2.2) (CR14) {Solanum tuberosum}; contains Pfam profile PF02271: Ubiquinol-cytochrome C reductase complex 14kD subunit | chr5:8857039-8858479 FORWARD | Aliases: F18G18.190, F18G18_190	7.8	7.3	0.6	1.4	100.0%	-3.8
4920	AT5G20885.1 | zinc finger (C3HC4-type RING finger) family protein, contains Pfam profile: PF00097 zinc finger, C3HC4 type (RING finger) | chr5:7083968-7084736 REVERSE | Aliases: None	5.8	5.3	0.5	1.4	100.0%	-4.2
4921	AT1G22690.1 | gibberellin-responsive protein, putative, similar to SP:P46688 Gibberellin-regulated protein 2 precursor {Arabidopsis thaliana}; contains Pfam profile PF02704: Gibberellin regulated protein | chr1:8027294-8028114 FORWARD | Aliases: T22J18.14, T22J18_14	2.6	2.4	0.1	1.4	100.0%	-5.0
4922	AT1G26110.1 | expressed protein | chr1:9024332-9027745 REVERSE | Aliases: F14G11.8, F14G11_8	8.8	7.8	0.9	1.4	100.0%	-3.8
4923	AT1G76540.1 | Symbol: CDKB2;1 | cell division control protein, putative, similar to SWISS-PROT:Q38775, cell division control protein 2 homolog D (Antirrhinum majus); contains protein kinase domain, Pfam:PF00069 | chr1:28725222-28727415 REVERSE | Aliases: CDKB2;1, Cyclin dependent kinase B2;1, F14G6.14, F14G6_14	4.3	4.8	-0.6	-1.4	100.0%	-4.0
4924	AT1G52220.2 | expressed protein, similar to expressed protein [Arabidopsis thaliana] (TAIR:At2g46820.1); similar to unknown protein [Oryza sativa (japonica cultivar-group)] (GB:XP_478022.1) | chr1:19457238-19458366 REVERSE | Aliases: F9I5.10, F9I5_10	6.5	5.9	0.7	1.4	100.0%	-3.5
4925	AT1G07790.1 | histone H2B, putative, strong similarity to histone H2B Arabidopsis thaliana GI:2407802, Gossypium hirsutum SP:O22582, Lycopersicon esculentum GI:3021489, Capsicum annuum SP:O49118; contains Pfam profile PF00125 Core histone H2A/H2B/H3/H4 | chr1:2412977-2413705 FORWARD | Aliases: F24B9.10, F24B9_10	6.6	7.4	-0.8	-1.4	100.0%	-3.6
4926	AT5G53760.2 | Symbol: MLO11 | similar to seven transmembrane MLO family protein / MLO-like protein 14 (MLO14) [Arabidopsis thaliana] (TAIR:At1g26700.1); similar to seven transmembrane protein Mlo9 [Zea mays] (GB:AAK38345.1); contains InterPro domain Mlo-related protein (InterPro:IPR004326) | chr5:21839760-21843677 FORWARD | Aliases: MGN6.12, MGN6_12	5.8	6.3	-0.5	-1.4	100.0%	-3.8
4927	AT4G05090.1 | inositol monophosphatase family protein, low similarity to SP:Q42546 3'(2'),5'-bisphosphate nucleotidase (EC 3.1.3.7) (DPNPase) {Arabidopsis thaliana}; contains Pfam profile PF00459: Inositol monophosphatase family | chr4:2609140-2611735 FORWARD | Aliases: C17L7.10, C17L7_10	5.7	5.5	0.3	1.4	100.0%	-4.4
4928	AT3G17930.1 | expressed protein | chr3:6141834-6143316 REVERSE | Aliases: MEB5.15	8.9	8.3	0.5	1.4	100.0%	-3.7
4929	AT1G67140.1 | expressed protein, similar to expressed protein [Arabidopsis thaliana] (TAIR:At2g22795.1); similar to hypothetical protein [Oryza sativa (japonica cultivar-group)] (GB:BAD45833.1) | chr1:25104571-25121035 REVERSE | Aliases: F5A8.12, F5A8_12	3.5	4.1	-0.6	-1.4	100.0%	-3.9
4930	AT5G17370.1 | WD-40 repeat family protein, contains 1 significant, 2 weak WD-40 repeats (PF00400); similar to transducin beta-like 1 protein.(SP:O60907) (Homo sapiens) | chr5:5721803-5724721 REVERSE | Aliases: MKP11.21, MKP11_21	3.7	4.0	-0.3	-1.4	100.0%	-4.3

Rank	Description	15.dpi	5.dpi	M	t	adj.q	B
4931	AT1G13950.1 | Symbol: EIF 5A |	6.2	5.6	0.6	1.4	100.0%	-4.0
4932	AT1G53645.1 | hydroxyproline-rich glycoprotein family protein | chr1:20029841-20032303 REVERSE | Aliases: None	7.0	7.5	-0.5	-1.3	100.0%	-3.8
4933	AT1G64170.1 | Symbol: ATCHX16 | cation/hydrogen exchanger, putative (CHX16), monovalent cation:proton antiporter family 2 (CPA2) member, PMID:11500563 | chr1:23818839-23821956 REVERSE | Aliases: CHX16, F22C12.7, F22C12_7	4.1	4.5	-0.4	-1.3	100.0%	-4.0
4934	AT5G20720.2 | Symbol: CPN20 | 20 kDa chaperonin, chloroplast (CPN21) (CHCPN10) (CPN20), identical to chloroplast 20 kDa chaperonin, chloroplast precursor (Protein Cpn21), chloroplast protein Cpn10, chloroplast chaperonin 10 (Ch-CPN10), SP:O65282 from (Arabidopsis thaliana); identical to cDNA chaperonin 20 GI:14587372 | chr5:7014688-7016476 FORWARD | Aliases: ATCPN21, CHAPERONIN 10, CHAPERONIN 20, CHCPN10, CHLOROPLAST CHAPERONIN 10, CPN10, CPN21, T1M15.120, T1M15_120	11.1	11.6	-0.5	-1.3	100.0%	-4.0
4935	AT5G11210.1 | Symbol: ATGLR2.5 | glutamate receptor family protein (GLR2.5), plant glutamate receptor family, PMID:11379626 | chr5:3571215-3574538 REVERSE | Aliases: F2I11.100, F2I11_100, GLR2.5	2.3	2.5	-0.2	-1.3	100.0%	-4.8
4936	AT5G05390.1 | laccase, putative / diphenol oxidase, putative, similar to laccase (Populus balsamifera subsp. trichocarpa)(GI:3805960) | chr5:1594754-1597043 FORWARD | Aliases: K18I23.20, K18I23_20	3.3	3.5	-0.2	-1.3	100.0%	-4.4
4937	AT5G59000.1 | zinc finger (C3HC4-type RING finger) family protein, contains Pfam profile: PF00097 zinc finger, C3HC4 type (RING finger) ;contains PROSITE PS00190: Cytochrome c family heme-binding site signature | chr5:23835980-23837463 FORWARD | Aliases: K19M22.19, K19M22_19	5.3	4.8	0.5	1.3	100.0%	-3.7
4938	AT1G62770.1 | invertase/pectin methylesterase inhibitor family protein, similar to pectinesterase from Arabidopsis thaliana SP:Q42534, Lycopersicon esculentum SP:Q43143, Phaseolus vulgaris SP:Q43111; contains Pfam profile PF04043: Plant invertase/pectin methylesterase inhibitor	3.6	3.8	-0.2	-1.3	100.0%	-4.7
4939	AT2G40540.1 | Symbol: KT2 | potassium transporter, putative (KT2), identical to putative potassium transporter AtKT2p (Arabidopsis thaliana) gi:2384671:gb:AAC49845, strong similarity to potassium transporter HAK2p (Mesembryanthemum crystallinum) GI:14091471; KUP/HAK/KT Transporter family member, PMID:11500563 | chr2:16937942-16941666 FORWARD | Aliases: ATKT2, ATKT2P, ATKUP2, KUP2, POTASSIUM TRANSPORTER 2, SHY3, T2P4.11, T2P4_11, TRK2	4.9	5.2	-0.4	-1.3	100.0%	-4.2
4940	AT1G29860.1 | Symbol: WRKY71 |	3.0	3.3	-0.3	-1.3	100.0%	-4.2
4941	AT5G42540.1 | Symbol: XRN2 | 5'-3' exoribonuclease (XRN2), identical to XRN2 (Arabidopsis thaliana) GI:11875630; contains Pfam domain PF03159: Putative 5'-3' exonuclease domain | chr5:17024661-17032029 FORWARD | Aliases: EXORIBONUCLEASE 2, K16E1.2, K16E1_2	5.4	6.1	-0.7	-1.3	100.0%	-3.9
4942	AT3G56910.1 | expressed protein | chr3:21080395-21081312 REVERSE | Aliases: T8M16.240	9.3	9.7	-0.4	-1.3	100.0%	-4.1
4943	AT1G64230.2 | similar to ubiquitin-conjugating enzyme E2-17 kDa 9 (UBC9) [Arabidopsis thaliana] (TAIR:At4g27960.1); similar to ubiquitin-conjugating enzyme E2-17 kDa 9 (UBC9) [Arabidopsis thaliana] (TAIR:At4g27960.2); similar to ubiquitin-conjugating enzyme OsUBC5b [Oryza sativa (japonica cultivar-group)] (GB:XP_464900.1); similar to ubiquitin-conjugating enzyme E2 [Gossypium raimondii] (GB:AAL99225.1); similar to ubiquitin-conjugating enzyme 8 [Capsicum annuum] (GB:AAR83891.1); similar to ubiquitin carrier protein (GB:AAA34125.1); contains InterPro domain Ubiquitin-conjugating enzymes (InterPro:IPR000608) | chr1:23837231-23839199 FORWARD | Aliases: F22C12.2, F22C12_2	11.9	11.5	0.4	1.3	100.0%	-4.5
4944	AT5G15470.1 | glycosyl transferase family 8 protein, contains Pfam profile: PF01501 glycosyl transferase family 8 | chr5:5021013-5024745 REVERSE | Aliases: T20K14.80, T20K14_80	3.0	3.3	-0.4	-1.3	100.0%	-4.1
4945	AT3G25520.1 | Symbol: ATL5 | 60S ribosomal protein L5, similar to 60S ribosomal protein L5 GB:P49625 from (Oryza sativa) | chr3:9270524-9272626 REVERSE | Aliases: A. THALIANA RIBOSOMAL PROTEIN L5, MWL2.17, RIBOSOMAL PROTEIN L5	9.6	10.5	-0.9	-1.3	100.0%	-3.7
4946	AT5G22280.1 | expressed protein | chr5:7373998-7375722 REVERSE | Aliases: T6G21.7	5.8	6.5	-0.7	-1.3	100.0%	-3.7
4947	AT5G66210.2 | Symbol: CPK28 | calcium-dependent protein kinase family protein / CDPK family protein, contains Pfam domains, PF00069: Protein kinase domain and PF00036: EF hand | chr5:26473517-26476696 REVERSE | Aliases: K2A18.29, K2A18_29	4.8	4.4	0.5	1.3	100.0%	-4.0
4948	AT5G15540.1 | expressed protein, low similarity to DNA repair and meiosis protein Rad9 (Coprinus cinereus) GI:1353390, SP:Q09725 Sister chromatid cohesion protein mis4 {Schizosaccharomyces pombe} | chr5:5047676-5057414 REVERSE | Aliases: T20K14.150, T20K14_150	3.6	4.0	-0.4	-1.3	100.0%	-4.2
4949	AT3G21210.1 | similar to DC1 domain-containing protein [Arabidopsis thaliana] (TAIR:At1g34480.1); similar to universal stress protein-like [Oryza sativa (japonica cultivar-group)] (GB:BAD53290.1); contains InterPro domain Zn-finger-like, PHD finger (InterPro:IPR001965); contains InterPro domain Usp domain (InterPro:IPR006016); contains InterPro domain DC1 domain (InterPro:IPR004146) | chr3:7437593-7440832 REVERSE | Aliases: MXL8.6	2.3	2.5	-0.2	-1.3	100.0%	-4.6
4950	AT4G12220.1 | expressed protein | chr4:7283242-7283733 REVERSE | Aliases: T4C9.60, T4C9_60	2.4	2.2	0.2	1.3	100.0%	-4.9

Rank	Description	15.dpi	5.dpi	M	t	adj.q	B
4951	AT5G44870.1 | disease resistance protein (TIR-NBS-LRR class), putative, domain signature TIR-NBS-LRR exists, suggestive of a disease resistance protein. | chr5:18131893-18135835 FORWARD | Aliases: K21C13.4, K21C13_4	3.0	2.9	0.2	1.3	100.0%	-4.7
4952	AT5G04560.1 | Symbol: DME | DEMETER protein (DME), identical to DEMETER protein (Arabidopsis thaliana) GI:21743571; contains Pfam profile PF00730: HhH-GPD superfamily base excision DNA repair protein | chr5:1309194-1318387 FORWARD | Aliases: AT5G04570, AT5G04580, DEMETER, DME1, T32M21.160, T32M21_160	3.9	4.2	-0.3	-1.3	100.0%	-4.5
4953	AT5G49590.1 | hypothetical protein, similar to unknown protein (pir::T02348) | chr5:20145962-20146780 FORWARD | Aliases: K6M13.15, K6M13_15	2.9	3.1	-0.2	-1.3	100.0%	-5.1
4954	AT1G56610.2 | similar to F-box family protein [Arabidopsis thaliana] (TAIR:At4g14096.1); contains InterPro domain FBD (InterPro:IPR006566) | chr1:21215620-21218227 FORWARD | Aliases: F25P12.94, F25P12_94	4.0	4.4	-0.4	-1.3	100.0%	-4.0
4955	AT4G11300.1 | expressed protein | chr4:6872182-6873537 REVERSE | Aliases: F8L21.90, F8L21_90	4.5	4.0	0.6	1.3	100.0%	-3.8
4956	AT3G05000.1 | transport protein particle (TRAPP) component Bet3 family protein, similar to Transport protein particle 33 kDa subunit (TRAPP 33 kDa subunit) (Swiss-Prot:Q99394) (Saccharomyces cerevisiae); contains Pfam profile PF04051: Transport protein particle (TRAPP) component, Bet3 | chr3:1387181-1388741 REVERSE | Aliases: T9J14.5, T9J14_5	7.0	6.4	0.6	1.3	100.0%	-3.9
4957	AT5G60260.1 | expressed protein, various predicted proteins, Arabidopsis thaliana | chr5:24272660-24273511 REVERSE | Aliases: F15L12.14, F15L12_14	3.1	2.9	0.3	1.3	100.0%	-4.5
4958	AT5G62740.1 | band 7 family protein, strong similarity to hypersensitive-induced response protein (Zea mays) GI:7716470; contains Pfam profile PF01145: SPFH domain / Band 7 family; supporting cDNA gi:17065547:gb:AY062850.1: | chr5:25218173-25219982 FORWARD | Aliases: MQB2.40, MQB2_40	8.1	7.4	0.7	1.3	100.0%	-4.0
4959	AT2G39810.1 | Symbol: HOS1 | expressed protein, similar to unknown protein [Oryza sativa (japonica cultivar-group)] (GB:XP_469639.1); contains domain CYS_RICH (PS50311) | chr2:16619883-16625135 FORWARD | Aliases: T5I7.11, T5I7_11	4.0	4.4	-0.3	-1.3	100.0%	-4.2
4960	AT5G07280.1 | Symbol: EMS1 | leucine-rich repeat protein kinase, putative / extra sporogenous cells (ESP), identical to extra sporogenous cells (Arabidopsis thaliana) gi:23304947:emb:CAD42912; contains Pfam domains PF00560: Leucine Rich Repeat and PF00069: Protein kinase domain | chr5:2285041-2288687 FORWARD | Aliases: EXCESS MICROSPOROCYTES1, EXS, EXTRA SPOROGENOUS CELL, T28J14.220, T28J14_220	2.8	3.2	-0.4	-1.3	100.0%	-4.2
4961	AT1G54440.1 | similar to 3'-5' exonuclease domain-containing protein / helicase and RNase D C-terminal domain-containing protein / HRDC domain-containing protein [Arabidopsis thaliana] (TAIR:At5g35910.1); similar to Putative nucleolar protein [Oryza sativa (japonica cultivar-group)] (GB:XP_470252.1); contains InterPro domain HRDC domain (InterPro:IPR002121); contains InterPro domain 3'-5' exonuclease (InterPro:IPR002562) | chr1:20326715-20331736 FORWARD | Aliases: F20D21.26, F20D21_26	5.6	6.1	-0.5	-1.3	100.0%	-4.1
4962	AT1G63780.1 | Symbol: IMP4 | brix domain-containing protein, contains Pfam domain, PF04427: Brix domain | chr1:23668536-23671015 REVERSE | Aliases: F24D7.3	6.4	6.8	-0.3	-1.3	100.0%	-4.1
4963	AT1G60420.1 | DC1 domain-containing protein, contains Pfam domain PF03107: DC1 domain | chr1:22265573-22268324 FORWARD | Aliases: T13D8.29, T13D8_29	9.0	9.6	-0.6	-1.3	100.0%	-4.2
4964	AT3G02720.1 | DJ-1 family protein / protease-related, similar to Intracellular Protease (Pyrococcus horikoshii) GI:11513902; contains Pfam profile: PF01965 ThiJ/PfpI family | chr3:586270-588270 FORWARD | Aliases: F16B3.36	8.1	7.8	0.3	1.3	100.0%	-4.2
4965	AT2G04740.1 | ankyrin repeat family protein, contains ankyrin repeats, Pfam:PF00023 | chr2:1657118-1659669 FORWARD | Aliases: F28I8.22, F28I8_22	5.3	5.7	-0.4	-1.3	100.0%	-4.0
4966	AT3G51630.1 | Symbol: WNK5 | protein kinase family protein, contains Pfam profile: PF00069 protein kinase domain | chr3:19159886-19163172 FORWARD | Aliases: T18N14.10, ZIK1	6.7	6.4	0.3	1.3	100.0%	-4.1
4967	AT5G20120.1 | expressed protein | chr5:6795891-6797155 FORWARD | Aliases: F5O24.10, F5O24_10	5.6	5.1	0.4	1.3	100.0%	-3.9
4968	AT1G61460.1 | S-locus protein kinase, putative, contains similarity to KI domain interacting kinase 1 (Zea mays) gi:2735017:gb:AAB93834; contains S-locus glycoprotein family domain, Pfam:PF00954 | chr1:22677863-22681378 REVERSE | Aliases: T1F9.5, T1F9_5	3.6	3.9	-0.3	-1.3	100.0%	-4.4
4969	AT3G46000.1 | Symbol: ADF2 | actin-depolymerizing factor, putative (ADF2), strong similarity to SP:Q9ZSK3 Actin-depolymerizing factor 4 (ADF-4) (AtADF4) {Arabidopsis thaliana}; contains Pfam profile PF00241: Cofilin/tropomyosin-type actin-binding protein | chr3:16918466-16919980 REVERSE | Aliases: ACTIN DEPOLYMERIZING FACTOR 2, F16L2.210	10.6	10.1	0.6	1.3	100.0%	-4.3
4970	AT5G47930.1 | 40S ribosomal protein S27 (RPS27D) | chr5:19423403-19424624 REVERSE | Aliases: K16F13.2, K16F13_2	11.9	12.3	-0.4	-1.3	100.0%	-4.5

Rank	Description	15.dpi	5.dpi	M	t	adj.q	B
4971	AT3G22620.1 | protease inhibitor/seed storage/lipid transfer protein (LTP) family protein, similar to pEARLI 1 (Accession No. L43080): an Arabidopsis member of a conserved gene family (PGF95-099), Plant Physiol. 109 (4), 1497 (1995); contains Pfam protease inhibitor/seed storage/LTP family domain PF00234 | chr3:8008541-8010024 FORWARD | Aliases: F16J14.17	3.3	4.0	-0.7	-1.3	100.0%	-3.6
4972	AT5G63450.1 | Symbol: CYP94B1 | cytochrome P450, putative | chr5:25426121-25427799 REVERSE | Aliases: MLE2.8, MLE2_8	3.9	3.6	0.2	1.3	100.0%	-4.6
4973	AT5G06550.1 | similar to transcription factor jumonji (jmjC) domain-containing protein [Arabidopsis thaliana] (TAIR:At1g78280.1); similar to hypothetical protein [Neurospora crassa] (GB:XP_327705.1); contains InterPro domain Cyclin-like F-box (InterPro:IPR001810); contains InterPro domain Transcription factor jumonji, jmjC (InterPro:IPR003347) | chr5:2001147-2003076 REVERSE | Aliases: F15M7.8, F15M7_8	4.5	4.9	-0.3	-1.3	100.0%	-4.0
4974	AT2G23530.1 | expressed protein, ; expression supported by MPSS | chr2:10027732-10029875 REVERSE | Aliases: F26B6.18, F26B6_18	7.4	6.8	0.6	1.3	100.0%	-3.7
4975	AT1G28960.5 | similar to MutT/nudix family protein [Arabidopsis thaliana] (TAIR:At2g33980.1); similar to unknown protein [Oryza sativa (japonica cultivar-group)] (GB:XP_476134.1); contains InterPro domain NUDIX hydrolase (InterPro:IPR000086) | chr1:10109580-10111646 REVERSE | Aliases: F1K23.5, F1K23_5	6.8	6.3	0.5	1.3	100.0%	-3.9
4976	AT5G23390.1 | expressed protein, contains Pfam profile: PF04842 plant protein of unknown function (DUF639) | chr5:7870516-7874508 REVERSE | Aliases: T32G24.2, T32G24_2	5.5	6.3	-0.8	-1.3	100.0%	-3.9
4977	AT4G31615.1 | transcriptional factor B3 family protein, low similarity to reproductive meristem gene 1 from (Brassica oleracea var. botrytis) GI:3170424, (Arabidopsis thaliana) GI:13604227; contains Pfam profile PF02362: B3 DNA binding domain | chr4:15320403-15322200 REVERSE | Aliases: None	2.4	2.3	0.1	1.3	100.0%	-5.2
4978	AT4G23410.1 | senescence-associated family protein, similar to senescence-associated protein 5 (Hemerocallis hybrid cultivar) gi:3551954:gb:AAC34855 | chr4:12223963-12225235 FORWARD | Aliases: F16G20.110, F16G20_110	3.8	3.3	0.5	1.3	100.0%	-3.7
4979	AT1G79690.1 | MutT/nudix family protein, contains Pfam NUDIX domain (PF00293); very low similarity to Chain A and Chain B of Escherichia coli isopentenyl diphosphate:dimethylallyl diphosphate isomerase (gi:15826361) (gi:15826360) | chr1:29990223-29996035 FORWARD | Aliases: F20B17.11, F20B17_11	9.5	9.8	-0.3	-1.3	100.0%	-4.4
4980	AT5G04160.1 | phosphate translocator-related, low similarity to SP:P52178 Triose phosphate/phosphate translocator, non-green plastid, chloroplast precursor (CTPT) {Brassica oleracea}, phosphoenolpyruvate/phosphate translocator precursor (Mesembryanthemum crystallinum) GI:9295275 | chr5:1142783-1144913 REVERSE | Aliases: F21E1.80, F21E1_80	4.6	5.1	-0.5	-1.3	100.0%	-4.0
4981	AT3G54060.2 | expressed protein, similar to expressed protein [Arabidopsis thaliana] (TAIR:At2g37960.1) | chr3:20029639-20031904 REVERSE | Aliases: F24B22.20	2.2	2.3	-0.1	-1.3	100.0%	-4.9
4982	AT4G01030.1 | pentatricopeptide (PPR) repeat-containing protein, contains INTERPRO:IPR002885 PPR repeats | chr4:448336-449838 REVERSE | Aliases: F3I3.50, F3I3_50	3.3	3.1	0.2	1.3	100.0%	-4.7
4983	AT5G23290.1 | c-myc binding protein, putative / prefoldin, putative, similar to Swiss-Prot:Q99471 prefoldin subunit 5 (C-myc binding protein Mm-1) (Myc modulator 1) (Homo sapiens) | chr5:7846084-7847626 FORWARD | Aliases: MKD15.15, MKD15_15	7.0	6.5	0.6	1.3	100.0%	-3.7
4984	AT1G33390.1 | helicase domain-containing protein, similar to kurz protein (Drosophila melanogaster) GI:5869803; contains Pfam profiles PF04408: Helicase associated domain (HA2), PF00271: Helicase conserved C-terminal domain | chr1:12099462-12104088 REVERSE | Aliases: F10C21.6	4.7	4.9	-0.3	-1.3	100.0%	-4.3
4985	AT5G38780.1 | S-adenosyl-L-methionine:carboxyl methyltransferase family protein, similar to defense-related protein cjs1 (Brassica carinata)(GI:14009292), caffeine synthase (Camellia sinensis)(GI:9967143), SAM:benzoic acid carboxyl methyltransferase (Antirrhinum majus)(GI:9789277) | chr5:15547245-15548752 FORWARD | Aliases: MKD10.80, MKD10_80	2.7	2.5	0.2	1.3	100.0%	-4.8
4986	AT3G46540.1 | epsin N-terminal homology (ENTH) domain-containing protein / clathrin assembly protein-related, contains Pfam PF01417: ENTH domain. ENTH (Epsin N-terminal homology) domain; similar to Chain A Of The Epsin N-Terminal Homology (Enth) Domain (GI:8569264) (Rattus Norvegicus); similar to Af10-protein (GI:1724114) (Avena fatua) | chr3:17145076-17146572 REVERSE | Aliases: F12A12.60	4.8	5.4	-0.6	-1.3	100.0%	-4.1
4987	AT1G12270.1 | stress-inducible protein, putative, similar to sti (stress inducible protein) (Glycine max) GI:872116; contains Pfam profile PF00515 TPR Domain | chr1:4172073-4174773 FORWARD | Aliases: F5O11.2, F5O11_2	6.0	7.0	-1.0	-1.3	100.0%	-4.0
4988	AT2G28320.1 | pleckstrin homology (PH) domain-containing protein / lipid-binding START domain-containing protein, contains Pfam profiles PF01852: START domain, PF00169: PH domain | chr2:12101823-12106721 FORWARD | Aliases: T1B3.16, T1B3_16	6.3	6.7	-0.4	-1.3	100.0%	-4.1
4989	AT3G25730.1 | AP2 domain-containing transcription factor, putative, contains Pfam profile: PF00847 AP2 domain; similar to RAV1 (DNA-binding protein) GB:BAA34250 (Arabidopsis thaliana) (Nucleic Acids Res. 27 (2), 470-478 (1999)) | chr3:9397657-9398896 FORWARD | Aliases: K13N2.14	8.0	7.6	0.3	1.3	100.0%	-4.3

Rank	Description	15.dpi	5.dpi	M	t	adj.q	B
4990	AT4G14690.1 | Symbol: ELIP2 | chlorophyll A-B binding family protein / early light-induced protein, putative, strong similarity to early light-induced protein; ELIP (Arabidopsis thaliana) GI:1872544; contains Pfam profile: PF00504 chlorophyll A-B binding protein | chr4:8418278-8419258 FORWARD | Aliases: DL3385W, EARLY LIGHT INDUCIBLE PROTEIN 2, FCAALL.232	8.2	8.8	-0.6	-1.3	100.0%	-3.5
4991	AT3G54400.1 | aspartyl protease family protein, contains Pfam profile: PF00026 eukaryotic aspartyl protease | chr3:20151036-20153620 REVERSE | Aliases: T14E10.1	6.8	5.9	0.9	1.3	100.0%	-3.7
4992	AT5G12900.1 | expressed protein | chr5:4071922-4074622 REVERSE | Aliases: T24H18.70, T24H18_70	3.8	4.1	-0.3	-1.3	100.0%	-4.3
4993	AT1G11730.1 | galactosyltransferase family protein, contains Pfam profile: PF01762 galactosyltransferase | chr1:3957473-3960113 FORWARD | Aliases: F25C20.12, F25C20_12	3.6	4.0	-0.4	-1.3	100.0%	-4.1
4994	AT1G07020.1 | expressed protein | chr1:2154900-2156007 REVERSE | Aliases: F10K1.34, F10K1_34	6.0	6.4	-0.4	-1.3	100.0%	-4.0
4995	AT5G10700.1 | expressed protein | chr5:3378054-3378868 REVERSE | Aliases: MAJ23.60, MAJ23_60	5.4	5.0	0.4	1.3	100.0%	-3.8
4996	AT1G35680.1 | 50S ribosomal protein L21, chloroplast / CL21 (RPL21), identical to 50S ribosomal protein L21, chloroplast precursor (CL21) (Arabidopsis thaliana) SWISS-PROT:P51412 | chr1:13209965-13211630 FORWARD | Aliases: F15O4.7	7.9	8.6	-0.7	-1.3	100.0%	-3.6
4997	AT2G18520.1 | pentatricopeptide (PPR) repeat-containing protein, low similarity to fertility restorer (Petunia x hybrida) GI:22128587; contains Pfam profile PF01535: PPR repeat | chr2:8040964-8042445 REVERSE | Aliases: F24H14.13, F24H14_13	3.5	3.8	-0.3	-1.3	100.0%	-4.3
4998	AT3G26110.1 | expressed protein | chr3:9542720-9543351 FORWARD | Aliases: MPE11.5	3.5	3.3	0.2	1.3	100.0%	-4.6
4999	AT2G07740.1 | zinc knuckle (CCHC-type) family protein, similar to zinc finger protein GB:AAA75253; some members of this protein family have a weak CCHC zinc fingers that is mostly from retroviral gag proteins (nucleocapsid) | chr2:3570258-3571570 REVERSE | Aliases: T12J2.6	3.4	3.0	0.4	1.3	100.0%	-4.4
5000	AT1G31550.2 | similar to lipase, putative [Arabidopsis thaliana] (TAIR:At1g28600.1); similar to lipase-like protein [Oryza sativa (japonica cultivar-group)] (GB:NP_917247.1); contains InterPro domain Lipolytic enzyme, G-D-S-L family (InterPro:IPR001087) | chr1:11295506-11297266 REVERSE | Aliases: T8E3.19, T8E3_19	3.7	3.4	0.3	1.3	100.0%	-4.3
5001	AT3G30460.1 | zinc finger (C3HC4-type RING finger) family protein, contains Pfam profile: PF00097 zinc finger, C3HC4 type (RING finger) | chr3:12106895-12107355 FORWARD | Aliases: MSJ3.9	3.6	3.2	0.3	1.3	100.0%	-4.2
5002	AT2G22740.2 | Symbol: SUVH6 | SET domain-containing protein (SUVH6), identical to SUVH6 (Arabidopsis thaliana) GI:13517753; contains Pfam profiles PF00856: SET domain, PF05033: Pre-SET motif, PF02182: YDG/SRA domain | chr2:9669718-9674061 REVERSE | Aliases: T9I22.18, T9I22_18	6.7	7.0	-0.4	-1.3	100.0%	-4.1
5003	AT1G66330.2 | senescence-associated family protein, similar to senescence-associated protein (GI:12836895) (Ipomoea batatas) | chr1:24733292-24736125 REVERSE | Aliases: T27F4.8, T27F4_8	6.1	5.5	0.7	1.3	100.0%	-3.9
5004	AT4G35750.1 | Rho-GTPase-activating protein-related, contains weak similarity to Rho-GTPase-activating protein 1 (GTPase-activating protein rhoOGAP) (Rho-related small GTPase protein activator) (CDC42 GTPase-activating protein) (p50-rhoGAP) (Swiss-Prot:Q07960) (Homo sapiens) | chr4:16940512-16941867 REVERSE | Aliases: F4B14.4	6.2	5.0	1.2	1.3	100.0%	-3.6
5005	AT5G42670.1 | agenet domain-containing protein, contains Pfam PF05641: Agenet domain | chr5:17125490-17127463 FORWARD | Aliases: MJB21.4, MJB21_4	6.0	6.6	-0.6	-1.3	100.0%	-3.8
5006	AT2G16760.1 | expressed protein | chr2:7282783-7284314 FORWARD | Aliases: T24I21.17, T24I21_17	4.7	5.0	-0.3	-1.3	100.0%	-4.0
5007	AT4G19550.1 | expressed protein | chr4:10659391-10661109 REVERSE | Aliases: F24J7.110, F24J7_110	3.9	3.4	0.4	1.3	100.0%	-4.1
5008	AT1G06550.1 | enoyl-CoA hydratase/isomerase family protein, similar to CHY1 (gi:8572760); contains Pfam profile PF00388 enoyl-CoA hydratase/isomerase family protein | chr1:2003653-2006563 REVERSE | Aliases: F12K11.12, F12K11_12	7.8	9.0	-1.2	-1.3	100.0%	-3.9
5009	AT1G75050.1 | thaumatin-like protein, putative / pathogenesis-related protein, putative, similar to thaumatin-like protein (Arabidopsis thaliana) GI:2435406, SP:P28493 Pathogenesis-related protein 5 precursor (PR-5) {Arabidopsis thaliana}; contains Pfam profile: PF00314 Thaumatin family | chr1:28183749-28184766 FORWARD | Aliases: F9E10.10, F9E10_10	3.6	3.8	-0.3	-1.3	100.0%	-4.3
5010	AT2G22650.1 | FAD-dependent oxidoreductase family protein | chr2:9631702-9634041 REVERSE | Aliases: T9I22.9, T9I22_9	5.5	5.9	-0.4	-1.3	100.0%	-4.2
5011	AT5G23140.1 | Symbol: CLPP2 | ATP-dependent Clp protease proteolytic subunit, putative, nClpP2/nClpP7; similar to SP:Q9X6W8 ATP-dependent Clp protease proteolytic subunit (EC 3.4.21.92) (Endopeptidase Clp) from (Azospirillum brasilense) | chr5:7783761-7785500 FORWARD | Aliases: MYJ24.13, MYJ24_13, NCLPP2	5.2	5.5	-0.3	-1.3	100.0%	-4.1

Rank	Description	15.dpi	5.dpi	M	t	adj.q	B
5012	AT2G45920.1 | U-box domain-containing protein, contains Pfam profile PF04564: U-box domain | chr2:18906288-18908376 FORWARD | Aliases: F4I18.10	4.0	4.6	-0.6	-1.3	100.0%	-3.9
5013	AT1G68160.1 | expressed protein | chr1:25549471-25552557 REVERSE | Aliases: T22E19.21, T22E19_21	5.3	5.0	0.3	1.3	100.0%	-4.1
5014	AT4G37000.1 | Symbol: ACD2 | accelerated cell death 2 (ACD2), identical to accelerated cell death 2 (ACD2) GI:12484129 from (Arabidopsis thaliana) | chr4:17442606-17443844 FORWARD | Aliases: ACCELERATED CELL DEATH 2, AP22.36, AP22_36	6.8	6.2	0.6	1.3	100.0%	-3.9
5015	AT2G26500.2 | cytochrome b6f complex subunit (petM), putative, nearly identical to cytochrome b6f complex subunit (GI:3090403) (Arabidopsis thaliana); alternative splice forms exist | chr2:11277353-11278031 FORWARD | Aliases: T9J22.17, T9J22_17	10.8	10.2	0.6	1.3	100.0%	-3.7
5016	AT4G32720.2 | similar to La domain-containing protein [Arabidopsis thaliana] (TAIR:At1g79880.1); similar to putative RNA recognition motif (RRM)-containing protein [Oryza sativa (japonica cultivar-group)] (GB:XP_466667.1); contains InterPro domain RNA-binding protein Lupus LaI (InterPro:IPR006630); contains InterPro domain Lupus La protein (InterPro:IPR002344); contains InterPro domain RNA-binding region RNP-1 (RNA recognition motif) (InterPro:IPR000504) | chr4:15787218-15789982 FORWARD | Aliases: F4D11.80, F4D11_80	7.5	8.0	-0.5	-1.3	100.0%	-3.8
5017	AT1G70750.1 | expressed protein, contains Pfam profile PF04576: Protein of unknown function, DUF593; supporting cDNA gi:4101563:gb:AF004556.1:AF004556	6.4	5.6	0.8	1.3	100.0%	-3.8
5018	AT2G01410.1 | expressed protein | chr2:174777-176398 REVERSE | Aliases: F10A8.30	6.7	6.4	0.3	1.3	100.0%	-4.5
5019	AT5G58540.3 | protein kinase family protein, contains Pfam domain, PF00069: Protein kinase domain | chr5:23680362-23682905 FORWARD | Aliases: MQJ2.16, MQJ2_16	3.2	2.9	0.3	1.3	100.0%	-4.1
5020	AT4G36650.1 | transcription factor IIB (TFIIB) family protein, contains Pfam domain, PF00382: Transcription factor TFIIB repeat | chr4:17283288-17285532 REVERSE | Aliases: AP22.23, AP22_23	3.2	3.0	0.2	1.3	100.0%	-4.5
5021	AT4G13820.1 | disease resistance family protein / LRR family protein, contains leucine rich-repeat (LRR) domains Pfam:PF00560, INTERPRO:IPR001611; similar to disease resistance protein (Lycopersicon esculentum) gi:3894383:gb:AAC78591 | chr4:8007990-8010739 REVERSE | Aliases: F18A5.210, F18A5_210	3.4	3.1	0.3	1.3	100.0%	-4.1
5022	AT3G05800.1 | expressed protein | chr3:1727347-1728246 FORWARD | Aliases: F10A16.9, F10A16_9	3.5	4.0	-0.5	-1.3	100.0%	-3.9
5023	AT1G73980.1 | phosphoribulokinase/uridine kinase family protein, weak similarity to SP:Q59190 Uridine kinase (EC 2.7.1.48) (Uridine monophosphokinase) (Cytidine monophosphokinase) {Borrelia burgdorferi}; contains Pfam profile PF00485: Phosphoribulokinase / Uridine kinase family	7.4	7.9	-0.5	-1.3	100.0%	-3.9
5024	AT3G22210.1 | expressed protein | chr3:7838791-7839474 REVERSE | Aliases: MKA23.19	4.5	5.1	-0.6	-1.3	100.0%	-3.8
5025	AT3G13550.2 | Symbol: FUS9 | similar to ubiquitin-conjugating enzyme, putative [Arabidopsis thaliana] (TAIR:At2g16740.1); similar to Ubiquitin-conjugating enzyme E2E 3 [Danio rerio] (GB:AAH67146.1); contains InterPro domain Ubiquitin-conjugating enzymes (InterPro:IPR000608) | chr3:4423697-4424890 REVERSE | Aliases: CIN4, CONSTITUTIVE PHOTOMORPHOGENIC 10, COP10, CYTOKININ INSENSITIVE 4, E2 UBIQUITIN CONJUGATING ENZYME, EMB144, EMBRYO DEFECTIVE 144, FUSCA 9, MRP15.21	6.7	5.9	0.7	1.3	100.0%	-3.8
5026	AT5G02020.2 | expressed protein | chr5:386079-388120 REVERSE | Aliases: T7H20.70, T7H20_70	5.8	5.2	0.5	1.3	100.0%	-3.7
5027	AT3G28230.1 | hypothetical protein | chr3:10530551-10531436 FORWARD | Aliases: MZF16.2	2.7	2.8	-0.1	-1.3	100.0%	-5.0
5028	AT4G35570.1 | Symbol: HMGB5 | high mobility group protein delta (HMGdelta) / HMG protein delta, identical to HMG protein (HMGdelta) (Arabidopsis thaliana) GI:2832363	7.6	8.0	-0.4	-1.3	100.0%	-4.0
5029	AT5G52590.1 | RabGAP/TBC domain-containing protein, contains similarity to SP:P48365 GTPase-activating protein GYP7 {Saccharomyces cerevisiae}; contains Pfam profile PF00566: TBC domain | chr5:21356854-21360210 FORWARD | Aliases: F6N7.7, F6N7_7	6.9	6.2	0.7	1.3	100.0%	-3.8
5030	AT5G11450.1 | oxygen-evolving complex-related, 23 kDa polypeptide of water-oxidizing complex of photosystem II, Nicotiana tabacum, EMBL:NT23WOP2B | chr5:3654431-3656556 FORWARD | Aliases: F15N18.40, F15N18_40	7.0	7.6	-0.6	-1.3	100.0%	-3.7
5031	AT2G01910.2 | similar to microtubule associated protein (MAP65/ASE1) family protein [Arabidopsis thaliana] (TAIR:At1g14690.1); similar to Unknown protein [Oryza sativa (japonica cultivar-group)] (GB:XP_470643.1); contains InterPro domain MAP65/ASE1 (InterPro:IPR007145) | chr2:417439-420586 FORWARD | Aliases: T23K3.10, T23K3_10	6.0	6.7	-0.7	-1.3	100.0%	-3.9
5032	AT3G04530.1 | Symbol: PPCK2 | phosphoenolpyruvate carboxylase kinase 2 (PPCK2), phosphoenolpyruvate carboxylase kinase 2 (Arabidopsis thaliana) gi:13877128:gb:AAK43710; contains protein kinase domain, Pfam:PF00069	4.7	3.9	0.7	1.3	100.0%	-3.6

Rank	Description	15.dpi	5.dpi	M	t	adj.q	B
5033	AT4G29340.1 | Symbol: PRF4 | profilin 3 (PRO3) (PFN3), identical to profilin 3 SP:Q38904 GI:1353765 from (Arabidopsis thaliana) | chr4:14447653-14448704 FORWARD | Aliases: F17A13.160, F17A13_160, PROFILIN 4	3.5	2.9	0.6	1.3	100.0%	-3.7
5034	AT1G04520.1 | 33 kDa secretory protein-related, contains Pfam PF01657: Domain of unknown function, duplicated in 33 KDa secretory proteins | chr1:1231577-1234005 FORWARD | Aliases: F19P19.1, F19P19_1	3.5	3.8	-0.3	-1.3	100.0%	-4.1
5035	AT3G01160.1 | expressed protein | chr3:54548-57469 REVERSE | Aliases: T4P13.15, T4P13_15	4.6	5.3	-0.7	-1.3	100.0%	-3.8
5036	AT2G01870.1 | expressed protein | chr2:389469-390376 REVERSE | Aliases: T23K3.6, T23K3_6	3.7	3.4	0.3	1.3	100.0%	-4.4
5037	AT4G29060.2 | Symbol: EMB2726 | similar to translation elongation factor Ts (EF-Ts), putative [Arabidopsis thaliana] (TAIR:At4g11120.1); similar to elongation factor TS [Synechocystis sp. PCC 6803] (GB:NP_441466.1); contains InterPro domain Elongation factor Ts (InterPro:IPR001816); contains InterPro domain Ubiquitin-associated domain (InterPro:IPR000449); contains InterPro domain RNA binding S1 (InterPro:IPR003029); contains InterPro domain Ribosomal protein S1 (InterPro:IPR000110) | chr4:14317262-14321573 FORWARD | Aliases: EMB2726, EMBRYO DEFECTIVE 2726, F19B15.90, F19B15_90	7.6	8.7	-1.0	-1.3	100.0%	-3.8
5038	AT2G27190.1 | Symbol: PAP1 | iron(III)-zinc(II) purple acid phosphatase (PAP12), identical to iron(III)-zinc(II) purple acid phosphatase (precursor) SP:Q38924 from (Arabidopsis thaliana) | chr2:11628304-11630534 REVERSE | Aliases: PURPLE ACID PHOSPHATASE 1, SECRETED PURPLE ACID PHOSPHATASE PRECURSOR, T22O13.4, T22O13_4	9.4	9.6	-0.2	-1.3	100.0%	-4.6
5039	AT1G04600.1 | Symbol: XIA | myosin, putative, similar to myosin (GI:499047) (Arabidopsis thaliana) | chr1:1262122-1272375 FORWARD | Aliases: ATXIA, T1G11.15, T1G11_15	3.0	3.2	-0.2	-1.3	100.0%	-4.6
5040	AT5G15010.1 | pentatricopeptide (PPR) repeat-containing protein, contains Pfam profile PF01535: PPR repeat | chr5:4857262-4858962 FORWARD | Aliases: F2G14.130, F2G14_130	3.3	3.7	-0.4	-1.3	100.0%	-4.6
5041	AT1G54850.1 | expressed protein, contains similarity to LMW heat shock protein GI:2326354 from (Arabidopsis thaliana) | chr1:20458056-20459887 FORWARD | Aliases: F14C21.42, F14C21_42	3.9	4.1	-0.2	-1.3	100.0%	-4.5
5042	AT2G25870.1 | haloacid dehalogenase-like hydrolase family protein, contains Pfam profiles PF00702: haloacid dehalogenase-like hydrolase, PF02130: Uncharacterized protein family UPF0054 | chr2:11038193-11041532 FORWARD | Aliases: AT2G25860, F17H15.10, F17H15_10	6.1	6.5	-0.4	-1.3	100.0%	-3.9
5043	AT5G57020.1 | Symbol: NMT1 | myristoyl-CoA:protein N-myristoyltransferase 1 (NMT1), identical to N-myristoyltransferase 1 (NMT1) (Arabidopsis thaliana) GI:7339834 | chr5:23092093-23094127 FORWARD | Aliases: ATNMT1, MHM17.15, MHM17_15, N MYRISTOYLTRANSFERASE 1	7.7	8.3	-0.6	-1.3	100.0%	-3.9
5044	AT5G17250.1 | phosphatidylinositolglycan class O (PIG-O) family protein, similar to Pig-o (Mus musculus) GI:8099973 | chr5:5668902-5673518 REVERSE | Aliases: MKP11.10, MKP11_10	3.5	3.2	0.3	1.3	100.0%	-4.2
5045	AT5G57180.3 | Symbol: CIA2 | similar to expressed protein [Arabidopsis thaliana] (TAIR:At4g25990.1); similar to expressed protein [Arabidopsis thaliana] (TAIR:At4g25990.2); similar to putative CIL [Oryza sativa (japonica cultivar-group)] (GB:BAD53999.1)	3.4	3.8	-0.4	-1.3	100.0%	-4.1
5046	AT5G02590.1 | chloroplast lumen common family protein, various predicted proteins, Arabidopsis thaliana | chr5:583084-584177 FORWARD | Aliases: T22P11.180, T22P11_180	4.8	5.3	-0.5	-1.3	100.0%	-3.8
5047	AT4G03990.1 | hypothetical protein, similar to At2g04970, At2g15200, At1g32830, At2g14140, At3g30450, At5g34895, At3g47270, At2g02200 | chr4:1916459-1917814 REVERSE | Aliases: T24H24.2, T24H24_2	2.7	2.9	-0.2	-1.3	100.0%	-5.0
5048	AT1G11430.1 | plastid developmental protein DAG, putative, similar to DAG protein, chloroplast precursor (Garden snapdragon) SWISS-PROT:Q38732	9.6	10.0	-0.4	-1.3	100.0%	-3.8
5049	AT2G40810.2 | WD-40 repeat protein family, similar to Gsa12p(GI:18307769)(Pichia pastoris); contains 3 Pfam PF00400: WD domain, G-beta repeats	5.5	5.0	0.5	1.3	100.0%	-4.0
5050	AT5G37980.1 | NADP-dependent oxidoreductase, putative, similar to probable NADP-dependent oxidoreductase (zeta-crystallin homolog) P1 (SP:Q39172)(gi:886428) and P2 (SP:Q39173)(gi:886430), Arabidopsis thaliana | chr5:15148590-15150315 FORWARD | Aliases: K18L3.140, K18L3_140	5.6	5.9	-0.4	-1.3	100.0%	-4.1
5051	AT2G42390.1 | protein kinase C substrate, heavy chain-related, similar to Protein kinase C substrate, 80 kDa protein, heavy chain (PKCSH) (80K-H protein) (Swiss-Prot:P14314) (Homo sapiens); contains 1 transmembrane domain;	7.3	6.9	0.3	1.3	100.0%	-4.0
5052	AT3G02910.1 | expressed protein, contains Pfam domain PF03674: Uncharacterised protein family (UPF0131) | chr3:649821-650928 FORWARD | Aliases: F13E7.14, F13E7_14	9.3	8.3	1.1	1.3	100.0%	-3.9

Rank	Description	15.dpi	5.dpi	M	t	adj.q	B
5053	AT4G29000.1 | tesmin/TSO1-like CXC domain-containing protein, similar to CXC domain containing TSO1-like protein 1 (SOL1) (Arabidopsis thaliana) GI:7767427, SP:Q9Y4I5 Tesmin (Metallothionein-like 5, testis-specific) {Homo sapiens}; contains Pfam profile PF03638: Tesmin/TSO1-like CXC domain | chr4:14293519-14297072 FORWARD | Aliases: F19B15.30, F19B15_30	2.4	2.5	-0.2	-1.3	100.0%	-4.9
5054	AT3G02990.1 | Symbol: ATHSFA1E | heat shock factor protein 2 (HSF2) / heat shock transcription factor 2 (HSTF2), identical to heat shock transcription factor 2 (HSF2) SP:Q96320 from	3.9	3.7	0.2	1.3	100.0%	-4.4
5055	AT1G48000.1 | Symbol: MYB112 |	3.6	3.3	0.3	1.3	100.0%	-4.5
5056	AT3G18490.1 | aspartyl protease family protein, contains Pfam domain, PF00026: eukaryotic aspartyl protease | chr3:6348761-6350674 REVERSE | Aliases: MYF24.39	9.1	9.7	-0.5	-1.3	100.0%	-4.0
5057	AT5G15390.1 | tRNA/rRNA methyltransferase (SpoU) family protein, similar to SP:P19396 tRNA (Guanosine-2'-O-)-methyltransferase (EC 2.1.1.34) {Escherichia coli O157:H7}; contains Pfam profile PF00588: SpoU rRNA Methylase (RNA methyltransferase, TrmH) family | chr5:4995158-4997398 FORWARD | Aliases: T20K14.1	7.1	6.7	0.5	1.3	100.0%	-3.8
5058	AT4G00200.1 | DNA-binding family protein, contains a AT hook motif (DNA binding motifs with a preference for A/T rich regions), Pfam:PF02178 | chr4:82445-84694 REVERSE | Aliases: F6N15.24, F6N15_24	4.4	4.2	0.2	1.3	100.0%	-4.7
5059	AT1G71830.1 | Symbol: SERK1 | leucine-rich repeat family protein / protein kinase family protein, contains Pfam domains PF00560: Leucine Rich Repeat and PF00069: Protein kinase domain | chr1:27021820-27025774 FORWARD | Aliases: ATSERK1, F14O23.21, F14O23_21, SOMATIC EMBRYOGENESIS RECEPTOR LIKE KINASE 1	6.5	7.0	-0.5	-1.3	100.0%	-4.2
5060	AT1G69680.1 | expressed protein, similar to MOG1 isoform A (GI:9864064) (Homo sapiens)	5.6	5.1	0.4	1.3	100.0%	-3.9
5061	AT1G34140.1 | Symbol: PAB1 | polyadenylate-binding protein, putative / PABP, putative, non-consensus splice donor TA at exon 1; similar to polyadenylate-binding protein (poly(A)-binding protein) from (Triticum aestivum) GI:1737492, (Nicotiana tabacum) GI:7673355, {Arabidopsis thaliana} SP:P42731; contains InterPro entry IPR000504: RNA-binding region RNP-1 (RNA recognition motif) (RRM). Only member of the class IV PABP family.	2.2	2.3	-0.1	-1.3	100.0%	-5.1
5062	AT5G51350.1 | leucine-rich repeat transmembrane protein kinase, putative | chr5:20885086-20887847 REVERSE | Aliases: MFG13.5, MFG13_5	5.4	5.8	-0.4	-1.3	100.0%	-4.1
5063	AT3G23820.1 | Symbol: GAE6 | NAD-dependent epimerase/dehydratase family protein, similar to nucleotide sugar epimerase from Vibrio vulnificus GI:3093975 (PID:g3093975), WbnF (Escherichia coli) GI:5739472, CAPI protein {Staphylococcus aureus} SP:P39858; contains Pfam profile: PF01370 NAD dependent epimerase/dehydratase family | chr3:8603451-8605469 FORWARD | Aliases: F14O13.9, UDP D GLUCURONATE 4 EPIMERASE 6	11.2	10.9	0.2	1.3	100.0%	-4.6
5064	AT2G40280.1 | dehydration-responsive family protein, similar to early-responsive to dehydration stress ERD3 protein (Arabidopsis thaliana) GI:15320410; contains Pfam profile PF03141: Putative methyltransferase | chr2:16832578-16835476 REVERSE | Aliases: T7M7.24	7.2	7.6	-0.5	-1.3	100.0%	-3.8
5065	AT2G18400.1 | ribosomal protein L6 family protein | chr2:7996561-7997668 REVERSE | Aliases: T30D6.9, T30D6_9	9.4	9.9	-0.4	-1.3	100.0%	-3.9
5066	AT5G67290.1 | FAD-dependent oxidoreductase family protein, contains Pfam profile PF01266: FAD dependent oxidoreductase	5.2	4.9	0.3	1.3	100.0%	-4.2
5067	AT5G53990.1 | glycosyltransferase family protein, contains Pfam profile: PF00201 UDP-glucoronosyl and UDP-glucosyl transferase	3.8	4.3	-0.5	-1.3	100.0%	-3.8
5068	AT1G64460.1 | phosphatidylinositol 3- and 4-kinase family protein, contains Pfam profile PF00454: Phosphatidylinositol 3- and 4-kinase	3.9	3.4	0.5	1.3	100.0%	-3.9
5069	AT3G53610.2 | Symbol: ATRAB8 |	6.1	6.5	-0.4	-1.3	100.0%	-3.9
5070	AT4G38870.1 | F-box family protein, contains Pfam profile: PF00646 F-box domain | chr4:18132577-18133857 REVERSE | Aliases: F19H22.2	2.2	2.0	0.2	1.3	100.0%	-5.0
5071	AT1G02500.2 | Symbol: SAM1 | S-adenosylmethionine synthetase 1 (SAM1), identical to S-adenosylmethionine synthetase 1 (Methionine adenosyltransferase 1, AdoMet synthetase 1) (Arabidopsis thaliana) SWISS-PROT:P23686 | chr1:518254-520437 FORWARD | Aliases: SAM 1, T14P4.17, T14P4_17	7.1	8.0	-0.9	-1.3	100.0%	-3.7
5072	AT1G13740.1 | expressed protein | chr1:4713671-4715404 FORWARD | Aliases: F21F23.17, F21F23_17	6.8	7.5	-0.7	-1.3	100.0%	-3.9
5073	AT1G04230.1 | expressed protein | chr1:1125571-1128167 FORWARD | Aliases: F19P19.34	5.1	4.7	0.3	1.3	100.0%	-4.1
5074	AT1G36390.2 | co-chaperone grpE family protein, similar to co-chaperone CGE1 precursor isoform b (Chlamydomonas reinhardtii) GI:15384279; contains Pfam profile PF01025: co-chaperone GrpE | chr1:13702613-13704782 REVERSE | Aliases: F7F23.11, F7F23_11	4.2	4.7	-0.5	-1.3	100.0%	-4.1
5075	AT5G52420.1 | expressed protein | chr5:21298999-21300507 FORWARD | Aliases: K24M7.17, K24M7_17	5.5	5.1	0.3	1.3	100.0%	-4.0

Rank	Description	15.dpi	5.dpi	M	t	adj.q	B
5076	AT2G43460.1 | 60S ribosomal protein L38 (RPL38A) | chr2:18053081-18054478 REVERSE | Aliases: T1O24.20	10.4	10.8	-0.4	-1.3	100.0%	-4.2
5077	AT3G01980.2 | short-chain dehydrogenase/reductase (SDR) family protein, contains Pfam profiles: PF00106 short chain dehydrogenase, PF00678 short chain dehydrogenase/reductase C-terminus | chr3:327634-328971 REVERSE | Aliases: F1C9.24, F1C9_24	5.0	5.8	-0.8	-1.3	100.0%	-4.0
5078	AT5G21070.1 | expressed protein | chr5:7156175-7157768 FORWARD | Aliases: T10F18.100, T10F18_100	4.8	4.3	0.5	1.3	100.0%	-4.2
5079	AT5G59600.1 | pentatricopeptide (PPR) repeat-containing protein, contains INTERPRO:IPR002885 PPR repeats | chr5:24028227-24030251 REVERSE | Aliases: F2O15.13, F2O15_13	3.5	3.7	-0.3	-1.3	100.0%	-4.2
5080	AT2G34570.1 | expressed protein, contains Pfam profile: PF04900 protein of unknown function, DUF652 | chr2:14569980-14571861 REVERSE | Aliases: T31E10.9, T31E10_9	6.1	6.4	-0.4	-1.3	100.0%	-4.0
5081	AT3G05570.1 | expressed protein | chr3:1616271-1616642 REVERSE | Aliases: F18C1.16, F18C1_16	7.5	7.9	-0.4	-1.3	100.0%	-3.8
5082	AT3G15260.2 | protein phosphatase 2C, putative / PP2C, putative, similar to protein phosphatase type 2C GB:AAD17805 from (Lotus japonicus) | chr3:5138703-5142651 FORWARD | Aliases: K7L4.6	8.0	7.5	0.4	1.3	100.0%	-4.1
5083	AT5G16830.1 | Symbol: SYP21 | syntaxin 21 (SYP21) / PEP12 homolog, identical to Syntaxin homolog (PEP12 homolog) (SP:Q39233) and syntaxin of plants 21 (GP:899122) {Arabidopsis thaliana}; contains Pfam profiles PF05739:SNARE domain and PF00804: Syntaxin	9.0	8.2	0.8	1.3	100.0%	-3.9
5084	AT1G01720.1 | Symbol: ATAF1 | no apical meristem (NAM) family protein, contains Pfam PF02365: No apical meristem (NAM) domain; similar to NAC domain protein NAM GB:AAD17313 GI:4325282 from (Arabidopsis thaliana) | chr1:268330-269819 FORWARD | Aliases: ANAC002, T1N6.12, T1N6_12	6.1	5.3	0.9	1.3	100.0%	-3.7
5085	AT1G77810.2 | galactosyltransferase family protein, contains Pfam profile PF01762: Galactosyltransferase | chr1:29265515-29267895 REVERSE | Aliases: T32E8.14	6.6	6.2	0.4	1.3	100.0%	-4.0
5086	AT2G31170.1 | tRNA synthetase class I (C) family protein, similar to cysteine-tRNA ligase (Escherichia coli) GI:41203; contains Pfam profile PF01406: tRNA synthetases class I (C) | chr2:13289458-13292722 REVERSE | Aliases: T16B12.16	3.0	3.2	-0.2	-1.3	100.0%	-4.5
5087	AT1G54200.1 | expressed protein | chr1:20239379-20240732 FORWARD | Aliases: F20D21.2, F20D21_2	3.6	3.9	-0.3	-1.3	100.0%	-4.1
5088	AT4G11340.1 | Toll-Interleukin-Resistance (TIR) domain-containing protein, domain signature TIR exists, suggestive of a disease resistance protein. | chr4:6894203-6899125 REVERSE | Aliases: F8L21.130, F8L21_130	2.6	2.8	-0.2	-1.3	100.0%	-4.7
5089	AT1G14990.1 | expressed protein | chr1:5167147-5168238 REVERSE | Aliases: T15D22.3, T15D22_3	7.4	6.8	0.6	1.3	100.0%	-4.1
5090	AT4G12740.1 | adenine-DNA glycosylase-related / MYH-related, similar to MYH (GI:18845094) (Rattus norvegicus); similar to adenine-DNA glycosylase (GI:12656850) (Mus musculus); contains TIGRFAM profile TIGR01084: A/G-specific adenine glycosylase (hits below the trusted cutoff) | chr4:7494575-7497593 REVERSE | Aliases: T20K18.90, T20K18_90	3.8	4.1	-0.3	-1.3	100.0%	-4.1
5091	AT3G45060.1 | Symbol: ATNRT2.6 | high-affinity nitrate transporter, putative, similar to trans-membrane nitrate transporter protein AtNRT2:1 (Arabidopsis thaliana) GI:3747058, high-affinity nitrate transporter ACH1 (Arabidopsis thaliana) GI:3608362 | chr3:16488656-16490467 REVERSE | Aliases: F14D17.130	4.9	4.5	0.4	1.3	100.0%	-4.0
5092	AT5G43850.1 | acireductone dioxygenase (ARD/ARD') family protein, similar to iron-deficiency induced gene (Hordeum vulgare) GI:14522834, SIPL (Homo sapiens) GI:16551383; contains Pfam profile PF03079: ARD/ARD' family | chr5:17644412-17646385 REVERSE | Aliases: MQD19.21, MQD19_21	10.0	9.7	0.4	1.3	100.0%	-4.4
5093	AT4G16820.1 | lipase class 3 family protein, similar to DEFECTIVE IN ANTHER DEHISCENCE1 (Arabidopsis thaliana) GI:16215706; contains Pfam profile PF01764: Lipase | chr4:9467582-9469135 FORWARD | Aliases: DL4435W, FCAALL.42	3.3	3.9	-0.6	-1.3	100.0%	-4.0
5094	AT3G02320.1 | similar to N2,N2-dimethylguanosine tRNA methyltransferase family protein [Arabidopsis thaliana] (TAIR:At5g15810.1); similar to putative N2,N2-dimethylguanosine tRNA methyltransferase [Oryza sativa (japonica cultivar-group)] (GB:AAT75242.1); contains InterPro domain N2,N2-dimethylguanosine tRNA methyltransferase (InterPro:IPR002905); contains InterPro domain SAM (and some other nucleotide) binding motif (InterPro:IPR000051) | chr3:469941-473695 FORWARD | Aliases: F11A12.1, F11A12_1	5.2	5.4	-0.3	-1.3	100.0%	-4.1
5095	AT1G63160.1 | replication factor C 40 kDa, putative, similar to SWISS-PROT:Q9WUK4 activator 1 40 kDa subunit (Replication factor C 40 kDa subunit, A1 40 kDa subunit, RF-C 40 kDa subunit, RFC40) (Mus musculus) | chr1:23425529-23427478 REVERSE | Aliases: F16M19.6, F16M19_6	3.5	3.9	-0.4	-1.3	100.0%	-4.0
5096	AT2G40460.1 | proton-dependent oligopeptide transport (POT) family protein, contains Pfam profile: PF00854 POT family | chr2:16903985-16908358 FORWARD | Aliases: T2P4.19, T2P4_19	5.5	6.0	-0.5	-1.3	100.0%	-3.7

Rank	Description	15.dpi	5.dpi	M	t	adj.q	B
5097	AT1G68710.1 | haloacid dehalogenase-like hydrolase family protein, similar to Potential phospholipid-transporting ATPase (EC 3.6.3.1) from {Mus musculus} SP:P98200, {Bos taurus} SP:Q29449, {Homo sapiens} SP:O43520; contains InterPro accession IPR005834: Haloacid dehalogenase-like hydrolase | chr1:25796870-25801638 REVERSE | Aliases: F24J5.6, F24J5_6	4.5	3.8	0.7	1.3	100.0%	-3.8
5098	AT3G45160.1 | expressed protein | chr3:16544436-16545063 REVERSE | Aliases: T14D3.100	8.9	9.7	-0.8	-1.3	100.0%	-4.0
5099	AT3G51580.1 | expressed protein | chr3:19141813-19144259 REVERSE | Aliases: T18N14.6	6.5	6.2	0.3	1.3	100.0%	-4.2
5100	AT4G26270.1 | phosphofructokinase family protein, similar to phosphofructokinase (Amycolatopsis methanolica) GI:17432243; contains Pfam profile PF00365: Phosphofructokinase | chr4:13300804-13304356 REVERSE | Aliases: T25K17.80, T25K17_80	7.4	6.9	0.5	1.3	100.0%	-4.0
5101	AT5G05780.1 | Symbol: ATHMOV34 | 26S proteasome non-ATPase regulatory subunit 7, putative / 26S proteasome regulatory subunit S12, putative / MOV34 protein, putative, contains similarity to 26s proteasome regulatory subunit s12 (proteasome subunit p40) (mov34 protein) SP:P26516 from (Mus musculus); contains Pfam profile PF01398: Mov34/MPN/PAD-1 family | chr5:1735734-1738493 FORWARD | Aliases: MJJ3.19, MJJ3_19, PUTATIVE 26S PROTEASOME SUBUNIT ATHMOV34	6.9	8.0	-1.0	-1.3	100.0%	-3.9
5102	AT1G63960.1 | hypothetical protein | chr1:23741864-23742169 FORWARD | Aliases: T12P18.2, T12P18_2	3.9	3.6	0.3	1.3	100.0%	-4.2
5103	AT5G22875.2 | expressed protein, similar to hypothetical protein [Ipomoea trifida] (GB:AAS79573.1) | chr5:7650585-7651779 FORWARD | Aliases: None	4.9	4.5	0.4	1.3	100.0%	-4.2
5104	AT5G20930.1 | Symbol: TSL | protein kinase, putative, nearly identical to protein kinase tousled gi:433052:gb:AAA32874 | chr5:7097910-7103200 FORWARD | Aliases: F22D1.100, F22D1_100, PROTEIN KINASE TSL, TOUSLED	6.5	6.9	-0.4	-1.3	100.0%	-4.2
5105	AT5G05100.1 | expressed protein | chr5:1505123-1507203 REVERSE | Aliases: MUG13.4, MUG13_4	9.6	9.4	0.2	1.3	100.0%	-4.7
5106	AT5G64030.1 | dehydration-responsive protein-related, similar to early-responsive to dehydration stress ERD3 protein (Arabidopsis thaliana) GI:15320410; contains Pfam profile PF03141: Putative methyltransferase | chr5:25641263-25645701 FORWARD | Aliases: MBM17.13, MBM17_13	8.1	8.6	-0.5	-1.3	100.0%	-4.2
5107	AT2G39920.1 | acid phosphatase class B family protein, weak similarity to pod storage protein (Phaseolus vulgaris GI:2627233 SP:P10743 STEM 31 kDa glycoprotein precursor (Vegetative storage protein B) {Glycine max}; contains Pfam profile PF03767: HAD superfamily (subfamily IIIB) phosphatase | chr2:16670102-16671616 REVERSE | Aliases: T28M21.8, T28M21_8	3.2	2.9	0.3	1.3	100.0%	-4.4
5108	AT3G19300.1 | protein kinase family protein, contains protein kinase domain, Pfam:PF00069 | chr3:6690124-6693290 REVERSE | Aliases: MLD14.2	4.4	5.0	-0.6	-1.3	100.0%	-3.9
5109	AT5G38020.1 | S-adenosyl-L-methionine:carboxyl methyltransferase family protein, similar to SAM:salicylic acid carboxyl methyltransferase (SAMT) (GI:6002712)(Clarkia breweri) and to SAM:benzoic acid carboxyl methyltransferase (BAMT)(GI:9789277)(Antirrhinum majus) | chr5:15182882-15184959 REVERSE | Aliases: F16F17.20, F16F17_20	2.9	3.2	-0.3	-1.3	100.0%	-4.3
5110	AT5G55050.1 | GDSL-motif lipase/hydrolase family protein, similar to family II lipases EXL3 GI:15054386, EXL1 GI:15054382, EXL2 GI:15054384 from (Arabidopsis thaliana); contains Pfam profile PF00657: GDSL-like Lipase/Acylhydrolase	4.7	5.3	-0.5	-1.3	100.0%	-3.9
5111	AT4G31090.1 | expressed protein | chr4:15122235-15123492 FORWARD | Aliases: F6E21.10, F6E21_10	4.8	4.0	0.8	1.3	100.0%	-4.0
5112	AT5G01470.1 | expressed protein | chr5:190927-192610 FORWARD | Aliases: T10O8.180, T10O8_180	4.9	5.3	-0.4	-1.3	100.0%	-3.9
5113	AT3G25870.1 | expressed protein | chr3:9464553-9465520 REVERSE | Aliases: MPE11.2	4.1	3.6	0.5	1.3	100.0%	-4.0
5114	AT5G59990.1 | expressed protein, ; expression supported by MPSS | chr5:24168432-24170310 REVERSE | Aliases: MMN10.24, MMN10_24	2.7	2.5	0.2	1.3	100.0%	-4.8
5115	AT1G19130.1 | expressed protein | chr1:6609360-6610593 REVERSE | Aliases: F14D16.29, F14D16_29	8.8	9.3	-0.4	-1.3	100.0%	-3.9
5116	AT1G10690.1 | expressed protein | chr1:3549993-3550633 REVERSE | Aliases: T16B5.17, T16B5_17	3.7	3.3	0.4	1.3	100.0%	-4.3
5117	AT5G50310.1 | kelch repeat-containing protein, similar to Kelch repeats protein 3 (SP:Q08979) (Saccharomyces cerevisiae); contains Pfam PF01344: Kelch motif (6 repeats) | chr5:20491713-20496641 FORWARD | Aliases: MXI22.1, MXI22_1	6.7	7.2	-0.5	-1.3	100.0%	-4.1
5118	AT3G23600.2 | similar to dienelactone hydrolase family protein [Arabidopsis thaliana] (TAIR:At3g23570.1); similar to 'putative endo-1,3;1,4-beta-D-glucanase' [Oryza sativa (japonica cultivar-group)] (GB:AAU10803.1); contains InterPro domain Dienelactone hydrolase (InterPro:IPR002925) | chr3:8473802-8475895 FORWARD | Aliases: MDB19.8	10.8	11.4	-0.6	-1.3	100.0%	-4.3
5119	AT2G20470.1 | protein kinase, putative, contains protein kinase domain, Pfam:PF00069 | chr2:8833358-8836578 REVERSE | Aliases: T13C7.6, T13C7_6	2.7	2.9	-0.2	-1.3	100.0%	-4.7

Rank	Description	15.dpi	5.dpi	M	t	adj.q	B
5120	AT1G64980.1 | expressed protein | chr1:24141180-24142924 REVERSE | Aliases: F13O11.28, F13O11_28	6.6	6.3	0.3	1.3	100.0%	-4.3
5121	AT5G06265.1 | hyaluronan mediated motility receptor-related, contains weak similarity to hyaluronan mediated motility receptor (Intracellular hyaluronic acid binding protein) (Receptor for hyaluronan-mediated motility) (CD168 antigen) (Swiss-Prot:O75330) (Homo sapiens) | chr5:1905293-1908168 FORWARD | Aliases: None	6.0	5.6	0.4	1.3	100.0%	-4.1
5122	AT3G51450.1 | strictosidine synthase family protein, similar to hemomucin (Drosophila melanogaster)(GI:1280434), strictosidine synthase (Rauvolfia serpentina)(SP:P15324) | chr3:19101947-19103482 FORWARD | Aliases: F26O13.90	5.8	6.1	-0.3	-1.3	100.0%	-4.2
5123	AT3G06240.1 | F-box family protein, contains F-box domain Pfam:PF00646	7.0	6.4	0.6	1.3	100.0%	-4.1
5124	AT5G26830.1 | threonyl-tRNA synthetase / threonine--tRNA ligase (THRRS), identical to SP:O04630 Threonyl-tRNA synthetase, mitochondrial precursor (EC 6.1.1.3) (Threonine--tRNA ligase) (ThrRS) {Arabidopsis thaliana}	8.1	8.4	-0.3	-1.3	100.0%	-4.3
5125	AT4G01450.3 | nodulin MtN21 family protein, similar to MtN21 GI:2598575 (root nodule development) from (Medicago truncatula) | chr4:608456-610705 FORWARD | Aliases: F11O4.14, F11O4_14	7.7	7.3	0.4	1.3	100.0%	-4.2
5126	AT2G44880.1 | pentatricopeptide (PPR) repeat-containing protein, contains Pfam profile PF01535: PPR repeat | chr2:18512314-18513981 FORWARD | Aliases: T13E15.11	2.7	2.5	0.1	1.3	100.0%	-4.8
5127	AT1G20270.1 | oxidoreductase, 2OG-Fe(II) oxygenase family protein, similar to prolyl 4-hydroxylase, alpha subunit, from Gallus gallus (GI:212530), Rattus norvegicus (GI:474940), Mus musculus (SP:Q60715); contains PF03171 2OG-Fe(II) oxygenase superfamily domain | chr1:7020742-7023057 REVERSE | Aliases: F14O10.12, F14O10_12	4.0	3.7	0.3	1.3	100.0%	-4.3
5128	AT3G48120.1 | expressed protein | chr3:17783122-17785724 FORWARD | Aliases: T24C20.2	4.7	4.3	0.4	1.3	100.0%	-4.0
5129	AT5G02200.1 | phytochrome A specific signal transduction component-related, contains weak similarity to phytochrome A specific signal transduction component PAT3 (Arabidopsis thaliana) gi:19421998:gb:AAL87850 and far-red elongated hypocotyl protein 1 (Arabidopsis thaliana) gi:17148773:gb:AAL35819 | chr5:437458-438892 FORWARD | Aliases: T7H20.250, T7H20_250	3.8	3.5	0.4	1.3	100.0%	-4.5
5130	AT1G06060.1 | RanBPM-related, similar to RANBPM {GI:13194576)(Homo sapiens) | chr1:1832942-1835576 FORWARD | Aliases: T21E18.11, T21E18_11	3.6	3.8	-0.3	-1.3	100.0%	-4.2
5131	AT4G17490.1 | Symbol: ATERF6 |	4.7	3.8	0.8	1.3	100.0%	-3.8
5132	AT5G49320.1 | expressed protein | chr5:20012251-20013440 REVERSE | Aliases: K21P3.22, K21P3_22	4.9	4.5	0.4	1.3	100.0%	-4.0
5133	AT5G13790.1 | Symbol: AGL15 | floral homeotic protein AGL-15 (AGL15) | chr5:4449017-4450846 REVERSE | Aliases: AGAMOUS LIKE 15, MADS BOX PROTEIN AGL15, MXE10.8, MXE10_8	3.0	3.2	-0.2	-1.3	100.0%	-4.7
5134	AT1G10360.1 | Symbol: ATGSTU18 | glutathione S-transferase, putative, similar to glutathione S-transferase (sp:Q03666:GTX4_TOBAC); similar to EST gb:H36275 gb:AB039930. | chr1:3395560-3396851 REVERSE | Aliases: F14N23.24, F14N23_24, GLUTATHIONE S TRANSFERASE 29, GST29	6.2	5.6	0.7	1.3	100.0%	-3.6
5135	AT4G34030.1 | Symbol: MCCB | methylcrotonyl-CoA carboxylase beta chain, mitochondrial / 3-methylcrotonyl-CoA carboxylase 2 (MCCB), identical to SP:Q9LDD8 Methylcrotonyl-CoA carboxylase beta chain, mitochondrial precursor (EC 6.4.1.4) (3-Methylcrotonyl-CoA carboxylase 2) (MCCase beta subunit) (3-methylcrotonyl-CoA:carbon dioxide ligase beta subunit) {Arabidopsis thaliana}; contains Pfam profile: PF01039 carboxyl transferase domain | chr4:16301198-16304031 FORWARD | Aliases: 3 METHYLCROTONYL COA CARBOXYLASE, F28A23.210, F28A23_210	10.2	10.5	-0.3	-1.3	100.0%	-4.5
5136	AT4G30580.1 | Symbol: ATS2 | phospholipid/glycerol acyltransferase family protein | chr4:14932347-14934525 REVERSE | Aliases: EMB1995, EMBRYO DEFECTIVE 1995, F17I23.80, F17I23_80	6.6	6.2	0.4	1.3	100.0%	-4.2
5137	AT3G21470.1 | pentatricopeptide (PPR) repeat-containing protein, contains INTERPRO:IPR002885 PPR repeats | chr3:7563614-7565254 FORWARD | Aliases: MIL23.2	2.7	2.9	-0.2	-1.3	100.0%	-4.7
5138	AT3G15020.1 | malate dehydrogenase (NAD), mitochondrial, putative, similar to mitochondrial NAD-dependent malate dehydrogenase GB:CAA10320 SP:Q9ZP06 (Arabidopsis thaliana); contains InterPro entry IPR001236: Lactate/malate dehydrogenase | chr3:5056083-5058255 FORWARD | Aliases: K15M2.16	9.3	10.1	-0.8	-1.3	100.0%	-3.9
5139	AT2G17930.1 | FAT domain-containing protein / phosphatidylinositol 3- and 4-kinase family protein, contains Pfam profiles PF02259 FAT domain, PF00454 Phosphatidylinositol 3- and 4-kinase, PF02260: FATC domain | chr2:7791458-7809312 REVERSE | Aliases: T13L16.5, T13L16_5	5.3	5.8	-0.5	-1.3	100.0%	-4.0

Rank	Description	15.dpi	5.dpi	M	t	adj.q	B
5140	AT3G57550.2 | Symbol: AGK2 | similar to guanylate kinase 1 (GK-1) [Arabidopsis thaliana] (TAIR:At2g41880.1); similar to guanylate kinase [Nicotiana tabacum] (GB:AAG12251.1); contains InterPro domain Guanylate kinase (InterPro:IPR008144); contains InterPro domain Guanylate kinase/L-type calcium channel region (InterPro:IPR008145) | chr3:21317700-21320441 FORWARD | Aliases: GK 2, GUANYLATE KINAS, T8H10.150	6.9	7.7	-0.8	-1.3	100.0%	-3.9
5141	AT3G01690.1 | expressed protein | chr3:256562-258554 FORWARD | Aliases: F4P13.23, F4P13_23	10.1	9.8	0.3	1.3	100.0%	-4.2
5142	AT4G00330.1 | Symbol: CRCK2 | protein kinase family protein, contains protein kinase domain, Pfam:PF00069 | chr4:142622-144523 REVERSE | Aliases: A_IG005I10.8, A_IG005I10_8, F5I10.8, F5I10_8	5.8	5.5	0.3	1.3	100.0%	-4.5
5143	AT3G09720.1 | DEAD/DEAH box helicase, putative, similar to RNA helicase involved in rRNA processing GB:6321267 from (Saccharomyces cerevisiae)c, ontains DEAD and DEAH box domain | chr3:2980236-2983578 REVERSE | Aliases: F11F8.31	4.2	4.4	-0.2	-1.3	100.0%	-4.3
5144	AT5G13020.1 | emsy N terminus domain-containing protein / ENT domain-containing protein, contains Pfam profile PF03735: ENT domain | chr5:4129362-4132092 REVERSE | Aliases: T19L5.3	5.8	5.2	0.6	1.3	100.0%	-3.9
5145	AT4G39280.1 | phenylalanyl-tRNA synthetase, putative / phenylalanine--tRNA ligase, putative, similar to SP:Q9Y285 Phenylalanyl-tRNA synthetase alpha chain (EC 6.1.1.20) (Phenylalanine- -tRNA ligase alpha chain) (PheRS) {Homo sapiens}; contains Pfam profile PF01409: tRNA synthetases class II core domain (F) | chr4:18281456-18284843 REVERSE | Aliases: T22F8.180, T22F8_180	8.8	9.6	-0.8	-1.3	100.0%	-3.8
5146	AT4G09730.1 | DEAD/DEAH box helicase, putative, RNA helicase -Mus musculus,PIR2:I84741	3.8	4.2	-0.4	-1.3	100.0%	-4.2
5147	AT2G33490.1 | hydroxyproline-rich glycoprotein family protein, Common family member:At3g26910 (Arabidopsis thaliana) | chr2:14190628-14194945 FORWARD | Aliases: F4P9.26, F4P9_26	3.8	4.1	-0.3	-1.3	100.0%	-4.2
5148	AT1G30300.1 | expressed protein, similar to putative hydrolase GI:7270684 from (Arabidopsis thaliana); similar to PhnP protein (GI:15620485) (Rickettsia conorii) | chr1:10673002-10675350 FORWARD | Aliases: F12P21.8, F12P21_8	4.0	4.2	-0.2	-1.3	100.0%	-4.3
5149	AT1G78810.2 | expressed protein | chr1:29633206-29635446 REVERSE | Aliases: F9K20.15, F9K20_15	7.2	7.7	-0.5	-1.3	100.0%	-3.9
5150	AT4G08670.1 | protease inhibitor/seed storage/lipid transfer protein (LTP) family protein, contains Pfam protease inhibitor/seed storage/LTP family domain PF00234 | chr4:5536696-5538207 REVERSE | Aliases: T3F12.2, T3F12_2	2.8	2.6	0.2	1.3	100.0%	-4.5
5151	AT2G24280.1 | serine carboxypeptidase S28 family protein, contains Pfam profile: PF05577 Serine carboxypeptidase S28 | chr2:10340364-10344161 FORWARD | Aliases: F27D4.19, F27D4_19	4.0	3.6	0.4	1.3	100.0%	-4.0
5152	AT1G51380.1 | eukaryotic translation initiation factor 4A, putative / eIF-4A, putative	4.6	5.0	-0.3	-1.3	100.0%	-4.0
5153	AT3G07160.1 | Symbol: ATGSL10 | glycosyl transferase family 48 protein, similar to glucan synthase GB:AAD11794 (Filobasidiella neoformans var. neoformans) | chr3:2264963-2279389 REVERSE | Aliases: GLUCAN SYNTHASE LIKE 10, GSL10, T1B9.18	6.8	7.3	-0.5	-1.3	100.0%	-4.1
5154	AT4G14930.1 | acid phosphatase survival protein SurE, putative, similar to Swiss-Prot:P36664 acid phosphatase surE (EC 3.1.3.2) (Stationary-phase survival protein surE) (Escherichia coli O157:H7); contains Pfam domain PF01975: Survival protein SurE | chr4:8538582-8541895 FORWARD | Aliases: DL3505W, FCAALL.200	6.6	7.2	-0.7	-1.3	100.0%	-3.9
5155	AT1G05960.1 | expressed protein, similar to hypothetical protein GB:AAF80120 GI:8810459 from (Arabidopsis thaliana) | chr1:1807972-1815463 REVERSE | Aliases: T21E18.21, T21E18_21	6.5	6.0	0.4	1.3	100.0%	-4.0
5156	AT1G64390.1 | endo-1,4-beta-glucanase, putative / cellulase, putative, similar to endo-beta-1,4-glucanase GI:4972236 from (Fragaria x ananassa) (Plant Mol. Biol. 40, 323-332 (1999)) | chr1:23914782-23918892 FORWARD | Aliases: F15H21.9, F15H21_9	3.9	4.7	-0.7	-1.3	100.0%	-4.1
5157	AT3G06770.3 | glycoside hydrolase family 28 protein / polygalacturonase (pectinase) family protein, weak similarity to polygalacturonase (Persea americana) GI:166951; contains PF00295: Glycosyl hydrolases family 28 (polygalacturonases) | chr3:2134806-2136918 REVERSE | Aliases: F3E22.9	4.0	4.6	-0.6	-1.3	100.0%	-4.2
5158	AT2G20450.1 | 60S ribosomal protein L14 (RPL14A) | chr2:8820947-8822332 FORWARD | Aliases: T13C7.4, T13C7_4	9.2	9.7	-0.5	-1.3	100.0%	-4.0
5159	AT1G17480.1 | calmodulin-binding family protein, low similarity to SF16 protein (Helianthus annuus) GI:560150; contains Pfam profile PF00612: IQ calmodulin-binding motif | chr1:6006454-6008259 REVERSE | Aliases: F28G4.3, F28G4_3	3.5	3.2	0.2	1.3	100.0%	-4.5
5160	AT5G67210.1 | expressed protein, contains Pfam profile PF04669: Protein of unknown function (DUF579) | chr5:26836245-26837198 FORWARD | Aliases: K21H1.17, K21H1_17	5.7	6.1	-0.4	-1.3	100.0%	-4.4

Rank	Description	15.dpi	5.dpi	M	t	adj.q	B
5161	AT1G70420.1 | expressed protein | chr1:26543237-26544425 REVERSE | Aliases: F17O7.4, F17O7_4	4.8	4.4	0.5	1.3	100.0%	-4.5
5162	AT5G04290.1 | KOW domain-containing transcription factor family protein | chr5:1196070-1202654 FORWARD | Aliases: T19N18.20, T19N18_20	8.7	8.0	0.6	1.3	100.0%	-4.5
5163	AT3G29280.1 | expressed protein, ; expression supported by MPSS | chr3:11237942-11239184 REVERSE | Aliases: MMF24.3	6.1	6.6	-0.5	-1.3	100.0%	-3.8
5164	AT4G02930.1 | elongation factor Tu, putative / EF-Tu, putative, similar to mitochondrial elongation factor Tu (Arabidopsis thaliana) gi:1149571:emb:CAA61511 | chr4:1295409-1298397 REVERSE | Aliases: T4I9.19	9.6	10.3	-0.7	-1.3	100.0%	-3.8
5165	AT4G39870.1 | expressed protein, hypothetical protein, Schizosaccharomyces cerevisae, Z99168 | chr4:18501889-18504422 FORWARD | Aliases: T5J17.40, T5J17_40	5.3	5.0	0.4	1.3	100.0%	-4.0
5166	AT1G21570.1 | zinc finger (CCCH-type) family protein, contains Pfam domain, PF00642: Zinc finger C-x8-C-x5-C-x3-H type (and similar) | chr1:7557251-7560272 REVERSE | Aliases: F24J8.21, F24J8_21	9.2	8.6	0.6	1.3	100.0%	-4.1
5167	AT2G39390.1 | 60S ribosomal protein L35 (RPL35B) | chr2:16457738-16458956 REVERSE | Aliases: F12L6.5, F12L6_5	10.6	11.0	-0.3	-1.3	100.0%	-4.3
5168	AT2G03420.1 | expressed protein | chr2:1034995-1035966 REVERSE | Aliases: T4M8.15, T4M8_15	4.5	5.0	-0.5	-1.3	100.0%	-3.7
5169	AT5G60630.1 | expressed protein, predicted protein, Arabidopsis thaliana | chr5:24386876-24387630 REVERSE | Aliases: MUP24.5, MUP24_5	4.8	5.3	-0.5	-1.3	100.0%	-3.8
5170	AT5G62940.1 | Dof-type zinc finger domain-containing protein, Dof zinc finger protein, Oryza sativa, EMBL:AB028129 | chr5:25274145-25275907 REVERSE | Aliases: MQB2.26, MQB2_26	4.3	4.5	-0.3	-1.3	100.0%	-4.3
5171	AT1G25290.1 | rhomboid family protein, contains PFAM domain PF01694, Rhomboid family | chr1:8867144-8869079 FORWARD | Aliases: F4F7.32, F4F7_32	5.0	4.7	0.2	1.3	100.0%	-4.4
5172	AT2G36410.3 | similar to expressed protein [Arabidopsis thaliana] (TAIR:At3g52920.2); similar to unknown [Oryza sativa (japonica cultivar-group)] (GB:AAO72703.1); contains InterPro domain Protein of unknown function DUF662 (InterPro:IPR007033) | chr2:15285848-15287683 FORWARD | Aliases: F1O11.4, F1O11_4	6.1	5.3	0.8	1.3	100.0%	-3.8
5173	AT1G01550.2 | Symbol: BPS1 | similar to expressed protein [Arabidopsis thaliana] (TAIR:At2g46080.1); similar to OSJNBa0018M05.6 [Oryza sativa (japonica cultivar-group)] (GB:XP_474319.1) | chr1:199792-201775 FORWARD | Aliases: BYPASS 1, F22L4.9, F22L4_9	10.2	9.6	0.6	1.3	100.0%	-4.1
5174	AT1G08970.4 | Symbol: HAP5C | CCAAT-box binding transcription factor Hap5a, putative | chr1:2882546-2884285 FORWARD | Aliases: F7G19.16, F7G19_16, HAP5C, HEME ACTIVATED PROTEIN 5C	9.8	8.9	0.8	1.3	100.0%	-3.9
5175	AT5G43960.2 | nuclear transport factor 2 (NTF2) family protein / RNA recognition motif (RRM)-containing protein, contains Pfam profiles PF02136: Nuclear transport factor 2 (NTF2) domain, PF00076: RNA recognition motif. (a.k.a. RRM, RBD, or RNP domain) | chr5:17706015-17709114 REVERSE | Aliases: MRH10.6, MRH10_6	5.8	6.2	-0.4	-1.3	100.0%	-4.0
5176	AT4G11410.1 | short-chain dehydrogenase/reductase (SDR) family protein, contains INTERPRO family IPR002198 Short-chain dehydrogenase/reductase (SDR) superfamily | chr4:6944919-6947328 REVERSE | Aliases: F25E4.30, F25E4_30	7.1	6.4	0.7	1.3	100.0%	-3.9
5177	AT1G69390.1 | chloroplast division protein, putative (MinE1), identical to chloroplast division protein homolog MinE1 GI:17511220 from (Arabidopsis thaliana) | chr1:26088317-26090144 FORWARD | Aliases: F23O10.25, F23O10_25	7.1	6.5	0.5	1.3	100.0%	-3.9
5178	AT5G49970.2 | pyridoxamine 5'-phosphate oxidase-related, contains weak similarity to Pyridoxamine 5'-phosphate oxidase (EC 1.4.3.5) (PNP/PMP oxidase) (PNPOx). (Swiss-Prot:P28225) (Shigella flexneri)	7.3	7.9	-0.6	-1.3	100.0%	-4.0
5179	AT3G21310.1 | expressed protein, contains Pfam profile PF03267: Arabidopsis protein of unknown function, DUF266 | chr3:7497296-7499158 FORWARD | Aliases: MXL8.19	3.2	3.1	0.2	1.3	100.0%	-5.0
5180	AT4G14465.1 | DNA-binding protein-related, contains Pfam domain PF03479: Domain of unknown function (DUF296), found in AT-hook motifs Pfam:PF02178 | chr4:8320626-8322185 FORWARD | Aliases: None	5.6	5.3	0.3	1.3	100.0%	-4.1
5181	AT4G31330.1 | expressed protein, contains Pfam profile PF04654: Protein of unknown function, DUF599 | chr4:15202302-15203437 FORWARD | Aliases: F8F16.150, F8F16_150	5.7	5.2	0.5	1.3	100.0%	-4.0
5182	AT5G36250.1 | protein phosphatase 2C, putative / PP2C, putative | chr5:14299333-14301811 FORWARD | Aliases: T30G6.11, T30G6_11	4.6	4.1	0.5	1.3	100.0%	-4.1
5183	AT5G24750.1 | expressed protein | chr5:8490575-8494590 REVERSE | Aliases: T4C12.20	5.2	5.5	-0.3	-1.3	100.0%	-4.1

Rank	Description	15.dpi	5.dpi	M	t	adj.q	B
5184	AT1G48280.1 | hydroxyproline-rich glycoprotein family protein | chr1:17838756-17841385 FORWARD | Aliases: F11A17.16, F11A17_16	4.3	4.7	-0.4	-1.3	100.0%	-4.0
5185	AT3G15160.1 | expressed protein | chr3:5104374-5108457 FORWARD | Aliases: F4B12.7	5.0	5.3	-0.3	-1.3	100.0%	-4.2
5186	AT3G08620.1 | KH domain-containing protein | chr3:2617774-2620579 FORWARD | Aliases: F17O14.9	4.5	4.1	0.4	1.3	100.0%	-4.0
5187	AT5G22360.1 | Symbol: ATVAMP714 | synaptobrevin family protein, similar to Synaptobrevin-like protein 1 (SP:P51809) (Homo sapiens) | chr5:7404014-7405657 REVERSE | Aliases: MWD9.16, MWD9_16, VAMP714	6.6	6.1	0.6	1.3	100.0%	-4.2
5188	AT1G72250.1 | kinesin motor protein-related | chr1:27196564-27201976 FORWARD | Aliases: T9N14.6, T9N14_6	3.1	2.9	0.2	1.3	100.0%	-4.4
5189	AT1G48170.1 | expressed protein | chr1:17792455-17793762 FORWARD | Aliases: F21D18.10	6.5	5.9	0.5	1.3	100.0%	-4.0
5190	AT5G51720.1 | expressed protein | chr5:21026822-21027571 FORWARD | Aliases: MIO24.14, MIO24_14	6.5	5.7	0.8	1.3	100.0%	-3.6
5191	AT1G32400.3 | Symbol: TOM2A | similar to expressed protein [Arabidopsis thaliana] (TAIR:At2g20230.1); similar to senescence-associated protein-like [Oryza sativa (japonica cultivar-group)] (GB:XP_480197.1); contains InterPro domain CD9/CD37/CD63 antigen (InterPro:IPR000301) | chr1:11689088-11691445 REVERSE | Aliases: F5D14.17, F5D14_17, TOBAMOVIRUS MULTIPLICATION 2A	9.4	8.8	0.6	1.3	100.0%	-4.2
5192	AT5G25070.1 | expressed protein | chr5:8641001-8643494 REVERSE | Aliases: T11H3.80, T11H3_80	5.7	6.2	-0.5	-1.3	100.0%	-4.0
5193	AT4G20480.1 | expressed protein, contains Pfam profile PF04396: Protein of unknown function, DUF537 | chr4:11034283-11035885 REVERSE | Aliases: F9F13.130, F9F13_130	8.4	7.8	0.5	1.3	100.0%	-4.1
5194	AT3G14770.1 | nodulin MtN3 family protein, similar to MtN3 GI:1619602 (root nodule development) from (Medicago truncatula) | chr3:4957363-4959709 REVERSE | Aliases: T21E2.6	2.7	2.4	0.3	1.3	100.0%	-4.1
5195	ATCG00350.1 | Symbol: PSAA | Encodes psaA protein comprising the reaction center for photosystem I along with psaB protein; hydrophobic protein encoded by the chloroplast genome. | chrC:39605-41857 REVERSE | Aliases: PSAA	4.7	4.3	0.4	1.3	100.0%	-4.3
5196	AT2G42780.1 | expressed protein | chr2:17807590-17809893 FORWARD | Aliases: F7D19.22, F7D19_22	7.6	7.9	-0.3	-1.3	100.0%	-4.2
5197	AT5G12850.1 | zinc finger (CCCH-type) family protein, contains Pfam domain, PF00642: Zinc finger C-x8-C-x5-C-x3-H type (and similar) and Pfam domain, PF00023: Ankyrin repeat | chr5:4056194-4059583 FORWARD | Aliases: T24H18.20, T24H18_20	4.3	4.7	-0.4	-1.3	100.0%	-4.1
5198	AT3G11040.1 | glycosyl hydrolase family 85 protein, contains similarity to endo-b-N-acetylglucosaminidase GI:13774138 from (Mucor hiemalis) | chr3:3460149-3463318 FORWARD | Aliases: F9F8.14	3.2	3.5	-0.3	-1.3	100.0%	-4.3
5199	AT3G23240.1 | Symbol: ATERF1/ERF1 | encodes a member of the ERF (ethylene response factor) subfamily B-3 of ERF/AP2 transcription factor family (ERF1). The protein contains one AP2 domain. There are 18 members in this subfamily including ATERF-1, ATERF-2, AND ATERF-5. EREBP like protein that binds GCC box of ethylene regulated promoters such as basic chitinases. Constitutive expression of ERF1 phenocopies ethylene over production. Involved in ethylene signaling cascade,downstream of EIN2 and EIN3. | chr3:8295651-8296611 FORWARD | Aliases: ATERF1, ERF1, ETHYLENE RESPONSE FACTOR 1, K14B15.4	3.6	3.4	0.2	1.3	100.0%	-4.7
5200	AT3G14690.1 | Symbol: CYP72A15 | cytochrome P450, putative, similar to GB:Q05047 from (Catharanthus roseus) | chr3:4937386-4939472 FORWARD | Aliases: MIE1.19	7.4	6.6	0.8	1.3	100.0%	-3.9
5201	AT2G37190.1 | 60S ribosomal protein L12 (RPL12A) | chr2:15626486-15627198 REVERSE | Aliases: T2N18.5, T2N18_5	8.8	9.4	-0.6	-1.3	100.0%	-3.8
5202	AT5G48230.2 | Symbol: EMB1276 | acetyl-CoA C-acyltransferase, putative / 3-ketoacyl-CoA thiolase, putative, strong similarity to Acetoacetyl-coenzyme A thiolase (E.C. 2.3.1.9) (Raphanus sativus) GI:1542941; contains InterPro accession IPR002155: Thiolase | chr5:19569241-19572755 REVERSE | Aliases: EMB1276, EMBRYO DEFECTIVE 1276, MIF21.12, MIF21_12	7.6	8.6	-0.9	-1.3	100.0%	-4.1
5203	AT4G04860.1 | Der1-like family protein / degradation in the ER-like family protein, contains Pfam profile: PF04511 Der1-like family | chr4:2459878-2461737 FORWARD | Aliases: T4B21.2, T4B21_2	6.1	5.6	0.5	1.3	100.0%	-4.2
5204	AT3G23605.1 | UBX domain-containing protein, contains Pfam profile PF00789: UBX domain	6.5	6.0	0.5	1.3	100.0%	-4.0
5205	AT5G38730.1 | pentatricopeptide (PPR) repeat-containing protein, contains Pfam profile PF01535: PPR repeat | chr5:15528131-15529921 FORWARD | Aliases: MKD10.5, MKD10_5	5.2	4.9	0.4	1.3	100.0%	-4.2

Rank	Description	15.dpi	5.dpi	M	t	adj.q	B
5206	AT4G16330.1 | oxidoreductase, 2OG-Fe(II) oxygenase family protein, similar to flavonone-3-hydroxylase (naringenin,2-oxoglutarate 3-dioxygenase) from Malus domestica (SP:Q06942), Pyrus communis (GI:20269881); contains Pfam domain PF03171, 2OG-Fe(II) oxygenase superfamily	10.8	10.4	0.4	1.3	100.0%	-4.5
5207	AT3G28050.1 | nodulin MtN21 family protein, similar to MtN21 (Medicago truncatula) GI:2598575; contains Pfam profile PF00892: Integral membrane protein | chr3:10444054-10446622 FORWARD | Aliases: MMG15.22	5.5	4.7	0.8	1.3	100.0%	-3.8
5208	AT5G16660.1 | expressed protein | chr5:5465490-5467066 REVERSE | Aliases: MTG13.11, MTG13_11	8.4	8.1	0.3	1.3	100.0%	-4.2
5209	AT5G66780.1 | expressed protein | chr5:26680799-26681629 FORWARD | Aliases: MUD21.2, MUD21_2	3.8	4.3	-0.5	-1.3	100.0%	-3.7
5210	AT5G09660.2 | Symbol: PMDH2 | similar to malate dehydrogenase, glyoxysomal, putative [Arabidopsis thaliana] (TAIR:At2g22780.1); similar to malate dehydrogenase 1 [Brassica napus] (GB:CAB43994.1); contains InterPro domain Malate dehydrogenase, active site (InterPro:IPR001252); contains InterPro domain Lactate/malate dehydrogenase (InterPro:IPR001236) | chr5:2993445-2995308 REVERSE | Aliases: F17I14.150, F17I14_150, PEROXISOMAL NAD MALATE DEHYDROGENASE 2, PMDH2	7.2	6.5	0.7	1.3	100.0%	-3.5
5211	AT3G06150.1 | expressed protein | chr3:1861803-1864122 REVERSE | Aliases: F28L1.9, F28L1_9	4.9	5.1	-0.2	-1.3	100.0%	-4.5
5212	AT1G73130.1 | expressed protein | chr1:27502696-27505557 FORWARD | Aliases: T18K17.21, T18K17_21	4.9	5.3	-0.4	-1.3	100.0%	-4.1
5213	AT1G67370.1 | Symbol: ASY1 | meiotic asynaptic mutant 1 (ASY1), identical to meiotic asynaptic mutant 1 (Arabidopsis thaliana) GI:7939627; contains Pfam profiles PF02301: DNA-binding HORMA domain, PF04433: SWIRM domain | chr1:25242780-25247500 REVERSE | Aliases: ASY1, ASYNAPTIC 1, F1N21.19	4.3	3.9	0.3	1.3	100.0%	-4.1
5214	AT5G05290.1 | Symbol: ATEXPA2 | expansin, putative (EXP2), identical to expansin At-EXP2 (Arabidopsis thaliana) gi:1041708:gb:AAB38073; alpha-expansin gene family, PMID:11641069 | chr5:1568695-1569865 FORWARD | Aliases: ARABIDOPSIS THALIANA EXPANSIN A2, ATEXP2, ATHEXP ALPHA 1.12, EXP2, EXPANSIN 2, K18I23.9, K18I23_9	3.8	3.5	0.3	1.3	100.0%	-4.3
5215	AT3G09150.3 | Symbol: HY2 | phytochromobilin:ferredoxin oxidoreductase, chloroplast / phytochromobilin synthase (HY2), identical to SP:Q9SR43 Phytochromobilin:ferredoxin oxidoreductase, chloroplast precursor (EC 1.3.7.4) (Phytochromobilin synthase) (PFB synthase) (PPhiB synthase) {Arabidopsis thaliana}; identical to cDNA for phytochromobilin synthase HY2 protein, GI:13359272 | chr3:2803615-2805569 FORWARD | Aliases: ELONGATED HYPOCOTYL 2, F3L24.1, GENOMES UNCOUPLED 3, GUN3, PHYTOCHROMOBILIN SYNTHASE	4.7	5.2	-0.5	-1.3	100.0%	-3.8
5216	AT5G08740.1 | pyridine nucleotide-disulphide oxidoreductase family protein, similar to DhnA (Synechococcus sp. PCC 7942) GI:15620546; contains Pfam profile PF00070: Pyridine nucleotide-disulphide oxidoreductase | chr5:2848507-2851424 REVERSE | Aliases: None	4.2	4.5	-0.3	-1.3	100.0%	-4.4
5217	AT5G65350.1 | histone H3, nearly identical to histone H3 from Zea mays SP:P05203, Medicago sativa GI:166384, Encephalartos altensteinii SP:P08903, Pisum sativum SP:P02300; contains Pfam profile PF00125 Core histone H2A/H2B/H3/H4 | chr5:26136437-26137051 REVERSE | Aliases: MNA5.8, MNA5_8	3.0	2.8	0.2	1.3	100.0%	-4.6
5218	AT4G08930.1 | Symbol: ATAPRL6 | Encodes a protein disulfide isomerase-like (PDIL) protein, a member of a multigene family within the thioredoxin (TRX) superfamily. This protein also belongs to the adenosine 5'-phosphosulfate reductase-like (APRL) group. | chr4:5729189-5730858 FORWARD | Aliases: APR LIKE 6, ATAPRL6, T3H13.4, T3H13_4	5.8	5.3	0.5	1.3	100.0%	-3.8
5219	AT3G01290.1 | band 7 family protein, similar to hypersensitive-induced response protein (Zea mays) GI:7716470; contains Pfam profile PF01145: SPFH domain / Band 7 family | chr3:88064-89429 REVERSE | Aliases: T22N4.8, T22N4_8	4.0	3.4	0.7	1.3	100.0%	-3.6
5220	AT5G11490.1 | adaptin family protein, similar to SP:Q9WV76 Adapter-related protein complex 4 beta 1 subunit (Beta subunit of AP- 4) {Mus musculus}, beta-adaptin Drosophila 1 {Drosophila melanogaster} GI:434902; contains Pfam profile: PF01602 Adaptin N terminal region | chr5:3671838-3676305 FORWARD | Aliases: F15N18.80, F15N18_80	6.3	6.7	-0.4	-1.3	100.0%	-3.9
5221	AT5G03740.1 | Symbol: HD2C | zinc finger (C2H2 type) family protein, contains Pfam domain, PF00096: Zinc finger, C2H2 type | chr5:981896-984201 FORWARD | Aliases: F17C15.160, F17C15_160, HISTONE DEACETYLASE 2C	9.2	9.5	-0.3	-1.3	100.0%	-4.3
5222	AT2G46750.1 | FAD-binding domain-containing protein, low similarity to SP:P58710 L-gulonolactone oxidase (EC 1.1.3.8) {Mus musculus}; contains Pfam profile PF01565: FAD binding domain | chr2:19215390-19218028 REVERSE | Aliases: F19D11.3	3.3	3.9	-0.5	-1.3	100.0%	-3.7
5223	AT1G66750.1 | Symbol: CDKD1;2 | cell division protein kinase, putative, similar to cell division protein kinase 7 (Homo sapiens) SWISS-PROT:P50613 | chr1:24898399-24900911 FORWARD | Aliases: CDKD1;2, Cyclin dependent kinase D1;2, F4N21.12, F4N21_12	5.2	5.6	-0.4	-1.3	100.0%	-4.2
5224	AT4G20460.1 | NAD-dependent epimerase/dehydratase family protein, similar to UDP-galactose 4-epimerase from Cyamopsis tetragonoloba GI:3021357 (EMBL:AJ005082), Bacillus subtilis SP:P55180; contains Pfam profile PF01370 NAD dependent epimerase/dehydratase family | chr4:11029747-11031583 REVERSE | Aliases: F9F13.110, F9F13_110	4.4	4.9	-0.5	-1.3	100.0%	-3.8

Rank	Description	15.dpi	5.dpi	M	t	adj.q	B
5225	AT3G55870.1 | anthranilate synthase, alpha subunit, putative, similar to anthranilate synthase alpha subunit, Ruta graveolens, EMBL:RGANTSYNB (gi:960291), Arabidopsis thaliana (SP:P32068) | chr3:20741837-20744882 FORWARD | Aliases: F27K19.50	3.2	3.1	0.2	1.3	100.0%	-4.7
5226	AT5G08139.1 | zinc finger (C3HC4-type RING finger) family protein, contains Pfam profile: PF00097 zinc finger, C3HC4 type (RING finger) | chr5:2616346-2617865 FORWARD | Aliases: None	5.9	5.5	0.3	1.3	100.0%	-4.3
5227	AT2G19290.1 | expressed protein | chr2:8373001-8374104 REVERSE | Aliases: F27F23.9, F27F23_9	2.5	2.4	0.2	1.3	100.0%	-5.0
5228	AT4G03410.2 | peroxisomal membrane protein-related, contains weak similarity to Swiss-Prot:P42925 22 kDa peroxisomal membrane protein (Mus musculus) | chr4:1501167-1503560 FORWARD | Aliases: F9H3.2	4.1	3.8	0.3	1.3	100.0%	-4.1
5229	AT3G49890.1 | expressed protein, lea32, Arabidopsis thaliana, EMBL:ATH131342 | chr3:18510221-18511705 FORWARD | Aliases: T16K5.240	4.9	5.6	-0.7	-1.3	100.0%	-4.0
5230	AT1G44810.1 | expressed protein, contains Pfam profile: PF04504 protein of unknown function, DUF573 | chr1:16925983-16927261 FORWARD | Aliases: T12C22.8, T12C22_8	6.1	5.8	0.3	1.3	100.0%	-4.3
5231	AT1G49700.1 | expressed protein, ; expression supported by MPSS | chr1:18392460-18393976 REVERSE | Aliases: F14J22.9, F14J22_9	3.5	3.2	0.3	1.3	100.0%	-4.4
5232	AT3G21780.1 | UDP-glucoronosyl/UDP-glucosyl transferase family protein, contains Pfam profile: PF00201 UDP-glucoronosyl and UDP-glucosyl transferase | chr3:7675058-7676353 REVERSE | Aliases: MSD21.11	3.8	3.4	0.4	1.3	100.0%	-3.9
5233	AT5G09930.1 | Symbol: ATGCN2 |	2.9	2.8	0.2	1.3	100.0%	-4.7
5234	AT5G13810.1 | glutaredoxin family protein | chr5:4455354-4457022 FORWARD | Aliases: MAC12.24, MAC12_24	6.6	6.2	0.4	1.3	100.0%	-4.2
5235	AT3G04840.1 | 40S ribosomal protein S3A (RPS3aA), similar to 40S ribosomal protein S3A (S phase specific protein GBIS289) GB:P49396 (Brassica rapa) | chr3:1329699-1331581 FORWARD | Aliases: T9J14.21, T9J14_21	10.1	10.6	-0.5	-1.3	100.0%	-4.2
5236	AT5G09850.1 | transcription elongation factor-related, low similarity to SP:P10712 Transcription elongation factor S-II (Transcription elongation factor A) {Mus musculus} | chr5:3063252-3065890 REVERSE | Aliases: MYH9.6, MYH9_6	4.9	5.5	-0.6	-1.3	100.0%	-4.0
5237	AT3G58450.2 | universal stress protein (USP) family protein, contains Pfam PF00582: universal stress protein family | chr3:21632979-21634131 FORWARD | Aliases: F14P22.40	3.4	3.1	0.3	1.3	100.0%	-4.4
5238	AT3G44520.1 | esterase/lipase/thioesterase family protein, similar to SP:Q02104 Lipase 1 precursor (EC 3.1.1.3) (Triacylglycerol lipase) {Psychrobacter immobilis}; contains Interpro entry IPR000379 | chr3:16121387-16122126 REVERSE | Aliases: F14L2.70	2.9	2.7	0.2	1.3	100.0%	-4.6
5239	AT5G19280.1 | Symbol: KAPP | kinase associated protein phosphatase (KAPP), identical to Kinase associated protein phosphatase (SP:P46014) (Arabidopsis thaliana); contains Pfam PF00481: Protein phosphatase 2C domain; contains Pfam PF00498: FHA domain | chr5:6488365-6493441 FORWARD | Aliases: F7K24.30, F7K24_30, KAPP, KINASE ASSOCIATED PROTEIN PHOSPHATASE, RAG1, ROOT ALTERNATED GROWTH 1, T24G5.3	6.4	6.0	0.4	1.3	100.0%	-4.0
5240	AT4G01320.1 | Symbol: ATSTE24 | CAAX protease, putative (STE24), contains Pfam domain, PF01435: Peptidase family M48 | chr4:545796-549261 FORWARD | Aliases: ATSTE24, F2N1.21, F2N1_21, STE24	8.5	9.0	-0.5	-1.3	100.0%	-4.2
5241	AT1G04830.1 | RabGAP/TBC domain-containing protein, low similarity to SP:Q08484 GTPase-activating protein GYP1 {Saccharomyces cerevisiae}; contains Pfam profile PF00566: TBC domain | chr1:1358916-1361980 REVERSE | Aliases: F13M7.18, F13M7_18	5.6	5.2	0.4	1.3	100.0%	-4.1
5242	AT2G04340.1 | expressed protein | chr2:1513555-1515400 FORWARD | Aliases: T23O15.19	5.9	5.5	0.5	1.3	100.0%	-4.1
5243	AT4G20440.3 | Symbol: SMB | similar to small nuclear ribonucleoprotein associated protein B, putative / snRNP-B, putative / Sm protein B, putative [Arabidopsis thaliana] (TAIR:At5g44500.1); similar to small nuclear ribonucleoprotein B' [Macropus eugenii] (GB:AAF40115.1); similar to small nuclear ribonucleoprotein-associated protein N - rat (GB:A35448); similar to small nuclear ribonucleoprotein polypeptide B/B' isoform B' [Bos taurus] (GB:AAX46330.1); contains InterPro domain Proline-rich region (InterPro:IPR000694); contains InterPro domain Small nuclear ribonucleoprotein (Sm protein) (InterPro:IPR001163)	4.7	5.5	-0.7	-1.3	100.0%	-3.9
5244	AT2G04430.1 | similar to MutT/nudix family protein [Arabidopsis thaliana] (TAIR:At2g04450.1); similar to OSJNBa0079A21.19 [Oryza sativa (japonica cultivar-group)] (GB:XP_473408.1); contains InterPro domain Anti-sense to fibroblast growth factor protein GFG (InterPro:IPR003293); contains InterPro domain NUDIX hydrolase (InterPro:IPR000086) | chr2:1538898-1541091 FORWARD | Aliases: T1O3.16, T1O3_16	4.5	4.1	0.5	1.3	100.0%	-3.9
5245	AT3G07350.1 | expressed protein, contains Pfam profile PF04720: Protein of unknown function (DUF506) | chr3:2347474-2348797 FORWARD | Aliases: F21O3.6	5.2	5.6	-0.4	-1.3	100.0%	-4.1

Rank	Description	15.dpi	5.dpi	M	t	adj.q	B
5246	AT5G01610.1 | expressed protein, contains Pfam profile PF04398: Protein of unknown function, DUF538 | chr5:231042-232198 FORWARD | Aliases: F7A7.130, F7A7_130	4.4	3.8	0.5	1.3	100.0%	-3.8
5247	AT3G03860.1 | Symbol: ATAPRL5 | Encodes a protein disulfide isomerase-like (PDIL) protein, a member of a multigene family within the thioredoxin (TRX) superfamily. This protein also belongs to the adenosine 5'-phosphosulfate reductase-like (APRL) group. | chr3:992135-994587 FORWARD | Aliases: APR LIKE 5, ATAPRL5, F20H23.9, F20H23_9	6.1	5.6	0.5	1.3	100.0%	-4.0
5248	AT1G07590.1 | pentatricopeptide (PPR) repeat-containing protein, low similarity to DNA-binding protein (Triticum aestivum) GI:6958202; contains Pfam profile PF01535: PPR repeat | chr1:2336520-2339388 REVERSE | Aliases: F22G5.3, F22G5_3	8.5	8.1	0.3	1.3	100.0%	-4.2
5249	AT1G79710.1 | integral membrane transporter family protein, similar to high affinity folic acid/methotrexate transporter 5 (GI:21898554) (Leishmania tarentolae); Interpro IPR001991/ PR00173 Sodium:dicarboxylater symporter family | chr1:30000030-30001881 FORWARD | Aliases: F19K16.31, F19K16_31	5.5	5.0	0.5	1.3	100.0%	-4.1
5250	AT2G15530.2 | zinc finger (C3HC4-type RING finger) family protein, similar to Pfam domain, PF00097: Zinc finger, C3HC4 type (RING finger) | chr2:6779996-6784353 FORWARD | Aliases: F9O13.8	6.1	6.3	-0.2	-1.3	100.0%	-4.3
5251	AT1G77580.2 | myosin heavy chain-related, low similarity to SP:P08799 Myosin II heavy chain, non muscle {Dictyostelium discoideum} | chr1:29148921-29152478 REVERSE | Aliases: T5M16.17, T5M16_17	3.1	3.3	-0.3	-1.3	100.0%	-4.5
5252	AT1G56660.1 | expressed protein | chr1:21241553-21244223 FORWARD | Aliases: F25P12.91, F25P12_91	6.9	6.0	1.0	1.3	100.0%	-4.0
5253	AT5G52660.2 | myb family transcription factor, contains PFAM profile: PF00249 myb-like DNA-binding domain | chr5:21376285-21379384 REVERSE | Aliases: F6N7.15, F6N7_15	3.9	3.4	0.4	1.3	100.0%	-4.0
5254	AT2G17260.1 | Symbol: GLR2 | glutamate receptor family protein (GLR3.1) (GLR2), identical to putative glutamate receptor GLR2 (Arabidopsis thaliana) gi:4185740:gb:AAD09174; plant glutamate receptor family, PMID:11379626 | chr2:7511838-7515476 FORWARD | Aliases: ATGLR2, ATGLR3.1, F5J6.2, F5J6_2, GLR3.1, GLUTAMATE RECEPTOR 2	4.9	4.6	0.3	1.3	100.0%	-4.1
5255	AT4G36870.2 | Symbol: BLH2 | similar to BEL1-like homeobox 4 protein (BLH4) [Arabidopsis thaliana] (TAIR:At2g23760.1); similar to BEL1-like homeobox 4 protein (BLH4) [Arabidopsis thaliana] (TAIR:At2g23760.2); similar to BEL1-related homeotic protein 13 [Solanum tuberosum] (GB:AAN03623.1); contains InterPro domain POX (InterPro:IPR006563); contains InterPro domain Homeobox (InterPro:IPR001356) | chr4:17368964-17373927 FORWARD | Aliases: AP22.90, AP22_90, BEL1 LIKE HOMEODOMAIN 2	2.6	2.3	0.3	1.3	100.0%	-4.4
5256	AT3G18180.1 | expressed protein, contains Pfam domain, PF04577: Protein of unknown function (DUF563) | chr3:6230276-6231884 FORWARD | Aliases: MRC8.18	2.7	2.4	0.2	1.3	100.0%	-4.4
5257	AT1G55970.1 | histone acetyltransferase 4 (HAC4), similar to CREB-binding protein GB:AAC51770 GI:2443859 from (Homo sapiens); contains Pfam PF02135: TAZ zinc finger profile; contains Pfam PF00569: Zinc finger, ZZ type domain; identical to histone acetyltransferase HAC4 (GI:14794966) {Arabidopsis thaliana} | chr1:20935794-20941955 REVERSE | Aliases: F14J16.27, F14J16_27	2.7	2.5	0.2	1.3	100.0%	-4.7
5258	AT1G69350.1 | pentatricopeptide (PPR) repeat-containing protein, contains Pfam profile: PF01535: PPR repeat | chr1:26073545-26075908 FORWARD | Aliases: F10D13.4, F10D13_4	2.7	3.0	-0.2	-1.3	100.0%	-4.6
5259	AT1G50740.1 | expressed protein, contains Pfam profile PF03647: Uncharacterised protein family (UPF0136) | chr1:18811340-18812667 FORWARD | Aliases: F4M15.3, F4M15_3	8.0	7.7	0.3	1.3	100.0%	-4.4
5260	AT4G23460.1 | beta-adaptin, putative, strong similarity to SP:Q10567 Adapter-related protein complex 1 beta 1 subunit (Beta-adaptin 1) (Homo sapiens), beta-adaptin (Drosophila melanogaster) GI:434902; contains Pfam profile: PF01602 Adaptin N terminal region | chr4:12243681-12248960 REVERSE | Aliases: F16G20.160, F16G20_160	6.0	6.6	-0.6	-1.3	100.0%	-3.9
5261	AT3G48960.1 | 60S ribosomal protein L13 (RPL13C), 60S ribosomal protein L13 (BBC1), Arabidopsis thaliana, gb:X75162 | chr3:18161429-18162397 REVERSE | Aliases: T2J13.200	2.7	2.9	-0.2	-1.3	100.0%	-4.6
5262	AT1G59980.1 | DNAJ heat shock N-terminal domain-containing protein, similar to Altered Response to Gravity (Arabidopsis thaliana) GI:4249662; contains Pfam profile PF00226 DnaJ domain | chr1:22084454-22087316 FORWARD | Aliases: T2K10.3, T2K10_3	3.4	3.7	-0.3	-1.3	100.0%	-4.2
5263	AT1G80420.2 | DNA repair protein, putative (XRCC1), identical to putative DNA repair protein XRCC1 (Arabidopsis thaliana) GI:11181954; contains Pfam domain, PF00533: BRCA1 C Terminus (BRCT) domain | chr1:30240115-30242576 REVERSE | Aliases: T21F11.25, T21F11_25	3.9	4.9	-1.0	-1.3	100.0%	-4.0
5264	AT2G40530.1 | expressed protein | chr2:16934580-16935274 FORWARD | Aliases: T2P4.12, T2P4_12	2.7	2.9	-0.2	-1.3	100.0%	-4.8

Rank	Description	15.dpi	5.dpi	M	t	adj.q	B
5265	AT4G18230.1 | expressed protein, predicted gene M02B7.4, Caenorhabditis elegans, PID:G1572781 | chr4:10080368-10081950 REVERSE | Aliases: T9A21.80, T9A21_80	4.8	4.3	0.4	1.3	100.0%	-3.9
5266	AT1G24575.1 | expressed protein | chr1:8711023-8711515 REVERSE | Aliases: None	2.5	2.3	0.1	1.3	100.0%	-5.2
5267	AT4G22753.1 | Symbol: SMO1 3 |	2.3	2.4	-0.2	-1.3	100.0%	-4.6
5268	AT5G20900.1 | expressed protein | chr5:7090751-7092541 FORWARD | Aliases: F22D1.70, F22D1_70	9.7	8.9	0.8	1.3	100.0%	-4.2
5269	AT1G09540.1 | Symbol: MYB61 | myb family transcription factor (MYB61), contains PFAM profile: myb DNA-binding domain PF00249 | chr1:3086163-3087914 FORWARD | Aliases: ATMYB61, F14J9.20, F14J9_20	3.6	4.1	-0.5	-1.3	100.0%	-4.0
5270	AT5G57220.1 | Symbol: CYP81F2 | cytochrome P450, putative, similar to	4.3	4.7	-0.3	-1.3	100.0%	-4.4
5271	AT5G51600.1 | Symbol: PLE | microtubule associated protein (MAP65/ASE1) family protein, low similarity to SP:P50275 Anaphase spindle elongation protein {Saccharomyces cerevisiae}, protein regulating cytokinesis 1 (PRC1) (Homo sapiens) GI:2865521; contains Pfam profile PF03999: Microtubule associated protein (MAP65/ASE1 family) | chr5:20978137-20981306 REVERSE | Aliases: ATMAP65 3, K17N15.15, K17N15_15, PLEIADE	4.3	4.0	0.4	1.3	100.0%	-4.0
5272	AT5G26600.2 | expressed protein, weak similarity to SP:P18549 Isopenicillin N epimerase (EC 5.-.-) {Streptomyces clavuligerus} | chr5:9377405-9379800 FORWARD | Aliases: None	7.4	7.1	0.3	1.3	100.0%	-4.3
5273	AT2G43760.1 | molybdopterin biosynthesis MoaE family protein, contains Pfam profile: PF02391 molybdopterin converting factor, subunit 2 | chr2:18140026-18141047 FORWARD | Aliases: F18O19.13	8.0	7.5	0.5	1.3	100.0%	-4.0
5274	AT1G07880.2 | Symbol: ATMPK13 | similar to mitogen-activated protein kinase, putative / MAPK, putative (MPK6) [Arabidopsis thaliana] (TAIR:At2g43790.1); similar to NRK1 MAPK [Nicotiana tabacum] (GB:BAB32406.1); similar to p43Nft6 serine/threonine protein kinase [Nicotiana tabacum] (GB:CAA58760.1); contains InterPro domain Serine/threonine protein kinase, active site (InterPro:IPR008271); contains InterPro domain MAP kinase (InterPro:IPR003527); contains InterPro domain Tyrosine protein kinase (InterPro:IPR001245); contains InterPro domain Serine/threonine protein kinase (InterPro:IPR002290); contains InterPro domain Protein kinase (InterPro:IPR000719) | chr1:2434056-2435742 REVERSE | Aliases: F24B9.3, F24B9_3	3.7	4.0	-0.3	-1.3	100.0%	-4.3
5275	AT2G32940.1 | PAZ domain-containing protein / piwi domain-containing protein, similar to SP:O04379 Argonaute protein (AGO1) {Arabidopsis thaliana}, SP:Q9XGW1 PINHEAD protein (ZWILLE protein) {Arabidopsis thaliana}; contains Pfam profiles PF02171: Piwi domain, PF02170: PAZ domain | chr2:13979295-13983933 REVERSE | Aliases: T21L14.12, T21L14_12	2.2	2.3	-0.1	-1.3	100.0%	-5.0
5276	AT3G52580.1 | 40S ribosomal protein S14 (RPS14C), ribosomal protein S14 -Zea mays,PIR2:A30097 | chr3:19514203-19515902 FORWARD | Aliases: F3C22.6	10.3	10.9	-0.6	-1.3	100.0%	-4.0
5277	AT4G22250.1 | zinc finger (C3HC4-type RING finger) family protein, contains Pfam profile: PF00097 zinc finger, C3HC4 type (RING finger) | chr4:11767525-11768464 REVERSE | Aliases: T10I14.80, T10I14_80	3.3	3.6	-0.3	-1.3	100.0%	-4.2
5278	AT1G23050.1 | hydroxyproline-rich glycoprotein family protein, contains proline-rich extensin domains, INTERPRO:IPR002965 | chr1:8168432-8169256 FORWARD | Aliases: F19G10.1	3.7	3.4	0.3	1.3	100.0%	-4.4
5279	AT3G07050.1 | GTP-binding family protein, contains Pfam domain, PF01926: GTPase of unknown function | chr3:2229608-2232352 REVERSE | Aliases: F17A9.21	6.9	8.1	-1.2	-1.3	100.0%	-3.9
5280	AT2G23250.1 | UDP-glucoronosyl/UDP-glucosyl transferase family protein, contains similarity to glucosyltransferases | chr2:9904889-9906205 REVERSE | Aliases: T20D16.12, T20D16_12	2.3	2.2	0.1	1.3	100.0%	-5.0
5281	AT3G06510.1 | Symbol: SFR2 | glycosyl hydrolase family 1 protein, similar to Beta-galactosidase (SP:P22498) (Sulfolobus solfataricus}; almost identical to beta-glucosidase GB:AAF23823 GI:6685165 from (Arabidopsis thaliana) | chr3:2016443-2019680 FORWARD | Aliases: F5E6.16, F5E6_16, SENSITIVE TO FREEZING 2	5.7	6.4	-0.6	-1.3	100.0%	-4.3
5282	AT1G72730.1 | eukaryotic translation initiation factor 4A, putative / eIF-4A, putative, similar to Eukaryotic initiation factor 4A-10 GB:P41382 (Nicotiana tabacum); identical to (putative) RNA helicase GB:CAA09211 (Arabidopsis thaliana) (Nucleic Acids Res. 27 (2), 628-636 (1999)) | chr1:27381460-27383844 REVERSE | Aliases: F28P22.8, F28P22_8	4.7	5.1	-0.3	-1.3	100.0%	-4.4
5283	AT1G64750.2 | DSS1/SEM1 family protein, contains Pfam profile PF05160: DSS1/SEM1 family | chr1:24056244-24057568 REVERSE | Aliases: F13O11.6, F13O11_6	8.5	9.0	-0.5	-1.3	100.0%	-3.9

Rank	Description	15.dpi	5.dpi	M	t	adj.q	B
5284	AT3G18130.1 | guanine nucleotide-binding family protein / activated protein kinase C receptor (RACK1), identical to guanine nucleotide-binding protein; activated protein kinase C receptor; RACK1 (GI:9294068) {Arabidopsis thaliana}; contains Pfam profile: PF00400 WD domain, G-beta repeat (7 copies) | chr3:6210912-6212439 REVERSE | Aliases: MRC8.11	9.6	10.3	-0.7	-1.3	100.0%	-3.8
5285	AT5G01400.1 | expressed protein, contains low similarity to symplekin SP:Q92797 from (Homo sapiens) | chr5:162549-171071 REVERSE | Aliases: T10O8.110, T10O8_110	4.9	5.5	-0.6	-1.3	100.0%	-4.0
5286	AT1G10570.2 | Ulp1 protease family protein, contains Pfam profile PF02902: Ulp1 protease family, C-terminal catalytic domain; similar to EST gb:N96456	5.3	5.7	-0.4	-1.3	100.0%	-4.1
5287	AT1G44835.2 | similar to 25.7 kDa protein [Cicer arietinum] (GB:CAB81546.1); contains InterPro domain YbaK/prolyl-tRNA synthetase associated region (InterPro:IPR007214) | chr1:16942246-16944949 FORWARD | Aliases: T12C22.11, T12C22_11	5.7	6.0	-0.4	-1.3	100.0%	-4.1
5288	AT1G43980.1 | pentatricopeptide (PPR) repeat-containing protein, contains INTERPRO:IPR002885 PPR repeats | chr1:16688272-16691942 REVERSE | Aliases: F9C16.15, F9C16_15	2.7	2.9	-0.2	-1.3	100.0%	-4.8
5289	AT1G18870.1 | isochorismate synthase, putative / isochorismate mutase, putative, similar to GI:17223087 | chr1:6515657-6519273 FORWARD | Aliases: F6A14.3, F6A14_3	2.8	3.0	-0.2	-1.3	100.0%	-4.7
5290	AT5G21105.1 | L-ascorbate oxidase, putative, similar to L-ascorbate oxidase from {Nicotiana tabacum} SP:Q40588, {Cucurbita pepo var. melopepo} SP:P37064; contains Pfam profile PF00394: Multicopper oxidase; supported by cDNA gi_15215753_gb_AY050406.1_; A false intron was added between exons 4 and 5 to circumvent the single nucleotide insertion in this BAC which, otherwise, causes a frameshift. | chr5:7172777-7177749 FORWARD | Aliases: None	8.3	7.1	1.1	1.3	100.0%	-3.6
5291	AT3G27740.1 | Symbol: CARA | carbamoyl-phosphate synthase (glutamine-hydrolyzing) (CARA) / glutamine-dependent carbamoyl-phosphate synthase small subunit, identical to carbamoyl phosphate synthetase small subunit GI:2462781 (Arabidopsis thaliana) | chr3:10282498-10285103 REVERSE | Aliases: CARBAMOYL PHOSPHATE SYNTHETASE A, CARBAMOYL PHOSPHATE SYNTHETASE SMALL SUBUNIT, MGF10.14	7.9	8.6	-0.8	-1.3	100.0%	-3.9
5292	AT3G02350.1 | glycosyl transferase family 8 protein, contains Pfam profile: PF01501 glycosyl transferase family 8 | chr3:479013-481481 FORWARD | Aliases: F11A12.4, F11A12_4	5.6	6.3	-0.6	-1.3	100.0%	-3.9
5293	AT1G21480.2 | exostosin family protein, contains Pfam profile: PF03016 Exostosin family | chr1:7518980-7521461 REVERSE | Aliases: F24J8.10, F24J8_10	5.7	6.0	-0.3	-1.3	100.0%	-4.3
5294	AT5G60710.1 | zinc finger (C3HC4-type RING finger) family protein, low similarity to SP:P97280 Inter-alpha-trypsin inhibitor heavy chain H3 precursor {Mesocricetus auratus}; contains Pfam profiles PF00097: Zinc finger, C3HC4 type (RING finger), PF00092: von Willebrand factor type A domain | chr5:24427944-24432294 REVERSE | Aliases: MUP24.2, MUP24_2	3.9	3.5	0.4	1.3	100.0%	-4.1
5295	AT4G04350.1 | Symbol: EMB2369 | leucyl-tRNA synthetase, putative / leucine--tRNA ligase, putative, similar to SP:P36430 Leucyl-tRNA synthetase (EC 6.1.1.4) (Leucine--tRNA ligase) (LeuRS) {Bacillus subtilis}; contains Pfam profile PF00133: tRNA synthetases class I (I, L, M and V) | chr4:2128088-2133126 FORWARD | Aliases: EMB2369, EMBRYO DEFECTIVE 2369, T19B17.7, T19B17_7	6.0	6.5	-0.4	-1.3	100.0%	-4.2
5296	AT5G57080.1 | expressed protein | chr5:23116185-23116594 FORWARD | Aliases: MUL3.2, MUL3_2	3.6	3.9	-0.4	-1.3	100.0%	-4.1
5297	AT1G09400.1 | 12-oxophytodienoate reductase, putative, similar to OPR1 (GI:3882355) and OPR2 (GI:3882356) | chr1:3033603-3035038 REVERSE | Aliases: F14J9.6, F14J9_6	2.7	2.5	0.2	1.3	100.0%	-4.5
5298	AT5G54095.1 | expressed protein | chr5:21969959-21970723 REVERSE | Aliases: None	2.7	2.5	0.2	1.3	100.0%	-4.6
5299	AT3G25230.1 | Symbol: ROF1 | peptidyl-prolyl cis-trans isomerase / FK506-binding protein (ROF1), identical to rotamase FKBP (ROF1) GB:U49453 (Arabidopsis thaliana) (Mol. Gen. Genet. 252 (5), 510-517 (1996)) | chr3:9189444-9192642 FORWARD | Aliases: MJL12.21, ROTAMASE FKBP 1	9.7	10.5	-0.8	-1.3	100.0%	-4.2
5300	AT3G12250.4 | Symbol: TGA6 | similar to bZIP transcription factor HBP-1b homolog [Arabidopsis thaliana] (TAIR:At5g06950.1); similar to bZIP transcription factor HBP-1b homolog [Arabidopsis thaliana] (TAIR:At5g06950.2); similar to TGA-type basic leucine zipper protein TGA2.1 [Phaseolus vulgaris] (GB:AAK84889.1); contains InterPro domain Basic-leucine zipper (bZIP) transcription factor (InterPro:IPR004827) | chr3:3906358-3908590 FORWARD | Aliases: BZIP45, F28J15.6	3.9	3.4	0.5	1.3	100.0%	-3.9
5301	AT1G64190.1 | 6-phosphogluconate dehydrogenase family protein, contains Pfam profiles: PF00393 6-phosphogluconate dehydrogenase C-terminal domain, PF03446 NAD binding domain of 6-phosphogluconate | chr1:23828991-23830799 REVERSE | Aliases: F22C12.5, F22C12_5	11.2	11.4	-0.3	-1.3	100.0%	-4.7
5302	AT1G60730.2 | aldo/keto reductase family protein, contains Pfam profile PF00248: oxidoreductase, aldo/keto reductase family | chr1:22361700-22363814 REVERSE | Aliases: F8A5.24, F8A5_24	4.4	4.9	-0.4	-1.3	100.0%	-4.0

Rank	Description	15.dpi	5.dpi	M	t	adj.q	B
5303	AT3G27050.1 | expressed protein | chr3:9979396-9980901 REVERSE | Aliases: MOJ10.12	8.6	8.2	0.5	1.3	100.0%	-4.2
5304	AT3G08660.1 | phototropic-responsive protein, putative, contains similarity to root phototropism RPT2 (Arabidopsis thaliana) gi:6959488:gb:AAF33112, a signal transducer of phototropic response PMID:10662859	3.2	3.0	0.1	1.3	100.0%	-5.0
5305	AT1G16820.1 | vacuolar ATP synthase catalytic subunit-related / V-ATPase-related / vacuolar proton pump-related, similar to Vacuolar ATP synthase catalytic subunit A (V-ATPase A subunit) (Vacuolar proton pump alpha subunit) (V-ATPase 69 kDa subunit) (SP:O23654) (Arabidopsis thaliana) | chr1:5756401-5758457 REVERSE | Aliases: F17F16.15	3.6	3.4	0.3	1.3	100.0%	-4.2
5306	AT1G20700.1 | homeobox-leucine zipper family protein, strong similarity to homeodomain protein PALE-2 (GI:20152544) (Arabidopsis thaliana); contains Pfam profile: PF00046 homeobox domain | chr1:7182548-7183999 FORWARD | Aliases: F2D10.19, F2D10_19	5.1	4.7	0.4	1.3	100.0%	-3.9
5307	AT1G23380.2 | Symbol: KNAT6 | homeobox transcription factor (KNAT6), nearly identical to homeodomain transcription factor KNAT6 (KNAT6L) GI:15991302 (Arabidopsis thaliana), homeodomain transcription factor KNAT6 (KNAT6S) (Arabidopsis thaliana) GI:15991300 | chr1:8297280-8302472 REVERSE | Aliases: F26F24.32, F26F24_32, KNAT6L, KNAT6S	5.3	4.8	0.6	1.3	100.0%	-4.2
5308	AT4G22720.2 | glycoprotease M22 family protein, similar to sialylglycoprotease (Haemophilus ducreyi) GI:6942294; contains Pfam profile PF00814: Glycoprotease family | chr4:11937075-11938977 FORWARD | Aliases: T12H17.110, T12H17_110	7.3	7.6	-0.3	-1.3	100.0%	-4.2
5309	AT3G44620.1 | similar to protein tyrosine phosphatase-like protein [Oryza sativa (japonica cultivar-group)] (GB:AAO72543.1); contains InterPro domain Low molecular weight phosphotyrosine protein phosphatase (InterPro:IPR000106) | chr3:16204816-16206820 FORWARD | Aliases: T18B22.20	4.0	4.4	-0.4	-1.3	100.0%	-4.1
5310	AT4G26130.1 | expressed protein | chr4:13240015-13241292 REVERSE | Aliases: F20B18.240, F20B18_240	2.9	2.7	0.2	1.3	100.0%	-5.0
5311	AT4G35560.1 | expressed protein | chr4:16881567-16887024 FORWARD | Aliases: F8D20.70, F8D20_70	5.1	5.7	-0.6	-1.3	100.0%	-3.8
5312	AT3G54300.1 | Symbol: ATVAMP727 | synaptobrevin family protein, similar to vesicle-associated membrane protein 7B (At VAMP7B), Arabidopsis thaliana, EMBL:AF025333 | chr3:20119187-20121624 REVERSE | Aliases: F24B22.260, VAMP727	9.3	8.3	1.0	1.3	100.0%	-3.7
5313	AT5G49010.1 | DNA replication protein-related, similar to Sld5 (Xenopus laevis) GI:29365477; contains Pfam profile PF05916: Eukaryotic protein of unknown function (DUF873) | chr5:19883690-19885367 FORWARD | Aliases: K19E20.14, K19E20_14	7.0	7.7	-0.7	-1.3	100.0%	-3.7
5314	AT3G09630.2 | similar to 60S ribosomal protein L4/L1 (RPL4D) [Arabidopsis thaliana] (TAIR:At5g02870.1); similar to PREDICTED OJ1014_E09.28 gene product [Oryza sativa (japonica cultivar-group)] (GB:XP_507356.1); contains InterPro domain Ribosomal protein L4/L1e (InterPro:IPR002136) | chr3:2953742-2955753 FORWARD | Aliases: F11F8.22	12.1	12.5	-0.4	-1.3	100.0%	-4.7
5315	AT3G14600.1 | 60S ribosomal protein L18A (RPL18aC), similar to GB:CAA08791 from (Podocoryne carnea) | chr3:4910704-4912180 FORWARD | Aliases: MIE1.10	10.0	10.6	-0.6	-1.3	100.0%	-3.9
5316	AT1G55690.3 | SEC14 cytosolic factor family protein / phosphoglyceride transfer family protein, similar to similar to phosphatidylinositol transfer-like protein IV (GI:14486707) (Lotus japonicus) and Phosphatidylinositol Transfer Protein GB:2780955 GI:2780955 (Saccharomyces cerevisiae)	4.1	4.4	-0.3	-1.3	100.0%	-4.1
5317	AT5G66440.1 | expressed protein | chr5:26547461-26548323 REVERSE | Aliases: K1F13.9, K1F13_9	9.4	8.7	0.7	1.3	100.0%	-3.9
5318	AT1G53340.1 | DC1 domain-containing protein, contains Pfam protein PF03107 DC1 domain | chr1:19902596-19904599 REVERSE | Aliases: F12M16.24, F12M16_24	4.8	4.0	0.9	1.3	100.0%	-3.8
5319	AT5G44020.1 | acid phosphatase class B family protein, similar to SP:P15490 STEM 28 kDa glycoprotein precursor (Vegetative storage protein A) {Glycine max}, acid phosphatase (Glycine max) GI:3341443; contains Pfam profile PF03767: HAD superfamily (subfamily IIIB) phosphatase | chr5:17729638-17731563 FORWARD | Aliases: MRH10.13, MRH10_13	3.5	3.2	0.3	1.3	100.0%	-4.1
5320	AT1G72160.1 | SEC14 cytosolic factor family protein / phosphoglyceride transfer family protein, similar to GI:807956 from (Saccharomyces cerevisiae)similar to polyphosphoinositide binding protein Ssh2p (GI:2739046) {Glycine max}; contains Pfam PF00650 : CRAL/TRIO domain; contains Pfam PF03765 : CRAL/TRIO, N-terminus | chr1:27157243-27159326 REVERSE | Aliases: T9N14.8, T9N14_8	6.1	6.8	-0.7	-1.3	100.0%	-3.8
5321	AT3G01770.1 | DNA-binding bromodomain-containing protein, contains bromodomain, INTERPRO:IPR001487 | chr3:275418-278792 REVERSE | Aliases: F28J7.10	10.0	10.4	-0.4	-1.3	100.0%	-4.3
5322	AT5G08640.1 | Symbol: FLS | flavonol synthase 1 (FLS1), identical to SP:Q96330; contains PF03171 2OG-Fe(II) oxygenase superfamily | chr5:2803959-2805448 FORWARD | Aliases: FLAVONOL SYNTHASE, T2K12.5	3.6	4.2	-0.6	-1.3	100.0%	-3.8

Rank	Description	15.dpi	5.dpi	M	t	adj.q	B
5323	AT4G14190.1 | similar to pentatricopeptide (PPR) repeat-containing protein [Arabidopsis thaliana] (TAIR:At3g42630.1); similar to pentatricopeptide (PPR) repeat-containing protein-like [Oryza sativa (japonica cultivar-group)] (GB:XP_483365.1); contains InterPro domain PPR repeat (InterPro:IPR002885) | chr4:8183539-8185182 REVERSE | Aliases: DL3135C, FCAALL.115	5.0	4.7	0.3	1.3	100.0%	-4.2
5324	AT2G46450.1 | Symbol: ATCNGC12 | cyclic nucleotide-regulated ion channel, putative (CNGC12), similar to cyclic nucleotide and calmodulin-regulated ion channel (cngc3) GI:4581201 from (Arabidopsis thaliana)	6.9	6.3	0.6	1.3	100.0%	-3.8
5325	AT1G51950.1 | Symbol: IAA18 | auxin-responsive protein / indoleacetic acid-induced protein 18 (IAA18), identical to SP:O24408:AXII_ARATH Auxin-responsive protein IAA18 (Indoleacetic acid-induced protein 18) {Arabidopsis thaliana}	6.2	5.8	0.4	1.3	100.0%	-4.0
5326	AT4G21850.2 | methionine sulfoxide reductase domain-containing protein / SelR domain-containing protein, low similarity to pilin-like transcription factor (Homo sapiens) GI:5059062, SP:P14930 Peptide methionine sulfoxide reductase msrA/msrB (EC 1.8.4.6) {Neisseria gonorrhoeae}; contains Pfam profile PF01641: SelR domain | chr4:11591128-11592250 REVERSE | Aliases: T8O5.60, T8O5_60	5.0	4.3	0.7	1.3	100.0%	-3.7
5327	AT1G12910.1 | Symbol: AN11 | flower pigmentation protein (AN11), contains 3 WD-40 repeats (PF00400); identical to GB:AAC18912 from (Arabidopsis thaliana) (Genes Dev. 11 (11), 1422-1434 (1997)) | chr1:4394899-4396289 REVERSE | Aliases: ATAN11, F13K23.16, F13K23_16	9.5	8.8	0.7	1.3	100.0%	-4.3
5328	AT3G09250.1 | expressed protein | chr3:2839007-2840871 FORWARD | Aliases: F3L24.12	6.0	5.7	0.3	1.3	100.0%	-4.1
5329	AT5G27650.1 | PWWP domain-containing protein, hypothetical protein F22F7.12 - Arabidopsis thaliana, EMBL:AC009606 | chr5:9785410-9789235 FORWARD | Aliases: T1G16.4	4.4	4.7	-0.3	-1.3	100.0%	-4.4
5330	AT5G67230.1 | glycosyl transferase family 43 protein, low similarity to Galactosylgalactosylxylosylprotein 3-beta-glucuronosyltransferase 1, Homo sapiens (SP:Q9P2W7), Rattus norvegicus (SP:O35789); contains Pfam domain Glycosyltransferase family 43 (PF03360) | chr5:26839704-26841905 FORWARD | Aliases: K21H1.19, K21H1_19	3.9	3.7	0.3	1.3	100.0%	-4.3
5331	AT1G24140.1 | matrixin family protein, similar to matrix metalloproteinase (Cucumis sativus) GI:7159629; contains InterPro accession IPR001818: Matrixin | chr1:8536034-8537376 REVERSE | Aliases: F3I6.6, F3I6_6	3.0	3.3	-0.2	-1.3	100.0%	-4.5
5332	AT3G14620.1 | Symbol: CYP72A8 | cytochrome P450, putative, similar to GB:Q05047 from (Catharanthus roseus) | chr3:4914921-4917083 FORWARD | Aliases: MIE1.12	5.0	4.5	0.5	1.3	100.0%	-3.8
5333	AT2G38100.1 | proton-dependent oligopeptide transport (POT) family protein, low similarity to SP:P46032 Peptide transporter PTR2-B (Histidine transporting protein) {Arabidopsis thaliana}; contains Pfam profile PF00854: POT family | chr2:15955562-15957306 REVERSE | Aliases: F16M14.3, F16M14_3	3.6	3.4	0.2	1.3	100.0%	-4.7
5334	AT3G25290.2 | similar to auxin-responsive protein, putative [Arabidopsis thaliana] (TAIR:At4g12980.1); similar to putative auxin-induced protein [Oryza sativa (japonica cultivar-group)] (GB:XP_483411.1); contains InterPro domain Cytochrome b561 / ferric reductase transmembrane (InterPro:IPR006593); contains InterPro domain Protein of unknown function DUF568 (InterPro:IPR007613); contains InterPro domain DOMON domain (InterPro:IPR005018) | chr3:9210178-9211824 FORWARD | Aliases: MJL12.25	6.4	7.0	-0.6	-1.3	100.0%	-3.8
5335	AT1G08110.4 | similar to lactoylglutathione lyase, putative / glyoxalase I, putative [Arabidopsis thaliana] (TAIR:At1g67280.1); similar to Glyoxalase I [Cicer arietinum] (GB:CAA12028.1); contains InterPro domain Glyoxalase I (InterPro:IPR004361); contains InterPro domain Glyoxalase/Bleomycin resistance protein/dioxygenase domain (InterPro:IPR004360) | chr1:2535368-2537928 FORWARD | Aliases: T6D22.20, T6D22_20	10.5	9.5	0.9	1.3	100.0%	-3.8
5336	AT5G14640.1 | protein kinase family protein, similar to glycogen synthase kinase-3 homolog MsK-3 SP:P51139 from (Medicago sativa); contains Pfam profile PF00069: Protein kinase domain | chr5:4719087-4722282 REVERSE | Aliases: T15N1.130, T15N1_130	6.1	5.7	0.4	1.3	100.0%	-4.3
5337	AT1G02640.1 | Symbol: BXL2 | glycosyl hydrolase family 3 protein, similar to beta-xylosidase GB:Z84377 GI:2102655 from (Aspergillus niger) | chr1:564205-567769 FORWARD | Aliases: ATBXL2, BETA XYLOSIDASE 2, T14P4.8, T14P4_8	4.9	5.7	-0.8	-1.3	100.0%	-4.0
5338	AT2G16390.1 | Symbol: DRD1 | SNF2 domain-containing protein / helicase domain-containing protein, low similarity to RAD54 (Drosophila melanogaster) GI:1765914; contains Pfam profiles PF00271: Helicase conserved C-terminal domain, PF00176: SNF2 family N-terminal domain | chr2:7104720-7108178 FORWARD | Aliases: CHR35, F16F14.11, F16F14_11	3.3	3.5	-0.2	-1.3	100.0%	-4.4
5339	AT1G71070.1 | glycosyltransferase family 14 protein / core-2/I-branching enzyme family protein, similar to glucosaminyl (N-acetyl) transferase GB:4758422 from (Homo sapiens) | chr1:26810850-26813166 REVERSE | Aliases: F23N20.6, F23N20_6	4.9	5.5	-0.6	-1.3	100.0%	-4.1
5340	AT5G35970.1 | DNA-binding protein, putative, similar to SWISS-PROT:Q60560 DNA-binding protein SMUBP-2 (Immunoglobulin MU binding protein 2, SMUBP-2) (Mesocricetus auratus) | chr5:14136124-14140570 REVERSE | Aliases: MEE13.8, MEE13_8	6.4	6.1	0.3	1.3	100.0%	-4.2

Rank	Description	15.dpi	5.dpi	M	t	adj.q	B
5341	AT2G18630.1 | expressed protein, unusual splice site at second intron; GA instead of conserved GT at donor site; similar to At14a GI:11994571 and GI:11994573 (Arabidopsis thaliana) | chr2:8087640-8089721 FORWARD | Aliases: F24H14.1	6.3	6.8	-0.6	-1.3	100.0%	-3.8
5342	AT5G57410.2 | similar to expressed protein [Arabidopsis thaliana] (TAIR:At2g18876.1); similar to unnamed protein product [Tetraodon nigroviridis] (GB:CAG08075.1) | chr5:23282130-23285030 REVERSE | Aliases: MSF19.7, MSF19_7	5.7	6.3	-0.6	-1.3	100.0%	-3.9
5343	AT1G68680.1 | expressed protein | chr1:25789169-25790587 FORWARD | Aliases: F24J5.21, F24J5_21	7.6	8.1	-0.5	-1.3	100.0%	-4.0
5344	AT5G64950.1 | mitochondrial transcription termination factor-related / mTERF-related, contains Pfam profile PF02536: mTERF | chr5:25971070-25972386 FORWARD | Aliases: MXK3.18, MXK3_18	6.1	6.4	-0.4	-1.3	100.0%	-4.1
5345	AT3G55090.1 | ABC transporter family protein, ATP-binding cassette-sub-family G-member 2, Mus musculus, EMBL:AF140218 | chr3:20427319-20429481 REVERSE | Aliases: T15C9.80	3.0	3.5	-0.4	-1.3	100.0%	-3.9
5346	AT1G03270.1 | expressed protein, contains Pfam profile PF01595: Domain of unknown function | chr1:799191-802436 FORWARD | Aliases: F15K9.13, F15K9_13	5.0	5.8	-0.8	-1.3	100.0%	-3.9
5347	AT3G27120.1 | spastin ATPase, putative, similar to SWISS-PROT:Q9QYY8 spastin (Fragment) (Mus musculus); contains Pfam domain, PF00004: ATPase, AAA family | chr3:10000931-10003372 REVERSE | Aliases: MOJ10.20	3.6	3.9	-0.2	-1.3	100.0%	-4.4
5348	AT1G14700.1 | purple acid phosphatase, putative, contains Pfam profile: PF00149 calcineurin-like phosphoesterase; similar to purple acid phosphatase (GI:20257479) (Arabidopsis thaliana) | chr1:5058675-5061052 FORWARD | Aliases: F10B6.10, F10B6_10	2.4	2.6	-0.2	-1.3	100.0%	-4.8
5349	AT1G06640.2 | 2-oxoglutarate-dependent dioxygenase, putative, similar to 2A6 (GI:599622) and tomato ethylene synthesis regulatory protein E8 (SP:P10967); contains Pfam profile: PF00671 Iron/Ascorbate oxidoreductase family | chr1:2032344-2034013 FORWARD | Aliases: F12K11.27, F12K11_27	10.9	10.5	0.4	1.3	100.0%	-4.6
5350	AT2G02660.1 | expressed protein | chr2:738503-739768 FORWARD | Aliases: T8K22.4, T8K22_4	2.6	2.7	-0.1	-1.3	100.0%	-5.3
5351	AT4G25960.1 | similar to multidrug resistance P-glycoprotein, putative [Arabidopsis thaliana] (TAIR:At1g28010.1); similar to multidrug resistance P-glycoprotein (PGP1) [Arabidopsis thaliana] (TAIR:At2g36910.1); similar to P-glycoprotein, putative [Arabidopsis thaliana] (TAIR:At1g10680.1); similar to multidrug resistance P-glycoprotein, putative [Arabidopsis thaliana] (TAIR:At3g28860.1); similar to multidrug resistance P-glycoprotein, putative [Arabidopsis thaliana] (TAIR:At1g27940.1); similar to OSJNBa0036B21.21 [Oryza sativa (japonica cultivar-group)] (GB:XP_472741.1); similar to MDR-like ABC transporter [Oryza sativa (japonica cultivar-group)] (GB:CAD59581.1); similar to P-glycoprotein [Solanum tuberosum] (GB:AAD10836.1); similar to MDR-like ABC transporter [Oryza sativa (japonica cultivar-group)] (GB:XP_467259.1); similar to OSJNBb0079B02.13 [Oryza sativa (japonica cultivar-group)] (GB:XP_474071.1); contains InterPro domain AAA ATPase (InterPro:IPR003593); contains InterPro domain ABC transporter (InterPro:IPR003439); contains InterPro domain ABC transporter, transmembrane region (InterPro:IPR001140); contains InterPro domain ATP/GTP-binding site motif A (P-loop) (InterPro:IPR001687) | chr4:13177418-13183640 FORWARD | Aliases: F20B18.70, F20B18_70	2.2	2.3	-0.1	-1.3	100.0%	-5.1
5352	AT2G30350.2 | endo/excinuclease amino terminal domain-containing protein, contains Pfam domain PF01541: Endo/excinuclease amino terminal domain | chr2:12941314-12942824 REVERSE | Aliases: T9D9.16, T9D9_16	6.2	6.9	-0.7	-1.3	100.0%	-3.7
5353	AT5G64360.4 | similar to DNAJ heat shock N-terminal domain-containing protein [Arabidopsis thaliana] (TAIR:At5g09540.1); similar to putative AT hook-containing MAR binding protein 1(AHM1) [Oryza sativa (japonica cultivar-group)] (GB:XP_482959.1); contains InterPro domain Heat shock protein DnaJ, N-terminal (InterPro:IPR001623) | chr5:25753566-25755828 REVERSE | Aliases: MSJ1.20, MSJ1_20	5.6	5.1	0.5	1.3	100.0%	-4.0
5354	AT3G24110.1 | calcium-binding EF hand family protein, contains Pfam profile: PF00036 EF hand, similar to calcium-modulated proteins | chr3:8704436-8705420 REVERSE | Aliases: MUJ8.1	4.1	4.5	-0.4	-1.3	100.0%	-4.0
5355	AT1G80900.1 | magnesium transporter CorA-like family protein (MGT1) (MRS2), low similarity to SP:Q01926 RNA splicing protein MRS2, mitochondrial precursor {Saccharomyces cerevisiae}; contains Pfam profile PF01544: CorA-like Mg2+ transporter protein | chr1:30403042-30405232 REVERSE | Aliases: F23A5.26, F23A5_26	4.4	4.2	0.2	1.3	100.0%	-4.5
5356	AT3G11500.1 | small nuclear ribonucleoprotein G, putative / snRNP-G, putative / Sm protein G, putative, similar to SWISS-PROT:Q15357 small nuclear ribonucleoprotein G (snRNP-G, Sm protein G, Sm-G, SmG) (Homo sapiens) | chr3:3621415-3622598 REVERSE | Aliases: F24K9.17	8.5	8.9	-0.3	-1.3	100.0%	-4.3
5357	AT3G21720.1 | isocitrate lyase, putative, similar to isocitrate lyase GI:167144 from (Brassica napus) | chr3:7652511-7655950 REVERSE | Aliases: MSD21.4	4.8	4.5	0.3	1.3	100.0%	-4.4
5358	AT2G32920.1 | Symbol: ATPDIL2 3 | Encodes a protein disulfide isomerase-like (PDIL) protein, a member of a multigene family within the thioredoxin (TRX) superfamily.	5.0	6.1	-1.1	-1.3	100.0%	-3.9

Rank	Description	15.dpi	5.dpi	M	t	adj.q	B
5359	AT2G33390.1 | expressed protein | chr2:14158655-14159820 FORWARD | Aliases: F4P9.16, F4P9_16	7.8	7.0	0.8	1.3	100.0%	-4.1
5360	AT3G23940.1 | dehydratase family, contains Pfam profile: PF00920 dehydratase family | chr3:8648712-8652564 FORWARD | Aliases: F14O13.18	10.9	11.3	-0.4	-1.3	100.0%	-4.0
5361	AT3G15520.1 | peptidyl-prolyl cis-trans isomerase TLP38, chloroplast / thylakoid lumen PPIase of 38 kDa / cyclophilin / rotamase, cyclophylin-type; identical to SP:P82869 Thylakoid lumenal 38 kDa protein, chloroplast precursor (P38) {Arabidopsis thaliana} | chr3:5248936-5252439 REVERSE | Aliases: MJK13.18	4.2	4.5	-0.3	-1.3	100.0%	-4.1
5362	AT5G02910.1 | F-box family protein, similar to ribosomal RNA apurinic site specific lyase (Triticum aestivum) GI:6505722; contains F-box domain Pfam:PF00646 | chr5:677085-679041 FORWARD | Aliases: F9G14.220, F9G14_220	5.0	4.6	0.3	1.3	100.0%	-4.0
5363	AT5G10070.2 | RNase L inhibitor protein-related, contains Pfam profiles PF04034: Domain of unknown function (DUF367), PF04068: Possible metal-binding domain in RNase L inhibitor, RLI	6.5	6.0	0.5	1.3	100.0%	-3.9
5364	AT1G76590.1 | zinc-binding family protein, similar to zinc-binding protein (Pisum sativum) GI:16117799; contains Pfam profile PF04640 : Protein of unknown function, DUF597 | chr1:28745585-28747101 FORWARD | Aliases: F14G6.19, F14G6_19	4.1	3.3	0.8	1.3	100.0%	-3.7
5365	AT3G56330.1 | N2,N2-dimethylguanosine tRNA methyltransferase family protein, low similarity to SP:Q9P804 N(2),N(2)-dimethylguanosine tRNA methyltransferase (EC 2.1.1.32) (tRNA(guanine-26,N(2)-N(2)) methyltransferase) {Schizosaccharomyces pombe}; contains Pfam profile PF02005: N2,N2-dimethylguanosine tRNA methyltransferase | chr3:20901229-20902923 FORWARD | Aliases: T5P19.2	3.8	4.1	-0.3	-1.3	100.0%	-4.3
5366	AT1G79940.2 | similar to DNAJ heat shock N-terminal domain-containing protein / sec63 domain-containing protein [Arabidopsis thaliana] (TAIR:At4g21180.1); similar to DNAJ-like Sec63 homologue [Plasmodium falciparum 3D7] (GB:NP_705096.1); contains InterPro domain Heat shock protein DnaJ (InterPro:IPR003095); contains InterPro domain Heat shock protein DnaJ, N-terminal (InterPro:IPR001623); contains InterPro domain Sec63 domain (InterPro:IPR004179) | chr1:30074497-30078232 FORWARD | Aliases: F19K16.10, F19K16_10	9.5	9.9	-0.4	-1.3	100.0%	-4.6
5367	AT4G11350.2 | similar to fringe-related protein [Arabidopsis thaliana] (TAIR:At4g23490.1); similar to fringe-related protein-like [Oryza sativa (japonica cultivar-group)] (GB:BAD29388.1) | chr4:6901677-6904220 FORWARD | Aliases: F8L21.140, F8L21_140	4.9	5.3	-0.4	-1.3	100.0%	-3.9
5368	AT5G63220.1 | expressed protein, contains Pfam PF04190: Protein of unknown function (DUF410) | chr5:25370285-25372707 REVERSE | Aliases: MDC12.19, MDC12_19	2.7	2.9	-0.2	-1.3	100.0%	-4.9
5369	AT2G44030.1 | kelch repeat-containing F-box family protein, low similarity to SKP1 interacting partner 6 (Arabidopsis thaliana) GI:10716957; contains Pfam profiles PF01344: Kelch motif, PF00646: F-box domain | chr2:18227162-18228304 FORWARD | Aliases: F6E13.16	4.0	3.7	0.3	1.3	100.0%	-4.3
5370	AT1G69690.1 | TCP family transcription factor, putative, similar to PCF1 (GI:2580438) and PCF2 ((GI:2580440) Oryza sativa) | chr1:26219887-26221514 FORWARD | Aliases: T6C23.11, T6C23_11	2.8	3.0	-0.2	-1.3	100.0%	-4.5
5371	AT2G36350.1 | protein kinase, putative, similar to protein kinase KIPK (KCBP-interacting protein kinase) (Arabidopsis thaliana) gi:7716430:gb:AAF68383	4.3	4.6	-0.4	-1.3	100.0%	-4.0
5372	AT4G28180.1 | expressed protein | chr4:13982917-13983687 REVERSE | Aliases: F26K10.60, F26K10_60	3.0	3.2	-0.2	-1.3	100.0%	-4.6
5373	AT5G23830.1 | MD-2-related lipid recognition domain-containing protein / ML domain-containing protein, contains Pfam profile PF02221: ML domain | chr5:8033743-8034892 FORWARD | Aliases: MRO11.13, MRO11_13	8.2	7.8	0.5	1.3	100.0%	-4.2
5374	AT5G41410.1 | Symbol: BEL1 | homeodomain protein (BEL1), identical to cDNA homeobox protein (BEL1) GI:28202124 | chr5:16597272-16601236 FORWARD | Aliases: BEL1, BELL 1, MYC6.12, MYC6_12	3.2	2.9	0.3	1.3	100.0%	-4.4
5375	AT5G47170.1 | expressed protein, similar to unknown protein (gb AAF26469.1) | chr5:19175735-19176187 REVERSE | Aliases: MQL5.2, MQL5_2	3.6	3.2	0.3	1.3	100.0%	-4.3
5376	AT1G79510.2 | expressed protein | chr1:29912906-29914881 REVERSE | Aliases: T8K14.7, T8K14_7	3.9	3.4	0.5	1.3	100.0%	-4.1
5377	AT3G19480.1 | D-3-phosphoglycerate dehydrogenase, putative / 3-PGDH, putative, similar to SP:O04130 from (Arabidopsis thaliana) | chr3:6752537-6754791 FORWARD | Aliases: MLD14.34	4.3	3.9	0.4	1.3	100.0%	-3.8
5378	AT2G03060.1 | MADS-box family protein | chr2:901613-903568 FORWARD | Aliases: T17M13.23, T17M13_23	2.9	2.7	0.2	1.3	100.0%	-4.8
5379	AT5G13600.1 | phototropic-responsive NPH3 family protein, contains NPH3 family domain, Pfam:PF03000; contains BTB/POZ domain, Pfam:PF00651 | chr5:4380435-4382500 FORWARD | Aliases: T6I14.9	3.9	3.6	0.3	1.3	100.0%	-4.3
5380	AT1G60620.1 | Symbol: ATRPAC43 | DNA-directed RNA polymerase, putative, identical to RNA polymerase subunit (Arabidopsis thaliana) GI:514324; contains Pfam profile PF01000: RNA polymerase Rpb3/RpoA insert domain | chr1:22334771-22337189 FORWARD | Aliases: RNA POLYMERASE I SUBUNIT 43	5.2	5.5	-0.3	-1.3	100.0%	-4.3

Rank	Description	15.dpi	5.dpi	M	t	adj.q	B
5381	AT1G19000.2 | myb family transcription factor, similar to MybSt1 GI:7705206 from (Solanum tuberosum) | chr1:6560783-6562772 REVERSE | Aliases: F14D16.15, F14D16_15	7.1	6.3	0.8	1.3	100.0%	-3.9
5382	AT3G02500.1 | expressed protein | chr3:519371-521754 FORWARD | Aliases: F16B3.13, F16B3_13	2.9	3.1	-0.3	-1.3	100.0%	-4.6
5383	AT5G06910.1 | Symbol: ATJ6 | DNAJ heat shock protein, putative (J6), identical to DnaJ homologue (Arabidopsis thaliana) GI:2689720; contains Pfam profile PF00226 DnaJ domain	7.1	7.9	-0.7	-1.3	100.0%	-3.9
5384	AT1G20300.1 | pentatricopeptide (PPR) repeat-containing protein, contains Pfam profile: PF01535 PPR repeat | chr1:7029619-7031525 FORWARD | Aliases: F14O10.10, F14O10_10	5.8	6.1	-0.4	-1.3	100.0%	-4.2
5385	AT2G26360.1 | similar to mitochondrial substrate carrier family protein [Arabidopsis thaliana] (TAIR:At2g35800.1); similar to MGC82075 protein [Xenopus laevis] (GB:AAH82409.1); contains InterPro domain Mitochondrial substrate carrier (InterPro:IPR001993); contains InterPro domain Mitochondrial carrier protein (InterPro:IPR002067) | chr2:11227795-11230291 REVERSE | Aliases: T9J22.3, T9J22_3	5.1	5.4	-0.3	-1.3	100.0%	-4.1
5386	AT1G48570.1 | zinc finger (Ran-binding) family protein, contains Pfam domain, PF00641: Zn-finger in Ran binding protein and others | chr1:17958528-17961387 REVERSE | Aliases: T1N15.19, T1N15_19	5.0	5.5	-0.5	-1.3	100.0%	-3.8
5387	AT3G20860.1 | protein kinase family protein, contains protein kinase domain, Pfam:PF00069; contains serine/threonine protein kinase domain, INTERPRO:IPR002290	3.1	4.1	-1.0	-1.3	100.0%	-3.6
5388	AT5G16270.1 | Rad21/Rec8-like family protein, weak similarity to cohesion family protein SYN2 (Arabidopsis thaliana) GI:12006360; contains Pfam profiles PF04824: Conserved region of Rad21 / Rec8 like protein, PF04825: N terminus of Rad21 / Rec8 like protein; supporting cDNA gi:18157648:gb:AF400129.1:AF400129 | chr5:5316302-5322699 FORWARD | Aliases: T21H19.190, T21H19_190	7.4	6.9	0.5	1.3	100.0%	-4.1
5389	AT2G35950.1 | expressed protein | chr2:15111863-15113167 FORWARD | Aliases: F11F19.14, F11F19_14	2.0	2.1	-0.1	-1.3	100.0%	-5.1
5390	AT3G10300.4 | similar to calcium-binding EF hand family protein [Arabidopsis thaliana] (TAIR:At5g04170.1); similar to putative fiber protein Fb1 [Oryza sativa (japonica cultivar-group)] (GB:XP_483756.1); contains InterPro domain Calcium-binding EF-hand (InterPro:IPR002048); contains InterPro domain Proline-rich region (InterPro:IPR000694) | chr3:3186326-3188232 FORWARD | Aliases: F14P13.10	5.6	5.2	0.4	1.3	100.0%	-4.2
5391	AT1G62290.2 | similar to aspartyl protease family protein [Arabidopsis thaliana] (TAIR:At1g11910.1); similar to aspartyl protease family protein [Arabidopsis thaliana] (TAIR:At4g04460.1); similar to aspartic proteinase (EC 3.4.23.-) - cowpea (GB:T11686); similar to ASPR_CUCPE Aspartic proteinase precursor (GB:O04057); similar to aspartic proteinase [Vigna unguiculata] (GB:AAB03843.2); similar to aspartic proteinase [Theobroma cacao] (GB:CAC86004.1); similar to aspartic proteinase 1 [Glycine max] (GB:BAB62890.1); contains InterPro domain Eukaryotic/viral aspartic protease, active site (InterPro:IPR001969); contains InterPro domain Saposin B subdomain (InterPro:IPR008140); contains InterPro domain Saposin-like type B, 2 (InterPro:IPR008138); contains InterPro domain Saposin B (InterPro:IPR008139); contains InterPro domain Aspartic protease A1, pepsin (InterPro:IPR001461); contains InterPro domain Saposin-like type B, 1 (InterPro:IPR007856) | chr1:23013576-23017193 REVERSE | Aliases: F19K23.21, F19K23_21	8.7	8.2	0.5	1.3	100.0%	-3.7
5392	AT4G05520.2 | calcium-binding EF hand family protein, similar to EH-domain containing protein 1 from {Mus musculus} SP:Q9WVK4, {Homo sapiens} SP:Q9H4M9, receptor-mediated endocytosis 1 from (Caenorhabditis elegans) GI:13487775, GI:13487777, GI:13487779; contains INTERPRO:IPR002048 calcium-binding EF-hand domain | chr4:2804448-2807926 FORWARD | Aliases: T1J24.10, T1J24_10	5.7	6.3	-0.6	-1.3	100.0%	-4.0
5393	AT2G26960.1 | myb family transcription factor (MYB81), contains PFAM profile: myb DNA binding domain PF00249; identical to cDNA putative transcription factor (MYB81) mRNA, partial cds GI:3941513 | chr2:11513143-11514503 REVERSE | Aliases: T20P8.1, T20P8_1	2.4	2.3	0.1	1.3	100.0%	-5.0
5394	AT5G26710.1 | glutamate-tRNA ligase, putative / glutamyl-tRNA synthetase, putatuve / GluRS, putative, identical to gi:3435196 | chr5:9305600-9308774 FORWARD | Aliases: None	10.5	11.0	-0.5	-1.3	100.0%	-4.4
5395	AT5G61240.1 | similar to leucine-rich repeat family protein [Arabidopsis thaliana] (TAIR:At1g13910.1); similar to Hcr2-0B [Lycopersicon esculentum] (GB:AAC78593.1); similar to putative leucine-rich repeat resistance protein [Solanum demissum] (GB:AAT38740.1); contains InterPro domain Leucine-rich repeat, typical subtype (InterPro:IPR003591); contains InterPro domain Leucine-rich repeat, plant specific (InterPro:IPR007090); contains InterPro domain Leucine-rich repeat (InterPro:IPR001611) | chr5:24646613-24649812 FORWARD | Aliases: MFB13.23, MFB13_23	7.2	7.9	-0.6	-1.3	100.0%	-3.9
5396	AT1G15410.1 | aspartate-glutamate racemase family, contains Pfam profile PF:01177 Aspartate-glutamate racemase family; contains TIGRFAM TIGR00035: aspartate racemase; similar to aspartate racemase (GI:5458794){Pyrococcus abyssi} | chr1:5298939-5300898 FORWARD | Aliases: F9L1.36, F9L1_36	5.3	5.0	0.3	1.3	100.0%	-4.2
5397	AT1G78200.2 | protein phosphatase 2C, putative / PP2C, putative, similar to protein phosphatase 2C GB:CAA72341 (Medicago sativa); contains Pfam profile: PF00481 Protein phosphatase 2C | chr1:29425231-29426755 FORWARD | Aliases: T11I11.14, T11I11_14	7.3	7.0	0.3	1.3	100.0%	-4.1

Rank	Description	15.dpi	5.dpi	M	t	adj.q	B
5398	AT5G48090.1 | expressed protein, ; expression supported by MPSS | chr5:19502460-19505609 FORWARD | Aliases: MDN11.17, MDN11_17	2.9	3.1	-0.2	-1.3	100.0%	-4.6
5399	AT5G06610.1 | expressed protein, contains Pfam profile PF04788: Protein of unknown function (DUF620) | chr5:2029898-2033724 FORWARD | Aliases: F15M7.14, F15M7_14	5.4	4.8	0.6	1.3	100.0%	-4.0
5400	AT2G19570.1 | Symbol: CDA1 | cytidine deaminase (CDD) / cytidine aminohydrolase, identical to cytidine deaminase (CDD) (Arabidopsis thaliana) GI:3046700 | chr2:8477460-8478726 REVERSE | Aliases: AT CDA1, CYTIDINE DEAMINASE 1, DESZ, F3P11.17, F3P11_17	7.1	7.4	-0.4	-1.3	100.0%	-4.2
5401	AT1G64530.1 | RWP-RK domain-containing protein, similar to nodule inception protein (Lotus japonicus) GI:6448579; contains Pfam profile: PF02042 RWP-RK domain	4.6	5.0	-0.4	-1.3	100.0%	-4.2
5402	AT5G39990.1 | glycosyltransferase family 14 protein / core-2/I-branching enzyme family protein, contains Pfam profile: PF02485 Core-2/I-Branching enzyme | chr5:16021520-16023968 FORWARD | Aliases: MYH19.150, MYH19_150	4.5	5.0	-0.5	-1.3	100.0%	-4.2
5403	AT2G35720.1 | DNAJ heat shock N-terminal domain-containing protein, low similarity to SP:O54946 DnaJ homolog subfamily B member 6 (Heat shock protein J2) Mus musculus; contains Pfam profile PF00226 DnaJ domain | chr2:15023749-15027090 FORWARD | Aliases: T20F21.9, T20F21_9	4.7	5.1	-0.3	-1.3	100.0%	-4.1
5404	AT2G47400.1 | Symbol: CP12 1 |	9.4	9.0	0.5	1.3	100.0%	-3.9
5405	AT1G60610.2 | expressed protein | chr1:22331577-22333941 REVERSE | Aliases: F8A5.13, F8A5_13	6.4	6.1	0.3	1.3	100.0%	-4.4
5406	AT3G22845.1 | emp24/gp25L/p24 protein-related, contains weak similarity to transmembrane protein (GI:1212965) (Homo sapiens) | chr3:8087314-8088754 FORWARD | Aliases: F5N5.1	8.6	9.1	-0.5	-1.3	100.0%	-4.1
5407	AT2G18900.1 | transducin family protein / WD-40 repeat family protein, contains 5 WD-40 repeats (PF00400); related to LACK protective antigen (GI:13625467) (Leishmania donovani) | chr2:8195339-8199567 REVERSE | Aliases: F19F24.10, F19F24_10	5.0	5.3	-0.3	-1.3	100.0%	-4.0
5408	AT1G80940.2 | expressed protein | chr1:30415667-30417021 FORWARD | Aliases: F23A5.30, F23A5_30	5.0	4.5	0.5	1.3	100.0%	-3.9
5409	AT2G01280.1 | transcription factor IIB (TFIIB) family protein, contains Pfam domain, PF00382: Transcription factor TFIIB repeat | chr2:145675-148783 FORWARD | Aliases: F10A8.16, F10A8_16	2.2	2.1	0.1	1.3	100.0%	-5.1
5410	AT4G32600.1 | zinc finger (C3HC4-type RING finger) family protein, low similarity to RING finger protein 38 (Homo sapiens) GI:21666412; contains Pfam profile PF00097: Zinc finger, C3HC4 type (RING finger) | chr4:15723468-15726105 FORWARD | Aliases: F4D11.200, F4D11_200	7.0	6.7	0.3	1.3	100.0%	-4.3
5411	AT5G11810.1 | expressed protein | chr5:3808721-3810749 FORWARD | Aliases: F14F18.1	7.3	7.9	-0.6	-1.3	100.0%	-4.3
5412	AT3G46720.1 | UDP-glucoronosyl/UDP-glucosyl transferase family protein, contains Pfam profile: PF00201 UDP-glucoronosyl and UDP-glucosyl transferase | chr3:17221840-17223333 REVERSE | Aliases: T6H20.250	4.0	4.2	-0.3	-1.3	100.0%	-4.5
5413	AT2G26660.1 | SPX (SYG1/Pho81/XPR1) domain-containing protein, low similarity to NUC-2 (Neurospora crassa) GI:1399532, xenotropic and polytropic murine leukemia virus receptor (Mus musculus castaneus) GI:6093320; contains Pfam profile PF03105: SPX domain	6.5	6.2	0.3	1.3	100.0%	-4.1
5414	AT5G27320.1 | expressed protein, similar to PrMC3 (Pinus radiata) GI:5487873 | chr5:9629095-9631213 FORWARD | Aliases: F21A20.30, F21A20_30	4.4	4.0	0.4	1.3	100.0%	-4.2
5415	AT1G07630.1 | protein phosphatase 2C family protein / PP2C family protein, similar to protein phosphatase-2c (GI:3608412) (Mesembryanthemum crystallinum); contains Pfam PF00481 : Protein phosphatase 2C domain | chr1:2349094-2351564 FORWARD | Aliases: F24B9.31, F24B9_31	4.7	5.2	-0.5	-1.3	100.0%	-3.8
5416	AT2G14900.1 | gibberellin-regulated family protein, similar to SP:P46690 Gibberellin-regulated protein 4 precursor {Arabidopsis thaliana} GASA4; contains Pfam profile PF02704: Gibberellin regulated protein | chr2:6411259-6412412 FORWARD | Aliases: T26I20.6, T26I20_6	3.3	4.0	-0.7	-1.3	100.0%	-3.6
5417	AT4G14270.2 | similar to dehydration-induced protein (ERD15) [Arabidopsis thaliana] (TAIR:At2g41430.2); similar to dehydration-induced protein (ERD15) [Arabidopsis thaliana] (TAIR:At2g41430.3); similar to dehydration-induced protein (ERD15) [Arabidopsis thaliana] (TAIR:At2g41430.4); similar to dehydration-induced protein (ERD15) [Arabidopsis thaliana] (TAIR:At2g41430.1); similar to early response to dehydration 15-like protein [Pseudotsuga menziesii var. menziesii] (GB:AAV92278.1); similar to early response to dehydration 15-like protein [Pseudotsuga menziesii var. menziesii] (GB:AAV92296.1)	8.0	7.6	0.4	1.3	100.0%	-4.2
5418	AT5G47440.1 | expressed protein, strong similarity to unknown protein (pir::G71442) | chr5:19260797-19262431 REVERSE | Aliases: MNJ7.2, MNJ7_2	3.1	2.9	0.2	1.3	100.0%	-4.7
5419	AT5G16290.2 | acetolactate synthase small subunit, putative, similar to gi:5931761 from Nicotiana plumbaginifolia | chr5:5333830-5337747 FORWARD | Aliases: MQK4.1, MQK4_1	7.2	7.8	-0.6	-1.3	100.0%	-3.8

Rank	Description	15.dpi	5.dpi	M	t	adj.q	B
5420	AT4G24280.1 | Symbol: CPHSC70 1 | heat shock protein 70, putative / HSP70, putative, strong similarity to heat shock protein 70 (Arabidopsis thaliana) GI:6746592; similar to heat shock 70 protein - Spinacia oleracea,PID:g2654208 | chr4:12589998-12593640 FORWARD | Aliases: CPHSC70 1, T22A6.110, T22A6_110	8.8	9.2	-0.4	-1.3	100.0%	-4.4
5421	AT4G19070.1 | cadmium-responsive protein / cadmium induced protein (AS8), identical to cadmium induced protein AS8 SP:P42735 from (Arabidopsis thaliana) | chr4:10446351-10448201 REVERSE | Aliases: T18B16.3	5.2	4.9	0.3	1.3	100.0%	-4.3
5422	AT2G24240.1 | potassium channel tetramerisation domain-containing protein, contains Pfam profile PF02214: K+ channel tetramerisation domain | chr2:10317728-10319408 FORWARD | Aliases: F27D4.15, F27D4_15	5.1	5.7	-0.6	-1.3	100.0%	-3.7
5423	AT2G46330.2 | Symbol: AGP16 | arabinogalactan-protein (AGP16), identical to gi:10880509:gb:AAG24284 | chr2:19025581-19026328 REVERSE | Aliases: ARABINOGALACTAN PROTEIN 16, F11C10.2	9.2	8.7	0.6	1.3	100.0%	-4.0
5424	AT5G09430.1 | hydrolase, alpha/beta fold family protein, low similarity to hydrolases from Rhodococcus sp. EtbD1 GI:3273239, EtbD2 GI:3273241; contains Pfam profile PF00561: hydrolase, alpha/beta fold family | chr5:2932163-2933363 FORWARD | Aliases: T5E8.230, T5E8_230	3.1	3.4	-0.3	-1.3	100.0%	-4.5
5425	AT5G61150.2 | Symbol: VIP4 | leo1-like family protein, weak similarity to SP:P38439 LEO1 protein {Saccharomyces cerevisiae}; contains Pfam profile PF04004: Leo1-like protein; supporting cDNA gi:21929714:gb:AF490422.1: | chr5:24620882-24624951 REVERSE | Aliases: MAF19.15, MAF19_15, VERNALIZATION INDEPENDENCE 4	6.2	6.8	-0.7	-1.3	100.0%	-4.1
5426	AT1G25470.2 | encodes a member of the ERF (ethylene response factor) subfamily B-6 of ERF/AP2 transcription factor family. The protein contains one AP2 domain. There are 12 members in this subfamily including RAP2.11. | chr1:8944596-8946601 REVERSE | Aliases: F2J7.8, F2J7_8	4.9	5.3	-0.4	-1.3	100.0%	-4.1
5427	AT1G10110.1 | F-box family protein, contains Pfam PF00646: F-box domain; similar to F-box protein family, AtFBX7 (GI:20197899) (Arabidopsis thaliana)	2.8	2.9	-0.1	-1.3	100.0%	-5.1
5428	AT1G21120.1 | O-methyltransferase, putative, similar to GI:2781394 | chr1:7395221-7396738 REVERSE | Aliases: T22I11.5, T22I11_5	3.8	4.3	-0.5	-1.3	100.0%	-4.1
5429	AT1G02840.3 | Symbol: SR1 | pre-mRNA splicing factor SF2 (SF2) / SR1 protein, identical to SP:O22315 Pre-mRNA splicing factor SF2 (SR1 protein) {Arabidopsis thaliana} | chr1:626741-629819 FORWARD | Aliases: ATSRP34, F22D16.16, F22D16_16, SRP34	10.1	9.8	0.3	1.3	100.0%	-4.5
5430	AT1G76040.2 | Symbol: CPK29 | similar to calcium-dependent protein kinase, putative / CDPK, putative [Arabidopsis thaliana] (TAIR:At1g50700.1); similar to calmodulin-domain protein kinase isoform 9 (CPK9) [Arabidopsis thaliana] (TAIR:At3g20410.1); similar to calcium-dependent protein kinase, putative / CDPK, putative [Arabidopsis thaliana] (TAIR:At4g04720.1); similar to calcium-dependent protein kinase, putative / CDPK, putative [Arabidopsis thaliana] (TAIR:At4g21940.1); similar to calcium-dependent protein kinase, putative / CDPK, putative [Arabidopsis thaliana] (TAIR:At1g61950.1); similar to calcium-dependent protein kinase [Oryza sativa (japonica cultivar-group)] (GB:CAA57157.1); similar to Ca2+-dependent protein kinase [Mesembryanthemum crystallinum] (GB:AAD17800.1); similar to calmodulin-like domain protein kinase isoenzyme gamma [Glycine max] (GB:AAB80693.1); similar to calcium-dependent protein kinase [Nicotiana tabacum] (GB:AAC25423.1); similar to PREDICTED P0048D08.105 gene product [Oryza sativa (japonica cultivar-group)] (GB:XP_506365.1); contains InterPro domain Serine/threonine protein kinase, active site (InterPro:IPR008271); contains InterPro domain Tyrosine protein kinase (InterPro:IPR001245); contains InterPro domain Calcium-binding EF-hand (InterPro:IPR002048); contains InterPro domain Serine/threonine protein kinase (InterPro:IPR002290); contains InterPro domain Protein kinase (InterPro:IPR000719); contains InterPro domain Proline-rich region (InterPro:IPR000694) | chr1:28542567-28545531 FORWARD | Aliases: T4O12.25, T4O12_25	6.2	5.5	0.7	1.3	100.0%	-4.0
5431	AT5G05370.1 | ubiquinol-cytochrome C reductase complex ubiquinone-binding protein, putative / ubiquinol-cytochrome C reductase complex 8.2 kDa protein, putative, strong similarity to SP:P46269 Ubiquinol-cytochrome C reductase complex ubiquinone-binding protein QP-C (EC 1.10.2.2) (Ubiquinol-cytochrome C reductase complex 8.2 kDa protein) {Solanum tuberosum}	10.9	11.1	-0.2	-1.3	100.0%	-4.5
5432	AT5G58160.1 | formin homology 2 domain-containing protein / FH2 domain-containing protein, low similarity to SP:Q05858 Formin (Limb deformity protein) {Gallus gallus}; contains Pfam profile PF02181: Formin Homology 2(FH2) Domain | chr5:23551169-23556691 FORWARD | Aliases: MCK7.3, MCK7_3	3.9	4.3	-0.4	-1.3	100.0%	-4.4
5433	AT3G52090.1 | Symbol: ATRPB13.6 | DNA-directed RNA polymerase II 13.6 kDa subunit (RPB13.6), identical to SP:Q38859 DNA-directed RNA polymerase II 13.6 kDa polypeptide (EC 2.7.7.6) {Arabidopsis thaliana} | chr3:19328832-19330030 REVERSE | Aliases: F4F15.200, RNA POLYMERASE II 13.6 KDA SUBUNIT	9.9	10.3	-0.4	-1.3	100.0%	-4.4
5434	AT3G18230.1 | octicosapeptide/Phox/Bem1p (PB1) domain-containing protein, contains Pfam profile PF00564: PB1 domain | chr3:6251489-6254227 FORWARD | Aliases: MIE15.2	5.9	5.6	0.3	1.3	100.0%	-4.3
5435	AT5G04170.1 | calcium-binding EF hand family protein, low similarity to peflin (Homo sapiens) GI:6015440; contains INTERPRO:IPR002048 calcium-binding EF-hand domain | chr5:1145432-1147782 FORWARD | Aliases: F21E1.90, F21E1_90	6.5	6.0	0.5	1.3	100.0%	-3.8
5436	AT1G01620.1 | Symbol: PIP1C | plasma membrane intrinsic protein 1C (PIP1C) / aquaporin PIP1.3 (PIP1.3) / transmembrane protein B (TMPB), identical to plasma membrane intrinsic protein 1c SP:Q08733 from (Arabidopsis thaliana) | chr1:225722-227302 REVERSE | Aliases: F22L4.16, F22L4_16, PIP1;3, TMP B	3.4	3.8	-0.5	-1.3	100.0%	-4.1
5437	AT3G16780.1 | 60S ribosomal protein L19 (RPL19B), similar to ribosomal protein L19 GB:CAA45090 from (Homo sapiens) | chr3:5708931-5710415 FORWARD | Aliases: MGL6.7	9.0	9.5	-0.5	-1.3	100.0%	-4.1

Rank	Description	15.dpi	5.dpi	M	t	adj.q	B
5438	AT2G20140.1 | 26S protease regulatory complex subunit 4, putative, similar to Swiss-Prot:P48601 26S protease regulatory subunit 4 (P26S4) (Drosophila melanogaster) | chr2:8699781-8702160 FORWARD | Aliases: T2G17.6, T2G17_6	7.1	7.8	-0.6	-1.3	100.0%	-3.9
5439	AT2G18320.1 | hypothetical protein | chr2:7963302-7964613 FORWARD | Aliases: T30D6.17, T30D6_17	3.1	3.4	-0.3	-1.3	100.0%	-4.6
5440	AT1G68750.1 | Symbol: ATPPC4 |	3.4	3.2	0.2	1.3	100.0%	-4.4
5441	AT5G47090.1 | expressed protein | chr5:19144486-19145996 FORWARD | Aliases: K14A3.4, K14A3_4	9.0	8.1	0.9	1.3	100.0%	-3.8
5442	AT3G61540.1 | peptidase family protein, similar to prolyl aminopeptidase (proline iminopeptidase) from Aeromonas sobria SP:P46547, Propionibacterium freudenreichii subsp. shermanii GI:2415704, Hafnia alvei GI:1754489 | chr3:22784361-22786802 FORWARD | Aliases: F2A19.140	6.8	7.4	-0.5	-1.3	100.0%	-4.3
5443	AT3G52040.1 | expressed protein | chr3:19315151-19315839 REVERSE | Aliases: F4F15.150	7.8	8.4	-0.5	-1.3	100.0%	-3.8
5444	AT4G20930.1 | 3-hydroxyisobutyrate dehydrogenase, putative, similar to SP:P29266 3-hydroxyisobutyrate dehydrogenase, mitochondrial precursor (EC 1.1.1.31) {Rattus norvegicus}; contains Pfam profile PF03446: NAD binding domain of 6-phosphogluconate dehydrogenase | chr4:11198417-11201084 REVERSE | Aliases: T13K14.90, T13K14_90	4.1	4.4	-0.3	-1.3	100.0%	-4.2
5445	AT4G40010.1 | serine/threonine protein kinase, putative, similar to serine-threonine protein kinase (Triticum aestivum) gi:2055374:gb:AAB58348	5.2	4.2	0.9	1.3	100.0%	-3.6
5446	AT4G32690.1 | Symbol: GLB3 | 2-on-2 hemoglobin (GLB3), identical to 2-on-2 hemoglobin (GLB3) GI:14165163 from (Arabidopsis thaliana) | chr4:15765436-15766990 FORWARD | Aliases: F4D11.1	7.6	6.8	0.8	1.3	100.0%	-4.0
5447	AT2G42370.1 | expressed protein | chr2:17650412-17652611 FORWARD | Aliases: MHK10.9, MHK10_9	3.5	4.2	-0.7	-1.3	100.0%	-4.2
5448	AT2G27170.1 | Symbol: TTN7 | similar to SMC2-like condensin, putative (SMC2) (TITAN3) [Arabidopsis thaliana] (TAIR:At5g62410.1); similar to cohesin complex subunit [Gallus gallus] (GB:NP_989848.1); similar to chondroitin sulfate proteoglycan 6 [Mus musculus] (GB:NP_031816.2); similar to putative SMC3 protein [Oryza sativa (japonica cultivar-group)] (GB:XP_464029.1); similar to putative SMC3 protein [Oryza sativa (japonica cultivar-group)] (GB:XP_464028.1); similar to chondroitin sulfate proteoglycan 6 (bamacan) [Homo sapiens] (GB:CAI16576.1); contains InterPro domain Structural maintenance of chromosome protein SMC, C-terminal (InterPro:IPR003405); contains InterPro domain SMC protein, N-terminal (InterPro:IPR003395) | chr2:11616397-11624509 REVERSE | Aliases: SMC3, T22O13.6, TITAN7	3.2	4.1	-1.0	-1.3	100.0%	-4.0
5449	AT2G27390.1 | proline-rich family protein, contains proline-rich extensin domains, INTERPRO:IPR002965 | chr2:11727562-11727966 REVERSE | Aliases: F12K2.3, F12K2_3	2.5	2.3	0.2	1.3	100.0%	-4.5
5450	AT1G53210.1 | sodium/calcium exchanger family protein / calcium-binding EF hand family protein, contains Pfam profiles: PF01699 sodium/calcium exchanger protein, PF00036 EF hand | chr1:19848300-19851504 FORWARD | Aliases: F12M16.12, F12M16_12	9.7	9.2	0.5	1.3	100.0%	-4.2
5451	AT1G63440.1 | copper-exporting ATPase, putative / responsive-to-antagonist 1, putative / copper-transporting ATPase, putative, similar to ATP dependent copper transporter SP:Q9S7J8 (Arabidopsis thaliana) | chr1:23531320-23534774 FORWARD | Aliases: F2K11.18, F2K11_18	7.3	7.6	-0.3	-1.3	100.0%	-4.2
5452	AT3G28345.1 | ABC transporter family protein, similar to P-glycoprotein (Arabidopsis thaliana) GI:3849833; contains Pfam profiles PF00005: ABC transporter, PF00664: ABC transporter transmembrane region | chr3:10595158-10600012 REVERSE | Aliases: MFJ20.7	2.4	2.3	0.1	1.3	100.0%	-5.1
5453	AT2G38185.4 | zinc finger (C3HC4-type RING finger) family protein, contains Pfam profile PF00097: Zinc finger, C3HC4 type (RING finger) | chr2:16007115-16013339 FORWARD | Aliases: None	2.7	2.6	0.2	1.3	100.0%	-4.9
5454	AT3G11630.1 | 2-cys peroxiredoxin, chloroplast (BAS1), identical to SP:Q96291 2-cys peroxiredoxin BAS1, chloroplast precursor {Arabidopsis thaliana}; contains Pfam profile: PF00578 AhpC/TSA (alkyl hydroperoxide reductase and thiol-specific antioxidant) family | chr3:3672126-3674081 FORWARD | Aliases: F24K9.28, F24K9_28, T19F11.3	10.0	10.9	-1.0	-1.3	100.0%	-4.0
5455	AT2G35940.3 | Symbol: BLH1 | similar to BEL1-like homeobox 4 protein (BLH4) [Arabidopsis thaliana] (TAIR:At2g23760.1); similar to BEL1-like homeobox 4 protein (BLH4) [Arabidopsis thaliana] (TAIR:At2g23760.2); similar to bell-like homeodomain protein 2 [Lycopersicon esculentum] (GB:AAP47025.1); contains InterPro domain POX (InterPro:IPR006563); contains InterPro domain Homeobox (InterPro:IPR001356) | chr2:15095911-15099027 REVERSE | Aliases: BEL1 like homeodomain 1, F11F19.15, F11F19_15	5.2	4.9	0.4	1.3	100.0%	-4.2
5456	AT4G36750.1 | quinone reductase family protein, similar to 1,4-benzoquinone reductase (Phanerochaete chrysosporium)(GI:4454993); similar to Trp repressor binding protein (Escherichia coli)(SP:P30849); contains flavodoxin domain PF00258 | chr4:17324575-17326462 FORWARD | Aliases: AP22.84, AP22_84	8.3	7.9	0.4	1.3	100.0%	-4.3
5457	AT4G32320.1 | peroxidase family protein, similar to L-ascorbate peroxidase (Arabidopsis thaliana) gi:1523789:emb:CAA66925; contains Pfam profile PF00141: Peroxidase | chr4:15602727-15605348 FORWARD | Aliases: F10M6.50, F10M6_50	4.9	4.6	0.3	1.3	100.0%	-4.3

Rank	Description	15.dpi	5.dpi	M	t	adj.q	B
5458	AT2G45350.1 | Symbol: CRR4 | Encodes a member of a PCMP (plant combinatorial and modular protein) family (PCMP-E subfamily) with 11 pentatricopeptide (PPR) repeats. The protein is involved in RNA editing of the initiation codon of ndhD in the chloroplast. | chr2:18701890-18703710 REVERSE | Aliases: CHLORORESPIRATORY REDUCTION 4, CRR4, F4L23.14	2.9	2.8	0.1	1.3	100.0%	-4.9
5459	AT5G22460.2 | esterase/lipase/thioesterase family protein, low similarity to 2-hydroxy-6-oxo-6-phenylhexa-2,4-dienoate hydrolase (Rhodococcus sp. RHA1) GI:8978311, SP:Q02104 Lipase 1 precursor (EC 3.1.1.3) (Triacylglycerol lipase) {Psychrobacter immobilis}; contains Interpro entry IPR000379 | chr5:7443511-7445403 REVERSE | Aliases: MWD9.26, MWD9_26	2.9	2.7	0.2	1.3	100.0%	-4.8
5460	AT1G10130.1 | Symbol: ECA3 | calcium-transporting ATPase 3, endoplasmic reticulum-type (ACA6) (ECA3), nearly identical to SP:Q9SY55 Calcium-transporting ATPase 3, endoplasmic reticulum-type (EC 3.6.3.8) {Arabidopsis thaliana}; contains InterPro Accession IPR006069: Cation transporting ATPase | chr1:3310932-3322215 FORWARD | Aliases: ATECA3, ENDOPLASMIC RETICULUM TYPE CALCIUM TRANSPORTING ATPASE 3, T27I1.16, T27I1_16	4.3	4.8	-0.5	-1.3	100.0%	-4.1
5461	AT1G52930.1 | brix domain-containing protein, contains Pfam domain, PF04427: Brix domain | chr1:19714905-19716898 FORWARD | Aliases: F14G24.20, F14G24_20	7.5	8.2	-0.7	-1.3	100.0%	-3.9
5462	AT1G15900.1 | expressed protein | chr1:5464350-5464691 FORWARD | Aliases: F7H2.24, F7H2_24	3.1	2.9	0.2	1.3	100.0%	-4.8
5463	AT1G18550.1 | kinesin motor protein-related, contains similarity to kinesin-related protein GI:4493964 from (Plasmodium falciparum) | chr1:6380922-6384333 REVERSE | Aliases: F25I16.11, F25I16_11	3.7	4.1	-0.4	-1.3	100.0%	-3.8
5464	AT3G15490.1 | expressed protein, contains Pfam profile: PF03398 eukaryotic protein of unknown function, DUF292	2.4	2.3	0.1	1.3	100.0%	-5.2
5465	AT1G61840.1 | DC1 domain-containing protein, similar to hypothetical protein GI:3184279 from (Arabidopsis thaliana); contains Pfam profile PF03107: DC1 domain	3.3	3.1	0.3	1.3	100.0%	-4.7
5466	AT5G52130.1 | expressed protein | chr5:21199694-21201101 REVERSE | Aliases: F17P19.2, F17P19_2	2.4	2.3	0.1	1.3	100.0%	-5.0
5467	AT3G15860.1 | expressed protein | chr3:5361214-5361726 REVERSE | Aliases: MSJ11.26	2.3	2.2	0.1	1.3	100.0%	-5.1
5468	AT1G69790.1 | protein kinase, putative, similar to protein kinase APK1A (Arabidopsis thaliana) SWISS-PROT:Q06548 | chr1:26270422-26272646 FORWARD | Aliases: T6C23.1, T6C23_1	4.1	3.4	0.7	1.3	100.0%	-3.8
5469	AT2G26160.1 | F-box family protein, contains F-box domain Pfam:PF00646	2.6	2.4	0.1	1.3	100.0%	-5.1
5470	AT4G31040.1 | proton extrusion protein-related, contains weak similarity to Proton extrusion protein pcxA (Swiss-Prot:P75028) (Synechocystis sp.) | chr4:15111491-15114132 REVERSE | Aliases: F6I18.50, F6I18_50	7.3	7.0	0.3	1.3	100.0%	-4.3
5471	AT3G22230.1 | 60S ribosomal protein L27 (RPL27B), similar to 60S RIBOSOMAL PROTEIN L27 GB:P41101 from (Solanum tuberosum) | chr3:7843974-7844604 REVERSE | Aliases: MKA23.22	8.9	9.5	-0.5	-1.3	100.0%	-4.0
5472	AT5G03380.2 | similar to heavy-metal-associated domain-containing protein [Arabidopsis thaliana] (TAIR:At2g36950.1); similar to GMFP5 [Glycine max] (GB:AAD09514.1); contains InterPro domain Heavy metal binding (InterPro:IPR006191); contains InterPro domain Heavy metal transport/detoxification protein (InterPro:IPR006121) | chr5:832103-834284 REVERSE | Aliases: F12E4.120, F12E4_120	8.9	8.2	0.8	1.3	100.0%	-3.9
5473	AT3G03490.1 | peroxisomal protein PEX19 family protein, contains Pfam profile: PF04614 Pex19 protein family | chr3:830172-831853 REVERSE | Aliases: T21P5.9, T21P5_9	9.4	9.1	0.3	1.3	100.0%	-4.3
5474	AT3G08860.1 | alanine--glyoxylate aminotransferase, putative / beta-alanine-pyruvate aminotransferase, putative / AGT, putative, similar to similar to SP:Q64565 Alanine--glyoxylate aminotransferase 2, mitochondrial precursor (EC 2.6.1.44) (AGT 2) (Beta-alanine-pyruvate aminotransferase) {Rattus norvegicus}; contains Pfam profile PF00202: aminotransferase, class III | chr3:2696565-2699164 REVERSE | Aliases: T16O11.21	2.6	2.8	-0.2	-1.3	100.0%	-4.9
5475	AT5G35320.1 | expressed protein | chr5:13539049-13540655 FORWARD | Aliases: T26D22.2, T26D22_2	4.6	4.2	0.4	1.3	100.0%	-4.4
5476	AT3G42670.1 | Symbol: CHR38 | SNF2 domain-containing protein / helicase domain-containing protein, low similarity to SP:P41410 DNA repair protein rhp54 (RAD54 homolog) {Schizosaccharomyces pombe}; contains PFam profiles PF00271: Helicase conserved C-terminal domain, PF00176: SNF2 family N-terminal domain | chr3:14766784-14771071 REVERSE | Aliases: CHR38, T12K4.120	3.8	4.1	-0.3	-1.3	100.0%	-4.2

Rank	Description	15.dpi	5.dpi	M	t	adj.q	B
5477	AT1G44350.1 | Symbol: ILL6 | IAA-amino acid hydrolase 6, putative (ILL6) / IAA-Ala hydrolase, putative, virtually identical to gr1-protein from (Arabidopsis thaliana) GI:3559811; similar to IAA-amino acid hydrolase GI:3421384 from (Arabidopsis thaliana); contains TIGRfam profile TIGR01891: amidohydrolase; contains Pfam profile PF01546: Peptidase family M20/M25/M40; identical to cDNA IAA-amino acid conjugate hydrolase-like protein (ILL6), partial cds GI:17978837 | chr1:16836650-16840726 REVERSE | Aliases: T18F15.9, T18F15_9	4.7	4.4	0.3	1.3	100.0%	-4.0
5478	AT1G75820.1 | Symbol: CLV1 | CLAVATA1 receptor kinase (CLV1), identical to receptor kinase (CLV1) GB:AAB58929 GI:2160756 (Arabidopsis thaliana) | chr1:28467123-28470632 REVERSE | Aliases: CLAVATA 1, CLV1 RECEPTOR KINASE PRECURSOR, FAS3, FASCIATA 3, FLO5, FLOWER DEVELOPMENT 5, T4O12.5, T4O12_5	4.5	4.0	0.6	1.3	100.0%	-3.6
5479	AT1G08580.1 | expressed protein | chr1:2715086-2716387 REVERSE | Aliases: F22O13.6, F22O13_6	8.6	9.1	-0.5	-1.3	100.0%	-3.9
5480	AT1G48560.1 | expressed protein | chr1:17956533-17958973 FORWARD | Aliases: T1N15.18, T1N15_18	4.5	4.8	-0.3	-1.3	100.0%	-4.0
5481	AT4G10810.1 | expressed protein | chr4:6645712-6646332 REVERSE | Aliases: F25I24.20, F25I24_20	7.3	6.6	0.7	1.3	100.0%	-3.9
5482	AT5G52180.1 | expressed protein | chr5:21217281-21218835 REVERSE | Aliases: F17P19.8, F17P19_8	6.6	6.2	0.4	1.3	100.0%	-4.3
5483	AT1G48830.2 | 40S ribosomal protein S7 (RPS7A), similar to 40S ribosomal protein S7 homolog GI:5532505 from (Brassica oleracea) | chr1:18063304-18064925 REVERSE | Aliases: F11I4.1, F11I4_1	12.0	12.4	-0.4	-1.3	100.0%	-4.7
5484	AT2G24420.2 | DNA repair ATPase-related, contains 2 transmembrane domains; similar to DNA double-strand break repair rad50 ATPase. (Swiss-Prot:O33600) (Sulfolobus acidocaldarius) | chr2:10387117-10390480 FORWARD | Aliases: T28I24.15, T28I24_15	5.2	4.6	0.7	1.3	100.0%	-4.1
5485	AT3G53970.2 | proteasome inhibitor-related, similar to proteasome inhibitor PI31 subunit (hPI31) SP:Q92530 from (Homo sapiens)	6.8	6.0	0.8	1.2	100.0%	-4.0
5486	AT2G35600.1 | expressed protein | chr2:14948171-14952407 REVERSE | Aliases: T32F12.2, T32F12_2	2.7	2.9	-0.1	-1.2	100.0%	-4.9
5487	AT4G21590.1 | bifunctional nuclease, putative, similar to bifunctional nuclease (Zinnia elegans) gi:4099833/gb:AAD00694. Activated by AGAMOUS in a cal-1, ap1-1 background. Expressed in the floral meristem and during stamen development. | chr4:11480379-11482726 FORWARD | Aliases: F17L22.50	3.2	3.0	0.2	1.2	100.0%	-4.5
5488	AT1G16780.1 | Symbol: AVPL1 | vacuolar-type H+-translocating inorganic pyrophosphatase, putative, similar to vacuolar-type H+-translocating inorganic pyrophosphatase GI:6901676 from (Arabidopsis thaliana) | chr1:5739292-5743562 REVERSE | Aliases: AVPL1, F17F16.2, F17F16_2, VACUOLAR PYROPHOSPHATASE LIKE PROTEIN 1	5.6	6.1	-0.5	-1.2	100.0%	-3.9
5489	AT4G04620.2 | autophagy 8b (APG8b), identical to autophagy 8b (Arabidopsis thaliana) GI:19912153; contains Pfam profile PF02991: Microtubule associated protein 1A/1B, light chain 3 | chr4:2328506-2330134 REVERSE | Aliases: F4H6.14, F4H6_14	8.2	7.7	0.5	1.2	100.0%	-4.4
5490	AT1G64820.1 | MATE efflux family protein, similar to ripening regulated protein DDTFR18 (Lycopersicon esculentum) GI:12231296; contains Pfam profile PF01554: Uncharacterized membrane protein family; contains 12 transmembrane domains, PMID: 11152613 | chr1:24092268-24094221 FORWARD | Aliases: F13O11.12, F13O11_12	3.9	3.7	0.2	1.2	100.0%	-4.7
5491	AT5G54160.1 | Symbol: ATOMT1 |	12.4	12.9	-0.5	-1.2	100.0%	-4.7
5492	AT1G10970.1 | Symbol: ZIP4 | metal transporter, putative (ZIP4), similar to Zn and Cd transporter ZNT1 (Thlaspi caerulescens) gi:7381054:gb:AAF61374; member of the Zinc (Zn2+)-Iron (Fe2+) permease (ZIP) family, PMID:11500563 | chr1:3665089-3667139 REVERSE | Aliases: T19D16.11, T19D16_11, ZRT, IRT LIKE PROTEIN	5.1	4.6	0.4	1.2	100.0%	-3.8
5493	AT3G18430.1 | calcium-binding EF hand family protein, similar to Calcineurin B subunit (Protein phosphatase 2B regulatory subunit) (Calcineurin regulatory subunit) SP:P42322 from (Naegleria gruberi); contains Pfam profile PF00036: EF hand	9.1	8.8	0.3	1.2	100.0%	-4.7
5494	AT3G23280.2 | zinc finger (C3HC4-type RING finger) family protein / ankyrin repeat family protein, contains Pfam profile: PF00097 zinc finger, C3HC4 type (RING finger) and Pfam profile: PF00023 ankyrin repeat | chr3:8321371-8324435 FORWARD | Aliases: K14B15.19	9.4	8.8	0.7	1.2	100.0%	-4.0
5495	AT5G60140.1 | transcriptional factor B3 family protein, contains Pfam profile PF02362: B3 DNA binding domain | chr5:24231303-24232581 REVERSE | Aliases: MGO3.2, MGO3_2	2.9	2.6	0.2	1.2	100.0%	-4.6
5496	AT1G22650.1 | beta-fructofuranosidase, putative / invertase, putative / saccharase, putative / beta-fructosidase, putative, similar to neutral invertase (Daucus carota) GI:4200165; contains Pfam profile PF04853: Plant neutral invertase | chr1:8013371-8015968 REVERSE | Aliases: T22J18.18, T22J18_18	3.8	4.2	-0.3	-1.2	100.0%	-4.3
5497	AT5G10830.1 | embryo-abundant protein-related, similar to embryo-abundant protein (Picea glauca) GI:1350531 | chr5:3423687-3425468 FORWARD | Aliases: T30N20.100, T30N20_100	6.8	7.2	-0.4	-1.2	100.0%	-4.2
5498	AT1G78960.1 | Symbol: ATLUP2 |	4.4	3.9	0.4	1.2	100.0%	-4.0

Rank	Description	15.dpi	5.dpi	M	t	adj.q	B
5499	AT4G28600.1 | Symbol: NPGR2 | calmodulin-binding protein, similar to pollen-specific calmodulin-binding protein MPCBP GI:10086260 from (Zea mays) | chr4:14129421-14132805 FORWARD | Aliases: NO POLLEN GERMINATION RELATED 2, T5F17.50, T5F17_50	6.0	5.5	0.5	1.2	100.0%	-4.3
5500	AT5G63560.1 | transferase family protein, similar to hypersensitivity-related gene product HSR201 - Nicotiana tabacum, EMBL:X95343; contains Pfam transferase family domain PF00248 | chr5:25466707-25468640 FORWARD | Aliases: MBK5.2, MBK5_2	4.0	4.3	-0.3	-1.2	100.0%	-4.2
5501	AT3G23060.1 | zinc finger (C3HC4-type RING finger) family protein, contains Pfam profile: PF00097 zinc finger, C3HC4 type (RING finger) | chr3:8200647-8203240 FORWARD | Aliases: MXC7.9	2.9	2.6	0.3	1.2	100.0%	-4.5
5502	AT1G48580.1 | expressed protein | chr1:17961931-17964278 REVERSE | Aliases: T1N15.20, T1N15_20	3.8	4.1	-0.3	-1.2	100.0%	-4.2
5503	AT3G49850.1 | myb family transcription factor, contains Pfam profile: PF00249 myb-like DNA-binding domain | chr3:18500030-18501981 FORWARD | Aliases: T16K5.200	4.8	5.3	-0.5	-1.2	100.0%	-3.9
5504	AT1G10200.1 | transcription factor LIM, putative, strong similarity to transcription factor Ntlim1 (Nicotiana tabacum) GI:5689136, LIM domain protein WLIM-1 (Helianthus annuus) GI:5070280; contains Pfam profile PF00412: LIM domain	7.2	6.5	0.7	1.2	100.0%	-4.3
5505	AT3G47340.3 | Symbol: ASN1 | asparagine synthetase 1 (glutamine-hydrolyzing) / glutamine-dependent asparagine synthetase 1 (ASN1), identical to SP:P49078 Asparagine synthetase (glutamine-hydrolyzing) (EC 6.3.5.4) (Glutamine- dependent asparagine synthetase) {Arabidopsis thaliana} | chr3:17448869-17452227 REVERSE | Aliases: AT ASN1, DARK INDUCIBLE 6, DIN6, GLUTAMINE DEPENDENT ASPARAGINE SYNTHETASE, T21L8.90	2.9	3.3	-0.4	-1.2	100.0%	-4.3
5506	AT4G19820.1 | glycosyl hydrolase family 18 protein, similar to chitinase/lysozyme GI:467689 from (Nicotiana tabacum) | chr4:10767446-10768624 REVERSE | Aliases: T16H5.180, T16H5_180	2.3	2.5	-0.2	-1.2	100.0%	-4.7
5507	AT4G38620.1 | Symbol: MYB4 | myb family transcription factor (MYB4), contains Pfam profile: PF00249 myb-like DNA-binding domain | chr4:18053545-18054993 FORWARD | Aliases: ATMYB4, T9A14.11	6.3	6.7	-0.4	-1.2	100.0%	-4.0
5508	AT4G39640.2 | gamma-glutamyltranspeptidase family protein, similar to SP:P19440 Gamma-glutamyltranspeptidase 1 precursor (EC 2.3.2.2) (Gamma-glutamyltransferase 1) (CD224 antigen) {Homo sapiens}; contains Pfam profilePF01019: Gamma-glutamyltranspeptidase	3.9	3.6	0.3	1.2	100.0%	-4.3
5509	AT2G42520.1 | DEAD box RNA helicase, putative, similar to SP:O00571 DEAD-box protein 3 (Helicase-like protein 2) {Homo sapiens}, DEAD box RNA helicase DDX3 (Homo sapiens) GI:3523150; contains Pfam profiles PF00270: DEAD/DEAH box helicase, PF00271: Helicase conserved C-terminal domain | chr2:17711913-17716025 FORWARD | Aliases: F14N22.21, F14N22_21	5.5	5.3	0.2	1.2	100.0%	-4.5
5510	AT4G08280.1 | expressed protein, hypothetical protein ssr1391 - Synechocystis sp. (strain PCC 6803),PIR2:S75571 | chr4:5230323-5232128 FORWARD | Aliases: T12G13.120, T12G13_120	6.1	5.4	0.7	1.2	100.0%	-3.9
5511	AT1G73640.1 | Ras-related GTP-binding family protein, contains Pfam profile: PF00071 ras family Pfam profile: PF00071 Ras family | chr1:27690653-27691788 FORWARD | Aliases: F25P22.5, F25P22_5	4.5	5.1	-0.6	-1.2	100.0%	-3.9
5512	AT4G33460.1 | Symbol: ATNAP13 | ABC transporter family protein, ABC-type transport protein sll1623 -Synechocystis,PIR2:S74812	6.0	5.5	0.5	1.2	100.0%	-4.0
5513	AT1G04430.1 | dehydration-responsive protein-related, similar to early-responsive to dehydration stress ERD3 protein (Arabidopsis thaliana) GI:15320410; contains Pfam profile PF03141: Putative methyltransferase | chr1:1198136-1201526 FORWARD | Aliases: F19P19.11, F19P19_11	9.5	10.0	-0.5	-1.2	100.0%	-4.2
5514	AT1G01140.3 | Symbol: CIPK9 | CBL-interacting protein kinase 9 (CIPK9), identical to CBL-interacting protein kinase 9 (Arabidopsis thaliana) gi:13249117:gb:AAK16684; contains Pfam profiles PF00069: Protein kinase domain and PF03822: NAF domain; identical to cDNA CBL-interacting protein kinase 9 (CIPK9) GI:13249116 | chr1:64167-67625 REVERSE | Aliases: CBL INTERACTING PROTEIN KINASE 9, F6F3.28, PKS6	6.4	5.9	0.5	1.2	100.0%	-3.9
5515	AT4G18250.1 | receptor serine/threonine kinase, putative, similar to to receptor serine/threonine kinase PR5K gi:1235680:gb:AAC49208 | chr4:10087354-10091974 REVERSE | Aliases: T9A21.100, T9A21_100	2.5	2.8	-0.2	-1.2	100.0%	-4.6
5516	AT4G04950.1 | thioredoxin family protein, similar to PKCq-interacting protein PICOT from (Mus musculus) GI:6840949, (Rattus norvegicus) GI:6840951; contains Pfam profile PF00085: Thioredoxin	6.3	7.1	-0.7	-1.2	100.0%	-4.0
5517	AT1G02690.2 | importin alpha-2 subunit, putative, similar to importin alpha-2 subunit (Karyopherin alpha-2 subunit) (KAP alpha) SP:O04294 from (Arabidopsis thaliana) | chr1:584260-587219 FORWARD | Aliases: T14P4.3, T14P4_3	5.9	6.3	-0.4	-1.2	100.0%	-4.2

Rank	Description	15.dpi	5.dpi	M	t	adj.q	B
5518	AT4G26090.1 | Symbol: RPS2 | disease resistance protein RPS2 (CC-NBS-LRR class), putative, domain signature CC-NBS-LRR exists, suggestive of a disease resistance protein. identical to RPS2 (gi:13661831) | chr4:13224347-13227881 FORWARD | Aliases: DISEASE RESISTANCE PROTEIN RPS2, F20B18.200, F20B18_200, RESISTANT TO P. SYRINGAE 2	3.3	3.0	0.3	1.2	100.0%	-4.2
5519	AT5G42760.1 | expressed protein, similar to hypothetical protein [Streptomyces sp. KO-3988] (GB:BAD86809.1); contains InterPro domain Protein of unknown function DUF142 (InterPro:IPR003455) | chr5:17166151-17167514 FORWARD | Aliases: MJB21.14, MJB21_14	4.8	4.2	0.5	1.2	100.0%	-4.1
5520	AT5G48020.1 | expressed protein | chr5:19479884-19482183 REVERSE | Aliases: MDN11.10, MDN11_10	8.5	9.1	-0.5	-1.2	100.0%	-3.9
5521	AT3G15250.1 | similar to protein kinase, putative [Arabidopsis thaliana] (TAIR:At1g53165.1); contains domain SER_RICH (PS50324) | chr3:5136269-5137202 REVERSE | Aliases: K7L4.5	2.1	2.0	0.1	1.2	100.0%	-5.0
5522	AT4G35760.1 | expressed protein | chr4:16942326-16944665 REVERSE | Aliases: F4B14.2	5.6	5.2	0.5	1.2	100.0%	-4.1
5523	AT5G06970.1 | expressed protein | chr5:2158229-2166399 REVERSE | Aliases: MOJ9.14, MOJ9_14	6.5	6.8	-0.3	-1.2	100.0%	-4.3
5524	AT5G67020.1 | expressed protein | chr5:26767076-26768475 FORWARD | Aliases: K8A10.9, K8A10_9	3.8	4.0	-0.2	-1.2	100.0%	-4.4
5525	AT3G42310.1 | expressed protein | chr3:14470804-14472483 FORWARD | Aliases: T14K23.20	5.5	4.8	0.8	1.2	100.0%	-3.5
5526	AT5G58490.1 | cinnamoyl-CoA reductase family, similar to cinnamoyl-CoA reductase from Pinus taeda (GI:17978649), Eucalyptus gunnii (GI:2058311) | chr5:23660248-23661824 FORWARD | Aliases: MQJ2.6, MQJ2_6	9.2	9.9	-0.7	-1.2	100.0%	-4.0
5527	AT3G26520.1 | Symbol: TIP2 | tonoplast intrinsic protein, putative, similar to tonoplast intrinsic protein GI:5081419 from (Brassica napus) | chr3:9723680-9725052 REVERSE | Aliases: GAMMA TIP2, MFE16.17, SALT STRESS INDUCIBLE PROTEIN, SITIP, TIP1;2, TONOPLAST INTRINSIC PROTEIN 2	4.1	3.6	0.5	1.2	100.0%	-3.9
5528	AT4G00960.1 | protein kinase family protein, contains Pfam domain PF00069: Protein kinase domain | chr4:414361-416180 FORWARD | Aliases: A_TM018A10.19, A_TM018A10_19, T18A10.6, T18A10_6	3.3	3.0	0.3	1.2	100.0%	-4.7
5529	AT3G04480.1 | endoribonuclease L-PSP family protein, contains Pfam domain PF01902: Domain of unknown function | chr3:1193636-1197403 REVERSE | Aliases: T27C4.13, T27C4_13	5.1	5.4	-0.4	-1.2	100.0%	-4.2
5530	AT1G71340.1 | glycerophosphoryl diester phosphodiesterase family protein, contains Pfam profile PF03009: Glycerophosphoryl diester phosphodiesterase family | chr1:26889162-26892000 REVERSE | Aliases: F3I17.1, F3I17_1	6.7	6.0	0.7	1.2	100.0%	-4.1
5531	AT1G32290.1 | expressed protein | chr1:11650202-11650547 REVERSE | Aliases: F27G20.4	8.8	8.4	0.4	1.2	100.0%	-4.5
5532	AT3G63240.1 | endonuclease/exonuclease/phosphatase family protein, similar to inositol polyphosphate 5-phosphatase I (GI:10444261) and II (GI:10444263) (Arabidopsis thaliana); contains Pfam profile PF03372: Endonuclease/Exonuclease/phosphatase family | chr3:23374859-23378567 REVERSE | Aliases: F16M2.90	2.5	3.1	-0.5	-1.2	100.0%	-4.2
5533	AT2G48070.2 | expressed protein | chr2:19669920-19671535 FORWARD | Aliases: T9J23.22	3.5	3.3	0.2	1.2	100.0%	-4.6
5534	AT3G27270.1 | expressed protein | chr3:10074517-10075397 FORWARD | Aliases: K17E12.9	4.3	4.6	-0.4	-1.2	100.0%	-4.1
5535	AT2G37320.1 | pentatricopeptide (PPR) repeat-containing protein, contains INTERPRO:IPR002885 PPR repeats | chr2:15674302-15675804 FORWARD | Aliases: F3G5.11, F3G5_11	3.2	3.1	0.1	1.2	100.0%	-4.8
5536	AT2G24320.1 | expressed protein | chr2:10352739-10353909 REVERSE | Aliases: T28I24.5, T28I24_5	2.6	2.8	-0.2	-1.2	100.0%	-4.7
5537	AT5G66170.2 | similar to senescence-associated family protein [Arabidopsis thaliana] (TAIR:At2g17850.1); similar to A Chain A, Solution Structure Of At5g66040, A Putative Protein From Arabidosis Thaliana (GB:1TQ1); contains InterPro domain Rhodanese-like (InterPro:IPR001763) | chr5:26464874-26465953 FORWARD | Aliases: K2A18.25, K2A18_25	7.6	7.2	0.5	1.2	100.0%	-4.0
5538	AT1G33360.1 | ATP-dependent Clp protease ATP-binding subunit ClpX, putative, similar to CLP protease regulatory subunit CLPX GI:2674203 from (Arabidopsis thaliana) | chr1:12091857-12096005 FORWARD | Aliases: F10C21.5	4.7	5.1	-0.4	-1.2	100.0%	-3.9
5539	AT1G04050.1 | SET domain-containing protein / suppressor of variegation related 1 (SUVR1), identical to suppressor of variegation related 1 (Arabidopsis thaliana) GI:15004614; contains Pfam profiles PF00856: SET domain, PF05033: Pre-SET motif; identical to cDNA trithorax 3 (ATX3) partial cds GI:15217142 | chr1:1045597-1048751 REVERSE | Aliases: AT1G04060, F21M11.35, F21M11_35	3.4	3.6	-0.2	-1.2	100.0%	-4.6

Rank	Description	15.dpi	5.dpi	M	t	adj.q	B
5540	AT1G61930.1 | expressed protein, contains Pfam profile PF04520: Protein of unknown function, DUF584 | chr1:22896766-22897635 FORWARD | Aliases: F8K4.12, F8K4_12	6.1	5.5	0.6	1.2	100.0%	-4.1
5541	AT4G30330.1 | small nuclear ribonucleoprotein E, putative / snRNP-E, putative / Sm protein E, putative, similar to SWISS-PROT:P08578 small nuclear ribonucleoprotein E (snRNP-E) (Sm protein E, Sm-E, SmE) (Chicken) | chr4:14836558-14838024 REVERSE | Aliases: F17I23.330, F17I23_330	7.4	7.9	-0.6	-1.2	100.0%	-3.9
5542	AT1G67780.1 | hypothetical protein | chr1:25416479-25419193 FORWARD | Aliases: F12A21.9, F12A21_9	2.6	2.7	-0.1	-1.2	100.0%	-5.3
5543	AT2G04620.1 | cation efflux family protein, potential member of the cation diffusion facilitator (CDF) family, or cation efflux (CE) family, see PMID:11500563	4.5	4.9	-0.4	-1.2	100.0%	-4.3
5544	AT3G63350.1 | Symbol: AT HSFA7B | heat shock transcription factor family protein, contains Pfam profile: PF00447 HSF-type DNA-binding domain | chr3:23410443-23411787 FORWARD | Aliases: HSFA7B, MAA21.4	2.6	2.4	0.2	1.2	100.0%	-4.7
5545	AT1G16270.1 | protein kinase family protein, contains PF:00069 Eukaryotic protein kinase domain. ESTs gb:H37741, gb:T43005 and gb:AI100340 come from this gene	3.9	4.2	-0.3	-1.2	100.0%	-4.4
5546	AT1G79570.1 | protein kinase family protein, low similarity to EDR1 (Arabidopsis thaliana) GI:11127925	3.9	4.2	-0.3	-1.2	100.0%	-4.4
5547	AT4G35010.1 | Symbol: BGAL11 |	2.4	2.6	-0.2	-1.2	100.0%	-4.9
5548	AT2G21410.1 | vacuolar proton ATPase, putative, similar to vacuolar proton ATPase 100-kDa subunit from Dictyostelium discoideum P:1384136:gb:AAB49621 | chr2:9169700-9175532 FORWARD | Aliases: F3K23.17, F3K23_17	7.0	6.7	0.2	1.2	100.0%	-4.5
5549	AT5G52560.1 | This gene was shown to have sequence similarities with pea PsUSP gene, encoding an UDP-sugar pyrophosphorylase. UDP-N-acetylglucosamine pyrophosphorylase-related, contains weak similarity to UDP-N-acetylglucosamine pyrophosphorylase (EC 2.7.7.23) (Swiss-Prot:O74933) (Candida albicans). | chr5:21347924-21352037 FORWARD | Aliases: F6N7.4, F6N7_4	7.0	7.8	-0.8	-1.2	100.0%	-4.0
5550	AT1G55110.1 | zinc finger (C2H2 type) family protein, contains Pfam domain, PF00096: Zinc finger, C2H2 type | chr1:20563951-20566838 REVERSE | Aliases: T7N22.5, T7N22_5	3.2	3.5	-0.2	-1.2	100.0%	-4.5
5551	AT3G54390.1 | expressed protein, similar to 6b-interacting protein 1 (NtSIP1) (Nicotiana tabacum) GI:18149189 | chr3:20148719-20150120 REVERSE | Aliases: T14E10.5	5.5	5.1	0.4	1.2	100.0%	-4.1
5552	AT3G16280.1 | encodes a member of the DREB subfamily A-4 of ERF/AP2 transcription factor family. The protein contains one AP2 domain. There are 17 members in this subfamily including TINY. | chr3:5518356-5519252 FORWARD | Aliases: MYA6.14	4.0	3.8	0.2	1.2	100.0%	-4.6
5553	AT5G47120.1 | Symbol: ATBI 1 |	10.0	9.2	0.8	1.2	100.0%	-4.4
5554	AT3G22740.1 | Symbol: HMT3 | homocysteine S-methyltransferase 3 (HMT-3), identical to homocysteine S-methyltransferase HMT-3 (Arabidopsis thaliana) GI:9966515; similar to homocysteine S-methyltransferase AtHMT-2 (GI:6685163) (Arabidopsis thaliana); similar to selenocysteine methyltransferase GB:P56707 from (Astragalus bisulcatus) | chr3:8032966-8035816 REVERSE | Aliases: MWI23.11	2.4	2.3	0.1	1.2	100.0%	-5.0
5555	AT3G01120.1 | Symbol: MTO1 | cystathionine gamma-synthase, chloroplast / O-succinylhomoserine (Thiol)-lyase (CGS), identical to SP:P55217 Cystathionine gamma-synthase, chloroplast precursor (EC 4.2.99.9) (CGS) (O-succinylhomoserine (Thiol)-lyase) {Arabidopsis thaliana}	12.2	11.7	0.5	1.2	100.0%	-4.5
5556	AT3G53770.1 | late embryogenesis abundant protein-related / LEA protein-related, contains weak similarity to Swiss-Prot:P46521 late embryogenesis abundant protein Lea5-A (Gossypium hirsutum)	2.2	2.3	-0.1	-1.2	100.0%	-5.2
5557	AT5G40970.1 | expressed protein | chr5:16431266-16431732 FORWARD | Aliases: MEE6.4, MEE6_4	6.4	5.6	0.8	1.2	100.0%	-3.9
5558	AT3G10050.1 | Symbol: OMR1 | threonine ammonia-lyase / threonine dehydratase / threonine deaminase (OMR1), identical to SP:Q9ZSS6 Threonine dehydratase biosynthetic, chloroplast precursor (EC 4.3.1.19, formerly EC 4.2.1.16) (Threonine deaminase) (TD) {Arabidopsis thaliana} | chr3:3098730-3101766 REVERSE | Aliases: L O METHYLTHREONINE RESISTANT 1, T22K18.12, THREONINE DEHYDRATASE/DEAMINASE	6.2	6.9	-0.7	-1.2	100.0%	-3.9
5559	AT1G79080.1 | pentatricopeptide (PPR) repeat-containing protein, contains Pfam profile PF01535: PPR repeat | chr1:29751865-29753837 REVERSE | Aliases: YUP8H12R.30, YUP8H12R_30	4.0	4.5	-0.5	-1.2	100.0%	-3.9
5560	AT3G21360.1 | expressed protein | chr3:7522810-7524586 FORWARD | Aliases: MHC9.4	6.7	6.3	0.4	1.2	100.0%	-4.1

Rank	Description	15.dpi	5.dpi	M	t	adj.q	B
5561	AT4G24330.1 | expressed protein, hypothetical protein - Caenorhabditis elegans,PID:e1350884 | chr4:12603531-12606338 REVERSE | Aliases: T22A6.160, T22A6_160	9.9	10.3	-0.4	-1.2	100.0%	-4.7
5562	AT1G76580.1 | similar to squamosa promoter-binding protein-like 1 (SPL1) [Arabidopsis thaliana] (TAIR:At2g47070.1); similar to putative SBP-domain protein [Oryza sativa (japonica cultivar-group)] (GB:XP_470314.1); contains domain SER_RICH (PS50324) | chr1:28740222-28743811 FORWARD | Aliases: F14G6.18, F14G6_18	4.1	3.9	0.2	1.2	100.0%	-4.7
5563	AT5G20700.1 | senescence-associated protein-related, similar to senescence-associated protein SAG102 (GI:22331931) (Arabidopsis thaliana); | chr5:7005860-7007119 REVERSE | Aliases: T1M15.100, T1M15_100	7.7	7.1	0.5	1.2	100.0%	-4.4
5564	AT5G08010.1 | expressed protein, condensin subunit SMC4, Drosophila melanogaster, EMBL:AF186472 | chr5:2569607-2571735 FORWARD | Aliases: F13G24.210, F13G24_210	6.6	7.0	-0.3	-1.2	100.0%	-4.4
5565	AT2G45770.1 | Symbol: CPFTSY |	4.6	4.9	-0.3	-1.2	100.0%	-4.2
5566	AT1G34300.1 | lectin protein kinase family protein, contains Pfam domains, PF01453: Lectin (probable mannose binding) and PF00069: Protein kinase domain | chr1:12503384-12506026 FORWARD | Aliases: F23M19.5, F23M19_5	6.6	6.3	0.3	1.2	100.0%	-4.1
5567	AT5G23710.1 | expressed protein | chr5:7996531-7997223 REVERSE | Aliases: MQM1.2, MQM1_2	5.9	5.6	0.4	1.2	100.0%	-4.0
5568	AT2G32460.1 | myb family transcription factor (MYB101), identical to putative transcription factor MYB101 GI:18087348 from (Arabidopsis thaliana) | chr2:13789285-13791548 REVERSE | Aliases: T32F6.1	2.4	2.6	-0.1	-1.2	100.0%	-4.9
5569	AT2G04350.2 | long-chain-fatty-acid--CoA ligase family protein / long-chain acyl-CoA synthetase family protein (LACS8), similar to LACS 4 (SP:O35547) from Rattus norvegicus, LACS 4 (SP:O60488) from Homo sapiens; contains Pfam HMM hit: AMP-binding enzymes PF00501	11.0	11.4	-0.4	-1.2	100.0%	-4.7
5570	AT3G17410.1 | serine/threonine protein kinase, putative, similar to Pto kinase interactor 1 GB:AAC61805 from (Lycopersicon esculentum)	7.3	6.7	0.6	1.2	100.0%	-4.0
5571	AT1G69480.1 | EXS family protein / ERD1/XPR1/SYG1 family protein, similar to PHO1 protein (Arabidopsis thaliana) GI:20069032; contains Pfam profiles PF03105: SPX domain, PF03124: EXS family	3.1	2.9	0.2	1.2	100.0%	-4.5
5572	AT2G01930.2 | expressed protein | chr2:427116-428682 REVERSE | Aliases: F23I14.2, F23I14_2	5.0	4.6	0.4	1.2	100.0%	-4.3
5573	AT1G22730.1 | MA3 domain-containing protein, contains Pfam profile PF02847: MA3 domain; low similarity to programmed cell death 4 protein (Gallus gallus) GI:12958564 | chr1:8046142-8049028 FORWARD | Aliases: T22J18.10, T22J18_10	4.8	5.2	-0.4	-1.2	100.0%	-4.2
5574	AT4G29830.1 | Symbol: VIP3 | transducin family protein / WD-40 repeat family protein, contains 7 WD-40 repeats (PF00400); G protein beta subunit-like protein, Schistosoma mansoni, gb:U30261	7.5	7.9	-0.4	-1.2	100.0%	-4.0
5575	AT3G20040.1 | similar to hexokinase, putative [Arabidopsis thaliana] (TAIR:At1g50460.1); similar to putative hexokinase [Oryza sativa (japonica cultivar-group)] (GB:XP_463697.1); contains InterPro domain Hexokinase (InterPro:IPR001312) | chr3:6994900-6998177 FORWARD | Aliases: MAL21.4	4.1	4.6	-0.5	-1.2	100.0%	-4.0
5576	AT3G18000.1 | Symbol: NMT1 | phosphoethanolamine N-methyltransferase 1 / PEAMT 1 (NMT1), identical to Phosphoethanolamine N-methyltransferase 1 (EC 2.1.1.103) (PEAMT 1) (AtNMT1) (SP:Q9FR44){Arabidopsis thaliana}; strong similarity to phosphoethanolamine N-methyltransferase from (Spinacia oleracea) GI:7407189, (Triticum aestivum) GI:17887465; contains Pfam profile PF01209: methlytransferase, UbiE/COQ5 family | chr3:6154369-6157558 FORWARD | Aliases: AT3G17990, MEB5.22, N METHYLTRANSFERASE 1, S ADENOSYLMETHIONINE:PHOSPHO ETHANOLAMINE	4.4	4.7	-0.3	-1.2	100.0%	-4.2
5577	AT5G47510.1 | SEC14 cytosolic factor family protein / phosphoglyceride transfer family protein, similar to phosphatidylinositol transfer-like protein IV (GI:14486707) (Lotus japonicus), SEC14 cytosolic factor (Phosphatidylinositol/phosphatidylcholine transfer protein) (PI/PCTP) (SP:P24859) (Kluyveromyces lactis) and to SEC14 cytosolic factor (SP:P53989) (Candida glabrata)	3.1	3.3	-0.2	-1.2	100.0%	-5.0
5578	AT5G47310.1 | expressed protein | chr5:19218088-19220148 FORWARD | Aliases: MQL5.17, MQL5_17	7.4	6.8	0.6	1.2	100.0%	-4.2
5579	AT3G26450.1 | major latex protein-related / MLP-related, low similarity to major latex protein {Papaver somniferum}(GI:294060) ; contains Pfam profile PF00407: Pathogenesis-related protein Bet v I family | chr3:9682535-9684605 REVERSE | Aliases: F20C19.24	3.7	3.9	-0.2	-1.2	100.0%	-4.4
5580	AT1G71740.1 | expressed protein | chr1:26990907-26991299 FORWARD | Aliases: F14O23.12, F14O23_12	3.6	4.3	-0.6	-1.2	100.0%	-3.6
5581	AT3G58050.1 | expressed protein | chr3:21503516-21508134 FORWARD | Aliases: T10K17.260	4.4	4.6	-0.3	-1.2	100.0%	-4.3

Rank	Description	15.dpi	5.dpi	M	t	adj.q	B
5582	AT3G02310.1 | Symbol: SEP2 | developmental protein SEPALLATA2 / floral homeotic protein (AGL4) (SEP2), identical to developmental protein SEPALLATA2 / floral homeotic protein AGL4 GB:P29384 (Arabidopsis thaliana), Pfam HMM hit: SRF-type transcription factors (DNA-binding and dimerization domain) | chr3:464286-467081 REVERSE | Aliases: AGL4, F14P3.4, F14P3_4, SEPALLATA2, TRANSCRIPTION FACTOR	2.9	2.8	0.2	1.2	100.0%	-4.8
5583	AT3G50620.1 | nodulation protein-related, contains weak similarity to nodulation protein H (EC 2.8.2.-) (Host-specificity of nodulation protein D) (Swiss-Prot:P06237) (Rhizobium meliloti) | chr3:18795777-18797864 REVERSE | Aliases: T20E23.220	4.4	4.7	-0.3	-1.2	100.0%	-4.2
5584	AT1G79470.1 | inosine-5'-monophosphate dehydrogenase, identical to inosine-5'-monophosphate dehydrogenase SP:P47996 {Arabidopsis thaliana} | chr1:29899491-29901650 REVERSE | Aliases: T8K14.11, T8K14_11	7.2	7.8	-0.5	-1.2	100.0%	-4.3
5585	AT4G22260.1 | Symbol: IM | alternative oxidase, putative / immutans protein (IM), identical to IMMUTANS from Arabidopsis thaliana (gi:4138855); contains Pfam profile PF01786 alternative oxidase | chr4:11769768-11772526 REVERSE | Aliases: IM1, IMMUTANS, T10I14.90, T10I14_90	6.9	6.4	0.5	1.2	100.0%	-4.1
5586	AT2G41800.1 | expressed protein, contains Pfam profile PF04862: Protein of unknown function, DUF642 | chr2:17443607-17445106 REVERSE | Aliases: T11A7.10, T11A7_10	4.6	4.9	-0.4	-1.2	100.0%	-4.0
5587	AT5G48720.1 | similar to expressed protein [Arabidopsis thaliana] (TAIR:At2g01990.1); similar to P0413C03.27 [Oryza sativa (japonica cultivar-group)] (GB:NP_914850.1)	4.1	3.7	0.3	1.2	100.0%	-4.3
5588	AT1G21980.1 | Symbol: ATPIP5K1 | 1-phosphatidylinositol-4-phosphate 5-kinase, putative / PIP kinase, putative / PtdIns(4)P-5-kinase, putative / diphosphoinositide kinase, putative, strong similarity to phosphatidylinositol-4-phosphate 5-kinase AtPIP5K1 (Arabidopsis thaliana) GI:3702691; contains Pfam profiles PF01504: Phosphatidylinositol-4-phosphate 5-Kinase, PF02493: MORN repeat | chr1:7734754-7738645 FORWARD | Aliases: PHOSPHATIDYLINOSITOL 4 PHOSPHATE 5 KINASE	7.7	8.2	-0.5	-1.2	100.0%	-4.2
5589	AT5G13700.1 | polyamine oxidase, putative, similar to SP:O64411 Polyamine oxidase precursor (EC 1.5.3.11) from Zea mays | chr5:4419924-4423036 REVERSE | Aliases: MSH12.17, MSH12_17	4.4	4.2	0.2	1.2	100.0%	-4.4
5590	AT1G19100.1 | ATP-binding region, ATPase-like domain-containing protein-related, low similarity to microrchidia (Homo sapiens) GI:5410257; contains non-consensus splice site (GC) at intron 8	4.1	4.4	-0.3	-1.2	100.0%	-4.2
5591	AT5G06720.1 | peroxidase, putative, identical to peroxidase (Arabidopsis thaliana) gi:1491617:emb:CAA68212 | chr5:2077430-2079006 REVERSE | Aliases: MPH15.8, MPH15_8	7.1	7.7	-0.6	-1.2	100.0%	-3.8
5592	AT4G21326.1 | subtilase family protein, contains similarity to subtilase; SP1 GI:9957714 from (Oryza sativa) | chr4:11346991-11349664 FORWARD | Aliases: None	2.9	3.1	-0.2	-1.2	100.0%	-4.4
5593	AT3G25110.1 | acyl-(acyl carrier protein) thioesterase / acyl-ACP thioesterase / oleoyl-(acyl-carrier protein) hydrolase / S-acyl fatty acid synthase thioesterase, identical to acyl-(acyl carrier protein) thioesterase (Arabidopsis thaliana) GI:804946 | chr3:9146281-9148493 REVERSE | Aliases: MJL12.9	4.5	5.0	-0.6	-1.2	100.0%	-4.3
5594	AT1G17440.2 | transcription initiation factor IID (TFIID) subunit A family protein, similar to SP:Q16514 Transcription initiation factor TFIID 20/15 kDa subunits (TAFII-20/TAFII-15) {Homo sapiens}; contains Pfam profile PF03847: Transcription initiation factor TFIID subunit A | chr1:5984154-5987764 REVERSE | Aliases: F1L3.13	4.7	4.3	0.4	1.2	100.0%	-4.1
5595	AT2G30800.1 | Symbol: HVT1 | DEIH-box RNA/DNA helicase, putative, similar to DEIH-box RNA/DNA helicase (Arabidopsis thaliana) GI:5881579; contains Pfam profiles PF04408: Helicase associated domain (HA2), PF00271: Helicase conserved C-terminal domain | chr2:13127430-13133846 REVERSE | Aliases: ATVT 1, HELICASE IN VASCULAR TISSUE AND TAPETUM, T11J7.19	3.8	4.2	-0.4	-1.2	100.0%	-4.3
5596	AT5G12410.1 | THUMP domain-containing protein, contains Pfam profile PF02926: THUMP domain | chr5:4021816-4023989 REVERSE | Aliases: None	6.9	7.5	-0.5	-1.2	100.0%	-4.1
5597	AT2G28000.1 | Symbol: CPN60A |	9.9	10.3	-0.4	-1.2	100.0%	-4.1
5598	AT5G15950.2 | similar to adenosylmethionine decarboxylase family protein [Arabidopsis thaliana] (TAIR:At3g02470.1); similar to S-adenosyl-L-methionine decarboxylase [Brassica juncea] (GB:AAB88273.1); contains InterPro domain S-adenosylmethionine decarboxylase (InterPro:IPR001985) | chr5:5206159-5208039 FORWARD | Aliases: F1N13.90, F1N13_90	4.4	4.0	0.3	1.2	100.0%	-4.2
5599	AT1G77460.1 | C2 domain-containing protein / armadillo/beta-catenin repeat family protein, similar to CCLS 65 (Silene latifolia) GI:2570102; contains Pfam profiles PF00514: Armadillo/beta-catenin-like repeat, PF00168: C2 domain | chr1:29109442-29116474 FORWARD | Aliases: T5M16.5, T5M16_5	3.9	4.2	-0.3	-1.2	100.0%	-4.3
5600	AT3G56160.1 | expressed protein | chr3:20848423-20852520 REVERSE | Aliases: F18O21.120	3.7	3.5	0.2	1.2	100.0%	-4.8
5601	AT5G45800.1 | leucine-rich repeat transmembrane protein kinase, putative | chr5:18592809-18596324 REVERSE | Aliases: MRA19.24, MRA19_24	3.8	4.3	-0.6	-1.2	100.0%	-4.0

Rank	Description	15.dpi	5.dpi	M	t	adj.q	B
5602	AT2G25450.1 | 2-oxoglutarate-dependent dioxygenase, putative, similar to 2A6 (GI:599622) and tomato ethylene synthesis regulatory protein E8 (SP:P10967) | chr2:10836995-10838733 REVERSE | Aliases: F13B15.11, F13B15_11	8.6	7.7	0.9	1.2	100.0%	-4.0
5603	AT1G34290.1 | leucine-rich repeat family protein, conatins leucine rich repeat (LRR) domains, Pfam:PF00560; contains similarity to disease resistance protein GI:3894383 from (Lycopersicon esculentum) | chr1:12497978-12498778 FORWARD | Aliases: F23M19.6, F23M19_6	2.3	2.4	-0.1	-1.2	100.0%	-5.0
5604	AT3G13800.1 | metallo-beta-lactamase family protein, similar to Metal Dependent Hydrolase GB:AAD18619 from (Chlamydophila pneumoniae)	4.9	4.6	0.3	1.2	100.0%	-4.3
5605	AT5G44580.1 | expressed protein | chr5:17996538-17997634 FORWARD | Aliases: K15C23.2, K15C23_2	2.8	2.5	0.3	1.2	100.0%	-4.6
5606	AT4G13010.1 | oxidoreductase, zinc-binding dehydrogenase family protein, low similarity to probable NADP-dependent oxidoreductase (zeta-crystallin homolog) P1 (SP:Q39172)(gi:886428) and P2 (SP:Q39173)(gi:886430); contains Pfam profile PF00107: oxidoreductase, zinc-binding dehydrogenase family | chr4:7600548-7602726 FORWARD | Aliases: F25G13.100, F25G13_100	10.5	9.8	0.6	1.2	100.0%	-4.1
5607	AT2G46700.1 | calcium-dependent protein kinase, putative / CDPK, putative, similar to calcium/calmodulin-dependent protein kinase homolog MCK1 (Zea mays) gi:1839597:gb:AAB47181 | chr2:19189794-19193648 REVERSE | Aliases: T3A4.8	5.3	5.6	-0.3	-1.2	100.0%	-4.2
5608	AT4G39520.1 | GTP-binding protein, putative, similar to SP:Q9Y295 Developmentally regulated GTP-binding protein 1 (DRG 1) {Homo sapiens}; contains Pfam profiles PF02824: TGS domain, PF01018: GTP1/OBG family	7.0	7.6	-0.7	-1.2	100.0%	-4.1
5609	AT3G48730.1 | glutamate-1-semialdehyde 2,1-aminomutase 2 (GSA 2) / glutamate-1-semialdehyde aminotransferase 2 (GSA-AT 2), identical to GSA2 (SP:Q42522) | chr3:18060608-18062687 FORWARD | Aliases: T8P19.240	8.4	9.3	-0.9	-1.2	100.0%	-3.7
5610	AT1G12120.1 | expressed protein, contains Pfam domain PF05904: Plant protein of unknown function (DUF863) | chr1:4114769-4117592 REVERSE | Aliases: T28K15.14, T28K15_14	7.5	7.9	-0.5	-1.2	100.0%	-4.2
5611	AT3G03780.2 | 5-methyltetrahydropteroyltriglutamate--homocysteine methyltransferase, putative / vitamin-B12-independent methionine synthase, putative / cobalamin-independent methionine synthase, putative, very strong similarity to SP:O50008 5-methyltetrahydropteroyltriglutamate--homocysteine methyltransferase (EC 2.1.1.14) (Vitamin-B12-independent methionine synthase isozyme) (Cobalamin-independent methionine synthase isozyme) {Arabidopsis thaliana}; contains Pfam profile PF01717: Methionine synthase, vitamin-B12 independent | chr3:957095-960985 FORWARD | Aliases: F20H23.19, F20H23_19	8.0	10.6	-2.5	-1.2	100.0%	-4.5
5612	AT5G17920.1 | Symbol: ATCIMS |	8.0	10.6	-2.5	-1.2	100.0%	-4.5
5613	AT5G47410.1 | expressed protein | chr5:19249442-19249813 FORWARD | Aliases: MQL5.27, MQL5_27	7.0	7.6	-0.7	-1.2	100.0%	-4.2
5614	AT1G18490.1 | expressed protein | chr1:6367061-6368842 FORWARD | Aliases: F15H18.26, F15H18_26	7.6	8.1	-0.5	-1.2	100.0%	-4.1
5615	AT3G57100.1 | expressed protein, similar to At14a (GI:11994571 and GI:11994573) (Arabidopsis thaliana); contains Pfam PF05055 : Protein of unknown function (DUF677); contains Prosite PS00453: FKBP-type peptidyl-prolyl cis-trans isomerase signature 1; contains one transmembrane domain | chr3:21144235-21145314 REVERSE | Aliases: F24I3.180	4.9	4.5	0.4	1.2	100.0%	-4.2
5616	AT5G64080.2 | protease inhibitor/seed storage/lipid transfer protein (LTP) family protein, contains Pfam protease inhibitor/seed storage/LTP family domain PF00234 | chr5:25662489-25663954 REVERSE | Aliases: MHJ24.6, MHJ24_6	7.1	7.9	-0.8	-1.2	100.0%	-3.5
5617	AT5G42280.1 | DC1 domain-containing protein, contains Pfam profile PF03107: DC1 domain	2.9	3.1	-0.2	-1.2	100.0%	-4.9
5618	AT5G24650.1 | mitochondrial import inner membrane translocase subunit Tim17/Tim22/Tim23 family protein, contains Pfam PF02466: Mitochondrial import inner membrane translocase subunit Tim17 | chr5:8437088-8439107 FORWARD | Aliases: K18P6.19, K18P6_19	7.1	7.9	-0.8	-1.2	100.0%	-3.9
5619	AT3G16640.1 | Symbol: TCTP | translationally controlled tumor family protein, similar to translationally controlled tumor protein GB:AAD10032 from (Hevea brasiliensis) | chr3:5669379-5670823 REVERSE | Aliases: MGL6.19, TRANSLATIONALLY CONTROLLED TUMOR PROTEIN	10.9	10.5	0.4	1.2	100.0%	-4.3
5620	AT5G66360.2 | ribosomal RNA adenine dimethylase family protein, similar to SP:P41819 Dimethyladenosine transferase (EC 2.1.1.-) (S-adenosylmethionine-6-N', N'-adenosyl(rRNA) dimethyltransferase) {Saccharomyces cerevisiae}; contains Pfam profile PF00398: ribosomal RNA adenine dimethylase family protein	5.9	6.4	-0.5	-1.2	100.0%	-4.1
5621	AT5G16320.1 | Symbol: FRL1 | expressed protein | chr5:5344505-5346022 FORWARD | Aliases: FRIGIDA LIKE 1, MQK4.4, MQK4_4	4.1	3.8	0.2	1.2	100.0%	-4.5
5622	AT2G32590.1 | barren family protein, low similarity to SP:Q9Y7R3 Condensin complex subunit 2 (p105) {Schizosaccharomyces pombe}; contains Pfam profile PF05786: Barren protein	4.4	4.8	-0.5	-1.2	100.0%	-4.3

Rank	Description	15.dpi	5.dpi	M	t	adj.q	B
5623	AT4G33500.1 | protein phosphatase 2C-related / PP2C-related, YHR077c (NMD2,IFS1) protein -Saccharomyces cerevisiae,PID:g555939	6.5	6.1	0.4	1.2	100.0%	-4.0
5624	AT1G10700.1 | ribose-phosphate pyrophosphokinase 3 / phosphoribosyl diphosphate synthetase 3 (PRS3), nearly identical to phosphoribosyl diphosphate synthase GI:4902470 from (Arabidopsis thaliana) | chr1:3554116-3556504 FORWARD | Aliases: T16B5.16	5.3	5.7	-0.3	-1.2	100.0%	-4.5
5625	AT2G25660.1 | Symbol: EMB2410 | expressed protein | chr2:10923281-10934468 REVERSE | Aliases: EMB2410, EMBRYO DEFECTIVE 2410, F3N11.11, F3N11_11	4.8	5.1	-0.3	-1.2	100.0%	-4.4
5626	AT3G54770.2 | similar to RNA recognition motif (RRM)-containing protein [Arabidopsis thaliana] (TAIR:At1g20880.1); similar to putative RRM-containing protein [Oryza sativa (japonica cultivar-group)] (GB:BAD37252.1); contains InterPro domain RNA-binding region RNP-1 (RNA recognition motif) (InterPro:IPR000504) | chr3:20284762-20286802 REVERSE | Aliases: T5N23.130	2.3	2.6	-0.2	-1.2	100.0%	-4.3
5627	AT3G02790.1 | zinc finger (C2H2 type) family protein, contains Pfam profile: PF00096 zinc finger, C2H2 type | chr3:604852-605428 FORWARD | Aliases: F13E7.27, F13E7_27	11.6	11.3	0.3	1.2	100.0%	-4.2
5628	AT5G14900.1 | helicase associated (HA2) domain-containing protein, similar to SP:P53131 Pre-mRNA splicing factor RNA helicase PRP43 (Helicase JA1) {Saccharomyces cerevisiae}; contains Pfam profile PF04408: Helicase associated domain (HA2)	2.0	2.1	-0.1	-1.2	100.0%	-5.4
5629	AT4G22560.1 | expressed protein | chr4:11880189-11880983 FORWARD | Aliases: F7K2.140, F7K2_140	5.2	4.6	0.5	1.2	100.0%	-4.1
5630	AT1G67500.1 | Symbol: ATREV3 |	6.8	7.3	-0.4	-1.2	100.0%	-4.3
5631	AT2G40935.2 | expressed protein, low similarity to PGPS/D12 (Petunia x hybrida) GI:4105794; contains Pfam profile PF04749: Protein of unknown function, DUF614	8.3	7.9	0.4	1.2	100.0%	-4.1
5632	AT3G62950.1 | glutaredoxin family protein, contains INTERPRO Domain IPR002109, Glutaredoxin (thioltransferase)	2.3	2.2	0.2	1.2	100.0%	-4.8
5633	AT5G66680.1 | Symbol: DGL1 | dolichyl-diphosphooligosaccharide-protein glycosyltransferase 48kDa subunit family protein, similar to SP:Q05052 Dolichyl-diphosphooligosaccharide--protein glycosyltransferase 48 kDa subunit precursor (EC 2.4.1.119) (Oligosaccharyl transferase 48 kDa subunit) {Canis familiaris}; contains Pfam profile PF03345: Dolichyl-diphosphooligosaccharide-protein glycosyltransferase 48kD subunit | chr5:26634770-26637893 REVERSE | Aliases: MSN2.7, MSN2_7	10.4	10.7	-0.4	-1.2	100.0%	-4.4
5634	AT2G35320.1 | Symbol: ATEYA | homologue of the animal Eys Absent genes. encodes a tyrosine-specific phosphatase. the protein sequence lacks the cys-containing signature of the classical tyrosine phosphatases. belongs to the aspartate-based phosphatases. The enzyme activity is strictly metal-dependent. | chr2:14873911-14875939 REVERSE | Aliases: ATEYA, T4C15.1, T4C15_1	6.0	6.3	-0.3	-1.2	100.0%	-4.2
5635	AT5G05250.1 | expressed protein | chr5:1557038-1557999 REVERSE | Aliases: K18I23.5, K18I23_5	8.4	7.8	0.6	1.2	100.0%	-4.0
5636	AT3G53140.1 | O-diphenol-O-methyl transferase, putative, similar to GI:6688808 (Medicago sativa subsp. x varia), caffeic acid O-methyltransferase (homt1), Populus kitakamiensis, EMBL:PKHOMT1A | chr3:19706621-19708520 FORWARD | Aliases: T4D2.70	3.4	3.7	-0.3	-1.2	100.0%	-4.6
5637	AT3G50560.1 | short-chain dehydrogenase/reductase (SDR) family protein, contains INTERPRO family IPR002198 short-chain dehydrogenase/reductase (SDR) superfamily | chr3:18772069-18774994 REVERSE | Aliases: T20E23.160	3.7	3.4	0.3	1.2	100.0%	-4.4
5638	AT5G61480.1 | leucine-rich repeat transmembrane protein kinase, putative | chr5:24741767-24745068 REVERSE | Aliases: MCI2.4, MCI2_4	5.4	5.7	-0.3	-1.2	100.0%	-4.5
5639	AT3G48425.1 | endonuclease/exonuclease/phosphatase family protein, similar to SP:P51173:APEA_DICDI DNA-(apurinic or apyrimidinic site) lyase (EC 4.2.99.18)(Class II apurinic/apyrimidinic(AP)-endonuclease) {Dictyostelium discoideum}; contains Pfam profile PF03372: Endonuclease/Exonuclease/phosphatase family | chr3:17942803-17945729 FORWARD | Aliases: None	5.0	5.4	-0.3	-1.2	100.0%	-4.1
5640	AT5G02220.1 | expressed protein | chr5:441705-442144 REVERSE | Aliases: T7H20.270, T7H20_270	6.3	5.7	0.6	1.2	100.0%	-3.9
5641	AT1G50670.1 | OTU-like cysteine protease family protein, contains Pfam profile PF02338: OTU-like cysteine protease | chr1:18778532-18780621 REVERSE | Aliases: F17J6.19, F17J6_19	9.3	9.7	-0.3	-1.2	100.0%	-4.6
5642	AT3G55760.2 | expressed protein | chr3:20711414-20714035 FORWARD | Aliases: F1I16.170	7.1	6.4	0.7	1.2	100.0%	-4.2
5643	AT5G65720.1 | cysteine desulfurase, mitochondrial (NIFS), identical to Cysteine desulfurase, mitochondrial precursor (SP:O49543) {Arabidopsis thaliana}; identical to cDNA GI:12656131; contains Pfam profile PF00266: aminotransferase, class V | chr5:26313479-26315298 FORWARD | Aliases: MPA24.7, MPA24_7	8.7	8.3	0.4	1.2	100.0%	-4.3
5644	AT5G24840.1 | expressed protein, contains Pfam profile PF02390: Putative methyltransferase | chr5:8533790-8535046 REVERSE | Aliases: F6A4.50, F6A4_50	7.7	8.1	-0.4	-1.2	100.0%	-3.9

Rank	Description	15.dpi	5.dpi	M	t	adj.q	B
5645	AT4G13235.1 | Encodes a defensin-like (DEFL) family protein. | chr4:7677520-7678243 REVERSE | Aliases: None	7.2	7.4	-0.2	-1.2	100.0%	-4.6
5646	AT5G57720.1 | transcriptional factor B3 family protein, contains Pfam profile PF02362: B3 DNA binding domain | chr5:23407153-23408752 REVERSE | Aliases: MRI1.8, MRI1_8	2.7	2.4	0.2	1.2	100.0%	-4.6
5647	AT4G35190.1 | expressed protein, contains Pfam profile PF03641: decarboxylase family protein | chr4:16746604-16748266 FORWARD | Aliases: T12J5.60, T12J5_60	4.4	4.7	-0.3	-1.2	100.0%	-4.3
5648	AT1G77350.2 | similar to Krtcap2 protein [Mus musculus] (GB:AAH43030.1); similar to keratinocytes associated protein 2 [Mus musculus] (GB:NP_079603.1); similar to ENSANGP00000016671 [Anopheles gambiae str. PEST] (GB:XP_312812.2) | chr1:29075214-29076793 FORWARD | Aliases: F2P24.6, F2P24_6	9.4	9.0	0.5	1.2	100.0%	-4.3
5649	AT3G62840.1 | small nuclear ribonucleoprotein D2, putative / snRNP core protein D2, putative / Sm protein D2, putative, similar to small nuclear ribonucleoprotein Sm D2 (snRNP core protein D2) (Sm-D2) (Mus musculus) SWISS-PROT:P43330 | chr3:23246086-23247791 REVERSE | Aliases: F26K9.270	10.9	11.2	-0.3	-1.2	100.0%	-4.3
5650	AT3G26590.1 | MATE efflux family protein, similar to ripening regulated protein DDTFR18 (Lycopersicon esculentum) GI:12231296; contains Pfam profile: PF01554 uncharacterized membrane protein family | chr3:9762938-9766552 REVERSE | Aliases: MFE16.12	4.7	4.9	-0.3	-1.2	100.0%	-4.6
5651	AT2G45700.1 | sterile alpha motif (SAM) domain-containing protein, similar to SNM1 protein (Mus musculus) GI:7595835; contains Pfam profile PF00536: SAM domain (Sterile alpha motif) | chr2:18834074-18837600 REVERSE | Aliases: F4I18.32	5.2	5.5	-0.3	-1.2	100.0%	-4.0
5652	AT2G46360.1 | expressed protein | chr2:19035561-19036088 FORWARD | Aliases: F11C10.5	2.4	2.2	0.1	1.2	100.0%	-5.2
5653	AT3G50150.1 | expressed protein, contains Pfam profile PF03140: Plant protein of unknown function; expression supported by MPSS | chr3:18606792-18608534 REVERSE | Aliases: F3A4.230	2.0	2.1	-0.1	-1.2	100.0%	-5.3
5654	AT4G25500.2 | Symbol: ATRSP35 | arginine/serine-rich splicing factor RSP40 (RSP40), identical to SP:P92965 Arginine/serine-rich splicing factor RSP40 {Arabidopsis thaliana} | chr4:13026028-13027422 FORWARD | Aliases: ARGININE/SERINE RICH SPLICING FACTOR, ATRSP40, SPLICING FACTOR AT SRP40, T30C3.9	7.9	7.3	0.6	1.2	100.0%	-4.0
5655	AT3G18680.2 | aspartate/glutamate/uridylate kinase family protein, similar to UMP-kinase GB:CAB38122 gi:4468612 from (Lactococcus lactis) ; contains Pfam profile PF00696: Amino acid kinase family | chr3:6427413-6429658 FORWARD | Aliases: MVE11.4	6.8	7.3	-0.6	-1.2	100.0%	-3.7
5656	AT5G47320.1 | Symbol: RPS19 | 30S ribosomal protein S19, mitochondrial (RPS19) | chr5:19220379-19222499 FORWARD | Aliases: MQL5.18, MQL5_18	8.3	8.9	-0.5	-1.2	100.0%	-4.0
5657	AT5G65710.1 | leucine-rich repeat transmembrane protein kinase, putative | chr5:26309598-26312666 FORWARD | Aliases: MPA24.6, MPA24_6	5.9	6.4	-0.5	-1.2	100.0%	-3.8
5658	AT1G06810.1 | expressed protein, contains Pfam PF04396: Protein of unknown function, DUF537 | chr1:2091920-2092527 REVERSE | Aliases: F4H5.25, F4H5_25	3.9	4.1	-0.3	-1.2	100.0%	-4.6
5659	AT4G18890.1 | brassinosteroid signalling positive regulator-related, contains similarity to BZR1 protein (Arabidopsis thaliana) gi:20270971:gb:AAM18490 | chr4:10352633-10355566 FORWARD | Aliases: F13C5.60, F13C5_60	3.4	3.1	0.2	1.2	100.0%	-4.4
5660	AT5G66530.1 | aldose 1-epimerase family protein, similar to apospory-associated protein C; APOC (Chlamydomonas reinhardtii) GI:6970044; contains Pfam profile PF01263: Aldose 1-epimerase | chr5:26570973-26572902 REVERSE | Aliases: K1F13.19, K1F13_19	8.9	9.4	-0.5	-1.2	100.0%	-4.0
5661	AT3G04570.1 | DNA-binding protein-related, contains Pfam domain PF03479: Domain of unknown function (DUF296), found in AT-hook motifs Pfam:PF02178 | chr3:1230992-1232334 FORWARD | Aliases: F7O18.4, F7O18_4	5.4	5.0	0.4	1.2	100.0%	-4.1
5662	AT5G61890.1 | encodes a member of the ERF (ethylene response factor) subfamily B-4 of ERF/AP2 transcription factor family. The protein contains one AP2 domain. There are 7 members in this subfamily.	4.5	4.9	-0.4	-1.2	100.0%	-3.8
5663	AT2G30950.1 | Symbol: VAR2 | FtsH protease (VAR2), identical to zinc dependent protease VAR2 GI:7650138 from (Arabidopsis thaliana) | chr2:13181402-13184300 FORWARD | Aliases: F7F1.16, F7F1_16, FTSH2, VAR2, VARIEGATED 2, YELLOW VARIEGATED	10.3	9.8	0.5	1.2	100.0%	-4.3
5664	AT5G65260.1 | polyadenylate-binding protein family protein / PABP family protein, low similarity to poly(A)-binding protein II (Drosophila melanogaster) GI:6007612; contains InterPro entry IPR000504: RNA-binding region RNP-1 (RNA recognition motif) (RRM) domain | chr5:26097457-26100243 REVERSE | Aliases: MQN23.21, MQN23_21	8.0	7.4	0.6	1.2	100.0%	-4.2
5665	AT2G25490.1 | Symbol: EBF1 | F-box family protein (FBL6), contains similarity to grr1 GI:2407790 from (Glycine max) | chr2:10854461-10857606 REVERSE | Aliases: EIN3 BINDING F BOX PROTEIN 1, F13B15.15, F13B15_15, FBL6	3.8	3.3	0.5	1.2	100.0%	-4.2

Rank	Description	15.dpi	5.dpi	M	t	adj.q	B
5666	AT2G25940.1 | Symbol: ALPHA VPE | vacuolar processing enzyme alpha / alpha-VPE, identical to SP:P49047 Vacuolar processing enzyme, alpha-isozyme precursor (EC 3.4.22.-) (Alpha-VPE) {Arabidopsis thaliana} | chr2:11069798-11073106 REVERSE | Aliases: ALPHAVPE, F17H15.4	5.2	4.6	0.7	1.2	100.0%	-3.8
5667	AT5G55130.2 | Symbol: CNX5 | similar to SUMO activating enzyme 2 (SAE2) [Arabidopsis thaliana] (TAIR:At2g21470.1); similar to SUMO activating enzyme 2 (SAE2) [Arabidopsis thaliana] (TAIR:At2g21470.2); similar to unnamed protein product [Tetraodon nigroviridis] (GB:CAG09882.1); contains InterPro domain UBA/THIF-type NAD/FAD binding fold (InterPro:IPR000594); contains InterPro domain Rhodanese-like (InterPro:IPR001763); contains InterPro domain MoeZ/MoeB (InterPro:IPR007901)	6.4	6.8	-0.4	-1.2	100.0%	-4.3
5668	AT5G59960.1 | expressed protein | chr5:24159240-24162559 REVERSE | Aliases: MMN10.21, MMN10_21	5.6	5.1	0.5	1.2	100.0%	-4.2
5669	AT1G16340.2 | similar to 2-dehydro-3-deoxyphosphooctonate aldolase / phospho-2-dehydro-3-deoxyoctonate aldolase / 3-deoxy-D-manno-octulosonic acid 8-phosphate synthetase (KDSA) [Arabidopsis thaliana] (TAIR:At1g79500.1); similar to 2-dehydro-3-deoxyphosphooctonate aldolase / phospho-2-dehydro-3-deoxyoctonate aldolase / 3-deoxy-D-manno-octulosonic acid 8-phosphate synthetase (KDSA) [Arabidopsis thaliana] (TAIR:At1g79500.3); similar to 2-dehydro-3-deoxyphosphooctonate aldolase / phospho-2-dehydro-3-deoxyoctonate aldolase / 3-deoxy-D-manno-octulosonic acid 8-phosphate synthetase (KDSA) [Arabidopsis thaliana] (TAIR:At1g79500.2); similar to 3-desoxy-D-manno octulosonic acid-8-phosphate synthase [Lycopersicon esculentum] (GB:CAC35366.1); similar to putative 2-dehydro-3-deoxyphosphooctonate aldolase [Oryza sativa (japonica cultivar-group)] (GB:AAO72599.1); contains InterPro domain DAHP synthetase I/KDSA superfamily (InterPro:IPR006218); contains InterPro domain 2-dehydro-3-deoxyphosphooctonate aldolase (InterPro:IPR006269)	8.9	9.5	-0.6	-1.2	100.0%	-3.8
5670	AT4G11150.1 | Symbol: TUF | vacuolar ATP synthase subunit E / V-ATPase E subunit / vacuolar proton pump E subunit (VATE), identical to SP:Q39258 Vacuolar ATP synthase subunit E (EC 3.6.3.14) (V-ATPase E subunit) (Vacuolar proton pump E subunit) {Arabidopsis thaliana} | chr4:6799958-6801927 FORWARD | Aliases: EMB2448, EMBRYO DEFECTIVE 2448, T22B4.130, T22B4_130, TUFF, VACUOLAR H+ ATPASE SUBUNIT E ISOFORM 1, VHA E1	10.3	10.7	-0.4	-1.2	100.0%	-4.1
5671	AT1G11800.1 | endonuclease/exonuclease/phosphatase family protein, contains Pfam profile PF03372: Endonuclease/Exonuclease/phosphatase family | chr1:3985224-3987289 REVERSE | Aliases: F25C20.3, F25C20_3	5.8	6.2	-0.4	-1.2	100.0%	-4.0
5672	AT5G27610.1 | similar to myb family transcription factor [Arabidopsis thaliana] (TAIR:At3g05380.1); similar to P0671B11.29 [Oryza sativa (japonica cultivar-group)] (GB:NP_914584.1); contains InterPro domain Myb DNA-binding domain (InterPro:IPR001005) | chr5:9764138-9769906 FORWARD | Aliases: F15A18.70, F15A18_70	3.9	3.7	0.2	1.2	100.0%	-4.6
5673	AT3G05690.1 | Symbol: HAP2B | CCAAT-binding transcription factor (CBF-B/NF-YA) family protein, contains Pfam profile: PF02045 CCAAT-binding transcription factor (CBF-B/NF-YA) subunit B | chr3:1676552-1678938 REVERSE | Aliases: ATHAP2B, F18C1.4, F18C1_4, HAP2	4.9	5.4	-0.5	-1.2	100.0%	-4.1
5674	AT2G23320.2 | Symbol: WRKY15 |	5.3	5.7	-0.4	-1.2	100.0%	-4.0
5675	AT3G14520.1 | terpene synthase/cyclase family protein, similar to terpene synthase GB:CAA72074 from (Arabidopsis thaliana) | chr3:4875755-4878340 REVERSE | Aliases: MIE1.2	3.0	3.1	-0.2	-1.2	100.0%	-4.9
5676	AT5G23760.1 | heavy-metal-associated domain-containing protein, Pfam profile PF00403: Heavy-metal-associated domain | chr5:8013090-8014231 REVERSE | Aliases: MRO11.20, MRO11_20	9.8	9.4	0.4	1.2	100.0%	-4.2
5677	AT4G25740.2 | similar to 40S ribosomal protein S10 (RPS10C) [Arabidopsis thaliana] (TAIR:At5g52650.1); similar to RS10_ORYSA 40S ribosomal protein S10 (GB:Q9AYP4); contains InterPro domain Plectin/S10, N-terminal (InterPro:IPR005326) | chr4:13107273-13108822 REVERSE | Aliases: F14M19.20, F14M19_20	9.7	10.2	-0.5	-1.2	100.0%	-3.9
5678	AT2G44230.1 | expressed protein | chr2:18293601-18295476 FORWARD | Aliases: F4I1.4	2.9	3.2	-0.2	-1.2	100.0%	-4.5
5679	AT3G14880.1 | DNA-binding protein-related, low similarity to tumor-related protein (Nicotiana glauca x Nicotiana langsdorffii) GI:688423, SP:O24160 TGACG-sequence specific DNA-binding protein TGA-2.1 {Nicotiana tabacum} | chr3:5006572-5007696 FORWARD | Aliases: K15M2.2	4.7	4.4	0.3	1.2	100.0%	-4.2
5680	AT3G19510.1 | homeobox protein (HAT 3.1), identical to homeotic protein HAT 3.1 (GI:11994474) (Arabidopsis thaliana) | chr3:6762770-6766570 REVERSE | Aliases: MLD14.37	4.6	5.1	-0.6	-1.2	100.0%	-4.2
5681	AT5G02500.1 | Symbol: HSC70 1 | heat shock cognate 70 kDa protein 1 (HSC70-1) (HSP70-1), identical to SP:P22953 Heat shock cognate 70 kDa protein 1 (Hsc70.1) {Arabidopsis thaliana} | chr5:553743-556437 REVERSE | Aliases: AT HSC70 1, HSC70, HSP70 1, T22P11.90, T22P11_90	10.9	11.2	-0.3	-1.2	100.0%	-4.7
5682	AT1G70290.1 | Symbol: ATTPS8 |	6.5	6.1	0.4	1.2	100.0%	-4.2
5683	AT4G23990.1 | Symbol: ATCSLG3 | cellulose synthase family protein, similar to cellulose synthase catalytic subunit from Arabidopsis thaliana (gi:5230423), cellulose synthase-5 from Zea mays (gi:9622882) | chr4:12456534-12460763 FORWARD | Aliases: CSLG3, T32A16.160, T32A16_160	6.0	5.4	0.6	1.2	100.0%	-3.8
5684	AT3G25020.1 | disease resistance family protein, contains leucine rich-repeat (LRR) domains Pfam:PF00560, INTERPRO:IPR001611; similar to Hcr2-0B (Lycopersicon esculentum) gi:3894387:gb:AAC78593 | chr3:9116875-9119547 REVERSE | Aliases: K3G3.7	3.0	3.2	-0.2	-1.2	100.0%	-4.6

Rank	Description	15.dpi	5.dpi	M	t	adj.q	B
5685	AT5G62000.3 | Symbol: ARF2 | transcriptional factor B3 family protein / auxin-responsive factor, putative (ARF1), contains Pfam profile: PF02362 B3 DNA binding domain; identical to cDNA ARF1 (auxin response factor) binding protein GI:2245393 | chr5:24927608-24932431 FORWARD | Aliases: ARF1 BINDING PROTEIN, ARF1 BP, AT5G62010, AUXIN RESPONSE FACTOR 2, HLS1 SUPPRESSOR, HSS, MTG10.1, MTG10_1	4.5	4.2	0.2	1.2	100.0%	-4.3
5686	AT5G11540.1 | FAD-binding domain-containing protein, similar to hypothetical protein (Oryza sativa (japonica))(GI:18844891); contains PF01565 FAD binding domain | chr5:3703003-3704982 REVERSE | Aliases: F15N18.130, F15N18_130	5.0	5.3	-0.3	-1.2	100.0%	-4.3
5687	AT1G62350.1 | pentatricopeptide (PPR) repeat-containing protein, contains Pfam profile PF01535: PPR repeat | chr1:23060318-23061600 FORWARD | Aliases: F24O1.37, F24O1_37	3.6	3.9	-0.4	-1.2	100.0%	-4.4
5688	AT2G04830.1 | F-box family protein, similar to F-box protein family, AtFBX7 (GI:20197899) (Arabidopsis thaliana); confirmed by FLcDNA GI:16604421; contains uncharacterized Arabidoppsis domain shared by 33 Arabidopsis proteins | chr2:1695352-1695853 FORWARD | Aliases: F28I8.13, F28I8_13	2.7	2.8	-0.1	-1.2	100.0%	-5.1
5689	AT1G30290.1 | pentatricopeptide (PPR) repeat-containing protein, contains Pfam profile PF01535: PPR repeat | chr1:10670304-10672722 REVERSE | Aliases: F12P21.10, F12P21_10	6.0	6.4	-0.4	-1.2	100.0%	-4.0
5690	AT5G02710.1 | expressed protein, contains Pfam PF03692: Uncharacterised protein family (UPF0153) | chr5:612606-614418 FORWARD | Aliases: F9G14.8	3.7	3.3	0.3	1.2	100.0%	-4.2
5691	AT1G36730.1 | eukaryotic translation initiation factor 5, putative / eIF-5, putative, similar to SP:P55876 Eukaryotic translation initiation factor 5 (eIF-5) {Zea mays}; contains Pfam profiles PF02020: eIF4-gamma/eIF5/eIF2-epsilon, PF01873: Domain found in IF2B/IF5 | chr1:13899422-13902025 REVERSE | Aliases: T15P17.7, T15P17_7	6.1	5.4	0.7	1.2	100.0%	-3.9
5692	AT3G09100.2 | mRNA capping enzyme family protein, similar to mRNA capping enzyme (Xenopus laevis) GI:7239232; contains Pfam profiles PF01331: mRNA capping enzyme catalytic domain, PF00782: Dual specificity phosphatase catalytic domain | chr3:2787986-2793537 REVERSE | Aliases: MZB10.13	4.9	5.2	-0.3	-1.2	100.0%	-4.5
5693	AT2G20940.1 | expressed protein | chr2:9009113-9009782 REVERSE | Aliases: F26H11.30, F26H11_30	6.2	6.9	-0.7	-1.2	100.0%	-3.7
5694	AT2G32560.1 | F-box family protein, contains Pfam PF00646: F-box domain; similar to SKP1 interacting partner 2 (SKIP2) TIGR_Ath1:At5g67250 | chr2:13831871-13834063 FORWARD | Aliases: AT2G32570, T26B15.12, T26B15_12	3.1	2.9	0.2	1.2	100.0%	-5.1
5695	AT5G45410.2 | expressed protein, similar to expressed protein [Arabidopsis thaliana] (TAIR:At4g25030.1); similar to expressed protein [Arabidopsis thaliana] (TAIR:At4g25030.2); similar to unknown protein [Oryza sativa (japonica cultivar-group)] (GB:XP_476693.1) | chr5:18419135-18421010 REVERSE | Aliases: MFC19.8, MFC19_8	9.6	8.2	1.4	1.2	100.0%	-3.8
5696	AT3G15110.1 | expressed protein | chr3:5084295-5085836 REVERSE | Aliases: F4B12.2	4.2	3.9	0.3	1.2	100.0%	-4.3
5697	AT3G26330.1 | Symbol: CYP71B37 | similar to cytochrome P450 family protein [Arabidopsis thaliana] (TAIR:At3g26300.1); similar to cytochrome P450 family protein [Arabidopsis thaliana] (TAIR:At3g26310.1); similar to cytochrome P450 71B26, putative (CYP71B26) [Arabidopsis thaliana] (TAIR:At3g26290.1); similar to cytochrome P450 family protein [Arabidopsis thaliana] (TAIR:At2g02580.1); similar to cytochrome P450 71B10 [Arabidopsis thaliana] (TAIR:At5g57260.1); similar to cytochrome P450 [Citrus sinensis] (GB:AAL24049.1); contains InterPro domain E-class P450, group I (InterPro:IPR002401); contains InterPro domain Cytochrome P450 (InterPro:IPR001128)	5.0	4.6	0.4	1.2	100.0%	-4.0
5698	AT5G03940.1 | Symbol: FFC | signal recognition particle 54 kDa protein, chloroplast / 54 chloroplast protein / SRP54 (FFC), identical to Swiss-Prot:P37107 signal recognition particle 54 kDa protein, chloroplast precursor (SRP54) (54 chloroplast protein) (54CP) (FFC) (Arabidopsis thaliana) | chr5:1059503-1063318 REVERSE | Aliases: 54CP, CPSRP54, F8F6.150, F8F6_150, FIFTY FOUR CHLOROPLAST HOMOLOGUE, SIGNAL RECOGNITION PARTICLE 54 KDA SUBUNIT, SRP54CP	7.7	8.3	-0.6	-1.2	100.0%	-4.1
5699	AT3G14840.2 | leucine-rich repeat family protein / protein kinase family protein, contains Pfam domains PF00560: Leucine Rich Repeat and PF00069: Protein kinase domain; contains 2 predicted transmembrane domains | chr3:4988278-4994065 FORWARD | Aliases: T21E2.2	6.6	7.0	-0.4	-1.2	100.0%	-4.2
5700	AT2G27285.1 | expressed protein, weak similarity to maebl (GI:20087019) (Plasmodium falciparum), chimeric erythrocyte-binding protein MAEBL (GI:22086284) (Plasmodium falciparum) | chr2:11683038-11685178 REVERSE | Aliases: None	8.7	9.4	-0.7	-1.2	100.0%	-4.2
5701	AT2G21060.1 | Symbol: ATGRP2B | cold-shock DNA-binding family protein / glycine-rich protein (GRP2), identical to Glycine-rich protein 2b (AtGRP2b) (Arabidopsis thaliana) SWISS-PROT:Q38896; contains Pfam domains PF00313: 'Cold-shock' DNA-binding domain and PF00098: Zinc knuckle | chr2:9043874-9044731 REVERSE | Aliases: F26H11.18, F26H11_18, GLYCINE RICH PROTEIN 2B	8.4	7.9	0.6	1.2	100.0%	-4.1
5702	AT5G01020.1 | protein kinase family protein, contains protein kinase domain, Pfam:PF00069 | chr5:5916-8443 REVERSE | Aliases: F7J8.5, F7J8_5	7.7	7.1	0.5	1.2	100.0%	-4.2
5703	AT1G44120.1 | C2 domain-containing protein / armadillo/beta-catenin repeat family protein, similar to CCLS 65 (Silene latifolia) GI:2570102; contains Pfam profiles PF00514: Armadillo/beta-catenin-like repeat, PF00168: C2 domain | chr1:16783050-16789854 FORWARD | Aliases: T7O23.25, T7O23_25	3.0	2.8	0.1	1.2	100.0%	-4.9

Rank	Description	15.dpi	5.dpi	M	t	adj.q	B
5704	AT2G41080.1 | pentatricopeptide (PPR) repeat-containing protein, contains INTERPRO:IPR002885 PPR repeats | chr2:17139931-17141671 FORWARD | Aliases: T3K9.15, T3K9_15	3.5	3.7	-0.2	-1.2	100.0%	-4.4
5705	AT4G30530.1 | defense-related protein, putative, strong similarity to defense-related protein (Brassica carinata) GI:14009290; contains Pfam profile PF00117: glutamine amidotransferase class-I | chr4:14920540-14922464 FORWARD | Aliases: F17I23.130, F17I23_130	8.1	8.8	-0.7	-1.2	100.0%	-4.0
5706	AT4G29910.1 | origin recognition complex subunit 5-related (ORC5), contains weak similarity to Origin recognition complex subunit 5 (Swiss-Prot:Q9WUV0) (Mus musculus)	4.6	5.0	-0.4	-1.2	100.0%	-3.9
5707	AT3G62240.1 | zinc finger (C2H2 type) family protein, contains Pfam PF00096: Zinc finger, C2H2 type | chr3:23044417-23047740 REVERSE | Aliases: T17J13.200	6.9	7.5	-0.6	-1.2	100.0%	-4.0
5708	AT2G15440.1 | expressed protein, contains Pfam profile PF04669: Protein of unknown function (DUF579) | chr2:6750695-6751912 REVERSE | Aliases: F26H6.4, F26H6_4	5.2	5.0	0.3	1.2	100.0%	-4.4
5709	AT3G47670.1 | similar to pectinesterase family protein [Arabidopsis thaliana] (TAIR:At1g53840.1); similar to pectin methylesterase [Lycopersicon esculentum] (GB:AAL02367.1); contains InterPro domain Plant invertase/pectin methylesterase inhibitor (InterPro:IPR007186); contains InterPro domain Pectinesterase inhibitor (InterPro:IPR006501) | chr3:17585770-17586865 REVERSE | Aliases: F1P2.220	4.0	3.7	0.3	1.2	100.0%	-4.4
5710	AT4G39850.1 | Symbol: PXA1 | peroxisomal ABC transporter (PXA1), identical to peroxisomal ABC transporter PXA1 GI:15320529 from (Arabidopsis thaliana); contains Pfam profile PF00005: ABC transporter; | chr4:18488513-18497130 FORWARD | Aliases: COMATOSE, CTS, PED3, PEROXISOMAL ABC TRANSPORTER 1, PEROXISOME DEFECTIVE 3, T5J17.20, T5J17_20	6.7	7.2	-0.4	-1.2	100.0%	-4.2
5711	AT4G23910.1 | expressed protein, various predicted proteins, Arabidopsis thaliana | chr4:12426718-12429297 FORWARD | Aliases: T32A16.80, T32A16_80	5.6	5.1	0.4	1.2	100.0%	-4.1
5712	AT1G68100.1 | Symbol: IAR1 | IAA-alanine resistance protein 1, putative, similar to IAA-alanine resistance protein 1 (Arabidopsis thaliana) SWISS-PROT:Q9M647; contains ZIP Zinc transporter domain, Pfam:PF02535; identical to cDNA IAA-alanine resistance protein 1 mRNA GI:6942042	6.1	5.8	0.3	1.2	100.0%	-4.1
5713	AT1G48050.1 | Symbol: KU80 | Ku80 family protein, identical to Ku80-like protein (Arabidopsis thaliana) GI:12006422; contains Pfam profiles: PF02197 Regulatory subunit of type II PKA R-subunit, PF02735: Ku70/Ku80 beta-barrel domain, PF03731: Ku70/Ku80 N-terminal alpha/beta domain, PF03730: Ku70/Ku80 C-terminal arm | chr1:17727121-17730712 FORWARD | Aliases: ATKU80, KU80 LIKE PROTEIN, T2J15.4	5.4	5.7	-0.3	-1.2	100.0%	-4.2
5714	AT3G27700.2 | RNA recognition motif (RRM)-containing protein, contains Pfam profile: PF00076 RNA recognition motif | chr3:10258354-10263211 REVERSE | Aliases: MGF10.11	4.1	4.4	-0.3	-1.2	100.0%	-4.4
5715	AT1G50660.1 | expressed protein, similar to liver stage antigen-1 (GI:510184) (Plasmodium falciparum); similar to Myosin II heavy chain, non muscle (Swiss-Prot:P08799) (Dictyostelium discoideum); similar to liver stage antigen (GI:9916) (Plasmodium falciparum); similar to Kinesin-like protein KLPA (Swiss-Prot:P28739) (Emericella nidulans) | chr1:18774605-18778354 FORWARD | Aliases: F17J6.18, F17J6_18	3.9	4.3	-0.4	-1.2	100.0%	-3.9
5716	AT5G51970.2 | sorbitol dehydrogenase, putative / L-iditol 2-dehydrogenase, putative, similar to NAD-dependent sorbitol dehydrogenase from Malus x domestica (gi:4519539) | chr5:21128820-21130629 FORWARD | Aliases: MSG15.7, MSG15_7	12.4	11.9	0.5	1.2	100.0%	-4.3
5717	AT1G71220.1 | UDP-glucose:glycoprotein glucosyltransferase, putative, similar to UDP-glucose:glycoprotein glucosyltransferase precursor GB:Q09332 (SP:Q09332) from Drosophila melanogaster, (gi:7670746) and (gi:11346464) from Homo sapiens | chr1:26845326-26855583 FORWARD | Aliases: F3I17.13, F3I17_13	6.5	7.0	-0.5	-1.2	100.0%	-4.1
5718	AT1G20630.1 | Symbol: CAT1 | catalase 1, identical to catalase 1 GI:2511725 from (Arabidopsis thaliana) | chr1:7146720-7149967 FORWARD | Aliases: CATALASE 1, F5M15.31, F5M15_31	8.0	8.8	-0.9	-1.2	100.0%	-4.0
5719	ATCG00420.1 | Symbol: NDHJ | NADH dehydrogenase subunit | chrC:48677-49153 REVERSE | Aliases: NDHJ	6.8	5.8	1.0	1.2	100.0%	-3.9
5720	AT1G22170.1 | phosphoglycerate/bisphosphoglycerate mutase family protein, similar to SP:P31217 Phosphoglycerate mutase 1 (EC 5.4.2.1) {Escherichia coli O157:H7}; contains Pfam profile PF00300: phosphoglycerate mutase family | chr1:7826278-7828307 FORWARD | Aliases: F16L1.10, F16L1_10	4.8	5.1	-0.3	-1.2	100.0%	-4.4
5721	AT2G38130.2 | Symbol: ATMAK3 |	7.4	7.1	0.3	1.2	100.0%	-4.3
5722	AT3G16810.1 | pumilio/Puf RNA-binding domain-containing protein, contains Pfam profile:PF00806 Pumilio-family RNA binding domains | chr3:5723255-5727626 REVERSE | Aliases: K20I9.3	5.9	6.2	-0.3	-1.2	100.0%	-4.3

Rank	Description	15.dpi	5.dpi	M	t	adj.q	B
5723	AT4G22590.1 | trehalose-6-phosphate phosphatase, putative, similar to trehalose-6-phosphate phosphatase (AtTPPA) GI:2944178; contains Pfam profile PF02358: Trehalose-phosphatase | chr4:11893559-11896464 REVERSE | Aliases: F7K2.170, F7K2_170	5.8	5.3	0.6	1.2	100.0%	-4.0
5724	AT5G26100.1 | hypothetical protein | chr5:9117293-9117820 REVERSE | Aliases: T1N24.107, T1N24_107	2.7	2.9	-0.2	-1.2	100.0%	-4.7
5725	AT2G43160.4 | similar to epsin N-terminal homology (ENTH) domain-containing protein [Arabidopsis thaliana] (TAIR:At3g59290.1); similar to OSJNBa0083N12.8 [Oryza sativa (japonica cultivar-group)] (GB:XP_473754.1); contains InterPro domain Epsin N-terminal homology (InterPro:IPR001026) | chr2:17955139-17960798 FORWARD | Aliases: F14B2.10	5.8	5.5	0.3	1.2	100.0%	-4.3
5726	AT2G35690.1 | Symbol: ACX5 | acyl-CoA oxidase, putative, strong similarity to acyl-CoA oxidase (Arabidopsis thaliana) GI:3044214 | chr2:15006833-15010145 FORWARD | Aliases: ACYL COA OXIDASE 5, T20F21.12, T20F21_12	4.0	4.4	-0.4	-1.2	100.0%	-4.3
5727	AT1G27930.1 | expressed protein, contains Pfam profile PF04669: Protein of unknown function (DUF579) | chr1:9731252-9732706 REVERSE | Aliases: F13K9.4, F13K9_4	7.3	6.9	0.4	1.2	100.0%	-4.3
5728	AT2G47680.1 | zinc finger (CCCH type) helicase family protein, similar to SP:Q28141 ATP-dependent RNA helicase A (Nuclear DNA helicase II) (DEAD-box protein 9) {Bos taurus}; contains Pfam profiles PF00271: Helicase conserved C-terminal domain, PF00642: Zinc finger C-x8-C-x5-C-x3-H type (and similar) | chr2:19552898-19557975 REVERSE | Aliases: F17A22.7	3.6	3.8	-0.3	-1.2	100.0%	-4.4
5729	AT5G26680.1 | endonuclease, putative, similar to Swiss-Prot:P39748 FLAP endonuclease-1 (Maturation factor 1) (MF1) (Homo sapiens) | chr5:9311885-9315478 REVERSE | Aliases: None	3.9	4.3	-0.4	-1.2	100.0%	-3.9
5730	AT2G43360.1 | Symbol: BIO2 | biotin synthase (BioB) (BIO2), identical to SP:P54967 Pfam profile PF04055: radical SAM domain protein | chr2:18017828-18020315 REVERSE | Aliases: BIOB, BIOTIN AUXOTROPH 2, BIOTIN AUXOTROPH B, BIOTIN SYNTHASE, T1O24.10	12.1	11.6	0.5	1.2	100.0%	-4.1
5731	AT2G38690.1 | expressed protein | chr2:16183554-16184741 REVERSE | Aliases: T6A23.11, T6A23_11	2.4	2.6	-0.2	-1.2	100.0%	-4.8
5732	AT5G50340.1 | DNA repair protein-related, similar to RadA (Pseudomonas aeruginosa) GI:1881700 | chr5:20508643-20512982 REVERSE | Aliases: MXI22.5, MXI22_5	4.0	3.8	0.3	1.2	100.0%	-4.3
5733	AT2G25800.1 | expressed protein | chr2:11012980-11016817 REVERSE | Aliases: F17H15.17, F17H15_17	4.6	5.1	-0.4	-1.2	100.0%	-4.2
5734	AT3G63150.1 | GTP-binding protein-related, low similarity to SP:Q38912 RAC-like GTP binding protein ARAC3 (GTP-binding protein ROP6) {Arabidopsis thaliana}; contains Pfam profile PF00036: EF hand (domain) | chr3:23339903-23343704 REVERSE | Aliases: T20O10.250	8.2	8.6	-0.3	-1.2	100.0%	-4.2
5735	AT2G04063.1 | glycine-rich protein | chr2:1349755-1350174 REVERSE | Aliases: F3L12.1, F3L12_1	4.9	4.4	0.5	1.2	100.0%	-4.2
5736	AT1G09130.2 | similar to ATP-dependent Clp protease proteolytic subunit (ClpR1) (nClpP5) [Arabidopsis thaliana] (TAIR:At1g49970.1); similar to COG0740: Protease subunit of ATP-dependent Clp proteases [Nostoc punctiforme PCC 73102] (GB:ZP_00108611.1); contains InterPro domain Clp protease (InterPro:IPR001907) | chr1:2939928-2942269 REVERSE | Aliases: F7G19.1, F7G19_1	9.3	8.5	0.8	1.2	100.0%	-3.8
5737	AT4G00810.2 | 60S acidic ribosomal protein P1 (RPP1B), similar to acidic ribosomal protein p1 | chr4:345952-347192 REVERSE | Aliases: A_TM018A10.9, A_TM018A10_9, T18A10.8, T18A10_8	10.0	10.7	-0.6	-1.2	100.0%	-3.8
5738	AT2G42610.2 | expressed protein, contains Pfam profile PF04852: Protein of unknown function (DUF640) | chr2:17754994-17757517 FORWARD | Aliases: F14N22.12, F14N22_12	2.9	2.8	0.2	1.2	100.0%	-4.8
5739	AT5G23640.1 | expressed protein | chr5:7968262-7968505 REVERSE | Aliases: MQM1.10, MQM1_10	3.1	3.0	0.2	1.2	100.0%	-5.2
5740	AT1G52740.1 | histone H2A, putative, similar to histone H2A.F/Z Arabidopsis thaliana GI:2407800; contains Pfam profile PF00125 Core histone H2A/H2B/H3/H4 | chr1:19648963-19650099 FORWARD | Aliases: F14G24.1, F14G24_1	9.7	9.2	0.5	1.2	100.0%	-4.4
5741	AT5G37480.1 | expressed protein | chr5:14901561-14903198 REVERSE | Aliases: MPA22.2, MPA22_2	7.7	7.4	0.4	1.2	100.0%	-4.3
5742	AT1G31250.1 | proline-rich family protein, contains proline-rich extensin domains, INTERPRO:IPR002965; similar to prolin rich protein GB:S44189 GI:433706 from (Zea mays) | chr1:11166538-11167493 REVERSE | Aliases: T19E23.3, T19E23_3	2.2	2.3	-0.1	-1.2	100.0%	-5.3
5743	AT2G41530.1 | esterase, putative, similar to SP:P10768 Esterase D (EC 3.1.1.1) {Homo sapiens}; contains Pfam profile: PF00756 putative esterase | chr2:17330573-17332689 REVERSE | Aliases: T32G6.5, T32G6_5	10.2	10.7	-0.6	-1.2	100.0%	-4.2

Rank	Description	15.dpi	5.dpi	M	t	adj.q	B
5744	AT3G55050.2 | serine/threonine protein phosphatase 2C (PP2C6), identical to Ser/Thr protein phosphatase 2C (PP2C6) (GI:15020818) (Arabidopsis thaliana); similar to protein phosphatase 2C (GI:3608412) (Mesembryanthemum crystallinum); contains Pfam PF00481 : Protein phosphatase 2C domain; contains TIGRFAM TIGR01573 : CRISPR-associated protein Cas2 | chr3:20411465-20413572 REVERSE | Aliases: T15C9.50	9.0	8.6	0.4	1.2	100.0%	-4.1
5745	AT4G09430.1 | disease resistance protein (TIR-NBS-LRR class), putative, domain signature TIR-NBS-LRR exists, suggestive of a disease resistance protein. | chr4:5970929-5975372 FORWARD | Aliases: T15G18.150, T15G18_150	2.7	2.9	-0.2	-1.2	100.0%	-4.8
5746	AT3G17380.1 | meprin and TRAF homology domain-containing protein / MATH domain-containing protein, similar to ubiquitin-specific protease 12 (Arabidopsis thaliana) GI:11993471; contains Pfam profile PF00917: MATH domain | chr3:5950206-5953699 FORWARD | Aliases: MGD8.22	7.0	7.9	-0.9	-1.2	100.0%	-3.9
5747	AT3G11850.2 | expressed protein, contains Pfam profile PF04576: Protein of unknown function, DUF593 | chr3:3738903-3741720 REVERSE | Aliases: F26K24.14	4.9	5.2	-0.3	-1.2	100.0%	-4.2
5748	AT1G78380.1 | Symbol: ATGSTU19 | glutathione S-transferase, putative, similar to glutathione transferase GI:2853219 from (Carica papaya) | chr1:29491306-29492799 REVERSE | Aliases: F3F9.11, F3F9_11, GLUTATHIONE TRANSFERASE 8, GST8	10.8	11.3	-0.5	-1.2	100.0%	-4.4
5749	AT1G31830.2 | amino acid permease family protein, weak similarity to y+LAT1a (amino acid transporter) (Mus musculus) GI:3970791; contains Pfam profile PF00324: Amino acid permease | chr1:11418606-11420703 REVERSE | Aliases: F5M6.16, F5M6_16	7.8	7.3	0.5	1.2	100.0%	-4.2
5750	AT2G40180.1 | protein phosphatase 2C, putative / PP2C, putative, contains PF00481: Protein phosphatase 2C domain; identical to protein phosphatase 2C (GI:4587992) (Arabidopsis thaliana) | chr2:16789600-16791225 FORWARD | Aliases: ATHPP2C5, T7M7.17	3.0	3.4	-0.4	-1.2	100.0%	-4.4
5751	AT2G01630.1 | glycosyl hydrolase family 17 protein / beta-1,3-glucanase, putative, similar to beta-1,3-glucanase GI:15150341 from (Camellia sinensis) | chr2:279283-282122 REVERSE | Aliases: T8O11.20, T8O11_20	4.3	4.6	-0.2	-1.2	100.0%	-4.5
5752	AT1G52980.1 | GTP-binding family protein, contains Pfam domain, PF01926: GTPase of unknown function | chr1:19741161-19744016 FORWARD | Aliases: F14G24.25, F14G24_25	7.3	7.7	-0.5	-1.2	100.0%	-3.9
5753	AT1G72900.1 | disease resistance protein (TIR-NBS class), putative, domain signature TIR-NBS exists, suggestive of a disease resistance protein. | chr1:27435226-27437443 FORWARD | Aliases: F3N23.10, F3N23_10	4.5	3.8	0.6	1.2	100.0%	-4.2
5754	AT4G29220.1 | phosphofructokinase family protein, similar to phosphofructokinase (Amycolatopsis methanolica) GI:17432243; contains Pfam profile PF00365: Phosphofructokinase | chr4:14403041-14406511 REVERSE | Aliases: F17A13.40, F17A13_40	4.3	5.0	-0.7	-1.2	100.0%	-3.7
5755	AT2G05755.1 | integral membrane family protein, contains Pfam PF00892: Integral membrane protein domain | chr2:2170890-2173757 FORWARD | Aliases: None	5.4	5.1	0.3	1.2	100.0%	-4.3
5756	AT3G22550.1 | senescence-associated protein-related, similar to senescence-associated protein SAG102 (GI:22331931) (Arabidopsis thaliana) | chr3:7991653-7993461 REVERSE | Aliases: F16J14.11	5.0	4.6	0.3	1.2	100.0%	-4.1
5757	AT1G79460.1 | Symbol: GA2 | ent-kaurene synthase / ent-kaurene synthetase B (KS) (GA2), identical to GI:3056725 (PMID:9536043); formerly called ent-kaurene synthetase B | chr1:29895285-29899428 FORWARD | Aliases: ATKS, ENT KAURENE SYNTHASE, GA REQUIRING 2, KS, T8K14.12, T8K14_12	5.2	5.4	-0.3	-1.2	100.0%	-4.4
5758	AT2G38770.1 | expressed protein | chr2:16210041-16217416 REVERSE | Aliases: T6A23.3, T6A23_3	3.6	3.4	0.2	1.2	100.0%	-4.5
5759	AT2G05840.2 | Symbol: PAA2 | similar to 20S proteasome alpha subunit A1 (PAA1) (PRC1) [Arabidopsis thaliana] (TAIR:At5g35590.1); similar to proteasome IOTA subunit [Glycine max] (GB:AAC28135.1); contains InterPro domain Proteasome subunit, A-type (InterPro:IPR000426); contains InterPro domain Multispecific proteasome protease (InterPro:IPR001353) | chr2:2234089-2236287 FORWARD | Aliases: 20S PROTEASOME SUBUNIT PAA2, T6P5.4, T6P5_4	11.1	11.5	-0.3	-1.2	100.0%	-4.7
5760	AT5G35590.1 | Symbol: PAA1 | 20S proteasome alpha subunit A1 (PAA1) (PRC1), identical to proteasome subunit alpha type 6-1 SP:O81146 GI:12643647 from (Arabidopsis thaliana); identical to cDNA proteasome subunit prc1 GI:2511587 | chr5:13782400-13785047 REVERSE | Aliases: 20S PROTEASOME SUBUNIT PAA1, K2K18.4, K2K18_4	11.1	11.5	-0.3	-1.2	100.0%	-4.7
5761	AT4G33540.1 | metallo-beta-lactamase family protein | chr4:16130876-16133963 FORWARD | Aliases: T16L1.30, T16L1_30	8.8	8.1	0.7	1.2	100.0%	-4.1
5762	AT1G22620.1 | phosphoinositide phosphatase family protein, contains similarity to phosphoinositide phosphatase SAC1 (Rattus norvegicus) gi:11095248:gb:AAG29810; contains Pfam domain, PF02383: SacI homology domain; identical to cDNA SAC domain protein 1 (SAC1) SAC1-FRA7 allele, GI:31415718 | chr1:7997665-8002933 REVERSE | Aliases: F12K8.3	5.7	5.9	-0.3	-1.2	100.0%	-4.4

Rank	Description	15.dpi	5.dpi	M	t	adj.q	B
5763	AT1G73260.1 | trypsin and protease inhibitor family protein / Kunitz family protein, similar to trypsin inhibitor propeptide (Brassica oleracea) GI:841208; contains Pfam profile PF00197: Trypsin and protease inhibitor | chr1:27550935-27551851 REVERSE | Aliases: T18K17.7, T18K17_7	5.0	5.6	-0.6	-1.2	100.0%	-3.6
5764	AT4G15975.1 | zinc finger (C3HC4-type RING finger) family protein, contains Pfam domain, PF00097: Zinc finger, C3HC4 type (RING finger) | chr4:9052187-9053164 FORWARD | Aliases: None	2.8	3.0	-0.1	-1.2	100.0%	-5.0
5765	AT1G05620.1 | inosine-uridine preferring nucleoside hydrolase family protein, similar to Chain A, Crystal Structure Of Nucleoside Hydrolase From Leishmania MajorGI:8569431; contains Pfam profile PF01156: Inosine-uridine preferring nucleoside hydrolase | chr1:1679165-1681705 FORWARD | Aliases: F3F20.7, F3F20_7	7.1	7.5	-0.4	-1.2	100.0%	-4.2
5766	AT3G14560.1 | expressed protein | chr3:4889079-4890166 FORWARD | Aliases: MIE1.6	4.1	3.3	0.8	1.2	100.0%	-3.7
5767	AT4G17420.1 | expressed protein, contains Pfam domain, PF01987: Protein of unknown function | chr4:9722451-9724652 REVERSE | Aliases: DL4745C, FCAALL.389	7.7	7.3	0.4	1.2	100.0%	-4.0
5768	AT2G17560.2 | Symbol: HMGB4 | similar to high mobility group protein delta (HMGdelta) / HMG protein delta [Arabidopsis thaliana] (TAIR:At4g35570.1); similar to HMG-1 like protein gene [Glycine max] (GB:CAA41200.1); similar to high mobility group protein [Solanum tuberosum] (GB:CAA05365.1); contains InterPro domain HMG1/2 (high mobility group) box (InterPro:IPR000910) | chr2:7649247-7650749 REVERSE | Aliases: HIGH MOBILITY GROUP B 4, HMG GAMMA, MJB20.12, MJB20_12, NFD04, NFD4	7.3	8.1	-0.8	-1.2	100.0%	-3.9
5769	AT3G08680.2 | leucine-rich repeat transmembrane protein kinase, putative, contains Pfam profile: PF00069 Eukaryotic protein kinase domain, PF00560 leucine Rich Repeat (5 copies) | chr3:2637603-2640844 FORWARD | Aliases: F17O14.15	5.4	5.8	-0.4	-1.2	100.0%	-4.4
5770	AT3G58990.1 | aconitase C-terminal domain-containing protein, contains Pfam profile PF00694: Aconitase C-terminal domain | chr3:21808035-21809280 REVERSE | Aliases: F17J16.40	4.2	3.6	0.6	1.2	100.0%	-3.6
5771	AT5G55200.1 | co-chaperone grpE protein, putative, similar to chaperone GrpE type 2 (Nicotiana tabacum) GI:3851640; contains Pfam profile PF01025: co-chaperone GrpE | chr5:22411860-22413777 FORWARD | Aliases: MCO15.15, MCO15_15	8.7	9.1	-0.4	-1.2	100.0%	-4.4
5772	AT1G14687.1 | zinc finger homeobox family protein / ZF-HD homeobox family protein | chr1:5047798-5048607 FORWARD | Aliases: None	4.0	3.6	0.3	1.2	100.0%	-4.2
5773	AT5G09800.1 | U-box domain-containing protein, low similarity to immediate-early fungal elicitor protein CMPG1 (Petroselinum crispum) GI:14582200; contains Pfam profile PF04564: U-box domain	5.5	5.2	0.3	1.2	100.0%	-4.4
5774	AT5G16850.1 | Symbol: ATTERT |	2.4	2.6	-0.2	-1.2	100.0%	-4.9
5775	AT5G50460.1 | protein transport protein SEC61 gamma subunit, putative, similar to Swiss-Prot:Q19967 protein transport protein SEC61 gamma subunit (Caenorhabditis elegans) | chr5:20568663-20569792 REVERSE | Aliases: MBA10.8	9.7	10.4	-0.7	-1.2	100.0%	-4.2
5776	AT5G26670.2 | pectinacetylesterase, putative, similar to pectinacetylesterase precursor GI:1431629 from (Vigna radiata) | chr5:9318283-9320990 FORWARD | Aliases: None	5.7	6.1	-0.4	-1.2	100.0%	-4.0
5777	AT4G10090.1 | expressed protein | chr4:6305439-6307514 FORWARD | Aliases: F28M11.10, F28M11_10	4.5	4.7	-0.2	-1.2	100.0%	-4.5
5778	AT5G61070.1 | histone deacetylase family protein (HDA18), identical to HDA18 (Arabidopsis thaliana) GI:21105769; similar to SP:Q9UBN7 Histone deacetylase 6 (HD6) {Homo sapiens}; contains Pfam profile PF00850: Histone deacetylase family | chr5:24588397-24591598 REVERSE | Aliases: MAF19.8, MAF19_8	3.1	3.3	-0.2	-1.2	100.0%	-4.6
5779	AT2G41090.1 | calmodulin-like calcium-binding protein, 22 kDa (CaBP-22), identical to SP:P30187 22 kDa calmodulin-like calcium-binding protein (CABP-22) (Arabidopsis thaliana) | chr2:17142862-17143930 FORWARD | Aliases: T3K9.14, T3K9_14	4.0	3.7	0.3	1.2	100.0%	-4.2
5780	AT4G03270.1 | Symbol: CYCD6;1 | cyclin family protein, similar to CycD3;2 (Lycopersicon esculentum) GI:6434199 ; contains Pfam profiles PF00134: Cyclin, N-terminal domain, PF02984: Cyclin, C-terminal domain | chr4:1432375-1433691 REVERSE | Aliases: CYCD6;1, Cyclin D6;1, F4C21.20, F4C21_20	2.9	3.1	-0.1	-1.2	100.0%	-4.9
5781	AT5G44320.1 | eukaryotic translation initiation factor 3 subunit 7, putative / eIF-3 zeta, putative / eIF3d, putative, similar to initiation factor 3d (Arabidopsis thaliana) GI:12407755, SP:O15371 Eukaryotic translation initiation factor 3 subunit 7 (eIF-3 zeta) (eIF3 p66) (eIF3d) {Homo sapiens}; contains Pfam profile PF05091: Eukaryotic translation initiation factor 3 subunit 7 (eIF-3) | chr5:17871816-17873907 REVERSE | Aliases: K9L2.10, K9L2_10	8.4	8.7	-0.3	-1.2	100.0%	-4.6
5782	AT3G26850.2 | expressed protein | chr3:9896779-9899243 REVERSE | Aliases: MDJ14.17	4.0	4.1	-0.1	-1.2	100.0%	-4.9

Rank	Description	15.dpi	5.dpi	M	t	adj.q	B
5783	AT1G05055.1 | basic transcription factor 2, 44kD subunit-related, contains weak similarity to Swiss-Prot:Q13888 TFIIH basal transcription factor complex p44 subunit (Basic transcription factor 2 44 kDa subunit, BTF2-p44, General transcription factor IIH polypeptide 2) (Homo sapiens) | chr1:1448718-1450855 REVERSE | Aliases: AT1G05050	6.2	6.5	-0.3	-1.2	100.0%	-4.2
5784	AT4G12540.1 | expressed protein | chr4:7429873-7431599 REVERSE | Aliases: T1P17.130, T1P17_130	3.1	3.4	-0.3	-1.2	100.0%	-4.3
5785	AT4G18850.1 | hypothetical protein | chr4:10342074-10342253 REVERSE | Aliases: F13C5.20, F13C5_20	2.4	2.3	0.1	1.2	100.0%	-5.2
5786	AT3G01630.1 | similar to nodulin-related [Arabidopsis thaliana] (TAIR:At1g31470.1); similar to P0031D02.33 [Oryza sativa (japonica cultivar-group)] (GB:NP_915291.1) | chr3:237246-239120 FORWARD | Aliases: F4P13.17, F4P13_17	3.0	2.8	0.2	1.2	100.0%	-4.8
5787	AT5G19100.1 | extracellular dermal glycoprotein-related / EDGP-related, low similarity to extracellular dermal glycoprotein EDGP precursor (Daucus carota) GI:285741, SP:P13917 Basic 7S globulin precursor {Glycine max} | chr5:6408244-6409419 REVERSE | Aliases: T16G12.140, T16G12_140	3.6	3.4	0.2	1.2	100.0%	-4.4
5788	AT1G55850.1 | Symbol: ATCSLE1 | cellulose synthase family protein, similar to cellulose synthase catalytic subunit (gi:13925881) from Nicotiana alata, cellulose synthase-5 (gi:9622882) from Zea mays | chr1:20880365-20883146 FORWARD | Aliases: CSLE1, F14J16.9, F14J16_9	7.1	7.5	-0.4	-1.2	100.0%	-4.1
5789	AT2G39990.1 | Symbol: EIF2 | eukaryotic translation initiation factor 3 subunit 5 / eIF-3 epsilon / eIF3f (TIF3F1), identical to SP:O04202 Eukaryotic translation initiation factor 3 subunit 5 (eIF-3 epsilon) (eIF3 p32 subunit) (eIF3f) {Arabidopsis thaliana}; contains Pfam profile PF01398: Mov34/MPN/PAD-1 family | chr2:16705174-16707090 REVERSE | Aliases: T28M21.15, T28M21_15, TRANSLATION INITIATION FACTOR EIF2 P47 SUBUNIT HOMOLOG	8.6	9.3	-0.8	-1.2	100.0%	-4.0
5790	AT4G13070.1 | group II intron splicing factor CRS1-related, contains weak similarity to CRS1 (Zea mays) gi:9837550:gb:AAG00595	4.3	4.7	-0.4	-1.2	100.0%	-4.3
5791	AT3G58560.1 | endonuclease/exonuclease/phosphatase family protein, similar to SP:P31384 Glucose-repressible alcohol dehydrogenase transcriptional effector (Carbon catabolite repressor protein 4) {Saccharomyces cerevisiae}; contains Pfam profile PF03372: Endonuclease/Exonuclease/phosphatase family | chr3:21661573-21665177 REVERSE | Aliases: F14P22.150	5.6	6.3	-0.7	-1.2	100.0%	-4.2
5792	AT5G42190.1 | Symbol: ASK2 | E3 ubiquitin ligase SCF complex subunit SKP1/ASK1 (At2) / UFO-binding protein (UIP2), E3 ubiquitin ligase; skp1b; identical to UIP2 GI:3719211 from (Arabidopsis thaliana); contains Pfam profiles PF01466: Skp1 family, dimerisation domain and PF03931:Skp1 family, tetramerisation domain; identical to cDNA UFO binding protein UIP2 mRNA, partial cds GI:3719210 | chr5:16870883-16872793 REVERSE | Aliases: ARABIDOPSIS SKP1 LIKE 2, MJC20.30, MJC20_30, SKP1B	11.3	10.6	0.7	1.2	100.0%	-4.0
5793	AT4G12640.1 | RNA recognition motif (RRM)-containing protein, contains InterPro entry IPR000504: RNA-binding region RNP-1 (RNA recognition motif) (RRM) | chr4:7462598-7467859 FORWARD | Aliases: T1P17.230, T1P17_230	3.7	3.9	-0.2	-1.2	100.0%	-4.7
5794	AT2G41710.2 | ovule development protein, putative, similar to ovule development protein AINTEGUMENTA (GI:1209099) (Arabidopsis thaliana);Pfam domain (PF00847)	6.0	5.6	0.4	1.2	100.0%	-4.1
5795	AT5G27820.1 | ribosomal protein L18 family protein, similar to SP:P52863 50S ribosomal protein L18 (Aeromonas proteolytica) {Vibrio proteolyticus} | chr5:9859858-9861133 FORWARD | Aliases: T1G16.150, T1G16_150	5.2	5.8	-0.6	-1.2	100.0%	-3.7
5796	AT5G06850.1 | C2 domain-containing protein, contains INTERPRO:IPR000008 C2 domain | chr5:2126976-2129325 REVERSE | Aliases: MOJ9.2, MOJ9_2	4.1	3.7	0.4	1.2	100.0%	-4.0
5797	AT5G64640.1 | pectinesterase family protein, contains Pfam profile: PF01095 pectinesterase | chr5:25853953-25856279 FORWARD | Aliases: MUB3.16, MUB3_16	4.4	4.8	-0.3	-1.2	100.0%	-4.2
5798	AT3G46580.1 | Symbol: MBD5 | methyl-CpG-binding domain-containing protein, weak similarity to SP:P51608 Methyl-CpG-binding protein 2 (MeCP-2 protein) (MeCP2) {Homo sapiens}; contains Pfam profile PF01429: Methyl-CpG binding domain | chr3:17159327-17160574 FORWARD | Aliases: F12A12.100	5.0	5.4	-0.4	-1.2	100.0%	-4.0
5799	AT1G31814.1 | Symbol: FRL2 | expressed protein | chr1:11412589-11414414 REVERSE | Aliases: F5M6.26, F5M6_26, FRIGIDA LIKE 2	3.4	3.2	0.2	1.2	100.0%	-4.6
5800	AT4G01860.2 | transducin family protein / WD-40 repeat family protein, contains ten G-protein beta-subunit (beta-transducin) WD-40 repeats | chr4:801032-808060 REVERSE | Aliases: T7B11.12, T7B11_12	2.6	2.9	-0.3	-1.2	100.0%	-4.7
5801	AT3G01850.2 | ribulose-phosphate 3-epimerase, cytosolic, putative / pentose-5-phosphate 3-epimerase, putative, strong similarity to D-ribulose-5-phosphate 3-epimerase (Oryza sativa) GI:6007803; contains Pfam profile PF00834: Ribulose-phosphate 3 epimerase family; contains non-consensus splice sites at exon 1 and exon2 | chr3:300047-302044 REVERSE | Aliases: F28J7.18, F28J7_18	5.5	4.9	0.6	1.2	100.0%	-3.9
5802	AT3G48030.1 | hypoxia-responsive family protein / zinc finger (C3HC4-type RING finger) family protein, similar to RING-H2 finger protein RHX1a (Arabidopsis thaliana) GI:3790591; contains Pfam profiles PF00097: Zinc finger C3HC4 type (RING finger), PF04588: Hypoxia induced protein conserved region | chr3:17736395-17738939 REVERSE | Aliases: T17F15.100	2.2	2.4	-0.2	-1.2	100.0%	-4.8

Rank	Description	15.dpi	5.dpi	M	t	adj.q	B
5803	AT3G25560.2 | protein kinase family protein, contains Prosite:PS00108: Serine/Threonine protein kinases active-site signature and PS00107: Protein kinases ATP-binding region signature | chr3:9280750-9283924 REVERSE | Aliases: MWL2.18	6.3	6.0	0.2	1.2	100.0%	-4.4
5804	AT5G34460.1 | replication protein-related, weak similarity to Replication Protein A 70	2.6	2.7	-0.1	-1.2	100.0%	-5.2
5805	AT3G59400.1 | Symbol: GUN4 | expressed protein | chr3:21959694-21960687 REVERSE | Aliases: F25L23.260	3.4	3.0	0.4	1.2	100.0%	-4.0
5806	AT3G16180.1 | proton-dependent oligopeptide transport (POT) family protein, contains Pfam profile: PF00854 POT family | chr3:5481331-5485100 REVERSE | Aliases: MSL1.22	5.2	4.7	0.5	1.2	100.0%	-4.1
5807	AT3G17465.1 | Symbol: RPL3P | ribosomal protein L3 family protein | chr3:5977823-5979578 REVERSE | Aliases: MKP6.7, RIBOSOMAL PROTEIN L3 PLASTID	6.4	7.0	-0.5	-1.2	100.0%	-3.8
5808	AT1G42580.1 | hypothetical protein, contains similarity to hypothetical proteins | chr1:15994136-15994615 REVERSE | Aliases: F8D11.4, F8D11_4	3.1	2.9	0.2	1.2	100.0%	-4.9
5809	AT4G21510.1 | F-box family protein, contains Pfam PF00646: F-box domain; similar to SKP1 interacting partner 2 (SKIP2) TIGR_Ath1:At5g67250 | chr4:11445868-11447175 REVERSE | Aliases: F18E5.130, F18E5_130	4.9	4.7	0.3	1.2	100.0%	-4.4
5810	AT2G45660.1 | Symbol: AGL20 | MADS-box protein (AGL20) | chr2:18814612-18818094 REVERSE | Aliases: AGAMOUS LIKE 20, AGL20, F17K2.19, MADS BOX PROTEIN AGL20, SOC1, SUPPRESSOR OF OVEREXPRESSION OF CO 1	5.4	5.1	0.3	1.2	100.0%	-4.4
5811	AT3G52940.2 | Symbol: FK | C-14 sterol reductase / delta(14)-sterol reductase / FACKEL (FK), identical to gi:9082182 | chr3:19641176-19644261 REVERSE | Aliases: ELL1, EXTRA LONG LIFESPAN 1, F8J2.111, FACKEL, HYD2	6.7	7.1	-0.4	-1.2	100.0%	-4.1
5812	AT4G11450.1 | expressed protein | chr4:6958676-6962041 REVERSE | Aliases: F25E4.70, F25E4_70	3.1	3.2	-0.2	-1.2	100.0%	-4.7
5813	AT3G29640.1 | hypothetical protein, contains similarity to hypothetical proteins | chr3:11490525-11491040 FORWARD | Aliases: T13J10.7	2.5	2.3	0.2	1.2	100.0%	-4.7
5814	AT4G36670.1 | mannitol transporter, putative, similar to mannitol transporter (Apium graveolens var. dulce) GI:12004316; contains Pfam profile PF00083: major facilitator superfamily protein | chr4:17287503-17289594 REVERSE | Aliases: AP22.86, AP22_86	4.4	5.1	-0.6	-1.2	100.0%	-3.9
5815	AT4G16420.3 | Symbol: ADA2B | transcriptional adaptor (ADA2b), identical to transcriptional adaptor ADA2b (Arabidopsis thaliana) gi:13591700:gb:AAK31320 | chr4:9262690-9266078 REVERSE | Aliases: DL4235C, FCAALL.57, PROPORZ1, PRZ1	7.9	7.6	0.3	1.2	100.0%	-4.3
5816	AT5G40150.1 | peroxidase, putative, identical to peroxidase ATP26a {Arabidopsis thaliana} GP:1890317:emb:CAA72487 | chr5:16076737-16078271 REVERSE | Aliases: MSN9.50, MSN9_50	3.8	4.1	-0.2	-1.2	100.0%	-4.5
5817	AT3G44950.1 | glycine-rich protein | chr3:16432030-16432271 FORWARD | Aliases: F14D17.20	7.2	6.8	0.4	1.2	100.0%	-4.0
5818	AT1G59960.1 | aldo/keto reductase, putative, similar to NADPH-dependent codeinone reductase GI:6478210 (Papaver somniferum), NAD(P)H dependent 6'-deoxychalcone synthase (Glycine max)(GI:18728) | chr1:22074961-22076812 REVERSE | Aliases: F23H11.27, F23H11_27	10.2	10.8	-0.5	-1.2	100.0%	-4.5
5819	AT4G23100.2 | Symbol: RML1 | similar to gamma-glutamylcysteine synthetase [Brassica juncea] (GB:CAA06613.1); similar to glutamate-cysteine ligase [Brassica juncea] (GB:CAD91714.1); similar to gamma-glutamylcysteine synthetase precursor [Pisum sativum] (GB:AAF22137.1); similar to gamma-glutamylcysteine synthetase [Lotus corniculatus var. japonicus] (GB:AAO27827.1); similar to glutamate-cysteine ligase [Brassica juncea] (GB:CAD91713.1); contains InterPro domain Glutamate--cysteine ligase, plant (InterPro:IPR006336) | chr4:12103172-12107310 REVERSE | Aliases: CAD2, CADMIUM SENSITIVE 2, F7H19.290, F7H19_290, GAMMA GLUTAMYL CYSTEINE SYNTHETASE, GAMMA GLUTAMYL CYSTEINE SYNTHETASE 1, GAMMA GLUTAMYLCYSTEINE SYNTHETASE, GSH1, ROOT MERISTEMLESS 1	11.6	10.6	1.0	1.2	100.0%	-3.9
5820	AT1G68070.1 | zinc finger (C3HC4-type RING finger) family protein, very low similarity to RING-H2 finger protein RHG1a (Arabidopsis thaliana) GI:3822225; contains Pfam profile: PF00097 zinc finger, C3HC4 type (RING finger)	5.6	5.4	0.2	1.2	100.0%	-4.5
5821	AT5G67110.1 | Symbol: ALC | basic helix-loop-helix (bHLH) family protein, contains Pfam profile: PF00010 helix-loop-helix DNA-binding domain | chr5:26802333-26803609 REVERSE | Aliases: ALCATRAZ, K21H1.7, K21H1_7	2.9	2.7	0.2	1.2	100.0%	-4.8
5822	AT5G21060.3 | similar to bifunctional aspartate kinase/homoserine dehydrogenase, putative / AK-HSDH, putative [Arabidopsis thaliana] (TAIR:At4g19710.2); similar to COG0460: Homoserine dehydrogenase [Rubrobacter xylanophilus DSM 9941] (GB:ZP_00199840.1); contains InterPro domain Homoserine dehydrogenase (InterPro:IPR001342) | chr5:7148980-7152866 REVERSE | Aliases: T10F18.90, T10F18_90	7.6	8.2	-0.6	-1.2	100.0%	-3.9
5823	AT5G17330.1 | Symbol: GAD | glutamate decarboxylase 1 (GAD 1), sp:Q42521 | chr5:5711070-5715077 FORWARD | Aliases: GAD1, GLUTAMATE DECARBOXYLASE, MKP11.30, MKP11_30	8.2	9.3	-1.1	-1.2	100.0%	-4.3

Rank	Description	15.dpi	5.dpi	M	t	adj.q	B
5824	AT2G21940.2 | shikimate kinase, putative, similar to shikimate kinase precursor from Lycopersicon esculentum (SP:Q00497); contains Pfam shikimate kinase domain PF01202 | chr2:9357926-9360235 FORWARD | Aliases: F7D8.26, F7D8_26	6.0	5.4	0.6	1.2	100.0%	-4.1
5825	AT4G19050.1 | mob1/phocein family protein, contains Pfam PF03637: Mob1/phocein family; contains Pfam F00560: Leucine Rich Repeats; contains TIGRFAMS profile TIGR01612: reticulocyte binding protein; hypothetical protein YIL106w, Saccharomyces cerevisiae, PIR2:S48466 | chr4:10438224-10443797 REVERSE | Aliases: T18B16.1	5.0	4.7	0.3	1.2	100.0%	-4.4
5826	AT2G13730.1 | hypothetical protein | chr2:5732639-5733085 REVERSE | Aliases: F13J11.8, F13J11_8	2.4	2.5	-0.1	-1.2	100.0%	-5.2
5827	AT4G00500.2 | lipase class 3 family protein / calmodulin-binding heat-shock protein-related | chr4:224992-227195 REVERSE | Aliases: F6N23.21, F6N23_21	4.4	4.1	0.3	1.2	100.0%	-4.1
5828	AT5G40530.2 | expressed protein, contains Pfam profile PF05148: Protein of unknown function (DUF691) | chr5:16251740-16253450 FORWARD | Aliases: MNF13.4, MNF13_4	5.6	6.1	-0.5	-1.2	100.0%	-4.4
5829	AT5G48590.1 | expressed protein | chr5:19715647-19717173 FORWARD | Aliases: K15N18.6, K15N18_6	6.0	5.7	0.2	1.2	100.0%	-4.6
5830	AT4G15100.1 | Symbol: SCPL30 |	2.7	2.6	0.1	1.2	100.0%	-4.9
5831	AT1G22890.1 | expressed protein | chr1:8102807-8103521 FORWARD | Aliases: F19G10.22, F19G10_22	2.8	3.0	-0.2	-1.2	100.0%	-5.2
5832	AT5G10290.1 | leucine-rich repeat family protein / protein kinase family protein, contains Pfam domains PF00560: Leucine Rich Repeat and PF00069: Protein kinase domain | chr5:3235296-3238720 REVERSE | Aliases: F18D22.60, F18D22_60	7.3	7.8	-0.5	-1.2	100.0%	-4.6
5833	AT5G14390.1 | expressed protein | chr5:4637315-4639759 REVERSE | Aliases: F18O22.180, F18O22_180	5.9	5.6	0.3	1.2	100.0%	-4.3
5834	AT5G01510.1 | expressed protein, contains Pfam domain, PF04884: Protein of unknown function, DUF647 | chr5:201605-205380 FORWARD | Aliases: F7A7.30, F7A7_30	3.6	3.8	-0.2	-1.2	100.0%	-4.6
5835	AT5G17640.1 | expressed protein | chr5:5810189-5813286 REVERSE | Aliases: K10A8.120, K10A8_120	5.3	4.8	0.5	1.2	100.0%	-4.0
5836	AT2G17250.1 | expressed protein, weak similarity to Ribosome biogenesis protein MAK21 (Swiss-Prot:Q12176) (Saccharomyces cerevisiae) | chr2:7506559-7510207 FORWARD | Aliases: T23A1.11, T23A1_11	6.9	7.3	-0.4	-1.2	100.0%	-4.2
5837	AT4G02130.3 | similar to glycosyl transferase family 8 protein [Arabidopsis thaliana] (TAIR:At1g02720.2); similar to glycosyl transferase family 8 protein [Arabidopsis thaliana] (TAIR:At1g02720.1); similar to putative glycosyltransferase [Oryza sativa (japonica cultivar-group)] (GB:XP_479332.1); similar to OSJNBa0074L08.24 [Oryza sativa (japonica cultivar-group)] (GB:XP_473276.1); contains InterPro domain Glycosyl transferase, family 8 (InterPro:IPR002495) | chr4:944360-947085 REVERSE | Aliases: T10M13.14, T10M13_14	4.5	4.2	0.3	1.2	100.0%	-4.2
5838	AT5G27000.1 | Symbol: ATK4 | kinesin motor protein-related, non-consensus AT donor splice site at exon 12; non-consensus AC acceptor splice site at exon 13 | chr5:9498055-9503137 FORWARD | Aliases: ARABIDOPSIS THALIANA KINESIN 4, F2P16.12, F2P16_12, KATD, KINESIN LIKE PROTEIN D	3.2	3.5	-0.3	-1.2	100.0%	-4.3
5839	AT3G15070.1 | zinc finger (C3HC4-type RING finger) family protein, similar to C-terminal zinc-finger (Glycine max) GI:558543; contains Pfam profile: PF00097 zinc finger, C3HC4 type (RING finger) | chr3:5069620-5073324 REVERSE | Aliases: K15M2.22	3.7	3.3	0.4	1.2	100.0%	-4.3
5840	AT4G32540.1 | Symbol: YUC | flavin-containing monooxygenase / FMO (YUCCA), identical to gi:16555352 | chr4:15700910-15702876 FORWARD | Aliases: L23H3.20, L23H3_20, YUCCA	5.1	5.6	-0.5	-1.2	100.0%	-3.9
5841	AT5G16750.1 | Symbol: TOZ | transducin family protein / WD-40 repeat family protein, contains 8 WD-40 repeats (PF00400); similar to transducin homolog sazD - Homo sapiens, EMBL:U02609 | chr5:5504349-5509345 REVERSE | Aliases: F5E19.90, F5E19_90, TORMOZ, TORMOZEMBRYO DEFECTIVE	5.2	5.5	-0.4	-1.2	100.0%	-4.2
5842	AT2G04890.1 | Symbol: SCL21 | scarecrow-like transcription factor 21 (SCL21) | chr2:1719778-1722378 REVERSE | Aliases: F1O13.2, F1O13_2, SCARECROW LIKE 21, SCL21	6.3	5.9	0.4	1.2	100.0%	-4.2
5843	AT4G04930.1 | Symbol: DES 1 LIKE | fatty acid desaturase family protein, similar to D. melanogaster Des-1 protein, GenBank accession number X94180; contains Pfam profile PF00487 Fatty acid desaturase domain | chr4:2509131-2511060 FORWARD | Aliases: DES 1 LIKE TRANSMEMBRANE PROTEIN, T1J1.1, T1J1_1	2.7	2.9	-0.2	-1.2	100.0%	-4.7
5844	AT1G14410.1 | DNA-binding protein-related, similar to DNA-binding protein p24 GI:9651810 from (Solanum tuberosum) | chr1:4929118-4930888 REVERSE | Aliases: F14L17.18, F14L17_18	5.8	6.3	-0.5	-1.2	100.0%	-4.0

Rank	Description	15.dpi	5.dpi	M	t	adj.q	B
5845	AT5G24340.1 | 3'-5' exonuclease domain-containing protein, contains Pfam profile PF01612: 3'-5' exonuclease | chr5:8297308-8300377 FORWARD | Aliases: K16H17.2, K16H17_2	3.5	3.3	0.2	1.2	100.0%	-4.7
5846	AT1G64185.1 | lactoylglutathione lyase family protein / glyoxalase I family protein, contains Pfam domain PF00903: glyoxalase family protein | chr1:23828177-23829447 FORWARD | Aliases: None	4.9	5.3	-0.4	-1.2	100.0%	-4.1
5847	AT4G39140.4 | expressed protein | chr4:18229325-18232034 REVERSE | Aliases: T22F8.40, T22F8_40	5.0	4.6	0.4	1.2	100.0%	-4.0
5848	AT3G54840.1 | Symbol: ARA6 | Rab GTPase (ARA6), identical to small GTPase Ara6 (Arabidopsis thaliana) GI:13160603 | chr3:20329480-20331970 FORWARD | Aliases: F28P10.180	9.2	8.2	1.0	1.2	100.0%	-4.1
5849	AT1G36030.1 | F-box family protein, similar to hypothetical protein GB:AAC61810 | chr1:13437798-13438040 FORWARD | Aliases: T22A15.5, T22A15_5	2.4	2.5	-0.1	-1.2	100.0%	-5.3
5850	AT5G62080.1 | protease inhibitor/seed storage/lipid transfer protein (LTP) family protein, similar to tapetum-specific protein a9 precursor {Brassica napus} SP:Q05772; contains Pfam protease inhibitor/seed storage/LTP family domain PF00234; supported by full-length cDNA Ceres:27795 | chr5:24950822-24951671 REVERSE | Aliases: MTG10.3, MTG10_3	2.3	2.4	-0.1	-1.2	100.0%	-4.9
5851	AT1G60850.3 | Symbol: ATRPAC42 | DNA-directed RNA polymerase, putative, identical to RNA polymerase subunit (Arabidopsis thaliana) GI:514322; contains Pfam profile PF01000: RNA polymerase Rpb3/RpoA insert domain | chr1:22401581-22403834 REVERSE | Aliases: AAC42, F23C21.3, F23C21_3, RNA POLYMERASE I SUBUNIT 42	6.3	6.8	-0.5	-1.2	100.0%	-4.0
5852	AT1G74800.1 | galactosyltransferase family protein, contains Pfam profile: PF01762 galactosyltransferase | chr1:28105629-28108773 REVERSE | Aliases: F25A4.23, F25A4_23	4.4	4.8	-0.4	-1.2	100.0%	-4.1
5853	AT3G60130.2 | similar to glycosyl hydrolase family 1 protein [Arabidopsis thaliana] (TAIR:At5g44640.1); similar to glycosyl hydrolase family 1 protein [Arabidopsis thaliana] (TAIR:At2g44450.1); similar to glycosyl hydrolase family 1 protein [Arabidopsis thaliana] (TAIR:At5g42260.1); similar to putative prunasin hydrolase precursor [Prunus serotina] (GB:AAL07490.1); similar to putative prunasin hydrolase isoform PH-L1 precursor [Prunus serotina] (GB:AAF34651.2); similar to prunasin hydrolase isoform PH B precursor [Prunus serotina] (GB:AAL39079.1); similar to prunasin hydrolase isoform PH B precursor [Prunus serotina] (GB:AAL06338.1); contains InterPro domain Glycoside hydrolase, family 1 (InterPro:IPR001360) | chr3:22221409-22224792 FORWARD | Aliases: T2O9.110	3.4	4.0	-0.6	-1.2	100.0%	-3.7
5854	AT2G25460.1 | expressed protein | chr2:10839838-10842561 REVERSE | Aliases: F13B15.12, F13B15_12	4.1	3.9	0.2	1.2	100.0%	-4.6
5855	AT2G20320.1 | DENN (AEX-3) domain-containing protein, contains Pfam domain PF02141: DENN (AEX-3) domain | chr2:8774187-8779217 FORWARD | Aliases: F11A3.13, F11A3_13	5.4	5.0	0.3	1.2	100.0%	-4.2
5856	AT1G08380.1 | expressed protein | chr1:2640858-2641825 REVERSE | Aliases: T27G7.25, T27G7_25	7.9	7.3	0.6	1.2	100.0%	-3.7
5857	AT2G32210.1 | expressed protein | chr2:13685063-13685907 FORWARD | Aliases: F22D22.4, F22D22_4	2.6	2.4	0.2	1.2	100.0%	-5.2
5858	AT3G57810.2 | OTU-like cysteine protease family protein, contains Pfam profile PF02338: OTU-like cysteine protease | chr3:21426805-21428925 FORWARD | Aliases: T10K17.20	6.7	7.1	-0.4	-1.2	100.0%	-4.0
5859	AT3G05650.1 | disease resistance family protein, contains leucine rich-repeat (LRR) domains Pfam:PF00560, INTERPRO:IPR001611; similar to Cf-4A protein (Lycopersicon esculentum) gi:3097197:emb:CAA73187 | chr3:1645686-1648579 REVERSE | Aliases: F18C1.8, F18C1_8	2.7	2.6	0.2	1.2	100.0%	-4.7
5860	AT4G05330.1 | zinc finger and C2 domain protein, putative, similar to zinc finger and C2 domain protein GI:9957238 from (Arabidopsis thaliana)	4.6	5.0	-0.4	-1.2	100.0%	-3.9
5861	AT3G47550.6 | zinc finger (C3HC4-type RING finger) family protein, contains Pfam domain, PF00097: Zinc finger, C3HC4 type (RING finger) | chr3:17534483-17536888 FORWARD | Aliases: F1P2.100	5.3	4.8	0.4	1.2	100.0%	-4.3
5862	AT4G35290.2 | Symbol: GLUR2 | glutamate receptor family protein (GLR3.2) (GLUR2), identical to putative glutamate receptor like-protein GLUR2 (Arabidopsis thaliana) gi:13160471:gb:AAK13248; plant glutamate receptor family, PMID:11379626 | chr4:16789726-16793685 FORWARD | Aliases: ATGLR3.2, ATGLUR2, F23E12.150, F23E12_150, GLR3.2	2.9	3.0	-0.2	-1.2	100.0%	-4.7
5863	AT2G42630.1 | protein kinase family protein, contains protein kinase domain, Pfam:PF00069 | chr2:17765609-17767786 REVERSE | Aliases: F14N22.10, F14N22_10	3.9	4.2	-0.3	-1.2	100.0%	-4.4
5864	AT5G50000.1 | protein kinase, putative, similar to protein kinase ATMRK1 (Arabidopsis thaliana) gi:2351097:dbj:BAA22079 | chr5:20359813-20362359 REVERSE | Aliases: MPF21.1, MPF21_1	7.4	8.1	-0.7	-1.2	100.0%	-4.0
5865	AT5G53020.1 | expressed protein | chr5:21517125-21519627 FORWARD | Aliases: MNB8.8, MNB8_8	4.5	4.2	0.3	1.2	100.0%	-4.3

Rank	Description	15.dpi	5.dpi	M	t	adj.q	B
5866	AT4G03150.1 | expressed protein | chr4:1393614-1394574 REVERSE | Aliases: F4C21.13	3.9	3.7	0.1	1.2	100.0%	-5.0
5867	AT1G53310.3 | Symbol: ATPPC1 |	8.5	9.8	-1.3	-1.2	100.0%	-4.3
5868	AT1G24610.1 | SET domain-containing protein, low similarity to SP:Q43088 Ribulose-1,5 bisphosphate carboxylase/oxygenase large subunit N- methyltransferase, chloroplast precursor (EC 2.1.1.127) {Pisum sativum}; contains Pfam profile PF00856: SET domain | chr1:8720177-8722732 REVERSE | Aliases: F21J9.27	8.8	8.3	0.5	1.2	100.0%	-4.0
5869	AT2G45120.1 | zinc finger (C2H2 type) family protein, contains Pfam domain, PF00096: Zinc finger, C2H2 type | chr2:18610693-18611826 FORWARD | Aliases: T14P1.7	2.6	2.5	0.1	1.2	100.0%	-4.9
5870	AT5G54310.1 | ARF GAP-like zinc finger-containing protein ZIGA3 (ZIGA3), nearly identical to ARF GAP-like zinc finger-containing protein ZIGA3 GI:10441352 from (Arabidopsis thaliana); contains InterPro accession IPR001164: Human Rev interacting-like protein (hRIP) | chr5:22074278-22078457 REVERSE | Aliases: MDK4.13, MDK4_13	7.4	7.9	-0.5	-1.2	100.0%	-4.3
5871	AT3G10080.1 | germin-like protein, putative, similar to germin-like protein 2 (Oryza sativa) GI:2655287 | chr3:3107281-3108182 REVERSE | Aliases: T22K18.9	3.5	3.8	-0.4	-1.2	100.0%	-4.0
5872	AT4G33780.1 | expressed protein | chr4:16201833-16203643 REVERSE | Aliases: T16L1.270, T16L1_270	8.8	8.3	0.5	1.2	100.0%	-4.1
5873	AT1G53970.1 | hypothetical protein | chr1:20152087-20152566 FORWARD | Aliases: T18A20.21	1.8	1.9	-0.1	-1.2	100.0%	-5.4
5874	AT3G05120.1 | expressed protein, low similarity to PrMC3 (Pinus radiata) GI:5487873 | chr3:1430483-1432784 FORWARD | Aliases: T12H1.8, T12H1_8	5.4	5.1	0.3	1.2	100.0%	-4.3
5875	AT3G14060.1 | expressed protein | chr3:4657053-4657608 REVERSE | Aliases: MDC16.20	3.8	4.3	-0.5	-1.2	100.0%	-3.8
5876	AT4G38370.1 | phosphoglycerate/bisphosphoglycerate mutase family protein, contains Pfam profile PF00300: phosphoglycerate mutase family | chr4:17969521-17971289 REVERSE | Aliases: F22I13.140, F22I13_140	8.4	8.9	-0.5	-1.2	100.0%	-3.9
5877	AT1G78750.1 | F-box family protein, contains F-box domain Pfam:PF00646	3.2	3.1	0.2	1.2	100.0%	-4.7
5878	AT4G39160.1 | similar to expressed protein [Arabidopsis thaliana] (TAIR:At3g28770.1); similar to myb domain-containing protein [Dictyostelium discoideum] (GB:EAL65687.1); contains InterPro domain Myb DNA-binding domain (InterPro:IPR001005) | chr4:18236822-18239738 FORWARD | Aliases: T22F8.60, T22F8_60	4.8	5.1	-0.4	-1.2	100.0%	-4.3
5879	AT5G47970.1 | nitrogen regulation family protein, strong similarity to unknown protein (emb:CAB87804.1) ; contains Pfam domain PF01207: Dihydrouridine synthase (Dus); similar to (SP:P45672) NIFR3-like protein (SP:P45672) (Azospirillum brasilense)	4.6	4.9	-0.3	-1.2	100.0%	-4.2
5880	AT4G27780.1 | Symbol: ACBP2 | acyl-CoA binding protein 2 (ACBP2), identical to acyl-CoA binding protein 2 (Arabidopsis thaliana) gi:12039034:gb:AAG46057 | chr4:13847555-13849893 FORWARD | Aliases: ACYL COA BINDING PROTEIN ACBP 2, ACYL COA BINDING PROTEIN ACBP2, T27E11.20, T27E11_20	6.6	6.1	0.4	1.2	100.0%	-4.2
5881	AT2G22840.1 | Symbol: AtGRF1 |	3.1	3.7	-0.5	-1.2	100.0%	-4.2
5882	AT2G17900.1 | Symbol: SDG37 | zinc finger (MYND type) family protein / SET domain-containing protein, contains Pfam profiles PF00856: SET domain, PF01753: MYND zinc finger (ZnF) domain; confirmed by cDNA (Paola Vittorioso--accession not assigned yet (paola.vittorioso@uniroma1.it) | chr2:7780410-7783765 REVERSE | Aliases: SET DOMAIN GROUP 37, T13L16.8, T13L16_8	4.5	4.8	-0.3	-1.2	100.0%	-4.3
5883	AT3G54630.1 | expressed protein, weak similarity to retinoblastoma-associated protein HEC (Homo sapiens) GI:2501873 | chr3:20232075-20234006 REVERSE | Aliases: T14E10.200	5.3	5.9	-0.5	-1.2	100.0%	-4.1
5884	AT1G18900.2 | pentatricopeptide (PPR) repeat-containing protein, low similarity to 67kD chloroplastic RNA-binding protein, P67 (Arabidopsis thaliana) GI:9755842; contains Pfam profile PF01535: PPR repeat | chr1:6529037-6532605 FORWARD | Aliases: F14D16.2, F14D16_2	5.1	5.6	-0.5	-1.2	100.0%	-4.2
5885	AT3G54030.1 | protein kinase family protein, contains protein kinase domain, Pfam:PF00069 | chr3:20021931-20024671 FORWARD | Aliases: F5K20.330	4.5	4.9	-0.4	-1.2	100.0%	-4.5
5886	AT5G10210.1 | expressed protein | chr5:3206237-3206830 FORWARD | Aliases: F18D22.2	4.2	3.6	0.6	1.2	100.0%	-4.0
5887	AT2G30810.1 | gibberellin-regulated family protein, similar to GASA5 (Arabidopsis thaliana) GI:1289320; contains Pfam profile PF02704: Gibberellin regulated protein | chr2:13134904-13135732 REVERSE | Aliases: F7F1.2, F7F1_2	2.5	2.4	0.1	1.2	100.0%	-5.0

Rank	Description	15.dpi	5.dpi	M	t	adj.q	B
5888	AT1G02960.3 | expressed protein, similar to hypothetical protein [Arabidopsis thaliana] (TAIR:At1g02965.1) | chr1:666872-669805 REVERSE | Aliases: F22D16.4, F22D16_4	6.6	6.2	0.4	1.2	100.0%	-4.2
5889	AT1G48840.1 | expressed protein, contains Pfam profile: PF04842 plant protein of unknown function (DUF639) | chr1:18065203-18068948 FORWARD | Aliases: T24P22.6, T24P22_6	4.3	4.6	-0.3	-1.2	100.0%	-4.2
5890	AT5G03555.1 | permease, cytosine/purines, uracil, thiamine, allantoin family protein, contains Pfam PF02133: permease, cytosine/purines, uracil, thiamine, allantoin family | chr5:898240-900158 REVERSE | Aliases: None	5.3	4.7	0.6	1.2	100.0%	-4.0
5891	AT4G10100.2 | Symbol: CNX7/SIR5 | molybdenum cofactor synthesis family protein, similar to Molybdenum cofactor synthesis protein 2 small subunit (Molybdopterin-synthase small subunit) (MOCS2A) (MOCO1-A) (Swiss-Prot:O96033) (Homo sapiens); contains TIGRFAM TIGR01682: molybdopterin converting factor, subunit 1; contains Pfam PF02597: ThiS family | chr4:6308712-6309504 FORWARD | Aliases: CNX7, F28M11.20, F28M11_20, MOLYBDOPTERIN SYNTHASE, SIR5	12.0	11.8	0.2	1.2	100.0%	-5.0
5892	AT4G02210.1 | expressed protein | chr4:973924-976129 REVERSE | Aliases: T2H3.2	4.7	4.5	0.2	1.2	100.0%	-4.6
5893	AT4G08460.2 | expressed protein | chr4:5377140-5380615 REVERSE | Aliases: T15F16.1, T15F16_1	5.9	5.7	0.3	1.2	100.0%	-4.4
5894	AT4G00760.1 | Symbol: APRR8 | two-component responsive regulator family protein / response regulator family protein, contains Pfam profile: PF00072 response regulator receiver domain | chr4:327025-329048 REVERSE | Aliases: A_TM018A10.20, A_TM018A10_20, PRR8, PSEUDO RESPONSE REGULATOR 8, T18A10.1, T18A10_1	4.5	4.1	0.4	1.2	100.0%	-4.3
5895	AT1G28760.1 | expressed protein, ; expression supported by MPSS | chr1:10103144-10104574 FORWARD | Aliases: F1K23.6, F1K23_6	4.4	4.7	-0.3	-1.2	100.0%	-4.1
5896	AT1G73200.1 | expressed protein | chr1:27527115-27530234 REVERSE | Aliases: T18K17.13, T18K17_13	4.3	4.6	-0.3	-1.2	100.0%	-4.4
5897	AT3G24050.1 | GATA transcription factor 1 (GATA-1), identical to GATA transcription factor 1 (AtGATA-1) GB:Y13648 (Arabidopsis thaliana) | chr3:8685929-8687684 FORWARD | Aliases: F14O13.7	4.9	4.1	0.8	1.2	100.0%	-3.9
5898	AT3G01520.1 | universal stress protein (USP) family protein, similar to ER6 protein (GI:5669654) (Lycopersicon esculentum); contains Pfam profile PF00582: universal stress protein family | chr3:208446-210110 FORWARD | Aliases: F4P13.7, F4P13_7	9.9	9.2	0.7	1.2	100.0%	-3.9
5899	AT1G70530.1 | protein kinase family protein, contains Pfam domain, PF00069: Protein kinase domain | chr1:26592413-26595042 REVERSE | Aliases: F24J13.10, F24J13_10	4.5	5.0	-0.4	-1.2	100.0%	-4.3
5900	AT2G33580.1 | protein kinase family protein / peptidoglycan-binding LysM domain-containing protein, protein kinase (Arabidopsis thaliana) GI:2852449; contains Pfam profiles PF01476: LysM domain, PF00069: Protein kinase domain | chr2:14226699-14228937 REVERSE | Aliases: F4P9.35, F4P9_35	5.7	5.3	0.4	1.2	100.0%	-4.3
5901	AT5G51300.3 | similar to KH domain-containing quaking protein, putative [Arabidopsis thaliana] (TAIR:At1g09660.1); similar to related to branch point bridging protein (MSL5) [Neurospora crassa] (GB:CAD36971.1); contains InterPro domain KH domain (InterPro:IPR004087); contains InterPro domain Zn-finger, CCHC type (InterPro:IPR001878); contains InterPro domain RNA-binding region RNP-1 (RNA recognition motif) (InterPro:IPR000504) | chr5:20866213-20869556 REVERSE | Aliases: MWD22.25, MWD22_25	3.6	4.2	-0.6	-1.2	100.0%	-4.1
5902	AT5G13730.1 | Symbol: SIGD | RNA polymerase sigma subunit SigD (sigD) / sigma-like factor (SIG4), identical to RNA polymerase sigma subunit SigD (Arabidopsis thaliana) GI:4972296, sigma-like factor (Arabidopsis thaliana) GI:3983260; contains Pfam profiles PF04545: Sigma-70, region 4, PF04539: Sigma-70 region 3, PF04542: Sigma-70 region 2 | chr5:4428700-4430837 REVERSE | Aliases: MSH12.20, MSH12_20, SIG4, SIGMA LIKE FACTOR 4	4.8	4.2	0.6	1.2	100.0%	-3.9
5903	AT2G39770.1 | Symbol: CYT1 | GDP-mannose pyrophosphorylase (GMP1), identical to GDP-mannose pyrophosphorylase from Arabidopsis thaliana (GI:3598958); updated per Conklin PL et al, PNAS 1999, 96(7):4198-203 | chr2:16595783-16598063 FORWARD | Aliases: CYTOKINESIS DEFECTIVE 1, EMB101, EMBRYO DEFECTIVE 101, GDP MANNOSE PYROPHOSPHORYLASE 1, GMP1, SENSITIVE TO OZONE 1, SOZ1, T5I7.7, T5I7_7, VITAMIN C DEFECTIVE 1, VTC1	8.1	7.4	0.7	1.2	100.0%	-4.3
5904	AT5G39520.1 | expressed protein, predicted protein, Synechocystis sp., PIR:S77152 | chr5:15840681-15842720 REVERSE | Aliases: MUL8.22, MUL8_22	3.0	3.2	-0.2	-1.2	100.0%	-4.8
5905	AT1G34065.1 | mitochondrial substrate carrier family protein, contains Pfam profile: PF00153 mitochondrial carrier protein | chr1:12398696-12400861 REVERSE | Aliases: None	3.9	4.1	-0.3	-1.2	100.0%	-4.5
5906	AT1G23100.1 | 10 kDa chaperonin, putative, similar to 10 kDa chaperonin SP:P34893 from (Arabidopsis thaliana) | chr1:8195769-8196464 FORWARD | Aliases: T26J12.12, T26J12_12	5.4	5.9	-0.4	-1.2	100.0%	-3.9
5907	AT2G41220.1 | Symbol: GLU2 | glutamate synthase, chloroplast (GLU2) / ferredoxin-dependent glutamate synthase (Fd-GOGAT 2), identical to SP:Q9T0P4 Ferredoxin-dependent glutamate synthase 2, chloroplast precursor (EC 1.4.7.1) (Fd-GOGAT 2) {Arabidopsis thaliana} | chr2:17185012-17195829 FORWARD | Aliases: F13H10.23, F13H10_23, GLUTAMATE SYNTHASE PRECURSOR	8.9	8.5	0.4	1.2	100.0%	-4.3

Rank	Description	15.dpi	5.dpi	M	t	adj.q	B
5908	AT3G23990.1 | Symbol: HSP60 | chaperonin (CPN60) (HSP60), identical to SWISS-PROT:P29197- chaperonin CPN60, mitochondrial precursor (HSP60) (Arabidopsis thaliana) | chr3:8668935-8672585 FORWARD | Aliases: F14O13.1	10.0	10.8	-0.7	-1.2	100.0%	-4.0
5909	AT5G04890.1 | Symbol: RTM2 | small heat shock-like protein (RTM2), similar to 17.9 kDa heat-shock protein (Helianthus annuus) GI:11990130; contains Pfam profile PF00011: Hsp20/alpha crystallin family; supporting cDNA gi:7407072:gb:AF208051.1:AF208051; identical to cDNA small heat shock-like protein (RTM2) GI:7407072, small heat shock-like protein (Arabidopsis thaliana) GI:7407073 | chr5:1427174-1428455 FORWARD | Aliases: MUK11.22, MUK11_22, RESTRICTED TEV MOVEMENT 2	5.6	4.9	0.7	1.2	100.0%	-3.7
5910	AT2G35490.1 | plastid-lipid associated protein PAP, putative, similar to plastid-lipid associated protein PAP3 (Brassica rapa) GI:14248552; contains Pfam profile PF04755: PAP_fibrillin | chr2:14919223-14920956 REVERSE | Aliases: T32F12.13, T32F12_13	8.5	8.0	0.5	1.2	100.0%	-4.0
5911	AT5G47435.2 | formyltetrahydrofolate deformylase, putative, similar to formyltetrahydrofolate deformylase (strain PCC 6803- Synechocystis sp.) SWISS-PROT:Q55135 | chr5:19258930-19261181 FORWARD | Aliases: None	11.2	10.8	0.3	1.2	100.0%	-4.6
5912	AT1G64020.1 | serpin-related / serine protease inhibitor-related, similar to phloem serpin-1 (Cucurbita maxima) GI:9937311 | chr1:23755419-23755784 REVERSE | Aliases: F22C12.27, F22C12_27	2.2	2.3	-0.2	-1.2	100.0%	-5.0
5913	AT5G10530.1 | lectin protein kinase, putative, similar to receptor lectin kinase 3 (Arabidopsis thaliana) gi:4100060:gb:AAD00733; contains protein kinase domain, Pfam:PF00069; contains legume lectins alpha and beta domains, Pfam:PF00138 and Pfam:PF00139 | chr5:3324979-3326934 REVERSE | Aliases: F12B17.120, F12B17_120	2.3	2.5	-0.2	-1.2	100.0%	-5.1
5914	AT5G22030.2 | ubiquitin-specific protease 8, putative (UBP8), similar to ubiquitin-specific protease 8 partial sequence GI:11993469 (Arabidopsis thaliana) | chr5:7289706-7293597 REVERSE | Aliases: None	4.7	4.4	0.3	1.2	100.0%	-4.2
5915	AT3G18880.1 | ribosomal protein S17 family protein, similar to 40S ribosomal protein S17 GB:Y08858 from (Nicotiana plumbaginifolia) | chr3:6509584-6510631 FORWARD | Aliases: MCB22.5	5.2	5.5	-0.3	-1.2	100.0%	-4.2
5916	AT5G46910.1 | transcription factor jumonji (jmj) family protein, contains Pfam domains PF02375: jmjN domain and PF02373: jmjC domain | chr5:19065007-19068107 FORWARD | Aliases: MQD22.4, MQD22_4	4.2	4.8	-0.5	-1.2	100.0%	-4.1
5917	AT1G76260.1 | transducin family protein / WD-40 repeat family protein, contains 6 WD-40 repeats (PF00400); similar to retinoblastoma A associated protein; RbAp48 (GI:3309245) (Xenopus laevis) | chr1:28615206-28618838 FORWARD | Aliases: T23E18.19, T23E18_19	8.7	9.4	-0.7	-1.2	100.0%	-4.1
5918	AT3G06190.2 | Symbol: ATBPM2 |	4.1	4.3	-0.3	-1.2	100.0%	-4.4
5919	AT3G12350.1 | F-box family protein, ; similar to SKP1 interacting partner 2 (SKIP2) TIGR_Ath1:At5g67250 | chr3:3931571-3933363 FORWARD | Aliases: T2E22.32	6.2	6.0	0.3	1.2	100.0%	-4.4
5920	AT1G74970.1 | Symbol: RPS9 | ribosomal protein S9 (RPS9), identical to ribosomal protein S9 (Arabidopsis thaliana) GI:5456946	5.6	6.2	-0.6	-1.2	100.0%	-3.7
5921	AT4G38690.1 | 1-phosphatidylinositol phosphodiesterase-related, contains weak similarity to 1-phosphatidylinositol phosphodiesterase precursor (EC 4.6.1.13) (Phosphatidylinositol diacylglycerol-lyase) (Phosphatidylinositol- specific phospholipase C) (PI-PLC). (Swiss-Prot:P34024) (Listeria monocytogenes) | chr4:18074469-18075759 REVERSE | Aliases: T9A14.2	3.1	2.9	0.2	1.2	100.0%	-4.7
5922	AT1G71720.1 | S1 RNA-binding domain-containing protein, contains Pfam domain, PF00575: S1 RNA binding domain | chr1:26987406-26989652 FORWARD | Aliases: F14O23.10, F14O23_10	8.2	8.7	-0.5	-1.2	100.0%	-3.9
5923	AT4G15440.1 | Symbol: HPL1 | hydroperoxide lyase (HPL1), identical to hydroperoxide lyase GI:3822403 from (Arabidopsis thaliana) | chr4:8835494-8838675 FORWARD | Aliases: CYP74B2, DL3766W, FCAALL.125, HYDROPEROXIDE LYASE 1	3.5	3.2	0.3	1.2	100.0%	-4.3
5924	AT1G15470.1 | transducin family protein / WD-40 repeat family protein, Strong similarity to gb AF096285 serine-threonine kinase receptor-associated protein from Mus musculus and contains 5 PF:00400 WD40, G-beta repeat domains. EST gb:F14050 comes from this gene | chr1:5315502-5317858 FORWARD | Aliases: F9L1.42, F9L1_42	7.1	7.6	-0.5	-1.2	100.0%	-4.4
5925	AT3G56630.1 | Symbol: CYP94D2 | cytochrome P450, putative, cytochrome P450 CYP94A1 - Vicia sativa, PIR:T08014	6.8	6.3	0.6	1.2	100.0%	-3.7
5926	AT5G54730.1 | expressed protein | chr5:22251015-22254030 REVERSE | Aliases: K5F14.9, K5F14_9	6.3	6.7	-0.4	-1.2	100.0%	-4.1
5927	AT1G72350.1 | MADS-box protein (AGL60), contains Pfam profile: PF00319 SRF-type transcription factor (DNA-binding and dimerisation domain) | chr1:27242935-27243609 REVERSE | Aliases: T10D10.18, T10D10_18	2.8	2.6	0.2	1.2	100.0%	-4.7

Rank	Description	15.dpi	5.dpi	M	t	adj.q	B
5928	AT4G35110.2 | expressed protein | chr4:16712467-16714958 REVERSE | Aliases: T12J5.14	3.5	3.6	-0.2	-1.2	100.0%	-4.6
5929	AT2G31670.1 | expressed protein, similar to NADH-ubiquinone oxidoreductase B16.6 subunit (EC 1.6.5.3) (EC 1.6.99.3) (Complex I-B16.6) (CI-B16.6) (Gene associated with retinoic-interferon-induced mortality 19 protein) (GRIM-19) (Cell death-regulatory protein GRIM-19) (Swiss-Prot:Q95KV7) (Bos taurus) | chr2:13479614-13480581 REVERSE | Aliases: T9H9.19, T9H9_19	7.4	8.0	-0.6	-1.2	100.0%	-4.2
5930	AT4G00230.1 | Symbol: XSP1 | subtilisin-like serine endopeptidase (XSP1), identical to subtilisin-type serine endopeptidase XSP1 GI:6708179 from (Arabidopsis thaliana) | chr4:93923-97449 FORWARD | Aliases: F6N15.3, F6N15_3, SUBTILISIN TYPE SERINE ENDOPEPTIDASE XSP1, XYLEM SERINE PEPTIDASE 1	4.1	3.6	0.5	1.2	100.0%	-4.0
5931	AT1G53920.1 | GDSL-motif lipase/hydrolase family protein, similar to Anther-specific proline-rich proteins SP:P40603 SP:P40602 from {Arabidopsis thaliana}; contains Pfam profile PF00657: GDSL-like Lipase/Acylhydrolase | chr1:20141380-20143305 FORWARD | Aliases: T18A20.15, T18A20_15	3.6	3.8	-0.2	-1.2	100.0%	-4.5
5932	AT4G30220.1 | small nuclear ribonucleoprotein F, putative / snRNP-F, putative / Sm protein F, putative, similar to SWISS-PROT:Q15356 small nuclear ribonucleoprotein F (snRNP-F, Sm protein F, Sm-F, SmF) (Mouse) | chr4:14802961-14804329 REVERSE | Aliases: F9N11.70, F9N11_70	8.1	8.6	-0.5	-1.2	100.0%	-3.8
5933	AT3G07960.1 | phosphatidylinositol-4-phosphate 5-kinase family protein, similar to phosphatidylinositol-4-phosphate 5-kinase AtPIP5K1 (Arabidopsis thaliana) GI:3702691; contains Pfam profiles PF01504: Phosphatidylinositol-4-phosphate 5-Kinase, PF02493: MORN repeat | chr3:2534893-2538342 FORWARD | Aliases: F17A17.30	4.3	4.7	-0.4	-1.2	100.0%	-4.2
5934	AT1G30080.1 | glycosyl hydrolase family 17 protein, similar to beta-1,3-glucanase precursor GI:4097948 from (Oryza sativa) | chr1:10550931-10553199 REVERSE | Aliases: T1P2.13, T1P2_13	5.8	5.4	0.3	1.2	100.0%	-4.0
5935	AT3G60910.1 | expressed protein, low similarity to PIR:I46078 endothelin converting enzyme from Bos primigenius taurus	7.9	7.7	0.2	1.2	100.0%	-4.5
5936	AT3G06710.1 | expressed protein, similar to expressed protein [Arabidopsis thaliana] (TAIR:At4g32670.1) | chr3:2118214-2120065 FORWARD | Aliases: T8E24.2	3.5	3.1	0.3	1.2	100.0%	-4.6
5937	AT1G57830.1 | Toll-Interleukin-Resistance (TIR) domain-containing protein, domain signature TIR exists, suggestive of a disease resistance protein. | chr1:21423974-21424468 FORWARD | Aliases: F12K22.13, F12K22_13	2.5	2.7	-0.2	-1.2	100.0%	-4.7
5938	AT1G34770.2 | MAGE-8 antigen-related, contains weak similarity to Swiss-Prot:P43361 melanoma-associated antigen 8 (MAGE-8 antigen) (Homo sapiens) | chr1:12746598-12748538 FORWARD | Aliases: F11O6.12	4.8	5.1	-0.3	-1.2	100.0%	-4.2
5939	AT3G13662.1 | disease resistance-responsive protein-related / dirigent protein-related, similar to pathogenesis-related protein (Pisum sativum) gi:4585273:gb:AAD25355; similar to dirigent protein (Forsythia x intermedia) gi:6694695:gb:AAF25358 | chr3:4467097-4467657 FORWARD | Aliases: MMM17.6	2.8	2.6	0.2	1.2	100.0%	-4.7
5940	AT2G27460.1 | sec23/sec24 transport family protein, weak similarity to SP:P53992 Protein transport protein Sec24C (SEC24-related protein C) {Homo sapiens}; contains Pfam domains PF04811: Sec23/Sec24 trunk domain, PF04815: Sec23/Sec24 helical domain and PF04810: Sec23/Sec24 zinc finger | chr2:11747732-11752465 FORWARD | Aliases: F10A12.14, F10A12_14	4.5	4.8	-0.3	-1.2	100.0%	-4.6
5941	AT3G56300.1 | tRNA synthetase class I (C) family protein, similar to cysteinyl-tRNA synthetase (Methanococcus maripaludis) GI:6599476; contains Pfam profile PF01406: tRNA synthetases class I (C) | chr3:20891148-20893345 FORWARD | Aliases: F18O21.260	4.2	4.5	-0.3	-1.2	100.0%	-4.2
5942	AT2G11370.1 | hypothetical protein | chr2:4538787-4539393 FORWARD | Aliases: F3K12.11, F3K12_11	2.4	2.3	0.1	1.2	100.0%	-5.1
5943	AT5G11890.1 | expressed protein | chr5:3831665-3832945 FORWARD | Aliases: F14F18.60, F14F18_60	3.5	4.0	-0.5	-1.2	100.0%	-4.1
5944	AT5G15850.1 | Symbol: COL1 | zinc finger protein CONSTANS-LIKE 1 (COL1), identical to Zinc finger protein CONSTANS-LIKE 1 SP:O50055 from (Arabidopsis thaliana) | chr5:5176094-5177900 REVERSE | Aliases: CONSTANS LIKE 1, F14F8.230, F14F8_230	4.2	3.9	0.3	1.2	100.0%	-4.3
5945	AT3G14330.1 | pentatricopeptide (PPR) repeat-containing protein, contains Pfam profile PF01535: PPR repeat | chr3:4779695-4782459 REVERSE | Aliases: MLN21.12	3.2	3.5	-0.2	-1.2	100.0%	-4.5
5946	AT3G26100.2 | regulator of chromosome condensation (RCC1) family protein, weak similarity to UVB-resistance protein UVR8 (Arabidopsis thaliana) GI:5478530; contains Pfam profile PF00415: Regulator of chromosome condensation (RCC1)	6.8	6.4	0.4	1.2	100.0%	-4.0
5947	AT5G01290.1 | similar to mRNA capping enzyme family protein [Arabidopsis thaliana] (TAIR:At3g09100.1); similar to mRNA capping enzyme family protein [Arabidopsis thaliana] (TAIR:At3g09100.2); similar to mRNA capping enzyme family protein [Arabidopsis thaliana] (TAIR:At5g28210.1); similar to similar to ENSANGP00000009960 [Apis mellifera] (GB:XP_397436.1); contains InterPro domain mRNA capping enzyme (InterPro:IPR001339); contains InterPro domain Tyrosine specific protein phosphatase and dual specificity protein phosphatase (InterPro:IPR000387) | chr5:117306-121568 FORWARD | Aliases: T10O8.2	5.5	5.8	-0.3	-1.2	100.0%	-4.2

Rank	Description	15.dpi	5.dpi	M	t	adj.q	B
5948	AT1G48630.1 | guanine nucleotide-binding family protein / activated protein kinase C receptor, putative / RACK, putative, contains 7 WD-40 repeats (PF00400); very similar to guanine nucleotide-binding protein; activated protein kinase C receptor; RACK1 (GI:9294068) {Arabidopsis thaliana}; similar to WD-40 repeat auxin-dependent protein ARCA (SP:O24456) (Arabidopsis thaliana); | chr1:17985448-17986995 REVERSE | Aliases: F11I4.18, F11I4_18	10.5	11.1	-0.5	-1.2	100.0%	-4.2
5949	AT5G53070.1 | ribosomal protein L9 family protein, contains similarity to ribosomal protein L9	4.3	4.8	-0.4	-1.2	100.0%	-3.9
5950	AT3G46750.1 | similar to expressed protein [Arabidopsis thaliana] (TAIR:At3g28770.1); similar to PREDICTED: similar to MATER protein isoform-H [Rattus norvegicus] (GB:XP_218237.3); similar to similar to ENSANGP00000017739 [Apis mellifera] (GB:XP_394403.1) | chr3:17230730-17232619 FORWARD | Aliases: T6H20.220	2.3	2.1	0.1	1.2	100.0%	-5.0
5951	AT3G24480.1 | leucine-rich repeat family protein / extensin family protein, similar to extensin-like protein (Lycopersicon esculentum) gi:5917664:gb:AAD55979; contains leucine-rich repeats, Pfam:PF00560; contains proline rich extensin domains, INTERPRO:IPR002965	5.5	6.1	-0.6	-1.2	100.0%	-4.0
5952	AT3G46910.1 | expressed protein | chr3:17289621-17290637 FORWARD | Aliases: T6H20.60	2.3	2.4	-0.1	-1.2	100.0%	-5.1
5953	AT5G26890.1 | hypothetical protein | chr5:9460468-9460719 FORWARD | Aliases: F2P16.150, F2P16_150	3.2	2.8	0.4	1.2	100.0%	-4.2
5954	AT2G29120.1 | Symbol: ATGLR2.7 | similar to glutamate receptor family protein (GLR2.8) (GLUR9) [Arabidopsis thaliana] (TAIR:At2g29110.1); similar to glutamate receptor family protein (GLR2.1) (GLR3) [Arabidopsis thaliana] (TAIR:At5g27100.1); similar to glutamate receptor family protein (GLR2.3) [Arabidopsis thaliana] (TAIR:At2g24710.1); similar to glutamate receptor family protein (GLR2.9) [Arabidopsis thaliana] (TAIR:At2g29100.1); similar to glutamate receptor family protein (GLR2.2) [Arabidopsis thaliana] (TAIR:At2g24720.1); similar to Avr9/Cf-9 rapidly elicited protein 141 [Nicotiana tabacum] (GB:AAP03877.1); similar to GLR21_ARATH Glutamate receptor 2.1 precursor (Ligand-gated ion channel 2.1) (AtGLR3) (GB:O04660); similar to GLR25_ARATH Glutamate receptor 2.5 precursor (Ligand-gated ion channel 2.5) (GB:Q9LFN5); similar to GLR24_ARATH Glutamate receptor 2.4 precursor (Ligand-gated ion channel 2.4) (GB:O81776); similar to GLR26_ARATH Glutamate receptor 2.6 precursor (Ligand-gated ion channel 2.6) (GB:Q9LFN8); contains InterPro domain Extracellular ligand-binding receptor (InterPro:IPR001828); contains InterPro domain Ionotropic glutamate receptor (InterPro:IPR001320)	2.7	2.5	0.2	1.2	100.0%	-4.9
5955	AT5G62020.1 | Symbol: AT HSFB2A | heat shock factor protein, putative (HSF6) / heat shock transcription factor, putative (HTSF6), identical to heat shock transcription factor 6 (HSF6) SP: Q9SCW4 from (Arabidopsis thaliana) ;contains Pfam profile: PF00447 HSF-type DNA-binding domain | chr5:24933315-24934869 FORWARD | Aliases: HSFB2A, MTG10.7, MTG10_7	5.3	5.0	0.3	1.2	100.0%	-4.2
5956	AT4G24090.1 | expressed protein | chr4:12512746-12514591 FORWARD | Aliases: T19F6.80, T19F6_80	5.5	5.1	0.3	1.2	100.0%	-4.1
5957	AT3G50310.1 | Symbol: MAPKKK20 | protein kinase-related, contains eukaryotic protein kinase domain, INTERPRO:IPR000719 | chr3:18659133-18660503 REVERSE | Aliases: F11C1.150	4.1	3.7	0.4	1.2	100.0%	-4.3
5958	AT2G47510.1 | Symbol: FUM1 | fumarate hydratase, putative / fumarase, putative, similar to SP:P55250 Fumarate hydratase, mitochondrial precursor (EC 4.2.1.2) (Fumarase) {Rhizopus oryzae}; contains Pfam profile PF00206: Lyase | chr2:19505458-19509316 FORWARD | Aliases: FUMARASE 1, T30B22.19	8.7	9.4	-0.7	-1.2	100.0%	-3.9
5959	AT5G50950.2 | fumarate hydratase, putative / fumarase, putative, similar to SP:P55250 Fumarate hydratase, mitochondrial precursor (EC 4.2.1.2) (Fumarase) {Rhizopus oryzae}; contains Pfam profile PF00206: Lyase | chr5:20746870-20751152 FORWARD | Aliases: K3K7.11, K3K7_11	8.7	9.4	-0.7	-1.2	100.0%	-3.9
5960	AT4G01370.1 | Symbol: ATMPK4 |	9.1	9.6	-0.6	-1.2	100.0%	-4.2
5961	AT1G11240.1 | expressed protein | chr1:3766754-3768294 REVERSE | Aliases: T28P6.24, T28P6_24	8.7	9.5	-0.8	-1.2	100.0%	-4.0
5962	AT1G06730.1 | pfkB-type carbohydrate kinase family protein, contains Pfam profile: PF00294 pfkB family carbohydrate kinase | chr1:2067528-2070445 FORWARD | Aliases: F4H5.18, F4H5_18	5.2	4.9	0.3	1.2	100.0%	-4.1
5963	AT5G19930.1 | integral membrane family protein, contains Pfam domain PF01940: Integral membrane protein | chr5:6737585-6739350 REVERSE | Aliases: F28I16.80, F28I16_80	7.2	6.6	0.5	1.2	100.0%	-4.1
5964	AT1G01290.1 | Symbol: CNX3 | molybdopterin biosynthesis CNX3 protein / molybdenum cofactor biosynthesis enzyme CNX3 (CNX3), identical to molybdopterin biosynthesis CNX3 protein SP:Q39056 from (Arabidopsis thaliana) | chr1:114286-115549 FORWARD | Aliases: COFACTOR OF NITRATE REDUCTASE AND XANTHINE DEHYDROGENASE 3, F6F3.9, F6F3_9	6.8	6.5	0.3	1.2	100.0%	-4.3
5965	AT1G48350.1 | ribosomal protein L18 family protein, similar to ribosomal protein L18 GI:3980238 from (Thermotoga maritima) | chr1:17870876-17872010 FORWARD | Aliases: F11A17.10, F11A17_10	9.6	10.1	-0.5	-1.2	100.0%	-4.0
5966	AT1G06010.1 | expressed protein | chr1:1822617-1823996 FORWARD | Aliases: T21E18.6, T21E18_6	6.1	6.6	-0.5	-1.2	100.0%	-4.0

Rank	Description	15.dpi	5.dpi	M	t	adj.q	B
5967	AT3G26380.1 | glycosyl hydrolase family protein 27 / alpha-galactosidase family protein / melibiase family protein, similar to alpha-galactosidase GI:10944326 from (Phanerochaete chrysosporium) | chr3:9661302-9664535 FORWARD | Aliases: F20C19.10	8.1	7.7	0.4	1.2	100.0%	-4.2
5968	AT1G08480.1 | expressed protein | chr1:2684237-2685620 FORWARD | Aliases: T27G7.26	9.3	9.8	-0.5	-1.2	100.0%	-4.4
5969	AT3G62650.2 | expressed protein, putative mitochondrial carrier protein At2g47490 - Arabidopsis thaliana, EMBL:AC002535 | chr3:23179037-23180163 FORWARD | Aliases: F26K9.80	8.3	7.9	0.4	1.2	100.0%	-4.3
5970	AT5G56140.1 | KH domain-containing protein | chr5:22742688-22745569 FORWARD | Aliases: MDA7.20, MDA7_20	4.3	3.8	0.5	1.2	100.0%	-4.0
5971	AT1G49760.1 | Symbol: PAB8 | polyadenylate-binding protein, putative / PABP, putative, similar to poly(A)-binding protein GB:AAF66825 GI:7673359 from (Nicotiana tabacum). Highly and ubiquitously expressed. Member of the class II PABP family. | chr1:18420148-18423715 FORWARD | Aliases: F14J22.3, F14J22_3, PAB8, POLY(A) BINDING PROTEIN 8	6.0	6.6	-0.6	-1.2	100.0%	-4.1
5972	AT1G69780.1 | Symbol: ATHB13 |	3.9	4.2	-0.3	-1.2	100.0%	-4.3
5973	AT3G47370.3 | similar to 40S ribosomal protein S20 (RPS20A) [Arabidopsis thaliana] (TAIR:At3g45030.1); similar to 40S ribosomal protein S20 (RPS20C) [Arabidopsis thaliana] (TAIR:At5g62300.1); similar to 40S subunit ribosomal protein [Oryza sativa (japonica cultivar-group)] (GB:XP_550614.1); contains InterPro domain Ribosomal protein S10, eukaryotic and archaeal form (InterPro:IPR005729); contains InterPro domain Ribosomal protein S10 (InterPro:IPR001848) | chr3:17464463-17465496 REVERSE | Aliases: T21L8.120	10.3	10.7	-0.4	-1.2	100.0%	-4.3
5974	AT1G65920.1 | regulator of chromosome condensation (RCC1) family protein / zinc finger protein-related, contains Pfam profiles: regulator of chromosome condensation (RCC1), PF01363 FYVE zinc finger | chr1:24528765-24532957 REVERSE | Aliases: F12P19.9, F12P19_9	2.6	2.8	-0.2	-1.2	100.0%	-4.7
5975	AT5G22510.1 | beta-fructofuranosidase, putative / invertase, putative / saccharase, putative / beta-fructosidase, putative, similar to neutral invertase (Daucus carota) GI:4200165; contains Pfam profile PF04853: Plant neutral invertase | chr5:7474813-7477922 REVERSE | Aliases: MQJ16.5, MQJ16_5	6.5	6.0	0.5	1.2	100.0%	-4.0
5976	AT4G37650.1 | Symbol: SHR | short-root transcription factor (SHR) | chr4:17691780-17693754 FORWARD | Aliases: F19F18.140, F19F18_140, SGR7, SHORT ROOT	5.1	5.5	-0.3	-1.2	100.0%	-4.2
5977	AT3G44320.1 | Symbol: NIT3 | nitrilase 3 (NIT3), identical to SP:P46010 Nitrilase 3 (EC 3.5.5.1) {Arabidopsis thaliana} | chr3:16004369-16006798 FORWARD | Aliases: NITRILASE 3, T10D17.110	6.5	7.0	-0.5	-1.2	100.0%	-4.3
5978	AT2G33290.1 | Symbol: SUVH2 | SET domain-containing protein (SUVH2), identical to SUVH2 (Arabidopsis thaliana) GI:13517745; contains Pfam profiles PF00856: SET domain, PF05033: Pre-SET motif, PF02182: YDG/SRA domain; identical to cDNA SUVH2 (SUVH2) GI:13517744	4.0	4.3	-0.3	-1.2	100.0%	-4.4
5979	AT4G00720.1 | shaggy-related protein kinase theta / ASK-theta (ASK8), identical to shaggy-related protein kinase theta (ASK-theta) (Arabidopsis thaliana) SWISS-PROT:Q96287 | chr4:293641-297297 REVERSE | Aliases: F6N23.11, F6N23_11	6.7	6.0	0.7	1.2	100.0%	-4.1
5980	AT3G61840.1 | expressed protein | chr3:22903456-22904244 FORWARD | Aliases: F21F14.10	2.5	2.4	0.1	1.2	100.0%	-5.1
5981	AT1G05500.1 | C2 domain-containing protein, similar to Ca2+-dependent lipid-binding protein (CLB1) GI:2789434 from (Lycopersicon esculentum) | chr1:1625097-1628939 FORWARD | Aliases: T25N20.15	5.8	6.2	-0.4	-1.2	100.0%	-4.3
5982	AT3G01170.1 | expressed protein | chr3:57675-58914 FORWARD | Aliases: T4P13.14, T4P13_14	5.2	5.1	0.2	1.2	100.0%	-4.6
5983	AT1G72740.1 | DNA-binding family protein / histone H1/H5 family protein, similar to DNA-binding protein PcMYB1 (Petroselinum crispum) GI:2224897; contains Pfam profiles PF00538: linker histone H1 and H5 family, PF00249: Myb-like DNA-binding domain | chr1:27383956-27386586 REVERSE | Aliases: F28P22.7, F28P22_7	7.0	6.5	0.6	1.2	100.0%	-4.1
5984	AT3G55620.1 | Symbol: EMB1624 | eukaryotic translation initiation factor 6, putative / eIF-6, putative, similar to SP:O55135 Eukaryotic translation initiation factor 6 (eIF-6) (B4 integrin interactor) {Mus musculus}; contains Pfam profile PF01912: eIF-6 family | chr3:20645182-20647485 FORWARD | Aliases: EMB1624, EMBRYO DEFECTIVE 1624, F1I16.30	9.8	10.3	-0.5	-1.2	100.0%	-4.3
5985	AT5G32470.1 | expressed protein | chr5:12102558-12105829 REVERSE | Aliases: F18O9.80, F18O9_80	6.0	6.4	-0.4	-1.2	100.0%	-4.1
5986	AT4G28230.1 | expressed protein | chr4:13995793-13997313 REVERSE | Aliases: F26K10.110, F26K10_110	8.0	7.4	0.6	1.2	100.0%	-4.1
5987	AT2G43320.1 | expressed protein | chr2:18003727-18007004 REVERSE | Aliases: F14B2.22	3.7	3.4	0.3	1.2	100.0%	-4.6
5988	AT1G27990.1 | expressed protein | chr1:9752786-9753906 REVERSE | Aliases: F13K9.9, F13K9_9	3.7	3.4	0.3	1.2	100.0%	-4.3

Rank	Description	15.dpi	5.dpi	M	t	adj.q	B
5989	AT5G42470.1 | expressed protein, low similarity to BRE alpha b isoform (Homo sapiens) GI:16326573 | chr5:16999544-17002112 FORWARD | Aliases: MDH9.17, MDH9_17	7.6	7.2	0.4	1.2	100.0%	-4.0
5990	AT3G03470.1 | Symbol: CYP89A9 | cytochrome P450, putative, similar to cytochrome P450 89A2 GB:Q42602 (Arabidopsis thaliana) | chr3:824559-826444 REVERSE | Aliases: T21P5.11, T21P5_11	3.4	3.1	0.3	1.2	100.0%	-4.4
5991	AT4G33760.1 | tRNA synthetase class II (D, K and N) family protein, similar to SP:P36419 Aspartyl-tRNA synthetase (EC 6.1.1.12) (Aspartate--tRNA ligase) (AspRS) {Thermus thermophilus}; contains Pfam profile PF00152: tRNA synthetases class II (D, K and N)	5.6	6.1	-0.4	-1.2	100.0%	-3.9
5992	AT3G63250.2 | Symbol: ATHMT 2/HMT 2 | homocysteine S-methyltransferase 2 (HMT-2), 99.7% identical to homocysteine S-methyltransferase AtHMT-2 (GI:6685163) (Arabidopsis thaliana) | chr3:23381313-23383591 REVERSE | Aliases: ATHMT 2, F16M2.100, HMT 2	10.1	9.6	0.4	1.2	100.0%	-4.0
5993	AT4G24220.1 | Symbol: VEP1 | expressed protein, protein induced upon wounding - Arabidopsis thaliana, PID:e257749 | chr4:12564955-12567274 FORWARD | Aliases: AWI31, T22A6.50, T22A6_50, VEIN PATTERNING	7.8	7.3	0.5	1.2	100.0%	-4.2
5994	AT2G17080.1 | hypothetical protein, contains Pfam profile PF03087: Arabidopsis protein of unknown function | chr2:7440408-7441199 REVERSE | Aliases: F6P23.10, F6P23_10	2.9	2.7	0.2	1.2	100.0%	-4.6
5995	AT3G24515.1 | ubiquitin-conjugating enzyme, putative, similar to Ubiquitin-conjugating enzyme E2 (Ubiquitin-protein ligase) (Ubiquitin carrier protein) from {Xenopus laevis} SP:P51669, {Schizosaccharomyces pombe} SP:P46595, {Caenorhabditis elegans} SP:P35129; contains Pfam profile PF00179: Ubiquitin-conjugating enzyme | chr3:8934479-8936286 REVERSE | Aliases: None	3.9	4.3	-0.4	-1.2	100.0%	-4.3
5996	AT1G62810.1 | copper amine oxidase, putative, similar to copper amine oxidase (Cicer arietinum) gi:3819099:emb:CAA08855 | chr1:23261391-23265631 REVERSE | Aliases: F23N19.18, F23N19_18	8.8	8.3	0.5	1.2	100.0%	-4.0
5997	AT5G51220.1 | ubiquinol-cytochrome C chaperone family protein, contains Pfam PF03981: Ubiquinol-cytochrome C chaperone	7.6	8.1	-0.4	-1.2	100.0%	-4.2
5998	AT3G29100.1 | Symbol: VTI13 | vesicle transport v-SNARE 13 (VTI13) / vesicle soluble NSF attachment protein receptor 13, identical to identical to Vesicle transport v-SNARE 13 (SP:Q9LVP9) {Arabidopsis thaliana}; similar to v-snare AtVTI1a (GI:6690274) (GB:AAF24061) (Arabidopsis thaliana) | chr3:11077073-11078725 REVERSE | Aliases: ATVTI13, MXE2.7, VTI13	4.4	4.1	0.3	1.2	100.0%	-4.3
5999	AT4G31450.1 | zinc finger (C3HC4-type RING finger) family protein, similar to Pfam domain, PF00097: Zinc finger, C3HC4 type (RING finger) | chr4:15255634-15258818 REVERSE | Aliases: F3L17.20, F3L17_20	5.0	4.7	0.4	1.2	100.0%	-4.0
6000	AT5G12420.1 | expressed protein | chr5:4024315-4026742 REVERSE | Aliases: None	2.3	2.8	-0.5	-1.2	100.0%	-3.7
6001	AT5G55500.1 | Symbol: AtXylT |	5.6	5.3	0.3	1.2	100.0%	-4.3
6002	AT1G11330.1 | S-locus lectin protein kinase family protein, contains Pfam domains, PF00954: S-locus glycoprotein family, PF00069: Protein kinase domain, and PF01453: Lectin (probable mannose binding) | chr1:3810221-3813607 FORWARD | Aliases: T28P6.2, T28P6_2	8.7	9.2	-0.5	-1.2	100.0%	-3.8
6003	AT3G20310.1 | Symbol: ATERF 7/ATERF7 |	6.3	5.7	0.6	1.2	100.0%	-4.1
6004	AT3G52340.3 | Symbol: SPP2 | similar to sucrose-phosphatase 1 (SPP1) [Arabidopsis thaliana] (TAIR:At2g35840.2); similar to sucrose-phosphatase 1 (SPP1) [Arabidopsis thaliana] (TAIR:At2g35840.1); similar to sucrose-6-phosphate phosphatase [Nicotiana tabacum] (GB:AAW32902.1); contains InterPro domain Sucrose-6F-phosphate phosphohydrolase, plant and cyanobacteria (InterPro:IPR006380); contains InterPro domain Sucrose-phosphate phosphatase (InterPro:IPR006378); contains InterPro domain HAD-superfamily hydrolase, subfamily IIB (InterPro:IPR006379) | chr3:19418001-19420311 FORWARD | Aliases: T25B15.110, sucrose phosphatase 2	5.4	5.7	-0.3	-1.2	100.0%	-4.6
6005	AT5G39830.2 | DegP protease, putative, contains similarity to DegP protease precursor GI:2565436 from (Arabidopsis thaliana) | chr5:15960048-15963051 FORWARD | Aliases: K13H13.1, K13H13_1	3.8	4.0	-0.3	-1.2	100.0%	-4.5
6006	AT3G53130.1 | Symbol: LUT1 | cytochrome P450 family protein, similar to Cytochrome P450 97B2 (SP:048921) (Glycine max) | chr3:19703749-19708520 FORWARD | Aliases: CYP97C1, LUTEIN DEFICIENT 1, T4D2.60	4.7	5.0	-0.3	-1.2	100.0%	-4.3
6007	AT1G21550.1 | calcium-binding protein, putative, contains similarity to calcium-binding protein GB:CAB63264 GI:6580549 from (Lotus japonicus) | chr1:7553090-7553865 REVERSE | Aliases: F24J8.15, F24J8_15	3.0	2.8	0.2	1.2	100.0%	-4.8
6008	AT3G19330.3 | polyadenylate-binding protein-related / PABP-related, similar to At14a (GI:11994571 and GI:11994573) (Arabidopsis thaliana); similar to Polyadenylate-binding protein 2 (Poly(A)-binding protein 2) (PABP 2) (Swiss-Prot:P42731) (Arabidopsis thaliana) | chr3:6699329-6701261 FORWARD | Aliases: MLD14.6	2.8	2.9	-0.1	-1.2	100.0%	-4.9

Rank	Description	15.dpi	5.dpi	M	t	adj.q	B
6009	AT5G64720.1 | similar to hypothetical protein [Arabidopsis thaliana] (TAIR:At2g21740.1); similar to ECA1 protein [Hordeum vulgare] (GB:AAF23356.1) | chr5:25889572-25889943 REVERSE | Aliases: MVP7.4, MVP7_4	2.8	2.6	0.2	1.2	100.0%	-4.6
6010	AT1G75210.1 | 5' nucleotidase family protein, contains Pfam profile: PF05761 5' nucleotidase family | chr1:28227530-28232636 FORWARD | Aliases: F22H5.7, F22H5_7	7.5	7.9	-0.4	-1.2	100.0%	-4.0
6011	AT1G08660.2 | glycosyl transferase family 29 protein / sialyltransferase family protein, contains Pfam profile: PF00777 sialyltransferase (Glycosyltransferase family 29) | chr1:2757046-2759767 REVERSE | Aliases: F22O13.14, F22O13_14	3.4	3.9	-0.5	-1.2	100.0%	-4.3
6012	AT3G27325.1 | expressed protein | chr3:10097461-10103324 FORWARD | Aliases: K17E12.15	2.3	2.5	-0.1	-1.2	100.0%	-5.0
6013	AT4G08170.2 | inositol 1,3,4-trisphosphate 5/6-kinase family protein, similar to inositol phosphate kinase (GI:27549256) (Zea mays); similar to inositol 1,3,4-trisphosphate 5/6-kinase (GI:3396079) (Arabidopsis thaliana) | chr4:5163396-5167176 REVERSE | Aliases: T12G13.10, T12G13_10	5.3	5.0	0.3	1.2	100.0%	-4.2
6014	AT1G50970.1 | membrane trafficking VPS53 family protein, contains Pfam domain PF04100: Vps53-like, N-terminal | chr1:18899991-18903936 FORWARD | Aliases: F8A12.19, F8A12_19	2.7	2.5	0.2	1.2	100.0%	-4.9
6015	AT5G07620.1 | protein kinase family protein, contains protein kinase domain, Pfam:PF00069 | chr5:2407402-2409067 REVERSE | Aliases: MBK20.5, MBK20_5	2.5	2.3	0.2	1.2	100.0%	-4.9
6016	AT1G32860.1 | glycosyl hydrolase family 17 protein, similar to beta-1,3-glucanase precursor GI:4097948 from (Oryza sativa) | chr1:11907089-11908908 REVERSE | Aliases: F9L11.6, F9L11_6	4.5	4.2	0.2	1.2	100.0%	-4.5
6017	AT2G07050.1 | Symbol: CAS1 | cycloartenol synthase (CAS1) / 2,3-epoxysqualene--cycloartenol cyclase / (S)-2,3-epoxysqualene mutase, identical to cycloartenol synthase (SP:P38605 : GI:452446) (PMID:7505443)	8.7	8.2	0.5	1.2	100.0%	-4.1
6018	AT1G47290.2 | 3-beta hydroxysteroid dehydrogenase/isomerase family protein, contains Pfam profile PF01073 3-beta hydroxysteroid dehydrogenase/isomerase domain; similar to NAD(P)-dependent steroid dehydrogenase from Homo sapiens (SP:Q15738), Mus musculus (SP:Q9R1J0) | chr1:17338294-17341713 FORWARD | Aliases: T3F24.9, T3F24_9	3.8	4.2	-0.4	-1.2	100.0%	-4.3
6019	AT5G22000.3 | Symbol: CIC7E11 | zinc finger (C3HC4-type RING finger) family protein, contains Pfam profile: PF00097 zinc finger, C3HC4 type (RING finger) | chr5:7277272-7280258 FORWARD | Aliases: CIC7E11, RHF2A, RING H2 FINGER PROTEIN RHF2A	7.9	7.5	0.3	1.2	100.0%	-4.3
6020	AT4G14990.1 | expressed protein | chr4:8565618-8569810 REVERSE | Aliases: DL3535C, FCAALL.26	5.2	4.8	0.5	1.2	100.0%	-3.9
6021	AT1G47980.1 | expressed protein | chr1:17695503-17697530 REVERSE | Aliases: T2J15.11, T2J15_11	3.6	3.9	-0.3	-1.2	100.0%	-4.2
6022	AT3G45620.1 | transducin family protein / WD-40 repeat family protein, contains 7 WD-40 repeats; similar to PC326 protein (GI:200241) (PIR2:S37694) (Mus musculus);Human (H326) translated mRNA - Homo sapiens, EMBL:HS06631	7.2	7.7	-0.5	-1.2	100.0%	-3.9
6023	AT2G34860.1 | chaperone protein dnaJ-related, contains Pfam PF00684 : DnaJ central domain (4 repeats); similar to Chaperone protein dnaJ (Heat shock protein 40) (SP:Q9UXR9) {Methanosarcina thermophila} | chr2:14715447-14717276 FORWARD | Aliases: F19I3.9, F19I3_9	7.4	7.0	0.4	1.2	100.0%	-4.1
6024	AT1G48940.1 | plastocyanin-like domain-containing protein | chr1:18109476-18110090 REVERSE | Aliases: F27K7.4	2.5	2.3	0.1	1.2	100.0%	-5.0
6025	AT1G23490.1 | Symbol: ATARF/ATARF1/ATARFA1A | Gene encoding ADP-ribosylation factor and similar to other ARFs and ARF-like proteins. Members of this family are known to be essential for vesicle coating and uncoating and functions in GTP-binding. The gene is shown to play a role in cell division, cell expansion and cellulose production using antisense construct. | chr1:8336693-8338661 FORWARD | Aliases: ATARF, ATARF1, ATARFA1A, F28C11.12, F5O8.5, F5O8_5	9.8	9.3	0.5	1.2	100.0%	-4.3
6026	AT4G20050.1 | Symbol: QRT3 | expressed protein, C65DMY30S | chr4:10849792-10853356 REVERSE | Aliases: F18F4.150, F18F4_150, QUARTET 3	3.7	4.0	-0.3	-1.2	100.0%	-4.4
6027	AT1G65440.1 | Symbol: GTB1 | glycine-rich protein | chr1:24310330-24318236 REVERSE | Aliases: GLOBAL TRANSCRIPTION FACTOR GROUP B, T8F5.22, T8F5_22	7.8	7.4	0.4	1.2	100.0%	-4.3
6028	AT1G32730.1 | expressed protein | chr1:11840890-11843237 FORWARD | Aliases: F6N18.11, F6N18_11	3.6	3.9	-0.3	-1.2	100.0%	-4.4
6029	AT4G27340.1 | Met-10+ like family protein, ; contains Pfam profile PF02475: Met-10+ like-protein | chr4:13687202-13690376 REVERSE | Aliases: F27G19.9	5.0	5.4	-0.3	-1.2	100.0%	-4.2
6030	AT3G26810.1 | transport inhibitor response protein, putative, E3 ubiquitin ligase SCF complex F-box subunit; similar to transport inhibitor response 1 GI:2352492 from (Arabidopsis thaliana) | chr3:9869082-9871877 FORWARD | Aliases: MDJ14.9	3.1	3.3	-0.3	-1.2	100.0%	-4.5

Rank	Description	15.dpi	5.dpi	M	t	adj.q	B
6031	AT3G27320.2 | similar to expressed protein [Arabidopsis thaliana] (TAIR:At5g14310.1); similar to putative esterase [Oryza sativa (japonica cultivar-group)] (GB:XP_469930.1) | chr3:10091302-10094028 FORWARD | Aliases: K17E12.14	3.6	3.8	-0.2	-1.2	100.0%	-4.6
6032	AT1G20580.1 | small nuclear ribonucleoprotein, putative / snRNP, putative / Sm protein, putative, similar to small nuclear ribonucleoprotein Sm D3 (snRNP core protein D3, Sm-D3) (Mus musculus) SWISS-PROT:P43331 | chr1:7128878-7130631 FORWARD | Aliases: F5M15.9, F5M15_9	9.4	9.0	0.4	1.2	100.0%	-4.2
6033	AT5G09740.1 | histone acetyltransferase, putative, similar to histone acetyltransferase (Homo sapiens) gi:8317213:gb:AAF72665 | chr5:3021981-3024726 REVERSE | Aliases: F17I14.70, F17I14_70	4.5	4.9	-0.4	-1.2	100.0%	-4.6
6034	AT3G11460.1 | pentatricopeptide (PPR) repeat-containing protein, contains Pfam profile PF01535: PPR repeat | chr3:3608256-3610127 FORWARD | Aliases: F24K9.13	3.9	4.0	-0.2	-1.2	100.0%	-4.9
6035	AT4G16280.3 | Symbol: FCA | flowering time control protein / FCA gamma (FCA), identical to SP:O04425 Flowering time control protein FCA {Arabidopsis thaliana}; four alternative splice variants, one splicing isoform contains a non-consensus CA donor splice site, based on cDNA: gi:2204090 | chr4:9207179-9214428 REVERSE | Aliases: DL4180C, FCA, FCAALL.331, FLOWERING TIME CONTROL PROTEIN FCA ALPHA, BETA, DELTA AND GAMMA	3.3	3.5	-0.3	-1.2	100.0%	-4.3
6036	AT5G06290.1 | 2-cys peroxiredoxin, chloroplast, putative, very strong similarity to SP:Q96291 2-cys peroxiredoxin BAS1, chloroplast precursor {Arabidopsis thaliana}; contains Pfam profile: PF00578 AhpC/TSA (alkyl hydroperoxide reductase and thiol-specific antioxidant) family | chr5:1919249-1921404 FORWARD | Aliases: MHF15.19, MHF15_19	9.3	9.9	-0.6	-1.2	100.0%	-3.7
6037	AT3G09880.1 | Symbol: ATB' BETA | serine/threonine protein phosphatase 2A (PP2A) regulatory subunit B' (B'beta), identical to B' regulatory subunit of PP2A (Arabidopsis thaliana) GI:2160692; similar to SWISS-PROT:Q28653 serine/threonine protein phosphatase 2A, 56 kDa regulatory subunit, delta isoform (PP2A, B subunit, B' delta isoform, PP2A, B subunit, B56 delta isoform, PP2A, B subunit, PR61 delta isoform, PP2A, B subunit, R5 delta isoform, PP2A, B subunit, B'-gamma) (Oryctolagus cuniculus); contains Pfam domain, PF01603: Protein phosphatase 2A regulatory B subunit (B56 family) | chr3:3029516-3032261 FORWARD | Aliases: F8A24.7	4.1	4.5	-0.3	-1.2	100.0%	-4.4
6038	AT1G02360.1 | chitinase, putative, similar to chitinase precursor GI:5880845 from (Petroselinum crispum) | chr1:471990-473140 REVERSE | Aliases: T6A9.5, T6A9_5	4.6	5.6	-0.9	-1.2	100.0%	-3.8
6039	AT1G09180.1 | Symbol: ATSAR1/ATSARA1A | GTP-binding protein, putative, strong similarity to SP:Q01474 GTP-binding protein SAR1B and SP:O04834 GTP-binding protein SAR1A (Arabidopsis thaliana) | chr1:2965025-2965974 FORWARD | Aliases: ATSAR1, ATSARA1A, T12M4.12, T12M4_12	4.7	4.4	0.3	1.2	100.0%	-4.5
6040	AT2G26110.1 | expressed protein, similar to expressed protein [Arabidopsis thaliana] (TAIR:At4g26130.1); similar to unknown protein [Oryza sativa (japonica cultivar-group)] (GB:BAD81521.1) | chr2:11127984-11129137 FORWARD | Aliases: T19L18.8, T19L18_8	3.1	3.4	-0.3	-1.2	100.0%	-4.2
6041	AT5G37060.1 | Symbol: ATCHX24 | cation/hydrogen exchanger, putative (CHX24), similar to Na+/H+-exchanging protein NapA - Enterococcus hirae, PIR:A42111; monovalent cation:proton antiporter family 2 (CPA2) member, PMID:11500563 | chr5:14659971-14662644 REVERSE | Aliases: CHX24, MJG14.5, MJG14_5	3.5	3.4	0.2	1.2	100.0%	-4.8
6042	AT3G54650.1 | F-box family protein (FBL17), contains similarity to F-box protein FBL2 GI:6010699 from (Rattus norvegicus) | chr3:20236764-20239899 REVERSE | Aliases: T5N23.10	4.0	4.4	-0.5	-1.2	100.0%	-4.3
6043	AT5G48580.1 | Symbol: FKBP15 2 | FK506-binding protein 2-2 (FKBP15-2) / immunophilin / peptidyl-prolyl cis-trans isomerase / rotamase, identical to SP:Q38936: FK506-binding protein 2-2 precursor (EC 5.2.1.8); | chr5:19713155-19714551 REVERSE | Aliases: IMMUNOPHILIN, K15N18.5, K15N18_5	8.0	8.5	-0.5	-1.2	100.0%	-4.0
6044	AT2G26000.2 | similar to zinc finger (ubiquitin-hydrolase) domain-containing protein [Arabidopsis thaliana] (TAIR:At2g42160.1); similar to BRAP2-like protein [Oryza sativa (japonica cultivar-group)] (GB:XP_464349.1); contains InterPro domain Zn-finger in ubiquitin thiolesterase (InterPro:IPR001607); contains InterPro domain Zn-finger, RING (InterPro:IPR001841) | chr2:11088380-11092406 FORWARD | Aliases: T19L18.19, T19L18_19	4.3	4.7	-0.4	-1.2	100.0%	-4.3
6045	AT1G67690.1 | peptidase M3 family protein / thimet oligopeptidase family protein, similar to SP:P42676 Neurolysin, mitochondrial precursor (EC 3.4.24.16) (Mitochondrial oligopeptidase M) {Rattus norvegicus}; contains Pfam profile PF01432: Peptidase family M3 | chr1:25372749-25377393 FORWARD | Aliases: F12A21.16	3.2	3.4	-0.2	-1.2	100.0%	-4.4
6046	AT3G09390.1 | Symbol: MT2A | metallothionein protein, putative (MT2A), identical to Swiss-Prot:P25860 metallothionein-like protein 2A (MT-2A) (MT-K) (MT-1G) (Arabidopsis thaliana) | chr3:2889492-2890235 REVERSE | Aliases: ATMT 1, ATMT K, F3L24.28, METALLOTHIONEIN 2A, MT2	12.9	12.4	0.5	1.2	100.0%	-3.9
6047	AT1G60060.1 | expressed protein | chr1:22142849-22145455 FORWARD | Aliases: T2K10.11, T2K10_11	2.6	2.4	0.2	1.2	100.0%	-5.1
6048	AT5G45780.1 | leucine-rich repeat transmembrane protein kinase, putative | chr5:18584173-18586852 REVERSE | Aliases: MRA19.22, MRA19_22	3.3	3.6	-0.3	-1.2	100.0%	-4.7

Rank	Description	15.dpi	5.dpi	M	t	adj.q	B
6049	AT1G20810.1 | immunophilin / FKBP-type peptidyl-prolyl cis-trans isomerase family protein, identical to Probable FKBP-type peptidyl-prolyl cis-trans isomerase 1, chloroplast precursor (Ppiase) (Rotamase) (SP:Q9LM71)(Arabidopsis thaliana); similar to SP:P25138 FK506-binding protein (Peptidyl-prolyl cis-trans isomerase) (PPiase) (EC 5.2.1.8) (Rotamase) {Neisseria meningitidis}; contains Pfam PF00254: peptidyl-prolyl cis-trans isomerase, FKBP-type | chr1:7232010-7233555 FORWARD | Aliases: F2D10.32, F2D10_32	4.5	4.3	0.3	1.2	100.0%	-4.3
6050	AT3G55005.1 | Symbol: TON1B | tonneau 1b (TON1b), identical to tonneau 1b (TON1b) GI:11494366 from (Arabidopsis thaliana) | chr3:20394979-20397355 FORWARD | Aliases: TONNEAU 1B	4.3	4.5	-0.2	-1.2	100.0%	-4.4
6051	AT4G31860.2 | protein phosphatase 2C, putative / PP2C, putative, protein phosphatase 2C, Schizosaccharomyces pombe, PIR2:S54297 | chr4:15405953-15408997 REVERSE | Aliases: F11C18.60, F11C18_60	7.9	7.5	0.4	1.2	100.0%	-4.5
6052	AT5G66520.1 | pentatricopeptide (PPR) repeat-containing protein, contains INTERPRO:IPR002885 PPR repeats | chr5:26569105-26570967 FORWARD | Aliases: K1F13.18, K1F13_18	2.7	2.9	-0.2	-1.2	100.0%	-4.8
6053	AT5G64610.1 | histone acetyltransferase, putative, similar to histone acetyltransferase (Homo sapiens) gi:8317213:gb:AAF72665 | chr5:25845386-25847750 REVERSE | Aliases: MUB3.13, MUB3_13	5.6	6.0	-0.4	-1.2	100.0%	-4.3
6054	AT2G46030.1 | Symbol: UBC6 | ubiquitin-conjugating enzyme 6 (UBC6), E2; identical to gi:431267, SP:P42750, PIR:S52661; contains a ubiquitin-conjugating enzymes active site (PDOC00163) | chr2:18938464-18940572 REVERSE | Aliases: T3F17.32, UBIQUITIN CONJUGATING ENZYME 6	5.7	5.1	0.6	1.2	100.0%	-4.2
6055	AT1G30130.2 | expressed protein, similar to hypothetical protein GI:1469227 from (Brassica oleracea) | chr1:10587587-10589606 FORWARD | Aliases: T2H7.7, T2H7_7	5.1	5.6	-0.5	-1.2	100.0%	-4.0
6056	AT1G56070.1 | Symbol: LOS1 | elongation factor 2, putative / EF-2, putative, similar to ELONGATION FACTOR 2 GB:O14460 from (Schizosaccharomyces pombe) | chr1:20971595-20975407 REVERSE | Aliases: AT1G56075, AT1G56075.1, T6H22.13, T6H22.24, T6H22_13	11.1	11.5	-0.4	-1.2	100.0%	-4.6
6057	AT5G64250.2 | 2-nitropropane dioxygenase family / NPD family, contains Pfam profile PF03060: oxidoreductase, 2-nitropropane dioxygenase (NPD) family | chr5:25714450-25716438 REVERSE | Aliases: MSJ1.9, MSJ1_9	9.4	9.1	0.3	1.2	100.0%	-4.7
6058	AT3G46880.1 | expressed protein, ; expression supported by MPSS | chr3:17276278-17277251 FORWARD | Aliases: T6H20.90	3.4	3.6	-0.2	-1.2	100.0%	-4.7
6059	AT5G60910.1 | Symbol: AGL8 | agamous-like MADS box protein AGL8 / FRUITFULL (AGL8), NAP1-1, Nicotiana tabacum, EMBL:AF009126; identical to SP:Q38876 Agamous-like MADS box protein AGL8 (Floral homeotic protein AGL8) (FRUITFULL){Arabidopsis thaliana} PMID:9502732, PMID:10648231; identical to cDNA agamous-like 8 (AGL8) GI:1004364 | chr5:24519708-24523339 REVERSE | Aliases: AGAMOUS LIKE 8, AGL8, FRUITFULL, FUL, MSL3.30, MSL3_30	3.5	3.3	0.3	1.2	100.0%	-4.4
6060	AT1G32490.1 | Symbol: EMB2733 | RNA helicase, putative, similar to ATP-dependent RNA helicase #3 (Homo sapiens) GI:3107913; contains Pfam profiles PF04408: Helicase associated domain (HA2), PF00271: Helicase conserved C-terminal domain | chr1:11742034-11749686 REVERSE | Aliases: EMB2733, EMBRYO DEFECTIVE 2733, F5D14.27, F5D14_27	3.5	3.8	-0.3	-1.2	100.0%	-4.4
6061	AT4G21880.1 | pentatricopeptide (PPR) repeat-containing protein, contains Pfam profile PF01535: PPR repeat | chr4:11605117-11611220 FORWARD | Aliases: T8O5.90, T8O5_90	3.3	3.6	-0.3	-1.2	100.0%	-4.1
6062	AT1G77800.1 | PHD finger family protein, contains Pfam domain, PF00628: PHD-finger | chr1:29258290-29265511 FORWARD | Aliases: T32E8.13, T32E8_13	6.2	5.8	0.3	1.2	100.0%	-4.2
6063	AT1G14230.1 | nucleoside phosphatase family protein / GDA1/CD39 family protein, low similarity to SP:P49961 Ectonucleoside triphosphate diphosphohydrolase 1 (EC 3.6.1.5) (Ecto-apyrase) {Homo sapiens}; contains Pfam profile PF01150: GDA1/CD39 (nucleoside phosphatase) family | chr1:4861262-4864089 FORWARD | Aliases: 7A19.33, 7A19_33, F7A19.33, F7A19_33	6.1	5.2	0.9	1.2	100.0%	-3.5
6064	AT1G14250.1 | nucleoside phosphatase family protein / GDA1/CD39 family protein, low similarity to SP:P97687 Ectonucleoside triphosphate diphosphohydrolase 1 (EC 3.6.1.5) (Ecto-apyrase) {Rattus norvegicus}; contains Pfam profile PF01150: GDA1/CD39 (nucleoside phosphatase) family	6.1	5.2	0.9	1.2	100.0%	-3.5
6065	AT1G11210.1 | expressed protein, contains similarity to cotton fiber expressed protein 1 (Gossypium hirsutum) gi:3264828:gb:AAC33276 | chr1:3755677-3756998 REVERSE | Aliases: T28P6.13, T28P6_13	4.4	3.8	0.6	1.2	100.0%	-4.1
6066	AT5G44820.1 | expressed protein, similar to unknown protein (pir::T04881) | chr5:18112940-18114812 REVERSE | Aliases: K23L20.17, K23L20_17	2.6	2.7	-0.1	-1.2	100.0%	-5.2
6067	AT3G27230.1 | expressed protein | chr3:10055166-10056928 FORWARD | Aliases: K17E12.5	7.4	7.9	-0.5	-1.2	100.0%	-4.1

Rank	Description	15.dpi	5.dpi	M	t	adj.q	B
6068	AT1G10390.2 | similar to nucleoporin family protein [Arabidopsis thaliana] (TAIR:At1g59660.1); similar to PREDICTED: nucleoporin 98 [Rattus norvegicus] (GB:XP_574504.1); contains InterPro domain Nucleoporin FG repeat (InterPro:IPR004325); contains InterPro domain Nucleoporin autopeptidase (InterPro:IPR007230)	5.7	5.2	0.5	1.2	100.0%	-4.1
6069	AT1G20410.1 | expressed protein | chr1:7078889-7082537 REVERSE | Aliases: F5M15.23, F5M15_23	3.4	3.7	-0.3	-1.2	100.0%	-4.3
6070	AT5G08340.2 | riboflavin biosynthesis protein-related, contains weak similarity to Riboflavin biosynthesis protein ribF. (Swiss-Prot:P08391) (Escherichia coli O157:H7) | chr5:2683838-2685998 FORWARD | Aliases: F8L15.70, F8L15_70	6.5	6.1	0.4	1.2	100.0%	-4.3
6071	AT1G19710.1 | glycosyl transferase family 1 protein, contains Pfam profile: PF00534 glycosyl transferases group 1 | chr1:6814828-6816707 FORWARD | Aliases: F14P1.19, F14P1_19	6.3	6.6	-0.3	-1.2	100.0%	-4.2
6072	AT1G66230.1 | Symbol: MYB20 | myb family transcription factor (MYB20), similar to myb-related transcription factor GI:1430846 from (Lycopersicon esculentum); contains PFAM profile: Myb DNA binding domain PF00249 | chr1:24680817-24682078 FORWARD | Aliases: MYB20, T6J19.5, T6J19_5	4.5	4.9	-0.4	-1.2	100.0%	-4.1
6073	AT1G67410.1 | exostosin family protein, contains Pfam profile: PF03016	2.8	3.0	-0.2	-1.2	100.0%	-4.5
6074	AT5G40940.1 | expressed protein | chr5:16423884-16425158 REVERSE | Aliases: MMG1.3, MMG1_3	2.3	2.2	0.1	1.2	100.0%	-5.2
6075	AT4G32810.1 | Symbol: CCD8 | Encodes a protein with similarity to carotenoid cleaving deoxygenases. Involved in the production of a graft transmissable signal to suppress axillary branching. Mutants have increased axillary branches. Expressed primarily in root apex. | chr4:15828232-15831481 FORWARD | Aliases: CAROTENOID CLEAVAGE DIOXYGENASE 8, MAX4, T16I18.20, T16I18_20	4.6	4.0	0.6	1.2	100.0%	-3.9
6076	AT1G05180.3 | Symbol: AXR1 | similar to auxin-resistance protein, putative [Arabidopsis thaliana] (TAIR:At2g32410.1); similar to putative ubiquitin-activating enzyme (alternative splicing product) [Oryza sativa (japonica cultivar-group)] (GB:XP_470133.1) | chr1:1498113-1501823 REVERSE | Aliases: AUXIN RESISTANT 1, AUXIN RESISTANT PROTEIN 1, YUP8H12.21, YUP8H12_21	9.5	9.8	-0.3	-1.2	100.0%	-4.9
6077	AT1G63200.1 | hypothetical protein | chr1:23437295-23438265 REVERSE | Aliases: F16M19.10, F16M19_10	2.5	2.4	0.1	1.2	100.0%	-5.1
6078	AT4G22280.2 | F-box family protein, contains Pfam profile PF00646: F-box domain | chr4:11776025-11778131 REVERSE | Aliases: T10I14.110, T10I14_110	3.3	3.1	0.2	1.2	100.0%	-4.6
6079	AT5G54860.1 | integral membrane transporter family protein, contains 10 transmembrane domains; contains Pfam PF03092: BT1 family; contains TIGRFAMS TIGR00788: folate/biopterin transporter; similar to high affinity folic acid/methotrexate transporter 5 (GI:21898554) (Leishmania tarentolae) | chr5:22301855-22304432 FORWARD | Aliases: MBG8.12, MBG8_12	5.0	5.4	-0.4	-1.2	100.0%	-4.1
6080	AT4G14050.1 | pentatricopeptide (PPR) repeat-containing protein, contains INTERPRO:IPR002885 PPR repeats | chr4:8103640-8105516 REVERSE | Aliases: DL3066C, FCAALL.80	2.6	2.8	-0.1	-1.2	100.0%	-4.9
6081	AT4G33905.1 | peroxisomal membrane protein 22 kDa, putative, similar to 22 kDa peroxisomal membrane protein PMP22 (Mus musculus) gi:10954089:gb:AAG25724 | chr4:16253820-16255617 REVERSE | Aliases: None	3.6	3.4	0.2	1.2	100.0%	-4.4
6082	AT5G51030.1 | short-chain dehydrogenase/reductase (SDR) family protein, contains INTERPRO family IPR002198 short chain dehydrogenase/reductase SDR family | chr5:20765088-20766578 REVERSE | Aliases: K3K7.21, K3K7_21	2.8	2.6	0.2	1.2	100.0%	-4.7
6083	AT5G24420.1 | glucosamine/galactosamine-6-phosphate isomerase-related, contains weak similarity to Swiss-Prot:O95336 6-phosphogluconolactonase (EC 3.1.1.31) (6PGL) (Homo sapiens) | chr5:8336617-8337971 REVERSE | Aliases: K16H17.13, K16H17_13	2.5	2.4	0.2	1.2	100.0%	-4.9
6084	AT1G04140.2 | transducin family protein / WD-40 repeat family protein, contains 4 WD-40 repeats (PF00400); similar to neural cell adhesion molecule 2, large isoform precursor gb:M76710 from Xenopus laevis, and beta transducin from S. cerevisiae gb:Q05946. ESTs gb:N65081 gb:Z30910, gb:Z34190, gb:Z34611, gb:R30101, gb:H36304, and gb:N65606 come from | chr1:1075544-1080629 REVERSE | Aliases: F20D22.9, F20D22_9	5.3	4.7	0.6	1.2	100.0%	-3.9
6085	AT5G41210.1 | Symbol: ATGSTT1 | glutathione S-transferase (GST10), identical to glutathione transferase AtGST 10 (Arabidopsis thaliana) GI:4049401 | chr5:16509603-16511126 REVERSE | Aliases: GST10, MEE6.28, MEE6_28	9.0	9.5	-0.5	-1.2	100.0%	-4.2
6086	AT3G13670.1 | protein kinase family protein, contains Pfam domains, PF00069: Protein kinase domain | chr3:4469229-4473696 FORWARD | Aliases: MMM17.17	6.1	6.9	-0.8	-1.1	100.0%	-4.3
6087	AT3G24770.1 | Symbol: CLE41 | CLE41, putative, CLAVATA/ESR-Related 41 (CLE41)	3.1	2.9	0.2	1.1	100.0%	-4.7
6088	ATCG00770.1 | Symbol: RPS8 | chloroplast 30S ribosomal protein S8 | chrC:80068-80472 REVERSE | Aliases: RPS8	4.9	6.6	-1.6	-1.1	100.0%	-4.0

Rank	Description	15.dpi	5.dpi	M	t	adj.q	B
6089	AT1G63970.2 | Symbol: ISPF | 2C-methyl-D-erythritol 2,4-cyclodiphosphate synthase, putative, similar to 2C-methyl-D-erythritol 2,4-cyclodiphosphate synthase GI:7621712 from (Catharanthus roseus) | chr1:23742354-23743999 REVERSE | Aliases: 2C METHYL D ERYTHRITOL 2,4 CYCLODIPHOSPHATE SYNTHASE, T12P18.1, T12P18_1	7.6	7.0	0.6	1.1	100.0%	-3.9
6090	AT5G04270.1 | zinc finger (DHHC type) family protein, low similarity to Golgi-specific DHHC zinc figer protein (Mus musculus) GI:21728103; contains Pfam profile PF01529: DHHC zinc finger domain | chr5:1182719-1184719 REVERSE | Aliases: T19N18.6	4.0	4.3	-0.2	-1.1	100.0%	-4.5
6091	AT1G58030.1 | Symbol: CAT2 | amino acid permease family protein, similar to SP:P18581 Low-affinity cationic amino acid transporter-2 (CAT-2) (T-cell early activation protein) {Mus musculus}; contains Pfam profile PF00324: Amino acid permease | chr1:21467671-21472085 REVERSE | Aliases: CATIONIC AMINO ACID TRANSPORTER 2	7.5	7.1	0.4	1.1	100.0%	-4.5
6092	AT4G33950.1 | Symbol: OST1 | protein kinase, putative, similar to abscisic acid-activated protein kinase (Vicia faba) gi:6739629:gb:AAF27340; contains protein kinase domain, Pfam:PF00069 | chr4:16272324-16274815 FORWARD | Aliases: F17I5.140, F17I5_140, OPEN STOMATA 1	5.3	4.8	0.6	1.1	100.0%	-4.2
6093	AT1G19680.1 | expressed protein | chr1:6805450-6808126 FORWARD | Aliases: F14P1.21, F14P1_21	6.9	6.7	0.2	1.1	100.0%	-4.5
6094	AT1G05860.1 | expressed protein | chr1:1769013-1771114 FORWARD | Aliases: T20M3.13, T20M3_13	3.6	3.8	-0.2	-1.1	100.0%	-4.8
6095	AT1G63610.2 | expressed protein | chr1:23587190-23589386 REVERSE | Aliases: F2K11.3, F2K11_3	6.2	6.9	-0.6	-1.1	100.0%	-4.0
6096	AT1G64950.1 | Symbol: CYP89A5 | cytochrome P450, putative, similar to cytochrome P450 89A2 (CYPLXXXIX) (SP:Q42602) (Arabidopsis thaliana);similar to cytochrome P450 (GI:438242) (Solanum melongena) | chr1:24131224-24133121 FORWARD | Aliases: F13O11.25, F13O11_25	10.4	9.9	0.5	1.1	100.0%	-5.1
6097	AT3G03670.1 | peroxidase, putative, similar to peroxidase GB:CAA66966 (Arabidopsis thaliana) | chr3:901862-903384 REVERSE | Aliases: T12J13.5, T12J13_5	3.1	3.4	-0.3	-1.1	100.0%	-4.5
6098	AT5G56490.1 | FAD-binding domain-containing protein, strong similarity to At1g32300, At2g46740, At2g46750, At2g46760; contains PF01565: FAD binding domain | chr5:22888689-22890670 FORWARD | Aliases: MCD7.26, MCD7_26	2.4	2.5	-0.1	-1.1	100.0%	-4.9
6099	AT3G51320.1 | pentatricopeptide (PPR) repeat-containing protein, contains INTERPRO:IPR002885 PPR repeats | chr3:19060832-19062480 REVERSE | Aliases: F24M12.360	5.3	5.7	-0.3	-1.1	100.0%	-4.1
6100	AT4G22620.1 | auxin-responsive family protein, auxin-induced protein 10A, Glycine max., PIR2:JQ1099 | chr4:11907642-11908124 FORWARD | Aliases: T12H17.10	4.7	4.4	0.3	1.1	100.0%	-4.3
6101	AT2G25190.1 | expressed protein | chr2:10741025-10742778 FORWARD | Aliases: F13D4.5	5.7	5.3	0.4	1.1	100.0%	-4.2
6102	AT5G10500.1 | kinase interacting family protein, similar to kinase interacting protein 1 (GI:13936326) (Petunia integrifolia) | chr5:3305332-3308110 FORWARD | Aliases: F12B17.150, F12B17_150	2.4	2.6	-0.2	-1.1	100.0%	-5.0
6103	AT1G60670.1 | expressed protein, similar to expressed protein [Arabidopsis thaliana] (TAIR:At1g10820.2); similar to unknown protein [Oryza sativa (japonica cultivar-group)] (GB:XP_466887.1) | chr1:22347320-22351273 FORWARD | Aliases: F8A5.19, F8A5_19	3.2	3.0	0.2	1.1	100.0%	-4.8
6104	AT3G12180.1 | cornichon family protein, contains Pfam profile: PF03311 cornichon protein | chr3:3883369-3884812 FORWARD | Aliases: F28J15.3	6.5	5.9	0.6	1.1	100.0%	-4.3
6105	AT1G74440.1 | expressed protein, similar to YGL010w-like protein GB:AAC32136 (Picea mariana) | chr1:27980163-27984065 REVERSE | Aliases: F1M20.12, F1M20_12	4.8	4.5	0.3	1.1	100.0%	-4.3
6106	AT4G14180.1 | expressed protein, ; expression supported by MPSS | chr4:8178325-8183510 FORWARD | Aliases: DL3130W, FCAALL.114	2.2	2.4	-0.1	-1.1	100.0%	-5.2
6107	AT2G30330.1 | GCN5L1 family protein, similar to GCN5-like protein 1 (RT14 protein) (Swiss-Prot:P78537) (Homo sapiens) | chr2:12935844-12936814 REVERSE | Aliases: T9D9.14, T9D9_14	4.5	4.2	0.3	1.1	100.0%	-4.5
6108	AT3G10960.1 | xanthine/uracil permease family protein, contains Pfam profile: PF00860 permease family | chr3:3424818-3427002 REVERSE | Aliases: F9F8.22	4.4	4.6	-0.2	-1.1	100.0%	-4.5
6109	AT3G16310.1 | mitotic phosphoprotein N' end (MPPN) family protein, contains Pfam profile PF05172: MPPN (Mitotic PhosphoProtein N' end) (rrm-like) domain | chr3:5526424-5528606 REVERSE | Aliases: MYA6.11	8.9	8.4	0.5	1.1	100.0%	-4.2
6110	AT5G04140.2 | Symbol: GLU1 | glutamate synthase (GLU1) / ferredoxin-dependent glutamate synthase (Fd-GOGAT 1), identical to ferredoxin-dependent glutamate synthase precursor (Arabidopsis thaliana) GI:3869251 | chr5:1130032-1138439 FORWARD | Aliases: F21E1.60, F21E1_60, FERREDOXIN DEPENDENT GLUAMATE SYNTHASE 1, GLS1, GLUAMATE SYNTHASE 1, GLUTAMATE SYNTHASE	6.5	6.0	0.5	1.1	100.0%	-3.9

Rank	Description	15.dpi	5.dpi	M	t	adj.q	B
6111	AT1G01440.1 | extra-large G-protein-related, weak similarity to extra-large G-protein (AtXLG1) (GI:3201682) (Arabidopsis thaliana) | chr1:159935-162219 REVERSE | Aliases: F6F3.24, F6F3_24	3.6	3.4	0.2	1.1	100.0%	-4.5
6112	AT1G11390.1 | ABC1 family protein, contains Pfam domain, PF03109: ABC1 family | chr1:3834459-3837498 REVERSE | Aliases: T23J18.5, T23J18_5	4.7	5.0	-0.2	-1.1	100.0%	-4.6
6113	ATMG00070.1 | Symbol: NAD9 | NADH dehydrogenase subunit 9 | chrM:23663-24235 REVERSE | Aliases: NAD9	3.2	4.0	-0.7	-1.1	100.0%	-4.3
6114	AT4G22750.1 | zinc finger (DHHC type) family protein, contains DHHC zinc finger domain PF01529	7.2	6.5	0.7	1.1	100.0%	-4.1
6115	AT1G20980.1 | Symbol: SPL14 | SPL1-Related2 protein (SPL1R2), strong similarity to SPL1-Related2 protein (Arabidopsis thaliana) GI:6006427; contains Pfam profile PF03110: SBP domain	6.1	6.4	-0.4	-1.1	100.0%	-4.1
6116	AT2G47070.1 | Symbol: SPL1 | squamosa promoter-binding protein-like 1 (SPL1), identical to squamosa promoter binding protein-like 1 (Arabidopsis thaliana) GI:5931655; contains Pfam profile PF03110: SBP domain | chr2:19343767-19347939 FORWARD | Aliases: F14M4.10, SQUAMOSA PROMOTER BINDING PROTEIN LIKE 1	5.2	5.0	0.2	1.1	100.0%	-4.6
6117	AT4G25080.3 | magnesium-protoporphyrin O-methyltransferase, putative / magnesium-protoporphyrin IX methyltransferase, putative, similar to SP:Q55467 Magnesium-protoporphyrin O-methyltransferase (EC 2.1.1.1) (Magnesium-protoporphyrin IX methyltransferase) {Synechocystis sp.} | chr4:12876958-12878639 FORWARD | Aliases: F24A6.6, MGPIXMT	8.6	8.2	0.4	1.1	100.0%	-4.2
6118	AT1G43880.1 | expressed protein, low similarity to protective antigen (Streptococcus pyogenes) GI:8996050, fibrinogen-binding protein (Streptococcus equi) GI:3093478 | chr1:16630750-16632385 FORWARD | Aliases: F28H19.18, F28H19_18	2.6	2.5	0.1	1.1	100.0%	-5.2
6119	AT2G26540.1 | uroporphyrinogen-III synthase family protein, contains Pfam PF02602: uroporphyrinogen-III synthase; weak similarity to Porphyrin biosynthesis protein HEMD)) (Uroporphyrinogen III methylase) (UROM); Uroporphyrinogen-III synthase (EC 4.2.1.75) (Swiss-Prot:Q59294) (Clostridium josui) | chr2:11294476-11297438 REVERSE | Aliases: T9J22.21, T9J22_21	5.5	5.2	0.3	1.1	100.0%	-4.2
6120	AT1G25450.1 | very-long-chain fatty acid condensing enzyme, putative, nearly identical to fatty acid condensing enzyme CUT1 GI:5001734 from (Arabidopsis thaliana) | chr1:8938466-8940374 REVERSE | Aliases: F2J7.9, F2J7_9	2.8	3.0	-0.2	-1.1	100.0%	-4.8
6121	AT5G14770.1 | pentatricopeptide (PPR) repeat-containing protein, contains Pfam profile PF01535: PPR repeat | chr5:4772884-4775700 REVERSE | Aliases: T9L3.70, T9L3_70	3.1	2.9	0.2	1.1	100.0%	-4.7
6122	AT1G35510.1 | expressed protein, contains Pfam PF03138: Plant protein family. The function of this family of plant proteins is unknown; previously annotated as 'growth regulator protein -related' based on similarity to axi 1 protein (GB:X80301) (GI:559920) from (Nicotiana tabacum), which, due to scienitific fraud was retracted. Retraction in: Schell J. EMBO J 1999 May 17;18(10):2908. PMID:10400497. | chr1:13071143-13074927 FORWARD | Aliases: F15O4.45	3.9	3.6	0.2	1.1	100.0%	-4.5
6123	AT1G70910.1 | zinc finger (C3HC4-type RING finger) family protein, contains Pfam profile: PF00097 zinc finger, C3HC4 type (RING finger) | chr1:26737817-26738302 FORWARD | Aliases: F15H11.25	3.1	3.3	-0.2	-1.1	100.0%	-5.0
6124	AT2G17972.1 | expressed protein | chr2:7828667-7829403 FORWARD | Aliases: None	6.3	5.8	0.5	1.1	100.0%	-3.7
6125	AT5G05600.1 | oxidoreductase, 2OG-Fe(II) oxygenase family protein, similar to flavonol synthase (Citrus unshiu)(gi:4126403), leucoanthocyanidin dioxygenase (Daucus carota)(gi:5924383); contains PF03171 2OG-Fe(II) oxygenase superfamily domain | chr5:1672121-1674740 FORWARD | Aliases: MOP10.14, MOP10_14	4.7	4.1	0.6	1.1	100.0%	-3.9
6126	AT5G58500.1 | expressed protein, contains Pfam profile PF04852: Protein of unknown function (DUF640) | chr5:23662761-23663309 REVERSE | Aliases: MQJ2.11, MQJ2_11	5.4	5.2	0.3	1.1	100.0%	-4.3
6127	AT2G42670.1 | expressed protein | chr2:17778901-17781481 REVERSE | Aliases: F14N22.6, F14N22_6	4.5	4.2	0.3	1.1	100.0%	-4.3
6128	AT5G49700.1 | DNA-binding protein-related, contains Pfam domain PF03479: Domain of unknown function (DUF296), found in AT-hook motifs Pfam:PF02178 | chr5:20209825-20210655 FORWARD | Aliases: K2I5.6, K2I5_6	3.9	3.6	0.3	1.1	100.0%	-4.4
6129	AT5G50370.1 | adenylate kinase, putative, similar to adenylate kinase (ATP-AMP transphosphorylase) (Arabidopsis thaliana) SWISS-PROT:O82514 | chr5:20526385-20527926 REVERSE | Aliases: MXI22.8, MXI22_8	8.4	9.4	-1.0	-1.1	100.0%	-4.0
6130	AT5G14230.1 | ankyrin repeat family protein, contains ankyrin repeats, Pfam:PF00023 | chr5:4593810-4595967 FORWARD | Aliases: F18O22.20, F18O22_20	4.1	4.4	-0.4	-1.1	100.0%	-4.3

Rank	Description	15.dpi	5.dpi	M	t	adj.q	B
6131	AT2G01950.1 | leucine-rich repeat transmembrane protein kinase, putative, similar to brassinosteroid insensitive protein | chr2:440635-444275 REVERSE | Aliases: F14H20.2, F14H20_2	4.3	4.8	-0.4	-1.1	100.0%	-4.2
6132	AT2G31250.1 | glutamyl-tRNA reductase, putative, similar to HEMA2 (SP:P49294), HEMA1 (SP:P42804) | chr2:13326698-13328603 REVERSE | Aliases: F16D14.9, F16D14_9	4.1	4.5	-0.4	-1.1	100.0%	-4.3
6133	AT1G28130.2 | Symbol: GH3.17 |	4.1	4.4	-0.3	-1.1	100.0%	-4.3
6134	AT4G14400.3 | Symbol: ACD6 | ankyrin repeat family protein, contains ankyrin repeats, Pfam domain PF00023 | chr4:8294448-8298598 FORWARD | Aliases: ACCELERATED CELL DEATH 6, DL3240W, FCAALL.190	3.1	3.0	0.2	1.1	100.0%	-4.9
6135	AT5G17660.1 | expressed protein, contains Pfam profile PF02390: Putative methyltransferase | chr5:5818550-5820866 REVERSE | Aliases: MVA3.1, MVA3_1	3.1	3.3	-0.2	-1.1	100.0%	-4.4
6136	AT1G63000.1 | Symbol: NRS/ER |	6.0	6.6	-0.6	-1.1	100.0%	-4.2
6137	AT2G40620.1 | bZIP transcription factor family protein, identical to b-Zip DNA binding protein GI:2246376 from (Arabidopsis thaliana); contains a bZIP transcription factor basic domain signature (PDOC00036)	3.2	3.0	0.2	1.1	100.0%	-4.6
6138	AT2G36360.2 | similar to kelch repeat-containing protein [Arabidopsis thaliana] (TAIR:At1g74150.1); similar to conjugation with cellular fusion-related protein, putative [Cryptococcus neoformans var. neoformans JEC21] (GB:AAW40898.1); contains InterPro domain Kelch repeat (InterPro:IPR006652) | chr2:15249983-15254694 REVERSE | Aliases: F2H17.3, F2H17_3	5.5	5.0	0.5	1.1	100.0%	-4.1
6139	AT1G76520.2 | auxin efflux carrier family protein, contains auxin efflux carrier domain, Pfam:PF03547 | chr1:28720049-28722308 FORWARD | Aliases: F14G6.12, F14G6_12	9.0	8.2	0.8	1.1	100.0%	-3.9
6140	AT1G21500.1 | expressed protein | chr1:7529871-7531014 REVERSE | Aliases: F24J8.11, F24J8_11	3.7	3.4	0.3	1.1	100.0%	-4.5
6141	AT1G51670.1 | expressed protein | chr1:19160810-19162417 FORWARD | Aliases: F19C24.12, F19C24_12	3.0	2.8	0.2	1.1	100.0%	-4.7
6142	AT5G48400.2 | Symbol: ATGLR1.2 | glutamate receptor family protein (GLR1.2), plant glutamate receptor family, PMID:11379626 | chr5:19633470-19636472 FORWARD | Aliases: GLR1.2, MJE7.3, MJE7_3	2.9	2.8	0.1	1.1	100.0%	-5.0
6143	AT5G01780.1 | oxidoreductase, 2OG-Fe(II) oxygenase family protein, low similarity to alkB protein - Escherichia coli, PIR:BVECKB, alkB (Caulobacter crescentus)(GI:2055386); contains Pfam domain PF03171 2OG-Fe(II) oxygenase superfamily | chr5:302362-304169 REVERSE | Aliases: T20L15.50, T20L15_50	6.2	5.6	0.6	1.1	100.0%	-4.1
6144	AT4G38510.4 | similar to vacuolar ATP synthase subunit B / V-ATPase B subunit / vacuolar proton pump B subunit / V-ATPase 57 kDa subunit [Arabidopsis thaliana] (TAIR:At1g76030.1); similar to vacuolar ATP synthase subunit B, putative / V-ATPase B subunit, putative / vacuolar proton pump B subunit, putative / V-ATPase 57 kDa subunit, putative [Arabidopsis thaliana] (TAIR:At1g20260.2); similar to vacuolar ATPase B subunit (GB:AAA81331.1); similar to H+-transporting two-sector ATPase (EC 3.6.3.14) chain B, vacuolar [imported] - Citrus unshiu (GB:T43789); similar to putative H+-transporting ATP synthase [Oryza sativa (japonica cultivar-group)] (GB:NP_916591.1); similar to vacuolar ATPase B subunit (GB:AAA81330.1); similar to VATB1_GOSHI Vacuolar ATP synthase subunit B isoform 1 (V-ATPase B subunit 1) (Vacuolar proton pump B subunit 1) (GB:Q43432); contains InterPro domain H+-transporting two-sector ATPase, alpha/beta subunit, C-terminal (InterPro:IPR000793); contains InterPro domain ATP synthase V-type, B subunit (InterPro:IPR005723); contains InterPro domain H+-transporting two-sector ATPase, alpha subunit, C-terminal (InterPro:IPR000790); contains InterPro domain H+-transporting two-sector ATPase, alpha/beta subunit, N-terminal (InterPro:IPR004100); contains InterPro domain H+-transporting two-sector ATPase, alpha/beta subunit, central region (InterPro:IPR000194)	6.8	7.8	-0.9	-1.1	100.0%	-4.1
6145	AT2G46470.1 | OXA1 protein, putative, similar to Oxa1 protein (Arabidopsis thaliana) GI:10176922 | chr2:19079385-19082195 FORWARD | Aliases: F11C10.16	5.0	5.4	-0.4	-1.1	100.0%	-4.0
6146	AT1G17040.1 | transcription factor-related, contains Pfam profile: PF00017 Src homology domain 2; similar to transcription factor (Danio rerio) (GI:3687402) | chr1:5824844-5828226 FORWARD | Aliases: F20D23.26, F20D23_26	4.3	4.6	-0.3	-1.1	100.0%	-4.4
6147	AT3G28700.1 | expressed protein, contains Pfam PF02636: Uncharacterized ACR, COG1565 | chr3:10760802-10763351 FORWARD | Aliases: MZN14.19	7.1	7.5	-0.4	-1.1	100.0%	-4.0
6148	AT3G53710.2 | similar to arabidopsis pde1 suppressor 1 protein (ASP1) [Arabidopsis thaliana] (TAIR:At2g37550.1); similar to CG4237-PA [Drosophila melanogaster] (GB:NP_524040.2); similar to putative ARF1 GTPase activating protein [Drosophila melanogaster] (GB:AAB64300.1); contains InterPro domain Human Rev interacting-like protein (hRIP) (InterPro:IPR001164) | chr3:19914291-19916494 REVERSE | Aliases: F5K20.10	7.1	6.9	0.2	1.1	100.0%	-4.5

Rank	Description	15.dpi	5.dpi	M	t	adj.q	B
6149	AT3G19960.1 | Symbol: ATATM | myosin (ATM), nearly identical to myosin (Arabidopsis thaliana) GI:6491702; similar to myosin GI:6491702 from (Arabidopsis thaliana) ;contains Pfam profiles: PF00063: myosin head (motor domain), PF00612: IQ calmodulin-binding motif; identical to cDNA myosin (ATM) GI:297068 | chr3:6948782-6956972 FORWARD | Aliases: ATM1, MZE19.3	4.9	5.2	-0.3	-1.1	100.0%	-4.3
6150	AT1G35220.1 | expressed protein, similar to unknown protein [Oryza sativa (japonica cultivar-group)] (GB:AAV43936.1) | chr1:12908892-12914780 FORWARD | Aliases: T9I1.1, T9I1_1	2.6	2.8	-0.2	-1.1	100.0%	-5.0
6151	AT4G07740.1 | expressed protein | chr4:4539635-4540679 FORWARD | Aliases: T6L9.5, T6L9_5	2.3	2.5	-0.2	-1.1	100.0%	-4.6
6152	AT3G21620.1 | early-responsive to dehydration protein-related / ERD protein-related, similar to ERD4 protein (early-responsive to dehydration stress) (Arabidopsis thaliana) GI:15375406; contains Pfam profile PF02714: Domain of unknown function DUF221 | chr3:7611051-7614048 REVERSE | Aliases: MIL23.19	2.5	2.7	-0.2	-1.1	100.0%	-4.7
6153	AT5G28010.1 | Bet v I allergen family protein, similar to Csf-2 (Cucumis sativus)(GI:5762258)(J Am Soc Hortic Sci 124, 136-139 (1999)); contains Pfam profile PF00407: Pathogenesis-related protein Bet v I family | chr5:10024817-10025386 FORWARD | Aliases: F15F15.80, F15F15_80	2.3	2.2	0.2	1.1	100.0%	-5.0
6154	AT5G20820.1 | auxin-responsive protein-related, similar to auxin-induced protein TGSAUR21 (GI:10185818) Tulipa gesneriana) | chr5:7046753-7047398 REVERSE | Aliases: T1M15.220, T1M15_220	4.0	4.7	-0.7	-1.1	100.0%	-3.6
6155	AT1G11610.1 | Symbol: CYP71A18 | cytochrome P450, putative, very strong similarity to cytochrome P450 (SP:Q9SAB6) (Arabidopsis thaliana); is a member of the PF:00067 Cytochrome P450 family | chr1:3907461-3909291 REVERSE | Aliases: F25C20.24, F25C20_24	2.3	2.5	-0.2	-1.1	100.0%	-5.2
6156	AT1G14470.1 | pentatricopeptide (PPR) repeat-containing protein, contains Pfam profile PF01535: PPR repeat | chr1:4954075-4955697 FORWARD | Aliases: F14L17.25, F14L17_25	2.9	2.8	0.2	1.1	100.0%	-4.9
6157	AT4G01310.1 | ribosomal protein L5 family protein, contains Pfam profiles PF00673: ribosomal L5P family C-terminus, PF00281: ribosomal protein L5 | chr4:543970-545535 REVERSE | Aliases: F2N1.5, F2N1_5	6.5	7.3	-0.8	-1.1	100.0%	-3.9
6158	AT5G06750.1 | protein phosphatase 2C family protein / PP2C family protein, similar to Ser/Thr protein phosphatase 2C (PP2C6) (GI:15020818) (Arabidopsis thaliana); similar to protein phosphatase 2C (GI:3608412) (Mesembryanthemum crystallinum); contains Pfam PF00481 : Protein phosphatase 2C domain; | chr5:2086267-2088529 REVERSE | Aliases: MPH15.11, MPH15_11	8.0	8.4	-0.4	-1.1	100.0%	-4.3
6159	AT3G55850.2 | Symbol: LAF3.1 |	4.4	4.7	-0.3	-1.1	100.0%	-4.2
6160	AT1G10680.1 | P-glycoprotein, putative, similar to P-glycoprotein GI:4204793 from (Solanum tuberosum) | chr1:3538471-3543783 REVERSE | Aliases: F20B24.12, F20B24_12	3.0	2.8	0.2	1.1	100.0%	-4.7
6161	AT5G17190.1 | expressed protein, similar to unknown protein (gb:AAF26109.1) | chr5:5652225-5652985 FORWARD | Aliases: MKP11.4, MKP11_4	9.8	9.1	0.7	1.1	100.0%	-4.4
6162	AT1G26340.1 | Symbol: B5 #6 | cytochrome b5, putative, similar to cytochrome b5 GB:BAA74839 GI:4240120 from (Arabidopsis thaliana) | chr1:9113887-9114958 FORWARD | Aliases: F28B23.1	4.6	4.4	0.2	1.1	100.0%	-4.9
6163	AT1G10460.1 | Symbol: GLP7 | germin-like protein (GLP7), identical to germin-like protein subfamily 1 member 1 (SP:P92998); similar to ESTs gb:T88481 and gb:AI099566	4.5	4.9	-0.4	-1.1	100.0%	-4.1
6164	AT1G24764.1 | Symbol: ATMAP70 2 | Member of the MAP70 protein family.	4.3	4.6	-0.3	-1.1	100.0%	-4.3
6165	AT1G58360.1 | Symbol: AAP1 | amino acid permease I (AAP1), identical to amino acid permease I GI:22641 from (Arabidopsis thaliana) | chr1:21680201-21684148 FORWARD | Aliases: F19C14.3, F19C14_3, NAT2, NEUTRAL AMINO ACID TRANSPORTER 2	9.0	7.8	1.2	1.1	100.0%	-4.0
6166	AT3G09700.1 | DNAJ heat shock N-terminal domain-containing protein, contains Pfam profile PF00226 DnaJ domain; similar to a region of DNAJ domain-containing protein MCJ GB:AAD38506 | chr3:2974886-2976282 REVERSE | Aliases: F11F8.29	6.4	6.7	-0.4	-1.1	100.0%	-4.1
6167	AT1G05690.1 | TAZ zinc finger family protein / BTB/POZ domain-containing protein, contains Pfam PF00651 : BTB/POZ domain; contains Pfam PF02135 : TAZ zinc finger; similar to p300/CBP acetyltransferase-related protein (GI:12597461) (Arabidopsis thaliana); similar to Speckle-type POZ protein (SP:O43791) (Homo sapiens) | chr1:1707082-1709346 FORWARD | Aliases: F3F20.14, F3F20_14	4.4	4.1	0.3	1.1	100.0%	-4.2
6168	AT5G05870.1 | Symbol: UGT76C1 | UDP-glucoronosyl/UDP-glucosyl transferase family protein, contains Pfam profile: PF00201 UDP-glucoronosyl and UDP-glucosyl transferase | chr5:1767640-1769263 FORWARD | Aliases: K18J17.2, K18J17_2, UGT76C1	5.7	5.9	-0.2	-1.1	100.0%	-4.7
6169	AT2G47980.1 | expressed protein | chr2:19638329-19644323 FORWARD | Aliases: T9J23.3	7.1	7.4	-0.3	-1.1	100.0%	-4.4

Rank	Description	15.dpi	5.dpi	M	t	adj.q	B
6170	ATCG00650.1 | Symbol: RPS18 | chloroplast-encoded ribosomal protein S18 | chrC:67917-68222 FORWARD | Aliases: RPS18	6.3	7.3	-0.9	-1.1	100.0%	-4.1
6171	AT5G15050.1 | glycosyltransferase family 14 protein / core-2/I-branching enzyme family protein, contains Pfam profile: PF02485 Core-2/I-Branching enzyme | chr5:4871642-4873601 REVERSE | Aliases: F2G14.170, F2G14_170	5.4	5.8	-0.4	-1.1	100.0%	-4.2
6172	AT1G68850.1 | peroxidase, putative, identical to peroxidase ATP23a GB:CAA70035 (Arabidopsis thaliana) | chr1:25887279-25888896 REVERSE | Aliases: T6L1.4, T6L1_4	2.9	3.2	-0.4	-1.1	100.0%	-4.0
6173	AT4G13260.1 | flavin-containing monooxygenase / FMO (YUCCA2), identical to gi:16555354	4.6	4.9	-0.3	-1.1	100.0%	-4.2
6174	AT1G64110.2 | AAA-type ATPase family protein, contains Pfam domain, PF00004: ATPase, AAA family | chr1:23800382-23805388 REVERSE | Aliases: F22C12.12, F22C12_12	11.2	10.7	0.4	1.1	100.0%	-4.3
6175	AT5G35160.1 | endomembrane protein 70, putative, p76, Homo sapiens, EMBL:HSU81006 | chr5:13432184-13434151 FORWARD | Aliases: T25C13.40, T25C13_40	6.3	6.8	-0.5	-1.1	100.0%	-4.3
6176	AT4G20150.1 | expressed protein | chr4:10888309-10889606 REVERSE | Aliases: F1C12.70, F1C12_70	12.7	13.0	-0.2	-1.1	100.0%	-4.8
6177	AT1G42960.1 | expressed protein localized to the inner membrane of the chloroplast. | chr1:16128235-16129727 FORWARD | Aliases: F13A11.2, F13A11_2	10.0	10.4	-0.4	-1.1	100.0%	-4.5
6178	AT5G25060.1 | RNA recognition motif (RRM)-containing protein, KIAA0332 - Homo sapiens, EMBL:AB002330 | chr5:8634077-8640220 REVERSE | Aliases: T11H3.70, T11H3_70	6.7	7.3	-0.6	-1.1	100.0%	-4.2
6179	AT1G72175.1 | zinc finger (C3HC4-type RING finger) family protein, contains Pfam profile: PF00097 zinc finger, C3HC4 type (RING finger) | chr1:27161537-27163219 FORWARD | Aliases: None	6.3	6.0	0.3	1.1	100.0%	-4.3
6180	AT4G03200.1 | expressed protein, contains Pfam PF03190: Protein of unknown function, DUF255 | chr4:1408159-1412756 FORWARD | Aliases: F4C21.12, F4C21_12	7.8	7.4	0.4	1.1	100.0%	-4.3
6181	AT3G05350.1 | similar to aminopeptidase P [Arabidopsis thaliana] (TAIR:At4g36760.1); similar to aminopeptidase-P [Aspergillus oryzae] (GB:BAD00702.1); contains InterPro domain Metallopeptidase family M24 (InterPro:IPR000994) | chr3:1526969-1530263 REVERSE | Aliases: T12H1.32, T12H1_32	5.9	5.6	0.3	1.1	100.0%	-4.4
6182	AT5G51620.2 | expressed protein | chr5:20984324-20985253 FORWARD | Aliases: K17N15.17, K17N15_17	9.1	8.5	0.5	1.1	100.0%	-4.3
6183	AT5G55630.2 | Symbol: KCO1 | outward rectifying potassium channel (KCO1), identical to kco1 (Arabidopsis thaliana) gi:2230761:emb:CAA69158 of the 2 pore, 4 transmembrane (2P/4TM) K+ channel family, PMID:11500563 | chr5:22548584-22550344 FORWARD | Aliases: MDF20.7, MDF20_7	4.1	4.4	-0.3	-1.1	100.0%	-4.2
6184	AT5G52310.1 | Symbol: COR78 | low-temperature-responsive protein 78 (LTI78) / desiccation-responsive protein 29A (RD29A) | chr5:21258075-21260839 FORWARD | Aliases: COLD REGULATED 78, K24M7.4, K24M7_4, LTI140, LTI78, RD29A, RESPONSIVE TO DESSICATION 29A	10.9	10.4	0.5	1.1	100.0%	-4.7
6185	AT2G21240.2 | expressed protein | chr2:9108397-9110127 REVERSE | Aliases: F7O24.4, F7O24_4	7.8	7.4	0.4	1.1	100.0%	-4.2
6186	AT5G46410.1 | NLI interacting factor (NIF) family protein, contains Pfam profile PF03031: NLI interacting factor | chr5:18842330-18846448 FORWARD | Aliases: MPL12.21, MPL12_21	5.3	5.0	0.3	1.1	100.0%	-4.1
6187	AT5G22220.3 | Symbol: E2F1 | similar to E2F transcription factor-3 (E2F3) [Arabidopsis thaliana] (TAIR:At2g36010.1); similar to transcription factor (E2F) [Chenopodium rubrum] (GB:CAC17702.1); contains InterPro domain Transcription factor E2F/dimerisation partner (TDP) (InterPro:IPR003316) | chr5:7360538-7364457 FORWARD | Aliases: ATE2FB, E2F TRANSCRIPTION FACTOR 1 E2F1, E2FB, E2FB TRANSCRIPTION FACTOR	4.0	3.7	0.2	1.1	100.0%	-4.4
6188	NA	6.2	5.7	0.5	1.1	100.0%	-4.7
6189	AT5G56730.1 | peptidase M16 family protein / insulinase family protein, contains Pfam domain, PF05193: Peptidase M16 inactive domain | chr5:22964132-22969802 REVERSE | Aliases: MIK19.18, MIK19_18	4.1	4.2	-0.2	-1.1	100.0%	-4.8
6190	AT1G11350.1 | S-locus lectin protein kinase family protein, contains Serine/Threonine protein kinases active-site signature, PROSITE:PS00108 | chr1:3817591-3820805 REVERSE | Aliases: T23J18.2, T23J18_2	2.5	2.7	-0.1	-1.1	100.0%	-5.0
6191	AT1G10960.1 | ferredoxin, chloroplast, putative, strong similarity to FERREDOXIN PRECURSOR GB:P16972 (SP:P16972) from (Arabidopsis thaliana) | chr1:3664386-3665039 FORWARD | Aliases: T19D16.12, T19D16_12	11.0	11.6	-0.6	-1.1	100.0%	-3.9

Rank	Description	15.dpi	5.dpi	M	t	adj.q	B
6192	AT2G31750.1 | UDP-glucoronosyl/UDP-glucosyl transferase family protein, contains Pfam profile: PF00201 UDP-glucoronosyl and UDP-glucosyl transferase | chr2:13504310-13507763 FORWARD | Aliases: F20M17.21, F20M17_21	3.5	3.9	-0.4	-1.1	100.0%	-4.1
6193	AT5G53540.1 | MSP1 protein, putative / intramitochondrial sorting protein, putative, similar to Swiss-Prot:P28737 MSP1 protein (TAT-binding homolog 4) (Saccharomyces cerevisiae); contains Pfam domain, PF00004: ATPase, AAA family | chr5:21766512-21768463 REVERSE | Aliases: MNC6.8, MNC6_8	8.6	8.9	-0.3	-1.1	100.0%	-4.4
6194	AT4G14760.1 | M protein repeat-containing protein, contains Pfam profile: PF02370 M protein repeat | chr4:8475964-8481089 FORWARD | Aliases: DL3420W, FCAALL.305	5.8	6.3	-0.5	-1.1	100.0%	-4.1
6195	AT4G28240.1 | wound-responsive protein-related, wound-induced protein - tomato (fragment), PIR2:S19773	8.2	7.8	0.4	1.1	100.0%	-4.4
6196	AT3G20960.1 | Symbol: CYP705A33 | cytochrome P450 family protein, similar to Cytochrome P450 93A1 (SP:Q42798)	3.0	2.6	0.4	1.1	100.0%	-4.1
6197	AT3G01460.1 | Symbol: MBD9 | PHD finger family protein / methyl-CpG binding domain-containing protein, contains Pfam profiles PF00628: PHD-finger (2 copies), PF01429: Methyl-CpG binding domain | chr3:173323-182971 FORWARD | Aliases: T13O15.10, T13O15_10	4.4	4.7	-0.3	-1.1	100.0%	-4.5
6198	AT5G07260.1 | homeobox protein-related, contains weak similarity to Homeobox protein FWA (Swiss-Prot:Q9FVI6) (Arabidopsis thaliana) | chr5:2277815-2280282 FORWARD | Aliases: T28J14.200, T28J14_200	3.1	3.2	-0.2	-1.1	100.0%	-4.9
6199	NA	2.8	2.7	0.1	1.1	100.0%	-5.1
6200	AT2G18910.1 | hydroxyproline-rich glycoprotein family protein | chr2:8199790-8201420 REVERSE | Aliases: F19F24.21	6.6	6.1	0.5	1.1	100.0%	-4.1
6201	AT2G41630.1 | Symbol: TFIIB | transcription initiation factor IIB-1 / general transcription factor TFIIB-1 (TFIIB1), identical to transcription initiation factor IIB-1 (TFIIB1) SP:P48512 from (Arabidopsis thaliana) | chr2:17362410-17364676 REVERSE | Aliases: T32G6.15, T32G6_15, TRANSCRIPTION FACTOR II B, TRANSCRIPTION FACTOR TFIIB	8.9	9.4	-0.6	-1.1	100.0%	-4.3
6202	AT3G50610.1 | expressed protein | chr3:18790704-18791393 REVERSE | Aliases: T20E23.210	8.5	7.8	0.7	1.1	100.0%	-3.7
6203	AT4G37380.1 | pentatricopeptide (PPR) repeat-containing protein, contains Pfam profile PF01535: PPR repeat | chr4:17572034-17573932 REVERSE | Aliases: F6G17.30, F6G17_30	6.0	6.4	-0.4	-1.1	100.0%	-4.1
6204	AT1G09660.2 | KH domain-containing quaking protein, putative, similar to GB:AAC67357 | chr1:3127783-3131049 REVERSE | Aliases: F21M12.5, F21M12_5	5.8	5.5	0.3	1.1	100.0%	-4.3
6205	AT3G33530.1 | transducin family protein / WD-40 repeat family protein, similar to WD-repeat protein 11 (SP:Q9BZH6) {Homo sapiens}; contains Pfam PF00400: WD domain, G-beta repeat (4 copies, 3 weak):2763187:gb:T46504.1:T46504 | chr3:14096340-14104656 FORWARD | Aliases: T4P3.9	5.2	5.5	-0.3	-1.1	100.0%	-4.3
6206	AT2G06210.2 | Symbol: ELF8 | phosphoprotein-related, low similarity to phosphoprotein from Mus musculus GI:1236239; contains Pfam profile PF00515 TPR Domain | chr2:2428900-2436684 REVERSE | Aliases: EARLY FLOWERING 8, F5K7.3, F5K7_3	5.3	6.0	-0.7	-1.1	100.0%	-4.3
6207	AT4G33000.2 | Symbol: CBL10 | calcineurin B-like protein 10 (CBL10), identical to calcineurin B-like protein 10 (Arabidopsis thaliana) GI:29150248 | chr4:15924759-15926631 FORWARD | Aliases: ATCBL10, F26P21.120, F26P21_120	3.4	3.7	-0.3	-1.1	100.0%	-4.3
6208	AT3G15710.1 | signal peptidase, putative, similar to SP:P13679 Microsomal signal peptidase 21 kDa subunit (EC 3.4.-.-) {Canis familiaris}; contains Pfam profile: PF00461 signal peptidase I | chr3:5323365-5324915 REVERSE | Aliases: MSJ11.11	8.1	8.5	-0.4	-1.1	100.0%	-4.3
6209	AT1G18910.1 | zinc finger (C3HC4-type RING finger) family protein, contains Pfam domain PF00097: Zinc finger, C3HC4 type (RING finger)	3.6	4.1	-0.5	-1.1	100.0%	-3.9
6210	AT5G60300.2 | lectin protein kinase family protein, contains Pfam domains, PF00069: Protein kinase domain and PF00139: Legume lectins beta domain | chr5:24281848-24284436 FORWARD | Aliases: F15L12.17, F15L12_17	6.1	5.7	0.4	1.1	100.0%	-4.0

Rank	Description	15.dpi	5.dpi	M	t	adj.q	B
6211	AT5G10240.2 | Symbol: ASN3 | similar to asparagine synthetase 1 (glutamine-hydrolyzing) / glutamine-dependent asparagine synthetase 1 (ASN1) [Arabidopsis thaliana] (TAIR:At3g47340.3); similar to asparagine synthetase 1 (glutamine-hydrolyzing) / glutamine-dependent asparagine synthetase 1 (ASN1) [Arabidopsis thaliana] (TAIR:At3g47340.2); similar to asparagine synthetase 2 (ASN2) [Arabidopsis thaliana] (TAIR:At5g65010.1); similar to asparagine synthetase 1 (glutamine-hydrolyzing) / glutamine-dependent asparagine synthetase 1 (ASN1) [Arabidopsis thaliana] (TAIR:At3g47340.1); similar to asparagine synthetase 2 (ASN2) [Arabidopsis thaliana] (TAIR:At5g65010.2); similar to asparagine synthetase [Helianthus annuus] (GB:AAF02776.1); similar to glutamine-dependent asparagine synthetase 1 [Hordeum vulgare subsp. vulgare] (GB:AAK49456.1); similar to glutamine-dependent asparagine synthetase [Triticum aestivum] (GB:AAU89392.1); similar to putative asparagine synthetase [Pinus sylvestris] (GB:CAD43058.1); similar to asparagine synthetase [Striga hermonthica] (GB:AAM94340.1); contains InterPro domain Glutamine amidotransferase, class-II (InterPro:IPR000583); contains InterPro domain Asparagine synthase, glutamine-hydrolyzing (InterPro:IPR006426); contains InterPro domain Asparagine synthase (InterPro:IPR001962)	8.2	8.7	-0.5	-1.1	100.0%	-3.9
6212	AT2G34790.1 | FAD-binding domain-containing protein, similar to SP:P30986 reticuline oxidase precursor (Berberine-bridge-forming enzyme) (BBE) (Tetrahydroprotoberberine synthase) (Eschscholzia californica); contains PF01565 FAD binding domain | chr2:14680444-14684349 REVERSE | Aliases: F19I3.2, F19I3_2	3.9	4.3	-0.4	-1.1	100.0%	-4.3
6213	AT2G22090.2 | UBP1 interacting protein 1a (UBA1a), nearly identical to UBP1 interacting protein 1a (Arabidopsis thaliana) GI:19574236; contains InterPro entry IPR000504: RNA-binding region RNP-1 (RNA recognition motif) (RRM); based on cDNA of partial mRNA for UBP1 interacting protein 1a (uba1a) GI:19574235 | chr2:9396080-9397586 REVERSE | Aliases: T16B14.6, T16B14_6	5.3	4.9	0.3	1.1	100.0%	-4.1
6214	AT4G02460.1 | DNA mismatch repair protein, putative, similar to SP:P54278 PMS1 protein homolog 2 (DNA mismatch repair protein PMS2) {Homo sapiens}; contains Pfam profiles PF02518: ATPase, histidine kinase-, DNA gyrase B-, and HSP90-like domain protein, PF01119: DNA mismatch repair protein, C-terminal domain | chr4:1076136-1080667 REVERSE | Aliases: T14P8.6, T14P8_6	3.5	3.8	-0.2	-1.1	100.0%	-4.6
6215	AT4G02680.1 | tetratricopeptide repeat (TPR)-containing protein, contains Pfam profile PF00515 TPR Domain | chr4:1181051-1184930 REVERSE | Aliases: T10P11.24, T10P11_24	7.9	8.5	-0.6	-1.1	100.0%	-4.1
6216	AT2G27310.1 | F-box family protein, contains Pfam PF00646: F-box domain;; similar to SKP1 interacting partner 2 (SKIP2) TIGR_Ath1:At5g67250 | chr2:11690940-11692045 REVERSE | Aliases: F12K2.11, F12K2_11	5.8	5.2	0.6	1.1	100.0%	-4.0
6217	AT1G13180.1 | Symbol: DIS1 | actin-related protein 3 (ARP3), identical to actin-related protein 3 (ARP3) (Arabidopsis thaliana) GI:21427461; contains Pfam profile PF00022: Actin | chr1:4495025-4498466 FORWARD | Aliases: ACTIN RELATED PROTEIN 3, ARP3, ATARP3, DISTORTED TRICHOMES 1, F3F19.20, F3F19_20	5.4	5.7	-0.2	-1.1	100.0%	-4.5
6218	AT1G34570.1 | expressed protein | chr1:12654918-12656464 REVERSE | Aliases: F12K21.27	3.8	3.2	0.6	1.1	100.0%	-3.9
6219	AT3G15750.1 | expressed protein | chr3:5335232-5336483 FORWARD | Aliases: MSJ11.15	3.8	3.2	0.6	1.1	100.0%	-3.9
6220	AT5G11180.1 | Symbol: ATGLR2.6 | glutamate receptor family protein (GLR2.6), plant glutamate receptor family, PMID:11379626 | chr5:3557262-3561576 REVERSE | Aliases: F2I11.70, F2I11_70, GLR2.6	2.6	2.4	0.1	1.1	100.0%	-5.0
6221	AT4G28450.1 | transducin family protein / WD-40 repeat family protein, SOF1 (involved in rRNA processing) protein-yeast | chr4:14061465-14064679 REVERSE | Aliases: F20O9.130, F20O9_130	5.4	5.8	-0.4	-1.1	100.0%	-4.4
6222	AT5G16650.1 | DNAJ heat shock N-terminal domain-containing protein, similar to SP:P30725 Chaperone protein dnaJ Clostridium acetobutylicum; contains Pfam profile PF00226: DnaJ domain	7.2	7.5	-0.3	-1.1	100.0%	-4.3
6223	AT1G74110.1 | Symbol: CYP78A10 | cytochrome P450 family protein, similar to Cytochrome P450 78A4 (SP:O65012) Cytochrome P450 78A4 (Pinus radiata); similar to cytochrome P-450 GB:AAB37231 from (Phalaenopsis sp. SM9108) | chr1:27870328-27872029 REVERSE | Aliases: F2P9.2, F2P9_2	2.6	2.4	0.2	1.1	100.0%	-4.8
6224	AT2G04040.1 | MATE efflux family protein, contains Pfam profile: PF01554 uncharacterized membrane protein family | chr2:1334515-1336485 REVERSE | Aliases: F3L12.13, F3L12_13	4.0	3.8	0.2	1.1	100.0%	-4.7
6225	AT1G32580.1 | plastid developmental protein DAG, putative, similar to DAG protein, chloroplast precursor (Garden snapdragon) SWISS-PROT:Q38732	5.9	6.4	-0.5	-1.1	100.0%	-4.0
6226	AT4G02250.1 | invertase/pectin methylesterase inhibitor family protein, low similarity to SP:P83326 Pectinesterase inhibitor (Pectin methylesterase inhibitor) (PMEI) {Actinidia chinensis}, pistil-specific gene sts15 (Solanum tuberosum) GI:1616628; contains Pfam profile PF04043: Plant invertase/pectin methylesterase inhibitor	2.5	2.4	0.2	1.1	100.0%	-4.8
6227	AT5G44510.1 | disease resistance protein (TIR-NBS-LRR class), putative, domain signature TIR-NBS-LRR exists, suggestive of a disease resistance protein. | chr5:17946900-17951415 REVERSE | Aliases: MFC16.19, MFC16_19	3.3	3.5	-0.2	-1.1	100.0%	-4.6
6228	AT5G50160.1 | ferric reductase-like transmembrane component family protein, contains Pfam profile PF01794: Ferric reductase like transmembrane component | chr5:20432997-20435930 FORWARD | Aliases: K6A12.2, K6A12_2	5.2	5.9	-0.7	-1.1	100.0%	-3.6

Rank	Description	15.dpi	5.dpi	M	t	adj.q	B
6229	AT2G31500.1 | Symbol: CPK24 | calcium-dependent protein kinase, putative / CDPK, putative, similar to calcium-dependent protein kinase (Arabidopsis thaliana) gi:836942:gb:AAA67655; contains protein kinase domain, Pfam:PF00069; contains EF hand domain (calcium-binding EF-hand), Pfam:PF00036, INTERPRO:IPR002048 | chr2:13420841-13423613 FORWARD | Aliases: T28P16.1	3.0	2.8	0.2	1.1	100.0%	-5.0
6230	AT2G23740.1 | similar to SET domain protein SDG117 [Zea mays] (GB:AAO32935.1); contains InterPro domain Nuclear protein Zn2+-binding (InterPro:IPR003606); contains InterPro domain Nuclear protein SET (InterPro:IPR001214); contains InterPro domain Zn-finger, C2H2 type (InterPro:IPR007087); contains InterPro domain Pre-SET (InterPro:IPR007728); contains InterPro domain SET-related region (InterPro:IPR003616) | chr2:10104639-10110478 FORWARD | Aliases: F27L4.8, F27L4_8	3.3	3.5	-0.2	-1.1	100.0%	-4.8
6231	AT1G07070.1 | 60S ribosomal protein L35a (RPL35aA), similar to ribosomal protein L35a GI:57118 from (Rattus norvegicus) | chr1:2168563-2169850 FORWARD | Aliases: F10K1.22, F10K1_22	8.6	9.1	-0.4	-1.1	100.0%	-4.1
6232	AT3G62930.1 | glutaredoxin family protein, contains glutaredoxin domain, INTERPRO:IPR002109 | chr3:23272513-23272821 REVERSE | Aliases: T20O10.30	3.2	3.0	0.2	1.1	100.0%	-4.7
6233	AT2G44840.1 | Symbol: ATERF13 | encodes a member of the ERF (ethylene response factor) subfamily B-3 of ERF/AP2 transcription factor family. The protein contains one AP2 domain. There are 18 members in this subfamily including ATERF-1, ATERF-2, AND ATERF-5. | chr2:18502416-18503347 FORWARD | Aliases: ATERF13, T13E15.15	3.9	3.6	0.2	1.1	100.0%	-4.5
6234	AT1G74270.1 | 60S ribosomal protein L35a (RPL35aC), similar to ribosomal protein L33B GB:NP_014877 from (Saccharomyces cerevisiae) | chr1:27931917-27933158 REVERSE | Aliases: F1O17.6, F1O17_6	9.8	10.3	-0.5	-1.1	100.0%	-4.1
6235	AT5G65380.1 | ripening-responsive protein, putative, similar to ripening regulated protein DDTFR18 (Lycopersicon esculentum) GI:12231296; contains Pfam profile PF01554: Uncharacterized membrane protein family | chr5:26140215-26143665 REVERSE | Aliases: MNA5.11, MNA5_11	3.8	3.4	0.4	1.1	100.0%	-4.1
6236	AT3G15720.1 | glycoside hydrolase family 28 protein / polygalacturonase (pectinase) family protein, similar to polygalacturonase (Cucumis sativus) GI:6624205; contains Pfam profile PF00295: Glycosyl hydrolases family 28 (polygalacturonases) | chr3:5325297-5327544 REVERSE | Aliases: MSJ11.12	3.0	3.4	-0.4	-1.1	100.0%	-4.5
6237	AT5G64120.1 | peroxidase, putative, identical to peroxidase (Arabidopsis thaliana) gi:1483222:emb:CAA67551 | chr5:25676532-25678228 REVERSE | Aliases: MHJ24.10, MHJ24_10	2.9	3.7	-0.8	-1.1	100.0%	-4.0
6238	AT3G62060.1 | pectinacetylesterase family protein, similar to pectinacetylesterase precursor GI:1431629 from (Vigna radiata); contains Pfam profile: PF03283 pectinacetylesterase | chr3:22991031-22993919 FORWARD | Aliases: T17J13.20	4.8	5.3	-0.4	-1.1	100.0%	-4.1
6239	AT3G06810.1 | acyl-CoA dehydrogenase-related, low similarity to acyl-CoA dehydrogenase (Acinetobacter sp. NCIMB9871) GI:14587418; contains Pfam profiles PF01636: Phosphotransferase enzyme family, PF00441: Acyl-CoA dehydrogenase C-terminal domain, PF02770: Acyl-CoA dehydrogenase middle domain | chr3:2146398-2150837 FORWARD | Aliases: AT3G06800, F3E22.5	4.4	4.7	-0.3	-1.1	100.0%	-4.4
6240	AT3G60410.3 | similar to expressed protein [Arabidopsis thaliana] (TAIR:At1g25370.1); similar to P0519D04.26 [Oryza sativa (japonica cultivar-group)] (GB:NP_917004.1); similar to unknown protein [Oryza sativa (japonica cultivar-group)] (GB:BAD82551.1) | chr3:22342460-22344571 FORWARD | Aliases: T8B10.70	4.4	4.1	0.2	1.1	100.0%	-4.4
6241	AT1G03250.1 | expressed protein | chr1:793353-795338 REVERSE | Aliases: F15K9.15, F15K9_15	5.5	5.1	0.4	1.1	100.0%	-4.2
6242	AT5G08200.1 | peptidoglycan-binding LysM domain-containing protein, contains Pfam profile PF01476: LysM domain	3.6	4.0	-0.4	-1.1	100.0%	-4.4
6243	AT5G06280.3 | expressed protein, similar to unknown protein (pir::T08447) | chr5:1918282-1918897 REVERSE | Aliases: MHF15.20, MHF15_20	5.2	4.8	0.4	1.1	100.0%	-4.4
6244	AT2G16790.1 | shikimate kinase family protein, similar to thermosensitive glucokinase from Escherichia coli (SP:P39208) (SP:P46859); contains Pfam profile PF01202: shikimate kinase | chr2:7291039-7292319 REVERSE | Aliases: T24I21.20, T24I21_20	7.2	6.7	0.6	1.1	100.0%	-4.2
6245	AT1G76200.1 | expressed protein | chr1:28598144-28599636 FORWARD | Aliases: T23E18.30, T23E18_30	13.0	13.1	-0.1	-1.1	100.0%	-5.2
6246	AT4G10640.1 | calmodulin-binding family protein, contains IQ calmodulin-binding motif, Pfam:PF00612 | chr4:6571895-6574423 FORWARD | Aliases: T4F9.100, T4F9_100	4.3	3.9	0.4	1.1	100.0%	-4.2
6247	AT4G19430.1 | expressed protein | chr4:10598456-10599155 REVERSE | Aliases: T5K18.210, T5K18_210	2.7	2.6	0.2	1.1	100.0%	-5.0
6248	AT1G65700.2 | similar to small nuclear ribonucleoprotein, putative / snRNP, putative / Sm protein, putative [Arabidopsis thaliana] (TAIR:At1g19120.1); similar to PREDICTED: similar to U6 snRNA-associated Sm-like protein LSm8 [Gallus gallus] (GB:XP_416009.1); contains InterPro domain Small nuclear ribonucleoprotein (Sm protein) (InterPro:IPR001163) | chr1:24437943-24439918 REVERSE | Aliases: F1E22.8, F1E22_8	9.2	9.6	-0.4	-1.1	100.0%	-4.1

Rank	Description	15.dpi	5.dpi	M	t	adj.q	B
6249	AT2G15270.1 | expressed protein | chr2:6644960-6646501 FORWARD | Aliases: F27O10.8, F27O10_8	7.4	8.2	-0.7	-1.1	100.0%	-4.1
6250	AT5G09380.1 | DNA-directed RNA polymerase III RPC4 family protein, low similarity to SP:P25441 DNA-directed RNA polymerase III 47 kDa polypeptide (EC 2.7.7.6) (C53) (RNA polymerase C subunit 4) {Saccharomyces cerevisiae}; contains Pfam profile PF05132: RNA polymerase III RPC4 | chr5:2911314-2913137 REVERSE | Aliases: T5E8.180, T5E8_180	4.7	4.5	0.2	1.1	100.0%	-4.4
6251	AT5G61820.1 | expressed protein, MtN19, Medicago truncatula, EMBL:MTY15367 | chr5:24851544-24853917 REVERSE | Aliases: MAC9.6, MAC9_6	10.3	10.0	0.3	1.1	100.0%	-4.5
6252	AT4G24680.1 | expressed protein | chr4:12733435-12739747 FORWARD | Aliases: F22K18.120, F22K18_120	6.9	6.6	0.3	1.1	100.0%	-4.4
6253	AT2G43860.1 | polygalacturonase, putative / pectinase, putative, similar to SP:P48979 Polygalacturonase precursor (EC 3.2.1.15) (PG) (Pectinase) {Prunus persica}; contains Pfam profile PF00295: Glycosyl hydrolases family 28 (polygalacturonases) | chr2:18170022-18171505 REVERSE | Aliases: F18O19.3	3.5	3.2	0.3	1.1	100.0%	-4.5
6254	AT3G45480.1 | zinc finger (C3HC4-type RING finger) family protein, contains a zinc finger, C3HC4 type (RING finger), signature, PROSITE:PS00518 | chr3:16694189-16695577 REVERSE | Aliases: F9K21.60	3.0	3.1	-0.1	-1.1	100.0%	-5.2
6255	AT1G61520.2 | Symbol: LHCA3*1 | similar to chlorophyll A-B binding protein, putative (LHCA5) [Arabidopsis thaliana] (TAIR:At1g45474.1); similar to chlorophyll A-B binding protein, putative (LHCA5) [Arabidopsis thaliana] (TAIR:At1g45474.2); similar to probable chlorophyll a/b-binding protein type III precursor - garden pea chloroplast (GB:T06411); contains InterPro domain Chlorophyll A-B binding protein (InterPro:IPR001344) | chr1:22703738-22705048 FORWARD | Aliases: LHCA3*1, T25B24.12, T25B24_12	10.7	10.2	0.5	1.1	100.0%	-3.9
6256	AT2G01590.1 | expressed protein | chr2:266648-267797 FORWARD | Aliases: F2I9.21, F2I9_21	3.4	3.7	-0.3	-1.1	100.0%	-4.6
6257	AT2G44950.1 | zinc finger (C3HC4-type RING finger) family protein, contains Pfam profile: PF00097 zinc finger, C3HC4 type (RING finger) | chr2:18549369-18555666 REVERSE | Aliases: AT2G44960, T14P1.25, T14P1_25	4.9	4.7	0.2	1.1	100.0%	-4.7
6258	AT3G26710.1 | expressed protein | chr3:9814763-9815987 FORWARD | Aliases: MLJ15.10	6.7	6.3	0.4	1.1	100.0%	-4.3
6259	AT1G02460.1 | glycoside hydrolase family 28 protein / polygalacturonase (pectinase) family protein, similar to polygalacturonase PG1 GI:5669846, PG2 GI:5669848 from (Glycine max); contains PF00295: Glycosyl hydrolases family 28 (polygalacturonases) | chr1:504673-507204 REVERSE | Aliases: T6A9.22	4.1	3.9	0.2	1.1	100.0%	-4.4
6260	AT1G74710.2 | Symbol: ICS1 | isochorismate synthase 1 (ICS1) / isochorismate mutase, identical to GI:17223087 and GB:AF078080; contains Pfam profile PF00425: chorismate binding enzyme; contains TIGRfam profile TIGR00543: isochorismate synthases; identical to cDNA isochorismate synthase 1 precursor (ICS1) nuclear gene for plastid product GI:17223086 | chr1:28073957-28077779 FORWARD | Aliases: EDS16, F25A4.31, ISOCHORISMATE SYNTHASE, ISOCHORISMATE SYNTHASEI, SALICYLIC ACID INDUCTION DEFICIENT 2, SID2	3.5	3.8	-0.4	-1.1	100.0%	-4.1
6261	AT4G24470.2 | Symbol: ZIM | zinc finger (GATA type) protein ZIM (ZIM), identical to zinc-finger protein expressed in Inflorescence Meristem, ZIM gi:8918533 from (Arabidopsis thaliana) | chr4:12645600-12647950 FORWARD | Aliases: T22A6.300, T22A6_300	10.2	9.7	0.5	1.1	100.0%	-4.3
6262	AT4G19640.1 | Symbol: ARA7 | Ras-related GTP-binding protein, putative, similar to GTP-binding protein RAB5A GI:1370178 from (Lotus japonicus) | chr4:10687258-10689621 REVERSE | Aliases: F24J7.190, F24J7_190	11.2	10.6	0.6	1.1	100.0%	-4.7
6263	AT1G12520.3 | Symbol: CCS1 | similar to superoxide dismutase (Cu-Zn), chloroplast (SODCP) / copper/zinc superoxide dismutase (CSD2) [Arabidopsis thaliana] (TAIR:At2g28190.1); similar to putative copper/zinc superoxide dismutase copper chaperone precursor [Lycopersicon esculentum] (GB:AAD12307.2); contains InterPro domain Copper/Zinc superoxide dismutase (InterPro:IPR001424) | chr1:4267104-4268884 REVERSE | Aliases: ATCCS, COPPER/ZINC SUPEROXIDE DISMUTASE COPPER CHAPERONE, F5O11.26, F5O11_26	9.0	9.5	-0.5	-1.1	100.0%	-4.4
6264	AT1G13910.1 | leucine-rich repeat family protein, contains leucine rich-repeat (LRR) domains Pfam:PF00560, INTERPRO:IPR001611; contains similarity to Hcr2-0A (Lycopersicon esculentum) gi:3894385:gb:AAC78592	4.3	4.0	0.3	1.1	100.0%	-4.1
6265	AT1G06500.2 | expressed protein | chr1:1989485-1990727 FORWARD | Aliases: F12K11.25, F12K11_25	7.6	7.2	0.5	1.1	100.0%	-4.1
6266	AT4G38220.2 | aminoacylase, putative / N-acyl-L-amino-acid amidohydrolase, putative, similar to aminoacylase-1 (N-acyl-L-amino-acid amidohydrolase, ACY-1)(Homo sapiens) SWISS-PROT:Q03154 | chr4:17925174-17927091 FORWARD | Aliases: F20D10.340, F20D10_340	9.5	9.2	0.4	1.1	100.0%	-4.4
6267	AT5G10730.1 | expressed protein | chr5:3390615-3393107 REVERSE | Aliases: MAJ23.90, MAJ23_90	8.4	8.9	-0.5	-1.1	100.0%	-4.1
6268	AT2G31980.1 | cysteine proteinase inhibitor-related, contains similarity to extracellular insoluble cystatin GI:2204077 from (Daucus carota) | chr2:13616174-13616979 REVERSE | Aliases: F22D22.27, F22D22_27	4.2	4.5	-0.3	-1.1	100.0%	-4.4

Rank	Description	15.dpi	5.dpi	M	t	adj.q	B
6269	AT5G09820.2 | similar to plastid-lipid associated protein PAP / fibrillin family protein [Arabidopsis thaliana] (TAIR:At3g26070.1); similar to fibrillin [Thermosynechococcus elongatus BP-1] (GB:NP_681209.1); contains InterPro domain PAP fibrillin (InterPro:IPR006843) | chr5:3055892-3057445 REVERSE | Aliases: MYH9.3, MYH9_3	3.7	4.0	-0.3	-1.1	100.0%	-4.5
6270	AT1G05380.1 | PHD finger transcription factor, putative | chr1:1578784-1582613 FORWARD | Aliases: T25N20.3, T25N20_3	5.5	5.1	0.4	1.1	100.0%	-4.3
6271	AT5G40640.1 | expressed protein | chr5:16294270-16297652 FORWARD | Aliases: MNF13.19, MNF13_19	3.9	4.1	-0.2	-1.1	100.0%	-4.5
6272	AT1G32070.2 | GCN5-related N-acetyltransferase (GNAT) family protein / nuclear shuttle interacting protein (NSI), very low similarity to SP:P09453 Ribosomal-protein-alanine acetyltransferase (EC 2.3.1.128) {Escherichia coli}; contains Pfam profile PF00583: acetyltransferase, GNAT family; PMID 12837950; identical to cDNA nuclear shuttle interacting protein (NSI) GI: 30790420 | chr1:11534664-11536318 REVERSE | Aliases: T12O21.3, T12O21_3	7.4	7.1	0.3	1.1	100.0%	-4.4
6273	AT1G76850.1 | expressed protein | chr1:28852845-28859393 FORWARD | Aliases: F7O12.2, F7O12_2	4.1	4.6	-0.5	-1.1	100.0%	-4.3
6274	AT3G57710.1 | protein kinase family protein, contains protein kinase domain, Pfam:PF00069 | chr3:21396936-21398376 REVERSE | Aliases: F15B8.100	4.2	4.4	-0.2	-1.1	100.0%	-4.6
6275	AT4G03230.1 | S-locus lectin protein kinase family protein, contains Pfam domins, PF00069: Protein kinase domain, PF00954: S-locus glycoprotein family and PF01453: Lectin (probable mannose binding) | chr4:1419278-1422828 REVERSE | Aliases: F4C21.16, F4C21_16	5.9	6.6	-0.6	-1.1	100.0%	-4.3
6276	AT1G68790.1 | expressed protein | chr1:25838372-25842982 REVERSE | Aliases: F14K14.10, F14K14_10	9.1	10.0	-0.8	-1.1	100.0%	-4.2
6277	AT2G01580.1 | expressed protein, and genefinder | chr2:265043-265904 REVERSE | Aliases: F2I9.20, F2I9_20	4.7	5.3	-0.6	-1.1	100.0%	-4.2
6278	AT4G25990.2 | Symbol: CIL | expressed protein	2.6	2.5	0.1	1.1	100.0%	-5.0
6279	AT4G16610.1 | zinc finger (C2H2 type) family protein, contains Pfam domain, PF00096: Zinc finger, C2H2 type | chr4:9354343-9355309 REVERSE | Aliases: DL4330C, FCAALL.418	3.6	3.8	-0.3	-1.1	100.0%	-4.3
6280	AT1G22050.1 | ubiquitin family protein, contains INTERPRO:IPR000626 ubiquitin domain | chr1:7771659-7773640 FORWARD | Aliases: F2E2.12, F2E2_12	8.6	8.0	0.5	1.1	100.0%	-4.3
6281	AT1G19240.1 | expressed protein | chr1:6649089-6650411 REVERSE | Aliases: T29M8.11, T29M8_11	8.5	8.1	0.4	1.1	100.0%	-4.1
6282	AT3G59100.1 | Symbol: ATGSL11 | glycosyl transferase family 48 protein, contains Pfam profile: PF02364 1,3-beta-glucan synthase | chr3:21854384-21864837 FORWARD | Aliases: F17J16.150, GLUCAN SYNTHASE LIKE 11, GSL11	6.1	5.8	0.3	1.1	100.0%	-4.5
6283	AT1G79950.1 | helicase-related, similar to BRCA1-binding helicase-like protein BACH1 (GI:13661819) Homo sapiens).; contains similarity to helicase-like protein NHL GI:6969265 from (Homo sapiens) | chr1:30078473-30084430 FORWARD | Aliases: F18B13.3, F18B13_3	7.1	7.8	-0.7	-1.1	100.0%	-4.4
6284	AT3G16530.1 | legume lectin family protein, contains Pfam domain, PF00139: Legume lectins beta domain | chr3:5624383-5625476 REVERSE | Aliases: MDC8.19	3.1	3.4	-0.2	-1.1	100.0%	-4.3
6285	AT4G04870.1 | CDP-alcohol phosphatidyltransferase family protein, similar to SP:Q07560 Cardiolipin synthetase (EC 2.7.8.-) {Saccharomyces cerevisiae; contains Pfam profile PF01066: CDP-alcohol phosphatidyltransferase | chr4:2461211-2464141 REVERSE | Aliases: T4B21.19, T4B21_19	8.3	8.7	-0.4	-1.1	100.0%	-4.1
6286	AT3G54890.3 | Symbol: LHCA1 | chlorophyll A-B binding protein / LHCI type I (CAB), identical to chlorophyll A/B-binding protein (Arabidopsis thaliana) GI:16207; contains Pfam profile: PF00504 chlorophyll A-B binding protein | chr3:20350482-20351968 REVERSE | Aliases: F28P10.130, F28P10_130, LHCA1	11.7	11.3	0.4	1.1	100.0%	-3.9
6287	AT4G19380.1 | alcohol oxidase-related, similar to long chain fatty alcohol oxidase from Candida cloacae (GI:6983581), Candida tropicalis (GI:6983594) | chr4:10568376-10572423 REVERSE | Aliases: T5K18.160, T5K18_160	2.7	2.5	0.2	1.1	100.0%	-4.7
6288	AT5G14080.1 | pentatricopeptide (PPR) repeat-containing protein, contains Pfam profile PF01535: PPR repeat | chr5:4543137-4545258 REVERSE | Aliases: MUA22.8, MUA22_8	5.2	5.5	-0.3	-1.1	100.0%	-4.5
6289	AT5G11820.1 | expressed protein, self-incompatibility protein S3 precursor, Papaver rhoeas cv., PIR:S69186 | chr5:3810650-3811177 REVERSE | Aliases: F14F18.2	2.0	1.9	0.1	1.1	100.0%	-5.2
6290	AT1G47970.1 | expressed protein | chr1:17690940-17692485 FORWARD | Aliases: T2J15.12, T2J15_12	7.9	7.5	0.4	1.1	100.0%	-4.6
6291	AT1G13940.1 | expressed protein | chr1:4762700-4766746 REVERSE | Aliases: F16A14.15	4.6	4.9	-0.3	-1.1	100.0%	-4.4

Rank	Description	15.dpi	5.dpi	M	t	adj.q	B
6292	AT4G00350.1 | MATE efflux family protein, similar to ripening regulated protein DDTFR18 (Lycopersicon esculentum) GI:12231296; contains Pfam profile PF01554 Uncharacterized membrane protein family | chr4:151919-154130 FORWARD | Aliases: A_IG005I10.20, A_IG005I10_20, F5I10.20, F5I10_20	2.6	2.4	0.2	1.1	100.0%	-4.8
6293	AT3G10010.1 | HhH-GPD base excision DNA repair family protein, similar to DEMETER protein (Arabidopsis thaliana) GI:21743571; contains Pfam profile PF00730: HhH-GPD superfamily base excision DNA repair protein | chr3:3081819-3088200 REVERSE | Aliases: T22K18.18	2.7	2.9	-0.2	-1.1	100.0%	-4.6
6294	AT3G46130.2 | Symbol: MYB111 |	7.6	7.1	0.4	1.1	100.0%	-4.1
6295	AT4G05080.1 | F-box family protein, contains Pfam PF00646: F-box domain; contains TIGRFAM TIGR01640 : F-box protein interaction domain	2.3	2.4	-0.1	-1.1	100.0%	-5.1
6296	AT2G30790.1 | photosystem II oxygen-evolving complex 23, putative, expression not detected; similar to SP:O49344 (GI:28800560 (OEC23) Arabidopsis; Non-identical EST and protein matches suggested a possible frameshift in exon 1 (a 4 base deletion between 73745 and 73746) and a different start for exon 2 (base 73645). | chr2:13126124-13127167 REVERSE | Aliases: T11J7.18, T11J7_18	3.4	3.2	0.2	1.1	100.0%	-4.5
6297	AT5G53960.1 | expressed protein | chr5:21924951-21925678 FORWARD | Aliases: K19P17.13, K19P17_13	2.2	2.1	0.1	1.1	100.0%	-5.5
6298	AT3G21110.2 | Symbol: PUR7 | similar to SAICAR synthetase [Nicotiana tabacum] (GB:AAR06292.1); contains InterPro domain SAICAR synthetase (InterPro:IPR001636) | chr3:7402141-7405614 REVERSE | Aliases: 5' PHOSPHORIBOSYL 4 (N SUCCINOCARBOXAMIDE) 5 AMINOIMIDAZOLE SYNTHETASE, MSA6.26, PURC, SAIRCAR SYNTHETASE	8.8	9.2	-0.4	-1.1	100.0%	-4.0
6299	AT1G77690.1 | amino acid permease, putative, similar to AUX1 (regulator of root gravitropism, putative permease) GI:1531758 GB:CAA67308 from (Arabidopsis thaliana); contains Pfam profile PF01490: Transmembrane amino acid transporter protein | chr1:29205835-29208234 REVERSE | Aliases: T32E8.2, T32E8_2	5.4	5.9	-0.5	-1.1	100.0%	-4.3
6300	ATCG00020.1 | Symbol: PSBA | Encodes chlorophyll binding protein D1, a part of the photosystem II reaction center core | chrC:383-1444 REVERSE | Aliases: PSBA	12.0	11.0	1.1	1.1	100.0%	-3.9
6301	AT2G29660.1 | zinc finger (C2H2 type) family protein, contains zinc finger, C2H2 type, domain, PROSITE:PS00028	6.4	6.1	0.3	1.1	100.0%	-4.4
6302	AT1G71750.1 | phosphoribosyltransferase family protein, similar to SP:Q02522 Hypoxanthine-guanine phosphoribosyltransferase (EC 2.4.2.8) (HGPRT) (HGPRTase) {Lactococcus lactis}; contains Pfam profile PF00156: Phosphoribosyl transferase domain | chr1:26991465-26992822 REVERSE | Aliases: F14O23.13, F14O23_13	5.4	5.8	-0.4	-1.1	100.0%	-4.3
6303	AT1G15810.1 | ribosomal protein S15 family protein, contains similarity to ribosomal protein S15 | chr1:5444413-5447229 FORWARD | Aliases: F7H2.15, F7H2_15	5.9	6.5	-0.6	-1.1	100.0%	-4.2
6304	AT5G02620.1 | ankyrin repeat family protein, contains ankyrin repeat domains, Pfam:PF00023 | chr5:589525-591675 FORWARD | Aliases: T22P11.210, T22P11_210	4.5	4.3	0.2	1.1	100.0%	-4.3
6305	AT5G49480.1 | Symbol: ATCP1 | sodium-inducible calcium-binding protein (ACP1) / sodium-responsive calcium-binding protein (ACP1), identical to NaCl-inducible Ca2+-binding protein GI:2352828 from (Arabidopsis thaliana) | chr5:20087969-20088864 FORWARD | Aliases: ACP1, K6M13.2, K6M13_2	8.2	7.8	0.4	1.1	100.0%	-4.2
6306	AT2G34780.1 | Symbol: EMB1611 | expressed protein | chr2:14675732-14680686 FORWARD | Aliases: EMB1611, EMBRYO DEFECTIVE 1611, F19I3.1, F19I3_1	4.7	4.9	-0.3	-1.1	100.0%	-4.5
6307	AT1G77670.1 | aminotransferase class I and II family protein, similar to kynurenine aminotransferase /glutamine transaminase K GI:1030066 (Rattus norvegicus) | chr1:29193795-29195869 REVERSE | Aliases: T5M16.26, T5M16_26	8.2	8.9	-0.7	-1.1	100.0%	-3.8
6308	AT3G56480.1 | myosin heavy chain-related, contains weak similarity to Myosin heavy chain, cardiac muscle alpha isoform (MyHC-alpha) (Alpha isomyosin) (Fragment) (Swiss-Prot:P04460) (Oryctolagus cuniculus) | chr3:20947077-20950609 REVERSE | Aliases: T5P19.130	2.3	2.5	-0.2	-1.1	100.0%	-4.9
6309	AT1G16180.1 | TMS membrane family protein / tumour differentially expressed (TDE) family protein, contains Pfam domain, PF03348: TMS membrane protein/tumour differentially expressed protein (TDE) | chr1:5540899-5542896 FORWARD | Aliases: T24D18.26, T24D18_26	9.7	9.1	0.7	1.1	100.0%	-4.5
6310	AT3G18420.1 | tetratricopeptide repeat (TPR)-containing protein, contains Pfam profile: PF00515 tetratricopeptide repeat domain (TPR domain) | chr3:6324639-6325799 REVERSE | Aliases: MYF24.14	7.1	7.7	-0.6	-1.1	100.0%	-4.0
6311	AT1G22720.1 | wall-associated kinase, putative, contains similarity to serine/threonine kinase gb:Y12531 from Brassica oleracea | chr1:8044221-8045654 REVERSE | Aliases: T22J18.11, T22J18_11	3.1	3.0	0.2	1.1	100.0%	-4.9
6312	AT3G28890.1 | leucine-rich repeat family protein, contains leucine rich-repeat (LRR) domains Pfam:PF00560, INTERPRO:IPR001611; contains similarity to Hcr2-0B (Lycopersicon esculentum) gi:3894387:gb:AAC78593	2.8	2.7	0.1	1.1	100.0%	-5.0

Rank	Description	15.dpi	5.dpi	M	t	adj.q	B
6313	AT5G44630.1 | Encodes a sesquiterpene synthase involved in generating all of the group B sesquiterpenes found in the Arabidopsis floral volatile blend. Strongly expressed in intrafloral nectaries. | chr5:18020507-18022843 FORWARD | Aliases: K15C23.7, K15C23_7	3.0	2.7	0.2	1.1	100.0%	-4.7
6314	AT1G67830.1 | GDSL-motif lipase/hydrolase family protein, similar to early nodulin ENOD8 (Medicago sativa) GI:304037, elicitor-induced glycoprotein iEP4 (Daucus carota) GI:1911765, lanatoside 15'-O-acetylesterase (Digitalis lanata) GI:3688284; contains Pfam profile PF00657: Lipase/Acylhydrolase with GDSL-like motif | chr1:25434992-25436635 REVERSE | Aliases: F12A21.4, F12A21_4	5.5	5.8	-0.3	-1.1	100.0%	-4.1
6315	AT4G11240.1 | Symbol: TOPP7 | serine/threonine protein phosphatase PP1 isozyme 6 (PP1BG) (TOPP6), identical to SP:P48486 Serine/threonine protein phosphatase PP1 isozyme 6 (EC 3.1.3.16) {Arabidopsis thaliana} | chr4:6847115-6849237 FORWARD | Aliases: F8L21.30, F8L21_30	5.1	5.6	-0.5	-1.1	100.0%	-4.1
6316	AT3G47540.1 | chitinase, putative, similar to basic endochitinase CHB4 precursor SP:Q06209 from (Brassica napus) | chr3:17532014-17533254 FORWARD | Aliases: F1P2.90	6.1	6.4	-0.4	-1.1	100.0%	-4.5
6317	AT2G40690.1 | Symbol: SFD1 | NAD-dependent glycerol-3-phosphate dehydrogenase family protein, low similarity to SP:Q26756 Glycerol-3-phosphate dehydrogenase (NAD+), glycosomal (EC 1.1.1.8) {Trypanosoma brucei rhodesiense}; contains Pfam profile PF01210: NAD-dependent glycerol-3-phosphate dehydrogenase | chr2:16981107-16983517 FORWARD | Aliases: SUPPRESSOR OF FATTY ACID DESATURASE DEFICIENCY 1, T7D17.13, T7D17_13	3.4	3.7	-0.3	-1.1	100.0%	-4.4
6318	ATCG00700.1 | Symbol: PSBN | PSII low MW protein | chrC:74249-74380 REVERSE | Aliases: PSBN	3.7	3.3	0.4	1.1	100.0%	-4.3
6319	AT5G09640.1 | Symbol: SNG2 | sinapoylglucose:choline sinapoyltransferase (SNG2), GC donor splice site at exon 11 and 13; TA donor splice site at exon 10; similar to serine carboxypeptidase I precursor (SP:P37890) (Oryza sativa); wound-inducible carboxypeptidase, Lycopersicon esculentum, EMBL:AF242849; contains Pfam profile PF00450: Serine carboxypeptidase; identical to cDNA sinapoylglucose:choline sinapoyltransferase (SNG2) GI:15418806 | chr5:2988315-2991157 FORWARD | Aliases: F17I14.170, F17I14_170, SCPL19, SINAPOYLGLUCOSE ACCUMULATOR 2	3.2	3.3	-0.2	-1.1	100.0%	-4.8
6320	AT4G29735.1 | expressed protein, contains Pfam domain PF05251: Uncharacterised protein family (UPF0197) | chr4:14562645-14564384 REVERSE | Aliases: None	10.9	10.4	0.5	1.1	100.0%	-4.3
6321	AT1G29940.1 | Symbol: NRPA2 | similar to DNA-directed RNA polymerase, putative [Arabidopsis thaliana] (TAIR:At5g45140.1); similar to hypothetical protein UM01133.1 [Ustilago maydis 521] (GB:EAK81692.1); similar to unnamed protein product [Debaryomyces hansenii CBS767] (GB:CAG89744.1); similar to SPBP23A10.07 [Schizosaccharomyces pombe] (GB:CAB66435.1); similar to hypothetical protein CaO19.7062 [Candida albicans SC5314] (GB:EAL01509.1); similar to putative DNA-directed RNA polymerase I subunit, 5'-partial [Oryza sativa (japonica cultivar-group)] (GB:AAM92814.1); contains InterPro domain RNA polymerase Rpb2, domain 3 (InterPro:IPR007645); contains InterPro domain RNA polymerase Rpb2, domain 7 (InterPro:IPR007641); contains InterPro domain RNA polymerase beta subunit (InterPro:IPR007644); contains InterPro domain RNA polymerase Rpb2, domain 2 (InterPro:IPR007642); contains InterPro domain RNA polymerase Rpb2, domain 6 (InterPro:IPR007120); contains InterPro domain RNA polymerase, beta subunit (InterPro:IPR007121) | chr1:10479170-10486803 REVERSE | Aliases: F1N18.2	7.2	7.7	-0.5	-1.1	100.0%	-4.0
6322	AT3G08980.1 | signal peptidase I family protein, similar to SP:P46972 Mitochondrial inner membrane protease subunit 2 (EC 3.4.99.-) {Saccharomyces cerevisiae}; contains Pfam profile PF00461: Signal peptidase I | chr3:2740813-2742690 FORWARD | Aliases: T16O11.6	4.6	5.0	-0.4	-1.1	100.0%	-4.0
6323	AT5G07950.1 | expressed protein, ferredoxin (2Fe-2S), Arabidopsis thaliana, PIR:B71412	4.2	4.0	0.2	1.1	100.0%	-4.6
6324	AT3G63220.2 | kelch repeat-containing F-box family protein, contains Pfam profiles PF01344: Kelch motif, PF00646: F-box domain	5.6	5.3	0.3	1.1	100.0%	-4.3
6325	AT3G18210.1 | oxidoreductase, 2OG-Fe(II) oxygenase family protein, contains Pfam domain PF03171 2OG-Fe(II) oxygenase superfamily | chr3:6237936-6240611 REVERSE | Aliases: MRC8.21	5.9	6.4	-0.5	-1.1	100.0%	-3.9
6326	AT4G16040.1 | hypothetical protein | chr4:9087194-9087466 REVERSE | Aliases: DL4060C, FCAALL.265	2.1	2.1	0.1	1.1	100.0%	-5.6
6327	AT2G42750.1 | DNAJ heat shock N-terminal domain-containing protein, low similarity to GFA2 (Arabidopsis thaliana) GI:21429604; contains Pfam profile PF00226: DnaJ domain | chr2:17800384-17802757 FORWARD | Aliases: F7D19.25, F7D19_25	5.5	5.1	0.4	1.1	100.0%	-4.1
6328	AT5G16550.1 | expressed protein | chr5:5405468-5406887 FORWARD | Aliases: MQK4.30, MQK4_30	6.3	5.8	0.6	1.1	100.0%	-4.3
6329	AT5G44290.3 | similar to protein kinase family protein [Arabidopsis thaliana] (TAIR:At1g03740.1); similar to putative CRK1 protein [Oryza sativa (japonica cultivar-group)] (GB:XP_479750.1); similar to putative CRK1 protein [Oryza sativa (japonica cultivar-group)] (GB:BAD89473.1); contains InterPro domain Serine/threonine protein kinase, active site (InterPro:IPR008271); contains InterPro domain Tyrosine protein kinase (InterPro:IPR001245); contains InterPro domain Serine/threonine protein kinase (InterPro:IPR002290); contains InterPro domain Protein kinase (InterPro:IPR000719) | chr5:17857651-17860824 REVERSE | Aliases: K9L2.5, K9L2_5	5.4	5.0	0.4	1.1	100.0%	-4.1
6330	AT5G49940.1 | Symbol: NFU2 | nitrogen fixation NifU-like family protein, similar to Nitrogen fixation protein nifU (SP:Q43885) {Anabaena azollae}; contains Pfam profile: PF01106 NifU-like domain | chr5:20332639-20334626 FORWARD | Aliases: ATCNFU2, K9P8.16	7.7	7.3	0.3	1.1	100.0%	-4.5

Rank	Description	15.dpi	5.dpi	M	t	adj.q	B
6331	AT4G23330.1 | eukaryotic translation initiation factor-related, contains weak similarity to Swiss-Prot:Q9LD55 eukaryotic translation initiation factor 3 subunit 10 (eIF-3 theta) (Eukaryotic translation initiation factor 3 large subunit) (eIF3a) (p114) (Arabidopsis thaliana) | chr4:12192146-12194620 FORWARD | Aliases: F16G20.30	3.6	3.3	0.3	1.1	100.0%	-4.2
6332	AT1G54570.1 | esterase/lipase/thioesterase family protein, contains Interpro entry IPR000379 | chr1:20384145-20388688 REVERSE | Aliases: T22H22.2, T22H22_2	7.2	6.9	0.3	1.1	100.0%	-4.2
6333	AT1G73920.2 | lipase family protein, similar to lipase GB:CAA74737 (SP:O46108) from (Drosophila melanogaster) | chr1:27794537-27798575 FORWARD | Aliases: F2P9.21, F2P9_21	5.8	6.3	-0.4	-1.1	100.0%	-4.0
6334	AT3G29320.1 | glucan phosphorylase, putative, similar to alpha-glucan phosphorylase, L isozyme 1 precursor GB:P04045 from (Solanum tuberosum) (J. Biochem. 106 (4), 691-695 (1989)) | chr3:11254059-11259051 FORWARD | Aliases: MUO10.17	9.1	9.6	-0.5	-1.1	100.0%	-4.0
6335	AT3G13480.1 | similar to expressed protein [Arabidopsis thaliana] (TAIR:At1g55475.1); similar to P0415C01.14 [Oryza sativa (japonica cultivar-group)] (GB:NP_917459.1)	7.5	6.9	0.6	1.1	100.0%	-4.4
6336	AT1G61700.1 | DNA-directed RNA polymerase II, putative (RPB10), identical to SP:Q9SYA6 DNA-directed RNA polymerase II 8.2 kDa polypeptide (EC 2.7.7.6) (RPB10) (RP10) (ABC10) {Arabidopsis thaliana}; very strong similarity to SP:Q39290 DNA-directed RNA polymerase II 8.2 kDa polypeptide {Brassica napus}; contains Pfam profile: PF01194 RNA polymerases N / 8 kDa subunit | chr1:22790770-22791777 FORWARD | Aliases: T13M11.6, T13M11_6	2.9	2.7	0.1	1.1	100.0%	-4.9
6337	AT2G37410.2 | Symbol: ATTIM17 2 | mitochondrial import inner membrane translocase (TIM17), nearly identical to SP:Q9SP35 Mitochondrial import inner membrane translocase subunit TIM17 {Arabidopsis thaliana}	10.5	9.8	0.7	1.1	100.0%	-4.3
6338	AT5G16930.1 | AAA-type ATPase family protein, contains Pfam domain, PF00004: ATPase, AAA family | chr5:5568396-5571958 FORWARD | Aliases: F2K13.80, F2K13_80	3.0	3.2	-0.2	-1.1	100.0%	-4.7
6339	AT1G70570.1 | anthranilate phosphoribosyltransferase, putative, similar to anthranilate phosphoribosyltransferase (EC 2.4.2.18) SP:O66576 from (Aquifex aeolicus) | chr1:26612290-26615947 FORWARD | Aliases: F24J13.14, F24J13_14	7.0	6.6	0.4	1.1	100.0%	-4.3
6340	AT4G15770.1 | 60S ribosome subunit biogenesis protein, putative, contains similarity to 60S ribosome subunit biogenesis protein NIP7 (Swiss-Prot:Q08962) (Saccharomyces cerevisiae) | chr4:8978009-8978877 FORWARD | Aliases: DL3925W, FCAALL.377	8.9	9.1	-0.2	-1.1	100.0%	-4.7
6341	AT3G61760.1 | dynamin-like protein B (DL1B), identical to dynamin-like protein B (Arabidopsis thaliana) GI:27543504; strong similarity to GTP-binding protein (Arabidopsis thaliana) GI:807577, phragmoplastin SDL5A (Glycine max) GI:1218004; contains Pfam profiles PF01031: Dynamin central region, PF00350: Dynamin family, PF02212: Dynamin GTPase effector domain | chr3:22871489-22875128 REVERSE | Aliases: ADL1B, F21F14.15	4.2	4.5	-0.3	-1.1	100.0%	-4.4
6342	AT4G01810.1 | protein transport protein-related, related to Sec23 protein (Homo sapiens) gi:1296664:emb:CAA65774 | chr4:776488-780331 REVERSE | Aliases: T7B11.7, T7B11_7	3.8	4.3	-0.4	-1.1	100.0%	-4.2
6343	AT2G37390.1 | heavy-metal-associated domain-containing protein, contains Pfam PF00403: Heavy-metal-associated domain; similar to copper homeostasis factor (CCH) (ATX1) (GB:U88711) (TIGR_Ath1:At3g56240) (Arabidopsis thaliana) | chr2:15701299-15702904 FORWARD | Aliases: F3G5.18, F3G5_18	3.0	2.8	0.2	1.1	100.0%	-4.8
6344	AT5G20640.1 | expressed protein, contains Pfam profile PF04525: Protein of unknown function (DUF567) | chr5:6984381-6985207 FORWARD | Aliases: T1M15.40, T1M15_40	5.9	5.5	0.4	1.1	100.0%	-4.4
6345	AT3G27310.1 | Symbol: PUX1 | expressed protein | chr3:10088438-10090338 REVERSE | Aliases: K17E12.13, PLANT UBX DOMAIN CONTAINING PROTEIN 1	7.0	6.7	0.3	1.1	100.0%	-4.4
6346	AT3G59060.4 | Symbol: PIL6 | similar to phytochrome-interacting factor 4 (PIF4) / basic helix-loop-helix protein 9 (bHLH9) / short under red-light 2 (SRL2) [Arabidopsis thaliana] (TAIR:At2g43010.1); similar to BP-5 protein [Oryza sativa] (GB:CAD32238.1); contains InterPro domain Basic helix-loop-helix dimerization domain bHLH (InterPro:IPR001092) | chr3:21838955-21841484 REVERSE | Aliases: F17J16.110, PHYTOCHROME INTERACTING FACTOR 3 LIKE 6, PHYTOCHROME INTERACTING FACTOR 5, PIF5	2.9	2.7	0.2	1.1	100.0%	-4.9
6347	AT3G53580.1 | diaminopimelate epimerase family protein, contains Pfam profile PF01678: Diaminopimelate epimerase | chr3:19875742-19878112 FORWARD | Aliases: F4P12.280	10.7	11.0	-0.2	-1.1	100.0%	-4.5
6348	AT4G36760.2 | Symbol: ATAPP1 |	6.9	7.5	-0.6	-1.1	100.0%	-4.3
6349	AT3G63000.1 | NPL4 family protein, contains Pfam domain, PF05021: NPL4 family | chr3:23294584-23296515 FORWARD | Aliases: T20O10.100	9.1	9.4	-0.3	-1.1	100.0%	-4.2

Rank	Description	15.dpi	5.dpi	M	t	adj.q	B
6350	AT5G25280.2 | serine-rich protein-related, contains some similarity to serine-rich proteins | chr5:8773795-8776003 FORWARD | Aliases: F18G18.20, F18G18_20	5.6	5.0	0.6	1.1	100.0%	-3.9
6351	AT5G54840.2 | GTP-binding family protein, similar to SP:P87027 Septum-promoting GTP-binding protein 1 (GTPase spg1)(Sid3 protein) {Schizosaccharomyces pombe} | chr5:22293856-22295643 REVERSE | Aliases: MBG8.10, MBG8_10	7.0	6.5	0.5	1.1	100.0%	-4.4
6352	AT1G76820.1 | expressed protein | chr1:28842467-28843745 FORWARD | Aliases: F28O16.19, F28O16_19	8.3	7.5	0.7	1.1	100.0%	-3.9
6353	AT4G24880.1 | expressed protein | chr4:12808530-12811383 FORWARD | Aliases: F13M23.20, F13M23_20	6.0	5.7	0.3	1.1	100.0%	-4.2
6354	AT2G30650.1 | 3-hydroxyisobutyryl-coenzyme A hydrolase, putative / CoA-thioester hydrolase, putative, strong similarity to gi:8572760; contains Pfam profile PF00388 enoyl-CoA hydratase/isomerase family protein | chr2:13060854-13063457 REVERSE | Aliases: T11J7.4, T11J7_4	2.4	2.2	0.1	1.1	100.0%	-5.2
6355	AT2G28610.1 | Symbol: PRS | homeobox-leucine zipper transcription factor (PRESSED FLOWER), identical to PRESSED FLOWER (GP:17907768) {Arabidopsis thaliana} | chr2:12269090-12270492 FORWARD | Aliases: PRESSED FLOWER, T8O18.10, T8O18_10	2.5	2.7	-0.2	-1.1	100.0%	-4.8
6356	AT1G43560.1 | thioredoxin family protein, contains Pfam profile: PF00085 Thioredoxin; similar to thioredoxin GI:142153 from (Synechococcus PCC6301) | chr1:16400539-16402318 REVERSE | Aliases: AT1G43565, T10P12.4, T10P12_4	6.2	6.8	-0.6	-1.1	100.0%	-3.8
6357	AT5G11680.1 | expressed protein, predicted proteins, Arabidopsis thaliana | chr5:3758859-3760848 FORWARD | Aliases: T22P22.70, T22P22_70	9.4	9.1	0.3	1.1	100.0%	-4.4
6358	AT3G30180.1 | Symbol: BR6OX2/CYP85A2 |	2.9	3.1	-0.2	-1.1	100.0%	-4.6
6359	AT4G24520.1 | Symbol: ATR1 | NADPH-cytochrome p450 reductase, putative / NADPH-ferrihemoprotein reductase, putative, similar to NADPH-ferrihemoprotein reductase NADPH-cytochrome P450 oxydoreductase isoform 1 (Populus balsamifera subsp. trichocarpa x Populus deltoides) GI:13183562, SP:P37116 NADPH-cytochrome P450 reductase (EC 1.6.2.4) (CPR) (Vigna radiata) {Phaseolus aureus} | chr4:12662851-12667165 REVERSE | Aliases: AR1, ARABIDOPSIS CYTOCHROME REDUCTASE, ARABIDOPSIS P450 REDUCTASE 1, F22K18.280, F22K18_280	5.7	6.2	-0.5	-1.1	100.0%	-4.0
6360	AT1G06390.2 | Symbol: GSK1 | shaggy-related protein kinase iota / ASK-iota (ASK9) (GSK1), identical to shaggy-related protein kinase iota (ASK-iota) (Arabidopsis thaliana) SWISS-PROT:Q39012	4.4	5.0	-0.6	-1.1	100.0%	-4.3
6361	AT5G08040.1 | expressed protein | chr5:2577203-2578120 REVERSE | Aliases: T22D6.2	10.0	10.5	-0.5	-1.1	100.0%	-4.3
6362	AT1G07260.1 | UDP-glucoronosyl/UDP-glucosyl transferase family protein, contains Pfam profile: PF00201 UDP-glucoronosyl and UDP-glucosyl transferase | chr1:2227593-2229318 REVERSE | Aliases: F10K1.3, F10K1_3	4.9	5.2	-0.3	-1.1	100.0%	-4.3
6363	AT5G17830.1 | expressed protein, contains Pfam domain, PF04515: Protein of unknown function, DUF580 | chr5:5893384-5895369 FORWARD | Aliases: MVA3.19, MVA3_19	2.2	2.4	-0.1	-1.1	100.0%	-5.0
6364	AT5G67370.1 | expressed protein, similar to unknown protein (gb:AAC18972.1) | chr5:26894794-26896768 REVERSE | Aliases: K8K14.9, K8K14_9	7.8	7.5	0.3	1.1	100.0%	-4.4
6365	AT3G11520.1 | Symbol: CYCB1;3 | cyclin, putative (CYC2), similar to cyclin (Arabidopsis thaliana) GI:1360646; contains Pfam profiles PF00134: Cyclin, N-terminal domain, PF02984: Cyclin, C-terminal domain; identical to cDNA cyclin box (cyc2) partial cds GI:456019 | chr3:3625330-3627236 REVERSE | Aliases: CYC2, CYCLIN B1;3, F24K9.20	3.3	3.8	-0.5	-1.1	100.0%	-4.2
6366	NA	11.1	10.9	0.2	1.1	100.0%	-4.7
6367	AT2G20560.1 | DNAJ heat shock family protein, SP:Q9UDY4 DnaJ homolog subfamily B member 4 (Heat shock 40 kDa protein 1 homolog) {Homo sapiens}; contains Pfam profile PF00226: DnaJ domain | chr2:8855211-8857051 REVERSE | Aliases: T13C7.15, T13C7_15	8.0	7.4	0.6	1.1	100.0%	-3.9
6368	AT5G37050.1 | expressed protein, hypothetical protein T28J14.60 - Arabidopsis thaliana, PIR:T48483 | chr5:14657740-14658651 REVERSE | Aliases: MJG14.3	2.4	2.3	0.1	1.1	100.0%	-5.2
6369	AT5G28920.1 | expressed protein | chr5:10940039-10943382 REVERSE | Aliases: F7P1.1	4.7	4.3	0.4	1.1	100.0%	-4.1
6370	AT3G19860.2 | similar to basic helix-loop-helix (bHLH) family protein [Arabidopsis thaliana] (TAIR:At4g36060.1); similar to putative amelogenin precursor [Oryza sativa] (GB:XP_470739.1); contains InterPro domain Proline-rich region (InterPro:IPR000694); contains InterPro domain Basic helix-loop-helix dimerization domain bHLH (InterPro:IPR001092) | chr3:6903839-6906127 FORWARD | Aliases: MPN9.10	6.1	5.6	0.5	1.1	100.0%	-4.2
6371	AT4G13140.1 | expressed protein | chr4:7650322-7651141 REVERSE | Aliases: F17N18.30, F17N18_30	8.6	8.1	0.4	1.1	100.0%	-4.1

Rank	Description	15.dpi	5.dpi	M	t	adj.q	B
6372	AT4G32260.1 | ATP synthase family, contains Pfam profile: PF00430 ATP synthase B/B' CF(0); identical to cDNA chloroplast ATP synthase beta chain precursor (atpG) GI:5730140 | chr4:15573643-15574743 REVERSE | Aliases: F10M6.100, F10M6_100	7.9	7.0	0.9	1.1	100.0%	-3.9
6373	AT1G14910.1 | epsin N-terminal homology (ENTH) domain-containing protein, contains Pfam PF01417: ENTH domain. ENTH (Epsin N-terminal homology) domain; similar to Chain A, Calm-N N-Terminal Domain Of Clathrin Assembly Lymphoid Myeloid Leukaemia Protein, Pi(4,5)p2 Complex (GP:13399999) {Homo sapiens} | chr1:5139707-5143680 REVERSE | Aliases: F10B6.32, F10B6_32	5.8	5.4	0.4	1.1	100.0%	-4.2
6374	AT5G02370.1 | kinesin motor protein-related, kinesin, Xenopus laevis, EMBL:XLA249840 | chr5:503406-506922 FORWARD | Aliases: T1E22.130, T1E22_130	4.5	4.9	-0.4	-1.1	100.0%	-4.3
6375	AT4G11220.1 | reticulon family protein (RTNLB2), similar to SP:Q64548 Reticulon 1 (Neuroendocrine-specific protein) {Rattus norvegicus}; contains Pfam profile PF02453: Reticulon | chr4:6837946-6839791 REVERSE | Aliases: F8L21.10, F8L21_10	9.0	8.6	0.5	1.1	100.0%	-4.4
6376	AT3G48720.1 | transferase family protein, similar to hypersensitivity-related hsr201 protein - Nicotiana tabacum,PIR2:T03274; contains Pfam transferase family domain PF00248 | chr3:18057308-18060437 FORWARD | Aliases: T8P19.230	3.3	3.1	0.2	1.1	100.0%	-4.6
6377	AT4G19710.2 | bifunctional aspartate kinase/homoserine dehydrogenase, putative / AK-HSDH, putative, similar to gb:X71364 (PIR:S46497) aspartate kinase / homoserine dehydrogenase from Arabidopsis thaliana | chr4:10724865-10729681 FORWARD | Aliases: T16H5.70, T16H5_70	8.6	9.1	-0.6	-1.1	100.0%	-3.9
6378	AT4G35130.1 | pentatricopeptide (PPR) repeat-containing protein, contains Pfam profile PF01535: PPR repeat | chr4:16721089-16723503 REVERSE | Aliases: T12J5.8	3.7	3.9	-0.2	-1.1	100.0%	-4.9
6379	AT4G01110.1 | expressed protein, similar to expressed protein [Arabidopsis thaliana] (TAIR:At2g46300.1); similar to hypothetical protein [Oryza sativa (japonica cultivar-group)] (GB:BAD72426.1) | chr4:479501-481160 REVERSE | Aliases: F2N1.3, F2N1_3	3.4	3.1	0.3	1.1	100.0%	-4.3
6380	AT3G55590.1 | GDP-mannose pyrophosphorylase, putative, strong similarity to GDP-mannose pyrophosphorylase from Arabidopsis thaliana (GI:3598958), Pichia angusta (GI:7331158); contains Pfam profile PF00483 Nucleotidyl transferase | chr3:20628458-20629860 FORWARD | Aliases: F1I16.2	3.2	3.0	0.2	1.1	100.0%	-4.6
6381	AT1G15720.1 | Symbol: TRFL5 | myb family transcription factor, contains Pfam PF00249: Myb-like DNA-binding domain | chr1:5406020-5407481 FORWARD | Aliases: F7H2.6, F7H2_6, TRF LIKE 5	3.9	4.2	-0.3	-1.1	100.0%	-4.5
6382	AT2G38970.1 | zinc finger (C3HC4-type RING finger) family protein, very low similarity to SP:P97280 Inter-alpha-trypsin inhibitor heavy chain H3 precursor {Mesocricetus auratus}; contains Pfam profiles PF00097: Zinc finger, C3HC4 type (RING finger), PF00092: von Willebrand factor type A domain	5.3	5.0	0.3	1.1	100.0%	-4.2
6383	AT3G51690.1 | expressed protein, low similarity to SP:P07271 DNA repair and recombination protein PIF1, mitochondrial precursor {Saccharomyces cerevisiae} | chr3:19187416-19189086 REVERSE | Aliases: T18N14.70	2.2	2.1	0.1	1.1	100.0%	-5.5
6384	AT1G11020.1 | zinc finger (C3HC4-type RING finger) family protein, contains Pfam profile: PF00097 zinc finger, C3HC4 type (RING finger) | chr1:3676691-3678524 FORWARD | Aliases: T19D16.25, T19D16_25	6.5	6.1	0.4	1.1	100.0%	-4.4
6385	AT3G09640.2 | Symbol: APX2 | similar to L-ascorbate peroxidase 1, cytosolic (APX1) [Arabidopsis thaliana] (TAIR:At1g07890.2); similar to L-ascorbate peroxidase 1, cytosolic (APX1) [Arabidopsis thaliana] (TAIR:At1g07890.3); similar to L-ascorbate peroxidase 1, cytosolic (APX1) [Arabidopsis thaliana] (TAIR:At1g07890.1); similar to cytosolic ascorbate peroxidase [Fragaria x ananassa] (GB:AAB94574.1); similar to cytosolic ascorbate peroxidase [Vigna unguiculata] (GB:AAB03844.1); similar to cytosolic ascorbate peroxidase 2 [Glycine max] (GB:BAC92740.1); contains InterPro domain Haem peroxidase (InterPro:IPR002016); contains InterPro domain Fungal lignin peroxidase (InterPro:IPR001621); contains InterPro domain Plant ascorbate peroxidase (InterPro:IPR002207) | chr3:2956161-2958332 FORWARD | Aliases: APX1B, ASCORBATE PEROXIDASE 2, CS2, F11F8.23	3.2	3.1	0.2	1.1	100.0%	-4.8
6386	AT2G01430.1 | homeobox-leucine zipper protein 17 (HB-17) / HD-ZIP transcription factor 17, identical to (GI:18857716) homeodomain-leucine zipper protein ATHB-17 (GI:18857716) (Arabidopsis thaliana) | chr2:187797-190368 REVERSE | Aliases: F2I9.5, F2I9_5	2.6	2.8	-0.2	-1.1	100.0%	-4.8
6387	AT1G12710.1 | Symbol: ATPP2 A12 | F-box family protein / SKP1 interacting partner 3-related, contains Pfam profile PF00646: F-box domain | chr1:4326737-4328479 REVERSE | Aliases: ATPP2 A12, T12C24.23, T12C24_23	3.8	3.7	0.2	1.1	100.0%	-4.8
6388	AT1G54030.1 | GDSL-motif lipase, putative, similar to myrosinase-associated proteins GI:1769968, GI:1769970, GI:1216391, GI:1216389 from (Brassica napus); contains Pfam profile PF00657: GDSL-like Lipase/Acylhydrolase | chr1:20171302-20173268 FORWARD | Aliases: F15I1.11, F15I1_11	7.4	8.2	-0.8	-1.1	100.0%	-4.2
6389	AT1G72410.1 | COP1-interacting protein-related, similar to COP1-Interacting ProteinI 7 (CIP7) (Arabidopsis thaliana) GI:3327870 | chr1:27255358-27259908 REVERSE | Aliases: T10D10.12, T10D10_12	3.5	3.6	-0.1	-1.1	100.0%	-5.1

Rank	Description	15.dpi	5.dpi	M	t	adj.q	B
6390	AT5G22750.1 | Symbol: RAD5 | SNF2 domain-containing protein / helicase domain-containing protein / RING finger domain-containing protein, similar to SP:P36607 DNA repair protein rad8 {Schizosaccharomyces pombe}; contains Pfam profiles PF00271: Helicase conserved C-terminal domain, PF00176: SNF2 family N-terminal domain, PF00097: Zinc finger, C3HC4 type (RING finger) | chr5:7565040-7571086 REVERSE | Aliases: MDJ22.17, MDJ22_17	3.5	3.7	-0.2	-1.1	100.0%	-4.8
6391	AT3G21500.2 | 1-deoxy-D-xylulose 5-phosphate synthase, putative / 1-deoxyxylulose-5-phosphate synthase, putative / DXP-synthase, putative, strong similarity to 1-D-deoxyxylulose 5-phosphate synthase (Lycopersicon esculentum) GI:5059160, DEF (deficient in photosynthesis) protein (Arabidopsis thaliana) GI:1399261; ; contains Pfam profiles PF02779: Transketolase, pyridine binding domain, PF02780: Transketolase, C-terminal domain | chr3:7573643-7576579 REVERSE | Aliases: MIL23.4	3.8	3.5	0.3	1.1	100.0%	-4.4
6392	AT1G17130.1 | cell cycle control protein-related, contains similarity to Swiss-Prot:Q9P7C5 cell cycle control protein cwf16 (Schizosaccharomyces pombe) | chr1:5854141-5856968 FORWARD | Aliases: F20D23.18, F20D23_18	6.6	7.1	-0.6	-1.1	100.0%	-4.0
6393	AT5G47770.1 | Symbol: FPS1 | farnesyl pyrophosphate synthetase 1, mitochondrial (FPS1) / FPP synthetase 1 / farnesyl diphosphate synthase 1, identical to SP:Q09152 Farnesyl pyrophosphate synthetase 1, mitochondrial precursor (FPP synthetase 1) (FPS 1) (Farnesyl diphosphate synthetase 1) (Includes: Dimethylallyltransferase (EC 2.5.1.1); Geranyltranstransferase (EC 2.5.1.10)) {Arabidopsis thaliana} | chr5:19362399-19364855 FORWARD | Aliases: FARNESYL DIPHOSPHATE SYNTHASE 1, MCA23.9, MCA23_9	6.5	7.0	-0.5	-1.1	100.0%	-4.4
6394	AT1G65860.1 | flavin-containing monooxygenase family protein / FMO family protein, similar to flavin-containing monooxygenase FMO3 (dimethylaniline monoxygenase (N-oxide forming) 3) GI:349533 (SP:P32417) from Oryctolagus cuniculus, (SP:P97501) from Mus musculus; contains Pfam profile PF00743 Flavin-binding monooxygenase-like domain | chr1:24502729-24506408 REVERSE | Aliases: F12P19.2, F12P19_2	3.1	3.3	-0.2	-1.1	100.0%	-4.9
6395	AT1G44160.1 | DNAJ chaperone C-terminal domain-containing protein, contains Pfam profile PF01556: DnaJ C terminal region | chr1:16797269-16798856 FORWARD | Aliases: T7O23.16, T7O23_16	3.0	3.6	-0.6	-1.1	100.0%	-3.7
6396	AT3G02470.2 | Symbol: SAMDC | similar to adenosylmethionine decarboxylase family protein [Arabidopsis thaliana] (TAIR:At5g15950.1); similar to S-adenosyl-L-methionine decarboxylase [Brassica juncea] (GB:AAF20160.1); contains InterPro domain S-adenosylmethionine decarboxylase (InterPro:IPR001985) | chr3:509428-511583 FORWARD | Aliases: ADOMETDC, F16B3.10, F16B3_10, S ADENOSYLMETHIONINE DECARBOXYLASE	11.7	11.9	-0.3	-1.1	100.0%	-5.0
6397	NA	8.1	7.5	0.5	1.1	100.0%	-4.5
6398	AT2G43930.1 | protein kinase family protein, contains similarity to NPK1-related protein kinase 2 GI:2342425 from (Arabidopsis thaliana) | chr2:18200227-18202658 FORWARD | Aliases: F6E13.6	5.7	5.5	0.2	1.1	100.0%	-4.4
6399	AT3G48880.2 | F-box family protein, N7 protein - Medicago truncatula, EMBL:CAA76808 ; similar to SKP1 interacting partner 2 (SKIP2) TIGR_Ath1:At5g67250 | chr3:18137897-18140157 FORWARD | Aliases: T21J18.150	5.2	4.9	0.4	1.1	100.0%	-4.3
6400	AT3G07870.1 | F-box family protein, contains F-box domain Pfam:PF00646	5.7	5.5	0.2	1.1	100.0%	-4.5
6401	AT4G12420.1 | Symbol: SKU5 | multi-copper oxidase, putative (SKU5), identical to multi-copper oxidase-related protein (SKU5)(GI:18158154) (Arabidopsis thaliana); similar to pollen-specific protein precursor - common tobacco, PIR2:S22495; contains Pfam profile: PF00394 Multicopper oxidase | chr4:7349938-7353020 REVERSE | Aliases: T1P17.10, T1P17_10	5.8	6.3	-0.5	-1.1	100.0%	-4.1
6402	AT3G08890.2 | expressed protein, contains Pfam profile PF04398: Protein of unknown function, DUF538 | chr3:2706642-2708753 FORWARD | Aliases: T16O11.17	4.5	4.3	0.3	1.1	100.0%	-4.5
6403	AT2G19800.1 | Symbol: MIOX2 | expressed protein, similar to myo-inositol oxygenase (Sus scrofa) gi:17432544:gb:AAL39076 | chr2:8537986-8540533 REVERSE | Aliases: F6F22.17, F6F22_17, MYO INOSITOL OXYGENASE 2	9.7	9.0	0.7	1.1	100.0%	-3.9
6404	AT1G50120.1 | expressed protein | chr1:18565497-18569804 FORWARD | Aliases: F2J10.3, F2J10_3	7.0	7.5	-0.5	-1.1	100.0%	-4.2
6405	AT1G25320.1 | leucine-rich repeat transmembrane protein kinase, putative, similar to putative receptor-like protein kinase GI:4262228 from (Arabidopsis thaliana) | chr1:8877844-8880297 FORWARD | Aliases: F4F7.29, F4F7_29	5.2	5.5	-0.3	-1.1	100.0%	-4.6
6406	AT4G10620.1 | expressed protein | chr4:6564275-6566500 FORWARD | Aliases: T4F9.80, T4F9_80	6.7	7.1	-0.3	-1.1	100.0%	-4.2
6407	AT5G46520.1 | disease resistance protein (TIR-NBS-LRR class), putative, domain signature TIR-NBS-LRR exists, suggestive of a disease resistance protein. | chr5:18884953-18889533 FORWARD | Aliases: K11I1.11, K11I1_11	2.8	3.3	-0.6	-1.1	100.0%	-3.9
6408	AT5G52500.1 | expressed protein, strong similarity to unknown protein (emb:CAB68146.1)	2.6	2.8	-0.2	-1.1	100.0%	-4.9

Rank	Description	15.dpi	5.dpi	M	t	adj.q	B
6409	AT1G22140.2 | expressed protein | chr1:7814830-7815620 REVERSE | Aliases: F2E2.21, F2E2_21	9.3	8.9	0.4	1.1	100.0%	-4.2
6410	AT5G06740.1 | lectin protein kinase family protein, contains Legume lectins beta-chain signature, PROSITE:PS00307 and Serine/Threonine protein kinases active-site signature, PROSITE:PS00108 | chr5:2084095-2086053 FORWARD | Aliases: MPH15.10, MPH15_10	4.1	4.4	-0.3	-1.1	100.0%	-4.3
6411	AT1G65820.1 | microsomal glutathione s-transferase, putative, similar to MGST3_HUMAN SP:O14880	10.7	10.3	0.4	1.1	100.0%	-5.0
6412	AT3G04750.1 | pentatricopeptide (PPR) repeat-containing protein, contains Pfam profile PF01535: PPR repeat | chr3:1301397-1303382 REVERSE | Aliases: F7O18.24, F7O18_24	4.3	4.0	0.3	1.1	100.0%	-4.6
6413	AT4G25570.1 | Symbol: ACYB 2 |	8.9	7.7	1.2	1.1	100.0%	-4.0
6414	AT4G27110.1 | phytochelatin synthetase-related, contains Pfam PF04833: Phytochelatin synthetase-like conserved region | chr4:13599242-13601527 REVERSE | Aliases: T24A18.60, T24A18_60	2.8	3.0	-0.2	-1.1	100.0%	-4.8
6415	AT5G56360.1 | calmodulin-binding protein, similar to alpha glucosidase II beta subunit from GI:2104691 (Mus musculus) | chr5:22840528-22845223 REVERSE | Aliases: MCD7.9, MCD7_9	8.8	9.2	-0.4	-1.1	100.0%	-4.4
6416	AT3G56200.1 | amino acid transporter family protein, low similarity to N system amino acids transporter NAT-1 (Mus musculus) GI:7406950; belongs to INTERPRO:IPR002422 amino acid/polyamine transporter, family II | chr3:20861023-20863001 FORWARD | Aliases: F18O21.160	6.1	5.6	0.5	1.1	100.0%	-4.1
6417	AT4G26890.1 | Symbol: MAPKKK16 | protein kinase family protein, contains protein kinase domain, Pfam:PF00069 | chr4:13511845-13513412 FORWARD | Aliases: F10M23.230, F10M23_230	3.9	3.5	0.4	1.1	100.0%	-4.1
6418	AT1G75910.1 | Symbol: EXL4 | family II extracellular lipase 4 (EXL4), EXL4 (PMID:11431566); similar to anter-specific proline-rich protein (APG) SP:P40602 (Arabidopsis thaliana (Mouse-ear cress)) | chr1:28505098-28506937 FORWARD | Aliases: T4O12.250, T4O12_250	2.7	2.9	-0.2	-1.1	100.0%	-4.8
6419	AT4G20090.1 | Symbol: EMB1025 | pentatricopeptide (PPR) repeat-containing protein, contains Pfam profile PF01535: PPR repeat | chr4:10868410-10870392 REVERSE | Aliases: EMB1025, EMBRYO DEFECTIVE 1025, F18F4.190, F18F4_190	3.7	3.8	-0.1	-1.1	100.0%	-4.9
6420	AT1G80720.1 | mitochondrial glycoprotein family protein / MAM33 family protein, similar to SUAPRGA1 (Emericella nidulans) GI:6562379; contains Pfam profile PF02330: Mitochondrial glycoprotein | chr1:30341030-30342450 FORWARD | Aliases: F23A5.7, F23A5_7	3.5	3.8	-0.3	-1.1	100.0%	-4.3
6421	AT3G02970.1 | phosphate-responsive 1 family protein, similar to phi-1 (phosphate-induced gene) (Nicotiana tabacum) GI:3759184; contains Pfam profile PF04674: Phosphate-induced protein 1 conserved region | chr3:669110-670502 REVERSE | Aliases: F13E7.8, F13E7_8	1.9	2.0	-0.1	-1.1	100.0%	-5.3
6422	AT3G22440.1 | hydroxyproline-rich glycoprotein family protein, identical to hydroxyproline-rich glycoprotein (Arabidopsis thaliana) gi:9293881:dbj:BAB01784 | chr3:7959761-7962207 FORWARD | Aliases: MCB17.3	6.1	6.6	-0.6	-1.1	100.0%	-4.1
6423	AT1G58430.1 | Symbol: RXF26 | GDSL-motif lipase/hydrolase family protein, similar to family II lipases EXL3 GI:15054386, EXL1 GI:15054382, EXL2 GI:15054384 from (Arabidopsis thaliana); contains Pfam profile PF00657: GDSL-like Lipase/Acylhydrolase | chr1:21715230-21716768 REVERSE | Aliases: F9K23.4, F9K23_4, RXF26	2.5	2.7	-0.1	-1.1	100.0%	-5.2
6424	AT4G31420.2 | zinc finger (C2H2 type) family protein, contains Pfam profile: PF00096 zinc finger, C2H2 type | chr4:15245822-15247930 REVERSE | Aliases: F3L17.9	8.6	8.3	0.3	1.1	100.0%	-4.5
6425	AT1G55260.1 | protease inhibitor/seed storage/lipid transfer protein (LTP) family protein, contains Pfam protease inhibitor/seed storage/LTP family domain PF00234 | chr1:20618418-20620034 FORWARD | Aliases: F7A10.16, F7A10_16	2.6	2.8	-0.2	-1.1	100.0%	-5.1
6426	AT5G53580.1 | aldo/keto reductase family protein, contains Pfam profile PF00248: oxidoreductase, aldo/keto reductase family | chr5:21782305-21784210 REVERSE | Aliases: MNC6.12, MNC6_12	9.7	9.3	0.4	1.1	100.0%	-4.0
6427	AT5G60520.1 | late embryogenesis abundant protein-related / LEA protein-related, similar to late embryogenesis abundant protein (Picea glauca) GI:1350543 | chr5:24349013-24350173 REVERSE | Aliases: MUF9.15, MUF9_15	3.4	3.2	0.2	1.1	100.0%	-4.8
6428	AT4G01880.1 | expressed protein, contains Pfam PF05206: Protein of unknown function (DUF715) | chr4:810699-812925 REVERSE | Aliases: T7B11.14, T7B11_14	3.8	4.2	-0.3	-1.1	100.0%	-4.2
6429	AT3G54660.1 | Symbol: GR | gluthatione reductase, chloroplast, nearly identical to SP:P42770 Glutathione reductase, chloroplast precursor (EC 1.8.1.7) (GR) (GRASE) {Arabidopsis thaliana}; identical to cDNA glutathione reductase GI:451197 | chr3:20240723-20244160 REVERSE | Aliases: EMB2360, EMBRYO DEFECTIVE 2360, GLUTATHIONE REDUCTASE, T5N23.20	7.7	7.1	0.6	1.1	100.0%	-4.0

Rank	Description	15.dpi	5.dpi	M	t	adj.q	B
6430	AT5G02040.2 | prenylated rab acceptor (PRA1) family protein, contains Pfam PF03208: PRA1 family protein | chr5:400896-402873 FORWARD | Aliases: T7H20.90, T7H20_90	4.5	4.1	0.4	1.1	100.0%	-4.2
6431	AT1G24150.1 | formin homology 2 domain-containing protein / FH2 domain-containing protein, contains formin homology 2 domain, Pfam:PF02181 | chr1:8549507-8551982 FORWARD | Aliases: F3I6.8, F3I6_8	2.9	3.7	-0.8	-1.1	100.0%	-4.3
6432	AT3G59760.3 | Symbol: OASC | cysteine synthase, mitochondrial, putative / O-acetylserine (thiol)-lyase, putative / O-acetylserine sulfhydrylase, putative, similar to SP:Q43725 Cysteine synthase, mitochondrial precursor (EC 4.2.99.8) (O- acetylserine sulfhydrylase) (O-acetylserine (Thiol)-lyase) {Arabidopsis thaliana} | chr3:22083451-22086393 REVERSE | Aliases: ATCS C, CSASE C, F24G16.30	10.1	10.8	-0.7	-1.1	100.0%	-4.1
6433	AT1G10930.1 | Symbol: ATSGS1 |	3.1	3.4	-0.3	-1.1	100.0%	-4.3
6434	AT3G45100.2 | Symbol: SETH2 | N-acetylglucosaminyl-phosphatidylinositol biosynthetic protein, putative, similar to PIG-A from Mus musculus (gi:577723(, Homo sapiens (SP:P37287); contains Pfam glycosyl transferase, group 1 family protein domain PF00534 | chr3:16515445-16517988 FORWARD | Aliases: T14D3.40	8.0	7.6	0.4	1.1	100.0%	-4.1
6435	AT2G20160.1 | Symbol: MEO | E3 ubiquitin ligase SCF complex subunit SKP1/ASK1 (At17), putative, E3 ubiquitin ligase; similar to Skp1 homolog Skp1b GI:3068809, UIP2 GI:3719211 from (Arabidopsis thaliana) | chr2:8706753-8707205 REVERSE | Aliases: ARABIDOPSIS SKP1 LIKE 17, ASK17, MEIDOS, T2G17.4, T2G17_4	3.3	3.1	0.2	1.1	100.0%	-4.6
6436	AT5G16800.2 | GCN5-related N-acetyltransferase (GNAT) family protein, very low similarity to SP:P39909 Spermine/spermidine acetyltransferase (EC 2.3.1.57) {Bacillus subtilis}; contains Pfam profile PF00583: acetyltransferase, GNAT family | chr5:5524317-5526566 REVERSE | Aliases: F5E19.140, F5E19_140	6.8	6.3	0.4	1.1	100.0%	-4.2
6437	AT2G42360.1 | zinc finger (C3HC4-type RING finger) family protein, contains Pfam domain, PF00097: Zinc finger, C3HC4 type (RING finger) | chr2:17647876-17648866 FORWARD | Aliases: MHK10.8, MHK10_8	2.3	2.5	-0.2	-1.1	100.0%	-4.8
6438	AT1G50500.1 | membrane trafficking VPS53 family protein, contains Pfam domain PF04100: Vps53-like, N-terminal | chr1:18711655-18719645 REVERSE | Aliases: F11F12.15	4.4	4.8	-0.3	-1.1	100.0%	-4.6
6439	AT5G58080.1 | Symbol: ARR18 | two-component responsive regulator family protein / response regulator family protein, contains Pfam profile: PF00072 response regulator receiver domain | chr5:23519011-23521227 REVERSE | Aliases: ARABIDOPSIS RESPONSE REGULATOR 18, K21L19.7, K21L19_7	2.6	2.4	0.2	1.1	100.0%	-4.9
6440	AT4G23950.1 | expressed protein, ; expression supported by MPSS | chr4:12442563-12444340 FORWARD | Aliases: T32A16.120, T32A16_120	4.3	4.6	-0.3	-1.1	100.0%	-4.4
6441	AT5G08130.1 | Symbol: BIM1 | basic helix-loop-helix (bHLH) family protein, contains Pfam profile: PF00010 helix-loop-helix DNA-binding domain	5.5	6.1	-0.6	-1.1	100.0%	-4.3
6442	AT1G04645.1 | self-incompatibility protein-related, similar to S3 self-incompatibility protein (Papaver rhoeas) GI:1107841 | chr1:1293769-1294262 REVERSE | Aliases: None	2.6	2.8	-0.1	-1.1	100.0%	-5.1
6443	AT5G62950.1 | expressed protein | chr5:25279458-25281047 REVERSE | Aliases: MQB2.27, MQB2_27	5.9	5.6	0.3	1.1	100.0%	-4.2
6444	AT4G14920.1 | PHD finger transcription factor, putative | chr4:8531153-8535838 REVERSE | Aliases: DL3500C, FCAALL.143	4.4	4.7	-0.2	-1.1	100.0%	-4.4
6445	AT2G26550.1 | Symbol: HO2 | heme oxygenase 2 (HO2), similar to heme oxygenase 2 (Arabidopsis thaliana) gi:4530595:gb:AAD22109	5.0	4.8	0.2	1.1	100.0%	-4.5
6446	AT1G26250.1 | proline-rich extensin, putative, similar to extensin gi:1165322:gb:AAB53156; contains proline-rich extensin domains, INTERPRO:IPR002965 | chr1:9083825-9085451 FORWARD | Aliases: F28B23.9, F28B23_9	3.3	3.8	-0.5	-1.1	100.0%	-4.2
6447	AT5G02210.1 | expressed protein, ; expression supported by MPSS | chr5:439251-439872 REVERSE | Aliases: T7H20.260, T7H20_260	4.5	4.0	0.5	1.1	100.0%	-4.0
6448	AT3G46390.1 | expressed protein | chr3:17082240-17082668 REVERSE | Aliases: F18L15.110	2.2	2.4	-0.2	-1.1	100.0%	-4.9
6449	AT1G05385.1 | photosystem II 11 kDa protein-related, similar to Photosystem II 11 kDa protein precursor (SP:P74367)(strain PCC 6803) {Synechocystis sp.} | chr1:1582673-1583717 REVERSE | Aliases: None	5.1	5.3	-0.3	-1.1	100.0%	-4.3
6450	AT1G23410.1 | ubiquitin extension protein, putative / 40S ribosomal protein S27A (RPS27aA), strong similarity to ubiquitin extension protein (UBQ5) GB:AAA32906 GI:166934 from (Arabidopsis thaliana) | chr1:8314913-8315520 FORWARD | Aliases: F26F24.28, F26F24_28	7.7	8.2	-0.5	-1.1	100.0%	-3.8
6451	AT3G47780.1 | Symbol: ATATH6 |	6.2	6.5	-0.4	-1.1	100.0%	-4.1
6452	AT2G02040.1 | Symbol: ATPTR2 B | peptide transporter (PTR2-B) / oligopeptide transporter 1-1, putative (OPT1-1), identical to peptide transporter PTR2-B SP:P46032 from (Arabidopsis thaliana); contains Pfam profile: PF00854 POT family; identical to cDNA NT1 GI:510237 | chr2:487422-489830 FORWARD | Aliases: F14H20.11, F14H20_11, HISTIDINE TRANSPORT PROTEIN, NITRATE TRANSPORTER 1, NTR1, PTR2 B	8.1	7.3	0.8	1.1	100.0%	-4.1

Rank	Description	15.dpi	5.dpi	M	t	adj.q	B
6453	AT1G02000.1 | Symbol: GAE2 | NAD-dependent epimerase/dehydratase family protein, similar to nucleotide sugar epimerase from Vibrio vulnificus GI:3093975 (PID:g3093975), WbnF (Escherichia coli) GI:5739472, CAPI protein {Staphylococcus aureus} SP:P39858; contains Pfam profile PF01370 NAD dependent epimerase/dehydratase family	5.9	5.5	0.3	1.1	100.0%	-4.5
6454	AT1G53420.1 | serine/threonine protein kinase-related, contains 1 predicted transmembrane domain; low similarity to receptor-like serine/threonine kinase (Arabidopsis thaliana) GI:2465923 | chr1:19930294-19935162 REVERSE | Aliases: F12M16.30	2.4	2.3	0.1	1.1	100.0%	-5.5
6455	AT4G13180.1 | short-chain dehydrogenase/reductase (SDR) family protein, similar to short-chain type dehydrogenase/reductase SP:Q08632 (Picea abies) | chr4:7657192-7658193 REVERSE | Aliases: F17N18.70, F17N18_70	10.7	9.5	1.2	1.1	100.0%	-3.9
6456	AT5G16080.1 | expressed protein, similar to PrMC3 (Pinus radiata) GI:5487873 | chr5:5252318-5253621 REVERSE | Aliases: F1N13.220, F1N13_220	3.3	3.7	-0.4	-1.1	100.0%	-4.2
6457	AT1G16670.1 | protein kinase family protein, contains protein kinase domain, Pfam:PF00069; similar to receptor-like serine/threonine kinase GI:2465923 from (Arabidopsis thaliana) | chr1:5697332-5699762 FORWARD | Aliases: F19K19.4, F19K19_4	6.1	5.8	0.3	1.1	100.0%	-4.3
6458	AT1G64050.1 | expressed protein | chr1:23766030-23769696 FORWARD | Aliases: F22C12.19, F22C12_19	2.9	3.3	-0.4	-1.1	100.0%	-4.5
6459	AT1G73490.1 | RNA recognition motif (RRM)-containing protein, contains INTERPRO:IPR000504 RNA-binding region RNP-1 (RNA recognition motif) domain | chr1:27636927-27638593 REVERSE | Aliases: T9L24.49, T9L24_49	7.2	6.9	0.3	1.1	100.0%	-4.6
6460	AT4G34400.1 | transcriptional factor B3 family protein, contains Pfam profile PF02362: B3 DNA binding domain | chr4:16445327-16447600 FORWARD | Aliases: F10M10.170, F10M10_170	3.1	2.9	0.2	1.1	100.0%	-4.7
6461	AT3G09180.1 | expressed protein | chr3:2819123-2821430 FORWARD | Aliases: MZB10.22	5.8	5.5	0.3	1.1	100.0%	-4.2
6462	AT5G23530.1 | expressed protein, contains similarity to PrMC3 (Pinus radiata) GI:5487873 | chr5:7932969-7934439 REVERSE | Aliases: MQM1.21, MQM1_21	3.0	3.3	-0.3	-1.1	100.0%	-4.1
6463	AT1G22070.1 | Symbol: TGA3 | bZIP family transcription factor (TGA3), identical to transcription factor GI:304113 from (Arabidopsis thaliana)	7.1	6.7	0.3	1.1	100.0%	-4.5
6464	AT1G77850.1 | Symbol: ARF17 | transcriptional factor B3 family protein, similar to auxin response factor 10 GI:6165644 from (Arabidopsis thaliana); contains Pfam profile PF02362: B3 DNA binding domain | chr1:29277207-29280313 FORWARD | Aliases: ARF17, AUXIN RESPONSE FACTOR 17, F28K19.6, F28K19_6	3.5	3.2	0.3	1.1	100.0%	-4.4
6465	AT1G16190.1 | DNA repair protein RAD23, putative, similar to DNA repair by nucleotide excision (NER) RAD23 protein, isoform II GI:1914685 from (Daucus carota)	3.0	3.3	-0.3	-1.1	100.0%	-4.6
6466	AT3G58540.1 | expressed protein | chr3:21659570-21660364 FORWARD | Aliases: F14P22.130	4.9	4.6	0.4	1.1	100.0%	-4.3
6467	AT2G22480.1 | phosphofructokinase family protein, similar to phosphofructokinase (Amycolatopsis methanolica) GI:17432243; contains Pfam profile PF00365: Phosphofructokinase | chr2:9552656-9555643 FORWARD | Aliases: F14M13.12, F14M13_12	8.8	9.4	-0.6	-1.1	100.0%	-4.2
6468	AT5G23440.1 | ferredoxin-thioredoxin reductase, putative, similar to ferredoxin-thioredoxin reductase, variable chain (FTR-V, Ferredoxin- thioredoxin reductase subunit A, FTR-A) (Zea mays) SWISS-PROT:P80680	4.6	4.3	0.3	1.1	100.0%	-4.4
6469	AT1G01130.1 | expressed protein, ; expression supported by MPSS | chr1:61963-63811 REVERSE | Aliases: F6F3.33	4.3	3.9	0.4	1.1	100.0%	-4.1
6470	AT5G58190.2 | expressed protein, contains Pfam profile PF04146: YT521-B-like family | chr5:23563599-23566775 FORWARD | Aliases: MCK7.6, MCK7_6	5.7	6.2	-0.5	-1.1	100.0%	-4.2
6471	AT5G16120.1 | hydrolase, alpha/beta fold family protein, similar to monoglyceride lipase from (Homo sapiens) GI:14594904, (Mus musculus) GI:2632162; contains Pfam profile PF00561: hydrolase, alpha/beta fold family | chr5:5265957-5267982 FORWARD | Aliases: T21H19.40, T21H19_40	7.8	7.6	0.3	1.1	100.0%	-4.4
6472	AT4G11690.1 | pentatricopeptide (PPR) repeat-containing protein, contains Pfam profile PF01535: PPR repeat | chr4:7056248-7057948 FORWARD | Aliases: T5C23.120, T5C23_120	2.9	3.2	-0.2	-1.1	100.0%	-4.8
6473	AT1G21350.3 | expressed protein | chr1:7476930-7479021 REVERSE | Aliases: F24J8.1, F24J8_1	4.2	4.5	-0.3	-1.1	100.0%	-4.2
6474	AT2G41380.1 | embryo-abundant protein-related, similar to embryo-abundant protein (Picea glauca) GI:1350531 | chr2:17259019-17260135 FORWARD | Aliases: F13H10.7, F13H10_7	3.8	3.6	0.2	1.1	100.0%	-4.6
6475	AT4G35940.1 | expressed protein | chr4:17021675-17023767 FORWARD | Aliases: T19K4.70	6.1	5.8	0.4	1.1	100.0%	-4.3

Rank	Description	15.dpi	5.dpi	M	t	adj.q	B
6476	AT1G11700.1 | expressed protein, contains Pfam profile PF04520: Protein of unknown function, DUF584 | chr1:3945737-3946613 FORWARD | Aliases: F25C20.15, F25C20_15	4.6	4.2	0.3	1.1	100.0%	-4.5
6477	AT4G03210.2 | Symbol: XTH9 | similar to xyloglucan:xyloglucosyl transferase / xyloglucan endotransglycosylase / endo-xyloglucan transferase (EXGT-A4) [Arabidopsis thaliana] (TAIR:At5g13870.1); similar to sadtomato protein [Capsicum annuum] (GB:AAS77347.1); contains InterPro domain Glycoside hydrolase, family 16 (InterPro:IPR000757); contains InterPro domain Beta-glucanase (InterPro:IPR008264) | chr4:1415953-1417352 FORWARD | Aliases: F4C21.14, F4C21_14, XYLOGLUCAN ENDOTRANSGLUCOSYLASE/HYDROLASE 9	10.2	9.8	0.4	1.1	100.0%	-4.2
6478	AT3G60190.1 | Symbol: ADL4 | dynamin-like protein E (DL1E), nearly identical to dynamin-like protein E (Arabidopsis thaliana) GI:19423872; contains Pfam profiles PF01031: Dynamin central region, PF00350: Dynamin family, PF02212: Dynamin GTPase effector domain | chr3:22255144-22258773 REVERSE | Aliases: ADL1E, ADL4, ADLP2, DYNAMIN LIKE PROTEIN 4, F27H5.4	6.9	7.2	-0.3	-1.1	100.0%	-4.5
6479	AT5G20940.1 | glycosyl hydrolase family 3 protein, beta-glucosidase, common nasturtium, PIR:T10521 | chr5:7103929-7106703 REVERSE | Aliases: F22D1.110, F22D1_110	3.8	3.6	0.2	1.1	100.0%	-4.6
6480	AT3G47910.1 | expressed protein, low similarity to nonmuscle myosin heavy chain (NMHC) (Homo sapiens) GI:189036; contains Pfam profiles PF04780: Protein of unknown function (DUF629), PF04781: Protein of unknown function (DUF627) | chr3:17687622-17692715 REVERSE | Aliases: T17F15.220	2.3	2.4	-0.1	-1.1	100.0%	-5.4
6481	AT5G58430.1 | exocyst subunit EXO70 family protein, leucine zipper-containing protein, Lycopersicon esculentum, PIR:S21495; contains Pfam domain PF03081: Exo70 exocyst complex subunit; similar to rexo70 (GI:2827160) {Rattus norvegicus} | chr5:23638409-23640670 REVERSE | Aliases: MQJ2.2, MQJ2_2	6.9	7.8	-1.0	-1.1	100.0%	-4.3
6482	AT1G30900.1 | vacuolar sorting receptor, putative, similar to BP-80 vacuolar sorting receptor (Pisum sativum) GI:1737222 | chr1:10997256-11000524 FORWARD | Aliases: F17F8.23	5.9	5.3	0.5	1.1	100.0%	-4.0
6483	AT3G26750.1 | expressed protein | chr3:9841247-9843316 REVERSE | Aliases: MDJ14.3	3.7	4.0	-0.3	-1.1	100.0%	-4.6
6484	AT3G59640.1 | glycine-rich protein | chr3:22039758-22041422 FORWARD | Aliases: T16L24.190	6.0	5.8	0.2	1.1	100.0%	-4.7
6485	AT1G78950.1 | beta-amyrin synthase, putative, similar to beta-Amyrin Synthase GI:3688600 from (Panax ginseng) and GI:8918271 from (Pisum sativum) | chr1:29689330-29693566 REVERSE | Aliases: YUP8H12R.44, YUP8H12R_44	4.5	4.2	0.3	1.1	100.0%	-4.3
6486	AT2G22270.1 | expressed protein | chr2:9470774-9472547 FORWARD | Aliases: T26C19.7, T26C19_7	4.0	3.7	0.3	1.1	100.0%	-4.4
6487	AT5G14570.2 | Symbol: ATNRT2.7 | transporter, putative, similar to trans-membrane nitrate transporter protein AtNRT2:1 (Arabidopsis thaliana) GI:3747058, high-affinity nitrate transporter ACH1 (Arabidopsis thaliana) GI:3608362; contains Pfam profile PF00083: Sugar (and other) transporter | chr5:4695189-4697081 REVERSE | Aliases: T15N1.60, T15N1_60	6.0	5.5	0.4	1.1	100.0%	-4.1
6488	AT4G30340.1 | diacylglycerol kinase family protein, contains INTERPRO domain, IPR001206, DAG-kinase catalytic domain | chr4:14838315-14841073 REVERSE | Aliases: F17I23.320, F17I23_320	2.3	2.4	-0.1	-1.1	100.0%	-5.2
6489	AT5G63970.2 | similar to copine-related [Arabidopsis thaliana] (TAIR:At3g01650.1); similar to P0401G10.23 [Oryza sativa (japonica cultivar-group)] (GB:NP_914244.1); contains InterPro domain von Willebrand factor, type A (InterPro:IPR002035) | chr5:25623997-25626213 REVERSE | Aliases: MBM17.7, MBM17_7	8.7	8.5	0.2	1.1	100.0%	-4.7
6490	AT2G35810.1 | expressed protein | chr2:15056229-15057324 FORWARD | Aliases: F11F19.28, F11F19_28	10.1	10.8	-0.8	-1.1	100.0%	-4.0
6491	AT2G46770.1 | Symbol: ANAC043/EMB2301 | no apical meristem (NAM) family protein, contains Pfam PF02365: No apical meristem (NAM) domain; | chr2:19227797-19229986 REVERSE | Aliases: ANAC043, EMB2301, EMBRYO DEFECTIVE 2301, F19D11.5	1.9	2.0	-0.1	-1.1	100.0%	-5.4
6492	AT1G47470.1 | hypothetical protein | chr1:17417051-17417521 REVERSE | Aliases: F16N3.31, F16N3_31	2.4	2.5	-0.1	-1.1	100.0%	-5.3
6493	AT1G80450.1 | VQ motif-containing protein, contains PF05678: VQ motif | chr1:30248847-30249580 REVERSE | Aliases: T21F11.22, T21F11_22	1.9	2.0	-0.2	-1.1	100.0%	-5.3
6494	AT1G18270.1 | ketose-bisphosphate aldolase class-II family protein, low similarity to KbaY (tagatose-1,6-bisphosphate aldolase) (Escherichia coli) GI:8895753; contains Pfam profile PF01116: Fructose-bisphosphate aldolase class-II | chr1:6283405-6293821 REVERSE | Aliases: T10O22.24, T10O22_24	8.1	7.7	0.4	1.1	100.0%	-4.0
6495	AT2G32810.1 | Symbol: BGAL9 | beta-galactosidase, putative / lactase, putative, similar to beta-galactosidase GI:7939617 from (Lycopersicon esculentum) | chr2:13925214-13932408 REVERSE | Aliases: F24L7.5, F24L7_5	6.7	7.2	-0.4	-1.1	100.0%	-4.5
6496	AT4G29770.1 | expressed protein | chr4:14575846-14577645 FORWARD | Aliases: F27B13.10, F27B13_10	6.2	6.9	-0.6	-1.1	100.0%	-3.6
6497	AT1G06510.1 | expressed protein | chr1:1992384-1993839 FORWARD | Aliases: F12K11.16, F12K11_16	4.6	4.3	0.3	1.1	100.0%	-4.3

Rank	Description	15.dpi	5.dpi	M	t	adj.q	B
6498	AT3G50780.1 | expressed protein | chr3:18886380-18888505 REVERSE | Aliases: F18B3.60	4.7	4.9	-0.3	-1.1	100.0%	-4.3
6499	AT1G13140.1 | Symbol: CYP86C3 | cytochrome P450 family protein, similar to Cytochrome P450 86A2 (SP:O23066) (Arabidopsis thaliana); contains Pfam PF:00067 Cytochrome P450 family | chr1:4478489-4480268 REVERSE | Aliases: F3F19.16, F3F19_16	2.9	3.2	-0.3	-1.1	100.0%	-4.6
6500	AT4G09980.1 | Symbol: EMB1691 | methyltransferase MT-A70 family protein, low similarity to SP:P25583 Karyogamy protein KAR4 {Saccharomyces cerevisiae}, (N6-adenosine)-methyltransferase (Mus musculus) GI:10179948; contains Pfam profile PF05063: MT-A70 (S-adenosylmethionine-binding subunit of human mRNA:m6A methyl-transferase (MTase)) | chr4:6247732-6252285 REVERSE | Aliases: EMB1691, EMBRYO DEFECTIVE 1691, T5L19.110, T5L19_110	2.7	2.5	0.1	1.1	100.0%	-5.0
6501	AT1G48760.3 | similar to gamma-adaptin, putative [Arabidopsis thaliana] (TAIR:At1g60070.1); similar to AP3D1 protein [Homo sapiens] (GB:AAH05142.1); contains InterPro domain Adaptin, N-terminal (InterPro:IPR002553)	3.9	4.3	-0.4	-1.1	100.0%	-4.3
6502	AT2G20850.1 | similar to leucine-rich repeat transmembrane protein kinase, putative [Arabidopsis thaliana] (TAIR:At4g03390.1); similar to putative leucine-rich repeat transmembrane protein kinase [Oryza sativa (japonica cultivar-group)] (GB:XP_464408.1); contains InterPro domain Tyrosine protein kinase (InterPro:IPR001245); contains InterPro domain Serine/threonine protein kinase (InterPro:IPR002290); contains InterPro domain Protein kinase (InterPro:IPR000719); contains InterPro domain Leucine-rich repeat, plant specific (InterPro:IPR007090); contains InterPro domain Leucine-rich repeat (InterPro:IPR001611) | chr2:8982429-8986460 REVERSE | Aliases: F5H14.18, F5H14_18	3.3	3.5	-0.2	-1.1	100.0%	-4.6
6503	AT1G28210.2 | Symbol: ATJ1 | DNAJ heat shock protein, putative, strong similarity to mitochondrial DnaJ protein (AtJ1) GI:564030 from (Arabidopsis thaliana); contains Pfam profiles PF00226: DnaJ domain, PF01556: DnaJ C terminal region, PF00684: DnaJ central domain (4 repeats); identical to cDNA DnaJ homolog AtJ1 (atj) GI:564029 | chr1:9854533-9860145 FORWARD | Aliases: ATJ, F3H9.13, F3H9_13	5.5	6.1	-0.5	-1.1	100.0%	-4.2
6504	AT3G53480.1 | ABC transporter family protein, PDR5-like ABC transporter, Spirodela polyrrhiza, EMBL:Z70524 | chr3:19836285-19842755 FORWARD | Aliases: F4P12.180	3.2	3.6	-0.3	-1.1	100.0%	-4.4
6505	AT5G37630.1 | Symbol: EMB2656 | chromosome condensation family protein, contains pfam profile: PF04154 chromosome condensation protein 3, C-terminal region | chr5:14964637-14969995 FORWARD | Aliases: EMB2656, EMBRYO DEFECTIVE 2217, EMBRYO DEFECTIVE 2656, K12B20.9, K12B20_9	3.7	4.0	-0.3	-1.1	100.0%	-4.3
6506	AT3G55360.1 | Symbol: CER10/ECR | Enoyl-CoA reductase (ECA) is involved in all very long chain fatty acids (VLCFA) elongation reactions that are required for cuticular wax, storage lipid and sphingolipid metabolism. The protein is located in the ER, but in contrast to its yeast homolog TSC13 is not particularly enriched in the nuclear envelope-vacuole junction. Mutants in this gene show abnormal organ morphology and stem glossiness. Cells in all tissues are only about 1/3 of the size of wild type cells. The morphological changes are most likely to result from the reduction in the VLCFA content of sphingolipids. Mutants also show abnormalities in the endocytic membrane organization and transport. | chr3:20531907-20533963 REVERSE | Aliases: CER10, ECR, T22E16.20	4.4	5.0	-0.6	-1.1	100.0%	-4.2
6507	AT4G00550.1 | Symbol: DGD2 | UDP-galactose:MGDG galactosyltransferase 2 / digalactosyldiacylglycerol synthase 2 (DGD2), identical to digalactosyldiacylglycerol synthase (DGD2) GI:18141112 (Arabidopsis thaliana) | chr4:237967-240500 REVERSE | Aliases: F6N23.24, F6N23_24	5.3	5.0	0.2	1.1	100.0%	-4.5
6508	AT3G49310.1 | expressed protein, contains PF05631: Protein of unknown function (DUF791) | chr3:18295849-18299037 REVERSE | Aliases: F2K15.170	4.4	4.1	0.3	1.1	100.0%	-4.6
6509	AT4G21270.1 | Symbol: ATK1 | kinesin-like protein A (KATA) | chr4:11329373-11334145 REVERSE | Aliases: ARABIDOPSIS THALIANA KINESIN 1, F7J7.210, F7J7_210, KATA, KATAP, KINESIN LIKE PROTEIN A	2.7	2.8	-0.2	-1.1	100.0%	-4.8
6510	AT2G17840.1 | Symbol: ERD7 | senescence/dehydration-associated protein-related (ERD7), similar to senescence-associated protein 12 (Hemerocallis hybrid cultivar) gi:3551958:gb:AAC34857; strong similarity to early-responsive to dehydration stress ERD7 protein (Arabidopsis thaliana) gi:15320412:dbj:BAB63916; identical to cDNA ERD7 partial cds GI:15320411 | chr2:7762687-7764934 REVERSE | Aliases: EARLY RESPONSIVE TO DEHYDRATION 7, T13L16.14, T13L16_14	8.3	7.7	0.6	1.1	100.0%	-4.2
6511	AT2G45500.1 | similar to spastin ATPase, putative [Arabidopsis thaliana] (TAIR:At3g27120.1); similar to Tobacco mosaic virus helicase domain-binding protein [Nicotiana tabacum] (GB:AAL25088.1); contains InterPro domain AAA ATPase (InterPro:IPR003593); contains InterPro domain AAA ATPase, central region (InterPro:IPR003959) | chr2:18756872-18759277 REVERSE | Aliases: F17K2.3	4.5	4.1	0.4	1.1	100.0%	-4.2
6512	AT1G49570.1 | peroxidase, putative, identical to peroxidase ATP5a (Arabidopsis thaliana) gi:1546702:emb:CAA67341; similar to peroxidase SWISS-PROT:P80679 from (Armoracia rusticana) | chr1:18350704-18352619 FORWARD | Aliases: F14J22.19, F14J22_19	3.3	2.8	0.6	1.1	100.0%	-4.3
6513	AT5G59290.1 | Symbol: UXS3 | UDP-glucuronic acid decarboxylase (UXS3), identical to UDP-glucuronic acid decarboxylase (Arabidopsis thaliana) GI:14595666; contains Pfam profile PF01370 NAD dependent epimerase/dehydratase family; identical to cDNA	7.7	8.4	-0.7	-1.1	100.0%	-4.3
6514	AT4G18920.1 | expressed protein | chr4:10368766-10370223 FORWARD | Aliases: F13C5.90, F13C5_90	3.4	3.7	-0.3	-1.1	100.0%	-4.5
6515	AT1G41830.1 | Symbol: SKS6 | multi-copper oxidase type I family protein, similar to pollen-specific BP10 protein (SP:Q00624)(Brassica napus); contains Pfam profile: PF00394 Multicopper oxidase | chr1:15606058-15610302 REVERSE | Aliases: F5A13.5, F5A13_5, SKS6	8.5	9.1	-0.6	-1.1	100.0%	-4.2

Rank	Description	15.dpi	5.dpi	M	t	adj.q	B
6516	AT3G11800.1 | expressed protein | chr3:3726114-3727734 FORWARD | Aliases: F26K24.9	8.3	7.7	0.6	1.1	100.0%	-4.4
6517	AT1G28240.1 | expressed protein | chr1:9868303-9872198 REVERSE | Aliases: F3H9.11, F3H9_11	4.2	3.9	0.3	1.1	100.0%	-4.7
6518	AT2G30620.1 | histone H1.2, nearly identical to SP:P26569 Histone H1.2 {Arabidopsis thaliana} | chr2:13052008-13053588 FORWARD | Aliases: T6B20.3, T6B20_3	10.0	10.3	-0.3	-1.1	100.0%	-4.4
6519	AT5G17230.2 | Symbol: PSY | similar to phytoene synthase [Capsicum annuum] (GB:CAA48155.1); contains InterPro domain Squalene/phytoene synthase (InterPro:IPR002060) | chr5:5659551-5662467 REVERSE | Aliases: MKP11.25, MKP11_25, PHYTOENE SYNTHASE	6.3	5.7	0.5	1.1	100.0%	-4.5
6520	AT4G25840.1 | haloacid dehalogenase-like hydrolase family protein, low similarity to SP:Q08623 GS1 protein {Homo sapiens}; contains InterPro accession IPR005834: Haloacid dehalogenase-like hydrolase	4.9	5.1	-0.3	-1.1	100.0%	-4.5
6521	AT1G15740.1 | leucine-rich repeat family protein | chr1:5410929-5415297 FORWARD | Aliases: F7H2.8, F7H2_8	7.0	6.3	0.7	1.1	100.0%	-4.2
6522	AT1G13810.1 | expressed protein, ; expression supported by MPSS | chr1:4734085-4735161 FORWARD | Aliases: F16A14.30, F16A14_30	5.7	6.1	-0.4	-1.1	100.0%	-4.0
6523	AT2G46510.1 | basic helix-loop-helix (bHLH) family protein, contains Pfam profile: PF00010 helix-loop-helix DNA-binding domain	4.1	3.8	0.3	1.1	100.0%	-4.6
6524	AT4G17310.2 | expressed protein, similar to expressed protein [Arabidopsis thaliana] (TAIR:At5g47455.3) | chr4:9685190-9686940 REVERSE | Aliases: DL4690C, FCAALL.398	4.7	4.4	0.3	1.1	100.0%	-4.4
6525	AT2G40970.1 | myb family transcription factor, contains Pfam profile: PF00249 myb-like DNA-binding domain | chr2:17104628-17105773 REVERSE | Aliases: T20B5.17, T20B5_17	7.1	6.7	0.4	1.1	100.0%	-4.3
6526	AT1G58300.1 | heme oxygenase, putative, similar to heme oxygenase 4 GI:14485565 from (Arabidopsis thaliana) | chr1:21631677-21633661 REVERSE | Aliases: F19C14.8, F19C14_8	2.7	2.6	0.2	1.1	100.0%	-4.9
6527	AT1G39350.1 | expressed protein | chr1:14610274-14611094 REVERSE | Aliases: F12G6.5	3.2	3.4	-0.2	-1.1	100.0%	-4.9
6528	AT3G21810.1 | zinc finger (CCCH-type) family protein, contains Pfam domain, PF00642: Zinc finger C-x8-C-x5-C-x3-H type (and similar) | chr3:7684437-7688623 FORWARD | Aliases: MSD21.12	4.7	4.2	0.5	1.1	100.0%	-4.3
6529	AT4G14670.1 | heat shock protein 101, putative / HSP101, putative, similar to heat shock protein 101 GI:6715468 GB:AAF26423 from (Arabidopsis thaliana) | chr4:8410049-8412552 FORWARD | Aliases: DL3375W, FCAALL.285	2.8	2.6	0.2	1.1	100.0%	-4.8
6530	AT5G63150.1 | expressed protein | chr5:25347426-25348070 FORWARD | Aliases: MDC12.12, MDC12_12	5.2	5.7	-0.4	-1.1	100.0%	-4.2
6531	AT5G28080.2 | Symbol: WNK9 | similar to protein kinase family protein [Arabidopsis thaliana] (TAIR:At3g04910.1); similar to mitogen activated protein kinase kinase [Oryza sativa] (GB:AAC32599.1); contains InterPro domain Serine/threonine protein kinase, active site (InterPro:IPR008271); contains InterPro domain Tyrosine protein kinase (InterPro:IPR001245); contains InterPro domain Serine/threonine protein kinase (InterPro:IPR002290); contains InterPro domain Protein kinase (InterPro:IPR000719) | chr5:10090074-10092406 REVERSE | Aliases: T24G3.10, T24G3_10	4.0	3.7	0.3	1.1	100.0%	-4.5
6532	AT5G19320.1 | Symbol: RANGAP2 | RAN GTPase activating protein 2 (RanGAP2), identical to RAN GTPase activating protein 2 GI:6708468 from (Arabidopsis thaliana) | chr5:6505112-6507410 REVERSE | Aliases: RAN GTPASE ACTIVATING PROTEIN 2	4.6	5.0	-0.4	-1.1	100.0%	-4.3
6533	AT2G32520.1 | dienelactone hydrolase family protein, low similarity to dienelactone hydrolase (Pseudomonas resinovorans) GI:13094163; contains Pfam profile PF01738: Dienelactone hydrolase family | chr2:13812596-13814613 REVERSE | Aliases: T26B15.8, T26B15_8	11.1	11.5	-0.4	-1.1	100.0%	-4.6
6534	AT2G14570.1 | SWIM zinc finger family protein | chr2:6228862-6230999 REVERSE | Aliases: T13P21.5, T13P21_5	4.3	4.4	-0.2	-1.1	100.0%	-4.7
6535	AT1G28070.1 | expressed protein, ; expression supported by MPSS | chr1:9783069-9783851 FORWARD | Aliases: F13K9.17, F13K9_17	5.9	5.5	0.4	1.1	100.0%	-4.3
6536	AT5G03900.2 | expressed protein, predicted protein, Synechocystis sp., PIR:S74969 | chr5:1048294-1052385 FORWARD | Aliases: F8F6.110	3.8	3.6	0.2	1.1	100.0%	-4.7
6537	AT5G24860.1 | Symbol: FPF1 | expressed protein | chr5:8541781-8542452 FORWARD | Aliases: F6A4.70, F6A4_70, FLOWERING PROMOTING FACTOR 1, FPF	3.2	3.0	0.1	1.1	100.0%	-4.9
6538	AT3G07510.1 | expressed protein | chr3:2393697-2394681 REVERSE | Aliases: F21O3.22	5.5	5.7	-0.2	-1.1	100.0%	-4.5
6539	AT1G01170.2 | similar to stress-related ozone-induced protein (OZI1) / stress-related ozone-responsive protein [Arabidopsis thaliana] (TAIR:At4g00860.1); similar to ozone-responsive stress-related protein-like [Oryza sativa (japonica cultivar-group)] (GB:NP_910318.1) | chr1:73824-74731 REVERSE | Aliases: F6F3.32	8.7	8.2	0.4	1.1	100.0%	-4.2

Rank	Description	15.dpi	5.dpi	M	t	adj.q	B
6540	AT1G02980.1 | Symbol: CUL2 | cullin family protein, similar to cullin 1 (Homo sapiens) GI:3139077; contains Pfam profile PF00888: Cullin family | chr1:677869-681212 FORWARD | Aliases: ATCUL2, F22D16.2, F22D16_2	2.8	3.0	-0.2	-1.1	100.0%	-5.0
6541	AT5G27680.1 | DEAD/DEAH box helicase, putative, similar to WRN (Werner syndrome) protein - Mus musculus, EMBL:AF241636; contains Pfam profiles PF00270: DEAD/DEAH box helicase, PF00271: Helicase conserved C-terminal domain, PF00627: UBA/TS-N domain | chr5:9793793-9798647 REVERSE | Aliases: T1G16.10, T1G16_10	2.5	2.7	-0.2	-1.1	100.0%	-5.0
6542	AT1G29070.1 | ribosomal protein L34 family protein, similar to plastid ribosomal protein L34 precursor GB:AAF64157 GI:7578860 from (Spinacia oleracea)	6.8	7.3	-0.5	-1.1	100.0%	-3.9
6543	AT5G67570.1 | Symbol: EMB1408 | pentatricopeptide (PPR) repeat-containing protein, contains Pfam profile PF01535: PPR repeat | chr5:26969551-26972073 FORWARD | Aliases: EMB1408, EMBRYO DEFECTIVE 1408, K9I9.14, K9I9_14	3.0	2.8	0.2	1.1	100.0%	-4.9
6544	AT3G44210.1 | hypothetical protein | chr3:15927257-15928319 FORWARD | Aliases: F26G5.160	2.3	2.4	-0.1	-1.1	100.0%	-5.4
6545	AT4G04960.1 | lectin protein kinase, putative, similar to receptor lectin kinase 3 (Arabidopsis thaliana) gi:4100060:gb:AAD00733; contains protein kinase domain, Pfam:PF00069; contains legume lectins alpha and beta domains, Pfam:PF00138 and Pfam:PF00139 | chr4:2533054-2535356 FORWARD | Aliases: T32N4.9, T32N4_9	5.2	4.9	0.4	1.1	100.0%	-4.3
6546	AT1G67950.4 | RNA recognition motif (RRM)-containing protein | chr1:25482030-25483839 REVERSE | Aliases: T23K23.20, T23K23_20	5.5	5.9	-0.4	-1.1	100.0%	-4.1
6547	AT3G47450.2 | expressed protein | chr3:17494053-17497332 REVERSE | Aliases: T21L8.200	5.2	5.7	-0.5	-1.1	100.0%	-3.9
6548	AT2G26240.1 | expressed protein, contains Pfam profile PF03647: Uncharacterised protein family (UPF0136) | chr2:11174335-11175535 REVERSE | Aliases: T1D16.12, T1D16_12	9.3	9.1	0.2	1.1	100.0%	-4.4
6549	AT4G37870.2 | similar to phosphoenolpyruvate carboxykinase (ATP), putative / PEP carboxykinase, putative / PEPCK, putative [Arabidopsis thaliana] (TAIR:At5g65690.1); similar to phosphoenolpyruvate carboxykinase [Flaveria pringlei] (GB:BAB43909.1); similar to phosphoenolpyruvate carboxykinase [Zoysia japonica] (GB:BAD88616.1); similar to phosphoenolpyruvate carboxykinase [Flaveria trinervia] (GB:BAB43907.1); similar to PEPCK [Cucumis sativus] (GB:AAM00814.1); similar to phosphoenolpyruvate carboxykinase [Lycopersicon esculentum] (GB:AAG01894.2); contains InterPro domain Phosphoenolpyruvate carboxykinase (ATP) (InterPro:IPR001272); contains InterPro domain Phosphoenolpyruvate carboxykinase, N-terminal (InterPro:IPR008210) | chr4:17802688-17806533 REVERSE | Aliases: T28I19.150, T28I19_150	11.3	12.1	-0.8	-1.1	100.0%	-4.2
6550	AT3G52910.1 | Symbol: AtGRF4 |	7.3	7.9	-0.6	-1.1	100.0%	-3.7
6551	AT5G02050.1 | mitochondrial glycoprotein family protein / MAM33 family protein, low similarity to SUAPRGA1 (Emericella nidulans) GI:6562379; contains Pfam profile PF02330: Mitochondrial glycoprotein	7.6	8.2	-0.6	-1.1	100.0%	-3.8
6552	AT5G65580.1 | expressed protein | chr5:26224880-26225188 FORWARD | Aliases: K21L13.9, K21L13_9	2.5	2.4	0.1	1.1	100.0%	-5.3
6553	ATCG00470.1 | Symbol: ATPE | ATPase epsilon subunit | chrC:52265-52663 REVERSE | Aliases: ATPE	13.3	13.5	-0.2	-1.1	100.0%	-4.9
6554	AT5G21130.1 | expressed protein | chr5:7185971-7186816 FORWARD | Aliases: T10F18.160, T10F18_160	3.1	2.9	0.2	1.1	100.0%	-4.7
6555	AT1G19170.1 | glycoside hydrolase family 28 protein / polygalacturonase (pectinase) family protein, low similarity to SP:P27644 Polygalacturonase (EC 3.2.1.15) (Pectinase) {Agrobacterium tumefaciens}; contains PF00295: Glycosyl hydrolases family 28 | chr1:6616634-6618977 FORWARD | Aliases: T29M8.4, T29M8_4	5.9	6.2	-0.3	-1.1	100.0%	-4.6
6556	AT1G73850.1 | expressed protein, similar to expressed protein [Arabidopsis thaliana] (TAIR:At3g20260.1); similar to unknown protein [Oryza sativa (japonica cultivar-group)] (GB:XP_483358.1) | chr1:27771557-27774118 FORWARD | Aliases: F2P9.28, F2P9_28	3.7	3.5	0.3	1.1	100.0%	-4.5
6557	AT1G17615.1 | disease resistance protein (TIR-NBS class), putative, domain signature TIR exists, suggestive of a disease resistance protein. | chr1:6059434-6060660 FORWARD | Aliases: F1L3.31, F1L3_31	2.4	2.5	-0.1	-1.1	100.0%	-5.4
6558	AT1G78790.1 | expressed protein | chr1:29628422-29629935 REVERSE | Aliases: F9K20.17, F9K20_17	7.1	6.7	0.4	1.1	100.0%	-3.9
6559	AT5G58380.1 | Symbol: CIPK10 |	8.3	8.9	-0.6	-1.1	100.0%	-4.4
6560	AT4G05200.1 | protein kinase family protein, contains Prosite:PS00108: Serine/Threonine protein kinases active-site signature | chr4:2679721-2682307 REVERSE | Aliases: C17L7.120, C17L7_120	2.7	3.1	-0.3	-1.1	100.0%	-4.1
6561	AT1G52410.2 | caldesmon-related, weak similarity to Caldesmon (CDM) (Swiss-Prot:P12957) (Gallus gallus) | chr1:19524403-19529293 FORWARD | Aliases: F19K6.14, F19K6_14	2.2	2.3	-0.1	-1.1	100.0%	-5.3

Rank	Description	15.dpi	5.dpi	M	t	adj.q	B
6562	AT1G54210.1 | autophagy 12a (APG12a), identical to autophagy 12a (Arabidopsis thaliana) GI:19912169 | chr1:20244912-20246230 FORWARD | Aliases: F20D21.38, F20D21_38	5.2	4.9	0.3	1.1	100.0%	-4.3
6563	AT5G40090.1 | disease resistance protein-related, contains Pfam domain, PF00931: NB-ARC domain, a novel signalling motif found in plant resistance gene products | chr5:16059147-16060680 REVERSE | Aliases: MUD12.70, MUD12_70	2.8	2.6	0.2	1.1	100.0%	-4.5
6564	AT3G11160.1 | expressed protein | chr3:3496073-3497169 REVERSE | Aliases: F11B9.9	3.7	3.4	0.3	1.1	100.0%	-4.3
6565	AT5G47940.1 | expressed protein | chr5:19426468-19429119 REVERSE | Aliases: K16F13.3, K16F13_3	4.2	4.5	-0.3	-1.1	100.0%	-4.3
6566	AT3G22760.1 | Symbol: SOL1 | CXC domain containing TSO1-like protein 1 (SOL1), identical to CXC domain containing TSO1-like protein 1 (SOL1) (Arabidopsis thaliana) GI:7767427; contains Pfam profile PF03638: Tesmin/TSO1-like CXC domain; supporting cDNA gi:7767426:gb:AF205142.1:AF205142	3.5	3.7	-0.3	-1.1	100.0%	-4.5
6567	AT1G61870.1 | pentatricopeptide (PPR) repeat-containing protein, contains Pfam profile PF01535: PPR repeat | chr1:22868669-22870280 REVERSE | Aliases: F8K4.8, F8K4_8	6.8	7.4	-0.6	-1.1	100.0%	-4.0
6568	AT5G43530.1 | SNF2 domain-containing protein / helicase domain-containing protein / RING finger domain-containing protein, similar to SP:P36607 DNA repair protein rad8 {Schizosaccharomyces pombe}; contains Pfam profiles PF00271: Helicase conserved C-terminal domain, PF00176: SNF2 family N-terminal domain, PF00097: Zinc finger, C3HC4 type (RING finger) | chr5:17506555-17512058 FORWARD | Aliases: K9D7.2, K9D7_2	2.8	3.0	-0.2	-1.1	100.0%	-4.6
6569	AT4G38580.1 | Symbol: ATFP6 | heavy-metal-associated domain-containing protein / copper chaperone (CCH)-related, low similarity to copper homeostasis factor (PMID:9701579)(GI:3168840); nearly identical to farnesylated protein TFP6 (GI:4097553); contains Heavy-metal-associated domain PF00403 | chr4:18034445-18035879 FORWARD | Aliases: F20M13.140, F20M13_140, FARNESYLATED PROTEIN 6	7.7	7.0	0.7	1.1	100.0%	-4.1
6570	AT2G23140.1 | armadillo/beta-catenin repeat family protein / U-box domain-containing protein, contains Pfam domain, PF00514: Armadillo/beta-catenin-like repeats and Pfam, PF04564: U-box domain | chr2:9852933-9855842 REVERSE | Aliases: T20D16.23, T20D16_23	3.5	3.7	-0.2	-1.1	100.0%	-4.8
6571	AT2G39840.1 | Symbol: TOPP4 | serine/threonine protein phosphatase PP1 isozyme 4 (TOPP4) / phosphoprotein phosphatase 1, identical to SP:P48484 Serine/threonine protein phosphatase PP1 isozyme 4 (EC 3.1.3.16) {Arabidopsis thaliana}, phosphoprotein phosphatase 1 GI:166801 (Arabidopsis thaliana); contains a Ser/Thr protein phosphatase signature (PDOC00115); contains a metallo-phosphoesterase motif (QDOC50185) | chr2:16634336-16636367 FORWARD | Aliases: None	5.2	4.8	0.4	1.1	100.0%	-4.5
6572	AT3G14860.2 | NHL repeat-containing protein, contains Pfam profile PF01436: NHL repeat	5.2	5.5	-0.4	-1.1	100.0%	-4.5
6573	AT3G27930.1 | expressed protein | chr3:10370536-10373673 REVERSE | Aliases: K24A2.1	6.6	6.4	0.2	1.1	100.0%	-4.4
6574	AT5G63390.1 | expressed protein, contains Pfam PF03138: Plant protein family. The function of this family of plant proteins is unknown; previously annotated as 'auxin-independent growth promoter-related protein' based on similarity to axi 1 protein (GB:X80301) (GI:559920) from (Nicotiana tabacum), which, due to scienitific fraud was retracted. Retraction in: Schell J. EMBO J 1999 May 17;18(10):2908. PMID:10400497.; expression supported by MPSS | chr5:25407738-25409817 REVERSE | Aliases: MLE2.2, MLE2_2	3.3	3.1	0.2	1.1	100.0%	-4.6
6575	AT4G14240.2 | similar to CBS domain-containing protein-related [Arabidopsis thaliana] (TAIR:At4g14230.1); similar to unknown protein [Oryza sativa (japonica cultivar-group)] (GB:AAT07635.1); contains InterPro domain CBS (InterPro:IPR002550) | chr4:8204342-8207340 REVERSE | Aliases: DL3160C, FCAALL.149	4.5	4.2	0.3	1.1	100.0%	-4.4
6576	AT1G56430.1 | nicotianamine synthase, putative, similar to nicotianamine synthase (Lycopersicon esculentum)(GI:4753801), nicotianamine synthase 2 (Hordeum vulgare)(GI:4894912) | chr1:21140609-21141752 FORWARD | Aliases: F13N6.10, F13N6_10	4.8	4.5	0.4	1.1	100.0%	-4.0
6577	AT3G23670.2 | similar to phragmoplast-associated kinesin-related protein (PAKRP1) [Arabidopsis thaliana] (TAIR:At4g14150.1); similar to OSJNBa0038P21.12 [Oryza sativa (japonica cultivar-group)] (GB:CAE05519.1); contains InterPro domain Kinesin, motor region (InterPro:IPR001752)	4.5	4.9	-0.4	-1.1	100.0%	-4.3
6578	AT1G66250.1 | glycosyl hydrolase family 17 protein, similar to beta-1,3-glucanase GI:15150341 from (Camellia sinensis)	5.0	5.3	-0.4	-1.1	100.0%	-4.1
6579	AT3G11640.1 | expressed protein | chr3:3674505-3675204 FORWARD | Aliases: T19F11.4	4.0	3.7	0.4	1.1	100.0%	-4.5
6580	AT1G22700.2 | tetratricopeptide repeat (TPR)-containing protein, contains Pfam profile PF00515 TPR Domain; ESTs gb:T43026, gb:R64902, gb:Z18169 and gb:N37374 come from this gene | chr1:8028181-8029956 REVERSE | Aliases: T22J18.13, T22J18_13	5.2	4.8	0.4	1.1	100.0%	-4.4
6581	AT4G04880.1 | adenosine/AMP deaminase family protein, low similarity to SP:P03958 Adenosine deaminase (EC 3.5.4.4) (Adenosine aminohydrolase) {Mus musculus}; contains Pfam profile PF00962: Adenosine/AMP deaminase | chr4:2464892-2467164 REVERSE | Aliases: T4B21.20, T4B21_20	4.6	5.0	-0.4	-1.1	100.0%	-4.2

Rank	Description	15.dpi	5.dpi	M	t	adj.q	B
6582	AT4G23710.1 | vacuolar ATP synthase subunit G 2 (VATG2) / V-ATPase G subunit 2 (VAG2) / vacuolar proton pump G subunit 2, identical to Swiss-Prot:O82629 vacuolar ATP synthase subunit G 2 (V-ATPase G subunit 2, Vacuolar proton pump G subunit 2) (Arabidopsis thaliana) | chr4:12350099-12351634 FORWARD | Aliases: F9D16.180, F9D16_180	8.4	7.9	0.6	1.1	100.0%	-4.2
6583	AT3G61590.1 | F-box family protein, contains weak hit to Pfam PF00646: F-box domain; stamina pistilloidia (Stp) - Pisum sativum, EMBL:AF004843	4.6	4.8	-0.2	-1.1	100.0%	-4.5
6584	AT5G23150.1 | Symbol: HUA2 | PWWP domain-containing protein, identical to cDNA putative transcription factor (HUA2) GI:4868119; contains Pfam profile	3.9	3.7	0.2	1.1	100.0%	-4.6
6585	AT1G31130.1 | expressed protein | chr1:11114682-11116176 REVERSE | Aliases: F28K20.6, F28K20_6	6.2	5.6	0.6	1.1	100.0%	-4.1
6586	AT5G57680.1 | expressed protein | chr5:23379971-23380920 REVERSE | Aliases: MRI1.3, MRI1_3	2.5	2.4	0.2	1.1	100.0%	-5.0
6587	AT3G17830.1 | DNAJ heat shock family protein, similar to SP:P35514 Chaperone protein dnaJ {Lactococcus lactis}; contains Pfam profiles PF00226: DnaJ domain, PF01556: DnaJ C terminal region, PF00684: DnaJ central domain (4 repeats)	3.6	3.9	-0.3	-1.1	100.0%	-4.2
6588	AT3G09070.1 | glycine-rich protein, similar to hypothetical protein GB:AAD32765 (Arabidopsis thaliana)	4.5	4.7	-0.2	-1.1	100.0%	-4.6
6589	AT2G31070.1 | TCP family transcription factor, putative, similar to TCP1 protein (GI:20269127) {Lupinus albus} and cycloidea (GI:12002867) (Lycopersicon esculentum) | chr2:13227555-13229686 REVERSE | Aliases: T16B12.12, T16B12_12	3.1	2.9	0.2	1.1	100.0%	-5.0
6590	AT5G56260.1 | dimethylmenaquinone methyltransferase family protein, similar to bacterial S-adenosylmethionine:2-demethylmenaquinone methyltransferases; contains Pfam profile PF03737: Dimethylmenaquinone methyltransferase | chr5:22792551-22794437 REVERSE | Aliases: K24C1.7, K24C1_7	7.8	7.4	0.4	1.1	100.0%	-4.3
6591	AT3G14440.1 | Symbol: NCED3 | 9-cis-epoxycarotenoid dioxygenase, putative / neoxanthin cleavage enzyme, putative / carotenoid cleavage dioxygenase, putative, similar to 9-cis-epoxycarotenoid dioxygenase GB:AAF26356 (GI:6715257)(Phaseolus vulgaris) | chr3:4831295-4833606 REVERSE | Aliases: 9 CIS EPOXYCAROTENOID DIOXYGENASE, ATNCED3, MOA2.4, NINE CIS EPOXYCAROTENOID DIOXYGENASE3, STO1	2.4	2.3	0.1	1.1	100.0%	-5.2
6592	AT1G14380.3 | similar to calmodulin-binding family protein [Arabidopsis thaliana] (TAIR:At2g02790.1); similar to 'unknown protein, contains IQ calmodulin-binding motif' [Oryza sativa (japonica cultivar-group)] (GB:XP_475770.1); contains InterPro domain IQ calmodulin-binding region (InterPro:IPR000048)	5.8	5.5	0.3	1.1	100.0%	-4.5
6593	AT5G09870.1 | Symbol: CESA5 | cellulose synthase, catalytic subunit, putative, similar to gi:2827141 cellulose synthase catalytic subunit (Ath-A), Arabidopsis thaliana | chr5:3073357-3077975 FORWARD | Aliases: CELLULASE SYNTHASE 5, MYH9.8, MYH9_8	6.1	5.9	0.2	1.1	100.0%	-4.4
6594	AT1G22410.1 | 2-dehydro-3-deoxyphosphoheptonate aldolase, putative / 3-deoxy-D-arabino-heptulosonate 7-phosphate synthase, putative / DAHP synthetase, putative, similar to 3-deoxy-D-arabino-heptulosonate 7-phosphate GI:170224 from (Nicotiana tabacum), SP:P21357 from Solanum tuberosum; contains Pfam Class-II DAHP synthetase family domain PF01474 | chr1:7912043-7914992 FORWARD | Aliases: F12K8.24, F12K8_24	11.0	11.4	-0.5	-1.1	100.0%	-5.0
6595	AT3G52110.1 | expressed protein | chr3:19334761-19336629 REVERSE | Aliases: F4F15.220	3.5	3.8	-0.2	-1.1	100.0%	-4.8
6596	AT4G26380.1 | DC1 domain-containing protein, contains Pfam profile PF03107: DC1 domain	3.7	3.4	0.3	1.1	100.0%	-4.2
6597	AT1G03860.3 | Symbol: ATPHB2 |	7.7	8.5	-0.8	-1.1	100.0%	-4.1
6598	AT4G12530.1 | protease inhibitor/seed storage/lipid transfer protein (LTP) family protein, similar to pEARLI 1 (Accession No. L43080): an Arabidopsis member of a conserved gene family (PGF95-099), Plant Physiol. 109 (4), 1497 (1995); contains Pfam protease inhibitor/seed storage/LTP family domain PF00234 | chr4:7428024-7428560 REVERSE | Aliases: T1P17.120, T1P17_120	2.7	2.5	0.2	1.1	100.0%	-5.0
6599	AT1G04690.1 | Symbol: KAB1 | potassium channel protein, putative, nearly identical to K+ channel protein (Arabidopsis thaliana) GI:1063415; contains Pfam profile PF00248: oxidoreductase, aldo/keto reductase family	8.8	9.4	-0.6	-1.1	100.0%	-4.6
6600	AT4G05400.2 | expressed protein, similar to expressed protein [Arabidopsis thaliana] (TAIR:At4g21140.1); similar to unknown protein [Oryza sativa (japonica cultivar-group)] (GB:BAD73259.1) | chr4:2741157-2743306 FORWARD | Aliases: C6L9.80, C6L9_80	6.7	7.3	-0.6	-1.1	100.0%	-4.1
6601	AT5G18610.1 | protein kinase family protein, contains eukaryotic protein kinase domain, PROSITE:PS00107 | chr5:6192738-6195373 FORWARD | Aliases: T28N17.90, T28N17_90	2.8	2.6	0.2	1.1	100.0%	-4.9

Rank	Description	15.dpi	5.dpi	M	t	adj.q	B
6602	AT4G14360.2 | similar to dehydration-responsive protein-related [Arabidopsis thaliana] (TAIR:At5g14430.2); similar to dehydration-responsive protein-related [Arabidopsis thaliana] (TAIR:At5g14430.1); similar to dehydration-responsive protein-related [Arabidopsis thaliana] (TAIR:At3g23300.1); similar to dehydration-responsive protein-related [Arabidopsis thaliana] (TAIR:At1g04430.1); similar to dehydration-responsive protein-like [Oryza sativa (japonica cultivar-group)] (GB:BAD46056.1); contains InterPro domain Putative methyltransferase DUF248 (InterPro:IPR004159) | chr4:8267285-8270989 REVERSE | Aliases: DL3220C, FCAALL.222	6.7	7.4	-0.7	-1.1	100.0%	-4.2
6603	ATMG00130.1 | Symbol: ORF121A | hypothetical protein | chrM:34190-34555 FORWARD | Aliases: ORF121A	2.1	2.2	-0.1	-1.1	100.0%	-5.3
6604	AT5G49060.1 | DNAJ heat shock N-terminal domain-containing protein, low similarity to SP:Q9QYI4 DnaJ homolog subfamily B member 12 {Mus musculus}; contains Pfam profile PF00226: DnaJ domain | chr5:19903724-19905576 FORWARD | Aliases: K20J1.3, K20J1_3	3.2	3.0	0.2	1.1	100.0%	-4.8
6605	AT4G32040.1 | Symbol: KNAT5 | homeobox protein knotted-1 like 5 (KNAT5) / homeodomain containing protein 1 (H1), identical to homeobox protein knotted-1 like 5 (KNAT5) SP:P48002 from (Arabidopsis thaliana)	5.2	4.7	0.5	1.1	100.0%	-4.0
6606	AT4G22756.1 | Symbol: SMO1 2 |	5.9	6.7	-0.8	-1.1	100.0%	-4.3
6607	AT1G69670.1 | Symbol: ATCUL3B/CUL3B | cullin, putative, contains similarity to Cullin homolog 3 (CUL-3) SP:Q13618, GI:3639052 from (Homo sapiens); contains Pfam profile PF00888: Cullin family. Interacts with members of AtBPM family and RBX1 suggesting it is part of an E3 ligase complex involved in RUB modification. | chr1:26205694-26207639 REVERSE | Aliases: ATCUL3B, CUL3B, T6C23.13, T6C23_13	3.9	4.3	-0.5	-1.1	100.0%	-4.4
6608	AT2G46270.2 | Symbol: GBF3 | G-box binding factor 3 (GBF3), identical to G-box binding factor 3 (GBF3) SP:P42776 from (Arabidopsis thaliana); contains Pfam profile: PF00170 bZIP transcription factor | chr2:19007264-19010476 FORWARD | Aliases: BZIP TRANSCRIPTION FACTOR, G BOX BINDING FACTOR 3, T3F17.8	5.5	5.0	0.5	1.1	100.0%	-4.2
6609	AT1G32500.1 | Symbol: ATNAP6 |	7.5	8.0	-0.5	-1.1	100.0%	-4.2
6610	AT1G01640.2 | speckle-type POZ protein-related, contains Pfam profile:PF00651 BTB/POZ domain; similar to Speckle-type POZ protein (SP:O43791) (Homo sapiens) | chr1:230994-232313 REVERSE | Aliases: T1N6.2, T1N6_2	7.7	6.9	0.8	1.1	100.0%	-4.1
6611	AT5G63520.1 | expressed protein, contains InterPro domain Cyclin-like F-box (InterPro:IPR001810) | chr5:25443403-25446338 REVERSE | Aliases: MLE2.15, MLE2_15	5.5	6.0	-0.5	-1.1	100.0%	-4.3
6612	AT3G06010.1 | homeotic gene regulator, putative, similar to SP:P25439 Homeotic gene regulator (Brahma protein) {Drosophila melanogaster}; contains Pfam profiles PF00271: Helicase conserved C-terminal domain, PF00176: SNF2 family N-terminal domain	3.6	4.5	-0.9	-1.1	100.0%	-4.3
6613	AT2G21300.2 | similar to kinesin motor family protein [Arabidopsis thaliana] (TAIR:At4g38950.1); similar to kinesin heavy chain [Zea mays] (GB:AAK91812.1); similar to putative kinesin heavy chain [Oryza sativa (japonica cultivar-group)] (GB:XP_467026.1); similar to OSJNBa0091D06.23 [Oryza sativa (japonica cultivar-group)] (GB:XP_473345.1); similar to putative kinesin heavy chain [Oryza sativa (japonica cultivar-group)] (GB:BAD46370.1); similar to kinesin heavy chain [Zea mays] (GB:AAK91822.1); contains InterPro domain Kinesin, motor region (InterPro:IPR001752)	2.3	2.5	-0.2	-1.1	100.0%	-4.9
6614	AT5G61060.1 | histone deacetylase family protein, similar to SP:Q9UBN7 Histone deacetylase 6 (HD6) {Homo sapiens}; contains Pfam profile PF00850: Histone deacetylase family | chr5:24584017-24588292 REVERSE | Aliases: MAF19.7, MAF19_7	4.7	5.2	-0.4	-1.1	100.0%	-4.1
6615	ATMG00060.1 | Symbol: NAD5C | Mitochondrial NADH dehydrogenase subunit 5. The gene is trans-spliced from the three different pre-cursors, NAD5a, NAD5b and NAD5c. | chrM:20571-22086 REVERSE | Aliases: NAD5, NAD5.3, NAD5C	3.5	3.9	-0.4	-1.1	100.0%	-4.4
6616	AT1G62660.1 | beta-fructosidase (BFRUCT3) / beta-fructofuranosidase / invertase, vacuolar, identical to beta-fructosidase GB:CAA67560 GI:1429209 (Arabidopsis thaliana); supported by full-length cDNA GI:14517549; identical to cDNA	2.7	3.2	-0.5	-1.1	100.0%	-4.2
6617	AT5G67010.1 | encodes a member of the ERF (ethylene response factor) subfamily B-6 of ERF/AP2 transcription factor family. The protein contains one AP2 domain. There are 12 members in this subfamily including RAP2.11. | chr5:26766284-26766842 REVERSE | Aliases: K8A10.8, K8A10_8	2.7	2.5	0.1	1.1	100.0%	-5.0
6618	AT3G06890.1 | expressed protein | chr3:2176862-2177592 FORWARD | Aliases: F17A9.4	5.2	4.8	0.4	1.1	100.0%	-4.3
6619	AT5G49250.1 | expressed protein | chr5:19982569-19983171 FORWARD | Aliases: K21P3.13, K21P3_13	2.8	2.6	0.2	1.1	100.0%	-4.6
6620	AT5G07400.1 | forkhead-associated domain-containing protein / FHA domain-containing protein | chr5:2340643-2345453 FORWARD | Aliases: T2I1.110, T2I1_110	4.3	4.6	-0.3	-1.1	100.0%	-4.6
6621	AT3G48020.1 | expressed protein | chr3:17735578-17735985 FORWARD | Aliases: T17F15.110	4.4	4.1	0.4	1.1	100.0%	-4.3

Rank	Description	15.dpi	5.dpi	M	t	adj.q	B
6622	AT3G50760.1 | similar to glycosyl transferase family 8 protein [Arabidopsis thaliana] (TAIR:At1g19300.1); similar to putative Avr9/Cf-9 rapidly elicited protein 231 [Oryza sativa (japonica cultivar-group)] (GB:BAD45664.1); contains InterPro domain Glycosyl transferase, family 8 (InterPro:IPR002495) | chr3:18879055-18880365 FORWARD | Aliases: F18B3.40	3.2	3.6	-0.4	-1.1	100.0%	-4.0
6623	AT4G29550.1 | expressed protein, contains Pfam profile PF04776: Protein of unknown function (DUF626) | chr4:14502218-14503853 FORWARD | Aliases: T16L4.60, T16L4_60	2.7	2.9	-0.2	-1.1	100.0%	-4.9
6624	AT1G35470.2 | SPla/RYanodine receptor (SPRY) domain-containing protein, similar to RanBPM (Homo sapiens) GI:15080674; contains Pfam profile PF00622: SPRY domain | chr1:13051436-13054965 REVERSE | Aliases: F12A4.1, F12A4_1	5.0	5.2	-0.3	-1.1	100.0%	-4.3
6625	AT1G44170.2 | Symbol: ALDH3H1 | aldehyde dehydrogenase, putative (ALDH), similar to aldehyde dehydrogenase ALDH (Craterostigma plantagineum) gi:17065918:emb:CAC84900	6.2	6.8	-0.6	-1.1	100.0%	-4.1
6626	AT1G54160.1 | CCAAT-binding transcription factor (CBF-B/NF-YA) family protein, contains Pfam profile: PF02045 CCAAT-binding transcription factor (CBF-B/NF-YA) subunit B | chr1:20221003-20223028 REVERSE | Aliases: F15I1.26, F15I1_26	6.9	6.4	0.5	1.1	100.0%	-3.8
6627	AT1G14300.1 | expressed protein, contains Pfam PF04063: Domain of unknown function (DUF383) and PF04064: Domain of unknown function (DUF384) | chr1:4882371-4884780 REVERSE | Aliases: F14L17.6, F14L17_6	6.0	6.3	-0.3	-1.1	100.0%	-4.4
6628	AT4G34430.4 | Symbol: ATSWI3C | DNA-binding family protein, contains Pfam domains PF04433: SWIRM domain, PF00249: Myb-like DNA-binding domain and PF00569: Zinc finger, ZZ type | chr4:16460924-16465327 FORWARD | Aliases: CHB03, CHB3, CHROMATIN REMODELING COMPLEX SUBUNIT B, F10M10.200, F10M10_200	3.3	3.1	0.1	1.1	100.0%	-4.9
6629	AT4G04640.1 | Symbol: ATPC1 | ATP synthase gamma chain 1, chloroplast (ATPC1), identical to SP:Q01908 ATP synthase gamma chain 1, chloroplast precursor (EC 3.6.3.14) {Arabidopsis thaliana} | chr4:2350496-2352016 REVERSE | Aliases: T19J18.4, T19J18_4	9.4	8.9	0.5	1.1	100.0%	-4.1
6630	AT1G26220.1 | GCN5-related N-acetyltransferase (GNAT) family protein, low similarity to SP:P09453 Ribosomal-protein-alanine acetyltransferase (EC 2.3.1.128) {Escherichia coli}; contains Pfam profile PF00583: acetyltransferase, GNAT family	3.2	3.0	0.2	1.1	100.0%	-4.6
6631	AT4G18430.1 | Ras-related GTP-binding protein, putative, similar to GTP-binding protein GI:303742 from (Pisum sativum)	2.8	3.2	-0.4	-1.1	100.0%	-3.9
6632	AT3G20190.1 | leucine-rich repeat transmembrane protein kinase, putative, similar to receptor kinase GB:AAA33715 (Petunia integrifolia) | chr3:7044950-7047396 FORWARD | Aliases: MAL21.23	3.1	3.3	-0.2	-1.1	100.0%	-4.9
6633	AT1G33050.2 | expressed protein | chr1:11968897-11971001 REVERSE | Aliases: T9L6.12, T9L6_12	5.7	5.2	0.4	1.1	100.0%	-4.2
6634	AT1G23090.1 | Symbol: AST91 | sulfate transporter, putative, similar to sulfate transporter (Arabidopsis thaliana) GI:2285885; contains Pfam profiles PF00916: Sulfate transporter family, PF01740: STAS domain | chr1:8184941-8189020 REVERSE | Aliases: SULFATE TRANSPORTER, SULFATE TRANSPORTER 91, SULTR3;3, T26J12.13, T26J12_13	4.2	4.5	-0.3	-1.1	100.0%	-4.4
6635	AT5G58800.2 | similar to quinone reductase family protein [Arabidopsis thaliana] (TAIR:At4g27270.1); similar to unknown [Prunus armeniaca] (GB:AAD38143.1); contains InterPro domain Flavodoxin/nitric oxide synthase (InterPro:IPR008254) | chr5:23763109-23764499 REVERSE | Aliases: MZN1.27, MZN1_27	8.4	8.0	0.4	1.1	100.0%	-4.3
6636	AT3G05970.1 | Symbol: LACS6 | long-chain-fatty-acid--CoA ligase / long-chain acyl-CoA synthetase (LACS6), strong similarity to AMP-binding protein (MF39P) gi:1617274 from Brassica napus, similar to putative long-chain-fatty-acid--CoA ligase (brain isozyme) GB:P33124 (Rattus norvegicus); contains Pfam AMP-binding enzyme domain PF00501; identical to cDNA AtLACS6 for long-chain acyl-CoA synthetase GI:22531705 | chr3:1786324-1791808 REVERSE | Aliases: F2O10.7, F2O10_7, LONG CHAIN ACYL COA SYNTHETASE 6	9.7	9.5	0.2	1.1	100.0%	-4.7
6637	AT1G18340.1 | basal transcription factor complex subunit-related, similar to TFIIH basal transcription factor complex p34 subunit (Basic transcription factor 2 34 kDa subunit) (BTF2-p34) (General transcription factor IIH polypeptide 3) (SP:Q8VD76) {Mus musculus}; contains Pfam PF03850: Transcription factor Tfb4 | chr1:6311476-6313924 REVERSE | Aliases: F15H18.15, F15H18_15	5.7	5.9	-0.2	-1.1	100.0%	-4.5
6638	AT5G57570.1 | expressed protein | chr5:23331010-23331420 REVERSE | Aliases: MUA2.15, MUA2_15	3.6	3.3	0.3	1.1	100.0%	-4.6
6639	AT5G19920.1 | transducin family protein / WD-40 repeat family protein, contains 5 WD-40 repeats (PF00400); related to TipD protein (SP:O15736, PIR:T08602) (Dictyostelium discoideum); related to WD-repeat protein RBAP1 (GI:9716495) (Zea mays) | chr5:6733218-6737375 REVERSE | Aliases: F28I16.70, F28I16_70	3.2	3.5	-0.3	-1.1	100.0%	-4.5
6640	AT3G17470.1 | RelA/SpoT domain-containing protein / calcium-binding EF-hand family protein, contains INTERPRO:IPR002048 calcium-binding EF-hand domain, Pfam profile PF04607: Region found in RelA / SpoT proteins | chr3:5979840-5982121 FORWARD | Aliases: MKP6.2	5.4	5.6	-0.2	-1.1	100.0%	-4.5

Rank	Description	15.dpi	5.dpi	M	t	adj.q	B
6641	AT1G52680.1 | late embryogenesis abundant protein-related / LEA protein-related, low similarity to late embryogenesis abundant proteins from (Brassica napus) SP:P13934, (Cicer arietinum) GI:2909420 | chr1:19621811-19622680 REVERSE | Aliases: F6D8.10, F6D8_10	2.5	2.4	0.1	1.1	100.0%	-5.1
6642	AT5G62150.1 | peptidoglycan-binding LysM domain-containing protein, contains Pfam profile PF01476: LysM domain	5.3	4.9	0.4	1.1	100.0%	-4.0
6643	AT5G06940.1 | leucine-rich repeat family protein, contains protein kinase domain, Pfam:PF00069; contains leucine-rich repeats, Pfam:PF00560 | chr5:2148079-2150772 REVERSE | Aliases: MOJ9.11, MOJ9_11	2.6	2.5	0.1	1.1	100.0%	-5.0
6644	AT4G26070.3 | Symbol: MEK1 | mitogen-activated protein kinase kinase (MAPKK) (MKK1) (MEK1), identical to MEK1 (Arabidopsis thaliana) gi:2196704:gb:AAB97145; mitogen-activated protein kinase kinase (MAPKK) family, PMID:12119167 | chr4:13217658-13219942 FORWARD | Aliases: ATMEK1, F20B18.180, F20B18_180, MITOGEN ACTIVATED PROTEIN KINASE KINASE, MKK1, NMAPKK	5.9	6.1	-0.3	-1.1	100.0%	-4.5
6645	AT4G24530.1 | expressed protein, contains Pfam PF03138: Plant protein family. The function of this family of plant proteins is unknown; previously annotated as 'PsRT17-1 like protein' based on similarity to PsRT17-1 (GP:1778376) (Pisum sativum) which was based upon similarity to axi 1 protein (GB:X80301) (GI:559920) from (Nicotiana tabacum), which, due to scienitific fraud was retracted. Retraction in: Schell J. EMBO J 1999 May 17;18(10):2908. PMID:10400497. | chr4:12667368-12670023 REVERSE | Aliases: F22K18.270, F22K18_270	6.0	5.7	0.3	1.1	100.0%	-4.3
6646	AT1G07380.1 | ceramidase family protein, contains similarity to mitochondrial ceramidase (Homo sapiens) gi:9246993:gb:AAF86240	5.6	5.3	0.3	1.1	100.0%	-4.2
6647	AT1G80920.1 | Symbol: J8 | DNAJ heat shock N-terminal domain-containing protein, similar to SP:Q05646 Chaperone protein dnaJ Erysipelothrix rhusiopathiae, SP:P45555 Chaperone protein dnaJ (HSP40) Staphylococcus aureus; contains Pfam profile PF00226 DnaJ domain | chr1:30408536-30409577 REVERSE | Aliases: F23A5.28, F23A5_28, J8	6.9	6.5	0.4	1.1	100.0%	-4.0
6648	AT1G27620.1 | transferase family protein, similar to hypersensitivity-related gene product HSR201 - Nicotiana tabacum, EMBL:X95343; contains Pfam transferase family domain PF00248 | chr1:9608406-9610529 FORWARD | Aliases: T22C5.6	3.9	4.3	-0.4	-1.1	100.0%	-4.1
6649	AT3G02555.1 | expressed protein | chr3:539712-541510 REVERSE | Aliases: None	4.5	4.3	0.2	1.1	100.0%	-4.5
6650	AT2G36900.1 | Symbol: MEMB11 |	8.2	8.0	0.2	1.1	100.0%	-4.7
6651	AT3G58660.1 | 60S ribosomal protein-related, contains weak similarity to 60S ribosomal protein L10A (CSA-19) (NEDD-6) (Swiss-Prot:P53026) (Mus musculus) | chr3:21712511-21714091 FORWARD | Aliases: T20N10.10	6.9	7.8	-0.9	-1.1	100.0%	-4.2
6652	AT1G35500.1 | expressed protein | chr1:13066065-13066574 FORWARD | Aliases: F12A4.10, F12A4_10	3.4	3.2	0.2	1.1	100.0%	-4.7
6653	AT1G52060.1 | jacalin lectin family protein, similar to myrosinase-binding protein homolog (Arabidopsis thaliana) GI:2997767; contains Pfam profile PF01419 jacalin-like lectin domain	4.1	3.8	0.3	1.1	100.0%	-4.4
6654	AT3G10810.1 | zinc finger (C3HC4-type RING finger) family protein, contains Pfam profile: PF00097 Zinc finger, C3HC4 type (RING finger) | chr3:3381853-3384510 REVERSE | Aliases: T7M13.11	5.5	5.8	-0.3	-1.1	100.0%	-4.7
6655	AT5G25220.2 | Symbol: KNAT3 | similar to homeobox protein knotted-1 like 4 (KNAT4) [Arabidopsis thaliana] (TAIR:At5g11060.1); similar to homeobox protein NTH23 - common tobacco (GB:T02220); contains InterPro domain Homeobox (InterPro:IPR001356); contains InterPro domain KNOX1 domain (InterPro:IPR005540); contains InterPro domain ELK domain (InterPro:IPR005539); contains InterPro domain KNOX2 domain (InterPro:IPR005541); contains InterPro domain Proline-rich region (InterPro:IPR000694) | chr5:8736077-8738587 FORWARD | Aliases: F21J6.18, F21J6_18, KNAT3 HOMEOBOX PROTEIN, KNOTTED1 LIKE HOMEOBOX GENE 3	6.7	6.2	0.4	1.1	100.0%	-4.3
6656	AT4G11100.1 | expressed protein | chr4:6768611-6770180 FORWARD | Aliases: T22B4.80, T22B4_80	3.3	3.5	-0.2	-1.1	100.0%	-4.6
6657	AT1G47210.2 | Symbol: CYCA3;2 | cyclin family protein, similar to A-type cyclin (Catharanthus roseus) GI:2190259; contains Pfam profile PF00134: Cyclin, N-terminal domain | chr1:17303396-17305306 FORWARD | Aliases: CYCA3;2, Cyclin A3;2, F8G22.8, F8G22_8	3.8	4.1	-0.3	-1.1	100.0%	-4.5
6658	AT2G35060.2 | Symbol: KUP11 | similar to potassium transporter family protein [Arabidopsis thaliana] (TAIR:At1g31120.1); similar to potassium transporter (KUP1) [Arabidopsis thaliana] (TAIR:At2g30070.1); similar to potassium transporter family protein [Arabidopsis thaliana] (TAIR:At1g60160.1); similar to potassium transporter family protein [Arabidopsis thaliana] (TAIR:At4g19960.1); similar to potassium transporter (KUP3) [Arabidopsis thaliana] (TAIR:At3g02050.1); similar to putative potasium transporter [Oryza sativa (japonica cultivar-group)] (GB:CAD21002.1); similar to OSJNBa0070C17.23 [Oryza sativa (japonica cultivar-group)] (GB:XP_473875.1); similar to putative potassium transporter [Oryza sativa (japonica cultivar-group)] (GB:BAD46101.1); similar to POT9_ARATH Putative potassium transporter 9 (AtPOT9) (GB:O49423); similar to putative potassium transporter [Oryza sativa (japonica cultivar-group)] (GB:XP_479449.1); contains InterPro domain K+ potassium transporter (InterPro:IPR003855) | chr2:14782142-14785949 REVERSE | Aliases: F19I3.29, F19I3_29	4.3	4.1	0.2	1.1	100.0%	-4.6

Rank	Description	15.dpi	5.dpi	M	t	adj.q	B
6659	AT5G40760.1 | Symbol: G6PD6 | Encodes a cytosolic glucose-6-phosphate dehydrogenase that is insensitive to reduction by DTT and whose mRNA is expressed ubiquitously.	9.0	9.6	-0.7	-1.1	100.0%	-4.3
6660	AT4G05060.1 | vesicle-associated membrane family protein / VAMP family protein, similar to VAP27 GI:6688926 (Nicotiana plumbaginifolia) | chr4:2590063-2592161 REVERSE | Aliases: C17L7.8	4.6	4.4	0.3	1.1	100.0%	-4.6
6661	AT3G01930.2 | nodulin family protein, similar to nodulin-like protein (Arabidopsis thaliana) GI:3329368, nodule-specific protein Nlj70 (Lotus japonicus) GI:3329366 | chr3:319068-321565 REVERSE | Aliases: F28J7.26	4.8	4.4	0.5	1.1	100.0%	-4.4
6662	AT2G01090.1 | ubiquinol-cytochrome C reductase complex 7.8 kDa protein, putative / mitochondrial hinge protein, putative, similar to SP:P48504 Ubiquinol-cytochrome C reductase complex 7.8 kDa protein (EC 1.10.2.2) (Mitochondrial hinge protein) (CR7) {Solanum tuberosum}; contains Pfam profile PF02320: Ubiquinol-cytochrome C reductase hinge protein | chr2:80014-81200 FORWARD | Aliases: F23H14.6, F23H14_6	9.8	9.3	0.4	1.1	100.0%	-4.2
6663	AT3G63130.1 | Symbol: RANGAP1 | RAN GTPase activating protein 1 (RanGAP1), contains Pfam PF00560: Leucine Rich Repeat domains; identical to RAN GTPase activating protein 1 (GI:6708466)(Arabidopsis thaliana) | chr3:23335596-23337888 FORWARD | Aliases: RAN GTPASE ACTIVATING PROTEIN 1, T20O10.230	3.6	3.8	-0.3	-1.1	100.0%	-4.5
6664	AT2G21220.1 | auxin-responsive protein, putative, similar to auxin-induced protein TGSAUR22 (GI:10185820) (Tulipa gesneriana) | chr2:9096407-9096894 FORWARD | Aliases: F7O24.6, F7O24_6	2.4	2.5	-0.1	-1.1	100.0%	-5.1
6665	AT1G14310.1 | haloacid dehalogenase-like hydrolase family protein, contains InterPro accession IPR005834: Haloacid dehalogenase-like hydrolase	7.7	8.0	-0.3	-1.1	100.0%	-4.3
6666	AT1G62740.1 | stress-inducible protein, putative, similar to sti (stress inducible protein) (Glycine max) GI:872116; contains Pfam profile PF00515 TPR Domain | chr1:23234626-23237449 FORWARD | Aliases: F23N19.10, F23N19_10	8.3	8.7	-0.4	-1.1	100.0%	-4.3
6667	AT2G44530.1 | ribose-phosphate pyrophosphokinase, putative / phosphoribosyl diphosphate synthetase, putative, very strong similarity to phosphoribosyl pyrophosphate synthase (Spinacia oleracea) GI:4902849; contains Pfam profile PF00156: Phosphoribosyl transferase domain	5.3	5.0	0.3	1.1	100.0%	-4.4
6668	AT1G71850.1 | expressed protein, ; expression supported by MPSS | chr1:27028537-27029949 REVERSE | Aliases: F14O23.23, F14O23_23	4.8	5.1	-0.3	-1.1	100.0%	-4.2
6669	AT1G28100.3 | expressed protein | chr1:9801069-9804413 FORWARD | Aliases: F13K9.27, F13K9_27	8.1	8.5	-0.4	-1.1	100.0%	-4.3
6670	AT2G37020.1 | translin family protein, similar to SP:Q62348 Translin {Mus musculus}; contains Pfam profile PF01997: Translin family | chr2:15553609-15556116 FORWARD | Aliases: T1J8.20, T1J8_20	5.7	6.3	-0.5	-1.1	100.0%	-4.1
6671	AT1G33440.1 | proton-dependent oligopeptide transport (POT) family protein, contains Pfam profile: PF00854 POT family | chr1:12127454-12130369 REVERSE | Aliases: F10C21.11, F10C21_11	3.1	2.9	0.2	1.1	100.0%	-4.6
6672	AT1G64640.1 | plastocyanin-like domain-containing protein, contains InterPro:IPR003245 plastocyanin-like domain | chr1:24025387-24026820 REVERSE | Aliases: F1N19.21, F1N19_21	2.7	2.9	-0.1	-1.1	100.0%	-5.2
6673	AT2G04220.1 | expressed protein | chr2:1445398-1446321 FORWARD | Aliases: T23O15.16	2.4	2.3	0.1	1.1	100.0%	-5.2
6674	AT4G39420.1 | expressed protein, ; expression supported by MPSS | chr4:18339725-18342147 FORWARD | Aliases: F23K16.50, F23K16_50	3.0	2.9	0.1	1.1	100.0%	-5.0
6675	AT1G15350.3 | expressed protein | chr1:5278286-5279860 REVERSE | Aliases: F9L1.29, F9L1_29	5.6	5.2	0.5	1.1	100.0%	-4.2
6676	AT1G55280.1 | expressed protein | chr1:20626627-20628297 REVERSE | Aliases: F7A10.4, F7A10_4	3.3	3.0	0.3	1.1	100.0%	-4.4
6677	AT5G03720.1 | Symbol: AT HSFA3 | heat shock transcription factor family protein, contains Pfam profile: PF00447 HSF-type DNA-binding domain | chr5:971912-973682 REVERSE | Aliases: F17C15.140, F17C15_140, HSFA3	6.2	5.7	0.5	1.1	100.0%	-4.1
6678	AT2G45470.1 | Symbol: FLA8 | fasciclin-like arabinogalactan-protein (FLA8) | chr2:18749614-18751185 REVERSE | Aliases: F4L23.2	4.4	5.2	-0.8	-1.1	100.0%	-4.2
6679	AT5G15200.1 | 40S ribosomal protein S9 (RPS9B), 40S ribosomal protein S9, Chlamydomonas sp., EMBL:AU066528 | chr5:4934886-4936384 REVERSE | Aliases: F8M21.90, F8M21_90	11.4	11.7	-0.3	-1.1	100.0%	-4.5
6680	AT5G07320.1 | mitochondrial substrate carrier family protein, similar to peroxisomal Ca-dependent solute carrier (Oryctolagus cuniculus) GI:2352427 (mitochondrial carrier superfamily); contains INTERPRO:IPR001993 Mitochondrial substrate carrier family, INTERPRO:IPR002048 calcium-binding EF-hand domain	6.1	6.6	-0.4	-1.1	100.0%	-4.1

Rank	Description	15.dpi	5.dpi	M	t	adj.q	B
6681	AT1G77960.1 | similar to octicosapeptide/Phox/Bem1p (PB1) domain-containing protein [Arabidopsis thaliana] (TAIR:At5g09620.1); similar to hypothetical protein DDB0216723 [Dictyostelium discoideum] (GB:EAL72846.1); contains domain HIS_RICH (PS50316); contains domain GLN_RICH (PS50322) | chr1:29315359-29317867 REVERSE | Aliases: F28K19.17, F28K19_17	2.7	2.8	-0.1	-1.1	100.0%	-5.3
6682	AT5G61530.2 | small G protein family protein / RhoGAP family protein, contains Pfam domain, PF00620: RhoGAP domain | chr5:24759311-24761985 FORWARD | Aliases: K11J9.10, K11J9_10	8.2	7.7	0.5	1.1	100.0%	-4.3
6683	AT1G70580.4 | Symbol: AOAT2 | similar to glutamate:glyoxylate aminotransferase 1 (GGT1) [Arabidopsis thaliana] (TAIR:At1g23310.1); similar to alanine aminotransferase [Oryza sativa (indica cultivar-group)] (GB:AAO84040.1); contains InterPro domain Aminotransferase, class I and II (InterPro:IPR004839); contains InterPro domain 1-aminocyclopropane-1-carboxylate synthase (InterPro:IPR001176) | chr1:26616525-26619732 FORWARD | Aliases: ALANINE 2 OXOGLUTARATE AMINOTRANSFERASE 2, F5A18.24, F5A18_24, GGT2, GLUTAMATE:GLYOXYLATE AMINOTRANSFERASE 2	7.3	7.9	-0.6	-1.1	100.0%	-4.1
6684	AT1G25350.1 | glutamine-tRNA ligase, putative / glutaminyl-tRNA synthetase, putative / GlnRS, putative, similar to tRNA-glutamine synthetase GI:2995454 from (Lupinus luteus) | chr1:8889097-8894245 REVERSE | Aliases: F4F7.26, F4F7_26	7.2	7.6	-0.4	-1.1	100.0%	-4.8
6685	AT2G38800.1 | calmodulin-binding protein-related, contains similarity to potato calmodulin-binding protein PCBP GI:17933110 from (Solanum tuberosum) | chr2:16224077-16226237 FORWARD | Aliases: F13I13.3, F13I13_3	8.4	8.0	0.4	1.1	100.0%	-4.2
6686	AT2G33440.1 | splicing factor family protein, similar to Splicing factor U2AF 65 kDa subunit (U2 snRNP auxiliary factor large subunit) {Homo sapiens} SP:P26368, {Mus musculus} SP:P26369; contains InterPro entry IPR000504: RNA-binding region RNP-1 (RNA recognition motif) (RRM) | chr2:14177716-14180513 FORWARD | Aliases: F4P9.21, F4P9_21	3.9	4.1	-0.2	-1.1	100.0%	-4.7
6687	AT1G55810.3 | uracil phosphoribosyltransferase, putative / UMP pyrophosphorylase, putative / UPRTase, putative, similar to SP:O65583 Uracil phosphoribosyltransferase (EC 2.4.2.9) (UMP pyrophosphorylase) (UPRTase) {Arabidopsis thaliana}; contains Pfam profile PF00485: Phosphoribulokinase / Uridine kinase family | chr1:20864294-20867932 FORWARD | Aliases: F20N2.21, F20N2_21	5.1	5.4	-0.3	-1.1	100.0%	-4.7
6688	AT5G05900.1 | UDP-glucoronosyl/UDP-glucosyl transferase family protein, contains Pfam profile: PF00201 UDP-glucoronosyl and UDP-glucosyl transferase | chr5:1774514-1776382 FORWARD | Aliases: K18J17.5, K18J17_5	3.2	3.4	-0.2	-1.1	100.0%	-4.5
6689	AT4G33150.2 | Symbol: LKR | lysine-ketoglutarate reductase/saccharopine dehydrogenase bifunctional enzyme, identical to lysine-ketoglutarate reductase/saccharopine dehydrogenase GI:2052508 from (Arabidopsis thaliana) | chr4:15985201-15991541 REVERSE | Aliases: F4I10.80, F4I10_80, LYSINE KETOGLUTARATE REDUCTASE/SACCHAROPINE DEHYDROGENASE, SACCHAROPINE DEHYDROGENASE	7.7	7.3	0.4	1.1	100.0%	-4.4
6690	AT3G29180.1 | expressed protein | chr3:11149452-11152797 FORWARD | Aliases: MUO22.2	8.6	8.9	-0.3	-1.1	100.0%	-4.5
6691	AT1G28150.1 | expressed protein | chr1:9834930-9835886 REVERSE | Aliases: F13K9.24	7.7	7.3	0.3	1.1	100.0%	-4.3
6692	AT2G16365.2 | F-box family protein, contains Pfam:PF00646 F-box domain	5.9	5.5	0.4	1.1	100.0%	-4.2
6693	AT1G47400.1 | expressed protein | chr1:17388157-17388626 FORWARD | Aliases: T3F24.21, T3F24_21	3.9	4.3	-0.3	-1.1	100.0%	-4.3
6694	AT5G02770.1 | expressed protein | chr5:627117-629253 REVERSE | Aliases: F9G14.80, F9G14_80	4.2	3.9	0.4	1.1	100.0%	-4.4
6695	AT3G16910.1 | Symbol: AAE7/ACN1 | Encodes a peroxisomal protein with acetyl-CoA synthetase activity that is responsible for the activation of acetate for entry into the glyoxylate cycle. | chr3:5773061-5775507 REVERSE | Aliases: AAE7, AC NON UTILIZING 1, ACN1, ACYL ACTIVATING ENZYME 7, K14A17.23	8.5	7.8	0.7	1.1	100.0%	-4.1
6696	AT4G33440.1 | glycoside hydrolase family 28 protein / polygalacturonase (pectinase) family protein, weak similarity to SP:P27644 Polygalacturonase (EC 3.2.1.15) (Pectinase) {Agrobacterium tumefaciens}; contains PF00295: Glycosyl hydrolases family 28 | chr4:16091943-16094961 FORWARD | Aliases: F17M5.200, F17M5_200	4.8	5.2	-0.4	-1.1	100.0%	-4.3
6697	AT2G40700.1 | DEAD/DEAH box helicase, putative (RH17), identical to GB:CAA09207, contains a DEAD/DEAH box family ATP-dependent helicas signature; identical to cDNA DEAD box RNA helicase, RH17 GI:3776008 | chr2:16983861-16986636 FORWARD | Aliases: T7D17.12, T7D17_12	3.8	4.1	-0.3	-1.1	100.0%	-4.4
6698	AT1G24490.1 | Symbol: ARTEMIS | 60 kDa inner membrane family protein, similar to chloroplast membrane protein (ALBINO3) (GI:3927828) (Arabidopsis thaliana) | chr1:8678846-8685262 FORWARD | Aliases: ARABIDOPSIS THALIANA ENVELOPE MEMBRANE INTEGRASE, F21J9.16	5.9	6.3	-0.4	-1.1	100.0%	-4.3
6699	AT3G24030.1 | hydroxyethylthiazole kinase family protein, contains Pfam profile: PF02110 hydroxyethylthiazole kinase family | chr3:8679196-8680819 REVERSE | Aliases: F14O13.24	8.0	7.6	0.4	1.1	100.0%	-4.1

Rank	Description	15.dpi	5.dpi	M	t	adj.q	B
6700	AT1G64140.1 | expressed protein, similar to putative disease resistance protein GB:CAB40943 GI:4586107 from (Arabidopsis thaliana); weak similarity to Loricrin (Swiss-Prot:P23490) (Homo sapiens) | chr1:23807456-23810822 REVERSE | Aliases: F22C12.10, F22C12_10	7.1	6.5	0.5	1.1	100.0%	-4.1
6701	AT4G05150.1 | octicosapeptide/Phox/Bem1p (PB1) domain-containing protein, various predicted proteins contains Pfam profile PF00564: PB1 domain	5.8	5.3	0.5	1.1	100.0%	-4.1
6702	AT5G46400.1 | expressed protein | chr5:18837259-18841877 FORWARD | Aliases: MPL12.20, MPL12_20	3.2	3.3	-0.1	-1.1	100.0%	-4.9
6703	AT3G05410.1 | expressed protein | chr3:1554779-1555405 FORWARD | Aliases: F22F7.15, F22F7_15	3.2	3.0	0.2	1.1	100.0%	-4.6
6704	AT4G09160.1 | SEC14 cytosolic factor family protein / phosphoglyceride transfer family protein, similar to polyphosphoinositide binding protein Ssh1p (GI:2739044) {Glycine max}; similar to polyphosphoinositide binding protein Ssh2, Glycine max, gb:T05953; contains Pfam PF00650 : CRAL/TRIO domain; contains Pfam PF03765 : CRAL/TRIO, N-terminus | chr4:5839680-5842391 FORWARD | Aliases: T8A17.90, T8A17_90	5.2	4.9	0.3	1.1	100.0%	-4.3
6705	AT4G37250.1 | leucine-rich repeat family protein / protein kinase family protein, contains protein kinase domain, Pfam:PF00069; contains leucine-rich repeats, Pfam:PF00560 | chr4:17527644-17530500 REVERSE | Aliases: AP22.22, AP22_22	6.6	6.8	-0.2	-1.1	100.0%	-4.6
6706	AT3G62040.1 | similar to haloacid dehalogenase-like hydrolase family protein [Arabidopsis thaliana] (TAIR:At5g59490.1); similar to expressed protein [Oryza sativa (japonica cultivar-group)] (GB:XP_470321.1); contains InterPro domain HAD-superfamily hydrolase, subfamily IA, variant 3 (InterPro:IPR006402); contains InterPro domain Haloacid dehalogenase-like hydrolase (InterPro:IPR005834) | chr3:22986251-22987558 REVERSE | Aliases: F21F14.210	4.1	3.5	0.6	1.1	100.0%	-3.8
6707	AT2G47840.1 | tic20 protein-related, similar to Tic20 (GI:3769673) (Pisum sativum) | chr2:19601257-19602071 REVERSE | Aliases: F17A22.23	7.9	7.7	0.2	1.1	100.0%	-4.5
6708	AT4G36790.1 | transporter-related, low similarity to spinster membrane proteins from (Drosophila melanogaster) GI:12003976, GI:12003972, GI:12003974, GI:12003970; contains Pfam profile PF00083: major facilitator superfamily protein | chr4:17336177-17338376 FORWARD | Aliases: AP22.91, AP22_91	5.9	6.1	-0.2	-1.1	100.0%	-4.6
6709	AT1G54770.1 | expressed protein | chr1:20438329-20439675 FORWARD | Aliases: T22H22.18, T22H22_18	4.8	5.1	-0.3	-1.1	100.0%	-4.2
6710	AT3G01220.1 | homeobox-leucine zipper protein, putative / HD-ZIP transcription factor, putative, similar to homeobox-leucine zipper protein, HAT7 (GB:Q00466) (Arabidopsis thaliana) | chr3:73488-75545 FORWARD | Aliases: T4P13.9, T4P13_9	3.9	4.1	-0.2	-1.1	100.0%	-4.6
6711	AT3G60360.1 | expressed protein | chr3:22323285-22325375 REVERSE | Aliases: T8B10.20	6.1	6.7	-0.5	-1.1	100.0%	-4.2
6712	AT1G54840.2 | expressed protein | chr1:20456117-20457916 REVERSE | Aliases: T22H22.23, T22H22_23	3.8	4.0	-0.2	-1.1	100.0%	-4.7
6713	AT2G11000.1 | Symbol: ATMAK10 | similar to Unknown (protein for MGC:64157) [Danio rerio] (GB:AAH53286.1); contains InterPro domain Mak10 subunit, NatC N(alpha)-terminal acetyltransferase (InterPro:IPR007244) | chr2:4348205-4354938 REVERSE | Aliases: F15K19.7, F15K19_7, MAK10	6.3	5.9	0.4	1.1	100.0%	-4.3
6714	AT5G18800.2 | NADH-ubiquinone oxidoreductase 19 kDa subunit (NDUFA8) family protein, contains Pfam profile: PF05850 NADH-ubiquinone oxidoreductase 19 kDa subunit | chr5:6267067-6268616 FORWARD | Aliases: F17K4.50, F17K4_50	12.3	12.6	-0.2	-1.1	100.0%	-5.0
6715	AT2G34970.1 | eIF4-gamma/eIF5/eIF2-epsilon domain-containing protein, similar to SP:Q64350 Translation initiation factor eIF-2B epsilon subunit (eIF-2B GDP-GTP exchange factor) {Rattus norvegicus}; contains Pfam profile PF02020: eIF4-gamma/eIF5/eIF2-epsilon | chr2:14753386-14755860 FORWARD | Aliases: F19I3.20, F19I3_20	5.5	5.8	-0.4	-1.1	100.0%	-4.7
6716	AT2G13960.1 | myb family transcription factor, contains PFAM profile: PF00249 myb-like DNA binding domain | chr2:5866667-5868393 FORWARD | Aliases: F9B22.7, F9B22_7	5.2	4.7	0.5	1.1	100.0%	-3.9
6717	AT5G02320.1 | Symbol: MYB3R 5 | myb family transcription factor (MYB3R5), contains Pfam profile: PF00249 myb-like DNA binding domain; identical to cDNA putative c-myb-like transcription factor MYB3R-5 (MYB3R5) GI:15375300 | chr5:483121-486432 REVERSE | Aliases: T1E22.80, T1E22_80	5.2	4.7	0.5	1.1	100.0%	-3.9
6718	AT1G61560.1 | Symbol: MLO6 | seven transmembrane MLO family protein / MLO-like protein 6 (MLO6), idenctical to membrane protein Mlo6 (Arabidopsis thaliana) gi:14091582:gb:AAK53799; similar to MLO protein SWISS-PROT:P93766, NCBI_gi:1877221 (Hordeum vulgare)(Barley); contains Pfam profile	2.6	2.7	-0.1	-1.1	100.0%	-4.8
6719	AT5G15330.1 | SPX (SYG1/Pho81/XPR1) domain-containing protein, similar to PHO1 protein (Arabidopsis thaliana) GI:20069032; contains Pfam profile PF03105: SPX domain | chr5:4980491-4982234 FORWARD | Aliases: F8M21.220, F8M21_220	3.6	3.4	0.2	1.1	100.0%	-4.7
6720	AT2G38250.1 | DNA-binding protein-related, contains similarity to DNA-binding protein GI:170271 from (Nicotiana tabacum) | chr2:16025462-16026578 FORWARD | Aliases: F16M14.18, F16M14_18	5.0	4.4	0.5	1.1	100.0%	-4.3
6721	AT5G53530.1 | vacuolar protein sorting-associated protein 26, putative / VPS26, putative, similar to vacuolar sorting protein 26 (Homo sapiens) GI:9622852; contains Pfam profile PF03643: Vacuolar protein sorting-associated protein 26 | chr5:21763269-21765790 REVERSE | Aliases: MNC6.7, MNC6_7	7.1	6.7	0.4	1.1	100.0%	-4.3

Rank	Description	15.dpi	5.dpi	M	t	adj.q	B
6722	AT4G22510.1 | expressed protein | chr4:11855059-11857083 REVERSE | Aliases: F7K2.90, F7K2_90	3.6	3.8	-0.2	-1.1	100.0%	-4.8
6723	AT1G27595.1 | expressed protein, similar to Symplekin (SP:Q92797) {Homo sapiens} | chr1:9595480-9603242 FORWARD | Aliases: T22C5.3	3.1	3.3	-0.2	-1.1	100.0%	-4.8
6724	AT2G25625.2 | expressed protein | chr2:10913538-10914357 FORWARD | Aliases: None	3.5	3.4	0.2	1.1	100.0%	-4.8
6725	AT1G64350.1 | Symbol: SEH1H | transducin family protein / WD-40 repeat family protein, contains 5 WD-40 repeats (PF00400); similar to nuclear pore protein SEH1 (SP:P53011) (Saccharomyces cerevisiae) | chr1:23885175-23886960 FORWARD | Aliases: F15H21.21, seh1 homolog	5.0	5.3	-0.3	-1.1	100.0%	-4.5
6726	NA	4.2	3.7	0.5	1.1	100.0%	-4.6
6727	AT5G61780.1 | tudor domain-containing protein / nuclease family protein, contains Pfam domains PF00567: Tudor domain and PF00565: Staphylococcal nuclease homologue | chr5:24838484-24844195 FORWARD | Aliases: MAC9.14, MAC9_14	6.0	5.6	0.5	1.1	100.0%	-4.2
6728	AT5G65550.1 | UDP-glucoronosyl/UDP-glucosyl transferase family protein, contains Pfam profile: PF00201 UDP-glucoronosyl and UDP-glucosyl transferase ;similar to flavonol 3-O-glucosyltransferase (anthocyanin rhamnosyl transferase) from Petunia hybrida (SP:Q43716)	4.0	3.7	0.3	1.1	100.0%	-4.5
6729	AT5G41850.1 | expressed protein | chr5:16773926-16775019 REVERSE | Aliases: K16L22.14, K16L22_14	3.8	4.1	-0.2	-1.1	100.0%	-4.6
6730	AT4G29600.1 | cytidine deaminase, putative / cytidine aminohydrolase, putative, identical to cytidine deaminase 7 (CDA7) (Arabidopsis thaliana) GI:3818574, cytidine deaminase homolog DesC (Arabidopsis thaliana) GI:4836445; similar to cytidine deaminase (CDD) (Arabidopsis thaliana) GI:3046700; contains Pfam profile PF00383: Cytidine and deoxycytidylate deaminase zinc-binding | chr4:14515584-14516507 FORWARD | Aliases: T16L4.110, T16L4_110	3.0	3.2	-0.2	-1.1	100.0%	-4.9
6731	AT1G71890.1 | sucrose transporter / sucrose-proton symporter (SUC5), nearly identical to sucrose transporter (Arabidopsis thaliana) GI:12057172 | chr1:27062101-27064447 FORWARD | Aliases: F17M19.4, F17M19_4	3.2	3.4	-0.2	-1.1	100.0%	-4.5
6732	AT3G09900.1 | Ras-related GTP-binding protein, putative, similar to GTP-binding protein GI:871510 from (Pisum sativum); contains Pfam profile: PF00071 Ras family | chr3:3034567-3036596 FORWARD | Aliases: F8A24.5	7.8	8.0	-0.2	-1.1	100.0%	-4.4
6733	AT4G37580.1 | Symbol: HLS1 | N-acetyltransferase, putative / hookless1 (HLS1), contains Pfam profile PF00583: acetyltransferase, GNAT family; identical to cDNA putative N-acetyltransferase hookless1 (HLS1) GI:1277089 | chr4:17658606-17660729 FORWARD | Aliases: CONSTITUTIVE PHOTOMORPHOGENIC 3, COP3, F19F18.70, F19F18_70, HOOKLESS 1, UNS2	4.5	4.2	0.2	1.1	100.0%	-4.5
6734	AT1G68990.1 | DNA-directed RNA polymerase, mitochondrial (RPOMT), identical to SP:P92969 DNA-directed RNA polymerase, mitochondrial precursor (EC 2.7.7.6) {Arabidopsis thaliana} | chr1:25938307-25944629 REVERSE | Aliases: T6L1.17, T6L1_17	3.9	4.1	-0.2	-1.1	100.0%	-4.6
6735	AT1G03950.1 | SNF7 family protein, contains Pfam domain, PF03357: SNF7 family | chr1:1011235-1013359 REVERSE | Aliases: F21M11.12, F21M11_12	5.6	5.0	0.6	1.1	100.0%	-4.0
6736	AT3G60950.1 | C2 domain-containing protein, similar to CLB1 (Lycopersicon esculentum) GI:2789434; contains Pfam profile PF00168: C2 domain | chr3:22535450-22540489 FORWARD | Aliases: T27I15.40	2.9	3.1	-0.3	-1.1	100.0%	-4.6
6737	AT3G61030.1 | C2 domain-containing protein, similar to CLB1 (Lycopersicon esculentum) GI:2789434; contains Pfam profile PF00168: C2 domain | chr3:22596630-22601670 FORWARD | Aliases: T27I15.120	2.9	3.1	-0.3	-1.1	100.0%	-4.6
6738	AT3G61050.2 | similar to C2 domain-containing protein [Arabidopsis thaliana] (TAIR:At3g60950.1); similar to C2 domain-containing protein [Arabidopsis thaliana] (TAIR:At3g61030.1); similar to putative CLB1 protein (calcium-dependent lipid binding) protein [Oryza sativa (japonica cultivar-group)] (GB:XP_477665.1); contains InterPro domain C2 domain (InterPro:IPR000008) | chr3:22608202-22612124 FORWARD | Aliases: T27I15.140	2.9	3.1	-0.3	-1.1	100.0%	-4.6
6739	AT5G41140.1 | expressed protein | chr5:16485675-16489774 FORWARD | Aliases: MEE6.21, MEE6_21	3.1	3.7	-0.6	-1.1	100.0%	-4.2
6740	AT2G44690.1 | Symbol: ARAC9 | Rac-like GTP-binding protein (ARAC9), identical to rac-like protein ARAC9 GI:5381419 from (Arabidopsis thaliana) | chr2:18436339-18437879 FORWARD | Aliases: F16B22.18	5.0	4.8	0.2	1.0	100.0%	-4.4
6741	AT2G33730.1 | DEAD box RNA helicase, putative, similar to SP:P23394 Pre-mRNA splicing factor RNA helicase PRP28 {Saccharomyces cerevisiae}; contains Pfam profiles PF00270: DEAD/DEAH box helicase, PF00271: Helicase conserved C-terminal domain | chr2:14272526-14275048 REVERSE | Aliases: T1B8.4, T1B8_4	8.0	8.2	-0.2	-1.0	100.0%	-4.8
6742	AT5G65180.2 | expressed protein, contains Pfam domain, PF04818: Protein of unknown function, DUF618 | chr5:26062602-26065257 FORWARD | Aliases: MQN23.12, MQN23_12	4.3	4.6	-0.4	-1.0	100.0%	-4.5
6743	AT5G59430.2 | telomere repeat-binding protein 1 (TRP1), identical to telomere repeat-binding protein TRP1 (Arabidopsis thaliana) GI:5459298 | chr5:23984541-23988171 FORWARD | Aliases: F2O15.20, F2O15_20	2.5	2.7	-0.2	-1.0	100.0%	-4.6

Rank	Description	15.dpi	5.dpi	M	t	adj.q	B
6744	AT1G76620.1 | expressed protein, contains Pfam profile PF04784: Protein of unknown function, DUF547 | chr1:28761453-28764191 FORWARD | Aliases: F14G6.22, F14G6_22	2.5	2.3	0.1	1.0	100.0%	-5.0
6745	AT1G15280.2 | glycine-rich protein | chr1:5252332-5256746 FORWARD | Aliases: F9L1.22, F9L1_22	3.7	3.5	0.2	1.0	100.0%	-4.5
6746	AT1G11160.1 | WD-40 repeat family protein / katanin p80 subunit, putative, similar to contains 6 WD-40 repeats (PF00400); katanin p80 subunit (GI:3005601) (Strongylocentrotus purpuratus) | chr1:3733925-3739703 FORWARD | Aliases: T28P6.17, T28P6_17	2.6	2.8	-0.2	-1.0	100.0%	-4.8
6747	AT1G04100.1 | Symbol: IAA10 | auxin-responsive protein / indoleacetic acid-induced protein 10 (IAA10), identical to SP:Q38828 Auxin-responsive protein IAA10 (Indoleacetic acid-induced protein 10) {Arabidopsis thaliana} | chr1:1059505-1061190 FORWARD | Aliases: AUXIN INDUCED PROTEIN 10, F20D22.13, F20D22_13	4.6	4.2	0.3	1.0	100.0%	-4.2
6748	AT1G21440.1 | mutase family protein, similar to carboxyvinyl-carboxyphosphonate phosphorylmutase GB:O49290 from (Arabidopsis thaliana); similar to carboxyphosphonoenolpyruvate mutase (GI:47149) (Streptomyces hygroscopicus); contains Prosite PS00161: Isocitrate lyase signature | chr1:7502159-7504140 REVERSE | Aliases: F24J8.7, F24J8_7	9.8	10.4	-0.6	-1.0	100.0%	-4.7
6749	AT5G52230.1 | Symbol: MBD13 | expressed protein | chr5:21225116-21229035 REVERSE | Aliases: F17P19.13, F17P19_13	2.4	2.6	-0.2	-1.0	100.0%	-4.9
6750	AT1G68110.1 | epsin N-terminal homology (ENTH) domain-containing protein / clathrin assembly protein-related, similar to clathrin assembly protein AP180 (GI:6492344) (Xenopus laevis); contains Pfam profile: PF01417 ENTH domain, suggesting involvement in endocytosis or cytoskeletal machinery | chr1:25528381-25529813 FORWARD | Aliases: T23K23.4, T23K23_4	3.0	2.8	0.2	1.0	100.0%	-4.8
6751	ATCG00140.1 | Symbol: ATPH | ATPase III subunit | chrC:13262-13507 REVERSE | Aliases: ATPH	9.3	8.7	0.5	1.0	100.0%	-4.3
6752	AT4G19900.1 | glycosyl transferase-related, contains Pfam profiles PF01535: PPR repeat, PF04572: Alpha 1,4-glycosyltransferase conserved region, PF04488: Glycosyltransferase sugar-binding region containing DXD motif; several hypothetical proteins - Arabidopsis thaliana | chr4:10786958-10791443 REVERSE | Aliases: F18F4.6, F18F4_6	3.6	3.9	-0.3	-1.0	100.0%	-4.5
6753	AT5G57710.1 | heat shock protein-related, contains similarity to 101 kDa heat shock protein; HSP101 (Triticum aestivum) gi:11561808:gb:AAC83689 | chr5:23402020-23405579 FORWARD | Aliases: MRI1.7, MRI1_7	6.8	6.4	0.3	1.0	100.0%	-4.4
6754	AT3G17365.1 | expressed protein, low similarity to PIR:I46078 endothelin converting enzyme from Bos primigenius taurus	5.9	6.3	-0.3	-1.0	100.0%	-4.7
6755	AT2G19880.1 | ceramide glucosyltransferase, putative, similar to ceramide glucosyltransferase (GI:14718995) (Gossypium arboreum); weak similarity to Ceramide glucosyltransferase (Glucosylceramide synthase) (GCS) (UDP-glucose:N-acylsphingosine D-glucosyltransferase) (GLCT-1) (Swiss-Prot:Q16739) (Homo sapiens)	3.8	3.6	0.2	1.0	100.0%	-4.6
6756	AT5G40490.1 | RNA recognition motif (RRM)-containing protein, ribonucleoprotein, Xenopus laevis, PIR:S40778; contains InterPro entry IPR000504: RNA-binding region RNP-1 (RNA recognition motif) (RRM)	9.1	8.6	0.5	1.0	100.0%	-4.3
6757	AT5G65440.1 | expressed protein | chr5:26168668-26174324 FORWARD | Aliases: MNA5.17, MNA5_17	5.1	5.4	-0.3	-1.0	100.0%	-4.5
6758	AT2G34470.2 | Symbol: UREG | similar to urease accessory protein G [Solanum tuberosum] (GB:CAC33002.1); similar to urease accessory protein G [Solanum tuberosum] (GB:CAC33000.1); contains InterPro domain Urease accessory protein UreG (InterPro:IPR004400); contains InterPro domain HypB/UreG, nucleotide-binding (InterPro:IPR002894) | chr2:14537814-14539584 REVERSE | Aliases: PSKF109, T31E10.19, T31E10_19, UREASE ACCESSORY PROTEIN UREG	10.1	10.4	-0.4	-1.0	100.0%	-4.7
6759	AT3G01320.1 | similar to paired amphipathic helix repeat-containing protein [Arabidopsis thaliana] (TAIR:At1g70060.1); similar to paired amphipathic helix repeat-containing protein [Arabidopsis thaliana] (TAIR:At1g59890.1); similar to paired amphipathic helix repeat-containing protein [Arabidopsis thaliana] (TAIR:At5g15020.1); similar to paired amphipathic helix repeat-containing protein [Arabidopsis thaliana] (TAIR:At1g24190.1); similar to transcriptional co-repressor -like [Oryza sativa (japonica cultivar-group)] (GB:XP_549869.1); contains InterPro domain Paired amphipathic helix (InterPro:IPR003822) | chr3:106662-113625 FORWARD | Aliases: T22N4.5, T22N4_5	5.6	6.3	-0.7	-1.0	100.0%	-4.4
6760	AT4G11920.1 | WD-40 repeat family protein, contains 6 WD repeats (PF00400); similar to Fzr1 (GI:6463679) {Homo sapiens}; similar to WD repeat protein Srw1 -Schizosaccharomyces pombe,PID:d1023012 | chr4:7160270-7163376 REVERSE | Aliases: T26M18.130, T26M18_130	4.7	4.4	0.2	1.0	100.0%	-4.5
6761	AT4G39660.1 | Symbol: AGT2 | alanine--glyoxylate aminotransferase, putative / beta-alanine-pyruvate aminotransferase, putative / AGT, putative, similar to SP:Q64565 Alanine--glyoxylate aminotransferase 2, mitochondrial precursor (EC 2.6.1.44) (AGT 2) (Beta-alanine-pyruvate aminotransferase) {Rattus norvegicus}; contains Pfam profile PF00202: aminotransferase, class III | chr4:18406767-18409466 FORWARD | Aliases: ALANINE:GLYOXYLATE AMINOTRANSFERASE 2, T19P19.50, T19P19_50	9.6	9.1	0.5	1.0	100.0%	-4.2
6762	AT4G16790.1 | hydroxyproline-rich glycoprotein family protein, contains proline-rich extensin domains, INTERPRO:IPR002965 | chr4:9451604-9453273 REVERSE | Aliases: DL4420C, FCAALL.137	4.0	3.8	0.2	1.0	100.0%	-4.8

Rank	Description	15.dpi	5.dpi	M	t	adj.q	B
6763	AT3G55520.1 | immunophilin, putative / FKBP-type peptidyl-prolyl cis-trans isomerase, putative, POSSIBLE PEPTIDYL-PROLYL CIS-TRANS ISOMERASE) (EC 5.2.1.8) (PPIASE) (ROTAMASE) SP:P30416(Mouse);P59 PROTEIN (HSP BINDING IMMUNOPHILIN), rabbit, SWISSPROT:P27124:FKB4_RABBIT | chr3:20604691-20606324 FORWARD | Aliases: T22E16.180	10.7	10.3	0.4	1.0	100.0%	-4.5
6764	AT2G21520.1 | similar to SEC14 cytosolic factor, putative / phosphoglyceride transfer protein, putative [Arabidopsis thaliana] (TAIR:At4g39170.1); similar to putative hosphatidylinositol/phophatidylcholine transfer protein [Oryza sativa (japonica cultivar-group)] (GB:XP_464026.1); contains InterPro domain Cellular retinaldehyde-binding)/triple function, C-terminal (InterPro:IPR001251); contains InterPro domain Cellular retinaldehyde binding/alpha-tocopherol transport (InterPro:IPR001071); contains InterPro domain Cellular retinaldehyde-binding/triple function, N-terminal (InterPro:IPR008273)	6.9	7.4	-0.5	-1.0	100.0%	-4.1
6765	AT4G33080.2 | similar to protein kinase, putative [Arabidopsis thaliana] (TAIR:At2g19400.1); similar to protein kinase [Raphanus sativus] (GB:BAC76895.1); similar to putative serine/threonine kinase 38 [Oryza sativa (japonica cultivar-group)] (GB:BAD72247.1); similar to unnamed protein product [Oryza sativa (japonica cultivar-group)] (GB:NP_914515.1); similar to protein kinase [Spinacia oleracea] (GB:CAA82991.1); contains InterPro domain Serine/threonine protein kinase, active site (InterPro:IPR008271); contains InterPro domain Tyrosine protein kinase (InterPro:IPR001245); contains InterPro domain Serine/threonine protein kinase (InterPro:IPR002290); contains InterPro domain Protein kinase (InterPro:IPR000719) | chr4:15959965-15963980 FORWARD | Aliases: F4I10.10, F4I10_10	5.1	4.8	0.3	1.0	100.0%	-4.4
6766	AT1G23740.1 | oxidoreductase, zinc-binding dehydrogenase family protein, contains Pfam profile PF00107: oxidoreductase, zinc-binding dehydrogenase family | chr1:8398115-8399717 REVERSE | Aliases: F5O8.29, F5O8_29	8.4	8.0	0.4	1.0	100.0%	-4.0
6767	AT1G14750.1 | Symbol: SDS | cyclin, putative (SDS), identical to cyclin-like protein (Arabidopsis thaliana) GI:20302467; low similarity to SP:P30278 G2/mitotic-specific cyclin 2 (B-like cyclin) (CycMs2 {Medicago sativa}; contains Pfam profiles PF00134: Cyclin, N-terminal domain, PF02984: Cyclin, C-terminal domain | chr1:5079402-5082515 REVERSE | Aliases: F10B6.15, F10B6_15, SOLO DANCERS	2.9	2.8	0.1	1.0	100.0%	-5.2
6768	AT3G45500.1 | expressed protein | chr3:16699403-16700678 REVERSE | Aliases: F9K21.80	2.6	2.9	-0.2	-1.0	100.0%	-4.8
6769	AT2G16340.1 | hypothetical protein | chr2:7080216-7080566 FORWARD | Aliases: F16F14.16, F16F14_16	6.6	6.2	0.4	1.0	100.0%	-4.4
6770	AT2G29420.1 | Symbol: ATGSTU7 | glutathione S-transferase, putative | chr2:12625013-12625976 REVERSE | Aliases: F16P2.20, F16P2_20, GLUTATHIONE S TRANSFERASE 25, GST25	9.2	8.8	0.4	1.0	100.0%	-4.5
6771	AT3G59810.1 | small nuclear ribonucleoprotein F, putative / U6 snRNA-associated Sm-like protein, putative / Sm protein F, putative, similar to SWISS-PROT:Q9Y4Y8 U6 snRNA-associated Sm-like protein LSm6 (Mus musculus) | chr3:22107492-22108885 FORWARD | Aliases: F24G16.80	8.2	7.8	0.4	1.0	100.0%	-4.3
6772	AT5G65160.1 | tetratricopeptide repeat (TPR)-containing protein, contains Pfam profile PF00515 TPR Domain | chr5:26048419-26051040 REVERSE | Aliases: MQN23.9, MQN23_9	4.4	4.1	0.3	1.0	100.0%	-4.3
6773	AT4G30810.1 | Symbol: SCPL29 |	8.9	9.3	-0.4	-1.0	100.0%	-4.4
6774	AT1G30160.2 | expressed protein, similar to expressed protein [Arabidopsis thaliana] (TAIR:At1g05540.1); contains InterPro domain Protein of unknown function DUF295 (InterPro:IPR005174) | chr1:10606235-10607768 FORWARD | Aliases: T2H7.4, T2H7_4	3.1	2.9	0.2	1.0	100.0%	-4.9
6775	AT5G32590.1 | myosin heavy chain-related, similar to Myosin heavy chain, non-muscle (Zipper protein) (Myosin II)(SP:Q99323) {Drosophila melanogaster} | chr5:12238819-12241552 REVERSE | Aliases: F3D18.80, F3D18_80	2.5	2.4	0.1	1.0	100.0%	-5.3
6776	AT2G01510.1 | pentatricopeptide (PPR) repeat-containing protein, contains Pfam profile PF01535: PPR repeat | chr2:230751-232505 REVERSE | Aliases: F2I9.13, F2I9_13	3.2	3.4	-0.2	-1.0	100.0%	-4.7
6777	AT5G47780.1 | glycosyl transferase family 8 protein, contains Pfam profile: PF01501 glycosyl transferase family 8; strong similarity to unknown protein (emb:CAB71043.1) | chr5:19365145-19367895 FORWARD | Aliases: MCA23.10, MCA23_10	7.7	8.0	-0.3	-1.0	100.0%	-4.6
6778	AT4G23010.1 | UDP-galactose transporter-related, contains weak similarity to UDP-galactose transporter related isozyme 1 (GI:1669562) (Mus musculus) | chr4:12060179-12062827 REVERSE | Aliases: F7H19.200, F7H19_200	6.5	6.0	0.5	1.0	100.0%	-4.3
6779	AT3G20150.1 | kinesin motor family protein, contains Pfam domain, PF00225: Kinesin motor domain | chr3:7031418-7036505 FORWARD | Aliases: MAL21.18	2.6	2.9	-0.3	-1.0	100.0%	-4.4
6780	AT1G67890.1 | protein kinase family protein, contains Pfam domain, PF00069: Protein kinase domain | chr1:25460842-25466455 FORWARD | Aliases: T23K23.26, T23K23_26	5.6	6.1	-0.5	-1.0	100.0%	-4.2
6781	AT1G66100.1 | thionin, putative, similar to thionin (Arabidopsis thaliana) GI:1181533 | chr1:24609334-24610200 REVERSE | Aliases: F15E12.20, F15E12_20	2.5	2.6	-0.2	-1.0	100.0%	-4.8

Rank	Description	15.dpi	5.dpi	M	t	adj.q	B
6782	AT1G31380.1 | hypothetical protein | chr1:11240432-11241080 REVERSE | Aliases: T8E3.3	2.7	2.8	-0.1	-1.0	100.0%	-5.1
6783	AT5G50410.1 | expressed protein | chr5:20545425-20547053 REVERSE | Aliases: MXI22.13, MXI22_13	6.9	6.4	0.4	1.0	100.0%	-3.9
6784	AT4G17170.1 | Symbol: AT RAB2 | Rab2-like GTP-binding protein (RAB2), identical to Rab2-like protein (At-RAB2) GI:1765896 from (Arabidopsis thaliana)	7.4	7.0	0.4	1.0	100.0%	-4.4
6785	AT4G20020.2 | expressed protein | chr4:10844141-10846133 REVERSE | Aliases: F18F4.120, F18F4_120	5.0	5.3	-0.2	-1.0	100.0%	-4.7
6786	AT4G18060.1 | SH3 domain-containing protein 3 (SH3P3), nearly identical to SH3 domain-containing protein 3 (Arabidopsis thaliana) GI:16974680; contains Pfam profile PF00018: SH3 domain | chr4:10027492-10029866 REVERSE | Aliases: F15J5.30, F15J5_30	5.5	5.9	-0.4	-1.0	100.0%	-4.1
6787	AT4G00240.1 | Symbol: PLDBETA2 | phospholipase D beta 2 / PLD beta 2 (PLDBETA2) / PLDdelta1, identical to SP:O23078 Phospholipase D beta 2 (EC 3.1.4.4) (AtPLDbeta2) (PLD beta 2) (PLDdelta1) (Arabidopsis thaliana); contains Pfam profiles: PF00614 phospholipase D.active site motif, PF00168 C2 domain | chr4:106380-110718 REVERSE | Aliases: F5I10.13	5.9	6.3	-0.3	-1.0	100.0%	-4.4
6788	AT1G55190.1 | prenylated rab acceptor (PRA1) family protein, contains Pfam profile PF03208: Prenylated rab acceptor (PRA1) | chr1:20592064-20592866 FORWARD | Aliases: F7A10.20, F7A10_20	5.3	5.6	-0.3	-1.0	100.0%	-4.3
6789	AT5G19720.1 | tRNA synthetase class I (E and Q) family protein, similar to tRNA-glutamine synthetase (Lupinus luteus) GI:2995455; contains Pfam profile PF03950: tRNA synthetases class I (E and Q), anti-codon binding domain	3.7	3.9	-0.2	-1.0	100.0%	-4.7
6790	AT1G06310.1 | Symbol: ACX6 | Encodes a putative acyl-CoA oxidase. However, no transcripts have been detected for this gene and no altered phenotypes have been detected in plants mutant for this gene. This suggests that ACX6 does not significantly contribute to seedling beta-oxidation of fatty acids or indole-3-butyric acid in vivo. | chr1:1926790-1930257 FORWARD | Aliases: ACX6, ACYL COA OXIDASE 6, T2D23.2, T2D23_2	2.3	2.5	-0.2	-1.0	100.0%	-5.0
6791	AT5G43860.1 | Symbol: ATCLH2 |	3.5	3.2	0.2	1.0	100.0%	-4.6
6792	AT4G33350.1 | chloroplast inner membrane import protein Tic22, putative, similar to Tic22 (Pisum sativum) gi:3769671:gb:AAC64606 | chr4:16064552-16066740 REVERSE | Aliases: F17M5.110, F17M5_110	6.0	6.5	-0.4	-1.0	100.0%	-4.2
6793	AT1G08490.1 | cysteine desulfurase, putative, similar to nitrogen fixation protein (nifS) GB:D64004 GI:1001701 from (Synechocystis sp); contains TIGRFAM TIGR01364: phosphoserine aminotransferase; contains Pfam PF00266: aminotransferase, class V | chr1:2685662-2688592 REVERSE | Aliases: T27G7.17, T27G7_17	5.9	5.6	0.3	1.0	100.0%	-4.2
6794	AT2G47960.1 | expressed protein | chr2:19632684-19636180 FORWARD | Aliases: F17A22.35	6.1	6.4	-0.4	-1.0	100.0%	-4.3
6795	AT2G17310.1 | Symbol: SON1 | F-box family protein, contains F-box domain Pfam:PF00646	3.3	3.1	0.2	1.0	100.0%	-4.6
6796	AT2G48130.1 | protease inhibitor/seed storage/lipid transfer protein (LTP) family protein, contains Pfam protease inhibitor/seed storage/LTP family domain PF00234 | chr2:19692138-19693063 REVERSE | Aliases: F11L15.3	2.7	3.1	-0.5	-1.0	100.0%	-3.8
6797	AT1G71080.1 | expressed protein | chr1:26813503-26815427 REVERSE | Aliases: F23N20.7, F23N20_7	8.7	8.1	0.6	1.0	100.0%	-4.2
6798	AT1G04340.1 | lesion inducing protein-related, similar to ORF, able to induce HR-like lesions (Nicotiana tabacum) | chr1:1163096-1164850 REVERSE | Aliases: F19P19.23, F19P19_23	7.3	6.9	0.4	1.0	100.0%	-4.4
6799	AT3G17620.1 | F-box family protein, contains Pfam profile: PF00646 F-box domain | chr3:6026452-6027700 REVERSE | Aliases: MKP6.18	3.3	3.4	-0.1	-1.0	100.0%	-4.9
6800	AT4G20910.1 | Symbol: HEN1 | double-stranded RNA binding protein-related / DsRBD protein-related, contains weak similarity to Pfam profile PF00035: Double-stranded RNA binding motif	3.4	3.7	-0.2	-1.0	100.0%	-4.5
6801	AT5G10930.1 | Symbol: CIPK5 | CBL-interacting protein kinase 5 (CIPK5), identical to CBL-interacting protein kinase 5 GP:9280632:gb:AAF86504 (Arabidopsis thaliana) | chr5:3445367-3447115 REVERSE | Aliases: CBL INTERACTING PROTEIN KINASE 5, T30N20.200, T30N20_200	5.3	5.6	-0.3	-1.0	100.0%	-4.5
6802	AT1G54510.1 | protein kinase family protein, contains protein kinase domain, Pfam:PF00069; contains serine/threonine protein kinase domain, INTERPRO:IPR002290	5.0	5.4	-0.3	-1.0	100.0%	-4.5
6803	AT3G06330.2 | similar to zinc finger (C3HC4-type RING finger) family protein [Arabidopsis thaliana] (TAIR:At5g18760.1); similar to zinc finger (C3HC4-type RING finger) protein-like [Oryza sativa (japonica cultivar-group)] (GB:XP_464222.1); contains InterPro domain Zn-finger, RING (InterPro:IPR001841) | chr3:1917246-1919931 FORWARD | Aliases: F24P17.21, F24P17_21	4.7	4.2	0.4	1.0	100.0%	-4.1

Rank	Description	15.dpi	5.dpi	M	t	adj.q	B
6804	AT3G05830.1 | Encodes alpha-helical IF (intermediate filament)-like protein. | chr3:1736686-1738670 FORWARD | Aliases: F10A16.12, F10A16_12	3.5	3.9	-0.5	-1.0	100.0%	-4.3
6805	AT1G64150.1 | expressed protein, contains Pfam profile PF01169: Uncharacterized protein family UPF0016	4.7	4.5	0.3	1.0	100.0%	-4.3
6806	AT5G55810.1 | nicotinamide-nucleotide adenylyltransferase, putative / NAD(+) pyrophosphorylase, putative, similar to nicotinamide mononucleotide adenylyl transferase (Homo sapiens) GI:11245478; contains Pfam profile PF01467: Cytidylyltransferase | chr5:22601294-22603520 REVERSE | Aliases: MDF20.25, MDF20_25	3.6	3.4	0.3	1.0	100.0%	-4.3
6807	AT3G42725.1 | expressed protein | chr3:14853888-14854695 REVERSE | Aliases: None	2.5	2.7	-0.1	-1.0	100.0%	-5.3
6808	AT5G14910.1 | heavy-metal-associated domain-containing protein, Pfam profile PF00403: Heavy-metal-associated domain | chr5:4823717-4825373 FORWARD | Aliases: F2G14.30, F2G14_30	6.3	6.8	-0.5	-1.0	100.0%	-4.0
6809	AT3G47430.1 | peroxisomal biogenesis factor 11 family protein / PEX11 family protein, contains Pfam PF05648: Peroxisomal biogenesis factor 11 (PEX11) | chr3:17491682-17492777 FORWARD | Aliases: T21L8.180	2.5	2.4	0.1	1.0	100.0%	-5.1
6810	AT5G44790.1 | Symbol: RAN1 | copper-exporting ATPase / responsive-to-antagonist 1 / copper-transporting ATPase (RAN1), identical to SP:Q9S7J8	9.2	8.7	0.5	1.0	100.0%	-4.4
6811	AT2G39080.1 | expressed protein | chr2:16316907-16319587 REVERSE | Aliases: T7F6.25, T7F6_25	6.3	6.5	-0.2	-1.0	100.0%	-4.7
6812	AT4G39530.1 | pentatricopeptide (PPR) repeat-containing protein, contains Pfam profile PF01535: PPR repeat | chr4:18374730-18377234 REVERSE | Aliases: F23K16.160, F23K16_160	2.9	3.1	-0.2	-1.0	100.0%	-4.8
6813	NA	6.2	5.7	0.5	1.0	100.0%	-4.7
6814	AT1G02305.1 | cathepsin B-like cysteine protease, putative, similar to cathepsin B-like cysteine proteinase (Nicotiana rustica) GI:609175; contains Pfam profile PF00112: Papain family cysteine protease | chr1:455778-458124 FORWARD | Aliases: None	10.5	9.8	0.6	1.0	100.0%	-4.7
6815	AT4G08430.1 | Ulp1 protease family protein, similar to At5g45570, At5g28235; contains Pfam profile PF02902: Ulp1 protease family, C-terminal catalytic domain | chr4:5347393-5350433 REVERSE | Aliases: C18G5.10, C18G5_10	2.7	2.6	0.1	1.0	100.0%	-5.3
6816	AT5G41330.1 | potassium channel tetramerisation domain-containing protein, contains Pfam profile PF02214: K+ channel tetramerisation domain | chr5:16552567-16555131 REVERSE | Aliases: MYC6.4, MYC6_4	4.4	3.9	0.5	1.0	100.0%	-4.2
6817	AT3G03300.1 | Symbol: DCL2 | DEAD/DEAH box helicase carpel factory-related, similar to RNA helicase GB:AAF03534 | chr3:768027-774834 REVERSE | Aliases: T17B22.28	4.8	5.3	-0.5	-1.0	100.0%	-4.6
6818	AT3G18910.1 | F-box family protein, contains Pfam:PF00646 F-box domain ; contains TIGRFAM TIGR01640: F-box protein interaction domain ; contains TIGRFAM TIGR01640: F-box protein interaction domain | chr3:6521766-6523049 FORWARD | Aliases: K13E13.1	5.2	5.0	0.2	1.0	100.0%	-4.5
6819	AT1G04120.1 | Symbol: ATMRP5 |	9.9	9.5	0.4	1.0	100.0%	-4.4
6820	AT1G70630.1 | expressed protein | chr1:26634895-26637759 FORWARD | Aliases: F24J13.20, F24J13_20	2.3	2.2	0.1	1.0	100.0%	-5.4
6821	AT1G20010.1 | Symbol: TUB5 | tubulin beta-5 chain (TUB5), nearly identical to SP:P29513 Tubulin beta-5 chain {Arabidopsis thaliana} | chr1:6937786-6940573 REVERSE | Aliases: T20H2.21, T20H2_21	6.4	6.0	0.4	1.0	100.0%	-4.5
6822	AT2G03690.1 | coenzyme Q biosynthesis Coq4 family protein / ubiquinone biosynthesis Coq4 family protein, contains Pfam profile PF05019: Coenzyme Q (ubiquinone) biosynthesis protein Coq4 | chr2:1121897-1123168 REVERSE | Aliases: F19B11.14, F19B11_14	6.3	5.9	0.4	1.0	100.0%	-4.2
6823	AT3G09380.1 | expressed protein, contains Pfam profile PF01883: Domain of unknown function | chr3:2885271-2886122 REVERSE | Aliases: F3L24.26	3.4	3.2	0.2	1.0	100.0%	-5.0
6824	AT1G29440.1 | auxin-responsive family protein, similar to auxin-induced protein 6B (SP:P33083) (Glycine max) | chr1:10304557-10304955 REVERSE | Aliases: F15D2.3, F15D2_3	2.3	2.2	0.1	1.0	100.0%	-5.7
6825	AT1G77510.1 | Symbol: ATPDIL1 2 | Encodes a protein disulfide isomerase-like (PDIL) protein, a member of a multigene family within the thioredoxin (TRX) superfamily.	8.1	8.8	-0.7	-1.0	100.0%	-4.3
6826	AT1G03060.1 | WD-40 repeat family protein / beige-related, similar to BEIGE (GI:3928547) (Rattus norvegicus); Similar to gb:U70015 lysosomal trafficking regulator from Mus musculus and contains 2 Pfam PF00400 WD-40, G-beta repeats. ESTs gb:T43386 and gb:AA395236 come from this gene | chr1:712473-726891 REVERSE | Aliases: F10O3.12, F10O3_12	4.5	4.9	-0.4	-1.0	100.0%	-4.4

Rank	Description	15.dpi	5.dpi	M	t	adj.q	B
6827	AT3G46920.1 | protein kinase family protein, similar to MAP3K delta-1 protein kinase (Arabidopsis thaliana) GI:2253010; contains Pfam profile: PF00069 Eukaryotic protein kinase domain | chr3:17291415-17295842 REVERSE | Aliases: T6H20.50	4.8	5.1	-0.4	-1.0	100.0%	-4.2
6828	AT3G06570.1 | kelch repeat-containing F-box family protein, contains F-box domain Pfam:PF00646 and Kelch motif Pfam:PF01344 | chr3:2047311-2048559 REVERSE | Aliases: F5E6.10, F5E6_10	6.6	6.3	0.3	1.0	100.0%	-4.3
6829	AT1G12400.1 | expressed protein | chr1:4221861-4222835 FORWARD | Aliases: F5O11.12, F5O11_12	7.9	8.3	-0.5	-1.0	100.0%	-4.1
6830	AT5G12470.1 | expressed protein | chr5:4044625-4047439 REVERSE | Aliases: None	10.8	10.4	0.4	1.0	100.0%	-4.7
6831	AT1G07140.1 | Symbol: SIRANBP | Ran-binding protein 1a (RanBP1a), identical to Ran-binding protein (atranbp1a) GI:2058282 from (Arabidopsis thaliana)	11.3	11.8	-0.4	-1.0	100.0%	-4.8
6832	AT1G55840.1 | SEC14 cytosolic factor (SEC14) / phosphoglyceride transfer protein, similar to polyphosphoinositide binding protein SEC14 homolog Ssh1p (GB:AAB94598) (Glycine max); identified in Eur J Biochem 1998 Dec 1;258(2):402-10 as AtSEC14, characterized by functional complementation in S. cerevisiae. | chr1:20877268-20879938 FORWARD | Aliases: F14J16.8, F14J16_8	9.2	9.9	-0.6	-1.0	100.0%	-4.5
6833	AT3G10740.1 | Symbol: ASD1 | glycosyl hydrolase family protein 51, similar to arabinoxylan arabinofuranohydrolase isoenzyme AXAH-II from GI:13398414 (Hordeum vulgare) | chr3:3360893-3365368 REVERSE | Aliases: T7M13.18	6.8	7.4	-0.6	-1.0	100.0%	-4.1
6834	AT1G20020.1 | ferredoxin--NADP(+) reductase, putative / adrenodoxin reductase, putative, strong similarity to Ferredoxin--NADP reductase, chloroplast precursor (EC 1.18.1.2) (FNR) from {Pisum sativum} SP:P10933, {Mesembryanthemum crystallinum} SP:P41343, {Spinacia oleracea} SP:P00455, (Capsicum annuum) GI:6899972 | chr1:6942796-6945006 FORWARD | Aliases: T20H2.20, T20H2_20	5.7	6.2	-0.5	-1.0	100.0%	-4.0
6835	AT5G14860.1 | UDP-glucoronosyl/UDP-glucosyl transferase family protein, contains Pfam profile: PF00201 UDP-glucoronosyl and UDP-glucosyl transferase | chr5:4805890-4807762 FORWARD | Aliases: T9L3.160, T9L3_160	3.9	4.3	-0.3	-1.0	100.0%	-4.4
6836	AT3G63010.1 | expressed protein, similar to PrMC3 (Pinus radiata) GI:5487873 | chr3:23300400-23302459 FORWARD | Aliases: T20O10.110	4.1	3.8	0.2	1.0	100.0%	-4.5
6837	AT5G15580.1 | expressed protein, unknown protein F14P3.18 - Arabidopsis thaliana, EMBL:AC009755 | chr5:5068304-5072495 REVERSE | Aliases: T20K14.190, T20K14_190	3.5	3.8	-0.3	-1.0	100.0%	-4.4
6838	AT1G48380.1 | Symbol: RHL1 | root hair initiation protein root hairless 1 (RHL1) | chr1:17881081-17882938 REVERSE | Aliases: F11A17.7, F11A17_7, ROOT HAIRLESS 1	6.4	6.1	0.3	1.0	100.0%	-4.1
6839	AT4G36900.1 | Symbol: RAP2.10 | encodes a member of the DREB subfamily A-5 of ERF/AP2 transcription factor family (RAP2.10). The protein contains one AP2 domain. There are 16 members in this subfamily including RAP2.9 and RAP2.1.	6.6	6.2	0.4	1.0	100.0%	-4.3
6840	AT5G18580.1 | Symbol: FASS | tonneau 2 (TON2), identical to tonneau 2 protein (TON2) GI:11494362 from (Arabidopsis thaliana); contains Pfam profile: PF00036 EF hand | chr5:6174998-6178569 FORWARD | Aliases: EMB40, EMBRYO DEFECTIVE 40, FASS 1, FS1, GDO, GORDO, T28N17.60, T28N17_60, TON2, TONNEAU 2	8.6	8.2	0.4	1.0	100.0%	-4.4
6841	AT5G11900.1 | eukaryotic translation initiation factor SUI1 family protein, similar to SP:O43583 Density-regulated protein (DRP1 protein) (Smooth muscle cell associated protein-3) {Homo sapiens}; contains Pfam profile PF01253: Translation initiation factor SUI1 | chr5:3834015-3836247 FORWARD | Aliases: F14F18.70, F14F18_70	6.9	6.5	0.4	1.0	100.0%	-4.0
6842	AT5G25080.1 | expressed protein | chr5:8643675-8647061 FORWARD | Aliases: T11H3.90, T11H3_90	4.7	4.2	0.5	1.0	100.0%	-4.5
6843	AT4G10850.1 | nodulin MtN3 family protein, similar to MtN3 GI:1619602 (root nodule development) from (Medicago truncatula) | chr4:6674994-6676976 FORWARD | Aliases: F25I24.60, F25I24_60	2.5	2.6	-0.1	-1.0	100.0%	-5.1
6844	AT4G14970.1 | expressed protein | chr4:8553851-8561661 FORWARD | Aliases: DL3525W, FCAALL.148	2.7	2.9	-0.2	-1.0	100.0%	-4.8
6845	AT2G36490.1 | Symbol: ROS1 | HhH-GPD base excision DNA repair family protein (ROS1), similar to DEMETER protein (Arabidopsis thaliana) GI:21743571; contains Pfam profile PF00730: HhH-GPD superfamily base excision DNA repair protein | chr2:15315100-15321351 REVERSE | Aliases: F1O11.12, F1O11_12	2.5	2.3	0.2	1.0	100.0%	-5.1
6846	AT3G18600.1 | DEAD/DEAH box helicase, putative, non-consensus acceptor splice site AT at exon 2; similar to DEAD box helicase protein GB:NP_006764 from (Homo sapiens), contains Pfam profile: PF00270	5.6	6.1	-0.4	-1.0	100.0%	-4.3
6847	AT5G30500.1 | galactinol synthase, putative, similar to galactinol synthase, isoform GolS-1 GI:5608497 from (Ajuga reptans) | chr5:11632575-11634156 FORWARD | Aliases: None	2.7	2.5	0.1	1.0	100.0%	-5.0
6848	AT5G09760.1 | pectinesterase family protein, contains Pfam profile: PF01095 pectinesterase | chr5:3032376-3034544 FORWARD | Aliases: F17I14.50, F17I14_50	8.7	9.3	-0.5	-1.0	100.0%	-4.4

Rank	Description	15.dpi	5.dpi	M	t	adj.q	B
6849	AT2G18180.1 | SEC14 cytosolic factor, putative / phosphoglyceride transfer protein, putative, contains Pfam PF00650 : CRAL/TRIO domain; contains Pfam PF03765 : CRAL/TRIO, N-terminussimilar to phosphatidylinositol transfer-like protein IV (GI:14486707) (Lotus japonicus);	3.4	3.2	0.2	1.0	100.0%	-4.6
6850	AT2G28930.3 | Symbol: APK1B | similar to protein kinase (APK1a) [Arabidopsis thaliana] (TAIR:At1g07570.2); similar to protein kinase (APK1a) [Arabidopsis thaliana] (TAIR:At1g07570.1); similar to putative protein kinase [Oryza sativa] (GB:XP_493889.1); similar to putative protein kinase [Oryza sativa (japonica cultivar-group)] (GB:NP_910058.1); contains InterPro domain Serine/threonine protein kinase, active site (InterPro:IPR008271); contains InterPro domain Tyrosine protein kinase (InterPro:IPR001245); contains InterPro domain Serine/threonine protein kinase (InterPro:IPR002290); contains InterPro domain Protein kinase (InterPro:IPR000719) | chr2:12431381-12434189 FORWARD | Aliases: T9I4.1, T9I4_1	6.3	6.7	-0.4	-1.0	100.0%	-4.2
6851	AT3G04910.2 | Symbol: WNK1 | similar to protein kinase family protein [Arabidopsis thaliana] (TAIR:At5g28080.1); similar to mitogen activated protein kinase kinase [Oryza sativa] (GB:AAC32599.1); contains InterPro domain Serine/threonine protein kinase, active site (InterPro:IPR008271); contains InterPro domain Tyrosine protein kinase (InterPro:IPR001245); contains InterPro domain Serine/threonine protein kinase (InterPro:IPR002290); contains InterPro domain Protein kinase (InterPro:IPR000719) | chr3:1354641-1358225 FORWARD | Aliases: T9J14.14, T9J14_14, WITH NO LYSINE (K) 1, ZIK4	5.8	6.1	-0.4	-1.0	100.0%	-4.2
6852	AT4G11200.1 | expressed protein, contains weak hit to Pfam profile PF03108: MuDR family transposase | chr4:6827754-6829492 REVERSE | Aliases: T22B4.180, T22B4_180	3.5	3.8	-0.3	-1.0	100.0%	-4.7
6853	AT3G13730.1 | Symbol: CYP90D1 | Encodes a cytochrome P-450 gene that is involved in brassinosteroid biosynthesis, most likely in the conversion step of teasterone (TE) to 3-dehydroteasterone (3DT), and/or 6-deoxoteasterone (6-deoxoTE) to 6-deoxo-3-dehydroteasterone (6-deoxo3DT); or the conversion of cathasterone (CT) to TE, and/or 6-deoxocathasterone (6-deoxoCT) to 6-deoxoTE. Member of the CYP90C CYP450 family. Similar to Cytochrome P450 90C1 (ROT3). | chr3:4497983-4500927 REVERSE | Aliases: MMM17.20	5.3	5.6	-0.3	-1.0	100.0%	-4.2
6854	AT1G64220.1 | preprotein translocase-related, similar to TOM7 protein (Solanum tuberosum) GI:3319774 | chr1:23836751-23836984 REVERSE | Aliases: F22C12.30, F22C12_30	2.9	2.8	0.1	1.0	100.0%	-5.0
6855	AT3G49360.1 | glucosamine/galactosamine-6-phosphate isomerase family protein, similar to SP:O95336 6-phosphogluconolactonase (EC 3.1.1.31) (6PGL) {Homo sapiens}; contains Pfam profile PF01182: Glucosamine-6-phosphate isomerase/6-phosphogluconolactonase | chr3:18314031-18315383 REVERSE | Aliases: F2K15.220	4.9	5.3	-0.4	-1.0	100.0%	-4.2
6856	AT1G75540.1 | zinc finger (B-box type) family protein, similar to zinc finger protein GB:BAA33202 GI:3618312 from (Oryza sativa) | chr1:28369720-28371313 FORWARD | Aliases: F10A5.24, F10A5_24	6.1	6.6	-0.6	-1.0	100.0%	-4.1
6857	AT1G72750.1 | Symbol: ATTIM23 2 | mitochondrial import inner membrane translocase subunit Tim17/Tim22/Tim23 family protein, contains Pfam PF02466: Mitochondrial import inner membrane translocase subunit Tim17 | chr1:27387027-27388186 FORWARD | Aliases: F28P22.6, F28P22_6	6.5	5.8	0.7	1.0	100.0%	-3.9
6858	AT2G24270.2 | Symbol: ALDH11A3 | NADP-dependent glyceraldehyde-3-phosphate dehydrogenase, putative, similar to NADP-dependent glyceraldehyde-3-phosphate dehydrogenase (NON-phosphorylating glyceraldehyde 3-phosphate; glyceraldehyde-3-phosphate dehydrogenase (NADP+)) (Nicotiana plumbaginifolia) SWISS-PROT:P93338 | chr2:10334132-10336826 REVERSE | Aliases: F27D4.18, F27D4_18	8.4	8.0	0.5	1.0	100.0%	-4.0
6859	AT1G15000.1 | Symbol: SCPL50 |	6.2	6.0	0.2	1.0	100.0%	-4.5
6860	AT4G09150.1 | T-complex protein 11, contains Pfam PF05794: T-complex protein 11 | chr4:5828622-5833657 FORWARD | Aliases: F23J3.180, F23J3_180	5.6	5.9	-0.3	-1.0	100.0%	-4.3
6861	AT1G06240.1 | expressed protein, contains Pfam domain, PF04305: Protein of unknown function (DUF455) | chr1:1911121-1913179 FORWARD | Aliases: F9P14.10, F9P14_10	4.9	4.6	0.4	1.0	100.0%	-4.1
6862	AT1G24040.2 | GCN5-related N-acetyltransferase (GNAT) family protein, contains Pfam profile PF00583: acetyltransferase, GNAT family | chr1:8503781-8506792 REVERSE | Aliases: T23E23.19, T23E23_19	5.6	5.3	0.3	1.0	100.0%	-4.3
6863	AT3G02930.1 | expressed protein, ; expression supported by MPSS | chr3:655313-658326 FORWARD | Aliases: F13E7.12, F13E7_12	6.1	7.1	-0.9	-1.0	100.0%	-4.0
6864	AT1G59840.2 | expressed protein | chr1:22030172-22033044 FORWARD | Aliases: F23H11.16, F23H11_16	6.1	5.5	0.6	1.0	100.0%	-3.7
6865	AT5G57980.1 | eukaryotic rpb5 RNA polymerase subunit family protein, similar to SP:P19388 DNA-directed RNA polymerase II 23 kDa polypeptide (EC 2.7.7.6) {Homo sapiens}; contains Pfam profiles PF03871: RNA polymerase Rpb5 N-terminal domain, PF01191: RNA polymerase Rpb5 C-terminal domain	3.0	3.3	-0.3	-1.0	100.0%	-4.6
6866	AT3G51520.1 | diacylglycerol acyltransferase family, contains Pfam PF03982: Diacylglycerol acyltransferase | chr3:19121628-19123739 FORWARD | Aliases: F26O13.160	6.5	5.9	0.6	1.0	100.0%	-4.1

Rank	Description	15.dpi	5.dpi	M	t	adj.q	B
6867	AT3G53600.1 | zinc finger (C2H2 type) family protein, contains Pfam domain, PF00096: Zinc finger, C2H2 type | chr3:19886510-19887037 FORWARD | Aliases: F4P12.300	2.8	2.7	0.1	1.0	100.0%	-5.2
6868	AT3G29400.1 | exocyst subunit EXO70 family protein, similar to EXO70 protein (GI:2352998) (Mus musculus); contains Pfam domain PF03081: Exo70 exocyst complex subunit | chr3:11298520-11300857 REVERSE | Aliases: MUO10.14	4.9	4.8	0.1	1.0	100.0%	-5.0
6869	AT3G25840.1 | protein kinase family protein, contains Pfam profile: PF00069 eukaryotic protein kinase domain | chr3:9453926-9458791 REVERSE | Aliases: K9I22.6	6.6	6.2	0.3	1.0	100.0%	-4.4
6870	AT2G30220.1 | GDSL-motif lipase/hydrolase family protein, similar to family II lipases EXL3 GI:15054386, EXL1 GI:15054382, EXL2 GI:15054384 from (Arabidopsis thaliana); contains Pfam profile PF00657: GDSL-like Lipase/Acylhydrolase	2.6	2.8	-0.2	-1.0	100.0%	-4.9
6871	AT2G30310.1 | GDSL-motif lipase/hydrolase family protein, similar to family II lipases EXL3 GI:15054386, EXL1 GI:15054382 from (Arabidopsis thaliana); contains Pfam profile PF00657: GDSL-like Lipase/Acylhydrolase | chr2:12930132-12931448 FORWARD | Aliases: T9D9.12, T9D9_12	2.6	2.8	-0.2	-1.0	100.0%	-4.9
6872	AT3G20730.1 | pentatricopeptide (PPR) repeat-containing protein, contains INTERPRO:IPR002885 PPR repeats | chr3:7247101-7249151 FORWARD | Aliases: MOE17.3	3.5	3.3	0.3	1.0	100.0%	-4.2
6873	AT5G42640.1 | zinc finger (C2H2 type) family protein, contains Pfam domain, PF00096: Zinc finger, C2H2 type | chr5:17105923-17106825 FORWARD | Aliases: MFO20.6, MFO20_6	2.2	2.3	-0.1	-1.0	100.0%	-5.6
6874	AT2G26530.2 | Symbol: AR781 | similar to calmodulin-binding protein [Arabidopsis thaliana] (TAIR:At2g15760.1); similar to unknown protein [Oryza sativa (japonica cultivar-group)] (GB:NP_914731.1) | chr2:11289853-11291289 REVERSE | Aliases: AR781, T9J22.20, T9J22_20	4.7	4.4	0.3	1.0	100.0%	-4.3
6875	AT2G45240.1 | Symbol: MAP1A | methionyl aminopeptidase, putative / methionine aminopeptidase, putative / peptidase M, putative, similar to SP:Q01662 Methionine aminopeptidase 1 precursor (EC 3.4.11.18) {Saccharomyces cerevisiae}; contains Pfam profile PF00557: metallopeptidase family M24 | chr2:18663040-18666151 FORWARD | Aliases: F4L23.25, METHIONINE AMINOPEPTIDASE 1A	5.5	5.9	-0.4	-1.0	100.0%	-4.3
6876	AT2G20350.1 | encodes a member of the ERF (ethylene response factor) subfamily B-6 of ERF/AP2 transcription factor family. The protein contains one AP2 domain. There are 12 members in this subfamily including RAP2.11. | chr2:8791850-8792477 FORWARD | Aliases: F11A3.10, F11A3_10	2.7	2.5	0.2	1.0	100.0%	-4.9
6877	AT5G62180.1 | expressed protein, similar to PrMC3, Pinus radiata, GI:5487873 | chr5:24996070-24997075 REVERSE | Aliases: MMI9.26	3.8	3.7	0.2	1.0	100.0%	-4.7
6878	AT1G23820.2 | Symbol: SPDS1 | spermidine synthase 1 (SPDSYN1) / putrescine aminopropyltransferase 1, identical to SP:Q9ZUB3 Spermidine synthase 1 (EC 2.5.1.16) (Putrescine aminopropyltransferase 1) (SPDSY 1) {Arabidopsis thaliana} | chr1:8420276-8422928 FORWARD | Aliases: F5O8.38, F5O8_38, SPERMIDINE SYNTHASE 1	12.0	12.4	-0.4	-1.0	100.0%	-5.0
6879	AT5G28800.1 | expressed protein, predicted protein, Arabidopsis thaliana | chr5:10822884-10823642 FORWARD | Aliases: T32B20.90, T32B20_90	3.3	3.2	0.2	1.0	100.0%	-5.0
6880	AT2G18990.1 | expressed protein | chr2:8245773-8247077 REVERSE | Aliases: F19F24.19, F19F24_19	4.3	4.6	-0.3	-1.0	100.0%	-4.3
6881	AT4G02690.1 | expressed protein, low similarity to N-methyl-D-aspartate receptor-associated protein (Drosophila melanogaster) GI:567104, NMDA receptor glutamate-binding subunit (Rattus sp.) GI:8248741; contains Pfam profile PF01027: Uncharacterized protein family UPF0005 | chr4:1186023-1187534 FORWARD | Aliases: T10P11.23, T10P11_23	3.9	3.6	0.2	1.0	100.0%	-4.5
6882	AT5G46190.1 | KH domain-containing protein, strong similarity to unknown protein (pir::T04533)	4.4	4.1	0.3	1.0	100.0%	-4.4
6883	AT2G44970.1 | lipase-related, contains weak similarity to lipase (Pseudomonas aeruginosa) gi:3550950:gb:AAC34733. | chr2:18555898-18559130 REVERSE | Aliases: T14P1.23, T14P1_23	8.4	8.7	-0.3	-1.0	100.0%	-4.4
6884	AT5G04480.1 | expressed protein | chr5:1271516-1277986 REVERSE | Aliases: T32M21.80, T32M21_80	3.9	3.7	0.2	1.0	100.0%	-4.4
6885	AT1G63680.1 | Symbol: PDE316 |	3.8	4.0	-0.3	-1.0	100.0%	-4.5
6886	AT5G59330.1 | expressed protein | chr5:23948640-23948903 FORWARD | Aliases: MNC17.24, MNC17_24	3.1	2.9	0.2	1.0	100.0%	-4.8
6887	AT4G25780.1 | pathogenesis-related protein, putative, similar to gene PR-1 protein - Medicago truncatula, SP:Q40374; contains Pfam profile PF00188: SCP-like extracellular protein | chr4:13121077-13121964 FORWARD | Aliases: F14M19.60, F14M19_60	5.2	4.9	0.3	1.0	100.0%	-4.1
6888	AT4G37110.1 | expressed protein | chr4:17484119-17486265 REVERSE | Aliases: AP22.72, AP22_72	4.4	4.7	-0.3	-1.0	100.0%	-4.3

Rank	Description	15.dpi	5.dpi	M	t	adj.q	B
6889	AT2G01470.1 | Symbol: STL2P | St12p protein (ST12p) / SEC12p protein, putative, 99.8% identical to St12p protein (GI:166878) {Arabidopsis thaliana} | chr2:211969-214464 REVERSE | Aliases: ATSEC12, F2I9.9, F2I9_9, SEC12P LIKE PROTEIN	10.2	10.5	-0.4	-1.0	100.0%	-4.4
6890	AT2G21800.1 | expressed protein, similar to expressed protein [Arabidopsis thaliana] (TAIR:At2g22140.1); similar to hypothetical protein DDB0205993 [Dictyostelium discoideum] (GB:EAL67624.1); contains domain LYS_RICH (PS50318) | chr2:9305235-9309004 FORWARD | Aliases: F7D8.12, F7D8_12	2.9	3.1	-0.2	-1.0	100.0%	-4.7
6891	AT2G38090.1 | myb family transcription factor, contains Pfam profile: PF00249 myb-like DNA-binding domain | chr2:15951789-15954163 FORWARD | Aliases: F16M14.2, F16M14_2	6.0	6.3	-0.3	-1.0	100.0%	-4.2
6892	AT3G18780.2 | Symbol: ACT2 | actin 2 (ACT2), identical to SP:Q96292 Actin 2 {Arabidopsis thaliana}; nearly identical to SP:Q96293 Actin 8 (Arabidopsis thaliana) GI:1669387 and to At1g49240 | chr3:6474877-6477210 FORWARD | Aliases: ACTIN 2, ACTIN2, DEFORMED ROOT HAIRS 1, DER1, LIGHT STRESS REGULATED 2, LSR2, MVE11.16	9.3	9.9	-0.6	-1.0	100.0%	-4.4
6893	AT2G02740.1 | transcription factor, putative, similar to DNA-binding protein p24 (Solanum tuberosum) GI:9651810, Plant Transcriptional Regulator Pbf-2 (Solanum tuberosum) (GI:21730639, GI:21730638, GI:21730640, GI:21730637) | chr2:769367-772312 FORWARD | Aliases: T20F6.12, T20F6_12	3.3	3.5	-0.2	-1.0	100.0%	-4.5
6894	AT1G01830.1 | armadillo/beta-catenin repeat family protein, armadillo/beta-catenin-like repeats, Pfam:PF00514 | chr1:298535-300729 REVERSE | Aliases: T1N6.25, T1N6_25	3.8	3.6	0.2	1.0	100.0%	-4.6
6895	AT5G12130.1 | Symbol: PDE149 |	5.5	5.8	-0.3	-1.0	100.0%	-4.5
6896	AT2G34800.1 | hypothetical protein | chr2:14689442-14689594 REVERSE | Aliases: F19I3.3, F19I3_3	2.4	2.5	-0.1	-1.0	100.0%	-5.6
6897	AT1G07960.3 | Symbol: ATPDIL5 1 | thioredoxin family protein, low similarity to protein disulfide isomerase 4 (Giardia intestinalis) GI:13489047; contains Pfam profile PF00085: Thioredoxin | chr1:2467174-2469151 FORWARD | Aliases: ATPDIL5 1, PDI LIKE 5 1, T6D22.5, T6D22_5	7.2	6.9	0.3	1.0	100.0%	-4.7
6898	AT4G39950.1 | Symbol: CYP79B2 | cytochrome P450 79B2, putative (CYP79B2), identical to cytochrome P450 (79B2) SP:O81346 from (Arabidopsis thaliana) | chr4:18525240-18527573 FORWARD | Aliases: T5J17.120, T5J17_120	3.8	4.3	-0.5	-1.0	100.0%	-3.9
6899	AT5G55030.2 | expressed protein | chr5:22344421-22347072 REVERSE | Aliases: K13P22.3, K13P22_3	4.2	4.5	-0.3	-1.0	100.0%	-4.6
6900	AT1G56350.1 | peptide chain release factor, putative, similar to SP:P28353 Peptide chain release factor 2 (RF-2). {Salmonella typhi}; contains Pfam profiles PF00472: Peptidyl-tRNA hydrolase domain, PF03462: PCRF domain | chr1:21098443-21101491 FORWARD | Aliases: F13N6.6, F13N6_6	5.9	5.5	0.3	1.0	100.0%	-4.1
6901	AT1G62390.1 | octicosapeptide/Phox/Bem1p (PB1) domain-containing protein / tetratricopeptide repeat (TPR)-containing protein, contains Pfam profiles PF00564: PB1 domain, PF00515: TPR Domain | chr1:23087975-23090792 REVERSE | Aliases: F24O1.41, F24O1_41	6.0	6.5	-0.5	-1.0	100.0%	-4.1
6902	AT2G39320.1 | OTU-like cysteine protease family protein, contains Pfam profile PF02338: OTU-like cysteine protease | chr2:16424670-16425595 REVERSE | Aliases: T16B24.4, T16B24_4	3.0	2.9	0.2	1.0	100.0%	-4.9
6903	AT1G18310.1 | glycosyl hydrolase family 81 protein, similar to beta-glucan binding protein GI:6625560 from (Phaseolus vulgaris) | chr1:6301193-6303949 REVERSE | Aliases: F15H18.17, F15H18_17	2.7	2.5	0.2	1.0	100.0%	-5.0
6904	AT4G31770.1 | calcineurin-like phosphoesterase family protein, contains Pfam profile: PF00149 calcineurin-like phosphoesterase	3.2	3.4	-0.2	-1.0	100.0%	-4.5
6905	AT5G18100.1 | Symbol: CSD3 | superoxide dismutase (Cu-Zn) / copper/zinc superoxide dismutase (CSD3), identical to copper/zinc superoxide dismutase GI:3273755	7.7	7.4	0.3	1.0	100.0%	-4.5
6906	AT4G16800.1 | enoyl-CoA hydratase, putative, similar to AU-binding protein/Enoyl-CoA hydratase (Homo sapiens) GI:780241, (Mus musculus)GI:6840920; contains Pfam profile PF00378 enoyl-CoA hydratase/isomerase family protein | chr4:9454835-9456328 REVERSE | Aliases: DL4425C, FCAALL.21	6.9	7.5	-0.6	-1.0	100.0%	-4.3
6907	AT1G06890.1 | transporter-related, low similarity to SP:Q9NTN3 UDP-glucuronic acid/UDP-N-acetylgalactosamine transporter {Homo sapiens}, SP:Q95YI5 UDP-sugar transporter UST74c {Drosophila melanogaster}, SP:Q18779 UDP-sugar transporter sqv-7 {Caenorhabditis elegans}; contains 8 predicted transmembrane domains | chr1:2111562-2114533 REVERSE | Aliases: F4H5.5, F4H5_5	8.2	7.9	0.3	1.0	100.0%	-4.5
6908	AT5G49945.1 | expressed protein, strong similarity to unknown protein (pir::T09896) | chr5:20334631-20337381 FORWARD | Aliases: None	6.6	7.0	-0.4	-1.0	100.0%	-4.3
6909	AT2G33560.1 | spindle checkpoint protein-related, similar to spindle checkpoint protein BubR1 (GI:22128593) (Xenopus laevis); similar to Mitotic checkpoint serine/threonine-protein kinase BUB1 beta (EC 2.7.1.-) (MAD3/BUB1-related protein kinase) (Mitotic checkpoint kinase MAD3L) (Swiss-Prot:Q9Z1S0) (Mus musculus) | chr2:14220680-14223075 REVERSE | Aliases: F4P9.33, F4P9_33	4.8	5.2	-0.5	-1.0	100.0%	-4.3
6910	AT2G10020.1 | expressed protein, and genefinder | chr2:3804313-3804513 FORWARD | Aliases: F7B19.16, F7B19_16	6.5	6.1	0.4	1.0	100.0%	-4.3

Rank	Description	15.dpi	5.dpi	M	t	adj.q	B
6911	AT3G15270.1 | Symbol: SPL5 | squamosa promoter-binding protein-like 5 (SPL5), identical to squamosa promoter binding protein-like 5 (Arabidopsis thaliana) GI:5931629; contains Pfam profile PF03110: SBP domain | chr3:5140396-5141353 REVERSE | Aliases: K7L4.7, SQUAMOSA PROMOTER BINDING PROTEIN LIKE 5	2.8	2.6	0.2	1.0	100.0%	-4.7
6912	AT3G25910.1 | expressed protein | chr3:9490486-9492729 FORWARD | Aliases: MPE11.8	8.7	8.2	0.5	1.0	100.0%	-4.2
6913	AT2G36300.1 | integral membrane Yip1 family protein, contains Pfam domain, PF04893: Yip1 domain | chr2:15220139-15221351 REVERSE | Aliases: F2H17.9, F2H17_9	7.4	7.0	0.3	1.0	100.0%	-4.3
6914	AT3G06410.1 | similar to zinc finger (CCCH-type) family protein [Arabidopsis thaliana] (TAIR:At5g18550.1); similar to putative floral homeotic protein HUA1 [Oryza sativa (japonica cultivar-group)] (GB:BAD81402.1); contains InterPro domain Zn-finger, C-x8-C-x5-C-x3-H type (InterPro:IPR000571) | chr3:1947310-1949775 REVERSE | Aliases: F24P17.12, F24P17_12	7.1	6.7	0.4	1.0	100.0%	-4.4
6915	AT4G27960.2 | Symbol: UBC9 | ubiquitin-conjugating enzyme E2-17 kDa 9 (UBC9), E2; identical to gi:297883, SP:P35132; identical to cDNA UBC9 for ubiquitin conjugating enzyme homolog GI:297883 | chr4:13915839-13917426 REVERSE | Aliases: T13J8.70, T13J8_70, UBIQUITIN CONJUGATING ENZYME 9, UBIQUITIN PROTEIN LIGASE	13.7	13.3	0.5	1.0	100.0%	-5.6
6916	AT5G53300.2 | Symbol: UBC10 | ubiquitin-conjugating enzyme 10 (UBC10), E2; identical to gi:297877, SP:P35133 | chr5:21649740-21651389 REVERSE | Aliases: None	13.7	13.3	0.5	1.0	100.0%	-5.6
6917	AT1G02850.3 | glycosyl hydrolase family 1 protein, contains Pfam PF00232 : Glycosyl hydrolase family 1 domain; TIGRFAM TIGR01233: 6-phospho-beta-galactosidase; similar to hydroxyisourate hydrolase (GI:19569603) (Glycine max) | chr1:630512-633259 FORWARD | Aliases: F22D16.15, F22D16_15	8.2	8.7	-0.6	-1.0	100.0%	-4.0
6918	AT2G01350.2 | quinolinate phosphoribosyl transferase family protein, contains Pfam profile: PF01729 quinolinate phosphoribosyl transferase, C-terminal domain | chr2:165145-167246 REVERSE | Aliases: F10A8.23, F10A8_23	9.7	10.0	-0.3	-1.0	100.0%	-4.6
6919	AT2G40790.1 | thioredoxin family protein, contains Pfam profile: PF00085 thioredoxin | chr2:17030041-17031346 REVERSE | Aliases: T7D17.3, T7D17_3	4.0	3.7	0.3	1.0	100.0%	-4.5
6920	AT3G25690.1 | Symbol: CHUP1 | hydroxyproline-rich glycoprotein family protein, Common family members: At4g18570, At4g04980, At5g61090 (Arabidopsis thaliana);	5.4	5.9	-0.5	-1.0	100.0%	-4.0
6921	AT5G05680.1 | nuclear pore complex protein-related, contains weak similarity to Nuclear pore complex protein Nup88 (Nucleoporin Nup88) (88 kDa nuclear pore complex protein) (Swiss-Prot:Q99567) (Homo sapiens) | chr5:1698271-1702616 FORWARD | Aliases: MJJ3.8, MJJ3_8	5.5	5.8	-0.3	-1.0	100.0%	-4.5
6922	AT2G37650.1 | scarecrow-like transcription factor 9 (SCL9), identical to cDNA scarecrow-like 9 (SCL9) mRNA, partial cds GI:4580524 | chr2:15799701-15802313 FORWARD | Aliases: F13M22.15, F13M22_15	3.7	3.4	0.3	1.0	100.0%	-4.5
6923	AT5G14590.1 | isocitrate dehydrogenase, putative / NADP+ isocitrate dehydrogenase, putative, strong similarity to isocitrate dehydrogenase (NADP+) (Nicotiana tabacum) GI:3021512; contains Pfam domain PF00180: dehydrogenase, isocitrate/isopropylmalate family | chr5:4703301-4706738 REVERSE | Aliases: T15N1.80, T15N1_80	10.1	10.5	-0.4	-1.0	100.0%	-4.4
6924	AT1G56520.1 | disease resistance protein (TIR-NBS-LRR class), putative, domain signature TIR-NBS-LRR exists, suggestive of a disease resistance protein. | chr1:21179279-21182585 REVERSE | Aliases: F25P12.102, F25P12_102	3.4	3.1	0.2	1.0	100.0%	-4.6
6925	AT1G74040.1 | Symbol: IMS1 | 2-isopropylmalate synthase 1 (IMS1), identical to 2-isopropylmalate synthase (IMS1) (Arabidopsis thaliana) GI:12330687; identical to cDNA 2-isopropylmalate synthase (IMS1) GI:12330686	7.8	8.2	-0.4	-1.0	100.0%	-4.2
6926	AT5G40950.1 | 50S ribosomal protein L27, chloroplast, putative (RPL27), identical to SP:Q9FLN4 ribosomal protein L27, chloroplast precursor {Arabidopsis thaliana}; similar to SP:P30155 50S ribosomal protein L27, chloroplast precursor (CL27) {Nicotiana tabacum} | chr5:16428037-16429284 FORWARD | Aliases: MMG1.7	6.8	7.4	-0.6	-1.0	100.0%	-4.0
6927	AT4G36060.2 | basic helix-loop-helix (bHLH) family protein, contains Pfam profile: PF00010 helix-loop-helix DNA-binding domain	2.5	2.4	0.1	1.0	100.0%	-4.8
6928	AT5G29050.1 | hypothetical protein, predicted protein, Arabidopsis thaliana | chr5:11111696-11112287 FORWARD | Aliases: F23C8.20, F23C8_20	2.3	2.2	0.1	1.0	100.0%	-5.3
6929	AT1G29660.1 | GDSL-motif lipase/hydrolase family protein, low similarity to family II lipase EXL1 (Arabidopsis thaliana) GI:15054382; contains InterPro Entry IPR001087 Lipolytic enzyme, G-D-S-L family | chr1:10371825-10373743 FORWARD | Aliases: F15D2.21, F15D2_21	2.0	1.9	0.1	1.0	100.0%	-5.5
6930	AT5G17210.2 | expressed protein | chr5:5656435-5658149 FORWARD | Aliases: MKP11.6, MKP11_6	7.5	6.9	0.6	1.0	100.0%	-4.1
6931	AT5G17720.1 | hydrolase, alpha/beta fold family protein, low similarity to SP:P27747 Dihydrolipoamide acetyltransferase component of acetoin cleaving system (EC 2.3.1.12) (Ralstonia eutropha) {Alcaligenes eutrophus}, SP:P24640 Lipase 3 precursor (EC 3.1.1.3) (Triacylglycerol lipase) {Moraxella sp}; contains Pfam profile PF00561: hydrolase, alpha/beta fold family | chr5:5845936-5847655 FORWARD | Aliases: MVA3.7, MVA3_7	3.5	3.8	-0.2	-1.0	100.0%	-4.6

Rank	Description	15.dpi	5.dpi	M	t	adj.q	B
6932	AT3G19450.1 | Symbol: CAD4 | cinnamyl-alcohol dehydrogenase (CAD), identical to SP:P48523 Cinnamyl-alcohol dehydrogenase (EC 1.1.1.195) (CAD) (Arabidopsis thaliana) | chr3:6744769-6747220 FORWARD | Aliases: ATCAD4, CAD, CAD C, CINNAMYL ALCOHOL DEHYDROGENASE 4, MLD14.30	7.9	8.5	-0.7	-1.0	100.0%	-4.0
6933	AT4G27430.1 | Symbol: CIP7 | COP1-interacting protein 7 (CIP7), identical to COP1-Interacting Protein 7 (CIP7) GI:3327868 from (Arabidopsis thaliana)	4.3	4.6	-0.3	-1.0	100.0%	-4.3
6934	AT3G07300.2 | similar to eukaryotic translation initiation factor 2B family protein / eIF-2B family protein [Arabidopsis thaliana] (TAIR:At2g44070.1); similar to putative translation initiation factor 2B beta subunit [Oryza sativa (japonica cultivar-group)] (GB:AAP53571.1); contains InterPro domain Initiation factor 2B (InterPro:IPR000649) | chr3:2324623-2327630 REVERSE | Aliases: T1B9.3	5.4	5.7	-0.3	-1.0	100.0%	-4.4
6935	AT5G48460.1 | fimbrin-like protein, putative, strong similarity to fimbrin-like protein AtFim2 (Arabidopsis thaliana) GI:2737926; contains Pfam profile PF00307: Calponin homology (CH) domain	6.0	6.2	-0.2	-1.0	100.0%	-4.7
6936	AT1G67820.1 | protein phosphatase 2C, putative / PP2C, putative, similar to protein phosphatase 2C emb:CAA72341.1 | chr1:25433516-25435725 FORWARD | Aliases: F12A21.5, F12A21_5	9.1	8.7	0.4	1.0	100.0%	-4.1
6937	AT3G25800.2 | Symbol: PDF1 | similar to serine/threonine protein phosphatase 2A (PP2A) regulatory subunit A (RCN1) [Arabidopsis thaliana] (TAIR:At1g25490.1); similar to serine/threonine protein phosphatase 2A (PP2A) 65 kDa regulatory subunit, putative [Arabidopsis thaliana] (TAIR:At1g13320.1); similar to protein phosphatase [Cicer arietinum] (GB:CAA10285.1); similar to serine/threonine protein phosphatase type 2A regulatory subunit A (GB:AAB60713.1); similar to Ser/Thr specific protein phosphatase 2A A regulatory subunit beta isoform [Medicago sativa subsp. x varia] (GB:AAG29594.1); similar to protein phosphatase 2A [Nicotiana tabacum] (GB:CAA66487.1); similar to phosphatase 2A regulatory A subunit [Oryza sativa (japonica cultivar-group)] (GB:XP_450276.1); contains InterPro domain HEAT repeat (InterPro:IPR000357) | chr3:9423825-9427281 REVERSE | Aliases: K13N2.2, PR 65, PR65	8.1	8.5	-0.4	-1.0	100.0%	-4.2
6938	AT5G18340.1 | U-box domain-containing protein, weak similarity to immediate-early fungal elicitor protein CMPG1 (Petroselinum crispum) GI:14582200; contains Pfam profile PF04564: U-box domain | chr5:6070642-6072200 REVERSE | Aliases: F20L16.60, F20L16_60	4.2	3.9	0.2	1.0	100.0%	-4.5
6939	AT3G13130.1 | expressed protein | chr3:4223015-4223620 FORWARD | Aliases: MJG19.8	3.2	3.4	-0.2	-1.0	100.0%	-4.9
6940	AT3G56720.1 | expressed protein | chr3:21022032-21024398 FORWARD | Aliases: T8M16.50	8.3	7.9	0.4	1.0	100.0%	-4.5
6941	AT3G09480.1 | histone H2B, putative, similar to histone from Arabidopsis thaliana GI:2407802, Gossypium hirsutum SP:O22582, H2B-3 GB:CAA12231 from (Lycopersicon esculentum); contains Pfam profile PF00125 Core histone H2A/H2B/H3/H4 | chr3:2914896-2915276 REVERSE | Aliases: F11F8.5	7.5	7.9	-0.5	-1.0	100.0%	-4.1
6942	AT4G12490.1 | protease inhibitor/seed storage/lipid transfer protein (LTP) family protein, similar to pEARLI 1 (Accession No. L43080): an Arabidopsis member of a conserved gene family (PGF95-099), Plant Physiol. 109 (4), 1497 (1995); contains Pfam protease inhibitor/seed storage/LTP family domain PF00234 | chr4:7409618-7410403 REVERSE | Aliases: T1P17.80, T1P17_80	2.6	2.5	0.1	1.0	100.0%	-4.9
6943	AT4G31750.1 | protein phosphatase 2C, putative / PP2C, putative, protein phosphatase 2C, Medicago sativa, PID:g2582800	8.3	8.0	0.3	1.0	100.0%	-4.6
6944	AT2G27750.1 | nucleolar matrix protein-related, contains Pfam domain, PF04935: Surfeit locus protein 6	2.9	3.0	-0.2	-1.0	100.0%	-5.0
6945	AT5G19750.1 | peroxisomal membrane 22 kDa family protein, similar to SP:P42925 22 kDa peroxisomal membrane protein {Mus musculus}; contains Pfam profile PF04117: Mpv17 / PMP22 family	4.2	4.5	-0.3	-1.0	100.0%	-4.6
6946	AT5G52100.1 | dihydrodipicolinate reductase family protein, weak similarity to dihydrodipicolinate reductase (Corynebacterium glutamicum) GI:311768; contains Pfam profiles PF01113: Dihydrodipicolinate reductase N-terminus, PF05173: Dihydrodipicolinate reductase C-terminus | chr5:21187371-21189571 FORWARD | Aliases: MSG15.20, MSG15_20	3.1	3.3	-0.2	-1.0	100.0%	-4.7
6947	AT4G02750.1 | pentatricopeptide (PPR) repeat-containing protein, contains INTERPRO:IPR002885 PPR repeats | chr4:1221116-1223461 REVERSE | Aliases: T5J8.5, T5J8_5	4.7	5.2	-0.5	-1.0	100.0%	-4.0
6948	AT1G64320.1 | myosin heavy chain-related, similar to myosin heavy chain (GI:4249703) (Rana catesbeiana); similar to smooth muscle myosin heavy chain SM2 (GI:2352945) (Homo sapiens) | chr1:23872699-23874211 FORWARD | Aliases: F15H21.20, F15H21_20	2.4	2.5	-0.1	-1.0	100.0%	-5.5
6949	AT4G11860.1 | expressed protein, contains Pfam domain PF04424: Protein of unknown function (DUF544) | chr4:7134078-7138497 REVERSE | Aliases: T26M18.70, T26M18_70	6.8	7.3	-0.5	-1.0	100.0%	-4.2
6950	AT1G20960.1 | Symbol: EMB1507 | U5 small nuclear ribonucleoprotein helicase, putative, similar to SP:O75643 U5 small nuclear ribonucleoprotein 200 kDa helicase {Homo sapiens}; contains Pfam profiles PF00270: DEAD/DEAH box helicase, PF00271: Helicase conserved C-terminal domain, PF02889: Sec63 domain | chr1:7302580-7309903 REVERSE | Aliases: EMB1507, EMBRYO DEFECTIVE 1507, F9H16.5, F9H16_5	7.2	7.6	-0.4	-1.0	100.0%	-4.2

Rank	Description	15.dpi	5.dpi	M	t	adj.q	B
6951	AT2G44560.1 | glycosyl hydrolase family 9 protein | chr2:18398990-18400730 REVERSE | Aliases: F16B22.5	3.1	3.3	-0.2	-1.0	100.0%	-4.9
6952	AT3G04690.1 | protein kinase family protein, contains protein kinase domain, Pfam:PF00069 | chr3:1273392-1275944 REVERSE | Aliases: F7O18.16, F7O18_16	2.6	2.4	0.1	1.0	100.0%	-5.0
6953	AT1G05410.2 | similar to tropomyosin-related [Arabidopsis thaliana] (TAIR:At5g48160.1); similar to unknown protein [Oryza sativa (japonica cultivar-group)] (GB:XP_469730.1); contains domain CYS_RICH (PS50311) | chr1:1585370-1587773 REVERSE | Aliases: T25N20.6, T25N20_6	6.3	6.6	-0.3	-1.0	100.0%	-4.3
6954	AT5G15600.1 | expressed protein | chr5:5078203-5079094 FORWARD | Aliases: T20K14.210, T20K14_210	7.3	6.9	0.4	1.0	100.0%	-4.0
6955	AT1G01080.2 | similar to 33 kDa ribonucleoprotein, chloroplast, putative / RNA-binding protein cp33, putative [Arabidopsis thaliana] (TAIR:At3g52380.1); similar to unnamed protein product [Nicotiana tabacum] (GB:CAA37879.1); contains InterPro domain RNA-binding region RNP-1 (RNA recognition motif) (InterPro:IPR000504) | chr1:45296-47019 REVERSE | Aliases: T25K16.19, T25K16_19	4.2	4.5	-0.3	-1.0	100.0%	-4.3
6956	AT3G43930.2 | similar to expressed protein [Arabidopsis thaliana] (TAIR:At2g26270.1); similar to unknown [Oryza sativa (japonica cultivar-group)] (GB:AAO72659.1); contains InterPro domain BRCT (InterPro:IPR001357) | chr3:15771885-15774317 REVERSE | Aliases: T15B3.70	2.7	2.6	0.2	1.0	100.0%	-4.9
6957	AT2G26830.1 | Symbol: EMB1187 | choline/ethanolamine kinase family protein, similar to SP:Q9HBU6 Ethanolamine kinase (EC 2.7.1.82) (EKI){Homo sapiens}, GmCK3p (Glycine max) GI:1438883; contains Pfam profile PF01633: Choline/ethanolamine kinase | chr2:11450639-11454184 FORWARD | Aliases: EMB1187, EMBRYO DEFECTIVE 1187, F12C20.13, F12C20_13	5.4	5.8	-0.5	-1.0	100.0%	-4.1
6958	AT2G10465.1 | replication protein-related, weak similarity to Replication Protein A 70	2.6	2.7	-0.1	-1.0	100.0%	-5.3
6959	AT5G67060.1 | basic helix-loop-helix (bHLH) family protein, contains Pfam profile: PF00010 helix-loop-helix DNA-binding domain	3.0	2.9	0.1	1.0	100.0%	-5.0
6960	AT5G45240.1 | disease resistance protein (TIR-NBS-LRR class), putative, domain signature TIR-NBS-LRR exists, suggestive of a disease resistance protein. | chr5:18330933-18336316 FORWARD | Aliases: K18C1.12, K18C1_12	2.6	2.7	-0.1	-1.0	100.0%	-5.1
6961	AT3G44330.1 | expressed protein | chr3:16017685-16021952 FORWARD | Aliases: T22K7.10	5.7	6.3	-0.5	-1.0	100.0%	-4.3
6962	AT2G34930.1 | disease resistance family protein, contains leucine rich-repeat domains Pfam:PF00560, INTERPRO:IPR001611; similar to Cf-2.1 (Lycopersicon pimpinellifolium) gi:1184075:gb:AAC15779 | chr2:14744145-14746983 REVERSE | Aliases: F19I3.16, F19I3_16	3.0	2.8	0.2	1.0	100.0%	-4.8
6963	AT1G20620.5 | Symbol: CAT3 | similar to catalase 1 [Arabidopsis thaliana] (TAIR:At1g20630.1); similar to catalase 2 [Arabidopsis thaliana] (TAIR:At4g35090.1); similar to catalase 1 [Nicotiana tabacum] (GB:AAB71764.1); similar to catalase [Brassica juncea] (GB:AAD17935.1); similar to catalase [Prunus persica] (GB:CAD42908.1); similar to catalase [Brassica napus] (GB:AAB53101.2); similar to catalase 2 [Raphanus sativus] (GB:AAD30291.2); contains InterPro domain Catalase (InterPro:IPR002226)	7.9	7.1	0.8	1.0	100.0%	-4.1
6964	AT1G63210.1 | expressed protein, contains Pfam profile: PF04283 protein of unknown function (DUF439) | chr1:23447353-23451019 FORWARD | Aliases: F16M19.22, F16M19_22	3.6	3.4	0.2	1.0	100.0%	-4.6
6965	AT5G65570.1 | pentatricopeptide (PPR) repeat-containing protein, contains INTERPRO:IPR002885 PPR repeats | chr5:26221194-26223410 FORWARD | Aliases: K21L13.8, K21L13_8	3.2	3.1	0.2	1.0	100.0%	-5.1
6966	AT1G73880.1 | UDP-glucoronosyl/UDP-glucosyl transferase family protein, contains Pfam profile: PF00201 UDP-glucoronosyl and UDP-glucosyl transferase | chr1:27788642-27790465 FORWARD | Aliases: F2P9.25, F2P9_25	10.8	10.0	0.8	1.0	100.0%	-4.0
6967	AT5G66540.1 | expressed protein, ; supported by full-Length cDNA gi:12057175 from (Arabidopsis thaliana) | chr5:26573576-26576653 REVERSE | Aliases: K1F13.21, K1F13_21	3.9	4.1	-0.2	-1.0	100.0%	-4.6
6968	AT5G48710.1 | ubiquitin-related, similar to SP:O13351 Ubiquitin-like protein smt3/pmt3 {Schizosaccharomyces pombe} | chr5:19772291-19772798 REVERSE | Aliases: K24G6.4, K24G6_4	4.2	3.9	0.3	1.0	100.0%	-4.6
6969	AT1G65370.1 | meprin and TRAF homology domain-containing protein / MATH domain-containing protein, contains Pfam profile PF00917: MATH domain | chr1:24288136-24289806 REVERSE | Aliases: T8F5.15, T8F5_15	6.9	7.4	-0.5	-1.0	100.0%	-3.9
6970	AT4G15570.1 | tRNA-splicing endonuclease positive effector-related, contains similarity to SEN1, a positive effector of tRNA-splicing endonuclease (Saccharomyces cerevisiae) gi:172574:gb:AAB63976 | chr4:8892637-8898994 FORWARD | Aliases: DL3825W, FCAALL.334	3.9	4.2	-0.3	-1.0	100.0%	-4.4
6971	AT4G22640.1 | expressed protein, various predicted proteins, Arabidopsis thaliana | chr4:11911611-11912019 FORWARD | Aliases: T12H17.30	3.6	4.2	-0.6	-1.0	100.0%	-4.2

Rank	Description	15.dpi	5.dpi	M	t	adj.q	B
6972	AT5G27150.1 | Symbol: NHX1 | sodium proton exchanger / Na+/H+ antiporter (NHX1), identical to Na+/H+ exchanger (Arabidopsis thaliana) gi:6650177:gb:AAF21755 and sodium proton exchanger Nhx1 (Arabidopsis thaliana) gi:4324597:gb:AAD16946; Member of The Monovalent Cation:Proton Antiporter (CPA1) Family, PMID:11500563 | chr5:9553441-9557516 FORWARD | Aliases: AT NHX1, ATNHX, ATNHX1, NA(+)/H(+) ANTIPORTER, NA+/H+ EXCHANGER, T21B4.60, T21B4_60	7.2	6.8	0.5	1.0	100.0%	-4.1
6973	AT1G02740.1 | MRG family protein, member of Pfam PF05712: MRG; similar to Transcription factor-like protein MRG15 (MORF-related gene 15 protein) (MSL3-1 protein) (Protein HSPC008/HSPC061) (SP:Q9UBU8) {Homo sapiens}	5.6	5.2	0.4	1.0	100.0%	-4.1
6974	AT3G19120.1 | expressed protein | chr3:6609418-6611065 REVERSE | Aliases: MVI11.2	4.0	4.3	-0.3	-1.0	100.0%	-4.3
6975	AT4G01990.1 | pentatricopeptide (PPR) repeat-containing protein, low similarity to DNA-binding protein (Triticum aestivum) GI:6958202; contains Pfam profile PF01535: PPR repeat | chr4:871145-872913 REVERSE | Aliases: T7B11.26, T7B11_26	4.5	4.9	-0.4	-1.0	100.0%	-4.4
6976	AT2G23240.2 | plant EC metallothionein-like family 15 protein, identical to EC protein homolog 2 (SP:Q42377) {Arabidopsis thaliana}; identical to an EST: GB:X92116:ATECPRHOM; contains a vertebrate metallothionein signature (PS00203); contains Pfam profile PF02068: Plant PEC family metallothionein | chr2:9902935-9903405 REVERSE | Aliases: T20D16.13, T20D16_13	3.5	3.2	0.3	1.0	100.0%	-4.6
6977	AT3G16010.1 | pentatricopeptide (PPR) repeat-containing protein, contains Pfam profile PF01535: PPR repeat | chr3:5434020-5436270 FORWARD | Aliases: MSL1.5	4.6	5.0	-0.5	-1.0	100.0%	-4.4
6978	AT4G12690.1 | expressed protein | chr4:7480804-7481867 FORWARD | Aliases: T20K18.40, T20K18_40	3.6	3.4	0.2	1.0	100.0%	-4.5
6979	AT1G33600.1 | leucine-rich repeat family protein, contains leucine rich-repeat (LRR) domains Pfam:PF00560, INTERPRO:IPR001611; contains similarity to gi:9294355:dbj:BAB02252 (Arabidopsis thaliana) | chr1:12180756-12182305 FORWARD | Aliases: T1E4.2, T1E4_2	2.4	2.6	-0.1	-1.0	100.0%	-5.1
6980	AT1G60010.1 | expressed protein | chr1:22099025-22100242 REVERSE | Aliases: T2K10.6, T2K10_6	5.0	4.5	0.5	1.0	100.0%	-4.1
6981	AT5G62610.1 | basic helix-loop-helix (bHLH) family protein, contains Pfam profile: PF00010 helix-loop-helix DNA-binding domain	9.4	9.1	0.3	1.0	100.0%	-4.3
6982	AT1G22590.2 | MADS-box family protein, similar to putative DNA-binding protein GI:6714399 from (Arabidopsis thaliana); MADS-box protein AGL87 | chr1:7983500-7984224 FORWARD | Aliases: F12K8.7, F12K8_7	3.4	3.3	0.2	1.0	100.0%	-4.9
6983	AT1G14850.1 | Symbol: NUP155 |	4.4	4.8	-0.4	-1.0	100.0%	-4.2
6984	AT1G10830.1 | sodium symporter-related, contains five transmembrane domains; Interpro IPR001991 Sodium:dicarboxylate symporter; EST gb:F13926 comes from this gene | chr1:3605590-3607494 REVERSE | Aliases: T16B5.3, T16B5_3	6.1	5.8	0.4	1.0	100.0%	-4.4
6985	AT5G53410.1 | hypothetical protein, similar to unknown protein (gb AAC73044.1)	3.9	3.5	0.4	1.0	100.0%	-4.6
6986	AT4G27080.1 | Symbol: ATPDIL5 4 | Encodes a protein disulfide isomerase-like (PDIL) protein, a member of a multigene family within the thioredoxin (TRX) superfamily.	6.6	7.2	-0.6	-1.0	100.0%	-4.3
6987	AT3G13610.1 | oxidoreductase, 2OG-Fe(II) oxygenase family protein, similar to desacetoxyvindoline 4-hydroxylase (Catharanthus roseus)(GI:1916643), flavonol synthase 1 (SP:Q96330); contains PF03171 2OG-Fe(II) oxygenase superfamily domain | chr3:4449455-4451184 FORWARD | Aliases: K20M4.9	2.8	3.2	-0.4	-1.0	100.0%	-4.0
6988	AT2G26250.1 | Symbol: FDH | beta-ketoacyl-CoA synthase family (FIDDLEHEAD) (FDH), identical to GB:AJ010713 (fiddlehead protein) | chr2:11177496-11180220 REVERSE | Aliases: FIDDLEHEAD, T1D16.11, T1D16_11	2.5	2.9	-0.4	-1.0	100.0%	-3.9
6989	AT1G67860.1 | expressed protein | chr1:25448468-25448926 REVERSE | Aliases: T23K23.29, T23K23_29	3.0	2.8	0.2	1.0	100.0%	-4.9
6990	AT1G49660.1 | expressed protein | chr1:18382365-18383467 REVERSE | Aliases: F14J22.11, F14J22_11	6.8	7.4	-0.6	-1.0	100.0%	-4.0
6991	AT5G47200.1 | Ras-related GTP-binding protein, putative, similar to GTP-binding protein GI:303750 from (Pisum sativum)	9.5	10.1	-0.6	-1.0	100.0%	-4.4
6992	AT3G57940.1 | expressed protein, contains Pfam profile PF05127: Putative ATPase (DUF699) | chr3:21460275-21466938 FORWARD | Aliases: T10K17.150	4.2	4.6	-0.4	-1.0	100.0%	-4.1
6993	AT2G32660.1 | disease resistance family protein / LRR family protein, contains leucine rich-repeat domains Pfam:PF00560, INTERPRO:IPR001611; similar to Cf-4 (Lycopersicon hirsutum) gi:2808683:emb:CAA05268 | chr2:13860836-13863189 REVERSE | Aliases: F24L7.20, F24L7_20	3.3	3.5	-0.2	-1.0	100.0%	-5.1
6994	AT1G75450.1 | Symbol: CKX6 | FAD-binding domain-containing protein / cytokinin oxidase family protein, similar to GB:CAA77151 from (Zea mays) (SP:Q9T0N8) (Plant J. 17 (6), 615-626 (1999)) | chr1:28318582-28321793 REVERSE | Aliases: CYTOKININ OXIDASE 6, F1B16.2, F1B16_2	4.7	4.3	0.4	1.0	100.0%	-4.1

Rank	Description	15.dpi	5.dpi	M	t	adj.q	B
6995	AT4G37370.1 | Symbol: CYP81D8 | cytochrome P450, putative, similar to Cytochrome P450 91A1 (SP:Q9FG65)(Arabidopsis thaliana); cytochrome P450, Glycyrrhiza echinata, AB001379 | chr4:17569822-17571698 REVERSE | Aliases: F6G17.20, F6G17_20	4.9	5.5	-0.6	-1.0	100.0%	-3.8
6996	AT1G50110.1 | branched-chain amino acid aminotransferase 6 / branched-chain amino acid transaminase 6 (BCAT6), contains Pfam profile: PF01063 aminotransferase class IV; identical to SP:Q9LPM9 Branched-chain amino acid aminotransferase 6 (EC 2.6.1.42) (Atbcat-6) {Arabidopsis thaliana} | chr1:18561685-18563940 REVERSE | Aliases: F2J10.4, F2J10_4	6.4	6.7	-0.4	-1.0	100.0%	-4.0
6997	AT4G33370.1 | DEAD-box protein abstrakt, putative, RNA helicase DBP2 - Saccharomyces cerevisiae, PID:g5272 | chr4:16069672-16071408 REVERSE | Aliases: F17M5.130, F17M5_130	3.8	3.5	0.2	1.0	100.0%	-4.6
6998	AT1G70770.1 | expressed protein | chr1:26691892-26694939 REVERSE | Aliases: F5A18.5, F5A18_5	8.2	8.7	-0.5	-1.0	100.0%	-4.3
6999	AT4G25434.2 | similar to MutT/nudix family protein [Arabidopsis thaliana] (TAIR:At5g47650.1); similar to OSJNBa0079A21.19 [Oryza sativa (japonica cultivar-group)] (GB:XP_473408.1); contains InterPro domain Anti-sense to fibroblast growth factor protein GFG (InterPro:IPR003293); contains InterPro domain NUDIX hydrolase (InterPro:IPR000086) | chr4:13003986-13005873 REVERSE | Aliases: None	4.0	4.1	-0.2	-1.0	100.0%	-4.8
7000	AT2G25930.1 | Symbol: ELF3 | hydroxyproline-rich glycoprotein family protein, identical to cDNA nematode responsive protein GI:2213418 | chr2:11066113-11070402 FORWARD | Aliases: EARLY FLOWERING 3, F17H15.25, PYK20	6.3	6.7	-0.4	-1.0	100.0%	-4.3
7001	AT5G24670.1 | similar to novel protein [Danio rerio] (GB:CAD43470.1); contains InterPro domain Cytidine/deoxycytidylate deaminase, zinc-binding region (InterPro:IPR002125) | chr5:8448100-8451221 FORWARD | Aliases: MXC17.5, MXC17_5	5.8	6.3	-0.5	-1.0	100.0%	-4.2
7002	AT3G57210.1 | expressed protein, contains Pfam profile PF04776: Protein of unknown function (DUF626) | chr3:21184341-21184670 REVERSE | Aliases: F28O9.60	2.7	2.5	0.1	1.0	100.0%	-4.9
7003	AT2G19330.1 | leucine-rich repeat family protein, contains leucine rich repeats, Pfam:PF00560 | chr2:8380029-8381535 FORWARD | Aliases: F27F23.13, F27F23_13	2.1	2.2	-0.1	-1.0	100.0%	-5.4
7004	AT1G58150.1 | expressed protein | chr1:21533768-21534123 FORWARD | Aliases: T15M6.2	6.1	5.6	0.5	1.0	100.0%	-4.2
7005	AT5G18920.1 | expressed protein | chr5:6310066-6310848 FORWARD | Aliases: F17K4.170, F17K4_170	3.9	4.2	-0.3	-1.0	100.0%	-4.2
7006	AT5G13610.1 | expressed protein | chr5:4383016-4385032 FORWARD | Aliases: T6I14.13	7.1	7.4	-0.4	-1.0	100.0%	-4.4
7007	AT1G68340.1 | expressed protein | chr1:25617860-25619066 REVERSE | Aliases: T22E19.3, T22E19_3	4.3	4.1	0.2	1.0	100.0%	-4.6
7008	AT1G24240.1 | ribosomal protein L19 family protein, similar to plastid ribosomal protein L19 precursor (Spinacia oleracea) gi:7582403:gb:AAF64312 | chr1:8586342-8588070 FORWARD | Aliases: F3I6.17, F3I6_17	4.9	4.7	0.2	1.0	100.0%	-4.5
7009	AT2G25430.1 | epsin N-terminal homology (ENTH) domain-containing protein, contains Pfam PF01417: ENTH domain. ENTH (Epsin N-terminal homology) domain;	6.0	5.6	0.4	1.0	100.0%	-4.3
7010	AT3G06700.3 | similar to 60S ribosomal protein L29 (RPL29B) [Arabidopsis thaliana] (TAIR:At3g06680.1); similar to P0475H04.11 [Oryza sativa (japonica cultivar-group)] (GB:NP_914175.1); contains InterPro domain Ribosomal L29e protein (InterPro:IPR002673) | chr3:2117361-2117960 REVERSE | Aliases: T8E24.8	10.9	11.4	-0.5	-1.0	100.0%	-4.6
7011	AT4G21990.1 | Symbol: APR3 | 5'-adenylylsulfate reductase (APR3) / PAPS reductase homolog (PRH26), identical to 5'-adenylylsulfate reductase (Arabidopsis thaliana) GI:2738760; identical to cDNA PAPS reductase homolog (PRH26) GI:1710113 | chr4:11657020-11659069 REVERSE | Aliases: 5' ADENYLYLPHOSPHOSULFATE REDUCTASE, APS REDUCTASE 3, ATAPR3, F1N20.90, F1N20_90, PAPS REDUCTASE HOMOLOG 26, PRH 26, PRH26	6.5	7.1	-0.6	-1.0	100.0%	-4.3
7012	AT1G24520.1 | Symbol: BCP1 | anther-specific protein agp1, identical to agp1 GI:780177 from (Arabidopsis thaliana) | chr1:8688617-8689251 FORWARD | Aliases: F21J9.18, F21J9_18	4.9	4.6	0.3	1.0	100.0%	-4.4
7013	AT1G12790.1 | expressed protein | chr1:4359149-4361267 REVERSE | Aliases: F13K23.4, F13K23_4	2.9	2.7	0.2	1.0	100.0%	-4.9
7014	AT1G69160.1 | expressed protein | chr1:26003877-26005251 FORWARD | Aliases: F4N2.12, F4N2_12	2.7	2.8	-0.1	-1.0	100.0%	-5.1
7015	AT1G24450.1 | ribonuclease III family protein, contains similarity to Swiss-Prot:P51837 ribonuclease III (EC 3.1.26.3) (RNase III) (Coxiella burnetii)	5.9	5.4	0.5	1.0	100.0%	-4.3
7016	AT2G15660.1 | similar to MADS-box family protein [Arabidopsis thaliana] (TAIR:At2g40210.1); similar to unnamed protein product [Oryza sativa (japonica cultivar-group)] (GB:NP_908461.1); similar to putative MADS-box protein AGL35 [Oryza sativa (japonica cultivar-group)] (GB:BAD86857.1) | chr2:6831328-6832658 REVERSE | Aliases: F9O13.21	2.8	2.9	-0.1	-1.0	100.0%	-5.2

Rank	Description	15.dpi	5.dpi	M	t	adj.q	B
7017	AT2G25730.1 | expressed protein | chr2:10963380-10979807 REVERSE | Aliases: F3N11.1, F3N11_1	7.1	7.3	-0.3	-1.0	100.0%	-4.6
7018	AT2G36990.1 | Symbol: SIGF | RNA polymerase sigma subunit SigF (sigF) / sigma-like factor (SIG6), identical to RNA polymerase sigma subunit SigF (Arabidopsis thaliana) GI:7209640; contains Pfam profiles PF04545: Sigma-70, region 4, PF04539: Sigma-70 region 3, PF04542: Sigma-70 region 2; identical to cDNA partial mRNA for putative sigma-like transcription factor (sig6 gene) GI:6273429 | chr2:15544337-15547218 REVERSE | Aliases: RNA POLYMERASE SIGMA 70 FACTOR, SIG6, T1J8.17, T1J8_17	5.8	6.2	-0.3	-1.0	100.0%	-4.2
7019	AT5G50170.1 | C2 domain-containing protein / GRAM domain-containing protein, low similarity to SP:P40748 Synaptotagmin III (SytIII) {Rattus norvegicus}; contains Pfam profiles PF00168: C2 domain, PF02893: GRAM domain | chr5:20438299-20442812 FORWARD | Aliases: K6A12.3, K6A12_3	5.3	5.7	-0.4	-1.0	100.0%	-4.1
7020	AT5G19830.1 | similar to peptidyl-tRNA hydrolase family protein [Arabidopsis thaliana] (TAIR:At5g16140.1); similar to CRS2 [Zea mays] (GB:AAF27939.1); similar to A Chain A, Crystal Structure Of The Chloroplast Group Ii Intron Splicing Factor Crs2 (GB:1RYB); contains InterPro domain Peptidyl-tRNA hydrolase (InterPro:IPR001328) | chr5:6703152-6705248 FORWARD | Aliases: T29J13.250, T29J13_250	6.8	6.4	0.4	1.0	100.0%	-3.9
7021	AT2G20490.2 | Symbol: NOP10 | expressed protein, similar to hypothetical protein LOC548917 [Xenopus tropicalis] (GB:NP_001016163.1); contains InterPro domain Nucleolar RNA-binding protein Nop10p (InterPro:IPR007264) | chr2:8838797-8839997 FORWARD | Aliases: NOP10, T13C7.8, T13C7_8	7.0	7.6	-0.6	-1.0	100.0%	-4.0
7022	AT2G18280.2 | similar to F-box family protein / tubby family protein [Arabidopsis thaliana] (TAIR:At1g47270.1); similar to F-box family protein / tubby family protein [Arabidopsis thaliana] (TAIR:At2g47900.1); similar to putative tubby-like protein [Oryza sativa (japonica cultivar-group)] (GB:XP_467371.1); contains InterPro domain Cyclin-like F-box (InterPro:IPR001810); contains InterPro domain Tubby (InterPro:IPR000007) | chr2:7953080-7955550 FORWARD | Aliases: T30D6.21, T30D6_21	8.8	8.3	0.5	1.0	100.0%	-4.2
7023	AT2G28330.1 | expressed protein | chr2:12106967-12107834 REVERSE | Aliases: T1B3.15, T1B3_15	3.8	3.6	0.2	1.0	100.0%	-4.7
7024	AT3G62080.1 | SNF7 family protein, contains Pfam domain, PF03357: SNF7 family | chr3:22997151-22999743 FORWARD | Aliases: T17J13.40	2.7	2.9	-0.3	-1.0	100.0%	-4.9
7025	ATCG00740.1 | Symbol: RPOA | RNA polymerase alpha subunit | chrC:77901-78890 REVERSE | Aliases: RPOA	9.5	10.2	-0.7	-1.0	100.0%	-4.2
7026	AT5G40180.1 | hypothetical protein, predicted protein, Arabidopsis thaliana | chr5:16085151-16085434 FORWARD | Aliases: MSN9.8, MSN9_8	2.9	2.8	0.1	1.0	100.0%	-5.2
7027	AT1G56230.1 | expressed protein | chr1:21049699-21052690 REVERSE | Aliases: F14G9.16, F14G9_16	6.6	7.1	-0.4	-1.0	100.0%	-4.3
7028	AT2G34310.3 | expressed protein | chr2:14484820-14487041 REVERSE | Aliases: F13P17.27	6.5	6.8	-0.3	-1.0	100.0%	-4.4
7029	AT4G05050.2 | Symbol: UBQ11 | similar to polyubiquitin (UBQ14) [Arabidopsis thaliana] (TAIR:At4g02890.1); similar to polyubiquitin (UBQ3) [Arabidopsis thaliana] (TAIR:At5g03240.2); similar to polyubiquitin (UBQ4) [Arabidopsis thaliana] (TAIR:At5g20620.1); similar to polyubiquitin (UBQ14) [Arabidopsis thaliana] (TAIR:At4g02890.2); similar to polyubiquitin (UBQ14) [Arabidopsis thaliana] (TAIR:At4g02890.3); similar to ubiquitin-like protein [Phaseolus vulgaris] (GB:AAB36545.1); similar to polyubiquitin [Fragaria x ananassa] (GB:AAB68045.1); similar to pentameric polyubiquitin (GB:AAA34124.1); contains InterPro domain Ubiquitin domain (InterPro:IPR000626) | chr4:2588002-2589137 REVERSE | Aliases: C17L7.6, UBIQUITIN 11	13.6	13.3	0.3	1.0	100.0%	-5.3
7030	AT5G40420.1 | Symbol: OLEO2 | glycine-rich protein / oleosin | chr5:16190621-16192011 REVERSE | Aliases: OLEOSIN TYPE 2, PA23	3.0	2.8	0.3	1.0	100.0%	-4.6
7031	AT3G52590.1 | Symbol: UBQ1 | ubiquitin extension protein 1 (UBQ1) / 60S ribosomal protein L40 (RPL40B), identical to GI:166929, GI:166930 | chr3:19516614-19517910 FORWARD | Aliases: EMB2167, EMBRYO DEFECTIVE 2167, F3C22.8, UBIQUITIN EXTENSION PROTEIN 1	12.2	12.4	-0.2	-1.0	100.0%	-4.6
7032	AT1G73530.1 | RNA recognition motif (RRM)-containing protein, low similarity to SP:Q03251 Glycine-rich RNA-binding protein 8 (CCR1 protein) {Arabidopsis thaliana}; contains InterPro entry IPR000504: RNA-binding region RNP-1 (RNA recognition motif) (RRM) | chr1:27647294-27649047 REVERSE | Aliases: T9L24.48, T9L24_48	4.7	5.1	-0.3	-1.0	100.0%	-4.1
7033	AT3G49910.1 | 60S ribosomal protein L26 (RPL26A), 60S RIBOSOMAL PROTEIN L26, Brassica rapa, EMBL:BRD495 | chr3:18515241-18515952 FORWARD | Aliases: F3A4.4	9.0	9.4	-0.4	-1.0	100.0%	-4.3
7034	AT2G12940.1 | similar to expressed protein [Arabidopsis thaliana] (TAIR:At2g13150.1); similar to transcription factor VSF-1 [Lycopersicon esculentum] (GB:CAA05898.1); similar to vsf-1 [Lycopersicon esculentum] (GB:CAA52015.1); similar to vsf-1 protein - tomato (GB:S52203); contains InterPro domain Basic-leucine zipper (bZIP) transcription factor (InterPro:IPR004827) | chr2:5324557-5325483 FORWARD | Aliases: T19K21.9, T19K21_9	2.3	2.2	0.1	1.0	100.0%	-5.2
7035	AT3G42550.1 | aspartyl protease family protein, weak similarity to CND41, chloroplast nucleoid DNA binding protein (Nicotiana tabacum) GI:2541876; contains Pfam profile PF00026: Eukaryotic aspartyl protease	2.2	2.4	-0.2	-1.0	100.0%	-5.2

Rank	Description	15.dpi	5.dpi	M	t	adj.q	B
7036	AT5G65940.2 | Symbol: CHY1 | similar to 3-hydroxyisobutyryl-coenzyme A hydrolase, putative / CoA-thioester hydrolase, putative [Arabidopsis thaliana] (TAIR:At2g30660.1); similar to putative enoyl-CoA-hydratase [Oryza sativa (japonica cultivar-group)] (GB:AAP54951.1); similar to enoyl-CoA hydratase [Prunus armeniaca] (GB:AAB88874.1); contains InterPro domain Enoyl-CoA hydratase/isomerase (InterPro:IPR001753) | chr5:26393917-26396460 REVERSE | Aliases: BETA HYDROXYISOBUTYRYL COA HYDROLASE 1, COA THIOESTER HYDROLASE, K14B20.11, K14B20_11	6.3	5.9	0.4	1.0	100.0%	-4.5
7037	AT1G10560.1 | armadillo/beta-catenin repeat family protein / U-box domain-containing protein, contains Pfam domain, PF00514: Armadillo/beta-catenin-like repeats and Pfam, PF04564: U-box domain | chr1:3484581-3486966 FORWARD | Aliases: T10O24.19, T10O24_19	4.9	4.5	0.4	1.0	100.0%	-4.4
7038	AT4G15040.1 | subtilase family protein, contains similarity to prepro-cucumisin GI:807698 from (Cucumis melo) | chr4:8581368-8584117 REVERSE | Aliases: DL3561C, FCAALL.176	2.4	2.3	0.1	1.0	100.0%	-5.3
7039	AT5G37380.2 | DNAJ heat shock N-terminal domain-containing protein, similar to SP:Q9QYI4 DnaJ homolog subfamily B member 12 {Mus musculus}; contains Pfam profile PF00226: DnaJ domain	5.4	6.2	-0.8	-1.0	100.0%	-4.3
7040	AT2G42620.1 | Symbol: MAX2 | F-box family protein (ORE9), E3 ubiquitin ligase SCF complex F-box subunit; identical to F-box containing protein ORE9 GI:15420162 from (Arabidopsis thaliana) | chr2:17763217-17765545 FORWARD | Aliases: F14N22.11, F14N22_11, MORE AXILLARY BRANCHES 2, ORE9, ORESARA 9	6.5	6.9	-0.5	-1.0	100.0%	-4.3
7041	AT4G17650.1 | aromatic-rich family protein, contains Pfam PF03654: Aromatic-Rich Protein Family | chr4:9827532-9829668 FORWARD | Aliases: DL4860W, FCAALL.381	4.6	5.0	-0.5	-1.0	100.0%	-4.3
7042	AT3G51300.1 | Symbol: ROP1 | Rac-like GTP-binding protein (ARAC11) / Rho-like GTP-binding protein (ROP1), identical to GTP binding protein Rop1At (Arabidopsis thaliana) GI:2558666, rac-like GTP binding protein Arac11 (Arabidopsis thaliana) GI:3603426 | chr3:19053866-19055330 FORWARD | Aliases: F24M12.340, ROP1AT	2.7	3.0	-0.3	-1.0	100.0%	-4.3
7043	AT2G18980.1 | peroxidase, putative, identical to peroxidase ATP22a (Arabidopsis thaliana) gi:1620369:emb:CAA70034 | chr2:8240417-8242394 REVERSE | Aliases: F19F24.18, F19F24_18	2.6	2.8	-0.1	-1.0	100.0%	-4.9
7044	AT4G18580.2 | expressed protein | chr4:10234421-10236028 REVERSE | Aliases: F28J12.240, F28J12_240	7.8	7.1	0.6	1.0	100.0%	-4.2
7045	AT1G80690.1 | expressed protein | chr1:30333961-30335695 REVERSE | Aliases: F23A5.4, F23A5_4	5.2	5.5	-0.3	-1.0	100.0%	-4.4
7046	AT4G35480.1 | Symbol: RHA3B | zinc finger (C3HC4-type RING finger) family protein, contains Pfam domain PF00097: Zinc finger, C3HC4 type (RING finger) | chr4:16852079-16852952 REVERSE | Aliases: F15J1.50, F15J1_50, RING H2 FINGER PROTEIN RHA3B	5.3	4.7	0.6	1.0	100.0%	-3.9
7047	AT4G39050.1 | kinesin-related protein (MKRP2), kinesin motor protein - Ustilago maydis, PID:g2062750; identical to cDNA MKRP2 mRNA for kinesin-related protein GI:16902293, kinesin-related protein (Arabidopsis thaliana) GI:16902294 | chr4:18193129-18200691 FORWARD | Aliases: F19H22.150, F19H22_150	3.4	4.2	-0.8	-1.0	100.0%	-4.3
7048	AT3G58760.1 | ankyrin protein kinase, putative, similar to ankyrin-kinase (Medicago truncatula) gi:18700701:gb:AAL78674 | chr3:21739691-21742904 FORWARD | Aliases: T20N10.110	4.5	4.2	0.3	1.0	100.0%	-4.6
7049	AT5G61865.1 | expressed protein | chr5:24864553-24867018 FORWARD | Aliases: None	3.5	3.7	-0.2	-1.0	100.0%	-4.7
7050	AT3G46070.1 | zinc finger (C2H2 type) family protein, contains zinc finger, C2H2 type, domain, PROSITE:PS00028	3.3	3.1	0.2	1.0	100.0%	-4.8
7051	AT4G31240.2 | expressed protein | chr4:15176461-15178514 REVERSE | Aliases: F8F16.60, F8F16_60	4.3	3.9	0.3	1.0	100.0%	-4.2
7052	AT5G38660.1 | Symbol: APE1 | expressed protein, similar to unknown protein (pir::S75762) | chr5:15490363-15492765 REVERSE | Aliases: ACCLIMATION OF PHOTOSYNTHESIS TO ENVIRONMENT, MBB18.21, MBB18_21	10.0	9.3	0.6	1.0	100.0%	-4.3
7053	AT2G26210.1 | ankyrin repeat family protein, contains ankyrin repeats, Pfam:PF00023 | chr2:11164230-11166369 FORWARD | Aliases: T1D16.15, T1D16_15	4.9	4.6	0.3	1.0	100.0%	-4.2
7054	AT1G63940.4 | monodehydroascorbate reductase, putative, similar to monodehydroascorbate reductase GB:AAD28178 (Brassica juncea) | chr1:23733769-23737555 FORWARD | Aliases: T12P18.4, T12P18_4	9.4	10.2	-0.7	-1.0	100.0%	-4.4
7055	AT5G48120.1 | expressed protein, low similarity to MMS19 (Mus musculus) GI:14029390 | chr5:19525177-19532000 REVERSE | Aliases: MDN11.21, MDN11_21	4.4	4.7	-0.2	-1.0	100.0%	-4.5
7056	AT3G52490.1 | heat shock protein-related, contains similarity to 101 kDa heat shock protein; HSP101 (Triticum aestivum) gi:11561808:gb:AAC83689 | chr3:19466677-19469897 REVERSE | Aliases: F22O6.130	5.3	5.8	-0.5	-1.0	100.0%	-4.2

Rank	Description	15.dpi	5.dpi	M	t	adj.q	B
7057	AT3G27300.2 | Symbol: G6PD5 | similar to glucose-6-phosphate 1-dehydrogenase / G6PD (ACG12) [Arabidopsis thaliana] (TAIR:At5g40760.1); similar to G6PD_MEDSA Glucose-6-phosphate 1-dehydrogenase, cytoplasmic isoform (G6PD) (GB:Q42919); similar to glucose-6-phosphate dehydrogenase [Nicotiana tabacum] (GB:CAA04992.1); similar to glucose-6-phosphate 1-dehydrogenase [Solanum tuberosum] (GB:CAA52442.1); similar to cytosolic glucose-6-phosphate dehydrogenase 2 [Petroselinum crispum] (GB:AAB69319.1); similar to glucose-6-phosphate dehydrogenase [Nicotiana tabacum] (GB:CAA04993.1); contains InterPro domain Glucose-6-phosphate dehydrogenase (InterPro:IPR001282) | chr3:10084305-10087889 REVERSE | Aliases: G6PD5, GLUCOSE 6 PHOSPHATE DEHYDROGENASE 5, K17E12.12	6.0	6.8	-0.9	-1.0	100.0%	-4.2
7058	AT1G28300.1 | Symbol: LEC2 | transcriptional factor B3 family protein / leafy cotyledon 2 (LEC2), nearly identical to LEAFY COTYLEDON 2 (Arabidopsis thaliana) GI:15987516; contains Pfam profile PF02362: B3 DNA binding domain | chr1:9896854-9899863 REVERSE | Aliases: F3H9.5, F3H9_5, LEAFY COTYLEDON 2	2.4	2.6	-0.2	-1.0	100.0%	-5.1
7059	AT5G41800.1 | amino acid transporter family protein, similar to amino acid permease 1 (Nicotiana sylvestris) GI:976402; belongs to INTERPRO:IPR002422 amino acid/polyamine transporter, family II | chr5:16751059-16753219 FORWARD | Aliases: K16L22.8, K16L22_8	6.2	5.9	0.3	1.0	100.0%	-4.3
7060	AT1G05400.1 | expressed protein, contains Pfam profile PF04396: Protein of unknown function, DUF537 | chr1:1584341-1585243 FORWARD | Aliases: T25N20.5, T25N20_5	4.0	3.7	0.4	1.0	100.0%	-4.5
7061	AT2G15790.1 | Symbol: SQN | peptidyl-prolyl cis-trans isomerase / cyclophilin-40 (CYP40) / rotamase, identical to cyclophilin-40 (Arabidopsis thaliana) GI:13442983; supporting cDNA gi:13442982:gb:AY026065.1: | chr2:6884857-6887980 REVERSE | Aliases: CYCLOPHILIN 40, CYP40, F19G14.21, F19G14_21, SQUINT	3.5	4.1	-0.6	-1.0	100.0%	-4.2
7062	AT5G58680.1 | armadillo/beta-catenin repeat family protein, contains Pfam profile: PF00514 armadillo/beta-catenin-like repeats (4 copies) | chr5:23725338-23726577 REVERSE | Aliases: MZN1.14, MZN1_14	4.6	4.4	0.3	1.0	100.0%	-4.6
7063	AT1G07000.1 | exocyst subunit EXO70 family protein, similar to leucine zipper protein GI:10177020 from (Arabidopsis thaliana) contains Pfam domain PF03081: Exo70 exocyst complex subunit; | chr1:2150193-2152324 REVERSE | Aliases: F10K1.28, F10K1_28	4.8	4.4	0.4	1.0	100.0%	-4.2
7064	AT2G22960.1 | serine carboxypeptidase S10 family protein, contains Pfam profile: PF00450 serine carboxypeptidase ;similar to sinapoylglucose:malate sinapoyltransferase GI:8699619 from (Arabidopsis thaliana)	2.6	2.4	0.1	1.0	100.0%	-5.4
7065	AT1G04000.1 | expressed protein, Location of ESTs 203I24T7, gb:H76794 and 203I24XP, gb:AA605510 | chr1:1028105-1029118 REVERSE | Aliases: F21M11.6, F21M11_6	3.7	3.4	0.2	1.0	100.0%	-4.6
7066	AT3G14630.1 | Symbol: CYP72A9 | cytochrome P450, putative, similar to GB:Q05047 from (Catharanthus roseus) | chr3:4917505-4919416 FORWARD | Aliases: MIE1.13	3.0	2.8	0.2	1.0	100.0%	-4.7
7067	AT4G39510.1 | Symbol: CYP96A12 | cytochrome P450 family protein, contains Pfam PF00067: Cytochrome P450; similar to Cytochrome P450 86A2 (SP:O23066) (Arabidopsis thaliana) | chr4:18368797-18370646 REVERSE | Aliases: F23K16.140, F23K16_140	4.7	4.3	0.4	1.0	100.0%	-4.4
7068	AT4G20740.1 | pentatricopeptide (PPR) repeat-containing protein, contains Pfam profile PF01535: PPR repeat | chr4:11126162-11128345 FORWARD | Aliases: F21C20.90, F21C20_90	3.8	3.9	-0.2	-1.0	100.0%	-4.6
7069	AT1G19320.1 | pathogenesis-related thaumatin family protein, similar to SP:P28493 Pathogenesis-related protein 5 precursor (PR-5) from (Arabidopsis thaliana), thaumatin-like protein (Arabidopsis thaliana) GI:2435406; contains Pfam profile PF00314: Thaumatin family | chr1:6679272-6680242 FORWARD | Aliases: F18O14.4, F18O14_4	2.7	3.0	-0.3	-1.0	100.0%	-4.3
7070	AT5G14180.1 | lipase family protein, similar to SP:Q64194 Lysosomal acid lipase/cholesteryl ester hydrolase precursor (EC 3.1.1.13) {Rattus norvegicus}; contains Pfam profile PF04083: ab-hydrolase associated lipase region | chr5:4571341-4574464 REVERSE | Aliases: MUA22.18, MUA22_18	3.1	3.0	0.1	1.0	100.0%	-5.1
7071	AT4G10950.1 | GDSL-motif lipase/hydrolase family protein, similar to family II lipase EXL1 GI:15054382 from (Arabidopsis thaliana); contains Pfam profile PF00657: GDSL-like Lipase/Acylhydrolase | chr4:6711498-6713508 REVERSE | Aliases: F25I24.160, F25I24_160	2.4	2.6	-0.1	-1.0	100.0%	-5.2
7072	AT5G43320.1 | casein kinase, putative, similar to casein kinase I (CKI2) (Arabidopsis thaliana) gi:1103322:emb:CAA55397; contains protein kinase domain, Pfam:PF00069 | chr5:17403095-17406629 REVERSE | Aliases: MWF20.1, MWF20_1	6.3	6.6	-0.4	-1.0	100.0%	-4.5
7073	AT3G31300.1 | hypothetical protein | chr3:12676906-12677097 FORWARD | Aliases: T22B15.1	2.2	2.3	-0.1	-1.0	100.0%	-5.7
7074	AT1G51700.1 | Symbol: ADOF1 | Dof-type zinc finger domain-containing protein (ADOF1), identical to cDNA adof1 mRNA for dof zinc finger protein, GI:3608260; contains Pfam profile PF02701: Dof domain, zinc finger | chr1:19177738-19178857 FORWARD | Aliases: ADOF1, F19C24.9, F19C24_9	6.3	6.0	0.3	1.0	100.0%	-4.3
7075	AT3G58840.1 | expressed protein | chr3:21768289-21769749 REVERSE | Aliases: T20N10.190	8.0	8.6	-0.6	-1.0	100.0%	-4.2
7076	AT3G57290.1 | Symbol: EIF3E | eukaryotic translation initiation factor 3E / eIF3e (TIF3E1), identical to eukaryotic initiation factor 3E subunit (Arabidopsis thaliana) gi:12407658:gb:AAG53613 | chr3:21207533-21210127 REVERSE | Aliases: ATEIF3E 1, EIF3E, F28O9.140, INT 6, TIF3E1	8.2	9.0	-0.7	-1.0	100.0%	-4.2

Rank	Description	15.dpi	5.dpi	M	t	adj.q	B
7077	AT1G16790.1 | ribosomal protein-related, similar to Chloroplast 30S ribosomal protein S8 (SP:P21508) (Euglena gracilis) | chr1:5744558-5745202 FORWARD | Aliases: F17F16.10, F17F16_10	5.3	5.7	-0.4	-1.0	100.0%	-4.2
7078	AT1G21170.1 | expressed protein | chr1:7413039-7419400 FORWARD | Aliases: T22I11.1	3.0	3.3	-0.2	-1.0	100.0%	-4.9
7079	AT1G05510.1 | expressed protein | chr1:1629452-1630862 FORWARD | Aliases: T25N20.16	2.8	2.7	0.2	1.0	100.0%	-4.9
7080	AT4G12750.1 | expressed protein | chr4:7497811-7503640 REVERSE | Aliases: T20K18.100, T20K18_100	4.5	4.7	-0.3	-1.0	100.0%	-4.5
7081	AT1G32570.1 | hypothetical protein | chr1:11777281-11777537 FORWARD | Aliases: T9G5.6, T9G5_6	4.6	4.2	0.4	1.0	100.0%	-4.3
7082	AT3G29260.1 | short-chain dehydrogenase/reductase (SDR) family protein, similar to 3-beta-hydroxysteroiddehydrogenase GI:15983819 from (Digitalis lanata) | chr3:11217189-11218103 REVERSE | Aliases: MXO21.13	4.1	3.8	0.3	1.0	100.0%	-4.4
7083	AT4G16900.1 | disease resistance protein (TIR-NBS-LRR class), putative, domain signature TIR-NBS-LRR exists, suggestive of a disease resistance protein. | chr4:9512026-9516560 REVERSE | Aliases: DL4480C, FCAALL.310	2.5	2.3	0.2	1.0	100.0%	-4.8
7084	AT3G20290.2 | similar to calcium-binding EF hand family protein [Arabidopsis thaliana] (TAIR:At4g05520.2); similar to calcium-binding EF hand family protein [Arabidopsis thaliana] (TAIR:At4g05520.1); similar to OSJNBa0043A12.19 [Oryza sativa (japonica cultivar-group)] (GB:XP_474282.1); similar to putative receptor Mediated Endocytosis RME-1 [Oryza sativa (japonica cultivar-group)] (GB:BAD28174.1); contains InterPro domain Calcium-binding EF-hand (InterPro:IPR002048); contains InterPro domain EPS15 homology (EH) (InterPro:IPR000261) | chr3:7074823-7078789 REVERSE | Aliases: MQC12.3	8.4	8.0	0.4	1.0	100.0%	-4.7
7085	AT1G21900.1 | emp24/gp25L/p24 family protein, similar to SP:O35587 Transmembrane protein Tmp21 precursor (21 kDa Transmembrane trafficking protein) {Mesocricetus auratus}; contains Pfam profile PF01105: emp24/gp25L/p24 family | chr1:7690859-7692370 REVERSE | Aliases: T26F17.12, T26F17_12	8.2	7.8	0.4	1.0	100.0%	-4.9
7086	AT4G25870.1 | expressed protein, contains Pfam profile PF03267: Arabidopsis protein of unknown function, DUF266 | chr4:13149700-13152286 REVERSE | Aliases: F14M19.150, F14M19_150	7.2	7.4	-0.2	-1.0	100.0%	-4.8
7087	AT5G56790.1 | protein kinase family protein, contains protein kinase domain, Pfam:PF00069 | chr5:22985165-22988756 FORWARD | Aliases: MIK19.26, MIK19_26	7.3	7.0	0.3	1.0	100.0%	-4.4
7088	AT1G42470.1 | patched family protein, similar to SP:O15118 Niemann-Pick C1 protein precursor from Homo sapiens (GB:AAB63982) (GI:2276463); contains Pfam profile PF02460 Patched family | chr1:15929427-15937648 FORWARD | Aliases: F7F22.1, F7F22_1	7.1	7.5	-0.4	-1.0	100.0%	-4.6
7089	AT5G15610.2 | proteasome family protein, contains Pfam domain, PF01399: PCI domain | chr5:5079516-5082208 FORWARD | Aliases: T20K14.220, T20K14_220	6.1	6.4	-0.3	-1.0	100.0%	-4.3
7090	AT2G35800.1 | mitochondrial substrate carrier family protein, contains INTERPRO:IPR001993 Mitochondrial substrate carrier family, INTERPRO:IPR002048 calcium-binding EF-hand domain | chr2:15051124-15055682 FORWARD | Aliases: F11F19.29, F11F19_29	4.9	5.2	-0.2	-1.0	100.0%	-4.5
7091	AT5G54070.1 | Symbol: AT HSFA9 | heat shock transcription factor family protein, contains Pfam profile: PF00447 HSF-type DNA-binding domain | chr5:21961209-21962545 FORWARD | Aliases: HSFA9, MJP23.4, MJP23_4	2.7	2.6	0.1	1.0	100.0%	-5.3
7092	AT2G28370.1 | expressed protein, contains Pfam PF04535 : Domain of unknown function (DUF588); similar to putative ethylene responsive element binding protein (GI:22135858) (Arabidopsis thaliana) | chr2:12138651-12140776 FORWARD | Aliases: T1B3.11, T1B3_11	5.6	4.8	0.8	1.0	100.0%	-4.2
7093	AT3G02810.1 | protein kinase family protein, contains protein kinase domain, Pfam:PF00069 | chr3:608467-610992 REVERSE | Aliases: F13E7.25, F13E7_25	2.5	2.4	0.1	1.0	100.0%	-5.1
7094	AT1G47730.1 | F-box family protein, contains Pfam PF00646: F-box domain; contains TIGRFAM TIGR01640 : F-box protein interaction domain; identical to hypothetical protein GB:AAD46016 GI:5668790 from (Arabidopsis thaliana) | chr1:17566371-17567546 REVERSE | Aliases: T2E6.20	2.5	2.7	-0.1	-1.0	100.0%	-5.4
7095	AT3G55420.1 | expressed protein | chr3:20558694-20559744 FORWARD | Aliases: T22E16.80	5.6	5.3	0.4	1.0	100.0%	-4.5
7096	AT3G11270.1 | 26S proteasome non-ATPase regulatory subunit 7, putative / 26S proteasome regulatory subunit S12, putative / MOV34 protein, putative, contains similarity to 26S proteasome regulatory subunit S12 (MOV34) SP:P26516 from (Mus musculus)	6.9	7.7	-0.8	-1.0	100.0%	-4.2
7097	AT4G32840.1 | phosphofructokinase family protein, similar to phosphofructokinase (Amycolatopsis methanolica) GI:17432243; contains Pfam profile PF00365: Phosphofructokinase | chr4:15844784-15848445 REVERSE | Aliases: T16I18.50, T16I18_50	4.9	5.2	-0.3	-1.0	100.0%	-4.7
7098	AT4G36020.1 | cold-shock DNA-binding family protein, contains Pfam domains, PF00313: 'Cold-shock' DNA-binding domain and PF00098: Zinc knuckle	9.5	9.1	0.3	1.0	100.0%	-4.4

Rank	Description	15.dpi	5.dpi	M	t	adj.q	B
7099	AT2G24120.2 | Symbol: PDE319 |	3.9	4.1	-0.2	-1.0	100.0%	-4.6
7100	AT2G14050.1 | minichromosome maintenance family protein / MCM family protein, low similarity to SP:P49736 DNA replication licensing factor MCM2 {Homo sapiens}; contains Pfam profile PF00493: MCM2/3/5 family	3.1	3.3	-0.2	-1.0	100.0%	-4.8
7101	AT5G02420.1 | expressed protein | chr5:523387-524074 FORWARD | Aliases: T22P11.10, T22P11_10	3.2	2.7	0.5	1.0	100.0%	-4.2
7102	AT3G06440.1 | galactosyltransferase family protein, contains Pfam profile: PF01762 galactosyltransferase | chr3:1972628-1975760 REVERSE | Aliases: F24P17.7, F24P17_7	4.2	4.5	-0.2	-1.0	100.0%	-4.5
7103	AT3G52260.2 | pseudouridine synthase family protein, similar to SP:P39219 Ribosomal large subunit pseudouridine synthase A (EC 4.2.1.70) (Pseudouridylate synthase) (Uracil hydrolyase) {Escherichia coli}; contains Pfam profile PF00849: RNA pseudouridylate synthase | chr3:19394254-19397453 REVERSE | Aliases: T25B15.30	4.2	3.9	0.3	1.0	100.0%	-4.3
7104	AT3G56090.1 | Symbol: ATFER3 |	7.2	7.9	-0.7	-1.0	100.0%	-3.8
7105	AT3G52220.1 | expressed protein | chr3:19380340-19382033 REVERSE | Aliases: T25B15.5	10.8	10.4	0.3	1.0	100.0%	-4.5
7106	AT1G48490.2 | similar to protein kinase, putative [Arabidopsis thaliana] (TAIR:At3g17850.1); similar to incomplete root hair elongation (IRE) / protein kinase, putative [Arabidopsis thaliana] (TAIR:At5g62310.1); similar to putative AGC family protein kinase [Dictyostelium discoideum] (GB:EAL71293.1); similar to similar to cell wall biosynthesis kinase; Cbk1p [Saccharomyces cerevisiae] [Dictyostelium discoideum] (GB:AAS45329.1); contains InterPro domain Serine/threonine protein kinase, active site (InterPro:IPR008271); contains InterPro domain Tyrosine protein kinase (InterPro:IPR001245); contains InterPro domain Serine/threonine protein kinase (InterPro:IPR002290); contains InterPro domain Protein kinase (InterPro:IPR000719)	7.8	6.9	0.9	1.0	100.0%	-4.3
7107	AT3G17850.1 | protein kinase, putative, similar to IRE (incomplete root hair elongation) (Arabidopsis thaliana) gi:6729346:dbj:BAA89783; contains protein kinase domain Pfam:PF00069 | chr3:6109711-6116464 REVERSE | Aliases: MEB5.7	7.8	6.9	0.9	1.0	100.0%	-4.3
7108	AT3G05890.1 | Symbol: RCI2B | hydrophobic protein (RCI2B) / low temperature and salt responsive protein (LTI6B), identical to SP:Q9ZNS6 Hydrophobic protein RCI2B (Low temperature and salt responsive protein LTI6B) {Arabidopsis thaliana} | chr3:1757637-1758498 REVERSE | Aliases: F2O10.15, F2O10_15, RARE COLD INDUCIBLE 2B	5.7	4.9	0.8	1.0	100.0%	-4.1
7109	AT5G64890.1 | expressed protein | chr5:25951798-25952735 FORWARD | Aliases: MXK3.12, MXK3_12	3.9	4.5	-0.6	-1.0	100.0%	-4.2
7110	AT1G79390.1 | expressed protein | chr1:29867946-29869214 REVERSE | Aliases: T8K14.19, T8K14_19	7.8	7.5	0.3	1.0	100.0%	-4.6
7111	AT5G64730.1 | transducin family protein / WD-40 repeat family protein, contains 7 WD-40 repeats (PF00400); similar to Will die slowly protein (SP:Q9V3J8) (Fruit fly) {Drosophila m.} | chr5:25890361-25892409 FORWARD | Aliases: MVP7.5, MVP7_5	6.0	5.6	0.4	1.0	100.0%	-4.4
7112	AT3G56580.3 | similar to zinc finger (C3HC4-type RING finger) family protein [Arabidopsis thaliana] (TAIR:At2g40830.1); similar to zinc finger (C3HC4-type RING finger) family protein [Arabidopsis thaliana] (TAIR:At2g40830.3); similar to zinc finger (C3HC4-type RING finger) family protein [Arabidopsis thaliana] (TAIR:At2g40830.2); similar to putative ring finger protein 126 isoform 1 [Oryza sativa (japonica cultivar-group)] (GB:BAD68141.1); contains InterPro domain Zn-finger, RING (InterPro:IPR001841) | chr3:20972568-20974728 FORWARD | Aliases: T5P19.5	3.0	3.3	-0.3	-1.0	100.0%	-4.7
7113	AT1G43260.1 | hypothetical protein, contains Pfam domain, PF04937: Protein of unknown function (DUF 659) | chr1:16320693-16321674 FORWARD | Aliases: F1I21.5, F1I21_5	2.5	2.4	0.1	1.0	100.0%	-5.2
7114	AT1G64370.1 | expressed protein | chr1:23892583-23893324 FORWARD | Aliases: F15H21.1, F15H21_1	3.9	3.6	0.3	1.0	100.0%	-4.3
7115	AT3G54270.1 | sucrose-phosphatase 3 (SPP3), nearly identical to sucrose-phosphatase (SPP3) (Arabidopsis thaliana) GI:16904077 | chr3:20098060-20100713 REVERSE | Aliases: F24B22.230	3.2	3.5	-0.2	-1.0	100.0%	-4.7
7116	AT3G13680.1 | F-box family protein, contains F-box domain Pfam:PF00646	4.1	3.7	0.4	1.0	100.0%	-4.5
7117	AT5G19260.1 | expressed protein, various predicted proteins | chr5:6479402-6480448 REVERSE | Aliases: F7K24.10, F7K24_10	4.9	4.6	0.3	1.0	100.0%	-4.4
7118	AT5G51850.1 | expressed protein, similar to unknown protein (emb:CAB81354.1) | chr5:21096478-21098704 FORWARD | Aliases: MIO24.1, MIO24_1	2.1	2.2	-0.1	-1.0	100.0%	-5.4
7119	AT4G17960.1 | expressed protein | chr4:9970859-9972468 FORWARD | Aliases: T6K21.140, T6K21_140	5.7	6.2	-0.5	-1.0	100.0%	-4.3
7120	AT5G11590.1 | encodes a member of the DREB subfamily A-4 of ERF/AP2 transcription factor family. The protein contains one AP2 domain. There are 17 members in this subfamily including TINY. | chr5:3727790-3728500 REVERSE | Aliases: T22P22.1	2.5	2.4	0.1	1.0	100.0%	-5.1

Rank	Description	15.dpi	5.dpi	M	t	adj.q	B
7121	AT1G14480.1 | similar to ankyrin repeat family protein [Arabidopsis thaliana] (TAIR:At1g14500.1); similar to ankyrin-like protein [Oryza sativa (japonica cultivar-group)] (GB:BAD30891.1); contains InterPro domain Ankyrin (InterPro:IPR002110) | chr1:4956252-4957966 FORWARD | Aliases: F14L17.26, F14L17_26	3.8	3.5	0.3	1.0	100.0%	-4.5
7122	AT2G22340.1 | expressed protein | chr2:9499959-9501035 REVERSE | Aliases: F14M13.26, F14M13_26	2.7	3.0	-0.2	-1.0	100.0%	-4.9
7123	AT5G59730.2 | similar to exocyst subunit EXO70 family protein [Arabidopsis thaliana] (TAIR:At2g28650.1); similar to putative leucine zipper protein [Oryza sativa (japonica cultivar-group)] (GB:BAB86177.1); contains InterPro domain Exo70 exocyst complex subunit (InterPro:IPR004140) | chr5:24081173-24083396 REVERSE | Aliases: MTH12.6, MTH12_6	5.1	4.9	0.2	1.0	100.0%	-4.6
7124	AT5G45530.1 | expressed protein, contains Pfam domain, PF04578: Protein of unknown function, DUF594 | chr5:18471543-18474504 REVERSE | Aliases: MFC19.20, MFC19_20	3.4	3.2	0.3	1.0	100.0%	-4.7
7125	AT1G32990.1 | Symbol: PRPL11 |	6.5	7.0	-0.6	-1.0	100.0%	-3.7
7126	AT5G58140.4 | Symbol: PHOT2 | protein kinase family protein / non phototropic hypocotyl 1-like protein (NPL1), contains Pfam domains, PF00069: Protein kinase domain and PF00785: PAC motif; similar to SP:O48963 Nonphototropic hypocotyl protein 1	4.0	3.8	0.2	1.0	100.0%	-4.8
7127	AT5G04720.1 | Symbol: ADR1 L2 | disease resistance protein (CC-NBS-LRR class), putative, domain signature CC-NBS-LRR exists, suggestive of a disease resistance protein. | chr5:1360713-1363885 FORWARD | Aliases: ADR1 L2, ADR1 LIKE 2, MUK11.4	7.3	6.9	0.5	1.0	100.0%	-4.2
7128	AT5G40850.1 | Symbol: UPM1 | urophorphyrin III methylase (UPM1), identical to urophorphyrin III methylase (GI:1146165) (Arabidopsis thaliana); similar to s-adenosyl-L-methionine-dependent uroporphyrinogen III methyltransferase (GI:1490606) (Arabidopsis thaliana); similar to Diphthine synthase (Diphtamide biosynthesis methyltransferase) (DPH5) (SP:P32469) (Saccharomyces cerevisiae); contains Pfam PF00590 : Tetrapyrrole (Corrin/Porphyrin) Methylases domain; contains TIGRFAM PF00590: Tetrapyrrole (Corrin/Porphyrin) Methylases | chr5:16383742-16386327 FORWARD | Aliases: MHK7.8, MHK7_8, UROPHORPHYRIN METHYLASE 1	7.8	7.5	0.4	1.0	100.0%	-4.5
7129	NA	2.2	2.2	-0.1	-1.0	100.0%	-5.4
7130	AT1G66740.1 | ASF1-like anti-silencing protein, putative, similar to SP:P32447 Anti-silencing protein 1 {Saccharomyces cerevisiae}; contains Pfam profile PF04729: Anti-silencing protein, ASF1-like; supporting cDNA gi:27530935:dbj:AB078339.1: | chr1:24896003-24897429 FORWARD | Aliases: F4N21.13, F4N21_13	4.3	4.7	-0.4	-1.0	100.0%	-4.4
7131	AT3G28760.2 | similar to conserved protein [Methanothermobacter thermautotrophicus str. Delta H] (GB:AAB85086.1); contains InterPro domain Protein of unknown function DUF109 (InterPro:IPR002812) | chr3:10794192-10796541 REVERSE | Aliases: T19N8.5	4.6	4.9	-0.4	-1.0	100.0%	-4.1
7132	AT1G43620.2 | UDP-glucose:sterol glucosyltransferase, putative, similar to UDP-glucose:sterol glucosyltransferase (Arabidopsis thaliana) GI:2462931; contains Pfam profile: PF03033 glycosyltransferase family 28 N-terminal domain | chr1:16427872-16432606 REVERSE | Aliases: T10P12.7, T10P12_7	4.5	5.0	-0.5	-1.0	100.0%	-4.3
7133	AT4G00360.1 | Symbol: CYP86A2 | cytochrome P450, putative | chr4:160768-163002 FORWARD | Aliases: A_IG005I10.21, A_IG005I10_21, F5I10.21, F5I10_21	4.2	3.8	0.5	1.0	100.0%	-4.0
7134	AT3G51680.1 | short-chain dehydrogenase/reductase (SDR) family protein, similar to short-chain alcohol dehydrogenase GI:1877480 from (Tripsacum dactyloides) | chr3:19184601-19185646 REVERSE | Aliases: T18N14.60	3.0	3.2	-0.1	-1.0	100.0%	-5.1
7135	AT1G76400.1 | ribophorin I family protein, similar to ribophorin I (Sus scrofa) GI:9857227; contains Pfam profile PF04597: Ribophorin I | chr1:28663126-28666604 REVERSE | Aliases: F15M4.10, F15M4_10	7.3	8.1	-0.8	-1.0	100.0%	-4.4
7136	AT1G31780.1 | similar to Component of oligomeric golgi complex 6 [Xenopus tropicalis] (GB:AAH75361.1) | chr1:11391210-11394729 FORWARD | Aliases: F5M6.21, F5M6_21	6.5	7.1	-0.5	-1.0	100.0%	-4.1
7137	AT3G09440.1 | heat shock cognate 70 kDa protein 3 (HSC70-3) (HSP70-3), identical to SP:O65719 Heat shock cognate 70 kDa protein 3 (Hsc70.3) {Arabidopsis thaliana} | chr3:2903205-2905728 REVERSE | Aliases: F3L24.33	7.3	7.8	-0.5	-1.0	100.0%	-4.2
7138	AT3G42630.1 | pentatricopeptide (PPR) repeat-containing protein, contains Pfam profile PF01535: PPR repeat | chr3:14733254-14734843 FORWARD | Aliases: T12K4.80	5.1	5.4	-0.3	-1.0	100.0%	-4.4
7139	AT1G79560.1 | Symbol: EMB1047/FTSH12 |	4.8	5.2	-0.4	-1.0	100.0%	-4.4
7140	AT5G64560.2 | magnesium transporter CorA-like family protein (MRS2-2), weak similarity to SP:Q01926 RNA splicing protein MRS2, mitochondrial precursor {Saccharomyces cerevisiae}; contains Pfam profile PF01544: CorA-like Mg2+ transporter protein | chr5:25824347-25826821 REVERSE | Aliases: MUB3.8, MUB3_8	4.3	4.7	-0.3	-1.0	100.0%	-4.5

Rank	Description	15.dpi	5.dpi	M	t	adj.q	B
7141	AT4G10610.2 | Symbol: RBP37 | similar to RNA-binding protein, putative [Arabidopsis thaliana] (TAIR:At1g32790.1); similar to putative RNA-binding protein RBP37 [Oryza sativa (japonica cultivar-group)] (GB:XP_479783.1); contains InterPro domain RNA-binding region RNP-1 (RNA recognition motif) (InterPro:IPR000504) | chr4:6557173-6559591 FORWARD | Aliases: ATRBP37, CID12, RNA BINDING PROTEIN 37, T4F9.70, T4F9_70	9.5	9.0	0.5	1.0	100.0%	-4.5
7142	AT5G13260.1 | expressed protein | chr5:4243031-4246880 FORWARD | Aliases: T31B5.80, T31B5_80	5.5	6.0	-0.5	-1.0	100.0%	-4.2
7143	AT5G02820.1 | Symbol: RHL2 | DNA topoisomerase VIA (SPO11), identical to topoisomerase 6 subunit A (spo11) (Arabidopsis thaliana) GI:12331186	7.2	7.6	-0.4	-1.0	100.0%	-4.1
7144	AT5G47060.1 | senescence-associated protein-related, similar to senescence-associated protein SAG102 (GI:22331931) (Arabidopsis thaliana); | chr5:19134034-19135273 FORWARD | Aliases: MQD22.20, MQD22_20	6.6	6.1	0.4	1.0	100.0%	-4.4
7145	AT1G24050.1 | expressed protein, similar to hypothetical protein [Arabidopsis thaliana] (TAIR:At1g70220.1); similar to hypothetical protein DDB0191031 [Dictyostelium discoideum] (GB:EAL72551.1) | chr1:8507009-8508853 FORWARD | Aliases: T23E23.20, T23E23_20	9.9	9.4	0.5	1.0	100.0%	-4.6
7146	AT2G21440.1 | RNA recognition motif (RRM)-containing protein, contains InterPro entry IPR000504: RNA-binding region RNP-1 (RNA recognition motif) (RRM) | chr2:9180640-9186246 REVERSE | Aliases: F3K23.20, F3K23_20	7.8	8.3	-0.5	-1.0	100.0%	-4.2
7147	AT3G13320.1 | Symbol: CAX2 | calcium exchanger (CAX2), almost identical to low affinity calcium antiporter CAX2 (GI:1488267) (Arabidopsis thaliana); Ca2+:Cation Antiporter (CaCA) Family member PMID:11500563 | chr3:4314536-4318358 FORWARD | Aliases: ATCAX2, CATION EXCHANGER 2, CAX2, MDC11.19	5.9	5.6	0.3	1.0	100.0%	-4.5
7148	AT3G44990.1 | Symbol: XTR8 | xyloglucan:xyloglucosyl transferase, putative / xyloglucan endotransglycosylase, putative / endo-xyloglucan transferase, putative | chr3:16457960-16459749 REVERSE | Aliases: ATXTR8, F14D17.60	6.6	6.2	0.4	1.0	100.0%	-4.3
7149	AT3G12380.1 | Symbol: ATARP5 |	6.8	7.0	-0.2	-1.0	100.0%	-4.6
7150	AT3G26060.1 | peroxiredoxin Q, putative, similar to peroxiredoxin Q (Sedum lineare) GI:6899842; contains Pfam profile: PF00578 AhpC/TSA (alkyl hydroperoxide reductase and thiol-specific antioxidant) family | chr3:9526013-9527611 FORWARD | Aliases: MPE11.21	7.2	7.9	-0.7	-1.0	100.0%	-3.7
7151	AT1G18150.2 | Symbol: ATMPK8 |	5.6	5.9	-0.4	-1.0	100.0%	-4.3
7152	AT5G14500.1 | aldose 1-epimerase family protein, similar to apospory-associated protein C, Chlamydomonas reinhardtii, EMBL:AF195243 Pfam profile PF01263: Aldose 1-epimerase | chr5:4674236-4676768 REVERSE | Aliases: T15N1.5	4.8	4.5	0.3	1.0	100.0%	-4.3
7153	AT3G19280.1 | Symbol: FUT11 | glycoprotein 3-alpha-L-fucosyltransferase A / FucTA / core alpha-(1,3)-fucosyltransferase (FUT11), identical to Glycoprotein 3-alpha-L-fucosyltransferase A (Core alpha-(1,3)-fucosyltransferase)(Fuc-T C3) (FucTA) (FucT1) (AtFUT11)(SP:Q9LJK1) from (Arabidopsis thaliana); contains Pfam profile PF00852: Fucosyl transferase; identical to cDNA alpha1,3-fucosyltransferase (FucTA) GI:13992482 | chr3:6681303-6683898 REVERSE | Aliases: ATFUT11, FUCT1, FUCTA, MVI11.20	4.4	4.6	-0.2	-1.0	100.0%	-4.7
7154	AT1G23000.1 | heavy-metal-associated domain-containing protein, similar to farnesylated protein ATFP3 (GI:4097547); contains PF00403 Heavy-metal-associated domain | chr1:8143393-8145059 FORWARD | Aliases: F19G10.6, F19G10_6	2.6	2.7	-0.1	-1.0	100.0%	-5.1
7155	AT3G55400.1 | methionyl-tRNA synthetase / methionine--tRNA ligase / MetRS (cpMetRS), identical to methionyl-tRNA synthetase MEtRS (Arabidopsis thaliana) GI:2266985 | chr3:20546608-20550044 REVERSE | Aliases: T22E16.60	8.0	8.4	-0.4	-1.0	100.0%	-3.9
7156	AT5G42890.1 | sterol carrier protein 2 (SCP-2) family protein, similar to sterol carrier protein-2 (Homo sapiens) GI:432973; contains Pfam profile PF02036: SCP-2 sterol transfer family | chr5:17211500-17213260 REVERSE | Aliases: MBD2.8, MBD2_8	9.7	10.0	-0.4	-1.0	100.0%	-4.4
7157	AT4G23630.1 | reticulon family protein (RTNLB1), weak similarity to Nogo-C protein (Rattus norvegicus) GI:6822251; contains Pfam profile PF02453: Reticulon | chr4:12317834-12319947 FORWARD | Aliases: F9D16.100, F9D16_100	9.2	8.5	0.8	1.0	100.0%	-4.3
7158	AT1G27680.1 | Symbol: APL2 | glucose-1-phosphate adenylyltransferase large subunit 2 (APL2) / ADP-glucose pyrophosphorylase, identical to SP:P55230 | chr1:9631272-9634584 FORWARD | Aliases: APL2, T22C5.13, T22C5_13	5.1	5.4	-0.3	-1.0	100.0%	-4.1
7159	AT5G49120.1 | senescence-associated protein-related, similar to senescence-associated protein SAG102 (GI:22331931) (Arabidopsis thaliana); | chr5:19926026-19926558 REVERSE | Aliases: K20J1.9, K20J1_9	2.6	2.5	0.1	1.0	100.0%	-5.2
7160	AT4G18930.1 | cyclic phosphodiesterase, identical to cyclic phosphodiesterase (Arabidopsis thaliana) gi:2065013:emb:CAA72363 | chr4:10370715-10372056 FORWARD | Aliases: F13C5.100, F13C5_100	9.2	9.5	-0.3	-1.0	100.0%	-4.4
7161	AT2G43020.1 | amine oxidase family protein, similar to polyamine oxidase SP:O64411 (Zea mays); contains Pfam profile PF01593 amine oxidase, flavin-containing | chr2:17898580-17901712 FORWARD | Aliases: MFL8.12, MFL8_12	7.3	7.0	0.3	1.0	100.0%	-4.6

Rank	Description	15.dpi	5.dpi	M	t	adj.q	B
7162	AT2G46540.1 | expressed protein | chr2:19117544-19118841 REVERSE | Aliases: F13A10.7, F13A10_7	12.3	12.4	-0.1	-1.0	100.0%	-5.4
7163	AT3G01960.1 | expressed protein | chr3:324588-325094 REVERSE | Aliases: F28J7.29, F28J7_29	2.3	2.2	0.1	1.0	100.0%	-5.3
7164	AT3G53430.1 | 60S ribosomal protein L12 (RPL12B), 60S RIBOSOMAL PROTEIN L12, Prunus armeniaca, SWISSPROT:RL12_PRUAR | chr3:19820665-19821447 REVERSE | Aliases: F4P12.130	10.0	10.5	-0.6	-1.0	100.0%	-4.2
7165	AT3G57680.1 | peptidase S41 family protein, similar to PSII D1 protein processing enzyme (GI::7268527) (Arabidopsis thaliana); similar to SP:Q55669 Carboxyl-terminal processing protease precursor (Photosystem II D1 protein processing peptidase) (EC 3.4.21.102) (strain PCC 6803) {Synechocystis sp.}; contains Pfam profile PF03572: Peptidase family S41B | chr3:21392031-21394606 FORWARD | Aliases: F15B8.130	5.8	5.1	0.6	1.0	100.0%	-4.1
7166	AT1G22360.2 | Symbol: AT2 | similar to UDP-glucoronosyl/UDP-glucosyl transferase family protein [Arabidopsis thaliana] (TAIR:At1g22380.1); similar to UDP-glucoronosyl/UDP-glucosyl transferase family protein [Arabidopsis thaliana] (TAIR:At1g22340.1); similar to UDP-glucoronosyl/UDP-glucosyl transferase family protein [Arabidopsis thaliana] (TAIR:At1g22400.1); similar to UDP-glucoronosyl/UDP-glucosyl transferase family protein [Arabidopsis thaliana] (TAIR:At1g22370.2); similar to glycosyltransferase NTGT5a [Nicotiana tabacum] (GB:BAD93689.1); contains InterPro domain UDP-glucoronosyl/UDP-glucosyl transferase (InterPro:IPR002213) | chr1:7894847-7897580 REVERSE | Aliases: None	5.2	5.9	-0.7	-1.0	100.0%	-3.9
7167	AT5G59250.1 | sugar transporter family protein, similar to D-xylose-H+ symporter from Lactobacillus brevis GI:2895856, sugar-porter family protein 2 (Arabidopsis thaliana) GI:14585701; contains Pfam profile PF00083: major facilitator superfamily protein | chr5:23921119-23924202 FORWARD | Aliases: MNC17.15, MNC17_15	6.9	6.6	0.3	1.0	100.0%	-4.4
7168	AT1G09820.1 | pentatricopeptide (PPR) repeat-containing protein, contains Pfam profile PF01535: PPR repeat | chr1:3190290-3192416 REVERSE | Aliases: F21M12.21, F21M12_21	4.1	4.3	-0.3	-1.0	100.0%	-4.4
7169	AT1G27400.1 | 60S ribosomal protein L17 (RPL17A), similar to GB:P51413 from (Arabidopsis thaliana); similar to ESTs gb:L33542 and gb:AA660016 | chr1:9515122-9516912 FORWARD | Aliases: F17L21.19, F17L21_19	9.9	10.5	-0.6	-1.0	100.0%	-4.1
7170	AT1G71060.1 | pentatricopeptide (PPR) repeat-containing protein, low similarity to CRP1 (Zea mays) GI:3289002; contains Pfam profile PF01535: PPR repeat | chr1:26809313-26810845 REVERSE | Aliases: F23N20.5, F23N20_5	3.6	3.4	0.3	1.0	100.0%	-4.3
7171	AT3G50270.1 | transferase family protein, similar to anthranilate N-hydroxycinnamoyl/benzoyltransferase, Dianthus caryophyllus (gi:2239091); contains Pfam transferase family domain PF002458 | chr3:18646902-18648323 FORWARD | Aliases: F11C1.110	3.4	3.6	-0.1	-1.0	100.0%	-5.0
7172	AT3G23880.1 | F-box family protein, contains F-box domain Pfam:PF00646	3.7	3.2	0.5	1.0	100.0%	-3.9
7173	AT1G13330.1 | expressed protein, similar to nuclear receptor coactivator GT198 (GI:16506273) {Rattus norvegicus}; similar to TBP-1 interacting protein (GI:7328534) (Homo sapiens) | chr1:4567932-4570565 FORWARD | Aliases: T6J4.9, T6J4_9	4.1	4.4	-0.3	-1.0	100.0%	-4.3
7174	AT2G45380.2 | similar to glycine-rich protein [Arabidopsis thaliana] (TAIR:At4g22740.1); similar to glycine-rich protein [Arabidopsis thaliana] (TAIR:At4g22740.2); similar to putative glycine-rich protein [Oryza sativa (japonica cultivar-group)] (GB:XP_479799.1)	2.8	2.6	0.3	1.0	100.0%	-4.2
7175	AT4G34460.3 | Symbol: AGB1 | guanine nucleotide-binding protein beta subunit (GB1) / GTP-binding protein beta subunit (AGB1) / transducin, contains 7 WD-40 repeats (PF00400); identical to Guanine nucleotide-binding protein beta subunit.SP:P49177 (Arabidopsis thaliana); Weiss, CA et al, PNAS 91:9954 (1994) | chr4:16477194-16479510 REVERSE | Aliases: ARABIDOPSIS G PROTEIN BETA SUBUNIT, ELK4, ERECTA LIKE 4, GTP BINDING PROTEIN BETA 1, T4L20.40, T4L20_40	3.7	3.1	0.6	1.0	100.0%	-4.2
7176	AT2G21640.1 | expressed protein | chr2:9261539-9262705 FORWARD | Aliases: F2G1.9, F2G1_9	4.2	4.0	0.3	1.0	100.0%	-4.3
7177	AT1G20290.1 | expressed protein | chr1:7025538-7029688 REVERSE | Aliases: F14O10.11, F14O10_11	2.5	2.4	0.1	1.0	100.0%	-5.4
7178	AT5G64420.1 | DNA polymerase V family, contains Pfam domain PF04931: DNA polymerase V | chr5:25773558-25778567 FORWARD | Aliases: MSJ1.26, MSJ1_26	5.7	6.0	-0.3	-1.0	100.0%	-4.5
7179	AT5G47600.1 | heat shock protein-related, contains weak similarity to Heat shock 22 kDa protein, mitochondrial precursor (Swiss-Prot:P46254) (Pisum sativum) | chr5:19317008-19317703 REVERSE | Aliases: MNJ7.19, MNJ7_19	4.2	3.7	0.5	1.0	100.0%	-4.4
7180	AT3G52570.1 | expressed protein, contains Interpro entry IPR000379 | chr3:19512108-19513977 FORWARD | Aliases: F3C22.3	7.3	7.0	0.3	1.0	100.0%	-4.5
7181	AT3G50120.1 | expressed protein, contains Pfam profile PF03140: Plant protein of unknown function; expression supported by MPSS | chr3:18596140-18598229 REVERSE | Aliases: F3A4.200	2.6	2.5	0.2	1.0	100.0%	-5.2

Rank	Description	15.dpi	5.dpi	M	t	adj.q	B
7182	AT1G62020.1 | coatomer protein complex, subunit alpha, putative, contains Pfam PF00400: WD domain, G-beta repeat; similar to Coatomer alpha subunit (Alpha-coat protein) (Alpha-COP) (HEPCOP) (HEP-COP) (SP:P53621) (Homo sapiens) | chr1:22923479-22927689 FORWARD | Aliases: F8K4.21, F8K4_21	8.1	8.6	-0.5	-1.0	100.0%	-4.3
7183	AT5G22290.1 | Symbol: ANAC089 | no apical meristem (NAM) family protein, contains Pfam PF02365: No apical meristem (NAM) domain | chr5:7375926-7377626 REVERSE | Aliases: ANAC089, T6G21.9	9.1	8.6	0.5	1.0	100.0%	-4.3
7184	AT2G38400.1 | Symbol: AGT3 | alanine--glyoxylate aminotransferase, putative / beta-alanine-pyruvate aminotransferase, putative / AGT, putative, similar to SP:Q64565 Alanine--glyoxylate aminotransferase 2, mitochondrial precursor (EC 2.6.1.44) (AGT 2) (Beta-alanine-pyruvate aminotransferase) {Rattus norvegicus}; contains Pfam profile PF00202: aminotransferase, class III | chr2:16090769-16093427 FORWARD | Aliases: ALANINE:GLYOXYLATE AMINOTRANSFERASE 2 HOMOLOG, ALANINE:GLYOXYLATE AMINOTRANSFERASE 3, T19C21.11, T19C21_11	7.2	7.6	-0.4	-1.0	100.0%	-4.4
7185	AT5G56910.1 | expressed protein | chr5:23040148-23041428 FORWARD | Aliases: MHM17.2, MHM17_2	8.8	8.2	0.5	1.0	100.0%	-4.1
7186	AT4G39590.1 | kelch repeat-containing F-box family protein, contains F-box domain Pfam:PF00646 and Kelch motif Pfam:PF01344 | chr4:18387325-18388726 REVERSE | Aliases: F23K16.220, F23K16_220	3.1	3.4	-0.3	-1.0	100.0%	-4.6
7187	AT4G22030.1 | F-box family protein, PF0064: F-box domain; similar to F-box protein family, AtFBX7 (GI:20197899) (Arabidopsis thaliana)	3.0	3.3	-0.2	-1.0	100.0%	-4.9
7188	AT3G03100.2 | similar to NADH:ubiquinone oxidoreductase B17.2-like subunit [Chlamydomonas reinhardtii] (GB:AAQ64638.1); contains InterPro domain NADH:ubiquinone oxidoreductase 17.2 kD subunit (InterPro:IPR007763) | chr3:705388-707657 REVERSE | Aliases: T17B22.21, T17B22_21	11.7	12.0	-0.3	-1.0	100.0%	-4.9
7189	AT5G26960.1 | kelch repeat-containing F-box family protein, predicted proteins, Arabidopsis thaliana and Oryza sativa; contains Pfam profiles PF01344: Kelch motif, PF00646: F-box domain | chr5:9484777-9486152 REVERSE | Aliases: F2P16.220, F2P16_220	6.3	5.9	0.4	1.0	100.0%	-4.4
7190	AT5G02410.1 | DIE2/ALG10 family, contains Pfam PF04922: DIE2/ALG10 family | chr5:517210-519754 REVERSE | Aliases: T1E22.170, T1E22_170	3.6	3.9	-0.3	-1.0	100.0%	-4.4
7191	AT3G10830.1 | hypothetical protein | chr3:3390255-3390698 FORWARD | Aliases: T7M13.9	2.2	2.1	0.1	1.0	100.0%	-5.4
7192	AT2G41330.1 | glutaredoxin family protein, contains Pfam profile PF00462: Glutaredoxin	3.6	3.3	0.3	1.0	100.0%	-4.4
7193	AT1G54370.1 | sodium proton exchanger, putative (NHX5), contains similarity to Na+/H+ antiporter GI:1655701 from (Xenopus laevis); Member of The Monovalent Cation:Proton Antiporter (CPA1) Family, PMID:11500563; contains non-consensus AT-AC splice sites at introns 3 and 10 | chr1:20296818-20300796 REVERSE | Aliases: F20D21.19, F20D21_19	2.9	3.3	-0.4	-1.0	100.0%	-4.0
7194	AT3G51530.1 | F-box family protein, various predicted proteins, Arabidopsis thaliana; contains Pfam profile PF00646: F-box domain | chr3:19123399-19125622 REVERSE | Aliases: F26O13.170	7.0	7.4	-0.4	-1.0	100.0%	-4.2
7195	AT2G18410.2 | expressed protein | chr2:7997847-7999856 FORWARD | Aliases: T30D6.8, T30D6_8	4.3	4.5	-0.2	-1.0	100.0%	-4.8
7196	AT3G59090.3 | similar to transmembrane protein-related (TOM1) [Arabidopsis thaliana] (TAIR:At4g21790.1); similar to P0046E05.19 [Oryza sativa (japonica cultivar-group)] (GB:NP_916134.1) | chr3:21850175-21853732 FORWARD | Aliases: F17J16.140	4.0	3.7	0.3	1.0	100.0%	-4.2
7197	AT5G20920.2 | Symbol: EIF2 BETA | eukaryotic translation initiation factor 2 subunit 2, putative / eIF-2-beta, putative, similar to SP:P41035 Eukaryotic translation initiation factor 2 subunit (eIF-2-beta) {Oryctolagus cuniculus}; contains Pfam profile PF01873: Domain found in IF2B/IF5 | chr5:7094721-7097077 REVERSE | Aliases: EMB1401, EMBRYO DEFECTIVE 1401, F22D1.90, F22D1_90	8.7	8.4	0.3	1.0	100.0%	-4.5
7198	AT2G47970.2 | NPL4 family protein, contains Pfam domain, PF05021: NPL4 family | chr2:19636544-19638208 FORWARD | Aliases: T9J23.1	7.5	7.2	0.3	1.0	100.0%	-4.6
7199	AT3G58670.1 | expressed protein | chr3:21714367-21717027 REVERSE | Aliases: T20N10.20	4.6	4.9	-0.3	-1.0	100.0%	-4.3
7200	AT2G46505.1 | Symbol: SDH4 | expressed protein | chr2:19096801-19097621 REVERSE | Aliases: None	8.5	9.2	-0.6	-1.0	100.0%	-4.2
7201	AT5G05080.1 | ubiquitin-conjugating enzyme, putative, similar to SP:Q16763 Ubiquitin-conjugating enzyme E2-24 kDa (EC 6.3.2.19) (Ubiquitin- protein ligase) (Ubiquitin carrier protein) {Homo sapiens}; contains Pfam profile PF00179: Ubiquitin-conjugating enzyme | chr5:1498556-1500780 REVERSE | Aliases: MUG13.6, MUG13_6	7.8	8.4	-0.7	-1.0	100.0%	-4.2
7202	AT1G50620.1 | PHD finger family protein, contains Pfam domain, PF00628: PHD-finger | chr1:18752205-18755006 FORWARD | Aliases: F17J6.14, F17J6_14	3.0	3.1	-0.1	-1.0	100.0%	-5.0

Rank	Description	15.dpi	5.dpi	M	t	adj.q	B
7203	AT3G62120.2 | tRNA synthetase class II (G, H, P and S) family protein, similar to SP:P07814 Bifunctional aminoacyl-tRNA synthetase (Includes: Glutamyl-tRNA synthetase (EC 6.1.1.17) (Glutamate--tRNA ligase); Prolyl-tRNA synthetase (EC 6.1.1.15) (Proline--tRNA ligase)) {Homo sapiens}; contains Pfam profiles PF00587: tRNA synthetase class II core domain (G, H, P, S and T), PF03129: Anticodon binding domain	8.7	9.4	-0.7	-1.0	100.0%	-4.2
7204	AT3G45130.1 | cycloartenol synthase, putative / 2,3-epoxysqualene--cycloartenol cyclase, putative / (S)-2,3-epoxysqualene mutase, putative, 77% similar to cycloartenol synthase (SP:P38605:gi:452446) (PMID: 7505443); oxidosqualene cyclase LcOSC2 - Luffa cylindrica, EMBL:AB033335 | chr3:16523256-16528507 REVERSE | Aliases: T14D3.70	3.4	3.1	0.3	1.0	100.0%	-4.3
7205	AT5G67630.1 | DNA helicase, putative, similar to RuvB-like DNA helicase reptin (Danio rerio) GI:27733814, reptin (Drosophila melanogaster) GI:7243682 | chr5:26984607-26986620 REVERSE | Aliases: K9I9.20, K9I9_20	3.2	3.6	-0.4	-1.0	100.0%	-4.6
7206	AT1G75640.1 | leucine-rich repeat family protein / protein kinase family protein, contains protein kinase domain, Pfam:PF00069; contains leucine-rich repeats, Pfam:PF00560 | chr1:28407261-28410683 REVERSE | Aliases: F10A5.16, F10A5_16	3.0	3.2	-0.2	-1.0	100.0%	-4.6
7207	AT5G17200.1 | glycoside hydrolase family 28 protein / polygalacturonase (pectinase) family protein, similar to polygalacturonase (Lycopersicon esculentum) GI:4325090; contains PF00295: Glycosyl hydrolases family 28 (polygalacturonases) | chr5:5653526-5655338 REVERSE | Aliases: MKP11.14, MKP11_14	3.0	2.8	0.2	1.0	100.0%	-4.8
7208	AT2G36090.1 | F-box family protein, contains Pfam PF00646: F-box domain; similar to SKP1 interacting partner 2 (SKIP2) TIGR_Ath1:At5g67250 | chr2:15165710-15166663 FORWARD | Aliases: F9C22.2, F9C22_2	6.4	5.8	0.6	1.0	100.0%	-4.0
7209	AT5G64500.1 | membrane protein-related, contains weak similarity to spinster type IV (GI:12003976) (Drosophila melanogaster) | chr5:25797608-25800880 FORWARD | Aliases: T12B11.9, T12B11_9	8.7	8.4	0.3	1.0	100.0%	-4.5
7210	AT5G18930.1 | adenosylmethionine decarboxylase family protein, contains Pfam profile: PF01536 adenosylmethionine decarboxylase	2.8	3.0	-0.1	-1.0	100.0%	-5.3
7211	AT5G15090.1 | porin, putative / voltage-dependent anion-selective channel protein, putative, similar to SP:P42055 34 kDa outer mitochondrial membrane protein porin (Voltage-dependent anion-selective channel protein) (VDAC) {Solanum tuberosum}; contains Pfam profile PF01459: Eukaryotic porin; identical to cDNA voltage-dependent anion-selective channel protein GI:4006940 | chr5:4889298-4891475 REVERSE | Aliases: F2G14.210, F2G14_210	8.3	8.9	-0.5	-1.0	100.0%	-4.7
7212	AT1G06145.1 | similar to pentatricopeptide (PPR) repeat-containing protein [Arabidopsis thaliana] (TAIR:At5g66520.1); similar to putative pentatricopeptide (PPR) repeat-containing protein [Oryza sativa (japonica cultivar-group)] (GB:XP_450548.1); contains InterPro domain PPR repeat (InterPro:IPR002885) | chr1:1867014-1868871 REVERSE | Aliases: None	4.3	4.5	-0.2	-1.0	100.0%	-4.6
7213	AT1G07770.2 | Symbol: RPS15A |	11.2	11.5	-0.2	-1.0	100.0%	-4.7
7214	AT2G20980.1 | expressed protein | chr2:9018735-9020937 REVERSE | Aliases: F26H11.26, F26H11_26	4.2	4.6	-0.3	-1.0	100.0%	-4.4
7215	AT4G22830.1 | expressed protein | chr4:11990149-11991379 REVERSE | Aliases: F7H19.1	3.1	2.9	0.2	1.0	100.0%	-4.5
7216	AT5G20410.1 | Symbol: MGD2 | 1,2-diacylglycerol 3-beta-galactosyltransferase, putative / monogalactosyldiacylglycerol synthase, putative / MGDG synthase, putative, identical to monogalactosyldiacylglycerol synthase (gi:3367638) from Arabidopsis thaliana, similar to MGDG synthase type A (gi:9884651) from Glycine max | chr5:6896679-6898785 FORWARD | Aliases: ATMGD2, F5O24.300, F5O24_300	5.4	5.0	0.4	1.0	100.0%	-4.1
7217	AT5G14530.1 | transducin family protein / WD-40 repeat family protein, similar to Will die slowly protein (SP:Q9V3J8) (Drosophila melanogaster) ; contains Pfam PF00400: WD domain, G-beta repeat (4 copies, 1 weak) | chr5:4684623-4686912 REVERSE | Aliases: T15N1.20, T15N1_20	6.0	5.6	0.4	1.0	100.0%	-4.1
7218	AT3G14350.3 | leucine-rich repeat transmembrane protein kinase, putative, similar to leucine-rich repeat transmembrane protein kinase 1 GB:AAC27894 from (Zea mays) | chr3:4782764-4787174 REVERSE | Aliases: MLN21.15	5.3	5.7	-0.4	-1.0	100.0%	-4.2
7219	AT1G03260.1 | expressed protein | chr1:795532-798463 REVERSE | Aliases: F15K9.14, F15K9_14	5.2	5.0	0.3	1.0	100.0%	-4.5
7220	AT5G09790.1 | PHD finger family protein / SET domain-containing protein, contains Pfam domain, PF00628: PHD-finger and PF00856: SET domain | chr5:3038979-3041045 REVERSE | Aliases: F17I14.20, F17I14_20	3.7	4.0	-0.3	-1.0	100.0%	-4.2
7221	AT4G00390.1 | expressed protein, contains Pfam profile: PF04504 protein of unknown function, DUF573 | chr4:171497-172843 REVERSE | Aliases: A_IG005I10.6, A_IG005I10_6, F5I10.6, F5I10_6	3.9	3.6	0.3	1.0	100.0%	-4.5
7222	AT4G16970.1 | protein kinase family protein, contains protein kinase domain, Pfam:PF00069 | chr4:9551478-9555839 REVERSE | Aliases: DL4515C, FCAALL.319	3.6	3.9	-0.3	-1.0	100.0%	-4.4

Rank	Description	15.dpi	5.dpi	M	t	adj.q	B
7223	AT1G76090.1 | Symbol: SMT3 | S-adenosyl-methionine-sterol-C-methyltransferase, identical to S-adenosyl-methionine-sterol-C-methyltransferase GI:2246456 from (Arabidopsis thaliana) | chr1:28555303-28556675 REVERSE | Aliases: SMT3, T23E18.40, T23E18_40	5.6	5.0	0.6	1.0	100.0%	-4.5
7224	AT1G34440.1 | expressed protein | chr1:12591326-12592715 REVERSE | Aliases: F12K21.23, F12K21_23	3.2	3.4	-0.2	-1.0	100.0%	-4.9
7225	AT1G09240.1 | nicotianamine synthase, putative, similar to nicotianamine synthase (Lycopersicon esculentum)(GI:4753801), nicotianamine synthase 2 (Hordeum vulgare)(GI:4894912) | chr1:2984883-2986132 FORWARD | Aliases: T12M4.5, T12M4_5	3.3	3.1	0.2	1.0	100.0%	-4.8
7226	AT4G29210.2 | gamma-glutamyltranspeptidase family protein, similar to SP:P07314 Gamma-glutamyltranspeptidase precursor (EC 2.3.2.2) (Gamma-glutamyltransferase) (GGT) {Rattus norvegicus}; contains Pfam profilePF01019: Gamma-glutamyltranspeptidase	5.6	5.9	-0.3	-1.0	100.0%	-4.3
7227	AT5G20400.1 | oxidoreductase, 2OG-Fe(II) oxygenase family protein, similar to naringenin,2-oxoglutarate 3-dioxygenase (flavonone-3-hydroxylase) (SP:Q06942)(Malus domestica); contains PF031712OG-Fe(II) oxygenase superfamily domain | chr5:6894856-6896351 FORWARD | Aliases: F5O24.290, F5O24_290	7.6	7.9	-0.3	-1.0	100.0%	-4.6
7228	AT3G13650.1 | disease resistance response protein-related/ dirigent protein-related, similar to dirigent protein (Thuja plicata) gi:6694699:gb:AAF25360; similar to pathogenesis-related protein (Pisum sativum) gi:4585273:gb:AAD25355 | chr3:4462899-4463879 FORWARD | Aliases: MMM17.15	5.5	6.0	-0.5	-1.0	100.0%	-4.1
7229	AT4G34200.1 | D-3-phosphoglycerate dehydrogenase, putative / 3-PGDH, putative, similar to phosphoglycerate dehydrogenase, Arabidopsis thaliana, SP:O04130 | chr4:16373850-16376626 REVERSE | Aliases: F10M10.7	8.3	9.2	-0.9	-1.0	100.0%	-4.0
7230	AT5G14105.1 | expressed protein | chr5:4551991-4553053 REVERSE | Aliases: None	8.9	9.2	-0.3	-1.0	100.0%	-4.3
7231	AT1G20540.1 | transducin family protein / WD-40 repeat family protein, contains 6 WD-40 repeats (PF00400); similar to Rbap46 polypeptide (GI:9454362) (Gallus gallus) | chr1:7112611-7115810 FORWARD | Aliases: F5M15.14, F5M15_14	8.6	9.0	-0.4	-1.0	100.0%	-4.4
7232	AT2G39170.1 | expressed protein | chr2:16341708-16343288 REVERSE | Aliases: T16B24.19, T16B24_19	6.4	6.1	0.3	1.0	100.0%	-4.3
7233	AT5G38150.1 | expressed protein | chr5:15240346-15242165 REVERSE | Aliases: MXA21.20, MXA21_20	4.3	4.7	-0.4	-1.0	100.0%	-4.2
7234	AT5G11850.1 | protein kinase family protein, contains Pfam domain, PF00069: Protein kinase domain; identical to cDNA MAP3K delta-1 protein kinase GI:2253009 | chr5:3816347-3821073 REVERSE | Aliases: F14F18.20, F14F18_20	4.9	5.1	-0.2	-1.0	100.0%	-4.6
7235	AT5G47070.1 | protein kinase, putative, similar to protein kinase (Lophopyrum elongatum) gi:13022177:gb:AAK11674 | chr5:19135770-19138136 REVERSE | Aliases: K14A3.2, K14A3_2	6.3	5.8	0.5	1.0	100.0%	-4.2
7236	AT1G55560.1 | Symbol: SKS14 | multi-copper oxidase type I family protein, similar to pollen-specific BP10 protein (SP:Q00624)(Brassica napus); contains Pfam profile: PF00394 Multicopper oxidase | chr1:20757989-20760320 REVERSE | Aliases: SKS14, T5A14.2, T5A14_2	2.2	2.3	-0.1	-1.0	100.0%	-5.6
7237	AT3G08930.2 | LMBR1 integral membrane family protein, contains 5 transmembrane domains; contains Pfam PF04791: LMBR1-like conserved region; similar to unknown protein GB:BAA83351 (Oryza sativa) | chr3:2713410-2717318 FORWARD | Aliases: T16O11.13	7.5	7.0	0.5	1.0	100.0%	-4.4
7238	AT3G17150.1 | invertase/pectin methylesterase inhibitor family protein, low similarity to SP:P83326 Pectinesterase inhibitor (Pectin methylesterase inhibitor) (PMEI) {Actinidia chinensis}; contains Pfam profile PF04043: Plant invertase/pectin methylesterase inhibitor | chr3:5847297-5849550 FORWARD | Aliases: K14A17.27	2.9	2.7	0.2	1.0	100.0%	-4.8
7239	AT5G63730.1 | IBR domain-containing protein, contains similarity to Swiss-Prot:Q94981 ariadne-1 protein (Ari-1) (Drosophila melanogaster) and Pfam:PF01485 IBR domain | chr5:25525326-25526932 REVERSE | Aliases: MBK5.21, MBK5_21	3.2	3.0	0.2	1.0	100.0%	-4.9
7240	AT3G60480.1 | expressed protein | chr3:22358426-22359213 REVERSE | Aliases: T8B10.140	9.1	8.8	0.2	1.0	100.0%	-4.5
7241	AT2G04700.1 | ferredoxin thioredoxin reductase catalytic beta chain family protein, contains Pfam profile: PF02943 ferredoxin thioredoxin reductase catalytic beta | chr2:1646805-1648554 FORWARD | Aliases: F28I8.26, F28I8_26	7.3	7.0	0.3	1.0	100.0%	-4.7
7242	AT3G24140.1 | basic helix-loop-helix (bHLH) family protein, contains Pfam profile: PF00010 Helix-loop-helix DNA-binding domain	2.8	2.7	0.2	1.0	100.0%	-4.9
7243	AT2G29250.1 | lectin protein kinase, putative, similar to receptor lectin kinase 3 (Arabidopsis thaliana) gi:4100060:gb:AAD00733; contains protein kinase domain, Pfam:PF00069; contains legume lectins alpha and beta domains, Pfam:PF00138 and Pfam:PF00139 | chr2:12585986-12587857 REVERSE | Aliases: F16P2.37, F16P2_37	2.6	2.5	0.1	1.0	100.0%	-5.2
7244	AT2G31820.1 | ankyrin repeat family protein, contains ankyrin repeat domains, Pfam:PF00023 | chr2:13537427-13539639 FORWARD | Aliases: F20M17.14, F20M17_14	8.0	7.8	0.2	1.0	100.0%	-4.9

Rank	Description	15.dpi	5.dpi	M	t	adj.q	B
7245	AT2G28590.1 | protein kinase family protein, contains protein kinase domain, Pfam:PF00069 | chr2:12256912-12258745 FORWARD | Aliases: T8O18.12, T8O18_12	5.1	5.4	-0.3	-1.0	100.0%	-4.4
7246	AT1G07010.1 | calcineurin-like phosphoesterase family protein, contains Pfam profile: PF00149 calcineurin-like phosphoesterase	6.4	6.0	0.4	1.0	100.0%	-4.1
7247	AT2G18710.1 | preprotein translocase secY subunit, chloroplast (CpSecY), Identical to SP:Q38885 Preprotein translocase secY subunit, chloroplast precursor (CpSecY) {Arabidopsis thaliana} | chr2:8119166-8121559 REVERSE | Aliases: MSF3.9, MSF3_9	7.6	6.8	0.7	1.0	100.0%	-4.2
7248	AT5G56380.1 | F-box family protein, similar to unknown protein (emb:CAB62440.1); contains Pfam profile PF00646: F-box domain | chr5:22855898-22858241 REVERSE | Aliases: MCD7.14, MCD7_14	4.0	4.3	-0.3	-1.0	100.0%	-4.5
7249	AT1G06820.1 | Symbol: CRTISO |	5.4	5.6	-0.3	-1.0	100.0%	-4.6
7250	AT3G15540.1 | Symbol: IAA19 | auxin-responsive protein / indoleacetic acid-induced protein 19 (IAA19), identical to SP:O24409 Auxin-responsive protein IAA19 (Indoleacetic acid-induced protein 19) {Arabidopsis thaliana} | chr3:5264031-5265683 FORWARD | Aliases: MJK13.22, MSG2	5.2	5.5	-0.3	-1.0	100.0%	-4.4
7251	AT3G46290.1 | protein kinase, putative, similar to receptor-like protein kinase (Catharanthus roseus) gi:1644291:emb:CAA97692 | chr3:17023994-17026772 FORWARD | Aliases: F12M12.260	2.8	2.7	0.1	1.0	100.0%	-4.9
7252	AT3G29185.2 | expressed protein | chr3:11156166-11158444 REVERSE | Aliases: MXO21.1	6.5	6.8	-0.3	-1.0	100.0%	-4.3
7253	AT5G04190.1 | phytochrome kinase substrate-related, contains similarity to Swiss-Prot:Q9SWI1 phytochrome kinase substrate 1 (Arabidopsis thaliana) | chr5:1150893-1152482 FORWARD | Aliases: F21E1.110, F21E1_110	2.3	2.4	-0.1	-1.0	100.0%	-5.3
7254	AT1G63870.1 | disease resistance protein (TIR-NBS-LRR class), putative, domain signature TIR-NBS-LRR exists, suggestive of a disease resistance protein. | chr1:23710794-23715564 REVERSE | Aliases: T12P18.11, T12P18_11	2.7	2.8	-0.1	-1.0	100.0%	-5.2
7255	AT1G59940.1 | Symbol: ARR3 | two-component responsive regulator / response regulator 3 (ARR3), identical to response regulator 3 GI:3953595 from (Arabidopsis thaliana) | chr1:22069282-22070638 REVERSE | Aliases: F23H11.25, F23H11_25, RESPONSE REGULATOR 3	3.9	4.1	-0.3	-1.0	100.0%	-4.3
7256	AT5G11500.1 | expressed protein, contains Pfam profile PF05670: Domain of unknown function (DUF814) | chr5:3676641-3679347 FORWARD | Aliases: F15N18.90, F15N18_90	11.1	10.8	0.3	1.0	100.0%	-5.0
7257	AT1G73320.2 | similar to Expressed protein [Arabidopsis thaliana] (TAIR:At1g08125.1); similar to P0684C02.12 [Oryza sativa (japonica cultivar-group)] (GB:NP_915768.1)	4.6	4.2	0.4	1.0	100.0%	-4.2
[truncated: 591,797 more chars]
